# What is the Relationship between Risky Outdoor Play and Health in Children? A Systematic Review

**DOI:** 10.3390/ijerph120606423

**Published:** 2015-06-08

**Authors:** Mariana Brussoni, Rebecca Gibbons, Casey Gray, Takuro Ishikawa, Ellen Beate Hansen Sandseter, Adam Bienenstock, Guylaine Chabot, Pamela Fuselli, Susan Herrington, Ian Janssen, William Pickett, Marlene Power, Nick Stanger, Margaret Sampson, Mark S. Tremblay

**Affiliations:** 1 British Columbia Injury Research & Prevention Unit, Child & Family Research Institute, University of British Columbia, British Columbia Children’s Hospital, F511-4480 Oak Street, Vancouver, BC V6H 3V4, Canada; E-Mail: takuro.ishikawa@cw.bc.ca (T.I.); 2 Department of Pediatrics, School of Population & Public Health, University of British Columbia, British Columbia Children’s Hospital, F511-4480 Oak Street, Vancouver, BC V6H 3V4, Canada; 3 School of Population & Public Health, University of British Columbia, 2206 East Mall, Vancouver, BC V6H 3V4, Canada; E-Mail: rlgibbons6@gmail.com; 4 Healthy Active Living and Obesity Research Group, Children’s Hospital of Eastern Ontario Research Institute, 401 Smyth Road, Ottawa, ON K1H 8L1, Canada; E-Mail: casgray@cheo.on.ca; 5 Department of Physical Education and Health, College of Early Childhood Education, Queen Maud University, Thrond Nergaards Vei 7, NO-7044 Trondheim, Norway; E-Mail: ebs@dmmh.no; 6 Bienenstock Natural Playgrounds, 64 Hatt Street, Dundas, ON L9H 7T6, Canada; E-Mail: adam@naturalplaygrounds.ca; 7 Evaluation Platform on Obesity Prevention, Quebec Heart and Lung Institute, Laval University, 2725 Chemin Ste-Foy, Local Y4283, QC G1V 4G5, Canada; E-Mail: guylaine.chabot@criucpq.ulaval.ca; 8 Parachute, 150 Eglinton Avenue East, Suite 300, Toronto, ON M4P 1E8, Canada; E-Mail: pfuselli@parachutecanada.org; 9 School of Architecture and Landscape Architecture, University of British Columbia, 379-2357 Main Mall, Vancouver, BC B6T 1Z4, Canada; E-Mail: susan.herrington@ubc.ca; 10 School of Kinesiology and Health Studies, Queen’s University, 99 University Avenue, Kingston, ON K7L 2P5, Canada; E-Mail: ian.janssen@queensu.ca; 11 Department of Public Health Sciences, Carruthers Hall, Queen’s University, Kingston, ON K7L 2P5, Canada; E-Mail: will.pickett@queensu.ca; 12 Forest School Canada, 411 Corkstown Road, Ottawa, ON K2K 2Y1, Canada; E-Mail: mpower@forestschoolcanada.ca; 13 Department of Environmental Studies, Huxley College of the Environment, Western Washington University, 416 High Street, Bellingham, Washington, DC 98225, USA; E-Mail: nick.stanger@wwu.edu; 14 Library Services, Children’s Hospital of Eastern Ontario, 401 Smyth Road, Ottawa, ON K1H 8L1, Canada; E-Mail: msampson@cheo.on.ca; 15 Department of Pediatrics, Children’s Hospital of Eastern Ontario Research Institute, 401 Smyth Road, Ottawa, ON K1H 8L1, Canada; E-Mail: mtremblay@cheo.on.ca

**Keywords:** risk taking, physical activity, supervision, injury, independent mobility, playground

## Abstract

Risky outdoor play has been associated with promoting children’s health and development, but also with injury and death. Risky outdoor play has diminished over time, concurrent with increasing concerns regarding child safety and emphasis on injury prevention. We sought to conduct a systematic review to examine the relationship between risky outdoor play and health in children, in order to inform the debate regarding its benefits and harms. We identified and evaluated 21 relevant papers for quality using the GRADE framework. Included articles addressed the effect on health indicators and behaviours from three types of risky play, as well as risky play supportive environments. The systematic review revealed overall positive effects of risky outdoor play on a variety of health indicators and behaviours, most commonly physical activity, but also social health and behaviours, injuries, and aggression. The review indicated the need for additional “good quality” studies; however, we note that even in the face of the generally exclusionary systematic review process, our findings support the promotion of risky outdoor play for healthy child development. These positive results with the marked reduction in risky outdoor play opportunities in recent generations indicate the need to encourage action to support children’s risky outdoor play opportunities. Policy and practice precedents and recommendations for action are discussed.

## 1. Introduction

The use of the word “risk” has changed over time, from a neutral term denoting the probability of a given outcome to being synonymous with “danger” and implying a negative value judgment [1,2,3,4]. In this article, we use the word “risk” in the context of risky play to denote a situation whereby a child can recognize and evaluate a challenge and decide on a course of action [3]. This is in contrast to common use of the word to describe hazards that children cannot assess for themselves and that have no clear benefit [3]. Accordingly, risky play is defined in this article as thrilling and exciting play that can include the possibility of physical injury [5]. Types of risky play include play at height, speed, near dangerous elements (e.g., water, fire), with dangerous tools, rough and tumble play (e.g., play fighting), and where there is the potential for disappearing or getting lost. These categories are based on Sandseter’s research observing children at play, and interviewing them regarding their perceptions of risky play [6,7]. Since publication, they have become commonly used internationally in research on this issue [8,9,10,11,12,13]. Detailed definitions and examples of each type of risky play are provided in Table 1.

**Table 1:** Definitions used to guide the systematic review (risky play behaviours).

**Risky Play**		
Thrilling and exciting forms of play that involve a risk of physical injury. The risk can be real or perceived [7,14]		
**Risky Play Categories** [5,6]	**Definition**	**Examples**
*Great heights*	Danger of injury from falling	Climbing/jumping from surfaces, balancing/playing on high objects (e.g., playground equipment), hanging/swinging at great heights
*High speed*	Uncontrolled speed and pace that can lead to collision with something (or someone)	Swinging at high speed
*Dangerous tools*	Can lead to injuries and wounds	Cutting tools (e.g., knives, saws, or axes), strangling tools (e.g., ropes)
*Dangerous elements*	Where children can fall into or from something	Cliffs, water, fire pits, trees
*Rough and Tumble Play*	Where children can be harmed	Wrestling or play fighting with other children or parents
*Disappear/get lost*	Where children can disappear from the supervision of adults or get lost alone	Exploring alone, playing alone in unfamiliar environments, general independent mobility, or unsupervised play

Some studies support the importance of risky play for children’s development, learning, mental health, and physical health, including physical activity, and healthy weights [5,12,15]. In one study, children in an experimental group exposed to a 14-week risky play intervention improved their risk detection and competence, increased self-esteem and decreased conflict sensitivity, relative to their pre-intervention performance, as well as when compared to a control group [16]. A cross-sectional study compared children with and without ready access to unsupervised outdoor play opportunities and found more developed motor skills, social behaviour, independence and conflict resolution in the former group [17]. Furthermore, experience with risks during childhood is believed to assist with developing risk management strategies, and the ability to negotiate decisions about substance use, relationships and sexual behaviour during adolescence [18,19].

Risky outdoor play opportunities have also been associated with negative health outcomes, such as injury or death. A study of 390 U.S. National Parks identified 46 injury-related fatalities to children and youth [20] among the 542 million visitors to the parks over a 2-year period [21]. While the most common cause was motor vehicle crashes (20%), other causes included risky outdoor play activities such as swimming (11%; play with dangerous elements), hiking and climbing (16%; play at height). Winter sports, such as skiing and snowboarding (play at speed) can also represent an important source of risky outdoor play-related injuries for children, with one review indicating rates of 2.86 to 6.6 injuries per skier days [22]. Playgrounds are a common arena for risky outdoor play. In Canada, approximately 2,500 children age 14 and under are hospitalized annually as a result of playground falls (play at height)—81% are for fractures [23]. Over a 10-year period in the U.S., there were over 2.1 million playground equipment related injuries to children treated in emergency departments, 75% of which were from falls [24]. Approximately 6,000 children were admitted to hospital annually, 92% for fractures [24].

The vast majority of risky outdoor play-related injury incidents result in minor injuries requiring minimal or no medical treatment [25,26,27]. The importance of preventing these minor injuries has been debated in the injury prevention field. Proponents for preventing all injuries cite the impossibility of predicting the consequences of most injury events, such as whether a fall will result in a bruise *vs.* a head injury [28,29]. Others point to the fact that injuries are an inevitable side effect of physical activity, which is necessary for a healthy and active lifestyle [12,30].

In many Western nations, prominent injury prevention strategies for children at play have included playground equipment safety standards and the promotion of close adult supervision [31,32]. Each strategy is described below, along with its potential influence on children’s engagement in risky outdoor play.

### 1.1. Playground Safety Standards

Playground safety standards exist in many nations and influence playground design. The Canadian Standards Association’s (CSA) standards for “Children’s Playspaces and Equipment” CAN/CSA-Z614 [33], originally published in 1990, are voluntary in Canada, but various local and provincial agencies mandate their adherence [34]. Standards can have an important role in ensuring the reduction of hazards on playgrounds that result in serious injuries. For example, head entrapment and strangulation were historically the main causes of death on playgrounds and have now become extremely rare [35,36]. One Canadian study compared injury rates in elementary schools that did and did not replace play equipment in order to meet new standard requirements [37]. Results indicated a decreasing but non-significant downward trend in injury incidents (ranging from minor incidents attended by school staff to a child sent home or to a health facility) in intervention schools; and a non-significant increasing injury trend in non-intervention schools, though they experienced less injuries overall than intervention schools.

Some standards are specifically designed to curtail risky play. For example, CSA standards set limits on the height of play equipment [38,39]. Concerns have been raised that standards have excessively restricted playground design options and resulted in Kit, Fence, Carpet (KFC) playgrounds with limited appeal and affordances for play [34,40,41]. KFC playgrounds have been rated as having inferior opportunities for promoting children’s emotional, social, physical and cognitive development [40]. CSA standards provide recommendations for surfacing materials, including sand, pea gravel, bark mulch and rubber surfacing. Rubber surfacing—the “C” in KFC—has become increasingly popular despite its relatively high cost, limited play affordance [40,41], and increased risk of fractures when compared to bark surfacing [42]. Ball [41] undertook a cost-benefit analysis of rubber surfacing to determine whether it warranted the investment in terms of injury reduction. He found that the relatively rare occurrence of serious injuries and fatalities on playgrounds might not warrant such an extensive and costly intervention that imposes substantial limits on children’s play. Ball [41] points to statistics showing that serious playground injuries in the UK have not decreased as standards have become more stringent and rubber surfacing more common, despite drops in children’s use of playgrounds as they have become less enticing.

### 1.2. Adult Supervision

Research has indicated that higher levels of direct supervision are associated with lower injury rates in children up to 10 years of age [43,44,45]. A study comparing parent supervision practices for children aged 2 to 6.5 years attending an emergency department for an injury with an age/sex matched control group attending for an illness, found that the control group received significantly higher levels of supervision [43]. Another study interviewed parents of children aged 0 to 4 years who were attending a hospital for an injury, regarding the level of supervision provided in the hour before and immediately prior to the injury event [46]. Children admitted to hospital had significantly lower supervision scores than children who were treated and released from the emergency department, indicating an association between quality of supervision and injury severity. Interventions to encourage increased and active caregiver supervision are an important focus for injury prevention [31,32,47,48].

As children grow and develop, parents’ supervision of children tends to transition toward less proximal forms [49]. Morrongiello, Corbett and Kane [50], distinguish between “monitoring” and “supervision” to illustrate this change, defining monitoring as a general awareness of child’s activities, as compared to supervision being a more active watching and listening (note that this distinction is not made in the clinical and developmental psychology literature [51]). Using these definitions, injury prevention research indicates that supervision, not monitoring, is related to lower rates of children’s injury; thus, researchers advocate high levels of active supervision extending throughout childhood and adolescence [50,52]. Morrongiello *et al.* [50] developed the Supervisions Attributes and Risk-Taking Questionnaire (SARTQ) and found that the SARTQ’s parental need for psychological control scale (e.g., “I often tell my child what s/he should do even when s/he has not asked my opinion”), and belief in supervision scale (e.g., “I don’t let my child out of my sight for too long”) were positively related to levels of direct supervision and negatively related to injuries for children aged 7 to 10 years. Schwebel *et al.* [52] found that parental monitoring was not a predictor of injury in 11 year olds, and hypothesized that this was because children were increasingly making decisions without parents’ input. They speculated that the decision-making skills of children and adolescents were not yet sufficiently developed and encouraged increased adult supervision for injury prevention.

Interventions to promote supervision have a direct impact on children’s opportunities for risky outdoor play. Many of the behaviours that are discouraged in interventions to promote caregiver supervision while children are at play (e.g., “Stamp-in-Safety” [47] and “Playground Safety Stars” [53]) are examples of risky outdoor play. Furthermore, caregivers are encouraged to actively supervise children, which would largely eliminate independent mobility and reduce opportunities for the other types of risky outdoor play.

### 1.3. Influence of Injury Prevention on Risky Outdoor Play and Injury Rates

Parental and societal attitudes placing ever-increasing emphasis on supervision and child injury prevention [54,55,56] have influenced children’s outdoor unsupervised activity, including independent mobility, and other opportunities to engage in risky outdoor play [54,57,58,59]. A study retrospectively comparing the play experiences of American mothers with those of their children aged 3 to 12 years found substantial decreases in time spent outdoors and in unstructured play, and increases in adult-structured activities [60]. Of the 830 respondents, 82% identified safety concerns, such as abduction and traffic, as limiting their children’s outdoor play. Similarly, while 75% of UK adults recalled playing in their local streets, 40% of children aged 7 to 11 years reported playing there in 2009 [61]. Adults reported local streets (29%) as their most favoured places to play in childhood; whereas children favoured playing inside a home (41%) [61]. Generational decreases in permission to travel to school without an adult between the ages of 7 to 11 years have been documented in England, from 86% in 1971, to 35% in 1990, and 25% in 2010 [58].

The influence of injury prevention strategies such as playground standards and supervision on injury rates is not clear. In Canada, playground related hospitalizations for children aged 1 to 13 years decreased from 45.8 to 32.7 per 100,000 population between 1994/1995 and 2011/2012 [35]. It is likely that a combination of factors influenced this trend, including injury prevention strategies, but also decreases in children’s time spent at playgrounds, possibly because playgrounds became less enticing, and/or resulting from increased parental fear for child safety, active supervision and reduced independent mobility.

Notable drops in playground injury hospitalization rates have not been documented in other nations with similar approaches to injury prevention. In the U.S., rates remained relatively stable between 1992 and 2005 [24,62]. Similarly, UK data show no consistent pattern between 1988 and 1999 [41]. In the Netherlands, there was an increase in injury rates between 1996 and 2009 [63].

### 1.4. What is the Relationship between Risky Outdoor Play and Health?

Child injury prevention programs have largely sought to limit risky play because of the possibility of physical injury. Societal and parental attitudes have also encouraged increasing supervision and diminishing independence, resulting from concerns about safety and abduction, as well as expectations that parents not appear to be neglectful of their children [64,65,66]. As efforts to keep children safe have expanded, their access to risky outdoor play has diminished [54,58,67]. There has been increasing discussion of children’s developmental need for risky outdoor play, and the potential for adverse consequences from a lack of risky outdoor play experiences on other aspects of children’s health and health behaviours [5,8,12,68]. Relevant literature has been published in a variety of disciplines but has not yet been synthesized to inform the discussion. The purpose of this systematic review is to examine the relationship between risky outdoor play and health related behaviours and outcomes in children, including physical activity, injuries, motor skill development, social health, mental health and spiritual health.

## 2. Methods

The current review is registered with the international prospective register of systematic reviews PROSPERO network (registration No. CRD42014006838).

### 2.1. Study Inclusion Criteria

The review aimed to identify all studies that examined the relationship between risky outdoor play and health related outcomes in children (aged 3.00–12.99 years). In studies that specified the school level of participants rather than the age, the standard age range for that grade level in the region where the study was conducted was used. Studies were included if risky play behaviours (see Table 1) identified by Sandseter and colleagues [5,6] were measured, or if environments that afford risky play (see Table 2) were observed or purposefully created. Eligible exposures of risky play included those obtained via objective (e.g., GIS, standard measuring tape measurement of vertical height of playground equipment) and subjective (e.g., researcher observations of rough and tumble play frequency, parent-reported permission for unsupervised play) measurement. Furthermore, studies were required to include a less risky or non-risky play behavioural or environmental comparison (including internal comparison) or control.

**Table 2:** Definitions used to guide the systematic review (risky play environments).

**Risky Play Environment** Environment that affords or accommodates risky play behaviours [69].		
**Affordances** Features of the environment can enable and invite children to engage in certain types of play behaviours [70]. Affordances are unique for each individual and can be influenced by personal characteristics (e.g., strength, fear) and other features that may inspire or constrain actions (e.g., trees with low branches afford climbing).		
**Risky Play Environments**	**Affordances for Risky Play**	**Risky Play Category**
*Climbable features* [69]	Affords climbing	Great heights
*Jump down-off-able features* [69]	Affords jumping down	Great heights
*Balance-on-able features* [69]	Affords balancing	Great heights
*Flat, relatively smooth surfaces* [69]	Affords running, RTP	High speed, RTP
*Slopes and slides* [69]	Affords sliding, running	High speed
*Swing-on-able features* [69]	Affords swinging	High speed, great heights
*Graspable/detached objects* [69]	Affords throwing, striking, and fencing	RTP
*Dangerous tools* [69]	Affords whittling, sawing, axing, and tying	Dangerous tools
*Dangerous elements* close to where the children play (e.g., lake/pond/sea, cliffs, fire pits, *etc.*) [69]	Affords falling into or from something	Dangerous elements
*Enclosure/restrictions* [69] (e.g., differently sized sub-spaces or private spaces where children can explore on their own or hide away from larger groups, mobility license [39,70])	Affords getting lost, disappearing	Disappear/get lost

RTP = rough and tumble play.

Positive and negative health related outcomes were considered in terms of the four domains of the expanded definition of health endorsed by the World Health Organization Executive Board in 1998 [71]: “Health is a dynamic state of complete physical, mental, spiritual and social well-being and not merely the absence of disease or infirmity”.

Restriction of children’s opportunities for risky play is increasingly being discussed in terms of a potential negative impact on physical activity behaviours (e.g., [68]). In light of being recognized by the World Health Organization’s Global Strategy to combat non-communicable diseases [72], physical activity and related behaviours (*i.e.*, sedentary behaviour) were included as outcomes in this review.

To allow for precision in our assessment of the relationships between risky play, physical activity and sedentary behaviour, we differentiated between acute (single bout) and habitual (usual) outcome behaviours. To be categorized as acute, the outcome behaviour must have been measured during exposure to the risky play activity and the comparator activity (e.g., sedentary behaviour measured during play in an adventure playground and during play on a traditional playground), such that it was possible to compare the behaviour in each setting. To be categorized as habitual, the assessment of the exposure and outcome must have been reported in generalities (e.g., play where children can disappear/get lost assessed as average amount of time children were allowed to play without supervision in a typical week and physical activity assessed as average reported minutes per week of MVPA) such that it was possible to determine the strength of association between engaging in a risky play behaviour and usual physical activity and sedentary behaviour levels.

Study designs eligible for inclusion were randomized controlled trial (RCT) and non-randomized controlled study (NRS) designs (e.g., cross sectional, retrospective, prospective, case control, longitudinal, controlled before-and-after studies). In longitudinal studies, data that aligned with our age criteria at a baseline or follow-up assessment were retained and earlier or later data assessments conducted while children were aged outside of that range were excluded.

### 2.2. Study Exclusion Criteria

As the volume of literature on risky play for most indicators was anticipated to be very low, we limited our exclusion criteria. Studies were excluded if they examined indoor play, structured/organized sport, the use of risky substances or risky sexual behaviours; if the mean age of participants was less than 3 years or greater than 12.99 years; and if the outcome of interest was not in line with one of the four categories included in the World Health Organization’s 1998 expanded definition of health [71]. Non-English studies were only excluded if they could not be translated using Google Translate. The volume of literature on injuries was anticipated to be very high, and to have been largely captured by existing systematic reviews dealing with falls and supervision (e.g., [73,74]). To extend—not duplicate—existing work, studies were excluded if the total number of children exposed to the risky play exposure was not identified, such that relative risk of injury could not be determined.

### 2.3. Search Strategy

The risky play electronic search strategy was created by Margaret Sampson and conducted in MEDLINE (1946–11 December 2013) and PsycInfo (1806–December 2013, week 2) using the Ovid interface. CINAHL, SportDiscus (EBSCOhost), and ERIC (Proquest) were searched from database inception to 12 December 2013. All co-authors were canvassed to nominate relevant studies to guide Margaret Sampson in the development of the search strategy. Reference lists of books on the topic of risky play, proceedings of the Risky Play Symposium “As safe as possible or as safe as necessary: Can injury prevention include healthy risk promotion?” [68], eligible studies and closely related articles were reviewed. PubMed “related citations” searching was conducted on eligible studies and closely related articles. Key content experts were contacted and asked to identify the most influential papers from their personal libraries examining risky play and health in (children) to ensure no key relevant articles were missed by the search. No new relevant articles were identified through key content experts.

The initial search identified that studies tended to cluster around disappear/get lost (*i.e.*, independent mobility, unsupervised play) and rough and tumble play behaviours. Supplemental searches were conducted to target these risky play behaviours specifically. The rough and tumble play targeted search was conducted 7–10 March 2014 and the disappear/get lost targeted search was conducted 17–18 March 2014 using the same sources as the initial search. The search strategies can be found in Supplementary File 1. References were imported into Reference Manager Software (Thompson Reuters, San Francisco, CA, USA) where duplicate references were removed (Margaret Sampson and Rebecca Gibbons).

Two reviewers screened titles and abstracts of potentially relevant articles (Rebecca Gibbons and Takuro Ishikawa). Two independent reviewers examined all full text articles (Rebecca Gibbons and Casey Gray). Any discrepancies were resolved by discussion and consensus between the two reviewers. Consensus was achieved for all eligibility decisions.

### 2.4. Data Extraction and Quality Assessment

Data extraction was completed by Rebecca Gibbons and checked by Casey Gray The quality of evidence for all studies was assessed by Casey Gray and a subset was checked by Takuro Ishikawa [36]. The Grading of Recommendations Assessment, Development and Evaluation (GRADE) framework was used to assess the quality of the evidence from this systematic review. Important features of each study were identified (*i.e.*, study design, risk of bias, consistency of results, directness of the intervention, precision of results, and possibility of a dose response gradient) and their potential influence on the estimate of effect for each outcome was judged [75]. Risk of bias for each individual study was examined in accordance with the Cochrane Handbook (http://handbook.cochrane.org/). The quality of evidence for each outcome of interest was examined separately for RCTs, which according to GRADE start as high quality evidence, and Non-Randomized Studies, which start as low quality evidence. The quality of each was rated down if most of the included studies were judged as having a high risk of bias. The quality of evidence was rated if there was evidence of a large effect or a dose response gradient [75].

The nature of risky active play interventions makes it impossible to blind participants and caregivers to group allocation. In addition, the frequent use of caregiver-, teacher-, and self-report measures to assess risky play type and health outcomes is likely to introduce a degree of social desirability bias. However, if these were the only potential sources of bias identified the quality of the evidence was not downgraded, following the guidance of Timmons *et al.* [76]. Studies were divided by type of risky play (e.g., rough and tumble play, great heights), and subdivided by health indicator and type of study design (RCT and Non-Randomized Studies). Details on data extraction follow in subsequent sections of this manuscript. Details on GRADE methodology can be found elsewhere [75].

### 2.5. Analysis

Meta-analysis was planned where data were sufficiently homogeneous in terms of statistical, clinical, and methodological characteristics. Otherwise, narrative syntheses were conducted. A priori comparisons for subgroup analysis were planned for gender if data reporting permitted. Studies that examined risky play supportive environments were grouped together as it would not be possible to disentangle or attribute specific health indicators to the various types of risky play behaviour affordances in these studies.

## 3. Results

**Figure 1:**
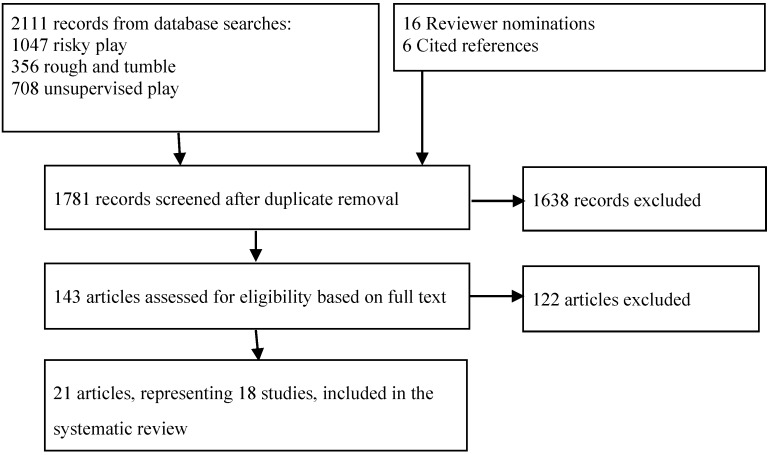
PRISMA flow diagram.

The PRISMA flow diagram for study inclusion and exclusion is included in Figure 1. All studies included in the review are summarized in Supplementary File 2, Table S1. All records that were screened at level 1 are included in Supplementary File 3. Eighteen eligible studies (21 articles) were identified from eight countries, with a cumulative sample of ~50,000 participants. The final sample included seven studies where children can disappear/get lost, one study involving great heights, five studies of rough and tumble play, and five studies of risky play supportive environments. No studies specifically examining the relationship between high speeds, dangerous elements or dangerous tools with indicators of health were found. Most studies included results for more than one health indicator and were presented accordingly. Due to heterogeneity in the measurement of risky play and health indicators used in each study, meta-analysis was not possible. In some cases, relative risk was not provided and these studies were retained for descriptive purposes. Results were summarized for all included studies, and where analytical methods were sufficiently homogenous, narrative synthesis was conducted. Quality of evidence is provided in the Summary of Findings in Table 3, Table 4, Table 5 and Table 6.

**Table 3:** Association between “play where the children can disappear/get lost” and health in children and youth.

Quality Assessment							No. of Participants	Absolute Effect (95% CI, SE)	Quality
No. of Studies	Design	Risk of Bias	Inconsistency	Indirectness	Imprecision	Other Considerations			
**Habitual physical activity (age range between 10 and 15 years, data collected over a single session up to a 5 year follow-up, habitual physical activity measured using accelerometry, pedometry, and scores on the Physical Activity Questionnaire for Children)**									
5	Observational studies ^**a**^	No serious risk of bias ^**b**^	No serious inconsistency ^**c**^	Serious indirectness ^**d**^	No serious imprecision	None	3915 ^**e**^	F_388_ = 6.2, *p* = 0.013	VERY LOW
								F_467_ = 7.3, *p* = 0.017	
								F_388_ = 6.2, *p* = 0.013	
								F_467_ = 5.8, *p* = 0.017	
								F_388_ = 3.7, *p* = 0.040	
								F_388_ = 3.4, *p* = 0.049	
								Boys % time LPA = 26.2 (7.3), MVPA 5.9 (3.6), *p* < 0.05	
								Girls % time LPA 23.7 (7.6), MVPA 3.9 (2.5), *p* < 0.05 ^**f**^	
								*b* = 29.3, SE^2^ ± 9.57	
								CI: 9.39–50.06, *p* < 0.01	
								*b* = 32.43 ± 13.53	
								CI: 3.23–61.62, *p* = 0.03 ^**g**^	
								P7 boys high IM = 87.4%, low IM = 74.8%, *p* = 0.012	
								OR = 2.44, CI: 1.10–5.41, *p* < 0.05	
								S2 girls high IM = 36.2%, low IM = 16.9%, *p* = 0.002	
								OR = 4.50, CI: 1.95–10.4, p < 0.05 ^**h**^	
								*r* = 0.180, *p* = 0.001; *r* = 0.112, *p* = 0.001; *r* = 0.188, *p* = 0.001	
								*r* = 0.092, *p* = 0.005	
								Beta = 33.55, CI: 19.23, 47.87, *x* = 4.59, *p* < 0.001	
								Beta = 17.89, CI: 6.20, 29.58, *x* = 3.00, *p* = 0.003	
								Beta = 24.13 (4.40, 43.78), *x* = 2.41, *p* = 0.016	
								Beta = 30.48 (16.73, 44.23), *x* = 4.35, *p* = 0.001	
								Beta = 21.03 (8.43, 33.64), *x* = 3.27, *p* = 0.001 ^**i**^	
								OR = 1.58 ± 0.228, CI: 1.19–2.10, *p* = 0.002	
								OR = 1.49 ± 0.194, CI: 1.16–1.93, *p* = 0.002	
								OR = 1.47 ± 0.236, CI: 1.08–2.02, *p* = 0.015 ^**j**^	
**Acute physical activity (age range between 0 and 18 years, data were collected over the course of one week, up to 2 months, acute PA measured through accelerometry and direct observation using SOPARC)**									
1	Observational studies ^**k**^	No. serious risk of bias	No serious inconsistency	No serious indirectness ^**l**^	Serious imprecision ^**m**^	None	2712	Estimate = −0.592, SE = 0.125, *t* = −4.73, *p* < 0.0001	VERY LOW
								OR = 0.55 (0.30–0.79);	
								Estimate = −0.592, SE = 0.125, *t* = −4.73, *p* < 0.0001	
								OR = 0.69 (0.42–0.95) ^**n**^	
**Social competence(age range between 7 and 12 years, data were collected during one session, social health was measured through semi-structured maternal interview)**									
1	Observational studies °	High risk of bias ^**p**^	No serious inconsistency	No serious indirectness	Serious imprecision ^**q**^	None	251	*r* = 0.37, *p* < 0.001; *r* = 0.15, *p* < 0.05; *r* = 0.16 *p* < 0.05; *r* = −0.15, *p* < 0.05 ^**r**^	VERY LOW

**Notes**: CI, 95% confidence interval; CPM, counts per minute; IM, independent mobility; LPA, light physical activity; MVPA, moderate to vigorous physical activity; OR, odds ratio; PA, physical activity; SOPARC, System for Observing Play and Recreation in Communities; **Habitual physical activity**: 0 Randomized trials, 5 observational studies [77,78,79,80,81,82]; **^a^** Includes 4 cross-sectional studies [77,78,79,80,81] and 1 longitudinal study [82]; **^b^** No psychometric testing of independent play measure [77]. Participants with excluded weekday PA data had significantly higher mean scores for Area-IM and minutes of daylight from 3:00 pm to sunrise. Participants with excluded weekend PA had significantly higher mean Local-IM and Area-IM, and lived in less deprived neighbourhoods [78]; 51% of recruited participants were included in the analysis as a result of attrition and missing data. A higher proportion of excluded children were from schools in outer suburban neighbourhoods (64.9% *vs.* 35.1%), and a lower proportion was from schools in inner urban (35.7% *vs.* 64.3%) and regional neighbourhoods (27.6% *vs.* 72.4%) [81]; **^c^** In a study of 927 children, in which 22.3% could play in any street, park, or go for a walk without an adult (parent reported data), IM was not associated with likelihood of achieving recommended pedometer based PA cut-points in boys or girls [82]; **^d^** Two studies used indirect measures of habitual PA [79,81]; Sample included 14 and 15 year old children [77]; **^e^** Page *et al.* [78,79] used the same sample of 1300 children from the PEACH Project. Study findings are reported for both papers however, participants are only counted once; **^f^** Boys with more IM were more active overall on weekdays (509,174.8 counts/day) than boys with less IM (472,530.2); girls with more IM were more active overall on weekdays (406,276.1) than girls with less IM (472,530.2 counts/day); boys with more IM engaged in more MVPA on weekdays (40.4 min) than boys with less IM (36.1 min); girls with more IM engaged in more MVPA on weekdays (27.5 min) than girls with less IM (24.9); Boys with more IM were more active overall on weekend days (395,607.5 counts/day) than boys with less IM (360,493.0 counts/day); girls with more IM were more active overall on weekend days (341,835.3 counts/day) than girls with less IM (28,722.3 counts/day), *p*’s < 0.05, F statistic was not reported; Boys with more IM had greater weekend MVPA (27.5 min) than boys with less IM (24.2 min), although neither group achieved recommended levels. Boys with more IM had more weekend light PA (165.5 min) than boys with less IM (164.2), *p* < 0.05, F statistic was not reported. Percentage of time boys granted high IM spent engaged in light PA and MVPA, respectively, was significantly higher than in boys with low IM (23.7 (7.2) and 4.2 (2.9) minutes in LPA and MVPA, respectively); Percentage of time girls granted high IM spent engaged in light PA and MVPA, respectively, compared with 22.2 (5.6) and 3.2 (2.2) for girls with low IM. F statistic was not reported [80]; **^g^** Frequent outdoor play ≥3 days per week. Children with frequent independent outdoor play had more daily minutes of light PA than children with less frequent independent outdoor play. Children with frequent independent outdoor play accumulated more total PA than children with less frequent independent outdoor play. For usual travel to non-school destinations, there were no significant differences in PA between the lower and higher IM groups [81]; **^h^** IM was positively associated with self-reported PA among boys in their final year of primary school (P7). P7 boys were more likely to be physically active in univariate (OR = 2.34, CI: 1.13–4.86, *p* < 0.05) and multivariate (modelled with peer support and peer socialisation) analyses. Girls in their second year of high-school (S2) were more likely to be physically active in univariate (OR = 2.80, CI: 1.56–5.03, *p* < 0.05) and multivariate (modelled with maternal support) after controlling for baseline PA. IM was not associated with PA for boys or girls during their final year of high-school. Sample included 641 children: unrestricted play included 58.9% of boys and 40.1% of girls in P7, 80.4% of boys and 69.1% of girls in S2, and 84.6% of boys and 80.3% of girls in S4. All sex differences and time trends were significant except in S4 where girls and boys did not differ in percentage of unrestricted play [77]; **^i^** Pairwise Pearson correlations between Local IM and weekday average CPM; Local IM and weekend average CPM, Area IM and weekday average CPM, Area IM and weekend average CPM. Sub-analyses by sex show significant cross-sectional associations between local IM and average weekday CPM for boys and girls; between Local IM and average weekend CPM for girls, but not boys; between Area IM and average weekday CPM for boys and girls; and no significant associations between Area IM and average weekend CPM for boys or girls [78]; ^**j**^ Boys’ Local IM associated with likelihood of playing outside every day; girls’ local IM not related to frequency of outdoor play. Boys’ Area IM associated with likelihood of playing outside every day; Girls’ Area IM associated with likelihood of playing outside every day; Outdoor play represents frequency of playing outside at unstructured activities ranging from 1: every day to 7: hardly ever. Frequency of outdoor play was related to beliefs about traffic (perceptions of safe places to cross, heavy traffic roads, pollution) and nuisance (perceptions of crime, noise, bullying in local neighbourhood) scores for girls, and social norm scores (*i.e.*, children to play with on streets, people walking and cycling around) for both boys and girls [79]; **Acute physical activity**: 0 Randomized trials; 1 observational study [79]; **^k^** Includes 1 cross sectional study [79]; **^l^** Sample included participants outside of the targeted age range (0–2 year olds and 14–18 year olds) however, mean age of participants permitted inclusion. Age break down was: 0–5 years (*n* = 1155), 6–12 years (*n* = 1111), 13–18 years (*n* = 446). Results were reported for total sample only [83]; **^m^** The magnitude of the number of included studies was small (N = 1) [79]; **^n^** Children’s PA activity on the playground was lower in the presence of a parent and non-parent supervising adult, respectively compared with when no adults were present [83]; **Social Competence:** 0 Randomized trials; 1 observational study [84]; Includes 1 cross-sectional study [84]; ^**p**^ Outcomes were self-reported [84]; ^**q**^ The magnitude of the median sample size was intermediate (N = 251). The magnitude of the number of included studies is small (N = 1) [84]; **^r^** Children with greater IM met more often to play with peers, play with school mates, and play with neighbourhood children. IM was negatively correlated with frequency of play with relatives or parents friends’ children [84].

**Table 4:** Association between risky play supportive environments and health in children and youth.

Quality Assessment							No. of Participants	Absolute Effect (95% CI, SE)		Quality
No. of Studies	Design	Risk of Bias	Inconsistency	Indirectness	Imprecision	Other Considerations				
**Acute physical activity (age range between 3 and 9.99 years, data collected over a single session up to a 2 year follow-up, acute physical activity measured through direct observation with observer behaviour mapping and accelerometry)**										
1	RCT	Low risk of bias ^**a**^	No serious inconsistency	No serious indirectness	Serious imprecision ^**b**^	None	221	11.2 ± 0.9 min/day MVPA, 10.0 ± 0.9 min/day MVPA		MODERATE
								Coefficient = 1.82		
								CI: 0.5–3.1, *p* = 0.006		
								72,100 ± 14,700 counts, 7200 ± 13,800 counts		
								Coefficient = 9.35		
								CI: 3.5–15.2, *p* = 0.002 ^**c**^		
4	Observational studies ^**d**^	Serious risk of bias ^**e**^	No serious inconsistency ^**f**^	No serious indirectness	Serious imprecision ^**g**^	None	552	1612 CPM (SD = 491), *p* = 0.014		VERY LOW
								es = 0.9 SD ^**h**^		
								39%, *p* < 0.05 ^**i**^		
								75 min; H = 26.6, *p* < 0.01 ^**j**^		
**Habitual physical activity (age range between 4.7 and 7.3 years, data collected at baseline, 13 weeks, and 2 years follow-up, habitual physical activity measured through accelerometry)**										
1	RCT	No serious risk of bias ^**a**^	No serious inconsistency ^**k**^	No serious indirectness	Serious imprecision ^**b**^	None	221		MODERATE	
**Habitual sedentary behaviour (age range between 4.7 and 7.3 years, data collected at baseline, 13 weeks, and 2 years follow-up, habitual sedentary behaviour measured through accelerometry)**										
1	RCT	No serious risk of bias ^**a**^	No serious inconsistency ^**l**^	No serious indirectness	Serious imprecision ^**b**^	None	221		MODERATE	
**Acute sedentary behaviour (age range between 4.7 and 7.3 years, data collected at baseline, 13 weeks, and 2 years follow-up, habitual physical activity measured through accelerometry)**										
1	RCT	No serious risk of bias ^**a**^	No serious inconsistency	No serious indirectness	Serious imprecision ^**b**^	None	221	22.7 ± 9.9 min/day, 23.2 ± 10.3 min/day;	MODERATE	
								coefficient = −2.13; CI: −3.8–(−0.5), *p* = 0.01 ^**m**^		
**Antisocial behaviour (age range between 5 and 9.99 years, distance between pre- and post-measures not reported, aggression measured through direct observation with observer behaviour mapping)**										
1	Observational study ^**n**^	Serious risk of bias ^**a**^	No serious inconsistency ^**p**^	No serious indirectness ^**q**^	No serious imprecision ^**r**^	None	~400		VERY LOW	

**Notes**: CI, 95% confidence interval; CPM, counts per minute; LPA, light physical activity; MVPA, moderate to vigorous physical activity; PA, physical activity; RCT, randomized controlled trial; **Acute physical activity**: 1 Randomized trial [15]; 4 Observational studies [85,86,87,88]; **^a^** The comparison condition was “usual care”. Following baseline testing outcome assessors were no longer blinded to group assignment [15]; **^b^** The magnitude of the median sample size is intermediate. The magnitude of the number of included studies is small (N = 1); **^c^** Children in the 13-week loose parts/adult risk reframing intervention had a larger increase in minutes/day of MVPA during break times than children in the comparison group at 13 weeks (pre-intervention minutes/day MVPA = 10.8 ± 0.9 and 11.4 ± 0.9, respectively). No difference between groups for LPA; Intervention children had a larger increase in total counts during break times than comparison group (pre-intervention counts = 69,700 ± 14,400 and 74,100 ± 15,200, respectively) [15]; **^d^** Includes 3 pre- and post-test studies [85,86,88] and 1 longitudinal study [87]; **^e^** Two studies assessed acute PA subjectively using observers to record “active play” occurrences [86,88]; **^f^** There was no difference in mean CPM when children played on a traditional playground in the spring, a traditional playground in the winter, or a nature setting in the spring. The traditional playground used for comparison included many built and natural elements that afford components of risky play and thus may not have allowed a true less risky comparison [87]; **^g^** The magnitude of the median sample size is intermediate. The magnitude of the number of included studies is small (N = 3); **^h^** Children had higher mean CPM after an 11 week loose parts playground intervention compared to baseline (Mean CPM = 1028, SD = 770) [85]; **^i^** The proportion of time children spent engaged in active play at post-test was significantly higher than at pre-test, 16%. Active play time was significantly higher following construction of a risky play affording playground environment than at pre-test. It is not clear how long after playground construction post-testing was conducted [86]; **^j^** Median length of stay on an adventure playground was higher than traditional playground and contemporary playground (21 and 32 min, respectively. Kruskal-Wallis one-way analysis of variance by ranks determined differences were significant at the 0.001 level [88]; **Habitual physical activity**: 1 Randomized trial [15]; 0 Observational studies; **^k^** No difference in whole day minutes of PA between children who participated in a 13 week playground based intervention with a 2 h risk-reframing intervention administered to parents and teachers compared with control group [15]; **Habitual sedentary behaviour**: 1 Randomized trial [15]; 0 Observational studies; **^l^** No difference between children who participated in a 13-week playground-based intervention with a 2-h risk-reframing adult intervention when compared to children in the control group for minutes per day sedentary [15]; **Acute sedentary behaviour**: 1 Randomized trial [15]; 0 Observational studies; **^m^** Post intervention time spent sedentary during break times in loose parts intervention and control group, respectively. Children in the 13 week loose parts intervention had a larger decrease in minutes/day of sedentary time during break times than the comparison group, whose sedentary time increased over the intervention period (pre-intervention min/day sedentary time = 23.8 ± 10.4 and 22.2 ± 9.9, respectively) [15]; **Antisocial behaviour**: 0 Randomized trials; 1 Observational study [86]; **^n^** Includes 1 pre-post test study; **°** Aggression was rated subjectively using direct observation [86]; **^p^** No change in aggression from pre- to post-risky play supportive playground construction [86]; **^q^** It is likely that the time frame (2 weeks, immediately after the new playground was built) was not sufficient to detect a difference in aggression from pre- to post-test [86]; **^r^** The magnitude of the number of included studies is small (N = 1).

**Table 5:** Association between great heights and health in children and youth.

Quality Assessment							No. of Participants	Absolute Effect (95% CI, SE)	Quality
No. of Studies	Design	Risk of Bias	Inconsistency	Indirectness	Imprecision	Other Considerations			
**Bone fractures (age range between 5 and 12 years, data collected over 1 year, bone fractures measured using incident reporting sheets)**									
1	Observational studies ^**a**^	No serious risk of bias	No serious inconsistency	No serious indirectness	Serious imprecision ^**b**^	None	25,782	58% ≤59”; 33% 60–79”; 9% >79” ^**c**^	VERY LOW

Notes: 0 Randomized trials; 1 observational study [89]; **^a^** Observational studies include 1 longitudinal study [89]; **^b^** The magnitude of included studies is small (N = 1); **^c^** During a 1 year observation period of all schools in a single school board, 57 fractures occurred (52 unaided falls,5 pushed) on the playground. Of those, the percentage of children who sustained a fracture from a fall at or below 59”, 60–79” and greater than 79” are reported here, respectively. There were no serious injuries from falls reported by any of the schools [89].

**Table 6:** Association between rough and tumble play and health in children and youth.

Quality Assessment							No. of Participants	Absolute Effect (95% CI, SE)	Quality
No. of Studies	Design	Risk of Bias	Inconsistency	Indirectness	Imprecision	Other Considerations			
**Social competence (age range between 42 months and 11.2 years, data collected over a single session up to 2 years, aspects of social competence were measured using teacher-report questionnaire, peer nominations of popularity and rejection, social cognitive problem solving task, observer rated)**									
5	Observational studies ^**a**^	Serious risk of bias ^**b**^	Serious inconsistency ^**c**^	No serious indirectness	Serious imprecision ^**d**^	None	359 ^**e**^	*r* = 0.30; *p* < 0.05; *R* = 0.09	VERY LOW
								*r* = 0.28; *p* < 0.05; *R* = 0.07	
								*r* = −0.28, *p* < 0.05; *R* = 0.07	
								*r* = 0.28, *p* < 0.05, *R* = 0.07	
								*r* = −0.32, *p* < 0.05; *R* = 0.10	
								*r* = −0.30, *p* < 0.05; *R* = 0.09 ^**f**^	
								*r* = 0.42, *p* = 0.37 ^**g**^	
								Year 1: *r* = 0.22 *p* < 0.05; *r* = −0.37, *p* < 0.01	
								year 2: *r* = 0.25, *p* < 0.05	
								Year 1 RTP to 2 social variables: *r* = 0.28, *p* < 0.01 ^**h**^	
								*r* = 0.34, *p* < 0.05; *r* = 0.54, *p* < 0.01	
								B = −0.87, *R*^2^ = 0.14, *p* = 0.03	
								B = 1.39, *R*^2^ = 0.32, *p* = 0.001	
								B = 3.30, *R*^2^ = 0.22, *p* = 0.006 ^**i**^	
								*r* = 0.30; *r* = 0.30, *p* < 0.05 ^**j**^	
								*r* = 0.56, *p* < 0.01 ^**k**^	
**Anti-social behavior (age range between 64 months and 13.5 years, data were collected over 8 months up to 22 months, aspects of anti-social behaviour were was measured using direct observation, teacher ratings, and a video behaviour discrimination task)**									
2	Observational studies ^**l,m**^	No serious risk of bias ^**n**^	Serious inconsistency °	No serious indirectness	Serious imprecision ^**p**^	None	176 ^**q**^	*P* (RTP leading to aggression) = 0.28%, *z* = 4.00, *p* < 0.05	VERY LOW
								χ^2^(40, *N* = 42) = 8.17, *p* < 0.004); *r* = 0.47, *p* < 0.01 ^**r**^	
								*r* = 0.29, *p* < 0.01	
								*P* (RTP rough leads to aggression) = 2.26%, *p* < 0.05^s^	

**Notes**: RTP, rough and tumble play; **Social competence**: 0 Randomized trials; 5 Observational studies [90,91,92,93,94,95,96]; **^a^** Observational studies include 1 longitudinal study [90] and 4 cross sectional studies [91,92,93,94,95,96]. Dewolf [91] was an unpublished graduate thesis; **^b^** It is unclear if participants were blinded to the outcomes assessed, and likely that their behaviour was affected by being observed. The research noted that after speaking to the children about their play the children were “distinctly aware of her presence” during later interactions. The outcome assessor (the researcher) was not blinded to the outcomes being assessed [91]; **^c^** RTP was not correlated with popularity [96]; For popular children, RTP was not correlated with antisocial behaviour. For rejected children, RTP was not correlated with interpersonal cognitive problem solving [93]; RTP in year 1 was not related to year 2 social problem solving scores for popular or rejected children [95]; RTP was not correlated with social impact, likes most nominations, likes least nominations, antisocial, or film for boys or girls. For girls, RTP also did not correlate with social preference, or interpersonal cognitive problem solving [94]; Boys’ engagement in RTP with other boys was not related to peer-acceptance by girls. Boys’ RTP with mixed-sex peer s was negatively related to peer acceptance by girls and teacher rated social competence [92]; Boys’ RTP chase was negatively correlated with peer nominations of likes least (*r* = −0.22, *p* < 0.05), and was not correlated with peer nominations of likes most, social impact, or social preference; RTP rough was negatively correlated with peer nominations of likes most (*r* = −0.37, *p* < 0.01) and was not correlated with peer nominations of likes least, social impact, or social preference [90]; **^d^** Low median sample size. Moderate number of included studies (N = 5). **^e^** Pellegrini [93,94,95] used the same sample. Results are reported separately but participants are counted once. Pellegrini [95] sample had 94 participants at year 1 and 72 participants at year 2; Pellegrini [90] sample consisted of 82 boys; Pellegrini [96] sample consisted of 42 boys; **^f^** Boys’ RTP with same sex peers was correlated with acceptance by same sex peers; Boys’ RTP+ pretend play with same sex peers was correlated with acceptance by same sex peers. Boys’ RTP with mixed sex peers was correlated with same sex peer acceptance. Boys’ RTP with same sex peers was related to teacher-rated social competence. Boys’ RTP with same sex peers was related to teacher-rated social competence. Boys’ RTP with mixed sex peers was negatively correlated with other sex peer acceptance and teacher rated social competence [92]; **^g^** Positive peer nominations was correlated with proportion of RTP events [91]; **^h^** RTP chase correlated with peer nominations of likes least, but not peer nominations of likes most, social impact, or social preference. RTP rough was negatively correlated with peer nominations of likes most, but was not related to peer nominations of likes least, social impact, or social preference [90]; **^i^** RTP flexibility was correlated with interpersonal cognitive problem solving (positive and negative solutions respectively). Popularity was not correlated with any aspect of RTP; RTP relative frequency negatively predicted popularity; RTP flexibility accounted for unique variance in the model to predict negative, and positive solutions to an interpersonal cognitive problem, respectively [96]; **^j^** For boys, RTP correlated with social preference and interpersonal cognitive problem solving, respectively, but not social impact, likes most or likes least peer ratings; For girls, RTP did not correlate with social preference, social impact, likes most, likes least, interpersonal cognitive problem solving [94]; **^k^** For popular children, RTP correlated with interpersonal cognitive problem solving [89]; **Antisocial behaviour**: 0 Randomized trials; 2 Observational studies [90,93,94]; **^l^** Includes 1 longitudinal study [90] and 1 cross sectional study [93,94]; **^m^** Pellegrini [90] is a longitudinal study, however only data from year 1 are included. Children in year 2 met age-based exclusion criteria; **^n^** It was not possible to blind assessors to outcomes, however assessors were blinded to children’s sociometric and dominance status [90,93,94]; The probability of RTP leading to observer rated aggression for popular children was not significant; For popular children, RTP was not correlated with anti-social behavior [93]; RTP frequency was not correlated with aggression frequency for boys or girls. RTP was not likely to lead to aggression for children in this study. For boys and girls RTP did not correlate with ability to discriminate between RTP and aggression on a film or with anti-social behaviour [94]; RTP (chasing) was not correlated with observed or teacher rated aggression. RTP (rough housing) was not correlated with teacher rated aggression [90]; **^p^** The magnitude of the median sample size was low; The magnitude of the number of included studies was small (N = 2); **^q^** The total sample includes 1 study of 82 Caucasian boys only [90]. Pellegrini [93,94] participants were from the same study. Results are reported separately but participants are only counted once. **^r^** The probability of RTP leading to observer rated aggression within the 3 min observation period was significant for rejected children. RTP was significantly more likely to lead to observer rated aggression for rejected children than with popular children. RTP positively correlated with anti-social behaviour for rejected children [93]. **^s^** RTP (rough housing) was correlated with observed aggression. The probability that RTP (rough housing) would lead to aggression within the 3 min observation period was 2.26% [90].

### 3.1. Play Where Children can Disappear/Get Lost

#### 3.1.1. Habitual Physical Activity

Six observational papers (one longitudinal, five cross sectional) from five studies examined the relationship between “play where the children can disappear/get lost” and habitual physical activity. The majority of the studies reported that independent mobility was positively related to physical activity [77,81], total activity counts [80], activity counts per minute [78], minutes of moderate to vigorous physical activity (MVPA), light activity [80], and self-reported likelihood of playing outside everyday [79]. Kirby *et al.* [77] found that boys (not girls) who were in their final year of primary school and whose parents did not restrict their independent outdoor play were more than twice as likely to be categorized as physically active than their peers with restricted independent play. Girls (not boys) in their second year of high school were more than four times as likely to be categorized as physically active compared with their peers with restricted independent play. Stone *et al.* [80] reported small differences between the children who had higher independent mobility and those with restricted independent mobility. Children with higher independent mobility had higher weekday and accelerometer counts per day, higher weekday MVPA minutes (boys and girls had 4.4 and 2.4 more minutes), and spent a greater percentage of the two hours immediately after school in light physical activity (boys and girls had 2.5% and 1.5% more time) and MVPA (boys and girls had 2.7% and 0.7% more time) than children with lower independent mobility. Furthermore, on weekend days boys (not girls) with higher independent mobility had 3.3 more minutes of MVPA and 4.3 more minutes of light physical activity than restricted children. Schoeppe *et al.* [81], found that children who played outside without supervision three or more days per week did not have higher MVPA, although they did accumulate significantly more daily minutes of light and total physical activity; sex-based analysis showed this relationship existed for girls, but not boys. Independent mobility to non-school destinations was not related to physical activity outcomes [81]. In one study, Page *et al.* showed that being allowed to visit locations without supervision in the local neighbourhood (Local) and in the wider area (Area) were each associated with more average weekday accelerometer counts per minute. Boys with higher Area and Local independent mobility and girls with higher Area independent mobility were approximately 1.5 times more likely to play outside everyday than children with lower independent mobility [79]. Local independent mobility was associated with higher weekday counts per minute for boys and girls, and higher weekend counts per minute for girls. Area independent mobility was associated with higher weekday counts per minute, but not weekend counts per minute for boys and girls. Unstandardized betas ranged from 17.89 to 33.5 [78].

In contrast, one study found that children with higher independent mobility did not have an increased likelihood of achieving pedometer based physical activity cut-points than children with lower independent mobility [82]. No studies showed a negative relationship between independent mobility and habitual physical activity.

#### 3.1.2. Acute Physical Activity

Two observational studies (one cross sectional; one repeated measures) examined the relationship between ‘play where the children can disappear/get lost’ and acute physical activity. The independent variable was assessed as presence or absence of a supervising adult [83], and as parent reported independent mobility [80]. Both studies showed that disappear/get lost was positively related to acute physical activity. Using a structured observer scoring system, Floyd *et al.* [83] observed that the presence of a parent or non-parent supervising adult was associated with a lower likelihood (ORs = 0.55 and 0.69) that children would engage in vigorous activity than when no adult was present. No studies demonstrated that disappear/get lost was unrelated or negatively related to acute physical activity.

#### 3.1.3. Social Competence

One observational study met the inclusion criteria. Prezza *et al.* [84] reported a largely positive relationship between disappear/get lost and social health. Specifically, small to moderate correlations suggest that that children with greater independent mobility met more often to play with peers; play with school mates; and play with neighbourhood children than their peers with less independent mobility. Children with greater independent mobility were less likely to play frequently with relatives or parents friends’ children [84].

### 3.2. Great Heights

One observational study that examined the relationship between height at which children play and the occurrence of injuries met our inclusion criteria. In a study that spanned one school year and included 25,782 students (all children registered with the participating school board), Rubie-Davies *et al.* [89] showed that fracture frequency and severity was not related to height of playground equipment. Ulna-radius fractures (most frequent type of fracture, accounting for 42% of playground fractures) were as likely to occur below 59” (54%) as they were above the mark (46%). The 6 reported tibia fractures occurred below 59”. No fractures to the head or spine occurred as a result from a fall from playground equipment.

### 3.3. Rough and Tumble Play

#### 3.3.1. Social Competence

Five observational studies examined the relationship between rough and tumble play and social competence in seven papers. Two studies examined the relationship between rough and tumble play and interpersonal cognitive problem solving. One study showed moderate to large positive correlations, suggesting that for popular children as assessed by number of peer ratings of “likes most” [93] and for boys [94], rough and tumble play was related to higher interpersonal cognitive problem solving scores. However, rough and tumble play and interpersonal cognitive problem solving were not related for rejected children [93], or for girls [94]. Moreover, rough and tumble play in year 1 was not related to interpersonal cognitive problem solving one year later for popular or rejected children [95]. In a second study, flexibility of rough and tumble play was positively predicted 32% and 22% of the variance in interpersonal cognitive problem solving (*i.e.*, the total number of positive and negative solutions to a problem solving task) [96].

Four studies examined the relationship between rough and tumble play and social status in 6 papers [90,91,92,93,94,96]. The results were inconsistent in showing a relationship between rough and tumble play and popularity among children and youth. Where correlations were significant, they were moderate in size). For boys, rough and tumble play positively correlated with social preference within one’s peer group (*i.e.*, the number of ‘likes most’ nominations minus “likes least” peer nominations) [94]. Boys’ rough and tumble play with other boys was moderately and positively correlated with acceptance among boys and teacher rated social competence (a composite of teacher-perceived aggression, peer acceptance and sensitivity). In same sex peer groups, boys’ rough and tumble play plus pretend play with other boys was positively correlated with acceptance among boys. In mixed sex peer groups, boys’ rough and tumble play was positively correlated with peer acceptance among boys [92]. In another study, peer nominations of “likes most” was positively correlated with the proportion of rough and tumble play events observed [91].

Two studies showed statistically negative relationships between rough and tumble play and social status. Boys’ rough and tumble play with mixed sex peer groups was negatively related to peer acceptance by girls, and to teacher rated social competence [92]. In a sample of boys, sub-analysis by type of rough and tumble play showed boys’ rough and tumble play that consisted of chasing (Chase) was negatively correlated with “likes least” nominations (r = −0.22, *p* < 0.05), and rough and tumble play that consisted of physical behaviours (Rough) was negatively correlated with “likes most” nominations (r = −0.37, *p* < 0.01) [90].

Half of the studies showed that rough and tumble play was not related to social status for particular forms of rough and tumble play and sex-based analyses. Rough and tumble play was not correlated with children’s popularity (peer nominations of “likes most” minus “likes least”) [96]. Sub-analysis by sex showed that rough and tumble play was not correlated with social impact (the total of “likes most” plus “likes least” nominations), “likes most” nominations, or “likes least” nominations for boys or girls [94]. For girls rough and tumble play did not correlate with social preference (“likes most” minus “likes least” nominations) [94]. Boys’ rough and tumble play with other boys was not correlated with peer acceptance by girls [92]. Sub-analysis by type showed boys’ rough and tumble play Chase was not correlated with peer nominations of “likes most”, social impact or social preference [90]. Boys’ rough and tumble play Rough was not correlated with peer nominations of “likes least”, social impact, or social preference [90].

#### 3.3.2. Anti-Social Behaviour

Two observational studies examined the relationship between rough and tumble play and aggression in three papers [90,93,94]. These studies showed somewhat inconsistent results. Specifically, rough and tumble play was not correlated with frequency of aggression for boys or girls [94], and was not likely to lead to aggression for popular children [93]. However, rough and tumble play was likely to lead to aggression for children whose peers had nominated them as being rejected, as assessed by number of “likes least” peer ratings [93]. Finally, rough and tumble play in the form of chasing was not correlated with observed or teacher rated aggression, and rough and tumble play in the form of rough housing was not correlated with teacher rated aggression in a sample of all boys [90]. In contrast, in the earlier study, Pellegrini [93] reported a significant probability that rough and tumble play in the form of rough housing would lead to aggression.

Two studies examined the relationship between rough and tumble play and anti-social behaviour. Rough and tumble play was correlated with anti-social behaviour for rejected children, but not for popular children [93], for boys or for girls [94].

One study examined the relationship between rough and tumble play and children’s ability to discriminate between rough and tumble play and aggression in a film. Pellegrini [94] showed that for boys and girls, rough and tumble play was not correlated with ability to distinguish rough and tumble play from aggression in a film.

### 3.4. Risky Play Supportive Environments

#### 3.4.1. Acute Physical Activity

One Cluster RCT and four observational studies examined the relationships between risky play supportive environments and acute physical activity. For their RCT, Engelen *et al.* [15] modified the playground environment of participating schools by introducing loose, primarily recycled materials (e.g., tires, milk crates) for use in play. They also hosted 2-h risk reframing sessions for parents and school staff where adults were encouraged to consider the benefits of play and consequences of limiting children’s opportunities for risk taking and physical activity. Engelen *et al.* [15] showed that 5–7 year old children in the loose parts/risk reframing intervention had a small but significant increase in minutes per day of MVPA (1.8 min, 95% CI 0.5–3.1) and in total activity counts (9400 counts, 95% CI 3.5–15.2) during break times, and engaged in 12% more MVPA than children in the control group schools after the 13 week intervention period. However, no difference was seen for light physical activity. The increased physical activity remained higher for a subset of the intervention group that was assessed 2 years later when they were 7–9 years old.

Two of the observational studies showed that children engaged in greater physical activity from pre- to post-test after being introduced to a risky play supportive environment. After 11 weeks on a loose parts playground there was a large effect size, with children reaching higher physical activity counts per minute on average compared to baseline (1612 *vs.* 1028) [85] and following construction of a “tire” playground children increased the proportion of time they engaged in active play from 16% to 39% [86]. Hayward *et al.* [88] observed that children spent significantly more time (75 min) at an adventure playground (supplies play material and not play equipment, with few to no observing adults) than at a traditional playground with pre-fabricated structures (21 min) and a contemporary playground (32 min) with several adults supervising.

One study did not show a difference in mean physical activity counts per minute when children played on a traditional playground in the spring, a traditional playground in the winter, or a nature setting in spring [87].

#### 3.4.2. Habitual Physical Activity

One Cluster RCT examined the relationship between risky play supportive environments and habitual physical activity. No difference was observed in whole day minutes of physical activity between children who participated in a 13 week playground based intervention with a 2 h risk-reframing intervention administered to parents and teachers compared to children in the control group [15].

#### 3.4.3. Habitual Sedentary Behaviour

One Cluster RCT examined the relationship between risky play supportive environments and habitual sedentary behaviour and showed there was no difference between children who participated in a 13 week playground based intervention with a 2 h risk-reframing adult intervention when compared to children in the control group for minutes per day sedentary [15].

#### 3.4.4. Acute Sedentary Behaviour

One Cluster RCT examined the relationship between risky play supportive environments and acute sedentary behaviour and showed that children in the loose parts intervention had a small but significant decrease in sedentary time per day (2.13 min, 95% CI −3.8 − (−0.5)) during break times after the 13 week intervention period [15].

#### 3.4.5. Anti-Social Behaviour

One observational study examined the relationship between risky play supportive environments and aggression and showed no change in aggression from pre- to post-risky play supportive playground construction [86].

#### 3.4.6. Social Behaviour

Although not quantitatively assessed, Hayward *et al.* [88] noted that at the two prebuilt playgrounds (*i.e.*, traditional and contemporary), use focused on the equipment, whereas interactive play was most common at the adventure playground. In addition, Bundy *et al.* [85] reported that according to teachers, children became more social, creative, and resilient after exposure to the loose parts intervention than they were before it was created.

### 3.5. Summary of Findings

Few studies met the inclusion criteria and there were no studies that investigated some subcategories of risky play, such as play with dangerous tools or play at speed. These types of risky play were often subsumed within studies examining broader concepts, such as risky play supportive environments. Only one RCT was included, and the bulk of other research was rated as having low methodological quality and subject to bias and confounding. The heterogeneity of risky play and outcome measures rendered meta-analysis impossible.

Overall, the systematic review revealed positive effects of risky outdoor play on health. Seven of eight papers examining play where children can disappear/get lost in children found increases in habitual and acute physical activity, and social health [77,78,79,80,81,82,83,84]. Floyd *et al.* [83] showed lower physical activity for children supervised by an adult. There was no association between play at height and injuries, with fracture frequency and severity being unrelated to height of playground equipment [89]. Notably, no serious injuries (e.g., to the head or spine) were reported during the year in which 25,782 children were followed. Studies examining rough and tumble play showed mixed results when examining effect of popularity, gender, and type of rough and tumble play. Overall, engaging in this type of risky play did not increase aggression, and was associated with increased social competence for boys and popular children. Risky play supportive environments generally led to an increase in physical activity and decrease in acute sedentary behaviours [15,85,86,88]. There was also an indication that these environments promoted increased play time, and behaviours, such as social interactions, creativity and resilience [85,88]. One study reported no relationship between physical activity and acute physical activity in a natural *vs.* traditional play environment [87]. However, the traditional playground included many built and natural elements that afford components of risky play and thus may not have facilitated a true comparison.

Gender analyses resulting from studies included in this systematic review did not find consistent gender patterns and, in some cases, gender comparisons were not conducted. McCormick *et al.* [82] found no gender differences in independent mobility and physical activity. Page *et al.* [78,79] found differences in sub-analyses. For example, Page *et al.* [78] found associations between local independent mobility and average weekend accelerometry counts per minute for girls, but not boys, but found no gender differences for other analyses. Page *et al.* [79] found that local independent mobility was associated with likelihood of daily outside play for boys but not girls, though gender differences were not found in examining area independent mobility. Frequency of outdoor play was associated with beliefs about traffic, neighbourhood nuisance and social norms for girls, but only social norms for boys [79]. Kirby *et al.* [77] found that boys aged 11 to 14 years, had greater independent mobility than girls. Pellegrini [94] found rough and tumble play was not correlated with aggression frequency for either gender. Colwell *et al.* [92], found that while boys’ engagement in rough and tumble play with same-sex peers was generally viewed positively by teachers and peers, engagement in rough and tumble play with girls was not.

## 4. Discussion

The growing discussion regarding the benefits and disadvantages of risky play on children’s health prompted this systematic review of the evidence [8,12,68,97,98,99]. We included 21 articles representing 18 studies that addressed the effect on health and health behaviors from three types of risky outdoor play (play where children can disappear/get lost, play at great heights, and rough and tumble play), as well as risky play supportive environments. The studies examined a variety of health behaviours and outcomes, with physical activity being the most common. Also examined were social competence and behaviours, injuries, and aggression. The findings overall suggest positive effects of risky outdoor play on health.

Only one study related to injury outcomes met inclusion criteria in our review because it was the only one to indicate the total number of children exposed to the risk. It found no association between fall height and injury [89]. Other studies have reported similar results (e.g., [100]). In contrast, a previous systematic review examining risk factors for unintentional fall-related injuries in children aged 0 to 6 years found an association between injuries and fall height and the quality of the surfacing [73]. Likewise, literature reviews that included examination of playground injuries reported similar findings [101,102]. Another systematic review found that absolute incidents of reported injuries of any severity from children’s unstructured physical activity (which included playground climbing frames, jungle gyms) were high relative to injuries from sport, and active transportation. However, the incidence rates per 1000 hours of unstructured physical activity for medically treated injuries was lower than among sports and active transportation, ranging from 0.15–0.17 injuries per 1000 hours of play [103]. Challenges in interpreting these heterogeneous findings relate to inconsistencies in defining injuries, lack of information on exposure to the risk, and limited examination of the interaction between fall height and absorption quality of playground surfacing material.

The broad research literature indicates that societal and familial gender role expectations shape boys’ and girls’ behaviours. Parents are more likely to encourage boys to engage in risk taking behaviours and girls are socialized to perceive themselves as more vulnerable than are boys [66,104,105,106]. Gendered aspects of parenting practices have been associated with greater exploratory and less restrictive behaviours among boys than among girls [105,107,108,109,110,111]. Of the studies included in our systematic review, only Kirby [77] specifically found greater independent mobility for boys than girls, and there were no consistent gender patterns discernable in other studies. Some studies did not conduct gender comparisons. Nevertheless, our results indicate the importance of continued efforts at systematic examination of gender differences.

Our review revealed the need for more studies that would be rated as “good quality evidence”, as they are most effective in influencing the positivist perspectives of the medical and health fields. However, we raise limitations inherent in the systematic review process that discounts large volumes of evidence as scientifically unsound [112,113]. These challenges are particularly relevant in research with children in natural settings, where randomized controlled scientific experiments can represent a “reality” with little applicability to community settings. Indeed, many research studies with compelling multi-disciplinary evidence for the importance of risky play were excluded from this review. We note that even in the face of this exclusionary process, the result of this review supported risky outdoor play for children’s health.

Generational differences indicating markedly decreasing access to risky outdoor play have been documented [54,57,58,60,114]. Safety concerns, such as injury or abduction, represent one of the main reasons for limiting children’s risky outdoor play [67,115,116]; and playground safety standards and active supervision are prominent safety strategies [31,32]. Our findings suggest a need to critically examine approaches to child injury prevention while at play, as these strategies can have unintended adverse consequences on children’s health. Children with opportunities for disappearing/getting lost had increased physical activity and social health, whereas supervised children had lower levels of physical activity [77,78,79,80,81,83]. The range of ages studied included 7 to 15 years (except Floyd *et al.* [83], which included all ages), suggesting that monitoring may be a more appropriate approach than active supervision for these age groups. In general, we recommend considering policy, practice and built environment approaches to risky outdoor play that balance safety with children’s other health outcomes.

Policy precedents can provide guidance and opportunities for action. For example, the British Government endorsed the UK Play Safety Forum’s policy statement that children’s need for risk be accommodated through stimulating and challenging environments that limit unacceptable risks of death or serious injury [68,117]. To operationalize this approach, the Play Safety Forum developed a practical tool for risk-benefit assessment of children’s play spaces, which considers the benefits of risky play and the reasonableness of safety measures [3]. This tool identifies hazards (potential sources of harm), the risk from that hazard (likelihood and severity of harm), and helps determine the need for modification or removal of the hazard. Some hazards are viewed as acceptable because they offer developmental benefit to children (e.g., changes in height; loose materials such as sticks). Hazards that have no benefit to children or are difficult for children to perceive are removed (e.g., sharp edges; head entrapment) [3]. Another example of an approach to risky play that combines policy and practice comes from Norway where the kindergarten curriculum emphasizes the importance of engaging in and mastering risk, ensuring that risky play remains a part of children’s lives from the early years [99].

Our findings that risky play supportive environments had numerous positive impacts on health, behaviour and development [15,85,86,88] make it clear that built environment solutions are also necessary. The papers included in our review suggest the quality of play spaces, determined by factors such as presence of natural elements (trees, plants), materials that can be manipulated by the children (e.g., wood, crates), and the freedom to engage in activities of their choosing influenced play affordances, children’s interest in playing there, and the play spaces’ value in health promotion [15,85,86,87,88]. This is supported by research in landscape architecture describing evidence-based criteria for playground design [70]. The inclusion of diverse high quality landscapes for children’s play is a criteria for the UNICEF Child Friendly Cities initiative [118].

Given the progressive decline of risky play opportunities, there is a need for action to slow or reverse the trend in order to promote and preserve children’s health.

## 5. Conclusions

The evidence from our systematic review indicates that the overall positive health effects of increased risky outdoor play provide greater benefit than the health effects associated with avoiding outdoor risky play. Although these findings are based on ‘very low’ to ‘moderate’ quality evidence, the evidence suggests overall positive effects of risky outdoor play on a variety of health indicators and behaviours in children aged 3-12 years. Specifically, play where children can disappear/get lost and risky play supportive environments were positively associated with physical activity and social health, and negatively associated with sedentary behaviour [77,78,79,80,81,82,83,84,85,88]. Play at height was not related to fracture frequency and severity [89]. Engaging in rough and tumble play did not increase aggression, and was associated with increased social competence for boys and popular children, however results were mixed for other children [15,85,86,88]. There was also an indication that risky play supportive environments promoted increased play time, social interactions, creativity and resilience [85,88]. These positive results reflect the importance supporting children’s risky outdoor play opportunities as a means of promoting children’s health and active lifestyles.

## Supplementary Files

Supplementary File 1
