# Supplementary material for: What is the Relationship between Risky Outdoor Play and Health in Children? A Systematic Review
Source: Int J Environ Res Public Health. 2015 Jun 8;12(6):6423–54. doi: 10.3390/ijerph120606423 (PMC4483710; doi:10.3390/ijerph120606423)
Supplement: Supplementary File 1 [file ijerph-12-06423-s001.pdf]

Supplementary Information

What is the Relationship between Risky Outdoor Play and Health in Children? A Systematic Review

---

Additional File 1. Search Strategy

Initial Search Strategy

MEDLINE (Ovid)

1. Risk taking/
2. Risk Management/
3. Safety/
4. Safety management/
5. or/1–4
6. “Play and Playthings”/
7. 5 and 6
8. ((risky or risk taking) adj3 play*).tw.
9. 7 or 8

PsycInfo (Ovid)

1. Risk taking/
2. Risk perception/
3. Safety/
4. or/1–3
5. Childhood Play Behavior/
6. Childhood Play Development/
7. Childrens Recreational Games/
8. Playgrounds/
9. or/5–8

CINAHL (EBSCOhost)

#	Query
S14	S12 or S13
S13	(S1 and S2) or S3 or S11
S12	((S1 and S2) or S3 or S11) and child*
S11	S9 and (S10 or S2)\
S10	TI play
S9	S7 and S8
S8	((risk* or adventur*) and play*)
S7	S4 or S5 or S6

S6 MH “Motor Skills+” or MH “Cognition+” or MH “Mental Processes+” or MH “Mental Health” or MH “Mental Disorders+” or MH “Substance-Related Disorders+” or MH “Personality Development+” or MH “Impulsive Behavior+” or MH “Social Behavior+” or MH “Psychological Well-Being”

S5 MH “Physical endurance” or MH “Physical exertion” or MH “musculoskeletal physiological processes” or MH “ exercise” or MH “movement” or MH “locomotion” or MH “running” or MH “swimming” or MH “walking” or MH “motor activity” or MH “Behavior and Behavior Mechanisms+” or MH “Psychological Processes and Principles+”

S4 MH “Physical Fitness” or MH “Sedentary Lifestyle” or MH “Obesity+” or MH “Overweight+” or MH “Body Fat Distribution” or MH “Body composition” or MH “Waist Circumference” or MH “Skinfold Thickness” or MH “Body weights and measures+”

S3 risky N3 play* or risk taking N3 play*

S2 (MH “Play and Playthings”)

S1 (MH “Risk Taking Behavior”) OR (MH “Attitude to Risk”) OR (MH “Risk Assessment”)

ERIC (Proquest)

(esu(Risk) AND esu(Play)) OR “risky play”

SportDiscus (EBSCOhost)

(((DE “Play”) and child*) OR (DE “Playgrounds”)) AND (DE “RISK-taking (Psychology)” or risky or (risk not (“at risk” or risk factor*)))

Secondary Search Strategy

MEDLINE (Ovid)

Unsupervised play—MEDLINE draft

PsycInfo (Ovid)

(((play AND (unsupervised OR no N2 supervision OR without n2 supervision OR unaccompanied OR unescorted OR adult* n2 presen* OR independent play OR autonomous play))) OR independent mobility) AND child*

CINAHL (EBSCOhost)

((MH “Play and Playthings+”) AND (unsupervised OR no N2 supervision OR without n2 supervision OR unaccompanied OR unescorted OR adult* n2 presen* OR independent play OR autonomous play)) OR (independent mobility AND child)

ERIC (Proquest)

(esu(play) AND (unsupervised OR no NEAR/2 supervision OR without NEAR/2 supervision OR unaccompanied OR unescorted OR adult* NEAR/2 presen* OR independent play OR autonomous play)) or (“independent mobility” and child*)

SportDiscus (EBSCOhost)

(DE “Play” AND (unsupervised OR no N2 supervision OR without n2 supervision OR unaccompanied OR unescorted OR adult* n2 presen* OR independent play OR autonomous play)) OR (independent mobility and child*)

Additional File 2. Summary of Included Studies.

Table S1. Summary of included studies.

First Author	Year	Country	Study Design	Population (*n*)	Age Group	Intervention or Exposure	Outcome (Measure)
Bundy [1]	2009	Australia	Experimental	12<br>Boys = 7<br>Girls = 5	Range = 5–7 years	Loose parts on playground (11 weeks)	Habitual and Acute PA (accelerometry)
Colwell [2]	2005	USA	Cross sectional	60<br>Boys = 33<br>Girls = 27	Range = 48–73 months<br>Mean = 57.61 months	RTP (direct observation)	social competence (peer rated, teacher rated)
Dewolf [3]	2000	USA	Cross sectional	9<br>Boys = 6<br>Girls = 3	Range = 42–62 months;<br>Mean = 51 months	RTP frequency (direct observation)	peer status (peer rated)
Engelen [4]	2013	Australia	Cluster RCT	221<br>Boys = 119<br>Girls = 102	Range = 4.7–7.3 years<br>Mean = 6.0 (0.6)	Loose parts on playground (school breaks, 13 weeks); 1 risk reframing session for parents and teachers	Habitual and acute PA, SB (accelerometry)
Floyd [5]	2011	USA	Cross sectional	2712<br>Boys = 1532<br>Girls = 1180	Range =~ 0–5 years (*n* = 1155), 6–12 years (*n* = 1111), 13–18 years (*n* = 446)	Independent mobility (direct observation)	PA, SB (SOPARC)
Hayward [6]	1974	USA	Cross sectional	16,419	6–13 years	Risky play supportive playground (observer rated)	Behavioural mapping
Kirby [7]	2011	Scotland	Longitudinal	641<br>Boys = 313<br>Girls = 328	Range = 11–15 years	Independent mobility (self-report)	PA (PAQ-C)
McCormack [8]	2011	Australia	Cross sectional	927<br>Boys = 424<br>Girls = 503	Range = 10–12 years	Independent mobility (Parent report)	PA (Pedometer)

Table S1. *Cont.*

First Author	Year	Country	Study Design	Population (*n*)	Age Group	Intervention or Exposure	Outcome (Measure)
Page [9]	2009	England	Cross sectional	1300<br>Male = 639<br>Female = 661	Range = 10–11 years<br>Mean = 10.95 (0.414)	Local independent mobility (self-report)<br>Area independent mobility (self-report)	PA (accelerometry)
Page [10]	2010	England	Cross sectional	1300<br>Male = 639<br>Female = 661	Range = 10–11 years<br>Mean = 10.95 (0.414)	Local independent mobility (self-report) Area independent mobility (self-report)	Outdoor active play (self-report)
Pellegrini [11]	1988	USA	Cross sectional	94<br>Boys = 48<br>Girls = 46	kindergartners: 14 months; second graders: 90 months; fourth graders: 117 months	RTP (direct observation)	Aggression, Antisocial behaviour (self-report) social competence (interpersonal cognitive problem solving procedure)
Pellegrini [12]	1989	USA	Cross sectional	94<br>Boys = 48<br>Girls = 46	kindergartners: 14 months; second graders: 90 months; fourth graders: 117 months	RTP (direct observation)	Aggression (observed and teacher rated) Social competence (peer rated; Interpersonal Cognitive Probem Solving procedure; video discrimination task)
Pellegrini [13]	1991	USA	Longitudinal	94<br>Boys = 48<br>Girls = 46	kindergartners: 14 months; second graders: 90 months; fourth graders: 117 months	RTP (direct observation)	social competence (interpersonal cognitive problem solving procedure)
Pellegrini [14]	1993	USA	Cross-sectional	Boys = 42	Mean = 11.2 years	RTP relative frequency, vigour and flexibility (direct observation)	Social competence (Interpersonal Cognitive Problem Solving procedure; Popularity (peer rated)

Table S1. *Cont.*

First Author	Year	Country	Study Design	Population (*n*)	Age Group	Intervention or Exposure	Outcome (Measure)
Pellegrini [15]	1995	USA	Longitudinal	Boys = 82	Range = 12.02–13.5 years<br>Mean = 12.93	RTP (rough and chase) relative frequency (direct observation)	social competence (peer-rated) aggression (peer rated, teacher rated)
Prezza [16]	2001	Italy	Cross sectional	251<br>Male = 133<br>Female = 118	Range = 7–12 years<br>Mean = 9.41 years	Global independent mobility score (mother semi-structured interview)	Social (frequency of play with peers)
Rubie Davies [17]	2007	New Zealand	Prospective cohort (1 year)	~25,782 students	5–12 years	Great heights (measuring playground equipment)	Number of students sustaining fractures (school report)
Schoeppe [18]	2014	Australia	Cross-sectional	191<br>Boys = 73<br>Girls = 118	Mean = 10.64 (0.89) years	Independent mobility (child self-report)	PA (combined HR and accelerometry)
Stone [19]	2014	Canada	Cross-sectional	856<br>Boys = 389<br>Girls = 467	Mean = 11.0 (0.6) years	Independent mobility (parent report questionnaire)	PA, SB (accelerometry)
Storli [20]	2010	Norway	Longitudinal	16<br>Boys = 9<br>Girls = 7	Range = 3–5 years	Nature (observed)	PA (accelerometry)
Weinstein [21]	1988	USA	Pre-post-test	~400	Range = 5–9.99 years (estimated according to national average for grades identified)	Risky play-supportive playground (observer rated)	PA (direct observation) Aggression (direct observation)

References

1. Bundy, A.C.; Luckett, T.; Tranter, P.J.; Naughton, G.A.; Wyver, S.R.; Ragen, J.; Spies, G. The risk is that there is "no risk": A simple, innovative intervention to increase children's activity levels. *Int. J. Early Years Educ.* **2009**, *17*, 33–45.
2. Colwell, M.J.; Lindsey, E.W. Preschool children's pretend and physical play and sex of play partner: connections to peer competence. *Sex Roles* **2005**, *52*, 497–509.
3. DeWolf, D. *Preschool Children's Negotiation of Intersubjectivity during Rough and Tumble Play*; Louisiana State University: Baton Rouge, LA, USA, 1999.
4. Engelen, L.; Bundy, A.C.; Naughton, G.; Simpson, J.M.; Bauman, A.; Ragen, J.; Baur, L.; Wyver, S.; Tranter, P.; Niehues, A.; *et al*. Increasing physical activity in young primary school children—It's child's play: A cluster randomised controlled trial. *Prev. Med.* **2013**, *56*, 319–325.
5. Floyd, M.F.; Bocarro, J.N.; Smith, W.R.; Baran, P.K.; Moore, R.C.; Cosco, N.G.; Edwards, M.B.; Suau, L.J.; Fang, K. Park-based physical activity among children and adolescents. *Amer. J. Prev. Med.* **2011**, *41*, 258–265.

6. Hayward, D.G.; Rothenberg, M.; Beasley, R.R. Children's play and urban playground environments: A comparison of traditional, contemporary, and adventure playground types. *Environ. Behav.* **1974**, *6*, 131–168.
7. Kirby, J.; Levin, K.; Inchley, J. Parental and peer influences on physical activity among Scottish adolescents: A longitudinal study. *J. Phys. Act. Health* **2011**, *8*, 785–793.
8. McCormack, G.; Giles-Corti, B. A cross-sectional study of the individual, social, and built environmental correlates of pedometer-based physical activity among elementary school children. *Int. J. Behav. Nutr. Phys. Act.* **2011**, *8*, 30, doi:10.1186/1479-5868-8-30.
9. Page, A.; Cooper, A.; Griew, P. Independent mobility in relation to weekday and weekend physical activity in children aged 10–11 years: The PEACH Project. *Int. J. Behav. Nutr. Phys. Act.* **2009**, *6*, doi:10.1186/1479-5868-6-2.
10. Page, A.S.; Cooper, A.R.; Griew, P.; Jago, R. Independent mobility, perceptions of the built environment and children's participation in play, active travel and structured exercise and sport: The PEACH Project. *Int. J. Behav. Nutr. Phys. Act.* **2010**, *7*, doi:10.1186/1479-5868-7-17.
11. Pellegrini, A. Elementary-school children's rough-and-tumble play and social competence. *Dev. Psychol.* **1988**, *24*, 802–806.
12. Pellegrini, A.D. Elementary school children's rough-and-tumble play. *Early Child. Res. Quart.* **1989**, *4*, 245–260.
13. Pellegrini, A.D. A longitudinal study of popular and rejected children's rough-and-tumble play. *Early Educ. Dev.* **1991**, *2*, 205–213.
14. Pellegrini, A.D. Boys' rough-and-tumble play, social competence and group composition. *Brit. J. Dev. Psychol.* **1993**, *11*, 237–248.
15. Pellegrini, A.D. A longitudinal study of boys' rough-and-tumble play and dominance during early adolescence. *J. Appl. Dev. Psychol.* **1995**, *16*, 77–93.
16. Prezza, M.; Pilloni, S.; Morabito, C.; Sersante, C.; Alparone, F.R.; Giuliani, M.V. The influence of psychosocial and environmental factors on children's independent mobility and relationship to peer frequentation. *J. Community Appl. Soc. Psychol.* **2001**, *11*, 435–450.
17. Rubie-Davies, C.M.; Townsend, M.A.R. Fractures in New Zealand elementary school settings. *J. Sch. Health* **2007**, *77*, 36–40.
18. Schoeppe, S.; Duncan, M.J.; Badland, H.M.; Oliver, M.; Browne, M. Associations between children's independent mobility and physical activity. *BMC Public Health* **2014**, *14*, doi:10.1186/1471-2458-14-91.
19. Stone, M.R.; Faulkner, G.E.; Mitra, R.; Buliung, R.N. The freedom to explore: Examining the influence of independent mobility on weekday, weekend and after-school physical activity behaviour in children living in urban and inner-suburban neighbourhoods of varying socioeconomic status. *Int. J. Behav. Nutr. Phys. Act.* **2014**, *11*, doi:10.1186/1479-5868-11-5.
20. Storli, R.; Hagen, T.L. Affordances in outdoor environments and children's physically active play in pre-school. *Eur. Early Child. Educ. Res. J.* **2010**, *18*, 445–456.
21. Weinstein, C.S.; Pinciotti, P. Changing a schoolyard: Intentions, design decisions, and behavioral outcomes. *Environ. Behav.* **1988**, *20*, 345–371.

Additional File 3: Records Screened

Please note that this file is encoded in RIS format to enable citation programs to exchange data.

TY - JOUR
ID - 1
T1 - A national study of neighborhood safety, outdoor play, television viewing, and obesity in preschool children
A1 - Burdette,H.L.
A1 - Whitaker,R.C.
Y1 - 2005/09//
N1 - Accession Number: 2009031131. Language: English. Entry Date: 20051028. Revision Date: 20091218. Publication Type: journal article; research; tables/charts. Commentary: Evenson K and Aytur S. DataBase: research and evaluation results. A national study of neighborhood safety, outdoor play, television viewing, and obesity in preschool children. AM J HEALTH PROMOT 2006 Sep-Oct; 21(1): 59 (commentary) . Journal Subset: Biomedical; Blind Peer Reviewed; Editorial Board Reviewed; Expert Peer Reviewed; Online/Print; Peer Reviewed; USA. Special Interest: Pediatric Care. Instrumentation: Neighborhood Environment for Children Rating Scales. Grant Information: National Institutes of Health (R01-HD41141). No. of Refs: 37 ref. NLM UID: 0376422. Email: burdette@email.chop.edu
KW - Obesity -- Epidemiology -- In Infancy and Childhood
KW - Obesity -- Risk Factors -- In Infancy and Childhood
KW - Physical Activity
KW - Residence Characteristics
KW - Television
KW - Adult
KW - Analysis of Variance
KW - Bivariate Statistics
KW - Body Height
KW - Body Mass Index
KW - Body Weight
KW - Chi Square Test
KW - Child Behavior
KW - Child,Preschool
KW - Cross Sectional Studies
KW - Descriptive Statistics
KW - Female
KW - Funding Source
KW - Interviews
KW - Male
KW - Mothers -- Psychosocial Factors
KW - One-Way Analysis of Variance
KW - Prevalence
KW - Safety
KW - Scales
KW - Socioeconomic Factors
KW - United States
KW - Human
KW - SportDiscus exploratory searching
RP - NOT IN FILE
SP - 657
EP - 662
JA - Pediatrics
VL - 116
IS - 3
U2 - PMID: 16140705

N2 - OBJECTIVE: To test the hypothesis that preschool children have a higher prevalence of obesity, spend less time playing outdoors, and spend more time watching television (TV) when they live in neighborhoods that their mothers perceive as unsafe. METHODS: In a cross-sectional survey in 20 large US cities, mothers reported the average daily time of outdoor play and TV viewing for their 3-year-old children, and the children's BMI was measured. Maternal perception of neighborhood safety was assessed with the Neighborhood Environment for Children Rating Scales; the scale score was used to divide children into tertiles of neighborhood safety. RESULTS: Of the 3141 children studied, 35% lived in households with incomes below the US poverty threshold. After adjustment for sociodemographic factors (household income and mothers' education, race/ethnicity, age, and marital status), obesity prevalence (BMI > or =95th percentile) did not differ in children from the least safe to the safest neighborhood safety tertile (18% vs 17% vs 20%) or in weekday (160 vs 151 vs 156 minutes/day) or weekend (233 vs 222 vs 222 minutes/day) outdoor play time. Children who lived in neighborhoods that were perceived by their mothers as the least safe watched more TV (201 vs 182 vs 185 minutes/day) and were more likely to watch >2 hours/day (66% vs 60% vs 62%). TV viewing and outdoor play minutes were not significantly correlated to each other or to BMI. CONCLUSIONS: In a national sample of preschool children, mothers' perception of neighborhood safety was related to their children's TV viewing time but not to their outdoor play time or risk for obesity
SN - 0031-4005
AD - Children's Hospital of Philadelphia, Division of Gastroenterology and Nutrition, 34th Street and Civic Center Boulevard, Philadelphia, PA 19104
UR - 16140705
ER -

TY - JOUR
ID - 2
T1 - Adolescent experimentation -- risky business or child's play?
A1 - Sweeney,C.T.
Y1 - 2002/03/25/
N1 - Accession Number: 2005069543. Language: English. Entry Date: 20050429. Revision Date: 20110916. Publication Type: journal article. Journal Subset: Nursing; USA. No. of Refs: 9 ref. NLM UID: 9892044
KW - Adolescent Behavior
KW - Risk Taking Behavior -- In Adolescence
KW - Adolescence
KW - Health Services Accessibility
KW - Sexuality
KW - Reviewer Nominated
RP - NOT IN FILE
EP - 4p
JA - Nursing Spectrum -- New York & New Jersey Edition
VL - 14A
IS - 6
SN - 1081-3101
AD - Clinical Director, Urban Solutions, Philadelphia
UR - http://search.ebscohost.com/login.aspx?direct=true&db=cin20&AN=2005069543&site=ehost-live
ER -

TY - JOUR
ID - 3
T1 - Clinical report---Injuries in youth soccer
A1 - Koutures,C.
A1 - Gregory,A.J.M.
Y1 - 2010/02//
N1 - Accession Number: 2010586357. Corporate Author: Council on Sports Medicine and Fitness. Language: English. Entry Date: 20100402. Publication Type: journal article; tables/charts. Journal Subset: Biomedical; Blind Peer Reviewed; Editorial Board Reviewed; Expert Peer Reviewed; Online/Print; Peer Reviewed; USA. Special Interest: Pediatric Care. No. of Refs: 48 ref. NLM UID: 0376422
KW - Athletic Injuries -- Prevention and Control -- In Adolescence

KW - Soccer
KW - Adolescence
KW - Athletic Injuries -- Risk Factors
KW - Brain Concussion
KW - Eye Injuries
KW - Facial Injuries
KW - Reviewer Nominated
RP - NOT IN FILE
SP - 410
EP - 414
JA - Pediatrics
VL - 125
IS - 2
U2 - PMID: 20100755
N2 - Injury rates in youth soccer, known as football outside the United States, are higher than in many other contact/collision sports and have greater relative numbers in younger, preadolescent players. With regard to musculoskeletal injuries, young females tend to suffer more knee injuries, and young males suffer more ankle injuries. Concussions are fairly prevalent in soccer as a result of contact/collision rather than purposeful attempts at heading the ball. Appropriate rule enforcement and emphasis on safe play can reduce the risk of soccer-related injuries. This report serves as a basis for encouraging safe participation in soccer for children and adolescents
SN - 0031-4005
UR - 20100755
ER -

TY - JOUR
ID - 4
T1 - Identification of risk factors and effective intervention strategies corresponding to the major causes of childhood death from injury
A1 - Rubsam,J.M.
Y1 - 2001/09//
N1 - Accession Number: 2002133026. Language: English. Entry Date: 20021011. Revision Date: 20060106. Publication Type: journal article; review; tables/charts. Journal Subset: Editorial Board Reviewed; Expert Peer Reviewed; Nursing; Peer Reviewed; USA. No. of Refs: 22 ref. NLM UID: 7507218
KW - Wounds and Injuries -- Risk Factors -- In Infancy and Childhood
KW - Accidents,Traffic -- Epidemiology -- In Infancy and Childhood
KW - Child Safety
KW - Age Factors
KW - Sex Factors
KW - Socioeconomic Factors
KW - Race Factors
KW - Child Passenger Safety
KW - Head Protective Devices
KW - Infant
KW - Child,Preschool
KW - Child
KW - Adolescence
KW - Male
KW - Female
KW - Reviewer Nominated
RP - NOT IN FILE
SP - 4
EP - 8
JA - Journal of the New York State Nurses Association
VL - 32
IS - 2

U2 - PMID: 16052901
N2 - Despite the dawn of a new millennium, unintentional injuries continue to be the leading cause of death in children greater than one year of age in the United States. This review outlines major risk factors that play a role in predicting injury, major causes of injury, and types of interventions successful in reducing childhood injury. This article describes community and national level intervention strategies that have been successful in reducing childhood injury
SN - 0028-7644
AD - Pediatric Trauma Nurse Coordinator, Babies & Children's Hospital of New York, New York Presbyterian Hospital, New York, NY
UR - 16052901
ER -

TY - JOUR
ID - 6
T1 - Psychological determinants of risk taking by children: an integrative model and implications for interventions
A1 - Morrongiello,B.A.
A1 - Lasenby-Lessard,J.
Y1 - 2007/02//
N1 - Accession Number: 2009522710. Language: English. Entry Date: 20080222. Publication Type: journal article. Journal Subset: Biomedical; Blind Peer Reviewed; Editorial Board Reviewed; Europe; Expert Peer Reviewed; Online/Print; Peer Reviewed; Public Health; UK & Ireland. Special Interest: Pediatric Care; Public Health. No. of Refs: 83 ref. NLM UID: 9510056
KW - Child Behavior
KW - Models,Psychological
KW - Risk Taking Behavior
KW - Age Factors
KW - Child
KW - Family
KW - Female
KW - Male
KW - Motivation
KW - Parenting
KW - Play and Playthings
KW - Safety -- Methods
KW - Sex Factors
KW - Social Behavior
KW - Reviewer Nominated
RP - NOT IN FILE
SP - 20
EP - 25
JA - Injury Prevention
VL - 13
IS - 1
U2 - PMID: 17296684
N2 - OBJECTIVES: To draw on empirical findings of the psychological factors that cause elementary-school children to engage in risky play behaviors that can lead to injury, with the aim of developing an integrative model that can support intervention-program planning. METHODS: An extensive review of literature on this topic was conducted, determinants of risk taking for which there was empirical support were identified, and results were synthesized to create an integrative model of children's risk taking. RESULTS: Research on risk taking in children is limited, but the findings support the importance of examining child, family and socio-environmental factors to understand children's risk-taking behaviors. CONCLUSIONS: Development of a model outlining the determinants of risk behaviors can provide a foundation for initiatives that aim to reduce such behaviors and prevent childhood injuries
SN - 1353-8047
UR - 17296684

ER -

TY - JOUR
ID - 7
T1 - The risk factor: play fills the void
A1 - Hopkins,G.
Y1 - 2006/06/15/
N1 - Accession Number: 2009225992. Language: English. Entry Date: 20060908. Publication Type: journal article; case study; pictorial. Journal Subset: Allied Health; Europe; UK & Ireland. Special Interest: Social Work. NLM UID: 9315530
KW - Adoption -- In Infancy and Childhood
KW - Attachment Behavior -- In Infancy and Childhood
KW - Child Behavior Disorders -- Prevention and Control
KW - Play Therapy -- In Infancy and Childhood
KW - Apraxia -- Diagnosis
KW - Child
KW - Male
KW - Multidisciplinary Care Team
KW - Risk Assessment
KW - SportDiscus exploratory searching
RP - NOT IN FILE
SP - 36
EP - 37
JA - Community Care
IS - 1627
SN - 0307-5508
UR - http://search.ebscohost.com/login.aspx?direct=true&db=cin20&AN=2009225992&site=ehost-live
ER -

TY - CHAP
ID - 8
T1 - Play as arousal seeking; or the quest for information
A1 - Ellis,M.J.
Y1 - 1976///
N1 - Accession Number: SPH40090; Author: Ellis, M.J. ; Conference: National Conference on the Mental Health Aspects of Sports, Exercise and Recreation, 17th, Atlantic City, 1975.; Language: English; Parent Item: SPH40067; Database Subset: Recreation and Leisure; Publication Type: Book Analytic; Update Code: 19940901; SIRC Article No.: 40090
Accession Number: SPH343803; Author: Butcher, J. ; Language: English; Parent Item: SPHP914; References: 8; General Notes: This research was supported by a research grant from the university of Manitoba SSHRC Fund Committee.; Publication Type: Journal article; Update Code: 19940501; SIRC Article No.: 343803; SportDiscus exploratory searching
KW - *PLAY
KW - *SPORTS
KW - *RISK-taking (Psychology)
KW - AROUSAL (Physiology)
KW - CHILDREN
KW - THEORY
KW - ADULTHOOD
KW - SportDiscus exploratory searching
RP - NOT IN FILE
T2 - In Craig, T.T. (ed.), Humanistic and mental health aspects of sports, exercise and recreation, Chicago, American Medical Association, 1976, p. 77-80
CY - United States

UR -
https://login.proxy.bib.uottawa.ca/login?url=http://search.ebscohost.com/login.aspx?direct=true&db=s3h&AN=SPH40090&site=ehost-live;http://articles.sirc.ca/search.cfm?id=40090
ER -

TY - CHAP
ID - 9
T1 - Play attitudes and risk taking as a function of achievement motivation
A1 - Levy,J.
A1 - Bishop,D.W.
Y1 - 1972///
N1 - Accession Number: SPH19641; Author: Levy, J. Author: Bishop, D.W. ; Conference: Canadian symposium on psycho-motor learning and sport psychology, 4th, Waterloo, Ont., 1972.; Language: English; Database Subset: SIRLS Database; Publication Type: Book Analytic; Update Code: 19940901; SIRC Article No.: 19641
Accession Number: SPH343803; Author: Butcher, J. ; Language: English; Parent Item: SPHP914; References: 8; General Notes: This research was supported by a research grant from the university of Manitoba SSHRC Fund Committee.; Publication Type: Journal article; Update Code: 19940501; SIRC Article No.: 343803; SportDiscus exploratory searching
KW - *PSYCHOLOGY
KW - *PERSONALITY
KW - *RISK-taking (Psychology)
KW - *PLAY
KW - *ACHIEVEMENT motivation
KW - ATTITUDE (Psychology)
KW - BOYS
KW - CHILDREN
KW - SportDiscus exploratory searching
RP - NOT IN FILE
T2 - In Proceedings - Canadian symposium, 4th - Psychomotor learning and sport psychology, Waterloo, Ont., Oct. 23-25, 1972; Ottawa, Dept. of National Health and Welfare, 1973, p. 483-504
CY - Canada
UR -
https://login.proxy.bib.uottawa.ca/login?url=http://search.ebscohost.com/login.aspx?direct=true&db=s3h&AN=SPH19641&site=ehost-live;http://articles.sirc.ca/search.cfm?id=19641
ER -

TY - CHAP
ID - 10
T1 - Play, stress and risk recreation
A1 - Wealand,J.
Y1 - 1982///
N1 - Accession Number: SPH120637; Author: Wealand, J. ; Language: English; Parent Item: SPH120627; References: 9; Database Subset: Recreation and Leisure; Publication Type: Book Analytic; Update Code: 19940901; SIRC Article No.: 120637
Accession Number: SPH343803; Author: Butcher, J. ; Language: English; Parent Item: SPHP914; References: 8; General Notes: This research was supported by a research grant from the university of Manitoba SSHRC Fund Committee.; Publication Type: Journal article; Update Code: 19940501; SIRC Article No.: 343803; SportDiscus exploratory searching
KW - *RECREATION
KW - *PLAY
KW - *STRESS (Physiology)
KW - *STRESS management
KW - *RISK-taking (Psychology)
KW - EXAMPLE
KW - SOCIAL psychology

KW - SportDiscus exploratory searching
RP - NOT IN FILE
T2 - In, Wais, E.G. (ed.), Seminar readings: play and stress in everyday life, Wembley, W.A., Dept. for Youth, Sport and Recreation, 1982, p. 24-32
UR -
https://login.proxy.bib.uottawa.ca/login?url=http://search.ebscohost.com/login.aspx?direct=true&db=s3h&AN=SPH120637&site=ehost-live;http://articles.sirc.ca/search.cfm?id=120637
ER -

TY - JOUR
ID - 11
T1 - SAFETY Sans SACRIFICES
A1 - Spencer,Anne Marie
Y1 - 2008/08//
N1 - Accession Number: 33653696; Author: Spencer, Anne-Marie ; No. of Pages: 4; Language: English; Publication Type: Journal Article; Update Code: 20080813
KW - *PLAYGROUNDS
KW - *LIFE skills
KW - DESIGN & construction
KW - PLAY environments -- Safety measures
KW - RISK-taking (Psychology) in children
KW - SELF-confidence in children
KW - SELF-esteem in children
KW - SportDiscus exploratory searching
RP - NOT IN FILE
SP - 54
EP - 57
JA - Parks & Recreation
VL - 43
IS - 8
N2 - The article discusses the balance between safety and enjoyability in playground design. The need for a certain amount of risk in children's play is discussed, citing children's disinterest in parks that are designed too conservatively. Benefits of healthy risk-taking are discussed, including self-confidence, prevention of fragility, and healthy development of life skills such as self-esteem
SN - 00312215
UR -
https://login.proxy.bib.uottawa.ca/login?url=http://search.ebscohost.com/login.aspx?direct=true&db=s3h&AN=33653696&site=ehost-live
ER -

TY - JOUR
ID - 12
T1 - Socialization of children's playground skill
A1 - Butcher,J.
Y1 - 1993/12//
N1 - Accession Number: SPH343803; Author: Butcher, J. ; Language: English; Parent Item: SPHP914; References: 8; General Notes: This research was supported by a research grant from the university of Manitoba SSHRC Fund Committee.; Publication Type: Journal article; Update Code: 19940501; SIRC Article No.: 343803; SportDiscus exploratory searching
KW - *PLAYGROUNDS
KW - *PLAY
KW - SOCIOLOGY
KW - CHILDREN
KW - ABILITY
KW - SOCIALIZATION
KW - SportDiscus exploratory searching

RP - NOT IN FILE
SP - 731
EP - 738
JA - Perceptual & Motor Skills
VL - 77
IS - 3 Part 1
CY - United States
N2 - This study analyzed the relationship between playground skill (measured on the Playground Skills Test) and several socialization variables, including opportunities for practice and parental involvement with playground play (measured on a parent's questionnaire). Data were collected for 64 children (24 girls, 40 boys), ages 7 to 9 years. Descriptive statistics were presented for availability of playground equipment, frequency of playground play outside of school hours, and amount of parental involvement. Playground proficiency was not related to frequency of playground play or to direct parental support (attendance and assistance while child was at a playground). Parental perceptions of a child's skill and attempts at risk-taking were significantly correlated with playground proficiency. A regression analysis of all socialization variables showed that the only significant predictor of playground proficiency was a child's risk-taking attempts
SN - 00315125
UR -
https://login.proxy.bib.uottawa.ca/login?url=http://search.ebscohost.com/login.aspx?direct=true&db=s3h&AN=SPH343803&site=ehost-live;http://articles.sirc.ca/search.cfm?id=343803
ER -

TY - JOUR
ID - 13
T1 - The effect of a developmental play program on the concept, risk-taking behaviors, and motoric proficiency of mildly handicapped children
A1 - Roswal,G.
A1 - Frith,G.
A1 - Dunleavy,A.O.
Y1 - 1984/03//
N1 - Accession Number: SPH150528; Author: Roswal, G. Author: Frith, G. Author: Dunleavy, A.O. ; Language: English; References: 36; Database Subset: Handicapped/Disabled Sport and Recreation Documents; Publication Type: Journal article; Update Code: 19940901; SIRC Article No.: 150528
Accession Number: SPH343803; Author: Butcher, J. ; Language: English; Parent Item: SPHP914; References: 8; General Notes: This research was supported by a research grant from the university of Manitoba SSHRC Fund Committee.; Publication Type: Journal article; Update Code: 19940501; SIRC Article No.: 343803; SportDiscus exploratory searching
KW - *MENTAL retardation
KW - *PLAY
KW - *RISK-taking (Psychology)
KW - *MOTOR ability
KW - CHILDREN
KW - PROGRAMS
KW - SELF-perception
KW - TESTING
KW - CHILDREN'S DEVELOPMENTAL PLAY PROGRAM
KW - REVIEW
KW - DISABILITIES-Mental retardation - research
KW - SportDiscus exploratory searching
RP - NOT IN FILE
SP - 43
EP - 50
JA - Physical Educator
VL - 41
IS - 1
CY - United States

N2 - The purpose of this study was to evaluate the effect of the Children's Developmental Play Program (CDPP) in the behavioral and neuromotor functioning of developmentally disabled children. 32 children between the ages of 5 and 13 years enrolled in educable mentally retarted classed, served as subjects. Data was collected on self concept, risk taking behaviors and motor proficiency of the students. Half of the children were enrolled in the CDPP. Overall improvements were observed in the experimental group, and this especially in both self concept and motor proficiency
SN - 00318981
UR -
https://login.proxy.bib.uottawa.ca/login?url=http://search.ebscohost.com/login.aspx?direct=true&db=s3h&AN=SPH150528&site=ehost-live;http://articles.sirc.ca/search.cfm?id=150528
ER -

TY - JOUR
ID - 14
T1 - Those daring young men (and women) on the flying trapeze: impetuous folly of calculated mastery?
A1 - Hofsess,L.
Y1 - 1986///Winter
N1 - Accession Number: SPH182250; Author: Hofsess, L. ; Language: English; References: 14; Publication Type: Journal article; Update Code: 19940901; SIRC Article No.: 182250
Accession Number: SPH343803; Author: Butcher, J. ; Language: English; Parent Item: SPHP914; References: 8; General Notes: This research was supported by a research grant from the university of Manitoba SSHRC Fund Committee.; Publication Type: Journal article; Update Code: 19940501; SIRC Article No.: 343803; SportDiscus exploratory searching
KW - *PLAY
KW - *RISK-taking (Psychology)
KW - *ACROBATICS
KW - *PSYCHOLOGY
KW - *PHYSICAL fitness
KW - *EXERCISE
KW - SURVEYS
KW - ABILITY
KW - MALES
KW - WOMEN
KW - COMPARATIVE studies
KW - SENSATION seeking
KW - SportDiscus exploratory searching
RP - NOT IN FILE
SP - 14
EP - 17
JA - Association for the Anthropological Study of Play Newsletter
VL - 12
IS - 2
CY - United States
UR -
https://login.proxy.bib.uottawa.ca/login?url=http://search.ebscohost.com/login.aspx?direct=true&db=s3h&AN=SPH182250&site=ehost-live;http://articles.sirc.ca/search.cfm?id=182250
ER -

TY - JOUR
ID - 54
T1 - Neural sensitivity to absolute and relative anticipated reward in adolescents
A1 - Vaidya,J.G.
A1 - Knutson,B.
A1 - O'Leary,D.S.
A1 - Block,R.I.
A1 - Magnotta,V.

Y1 - 2013///
N1 - DA - 20130401
IS - 1932-6203 (Electronic)
IS - 1932-6203 (Linking)
LA - eng
PT - Journal Article
PT - Research Support, N.I.H., Extramural
SB - IM
KW - Adolescent
KW - Adult
KW - Anticipation,Psychological
KW - Basal Ganglia
KW - physiopathology
KW - Brain Mapping
KW - Child
KW - Female
KW - Humans
KW - Impulsive Behavior
KW - Male
KW - Motivation
KW - Nervous System
KW - Prefrontal Cortex
KW - Reward
KW - Task Performance and Analysis
KW - Reviewer Nominated
RP - NOT IN FILE
SP - e58708
JF - PLoS ONE [Electronic Resource]
JA - PLoS One
VL - 8
IS - 3
N2 - Adolescence is associated with a dramatic increase in risky and impulsive behaviors that have been attributed to developmental differences in neural processing of rewards. In the present study, we sought to identify age differences in anticipation of absolute and relative rewards. To do so, we modified a commonly used monetary incentive delay (MID) task in order to examine brain activity to relative anticipated reward value (neural sensitivity to the value of a reward as a function of other available rewards). This design also made it possible to examine developmental differences in brain activation to absolute anticipated reward magnitude (the degree to which neural activity increases with increasing reward magnitude). While undergoing fMRI, 18 adolescents and 18 adult participants were presented with cues associated with different reward magnitudes. After the cue, participants responded to a target to win money on that trial. Presentation of cues was blocked such that two reward cues associated with $.20, $1.00, or $5.00 were in play on a given block. Thus, the relative value of the $1.00 reward varied depending on whether it was paired with a smaller or larger reward. Reflecting age differences in neural responses to relative anticipated reward (i.e., reference dependent processing), adults, but not adolescents, demonstrated greater activity to a $1 reward when it was the larger of the two available rewards. Adults also demonstrated a more linear increase in ventral striatal activity as a function of increasing absolute reward magnitude compared to adolescents. Additionally, reduced ventral striatal sensitivity to absolute anticipated reward (i.e., the difference in activity to medium versus small rewards) correlated with higher levels of trait Impulsivity. Thus, ventral striatal activity in anticipation of absolute and relative rewards develops with age. Absolute reward processing is also linked to individual differences in Impulsivity
AD - Department of Psychiatry, University of Iowa Carver College of Medicine, Iowa City, Iowa, United States of America. jatin-vaidya@uiowa.edu
UR - 23544046
ER -

TY - JOUR
ID - 56

T1 - Mindfulness for adolescents: a promising approach to supporting emotion regulation and preventing risky behavior
A1 - Broderick,P.C.
A1 - Jennings,P.A.
Y1 - 2012///
N1 - DA - 20130129
IS - 1537-5781 (Electronic)
IS - 1533-8916 (Linking)
LA - eng
PT - Journal Article
SB - IM
KW - Adolescent
KW - Adolescent Behavior
KW - Belgium
KW - Humans
KW - Netherlands
KW - Personal Autonomy
KW - Social Identification
KW - Reviewer Nominated
RP - NOT IN FILE
SP - 111
EP - 26, 11
JA - New Dir.Youth Dev
VL - 2012
IS - 136
N2 - This article reviews the contextual and neuropsychological challenges of the adolescent period with particular attention to the role that universal prevention can play in moderating the harmful effects of stress. The centrality of emotion regulation skills to long-term health and wellness suggests their importance in prevention and intervention efforts for youth. Mindfulness has been shown to be an effective means of reducing stress and improving emotion balance in research with adults, although research on mindfulness with adolescents is limited. The authors present available data and describe one potentially effective program for adolescent mindfulness: Learning to BREATHE
AD - West Chester University of Pennsylvania, USA
UR - 23359447
ER -

TY - JOUR
ID - 60
T1 - The effects of risk-glorifying media exposure on risk-positive cognitions, emotions, and behaviors: a meta-analytic review
A1 - Fischer,P.
A1 - Greitemeyer,T.
A1 - Kastenmuller,A.
A1 - Vogrincic,C.
A1 - Sauer,A.
Y1 - 2011/05//
N1 - DA - 20110426
IS - 1939-1455 (Electronic)
IS - 0033-2909 (Linking)
LA - eng
PT - Journal Article
PT - Meta-Analysis
PT - Review
SB - IM
KW - Adolescent
KW - Adolescent Behavior

KW - psychology
KW - Adult
KW - Advertising as Topic
KW - Age Distribution
KW - Alcohol Drinking
KW - Attitude to Health
KW - Automobile Driving
KW - Cognition
KW - Cross-Sectional Studies
KW - Emotions
KW - Female
KW - Humans
KW - Male
KW - Mass Media
KW - Music
KW - Risk-Taking
KW - Sexual Behavior
KW - Smoking
KW - Social Behavior
KW - Sports
KW - Video Games
KW - Young Adult
RP - NOT IN FILE
SP - 367
EP - 390
JA - Psychol Bull
VL - 137
IS - 3
N2 - In recent years, there has been a surge in the quantity of media content that glorifies risk-taking behavior, such as risky driving, extreme sports, or binge drinking. The authors conducted a meta-analysis involving more than 80,000 participants and 105 independent effect sizes to examine whether exposure to such media depictions increased their recipients' risk-taking inclinations. A positive connection was found for overall, combined risk taking (g=.41); as well as its underlying dimensions: risk-taking behaviors (g=.41), risk-positive cognitions and attitudes (g=.35), and risk-positive emotions (g=.56). This effect was observed across varying research methods (experimental, correlational, longitudinal); types of media (video games, movies, advertising, TV, music); and differing risk-related outcome measures (e.g., smoking, drinking, risky driving, sexual behavior). Multiple moderator analyses revealed 2 theoretically new boundary conditions for sociocognitive models. First, the effect was stronger for active (i.e., video games) than for passive (e.g., film, music) exposure to risk-glorifying media content. Second, the effect was stronger when there was a high degree of contextual fit between the media content and type of risk-taking measure. The theoretical, practical, and societal implications of the present research synthesis are discussed
AD - Department of Psychology, University of Graz, Graz, Austria. peter.fischer@uni-graz.at
UR - 21341887
ER -

TY - JOUR
ID - 61
T1 - Children's risky play from an evolutionary perspective: the anti-phobic effects of thrilling experiences
A1 - Sandseter,E.B.
A1 - Kennair,L.E.
Y1 - 2011///
N1 - DA - 20120905
IS - 1474-7049 (Electronic)
IS - 1474-7049 (Linking)
LA - eng

PT - Journal Article
SB - IM
KW - Adaptation,Psychological
KW - Anxiety Disorders
KW - prevention & control
KW - psychology
KW - Biological Evolution
KW - Child
KW - Child Development
KW - Child,Preschool
KW - Fear
KW - Humans
KW - Parenting
KW - Phobic Disorders
KW - Play and Playthings
KW - Risk-Taking
KW - Reviewer Nominated
RP - NOT IN FILE
SP - 257
EP - 284
JA - Evol.Psychol
VL - 9
IS - 2
N2 - This theoretical article views children's risky play from an evolutionary perspective, addressing specific evolutionary functions and especially the anti-phobic effects of risky play. According to the non-associative theory, a contemporary approach to the etiology of anxiety, children develop fears of certain stimuli (e.g., heights and strangers) that protect them from situations they are not mature enough to cope with, naturally through infancy. Risky play is a set of motivated behaviors that both provide the child with an exhilarating positive emotion and expose the child to the stimuli they previously have feared. As the child's coping skills improve, these situations and stimuli may be mastered and no longer be feared. Thus fear caused by maturational and age relevant natural inhibition is reduced as the child experiences a motivating thrilling activation, while learning to master age adequate challenges. It is concluded that risky play may have evolved due to this anti-phobic effect in normal child development, and it is suggested that we may observe an increased neuroticism or psychopathology in society if children are hindered from partaking in age adequate risky play
AD - Department of Physical Education, Queen Maud University College of Early Childhood Education (DMMH), Trondheim, Norway. ebs@dmmh.no
UR - 22947972
ER -

TY - JOUR
ID - 62
T1 - Parenting by anxious mothers: effects of disorder subtype, context and child characteristics
A1 - Murray,L.
A1 - Lau,P.Y.
A1 - Arteche,A.
A1 - Creswell,C.
A1 - Russ,S.
A1 - Zoppa,L.D.
A1 - Muggeo,M.
A1 - Stein,A.
A1 - Cooper,P.
Y1 - 2012/02//
N1 - DA - 20120113
IS - 1469-7610 (Electronic)
IS - 0021-9630 (Linking)
LA - eng

PT - Comparative Study
PT - Journal Article
PT - Research Support, Non-U.S. Gov't
SB - IM
KW - Adult
KW - Anxiety Disorders
KW - psychology
KW - Child Behavior
KW - Child,Preschool
KW - Female
KW - Humans
KW - Inhibition (Psychology)
KW - Mother-Child Relations
KW - Mothers
KW - Neuropsychological Tests
KW - Parenting
KW - Phobic Disorders
KW - Play and Playthings
KW - Reviewer Nominated
RP - NOT IN FILE
SP - 188
EP - 196
JA - J Child Psychol Psychiatry
VL - 53
IS - 2
N2 - BACKGROUND: There has been increasing research interest in parenting by anxious adults; however, little is known about anxiety-subtype effects, or effects of the context in which parenting is assessed. METHODS: Two groups of anxious mothers, social phobia (N = 50), generalised anxiety disorder (N = 38), and nonanxious controls (N = 62) were assessed with their 4.9-year-old children in three tasks: two presented threat specifically relevant to each maternal disorder, namely, a social threat task where the child had to give a speech, and a nonsocial threat task where the child had to explore potentially scary objects; the third was a nonthreat task (playing with play dough). Seven parenting dimensions were scored. Effects on parenting of maternal anxiety subgroup and task, and their interactions, were examined, as were effects of earlier child behavioural inhibition and currently manifest anxiety. RESULTS: There were no parenting differences between maternal groups in the nonthreat play-dough task; parenting difficulties in the two anxious groups were principally evident in the disorder-specific challenge. Parenting differences between nonanxious and anxious mothers occurred independently of child characteristics. There was little evidence for particular forms of parenting difficulty being unique to maternal disorder. CONCLUSIONS: Anxious mothers' parenting difficulties emerge when occurring under challenge, especially when this is disorder-specific. These effects should be considered in research and clinical practice
AD - Winnicott Research Unit, School of Psychology, University of Reading, Reading, UK. lynne.murray@rdg.ac.uk
UR - 21985622
ER -

TY - BOOK
ID - 63
T1 - Risk and Our Pedagogical Relation to Children: On the Playground and Beyond. SUNY Series, Early Childhood Education: Inquiries and Insights
A1 - Smith,Stephen J.
Y1 - 1998///
KW - Reviewer Nominated
RP - NOT IN FILE
PB - State University of New York Press, State University Plaza, Albany, NY 12246 (paperback: ISBN-0-7914-3594-6, $19.95; hardcover: ISBN-0-7914-3593-8, $59.50).

N2 - This book uses the playground as a reference point for a phenomenological examination of risk in children's lives and the development of a pedagogy of risk. Chapter 1 defines risk and discusses the use of anecdotes as a methodological device. Chapter 2 examines how considering risk as challenge and adventure leads to questions concerning adults' relationship to children and enabling children to take risks in relative safety. Chapter 3 examines adults' actions with children and the relationality of risk, while chapter 4 focuses on helping children to take risks in relative safety through adult encouragement. Chapter 5 considers direct and indirect social challenges to take risks, distinguishes positive and negative challenges, and examines how the visibility of risk allows the construction of a pedagogy. Chapter 6 maintains that a pedagogy of risk rests upon acknowledging one's own ability to take risks and focuses on how working through apprehensiveness leads to reconciling adults' need to lend security to children's explorations with children's need to test the security of their world. Chapter 7 maintains that a child-oriented language of risk gives a fuller sense of the meaning of young children's physical activity than does the language of skill development, and discusses the importance of attending to the riskiness of children's activity over the course of successive playground encounters. Chapter 8 focuses on implications of the practice of risk and their application to other situations. Chapter 9 makes recommendations for implementing a pedagogical relation sensitive to risk. (Contains approximately 275 references.) (KB)
SN - ISBN-0-7914-3594-6
ER -

TY - JOUR
ID - 64
T1 - Risky play and children's safety: balancing priorities for optimal child development
A1 - Brussoni,M.
A1 - Olsen,L.L.
A1 - Pike,I.
A1 - Sleet,D.A.
Y1 - 2012/09//
N1 - DA - 20121203
IS - 1660-4601 (Electronic)
IS - 1660-4601 (Linking)
LA - eng
PT - Journal Article
PT - Research Support, Non-U.S. Gov't
PT - Review
SB - IM
KW - Reviewer Nominated
KW - Child
KW - Child Development
KW - Child,Preschool
KW - Female
KW - Humans
KW - Male
KW - Play and Playthings
KW - Public Health
KW - Risk-Taking
KW - Safety Management
KW - standards
KW - Wounds and Injuries
KW - prevention & control
RP - NOT IN FILE
SP - 3134
EP - 3148
JF - International Journal of Environmental Research & Public Health [Electronic Resource]
JA - Int.J Environ.Res.Public Health
VL - 9
IS - 9

N2 - Injury prevention plays a key role in keeping children safe, but emerging research suggests that imposing too many restrictions on children's outdoor risky play hinders their development. We explore the relationship between child development, play, and conceptions of risk taking with the aim of informing child injury prevention. Generational trends indicate children's diminishing engagement in outdoor play is influenced by parental and societal concerns. We outline the importance of play as a necessary ingredient for healthy child development and review the evidence for arguments supporting the need for outdoor risky play, including: (1) children have a natural propensity towards risky play; and, (2) keeping children safe involves letting them take and manage risks. Literature from many disciplines supports the notion that safety efforts should be balanced with opportunities for child development through outdoor risky play. New avenues for investigation and action are emerging seeking optimal strategies for keeping children "as safe as necessary," not "as safe as possible." This paradigm shift represents a potential for epistemological growth as well as cross-disciplinary collaboration to foster optimal child development while preserving children's safety
AD - Department of Pediatrics, School of Population and Public Health, British Columbia Injury Research and Prevention Unit, Child and Family Research Institute, University of British Columbia, British Columbia Children's Hospital, L408-4480 Oak Street, Vancouver, V6H 3V4 BC, Canada. mbrussoni@cw.bc.ca
UR - 23202675
ER -

TY - BOOK
ID - 66
T1 - Managing risk in play provision : implementation guide
A1 - Ball,D.
A1 - Gill,T.
A1 - Spiegal,B..
Y1 - 2008///
KW - Reviewer Nominated
RP - IN FILE
CY - [Annesley]
PB - DCSF Publications
SN - 9781847752895 1847752896
ER -

TY - BOOK
ID - 67
T1 - Nothing ventured : balancing risks and benefits in the outdoors
A1 - Gill,Tim.,English Outdoor Council.
Y1 - 2010///
KW - Reviewer Nominated
RP - NOT IN FILE
CY - [England]
PB - English Outdoor Council
SN - 9780956663603 0956663605
ER -

TY - JOUR
ID - 68
T1 - Everyday uncertainties: reframing perceptions of risk in outdoor free play
A1 - Niehues,Anita Nelson
A1 - Bundy,Anita
A1 - Broom,Alex
A1 - Tranter,Paul
A1 - Ragen,Jo
A1 - Engelen,Lina
Y1 - 2013/06/10/
N1 - doi: 10.1080/14729679.2013.798588
KW - Reviewer Nominated

RP - NOT IN FILE
SP - 223
EP - 237
JF - Journal of Adventure Education & Outdoor Learning
VL - 13
IS - 3
PB - Routledge
N2 - This paper reports the results of risk reframing, an intervention to offer parents and educators a context for building new and complex perceptions of risk in children's outdoor free play. Our objective was to alter these adults' perceptions of risk to increase the sustainability of an innovative child-centred playground intervention. Qualitative data in the form of audio-recordings of risk-reframing sessions, brief participant evaluations and field notes kept by project staff were collected and either transcribed in their entirety or summarised in brief written reports. These data were subjected to constant comparative analysis to identify emergent themes. Results suggest that educators and parents benefit from opportunities to share risk perceptions and discuss the costs and benefits for offering outdoor free play to children to achieve their common goals for children: health, happiness and resilience.
SN - 1472-9679
Y2 - 2013/12/04/
M3 - doi: 10.1080/14729679.2013.798588
UR - http://dx.doi.org/10.1080/14729679.2013.798588
ER -

TY - BOOK
ID - 75
T1 - Play: How it shapes the brain, opens the imagination, and invigorates the soul
A1 - Brown,S.
Y1 - 2009///
N1 - Brown, S. (2009). Play: How it shapes the brain, opens the imagination, and invigorates the soul . New York: Avery.
KW - Cited Reference
RP - NOT IN FILE
SP - null
VL - null
A2 - null
CY - New York
PB - Avery
T3 - null
SN - null
ER -

TY - BOOK
ID - 92
T1 - Risk: The science and politics of fear
A1 - Gardner,D.
Y1 - 2008///
N1 - Gardner, D. (2008). Risk: The science and politics of fear . Carlton North, VIC: Scribe Publications.
KW - Cited Reference
RP - NOT IN FILE
CY - Carleton North
PB - Scribe Publications
ER -

TY - BOOK
ID - 98
T1 - Understanding and managing risk attitudes
A1 - Hillson,D.

A1 - Murray-Webster,R.
Y1 - 2007///
KW - Cited Reference
RP - NOT IN FILE
CY - Aldershot
PB - Gower
ER -

TY - BOOK
ID - 113
T1 - Free-range kids: Giving our children the freedom we had without going nuts with worry
A1 - Skenazy,L.
Y1 - 2009///
KW - Cited Reference
RP - NOT IN FILE
CY - San Francisco
PB - Jossey-Bass
ER -

TY - BOOK
ID - 117
T1 - Playing outdoors: Spaces and places, risk and challenge
A1 - Tovey,H.
Y1 - 2007///
KW - Cited Reference
RP - NOT IN FILE
CY - Maidenhead
PB - Open University Press
T3 -

ER -

TY - BOOK
ID - 119
T1 - Too safe for their own good: How risk and responsibility help teens thrive
A1 - Ungar,M.
Y1 - 2007///
KW - Cited Reference
RP - NOT IN FILE
CY - Crow's Nest, NSW
PB - Inspired Living
ER -

TY - JOUR
ID - 120
T1 - The Sydney playground project: popping the bubblewrap--unleashing the power of play: a cluster randomized controlled trial of a primary school playground-based intervention aiming to increase children's physical activity and social skills
A1 - Bundy,A.C.
A1 - Naughton,G.
A1 - Tranter,P.
A1 - Wyver,S.
A1 - Baur,L.
A1 - Schiller,W.
A1 - Bauman,A.
A1 - Engelen,L.

A1 - Ragen,J.
A1 - Luckett,T.
A1 - Niehues,A.
A1 - Stewart,G.
A1 - Jessup,G.
A1 - Brentnall,J.
Y1 - 2011///
N1 - DA - 20111007
IS - 1471-2458 (Electronic)
IS - 1471-2458 (Linking)
LA - eng
PT - Journal Article
PT - Randomized Controlled Trial
PT - Research Support, Non-U.S. Gov't
SB - IM
KW - PubMed related articles - informal
KW - Bullying
KW - Child
KW - Child,Preschool
KW - Cluster Analysis
KW - Exercise
KW - Female
KW - Health Promotion
KW - methods
KW - Humans
KW - Male
KW - Mental Health
KW - New South Wales
KW - Overweight
KW - prevention & control
KW - Play and Playthings
KW - psychology
KW - Schools
KW - SOCIALIZATION
RP - NOT IN FILE
SP - 680
JA - BMC Public Health
VL - 11
N2 - BACKGROUND: In the Westernised world, numerous children are overweight and have problems with bullying and mental health. One of the underlying causes for all three is postulated to be a decrease in outdoor free play. The aim of the Sydney Playground Project is to demonstrate the effectiveness of two simple interventions aimed to increase children's physical activity and social skills. METHODS/DESIGN: This study protocol describes the design of a 3-year cluster randomised controlled trial (CRCT), in which schools are the clusters. The study consists of a 13-week intervention and 1 week each of pre-and post-testing. We are recruiting 12 schools (6 control; 6 intervention), with 18 randomly chosen participants aged 5 to 7 years in each school. The two intervention strategies are: (1) Child-based intervention: Unstructured materials with no obvious play value introduced to the playground; and (2) Adult-based intervention: Risk reframing sessions held with parents and teachers with the aim of exploring the benefits of allowing children to engage in activities with uncertain outcomes. The primary outcome of the study, physical activity as measured by accelerometer counts, is assessed at baseline and post-intervention. Additional assessments include social skills and interactions, self-concept, after school time use and anthropometric data. Qualitative data (i.e., transcriptions of audio recordings from the risk reframing sessions and of interviews with selected teacher and parent volunteers) are analysed to understand their perceptions of risk in play. The control schools have recess as usual. In addition to outcome evaluation, regular process evaluation sessions are held to monitor fidelity to the treatment. DISCUSSION: These simple interventions, which could be adopted in every primary school, have the potential of initiating a

self-sustaining cycle of prevention for childhood obesity, bullying and mental ill health. TRIAL REGISTRATION: Australian and New Zealand Clinical Trials Registration Number ACTRN12611000089932
AD - Faculty of Health Sciences, University of Sydney, Lidcombe, Australia. anita.bundy@sydney.edu.au
UR - 21884603
ER -

TY - JOUR
ID - 121
T1 - Jumping off and being careful: children's strategies of risk management in everyday life
A1 - Christensen,P.
A1 - Mikkelsen,M.R.
Y1 - 2008/01//
N1 - DA - 20080207
IS - 0141-9889 (Print)
IS - 0141-9889 (Linking)
LA - eng
PT - Journal Article
SB - IM
KW - PubMed related articles - informal
KW - Accidents
KW - Activities of Daily Living
KW - Anthropology,Cultural
KW - Child
KW - Denmark
KW - Female
KW - Humans
KW - Interviews as Topic
KW - Male
KW - Play and Playthings
KW - Risk Management
KW - Risk-Taking
KW - Sex Factors
RP - NOT IN FILE
SP - 112
EP - 130
JF - Sociology of Health & Illness
JA - Sociol.Health Illn.
VL - 30
IS - 1
N2 - This article addresses the complexity of children's risk landscapes through an ethnography of 10- to 12-year-old Danish children. The data revealed how children individually and collectively engaged with risk in their everyday activities. The children assessed risks in relation to their perceptions of their health as strength and control, negotiated the conditions of playing, and attuned their responses to situations of potential social and physical conflict. In the paper this risk engagement is illustrated in a variety of contexts: children's decisions to wear or not to wear a bicycle helmet; playing and games and routine pushing and shoving at school. In looking after themselves, children negotiate rules of participation and they safeguard personal and collective interests. Gender differences in these processes are addressed and discussed. The article argues that risk engagement is an important resource through which children also learn from their own mistakes. This is a necessary learning process when children engage with their personal health and safety. The article critically discusses different sociological frameworks and shows the significance of the study for the growing literature on understanding the meaning of risk in childhood
AD - Institute of Education, University of Warwick, Coventry. P.H.Christensen@warwick.ac.uk
UR - 18254836
ER -

TY - JOUR

ID - 122
T1 - Understanding children's injury-risk behaviors: the independent contributions of cognitions and emotions
A1 - Morrongiello,B.A.
A1 - Lasenby-Lessard,J.
A1 - Matheis,S.
Y1 - 2007/09//
N1 - DA - 20070910
IS - 0146-8693 (Print)
IS - 0146-8693 (Linking)
LA - eng
PT - Journal Article
PT - Research Support, Non-U.S. Gov't
SB - IM
KW - PubMed related articles - informal
KW - Affect
KW - Child
KW - Cognition
KW - Decision Making
KW - Female
KW - Humans
KW - Male
KW - Risk-Taking
KW - Wounds and Injuries
KW - epidemiology
RP - NOT IN FILE
SP - 926
EP - 937
JA - J Pediatr.Psychol.
VL - 32
IS - 8
N2 - OBJECTIVE: Unintentional injuries are a leading threat to the health of elementary-school children, with many injuries happening when children are left to make their own decisions about risk taking during play. The present study sought to identify determinants of children's physical taking. METHODS: An ecologically valid task that posed some threat of injury was used (i.e., highest height of a balance beam they would walk across). Ratings of cognitions (extent of danger, perceived vulnerability for personal injury, potential severity of injury) and emotional reactions (fear, excitement) were taken when on the beam, just before the children walked across. RESULTS: Regression analysis, controlling for age and sex, revealed that risk taking was predicted from ratings of danger, fear, and excitement. CONCLUSIONS: Both cognitive and emotional factors independently contribute to predict children's physical risk taking. Theoretical and practical implications of these findings are discussed
AD - Psychology Department, University of Guelph, Guelph, Ontario N1G 2W1, Canada. bmorrong@uoguelph.ca
UR - 17522114
ER -

TY - JOUR
ID - 123
T1 - Are children and adolescents less active if parents restrict their physical activity and active transport due to perceived risk?
A1 - Carver,A.
A1 - Timperio,A.
A1 - Hesketh,K.
A1 - Crawford,D.
Y1 - 2010/06//
N1 - DA - 20100503
IS - 1873-5347 (Electronic)

IS - 0277-9536 (Linking)
LA - eng
PT - Journal Article
PT - Research Support, Non-U.S. Gov't
SB - IM
KW - PubMed related articles - informal
KW - Adolescent
KW - Age Factors
KW - Bicycling
KW - statistics & numerical data
KW - Child
KW - Cross-Sectional Studies
KW - Exercise
KW - Female
KW - Humans
KW - Linear Models
KW - Male
KW - Parent-Child Relations
KW - Parents
KW - psychology
KW - Residence Characteristics
KW - Risk
KW - Safety
KW - Sex Factors
KW - Time Factors
KW - Victoria
KW - Walking
RP - NOT IN FILE
SP - 1799
EP - 1805
JA - Soc.Sci.Med.
VL - 70
IS - 11
N2 - The study examined parental restriction of children's active transport and physical activity outside school hours due to safety concerns, and how this restriction was associated with perceived risk and with youth physical activity levels. We used a cross-sectional design with data from children aged 10-11 years (n=170) and adolescents aged 15-17 years (n=270) who participated in the five-year follow-up of the Children Living in Active Neighbourhoods longitudinal study in Melbourne, Australia. Walking/cycling to local destinations was survey-reported. Moderate-to-vigorous physical activity (MVPA) was recorded during non-school hours using accelerometers. Using surveys, parents reported perceived risk of their children being harmed in their neighbourhood, and restrictions they placed on their children's physical activity. Linear regression analyses examined how perceived risk, constrained behaviour, and physical activity were related. We found that perceived risk did not vary by age-group and was positively associated with constrained behaviour among adolescents but not children. Children were subject to higher levels of constrained behaviour than adolescents. Constrained behaviour was negatively associated with active transport among younger boys and among girls in both age-groups. Furthermore, it was negatively associated with younger boys' MVPA on weekends and with adolescent girls' MVPA during evenings. There were no associations between constrained behaviour and active transport or MVPA among adolescent boys. The findings demonstrate that constrained behaviour exhibited by parents may result in lower levels of active transport and of MVPA outside school hours. In particular, this was true for children and adolescent girls. Social interventions to improve perceived safety and physical interventions involving redesign of the built environment to improve actual safety may help to ease parental restriction of their children's active transport and physical activity in their neighbourhood
AD - Deakin University, Burwood, VIC 3125, Australia. alison.carver@deakin.edu.au
UR - 20347200
ER -

TY - JOUR
ID - 124
T1 - Increasing physical activity in young primary school children--it's child's play: a cluster randomised controlled trial
A1 - Engelen,L.
A1 - Bundy,A.C.
A1 - Naughton,G.
A1 - Simpson,J.M.
A1 - Bauman,A.
A1 - Ragen,J.
A1 - Baur,L.
A1 - Wyver,S.
A1 - Tranter,P.
A1 - Niehues,A.
A1 - Schiller,W.
A1 - Perry,G.
A1 - Jessup,G.
A1 - van der Ploeg,H.P.
Y1 - 2013/05//
N1 - DA - 20130422
IS - 1096-0260 (Electronic)
IS - 0091-7435 (Linking)
LA - eng
PT - Journal Article
PT - Randomized Controlled Trial
PT - Research Support, Non-U.S. Gov't
SB - IM
KW - PubMed related articles - informal
KW - Accelerometry
KW - Adult
KW - Child
KW - Child,Preschool
KW - Early Intervention (Education)
KW - Female
KW - Health Promotion
KW - methods
KW - Humans
KW - Male
KW - Motor Activity
KW - Play and Playthings
KW - School Health Services
KW - Students
KW - statistics & numerical data
RP - NOT IN FILE
SP - 319
EP - 325
JA - Prev.Med.
VL - 56
IS - 5
N2 - OBJECTIVE: To explore the effects of an innovative school-based intervention for increasing physical activity. METHODS: 226 children (5-7 years old) randomly selected from 12 Australian primary schools were recruited to a cluster randomised trial with schools randomly allocated to intervention or control conditions. The 13-week intervention comprised: (1) altering the school playground by introducing loose materials and (2) a teacher-parent intervention exploring perceptions of risk associated with children's free play. The primary outcomes were total accelerometer counts and moderate-vigorous physical activity during break times. Testing took place in Sydney, 2009-2010. RESULTS: 221 participants were tested at baseline. Mixed-effect multilevel

regression revealed a small but significant increase from the intervention on total counts (9400 counts, 95% CI 3.5-15.2, p=0.002) and minutes of MVPA (1.8 min, 95% CI 0.5-3.1, p=0.006); and a decrease in sedentary activity (2.1 min, 95% CI 0.5-3.8, p=0.01) during break times. We retested children in one intervention school after 2 years; they maintained the gains. CONCLUSIONS: Capturing children's intrinsic motivations to play while simultaneously helping adults reconsider views of free play as risky provided increases in physical activity during break times. Using accelerometry as the sole measure of physical activity may underestimate the effect. Trial registration: ACTRN12611000089932
AD - Faculty of Health Sciences, University of Sydney, Lidcombe, Australia. lina.engelen@sydney.edu.au
UR - 23462477
ER -

TY - JOUR
ID - 125
T1 - Supporting the development of risk-taking behaviours in the early years: an exploratory study
A1 - Waters,Jane
A1 - Begley,Sharon
Y1 - 2007/11//
KW - Reviewer Nominated
RP - NOT IN FILE
SP - 365
EP - 377
JF - Education 3-13
VL - 35
IS - 4
N2 - Children's opportunities for independent play in natural outdoor spaces, and the associated opportunities to take and negotiate risk, are being eroded despite potential links between such play and the development of positive learning dispositions. This paper reports the findings of an exploratory study that documented the risk-taking behaviours displayed by four-year-old children in the natural environment of a Forest School and within their school outdoor play-space. The data indicated that the Forest School environment was better able to support the development of positive risk-taking behaviours. The environmental features that may have contributed to the observed behaviours are considered.
UR - http://www.tandfonline.com/doi/abs/10.1080/03004270701602632
L1 - file:///J:/_Searches/Trembly/Risku Play (Casey Gray)/new articles/Waters-2007-Supporting the devel.pdf
ER -

TY - JOUR
ID - 126
T1 - Physical Risk-taking: Dangerous or endangered?
A1 - Stephenson,Alison
Y1 - 2003/03//
KW - Reviewer Nominated
RP - NOT IN FILE
SP - 35
EP - 43
JA - Early Years
VL - 23
IS - 1
N2 - Young children's enthusiasm for undertaking challenging physical activities is described, and examples are given of physical risk-taking by 4 year olds and by younger children. Having acknowledged the prevalence of such play, three emerging questions are discussed: what are acceptable physical challenges for 4 year olds, how can we juggle the need for challenge with the increasingly restrictive safety requirements, and what may be the wider implications of restricting children's access to physical challenges?
UR - http://www.tandfonline.com/doi/abs/10.1080/0957514032000045573

L1 - file:///J:/_Searches/Trembly/Risku Play (Casey Gray)/new articles/Stephenson Physical risk taking.pdf
ER -

TY - JOUR
ID - 127
T1 - "If Only" : Outdoor and adventurous activities and generalised academic development
A1 - Dismore,Harriet
A1 - Bailey,Richard
Y1 - 2005/01//
KW - Reviewer Nominated
RP - NOT IN FILE
SP - 9
EP - 19
JF - Journal of Adventure Education & Outdoor Learning
VL - 5
IS - 1
N2 - This paper reports on research conducted as part of a project, the aim of which was to utilise outdoor and adventurous experiences to help schools address their 'if only'factor -- that aspect of learning perceived to be key to raising standards, whether it be the acquisition of knowledge, skills or a change in attitudes to learning. The project involved nine schools and 671 children, all engaged in the "I Can~" project and the follow-up activities that took place at schools. The research was carried out with children, teachers and parents and consisted of a series of focus groups and analysis of children's work. Limitations inherent within the research opportunity prevent the confident attribution of causation. However, the study's findings do offer insight into an under-researched, yet potentially valuable educational experience. The findings suggest that outdoor and adventurous activities can impact upon children's learning in school by addressing their intellectual, affective and social development.
SN - 1472967058520
UR - http://www.tandfonline.com/doi/abs/10.1080/14729670585200561
L1 - file:///J:/_Searches/Trembly/Risku Play (Casey Gray)/new articles/Dismore-2005-If only_ Outdoor and adventurous.pdf
ER -

TY - JOUR
ID - 128
T1 - Development of T-STAT for early autism screening
A1 - Chiang,C.H.
A1 - Wu,C.C.
A1 - Hou,Y.M.
A1 - Chu,C.L.
A1 - Liu,J.H.
A1 - Soong,W.T.
Y1 - 2013/05//
N1 - Chiang, Chung-Hsin. Wu, Chin-Chin. Hou, Yuh-Ming. Chu, Ching-Lin. Liu, Jiun-Horng. Soong, Wei-Tsuen
Journal of autism and developmental disorders
7904301, hgw
IM
Journal Article. Validation Studies
English
KW - Medline
KW - Autistic Disorder/di [Diagnosis]
KW - Child Behavior
KW - Child
KW - Preschool
KW - Early Diagnosis

KW - Female
KW - Humans
KW - Male
KW - Mass Screening/mt [Methods]
KW - Play and Playthings
KW - Questionnaires
KW - Sensitivity and Specificity
KW - Taiwan
RP - NOT IN FILE
SP - 1028
EP - 1037
JF - Journal of Autism & Developmental Disorders
JA - J Autism Dev.Disord
VL - 43
IS - 5
CY - United States
N2 - This study's purpose was to modify the Screening Tool for Autism in Two-Year-Olds (STAT) into a Taiwanese version called T-STAT. Study 1 included 15children with Autism and 15 children with Developmental Delay (DD) or language impairment (LI) aged between 24 and 35months. Study 2 had 77 young children with Autism, PDD-NOS, or DD/LI as a clinical-based validation sample. In Study 1, the signal detection procedure found that a cutoff score of 2 would yield high sensitivity and specificity in T-STAT. In Study 2, using a score of 2 as a cutoff, the agreement between T-STAT risk and ADOS classification was highly acceptable. Results were promising as a Level 2 screening tool for Autism for ages two to three
SN - 1573-3432
AD - Department of Psychology, National Chengchi University, No.64, Sec.2, ZhiNan Rd., Wenshan District, Taipei, 11605, Taiwan. chchiang@nccu.edu.tw
UR - 22976373
ER -

TY - JOUR
ID - 129
T1 - Quality of interaction between at-risk infants and caregiver at 12-15 months is associated with 3-year autism outcome
A1 - Wan,M.W.
A1 - Green,J.
A1 - Elsabbagh,M.
A1 - Johnson,M.
A1 - Charman,T.
A1 - Plummer,F.
A1 - BASIS Team.
Y1 - 2013/07//
N1 - Wan, Ming Wai. Green, Jonathan. Elsabbagh, Mayada. Johnson, Mark. Charman, Tony. Plummer, Faye. BASIS Team
Journal of child psychology and psychiatry, and allied disciplines
hp3, 0375361
IM
Journal Article. Research Support, Non-U.S. Gov't
English
KW - Medline
KW - Age Factors
KW - Child Development Disorders
KW - Pervasive/di [Diagnosis]
KW - Pervasive/ge [Genetics]
KW - Pervasive/px [Psychology]
KW - Child
KW - Preschool

KW - Cohort Studies
KW - Cross-Sectional Studies
KW - Female
KW - Genetic Predisposition to Disease/ge [Genetics]
KW - Humans
KW - Infant
KW - Longitudinal Studies
KW - Male
KW - Mother-Child Relations
KW - Neuropsychological Tests
KW - Parenting/px [Psychology]
KW - Play and Playthings
KW - Prognosis
KW - Risk Factors
KW - Siblings
KW - Social Adjustment
KW - Social Environment
RP - NOT IN FILE
SP - 763
EP - 771
JF - Journal of Child Psychology & Psychiatry & Allied Disciplines
JA - J Child Psychol.Psychiatry
VL - 54
IS - 7
CY - England
N2 - BACKGROUND: Recent models of the early emergence of autism spectrum disorder (ASD) propose that infant intrinsic risk susceptibilities in behaviour may be amplified by interaction within the early social environment into an increasingly atypical developmental trajectory. This study examines whether 6- and 12-month parent-infant interactions in at-risk siblings differ from those with low-risk and whether--in at-risk siblings--such interactions predict later 3-year classification of ASD or no ASD. METHOD: Within the British Autism Study of Infant Siblings (BASIS), 6-min videotaped episodes of parent-infant free play in infants at 6-10months (45 at-risk siblings and 47 low-risk siblings) and 12-15months (43 at-risk siblings and 48 low-risk siblings) in a laboratory setting were rated on the Manchester Assessment of Caregiver-Infant Interaction (MACI), blind to participant information. Standard tests were administered for concurrent behavioural signs of ASD features and developmental level. Systematic consensus diagnostic classification of ASD was made at 3years for the at-risk siblings. RESULTS: Parent nondirectiveness and sensitive responsiveness differed in relation to ASD/risk status (at-risk ASD, at-risk no-ASD and low-risk) at both 6 and 12months. At 6months, infant liveliness was lower in the at-risk groups; at 12months, infant attentiveness to parent and positive affect were lower in the at-risk group later diagnosed with ASD. Dyadic mutuality and intensity of engagement showed a group effect at 12months. Dyadic mutuality, infant positive affect and infant attentiveness to parent at 12months (but not 6months) predicted 3-year ASD outcome, whereas infant ASD-related behavioural atypicality did not. CONCLUSIONS: This is the first prospective evidence that early dyadic interaction between at-risk infants and their parents is associated with later diagnostic outcome in ASD. Possible explanations for these findings and their theoretical implications are considered. 2012 The Authors. Journal of Child Psychology and Psychiatry 2012 Association for Child and Adolescent Mental Health
SN - 1469-7610
AD - Institute of Brain, Behaviour and Mental Health, University of Manchester, Manchester, UK. m.w.wan@manchester.ac.uk
UR - 23227853
ER -

TY - JOUR
ID - 130
T1 - Modifiable lifestyle behavior patterns, sedentary time and physical activity contexts: a cluster analysis among middle school boys and girls in the SALTA study
A1 - Marques,E.A.

A1 - Pizarro,A.N.
A1 - Figueiredo,P.
A1 - Mota,J.
A1 - Santos,M.P.
Y1 - 2013/06//
N1 - Marques, Elisa A. Pizarro, Andreia N. Figueiredo, Pedro. Mota, Jorge. Santos, Maria P
Preventive medicine
pm4, 0322116
IM
Journal Article. Research Support, Non-U.S. Gov't
English
KW - Medline
KW - Body Mass Index
KW - Child
KW - Cholesterol
KW - HDL/bl [Blood]
KW - Cluster Analysis
KW - Exercise Test
KW - Female
KW - Health Behavior
KW - Humans
KW - Male
KW - Motor Activity
KW - Physical Fitness
KW - Portugal
KW - Recreation
KW - Risk Assessment
KW - Risk Factors
KW - Sedentary Lifestyle
KW - Students/sn [Statistics & Numerical Data]
KW - Travel
RP - NOT IN FILE
SP - 413
EP - 415
JA - Preventive Medicine
VL - 56
IS - 6
CY - United States
N2 - OBJECTIVE: To analyze how modifiable health-related variables are clustered and associated with children's participation in play, active travel and structured exercise and sport among boys and girls. METHODS: Data were collected from 9 middle-schools in Porto (Portugal) area. A total of 636 children in the 6th grade (340 girls and 296 boys) with a mean age of 11.64 years old participated in the study. Cluster analyses were used to identify patterns of lifestyle and healthy/unhealthy behaviors. Multinomial logistic regression analysis was used to estimate associations between cluster allocation, sedentary time and participation in three different physical activity (PA) contexts: play, active travel, and structured exercise/sport. RESULTS: Four distinct clusters were identified based on four lifestyle risk factors. The most disadvantaged cluster was characterized by high body mass index, low high-density lipoprotein cholesterol and cardiorespiratory fitness and a moderate level of moderate to vigorous PA. Everyday outdoor play (OR=1.85, 95%CI 0.318-0.915) and structured exercise/sport (OR=1.85, 95%CI 0.291-0.990) were associated with healthier lifestyle patterns. There were no significant associations between health patterns and sedentary time or travel mode. CONCLUSION: Outdoor play and sport/exercise participation seem more important than active travel from school in influencing children's healthy cluster profiles. Copyright 2013 Elsevier Inc. All rights reserved
SN - 1096-0260
AD - Research Centre in Physical Activity, Health and Leisure (CIAFEL), Faculty of Sport Science, University of Porto, Porto, Portugal. emarques@fade.up.pt
UR - 23567246

ER -

TY - JOUR
ID - 132
T1 - Environmental lead exposure risks associated with children's outdoor playgrounds
A1 - Taylor,M.P.
A1 - Camenzuli,D.
A1 - Kristensen,L.J.
A1 - Forbes,M.
A1 - Zahran,S.
Y1 - 2013/07//
N1 - Taylor, Mark Patrick. Camenzuli, Danielle. Kristensen, Louise Jane. Forbes, Miriam. Zahran, Sammy
Environmental pollution (Barking, Essex : 1987)
dvl, 8804476
IM
Journal Article. Research Support, Non-U.S. Gov't
English
KW - Medline
KW - Air Pollutants/an [Analysis]
KW - Child
KW - Child Care
KW - Environmental Exposure/an [Analysis]
KW - Environmental Exposure/sn [Statistics & Numerical Data]
KW - Environmental Pollution/sn [Statistics & Numerical Data]
KW - Humans
KW - Lead/an [Analysis]
KW - Lead Poisoning/ep [Epidemiology]
KW - Metallurgy
KW - Play and Playthings
KW - Risk Assessment
KW - Soil Pollutants/an [Analysis]
KW - South Australia
RP - NOT IN FILE
SP - 447
EP - 454
JF - Environmental Pollution
JA - Environ.Pollut.
VL - 178
CY - England
N2 - This study examines exposure risks associated with lead smelter emissions at children's public playgrounds in Port Pirie, South Australia. Lead and other metal values were measured in air, soil, surface dust and on pre- and post-play hand wipes. Playgrounds closest to the smelter were significantly more lead contaminated compared to those further away (t(27.545)=3.76; p=.001). Port Pirie post-play hand wipes contained significantly higher lead loadings (maximum hand lead value of 49,432mug/m(2)) than pre-play hand wipes (t(27)=3.57, p=.001). A 1% increase in air lead (mug/m(3)) was related to a 0.713% increase in lead dust on play surfaces (95% CI, 0.253-1.174), and a 0.612% increase in post-play wipe lead (95% CI, 0.257-0.970). Contaminated dust from smelter emissions is determined as the source and cause of childhood lead poisoning at a rate of approximately one child every third day. Copyright 2013 Elsevier Ltd. All rights reserved
SN - 1873-6424
AD - Environmental Science, Department of Environment and Geography, Faculty of Science, Macquarie University, North Ryde, Sydney, NSW 2109, Australia. mark.taylor@mq.edu.au
UR - 23643852
ER -

TY - JOUR
ID - 133

T1 - Systematic assessment of noise amplitude generated by toys intended for young children
A1 - Mahboubi,H.
A1 - Oliaei,S.
A1 - Badran,K.W.
A1 - Ziai,K.
A1 - Chang,J.
A1 - Zardouz,S.
A1 - Shahriari,S.
A1 - Djalilian,H.R.
Y1 - 2013/06//
N1 - Mahboubi, Hossein. Oliaei, Sepehr. Badran, Karam W. Ziai, Kasra. Chang, Janice. Zardouz, Shawn. Shahriari, Shawn. Djalilian, Hamid R
Otolaryngology--head and neck surgery : official journal of American Academy of Otolaryngology-Head and Neck Surgery
7909794, 8508176, on7, on8
IM
Comparative Study. Journal Article
English
KW - Medline
KW - Age Factors
KW - Analysis of Variance
KW - Audiometry/mt [Methods]
KW - Auditory Threshold
KW - California
KW - Child Welfare
KW - Child
KW - Preschool
KW - Consumer Product Safety
KW - Female
KW - Hearing Loss
KW - Noise-Induced/et [Etiology]
KW - Noise-Induced/pc [Prevention & Control]
KW - Humans
KW - Infant
KW - Male
KW - Marketing
KW - Needs Assessment
KW - Noise/ae [Adverse Effects]
KW - Play and Playthings
KW - Retrospective Studies
KW - Risk Assessment
RP - NOT IN FILE
SP - 1043
EP - 1047
JF - Otolaryngology - Head & Neck Surgery
JA - Otolaryngol.Head Neck Surg.
VL - 148
IS - 6
CY - England
N2 - OBJECTIVE: To systematically evaluate the noise generated by toys targeted for children and to compare the results over the course of 4 consecutive holiday shopping seasons. STUDY DESIGN: Experimental study. SETTING: Academic medical center. SUBJECTS AND METHODS: During 2008-2011, more than 200 toys marketed for children older than 6 months were screened for loudness. The toys with sound output of more than 80 dBA at speaker level were retested in a soundproof audiometry booth. The generated sound amplitude of each toy was measured at speaker level and at 30 cm away from the speaker. RESULTS: Ninety different toys were analyzed. The mean (SD) noise amplitude was 100 (8) dBA (range, 80-121 dBA) at the speaker level and

80 (11) dBA (range, 60-109 dBA) at 30 cm away from the speaker. Eighty-eight (98%) had more than an 85-dBA noise amplitude at speaker level, whereas 19 (26%) had more than an 85-dBA noise amplitude at a 30-cm distance. Only the mean noise amplitude at 30 cm significantly declined during the studied period (P < .001). There was no significant difference in mean noise amplitude of different toys specified for different age groups. CONCLUSION: Our findings demonstrate the persistence of extremely loud toys marketed for very young children. Acoustic trauma from toys remains a potential risk factor for noise-induced hearing loss in this age group, warranting promotion of public awareness and regulatory considerations for manufacture and marketing of toys
SN - 1097-6817
AD - Division of Neurotology and Skull Base Surgery, Department of Otolaryngology-Head and Neck Surgery, University of California, Irvine, California 92868, USA
UR - 23525849
ER -

TY - JOUR
ID - 134
T1 - Do features of public open spaces vary between urban and rural areas?
A1 - Veitch,J.
A1 - Salmon,J.
A1 - Ball,K.
A1 - Crawford,D.
A1 - Timperio,A.
Y1 - 2013/02//
N1 - Veitch, Jenny. Salmon, Jo. Ball, Kylie. Crawford, David. Timperio, Anna
Preventive medicine
pm4, 0322116
IM
Comparative Study. Journal Article. Research Support, Non-U.S. Gov't
English
KW - Medline
KW - Adult
KW - Australia
KW - Child
KW - Environment Design
KW - Health Promotion/st [Standards]
KW - Humans
KW - Lighting
KW - Motor Activity
KW - Obesity/pc [Prevention & Control]
KW - Play and Playthings
KW - Public Facilities
KW - Rural Population/sn [Statistics & Numerical Data]
KW - Safety
KW - Social Planning
KW - Urban Population/sn [Statistics & Numerical Data]
KW - Urban Renewal/st [Standards]
RP - NOT IN FILE
SP - 107
EP - 111
JA - Preventive Medicine
VL - 56
IS - 2
CY - United States
N2 - OBJECTIVE: Parks are an important setting for physical activity and specific park features have been shown to be associated with park visitation and physical activity. Most park-based research has been conducted in urban settings with few studies examining rural parks. This study examined differences in features of parks in

urban compared with rural areas. METHODS: In 2009/10 a tool was developed to audit 433 urban and 195 rural parks located in disadvantaged areas of Victoria, Australia. Features assessed included: access; lighting/safety; aesthetics; amenities; paths; outdoor courts/ovals; informal play spaces; and playgrounds (number, diversity, age appropriateness and safety of play equipment). RESULTS: Rural parks scored higher for aesthetics compared with urban parks (5.08 vs 4.44). Urban parks scored higher for access (4.64 vs 3.89), lighting/safety (2.01 vs 1.76), and diversity of play equipment (7.37 vs 6.24), and were more likely to have paths suitable for walking/cycling (58.8% vs 40.9%) and play equipment for older children (68.2% vs 17.1%). CONCLUSION: Although the findings cannot be generalized to all urban and rural parks, the results may be used to inform advocacy for park development in rural areas to create parks that are more supportive of physical activity for children and adults. Copyright 2012 Elsevier Inc. All rights reserved
SN - 1096-0260
AD - Centre for Physical Activity and Nutrition Research, Deakin University, Burwood, Victoria 3125, Australia. jenny.veitch@deakin.edu.au
UR - 23201001
ER -

TY - JOUR
ID - 135
T1 - JAMA patient page. Summer safety for kids
A1 - Goodman,D.M.
A1 - Lynm,C.
A1 - Livingston,E.H.
Y1 - 2013/06//
N1 - Goodman, Denise M. Lynm, Cassio. Livingston, Edward H
JAMA : the journal of the American Medical Association
7501160
AIM, IM
Patient Education Handout
English
KW - Medline
KW - Child
KW - Humans
KW - Play and Playthings
KW - Risk
KW - Safety
KW - Seasons
KW - United States/ep [Epidemiology]
KW - Wounds and Injuries/mo [Mortality]
KW - Wounds and Injuries/pc [Prevention & Control]
RP - NOT IN FILE
SP - 2505
JF - JAMA
VL - 309
IS - 23
CY - United States
SN - 1538-3598
UR - 23780466
ER -

TY - JOUR
ID - 136
T1 - Children's physical activity and parents' perception of the neighborhood environment: neighborhood impact on kids study
A1 - Tappe,K.A.
A1 - Glanz,K.
A1 - Sallis,J.F.

A1 - Zhou,C.
A1 - Saelens,B.E.
Y1 - 2013///
N1 - Tappe, Karyn A. Glanz, Karen. Sallis, James F. Zhou, Chuan. Saelens, Brian E
The international journal of behavioral nutrition and physical activity
101217089
IM
Journal Article. Research Support, N.I.H., Extramural
English
KW - Medline
KW - Actigraphy
KW - California
KW - Child
KW - Child Behavior
KW - Crime
KW - Data Collection
KW - Environment
KW - Environment Design
KW - Esthetics
KW - Exercise
KW - Female
KW - Health Behavior
KW - Humans
KW - Male
KW - Motor Activity
KW - Multivariate Analysis
KW - Obesity/pc [Prevention & Control]
KW - Parents
KW - Perception
KW - Recreation
KW - Residence Characteristics
KW - Safety
KW - Washington
RP - NOT IN FILE
SP - 39
JF - International Journal of Behavioral Nutrition & Physical Activity
JA - Int.
VL - 10
CY - England
N2 - BACKGROUND: Physical activity is important to children's physical health and well-being. Many factors contribute to children's physical activity, and the built environment has garnered considerable interest recently, as many young children spend much of their time in and around their immediate neighborhood. Few studies have identified correlates of children's activity in specific locations. This study examined associations between parent report of their home neighborhood environment and children's overall and location-specific physical activity. METHODS: Parents and children ages 6 to 11 (n=724), living in neighborhoods identified through objective built environment factors as high or low in physical activity environments, were recruited from Seattle and San Diego metropolitan areas, 2007-2009. Parents completed a survey about their child's activity and perceptions of home neighborhood environmental attributes. Children wore an accelerometer for 7 days. Multivariate regression models explored perceived environment correlates of parent-reported child's recreational physical activity in their neighborhood, in parks, and in general, as well as accelerometry-based moderate-to-vigorous activity (MVPA) minutes. RESULTS: Parent-reported proximity to play areas correlated positively with both accelerometery MVPA and parent-reported total child physical activity. Lower street connectivity and higher neighborhood aesthetics correlated with higher reported child activity in the neighborhood, while reported safety from crime and walk and cycle facilities correlated positively with reported child activity in public recreation spaces. CONCLUSIONS: Different aspects of parent's perceptions of the neighborhood environment appear to correlate with different aspects of children's activity. However, prioritizing closer

proximity to safe play areas may best improve children's physical activity and, in turn, reduce their risk of obesity and associated chronic diseases
SN - 1479-5868
AD - Department of Biostatistics and Epidemiology, Perelman School of Medicine, University of Pennsylvania, Philadelphia, USA
UR - 23531282
ER -

TY - JOUR
ID - 137
T1 - Determinants of vitamin D status in young children: results from the Belgian arm of the IDEFICS (Identification and Prevention of Dietary- and Lifestyle-Induced Health Effects in Children and Infants) Study
A1 - Sioen,I.
A1 - Mouratidou,T.
A1 - Kaufman,J.M.
A1 - Bammann,K.
A1 - Michels,N.
A1 - Pigeot,I.
A1 - Vanaelst,B.
A1 - Vyncke,K.
A1 - De,Henauw S.
A1 - IDEFICS consortium.
Y1 - 2012/06//
N1 - Sioen, Isabelle. Mouratidou, Theodora. Kaufman, Jean-Marc. Bammann, Karin. Michels, Nathalie. Pigeot, Iris. Vanaelst, Barbara. Vyncke, Krishna. De Henauw, Stefaan. IDEFICS consortium
Public health nutrition
9808463, dgy
IM
Journal Article. Research Support, Non-U.S. Gov't
English
KW - Medline
KW - Adipose Tissue
KW - Belgium/ep [Epidemiology]
KW - Body Constitution
KW - Body Mass Index
KW - Child
KW - Cross-Sectional Studies
KW - Female
KW - Health Status
KW - Humans
KW - Male
KW - Obesity
KW - Abdominal/co [Complications]
KW - Play and Playthings
KW - Prevalence
KW - Reference Values
KW - Regression Analysis
KW - Risk Factors
KW - Seasons
KW - Sunlight
KW - Vitamin D/aa [Analogs & Derivatives]
KW - Vitamin D/bl [Blood]
KW - Vitamin D Deficiency/bl [Blood]
KW - Vitamin D Deficiency/ep [Epidemiology]
KW - Vitamin D Deficiency/et [Etiology]
RP - NOT IN FILE

SP - 1093
EP - 1099
JF - Public Health Nutrition
JA - Public Health Nutr
VL - 15
IS - 6
CY - England
N2 - OBJECTIVE: To describe the vitamin D status of Belgian children and examine the influence of non-nutritional determinants, in particular of anthropometric variables. DESIGN: Cross-sectional data of Belgian participants of the EU 6th Framework Programme IDEFICS (Identification and Prevention of Dietary- and Lifestyle-Induced Health Effects in Children and Infants) Study. SETTING: 25-Hydroxyvitamin D (25(OH)D) was measured using RIA. Vitamin D status was categorized as deficient (<25 nmol/l), insufficient (25-50 nmol/l), sufficient (50-75 nmol/l) and optimal (>=75 nmol/l). Anthropometric measurements included height, weight, waist and hip circumferences and triceps and subscapular skinfold thicknesses. SUBJECTS: Children (n 357) aged 4-11 years. RESULTS: Serum 25(OH)D ranged from 13.6 to 123.5 nmol/l (mean 47.2 (sd 14.6) nmol/l); with 5 % deficient, 53 % insufficient, 40 % sufficient and 2 % optimal. No significant differences were found by age and gender. Significant differences in 25(OH)D were observed for month of sampling (P < 0.001), number of hours playing outside per week (r = 0.140), weight (r = -0.121), triceps (r = -0.112) and subscapular (r = -0.119) skinfold thickness, sum of two skinfold thicknesses (r = -0.125) and waist circumference (r = -0.108). Linear regression analysis of 25(OH)D adjusted for age, month of sampling and hours playing outside per week suggested that (i) weight, (ii) BMI Z-score, (iii) waist circumference and (iv) triceps and subscapular skinfold thickness (as well as the sum of both) independently influenced 25(OH)D. CONCLUSIONS: The majority of Belgian children had a suboptimal vitamin D status, with more than half having an insufficient status in winter and spring. Month of the year, weekly number of hours playing outside and body composition - both central and abdominal obesity - were identified as important determinants of vitamin D status in Belgian children
SN - 1475-2727
AD - Department of Public Health, Ghent University, UZ 2 Blok A, De Pintelaan 185, B-9000 Ghent, Belgium. Isabelle.Sioen@UGent.be
UR - 22152637
ER -

TY - JOUR
ID - 138
T1 - Elevated serum levels of interleukin-17A in children with autism
A1 - Al-Ayadhi,L.Y.
A1 - Mostafa,G.A.
Y1 - 2012///
N1 - Al-Ayadhi, Laila Yousef. Mostafa, Gehan Ahmed
Journal of neuroinflammation
101222974
IM
Journal Article. Research Support, Non-U.S. Gov't
English
KW - Medline
KW - Autistic Disorder/bl [Blood]
KW - Autistic Disorder/di [Diagnosis]
KW - Autistic Disorder/et [Etiology]
KW - Biological Markers/bl [Blood]
KW - Child
KW - Cross-Sectional Studies
KW - Female
KW - Humans
KW - Interleukin-17/bl [Blood]
KW - Male
KW - Risk Factors

KW - Serum/me [Metabolism]
RP - NOT IN FILE
SP - 158
JF - Journal of Neuroinflammation
JA - J Neuroinflammation
VL - 9
CY - England
N2 - BACKGROUND: The T-helper (Th)1/Th2 dichotomy dominated the field of immune regulation until interleukin (IL)-17-expressing T cells (Th17) were proposed to be a third lineage of helper T cells, the key players in the pathogenesis of autoimmune disorders. Autoimmunity to brain tissue may play a pathogenic role in autism. IL-17A is a pro-inflammatory cytokine that has been shown to play an important role in various autoimmune neuroinflammatory diseases. The aim of this study was to measure serum levels of IL-17A in relation to the degree of the severity of autism. METHODS: Serum IL-17A levels were measured by ELISA in 45 children with autism and 40 matched healthy controls. RESULTS: Children with autism had significantly higher serum IL-17A levels than healthy controls (P <0.001), with increased serum levels of IL-17A found in 48.9% of the autism group. Patients with severe autism had significantly higher serum IL-17A levels than those with mild to moderate autism (P=0.01), and raised serum IL-17A levels were significantly more common in children with severe autism (67.9%) than in those with mild to moderate autism (17.6%), P=0.001. CONCLUSIONS: Serum IL-17A levels were raised in the group with autism, and the levels correlated significantly with the severity of autism. This is the first study to measure levels of IL-17A in relation to the severity of autism, to our knowledge. Further research, with a larger subject population, is warranted to determine whether the increase of serum IL-17A levels plasma has a pathogenic role in autism, and whether anti- IL-17A therapy could be useful
SN - 1742-2094
AD - Department of Physiology, Autism Research and Treatment Center, Al-Amodi Autism Research Chair, Faculty of Medicine, King Saud University, Riyadh, Saudi Arabia
UR - 22748016
ER -

TY - JOUR
ID - 139
T1 - Effect of play-based summer break exercise on cardiovascular function in adolescents
A1 - Meucci,M.
A1 - Curry,C.D.
A1 - Baldari,C.
A1 - Guidetti,L.
A1 - Cook,C.
A1 - Collier,S.R.
Y1 - 2013/01//
N1 - Meucci, Marco. Curry, Chelsea D. Baldari, Carlo. Guidetti, Laura. Cook, Carol. Collier, Scott R
Acta paediatrica (Oslo, Norway : 1992)
bgc, 9205968
IM
Comparative Study. Journal Article. Research Support, Non-U.S. Gov't
English
KW - Medline
KW - Adolescent
KW - Cardiovascular Diseases/ep [Epidemiology]
KW - Cardiovascular Physiological Phenomena
KW - Child
KW - Exercise/ph [Physiology]
KW - Female
KW - Humans
KW - Male
KW - Play and Playthings
KW - Risk Factors

KW - Seasons
RP - NOT IN FILE
SP - e24
EP - e28
JF - Acta Paediatrica
JA - Acta Paediatr
VL - 102
IS - 1
CY - Norway
N2 - AIM: To compare the effects of 4 and 8 weeks of play-based, supervised exercise during summer break versus an unsupervised break on cardiovascular function in adolescent children. METHODS: Twenty-two subjects were divided into a 4-week exercise group (age 10.1 +/- 1.3 years), an 8-week exercise group (age 9.4 +/- 1.7 years) or a control group (age 10.0 +/- 1.3 years). The activity groups participated in a supervised summer camp for 6 h/day, 5 days/week including a discontinuous play-based physical activity program and a healthy lifestyle, while the control group were told to keep their regular summer break routines. Anthropometrics, pulse wave velocity, augmentation index, blood pressure and peak oxygen consumption were evaluated before and after the intervention. RESULTS: Normalized augmentation index (75 beats/min) significantly decreased after 4 and 8 weeks in the active groups (p = 0.04) while pulse wave velocity showed no significant changes in all groups. Mean arterial pressure decreased (p = 0.003) and peak oxygen consumption increased (p = 0.001) significantly in the 8 week group. CONCLUSION: These data suggest that 8 weeks of supervised play-based activity yield several cardio-beneficial results in adolescents, which may act as a clinical prophylaxis throughout their lifetime. 2012 The Author(s)/Acta Paediatrica 2012 Foundation Acta Paediatrica
SN - 1651-2227
AD - University of Rome Foro Italico, Rome, Italy
UR - 23062246
ER -

TY - JOUR
ID - 141
T1 - Doll play narratives about starting school in children of socially anxious mothers, and their relation to subsequent child school-based anxiety
A1 - Pass,L.
A1 - Arteche,A.
A1 - Cooper,P.
A1 - Creswell,C.
A1 - Murray,L.
Y1 - 2012/11//
N1 - Pass, Laura. Arteche, Adriane. Cooper, Peter. Creswell, Cathy. Murray, Lynne
Journal of abnormal child psychology
0364547, h33
IM
Journal Article. Research Support, Non-U.S. Gov't
English
KW - Medline
KW - Adult
KW - Anxiety Disorders/px [Psychology]
KW - Child of Impaired Parents
KW - Child
KW - Preschool
KW - Depression
KW - Female
KW - Forecasting
KW - Humans
KW - Longitudinal Studies
KW - Male
KW - Mother-Child Relations

KW - Narration
KW - Play and Playthings
KW - Schools
KW - Social Behavior
KW - Students
RP - NOT IN FILE
SP - 1375
EP - 1384
JF - Journal of Abnormal Child Psychology
JA - J Abnorm.Child Psychol.
VL - 40
IS - 8
CY - United States
N2 - Child social anxiety is common, and predicts later emotional and academic impairment. Offspring of socially anxious mothers are at increased risk. It is important to establish whether individual vulnerability to disorder can be identified in young children. The responses of 4.5 year-old children of mothers with social phobia (N=62) and non-anxious mothers (N=60) were compared, two months before school entry, using a Doll Play (DP) procedure focused on the social challenge of starting school. DP responses were examined in relation to teacher reports of anxious-depressed symptoms and social worries at the end of the child's first school term. The role of earlier child behavioral inhibition and attachment, assessed at 14 months, was also considered. Compared to children of non-anxious mothers, children of mothers with social phobia were significantly more likely to give anxiously negative responses in their school DP (OR=2.57). In turn, negative DP predicted teacher reported anxious-depressed and social worry problems. There were no effects of infant behavioral inhibition or attachment. Vulnerability in young children at risk of anxiety can be identified using Doll Play narratives
SN - 1573-2835
AD - Winnicott Research Unit, School of Psychology, University of Reading, Reading, West Berkshire, RG6 6AL, UK
UR - 22588362
ER -

TY - JOUR
ID - 142
T1 - Prevalences of overweight and obesity and their association with physical activity pattern among Iranian adolescents aged 12-17 years
A1 - Hajian-Tilaki,K.
A1 - Heidari,B.
Y1 - 2012/12//
N1 - Hajian-Tilaki, Karimollah. Heidari, Behzad
Public health nutrition
9808463, dgy
IM
Journal Article. Research Support, Non-U.S. Gov't
English
KW - Medline
KW - Adolescent
KW - Adolescent Behavior
KW - Child
KW - Computers
KW - Cross-Sectional Studies
KW - Exercise
KW - Female
KW - Health Behavior
KW - Humans
KW - Iran/ep [Epidemiology]
KW - Leisure Activities
KW - Male

KW - Obesity/ep [Epidemiology]
KW - Overweight/ep [Epidemiology]
KW - Play and Playthings
KW - Prevalence
KW - Schools
KW - Sedentary Lifestyle
KW - Sex Factors
KW - Sports
RP - NOT IN FILE
SP - 2246
EP - 2252
JF - Public Health Nutrition
JA - Public Health Nutr
VL - 15
IS - 12
CY - England
N2 - OBJECTIVE: The objective of the present study was to investigate the prevalences of overweight and obesity and their association with physical activity pattern among adolescents in northern Iran. DESIGN: A cross-sectional study. SETTING: Babol, northern Iran. SUBJECTS: A representative sample of 1200 adolescents aged 12-17 years was recruited. All selected adolescents were interviewed, their leisure-time and sport physical activities were collected by a standard Baecke questionnaire and their height and weight were measured by standard methods. The diagnosis of overweight and obesity was determined by comparing BMI values with the BMI index for age and sex percentiles set by the US Centers for Disease Control and Prevention in 2000. RESULTS: The prevalences of overweight and obesity were 15.1 % and 8.3 %, respectively. The proportion of obesity was significantly higher in boys than in girls (10.2 % v. 6.5 %, P = 0.028). Overall, the levels of high, moderate and low leisure-time physical activity were 2.9 %, 79.4 % and 17.7 %, respectively. The corresponding levels of high, moderate and low sport physical activity were 17.4 %, 75.2 % and 7.4 %. The levels of leisure-time and sport physical activities were significantly higher among the boys than the girls (P < 0.001). There was also a significant association of studying in private schools and playing computer games with overweight/obesity (P < 0.001). Moderate leisure-time physical activity was unexpectedly associated with a greater risk of overweight/obesity compared with the low level (P = 0.002). CONCLUSIONS: The findings indicate a high prevalence of overweight/obesity in these Iranian adolescents, particularly the boys. The influence of physical activity and diet on overweight/obesity requires further prospective studies
SN - 1475-2727
AD - Department of Social Medicine and Health, Babol University of Medical Sciences, Babol, Islamic Republic of Iran. drhajian@yahoo.com
UR - 22578771
ER -

TY - JOUR
ID - 143
T1 - Outdoor play among children in relation to neighborhood characteristics: a cross-sectional neighborhood observation study
A1 - Aarts,M.J.
A1 - de Vries,S.I.
A1 - van Oers,H.A.
A1 - Schuit,A.J.
Y1 - 2012///
N1 - Aarts, Marie-Jeanne. de Vries, Sanne I. van Oers, Hans Am. Schuit, Albertine J
The international journal of behavioral nutrition and physical activity
101217089
IM
Journal Article. Research Support, Non-U.S. Gov't
English
KW - Medline
KW - Child

KW - Preschool
KW - Cross-Sectional Studies
KW - Environment Design
KW - Evaluation Studies as Topic
KW - Female
KW - Humans
KW - Male
KW - Motor Activity
KW - Multivariate Analysis
KW - Netherlands
KW - Parents
KW - Play and Playthings
KW - Questionnaires
KW - Residence Characteristics
KW - Safety
RP - NOT IN FILE
SP - 98
JF - International Journal of Behavioral Nutrition & Physical Activity
JA - Int.
VL - 9
CY - England
N2 - BACKGROUND: Although environmental characteristics as perceived by parents are known to be related to children's outdoor play behavior, less is known about the relation between independently measured neighborhood characteristics and outdoor play among children. The purpose of this study was to identify quantitative as well as qualitative neighborhood characteristics related to outdoor play by means of neighborhood observations. METHODS: Questionnaires including questions on outdoor play behavior of the child were distributed among 3,651 parents of primary school children (aged 4-12 years). Furthermore, neighborhood observations were conducted in 33 Dutch neighborhoods to map neighborhood characteristics such as buildings, formal outdoor play facilities, public space, street pattern, traffic safety, social neighborhood characteristics, and general impression. Data of the questionnaires and the neighborhood observations were coupled via postal code of the respondents. Multilevel GEE analyses were performed to quantify the correlation between outdoor play and independently measured neighborhood characteristics. RESULTS: Parental education was negatively associated with outdoor play among children. Neither the presence nor the overall quality of formal outdoor play facilities were (positively) related to outdoor play among children in this study. Rather, informal play areas such as the presence of sidewalks were related to children's outdoor play. Also, traffic safety was an important characteristic associated with outdoor play. CONCLUSIONS: This study showed that, apart from individual factors such as parental education level, certain modifiable characteristics in the neighborhood environment (as measured by neighborhood observations) were associated with outdoor play among boys and girls of different age groups in The Netherlands. Local policy makers from different sectors can use these research findings in creating more activity-friendly neighborhoods for children
SN - 1479-5868
AD - Department Tranzo, Scientific Center for Care and Welfare, Tilburg University, Tilburg, The Netherlands. mj.aarts@maastrichtuniversity.nl
UR - 22901102
ER -

TY - JOUR
ID - 144
T1 - Playground safety and quality in chicago
A1 - Allen,E.M.
A1 - Hill,A.L.
A1 - Tranter,E.
A1 - Sheehan,K.M.
Y1 - 2013/02//
N1 - Allen, Erin M. Hill, Amy L. Tranter, Erma. Sheehan, Karen M
Pediatrics

oxv, 0376422
AIM, IM
Journal Article. Research Support, Non-U.S. Gov't
English
KW - Medline
KW - Accidental Falls/pc [Prevention & Control]
KW - Chicago
KW - Child
KW - Preschool
KW - Cross-Sectional Studies
KW - Equipment Design/st [Standards]
KW - Female
KW - Health Surveys
KW - Humans
KW - Infant
KW - Newborn
KW - Male
KW - Motor Activity
KW - Play and Playthings/in [Injuries]
KW - Public Sector
KW - Safety/st [Standards]
KW - Social Environment
KW - Socioeconomic Factors
KW - Wounds and Injuries/ep [Epidemiology]
KW - Wounds and Injuries/pc [Prevention & Control]
RP - NOT IN FILE
SP - 233
EP - 241
JA - Pediatrics
VL - 131
IS - 2
CY - United States
N2 - OBJECTIVES: To assess playground safety and quality in Chicago, Illinois, identify disparities in access, and use the data to inform collaborative improvement. METHODS: A cross-sectional survey of public park playgrounds in Chicago, Illinois, was conducted in 2009, 2010, and 2011 by using the National Program for Playground Safety Standardized Survey. All playgrounds were surveyed in 2009 and 2010; those that failed in 2010 were resurveyed in 2011. Playgrounds were assessed in 4 main categories: age-appropriate design, fall surfacing, equipment maintenance, and physical environment. Safety scores were generated from the assessment. Geographic information system mapping provided a visual description of the playground pass/fail rate based on neighborhood, child population, race/ethnicity, and poverty level. RESULTS: Of the ~500 playgrounds, 467 were assessed in 2009, and 459 were assessed in 2010. In 2009, half of all playgrounds (55%) and in 2010, nearly two-thirds (61%) earned scores consistent with safe playgrounds (P < .001). Playgrounds scored poorest in fall surfacing and equipment maintenance. Geographic information system mapping showed neighborhoods with a higher percentage of children and impoverished families had fewer playgrounds and more failing playgrounds. In 2011, 154 (85%) of the playgrounds that failed in 2010 were surveyed. The mean playground score among failing playgrounds improved significantly between 2010 (61%) and 2011 (67%, P < .001). CONCLUSIONS: Since the playground improvement initiative began in 2009, considerable progress has been made in the safety scores, although access to high-quality playgrounds varies by neighborhood. Many failing playgrounds can be brought up to standard with improvement in fall surfacing and equipment maintenance
SN - 1098-4275
AD - Baylor College of Medicine, 3925 Fairmont Parkway, Pasadena, TX 77504, USA. emallen@bcm.edu
UR - 23339221
ER -

TY - JOUR

ID - 146
T1 - Protecting children or creating vulnerability?
A1 - Hewitt-Taylor,J.
A1 - Heaslip,V.
Y1 - 2012/12//
N1 - Hewitt-Taylor, Jaqui. Heaslip, Vanessa
Community practitioner : the journal of the Community Practitioners' & Health Visitors' Association
9809060
N
Journal Article
English
KW - Medline
KW - Child
KW - Child Welfare
KW - Community Health Nursing/mt [Methods]
KW - Harm Reduction
KW - Humans
KW - Nurse's Role
KW - Play and Playthings
KW - Risk-Taking
KW - Social Environment
KW - Vulnerable Populations
RP - NOT IN FILE
SP - 31
EP - 33
JF - Community Practitioner
JA - Community Pract
VL - 85
IS - 12
CY - England
N2 - Children are, rightly, viewed as vulnerable and in need of safeguarding. However, protecting children from harm should not eclipse their need to learn about managing risky situations and those in which they are vulnerable. How adults view risk and vulnerability will affect the way in which they enable children to learn through activities which carry a degree of risk. Community practitioners may be well placed to discuss with parents how the risk and benefit of various activities can be considered, and children enabled to learn and develop resilience through acceptable risk taking activities. This paper discusses the challenges which community practitioners may encounter when working with parents on enabling them to achieve a balance between risk and safety for their children. It presents some key concepts and perspectives which may assist them to facilitate discussions with children and families
SN - 1462-2815
AD - School of Health and Social Care, Bournemouth University. jhtaylor@bournemouth.ac.uk
UR - 23304892
ER -

TY - JOUR
ID - 147
T1 - Randomized trial of a peer resistance skill-building game for Hispanic early adolescent girls
A1 - Norris,A.E.
A1 - Hughes,C.
A1 - Hecht,M.
A1 - Peragallo,N.
A1 - Nickerson,D.
Y1 - 2013/01//
N1 - Norris, Anne E. Hughes, Charles. Hecht, Michael. Peragallo, Nilda. Nickerson, David
Nursing research
o9k, 0376404

AIM, IM, N
Journal Article. Randomized Controlled Trial. Research Support, N.I.H., Extramural. Research Support, Non-U.S. Gov't
English
KW - Medline
KW - Adaptation
KW - Psychological
KW - Adolescent
KW - Adolescent Behavior/px [Psychology]
KW - Child
KW - Feasibility Studies
KW - Female
KW - Hispanic Americans/px [Psychology]
KW - Humans
KW - Interpersonal Relations
KW - Peer Group
KW - Risk-Taking
KW - Role Playing
KW - Self Efficacy
KW - Sex Factors
KW - Video Games/px [Psychology]
RP - NOT IN FILE
SP - 25
EP - 35
JF - Nursing Research
JA - Nurs Res.
VL - 62
IS - 1
CY - United States
N2 - BACKGROUND: Adolescents can use peer resistance skills to avoid being pressured into risky behavior, such as early sexual behavior. Avatar-based virtual reality technology offers a novel way to help build these skills. OBJECTIVES: The aims of this study were to evaluate the feasibility of an avatar-based virtual reality peer resistance skill building game (DRAMA-RAMA), to explore the impact of game play on peer resistance self-efficacy, and to assess how positively the game was perceived. METHODS: Forty-four low-income early adolescent Hispanic girls were assigned randomly to either the intervention (DRAMA-RAMA) or attention control game (Wii Dancing With the Stars) condition. All participants were offered a five-session curriculum that included peer resistance skill content before playing their respective game for 15 minutes, once a week, for 2 weeks. Participants completed electronic surveys at baseline, after game play, and at 2 months to assess demographics, peer resistance self-efficacy, and sexual behavior. They also completed a paper-pencil game experience questionnaire immediately after game play. Data were analyzed using descriptive statistics, t test, chi-square, and analyses of covariance. RESULTS: Separate analyses of covariance showed a significant game effect at posttest for the peer resistance self-efficacy measure ($F = 4.21$, $p < .05$), but not at follow-up ($F = 0.01$, $p = .92$). DRAMA-RAMA was rated as positively as the Wii Dancing With the Stars ($p > .26$). DISCUSSION: This randomized control trial provides preliminary support for the hypothesis that playing an avatar-based virtual reality technology game can strengthen peer resistance skills, and early adolescent Hispanic girls will have a positive response to this game
SN - 1538-9847
AD - College of Nursing, University of Central Florida, Orlando, FL 32826, USA. Anne.Norris@ucf.edu
UR - 23150043
ER -

TY - JOUR
ID - 148
T1 - Concussions in soccer: a current understanding
A1 - Levy,M.L.
A1 - Kasasbeh,A.S.

A1 - Baird,L.C.
A1 - Amene,C.
A1 - Skeen,J.
A1 - Marshall,L.
Y1 - 2012/11//
N1 - Levy, Michael L. Kasasbeh, Aimen S. Baird, Lissa Catherine. Amene, Chiazo. Skeen, Jeff. Marshall, Larry
World neurosurgery
101528275
IM
Journal Article
English
KW - Medline
KW - Adolescent
KW - Adult
KW - Athletic Injuries/ep [Epidemiology]
KW - Athletic Injuries/pc [Prevention & Control]
KW - Brain Concussion/ep [Epidemiology]
KW - Brain Concussion/pc [Prevention & Control]
KW - Child
KW - Cognition Disorders/ep [Epidemiology]
KW - Cognition Disorders/pc [Prevention & Control]
KW - Female
KW - Head Protective Devices
KW - Humans
KW - Incidence
KW - Male
KW - Recovery of Function
KW - Risk Factors
KW - Soccer/in [Injuries]
KW - Soccer/sn [Statistics & Numerical Data]
KW - United States/ep [Epidemiology]
KW - Young Adult
RP - NOT IN FILE
SP - 535
EP - 544
JF - World Neurosurgery
JA - World Neurosurg.
VL - 78
IS - 5
CY - United States
N2 - OBJECTIVE: Traumatic brain injury (TBI) is a significant public health problem in the United States, with approximately 1.5-2 million TBIs occurring each year. However, it is believed that these figures underestimate the true toll of TBI. Soccer is the most popular sport in the world and has a following of millions in the United States. Soccer is a sport not traditionally identified as high-risk for concussions, yet several studies have shown that concussion rates in soccer are comparable to, and often exceed those of, other contact sports. As many as 22% of all soccer injuries are concussions. METHODS: Soccer is a sport not traditionally identified as high risk for concussions, yet several studies have shown that concussion rates in soccer are comparable to, and often exceed those of, other contact sports. As many as 22% of all soccer injuries are concussions. Head injury during soccer is usually the result of either "direct contact" or contact with the ball while "heading" the ball. Relationships between the number of headers sustained in a single season and the degree of cognitive impairment (attention and visual/verbal memory) have been demonstrated. It is also likely that multiple concussions may cause cumulative neuropsychologic impairment in soccer players. RESULTS: Although our understanding of risk factors for sports-related concussions is far from complete, there is great potential for prevention in sports-related concussions. Several measures must be taken to avert the development of concussions in soccer and, when they take place, reduce their effects. These include the development and testing

of effective equipment during play, the maintenance of regulatory standards for all such equipment, educating young athletes on the safe and appropriate techniques used during play, and strict adherence to the rules of competition. CONCLUSIONS: In spite of such preventive measures, concussions in soccer will continue to occur. Considering the frequency of concussions in soccer, the serious sequelae of these concussions, and because almost half of concussed soccer players were noncompliant with recommended American Academy of Neurology return-to-play guidelines, further measures must be taken to protect players, in addition to understanding those criteria that result in removing an injured player from competition and the steps by which to safely return an athlete to competition after injury. Copyright 2012 Elsevier Inc. All rights reserved
SN - 1878-8750
AD - Division of Pediatric Neurosurgery Rady Children's Hospital of San Diego, San Diego, CA, USA. mlevy@rchsd.org
UR - 22120567
ER -

TY - JOUR
ID - 149
T1 - What role does nutrition play in the prevention or treatment of childhood lead poisoning?
A1 - Cunningham,E.
Y1 - 2012/11//
N1 - Cunningham, Eleese
Journal of the Academy of Nutrition and Dietetics
101573920
AIM, IM
Journal Article
English
KW - Medline
KW - Child
KW - Preschool
KW - Drinking Water/ch [Chemistry]
KW - Environmental Exposure/ae [Adverse Effects]
KW - Female
KW - Food Contamination/pc [Prevention & Control]
KW - Health Policy
KW - Humans
KW - Infant
KW - Lead/ad [Administration & Dosage]
KW - Lead/bl [Blood]
KW - Lead Poisoning/pc [Prevention & Control]
KW - Lead Poisoning
KW - Nervous System
KW - Childhood/pc [Prevention & Control]
KW - Male
KW - Nutritional Status
KW - Primary Prevention/mt [Methods]
KW - Risk Assessment
KW - Risk Factors
KW - United States
RP - NOT IN FILE
SP - 1916
JF - Journal of the Academy of Nutrition & Dietetics
JA - J Acad.Nutr Diet
VL - 112
IS - 11
CY - United States
SN - 2212-2672
AD - Academy of Nutrition and Dietetics' Knowledge Center Team. Chicago, IL, USA

UR - 23102188
ER -

TY - JOUR
ID - 150
T1 - Trampoline safety in childhood and adolescence
A1 - Council on Sports Medicine and Fitness,American Academy of Pediatrics
A1 - Briskin,S.
A1 - LaBotz,M.
Y1 - 2012/10//
N1 - Council on Sports Medicine and Fitness, American Academy of Pediatrics. Briskin, Susannah. LaBotz, Michele
Pediatrics
oxv, 0376422
AIM, IM
Journal Article. Practice Guideline
English
KW - Medline
KW - Adolescent
KW - Athletic Injuries/ep [Epidemiology]
KW - Athletic Injuries/pc [Prevention & Control]
KW - Child
KW - Humans
KW - Play and Playthings/in [Injuries]
KW - Safety
KW - Sports Equipment/ae [Adverse Effects]
KW - United States/ep [Epidemiology]
RP - NOT IN FILE
SP - 774
EP - 779
JA - Pediatrics
VL - 130
IS - 4
CY - United States
N2 - Despite previous recommendations from the American Academy of Pediatrics discouraging home use of trampolines, recreational use of trampolines in the home setting continues to be a popular activity among children and adolescents. This policy statement is an update to previous statements, reflecting the current literature on prevalence, patterns, and mechanisms of trampoline-related injuries. Most trampoline injuries occur with multiple simultaneous users on the mat. Cervical spine injuries often occur with falls off the trampoline or with attempts at somersaults or flips. Studies on the efficacy of trampoline safety measures are reviewed, and although there is a paucity of data, current implementation of safety measures have not appeared to mitigate risk substantially. Therefore, the home use of trampolines is strongly discouraged. The role of trampoline as a competitive sport and in structured training settings is reviewed, and recommendations for enhancing safety in these environments are made
SN - 1098-4275
UR - 23008455
ER -

TY - JOUR
ID - 151
T1 - The effects of a responsive parenting intervention on parent-child interactions during shared book reading
A1 - Landry,S.H.
A1 - Smith,K.E.
A1 - Swank,P.R.
A1 - Zucker,T.
A1 - Crawford,A.D.

A1 - Solari,E.F.
Y1 - 2012/07//
N1 - Landry, Susan H. Smith, Karen E. Swank, Paul R. Zucker, Tricia. Crawford, April D. Solari, Emily F
Developmental psychology
0260564, cn8
IM
Journal Article. Randomized Controlled Trial
English
KW - Medline
KW - Attention
KW - Books
KW - Child Behavior
KW - Child Development
KW - Child Language
KW - Child
KW - Preschool
KW - Cooperative Behavior
KW - Education/mt [Methods]
KW - Female
KW - Humans
KW - Male
KW - Mother-Child Relations
KW - Reading
KW - Statistics as Topic
RP - NOT IN FILE
SP - 969
EP - 986
JF - Developmental Psychology
JA - Dev.Psychol.
VL - 48
IS - 4
CY - United States
N2 - This study examined mother-child shared book reading behaviors before and after participation in a random-assignment responsive parenting intervention called Play and Learning Strategies (PALS) that occurred during infancy (PALS I), the toddler-preschool (PALS II) period, or both as compared with a developmental assessment (DAS) intervention (DAS I and/or II). The efficacy of PALS was previously demonstrated for improving mother and child behaviors within play contexts, everyday activities, and standardized measures of child language. We hypothesized that PALS effects would generalize to influence maternal and child behaviors during a shared reading task even though this situation was not a specific focus of the intervention and that this would be similar for children who varied in biological risk. Participation in at least PALS II was expected to have a positive effect due to children's increased capacity to engage in book reading at this age. Four groups of randomized mothers and their children (PALS I-II, PALS I-DAS II, DAS I-PALS II, DAS I-II) were observed in shared reading interactions during the toddler-preschool period and coded for (a) mother's affective and cognitive-linguistic supports and (b) child's responses to maternal requests and initiations. Support was found for significant changes in observed maternal and child behaviors, and evidence of mediation was found for the intervention to affect children's behaviors through change in maternal responsiveness behaviors. These results add to other studies supporting the importance of targeting a broad range of responsive behaviors across theoretical frameworks in interventions to facilitate children's development
SN - 1939-0599
AD - Children's Learning Institute, University of Texas Health Science Center, 7000 Fannin, Suite 2300, Houston, TX 77030, USA.susan.landry@uth.tmc.edu
UR - 22122475
ER -

TY - JOUR
ID - 152

T1 - Supporting vocationally oriented learning in the high school years: rationale, tasks, challenges
A1 - Halpern,R.
Y1 - 2012/10//
N1 - Halpern, Robert
New directions for youth development
101090644
IM
Journal Article
English
KW - Medline
KW - Child
KW - Child Development
KW - Humans
KW - Learning
KW - Risk Factors
KW - Role
KW - Schools/og [Organization & Administration]
KW - Schools
KW - United States
KW - Vocational Education/mt [Methods]
KW - Vocational Education/og [Organization & Administration]
KW - Vocational Education
RP - NOT IN FILE
SP - 85
EP - 106
JF - New Directions for Youth Development
JA - New Dir.Youth Dev.
VL - 2012
IS - 134
CY - United States
N2 - This article highlights the limitations of our current educational system in terms of vocational learning and highlights the role that vocational learning can play in supporting youth development and improving youth outcomes. It discusses the role that nonschool settings can play in supporting vocational learning and suggests strategies to improve our in-school and out-of-school systems to build a more coherent whole that promotes youth development across various settings. Copyright 2012 Wiley Periodicals, Inc., A Wiley Company
SN - 1537-5781
AD - Research Council, Erikson Institute in Chicago, USA
UR - 22826169
ER -

TY - JOUR
ID - 153
T1 - Evaluation of a coping kit for children with challenging behaviors in a pediatric hospital
A1 - Drake,J.
A1 - Johnson,N.
A1 - Stoneck,A.V.
A1 - Martinez,D.M.
A1 - Massey,M.
Y1 - 2012/07//
N1 - Drake, Jennifer. Johnson, Norah. Stoneck, Alice V. Martinez, Deb M. Massey, Megan
Pediatric nursing
oun, 7505804
N
Evaluation Studies. Journal Article
English
KW - Medline

KW - Adaptation
KW - Psychological
KW - Anxiety Disorders/px [Psychology]
KW - Anxiety Disorders/th [Therapy]
KW - Child
KW - Hospitalized/px [Psychology]
KW - Cross-Sectional Studies
KW - Disabled Children/px [Psychology]
KW - Female
KW - Hospitals
KW - Pediatric
KW - Humans
KW - Male
KW - Midwestern United States
KW - Pediatric Nursing/mt [Methods]
KW - Play and Playthings
RP - NOT IN FILE
SP - 215
EP - 221
JF - Pediatric Nursing
JA - Pediatr Nurs
VL - 38
IS - 4
CY - United States
N2 - This study attempted to answer the question, "Do nurses perceive coping kits to be effective at meeting the needs of hospitalized children with developmental disabilities who are at increased risk for challenging behaviors?" A cross-sectional post-test survey study design was used, with a convenience sample of 24 registered nurses at a Midwestern free-standing children's hospital. A coping kit with simple communication cards, social script book, and distraction items (toys) was developed to enhance communication and distract children with developmental disabilities (including autism spectrum disorder) undergoing procedures in the hospital. A modified version of Hudson's (2006) intervention effectiveness survey was used to measure the nurse's perception of the effectiveness of the coping kit. Nurses perceived the coping kits to be effective for decreasing their patient's anxiety, calming the child's behavior, and increasing cooperation during procedures. The nurse can develop a plan of care that includes a coping kit to help gain cooperation with the hospitalized child with challenging behaviors
SN - 0097-9805
AD - Children's Hospital of Wisconsin, Milwaukee, WI, USA
UR - 22970487
ER -

TY - JOUR
ID - 154
T1 - Home environment relationships with children's physical activity, sedentary time, and screen time by socioeconomic status
A1 - Tandon,P.S.
A1 - Zhou,C.
A1 - Sallis,J.F.
A1 - Cain,K.L.
A1 - Frank,L.D.
A1 - Saelens,B.E.
Y1 - 2012///
N1 - Tandon, Pooja S. Zhou, Chuan. Sallis, James F. Cain, Kelli L. Frank, Lawrence D. Saelens, Brian E
The international journal of behavioral nutrition and physical activity
101217089
IM

Comparative Study. Journal Article. Research Support, N.I.H., Extramural. Research Support, Non-U.S. Gov't English
KW - Medline
KW - Adult
KW - Bicycling
KW - Child
KW - Child Behavior
KW - Data Collection
KW - Educational Status
KW - Environment
KW - Exercise
KW - Female
KW - Health Behavior
KW - Humans
KW - Income
KW - Male
KW - Middle Aged
KW - Movement
KW - Obesity/et [Etiology]
KW - Parent-Child Relations
KW - Parenting
KW - Parents
KW - Play and Playthings
KW - Sedentary Lifestyle
KW - Social Class
KW - Social Environment
KW - Television
KW - Video Games
RP - NOT IN FILE
SP - 88
JF - International Journal of Behavioral Nutrition & Physical Activity
JA - Int.
VL - 9
CY - England
N2 - BACKGROUND: Children in households of lower socioeconomic status (SES) are more likely to be overweight/obese. We aimed to determine if home physical activity (PA) environments differed by SES and to explore home environment mediators of the relation of family SES to children's PA and sedentary behavior. METHODS: Participants were 715 children aged 6 to 11 from the Neighborhood Impact on Kids (NIK) Study. Household SES was examined using highest educational attainment and income. Home environment was measured by parent report on a survey. Outcomes were child's accelerometer-measured PA and parent-reported screen time. Mediation analyses were conducted for home environment factors that varied by SES. RESULTS: Children from lower income households had greater media access in their bedrooms (TV 52% vs. 14%, DVD player 39% vs. 14%, video games 21% vs. 9%) but lower access to portable play equipment (bikes 85% vs. 98%, jump ropes 69% vs. 83%) compared to higher income children. Lower SES families had more restrictive rules about PA (2.5 vs. 2.0). Across SES, children watched TV/DVDs with parents/siblings more often than they engaged in PA with them. Parents of lower SES watched TV/DVDs with their children more often (3.1 vs. 2.5days/week). Neither total daily and home-based MVPA nor sedentary time differed by SES. Children's daily screen time varied from 1.7 hours/day in high SES to 2.4 in low SES families. Media in the bedroom was related to screen time, and screen time with parents was a mediator of the SES--screen time relationship. CONCLUSIONS: Lower SES home environments provided more opportunities for sedentary behavior and fewer for PA. Removing electronic media from children's bedrooms has the potential to reduce disparities in chronic disease risk
SN - 1479-5868
AD - Seattle Children's Research Institute, M/S CW8-6, P,O, Box 5371, Seattle, WA 98145-5005, USA. pooja@uw.edu
UR - 22835155

ER -

TY - JOUR
ID - 155
T1 - Risk factors predisposing to pedestrian road traffic injury in children living in Lima, Peru: a case-control study
A1 - Pernica,J.M.
A1 - LeBlanc,J.C.
A1 - Soto-Castellares,G.
A1 - Donroe,J.
A1 - Carhuancho-Meza,B.A.
A1 - Rainham,D.G.
A1 - Gilman,R.H.
Y1 - 2012/08//
N1 - Pernica, Jeffrey M. LeBlanc, John C. Soto-Castellares, Giselle. Donroe, Joseph. Carhuancho-Meza, Bristan A. Rainham, Daniel G C. Gilman, Robert H
Archives of disease in childhood
6xg, 0372434
AIM, IM
Journal Article. Research Support, Non-U.S. Gov't
English
KW - Medline
KW - Accidents
KW - Traffic/sn [Statistics & Numerical Data]
KW - Case-Control Studies
KW - Child
KW - Family
KW - Female
KW - Humans
KW - Male
KW - Peru/ep [Epidemiology]
KW - Recreation
KW - Risk Factors
KW - Schools
KW - Walking/in [Injuries]
KW - Wounds and Injuries/ep [Epidemiology]
RP - NOT IN FILE
SP - 709
EP - 713
JF - Archives of Disease in Childhood
JA - Arch.Dis.Child
VL - 97
IS - 8
CY - England
N2 - OBJECTIVE: To describe the epidemiology of pedestrian road traffic injury in Lima and to identify associated child-level, family-level, and school travel-related variables. DESIGN: Case-control study. SETTING: The Instituto Nacional de Salud del Nino, the largest paediatric hospital in the city. PARTICIPANTS: Cases were children who presented because of pedestrian road traffic injury. Controls presented with other diagnoses and were matched on age, sex and severity of injury. RESULTS: Low socioeconomic status, low paternal education, traffic exposure during the trip to school, lack of supervision during outside play, and duration of outside play were all statistically significantly associated with case-control status. In multivariate logistic regression, a model combining the lack of supervision during outside play and the number of the streets crossed walking to school best predicted case-control status ($p<0.001$). CONCLUSIONS: These results emphasise that an assessment of children's play behaviours and school locations should be considered and integrated into any plan for an intervention designed to reduce pedestrian road traffic injury. A

child-centred approach will ensure that children derive maximum benefit from sorely needed public health interventions
SN - 1468-2044
AD - Department of Pediatrics, McMaster University, Hamilton, Canada
UR - 22685050
ER -

TY - JOUR
ID - 156
T1 - Behavioral risk factors for overweight in early childhood; the 'Be active, eat right' study
A1 - Veldhuis,L.
A1 - Vogel,I.
A1 - Renders,C.M.
A1 - van,Rossem L.
A1 - Oenema,A.
A1 - HiraSing,R.A.
A1 - Raat,H.
Y1 - 2012///
N1 - Veldhuis, Lydian. Vogel, Ineke. Renders, Carry M. van Rossem, Lenie. Oenema, Anke. HiraSing, Remy A. Raat, Hein
The international journal of behavioral nutrition and physical activity
101217089
IM
Journal Article. Research Support, Non-U.S. Gov't
English
KW - Medline
KW - Beverages
KW - Child Behavior
KW - Child
KW - Preschool
KW - Confidence Intervals
KW - Cross-Sectional Studies
KW - Diet
KW - Dietary Sucrose/ad [Administration & Dosage]
KW - Exercise
KW - Feeding Behavior
KW - Female
KW - Health Behavior
KW - Humans
KW - Male
KW - Obesity/et [Etiology]
KW - Odds Ratio
KW - Overweight
KW - Play and Playthings
KW - Risk Factors
KW - Risk-Taking
KW - Sedentary Lifestyle
KW - Television
RP - NOT IN FILE
SP - 74
JF - International Journal of Behavioral Nutrition & Physical Activity
JA - Int.
VL - 9
CY - England
N2 - BACKGROUND: The lifestyle-related behaviors having breakfast, drinking sweet beverages, playing outside and watching TV have been indicated to have an association with childhood overweight, but research

among young children (below 6 years old) is limited. The aim of the present study was to assess the associations between these four behaviors and overweight among young children. METHODS: This cross-sectional study used baseline data on 5-year-old children (n = 7505) collected for the study 'Be active, eat right'. Age and sex-specific cut-off points for body mass index of the International Obesity Task Force were used to assess overweight/obesity. Multivariable logistic regression analyses were applied. RESULTS: For children whom had breakfast <7 days/week and watched TV >2 hours/day, the odds ratio (OR) for having overweight (obesity included) was, respectively, 1.49 (95% confidence interval (CI): 1.13-1.95), and 1.25 (95% CI: 1.03-1.51). There was a positive association between the number of risk behaviors present and the risk for having overweight. For children with 3 or all of the risk behaviors having breakfast <7 days/week, drinking sweet beverages >2 glasses/day, playing outside <1 h/day, watching TV >2 hs/day, the OR for overweight was 1.73 (95% CI: 1.11-2.71) (all models adjusted for children's sex and sociodemographic characteristics). CONCLUSION: Given the positive association between the number of behavioral risk factors and overweight, further studies are needed to evaluate the effectiveness of behavioral counseling of parents of toddlers in preventing childhood overweight. In the meantime we recommend physicians to target all four behaviors for counseling during well-child visits
SN - 1479-5868
AD - Department of Public Health, Erasmus MC-University Medical Centre Rotterdam, Rotterdam, The Netherlands. l.veldhuis@erasmusmc.nl
UR - 22704042
ER -

TY - JOUR
ID - 157
T1 - Combined influence of media use on subjective health in elementary school children in Japan: a population-based study
A1 - Nakamura,H.
A1 - Ohara,K.
A1 - Kouda,K.
A1 - Fujita,Y.
A1 - Mase,T.
A1 - Miyawaki,C.
A1 - Okita,Y.
A1 - Ishikawa,T.
Y1 - 2012///
N1 - Nakamura, Harunobu. Ohara, Kumiko. Kouda, Katsuyasu. Fujita, Yuki. Mase, Tomoki. Miyawaki, Chiemi. Okita, Yoshimitsu. Ishikawa, Tetsuya
BMC public health
100968562
IM
Journal Article
English
KW - Medline
KW - Child
KW - Choice Behavior
KW - Computers/ut [Utilization]
KW - Cross-Sectional Studies
KW - Female
KW - Food Habits/px [Psychology]
KW - Health Status Indicators
KW - Humans
KW - Japan
KW - Logistic Models
KW - Male
KW - Mass Media/ut [Utilization]
KW - Play and Playthings/px [Psychology]
KW - Population Surveillance

KW - Psychophysiologic Disorders/et [Etiology]
KW - Quality of Life
KW - Questionnaires
KW - Risk Factors
KW - Schools
KW - Sedentary Lifestyle
KW - Sleep/ph [Physiology]
KW - Students/px [Psychology]
KW - Students/sn [Statistics & Numerical Data]
KW - Television/ut [Utilization]
KW - Time Factors
KW - Walking/ph [Physiology]
KW - Walking/px [Psychology]
RP - NOT IN FILE
SP - 432
JA - BMC Public Health
VL - 12
CY - England
N2 - BACKGROUND: In recent years in Japan, electronic games, home computers, and the internet have assumed an important place in people's lives, even for elementary school children. Subjective health complaints have also become a problem among children. In the present study, we investigated the relationship between media use and health status in elementary school children in Japan. METHODS: A cross-sectional school-based population survey was conducted in 2009 with a sample of fourth-, fifth-, and sixth-grade children (age range: 10-12years old) in elementary schools in Japan (n=3,464). Self-reported health, lifestyle habits, and time spent using media were assessed. RESULTS: The use of games, television, and personal computers was significantly associated with lifestyle ($p<0.05$) and subjective health ($p<0.05$). In addition, the use of games, the use of television, and the use of personal computers were mutually associated. The greater the number of media used for more than 1 hour was, the higher the odds ratio of the association of media use with unhealthy lifestyle and subjective health complaints was. The plural use of these media had stronger associations with unhealthy lifestyle and subjective health complaints. CONCLUSIONS: Game, television, and personal-computer use were mutually associated, and the plural use of these media had stronger associations with unhealthy lifestyle and subjective health complaints. Excessive use of media might be a risk for unhealthy lifestyle and subjective health complaints
SN - 1471-2458
AD - Graduate School of Human Development and Environment, Kobe University, 3-11 Tsurukabuto, Nada, Kobe, Japan. hal@kobe-u.ac.jp
UR - 22694807
ER -

TY - JOUR
ID - 158
T1 - Affective dynamics in triadic peer interactions in early childhood
A1 - Lavictoire,L.A.
A1 - Snyder,J.
A1 - Stoolmiller,M.
A1 - Hollenstein,T.
Y1 - 2012/07//
N1 - Lavictoire, Lindsay A. Snyder, James. Stoolmiller, Mike. Hollenstein, Tom
Nonlinear dynamics, psychology, and life sciences
9704130
IM
Journal Article
English
KW - Medline
KW - Affect
KW - Analysis of Variance

KW - Child
KW - Child Behavior/px [Psychology]
KW - Child Behavior Disorders/px [Psychology]
KW - Preschool
KW - Cohort Studies
KW - Female
KW - Humans
KW - Internal-External Control
KW - Interpersonal Relations
KW - Male
KW - Parent-Child Relations
KW - Peer Group
KW - Play and Playthings/px [Psychology]
KW - Socioeconomic Factors
RP - NOT IN FILE
SP - 293
EP - 312
JF - Nonlinear Dynamics, Psychology, & Life Sciences
JA - Nonlinear Dynamics Psychol.Life Sci
VL - 16
IS - 3
CY - United States
N2 - In interpersonal interaction research, moving beyond dyadic to triadic dynamics can be analytically daunting. We explored the affective states expressed during triadic peer interactions to understand how patterns were associated with childhood psychopathology and sociometric status. High-risk kindergarten children (N = 216) were observed in several groups of three during a free play task. We extended the state space grid technique to 3-dimensional analysis in order to derive variables of interest. Unlike results from parent-child dyadic interactions, triadic affective variability was not strongly associated with externalizing or internalizing problems. However, several triadic affective states were distinguished by externalizing, internalizing, and sociometric status, most commonly mutually aversive affect. Strengths and limitations of this methodology in relation to understanding peer triadic interactions are discussed
SN - 1090-0578
AD - Queen's University, Kingston, ON, Canada
UR - 22695150
ER -

TY - JOUR
ID - 159
T1 - Geographic variations in elementary school-based physical activity practices
A1 - Turner,L.
A1 - Chaloupka,F.J.
A1 - Slater,S.J.
Y1 - 2012/07//
N1 - Turner, Lindsey. Chaloupka, Frank J. Slater, Sandy J
The Journal of school health
k13, 0376370
IM, N
Journal Article
English
KW - Medline
KW - Child
KW - Data Collection
KW - Geography
KW - Health Promotion/mt [Methods]
KW - Health Promotion/sn [Statistics & Numerical Data]
KW - Humans

KW - Illinois
KW - Logistic Models
KW - Male
KW - Motor Activity/ph [Physiology]
KW - Physical Education and Training/sn [Statistics & Numerical Data]
KW - Play and Playthings
KW - Risk Factors
KW - School Health Services
KW - Schools
KW - Students
RP - NOT IN FILE
SP - 307
EP - 310
JF - Journal of School Health
JA - J Sch Health
VL - 82
IS - 7
CY - United States
N2 - BACKGROUND: Physical activity (PA) is associated with health and academic benefits among children, but many schools do not meet national recommendations. This study examined school-based PA practices in nationally representative samples of public elementary schools, and geographic variations in those practices. METHODS: Mail-back surveys were used to gather data from 578 public elementary schools during the 2006-2007 school year and 680 public elementary schools during the 2009-2010 school year. RESULTS: Nationwide, only 1 in 5 schools met the national recommendation of at least 150 minutes of physical education (PE) per week, although PE was more prevalent in the South. Nearly three-fourths of schools nationwide met recommendations for 20 or more minutes of recess daily, and this was more prevalent in the Midwest but less so in the South. Approximately 40% of schools offered sports and approximately 40% offered other opportunities for PA during the school day. The only practice that increased over time was formal classroom instruction on PA. CONCLUSIONS: There has been little change in elementary school-based PA practices nationwide in recent years. With the upcoming renewal of the Elementary and Secondary Education Act, policymakers have an important opportunity to promote increased PA in schools. 2012, American School Health Association
SN - 1746-1561
AD - Institute for Health Research and Policy, University of Illinois at Chicago, 1747 W. Roosevelt Road, M/C 275, Room 558, Chicago, IL 60608, USA. lindseyt@uic.edu
UR - 22671946
ER -

TY - JOUR
ID - 160
T1 - The road user behaviour of school students in Belgium
A1 - Sullman,M.J.
A1 - Thomas,A.
A1 - Stephens,A.N.
Y1 - 2012/09//
N1 - Sullman, Mark J M. Thomas, Abigail. Stephens, Amanda N
Accident; analysis and prevention
acs, 1254476
IM
Journal Article. Validation Studies
English
KW - Medline
KW - Accidents
KW - Traffic/pc [Prevention & Control]
KW - Traffic/px [Psychology]
KW - Traffic/sn [Statistics & Numerical Data]
KW - Adolescent

KW - Adolescent Behavior
KW - Age Factors
KW - Belgium
KW - Bicycling
KW - Child
KW - Dangerous Behavior
KW - Female
KW - Health Surveys
KW - Humans
KW - Male
KW - Play and Playthings
KW - Psychological Tests
KW - Questionnaires
KW - Risk Reduction Behavior
KW - Running
KW - Safety
KW - Self Report
KW - Sex Factors
KW - Walking
RP - NOT IN FILE
SP - 495
EP - 504
JF - Accident Analysis & Prevention
JA - Accid.Anal.Prev.
VL - 48
CY - England
N2 - The present study aimed to investigate both the on road behaviour of Belgian school students and the validity of the Adolescent Road User Behaviour Questionnaire (ARBQ) in a sample of students attending school in Belgium. In total, 294 adolescents completed the ARBQ along with measures of their self-reported accident involvement and sensation seeking behaviour. Confirmatory Factor Analysis supported the original factor structure of: "unsafe road crossing", "playing on the road" and "planned protective behaviour" for the 21-item version of the questionnaire, but not for the full scale. Males were found to engage more often in unsafe crossing behaviour and playing on the roads. There were also age differences, with unsafe road crossing increasing with age and engagement in planned protective behaviours improving with age. Those who reported being involved in an accident also reported more frequent engagement in unsafe crossing, playing on the roads, thrill seeking behaviour and lower levels of behaviour inhibition. Therefore, this study confirms that the ARBQ is a useful tool for investigating safety-related behaviours that contribute to accident involvement. Copyright 2012 Elsevier Ltd. All rights reserved
SN - 1879-2057
AD - Cranfield University, UK. M.Sullman@cranfield.ac.uk
UR - 22664716
ER -

TY - JOUR
ID - 161
T1 - Developmental Coordination Disorder, gender, and body weight: examining the impact of participation in active play
A1 - Cairney,J.
A1 - Kwan,M.Y.
A1 - Hay,J.A.
A1 - Faught,B.E.
Y1 - 2012/09//
N1 - Cairney, John. Kwan, Matthew Y W. Hay, John A. Faught, Brent E
Research in developmental disabilities
8709782, rid
IM

Journal Article
English
KW - Medline
KW - Adipose Tissue/ph [Physiology]
KW - Adolescent
KW - Body Mass Index
KW - Body Weight/ph [Physiology]
KW - Child
KW - Cross-Sectional Studies
KW - Female
KW - Humans
KW - Male
KW - Motor Activity/ph [Physiology]
KW - Motor Skills Disorders/ep [Epidemiology]
KW - Motor Skills Disorders/pp [Physiopathology]
KW - Obesity/ep [Epidemiology]
KW - Obesity/pp [Physiopathology]
KW - Overweight/ep [Epidemiology]
KW - Overweight/pp [Physiopathology]
KW - Play and Playthings
KW - Risk Factors
KW - Sex Distribution
KW - Sports/sn [Statistics & Numerical Data]
RP - NOT IN FILE
SP - 1566
EP - 1573
JF - Research in Developmental Disabilities
JA - Res.Dev.Disabil.
VL - 33
IS - 5
CY - United States
N2 - BACKGROUND: To examine whether differences in participation in active play (PAP) can account for gender differences in the relationship between Developmental Coordination Disorder (DCD) and body weight/fat (BMI and percentage fat) in youth. METHODS: A cross-sectional investigation of students in grades four through eight (n = 590). Height, weight (BMI), and percentage body fat using bioelectrical impedance analysis (BIA; RJL Systems, MI) were collected. Motor proficiency and physical activity levels were also evaluated. RESULTS: We found gender specific patterns in the relationship between PAP, DCD, and BMI and body fat. Among boys with DCD, greater participation in active play is associated with higher BMI and percentage body fat. For girls with the disorder, the opposite relationship is observed. CONCLUSIONS: Participation in active play moderates the association between DCD, gender and body weight. Three possible explanations for why PAP is associated with higher BMI and percentage body fat in boys with DCD are provided. Copyright 2012 Elsevier Ltd. All rights reserved
SN - 1873-3379
AD - Department of Family Medicine, McMaster University, Hamilton, ON, Canada
UR - 22522216
ER -

TY - JOUR
ID - 162
T1 - Height, fun and safety in the design of children's playground equipment
A1 - Wakes,S.
A1 - Beukes,A.
Y1 - 2012///
N1 - Wakes, Sarah. Beukes, Amanda
International journal of injury control and safety promotion
101247254

IM
Journal Article. Research Support, Non-U.S. Gov't
English
KW - Medline
KW - Accidental Falls
KW - Child
KW - Equipment Design
KW - Female
KW - Humans
KW - Male
KW - Observation
KW - Parents/px [Psychology]
KW - Perception
KW - Play and Playthings/in [Injuries]
KW - Play and Playthings/px [Psychology]
KW - Questionnaires
KW - Risk Factors
KW - Safety
KW - Wounds and Injuries/pc [Prevention & Control]
RP - NOT IN FILE
SP - 101
EP - 108
JF - International Journal of Injury Control & Safety Promotion
JA - Int.J Inj.Contr.Saf Promot
VL - 19
IS - 2
CY - England
N2 - The study reported in this paper adopted a holistic design approach to investigate issues associated with height related playground injuries from a users' perspective. The main objective was to gain an understanding of the relationship between height and fun so as to establish practical guidelines for addressing the causes of height related injuries whilst maintaining the attributes of playground equipment that children find fun and challenging. Results show that, on the one hand, the risk of injury increases when height is coupled with the use of upper body strength and, on the other hand, that coordination is a greater source of fun and challenge than height for children. Accordingly, it is suggested that the level of risk of injury attached to children's playground equipment can be reduced when the use of lower body strength and coordination are combined with lower free fall heights
SN - 1745-7319
AD - Department of Applied Sciences, University of Otago, Dunedin, New Zealand. sarah@design.otago.ac.nz
UR - 21838539
ER -

TY - JOUR
ID - 163
T1 - Health risks of Oregon eighth-grade participants in the "choking game": results from a population-based survey
A1 - Ramowski,S.K.
A1 - Nystrom,R.J.
A1 - Rosenberg,K.D.
A1 - Gilchrist,J.
A1 - Chaumeton,N.R.
Y1 - 2012/05//
N1 - Ramowski, Sarah K. Nystrom, Robert J. Rosenberg, Kenneth D. Gilchrist, Julie. Chaumeton, Nigel R
Pediatrics
oxv, 0376422
AIM, IM
Comparative Study. Journal Article
English

KW - Medline
KW - Adolescent
KW - Airway Obstruction/ep [Epidemiology]
KW - Airway Obstruction/eh [Ethnology]
KW - Airway Obstruction/px [Psychology]
KW - Brain/bs [Blood Supply]
KW - Brain Ischemia/eh [Ethnology]
KW - Brain Ischemia/px [Psychology]
KW - Child
KW - Comorbidity
KW - Cross-Sectional Studies
KW - Ethnic Groups/px [Psychology]
KW - Ethnic Groups/sn [Statistics & Numerical Data]
KW - Euphoria
KW - Female
KW - Health Surveys
KW - Humans
KW - Hypoxia
KW - Brain/eh [Ethnology]
KW - Brain/px [Psychology]
KW - Male
KW - Mental Disorders/ep [Epidemiology]
KW - Mental Disorders/eh [Ethnology]
KW - Mental Disorders/px [Psychology]
KW - Motivation
KW - Oregon
KW - Play and Playthings/px [Psychology]
KW - Risk-Taking
KW - Sex Factors
KW - Substance-Related Disorders/ep [Epidemiology]
KW - Substance-Related Disorders/eh [Ethnology]
KW - Substance-Related Disorders/px [Psychology]
RP - NOT IN FILE
SP - 846
EP - 851
JA - Pediatrics
VL - 129
IS - 5
CY - United States
N2 - OBJECTIVE: To examine the risk behaviors associated with participation in the "choking game" by eighth-graders in Oregon. METHODS: We obtained data from the 2009 Oregon Healthy Teens survey, a cross-sectional weighted survey of 5348 eighth-graders that questioned lifetime prevalence and frequency of choking game participation. The survey also included questions about physical and mental health, gambling, sexual activity, nutrition, physical activity/body image, exposure to violence, and substance use. RESULTS: Lifetime prevalence of choking game participation was 6.1% for Oregon eighth-graders, with no differences between males and females. Of the eighth-grade choking game participants, 64% had engaged in the activity more than once and 26.6% >5 times. Among males, black youth were more likely to participate than white youth. Among both females and males, Pacific Islander youth were much more likely to participate than white youth. Multivariate logistic regression revealed that sexual activity and substance use were significantly associated with choking game participation for both males and females. CONCLUSIONS: At >6%, the prevalence of choking game participation among Oregon youth is consistent with previous findings. However, we found that most of those who participate will put themselves at risk more than once. Participants also have other associated health risk behaviors. The comprehensive adolescent well visit, as recommended by the American Academy of Pediatrics, is a good opportunity for providers to conduct a health behavior risk assessment and, if appropriate, discuss the dangers of engaging in this activity
SN - 1098-4275

AD - Oregon Health Authority, Public Health Division, Portland, Oregon 97232, USA
UR - 22508913
ER -

TY - JOUR
ID - 164
T1 - Child, family, and neighborhood associations with parent and peer interactive play during early childhood
A1 - Kenney,M.K.
Y1 - 2012/04//
N1 - Kenney, Mary Kay
Maternal and child health journal
9715672, di8
IM
Journal Article
English
KW - Medline
KW - Child Behavior
KW - Child Development
KW - Child
KW - Preschool
KW - Cross-Sectional Studies
KW - Family Characteristics
KW - Female
KW - Humans
KW - Infant
KW - Logistic Models
KW - Male
KW - Parent-Child Relations
KW - Parenting
KW - Parents
KW - Peer Group
KW - Play and Playthings
KW - Residence Characteristics
KW - Social Behavior
KW - Social Environment
KW - Socioeconomic Factors
KW - United States
RP - NOT IN FILE
SP - S88
EP - 101
JF - Maternal & Child Health Journal
JA - Matern.Child Health J
VL - 16 Suppl 1
CY - United States
N2 - To examine national patterns of peer and parent interactive play opportunities that enhance early learning/socialization. Bivariate and multivariable analyses of cross-sectional data on 22,797 children aged 1-5 years from the National Survey of Children's Health 2007 were performed to determine the child, family, and neighborhood factors associated with four parent-initiated activities. Outcomes measures included time (days/week) children spent: participating in peer play; being read to; sung to/told stories; and taken on family outings. Covariates included race/ethnicity, poverty, TV watching, childcare, child and maternal physical and mental health, family factors (structure, size, language, stress, education), and neighborhood factors (amenities, support, physical condition, safety). According to adjusted regression models, minority children from lower income, non-English-speaking households with limited education, poorer maternal health and greater parenting stress were read to/told stories less than children without these characteristics, while neighborhood factors exerted less influence. In contrast, significant reductions in days/week of peer play were associated with unsupportive neighborhoods and those with the poorest physical conditions and limited amenities. Likewise,

reductions in outings were associated with fewer neighborhood amenities. The findings of this study indicate that a variety of child, family, and neighborhood factors are associated with parent-initiated behaviors such as reading, storytelling, peer interactive play, and family outings. Appropriate evidence-based home visiting interventions targeting child health, parenting skills, early childhood education, and social services in at-risk communities would appear to be appropriate vehicles for addressing such parent-initiated play activities that have the potential to enhance development
SN - 1573-6628
AD - U.S. Department of Health and Human Services, Health Resources and Services Administration, Maternal and Child Health Bureau, 5600 Fishers Lane, Rm 18-41, Rockville, MD 20857, USA. mkenney@hrsa.gov
UR - 22453331
ER -

TY - JOUR
ID - 165
T1 - Canadian Physical Activity Guidelines for the Early Years (aged 0-4 years)
A1 - Tremblay,M.S.
A1 - Leblanc,A.G.
A1 - Carson,V.
A1 - Choquette,L.
A1 - Connor,Gorber S.
A1 - Dillman,C.
A1 - Duggan,M.
A1 - Gordon,M.J.
A1 - Hicks,A.
A1 - Janssen,I.
A1 - Kho,M.E.
A1 - Latimer-Cheung,A.E.
A1 - Leblanc,C.
A1 - Murumets,K.
A1 - Okely,A.D.
A1 - Reilly,J.J.
A1 - Spence,J.C.
A1 - Stearns,J.A.
A1 - Timmons,B.W.
A1 - Canadian Society for Exercise Physiology.
Y1 - 2012/04//
N1 - Tremblay, Mark S. Leblanc, Allana G. Carson, Valerie. Choquette, Louise. Connor Gorber, Sarah. Dillman, Carrie. Duggan, Mary. Gordon, Mary Jane. Hicks, Audrey. Janssen, Ian. Kho, Michelle E. Latimer-Cheung, Amy E. Leblanc, Claire. Murumets, Kelly. Okely, Anthony D. Reilly, John J. Spence, John C. Stearns, Jodie A. Timmons, Brian W. Canadian Society for Exercise Physiology
Applied physiology, nutrition, and metabolism = Physiologie appliquee, nutrition et metabolisme
101264333
IM
Guideline. Journal Article. Research Support, Non-U.S. Gov't
English French
KW - Medline
KW - Canada
KW - Child Behavior/ph [Physiology]
KW - Child Welfare
KW - Child
KW - Preschool
KW - Humans
KW - Infant
KW - Life Style
KW - Motor Activity
KW - Public Health

RP - NOT IN FILE
SP - 345
EP - 369
JF - Applied Physiology, Nutrition, & Metabolism = Physiologie Appliquee, Nutrition et Metabolisme
JA - Appl.Physiol Nutr Metab
VL - 37
IS - 2
CY - Canada
N2 - The Canadian Society for Exercise Physiology (CSEP), with assistance from multiple partners, stakeholders, and researchers, developed the first Canadian Physical Activity Guidelines for the Early Years (aged 0-4 years). These national guidelines were created in response to an urgent call from public health, health care, child care, and fitness practitioners for healthy active living guidance for the early years. The guideline development process was informed by the Appraisal of Guidelines for Research Evaluation (AGREE) II instrument and the evidence assessed using the Grading of Recommendations Assessment, Development, and Evaluation (GRADE) system. The recommendations are informed by evidence from a systematic review that examined the relationships between physical activity and health indicators (healthy body weight, bone and skeletal health, motor skill development, psychosocial health, cognitive development, and cardio-metabolic disease risk factors) for three age groups (infants aged <1 year; toddlers aged 1-2 years; preschoolers aged 3-4 years). The new guidelines include a preamble to provide context, followed by the specific recommendations. The final guidelines benefitted from an extensive on-line consultation process with input from over 900 domestic and international stakeholders, end-users, and key informants. The final guideline recommendations state that for healthy growth and development, infants (aged <1 year) should be physically active several times daily - particularly through interactive floor-based play. Toddlers (aged 1-2 years) and preschoolers (aged 3-4 years) should accumulate at least 180 min of physical activity at any intensity spread throughout the day, including a variety of activities in different environments, activities that develop movement skills, and progression toward at least 60 min of energetic play by 5 years of age. More daily physical activity provides greater benefits
SN - 1715-5312
AD - University of Ottawa, Ottawa, ON, Canada. mtremblay@cheo.on.ca
UR - 22448608
ER -

TY - JOUR
ID - 166
T1 - Maternal play behaviors, child negativity, and preterm or low birthweight toddlers' visual-spatial outcomes: testing a differential susceptibility hypothesis
A1 - lworth-Bart,J.E.
A1 - Miller,K.E.
A1 - Hane,A.
Y1 - 2012/04//
N1 - Dilworth-Bart, Janean E. Miller, Kyle E. Hane, Amanda
Infant behavior & development
7806016
IM
Journal Article. Research Support, N.I.H., Extramural. Research Support, Non-U.S. Gov't
English
KW - Medline
KW - Body Temperature
KW - Child Development
KW - Child
KW - Preschool
KW - Cognition Disorders/et [Etiology]
KW - Disease Susceptibility
KW - Female
KW - Humans
KW - Infant

KW - Low Birth Weight
KW - Newborn
KW - Longitudinal Studies
KW - Male
KW - Maternal Behavior
KW - Mother-Child Relations
KW - Neuropsychological Tests
KW - Photic Stimulation
KW - Play and Playthings
KW - Premature Birth/pp [Physiopathology]
KW - Psychiatric Status Rating Scales
KW - Regression Analysis
KW - Risk Factors
KW - Sex Factors
KW - Space Perception/ph [Physiology]
RP - NOT IN FILE
SP - 312
EP - 322
JF - Infant Behavior & Development
JA - Infant behav
VL - 35
IS - 2
CY - United States
N2 - OBJECTIVE: We examined the joint roles of child negative emotionality and parenting in the visual-spatial development of toddlers born preterm or with low birthweights (PTLBW). METHOD: Neonatal risk data were collected at hospital discharge, observer- and parent-rated child negative emotionality was assessed at 9-months postterm, and mother-initiated task changes and flexibility during play were observed during a dyadic play interaction at 16-months postterm. Abbreviated IQ scores, and verbal/nonverbal and visual-spatial processing data were collected at 24-months postterm. RESULTS: Hierarchical regression analyses did not support our hypothesis that the visual-spatial processing of PTLBW toddlers with higher negative emotionality would be differentially susceptible to parenting behaviors during play. Instead, observer-rated distress and a negativity composite score were associated with less optimal visual-spatial processing when mothers were more flexible during the 16-month play interaction. Mother-initiated task changes did not interact with any of the negative emotionality variables to predict any of the 24-month neurocognitive outcomes, nor did maternal flexibility interact with mother-rated difficult temperament to predict the visual-spatial processing outcomes. Copyright A 2011 Elsevier Inc. All rights reserved
SN - 1934-8800
AD - Department of Human Development and Family Studies, University of Wisconsin, Madison, WI, United States. jedilworth@wisc.edu
UR - 22209050
ER -

TY - JOUR
ID - 167
T1 - Exposure to open-fire cooking and cognitive performance in children
A1 - Munroe,R.L.
A1 - Gauvain,M.
Y1 - 2012///
N1 - Munroe, Robert L. Gauvain, Mary
International journal of environmental health research
9106628, dus
IM
Journal Article. Research Support, U.S. Gov't, Non-P.H.S.
English
KW - Medline
KW - Air Pollution

KW - Indoor/ae [Adverse Effects]
KW - Belize/ep [Epidemiology]
KW - Child
KW - Preschool
KW - Cognition/de [Drug Effects]
KW - Cooking
KW - Environmental Exposure/ae [Adverse Effects]
KW - Female
KW - Fires
KW - Humans
KW - Kenya/ep [Epidemiology]
KW - Male
KW - Memory/de [Drug Effects]
KW - Nepal/ep [Epidemiology]
KW - Pattern Recognition
KW - Visual/de [Drug Effects]
KW - Play and Playthings
KW - Samoa/ep [Epidemiology]
KW - Smoke/ae [Adverse Effects]
KW - Wood
RP - NOT IN FILE
SP - 156
EP - 164
JF - International Journal of Environmental Health Research
JA - Int.J Environ.Health Res.
VL - 22
IS - 2
CY - England
N2 - We reexamined field data on cognitive performance in light of recent research that shows open-fire cooking--with its emission of harmful substances--to pose a risk to healthy physical development. Tests of three- to nine-year-old children in four communities around the world yielded evidence concerning block-building skills, memory, and the discernment of embedded figures. Naturalistic observations of these children were also undertaken in everyday settings. Open-fire cooking (as opposed to cooking on kerosene stoves) was associated with both lower cognitive performance and less frequent structured play at all ages. Although these correlational results do not reveal causal mechanisms, they are consistent with ideas about negative developmental consequences of exposure to open-fire cooking and suggest that research is needed on the effect on brain development of practices involving production of indoor smoke
SN - 1369-1619
AD - Pitzer College, Claremont, California, USA
UR - 22128885
ER -

TY - JOUR
ID - 168
T1 - Societal values and policies may curtail preschool children's physical activity in child care centers
A1 - Copeland,K.A.
A1 - Sherman,S.N.
A1 - Kendeigh,C.A.
A1 - Kalkwarf,H.J.
A1 - Saelens,B.E.
Y1 - 2012/02//
N1 - Copeland, Kristen A. Sherman, Susan N. Kendeigh, Cassandra A. Kalkwarf, Heidi J. Saelens, Brian E
Pediatrics
oxv, 0376422
AIM, IM

Journal Article. Research Support, N.I.H., Extramural. Research Support, Non-U.S. Gov't
English
KW - Medline
KW - Child Day Care Centers
KW - Child
KW - Preschool
KW - Female
KW - Focus Groups
KW - Health Surveys
KW - Humans
KW - Male
KW - Motor Activity
KW - Ohio
KW - Play and Playthings
KW - Public Policy
KW - Safety Management
KW - Sedentary Lifestyle
KW - Social Values
KW - Wounds and Injuries/et [Etiology]
KW - Wounds and Injuries/pc [Prevention & Control]
RP - NOT IN FILE
SP - 265
EP - 274
JA - Pediatrics
VL - 129
IS - 2
CY - United States
N2 - BACKGROUND AND OBJECTIVES: Three-fourths of US preschool-age children are in child care centers. Children are primarily sedentary in these settings, and are not meeting recommended levels of physical activity. Our objective was to identify potential barriers to children's physical activity in child care centers. METHODS: Nine focus groups with 49 child care providers (55% African American) were assembled from 34 centers (inner-city, suburban, Head Start, and Montessori) in Cincinnati, Ohio. Three coders independently analyzed verbatim transcripts for themes. Data analysis and interpretation of findings were verified through triangulation of methods. RESULTS: We identified 3 main barriers to children's physical activity in child care: (1) injury concerns, (2) financial, and (3) a focus on "academics." Stricter licensing codes intended to reduce children's injuries on playgrounds rendered playgrounds less physically challenging and interesting. In addition, some parents concerned about potential injury, requested staff to restrict playground participation for their children. Small operating margins of most child care centers limited their ability to install abundant playground equipment. Child care providers felt pressure from state mandates and parents to focus on academics at the expense of gross motor play. Because children spend long hours in care and many lack a safe place to play near their home, these barriers may limit children's only opportunity to engage in physical activity. CONCLUSIONS: Societal priorities for young children--safety and school readiness--may be hindering children's physical development. In designing environments that optimally promote children's health and development, child advocates should think holistically about potential unintended consequences of policies
SN - 1098-4275
AD - Division of General and Community Pediatrics, Cincinnati Children's Hospital Medical Center, Cincinnati, OH 45229, USA. kristen.copeland@cchmc.org
UR - 22218842
ER -

TY - JOUR
ID - 169
T1 - "We don't need no education": Video game preferences, video game motivations, and aggressiveness among adolescent boys of different educational ability levels
A1 - Nije,Bijvank M.
A1 - Konijn,E.A.

A1 - Bushman,B.J.
Y1 - 2012/02//
N1 - Nije Bijvank, Marije. Konijn, Elly A. Bushman, Brad J
Journal of adolescence
hwt, 7808986
IM
Journal Article
English
KW - Medline
KW - Adolescent
KW - Adolescent Behavior/px [Psychology]
KW - Aggression/px [Psychology]
KW - Child
KW - Educational Status
KW - Humans
KW - Male
KW - Motivation
KW - Risk Factors
KW - Video Games/px [Psychology]
KW - Violence/px [Psychology]
RP - NOT IN FILE
SP - 153
EP - 162
JF - Journal of Adolescence
JA - J Adolesc
VL - 35
IS - 1
CY - England
N2 - This research focuses on low educational ability as a risk factor for aggression and violent game play. We propose that boys of lower educational ability are more attracted to violent video games than other boys are, and that they are also higher in trait aggressiveness and sensation seeking. Participants were Dutch boys in public schools (N = 830, age-range 11-17). In the Netherlands, standardized tests are used to place students into lower, medium, and higher educational ability groups. Results showed that boys in the lower educational ability group preferred to play violent, stand-alone games, identified more with video game characters, and perceived video games to be more realistic than other boys did. Lower levels of education were also related to higher levels of aggressiveness and sensation seeking. Higher educational ability boys preferred social, multiplayer games. Within a risk and resilience model, boys with lower educational ability are at greater risk for aggression. Copyright 2011 The Foundation for Professionals in Services for Adolescents. All rights reserved
SN - 1095-9254
AD - Department of Communication Science, School of Social Sciences, VU University Amsterdam, Amsterdam, The Netherlands. m.nijebijvank@saxion.nl
UR - 21529925
ER -

TY - JOUR
ID - 170
T1 - Security of attachment and quality of mother-toddler social interaction in a high-risk sample
A1 - Haltigan,J.D.
A1 - Lambert,B.L.
A1 - Seifer,R.
A1 - Ekas,N.V.
A1 - Bauer,C.R.
A1 - Messinger,D.S.
Y1 - 2012/02//
N1 - Haltigan, John D. Lambert, Brittany L. Seifer, Ronald. Ekas, Naomi V. Bauer, Charles R. Messinger, Daniel S

Infant behavior & development
7806016
IM
Comparative Study. Journal Article. Multicenter Study. Research Support, N.I.H., Extramural
English
KW - Medline
KW - Adolescent
KW - Adult
KW - Child
KW - Preschool
KW - Female
KW - Humans
KW - Interpersonal Relations
KW - Male
KW - Middle Aged
KW - Mother-Child Relations
KW - Object Attachment
KW - Play and Playthings/px [Psychology]
KW - Risk-Taking
KW - Young Adult
RP - NOT IN FILE
SP - 83
EP - 93
JF - Infant Behavior & Development
JA - Infant behav
VL - 35
IS - 1
CY - United States
N2 - The quality of children's social interactions and their attachment security with a primary caregiver are two widely studied indices of socioemotional functioning in early childhood. Although both Bowlby and Ainsworth suggested that the parent-child interactions underlying the development of attachment security could be distinguished from other aspects of parent-child interaction (e.g., play), relatively little empirical research has examined this proposition. The aim of the current study was to explore this issue by examining concurrent relations between toddler's attachment security in the Strange Situation Procedure and quality of mother-child social interaction in a high-risk sample of toddlers characterized by prenatal cocaine exposure and low levels of maternal education. Analyses of variance suggested limited relations between attachment security and quality of social interaction. Further research examining the interrelations among various components of the parent-child relationship is needed. Copyright 2011 Elsevier Inc. All rights reserved
SN - 1934-8800
AD - University of North Carolina at Greensboro, USA. jdhaltig@uncg.edu
UR - 21981901
ER -

TY - JOUR
ID - 171
T1 - Physical activity and screen time behaviour in metropolitan, regional and rural adolescents: a -sectional study of Australians aged 9-16 years
A1 - Dollman,J.
A1 - Maher,C.
A1 - Olds,T.S.
A1 - Ridley,K.
Y1 - 2012/01//
N1 - Dollman, James. Maher, Carol. Olds, Tim S. Ridley, Kate
Journal of science and medicine in sport / Sports Medicine Australia
c54, 9812598
IM

Journal Article. Research Support, Non-U.S. Gov't
English
KW - Medline
KW - Adolescent
KW - Australia
KW - Child
KW - Cross-Sectional Studies
KW - Female
KW - Humans
KW - Male
KW - Motor Activity/ph [Physiology]
KW - Rural Population
KW - Sedentary Lifestyle
KW - Urban Population
RP - NOT IN FILE
SP - 32
EP - 37
JF - Journal of Science & Medicine in Sport
JA - J Sci Med Sport
VL - 15
IS - 1
CY - Australia
N2 - OBJECTIVES: While there is consistent evidence that rural adults in Australia are less active than their urban counterparts, studies relating geographical remoteness to activity patterns in Australian adolescents have yielded inconsistent results. The aim of this study was to describe objectively and subjectively measured patterns of physical activity and sedentary behaviours across remoteness categories in a representative sample of 9-16 year old Australians. DESIGN: Cross-sectional observational study. METHODS: 2071 Australian adolescents provided self-report use of time data on four days and wore a pedometer for at least 6 days within the 2007 Australian National Children's Nutrition and Physical Activity Survey. Comparisons of activity patterns were made across four objectively-determined remoteness categories (Major City, Inner Regional, Outer Regional and Remote), adjusting for household income, parental education and age. RESULTS: Adolescents living in major cities self-reported 11-29 min less moderate to vigorous physical activity each day than their counterparts living in geographically more remote areas, and took 150-850 fewer steps each day. While there were no differences in time spent in sport or active transport, differences in free play participation were significant. Males in major cities also reported higher levels of screen time. Differences were somewhat more marked among males than among females. CONCLUSIONS: Activity levels among Australian adolescents show contrasting patterns of geographical differences to those found in Australian adults. Higher levels of free play among rural Australian adolescents may be due to more available space and less fear of traffic and stranger risks. Copyright 2011 Sports Medicine Australia. Published by Elsevier Ltd. All rights reserved
SN - 1878-1861
AD - Nutritional Physiology Research Centre, University of South Australia, Adelaide, South Australia, Australia. james.dollman@unisa.edu.au
UR - 21742553
ER -

TY - JOUR
ID - 172
T1 - Imperial boyhood: piracy and the play ethic
A1 - Deane,B.
Y1 - 2011///
N1 - Deane, Bradley
Victorian studies
9426344
Q

Historical Article. Journal Article
English
KW - Medline
KW - Aggression/ph [Physiology]
KW - Aggression/px [Psychology]
KW - Child
KW - Colonialism/hi [History]
KW - Colonialism
KW - Competitive Behavior/ph [Physiology]
KW - Competitive Behavior
KW - Fantasy
KW - Great Britain/eh [Ethnology]
KW - Hierarchy
KW - Social/hi [History]
KW - History
KW - 19th Century
KW - 20th Century
KW - Humans
KW - Literature/hi [History]
KW - Literature
KW - Male
KW - Masculinity/hi [History]
KW - Morals
KW - Play and Playthings/px [Psychology]
KW - Play and Playthings
KW - Power (Psychology)
RP - NOT IN FILE
SP - 689
EP - 714
JF - Victorian Studies
JA - Vic.stud.
VL - 53
IS - 4
CY - United States
N2 - Representations of perpetual boyhood came to fascinate the late Victorians, partly because such images could naturalize a new spirit of imperial aggression and new policies of preserving power. This article traces the emergence of this fantasy through a series of stories about the relationship of the boy and the pirate, figures whose opposition in mid-Victorian literature was used to articulate the moral legitimacy of colonialism, but who became doubles rather than antitheses in later novels, such as R.L. Stevenson's "Treasure Island" and Joseph Conrad's "Lord Jim." Masculine worth needed no longer to be measured by reference to transcendent, universal laws, but by a morally flexible ethic of competitive play, one that bound together boyishness and piracy in a satisfying game of international adventure
SN - 0042-5222
AD - University of Minnesota, Morris
UR - 22355829
ER -

TY - JOUR
ID - 173
T1 - Where they live, how they play: neighborhood greenness and outdoor physical activity among preschoolers
A1 - Grigsby-Toussaint,D.S.
A1 - Chi,S.H.
A1 - Fiese,B.H.
A1 - STRONG Kids Project Writing Group.
Y1 - 2011///

N1 - Grigsby-Toussaint, Diana S. Chi, Sang-Hyun. Fiese, Barbara H. STRONG Kids Project Writing Group
International journal of health geographics
101152198
IM
Journal Article. Research Support, Non-U.S. Gov't
English
KW - Medline
KW - Child Behavior
KW - Child Welfare
KW - Child
KW - Preschool
KW - Cohort Studies
KW - Environment
KW - Exercise/ph [Physiology]
KW - Female
KW - Humans
KW - Linear Models
KW - Male
KW - Motor Activity/ph [Physiology]
KW - Multivariate Analysis
KW - Parent-Child Relations
KW - Play and Playthings
KW - Residence Characteristics
KW - Risk Assessment
KW - Time Factors
KW - United States
KW - Urban Population
RP - NOT IN FILE
SP - 66
JF - International Journal of Health Geographics [Electronic Resource]
JA - Int.J Health Geogr.
VL - 10
CY - England
N2 - BACKGROUND: Emerging empirical evidence suggests exposure to "green" environments may encourage higher levels of physical activity among children. Few studies, however, have explored this association exclusively in pre-school aged children in the United States. We examined whether residing in neighborhoods with higher levels of greenness was associated with higher levels of outdoor physical activity among preschoolers. In addition, we also explored whether outdoor playing behaviors (e.g., active vs. quiet) were influenced by levels of neighborhood greenness independent of demographic and parental support factors. RESULTS: Higher levels of neighborhood greenness as measured by the Normalized Difference Vegetation Index (NDVI) was associated with higher levels of outdoor playing time among preschool-aged children in our sample. Specifically, a one unit increase in neighborhood greenness increased a child's outdoor playing time by approximately 3 minutes. A dose-response relationship was observed between increasing levels of parental support for physical activity (e.g., time spent playing with children) and child outdoor physical activity ($p < 0.01$). CONCLUSIONS: Consistent with previous studies, neighborhood greenness influences physical activity behavior. However, for preschoolers, parental involvement may be more critical for improving physical activity levels
SN - 1476-072X
AD - Department of Kinesiology and Community Health, Division of Nutritional Sciences, University of Illinois at Urbana Champaign, USA. dgrigs1@illinois.edu
UR - 22165919
ER -

TY - JOUR
ID - 174
T1 - Home, school, and neighborhood environment factors and youth physical activity

A1 - Millstein,R.A.
A1 - Strobel,J.
A1 - Kerr,J.
A1 - Sallis,J.F.
A1 - Norman,G.J.
A1 - Durant,N.
A1 - Harris,S.
A1 - Saelens,B.E.
Y1 - 2011/11//
N1 - Millstein, Rachel A. Strobel, Joe. Kerr, Jacqueline. Sallis, James F. Norman, Gregory J. Durant, Nefertiti. Harris, Sion. Saelens, Brian E
Pediatric exercise science
8909729
IM
Journal Article
English
KW - Medline
KW - Adolescent
KW - Child
KW - Preschool
KW - Data Collection
KW - Environment Design
KW - Female
KW - Humans
KW - Linear Models
KW - Male
KW - Motor Activity
KW - Multivariate Analysis
KW - Parents
KW - Play and Playthings
KW - Public Facilities
KW - Residence Characteristics
KW - Safety
KW - Schools
KW - Socioeconomic Factors
KW - Sports Equipment
RP - NOT IN FILE
SP - 487
EP - 503
JF - Pediatric Exercise Science
JA - Pediatr Exerc.Sci
VL - 23
IS - 4
CY - United States
N2 - This study examined the contributions of home, school, and neighborhood factors related to youth physical activity (PA). Adolescents (ages 12-18; N = 137) and parents of younger children (ages 5-11; N = 104) from three US metropolitan areas completed surveys. Youth PA was estimated from six items assessing overall physical activity. Bivariate analyses between environment factors and PA were significant correlations in each environmental setting for adolescents (r's:0.16-0.28), but for parents of children, only for the home and neighborhood settings (r's: 0.14-0.39). For adolescents, pieces of equipment at home, family recreation membership, equipment at school, and neighborhood aesthetics explained 15.8% of variance in PA. For younger children (based on parent report), pieces of equipment at home, neighborhood traffic safety, walking/cycling facilities, and street connectivity explained 21.4% of the variance in PA. Modifiable factors like increasing access to equipment at home and school, and improving neighborhood aesthetics may impact youth PA. To optimize explanation of youth PA, factors from multiple environments need to be considered
SN - 1543-2920

AD - SDSU/UCSD Joint Doctoral Program in Clinical Psychology, San Diego, CA, USA
UR - 22109776
ER -

TY - JOUR
ID - 175
T1 - High frequency of rare copy number variants affecting functionally related genes in patients with structural brain malformations
A1 - Kariminejad,R.
A1 - Lind-Thomsen,A.
A1 - Tumer,Z.
A1 - Erdogan,F.
A1 - Ropers,H.H.
A1 - Tommerup,N.
A1 - Ullmann,R.
A1 - Moller,R.S.
Y1 - 2011/12//
N1 - Kariminejad, Roxana. Lind-Thomsen, Allan. Tumer, Zeynep. Erdogan, Fikret. Ropers, Hans H. Tommerup, Niels. Ullmann, Reinhard. Moller, Rikke S
Human mutation
brd, 9215429
IM
Journal Article. Research Support, Non-U.S. Gov't
English
KW - Medline
KW - Agenesis of Corpus Callosum/ge [Genetics]
KW - Agenesis of Corpus Callosum/ra [Radiography]
KW - Brain/ra [Radiography]
KW - Child
KW - Preschool
KW - Cohort Studies
KW - Comparative Genomic Hybridization
KW - DNA Copy Number Variations/ge [Genetics]
KW - Epilepsy/ge [Genetics]
KW - Epilepsy/ra [Radiography]
KW - Female
KW - Gene Dosage/ge [Genetics]
KW - Gene Frequency
KW - Humans
KW - Intellectual Disability/ge [Genetics]
KW - Intellectual Disability/ra [Radiography]
KW - Magnetic Resonance Imaging
KW - Male
KW - Nervous System Malformations/ge [Genetics]
KW - Nervous System Malformations/ra [Radiography]
KW - Phenotype
KW - Proteins/ge [Genetics]
KW - Tomography Scanners
KW - X-Ray Computed
RP - NOT IN FILE
SP - 1427
EP - 1435
JF - Human Mutation
JA - Hum.Mutat.
VL - 32
IS - 12

CY - United States
N2 - During the past years, significant advances have been made in our understanding of the development of the human brain, and much of this knowledge comes from genetic studies of disorders associated with abnormal brain development. We employed array-comparative genomic hybridization (CGH) to investigate copy number variants (CNVs) in a cohort of 169 patients with various structural brain malformations including lissencephaly, polymicrogyria, focal cortical dysplasia, and corpus callosum agenesis. The majority of the patients had intellectual disabilities (ID) and suffered from symptomatic epilepsy. We detected at least one rare CNV in 38 patients (22.5%). All genes located within the rare CNVs were subjected to enrichment analysis for specific Gene Ontology Terms or Kyoto Encyclopedia of Genes and Genomes pathways and to protein-protein network analysis. Based on these analyses, we propose that genes involved in "axonal transport," "cation transmembrane transporter activity," and the "c-Jun N-terminal kinase (JNK) cascade" play a significant role in the etiology of brain malformations. This is to the best of our knowledge the first systematic study of CNVs in patients with structural brain malformations and our data show that CNVs play an important role in the etiology of these malformations, either as direct causes or as genetic risk factors. 2011 Wiley Periodicals, Inc
SN - 1098-1004
AD - Kariminejad-Najmabadi Pathology and Genetics Center, Tehran, Iran
UR - 21882292
ER -

TY - JOUR
ID - 176
T1 - Association of maternal and child blood lead and hemoglobin levels with maternal perceptions of parenting their young children
A1 - Kordas,K.
A1 - Ardoino,G.
A1 - Ciccariello,D.
A1 - Manay,N.
A1 - Ettinger,A.S.
A1 - Cook,C.A.
A1 - Queirolo,E.I.
Y1 - 2011/12//
N1 - Kordas, Katarzyna. Ardoino, Graciela. Ciccariello, Daniela. Manay, Nelly. Ettinger, Adrienne S. Cook, Casey A. Queirolo, Elena I
Neurotoxicology
oap, 7905589
IM
Journal Article
English
KW - Medline
KW - Adult
KW - Anemia/bl [Blood]
KW - Anemia/px [Psychology]
KW - Biological Markers/bl [Blood]
KW - Child Behavior
KW - Child Development
KW - Child
KW - Preschool
KW - Depression/bl [Blood]
KW - Depression/px [Psychology]
KW - Emotions
KW - Environmental Pollutants/ae [Adverse Effects]
KW - Environmental Pollutants/bl [Blood]
KW - Exploratory Behavior
KW - Factor Analysis
KW - Statistical
KW - Female

KW - Hemoglobins/an [Analysis]
KW - Humans
KW - Infant
KW - Lead/ae [Adverse Effects]
KW - Lead/bl [Blood]
KW - Lead Poisoning
KW - Nervous System
KW - Adult/bl [Blood]
KW - Adult/di [Diagnosis]
KW - Adult/px [Psychology]
KW - Childhood/bl [Blood]
KW - Childhood/di [Diagnosis]
KW - Childhood/px [Psychology]
KW - Male
KW - Maternal Behavior
KW - Mother-Child Relations
KW - Mothers/px [Psychology]
KW - Multivariate Analysis
KW - Neuropsychological Tests
KW - Parenting
KW - Perception
KW - Play and Playthings
KW - Questionnaires
KW - Risk Assessment
KW - Risk Factors
KW - Stress
KW - Psychological/bl [Blood]
KW - Psychological/px [Psychology]
KW - Uruguay
KW - Young Adult
RP - NOT IN FILE
SP - 693
EP - 701
JF - Neurotoxicology
VL - 32
IS - 6
CY - Netherlands
N2 - Biological and psychosocial factors affect child development and behavior. Whereas biological underpinnings behind the neurotoxic effects of lead are studied extensively, the effects of psychosocial factors contributing to poor behavioral outcomes in lead-exposed children are not well understood. Parental attributes and practices may moderate or mediate the effects of lead on children's behavioral outcomes. We investigated the hypothesis that maternal and child lead and hemoglobin levels are associated with maternal perceptions of their parenting. Specifically, we hypothesized that children with higher blood lead (BLL) and lower hemoglobin concentrations would be associated with poorer maternal self-assessments of their parenting skills or the mother-child relationship. Children aged 13-55 months and their mothers (n=109) were recruited from among the participants of a previous lead and anemia screening study and from preschools in Montevideo, Uruguay. The mother-child pair attended two study visits: one to collect biological samples and answer demographic and child questionnaires, including statements regarding parenting; and a second to evaluate maternal IQ, depression and stress, and child development. Of the children, 51.6% had blood lead concentrations (BLLs) >= 5 ug/dL, 18.0% had anemia, and 8% had both conditions. Among mothers, 48.4% had BLLs >= 5 ug/dL, 16.0% had anemia, and 11% had both. BLLs >= 5 ug/dL in mother or child were associated with lower maternal perceptions of being skilled at discipline (p<0.05). Maternal anemia was associated with lower likelihood that mothers would let their children explore and play (p<0.05), whereas child anemia was associated with maternal perception of lower emotional support (p<0.01). In addition to shared environmental exposures, parenting and family interactions need to be considered as potentially contributing factors to poorer outcomes in lead-exposed children. Copyright A 2011 Elsevier Inc. All rights reserved

SN - 1872-9711
AD - Department of Nutritional Sciences, Pennsylvania State University, University Park, PA 16802, USA. kxk48@psu.edu
UR - 21925208
ER -

TY - JOUR
ID - 178
T1 - Development and results of a new methodology to perform focus group with preschool children on their beliefs and attitudes on physical activity
A1 - Cammisa,M.
A1 - Montrone,R.
A1 - Caroli,M.
Y1 - 2011/10//
N1 - Cammisa, Maria. Montrone, Rosa. Caroli, Margherita
International journal of pediatric obesity : IJPO : an official journal of the International Association for the Study of Obesity
101256330
IM
Journal Article. Research Support, Non-U.S. Gov't
English
KW - Medline
KW - Art
KW - Child
KW - Preschool
KW - Environment
KW - Faculty
KW - Female
KW - Focus Groups/mt [Methods]
KW - Health Behavior
KW - Health Knowledge
KW - Attitudes
KW - Practice
KW - Humans
KW - Male
KW - Motor Activity
KW - Parents
KW - Program Development/mt [Methods]
KW - Questionnaires
RP - NOT IN FILE
SP - 22
EP - 27
JF - International Journal of Pediatric Obesity
JA - Int.J Pediatr Obes.
VL - 6 Suppl 2
CY - England
N2 - The aim of this study is to develop and test a method to perform focus groups (FGs) and to elicit the subjective views of preschool-age children on physical activity and perceived kindergarten barriers to practice it. FGs have been held in three different kindergarten classes with 49 children who were 4-5 years old. Children were asked to draw themselves in their preferred way of playing and were asked few questions about their drawings to understand their behaviours and ideas. In class A and B, 67% and 75% of the children, respectively, drew sedentary plays (table and impersonation games). Children referred that the main obstacle to perform active games outside home/kindergarten was the parents' and teachers' perceived risk that they could be hurt or catch a cold. The children would like to have more table games in the kindergarten. 81% of children in class C drew active group games. All these children were well satisfied with their kindergarten environment and did not refer to any adults' fear regarding active play. This class teacher spent a lot of time to develop children's motor

abilities through active games and often used the garden to let the children to play freely. The use of drawings to understand children's habits on physical activity has turned out to be a reliable and easy tool in preschool children. The different results obtained in the two children groups show the need to change the beliefs and the behaviours of teachers and parents who seem to be non-architectural "invisible" barriers to be knocked down. This protocol has been developed by ASL Brindisi within the framework of PERISCOPE's objective to develop new methodologies
SN - 1747-7174
AD - Nutrition Unit, Department of Prevention, Azienda Sanitaria Locale Brindisi, Brindisi, Italy
UR - 21923291
ER -

TY - JOUR
ID - 179
T1 - Update on environmental risk factors for attention-deficit/hyperactivity disorder. [Review]
A1 - Froehlich,T.E.
A1 - Anixt,J.S.
A1 - Loe,I.M.
A1 - Chirdkiatgumchai,V.
A1 - Kuan,L.
A1 - Gilman,R.C.
Y1 - 2011/10//
N1 - Froehlich, Tanya E. Anixt, Julia S. Loe, Irene M. Chirdkiatgumchai, Vilawan. Kuan, Lisa. Gilman, Richard C
Current psychiatry reports
100888960, dym
IM
Journal Article. Research Support, N.I.H., Extramural. Research Support, Non-U.S. Gov't. Review
English
KW - Medline
KW - Attention Deficit Disorder with Hyperactivity/et [Etiology]
KW - Child
KW - Preschool
KW - Environmental Exposure/ae [Adverse Effects]
KW - Female
KW - Humans
KW - Life Style
KW - Pregnancy
KW - Prenatal Exposure Delayed Effects
KW - Risk Factors
RP - NOT IN FILE
SP - 333
EP - 344
JF - Current Psychiatry Reports
JA - Curr.Psychiatry Rep.
VL - 13
IS - 5
CY - United States
N2 - Attention-deficit/hyperactivity disorder (ADHD) is a prevalent neurobehavioral disorder affecting 5% to 10% of children. Although considered to be a highly familial disorder, ADHD heritability estimates of 60% to 80% highlight the considerable role that environmental factors may still play in disorder susceptibility. Proposed ADHD environmental risk factors include prenatal substance exposures, heavy metal and chemical exposures, nutritional factors, and lifestyle/psychosocial factors. This paper reviews the literature published in 2010 investigating the association between environmental risk factors and ADHD or related symptomatology. Sources of risk factor exposure and the proposed mechanism by which each exposure is linked to ADHD-related neurobehavioral changes are also reported. Methodologic limitations of the current literature are discussed, and

guidelines for future study are proposed. An improved understanding of the role that environmental factors play in ADHD etiology is critical to future ADHD prevention efforts
SN - 1535-1645
AD - Cincinnati Children's Hospital, Cincinnati, OH 45229, USA. tanya.froehlich@cchmc.org
UR - 21779823
ER -

TY - JOUR
ID - 180
T1 - Observations of caregiver supervision of children at beaches: identification of factors associated with high supervision
A1 - Petrass,L.A.
A1 - Blitvich,J.D.
A1 - Finch,C.F.
Y1 - 2011/08//
N1 - Petrass, Lauren A. Blitvich, Jennifer D. Finch, Caroline F
Injury prevention : journal of the International Society for Child and Adolescent Injury Prevention
crz, 9510056
IM
Journal Article. Research Support, Non-U.S. Gov't
English
KW - Medline
KW - Adolescent
KW - Adult
KW - Australia
KW - Bathing Beaches
KW - Caregivers/sn [Statistics & Numerical Data]
KW - Child
KW - Preschool
KW - Female
KW - Humans
KW - Infant
KW - Linear Models
KW - Male
KW - Middle Aged
KW - Play and Playthings
KW - Questionnaires
KW - Risk Factors
KW - Safety
KW - Social Environment
KW - Young Adult
RP - NOT IN FILE
SP - 244
EP - 249
JA - Injury Prevention
VL - 17
IS - 4
CY - England
N2 - OBJECTIVE: To identify independent factors associated with caregiver supervision levels at beaches. METHODS: Children (aged 1-14 years) engaged in beach play and their caregivers were observed at Australian beaches during September-April, 2008/09. Caregiver, child, and environmental factor data were collected and recorded on a validated observation instrument. The main outcome of interest was a continuous measure of supervision. After adjusting for potential clustering by beach/state, multivariable linear regression was used to identify independent factors associated with caregiver supervision. RESULTS: Four independent predictors of supervision and one interaction effect (child age by the caregiver and child position on beach) were identified. Caregivers who were supervising from a different position to where the child was playing were less likely to

provide high supervision, as were older caregivers (>35 years) compared to younger caregivers (<=34 years). Compared to children playing alone, children playing with one, or two or more others were less likely to receive high supervision, and children aged 1-4 years were likely to receive higher supervision than older children. CONCLUSION: This study provides new knowledge about underlying factors associated with the level of caregiver supervision in beach settings. Future studies should continue to explore independent predictors of supervision so that effective programmes, which focus on caregiver supervision, can be targeted to address poor supervision practices
SN - 1475-5785
AD - School of Human Movement and Sport Sciences, University of Ballarat, Mt Helen, Ballarat, Victoria, Australia. l.petrass@ballarat.edu.au
UR - 21335448
ER -

TY - JOUR
ID - 181
T1 - Testing and beyond: strategies and tools for evaluating and assessing infants and toddlers
A1 - Crais,E.R.
Y1 - 2011/07//
N1 - Crais, Elizabeth R
Language, speech, and hearing services in schools
0323431
IM
Journal Article
English
KW - Medline
KW - Child
KW - Preschool
KW - Cooperative Behavior
KW - Evidence-Based Practice
KW - Guidelines as Topic
KW - Humans
KW - Infant
KW - Language Development Disorders/di [Diagnosis]
KW - Language Development Disorders/th [Therapy]
KW - Language Tests
KW - Mass Screening
KW - Multilingualism
KW - Phonetics
KW - Play and Playthings
KW - Professional-Family Relations
KW - Risk Factors
KW - Semantics
RP - NOT IN FILE
SP - 341
EP - 364
JF - Language, Speech & Hearing Services in the Schools
JA - LANG SPEECH HEAR SERV SCH
VL - 42
IS - 3
CY - United States
N2 - PURPOSE: This article is a condensation of the recent American Speech-Language-Hearing Association (ASHA) document entitled Roles and Responsibilities of Speech-Language Pathologists in Early Intervention: Guidelines (ASHA, 2008). The article presents information on recommended and evidence-based practices related to the screening, evaluation, and assessment of infants and toddlers with, or at risk for, communication deficits. It is intended to be used by clinicians to enhance their practices. METHOD: The Guidelines document was developed using the available research evidence, expert clinical perspectives, and, when possible, family

perspectives related to the screening, evaluation, and assessment process. The current article includes key definitions and an overview of recommended practices in screening, evaluation, assessment, and results interpretation. IMPLICATIONS: The article provides important and timely information for professionals and families related to the screening, evaluation, and assessment of infants and toddlers with, or at risk for, communication deficits. Speech-language pathologists, other professionals, and family members can use the information to implement recommended and evidence-based practices when screening, evaluating, and assessing young children for communication deficits
SN - 1558-9129
AD - University of North Carolina at Chapel Hill, NC, USA. bcrais@med.unc.edu
UR - 20679409
ER -

TY - JOUR
ID - 182
T1 - "I think he is in his room playing a video game": parental supervision of young elementary-school children at home
A1 - Morrongiello,B.A.
A1 - Kane,A.
A1 - Zdzieborski,D.
Y1 - 2011/07//
N1 - Morrongiello, Barbara A. Kane, Alexa. Zdzieborski, Daniel
Journal of pediatric psychology
jl8, 7801773
IM
Journal Article. Research Support, Non-U.S. Gov't
English
KW - Medline
KW - Accident Prevention
KW - Accidents
KW - Adult
KW - Child
KW - Female
KW - Humans
KW - Male
KW - Parent-Child Relations
KW - Parents
KW - Play and Playthings
KW - Prospective Studies
KW - Questionnaires
KW - Risk
KW - Risk-Taking
KW - Safety
KW - Social Environment
KW - Wounds and Injuries
RP - NOT IN FILE
SP - 708
EP - 717
JF - Journal of Pediatric Psychology
JA - J Pediatr Psychol.
VL - 36
IS - 6
CY - United States
N2 - OBJECTIVES: Using a prospective design, this research examined supervision of young elementary-school children at home and how this relates to child injury, parent permissiveness, and children's risk-taking propensity. METHODS: Mothers reported children's history of injuries and recorded home supervision over a 2-month interval on a weekly basis. Children independently completed diaries about daily events, including

injuries. RESULTS: Children spent 24% of time alone, mostly supervised intermittently or not at all. Parent permissiveness was associated with increased time unsupervised, while children's risk-taking propensity was associated with decreased time unsupervised. Greater direct supervision was associated with fewer injuries, while more indirect and non-supervision time emerged as risk factors and were associated with more frequent injury. CONCLUSIONS: These results extend those from preschool-aged children and suggest that caregiver supervision influences risk of injury across a broad age range throughout childhood. Implications for children's safety are discussed
SN - 1465-735X
AD - Psychology Department, University of Guelph, Ontario, Canada. bmorrong@uoguelph.ca
UR - 20693264
ER -

TY - JOUR
ID - 183
T1 - Playground renovations and quality at public parks in Boston,Massachusetts, 1996-2007
A1 - Barrett,J.L.
A1 - Hannon,C.
A1 - Keefe,L.
A1 - Gortmaker,S.L.
A1 - Cradock,A.L.
Y1 - 2011/07//
N1 - Barrett, Jessica L. Hannon, Cynthia. Keefe, Linda. Gortmaker, Steven L. Cradock, Angie L
Preventing chronic disease
101205018
IM
Journal Article. Research Support, U.S. Gov't, P.H.S.
English
KW - Medline
KW - Activities of Daily Living
KW - Adolescent
KW - Architectural Accessibility/st [Standards]
KW - Boston
KW - Child
KW - Humans
KW - Play and Playthings
KW - Retrospective Studies
KW - Safety Management/st [Standards]
RP - NOT IN FILE
SP - A72
JF - Preventing Chronic Disease
JA - Prev.Chronic Dis.
VL - 8
IS - 4
CY - United States
N2 - INTRODUCTION: Recreational and transportation infrastructure can promote physical activity among children and adolescents. The Play Across Boston community-based research project sought to estimate and compare playground renovation rates across Boston areas before and after a playground quality assessment, to describe changes in playground quality among a subset of parks, and to document features of local transportation infrastructure around parks. METHODS: We used an observational pretest-posttest design to estimate playground renovation rates among 103 city-operated parks. Renovation rates were calculated on the basis of annual city Parks Department capital budgets from fiscal years 1996 through 2007. We used the same design to describe changes between a 2000 to 2001 baseline assessment of playground quality and a 2007 follow-up measured via observation of a subsample of 18 low-scoring parks in disadvantaged areas. We used (2) analysis to compare percentages of playgrounds renovated across city areas before and after baseline assessment, logistic regression analysis to calculate odds ratios comparing renovation rates after baseline by city area, and paired t tests to compare playground quality at baseline and follow-up. RESULTS: Overall playground

renovation rates before (29%) and after (34%) baseline assessment were similar. Parks scoring low on playground quality at baseline were renovated after baseline at a higher rate than high-scoring playgrounds. After accounting for baseline playground quality, parks in disadvantaged areas were renovated at a rate similar to those in other areas. Playground quality scores improved between baseline (mean, 38.3; 95% confidence interval, 35.3-41.3) and 2007 in a subsample of previously low-scoring parks in disadvantaged areas. CONCLUSION: The findings of the 2007 follow-up assessment indicate an equitable rate of playground renovation across city areas according to need
SN - 1545-1151
AD - Department of Society, Human Development, and Health, Harvard School of Public Health, Landmark Center, 401 Park Drive, Boston, MA 02215, USA. jbarrett@hsph.harvard.edu
UR - 21672396
ER -

TY - JOUR
ID - 184
T1 - Investigating children's physical activity and sedentary behavior using ecological momentary assessment with mobile phones
A1 - Dunton,G.F.
A1 - Liao,Y.
A1 - Intille,S.S.
A1 - Spruijt-Metz,D.
A1 - Pentz,M.
Y1 - 2011/06//
N1 - Dunton, Genevieve F. Liao, Yue. Intille, Stephen S. Spruijt-Metz, Donna. Pentz, Maryann
Obesity (Silver Spring, Md.)
101264860
IM
Clinical Trial. Journal Article. Research Support, N.I.H., Extramural. Research Support, Non-U.S. Gov't. Validation Studies
English
KW - Medline
KW - Adolescent
KW - Adolescent Behavior
KW - Body Mass Index
KW - California
KW - Cellular Phone
KW - Child
KW - Child Behavior
KW - Feasibility Studies
KW - Humans
KW - Leisure Activities
KW - Male
KW - Monitoring
KW - Ambulatory/mt [Methods]
KW - Motor Activity
KW - Patient Compliance
KW - Patient Preference
KW - Pilot Projects
KW - Research Design
KW - Sedentary Lifestyle
KW - Self Report
KW - Time Factors
RP - NOT IN FILE
SP - 1205
EP - 1212
JF - Obesity

JA - Obesity (Silver.Spring)
VL - 19
IS - 6
CY - United States
N2 - The risk of obesity during childhood can be significantly reduced through increased physical activity and decreased sedentary behavior. Recent technological advances have created opportunities for the real-time measurement of these behaviors. Mobile phones are ubiquitous and easy to use, and thus have the capacity to collect data from large numbers of people. The present study tested the feasibility, acceptability, and validity of an electronic ecological momentary assessment (EMA) protocol using electronic surveys administered on the display screen of mobile phones to assess children's physical activity and sedentary behaviors. A total of 121 children (ages 9-13, 51% male, 38% at risk for overweight/overweight) participated in EMA monitoring from Friday afternoon to Monday evening during children's nonschool time, with 3-7 surveys/day. Items assessed current activity (e.g., watching TV/movies, playing video games, active play/sports/exercising). Children simultaneously wore an Actigraph GT2M accelerometer. EMA survey responses were time-matched to total step counts and minutes of moderate-to-vigorous physical activity (MVPA) occurring in the 30 min before each EMA survey prompt. No significant differences between answered and unanswered EMA surveys were found for total steps or MVPA. Step counts and the likelihood of 5+ min of MVPA were significantly higher during EMA-reported physical activity (active play/sports/exercising) vs. sedentary behaviors (reading/computer/homework, watching TV/movies, playing video games, riding in a car) ($P < 0.001$). Findings generally support the acceptability and validity of a 4-day EMA protocol using mobile phones to measure physical activity and sedentary behavior in children during leisure time
SN - 1930-739X
AD - Department of Preventive Medicine, University of Southern California, Alhambra, California, USA. dunton@usc.edu
UR - 21164502
ER -

TY - JOUR
ID - 185
T1 - The association between overweight and opportunity structures in the built environment: a multi-level analysis among elementary school youth in the PLAY-ON study
A1 - Leatherdale,S.T.
A1 - Pouliou,T.
A1 - Church,D.
A1 - Hobin,E.
Y1 - 2011/06//
N1 - Leatherdale, Scott T. Pouliou, Theodora. Church, Dana. Hobin, Erin
International journal of public health
101304551
IM
Journal Article. Multicenter Study. Research Support, Non-U.S. Gov't
English
KW - Medline
KW - Body Mass Index
KW - Child
KW - Cross-Sectional Studies
KW - Environment
KW - Environment Design
KW - Exercise
KW - Humans
KW - Logistic Models
KW - Ontario
KW - Overweight/ep [Epidemiology]
KW - Prevalence
KW - Risk Factors
KW - Schools

KW - Self Report
KW - Students
RP - NOT IN FILE
SP - 237
EP - 246
JF - International Journal of Public Health
JA - Int.J Public Health
VL - 56
IS - 3
CY - Switzerland
N2 - OBJECTIVE: To examine school-level opportunity structures of the built environment and student characteristics associated with being overweight. METHODS: Multi-level logistic regression analysis were used to examine the school- and student-level characteristics associated with the odds of a student being overweight among grade 5-8 students attending 30 elementary schools in Ontario, Canada, as part of the Play-Ontario (PLAY-ON) study. RESULTS: Significant between school random variation in overweight was identified [2(u0)= 0.187 (0.084), P < 0.001]; school-level differences accounted for 5.4% of the variability in the odds of a student being overweight. The more fast-food retailers there were surrounding a school, the more likely a student was to be overweight; students in grade 5 were at increased risk relative to students in grades 6-8. The more grocery stores there were surrounding a school, the more likely a student was to be overweight; students in grade 5 were at increased risk relative to students in grades 6-8. CONCLUSIONS: Developing a better understanding of the school- and student-level characteristics associated with overweight among youth is critical for informing intervention programs and policies
SN - 1661-8564
AD - Department of Population Studies and Surveillance, Cancer Care Ontario, 620 University Avenue, Toronto, ON M5G 2L7, Canada. scott.leatherdale@cancercare.on.ca
UR - 21046190
ER -

TY - JOUR
ID - 186
T1 - Do mothers' play behaviors moderate the associations between socioeconomic status and 24-month neurocognitive outcomes of toddlers born preterm or with low birth weight?
A1 - lworth-Bart,J.E.
A1 - Poehlmann,J.A.
A1 - Miller,K.E.
A1 - Hilgendorf,A.E.
Y1 - 2011/04//
N1 - Dilworth-Bart, Janean E. Poehlmann, Julie A. Miller, Kyle E. Hilgendorf, Amy E
Journal of pediatric psychology
jl8, 7801773
IM
Journal Article. Research Support, N.I.H., Extramural. Research Support, Non-U.S. Gov't
English
KW - Medline
KW - Attention
KW - Child Development
KW - Child
KW - Preschool
KW - Cognition
KW - Female
KW - Humans
KW - Infant
KW - Low Birth Weight/px [Psychology]
KW - Newborn
KW - Male
KW - Memory

KW - Short-Term
KW - Mother-Child Relations
KW - Mothers/px [Psychology]
KW - Neuropsychological Tests
KW - Play and Playthings/px [Psychology]
KW - Premature Birth/px [Psychology]
KW - Social Class
KW - Social Environment
RP - NOT IN FILE
SP - 289
EP - 300
JF - Journal of Pediatric Psychology
JA - J Pediatr Psychol.
VL - 36
IS - 3
CY - United States
N2 - OBJECTIVE: We examined whether neonatal risks and maternal scaffolding (i.e., task changes and flexibility) during a 16-month post-term play interaction moderated the association between socioeconomic status (SES), visual-spatial processing and emerging working memory assessed at 24 months post-term among 75 toddlers born preterm or low birth weight. METHOD: SES and neonatal risk data were collected at hospital discharge and mother-child play interactions were observed at 16-month post-term. General cognitive abilities, verbal/nonverbal working memory and visual-spatial processing data were collected at 24 months. RESULTS: Neonatal risks did not moderate the associations between SES and 24-month outcomes. However, lower mother-initiated task changes were related to better 24-month visual-spatial processing among children living in higher SES homes. Mothers' flexible responses to child initiated task changes similarly moderated the impact of SES on 24-month visual-spatial processing. CONCLUSION: Our results suggest that mothers' play behaviors differentially relate to child outcomes depending on household SES
SN - 1465-735X
AD - Department of Human Development and Family Studies, University of Wisconsin, 1305 Linden Drive, Madison, WI 53706-1523, USA. jedilworth@wisc.edu
UR - 20656763
ER -

TY - JOUR
ID - 187
T1 - Adolescents risky MP3-player listening and its psychosocial correlates
A1 - Vogel,I.
A1 - Brug,J.
A1 - Van der Ploeg,C.P.
A1 - Raat,H.
Y1 - 2011/04//
N1 - Vogel, Ineke. Brug, Johannes. Van der Ploeg, Catharina P B. Raat, Hein
Health education research
bqp, 8608459
T
Journal Article. Research Support, Non-U.S. Gov't
English
KW - Medline
KW - Adolescent
KW - Adolescent Behavior/px [Psychology]
KW - Child
KW - Female
KW - Habits
KW - Hearing Loss
KW - Noise-Induced/et [Etiology]
KW - Noise-Induced/pc [Prevention & Control]

KW - Humans
KW - MP3-Player/sn [Statistics & Numerical Data]
KW - MP3-Player/td [Trends]
KW - Male
KW - Netherlands
KW - Risk-Taking
KW - Young Adult
RP - NOT IN FILE
SP - 254
EP - 264
JF - Health Education Research
JA - Health Educ Res.
VL - 26
IS - 2
CY - England
N2 - Analogue to occupational noise-induced hearing loss, MP3-induced hearing loss may be evolving into a significant social and public health problem. To inform prevention strategies and interventions, this study investigated correlates of adolescents' risky MP3-player listening behavior primarily informed by protection motivation theory. We invited 1687 adolescents (12- to 19-year old) of Dutch secondary schools to complete questionnaires about their MP3-player listening, sociodemographic characteristics and presumed psychosocial determinants of MP3-player listening. Of all participants, 90% reported listening to music through earphones on MP3 players; 28.6% were categorized as listeners at risk for hearing loss due to estimated exposure of 89 dBA for >=1 hour per day. Compared with listeners not at risk for hearing loss, listeners at risk were more likely not to live with both parents, to experience rewards of listening to high-volume levels, to report a high habit strength related to risky MP3 listening, and were less likely to be motivated to protect their hearing. Habit strength was the strongest correlate of risky listening behavior, suggesting that voluntary behavior change among adolescents might be difficult to achieve and that a multiple strategy approach may be needed to prevent MP3-induced hearing loss
SN - 1465-3648
AD - Department of Public Health, Erasmus MC, University Medical Center, PO Box 2040, Rotterdam 3000 CA, The Netherlands. i.vogel@erasmusmc.nl
UR - 21321009
ER -

TY - JOUR
ID - 188
T1 - Peer-related social competence of young children with Down syndrome
A1 - Guralnick,M.J.
A1 - Connor,R.T.
A1 - Johnson,L.C.
Y1 - 2011/01//
N1 - Guralnick, Michael J. Connor, Robert T. Johnson, L Clark
American journal on intellectual and developmental disabilities
101492916
IM
Journal Article. Research Support, N.I.H., Extramural
English
KW - Medline
KW - Child
KW - Child Psychology
KW - Preschool
KW - Down Syndrome/px [Psychology]
KW - Family Health
KW - Female
KW - Friends/px [Psychology]
KW - Humans

KW - Male
KW - Peer Group
KW - Play and Playthings/px [Psychology]
KW - Social Behavior
RP - NOT IN FILE
SP - 48
EP - 64
JF - American Journal on Intellectual & Developmental Disabilities
JA - Am J Intellect.Dev.Disabil.
VL - 116
IS - 1
CY - United States
N2 - The peer-related social competence of children with Down syndrome was examined in an observational study. Dyadic interactions with peers of children with Down syndrome were compared with the dyadic interactions of matched groups of typically developing children and with playmates differing in both familiarity and social skills. Results suggested that both risk and protective factors influenced the peer interactions of children with Down syndrome. Recommendations are made for applying contemporary models of peer-related social competence to etiologic subgroups to better understand the mechanisms involved and to provide direction for the design of intervention programs
SN - 1944-7515
AD - Center on Human Development and Disability, University of Washington, Seattle, WA, USA. mjgural@u.washington.edu
UR - 21291310
ER -

TY - JOUR
ID - 189
T1 - Suspected motor problems and low preference for active play in childhood are associated with physical inactivity and low fitness in adolescence
A1 - Kantomaa,M.T.
A1 - Purtsi,J.
A1 - Taanila,A.M.
A1 - Remes,J.
A1 - Viholainen,H.
A1 - Rintala,P.
A1 - Ahonen,T.
A1 - Tammelin,T.H.
Y1 - 2011///
N1 - Kantomaa, Marko T. Purtsi, Jarno. Taanila, Anja M. Remes, Jouko. Viholainen, Helena. Rintala, Pauli. Ahonen, Timo. Tammelin, Tuija H
PloS one
101285081
IM
Journal Article. Research Support, N.I.H., Extramural. Research Support, Non-U.S. Gov't
English
KW - Medline
KW - Adolescent
KW - Child
KW - Choice Behavior
KW - Finland
KW - Humans
KW - Longitudinal Studies
KW - Motor Activity
KW - Physical Fitness/ph [Physiology]
KW - Physical Fitness/px [Psychology]
KW - Physical Fitness

KW - Prospective Studies
RP - NOT IN FILE
SP - e14554
JF - PLoS ONE [Electronic Resource]
JA - PLoS One
VL - 6
IS - 1
CY - United States
N2 - BACKGROUND: This prospective longitudinal study investigates whether suspected motor problems and low preference for active play in childhood are associated with physical inactivity and low cardiorespiratory fitness in adolescence. METHODOLOGY/PRINCIPAL FINDINGS: The study sample consisted of the Northern Finland Birth Cohort 1986 (NFBC 1986) composed of 5,767 children whose parents responded to a postal inquiry concerning their children's motor skills at age 8 years and who themselves reported their physical activity at age 16 years. Cardiorespiratory fitness was measured with a cycle ergometer test at age 16 years. Odds ratios (OR) and their 95% confidence intervals (95% CI) for the level of physical activity and fitness were obtained from multinomial logistic regression and adjusted for socio-economic position and body mass index. Low preference for active play in childhood was associated with physical inactivity (boys: OR 3.31, 95% CI 2.42-4.53; girls: OR 1.79, 95% CI 1.36-2.36) and low cardiorespiratory fitness (boys: OR 1.87, 95% CI 1.27-2.74; girls: OR 1.52, 95% CI 1.09-2.11) in adolescence. Suspected gross (OR 2.16, 95% CI 1.33-3.49) and fine (OR 1.88, 95% CI 1.35-2.60) motor problems were associated with physical inactivity among boys. Children with suspected motor problems and low preference for active play tended to have an even higher risk of physical inactivity in adolescence. CONCLUSIONS/SIGNIFICANCE: Low preference for active play in childhood was associated with physical inactivity and low cardiorespiratory fitness in adolescence. Furthermore, children with suspected motor problems and low preference for active play tended to have an even higher risk of physical inactivity in adolescence. Identification of children who do not prefer active play and who have motor problems may allow targeted interventions to support their motor learning and participation in active play and thereby promote their physical activity and fitness in later life
SN - 1932-6203
AD - LIKES - Research Center for Sport and Health Sciences, Jyvaskyla, Finland. marko.kantomaa@likes.fi
UR - 21267447
ER -

TY - JOUR
ID - 190
T1 - Primary care strategies for promoting parent-child interactions and school readiness in at-risk families: the Bellevue Project for Early Language, Literacy, and Education Success
A1 - Mendelsohn,A.L.
A1 - Huberman,H.S.
A1 - Berkule,S.B.
A1 - Brockmeyer,C.A.
A1 - Morrow,L.M.
A1 - Dreyer,B.P.
Y1 - 2011/01//
N1 - Mendelsohn, Alan L. Huberman, Harris S. Berkule, Samantha B. Brockmeyer, Carolyn A. Morrow, Lesley M. Dreyer, Benard P
Archives of pediatrics & adolescent medicine
9422751, bwf
AIM, IM
Comparative Study. Journal Article. Randomized Controlled Trial. Research Support, N.I.H., Extramural. Research Support, Non-U.S. Gov't. Research Support, U.S. Gov't, Non-P.H.S.
English
KW - Medline
KW - Child Development/ph [Physiology]
KW - Child
KW - Preschool
KW - Confidence Intervals

KW - Early Intervention (Education)/og [Organization & Administration]
KW - Female
KW - Health Promotion/og [Organization & Administration]
KW - Health Status Disparities
KW - Hospitals
KW - Urban
KW - Humans
KW - Infant
KW - Newborn
KW - Language Development
KW - Learning
KW - Male
KW - New York City
KW - Parent-Child Relations
KW - Pediatrics/mt [Methods]
KW - Play Therapy/mt [Methods]
KW - Play and Playthings
KW - Poverty
KW - Primary Health Care/og [Organization & Administration]
KW - Program Evaluation
KW - Reference Values
KW - Risk Assessment
KW - Socioeconomic Factors
KW - Teaching Materials
KW - Time Factors
KW - Video Recording/mt [Methods]
RP - NOT IN FILE
SP - 33
EP - 41
JF - Archives of Pediatrics & Adolescent Medicine
JA - Arch.Pediatr Adolesc Med
VL - 165
IS - 1
CY - United States
N2 - OBJECTIVE: To determine the effects of pediatric primary care interventions on parent-child interactions in families with low socioeconomic status. DESIGN: In this randomized controlled trial, participants were randomized to 1 of 2 interventions (Video Interaction Project [VIP] or Building Blocks [BB]) or the control group. SETTING: Urban public hospital pediatric primary care clinic. PARTICIPANTS: Mother-newborn dyads enrolled post partum from November 1, 2005, through October 31, 2008. INTERVENTIONS: In the VIP group, mothers and newborns participated in 1-on-1 sessions with a child development specialist who facilitated interactions in play and shared reading by reviewing videos made of the parent and child on primary care visit days; learning materials and parenting pamphlets were also provided. In the BB group, parenting materials, including age-specific newsletters suggesting interactive activities, learning materials, and parent-completed developmental questionnaires, were mailed to the mothers. MAIN OUTCOME MEASURES: Parent-child interactions were assessed at 6 months with the StimQ-Infant and a 24-hour shared reading recall diary. RESULTS: A total of 410 families were assessed. The VIP group had a higher increased StimQ score (mean difference, 3.6 points; 95% confidence interval, 1.5 to 5.6 points; Cohen d, 0.51; 0.22 to 0.81) and more reading activities compared to the control group. The BB group also had an increased overall StimQ score compared with the control group (Cohen d, 0.31; 95% confidence interval, 0.03 to 0.60). The greatest effects for the VIP group were found for mothers with a ninth-grade or higher reading level (Cohen d, 0.68; 95% confidence interval, 0.33 to 1.03). CONCLUSIONS: The VIP and BB groups each led to increased parent-child interactions. Pediatric primary care represents a significant opportunity for enhancing developmental trajectories in at-risk children. TRIAL REGISTRATION: clinicaltrials.gov Identifier: NCT00212576
SN - 1538-3628
AD - Division of Developmental-Behavioral Pediatrics, Department of Pediatrics, New York University School of Medicine and Bellevue Hospital Center, New York, NY 10016, USA. alm5@nyu.edu

UR - 21199978
ER -

TY - JOUR
ID - 191
T1 - A potential new health risk from lead in used consumer products purchased in the United States
A1 - Sharmer,L.
A1 - Shackley,M.S.
A1 - Harding,A.K.
Y1 - 2010/12//
N1 - Sharmer, Laurel. Shackley, M Steven. Harding, Anna K
Journal of environmental health
iac, 0405525
IM
Journal Article. Multicenter Study. Research Support, Non-U.S. Gov't
English
KW - Medline
KW - Child
KW - Child Welfare
KW - Consumer Product Safety/lj [Legislation & Jurisprudence]
KW - Consumer Product Safety
KW - Environmental Pollutants/an [Analysis]
KW - Household Products/an [Analysis]
KW - Humans
KW - Lead/an [Analysis]
KW - Lead Poisoning/pc [Prevention & Control]
KW - Maximum Allowable Concentration
KW - Paint/an [Analysis]
KW - Play and Playthings
KW - United States
RP - NOT IN FILE
SP - 8
EP - 12
JF - Journal of Environmental Health
JA - J Environ.Health
VL - 73
IS - 5
CY - United States
N2 - The Lead Renovation, Repair, and Painting Rule and the Consumer Product Safety Improvement Act, both enacted in 2008, were intended to protect children from exposure to lead by setting federal limits on lead content. Neither of these federal actions, however, addresses a newly recognized pathway of exposure to lead from the use of used consumer products in the home. In the study described in this article, the authors purchased 28 used consumer items in the United States in 2004 and analyzed them for lead content using X-ray fluorescence technology. Nineteen of the items exceeded the federal standards for lead. The amount of lead in the items ranged from 745 parts per million (ppm) to 428,525 ppm. The authors' research shows that such items, which are easily purchased throughout the U.S., may contain surface lead concentrations in amounts greater than 700 times current federal limits. This article reveals an ongoing public health threat involved in exposure to lead that is not addressed by current laws or regulations. Addressing the risk involved in this threat requires continued research, public education, and targeted regulatory action
SN - 0022-0892
AD - Department of Community Health, State University of New York, Postdam, NY 13676, USA. sharmela@potsdam.edu
UR - 21189787
ER -

TY - JOUR

ID - 192
T1 - Association of adolescent choking game activity with selected risk behaviors
A1 - Dake,J.A.
A1 - Price,J.H.
A1 - Kolm-Valdivia,N.
A1 - Wielinski,M.
Y1 - 2010/11//
N1 - Dake, Joseph A. Price, James H. Kolm-Valdivia, Nicole. Wielinski, Margaret
Academic pediatrics
101499145
IM
Journal Article. Multicenter Study
English
KW - Medline
KW - Adolescent
KW - Adolescent Behavior
KW - Age Factors
KW - Airway Obstruction/px [Psychology]
KW - Child
KW - Female
KW - Humans
KW - Male
KW - Mental Disorders/ep [Epidemiology]
KW - Mental Disorders/px [Psychology]
KW - Midwestern United States/ep [Epidemiology]
KW - Play and Playthings
KW - Risk Factors
KW - Risk-Taking
KW - Substance-Related Disorders/ep [Epidemiology]
KW - Substance-Related Disorders/px [Psychology]
KW - Violence/px [Psychology]
KW - Violence/sn [Statistics & Numerical Data]
RP - NOT IN FILE
SP - 410
EP - 416
JF - Academic pediatrics
JA - Acad.Pediatr
VL - 10
IS - 6
CY - United States
N2 - OBJECTIVE: Previous research has recommended education for parents, teachers, and anticipatory guidance by pediatricians regarding participation in the so-called choking game, a potentially fatal behavior. The purpose of this study was to examine possible associations between selected demographic variables and risk behaviors with youth engagement in the choking game on the basis of secondary data analysis from a general adolescent health risk behavior survey. METHODS: Self-administered survey data from an adolescent needs assessment was used to assess choking game behavior between fall 2008 and fall 2009. The sample included 192 classrooms across 88 schools in a Midwestern state. RESULTS: Of the 3598 questionnaires distributed to middle and high school students, 3408 (95%) were returned completed. Participation rate in the choking game was 9%, with male participation (11%) greater than female participation (7%), and high school students (11%) more likely than middle school students (5%) to participate. Adjusted odds ratios found that the likelihood of middle school students engaging in the choking game were higher for older students, substance users, and those having lower grades. For high school students, adjusted odds ratios found that being older, substance use, and selected mental health issues (forced sex and attempted suicide) were most associated with choking activities. CONCLUSIONS: Engaging in the choking game was highly associated with abuse of substances, suggesting that youth engage in the choking game for the thrill-seeking experience of brief euphoria, a drug-related feeling. To reduce the potentially fatal consequences associated with this behavior, pediatricians should screen youths

and provide anticipatory guidance for higher-risk youths and their parents. Copyright 2010 Academic Pediatric Association. Published by Elsevier Inc. All rights reserved
SN - 1876-2867
AD - Department of Health and Recreation Professions, University of Toledo, Toledo, Ohio 43606, USA. jdake@utnet.utoledo.edu
UR - 21075323
ER -

TY - JOUR
ID - 193
T1 - The crucial role of recess in schools
A1 - Ramstetter,C.L.
A1 - Murray,R.
A1 - Garner,A.S.
Y1 - 2010/11//
N1 - Ramstetter, Catherine L. Murray, Robert. Garner, Andrew S
The Journal of school health
k13, 0376370
IM, N
Journal Article
English
KW - Medline
KW - Adolescent
KW - Body Mass Index
KW - Child
KW - Child Welfare/sn [Statistics & Numerical Data]
KW - Exercise
KW - Female
KW - Health Promotion/og [Organization & Administration]
KW - Humans
KW - Male
KW - Obesity/ep [Epidemiology]
KW - Obesity/pc [Prevention & Control]
KW - Physical Education and Training/sn [Statistics & Numerical Data]
KW - Play and Playthings
KW - Public Policy
KW - School Health Services/og [Organization & Administration]
KW - Schools/og [Organization & Administration]
KW - United States/ep [Epidemiology]
RP - NOT IN FILE
SP - 517
EP - 526
JF - Journal of School Health
JA - J Sch Health
VL - 80
IS - 11
CY - United States
N2 - BACKGROUND: Recess is at the heart of a vigorous debate over the role of schools in promoting optimal child development and well-being. Reallocating time to accentuate academic concerns is a growing trend and has put recess at risk. Conversely, pressure to increase activity in school has come from efforts to combat childhood obesity. The purpose of this review was to examine the value of recess as an integral component of the school day. METHODS: A comprehensive review of recess-specific literature was conducted, beginning with a Google Scholar search, to cull definitions, position statements, and policy recommendations from national/international associations and organizations. A multi-database search followed. Additional articles were selected from reference lists. RESULTS: The search yielded a range of articles, from those focused on specific aspects of recess to those that examined multiple factors, including how to structure and conduct recess. Several

themes emerged supporting recess as beneficial for children's cognitive, social, emotional, and physical functioning. Optimal recess was well-supervised and safe. Crucial components were well-maintained playground equipment and well-trained supervisors. CONCLUSION: Recess serves a critical role in school as a necessary break from the rigors of academic challenges. Recess is a complement to, not a replacement for, physical education. Both promote activity and a healthy lifestyle; however, recess--particularly unstructured recess and free play--provides a unique contribution to a child's creative, social, and emotional development. From the perspective of children's health and well-being, recess time should be considered a child's personal time and should not be withheld for academic or punitive reasons. 2010, American School Health Association
SN - 1746-1561
AD - Center for the Enhancement of Teaching and Learning, Health Promotion and Education, University of Cincinnati, Langsam 480, Mail Location 0033, PO Box 210033, Cincinnati, OH 45221-0033, USA. ramstecl@ucmail.uc.edu
UR - 21039550
ER -

TY - JOUR
ID - 194
T1 - Trajectories of antisocial behaviour towards siblings predict antisocial behaviour towards peers
A1 - Ensor,R.
A1 - Marks,A.
A1 - Jacobs,L.
A1 - Hughes,C.
Y1 - 2010/11//
N1 - Ensor, Rosie. Marks, Alex. Jacobs, Lorna. Hughes, Claire
Journal of child psychology and psychiatry, and allied disciplines
hp3, 0375361
IM
Comparative Study. Journal Article. Research Support, Non-U.S. Gov't
English
KW - Medline
KW - Aggression
KW - Antisocial Personality Disorder/ep [Epidemiology]
KW - Antisocial Personality Disorder/px [Psychology]
KW - Bullying
KW - Child
KW - Child Behavior/px [Psychology]
KW - Preschool
KW - Educational Status
KW - Female
KW - Humans
KW - Interpersonal Relations
KW - Male
KW - Mothers
KW - Neuropsychological Tests
KW - Peer Group
KW - Personality Assessment
KW - Personality Development
KW - Play and Playthings/px [Psychology]
KW - Risk Factors
KW - Sex Factors
KW - Siblings/px [Psychology]
KW - Social Behavior
RP - NOT IN FILE
SP - 1208
EP - 1216
JF - Journal of Child Psychology & Psychiatry & Allied Disciplines

JA - J Child Psychol.Psychiatry
VL - 51
IS - 11
CY - England
N2 - BACKGROUND: Young siblings' antisocial behaviour is common yet its impact has received relatively little research attention. METHODS: We examined trajectories of antisocial behaviour for a socially diverse sample (n = 99, 58 boys and 41 girls) who were filmed with their older siblings (52 boys and 47 girls) at ages 3 and 6 and with unfamiliar peers at age 6. Latent growth models were used to analyse three indicators of antisocial behaviour (refusal to share/interact, bullying and harming). RESULTS: The average trajectory of antisocial behaviour towards siblings was stable and particularly high for boys with brothers and for children of mothers with no educational qualifications. Sustained and escalating antisocial behaviours towards siblings predicted bullying and refusals to share/interact with unfamiliar peers, independent of associations with concurrent antisocial behaviour towards sibling. CONCLUSIONS: These findings highlight the importance of a developmental perspective when examining antisocial behaviour between young siblings. 2010 The Authors. Journal of Child Psychology and Psychiatry 2010 Association for Child and Adolescent Mental Health
SN - 1469-7610
AD - Centre for Family Research, University of Cambridge, Free School Lane, Cambridge, UK. rad35@cam.ac.uk
UR - 20584100
ER -

TY - JOUR
ID - 195
T1 - Outdoor play: A survey of parent's perceptions of their child's safety
A1 - Kalish,M.
A1 - Banco,L.
A1 - Burke,G.
A1 - Lapidus,G.
Y1 - 2010/10//
N1 - Kalish, Meredith. Banco, Leonard. Burke, Georgine. Lapidus, Garry
The Journal of trauma
kaf, 0376373
AIM, IM
Journal Article
English
KW - Medline
KW - Adult
KW - Attitude
KW - Child
KW - Preschool
KW - Crime
KW - Environment Design
KW - Female
KW - Humans
KW - Male
KW - Middle Aged
KW - Parents/px [Psychology]
KW - Play and Playthings
KW - Residence Characteristics
KW - Safety
KW - Socioeconomic Factors
KW - Young Adult
RP - NOT IN FILE
SP - S218
EP - S222
JF - Journal of Trauma-Injury Infection & Critical Care

JA - J Trauma
VL - 69
IS - 4 Suppl
CY - United States
N2 - BACKGROUND: Anecdotal reports suggest that parents may be restricting outdoor play in response to a perceived lack of safety and fear of violence and crime in urban environments. The study objective was to determine whether parents who perceived their neighborhood as unsafe would be most likely to restrict their child's outdoor play and report the greatest worries related to neighborhood characteristics. METHODS: A convenience sample of primary caregivers of children aged 5 years to 7 years were recruited from a pediatric primary care center based at an urban academic children's hospital. Study participants completed a 23-question survey available in English and Spanish. RESULTS: Two hundred fifty-four parents completed the survey. Most were English speakers (69%), female (89%), and aged between 21 years and 35 years (76%). Sixty-two percent let their child play outside often or sometimes; whereas 19% reported never allowing their child to play outside. Parents were less likely to allow outside play as their degree of worry increased about traffic ($p < 0.0001$), rundown parks ($p < 0.003$), crime ($p < 0.0001$), witnessing violence ($p < 0.0001$), being a victim of violence ($p < 0.0009$), drugs ($p < 0.0001$), gangs ($p < 0.004$), and weapons ($p < 0.003$). Frequency of outdoor play was not correlated with the sex or age of the child or number of children in the family. Spanish speakers were less likely to allow outside play ($p < 0.008$) but more likely to allow unsupervised play ($p < 0.03$). CONCLUSIONS: Decreased frequency of outdoor play correlated with increased parental concerns about safety, however, many parents still report allowing outdoor play at least sometimes
SN - 1529-8809
AD - Connecticut Children's Medical Center, University of Connecticut School of Medicine, Hartford, USA
UR - 20938312
ER -

TY - JOUR
ID - 196
T1 - Playing with a child with ADHD: a focus on the playmates
A1 - Cordier,R.
A1 - Bundy,A.
A1 - Hocking,C.
A1 - Einfeld,S.
Y1 - 2010/09//
N1 - Cordier, Reinie. Bundy, Anita. Hocking, Clare. Einfeld, Stewart
Scandinavian journal of occupational therapy
9502210
IM
Journal Article. Research Support, Non-U.S. Gov't
English
KW - Medline
KW - Age Factors
KW - Attention Deficit Disorder with Hyperactivity/px [Psychology]
KW - Child
KW - Preschool
KW - Empathy
KW - Female
KW - Health Status Indicators
KW - Humans
KW - Interpersonal Relations
KW - Male
KW - Peer Group
KW - Play and Playthings
KW - Sex Factors
KW - Siblings
RP - NOT IN FILE
SP - 191

EP - 199
JF - Scandinavian Journal of Occupational Therapy
JA - Scand.J Occup Ther
VL - 17
IS - 3
CY - England
N2 - Play is the context for acquiring social skills. While it is logical to suspect that the difficulties associated with ADHD would affect play, there is surprisingly little work in that area. Further, there is almost no previous research describing the playmates of children with ADHD. This study involved children with ADHD (n = 112) playing with a usual playmate (n = 112), and pairs of age-, ethnicity-, and sex-matched children (n = 126) playing together. In this paper, the authors interpret the data from the perspective of the playmates. The overall finding was that the play behavior of the playmates closely resembled that of the children with ADHD; it was characterized by low levels of empathy. A primary purpose of this paper is to explore possible explanations for this surprising finding. Two possible explanations are explored. First, children with ADHD are demanding playmates so that children who play with them mirror their negative behaviors. Second, although the playmates did not have ADHD, their play behaviors might reflect the known risk of negative behaviors reported amongst siblings and peers of children with ADHD. Evidence to support both explanations is presented and new lines of research are proposed to examine each possibility
SN - 1651-2014
AD - Faculty of Health Sciences, Discipline of Occupational Therapy, The University of Sydney, Australia
UR - 19670080
ER -

TY - JOUR
ID - 197
T1 - The Stamp-in-Safety programme, an intervention to promote better supervision of children on childcare centre playgrounds: an evaluation in an urban setting
A1 - Chelvakumar,G.
A1 - Sheehan,K.
A1 - Hill,A.L.
A1 - Lowe,D.
A1 - Mandich,N.
A1 - Schwebel,D.C.
Y1 - 2010/10//
N1 - Chelvakumar, Gayathri. Sheehan, Karen. Hill, Amy L. Lowe, Danita. Mandich, Nicole. Schwebel, David C
Injury prevention : journal of the International Society for Child and Adolescent Injury Prevention
crz, 9510056
IM
Journal Article. Research Support, Non-U.S. Gov't
English
KW - Medline
KW - Accident Prevention
KW - Accidental Falls/pc [Prevention & Control]
KW - Accidental Falls/sn [Statistics & Numerical Data]
KW - Behavior Control
KW - Child Day Care Centers
KW - Child
KW - Preschool
KW - Equipment Design
KW - Female
KW - Humans
KW - Male
KW - Play and Playthings/in [Injuries]
KW - Social Environment
KW - United States/ep [Epidemiology]

RP - NOT IN FILE
SP - 352
EP - 354
JA - Injury Prevention
VL - 16
IS - 5
CY - England
N2 - Using a non-equivalent control group design, this report evaluated a previously studied behavioural intervention, the Stamp-in-Safety programme, which is designed to reduce the injury risk for young children on playgrounds at childcare centres by increasing the quality of adult supervision and rewarding children for safe play. In an urban, commercial childcare centre, 71 children aged 3-5 years and 15 teachers participated. Primary outcome measures were teacher verbalisations (warnings, explanations, redirects), teacher location (core, outskirt, or fringe of playground), child risk-taking behaviours (using equipment appropriately) and the number of injuries on the playground. Analyses revealed that the intervention had a modest positive effect in promoting safer teacher and child playground behaviours. This study reaffirms previous results that the Stamp-in-Safety programme is an effective method to decrease the risk of playground injuries at childcare centres
SN - 1475-5785
AD - Medical College of Wisconsin, Milwaukee, Wisconsin, USA
UR - 20587809
ER -

TY - JOUR
ID - 198
T1 - Safety standards and socioeconomic disparities in school playground injuries: a retrospective cohort study
A1 - Macpherson,A.K.
A1 - Jones,J.
A1 - Rothman,L.
A1 - Macarthur,C.
A1 - Howard,A.W.
Y1 - 2010///
N1 - Macpherson, Alison K. Jones, Jennifer. Rothman, Linda. Macarthur, Colin. Howard, Andrew W
BMC public health
100968562
IM
Journal Article
English
KW - Medline
KW - Child
KW - Cohort Studies
KW - Guidelines as Topic
KW - Humans
KW - Ontario/ep [Epidemiology]
KW - Play and Playthings/in [Injuries]
KW - Program Evaluation
KW - Retrospective Studies
KW - Safety/st [Standards]
KW - Social Class
KW - Wounds and Injuries/ep [Epidemiology]
KW - Wounds and Injuries/pc [Prevention & Control]
RP - NOT IN FILE
SP - 542
JA - BMC Public Health
VL - 10
CY - England
N2 - BACKGROUND: Playground injuries are fairly common and can require hospitalization and or surgery. Previous research has suggested that compliance with guidelines or standards can reduce the incidence of such

injuries, and that poorer children are at increased risk of playground injuries. OBJECTIVE: The objective of this study was to determine the association between playground injury and school socioeconomic status before and after the upgrading of playground equipment to meet CSA guidelines. METHODS: Injury data were collected from January 1998-December 1999 and January 2004 - June 2007 for 374 elementary schools in Toronto, Canada. The objective of this study was to investigate the effect of a program of playground assessment, upgrading, and replacement on school injury rates and socio-economic status. Injury rates were calculated for all injuries, injuries that did not occur on equipment, and injuries on play equipment. Poisson regression was performed to determine the relationship between injury rates and school socio-economic status. RESULTS: Prior to upgrading the equipment there was a significant relationship between socio-economic status and equipment-related injuries with children at poorer schools being at increased risk (Relative risk: 1.52 [95% CI = 1.24-1.86]). After unsafe equipment was upgraded, the relationship between injury and SES decreased and was no longer significant (RR 1.13 [95% CI = 0.95-1.32]). CONCLUSIONS: Improvements in playground equipment can result in an environment in which students from schools in poorer neighbourhoods are no longer at increased risk of injuries on play equipment
SN - 1471-2458
AD - York University, 4700 Keele Street, Toronto ON, Canada. alison3@yorku.ca
UR - 20825679
ER -

TY - JOUR
ID - 199
T1 - Negative and atypical story content themes depicted by children with behaviour problems
A1 - Wan,M.W.
A1 - Green,J.
Y1 - 2010/10//
N1 - Wan, Ming Wai. Green, Jonathan
Journal of child psychology and psychiatry, and allied disciplines
hp3, 0375361
IM
Journal Article
English
KW - Medline
KW - Adult
KW - Aggression/px [Psychology]
KW - Anger
KW - Child
KW - Child Behavior Disorders/px [Psychology]
KW - Depression/px [Psychology]
KW - Female
KW - Humans
KW - Internal-External Control
KW - Male
KW - Maternal Behavior/px [Psychology]
KW - Mother-Child Relations
KW - Mothers/px [Psychology]
KW - Play and Playthings/px [Psychology]
KW - Questionnaires
KW - Risk Factors
KW - Sex Factors
RP - NOT IN FILE
SP - 1125
EP - 1131
JF - Journal of Child Psychology & Psychiatry & Allied Disciplines
JA - J Child Psychol.Psychiatry
VL - 51
IS - 10

CY - England
N2 - BACKGROUND: Specific thematic content arising from children's doll play is often considered to give clinically meaningful information regarding their mental state, but has received little systematic enquiry. This exploratory study examined the negative and atypical content themes in the attachment story narratives of children with behaviour problems, and their associations with child symptomatology and parental depressed mood. METHOD: Videotaped mother-child attachment doll play completions of 77 children from a clinical sample were blind coded for negative content themes. Mothers completed questionnaires for maternal depression and child behaviour. RESULTS: Children rating positive for behaviour disorder were more likely to represent maternal injury, role reversal and marked maternal sadness. Severe behavioural symptoms were associated with more pervasive negative themes. Half of the overall sample depicted child accidents and maternal unresponsiveness, whereas child aggression, child anger and maternal absence were rare. Girls with depressed mothers showed an absence of particular negative representations (e.g., child sadness) compared to boys. CONCLUSIONS: Specific atypical themes are associated with the extent of child psychopathology and may be modified by exposure to maternal depressed mood. The methodological limitations in this exploratory study are also discussed
SN - 1469-7610
AD - Psychiatry Research Group, University of Manchester, UK. m.w.wan@manchester.ac.uk
UR - 20331493
ER -

TY - JOUR
ID - 200
T1 - Diabulimia and the role of school health personnel. [Review]
A1 - Hasken,J.
A1 - Kresl,L.
A1 - Nydegger,T.
A1 - Temme,M.
Y1 - 2010/10//
N1 - Hasken, Julie. Kresl, Laura. Nydegger, Teresa. Temme, Megan
The Journal of school health
k13, 0376370
IM, N
Journal Article. Review
English
KW - Medline
KW - Adolescent
KW - Child
KW - Child Welfare
KW - Diabetes Mellitus
KW - Type 1
KW - Directive Counseling
KW - Eating Disorders/pc [Prevention & Control]
KW - Female
KW - Health Education
KW - Health Personnel
KW - Humans
KW - Insulin/pd [Pharmacology]
KW - Male
KW - Nutritional Status
KW - Professional Role
KW - Risk-Taking
KW - School Health Services
KW - Weight Loss
RP - NOT IN FILE
SP - 465
EP - 469

JF - Journal of School Health
JA - J Sch Health
VL - 80
IS - 10
CY - United States
N2 - BACKGROUND: Diabulimia, the omission or reduction of insulin use by persons with type 1 diabetes, is a harmful method of weight control. The purpose of this article is to present school health personnel with the information they may need to become more aware of the possibility of diabulimia in their students-especially females-with type 1 diabetes. METHODS: A review of the somewhat limited medical and diabetes-related organizations' literature on diabulimia was conducted to establish the role that school health personnel could play in raising awareness of students with this condition as well as education for diabulimia prevention. RESULTS: Since insulin encourages fat storage, many with type 1 diabetes have discovered the relationship between reducing the amount of insulin they take and corresponding weight loss. Improper regulation of needed insulin treatments poses serious health problems that may require immediate medical attention. CONCLUSION: School personnel, especially those in the Coordinated School Health Program areas of comprehensive school health education, school health services, and guidance and counseling services have key roles to play in the prevention and recognition of diabulimia in students with diabetes. 2010, American School Health Association
SN - 1746-1561
AD - Truman State University, Kirksville, MO 63501, USA. jmh588@truman.edu
UR - 20840655
ER -

TY - JOUR
ID - 201
T1 - Building a better playground
A1 - Barovick,H.
Y1 - 2010/08/09/
N1 - Barovick, Harriet
Time
9877130, bqn
K
News
English
KW - Medline
KW - Child
KW - Preschool
KW - Equipment Design
KW - Humans
KW - Play and Playthings
KW - Safety
KW - United States
RP - NOT IN FILE
SP - 45
EP - 46
JF - Time
VL - 176
IS - 6
CY - United States
SN - 0040-781X
UR - 20722333
ER -

TY - JOUR
ID - 202
T1 - Permanent play facilities in school playgrounds as a determinant of children's activity
A1 - Nielsen,G.

A1 - Taylor,R.
A1 - Williams,S.
A1 - Mann,J.
Y1 - 2010/07//
N1 - Nielsen, Glen. Taylor, Rachael. Williams, Sheila. Mann, Jim
Journal of physical activity & health
101189457
IM
Journal Article
English
KW - Medline
KW - Child
KW - Preschool
KW - Environment Design
KW - Exercise
KW - Humans
KW - Monitoring
KW - Ambulatory
KW - Play and Playthings
KW - Schools
RP - NOT IN FILE
SP - 490
EP - 496
JF - Journal of Physical Activity & Health
JA - J Phys Act.Health
VL - 7
IS - 4
CY - United States
N2 - BACKGROUND: To investigate whether the number of permanent playground facilities in schools influences objectively measured physical activity. METHODS: Physical activity was measured using Actical accelerometers over 2 to 5 days in 417 children (5-12 years) from 7 schools. The number of permanent play facilities likely to encourage physical activity in individuals or groups of children (eg, adventure playgrounds, swings, trees, playground markings, courts, sandpits) were counted on 2 occasions in each school. The surface area of each playground (m(2)) was also measured. RESULTS: The number of permanent play facilities in schools ranged from 14 to 35 and was positively associated with all measures of activity. For each additional play facility, average accelerometry counts were 3.8% (P < .001) higher at school and 2.7% (P < .001) higher overall. Each additional play facility was also associated with 2.3% (P = .001) or 4 minutes more moderate/vigorous activity during school hours and 3.4% (P < .001) more (9 minutes) over the course of the day. School playground area did not affect activity independent of the number of permanent play facilities. Findings were consistent across age and sex groups. CONCLUSION: Increasing the number of permanent play facilities at schools may offer a cost-effective and sustainable option for increasing physical activity in young children
SN - 1543-3080
AD - Dept of Exercise and Sport Sciences, University of Copenhagen, Denmark
UR - 20683091
ER -

TY - JOUR
ID - 203
T1 - Associations between neighborhood safety, availability of recreational facilities, and adolescent physical activity among Canadian youth
A1 - Nichol,M.
A1 - Janssen,I.
A1 - Pickett,W.
Y1 - 2010/07//

N1 - Nichol, Marianne. Janssen, Ian. Pickett, William
Journal of physical activity & health
101189457
IM
Journal Article
English
KW - Medline
KW - Adolescent
KW - Canada
KW - Child
KW - Exercise
KW - Female
KW - Health Behavior
KW - Humans
KW - Male
KW - Play and Playthings
KW - Residence Characteristics
KW - Safety
RP - NOT IN FILE
SP - 442
EP - 450
JF - Journal of Physical Activity & Health
JA - J Phys Act.Health
VL - 7
IS - 4
CY - United States
N2 - BACKGROUND: The safety of neighborhoods and availability of parks and facilities may influence adolescent physical activity independently or interactively. METHODS: 9114 Canadians in grades 6 to 10 completed the 2006 Health Behavior in School-Aged Children Survey. The outcome of interest was students' self-reported participation in moderate-to-vigorous physical activity outside of school. A composite scale based on questions measuring student perceptions of safety was used to capture individual perceptions of safety. In addition, schools were grouped into quintiles based on the mean of the perceived safety scale, used as a proxy for peer perceptions. The number of parks and recreational facilities within 5 km of schools was abstracted from a geographical information system. RESULTS: Moderate gradients in physical activity were observed according to individual and group perceptions of safety. Boys and girls with the highest perceptions of safety were 1.31 (95% CI: 1.17-1.45) and 1.45 (1.26-1.65) times more likely to be physically active, respectively, than those with the lowest perceptions. Compared with those who perceived the neighborhood as least safe, elementary students in higher quintiles were 1.31, 1.39, 1.37, and 1.56 times more likely to be physically active (P(trend) = 0.012). Increased numbers of recreational features were not related to physical activity irrespective of neighborhood safety. CONCLUSIONS: Individual and group perceptions of neighborhood safety were modestly associated with adolescents' physical activity
SN - 1543-3080
AD - Dept of Community Health and Epidemiology, Queen's University, Kingston, Ontario, Canada
UR - 20683085
ER -

TY - JOUR
ID - 204
T1 - Noninvasive screening for risk factors of type 2 diabetes in young, rural, caucasian children
A1 - Peterson,S.
A1 - Sheffer,S.
A1 - Roth,S.L.
A1 - Bennett,P.A.
A1 - Lloyd,L.
Y1 - 2010/08//

N1 - Peterson, Sharon. Sheffer, Sarah. Roth, Sara Long. Bennett, Paul A. Lloyd, Les
The Journal of school nursing : the official publication of the National Association of School Nurses
bgs, 9206498
N
Journal Article
English
KW - Medline
KW - Adolescent
KW - Age Factors
KW - Body Mass Index
KW - Child
KW - Preschool
KW - Confidence Intervals
KW - Cross-Sectional Studies
KW - Diabetes Mellitus
KW - Type 2/di [Diagnosis]
KW - Type 2/ep [Epidemiology]
KW - Type 2/nu [Nursing]
KW - European Continental Ancestry Group/sn [Statistics & Numerical Data]
KW - Female
KW - Humans
KW - Illinois/ep [Epidemiology]
KW - Male
KW - Mass Screening/mt [Methods]
KW - Odds Ratio
KW - Prevalence
KW - Risk Factors
KW - Rural Population/sn [Statistics & Numerical Data]
KW - School Nursing
RP - NOT IN FILE
SP - 301
EP - 309
JF - Journal of School Nursing
JA - J Sch Nurs
VL - 26
IS - 4
CY - United States
N2 - School nurses play an important role in identifying students who are at risk for Type 2 diabetes mellitus (T2DM). Few studies have screened Caucasian students, and none have targeted rural, low-income, elementary children. The five noninvasive risk factors used for this study were family history, high body mass index (BMI) for age/sex, racial/ethnic background, hypertension, and acanthosis nigricans. Two thirds of those screened (n = 299) had at least one of the five risk factors for T2DM. Seventeen students (5.6% of those screened) had three or more of the five risk factors and were considered at risk for T2DM. Fifteen percent (n = 43) had hypertension or prehypertension, and 18% (n = 53) were morbidly obese. Hypertension and acanthosis nigricans were significantly associated with being at risk in morbidly obese students. School nurses play an important role as frontline health professionals who are aware that risk factors for T2DM exist in all schools, regardless of students' age, size of community, or ethnic background
SN - 1546-8364
AD - Department of Animal Science, Food and Nutrition, Southern Illinois University, Carbondale, Illinois, USA
UR - 20335231
ER -

TY - JOUR
ID - 205
T1 - A pilot study of maternal sensitivity in the context of emergent autism

A1 - Baker,J.K.
A1 - Messinger,D.S.
A1 - Lyons,K.K.
A1 - Grantz,C.J.
Y1 - 2010/08//
N1 - Baker, Jason K. Messinger, Daniel S. Lyons, Kara K. Grantz, Caroline J
Journal of autism and developmental disorders
7904301, hgw
IM
Journal Article. Research Support, N.I.H., Extramural. Research Support, Non-U.S. Gov't
English
KW - Medline
KW - Age Factors
KW - Child Behavior Disorders/px [Psychology]
KW - Child Development
KW - Child Development Disorders
KW - Pervasive/di [Diagnosis]
KW - Pervasive/px [Psychology]
KW - Child
KW - Preschool
KW - Humans
KW - Infant
KW - Language Development
KW - Mother-Child Relations
KW - Mothers/px [Psychology]
KW - Pilot Projects
KW - Play and Playthings/px [Psychology]
KW - Risk Factors
RP - NOT IN FILE
SP - 988
EP - 999
JF - Journal of Autism & Developmental Disorders
JA - J Autism Dev.Disord
VL - 40
IS - 8
CY - United States
N2 - Unstructured mother-toddler interactions were examined in 18-month-old high- and low-risk children subsequently diagnosed (n = 12) or not diagnosed (n = 21) with autism spectrum disorders (ASD) at 36 months. Differences in maternal sensitivity were not found as a function of emergent ASD status. A differential-susceptibility moderation model of child risk guided investigations linking maternal sensitivity to child behavior and language growth. Group status moderated the relation between sensitivity and concurrent child behavior problems, with a positive association present for children with emergent ASD. Maternal sensitivity at 18 months predicted expressive language growth from age 2 to 3 years among children with emergent ASD only. Findings underscore the importance of understanding parent-child interaction during this key period in the development of autism symptomatology
SN - 1573-3432
AD - Waisman Center, University of Wisconsin-Madison, Rm 533, Madison, WI, USA. jbaker@waisman.wisc.edu
UR - 20130975
ER -

TY - JOUR
ID - 206
T1 - Clinical management of musculoskeletal injuries in active children and youth
A1 - Mazer,B.
A1 - Shrier,I.

A1 - Feldman,D.E.
A1 - Swaine,B.
A1 - Majnemer,A.
A1 - Kennedy,E.
A1 - Chilingaryan,G.
Y1 - 2010/07//
N1 - Mazer, Barbara. Shrier, Ian. Feldman, Debbie Ehrmann. Swaine, Bonnie. Majnemer, Annette. Kennedy, Eileen. Chilingaryan, Gevorg
Clinical journal of sport medicine : official journal of the Canadian Academy of Sport Medicine
b9t, 9103300
IM
Journal Article. Research Support, Non-U.S. Gov't
English
KW - Medline
KW - Adolescent
KW - Athletic Injuries/th [Therapy]
KW - Attitude of Health Personnel
KW - Child
KW - Decision Making
KW - Humans
KW - Parents
KW - Physical Therapy Specialty
KW - Physician's Practice Patterns
KW - Questionnaires
KW - Risk Assessment
KW - Sports Equipment
KW - Sports Medicine
RP - NOT IN FILE
SP - 249
EP - 255
JF - Clinical Journal of Sport Medicine
JA - Clin.J Sport Med
VL - 20
IS - 4
CY - United States
N2 - OBJECTIVE: To describe how different health care specialists manage musculoskeletal injury in children and examine factors influencing return to play decisions. DESIGN: National survey. SETTING: Secure Web site hosting online questionnaire. PARTICIPANTS: Medical doctors, physical therapists, and athletic therapists who were members of their respective sport medicine specialty organizations. INDEPENDENT VARIABLES: Professional affiliation and the effect of the following factors were examined: pushy parent, cautious parent, protective equipment, previous injury, musculoskeletal maturity, game importance, position played, team versus individual sport, and time since injury. MAIN OUTCOME MEASURES: Recommendation of return to activity after common injuries seen in children and adolescents as described in 5 vignettes; consistency of responses across vignettes. RESULTS: The survey was completed by 464 respondents (34%). There were several differences between the professional groups in their recommendations to return to activity. Most factors studied did not tend to influence the decision to return to activity, although protective equipment often increased the response to return sooner. The number of participants who would return a child to activity sooner or later for each factor varied greatly across the 5 vignettes, except for pushy parent or cautious parent. CONCLUSIONS: Management practices of sport medicine clinicians vary according to profession, child, clinical factors, and sport-related factors. Decisions regarding return to play vary according to 5 specific characteristics of each clinical case. These findings help establish areas of consensus and disagreement in the management of children with injuries and safe return to physical activity
SN - 1536-3724
AD - McGill University, School of Physical and Occupational Therapy, Montreal, Quebec, Canada. barbara.mazer@mcgill.ca
UR - 20606509

ER -

TY - JOUR
ID - 207
T1 - Children's play after anaesthesia and surgery: background factors and associations to behaviour during anaesthetic induction
A1 - Proczkowska-Bjorklund,M.
A1 - Gustafsson,P.A.
A1 - Svedin,C.G.
Y1 - 2010/06//
N1 - Proczkowska-Bjorklund, Marie. Gustafsson, Per A. Svedin, Carl Goran
Journal of child health care : for professionals working with children in the hospital and community
9806360, dcl
N
Journal Article. Research Support, Non-U.S. Gov't
English
KW - Medline
KW - Age Factors
KW - Anesthesia/px [Psychology]
KW - Child
KW - Child Behavior/px [Psychology]
KW - Child Psychology
KW - Preschool
KW - Female
KW - General Surgery
KW - Humans
KW - Male
KW - Patient Compliance
KW - Play and Playthings/px [Psychology]
KW - Preanesthetic Medication
KW - Shyness
KW - Videotape Recording
RP - NOT IN FILE
SP - 170
EP - 178
JF - Journal of Child Health Care
JA - J Child Health Care
VL - 14
IS - 2
CY - England
N2 - Children can experience anaesthetic induction as fearful and frightening and this can lead to postoperative behaviour changes and symptoms of high anxiety. A fearful experience can also lead to avoidant reactions due to raised negative emotions in situations similar to that, which evoked the fear. To analyse children's reactions after anaesthesia to anaesthetic play equipment, 49 children (three-six years old) were video-filmed during play with anaesthetic equipment 14 days after anaesthesia and surgery. The risk that the child avoided playing with anaesthetic equipment was increased if the child took the premedication unwillingly and if the child was younger. The risk for not telling about the experience was increased if the child took the premedication unwillingly and if the child showed signs of shyness. The risk for telling mostly unspecific memories increased if the child was younger and if the child showed signs of shyness. Avoidant reactions could bee seen in 50 percent of the children. It is important to be aware of the characteristics of a vulnerable child (age, shyness) and to meet the child without raising negative emotions in any part of the anaesthetic process, in order to avoid negative reactions in future encounters
SN - 1741-2889
AD - Linkoping University, Sweden. marie.proczkowska.bjorklund@lj.se
UR - 20212059
ER -

TY - JOUR
ID - 208
T1 - Built environment, parents' perception, and children's vigorous outdoor play
A1 - Bringolf-Isler,B.
A1 - Grize,L.
A1 - Mader,U.
A1 - Ruch,N.
A1 - Sennhauser,F.H.
A1 - Braun-Fahrlander,C.
A1 - SCARPOL team.
Y1 - 2010/05//
N1 - Bringolf-Isler, Bettina. Grize, Leticia. Mader, Urs. Ruch, Nicole. Sennhauser, Felix H. Braun-Fahrlander, Charlotte. SCARPOL team
Preventive medicine
pm4, 0322116
IM
Journal Article. Research Support, Non-U.S. Gov't
English
KW - Medline
KW - Accidents
KW - Traffic
KW - Adolescent
KW - Age Factors
KW - Attitude to Health/eh [Ethnology]
KW - Attitude to Health
KW - Child
KW - Child Welfare/eh [Ethnology]
KW - Child Welfare/sn [Statistics & Numerical Data]
KW - Child Welfare
KW - Cross-Sectional Studies
KW - Environment Design
KW - Family Characteristics
KW - Female
KW - Geographic Information Systems
KW - Humans
KW - Male
KW - Multivariate Analysis
KW - Parents/ed [Education]
KW - Parents/px [Psychology]
KW - Play and Playthings
KW - Population Density
KW - Questionnaires
KW - Regression Analysis
KW - Residence Characteristics
KW - Safety
KW - Sex Factors
KW - Social Environment
KW - Statistics
KW - Nonparametric
KW - Switzerland
KW - Time Factors
KW - Urbanization
RP - NOT IN FILE
SP - 251
EP - 256

JA - Preventive Medicine
VL - 50
IS - 5-6
CY - United States
N2 - OBJECTIVES: To evaluate the combined effects and relative importance of socio-cultural factors as well as parents' subjectively perceived and objectively assessed environment on time children spent vigorously playing outdoors. METHODS: Cross-sectional study conducted in Berne, Biel-Bienne, and Payerne (Switzerland) during the school year 2004/2005. Included 1345 parental questionnaires from children out of three age groups (6/7, 9/10, and 13/14 years). A total of 1081 (80%) provided a home address, which could be linked to environmental data using a geographic information system (GIS). RESULTS: GIS-derived main street density in a buffer of 100 m around the home was inversely associated with time playing outdoors in adolescents and younger children, but only in more urbanized areas. In addition and independently of GIS-based main street density, parental concern about traffic safety was associated with less time playing outdoors in primary school children. Girls, adolescents, and children from the French speaking part of the country spent less time playing outdoors. A non-Swiss nationality and having younger siblings increased time playing vigorously outdoors in adolescents. CONCLUSION: In addition to socio-cultural factors, parents' perceptions and objectively measured environmental factors were significantly associated with the time spent vigorously playing outdoors. These associations differed by age group. Copyright (c) 2010 The Institute For Cancer Prevention. Published by Elsevier Inc. All rights reserved
SN - 1096-0260
AD - Swiss Tropical and Public Health Institute, Basel, Switzerland. Bettina.Bringolf@unibas.ch
UR - 20346370
ER -

TY - JOUR
ID - 209
T1 - Early identification of children at risk of unintentional injury: a sensation seeking scale for children 2-5 years of age
A1 - Morrongiello,B.A.
A1 - Sandomierski,M.
A1 - Valla,J.
Y1 - 2010/07//
N1 - Morrongiello, Barbara A. Sandomierski, Megan. Valla, Jasmina
Accident; analysis and prevention
acs, 1254476
IM
Journal Article. Research Support, Non-U.S. Gov't. Research Support, U.S. Gov't, P.H.S.
English
KW - Medline
KW - Accident Proneness
KW - Child Behavior/px [Psychology]
KW - Child
KW - Preschool
KW - Cross-Sectional Studies
KW - Female
KW - Humans
KW - Male
KW - Play and Playthings/px [Psychology]
KW - Pleasure
KW - Psychometrics
KW - Questionnaires
KW - Reproducibility of Results
KW - Risk Assessment
KW - Risk-Taking
KW - Wounds and Injuries/ep [Epidemiology]
KW - Wounds and Injuries/px [Psychology]

RP - NOT IN FILE
SP - 1332
EP - 1337
JF - Accident Analysis & Prevention
JA - Accid.Anal.Prev.
VL - 42
IS - 4
CY - England
N2 - OBJECTIVE: Sensation seeking is a personality attribute associated with injury in school-age children, adolescents, and adults. This study aimed to develop and evaluate the psychometric properties of a questionnaire measure of sensation seeking for young children 2-5 years of age. METHODS: Items tapping aspects of sensation seeking (Novelty Seeking, Behavioral Intensity, Thrill Seeking) were developed, content validation was completed by child development experts, and 72 parents then completed the Sensation Seeking Scale for Young Children (SSSYC) twice (3 months intervening) to establish internal and test-retest reliabilities. To assess criterion validity, scores were related to children's preferences for high versus low sensation seeking activities, their free play behavior in an indoor playroom, and to children's history of unintentional injuries. RESULTS: Indices of internal reliability and test-retest reliability were good and questionnaire scores positively correlated with sensation seeking behaviors during free play, preferences for high versus low sensation seeking activities, and injury measures, providing support for criterion validity. CONCLUSION: This initial evaluation of the SSSYC suggests that it is a psychometrically sound measure of sensation seeking. This scale may be useful for identifying high sensation seekers at young ages, providing the opportunity for early intervention to prevent unintentional injuries. Copyright 2010 Elsevier Ltd. All rights reserved
SN - 1879-2057
AD - Psychology Department, University of Guelph, Guelph, Ontario N1G 2W1, Canada. bmorrong@uoguelph.ca
UR - 20441850
ER -

TY - JOUR
ID - 210
T1 - A review of state regulations to promote physical activity and safety on playgrounds in child care centers and family child care homes
A1 - Cradock,A.L.
A1 - O'Donnell,E.M.
A1 - Benjamin,S.E.
A1 - Walker,E.
A1 - Slining,M.
Y1 - 2010/03//
N1 - Cradock, Angie L I. O'Donnell, Emily M. Benjamin, Sara E. Walker, Elizabeth. Slining, Meghan
Journal of physical activity & health
101189457
IM
Comparative Study. Journal Article. Research Support, U.S. Gov't, P.H.S.
English
KW - Medline
KW - Caregivers/lj [Legislation & Jurisprudence]
KW - Caregivers/st [Standards]
KW - Child
KW - Child Care/lj [Legislation & Jurisprudence]
KW - Child Care/st [Standards]
KW - Child Welfare
KW - District of Columbia
KW - Government Regulation
KW - Health Promotion
KW - Humans
KW - Motor Activity

KW - Play and Playthings
KW - Safety/sn [Statistics & Numerical Data]
KW - State Government
KW - United States
KW - Virgin Islands of the United States
KW - Wounds and Injuries/pc [Prevention & Control]
RP - NOT IN FILE
SP - S108
EP - S119
JF - Journal of Physical Activity & Health
JA - J Phys Act.Health
VL - 7 Suppl 1
CY - United States
N2 - BACKGROUND: As interventions increasingly emphasize early child care settings, it is necessary to understand the state regulatory context that provides guidelines for outdoor physical activity and safety and sets standards for child care environments. METHODS: Researchers reviewed regulations for child care facilities for 50 states, the District of Columbia and the Virgin Islands. We compared state regulations with national standards for 17 physical activity- and safety-related items for outdoor playground settings outlined in Caring for Our Children: National Health and Safety Performance Standards: Guidelines for Out-of-Home Child Care Programs (CFOC). State regulations were coded as fully, partially or not addressing the CFOC standard and state-level summary scores were calculated. RESULTS: On average, state regulations fully addressed one-third of 17 CFOC standards in regulations for centers (34%) and family child care homes (27%). Data suggest insufficient attention to outdoor play area proximity and size, equipment height, surfacing, and inspections. CONCLUSIONS: Considerable variation exists among state regulations related to physical activity promotion and injury prevention within outdoor play areas. Many states' regulations do not comply with published national health and safety standards. Enhancing regulations is one component of a policy approach to promoting safe, physically active child care settings
SN - 1543-3080
AD - Dept. of Society, Human Development, and Health, Harvard School of Public Health, Boston, MA, USA
UR - 20440004
ER -

TY - JOUR
ID - 211
T1 - Association of anemia, child and family characteristics with elevated blood lead concentrations in preschool children from Montevideo, Uruguay
A1 - Queirolo,E.I.
A1 - Ettinger,A.S.
A1 - Stoltzfus,R.J.
A1 - Kordas,K.
Y1 - 2010/04//
N1 - Queirolo, Elena I. Ettinger, Adrienne S. Stoltzfus, Rebecca J. Kordas, Katarzyna
Archives of environmental & occupational health
101282564
AIM, IM
Journal Article. Research Support, Non-U.S. Gov't
English
KW - Medline
KW - Age Factors
KW - Anemia/ci [Chemically Induced]
KW - Child
KW - Preschool
KW - Educational Status
KW - Environmental Exposure/ae [Adverse Effects]
KW - Family Characteristics
KW - Female

KW - Hemoglobins/an [Analysis]
KW - Humans
KW - Infant
KW - Lead/bl [Blood]
KW - Lead Poisoning/co [Complications]
KW - Male
KW - Maternal Age
KW - Occupational Exposure/ae [Adverse Effects]
KW - Play and Playthings
KW - Uruguay/ep [Epidemiology]
RP - NOT IN FILE
SP - 94
EP - 100
JF - Archives of Environmental & Occupational Health
JA - Arch.Environ.Occup Health
VL - 65
IS - 2
CY - United States
N2 - Elevated blood lead levels (BPbs) have been identified in Uruguayan children in the La Teja neighborhood of Montevideo, but the extent of lead exposure in other city areas is unknown. Sources and predictors of exposure also remain understudied in this population. In 2007, the authors screened lead and hemoglobin levels in capillary blood of 222 preschool children from several areas of Montevideo, Uruguay, and identified predictors of elevated BPbs. Mean BPb was 9.0 +/- 6.0 microg/dL and 32.9% of children had levels >or= 10microg/dL. Mean hemoglobin level was 10.5 +/- 1.5 g/dL, with 44.1% having levels <10.5g/dL. Older child age, hemoglobin <10.5g/dL, and putting fingers/toys in the mouth were associated with higher BPbs. Young maternal age, less education, father's job with potential risk of lead exposure, and fewer family possessions were also associated with higher BPbs. Pediatric lead exposure is a public health problem in Uruguay, with children experiencing elevated BPbs at a young age
SN - 1933-8244
AD - Center for Research, Catholic University of Uruguay, Montevideo, Uruguay Clinic for Environmental Contaminants, Pereira Rossell Hospital, Montevideo, Uruguay
UR - 20439228
ER -

TY - JOUR
ID - 212
T1 - The representation of fathers by children of depressed mothers: refining the meaning of parentification in high-risk samples
A1 - Woolgar,M.
A1 - Murray,L.
Y1 - 2010/05//
N1 - Woolgar, Matthew. Murray, Lynne
Journal of child psychology and psychiatry, and allied disciplines
hp3, 0375361
IM
Journal Article. Research Support, Non-U.S. Gov't
English
KW - Medline
KW - Child Behavior/px [Psychology]
KW - Child Development
KW - Child of Impaired Parents/px [Psychology]
KW - Child
KW - Preschool
KW - Depressive Disorder/px [Psychology]
KW - Family Conflict/px [Psychology]
KW - Father-Child Relations

KW - Fathers/px [Psychology]
KW - Female
KW - Humans
KW - Male
KW - Mothers/px [Psychology]
KW - Risk Factors
KW - Sex Factors
RP - NOT IN FILE
SP - 621
EP - 629
JF - Journal of Child Psychology & Psychiatry & Allied Disciplines
JA - J Child Psychol.Psychiatry
VL - 51
IS - 5
CY - England
N2 - BACKGROUND: Children's representations of mothers in doll-play are associated with child adjustment. Despite the importance of fathers for children's adjustment, especially in the context of maternal psychopathology, few studies have considered children's representations of their fathers. METHOD: We examined the portrayal of fathers by 5-year-old children of depressed (N = 55) and non-depressed (N = 39) mothers in a doll-play procedure concerning family experience. RESULTS: Children gave equal prominence in their play to mothers and fathers. Representations of fathers were unrelated to maternal mood, but were associated with parental conflict. Representations of child care for the father that was unreciprocated predicted poor child adjustment in school, but only in children exposed to maternal postnatal depression. CONCLUSIONS: It may be clinically useful to consider children's distinctive representations of their mother and father; but the concept of parentification in relation to risk and resilience effects requires refinement
SN - 1469-7610
AD - Institute of Psychiatry, Denmark Hill, London, SE5 8AF, UK. Matt.Woolgar@iop.kcl.ac.uk
UR - 19788554
ER -

TY - JOUR
ID - 213
T1 - Short-term effects of playing computer games on attention
A1 - Tahiroglu,A.Y.
A1 - Celik,G.G.
A1 - Avci,A.
A1 - Seydaoglu,G.
A1 - Uzel,M.
A1 - Altunbas,H.
Y1 - 2010/05//
N1 - Tahiroglu, Aysegul Yolga. Celik, Gonca Gul. Avci, Ayse. Seydaoglu, Gulsah. Uzel, Mehtap. Altunbas, Handan
Journal of attention disorders
9615686
IM
Journal Article. Research Support, Non-U.S. Gov't
English
KW - Medline
KW - Attention
KW - Child
KW - Cognition
KW - Female
KW - Humans
KW - Learning
KW - Male
KW - Play and Playthings

KW - Time Factors
KW - Video Games
RP - NOT IN FILE
SP - 668
EP - 676
JF - Journal of Attention Disorders
JA - J Atten.Disord
VL - 13
IS - 6
CY - United States
N2 - OBJECTIVE: The main aim of the present study is to investigate the short-term cognitive effects of computer games in children with different psychiatric disorders and normal controls. METHOD: One hundred one children are recruited for the study (aged between 9 and 12 years). All participants played a motor-racing game on the computer for 1 hour. The TBAG form of the Stroop task was administered to all participants twice, before playing and immediately after playing the game. RESULTS: Participants with improved posttest scores, compared to their pretest scores, used the computer on average 0.67 +/- 1.1 hr/day, while the average administered was measured at 1.6 +/- 1.4 hr/day and 1.3 +/- 0.9 hr/day computer use for participants with worse or unaltered scores, respectively. According to the regression model, male gender, younger ages, duration of daily computer use, and ADHD inattention type were found to be independent risk factors for worsened posttest scores. CONCLUSION: Time spent playing computer games can exert a short-term effect on attention as measured by the Stroop test
SN - 1557-1246
AD - Cukurova University, Adana, Turkey. ayolga@gmail.com
UR - 19773602
ER -

TY - JOUR
ID - 214
T1 - An analysis of risk factors of non-fatal drowning among children in rural areas of Guangdong Province, China: a case-control study
A1 - Ma,W.J.
A1 - Nie,S.P.
A1 - Xu,H.F.
A1 - Xu,Y.J.
A1 - Song,X.L.
A1 - Guo,Q.Z.
A1 - Zhang,Y.R.
Y1 - 2010///
N1 - Ma, Wen Jun. Nie, Shao Ping. Xu, Hao Feng. Xu, Yan Jun. Song, Xiu Ling. Guo, Qiao Zhi. Zhang, Yu Run
BMC public health
100968562
IM
Journal Article. Research Support, Non-U.S. Gov't
English
KW - Medline
KW - Accident Prevention/mt [Methods]
KW - Adult
KW - Case-Control Studies
KW - Child
KW - China/ep [Epidemiology]
KW - Drowning/ep [Epidemiology]
KW - Drowning/et [Etiology]
KW - Female
KW - Fresh Water
KW - Humans

KW - Male
KW - Play and Playthings
KW - Risk Factors
KW - Rural Population/sn [Statistics & Numerical Data]
KW - Sex Factors
KW - Swimming
RP - NOT IN FILE
SP - 156
JA - BMC Public Health
VL - 10
CY - England
N2 - BACKGROUND: Drowning is a major cause of morbidity and mortality for children, yet non-fatal drowning remains poorly understood. The aim of this study was to explore potential modifiable risk factors of non-fatal drowning among children in rural areas of China. METHODS: A cross-sectional survey was first conducted to obtain non-fatal drowning cases, and 7432 students in grades three to eight from 17 schools participated in the cross sectional survey. Of these, 805 students reported that they experienced non-fatal drowning in the previous year. Then 368 cases were selected randomly to participate in a 1:1 matched case-control study. Each drowning case was matched by one control with the same sex and similar age (the gap less than 2 years) who was selected randomly from the same class. RESULTS: Boys were more likely to be involved in non-fatal drowning. Non-fatal drowning most often happened in the afternoon (65.1%) and natural bodies of water were the most common sites of drowning (71.1%). Swimming, diving and playing in natural waters were the leading activities that preceded non-fatal drowning. The significant risk factors for non-fatal drowning were swimming in natural waters without adult supervision (OR = 3.40, 95% CI: 1.92-6.03), playing in or beside natural waters (OR = 2.08, 95% CI: 1.17-3.70) and poor swimming skills (OR = 2.74, 95% CI: 1.14-6.62). However, the following variables were protective factors: supervisor aged 30 years or over (OR = 0.20, 95% CI: 0.09-0.49) and no water activities (OR = 0.36, 95% CI: 0.18-0.70). CONCLUSIONS: The reduction in dangerous water activities, swimming training and enhancement in supervision among children might decrease the risk of non-fatal drowning
SN - 1471-2458
AD - Department of Non-communicable Disease Control and Prevention, Center for Disease Control and Prevention of Guangdong Province, China. mwj68@tom.com
UR - 20334694
ER -

TY - JOUR
ID - 215
T1 - [Are children and adolescents of today more sedentary?]. [German]
A1 - Staub,A.
Y1 - 2010/03//
N1 - Staub, Angelika
Kinderkrankenschwester : Organ der Sektion Kinderkrankenpflege / Deutsche Gesellschaft fur Sozialpadiatrie und Deutsche Gesellschaft fur Kinderheilkunde
awq, 8305989
N
Journal Article
German
KW - Medline
KW - Adolescent
KW - Child
KW - Child Development
KW - Preschool
KW - Chronic Disease/nu [Nursing]
KW - Chronic Disease/pc [Prevention & Control]
KW - Germany
KW - Humans
KW - Motor Activity

KW - Motor Skills
KW - Physical Fitness
KW - Play and Playthings
KW - Psychosocial Deprivation
KW - Risk Factors
RP - NOT IN FILE
SP - 118
JF - Kinderkrankenschwester
VL - 29
IS - 3
CY - Germany
SN - 0723-2276
AD - AngelikaStaub@web.de
UR - 20364661
ER -

TY - JOUR
ID - 216
T1 - Childhood obesity and the built environment.[Erratum appears in Pediatr Ann. 2010 Jun;39(6):331]
A1 - Razani,N.
A1 - Tester,J.
Y1 - 2010/03//
N1 - Razani, Nooshin. Tester, June
Pediatric annals
oub, 0356657
IM
Journal Article
English
KW - Medline
KW - Acanthosis Nigricans
KW - Child
KW - Environment Design
KW - Health Promotion
KW - Humans
KW - Life Style
KW - Motor Activity
KW - Obesity/ep [Epidemiology]
KW - Obesity/pc [Prevention & Control]
KW - Pediatrics
KW - Physician's Role
KW - Play and Playthings
KW - Poverty
KW - Safety
KW - Social Environment
KW - Suburban Population
RP - NOT IN FILE
SP - 133
EP - 139
JF - Pediatric Annals
JA - Pediatr Ann.
VL - 39
IS - 3
CY - United States
SN - 0090-4481
AD - Adolescent Medicine Department, Children's Hospital & Research Center, Oakland, CA, USA.
nrazani@mail.cho.org

UR - 20302244
ER -

TY - JOUR
ID - 217
T1 - Television viewing, computer game play and book reading during meals are predictors of meal skipping in a cross-sectional sample of 12-, 14- and 16-year-olds
A1 - Custers,K.
A1 - Van den,Bulck J.
Y1 - 2010/04//
N1 - Custers, Kathleen. Van den Bulck, Jan
Public health nutrition
9808463, dgy
IM
Journal Article
English
KW - Medline
KW - Adolescent
KW - Belgium
KW - Body Mass Index
KW - Books
KW - Child
KW - Computers
KW - Cross-Sectional Studies
KW - Feeding Behavior
KW - Female
KW - Humans
KW - Male
KW - Play and Playthings
KW - Prevalence
KW - Questionnaires
KW - Television
KW - Video Games
RP - NOT IN FILE
SP - 537
EP - 543
JF - Public Health Nutrition
JA - Public Health Nutr
VL - 13
IS - 4
CY - England
N2 - OBJECTIVE: To examine whether television viewing, computer game playing or book reading during meals predicts meal skipping with the aim of watching television, playing computer games or reading books (media meal skipping). DESIGN: A cross-sectional study was conducted using a standardized self-administered questionnaire. Analyses were controlled for age, gender and BMI. SETTING: Data were obtained from a random sample of adolescents in Flanders, Belgium. SUBJECTS: Seven hundred and ten participants aged 12, 14 and 16 years. RESULTS: Of the participants, 11.8 % skipped meals to watch television, 10.5 % skipped meals to play computer games and 8.2 % skipped meals to read books. Compared with those who did not use these media during meals, the risk of skipping meals in order to watch television was significantly higher for those children who watched television during meals (2.9 times higher in those who watched television during at least one meal a day). The risk of skipping meals for computer game playing was 9.5 times higher in those who played computer games weekly or more while eating, and the risk of meal skipping in order to read books was 22.9 times higher in those who read books during meals less than weekly. The more meals the respondents ate with the entire family, the less likely they were to skip meals to watch television. CONCLUSIONS: The use of media during meals predicts meal skipping for using that same medium. Family meals appear to be inversely related to meal skipping for television viewing

SN - 1475-2727
AD - Leuven School for Mass Communication Research and Leuven Food Science and Nutrition Research Centre (LFoRCe), Katholieke Universiteit Leuven, Leuven, Belgium. kathleen.custers@soc.kuleuven.be
UR - 19772688
ER -

TY - JOUR
ID - 218
T1 - Nonspecific low back pain during childhood: a retrospective epidemiological study of risk factors
A1 - Kaspiris,A.
A1 - Grivas,T.B.
A1 - Zafiropoulou,C.
A1 - Vasiliadis,E.
A1 - Tsadira,O.
Y1 - 2010/03//
N1 - Kaspiris, Angelos. Grivas, Theodoros B. Zafiropoulou, Crisi. Vasiliadis, Elias. Tsadira, Olia
Journal of clinical rheumatology : practical reports on rheumatic & musculoskeletal diseases
9518034
IM
Journal Article
English
KW - Medline
KW - Adolescent
KW - Age Factors
KW - Body Height
KW - Child
KW - Female
KW - Greece/ep [Epidemiology]
KW - Health Surveys
KW - Humans
KW - Low Back Pain/ep [Epidemiology]
KW - Low Back Pain/et [Etiology]
KW - Male
KW - Overweight
KW - Prevalence
KW - Retrospective Studies
KW - Sex Factors
RP - NOT IN FILE
SP - 55
EP - 60
JF - JCR: Journal of Clinical Rheumatology
JA - J
VL - 16
IS - 2
CY - United States
N2 - OBJECTIVES: In contrast to what was believed in the past, nonspecific low back pain is a fairly frequent condition in children, whose pathophysiology remains unclear as yet. Although many factors have been implicated in its development, results are often contradictory. METHODS: Our study aims to examine most of the reasons investigated in the international literature, as well as the previously unexamined impact of passive smoking in its clinical appearance. It is a retrospective study that investigates the symptom of nonspecific low back pain during a 12-month period before the visit of children to our department. The research included 692 children aged 7.5 to 14 years. The data were collected using a semi-structured questionnaire, which included a mix of open and closed questions, followed by physical examination during their visit. RESULTS: A total of 153 children were considered to present nonspecific low back pain during the previous year. The determinant factors appear to be greater age, the male sex, larger height, increased weight, dissatisfaction with school chairs, the clinical presentation of back pain in at least 1 parent, and coexisting anatomic orthopedic conditions. On the

contrary, the weight of the school bag, the way in which it was carried and participation in sports, as well as the time spent by children in front of the TV or PC playing video or play station games, did not appear to have a statistically significant correlation with its appearance. In general, passive smoking does not appear to be a risk factor (P[r] = 0.341), and does not seem to play a leading role in the etiology of the condition. Furthermore, even the heaviness of parental smoking (over 20 cigarettes a day) does not seem to alter the appearance of the disease. The effect of nonspecific low back pain in children's activities was measured using Hannover Functional Ability and Rolland Morris questionnaires, appropriately modified to childhood, where he found a moderate or severe restriction of activity in 23.52% (score >5) and 19.61% (score >6), respectively. CONCLUSIONS: The data analysis shows that nonspecific low back pain in children is a benign disorder with an unknown pathophysiological mechanism. Many anthropometric characteristics and environmental factors are implicated, but to a different degree each time. Passive smoking as well as the heaviness thereof does not appeal to play in important role in its clinical presentation. Further investigation is deemed necessary to determine the existence of other risk factors, as well as the level of their participation in the condition's pathophysiology
SN - 1536-7355
AD - Department of Trauma and Orthopaedics, Thriasio General Hospital-NHS, Attica, Greece.
angkaspiris@upatras.gr
UR - 20130481
ER -

TY - JOUR
ID - 219
T1 - Constituent year: a new consideration for injury risk in Canadian youth ice hockey
A1 - Wattie,N.
A1 - Cobley,S.P.
A1 - Macpherson,A.
A1 - Montelpare,W.J.
A1 - McKenna,J.
A1 - Howard,A.
A1 - Baker,J.
Y1 - 2010/03//
N1 - Wattie, Nick. Cobley, Stephen P. Macpherson, Alison. Montelpare, William J. McKenna, Jim. Howard, Andrew. Baker, Joseph
Clinical journal of sport medicine : official journal of the Canadian Academy of Sport Medicine
b9t, 9103300
IM
Journal Article
English
KW - Medline
KW - Adolescent
KW - Age Factors
KW - Canada
KW - Child
KW - Competitive Behavior
KW - Databases
KW - Factual
KW - Hockey/in [Injuries]
KW - Humans
KW - Logistic Models
KW - Retrospective Studies
KW - Risk Assessment
RP - NOT IN FILE
SP - 113
EP - 116
JF - Clinical Journal of Sport Medicine
JA - Clin.J Sport Med
VL - 20

IS - 2
CY - United States
N2 - OBJECTIVE: To examine if chronological age within Canadian youth ice hockey's 2-year age bands influences the proportion of injury. DESIGN: Retrospective secondary data analyses. PARTICIPANTS: Information on 4736 injured youth ice hockey players (10-15 years old) reported by the Canadian Hospitals Injury Reporting Prevention Program (CHIRPP) and 4959 (12-15 years old) injured players reported by the Hockey Canada Insurance Database (HCID). MAIN OUTCOME MEASURES: Proportions of injuries according to constituent year (first vs second year of participation within 2-year youth ice hockey age bands). INDEPENDENT VARIABLES: The influence of age band (Atom, 10/11; Peewee, 12/13; Bantam, 14/15) and level of competitive play on constituent year injury proportions were examined. RESULTS: Injured Atom and Peewee players (CHIRPP) were more likely to be in constituent year 2 (Atom: odds ratio [OR], 1.72; 95% confidence interval [CI], 1.46-2.03; Peewee: OR, 1.25; 95% CI, 1.10-1.42). Injured players (HCID) at the highest tiers of competitive play were more likely to be in constituent year 2 (eg, Peewee: OR, 2.91; 95% CI, 1.92-4.41; Bantam: 1.89; 95% CI, 1.46-2.46). CONCLUSION: Constituent year may be a factor in determining injury risk and may be relevant to those managing the risk of injury for youth ice hockey players
SN - 1536-3724
AD - Carnegie Faculty of Sport and Education, Leeds Metropolitan University, Leeds, United Kingdom. n.wattie@leedsmet.ac.uk
UR - 20215893
ER -

TY - JOUR
ID - 220
T1 - The elephant in the playground: confronting lead-contaminated soils as an important source of lead burdens to urban populations
A1 - Filippelli,G.M.
A1 - Laidlaw,M.A.
Y1 - 2010///
N1 - Filippelli, Gabriel M. Laidlaw, Mark A
Perspectives in biology and medicine
ozo, 0401132
IM
Journal Article
English
KW - Medline
KW - Child
KW - Preschool
KW - Environmental Exposure/ae [Adverse Effects]
KW - Environmental Exposure/an [Analysis]
KW - Geography
KW - Health Policy
KW - Humans
KW - Lead/an [Analysis]
KW - Lead/bl [Blood]
KW - Lead Poisoning/bl [Blood]
KW - Lead Poisoning/et [Etiology]
KW - Play and Playthings
KW - Poverty
KW - Soil Pollutants/ae [Adverse Effects]
KW - Soil Pollutants/an [Analysis]
KW - United States
KW - Urban Population
RP - NOT IN FILE
SP - 31
EP - 45
JF - Perspectives in Biology & Medicine

JA - Perspect.Biol.Med
VL - 53
IS - 1
CY - United States
N2 - Although significant headway has been made over the past 50 years in understanding and reducing the sources and health risks of lead, the incidence of lead poisoning remains shockingly high in urban regions of the United States. At particular risk are poor people who inhabit the polluted centers of our older cities without the benefits of adequate nutrition, education, and access to health care. To provide a future with fewer environmental and health burdens related to lead, we need to consider the multiple pathways of lead exposure in children, including their continued contact with dust derived from inner-city soils. Recent research into the causes of seasonal variations in blood-lead levels among children has confirmed the importance of soil in lead exposure. "Capping" lead-contaminated soil with lead-free soil or soil amendment appears to be a simple and cost-effective way to reduce the lead load for urban youth
SN - 1529-8795
AD - Department of Earth Sciences, Indiana University-Purdue University Indianapolis (IUPUI), Indianapolis, IN 46205, USA. gfilippe@iupui.edu
UR - 20173294
ER -

TY - JOUR
ID - 221
T1 - Violence in youth sports: hazing, brawling and foul play. [Review]
A1 - Fields,S.K.
A1 - Collins,C.L.
A1 - Comstock,R.D.
Y1 - 2010/01//
N1 - Fields, S K. Collins, C L. Comstock, R D
British journal of sports medicine
0432520
IM
Journal Article. Review
English
KW - Medline
KW - Adolescent
KW - Aggression/px [Psychology]
KW - Child
KW - Competitive Behavior
KW - Dangerous Behavior
KW - Humans
KW - Sports/px [Psychology]
KW - Sports/sn [Statistics & Numerical Data]
KW - Violence/px [Psychology]
KW - Violence/sn [Statistics & Numerical Data]
RP - NOT IN FILE
SP - 32
EP - 37
JF - British Journal of Sports Medicine
JA - BJSM.online.
VL - 44
IS - 1
CY - England
N2 - By separating hazing, brawling, and foul play and failing to recognise that their connection to sport binds them together into a cohesive subset of sport injury and youth violence, past research has failed to show how sports-related violence is a broad example of interpersonal violence. The acceptance of violence within the sporting culture may, in part, explain why sports-related violence has not yet been widely recognised as a public health concern. This review shows that sports-related violence, including hazing, brawling and foul play, occurs

among youth athletes of all ages and in a variety of different sports. The few studies to address this issue have all acknowledged the dangers of sports-related violence; however, no incident tracking method has been developed. Future research must provide accurate national estimates of the incidence of sports-related violence among youth, identify associated risk factors, evaluate preventive interventions and identify effective methods of distributing and implementing evidence-based interventions. Monitoring the magnitude and distribution of the burden of sports-related violence and building the scientific infrastructure necessary to support the development and widespread application of effective sports-related prevention interventions are essential first steps toward a reduction in the incidence of sports-related violence
SN - 1473-0480
AD - College of Education, School of Physical Activity and Educational Services, The Ohio State University, Columbus, OH 43205, USA
UR - 19858113
ER -

TY - JOUR
ID - 222
T1 - Test-retest reliability of the National Program for Playground Safety Report Card
A1 - Xethali,V.
A1 - Christoforidis,C.
A1 - Kambas,A.
A1 - Aggelousis,N.
A1 - Fatouros,I.
Y1 - 2009/12//
N1 - Xethali, Vasiliki. Christoforidis, Christos. Kambas, Antonis. Aggelousis, Nikolaos. Fatouros, Ioannis
International journal of injury control and safety promotion
101247254
IM
Journal Article
English
KW - Medline
KW - Child
KW - Equipment Safety
KW - Humans
KW - Observer Variation
KW - Play and Playthings
KW - Reproducibility of Results
KW - Risk Assessment/st [Standards]
KW - Safety/st [Standards]
RP - NOT IN FILE
SP - 249
EP - 251
JF - International Journal of Injury Control & Safety Promotion
JA - Int.J Inj.Contr.Saf Promot
VL - 16
IS - 4
CY - England
SN - 1745-7319
AD - Department of Physical Education and Sport Science, Democritus University of Thrace, Thrace, Komotini, Greece
UR - 20183704
ER -

TY - JOUR
ID - 223
T1 - Update on "the choking game"
A1 - Andrew,T.A.

A1 - Macnab,A.
A1 - Russell,P.
Y1 - 2009/12//
N1 - Andrew, Thomas A. Macnab, Andrew. Russell, Patricia
The Journal of pediatrics
jlz, 0375410
AIM, IM
Journal Article
English
KW - Medline
KW - Adolescent
KW - Airway Obstruction/co [Complications]
KW - Airway Obstruction/ep [Epidemiology]
KW - Airway Obstruction/px [Psychology]
KW - Asphyxia/co [Complications]
KW - Asphyxia/ep [Epidemiology]
KW - Asphyxia/px [Psychology]
KW - Child
KW - Child Behavior/px [Psychology]
KW - Female
KW - Humans
KW - Male
KW - Play and Playthings
KW - Risk-Taking
KW - United States/ep [Epidemiology]
RP - NOT IN FILE
SP - 777
EP - 780
JF - Journal of Pediatrics
JA - J Pediatr
VL - 155
IS - 6
CY - United States
SN - 1097-6833
AD - Office of the Chief Medical Examiner, Concord, NH 03301, USA. thomas.andrew@doj.nh.gov
UR - 19914429
ER -

TY - JOUR
ID - 224
T1 - Brief report: Young children's risk of unintentional injury: a comparison of mothers' and fathers' supervision beliefs and reported practices
A1 - Morrongiello,B.A.
A1 - Walpole,B.
A1 - McArthur,B.A.
Y1 - 2009/11//
N1 - Morrongiello, Barbara A. Walpole, Beverly. McArthur, Brae Anne
Journal of pediatric psychology
jl8, 7801773
IM
Journal Article. Research Support, Non-U.S. Gov't
English
KW - Medline
KW - Child
KW - Preschool
KW - Culture

KW - Female
KW - Gender Identity
KW - Health Surveys
KW - Humans
KW - Male
KW - Maternal Behavior/px [Psychology]
KW - Ontario
KW - Parenting/px [Psychology]
KW - Paternal Behavior
KW - Play and Playthings
KW - Questionnaires
KW - Risk-Taking
KW - Safety
KW - Self Care/px [Psychology]
KW - Wounds and Injuries/ep [Epidemiology]
KW - Wounds and Injuries/pc [Prevention & Control]
KW - Wounds and Injuries/px [Psychology]
RP - NOT IN FILE
SP - 1063
EP - 1068
JF - Journal of Pediatric Psychology
JA - J Pediatr Psychol.
VL - 34
IS - 10
CY - United States
N2 - OBJECTIVE: There is increasing interest in understanding how parent supervision influences young children's risk of injury, but nearly all of this research has been conducted with mothers. The present study compared first-time mothers' and fathers' supervisory beliefs and reported practices, and related these scores to parental reports of their child's history of injuries. METHODS: Mothers and fathers of children 2-5 years each independently completed a telephone interview and previously validated questionnaires about their supervisory beliefs and practices and their child's history of injuries. RESULTS: Mothers and fathers provided similar reports of their child's injuries (minor, medically attended) and scored similarly on various supervision indices. Despite these similarities, the way mothers' and fathers' supervision indices related to children's injury history scores differed. Children's frequency of minor and medically attended injuries was predicted from maternal supervisory scores but not from paternal scores. CONCLUSIONS: Maternal supervision has more impact on children's risk of injury than paternal supervision, possibly because mothers spend more time with children than fathers
SN - 1465-735X
AD - Psychology Department, University of Guelph, Guelph, Ontario, Canada. bmorrong@uoguelph.ca
UR - 19276175
ER -

TY - JOUR
ID - 225
T1 - Design of the iPlay study: systematic development of a physical activity injury prevention programme for primary school children
A1 - Collard,D.C.
A1 - Chinapaw,M.J.
A1 - van,Mechelen W.
A1 - Verhagen,E.A.
Y1 - 2009///
N1 - Collard, Dorine C M. Chinapaw, Mai J M. van Mechelen, Willem. Verhagen, Evert A L M
Sports medicine (Auckland, N.Z.)
8412297, spo
IM

Journal Article. Research Support, Non-U.S. Gov't
English
KW - Medline
KW - Accident Prevention/mt [Methods]
KW - Accidents
KW - Athletic Injuries/ec [Economics]
KW - Athletic Injuries/pc [Prevention & Control]
KW - Child
KW - Faculty
KW - Female
KW - Focus Groups
KW - Health Promotion
KW - Health Services Needs and Demand
KW - Humans
KW - Interviews as Topic
KW - Male
KW - Motor Activity
KW - Needs Assessment
KW - Netherlands
KW - Play and Playthings/in [Injuries]
KW - Play and Playthings
KW - Program Development
KW - Program Evaluation
KW - Questionnaires
KW - Schools
KW - Social Marketing
KW - Students
RP - NOT IN FILE
SP - 889
EP - 901
JF - Sports Medicine
JA - Sports Med
VL - 39
IS - 11
CY - New Zealand
N2 - Health benefits of physical activity in children are well known. However, a drawback is the risk of physical activity-related injuries. Children are at particular risk for these injuries, because of a high level of exposure. Because of the high prevalence of physical activity injuries and the negative short- and long-term consequences, prevention of these injuries in children is important. This article describes how we systematically developed a school-based physical activity injury prevention programme using the intervention mapping (IM) protocol. IM describes a process for developing theory- and evidence-based health promotion programmes. The development can be described in six steps: (i) perform a needs assessment; (ii) identify programme and performance objectives; (iii) select methods and strategies; (iv) develop programme; (v) adopt and implement; and (vi) evaluate. First, the results of the needs assessment showed the injury problem in children and the different risk factors for physical activity injuries. Based on the results of the needs assessment the main focus of the injury prevention programme was described. Second, the overall programme objective of the injury prevention programme was defined as reducing the incidence of lower extremity physical activity injuries. Third, theoretical methods and practical strategies were selected to accomplish a decrease in injury incidence. The theoretical methods used were active learning, providing cues and scenario-based risk information, and active processing of information. The practical strategy of the injury prevention programme was an 8-month course about injury prevention to be used in physical education classes in primary schools. Fourth, programme materials that were used in the injury prevention programme were developed, including newsletters for children and parents, posters, exercises to improve motor fitness, and an information website. Fifth, an implementation plan was designed in order to ensure that the prevention programme would be implemented, adopted and sustained over time. Finally, an evaluation plan was designed. The injury prevention programme is being evaluated in a cluster randomized controlled trial with more than 2200 children from 40 primary schools

throughout the Netherlands. The IM process is a useful process for developing an injury prevention programme. Based on the steps of the IM we developed an 8-month injury prevention programme to be used in physical education classes of primary schools
SN - 0112-1642
AD - EMGO Institute for Health and Care Research and Department of Public and Occupational Health, VU University Medical Center, Amsterdam, the Netherlands
UR - 19827858
ER -

TY - JOUR
ID - 226
T1 - Return to play? Practical considerations for young athletes with cardiovascular disease. [Review] [60 refs]
A1 - Anderson,B.R.
A1 - Vetter,V.L.
Y1 - 2009/09//
N1 - Anderson, B R. Vetter, V L
British journal of sports medicine
0432520
IM
Journal Article. Review
English
KW - Medline
KW - Adolescent
KW - Adult
KW - Child
KW - Death
KW - Sudden
KW - Cardiac/et [Etiology]
KW - Cardiac/pc [Prevention & Control]
KW - Defibrillators
KW - Exercise/ph [Physiology]
KW - Exercise/px [Psychology]
KW - Heart Diseases/co [Complications]
KW - Heart Diseases/px [Psychology]
KW - Heart Diseases/th [Therapy]
KW - Humans
KW - Physical Education and Training/mt [Methods]
KW - Practice Guidelines as Topic
KW - Risk Factors
KW - Schools
KW - Sports/ph [Physiology]
KW - Sports/px [Psychology]
KW - Young Adult
RP - NOT IN FILE
SP - 690
EP - 695
JF - British Journal of Sports Medicine
JA - BJSM.online.
VL - 43
IS - 9
CY - England
N2 - Sports activities are an important part of the daily life of young persons. For a small proportion of those with cardiac conditions, sports participation can result in sudden cardiac arrest. Children and adolescents often participate in both organised and spontaneous play, for which activity guidelines are needed when a condition known to cause sudden cardiac death is identified. This article examines the current guidelines that provide recommendations for sports participation by young persons with cardiovascular disease and reviews the current

information on risks of physical activity for those with specific cardiac conditions. The goal of the practitioner is to balance the risks of restriction from physical activity with the benefits of participation. Some practical recommendations are offered for consideration, along with potential alternatives to absolute restriction. [References: 60]
SN - 1473-0480
AD - University of Pennsylvania School of Medicine, Pennsylvania, USA
UR - 19734504
ER -

TY - JOUR
ID - 227
T1 - Through the eyes of children: engaging primary school-aged children in creating supportive school environments for physical activity and nutrition
A1 - Fitzgerald,E.
A1 - Bunde-Birouste,A.
A1 - Webster,E.
Y1 - 2009/08//
N1 - Fitzgerald, Emma. Bunde-Birouste, Anne. Webster, Emma
Health promotion journal of Australia : official journal of Australian Association of Health Promotion Professionals
9710936
IM
Journal Article
English
KW - Medline
KW - Child
KW - Preschool
KW - Consumer Participation
KW - Exercise
KW - Female
KW - Focus Groups
KW - Food Habits
KW - Health Behavior
KW - Health Promotion
KW - Humans
KW - Male
KW - Play and Playthings
KW - Risk Reduction Behavior
KW - School Health Services
KW - Schools
RP - NOT IN FILE
SP - 127
EP - 132
JF - Health Promotion Journal of Australia
JA - HEALTH PROMOT J AUST
VL - 20
IS - 2
CY - Australia
N2 - ISSUES ADDRESSED: This paper presents a qualitative study of physical activity, eating and drinking experiences of children at Wellington Public School, NSW. The study was designed and implemented to inform school programs and policies with a goal of fostering a supportive and healthy environment at the school. METHODS: The study involved use of focus groups, drawings and photovoice with 37 students from Kindergarten to Year 6 to determine problems, underlying causes and possible student-led interventions to enhance the school physical activity and nutritional environment. RESULTS: Students considered play as related to fun games with friends. Sport was associated with structured, competitive types of physical activity. Infants class students preferred play, while primary students reported a preference for semi-structured and

structured activities. Suggestions to improve school playing areas mirrored these preferences. The children suggested strategies for increasing fruit, vegetable and water consumption during the school day. CONCLUSION: The students were keen to explore ways to increase physical activity opportunities and develop a supportive nutritional environment at school. Simple, easy to implement suggestions were among the outcomes of the study, reinforcing the importance of including students' views in healthy school program and policy development
SN - 1036-1073
AD - Greater Western Area Health Service, New South Wales. Emma.Fitzgerald@gwahs.health.nsw.gov.au
UR - 19642961
ER -

TY - JOUR
ID - 228
T1 - Community-based interventions to optimize early childhood development in low resource settings. [Review] [90 refs]
A1 - Maulik,P.K.
A1 - Darmstadt,G.L.
Y1 - 2009/08//
N1 - Maulik, P K. Darmstadt, G L
Journal of perinatology : official journal of the California Perinatal Association
jfp, 8501884
IM
Journal Article. Review
English
KW - Medline
KW - Child
KW - Preschool
KW - Delivery of Health Care
KW - Developing Countries
KW - Developmental Disabilities/th [Therapy]
KW - Early Intervention (Education)/ec [Economics]
KW - Early Intervention (Education)/mt [Methods]
KW - Humans
KW - Infant
KW - Newborn
KW - Mother-Child Relations
KW - Play and Playthings
KW - Poverty Areas
RP - NOT IN FILE
SP - 531
EP - 542
JF - Journal of Perinatology
JA - J Perinatol.
VL - 29
IS - 8
CY - United States
N2 - OBJECTIVE: Interventions targeting the early childhood period (0 to 3 years) help to improve neuro-cognitive functioning throughout life. Some of the more low cost, low resource-intensive community practices for this age-group are play, reading, music and tactile stimulation. This research was conducted to summarize the evidence regarding the effectiveness of such strategies on child development, with particular focus on techniques that may be transferable to developing countries and to children at risk of developing secondary impairments. STUDY DESIGN: PubMed, PsycInfo, Embase, ERIC, CINAHL and Cochrane were searched for studies involving the above strategies for early intervention. Reference lists of these studies were scanned and other studies were incorporated based on snow-balling. RESULT: Overall, 76 articles corresponding to 53 studies, 24 of which were randomized controlled trials, were identified. Sixteen of those studies were from low- and middle-income countries. Play and reading were the two commonest interventions and showed positive

impact on intellectual development of the child. Music was evaluated primarily in intensive care settings. Kangaroo Mother Care, and to a lesser extent massage, also showed beneficial effects. Improvement in parent-child interaction was common to all the interventions. CONCLUSION: Play and reading were effective interventions for early childhood interventions in low- and middle-income countries. More research is needed to judge the effectiveness of music. Kangaroo Mother Care is effective for low birth weight babies in resource poor settings, but further research is needed in community settings. Massage is useful, but needs more rigorous research prior to being advocated for community-level interventions. [References: 90]
SN - 1476-5543
AD - Department of Mental Health, Bloomberg School of Public Health, Johns Hopkins University, Baltimore, MD 21205, USA
UR - 19404276
ER -

TY - JOUR
ID - 229
T1 - Head Start children's lifestyle behaviors, parental perceptions of weight, and body mass index
A1 - Hudson,C.E.
A1 - Cherry,D.J.
A1 - Ratcliffe,S.J.
A1 - McClellan,L.C.
Y1 - 2009/08//
N1 - Hudson, Cindy E. Cherry, Donna J. Ratcliffe, Sarah J. McClellan, Lynx C
Journal of pediatric nursing
jns, 8607529
IM, N
Journal Article. Research Support, N.I.H., Extramural. Research Support, Non-U.S. Gov't
English
KW - Medline
KW - Alabama/ep [Epidemiology]
KW - Body Mass Index
KW - Body Weight
KW - Child Welfare
KW - Child
KW - Preschool
KW - Cross-Sectional Studies
KW - Diet
KW - Early Intervention (Education)
KW - Female
KW - Humans
KW - Life Style
KW - Male
KW - Obesity/ep [Epidemiology]
KW - Parents/px [Psychology]
KW - Prevalence
KW - Questionnaires
KW - Risk Factors
RP - NOT IN FILE
SP - 292
EP - 301
JF - Journal of Pediatric Nursing
JA - J Pediatr Nurs
VL - 24
IS - 4
CY - United States
N2 - This study examined lifestyle behaviors (food intake, active play, and screen time), parental perception of children's weight, and body mass index (BMI) of children enrolled in Head Start. Ninety-six parent-child dyads

participated. Obesity prevalence (15.6%) was higher than the national average (10.4%); however, most parents (86.5%) of obese children did not perceive their children as obese. Regardless of BMI, food intake and active play levels generally did not meet recommended guidelines. Further, children who were less active were more likely to eat snacks (chi(2) = 6.24, p < or = .04). The role of pediatric nurses in counseling and referring Head Start families is explored
SN - 1532-8449
AD - College of Nursing, Medical University of South Carolina, Charleston, SC, USA. hudsonc@musc.edu
UR - 19632506
ER -

TY - JOUR
ID - 230
T1 - Predicting preschoolers' social-cognitive play behavior: attachment, peers, temperament, and physiological regulation
A1 - Porter,C.L.
Y1 - 2009/04//
N1 - Porter, Christin L
Psychological reports
qf6, 0376475
IM
Comparative Study. Journal Article
English
KW - Medline
KW - Adolescent
KW - Adult
KW - Biological Markers
KW - Child Behavior/px [Psychology]
KW - Child
KW - Preschool
KW - Cognition
KW - Discriminant Analysis
KW - Female
KW - Heart/ir [Innervation]
KW - Heart/ph [Physiology]
KW - Humans
KW - Interpersonal Relations
KW - Male
KW - Object Attachment
KW - Peer Group
KW - Personality Assessment
KW - Play and Playthings
KW - Probability
KW - Q-Sort
KW - Questionnaires
KW - Shyness
KW - Social Behavior
KW - Social Support
KW - Sociometric Techniques
KW - Temperament
KW - Vagus Nerve/ph [Physiology]
RP - NOT IN FILE
SP - 517
EP - 528
JF - Psychological Reports
JA - Psychol.Rep.
VL - 104

IS - 2
CY - United States
N2 - Research on children's social-cognitive play typologies (i.e., active and passive forms of solitary and social play) suggests links of early play behaviors and later social development and risk status. To date, few studies have examined simultaneously suspected links between children's social-cognitive play types and factors believed to shape these early social-play behaviors. This study examined a simultaneous model of individual (temperament, physiology) and relational variables (attachment, peer networks) believed to influence children's social-cognitive play types, including individual characteristics drawn from the Child Behavior Questionnaire which measures dimensions of shyness and impulsivity, a lab-based assessment of social withdrawal, and physiological markers linked to social regulation (cardiac vagal tone and vagal regulation). Children's attachment status to parents was gathered using Q-Sort methodology, and a measure of previous peer network size was obtained from parents' reports to examine potential links between relational history and social-cognitive play types. Predictive discriminant function analysis showed that children's (N = 54, age range 35 to 58 months) social-cognitive play was better predicted on the basis of multiple independent variables than individual, zero-order relations. When predicting children's social-cognitive play typologies, a multidimensional view which encompasses both individual characteristics and social-relational variables may best predict social - cognitive play types and help understanding of children's social trajectories
SN - 0033-2941
AD - 2093 JFSB, School of Family Life, Brigham Young University, Provo, UT 84602, USA.
chris_porter@byu.edu
UR - 19610482
ER -

TY - JOUR
ID - 231
T1 - Rationale, design and methods for a randomised and controlled trial to investigate whether home access to electronic games decreases children's physical activity
A1 - Straker,L.M.
A1 - Abbott,R.A.
A1 - Piek,J.P.
A1 - Pollock,C.M.
A1 - Davies,P.S.
A1 - Smith,A.J.
Y1 - 2009///
N1 - Straker, Leon M. Abbott, Rebecca A. Piek, Jan P. Pollock, Clare M. Davies, Peter S. Smith, Anne J
BMC public health
100968562
IM
Journal Article. Randomized Controlled Trial. Research Support, Non-U.S. Gov't
English
KW - Medline
KW - Attitude to Health
KW - Child
KW - Electrical Equipment and Supplies
KW - Energy Metabolism
KW - Exercise
KW - Female
KW - Humans
KW - Male
KW - Motor Activity
KW - Video Games/px [Psychology]
KW - Video Games
RP - NOT IN FILE
SP - 212
JA - BMC Public Health
VL - 9

CY - England
N2 - BACKGROUND: Many children are reported to have insufficient physical activity (PA) placing them at greater risk of poor health outcomes. Participating in sedentary activities such as playing electronic games is widely believed to contribute to less PA. However there is no experimental evidence that playing electronic games reduces PA. There is also no evidence regarding the effect of different types of electronic games (traditional sedentary electronic games versus new active input electronic games) on PA. Further, there is a poor understanding about how characteristics of children may moderate the impact of electronic game access on PA and about what leisure activities are displaced when children play electronic games. Given that many children play electronic games, a better understanding of the effect of electronic game use on PA is critical to inform child health policy and intervention. METHODS: This randomised and controlled trial will examine whether PA is decreased by access to electronic games and whether any effect is dependent on the type of game input or the child's characteristics. Children aged 10-12 years (N = 72, 36 females) will be recruited and randomised to a balanced ordering of 'no electronic games', 'traditional' electronic games and 'active' electronic games. Each child will participate in each condition for 8 weeks, and be assessed prior to participation and at the end of each condition. The primary outcome is PA, assessed by Actical accelerometers worn for 7 days on the wrist and hip. Energy expenditure will be assessed by the doubly labelled water technique and motor coordination, adiposity, self-confidence, attitudes to technology and PA and leisure activities will also be assessed. A sample of 72 will provide a power of > 0.9 for detecting a 15 mins difference in PA (sd = 30 mins). DISCUSSION: This is the first such trial and will provide critical information to understand whether access to electronic games affects children's PA. Given the vital importance of adequate PA to a healthy start to life and establishing patterns which may track into adulthood, this project can inform interventions which could have a profound impact on the long term health of children. TRIAL REGISTRATION: This trial is registered in the Australia and New Zealand Clinical Trials Registry (ACTRN 12609000279224)
SN - 1471-2458
AD - School of Physiotherapy, Curtin University of Technology, Perth, Australia. L.Straker@curtin.edu.au
UR - 19563680
ER -

TY - JOUR
ID - 232
T1 - The impact of community-built playgrounds on the community
A1 - Daniels,D.M.
A1 - Johnson,E.L.
Y1 - 2009/07//
N1 - Daniels, Dawn Marie. Johnson, Estell Lenita
The Journal of trauma
kaf, 0376373
AIM, IM
Journal Article
English
KW - Medline
KW - Accident Prevention
KW - Child
KW - Community Networks
KW - Consumer Participation
KW - Data Collection
KW - Humans
KW - Play and Playthings
KW - Safety Management
RP - NOT IN FILE
SP - S16
EP - S19
JF - Journal of Trauma-Injury Infection & Critical Care
JA - J Trauma
VL - 67
IS - 1 Suppl

CY - United States
N2 - BACKGROUND: Community-built playgrounds have been an integral component of Injury Free Coalition for Kids sites as they work with communities to provide safe places for children to play. The purpose of this pilot survey was to explore the potential impact of the community-built playground on the community. METHODS: A survey was developed and sent electronically via an Injury Free listserve to the Injury Free sites that had built one of the 29 Injury Free playgrounds between 2002 and 2007. Variables examined included condition and maintenance of the playground, vandalism, and community participation. Open-ended questions were used to examine the impact of the playground on the community and school. Descriptive statistics were used to describe quantitative data. Open-ended responses were collated and grouped into themes. RESULTS: Surveys were returned from 28 Allstate playground sites. The playgrounds were built within the last 6 years with the majority (64%) built 3 or more years ago. Although 17 of the playgrounds sustained minor vandalism, 11 of the sites reported the vandalism was corrected by the community. Community impact themes centered on revitalization and empowerment, safety, and social capital changes whereas school impact themes centered on socialization and health. CONCLUSIONS: From the perspective of the program coordinators, the playgrounds had a positive impact on the communities. Further research within this arena is needed to explore the relationship of community-built playgrounds and community development
SN - 1529-8809
AD - Riley Hospital for Children, Indianapolis, Indiana 46202, USA. dmdaniels@clarian.org
UR - 19590347
ER -

TY - JOUR
ID - 233
T1 - From genetics to genomics: ethics, policy, and parental decision-making
A1 - Wilfond,B.
A1 - Ross,L.F.
Y1 - 2009/07//
N1 - Wilfond, Benjamin. Ross, Lainie Friedman
Journal of pediatric psychology
jl8, 7801773
IM
Journal Article
English
KW - Medline
KW - Adolescent
KW - Child
KW - Child Advocacy/es [Ethics]
KW - Decision Making/es [Ethics]
KW - Disclosure/es [Ethics]
KW - Ethics
KW - Medical
KW - Genetic Counseling/es [Ethics]
KW - Genetic Diseases
KW - Inborn/di [Diagnosis]
KW - Inborn/ge [Genetics]
KW - Genetic Testing/es [Ethics]
KW - Genomics/es [Ethics]
KW - Heterozygote Detection
KW - Humans
KW - Parental Consent/es [Ethics]
KW - Risk Assessment/es [Ethics]
RP - NOT IN FILE
SP - 639
EP - 647
JF - Journal of Pediatric Psychology
JA - J Pediatr Psychol.

VL - 34
IS - 6
CY - United States
N2 - OBJECTIVE: Ethical evaluation of genetic testing in children is traditionally based on balancing clinical benefits and risks. However, this focus can be inconsistent with the general practice of respecting parental decision-making about their children's health care. We argue that respect for parental decision-making should play a larger role in shaping pediatric genetic testing practices, and play a similar role regarding decisions to use emerging genomic technologies. METHODS: Genomic testing involves the examination of thousands of DNA markers spanning genes throughout the genome and their interrelationships, yielding virtually limitless interpretations. We presume that parents and providers should proceed cautiously in applying genomic testing in children, as we explore how genomic testing will stress the fault lines of the traditional ethical analysis. RESULTS: Empirical data about the psychosocial risks and benefits of genetic testing of children do not reveal serious harms, yet virtually no such data exist yet about genomic testing. Unless empirical social and behavioral data indicate that genomic testing is highly likely to cause serious harms to the children, parental decisions to obtain comprehensive genomic testing in their children should be respected. Once comprehensive genomic testing of children becomes routine, resultant information may be more easily integrated by families than anticipated. CONCLUSIONS: Research on the social and behavioral impact of comprehensive genomic testing on children and their families is needed to further inform parents, clinicians, and policy makers
SN - 1465-735X
AD - Treuman Katz Center for Pediatric Bioethics, Seattle Children's Hospital, Seattle, WA 98101, USA. benjamin.wilfond@seattlechildrens.org
UR - 18647793
ER -

TY - JOUR
ID - 234
T1 - [ADHD and attachment processes: are they related?]. [Review] [39 refs] [French]
A1 - Franc,N.
A1 - Maury,M.
A1 - Purper-Ouakil,D.
Y1 - 2009/06//
N1 - Franc, N. Maury, M. Purper-Ouakil, D
L'Encephale
efb, 7505643
IM
English Abstract. Journal Article. Review
French
KW - Medline
KW - Attention Deficit Disorder with Hyperactivity/di [Diagnosis]
KW - Attention Deficit Disorder with Hyperactivity/ge [Genetics]
KW - Attention Deficit Disorder with Hyperactivity/px [Psychology]
KW - Child
KW - Comorbidity
KW - Epigenesis
KW - Genetic/ge [Genetics]
KW - Female
KW - Genetic Predisposition to Disease/ge [Genetics]
KW - Genetic Predisposition to Disease/px [Psychology]
KW - Humans
KW - Infant
KW - Newborn
KW - Internal-External Control
KW - Pregnancy
KW - Prenatal Exposure Delayed Effects
KW - Reactive Attachment Disorder/di [Diagnosis]
KW - Reactive Attachment Disorder/px [Psychology]

KW - Risk Factors
KW - Social Environment
RP - NOT IN FILE
SP - 256
EP - 261
JF - Encephale
VL - 35
IS - 3
CY - France
N2 - Attention-deficit/hyperactivity disorder (ADHD) is defined on the basis of developmentally inappropriate inattention, motor activity and impulsivity that emerges early in development and causes impairment in social and academic functioning. ADHD is described as a multifactorial disease, with a well studied genetic vulnerability, and early environmental factors also playing an important role in the development and course of the disorder. Current aetiological models emphasize interaction between genes and environment. The concept of attachment, as proposed by John Bowlby, reflects quality of early interactions, and should therefore be considered as an early developmental factor. First, clinical findings emphasize similitude between both disorders; emotional dysregulation is an important feature in reactive attachment disorder as well as in ADHD. Emotion regulation is highly related to attachment security in young children and could play a part in the development of early attention processes. Moreover, difficult temperament is associated with higher risk for ADHD on the one hand, and can disturb the process of attachment on the other. Parental caregiving - including maternal sensitivity, positive parenting practices - is a main factor involved in the development of attachment, and has shown to be associated with better outcomes in ADHD children, especially with less oppositional/conduct disorders. Second, the aim of our review is to present clinical studies that have looked for a link between ADHD and attachment: the type of attachment could play a part in the course of the disorder: insecure and disorganised attachment types tend to be associated with a higher risk of externalised behaviors in children. For ADHD, this effect seems to be weaker than for other externalised disorders, and has been shown only in populations of at-risk children. Clinical studies also raise the question of possible links between reactive attachment disorder and ADHD. In children suffering severe early deprivon such as institution-rearing, inattention/hyperactivity symptoms were shown to be high, but these findings may not be valid in less severely deprived children. Third, another link could depend on a common vulnerability for ADHD and attachment disorder. Some perinatal factors, such as smoking during pregnancy or prematurity, have been shown to increase the risk of hyperactive symptoms in children. These variables may also be associated with a higher risk of impaired early interactions. Recent animal studies have raised interest in the role of prenatal stress in the emotional and behavioral development of the offspring, particularly as regards vulnerability to stress. Epigenetic mechanisms may be involved in durable alterations of the hypothalamo-pituitary-adrenergic axis. Preliminary findings in humans show that prenatal stress or maternal depression may also influence the development of the child. The understanding of the relationship between attachment and ADHD may help to better target prevention and intervention efforts. As the perinatal period seems to be particularly involved in both ADHD and attachment disorders, early guidance and possibly prenatal interventions should be developed and assessed for mothers and caregivers with risk-factors. [References: 39]
SN - 0013-7006
AD - MPEA Saint-Eloi, universite Montpellier-I, CHU de Montpellier, 80, avenue Augustin-Fliche, 34295 Montpellier cedex 5, France. n-franc@chu-montpellier.fr
UR - 19540412
ER -

TY - JOUR
ID - 235
T1 - How should risk be communicated to children: a cross-sectional study comparing different formats of probability information
A1 - Ulph,F.
A1 - Townsend,E.
A1 - Glazebrook,C.
Y1 - 2009///
N1 - Ulph, Fiona. Townsend, Ellen. Glazebrook, Cris
BMC medical informatics and decision making

101088682
IM
Journal Article. Research Support, Non-U.S. Gov't
English
KW - Medline
KW - Child
KW - Communication
KW - Comprehension
KW - Cross-Sectional Studies
KW - Female
KW - Genetic Predisposition to Disease/ge [Genetics]
KW - Genetic Predisposition to Disease/px [Psychology]
KW - Genetic Testing/sn [Statistics & Numerical Data]
KW - Great Britain
KW - Heterozygote Detection
KW - Humans
KW - Judgment
KW - Male
KW - Models
KW - Statistical
KW - Patient Education as Topic/mt [Methods]
KW - Patient Participation
KW - Play and Playthings
KW - Probability Learning
KW - Risk
RP - NOT IN FILE
SP - 26
JF - BMC Medical Informatics & Decision Making
JA - BMC Med Inf.Decis.Mak.
VL - 9
CY - England
N2 - BACKGROUND: Newborn screening, which identifies inherited disorders and sometimes carrier status, will increasingly involve health professionals in the provision of appropriate information and support to children and their families. The ability to understand carrier results relies on an understanding of probabilistic terms. However, little is known about how best to convey probabilistic medical information to children. Research with adult populations suggests information format significantly affects comprehension. This study aimed to explore which presentation format is most effective in conveying probabilistic information to children. METHODS: A probabilistic task based on the cup game was used to measure which of five different formats was associated with greatest understanding in children aged 7-11 years old (n = 106). Formats used were verbal labels (e.g. rarely, sometimes), percentages, proportion-word (e.g. 1 in X), proportion-notation (e.g. 1:X) and pie charts. There was also an additional mixed format condition. In each trial a picture was presented of three cups, each with a different probability depicted beneath it, and the child was asked to select which cup was most likely to contain the ball. Three trials were presented per format. Children also rated how certain they were that they had answered correctly. RESULTS: There was a significant relationship between format and comprehension scores. Post hoc tests showed children performed significantly better when probability was presented as a pie chart, in comparison to percentages, proportion-notation, proportion-word and mixed format trials. Furthermore, most children (84%) got all trials correct for this format and children were significantly more certain that their response was correct in the pie chart trials compared to all the other formats ($p < 0.001$). Significant positive correlations were found between self-ratings of certainty and comprehension of verbal labels, percentages and pie charts. Older age was also associated with better performance on all formats except percentages. Overall comprehension was calculated by summing the scores for the individual trials and this was independently associated with older age and higher IQ. CONCLUSION: The results suggest that 7-11 year olds can understand probability information, but that the format used will significantly affect the accuracy and confidence with which children in this age group make judgements about the likelihood of an event. Of the formats studied, pie charts appear to be the optimal method of presenting probabilistic information to children in this age group.

Health professionals and designers of health messages should be cognisant of this when communicating medical information to children aged 7-11 years old
SN - 1472-6947
AD - School of Psychological Sciences, University of Manchester, Oxford Road, Manchester, UK. fiona.ulph@manchester.ac.uk
UR - 19500337
ER -

TY - JOUR
ID - 236
T1 - Youth sports in the heat: recovery and scheduling considerations for tournament play
A1 - Bergeron,M.F.
Y1 - 2009///
N1 - Bergeron, Michael F
Sports medicine (Auckland, N.Z.)
8412297, spo
IM
Journal Article
English
KW - Medline
KW - Adolescent
KW - Body Temperature Regulation/ph [Physiology]
KW - Child
KW - Competitive Behavior
KW - Dehydration/pc [Prevention & Control]
KW - Female
KW - Heat Stress Disorders/pc [Prevention & Control]
KW - Hot Temperature
KW - Humans
KW - Leadership
KW - Male
KW - Sports
KW - Sports Medicine
RP - NOT IN FILE
SP - 513
EP - 522
JF - Sports Medicine
JA - Sports Med
VL - 39
IS - 7
CY - New Zealand
N2 - One of the biggest challenges facing numerous young athletes is attempting to perform safely and effectively in the heat. An even greater performance challenge and risk for incurring exertional heat injury is encountered when a young athlete has to compete multiple times on the same day, with only a short rest period between rounds of play, during a hot-weather tournament. Within the scope of the rules, tournament directors frequently provide athletes with only the minimum allowable time between same-day matches or games. Notably, prior same-day exercise has been shown to increase cardiovascular and thermal strain and perception of effort in subsequent activity bouts, and the extent of earlier exercise-heat exposure can affect performance and competition outcome. Incurred water and other nutrient deficits are often too great to offset during short recovery periods between competition bouts, and the athletes are sometimes 'forced' to compete again not sufficiently replenished. Providing longer rest periods between matches and games can significantly improve athlete safety and performance, by enhancing recovery and minimizing the 'carryover' effects from previous competition-related physical activity and heat exposure that can negatively affect performance and safety. Governing bodies of youth sports need to address this issue and provide more specific, appropriate and evidence-based guidelines for minimum rest periods between same-day contests for all levels of tournament play in the heat. Youth athletes are capable of tolerating the heat and performing reasonably well and safely in a

range of hot environments if they prepare well, manage hydration sufficiently, and are provided the opportunity to recover adequately between contests
SN - 0112-1642
AD - National Institute for Athletic Health and Performance and Center for Youth Sports and Health, Sanford USD Medical Center, Sioux Falls, South Dakota, USA. BergeroM@sanfordhealth.org
UR - 19530749
ER -

TY - JOUR
ID - 237
T1 - Rough-and-tumble play and the regulation of aggression: an observational study of father-child play dyads
A1 - Flanders,J.L.
A1 - Leo,V.
A1 - Paquette,D.
A1 - Pihl,R.O.
A1 - Seguin,J.R.
Y1 - 2009/07//
N1 - Flanders, Joseph L. Leo, Vanessa. Paquette, Daniel. Pihl, Robert O. Seguin, Jean R
Aggressive behavior
7502265
IM
Journal Article. Research Support, Non-U.S. Gov't
English
KW - Medline
KW - Aggression/px [Psychology]
KW - Child
KW - Preschool
KW - Dominance-Subordination
KW - Father-Child Relations
KW - Female
KW - Humans
KW - Internal-External Control
KW - Male
KW - Play and Playthings
KW - Questionnaires
KW - Risk Factors
KW - SOCIALIZATION
KW - Videotape Recording
RP - NOT IN FILE
SP - 285
EP - 295
JF - Aggressive Behavior
JA - Aggress.Behav
VL - 35
IS - 4
CY - United States
N2 - Rough-and-tumble play (RTP) is a common form of play between fathers and children. It has been suggested that RTP can contribute to the development of selfregulation. This study addressed the hypothesis that the frequency of father-child RTP is related to the frequency of physically aggressive behavior in early childhood. This relationship was expected to be moderated by the dominance relationship between father and son during play. Eighty-five children between the ages of 2 and 6 years were videotaped during a free-play session with their fathers in their homes and questionnaire data was collected about father-child RTP frequency during the past year. The play dyads were rated for the degree to which the father dominated play interactions. A significant statistical interaction revealed that RTP frequency was associated with higher levels of physical aggression in children whose fathers were less dominant. These results indicate that RTP is indeed related to

physical aggression, though this relationship is moderated by the degree to which the father is a dominant playmate. Copyright 2009 Wiley-Liss, Inc
SN - 1098-2337
AD - Department of Psychology, McGill University, Montreal, Quebec, Canada. joe.flanders@mail.mcgill.ca
UR - 19431190
ER -

TY - JOUR
ID - 238
T1 - [Scientificaly sound methodological approaches to safety evaluation of toys]. [Review] [5 refs] [Russian]
A1 - Teksheva,L.M.
A1 - Barsukova,N.K.
A1 - Markelova,S.V.
Y1 - 2009///
N1 - Teksheva, L M. Barsukova, N K. Markelova, S V
Vestnik Rossiiskoi akademii meditsinskikh nauk / Rossiiskaia akademiia meditsinskikh nauk
bl9, 9215641
IM
English Abstract. Journal Article. Review
Russian
KW - Medline
KW - Child
KW - Child Welfare
KW - Environmental Exposure/ae [Adverse Effects]
KW - Environmental Health/st [Standards]
KW - Humans
KW - Play and Playthings
KW - Russia
KW - Safety Management/og [Organization & Administration]
RP - NOT IN FILE
SP - 44
EP - 47
JF - Vestnik Rossiiskoi Akademii Meditsinskikh Nauk
JA - Vestn.Ross.Akad.Med Nauk
IS - 5
CY - Russia (Federation)
N2 - Sanitary-epidemiologic safety of toys is an important aspect of health protection and promotion for the rising generation. The authors propose scientifically substantiated methods allowing to improve quality and efficiency of safety evaluation of toys. [References: 5]
SN - 0869-6047
UR - 19514126
ER -

TY - JOUR
ID - 239
T1 - [Children with ADHD: a retrospective description of their behavioural features as toddlers]. [Spanish]
A1 - Magallon,S.
A1 - Crespo-Eguilaz,N.
A1 - Ecay,M.
A1 - Poch-Olive,M.L.
A1 - Narbona,J.
Y1 - 2009/06//
N1 - Magallon, S. Crespo-Eguilaz, N. Ecay, M. Poch-Olive, M L. Narbona, J
Anales de pediatria (Barcelona, Spain : 2003)
101162596
IM

English Abstract. Journal Article. Research Support, Non-U.S. Gov't
Spanish
KW - Medline
KW - Age Factors
KW - Attention Deficit Disorder with Hyperactivity/px [Psychology]
KW - Child
KW - Child Behavior
KW - Preschool
KW - Female
KW - Humans
KW - Male
KW - Questionnaires
KW - Retrospective Studies
RP - NOT IN FILE
SP - 562
EP - 569
JF - Anales de Pediatria
JA - An Pediatr (Barc.)
VL - 70
IS - 6
CY - Spain
N2 - OBJECTIVES: To study the relationship between behavioural profile of children suffering from Attention Deficit Hyperactivity Disorder (ADHD) and the previous behavioural style of these patients as toddlers. SUBJECTS AND METHODS: We asked the parents of 50 schoolchildren with ADHD, and those of 30 controls, to fill in a Spanish version of the Toddler Behaviour Questionnaire (TBQ) from their retrospective perception of their children's behaviour as toddlers. TBQ items were grouped by factor analysis; t-Student between the scores of both groups and a multiple correlation analysis of TBQ and DSM-IV-ADHD-RS in each of the groups were used. RESULTS: Children in the ADHD group were reported by parents to have had a different toddler behavioural profile in comparison to that of control children (P<0.05). These differences were associated with adapting to new environments, mood, regularity and stability of play behaviour. A correlation was found between behavioural profile in DSM-IV-ADHD- RS and TBQ. CONCLUSIONS: The results of this study should be interpreted with caution. However, they suggest that in the fifth trimester of life a particular behavioural style as regards regularity, stability of play, and mood, could indicate a risk of developing ADHD in the future. This behavioural style should be taken into consideration in rearing and early education prospective studies
SN - 1695-4033
AD - Unidad de Neurologia Pediatrica, Departamento de Pediatria, Clinica Universitaria de Navarra, Universidad de Navarra, Pamplona, Spain
UR - 19423412
ER -

TY - JOUR
ID - 240
T1 - Age at onset of anorexia nervosa and breast cancer risk
A1 - Papadopoulos,F.C.
A1 - Pantziaras,I.
A1 - Lagiou,P.
A1 - Brandt,L.
A1 - Ekselius,L.
A1 - Ekbom,A.
Y1 - 2009/06//
N1 - Papadopoulos, Fotios C. Pantziaras, Ioannis. Lagiou, Pagona. Brandt, Lena. Ekselius, Lisa. Ekbom, Anders
European journal of cancer prevention : the official journal of the European Cancer Prevention Organisation (ECP)
bnn, 9300837

IM
Journal Article. Research Support, Non-U.S. Gov't
English
KW - Medline
KW - Adolescent
KW - Adult
KW - Age Factors
KW - Age of Onset
KW - Anorexia Nervosa/co [Complications]
KW - Breast Neoplasms/co [Complications]
KW - Breast Neoplasms/ep [Epidemiology]
KW - Caloric Restriction
KW - Child
KW - Cohort Studies
KW - Energy Intake/ph [Physiology]
KW - Female
KW - Follow-Up Studies
KW - Humans
KW - Middle Aged
KW - Registries
KW - Retrospective Studies
KW - Risk Factors
KW - Sweden/ep [Epidemiology]
KW - Young Adult
RP - NOT IN FILE
SP - 207
EP - 211
JF - European Journal of Cancer Prevention
JA - Eur.J Cancer Prev.
VL - 18
IS - 3
CY - England
N2 - The objective of this study was to investigate breast cancer occurrence among women treated for anorexia nervosa (AN), with emphasis on age at the onset of this disorder. We conducted a register-based retrospective cohort with a total of 6009 women with at least one admission with an AN diagnosis during the period 1973-2003 in Sweden. During a mean follow-up of 13.4 years, information on 80 057 women-years was generated. The standardized incidence ratio (SIR)--the ratio of observed-to-expected number of cases--was used as the measure of relative risk. Overall, 16 women developed breast cancer versus 25.5 expected cases [SIR: 0.6, 95% confidence interval (CI): 0.4-0.9]. Among women who were first admitted for AN between the age of 10 and 24 years, four developed breast cancer versus 11.3 expected (SIR: 0.4, 95% CI: 0.1-0.9). In this group of women with early onset AN, only one parous woman developed breast cancer versus 6.3 expected (SIR: 0.2, 95% CI: 0-0.9). Among women first hospitalized for AN between the age of 25 and 40 years, 12 developed breast cancer, whereas the expected number was 14.2, a nonsignificant deficit. Our results suggest that early onset AN may play an important role in the development of breast cancer, possibly because of the extreme restriction of energy intake at a crucial period for mammary gland development. Late onset AN is likely to play a relatively less important role
SN - 1473-5709
AD - Department of Neuroscience, Psychiatry, Uppsala University Hospital, Uppsala, Sweden. fotis.papadopoulos@gmail.com
UR - 19491607
ER -

TY - JOUR
ID - 241
T1 - The built environment: designing communities to promote physical activity in children
A1 - Committee on Environmental Health

A1 - Tester,J.M.
Y1 - 2009/06//
N1 - Committee on Environmental Health. Tester, June M
Pediatrics
oxv, 0376422
AIM, IM
Journal Article
English
KW - Medline
KW - Accidents
KW - Traffic/pc [Prevention & Control]
KW - Adolescent
KW - Child
KW - City Planning
KW - Crime/pc [Prevention & Control]
KW - Environment Design
KW - Health Promotion
KW - Humans
KW - Life Style
KW - Motor Activity
KW - Play and Playthings
KW - Recreation
KW - Residence Characteristics
KW - Safety Management
KW - United States
KW - Walking
RP - NOT IN FILE
SP - 1591
EP - 1598
JA - Pediatrics
VL - 123
IS - 6
CY - United States
N2 - An estimated 32% of American children are overweight, and physical inactivity contributes to this high prevalence of overweight. This policy statement highlights how the built environment of a community affects children's opportunities for physical activity. Neighborhoods and communities can provide opportunities for recreational physical activity with parks and open spaces, and policies must support this capacity. Children can engage in physical activity as a part of their daily lives, such as on their travel to school. Factors such as school location have played a significant role in the decreased rates of walking to school, and changes in policy may help to increase the number of children who are able to walk to school. Environment modification that addresses risks associated with automobile traffic is likely to be conducive to more walking and biking among children. Actions that reduce parental perception and fear of crime may promote outdoor physical activity. Policies that promote more active lifestyles among children and adolescents will enable them to achieve the recommended 60 minutes of daily physical activity. By working with community partners, pediatricians can participate in establishing communities designed for activity and health
SN - 1098-4275
UR - 19482771
ER -

TY - JOUR
ID - 242
T1 - Dying to play video games: carbon monoxide poisoning from electrical generators used after hurricane Ike
A1 - Fife,C.E.
A1 - Smith,L.A.
A1 - Maus,E.A.
A1 - McCarthy,J.J.

A1 - Koehler,M.Z.
A1 - Hawkins,T.
A1 - Hampson,N.B.
Y1 - 2009/06//
N1 - Fife, Caroline E. Smith, Latisha A. Maus, Erik A. McCarthy, James J. Koehler, Michelle Z. Hawkins, Trina. Hampson, Neil B
Pediatrics
oxv, 0376422
AIM, IM
Journal Article. Research Support, U.S. Gov't, P.H.S.
English
KW - Medline
KW - Academic Medical Centers/sn [Statistics & Numerical Data]
KW - Adolescent
KW - Adult
KW - Aged
KW - 80 and over
KW - Carbon Monoxide Poisoning/di [Diagnosis]
KW - Carbon Monoxide Poisoning/et [Etiology]
KW - Carbon Monoxide Poisoning/mo [Mortality]
KW - Carbon Monoxide Poisoning/pc [Prevention & Control]
KW - Cause of Death
KW - Child
KW - Preschool
KW - Cross-Sectional Studies
KW - Cyclonic Storms
KW - Disease Outbreaks
KW - Electric Power Supplies/ae [Adverse Effects]
KW - Electric Power Supplies/sn [Statistics & Numerical Data]
KW - Female
KW - Gasoline
KW - Health Education
KW - Humans
KW - Incidence
KW - Infant
KW - Male
KW - Middle Aged
KW - Risk Factors
KW - Television/sn [Statistics & Numerical Data]
KW - Texas
KW - Video Games/px [Psychology]
KW - Video Games/sn [Statistics & Numerical Data]
KW - Young Adult
RP - NOT IN FILE
SP - e1035
EP - e1038
JA - Pediatrics
VL - 123
IS - 6
CY - United States
N2 - BACKGROUND: Carbon monoxide (CO) poisoning is common after major storms because of loss of electrical power and use of alternate fuel sources for heat and electricity. In past epidemics of hurricane-related CO poisoning, the source has typically been gasoline-powered electrical generators. Although it is typically believed that generators were used to power air conditioning and refrigeration, this report demonstrates an unsuspected reason for their use. PATIENTS AND METHODS: After Hurricane Ike's landfall in September 2008, major power outages were associated with an epidemic of CO poisoning from electrical generators, as

expected. Staff at Memorial Hermann Hospital-Texas Medical Center treated or telephone-triaged cases from the Houston area. A review of the details of those cases forms the basis of this report. RESULTS: Memorial Hermann Hospital-Texas Medical Center staff treated or triaged 37 individuals exposed to CO from gasoline-powered electrical generators in 13 incidents in the first 36 hours after landfall of the hurricane. Notably, 54% (20 of 37) of the patients were under the age of 18 years. Symptoms ranged from mild to severe, with 1 child dying at the scene. Eleven patients were treated with hyperbaric oxygen. Among 9 incidents in which the reason for generator use was determined, 5 were due to generators powering video games or televisions to watch movies or programs. These 5 incidents in which video games were being powered accounted for 75% (15 of 20) of the pediatric poisonings. CONCLUSIONS: Generator-related CO poisoning is indeed common during power outages after hurricanes. However, generators are commonly being used to provide electricity to power entertainment devices for children, such as video games. Additional public education about CO risk is needed, perhaps directed at older children and teenagers through the schools in regions susceptible to hurricanes
SN - 1098-4275
AD - Department of Medicine, Division of Cardiology, University of Texas Health Science Center, 6431 Fannin St, MSB 1.247, Houston, TX 77030, USA. caroline.e.fife@uth.tmc.edu
UR - 19482736
ER -

TY - JOUR
ID - 243
T1 - A deadly game for boys
A1 - Thomas,S.P.
Y1 - 2009/05//
N1 - Thomas, Sandra P
Issues in mental health nursing
gy7, 7907126
N
Journal Article
English
KW - Medline
KW - Adolescent
KW - Airway Obstruction/mo [Mortality]
KW - Airway Obstruction/nu [Nursing]
KW - Airway Obstruction/px [Psychology]
KW - Asphyxia/mo [Mortality]
KW - Asphyxia/nu [Nursing]
KW - Asphyxia/px [Psychology]
KW - Brain Damage
KW - Chronic/mo [Mortality]
KW - Chronic/nu [Nursing]
KW - Chronic/px [Psychology]
KW - Cause of Death
KW - Child
KW - Euphoria/ph [Physiology]
KW - Humans
KW - Male
KW - Motivation
KW - Nursing Diagnosis
KW - Play and Playthings
KW - Risk-Taking
RP - NOT IN FILE
SP - 287
JF - Issues in Mental Health Nursing
JA - Issues Ment.Health Nurs
VL - 30
IS - 5

CY - United States
SN - 1096-4673
UR - 19437246
ER -

TY - JOUR
ID - 244
T1 - The effects of prosocial video games on prosocial behaviors: international evidence from correlational, longitudinal, and experimental studies
A1 - Gentile,D.A.
A1 - Anderson,C.A.
A1 - Yukawa,S.
A1 - Ihori,N.
A1 - Saleem,M.
A1 - Ming,L.K.
A1 - Shibuya,A.
A1 - Liau,A.K.
A1 - Khoo,A.
A1 - Bushman,B.J.
A1 - Rowell,Huesmann L.
A1 - Sakamoto,A.
Y1 - 2009/06//
N1 - Gentile, Douglas A. Anderson, Craig A. Yukawa, Shintaro. Ihori, Nobuko. Saleem, Muniba. Ming, Lim Kam. Shibuya, Akiko. Liau, Albert K. Khoo, Angeline. Bushman, Brad J. Rowell Huesmann, L. Sakamoto, Akira
Personality & social psychology bulletin
7809042
IM
Comparative Study. Journal Article. Research Support, N.I.H., Extramural. Research Support, Non-U.S. Gov't
English
KW - Medline
KW - Adolescent
KW - Adolescent Behavior/px [Psychology]
KW - Aggression/px [Psychology]
KW - Child
KW - Child Behavior/px [Psychology]
KW - Empathy
KW - Female
KW - Helping Behavior
KW - Humans
KW - Internal-External Control
KW - Longitudinal Studies
KW - Male
KW - Models
KW - Psychological
KW - Research Design
KW - Risk Factors
KW - Sex Factors
KW - Social Behavior
KW - Students/px [Psychology]
KW - Video Games/ae [Adverse Effects]
KW - Video Games/px [Psychology]
KW - Violence/px [Psychology]
KW - Visual Perception
RP - NOT IN FILE
SP - 752

EP - 763
JF - Personality & Social Psychology Bulletin
JA - Pers.Soc Psychol.Bull.
VL - 35
IS - 6
CY - United States
N2 - Although dozens of studies have documented a relationship between violent video games and aggressive behaviors, very little attention has been paid to potential effects of prosocial games. Theoretically, games in which game characters help and support each other in nonviolent ways should increase both short-term and long-term prosocial behaviors. We report three studies conducted in three countries with three age groups to test this hypothesis. In the correlational study, Singaporean middle-school students who played more prosocial games behaved more prosocially. In the two longitudinal samples of Japanese children and adolescents, prosocial game play predicted later increases in prosocial behavior. In the experimental study, U.S. undergraduates randomly assigned to play prosocial games behaved more prosocially toward another student. These similar results across different methodologies, ages, and cultures provide robust evidence of a prosocial game content effect, and they provide support for the General Learning Model
SN - 0146-1672
AD - Iowa State University, Ames, IA 50011, USA. dgentile@iastate.edu
UR - 19321812
ER -

TY - JOUR
ID - 245
T1 - Soft landings: encouraging compliance with safety standards in Local Government Authority playgrounds
A1 - Sherker,S.
A1 - Ritchie,J.
A1 - Eager,D.
A1 - Dennis,R.
Y1 - 2009/04//
N1 - Sherker, Shauna. Ritchie, Jan. Eager, David. Dennis, Rebecca
Health promotion journal of Australia : official journal of Australian Association of Health Promotion Professionals
9710936
IM
Journal Article. Research Support, Non-U.S. Gov't
English
KW - Medline
KW - Child
KW - Preschool
KW - Data Collection
KW - Databases as Topic
KW - Humans
KW - Local Government
KW - Maintenance
KW - New South Wales
KW - Play and Playthings/in [Injuries]
KW - Policy Making
KW - Safety/st [Standards]
KW - Wounds and Injuries/pc [Prevention & Control]
RP - NOT IN FILE
SP - 31
EP - 36
JF - Health Promotion Journal of Australia
JA - HEALTH PROMOT J AUST
VL - 20
IS - 1

CY - Australia
N2 - ISSUES ADDRESSED: Consistent with health promotion principles of good practice, addressing playground injury necessitates the creation of a supportive environment for the enhancement of wellbeing and the prevention of injuries. This study aims to survey local governments to: determine compliance with playground safety standards; establish frequency of playground inspections and maintenance; and identify motivators and barriers to compliance with safety standards. METHODS: A survey of key informants for playground safety in all 152 local government councils in New South Wales (NSW) was undertaken. RESULTS: Of 152 local councils in NSW (43 metropolitan and 109 non-metropolitan), 71.7% (n=109) completed the survey, 12.5% (n=19) refused to participate and no response was received by 15.8% (n=24). Self-reported compliance with key aspects of the standard was generally high. However, only 55% of councils complied with surface impact attenuation <200 gmax and <1,000 HIC. Further, only 14.7% of councils reported impact testing the playground surface during inspections. The main motivators to compliance included: reducing risk of litigation or liability; enhancing community and child safety, and minimising the risk of injury. The main barriers included a lack of: time; personnel, and a lack of adequate and appropriate funding. CONCLUSIONS: Local Government Authorities have a duty of care to ensure the safety of playgrounds in their jurisdiction. They require time, personnel and adequate and appropriate funding in order to achieve this aim
SN - 1036-1073
AD - NSW Injury Risk Management Research Centre, University of New South Wales, Australia. Shauna.Sherker@unsw.edu.au
UR - 19402813
ER -

TY - JOUR
ID - 246
T1 - Parent/Caregiver supervision and child injury: a systematic review of critical dimensions for understanding this relationship. [Review] [47 refs]
A1 - Petrass,L.
A1 - Blitvich,J.D.
A1 - Finch,C.F.
Y1 - 2009/04//
N1 - Petrass, Lauren. Blitvich, Jenny D. Finch, Caroline F
Family & community health
ese, 7809641
IM
Journal Article. Research Support, Non-U.S. Gov't. Review
English
KW - Medline
KW - Accident Prevention/mt [Methods]
KW - Accident Prevention/sn [Statistics & Numerical Data]
KW - Caregivers/sn [Statistics & Numerical Data]
KW - Child
KW - Child Care/sn [Statistics & Numerical Data]
KW - Evidence-Based Medicine
KW - Helping Behavior
KW - Humans
KW - Parent-Child Relations
KW - Parenting/px [Psychology]
KW - Parents/px [Psychology]
KW - Play and Playthings/in [Injuries]
KW - Safety/sn [Statistics & Numerical Data]
KW - Wounds and Injuries/ep [Epidemiology]
KW - Wounds and Injuries/pc [Prevention & Control]
RP - NOT IN FILE
SP - 123
EP - 135
JF - Family & Community Health

JA - Fam.Community Health
VL - 32
IS - 2
CY - United States
N2 - This study reviewed the relationship between recognized dimensions of supervision and children's injuries based on Saluja et al's (Injury Control and Safety Promotion. 2004;11:17-22) hierarchal model of supervision strategies. A systematic review of peer-review studies was done with the earliest records available to 2007. There were 112 potentially relevant articles identified; 31 studies met all inclusion criteria. Reported studies were categorized according to the dimensions of supervision they addressed. Studies were not evenly distributed across the dimensions. There was evidence from the study that directly linking dimensions of supervision to child injury risk and outcomes is scarce. Future studies should consider attention, proximity, and continuity of supervision to provide a holistic understanding of the relationship between supervision and injury. [References: 47]
SN - 1550-5057
AD - School of Human Movement and Sport Sciences, University of Ballarat, Mt Helen, Ballarat, Victoria, Australia
UR - 19305211
ER -

TY - JOUR
ID - 247
T1 - Disorganized attachment and social skills as indicators of Head Start children's school readiness skills
A1 - Stacks,A.M.
A1 - Oshio,T.
Y1 - 2009/03//
N1 - Stacks, Ann M. Oshio, Toko
Attachment & human development
100901315
IM
Journal Article. Research Support, N.I.H., Extramural. Research Support, Non-U.S. Gov't
English
KW - Medline
KW - Child Development
KW - Child
KW - Preschool
KW - Early Intervention (Education)
KW - Female
KW - Humans
KW - Internal-External Control
KW - Male
KW - Object Attachment
KW - Parent-Child Relations
KW - Play and Playthings
KW - Poverty
KW - Schools
KW - Social Behavior
RP - NOT IN FILE
SP - 143
EP - 164
JF - Attachment & Human Development
JA - Attach.Hum.Dev.
VL - 11
IS - 2
CY - England
N2 - The relationships among social skills, dysregulation of symbolic representations of attachment, and school readiness were examined. Participants were 74 preschool children from low-income families in Midwest

America. Attachment representations and dysregulation of symbolic representations of attachment were assessed using a story completion task (George & Solomon, 2000) and teachers completed a survey of child behavior, which was used to assess social skills and school readiness skills. Dysregulated content in children's narratives and social skills were significant negative correlates of school readiness. There was also a marginally significant negative association between defensive dysregulation and school readiness skills for children classified as disorganized. Furthermore, a specific marker of dysregulation, controlling behavior toward the administrator, was negatively associated with school readiness, but only for children classified as disorganized. Results from this study suggest that a breakdown in the strategies of insecure/organized children may be a risk factor for low levels of school readiness and that different forms of disorganization may be associated with different types of risk. It may be useful for future studies to account for different forms of disorganization and evidence of a breakdown of strategy
SN - 1469-2988
AD - Merrill Palmer Skillman Institute Wayne State University, MI, USA. amstacks@wayne.edu
UR - 19266363
ER -

TY - JOUR
ID - 248
T1 - Ocular trauma resulting from paintball injury
A1 - Pahk,P.J.
A1 - Adelman,R.A.
Y1 - 2009/04//
N1 - Pahk, Patricia J. Adelman, Ron A
Graefe's archive for clinical and experimental ophthalmology = Albrecht von Graefes Archiv fur klinische und experimentelle Ophthalmologie
fpr, 8205248
IM
Journal Article. Research Support, Non-U.S. Gov't
English
KW - Medline
KW - Adolescent
KW - Adult
KW - Athletic Injuries/et [Etiology]
KW - Athletic Injuries/su [Surgery]
KW - Child
KW - Eye Injuries/et [Etiology]
KW - Eye Injuries/su [Surgery]
KW - Eye Protective Devices
KW - Female
KW - Follow-Up Studies
KW - Humans
KW - Hyphema/et [Etiology]
KW - Hyphema/su [Surgery]
KW - Male
KW - Paint
KW - Play and Playthings/in [Injuries]
KW - Retinal Diseases/et [Etiology]
KW - Retinal Diseases/su [Surgery]
KW - Retrospective Studies
KW - Visual Acuity
KW - Wounds
KW - Nonpenetrating/et [Etiology]
RP - NOT IN FILE
SP - 469
EP - 475
JF - Graefes Archive for Clinical & Experimental Ophthalmology

JA - Graefes Arch.Clin.Exp.Ophthalmol.
VL - 247
IS - 4
CY - Germany
N2 - BACKGROUND: Paintball-related ocular injuries result in severe damage and loss of vision. Despite efforts to increase public awareness and improve safety features, the incidence of eye injuries has increased over time. We examined the characteristics and ocular effects of paintball injury at our tertiary referral center. METHODS: Retrospective review of charts of patients with paintball injury between 1998-2005. RESULTS: Fourteen patients were evaluated for paintball injury; 13 (93%) were male and one (7%) was female. Average age was 17 years (range from 9 to 30). Thirteen patients (95%) did not wear eye protection; one is unknown. Injuries occurred while playing paintball in three patients (21%); ten (71%) were injured in either accidental (four patients) or intentional (six patients) shootings not associated with play. Minimum follow-up was 6 weeks, except for one patient who was lost to follow-up. Seven patients (50%) had final visual acuity (VA) better than 20/200, six patients (43%) were 20/200 or worse, and information on one patient was not available. VA, except in one case, improved with treatment. Decreased VA in this case was due to proliferative vitreoretinopathy (PVR). Hyphema was noted in ten patients (71%), vitreous hemorrhage in eight (57%), retinal hemorrhage in six (43%), retinal tear or detachment in six (43%), commotio retinae in six (43%), iris injury in five (36%), keratopathy in four (29%), lens injury in two (14%), subluxation of lens in two (14%), secondary glaucoma in two (14%), open globe/intraocular foreign body (IOFB) in one (7%), choroidal rupture in one (7%), and proliferative vitreoretinopathy (PVR) in one (7%). Ten patients (71%) required intervention (surgery, laser retinopexy or cryotherapy). CONCLUSION: Paintball trauma results in significant ocular injury and loss of vision. Most injuries occur in unsupervised settings without proper eye protection. Ten patients (71%) were injured in accidental or intentional shootings. Lack of supervision and use of paintball materials as assault weapons make the risk for ocular injury more significant. Improved safety features of paintball equipment, along with continued education of proper eye protection, may reduce the incidence of severe ocular injuries
SN - 1435-702X
AD - Department of Ophthalmology, Lahey Clinic, Burlington, MA, USA
UR - 19034480
ER -

TY - JOUR
ID - 249
T1 - Relationships between weight status and child, parent and community characteristics in preschool children
A1 - Jones,R.A.
A1 - Okely,A.D.
A1 - Gregory,P.
A1 - Cliff,D.P.
Y1 - 2009///
N1 - Jones, Rachel A. Okely, Anthony D. Gregory, Parriel. Cliff, Dylan P
International journal of pediatric obesity : IJPO : an official journal of the International Association for the Study of Obesity
101256330
IM
Journal Article. Research Support, Non-U.S. Gov't
English
KW - Medline
KW - Body Weight
KW - Child Development
KW - Child
KW - Preschool
KW - Cross-Sectional Studies
KW - Exercise
KW - Family Characteristics
KW - Female
KW - Humans
KW - Male

KW - Models
KW - Biological
KW - Motor Activity
KW - Motor Skills
KW - New South Wales
KW - Overweight/et [Etiology]
KW - Overweight/pp [Physiopathology]
KW - Parenting
KW - Public Facilities
KW - Recreation
KW - Risk Assessment
KW - Risk Factors
KW - Television
RP - NOT IN FILE
SP - 54
EP - 60
JF - International Journal of Pediatric Obesity
JA - Int.J Pediatr Obes.
VL - 4
IS - 1
CY - England
N2 - OBJECTIVE: To examine, using an Ecological Systems Theoretical framework, relationships between weight status and child, parent and community characteristics and risk factors among preschool children. METHODS: Cross-sectional data was collected from 140 children and their parents from 11 randomly selected preschools. Outcome variables included: motor development; perceived competence; objectively measured physical activity; time spent in active and quiet play; location and number of televisions; parental rules around physical activity and time spent watching television; availability of sport and physical activity programs; and parks and open spaces and access to footpaths. RESULTS: Overweight children spent more time in quiet play and watching television and less time in active play and physical activity. Perceived competence and motor development were similar for both overweight and non-overweight children. Associations between weight status and several parent and community characteristics were not evident, except for access to footpaths. Overweight children had greater access to footpaths compared with non-overweight children (p=0.046). CONCLUSION: The results reported here showed little difference between overweight and non-overweight children in relation to a variety of child, parent and community variables. However, for some characteristics, differences in older children have been reported. Longitudinal studies are required to confirm when these characteristics begin to differ, what effects these differences have on behaviour and weight-status, and therefore when targeted treatment should be provided during a child's development
SN - 1747-7174
AD - Child Obesity Research Centre, Faculty of Education, University of Wollongong, Wollongong NSW, Australia. rachelj@uow.edu.au
UR - 18608633
ER -

TY - JOUR
ID - 250
T1 - Once bitten, twice shy? Medically-attended injuries can sensitise parents to children's risk of injuries on playgrounds
A1 - Morrongiello,B.A.
A1 - Howard,A.W.
A1 - Rothman,L.
A1 - Sandomierski,M.
Y1 - 2009/02//
N1 - Morrongiello, B A. Howard, A W. Rothman, L. Sandomierski, M
Injury prevention : journal of the International Society for Child and Adolescent Injury Prevention
crz, 9510056
IM

Journal Article
English
KW - Medline
KW - Adaptation
KW - Psychological
KW - Analysis of Variance
KW - Attitude of Health Personnel
KW - Case-Control Studies
KW - Child
KW - Female
KW - Humans
KW - Male
KW - Parents/px [Psychology]
KW - Perception
KW - Play and Playthings
KW - Risk Assessment
KW - Risk Factors
KW - Wounds and Injuries/px [Psychology]
RP - NOT IN FILE
SP - 50
EP - 54
JA - Injury Prevention
VL - 15
IS - 1
CY - England
N2 - OBJECTIVE: To compare risk perceptions of parents whose child sustained a medically attended playground injury (cases) with those of parents whose child had not (controls) to address two questions. Does having a child experience a medically attended injury: (1) sensitise parents to children's injury vulnerability and severity; (2) influence parents' appraisal of the injury mechanism (child's behaviour), attributions for injuries or beliefs about strategies for prevention? METHOD: Each case-control parent dyad was assigned to one of two conditions: (1) being presented with 10 common injury-risk playground behaviours specific to the equipment on which their child had been hurt, and asked to appraise injury vulnerability and severity; or (2) being presented with scenarios about playground injuries that varied in severity but were all based on the same child behaviour, and asked questions about this behaviour, attributions for injury and strategies for prevention. RESULTS: The results support the occurrence of a sensitisation process. Compared with control parents, case parents showed higher ratings of injury severity and children's vulnerability to injury, made fewer attributions for injuries to bad luck, and endorsed a greater diversity of prevention strategies, including parent (closer supervision), child (teaching rules about safe play on playgrounds) and environmental (modifications to playgrounds). CONCLUSIONS: A child's medically attended injury can create a "teachable moment" for the parent. Linking injury-prevention programming to this teachable moment may increase the likelihood of evoking changes in parental supervisory behaviour and their setting of rules limiting their child's risk behaviours to reduce the occurrence of childhood injury
SN - 1475-5785
AD - Psychology Department, University of Guelph, Guelph, Canada. bmorrong@uoguelph.ca
UR - 19190277
ER -

TY - JOUR
ID - 251
T1 - Exuberant and inhibited toddlers: stability of temperament and risk for problem behavior
A1 - Stifter,C.A.
A1 - Putnam,S.
A1 - Jahromi,L.
Y1 - 2008///
N1 - Stifter, Cynthia A. Putnam, Samuel. Jahromi, Laudan
Development and psychopathology

cn7, 8910645
IM
Journal Article. Research Support, N.I.H., Extramural
English
KW - Medline
KW - Adaptation
KW - Psychological
KW - Arousal
KW - Child Behavior Disorders/di [Diagnosis]
KW - Child Behavior Disorders/px [Psychology]
KW - Child
KW - Preschool
KW - Emotions
KW - Female
KW - Frustration
KW - Humans
KW - Inhibition (Psychology)
KW - Internal-External Control
KW - Longitudinal Studies
KW - Male
KW - Personality Assessment
KW - Personality Development
KW - Play and Playthings
KW - Risk Factors
KW - Shyness
KW - Social Behavior
KW - Social Environment
KW - Temperament
RP - NOT IN FILE
SP - 401
EP - 421
JF - Development & Psychopathology
JA - Dev.Psychopathol.
VL - 20
IS - 2
CY - United States
N2 - Temperament, effortful control, and problem behaviors at 4.5 years were assessed in 72 children classified as exuberant, inhibited, and low reactive as 2-year-olds. Exuberant toddlers were more positive, socially responsive to novel persons, less shy, and rated as having more problem behaviors, including externalizing and internalizing behaviors, than other children as preschoolers. Two forms of effortful control, the ability to delay a response and the ability to produce a subdominant response, were associated with fewer externalizing behaviors, whereas expressing more negative affect (relative to positive/neutral affect) when disappointed was related to more internalizing behaviors. Interaction effects implicated high levels of unregulated emotion during disappointment as a risk factor for problem behaviors in exuberant children
SN - 1469-2198
AD - Pennsylvania State University, University Park, PA 16803, USA. tvr@psu.edu
UR - 18423086
ER -

TY - JOUR
ID - 252
T1 - Exposure to externalizing peers in early childhood: homophily and peer contagion processes
A1 - Hanish,L.D.
A1 - Martin,C.L.
A1 - Fabes,R.A.
A1 - Leonard,S.

A1 - Herzog,M.
Y1 - 2005/06//
N1 - Hanish, Laura D. Martin, Carol Lynn. Fabes, Richard A. Leonard, Stacie. Herzog, Melissa
Journal of abnormal child psychology
0364547, h33
IM
Journal Article. Research Support, N.I.H., Extramural. Research Support, Non-U.S. Gov't. Research Support, U.S. Gov't, P.H.S.
English
KW - Medline
KW - Child
KW - Child Behavior/px [Psychology]
KW - Child Development
KW - Preschool
KW - Choice Behavior
KW - Female
KW - Humans
KW - Imitative Behavior
KW - Male
KW - Peer Group
KW - Play and Playthings
KW - Psychological Theory
KW - Sex Factors
KW - Sexual Behavior/px [Psychology]
KW - Social Behavior
RP - NOT IN FILE
SP - 267
EP - 281
JF - Journal of Abnormal Child Psychology
JA - J Abnorm.Child Psychol.
VL - 33
IS - 3
CY - United States
N2 - Guided by a transactional model, we examined the predictors and effects of exposure to externalizing peers in a low-risk sample of preschoolers and kindergarteners. On the basis of daily observations of peer interactions, we calculated measures of total exposure to externalizing peers and measures of exposure to same- and other-sex externalizing peers. Analyses of predictors of externalizing peer exposure supported a homophily hypothesis for girls. Tests of peer contagion effects varied by sex, and exposure to externalizing peers predicted multiple problem behaviors for girls but not for boys. Sex differences were a function of children's own sex, but not of peers' sex. The study provides evidence of externalizing peer exposure effects in a low-risk sample of young children, notably for girls
SN - 0091-0627
AD - Department of Family and Human Development, Arizona State University, Tempe, Arizona 85287-2502, USA. laura.hanish@asu.edu
UR - 15957556
ER -

TY - JOUR
ID - 253
T1 - Dose-response effect of mother-infant clinical home visiting on aggressive behavior problems in kindergarten
A1 - Lyons-Ruth,K.
A1 - Melnick,S.
Y1 - 2004/06//
N1 - Lyons-Ruth, Karlen. Melnick, Sharon
Journal of the American Academy of Child and Adolescent Psychiatry

hg5, 8704565
IM
Clinical Trial. Controlled Clinical Trial. Journal Article. Research Support, Non-U.S. Gov't. Research Support, U.S. Gov't, P.H.S.
English
KW - Medline
KW - Aggression
KW - Analysis of Variance
KW - Child Behavior Disorders/ep [Epidemiology]
KW - Child Behavior Disorders/pc [Prevention & Control]
KW - Child
KW - Preschool
KW - Community Mental Health Services
KW - Female
KW - Follow-Up Studies
KW - Home Care Services
KW - Humans
KW - Infant
KW - Linear Models
KW - Male
KW - Poverty
KW - Time Factors
KW - United States/ep [Epidemiology]
RP - NOT IN FILE
SP - 699
EP - 707
JF - Journal of the American Academy of Child & Adolescent Psychiatry
JA - J Am Acad.Child Adolesc Psychiatry
VL - 43
IS - 6
CY - United States
N2 - OBJECTIVE: The objective of this follow-up study was to assess the long-term effects of clinical infant home-visiting services on child outcomes at school entry. METHOD: Participants were 63 five-year-olds from low-income families, half of whom were referred to parent-infant home-visiting services during the first 18 months of life due to concerns about the caretaking environment. Families received between 0 and 18 months of weekly home visits based on infant age at entry into the study. At age 5, children were rated by teachers on the Preschool Behavior Questionnaire for behavior problems in the classroom and by parents both on the Simmons Behavior Checklist for behavior problems at home and on the Achenbach Social Competence Items for positive play behaviors with friends. RESULTS: With initial family risk status and child gender controlled, teacher-rated hostile behavior problems decreased in dose-response relation to the duration of early home-visiting services, which accounted for 15% of the variance in child hostile behavior. Parents' reports of positive play behaviors were positively linearly related to service duration. Parents' reports of behavior problems were less reliably related to service duration than teacher reports. CONCLUSIONS: Early home-visiting services reduced the incidence of aggressive behavior problems among socially at-risk children for up to 3.5 years after the end of services
SN - 0890-8567
AD - Department of Psychiatry, Harvard Medical School at the Cambridge Hospital, Cambridge, MA 02139, USA. klruth@hms.harvard.edu
UR - 15167086
ER -

TY - JOUR
ID - 254
T1 - Modern sports eye injuries
A1 - Capao Filipe,J.A.
A1 - Rocha-Sousa,A.

A1 - Falcao-Reis,F.
A1 - Castro-Correia,J.
Y1 - 2003/11//
N1 - Capao Filipe, J A. Rocha-Sousa, A. Falcao-Reis, F. Castro-Correia, J
The British journal of ophthalmology
azk, 0421041
IM
Journal Article
English
KW - Medline
KW - Adolescent
KW - Adult
KW - Athletic Injuries/et [Etiology]
KW - Athletic Injuries/pp [Physiopathology]
KW - Athletic Injuries/th [Therapy]
KW - Child
KW - Eye Injuries/et [Etiology]
KW - Eye Injuries/pp [Physiopathology]
KW - Eye Injuries/th [Therapy]
KW - Eye Protective Devices
KW - Eyelids/in [Injuries]
KW - Eyelids/su [Surgery]
KW - Female
KW - Follow-Up Studies
KW - Humans
KW - Laser Coagulation
KW - Male
KW - Middle Aged
KW - Play and Playthings/in [Injuries]
KW - Portugal
KW - Prospective Studies
KW - Racquet Sports/in [Injuries]
KW - Retinal Perforations/et [Etiology]
KW - Retinal Perforations/su [Surgery]
KW - Soccer/in [Injuries]
KW - Visual Acuity
RP - NOT IN FILE
SP - 1336
EP - 1339
JF - British Journal of Ophthalmology
JA - Br J Ophthalmol.
VL - 87
IS - 11
CY - England
N2 - AIMS: To determine the severity and long term sequelae of eye injuries caused by modern sports that could be responsible for significant ocular trauma in the future. METHODS: Prospective observational study of 24 (25 eyes) athletes with sports related ocular injuries from health clubs, war games, adventure, radical and new types of soccer, presenting to an eye emergency department between 1992 and 2002 (10 years). RESULTS: Modern sports were responsible for 8.3% of the 288 total sports eye injuries reported. Squash (29.2%) was the most common cause, followed by paintball (20.8%) and motocross (16.6%). The most common diagnosis during the follow up period was retinal breaks (20%). 18 (75%) patients sustained a severe injury. The final visual acuity remained <20/100 in two paintball players. CONCLUSIONS: Ocular injuries resulting from modern sports are often severe. Adequate instruction of the participants in the games, proper use of eye protectors, and a routine complete ophthalmological examination after an eye trauma should be mandatory
SN - 0007-1161

AD - Department of Ophthalmology, University of Porto School of Medicine, S Joao Hospital, Porto, Portugal. jacapaofilipe@netcabo.pt
UR - 14609827
ER -

TY - JOUR
ID - 255
T1 - Mother-infant and father-infant attachment among alcoholic families
A1 - Eiden,R.D.
A1 - Edwards,E.P.
A1 - Leonard,K.E.
Y1 - 2002///
N1 - Eiden, Rina Das. Edwards, Ellen Peterson. Leonard, Kenneth E
Development and psychopathology
cn7, 8910645
IM
Journal Article. Research Support, U.S. Gov't, P.H.S.
English
KW - Medline
KW - Adult
KW - Alcoholism/px [Psychology]
KW - Antisocial Personality Disorder/di [Diagnosis]
KW - Antisocial Personality Disorder/px [Psychology]
KW - Child
KW - Child of Impaired Parents/px [Psychology]
KW - Preschool
KW - Conflict (Psychology)
KW - Depression/px [Psychology]
KW - Father-Child Relations
KW - Female
KW - Humans
KW - Infant
KW - Longitudinal Studies
KW - Male
KW - Middle Aged
KW - Mother-Child Relations
KW - New York
KW - Object Attachment
KW - Reactive Attachment Disorder/di [Diagnosis]
KW - Reactive Attachment Disorder/px [Psychology]
KW - Risk Factors
KW - Temperament
RP - NOT IN FILE
SP - 253
EP - 278
JF - Development & Psychopathology
JA - Dev.Psychopathol.
VL - 14
IS - 2
CY - United States
N2 - This study examined the association between fathers' alcoholism and other risk factors such as parental depression, family conflict, infant temperament, and parent-infant attachment. The quality of parent-infant interactions was hypothesized to be a proximal mediator of the associations among alcoholism and other risk factors and attachment. The participants were 223 families (104 nonalcoholic families and 119 alcoholic families) with 12-month-old infants recruited through birth records. Infants in families with two parents with alcohol problem had significantly higher rates of insecure attachment with both parents. Structural Equations

Modeling indicated that the fathers' alcohol problem was associated with lower paternal sensitivity (higher negative affect, lower positive engagement, and lower sensitive responding) during father-infant play interactions, and this in tum was associated with higher risk for infant attachment insecurity with fathers. The association between the fathers' alcohol problem and infant attachment security with the mother was mediated by matemal depression, and matemal alcohol problems and family conflict were associated with maternal sensitivity during play interactions. These results indicate that the fathers' alcoholism is associated with higher family risk including the quality of the parent-infant relationship; infant attachment develops in a family context; and this context has a significant association with attachment security
SN - 0954-5794
AD - University at Buffalo, State University of New York, USA. eiden@ria.buffalo.edu
UR - 12030691
ER -

TY - JOUR
ID - 257
T1 - Resilient adolescent adjustment among girls: buffers of childhood peer rejection and attention-deficit/hyperactivity disorder
A1 - Mikami,A.Y.
A1 - Hinshaw,S.P.
Y1 - 2006/12//
N1 - Mikami, Amori Yee. Hinshaw, Stephen P
Journal of abnormal child psychology
0364547, h33
IM
Journal Article. Research Support, N.I.H., Extramural. Research Support, Non-U.S. Gov't
English
KW - Medline
KW - Adolescent
KW - Attention Deficit Disorder with Hyperactivity/di [Diagnosis]
KW - Attention Deficit Disorder with Hyperactivity/px [Psychology]
KW - Child
KW - Female
KW - Follow-Up Studies
KW - Humans
KW - Interpersonal Relations
KW - Life Change Events
KW - Peer Group
KW - Rejection (Psychology)
KW - Severity of Illness Index
KW - Social Adjustment
KW - Social Behavior
RP - NOT IN FILE
SP - 825
EP - 839
JF - Journal of Abnormal Child Psychology
JA - J Abnorm.Child Psychol.
VL - 34
IS - 6
CY - United States
N2 - Examined a risk-resilience model of peer rejection and attention-deficit/hyperactivity disorder (ADHD) in a 5-year longitudinal study of 209 ethnically and socioeconomically diverse girls aged 6-13 at baseline and 11-18 at follow-up. Risk factors were childhood ADHD diagnosis and peer rejection; hypothesized protective factors were childhood measures of self-perceived scholastic competence, engagement in goal-directed play when alone, and popularity with adults. Adolescent criterion measures were multi-informant composites of externalizing and internalizing behavior plus indicators of academic achievement, eating pathology, and substance use. ADHD and peer rejection predicted risk for all criterion measures except for substance use,

which was predicted by ADHD only. ADHD and peer rejection predicted lower adolescent academic achievement controlling for childhood achievement, but they did not predict adolescent externalizing and internalizing behavior after controlling for baseline levels of these constructs. Regarding buffers, self-perceived scholastic competence in childhood (with control of academic achievement) predicted resilient adolescent functioning. Contrary to hypothesis, goal-directed play in childhood was associated with poor adolescent outcomes. Buffers were not found to have differential effectiveness among girls with ADHD relative to comparison girls
SN - 0091-0627
AD - Department of Psychology, University of Virginia, 102 Gilmer Hall, P.O. Box 400400, Charlottesville, VA 22904-4400, USA. mikami@virginia.edu
UR - 17051436
ER -

TY - JOUR
ID - 258
T1 - Understanding high traffic injury risks for children in low socioeconomic areas: a qualitative study of parents' views
A1 - Christie,N.
A1 - Ward,H.
A1 - Kimberlee,R.
A1 - Towner,E.
A1 - Sleney,J.
Y1 - 2007/12//
N1 - Christie, N. Ward, H. Kimberlee, R. Towner, E. Sleney, J
Injury prevention : journal of the International Society for Child and Adolescent Injury Prevention
crz, 9510056
IM
Journal Article. Multicenter Study
English
KW - Medline
KW - Accidents
KW - Traffic/px [Psychology]
KW - Traffic/sn [Statistics & Numerical Data]
KW - Adolescent
KW - Attitude to Health
KW - Child
KW - England
KW - Focus Groups
KW - Humans
KW - Motor Vehicles
KW - Parents/px [Psychology]
KW - Play and Playthings/in [Injuries]
KW - Play and Playthings/px [Psychology]
KW - Risk Factors
KW - Safety
KW - Socioeconomic Factors
KW - Wounds and Injuries/px [Psychology]
RP - NOT IN FILE
SP - 394
EP - 397
JA - Injury Prevention
VL - 13
IS - 6
CY - England
N2 - OBJECTIVE: To gain an in-depth qualitative understanding of parents' views about their children's exposure to road traffic injury risk in low socioeconomic areas. DESIGN: Focus groups facilitated by a

moderator with content analysis of data. SETTING: Focus groups were conducted in 10 low socioeconomic English districts that also have high rates of child pedestrian injury. Research was conducted in community venues within each area. SUBJECTS: Parents of children aged 9-14 years living in low socioeconomic areas. RESULTS: Parents believe that children play in their local streets for the following reasons: they like playing out with friends near home; there are few safe, secure, and well-maintained public spaces for children; children are excluded from affordable leisure venues because of their costs; insufficient parental responsibility. For children that play in the street, the key sources of risk identified by parents were: illegal riding and driving around estates and on the pavements; the speed and volume of traffic; illegal parking; drivers being poorly informed about where children play; children's risk-taking behavior. CONCLUSIONS: Intervention programs need to take into account multiple reasons why children in low socioeconomic areas become exposed to hazardous environments thereby increasing their risk of injury. Multi-agency partnerships involving the community are increasingly needed to implement traditional road safety approaches, such as education, engineering, and enforcement, and provide safe and accessible public space, affordable activities for children, and greater support for parents
SN - 1353-8047
AD - Postgraduate Medical School, University of Surrey, Guildford, Surrey, UK. n.christie@surrey.ac.uk
UR - 18056316
ER -

TY - JOUR
ID - 259
T1 - Resale of recalled children's products online: an examination of the world's largest yard sale
A1 - Brown,Kirschman K.
A1 - Smith,G.A.
Y1 - 2007/08//
N1 - Brown Kirschman, Keri. Smith, Gary A
Injury prevention : journal of the International Society for Child and Adolescent Injury Prevention
crz, 9510056
IM
Journal Article
English
KW - Medline
KW - Child
KW - Preschool
KW - Commerce/st [Standards]
KW - Consumer Product Safety
KW - Female
KW - Humans
KW - Infant
KW - Internet
KW - Male
KW - Play and Playthings/in [Injuries]
KW - Risk Management
RP - NOT IN FILE
SP - 228
EP - 231
JA - Injury Prevention
VL - 13
IS - 4
CY - England
N2 - OBJECTIVE: To examine an online auction site for the presence and sale of children's products and toys previously recalled because of safety concerns. METHODS: Targeted items were randomly selected from US Consumer Product Safety Commission (CPSC) press releases of recalled children's products dated 1992-2004. Auction listings from eBay were searched for the 150 targeted recalled items for 30 days. Item, seller, and buyer information were recorded from the auction listings. RESULTS: 190 auctions contained or were suspected to contain a recalled children's item from the target list. Most of the recalled items were listed for sale from

addresses within the United States, with sellers from Canada, Australia, Great Britain, and Ireland also represented. On average, six bids were placed on each recalled item, with 70% of auctions eventuating in a sale. CONCLUSIONS: Recalled children's products were found to be available for sale online and were sold most of the time, presenting a risk of injury to children. Although the CPSC is charged with notifying the public of recalled items, these results suggest that potentially hazardous products are recirculating online. A multi-front initiative to decrease the presence of hazards in online auctions is needed. This initiative should include increased manufacturer efforts to improve recall return rates, a requirement by online auction sites that sellers verify non-recall status before item posting, and parental checks of government recall websites before item purchase. Investigation of parental understanding and awareness of recalls and the potential risks associated with recall announcements is needed
SN - 1353-8047
AD - Department of Psychology, University of Dayton, Dayton, OH 45409, USA.
kirschKe@notes.udayton.edu
UR - 17686931
ER -

TY - JOUR
ID - 260
T1 - Superhero-related injuries in paediatrics: a case series
A1 - Davies,P.
A1 - Surridge,J.
A1 - Hole,L.
A1 - Munro-Davies,L.
Y1 - 2007/03//
N1 - Davies, Patrick. Surridge, Julia. Hole, Laura. Munro-Davies, Lisa
Archives of disease in childhood
6xg, 0372434
AIM, IM
Case Reports. Journal Article
English
KW - Medline
KW - Accidental Falls
KW - Child
KW - Clothing
KW - Fractures
KW - Bone/et [Etiology]
KW - Humans
KW - Imitative Behavior
KW - Infant
KW - Male
KW - Play and Playthings/in [Injuries]
KW - Risk-Taking
RP - NOT IN FILE
SP - 242
EP - 243
JF - Archives of Disease in Childhood
JA - Arch.Dis.Child
VL - 92
IS - 3
CY - England
N2 - Five cases of serious injuries to children wearing superhero costumes, involving extreme risk-taking behaviour, are presented here. Although children have always displayed behaviour seemingly unwise to the adult eye, the advent of superhero role models can give unrealistic expectations to the child, which may lead to serious injury. The children we saw have all had to contemplate on their way to hospital that they do not in fact possess superpowers. The inbuilt injury protection which some costumes possess is also discussed
SN - 1468-2044

AD - Department of Paediatrics, Queen's Medical Centre, Nottingham, UK
UR - 17337680
ER -

TY - JOUR
ID - 261
T1 - The mercury emergency and Hamilton school children: a follow-up analysis
A1 - George,L.
A1 - Scott,F.E.
A1 - Cole,D.
A1 - Siracusa,L.
A1 - Buffett,C.
A1 - Hunter,W.
A1 - Zinkewich,R.
Y1 - 1996/07//
N1 - George, L. Scott, F E. Cole, D. Siracusa, L. Buffett, C. Hunter, W. Zinkewich, R
Canadian journal of public health = Revue canadienne de sante publique
ck6, 0372714
IM
Journal Article
English
KW - Medline
KW - Adolescent
KW - Child
KW - Emergencies
KW - Environmental Exposure/an [Analysis]
KW - Female
KW - Humans
KW - Male
KW - Mercury/ae [Adverse Effects]
KW - Mercury
KW - Mercury Poisoning/co [Complications]
KW - Mercury Poisoning/ep [Epidemiology]
KW - Mercury Poisoning/pc [Prevention & Control]
KW - Ontario/ep [Epidemiology]
KW - Play and Playthings
KW - Risk Factors
RP - NOT IN FILE
SP - 224
EP - 226
JF - Canadian Journal of Public Health
JA - Can J Public Health
VL - Revue Canadienne de Sante Publique. 87
IS - 4
CY - CANADA
N2 - In September 1993, children broke into a laboratory in an abandoned scrap metal recycling plant in Hamilton and removed mercury and other materials from the site. They began distribution of the mercury to their peers but this activity was halted once an emergency response was established. This paper focuses on the identification and assessment of children exposed to mercury and the use of Epi Info 6 in the analysis. Approximately 6,000 children were screened and 269 children identified as being exposed or at risk of exposure. We found that distribution of mercury within the school system was swift. Children exhibited a number of risky behaviours with mercury. There were no significant gender differences in these behaviours. The majority of children involved were 10 to 13 years of age. No child exhibited health effects from exposure
SN - 0008-4263
AD - McMaster University, Faculty of Health Sciences, Hamilton, ON
UR - 8870298

ER -

TY - JOUR
ID - 262
T1 - Does play equipment conform to the Canadian standard?
A1 - Lesage,D.
A1 - Robitaille,Y.
A1 - Dorval,D.
A1 - Beaulne,G.
Y1 - 1995/07//
N1 - Lesage, D. Robitaille, Y. Dorval, D. Beaulne, G
Canadian journal of public health = Revue canadienne de sante publique
ck6, 0372714
IM
Journal Article
English
KW - Medline
KW - Child
KW - Child Welfare
KW - Equipment Safety
KW - Humans
KW - Play and Playthings
KW - Quebec
KW - Safety/st [Standards]
RP - NOT IN FILE
SP - 279
EP - 283
JF - Canadian Journal of Public Health
JA - Can J Public Health
VL - Revue Canadienne de Sante Publique. 86
IS - 4
CY - CANADA
N2 - In the summer of 1991, play equipment in 254 playgrounds located on the island of Montreal were inspected, using a checklist made up of items drawn from the Canadian standard for the safety of children's playspaces and equipment. The results of the study, covering 605 climbers, 522 swings and 181 slides, made it possible to identify the most and least respected aspect of safety. For example, one out of two pieces of play equipment was installed on a protective surface that did not conform to the Canadian standard; seven out of ten swings had seats made of non-impact-absorbing materials; and six out of ten pieces of equipment had head entrapment openings. Knowing the physical shortcomings of play equipment is an important step in reducing injuries sustained on it. However, to be effective, the prevention of injuries related to play equipment requires a concerted effort on the part of several partners
SN - 0008-4263
AD - Direction de la sante publique de Montreal-Centre, Christophe-Colomb
UR - 7497417
ER -

TY - JOUR
ID - 263
T1 - Influencing children's pregambling game playing via conditional discrimination training
A1 - Johnson,T.E.
A1 - Dixon,M.R.
Y1 - 2009///
N1 - Johnson, Taylor E. Dixon, Mark R
Journal of applied behavior analysis
0174763, hdr
IM

Journal Article
English
KW - Medline
KW - Child
KW - Choice Behavior
KW - Discrimination Learning
KW - Female
KW - Gambling/px [Psychology]
KW - Humans
KW - Male
KW - Play and Playthings
KW - Transfer (Psychology)
RP - NOT IN FILE
SP - 73
EP - 81
JF - Journal of Applied Behavior Analysis
JA - J Appl.Behav Anal.
VL - 42
IS - 1
CY - United States
N2 - Past research has demonstrated a transformation of stimulus functions under similar conditions using gambling tasks and adults (e.g., Zlomke & Dixon, 2006), and the present study attempted to extend this research. Experimenters exposed 7 children (ages 7 to 10 years) to a simulated board game with concurrently available dice differing only by color. Following initial exposure to the game, participants were trained to discriminate between two contextual cues representing the relational frames of more than and less than. Following the training procedure, experimenters reexposed participants to the simulated board game. Six of the 7 participants demonstrated an increased preference toward the die with the color that had been paired with more than during the conditional discrimination training
SN - 1938-3703
AD - Southern Illinois University, IL, USA
UR - 19721731
ER -

TY - JOUR
ID - 264
T1 - Factors associated with obesity in Indonesian adolescents
A1 - Collins,A.E.
A1 - Pakiz,B.
A1 - Rock,C.L.
Y1 - 2008///
N1 - Collins, Allison E. Pakiz, Bilge. Rock, Cheryl L
International journal of pediatric obesity : IJPO : an official journal of the International Association for the Study of Obesity
101256330
IM
Journal Article. Research Support, Non-U.S. Gov't
English
KW - Medline
KW - Adolescent
KW - Adolescent Behavior/eh [Ethnology]
KW - Age Factors
KW - Asian Continental Ancestry Group/sn [Statistics & Numerical Data]
KW - Body Mass Index
KW - Chi-Square Distribution
KW - Child
KW - Child Behavior/eh [Ethnology]

KW - Computers
KW - Cross-Sectional Studies
KW - Eating/eh [Ethnology]
KW - Fast Foods
KW - Feeding Behavior
KW - Female
KW - Food Habits/eh [Ethnology]
KW - Health Surveys
KW - Humans
KW - Indonesia/ep [Epidemiology]
KW - Life Style/eh [Ethnology]
KW - Logistic Models
KW - Male
KW - Motor Activity
KW - Obesity/di [Diagnosis]
KW - Obesity/eh [Ethnology]
KW - Obesity/px [Psychology]
KW - Questionnaires
KW - Risk Assessment
KW - Risk Factors
KW - Sedentary Lifestyle/eh [Ethnology]
KW - Socioeconomic Factors
KW - Video Games
RP - NOT IN FILE
SP - 58
EP - 64
JF - International Journal of Pediatric Obesity
JA - Int.J Pediatr Obes.
VL - 3
IS - 1
CY - England
N2 - OBJECTIVE. To investigate adolescent eating, activity, and behavioral patterns, and attitudes and their associations with obesity in selected communities in Indonesia. DESIGN. A cross-sectional questionnaire and physical measurement-based study in three communities of varying modernization levels in Indonesia (Yogyakarta, Kuta, and Jakarta). Subjects. 1758 middle school students (aged 12-15 years) recruited at six different schools: one public and one private school in each of the three target communities. MEASUREMENTS. The questionnaire collected data on demographic characteristics (age, gender, ethnicity, estimated household income); fast food eating habits (frequency, types of food/restaurant, general beliefs about fast foods); television, computer, and Play Station usage; physical activity (hrs/wk of participation in physical activity, transportation means for attending school); and eating habits (frequency of consuming selected foods and beverages). Student's height and weights were obtained, body mass index (BMI: weight [kg]/height [m(2)]) was calculated, and obesity was defined as >=95%, using the BMI-for-age cut-offs from the Centers for Disease Control and Prevention (CDC) charts. RESULTS. Chi-square analysis revealed associations between obesity and community setting, family income, use of a computer or Play Station, type of transportation to school, and beliefs about fast foods (P<0.01). According to the logistic regression analysis, adolescents from families with incomes over Rp 2 mil were three times as likely to be obese (95% CI 1.9, 4.9) and boys were 2.6 times more likely to be obese (95% CI 1.5, 4.5). CONCLUSION. Greater likelihood of obesity among Indonesian adolescents who spend greater amounts of time using a computer or Play Station suggests that such sedentary activities may be replacing physical activity, promoting an energy imbalance, and subsequently, an increased risk for obesity
SN - 1747-7174
AD - Department of Family and Preventive Medicine, University of California, San Diego, La Jolla, USA
UR - 17852550
ER -

TY - JOUR

ID - 265
T1 - A place to play: socioeconomic and spatial factors in children's physical activity
A1 - Ziviani,J.
A1 - Wadley,D.
A1 - Ward,H.
A1 - Macdonald,D.
A1 - Jenkins,D.
A1 - Rodger,S.
Y1 - 2008/03//
N1 - Ziviani, Jenny. Wadley, David. Ward, Heather. Macdonald, Doune. Jenkins, David. Rodger, Sylvia
Australian occupational therapy journal
15420200r
IM
Comparative Study. Journal Article. Research Support, Non-U.S. Gov't
English
KW - Medline
KW - Attitude to Health
KW - Australia/ep [Epidemiology]
KW - Child
KW - Child Welfare/sn [Statistics & Numerical Data]
KW - Exercise
KW - Female
KW - Health Behavior
KW - Health Promotion/og [Organization & Administration]
KW - Humans
KW - Income/sn [Statistics & Numerical Data]
KW - Male
KW - Obesity/ep [Epidemiology]
KW - Obesity/pc [Prevention & Control]
KW - Parent-Child Relations
KW - Play and Playthings
KW - Risk Factors
KW - Socioeconomic Factors
RP - NOT IN FILE
SP - 2
EP - 11
JF - Australian Occupational Therapy Journal
JA - Aust Occup Ther J
VL - 55
IS - 1
CY - Australia
N2 - BACKGROUND AND AIMS: Concerns about physical inactivity in children and growing levels of obesity are expressed by politicians, health economists and those involved with the health and well-being of children. As this has the potential to be a major health issue, the aim of this investigation was to explore any contributing socioenvironmental considerations. METHODS AND RESULTS: Census-matched survey data were analysed from 318 parents of 6- to 7-year-old children, revealing that family socioeconomic status (SES) influenced the places where children engaged in physical activity. Children from low SES backgrounds spent significantly more time playing close to their homes, and their families were less able to afford access to commercial physical-activity facilities, than those from middle and high SES families. Although neighbourhood-based activities are generally associated with more spontaneous free play, such activities may not provide the same opportunities for supervision and physical skill building available through commercial-based activities. CONCLUSIONS: Given that access to 'enriching' physical-activity spaces may be limited by the capacity to pay, these findings have implications for professionals such as occupational therapists who can take on a role in advocating for equity in access and promotion of a more engaging urban design. Dialogue with urban planners is central to this process
SN - 1440-1630

AD - School of Rehabilitation Sciences, The University of Queensland, St Lucia, Queensland, Australia
UR - 20887428
ER -

TY - JOUR
ID - 266
T1 - Atypical sources of childhood lead poisoning in the United States: a systematic review from 1966-2006. [Review] [49 refs]
A1 - Gorospe,E.C.
A1 - Gerstenberger,S.L.
Y1 - 2008/09//
N1 - Gorospe, Emmanuel C. Gerstenberger, Shawn L
Clinical toxicology (Philadelphia, Pa.)
101241654
AIM, IM
Journal Article. Review
English
KW - Medline
KW - Candy/ae [Adverse Effects]
KW - Child
KW - Condiments/ae [Adverse Effects]
KW - Cosmetics/ae [Adverse Effects]
KW - Environmental Exposure
KW - Environmental Pollutants/ae [Adverse Effects]
KW - Female
KW - Health Knowledge
KW - Attitudes
KW - Practice
KW - Household Articles
KW - Humans
KW - Infant
KW - Lead Poisoning/di [Diagnosis]
KW - Lead Poisoning/ep [Epidemiology]
KW - Lead Poisoning/et [Etiology]
KW - Male
KW - Medicine
KW - Traditional
KW - Play and Playthings
KW - Practice Guidelines as Topic
KW - Predictive Value of Tests
KW - Questionnaires
KW - Risk Factors
KW - United States/ep [Epidemiology]
RP - NOT IN FILE
SP - 728
EP - 737
JF - Clinical Toxicology: The Official Journal of the American Academy of Clinical Toxicology & European Association of Poisons Centres & Clinical Toxicologists
JA - Clin.Toxicol.(Phila)
VL - 46
IS - 8
CY - United States
N2 - BACKGROUND: Lead poisoning from atypical sources, which excludes the well-established lead-based paint ingestions and exposure in occupational settings, are increasingly reported in medical literature. Our objective is to increase awareness on atypical sources of lead exposure and to formulate recommendations for their detection based on actual reported cases. METHODS: We systematically retrieved and reviewed reports on

pediatric lead poisoning in the U.S. from atypical sources by searching Medline, Embase, CINAHL, Academic Search Premier, AltHealth, websites of state lead poisoning prevention programs, and the U.S. Consumer Product Safety Commission database for reports published from January 1966 to December 2006. RESULTS: We retrieved 28 published reports that met our inclusion criteria. Of these reports, 20 are case reports and 8 case series, documenting a total of 82 incidents of lead poisoning in children from atypical sources. CONCLUSION: There are varied sources of atypical lead exposure among U.S. children. The sources were grouped in the following categories based on their utility: fashion accessories, folk remedies, imported condiments & candies, pellets & bullets, and lastly, recreational & domestic items. Based on these findings, we have formulated a questionnaire that may assist in the identification of atypical lead sources in the home. [References: 49]
SN - 1556-3650
AD - University ofNevada Las Vegas, School of Public Health, Department of Environmental & Occupational Health, Las Vegas, Nevada 89154-3064, USA
UR - 18608287
ER -

TY - JOUR
ID - 267
T1 - Assessment of the current status of playground safety in the midwestern region of Turkey: an effort to provide a safe environment for children
A1 - Uskun,E.
A1 - Kisioglu,A.N.
A1 - Altay,T.
A1 - Cikinlar,R.
A1 - Kocakaya,A.
Y1 - 2008/11//
N1 - Uskun, Ersin. Kisioglu, Ahmet Nesimi. Altay, Tulin. Cikinlar, Rengul. Kocakaya, Asuman
The Turkish journal of pediatrics
wkv, 0417505
IM
Journal Article
English
KW - Medline
KW - Accident Prevention
KW - Child
KW - Preschool
KW - Equipment Design
KW - Equipment Safety
KW - Guideline Adherence
KW - Humans
KW - Play and Playthings
KW - Safety
KW - Turkey
KW - Wounds and Injuries/pc [Prevention & Control]
RP - NOT IN FILE
SP - 559
EP - 565
JF - Turkish Journal of Pediatrics
JA - Turk.J Pediatr
VL - 50
IS - 6
CY - Turkey
N2 - This study aimed to identify and evaluate the degree of conformity to the playground standards and the level of compliance with current safety specifications of the playgrounds in the midwestern region of Turkey. An observational technique was used at a total of 57 public playgrounds. A playground safety control form was prepared based on the United States National Program for Playground Safety and the Consumer Product Safety Commission security standards, since there is no national law covering playground equipment and safety in

Turkey. The study evaluated the surroundings of the playground, arrangement of equipment in the playground, and characteristics of the equipment. The percentage of playgrounds surveyed with inadequate or hard surfacing was 80.7%. Fifty-two percent of the equipment was found to be inappropriate. Equipment was higher than the recommended heights. The results of our study unfortunately point out that playgrounds for children do not meet many of the safety criteria
SN - 0041-4301
AD - Department of Public Health, Faculty of Medicine, Suleyman Demirel University, Isparta, Turkey
UR - 19227420
ER -

TY - JOUR
ID - 268
T1 - River tree rope swing injuries
A1 - Sorey,W.H.
A1 - Cassidy,L.D.
A1 - Crout,J.
A1 - Blount,P.
Y1 - 2008/07//
N1 - Sorey, William H. Cassidy, Laura D. Crout, Jeffery. Blount, Phillip
Southern medical journal
uvh, 0404522
AIM, IM
Journal Article
English
KW - Medline
KW - Adolescent
KW - Adult
KW - Athletic Injuries/ep [Epidemiology]
KW - Athletic Injuries/et [Etiology]
KW - Child
KW - Preschool
KW - Drowning/ep [Epidemiology]
KW - Drowning/et [Etiology]
KW - Female
KW - Humans
KW - Male
KW - Play and Playthings/in [Injuries]
KW - Prospective Studies
KW - Registries
KW - Retrospective Studies
KW - Swimming/in [Injuries]
KW - United States/ep [Epidemiology]
KW - Young Adult
RP - NOT IN FILE
SP - 699
EP - 702
JF - Southern Medical Journal
JA - South Med J
VL - 101
IS - 7
CY - United States
N2 - OBJECTIVE: To review injuries associated with falls from river tree rope swings (RTRS) and identify points of action for prevention. METHODS: This study was a retrospective analysis of injury reports directly related to falls from RTRS. Case reports for 2002-06 were extracted from the National Electronic Injury Surveillance System (NEISS), US legal literature, and news reports. Direct observation of river tree rope swings was performed with kayak and camera to identify potential hazards. A laboratory study was performed on

samples of rope used in RTRS to assess entanglement risk. RESULTS: NEISS detailed 73 injuries directly related to falls from RTRS in ages 5-25 years. Finger fractures were the most commonly reported injury. Based on NEISS data, an estimated 700 injuries may occur related to RTRS each year. RTRS share risk of severe injuries with traditional rope swings such as lower extremity fractures, concussions, and spinal cord injuries. Drowning, finger avulsion and genital lacerations appear more common with river tree rope swings. CONCLUSIONS: RTRS are dangerous and are associated with a variety of serious injuries from falls and entanglement. The most hazardous risk factors of RTRS are use by a non-swimmer, shallow water, extreme fall distance, and presence of a small diameter retrieval line. RTRS injuries occurring in remote locations are challenges to rural physicians and EMS providers. Prevention of recreational injuries, including injuries sustained from RTRS, remains a daunting task
SN - 1541-8243
AD - Department of Pediatrics, University of Mississippi School of Medicine, Jackson, MS 39216, USA. wsorey@ped.umsmed.edu
UR - 18580723
ER -

TY - JOUR
ID - 269
T1 - Active play and screen time in US children aged 4 to 11 years in relation to sociodemographic and weight status characteristics: a nationally representative cross-sectional analysis
A1 - Anderson,S.E.
A1 - Economos,C.D.
A1 - Must,A.
Y1 - 2008///
N1 - Anderson, Sarah E. Economos, Christina D. Must, Aviva
BMC public health
100968562
IM
Journal Article
English
KW - Medline
KW - Body Weight/eh [Ethnology]
KW - Body Weight/ph [Physiology]
KW - Child
KW - Preschool
KW - Continental Population Groups
KW - Cross-Sectional Studies
KW - Ethnic Groups
KW - Female
KW - Humans
KW - Male
KW - Motor Activity
KW - Obesity/ep [Epidemiology]
KW - Obesity/eh [Ethnology]
KW - Play and Playthings
KW - Risk Factors
KW - Social Class
KW - United States/ep [Epidemiology]
RP - NOT IN FILE
SP - 366
JA - BMC Public Health
VL - 8
CY - England
N2 - BACKGROUND: The high prevalence of childhood obesity underscores the importance of monitoring population trends in children's activity and screen time, and describing associations with child age, gender, race/ethnicity, and weight status. Our objective was to estimate the proportion of young children in the US who

have low levels of active play or high levels of screen time, or who have both these behaviors, and to describe associations with age, gender, race/ethnicity, and weight status. METHODS: We analyzed data collected during the National Health and Nutrition Examination Surveys 2001-2004, a US nationally representative cross-sectional study. We studied 2964 children aged 4.00 to 11.99 years. Our main outcomes were reported weekly times that the child played or exercised hard enough to sweat or breathe hard (active play), daily hours the child watched television/videos, used computers, or played computer games (screen time), and the combination of low active play and high screen time. Low active play was defined as active play 6 times or less per week. High screen time was defined as more than 2 hours per day. We accounted for the complex survey design in analyses and report proportions and 95% confidence intervals. We used Wald Chi-square to test for differences between proportions. To identify factors associated with low active play and high screen time, we used multivariate logistic regression. RESULTS: Of US children aged 4 to 11 years, 37.3% (95% confidence interval, 34.1% to 40.4%) had low levels of active play, 65.0% (95% CI, 61.4% to 68.5%) had high screen time, and 26.3% (95% CI, 23.8% to 28.9%) had both these behaviors. Characteristics associated with a higher probability of simultaneously having low active play and high screen time were older age, female gender, non-Hispanic black race/ethnicity, and having a BMI-for-age > or =95th percentile of the CDC growth reference. CONCLUSION: Many young children in the US are reported to have physical activity and screen time behaviors that are inconsistent with recommendations for healthy pediatric development. Children who are overweight, approaching adolescence, girls, and non-Hispanic blacks may benefit most from public health policies and programs aimed at these behaviors
SN - 1471-2458
AD - Division of Epidemiology, The Ohio State University College of Public Health, Columbus, Ohio, USA. sanderson@cph.osu.edu
UR - 18945351
ER -

TY - JOUR
ID - 270
T1 - Neighborhood characteristics in relation to diet, physical activity and overweight of Canadian children
A1 - Veugelers,P.
A1 - Sithole,F.
A1 - Zhang,S.
A1 - Muhajarine,N.
Y1 - 2008///
N1 - Veugelers, Paul. Sithole, Fortune. Zhang, Sharon. Muhajarine, Nazeem
International journal of pediatric obesity : IJPO : an official journal of the International Association for the Study of Obesity
101256330
IM
Journal Article. Research Support, Non-U.S. Gov't
English
KW - Medline
KW - Child
KW - Crime
KW - Cross-Sectional Studies
KW - Diet
KW - Exercise
KW - Health Behavior
KW - Health Surveys
KW - Humans
KW - Life Style
KW - Nova Scotia
KW - Odds Ratio
KW - Overweight/et [Etiology]
KW - Overweight/pp [Physiopathology]
KW - Overweight/pc [Prevention & Control]
KW - Play and Playthings

KW - Public Facilities
KW - Questionnaires
KW - Residence Characteristics
KW - Risk Assessment
KW - Risk Factors
KW - Socioeconomic Factors
KW - Sports
KW - Time Factors
RP - NOT IN FILE
SP - 152
EP - 159
JF - International Journal of Pediatric Obesity
JA - Int.J Pediatr Obes.
VL - 3
IS - 3
CY - England
N2 - BACKGROUND: Neighborhood infrastructure may provide an important opportunity to prevent overweight among children. In the present study we investigated whether access to shops for modestly priced fresh produce, access to parks and playgrounds, access to recreational facilities and neighborhood safety are related to children's diet, physical and sedentary activities, and body weights. METHODS: Data were obtained from the Children's Lifestyle and School-performance Study, a survey including 5,471 grade five students and their parents in the province of Nova Scotia, Canada. Students completed the Harvard Food Frequency Questionnaire and had their height and weight measured. Parents completed questions on socio-economic background and how they perceived their neighborhood. We applied multilevel regression methods to relate these neighborhood characteristics with children's fruit and vegetable consumption, dietary fat intake, diet quality, frequency of engaging in sports with and without a coach, screen time, overweight and obesity. RESULTS: Children in neighborhoods with greater perceived access to shops had healthier diets and were less likely to be overweight or obese. Children in neighborhoods with good access to playgrounds, parks and recreational facilities were reportedly more active and were less likely to be overweight or obese, whereas children in safe neighborhoods engaged more in unsupervised sports. CONCLUSIONS: The study demonstrated associations between neighborhood characteristics, health behaviors and childhood overweight. This contributes to the knowledge base that is still too narrow to justify informed preventative public health policy. We advocate the evaluation of natural experiments created by new policy that affect neighborhood infrastructures as the optimal opportunity to enlarge this knowledge base
SN - 1747-7166
AD - School of Public Health, University of Alberta, Alberta, Canada. paul.veugelers@ualberta.ca
UR - 19086297
ER -

TY - JOUR
ID - 271
T1 - Patient's page. Toy safety: thinking inside the box
A1 - Bright,M.
Y1 - 2008/12//
N1 - Bright, Melissa
Southern medical journal
uvh, 0404522
AIM, IM
Journal Article
English
KW - Medline
KW - Age Factors
KW - Child
KW - Preschool
KW - Consumer Product Safety/lj [Legislation & Jurisprudence]
KW - Consumer Product Safety/st [Standards]

KW - Humans
KW - Infant
KW - Lead Poisoning/di [Diagnosis]
KW - Lead Poisoning/pc [Prevention & Control]
KW - Play and Playthings/in [Injuries]
KW - Risk Assessment/lj [Legislation & Jurisprudence]
RP - NOT IN FILE
SP - 1191
EP - 1192
JF - Southern Medical Journal
JA - South Med J
VL - 101
IS - 12
CY - United States
SN - 1541-8243
UR - 19005428
ER -

TY - JOUR
ID - 272
T1 - Sensory modulation disorder: a risk factor for participation in daily life activities
A1 - Bar-Shalita,T.
A1 - Vatine,J.J.
A1 - Parush,S.
Y1 - 2008/12//
N1 - Bar-Shalita, Tami. Vatine, Jean-Jacques. Parush, Shula
Developmental medicine and child neurology
0006761, e83
IM
Journal Article. Research Support, Non-U.S. Gov't
English
KW - Medline
KW - Achievement
KW - Activities of Daily Living/px [Psychology]
KW - Arousal
KW - Avoidance Learning
KW - Child
KW - Child Behavior Disorders/di [Diagnosis]
KW - Child Behavior Disorders/px [Psychology]
KW - Defense Mechanisms
KW - Female
KW - Habits
KW - Humans
KW - Israel
KW - Male
KW - Motivation
KW - Neurologic Examination/sn [Statistics & Numerical Data]
KW - Personality Assessment/sn [Statistics & Numerical Data]
KW - Play and Playthings
KW - Psychometrics
KW - Sensation Disorders/di [Diagnosis]
KW - Sensation Disorders/px [Psychology]
KW - Sensory Gating
KW - Social Adjustment
KW - Social Environment
RP - NOT IN FILE

SP - 932
EP - 937
JF - Developmental Medicine & Child Neurology
JA - Dev.Med Child Neurol.
VL - 50
IS - 12
CY - England
N2 - Participation in childhood daily functional performance was examined in 78 children: 44 with sensory modulation disorder (SMD); (33 males, 11 females; mean age 7y 6mo [SD 1.20]) and 34 without SMD (18 males, 16 females; mean age 7y 8mo [SD 1.33]). Group classification was determined using two measures: the Short Sensory Profile (SSP) and the Full-form Sensory Profile. Parents completed a battery of caregiver questionnaires. Children with SMD scored significantly lower on all three participation scales than the control group. A high correlation was observed between level of activity performance of the Participation in Childhood Occupations Questionnaire (PICO-Q) and the SSP, and a moderate correlation was observed between the Enjoyment of Performance of the PICO-Q and the SSP. A low correlation was observed between Frequency of Performance of the PICO-Q and the SSP. Logistic regression indicated that all three participation scales (level of activity performance, level of enjoyment of the activity, and frequency of performance of the activity) are significantly associated with group classification, with odds ratios of 3.13, 2.05, and 1.73 respectively. These findings are the first, to our knowledge, to confirm claims of limited participation in daily activities among children with SMD. Our results have significant clinical implications and provide support for the need for practitioners and caregivers of children with SMD to facilitate participation
SN - 1469-8749
AD - School of Occupational Therapy, Faculty of Medicine of Hadassah and the Hebrew University of Jerusalem, Israel
UR - 19046186
ER -

TY - JOUR
ID - 273
T1 - Food, fun, and fitness internet program for girls: pilot evaluation of an e-Health youth obesity prevention program examining predictors of obesity
A1 - Thompson,D.
A1 - Baranowski,T.
A1 - Cullen,K.
A1 - Watson,K.
A1 - Liu,Y.
A1 - Canada,A.
A1 - Bhatt,R.
A1 - Zakeri,I.
Y1 - 2008/11//
N1 - Thompson, Debbe. Baranowski, Tom. Cullen, Karen. Watson, Kathy. Liu, Yan. Canada, Ashanti. Bhatt, Riddhi. Zakeri, Issa
Preventive medicine
pm4, 0322116
IM
Evaluation Studies. Journal Article. Research Support, N.I.H., Extramural. Research Support, Non-U.S. Gov't. Research Support, U.S. Gov't, Non-P.H.S.
English
KW - Medline
KW - African Americans
KW - Child
KW - Diet
KW - Exercise
KW - Female
KW - Fruit
KW - Humans

KW - Internet
KW - Obesity/et [Etiology]
KW - Obesity/pc [Prevention & Control]
KW - Pilot Projects
KW - Play and Playthings
KW - Self Efficacy
KW - Texas
KW - Vegetables
RP - NOT IN FILE
SP - 494
EP - 497
JA - Preventive Medicine
VL - 47
IS - 5
CY - United States
N2 - OBJECTIVE: This pilot study tested whether an Internet-based intervention could achieve change in fruit, juice, and vegetable consumption, physical activity, and self-efficacy in youth at-risk of obesity. METHOD: Participants were 80 8-to-10-year-old African American girls at-risk of obesity, with a home computer, Internet access, and an e-mail address. A two-group design was followed. Groups differed only on incentive schedule (immediate, delayed). The 8-week home-based program, conducted entirely over the Internet, promoted fruit, juice, vegetables, and water intake and physical activity. Pre-post measures were collected through self-report via the program website. The study was conducted in the greater Houston, TX, area September through November, 2004. RESULTS: Statistically significant pre-to-post differences were observed in fruit, juice, and vegetable consumption (p=.002), physical activity--yesterday (p<.001), physical activity--usually (p=.001), and fruit, juice, and vegetable self-efficacy (p=.003). CONCLUSION: Internet-based obesity prevention programs may be an effective channel for promoting healthy diet and physical activity behaviors to youth at-risk of obesity. Additional research is needed to more fully examine their effectiveness at promoting and maintaining diet and physical activity change
SN - 1096-0260
AD - USDA/ARS Children's Nutrition Research Center, Baylor College of Medicine, Department of Pediatrics, Houston, TX, USA. dit@bcm.tmc.edu
UR - 18718846
ER -

TY - JOUR
ID - 274
T1 - Longitudinal effects of violent video games on aggression in Japan and the United States
A1 - Anderson,C.A.
A1 - Sakamoto,A.
A1 - Gentile,D.A.
A1 - Ihori,N.
A1 - Shibuya,A.
A1 - Yukawa,S.
A1 - Naito,M.
A1 - Kobayashi,K.
Y1 - 2008/11//
N1 - Anderson, Craig A. Sakamoto, Akira. Gentile, Douglas A. Ihori, Nobuko. Shibuya, Akiko. Yukawa, Shintaro. Naito, Mayumi. Kobayashi, Kumiko
Pediatrics
oxv, 0376422
AIM, IM
Journal Article. Research Support, N.I.H., Extramural. Research Support, Non-U.S. Gov't. Research Support, U.S. Gov't, P.H.S.
English
KW - Medline
KW - Adolescent

KW - Aggression
KW - Child
KW - Culture
KW - Female
KW - Humans
KW - Japan
KW - Male
KW - United States
KW - Video Games/ae [Adverse Effects]
KW - Video Games/px [Psychology]
KW - Violence
RP - NOT IN FILE
SP - e1067
EP - e1072
JA - Pediatrics
VL - 122
IS - 5
CY - United States
N2 - CONTEXT: Youth worldwide play violent video games many hours per week. Previous research suggests that such exposure can increase physical aggression. OBJECTIVE: We tested whether high exposure to violent video games increases physical aggression over time in both high- (United States) and low- (Japan) violence cultures. We hypothesized that the amount of exposure to violent video games early in a school year would predict changes in physical aggressiveness assessed later in the school year, even after statistically controlling for gender and previous physical aggressiveness. DESIGN: In 3 independent samples, participants' video game habits and physically aggressive behavior tendencies were assessed at 2 points in time, separated by 3 to 6 months. PARTICIPANTS: One sample consisted of 181 Japanese junior high students ranging in age from 12 to 15 years. A second Japanese sample consisted of 1050 students ranging in age from 13 to 18 years. The third sample consisted of 364 United States 3rd-, 4th-, and 5th-graders ranging in age from 9 to 12 years. RESULTS. Habitual violent video game play early in the school year predicted later aggression, even after controlling for gender and previous aggressiveness in each sample. Those who played a lot of violent video games became relatively more physically aggressive. Multisample structure equation modeling revealed that this longitudinal effect was of a similar magnitude in the United States and Japan for similar-aged youth and was smaller (but still significant) in the sample that included older youth. CONCLUSIONS: These longitudinal results confirm earlier experimental and cross-sectional studies that had suggested that playing violent video games is a significant risk factor for later physically aggressive behavior and that this violent video game effect on youth generalizes across very different cultures. As a whole, the research strongly suggests reducing the exposure of youth to this risk factor
SN - 1098-4275
AD - Department of Psychology,Center for the Study of Violence, Iowa State University, Ames, Iowa 50011, USA. caa@iastate.edu
UR - 18977956
ER -

TY - JOUR
ID - 275
T1 - "Practice what you preach": induced hypocrisy as an intervention strategy to reduce children's intentions to risk take on playgrounds
A1 - Morrongiello,B.A.
A1 - Mark,L.
Y1 - 2008/11//
N1 - Morrongiello, Barbara A. Mark, Landa
Journal of pediatric psychology
jl8, 7801773
IM
Journal Article. Multicenter Study. Randomized Controlled Trial. Research Support, Non-U.S. Gov't
English

KW - Medline
KW - Accidental Falls/pc [Prevention & Control]
KW - Adolescent
KW - Age Factors
KW - Behavior Therapy/mt [Methods]
KW - Character
KW - Child
KW - Cognitive Dissonance
KW - Exploratory Behavior
KW - Fear
KW - Female
KW - Generalization (Psychology)
KW - Health Education
KW - Health Knowledge
KW - Attitudes
KW - Practice
KW - Humans
KW - Intention
KW - Judgment
KW - Male
KW - Motivation
KW - Ontario
KW - Play and Playthings
KW - Risk-Taking
KW - Safety
KW - Self Concept
KW - Wounds and Injuries/pc [Prevention & Control]
KW - Wounds and Injuries/px [Psychology]
RP - NOT IN FILE
SP - 1117
EP - 1128
JF - Journal of Pediatric Psychology
JA - J Pediatr Psychol.
VL - 33
IS - 10
CY - United States
N2 - OBJECTIVE: An intervention based on induced hypocrisy was conducted to reduce children's intentions to show fall-risk behaviors on playground equipment. METHODS: To induce hypocrisy participants (7-12 years) advocated for safe-play behaviors while being made mindful about past failures to play safely on playgrounds. To measure the impact of the intervention, children created posters indicating which playground behaviors they Would Do and Would Not Do; children believed they would have to demonstrate the behaviors endorsed on their posters. RESULTS: The intervention resulted in significant reductions in the risk behaviors children endorsed both for risk behaviors targeted (specific effects) and nontargeted risk behaviors (generalized effects). Assessing the mechanism by which the intervention produced changes in behavioral intentions revealed it yielded changes in cognitions that predicted subsequent reductions in risk-taking intentions. CONCLUSIONS: The present intervention was successful in reducing children's intentions to engage in risky playground behaviors and it did so by changing cognitions
SN - 1465-735X
AD - Department of Psychology, University of Guelph, MacKinnon Building, 5th Floor, Guelph, ONT, N1G 2W1, Canada. bmorrong@uoguelph.ca
UR - 18296456
ER -

TY - JOUR
ID - 276

T1 - I am almost like a fish: an investigation of how children with congenital heart disease experience and perform movement in daily life
A1 - Bjorbaekmo,W.
A1 - Engelsrud,G.
Y1 - 2008/11//
N1 - Bjorbaekmo, W. Engelsrud, G
Child: care, health and development
7602632, c9x
IM
Journal Article
English
KW - Medline
KW - Adaptation
KW - Psychological
KW - Child
KW - Energy Metabolism
KW - Heart Defects
KW - Congenital/me [Metabolism]
KW - Congenital/pp [Physiopathology]
KW - Congenital/px [Psychology]
KW - Humans
KW - Motor Activity
KW - Parents
KW - Play and Playthings/px [Psychology]
KW - Risk Factors
RP - NOT IN FILE
SP - 781
EP - 788
JF - Child: Care, Health & Development
JA - Child Care Health Dev.
VL - 34
IS - 6
CY - England
N2 - BACKGROUND: Because of dramatic medical improvements, most children with congenital heart disease (CHD) survive into adulthood. Nevertheless, they remain in need of long-term health care. Living with CHD implies having diminished aerobic capacity. As far as we know, no previous study within healthcare research has focused on how children with CHD experience movement and activity in daily life. METHODS: In order to examine this topic, a qualitative approach was employed that combined both interviews and observation of 11 children between 7 and 12 years of age and interviews with their parents. The theoretical base of the article is inspired by the philosopher Maurice Merleau-Ponty and his theory of movement. We use his descriptions of movement as intentional expressions to illuminate how children with CHD move in daily life. RESULTS: The study shows how the children use different strategies to participate in play and that they move to fulfill their first priority: to be together with other children. Despite having limited physical endurance, the children perceive themselves as having the same capabilities as other children and as acting as they do. At the same time, they are not unaware of their own limitations. They adjust and respond to the challenges they face in the different situations to which they are exposed. On one hand, they want others to take their limitations into consideration, while, on the other hand, they do not want others to know about these limitations. CONCLUSIONS: In our analysis, we interpret that living with CHD creates situations where the children constantly face their limitations, the gazes of others and their own wish to participate
SN - 1365-2214
AD - University of Oslo, Institute of Nursing and Health Sciences, Section of Health Sciences, Oslo, Norway. w.s.bjorbakmo@medisin.uio.no
UR - 18947344
ER -

TY - JOUR

ID - 277
T1 - Places to play: association of park space and facilities with healthy weight status among children
A1 - Potwarka,L.R.
A1 - Kaczynski,A.T.
A1 - Flack,A.L.
Y1 - 2008/10//
N1 - Potwarka, Luke R. Kaczynski, Andrew T. Flack, Andrea L
Journal of community health
7600747, hut
IM
Journal Article
English
KW - Medline
KW - Adolescent
KW - Body Weight
KW - Child
KW - Preschool
KW - Female
KW - Humans
KW - Male
KW - Ontario
KW - Play and Playthings
KW - Public Facilities
KW - Urban Population
RP - NOT IN FILE
SP - 344
EP - 350
JF - Journal of Community Health
JA - J Community Health
VL - 33
IS - 5
CY - United States
N2 - The purpose of this study was to examine how healthy weight status among youth was related to (i) three proximity-based park variables: number of parks within 1 km of home, total area of parkland within 1 km, and distance to the closest park from home, and (ii) the availability of 13 specific park facilities within 1 km of the home. Data were collected from parents of children living in four neighborhoods of a medium-sized Canadian city. Logistic regression analyses revealed that none of the three proximity-based park variables was significantly associated with healthy weight status among children in the sample. However, when availability of the 13 park facilities was examined, children with a park playground within 1 km were almost five times more likely to be classified as being of a healthy weight rather then at risk or overweight compared to those children without playgrounds in nearby parks. Results suggest that availability of certain park facilities may play a more important role in promoting physical activity and healthy weight status among children than availability of park space in general. Implications for park design are discussed
SN - 0094-5145
AD - Department of Recreation and Leisure Studies, University of Waterloo, Waterloo, ON, Canada N2L 3G1. lrpotwar@ahsmail.uwaterloo.ca
UR - 18484170
ER -

TY - JOUR
ID - 278
T1 - Exercise aids, neighborhood safety, and physical activity in adolescents and parents
A1 - Kerr,J.
A1 - Norman,G.J.
A1 - Sallis,J.F.
A1 - Patrick,K.

Y1 - 2008/07//
N1 - Kerr, Jacqueline. Norman, Gregory J. Sallis, James F. Patrick, Kevin
Medicine and science in sports and exercise
8005433, mg8
IM, S
Journal Article. Research Support, N.I.H., Extramural
English
KW - Medline
KW - Adolescent
KW - Adolescent Behavior
KW - Adult
KW - Child
KW - Confidence Intervals
KW - Environment Design
KW - Equipment and Supplies
KW - Exercise
KW - Female
KW - Health Behavior
KW - Humans
KW - Logistic Models
KW - Male
KW - Motor Activity
KW - Odds Ratio
KW - Parents
KW - Play and Playthings
KW - Questionnaires
KW - Residence Characteristics
KW - Safety
KW - Sports
RP - NOT IN FILE
SP - 1244
EP - 1248
JF - Medicine & Science in Sports & Exercise
JA - Med Sci Sports Exerc.
VL - 40
IS - 7
CY - United States
N2 - PURPOSE: To investigate the relationships among exercise aids available at home, physical activity, and perceived neighborhood safety. METHODS: Physical activity was assessed using the 7-d recall interview for adolescents (n = 878) and the International Physical Activity Questionnaire for parents (n = 853). Parents reported exercise aids such as fitness equipment, running shoes, and dogs in their household using a 16-item checklist and perceptions of neighborhood safety using the Neighborhood Environment Walkability Scale. Physical activity scores were dichotomized to represent meeting weekly guidelines for children (300 min) and adults (150 min). Logistic regression analyses investigated the interaction between exercise equipment and neighborhood safety in relation to the two physical activity outcomes, controlling for participant demographics. RESULTS: The number of home-use (OR = 1.27) and outdoor-use (OR = 1.24) exercise aids was significantly related to physical activity in adolescent girls but not boys. An interaction effect indicated that the relationship between home-use exercise equipment and physical activity levels was specific for girls in neighborhoods perceived as less-safe (OR = 4.40), rather than those perceived as safe (OR = 1.07, P < 0.01). In the parent sample, home-use (OR = 1.24) and outdoor use (OR = 1.16) exercise aids were significantly related to physical activity levels. An interaction between outdoor exercise aids and safety indicated that the effect was specific to parents who lived in neighborhoods perceived as safe (OR = 2.43) compared to those perceived as less-safe (OR = 0.91, P < 0.01). CONCLUSION: Girls living in neighborhoods their parents perceive to be less-safe may benefit from having exercise equipment they can use in the home. Parents living in neighborhoods perceived to be safe may benefit from having exercise aids that they can use outside
SN - 0195-9131

AD - 1Department of Psychology, San Diego State University, University of California, San Diego, CA 92103, USA. jkerr@projects.sdsu.edu
UR - 18580403
ER -

TY - JOUR
ID - 279
T1 - An investigation of school playground safety practices as reported by school nurses
A1 - Hudson,S.D.
A1 - Olsen,H.M.
A1 - Thompson,D.
Y1 - 2008/06//
N1 - Hudson, Susan D. Olsen, Heather M. Thompson, Donna
The Journal of school nursing : the official publication of the National Association of School Nurses
bgs, 9206498
N
Evaluation Studies. Journal Article
English
KW - Medline
KW - Attitude of Health Personnel
KW - Child
KW - Communication
KW - Documentation
KW - Equipment Safety
KW - Health Knowledge
KW - Attitudes
KW - Practice
KW - Health Services Needs and Demand
KW - Human Engineering
KW - Humans
KW - Nurse's Role/px [Psychology]
KW - Nurse's Role
KW - Nursing Evaluation Research
KW - Nursing Methodology Research
KW - Nursing Staff/ed [Education]
KW - Nursing Staff/og [Organization & Administration]
KW - Nursing Staff/px [Psychology]
KW - Play and Playthings
KW - Questionnaires
KW - Safety Management/og [Organization & Administration]
KW - School Nursing/ed [Education]
KW - School Nursing/og [Organization & Administration]
KW - United States
RP - NOT IN FILE
SP - 138
EP - 144
JF - Journal of School Nursing
JA - J Sch Nurs
VL - 24
IS - 3
CY - United States
N2 - The purpose of this study was to investigate school playground safety practices. The study used a purposeful sample of school nurses who attended a playground safety workshop at the 2006 National Association of School Nurses annual conference. Seventy-five questionnaires were distributed, and 64 useable questionnaires were returned. The responses indicated that little attention is being given to providing safe playground environments in schools as measured by best practices of supervision, age-appropriate design, fall

surfacing, and equipment maintenance. Participants pointed to the need for better supervision and supervision training, careful selection of age-appropriate equipment, maintaining adequate fall surfaces under the equipment, and ensuring that equipment is properly maintained and repaired. The study also revealed that school nurses believe they could play a role in playground injury prevention through the collection and analysis of injury data, communication to administrators about the need for comprehensive planning of the play environment, and becoming active members of playground safety committees
SN - 1059-8405
AD - National Program for Playground Safety, University of Northern Iowa, School of Health, Physical Education, and Leisure Services, Cedar Falls, Iowa, USA
UR - 18557672
ER -

TY - JOUR
ID - 280
T1 - Sex-typical play: masculinization/defeminization in girls with an autism spectrum condition
A1 - Knickmeyer,R.C.
A1 - Wheelwright,S.
A1 - Baron-Cohen,S.B.
Y1 - 2008/07//
N1 - Knickmeyer, Rebecca C. Wheelwright, Sally. Baron-Cohen, Simon B
Journal of autism and developmental disorders
7904301, hgw
IM
Journal Article. Research Support, Non-U.S. Gov't
English
KW - Medline
KW - Adolescent
KW - Autistic Disorder/di [Diagnosis]
KW - Autistic Disorder/pp [Physiopathology]
KW - Autistic Disorder/px [Psychology]
KW - Brain/pp [Physiopathology]
KW - Child
KW - Preschool
KW - Fantasy
KW - Female
KW - Gender Identity
KW - Humans
KW - Imagination/ph [Physiology]
KW - Male
KW - Personality Assessment
KW - Play and Playthings
KW - Pregnancy
KW - Prenatal Exposure Delayed Effects/pp [Physiopathology]
KW - Prenatal Exposure Delayed Effects/px [Psychology]
KW - Reference Values
KW - Testosterone/ph [Physiology]
RP - NOT IN FILE
SP - 1028
EP - 1035
JF - Journal of Autism & Developmental Disorders
JA - J Autism Dev.Disord
VL - 38
IS - 6
CY - United States
N2 - We tested the hypothesis that prenatal masculinization of the brain by androgens increases risk of developing an autism spectrum condition (ASC). Sex-typical play was measured in n = 66 children diagnosed

with an ASC and n = 55 typically developing age-matched controls. Consistent with the hypothesis, girls with autism did not show the female-typical play preferences, though this was only seen on non-pretence items. Boys with autism showed a preference for male play on non-pretence items, in keeping with their sex. Girls with autism engaged in more pretend play than boys with autism, suggesting that pretence is relatively more protected in females with autism. We conclude that play preference studies in ASC provide partial support for the fetal androgen theory
SN - 0162-3257
AD - Department of Psychiatry, Autism Research Center, University of Cambridge, Douglas House, Cambridge, UK. rebecca_knickmeyer@med.unc.edu
UR - 17985222
ER -

TY - JOUR
ID - 281
T1 - Attachment narratives and behavioral and emotional symptoms in an ethnically diverse, at-risk sample
A1 - Futh,A.
A1 - O'Connor,T.G.
A1 - Matias,C.
A1 - Green,J.
A1 - Scott,S.
Y1 - 2008/06//
N1 - Futh, Annabel. O'Connor, Thomas G. Matias, Carla. Green, Jonathan. Scott, Stephen
Journal of the American Academy of Child and Adolescent Psychiatry
hg5, 8704565
IM
Journal Article. Research Support, Non-U.S. Gov't
English
KW - Medline
KW - Affective Symptoms/di [Diagnosis]
KW - Affective Symptoms/eh [Ethnology]
KW - Affective Symptoms/px [Psychology]
KW - Antisocial Personality Disorder/di [Diagnosis]
KW - Antisocial Personality Disorder/eh [Ethnology]
KW - Antisocial Personality Disorder/px [Psychology]
KW - Child
KW - Preschool
KW - England
KW - Ethnic Groups/px [Psychology]
KW - Female
KW - Health Surveys
KW - Humans
KW - Male
KW - Narration
KW - Peer Group
KW - Personality Assessment/sn [Statistics & Numerical Data]
KW - Play and Playthings
KW - Poverty Areas
KW - Psychometrics/sn [Statistics & Numerical Data]
KW - Reactive Attachment Disorder/di [Diagnosis]
KW - Reactive Attachment Disorder/eh [Ethnology]
KW - Reactive Attachment Disorder/px [Psychology]
KW - Reproducibility of Results
KW - Social Adjustment
KW - Sociometric Techniques
RP - NOT IN FILE
SP - 709

EP - 718
JF - Journal of the American Academy of Child & Adolescent Psychiatry
JA - J Am Acad.Child Adolesc Psychiatry
VL - 47
IS - 6
CY - United States
N2 - OBJECTIVE: To examine the clinical correlates and predictions from attachment narratives in a sample of early school-age children from a high psychosocial risk, ethnically diverse sample. METHOD: A total of 113 children were assessed using the Manchester Child Attachment Story Task, a semistructured assessment of children's attachment representations. Parents and teachers provided data using the Strengths and Difficulties Questionnaire. Peer nominations of popularity and antisocial behavior were also obtained. RESULTS: Significant associations that were modest to moderate in magnitude were obtained between attachment narrative scales indexing security, coherence, and disorganization with multiple indices of children's behavioral and emotional adjustment, prosocial behavior and competence; these associations held across ethnic groups and were independent of psychosocial risk. CONCLUSIONS: The findings build on and extend support for the use of attachment narrative assessments in the clinical context and demonstrate their applicability and validity across a broad range of ethnicity and social contexts
SN - 1527-5418
AD - King's College London, Institute of Psychiatry, UK
UR - 18434917
ER -

TY - JOUR
ID - 282
T1 - Peer tutoring to prevent firearm play acquisition, generalization, and long-term maintenance of safety skills
A1 - Jostad,C.M.
A1 - Miltenberger,R.G.
A1 - Kelso,P.
A1 - Knudson,P.
Y1 - 2008///
N1 - Jostad, Candice M. Miltenberger, Raymond G. Kelso, Pamela. Knudson, Peter
Journal of applied behavior analysis
0174763, hdr
IM
Journal Article
English
KW - Medline
KW - Behavior Therapy/mt [Methods]
KW - Child
KW - Child Day Care Centers
KW - Preschool
KW - Feedback
KW - Psychological
KW - Female
KW - Firearms
KW - Follow-Up Studies
KW - Generalization (Psychology)
KW - Humans
KW - Imitative Behavior
KW - Male
KW - Peer Group
KW - Play and Playthings
KW - Practice (Psychology)
KW - Reinforcement
KW - Social

KW - Safety
KW - Social Environment
KW - Transfer (Psychology)
RP - NOT IN FILE
SP - 117
EP - 123
JF - Journal of Applied Behavior Analysis
JA - J Appl.Behav Anal.
VL - 41
IS - 1
CY - United States
N2 - Hundreds of accidental injuries and deaths to children occur annually in the United States as a result of firearm play. Behavioral skills training (BST) and in situ training have been found to be effective in teaching children the skills to use if they find a firearm, but training requires substantial time and effort. The current study examined the use of peers as tutors as a potential way to decrease the time and resources needed to teach these safety skills to youngsters. Peer trainers conducted BST and in situ training with other children. Children taught by the peer trainers acquired the safety skills and demonstrated them in naturalistic situations in which the skills were needed. Furthermore, all of the peer trainers acquired and maintained the skills. These results support the use of peer tutoring for teaching safety skills to other children
SN - 0021-8855
AD - North Dakota State University, USA
UR - 18468285
ER -

TY - JOUR
ID - 283
T1 - Safe play and its effect on childhood obesity
A1 - Mathieu,J.
Y1 - 2008/05//
N1 - Mathieu, Jennifer
Journal of the American Dietetic Association
h6f, 7503061
AIM, IM
Journal Article
English
KW - Medline
KW - Child
KW - Exercise/ph [Physiology]
KW - Female
KW - Health Promotion
KW - Humans
KW - Life Style
KW - Male
KW - Obesity/ep [Epidemiology]
KW - Obesity/pc [Prevention & Control]
KW - Play and Playthings
KW - Risk Factors
KW - Safety
KW - Television
RP - NOT IN FILE
SP - 774
EP - 775
JF - Journal of the American Dietetic Association
JA - J Am Diet Assoc.
VL - 108
IS - 5

CY - United States
SN - 0002-8223
UR - 18442496
ER -

TY - JOUR
ID - 284
T1 - Physical activity among adolescents. When do parks matter?
A1 - Babey,S.H.
A1 - Hastert,T.A.
A1 - Yu,H.
A1 - Brown,E.R.
Y1 - 2008/04//
N1 - Babey, Susan H. Hastert, Theresa A. Yu, Hongjian. Brown, E Richard
American journal of preventive medicine
8704773, apl
IM
Journal Article. Research Support, Non-U.S. Gov't
English
KW - Medline
KW - Adolescent
KW - Adolescent Behavior
KW - California
KW - Child
KW - Environment Design
KW - Female
KW - Health Surveys
KW - Humans
KW - Logistic Models
KW - Male
KW - Motor Activity
KW - Play and Playthings
KW - Public Facilities
KW - Residence Characteristics
KW - Safety
KW - Socioeconomic Factors
RP - NOT IN FILE
SP - 345
EP - 348
JA - American Journal of Preventive Medicine
VL - 34
IS - 4
CY - Netherlands
N2 - BACKGROUND: The availability of places to engage in physical activity may influence physical activity levels. This study examined whether the relationship between physical activity and access to parks differs depending on adolescents' sociodemographic, housing, and neighborhood characteristics. METHODS: Data were analyzed from 4010 adolescents who responded to the 2003 California Health Interview Survey (CHIS). Analyses were conducted in 2005-2006. Five sets of logistic regressions were conducted to examine the relationship between physical activity and access to a safe park among adolescents living in (1) urban versus rural areas; (2) apartment buildings versus houses, (3) neighborhoods perceived as unsafe versus safe; (4) lower- versus higher-income families; and (5) adolescents who were Latino, African American, Asian, or white. Analyses also examined interactions between park access and these factors. RESULTS: Access to a safe park was positively associated with regular physical activity and negatively associated with inactivity for adolescents in urban areas, but not rural areas. Additionally, adolescents with access to a safe park were less likely to be inactive than those without access among those living in (1) apartment buildings, (2) unsafe neighborhoods, and (3) lower-income families. Park access was not associated with regular physical activity for these groups. The

association between park access and physical activity varied by race/ethnicity. CONCLUSIONS: These findings suggest that the relationship between physical activity and access to parks differs depending on adolescents' sociodemographic, housing, and neighborhood characteristics, and that parks may be particularly important for promoting physical activity among urban adolescents
SN - 0749-3797
AD - Center for Health Policy Research, University of California at Los Angeles, Los Angeles, California 90024, USA. sbabey@ucla.edu
UR - 18374249
ER -

TY - JOUR
ID - 285
T1 - Neighborhood disorder, perceived safety, and readiness to encourage use of local playgrounds
A1 - Miles,R.
Y1 - 2008/04//
N1 - Miles, Rebecca
American journal of preventive medicine
8704773, apl
IM
Journal Article
English
KW - Medline
KW - Adult
KW - Child
KW - Cross-Sectional Studies
KW - Environment Design
KW - Europe
KW - Female
KW - Humans
KW - Male
KW - Middle Aged
KW - Motor Activity
KW - Motor Vehicles
KW - Parents/px [Psychology]
KW - Perception
KW - Play and Playthings
KW - Public Facilities
KW - Residence Characteristics
KW - Safety
KW - Walking
RP - NOT IN FILE
SP - 275
EP - 281
JA - American Journal of Preventive Medicine
VL - 34
IS - 4
CY - Netherlands
N2 - BACKGROUND: Knowledge of the association between the neighborhood physical environment and adults' readiness to encourage children's use of local playgrounds, and the extent to which perceived safety acts as a mediator, can inform efforts to increase children's physical activity. METHODS: Data were obtained from seven European cities based on a cross-sectional household survey conducted between 2001 and 2002. The sample included 2123 household informants (from a total of 2782 households) with a median age of 48 years; 65% were women, 66% were married, and 33% had achieved a secondary education. Indicators of local neighborhood physical disorder (litter, graffiti, lack of greenery), traffic volume, and land use were directly observed by trained surveyors. Perceived safety, encouragement of playground use, and physical activity levels were assessed with self-reported measures. Analyses were conducted in 2007. RESULTS: Respondents in

neighborhoods showing signs of low or moderate physical disorder compared to high physical disorder had slightly over twice the odds of encouraging children to use local playgrounds (p<0.01). The percentage of the effect of neighborhood physical disorder accounted for by perceived safety was between 15% and 20%. Neighborhood physical disorder was associated only with adults' occasional involvement in sports or exercise and only among women (p<0.05); perceived safety was not significantly associated with physical activity for either men or women. CONCLUSIONS: Neighborhood physical environments and perceived safety influence adults' readiness to encourage children's physical activity and women's occasional involvement in sports or exercise. Health promotion strategies designed to upgrade the environments near where children live and to address parental safety concerns merit further exploration
SN - 0749-3797
AD - Department of Urban and Regional Planning, Florida State University, Tallahassee, Florida 32306-2280, USA. rebecca.miles@fsu.edu
UR - 18374240
ER -

TY - JOUR
ID - 286
T1 - Parenting behaviors and attitudes about supervision among parents of acutely poisoned children
A1 - Sinclair,K.A.
A1 - Morrongiello,B.A.
A1 - Dowd,M.D.
Y1 - 2008/03//
N1 - Sinclair, Kelly A. Morrongiello, Barbara A. Dowd, M Denise
Ambulatory pediatrics : the official journal of the Ambulatory Pediatric Association
101089367
IM
Journal Article
English
KW - Medline
KW - Adolescent
KW - Adult
KW - Attitude
KW - Child
KW - Preschool
KW - Female
KW - Humans
KW - Infant
KW - Male
KW - Middle Aged
KW - Parenting/px [Psychology]
KW - Parents/px [Psychology]
KW - Play and Playthings
KW - Poisoning
KW - Safety
RP - NOT IN FILE
SP - 135
EP - 138
JF - Ambulatory Pediatrics
JA - Ambul.Pediatr
VL - 8
IS - 2
CY - United States
N2 - OBJECTIVE: This study describes the supervision beliefs of parents of children with unintentional poisonings and examines the relationship between supervision beliefs, household composition, and parental age. METHODS: Children aged <60 months presenting with an unintentional poisoning to an emergency department or urgent care of an urban, academic medical center in Kansas City, Missouri, were identified. Parents

completed the Beliefs About Supervision Questionnaire (BAS-Q) by phone within 2 weeks of their child's visit. The questionnaire consisted of 14 child activity scenarios, each followed by 2 questions: What age would you permit your child to perform this activity without constant supervision? How often would you check on a child of this age while engaged in the activity? Two scores were derived: mean age across all scenarios (BAS-Age) and mean time until child is checked on across all scenarios (BAS-Time). RESULTS: One hundred parents completed the BAS-Q. The majority of respondents were mothers (82%), 40% had 2 or more children in the home aged 5 years or younger, and 61% had 2 or more adults living in the home. Pearson correlations revealed associations for BAS-Time with total children within the home (r = .28, P < .005) and number of children aged 6 to 12 years in the home (r = .28, P < .005). BAS-Age was not related to any aspect of household composition. CONCLUSION: Less vigorous parental supervision was associated with an increased number of children in the home. Parental supervision may be influenced by reliance on other children in the home
SN - 1539-4409
AD - Division of Emergency Medical Services, The Children's Mercy Hospital, Kansas City, Missouri 64113, USA. ksinclair@cmh.edu
UR - 18355743
ER -

TY - JOUR
ID - 287
T1 - Physical activity levels among children attending after-school programs
A1 - Trost,S.G.
A1 - Rosenkranz,R.R.
A1 - Dzewaltowski,D.
Y1 - 2008/04//
N1 - Trost, Stewart G. Rosenkranz, Richard R. Dzewaltowski, David
Medicine and science in sports and exercise
8005433, mg8
IM, S
Journal Article. Research Support, U.S. Gov't, Non-P.H.S.
English
KW - Medline
KW - Acceleration
KW - Anthropometry
KW - Body Mass Index
KW - Child
KW - Child Welfare
KW - Exercise/ph [Physiology]
KW - Female
KW - Health Status
KW - Humans
KW - Male
KW - Motor Activity
KW - School Health Services
KW - Schools
KW - Time Factors
RP - NOT IN FILE
SP - 622
EP - 629
JF - Medicine & Science in Sports & Exercise
JA - Med Sci Sports Exerc.
VL - 40
IS - 4
CY - United States
N2 - PURPOSE: To describe the physical activity (PA) levels of children attending after-school programs, 2) examine PA levels in specific after-school sessions and activity contexts, and 3) evaluate after-school PA differences in groups defined by sex and weight status. METHODS: One hundred forty-seven students in grades

3-6 (mean age: 10.1 +/- 0.7, 54.4% male, 16.5 % overweight (OW), 22.8% at-risk for OW) from seven after-school programs in the midwestern United States wore Actigraph GT1M accelerometers for the duration of their attendance to the program. PA was objectively assessed on six occasions during an academic year (three fall and three spring). Stored activity counts were uploaded to a customized data-reduction program to determine minutes of sedentary (SED), light (LPA), moderate (MPA), vigorous (VPA), and moderate-to-vigorous (MVPA) physical activity. Time spent in each intensity category was calculated for the duration of program attendance, as well as specific after-school sessions (e.g., free play, snack time). RESULTS: On average, participants exhibited 42.6 min of SED, 40.8 min of LPA, 13.4 min of MPA, and 5.3 min of VPA. The average accumulation of MVPA was 20.3 min. Boys exhibited higher levels of MPA, VPA, and MVPA, and lower levels of SED and LPA, than girls. OW and at-risk-for-OW students exhibited significantly less VPA than nonoverweight students, but similar levels of LPA, MPA, and MVPA. MVPA levels were significantly higher during free-play activity sessions than during organized or structured activity sessions. CONCLUSION: After-school programs seem to be an important contributor to the PA of attending children. Nevertheless, ample room for improvement exists by making better use of existing time devoted to physical activity
SN - 0195-9131
AD - Department of Nutrition and Exercise Sciences, Oregon State University, Corvallis, OR 97333, USA. stewart.trost@oregonstate.edu
UR - 18317385
ER -

TY - JOUR
ID - 289
T1 - Playing it safe: the influence of neighbourhood safety on children's physical activity. A review. [Review] [72 refs]
A1 - Carver,A.
A1 - Timperio,A.
A1 - Crawford,D.
Y1 - 2008/06//
N1 - Carver, Alison. Timperio, Anna. Crawford, David
Health & place
din, 9510067
T
Journal Article. Review
English
KW - Medline
KW - Child
KW - Preschool
KW - Exercise
KW - Humans
KW - Play and Playthings
KW - Residence Characteristics
KW - Safety
KW - Victoria
RP - NOT IN FILE
SP - 217
EP - 227
JF - Health & Place
JA - Health Place
VL - 14
IS - 2
CY - England
N2 - Compared with previous generations, children spend less time playing outdoors and have lower participation rates in active transport. Many studies have identified lack of neighbourhood safety as a potential barrier to children's physical activity. This review describes concerns regarding 'stranger danger' and road safety, and discusses empirical studies that examine associations between neighbourhood safety and physical activity among youth. Variability of perceptions of safety between parents and youth are examined; 'social traps'

are identified; and physical/social environmental interventions aimed at improving neighbourhood safety are discussed. A research agenda is suggested for further study of perceived and objective measures of neighbourhood safety and their associations with children's physical activity. [References: 72]
SN - 1353-8292
AD - Centre for Physical Activity and Nutrition Research, School of Exercise and Nutrition Sciences, Deakin University, VIC, Australia. alison.carver@deakin.edu.au
UR - 17662638
ER -

TY - JOUR
ID - 290
T1 - Product recalls. Recalls: a bad summer for toys from China...but risks should be kept in perspective
Y1 - 2007/09//
N1 - Child health alert
9881972
K
Journal Article
English
KW - Medline
KW - Child
KW - China
KW - Consumer Product Safety
KW - Humans
KW - Lead
KW - Lead Poisoning/et [Etiology]
KW - Paint/to [Toxicity]
KW - Play and Playthings
RP - NOT IN FILE
SP - 5
EP - 6
JF - Child Health Alert
VL - 25
CY - United States
SN - 1064-4849
UR - 18273937
ER -

TY - JOUR
ID - 291
T1 - Maternal secure base support and preschoolers' secure base behavior in natural environments
A1 - Posada,G.
A1 - Kaloustian,G.
A1 - Richmond,M.K.
A1 - Moreno,A.J.
Y1 - 2007/12//
N1 - Posada, German. Kaloustian, Garene. Richmond, Melissa K. Moreno, Amanda J
Attachment & human development
100901315
IM
Journal Article. Research Support, Non-U.S. Gov't
English
KW - Medline
KW - Adult
KW - Child Behavior
KW - Child
KW - Preschool

KW - Female
KW - Humans
KW - Male
KW - Meta-Analysis as Topic
KW - Middle Aged
KW - Mother-Child Relations
KW - Object Attachment
KW - Play and Playthings
KW - Safety
KW - Social Environment
KW - United States
RP - NOT IN FILE
SP - 393
EP - 411
JF - Attachment & Human Development
JA - Attach.Hum.Dev.
VL - 9
IS - 4
CY - England
N2 - Bowlby and Ainsworth's theory of attachment poses that concurrent caregiving behavior is a key factor in influencing and maintaining a child's organization of secure-base behavior, and ultimately, security throughout childhood. Empirical demonstrations of the relation between the constructs after infancy are relatively scant and research is needed to examine the relation between the variables across a wide range of contexts, over longer observational periods, and in developmentally appropriate ways. Two studies of preschoolers and their mothers were conducted in naturalistic settings. Fifty child-mother middle-class dyads, predominantly Caucasian, participated in Study 1 and 40 in Study 2. The mean age for children was 52 months (Study 1) and 36 months (Study 2). In Study 1, a home and a playground visits were conducted. In Study 2, two home and a playground visits were conducted. Observers used the Maternal Behavior for Preschoolers Q-Set to provide age-relevant descriptions of maternal behavior, and the Attachment Q-Set to provide descriptions of child behavior. Overall, findings indicated that maternal secure base support was significantly related to the organization of child secure base behavior (r = .31 and .49 for Study 1 and Study 2, respectively). Results are discussed in terms of the importance of specifying caregiving domains and contexts of assessments, and their implications for attachment theory
SN - 1461-6734
AD - Purdue University, West Lafayette, IN 47907, USA. gposada@purdue.edu
UR - 18049935
ER -

TY - JOUR
ID - 292
T1 - When lead lurks in your nursery
A1 - Wallis,C.
Y1 - 2007/09/17/
N1 - Wallis, Claudia
Time
9877130, bqn
K
News
English
KW - Medline
KW - Child
KW - Preschool
KW - Humans
KW - Lead Poisoning/pc [Prevention & Control]
KW - Play and Playthings
KW - Safety Management/mt [Methods]

RP - NOT IN FILE
SP - 51
JF - Time
VL - 170
IS - 12
CY - United States
SN - 0040-781X
UR - 17969970
ER -

TY - JOUR
ID - 293
T1 - The food insecurity-obesity paradox: a review of the literature and the role food stamps may play. [Review] [47 refs]
A1 - Dinour,L.M.
A1 - Bergen,D.
A1 - Yeh,M.C.
Y1 - 2007/11//
N1 - Dinour, Lauren M. Bergen, Dara. Yeh, Ming-Chin
Journal of the American Dietetic Association
h6f, 7503061
AIM, IM
Journal Article. Research Support, Non-U.S. Gov't. Review
English
KW - Medline
KW - Adolescent
KW - Adult
KW - Body Mass Index
KW - Child
KW - Preschool
KW - Diet/st [Standards]
KW - Female
KW - Food Services/sn [Statistics & Numerical Data]
KW - Food Services/ut [Utilization]
KW - Food Services
KW - Food Supply/sn [Statistics & Numerical Data]
KW - Health Status
KW - Humans
KW - Hunger
KW - Male
KW - Middle Aged
KW - Obesity/ep [Epidemiology]
KW - Poverty
KW - Risk Factors
KW - Sex Factors
RP - NOT IN FILE
SP - 1952
EP - 1961
JF - Journal of the American Dietetic Association
JA - J Am Diet Assoc.
VL - 107
IS - 11
CY - United States
N2 - During the past decade, rates of food insecurity and obesity have risen, and an association has been made between these two seemingly paradoxical states. Although this relationship has not been repeatedly seen in men, research suggests a correlation in women. Studies have not been able to consistently show a relationship in

children, because findings differ based on age, race/ethnicity, household income, and sex. Several proposed hypotheses explain why a correlation between food insecurity and obesity exists in adults - especially women - but not in children. This review proposes a conceptual framework linking the Food Stamp Program and other coping strategies to the food insecurity-obesity relationship. This link has implications for Food Stamp Program policy changes, welfare reform, and poverty prevention. [References: 47]
SN - 0002-8223
AD - Graduate Center, City University of New York, New York, USA. LMDnutrition@gmail.com
UR - 17964316
ER -

TY - JOUR
ID - 294
T1 - Positive and protective: effects of early theory of mind on problem behaviors in at-risk preschoolers
A1 - Hughes,C.
A1 - Ensor,R.
Y1 - 2007/10//
N1 - Hughes, Claire. Ensor, Rosie
Journal of child psychology and psychiatry, and allied disciplines
hp3, 0375361
IM
Journal Article
English
KW - Medline
KW - Adaptation
KW - Psychological
KW - Affect
KW - Affective Symptoms/di [Diagnosis]
KW - Affective Symptoms/px [Psychology]
KW - Attention Deficit Disorder with Hyperactivity/di [Diagnosis]
KW - Attention Deficit Disorder with Hyperactivity/px [Psychology]
KW - Attention Deficit and Disruptive Behavior Disorders/di [Diagnosis]
KW - Attention Deficit and Disruptive Behavior Disorders/px [Psychology]
KW - Child Behavior Disorders/di [Diagnosis]
KW - Child Behavior Disorders/px [Psychology]
KW - Child Development Disorders
KW - Pervasive/di [Diagnosis]
KW - Pervasive/px [Psychology]
KW - Child
KW - Preschool
KW - Conduct Disorder/di [Diagnosis]
KW - Conduct Disorder/px [Psychology]
KW - Culture
KW - Female
KW - Follow-Up Studies
KW - Humans
KW - Male
KW - Mother-Child Relations
KW - Parenting/px [Psychology]
KW - Personal Construct Theory
KW - Personality Assessment
KW - Play and Playthings
KW - Prognosis
KW - Psychosocial Deprivation
KW - Risk Factors
RP - NOT IN FILE
SP - 1025

EP - 1032
JF - Journal of Child Psychology & Psychiatry & Allied Disciplines
JA - J Child Psychol.Psychiatry
VL - 48
IS - 10
CY - England
N2 - BACKGROUND: Exposure to harsh parenting and children's skills in 'Theory of Mind' (ToM) show independent and interacting associations with problem behaviors at age 2 (Hughes & Ensor, 2006). This study examined whether these age-2 measures also predict age-4 problem behaviors. METHOD: In a socially diverse sample (N = 120), multi-informant, multi-measure, multi-setting ratings indexed problem behaviors at ages 2, 3 and 4; children completed both ToM and verbal-ability tasks at age 2, while video-based ratings of maternal negative affect and control within dyadic mother-child play indexed harsh parenting. RESULTS: Age-2 harsh parenting and ToM were independent and interacting predictors of age-4 problem behaviors, even with age-2 problem behaviors, verbal ability and social disadvantage all controlled. The interaction between harsh parenting and ToM distinguished persistent vs. diminishing problem behaviors. CONCLUSIONS: Both child and family characteristics predict increases in problem behaviors from 2 to 4; adverse effects of harsh parenting are attenuated for children with good ToM skills
SN - 0021-9630
AD - Centre for Family Research, University of Cambridge, UK. ch288@cam.ac.uk
UR - 17915003
ER -

TY - JOUR
ID - 295
T1 - Making an "A" in playground injury prevention
A1 - Roper,J.D.
Y1 - 2007/09//
N1 - Roper, Jean Dobson
School nurse news
100956395
N
Journal Article
English
KW - Medline
KW - Adolescent
KW - Child
KW - Child Welfare
KW - Equipment Safety
KW - Humans
KW - Play and Playthings
KW - Program Development
KW - Risk Factors
KW - Safety/st [Standards]
KW - School Health Services
KW - School Nursing
KW - Social Marketing
KW - United States
KW - Wounds and Injuries/pc [Prevention & Control]
RP - NOT IN FILE
SP - 33
EP - 35
JF - School Nurse News
VL - 24
IS - 4
CY - United States
SN - 1080-7543

AD - Kingston City School District, NY, USA. jroper@kingstoncityschools.org
UR - 17913105
ER -

TY - JOUR
ID - 297
T1 - Trajectories of maternal depressive symptoms, maternal sensitivity, and children's functioning at school entry
A1 - Campbell,S.B.
A1 - Matestic,P.
A1 - von,Stauffenberg C.
A1 - Mohan,R.
A1 - Kirchner,T.
Y1 - 2007/09//
N1 - Campbell, Susan B. Matestic, Patricia. von Stauffenberg, Camilla. Mohan, Roli. Kirchner, Thomas
Developmental psychology
0260564, cn8
IM
Journal Article. Multicenter Study. Research Support, N.I.H., Extramural
English
KW - Medline
KW - Child
KW - Child of Impaired Parents/px [Psychology]
KW - Preschool
KW - Depression/di [Diagnosis]
KW - Depression/px [Psychology]
KW - Female
KW - Humans
KW - Infant
KW - Internal-External Control
KW - Longitudinal Studies
KW - Male
KW - Maternal Behavior/px [Psychology]
KW - Mother-Child Relations
KW - Mothers/px [Psychology]
KW - Personality Assessment
KW - Play and Playthings
KW - Social Adjustment
KW - Socioeconomic Factors
RP - NOT IN FILE
SP - 1202
EP - 1215
JF - Developmental Psychology
JA - Dev.Psychol.
VL - 43
IS - 5
CY - United States
N2 - Using data from the National Institute of Child Health and Human Development Study of Early Child Care and Youth Development, the authors modeled trajectories of maternal depressive symptoms from infant age 1 month to 7 years. The authors identified 6 trajectories of maternal depressive symptoms: high-chronic, moderate-increasing, high-decreasing, intermittent, moderate-stable, and low-stable. Women on these depression trajectories varied in sociodemographic risk and in changes in observed maternal sensitivity over time. Maternal sensitivity was generally higher and increased when depressive symptoms were low; sensitivity was lower and decreased when depressive symptoms were either high or increasing. Child outcomes at 1st grade were examined by trajectory group. The authors discuss the complexity of disentangling maternal symptoms

from maternal sensitivity and sociodemographic risk when predicting children's functioning. PsycINFO Database Record (c) 2007 APA, all rights reserved
SN - 0012-1649
AD - Department of Psychology, University of Pittsburgh, PA 15260, USA. sbcamp@pitt.edu
UR - 17723045
ER -

TY - JOUR
ID - 298
T1 - Falls from playground equipment: will the new Australian playground safety standard make a difference and how will we tell?
A1 - Mitchell,R.
A1 - Sherker,S.
A1 - Cavanagh,M.
A1 - Eager,D.
Y1 - 2007/08//
N1 - Mitchell, Rebecca. Sherker, Shauna. Cavanagh, Margaret. Eager, David
Health promotion journal of Australia : official journal of Australian Association of Health Promotion Professionals
9710936
IM
Comparative Study. Journal Article. Research Support, Non-U.S. Gov't
English
KW - Medline
KW - Accidental Falls/pc [Prevention & Control]
KW - Accidental Falls/sn [Statistics & Numerical Data]
KW - Adolescent
KW - Age Distribution
KW - Child
KW - Preschool
KW - Craniocerebral Trauma/ep [Epidemiology]
KW - Craniocerebral Trauma/et [Etiology]
KW - Female
KW - Hospitalization/sn [Statistics & Numerical Data]
KW - Humans
KW - Incidence
KW - Infant
KW - Newborn
KW - Length of Stay
KW - Male
KW - New South Wales/ep [Epidemiology]
KW - Play and Playthings/in [Injuries]
KW - Risk Assessment
KW - Safety/st [Standards]
RP - NOT IN FILE
SP - 98
EP - 104
JF - Health Promotion Journal of Australia
JA - HEALTH PROMOT J AUST
VL - 18
IS - 2
CY - Australia
N2 - ISSUE ADDRESSED: This study describes the trend in incidence of hospitalised falls from playground equipment of children aged 14 years or less in New South Wales (NSW) and considers the potential effectiveness of playground safety standards in reducing the impact of playground-related injuries. METHOD: Hospitalisations of children aged 14 years or less following a fall from playground equipment were identified

from the NSW hospitalisation data for the financial years 1992/93 to 2003/04 and described. RESULTS: During 1992/93 to 2003/04 there were 16,828 hospitalisations of children aged 0-14 years as a result of a fall from playground equipment, at a rate of 106.6 per 100,000 children. The incidence of hospitalisation increased from 83.3 to 130.3 per 100,000 children between 1992/93 to 2003/04. Males aged 5-9 years had the highest rate of hospitalisation at 198.4 per 100,000 children. Injury type varied by age group, but injuries to the elbow and forearm were common for all age groups. The rate of upper limb fractures that resulted in hospitalisation increased, while the rate of serious head injuries decreased. CONCLUSIONS: While severe head injuries have declined between 1992/93 to 2003/04, the increasing trend of upper limb fractures is of concern. Many factors need to be taken into account to assess the effectiveness of playground safety standards. The collection of exposure data is also crucial to be able to calculate the true risk associated with childhood falls from playground equipment
SN - 1036-1073
AD - NSW Injury Risk Management Research Centre, University of New South Wales.
r.mitchell@unsw.edu.au
UR - 17663656
ER -

TY - JOUR
ID - 299
T1 - Persistence of overweight among young children living in low income communities in Ontario
A1 - Evers,S.
A1 - Arnold,R.
A1 - Hamilton,T.
A1 - Midgett,C.
Y1 - 2007/06//
N1 - Evers, Susan. Arnold, Robert. Hamilton, Theresa. Midgett, Corina
Journal of the American College of Nutrition
h51, 8215879
IM
Journal Article. Research Support, Non-U.S. Gov't
English
KW - Medline
KW - Body Height/ph [Physiology]
KW - Body Mass Index
KW - Body Weight/ph [Physiology]
KW - Child
KW - Child Development
KW - Child Nutritional Physiological Phenomena/ph [Physiology]
KW - Preschool
KW - Diet
KW - Exercise/ph [Physiology]
KW - Female
KW - Humans
KW - Male
KW - Obesity/ep [Epidemiology]
KW - Obesity/et [Etiology]
KW - Obesity/pc [Prevention & Control]
KW - Ontario
KW - Overweight
KW - Play and Playthings
KW - Poverty
KW - Predictive Value of Tests
KW - Prevalence
KW - Risk Assessment
KW - Risk Factors
KW - Weight Gain/ph [Physiology]

RP - NOT IN FILE
SP - 219
EP - 224
JF - Journal of the American College of Nutrition
JA - J Am Coll.Nutr
VL - 26
IS - 3
CY - United States
N2 - OBJECTIVE: The rise in the prevalence of overweight in pediatric populations is a major health concern. Little is known however, about the prevalence of overweight in younger children. Our objectives were to determine the annual prevalence of overweight in children from junior kindergarten (JK) to grade 3; to assess the persistence of overweight over this time period; and, to identify factors associated with overweight in this group. METHODS: Annual interviews were completed with parents (primarily the mother) living in economically disadvantaged communities in Ontario who are participating in the Better Beginnings, Better Futures project. Weight and height were measured annually for the children (n=760) beginning in JK. Risk of overweight was defined as body mass index (BMI) >or= 85th to < 95th percentile; overweight was BMI >or= 95th percentile. Parents' height and weight were self-reported; BMI >or= 25 was considered overweight. RESULTS: The risk of overweight among children ranged from 14.1% to 17.5%; the prevalence of overweight increased from 9.9% to 15.2%; 68.2% (15/22) of the children who were overweight in JK were >95th percentile in grade 3. BMI >or= 85th to < 95th percentile or >or= 95th percentile in JK were strongly predictive of overweight in grade 3. Almost 50% of the mothers were overweight. CONCLUSIONS: A high prevalence of overweight was found in young children; and, for a large proportion, their early weight status persisted. Strategies promoting healthy eating and physical activity for both children and parents are essential
SN - 0731-5724
AD - Department of Family Relations and Applied Nutrition, University of Guelph, Guelph, ON N1G 2W1, Canada. severs@uoguelph.ca
UR - 17634166
ER -

TY - JOUR
ID - 300
T1 - Waterslide exit velocities, user behaviours and injury prevention
A1 - Blitvich,J.D.
A1 - McElroy,G.K.
Y1 - 2007/03//
N1 - Blitvich, J D. McElroy, G K
International journal of injury control and safety promotion
101247254
IM
Journal Article
English
KW - Medline
KW - Acceleration
KW - Australia/ep [Epidemiology]
KW - Child
KW - Child Behavior
KW - Humans
KW - Life Style
KW - Pilot Projects
KW - Play and Playthings/in [Injuries]
KW - Posture
KW - Risk Assessment
KW - Risk Factors
KW - Risk-Taking
KW - Safety
KW - Swimming Pools/sn [Statistics & Numerical Data]

KW - Wounds and Injuries/ep [Epidemiology]
KW - Wounds and Injuries/et [Etiology]
KW - Wounds and Injuries/pc [Prevention & Control]
RP - NOT IN FILE
SP - 54
EP - 56
JF - International Journal of Injury Control & Safety Promotion
JA - Int.J Inj.Contr.Saf Promot
VL - 14
IS - 1
CY - England
SN - 1745-7300
AD - School of Human Movement and Sport Sciences, University of Ballarat, PO Box 663, Ballarat, Vic 3350, Australia. j.blitvich@ballarat.edu.au
UR - 17624012
ER -

TY - JOUR
ID - 301
T1 - Trends in fall injuries associated with children's outdoor climbing frames
A1 - Ball,D.J.
Y1 - 2007/03//
N1 - Ball, David J
International journal of injury control and safety promotion
101247254
IM
Journal Article. Research Support, Non-U.S. Gov't
English
KW - Medline
KW - Accidental Falls/sn [Statistics & Numerical Data]
KW - Adolescent
KW - Child
KW - Preschool
KW - Equipment Safety
KW - Great Britain/ep [Epidemiology]
KW - Humans
KW - Pilot Projects
KW - Play and Playthings/in [Injuries]
KW - Risk Assessment
KW - Risk Factors
KW - Safety
KW - Wounds and Injuries/ep [Epidemiology]
KW - Wounds and Injuries/et [Etiology]
RP - NOT IN FILE
SP - 49
EP - 53
JF - International Journal of Injury Control & Safety Promotion
JA - Int.J Inj.Contr.Saf Promot
VL - 14
IS - 1
CY - England
N2 - Falls from publicly owned climbing equipment are often cited as the major cause of injury on children's outdoor playgrounds and have been the focus of substantial interventions in the UK since the early 1980s. Analysis of national data on falls from climbing frames for 1988 to 2002 shows that the main discernible trend during this period is an increase in the occurrence of injuries to the lower arm. Whether this is attributable to a behavioural response to some of the interventions, or to some other factor, is unknown

SN - 1745-7300
AD - Centre for Decision Analysis and Risk Management, School of Health and Social Sciences, Middlesex University, Queensway, Enfield, Middlesex, UK. D.Ball@mdx.ac.uk
UR - 17624011
ER -

TY - JOUR
ID - 302
T1 - Addressing the issue of falls off playground equipment: an empirically-based intervention to reduce fall-risk behaviors on playgrounds
A1 - Morrongiello,B.A.
A1 - Matheis,S.
Y1 - 2007/08//
N1 - Morrongiello, Barbara A. Matheis, Shawn
Journal of pediatric psychology
jl8, 7801773
IM
Journal Article. Research Support, Non-U.S. Gov't
English
KW - Medline
KW - Accidental Falls/pc [Prevention & Control]
KW - Accidental Falls/sn [Statistics & Numerical Data]
KW - Child
KW - Child Behavior
KW - Empirical Research
KW - Female
KW - Humans
KW - Leisure Activities
KW - Male
KW - Play and Playthings
KW - Questionnaires
KW - Risk-Taking
KW - Safety
KW - Technology
KW - Wounds and Injuries/ep [Epidemiology]
KW - Wounds and Injuries/pc [Prevention & Control]
RP - NOT IN FILE
SP - 819
EP - 830
JF - Journal of Pediatric Psychology
JA - J Pediatr Psychol.
VL - 32
IS - 7
CY - United States
N2 - OBJECTIVE: The present study evaluated the impact of an intervention to reduce fall-risk behaviors on playgrounds among children 6-11 years of age. METHODS: Children completed posters indicating risky playground behaviors they would and would not do. In the intervention group, video and audio presentations were used to expose children to injury occurrences so that injury vulnerability was communicated in a fear-evoking way. In the control group, children only completed the pre- and post-intervention measures. RESULTS: Significant decreases in intentions to risk-take were obtained in the intervention, but not the control group. Effectiveness did not vary with children's age or sex, but was greater for those scoring high in sensation-seeking. CONCLUSIONS: A fear-appeals approach proved successful to reduce intended fall-risk behaviors, particularly for children high in sensation-seeking whose risk-taking is motivated by affect arousal
SN - 0146-8693
AD - Psychology Department, University of Guelph, Guelph, Ontario, N1G 2W1, Canada. bmorrong@uoguelph.ca

UR - 17522112
ER -

TY - JOUR
ID - 303
T1 - Social and communication development in toddlers with early and later diagnosis of autism spectrum disorders
A1 - Landa,R.J.
A1 - Holman,K.C.
A1 - Garrett-Mayer,E.
Y1 - 2007/07//
N1 - Landa, Rebecca J. Holman, Katherine C. Garrett-Mayer, Elizabeth
Archives of general psychiatry
72c, 0372435
AIM, IM
Comparative Study. Journal Article. Research Support, N.I.H., Extramural
English
KW - Medline
KW - Age Factors
KW - Autistic Disorder/di [Diagnosis]
KW - Autistic Disorder/ge [Genetics]
KW - Autistic Disorder/px [Psychology]
KW - Child Development/ph [Physiology]
KW - Child
KW - Preschool
KW - Communication Disorders/di [Diagnosis]
KW - Communication Disorders/ge [Genetics]
KW - Communication Disorders/px [Psychology]
KW - Developmental Disabilities/di [Diagnosis]
KW - Developmental Disabilities/ge [Genetics]
KW - Developmental Disabilities/px [Psychology]
KW - Female
KW - Genetic Predisposition to Disease/ge [Genetics]
KW - Humans
KW - Infant
KW - Language Tests/sn [Statistics & Numerical Data]
KW - Longitudinal Studies
KW - Male
KW - Nonverbal Communication/ph [Physiology]
KW - Nonverbal Communication
KW - Personality Inventory/sn [Statistics & Numerical Data]
KW - Phenotype
KW - Play and Playthings/px [Psychology]
KW - Prospective Studies
KW - Psychiatric Status Rating Scales/sn [Statistics & Numerical Data]
KW - Risk Factors
KW - Siblings/px [Psychology]
KW - Social Behavior
KW - SOCIALIZATION
KW - Statistics
KW - Nonparametric
RP - NOT IN FILE
SP - 853
EP - 864
JF - Archives of General Psychiatry
JA - Arch.Gen.Psychiatry

VL - 64
IS - 7
CY - United States
N2 - CONTEXT: To our knowledge, no prospective studies of the developmental course of early and later diagnosis of autism spectrum disorders from 14 months of age exist. OBJECTIVE: To examine patterns of development from 14 to 24 months in children with early and later diagnosis of autism spectrum disorders. DESIGN: Prospective, longitudinal design in which 125 infants at high and low risk for autism were tested from age 14 to 36 months. Comprehensive standardized assessments included measures of social, communication, and play behavior. SETTING: Testing occurred at a major medical and research institution as part of a large, ongoing longitudinal study. PARTICIPANTS: Low-risk controls (n = 18) and siblings of children with autism, grouped on the basis of outcome diagnostic classification at 30 or 36 months: autism spectrum disorders (early diagnosis, n = 16; later diagnosis, n = 14), broader autism phenotype (n = 19), and non-broader autism phenotype (n = 58). MAIN OUTCOME MEASURES: Social, communication, and symbolic abilities were assessed. RESULTS: Social, communication, and play behavior in the early-diagnosis group differed from that in all other groups by 14 months of age. By 24 months, the later-diagnosis group differed from the non-autism spectrum disorder groups in social and communication behavior, but not from the early-diagnosis group. Examination of growth trajectories suggests that autism may involve developmental arrest, slowing, or even regression. CONCLUSION: This study provides insight into different patterns of development of children with early vs later diagnosis of autism spectrum disorders
SN - 0003-990X
AD - Kennedy Krieger Institute, Center for Autism and Related Disorders, The Johns Hopkins School of Medicine, Baltimore, Maryland 21211, USA
UR - 17606819
ER -

TY - JOUR
ID - 304
T1 - A meta-analysis of children's hand-to-mouth frequency data for estimating nondietary ingestion exposure
A1 - Xue,J.
A1 - Zartarian,V.
A1 - Moya,J.
A1 - Freeman,N.
A1 - Beamer,P.
A1 - Black,K.
A1 - Tulve,N.
A1 - Shalat,S.
Y1 - 2007/04//
N1 - Xue, Jianping. Zartarian, Valerie. Moya, Jacqueline. Freeman, Natalie. Beamer, Paloma. Black, Kathy. Tulve, Nicolle. Shalat, Stuart
Risk analysis : an official publication of the Society for Risk Analysis
8109978, ria
IM
Journal Article. Meta-Analysis. Research Support, U.S. Gov't, Non-P.H.S.
English
KW - Medline
KW - Age Factors
KW - Child
KW - Preschool
KW - Eating
KW - Environmental Exposure
KW - Environmental Monitoring
KW - Environmental Pollutants/to [Toxicity]
KW - Face
KW - Humans
KW - Infant
KW - Motor Activity

KW - Mouth
KW - Play and Playthings
KW - Risk Assessment
KW - Sex Factors
RP - NOT IN FILE
SP - 411
EP - 420
JF - Risk Analysis
JA - Risk Anal.
VL - 27
IS - 2
CY - United States
N2 - Because of their mouthing behaviors, children have a higher potential for exposure to available chemicals through the nondietary ingestion route; thus, frequency of hand-to-mouth activity is an important variable for exposure assessments. Such data are limited and difficult to collect. Few published studies report such information, and the studies that have been conducted used different data collection approaches (e.g., videography versus real-time observation), data analysis and reporting methods, ages of children, locations, and even definitions of "mouthing." For this article, hand-to-mouth frequency data were gathered from 9 available studies representing 429 subjects and more than 2,000 hours of behavior observation. A meta-analysis was conducted to study differences in hand-to-mouth frequency based on study, age group, gender, and location (indoor vs. outdoor), to fit variability and uncertainty distributions that can be used in probabilistic exposure assessments, and to identify any data gaps. Results of this analysis indicate that age and location are important for hand-to-mouth frequency, but study and gender are not. As age increases, both indoor and outdoor hand-to-mouth frequencies decrease. Hand-to-mouth behavior is significantly greater indoors than outdoors. For both indoor and outdoor hand-to-mouth frequencies, interpersonal, and intra-personal variability are approximately 60% and approximately 30%, respectively. The variance difference among different studies is much bigger than its mean, indicating that different studies with different methodologies have similar central values. Weibull distributions best fit the observed data for the different variables considered and are presented in this article by study, age group, and location. Average indoor hand-to-mouth behavior ranged from 6.7 to 28.0 contacts/hour, with the lowest value corresponding to the 6 to <11 year olds and the highest value corresponding to the 3 to <6 month olds. Average outdoor hand-to-mouth frequency ranged from 2.9 to 14.5 contacts/hour, with the lowest value corresponding to the 6 to <11 year olds and the highest value corresponding to the 6 to <12 month olds. The analysis highlights the need for additional hand-to-mouth data for the <3 months, 3 to <6 months, and 3 to <6 year age groups using standardized collection and analysis because of lack of data or high uncertainty in available data. This is the first publication to report Weibull distributions as the best fitting distribution for hand-to-mouth frequency; using the best fitting exposure factor distribution will help improve estimates of exposure. The analyses also represent a first comprehensive effort to fit hand-to-mouth frequency variability and uncertainty distributions by indoor/outdoor location and by age groups, using the new standard set of age groups recommended by the U.S. Environmental Protection Agency for assessing childhood exposures. Thus, the data presented in this article can be used to update the U.S. EPA's Child-Specific Exposure Factors Handbook and to improve estimates of nondietary ingestion in probabilistic exposure modeling
SN - 0272-4332
AD - U.S. Environmental Protection Agency, Boston, MA, USA
UR - 17511707
ER -

TY - JOUR
ID - 305
T1 - Frequency of caregiver supervision of young children during play
A1 - Chen,X.
A1 - Beran,M.
A1 - Altkorn,R.
A1 - Milkovich,S.
A1 - Gruaz,K.
A1 - Rider,G.
A1 - Kanti,A.

A1 - Ochsenhirt,J.
Y1 - 2007/06//
N1 - Chen, Xiao. Beran, Melissa. Altkorn, Robert. Milkovich, Scott. Gruaz, Kristin. Rider, Gene. Kanti, Amita. Ochsenhirt, Jen
International journal of injury control and safety promotion
101247254
IM
Journal Article
English
KW - Medline
KW - Caregivers
KW - Child
KW - Child Care/mt [Methods]
KW - Preschool
KW - Data Collection
KW - Female
KW - Humans
KW - Male
KW - Ohio
KW - Play and Playthings
KW - Safety
RP - NOT IN FILE
SP - 122
EP - 124
JF - International Journal of Injury Control & Safety Promotion
JA - Int.J Inj.Contr.Saf Promot
VL - 14
IS - 2
CY - England
SN - 1745-7300
AD - Intertek Risk Assessment and Management, Oak Brook, IL, USA. xiao.chen@intertek.com
UR - 17510849
ER -

TY - JOUR
ID - 306
T1 - Survey of primary school educators regarding burn-risk behaviors and fire-safety education
A1 - Dougherty,J.
A1 - Pucci,P.
A1 - Hemmila,M.R.
A1 - Wahl,W.L.
A1 - Wang,S.C.
A1 - Arbabi,S.
Y1 - 2007/06//
N1 - Dougherty, Joseph. Pucci, Pamela. Hemmila, Mark R. Wahl, Wendy L. Wang, Stewart C. Arbabi, Saman
Burns : journal of the International Society for Burn Injuries
afc, 8913178
IM
Journal Article. Multicenter Study. Research Support, Non-U.S. Gov't
English
KW - Medline
KW - Adolescent
KW - Burns/et [Etiology]
KW - Burns/pc [Prevention & Control]
KW - Child
KW - Preschool

KW - Faculty
KW - Fires/pc [Prevention & Control]
KW - Firesetting Behavior/et [Etiology]
KW - Health Education/st [Standards]
KW - Health Knowledge
KW - Attitudes
KW - Practice
KW - Humans
KW - Infant
KW - Perception
KW - Professional Competence/st [Standards]
KW - Risk Assessment
KW - Risk-Taking
KW - Safety Management/st [Standards]
KW - Schools
KW - Teaching/st [Standards]
RP - NOT IN FILE
SP - 472
EP - 476
JF - Burns
VL - 33
IS - 4
CY - England
N2 - OBJECTIVE: To assess primary school educator knowledge and opinions regarding fire-setting behaviors and burn-injury prevention education. DESIGN AND SETTING: A written survey, consisting of 24 questions, was distributed to 8 primary schools. The survey was designed by our burn center personnel to assess basic knowledge and opinions. It was distributed to teachers by the school principals and completed surveys were collected by our staff. RESULTS: Twenty percent of elementary school educators surveyed had experience teaching burned children (mean age: 7+/-3; range: 2-17); 8% had experience with children that were injured due to fire-play. Fire experimentation begins at 6.1+/-2 years of age (range: 2-13). Educators believe students can benefit from a fire prevention curriculum beginning at 7.3+/-1.8 years (range: 5-12). The average time requested for an elementary school burn-injury prevention program was 4+/-3h. Kindergarten teachers requested 8h and fifth grade teachers asked for 3.3h. Over 97% of elementary school teachers agree that discussions about the medical and social consequences of burns will reduce fire-play behavior in children. CONCLUSIONS: Nearly all primary school educators surveyed agreed that burn injuries and attempting to curb fire-play are important societal issues. There was wide agreement that including a description of the medical and social consequences of burns in a preventive curriculum would enhance its efficacy. The younger students are, the more time teachers require to adequately convey fire safety instruction
SN - 0305-4179
AD - The University of Michigan Health System Burn Center, Ann Arbor, MI, United States
UR - 17467910
ER -

TY - JOUR
ID - 307
T1 - Safe and healthy school environments. [Review] [72 refs]
A1 - Geller,R.J.
A1 - Rubin,I.L.
A1 - Nodvin,J.T.
A1 - Teague,W.G.
A1 - Frumkin,H.
Y1 - 2007/04/20/
N1 - Geller, Robert J. Rubin, I Leslie. Nodvin, Janice T. Teague, W Gerald. Frumkin, Howard
Pediatric clinics of North America
oum, 0401126
AIM, IM

Journal Article. Review
English
KW - Medline
KW - Child
KW - Child Welfare/sn [Statistics & Numerical Data]
KW - Child Welfare
KW - Crowding
KW - Emergency Medical Services
KW - Environmental Exposure/pc [Prevention & Control]
KW - Environmental Health/og [Organization & Administration]
KW - Food Services
KW - Health Planning
KW - Health Promotion
KW - Humans
KW - Humidity/ae [Adverse Effects]
KW - Interior Design and Furnishings
KW - Lighting/ae [Adverse Effects]
KW - Noise/ae [Adverse Effects]
KW - Pediatrics/og [Organization & Administration]
KW - Play and Playthings
KW - Safety Management/og [Organization & Administration]
KW - School Health Services/og [Organization & Administration]
KW - Temperature
KW - Transportation
KW - United States/ep [Epidemiology]
KW - Wounds and Injuries/pc [Prevention & Control]
RP - NOT IN FILE
SP - 351
EP - 373
JF - Pediatric Clinics of North America
JA - Pediatr Clin.North Am
VL - 54
IS - 2
CY - United States
N2 - Children spend much of their waking time at school. Many of the factors in the school environment can be improved with careful planning and allocation of resources. The pediatrician, as a child advocate, is in an excellent position to influence the allocation of school resources to improve the educational outcome. This article summarizes some of the current understanding gathered from applying an environmental health approach to the school setting and provides a basis for the interested physician and other child advocate to learn more and get involved. [References: 72]
SN - 0031-3955
AD - Emory Southeast Pediatric Environmental Health Specialty Unit, 49 Jesse Hill Jr Drive SE, Atlanta, GA 30303, USA. rgeller@georgiapoisoncenter.org
UR - 17448364
ER -

TY - JOUR
ID - 308
T1 - [Bedtime, television and computer habits of primary school children in Germany]. [German]
A1 - Heins,E.
A1 - Seitz,C.
A1 - Schuz,J.
A1 - Toschke,A.M.
A1 - Harth,K.
A1 - Letzel,S.
A1 - Bohler,E.

Y1 - 2007/03//
N1 - Heins, E. Seitz, C. Schuz, J. Toschke, A M. Harth, K. Letzel, S. Bohler, E
Gesundheitswesen (Bundesverband der Arzte des Offentlichen Gesundheitsdienstes (Germany))
bfd, 9204210
IM
English Abstract. Journal Article
German
KW - Medline
KW - Adolescent
KW - Child
KW - Computers/sn [Statistics & Numerical Data]
KW - Female
KW - Germany/ep [Epidemiology]
KW - Habits
KW - Humans
KW - Leisure Activities
KW - Male
KW - Play and Playthings
KW - Risk Assessment/mt [Methods]
KW - Risk Factors
KW - Schools/sn [Statistics & Numerical Data]
KW - Sleep Deprivation/ep [Epidemiology]
KW - Students/sn [Statistics & Numerical Data]
KW - Television/sn [Statistics & Numerical Data]
KW - Video Games
RP - NOT IN FILE
SP - 151
EP - 157
JF - Gesundheitswesen
VL - 69
IS - 3
CY - Germany
N2 - BACKGROUND: Pediatricians recommend daily sleep of at least ten hours for children aged 9 and 10 years. Sufficient sleep is necessary for maintaining the body's homeostasis, as well as for fixing memories and learning. Lack of sleep in children has been associated with a diminished school performance, reduced attention span, and obesity. Adulthood is influenced by childhood lifestyle habits. Data from 4th graders in 34 schools in Mainz and its surroundings were analysed in order to determine negative health lifestyle factors in German primary school children, such as lack of sleep and increased leisure time spent watching television and computer gaming. METHODS: Data from a cross-sectional study regarding cellular phone use in fourth-graders in Mainz were used for this analysis. Bedtime, television and computer use habits, as well as other factors were explored. A total of 1933 children from 34 schools participated by answering a questionnaire in the 2002-2003 school year (participation rate: 88%). Complete data for the secondary analysis were available from 1889 students (51% male, median age 10 years). RESULTS: Overall, 28% of the children reported going to bed after 9 pm on week nights, 16% reported watching television more than three hours daily, and 11% played computer or video games more than three hours daily. In the adjusted binary logistic regression model, being older, male, having older siblings, watching television (OR 1.82, 95% CI 1.28-2.61) and playing computer games (OR 3.23; 95% CI 2.24-4.67) for more than three hours daily, owning a mobile phone, and being in a school in the city were associated with going to bed after 9 pm. CONCLUSION: Every fourth child does not obtain a sleep duration of 10 hours, under the assumption that primary schoolchildren need to be awake at 7 am on weekdays. Lifestyle factors that may negatively influence a child's development determine their actual and future habits. Sufficient sleep and less television and computer leisure times should be assertively emphasized to parents and carers of primary school students, in order to prevent the negative consequences of lack of sleep, such as diminished school performance. Developing sex-specific prevention programs, which take into account the complex lifestyles and habits of primary schoolchildren, seems to be relevant
SN - 1439-4421

AD - Institut fur Arbeits-, Sozial- und Umweltmedizin der Johannes Gutenberg-Universitat Mainz, Mainz, Germany. heins@uni-mainz.de
UR - 17440845
ER -

TY - JOUR
ID - 309
T1 - Is work in education child's play? Understanding risks to educators arising from work organization and design of work spaces
A1 - Seifert,A.M.
Y1 - 2007///
N1 - Seifert, Ana Maria
New solutions : a journal of environmental and occupational health policy : NS
9100937
IM
Journal Article
English
KW - Medline
KW - Adult
KW - Burnout
KW - Professional/px [Psychology]
KW - Child
KW - Child Behavior Disorders
KW - Developmental Disabilities
KW - Education
KW - Special/ec [Economics]
KW - Special/ma [Manpower]
KW - Special/mt [Methods]
KW - Faculty/sd [Supply & Distribution]
KW - Faculty
KW - Female
KW - Human Engineering
KW - Humans
KW - Interprofessional Relations
KW - Interviews as Topic
KW - Job Satisfaction
KW - Middle Aged
KW - Occupational Health
KW - Organizational Culture
KW - Quebec
KW - Risk Factors
KW - Safety
KW - Schools/st [Standards]
KW - Urban Health
KW - Women's Health
KW - WOMEN
KW - Working
KW - Workplace/px [Psychology]
RP - NOT IN FILE
SP - 97
EP - 110
JF - New Solutions
JA - New solut.
VL - 17
IS - 1-2
CY - United States

N2 - The educational sector exposes its primarily female work force to numerous psychosocial risk factors. At the request of the education workers', ergonomists developed a participatory research project in order to understand the determinants of the difficulties experienced by special education technicians. These technicians work with students presenting behavioral and learning difficulties as well as developmental and mental health problems. Eighteen technicians were interviewed and the work of seven technicians and two teachers was observed. Technicians prevent and manage crisis situations and help students acquire social skills. Coordination with teachers is made difficult by the fact that most technicians work part time, part year, and many technicians' work areas and classrooms are physically distant one from another. Most technicians change schools each year and must continually reconstruct work teams. Management strategies and poorly adapted working spaces can have important repercussions on coordination among educators and on technicians' capacity to help students and prevent aggressive behavior
SN - 1048-2911
AD - CINBIOSE-UQAM, CP 8888 Succ Centre Ville Montreal H3C 3P8 Quebec, Canada. seifert.ana-maria@uqam.ca
UR - 17434862
ER -

TY - JOUR
ID - 310
T1 - Fractures in New Zealand elementary school settings
A1 - Rubie-Davies,C.M.
A1 - Townsend,M.A.
Y1 - 2007/01//
N1 - Rubie-Davies, Christine M. Townsend, Michael A R
The Journal of school health
k13, 0376370
IM, N
Journal Article
English
KW - Medline
KW - Child
KW - Equipment Design/st [Standards]
KW - Female
KW - Fractures
KW - Bone/ep [Epidemiology]
KW - Bone/et [Etiology]
KW - Humans
KW - Male
KW - New Zealand/ep [Epidemiology]
KW - Play and Playthings/in [Injuries]
KW - Safety/sn [Statistics & Numerical Data]
KW - School Health Services/sn [Statistics & Numerical Data]
KW - Schools/st [Standards]
RP - NOT IN FILE
SP - 36
EP - 40
JF - Journal of School Health
JA - J Sch Health
VL - 77
IS - 1
CY - United States
N2 - BACKGROUND: There is a need for greater international understanding of student safety in schools. This New Zealand study investigated the causes and school location of fractures sustained by students attending elementary school, with special emphasis on the types of fractures sustained following falls from playground equipment of various heights. METHODS: Over a 1-year period, 76 participating schools (with a combined roll of over 25,000 students) completed a questionnaire about the nature and circumstances of student fractures

sustained during regular school hours. RESULTS: Some 118 students sustained a total of 131 fractures. Injuries from playground equipment were no more frequent than those from general activities at school, and most were sustained in falls from heights less than 59 inches. Most fractures were to the upper limbs. Fractures were found to vary by gender and school size. CONCLUSIONS: The results are discussed in terms of the conditions and policies present in local schools, and the tension that exists in maintaining safety while offering appropriate challenges to students. Improvements in school safety may be more likely to result from a greater focus on the way that students interact at school, rather than on modifications to playground equipment
SN - 0022-4391
AD - Faculty of Education, The University of Auckland, Private Bag 92019, Auckland, New Zealand. c.rubie@auckland.ac.nz
UR - 17212758
ER -

TY - JOUR
ID - 311
T1 - Television watching and frequency of family meals are predictive of overweight onset and persistence in a national sample of school-aged children
A1 - Gable,S.
A1 - Chang,Y.
A1 - Krull,J.L.
Y1 - 2007/01//
N1 - Gable, Sara. Chang, Yiting. Krull, Jennifer L
Journal of the American Dietetic Association
h6f, 7503061
AIM, IM
Journal Article. Research Support, U.S. Gov't, Non-P.H.S.
English
KW - Medline
KW - Body Mass Index
KW - Child
KW - Child Development
KW - Preschool
KW - Cohort Studies
KW - Confidence Intervals
KW - Eating
KW - Exercise/ph [Physiology]
KW - Female
KW - Humans
KW - Logistic Models
KW - Longitudinal Studies
KW - Male
KW - Multivariate Analysis
KW - Obesity/ep [Epidemiology]
KW - Obesity/et [Etiology]
KW - Odds Ratio
KW - Overweight
KW - Play and Playthings
KW - Predictive Value of Tests
KW - Risk Assessment
KW - Social Class
KW - Television
KW - Time Factors
KW - United States/ep [Epidemiology]
KW - Weight Gain/ph [Physiology]
RP - NOT IN FILE
SP - 53

EP - 61
JF - Journal of the American Dietetic Association
JA - J Am Diet Assoc.
VL - 107
IS - 1
CY - United States
N2 - OBJECTIVE: To identify eating and activity factors associated with school-aged children's onset of overweight and persistent overweight. DESIGN: Data were gathered at four time points between kindergarten entry and spring of third grade. Children were directly weighed and measured and categorized as not overweight (<95th percentile of body mass index) or overweight (> or =95th percentile body mass index); parents were interviewed by telephone or in person. SUBJECTS/SETTING: Subjects were participants in the Early Childhood Longitudinal Study-Kindergarten Cohort, a nationally representative sample of children who entered kindergarten during 1998-1999. Children who weighed <2,000 g at birth, received therapeutic services before kindergarten, skipped or repeated a grade, or without complete height and weight data were excluded, resulting in 8,459 participants. Children with intermittent overweight were not examined (n=459); analyses addressed 8,000 children. MAIN OUTCOME MEASURES: Three mutually exclusive groups of children were identified: never overweight, overweight onset, and persistent overweight. STATISTICAL ANALYSES PERFORMED: Multilevel, multivariate logistic regression analyses estimated the effects of eating and activity factors on the odds of overweight onset and persistent overweight above child sex, race, and family socioeconomic status. RESULTS: Children who watched more television (odds ratio [OR] 1.02) and ate fewer family meals (OR 1.08) were more likely to be overweight for the first time at spring semester of third grade. Children who watched more television (OR 1.03), ate fewer family meals (OR 1.08), and lived in neighborhoods perceived by parents as less safe for outdoor play (OR 1.32) were more likely to be persistently overweight. Child aerobic exercise and opportunities for activity were not associated with a greater likelihood of weight problems. CONCLUSIONS: This study supports theories regarding the contributions of television watching, family meals, and neighborhood safety to childhood weight status. When working with families to prevent and treat childhood weight problems, food and nutrition professionals should attend to children's time spent with screen media, the frequency of family mealtimes, and parents' perceptions of neighborhood safety for children's outdoor play
SN - 0002-8223
AD - Department of Psychological Sciences, University of Missouri, Columbia, MO 65211-7700, USA. gables@missouri.edu
UR - 17197271
ER -

TY - JOUR
ID - 312
T1 - BB guns--a dangerous toy
A1 - Chakravarty,D.
A1 - Chopra,S.
A1 - Jones,J.
Y1 - 2006/12//
N1 - Chakravarty, D. Chopra, S. Jones, J
Journal of the Royal Army Medical Corps
jv6, 7505627
IM
Case Reports. Journal Article
English
KW - Medline
KW - Accidents
KW - Home
KW - Child
KW - Child Behavior
KW - Finger Injuries/di [Diagnosis]
KW - Finger Injuries/et [Etiology]
KW - Firearms
KW - Hand Injuries/di [Diagnosis]

KW - Hand Injuries/et [Etiology]
KW - Humans
KW - Male
KW - Play and Playthings
KW - Safety
RP - NOT IN FILE
SP - 239
EP - 241
JF - Journal of the Royal Army Medical Corps
JA - J R Army Med Corps
VL - 152
IS - 4
CY - England
SN - 0035-8665
AD - Department of Orthopaedics, Peterborough Hospital, NHS Trust, Thorpe Road, Peterborough PE3 6DA.
debasischakravarty@hotmail.com
UR - 17508645
ER -

TY - JOUR
ID - 313
T1 - Some reflections on infancy-onset trichotillomania
A1 - Keren,M.
A1 - Ron-Miara,A.
A1 - Feldman,R.
A1 - Tyano,S.
Y1 - 2006///
N1 - Keren, Miri. Ron-Miara, Adi. Feldman, Ruth. Tyano, Samuel
The Psychoanalytic study of the child
qdi, 0376472
IM
Case Reports. Journal Article
English
KW - Medline
KW - Child
KW - Preschool
KW - Communication
KW - Female
KW - Follow-Up Studies
KW - Humans
KW - Infant
KW - Life Change Events
KW - Maternal Behavior
KW - Mother-Child Relations
KW - Object Attachment
KW - Play and Playthings
KW - Psychoanalytic Theory
KW - Psychoanalytic Therapy
KW - Risk Factors
KW - Treatment Outcome
KW - Trichotillomania/px [Psychology]
KW - Trichotillomania/th [Therapy]
RP - NOT IN FILE
SP - 254
EP - 272
JF - Psychoanalytic Study of the Child

JA - Psychoanal.Study Child
VL - 61
CY - United States
N2 - Whether infancy-onset trichotillomania is best regarded as a habit, an early sign of obsessive compulsive disorder, a symptom of anxiety, or a sign of severe deprivation has been a topic of continuous debate. In this paper, we describe our clinical experience with nine consecutive cases of infancy-onset trichotillomania and detail the evaluation process and treatment course in one case. A distinct psychosocial stressor was identified in all cases, often accompanied by loss in the parents' histories. Most of the children had no transitional object. In six infants, the symptom resolved after treatment and did not recur, while in three others improvement was partial. Length of treatment varied from four to twenty-one sessions and outcome was unrelated to treatment duration. In all cases, mother-child interactions were characterized by a lack of maternal physical contact and warmth, sharp maternal transitions between under-involvement and intrusiveness, lack of mutual engagement, and no elaboration of symbolic play. The infant's behavior during play was marked by anxiety, irritability, and momentary withdrawal from the interaction. Our cases reveal an impaired affective interpersonal communication between mother and child, often masked by a fair overallfamily instrumental functioning. It is tenta- tively suggested that infancy-onset trichotillomania represents an end-point symptom of several factors, such as a disturbed parent-infant relationship, a low pain threshold in the infant, and a parental hypersensitivity to overt expressions of aggressive impulses and negative affects. Issues related to treatment modalities are also addressed. Discussion focused on our experience that early-onset cases of trichotillomania are often not benign or homogenous in terms of etiology, course, or response to treatment and require much further study
SN - 0079-7308
AD - Infant Mental Health Unit, Geha Mental Health Center, Petah Tiqva, Israel
UR - 17370463
ER -

TY - JOUR
ID - 314
T1 - Resilience among at-risk Hispanic American preschool children
A1 - Oades-Sese,G.V.
A1 - Esquivel,G.B.
Y1 - 2006/12//
N1 - Oades-Sese, Geraldine V. Esquivel, Giselle B
Annals of the New York Academy of Sciences
5nm, 7506858
IM
Journal Article
English
KW - Medline
KW - Adaptation
KW - Psychological
KW - Child
KW - Preschool
KW - Female
KW - Hispanic Americans/px [Psychology]
KW - Humans
KW - Male
KW - Multilingualism
KW - Risk Factors
KW - Social Adjustment
RP - NOT IN FILE
SP - 335
EP - 339
JF - Annals of the New York Academy of Sciences
JA - Ann.N.Y.Acad.Sci
VL - 1094
CY - United States

N2 - This study combines cognitive (i.e., intelligence), psychosocial (i.e., inhibition, activity level, negative emotionality, emotion regulation, autonomy), and cultural-linguistic factors (i.e., level of acculturation and bilingualism) to determine patterns of resilience and vulnerability among 207 economically disadvantaged Hispanic American preschool children, from 50 early childhood classrooms, as gauged by their social competence during peer play. Person-oriented analysis yielded six distinct profiles, two profiles of which were resilient and one identified as vulnerable. Results of this study revealed within-group differences in resilience among these children and the significant role bilingualism and maintenance of the home language play in their social-emotional development
SN - 0077-8923
AD - Graduate School of Education, Division of Psychological and Educational Services, Fordham University, New York, New York, USA. oadessese@fordham.edu
UR - 17347372
ER -

TY - JOUR
ID - 315
T1 - Toys and safety
A1 - Scowen,P.
Y1 - 2006///
N1 - Scowen, Pat
The journal of family health care
101142028
N
Journal Article
English
KW - Medline
KW - Child
KW - Preschool
KW - Great Britain
KW - Humans
KW - Infant
KW - Play and Playthings
KW - Safety/lj [Legislation & Jurisprudence]
KW - Safety
RP - NOT IN FILE
SP - 134
EP - 135
JF - Journal of Family Health Care
JA - J Fam.Health Care
VL - 16
IS - 5
CY - England
N2 - Christmas is coming and with it the purchase of toys for the children. But toys are also given all year round, and educating each new generation of parents about toy and play safety is part of the public health role for health professionals working with families
SN - 1474-9114
UR - 17139973
ER -

TY - JOUR
ID - 316
T1 - Children's indoor and outdoor play patterns in Ahwaz City: implications for injury prevention
A1 - Soori,H.
Y1 - 2006/05//
N1 - Soori, H
Eastern Mediterranean health journal = La revue de sante de la Mediterranee orientale = al-Majallah al-sihhiyah

li-sharq al-mutawassit
daq, 9608387
IM
Journal Article
English
KW - Medline
KW - Accident Prevention
KW - Age Factors
KW - Bicycling/in [Injuries]
KW - Bicycling/sn [Statistics & Numerical Data]
KW - Child
KW - Cross-Sectional Studies
KW - Female
KW - Humans
KW - Iran/ep [Epidemiology]
KW - Leisure Activities
KW - Male
KW - Needs Assessment/og [Organization & Administration]
KW - Parents/px [Psychology]
KW - Play and Playthings/in [Injuries]
KW - Public Facilities/sn [Statistics & Numerical Data]
KW - Questionnaires
KW - Risk Assessment
KW - Risk Factors
KW - Safety Management/og [Organization & Administration]
KW - Sex Factors
KW - Soccer/in [Injuries]
KW - Soccer/sn [Statistics & Numerical Data]
KW - Socioeconomic Factors
KW - Television/sn [Statistics & Numerical Data]
KW - Urban Population/sn [Statistics & Numerical Data]
KW - Wounds and Injuries/ep [Epidemiology]
KW - Wounds and Injuries/et [Etiology]
KW - Wounds and Injuries/pc [Prevention & Control]
RP - NOT IN FILE
SP - 372
EP - 381
JF - Eastern Mediterranean Health Journal
JA - East Mediterr.Health J
VL - 12
IS - 3-4
CY - Egypt
N2 - A cross-sectional study in Ahwaz city, Islamic Republic of Iran, described children's indoor and outdoor play patterns. A total of 952 pupils aged 7 and 9 years and 942 parents completed questionnaires. Children's favourite activities were: playing football (22.3%), cycling (13.2%) and watching television (12.3%). This differed from those perceived by the parents who reported watching television (20.3%), playing football (13.3%) and playing in street (11.4%). Children reported their favourite places to play were: street (27.4%), park/playground (18.3%) and home (17.8%). There were significant differences in play patterns by age and sex. The play patterns of older children and boys exposed them to more hazardous situations than younger children and girls
SN - 1020-3397
AD - Department of Epidemiology and Biostatistics, Faculty of Public Health, Shaheed Beheshti University of Medical Sciences, Tehran, Islamic Republic of Iran. hsoori@yahoo.com
UR - 17037706
ER -

TY - JOUR
ID - 317
T1 - Early identification of autism: how early can we go?. [Review] [127 refs]
A1 - Crais,E.R.
A1 - Watson,L.R.
A1 - Baranek,G.T.
A1 - Reznick,J.S.
Y1 - 2006/08//
N1 - Crais, Elizabeth R. Watson, Linda R. Baranek, Grace T. Reznick, J Steven
Seminars in speech and language
8405117, cfb, 8405117
IM
Journal Article. Review
English
KW - Medline
KW - Age Factors
KW - Autistic Disorder/di [Diagnosis]
KW - Autistic Disorder/ep [Epidemiology]
KW - Child
KW - Child Behavior
KW - Child Development
KW - Preschool
KW - Communication
KW - Early Intervention (Education)/sn [Statistics & Numerical Data]
KW - Humans
KW - Play and Playthings
KW - Risk Assessment
KW - Social Behavior
RP - NOT IN FILE
SP - 143
EP - 160
JF - Seminars in Speech & Language
JA - Semin Speech Lang
VL - 27
IS - 3
CY - United States
N2 - Identification of young children at risk for Autism Spectrum Disorders (ASD) depends on early behavioral symptomatology and yet conventional criteria provide little guidance for use with infants and toddlers. Recent research, however, has demonstrated that there are patterns of behavior below 2 years of age that distinguish children with autism from those who are developing typically or those with other developmental disabilities. Skill areas with particular promise for early identification include social communication, sensory regulation, and play. This article previews current innovative methodologies, presents a synthesis of recent research findings related to these three key areas, and provides clinicians with practical guidelines for early identification of infants and toddlers at risk for ASD and other disorders. [References: 127]
SN - 0734-0478
AD - Division of Speech and Hearing Sciences, University of North Carolina at Chapel Hill, Chapel Hill, North Carolina 27599, USA. bcrais@med.unc.edu
UR - 16941286
ER -

TY - JOUR
ID - 318
T1 - Bubbles to Wubbles: an investigation involving the contamination of soap bubble products at a pediatric hospital
A1 - Dolan,S.A.
A1 - Eberhart,T.

A1 - James,J.F.
Y1 - 2006/07//
N1 - Dolan, Susan A. Eberhart, Teresa. James, John F
Journal for specialists in pediatric nursing : JSPN
101142025
IM, N
Journal Article
English
KW - Medline
KW - Air
KW - Child
KW - Clinical Nursing Research
KW - Colony Count
KW - Microbial
KW - Colorado
KW - Drug Contamination/pc [Prevention & Control]
KW - Drug Contamination/sn [Statistics & Numerical Data]
KW - Equipment Contamination/pc [Prevention & Control]
KW - Equipment Contamination/sn [Statistics & Numerical Data]
KW - Equipment Contamination
KW - Hair Preparations/sd [Supply & Distribution]
KW - Hair Preparations
KW - Hospitals
KW - Pediatric
KW - Humans
KW - Infection Control/mt [Methods]
KW - Play and Playthings
KW - Safety Management
KW - Soaps/sd [Supply & Distribution]
KW - Soaps
KW - Solutions
RP - NOT IN FILE
SP - 189
EP - 195
JF - Journal for Specialists in Pediatric Nursing: JSPN
JA - J Spec.Pediatr Nurs
VL - 11
IS - 3
CY - United States
SN - 1539-0136
AD - Nurse Epidemiologist. dolan.susan@tchden.org
UR - 16774529
ER -

TY - JOUR
ID - 319
T1 - Not all risk is bad, playgrounds as a learning environment for children
A1 - Mitchell,R.
A1 - Cavanagh,M.
A1 - Eager,D.
Y1 - 2006/06//
N1 - Mitchell, Rebecca. Cavanagh, Margaret. Eager, David
International journal of injury control and safety promotion
101247254
IM

Journal Article
English
KW - Medline
KW - Adolescent
KW - Child
KW - Child Behavior
KW - Child Development
KW - Preschool
KW - Hospitalization/sn [Statistics & Numerical Data]
KW - Humans
KW - Infant
KW - Learning
KW - New South Wales/ep [Epidemiology]
KW - Play and Playthings/in [Injuries]
KW - Play and Playthings/px [Psychology]
KW - Risk-Taking
KW - Wounds and Injuries/ep [Epidemiology]
RP - NOT IN FILE
SP - 122
EP - 124
JF - International Journal of Injury Control & Safety Promotion
JA - Int.J Inj.Contr.Saf Promot
VL - 13
IS - 2
CY - England
SN - 1745-7300
AD - NSW Injury Risk Management Research Centre, formerly, Injury Prevention and Policy Branch, NSW Health Department, Australia. r.mitchell@unsw.edu.au
UR - 16707350
ER -

TY - JOUR
ID - 320
T1 - Early language and communication development of infants later diagnosed with autism spectrum disorder
A1 - Mitchell,S.
A1 - Brian,J.
A1 - Zwaigenbaum,L.
A1 - Roberts,W.
A1 - Szatmari,P.
A1 - Smith,I.
A1 - Bryson,S.
Y1 - 2006/04//
N1 - Mitchell, Shelley. Brian, Jessica. Zwaigenbaum, Lonnie. Roberts, Wendy. Szatmari, Peter. Smith, Isabel. Bryson, Susan
Journal of developmental and behavioral pediatrics : JDBP
htf, 8006933
IM
Journal Article. Research Support, Non-U.S. Gov't
English
KW - Medline
KW - Autistic Disorder/di [Diagnosis]
KW - Autistic Disorder/px [Psychology]
KW - Child
KW - Preschool
KW - Communication
KW - Developmental Disabilities/et [Etiology]

KW - Developmental Disabilities/px [Psychology]
KW - Female
KW - Humans
KW - Infant
KW - Language
KW - Language Disorders/di [Diagnosis]
KW - Language Disorders/et [Etiology]
KW - Male
KW - Play and Playthings
KW - Siblings
KW - Social Behavior
RP - NOT IN FILE
SP - S69
EP - S78
JF - Journal of Developmental & Behavioral Pediatrics
JA - J Dev.Behav Pediatr
VL - 27
IS - 2 Suppl
CY - United States
N2 - It is well recognized that delayed "first words" is among the most common presenting symptoms of autistic spectrum disorders (ASD). However, data on earlier language and communication development in children with ASD are limited to retrospective reports from parents and from home videos. In this study, we prospectively collected parent report data on early communication and language development in 97 infant siblings of children with ASD and 49 low-risk controls. Parents completed the MacArthur Communicative Development Inventory--Infant Form at 12 and 18 months. Analysis compared 3 groups defined on the basis of diagnostic assessment at 24 months: (1) siblings with ASD (n = 15), (2) siblings not meeting diagnostic criteria for ASD (n = 82), and (3) low-risk controls, none of whom had ASD (n = 49). Children with ASD showed delays in early language and communication compared with non-ASD siblings and controls. At 12 months, the ASD group was reported to understand significantly fewer phrases and to produce fewer gestures. At 18 months, they showed delays in their understanding of phrases, comprehension and production of single words, and use of gestures. Siblings not diagnosed with ASD also used fewer play-related gestures at 18 months than low-risk controls, even when children with identified language delays were excluded. Overall, this prospective study confirms that delays in communication and language development are apparent early in life in children with ASD, and emphasizes that developmental surveillance should include monitoring for delays in gesture, which may be among the earliest signs of ASD
SN - 0196-206X
AD - Autism Research Unit, The Hospital for Sick Children, Toronto, Ontario, Canada
UR - 16685188
ER -

TY - JOUR
ID - 321
T1 - Estimation of daily bisphenol a intake of Japanese individuals with emphasis on uncertainty and variability
A1 - Miyamoto,K.
A1 - Kotake,M.
Y1 - 2006///
N1 - Miyamoto, Ken-ichi. Kotake, Mari
Environmental sciences : an international journal of environmental physiology and toxicology
9209787
IM
Journal Article. Research Support, Non-U.S. Gov't
English
KW - Medline
KW - Adolescent
KW - Adult
KW - Bottle Feeding

KW - Child
KW - Preschool
KW - Environmental Exposure/an [Analysis]
KW - Environmental Monitoring
KW - Environmental Pollutants/an [Analysis]
KW - Female
KW - Food Contamination
KW - Glucuronides/ur [Urine]
KW - Humans
KW - Infant
KW - Newborn
KW - Inhalation Exposure/an [Analysis]
KW - Male
KW - Monte Carlo Method
KW - Phenols/an [Analysis]
KW - Phenols/ur [Urine]
KW - Play and Playthings
KW - Risk Assessment
KW - Uncertainty
KW - Water Supply
RP - NOT IN FILE
SP - 15
EP - 29
JF - Environmental Sciences
JA - Environ.
VL - 13
IS - 1
CY - Japan
N2 - The purpose of this study was to comprehensively assess the exposure of Japanese individuals to bisphenol A (BPA) with emphasis on uncertainty and variability in available information. The uncertainty and variability in parameters were numerically analyzed using Monte Carlo simulation. The uncertainty in the functional relationship between sources and exposure was treated by comparing two approaches: one was to aggregate ingestion and inhalation through all possible exposure pathways and the other was to estimate the intake from urinary excretion by backward calculation. For individuals aged 6 months or above, food was the most significant source of intake. The alteration of the method used in inactivating the inside surface of drink cans slightly contributed to the decrease in daily intake. By the backward calculation approach based on urinary excretion, 95% confidence intervals for the daily intake for high-exposure populations were estimated to be 0.037-0.064 microg/kg/day for males and 0.043-0.075 microg/kg/day for females. Even conservatively estimated daily intakes were lower than the EU's temporary tolerable daily intake (TDI) of 10 microg/kg/day as well as the U.S. Environmental Protection Agency (US EPA)'s reference dose (RfD) of 50 microg/kg/day. Thus, it is unlikely that humans, including infants and young children, are at unacceptable risk from possible BPA exposure
SN - 0915-955X
AD - Research Center for Chemical Risk Management, National Institute of Advanced Industrial Science and Technology, Tsukuba, Ibaraki, Japan. ken-ichi.miyamoto@aist.go.jp
UR - 16685249
ER -

TY - JOUR
ID - 322
T1 - Does children's screen time predict requests for advertised products? Cross-sectional and prospective analyses
A1 - Chamberlain,L.J.
A1 - Wang,Y.
A1 - Robinson,T.N.
Y1 - 2006/04//

N1 - Chamberlain, Lisa J. Wang, Yun. Robinson, Thomas N
Archives of pediatrics & adolescent medicine
9422751, bwf
AIM, IM
Journal Article. Research Support, N.I.H., Extramural
English
KW - Medline
KW - Advertising as Topic/sn [Statistics & Numerical Data]
KW - Advertising as Topic
KW - Beverages
KW - California/ep [Epidemiology]
KW - Child
KW - Confounding Factors (Epidemiology)
KW - Cross-Sectional Studies
KW - Environmental Exposure
KW - Female
KW - Follow-Up Studies
KW - Food
KW - Humans
KW - Life Style
KW - Male
KW - Multivariate Analysis
KW - Play and Playthings
KW - Predictive Value of Tests
KW - Prospective Studies
KW - Risk Factors
KW - Self Disclosure
KW - Television/sn [Statistics & Numerical Data]
KW - Television
KW - Time Factors
RP - NOT IN FILE
SP - 363
EP - 368
JF - Archives of Pediatrics & Adolescent Medicine
JA - Arch.Pediatr Adolesc Med
VL - 160
IS - 4
CY - United States
N2 - OBJECTIVE: To examine children's screen media exposure and requests for advertised toys and food/drinks. DESIGN: Prospective cohort study. SETTING: Twelve elementary schools in northern California. PARTICIPANTS: Eight hundred twenty-seven third grade children participated at baseline; 386 students in 6 schools were followed up for 20 months. INTERVENTION: None. MAIN OUTCOME MEASURES: Child self-reported requests for advertised toys and foods/drinks. RESULTS: At baseline, children's screen media time was significantly associated with concurrent requests for advertised toys (Spearman r = 0.15 [TV viewing] and r = 0.20 [total screen time]; both P<.001) and foods/drinks (Spearman r = 0.16 [TV viewing] and r = 0.18 [total screen time]; both P<.001). In prospective analysis, children's screen media time at baseline was significantly associated with their mean number of toy requests 7 to 20 months later (Spearman r = 0.21 [TV viewing] and r = 0.24 [total screen time]; both P<.001) and foods/drinks requests (Spearman r = 0.14 [TV viewing] and r = 0.16 [total screen time]; both P<.01). After adjusting for baseline requests and sociodemographic variables, the relationship between screen media exposure and future requests for advertised foods/drinks remained significant for total TV viewing and total screen media exposure. The relationship with future requests for toys remained significant for total screen media exposure. CONCLUSIONS: Screen media exposure is a prospective risk factor for children's requests for advertised products. Future experimental studies on children's health- and consumer-related outcomes are warranted
SN - 1072-4710

AD - Division of General Pediatrics, Department of Pediatrics and Stanford Prevention Research Center, Stanford University School of Medicine, 750 Welch Road, Palo Alto, CA 94304, USA. lchamberlain@stanford.edu
UR - 16585480
ER -

TY - JOUR
ID - 323
T1 - A probabilistic arsenic exposure assessment for children who contact chromated copper arsenate (CCA)-treated playsets and decks, Part 2: Sensitivity and uncertainty analyses
A1 - Xue,J.
A1 - Zartarian,V.G.
A1 - Ozkaynak,H.
A1 - Dang,W.
A1 - Glen,G.
A1 - Smith,L.
A1 - Stallings,C.
Y1 - 2006/04//
N1 - Xue, Jianping. Zartarian, Valerie G. Ozkaynak, Haluk. Dang, Winston. Glen, Graham. Smith, Luther. Stallings, Casson
Risk analysis : an official publication of the Society for Risk Analysis
8109978, ria
IM
Journal Article
English
KW - Medline
KW - Arsenates/ae [Adverse Effects]
KW - Arsenic/ad [Administration & Dosage]
KW - Arsenic/ae [Adverse Effects]
KW - Child
KW - Environmental Exposure
KW - Housing
KW - Humans
KW - Models
KW - Statistical
KW - Monte Carlo Method
KW - Play and Playthings
KW - Risk Assessment/sn [Statistics & Numerical Data]
KW - Risk Assessment
KW - Sensitivity and Specificity
KW - Soil Pollutants/ae [Adverse Effects]
KW - Uncertainty
KW - United States
KW - United States Environmental Protection Agency
KW - Wood
RP - NOT IN FILE
SP - 533
EP - 541
JF - Risk Analysis
JA - Risk Anal.
VL - 26
IS - 2
CY - United States
N2 - A probabilistic model (SHEDS-Wood) was developed to examine children's exposure and dose to chromated copper arsenate (CCA)-treated wood, as described in Part 1 of this two-part article. This Part 2 article discusses sensitivity and uncertainty analyses conducted to assess the key model inputs and areas of needed

research for children's exposure to CCA-treated playsets and decks. The following types of analyses were conducted: (1) sensitivity analyses using a percentile scaling approach and multiple stepwise regression; and (2) uncertainty analyses using the bootstrap and two-stage Monte Carlo techniques. The five most important variables, based on both sensitivity and uncertainty analyses, were: wood surface residue-to-skin transfer efficiency; wood surface residue levels; fraction of hand surface area mouthed per mouthing event; average fraction of nonresidential outdoor time a child plays on/around CCA-treated public playsets; and frequency of hand washing. In general, there was a factor of 8 for the 5th and 95th percentiles and a factor of 4 for the 50th percentile in the uncertainty of predicted population dose estimates due to parameter uncertainty. Data were available for most of the key model inputs identified with sensitivity and uncertainty analyses; however, there were few or no data for some key inputs. To evaluate and improve the accuracy of model results, future measurement studies should obtain longitudinal time-activity diary information on children, spatial and temporal measurements of residue and soil concentrations on or near CCA-treated playsets and decks, and key exposure factors. Future studies should also address other sources of uncertainty in addition to parameter uncertainty, such as scenario and model uncertainty
SN - 0272-4332
AD - U.S. Environmental Protection Agency, Office of Research and Development, National Exposure Research Laboratory, Research Triangle Park, NC, USA
UR - 16573638
ER -

TY - JOUR
ID - 324
T1 - The Stamp-in-Safety program: a behavioral intervention to reduce behaviors that can lead to unintentional playground injury in a preschool setting
A1 - Schwebel,D.C.
A1 - Summerlin,A.L.
A1 - Bounds,M.L.
A1 - Morrongiello,B.A.
Y1 - 2006/03//
N1 - Schwebel, David C. Summerlin, Adam L. Bounds, Marjorie L. Morrongiello, Barbara A
Journal of pediatric psychology
jl8, 7801773
IM
Clinical Trial. Journal Article
English
KW - Medline
KW - Adult
KW - Alabama
KW - Analysis of Variance
KW - Behavior Control/mt [Methods]
KW - Child Day Care Centers
KW - Child
KW - Preschool
KW - Female
KW - Humans
KW - Male
KW - Play and Playthings/in [Injuries]
KW - Play and Playthings
KW - Token Economy
KW - Wounds and Injuries/pc [Prevention & Control]
RP - NOT IN FILE
SP - 152
EP - 162
JF - Journal of Pediatric Psychology
JA - J Pediatr Psychol.
VL - 31

IS - 2
CY - United States
N2 - OBJECTIVE: To introduce and evaluate the Stamp-in-Safety program, a behavioral intervention designed to increase the quality of supervision by teachers on preschool playgrounds and to reduce the risk of unintentional child playground injury. METHODS: A quasi-experimental time series design was used, with observational techniques, to evaluate behavior on the playground before, during, and after the intervention as well as during a 6-month post-intervention assessment. RESULTS: Both applied behavior analysis techniques and inferential statistics suggest that the Stamp-in-Safety program resulted in behavioral changes likely to reduce the risk of child injury on the playgrounds of childcare centers. CONCLUSION: Results indicate promise for the Stamp-in-Safety program as a low-cost, easily implemented intervention to reduce pediatric playground injury risk at childcare centers. Suggestions for future research are offered
SN - 0146-8693
AD - Department of Psychology, University of Alabama at Birmingham, 1300 University Boulevard, CH 415, Birmingham, Alabama 35294, USA. schwebel@uab.edu
UR - 16467315
ER -

TY - JOUR
ID - 325
T1 - Smoking among secondary school students in Negeri Sembilan, Malaysia
A1 - Lee,L.K.
A1 - Paul,C.Y.
A1 - Kam,C.W.
A1 - Jagmohni,K.
Y1 - 2005///
N1 - Lee, L K. Paul, C Y C. Kam, C W. Jagmohni, K
Asia-Pacific journal of public health / Asia-Pacific Academic Consortium for Public Health
asj, 8708538
IM
Journal Article
English
KW - Medline
KW - Adolescent
KW - Adult
KW - Child
KW - Cross-Sectional Studies
KW - Female
KW - Humans
KW - Malaysia/ep [Epidemiology]
KW - Male
KW - Questionnaires
KW - Schools
KW - Smoking/ep [Epidemiology]
RP - NOT IN FILE
SP - 130
EP - 136
JF - Asia-Pacific Journal of Public Health
JA - Asia Pac.J Public Health
VL - 17
IS - 2
CY - China
N2 - This study was done to determine the prevalence of smoking and factors influencing cigarette smoking among secondary school students in Negeri Sembilan, Malaysia. This is a cross-sectional school survey conducted on 4500 adolescent students based on a structured questionnaire. Data was collected using the supervised self-administered questionnaire the Youth Risk Behaviour Surveillance in the Malaysian National Language Bahasa Malaysia. The prevalence of smoking among the students was 14.0%. About a third of the

students (37.8%) started smoking at 13 to 14 years of age. The prevalence of smoking among the male students was higher (26.6%) compared to the female students (3.1%). Adolescent smoking was associated with (1) sociodemographic factors (age, ethnicity, rural/urban status); (2) environmental factors (parental smoking, staying with parents); (3) behavioural factors (playing truant and risk-taking behaviours such as physical fighting, drug use, alcohol use, sexual activity, lack of seatbelt use, riding with a drunk driver); (4) lifestyle behaviours (being on diet and lack of exercise); (5) personal factors (feeling sad and suicidal behaviours). In conclusion, smoking is a major problem among Malaysian adolescents. Certain groups of adolescents tend to be at higher risk of smoking. This problem should be curbed early by targeting these groups of high risk adolescents
SN - 1010-5395
AD - Department of Community Medicine, International Medical University, Sesama Centre, Plaza Komanwel, Bukit Jalil, 57000 Kuala Lumpur, Malaysia
UR - 16425658
ER -

TY - JOUR
ID - 326
T1 - Playground safety in South Western Sydney
A1 - Martin,J.
A1 - Cooper,C.D.
Y1 - 2005/11//
N1 - Martin, J. Cooper, C D
Journal of paediatrics and child health
arp, 9005421
IM
Journal Article. Research Support, Non-U.S. Gov't
English
KW - Medline
KW - Accident Prevention
KW - Accidental Falls/pc [Prevention & Control]
KW - Child
KW - Data Collection
KW - Equipment Design/st [Standards]
KW - Guideline Adherence
KW - Humans
KW - New South Wales
KW - Play and Playthings
KW - Safety
RP - NOT IN FILE
SP - 587
EP - 591
JF - Journal of Paediatrics & Child Health
JA - J Paediatr Child Health
VL - 41
IS - 11
CY - Australia
N2 - OBJECTIVE: Recognition of the cost of injuries sustained in playgrounds has led to the development of Australian/New Zealand Standards for playground safety. This study assesses compliance of public playgrounds in South Western Sydney with the current Standards. METHODS: Playgrounds in South Western Sydney were randomly audited for compliance with Australian/New Zealand Standards for playground equipment pertaining to fall height, type of undersurface material, adequacy of maintenance of undersurface materials and adequacy of the safe fall zone. Comparisons were made with an audit of playgrounds in New South Wales undertaken in 1995 by Kidsafe. RESULTS: Of the 257 pieces of equipment from 87 playgrounds audited, only three (3.4%) playgrounds and 65 (25.3%) pieces of equipment complied with all criteria assessed. Compared with the 1995 study, there has been a significant improvement in equipment complying with height standards (84.4% vs 72.2%, $P < 0.0001$) and appropriate undersurface material (66.8% vs 45.4%, $P < 0.0001$). Pinebark is now the

most common appropriate undersurface material used but is poorly maintained, particularly in high-traffic areas. Of 131 pieces of equipment with an appropriate undersurface material, only 38 (29%) had an adequate safe fall zone. CONCLUSIONS: Improvements in playground safety have occurred; however, very few playgrounds meet the Australian/New Zealand Standards. Compliance with Standards pertaining to maintenance of undersurface materials and safe fall zone is poor. Mechanisms need to be developed to support councils in the upgrading and maintenance of existing playgrounds
SN - 1034-4810
AD - The Children's Hospital at Westmead, New South Wales, Australia. janem3@chw.edu.au
UR - 16398844
ER -

TY - JOUR
ID - 327
T1 - Teaching safety skills to children to prevent gun play: an evaluation of in situ training
A1 - Miltenberger,R.G.
A1 - Gatheridge,B.J.
A1 - Satterlund,M.
A1 - Egemo-Helm,K.R.
A1 - Johnson,B.M.
A1 - Jostad,C.
A1 - Kelso,P.
A1 - Flessner,C.A.
Y1 - 2005///
N1 - Miltenberger, Raymond G. Gatheridge, Brian J. Satterlund, Melisa. Egemo-Helm, Kristin R. Johnson, Brigitte M. Jostad, Candice. Kelso, Pamela. Flessner, Christopher A
Journal of applied behavior analysis
0174763, hdr
IM
Evaluation Studies. Journal Article
English
KW - Medline
KW - Child
KW - Preschool
KW - Female
KW - Firearms
KW - Humans
KW - Male
KW - Play and Playthings
KW - Safety
KW - Teaching/mt [Methods]
KW - Wounds
KW - Gunshot/pc [Prevention & Control]
RP - NOT IN FILE
SP - 395
EP - 398
JF - Journal of Applied Behavior Analysis
JA - J Appl.Behav Anal.
VL - 38
IS - 3
CY - United States
N2 - This study evaluated behavioral skills training with added in situ training for teaching safety skills to prevent gun play. Following baseline, each child received two sessions of behavioral skills training and one in situ training session. Additional in situ training sessions were conducted until the child exhibited the safety skills (don't touch the gun, get away, and tell an adult). All children acquired and maintained the safety skills at a 3-month follow-up. In addition, of the 7 children assessed in a dyad situation, all exhibited the correct skills in the presence of another child

SN - 0021-8855
AD - Department of Psychology, North Dakota State University, Fargo, North Dakota 58105, USA.
ray.miltenberger@ndsu.edu
UR - 16270848
ER -

TY - JOUR
ID - 328
T1 - Assessing peer entry and play in preschoolers at risk for maladjustment
A1 - Brotman,L.M.
A1 - Gouley,K.K.
A1 - Chesir-Teran,D.
Y1 - 2005/12//
N1 - Brotman, Laurie Miller. Gouley, Kathleen Kiely. Chesir-Teran, Daniel
Journal of clinical child and adolescent psychology : the official journal for the Society of Clinical Child and Adolescent Psychology, American Psychological Association, Division 53
101133858
IM
Journal Article. Validation Studies
English
KW - Medline
KW - Child Behavior Disorders/di [Diagnosis]
KW - Child
KW - Preschool
KW - Female
KW - Humans
KW - Male
KW - Observer Variation
KW - Peer Group
KW - Play and Playthings
KW - Psychiatric Status Rating Scales
KW - Psychometrics
KW - Risk Factors
RP - NOT IN FILE
SP - 671
EP - 680
JF - Journal of Clinical Child & Adolescent Psychology
JA - J Clin.Child Adolesc Psychol.
VL - 34
IS - 4
CY - United States
N2 - This study evaluated the psychometric properties of an observational rating system for assessing preschoolers' peer entry and play skills: Observed Peer Play in Unfamiliar Settings (OPPUS). Participants were 84 preschoolers at risk for psychopathology. Reliability and concurrent validity are reported. The 30-min paradigm yielded reliable indexes of engaging and disruptive behavior. Adequate interrater reliability and stability were obtained with minimally trained observers. OPPUS scores were related in expected ways to independent observations of disruptive and engaging behavior during parent-child interactions. OPPUS scores were associated with parent-rated self-control and internalizing behaviors. OPPUS scores were associated with parent ratings of play skills for children with school experience and assertiveness for children without school experience. The OPPUS is a useful, brief method for assessing peer entry and play behaviors in preschoolers
SN - 1537-4416
AD - NYU Child Study Center, New York University School of Medicine, NY 10016, USA.
laurie.brotman@med.nyu.edu
UR - 16232064
ER -

TY - JOUR
ID - 329
T1 - [MusicPlayTherapy--a parent-child psychotherapy for children 0-4 years old]. [German]
A1 - Stumptner,K.
A1 - Thomsen,C.
Y1 - 2005/10//
N1 - Stumptner, Katrin. Thomsen, Cornelia
Praxis der Kinderpsychologie und Kinderpsychiatrie
pj6, 0404246
IM
English Abstract. Journal Article
German
KW - Medline
KW - Child
KW - Preschool
KW - Family Therapy/mt [Methods]
KW - Female
KW - Humans
KW - Infant
KW - Male
KW - Music Therapy/mt [Methods]
KW - Parent-Child Relations
KW - Personality Development
KW - Play Therapy/mt [Methods]
KW - Psychotherapeutic Processes
RP - NOT IN FILE
SP - 684
EP - 699
JF - Praxis der Kinderpsychologie und Kinderpsychiatrie
JA - Prax.Kinderpsychol.Kinderpsychiatr.
VL - 54
IS - 8
CY - Germany
N2 - The early stage of building up the parent-child relationship is especially important. It is the basis for the child's development of the ability to relate to others and his or her further emotional, social and cognitive development. In this important early phase various risk factors may alienate parents from their intuitive parental competence towards their children. Such interaction problems indicate an intervention in the form of parent-children psychotherapy. This constitutes an entry point for the concept of MusicPlayTherapy (MPT): The early relationship is characterized mainly by complex communication sequences that address the senses at all levels. Therefore, the MPT concept integrates music as medium to communicate and opens up a playing space for play that allows emotions and experiences to be expressed. The components of music such as rhythm, sound, and melody stimulate babies and toddlers to express, play, and communicate preverbally. We work with the child and a parent in the MusicPlayTherapy sessions. Parents learn again to play and thereby learn to reach their children emotionally and to communicate with them. We complement the therapy sessions by counselling sessions with both parents
SN - 0032-7034
AD - Institut fur Musik und Psychotherapie, Berlin. katrin.stumptner@t-online.de
UR - 16231598
ER -

TY - JOUR
ID - 330
T1 - Use of cigarettes and alcohol by preschoolers while role-playing as adults: "Honey, have some smokes"
A1 - Dalton,M.A.
A1 - Bernhardt,A.M.
A1 - Gibson,J.J.

A1 - Sargent,J.D.
A1 - Beach,M.L.
A1 - chi-Mejia,A.M.
A1 - Titus-Ernstoff,L.T.
A1 - Heatherton,T.F.
Y1 - 2005/09//
N1 - Dalton, Madeline A. Bernhardt, Amy M. Gibson, Jennifer J. Sargent, James D. Beach, Michael L. Adachi-Mejia, Anna M. Titus-Ernstoff, Linda T. Heatherton, Todd F
Archives of pediatrics & adolescent medicine
9422751, bwf
AIM, IM
Journal Article. Research Support, Non-U.S. Gov't
English
KW - Medline
KW - Adult
KW - Age Factors
KW - Alcohol Drinking/px [Psychology]
KW - Attitude to Health
KW - Child
KW - Child Behavior/px [Psychology]
KW - Preschool
KW - Female
KW - Humans
KW - Learning
KW - Male
KW - New Hampshire
KW - Parents/px [Psychology]
KW - Play and Playthings
KW - Questionnaires
KW - Risk-Taking
KW - Role Playing
KW - Smoking/px [Psychology]
KW - Social Conformity
KW - Social Environment
KW - Social Perception
RP - NOT IN FILE
SP - 854
EP - 859
JF - Archives of Pediatrics & Adolescent Medicine
JA - Arch.Pediatr Adolesc Med
VL - 159
IS - 9
CY - United States
N2 - OBJECTIVE: To examine preschoolers' attitudes, expectations, and perceptions of tobacco and alcohol use. DESIGN: Structured observational study. Children used props and dolls to act out a social evening for adults. As part of the role play, each child selected items from a miniature grocery store stocked with 73 different products, including beer, wine, and cigarettes, for an evening with friends. SETTING: A behavioral laboratory at the Department of Psychological and Brain Sciences, Dartmouth College. PATIENTS: One hundred twenty children, 2 to 6 years old, participated individually in the role-playing. MAIN OUTCOME MEASURE: Whether or not a child purchased cigarettes or alcohol at the store. RESULTS: Children purchased a mean of 17 of the 73 products in the store. Thirty-four children (28.3%) bought cigarettes and 74 (61.7%) bought alcohol. Children were more likely to buy cigarettes if their parents smoked (adjusted odds ratio [OR], 3.90; 95% confidence interval [CI], 1.20-12.63). Children were more likely to buy beer or wine if their parents drank alcohol at least monthly (adjusted OR, 3.04; 95% CI, 1.02-9.10) or if they viewed PG-13- or R-rated movies (adjusted OR, 5.10; 95% CI, 1.14-22.90). Children's play behavior suggests that they are highly attentive to the use and enjoyment of alcohol and tobacco and have well-established expectations about how cigarettes

and alcohol fit into social settings. CONCLUSIONS: The data suggest that observation of adult behavior, especially parental behavior, may influence preschool children to view smoking and drinking as appropriate or normative in social situations. These perceptions may relate to behaviors adopted later in life
SN - 1072-4710
AD - Department of Pediatrics, Dartmouth Medical School, Norris Cotton Cancer Center, Lebanon, NH 03756, USA. Madeline.Dalton@Dartmouth.edu
UR - 16143745
ER -

TY - JOUR
ID - 332
T1 - The effect of safer play equipment on playground injury rates among school children
A1 - Howard,A.W.
A1 - Macarthur,C.
A1 - Willan,A.
A1 - Rothman,L.
A1 - Moses-McKeag,A.
A1 - Macpherson,A.K.
Y1 - 2005/05/24/
N1 - Howard, Andrew W. MacArthur, Colin. Willan, Andrew. Rothman, Linda. Moses-McKeag, Alexandra. MacPherson, Alison K
CMAJ : Canadian Medical Association journal = journal de l'Association medicale canadienne
9711805
AIM, IM
Evaluation Studies. Journal Article
English
KW - Medline
KW - Accident Prevention
KW - Child
KW - Equipment Design
KW - Guideline Adherence
KW - Humans
KW - Ontario
KW - Play and Playthings
KW - Risk Assessment
KW - Safety
KW - Schools
KW - Wounds and Injuries/pc [Prevention & Control]
RP - NOT IN FILE
SP - 1443
EP - 1446
JF - CMAJ Canadian Medical Association Journal
JA - CMAJ
VL - 172
IS - 11
CY - Canada
N2 - BACKGROUND: Changes to Canadian Standards Association (CSA) standards for playground equipment prompted the removal of hazardous equipment from 136 elementary schools in Toronto. We conducted a study to determine whether applying these new standards and replacing unsafe playground equipment with safe equipment reduced the number of school playground injuries. METHODS: A total of 86 of the 136 schools with hazardous play equipment had the equipment removed and replaced with safer equipment within the study period (intervention schools). Playground injury rates before and after equipment replacement were compared in intervention schools. A database of incident reports from the Ontario School Board Insurance Exchange was used to identify injury events. There were 225 schools whose equipment did not require replacement (nonintervention schools); these schools served as a natural control group for background injury rates during the study period. Injury rates per 1000 students per month, relative risks (RRs) and 95% confidence

intervals (CIs) were calculated, adjusting for clustering within schools. RESULTS: The rate of injury in intervention schools decreased from 2.61 (95% CI 1.93-3.29) per 1000 students per month before unsafe equipment was removed to 1.68 (95% CI 1.31-2.05) after it was replaced (RR 0.70, 95% CI 0.62-0.78). This translated into 550 injuries avoided in the post-intervention period. In nonintervention schools, the rate of injury increased from 1.44 (95% CI 1.07-1.81) to 1.81 (95% CI 1.07-2.53) during the study period (RR 1.40, 95% CI 1.29-1.52). INTERPRETATION: The CSA standards were an effective tool in identifying hazardous playground equipment. Removing and replacing unsafe equipment is an effective strategy for preventing playground injuries
SN - 1488-2329
AD - Department of Population Health Sciences, The Hospital for Sick Children, Toronto, Ont
UR - 15911858
ER -

TY - JOUR
ID - 333
T1 - Danger in the toy box
A1 - Stephenson,M.
Y1 - 2005/05//
N1 - Stephenson, Miranda
Journal of pediatric health care : official publication of National Association of Pediatric Nurse Associates & Practitioners
jml, 8709735
N
Journal Article
English
KW - Medline
KW - Accident Prevention
KW - Accidents
KW - Home/pc [Prevention & Control]
KW - Home/sn [Statistics & Numerical Data]
KW - Child
KW - Child Welfare
KW - Choice Behavior
KW - Consumer Product Safety
KW - Health Education/mt [Methods]
KW - Humans
KW - Nurse Practitioners
KW - Nurse's Role
KW - Parents/ed [Education]
KW - Parents/px [Psychology]
KW - Pediatric Nursing/mt [Methods]
KW - Play and Playthings/in [Injuries]
KW - Safety Management/mt [Methods]
KW - United States/ep [Epidemiology]
RP - NOT IN FILE
SP - 187
EP - 189
JF - Journal of Pediatric Health Care
JA - J Pediatr Health Care
VL - 19
IS - 3
CY - United States
SN - 0891-5245
AD - Emergency Department, Children's Medical Center Dallas, Dallas, Texas, USA
UR - 15867837
ER -

TY - JOUR
ID - 334
T1 - Child safety. Mary Ucci: getting the playground ready for spring
Y1 - 2005/04//
N1 - Child health alert
9881972
K
Journal Article
English
KW - Medline
KW - Child
KW - Humans
KW - Play and Playthings
KW - Safety Management
KW - Seasons
RP - NOT IN FILE
SP - 5
EP - 6
JF - Child Health Alert
VL - 23
CY - United States
SN - 1064-4849
UR - 15822212
ER -

TY - JOUR
ID - 335
T1 - The in situ performance of playground surfacing: implications for maintenance and injury prevention
A1 - Sherker,S.
A1 - Short,A.
A1 - Ozanne-Smith,J.
Y1 - 2005/03//
N1 - Sherker, Shauna. Short, Andrew. Ozanne-Smith, Joan
International journal of injury control and safety promotion
101247254
IM
Journal Article
English
KW - Medline
KW - Australia
KW - Child
KW - Craniocerebral Trauma/pc [Prevention & Control]
KW - Deceleration
KW - Humans
KW - Maintenance
KW - Materials Testing
KW - Play and Playthings
KW - Safety/st [Standards]
KW - Surface Properties
KW - Time Factors
KW - Wood
RP - NOT IN FILE
SP - 63
EP - 66
JF - International Journal of Injury Control & Safety Promotion

JA - Int.J Inj.Contr.Saf Promot
VL - 12
IS - 1
CY - Netherlands
SN - 1745-7300
AD - Accident Research Centre, Monash University, Victoria, Australia. Shauna.Sherker@UNSW.edu.au
UR - 15814379
ER -

TY - JOUR
ID - 336
T1 - Keeping all children healthy: challenges to leading an active lifestyle for preschool children qualifying for at-risk programs
A1 - Goodway,J.D.
A1 - Smith,D.W.
Y1 - 2005/04//
N1 - Goodway, Jacqueline D. Smith, Dennis W
Family & community health
ese, 7809641
IM
Journal Article
English
KW - Medline
KW - African Americans
KW - Child Welfare
KW - Child
KW - Preschool
KW - Environment
KW - Female
KW - Humans
KW - Life Style
KW - Male
KW - Motor Activity
KW - Play and Playthings
KW - Poverty
KW - Residence Characteristics
KW - Urban Population
RP - NOT IN FILE
SP - 142
EP - 155
JF - Family & Community Health
JA - Fam.Community Health
VL - 28
IS - 2
CY - United States
N2 - This study examined contextual factors associated with physical activity of urban African American preschoolers (N = 59). Qualitative research methodologies utilized data from home visits, caregiver and child interviews, field notes, and document collection. The primary finding suggested, "In underserved communities fundamental barriers exist that obstruct young children's ability to be physically active." Four themes were developed revealing that outside environments limited opportunities for physical activity, home environments resulted in sedentary behaviors, and communities had limited physical activity role models. Despite this, young children enjoyed being physically active
SN - 0160-6379
AD - School of Physical Activity and Educational Services, The Ohio State University, 303 Pomerene Hall, 1760 Neil Avenue, Columbus, OH 43026, USA. goodway-shiebler.1@osu.edu
UR - 15778628

ER -

TY - JOUR
ID - 337
T1 - Are current playground safety standards adequate for preventing arm fractures?
A1 - Goulding,A.
A1 - Grant,A.M.
A1 - Davidson,P.L.
Y1 - 2005/01/03/
N1 - Goulding, Ailsa. Grant, Andrea M. Davidson, Peter L
The Medical journal of Australia
0400714, m26
IM
Comment. Letter
English
KW - Medline
KW - Arm Injuries/ep [Epidemiology]
KW - Arm Injuries/pc [Prevention & Control]
KW - Australia/ep [Epidemiology]
KW - Body Mass Index
KW - Child
KW - Female
KW - Fractures
KW - Bone/ep [Epidemiology]
KW - Bone/pc [Prevention & Control]
KW - Guidelines as Topic
KW - Humans
KW - Male
KW - Obesity/ep [Epidemiology]
KW - Play and Playthings/in [Injuries]
KW - Risk Factors
KW - Safety/st [Standards]
RP - NOT IN FILE
SP - 46
EP - 47
JF - Medical Journal of Australia
JA - Med J Aust
VL - 182
IS - 1
CY - Australia
SN - 0025-729X
UR - 15651954
ER -

TY - JOUR
ID - 338
T1 - Randomized controlled trial of a family problem-solving intervention
A1 - Drummond,J.
A1 - Fleming,D.
A1 - McDonald,L.
A1 - Kysela,G.M.
Y1 - 2005/02//
N1 - Drummond, Jane. Fleming, Darcy. McDonald, Linda. Kysela, Gerard M
Clinical nursing research
9208508, bnz
N

Clinical Trial. Journal Article. Randomized Controlled Trial
English
KW - Medline
KW - Adaptation
KW - Psychological
KW - Adult
KW - Attitude to Health
KW - Child
KW - Preschool
KW - Conflict (Psychology)
KW - Cooperative Behavior
KW - Cross-Over Studies
KW - Cues
KW - Developmental Disabilities/pc [Prevention & Control]
KW - Developmental Disabilities/px [Psychology]
KW - Developmental Disabilities
KW - Family/px [Psychology]
KW - Family Therapy/og [Organization & Administration]
KW - Female
KW - Health Knowledge
KW - Attitudes
KW - Practice
KW - Humans
KW - Male
KW - Middle Aged
KW - Parent-Child Relations
KW - Play and Playthings/px [Psychology]
KW - Problem Solving
KW - Program Evaluation
KW - Self Efficacy
KW - Social Support
KW - Stress
KW - Psychological/et [Etiology]
KW - Psychological/pc [Prevention & Control]
KW - Psychological/px [Psychology]
KW - Videotape Recording
RP - NOT IN FILE
SP - 57
EP - 80
JF - Clinical Nursing Research
JA - Clin.Nurs Res.
VL - 14
IS - 1
CY - United States
N2 - Adaptive problem solving contributes to individual and family health and development. In this article, the effect of the cooperative family learning approach (CFLA) on group family problem solving and on cooperative parenting communication is described. A pretest or posttest experimental design was used. Participant families were recruited from Head Start programs and exhibited two or more risk factors. Participant preschool children were screened to have two or more developmental delays. Direct behavioral observation measures were used to determine group family problem solving and cooperative parenting communication outcomes. Few group family problem-solving behaviors were coded, and they displayed little variability. However, intervention parents increased the length of time they played and extended the cooperative parent-child interactions. The evidence shows that CFLA has the potential to enhance parental-modeling of cooperative behavior while engaged in play activities with preschoolers. Direct measurement of group family problem solving was difficult. Solutions are suggested
SN - 1054-7738

AD - University of Alberta, Edmonton, Canada
UR - 15604228
ER -

TY - JOUR
ID - 339
T1 - [Epidemiology of eye injuries in Abidjanian children]. [French]
A1 - Mensah,A.
A1 - Fany,A.
A1 - Adjorlolo,C.
A1 - Toure,M.L.
A1 - Kasieu,Gbe M.
A1 - Mihluedo,K.A.
A1 - Diallo,A.J.
A1 - Coulibaly,F.
A1 - Berete,R.
Y1 - 2004/10//
N1 - Mensah, Adama. Fany, Adama. Adjorlolo, Christiane. Toure, Marie-Louise. Kasieu Gbe, Maxime. Mihluedo, K A. Diallo, A J W. Coulibaly, F. Berete, R
Sante (Montrouge, France)
b1i, 9212437
IM
Comparative Study. English Abstract. Journal Article
French
KW - Medline
KW - Adolescent
KW - Age Factors
KW - Blindness/et [Etiology]
KW - Child
KW - Preschool
KW - Contusions/co [Complications]
KW - Cote d'Ivoire/ep [Epidemiology]
KW - Eye Burns/co [Complications]
KW - Eye Burns/ep [Epidemiology]
KW - Eye Burns/th [Therapy]
KW - Eye Foreign Bodies/co [Complications]
KW - Eye Foreign Bodies/ep [Epidemiology]
KW - Eye Foreign Bodies/th [Therapy]
KW - Eye Injuries/co [Complications]
KW - Eye Injuries/ep [Epidemiology]
KW - Eye Injuries/et [Etiology]
KW - Eye Injuries/th [Therapy]
KW - Eye Injuries
KW - Penetrating/co [Complications]
KW - Penetrating/ep [Epidemiology]
KW - Penetrating/th [Therapy]
KW - Female
KW - Hospitalization
KW - Humans
KW - Infant
KW - Newborn
KW - Male
KW - Primary Health Care
KW - Prospective Studies
KW - Retinal Detachment/et [Etiology]
KW - Sex Factors

KW - Visual Acuity
RP - NOT IN FILE
SP - 239
EP - 243
JF - Sante
VL - 14
IS - 4
CY - France
N2 - Eye injuries, most of them preventable, are particularly severe in Africa, because of the risks of infection and delay in treatment. We report a 16-month (March 1997-June 1998) prospective observational survey of eye injuries in children up to the age of 15 years at Treichville-Abidjan University Hospital. During this period, 62 children were treated for these injuries. Ocular traumas represented 4% (n = 245) of new admissions in ophthalmology, and 29% of these injuries occurred in children. The sex ratio was 5:2 boys to girls, and their mean age was 8.66 +/- 3.56 years. Eye injuries occurred most often during play (84%, n = 52). More than 85% (n = 53) of children were alone or without adult supervision at the time of the injury. The causal agent was most often wood (35%) followed by metal (29%). The mean time from injury to hospital admission was 1.8 +/- 0.77 day. Only 19% of patients were admitted the day of injury. Most patients (66%) first sought treatment in a primary care centre. More than 70% (n = 44) of injuries necessitated hospitalisation. Open eyeball wounds were the most common injury (53%) and were associated with endophthalmitis in 16%. The mean recovery of visual acuity was 0.1. At admission, 40 of the injured eyes (64.5%) had monocular blindness; six recovered, for a final blindness rate of 55%. Injuries associated with wooden objects had a final blindness rate of 77% and play-related injuries 63%. The primary posttraumatic sequelae were corneal scars. Eyeball phthysis (14.5%) was secondary to 8 open wounds of the eyeball and one postcontusion retinal detachment. Eye injuries remain a major cause of monocular blindness in children in Cote d'Ivoire. Most of them can be prevented by relatively simple measures including supervision of children and rapid hospitalisation when injury occurs
SN - 1157-5999
AD - Centre hospitalier universitaire de Treichville, 01 BP V3 Abidjan 01 Cote d'Ivoire
UR - 15745874
ER -

TY - JOUR
ID - 340
T1 - Rearing behavior and rearing stress of fathers with children of preschool and school age
A1 - Lee,J.H.
A1 - Kim,H.Y.
A1 - Park,Y.A.
Y1 - 2004/12//
N1 - Lee, Ja-Hyung. Kim, Hye-Young. Park, Young-Ae
Taehan Kanho Hakhoe chi
101191388
IM, N
Comparative Study. Journal Article
English
KW - Medline
KW - Adolescent
KW - Adult
KW - Age Factors
KW - Analysis of Variance
KW - Child
KW - Child Rearing/eh [Ethnology]
KW - Child Rearing/px [Psychology]
KW - Preschool
KW - Father-Child Relations/eh [Ethnology]
KW - Fathers/ed [Education]
KW - Fathers/px [Psychology]
KW - Gender Identity

KW - Health Services Needs and Demand
KW - Health Surveys
KW - Humans
KW - Korea/ep [Epidemiology]
KW - Middle Aged
KW - Nursing Methodology Research
KW - Paternal Behavior/eh [Ethnology]
KW - Play and Playthings
KW - Questionnaires
KW - Risk Factors
KW - Severity of Illness Index
KW - Social Support
KW - Stress
KW - Psychological/eh [Ethnology]
KW - Psychological/pc [Prevention & Control]
KW - Psychological/px [Psychology]
RP - NOT IN FILE
SP - 1491
EP - 1498
JF - Daehan Ganho Haghoeji
JA - Taehan Kanho.Hakhoe.Chi
VL - 34
IS - 8
CY - Korea (South)
N2 - PURPOSE: This study was conducted to compare the paternal rearing behavior and rearing stress level between fathers with a preschooler and fathers with school children so that it can be utilized as a basic source for developing parental rearing education programs. METHODS: A descriptive comparative method was conducted to identify the paternal rearing behavior and paternal rearing stress. Respondents were 361 fathers who had either preschoolers (n=189) or children of elementary age (n=172). RESULTS: Comparing the two group's means, the rearing activity score and rearing stress there were significant differences. In the school children's group's father, 'outdoor activity' and 'guidance on discipline activity' were significantly higher than the other group. In the preschool children's fathers group, 'play interaction activity' was statistically significant higher than the other, and the child-part mean score of paternal rearing stress was significantly higher than the other group. The correlation between paternal rearing behavior and paternal rearing stress, indicates that more paternal rearing behavior means less paternal rearing stress. CONCLUSION: These results of this study will help design more effective rearing programs for fathers that have either preschool children or school children by providing the basic data for paternal rearing behaviors and paternal rearing stress
SN - 1598-2874
AD - College of Nursing Science, Ewha Womans University, Seoul 120-750, Korea. jahlee@ewha.ac.kr
UR - 15687792
ER -

TY - JOUR
ID - 341
T1 - Which factors may play a pivotal role on determining the type of psychiatric disorder in children and adolescents with epilepsy?
A1 - Thome-Souza,S.
A1 - Kuczynski,E.
A1 - Assumpcao,F.,Jr.
A1 - Rzezak,P.
A1 - Fuentes,D.
A1 - Fiore,L.
A1 - Valente,K.D.
Y1 - 2004/12//
N1 - Thome-Souza, Sigride. Kuczynski, Evelyn. Assumpcao, Francisco Jr. Rzezak, Patricia. Fuentes, Daniel. Fiore, Lia. Valente, Kette D

Epilepsy & behavior : E&B
100892858
IM
Comparative Study. Journal Article
English
KW - Medline
KW - Adolescent
KW - Age Factors
KW - Chi-Square Distribution
KW - Child
KW - Preschool
KW - Demography
KW - Diagnosis
KW - Differential
KW - Electroencephalography/mt [Methods]
KW - Epilepsy/cl [Classification]
KW - Epilepsy/co [Complications]
KW - Epilepsy/di [Diagnosis]
KW - Family
KW - Female
KW - Humans
KW - Magnetic Resonance Imaging/mt [Methods]
KW - Male
KW - Mental Disorders/cl [Classification]
KW - Mental Disorders/di [Diagnosis]
KW - Mental Disorders/ep [Epidemiology]
KW - Mental Disorders/et [Etiology]
KW - Neuropsychological Tests
KW - Psychiatric Status Rating Scales
KW - Risk Factors
KW - Sex Factors
KW - Statistics
KW - Nonparametric
RP - NOT IN FILE
SP - 988
EP - 994
JF - Epilepsy & Behavior
JA - Epilepsy Behav
VL - 5
IS - 6
CY - United States
N2 - Physicians have become aware of the high prevalence of psychiatric disorders (PDs) in children and adolescents with epilepsy; however, there are many controversies as to which factors may have an important role in the different types of PD. This study was designed to assess the main PD; verify the age of onset compared with the age of diagnosis of the PD; and determine which factors may be correlated with the type of PD described. For this purpose, a multidisciplinary team evaluated children and adolescents (4-18 years) with epilepsy and analyzed patient-related factors such as age (grouped according to Piaget's cognitive scale: <6 years, 7-13 years, >13 years), sex, family history of PDs, and cognitive status. With respect to epilepsy features, we considered age of onset, duration, seizure control at the time of psychiatric evaluation, refractoriness, antiepileptic drugs (mono- vs polytherapy), seizure type (generalized vs focal), and epilepsy type (idiopathic vs symptomatic/probably symptomatic). Depression occurred in 36.4% and attention-deficit hyperactivity disorder (ADHD) in 29.1%, these being the most frequent PDs in this series. Focal epilepsy was significantly more frequent in children and adolescents with PDs. As to the type of PD, age was an important factor, with a predominance of ADHD in children and depression in adolescents (P<0.0001). Family history was contributory for depression, but not for others PDs (P<0.0001). Depression remained underdiagnosed and untreated for a longer period. Impact of early diagnosis and treatment remains unknown

SN - 1525-5050
AD - Laboratory of Clinical Neurophysiology, Institute and Department of Psychiatry, Clinic's Hospital University of Sao Paulo Medical School, Sao Paulo, Brazil
UR - 15582849
ER -

TY - JOUR
ID - 342
T1 - Hazards in our environment: the continuing problem of lead in toys...and pressure-treated playground equipment may not pose a risk
Y1 - 2004/11//
N1 - Child health alert
9881972
K
Journal Article
English
KW - Medline
KW - Arsenic/an [Analysis]
KW - Child
KW - Environmental Exposure/an [Analysis]
KW - Humans
KW - Lead Poisoning/et [Etiology]
KW - Play and Playthings
RP - NOT IN FILE
SP - 4
EP - 5
JF - Child Health Alert
VL - 22
CY - United States
SN - 1064-4849
UR - 15612096
ER -

TY - JOUR
ID - 343
T1 - Relation between increased numbers of safe playing areas and decreased vehicle related child mortality rates in Japan from 1970 to 1985: a trend analysis
A1 - Nakahara,S.
A1 - Nakamura,Y.
A1 - Ichikawa,M.
A1 - Wakai,S.
Y1 - 2004/12//
N1 - Nakahara, Shinji. Nakamura, Yasuhide. Ichikawa, Masao. Wakai, Susumu
Journal of epidemiology and community health
i1p, 7909766
IM
Journal Article
English
KW - Medline
KW - Accidents
KW - Traffic/mo [Mortality]
KW - Traffic/pc [Prevention & Control]
KW - Adolescent
KW - Child
KW - Child Mortality/td [Trends]
KW - Preschool

KW - Environment Design
KW - Humans
KW - Infant
KW - Newborn
KW - Japan/ep [Epidemiology]
KW - Play and Playthings
KW - Regression Analysis
KW - Safety/sn [Statistics & Numerical Data]
RP - NOT IN FILE
SP - 976
EP - 981
JF - Journal of Epidemiology & Community Health
JA - J Epidemiol.Community Health
VL - 58
IS - 12
CY - England
N2 - OBJECTIVES: To examine vehicle related mortality trends of children in Japan; and to investigate how environmental modifications such as the installation of public parks and pavements are associated with these trends. DESIGN: Poisson regression was used for trend analysis, and multiple regression modelling was used to investigate the associations between trends in environmental modifications and trends in motor vehicle related child mortality rates. SETTING: Mortality data of Japan from 1970 to 1994, defined as E-code 810-23 from 1970 to 1978 and E810-25 from 1979 to 1994, were obtained from vital statistics. Multiple regression modelling was confined to the 1970-1985 data. Data concerning public parks and other facilities were obtained from the Ministry of Land, Infrastructure, and Transport. SUBJECTS: Children aged 0-14 years old were examined in this study and divided into two groups: 0-4 and 5-14 years. MAIN RESULTS: An increased number of public parks was associated with decreased vehicle related mortality rates among children aged 0-4 years, but not among children aged 5-14. In contrast, there was no association between trends in pavements and mortality rates. CONCLUSIONS: An increased number of public parks might reduce vehicle related preschooler deaths, in particular those involving pedestrians. Safe play areas in residential areas might reduce the risk of vehicle related child death by lessening the journey both to and from such areas as well as reducing the number of children playing on the street. However, such measures might not be effective in reducing the vehicle related mortalities of school age children who have an expanded range of activities and walk longer distances
SN - 0143-005X
AD - Department of International Community Health, Graduate School of Medicine, The University of Tokyo, 7-3-1 Hongo, Bunkyo-ku, Tokyo 113-0033, Japan. shinji@m.u-tokyo.ac.jp
UR - 15547055
ER -

TY - JOUR
ID - 344
T1 - Utilization of juvenile animal studies to determine the human effects and risks of environmental toxicants during postnatal developmental stages. [Review] [130 refs]
A1 - Brent,R.L.
Y1 - 2004/10//
N1 - Brent, Robert L
Birth defects research. Part B, Developmental and reproductive toxicology
101155115
IM
Journal Article. Review
English
KW - Medline
KW - Adolescent
KW - Adult
KW - Animals
KW - Newborn
KW - Child

KW - Child Development/de [Drug Effects]
KW - Child Development/re [Radiation Effects]
KW - Preschool
KW - Environmental Exposure
KW - Humans
KW - Infant
KW - Intellectual Disability/et [Etiology]
KW - Models
KW - Animal
KW - Neoplasms/et [Etiology]
KW - Radiation
KW - Ionizing
KW - Risk Assessment
KW - Teratogens/pd [Pharmacology]
KW - Teratogens/to [Toxicity]
RP - NOT IN FILE
SP - 303
EP - 320
JF - Birth Defects Research
JA - Birth Defects Res.Part B Dev.Reprod.Toxicol.
VL - Part B, Developmental and Reproductive Toxicology. 71
IS - 5
CY - United States
N2 - BACKGROUND: Toxicology studies utilizing animals and in vitro cellular or tissue preparations have been used to study the toxic effects and mechanism of action of drugs and chemicals and to determine the effective and safe dose of drugs in humans and the risk of toxicity from chemical exposures. Testing in animals could be improved if animal dosing using the mg/kg basis was abandoned and drugs and chemicals were administered to compare the effects of pharmacokinetically and toxicokinetically equivalent serum levels in the animal model and human. Because alert physicians or epidemiology studies, not animal studies, have discovered most human teratogens and toxicities in children, animal studies play a minor role in discovering teratogens and agents that are deleterious to infants and children. In vitro studies play even a less important role, although they are helpful in describing the cellular or tissue effects of the drugs or chemicals and their mechanism of action. One cannot determine the magnitude of human risks from in vitro studies when they are the only source of toxicology data. METHODS: Toxicology studies on adult animals is carried out by pharmaceutical companies, chemical companies, the Food and Drug Administration (FDA), many laboratories at the National Institutes of Health, and scientific investigators in laboratories throughout the world. Although there is a vast amount of animal toxicology studies carried out on pregnant animals and adult animals, there is a paucity of animal studies utilizing newborn, infant, and juvenile animals. This deficiency is compounded by the fact that there are very few toxicology studies carried out in children. That is one reason why pregnant women and children are referred to as "therapeutic orphans." RESULTS: When animal studies are carried out with newborn and developing animals, the results demonstrate that generalizations are less applicable and less predictable than the toxicology studies in pregnant animals. Although many studies show that infants and developing animals may have difficulty in metabolizing drugs and are more vulnerable to the toxic effects of environmental chemicals, there are exceptions that indicate that infants and developing animals may be less vulnerable and more resilient to some drugs and chemicals. In other words, the generalization indicating that developing animals are always more sensitive to environmental toxicants is not valid. For animal toxicology studies to be useful, animal studies have to utilize modern concepts of pharmacokinetics and toxicokinetics, as well as "mechanism of action" (MOA) studies to determine whether animal data can be utilized for determining human risk. One example is the inability to determine carcinogenic risks in humans for some drugs and chemicals that produce tumors in rodents, When the oncogenesis is the result of peroxisome proliferation, a reaction that is of diminished importance in humans. CONCLUSIONS: Scientists can utilize animal studies to study the toxicokinetic and toxicodynamic aspects of drugs and environmental toxicants. But they have to be carried out with the most modern techniques and interpreted with the highest level of scholarship and objectivity. Threshold exposures, no-adverse-effect level (NOAEL) exposures, and toxic effects can be determined in animals, but have to be interpreted with caution when applying them to the human. Adult problems in growth, endocrine dysfunction, neurobehavioral abnormalities, and oncogenesis may be related to exposures to drugs, chemicals, and physical

agents during development and may be fruitful areas for investigation. Maximum permissible exposures have to be based on data, not on generalizations that are applied to all drugs and chemicals. Epidemiology studies are still the best methodology for determining the human risk and the effects of environmental toxicants. Carrying out these focused studies in developing humans will be difficult. Animal studies may be our only alternative for answering many questions with regard to specific postnatal developmental vulnerabilities. 2004 Wiley-Liss, Inc. [References: 130]
SN - 1542-9733
AD - Thomas Jefferson University, Alfred I. duPont Hospital for Children, Laboratory of Clinical and Environmental Teratology, Wilmington, Delaware 19899, USA. rbrent@nemours.org
UR - 15505806
ER -

TY - JOUR
ID - 345
T1 - Testing the prenatal hormone hypothesis of tic-related disorders: gender identity and gender role behavior
A1 - Alexander,G.M.
A1 - Peterson,B.S.
Y1 - 2004///
N1 - Alexander, Gerianne M. Peterson, Bradley S
Development and psychopathology
cn7, 8910645
IM
Comparative Study. Journal Article. Research Support, Non-U.S. Gov't. Research Support, U.S. Gov't, P.H.S.
English
KW - Medline
KW - Adolescent
KW - Adult
KW - Androgens/ph [Physiology]
KW - Brain/pp [Physiopathology]
KW - Child
KW - Female
KW - Gender Identity
KW - Humans
KW - Male
KW - Neuropsychological Tests
KW - Obsessive-Compulsive Disorder/di [Diagnosis]
KW - Obsessive-Compulsive Disorder/pp [Physiopathology]
KW - Personality Assessment
KW - Pregnancy
KW - Prenatal Exposure Delayed Effects
KW - Risk Factors
KW - Sex Differentiation/ph [Physiology]
KW - Social Behavior
KW - Tourette Syndrome/di [Diagnosis]
KW - Tourette Syndrome/pp [Physiopathology]
RP - NOT IN FILE
SP - 407
EP - 420
JF - Development & Psychopathology
JA - Dev.Psychopathol.
VL - 16
IS - 2
CY - United States
N2 - The hypothesis that prenatal masculinization of the brain increases risk of tic disorders in postnatal life was tested by measuring gender and gender role behavior in 89 children and adults with a clinical diagnosis of Tourette syndrome or obsessive compulsive disorder and 67 healthy, unaffected children and adults. Consistent

with this hypothesis, a tic disorder in females was associated with more gender dysphoria, increased masculine play preferences, and a more typically "masculine" pattern of performance on two sex-typed spatial tasks. Males with tic disorders reported increased masculine play preferences, and the strength of these preferences was positively associated with the severity of tic symptoms. In addition, unlike their female counterparts, males with tic disorders showed a relative impairment in mental rotation ability. These behavioral profiles are consistent with those of children who have verifiable elevations in prenatal androgen levels. These findings therefore support the hypothesis that an altered androgen-dependent process of sexual differentiation during prenatal life may contribute to the development of tic-related disorders
SN - 0954-5794
AD - Texas A&M University, Department of Psychology, College Station, 77843, USA. gma@psyc.tamu.edu
UR - 15487603
ER -

TY - JOUR
ID - 346
T1 - Infant joint attention skill and preschool behavioral outcomes in at-risk children
A1 - Sheinkopf,S.J.
A1 - Mundy,P.
A1 - Claussen,A.H.
A1 - Willoughby,J.
Y1 - 2004///
N1 - Sheinkopf, Stephen J. Mundy, Peter. Claussen, Angelika H. Willoughby, Jennifer
Development and psychopathology
cn7, 8910645
IM
Journal Article. Research Support, Non-U.S. Gov't. Research Support, U.S. Gov't, P.H.S.
English
KW - Medline
KW - Attention/de [Drug Effects]
KW - Attention Deficit and Disruptive Behavior Disorders/ci [Chemically Induced]
KW - Attention Deficit and Disruptive Behavior Disorders/di [Diagnosis]
KW - Attention Deficit and Disruptive Behavior Disorders/px [Psychology]
KW - Child Behavior Disorders/ci [Chemically Induced]
KW - Child Behavior Disorders/di [Diagnosis]
KW - Child Behavior Disorders/px [Psychology]
KW - Child
KW - Preschool
KW - Cocaine/to [Toxicity]
KW - Female
KW - Humans
KW - Infant
KW - Newborn
KW - Intelligence/de [Drug Effects]
KW - Language Development Disorders/ci [Chemically Induced]
KW - Language Development Disorders/di [Diagnosis]
KW - Language Development Disorders/px [Psychology]
KW - Male
KW - Mother-Child Relations
KW - Nonverbal Communication
KW - Peer Group
KW - Personality Assessment
KW - Play and Playthings/px [Psychology]
KW - Pregnancy
KW - Prenatal Exposure Delayed Effects
KW - Risk Factors
KW - Social Behavior

KW - Social Isolation
RP - NOT IN FILE
SP - 273
EP - 291
JF - Development & Psychopathology
JA - Dev.Psychopathol.
VL - 16
IS - 2
CY - United States
N2 - This study examined whether infant joint attention (JA) skills predicted social behaviors in a sample of at-risk preschool children (n = 30) with a history of prenatal exposure to cocaine. JA behaviors were assessed with the Early Social and Communication Scales at 12, 15, and 18 months of age. Three classes of JA were measured: Initiating JA (IJA), Responding to JA (RJA), and Requests. Behavioral outcomes were measured at 36 months and included ratings of disruptive and withdrawn behaviors and social competence. JA behaviors were related to behavioral outcomes after controlling for language and cognitive ability. The functionally distinct uses of JA were differentially related to behavioral outcome. IJA negatively predicted disruptive behaviors, whereas Requests positively predicted disruptive behaviors. Infant RJA negatively predicted withdrawn behaviors and positively predicted social competence. These results are interpreted in the context of competing theories that attempt to explain variability in the expression of JA skills in the second year of life
SN - 0954-5794
AD - E P Bradley Hospital, Brown Medical School, East Providence, RI 02915, USA. Stephen_Sheinkopf@brown.edu
UR - 15487596
ER -

TY - JOUR
ID - 347
T1 - Comparison of two programs to teach firearm injury prevention skills to 6- and 7-year-old children
A1 - Gatheridge,B.J.
A1 - Miltenberger,R.G.
A1 - Huneke,D.F.
A1 - Satterlund,M.J.
A1 - Mattern,A.R.
A1 - Johnson,B.M.
A1 - Flessner,C.A.
Y1 - 2004/09//
N1 - Gatheridge, Brian J. Miltenberger, Raymond G. Huneke, Daniel F. Satterlund, Melisa J. Mattern, Amanda R. Johnson, Brigette M. Flessner, Christopher A
Pediatrics
oxv, 0376422
AIM, IM
Clinical Trial. Comparative Study. Journal Article. Randomized Controlled Trial
English
KW - Medline
KW - Child
KW - Child Behavior
KW - Female
KW - Firearms
KW - Humans
KW - Male
KW - Play and Playthings
KW - Role Playing
KW - Safety
KW - Teaching/mt [Methods]
KW - Wounds
KW - Gunshot/pc [Prevention & Control]

RP - NOT IN FILE
SP - e294
EP - e299
JA - Pediatrics
VL - 114
IS - 3
CY - United States
N2 - BACKGROUND: Each year, hundreds of children unintentionally kill or injure other children while playing with firearms in the United States. Although the numbers of these deaths and injuries are distressing, few prevention programs have been developed to prevent gun play among children. OBJECTIVE: This study compared the efficacy of 2 programs designed to prevent gun play among young children. DESIGN: A posttest-only, control group design with 2 treatment groups was used. Children were randomly assigned to 1 of 2 treatment groups or a control group. For all children who did not exhibit the skills after training, 1 in situ (ie, real-life situation) training session was conducted. SETTING: Participant recruitment, training sessions, and assessments were all conducted in the children's after-school program facility. PARTICIPANTS: Forty-five children, 6 or 7 years of age, were recruited for participation. INTERVENTIONS: The efficacy of the Eddie Eagle GunSafe Program, developed by the National Rifle Association, and a behavioral skills training program that emphasized the use of instruction, modeling, rehearsal, and feedback was evaluated. MAIN OUTCOME MEASURES: The criterion firearm safety behaviors included both motor and verbal responses, which were assessed in a naturalistic setting and then assigned a numerical value based on a scale of 0 to 3. RESULTS: Both programs were effective for teaching children to verbalize the safety skills message (don't touch the gun, get away, and tell an adult). However, children who received behavioral skills training were significantly more likely to demonstrate the desired safety skills in role-playing assessments and in situ assessments than were children who received Eddie Eagle program training. In addition, in situ training was found to be effective for teaching the desired safety skills for both groups of children. CONCLUSIONS: Injury prevention programs using education-based learning materials are less effective for teaching children the desired safety skills, compared with programs incorporating active learning approaches (eg, modeling, rehearsal, and feedback). The efficacy of both types of injury prevention programs for teaching the desired skills could be significantly enhanced with the use of in situ training. This program, when implemented with 6- and 7-year-old children, was effective in teaching the desired safety skills
SN - 1098-4275
AD - Department of Psychology, North Dakota State University, Fargo, ND 58105, USA
UR - 15342889
ER -

TY - JOUR
ID - 348
T1 - Mother-Child touch patterns in infant feeding disorders: relation to maternal, child, and environmental factors
A1 - Feldman,R.
A1 - Keren,M.
A1 - Gross-Rozval,O.
A1 - Tyano,S.
Y1 - 2004/09//
N1 - Feldman, Ruth. Keren, Miri. Gross-Rozval, Orna. Tyano, Sam
Journal of the American Academy of Child and Adolescent Psychiatry
hg5, 8704565
IM
Journal Article. Research Support, Non-U.S. Gov't
English
KW - Medline
KW - Case-Control Studies
KW - Child
KW - Preschool
KW - Depression/di [Diagnosis]
KW - Depression/px [Psychology]

KW - Failure to Thrive/di [Diagnosis]
KW - Failure to Thrive/px [Psychology]
KW - Feeding Behavior
KW - Feeding and Eating Disorders of Childhood/di [Diagnosis]
KW - Feeding and Eating Disorders of Childhood/px [Psychology]
KW - Female
KW - Humans
KW - Infant
KW - Male
KW - Mother-Child Relations
KW - Personal Space
KW - Play and Playthings
KW - Risk Factors
KW - Social Environment
KW - Touch
RP - NOT IN FILE
SP - 1089
EP - 1097
JF - Journal of the American Academy of Child & Adolescent Psychiatry
JA - J Am Acad.Child Adolesc Psychiatry
VL - 43
IS - 9
CY - United States
N2 - OBJECTIVE: To examine mother and child's touch patterns in infant feeding disorders within a transactional framework. METHOD: Infants (aged 9-34 months) referred to a community-based clinic were diagnosed with feeding disorders (n = 20) or other primary disorder (n = 27) and were case matched with nonreferred controls (n = 47). Mother-child play and feeding were observed and the home environment was assessed. Microcoding detected touch patterns, response to partner's touch, and proximity at play. Relational behaviors were coded during feeding. RESULTS: Compared with infants with other primary disorder and case-matched controls, less maternal affectionate, proprioceptive, and unintentional touch was observed in those with feeding disorders. Children with feeding disorders displayed less affectionate touch, more negative touch, and more rejection of the mother's touch. More practical and rejecting maternal responses to the child's touch were observed, and children were positioned more often out of reach of the mothers' arms. Children with feeding disorders exhibited more withdrawal during feeding and the home environment was less optimal. Feeding efficacy was predicted by mother-child touch, reduced maternal depression and intrusiveness, easy infant temperament, and less child withdrawal, controlling for group membership. CONCLUSIONS: Proximity and touch are especially disturbed in feeding disorders, suggesting fundamental relationship difficulties. Mothers provide less touch that supports growth, and children demonstrate signs of touch aversion. Touch patterns may serve as risk indicators of potential growth failure. Copyright 2004 American Academy of Child and Adolescent Psychiatry
SN - 0890-8567
AD - Department of Psychology, Bar-Ilan University, Israel. feldman@mail.biu.ac.il
UR - 15322412
ER -

TY - JOUR
ID - 349
T1 - The ergonomics of children in playground equipment safety
A1 - Roderick,L.M.
Y1 - 2004///
N1 - Roderick, Larry M
Journal of safety research
1264241
IM
Editorial
English

KW - Medline
KW - Child
KW - Equipment Design
KW - Human Engineering
KW - Humans
KW - Play and Playthings
KW - Safety Management
KW - United States
KW - Wounds and Injuries/pc [Prevention & Control]
RP - NOT IN FILE
SP - 249
EP - 254
JF - Journal of Safety Research
JA - J Safety Res.
VL - 35
IS - 3
CY - United States
N2 - INTRODUCTION: Annually, some 200,000 U.S. children require hospital emergency room treatment from injuries suffered on playground equipment. Most of these injuries (70%) are the result of falls. While standards for protective surfacing within the play zone areas are very adequate, standards for the prevention of falls are very inadequate. "Climbers" are responsible for the greatest percentage of injuries with most of these injuries the result of falls. Current standards are woefully lacking in regard to these climbers. METHOD: This paper reviews the history of climber research and climber standards. RESULTS: It then presents recommendations for improved standards in this area based on the ergonomics of the users, children
SN - 0022-4375
UR - 15288558
ER -

TY - JOUR
ID - 350
T1 - Predictors of effortful control among children of alcoholic and nonalcoholic fathers
A1 - Eiden,R.D.
A1 - Edwards,E.P.
A1 - Leonard,K.E.
Y1 - 2004/05//
N1 - Eiden, Rina D. Edwards, Ellen P. Leonard, Kenneth E
Journal of studies on alcohol
7503813
IM
Journal Article. Research Support, U.S. Gov't, P.H.S.
English
KW - Medline
KW - Alcoholism/ep [Epidemiology]
KW - Child Behavior Disorders/ep [Epidemiology]
KW - Child Psychology
KW - Child of Impaired Parents/px [Psychology]
KW - Child
KW - Preschool
KW - Fathers/sn [Statistics & Numerical Data]
KW - Female
KW - Humans
KW - Infant
KW - Male
KW - Parenting
KW - Play and Playthings
KW - Prospective Studies

KW - Questionnaires
KW - Temperament
RP - NOT IN FILE
SP - 309
EP - 319
JF - Journal of Studies on Alcohol
VL - 65
IS - 3
CY - United States
N2 - OBJECTIVE: The purpose of this study was (1) to examine the association between fathers' alcoholism and children's effortful control and (2) to examine the role of parental warmth and toddler temperament as mediators or moderators of this relationship. METHOD: Families were recruited through New York State birth records when their infant was age 12 months. The final sample consisted of 226 families (116 boys) constituting two major groups: a nonalcoholic group consisting of parents with no or few current alcohol problems (n = 102) and a father alcoholic group (n = 124). Families were assessed when their child was ages 12, 18, 24 and 36 months. RESULTS: Results indicate that boys of alcoholic fathers exhibit lower overall levels of effortful control than boys of nonalcoholic fathers. For boys, fathers' warmth over the second year of life mediated the association between fathers' alcoholism and effortful control. Maternal warmth was a unique predictor of effortful control for boys. For girls, fathers' alcoholism was associated with lower paternal warmth, which was in turn a significant predictor of effortful control. Child activity level and negative affect were associated with effortful control for boys but did not account for significant variance when entered in regression models with fathers' alcoholism and parenting variables. CONCLUSIONS: Sons of alcoholic fathers are at an increased risk of problems in self-regulation when they are ages 2 to 3 years. Paternal warmth mediates the association between fathers' alcoholism and self-regulation for both boys and girls, although the nature of mediation may vary by child gender
SN - 0096-882X
AD - Research Institute on Addictions, University at Buffalo, State University of New York, 1021 Main Street, Buffalo, New York 14203, USA. eiden@ria.buffalo.edu
UR - 15222587
ER -

TY - JOUR
ID - 351
T1 - Are current playground safety standards adequate for preventing arm fractures?.[Erratum appears in Med J Aust. 2004 Nov 15;181(10):532]
A1 - Sherker,S.
A1 - Ozanne-Smith,J.
Y1 - 2004/06/07/
N1 - Sherker, Shauna. Ozanne-Smith, Joan
The Medical journal of Australia
0400714, m26
IM
Journal Article. Research Support, Non-U.S. Gov't
English
KW - Medline
KW - Accidental Falls/pc [Prevention & Control]
KW - Arm Injuries/pc [Prevention & Control]
KW - Child
KW - Female
KW - Fractures
KW - Bone/pc [Prevention & Control]
KW - Hospitalization/td [Trends]
KW - Humans
KW - Humeral Fractures/pc [Prevention & Control]
KW - Male
KW - Play and Playthings

KW - Radius Fractures/pc [Prevention & Control]
KW - Safety/st [Standards]
KW - Ulna Fractures/pc [Prevention & Control]
KW - Victoria
RP - NOT IN FILE
SP - 562
EP - 565
JF - Medical Journal of Australia
JA - Med J Aust
VL - 180
IS - 11
CY - Australia
N2 - OBJECTIVE: To assess compliance with current standards of playgrounds where children have sustained a fall-related arm fracture. DESIGN, SETTING AND PARTICIPANTS: Between October 2000 and December 2002, a consecutive prospective series of 402 children aged under 13 years who fell from playground equipment and sustained an arm fracture was identified by emergency department staff in five Victorian hospitals. Trained field testers measured playground equipment height, surface type and depth, and surface impact attenuation factors to determine compliance with safety standards. MAIN OUTCOME MEASURES: Playground compliance with current Australian safety standards. RESULTS: Ninety-eight percent of playgrounds had a recommended type of surface material. The mean surface depth was 11.1 cm (SD, 5.0 cm) and the mean equipment height was 2.04 m (SD, 0.43 m). Although over 85% of playgrounds complied with recommended maximum equipment height and surface impact attenuation characteristics, only 4.7% complied with recommended surface depth. CONCLUSION: Playgrounds where children have sustained an arm fracture generally comply with all important safety recommendations except surface depth. Playground fall-related arm fracture requires specific countermeasures for prevention, distinct from head injury prevention guidelines
SN - 0025-729X
AD - Accident Research Centre, Building 70, Monash University, Melbourne, VIC 3800, Australia. ShaunaSherker@yahoo.com.au
UR - 15174986
ER -

TY - JOUR
ID - 352
T1 - Creating opportunities for parent empowerment: program effects on the mental health/coping outcomes of critically ill young children and their mothers
A1 - Melnyk,B.M.
A1 - pert-Gillis,L.
A1 - Feinstein,N.F.
A1 - Crean,H.F.
A1 - Johnson,J.
A1 - Fairbanks,E.
A1 - Small,L.
A1 - Rubenstein,J.
A1 - Slota,M.
A1 - Corbo-Richert,B.
Y1 - 2004/06//
N1 - Melnyk, Bernadette Mazurek. Alpert-Gillis, Linda. Feinstein, Nancy Fischbeck. Crean, Hugh F. Johnson, Jean. Fairbanks, Eileen. Small, Leigh. Rubenstein, Jeffrey. Slota, Margaret. Corbo-Richert, Beverly
Pediatrics
oxv, 0376422
AIM, IM
Clinical Trial. Journal Article. Randomized Controlled Trial. Research Support, U.S. Gov't, P.H.S.
English
KW - Medline
KW - Adaptation
KW - Psychological

KW - Adult
KW - Child
KW - Child Behavior/px [Psychology]
KW - Hospitalized/px [Psychology]
KW - Preschool
KW - Critical Illness/px [Psychology]
KW - Depression/pc [Prevention & Control]
KW - Female
KW - Hospitalization
KW - Humans
KW - Infant
KW - Intensive Care Units
KW - Pediatric
KW - Male
KW - Mother-Child Relations
KW - Mothers/px [Psychology]
KW - Stress Disorders
KW - Post-Traumatic/pc [Prevention & Control]
KW - Stress
KW - Psychological/th [Therapy]
RP - NOT IN FILE
SP - e597
EP - e607
JA - Pediatrics
VL - 113
IS - 6
CY - United States
N2 - OBJECTIVE: Increasing numbers of children in the United States (ie, approximately 200 children per 100,000 population) require intensive care annually, because of advances in pediatric therapeutic techniques and a changing spectrum of pediatric disease. These children are especially vulnerable to a multitude of short- and long-term negative emotional, behavioral, and academic outcomes, including a higher risk of posttraumatic stress disorder (PTSD) and a greater need for psychiatric treatment, compared with matched hospitalized children who do not require intensive care. In addition, the parents of these children are at risk for the development of PTSD, as well as other negative emotional outcomes (eg, depression and anxiety disorders). There has been little research conducted to systematically determine the effects of interventions aimed at improving psychosocial outcomes for critically ill children and their parents, despite recognition of the adverse effects of critical care hospitalization on the nonphysiologic well-being of patients and their families. The purpose of this study was to evaluate the effects of a preventive educational-behavioral intervention program, the Creating Opportunities for Parent Empowerment (COPE) program, initiated early in the intensive care unit hospitalization on the mental health/psychosocial outcomes of critically ill young children and their mothers. DESIGN: A randomized, controlled trial with follow-up assessments 1, 3, 6, and 12 months after hospitalization was conducted with 174 mothers and their 2- to 7-year-old children who were unexpectedly hospitalized in the pediatric intensive care units (PICUs) of 2 children's hospitals. The final sample of 163 mothers ranged in age from 18 to 52 years, with a mean of 31.2 years. Among the mothers reporting race/ethnicity, the sample included 116 white (71.2%), 33 African American (20.3%), 3 Hispanic (1.8%), and 2 Indian (1.2%) mothers. The mean age of the hospitalized children was 50.3 months. Ninety-nine children (60.7%) were male and 64 (39.3%) were female. The major reasons for hospitalization were respiratory problems, accidental trauma, neurologic problems, and infections. Fifty-seven percent (n = 93) of the children had never been hospitalized overnight, and none had experienced a previous PICU hospitalization. INTERVENTIONS: Mothers in the experimental (COPE) group received a 3-phase educational-behavioral intervention program 1) 6 to 16 hours after PICU admission, 2) 2 to 16 hours after transfer to the general pediatric unit, and 3) 2 to 3 days after their children were discharged from the hospital. Control mothers received a structurally equivalent control program. The COPE intervention was based on self-regulation theory, control theory, and the emotional contagion hypothesis. The COPE program, which was delivered with audiotapes and matching written information, as well as a parent-child activity workbook that facilitated implementing the audiotaped information, focused on increasing 1) parents' knowledge and understanding of the range of behaviors and emotions that young children

typically display during and after hospitalization and 2) direct parent participation in their children's emotional and physical care. The COPE workbook, which was provided to parents and children after transfer from the PICU to the general pediatric unit, contained 3 activities to be completed before discharge from the hospital, ie, 1) puppet play to encourage expression of emotions in a nonthreatening manner, 2) therapeutic medical play to assist children in obtaining some sense of mastery and control over the hospital experience, and 3) reading and discussing Jenny's Wish, a story about a young child who successfully copes with a stressful hospitalization. OUTCOME MEASURES: Primary outcomes included maternal anxiety, negative mood state, depression, maternal beliefs, parental stress, and parent participation in their children's care, as well as child adjustment, which was assessed with the Behavioral Assessment System for Children (parent form). RESn (parent form). RESULTS: COPE mothers reported significantly less parental stress and participated more in their children's physical and emotional care on the pediatric unit, compared with control mothers, as rated by nurses who were blinded with respect to study group. In comparison with control mothers, COPE mothers reported less negative mood state, less depression, and fewer PTSD symptoms at certain follow-up assessments after hospitalization. In addition, COPE mothers reported stronger beliefs regarding their children's likely responses to hospitalization and how they could enhance their children's adjustment, compared with control mothers. COPE children, in comparison with control children, exhibited significantly fewer withdrawal symptoms 6 months after discharge, as well as fewer negative behavioral symptoms and externalizing behaviors at 12 months. COPE mothers also reported less hyperactivity and greater adaptability among their children at 12 months, compared with control mothers. One year after discharge, a significantly higher percentage of control group children (25.9%) exhibited clinically significant behavioral symptoms, compared with COPE children (2.3%). In addition, 6 and 12 months after discharge, significantly higher percentages of control group children exhibited clinically significant externalizing symptoms (6 months, 14.3%; 12 months, 22.2%), compared with COPE children (6 months, 1.8%; 12 months, 4.5%). CONCLUSIONS: The findings of this study indicated that mothers who received the COPE program experienced improved maternal functional and emotional coping outcomes, which resulted in significantly fewer child adjustment problems, in comparison with the control group. With the increasing prevalence of attention-deficit/hyperactivity disorder and externalizing problems among children and the documented lack of mental health screening and early intervention services for children in this country, the COPE intervention could help protect this high-risk population of children from developing these troublesome problems. As a result, the mental health status of children after critical care hospitalization could be improved. With routine provision of the COPE program in PICUs throughout the country, family burdens and costs associated with the mental health treatment of these problems might be substantially reduced
SN - 1098-4275
AD - School of Nursing, University of Rochester, Rochester, New York 14642, USA. bernadette_melnyk@urmc.rochester.edu
UR - 15173543
ER -

TY - JOUR
ID - 353
T1 - Safe play takes planning: what role would you have?
A1 - Ilardi,D.
Y1 - 2004/05//
N1 - Ilardi, Deb
School nurse news
100956395
N
Journal Article
English
KW - Medline
KW - Child
KW - Clothing/ae [Adverse Effects]
KW - Equipment Safety
KW - Humans
KW - Needs Assessment
KW - Nurse's Role
KW - Nursing Assessment

KW - Patient Care Planning/og [Organization & Administration]
KW - Planning Techniques
KW - Play and Playthings/in [Injuries]
KW - Play and Playthings
KW - Safety Management/og [Organization & Administration]
KW - School Health Services
KW - School Nursing/og [Organization & Administration]
KW - Sunburn/pc [Prevention & Control]
RP - NOT IN FILE
SP - 26
EP - 31
JF - School Nurse News
VL - 21
IS - 3
CY - United States
SN - 1080-7543
UR - 15171092
ER -

TY - JOUR
ID - 354
T1 - Unsafe to play? Neighborhood disorder and lack of safety predict reduced physical activity among urban children and adolescents
A1 - Molnar,B.E.
A1 - Gortmaker,S.L.
A1 - Bull,F.C.
A1 - Buka,S.L.
Y1 - 2004/05//
N1 - Molnar, Beth E. Gortmaker, Steven L. Bull, Fiona C. Buka, Stephen L
American journal of health promotion : AJHP
amj, 8701680
T
Journal Article. Research Support, Non-U.S. Gov't. Research Support, U.S. Gov't, P.H.S.
English
KW - Medline
KW - Adolescent
KW - Adolescent Behavior/eh [Ethnology]
KW - Anomie
KW - Censuses
KW - Chicago
KW - Child
KW - Child Behavior/eh [Ethnology]
KW - Environment Design
KW - Exercise
KW - Female
KW - Health Behavior/eh [Ethnology]
KW - Health Behavior
KW - Humans
KW - Longitudinal Studies
KW - Male
KW - Residence Characteristics/sn [Statistics & Numerical Data]
KW - Safety/sn [Statistics & Numerical Data]
KW - Social Conditions
KW - Urban Health/sn [Statistics & Numerical Data]
RP - NOT IN FILE
SP - 378

EP - 386
JF - American Journal of Health Promotion
JA - Am J Health Promot
VL - 18
IS - 5
CY - United States
N2 - PURPOSE: Lack of physical activity is associated with increased risk of overweight and cardiovascular disease, conditions associated with lower socioeconomic status (SES). Associations between activity levels of urban youth and limited access to safe recreation areas in their neighborhoods of residence were investigated. DESIGN: Analyses of data from the Project on Human Development in Chicago Neighborhoods, a multilevel longitudinal study of families and communities, are reported. SETTING: Chicago, Illinois. SUBJECTS: Individual-level data were obtained from 1378 youth 11 to 16 years old and caregivers living in 80 neighborhood clusters. Neighborhood-level data were collected from 8782 community residents and videotapes of 15,141 block faces. MEASURES: Parental estimates of hours youth spent in recreational programming were used to estimate physical activity. A scale of residents' assessment of neighborhood safety for children's play was created; disorder measures came from videotaped observations. RESULTS: Physical activity averaged 2.7 hours/week (SD = 5.0), varying significantly across neighborhoods. Using hierarchical linear regression, SES, age, and male gender, but not body mass index, were independently associated with physical activity. Lower neighborhood safety and social disorder were significantly associated with less activity, controlling for demographics. CONCLUSIONS: One mechanism for reduced physical activity among youth may be the influence of unsafe neighborhoods. Neighborhood interventions to increase safety and reduce disorder may be efficacious in increasing physical activity, thereby reducing risk of overweight and cardiovascular disease
SN - 0890-1171
AD - Department of Society, Human Development, and Health, Harvard School of Public Health, Boston, Massachusetts 02115, USA
UR - 15163139
ER -

TY - JOUR
ID - 355
T1 - Teaching safety skills to children to prevent gun play
A1 - Himle,M.B.
A1 - Miltenberger,R.G.
A1 - Flessner,C.
A1 - Gatheridge,B.
Y1 - 2004///
N1 - Himle, Michael B. Miltenberger, Raymond G. Flessner, Christopher. Gatheridge, Brian
Journal of applied behavior analysis
0174763, hdr
IM
Journal Article
English
KW - Medline
KW - Child Behavior/px [Psychology]
KW - Child
KW - Preschool
KW - Firearms
KW - Generalization (Psychology)
KW - Humans
KW - Play and Playthings
KW - Safety
KW - Teaching/mt [Methods]
RP - NOT IN FILE
SP - 1
EP - 9
JF - Journal of Applied Behavior Analysis

JA - J Appl.Behav Anal.
VL - 37
IS - 1
CY - United States
N2 - Research has shown that children often engage in gun play when they find a firearm and that this behavior is often involved in unintentional firearm injuries. Previous research has shown existing programs to be ineffective for teaching children safety skills to reduce gun play. This study examined the effectiveness of a behavioral skills training (BST) program supplemented with in situ training for teaching children safety skills to use when they find a gun (i.e., don't touch, leave the area, tell an adult). Eight 4- to 5-year-old children were trained and assessed in a naturalistic setting and in a generalized setting in a multiple baseline across subjects design. Results showed that 3 of the children performed the skills after receiving BST, whereas 5 of the children required supplemental in situ training. All children in the study learned to perform the skills when assessed in a naturalistic setting and when assessed in a generalization setting. Performance was maintained at 2- to 8-week follow-up assessments
SN - 0021-8855
AD - North Dakota State University, Fargo, North Dakota 58105, USA
UR - 15154211
ER -

TY - JOUR
ID - 356
T1 - Policy issues and risk-benefit trade-offs of 'safer surfacing' for children's playgrounds
A1 - Ball,D.J.
Y1 - 2004/07//
N1 - Ball, David J
Accident; analysis and prevention
acs, 1254476
IM
Journal Article. Research Support, Non-U.S. Gov't
English
KW - Medline
KW - Adolescent
KW - Child
KW - Preschool
KW - Construction Materials
KW - Environment Design
KW - Great Britain/ep [Epidemiology]
KW - Health Policy
KW - Humans
KW - Infant
KW - Models
KW - Theoretical
KW - Play and Playthings
KW - Risk Assessment
KW - Risk Management
KW - Wounds and Injuries/mo [Mortality]
KW - Wounds and Injuries/pc [Prevention & Control]
RP - NOT IN FILE
SP - 661
EP - 670
JF - Accident Analysis & Prevention
JA - Accid.Anal.Prev.
VL - 36
IS - 4
CY - England

N2 - The case for the universal application of 'safer surfacing' in playgrounds is assessed in terms of absolute risk, cost-benefit, and qualitative factors, and found to be open to question on each front. In parallel, it is noted that members of the child welfare and play communities are increasingly of the view that playgrounds are losing their appeal for children, which in turn has its own health, safety and developmental consequences. In part, this loss of attractiveness may have linkages with the recent concern over play safety and the imposition of measures such as 'safer surfacing'. It is proposed that the drive for play safety, which has existed for some 20 years, should be succeeded by a more holistic approach which, rather than considering play safety in isolation, acknowledges the importance of all attributes of play including child safety both on and off playgrounds, potential beneficial aspects of childhood risk exposure, adventure, and play value
SN - 0001-4575
AD - Centre for Decision Analysis and Risk Management, School of Health and Social Sciences, Middlesex University, Queensway, Enfield, Middlesex EN3 4SF, UK. d.ball@mdx.ac.uk
UR - 15094421
ER -

TY - JOUR
ID - 357
T1 - Measuring parent attributes and supervision behaviors relevant to child injury risk: examining the usefulness of questionnaire measures
A1 - Morrongiello,B.A.
A1 - House,K.
Y1 - 2004/04//
N1 - Morrongiello, B A. House, K
Injury prevention : journal of the International Society for Child and Adolescent Injury Prevention
crz, 9510056
IM
Journal Article. Research Support, Non-U.S. Gov't
English
KW - Medline
KW - Accident Prevention
KW - Attitude to Health
KW - Child Behavior
KW - Child Care/mt [Methods]
KW - Child
KW - Preschool
KW - Female
KW - Humans
KW - Male
KW - Parent-Child Relations
KW - Parenting
KW - Parents/px [Psychology]
KW - Parents
KW - Play and Playthings
KW - Questionnaires
KW - Risk Factors
KW - Safety
KW - Self-Assessment
KW - Wounds and Injuries/et [Etiology]
KW - Wounds and Injuries/pc [Prevention & Control]
RP - NOT IN FILE
SP - 114
EP - 118
JA - Injury Prevention
VL - 10
IS - 2
CY - England

N2 - OBJECTIVE: This study aimed to identify self report questionnaire measures of parent attributes and behaviors that have relevance for understanding injury risk among children 2-5 years of age, and test a new Parent Supervision Attributes Profile Questionnaire (PSAPQ) that was developed to measure aspects of protectiveness and parent supervision. METHODS: Naturalistic observations were conducted of parents' supervision of children on playgrounds, with questionnaires subsequently completed by the parent to measure parent education, family income, parent personality attributes, attributes relevant to parent supervision, and beliefs about parents' control over the child's health status. These measures were then related to children's risk taking and injury history. RESULTS: Visual supervision, auditory supervision, and physical proximity were highly intercorrelated, indicating that parents employed all types of behaviors in service of supervision, rather than relying predominantly on one type of supervisory behavior. Physical proximity was the only aspect of supervision behavior that served a protective function and related to children's risk taking behaviors: parents who remained close to their children had children who engaged in less risk taking. On questionnaires, parents who reported more conscientiousness, protectiveness, worry about safety, vigilance in supervision, confidence in their ability to keep their child safe, and belief in control over their child's health had children who showed less risk taking and/or experienced fewer injuries. The new PSAPQ measure was associated with specific aspects of supervision as well as children's risk taking and injury history. CONCLUSIONS: This study reveals several parent attributes and behaviors with relevance for child injury risk that can be measured via self report questionnaires, including the new PSAPQ
SN - 1353-8047
AD - Psychology Department, University of Guelph, Guelph, Ontario, Canada. bmorrong@uoguelph.ca
UR - 15066978
ER -

TY - JOUR
ID - 358
T1 - Head injury and limb fracture in modern playgrounds
A1 - Norton,C.
A1 - Rolfe,K.
A1 - Morris,S.
A1 - Evans,R.
A1 - James,R.
A1 - Jones,M.D.
A1 - Cory,C.
A1 - Dunstan,F.
A1 - Sibert,J.R.
Y1 - 2004/02//
N1 - Norton, C. Rolfe, K. Morris, S. Evans, R. James, R. Jones, M D. Cory, C. Dunstan, F. Sibert, J R
Archives of disease in childhood
6xg, 0372434
AIM, IM
Journal Article. Research Support, Non-U.S. Gov't
English
KW - Medline
KW - Accident Prevention
KW - Accidental Falls
KW - Adolescent
KW - Arm Injuries/ep [Epidemiology]
KW - Child
KW - Preschool
KW - Craniocerebral Trauma/ep [Epidemiology]
KW - Female
KW - Fractures
KW - Bone/ep [Epidemiology]
KW - Humans
KW - Infant
KW - Male

KW - Play and Playthings/in [Injuries]
KW - Prevalence
KW - Safety Management
KW - Wales/ep [Epidemiology]
RP - NOT IN FILE
SP - 152
EP - 153
JF - Archives of Disease in Childhood
JA - Arch.Dis.Child
VL - 89
IS - 2
CY - England
N2 - There were no serious head injuries in modern Cardiff municipal playgrounds with safety surfaces over five years injury surveillance. The literature suggests serious head injuries did occur before the introduction of safety surfaces
SN - 1468-2044
AD - Accident and Emergency Department, Cardiff and Vale NHS Trust, UK
UR - 14736633
ER -

TY - JOUR
ID - 359
T1 - Investigation of the level of safety and appropriateness of playgrounds in Elazig city in Turkey
A1 - AcIk,Y.
A1 - Gulbayrak,C.
A1 - Turaci,Celik G.
Y1 - 2004/02//
N1 - AcIk, Yasemin. Gulbayrak, Canan. Turaci Celik, Gulen
International journal of environmental health research
9106628, dus
IM
Journal Article
English
KW - Medline
KW - Accident Prevention
KW - Child
KW - Preschool
KW - Equipment Design
KW - Guideline Adherence
KW - Humans
KW - Play and Playthings
KW - Safety
KW - Turkey
KW - Wounds and Injuries/pc [Prevention & Control]
RP - NOT IN FILE
SP - 75
EP - 82
JF - International Journal of Environmental Health Research
JA - Int.J Environ.Health Res.
VL - 14
IS - 1
CY - England
N2 - This study was conducted to identify and evaluate the degree of appropriateness of children's playground standards and the level of compliance with the current safety specifications of the playgrounds in Elazig city in Turkey. This was accomplished by on-site observation and measurement in the total of 24 playgrounds present in this city centre. It was determined that 83.3% of the playgrounds were next to a street and in 60% of these

playgrounds there were no fences or any barrier to keep children from running into the street. In 87.5% of playgrounds the surfacing was not in proper depth and good order. Separate playgrounds for different ages were provided in only 12.5% of the playgrounds. In 62.5% of the playgrounds children were supervised to make sure they were safe. There was unsafe equipment in 95.8% of the playgrounds. In 83.3% of the playgrounds there were sharp edges, pinch points, or playground debris that might injure a child. The findings of the present study indicate that nearly all of the playgrounds in Elazig city center do not meet the criteria for playground standards and are not in compliance with the current safety specifications and need improvement
SN - 0960-3123
AD - Department of Public Health, Faculty of Medicine, Firat University, Elazig, Turkey. yacik@firat.edu.tr
UR - 14660120
ER -

TY - JOUR
ID - 360
T1 - The play and language behavior of mothers with and without dyslexia and its association to their toddlers' language development
A1 - Lyytinen,P.
A1 - Eklund,K.
A1 - Lyytinen,H.
Y1 - 2003/01//
N1 - Lyytinen, Paula. Eklund, Kenneth. Lyytinen, Heikki
Journal of learning disabilities
0157312, iwo
IM
Journal Article. Research Support, Non-U.S. Gov't
English
KW - Medline
KW - Child Language
KW - Child
KW - Preschool
KW - Dyslexia/di [Diagnosis]
KW - Dyslexia/ep [Epidemiology]
KW - Female
KW - Follow-Up Studies
KW - Humans
KW - Infant
KW - Language
KW - Male
KW - Maternal Behavior
KW - Mother-Child Relations
KW - Mothers/sn [Statistics & Numerical Data]
KW - Play and Playthings
KW - Questionnaires
KW - Verbal Behavior
RP - NOT IN FILE
SP - 74
EP - 86
JF - Journal of Learning Disabilities
JA - J Learn.Disabil.
VL - 36
IS - 1
CY - United States
N2 - The play and language behavior of mothers with (n = 49) and without (n = 49) specific reading disabilities (RD) was investigated during play with their 14-month-old children. The contribution of maternal behavior to the language development of their children was examined. The children's receptive and expressive language skills were assessed longitudinally at 14, 18, and 30 months, using the MacArthur Communicative Development

Inventories and the Reynell Developmental Language Scales. Children with and without familial risk for RD did not differ from each other in any play or language measures at these ages. No group differences were found for mothers' manifestations of nonsymbolic play and language. However, the typically reading (TR) mothers produced significantly more symbolic play and language in play interactions with their child than did the mothers with RD. The correspondence between mother-child symbolic play and maternal play-related language was also higher for the TR group than it was for the RD group. The mothers' symbolic play did not show any relation to their children's language development, but their expressions of symbolic language did show a relationship. The mothers' child-directed symbolic language contributed significantly toward the comprehension skills of 14- and 18-month-olds in the RD group. This association, however, was lower at 30 months, at which point it was reliably present for the first time in the TR group
SN - 0022-2194
AD - Department of Psychology, University of Jyvaskyla, Agora, Finland. plyytine@psyka.jyu.fi
UR - 15490894
ER -

TY - JOUR
ID - 361
T1 - Effects of gun admonitions on the behaviors and attitudes of school-aged boys
A1 - Hardy,M.S.
Y1 - 2003/10//
N1 - Hardy, Marjorie S
Journal of developmental and behavioral pediatrics : JDBP
htf, 8006933
IM
Journal Article
English
KW - Medline
KW - Adolescent
KW - Adolescent Behavior
KW - Attitude
KW - Child
KW - Child Behavior
KW - Firearms
KW - Health Education
KW - Humans
KW - Male
KW - Play and Playthings/px [Psychology]
KW - Safety
KW - Schools
RP - NOT IN FILE
SP - 352
EP - 358
JF - Journal of Developmental & Behavioral Pediatrics
JA - J Dev.Behav Pediatr
VL - 24
IS - 5
CY - United States
N2 - Gun-safety programs frequently warn children not to touch guns with little consideration that the messages may enhance the allure of the guns. In the current study, boys rated and ranked their preference for a gun in comparison with other desirable objects. One week later, when left alone in the room with the objects, they were forbidden to touch either the gun or some other object. Observations of their behavior and reevaluation of their attitudes followed. Although the boys forbidden to touch the gun did not report an increased preference for it, all participants were significantly more likely to touch the forbidden gun than to touch other forbidden objects. Age, parent- and coach-reported simulated gun-play, and coach-reported risky behavior predicted gun-touching behavior. The results of the current study indicate that guns hold a unique allure and cast further doubt on the ability of gun admonitions to keep children safe around guns

SN - 0196-206X
AD - Department of Psychology, Eckerd College, St. Petersburg, FL 33711, USA
UR - 14578696
ER -

TY - JOUR
ID - 362
T1 - Evaluation of North Carolina child care safety regulations
A1 - Kotch,J.B.
A1 - Hussey,J.M.
A1 - Carter,A.
Y1 - 2003/09//
N1 - Kotch, J B. Hussey, J M. Carter, A
Injury prevention : journal of the International Society for Child and Adolescent Injury Prevention
crz, 9510056
IM
Evaluation Studies. Journal Article. Research Support, Non-U.S. Gov't
English
KW - Medline
KW - Child
KW - Child Care/lj [Legislation & Jurisprudence]
KW - Child Day Care Centers/lj [Legislation & Jurisprudence]
KW - Child Day Care Centers/st [Standards]
KW - Child Day Care Centers/td [Trends]
KW - Environment Design/st [Standards]
KW - Humans
KW - North Carolina
KW - Patient Acceptance of Health Care
KW - Play and Playthings/in [Injuries]
KW - Questionnaires
KW - Risk Factors
KW - Safety/lj [Legislation & Jurisprudence]
RP - NOT IN FILE
SP - 220
EP - 225
JA - Injury Prevention
VL - 9
IS - 3
CY - England
N2 - INTRODUCTION: The goal of this study was to track any changes in injuries and injury hazards during the first 3.5 years of implementation of the North Carolina Child Care Commission's 1996 playground safety regulations. METHODS: All reports (n=5402) of medically attended injuries in regulated child care settings in North Carolina during the period 1 January 1997 through 30 June 2000 were reviewed and analyzed. A total of 294 playground safety inspections were conducted in November and December 1998 in randomly selected North Carolina child care centers, and the playground safety inspections were repeated in 76 child care centers in August 2000. Finally, in 1999 a 1992 child care center director self assessment of safety features in classrooms was sent to the directors of 291 of the 294 centers. RESULTS: The annual rate of reported, medically attended injuries occurring in regulated child care facilities in North Carolina declined by 22% from 1997 to 1999. The playground safety inspections in the year 2000 revealed that, for nine of 10 playground concerns and 12 of 18 playground safety hazards, average ratings were equal to or better than those of 1998. Finally, the director surveys revealed no dramatic changes in classroom safety hazards since 1992. DISCUSSION: This study represents the first time that the authors are aware of that a significant decline in state-wide child care injury rates has been associated with improved regulation of playground safety in the US
SN - 1353-8047
AD - Department of Maternal and Child Health, School of Public Health, University of North Carolina at Chapel Hill, 27599, USA. jonathan_kotch@unc.edu

UR - 12966009
ER -

TY - JOUR
ID - 363
T1 - Injury and frequency of use of playground equipment in public schools and parks in Brisbane, Australia
A1 - Nixon,J.W.
A1 - Acton,C.H.
A1 - Wallis,B.
A1 - Ballesteros,M.F.
A1 - Battistutta,D.
Y1 - 2003/09//
N1 - Nixon, J W. Acton, C H C. Wallis, B. Ballesteros, M F. Battistutta, D
Injury prevention : journal of the International Society for Child and Adolescent Injury Prevention
crz, 9510056
IM
Journal Article. Research Support, Non-U.S. Gov't
English
KW - Medline
KW - Australia/ep [Epidemiology]
KW - Child
KW - Emergencies/ep [Epidemiology]
KW - Environmental Exposure
KW - Equipment Design
KW - Humans
KW - Play and Playthings/in [Injuries]
KW - Recreation
KW - Risk Factors
KW - Safety
KW - Schools
KW - Urban Health
RP - NOT IN FILE
SP - 210
EP - 213
JA - Injury Prevention
VL - 9
IS - 3
CY - England
N2 - OBJECTIVE: The purpose of this study was to determine the frequency of use of play equipment in public schools and parks in Brisbane, Australia, and to estimate an annual rate of injury per use of equipment, overall and for particular types of equipment. METHODS: Injury data on all children injured from playground equipment and seeking medical attention at the emergency department of either of the two children's hospitals in the City of Brisbane were obtained for the years 1996 and 1997. Children were observed at play on five different pieces of play equipment in a random sample of 16 parks and 16 schools in the City of Brisbane. Children injured in the 16 parks and schools were counted, and rates of injury and use were calculated. RESULTS: The ranked order for equipment use in the 16 schools was climbing equipment (3762 uses), horizontal ladders (2309 uses), and slides (856 uses). Each horizontal ladder was used 2.6 times more often than each piece of climbing equipment. Each horizontal ladder was used 7.8 times more than each piece of climbing equipment in the sample of public parks. Slides were used 4.6 times more than climbing equipment in parks and 1.2 times more in public schools. The annual injury rate for the 16 schools and 16 parks under observation was 0.59/100 000 and 0.26/100 000 uses of equipment, respectively. CONCLUSIONS: This study shows that annual number of injuries per standardized number of uses could be used to determine the relative risk of particular pieces of playground equipment. The low overall rate of injuries/100 000 uses of equipment in this study suggests that the benefit of further reduction of injury in this community may be marginal and outweigh the economic costs in addition to reducing challenging play opportunities
SN - 1353-8047

AD - Department of Paediatrics and Child Health, University of Queensland, Brisbane, Australia. j.nixon@uq.edu.au
UR - 12966007
ER -

TY - JOUR
ID - 364
T1 - Association of maternal obesity and depressive symptoms with television-viewing time in low-income preschool children
A1 - Burdette,H.L.
A1 - Whitaker,R.C.
A1 - Kahn,R.S.
A1 - Harvey-Berino,J.
Y1 - 2003/09//
N1 - Burdette, Hillary L. Whitaker, Robert C. Kahn, Robert S. Harvey-Berino, Jean
Archives of pediatrics & adolescent medicine
9422751, bwf
AIM, IM
Journal Article. Research Support, Non-U.S. Gov't. Research Support, U.S. Gov't, Non-P.H.S.. Research Support, U.S. Gov't, P.H.S.
English
KW - Medline
KW - Adult
KW - Body Mass Index
KW - Chi-Square Distribution
KW - Child
KW - Preschool
KW - Cross-Sectional Studies
KW - Depression/ep [Epidemiology]
KW - Female
KW - Humans
KW - Male
KW - Mothers/px [Psychology]
KW - Obesity/ep [Epidemiology]
KW - Poverty
KW - Questionnaires
KW - Risk Factors
KW - Television
KW - Vermont/ep [Epidemiology]
RP - NOT IN FILE
SP - 894
EP - 899
JF - Archives of Pediatrics & Adolescent Medicine
JA - Arch.Pediatr Adolesc Med
VL - 157
IS - 9
CY - United States
N2 - BACKGROUND: Decreasing television (TV)-viewing time may improve child health and well-being. These viewing patterns are shaped during the preschool years. Because mothers play an important role in determining how much TV their preschool children watch, a better understanding is needed of the maternal factors that influence children's TV viewing. OBJECTIVE: To examine the relationship of depressive symptoms and obesity in low-income mothers with TV-viewing time in their preschool children. METHODS: Cross-sectional, self-administered survey of 295 low-income mothers of 3- and 4-year-old children (92% white) enrolled in the Vermont Special Supplemental Nutrition Program for Women, Infants, and Children. Mothers reported children's usual weekday and weekend-day TV-viewing time. Maternal depressive symptoms were measured with the Center for Epidemiologic Studies Depression Scale (CES-D). Maternal body mass index was

calculated from self-reported height and weight measurements (weight in kilograms divided by height in meters squared). RESULTS: Children watched a mean of 2.2 +/-1.2 hours of TV per day. Those in the upper quartile of TV-viewing time (high TV viewers) watched 3 or more hours of TV per day. Of the mothers, 12% had both obesity (BMI > or =30) and depressive symptoms (CES-D score > or =16), 19% were obese only, and 18% had depressive symptoms only. Children were more likely to be high TV viewers if their mothers had clinically significant depressive symptoms (35% vs 23%; P =.03) or if their mothers were obese (35% vs 22%; P =.03). Forty-two percent of children were high TV viewers if the mother had both depressive symptoms and obesity, 30% if the mother had only depressive symptoms, 29% if the mother had only obesity, and 20% if the mother had neither depressive symptoms nor obesity (P =.06 overall; P for trend =.009 using the chi2 test). CONCLUSIONS: Among low-income preschool children, those whose mothers had either depressive symptoms or obesity were more likely to watch 3 or more hours of TV a day. Strategies to reduce TV viewing in young children should consider the role that maternal obesity and depressive symptoms may play in how preschool children spend their time
SN - 1072-4710
AD - Division of General and Community Pediatrics, Cincinnati Children's Hospital Medical Center, OH 45229-3039, USA. hillary.burdette@chmcc.org
UR - 12963595
ER -

TY - JOUR
ID - 365
T1 - [Playground safety: an introduction to the problem]. [Portuguese]
A1 - Harada,Mde J.
A1 - Pedreira,Mda L.
A1 - Andreotti,J.T.
Y1 - 2003/05//
N1 - Harada, Maria de Jesus C S. Pedreira, Mavilde da L G. Andreotti, Janaina Trevizan
Revista latino-americana de enfermagem
bxx, 9420934
N
English Abstract. Journal Article
Portuguese
KW - Medline
KW - Accident Prevention
KW - Accidents/mo [Mortality]
KW - Child
KW - Humans
KW - Play and Playthings
KW - Safety
RP - NOT IN FILE
SP - 383
EP - 386
JF - Revista Latino-Americana de Enfermagem
JA - Rev Lat.Am Enfermagem
VL - 11
IS - 3
CY - Brazil
N2 - This study aims to approach the theme of accident prevention in relation to the use of playground toys, which is little explored in national literature. It is estimated that, annually, more than 200,000 accidents with children happen in playgrounds in the United States of America. Besides discussing this problem, we present some general recommendations about safety in these places and a reflection on the need to alert and educate society about the importance of prevention
SN - 0104-1169
AD - Departamento de Enfermagen da Disciplina de enfermagem Pediatrica, Universidade Federal de Sao Paulo, Brazil. jjharada@uol.com.br
UR - 12958645

ER -

TY - JOUR
ID - 366
T1 - Psychological aspects of serious head injury in the competitive athlete. [Review] [37 refs]
A1 - Putukian,M.
A1 - Echemendia,R.J.
Y1 - 2003/07/20/
N1 - Putukian, Margot. Echemendia, Ruben J
Clinics in sports medicine
csm, 8112473
IM
Journal Article. Review
English
KW - Medline
KW - Adaptation
KW - Psychological
KW - Adolescent
KW - Adult
KW - Athletic Injuries/co [Complications]
KW - Athletic Injuries/px [Psychology]
KW - Child
KW - Preschool
KW - Competitive Behavior
KW - Craniocerebral Trauma/co [Complications]
KW - Craniocerebral Trauma/px [Psychology]
KW - Humans
KW - Mood Disorders/et [Etiology]
KW - Personality
KW - Post-Concussion Syndrome/di [Diagnosis]
KW - Post-Concussion Syndrome/et [Etiology]
KW - Risk Factors
KW - Self Concept
RP - NOT IN FILE
SP - 617
EP - 630
JF - Clinics in Sports Medicine
JA - Clin.Sports Med
VL - 22
IS - 3
CY - United States
N2 - In addition to the physical aspects of athletic injury, there are psychological aspects to consider. These are important during all phases of injury management: the acute injury, the rehabilitative process, and the return-to-play progression. Emotional disturbances are often associated with mild traumatic brain injury (mTBI) both directly and indirectly. Evaluating the athlete with mTBI can be made more difficult by the need to distinguish the physical and the emotional effects of injury and to differentiate the emotional factors directly related to the brain injury from those that arise indirectly (eg, reaction to being held out of play). This article discusses some of the psychological issues related to evaluating the head injured athlete. [References: 37]
SN - 0278-5919
AD - Primary Care Sports Medicine, The Pennsylvania State University, 1850 East Park Avenue, Suite 112, University Park, PA 16803, USA. mxp19@psu.edu
UR - 12852690
ER -

TY - JOUR
ID - 367

T1 - Effects of pairing aggressive and nonaggressive children in strategic peer affiliation
A1 - Hektner,J.M.
A1 - August,G.J.
A1 - Realmuto,G.M.
Y1 - 2003/08//
N1 - Hektner, Joel M. August, Gerald J. Realmuto, George M
Journal of abnormal child psychology
0364547, h33
IM
Journal Article. Research Support, Non-U.S. Gov't. Research Support, U.S. Gov't, P.H.S.
English
KW - Medline
KW - Aggression/px [Psychology]
KW - Child
KW - Female
KW - Follow-Up Studies
KW - Humans
KW - Male
KW - Peer Group
KW - Play and Playthings
KW - Sex Factors
KW - Social Perception
KW - Socioeconomic Factors
RP - NOT IN FILE
SP - 399
EP - 412
JF - Journal of Abnormal Child Psychology
JA - J Abnorm.Child Psychol.
VL - 31
IS - 4
CY - United States
N2 - Examined the behavior of 118 second graders who participated in a 6-week summer school program that incorporated strategic peer affiliation (a "buddy system"). Moderately aggressive children (the targets of the intervention) were paired with nonaggressive peers throughout the program. All participants were observed playing foosball with their buddies and with aggressive and nonaggressive nonbuddies as teammates. Aggressive children had lower levels of disruptive behavior when their teammate was nonaggressive, regardless of whether the teammate was a buddy. Nonaggressive children showed elevated disruptive behavior when playing with an aggressive nonbuddy, but not when playing with an aggressive buddy. The highest level of aggressive behavior was seen in pairs of aggressive teammates who were friends. One year later, no increase in peer-rated aggressive behavior was found in either group. Results suggest that unidirectional peer influence is possible and that strategic peer affiliation can be an effective intervention that does not put nonaggressive children at risk for acquiring undesired behaviors
SN - 0091-0627
AD - Department of Child Development and Family Science, North Dakota State University, Fargo, North Dakota 58105, USA. joel.hektner@ndsu.nodak.edu
UR - 12831229
ER -

TY - JOUR
ID - 368
T1 - Buffers of peer rejection among girls with and without ADHD: the role of popularity with adults and goal-directed solitary play
A1 - Mikami,A.Y.
A1 - Hinshaw,S.P.
Y1 - 2003/08//

N1 - Mikami, Amori Yee. Hinshaw, Stephen P
Journal of abnormal child psychology
0364547, h33
IM
Journal Article. Research Support, U.S. Gov't, P.H.S.
English
KW - Medline
KW - Aggression/px [Psychology]
KW - Anxiety/ep [Epidemiology]
KW - Anxiety/px [Psychology]
KW - Attention Deficit Disorder with Hyperactivity/ep [Epidemiology]
KW - Attention Deficit Disorder with Hyperactivity/px [Psychology]
KW - Child
KW - Depression/ep [Epidemiology]
KW - Depression/px [Psychology]
KW - Female
KW - Goals
KW - Humans
KW - Male
KW - Peer Group
KW - Play and Playthings
KW - Rejection (Psychology)
KW - Risk Factors
KW - Social Desirability
KW - Social Isolation
RP - NOT IN FILE
SP - 381
EP - 397
JF - Journal of Abnormal Child Psychology
JA - J Abnorm.Child Psychol.
VL - 31
IS - 4
CY - United States
N2 - We investigated a risk-resilience model in 91 girls with ADHD and 58 age- and ethnicity-equated comparison girls, who participated in all-female naturalistic summer research camps. The hypothesized risk factor was peer rejection (assessed via sociometric nominations), with criterion measures including multiinformant composites of aggressive behavior and anxious/depressed symptoms. The two hypothesized protective factors were the girls' popularity with adult staff (assessed via staff ratings) and objective observations of goal-directed solitary play. Peer rejection was related to higher levels of aggressive behavior and depressed/anxious behavior, confirming its status as a risk factor. Next, for all girls, popularity with adults predicted lower levels of aggression and goal-directed solitary play predicted lower levels of anxiety/depression. Whereas popularity with adults was most protective among the peer-accepted subgroup, solitary play was most protective among the peer-rejected subgroup. Diagnostic status (ADHD versus comparison) moderated the findings such that engaging in meaningful solitary play was a stronger predictor of lower levels of anxious/depressed behavior in girls with ADHD than in comparison girls. We discuss the need for replication in prospective research and implications for research and intervention regarding the social functioning of peer-rejected children, particularly those with behavior disorders
SN - 0091-0627
AD - University of California, Berkeley, California 94720-1650, USA
UR - 12831228
ER -

TY - JOUR
ID - 369
T1 - Comparing unintentional and intentional injuries in a school setting
A1 - Limbos,M.A.

A1 - Peek-Asa,C.
Y1 - 2003/03//
N1 - Limbos, Mary Ann P. Peek-Asa, Corinne
The Journal of school health
k13, 0376370
IM, N
Comparative Study. Journal Article
English
KW - Medline
KW - Accident Prevention
KW - Accidents/sn [Statistics & Numerical Data]
KW - Adolescent
KW - Age Distribution
KW - Child
KW - Female
KW - Humans
KW - Incidence
KW - Los Angeles/ep [Epidemiology]
KW - Male
KW - Play and Playthings
KW - Population Surveillance
KW - Risk Factors
KW - Risk Management
KW - Schools/sn [Statistics & Numerical Data]
KW - Sex Distribution
KW - Students/sn [Statistics & Numerical Data]
KW - Time Factors
KW - Urban Health/sn [Statistics & Numerical Data]
KW - Violence/pc [Prevention & Control]
KW - Violence/sn [Statistics & Numerical Data]
KW - Wounds and Injuries/ep [Epidemiology]
KW - Wounds and Injuries/et [Etiology]
KW - Wounds and Injuries/pc [Prevention & Control]
KW - Wounds and Injuries/px [Psychology]
RP - NOT IN FILE
SP - 101
EP - 106
JF - Journal of School Health
JA - J Sch Health
VL - 73
IS - 3
CY - United States
N2 - This study determined the incidence of violence-related injuries in an urban school district, and compared characteristics of unintentional and intentional school injuries. A sample of student Accident Report Forms completed for a school district in 1997 were reviewed for demographic characteristics of the student and injury characteristics. Injuries were categorized as unintentional, intentional, or of unknown intent. Annual incidence rates of injury per 100 students were calculated by intention, grade, and gender. Comparisons between unintentional and intentional injuries were made using odds ratios and 95% confidence intervals. Of 11,674 annualized injuries, 77.2% were unintentional, 16.8% were intentional, and 6.0% were of unknown intent. The overall annualized injury rate was 1.74 injuries per 100 students/year. The unintentional injury rate was almost five times the intentional injury rate of 0.29 injuries per 100 students/year. High school students had both the highest unintentional and intentional injury rates. Males in all grade levels had the highest rates of injury. Most injuries occurred during school hours. Intentional injuries were almost three times more likely to be associated with unstructured play or after school playground hours; were less likely to be witnessed events; and were more likely to occur on the surrounding school grounds than unintentional injuries. Unintentional injuries represent a greater risk to school children than do intentional injuries. A potential area to focus interventions for intentional

injuries are modifications of the school environment and surrounding grounds to improve supervision and monitoring
SN - 0022-4391
AD - USC Keck School of Medicine, Division of General Pediatrics, Childrens Hospital of Los Angeles, 4650 Sunset Blvd., MS# 76, Los Angeles, CA 90027, USA. mlimbos@chla.usc.edu
UR - 12677728
ER -

TY - JOUR
ID - 370
T1 - The relation between attention deficit hyperactivity disorder and substance abuse: what role does conduct disorder play?. [Review] [72 refs]
A1 - Flory,K.
A1 - Lynam,D.R.
Y1 - 2003/03//
N1 - Flory, Kate. Lynam, Donald R
Clinical child and family psychology review
dw5, 9807947
IM
Journal Article. Research Support, Non-U.S. Gov't. Research Support, U.S. Gov't, P.H.S.. Review
English
KW - Medline
KW - Adult
KW - Attention Deficit Disorder with Hyperactivity/ep [Epidemiology]
KW - Child
KW - Comorbidity
KW - Conduct Disorder/ep [Epidemiology]
KW - Humans
KW - Substance-Related Disorders/ep [Epidemiology]
RP - NOT IN FILE
SP - 1
EP - 16
JF - Clinical Child & Family Psychology Review
JA - Clin.Child Fam.Psychol.Rev
VL - 6
IS - 1
CY - United States
N2 - This paper presents a comprehensive review of the literature examining the relation between childhood attention deficit hyperactivity disorder (ADHD) and substance abuse. A number of researchers have statistically controlled for conduct disorder (CD) when examining the ADHD-substance-abuse relation. These studies have generally found that the ADHD-substance-abuse relation disappears when the high overlap between ADHD and CD is taken into account. On this basis, one may conclude that ADHD is unimportant to substance abuse. However, there is some evidence from both empirical studies and related fields that ADHD and CD may interact to afford a higher risk for substance abuse than may either disorder alone. Thus, ADHD appears to be important to substance abuse in that it interacts with CD to increase the risk. This paper calls for more research that directly examines the joint effects of ADHD and CD on risk for substance abuse. There is also a need for more research that examines gender differences, and mechanisms of the relations among ADHD, CD, and substance abuse. [References: 72]
SN - 1096-4037
AD - Department of Psychology, University of Kentucky, Lexington, Kentucky 40506-0044, USA. khflor0@uky.edu
UR - 12659448
ER -

TY - JOUR
ID - 371

T1 - Inner ear damage in children due to noise exposure from toy cap pistols and firecrackers: a retrospective review of 53 cases
A1 - Segal,S.
A1 - Eviatar,E.
A1 - Lapinsky,J.
A1 - Shlamkovitch,N.
A1 - Kessler,A.
Y1 - 2003/01//
N1 - Segal, S. Eviatar, E. Lapinsky, J. Shlamkovitch, N. Kessler, A
Noise & health
9815620
IM
Journal Article
English
KW - Medline
KW - Adolescent
KW - Audiometry
KW - Pure-Tone
KW - Bone Conduction
KW - Child
KW - Child Welfare
KW - Dizziness/di [Diagnosis]
KW - Dizziness/et [Etiology]
KW - Dizziness/pc [Prevention & Control]
KW - Electronystagmography
KW - Female
KW - Firearms
KW - Hearing Loss
KW - Noise-Induced/di [Diagnosis]
KW - Noise-Induced/et [Etiology]
KW - Noise-Induced/pc [Prevention & Control]
KW - Humans
KW - Male
KW - Play and Playthings/in [Injuries]
KW - Retrospective Studies
KW - Safety
KW - Severity of Illness Index
KW - Speech Reception Threshold Test
KW - Tinnitus/di [Diagnosis]
KW - Tinnitus/et [Etiology]
KW - Tinnitus/pc [Prevention & Control]
KW - Tympanic Membrane Perforation/di [Diagnosis]
KW - Tympanic Membrane Perforation/et [Etiology]
KW - Tympanic Membrane Perforation/pc [Prevention & Control]
RP - NOT IN FILE
SP - 13
EP - 18
JF - Noise & Health
JA - Noise Health
VL - 5
IS - 18
CY - England
N2 - This retrospective study presents the findings of inner ear damage documented in 53 children exposed to impulsive sound emitted by toy weapons and firecrackers. There were 49 boys and four girls aged between four and fourteen years. Thirty-nine children were affected unilaterally while fourteen had bilateral hearing loss (total of 67 ears). Most of the hearing loss (>70%) was sensorineural high frequency hearing loss, while only

nine out of the 67 injured ears had sensorineural mid frequency hearing loss. Seven children sustained a traumatic ear drum perforation. Dizziness or tinnitus was reported by twenty children, with pathological ENG findings in four of them. This paper re-emphasizes the possibility of inner ear damage in children from exposure to noisy toys
SN - 1463-1741
AD - Department of Otolaryngology, Assaf Harofeh Medical Center, Affiliated to Sackler Faculty of Medicine, Tel Aviv University, Israel
UR - 12631431
ER -

TY - JOUR
ID - 372
T1 - Using an injury diary to describe the ecology of children's daily injuries
A1 - Schwebel,D.C.
A1 - Binder,S.C.
A1 - Plumert,J.M.
Y1 - 2002///
N1 - Schwebel, David C. Binder, Stephanie C. Plumert, Jodie M
Journal of safety research
1264241
IM
Journal Article. Research Support, Non-U.S. Gov't
English
KW - Medline
KW - Accident Prevention
KW - Accidental Falls/pc [Prevention & Control]
KW - Accidental Falls/sn [Statistics & Numerical Data]
KW - Accidents
KW - Home/pc [Prevention & Control]
KW - Home/sn [Statistics & Numerical Data]
KW - Analysis of Variance
KW - Causality
KW - Child
KW - Child Welfare
KW - Data Collection/mt [Methods]
KW - Data Interpretation
KW - Statistical
KW - Female
KW - Human Engineering
KW - Humans
KW - Male
KW - Medical Records/st [Standards]
KW - Parents
KW - Play and Playthings
KW - Safety Management
KW - Wounds and Injuries/et [Etiology]
RP - NOT IN FILE
SP - 301
EP - 319
JF - Journal of Safety Research
JA - J Safety Res.
VL - 33
IS - 3
CY - United States
N2 - PROBLEM: Two problems were addressed in this study. First, are daily injury diaries an effective means to gather information about children's unintentional injuries? Second, what are the circumstances of children's

injuries as recorded through the diary method? METHOD: Two studies were conducted with a total of 172 children to describe the ecology of children's daily unintentional injuries. Families completed a daily diary for 14 days, describing the circumstances surrounding the injuries children experienced each day. RESULTS: Descriptive data is provided on the locations, causes, and types of injuries children incurred, as well as who was present when they were injured. Daily injury rate was modestly related to the number of major injuries children had incurred in their lifetimes. DISCUSSION: The diary methodology was an effective means to study the ecology of children's daily injuries. Children's injuries occur in a wide range of circumstances that can be quantified through diary techniques. IMPACT ON INDUSTRY: Data obtained from daily injury diaries may be useful in a variety of areas, including study of the etiology of childhood injury, design of interventions to prevent injury, and engineering of toys and playground equipment for children
SN - 0022-4375
AD - Department of Psychology, University of Alabama at Birmingham, 1300 University Boulevard, 415 CH, Birmingham, AL 35294, USA. schwebel@uab.edu
UR - 12404995
ER -

TY - JOUR
ID - 373
T1 - ADHD outside the laboratory: boys' executive function performance on tasks in videogame play and on a visit to the zoo
A1 - Lawrence,V.
A1 - Houghton,S.
A1 - Tannock,R.
A1 - Douglas,G.
A1 - Durkin,K.
A1 - Whiting,K.
Y1 - 2002/10//
N1 - Lawrence, Vivienne. Houghton, Stephen. Tannock, Rosemary. Douglas, Graham. Durkin, Kevin. Whiting, Ken
Journal of abnormal child psychology
0364547, h33
IM
Journal Article. Research Support, Non-U.S. Gov't
English
KW - Medline
KW - Analysis of Variance
KW - Attention Deficit Disorder with Hyperactivity/px [Psychology]
KW - Child
KW - Cohort Studies
KW - Humans
KW - Inhibition (Psychology)
KW - Internal-External Control
KW - Male
KW - Memory/ph [Physiology]
KW - Neuropsychological Tests/sn [Statistics & Numerical Data]
KW - Psychomotor Performance/ph [Physiology]
KW - Task Performance and Analysis
KW - Video Games/px [Psychology]
RP - NOT IN FILE
SP - 447
EP - 462
JF - Journal of Abnormal Child Psychology
JA - J Abnorm.Child Psychol.
VL - 30
IS - 5
CY - United States

N2 - One current theory of attention-deficit/hyperactivity disorder (ADHD) proposes that a primary deficit in behavioral inhibition gives rise to secondary deficits in 4 executive functions and motor control. To date, empirical support for this model is based primarily on laboratory-based cognitive methods. This study assessed behavioral inhibition and executive functioning in children with ADHD in 2 real-life contexts: videogames (motor-skill target game, cognitively demanding adventure game) and an outing at the zoo (route tasks). Participants were a community sample of 57 boys diagnosed with ADHD (20 inattentive, 37 combined type) and 57 normally developing control boys, matched individually for age and nonverbal IQ. Operationally defined measures of behavioral inhibition and specific executive functions were derived from these activities and assessed under contrasting conditions of low or high working memory and distractor loads. There were no group differences in basic motor skills on the target game, nor in terms of the ability to inhibit a prepotent or ongoing response in the adventure videogame. However, boys with ADHD exhibited more self-talk, more effortful response preparation, and completed fewer challenges in the latter videogame. Also, they manifested inhibition deficits in terms of interference control during the route task at the zoo and took longer to complete the tasks. Typically, these differences were greatest under conditions of high working memory and distractor loads. Findings from this study suggest that cognitive difficulties in ADHD may be context dependent and that ADHD is associated with deficits in some but not all aspects of behavioral inhibition
SN - 0091-0627
AD - The Graduate School of Education, The University of Western Australia, Perth
UR - 12403149
ER -

TY - JOUR
ID - 374
T1 - Arsenic and barbecue
A1 - Lavelle,M.
Y1 - 2002/09/16/
N1 - Lavelle, Marianne
U.S. news & world report
9877797, wsl
K
News
English
KW - Medline
KW - Arsenic/an [Analysis]
KW - Arsenic/ec [Economics]
KW - Arsenic Poisoning/pc [Prevention & Control]
KW - Child
KW - Construction Materials/ae [Adverse Effects]
KW - Construction Materials/an [Analysis]
KW - Consumer Product Safety
KW - Humans
KW - Play and Playthings
KW - Public Health
KW - Recreation
KW - Risk Factors
KW - United States
KW - United States Environmental Protection Agency
KW - Wood
RP - NOT IN FILE
SP - 58
EP - 59
JF - US News & World Report
JA - US News World Rep.
VL - 133
IS - 10
CY - United States

SN - 0041-5537
UR - 12298350
ER -

TY - JOUR
ID - 375
T1 - Exposure to traffic among urban children injured as pedestrians
A1 - Posner,J.C.
A1 - Liao,E.
A1 - Winston,F.K.
A1 - Cnaan,A.
A1 - Shaw,K.N.
A1 - Durbin,D.R.
Y1 - 2002/09//
N1 - Posner, J C. Liao, E. Winston, F K. Cnaan, A. Shaw, K N. Durbin, D R
Injury prevention : journal of the International Society for Child and Adolescent Injury Prevention
crz, 9510056
IM
Journal Article. Research Support, Non-U.S. Gov't
English
KW - Medline
KW - Accidents
KW - Traffic/sn [Statistics & Numerical Data]
KW - Adolescent
KW - Age Factors
KW - Child
KW - Preschool
KW - Cross-Sectional Studies
KW - Female
KW - Humans
KW - Male
KW - Risk Factors
KW - Urban Health/sn [Statistics & Numerical Data]
KW - Walking/sn [Statistics & Numerical Data]
RP - NOT IN FILE
SP - 231
EP - 235
JA - Injury Prevention
VL - 8
IS - 3
CY - England
N2 - OBJECTIVES: To explore the immediate pre-crash activities and the routine traffic exposure (street crossing and play) in a sample of urban children struck by automobiles. In particular, the traffic exposure of children who were struck while playing was compared with that of those struck while crossing streets. DESIGN: Cross sectional survey. SETTING: Urban pediatric emergency department. PATIENTS: A total of 139 children ages 4-15 years evaluated for acute injuries resulting from pedestrian-motor vehicle collisions during a 14 month period. MAIN OUTCOME MEASURES: Sites of outdoor play, daily time in outdoor play, weekly number of street crossings, pre-crash circumstance (play v walking). RESULTS: Altogether 39% of the children routinely used the street and 64% routinely used the sidewalks as play areas. The median number of street crossings per week per child was 27. There were no differences in exposures for the 29% who were hit while playing compared with the 71% who were hit while walking. Although 84% of the children walked to or from school at least one day per week, only 15% of the children were struck while on the school walking trip. The remainder were injured either while playing outdoors or while walking to other places. CONCLUSIONS: Urban children who are victims of pedestrian crashes have a high level of traffic exposure from a variety of circumstances related to their routine outdoor playing and street crossing activities. The distributions of traffic exposures were similar across the sample, indicating that the sample as a whole had high traffic exposure,

regardless of the children's activity preceding the crash. Future pedestrian injury programs should address the pervasive nature of children's exposure to traffic during their routine outdoor activities
SN - 1353-8047
AD - Department of Pediatrics, Children's Hospital of Philadelphia, University of Pennsylvania School of Medicine, Philadelphia 19104, USA. posner@email.chop.edu
UR - 12226122
ER -

TY - JOUR
ID - 376
T1 - Profiles of social competence among low-income African American preschool children
A1 - Mendez,J.L.
A1 - Fantuzzo,J.
A1 - Cicchetti,D.
Y1 - 2002/07//
N1 - Mendez, Julia L. Fantuzzo, John. Cicchetti, Dante
Child development
0372725, d28
IM
Journal Article. Research Support, U.S. Gov't, P.H.S.
English
KW - Medline
KW - African Americans/px [Psychology]
KW - Attention Deficit and Disruptive Behavior Disorders/di [Diagnosis]
KW - Attention Deficit and Disruptive Behavior Disorders/px [Psychology]
KW - Child
KW - Preschool
KW - Early Intervention (Education)
KW - Female
KW - Humans
KW - Language Development
KW - Male
KW - Peer Group
KW - Play and Playthings
KW - Poverty/px [Psychology]
KW - Risk Factors
KW - Social Behavior
KW - SOCIALIZATION
KW - Vocabulary
RP - NOT IN FILE
SP - 1085
EP - 1100
JF - Child Development
JA - Child Dev.
VL - 73
IS - 4
CY - United States
N2 - Relations between children's personal attributes and peer play competence were investigated in a sample of 141 African American preschool children who participated in Head Start. Variable-oriented analyses confirmed that dispositions of temperament, emotion regulation, autonomy, and language were related to children's peer play competence in the classroom. Person-oriented analyses revealed distinctive profiles of personal attributes linked to adaptive preschool social functioning. A small group of resilient children whose profile was characterized by highly adaptable temperament, ability to approach new situations, and above average vocabulary development evidenced the greatest social competence with peers. Children who were disruptive with peers were equally divided between two profiles characterized by inattention and activity, but with differential performance on vocabulary tasks. A profile containing calm, reticent children was the group

least likely to engage in disruptive peer play. Inspection of the six profiles revealed the within-group variability for this economically disadvantaged sample and illustrated the differential importance of temperament, regulation, and language constructs. Findings from the profile analyses and relations with peer competence inform the study of resilience in social development for urban African American children who participate in early intervention preschool programs
SN - 0009-3920
AD - Department of Psychology, University of South Carolina, Columbia 29208, USA. mendez@sc.edu
UR - 12146735
ER -

TY - JOUR
ID - 377
T1 - Child safety. Getting the playground ready for spring...and a good resource for playground safety materials
A1 - Ucci,M.
Y1 - 2002/04//
N1 - Ucci, Mary
Child health alert
9881972
K
Journal Article
English
KW - Medline
KW - Child
KW - Humans
KW - Play and Playthings
KW - Safety
KW - United States
RP - NOT IN FILE
SP - 5
JF - Child Health Alert
VL - 20
CY - United States
SN - 1064-4849
UR - 12143776
ER -

TY - JOUR
ID - 378
T1 - Childhood social arena and cognitive sets in eating disorders
A1 - Troop,N.A.
A1 - Bifulco,A.
Y1 - 2002/06//
N1 - Troop, Nicholas A. Bifulco, Antonia
The British journal of clinical psychology / the British Psychological Society
bok, 8105533
IM
Journal Article
English
KW - Medline
KW - Adolescent
KW - Adult
KW - Anorexia Nervosa/px [Psychology]
KW - Attitude
KW - Bulimia/px [Psychology]
KW - Child
KW - Female

KW - Humans
KW - Personality Development
KW - Risk Factors
KW - Social Adjustment
KW - Social Environment
RP - NOT IN FILE
SP - 205
EP - 211
JF - British Journal of Clinical Psychology
JA - Br J Clin.Psychol.
VL - 41
IS - Pt 2
CY - England
N2 - OBJECTIVE: While there is much evidence to suggest that women with eating disorders experience difficulties in the social domain, little has been done to establish whether such difficulties play a causal role or the extent to which these involve cognitive factors. The purpose of this report is to determine whether difficulties in certain aspects of the childhood social arena are reported as existing prior to developing an eating disorder. METHOD: A sample of 43 women with a history of eating disorders and 20 women with no such history were interviewed retrospectively about their feelings and experiences of loneliness, shyness and inferiority in childhood and adolescence. RESULTS: Women with a history of anorexia nervosa of the binge/purge subtype reported higher levels of loneliness, shyness and feelings of inferiority in adolescence than did women with no history of an eating disorder, and women with a history of bulimia nervosa reported higher levels of shyness. However, this was not true for earlier childhood where such feelings did not differ significantly between groups. This difference could not be accounted for by current depressive disorder, recovery from the eating disorder or level of victimization in adolescence. CONCLUSION: There are a number of differences in the aetiology of subtypes of eating disorder. The present results suggest that cognitive styles pertaining to the social arena in adolescence, and prior to the onset of any eating disorders, may play a causal role in the development of anorexia nervosa of the binge/purge subtype, but not anorexia nervosa of the restricting subtype
SN - 0144-6657
AD - Department of Psychology, London Guildhall University, London, UK. ntroop@lgu.ac.uk
UR - 12034006
ER -

TY - JOUR
ID - 379
T1 - Teaching firearm safety to children: failure of a program
A1 - Hardy,M.S.
Y1 - 2002/04//
N1 - Hardy, Marjorie S
Journal of developmental and behavioral pediatrics : JDBP
htf, 8006933
IM
Clinical Trial. Journal Article. Randomized Controlled Trial
English
KW - Medline
KW - Aggression/px [Psychology]
KW - Child
KW - Preschool
KW - Female
KW - Firearms
KW - Health Education
KW - Health Knowledge
KW - Attitudes
KW - Practice
KW - Humans

KW - Male
KW - Parent-Child Relations
KW - Play and Playthings
KW - Program Evaluation
KW - Safety
KW - Wounds
KW - Gunshot/pc [Prevention & Control]
KW - Gunshot/px [Psychology]
RP - NOT IN FILE
SP - 71
EP - 76
JF - Journal of Developmental & Behavioral Pediatrics
JA - J Dev.Behav Pediatr
VL - 23
IS - 2
CY - United States
N2 - The present study investigated the effectiveness of a skills-based firearm safety program on reducing children's play with firearms. In a randomized control study, 34 children aged 4 to 7 years participated in a week-long firearm safety program; the Control Group was composed of 36 children. After the program, pairs of children were observed playing in a structured setting in which they had access to a semiautomatic pistol. A total of 53% of the pairs played with the gun, and there was no difference in gun-play behavior between those children who did and did not receive the intervention. Interview data revealed significant discrepancies in parent and child reports of parental gun ownership and inaccurate parental predictions of their children's interest in guns. The results of the current study cast doubt on the potential effectiveness of skills-based gun safety programs for children
SN - 0196-206X
AD - Department of Psychology, Eckerd College, St. Petersburg, Florida 33711, USA. hardyms.eckerd.edu
UR - 11943968
ER -

TY - JOUR
ID - 380
T1 - Decreasing children's risk taking on the playground
A1 - Heck,A.
A1 - Collins,J.
A1 - Peterson,L.
Y1 - 2001///
N1 - Heck, A. Collins, J. Peterson, L
Journal of applied behavior analysis
0174763, hdr
IM
Journal Article. Research Support, Non-U.S. Gov't. Research Support, U.S. Gov't, P.H.S.
English
KW - Medline
KW - Child
KW - Humans
KW - Play and Playthings
KW - Random Allocation
KW - Risk-Taking
KW - Teaching
KW - Wounds and Injuries/pc [Prevention & Control]
RP - NOT IN FILE
SP - 349
EP - 352
JF - Journal of Applied Behavior Analysis
JA - J Appl.Behav Anal.

VL - 34
IS - 3
CY - United States
N2 - Playground mishaps are some of the most common sources of injury and are the leading killer of children. The present study used a multiple baseline design across three classrooms (N = 379 children). With minimal teaching and rewards, children decreased and maintained decreased risky playground behaviors on slides. Floor effects on climbers prevented the demonstration of similar effects. The decreases seen in risky slide behavior are discussed within the context of preventive safety training for playground injuries
SN - 0021-8855
AD - University of Missouri-Columbia, 65211, USA
UR - 11678532
ER -

TY - JOUR
ID - 381
T1 - Planar asymmetry tips the phonological playground and environment raises the bar
A1 - Eckert,M.A.
A1 - Lombardino,L.J.
A1 - Leonard,C.M.
Y1 - 2001/07//
N1 - Eckert, M A. Lombardino, L J. Leonard, C M
Child development
0372725, d28
IM
Journal Article. Research Support, Non-U.S. Gov't. Research Support, U.S. Gov't, P.H.S.
English
KW - Medline
KW - Brain Mapping
KW - Child
KW - Dominance
KW - Cerebral/ph [Physiology]
KW - Dyslexia/ge [Genetics]
KW - Dyslexia/pp [Physiopathology]
KW - Female
KW - Humans
KW - Intelligence/ge [Genetics]
KW - Intelligence/ph [Physiology]
KW - Longitudinal Studies
KW - Magnetic Resonance Imaging
KW - Male
KW - Phonetics
KW - Play and Playthings
KW - Reading
KW - Risk Factors
KW - Social Environment
KW - Socioeconomic Factors
KW - Temporal Lobe/pp [Physiopathology]
RP - NOT IN FILE
SP - 988
EP - 1002
JF - Child Development
JA - Child Dev.
VL - 72
IS - 4
CY - United States

N2 - Reading readiness varies as a function of family and environmental variables. This study of 11-year-old children (N = 39) was designed to determine if there was an additional or interactive contribution of brain structure. Evidence is presented that both environmental and biological variables predict phonological development. Temporal lobe (planar) asymmetry, hand preference, family history of reading disability, and SES explained over half of the variance in phonological and verbal performance. The results demonstrate a linear association between cerebral organization and phonological skill within socioeconomic groups. These data provide concrete evidence to support the commonly held assumption that both environmental and biological factors are independent determinants of a child's ability to process linguistic information
SN - 0009-3920
AD - Department of Neuroscience, McKnight Brain Institute, University of Florida, Gainesville 32610, USA. eckert@ufl.edu
UR - 11480950
ER -

TY - JOUR
ID - 382
T1 - Return to play after cervical spine injury
A1 - Morganti,C.
A1 - Sweeney,C.A.
A1 - Albanese,S.A.
A1 - Burak,C.
A1 - Hosea,T.
A1 - Connolly,P.J.
Y1 - 2001/05/15/
N1 - Morganti, C. Sweeney, C A. Albanese, S A. Burak, C. Hosea, T. Connolly, P J
Spine
7610646, uxk, 7610649
IM
Journal Article
English
KW - Medline
KW - Adolescent
KW - Adult
KW - Athletic Injuries/pp [Physiopathology]
KW - Athletic Injuries/px [Psychology]
KW - Athletic Injuries/th [Therapy]
KW - Cervical Vertebrae/in [Injuries]
KW - Child
KW - Decision Making
KW - Humans
KW - Medicine
KW - Physicians
KW - Postoperative Period
KW - Practice Guidelines as Topic
KW - Professional Practice
KW - Questionnaires
KW - Risk Factors
KW - Specialization
KW - Sports
KW - Time Factors
RP - NOT IN FILE
SP - 1131
EP - 1136
JF - Spine
VL - 26
IS - 10

CY - United States
N2 - STUDY DESIGN: A questionnaire survey was mailed to members of the Cervical Spine Research Society, the Herodiuus Sports Medicine Society, and to members of the authors' Department of Orthopaedics. OBJECTIVES: The purpose of our study was to evaluate what influence, if any, factors such as published guidelines, type of sport of the patient, number of years in practice, subspecialty interest, and sports participation of the respondent held in the "return to play" decision-making process after a cervical spine injury. SUMMARY OF BACKGROUND DATA: The consequences of cervical spine injury are potentially catastrophic, and return to play decisions in athletes with a history of neck injury can be agonizing. Although recent publications have addressed some of the concerns regarding cervical spine injuries in the athletic population, many questions remain unanswered. Factors such as published guidelines, type of sport of the patient, number of years in practice, subspecialty interest, and sports participation of the respondent have all been suggested as having a possible role in return to play decisions. METHODS: Representative radiographs and case histories of 10 athletes who had sustained neck injury were mailed to 346 physicians. For each case physicians selected every type of play (of six categories) that they felt comfortable recommending. Type of play was divided into six categories: Type 1, collision sports; Type 2, contact sports; Type 3, noncontact, high velocity sports; Type 4, noncontact, repetitive load sports (e.g., running); Type 5, noncontact, low impact sports; Type 6, no sports. In addition, demographic data regarding board certification, subspecialty interest, number of years in practice, use of guidelines in return to play decisions, and personal participation in sports were queried from all respondents. Statistical analysis was completed with Statview (Berkeley, CA). Basic descriptive statistics, chi2, and ANOVA were used where appropriate. RESULTS: Three hundred forty-six questionnaires were mailed and 113 were returned (response rate 32.7%). One hundred ten (97%) of the respondents who completed the questionnaire were board certified. Seventy-five were subspecialists in spine, 22 were subspecialists in sportsmedicine, and 13 reported interests in both sports medicine and spine. Use of Published Guidelines. Although 49% of respondents reported using guidelines in decision-making, the use of guidelines was statistically significant in only one case (P = 0.04). Hierarchy of Risk. In general, those physicians who participated in the study followed the hierarchy of risk that we established in this study (Type 1 [collision sports; highest level of risk] through Type 6 [no sports; lowest level of risk]). Twelve (10.6%) respondents, however, deviated from it in one or more cases. Years in Practice. In three cases there was a statistically significant association between the number of years a physician was in practice and the type of play selected (P < 0.05). In each case a lower level of play tended to be recommended by more senior physicians. Subspecialty Interest. In three cases those respondents with a spine subspecialty interest recommended returned to a higher level of play (P < 0.05). CONCLUSIONS: There is no consensus on the postinjury management of many cervical spine-injured patients. Further research, education, and discussion on this topic are needed
SN - 0362-2436
AD - Department of Orthopedic Surgery, State University of New York, Upstate Medical University, Syracuse, New York 13202, USA
UR - 11413424
ER -

TY - JOUR
ID - 383
T1 - Seeing is believing: what do boys do when they find a real gun?
A1 - Jackman,G.A.
A1 - Farah,M.M.
A1 - Kellermann,A.L.
A1 - Simon,H.K.
Y1 - 2001/06//
N1 - Jackman, G A. Farah, M M. Kellermann, A L. Simon, H K
Pediatrics
oxv, 0376422
AIM, IM
Journal Article. Research Support, Non-U.S. Gov't
English
KW - Medline
KW - Adult
KW - Age Factors

KW - Attitude
KW - Child
KW - Child Behavior/px [Psychology]
KW - Exploratory Behavior
KW - Female
KW - Firearms/st [Standards]
KW - Firearms
KW - Humans
KW - Male
KW - Parent-Child Relations
KW - Parents/ed [Education]
KW - Parents/px [Psychology]
KW - Play and Playthings/px [Psychology]
KW - Safety/st [Standards]
KW - Sampling Studies
KW - Sex Factors
RP - NOT IN FILE
SP - 1247
EP - 1250
JA - Pediatrics
VL - 107
IS - 6
CY - United States
N2 - OBJECTIVES: To determine how boys behave when they find a handgun in a presumably safe environment and to compare parental expectations of their child's interest in real guns with this observed behavior. METHODS: A convenience sample of 8- to 12-year-old boys was recruited from families that completed a survey on firearm ownership, storage practices, and parental perceptions. Parents were asked to rate their child's interest in real guns on a scale from 1 to 5: 1-2 = low interest, 3 = moderate interest, and 4-5 = high interest. Parents of an eligible child were asked to bring to the exercise 1 of their son's playmates and/or a sibling in the same age range. After informed parental consent was obtained, each pair or trio of boys was placed in a room with a 1-way mirror and observed for up to 15 minutes. Two water pistols and an actual.380 caliber handgun were concealed in separate drawers. The handgun contained a radio transmitter that activated a light whenever the trigger was depressed with sufficient force to discharge the firearm. After the exercise, each boy was asked whether he thought that the pistol was real or a toy. Before leaving, each child was counseled about safe behavior around guns. RESULTS: Twenty-nine groups of boys (n = 64) took part in the study. The mean age of participants was 9.8 years. Twenty-one of the groups (72%) discovered the handgun (n = 48 boys); 16 groups (76%) handled it (n = 30 boys). One or more members in 10 of the groups (48%) pulled the trigger (n = 16 boys). Approximately half of the 48 boys who found the gun thought that it was a toy or were unsure whether it was real. Parental estimates of their child's interest in guns did not predict actual behavior on finding the handgun. Boys who were believed to have a low interest in real guns were as likely to handle the handgun or pull the trigger as boys who were perceived to have a moderate or high interest in guns. More than 90% of the boys who handled the gun or pulled the trigger reported that they had previously received some sort of gun safety instruction. CONCLUSION: Many 8- to 12-year-old boys will handle a handgun if they find one. Guns that are kept in homes should be stored in a manner that renders them inaccessible to children.guns, weapons, firearms, children, childhood behavior, injury prevention
SN - 1098-4275
AD - Division of Pediatric Emergency Medicine, Department of Pediatrics, Emory University School of Medicine and the Children's Healthcare of Atlanta, Atlanta, Georgia, USA. geoffrey.jackman@hsc.utah.edu
UR - 11389238
ER -

TY - JOUR
ID - 384
T1 - Playground injuries in children: a review and Pennsylvania Trauma Center experience. [Review] [23 refs]
A1 - Bernardo,L.M.
A1 - Gardner,M.J.

A1 - Seibel,K.
Y1 - 2001/01//
N1 - Bernardo, L M. Gardner, M J. Seibel, K
Journal of the Society of Pediatric Nurses : JSPN
cky, 9615684
IM, N
Journal Article. Review
English
KW - Medline
KW - Accidental Falls/sn [Statistics & Numerical Data]
KW - Child
KW - Child Welfare
KW - Preschool
KW - Female
KW - Humans
KW - Length of Stay/sn [Statistics & Numerical Data]
KW - Male
KW - Patient Education as Topic
KW - Pediatric Nursing
KW - Pennsylvania/ep [Epidemiology]
KW - Play and Playthings/in [Injuries]
KW - Population Surveillance
KW - Primary Prevention
KW - Registries
KW - Retrospective Studies
KW - Risk Factors
KW - Safety Management
KW - Seasons
KW - Time Factors
KW - Trauma Centers/sn [Statistics & Numerical Data]
KW - Wounds and Injuries/ep [Epidemiology]
KW - Wounds and Injuries/et [Etiology]
KW - Wounds and Injuries/pc [Prevention & Control]
RP - NOT IN FILE
SP - 11
EP - 20
JF - Journal of the Society of Pediatric Nurses
JA - J Soc Pediatr Nurs
VL - 6
IS - 1
CY - United States
N2 - ISSUES AND PURPOSE: To describe patient demographics, injury characteristics, and circumstances of playground injuries in children admitted to Pennsylvania trauma centers and to identify injury prevention strategies. DESIGN AND METHODS: Retrospective, descriptive study of 234 children ages 1 to 18 years sustaining playground-related injuries and whose hospital data were entered into the Pennsylvania Trauma Outcome Study. RESULTS: Most of the injuries occurred between April and September (77%), and noon to 6 P.M. (69%). Falls from playground equipment constituted the highest proportion of incidents (73%). Of 421 injuries (M = 1.8/patient), most were upper extremity (n = 117) and head (n = 110) injuries. PRACTICE IMPLICATIONS: Nurses can advocate for playground safety by teaching children to play safely and recommending age-appropriate equipment and protective surfacing. [References: 23]
SN - 1088-145X
AD - School of Nursing, University of Pittsburgh, PA, USA
UR - 11288499
ER -

TY - JOUR

ID - 385
T1 - Maternal attachment and sensitivity in an at-risk sample
A1 - Oyen,A.S.
A1 - Landy,S.
A1 - Hilburn-Cobb,C.
Y1 - 2000/09//
N1 - Oyen, A S. Landy, S. Hilburn-Cobb, C
Attachment & human development
100901315
IM
Journal Article
English
KW - Medline
KW - Adult
KW - Child
KW - Preschool
KW - Female
KW - Humans
KW - Infant
KW - Male
KW - Maternal Behavior
KW - Mother-Child Relations
KW - Object Attachment
KW - Ontario
KW - Personality Assessment
KW - Personality Development
KW - Play and Playthings
KW - Risk Factors
KW - Social Welfare
KW - Urban Population
RP - NOT IN FILE
SP - 203
EP - 217
JF - Attachment & Human Development
JA - Attach.Hum.Dev.
VL - 2
IS - 2
CY - England
N2 - This study investigated the relationship between adult attachment status and maternal sensitivity in a sample of 30 at-risk mother-child dyads. The children were 18-42 months old with an equal distribution of boys and girls that were at risk for compromised development due to a number of social, emotional and environmental factors. Using the Adult Attachment Interview (AAI; George, Kaplan, & Main, 1985) it was found that only 17% of the mothers were classified as autonomous, while 83% were classified as anxious regarding attachment. Sensitivity was measured using the Emotional Availability Scales (EAS) (Biringen, Robinson, & Emde, 1993). Each mother-child pair was video-taped in their home during a 30-minute interactional sequence in which they played with a given set of toys. Only the results from the Maternal Sensitivity Scale are reported here. Mothers who were free to evaluate their attachment experiences on the AAI were most sensitive. Mothers who showed anxiety in evaluating attachment, as a group, while undoubtedly caring and concerned, were less sensitive. There was, however, wide variability within the group of mothers with anxious attachment representations, Preoccupied mothers showing the least optimal interactions
SN - 1461-6734
AD - Barne- og ungdomspsykiatrisk avdeling (BUPA), Ullevaal sykehus, 0407 Oslo, Norway. asoyen@online.no
UR - 11707911
ER -

TY - JOUR
ID - 386
T1 - Maternal substance use during pregnancy and developmental outcome at age three
A1 - Faden,V.B.
A1 - Graubard,B.I.
Y1 - 2000///
N1 - Faden, V B. Graubard, B I
Journal of substance abuse
bjp, 9001404
IM
Journal Article
English
KW - Medline
KW - Cannabinoids/ae [Adverse Effects]
KW - Child
KW - Preschool
KW - Developmental Disabilities/di [Diagnosis]
KW - Developmental Disabilities/et [Etiology]
KW - Female
KW - Fetal Alcohol Syndrome/di [Diagnosis]
KW - Fetal Alcohol Syndrome/et [Etiology]
KW - Follow-Up Studies
KW - Humans
KW - Infant
KW - Newborn
KW - Logistic Models
KW - Male
KW - Personality Assessment
KW - Pregnancy
KW - Prenatal Exposure Delayed Effects
KW - Risk Factors
KW - Smoking/ae [Adverse Effects]
RP - NOT IN FILE
SP - 329
EP - 340
JF - Journal of Substance Abuse
JA - J Subst Abuse
VL - 12
IS - 4
CY - United States
N2 - PURPOSE: The data from the National Maternal and Infant Health Survey (NMIHS) and its 3-year follow-up offer a unique opportunity to study the effects of substance (alcohol, marijuana, and tobacco) use during gestation on development at age three in a nationally representative sample. Using this data, the relationship of development (language, gross motor, fine motor, and adaptive behavior) and specific behaviors (eating problems, length of play, activity level, difficulty of management, level of happiness, fearfulness, ability to get along with peers, tantrums, eating nonfood) and maternal drinking, marijuana use, and cigarette smoking was studied in a sample of live births who had been followed up at age three. METHODS: The data were analyzed using a cumulative logit model of ordinal responses. RESULTS: Higher activity level, greater difficulty of management, tantrums, eating problems, and eating nonfood were related to maternal drinking during pregnancy. Increased fearfulness, poorer motor skills, and shorter length of play were associated with maternal marijuana use during pregnancy. Less well developed language, higher activity level, greater difficulty of management, fearfulness, decreased ability to get along with peers, and increased tantrums were associated with maternal cigarette smoking during pregnancy. The preponderance of significant effects involved the behaviors studied rather than the developmental indices. IMPLICATIONS: It may be that the effects of substance use during pregnancy, especially more subtle ones, show up in behavior before they can be measured by developmental scales

SN - 0899-3289
AD - Division of Biometry and Epidemiology, National Institute on Alcohol Abuse and Alcoholism, 6000 Executive Boulevard, Bethesda, MD 20892, USA. vfaden@willco.niaaa.nih.gov
UR - 11452837
ER -

TY - JOUR
ID - 387
T1 - Clinical trials. Enforcers halt NIH study called less risky than outdoor play
A1 - Marshall,E.
Y1 - 2000/11/17/
N1 - Marshall, E
Science (New York, N.Y.)
0404511, uj7
IM
News
English
KW - Medline
KW - Child
KW - Clinical Trials as Topic
KW - Glucose Clamp Technique
KW - Humans
KW - National Institutes of Health (U.S.)
KW - Obesity/me [Metabolism]
KW - Risk
KW - United States
RP - NOT IN FILE
SP - 1281
JF - Science
VL - 290
IS - 5495
CY - UNITED STATES
N2 - How much risk should healthy children face in a research experiment? The answer can land even the best clinicians in hot water, especially if they read the rules differently from the government officials who must enforce them
SN - 0036-8075
UR - 11185397
ER -

TY - JOUR
ID - 388
T1 - [Physical activity in children]. [Review] [9 refs] [Japanese]
A1 - Okada,T.
A1 - Shimomitsu,K.
Y1 - 2000/09//
N1 - Okada, T. Shimomitsu, K
Nihon rinsho. Japanese journal of clinical medicine
kim, 0420546
IM
Journal Article. Review
Japanese
KW - Medline
KW - Adolescent
KW - Child
KW - Child Development
KW - Preschool

KW - Coronary Disease/pc [Prevention & Control]
KW - Exercise/ph [Physiology]
KW - Humans
KW - Obesity/pc [Prevention & Control]
KW - Play and Playthings
KW - Risk
RP - NOT IN FILE
SP - 290
EP - 293
JF - Nippon Rinsho - Japanese Journal of Clinical Medicine
JA - Nippon Rinsho
VL - 58 Suppl
CY - JAPAN
SN - 0047-1852
AD - Department of Pediatrics, Nihon University School of Medicine
UR - 11085130
ER -

TY - JOUR
ID - 389
T1 - Ocular injuries by elastic cords in children
A1 - Da,Pozzo S.
A1 - Pensiero,S.
A1 - Perissutti,P.
Y1 - 2000/11//
N1 - Da Pozzo, S. Pensiero, S. Perissutti, P
Pediatrics
oxv, 0376422
IM
Journal Article
English
KW - Medline
KW - Adult
KW - Age Factors
KW - Child
KW - Child Behavior
KW - Elastomers/ae [Adverse Effects]
KW - Eye Injuries/ep [Epidemiology]
KW - Eye Injuries/et [Etiology]
KW - Eye Injuries/pc [Prevention & Control]
KW - Follow-Up Studies
KW - Hospitalization
KW - Humans
KW - Play and Playthings/in [Injuries]
KW - Primary Prevention
KW - Retinal Detachment/et [Etiology]
KW - Transportation/is [Instrumentation]
KW - Transportation/mt [Methods]
KW - Trauma Severity Indices
KW - Visual Acuity
KW - Wounds
KW - Nonpenetrating/ep [Epidemiology]
KW - Nonpenetrating/et [Etiology]
KW - Nonpenetrating/pc [Prevention & Control]
RP - NOT IN FILE
SP - E65

JA - Pediatrics
VL - 106
IS - 5
CY - UNITED STATES
N2 - BACKGROUND: Elastic cords hitting the eyeball as high-speed projectiles can severely damage ocular structures and can produce permanent visual function impairment. OBJECTIVES: To evaluate the frequency, mechanics, and severity of eye injuries caused by elastic cords in children to adopt the most appropriate preventive measures. METHODS: A retrospective medical records review of hospital admissions secondary to ocular trauma between 1991 and 1997 in a pediatric ophthalmology unit at an urban tertiary care pediatric hospital was performed to select all children admitted for ocular injury caused by an elastic cord. RESULTS: Eight children fulfilled the inclusion criteria; the prevalence ratio was 2% of all pediatric trauma admissions. In all cases the mechanics of trauma was a combination of blunt and high-speed projectile injury. The mechanism of trauma in younger patients was typically a cord that was misused during unsupervised playtime, whereas cord slipping from car roof racks was noted in older patients. One patient suffered a severe permanent visual impairment caused by retinal detachment. All other children regained full visual acuity at the time of discharge and maintained it through a mean follow-up of 22 months (range: 18-29). CONCLUSION: Circumstances of injury in younger children are different from those found in older children, the latter being similar to those reported for adults. Prevention is the primary measure to be taken to reduce the prevalence of this injury and to lower the risk for ocular severe anatomic damage as much as possible. This can be achieved primarily by modifying the design of the hooks, intensifying educational campaigns, and keeping elastic cords out of children's reach
SN - 1098-4275
AD - Ophthalmology Unit, Children's Hospital, Trieste, Italy. dapozzo@onenet.it
UR - 11061802
ER -

TY - JOUR
ID - 390
T1 - Physicians play an important role in helping to eliminate childhood lead poisoning
A1 - Tillman,U.J.
Y1 - 2000/08//
N1 - Tillman, U J
Delaware medical journal
e0b, 0370077
IM
Journal Article
English
KW - Medline
KW - Child
KW - Preschool
KW - Delaware
KW - Female
KW - Guidelines as Topic
KW - Humans
KW - Lead Poisoning/pc [Prevention & Control]
KW - Male
KW - Mass Screening/st [Standards]
KW - Physician's Role
KW - Risk Factors
RP - NOT IN FILE
SP - 357
EP - 360
JF - Delaware Medical Journal
JA - Del.Med J
VL - 72
IS - 8

CY - UNITED STATES
SN - 0011-7781
AD - Division of Public Health, Delaware Health and Social Services, USA
UR - 10984979
ER -

TY - JOUR
ID - 391
T1 - Gender-specific characteristics of 5-year-olds' play narratives and associations with behavior ratings
A1 - von,Klitzing K.
A1 - Kelsay,K.
A1 - Emde,R.N.
A1 - Robinson,J.
A1 - Schmitz,S.
Y1 - 2000/08//
N1 - von Klitzing, K. Kelsay, K. Emde, R N. Robinson, J. Schmitz, S
Journal of the American Academy of Child and Adolescent Psychiatry
hg5, 8704565
IM
Comparative Study. Journal Article. Research Support, Non-U.S. Gov't. Research Support, U.S. Gov't, P.H.S..
Twin Study
English
KW - Medline
KW - Aggression/px [Psychology]
KW - Child
KW - Child Behavior
KW - Child Behavior Disorders/di [Diagnosis]
KW - Child Behavior Disorders/px [Psychology]
KW - Child Psychology
KW - Preschool
KW - Female
KW - Gender Identity
KW - Humans
KW - Internal-External Control
KW - Male
KW - Projective Techniques
KW - Psychiatric Status Rating Scales
KW - Sex Distribution
RP - NOT IN FILE
SP - 1017
EP - 1023
JF - Journal of the American Academy of Child & Adolescent Psychiatry
JA - J Am Acad.Child Adolesc Psychiatry
VL - 39
IS - 8
CY - UNITED STATES
N2 - OBJECTIVES: To examine the content and structure of children's play narratives in a large sample of 5-year-olds in order to replicate previous findings, explore the role of gender differences, and identify a pattern that can provide useful information about children's behavior. METHOD: The MacArthur Story Stem Battery and coding system was used to code content themes and coherence from play narratives of 652 twins in a nonclinical sample. To measure behavior problems, parents completed the Child Behavior Checklist when their children were aged 5 and 7 years; teachers completed the Teacher's Report Form when the children were aged 7 years. RESULTS: Girls told more coherent narratives with less aggression than boys. Aggressive themes were found to correlate with behavior problems as in previous studies. Upon further examination, this correlation held for girls but not boys. Children who told repeated aggressive/incoherent narratives had more behavior problems than those who did not show this narrative pattern. CONCLUSIONS: The Story Stem methodology is useful for

gaining access into the young child's inner world. The gender of the child, content of the story, and coherence of the story all provide useful information in identifying narratives that may indicate more risk for behavior problems
SN - 0890-8567
AD - Department of Child and Adolescent Psychiatry, University of Basel, Switzerland
UR - 10939230
ER -

TY - JOUR
ID - 392
T1 - Children are dying for some exercise
A1 - Egoscue,P.
Y1 - 2000/06//
N1 - Egoscue, P
Minnesota medicine
nby, 8000173
IM
Editorial
English
KW - Medline
KW - Adolescent
KW - Adult
KW - Athletic Injuries/et [Etiology]
KW - Athletic Injuries/mo [Mortality]
KW - Child
KW - Chronic Disease
KW - Exercise
KW - Female
KW - Humans
KW - Life Style
KW - Male
KW - Pain/et [Etiology]
KW - Play and Playthings
KW - Risk Factors
RP - NOT IN FILE
SP - 42
EP - 44
JF - Minnesota Medicine
JA - Minn.Med
VL - 83
IS - 6
CY - UNITED STATES
SN - 0026-556X
UR - 10881572
ER -

TY - JOUR
ID - 393
T1 - Gender-related behavior during childhood and associations with adult abdominal obesity: a nested case-control study in women
A1 - Rosmond,R.
A1 - Baghei,F.
A1 - Holm,G.
A1 - Bjorntorp,P.
Y1 - 2000/05//

N1 - Rosmond, R. Baghei, F. Holm, G. Bjorntorp, P
Journal of women's health & gender-based medicine
dfz, 100888719
IM
Journal Article. Research Support, Non-U.S. Gov't
English
KW - Medline
KW - Abdomen
KW - Adult
KW - Body Composition
KW - Case-Control Studies
KW - Child
KW - Child Behavior
KW - Female
KW - Humans
KW - Hyperandrogenism/ep [Epidemiology]
KW - Male
KW - Obesity/ep [Epidemiology]
KW - Questionnaires
KW - Sex
KW - Sweden/ep [Epidemiology]
KW - Women's Health
RP - NOT IN FILE
SP - 413
EP - 419
JF - Journal of Womens Health & Gender-Based Medicine
JA - J Womens Health Gend.Based Med
VL - 9
IS - 4
CY - UNITED STATES
N2 - Abdominal obesity affects many aspects of women's health, and recent studies indicate that hyperandrogenicity (HA) may contribute to the excess of body fat in women. As hormone behavior research attributes male-like play patterns in childhood to the effects of androgens, the aim of the present study was to assess the potential association of such behavior with obesity in adult women. In a randomly selected sample of 40-year-old women (n = 1464), 78% volunteered to respond to a questionnaire collecting information on the effect of other variables on childhood behavior. Self-reported body weight, height, and waist and hip circumferences were used to calculate body mass index (BMI) and waist/hip ratio (WHR). Age at menarche showed an inverse association with overweight (BMI > or = 25) (odds ratio [OR] = 0.82). Reports of gender-related behavior as a child showed that playing with girls and girl toys was negatively related to both overweight and abdominal obesity (WHR > or = 0.85). Among respondents who were overweight, relationships were found for playing with boys (OR = 0.90) and fighting (OR = 1.70). The OR of playing with boy toys and fighting among respondents with abdominal obesity were increased 1.12 and 1.65, respectively. Interests in athletics as a child seemed to decrease the risk for overweight (OR = 0.89) and abdominal obesity (OR = 0.91). Furthermore, dose-response analysis between the individual exposure levels and the OR for overweight showed a negative trend for playing with girls (p = 0.002) and girl toys (p = 0.017) and a positive trend for playing with boys (p = 0.011) and fighting (p = 0.031). Among respondents with abdominal obesity, positive dose-response effects were found for playing with boys (p = 0.026) and boy toys (p = 0.036) and fighting (p = 0.008). Thus, women with an elevated WHR showed a preference to play with boys and boy toys and also fought frequently as children. This might be a sign of a relative HA in childhood ("tomboyism"). These preliminary observations suggest that HA may originate in childhood
SN - 1524-6094
AD - Institute of Heart and Lung Diseases, Department of Cardiovascular Prevention, Sahlgrenska University Hospital, Goteborg, Sweden
UR - 10868614
ER -

TY - JOUR
ID - 394
T1 - Mothers' responses to sons and daughters engaging in injury-risk behaviors on a playground: implications for sex differences in injury rates
A1 - Morrongiello,B.A.
A1 - Dawber,T.
Y1 - 2000/06//
N1 - Morrongiello, B A. Dawber, T
Journal of experimental child psychology
2985128r, i2n
IM
Journal Article. Research Support, Non-U.S. Gov't
English
KW - Medline
KW - Child
KW - Female
KW - Gender Identity
KW - Humans
KW - Male
KW - Mother-Child Relations
KW - Parenting/px [Psychology]
KW - Play and Playthings
KW - Reinforcement
KW - Verbal
KW - Risk-Taking
KW - SOCIALIZATION
KW - Wounds and Injuries/pc [Prevention & Control]
KW - Wounds and Injuries/px [Psychology]
RP - NOT IN FILE
SP - 89
EP - 103
JF - Journal of Experimental Child Psychology
JA - J Exp.Child Psychol.
VL - 76
IS - 2
CY - UNITED STATES
N2 - Videotapes of children engaging in injury-risk activities on a playground were shown to mothers, who were asked to intervene by stopping the tape and saying whatever they would to their child in the situation shown. Results revealed that mothers of daughters were more likely to judge behaviors as posing some degree of injury risk, and they intervened more frequently and quickly than mothers of sons. Mothers' speed to intervene positively correlated with both children's injury history and their risk-taking tendencies, indicating that mothers of children who were previously injured and who often engaged in injury-risk behaviors had a higher degree of tolerance for children's risk taking than mothers of children who experienced fewer injuries and less frequently engaged in injury-risk behaviors. Mothers' verbalizations to children's risk taking revealed that daughters received more cautions and statements communicating vulnerability for injury, whereas sons received more statements encouraging risk-taking behavior. Copyright 2000 Academic Press
SN - 0022-0965
AD - University of Guelph, Ontario, Canada
UR - 10788304
ER -

TY - JOUR
ID - 395
T1 - The influence of physical activity, socioeconomic status, and ethnicity on the weight status of adolescents
A1 - McMurray,R.G.
A1 - Harrell,J.S.

A1 - Deng,S.
A1 - Bradley,C.B.
A1 - Cox,L.M.
A1 - Bangdiwala,S.I.
Y1 - 2000/03//
N1 - McMurray, R G. Harrell, J S. Deng, S. Bradley, C B. Cox, L M. Bangdiwala, S I
Obesity research
cde, 9305691
IM
Journal Article. Research Support, U.S. Gov't, P.H.S.
English
KW - Medline
KW - Adolescent
KW - African Americans
KW - Body Weight
KW - Child
KW - Ethnic Groups
KW - European Continental Ancestry Group
KW - Exercise
KW - Female
KW - Humans
KW - Logistic Models
KW - Male
KW - Rural Population
KW - Sex Characteristics
KW - Socioeconomic Factors
KW - Television
KW - Urban Population
KW - Video Games
RP - NOT IN FILE
SP - 130
EP - 139
JF - Obesity Research
JA - Obes.Res.
VL - 8
IS - 2
CY - UNITED STATES
N2 - OBJECTIVE: This study examined the effects of physical activity, television viewing, video game play, socioeconomic status (SES), and ethnicity on body mass index (BMI). RESEARCH METHODS AND PROCEDURES: The sample was 2389 adolescents, 10 to 16 years of age (12.7 +/- 1.0 years); 1240 (52%) females and 1149 (48%) males; 77% white and 23% African American; from rural (77%) and urban (23%) settings. BMI and skinfolds were directly assessed. All other data were obtained from questionnaires. RESULTS: Watching television on non-school days was related to being overweight ($p < 0.005$). However, when BMI analyses were adjusted for ethnicity and SES, there were no significant effects of television viewing on BMI ($p > 0.061$). Increased hours of video game play enhanced the risk of being overweight for both genders when analyses were adjusted for ethnicity and SES ($p < 0.019$). In males, participation in as little as one high-intensity physical activity 3 to 5 days a week decreased the ethnic- and SES-adjusted relative risk of being overweight (RR = 0.646; CI: 0.427 to 0.977). For females, the ethnic- and SES-adjusted relative risk for being overweight was not significantly altered by physical activity. The logistic analyses further indicated the influence of low SES and African American ethnicity overshadowed any direct effect of television or videos. DISCUSSION: Because weight status of male adolescents appears to be more related to exercise habits than to television or video game habits, increased participation in high-intensity exercise appears to be important. For females, neither videos nor exercise habits appear to be related to risk of being overweight. However, ethnicity and SES may be important factors that can influence body weight status, while television viewing may be of some importance. Thus, programs to reduce obesity in female adolescent should focus their efforts in lower SES communities

SN - 1071-7323
AD - Department of Exercise and Sport Science, University of North Carolina, Chapel Hill 27599-8700, USA. exphys@email.unc.edu
UR - 10757199
ER -

TY - JOUR
ID - 396
T1 - "Troo, the Traumaroo" bicycle and playground safety program: a pilot study of kindergarten through second graders in the southwest
A1 - Knight-Bohnhoff,K.
A1 - Smith,J.
A1 - Deis,J.
A1 - Chavez,Y.
A1 - Horne-Lucero,L.
Y1 - 1999/04//
N1 - Knight-Bohnhoff, K. Smith, J. Deis, J. Chavez, Y. Horne-Lucero, L
Journal of trauma nursing : the official journal of the Society of Trauma Nurses
cfl, 9512997, 9512997
N
Journal Article. Research Support, Non-U.S. Gov't
English
KW - Medline
KW - Bicycling/in [Injuries]
KW - Child
KW - Preschool
KW - Health Education/og [Organization & Administration]
KW - Health Knowledge
KW - Attitudes
KW - Practice
KW - Humans
KW - Pilot Projects
KW - Play and Playthings
KW - Program Evaluation
KW - Safety Management/og [Organization & Administration]
KW - School Health Services/og [Organization & Administration]
KW - Southwestern United States
RP - NOT IN FILE
SP - 28
EP - 36
JF - Journal of Trauma Nursing
JA - J Trauma Nurs
VL - 6
IS - 2
CY - UNITED STATES
N2 - "Troo, the Traumaroo" bicycle and playground safety education program created for kindergarten, first, and second grade students, was provided to a convenience sample of seven elementary schools in a Southwestern city. Favorable principal, classroom teacher, and school nurse evaluations indicated that the "Troo, the Traumaroo" program was successful in providing young children with bicycle and playground safety in a fun and entertaining way. Results of this pilot study indicated that kindergarten children pre-tested for bicycle safety knowledge prior to participating in the program, had significantly higher bicycle safety knowledge scores 30 days after participating in the program ($p < .0001$)
SN - 1078-7496
AD - Troo, the Traumaroo Bicycle and Playground Safety Program, Albuquerque, N.M., USA
UR - 10524047
ER -

TY - JOUR
ID - 397
T1 - [Documentation of the effects of child cap pistols]. [German]
A1 - Fleischer,G.
A1 - Hoffmann,E.
A1 - Lang,R.
A1 - Muller,R.
Y1 - 1999/06//
N1 - Fleischer, G. Hoffmann, E. Lang, R. Muller, R
HNO
g9p, 2985099r
IM
English Abstract. Journal Article. Research Support, Non-U.S. Gov't
German
KW - Medline
KW - Audiometry
KW - Pure-Tone
KW - Auditory Threshold
KW - Child
KW - Female
KW - Firearms
KW - Germany
KW - Hearing Loss
KW - Noise-Induced/di [Diagnosis]
KW - Noise-Induced/et [Etiology]
KW - Humans
KW - Male
KW - Play and Playthings
KW - Risk Factors
KW - Tinnitus/di [Diagnosis]
KW - Tinnitus/et [Etiology]
RP - NOT IN FILE
SP - 535
EP - 540
JF - HNO
VL - 47
IS - 6
CY - GERMANY
N2 - The study presents an analysis of 20 cases of acute acoustic trauma caused by toy pistols. The problem generally involves boys within the age range from 6 to 11 years. Half of the incidents involved attacks with toy pistols used as weapons. Auditory damage appeared in two types, each of which had a notch in the audiogram at the frequency range between 10 and 14 kHz. In children, a shot close to one ear can damage both ears. Great differences in the severity of tinnitus were apparent. The most severe cases of tinnitus were observed in the children with no or minor permanent auditory damage. Children experiencing long periods of uninterrupted tinnitus suffer significantly. This is not only detrimental to family life, but also to the performance of the children at school. The total expenses of treatment for these 20 cases were estimated to be approximately 80,000 DM (40,000 Euro)
SN - 0017-6192
AD - Arbeitsgruppe Horforschung, Medizinisches Zentrum fur Okologie, Justus-Liebig-Universitat Giessen
UR - 10427522
ER -

TY - JOUR
ID - 398
T1 - Behavioral inhibition in children from families at high risk for developing alcoholism

A1 - Hill,S.Y.
A1 - Lowers,L.
A1 - Locke,J.
A1 - Snidman,N.
A1 - Kagan,J.
Y1 - 1999/04//
N1 - Hill, S Y. Lowers, L. Locke, J. Snidman, N. Kagan, J
Journal of the American Academy of Child and Adolescent Psychiatry
hg5, 8704565
IM
Journal Article. Research Support, U.S. Gov't, P.H.S.
English
KW - Medline
KW - Adult
KW - Alcoholism/di [Diagnosis]
KW - Alcoholism/px [Psychology]
KW - Child
KW - Child Behavior/px [Psychology]
KW - Child Psychology
KW - Child of Impaired Parents/px [Psychology]
KW - Preschool
KW - Female
KW - Humans
KW - Inhibition (Psychology)
KW - Male
KW - Parents/px [Psychology]
KW - Risk Assessment
KW - Temperament
RP - NOT IN FILE
SP - 410
EP - 417
JF - Journal of the American Academy of Child & Adolescent Psychiatry
JA - J Am Acad.Child Adolesc Psychiatry
VL - 38
IS - 4
CY - UNITED STATES
N2 - OBJECTIVE: To determine whether children at risk for the development of adult alcohol dependence would show greater "behavioral inhibition" to the unfamiliar, an early childhood temperament characteristic. METHOD: One hundred peer play evaluations were conducted blindly with preschool children from families selected to be at high or low risk for developing alcohol dependence. Each child was paired with different children (same-sex pairs) in independent sessions to determine the stability of the behavioral response. RESULTS: High-risk children spent significantly more time staring at the other child during the peer play session while refraining from engaging in play, and significantly less time speaking to the other child. Significantly more time was spent proximal to the parent, but only on the first peer play session. These behaviors have been shown to be indicators of behavioral inhibition. CONCLUSIONS: These findings suggest that the presence of behavioral inhibition to the unfamiliar in childhood may be a risk factor for later development of alcohol dependence. While there is abundant evidence that childhood externalizing behaviors are risk factors for later development of substance dependence, the present results suggest that internalizing behaviors may be a pathway as well
SN - 0890-8567
AD - Department of Psychiatry, University of Pittsburgh Medical Center, Western Psychiatric Institute and Clinic, USA
UR - 10199112
ER -

TY - JOUR

ID - 399
T1 - Ocular injuries from paintball pellets
A1 - Thach,A.B.
A1 - Ward,T.P.
A1 - Hollifield,R.D.
A1 - Dugel,P.U.
A1 - Sipperley,J.O.
A1 - Marx,J.L.
A1 - Abrams,D.A.
A1 - Wroblewski,K.J.
A1 - Sonkin,P.L.
A1 - Birdsong,R.H.
A1 - Dunlap,W.A.
Y1 - 1999/03//
N1 - Thach, A B. Ward, T P. Hollifield, R D. Dugel, P U. Sipperley, J O. Marx, J L. Abrams, D A. Wroblewski, K J. Sonkin, P L. Birdsong, R H. Dunlap, W A
Ophthalmology
oi5, 7802443
IM
Journal Article
English
KW - Medline
KW - Adolescent
KW - Adult
KW - Child
KW - Cornea/in [Injuries]
KW - Eye Injuries/et [Etiology]
KW - Eye Injuries/pa [Pathology]
KW - Eye Injuries/su [Surgery]
KW - Eye Protective Devices
KW - Female
KW - Humans
KW - Hyphema/et [Etiology]
KW - Hyphema/pa [Pathology]
KW - Male
KW - Paint
KW - Play and Playthings/in [Injuries]
KW - Retinal Detachment/et [Etiology]
KW - Retinal Detachment/pa [Pathology]
KW - Retinal Perforations/et [Etiology]
KW - Retinal Perforations/pa [Pathology]
KW - Retrospective Studies
KW - Rupture
KW - Sclera/in [Injuries]
KW - Visual Acuity
KW - Vitreous Hemorrhage/et [Etiology]
KW - Vitreous Hemorrhage/pa [Pathology]
KW - Wounds
KW - Nonpenetrating/et [Etiology]
KW - Nonpenetrating/pa [Pathology]
KW - Nonpenetrating/su [Surgery]
RP - NOT IN FILE
SP - 533
EP - 537
JF - Ophthalmology
VL - 106

IS - 3
CY - UNITED STATES
N2 - OBJECTIVE: To evaluate the ocular effects of blunt trauma due to injury from a paintball pellet. DESIGN: Noncomparative case series. PARTICIPANTS: Thirteen patients who suffered ocular injury from paintballs are described. The patients presented to six different civilian and military emergency departments in tertiary care medical centers. INTERVENTION: Patients were treated for the ocular injury. MAIN OUTCOME MEASURES: Patients were evaluated for initial and final visual acuity. The reason for persistent loss of vision was delineated. RESULTS: There were 12 males and 1 female with an average age of 21 years (range, 12-33 years). Eleven of the 13 had no ocular protection at the time of the ocular injury. On initial examination, nine patients had a hyphema, nine had a vitreous hemorrhage, six had a retinal tear or detachment, three had corneal or corneal-scleral ruptures, and one had traumatic optic neuropathy. The final visual acuity was 20/40 or better in two patients, 20/50 to 20/150 in three patients, and 20/200 or worse in eight patients. CONCLUSION: Injuries due to paintball pellets can result in severe ocular damage and significant loss of vision. Eyecare professionals should be aware of the risks of this sport and must strongly advise participants to wear adequate protection when involved in this activity
SN - 0161-6420
AD - Ophthalmology Service, Walter Reed Army Medical Center, Washington, DC 20307, USA
UR - 10080210
ER -

TY - JOUR
ID - 400
T1 - The prevention of playground injuries
A1 - Hudson,S.
A1 - Thompson,D.
A1 - Mack,M.G.
Y1 - 1999/08//
N1 - Hudson, S. Thompson, D. Mack, M G
The Journal of school nursing : the official publication of the National Association of School Nurses
bgs, 9206498
N
Journal Article. Research Support, U.S. Gov't, P.H.S.
English
KW - Medline
KW - Accidental Falls/pc [Prevention & Control]
KW - Accidental Falls/sn [Statistics & Numerical Data]
KW - Child
KW - Child Welfare
KW - Humans
KW - Nursing Assessment
KW - Play and Playthings
KW - Primary Prevention/mt [Methods]
KW - Risk Assessment
KW - Risk Factors
KW - Safety Management/mt [Methods]
KW - School Nursing/mt [Methods]
KW - United States/ep [Epidemiology]
KW - Wounds and Injuries/ep [Epidemiology]
KW - Wounds and Injuries/pc [Prevention & Control]
RP - NOT IN FILE
SP - 30
EP - 33
JF - Journal of School Nursing
JA - J Sch Nurs
VL - 15
IS - 3

CY - UNITED STATES
N2 - Playground accidents are the most common cause of injury to children in school. As the health professional who attends to these injured children, school nurses should be aware of safety practices that can help eliminate the risks that children encounter. The National Program for Playground Safety has identified four areas that can reduce the level of risk on school playgrounds: supervision, age-appropriate design of the play area, fall surfaces under and around playground equipment, and equipment and surface maintenance. The article discusses each of these, and recommends leadership methods and practices that nurses can implement that will eliminate or reduce risk factors. In addition, a comprehensive playground risk model is described, and suggestions for policy areas are outlined. Policy areas include assessment, education, and enforcement in relation to each of the risk factors
SN - 1059-8405
AD - National Program for Playground Safety, School of Health, Physical Education and Leisure Services, University of Northern Iowa, Cedar Falls, USA
UR - 10745799
ER -

TY - JOUR
ID - 401
T1 - Problem-solving ability of inner-city children with and without in utero cocaine exposure
A1 - Betancourt,L.
A1 - Fischer,R.
A1 - Giannetta,J.
A1 - Malmud,E.
A1 - Brodsky,N.L.
A1 - Hurt,H.
Y1 - 1999/12//
N1 - Betancourt, L. Fischer, R. Giannetta, J. Malmud, E. Brodsky, N L. Hurt, H
Journal of developmental and behavioral pediatrics : JDBP
htf, 8006933
IM
Comparative Study. Journal Article. Research Support, Non-U.S. Gov't. Research Support, U.S. Gov't, P.H.S.
English
KW - Medline
KW - Child
KW - Preschool
KW - Cocaine
KW - Female
KW - Humans
KW - Pregnancy
KW - Prenatal Exposure Delayed Effects
KW - Problem Solving
KW - Socioeconomic Factors
KW - Urban Population/sn [Statistics & Numerical Data]
RP - NOT IN FILE
SP - 418
EP - 424
JF - Journal of Developmental & Behavioral Pediatrics
JA - J Dev.Behav Pediatr
VL - 20
IS - 6
CY - UNITED STATES
N2 - To evaluate the problem-solving abilities of a cohort of inner-city cocaine-exposed children and controls, children were invited to play with the Goodman Lock Box, a large red and blue box with 10 compartments, each having a different lock and toy. Examiners, blinded to the children's group status, coded the children's activities during the 6.5-minute play period. Nineteen behaviors were collapsed into three outcomes: Aimless Actions, Competence, and Mental Organization. Groups' scores were compared with scores of the Goodman

standardization sample of mixed socioeconomic status preschoolers. Seventy-three cocaine-exposed children and 82 controls were evaluated at age 3.5 years; of these, 58 cocaine-exposed and 63 controls were reevaluated at age 4.5 years. The groups' scores did not differ on any outcome at either time point (p > or = .22). However, both groups' Mental Organization scores were consistently lower than the Goodman group at each age (p < .01). This high-risk cohort may experience problems functioning in more complex environments such as the classroom
SN - 0196-206X
AD - Division of Neonatology, Albert Einstein Medical Center, Philadelphia, Pennsylvania 19141, USA
UR - 10608371
ER -

TY - JOUR
ID - 402
T1 - The socioemotional development of 5-year-old children of postnatally depressed mothers
A1 - Murray,L.
A1 - Sinclair,D.
A1 - Cooper,P.
A1 - Ducournau,P.
A1 - Turner,P.
A1 - Stein,A.
Y1 - 1999/11//
N1 - Murray, L. Sinclair, D. Cooper, P. Ducournau, P. Turner, P. Stein, A
Journal of child psychology and psychiatry, and allied disciplines
hp3, 0375361
IM
Journal Article. Research Support, Non-U.S. Gov't
English
KW - Medline
KW - Child Behavior Disorders/di [Diagnosis]
KW - Child Behavior Disorders/px [Psychology]
KW - Child of Impaired Parents/px [Psychology]
KW - Child
KW - Preschool
KW - Depression
KW - Postpartum/px [Psychology]
KW - Female
KW - Humans
KW - Male
KW - Marriage/px [Psychology]
KW - Object Attachment
KW - Parenting/px [Psychology]
KW - Personality Assessment
KW - Personality Development
KW - Risk Factors
KW - Social Adjustment
RP - NOT IN FILE
SP - 1259
EP - 1271
JF - Journal of Child Psychology & Psychiatry & Allied Disciplines
JA - J Child Psychol.Psychiatry
VL - 40
IS - 8
CY - ENGLAND
N2 - A community sample of depressed and well mothers, recruited at 2 months postpartum and assessed through to 18 months, was followed up at 5 years. The quality of mother-child interactions was assessed, as was the children's behavioural and social adjustment, using maternal reports and observations of child behaviour

during free play at school. Several aspects of child outcome were found to be associated with postnatal depression, even when taking account of current adverse circumstances (maternal depression and parental conflict). These included the child's behaviour with the mother, the presence of behavioural disturbance at home, and the content and social patterning of play at school. These associations with postnatal depression were independent of the child's gender. The child's relationship with the mother appeared to be mediated by the quality of infant attachment at 18 months. The mother's behaviour with her child was more affected by current difficulties, in particular by conflict with the child's father. Together these findings suggest that, while maternal behaviour varies with changing circumstances, exposure to maternal depression in the early postpartum months may have an enduring influence on child psychological adjustment
SN - 0021-9630
AD - Winnicott Research Unit, Department of Psychology, The University of Reading, UK
UR - 10604404
ER -

TY - JOUR
ID - 403
T1 - Childhood injury prevention at home and play. [Review] [39 refs]
A1 - Dowd,M.D.
Y1 - 1999/12//
N1 - Dowd, M D
Current opinion in pediatrics
but, 9000850
IM
Journal Article. Review
English
KW - Medline
KW - Accidents
KW - Home/pc [Prevention & Control]
KW - Bicycling/in [Injuries]
KW - Burns/pc [Prevention & Control]
KW - Child
KW - Head Protective Devices
KW - Humans
KW - Play and Playthings
KW - Poisoning/pc [Prevention & Control]
KW - Wounds
KW - Gunshot/pc [Prevention & Control]
RP - NOT IN FILE
SP - 578
EP - 582
JF - Current Opinion in Pediatrics
JA - Curr.Opin.Pediatr
VL - 11
IS - 6
CY - UNITED STATES
N2 - Unintentional injuries are the leading cause of death for children and adolescents in the United States, and they create a significant burden of disability and financial cost. If motor vehicle-associated injuries are not considered, children are most commonly injured in their home and play environments. The reduction over the past 20 years in childhood deaths related to motor vehicle injury has been significant, but rates of childhood death due to other causes, such as firearms, have increased. This review focuses on several categories of injuries other than motor vehicle injuries and highlights a few recent successful community- and practice-based injury-prevention programs. In addition, recent epidemiologic studies describing risk factors for injury-related death are discussed. Injuries due to interpersonal violence and motor vehicles are covered elsewhere. [References: 39]
SN - 1040-8703
AD - Division of Emergency Medicine, Children's Mercy Hospital, Kansas City, Missouri 64108, USA
UR - 10590919

ER -

TY - JOUR
ID - 404
T1 - Are ball pits the playground for potentially harmful bacteria?
A1 - Davis,S.G.
A1 - Corbitt,A.M.
A1 - Everton,V.M.
A1 - Grano,C.A.
A1 - Kiefner,P.A.
A1 - Wilson,A.S.
A1 - Gray,M.
Y1 - 1999/03//
N1 - Davis, S G. Corbitt, A M. Everton, V M. Grano, C A. Kiefner, P A. Wilson, A S. Gray, M
Pediatric nursing
oun, 7505804
N
Journal Article. Research Support, Non-U.S. Gov't
English
KW - Medline
KW - Bacterial Infections/et [Etiology]
KW - Bacterial Infections/pc [Prevention & Control]
KW - Child
KW - Preschool
KW - Colony Count
KW - Microbial
KW - Disinfection/mt [Methods]
KW - Equipment Contamination/pc [Prevention & Control]
KW - Equipment Contamination/sn [Statistics & Numerical Data]
KW - Health Education
KW - Humans
KW - Pediatric Nursing
KW - Pilot Projects
KW - Play and Playthings
KW - Restaurants
KW - Safety Management/mt [Methods]
KW - Urban Health/sn [Statistics & Numerical Data]
KW - Virginia
RP - NOT IN FILE
SP - 151
EP - 155
JF - Pediatric Nursing
JA - Pediatr Nurs
VL - 25
IS - 2
CY - UNITED STATES
N2 - Ball pits, enclosed play areas with padded floors and pits of small plastic balls, have become popular features for children at fast food restaurants. This pilot study sought to identify and confirm bacterial organisms that place children at a potential health risk in three play pits within fast food restaurants. Data for this descriptive study were randomly collected from restaurants offering play pits with multicolored, round, hollow, plastic balls within urban communities of the Tidewater region of Virginia. Specimens were collected from entrances into the ball pits as well as various areas of the bottom lining to incur a representative sample. Results indicated an increased level of normal flora as well as nonhuman flora, demonstrating that bacteria are present within the ball pits. The results question the safety of these play pits for both health care providers and parents. Nurses play a vital role in public awareness through health education. Disinfection protocol and proper handwashing are the keys to making ball pit play areas safe for children

SN - 0097-9805
AD - Coronary Care Unit, Riverside Regional Medical Center, Newport News, VA, USA
UR - 10532011
ER -

TY - JOUR
ID - 405
T1 - Initial assessment of children's ability to climb stair guarding
A1 - Riley,J.E.
A1 - Roys,M.S.
A1 - Cayless,S.M.
Y1 - 1998/12//
N1 - Riley, J E. Roys, M S. Cayless, S M
The journal of the Royal Society for the Promotion of Health
IM
Journal Article. Research Support, Non-U.S. Gov't
English
KW - Medline
KW - Accidents
KW - Home/pc [Prevention & Control]
KW - Home
KW - Biomechanics
KW - Body Height
KW - Child
KW - Child Behavior
KW - Preschool
KW - Female
KW - Hand Strength/ph [Physiology]
KW - Humans
KW - Leg/ah [Anatomy & Histology]
KW - Male
KW - Play and Playthings
KW - Posture
KW - Protective Devices/st [Standards]
KW - Safety Management/mt [Methods]
KW - Sex Characteristics
KW - Walking/in [Injuries]
KW - Walking/ph [Physiology]
RP - NOT IN FILE
SP - 331
EP - 337
JF - Journal of the Royal Society for the Promotion of Health
JA - J R SOC PROMOT HEALTH
VL - 118
IS - 6
CY - ENGLAND
N2 - This study examined the ability of children aged between four and six years to climb stair guarding set at the minimum height for domestic handrails recommended in the guidance given in Approved Document K that supports the Building Regulations in England and Wales. The purpose was to assess the adequacy of such safety guidance. Simulated guarding was set up at an indoor play centre and behaviours examined in two groups of children. Observations were made during free play and during solicited climbing. Results indicate that the majority of children aged between four and six years can climb onto or over stair guarding unassisted. Younger children are influenced and frequently abetted by their elders in climbing, behaviour which may be replicated in the home environment. Three distinct climbing strategies are observed: the body hoisted in a semi-prone position onto the top of the guarding; the body lifted by arms above the height of the guarding enabling a kneeling position on the top of the guarding; and, the knee bent against the side of the guarding to gain the

added height required to make the climb. It was noted that older or taller children tend to rely on height, leg length or each whilst climbing whereas younger children appear to depend more on strength. A re-examination of safety guidance in relation to barrier climbability by children is recommended, and has started at BRE
SN - 1466-4240
AD - Healthy Building Centre, Garston, Watford
UR - 10076694
ER -

TY - JOUR
ID - 406
T1 - [Toy cap pistols and their effect on hearing]. [German]
A1 - Fleischer,G.
A1 - Hoffmann,E.
A1 - Muller,R.
A1 - Lang,R.
Y1 - 1998/09//
N1 - Fleischer, G. Hoffmann, E. Muller, R. Lang, R
HNO
g9p, 2985099r
IM
English Abstract. Journal Article
German
KW - Medline
KW - Auditory Fatigue/ph [Physiology]
KW - Child
KW - Consumer Product Safety
KW - Europe
KW - Firearms
KW - Hearing Loss
KW - Noise-Induced/et [Etiology]
KW - Humans
KW - Play and Playthings
KW - Risk Factors
KW - Sound Spectrography
RP - NOT IN FILE
SP - 815
EP - 820
JF - HNO
VL - 46
IS - 9
CY - GERMANY
N2 - In order to understand the repeated occurrences of auditory impairments caused by toy pistols, an analysis of the circumstances involved was performed at Justus-Liebig University of Giessen. The acoustic impact of toy pistols on the ear was determined with a special measuring system for impulse noise. Results were compared with the acoustic impacts of the G3 common rifle of the German military on the ear of the soldier. It was apparent that all of the five randomly selected types of toy pistols were much louder than the military rifle, if fired close to the ear. The current standard of the European Union related to the safety of toys (EN 71-1) tolerates peaks of impulse noise from toy pistols that are actually illegal for workplaces without auditory protection. Measurements showed that the toy pistols tested were even louder than that tolerated by the EU standard. Problems related to acute acoustic trauma caused by these toys are discussed using recent examples. In order to prevent such injuries, we suggest limiting the loudness of the "worst case" instead of using unrealistic measurements
SN - 0017-6192
AD - Arbeitsgruppe Horforschung, Medizinisches Zentrum fur Okologie, Klinikum der Universitat Giessen
UR - 9816536
ER -

TY - JOUR
ID - 407
T1 - Working with avoidant children: a clinical challenge
A1 - Berson,N.
A1 - Meisburger,D.
Y1 - 1998/07//
N1 - Berson, N. Meisburger, D
Child welfare
0372735, d2u, 0372735
IM
Journal Article
English
KW - Medline
KW - Art
KW - Avoidance Learning
KW - Child
KW - Child Abuse/di [Diagnosis]
KW - Child Abuse/px [Psychology]
KW - Child Psychology
KW - Child Welfare
KW - Female
KW - Humans
KW - Interviews as Topic/mt [Methods]
KW - Male
KW - Photography
KW - Play and Playthings
KW - Professional-Patient Relations
KW - Risk Factors
KW - Truth Disclosure
RP - NOT IN FILE
SP - 427
EP - 439
JF - Child Welfare
VL - 77
IS - 4
CY - UNITED STATES
N2 - Clinicians have become increasingly aware of the difficulty inherent in interviewing avoidant children for possible maltreatment. Evaluations of avoidant children in cases involving multiple risk factors for maltreatment are often the most complex. The dilemma lies in balancing forensic standards while ensuring that every effort is made to enable children to relate their experiences. It is incumbent upon the evaluator to be aware of the reasons children may resist sharing their experiences. Initial stages of the interview process are discussed, including techniques for dealing with avoidant children
SN - 0009-4021
AD - Department of Psychiatry, University of North Carolina, Chapel Hill, USA
UR - 9666553
ER -

TY - JOUR
ID - 408
T1 - Toys within chocolate eggs--an ingestion hazard
A1 - Weizman,Z.
A1 - Krugliak,P.
Y1 - 1998/04//
N1 - Weizman, Z. Krugliak, P
Acta paediatrica (Oslo, Norway : 1992)

bgc, 9205968
IM
Letter
English
KW - Medline
KW - Accidents
KW - Cacao
KW - Candy/ae [Adverse Effects]
KW - Candy
KW - Child
KW - Preschool
KW - Foreign Bodies
KW - Humans
KW - Infant
KW - Play and Playthings/in [Injuries]
KW - Safety
RP - NOT IN FILE
SP - 478
EP - 479
JF - Acta Paediatrica
JA - Acta Paediatr
VL - 87
IS - 4
CY - NORWAY
SN - 0803-5253
UR - 9628313
ER -

TY - JOUR
ID - 409
T1 - The rocking horse risk
A1 - Woodward,S.
Y1 - 1998/04//
N1 - Woodward, S
Nursing management (Harrow, London, England : 1994)
b2k, 9433248
N
Journal Article
English
KW - Medline
KW - Accidental Falls/pc [Prevention & Control]
KW - Child
KW - Hospitals
KW - Pediatric/og [Organization & Administration]
KW - Public/og [Organization & Administration]
KW - Humans
KW - London
KW - Nursing
KW - Supervisory/og [Organization & Administration]
KW - Pediatric Nursing
KW - Play and Playthings
KW - Risk Management/og [Organization & Administration]
KW - State Medicine
RP - NOT IN FILE
SP - 10
EP - 13

JF - Nursing Management (Harrow)
JA - Nurs Manage.(London)
VL - 5
IS - 1
CY - ENGLAND
SN - 1354-5760
AD - Department of Nursing, Great Ormond Street Hospital for Children NHS Trust, London
UR - 9611595
ER -

TY - JOUR
ID - 410
T1 - Why do boys engage in more risk taking than girls? The role of attributions, beliefs, and risk appraisals
A1 - Morrongiello,B.A.
A1 - Rennie,H.
Y1 - 1998/02//
N1 - Morrongiello, B A. Rennie, H
Journal of pediatric psychology
jl8, 7801773
IM
Comparative Study. Journal Article. Research Support, Non-U.S. Gov't
English
KW - Medline
KW - Analysis of Variance
KW - Child
KW - Culture
KW - Female
KW - Humans
KW - Male
KW - Ontario
KW - Risk-Taking
KW - Sex Factors
KW - Social Perception
KW - Wounds and Injuries/pc [Prevention & Control]
KW - Wounds and Injuries/px [Psychology]
RP - NOT IN FILE
SP - 33
EP - 43
JF - Journal of Pediatric Psychology
JA - J Pediatr Psychol.
VL - 23
IS - 1
CY - UNITED STATES
N2 - OBJECTIVE: Assessed for age and sex differences in school-age children's reporting of injury-risk behaviors, ratings of injury-risk in various play situations, attributions for injuries (self, other, bad luck), and beliefs about their vulnerability to injury in comparison to their peers (more, less, comparable vulnerability). METHODS: We used a structured interview and drawings that depicted children showing wary or confident facial expressions when engaged in injury-risk play activities. RESULTS: Children's reported risk taking could be predicted from their risk appraisals, beliefs about the likelihood of injury, and attributions of injuries to bad luck, and these factors resulted 80% correct assignment of cases by sex in a discriminant analysis. The wary affect display resulted in higher injury-risk ratings than the confident display, with this effect being greater for girls than boys. CONCLUSIONS: Cognitive-based factors differentiate boys from girls and contribute to sex differences in children's injury-risk behaviors
SN - 0146-8693
AD - Case Western Reserve University, USA. bethc@pcicirs.pci.pitt.edu
UR - 9564127

ER -

TY - JOUR
ID - 411
T1 - From the Centers for Disease Control and Prevention. Toy-related injuries among children and teenagers--United States, 1996
Y1 - 1998/01/28/
N1 - JAMA : the journal of the American Medical Association
7501160
AIM, IM
Case Reports. Journal Article
English
KW - Medline
KW - Accidents
KW - Home
KW - Adolescent
KW - Child
KW - Preschool
KW - Consumer Product Safety
KW - Female
KW - Humans
KW - Infant
KW - Male
KW - Play and Playthings
KW - Safety
KW - United States/ep [Epidemiology]
KW - Wounds and Injuries/ep [Epidemiology]
KW - Wounds and Injuries/et [Etiology]
KW - Wounds and Injuries/mo [Mortality]
RP - NOT IN FILE
SP - 265
JF - JAMA
VL - 279
IS - 4
CY - UNITED STATES
SN - 0098-7484
UR - 9450699
ER -

TY - JOUR
ID - 412
T1 - Childhood eye injuries in North Jordan
A1 - Al-Bdour,M.D.
A1 - Azab,M.A.
Y1 - 1998///
N1 - Al-Bdour, M D. Azab, M A
International ophthalmology
gsf, 7904294
IM
Journal Article
English
KW - Medline
KW - Adolescent
KW - Age Distribution
KW - Athletic Injuries/ep [Epidemiology]
KW - Child

KW - Preschool
KW - Eye Injuries/ep [Epidemiology]
KW - Eye Injuries/et [Etiology]
KW - Eye Injuries/pp [Physiopathology]
KW - Female
KW - Humans
KW - Infant
KW - Newborn
KW - Jordan
KW - Male
KW - Play and Playthings
KW - Sex Distribution
KW - Time Factors
KW - Visual Acuity
KW - Wounds
KW - Nonpenetrating/ep [Epidemiology]
KW - Penetrating/ep [Epidemiology]
RP - NOT IN FILE
SP - 269
EP - 273
JF - International Ophthalmology
JA - Int.Ophthalmol.
VL - 22
IS - 5
CY - NETHERLANDS
N2 - AIMS: To provide epidemiological data on serious eye injuries among children that can be utilized in planning health education and safety strategies for preventing such injuries. MATERIALS AND METHODS: Records were reviewed of 116 children who sustained serious eye injuries which required admission to Princess Basma Teaching Hospital between October 1995 and November 1998. The material was analyzed retrospectively with respect to various epidemiological features. RESULTS: 71.5% of the injured children were male and 28.5% were female. There was a marked preponderance of injuries in the age group 6-10 years. The majority of injuries occurred during play and sport (74.1%). Stones and sharp objects were the most common causes accounting for 18.1% and 17.2% respectively. Most of the sharp objects were household instruments. Perforating injuries were more common than non-perforating injuries. 56% of injured eyes had a low vision with visual acuity between 6/24 and 3/60, and 13% had a blinding outcome with visual acuity less than 3/60. CONCLUSION: The major causes of serious eye injuries among these children were unnecessary risks during play, the use of dangerous objects and insufficient supervision by adults, especially at home. Health education should be directed towards prevention of such serious injuries
SN - 0165-5701
AD - Department of Ophthalmology, School of Medicine, Jordan University of Science and Technology, Irbid. mdb@firstnet.com.jo
UR - 10826542
ER -

TY - JOUR
ID - 413
T1 - Transorbital penetrating brain injury caused by a toy arrow: a case report. [Review] [17 refs]
A1 - Paucic-Kirincic,E.
A1 - Prpic,I.
A1 - Gazdik,M.
A1 - Kriz,M.
A1 - Vojnikovic,B.
A1 - Golubovic,V.
Y1 - 1997/07//
N1 - Paucic-Kirincic, E. Prpic, I. Gazdik, M. Kriz, M. Vojnikovic, B. Golubovic, V
Pediatric rehabilitation

c5h, 9709256
IM
Case Reports. Journal Article. Review
English
KW - Medline
KW - Brain Injuries/et [Etiology]
KW - Cerebral Hemorrhage/et [Etiology]
KW - Child
KW - Cognition Disorders/et [Etiology]
KW - Coma/et [Etiology]
KW - Eye Enucleation
KW - Eye Injuries
KW - Penetrating/et [Etiology]
KW - Follow-Up Studies
KW - Glasgow Coma Scale
KW - Hemiplegia/et [Etiology]
KW - Humans
KW - Male
KW - Orbital Fractures/et [Etiology]
KW - Play and Playthings/in [Injuries]
KW - Prolapse
KW - Tomography
KW - X-Ray Computed
KW - Wounds
RP - NOT IN FILE
SP - 191
EP - 193
JF - Pediatric Rehabilitation
JA - Pediatr Rehabil.
VL - 1
IS - 3
CY - ENGLAND
N2 - A case of a 9-year-old boy with a transorbital toy-arrow injury to the brain is presented. At admission he was in coma (Glasgow Coma Scale of 6) with right hemiparesis and had a completely prolapsed left eye. Computerized tomography revealed intracranial haemorrhage and fracture of the orbital wall, which were treated conservatively. His left eye was enucleated due to massive injury. At the 6-month check-up the boy still show neurological signs of latent right hemiparesis. Disturbances, mostly cognitive, were noted on his psychological tests. A survey of the literature reveals no report of this nature in the paediatric age group. The necessity of continuous monitoring of new environmental risks as they occur, and the requirement for the prevention of recreational brain injuries in children, is stressed. [References: 17]
SN - 1363-8491
AD - Department of Pediatrics Children's Hospital Kantrida, Rijeka, Croatia
UR - 9689255
ER -

TY - JOUR
ID - 414
T1 - Childhood obesity: a risk factor for traumatic injuries to anterior teeth
A1 - Petti,S.
A1 - Cairella,G.
A1 - Tarsitani,G.
Y1 - 1997/12//
N1 - Petti, S. Cairella, G. Tarsitani, G
Endodontics & dental traumatology
ehd, 8508054
D

Journal Article
English
KW - Medline
KW - Accident Proneness
KW - Age Distribution
KW - Child
KW - Child Behavior
KW - Cross-Sectional Studies
KW - Female
KW - Humans
KW - Life Style
KW - Male
KW - Obesity/co [Complications]
KW - Obesity/px [Psychology]
KW - Prevalence
KW - Questionnaires
KW - Risk Factors
KW - Rome/ep [Epidemiology]
KW - Sex Distribution
KW - Tooth Injuries/ep [Epidemiology]
KW - Tooth Injuries/et [Etiology]
RP - NOT IN FILE
SP - 285
EP - 288
JF - Endodontics & Dental Traumatology
JA - Endod.Dent.Traumatol.
VL - 13
IS - 6
CY - DENMARK
N2 - The relationship between obesity and traumatic dental injuries was evaluated in a study population of 938 6- to 11-year-old schoolchildren from Rome, Italy, and it was suggested by the lifestyle and the physical activity of obese children, which, according to many authors, is different from the lifestyle of other children. The subjects were examined at school: presence and type of dental injury, overjet, incisor protrusion, upper lip incompetence and presence of obesity were recorded. Trauma predisposition was evaluated with a questionnaire investigating the behaviour which may predispose to impacts. Six out of eight questions in this questionnaire concerned the children's lifestyle and the trauma predisposition score was therefore also considered an estimate of lifestyle and physical activity. The dental injury prevalence of the study population was 21.3% and obesity prevalence was 11.4%. Of the obese children 30.8% had dental injuries vs. only 20.0% of non-obese subjects (P = 0.007). Obese children had only enamel and enamel-dentine fractures and the main reported trauma cause was indoor play; conversely, lean children also had other types of injuries and the most frequently reported cause was outdoor play. The main predisposing factors significantly affecting the probability of dental injury, evaluated with multiple logistic regression, were upper lip uncoverage (OR: 1.23; P = 0.02), overjet larger than 3 mm (OR: 1.68; P = 0.001) and obesity (OR: 1.45; P = 0.01). Surprisingly, trauma predisposition was a protective factor (OR: 0.50; P = 0.00001). The results of this study were explained by the significant inverse correlation between obesity and trauma-predisposing behaviour, thus suggesting that subjects frequently playing sports and lively games were not only less obese but also more skillful and, for this reason, less prone to trauma when they fell or sustained impacts
SN - 0109-2502
AD - G. Sanarelli Hygiene Institute, La Sapienza University, Rome, Italy
UR - 9558511
ER -

TY - JOUR
ID - 415
T1 - Council playgrounds in New South Wales: compliance with safety guidelines
A1 - Witheaneachi,D.

A1 - Meehan,T.
Y1 - 1997/10//
N1 - Witheaneachi, D. Meehan, T
Australian and New Zealand journal of public health
ck2, 9611095
IM
Journal Article
English
KW - Medline
KW - Child
KW - Child Welfare
KW - Humans
KW - New South Wales
KW - Play and Playthings
KW - Safety
KW - Wounds and Injuries/pc [Prevention & Control]
RP - NOT IN FILE
SP - 577
EP - 580
JF - Australian & New Zealand Journal of Public Health
JA - Aust N.Z.J Public Health
VL - 21
IS - 6
CY - AUSTRALIA
N2 - Despite initiatives to improve the safety of play environments, playground-related injuries continue to be a major public health problem. Efforts to address playground safety in New South Wales were consolidated during 1994 through the Kidsafe Playground Safety Project. In stage 1 of the project (described here), 240 council playgrounds were assessed to determine the extent to which playground equipment complied with safety guidelines. On-site inspection of the selected playgrounds provided data on 862 separate pieces of playground equipment. Of the 723 pieces requiring undersurfacing, less than half (45.4 per cent) had the recommended type of undersurfacing while only 42 of those pieces had undersurfacing to the recommended depth. However, when the fall height of equipment was considered in addition to the undersurfacing guidelines, only 13 (1.8 per cent) of the 723 pieces of equipment simultaneously satisfied all of the safety guidelines. Regrettably, none of the 240 council playgrounds assessed complied fully with the key safety guidelines. The results underscore the need for a collaborative effort on the part of local government, Kidsafe and health promotion and regional public health units to ensure that council playgrounds in New South Wales comply with recommended safety guidelines
SN - 1326-0200
AD - Kidsafe NSW, The New Children's Hospital, Sydney
UR - 9470261
ER -

TY - JOUR
ID - 416
T1 - Talking about self and other: emergence of an internal state lexicon in young children with Down syndrome
A1 - Beeghly,M.
A1 - Cicchetti,D.
Y1 - 1997///
N1 - Beeghly, M. Cicchetti, D
Development and psychopathology
cn7, 8910645
IM
Journal Article. Research Support, Non-U.S. Gov't
English
KW - Medline
KW - Adult

KW - Affect
KW - Child
KW - Preschool
KW - Cognition
KW - Cohort Studies
KW - Down Syndrome/px [Psychology]
KW - Emotions
KW - Female
KW - Humans
KW - Infant
KW - Interpersonal Relations
KW - Judgment
KW - Language Development
KW - Male
KW - Maternal Age
KW - Reference Values
KW - Self Concept
KW - Socioeconomic Factors
KW - Speech
RP - NOT IN FILE
SP - 729
EP - 748
JF - Development & Psychopathology
JA - Dev.Psychopathol.
VL - 9
IS - 4
CY - UNITED STATES
N2 - The ability to talk about the internal states (IS) of self and other is an age-typical development of early childhood that is thought to reflect young children's emergent self-other understanding. This study examined the emergence of an IS lexicon in a cross-sectional sample of young children with Down syndrome (DS) and a cognitively and demographically comparable group of normally developing (ND) children. Children's IS lexicons were derived from transcripts of their spontaneous utterances during two laboratory contexts: a mother-child emotions picture book task and semistructured play. Children with DS produced significantly fewer IS words and fewer IS word types than their MA-matched counterparts. Controlling for corpus size, children with DS also were less likely to attribute internal states to themselves and were more context bound in their use of IS language. In addition, children with DS also differed from ND children in the semantic content of their IS language, with proportionately higher rates of affective words and lower rates for words about volition ability, and cognition. For both the DS and ND groups, individual differences in IS language production were significantly related to general expressive language skills. However, dissociations were observed for the relation between children's IS word production and nonverbal symbolic play skills in the two groups. These findings suggest some degree of disorganization at the interface among symbolic domains for children with DS. Because IS language is critical to the regulation of social interaction and an early index of self-other differentiation and understanding, children with DS may be at risk for later compromises in self-organization
SN - 0954-5794
AD - Harvard Medical School, Boston, MA, USA. beeghly@a1.tch.harvard.edu
UR - 9449003
ER -

TY - JOUR
ID - 417
T1 - An analysis of playground surface injuries
A1 - Mack,M.G.
A1 - Thompson,D.
A1 - Hudson,S.
Y1 - 1997/12//

N1 - Mack, M G. Thompson, D. Hudson, S
Research quarterly for exercise and sport
r6y, 8006373
IM
Journal Article. Research Support, U.S. Gov't, P.H.S.
English
KW - Medline
KW - Accidental Falls/sn [Statistics & Numerical Data]
KW - Child
KW - Preschool
KW - Humans
KW - Play and Playthings
KW - Safety
KW - United States/ep [Epidemiology]
KW - Wounds and Injuries/ep [Epidemiology]
RP - NOT IN FILE
SP - 368
EP - 372
JF - Research Quarterly for Exercise & Sport
JA - Res.Q Exerc.Sport
VL - 68
IS - 4
CY - UNITED STATES
SN - 0270-1367
AD - School of Health, Physical Education, and Leisure Services, University of Northern Iowa, USA
UR - 9421850
ER -

TY - JOUR
ID - 418
T1 - Toy-related injuries among children and teenagers--United States, 1996
A1 - Centers for Disease Control and Prevention (CDC)
Y1 - 1997/12//
N1 - Centers for Disease Control and Prevention (CDC)
MMWR. Morbidity and mortality weekly report
ne8, 7802429
IM
Case Reports. Journal Article
English
KW - Medline
KW - Accidents
KW - Home
KW - Adolescent
KW - Child
KW - Preschool
KW - Consumer Product Safety
KW - Female
KW - Humans
KW - Infant
KW - Male
KW - Play and Playthings
KW - Safety
KW - United States/ep [Epidemiology]
KW - Wounds and Injuries/ep [Epidemiology]
KW - Wounds and Injuries/et [Etiology]
KW - Wounds and Injuries/mo [Mortality]

RP - NOT IN FILE
SP - 1185
EP - 1189
JF - MMWR - Morbidity & Mortality Weekly Report
JA - MMWR Morb.Mortal.Wkly.Rep.
VL - 46
IS - 50
CY - UNITED STATES
N2 - Each year, approximately two billion toys and games are sold in the United States. Although most toys are safe when risks are measured against the frequency of their use, children are at risk for some toy-related injuries and deaths. To characterize the magnitude of this problem, CDC analyzed data from the U.S. Consumer Product Safety Commission (CPSC) for 1996. This report summarizes this analysis and underscores the importance of parental participation in the selection and use of toys
SN - 0149-2195
UR - 9414147
ER -

TY - JOUR
ID - 419
T1 - Behavioural changes in children following day-case surgery: a 4-week follow-up of 551 children
A1 - Kotiniemi,L.H.
A1 - Ryhanen,P.T.
A1 - Moilanen,I.K.
Y1 - 1997/10//
N1 - Kotiniemi, L H. Ryhanen, P T. Moilanen, I K
Anaesthesia
4mc, 0370524
AIM, IM
Journal Article. Multicenter Study
English
KW - Medline
KW - Adolescent
KW - Age Factors
KW - Ambulatory Surgical Procedures/px [Psychology]
KW - Anesthesia
KW - General
KW - Child
KW - Child Behavior
KW - Child Behavior Disorders/et [Etiology]
KW - Preschool
KW - Female
KW - Follow-Up Studies
KW - Humans
KW - Infant
KW - Male
KW - Pain
KW - Postoperative/px [Psychology]
KW - Play and Playthings
KW - Postoperative Complications
KW - Preanesthetic Medication/px [Psychology]
KW - Prospective Studies
KW - Risk Factors
RP - NOT IN FILE
SP - 970
EP - 976
JF - Anaesthesia

VL - 52
IS - 10
CY - ENGLAND
N2 - The purpose of this prospective multicentre survey was to evaluate the occurrence and the type of changes in children's behaviour during the first 4 weeks following the day of surgery, and to assess the significance of some patient-related factors on the incidence. Pre- and postoperative questionnaires were completed by the parents of 551 children aged 4 months to 13.4 years in five hospitals incorporating nine operative units in Northern Finland. The overall incidence of problematical behavioural changes was 47% and that of beneficial changes 17%. Problematical changes were most common in the 1.0 to 2.9 year olds and the incidence decreased significantly from 46% on the day of the operation to 9% 4 weeks later ($p < 0.0001$). Predictors by multiple logistic regression analysis were age, mild pain at home following surgery, severe pain and a previous bad experience of health care which had adversely affected the attitude of the child towards doctors or nurses. Hospital influenced playing was a significant factor 3 and 4 weeks after the operation. By the 4th week, beneficial and problematical changes were equally common (9%). Gender, previous operations and experience of repeated paracenteses (for treatment of middle ear infection) did not have a significant effect on the incidence. Pain on the day of the operation predicted the occurrence of behavioural problems up to the 4th week, 2-4 weeks longer than the duration of pain itself. The results emphasise the importance of effective prevention of postoperative pain as well as the importance of avoiding unpleasant experiences in all contacts children have with health care. Playing could perhaps be used to help children cope with a short hospital experience
SN - 0003-2409
AD - Department of Anaesthesiology, University of Oulu, Finland
UR - 9370839
ER -

TY - JOUR
ID - 420
T1 - Safety of surfaces and equipment for children in playgrounds
A1 - Mott,A.
A1 - Rolfe,K.
A1 - James,R.
A1 - Evans,R.
A1 - Kemp,A.
A1 - Dunstan,F.
A1 - Kemp,K.
A1 - Sibert,J.
Y1 - 1997/06/28/
N1 - Mott, A. Rolfe, K. James, R. Evans, R. Kemp, A. Dunstan, F. Kemp, K. Sibert, J
Lancet
2985213r, l0s, 0053266
AIM, IM
Journal Article. Research Support, Non-U.S. Gov't
English
KW - Medline
KW - Accidental Falls/sn [Statistics & Numerical Data]
KW - Adolescent
KW - Arm Injuries/ep [Epidemiology]
KW - Arm Injuries/et [Etiology]
KW - Child
KW - Child Welfare
KW - Preschool
KW - Construction Materials
KW - Fractures
KW - Bone/ep [Epidemiology]
KW - Bone/et [Etiology]
KW - Humans
KW - Infant

KW - Newborn
KW - Play and Playthings
KW - Rubber
KW - Safety
KW - Surface Properties
KW - Wales/ep [Epidemiology]
KW - Wounds and Injuries/ep [Epidemiology]
KW - Wounds and Injuries/et [Etiology]
KW - Wounds and Injuries/pc [Prevention & Control]
RP - NOT IN FILE
SP - 1874
EP - 1876
JF - Lancet
VL - 349
IS - 9069
CY - ENGLAND
N2 - BACKGROUND: The safety of playgrounds is important to protect children from injury, but studies are mostly done mainly under laboratory conditions without epidemiological data. We investigated the safety of different playground surfaces, and types and heights of equipment in public playgrounds in the City of Cardiff, UK. METHODS: We did a correlational study of 330 children aged between 0 and 14 years. All children were hurt when playing in playgrounds in Cardiff and presented to the Accident and Emergency Department in Cardiff Royal Infirmary during summer (April to September) 1992 and 1993, and the whole of 1994. We studied the children's hospital records to establish the type of injury and interviewed their parents to find out the playground and type of equipment involved. The main outcome measures were the number of children injured whilst playing, and injury rates per observed number of children on different surfaces, types, and heights of equipment. FINDINGS: Children sustained significantly more injuries in playgrounds with concrete surfaces than in those with bark or rubberised surfaces ($p < 0.001$). Playgrounds with rubber surfaces had the lowest rate of injury, with a risk half that of bark and a fifth of that of concrete. Bark surfaces were not significantly more protective against arm fractures than concrete. Most injuries were equipment related. Injury risk due to falls from monkey bars (suspended parallel bars or rings between which children swing) was twice that for climbing-frames and seven times that for swings or slides. The height of the equipment correlated significantly with the number of fractures ($p = 0.005$) from falls. INTERPRETATION: Rubber or bark surfacing is associated with a low rate of injuries and we support their use in all public playgrounds. Bark alone is insufficient, however, to prevent all injuries, particularly arm fractures. Rubberised impact-absorbing surfaces are safer than bark. We believe that playing on monkeys bars increases the risk of injury in playgrounds and that they should generally not be installed. Safety standards should be based on physical and epidemiological data. Our data suggest that the proposed raising of the maximum fall height from 2.5 m to 3.0 m in Europe is worrying
SN - 0140-6736
AD - Department of Child Health, University of Wales College of Medicine Academic Centre, Llandough Hospital, Penarth, Vale of Glamorgan, UK
UR - 9217759
ER -

TY - JOUR
ID - 421
T1 - A descriptive analysis of children's playground injuries in the United States 1990-4
A1 - Mack,M.G.
A1 - Hudson,S.
A1 - Thompson,D.
Y1 - 1997/06//
N1 - Mack, M G. Hudson, S. Thompson, D
Injury prevention : journal of the International Society for Child and Adolescent Injury Prevention
crz, 9510056
IM
Journal Article. Research Support, U.S. Gov't, P.H.S.
English

KW - Medline
KW - Adolescent
KW - Age Distribution
KW - Child
KW - Preschool
KW - Data Collection
KW - Equipment and Supplies/sn [Statistics & Numerical Data]
KW - Female
KW - Hospitalization/sn [Statistics & Numerical Data]
KW - Humans
KW - Infant
KW - Newborn
KW - Injury Severity Score
KW - Male
KW - Play and Playthings/in [Injuries]
KW - Risk Factors
KW - Safety
KW - Sampling Studies
KW - Sex Distribution
KW - Survival Rate
KW - United States/ep [Epidemiology]
KW - Wounds and Injuries/cl [Classification]
KW - Wounds and Injuries/ep [Epidemiology]
KW - Wounds and Injuries/et [Etiology]
KW - Wounds and Injuries/pc [Prevention & Control]
RP - NOT IN FILE
SP - 100
EP - 103
JA - Injury Prevention
VL - 3
IS - 2
CY - ENGLAND
N2 - OBJECTIVES: To review playground injury statistics over a five year period in order to develop an awareness of how and where children in the United States are being injured. METHODS: All data are based on the United States Consumer Product Safety Commission's National Electronic Injury Surveillance System (NEISS) for playground related injuries during 1990-4. The surveillance data includes injuries recorded in more than 90 hospital emergency departments located throughout the United States. RESULTS: Each year there are roughly 211,000 preschool or elementary school-children in the United States who receive emergency department care for injuries associated with playground equipment. On average, 17 of these cases result in death. 70% of all injuries occur on public playgrounds, with nearly one third classified as severe. Swings, climbers, and slides are the pieces of playground equipment associated with 88% of all NEISS reported injuries. Falls to the surface are responsible for 70%. CONCLUSIONS: NEISS playground injury statistics contribute to our understanding of playground injuries. By identifying where and how children are injured, suggestions can be made in an attempt to make playgrounds safer
SN - 1353-8047
AD - National Program for Playground Safety, University of Northern Iowa, School of Health, Physical Education, and Leisure Services, Cedar Falls 50614-0161, USA
UR - 9213154
ER -

TY - JOUR
ID - 422
T1 - Factors parents use in selecting play spaces for young children
A1 - Sallis,J.F.
A1 - McKenzie,T.L.
A1 - Elder,J.P.

A1 - Broyles,S.L.
A1 - Nader,P.R.
Y1 - 1997/04//
N1 - Sallis, J F. McKenzie, T L. Elder, J P. Broyles, S L. Nader, P R
Archives of pediatrics & adolescent medicine
9422751, bwf
AIM, IM
Comparative Study. Journal Article. Research Support, U.S. Gov't, P.H.S.
English
KW - Medline
KW - Adult
KW - California
KW - Child
KW - Preschool
KW - Data Collection
KW - Decision Making
KW - European Continental Ancestry Group/px [Psychology]
KW - Female
KW - Humans
KW - Male
KW - Mexican Americans/px [Psychology]
KW - Parents/px [Psychology]
KW - Play and Playthings
KW - Safety
KW - Socioeconomic Factors
RP - NOT IN FILE
SP - 414
EP - 417
JF - Archives of Pediatrics & Adolescent Medicine
JA - Arch.Pediatr Adolesc Med
VL - 151
IS - 4
CY - UNITED STATES
N2 - BACKGROUND: The amount of time children spend in play spaces (ie, physical locations that are appropriate for children's physical activity) near their homes is correlated with their level of physical activity. OBJECTIVE: To examine factors used in parents' decisions about the selection of play spaces for their children. SUBJECTS: Parents (primarily mothers) of 178 Mexican American and 122 white children who were a mean age of 4.9 years old at the first measurement. MEASURES: In individual interviews, parents rated 24 factors on their importance in selecting for their children a play space that is away from their home or yard. Decision factors were rated from 1 (ie, not important at all) to 5 (ie, very important). RESULTS: The most important factors, with ratings ranging from 4.8 to 4.2, were safety and availability of toilets, drinking water, lighting, and shade. Mexican American parents rated 8 of 24 items significantly higher than did white parents, including lighted at night, organized activities, play supplies, and drinking water. White parents rated 5 of 24 items significantly higher than did Mexican American parents, including distance from home, cost of admission, and child's friends go there. The rated importance of 7 of 24 items increased during 1 year, including play supplies, drinking water, distance from home, and parents' friends or relatives go there. CONCLUSIONS: These results indicate that parents can identify factors they use in selecting places for their young children to play, and selection factors differ somewhat by ethnicity or socioeconomic status. Further studies are needed to determine whether improvements on the most important selection factors might be effective in increasing the use of play spaces by children and their parents. Clinicians may be able to use the most highly rated decision factors to help parents assess the acceptability of play spaces in their areas
SN - 1072-4710
AD - Department of Psychology, San Diego State University, USA
UR - 9111442
ER -

TY - JOUR
ID - 423
T1 - Peer assessment of social reputation in community samples of disruptive and nondisruptive children: utility of the revised class play method
A1 - Realmuto,G.M.
A1 - August,G.J.
A1 - Sieler,J.D.
A1 - Pessoa-Brandao,L.
Y1 - 1997/03//
N1 - Realmuto, G M. August, G J. Sieler, J D. Pessoa-Brandao, L
Journal of clinical child psychology
co7, 7603095
IM
Comparative Study. Journal Article. Research Support, U.S. Gov't, P.H.S.
English
KW - Medline
KW - Child
KW - Female
KW - Humans
KW - Longitudinal Studies
KW - Male
KW - Peer Group
KW - Social Behavior Disorders/di [Diagnosis]
KW - Social Behavior Disorders/px [Psychology]
KW - Social Desirability
RP - NOT IN FILE
SP - 67
EP - 76
JF - Journal of Clinical Child Psychology
JA - J Clin.Child Psychol.
VL - 26
IS - 1
CY - UNITED STATES
N2 - Examined the psychometric properties of the Revised Class Play (RCP), a peer assessment measure of social reputation, in epidemiological samples of disruptive (n = 220) and nondisruptive (n = 104) children in Grades 2 through 5. Principal components analyses yielded a four-factor solution that was similar for disruptive and nondisruptive children and to previous research with this instrument. Discriminative function analyses demonstrated that the four RCP dimensions were each successful in predicting group membership, with the leadership and social etiquette dimensions best able to differentiate disruptive and nondisruptive groups. Regression modeling showed that the variance accounted for by the four RCP dimensions was large and varied for specific dimensions based on the criterion variable chosen. The advantages of the RCP as a devise for tracking social competence and peer reputation in high-risk disruptive children are discussed
SN - 0047-228X
AD - Division of Child and Adolescent Psychiatry, University of Minnesota, Minneapolis, USA
UR - 9118177
ER -

TY - JOUR
ID - 424
T1 - Prevention and management of aerodigestive foreign body injuries in childhood. [Review] [13 refs]
A1 - Reilly,J.S.
A1 - Cook,S.P.
A1 - Stool,D.
A1 - Rider,G.
Y1 - 1996/12//

N1 - Reilly, J S. Cook, S P. Stool, D. Rider, G
Pediatric clinics of North America
oum, 0401126
AIM, IM
Journal Article. Review
English
KW - Medline
KW - Age Distribution
KW - Bronchoscopy
KW - Child
KW - Preschool
KW - Computer Simulation
KW - Esophagoscopy
KW - Esophagus/in [Injuries]
KW - Esophagus
KW - Foreign Bodies/di [Diagnosis]
KW - Foreign Bodies/ep [Epidemiology]
KW - Foreign Bodies/th [Therapy]
KW - Humans
KW - Infant
KW - Newborn
KW - Larynx/in [Injuries]
KW - Larynx
KW - Play and Playthings
KW - Risk Factors
KW - Safety
RP - NOT IN FILE
SP - 1403
EP - 1411
JF - Pediatric Clinics of North America
JA - Pediatr Clin.North Am
VL - 43
IS - 6
CY - UNITED STATES
N2 - Pediatricians have a critical role in the evaluation of children who experience a choking episode and foreign body injury. Familiarity with important symptoms and signs improves diagnostic skills and complements radiographic evaluation. Further reduction in injury prevention is assisted by parental education and avoidance of objects and foods that produce the greatest risk. Design modifications of toys and other products for children's use is the next important step in this safety process. [References: 13]
SN - 0031-3955
AD - Department of Pediatrics, Alfred I. duPont Hospital for Children, Wilmington, Delaware, USA
UR - 8973519
ER -

TY - JOUR
ID - 425
T1 - A nursing intervention to ensure a safe playground environment
A1 - McEvoy,M.
A1 - Montana,B.
A1 - Panettieri,M.
Y1 - 1996/09//
N1 - McEvoy, M. Montana, B. Panettieri, M
Journal of pediatric health care : official publication of National Association of Pediatric Nurse Associates & Practitioners
jml, 8709735
N

Journal Article
English
KW - Medline
KW - Child
KW - Child Behavior
KW - Humans
KW - Nurse Practitioners/og [Organization & Administration]
KW - Nursing Assessment
KW - Pediatric Nursing/og [Organization & Administration]
KW - Pilot Projects
KW - Play and Playthings
KW - Safety
KW - School Nursing/og [Organization & Administration]
KW - Wounds and Injuries/pc [Prevention & Control]
RP - NOT IN FILE
SP - 209
EP - 216
JF - Journal of Pediatric Health Care
JA - J Pediatr Health Care
VL - 10
IS - 5
CY - UNITED STATES
N2 - Unintentional injuries sustained on school playgrounds are a continual problem not only for children but also for the school nurses who are concerned with their safety. A group of school nurses organized by the local health department's pediatric nurse practitioner responded proactively to this problem by enlisting both school officials and students to accept joint responsibility for a safe play environment. The school nurses assisted the staff in providing a safe play environment and encouraged the students to behave in a safe and acceptable manner on the playground. The nurses developed (a) a Playground Safety Assessment Guide for the school staff to establish and maintain a safe play environment, (b) an in-service session for playground monitors to learn how to supervise children at recess, and (c) a student-oriented program for the children to improve behavior on the playground based on a program conceived by Utah State University (Daug & Fukui, 1988). School nurses and pediatric nurse practitioners can use this program as a model for other schools to improve the physical play environment and behavior of the children at recess
SN - 0891-5245
UR - 8920353
ER -

TY - JOUR
ID - 426
T1 - Can emotions and themes in children's play predict behavior problems?
A1 - Warren,S.L.
A1 - Oppenheim,D.
A1 - Emde,R.N.
Y1 - 1996/10//
N1 - Warren, S L. Oppenheim, D. Emde, R N
Journal of the American Academy of Child and Adolescent Psychiatry
hg5, 8704565
IM
Journal Article. Research Support, Non-U.S. Gov't. Research Support, U.S. Gov't, P.H.S.
English
KW - Medline
KW - Child Behavior Disorders/di [Diagnosis]
KW - Child Behavior Disorders/px [Psychology]
KW - Child
KW - Preschool
KW - Emotions

KW - Female
KW - Humans
KW - Internal-External Control
KW - Male
KW - Personality Assessment
KW - Play and Playthings
KW - Risk Factors
RP - NOT IN FILE
SP - 1331
EP - 1337
JF - Journal of the American Academy of Child & Adolescent Psychiatry
JA - J Am Acad.Child Adolesc Psychiatry
VL - 35
IS - 10
CY - UNITED STATES
N2 - OBJECTIVE: To empirically test whether systematic examination of emotions and themes in children's play can provide useful information about childhood problems. METHOD: Using the MacArthur Story-Stem Battery and coding system, distress and destructive themes (aggression, personal injury, and atypical negative responses) were coded from the play of 51 children at ages 3, 4, and 5 years, in a low-risk, nonclinical volunteer sample. To measure behavior problems, both parents completed the Child Behavior Checklist at all ages, and teachers completed the Teacher's Report Form when the children reached 5 years of age. RESULTS: Both distress and destructive themes in the play of 4- and 5-year-olds were found to correlate with externalizing behavior problems as rated by parents and teachers. CONCLUSIONS: Children who display more distress during play at 4 and 5 years of age and who demonstrate destructive themes at 4 and 5 years of age appear to have more externalizing behavior problems, as rated by their parents and teachers. These results provide empirical support for the use of play as an assessment tool in young children. The findings suggest approaches to and limitations of play interpretation
SN - 0890-8567
AD - Division of Child and Adolescent Psychiatry, University of Minnesota, Minneapolis 55455, USA
UR - 8885587
ER -

TY - JOUR
ID - 427
T1 - Effects of prenatal exposure to opioids on focused attention in toddlers during free play
A1 - Schneider,J.W.
A1 - Hans,S.L.
Y1 - 1996/08//
N1 - Schneider, J W. Hans, S L
Journal of developmental and behavioral pediatrics : JDBP
htf, 8006933
IM
Journal Article
English
KW - Medline
KW - Attention/de [Drug Effects]
KW - Child
KW - Preschool
KW - Female
KW - Heroin/ae [Adverse Effects]
KW - Heroin Dependence/rh [Rehabilitation]
KW - Humans
KW - Male
KW - Methadone/ae [Adverse Effects]
KW - Methadone/tu [Therapeutic Use]
KW - Mother-Child Relations

KW - Narcotics/ae [Adverse Effects]
KW - Narcotics/tu [Therapeutic Use]
KW - Play and Playthings
KW - Pregnancy
KW - Prenatal Exposure Delayed Effects
RP - NOT IN FILE
SP - 240
EP - 247
JF - Journal of Developmental & Behavioral Pediatrics
JA - J Dev.Behav Pediatr
VL - 17
IS - 4
CY - UNITED STATES
N2 - The goals of this study were: (1) to determine if 24-month-old children exposed to opioids show decreased focused attention during free play compared with children of the same age who were not prenatally exposed; (2) to identify medical and social risk factors other than drug exposure that are related to focused attention; and (3) to determine if mothers' teaching ability had an effect on attention. Focused attention was rated during a 3-minute free play session for 30 toddlers who were methadone-exposed and for 44 comparison toddlers. The mother teaching the child to use a toy was also rated separately from the free play session. There was no difference in focused attention of 24 month olds during free play based only on prenatal exposure. Despite group differences in medical and social risk factors, only maternal IQ was significantly related to focused attention. Maternal instruction was strongly related to focused attention and mediated the effects of maternal IQ on attention
SN - 0196-206X
AD - Northwestern University Medical Center, Program in Physical Therapy, Children's Memorial Medical Center, Chicago, IL 60611, USA
UR - 8856519
ER -

TY - JOUR
ID - 428
T1 - A firearm safety program for children: they just can't say no
A1 - Hardy,M.S.
A1 - Armstrong,F.D.
A1 - Martin,B.L.
A1 - Strawn,K.N.
Y1 - 1996/08//
N1 - Hardy, M S. Armstrong, F D. Martin, B L. Strawn, K N
Journal of developmental and behavioral pediatrics : JDBP
htf, 8006933
IM
Clinical Trial. Journal Article. Randomized Controlled Trial. Research Support, Non-U.S. Gov't
English
KW - Medline
KW - Aggression/px [Psychology]
KW - Child
KW - Preschool
KW - Female
KW - Firearms
KW - Health Education
KW - Humans
KW - Male
KW - Play and Playthings
KW - Risk Factors
KW - Safety
KW - Treatment Outcome

KW - Wounds
KW - Gunshot/pc [Prevention & Control]
KW - Gunshot/px [Psychology]
RP - NOT IN FILE
SP - 216
EP - 221
JF - Journal of Developmental & Behavioral Pediatrics
JA - J Dev.Behav Pediatr
VL - 17
IS - 4
CY - UNITED STATES
N2 - The purpose of this study was to compare children's play and aggressive behavior with firearms before and after an information-based intervention. Correlates of aggression and gun play were also analyzed. Subjects were 24 pairs of preschool children videotaped for 10 minutes in a structured play setting, in which they had access to a variety of toys and to real and toy guns. One child from each dyad was then exposed to an information-based intervention and told not to play with guns. The children were again videotaped in the same setting approximately 1 week later. Results indicated that the intervention was ineffective in modifying the behavior of the children. Regression analyses revealed that access to a parent's firearm was correlated with gun play and that gun play and handling of firearms in the home were correlated with aggressive behavior. The findings in this study represent the first systematic attempt to decrease gun play in children and suggest that information provision alone is an insufficient intervention
SN - 0196-206X
AD - Department of Psychology, University of North Carolina at Charlotte 28223, USA
UR - 8856516
ER -

TY - JOUR
ID - 429
T1 - Foster home environments: a preliminary report
A1 - Simms,M.D.
A1 - Horwitz,S.M.
Y1 - 1996/06//
N1 - Simms, M D. Horwitz, S M
Journal of developmental and behavioral pediatrics : JDBP
htf, 8006933
IM
Journal Article. Research Support, U.S. Gov't, P.H.S.
English
KW - Medline
KW - Adult
KW - Affective Symptoms/di [Diagnosis]
KW - Affective Symptoms/px [Psychology]
KW - Child
KW - Child Behavior Disorders/di [Diagnosis]
KW - Child Behavior Disorders/px [Psychology]
KW - Preschool
KW - Connecticut
KW - Developmental Disabilities/di [Diagnosis]
KW - Developmental Disabilities/px [Psychology]
KW - Female
KW - Foster Home Care/px [Psychology]
KW - Humans
KW - Longitudinal Studies
KW - Male
KW - Middle Aged
KW - Parenting/px [Psychology]

KW - Prospective Studies
KW - Psychosocial Deprivation
KW - Risk Factors
KW - Social Environment
KW - Socioeconomic Factors
RP - NOT IN FILE
SP - 170
EP - 175
JF - Journal of Developmental & Behavioral Pediatrics
JA - J Dev.Behav Pediatr
VL - 17
IS - 3
CY - UNITED STATES
N2 - This study evaluated the quality of developmental and emotional stimulation available to young children in their first foster home placement. Visits were made to 28 of 34 (82%) homes of children ages 2 to 6 years who were enrolled in a prospective study of foster care. Based on the results of the Home Observation for Measurement of the Environment (HOME) scale, 18% of homes were found to be understimulating. Multivariate analysis revealed that low HOME scores were related only to income level and not to other foster parent characteristics. Subscale scores showed that low HOME scores were due largely to lack of adequate play materials and limited interaction between the foster parents and the children. These results demonstrate great variability in the quality of foster home environments. Adjusting foster care subsidies according to foster parents' income levels, providing age-appropriate books and play materials, and additional training of foster parents should increase the developmental and emotional stimulation available to foster children
SN - 0196-206X
AD - Department of Pediatrics, Medical College of Wisconsin, Milwaukee, USA
UR - 8783063
ER -

TY - JOUR
ID - 430
T1 - Ethical principles in federal regulations: the case of children and research risks
A1 - Williams,P.C.
Y1 - 1996/04//
N1 - Williams, P C
The Journal of medicine and philosophy
izd, 7610512
E, IM
Journal Article
English
KW - Medline
KW - Adolescent
KW - Adult
KW - Advisory Committees
KW - Body Height/de [Drug Effects]
KW - Child
KW - Preschool
KW - Dwarfism/dt [Drug Therapy]
KW - Dwarfism/et [Etiology]
KW - Ethical Review
KW - Ethics Committees
KW - Research
KW - Ethics
KW - Medical
KW - Federal Government
KW - Female
KW - Government Regulation

KW - Growth Hormone/tu [Therapeutic Use]
KW - Health
KW - Humans
KW - Informed Consent/lj [Legislation & Jurisprudence]
KW - Male
KW - Nontherapeutic Human Experimentation
KW - Placebos/tu [Therapeutic Use]
KW - Randomized Controlled Trials as Topic/lj [Legislation & Jurisprudence]
KW - Recombinant Proteins/tu [Therapeutic Use]
KW - Risk Assessment
KW - Social Responsibility
KW - Social Values
KW - Treatment Outcome
RP - NOT IN FILE
SP - 169
EP - 186
JF - Journal of Medicine & Philosophy
JA - J Med Philos.
VL - 21
IS - 2
CY - NETHERLANDS
N2 - Ethical principles play an important part not only in the promulgation of regulations but also in their application, i.e., enforcement and adjudication. while traditional ethical principles--promotion of welfare, freedom, and fairness--play an important role in both elements of regulation, some other kinds of ethical principles are significant as well. Principles governing the structure of decision processes should shape the structure and actions of agencies; principles of wise application should govern the work of those whose responsibility it is to apply regulatory language to particular situations. These points are demonstrated by investigating a case study: federal regulations designed to protect children involved in scientific research applied to a placebo study of the effects of recombinant human growth hormone on children of extremely short stature
SN - 0360-5310
AD - Department of Preventive Medicine, School of Medicine, S.U.N.Y.- Stony Brook, USA
UR - 8739071
ER -

TY - JOUR
ID - 431
T1 - Small parts aspiration, ingestion, and choking in small children: findings of the small parts research project
A1 - Rider,G.
A1 - Wilson,C.L.
Y1 - 1996/06//
N1 - Rider, G. Wilson, C L
Risk analysis : an official publication of the Society for Risk Analysis
8109978, ria
IM
Journal Article
English
KW - Medline
KW - Adolescent
KW - Age Distribution
KW - Airway Obstruction/et [Etiology]
KW - Airway Obstruction/mo [Mortality]
KW - Child
KW - Preschool
KW - Consumer Product Safety
KW - Data Interpretation
KW - Statistical

KW - Foreign Bodies
KW - Humans
KW - Infant
KW - Newborn
KW - Inhalation
KW - Play and Playthings/in [Injuries]
KW - Risk Assessment
KW - Safety
KW - United States/ep [Epidemiology]
RP - NOT IN FILE
SP - 321
EP - 330
JF - Risk Analysis
JA - Risk Anal.
VL - 16
IS - 3
CY - UNITED STATES
N2 - Obtaining information on current child injury trends and their associated issues is an important factor in developing products that meet or surpass acceptable toy safety boundaries. Understanding these boundaries helps determine safe product design characteristics that reduce the risk of product-related injury. Inchcape Testing Services developed a Small Parts Aspiration, Ingestion, and Choking Hazards Research Project, independent of an ongoing consumer Product Safety Commission (CPSC) project. The project's purpose was to determine, independent of CPSC, whether a more stringent small parts regulatory standard was necessary and, if so, to ensure that the standard was determined objectively. This article reports on the project's findings relating to critical characteristics (size, shape, and consistency) relative to the victim's age, of objects responsible for child choking injuries and fatalities
SN - 0272-4332
AD - Inchcape Testing Services, Risk Analysis & Management, Moonachie, New Jersey 07074, USA
UR - 8693159
ER -

TY - JOUR
ID - 432
T1 - The role of dietary fiber in the development and treatment of childhood obesity. [Review] [51 refs]
A1 - Kimm,S.Y.
Y1 - 1995/11//
N1 - Kimm, S Y
Pediatrics
oxv, 0376422
AIM, IM
Comparative Study. Journal Article. Review
English
KW - Medline
KW - Child
KW - Chronic Disease
KW - Developing Countries
KW - Diet
KW - Reducing
KW - Dietary Fats/ad [Administration & Dosage]
KW - Dietary Fiber/ad [Administration & Dosage]
KW - Dietary Fiber/tu [Therapeutic Use]
KW - Digestion
KW - Educational Status
KW - Energy Intake
KW - Environment
KW - Female

KW - Health Promotion
KW - Humans
KW - Obesity/dh [Diet Therapy]
KW - Obesity/et [Etiology]
KW - Obesity/ge [Genetics]
KW - Poverty
KW - Preventive Medicine
KW - Research Design
KW - United States
KW - Weight Loss
RP - NOT IN FILE
SP - 1010
EP - 1014
JA - Pediatrics
VL - 96
IS - 5 Pt 2
CY - UNITED STATES
N2 - Childhood obesity is viewed as a public health problem in the United States because of its assumed high prevalence and increasing secular trend. The best estimate of the genetic contribution to obesity ranges from 5% to 25%. Environmental factors play a major role in obesity development. Low income and a low level of education have been associated with obesity, particularly among white women. Caloric intake as a risk factor for obesity has not been clearly established. This lack of a clear-cut association may be attributable to the problem of accurately measuring caloric intake. Several studies have linked increased total fat intake, rather than caloric intake, with obesity. Some studies have linked television viewing to obesity in children. Obesity is rare among the populations of developing countries, where dietary fiber intake is high. Explanations for the role of dietary fiber in obesity include a reduced caloric density of the foods, a slower rate of food ingestion, and possible effects on satiety. Most studies on the role of fiber in the treatment of obesity have been somewhat limited by lack of comparison groups, inadequate sample sizes, and short durations of the observations. However, although limited, the available evidence suggests that fiber potentially could play a useful role in weight reduction. For children, fiber administration should be considered as an adjuvant therapy rather than a primary modality, because fiber might aid in promoting satiety during meals and curbing hunger between meals.(ABSTRACT TRUNCATED AT 250 WORDS) [References: 51]
SN - 0031-4005
AD - Department of Family Medicine and Clinical Epidemiology, School of Medicine, University of Pittsburgh, USA
UR - 7494672
ER -

TY - JOUR
ID - 433
T1 - Firesetting during the preschool period: assessment and intervention issues
A1 - Hanson,M.
A1 - Mackay,S.
A1 - Atkinson,L.
A1 - Staley,S.
A1 - Pignatiello,A.
Y1 - 1995/08//
N1 - Hanson, M. Mackay, S. Atkinson, L. Staley, S. Pignatiello, A
Canadian journal of psychiatry. Revue canadienne de psychiatrie
clr, 7904187
IM
Case Reports. Journal Article. Research Support, Non-U.S. Gov't
English
KW - Medline
KW - Attention Deficit Disorder with Hyperactivity/di [Diagnosis]
KW - Attention Deficit Disorder with Hyperactivity/px [Psychology]

KW - Attention Deficit Disorder with Hyperactivity/th [Therapy]
KW - Behavior Therapy
KW - Burns/pc [Prevention & Control]
KW - Burns/px [Psychology]
KW - Child Behavior Disorders/di [Diagnosis]
KW - Child Behavior Disorders/px [Psychology]
KW - Child Behavior Disorders/th [Therapy]
KW - Child
KW - Preschool
KW - Family/px [Psychology]
KW - Fatal Outcome
KW - Firesetting Behavior/pc [Prevention & Control]
KW - Firesetting Behavior/px [Psychology]
KW - Humans
KW - Male
KW - Personality Assessment
KW - Play and Playthings
KW - Sibling Relations
RP - NOT IN FILE
SP - 299
EP - 303
JF - Canadian Journal of Psychiatry - Revue Canadienne de Psychiatrie
JA - Can J Psychiatry
VL - 40
IS - 6
CY - CANADA
N2 - OBJECTIVE: To describe a methodology of assessing preschoolers involved in firesetting incidents, and outline the psychiatric implications of firesetting incidents in young children. METHOD: To outline The Arson Prevention Program for Children and present case vignettes. RESULTS: The heightened risk of burn injury or fatality in fires caused by young children is highlighted and practical suggestions for facilitating the immediate safety of the child and family are presented. CONCLUSION: Despite the popular notion that fire interest and play is relatively benign in young children, the cases show that, as with older children, firesetting in preschoolers can be associated with serious child and/or family psychopathology
SN - 0706-7437
AD - Family Court Clinic, Clarke Institute of Psychiatry, Toronto, Ontario
UR - 7585398
ER -

TY - JOUR
ID - 434
T1 - Developmental testing. [Review] [25 refs]
A1 - Gilbride,K.E.
Y1 - 1995/09//
N1 - Gilbride, K E
Pediatrics in review / American Academy of Pediatrics
peo, 8103046
IM
Journal Article. Review
English
KW - Medline
KW - Aptitude Tests
KW - Child
KW - Child Behavior Disorders/di [Diagnosis]
KW - Preschool
KW - Developmental Disabilities/di [Diagnosis]
KW - Humans

KW - Infant
KW - Learning Disorders/di [Diagnosis]
KW - Psychological Tests
KW - Psychometrics/mt [Methods]
RP - NOT IN FILE
SP - 338
EP - 345
JF - Pediatrics in Review
JA - Pediatr Rev
VL - 16
IS - 9
CY - UNITED STATES
N2 - Pediatricians play a central role in monitoring the development of infants and children during the course of providing well child care. Parents turn to pediatricians for help in determining whether their child has a temporary lag in development, a serious delay or disorder, or a significant behavior problem that should be addressed. With the passage of PL 99-457, pediatricians also play a key role in referring children at risk to early intervention services. By employing a strategy of developmental surveillance, with periodic developmental screening, the pediatrician can determine when a child should be referred for more extensive developmental or psychological testing, which will aid in the process of diagnosis and treatment of developmental disabilities and behavioral disturbances. Knowledge of the screening and testing measures used commonly, as well as their limitations, will result in more accurate interpretation of the data derived from such measures. Once delays are diagnosed and treatment is initiated, repeated assessments over time will serve to identify areas in need of continuing intervention while indicating gains made in specific areas of developmental functioning. Throughout this process, the pediatrician's role as advocate for the child and family serves as a bridge to other professionals and services, with the ultimate goal of facilitating the optimal development of the child. [References: 25]
SN - 0191-9601
AD - Harbor-UCLA Medical Center, Torrance, USA
UR - 7567708
ER -

TY - JOUR
ID - 435
T1 - Injury prevention in an urban setting: challenges and successes
A1 - Laraque,D.
A1 - Barlow,B.
A1 - Durkin,M.
A1 - Heagarty,M.
Y1 - 1995///
N1 - Laraque, D. Barlow, B. Durkin, M. Heagarty, M
Bulletin of the New York Academy of Medicine
bqo, 7505398
IM
Journal Article. Research Support, Non-U.S. Gov't. Research Support, U.S. Gov't, P.H.S.
English
KW - Medline
KW - Accident Prevention
KW - Adolescent
KW - Adult
KW - Burns/pc [Prevention & Control]
KW - Child
KW - Community-Institutional Relations
KW - Consumer Participation
KW - Environment
KW - Government Agencies
KW - Health Care Coalitions
KW - Health Education

KW - Humans
KW - New York City
KW - Organizational Objectives
KW - Outcome Assessment (Health Care)
KW - Parents
KW - Personnel
KW - Hospital
KW - Play and Playthings
KW - Program Development
KW - Program Evaluation
KW - Safety
KW - Schools
KW - Stress
KW - Physiological/pc [Prevention & Control]
KW - Urban Health Services
KW - Violence
KW - Wounds and Injuries/pc [Prevention & Control]
RP - NOT IN FILE
SP - 16
EP - 30
JF - Bulletin of the New York Academy of Medicine
JA - Bull.N.Y.Acad.Med
VL - 72
IS - 1
CY - UNITED STATES
N2 - The Harlem Hospital Injury Prevention Program (HHIPP) was established in 1988 with the goal of reducing injuries to children in central Harlem by providing safe play areas, supervised activities, and injury prevention education. To achieve this goal, a broad-based coalition was formed with state and local governmental agencies interested in injury prevention and with community groups, schools, parents, and hospital staff. An evaluation of the program in terms of both process and outcome formed a critical element of this effort. Since 1988 the HHIPP, as the lead agency for the Healthy Neighborhoods/Safe Kids Coalition, developed or participated in two types of programs: injury-prevention education programs and programs that provide safe activities and/or environments for children. The educational programs included Window Guards campaign; Safety City Program; Kids, Injuries and Street Smarts Program (KISS); Burn Prevention Curriculum and Smoke Detector Distribution; Harlem Alternative to Violence Program; Adolescent Outreach Program; and Critical Incident Stress Management Teams. The safe activities and environmental programs included the Bicycle Safety Program/Urban Youth Bike Corps; Playground Injury Prevention Program; the Greening of Harlem Program; the Harlem Horizon Art Studio; Harlem Hospital Dance Clinic; Unity through Murals project; baseball at the Harlem Little League; winter baseball clinic; and the soccer league. Each program was conceived using injury data, coupled with parental concern and activism, which acted as catalysts to create a community coalition to respond to a specific problem. Data systems developed over time, which monitored the prevalence and incidence of childhood injuries in northern Manhattan, including central Harlem, became essential not only to identify specific types of childhood injuries in this community but also to evaluate these programs for the prevention of injuries in children
SN - 0028-7091
AD - Harlem Hospital Center, Columbia University College of Physicians and Surgeons, USA
UR - 7581311
ER -

TY - JOUR
ID - 436
T1 - Parent-child interactions in alcoholic and nonalcoholic families
A1 - Whipple,E.E.
A1 - Fitzgerald,H.E.
A1 - Zucker,R.A.
Y1 - 1995/01//

N1 - Whipple, E E. Fitzgerald, H E. Zucker, R A
The American journal of orthopsychiatry
3r6, 0400640
IM
Journal Article. Research Support, Non-U.S. Gov't. Research Support, U.S. Gov't, P.H.S.
English
KW - Medline
KW - Child of Impaired Parents/px [Psychology]
KW - Child
KW - Preschool
KW - Humans
KW - Male
KW - Parent-Child Relations
KW - Personality Assessment
KW - Personality Development
KW - Play and Playthings
KW - Risk Factors
RP - NOT IN FILE
SP - 153
EP - 159
JF - American Journal of Orthopsychiatry
JA - Am J Orthopsychiatry
VL - 65
IS - 1
CY - UNITED STATES
N2 - A community-recruited sample of alcoholic and nonalcoholic families was videotaped in a standardized play task involving parents and their preschool-aged sons. Microanalyses revealed that alcoholic families had lower dyadic synchrony, that parents were viewed as less able to engage their children, and that the coders liked the control parents and children more. Variations in the parent-child interactions are discussed
SN - 0002-9432
AD - School of Social Work, Michigan State University, East Lansing, USA
UR - 7733211
ER -

TY - JOUR
ID - 437
T1 - [Sensorineural hearing loss in children starting school. Results of a representative hearing screening study]. [German]
A1 - Kruppa,B.
A1 - Dieroff,H.G.
A1 - Ising,H.
Y1 - 1995/01//
N1 - Kruppa, B. Dieroff, H G. Ising, H
HNO
g9p, 2985099r
IM
English Abstract. Journal Article
German
KW - Medline
KW - Audiometry
KW - Pure-Tone
KW - Auditory Threshold
KW - Child
KW - Cross-Sectional Studies
KW - Female
KW - Germany/ep [Epidemiology]

KW - Hearing Loss/di [Diagnosis]
KW - Hearing Loss/ep [Epidemiology]
KW - Hearing Loss/et [Etiology]
KW - Hearing Loss
KW - Bilateral/di [Diagnosis]
KW - Bilateral/ep [Epidemiology]
KW - Bilateral/et [Etiology]
KW - High-Frequency/di [Diagnosis]
KW - High-Frequency/ep [Epidemiology]
KW - High-Frequency/et [Etiology]
KW - Sensorineural/di [Diagnosis]
KW - Sensorineural/ep [Epidemiology]
KW - Sensorineural/et [Etiology]
KW - Humans
KW - Incidence
KW - Male
KW - Mass Screening
KW - Play and Playthings
KW - Risk Factors
RP - NOT IN FILE
SP - 31
EP - 34
JF - HNO
VL - 43
IS - 1
CY - GERMANY
N2 - Audiograms in the 0.5-6 kHz frequency range were taken, using improved audiometric screening methods, in 2032 children, 6-7 years old, who were undergoing their pre-school medical examinations. In 151 children (7.4%), the threshold for hearing was above 20 dB for at least one frequency. Of these, 82 children (4% of the overall sample) had a hearing impairment defined as a difference of > or = 10 dB between at least one frequency (3, 4 or 6 kHz) and the mean value from 1+2 kHz. This was evaluated as a sensorineural hearing loss. Unilateral losses (lateral difference > or = 10 dB) were more common than bilateral losses (2.5% vs 1.6% of the overall sample). These unilateral hearing losses in the high-frequency range are essentially regarded as the result of playing with impulse-noise producing toys, such as toy guns with caps
SN - 0017-6192
AD - Umweltbundesamt, Institut fur Wasser-, Boden- und Lufthygiene, Berlin
UR - 7890549
ER -

TY - JOUR
ID - 438
T1 - Identifying video game addiction in children and adolescents
A1 - Fisher,S.
Y1 - 1994/09//
N1 - Fisher, S
Addictive behaviors
2gw, 7603486
IM
Journal Article. Research Support, Non-U.S. Gov't
English
KW - Medline
KW - Adolescent
KW - Behavior
KW - Addictive/di [Diagnosis]
KW - Addictive/px [Psychology]
KW - Child

KW - Female
KW - Gambling/px [Psychology]
KW - Humans
KW - Male
KW - Personality Inventory/sn [Statistics & Numerical Data]
KW - Play and Playthings
KW - Psychometrics
KW - Reproducibility of Results
KW - Risk Factors
RP - NOT IN FILE
SP - 545
EP - 553
JF - Addictive Behaviors
JA - Addict.Behav
VL - 19
IS - 5
CY - ENGLAND
N2 - There is a current trend of thought among some scholars of gambling that arcade video game playing in some adolescents may develop into a behavior which resembles a gambling addiction. A scale, developed to identify arcade video game addiction in adolescents, was administered to 467 secondary school children in a coastal town in the UK. Initial psychometric tests show that the scale has acceptable internal consistency reliability and factorial validity, and is significantly related to alternative means of assessing excessive video game play. The implications of the study findings are discussed together with its limitations and suggestions for future research
SN - 0306-4603
AD - Department of Applied Social Science, University of Plymouth, Devon, UK
UR - 7832013
ER -

TY - JOUR
ID - 439
T1 - [Early indications for partial learning disorders--anamnestic and diagnostic strategies]. [German]
A1 - Michaelis,R.
A1 - Niemann,G.
A1 - Schoning,M.
Y1 - 1994/10//
N1 - Michaelis, R. Niemann, G. Schoning, M
Gesundheitswesen (Bundesverband der Arzte des Offentlichen Gesundheitsdienstes (Germany))
bfd, 9204210
IM
English Abstract. Journal Article
German
KW - Medline
KW - Child
KW - Preschool
KW - Developmental Disabilities/di [Diagnosis]
KW - Developmental Disabilities/pc [Prevention & Control]
KW - Dyslexia/di [Diagnosis]
KW - Dyslexia/pc [Prevention & Control]
KW - Female
KW - Humans
KW - Language Development Disorders/di [Diagnosis]
KW - Language Development Disorders/pc [Prevention & Control]
KW - Learning Disorders/di [Diagnosis]
KW - Learning Disorders/pc [Prevention & Control]
KW - Male

KW - Medical History Taking
KW - Neuropsychological Tests
KW - Play and Playthings
KW - Risk Factors
RP - NOT IN FILE
SP - 534
EP - 536
JF - Gesundheitswesen
VL - 56
IS - 10
CY - GERMANY
N2 - A surprisingly small amount of information exists about early symptoms of learning disorders becoming evident during the first years at school. Only one relation seems to carry some degree of reliability: Early language deficits and/or retardation of language acquisition and later learning disorders including dyslexia. Because of these limitations, a more pragmatic approach is recommended. Questionnaires can be used asking for specific and age-related skills, involving the existence and good functioning of strategically working and concept-building central structures. Children with deficits in these structures will develop and experience dysfunctions in their cognitive, social and motor abilities. Consequently, they present behavioural problems. Children whose questionnaires offer suspicion in respect of specific dysfunctions should be tested by means of structured play tests or by appropriate developmental tests, to determine their individual patterns of talents and specific shortcomings. An early therapeutic intervention, aimed at the child's specific and individual deficits, may prevent or reduce learning problems at school
SN - 0941-3790
AD - Abteilung Neuropadiatrie, Entwicklungsneurologie, Sozialpadiatrie Universitatskinderklinik Tubingen
UR - 7528582
ER -

TY - JOUR
ID - 440
T1 - The Central Harlem playground injury prevention project: a model for change
A1 - Laraque,D.
A1 - Barlow,B.
A1 - Davidson,L.
A1 - Welborn,C.
Y1 - 1994/10//
N1 - Laraque, D. Barlow, B. Davidson, L. Welborn, C
American journal of public health
1254074, 3xw
AIM, IM
News
English
KW - Medline
KW - Adolescent
KW - Child
KW - Humans
KW - New York City
KW - Play and Playthings
KW - Safety
KW - Urban Health
KW - Wounds and Injuries/pc [Prevention & Control]
RP - NOT IN FILE
SP - 1691
EP - 1692
JF - American Journal of Public Health
JA - Am J Public Health
VL - 84

IS - 10
CY - UNITED STATES
SN - 0090-0036
UR - 7943500
ER -

TY - JOUR
ID - 441
T1 - Activity-based intervention and direct instruction: a comparison study
A1 - Losardo,A.
A1 - Bricker,D.
Y1 - 1994/05//
N1 - Losardo, A. Bricker, D
American journal of mental retardation : AJMR
ame, 8800463
IM
Journal Article
English
KW - Medline
KW - Attention
KW - Child
KW - Preschool
KW - Education of Intellectually Disabled/mt [Methods]
KW - Female
KW - Generalization (Psychology)
KW - Humans
KW - Male
KW - Mental Recall
KW - Play and Playthings
KW - Verbal Learning
KW - Vocabulary
RP - NOT IN FILE
SP - 744
EP - 765
JF - American Journal of Mental Retardation
JA - Am J Ment.Retard.
VL - 98
IS - 6
CY - UNITED STATES
N2 - An alternating treatments design was used to compare the effectiveness of two intervention procedures--direct instruction and activity-based instruction--on the acquisition and generalization of object names by preschool-age children with developmental delays or who were at-risk for such delays. Subjects were given a pretest to determine unknown object names. A systematic alternation of treatments was subsequently provided by trained interventionists. After a baseline period of 1 week, two 15-minute treatment sessions employing activity-based intervention and direct instruction were followed by a 15-minute free-play generalization session, 3 days a week, for 6 weeks. A return to baseline phase was then conducted for 1 week. Structured generalization probes were also administered throughout treatment and 4 weeks after the study ended. Results indicated differential effects for both treatments
SN - 0895-8017
AD - Center on Human Development, University of Oregon, Eugene 97403-1211
UR - 8054203
ER -

TY - JOUR
ID - 442
T1 - Analysis of environmental deprivation: cognitive and social development in Romanian orphans

A1 - Kaler,S.R.
A1 - Freeman,B.J.
Y1 - 1994/05//
N1 - Kaler, S R. Freeman, B J
Journal of child psychology and psychiatry, and allied disciplines
hp3, 0375361
IM
Journal Article. Research Support, U.S. Gov't, P.H.S.
English
KW - Medline
KW - Child
KW - Abandoned/px [Psychology]
KW - Institutionalized/px [Psychology]
KW - Preschool
KW - Developing Countries
KW - Developmental Disabilities/et [Etiology]
KW - Developmental Disabilities/px [Psychology]
KW - Female
KW - Humans
KW - Infant
KW - Intellectual Disability/px [Psychology]
KW - Intelligence Tests/sn [Statistics & Numerical Data]
KW - Male
KW - Personality Assessment/sn [Statistics & Numerical Data]
KW - Personality Development
KW - Play and Playthings
KW - Psychometrics
KW - Psychosocial Deprivation
KW - Risk Factors
KW - Romania
KW - Social Adjustment
KW - SOCIALIZATION
RP - NOT IN FILE
SP - 769
EP - 781
JF - Journal of Child Psychology & Psychiatry & Allied Disciplines
JA - J Child Psychol.Psychiatry
VL - 35
IS - 4
CY - ENGLAND
N2 - The cognitive and social developmental status of a representative group of Romanian orphans between the ages of 23 and 50 months living in the Leagan de Copii in Timisoara, Romania was assessed using a variety of traditional and nontraditional measures. Results indicated that the orphanage sample all exhibited deficits in cognitive and social functioning; the majority were severely delayed. Correlations between the traditional and nontraditional measures indicated that children's delays occurred across domains. Deficits were not related to length of time in the orphanage, age at entrance, Apgar scores, or birthweight. The children's greatest capability was in peer social interaction
SN - 0021-9630
AD - Neuropsychiatric Institute, University of California, Los Angeles
UR - 7518826
ER -

TY - JOUR
ID - 443
T1 - Affective interactions of depressed and nondepressed mothers and their children
A1 - Radke-Yarrow,M.

A1 - Nottelmann,E.
A1 - Belmont,B.
A1 - Welsh,J.D.
Y1 - 1993/12//
N1 - Radke-Yarrow, M. Nottelmann, E. Belmont, B. Welsh, J D
Journal of abnormal child psychology
0364547, h33
IM
Journal Article. Research Support, Non-U.S. Gov't. Research Support, U.S. Gov't, P.H.S.
English
KW - Medline
KW - Affect
KW - Bipolar Disorder/di [Diagnosis]
KW - Bipolar Disorder/px [Psychology]
KW - Child of Impaired Parents/px [Psychology]
KW - Child
KW - Preschool
KW - Depressive Disorder/di [Diagnosis]
KW - Depressive Disorder/px [Psychology]
KW - Female
KW - Gender Identity
KW - Humans
KW - Infant
KW - Male
KW - Mother-Child Relations
KW - Personality Assessment
KW - Personality Development
KW - Play and Playthings
KW - Risk Factors
RP - NOT IN FILE
SP - 683
EP - 695
JF - Journal of Abnormal Child Psychology
JA - J Abnorm.Child Psychol.
VL - 21
IS - 6
CY - UNITED STATES
N2 - The expressed affect of clinically depressed and nondepressed mothers as measured by the Schedule for Affective Disorders and Schizophrenia: Lifetime Version (SADS-L) and their children (1 1/2 to 3 1/2 years) was observed in seminatural situations. The objectives were to investigate how maternal depression enters into affective interactions between mother and child and how the affect patterns of mother and child are related. Forty-nine unipolar and 24 bipolar depressed mothers and 45 nondepressed mothers were observed on 2 days, 2 weeks apart, for a total of 5 h. Each minute was coded for the predominant affect of mother and child. Affects relevant to depression (anxious--said, irritable--angry, downcast, pleasant, tender-affectionate) were coded. Depressed mothers expressed significantly more negative affect than did control mothers. Mothers' expressed affect and their self-reports of affect on days of observation were unrelated. Mother's and child's affects, measured on different days, were significantly correlated. Unipolar mothers and mothers severely depressed spent significantly more time in prolonged bouts of negative affect. There was significant synchrony between their bouts and the negative bouts of their daughters. Gender of child was related to mother's and child's affect, and to relations between mother's and child's affect
SN - 0091-0627
AD - National Institute of Mental Health, Bethesda, Maryland 20892
UR - 8126320
ER -

TY - JOUR

ID - 444
T1 - Playground safety
A1 - Leung,A.K.
A1 - Robson,W.L.
A1 - Lim,S.H.
A1 - Chopra,S.
Y1 - 1993/12//
N1 - Leung, A K. Robson, W L. Lim, S H. Chopra, S
Journal of the Royal Society of Health
jx3, 8303144
IM
Journal Article
English
KW - Medline
KW - Accident Prevention
KW - Child
KW - Child Welfare
KW - Equipment Design
KW - Health Education
KW - Humans
KW - Play and Playthings
KW - Safety
KW - Surface Properties
RP - NOT IN FILE
SP - 320
EP - 323
JF - Journal of the Royal Society of Health
JA - J R Soc Health
VL - 113
IS - 6
CY - ENGLAND
SN - 0264-0325
AD - University of Calgary, Canada
UR - 8308853
ER -

TY - JOUR
ID - 445
T1 - Risk of noise-induced hearing loss following exposure to Chinese firecrackers. [Review] [26 refs]
A1 - Smoorenburg,G.F.
Y1 - 1993/11//
N1 - Smoorenburg, G F
Audiology : official organ of the International Society of Audiology
9dv, 1273752
IM
Journal Article. Research Support, Non-U.S. Gov't. Review
English
KW - Medline
KW - Adolescent
KW - Adult
KW - Auditory Threshold
KW - Child
KW - Preschool
KW - Explosions
KW - Hearing Loss
KW - Noise-Induced/ep [Epidemiology]

KW - Noise-Induced/et [Etiology]
KW - Humans
KW - Incidence
KW - Maximum Allowable Concentration
KW - Noise/ae [Adverse Effects]
KW - Play and Playthings
KW - Risk Factors
KW - Sound Spectrography
KW - Time Factors
KW - Tinnitus/et [Etiology]
RP - NOT IN FILE
SP - 333
EP - 343
JF - Audiology
VL - 32
IS - 6
CY - SWITZERLAND
N2 - Firecrackers produce sound impulses reaching peak levels measured at the ear sometimes in excess of 160 dB when fired at 2 m distance. These sound levels are potentially hazardous to the ear. Current damage risk criteria for impulse sounds show that for 10 impulses the peak levels should not exceed 149 dB(lin,peak) at the ear. The A-weighted, impulse (integration time 35 ms) levels should not exceed 125 dB(A,imp) at the ear for 10 impulses. [References: 26]
SN - 0020-6091
AD - TNO Institute for Human Factors, Soesterberg, The Netherlands
UR - 8267524
ER -

TY - JOUR
ID - 446
T1 - Risk of sports activities in children with Down's syndrome and atlantoaxial instability
A1 - Cremers,M.J.
A1 - Bol,E.
A1 - de,Roos F.
A1 - van,Gijn J.
Y1 - 1993/08/28/
N1 - Cremers, M J. Bol, E. de Roos, F. van Gijn, J
Lancet
2985213r, l0s, 0053266
AIM, IM
Clinical Trial. Comparative Study. Journal Article. Randomized Controlled Trial. Research Support, Non-U.S. Gov't
English
KW - Medline
KW - Adolescent
KW - Adult
KW - Atlanto-Axial Joint/ra [Radiography]
KW - Atlanto-Axial Joint
KW - Child
KW - Preschool
KW - Down Syndrome/co [Complications]
KW - Down Syndrome/pp [Physiopathology]
KW - Female
KW - Humans
KW - Joint Instability/co [Complications]
KW - Male
KW - Motor Skills

KW - Nervous System Diseases/et [Etiology]
KW - Risk Factors
KW - Sports
RP - NOT IN FILE
SP - 511
EP - 514
JF - Lancet
VL - 342
IS - 8870
CY - ENGLAND
N2 - 10-40% of children with Down's syndrome have atlantoaxial instability. These children might run the risk of spinal cord compression if they play sport. The aim of our study was to assess this presumed risk. We obtained 282 radiographs of the cervical spine from a cohort of 400 children and young adults with Down's syndrome who attended special schools and who were between 4 and 20 years old (about 25% of all such children in the Netherlands). The atlantoaxial distance was more than 4 mm in 91 children. These children were randomly assigned to two groups, with the provision that all children at any particular school were assigned to the same group. Children of one group were allowed to continue their habitual sports and exercise activities, whereas those in the other group were advised not to play "risky" sports (as defined by a panel of four experts) and not to make "risky" movements during physical education lessons. The compliance of the experimental group was good. After a year, there were no differences between the groups in scores on a functional motor scale, the frequency of neurological signs, or changes in the atlantoaxial distance. The motor function of a third group of 44 children with Down's syndrome but normal atlantoaxial distances was similar to that of children in the other two groups, as was the frequency of neurological signs. These findings suggest there is no reason to stop children with Down's syndrome from playing certain sports and no need to screen them by radiography before they take up such sports activities
SN - 0140-6736
AD - Janus Jongbloed Research Center, University Department of Physiology and Sports, Utrecht, Netherlands
UR - 8102665
ER -

TY - JOUR
ID - 447
T1 - Pretend play in high-risk and developmentally delayed children. [Review] [46 refs]
A1 - Sigman,M.
A1 - Sena,R.
Y1 - 1993///
N1 - Sigman, M. Sena, R
New directions for child development
n6g, 8104414
IM
Journal Article. Research Support, U.S. Gov't, Non-P.H.S.. Research Support, U.S. Gov't, P.H.S.. Review
English
KW - Medline
KW - Child
KW - Preschool
KW - Cognition
KW - Developmental Disabilities
KW - Exploratory Behavior
KW - Humans
KW - Infant
KW - Newborn
KW - Play and Playthings
RP - NOT IN FILE
SP - 29
EP - 42
JF - New Directions for Child Development

JA - New Dir.Child Dev.
IS - 59
CY - UNITED STATES
SN - 0195-2269
AD - University of California, Los Angeles
UR - 7683392
ER -

TY - JOUR
ID - 448
T1 - Family experiences, attitudes, and household safety practices regarding firearms
A1 - Wiley,C.C.
A1 - Casey,R.
Y1 - 1993/02//
N1 - Wiley, C C. Casey, R
Clinical pediatrics
dhe, 0372606, 8407647
AIM, IM
Journal Article
English
KW - Medline
KW - Adult
KW - Child
KW - Preschool
KW - Continental Population Groups
KW - Educational Status
KW - Employment
KW - Family/px [Psychology]
KW - Family
KW - Fear
KW - Female
KW - Firearms/st [Standards]
KW - Firearms/sn [Statistics & Numerical Data]
KW - Health Knowledge
KW - Attitudes
KW - Practice
KW - Hospitals
KW - Pediatric
KW - Humans
KW - Income
KW - Interviews as Topic
KW - Male
KW - Marital Status
KW - Maternal Age
KW - Mothers/ed [Education]
KW - Mothers/px [Psychology]
KW - Ownership
KW - Philadelphia
KW - Play and Playthings
KW - Prospective Studies
KW - Questionnaires
KW - Safety
KW - Sex Factors
KW - Socioeconomic Factors
KW - Television
KW - Urban Population

KW - Violence
RP - NOT IN FILE
SP - 71
EP - 76
JF - Clinical Pediatrics
JA - Clin.Pediatr (Phila)
VL - 32
IS - 2
CY - UNITED STATES
N2 - To assess families' experiences with shootings and firearm ownership and attitudes, 208 children aged 5 to 12 years were interviewed and 242 parents filled out a questionnaire, all at an urban hospital. Forty-three percent of the parents and 25% of the children had personal knowledge of shootings. Of the parents, 10% owned guns, of which only 21% were stored safely. Gun ownership was more common in homes with resident adult males (14%) than in those with no adult males (3%). Most children (81%) played with guns, and 20% to 25% preferred violent TV shows, games, and toys. Parents' and children's attitudes toward firearms and children's gun play did not differ by level of mother's education; method of payment for, or site of, health care; income; or exposure to shootings. Children with personal knowledge of shootings more often expressed fears of being shot than did other children (52% vs 31%). The children in our study population were similar in their gun play and attitudes about gun use despite disparate socioeconomic backgrounds and exposure to shootings
SN - 0009-9228
AD - Division of General Pediatrics, Children's Hospital of Philadelphia, Pennsylvania 19104
UR - 8432082
ER -

TY - JOUR
ID - 449
T1 - Parenting provided by adults with mental retardation. [Review] [86 refs]
A1 - Dowdney,L.
A1 - Skuse,D.
Y1 - 1993/01//
N1 - Dowdney, L. Skuse, D
Journal of child psychology and psychiatry, and allied disciplines
hp3, 0375361
IM
Journal Article. Review
English
KW - Medline
KW - Adult
KW - Child
KW - Child Abuse/ep [Epidemiology]
KW - Female
KW - Humans
KW - Intellectual Disability/ep [Epidemiology]
KW - Intellectual Disability/rh [Rehabilitation]
KW - Intellectual Disability
KW - Male
KW - Middle Aged
KW - Parenting
KW - Risk Factors
RP - NOT IN FILE
SP - 25
EP - 47
JF - Journal of Child Psychology & Psychiatry & Allied Disciplines
JA - J Child Psychol.Psychiatry
VL - 34
IS - 1

CY - ENGLAND
N2 - Studies assessing the quality of parenting provided by adults with mental retardation present conflicting conclusions. Some consider the majority to be doing reasonably well, whilst others report frequently unsatisfactory caretaking. There are a number of reasons for such different views. First, inconsistent selection criteria make it hard to compare across studies. In particular, sample composition will be influenced by the recruitment source. For example, if parents have been chosen from voluntary educational programmes a rather different picture is likely to be found than if they have been selected from individuals known to, or referred by, statutory agencies. On the whole, authors working with subjects from the former source have been rather more optimistic than those working with parents referred because there were already serious concerns about parenting difficulties or about delayed child development. Secondly, the majority of studies have used poorly defined global measures of parenting, with variable criteria of what constitutes adequate care. Some have concentrated on physical care and hygiene, whilst others have looked for the presence of affection and warmth. A child's reception into care as the sole measure of the quality of parenting is an unsatisfactory criterion because parental retardation has itself occasionally been used as the basis for removal of a child into care, even in the absence of other evidence of neglect or abuse. Thirdly, methodological flaws are found in studies that have used observational assessments of parenting. Such studies have suggested mothers with mental retardation tend to lack interactive skills (such as high levels of praise and imitation, and low restrictiveness) which are known to be associated with optimal child development. Control groups have often not been matched on social and other variables which might be expected to exert a significant influence upon parenting practices. In addition, the generalisability of these observational studies is open to question as parenting style has been adduced from brief play sessions, lasting at the most 10 minutes. The extent to which mothers with mental retardation play spontaneously with their children at home in a stimulating and age-appropriate fashion has not been measured. With regard to the evidence on abuse and neglect, questions have been raised about whether the children of parents with mental retardation are at increased risk. This problem has rarely been addressed in a methodologically satisfactory way. First, most studies have drawn their samples from referrals to medical or psychiatric departments. Secondly, as indicated earlier, reception into care cannot by itself be taken to be an indicator of child abuse.(ABSTRACT TRUNCATED AT 400 WORDS) [References: 86]
SN - 0021-9630
AD - Department of Child Health, Kingston Hospital, Kingston upon Thames, U.K
UR - 8421068
ER -

TY - JOUR
ID - 450
T1 - Child care center's new playground proves that safety isn't child's play
A1 - Minnich,J.S.
Y1 - 1993/01//
N1 - Minnich, J S
Health facilities management
8906517, hfm
H
Journal Article
English
KW - Medline
KW - Child Day Care Centers
KW - Child
KW - Preschool
KW - Facility Design and Construction
KW - Hospital Bed Capacity
KW - 500 and over
KW - Humans
KW - Infant
KW - Play and Playthings
KW - Safety
KW - Virginia
RP - NOT IN FILE

SP - 14
EP - 15
JF - Health Facilities Management
JA - Health Facil.Manage.
VL - 6
IS - 1
CY - UNITED STATES
SN - 0899-6210
UR - 10123278
ER -

TY - JOUR
ID - 451
T1 - Inhibition in toddlerhood and the dynamics of the child's interaction with an unfamiliar peer at age five
A1 - Kochanska,G.
A1 - Radke-Yarrow,M.
Y1 - 1992/04//
N1 - Kochanska, G. Radke-Yarrow, M
Child development
0372725, d28
IM
Journal Article. Research Support, Non-U.S. Gov't. Research Support, U.S. Gov't, P.H.S.
English
KW - Medline
KW - Age Factors
KW - Child Behavior
KW - Child
KW - Preschool
KW - Female
KW - Forecasting
KW - Humans
KW - Inhibition (Psychology)
KW - Interpersonal Relations
KW - Male
KW - Peer Group
RP - NOT IN FILE
SP - 325
EP - 335
JF - Child Development
JA - Child Dev.
VL - 63
IS - 2
CY - UNITED STATES
N2 - Measures of inhibition to social and nonsocial unfamiliar events, obtained in toddlerhood, were studied as predictors of social behaviors during an interaction with an unfamiliar peer in 100 5-year-old children. Social inhibition predicted a highly shy and inhibited behavioral pattern with peer and less frequent expression of affect during fantasy play; nonsocial inhibition predicted decreased involvement in group play. Analysis of the changing dynamics of the ongoing peer interaction revealed that the role of child inhibition as a predictor of social behavior may be mostly evident during the initial encounter with the peer. Children who as toddlers were particularly socially inhibited, during the initial phase of peer interaction showed a significantly stronger pattern of shy and inhibited behavior and proximity to mother. In contrast to existing evidence that maternal depression may be a risk factor for the child's long-term peer relationships, no differences in social behavior were found between children of normal and affectively ill mothers during a brief encounter with unfamiliar peers
SN - 0009-3920
AD - National Institute of Mental Health, Bethesda, MD 20892
UR - 1611937

ER -

TY - JOUR
ID - 452
T1 - The role of after-school programs in the lives of inner-city children: a study of the "urban youth network"
A1 - Halpern,R.
Y1 - 1992/05//
N1 - Halpern, R
Child welfare
0372735, d2u, 0372735
IM
Journal Article
English
KW - Medline
KW - Chicago
KW - Child
KW - Child Day Care Centers/ec [Economics]
KW - Child Day Care Centers/ma [Manpower]
KW - Child Day Care Centers/og [Organization & Administration]
KW - Child Welfare
KW - Community Mental Health Services/ec [Economics]
KW - Community Mental Health Services/ma [Manpower]
KW - Community Mental Health Services/og [Organization & Administration]
KW - Group Processes
KW - Health Services Research
KW - Humans
KW - Play and Playthings
KW - Safety
KW - Student Dropouts
KW - Urban Health
KW - Violence
RP - NOT IN FILE
SP - 215
EP - 230
JF - Child Welfare
VL - 71
IS - 3
CY - UNITED STATES
N2 - A study of a network of agency centers located in eight of Chicago's most deprived neighborhoods reveals a great deal about inner-city children and the difficulties of reaching them and holding their attendance. It also points up sharply the funding and staffing necessary to enable the centers to realize their potential for services to these children
SN - 0009-4021
AD - Erikson Institute, Chicago, IL
UR - 1606847
ER -

TY - JOUR
ID - 453
T1 - Self-reported safety practices in child care facilities
A1 - O'Connor,M.A.
A1 - Boyle,W.E.,Jr.
A1 - O' Connor,G.T.
A1 - Letellier,R.
Y1 - 1992/01//

N1 - O'Connor, M A. Boyle, W E Jr. O' Connor, G T. Letellier, R
American journal of preventive medicine
8704773, apl
IM
Journal Article. Research Support, U.S. Gov't, P.H.S.
English
KW - Medline
KW - Accidental Falls/pc [Prevention & Control]
KW - Age Factors
KW - Child
KW - Child Day Care Centers/st [Standards]
KW - Preschool
KW - Humans
KW - Interviews as Topic
KW - Licensure/lj [Legislation & Jurisprudence]
KW - New Hampshire
KW - Play and Playthings
KW - Safety
KW - Wounds and Injuries/mo [Mortality]
KW - Wounds and Injuries/pc [Prevention & Control]
RP - NOT IN FILE
SP - 14
EP - 18
JA - American Journal of Preventive Medicine
VL - 8
IS - 1
CY - UNITED STATES
N2 - To determine the prevalence of safety hazards and current injury prevention practices in child care settings, we administered a structured telephone interview to a geographically stratified, randomly selected sample of licensed child care facilities. Representatives of 130 child care facilities responded to questions about current injury prevention practices. Specific hazards assessed were related to burns, falls, poisoning, playgrounds, and emergency telephone numbers. Results indicated that 26.8% of providers who knew the temperature of their tap water stated that it was over 130 degrees F.; 14.1% had space heaters accessible to children; 30.3% of those with stairs accessible to children lacked safety gates; 61.4% of those with playgrounds did not have an impact-absorbing surface under playground equipment; 16.9% of respondents had an unexpired bottle of syrup of ipecac; 55.8% demonstrated that a poison control center telephone number was available to them; and 80% of providers could demonstrate the availability of the telephone number of the local ambulance. We conclude that potential and remedial injury hazards exist in some licensed child care centers and that providers of child care within licensed facilities are a promising target for childhood injury prevention interventions
SN - 0749-3797
AD - Department of Maternal and Child Health, Injury Prevention Resource and Research Center, Dartmouth Medical School, Hanover, NH 03756
UR - 1575995
ER -

TY - JOUR
ID - 454
T1 - AAOS launches campaign to prevent injury
A1 - Hensinger,R.
Y1 - 1992/02//
N1 - Hensinger, R
Minnesota medicine
nby, 8000173
IM

Letter
English
KW - Medline
KW - Adolescent
KW - Child
KW - Health Education
KW - Humans
KW - Play and Playthings
KW - Safety
KW - Wounds and Injuries/pc [Prevention & Control]
RP - NOT IN FILE
SP - 8
EP - 9
JF - Minnesota Medicine
JA - Minn.Med
VL - 75
IS - 2
CY - UNITED STATES
SN - 0026-556X
UR - 1565079
ER -

TY - JOUR
ID - 455
T1 - Evaluation of an intervention to reduce playground hazards in Atlanta child-care centers
A1 - Sacks,J.J.
A1 - Brantley,M.D.
A1 - Holmgreen,P.
A1 - Rochat,R.W.
Y1 - 1992/03//
N1 - Sacks, J J. Brantley, M D. Holmgreen, P. Rochat, R W
American journal of public health
1254074, 3xw
AIM, IM
Journal Article
English
KW - Medline
KW - Accident Prevention
KW - Administrative Personnel/ed [Education]
KW - Child Day Care Centers/og [Organization & Administration]
KW - Child Day Care Centers/st [Standards]
KW - Child Day Care Centers/sn [Statistics & Numerical Data]
KW - Child
KW - Preschool
KW - Evaluation Studies as Topic
KW - Georgia
KW - Humans
KW - Play and Playthings
KW - Safety
RP - NOT IN FILE
SP - 429
EP - 431
JF - American Journal of Public Health
JA - Am J Public Health
VL - 82
IS - 3

CY - UNITED STATES
N2 - In 1988, we identified playground hazards at 58 child-care centers (CCCs) and intervened by showing the director the hazards and distributing safety information. In 1990, we evaluated the 58 intervention CCCs as well as 71 randomly selected control CCCs. Intervention centers had 9.4 hazards per playground; control centers had 8.0. We conclude that the intervention was ineffective
SN - 0090-0036
AD - Division of Injury Control, Centers for Disease Control, Atlanta, GA 30333
UR - 1536361
ER -

TY - JOUR
ID - 456
T1 - Heterogeneities in water contact patterns and the epidemiology of Schistosoma haematobium
A1 - Chandiwana,S.K.
A1 - Woolhouse,M.E.
Y1 - 1991/12//
N1 - Chandiwana, S K. Woolhouse, M E
Parasitology
or0, 0401121
IM
Journal Article. Research Support, Non-U.S. Gov't
English
KW - Medline
KW - Adolescent
KW - Adult
KW - Age Factors
KW - Animals
KW - Baths
KW - Child
KW - Preschool
KW - Disease Vectors
KW - Female
KW - Humans
KW - Laundering
KW - Male
KW - Play and Playthings
KW - Schistosoma haematobium/ip [Isolation & Purification]
KW - Schistosomiasis haematobia/ep [Epidemiology]
KW - Schistosomiasis haematobia/tm [Transmission]
KW - Sex Factors
KW - Snails/ps [Parasitology]
KW - Swimming
KW - Water
KW - Zimbabwe/ep [Epidemiology]
RP - NOT IN FILE
SP - 363
EP - 370
JF - Parasitology
VL - 103 Pt 3
CY - ENGLAND
N2 - Variations in the amount of water contact made by individuals and in the amount of water contact made at different sites may have significant impacts on patterns of human schistosome infection. Previous studies have reported variations in the rate of water contact and differences in the sites used between age/sex classes, but there is limited information on variations in individual water contact behaviour. In this paper we report and analyse observations of essentially all water contacts made over a two week period by all individuals in a rural community in eastern Zimbabwe. The mean rate of water contact was 0.43 contacts/person/day. These data were

over-dispersed, ranging from zero to 3.3 contacts/person/day; 90% of contacts were made by only 37% of the population. Contact rates were related to age (highest in 8 to 10-year-olds) but not sex, with substantial variation unaccounted for by these variables. Age and sex classes differed in types of water-related activities and the time of day of contact. A greater diversity of sites was used by children than by adults and by males than by females. Individual contact rates were correlated with intensities of infection, although the risk of infection per contact was estimated to be highest in 2 to 4-year-old children and higher for males than females. Five contact sites were used during the study period, with more than 50% of contacts occurring at just 2 sites. Different age and sex classes used different sites and there were additional site-related differences in types of activity and the time of day of use. The implications of these water contact patterns for schistosome epidemiology are discussed. In particular the results provide strong quantitative support for control programmes aimed at heavily used sites (e.g. focal molluscicidal) or at the minority of individuals making most water contacts (e.g. targeted chemotherapy)
SN - 0031-1820
AD - Blair Research Laboratory, Harare, Zimbabwe
UR - 1780173
ER -

TY - JOUR
ID - 457
T1 - The Play Activity Questionnaire: a parent report measure of children's play preferences
A1 - Finegan,J.A.
A1 - Niccols,G.A.
A1 - Zacher,J.E.
A1 - Hood,J.E.
Y1 - 1991/08//
N1 - Finegan, J A. Niccols, G A. Zacher, J E. Hood, J E
Archives of sexual behavior
8gr, 1273516
IM
Journal Article. Research Support, Non-U.S. Gov't
English
KW - Medline
KW - Child
KW - Child Psychology
KW - Preschool
KW - Choice Behavior
KW - Evaluation Studies as Topic
KW - Factor Analysis
KW - Statistical
KW - Female
KW - Gender Identity
KW - Humans
KW - Male
KW - Parents/px [Psychology]
KW - Play and Playthings
KW - Questionnaires/st [Standards]
KW - Reproducibility of Results
KW - Sex Factors
RP - NOT IN FILE
SP - 393
EP - 408
JF - Archives of Sexual Behavior
JA - Arch.Sex Behav
VL - 20
IS - 4
CY - UNITED STATES

N2 - Three studies describe the development and validation of the Play Activity Questionnaire (PQ), a parent report measure of children's play preferences. In Study 1, the 15-item PQ was completed by parents of 239 6- to 8-year-old children, and exploratory factor analysis revealed four play factors: Active and Adventurous, Athletic, Rough-and-Tumble, and Quiet. In Study 2, the factor structure was replicated with PQ data on 203 children of a broader age range (4 to 12 years). The play factors accounted for 65.7% of the variance. Boys' scores exceeded girls' scores on the Athletic and Rough-and-Tumble play factors. Girls' scores exceeded boys' scores on the Quiet play factor. Sex differences were not observed on the Active and Adventurous play factor. Interparent agreement was moderately high, and PQ factor scores were unrelated to social class or nationality. Individual factor scores showed moderate to high stability over 5 months. Validity of the play factors was examined in Study 3 by relating factor scores to measures of activity level, aggression, and sex-typed play. Play factor scores were associated with several of these measures, suggesting that the PQ is a valid measure of children's play behavior with potential applications in the fields of developmental psychopathology and psychoneuroendocrinology, and in studies of normal play and sex-role development
SN - 0004-0002
AD - Department of Psychology, Hospital for Sick Children, Toronto, Canada
UR - 1953330
ER -

TY - JOUR
ID - 458
T1 - 'To be the best or not to be, that is the question ...' On enactment, play and acting out
A1 - Van,Waning A.
Y1 - 1991///
N1 - Van Waning, A
The International journal of psycho-analysis
gsl, 2985179r
IM
Case Reports. Journal Article
English
KW - Medline
KW - Acting Out
KW - Child
KW - Fantasy
KW - Humans
KW - Male
KW - Play Therapy/mt [Methods]
KW - Psychoanalytic Interpretation
KW - Psychoanalytic Theory
KW - Psychoanalytic Therapy/mt [Methods]
KW - Risk-Taking
KW - Symbolism
KW - Transference (Psychology)
RP - NOT IN FILE
SP - 539
EP - 551
JF - International Journal of Psycho-Analysis
JA - Int.J Psychoanal.
VL - 72
IS - Pt 3
CY - ENGLAND
SN - 0020-7578
UR - 1718915
ER -

TY - JOUR
ID - 459

T1 - Sexualized doll play
A1 - Sonnenschein,K.
A1 - Licamele,W.
Y1 - 1991/07//
N1 - Sonnenschein, K. Licamele, W
Journal of the American Academy of Child and Adolescent Psychiatry
hg5, 8704565
IM
Comment. Letter
English
KW - Medline
KW - Child
KW - Child Abuse
KW - Sexual/di [Diagnosis]
KW - Sexual/lj [Legislation & Jurisprudence]
KW - Sexual/px [Psychology]
KW - Preschool
KW - Humans
KW - Play and Playthings
KW - Psychosexual Development
KW - Risk Factors
RP - NOT IN FILE
SP - 694
EP - 695
JF - Journal of the American Academy of Child & Adolescent Psychiatry
JA - J Am Acad.Child Adolesc Psychiatry
VL - 30
IS - 4
CY - UNITED STATES
SN - 0890-8567
UR - 1890108
ER -

TY - JOUR
ID - 460
T1 - Focus on summertime
A1 - Rollins,J.A.
Y1 - 1991/05//
N1 - Rollins, J A
Pediatric nursing
oun, 7505804
N
Journal Article
English
KW - Medline
KW - Child
KW - Preschool
KW - Drowning/pc [Prevention & Control]
KW - Humans
KW - Patient Advocacy
KW - Play and Playthings
KW - Safety
KW - Seat Belts
RP - NOT IN FILE
SP - 317
EP - 319

JF - Pediatric Nursing
JA - Pediatr Nurs
VL - 17
IS - 3
CY - UNITED STATES
SN - 0097-9805
UR - 2062597
ER -

TY - JOUR
ID - 461
T1 - Sociometric status: its stability and validity among neglected, rejected and popular children
A1 - Ollendick,T.H.
A1 - Greene,R.W.
A1 - Francis,G.
A1 - Baum,C.G.
Y1 - 1991/03//
N1 - Ollendick, T H. Greene, R W. Francis, G. Baum, C G
Journal of child psychology and psychiatry, and allied disciplines
hp3, 0375361
IM
Journal Article
English
KW - Medline
KW - Aggression/px [Psychology]
KW - Child
KW - Female
KW - Humans
KW - Internal-External Control
KW - Male
KW - Peer Group
KW - Personality Development
KW - Play and Playthings
KW - Rejection (Psychology)
KW - Social Behavior
KW - Social Desirability
KW - Social Isolation/px [Psychology]
KW - Sociometric Techniques
RP - NOT IN FILE
SP - 525
EP - 534
JF - Journal of Child Psychology & Psychiatry & Allied Disciplines
JA - J Child Psychol.Psychiatry
VL - 32
IS - 3
CY - ENGLAND
N2 - Although important distinctions among sociometrically rejected, neglected, and popular children have been reported in the literature, concerns have been raised about use of negative peer nominations in identifying these children. A revised procedure developed by Asher and Dodge [Developmental Psychology, 22, 444-449 (1986)] eliminates the need to obtain negative peer nominations. In the present study, the construct validity of this revised procedure was explored and its long-term stability examined over 6, 12 and 18 months. Our findings establish significant differences on several indices among rejected, neglected and popular children. However, the differences between rejected and popular children were more robust than those between neglected and popular children, who failed to differ from one another on most of the measures. Moreover, fairly good long-term stability was found for popular and rejected status, whereas the long-term stability for neglected status was

quite poor. These findings were addressed in the context of similar research employing negative nomination methods. Implications for the identification of at-risk children were also discussed
SN - 0021-9630
AD - Department of Psychology, Virginia Polytechnic Institute and State University, Blacksburg 24061-0436
UR - 2061371
ER -

TY - JOUR
ID - 462
T1 - Video analysis of playground injury-risk situations
A1 - Coppens,N.M.
A1 - Gentry,L.K.
Y1 - 1991/04//
N1 - Coppens, N M. Gentry, L K
Research in nursing & health
7806136, raq
IM, N
Journal Article. Research Support, Non-U.S. Gov't
English
KW - Medline
KW - Aggression
KW - Child
KW - Female
KW - Humans
KW - Male
KW - New England/ep [Epidemiology]
KW - Play and Playthings
KW - Risk Factors
KW - Schools
KW - Sex Factors
KW - Videotape Recording
KW - Wounds and Injuries/ep [Epidemiology]
KW - Wounds and Injuries/px [Psychology]
RP - NOT IN FILE
SP - 129
EP - 136
JF - Research in Nursing & Health
JA - Res.Nurs Health
VL - 14
IS - 2
CY - UNITED STATES
N2 - An observational study was conducted to examine injury-risk situations as they occurred during playground recess periods. A process analysis approach was used to describe antecedent conditions, response, consequence, and reaction depicted in the video scenes. Results indicate that boys were more involved than girls, playground equipment was frequently used improperly, aggressive behaviors were sometimes a factor, situations occurred more frequently with nonteacher than teacher monitors, and children infrequently took action to stop the situations. Other children were more likely to react to the target child than the monitor. Direction is provided for child and monitor safety promotion programs
SN - 0160-6891
AD - Nursing Department, College of Health Professions, University of Lowell, Massachusetts 01854
UR - 2047534
ER -

TY - JOUR
ID - 463

T1 - [Health and hygiene research on constructive toys intended for children over 7 under natural conditions]. [Bulgarian]
A1 - Uzunova,S.
A1 - Mineva,T.
Y1 - 1990///
N1 - Uzunova, S. Mineva, T
Problemi na khigienata
ppc, 7703299
IM
English Abstract. Journal Article
Bulgarian
KW - Medline
KW - Child
KW - Child Development
KW - Evaluation Studies as Topic
KW - Humans
KW - Hygiene
KW - Play and Playthings
KW - Questionnaires
KW - Safety
RP - NOT IN FILE
SP - 38
EP - 47
JF - Problemi na Khigienata
JA - Probl.Khig.
VL - 15
CY - BULGARIA
N2 - Physiological and questionnaire investigations are carried out on 10 constructive toys, with the participation of 38 children 7 to 12 years old. A testimonial of the toys is made according to mass, dimensions, technological treatment, packing etc. A number of discrepancies with the hygienic requirements were established: 1) imperfection of the technological treatment (sharp edges, tops and holes, difficult assembling and disassembling of details, not fitting some details at construction, presence of odour, dust in the packings); 2) discrepancy with the announced age destination; 3) faults (incongruity of colours, small dimensions of models and not clear and contrast models, absence of age destination); 4) unphysiological strain and fatigue during play with part of the toys. On the basis of the results are developed hygiene recommendations to the manufacturing of constructive toys for children over 7 years
SN - 0323-9179
UR - 2099461
ER -

TY - JOUR
ID - 464
T1 - Children with congenital anomalies: the preschool period
A1 - Allen,R.
A1 - Wasserman,G.A.
A1 - Seidman,S.
Y1 - 1990/06//
N1 - Allen, R. Wasserman, G A. Seidman, S
Journal of pediatric psychology
jl8, 7801773
IM
Journal Article. Research Support, Non-U.S. Gov't. Research Support, U.S. Gov't, P.H.S.
English
KW - Medline
KW - Child
KW - Preschool

KW - Congenital Abnormalities/px [Psychology]
KW - Cooperative Behavior
KW - Disabled Persons/px [Psychology]
KW - Female
KW - Humans
KW - Male
KW - Mother-Child Relations
KW - Personality Development
KW - Play and Playthings
RP - NOT IN FILE
SP - 327
EP - 345
JF - Journal of Pediatric Psychology
JA - J Pediatr Psychol.
VL - 15
IS - 3
CY - UNITED STATES
N2 - To address a gap regarding the development of preschool children with physical congenital anomalies and the adaptational responses of their caretakers, 37 children with anomalies and 44 control children were assessed. Children were administered the Stanford-Binet and the Preschool Language Scale, and the children and their mothers were observed in a series of semi-structured situations. Performance on linguistic and intellectual tests was related to socioeconomic status, not to medical risk. Children with anomalies were more socially reticent and compliant than peers, and their mothers were more controlling and nonverbally active during a teaching task. Results are discussed in terms of Bell's lower-limit adaptational pattern
SN - 0146-8693
AD - Department of Psychology, Long Island University, Brooklyn, New York 11201
UR - 2143228
ER -

TY - JOUR
ID - 465
T1 - Influences of TV games on physical and psychological development of Japanese kindergarten children
A1 - Shimai,S.
A1 - Masuda,K.
A1 - Kishimoto,Y.
Y1 - 1990/06//
N1 - Shimai, S. Masuda, K. Kishimoto, Y
Perceptual and motor skills
ozb, 0401131
IM
Comparative Study. Journal Article
English
KW - Medline
KW - Child Behavior Disorders/px [Psychology]
KW - Child Development
KW - Child
KW - Preschool
KW - Cross-Cultural Comparison
KW - Female
KW - Humans
KW - Japan
KW - Male
KW - Personality Development
KW - Play and Playthings
KW - Risk Factors
KW - Sex Factors

KW - Television
RP - NOT IN FILE
SP - 771
EP - 776
JA - Perceptual & Motor Skills
VL - 70
IS - 3 Pt 1
CY - UNITED STATES
N2 - The present study was an investigation of playing a TV game and its influences on physical and psychological development of kindergarten children. A self-completed questionnaire was delivered to the parents of 426 children. Of these, 408 had fully completed answers. Questions regarded the amounts of TV game play, its influences on health and on development of children and the attitudes of parents toward the game. Analysis indicated that the kindergarten children, especially boys, played frequently and both the percentage of players and the number of playing hours increased with age. Few adverse effects were reported on vision or other aspects of physical health. Children who played the game had much higher scores on sociability than those with no experience. This seemed to reflect that children acquired a new repertoire of plays from the TV game
SN - 0031-5125
AD - Department of Hygiene and Preventive Medicine, Fukushima Medical College, Japan
UR - 2377408
ER -

TY - JOUR
ID - 466
T1 - Effects of obesity, social interactions, and physical environment on physical activity in preschoolers
A1 - Klesges,R.C.
A1 - Eck,L.H.
A1 - Hanson,C.L.
A1 - Haddock,C.K.
A1 - Klesges,L.M.
Y1 - 1990///
N1 - Klesges, R C. Eck, L H. Hanson, C L. Haddock, C K. Klesges, L M
Health psychology : official journal of the Division of Health Psychology, American Psychological Association
ejl, 8211523
IM
Journal Article. Research Support, Non-U.S. Gov't. Research Support, U.S. Gov't, P.H.S.
English
KW - Medline
KW - Child
KW - Preschool
KW - Female
KW - Humans
KW - Male
KW - Motor Activity
KW - Obesity/ge [Genetics]
KW - Obesity/px [Psychology]
KW - Parent-Child Relations
KW - Play and Playthings
KW - Risk Factors
KW - Sex Factors
KW - Social Environment
RP - NOT IN FILE
SP - 435
EP - 449
JF - Health Psychology
JA - Health Psychol.
VL - 9

IS - 4
CY - UNITED STATES
N2 - Examined demographic, environmental, and parent-child interactional correlates of physical activity in a group of 222 preschoolers. Activity levels were assessed with a system that quantified directly observed physical activity in the natural environment. Using regression-modeling procedures, results revealed a significant relationship between (a) child's relative weight, parental weight status, and percentage of time spent outdoors (environment) and (b) children's activity levels. Parental obesity was associated with lower levels of physical activity in children, childhood relative weight was associated with slightly higher levels of physical activity, and more outdoor activity was associated with higher activity levels. Parental participation in children's activities also significantly interacted with levels of parental obesity in predicting activity levels. Those children with a 50% risk for obesity (as defined by both, one, or neither parent being overweight) had small changes in activity across levels of parent-child interaction, whereas those at higher risk for obesity responded with increased activity as parent-child interactions increased. Results are discussed, and the implications of these findings for future intervention efforts are examined
SN - 0278-6133
AD - Department of Psychology, Memphis State University, TN 38152
UR - 2373068
ER -

TY - JOUR
ID - 467
T1 - Safe care/safe play: child care as a site for injury prevention
A1 - Finn-Stevenson,M.
A1 - Stevenson,J.J.
Y1 - 1990///
N1 - Finn-Stevenson, M. Stevenson, J J
Children today
d2e, 0320227, 0320227
IM
Journal Article
English
KW - Medline
KW - Accident Prevention
KW - Child
KW - Child Care/mt [Methods]
KW - Preschool
KW - Connecticut
KW - Humans
KW - Play and Playthings
KW - Risk Factors
KW - Safety
KW - Wounds and Injuries/ep [Epidemiology]
KW - Wounds and Injuries/pc [Prevention & Control]
RP - NOT IN FILE
SP - 16
EP - 20, 32
JF - Children Today
JA - Child Today
VL - 19
IS - 2
CY - UNITED STATES
SN - 0361-4336
AD - Bush Center in Child Development and Social Policy, Yale University
UR - 2347253
ER -

TY - JOUR
ID - 468
T1 - Playroom injury leads to hospital liability
Y1 - 1990/01//
N1 - Hospital law newsletter
hln, 8911901
H
Journal Article
English
KW - Medline
KW - Child Care/lj [Legislation & Jurisprudence]
KW - Child
KW - Preschool
KW - Humans
KW - Louisiana
KW - Malpractice/lj [Legislation & Jurisprudence]
KW - Outpatient Clinics
KW - Hospital/lj [Legislation & Jurisprudence]
KW - Play and Playthings
KW - Safety
RP - NOT IN FILE
SP - 3
EP - 5
JF - Hospital Law Newsletter
JA - Hosp.Law Newsl.
VL - 7
IS - 3
CY - UNITED STATES
SN - 0738-0984
UR - 10104397
ER -

TY - JOUR
ID - 469
T1 - Roulette of the road: a new kind of suicide attempt? A preliminary report
A1 - Hes,J.P.
Y1 - 1989/10//
N1 - Hes, J P
Crisis
dt9, 8218602
IM
Journal Article
English
KW - Medline
KW - Accidents
KW - Traffic/px [Psychology]
KW - Adolescent
KW - Child
KW - Gambling
KW - Humans
KW - Israel
KW - Play and Playthings
KW - Risk-Taking
KW - Suicide
KW - Attempted/px [Psychology]
RP - NOT IN FILE

SP - 179
EP - 180
JF - Crisis: Journal of Crisis Intervention & Suicide
JA - Crisis
VL - 10
IS - 2
CY - CANADA
N2 - The author reports on children aged 7-14 who throw an object onto the highway shortly before a car comes and run to fetch it in front of the approaching automobile. He discusses the problems linked with that roulette-style play with death
SN - 0227-5910
UR - 2591251
ER -

TY - JOUR
ID - 470
T1 - Attention deficit disorder and allergy: a neurochemical model of the relation between the illnesses. [Review] [110 refs]
A1 - Marshall,P.
Y1 - 1989/11//
N1 - Marshall, P
Psychological bulletin
0376473, qe8
IM
Journal Article. Review
English
KW - Medline
KW - Arousal/ph [Physiology]
KW - Attention Deficit Disorder with Hyperactivity/pp [Physiopathology]
KW - Brain/pp [Physiopathology]
KW - Child
KW - Humans
KW - Hypersensitivity/pp [Physiopathology]
KW - Receptors
KW - Adrenergic/ph [Physiology]
KW - Cholinergic/ph [Physiology]
KW - Risk Factors
RP - NOT IN FILE
SP - 434
EP - 446
JF - Psychological Bulletin
JA - Psychol.Bull.
VL - 106
IS - 3
CY - UNITED STATES
N2 - Empirical studies suggest that allergies play an etiological role in a small subgroup of children who suffer from attention deficit-hyperactivity disorder (ADHD). Research indicates that allergic reactions results in cholinergic hyperresponsiveness and beta-adrenergic hyporesponsiveness in the autonomic nervous system. Evidence is reviewed that similar imbalances in central nervous system cholinergic/adrenergic activity play a causal role in manic and depressive behaviors. It is hypothesized that allergic reactions engender cholinergic/adrenergic activity imbalances in the central nervous system, leading to poorly regulated arousal levels and ADHD behaviors in some children. [References: 110]
SN - 0033-2909
UR - 2682719
ER -

TY - JOUR
ID - 471
T1 - Sociability and social withdrawal in childhood: stability and outcomes
A1 - Rubin,K.H.
A1 - Hymel,S.
A1 - Mills,R.S.
Y1 - 1989/06//
N1 - Rubin, K H. Hymel, S. Mills, R S
Journal of personality
jmv, 2985194r
IM
Journal Article. Research Support, Non-U.S. Gov't
English
KW - Medline
KW - Child Behavior Disorders/px [Psychology]
KW - Child
KW - Preschool
KW - Depression/px [Psychology]
KW - Female
KW - Humans
KW - Interpersonal Relations
KW - Loneliness
KW - Longitudinal Studies
KW - Male
KW - Peer Group
KW - Personality Development
KW - Play and Playthings
KW - Self Concept
KW - Shyness
KW - Social Isolation
KW - Teaching
RP - NOT IN FILE
SP - 237
EP - 255
JF - Journal of Personality
JA - J Pers.
VL - 57
IS - 2
CY - UNITED STATES
N2 - The stability and long-term correlates of both social withdrawal and sociability were investigated in a longitudinal study of normal children in kindergarten through the fifth grade. Both observational and peer assessment indices of withdrawal and sociability were considered; furthermore, a distinction was made between observed active versus passive solitude. Of particular interest was whether social withdrawal in early childhood was predictive of subsequent internalizing problems, as assessed by self-reports of social competence, overall self-worth, loneliness, and depression as well as teacher ratings of shy/anxious behavior, in later childhood. Results indicated a modest degree of stability for observed social withdrawal but not for observed sociability, nor for active or passive forms of solitude. Somewhat higher stability correlations were obtained for peer assessments of both sociability and withdrawal. In terms of predictive outcomes, significant relations were found between early social withdrawal in kindergarten and Grade 2 and subsequent internalizing problems in Grades 4 and 5, although the pattern of results was mixed. These data suggest that social withdrawal in early childhood may be predictive of risk for internalizing difficulties in later childhood
SN - 0022-3506
AD - Department of Psychology, University of Waterloo, Ontario, Canada
UR - 2769556
ER -

TY - JOUR
ID - 472
T1 - Intelligence and behavior problems in 5-year-olds in relation to representational abilities in the second year of life
A1 - McDonald,M.A.
A1 - Sigman,M.
A1 - Ungerer,J.A.
Y1 - 1989/04//
N1 - McDonald, M A. Sigman, M. Ungerer, J A
Journal of developmental and behavioral pediatrics : JDBP
htf, 8006933
IM
Journal Article. Research Support, Non-U.S. Gov't. Research Support, U.S. Gov't, P.H.S.
English
KW - Medline
KW - Child Behavior Disorders/di [Diagnosis]
KW - Child Language
KW - Child
KW - Preschool
KW - Cognition
KW - Humans
KW - Infant
KW - Newborn
KW - Premature
KW - Intelligence
KW - Language Development
KW - Male
KW - Play and Playthings
KW - Predictive Value of Tests
KW - Psychomotor Performance
KW - Risk Factors
KW - Time Factors
RP - NOT IN FILE
SP - 86
EP - 91
JF - Journal of Developmental & Behavioral Pediatrics
JA - J Dev.Behav Pediatr
VL - 10
IS - 2
CY - UNITED STATES
N2 - Assessments of sensorimotor skill, play, language, and developmental level were administered to 20 preterm and 20 full-term infants during sessions at 13 1/2 and 22 months of age. At 5-year follow-up, children were tested with the Wechsler Preschool and Primary Scale of Intelligence (WIPPSI) and the Developmental Test of Visual-Motor Integration (VMI). Behavior problems were also assessed. At 5 years, the preterm sample showed slight delays in cognitive functioning. Measures of development and language at 13 1/2 and 22 months and relational play at 22 months were most predictive of cognitive outcome. Children with behavior problems at 5 years differed from those without problems in terms of their play and language skills. These data suggest that measures of development, particularly language development, and of play are predictive of later cognitive and behavioral functioning
SN - 0196-206X
AD - Department of Psychiatry, UCLA School of Medicine 90024
UR - 2708542
ER -

TY - JOUR
ID - 473

T1 - Toy weapons and firecrackers: a source of hearing loss
A1 - Gupta,D.
A1 - Vishwakarma,S.K.
Y1 - 1989/03//
N1 - Gupta, D. Vishwakarma, S K
The Laryngoscope
8607378, l1w
IM
Journal Article
English
KW - Medline
KW - Adolescent
KW - Adult
KW - Auditory Threshold
KW - Child
KW - Preschool
KW - Firearms
KW - Hearing Loss
KW - Noise-Induced/et [Etiology]
KW - Humans
KW - Play and Playthings
KW - Risk Factors
KW - Sound Spectrography
RP - NOT IN FILE
SP - 330
EP - 334
JF - Laryngoscope
VL - 99
IS - 3
CY - UNITED STATES
N2 - Although acoustic injury as a result of exposure to noisy toys and firecrackers has been reported previously, most of these studies have been conducted on adults. The purpose of this prospective study, conducted at the time of Deepawali, an Indian festival of fireworks, was two-fold: 1. to measure the acoustic output of representative samples of toy weapons and firecrackers and the intensity level at critical spectator points from the site of emission; and 2. to determine the auditory status of a cross section of the target population, involving 600 participants from various age groups, before and after exposure to firecracker noise at Deepawali. The average sound level at a distance of 3 m was 150 dB, thus exceeding the damage risk criterion for adults (i.e., 130 dB peak level). An average 30 dB persistent sensorineural hearing loss was found in 2.5% of the target population as a result of toy weapon/firecracker noise during Deepawali. The 9- to 15-year-old age group was most affected. A judicious approach in the manufacture and use of toy weapons and firecrackers, in addition to legal restraints, is advocated
SN - 0023-852X
AD - Department of ENT, J.N. Medical College, Aligarh Muslim University, India
UR - 2918804
ER -

TY - JOUR
ID - 474
T1 - Epidemiology of severe eye injuries in childhood
A1 - LaRoche,G.R.
A1 - McIntyre,L.
A1 - Schertzer,R.M.
Y1 - 1988/12//
N1 - LaRoche, G R. McIntyre, L. Schertzer, R M
Ophthalmology
oi5, 7802443

IM
Journal Article. Research Support, Non-U.S. Gov't
English
KW - Medline
KW - Blindness/ep [Epidemiology]
KW - Blindness/et [Etiology]
KW - Child
KW - Preschool
KW - Cross-Sectional Studies
KW - Eye Injuries/ep [Epidemiology]
KW - Eye Injuries/et [Etiology]
KW - Female
KW - Humans
KW - Male
KW - Nova Scotia
KW - Play and Playthings
KW - Risk Factors
KW - Visual Acuity
RP - NOT IN FILE
SP - 1603
EP - 1607
JF - Ophthalmology
VL - 95
IS - 12
CY - UNITED STATES
N2 - The authors conducted a cross-sectional study of all ocular injury cases admitted to a children's hospital between January 1978 and December 1984. Of 222 injuries reviewed, 77 (35%) resulted in some visual deficit. Males were significantly overrepresented in all age groups with an average male:female ratio of 3.5:1. The distribution of injuries was: contusions, 114 (51%); penetrating lacerations, 62 (28%); foreign bodies and burns, 11 (5%); and nonpenetrating lacerations, 35 (16%). Sixteen (7%) ocular injuries were associated with BB gun pellets and six of these children (42%) were blinded in the injured eye as a result. Two other cases of blindness resulted from ocular penetration by homemade "Jinsang Stars," underscoring the adverse influence of media on children's games. Adult supervision could have potentially prevented most cases of permanent visual deficit. The authors suggest that legislation restricting the use of BB guns be passed and that a program of adult and child eye safety education including "eye watch" warnings on potentially hazardous toys be developed
SN - 0161-6420
AD - Department of Ophthalmology, Izaak Walton Killam Hospital for Children, Dalhousie University, Halifax, NS, Canada
UR - 3265999
ER -

TY - JOUR
ID - 475
T1 - Planning playgrounds for children with disabilities. [Review] [33 refs]
A1 - Stout,J.
Y1 - 1988/10//
N1 - Stout, J
The American journal of occupational therapy : official publication of the American Occupational Therapy Association
7705978, 3o4
IM
Journal Article. Review
English
KW - Medline
KW - Child
KW - Disabled Persons

KW - Environment Design
KW - Equipment Design
KW - Humans
KW - Occupational Therapy
KW - Play and Playthings
KW - Safety
RP - NOT IN FILE
SP - 653
EP - 657
JA - American Journal of Occupational Therapy
VL - 42
IS - 10
CY - UNITED STATES
N2 - Occupational therapists are well qualified for involvement in playground planning. They know how to eliminate architectural barriers, adapt equipment to maximize independence and functional levels, and use play in facilitating children's development. By participating in playground planning or adaptation, the occupational therapist can present play opportunities for children with disabilities, increase public awareness of occupational therapy, and extend the use of treatment modalities. This paper makes specific suggestions for planning and building to make the idea for a playground a realistic goal. A case example illustrates the planning of a playground in a hospital setting. [References: 33]
SN - 0272-9490
AD - Indiana University, Indianapolis 46223
UR - 2974238
ER -

TY - JOUR
ID - 476
T1 - Toddlers' concentration: does maternal depression make a difference?
A1 - Breznitz,Z.
A1 - Friedman,S.L.
Y1 - 1988/05//
N1 - Breznitz, Z. Friedman, S L
Journal of child psychology and psychiatry, and allied disciplines
hp3, 0375361
IM
Journal Article. Research Support, Non-U.S. Gov't. Research Support, U.S. Gov't, P.H.S.
English
KW - Medline
KW - Adult
KW - Arousal
KW - Attention
KW - Child
KW - Preschool
KW - Depressive Disorder/ge [Genetics]
KW - Depressive Disorder/px [Psychology]
KW - Female
KW - Humans
KW - Infant
KW - Male
KW - Mother-Child Relations
KW - Play and Playthings
KW - Risk Factors
RP - NOT IN FILE
SP - 267
EP - 279
JF - Journal of Child Psychology & Psychiatry & Allied Disciplines

JA - J Child Psychol.Psychiatry
VL - 29
IS - 3
CY - ENGLAND
N2 - Twenty-five mother-toddler dyads with depressed mothers were compared with 25 dyads with well mothers on measures of attention during 20 min of spontaneous play in a home-like setting. Children of depressed women focused attention on more objects for shorter durations. Group differences could be accounted for by mothers' involvement in their children's play. Depressed women initiated and terminated more instances of attention to objects than well mothers. Correlations between maternal behaviors and children's attention were statistically significant. Results support the hypothesis that poorer attention of children of depressed women is at least in part mediated by inculcation
SN - 0021-9630
AD - Department of Education, Haifa University, Israel
UR - 3417803
ER -

TY - JOUR
ID - 477
T1 - Family variables and physical activity in preschool children
A1 - Sallis,J.F.
A1 - Patterson,T.L.
A1 - McKenzie,T.L.
A1 - Nader,P.R.
Y1 - 1988/04//
N1 - Sallis, J F. Patterson, T L. McKenzie, T L. Nader, P R
Journal of developmental and behavioral pediatrics : JDBP
htf, 8006933
IM
Journal Article. Research Support, U.S. Gov't, P.H.S.
English
KW - Medline
KW - Body Weight
KW - Child
KW - Preschool
KW - Coronary Disease/ge [Genetics]
KW - Female
KW - Humans
KW - Imitative Behavior
KW - Male
KW - Motor Activity
KW - Obesity/ge [Genetics]
KW - Parents
KW - Physical Exertion
KW - Play and Playthings
KW - Schools
KW - Nursery
KW - Type A Personality
RP - NOT IN FILE
SP - 57
EP - 61
JF - Journal of Developmental & Behavioral Pediatrics
JA - J Dev.Behav Pediatr
VL - 9
IS - 2
CY - UNITED STATES

N2 - Correlates of the physical activity habits of preschool children were studied in a multiethnic sample. Physical activity levels of 33 low-income children were observed systematically during free-play periods at preschool. Children spent 58% of free-play time in sedentary activities (e.g., sitting), and were vigorously active only 11% of the time. Independent variables studied were child body mass index (BMI), teacher-rated Type A behavior, and parent-reported mother BMI, father BMI, parent vigorous activity, and family cardiovascular disease (CVD) risk. The multiple regression of moderate-intensity activity was significant, with family CVD risk, parent vigorous activity, and father BMI accounting for significant amounts of variance. The results suggest that the effects of parental role modeling on child physical activity levels may extend to free-play settings far beyond the confines of the home environment
SN - 0196-206X
AD - Department of Pediatrics, University of California, San Diego, La Jolla 92093-0631
UR - 3366911
ER -

TY - JOUR
ID - 478
T1 - Recreational waterslide injuries
A1 - Saunders,C.E.
Y1 - 1988/02//
N1 - Saunders, C E
Southern medical journal
uvh, 0404522
AIM, IM
Journal Article
English
KW - Medline
KW - Adolescent
KW - Adult
KW - Child
KW - Preschool
KW - Female
KW - Humans
KW - Male
KW - Middle Aged
KW - Play and Playthings
KW - Safety
KW - Wounds and Injuries/ep [Epidemiology]
KW - Wounds and Injuries/et [Etiology]
KW - Wounds and Injuries/pc [Prevention & Control]
RP - NOT IN FILE
SP - 189
EP - 192
JF - Southern Medical Journal
JA - South Med J
VL - 81
IS - 2
CY - UNITED STATES
N2 - Recreational waterslide facilities are a growing phenomenon in the United States, and injuries associated with their use are beginning to appear. Although there have been fatalities, the vast majority of injuries are minor, and occur over a large denominator of slide rides. The overall incidence of injuries is low, though the incidence varies from one facility to another. If safety practices and design features are implemented, waterslide use may become safer. Epidemiologic data will be required to clearly identify the optimal features of injury prevention
SN - 0038-4348
AD - Department of Surgery, University of Colorado Health Sciences Center, Denver
UR - 3340872

ER -

TY - JOUR
ID - 479
T1 - Toys. Good, bad, but never indifferent
A1 - Osterhout,S.K.
Y1 - 1987/12//
N1 - Osterhout, S K
North Carolina medical journal
ntx, 2984805r
IM
Journal Article
English
KW - Medline
KW - Child
KW - Consumer Advocacy
KW - Consumer Product Safety
KW - Humans
KW - Play and Playthings/st [Standards]
KW - Safety
KW - United States
RP - NOT IN FILE
SP - 645
EP - 649
JF - North Carolina Medical Journal
JA - N.C.Med J
VL - 48
IS - 12
CY - UNITED STATES
SN - 0029-2559
UR - 3480437
ER -

TY - JOUR
ID - 480
T1 - Sexual abuse in vulnerable and high risk children
A1 - Cohen,T.B.
A1 - Galenson,E.
A1 - van,Leeuwen K.
A1 - Steele,B.F.
A1 - Sherkow,S.P.
A1 - Etezady,M.H.
Y1 - 1987///
N1 - Cohen, T B. Galenson, E. van Leeuwen, K. Steele, B F. Sherkow, S P. Etezady, M H
Child abuse & neglect
can, 7801702
IM
Case Reports. Journal Article
English
KW - Medline
KW - Anxiety
KW - Castration
KW - Child
KW - Child Abuse
KW - Sexual/px [Psychology]
KW - Preschool

KW - Female
KW - Humans
KW - Incest
KW - Male
KW - Mother-Child Relations
KW - Play and Playthings
KW - Psychosexual Development
KW - Risk Factors
KW - Sexual Dysfunctions
KW - Psychological/et [Etiology]
RP - NOT IN FILE
SP - 461
EP - 474
JF - Child Abuse & Neglect
JA - Child Abuse Negl.
VL - 11
IS - 3
CY - ENGLAND
SN - 0145-2134
UR - 3676899
ER -

TY - JOUR
ID - 481
T1 - Home Screening Questionnaire: its validity in assessing home environment
A1 - Frankenburg,W.K.
A1 - Coons,C.E.
Y1 - 1986/04//
N1 - Frankenburg, W K. Coons, C E
The Journal of pediatrics
jlz, 0375410
AIM, IM
Journal Article
English
KW - Medline
KW - Child
KW - Child Development
KW - Preschool
KW - Cognition
KW - Educational Status
KW - Evaluation Studies as Topic
KW - Hispanic Americans
KW - Humans
KW - Infant
KW - Newborn
KW - Male
KW - Parent-Child Relations
KW - Play and Playthings
KW - Questionnaires
KW - Risk
KW - Social Environment
KW - Socioeconomic Factors
RP - NOT IN FILE
SP - 624
EP - 626
JF - Journal of Pediatrics

JA - J Pediatr
VL - 108
IS - 4
CY - UNITED STATES
N2 - To develop a quick, practical tool to identify home environments likely to be suboptimal for the development of children, we adapted segments of the Home Observation for Measurement of the Environment (HOME) Inventory, which assesses the environments of children from birth to 6 years, and devised the Home Screening Questionnaire (HSQ). Whereas the HOME Inventory requires a home visit, the HSQ consists of two forms, one for children from birth to 3 years, another for 3 to 6 years, that are completed by parents without a home visit. The questions are formulated at the third to sixth grade reading level and can be completed in 15 to 20 minutes. Scoring and interpretation by a professional takes 5 minutes. HSQ questions were answered by more than 1500 parents of low-income families. Between 81% and 86% of the environments determined to be of concern by the HOME Inventory were identified by the briefer and less expensive HSQ. Thus the HSQ readily lends itself to use by health care providers who serve low-income families
SN - 0022-3476
UR - 3958839
ER -

TY - JOUR
ID - 482
T1 - Mediating influences of social support: personal, family, and child outcomes
A1 - Dunst,C.J.
A1 - Trivette,C.M.
A1 - Cross,A.H.
Y1 - 1986/01//
N1 - Dunst, C J. Trivette, C M. Cross, A H
American journal of mental deficiency
3ma, 0372647
IM
Journal Article. Research Support, Non-U.S. Gov't. Research Support, U.S. Gov't, P.H.S.
English
KW - Medline
KW - Attitude
KW - Child Development
KW - Child
KW - Preschool
KW - Developmental Disabilities/px [Psychology]
KW - Disabled Persons/px [Psychology]
KW - Family
KW - Female
KW - Humans
KW - Intellectual Disability/px [Psychology]
KW - Male
KW - Parent-Child Relations
KW - Social Class
KW - Social Environment
KW - Social Support
RP - NOT IN FILE
SP - 403
EP - 417
JF - American Journal of Mental Deficiency
JA - Am J Ment.Defic.
VL - 90
IS - 4
CY - UNITED STATES

N2 - The mediating influences of social support were examined in a study of 137 parents of mentally retarded, physically impaired, and developmentally at-risk children. Social system theory was used as a conceptual framework for assessing the effects of social support on personal well-being, parental attitudes toward their child, family integrity, parental perceptions of child functioning, parent-child play opportunities, and child behavior and development. A series of hierarchical multiple regression analyses by sets, controlling for family SES and income, child sex and age, and child developmental quotient and diagnosis showed that both satisfaction with support and number of sources of support had main and/or interactive effects in all sets of outcome measures. More supportive social networks were associated with better personal well-being, more positive attitudes, and more positive influences on parent-child play opportunities and child behavior and development. Findings were discussed in terms of both methodological and conceptual contributions to understanding the broad-based influences of social support
SN - 0002-9351
UR - 2418680
ER -

TY - JOUR
ID - 483
T1 - Leads from the MMWR. Toy safety--United States, 1984
Y1 - 1986/01/17/
N1 - JAMA : the journal of the American Medical Association
7501160
AIM, IM
Journal Article
English
KW - Medline
KW - Accidents
KW - Adolescent
KW - Child
KW - Preschool
KW - Consumer Product Safety
KW - Humans
KW - Mortality
KW - Play and Playthings
KW - Safety
KW - United States
KW - Wounds and Injuries/ep [Epidemiology]
KW - Wounds and Injuries/et [Etiology]
RP - NOT IN FILE
SP - 312
EP - 313
JF - JAMA
VL - 255
IS - 3
CY - UNITED STATES
SN - 0098-7484
UR - 3941508
ER -

TY - JOUR
ID - 484
T1 - Toy safety--United States, 1984
A1 - Centers for Disease Control (CDC)
Y1 - 1984///
N1 - Centers for Disease Control (CDC)
MMWR. Morbidity and mortality weekly report
ne8, 7802429

IM
Journal Article
English
KW - Medline
KW - Accidents
KW - Adolescent
KW - Child
KW - Preschool
KW - Consumer Product Safety
KW - Humans
KW - Mortality
KW - Play and Playthings
KW - Safety
KW - United States
KW - Wounds and Injuries/ep [Epidemiology]
KW - Wounds and Injuries/et [Etiology]
RP - NOT IN FILE
SP - 755
EP - 756
JF - MMWR - Morbidity & Mortality Weekly Report
JA - MMWR Morb.Mortal.Wkly.Rep.
VL - 34
IS - 50
CY - UNITED STATES
SN - 0149-2195
UR - 3934519
ER -

TY - JOUR
ID - 485
T1 - At-risk toddlers and their mothers: the special case of physical handicap
A1 - Wasserman,G.A.
A1 - Allen,R.
A1 - Solomon,C.R.
Y1 - 1985/02//
N1 - Wasserman, G A. Allen, R. Solomon, C R
Child development
0372725, d28
IM
Journal Article. Research Support, Non-U.S. Gov't. Research Support, U.S. Gov't, P.H.S.
English
KW - Medline
KW - Child Behavior
KW - Child Development
KW - Child
KW - Preschool
KW - Congenital Abnormalities/px [Psychology]
KW - Disabled Persons/px [Psychology]
KW - Female
KW - Humans
KW - Infant
KW - Newborn
KW - Premature
KW - Diseases/px [Psychology]
KW - Male
KW - Maternal Behavior

KW - Mother-Child Relations
KW - Play and Playthings
KW - Risk
KW - Social Behavior
RP - NOT IN FILE
SP - 73
EP - 83
JF - Child Development
JA - Child Dev.
VL - 56
IS - 1
CY - UNITED STATES
N2 - Previous studies of school-aged children with physical handicaps indicate passivity, inhibition, and deficits in cognitive-linguistic functioning, sometimes interpreted as consequent to parent-child problems. The present study examined the functioning of toddlers with physical anomalies (but without CNS damage) in comparison to premature toddlers (who share deviant early experience but not deviant physical appearance) and to normal toddlers. 14 mother-child pairs in each group were videotaped at 9, 12, 18, and 24 months in a variety of semistructured situations. Both premature and handicapped toddlers performed more poorly in measures of social initiative, focused play, language production, and 2-year IQ. In addition, handicapped toddlers manifested increased distractibility, decreased compliance, and reluctance to separate from their mothers. Mothers of both prematures and handicapped toddlers were more initiating and less responsive than controls, while mothers of handicapped children were also more likely to both encourage and ignore their toddlers. Patterns previously reported for older children are therefore found here with toddlers. Maternal patterns are interpreted as supporting Bell's model of compensatory "lower-limit" effects
SN - 0009-3920
UR - 3157552
ER -

TY - JOUR
ID - 486
T1 - Legal approaches to injury prevention
A1 - Swartz,E.M.
Y1 - 1985/02//
N1 - Swartz, E M
Pediatric clinics of North America
oum, 0401126
AIM, IM
Journal Article
English
KW - Medline
KW - Child
KW - Child Advocacy/lj [Legislation & Jurisprudence]
KW - Expert Testimony
KW - Humans
KW - Legislation as Topic
KW - Physician's Role
KW - Play and Playthings
KW - Safety
KW - United States
KW - Wounds and Injuries/pc [Prevention & Control]
RP - NOT IN FILE
SP - 213
EP - 219
JF - Pediatric Clinics of North America
JA - Pediatr Clin.North Am
VL - 32

IS - 1
CY - UNITED STATES
N2 - Injury prevention can be achieved, but it will require a fundamental reexamination of our approach to injury causation. We must learn to examine the manufacturing and marketing practices of companies that produce the products associated with children's injuries, for these are the real culprits in our national childhood injury plague. Most importantly, we must learn to demand from industry that it take injury prevention seriously. If it refuses to do so it must face the consequences before the American system of justice. Legal advocacy can be a valuable tool in this effort. The legal system provides the means to pierce the corporate veil of secrecy and to learn how and why products are made of hazardous design. Under the light of public scrutiny, culprit companies can be made to pay the price for producing hazardous products. Only in this manner will industry be given the incentive to increase product safety
SN - 0031-3955
UR - 3975090
ER -

TY - JOUR
ID - 487
T1 - Injuries at play
A1 - Greensher,J.
A1 - Mofenson,H.C.
Y1 - 1985/02//
N1 - Greensher, J. Mofenson, H C
Pediatric clinics of North America
oum, 0401126
AIM, IM
Journal Article
English
KW - Medline
KW - Child
KW - Preschool
KW - Humans
KW - Infant
KW - Play and Playthings
KW - Risk
KW - Safety
KW - Walkers/ae [Adverse Effects]
KW - Wounds and Injuries/et [Etiology]
KW - Wounds and Injuries/pc [Prevention & Control]
RP - NOT IN FILE
SP - 127
EP - 139
JF - Pediatric Clinics of North America
JA - Pediatr Clin.North Am
VL - 32
IS - 1
CY - UNITED STATES
N2 - The problem of toy-related injuries is a complicated one. The authors detail specific hazards related to toys, playgrounds, skateboards, and baby walkers. Guidelines are provided for toy suitability for children of all ages
SN - 0031-3955
UR - 3975083
ER -

TY - JOUR
ID - 488
T1 - Are toy guns too dangerous?

A1 - Tanz,R.
A1 - Christoffel,K.K.
A1 - Sagerman,S.
Y1 - 1985/02//
N1 - Tanz, R. Christoffel, K K. Sagerman, S
Pediatrics
oxv, 0376422
AIM, IM
Journal Article. Research Support, Non-U.S. Gov't
English
KW - Medline
KW - Accident Prevention
KW - Adolescent
KW - Adult
KW - Child
KW - Preschool
KW - Consumer Product Safety
KW - Female
KW - Humans
KW - Infant
KW - Newborn
KW - Legislation as Topic
KW - Male
KW - Play and Playthings
KW - Safety
KW - United States
KW - Wounds and Injuries/ep [Epidemiology]
KW - Wounds and Injuries/pc [Prevention & Control]
RP - NOT IN FILE
SP - 265
EP - 268
JA - Pediatrics
VL - 75
IS - 2
CY - UNITED STATES
N2 - Toy guns with projectiles may injure young children. A study was undertaken to assess the danger posed by these toys and the regulations governing them. US Consumer Product Safety Commission (CPSC) data indicate that toy guns with projectiles cause relatively few injuries, mostly to young boys. The magnitude of the problem is smaller than that of nonpowder and powder firearms. Most often, toy gun injuries are to the face and eyes. Of the estimated 818 injuries in 1980 and 1981, 2.9% required hospitalization. More than 400 days were lost from school and work, and there were nearly 3,000 days of restricted activity due to these toy gun injuries. The hazard posed by toy guns with projectiles can be reduced through the legitimate regulatory authority of the Consumer Product Safety Commission with minor impact on retail cost, toy industry sales, and the play value of the toys. The yearly cost to consumers from increased regulation would probably be similar to the annual cost of the injuries. Regulation is warranted to protect young children who use these toys
SN - 0031-4005
UR - 3969326
ER -

TY - JOUR
ID - 489
T1 - Behavioral assessment and treatment of chronic food refusal in handicapped children
A1 - Riordan,M.M.
A1 - Iwata,B.A.
A1 - Finney,J.W.
A1 - Wohl,M.K.

A1 - Stanley,A.E.
Y1 - 1984///
N1 - Riordan, M M. Iwata, B A. Finney, J W. Wohl, M K. Stanley, A E
Journal of applied behavior analysis
0174763, hdr
IM
Case Reports. Journal Article. Research Support, U.S. Gov't, P.H.S.
English
KW - Medline
KW - Behavior Therapy/mt [Methods]
KW - Cerebral Palsy/co [Complications]
KW - Child
KW - Preschool
KW - Disabled Persons
KW - Eating Disorders/co [Complications]
KW - Eating Disorders/di [Diagnosis]
KW - Eating Disorders/th [Therapy]
KW - Failure to Thrive/co [Complications]
KW - Female
KW - Food Preferences
KW - Hemiplegia/co [Complications]
KW - Humans
KW - Hydrocephalus/co [Complications]
KW - Infant
KW - Male
KW - Play and Playthings
KW - Reinforcement
KW - Social
KW - Seizures/co [Complications]
KW - Vision Disorders/co [Complications]
RP - NOT IN FILE
SP - 327
EP - 341
JF - Journal of Applied Behavior Analysis
JA - J Appl.Behav Anal.
VL - 17
IS - 3
CY - UNITED STATES
N2 - In this study, we examined the eating behavior of four handicapped children, none of whom exhibited self-feeding skills. All children had a history of food refusal and were nutritionally at risk; one child received all nourishment by way of gastrostomy tube. Baseline data taken during mealtimes indicated that all children accepted very little food, expelled food frequently, and engaged in a number of disruptive behaviors. Treatments consisted of one or more of the following contingent events: social praise, access to preferred foods, brief periods of toy play, and forced feeding. Results of multiple-baseline and reversal designs showed marked behavioral improvement for each child and increases in the amount of food consumed. Further improvements were noted at follow-up, which ranged from 7 to 30 months posttreatment
SN - 0021-8855
UR - 6239853
ER -

TY - JOUR
ID - 490
T1 - Playing it safe. Three. Making a sensible choice
A1 - Dann,C.
Y1 - 1984/11/07/

N1 - Dann, C
Nursing times
0423236, o9u
N
Journal Article
English
KW - Medline
KW - Accident Prevention
KW - Child
KW - Great Britain
KW - Humans
KW - Legislation as Topic
KW - Play and Playthings
KW - Safety
RP - NOT IN FILE
SP - 56
EP - 57
JF - Nursing Times
JA - Nurs Times
VL - 80
IS - 45
CY - ENGLAND
SN - 0954-7762
UR - 6568626
ER -

TY - JOUR
ID - 491
T1 - Playing it safe. Two. Toy libraries
A1 - Swaffield,L.
Y1 - 1984/11/07/
N1 - Swaffield, L
Nursing times
0423236, o9u
N
Bibliography. Journal Article
English
KW - Medline
KW - Accident Prevention
KW - Bibliography as Topic
KW - Child
KW - Preschool
KW - Humans
KW - Play and Playthings
KW - Safety
RP - NOT IN FILE
SP - 55
EP - 56
JF - Nursing Times
JA - Nurs Times
VL - 80
IS - 45
CY - ENGLAND
SN - 0954-7762
UR - 6390351
ER -

TY - JOUR
ID - 492
T1 - Playing it safe: top ten toys 1985
A1 - Swaffield,L.
Y1 - 1984/11/07/
N1 - Swaffield, L
Nursing times
0423236, o9u
N
Journal Article
English
KW - Medline
KW - Accident Prevention
KW - Child
KW - Preschool
KW - Humans
KW - Infant
KW - Play and Playthings
KW - Safety
RP - NOT IN FILE
SP - 53
EP - 54
JF - Nursing Times
JA - Nurs Times
VL - 80
IS - 45
CY - ENGLAND
SN - 0954-7762
UR - 6568625
ER -

TY - JOUR
ID - 493
T1 - [Methodological approach to the hygienic evaluation of toys made of polymer materials]. [Russian]
A1 - Stan,V.V.
Y1 - 1984/08//
N1 - Stan, V V
Gigiena i sanitariia
fpz, 0412700
IM
Journal Article
Russian
KW - Medline
KW - Child
KW - Preschool
KW - Humans
KW - Hygiene
KW - Infant
KW - methods
KW - Play and Playthings
KW - Polymers/an [Analysis]
KW - Polymers/to [Toxicity]
KW - Safety
RP - NOT IN FILE
SP - 32

EP - 35
JF - Gigiena i Sanitariia
JA - Gig.Sanit.
IS - 8
CY - USSR
SN - 0016-9900
UR - 6489777
ER -

TY - JOUR
ID - 494
T1 - Are test materials toys? A reply to Ridenour and Reid
A1 - Bayley,N.
A1 - Hunt,J.V.
Y1 - 1983/12//
N1 - Bayley, N. Hunt, J V
Perceptual and motor skills
ozb, 0401131
IM
Journal Article
English
KW - Medline
KW - Accident Prevention
KW - Child
KW - Preschool
KW - Humans
KW - Infant
KW - Play and Playthings
KW - Psychological Tests/is [Instrumentation]
KW - Safety
RP - NOT IN FILE
SP - 1270
JA - Perceptual & Motor Skills
VL - 57
IS - 3 Pt 2
CY - UNITED STATES
SN - 0031-5125
UR - 6664803
ER -

TY - JOUR
ID - 495
T1 - [Development of methodical principles and approval of new forms of contact with school children (ages 12-13 years) in carrying out preventive measures (results of a pilot study)]. [Russian]
A1 - Mukhamedzhanova,R.F.
A1 - Suslova,E.A.
A1 - Gusev,G.V.
A1 - Chazova,L.V.
A1 - Dobo,S.S.
Y1 - 1983///
N1 - Mukhamedzhanova, R F. Suslova, E A. Gusev, G V. Chazova, L V. Dobo, S S
Biulleten' Vsesoiuznogo kardiologicheskogo nauchnogo tsentra AMN SSSR
ahs, 8003723
IM
English Abstract. Journal Article
Russian

KW - Medline
KW - Adolescent
KW - Blood Pressure
KW - Body Height
KW - Body Weight
KW - Cardiovascular Diseases/pc [Prevention & Control]
KW - Child
KW - Female
KW - Health Promotion
KW - Humans
KW - Male
KW - Mass Screening
KW - Moscow
KW - Physical Exertion
KW - Pilot Projects
KW - Play and Playthings
KW - Preventive Medicine/mt [Methods]
KW - Risk
KW - Sex Factors
RP - NOT IN FILE
SP - 82
EP - 88
JF - Biulleten Vsesoiuznogo Kardiologicheskogo Nauchnogo Tsentra Amn SSSR
JA - Biull.Vsesoiuznogo Kardiol.Nauchn.Tsentra AMN SSSR
VL - 6
IS - 2
CY - USSR
N2 - The results of the screening and 1-year follow-up of pupils from two Moscow schools of general education (Moscow secondary schools) are presented. The program on preventive measures was conducted by using the playing forms of contact and influence, at one of the above schools (experimental school). Most of the adolescents of the experimental school (mean 80%) participated in the preventive measures. By the completion of the program the number of the participants increased up to 94%. A reliably significant increase in the education level on the problems about the adverse effects of smoking and beneficial effects of regular nutrition in adolescents of the experimental school was observed. The analysis of the results of the screening and follow-up of schoolchildren aged 12-13 years enables us to conclude that it is necessary to work out new criteria for the assessment of preventive measures among adolescents
SN - 0201-7369
UR - 6639762
ER -

TY - JOUR
ID - 496
T1 - Intervention approaches to conduct disorders in young children. [Review] [41 refs]
A1 - Webster-Stratton,C.
Y1 - 1983///
N1 - Webster-Stratton, C
The Nurse practitioner
oa1, 7603663
IM, N
Journal Article. Review
English
KW - Medline
KW - Adult
KW - Aggression
KW - Behavior Therapy
KW - Child

KW - Child Behavior
KW - Child Behavior Disorders/di [Diagnosis]
KW - Child Behavior Disorders/px [Psychology]
KW - Child Behavior Disorders/th [Therapy]
KW - Child Rearing
KW - Preschool
KW - Cooperative Behavior
KW - Humans
KW - Nursing Assessment
KW - Nursing Process
KW - Parent-Child Relations
KW - Parents/ed [Education]
KW - Play and Playthings
KW - Reinforcement
KW - Social
RP - NOT IN FILE
SP - 23
EP - 24. 33-34
JF - Nurse Practitioner
JA - Nurse Pract
VL - 8
IS - 5
CY - UNITED STATES
N2 - The scope of the problem in regard to childhood aggression or conduct disorders is large and far exceeds the resources for dealing with them. Moreover, such children are at high risk for developing serious adolescent and adult psychological problems. Nurse practitioners are placed in strategic positions to be able to screen for, identify and treat early signs of conduct disorders in young children. This review describes assessment procedures and the essential components of a parent training program designed to help families reduce childhood aggression. By intervening with families early, nurse practitioners may be able to design effective preventive programs and improve the prognosis for these children. [References: 41]
SN - 0361-1817
UR - 6346154
ER -

TY - JOUR
ID - 497
T1 - [Hygienic requirements for children's toys]. [Russian]
A1 - Prokof'eva,N.G.
A1 - Koval'chuk,E.N.
Y1 - 1982///
N1 - Prokof'eva, N G. Koval'chuk, E N
Fel'dsher i akusherka
eva, 16930040r
N
Journal Article
Russian
KW - Medline
KW - Age Factors
KW - Child
KW - Child Development
KW - Preschool
KW - Humans
KW - Hygiene
KW - Infant
KW - Play and Playthings
KW - Safety

RP - NOT IN FILE
SP - 11
EP - 14
JF - Feldsher i Akusherka
JA - Feldsher Akush.
VL - 47
IS - 3
CY - USSR
SN - 0014-9772
UR - 6918301
ER -

TY - JOUR
ID - 498
T1 - Childhood injuries: recognition and prevention
A1 - Berger,L.R.
Y1 - 1981/11//
N1 - Berger, L R
Current problems in pediatrics
dvf, 1272515
IM
Journal Article
English
KW - Medline
KW - Accident Prevention
KW - Accidents
KW - Traffic/pc [Prevention & Control]
KW - Athletic Injuries/pc [Prevention & Control]
KW - Automobile Driving
KW - Burns/pc [Prevention & Control]
KW - Child
KW - Female
KW - Health Education
KW - Humans
KW - Male
KW - Play and Playthings
KW - Risk
KW - Safety
KW - Seat Belts
KW - Wounds and Injuries/ep [Epidemiology]
KW - Wounds and Injuries/pc [Prevention & Control]
RP - NOT IN FILE
SP - 1
EP - 59
JF - Current Problems in Pediatrics
JA - Curr.Probl.Pediatr
VL - 12
IS - 1
CY - UNITED STATES
SN - 0045-9380
UR - 7318498
ER -

TY - JOUR
ID - 499
T1 - Attentiveness and responsiveness to auditory stimuli of children at risk for mental retardation

A1 - Finkelstein,N.W.
A1 - Gallagher,J.J.
A1 - Farran,D.C.
Y1 - 1980/09//
N1 - Finkelstein, N W. Gallagher, J J. Farran, D C
American journal of mental deficiency
3ma, 0372647
IM
Journal Article. Research Support, U.S. Gov't, P.H.S.
English
KW - Medline
KW - Attention
KW - Auditory Perception
KW - Child
KW - Preschool
KW - Female
KW - Humans
KW - Intellectual Disability/px [Psychology]
KW - Intellectual Disability/rh [Rehabilitation]
KW - Male
KW - Play and Playthings
KW - Psychological Tests
KW - Risk
KW - Speech Perception
KW - Visual Perception
RP - NOT IN FILE
SP - 135
EP - 144
JF - American Journal of Mental Deficiency
JA - Am J Ment.Defic.
VL - 85
IS - 2
CY - UNITED STATES
N2 - The major hypothesis in the present study was that differences between high-risk and low-risk children in performance on standardized tests of cognitive ability would be explained, in part, by differences in attentiveness to auditory stimuli and differences in task orientation. The data collected revealed the anticipated higher standardized test scores for low-risk children. Within the high-risk sample, few differences were found between children who attended an intervention day-care program and those who did not. Measures from a laboratory task and a free-play observation suggested that attention is a key factor in understanding the high-risk child's development and also that differences in attention between high- and low-risk children can be reliably obtained as early as 3 years of age. A lessened attention span was demonstrated by high-risk children to both auditory and visual stimuli if either were complex or demanding
SN - 0002-9351
UR - 7446581
ER -

TY - JOUR
ID - 500
T1 - Strangulation in childhood: epidemiology and clinical course
A1 - Feldman,K.W.
A1 - Simms,R.J.
Y1 - 1980/06//
N1 - Feldman, K W. Simms, R J
Pediatrics
oxv, 0376422
AIM, IM

Journal Article
English
KW - Medline
KW - Accident Prevention
KW - Accidents
KW - Home
KW - Adolescent
KW - Asphyxia/co [Complications]
KW - Asphyxia/ep [Epidemiology]
KW - Asphyxia/et [Etiology]
KW - Beds/st [Standards]
KW - Brain Damage
KW - Chronic/et [Etiology]
KW - Child
KW - Preschool
KW - Clothing
KW - Equipment and Supplies/st [Standards]
KW - Female
KW - Government Agencies
KW - Humans
KW - Hypoxia
KW - Brain/et [Etiology]
KW - Infant
KW - Newborn
KW - Male
KW - Play and Playthings
KW - Safety
KW - Sudden Infant Death/et [Etiology]
KW - Suicide
KW - United States
RP - NOT IN FILE
SP - 1079
EP - 1085
JA - Pediatrics
VL - 65
IS - 6
CY - UNITED STATES
N2 - A review of 233 cases of childhood strangulation was made to determine injury epidemiology. Consistent patterns of injury were observed. Crib and playground equipment strangulations are already the subject of Consumer Product Safety Commission (CPSC) preventative regulations. High chair-, playpen mesh-, pacifier cord-, and clothing-related injuries would be amenable to prevention by improved product safety regulation. The CPSC has been petitioned to remedy the first two of these hazards. Many crib and rope strangulations can only be prevented by direct patient counseling. Although Congress empowered and required the CPSC to recognize and correct unsafe products, its surveillance systems may obscure the very design defects that cause injury. Likewise, political considerations may slow enactment of new design regulations. Physicians who treat childhood accident victims are in a position to recognize hazardous products and lobby for their improvement. Of the 233 cases 38 were further evaluated to delineate the clinical course of childhood strangulation. Victims who fail to resume normal cardiopulmonary function by the time of hospitalization have a poor prognosis for neurologic recovery with current modes of therapy. Improved treatment of hypoxic and ischemic cerebral injury might prevent some of the deaths and handicaps resulting from strangulation
SN - 0031-4005
UR - 7375230
ER -

TY - JOUR
ID - 501

T1 - Migration and mental illness: what role do traditional childhood socialization practices play?
A1 - Danna,J.J.
Y1 - 1980/03//
N1 - Danna, J J
Culture, medicine and psychiatry
dt5, 7707467
IM, J
Comparative Study. Journal Article
English
KW - Medline
KW - Australia
KW - Child
KW - Child Rearing
KW - Cross-Cultural Comparison
KW - Female
KW - Humans
KW - Male
KW - Mental Disorders/et [Etiology]
KW - Sicily/eh [Ethnology]
KW - SOCIALIZATION
KW - Transients and Migrants
KW - United States
KW - Urbanization
RP - NOT IN FILE
SP - 25
EP - 42
JF - Culture, Medicine & Psychiatry
JA - Cult.Med Psychiatry
VL - 4
IS - 1
CY - NETHERLANDS
N2 - Interpretations of studies which show a correlation between migration and mental illness generally focus on sociocultural variables while giving scant attention to determinants of cognitive-personality orientations. This article examines childhood socialization practices as a key variable for comparing points of psychological stress between the traditional rural culture and the post-industrial urban culture. The hypothesis that harsh and restrictive childhood socialization practices foster the development of a cognitive-personality orientation which increases the likelihood of maladaptive responses to culture change is illustrated by anthropological and psychological data obtained from Sicilians living in Sicily, the U.S.A. and Australia. Suggestions are given for minimizing the mental health risks involved during the critical transition period following migration
SN - 0165-005X
UR - 7371420
ER -

TY - JOUR
ID - 502
T1 - Play aids for the handicapped: children - safely - at play
A1 - Marais,E.
Y1 - 1980/02/07/
N1 - Marais, E
Nursing mirror
o98, 7708429
N
Journal Article
English
KW - Medline
KW - Child

KW - Disabled Persons
KW - Humans
KW - Play and Playthings
KW - Safety
RP - NOT IN FILE
SP - 26
EP - 27
JF - Nursing Mirror
JA - Nurs Mirror
VL - 150
IS - 6
CY - ENGLAND
SN - 0029-6511
UR - 6444716
ER -

TY - JOUR
ID - 503
T1 - Peer preferences of at-risk and normally developing children in a preschool mainstream classroom
A1 - Cavallaro,S.A.
A1 - Porter,R.H.
Y1 - 1980/01//
N1 - Cavallaro, S A. Porter, R H
American journal of mental deficiency
3ma, 0372647
IM
Journal Article. Research Support, U.S. Gov't, P.H.S.
English
KW - Medline
KW - Child Development
KW - Child
KW - Preschool
KW - Education of Intellectually Disabled
KW - Eye Movements
KW - Female
KW - Group Processes
KW - Humans
KW - Intellectual Disability/px [Psychology]
KW - Interpersonal Relations
KW - Male
KW - Peer Group
KW - Play and Playthings
KW - Risk
KW - Social Adjustment
RP - NOT IN FILE
SP - 357
EP - 366
JF - American Journal of Mental Deficiency
JA - Am J Ment.Defic.
VL - 84
IS - 4
CY - UNITED STATES
N2 - Social interactions and peer preferences in a preschool mainstream classroom containing normally developing and at-risk children were studied using the ethological method of direct observation. Data on social play and on gaze orientation indicated that normally developing children and at-risk children interacted primarily with children from the same group (other normally developing and at-risk children, respectively). Data

on selection on game partner and on preference of seat neighbor, however, revealed peer preference by normally developing children only. The findings were discussed as having theoretical implications for using direct observation vs. the more traditional sociometric methods for studying social adaptation in the mainstream classroom and as having practical implications for mainstreaming at risk-preschoolers
SN - 0002-9351
UR - 7355909
ER -

TY - JOUR
ID - 504
T1 - The child at risk in play
A1 - Morris,B.
Y1 - 1979/09//
N1 - Morris, B
The New Zealand nursing journal. Kai tiaki
19110850r, obt
N
Journal Article
English
KW - Medline
KW - Accident Prevention
KW - Child
KW - Child Advocacy
KW - Humans
KW - Play and Playthings
KW - Recreation
KW - Safety
RP - NOT IN FILE
SP - 5
EP - 6
JF - New Zealand Nursing Journal
JA - N.Z.Nurs J
VL - 72
IS - 9
CY - NEW ZEALAND
SN - 0028-8535
UR - 291817
ER -

TY - JOUR
ID - 505
T1 - Providing a safe environment for children
A1 - Brown,V.
Y1 - 1978/01//
N1 - Brown, V
MCN. The American journal of maternal child nursing
ma3, 7605941
N
Journal Article
English
KW - Medline
KW - Accident Prevention
KW - Beds/st [Standards]
KW - Child
KW - Preschool
KW - Environment

KW - Humans
KW - Infant
KW - Play and Playthings
KW - Safety
RP - NOT IN FILE
SP - 53
EP - 55
JF - MCN, American Journal of Maternal Child Nursing
JA - MCN Am J Matern.Child Nurs
VL - 3
IS - 1
CY - UNITED STATES
SN - 0361-929X
UR - 414042
ER -

TY - JOUR
ID - 506
T1 - Toy safety in hospitals--or beware of parents bearing gifts
A1 - Frank,D.J.
A1 - Drobish,N.L.
Y1 - 1975/04//
N1 - Frank, D J. Drobish, N L
Clinical pediatrics
dhe, 0372606, 8407647
AIM, IM
Journal Article
English
KW - Medline
KW - Accident Prevention
KW - Child
KW - Hospitalized
KW - Preschool
KW - Humans
KW - Male
KW - Parents
KW - Play and Playthings
KW - Safety
RP - NOT IN FILE
SP - 400
EP - 402
JF - Clinical Pediatrics
JA - Clin.Pediatr (Phila)
VL - 14
IS - 4
CY - UNITED STATES
N2 - Toys are an indispensable component of play therapy for hospitalized children. They can also be dangerous and may result in catastrophic accidents. Extreme care should be exercised in the purchase of "safe toys" for all infants and children in and out of the hospital. The guidelines of the Bureau of Product Safety of the Federal Drug Administration (FDA) dealing with toy safely can be very helpful for child card professionals as well as parents who purchase toys. An explanation of the additional environmental hazards of toys in hospital settings should be an important part of the total in-service training of all personnel who care for infants and children in hospitals. In addition, it might be well for the Division of Product Safety of the FDA to consider criteria for toy safety in hospitals as a separate category in future Bulletins dealing with this problem
SN - 0009-9228
UR - 1120384

ER -

TY - JOUR
ID - 507
T1 - Follow-up of infants at risk of minor brain dysfunction
A1 - Kalverboer,A.F.
A1 - Touwen,B.C.
A1 - Prechtl,H.F.
Y1 - 1973/02/28/
N1 - Kalverboer, A F. Touwen, B C. Prechtl, H F
Annals of the New York Academy of Sciences
5nm, 7506858
IM
Journal Article
English
KW - Medline
KW - Attention Deficit Disorder with Hyperactivity/co [Complications]
KW - Attention Deficit Disorder with Hyperactivity
KW - Child Behavior
KW - Child
KW - Preschool
KW - Exploratory Behavior
KW - Female
KW - Fixation
KW - Ocular
KW - Follow-Up Studies
KW - Humans
KW - Hyperkinesis/et [Etiology]
KW - Male
KW - Mother-Child Relations
KW - Motor Activity
KW - Motor Skills
KW - Neurologic Examination
KW - Play and Playthings
KW - Prognosis
KW - Psychometrics
KW - Sex Factors
KW - Spatial Behavior
RP - NOT IN FILE
SP - 173
EP - 187
JF - Annals of the New York Academy of Sciences
JA - Ann.N.Y.Acad.Sci
VL - 205
CY - UNITED STATES
SN - 0077-8923
UR - 4511278
ER -

TY - JOUR
ID - 508
T1 - "If you ever see this video, we're probably dead"-A boy's own heterotopia (notes from an inner London playground). [References]
A1 - Richards,Chris
Y1 - 2013///

N1 - Peer Reviewed Journal: 2013-24587-007
English
Journal; Peer Reviewed Journal
KW - PsycInfo
KW - epidemiology
KW - Games
KW - Playgrounds
KW - Pretend Play
KW - Age Differences
KW - Childhood Development
KW - Elementary Schools
RP - NOT IN FILE
SP - 383
EP - 398
JF - Journal of Children and Media
VL - 7:
IS - 3
N2 - This article draws on a 2-year research project focusing on children's playground games, and their relationship to popular media, in an inner London primary school. Extensive video recording of playground events was employed in the research. In addition, the children were invited to participate in documenting their own play using video camcorders. The article examines two interrelated videos made by one 11-year-old boy. Together, they provide one example of pretend play scenarios rendering the "safe" playground a location for the experience of threat and danger, attack and pursuit. Play of this kind is interpreted as a form of dramatic staging of the self at risk. The analysis suggests that in constructing a narrative fantasy of risk and vulnerability the boy also explores and exercises control. Emotionally, such a "heterotopian" transformation of the playground is exciting and pleasurable. Drawing eclectically and constructively on popular media sources, the play scenario further enables him to dramatise the social relations of the playground and, in particular, to negotiate power and control between adults and children and between children of different ages. (PsycINFO Database Record (c) 2013 APA, all rights reserved) (journal abstract)
SN - 1748-2798
AD - Richards, Chris: Department of Culture, Communication and Media, Institute of Education, London, United Kingdom
ER -

TY - JOUR
ID - 509
T1 - My brother, my friend: Positive sibling relationships, peer acceptance, and internalizing problems in low-income boys
A1 - Nichols,Sara R.
Y1 - 2013///
N1 - Dissertation Abstract: 2013-99181-189
English
Dissertation Abstract
KW - PsycInfo
KW - Emotional Adjustment
KW - Internalization
KW - Peers
KW - Sibling Relations
KW - Human Males
KW - Lower Income Level
RP - NOT IN FILE
SP - No
JF - Dissertation Abstracts International: Section B: The Sciences and Engineering
VL - 74:
IS - 3-B(E

N2 - 80% of American children grow up in a household with one or more siblings (Dunn, 2000). These relationships are known to be intense and highly affectively-charged (Dunn, 1983) and are many individuals' longest-duration relationships, extending across the lifespan farther than most friendships, marital, or parental relationships (Dunn, 1998; Sroufe et al, 2005). A growing body of work suggests that sibling relationships contribute to children's social, cognitive and emotional development, as well as to eventual psychopathology outcomes (Brody, 1998). The current study examines low-income boys' sibling play interactions at age five as a predictor of their subsequent psychological adjustment in later childhood (N = 133). In particular, positive play is examined as a marker of high-quality sibling relationships. The study makes four primary contributions to the field: 1) Identifying child and family predictors of positive sibling interaction in a high-risk sample of young children observed during regular play; 2) Finding differences in positive sibling interactions between siblings with small and large age differences, older versus younger siblings, and African-American and European-American sibling dyads; 3) Demonstrating a relationship between positive sibling interaction at five years of age and absence of psychiatric diagnoses and fewer symptoms up to seven years later in boys at risk for psychopathology; and 4) Identifying the unique contributions of positive sibling relationships, independent of family functioning and peer relationships, in buffering against the development of psychopathology in this high-risk sample. Results suggest that sibling interactions and in particular, resolution of conflict and negative affect during sibling play, are important directions for continued examination and intervention. (PsycINFO Database Record (c) 2013 APA, all rights reserved)
SN - 0419-4217
AD - Nichols, Sara R.: U Pittsburgh, US
ER -

TY - JOUR
ID - 511
T1 - Outdoor play and learning in early childhood from different cultural perspectives. [References]
A1 - Prince,Heather; Allin,Linda; Sandseter,Ellen Beate Hansen; Arlemalm-Hagser,Eva
Y1 - 2013///
N1 - Peer Reviewed Journal: 2013-28756-002
English
Journal; Peer Reviewed Journal
KW - PsycInfo
KW - Childhood Development
KW - Childhood Play Behavior
KW - Cross Cultural Differences
KW - Early Childhood Development
RP - NOT IN FILE
SP - 183
EP - 188
JF - Journal of Adventure Education and Outdoor Learning
VL - 13:
IS - 3
N2 - This editorial provides an overview of the present issue of Journal of Adventure Education and Outdoor Learning. The Journal focuses on outdoor play and learning in early childhood through a lens of cultural differences and similarities. Five articles are included in this issue and are preceded by a discussion of the contemporary challenges in this area of research. The first two papers in this issue examine the environments of play in different parts of Europe, the value placed on them and their optimization. The next paper shows how altering adults' perception of risk could enhance the sustainability of outdoor play. The next two papers focus more on the processes and outcomes that can be achieved through outdoor play. This is an important time for early childhood practitioners and researchers to re-evaluate approaches to outdoor play, and to consider the different opportunities available for outdoor play and play in natural environments in early childhood. It is hoped that this issue will make a significant contribution. (PsycINFO Database Record (c) 2013 APA, all rights reserved)
SN - 1472-9679
AD - Prince, Heather: University of Cumbria, Ambleside, United Kingdom Allin, Linda: Northumbria University, United Kingdom
ER -

TY - JOUR
ID - 512
T1 - Predicting autism from social communication skills, early play and engagement of children showing high risk for autism
A1 - Huynh,Linh Ngoc
Y1 - 2013///
N1 - Dissertation Abstract: 2013-99170-276
English
Dissertation Abstract
KW - PsycInfo
KW - At Risk Populations
KW - Autism
KW - Childhood Development
KW - Communication Skills
KW - Social Skills
KW - Childhood Play Behavior
RP - NOT IN FILE
SP - No
JF - Dissertation Abstracts International Section A: Humanities and Social Sciences
VL - 74:
IS - 3-A(E
N2 - The substantial increase in the number of children with ASD has stressed the importance of early detection and identification of ASD in the research community. Forty-nine children between the ages of 15 and 30 months were identified as high risk for autism in this study. The study examined the contribution of early social communication; early play behaviors and engagement with caregivers to later language development and autism diagnosis. Joint attention skills, play skills, restricted and repetitive behaviors, and engagement with caregiver were coded in this study. The results of this study showed early deficits in initiating joint attention skills was more related to receiving a later diagnosis of autism. (PsycINFO Database Record (c) 2013 APA, all rights reserved)
SN - 0419-4209
AD - Huynh, Linh Ngoc: U California, Los Angeles, US
ER -

TY - JOUR
ID - 513
T1 - Shy toddlers' responses to an unfamiliar social setting: The effect of encouragement of independence and high warmth behaviors
A1 - Grady,Jessica Stoltzfus
Y1 - 2013///
N1 - Dissertation Abstract: 2013-99160-106
English
Dissertation Abstract
KW - PsycInfo
KW - Peers
KW - Social Anxiety
KW - Social Behavior
RP - NOT IN FILE
SP - No
JF - Dissertation Abstracts International: Section B: The Sciences and Engineering
VL - 74:
IS - 2-B(E
N2 - Toddlers who are shy may be at risk for poor peer relations, social withdrawal, and social anxiety. A large body of literature supports that parenting behaviors may mitigate this risk, however findings are seemingly mixed regarding whether high warmth parenting and encouraging parenting facilitate or hinder children's social behaviors. The present study employed an experimental design to determine the specific influence of high

warmth maternal parenting and encouragement of independence maternal parenting on shy toddlers' approach and avoidance behaviors in a play session with unfamiliar peers. One hundred and nineteen mothers of toddlers between 21 and 27 months of age completed temperament and other questionnaires. The toddlers were classified as shy or non-shy based on mothers' responses on a social fear subscale of the temperament questionnaire. One shy and two non-shy toddlers and their mothers participated in each of a total of 32 25-minute play session, during which time shy toddlers' mothers' behaviors were manipulated and children's approach and avoidance behaviors were recorded for later coding. Results indicated that shy toddlers demonstrated less approach and more avoidance during the initial baseline of the study. However, shy toddlers did not increase their approach or decrease their avoidance following either manipulation. Exploratory analyses provide some support for the beneficial impact of encouragement of independence parenting; shy children were indistinguishable from their non-shy peers in their avoidance behaviors after the encouragement of independence intervention, but not after the high warmth intervention. Despite the limited findings in the present study, the broader literature base suggests that the maternal high warmth and encouragement of independence behaviors that were the focus of the present study have the potential to teach shy toddlers to approach instead of avoid social novelty and merit further study. (PsycINFO Database Record (c) 2013 APA, all rights reserved)
SN - 0419-4217
AD - Grady, Jessica Stoltzfus: West Virginia U., US
ER -

TY - JOUR
ID - 514
T1 - Identification of early behavioral markers of anxiety and social withdrawal in preschool children
A1 - Ale,Chelsea M.
Y1 - 2013///
N1 - Dissertation Abstract: 2013-99161-106
English
Dissertation Abstract
KW - PsycInfo
KW - Behavioral Sciences
KW - Preschool Students
KW - Social Anxiety
KW - Withdrawal (Defense Mechanism)
KW - Social Skills
RP - NOT IN FILE
SP - No
JF - Dissertation Abstracts International: Section B: The Sciences and Engineering
VL - 74:
IS - 2-B(E
N2 - This study examined an observational method for identifying specific behaviors to indicate social anxiety and social withdrawal in preschool children. During an interaction task with a novel adult, 28 children (ages 4 and 5) were observed for specific verbal and non-verbal behaviors (i.e., total utterances, commands, questions, unsolicited conversation, direct responses, eye gaze aversion, non-verbal response, physical distancing, freezing, smiling) and global behaviors (i.e., shyness, volume or speech, and postural rigidity), theoretically relevant to early childhood social anxiety. Behaviors observed during the interaction task were examined in relation to the parent-reported Spence Preschool Anxiety Scale's social anxiety subscale (Spence, Rapee, Edwards, & Ingram, 2001) and the percentage of solitary behavior observed during unstructured play at preschool (Morris, Messer, & Gross, 1995). Four years later, participants were re-contacted and 15 parents and children completed the Social Phobia and Anxiety Inventory for Children, Parent Report (P-SPAIC; Beidel, Turner, Hamlin, & Morris, 2000), the Child Behavior Checklist Social Competence Subscale (CBCL, Social Competence; Achenbach, 1991), and the Social Phobia and Anxiety Inventory for Children, Item 10 self-report (SPAIC-10, T. Morris, personal communication). Preliminary analyses revealed that the specific verbal and non-verbal behaviors (with the exception of physical distancing and commands) were significantly correlated with the global behaviors. The global behaviors did not predict a significant amount of variance in solitary behavior during unstructured play. None of the specific verbal or non-verbal behaviors were significantly related to solitary behavior during unstructured play or the Spence Preschool Anxiety Scale's social anxiety subscale. Although solitary behavior during unstructured play and the Spence Preschool Anxiety Scale's social anxiety subscale were significantly

related, neither one was significantly differentially related to any of the specific verbal or non-verbal behaviors observed during the interaction task. Unsolicited conversation and direct responses were significantly related to CBCL Social Competence at follow-up. Although a significant difference was not detected, a large effect was seen for total utterances, commands, and non-verbal responses to direct questions for those children who were elevated on one or more of the follow-up measures compared to children who were not elevated on any follow-up measure. Although limited by a lack of adequate statistical power and a restricted range of social anxiety at baseline, the study has important implications for the identification of specific behaviors in preschool that may be risk-factors for social anxiety childhood. (PsycINFO Database Record (c) 2013 APA, all rights reserved)
SN - 0419-4217
AD - Ale, Chelsea M.: West Virginia U., US
ER -

TY - JOUR
ID - 515
T1 - Is father-child rough-and-tumble play associated with attachment or activation relationships? [References]
A1 - Paquette,Daniel
Y1 - 2013///
N1 - Peer Reviewed Journal: 2013-18617-003
English
Journal; Peer Reviewed Journal
KW - PsycInfo
KW - Attachment Behavior
KW - Childhood Play Behavior
KW - Competition
KW - Father Child Relations
RP - NOT IN FILE
SP - 760
EP - 773
JF - Early Child Development and Care
VL - 183:
IS - 6
N2 - The activation relationship theory, primarily focused on parental stimulation of risk-taking along with parental control during exploration, predicts that boys will be activated more than girls by their fathers. This theory may explain why fathers engage in rough-and-tumble play (RTP) with children more frequently than mothers, especially with boys. Thus, the main objective of this study is to verify the existence of a positive association between the father-son activation relationship and the frequency of father-son RTP. Given that RTP is thought to encourage the development of competition skills, it was anticipated that no association would be found between the father-child attachment relationship and the frequency of father-child RTP. The Strange Situation (SS) and Risky Situation procedures were conducted with 58 father-child dyads (22 boys and 36 girls) during toddlerhood to evaluate the attachment relationship and the activation relationship, respectively. The fathers completed a play questionnaire when children were three years old to assess RTP. Results show that the more optimally activated boys are by their fathers in toddlerhood, the more they engage in RTP with them at the age of three years. As it was anticipated, there is no association between the father-child attachment relationship in toddlerhood and the frequency of father-child RTP in the preschool period. In addition, the tendency of avoidant children to engage in more RTP with their fathers supports the hypothesis that the SS evaluates something different when used with father-child dyads instead of mother-child dyads. (PsycINFO Database Record (c) 2013 APA, all rights reserved) (journal abstract)
SN - 0300-4430
AD - Paquette, Daniel: Ecole de Psychoeducation, Universite de Montreal, Montreal, PQ, Canada Dumont, Caroline: Departement de Psychologie, Universite de Montreal, Montreal, PQ, Canada
ER -

TY - JOUR
ID - 516
T1 - Developmental coordination disorder and internalizing problems in children: The environmental stress hypothesis elaborated. [References]

A1 - Cairney,John; Rigoli,Daniela; Piek,Jan
Y1 - 2013///
N1 - Peer Reviewed Journal: 2013-28507-001
English
Journal; Peer Reviewed Journal
KW - PsycInfo
KW - Environmental Stress
KW - Internalization
KW - Motor Coordination
KW - Dyspraxia
KW - Anxiety
KW - Mental Health
RP - NOT IN FILE
SP - 224
EP - 238
JF - Developmental Review
VL - 33:
IS - 3
N2 - There is a growing literature connecting poor motor coordination to physical and mental health outcomes in children and adolescents. These studies suggest that children with disorders such as developmental coordination disorder (DCD) are at greater risk for depression and anxiety, as well as obesity, and poor physical fitness. With regard to internalizing problems (symptoms of depression and anxiety), there is also evidence to suggest that the environment may play an important role in the etiology of psychological distress in this population. Cairney, Veldhuizen, & Szatmari, 2010 used the phrase "environmental stress hypothesis" to highlight the role that negative exposure to personal and interpersonal stressors might play in accounting for higher rates of internalizing symptoms in children with DCD. In this paper, we elaborate further on this basic premise, offering a model linking DCD to internalizing problems based on Pearlin's stress process framework. In addition to stressors (risk) and protective factors, we incorporate both physical activity and obesity into our stress model. Next, we review the existing literature to see if there is evidence supporting specific components (pathways) of the model. In doing so, areas in need of further research are identified. Implications for intervention are also provided. (PsycINFO Database Record (c) 2013 APA, all rights reserved) (journal abstract)
SN - 0273-2297
AD - Cairney, John: Department of Family Medicine, McMaster University, Hamilton, ON, Canada Rigoli, Daniela: School of Psychology and Speech Pathology, Curtin Health Innovation Research Institute (CHIRI), Curtin University, Perth, WAU, Australia Piek, Jan: School of Psychology and Speech Pathology, Curtin Health Innovation Research Institute (CHIRI), Curtin University, Perth, WAU, Australia
ER -

TY - JOUR
ID - 517
T1 - Examining the etiology of depression in adolescents: Exploring cognitive and intrapersonal vulnerability models
A1 - McWhinnie,Chad M.
Y1 - 2013///
N1 - Dissertation Abstract: 2013-99140-172
English
Dissertation Abstract
KW - PsycInfo
KW - Adolescent Psychiatry
KW - Cognitive Processes
KW - Etiology
KW - Major Depression
KW - Mental Health
RP - NOT IN FILE
SP - No
JF - Dissertation Abstracts International: Section B: The Sciences and Engineering

VL - 74:
IS - 1-B(E
N2 - Recent epidemiological studies suggest that the rates of depressive disorders are increasing among Chinese adolescents, and are now comparable to Western samples of adolescents. An estimated 16-26 million of Mainland Chinese children and adolescents suffer from depression, prompting increased public concern. As societal and cultural changes play a profound role in the development of mental health problems in adolescence, researchers have recently begun to examine how such changes have influenced the etiology of depression amongst Chinese adolescents. However, given profound cultural, social, political, and economic differences, Western theoretical models of depression cannot automatically be extended to Chinese adolescents. At the same time, there are several reasons why Western theoretical models of depression may be relevant to Chinese youth, including: (1) an influx of individualistic values may challenge long-standing collectivistic values; (2) similar epidemiological increases in the prevalence of depression among adolescents have occurred across cultures; and (3) Chinese adolescents experience similar stressors to those of Western adolescents. The purpose of the dissertation is to examine vulnerability factors to depression among Chinese adolescents within a stress generation framework using a multi-wave longitudinal design. The research described in Chapter 2 investigates the role that autonomy and stress play in the development of depressive symptoms. More specifically, the research examines whether dependent interpersonal stressors mediate the relationship between lower levels of perceived choice in one's actions or awareness of oneself, and subsequent increases in depressive symptoms. Additionally, we examine model specificity to better discriminate etiological risk factors for depression and anxiety, and the results of hierarchical linear modeling indicate that the relationship between lower levels of self-awareness and higher levels of depressive symptoms is mediated by a greater occurrence of dependent, but not independent, interpersonal stress. The results also indicate non-specificity. Overall, findings suggest that lack of autonomy in Chinese adolescents generates stress, and increases one's vulnerability to develop depressive and anxious symptoms. The research described in Chapter 3 examines underlying mechanisms that mediate the prospective relationship between self-concept clarity, stress, and depressive symptoms. Main effect analyses indicate that lower self-concept clarity predicted higher levels of depressive symptoms over time. Further, multilevel analyses reveal that stress mediates the relationship between self-concept clarity and subsequent depressive symptoms. At the same time, gender analyses indicate that the mediation effect may be driven by boys as opposed to girls indicating that there may be other pathways that contribute to depression among adolescent girls. The research presented in the current dissertation has important implications. Results from Chapters 2 and 3 suggest the etiological models developed in Western contexts may be relevant to understanding psychopathology among Chinese youth. Such insight may, ultimately, provide insight about which vulnerability factors to target when developing prevention, intervention, and treatment programs for adolescents in mainland China. (PsycINFO Database Record (c) 2013 APA, all rights reserved)
SN - 0419-4217
AD - McWhinnie, Chad M.: McGill U., Canada
ER -

TY - JOUR
ID - 518
T1 - An auditory-perceptual intervention program for fricatives: Effects and implications for toddlers without fricatives
A1 - Bandaranayake,Dakshika W.
Y1 - 2013///
N1 - Dissertation Abstract: 2013-99120-104
English
Dissertation Abstract
KW - PsycInfo
KW - Age Differences
KW - Childhood Development
KW - Intervention
KW - Program Development
KW - Auditory Perception
RP - NOT IN FILE
SP - No
JF - Dissertation Abstracts International: Section B: The Sciences and Engineering

VL - 73:
IS - 12-B(E
N2 - Purpose. Fricatives are a class of sounds that is considered difficult to produce, and are generally developed later than stops, nasals and glides in speech sound development. Current understanding of fricative development suggests that children who develop fricatives at an early age may have better expressive language skills than children who do not develop fricatives early. The purpose of this study was to investigate the changes in fricative production, overall consonant production, and vocabulary growth in a group of toddlers who participated in a short-term, intensive auditory-perceptual intervention program. Methods. The study sample consisted of eight typically developing 18-month-old toddlers with half (n=4) in the treatment group and half (n=4) on the control group. Each toddler in both groups was visited 3 days a week for a period of 3 weeks. During each visit, a 15-minute session with a book-reading activity with a specially designed book and a play activity using selected toys and a play-script was conducted. The treatment group toddlers participated in an auditory-perceptual intervention with the book and the script designed to provide stimulation for the sounds /s/ and /z/. The control group toddlers participated in similar activities, however with materials designed to avoid stimulation of /s/ and /z/ sounds. Children's performance was assessed with speech samples and the vocabulary scores obtained at pre-treatment, post-treatment, and when the child reached 20 months of age (follow-up). A speech sample was also collected from each child before every treatment session. The assessment data were statistically analyzed to look at the differences in speech sound production and vocabulary over time for both groups of toddlers. Results. The number of fricative /s/ and /z/ productions, number of fricatives, number of fricative types, percentage of fricative types and vocabulary raw scores significantly changed over time for toddlers of both groups combined from pre-treatment to post-treatment and/or follow-up sessions. No significant group effects or interactions between group and time were found for any of the outcome measures. Individual data revealed that 7/8 participants produced fricatives and all 8 of the toddlers increased their vocabulary raw scores from pre-treatment to post-treatment and/or follow-up session. Conclusions. All toddlers enrolled in the intervention completed the intervention as planned. Fricative production and overall vocabulary changed over the study period of 2 months (between 18-20 months) for toddlers of both groups, regardless of stimulated for fricatives or not. There is no evidence from this study that targeting fricatives using an auditory-perceptual method was helpful in phonological and vocabulary development. The lack of differences between the groups suggests that general language stimulation rather than specific fricative stimulation may result in the development of fricatives. Hence, early intervention programs with more general rather than specific stimulation may be appropriate and beneficial for toddlers identified with or at-risk for speech and language delay. The evidence that toddlers as young as 18 months of age are able to participate in a structured intervention program suggests that such programs might be viable for children who are at risk for speech and language delay. (PsycINFO Database Record (c) 2013 APA, all rights reserved)
SN - 0419-4217
AD - Bandaranayake, Dakshika W.: U Cincinnati, US
ER -

TY - JOUR
ID - 519
T1 - Assessing problematic video gaming using the Theory of Planned Behavior: A longitudinal study of Dutch young people. [References]
A1 - Haagsma,Maria C.
Y1 - 2013///
N1 - Peer Reviewed Journal: 2013-10061-004
English
Journal; Peer Reviewed Journal
KW - PsycInfo
KW - Adolescent Attitudes
KW - Computer Games
KW - Pathological Gambling
KW - Planned Behavior
KW - Reasoned Action
KW - Age Differences
KW - Human Sex Differences
KW - Self Control

RP - NOT IN FILE
SP - 172
EP - 185
JF - International Journal of Mental Health and Addiction
VL - 11:
IS - 2
N2 - Although excessive video gaming has been linked to a range of psychological problems in young people, there have been few systematic attempts to conceptualize problem gaming using established psychological theory. The aim of this study was to examine problematic game use (PGU) using the Theory of Planned Behavior (TPB). A two-wave, six-month longitudinal study examined relationships between core components of the TPB model, video gaming activity and problematic video-game play. Respondents were recruited from nine pre-vocational and senior vocational schools in the western region of the Netherlands. The sample consisted of 810 video game-playing adolescents and young adults (72.8 % boys) aged 12 to 22 years. The results showed that TPB predictors, including subjective norm, perceived behavioral control (PBC) and descriptive norm, explained 13 % of the variance in video gaming intention. Although TBP variables accounted for a significant amount of variance in PGU scores at baseline, the TPB model was less useful in predicting future gaming behavior and PGU. Perceived behavioral control was found to be the most important factor in predicting problem video-gaming behavior, this has some practical implications with regard to the treatment of problem video-gaming among young people. For example, assessing a client's perceived lack of control over gaming may be a simple but useful screening measure to evaluate risk of future problem play. Furthermore, treatment strategies may be aimed at helping the client to rebuild self-control. (PsycINFO Database Record (c) 2013 APA, all rights reserved) (journal abstract)
SN - 1557-1874
AD - Haagsma, Maria C.: Department of Psychology, Health & Technology, Faculty of Behavioral Sciences, University of Twente, Enschede, Netherlands King, Daniel L.: School of Psychology, University of Adelaide, Adelaide, SA, Australia Pieterse, Marcel E.: Department of Psychology, Health & Technology, Faculty of Behavioral Sciences, University of Twente, Enschede, Netherlands Peters, Oscar: Department of Media, Communication & Organization, Faculty of Behavioral Sciences, University of Twente, Enschede, Netherlands
ER -

TY - JOUR
ID - 520
T1 - Wigs, disguises and child's play: Solidarity in teacher education. [References]
A1 - Heilbronn,Ruth
Y1 - 2013///
N1 - Peer Reviewed Journal: 2013-22161-004
English
Journal; Peer Reviewed Journal
KW - PsycInfo
KW - Educational Psychology
KW - Ethics
KW - Teacher Education
KW - Social Support
RP - NOT IN FILE
SP - 31
EP - 41
JF - Ethics and Education
VL - 8:
IS - 1
N2 - It is generally acknowledged that much contemporary education takes place within a dominant audit culture, in which accountability becomes a powerful driver of educational practices. In this culture, both pupils and teachers risk being configured as a means to an assessment and target-driven end: pupils are schooled within a particular paradigm of education. The article discusses some ethical issues raised by such schooling, particularly the tensions arising for teachers, and by implication, teacher educators who prepare and support teachers for work in situations where vocational aims and beliefs may be in conflict with instrumental aims. The article offers De Certeau's concept of la perruque to suggest an opening to playful engagement for human ends

in education, as a way of contending with and managing the tensions generated. I use the concept to recover an idea of solidarity for teacher educators and teachers to enable ethical teaching in difficult times. (PsycINFO Database Record (c) 2013 APA, all rights reserved) (journal abstract)
SN - 1744-9642
AD - Heilbronn, Ruth: Institute of Education, University of London, London, United Kingdom
ER -

TY - JOUR
ID - 521
T1 - Childhood sexual abuse and sexual risk-taking among men who have sex with women: The roles of alcohol, mental health symptoms, and sex motives
A1 - Schraufnagel,Trevor J.
Y1 - 2013///
N1 - Dissertation Abstract: 2013-99180-283
English
Dissertation Abstract
KW - PsycInfo
KW - Child Abuse
KW - Mental Health
KW - Sexual Abuse
KW - Sexual Risk Taking
KW - Symptoms
KW - Human Females
KW - Sexual Intercourse (Human)
KW - Sexual Partners
RP - NOT IN FILE
SP - No
JF - Dissertation Abstracts International: Section B: The Sciences and Engineering
VL - 74:
IS - 3-B(E
N2 - Several studies have suggested that those who have experienced childhood sexual abuse (CSA) are at greater risk for sexually-transmitted infections later in life than are their non-abused counterparts. For the most part, the populations investigated to date have consisted of women, men who have sex with men, and high-risk clinical or homeless populations. As such, little is known in this domain about men who have sex with women (MSW). Furthermore, even among the most frequently studied populations, research that has been devoted to identifying and examining potential mediators in the CSA - sexual risk relationship is scarce. To address these gaps, the current studies evaluated the presence and possible mediators of the CSA - sexual risk relationship among a community sample of MSW. Paper I reviews the research on CSA and sexual risk-taking that has been conducted to date among MSW and highlights the role that alcohol consumption may play as a mediator in such a relationship. Paper I goes on to present a longitudinal study that examined the effect of CSA on several later sexual risk indices and evaluates the extent to which alcohol-related variables factor into this relationship. Paper I also presents findings related to idiosyncratic CSA characteristics and how such characteristics may affect alcohol- and sexual risk-related outcomes. Paper II extends Paper I by introducing and reviewing additional, non-alcohol-related variables that may play a mediational role in the CSA - sexual risk relationship. Paper II then proceeds to present a longitudinal study that examined mood and anxiety symptoms and sex motives as mediators in the relationship between CSA and number of sexual partners. The results from these studies extend previous findings concerning the relationship between one's history of CSA and one's subsequent sexual risk-related behaviors. Furthermore, the current studies underscore the importance of ongoing research to further refine our understanding of sexual risk-taking in the wake of sexual abuse. (PsycINFO Database Record (c) 2013 APA, all rights reserved)
SN - 0419-4217
AD - Schraufnagel, Trevor J.: U Washington, US
ER -

TY - JOUR
ID - 523

T1 - The effects of alcohol advertising on college students' behaviors: Using family communication as a protective factor against heavy drinking and risky sexual behaviors
A1 - Radanielina-Hita,Marie Louise
Y1 - 2013///
N1 - Dissertation Abstract: 2013-99090-390
English
Dissertation Abstract
KW - PsycInfo
KW - Advertising
KW - Alcohols
KW - Family Relations
KW - Sexual Risk Taking
KW - Student Attitudes
KW - Alcohol Drinking Attitudes
KW - College Students
KW - Communication
KW - Decision Making
RP - NOT IN FILE
SP - No
JF - Dissertation Abstracts International Section A: Humanities and Social Sciences
VL - 73:
IS - 11-A(E
N2 - An online survey of undergraduates explored the effects of recalled parent-child interaction regarding media on their critical thinking skills, beliefs about alcohol and sex and current reports of risky behaviors. Students completed the questionnaire three times during fall 2011. The SEM analyses were based on 676 students. The longitudinal data set was used to model a latent trajectory of drinking and risky behaviors. Students whose parents critiqued media content reported a higher level of critical thinking toward media sources and media content. More critical thinking toward media sources decreased the effects of advertising on alcohol-related expectancies. More critical thinking toward media content decreased the effects of advertising on sex-related expectancies, alcohol-related behaviors and risky sexual behaviors. Parents' mediation served as protective factor against the effects of advertising on drinking via their effects on critical thinking toward media sources and alcohol-related expectancies. Negative mediation decreased risky sexual behaviors via its prior effects on critical thinking toward media content and sex-related expectancies. On the other hand, students whose parents endorsed media portrayal reported lower levels of critical thinking. Positive mediation predicted more risky sexual behaviors. Therefore, critical thinking toward media directly and consistently affected risky behaviors. Alcohol-related expectancies were related to sex-related expectancies. At time 1, more drinking predicted more risky sexual behaviors. However, the relationship did not stand the test of time. The analyses also revealed a linear decrease of drinking from time 1 to time 3. Developing critical thinking toward media is an effective approach to helping young people make good decision about their health. Although students' understanding of advertising intent may be biased by the emotional aspect of decision making, critical thinking decreased the appeal of advertising on risky behaviors. Parents play an important role in developing their children's critical thinking skills. As the positive effect of parent-child communication influenced current behaviors, prevention programs targeting college students may still benefit from an inclusion of family communication practices. Although preliminary, the non-significant association between alcohol-related behaviors and risky sexual behaviors across time buttressed previous research, which established a more correlational nature rather than directional relationship. (PsycINFO Database Record (c) 2013 APA, all rights reserved)
SN - 0419-4209
AD - Radanielina-Hita, Marie Louise: Washington State U., US
ER -

TY - JOUR
ID - 524
T1 - Exploring individual trajectories of social communicative development in toddlers at risk for autism spectrum disorders. [References]
A1 - Dereu,Mieke; Roeyers,Herbert; Raymaekers,Ruth; Warreyn,Petra

Y1 - 2012///
N1 - Peer Reviewed Journal: 2012-13564-011
English
Journal; Peer Reviewed Journal
KW - PsycInfo
KW - Autism
KW - Communication
KW - Risk Factors
RP - NOT IN FILE
SP - 1038
EP - 1052
JF - Research in Autism Spectrum Disorders
VL - 6:
IS - 3
N2 - Whilst impairments in joint attention, imitation, and pretend play are well documented in children with autism spectrum disorder (ASD), the developmental trajectories of these symptoms remain unknown. The main objective was to explore these trajectories in a sample of children at risk for ASD between the ages of 2 and 4 years. After screening positive for ASD, 17 children were prospectively followed with 6-monthly assessments of social communicative skills and general development. During this study, 9 children were diagnosed with ASD. Results showed that there was growth in imitation skills and in pretend play ability. Also, a slightly increased amount of initiation of joint attention was noted. However, the quality of joint attention behaviours did not significantly improve and growth rates did not significantly differ between children with ASD and non-ASD cases. There was a great intra-individual variability, especially in the performances on the imitation tasks. Also interindividual variability was apparent: some children with ASD showed a delayed development of imitation or pretend play, whereas others had a more clearly deviant developmental profile. The importance of looking at individual trajectories and variability in the study of social communicative development in children with ASD is discussed. (PsycINFO Database Record (c) 2013 APA, all rights reserved) (journal abstract)
SN - 1750-9467
AD - Dereu, Mieke: Research Group Developmental Disorders, Department of Experimental Clinical and Health Psychology, Ghent University, Ghent, Belgium Roeyers, Herbert: Research Group Developmental Disorders, Department of Experimental Clinical and Health Psychology, Ghent University, Ghent, Belgium Raymaekers, Ruth: Research Group Developmental Disorders, Department of Experimental Clinical and Health Psychology, Ghent University, Ghent, Belgium Warreyn, Petra: Research Group Developmental Disorders, Department of Experimental Clinical and Health Psychology, Ghent University, Ghent, Belgium
ER -

TY - JOUR
ID - 527
T1 - Romeo and Juliet and dramatic distancing: Chaos and anger contained for inner-city adolescents in multicultural schools. [References]
A1 - Carr,Mandy
Y1 - 2012///
N1 - Book: 2012-04044-009
English
Book; Edited Book
KW - PsycInfo
KW - Pervasive Developmental Disorders
KW - Psychodrama
KW - School Based Intervention
KW - Theatre
KW - Bullying
KW - Adolescent Development
KW - Anger
KW - Coping Behavior
KW - Education
KW - Mental Confusion

KW - Multicultural Education
KW - Parents
KW - Resilience (Psychological)
KW - Schools
KW - Urban Environments
RP - NOT IN FILE
SP - 91
EP - 97
JF - Leigh, Lauraine [Ed]; Gersch, Irvine [Ed]; Dix, Ann [Ed]; Haythorne, Deborah [Ed]
VL - :
IS - 2012
N2 - (from the chapter) Adolescence can be viewed as a time of transition from child to adulthood, where a cocktail of hormones and changes to body and mind can create anger and confusion. Although this may seem a modern problem, teenagers through the ages in different ways have shared the same fears and difficulties. In this chapter I intend to examine how Romeo and Juliet, Shakespeare's play about teenage love and family tensions, may be used in dramatherapy sessions to help reflect issues in the twenty-first century. I will examine how Shakespeare reaches out to troubled young people whose unexpressed anger may be impeding their educational and emotional development. Karkou (2010: 13) notes that children at risk of developing mental health problems can have their initial contact with qualified professionals in school. Difficulties can thus be identified and addressed early, without resorting to specialized services outside the school environment. This chapter aims to show the impact of the work on the resilience of young people from a variety of cultures as well as the importance of providing therapeutic interventions within a school context. Two case studies are used to demonstrate how issues in the play Romeo and Juliet can support teenagers in expressing and processing their anger in a therapeutic context, enabling them to develop the coping skills and emotional resilience to function and fulfil their educational potential. The first case study will explore the use of parental themes from Romeo and Juliet with an adolescent boy on the autistic spectrum, in a special school. The second will describe a story devised by a 12-year-old girl to process bullying issues in an inner-city secondary school, following her engagement with the theme of tension between the Montagues and Capulets. Whilst these cases are fictitious, they are based on an amalgam of young people's experiences within dramatherapy. (PsycINFO Database Record (c) 2013 APA, all rights reserved)
AD - Carr, Mandy: British Association of Dramatherapists, Equality and Diversity Sub-Committee, United Kingdom
ER -

TY - JOUR
ID - 528
T1 - Increased corticosterone in peripubertal rats leads to long-lasting alterations in social exploration and aggression. [References]
A1 - Veenit,Vandana
Y1 - 2013///
N1 - Peer Reviewed Journal: 2013-15009-001
English
Journal; Peer Reviewed Journal
KW - PsycInfo
KW - Animal Aggressive Behavior
KW - Animal Social Behavior
KW - Corticosterone
KW - Stress
KW - Rats
RP - NOT IN FILE
JF - Frontiers in Behavioral Neuroscience
VL - 7 Apr 2013, ArtID 26.
N2 - Stress during childhood and adolescence enhances the risk of psychopathology later in life. We have previously shown that subjecting male rats to stress during the peripubertal period induces long-lasting effects on emotion and social behaviors. As corticosterone is increased by stress and known to exert important programming effects, we reasoned that increasing corticosterone might mimic the effects of peripubertal stress.

To this end, we injected corticosterone (5mg/kg) on 7 scattered days during the peripuberty period (P28-P30, P34, P36, P40, and P42), following the same experimental schedule as for stress administration in our peripubertal paradigm. We measured play behavior in the homecage and, at adulthood, the corticosterone response to novelty and behavioral responses in tests for anxiety- and depression-like behaviors, aggression, and social exploration. As compared to vehicle, corticosterone-treated animals exhibit more aggressive play behavior during adolescence, increased aggressive behavior in a resident-intruder (RI) test while reduced juvenile exploration and corticosterone reactivity at adulthood. Whereas the corticosterone treatment mimicked alterations induced by the peripuberty stress protocol in the social domain, it did not reproduce previously observed effects of peripuberty stress on increasing anxiety-like and depression-like behaviors, respectively evaluated in the elevated plus maze (EPM) and the forced swim tests. Our findings indicate that increasing corticosterone levels during peripuberty might be instrumental to program alterations in the social domain observed following stress, whereas other factors might need to be recruited for the programming of long-term changes in emotionality. Our study opens the possibility that individual differences on the degree of glucocorticoid activation during peripuberty might be central to defining differences in vulnerability to develop psychopathological disorders coursing with alterations in the social realm. (PsycINFO Database Record (c) 2013 APA, all rights reserved) (journal abstract)
SN - 1662-5153
AD - Veenit, Vandana: Laboratory of Behavioral Genetics, School of Life Sciences, Brain Mind Institute, Ecole Polytechnique Federale de Lausanne, Lausanne, Switzerland Cordero, Maria I.: Laboratory of Behavioral Genetics, School of Life Sciences, Brain Mind Institute, Ecole Polytechnique Federale de Lausanne, Lausanne, Switzerland Tzanoulinou, Stamatina: Laboratory of Behavioral Genetics, School of Life Sciences, Brain Mind Institute, Ecole Polytechnique Federale de Lausanne, Lausanne, Switzerland Sandi, Carmen: Laboratory of Behavioral Genetics, School of Life Sciences, Brain Mind Institute, Ecole Polytechnique Federale de Lausanne, Lausanne, Switzerland
ER -

TY - JOUR
ID - 529
T1 - Cul-de-sac kids. [References]
A1 - Hochschild,Thomas R.J.
Y1 - 2013///
N1 - Peer Reviewed Journal: 2013-14643-007
English
Journal; Peer Reviewed Journal
KW - PsycInfo
KW - Childhood Development
KW - Childhood Play Development
KW - Neighborhoods
KW - Safety
KW - Social Interaction
RP - NOT IN FILE
SP - 229
EP - 243
JF - Childhood: A Global Journal of Child Research
VL - 20:
IS - 2
N2 - Previous research indicates that adults who live on cul-de-sac streets are more likely to have positive experiences with neighbors than residents of other street types (Brown and Werner, 1985; Hochschild Jr, 2011; Mayo Jr, 1979; Willmott, 1963). The present research ascertains whether street design has an impact on children's neighborhood experiences. The author interviewed 73 adults who live on cul-de-sacs and 37 adults who live on through-streets (N = 110) to determine adults' understandings of children's neighborhood experiences. The data reveal three primary benefits for children raised on cul-de-sacs. First, because cul-de-sacs tend to be territorial streets, parents are more likely to let their children play outside unattended under the watchful eyes of neighbors. These neighbors provide a social safety net for cul-de-sac children and their friends. Second, because they are aware that they are likely being watched, cul-de-sac children are less likely to partake in deviant activities while on their street. The author refers to this internalized form of self-discipline as street

panopticism. Third, the low traffic levels on cul-de-sacs create greater opportunity for uninterrupted play, thus drawing cul-de-sac kids outside for individual and group recreation. The author argues that cul-de-sacs, as well as other low-traffic streets, can enhance children's neighborhood experiences and create more vibrant neighborhoods. (PsycINFO Database Record (c) 2013 APA, all rights reserved) (journal abstract)
SN - 0907-5682
AD - Hochschild, Thomas R.: Valdosta State University, Valdosta, GA, US
ER -

TY - JOUR
ID - 530
T1 - Developmental changes in the free play and programmed play of children at risk for developmental disabilities in an early child-parent support group. [Japanese]. [References]
A1 - Saita,Yasuaki
Y1 - 2012///
N1 - Peer Reviewed Journal: 2012-23397-004
Japanese
Journal; Peer Reviewed Journal
KW - PsycInfo
KW - At Risk Populations
KW - Childhood Development
KW - Childhood Play Behavior
KW - Developmental Disabilities
KW - Parent Child Relations
RP - NOT IN FILE
SP - 31
EP - 43
JF - Japanese Journal of Special Education
VL - 50:
IS - 1
N2 - The present study examined effects of an early treatment group for parents and children through a longitudinal assessment of the children's behavior. The group's purpose was for developmental support and child-care support for parents and their children who had delays in communication skills or behavior problems. The parent-child pairs (N = 30) were assessed at the beginning and end of a 6-month period. Assessment sheets were used to record their behavior in free play and programmed play; other domains assessed included understanding skills, expression skills, activity, behavior problems, and the parent-child relationship. The mean age of the children at the first assessment was 30.9 months (SD = 8.6). Assessments of both types of play and all the domains were higher in the second assessment than in the first. Structural equation modeling (SEM) analysis revealed the following: (a) the growth in expression skills and in the relations between the parents and their children was related to the children's growth in free play and programmed play, but (b) the children's growth in understanding skills was related only to their growth in free play. It was concluded that important skills for the two types of play are different. These findings and other findings from follow-up done after the group and case studies suggest that early group activities can be useful for assessment and early treatment. (PsycINFO Database Record (c) 2013 APA, all rights reserved) (journal abstract)
SN - 0387-3374
AD - Saita, Yasuaki: Faculty of Humanities, University of Kitakyushu, Kitakyushu-Shi, Japan
ER -

TY - JOUR
ID - 531
T1 - Adhd symptoms and risk for alcohol use and cigarette smoking among college students: What role do social-cognitive factors play?
A1 - Glass,Kerrie
Y1 - 2013///
N1 - Dissertation Abstract: 2013-99060-295
English
Dissertation Abstract

KW - PsycInfo
KW - Alcohols
KW - Attention Deficit Disorder with Hyperactivity
KW - Risk Factors
KW - Tobacco Smoking
KW - Social Cognition
RP - NOT IN FILE
SP - No
JF - Dissertation Abstracts International: Section B: The Sciences and Engineering
VL - 73:
IS - 9-B(E
N2 - Attention-deficit/hyperactivity disorder (ADHD) is a common childhood disorder which often persists into adulthood and has been associated with an increased risk for substance use. Although more college students with high levels of ADHD symptoms are attending college, little research has examined whether ADHD symptoms are associated with substance use or whether social and cognitive factors help to explain whether ADHD symptoms are linked to this increased substance use risk. The aims of the current study were: (1) To examine whether ADHD symptoms were associated with increasing trajectories of cigarette smoking, alcohol use, and alcohol-related problems among first-year college students, and (2) To examine whether constructs related to Social Cognitive Theory-perceived social norms, positive outcome expectancies, or self-efficacy-mediated the proposed relation between ADHD symptoms and cigarette smoking, alcohol use, and alcohol-related problems trajectories. It was hypothesized that greater ADHD symptomatology would be related to increasing levels of cigarette smoking, alcohol use, and alcohol problems during the study. Further, it was hypothesized that perceived social norms, positive outcome expectancies, and self-efficacy would partially mediate these relations. Participants were 449 college freshmen who completed three online surveys during their first year of college. Hypotheses were largely unsupported, but results suggested that ADHD symptoms were positively associated with baseline alcohol problems after controlling for conduct disorder (CD) symptoms. ADHD was not significantly associated with changes in any of the substance use trajectories, and there was no evidence for the proposed social cognitive factors mediating the relation between ADHD and changes in substance use. Possible reasons for the null findings are discussed. Future directions and clinical implications are also presented. (PsycINFO Database Record (c) 2013 APA, all rights reserved)
SN - 0419-4217
AD - Glass, Kerrie: U South Carolina, US
ER -

TY - JOUR
ID - 533
T1 - Temperament and risk: Resilient and vulnerable responses to adversity. [References]
A1 - Lengua,Liliana J.
Y1 - 2012///
N1 - Book: 2012-22484-025
English
Book; Edited Book
KW - PsycInfo
KW - Personality
KW - Resilience (Psychological)
KW - Risk Factors
KW - Stress Reactions
KW - Childhood Development
KW - Individual Differences
RP - NOT IN FILE
SP - 519
EP - 540
JF - Zentner, Marcel [Ed]; Shiner, Rebecca L [Ed]
VL - :
IS - 2012

N2 - (from the chapter) In a bioecological model it is posited that individuals develop within multiple contexts, and that development is affected by transactions and interactions of factors at many levels, including distal and proximal influences (Bronfenbrenner & Morris, 1998). Distal factors, such as cultural-, socioeconomic-, and community-level influences provide the contexts in which proximal factors, such as family relationships and parenting, influence individual development. Risk factors present at these varying contextual levels can result in children developing cognitive, social-emotional or behavioral problems. However, individual-level factors also play a critical role, both directly by contributing to developmental outcomes, and indirectly by filtering or altering the impact of contextual risk on development. One essential individual difference factor is temperament, which is a key contributor to children's vulnerable or resilient responses to the experience of adversity or risk. The conceptual model shown in Figure 25.1, on which this chapter is based, posits that risk and promotive factors shape the biological underpinnings and behavioral manifestations of temperament. In turn, temperament influences the degree to which a child is exposed to risk and promotive factors in a bioecological model it is posited that individuals develop within multiple contexts, and that development is affected by transactions and interactions of factors at many levels, including distal and proximal influences (Bronfenbrenner & Morris, 1998). Distal factors, such as cultural-, socioeconomic-, and community-level influences provide the contexts in which proximal factors, such as family relationships and parenting, influence individual development. Risk factors present at these varying contextual levels can result in children developing cognitive, social-emotional or behavioral problems. However, individual-level factors also play a critical role, both directly by contributing to developmental outcomes, and indirectly by filtering or altering the impact of contextual risk on development. One essential individual difference factor is temperament, which is a key contributor to children's vulnerable or resilient responses to the experience of adversity or risk. The conceptual model shown in Figure 25.1, on which this chapter is based, posits that risk and promotive factors shape the biological underpinnings and behavioral manifestations of temperament. In turn, temperament influences the degree to which a child is exposed to risk and promotive factors, and contributes to the likelihood of children developing problems. Temperament also moderates experiences of adversity or risk. This chapter first provides a brief overview of the conceptual frameworks of risk, resilience, and temperament employed in our discussion. We then present evidence for risk and promotive factors shaping temperament, the role of temperament in influencing exposure to risk and promotive factors, and the moderating effects of temperament on experiences of adversity or risk. (PsycINFO Database Record (c) 2013 APA, all rights reserved)
AD - Lengua, Liliana J.: Department of Psychology, University of Washington, Seattle, WA, US Wachs, Theodore D.: Departmentof Psychological Sciences, Purdue University, West Lafayette, IN, US
ER -

TY - JOUR
ID - 534
T1 - Facilitating prosocial behavior in at-risk children through cooperative media-based play with peers
A1 - Lichtenberg,Jesse D.
Y1 - 2013///
N1 - Dissertation Abstract: 2013-99020-138
English
Dissertation Abstract
KW - PsycInfo
KW - Peers
KW - Prosocial Behavior
KW - Recreation
KW - Media Exposure
KW - At Risk Populations
RP - NOT IN FILE
SP - No
JF - Dissertation Abstracts International: Section B: The Sciences and Engineering
VL - 73:
IS - 7-B(E
N2 - The study sought to combine the benefits of both the play of children and the use of interactive media in an effort to increase comradery and prosocial behavior between pairs of participants composed of typically developing students and those deemed at-risk concerning social development. Over the course of a four-week intervention emphasizing cooperative video game play, an increased presence of effective, appropriate social

interaction within participant pairs was observed in addition to a pattern regarding devotion to certain recess activities by those participants observed on multiple occasions. Considerations regarding study results, methodology, and resources are discussed. (PsycINFO Database Record (c) 2013 APA, all rights reserved)
SN - 0419-4217
AD - Lichtenberg, Jesse D.: U Nebraska at Omaha, US
ER -

TY - JOUR
ID - 535
T1 - A hop, skip, and a jump: A case study examining playground safety practices and policies within a mid-size school district
A1 - Thorsen,Lisa
Y1 - 2013///
N1 - Dissertation Abstract: 2013-99010-368
English
Dissertation Abstract
KW - PsycInfo
KW - Playgrounds
KW - Safety
KW - Schools
KW - Best Practices
KW - Occupational Safety
KW - Health Care Policy
RP - NOT IN FILE
SP - No
JF - Dissertation Abstracts International Section A: Humanities and Social Sciences
VL - 73:
IS - 7-A(E
N2 - Although national playground safety standards and guidelines exist, more than 200,000 children are injured on playgrounds each year seriously enough to warrant emergency medical treatment. The most frequent site for these injuries is on the school playground. This qualitative case study discusses the schools conformance to national playground safety standards and guidelines and discusses the factors that affect alignment to these standards. Limited stakeholder knowledge and accountability for playground safety, and splintered communication processes within the school create unsafe play environments for children. Through the comprehensive formulation and implementation of playground safety policy, schools can take proactive roles in keeping children safe. (PsycINFO Database Record (c) 2013 APA, all rights reserved)
SN - 0419-4209
AD - Thorsen, Lisa: Hofstra U., US
ER -

TY - JOUR
ID - 536
T1 - Observing young children's creative thinking: Engagement, involvement and persistence. [References]
A1 - Robson,Sue
Y1 - 2012///
N1 - Peer Reviewed Journal: 2012-33766-005
English
Journal; Peer Reviewed Journal
KW - PsycInfo
KW - Creativity
KW - Involvement
KW - Persistence
KW - Student Engagement
KW - Thinking
RP - NOT IN FILE
SP - 349

EP - 364
JA - International Journal of Early Years Education
VL - 20:
IS - 4
N2 - This paper looks at young children's creative thinking as inferred through observations of their activities. A total of 52 episodes of child-initiated and adult-initiated activities in 3- to 4-year-olds in an English Children's Centre were analysed using the Analysing Children's Creative Thinking (ACCT) Framework. Results showed that activities such as gardening and construction were as valuable for supporting creative thinking as ones traditionally associated with creativity, for example, music and painting. Outdoor play of all kinds and sociodramatic play were particularly effective contexts. All adults played a significant role in facilitating children's initial engagement in activities, and at supporting their speculative thinking and use of prior knowledge. Teachers were often more successful than other adults in supporting the acquisition of new knowledge. Child-initiated activities featured the highest levels of involvement, and were associated with trying out and analysing ideas, flexibility and originality, imagining and hypothesising. This was particularly evident in group or pair play. Children were also more persistent in child-initiated activities. Evidence of risk-taking behaviour was low, although more apparent in child-initiated activities than adult-initiated activities, or activities in which adults were present. (PsycINFO Database Record (c) 2013 APA, all rights reserved) (journal abstract)
SN - 0966-9760
AD - Robson, Sue: School of Education, University of Roehampton, London, United Kingdom Rowe, Victoria: School of Education, University of Roehampton, London, United Kingdom
ER -

TY - JOUR
ID - 537
T1 - Typology of dangerous games among middle and high school students: Towards a study of the psychological profiles. [French]. [References]
A1 - Bernadet,Sabrina; Purper-Ouakil,Diane; Michel,Gregory
Y1 - 2012///
N1 - Peer Reviewed Journal: 2012-31226-011
French
Journal; Peer Reviewed Journal
KW - PsycInfo
KW - Adolescent Psychopathology
KW - Childhood Play Behavior
KW - Dangerousness
KW - Games
KW - Risk Taking
KW - Aggressive Behavior
KW - Anoxia
KW - Competition
KW - Coping Behavior
KW - High School Students
KW - Middle School Students
KW - Risk Factors
RP - NOT IN FILE
SP - 654
EP - 658
JF - Annales Medico-Psychologiques
VL - 170:
IS - 9
N2 - Twelve percent of seven to 17 year-old children have been involved in a dangerous game [23]. Nowadays, it distinguishes non-oxygenation games, aggression games and challenge games. Often mixed together, they are part of the broad spectrum of risk taking behaviors. The objective of this research is to identify psychological factors associated with each type of dangerous games. A multisource assessment (832 adolescents and 416 parents) found that asphyxial games could become addictive; challenge games would be the response to a high

novelty-seeking and that aggression games could be the indicator of an emotional detachment. (PsycINFO Database Record (c) 2013 APA, all rights reserved) (journal abstract)
SN - 0003-4487
AD - Bernadet, Sabrina: Laboratoire Psychologie Sante et Qualite de Vie, EA 4139, Universite Bordeaux-Segalen, UFR Sciences de l'Homme, Bordeaux, France Purper-Ouakil, Diane: Centre Hospitalier Universitaire Saint-Eloi, Montpellier, France Michel, Gregory: Laboratoire Psychologie Sante et Qualite de Vie, EA 4139, Universite Bordeaux-Segalen, UFR Sciences de l'Homme, Bordeaux, France
ER -

TY - JOUR
ID - 538
T1 - Using a play intervention to improve the play skills of children with a language delay. [References]
A1 - Sualy,Abbey; Yount,Sara; Kelly-Vance,Lisa; Ryalls,Brigette
Y1 - 2011///
N1 - Peer Reviewed Journal: 2012-03151-006
English
Journal; Peer Reviewed Journal
KW - PsycInfo
KW - At Risk Populations
KW - Childhood Development
KW - Childhood Play Behavior
KW - Language Disorders
KW - Social Skills
KW - Language Delay
RP - NOT IN FILE
SP - 105
EP - 122
JF - International Journal of Psychology: A Biopsychosocial Approach / Tarptautinis psichilogijos zurnalas: Biopsichosocialinis poziuris
VL - 9 2011, pp. 105-122.
N2 - Background: Children with language delays are at risk for difficulty with social skills because their play skill repertoire may be limited. Early interventions can help students learn play skills and prevent future social difficulties. Purpose: Minimal studies have addressed the impact of a play intervention, but the few that exist have found positive effects on typically developing and at-risk children. The effect of a play intervention on the play skills of preschool age children verified with a language delay was examined in this study because of the importance in improving play skills for this group. Materials and method: Children's free play skills were assessed using the PIECES and then they were assigned to either an intervention or comparison group. The intervention consisted of short-term facilitated play instruction from adults and took place in the early intervention classroom. A pretest posttest design was used to evaluate the effect of the intervention, also using the PIECES. Results: Five of six children in the intervention group improved their play skills while the five children in the comparison group remained at or below their pretest levels. Conclusions: The children's play increased in complexity by moving from exploratory to complex pretend play. Prompting, modeling and reinforcement are methods which effectively encourage play skill development. (PsycINFO Database Record (c) 2013 APA, all rights reserved) (journal abstract)
SN - 1941-7233
AD - Sualy, Abbey: University of Nebraska at Omaha, Omaha, NE, US Yount, Sara: University of Nebraska at Omaha, Omaha, NE, US Kelly-Vance, Lisa: University of Nebraska at Omaha, Omaha, NE, US Ryalls, Brigette: University of Nebraska at Omaha, Omaha, NE, US
ER -

TY - JOUR
ID - 539
T1 - Association of maternal scaffolding to maternal education and cognition in toddlers born preterm and full term. [References]
A1 - Lowe,Jean R.
Y1 - 2013///

N1 - Peer Reviewed Journal: 2012-33680-015
English
Journal; Peer Reviewed Journal
KW - PsycInfo
KW - At Risk Populations
KW - Educational Attainment Level
KW - Parental Characteristics
KW - Premature Birth
KW - Scaffolding
KW - Cognitive Development
KW - Neonatal Development
RP - NOT IN FILE
SP - 72
EP - 77
JF - Acta Paediatrica
JA - Acta Paediatr
VL - 102:
IS - 1
N2 - Aim: Parental behaviour described as 'scaffolding' has been shown to influence outcomes in at-risk children. The purpose of this study was to compare maternal verbal scaffolding in toddlers born preterm and full term. Methods: The scaffolding behaviour of mothers of toddlers born preterm and healthy full term was compared during a 5-min videotaped free play session with standardized toys. We compared two types of scaffolding and their associations with socio-demographic, neonatal medical factors and cognition. Results: The mothers of toddlers born full term used more complex scaffolding. Maternal education was associated with complex scaffolding scores for the preterm children only. Specifically, the preterm children who were sicker in the neonatal period, and whose mothers had higher education, used more complex scaffolding. In addition, children born preterm, who had less days of ventilation, had higher cognitive scores when their mothers used more complex scaffolding. Similarly, cognitive and scaffolding scores were higher for children born full term. Conclusion: Our findings highlight early differences in mother-child interactive styles of toddlers born preterm compared with full term. Teaching parents play methods that support early problem-solving skills may support a child's method of exploration and simultaneously their language development. (PsycINFO Database Record (c) 2013 APA, all rights reserved) (journal abstract)
SN - 0803-5253
AD - Lowe, Jean R.: School of Medicine, University of New Mexico, Albuquerque, NM, US Erickson, Sarah J.: Department of Psychology, University of New Mexico, Albuquerque, NM, US MacLean, Peggy: School of Medicine, University of New Mexico, Albuquerque, NM, US Schrader, Ron: Clinical and Translational Science Center, University of New Mexico, Albuquerque, NM, US Fuller, Janell: School of Medicine, University of New Mexico, Albuquerque, NM, US
ER -

TY - JOUR
ID - 540
T1 - Head start early mental health intervention: Effects of child-centered play therapy on disruptive behaviors. [References]
A1 - Bratton,Sue C.
Y1 - 2013///
N1 - Peer Reviewed Journal: 2012-32334-001
English
Journal; Peer Reviewed Journal
KW - PsycInfo
KW - Behavior Problems
KW - Client Centered Therapy
KW - Play Therapy
KW - Project Head Start
KW - Intervention
KW - Mental Health

KW - Preschool Students
RP - NOT IN FILE
SP - 28
EP - 42
JF - International Journal of Play Therapy
VL - 22:
IS - 1
N2 - This pilot study examined the effectiveness of child-centered play therapy (CCPT) with 54 low income preschool children (42% African American, 39% Hispanic, and 18% Caucasian) identified with clinical levels of disruptive behaviors. Children were assigned to the experimental treatment, CCPT, or to the active control, reading mentoring (RM), through block randomization. Results from a 2 (group) x 3 (measures) split plot analysis of variance indicated that according to teachers blinded to children's treatment group assignment, experimental group children demonstrated a statistically significant decrease (p < .05) in disruptive behaviors compared with the RM group across 3 points of measurement. Post hoc analysis showed that children receiving CCPT demonstrated statistically significant decreases (p < .025) in aggression and attention problems. Regarding practical significance of the findings, CCPT demonstrated large p2 treatment effects ranging from .17 to .34. Of the 27 children receiving CCPT, 21 children moved from clinical levels of behavioral concern at pretest to more normative levels of functioning following treatment, thus establishing the clinical significance of CCPT as an early mental health intervention for at-risk children in Head Start. (PsycINFO Database Record (c) 2013 APA, all rights reserved) (journal abstract)
SN - 1555-6824
AD - Bratton, Sue C.: Department of Counseling and Higher Education, University of North Texas, Denton, TX, US Ceballos, Peggy L.: Department of Counseling, University of North Carolina at Charlotte, Charlotte, NC, US Sheely-Moore, Angela I.: Department of Counseling and Educational Leadership, Montclair State University, Upper Montclair, NJ, US Meany-Walen, Kristin: Clinical Mental Health and School Counseling Program, University of Northern Iowa, Cedar Falls, IA, US Pronchenko, Yulia: Department of Counseling and Higher Education, University of North Texas, Denton, TX, US Jones, Leslie D.: Department of Counseling and Higher Education, University of North Texas, Denton, TX, US
ER -

TY - JOUR
ID - 542
T1 - New reference values must be established for the Alberta Infant Motor Scales for accurate identification of infants at risk for motor developmental delay in Flanders
A1 - De Kegel,A.
Y1 - 2013///
N1 - Peer Reviewed Journal: 2013-02675-014
English
Journal; Peer Reviewed Journal
KW - PsycInfo
KW - At Risk Populations
KW - Infant Development
KW - Motor Development
KW - Test Construction
KW - Test Validity
RP - NOT IN FILE
SP - 260
EP - 267
JF - Child: Care, Health and Development
VL - 39:
IS - 2
N2 - Background: The Alberta Infant Motor Scales (AIMS) is a reliable and valid assessment tool to evaluate the motor performance from birth to independent walking. This study aimed to determine whether the Canadian reference values on the AIMS from 1990-1992 are still useful to Flemish infants, assessed in 2007-2010. Additionally, the association between motor performance and sleep and play positioning will be determined. Methods: A total of 270 Flemish infants between 0 and 18 months, recruited by formal day care services, were

assessed with the AIMS by four trained physiotherapists. Information about sleep and play positioning was collected by mean of a questionnaire. Results: Flemish infants perform significantly lower on the AIMS compared with the reference values (P < 0.001). Especially, infants from the age groups of 4, 5, 6, 7, 8, 9, 10, 11, 12, 13 and of 15 months showed significantly lower scores. From the information collected by parental questionnaires, the lower motor scores seem to be related to the sleep position, the amount of play time in prone, in supine and in a sitting device. Infants who are exposed often to frequently to prone while awake showed a significant higher motor performance than infants who are exposed less to prone (<6 m: P = 0.002; >6 m: P = 0.013). Infants who are placed often to frequently in a sitting device in the first 6 months of life (P = 0.010) and in supine after 6 months (P = 0.001) performed significantly lower than those who are placed less in it. Conclusion: Flemish infants recruited by formal day care services, show significantly lower motor scores than the Canadian norm population. New reference values should be established for the AIMS for accurate identification of infants at risk. Prevention of sudden infant death syndrome by promoting supine sleep position should go together with promotion of tummy time when awake and avoiding to spent too much time in sitting devices when awake. (PsycINFO Database Record (c) 2013 APA, all rights reserved) (journal abstract)
SN - 0305-1862
AD - De Kegel, A.: Department of Rehabilitation Sciences and Physiotherapy Ghent, Artevelde University College - Ghent University, Ghent, Belgium Peersman, W.: Department of Rehabilitation Sciences and Physiotherapy Ghent, Artevelde University College - Ghent University, Ghent, Belgium Onderbeke, K.: Department of Rehabilitation Sciences and Physiotherapy Ghent, Artevelde University College - Ghent University, Ghent, Belgium Baetens, T.: Department of Rehabilitation Sciences and Physiotherapy Ghent, Artevelde University College - Ghent University, Ghent, Belgium Dhooge, I.: Faculty of Medicine and Health Sciences, Department of Oto-rhino-laryngology, Ghent University, Ghent, Belgium Van Waelvelde, H.: Department of Rehabilitation Sciences and Physiotherapy Ghent, Artevelde University College - Ghent University, Ghent, Belgium
ER -

TY - JOUR
ID - 543
T1 - Pedometers, accelerometers, and observational methods: A comparison of measurements of physical activity in fourth-grade students
A1 - Greene,Amanda
Y1 - 2012///
N1 - Dissertation Abstract: 2012-99210-063
English
Dissertation Abstract
KW - PsycInfo
KW - Exercise
KW - Methodology
KW - Observation Methods
KW - Physical Activity
KW - Schools
KW - Activity Level
KW - Grade Level
KW - Measurement
RP - NOT IN FILE
SP - 1726
JF - Dissertation Abstracts International Section A: Humanities and Social Sciences
VL - 73:
IS - 5-A
N2 - In recent years physical inactivity among students has become a matter of great concern. Nearly 65% of students do not meet the daily recommended level of physical activity, which is 60 minutes or more of moderate to vigorous physical activity each day, with 50% of that time being spent in moderate to vigorous levels of activity (CDC, 2010b). As a result, the 21 st century has shown to be a time of many health problems such as, obesity, diabetes, and heart disease. In fact, nearly one third of all children are considered obese or overweight (Slawta & DeNeui, 2009). Researchers suggest that these health problems are directly related to students' sedentary lifestyles (Pate et al., 2006). Schools play a pivotal role in addressing and increasing physical activity

during the school day. The purpose of this research study was to measure levels of physical activity in elementary students during school hours. Specifically, the study sought to discover if there were increased levels of physical activity while students were using a cross-curricular adventure playground, as compared to when they were engaged in free play or physical education class. The study also compared the different measurement types (pedometers, accelerometers, and the observational method) used to assess physical activity, to indicate which measurement types were most feasible in the elementary school setting. Schools are ideal locations for assessing levels of physical activity, as 95% of all children are enrolled in these learning institutions (National Center for Educational Statistics, 2005). The data indicate that physical education provided for the highest levels of moderate to vigorous activity, while both free play and cross-curricular activity still rendered adequate levels of physical activity. After studying the different measurement protocols (pedometers, accelerometers, and the observational method) used in this study, results suggested the pedometer to be the most feasible device to use for measuring children in these types of physical activity settings. One implication for practice was implementing cross-curricular physical activity as a supplement to other physical activities or as an addition to physical education classes in an effort to allow more time for academic instruction while having students engaged in physical activity. Another recommendation for practice was to use pedometers as a cost-effective physical activity measurement device for elementary students. (PsycINFO Database Record (c) 2013 APA, all rights reserved)
SN - 0419-4209
AD - Greene, Amanda: East Tennessee State U., US
ER -

TY - JOUR
ID - 544
T1 - Product-based television and young children's pretend play in Australia. [References]
A1 - Fletcher,Renee
Y1 - 2012///
N1 - Peer Reviewed Journal: 2012-32006-002
English
Journal; Peer Reviewed Journal
KW - PsycInfo
KW - Childhood Development
KW - Pretend Play
KW - Product Design
KW - Television
RP - NOT IN FILE
SP - 5
EP - 17
JF - Journal of Children and Media
VL - 6:
IS - 1
N2 - Product-based television refers to programming that implicitly promotes toys to children in the guise of a television show. The aim of the present study was to examine how exposure to such programs might impact on one of the most important domains of young children's development: pretend play. Thirty-six preschoolers in Australia watched 1 of 3 different types of television program (product-based with action-adventure themes; product-based with educational themes; or non-product-based) and were given a range of product-based and non-product-based toys to play with. Contrary to expectation, program type had minimal impact on the overall frequency of pretend play children engaged in. However, compared to educational product-based programs and non-product-based programs, children who watched action-adventure product-based programs exhibited significantly less acts of novel pretense and significantly more acts of imitative pretense. Findings such as this are critical to the ongoing debate surrounding the regulation of television content aimed at children. (PsycINFO Database Record (c) 2013 APA, all rights reserved) (journal abstract)
SN - 1748-2798
AD - Fletcher, Renee: University of Queensland, St Lucia, QLD, Australia
ER -

TY - JOUR

ID - 545
T1 - Evaluating the healing power of art and play: A cross-cultural investigation of psychosocial resilience in child and adolescent survivors of the 2008 Sichuan, China earthquake
A1 - Fu,Christine Yu-Heng
Y1 - 2012///
N1 - Dissertation Abstract: 2012-99200-557
English
Dissertation Abstract
KW - PsycInfo
KW - Art Therapy
KW - Cross Cultural Differences
KW - Natural Disasters
KW - Play Therapy
KW - Survivors
KW - Mental Health
KW - Resilience (Psychological)
RP - NOT IN FILE
SP - 2152
JF - Dissertation Abstracts International: Section B: The Sciences and Engineering
VL - 73:
IS - 4-B
N2 - This dissertation builds upon an evaluation that was conducted at the end of a psychosocial intervention that was implemented to improve mental health and foster resilience among child and adolescent survivors after the 2008 Sichuan, China earthquake. First, a comprehensive review was conducted of universal interventions conducted with children and adolescents after a disaster to examine theoretical and empirical findings. Of the 27 studies identified, only seven were informed by theory. Twenty-five studies demonstrated evidence that the intervention decreased distress symptoms and improved multiple functioning domains. However, the lack of theory creates a black box and presents challenges to program fidelity and replication as well as measurement of specific program component effects and cost-effectiveness. Second, exploratory factor analysis of the Connor-Davidson Resilience Scale (CD-RISC) among a sample of 2,132 students in the control group found two resilience factors termed by Chinese scholars as: Rational Thinking: logic based on socially accepted cultural norms and Self-Awareness: rooted in an interdependent view of the self in relation to others and environmental factors. Consistent with previous studies, the CD-RISC demonstrated strong construct validity and adequate internal consistency. The stability of the model was tested using confirmatory factor analysis with data from 1,988 intervention participants. Lastly, direct and indirect effects of the intervention were examined. Mean scores in CD-RISC did not differ between groups (p=0.67). Mean Rational Thinking was marginally higher (p=0.05) but lower for Self-Awareness (p=0.02) intervention versus controls. Mean PTSD was lower in the intervention than controls (p<0.001). The log odds of PTSD did not differ between groups (OR: 1.16, p=0.09). Rational Thinking and PTSD had similar parameter estimates that were negatively related in both groups. Self-Awareness was positively related with PTSD in both groups, but was larger in the intervention. The program focused mainly on strengthening individual traits, which may have heightened perceived variability of external forces that supersede internal capacities. The relationship between Risk Exposure and PTSD was negative for the intervention group. This may be attributed to attachment relationships with intervention caregivers and peers. Findings support the need for more cross-cultural approaches to understanding resilience and adaptive outcomes. (PsycINFO Database Record (c) 2012 APA, all rights reserved)
SN - 0419-4217
AD - Fu, Christine Yu-Heng: The Johns Hopkins U., US
ER -

TY - JOUR
ID - 546
T1 - Variables associated with children's physical activity levels during recess: The A-CLASS project. [References]
A1 - Ridgers,Nicola D.
Y1 - 2010///

N1 - Peer Reviewed Journal: 2012-32493-001
English
Journal; Peer Reviewed Journal
KW - PsycInfo
KW - Activity Level
KW - Childhood Play Behavior
KW - Health Behavior
KW - Physical Activity
KW - School Environment
KW - Playgrounds
KW - Risk Factors
RP - NOT IN FILE
JF - The International Journal of Behavioral Nutrition and Physical Activity
VL - 7 Oct 2010, ArtID 74.
N2 - Background: School recess provides a daily opportunity for children to engage in physically active behaviors. However, few studies have investigated what factors may influence children's physical activity levels in this context. Such information may be important in the development and implementation of recess interventions. The aim of this study was to investigate the association between a range of recess variables and children's sedentary, moderate and vigorous physical activity in this context. Methods: One hundred and twenty-eight children (39% boys) aged 9-10 years old from 8 elementary schools had their physical activity levels observed during school recess using the System for Observing Children's Activity and Relationships during Play (SOCARP). Playground variables data were also collected at this time. Multilevel prediction models identified variables that were significantly associated with children's sedentary, moderate and vigorous physical activity during recess. Results: Girls engaged in 13.8% more sedentary activity and 8.2% less vigorous activity than boys during recess. Children with no equipment provision during recess engaged in more sedentary activity and less moderate activity than children provided with equipment. In addition, as play space per child increased, sedentary activity decreased and vigorous activity increased. Temperature was a significant negatively associated with vigorous activity. Conclusions: Modifiable and unmodifiable factors were associated with children's sedentary, moderate and vigorous physical activity during recess. Providing portable equipment and specifying areas for activities that dominate the elementary school playground during recess may be two approaches to increase recess physical activity levels, though further research is needed to evaluate the short and long-term impact of such strategies. (PsycINFO Database Record (c) 2012 APA, all rights reserved) (journal abstract)
SN - 1479-5868
AD - Ridgers, Nicola D.: Centre for Physical Activity and Nutrition Research, Deakin University, Burwood, VIC, Australia Fairclough, Stuart J.: Faculty of Education, Community and Leisure, Liverpool John Moores University, Liverpool, United Kingdom Stratton, Gareth: Research Institute for Sport and Exercise Sciences, Liverpool John Moores University, Liverpool, United Kingdom
ER -

TY - JOUR
ID - 547
T1 - Features and amenities of school playgrounds: A direct observation study of utilization and physical activity levels outside of school time. [References]
A1 - Colabianchi,Natalie
Y1 - 2011///
N1 - Peer Reviewed Journal: 2012-32528-001
English
Journal; Peer Reviewed Journal
KW - PsycInfo
KW - Activity Level
KW - Physical Activity
KW - Playgrounds
KW - Recreation Areas
KW - School Facilities
KW - Safety

RP - NOT IN FILE
JF - The International Journal of Behavioral Nutrition and Physical Activity
VL - 8 Apr 2011, ArtID 32.
N2 - Background: A significant amount of research has examined whether park or playground availability is associated with physical activity. However, little research has examined whether specific features or amenities of parks or playgrounds, such as the number of unique types of playground equipment or the safety of the equipment is associated with utilization of the facility or physical activity levels while at the facility. There are no studies that use direct observation and a detailed park assessment to examine these associations. Methods: Twenty urban schoolyards in the Midwest, ten of which were renovated, were included in this study. Using a detailed environmental assessment tool (i.e., Environmental Assessment of Public Recreation Spaces), information on a variety of playground attributes was collected. Using direct observation (i.e., System for Observing Play and Leisure Activity in Youth), the number of adults, girls and boys attending each schoolyard and their physical activity levels were recorded. Each schoolyard was observed ten times for 90 minutes each time outside of school hours. Clustered multivariable negative binomial regressions and linear regressions were completed to examine the association between playground attributes and utilization of the schoolyard and the proportion active on the playground, respectively. Effect modification by renovation status was also examined. Results: At renovated schoolyards, the total number of play features was significantly associated with greater utilization in adults and girls; overall cleanliness was significantly associated with less utilization in girls and boys; and coverage/ shade for resting features was significantly associated with greater utilization in adults and boys. At unrenovated schoolyards, overall safety was significantly associated with greater utilization in boys. No playground attribute was associated with the proportion active on the playground after adjusting for all other significant playground attributes. Conclusions: Having a large quantity of play features and shade at renovated playgrounds were positively associated with utilization of the schoolyard. Modifying playgrounds to have these features may increase the utilization of these facilities outside of school time. Additional research should explore what features and amenities are associated with increased physical activity levels of children and adults who utilize the facilities. (PsycINFO Database Record (c) 2012 APA, all rights reserved) (journal abstract)
SN - 1479-5868
AD - Colabianchi, Natalie: Department of Epidemiology and Biostatistics, Arnold School of Public Health, University of School Carolina, Columbia, SC, US Maslow, Andrea L.: R. Stuart Dickson Institute for Health Studies, Carolinas HealthCare System, Charlotte, NC, US Swayampakala, Kamala: Department of Epidemiology and Biostatistics, Arnold School of Public Health, University of School Carolina, Columbia, SC, US
ER -

TY - JOUR
ID - 548
T1 - A multi-level examination of school programs, policies and resources associated with physical activity among elementary school youth in the PLAY-ON study. [References]
A1 - Leatherdale,Scott T.
Y1 - 2010///
N1 - Peer Reviewed Journal: 2012-31438-001
English
Journal; Peer Reviewed Journal
KW - PsycInfo
KW - Elementary School Students
KW - Physical Activity
KW - Policy Making
KW - School Based Intervention
KW - School Environment
RP - NOT IN FILE
JF - The International Journal of Behavioral Nutrition and Physical Activity
VL - 7 Jan 2010, ArtID 6.
N2 - Background: Given the decline in physical activity (PA) levels among youth populations it is vital to understand the factors that are associated with PA in order to inform the development of new prevention programs. Many studies have examined individual characteristics associated with PA among youth yet few have studied the relationship between the school environment and PA despite knowing that there is variability in

student PA levels across schools. Methods: Using multi-level logistic regression analyses we explored the school- and student-level characteristics associated with PA using data from 2,379 grade 5 to 8 students attending 30 elementary schools in Ontario, Canada as part of the PLAY-Ontario study. Results: Findings indicate that there was significant between-school random variation for being moderately and highly active; school-level differences accounted for 4.8% of the variability in the odds of being moderately active and 7.3% of the variability in the odds of being highly active. Students were more likely to be moderately active if they attended a school that used PA as a reward and not as discipline, and students were more likely to be highly active if they attended a school with established community partnerships. Important student characteristics included screen time sedentary behavior, participating in team sports, and having active friends. Conclusion: Future research should evaluate if the optimal population level impact for school-based PA promotion programming might be achieved most economically if intervention selectively targeted the schools that are putting students at the greatest risk for inactivity. (PsycINFO Database Record (c) 2012 APA, all rights reserved) (journal abstract)
SN - 1479-5868
AD - Leatherdale, Scott T.: Department of Population Studies and Surveillance, Cancer Care, Ontario, Toronto, ON, Canada Manske, Steve: Propel Centre for Population Health Impact, Canadian Cancer Society and the University of Waterloo, Waterloo, ON, Canada Faulkner, Guy: Faculty of Physical Education and Health, University of Toronto, Toronto, ON, Canada Arbour, Kelly: Faculty of Physical Education and Health, University of Toronto, Toronto, ON, Canada Bredin, Chad: Propel Centre for Population Health Impact, Canadian Cancer Society and the University of Waterloo, Waterloo, ON, Canada
ER -

TY - JOUR
ID - 549
T1 - Time spent playing outdoors after school and its relationship with independent mobility: A cross-sectional survey of children aged 10-12 years in Sydney, Australia. [References]
A1 - Wen,Li Ming; Kite,James; Merom,Dafna; Rissel,Chris
Y1 - 2009///
N1 - Peer Reviewed Journal: 2012-30462-001
English
Journal; Peer Reviewed Journal
KW - PsycInfo
KW - Childhood Play Behavior
KW - Health
KW - Leisure Time
KW - Physical Activity
KW - Physical Mobility
KW - At Risk Populations
KW - Obesity
KW - Prevention
RP - NOT IN FILE
JF - The International Journal of Behavioral Nutrition and Physical Activity
VL - 6 Mar 2009, ArtID 15.
N2 - Background: Time spent outdoors is positively associated with physical activity and has been suggested as a proxy for physical activity of children. The role of children's independence in physical activity and time spent outdoors is less understood. This study aimed to assess how much time children spent playing outdoors after school, and to explore the relationship between outdoor play and independence among children aged 10-12 years. Method: Children recorded how much time they spent playing outdoors or watching TV/videos or playing computer games after school using a five-day diary, and also reported whether they were allowed to walk on their own in their neighbourhood as an indicator of their independent mobility. Parents were surveyed on family demographics and perception of neighbourhood safety. The surveys were conducted in late 2006 as part of the Central Sydney Walk to School program which involved 1975 children and their parents from 24 primary schools. Factors associated with time spent playing outdoors were determined by logistic regression modelling. Results: Thirty-seven per cent of children spent less than half an hour a day playing outdoors after school, and 43% spent more than 2 hours a day watching TV, videos or playing computer games. Forty-eight per cent of children were allowed to walk on their own near where they lived. Children's independent mobility was

significantly associated with outdoor play after adjusting for other confounders. Compared with those who were never allowed to walk on their own near where they lived, students who were allowed to walk on their own were significantly more likely to spend more than half an hour a day playing outdoors after school with an adjusted odds ratio of 2.6, 95% CI 1.84-3.58, P < 0.001. Conclusion: The findings that a significant proportion of children spend less than half an hour a day playing outdoors after school and have excessive screen time have important implications for physical activity promotion and obesity prevention. The study also suggests that children's independent mobility should be considered in research and evaluation into children's play and physical activity. Environments that promote greater independent mobility in children may increase their physical activity levels and hence reduce their risk of overweight/obesity. (PsycINFO Database Record (c) 2012 APA, all rights reserved) (journal abstract)
SN - 1479-5868
AD - Wen, Li Ming: Health Promotion Service, Sydney South West Area Health Service, Syndey, NSW, Australia Kite, James: Health Promotion Service, Sydney South West Area Health Service, Syndey, NSW, Australia Merom, Dafna: School of Public Health, University of Sydney, Sydney, NSW, Australia Rissel, Chris: Health Promotion Service, Sydney South West Area Health Service, Syndey, NSW, Australia
ER -

TY - JOUR
ID - 550
T1 - Review of Sexual sabotage: How one mad scientist unleashed a plague of corruption and contagion on America. [References]
A1 - Janssen,Diederik F.
Y1 - 2012///
N1 - Peer Reviewed Journal: 2012-28578-026
English
Journal; Peer Reviewed Journal
KW - PsycInfo
KW - Communities
KW - Contagion
KW - Family
KW - Sexuality
RP - NOT IN FILE
SP - 1315
EP - 1318
JF - Archives of Sexual Behavior
JA - Arch.Sex Behav
VL - 41:
IS - 5
N2 - Reviews the book, Sexual Sabotage: How One Mad Scientist Unleashed a Plague of Corruption and Contagion on America by Judith A. Reisman (2010). Reisman's recent work, with 1,103 footnotes, is a recapitulation of her 35-year anti-Kinsey campaign, already featuring book-length studies published in 1990, 1998, and 2006, as well as documentary appearances in 1995, 1998, and 2009. Reisman's ever more repetitive polemic, attacking, for instance, each and every contributor daring to appear in The Kinsey Institute's books on childhood, clearly aspires to spearhead American ultra-conservative resentment of sexology as a field "led by mad sexual psychopaths" who "sexualize everything-as the lugubrious child-harming front piece of an entire "postmodern" degeneration of Family and Nation. Reisman's book makes perfect sense where she engages this familiar calculus, in fact honoring Kinsey's own solicitation to play truth games. But the predicament of American sexologists must be clear: not to play taboo's game of generalizations and proclamations will make of you a sufferer of precisely qualified "distorted cognitions". In today's regime of fear, American sexologists will typically end up "uncovering" and soliciting the sour evidence American populists, eerily, seem to crave. Reisman can afford to be honest at sporadic junctures in her text, where she does not attempt to hide her moral concerns behind "35 years" of scientific posturing. To dismiss on an empirical basis her pitching of value, in any case, will risk complicity with her darker, precisely her "scientific," moments: exactly the scientific cover-up of moral dilemmas she accuses her nemesis Kinsey of. There's nothing wrong with moral conventions, obviously: incest taboos have ranked among the few universal traits of Man, and their particular, late modern transcriptions have convinced a whole generation of Child-savers, Woman-liberators, Family-protectors, Community-

guardians, and Nation-builders. Conversely: the moment taboos begin to require scientific (medical) approval, their days may be numbered. (PsycINFO Database Record (c) 2012 APA, all rights reserved)
SN - 0004-0002
ER -

TY - JOUR
ID - 552
T1 - Do theory and pedagogy have an impact on provisions for outdoor learning? A comparison of approaches in Australia and Norway. [References]
A1 - Sandseter,Ellen Beate Hansen; Little,Helen; Wyver,Shirley
Y1 - 2012///
N1 - Peer Reviewed Journal: 2012-22833-003
English
Journal; Peer Reviewed Journal
KW - PsycInfo
KW - Cross Cultural Differences
KW - Curriculum
KW - Learning Environment
KW - Teaching
KW - Early Childhood Development
KW - Education
KW - Theories
RP - NOT IN FILE
SP - 167
EP - 182
JF - Journal of Adventure Education and Outdoor Learning
VL - 12:
IS - 3
N2 - In this paper, we contrast the early childhood education and care aims and curriculum in Norway and Australia and the theoretical underpinnings of Norwegian and Australian pedagogy in an attempt to partially explain the different approaches to (risky) outdoor play in these two countries. We argue that Norwegian pedagogical approaches may allow teachers to theorize more about, and place greater emphasis on, physical and motor development than teachers in countries such as Australia. Evidence includes reference to theories typically used in Norwegian and Australian writing. Additionally, we examine interview data from teachers, which reveals that both groups of teachers share similar understandings of the importance of risk-taking in play, but there is more consistency between belief and practice for Norwegian than Australian teachers. We argue that the differences in alignment of belief and practice in the two countries are partly attributable to the differing theoretical bases of teachers' pedagogical approaches in each country. These findings have implications for theorizing about outdoor play and learning. In particular, we argue that early childhood teacher training programmes should include approaches based on Gibsonian and Dynamic Systems theories. (PsycINFO Database Record (c) 2012 APA, all rights reserved) (journal abstract)
SN - 1472-9679
AD - Sandseter, Ellen Beate Hansen: Queen Maud University College of Early Childhood Education (DMMH), Trondheim, Norway Little, Helen: Macquarie University, Macquarie Park, NSW, Australia Wyver, Shirley: Macquarie University, Macquarie Park, NSW, Australia
ER -

TY - JOUR
ID - 553
T1 - Temporal processing in children with language disorders. [References]
A1 - Burns,Martha
Y1 - 2011///
N1 - Book: 2010-22258-017
English
Book; Edited Book
KW - PsycInfo

KW - Auditory Perception
KW - Language Development
KW - Language Disorders
KW - Psycholinguistics
KW - Cognition
KW - Cognitions
KW - Cognitive Science
KW - Language
RP - NOT IN FILE
SP - 361
EP - 372
JF - Guendouzi, Jackie [Ed]; Loncke, Filip [Ed]; Williams, Mandy J [Ed]
VL - :
IS - 2011
N2 - (from the chapter) Noam Chomsky's theories ultimately led to the emergence of a new scientific discipline, psycholinguistics, which focused the fields of cognitive science and psychology on the importance of language to human cognition. This in turn, provided an enormous boost to the field of speech pathology by emphasizing the need for identification and remediation of language problems in children; ultimately leading in 1978 to the addition of Language in the name of the professional organization ASHA (from the American Speech and Hearing Association to the American Speech-Language-Hearing Association), and resulting in an approximate fivefold increase in certified ASHA members during the same time period. Yet, because of the profound influence of Chomsky during the period and the prevailing viewpoint that language was an innate human capacity, many psycholinguists originally dismissed the findings of other scientists, maintaining that nonverbal processing could not play a significant part in a neurological faculty innately derived through a "language acquisition device". Today, although some controversy persists regarding the importance of auditory processing in language acquisition, the prevalence of interdisciplinary research involving developmental psycholinguistics, clinicians and neuroscientists has led to a resurgence of interest in causal mechanisms of language disturbance and recognition of the importance of timing and synchrony in neurological cognitive systems. In this regard, what is now often referred to as the "temporal processing hypothesis," stemming from the research of Tallal and Piercy in the 1970s, has been refined and studied extensively in recent years. Essentially the hypothesis asserts that some language impaired children have difficulty processing rapidly changing acoustic details, which interferes with their ability to adequately parse incoming language signals into phonemes and thereby have an increased risk of developing speech, language, and/or reading problems. This explanation essentially views the capacity to process rapidly changing sensory inputs as a probable core neurological component of phonological processing. How this core capacity is understood by neuroscience and the role it may play in language acquisition is an unfolding scientific inquiry. This chapter will attempt to review and clarify the current state of neuroscience research that led to and supports the temporal processing hypothesis and will review research on assessment and the remediation value to auditory training of temporal processing skills. However, the viewpoint of this chapter is that the consideration of the role of temporal processing in language learning does not necessitate rejecting Chomsky or the vast accumulation of psycholinguistic research on language learning and language disorders. Rather, it is the perspective of this author, that understanding temporal processing adds to what some might consider the top-down influence of language and conceptual knowledge on learning by specifying co-occurring bottom-up processes that influence language learning as well. (PsycINFO Database Record (c) 2012 APA, all rights reserved)
AD - Burns, Martha: Department of Communication Sciences and Disorders, Northwestern University, Evanston, IL, US
ER -

TY - JOUR
ID - 554
T1 - Correlates of pupils' sense of futility in primary education in Flanders: The role of the teacher. [References]
A1 - Van Houtte,Mieke; Van Maele,Dimitri; Agirdag,Orhan
Y1 - 2012///

N1 - Book: 2012-25935-008
English
Book; Edited Book
KW - PsycInfo
KW - Elementary School Students
KW - Elementary School Teachers
KW - Hopelessness
KW - Roles
KW - Student Attitudes
KW - Academic Achievement
KW - Elementary Education
KW - Elementary Schools
KW - Parent Child Relations
KW - School Environment
KW - Social Support
KW - Socioeconomic Status
KW - Trust (Social Behavior)
RP - NOT IN FILE
SP - 149
EP - 170
JF - DiPaola, Michael F [Ed]; Forsyth, Patrick B [Ed]
VL - :
IS - 2012
N2 - (from the chapter) Pupils' sense of futility with respect to school is an important predictor of lower achievement, lower study involvement, and school misconduct. Feelings of futility regarding school are particularly prevalent among pupils from lower socioeconomic status (SES) backgrounds. The present study examines which school and pupil features are associated with pupils' sense of futility. Furthermore, it aims to investigate the role teachers might play in either enhancing or buffering these feelings of futility, especially in low SES pupils, by taking into account the effect of faculty trust and pupils' perceived teacher support. By means of multilevel analysis of data collected during the 2008-2009 school year from 2,845 pupils and 706 teachers across a sample of 68 primary schools in Flanders (Belgium), this study confirms that higher feelings of futility associate with pupils' low SES background, low ability, and low perceived parental support. These associations result in higher levels of sense of futility in low SES and low ability schools. The findings also indicate the crucial role teachers might play. Although neither faculty trust nor perceived teacher support seem able to buffer the development of feelings of futility in low SES and low ability pupils, having trusting and supportive teachers lowers the risk of strong feelings of futility. An important policy implication of this study is therefore that it might be rewarding to improve faculty trust in pupils in order to fight pupils' feelings of futility. Other strategies are, however, advisable in order to buffer the higher feelings of futility in low SES pupils in particular. (PsycINFO Database Record (c) 2012 APA, all rights reserved)
AD - Van Houtte, Mieke: Ghent University, Department of Sociology, Research Group CuDOS, Belgium Van Maele, Dimitri: Ghent University, Department of Sociology, Research Group CuDOS, Belgium Agirdag, Orhan: Ghent University, Department of Sociology, Research Group CuDOS, Belgium
ER -

TY - JOUR
ID - 555
T1 - A dynamic systems approach to co-emotion regulation in preschoolers and mothers in the context of strange situation: Predictors and developmental consequences
A1 - Guo,Yuqing
Y1 - 2012///
N1 - Dissertation Abstract: 2012-99160-397
English
Dissertation Abstract
KW - PsycInfo
KW - Childhood Development
KW - Emotional Regulation

KW - Mother Child Relations
KW - Affective Valence
KW - Developmental Psychology
RP - NOT IN FILE
SP - 876
JF - Dissertation Abstracts International: Section B: The Sciences and Engineering
VL - 73:
IS - 2-B
N2 - Co-emotion regulation has been recognized as playing a crucial role in the developmental outcomes of young children. Despite many fruitful studies in this area, measuring the process of change within co-emotion regulation remains challenging. This study applied dynamic systems to define co-emotion regulation and introduced the State Space Grid to quantify its process of change. The study combined new behavioral coding of existing video archives of Strange Situation with secondary Seattle data from NICHD Study of Early Child Care and Young Development. Affect was coded in preschoolers and mothers in the first play and the second reunion episodes of Strange Situation. Repeated measure analysis mixed models were used to describe how co-emotion regulation changed between secure and insecure dyads from the first play to the second reunion. The results show that secure dyads had a stable affective regulation structure and were thus not prone to being impacted by separation of Strange Situation. Conversely, insecure dyads had an unstable affective regulation structure and were vulnerable to influence by the separation. The difference of co-emotion regulation between secure and insecure dyads existed before the separation and remained after the separation. Hierarchical multiple regression analyses were used to explore predictors of shared positive affect and its developmental outcomes after controlling for child, mother, and family covariates. Results found that maternal sensitivity mediated family risks and shared positive affect in the second reunion, but not in the first play, suggesting that history of maternal sensitivity plays a greater role in maintaining mutual positive affect under stressful situations than it does in non-stress or low-stress situations. Results also revealed that shared positive affect within a non-stressful context promotes cognitive competency and self-regulation development, while maintaining or quickly resuming shared positive affect after a stressful context is a protective factor for internalizing problems. Findings provide evidence that dynamic systems is a promising theoretical paradigm for conceptualizing co-emotion regulation and that State Space Grid is a valid method to operationalize its process of change. Applications for early prevention or intervention are discussed, as well as implications for nursing science. (PsycINFO Database Record (c) 2012 APA, all rights reserved)
SN - 0419-4217
AD - Guo, Yuqing: U Washington, US
ER -

TY - JOUR
ID - 556
T1 - Adult opinions about the age at which children can be left home alone, bathe alone, or bike alone: Second Injury Control and Risk Survey (ICARIS-2). [References]
A1 - Mack,Karin A.
Y1 - 2012///
N1 - Peer Reviewed Journal: 2012-19793-001
English
Journal; Peer Reviewed Journal
KW - PsycInfo
KW - Child Self Care
KW - Childhood Development
KW - Parental Attitudes
KW - Injuries
KW - Prevention
RP - NOT IN FILE
SP - 223
EP - 226
JF - Journal of Safety Research
JA - J Safety Res.
VL - 43:

IS - 3
N2 - Problem: This study describes adult opinions about child supervision during various activities. Methods: Data come from a survey of U.S. adults. Respondents were asked the minimum age a child could safely: stay home alone; bathe alone; or ride a bike alone. Respondents with children were asked if their child had ever been allowed to: play outside alone; play in a room at home for more than 10 minutes alone; bathe with another child; or bathe alone. Results: The mean age that adults believed a child could be home alone was 13.0 years (95% CI=12.9-13.1), bathe alone was 7.5 years (95% CI=7.4-7.6), or bike alone was 10.1 years (95% CI=10.0-10.3). There were significant differences by income, education, and race. Discussion: Assessing adult's understanding of the appropriate age for independent action helps set a context for providing guidance on parental supervision. Guidelines for parents should acknowledge social norms and child development stages. Impact on Industry: Knowledge of social norms can help guide injury prevention messages for parents. (PsycINFO Database Record (c) 2012 APA, all rights reserved) (journal abstract)
SN - 0022-4375
AD - Mack, Karin A.: Division of Unintentional Injury Prevention, National Center for Injury Prevention and Control, Centers for Disease Control and Prevention, Atlanta, GA, US Dellinger, Ann: National Center for Injury Prevention and Control, Centers for Disease Control and Prevention, Atlanta, GA, US West, Bethany A.: Division of Unintentional Injury Prevention, National Center for Injury Prevention and Control, Centers for Disease Control and Prevention, Atlanta, GA, US
ER -

TY - JOUR
ID - 557
T1 - A diathesis-stress model of victimization: Relations among temperament, peer victimization, psychopathology, and health problems
A1 - Biebl,Sara J.
Y1 - 2012///
N1 - Dissertation Abstract: 2012-99140-347
English
Dissertation Abstract
KW - PsycInfo
KW - Life Experiences
KW - Personality
KW - Psychopathology
KW - Stress
KW - Victimization
KW - Health
KW - Mathematical Modeling
KW - Peers
RP - NOT IN FILE
SP - 608
JF - Dissertation Abstracts International: Section B: The Sciences and Engineering
VL - 73:
IS - 1-B
N2 - Youth who are victimized by their peers are more likely to develop symptoms of psychopathology and health problems in adolescence and adulthood. Also, temperamental traits of behavioral inhibition and emotional reactivity have been found to be significant risk factors for the development of mental and physical health problems. Diathesis-stress models of psychopathology indicate that the interaction between temperament and experiences of trauma may lead an individual to develop psychopathology. Most studies using a biosocial model of psychopathology, however, have focused more on experiences of physical and sexual abuse rather than experiences of peer abuse. No studies to date have examined how experiences of peer victimization moderate the relationship between temperament and later experiences of psychopathology and health problems, which was the primary focus of the present study. A second aim of the present study was to examine how the stability of victimization throughout childhood related to health problems in adolescence. Seventy 12- to 20-year-old youth participated in the present longitudinal study. This longitudinal study included three specific time points. At time 1, when participants were five years of age, they engaged in a 20-minute peer play interaction and their parents completed temperament measures. Tapes of the peer play interaction were rated by trained

coders for instances of peer victimization. During time 2, these same youth were between the ages of 10 and 18 and were contacted again and asked to complete measures related to peer victimization. Finally, at time 3, which was the present study, participants were between the ages of 12 and 20 and completed a 1-hour telephone interview which was used to obtain information about experiences of peer victimization as well as mental and physical health outcomes. Results from the present study indicated that youth with specific early temperamental traits (behaviorally inhibited and difficult) and who experienced peer victimization that is impairing appeared to be at multiplicatively greater risk for developing mental and physical health problems in adolescence compared to youth who did not have these same biological vulnerabilities and environmental experiences. Additionally, youth who chronically experienced peer victimization had significantly more mental and physical health problems than youth who experienced less stable victimization. This study adds to the current research on peer victimization by using a diathesis-stress model to examine the interactive effects of peer victimization and temperament on negative mental health and physical health outcomes. Moreover, findings from this study will provide researchers and professionals with information that can aide in the development of interventions for children who experience peer victimization and who suffer from different types of health problems and symptoms of psychopathology. More specifically, by understanding how innate factors such as temperament interact with a child's experience of peer victimization, researchers and clinicians may be able to design more specialized interventions for children based on the patterns of their victimization experiences and temperamental traits. (PsycINFO Database Record (c) 2012 APA, all rights reserved)
SN - 0419-4217
AD - Biebl, Sara J. W.: Southern Illinois U Carbondale, US
ER -

TY - JOUR
ID - 558
T1 - Orangutans (Pongo abelii) "play the odds": Information-seeking strategies in relation to cost, risk, and benefit. [References]
A1 - Marsh,Heidi L.
Y1 - 2012///
N1 - Peer Reviewed Journal: 2011-24369-001
English
Journal; Peer Reviewed Journal
KW - PsycInfo
KW - Information Seeking
KW - Metacognition
KW - Primates (Nonhuman)
KW - Animal Cognition
RP - NOT IN FILE
SP - 263
EP - 278
JF - Journal of Comparative Psychology
JA - J Comp Psychol.
VL - 126:
IS - 3
N2 - Recent research has examined whether animals possess metacognition, or the ability to monitor their knowledge states. However, the extent to which animals actively control their knowledge states is still not well delineated. Although organisms might be capable of seeking information when it is lacking, it does not mean that it is always adaptive to do so. In the present set of experiments, we examined the flexibility of this behavior in captive orangutans (Pongo abelii; two adults and one juvenile) in a foraging task, by varying the necessity of information-seeking, the cost associated with it, the likelihood of error, and the value of the reward. In Experiment 1, subjects searched for information most often when it was "cheapest" energetically. In Experiment 2, subjects searched for information most often when the odds of making an error were the greatest. In Experiment 3, subjects searched for information more when the reward was doubled in value. In Experiment 4, adult subjects adapted to risk/benefit trade-offs in their searching behavior. In every experiment, subjects sought information more often when they needed it than when they already knew the solution to the problem. Therefore, the current research suggests that information-seeking behavior in orangutans shows a sophisticated level of flexibility, comparable to that seen in human children, as they appear to "play the odds" when making

the decision to seek information or not. (PsycINFO Database Record (c) 2012 APA, all rights reserved) (journal abstract)
SN - 0735-7036
AD - Marsh, Heidi L.: Department of Psychology, York University, Toronto, ON, Canada MacDonald, Suzanne E.: Department of Psychology, York University, Toronto, ON, Canada
ER -

TY - JOUR
ID - 559
T1 - Parents as HIV/AIDS educators. [References]
A1 - Krauss,Beatrice J.
Y1 - 2012///
N1 - Book: 2012-15887-004
English
Book; Edited Book
KW - PsycInfo
KW - AIDS Prevention
KW - Education
KW - HIV
KW - Parent Training
KW - Parents
KW - AIDS
KW - Caregivers
KW - Evidence Based Practice
KW - Intervention
KW - Parent Child Communication
RP - NOT IN FILE
SP - 97
EP - 120
JF - Pequegnat, Willo [Ed]; Bell, Carl C [Ed]
VL - :
IS - 2012
N2 - (from the chapter) Parents and caregivers play a special role in HIV prevention efforts for youth. Parents are able to reach youth early and in a non-controversial way. Parents can engage in continuous discussions about sex and sexuality, HIV, substance use, and sexual risk prevention. Having frequent contact with their children allows them to provide sequential and time-sensitive information that is immediately responsive to the child's questions and anticipated needs. Parents and caregivers help youth shape and form healthy attitudes and behaviors, and support youth with supervision, positive reinforcement and skills building. Given the proper tools to harness their parenting and communication skills, parents and caregivers are a force to be reckoned with. There is a growing literature that highlights the important role parents and caregivers play in addressing teen substance use and sexual risk behavior; however, evidence-based interventions to strengthen parents' role in HIV prevention or even in reproductive health promotion are rare and not widely disseminated. This chapter describes two evidence-based interventions. Parents Matter! and the Parent/Preadolescent Training for HIV (PATH) Prevention. Both are based on research addressing the need to intervene early, child-parent communication, and risk reduction science and strategies. Data on outcomes and description of the dissemination of these interventions are presented. Among the intriguing findings are that both projects were easily accepted by communities, both led to reported risk reduction or intention to reduce risk, and that improved communication may have generalized to create positive outcomes for risks other than those associated with HIV. Each intervention has found new audiences, through formal and informal pathways. A continuing challenge is to maintain and update interventions as new risks emerge and as new populations are at risk as the HIV epidemic changes. (PsycINFO Database Record (c) 2012 APA, all rights reserved)
AD - Krauss, Beatrice J.: City University of New York, School of Public Health, Hunter College, New York, NY, US Miller, Kim S.: Centers for Disease Control and Prevention, Center for Global Health, Division of Global HIV/AIDS Prevention, US
ER -

TY - JOUR
ID - 560
T1 - The development of self-representations across the life span. [References]
A1 - Diehl,Manfred
Y1 - 2011///
N1 - Book: 2011-03366-024
English
Book; Edited Book
KW - PsycInfo
KW - Developmental Psychology
KW - Life Span
KW - Adaptive Behavior
KW - Cognitive Development
KW - Culture (Anthropological)
KW - Life Expectancy
KW - Meaning
KW - Psychosocial Development
KW - Psychosocial Factors
KW - Resilience (Psychological)
KW - Self Concept
KW - Self Regulation
RP - NOT IN FILE
SP - 611
EP - 646
JF - Fingerman, Karen L [Ed]; Berg, Cynthia A [Ed]; Smith, Jacqui [Ed]; Antonucci, Toni C [Ed]
VL - :
IS - 2011
N2 - (from the chapter) This chapter focuses on the development of self-representations across the life span. The main questions addressed in this chapter are as follows: How do individuals develop an understanding of their own person and what are the developmental milestones in this process? How does the content and structural organization of self-representations change across the life span? How is individuals' development of self-representations linked to basic developmental processes in cognitive and social-emotional functioning? Are there qualitative differences in the organization and the functions of self-representations at different parts of the life span? What role do different social and cultural contexts play in the development of self-representations across the life span? These questions will be addressed within the meta-theoretical framework of life-span developmental psychology and from a perspective that conceives individuals as producers of their own development. These perspectives are chosen as guiding frameworks for several reasons. First, self-representations constitute what James defined as the self-concept, the Me-self, the self as object, or the self as known. Second, since the mid-1980s theory and research have conceptualized the self concept as a contextualized and dynamic knowledge structure with adaptive and self-regulatory functions. Third, life-span psychologists have emphasized that the self-concept gives individuals a sense of continuity and permanence, allows them to distinguish themselves and their developmental history from others, and gives their experiences meaning within a larger biography. For purposes of terminological clarity, it is important to provide a working definition of the key construct. Specifically, in this chapter the terms self-representations and self-concept are used interchangeably to refer to those attributes that are (1) part of a person's self-understanding and self-knowledge; (2) the focus of self-awareness and self-reflection; and (3) consciously acknowledged by the person through language or other means of communication. This chapter has three major parts. The first major part focuses on the development of self-representations in childhood and adolescence. Specifically, this section describes the developmental milestones from early childhood to late adolescence, the part of the life span for which the greatest amount of research exists. The second major part addresses the development of self-representations across the adult years, describing findings that cover the life span from early adulthood to late life. The third major section focuses on several key issues in the study of self-representations across the life span. These issues address to what extent and under what circumstances self-representations may serve as risk or resilience factors, what role social and cultural contexts play vis-a-vis self-representations, and whether psychosocial interventions can optimize the adaptive function of self-representations. This section also identifies

the major gaps in our current knowledge and outlines future directions for the study of self-representations. (PsycINFO Database Record (c) 2012 APA, all rights reserved)
AD - Diehl, Manfred: Department of Human Development and Family Studies, Colorado State University, Fort Collins, CO, US Youngblade, Lise M.: Department of Human Development and Family Studies, Colorado State University, Fort Collins, CO, US Hay, Elizabeth L.: Department of Human Development and Family Studies, Colorado State University, Fort Collins, CO, US Chui, Helena: Department of Human Development and Family Studies, Colorado State University, Fort Collins, CO, US
ER -

TY - JOUR
ID - 561
T1 - Sociodramatic play, family socioeconomic risk, and emotional competence in urban, preschool children
A1 - Muljat Bailey,Alesha
Y1 - 2012///
N1 - Dissertation Abstract: 2012-99100-550
English
Dissertation Abstract
KW - PsycInfo
KW - Competence
KW - Family
KW - Preschool Students
KW - Socioeconomic Status
KW - Urban Environments
KW - Literacy
KW - Peers
KW - Pretend Play
KW - Risk Factors
KW - Social Skills
RP - NOT IN FILE
SP - 7092
JF - Dissertation Abstracts International: Section B: The Sciences and Engineering
VL - 72:
IS - 11-B
N2 - Children's sociodramatic play (also known as make-believe or pretend play with peers) is positively related to numerous aspects of their cognitive and social competence, however, few studies have investigated its role in emotional competence. Family socioeconomic risk is associated with greater overall socioemotional and behavioral problems yet few studies have examined its relation to specific aspects of emotional competence. Further, the relation between family risk and sociodramatic play is equivocal. The present study investigated relations among children's frequency of sociodramatic play, children's emotional competence (emotional understanding and empathy), and family risk. Data were collected from 81 children, aged 4 to 5 years, and their primary parent/guardian and teacher. Participants were recruited from preschool classrooms in the greater Pacific Northwest serving families from diverse socioeconomic and ethnic backgrounds. Evidence was found for significant relations between children's sociodramatic play and emotional competence, as well as family risk and emotional competence. Children who engaged in more sociodramatic play had higher levels of anger attribution bias but not higher levels of emotion knowledge or empathy. Children with higher family risk had lower levels of emotion knowledge. No evidence was found for the relation between family risk and sociodramatic play. Further, the hypothesis that family risk would moderate the relation between sociodramatic play and emotional competence was partially supported. Family risk moderated the relation between sociodramatic play and anger attribution bias and showed a trend for moderating the relation between risk and emotion knowledge. Children with high levels of both family risk and sociodramatic play had higher levels of anger bias and lower levels of overall emotion knowledge than children with high risk and low levels of sociodramatic play. Conversely, the emotion knowledge of children with low levels of family risk was higher when they engaged in more sociodramatic play. These results suggest the benefits of sociodramatic play may differ for children from different risk backgrounds. There may be important qualitative differences in the play of children with varying risk levels. Future research should explicate these relations further and examine in more

detail the qualitative nature of play of children from different risk backgrounds. (PsycINFO Database Record (c) 2012 APA, all rights reserved)
SN - 0419-4217
AD - Muljat Bailey, Alesha: Seattle Pacific U., US
ER -

TY - JOUR
ID - 562
T1 - Adolescent motherhood and developmental outcomes of children in early Head Start: The influence of maternal parenting behaviors, well-being, and risk factors within the family setting. [References]
A1 - Rafferty,Yvonne
Y1 - 2011///
N1 - Peer Reviewed Journal: 2012-11012-011
English
Journal; Peer Reviewed Journal
KW - PsycInfo
KW - Adolescent Mothers
KW - Developmental Stages
KW - Mother Child Relations
KW - Risk Factors
KW - Well Being
KW - Cognitive Ability
KW - Family
KW - Language
KW - Lower Income Level
KW - Project Head Start
KW - Parenting
RP - NOT IN FILE
SP - 228
EP - 245
JF - American Journal of Orthopsychiatry
JA - Am J Orthopsychiatry
VL - 81:
IS - 2
N2 - This longitudinal study examined the influence of parenting behaviors, well-being, and risk factors of low-income adolescent mothers on the cognitive and language abilities of children from infancy to age 3. Participants consisted of 1,240 mother-child dyads enrolled in the Early Head Start Research and Evaluation Project. Data were collected using structured interviews with the mothers and from videotaped mother-child interactions during play activities when children were approximately 14 months old and again at 36 months of age. Positive parenting behaviors exhibited toward the 14-month-old children predicted gains in both cognitive and language abilities more so than did maternal well-being, risk factors within the family setting, and demographic risk factors. Gains in cognitive abilities from infancy to age 3 were predicted by supportive parenting, higher family resources, and lower family conflict when children were infants. Gains in language abilities were predicted by supportive parenting, support for language and learning in the home environment, and higher family resources when children were infants. Finally, path analyses showed that maternal age had an indirect effect on child cognitive and language abilities at age 3 through effects on parenting behaviors. Older mothers were more likely to be supportive during play at age 14 months, which in turn promoted enhanced developmental outcomes at age 3. Implications for intervention and future research are discussed. (PsycINFO Database Record (c) 2012 APA, all rights reserved) (journal abstract)
SN - 0002-9432
AD - Rafferty, Yvonne: Pace University, New York, NY, US Griffin, Kenneth W.: Cornell University, Ithaca, NY, US Lodise, Michelle: Pace University, New York, NY, US
ER -

TY - JOUR
ID - 564

T1 - Directive and responsive behaviors of mothers in familiar and unfamiliar toy play with and without a single message voice output communication aid
A1 - Medeiros,Kara F.
Y1 - 2012///
N1 - Dissertation Abstract: 2012-99090-100
English
Dissertation Abstract
KW - PsycInfo
KW - Dyads
KW - Mother Child Communication
KW - Mother Child Relations
KW - Toys
KW - Messages
KW - Mothers
RP - NOT IN FILE
SP - 4000
JF - Dissertation Abstracts International Section A: Humanities and Social Sciences
VL - 72:
IS - 11-A
N2 - Twenty-five mother-child dyads participated in three types of toy play both with and without a BIGmack, a single-message voice-output communication aid (VOCA). Participants were mothers of children who were considered at-risk for being nonspeaking. A quantitative within-group research design was used for this project comparing maternal directive and responsive behaviors across toy play activities. The mothers were asked to play with their children using three types of toys: a) familiar, b) unfamiliar, and c) complex toys, both with and without a BIGmack VOCA that had been programmed to say "more" when pressed. Dyads participated in two experimental sessions in their home. During the first home visit, dyads were asked to "play and have fun" and asked to play with all three types of toys without the BIGmack, allowing for collection of baseline data of responsive and directive maternal behaviors in each type of toy play. At the next home visit, children and mothers were separated within the house and then children were taught to activate the BIGmack to request "more" in play. Children and mothers were then reunited and again asked to "play and have fun" with the same three types of toys with the BIGmack present. The dyad's play was recorded for a target of 10 minutes for each type of toy, and the mother's interactions using each type of toy were coded for responsive and directive behaviors. Rate-per-minute of maternal directive and responsive behaviors was analyzed using a repeated measures analysis of variance (ANOVA). Results indicated that mothers used significantly more responsive behaviors when playing with their own toys than with unfamiliar or complex toys. (PsycINFO Database Record (c) 2012 APA, all rights reserved)
SN - 0419-4209
AD - Medeiros, Kara F.: U Nebraska - Lincoln, US
ER -

TY - JOUR
ID - 566
T1 - Primary care management of child & adolescent depressive disorders. [References]
A1 - Wren,Frances J.
Y1 - 2012///
N1 - Peer Reviewed Journal: 2012-11600-015
English
Journal; Peer Reviewed Journal
KW - PsycInfo
KW - epidemiology
KW - Health Care Services
KW - Major Depression
KW - Primary Health Care
KW - Risk Factors
RP - NOT IN FILE
SP - 401

EP - 419
JF - Child and Adolescent Psychiatric Clinics of North America
VL - 21:
IS - 2
N2 - Depressive disorders are a problem with profound societal impact, whether measured by prevalence, burden of disability and suffering across the life span, mortality, or financial costs for health care and for society at large. As such, they merit strategic public health intervention. The World Health Organization estimates that 120 million people live with depressive disorders, of whom fewer than 25% receive any treatment. Integrating mental health services into primary care has been cited by World Health Organization as the most viable way of closing the treatment gap in mental health care. With this in mind, we discuss how recent work on the primary care management of mental health and psychosocial problems might be applied to pediatric depressive disorders. The general clinical effectiveness of the collaborative care approach is now viewed as established, at least compared with usual care in the integrated US health care settings, where the dominant model was developed. The task of preparing primary care providers to play an effective role in the management of pediatric depressive disorders is not small. The American Academy of Pediatrics (AAP) has acknowledged that it represents systemic change that will take time and involve transformational changes in pediatric primary care practice, requiring new knowledge and skills, payment structures, collaborative relationships, office systems, and resources. Less overtly acknowledged is that this will involve complementary changes in child mental health practice. Pediatric primary care providers have the potential to play powerful roles in reducing the burden of depression in further areas, outside the scope of this paper. We have mentioned the potential for using the longitudinal and early established relationship with the primary care clinician to identify and intervene early with conditions that are risk factors for later depressive disorders. In addition, primary care clinicians often see new mothers more than any other professional during the first year of a child's life. Finally, there are no data on how primary care clinicians will actually use the newly developed tools and on what impact any such changes in practice will have on the likelihood that depressed youth will receive treatment, on the types and adequacy of treatment provided and on clinical or cost outcomes. Dissemination must be accompanied by evaluation. (PsycINFO Database Record (c) 2012 APA, all rights reserved)
SN - 1056-4993
AD - Wren, Frances J.: Department of Psychiatry and Behavioral Sciences, Stanford University School of Medicine, Stanford, CA, US Foy, Jane Meschan: Department of Pediatrics, Wake Forest University School of Medicine, Medical Center Boulevard, Winston-Salem, NC, US Ibeziako, Patricia I.: Harvard Medical School, Boston, MA, US
ER -

TY - JOUR
ID - 568
T1 - Focused attention, heart rate deceleration, and cognitive development in preterm and full-term infants. [References]
A1 - Thomas,Julianne H.
Y1 - 2012///
N1 - Peer Reviewed Journal: 2012-09177-003
English
Journal; Peer Reviewed Journal
KW - PsycInfo
KW - Attention
KW - Cognitive Development
KW - Heart Rate
KW - Infant Development
KW - Premature Birth
RP - NOT IN FILE
SP - 383
EP - 400
JF - Developmental Psychobiology
VL - 54:
IS - 4

N2 - The majority of children who are born very preterm escape major impairment, yet more subtle cognitive and attention problems are very common in this population. Previous research has linked infant focused attention during exploratory play to later cognition in children born full-term and preterm. Infant focused attention can be indexed by sustained decreases in heart rate (HR). However there are no preterm studies that have jointly examined infant behavioral attention and concurrent HR response during exploratory play in relation to developing cognition. We recruited preterm infants free from neonatal conditions associated with major adverse outcomes, and further excluded infants with developmental delay (Bayley Mental Development Index [MDI < 70]) at 8 months corrected age (CA). During infant exploratory play at 8 months CA, focused attention and concurrent HR response were compared in 83 preterm infants (born 23-32 weeks gestational age [GA]) who escaped major impairment to 46 fullterm infants. Focused attention and HR response were then examined in relation to Bayley MDI, after adjusting for neonatal risk. MDI did not differ by group, yet full-term infants displayed higher global focused attention ratings. Among the extremely preterm infants born < 29 weeks, fewer days on mechanical ventilation, mean longest focus, and greater HR deceleration during focused attention episodes, accounted for 49% of adjusted variance in predicting concurrent MDI. There were no significant associations for later-born gestational age (29-32 weeks) or full-term infants. Among extremely preterm infants who escape major impairment, our findings suggest unique relationships between focused attention, HR deceleration, and developing cognition. (PsycINFO Database Record (c) 2012 APA, all rights reserved) (journal abstract)
SN - 0012-1630
AD - Thomas, Julianne H. Petrie: Neonatal Follow-Up Program, Children's and Women's Health Centre of British Columbia, Vancouver, BC, Canada Whitfield, Michael F.: Neonatal Follow-Up Program, Children's and Women's Health Centre of British Columbia, Vancouver, BC, Canada Oberlander, Tim F.: Neonatal Follow-Up Program, Children's and Women's Health Centre of British Columbia, Vancouver, BC, Canada Synnes, Anne R.: Neonatal Follow-Up Program, Children's and Women's Health Centre of British Columbia, Vancouver, BC, Canada Grunau, Ruth E.: Neonatal Follow-Up Program, Children's and Women's Health Centre of British Columbia, Vancouver, BC, Canada
ER -

TY - JOUR
ID - 569
T1 - Restorative magical adventure or warcrack? Motivated MMO play and the pleasures and perils of online experience. [References]
A1 - Snodgrass,Jeffrey G.
Y1 - 2012///
N1 - Peer Reviewed Journal: 2012-08438-001
English
Journal; Peer Reviewed Journal
KW - PsycInfo
KW - Computer Games
KW - Experiences (Events)
KW - Internet
KW - Motivation
KW - Online Social Networks
KW - Addiction
RP - NOT IN FILE
SP - 3
EP - 28
JF - Games and Culture: A Journal of Interactive Media
VL - 7:
IS - 1
N2 - Combining perspectives from the new science of happiness with discussions regarding "problematic" and "addictive" play in multiplayer online games, the authors examine how player motivations pattern both positive and negative gaming experiences. Specifically, using ethnographic interviews and a survey, the authors explore the utility of Yee's three-factor motivational framework for explaining the positive or negative quality of experiences in the popular online game World of War-craft (2004-2012). The authors find that playing to Achieve is strongly associated with distressful play, results that support findings from other studies. By contrast,

Social and Immersion play lead more typically to positive gaming experiences, conclusions diverging from those frequently reported in the literature. Overall, the authors suggest that paying attention to the positive as well as negative dimensions of inhabiting these online worlds will provide both for more balanced portraits of gamers' experiences and also potentially clarify pathways toward problematic and addictive play. (PsycINFO Database Record (c) 2012 APA, all rights reserved) (journal abstract)
SN - 1555-4120
AD - Snodgrass, Jeffrey G.: Colorado State University, Fort Collins, CO, US Dengah, H. J. Francois: University of Alabama, Tuscaloosa, AL, US Lacy, Michael G.: Colorado State University, Fort Collins, CO, US Fagan, Jesse: Colorado State University, Fort Collins, CO, US Most, David: Colorado State University, Fort Collins, CO, US Blank, Michael: Colorado State University, Fort Collins, CO, US Howard, Lahoma: Colorado State University, Fort Collins, CO, US Kershner, Chad R.: Colorado State University, Fort Collins, CO, US Krambeer, Gregory: Colorado State University, Fort Collins, CO, US Leavitt-Reynolds, Alissa: Colorado State University, Fort Collins, CO, US Reynolds, Adam: Colorado State University, Fort Collins, CO, US Vyvial-Larson, Jessica: Colorado State University, Fort Collins, CO, US Whaley, Josh: Colorado State University, Fort Collins, CO, US Wintersteen, Benjamin: Colorado State University, Fort Collins, CO, US
ER -

TY - JOUR
ID - 571
T1 - Restrictive safety or unsafe freedom? Norwegian ECEC practitioners' perceptions and practices concerning children's risky play. [References]
A1 - Sandseter,Ellen Beate Hansen
Y1 - 2012///
N1 - Peer Reviewed Journal: 2011-28752-006
English
Journal; Peer Reviewed Journal
KW - PsycInfo
KW - Child Care
KW - Childhood Play Behavior
KW - Professional Personnel
KW - Risk Taking
KW - Safety
KW - Education
RP - NOT IN FILE
SP - 83
EP - 101
JF - Child Care in Practice
VL - 18:
IS - 1
N2 - Many countries face a growing debate on the balance between ensuring children's safety and allowing children to play in physically and emotionally stimulating and challenging environments. This study is theoretically and conceptually situated within this debate, focusing on how early childhood education and care (ECEC) practitioners perceive the risk in children's play, and how they handle children's physical risk-taking given their dual responsibilities to keep children safe and to provide enough developmental challenges. Qualitative semi-structured interviews were conducted with seven ECEC practitioners with the goal of gaining insight into their opinions and reflections on children's risky play in kindergarten. The results show that Norwegian ECEC practitioners assess children's opportunities to engage in risky play in kindergarten as good: play environments provide opportunities for risky play, and practitioners allow and encourage children's risky play. The practitioners have a positive attitude toward risky play and state that they think it is important for children's development. The interviews show that the strategies for handling risky play are adapted instinctively in the play situation, considering each child individually, and common boundaries or rules are rare. The results of this study are a contribution to the discussion on children's play safety and how to handle risk-taking play in ECEC settings, and would hopefully be inspiring to ECEC practitioners and politicians in countries other than Norway. (PsycINFO Database Record (c) 2012 APA, all rights reserved) (journal abstract)
SN - 1357-5279

AD - Sandseter, Ellen Beate Hansen: Queen Maud University College of Early Childhood Education, Trondheim, Norway
ER -

TY - JOUR
ID - 572
T1 - Childcare practices among construction workers in Chira Chas, Jharkhand. [References]
A1 - Dhar,Rajib
Y1 - 2012///
N1 - Peer Reviewed Journal: 2011-28752-003
English
Journal; Peer Reviewed Journal
KW - PsycInfo
KW - Blue Collar Workers
KW - Child Care
KW - Lower Income Level
KW - Parents
RP - NOT IN FILE
SP - 23
EP - 50
JF - Child Care in Practice
VL - 18:
IS - 1
N2 - This study aims at examining the childcare practices and issues experienced by the low-income construction workers in India. It is concerned with understanding varied aspects relating to problems that construction workers, as parents, face while bringing up their children in one of the small construction companies of eastern India, in the state of Jharkhand. Qualitative methods have been used in order to collect the data based upon phenomenological principles. Ethnography and photo-elicitation were used as a primary method of data collection. Apart from this, in-depth interviews were also conducted with the workers of the construction company. Discussion with the participants led to the emergence of four themes: children left alone; playing at risky site areas; poor health of children; and children working along with parents. The research findings indicate that infants are taken by their parents to their place of work whereas children between five and 10 years had to be left alone at home. It was also found that acute poverty was a major cause leading to the consumption of inadequate and low nutritional food, leading to poor health of the children. Based on the study findings, there is an imperative that societal forerunners and philanthropists continue to use research findings to understand the childcare practices amongst the low-income workers and draft strategies accordingly, to improve the situation. (PsycINFO Database Record (c) 2012 APA, all rights reserved) (journal abstract)
SN - 1357-5279
AD - Dhar, Rajib: Symbiosis International University, India
ER -

TY - JOUR
ID - 574
T1 - Reviews of Risk and adventure in early years outdoor play: Learning from forest schools and Forest schools for all. [References]
A1 - Huggins,Valerie
Y1 - 2012///
N1 - Peer Reviewed Journal: 2012-03732-009
English
Journal; Peer Reviewed Journal
KW - PsycInfo
KW - Childhood Play Behavior
KW - Education
KW - Learning
KW - Schools
KW - Wilderness Experience

RP - NOT IN FILE
SP - 99
EP - 100
JF - Early Years: An International Journal of Research and Development
VL - 32:
IS - 1
N2 - Reviews the books, Risk and adventure in early years outdoor play: Learning from forest schools by Sara Knight (2011) and Forest schools for all by by Sara Knight (2011). The first book draws on the author's experience as a lead forest school practitioner to argue the necessity for young children to have adventurous, challenging outdoor activities, whilst recognizing that many early years workers lack expertise in this area and are nervous about providing what they see as dangerous play activities outdoors. So, the focus of the book is reassuringly practical, and discussion about the management of any risks involved is prominent throughout. The book has considerable strengths as a resource for early years practitioners, and the staff of a setting could well use it to support the systematic development of their outdoor provision, discussing and using the activities and points for practice incorporated into each chapter. The second book extends the focus in a most interesting and helpful way by analyzing how forest schools in the UK have developed and shows how, through the forest schools for all approach, their innovative provision and practice in outdoor learning are being adopted within later stages of education. So, in both books the focus is very practical. (PsycINFO Database Record (c) 2012 APA, all rights reserved)
SN - 0957-5146
AD - Huggins, Valerie: Plymouth University, Plymouth, United Kingdom
ER -

TY - JOUR
ID - 576
T1 - Participation in play activities of children with cerebral palsy
A1 - Malkawi,Somaya H.
Y1 - 2011///
N1 - Dissertation Abstract: 2011-99220-040
English
Dissertation Abstract
KW - PsycInfo
KW - ABILITY
KW - Cerebral Palsy
KW - Childhood Play Behavior
KW - Motor Processes
KW - At Risk Populations
RP - NOT IN FILE
SP - 2715
JF - Dissertation Abstracts International: Section B: The Sciences and Engineering
VL - 72:
IS - 5-B
N2 - The aim of this study was to describe the overall play participation of children with cerebral palsy (CP) aged 9-13 years by measuring what activities they participate in using the Preteen Play Profile (Henry, 2000); and to explore variations in participation in different play categories based on each child's manual ability, gross motor function, CP distribution, age and gender. A mail-based survey was conducted of children with CP from three facilities in the United States. Of 252 surveys mailed, 15 were undeliverable because of the wrong addresses and 93 completed surveys were returned (39.2%). The children (58 males, 35 females) were aged between 9 and 13 years (mean age 11 years 1 month, SD= 1.06). Findings showed that children with CP participated in a wide range of activities in the past year. The majority of play activities were indoor rather than outdoor, sedentary rather than active, and lack structure. Watching television and listening to music was by far the children's major play activity. Play categories were ranked from highest to lowest in play participation as follows: indoor, creative, social, summer, outdoor, winter, sports and lessons. It was found that children with quadriplegia who are in Levels IV and V on both the MACS and GMFCS are the most at risk groups of decreased play participation. The children's manual ability and gross motor function were the main predictors of play participation indicating the need for using both classification systems in practice and research. Children of

both genders participated in similar play activities but when analyzing separate play categories, females achieved significantly higher scores than males in creative, lessons and social activities. The results of this study pointed to the need for integration of more outdoor, sports, and social play into the daily routines and curricula of centers, and schools that serve children with CP. The study concluded with some recommendations on how deficiencies and gaps in play participation can be filled through several proactive approaches that pediatric occupational therapists can take in conjunction with caregivers and community members to improve the lives of children with CP. (PsycINFO Database Record (c) 2012 APA, all rights reserved)
SN - 0419-4217
AD - Malkawi, Somaya H.: U Kentucky, US
ER -

TY - JOUR
ID - 577
T1 - Young children and educators engagement and learning outdoors: A basis for rights-based programming. [References]
A1 - Blanchet-Cohen,Natasha; Elliot,Enid
Y1 - 2011///
N1 - Peer Reviewed Journal: 2011-23994-006
English
Journal; Peer Reviewed Journal
KW - PsycInfo
KW - Early Childhood Development
KW - Learning Environment
KW - Student Engagement
KW - Teachers
RP - NOT IN FILE
SP - 757
EP - 777
JF - Early Education and Development
VL - 22:
IS - 5
N2 - Research Findings: This article reports on a study undertaken with 4 early childhood programs in a medium-size city in Canada investigating young children's and educators' perspectives on engagement and learning possibilities outdoors. A rights-based methodology including participant observations and interactive activities with children as well as focus groups and discussion groups with educators reveals the diversity and richness of young children's learning opportunities in the natural outdoor space. Educators also talk about forming more egalitarian and fulfilling relationships with children in outdoor activities. The value educators placed on play in natural spaces led to the creation of opportunities for play outside and motivated educators to support children's interactions outdoors by mediating policy and societal fear of the risk of outdoor play. Practice or Policy: The results of the study highlight the value of a learning community for early childhood educators so that they might support children's full use of outdoor space and the critical role of adult allies in advocating for rights-based programming. (PsycINFO Database Record (c) 2012 APA, all rights reserved) (journal abstract)
SN - 1040-9289
AD - Blanchet-Cohen, Natasha: Department of Applied Human Sciences, Concordia University, Montreal, PQ, Canada Elliot, Enid: School of Child and Youth Care, University of Victoria, Victoria, BC, Canada
ER -

TY - JOUR
ID - 579
T1 - Exploring the effects of a social communication intervention on the interactive play of young at-risk children
A1 - Denning,Christopher B.
Y1 - 2011///

N1 - Dissertation Abstract: 2011-99230-165
English
Dissertation Abstract
KW - PsycInfo
KW - At Risk Populations
KW - Childhood Development
KW - Communication Skills Training
KW - School Based Intervention
RP - NOT IN FILE
SP - 1958
JF - Dissertation Abstracts International Section A: Humanities and Social Sciences
VL - 72:
IS - 6-A
N2 - The purpose of this study was to provide a retrospective analysis of data collected during a series of single subject design studies of a social communication intervention targeting skills such as initiations, responses, turn-takes, and name use (Stanton-Chapman, Denning, & Jamison, 2008; Stanton-Chapman, Denning, & Jamison, in review; Stanton-Chapman, Jamison, & Denning, 2008). The present study investigated the children.s interactive play exhibited throughout a 10-minute period of free-play during a social communication intervention that consisted of five baseline and fifteen intervention sessions. Additionally, the play behaviors of the children were examined using percent non-overlapping data (PND) and multilevel modeling (MLM). To achieve these purposes the following research questions were addressed: (a) whether solitary play (e.g., solitary, onlooker, parallel) and interactive play differed across measurement occasions (e.g., baseline, intervention)? And whether children differed in their response to the intervention based upon presence of an identified disability? The children in the study were preschool students (n = 34) enrolled in Head Start and collaborative classrooms and kindergarten students. Their ages ranged from 44 months to 70 months. Participants had poor language skills, poor social skills, problem behavior, or all of the above. The results indicated that children's parallel play decreased and their interactive play increased as a result of the intervention based upon PND values and MLM analysis. In addition, children with SLI had better outcomes than other children in the study, and children with DD had poorer outcomes than other children in the study. Males and all male dyads appeared to outperform females and all female dyads, and mixed dyads appeared to have the poorest results based upon PND and visual analysis. Implications from the present study include the following. When planning centers in preschool classrooms teachers may need to consider the purpose of the center and how children respond to different themes. Second, the present study highlighted that teachers should attend to the gender pairings of children during a social skill intervention. Future research needs to examine whether the effects of treatment diminish over time. Lastly, additional research is needed to understand how different groups respond to social communication interventions. (PsycINFO Database Record (c) 2012 APA, all rights reserved)
SN - 0419-4209
AD - Denning, Christopher B.: U Virginia, US
ER -

TY - JOUR
ID - 580
T1 - Football pitches and Barbie dolls: Young children's perceptions of their school playground. [References]
A1 - Pearce,Gemma
Y1 - 2011///
N1 - Peer Reviewed Journal: 2011-25781-006
English
Journal; Peer Reviewed Journal
KW - PsycInfo
KW - Childhood Development
KW - Childhood Play Behavior
KW - Playgrounds
KW - Football
RP - NOT IN FILE
SP - 1361
EP - 1379

JF - Early Child Development and Care
VL - 181:
IS - 10
N2 - Playgrounds and play times offer valuable contexts for children to explore and learn about themselves and their social lives. This study sought to gather evidence of children's perceptions of their playgrounds and play times, specifically whether the playgrounds were seen positively or negatively and the types of activities in which they engaged. Child-oriented methods were adopted inspired by the Mosaic approach. The main themes from the focus group interviews were social play (friendship, loneliness/solitude and fair play); physical activity play (activities and rationale); risk (injuries and bullying); and gender (action/stillness and gendered roles). Research suggests that segregation declines when adult supervision supports shared play, and our experiences suggest that such intervention is the most likely solution to the 'problem' of gender play in this setting. (PsycINFO Database Record (c) 2012 APA, all rights reserved) (journal abstract)
SN - 0300-4430
AD - Pearce, Gemma: School of Sport and Exercise Sciences, University of Birmingham, Birmingham, United Kingdom Bailey, Richard P.: RBES Ltd., Birmingham, United Kingdom
ER -

TY - JOUR
ID - 581
T1 - Using a framework to explore associations between parental substance use and the health outcomes of their adolescent children. [References]
A1 - Francis,Shelley A.
Y1 - 2011///
N1 - Peer Reviewed Journal: 2010-26854-001
English
Journal; Peer Reviewed Journal
KW - PsycInfo
KW - Adolescent Development
KW - Drug Abuse
KW - Parental Characteristics
KW - Risk Taking
KW - Health
KW - Sexual Risk Taking
RP - NOT IN FILE
SP - 1
EP - 14
JF - Journal of Child & Adolescent Substance Abuse
VL - 20:
IS - 1
N2 - Risk-taking behavior plays a significant role in the lives of adolescents. Adolescents engaging in risk behaviors such as substance use and risky sexual activity are at increased risk for contracting STDs, unplanned pregnancy, and other health problems. Consequently, children of substance abusers are at even greater risk for engaging in maladaptive behaviors given their social environment and the potential exposure to parental substance use/abuse. Although the empirical literature on adolescent risk-taking behavior is vast, less empirical research has examined how parental substance use is associated with the health outcomes of adolescent children of substance abusers, especially children of parents who use illicit substances. This study proposes a framework to synthesize the research on the health outcomes of adolescent children of substance abusers and provides recommendations for future study. (PsycINFO Database Record (c) 2012 APA, all rights reserved) (journal abstract)
SN - 1067-828X
AD - Francis, Shelley A.: Ohio State University, Columbus, OH, US
ER -

TY - JOUR
ID - 582
T1 - Children's choices and strategies in video games. [References]

A1 - Hamlen,Karla R.
Y1 - 2011///
N1 - Peer Reviewed Journal: 2010-24267-059
English
Journal; Peer Reviewed Journal
KW - PsycInfo
KW - Computer Games
KW - Decision Making
KW - Learning Environment
KW - Schools
RP - NOT IN FILE
SP - 532
EP - 539
JF - Computers in Human Behavior
VL - 27:
IS - 1
N2 - It is important to develop an understanding of children's engagement and choices in learning experiences outside of school as this has implications for their development and orientations to other learning environments. This mixed-methods study examines relationships between the genres of video games children choose to play and the learning strategies they employ to improve at these games. It also explores students' motivations for playing the games they choose to play. One hundred eighteen fourth- and fifth-grade students participated in this study. Qualitative analyses of student responses resulted in a model for classifying motivation for game choices. Children primarily cite reasons that can be classified as psychological or cognitive reasons for choosing to play certain video games, and are motivated by the challenge and thinking required in the games. Analyses using Chi-square tests of association demonstrated significant relationships between video game genre and learning strategy used for two of the six learning strategies ($p < .05$). Children playing action games are more likely to use repetition to learn the game and children playing adventure games are more likely to use their imaginations to take on the role of the character in the game and think the way the character would to make decisions in the game. There were also several gender differences in learning preferences. (PsycINFO Database Record (c) 2012 APA, all rights reserved) (journal abstract)
SN - 0747-5632
AD - Hamlen, Karla R.: Department of Curriculum & Foundations, Cleveland State University, Cleveland, OH, US
ER -

TY - JOUR
ID - 583
T1 - Measuring safety levels in playgrounds using environment assessment scales: The issue of playground safety in Greece. [References]
A1 - Botsoglou,Kafenia; Hrisikou,Spyridoula; Kakana,Domna Mika
Y1 - 2011///
N1 - Peer Reviewed Journal: 2011-10247-003
English
Journal; Peer Reviewed Journal
KW - PsycInfo
KW - Playgrounds
KW - Safety
KW - Risk Assessment
RP - NOT IN FILE
SP - 749
EP - 760
JF - Early Child Development and Care
VL - 181:
IS - 6
N2 - Playgrounds beget an unrivalled context which, through play activity, can foster children's growth. The foremost function of all playgrounds is to provide for safety. In the present study, our primary focus is to

determine the degree of adequacy as far as playground equipment is concerned, including estimates of imminent dangers and the level of compliance with the Greek safety standards. Thus, a relevant assessment scale was constructed based on the standards of the US National Program for Playground Safety and the Consumer Product Safety Commission that include all criteria relevant to safety. Our observations were rated as 'adequate' or 'inadequate' according to predetermined characteristics, and they were subsequently recoded into 'yes' or 'no', respectively, stating the existence or the absence of danger imminence, and finally each kind of equipment was measured in numbers. Results show that approximately all of our sample playgrounds failed to meet current specification requirements for playground safety. The practical implications of the present study entail that a plan for the supervision and upkeep of playground quality should be developed at a national level. In this way, playgrounds in Greece would guarantee for a safer context and foster entertainment, learning and general well-being. (PsycINFO Database Record (c) 2012 APA, all rights reserved) (journal abstract)
SN - 0300-4430
AD - Botsoglou, Kafenia: Special Education Department, University of Thessaly, Volos, Greece Hrisikou, Spyridoula: Early Childhood Education Department, University of Thessaly, Volos, Greece Kakana, Domna Mika: Early Childhood Education Department, University of Thessaly, Volos, Greece
ER -

TY - JOUR
ID - 584
T1 - Promoting turn-taking skills in preschool children with disabilities: The effects of a peer-based social communication intervention. [References]
A1 - Stanton-Chapman,Tina L.
Y1 - 2011///
N1 - Peer Reviewed Journal: 2010-25929-001
English
Journal; Peer Reviewed Journal
KW - PsycInfo
KW - Communication Skills Training
KW - Developmental Disabilities
KW - Interpersonal Communication
KW - Peers
KW - Social Interaction
RP - NOT IN FILE
SP - 303
EP - 319
JF - Early Childhood Research Quarterly
VL - 26:
IS - 3
N2 - The current study evaluated the turn-taking skills of preschoolers with disabilities who participated in a social communication intervention that targeted initiations, responses, and turn-taking skills, and taught children to repair and revise and to avoid interruptions and overlaps. Ten children who enrolled in an inclusive at-risk classroom met the inclusion criteria. A multiple baseline design across two dyads replicated across three additional dyads was used to determine the effects of the intervention. The social communication intervention was highly effective for five children, moderately effective for three children, and mildly effective for two children in increasing the rate of initiations with an immediate peer response from baseline to intervention. The social communication intervention was highly effective for one child, moderately effective for three children, mildly effective for two children, and ineffective for four children in improving turn-taking skills. Generalization results showed that nine out of ten participants demonstrated increased peer play following the intervention, increased levels of child-initiated interactions with a positive peer response, and decreased levels of solitary play. Several implications for practice were derived from the findings. By teaching children turn-taking strategies, the quality of social interactions that children have with their peers is likely to improve. The intervention offers a more systematic technique for teaching social communication and play skills than do informal strategies commonly used by teachers. Social validity assessments indicated that teachers found the intervention acceptable and produced important changes in behavior. (PsycINFO Database Record (c) 2012 APA, all rights reserved) (journal abstract)
SN - 0885-2006

AD - Stanton-Chapman, Tina L.: University of Virginia, Charlottesville, VA, US Snell, Martha E.: University of Virginia, Charlottesville, VA, US
ER -

TY - JOUR
ID - 585
T1 - Playing with the elephant in the room: A group play therapy treatment manual for young children of parents with alcoholism
A1 - Shores,Jessica Sunny
Y1 - 2011///
N1 - Dissertation Abstract: 2011-99060-125
English
Dissertation Abstract
KW - PsycInfo
KW - Alcoholism
KW - Group Psychotherapy
KW - Mental Health
KW - Parents
KW - Play Therapy
RP - NOT IN FILE
SP - 5805
JF - Dissertation Abstracts International: Section B: The Sciences and Engineering
VL - 71:
IS - 9-B
N2 - Due to the increase in the large numbers of children growing up in homes with parents who struggle with alcoholism, more attention has recently been given to the physical and mental health of children of alcohol-dependent parents. Even though the substance abuse, family, and child development research have more recently begun to notice and understand the psychopathology of children of parents with alcoholism, there continues to be a lack in evidence based approaches to treating these children's specific problems. An extensive review of the literature reveals that children of parents with alcoholism suffer from a variety of emotional, behavioral, and social problems due to the increased chance of abuse, neglect, and chaos provided by the family environment impacted by alcoholism. Research supports that the family dynamics, behaviors of parents with alcoholism, and the parent-child relationship are all major factors in determining how a child might develop specific characteristics for relating to others and develop particular problematic thought processes, behavioral issues, and difficulty regulating emotion evident at both home and school. Young children of parents living with alcoholism are at risk for developing substance abuse and other unhealthy ways for coping as a result of their chaotic and dysfunctional home environment. These children are observed in schools, extracurricular activities, and home environments as typically insecure, fearful, anxious, sad, aggressive, withdrawn, or overachieving. While these characteristics have been noted throughout the literature, mental health professionals continue to lack specialized treatment programs targeting children affected by alcoholism. The current dissertation offers professionals the opportunity to gain knowledge regarding the psychosocial functioning of children of parents suffering from alcoholism in relation to their family and peer relationships, while also providing information on the small number of treatment modalities described in the literature today. The included manual provides practitioners with a step-by-step group play therapy program designed for young children of parents with alcoholism. The goals of this intervention are: improving social functioning, increasing trust, security, and self-esteem, enhancing coping skills for emotion regulation, and facilitating disclosure of trauma and distress. Additional benefits include decreasing aggression, secrecy, shame, and guilt that may be associated with the family's struggle with alcoholism. (PsycINFO Database Record (c) 2012 APA, all rights reserved)
SN - 0419-4217
AD - Shores, Jessica Sunny: U Hartford, US
ER -

TY - JOUR
ID - 586
T1 - Maternal scaffolding and preterm toddlers' visual-spatial processing and emerging working memory. [References]

A1 - Dilworth-Bart,Janean; Poehlmann,Julie; Hilgendorf,Amy E; Miller,Kyle; Lambert,Heather
Y1 - 2010///
N1 - Peer Reviewed Journal: 2010-03981-014
English
Journal; Peer Reviewed Journal
KW - PsycInfo
KW - Infant Development
KW - Neonatal Period
KW - Premature Birth
KW - Short Term Memory
KW - Visuospatial Ability
KW - At Risk Populations
KW - Mothers
KW - Scaffolding
KW - Socioeconomic Status
RP - NOT IN FILE
SP - 209
EP - 220
JF - Journal of Pediatric Psychology
JA - J Pediatr Psychol.
VL - 35:
IS - 2
N2 - Objective: We examined longitudinal associations among neonatal and socioeconomic risks, maternal scaffolding behaviors, and 24-month visual-spatial processing and working memory in a sample of 73 toddlers born preterm or low birthweight (PT LBW). Methods: Risk data were collected at hospital discharge and dyadic play interactions were observed at 16-months postterm. Abbreviated IQ scores, verbal/nonverbal working memory, and verbal/nonverbal visual-spatial processing data were collected at 24-months postterm. Results: Higher attention scaffolding and lower emotion scaffolding during 16-month play were associated with 24-month verbal working memory scores. A joint significance test revealed that maternal attention and emotion scaffolding during 16-month play mediated the relationship between socioeconomic risk and 24-month verbal working memory. Conclusions: These findings suggest areas for future research and intervention with children born PT LBW who also experience high socioeconomic risk. (PsycINFO Database Record (c) 2013 APA, all rights reserved) (journal abstract)
SN - 0146-8693
AD - Dilworth-Bart, Janean: University of Wisconsin, Madison, WI, US Poehlmann, Julie: University of Wisconsin, Madison, WI, US Hilgendorf, Amy E.: University of Wisconsin, Madison, WI, US Miller, Kyle: University of Wisconsin, Madison, WI, US Lambert, Heather: University of Wisconsin, Madison, WI, US
ER -

TY - JOUR
ID - 589
T1 - Long-term effects of interparental violence and child physical maltreatment experiences on PTSD and behavior problems: A national survey of Taiwanese college students. [References]
A1 - Shen,April Chiung-Tao
Y1 - 2009///
N1 - Peer Reviewed Journal: 2009-06228-004
English
Journal; Peer Reviewed Journal
KW - PsycInfo
KW - Behavior Problems
KW - Child Abuse
KW - Domestic Violence
KW - Physical Abuse
KW - Posttraumatic Stress Disorder
KW - Parents
RP - NOT IN FILE

SP - 148
EP - 160
JF - Child Abuse & Neglect
JA - Child Abuse Negl.
VL - 33:
IS - 3
N2 - Objectives: This study investigated the joint long-term impact of witnessing interparental violence and experiencing child physical maltreatment on young adults' trauma symptoms and behavior problems. It also explored Chinese traditional beliefs as a possible contributor to young adults' trauma and behavior. Methods: This study used self-reporting measures to collect data from a national proportionate stratified sample of 1,924 college students in Taiwan. The sample was divided into four groups: no violence; interparental violence only; child physical maltreatment only and dual violence, to compare the combined effect of dual violence on long-term outcome with the no violence group and the one type of violence group. Results: The results indicated a significant association of interparental violence and child physical maltreatment, and 11.3% of participants reported witnessing partner violence between parents and experiencing physical maltreatment during childhood. Participants experiencing dual violence reported more trauma symptoms and behavior problems than did those experiencing only one form of violence or none at all. Exposure to both interparental violence and child physical maltreatment during childhood is a significant predictor of young adults' trauma symptoms and behavior problems, after controlling for other potentially confounding risk factors. Cultural factors also play a significant role in predicting young adults' trauma symptoms and internalizing behavior problems, after accounting for control variables and violence-related variables. Moreover, cultural factors interact significantly with dual violence experiences in predicting young adults' externalizing behavior problems. Conclusions: This study extended Western co-occurrence study findings with large Taiwanese community samples. The results demonstrated that dual violence experiences during childhood have long-term detrimental impact on young adults' trauma symptoms and behavior problems. Cultural beliefs and their interaction with dual violence experiences play a significant role in young adults' trauma symptoms and behavior problems as well. Practice implications: The present findings underscore the need for interventions for young adults exposed to childhood dual violence. Moreover, the findings highlight the need for culturally sensitive interventions to address the cultural factor impact on young adults' trauma symptoms and behavior problems. (PsycINFO Database Record (c) 2013 APA, all rights reserved) (journal abstract)
SN - 0145-2134
AD - Shen, April Chiung-Tao: National Taiwan University, Department of Social Work, Taipei, Taiwan
ER -

TY - JOUR
ID - 590
T1 - Individual differences in children's risk perception and appraisals in outdoor play environments. [References]
A1 - Little,Helen
Y1 - 2010///
N1 - Peer Reviewed Journal: 2010-26649-004
English
Journal; Peer Reviewed Journal
KW - PsycInfo
KW - Childhood Play Behavior
KW - Individual Differences
KW - Risk Perception
KW - SENSATION seeking
KW - Risk Taking
RP - NOT IN FILE
SP - 297
EP - 313
JA - International Journal of Early Years Education
VL - 18:
IS - 4

N2 - Child characteristics including age, gender, risk-taking behaviour and sensation seeking are thought to influence children's ability to appraise risks. The present study investigated children's risk perceptions and appraisals in the context of common outdoor physical play activities. Risk perceptions and appraisal of four- and five-year olds were assessed using a pictorial risk appraisal measure and through individual semi-structured interviews examining children's decision-making. Sensation seeking and risk-taking behaviour were assessed using self-and parent-report measures. Results revealed that the children were able to identify injury risk behaviours however differentiating the severity of the potential injury was less accurate. Furthermore, the children appeared to use these judgements in their play to inform their behaviour whilst using playground equipment. Whilst they actively sought out those activities that offered challenge and excitement, they were aware of their abilities and showed caution in engaging in activities that were beyond their current capabilities. The results have implications for the provision of calculated risk-taking in outdoor play and for adult responses to the risk-taking behaviour of children. (PsycINFO Database Record (c) 2013 APA, all rights reserved) (journal abstract)
SN - 0966-9760
AD - Little, Helen: Institute of Early Childhood, Macquarie University, Sydney, NSW, Australia Wyver, Shirley: Institute of Early Childhood, Macquarie University, Sydney, NSW, Australia
ER -

TY - JOUR
ID - 591
T1 - High school students as therapeutic agents with young children. [References]
A1 - Jones,Leslie
Y1 - 2010///
N1 - Book: 2010-05340-021
English
Book; Edited Book
KW - PsycInfo
KW - Family Therapy
KW - Kindergarten Students
KW - Peer Counseling
KW - Peer Relations
KW - Play Therapy
KW - At Risk Populations
KW - Behavior Problems
KW - High School Students
KW - Leadership
KW - Preschool Students
KW - School Adjustment
KW - School Based Intervention
RP - NOT IN FILE
SP - 373
EP - 387
JF - Baggerly, Jennifer N [Ed]; Ray, Dee C [Ed]; Bratton, Sue C [Ed]
VL - :
IS - 2010
N2 - (from the chapter) This chapter examines whether the use of a filial therapy model adapted to train high school students in a peer assistance program in developmentally appropriate methods could enhance their skills when working with children. A pre-test-post-test control group design was used to measure the adjustment of pre-kindergarten and kindergarten children identified as at risk. The experimental group children received 20-minute structured play sessions for an average of 20 weeks over seven months. Play sessions were facilitated by 32 high school juniors and seniors in a Peer Assistance and Leadership (PALs) class trained in child-centered play therapy skills and procedures. Comparison group high school students were trained in traditional PALs curriculum during their class time. The students were assigned to treatment group by their classroom assignment. There were two regularly scheduled PALS classes in the participating school. One class was randomly drawn to participate in the experimental treatment, and the other class was drawn to participate in the control treatment. Children in the experimental group demonstrated a significant reduction in their total

behavior problems. Filial-trained PALSs students demonstrated a significant increase in empathy play behaviors and interactions during playtimes with children. The results of this study prove that using a filial therapy model to train high school students as therapeutic agents is effective with prekindergarten and kindergarten children experiencing school adjustment difficulties and suggest the use of a filial therapy training model as an effective method of training peer helpers. (PsycINFO Database Record (c) 2012 APA, all rights reserved)
AD - Jones, Leslie: Private Practice, Prosper, TX, US
ER -

TY - JOUR
ID - 592
T1 - Impact of school-based child-centered play therapy on academic achievement, self-concept, and teacher-child relationships. [References]
A1 - Blanco,Pedro J.
Y1 - 2010///
N1 - Book: 2010-05340-007
English
Book; Edited Book
KW - PsycInfo
KW - Academic Achievement
KW - Client Centered Therapy
KW - Play Therapy
KW - Self Concept
KW - Teacher Student Interaction
KW - At Risk Populations
KW - Childhood Development
KW - Mental Health Programs
KW - Schools
RP - NOT IN FILE
SP - 126
EP - 144
JF - Baggerly, Jennifer N [Ed]; Ray, Dee C [Ed]; Bratton, Sue C [Ed]
VL - :
IS - 2010
N2 - (from the chapter) This chapter discusses a study, the purpose of which was to concentrate improving academic progress by using a mental health program intended to address the developmental needs of children. Child-centered play therapy (CCPT) is one possibility for providing a mental health program in public schools. The study investigated the effectiveness of CCPT on at-risk first-grade students' academic achievement, self-concept, and student-teacher relationships. Specifically, the study examined the effect of CCPT on increasing the child's early achievement composite as measured by the Young Children's Achievement Test (YCAT), the global self-concept scale of the Pictorial Scale of Perceived Competence and Social Acceptance for Young Children (PSPCSAYC), and the total scale of the Student-Teacher Relationship Scale (STRS). (PsycINFO Database Record (c) 2012 APA, all rights reserved)
AD - Blanco, Pedro J.: Delta State University, Cleveland, MS, US
ER -

TY - JOUR
ID - 593
T1 - Child-centered play therapy research: The evidence base for effective practice. [References]
A1 - Baggerly,Jennifer N.
Y1 - 2010///
N1 - Book: 2010-05340-000
English
Book; Edited Book
(Created by PsycINFO) Foreword [by] Garry L. Landreth Preface About the editors Contributors Section I: Overview of play therapy research * What the research shows about play therapy: Twenty-first century update / Dee C. Ray and Sue C. Bratton / 3-33 Section II: Research in play therapy * Increased self-efficacy: One reason

for play therapy success / Marijane Fall / 37-50 * The efficacy of intensive individual child-centered play therapy for chronically ill children / Elizabeth Murphy Jones and Kara Carnes-Holt / 51-67 * Intensive sibling group play therapy with child witnesses of domestic violence / Ashley Tyndall-Lind / 69-83 * Effects of postearthquake group play therapy with Chinese children / Yih-Jiun Shen / 85-103 * Play therapy effect on relationship stress / Dee C. Ray and Natalya A. Edwards / 105-123 * Impact of school-based child-centered play therapy on academic achievement, self-concept, and teacher-child relationships / Pedro J. Blanco / 126-144 * Play therapy with children exhibiting ADHD / Dee C. Ray / 145-162 * A school-based group activity therapy intervention with learning-disabled preadolescents exhibiting behavior problems / Jill Packman and Ireon Lebeauf / 163-176 * School-based child-centered play therapy with Hispanic children / Yvonne Garza / 177-191 * Effectiveness of child-centered play therapy for children referred for aggression / Brandy Schumann / 193-208 * Effectiveness of child-centered play therapy and person-centered teacher consultation on ADHD: A single-case study design / April Schottelkorb / 209-230 * Child-centered play therapy and child development: A single-case analysis / April Garofano-Brown / 231-248 * Children's perceptions of play therapy / Eric J. Green / 249-263 Section III: Research in filial therapy * Child parent relationship therapy: A review of controlled-outcome research / Sue C. Bratton, Garry L. Landreth and Yung-Wei Dennis Lin / 267-293 * Filial therapy with parents of chronically ill children / Kristi Tew / 295-309 * Filial therapy with Native Americans on the Flathead Reservation / Geri Glover / 311-321 * Filial therapy with Chinese parents / Tom Yuen / 323-337 * Child parent relationship therapy with African American parents / Angela I. Sheely-Moore / 339-353 * Child Parent Relationship Therapy with Hispanic parents / Peggy Ceballos / 355-372 * High school students as therapeutic agents with young children / Leslie Jones / 373-387 * Filial therapy with teachers of deaf and hard of hearing preschool children / D. Michael Smith / 389-407 * Child-centered kinder training for teachers of preschool children deemed at risk / Phyllis Post / 409-426 * An early mental health intervention for disadvantaged preschool children / Mary O. Morrison and Wendy P. Helker / 427-446 * A qualitative study of parents' perceptions of filial therapy in a public school / Yuehong Chen Foley / 447-464 Section IV: Future research directions for play therapy * Evidence-based standards and tips for play therapy researchers / Jennifer N. Baggerly / 467-479 Author index Subject index
KW - PsycInfo
KW - Client Centered Therapy
KW - Evidence Based Practice
KW - Experimentation
KW - Play Therapy
KW - Chronic Illness
KW - Clinicians
KW - Domestic Violence
KW - Empirical Methods
KW - History
KW - Natural Disasters
KW - Witnesses
KW - Best Practices
RP - NOT IN FILE
SP - Hoboken, NJ, US
JF - (2010)
VL - Child-centered play therapy research: The evidence base for effective practice. xxiii, 502 pp. Hoboken, NJ, US: John Wiley & Sons Inc; US.
N2 - (from the jacket) This book offers mental health professionals, school district administrators, community agency administrators, judges, lawyers, child protection caseworkers, and medical professionals a comprehensive discussion of play therapy research studies. Guidance is provided on evidence-based methods, as well as on how future play therapy research should be conducted. Edited by experts in the field of play therapy, this compilation features contributions by child-centered play therapy researchers, with relevant discussion of: the history of play therapy research; a synopsis of current empirical support; and play therapy research on chronically ill children, child witnesses of domestic violence, and victims of natural disasters, among many other topics. With coverage of important practice guidelines, this book identifies the most prominent and current play therapy research studies, as well as research directions for clinicians to design evidence-based research studies of their own. (PsycINFO Database Record (c) 2012 APA, all rights reserved)

AD - Baggerly, Jennifer N.: University of South Florida, Tampa, FL, US Ray, Dee C.: Counseling Program, University of North Texas, Denton, TX, US Bratton, Sue C.: Center for Play Therapy, University of North Texas, Denton, TX, US
ER -

TY - JOUR
ID - 594
T1 - Emotion regulation, language ability, and the stability of preschool children's peer play behavior. [References]
A1 - Cohen,Jeremy S; Mendez,Julia
Y1 - 2009///
N1 - Peer Reviewed Journal: 2009-24145-008
English
Journal; Peer Reviewed Journal
KW - PsycInfo
KW - Childhood Play Behavior
KW - Emotional Development
KW - Language Development
KW - Preschool Students
KW - Peer Relations
KW - Social Skills
KW - Vocabulary
RP - NOT IN FILE
SP - 1016
EP - 1037
JF - Early Education and Development
VL - 20:
IS - 6
N2 - Research Findings: This study examined the stability of preschoolers' peer play behavior across the school year and the relations between emotion regulation, receptive vocabulary, and the trajectory of social competence deficits. Participants were 331 preschool children attending Head Start; they were primarily African American and from a low-SES background. Peer play behavior was moderately stable from fall to spring. Analyses revealed that emotional lability in the fall was associated with consistently maladaptive and declining social competence. Furthermore, children who exhibited stable maladaptive behavior had lower receptive language skills and emotion regulation in the fall than children who exhibited consistently adaptive behavior. Preschool children with comorbid externalizing and internalizing behaviors during peer play were at the greatest risk for consistent peer play difficulties or declining social competence over the course of the year compared to their peers. Practice: The present study informs practices for identifying at-risk preschoolers shortly after entry into an early education experience. Moreover, the findings suggest that without effective interventions, those at-risk children are likely to exhibit consistently poor social competence over time. Implications for the use of early intervention and prevention targeting specific behavioral and peer problems are discussed. (PsycINFO Database Record (c) 2012 APA, all rights reserved) (journal abstract)
SN - 1040-9289
AD - Cohen, Jeremy S.: Department of Psychology, Temple University, Philadelphia, PA, US Mendez, Julia L.: Department of Psychology, University of North Carolina at Greensboro, Greensboro, NC, US
ER -

TY - JOUR
ID - 596
T1 - Infant symbolic play as an early indicator of fetal alcohol-related deficit. [References]
A1 - Molteno,Christopher D.
Y1 - 2010///
N1 - Peer Reviewed Journal: 2011-06687-002
English
Journal; Peer Reviewed Journal
KW - PsycInfo

KW - Fetal Alcohol Syndrome
KW - Infant Development
KW - Prenatal Exposure
KW - Recreation
KW - Family Background
KW - Milieu Therapy
KW - Sociocultural Factors
RP - NOT IN FILE
SP - 586
EP - 607
JF - Infancy
VL - 15:
IS - 6
N2 - Infant symbolic play was examined in relation to prenatal alcohol exposure and socioenvironmental background and to predict which infants met criteria for fetal alcohol syndrome (FAS) at 5 years. A total of 107 Cape-Colored, South African infants born to heavy drinking mothers and abstainers/light drinkers were recruited prenatally. Complexity of play, sociodemographic and psychological correlates of maternal alcohol use, and quality of parenting were assessed at 13 months, and intelligence quotient and FAS diagnosis at 5 years. The effect of drinking on spontaneous play was not significant after control for social environment. In contrast, prenatal alcohol and quality of parenting related independently to elicited play. Elicited play predicted 5-year Digit Span and was poorer in infants subsequently diagnosed with FAS/ partial FAS and in nonsyndromal heavily exposed infants, compared with abstainers/light drinkers. Thus, symbolic play may provide an early indicator of risk for alcohol-related deficits. The independent effects of prenatal alcohol and quality of parenting suggest that infants whose symbolic play is adversely affected by alcohol exposure may benefit from stimulation from a responsive caregiver. (PsycINFO Database Record (c) 2012 APA, all rights reserved) (journal abstract)
SN - 1525-0008
AD - Molteno, Christopher D.: Department of Psychiatry and Mental Health, University of Cape Town, Faculty of Health Sciences, Cape Town, South Africa Jacobson, Sandra W.: Department of Psychiatry and Behavioral Neurosciences, Wayne State University School of Medicine, Detroit, MI, US Carter, R. Colin: Department of Pediatrics, Children's Hospital Boston, Boston, MA, US Jacobson, Joseph L.: Department of Psychiatry and Behavioral Neurosciences, Wayne State University School of Medicine, Detroit, MI, US
ER -

TY - JOUR
ID - 597
T1 - Cognitive correlates in toddlers born very low birth weight and full-term. [References]
A1 - Lowe,Jean
Y1 - 2010///
N1 - Peer Reviewed Journal: 2010-25362-026
English
Journal; Peer Reviewed Journal
KW - PsycInfo
KW - Birth Weight
KW - Cognitive Development
KW - Infant Development
KW - Mother Child Communication
KW - Premature Birth
RP - NOT IN FILE
SP - 629
EP - 634
JF - Infant Behavior & Development
JA - Infant behav
VL - 33:
IS - 4

N2 - Understanding what contributes to children's cognitive development can improve our ability to identify those children at risk for later developmental disorders. We hypothesized that cognition would be more strongly associated with child and mother interaction variables such as communication, sensitivity and affect during play in contrast to medical variables in preterm children, and that these same variables would also be correlates of cognition in children born full-term. Cognition was measured by the Bayley Scales of Infant Development-II Mental Developmental Index and mother-toddler play interactions were coded with the Caregiver-Child Affect, Responsiveness and Engagement Scales (C-CARES) for child and mother affect, communication, and sensitivity in 40 very low birth weight (VLBW) and 54 full-term toddlers at 18-22 months of age, adjusted for gestational age. Two different multivariate models were identified that best predicted cognition in the two sets of toddlers. For the toddlers born VLBW, days on ventilation, maternal education and the three C-CARES Child Play scales (sensitivity, affect and communication) were the best predictors of cognition. In contrast, the multivariate model that best correlated with cognition for the children born full-term included the Maternal Communication scale of the C-CARES. The different multivariate models identified for toddlers born preterm compared to those born full-term emphasizes the importance of using identification and cognitive intervention techniques that are uniquely tailored for children born very low birth weight. Findings highlight the importance of investigating beyond more traditional measures of cognition by incorporating play-based socio-emotional measures. (PsycINFO Database Record (c) 2012 APA, all rights reserved) (journal abstract)
SN - 0163-6383
AD - Lowe, Jean: University of New Mexico Children's Hospital, Department of Pediatrics, Division of Neonatology, Albuquerque, NM, US Erickson, Sarah J.: University of New Mexico, Department of Psychology, Albuquerque, NM, US MacLean, Peggy: University of New Mexico, Department of Psychology, Albuquerque, NM, US
ER -

TY - JOUR
ID - 598
T1 - Obesity in early childhood and working in pre-school settings. [References]
A1 - de Silva-Sanigorski,Andrea M.
Y1 - 2010///
N1 - Book: 2010-10942-029
English
Book; Edited Book
KW - PsycInfo
KW - Childhood Development
KW - Health Promotion
KW - Obesity
KW - Prevention
KW - Weight Gain
KW - Critical Period
KW - Preschool Education
KW - Risk Factors
RP - NOT IN FILE
SP - 253
EP - 262
JF - Waters, Elizabeth [Ed]; Swinburn, Boyd [Ed]; Seidell, Jacob [Ed]; Uauy, Ricardo [Ed]
VL - :
IS - 2010
N2 - (from the chapter) This chapter examines several issues on obesity in early childhood that have direct practical relevance to supporting healthy weight gain in this critical period of life. The first fundamental issue for child health nursing practice is the definition of normal growth in early childhood, which leads directly into an examination of the assessment of at-risk growth patterns and other risk factors for later childhood (and adult) obesity. Appropriate feeding practices are fundamental to healthy weight gain in early childhood and these recommendations are reviewed as well as the importance of active play, motor skill development and reduced television viewing time. Patterns for all human behaviors are highly influenced by environments, and this is most true in the early childhood years. Thus, the many preschool settings outside the home environment are also

examined as opportunities for implementing the policies and practices needed to promote healthy eating and active play. (PsycINFO Database Record (c) 2012 APA, all rights reserved)
AD - de Silva-Sanigorski, Andrea M.: WHO Collaborating Centre for Obesity Prevention, Deakin University, Geelong, VIC, Australia Corvalan, Camila: School of Public Health, Faculty of Medicine, University of Chile, Santiago, Chile Uauy, Ricardo: London School of Hygiene and Tropical Medicine, London, England
ER -

TY - JOUR
ID - 599
T1 - Infant physiological response to the still-face paradigm: Contributions of maternal sensitivity and infants' early regulatory behavior. [References]
A1 - Conradt,Elisabeth
Y1 - 2010///
N1 - Peer Reviewed Journal: 2010-10509-002
English
Journal; Peer Reviewed Journal
KW - PsycInfo
KW - Facial Expressions
KW - Mother Child Relations
KW - Physiological Arousal
KW - Self Regulation
KW - Sensitivity (Personality)
KW - Emotional Regulation
KW - Environmental Effects
KW - Infant Development
KW - Poverty
KW - Risk Factors
KW - Social Stress
RP - NOT IN FILE
SP - 251
EP - 265
JF - Infant Behavior & Development
JA - Infant behav
VL - 33:
IS - 3
N2 - The current study examined the independent and additive contributions of maternal sensitivity measured prior to and following a social stressor, and infant behaviors to infants' physiological response to the still-face paradigm (SFP) in a sample characterized by poverty-related environmental risk. Ninety-one mother/infant dyads participated in the SFP when their infants were 5 months old. Maternal sensitivity was coded during the play and reunion episodes. Infant behaviors were coded during the reunion episode of the SFP while measures of heart rate (HR) and Respiratory Sinus Arrhythmia (RSA) were collected from the infants at baseline and across the SFP. Maternal sensitivity during the reunion episode predicted infants' biobehavioral reactivity and regulation, over and above maternal sensitivity during the play episode. Infants' HR decreased, and RSA increased, with greater levels of maternal sensitivity during the reunion episode. Infants also exhibited greater attentional engagement and fewer resistant behaviors with greater levels of maternal sensitivity during the reunion episode. Finally, infant behaviors predicted change in HR and RSA from the still-face to the reunion episode, above and beyond that of maternal behaviors. These findings further our understanding of the dyadic basis for the development of emotion regulation in infancy. (PsycINFO Database Record (c) 2012 APA, all rights reserved) (journal abstract)
SN - 0163-6383
AD - Conradt, Elisabeth: Psychology Department, University of Oregon, Eugene, OR, US Ablow, Jennifer: Psychology Department, University of Oregon, Eugene, OR, US
ER -

TY - JOUR
ID - 600

T1 - In reply: "Nutritional-assistance programs play a critical role in reducing food insecurity." [References]
A1 - Melchior,Maria; Caspi,Avshalom; Belsky,Daniel; Moffitt,Terrie
Y1 - 2010///
N1 - Peer Reviewed Journal: 2011-14269-035
English
Journal; Peer Reviewed Journal
KW - PsycInfo
KW - Food
KW - Lower Income Level
KW - Mental Health
KW - Nutritional Deficiencies
KW - Socioeconomic Status
KW - Alcoholism
KW - At Risk Populations
KW - Domestic Violence
KW - Drug Abuse
KW - Family
KW - Major Depression
KW - Mothers
RP - NOT IN FILE
SP - e1267
EP - e1268
JA - Pediatrics
VL - 125:
IS - 5
N2 - Reply by the current authors to the comments made by Deborah A. Frank et al. (see record 2011-14269-034) on the original article (see record 2010-00879-002). Frank et al. point out that food assistance programs are not sufficiently accessible to eligible families and argue that such programs are key in eliminating food insecurity. We agree that programs that provide income supports or food assistance to deprived families should be strengthened. However, the results of our study indicate that these interventions may not reach children of families in which the mother suffers from mental illness. Mothers with mental health problems may not apply for assistance, may do so incorrectly, or may struggle to translate assistance into a healthy diet for their children. Safety net policies that protect children from food insecurity depend on parents, and in many cases mothers, to seek out resources and to effectively pass them on to their children. Mothers, therefore, are the key conduit link between government policies and children, and mental health problems can weaken this link. If mothers' mental health is neglected, then there will always be a substantial segment of the population of poor children whose nutritional needs are left unmet by even the best government-assistance programs. Why not do whatever it takes to enable every child to benefit? (PsycINFO Database Record (c) 2012 APA, all rights reserved)
SN - 0031-4005
AD - Melchior, Maria: Inserm U1018, Centre for Research in Epidemiology and Population Health, Epidemiology of Occupational and Social Determinants of Health, Villejuif, France Caspi, Avshalom: Department of Psychology, Duke University, Durham, NC, US Belsky, Daniel: Department of Health Policy and Management, Gillings School of Global Public Health, University of North Carolina, Chapel Hill, NC, US Moffitt, Terrie E.: Department of Psychology, Duke University, Durham, NC, US
ER -

TY - JOUR
ID - 601
T1 - The influence of parent attributional style and family structure on adolescent adjustment to chronic illness
A1 - Guion,Kimberly
Y1 - 2010///
N1 - Dissertation Abstract: 2010-99240-423
English
Dissertation Abstract
KW - PsycInfo
KW - Attribution

KW - Chronic Illness
KW - Coping Behavior
KW - Family Structure
KW - Parents
KW - Adjustment
KW - Chronicity (Disorders)
RP - NOT IN FILE
SP - 3934
JF - Dissertation Abstracts International: Section B: The Sciences and Engineering
VL - 71:
IS - 6-B
N2 - Adolescents diagnosed with a variety of chronic diseases are at increased risk for developing internalizing and externalizing problems. Various family factors may play an important role in adolescent adjustment, particularly in the chronic illness population given the important role that family members play in medical care and coping. Previous literature has demonstrated the separate contributions of parental attributions and adolescent attributions to adolescent adjustment. However, it is unknown whether parental coping affects adolescent mental health outcomes directly or indirectly by influencing children's attributional style. Further, there is a dearth of research examining the role of family structure in coping in pediatric populations. The current project examined the relationships between parental coping style and family structure to adolescent adjustment. Adolescents (N=123; M= 14.4 yrs.) diagnosed with cystic fibrosis, diabetes, or muscular dystrophy and a caregiver completed measures of attributional style and adolescent adjustment. Mediation analyses indicated that adolescent attributions did not mediate the relationship between parent attributions and adolescent adjustment. However, both adolescent and parent attributions predicted teen externalizing problems, and adolescent attributions predicted depression and anxiety. Regression analyses indicated the number of active caregivers reported, but not family structure, was related to adolescent externalizing problems. Age and parental distress were also significant predictors of adolescent outcomes. These data represent a novel look at complex relations between family factors and adolescent adjustment. Results suggest that a key entry point for intervention may relate to parental mental health and adolescent coping. (PsycINFO Database Record (c) 2012 APA, all rights reserved)
SN - 0419-4217
AD - Guion, Kimberly: U Alabama at Birmingham, US
ER -

TY - JOUR
ID - 602
T1 - Should children with Down's syndrome who have asymptomatic atlantoaxial instability avoid horse riding? [References]
A1 - Rao,Srinidhi J.
Y1 - 2010///
N1 - Peer Reviewed Journal: 2011-00914-016
English
Journal; Peer Reviewed Journal
KW - PsycInfo
KW - Down's Syndrome
KW - epidemiology
KW - Physical Activity
KW - Spinal Cord
KW - Horses
KW - Schools
RP - NOT IN FILE
SP - 774
EP - 776
JF - Journal of Paediatrics and Child Health
VL - 46:
IS - 12

N2 - A 5-year-old girl with Down's syndrome wishes to take horse riding lessons and was asked by the riding school to be screened for atlantoaxial instability (AAI). AAI is a known association with Down's syndrome with a prevalence of 10-30% depending on the criteria used to define it. In children with Down's syndrome and asymptomatic AAI, do strenuous physical activities like horse riding increase the risk of symptomatic AAI or spinal cord compression? We searched Medline from 1950 to January week 2 2010 for the key words: (Atlantoaxial instability OR Atlantoaxial joint) AND Down Syndrome, and limited the studies to children 0-18 years and written in the English language. A total of 86 articles were found, of which four were relevant. Among the relevant articles identified by the search, there was one randomised controlled trial and three observational studies. The paper that best answers the question was by Cremers et al. While the authors conclude that it is safe for children with Down's syndrome and AAI to play risky sport, we do not think that the evidence presented in the study is sufficient to reach that conclusion. Hence, we would advise the parents of our patient with an atlanto dens interval of 6.5 mm that there is insufficient information to determine whether horse riding may increase the risk for symptomatic AAI in her situation. The decision to participate in horse riding is to be based on the balance of the risks of suffering the rare chance of spinal cord compression to the benefits of engaging in a rewarding outdoor activity. (PsycINFO Database Record (c) 2012 APA, all rights reserved)
SN - 1034-4810
AD - Rao, Srinidhi J. V.: Department of Paediatrics, Children's Hospital at Westmead, Westmead, NSW, Australia Caldwell, Patrina: Discipline of Paediatrics and Child Health, University of Sydney, Sydney, NSW, Australia
ER -

TY - JOUR
ID - 603
T1 - Incorporating divergent thinking training into play interventions for preschool children with developmental risk factors. [References]
A1 - Mallory,Jessica M.
Y1 - 2010///
N1 - Peer Reviewed Journal: 2010-23177-005
English
Journal; Peer Reviewed Journal
KW - PsycInfo
KW - Cognitive Development
KW - Divergent Thinking
KW - Preschool Students
KW - Recreation
KW - School Based Intervention
KW - Risk Factors
RP - NOT IN FILE
SP - 57
EP - 71
JF - The International Journal of Creativity & Problem Solving
VL - 20:
IS - 2
N2 - Cognitive development and play development are mutually reinforcing. The present study measured the effect of an intervention intended to address both play skills and cognitive skills directly by incorporating divergent thinking prompts into a play-based intervention. The play of six at-risk children was observed and video recorded during independent pretend play. Qualitative and quantitative aspects of this play were recorded. Three at-risk children participated in six week divergent thinking intervention, while three comparison children participated in the general preschool curriculum. Children's play was observed and recorded pre- and postintervention. Results indicated that children who participated in the intervention did improve on measures of play quality, quantity, or both more than children who did not participate in the intervention. Implications for educators are discussed. (PsycINFO Database Record (c) 2012 APA, all rights reserved) (journal abstract)
SN - 1598-723X
AD - Mallory, Jessica M.: University of Nebraska at Omaha, Omaha, NE, US Kelly-Vance, Lisa: University of Nebraska at Omaha, Omaha, NE, US Ryalls, Brigette: University of Nebraska at Omaha, Omaha, NE, US
ER -

TY - JOUR
ID - 604
T1 - Temperament and maternal emotion socialization beliefs as predictors of early childhood social behavior in the laboratory and classroom. [References]
A1 - Root,Amy Kennedy; Stifter,Cynthia
Y1 - 2010///
N1 - Peer Reviewed Journal: 2010-24343-001
English
Journal; Peer Reviewed Journal
KW - PsycInfo
KW - Early Childhood Development
KW - Psychosocial Development
KW - Risk Factors
KW - Social Behavior
KW - SOCIALIZATION
KW - Classrooms
KW - Emotional States
KW - Mother Child Relations
RP - NOT IN FILE
SP - 241
EP - 257
JF - Parenting: Science and Practice
VL - 10:
IS - 4
N2 - Objective: The authors examined the roles of children's approach behavior and maternal emotion socialization practices in the development of social behavior in unfamiliar and familiar contexts from preschool to early childhood years. Design: At 4.5 years of age, children were observed, and an assessment of approach behavior was obtained; at this time, mothers reported about their emotion socialization beliefs. Two years later, children returned to the laboratory to participate in a peer play paradigm. When children were 7 years of age, teachers completed a questionnaire about children's social behaviors in the classroom. Results: Mothers' emotion socialization beliefs contribute to the developmental outcomes of approach behavior. For instance, observations of approach behaviors predicted a greater proportion of group play in the unfamiliar peer group when mothers reported highly supportive emotion socialization beliefs. Conclusion: Mothers' emotion socialization beliefs appear to play an important role in modifying the developmental course of approach behavior during early childhood. (PsycINFO Database Record (c) 2012 APA, all rights reserved) (journal abstract)
SN - 1529-5192
AD - Root, Amy Kennedy: Department of Technology, Learning and Culture, Morgantown, WV, US Stifter, Cynthia: Pennsylvania State University, PA, US
ER -

TY - JOUR
ID - 605
T1 - Review of Playing outdoors, spaces and places, risk and challenge. [References]
A1 - Garrick,Ros
Y1 - 2010///
N1 - Peer Reviewed Journal: 2010-21379-012
English
Journal; Peer Reviewed Journal
KW - PsycInfo
KW - Childhood Play Behavior
KW - Early Childhood Development
KW - Learning
KW - Social Environments
RP - NOT IN FILE

SP - 283
EP - 285
JF - Early Years: An International Journal of Research and Development
VL - 30:
IS - 3
N2 - Reviews the book, Playing outdoors, spaces and places, risk and challenge by Helen Tovey (2007). This book has been written as part of a series of books in the 'Debating Play' series, edited by Tina Bruce. The series sets out to promote critical reflection on young children's play across a wide range of contexts, and to support those working with children to provide play opportunities with rich learning potential. Tovey's book focuses on the opportunities for outdoor play in settings for young children, primarily children from one to five years, in the UK. The book begins, as some similar books have done, with an evocation of the rich sensory experience of outdoor play in a previous era when free play in natural environments was a relatively common experience. Tovey then considers some broad issues for young children today that have led to diminished opportunities for outdoor play, and she examines the unintended, negative consequences that flow from this. This challenge leads to the heart of the book and to the chapter where Tovey sets out what she sees as the key design principles of outdoor spaces and places. Like the rest of the book, this chapter is richly informed by a range of research, drawing for example on the research of landscape architects, sociologists and geographers as well as educationists. (PsycINFO Database Record (c) 2012 APA, all rights reserved)
SN - 0957-5146
AD - Garrick, Ros: Sheffield Hallam University, Sheffield, United Kingdom
ER -

TY - JOUR
ID - 606
T1 - The effects of information sharing and modeling on teacher talk and children's language during dramatic play
A1 - Combs,Sandra Genevieve Patterson
Y1 - 2010///
N1 - Dissertation Abstract: 2010-99040-016
English
Dissertation Abstract
KW - PsycInfo
KW - Childhood Development
KW - Language Development
KW - Project Head Start
KW - Simulation
KW - Teachers
KW - Language
RP - NOT IN FILE
SP - 4800
JF - Dissertation Abstracts International: Section B: The Sciences and Engineering
VL - 70:
IS - 8-B
N2 - The goal of preschool is to prepare children for later schooling. In the case of Head Start, the goal is, more specifically, to prepare children at-risk for later language and academic difficulties due to poverty for school. Children with strong language skills enter school able to handle the input from teachers and are more likely to do well in school. The quality of language interactions a teacher and child have in preschool is important to Head Start children. Teachers can have a positive impact on the language development of preschool children, thereby increasing these children's chances of later academic success. One possible means of supporting children in Head Start is supporting teachers in development and use of effective language enhancement strategies. Speech-language pathologists (SLPs) have knowledge of effective strategies for increasing the language skills of young children. It would therefore be helpful for SLPs to collaborate in trainings with teachers in Head Start. This project was a quasi-experimental study of an SLP professional development program involving teachers, children and two SLPs in two urban Head Start centers. This study examined the changes in teachers' use of language enhancement strategies following ongoing, in class information sharing and modeling by an SLP during play in the dramatic play center. This study also examined the subsequent changes

in the language of specific children in these classrooms following their teachers' participation in the program. The specific aims were to determine: (1) if teachers' use of language enhancement strategies during play in a dramatic play center increased immediately following ongoing, information sharing and modeling by an SLP, (2) if teachers continued to used the strategies longitudinally, (3) if there was a difference between the number of strategies use by the experimental group and control group at baseline and final taping, (4) if children's language complexity changed as a result of changes in their teachers' language use, and (5) if there was a difference between the language complexity of the children in the experimental group and control group at baseline and final taping. The results of this study indicated that teachers significantly increased the number of open-ended questions and expansions/extensions immediately following information sharing and modeling by the SLP. The teachers did not significantly increase the number of targeted and specific vocabulary they used immediately following the modeling by the SLP. The teachers did not demonstrate maintenance of changes for either variable at final follow-up taping. The children's language complexity was not significantly different on any of the measures throughout the project. One of the implications of this project is that SLPs may consider using short informational meetings and modeling of specific targeted language strategies as a means of supporting teachers in providing a language rich classroom. (PsycINFO Database Record (c) 2012 APA, all rights reserved)
SN - 0419-4217
AD - Combs, Sandra Genevieve Patterson: U Cincinnati, US
ER -

TY - JOUR
ID - 607
T1 - Case study: Parent-implemented prelinguistic milieu teaching with a high risk dyad. [References]
A1 - McCathren,Rebecca B.
Y1 - 2010///
N1 - Peer Reviewed Journal: 2010-16284-005
English
Journal; Peer Reviewed Journal
KW - PsycInfo
KW - Developmental Disabilities
KW - Linguistics
KW - Parents
KW - Dyads
KW - Intentional Learning
KW - Interpersonal Communication
KW - Poverty
KW - Teaching
RP - NOT IN FILE
SP - 243
EP - 252
JF - Communication Disorders Quarterly
VL - 31:
IS - 4
N2 - The purpose of this study was twofold. The first purpose was to determine if a mother with mild developmental disabilities living in poverty was able to implement Prelinguistic Milieu Teaching (PMT) strategies. The strategies included following the child's lead, arranging the environment to increase opportunities for communication, imitating the child's play and communication behaviors, modeling play and communication, and using words to map the child's experience and actions. The second purpose was to see if using these strategies resulted in increased intentional communication in her daughter. Intentional communication is when the young child coordinates the use of eye gaze, vocalizations, and/or gestures to achieve a particular communication outcome. The child is demonstrating intentional communication when she persistently alternates gaze between the adult and the object or event of interest or includes attention to both the adult and object in an interaction. For example, a child might reach toward an out-of-reach toy and look back and forth between the toy and the parent, communicating that she wants her parent to get the toy for her. The results indicated that the mother was able to implement the PMT strategies, and the use of these strategies increased her daughter's intentional communication. (PsycINFO Database Record (c) 2012 APA, all rights reserved) (journal abstract)

SN - 1525-7401
AD - McCathren, Rebecca B.: University of Missouri-Columbia, Columbia, MO, US
ER -

TY - JOUR
ID - 608
T1 - An activating mechanism of aggressive behaviour in disorganised attachment: A moment-to-moment case analysis of a three-year-old. [References]
A1 - Kim,Eun Young
Y1 - 2010///
N1 - Peer Reviewed Journal: 2010-15394-005
English
Journal; Peer Reviewed Journal
KW - PsycInfo
KW - Aggressive Behavior
KW - Attachment Behavior
KW - Cognitive Ability
KW - Risk Factors
KW - Therapeutic Processes
RP - NOT IN FILE
SP - 152
EP - 167
JF - Journal of Child Psychotherapy
VL - 36:
IS - 2
N2 - This study examines an activating mechanism of aggressive behaviour in young children. Many studies on attachment theories have indicated disorganised attachment as a significant risk factor for externalising problems and have explained the aetiology of disorganised attachment in terms of deficits in affect, behaviour and cognitive functions from a traumatic or inconsistent environment. Via the moment-to-moment analysis of the play therapy process with a three-year-old, this paper attempts to identify how these emotional, behavioural and cognitive factors interact in actual situations and can lead to an observable behaviour: aggressive behaviour. The findings from this study indicate that the child with disorganised attachment was going through a dual process, one with her therapist and the other with her internalised caregivers. When anxiety and an ambiguous social cue from an unpredictable situation were added to the child's repetition of previous attachment experience, this 'click' lowered the threshold of regulation of affect, behaviour and cognition. The child turned to disorganised and aggressive behaviour, relying on her physiological signals regarding the perceived threat. Finally, this paper describes how these difficulties can be channelled by symbolic play via displacement and containment. (PsycINFO Database Record (c) 2012 APA, all rights reserved) (journal abstract)
SN - 0075-417X
AD - Kim, Eun Young: Portia Bell Hume Center, Fremont, CA, US
ER -

TY - JOUR
ID - 609
T1 - Assessment and diagnosis of autism and spectrum disorders in children. [References]
A1 - Rojahn,Johannes
Y1 - 2010///
N1 - Peer Reviewed Journal: 2010-13545-001
English
Journal; Peer Reviewed Journal
KW - PsycInfo
KW - Adaptive Behavior
KW - Autism
KW - Comorbidity
KW - Diagnosis
KW - Measurement

RP - NOT IN FILE
SP - 313
EP - 315
JF - Journal of Developmental and Physical Disabilities
VL - 22:
IS - 4
N2 - This special issue of the Journal of Developmental and Physical Disabilities, which includes both comprehensive review papers as well as data based papers can be grouped into three categories: (1) identification and assessment of ASD core symptoms, (2) diagnosis and assessment of comorbidity in children with ASD, and (3) assessment of adaptive behavior (play skills and verbalizations) in children with ASD. In light of the stunning increase in reported prevalence rates in most countries the authors of the review paper conclude the most widely used and intensively scrutinized measures at present (ADI-R, ADOS, CHAT and its variants, and the BISCUIT, Social and Communication Questionnaire). Three papers of this Special Issue focus on the assessment of concurrent behavioral and psychiatric conditions in children with ASD. These are Validity of Comorbid Psychiatric Disorders in Youngsters with Autism Spectrum Disorders,Emergent Comorbidity in At Risk Children with and without Autism Spectrum Disorder-A Cross-Sectional Study, and Assessment of Feeding Problems in Children with Autism Spectrum Disorders. Last paper presents an assessment method for play skills and verbalizations in a study that explored the efficacy of video modeling for teaching play skills to two four-year-old boys with ASD. (PsycINFO Database Record (c) 2012 APA, all rights reserved)
SN - 1056-263X
AD - Rojahn, Johannes: George Mason University, Fairfax, VA, US Matson, Johnny L.: Louisiana State University, Baton Rouge, LA, US
ER -

TY - JOUR
ID - 610
T1 - Temperament at 5 years of age predicts amygdala and orbitofrontal volume in the right hemisphere in adolescence. [References]
A1 - Hill,Shirley Y.
Y1 - 2010///
N1 - Peer Reviewed Journal: 2010-06509-005
English
Journal; Peer Reviewed Journal
KW - PsycInfo
KW - Amygdala
KW - Brain Size
KW - Inhibition (Personality)
KW - Personality
KW - Prefrontal Cortex
KW - Adolescent Development
KW - Childhood Development
KW - Magnetic Resonance Imaging
KW - Right Hemisphere
RP - NOT IN FILE
SP - 14
EP - 21
JF - Psychiatry Research: Neuroimaging
VL - 182:
IS - 1
N2 - It was of interest to determine if hemispheric differences in orbitofrontal cortex (OFC) volume would be related to behavioral inhibition observed in a peer-play setting. Magnetic resonance imaging (MRI) was carried out in 23 individuals (19 males and 4 females) at an average age of 14.87 +/- 1.14 years who were either at high or low risk for alcohol dependence. All subjects had previously been evaluated in a preschool peer play paradigm (5.03 +/- 0.78 years) assessing behavioral inhibition. Region of interest measures were traced for the OFC and the amygdala, and confirmed with voxel based morphometry. Behavioral inhibition, a behavioral tendency that often occurs in a novel setting in reaction to strangers, includes the following: greater time spent

next to the mother, greater time staring at another child, and longer latency to begin play with another child. A significant relationship was seen between greater right OFC volume and indicators of behavioral inhibition including greater time spent proximal to their mother and greater time staring at the other child. Also, larger amygdala volume was associated with more time spent proximal to the mother. Behavioral control, including both over- and under-control, is likely to be subserved by neural circuitry associated with emotion regulation including the right OFC and the amygdala. (PsycINFO Database Record (c) 2012 APA, all rights reserved) (journal abstract)
SN - 0925-4927
AD - Hill, Shirley Y.: Department of Psychiatry, University of Pittsburgh School of Medicine, Pittsburgh, PA, US Tessner, Kevin: Department of Psychiatry, University of Pittsburgh School of Medicine, Pittsburgh, PA, US Wang, Shuhui: Department of Psychiatry, University of Pittsburgh School of Medicine, Pittsburgh, PA, US Carter, Howard: Department of Psychiatry, University of Pittsburgh School of Medicine, Pittsburgh, PA, US McDermott, Michael: Department of Psychiatry, University of Pittsburgh School of Medicine, Pittsburgh, PA, US
ER -

TY - JOUR
ID - 611
T1 - Family-focused trauma intervention: Using metaphor and play with victims of abuse and neglect. [References]
A1 - Pernicano,Pat
Y1 - 2010///
N1 - Book: 2010-06089-000
English
Book; Authored Book
(Created by PsycINFO) (Abbreviated) Acknowledgments Introduction Impact of trauma and overview of treatment Establishing appropriate roles and boundaries in families The fight of fight or flight: Self-control and modulation of affect Reducing hyperactivity and agitation Flight: Avoidance, withdrawal, and dissociation Reducing worry, fear, and anxiety Disrupted attachment: Coping with loss and renewing trust Changing depressed mood Empathy: Caring for yourself and others Responsibility and accountability Safety and protection The family journey: Pathways to change Coming to terms with out-of-home care Self-acceptance and hope Appendix: Squiggles stories References Index About the Author
KW - PsycInfo
KW - Child Abuse
KW - Child Neglect
KW - Family Intervention
KW - Trauma
KW - Victimization
KW - At Risk Populations
KW - Attachment Behavior
KW - Relapse (Disorders)
KW - Transgenerational Patterns
RP - NOT IN FILE
SP - Lanham, MD, US
JF - (2010)
VL - Family-focused trauma intervention: Using metaphor and play with victims of abuse and neglect. xi, 199 pp. Lanham, MD, US: Jason Aronson; US.
N2 - (from the cover) Treatment of abuse and neglect needs to be family-focused in order to reduce troubling symptoms, address family risk and relapse potential, treat cross-generational patterns, and remediate attachment deficits. Family-Focused Trauma Intervention translates issues central to abuse and neglect recovery into metaphorical stories and family-based interventions. Each chapter provides a summary of an issue or theme; one or more pertinent stories; and examples of parallel family, group, and individual interventions. These stories and family-focused interventions, which may be used within any theoretical framework, provide a wide variety of practitioners with a needed bridge between theory and practice. (PsycINFO Database Record (c) 2012 APA, all rights reserved)
AD - Pernicano, Pat: Providence Self Sufficiency Ministries, Georgetown, IN, US

ER -

TY - JOUR
ID - 612
T1 - Maternal anxiety, mother-infant interactions, and infants' response to challenge. [References]
A1 - Kaitz,Marsha; Maytal,Hilla Rubin; Devor,Noa; Bergman,Liat; Mankuta,David
Y1 - 2010///
N1 - Peer Reviewed Journal: 2010-05773-004
English
Journal; Peer Reviewed Journal
KW - PsycInfo
KW - Anxiety
KW - Emotional Regulation
KW - Mother Child Relations
KW - Stress
KW - Infant Development
KW - Mothers
RP - NOT IN FILE
SP - 136
EP - 148
JF - Infant Behavior & Development
JA - Infant behav
VL - 33:
IS - 2
N2 - Children of anxious mothers are at risk for social-emotional difficulties and disturbed, early interactions with their mother may account for some of the risk. This study evaluated the association between maternal anxiety, features of mother-infant interactions, and infants' emotion regulation during stressful situations (still-face, play with a stranger). Thirty-four anxiety-disordered mothers of 6-month-old infants and 59 typical dyads comprised the sample. Dyads were filmed during free play, teaching, care giving, and face-to-face play; and monadic (e.g., maternal sensitivity, infant affect) and dyadic measures (e.g., synchrony) were derived by global or time-event coding of the films. Results indicate that, compared to controls, more anxious mothers showed exaggerated behavior with their infant during free play and teaching, and infants of anxious mothers were less likely to show negative affect during the still-face and stranger challenges. We conclude that anxious maternal behavior reflects the hyperarousal that is characteristic of most anxiety disorders; and infants of anxious mothers and controls show differences in the manner in which they cope with social challenges. (PsycINFO Database Record (c) 2012 APA, all rights reserved) (journal abstract)
SN - 0163-6383
AD - Kaitz, Marsha: Department of Psychology, Hebrew University, Jerusalem, Israel Maytal, Hilla Rubin: Department of Psychology, Hebrew University, Jerusalem, Israel Devor, Noa: Department of Psychology, Hebrew University, Jerusalem, Israel Bergman, Liat: Department of Psychology, Hebrew University, Jerusalem, Israel Mankuta, David: Hadassah Hospital-Hebrew University Medical School, Jerusalem, Israel
ER -

TY - JOUR
ID - 613
T1 - Problematic dyadic interaction among toddlers and their polydrug-cocaine-using mothers. [References]
A1 - Molitor,Adriana
Y1 - 2010///
N1 - Peer Reviewed Journal: 2010-05689-001
English
Journal; Peer Reviewed Journal
KW - PsycInfo
KW - Cocaine
KW - Dyads
KW - Mother Child Relations
KW - Mothers

KW - Polydrug Abuse
KW - Early Childhood Development
KW - Social Interaction
RP - NOT IN FILE
SP - 121
EP - 140
JF - Infant Mental Health Journal
VL - 31:
IS - 2
N2 - Dyadic interactions between 84 at-risk toddlers and their nondrug-, polydrug-non-cocaine-, or polydrug-cocaine-using mothers were examined during unstructured play. Coded videotaped behaviors revealed two maternal constructs (interactive competence, maladaptive engagement) and three toddler constructs (responsiveness, active positive initiation, play). Cocaine-using mothers displayed more maladaptive engagement than did prenatal nondrug users; however, poorer interactive competence further distinguished them from polydrug-but-noncocaine users. Toddlers of cocaine-using mothers displayed less responsiveness and positive initiation than non-drug-group peers; however, these behaviors did not reliably differ from polydrug-non-cocaine counterparts. Play interest remained relatively similar across groups. Results suggest that difficulties in social interaction appear to continue among cocaine-using mothers during dyadic exchanges with their offspring in the second year; however, toddler deficits do not appear to be greater than those experienced by polydrug-non-cocaine counterparts. (PsycINFO Database Record (c) 2012 APA, all rights reserved) (journal abstract)
SN - 0163-9641
AD - Molitor, Adriana: University of San Diego, San Diego, CA, US Mayes, Linda C.: Yale University Child Study Center, New Haven, CT, US
ER -

TY - JOUR
ID - 614
T1 - Play therapy for anger management in the schools. [References]
A1 - Fischetti,Barbara A.
Y1 - 2010///
N1 - Book: 2010-04666-014
English
Book; Edited Book
KW - PsycInfo
KW - Anger Control
KW - Play Therapy
KW - Psychotherapeutic Techniques
KW - School Based Intervention
KW - Group Counseling
KW - Individual Psychotherapy
RP - NOT IN FILE
SP - 283
EP - 305
JF - Drewes, Athena A [Ed]; Schaefer, Charles E [Ed]
VL - :
IS - 2010
N2 - (from the chapter) This chapter reviewed play therapy techniques and approaches useful for anger management issues. Individual counseling techniques suggested for therapeutic intervention included client-centered play therapy, release play therapy, cognitive-behavioral play therapy, and developing an individual treatment approach incorporating play therapy techniques based on individual student treatment goals. Group counseling techniques included client-centered play therapy, cognitive-behavioral play therapy, structured social games and adventure play, therapeutic games, and the use of specific programs designed to ameliorate anger management difficulties that incorporate play techniques. A prescriptive approach to play therapy was recommended for addressing a student's needs specific to anger management difficulties. Interventions may include individual play therapy, group play therapy, and family counseling. Additionally, special play sessions

between parent and child were discussed to assist with anger management difficulties at home. Specific case examples were shared to illustrate the play techniques. Finally, specific play therapy techniques were identified for potential inclusion in a treatment program. (PsycINFO Database Record (c) 2012 APA, all rights reserved)
AD - Fischetti, Barbara A.: Westport Public Schools, Westport, CT, US
ER -

TY - JOUR
ID - 615
T1 - Play therapy for preschool children. [References]
A1 - Schaefer,Charles E [Ed]
Y1 - 2010///
N1 - Book: 2009-08925-000
English
Book; Edited Book
(Created by PsycINFO) Contributors Introduction I. Play-based prevention programs * Clinical and developmental issues in psychotherapy with preschool children: Laying the groundwork for play therapy / Julie Blundon Nash and Charles E. Schaefer / 15-28 * Evidence supporting the benefit of play for mild to moderate behavior problems of preschool children / Charles E. Schaefer / 31-45 * Play therapy for insecurely attached preschool children / Helen E. Benedict and Diana Schofield / 47-66 II. Play interventions for internalizing disorders * Posttraumatic parenting: A parent-child dyadic treatment for young children's posttraumatic adjustment / Janine Shelby, Claudia Avina, and Heather Warnick / 69-87 * Filial play therapy for grieving preschool children / Hilda R. Glazer / 89-105 * Strategic play therapy techniques for anxious preschoolers / Paris Goodyear-Brown / 107-129 * Use of play in child-parent psychotherapy with preschoolers traumatized by domestic violence / Manuela A. Diaz and Alicia F. Lieberman / 131-156 * Cognitive-behavioral play therapy for preschoolers: Integrating play and cognitive-behavioral interventions / Susan M. Knell and Meena Dasari / 157-178 III. Play interventions for externalizing disorders * Role of sociodramatic play in promoting self-regulation / Katherine A. Gioia and Renee M. Tobin / 181-198 * Parent-child interaction therapy / Corissa L. Callahan, Monica L. Stevens, and Sheila Eyberg / 199-221 * Jungian sandplay therapy for preschoolers with disruptive behavior problems / Eric J. Green and Kristi Gibbs / 223-244 * Parents, teachers, and therapists using child-directed play therapy and coaching skills to promote children's social and emotional competence and build positive relationships / Carolyn Webster-Stratton and M. Jamila Reid / 245-273 IV. Play interventions for developmental disorders * From novice to expert: Guiding children on the autism spectrum in integrated play groups / David Neufeld and Pamela Wolfberg / 277-299 * Teaching social skills to developmentally delayed preschoolers / Johnny L. Matson and Jill C. Fodstad / 301-322 Index About the editor
KW - PsycInfo
KW - At Risk Populations
KW - Disorders
KW - Play Therapy
KW - Prevention
KW - Developmental Disabilities
KW - Mental Disorders
RP - NOT IN FILE
SP - Washington, DC, US
JF - (2010)
VL - Play therapy for preschool children. x, 333 pp. Washington, DC, US: American Psychological Association; US.
N2 - (from the jacket) Play Therapy for Preschool Children is a comprehensive sourcebook of play interventions for preventing and resolving the most common disorders of children ages 3 to 5 years. Leading clinicians and researchers present both the research background and recent advances in play therapy adapted for use with children who exhibit a variety of internalizing, externalizing, and developmental disorders as well as prevention programs for at-risk children. Case illustrations are used to demonstrate interventions for children with mild to moderate behavior problems, preschool children with insecure attachment, and children with posttraumatic issues. Contributors demonstrate how to use play therapy in conjunction with parent psychotherapy and cognitive-behavioral therapy, discuss interventions for autistic and developmentally delayed children, and illustrate the use of play interventions in classroom and parent-child settings. Throughout, chapter authors provide practical guidance for the full implementation of clinical ideas, from intake through termination.

Clearly written and comprehensive in scope, this book is a must-have for any student or mental health professional who seeks a foil understanding of the latest theory and practice of play therapy with this uniquely vulnerable and often misunderstood population. (PsycINFO Database Record (c) 2012 APA, all rights reserved)
AD - Schaefer, Charles E.: Fairleigh Dickinson University, Teaneck, NJ, US
ER -

TY - JOUR
ID - 617
T1 - Characteristics of risky play. [References]
A1 - Sandseter,Ellen Beate Hansen
Y1 - 2009///
N1 - Peer Reviewed Journal: 2010-11391-002
English
Journal; Peer Reviewed Journal
KW - PsycInfo
KW - Childhood Play Behavior
KW - Injuries
KW - Risk Taking
RP - NOT IN FILE
SP - 3
EP - 21
JF - Journal of Adventure Education and Outdoor Learning
VL - 9:
IS - 1
N2 - This paper explores what makes children's risky play risky. Risky play can generally be defined as thrilling and exciting forms of play that involve a risk of physical injury. Few, if any, studies have been conducted to explore what identifies play activity as risky. The present study aims to determine what characteristics to judge risky play by. Risky play in two Norwegian preschools was observed and videotaped during 18 observation days over five months. The results reveal two categories of risk characteristics in children's play: (1) environmental characteristics (features of the play environment), and (2) individual characteristics (how the play was carried out by the child). These two categories include several different risk characteristics that identify the risk in risky play. The results indicate that both features of the play environment as well as children's risk-taking actions based on their subjective risk perception influence the objective risk present in the play situation. The study contributes to an increased focus on children's natural urge for risky play, and to what characterizes such play. (PsycINFO Database Record (c) 2012 APA, all rights reserved) (journal abstract)
SN - 1472-9679
AD - Sandseter, Ellen Beate Hansen: Department Physical Education, Queen Maud University, College of Early Childhood Education (DMMH), Trondheim, Norway
ER -

TY - JOUR
ID - 618
T1 - Pretend play, emotional processes, and developing narratives. [References]
A1 - Russ,Sandra W.
Y1 - 2009///
N1 - Book: 2010-05424-015
English
Book; Edited Book
KW - PsycInfo
KW - Creative Writing
KW - Emotions
KW - Narratives
KW - Pretend Play
KW - Creativity
KW - Imagination

KW - Writers
RP - NOT IN FILE
SP - 247
EP - 263
JF - Kaufman, Scott Barry [Ed]; Kaufman, James C [Ed]
VL - :
IS - 2009
N2 - (from the chapter) Pretend play sets the stage for the creative writer. So many processes that are important in creative writing occur in the play of childhood and are developed in the arena of pretend play. Pretend play involves imagination, fantasy, storytelling, emotional expression, becoming absorbed in the moment, being spontaneous, taking risks, understanding the perspectives of others, and experiencing the joy of creation. Individuals who are able to engage in pretend play as children should be able to access these processes as adults during the writing process. This chapter reviews processes in pretend play that are important in creativity, with a specific focus on emotional processes and creative writing. Although pretend play in children is a long way from creative writing in adults, nevertheless basic elements of the creative process occur in play. We can study these processes and learn about how play can help foster these elements. As in many areas of creativity, both case studies of creative writers (Wallace, 1989) and research studies offer valuable information. This chapter includes theory, research, and case examples that illustrate creative processes in creative authors. (PsycINFO Database Record (c) 2012 APA, all rights reserved)
AD - Russ, Sandra W.: Department of Psychology, Case Western Reserve University, Cleveland, OH, US
ER -

TY - JOUR
ID - 619
T1 - Child-centered play therapy for children with low vision: A multiple case study
A1 - Filaccio,MaryLynn D.
Y1 - 2009///
N1 - Dissertation Abstract: 2009-99180-016
English
Dissertation Abstract
KW - PsycInfo
KW - Childhood Development
KW - Play Therapy
KW - Risk Factors
KW - Social Isolation
KW - Vision
RP - NOT IN FILE
SP - 1941
JF - Dissertation Abstracts International: Section B: The Sciences and Engineering
VL - 70:
IS - 3-B
N2 - This qualitative study was designed to explore the impact of child-centered play therapy for children with low vision. Because children with low vision are at risk for social isolation and misperceptions by others about their abilities, the current study sought to understand the impact of participation in play therapy on self-confidence and self-efficacy in children with low vision. The current study used qualitative methods including participant observation, narrative, and expert check-in/supervision to track the progress of three children, ages 4, 6, and 9 years, with low vision. Each child participated in six sessions of child-centered play therapy in their school settings. A mobile play therapy room supplied with toys appropriate for children with visual impairment was created and established for each session in the schools. Transcripts (videotape and audiotape) of each session were analyzed for themes, and cross-case analyses were conducted. Classroom teachers and visual impairment teachers were consulted weekly regarding their observations on behavior change during the course of therapy. In addition, classroom teachers and visual impairment teachers were asked to complete a survey to assess their perception of the child's self-efficacy before therapy and after therapy. The findings suggest that children with low vision may benefit from play therapy as evidenced by their progress in therapy and teacher reports. The findings also suggest that children with low vision seem to be capable of engaging in a wide variety of play behaviors including exploratory, functional/manipulative, make-believe, and games with rules. Further,

the findings remind us that the development of children with disabilities are also influenced by many of the same factors as children without disability, that is, age, gender, family characteristics, and socioeconomic status. Implications and suggestions for future research are provided. (PsycINFO Database Record (c) 2012 APA, all rights reserved)
SN - 0419-4217
AD - Filaccio, MaryLynn D.: U Northern Colorado, US
ER -

TY - JOUR
ID - 620
T1 - The relationship between individual differences in cognitive, social and personality development and the increase in complexity of children's alcohol expectancies
A1 - Bekman,Nicole M.
Y1 - 2009///
N1 - Dissertation Abstract: 2009-99160-142
English
Dissertation Abstract
KW - PsycInfo
KW - Cognitive Complexity
KW - Cognitive Development
KW - Individual Differences
KW - Personality Development
KW - Psychosocial Development
KW - Alcohols
RP - NOT IN FILE
SP - 1333
JF - Dissertation Abstracts International: Section B: The Sciences and Engineering
VL - 70:
IS - 2-B
N2 - The current study aimed to simultaneously examine cognitive, social and personality development in a cross-sectional sample of 3rd, 4th and 5th grade children to explore the interplay among these processes and how they relate to changes in children's understanding of alcohol. To replicate previous work, this study comprehensively examined relative increases in types of expectancies as a function of development. Results demonstrated that children in higher grades held more positive, negative and sedating expectancies of alcohol and positive alcohol expectancies increased more than negative alcohol expectancies. Improved performance on cognitive measures were associated with positive alcohol expectancy endorsement, indicating that children's ability to incorporate positive beliefs about alcohol, which are conflicting with information typically taught to children in this age range, may be related to their ability to form and articulate concepts with age. Among male participants, sensation seeking increased with age and was strongly associated with positive ideas about alcohol use, such as wanting to experiment with alcohol or planning to drink as an adult. Social influences on alcohol expectancies included exposure to drinking. When children's parents drank more, they had higher positive, negative and sedating alcohol expectancies, indicating that they had a greater understanding of all potential consequences of drinking, while children whose friends drank had higher positive but not other types of expectancies. Additionally, children who turned to adults for advice held increasing levels of negative and sedating alcohol expectancies across age, while children who sought support from their peers showed higher levels of positive and arousing expectancies across age groups. The interplay between cognitive development and risk factors such as social awareness of alcohol, source of social influence, and sensation seeking personality begins to demonstrate key relationships to alcohol expectancies in late childhood. These social and personality risk factors are likely to play an even greater role in early adolescence as children move to middle school and experience puberty. This study provides a basis for future elaboration of the roles these constructs play in an individual's ability to understand the multifaceted expectations that are held in our society about the effects of alcohol on human behavior. (PsycINFO Database Record (c) 2012 APA, all rights reserved)
SN - 0419-4217
AD - Bekman, Nicole M.: U South Florida, US
ER -

TY - JOUR
ID - 621
T1 - The communicative accomplishment of mutuality during father-son play in early childhood
A1 - Sweet,Dawn M.
Y1 - 2009///
N1 - Dissertation Abstract: 2009-99070-101
English
Dissertation Abstract
KW - PsycInfo
KW - Childhood Play Behavior
KW - Father Child Communication
KW - Father Child Relations
KW - Fathers
KW - Sons
KW - Individual Differences
RP - NOT IN FILE
SP - 3803
JF - Dissertation Abstracts International Section A: Humanities and Social Sciences
VL - 69:
IS - 10-A
N2 - This dissertation reports a three-study investigation of mutuality within self-directed play sessions of four African-American father-son dyads at two points in time. These three studies use multiple methods for systematically analyzing face-to-face interaction occurring within a finite space, namely a play area of a daycare center, during a fixed period of time, approximately 15 minutes. This dissertation develops a systematic approach for studying mutuality as it links to well-being as a quality of every day life and individual development. This research offers communication explanations for how relationships in the early stages of life are formed and a way of thinking about well-being across psychological, cognitive, emotional, and social domains from a communication perspective. Study 1 uses exploratory microanalytic techniques (Mokros, 2003) to identify units of decision making during father-son play. Through its in-depth and systematic examination of decision making, Study 1 provides a vocabulary for talking about decision making from a communication perspective and ultimately provides a conceptual framework and coding system for identifying features of mutuality within father-son interaction. From insights gleaned from this first study, the research in Study 2 reports a comparative study that focuses on understanding individual differences in the amount and quality of mutuality exhibited within and across four father-son dyads at two points in time. This research concludes with Study 3, a comparative study designed to link Interaction States identified in Study 2 with Command Sequences identified by using the Dyadic Parent-Child Interaction Coding System (DPICS) (Eyeberg & Robinson, 1983). This study contributes to communication theory and research because it examines the moment-to-moment child-rearing practices in families at risk and speaks to how through communicative practices, fathers and sons are able to construct and sustain moments of mutual focus on a task or each other and what this says about not only how relationships in the early stages of life are developed but also about well-being as a quality of everyday life and individual development. (PsycINFO Database Record (c) 2012 APA, all rights reserved)
SN - 0419-4209
AD - Sweet, Dawn M.: Rutgers The State U New Jersey - New Brunswick, US
ER -

TY - JOUR
ID - 622
T1 - Beyond the words of a storyteller: The cine-semiotic play of the abject, terror and community in the anti-hunting trilogy of Thornton W. Burgess
A1 - Connor,Kathleen Marie
Y1 - 2009///
N1 - Dissertation Abstract: 2009-99030-028
English
Dissertation Abstract
KW - PsycInfo
KW - Communities

KW - Language
KW - Semiotics
KW - Terrorism
RP - NOT IN FILE
SP - 3073
JF - Dissertation Abstracts International Section A: Humanities and Social Sciences
VL - 69:
IS - 8-A
N2 - Thornton W. Burgess (b. 1874, d. 1965) was a children's writer who attained great popularity and commercial success, with stories such as Old Mother West Wind (1910) and The Adventures of Peter Cottontail (1914) published by Little, Brown and Company, and with reprinted titles now published by Dover Press. His stories have been read from their initial publication in the early 1900s until the present day, and numerous titles have been translated to languages such as Japanese, French, Italian, and Gaelic. However, at times his works were not well-reviewed by children's literary critics, and so his mixed reception creates a mystery around "What is it about Burgess that readers either loved or hated?" My conceptual framework grounded in ideas of New Historicism and cultural studies draws upon psychoanalytic concepts (after Kristeva, others) and film theory (after Bordwell, others) to explore the narrative appeal of three related Burgess works: The Adventures of Poor Mrs. Quack (1917), The Adventures of Bob White (1919), and Lightfoot the Deer (1921). I was able to trace how Mr. Burgess conveyed his inner-most thoughts of primal drama (after Freud, Kristeva and Adler) of the lived and imagined stories of family and domestic affairs of friends and family, co joined with a political message of the early 20th century show-down between nature and encroaching urbanization. I found that Burgess practiced an economy of story-telling known for cinema and film (after Heath). The result in his well-known stories was an emergent aesthetic experience of seeing/feeling/meaning I term the cine-semiotic, evoking notions of le semiotique = psychoanalytic semiotics and la semiotique = filmic or sign operation semiotics (Rosen, 1986). It seems that Burgess shares a visual aesthetic with other children's writers such as Beatrix Potter, the English creator of Peter Cottontail (Carpenter, 1989). Through a close reading, documentation, and interpretation of three selected stories of Burgess which I term his Anti-Hunting Trilogy, this study seeks to understand the enigmatic nature of his mixed reception in early 20 th century children's literature. I have used archival-historical primary research materials along with readings of his stories to provide data around the nature of his popularly appealing narrative aesthetic. I became aware of a mode of address to the reader that I have termed the "cine-semiotic," which speaks to the language of Mr. Burgess's stories based in deep renditions of psychic dynamics which emanate through: (a) scripted movements of the abject and abjection, terror and anxiety, and community and wholeness; and (b) telling and showing aspects of narrative that play out through visual iconicity and other cinematic operations of the story language (after Bordwell, 1985). The results of my study provide a new means for interpreting, understanding and expressing the social, cultural and psychic effects resident within three stories written and published by Mr. Burgess from 1917 to 1921, and republished into the 21st century by Dover Publications for an avid and devoted contemporary readership. The implications of the study are that, under the conditions of language looking in at language, scholars looking in at children's stories can begin to discern meaningful patterns of cultural discourse that may otherwise go undiscerned. (PsycINFO Database Record (c) 2012 APA, all rights reserved)
SN - 0419-4209
AD - Connor, Kathleen-Marie: U Ottawa, Canada
ER -

TY - JOUR
ID - 623
T1 - Yoplay: Yoga based play therapy as a model of early intervention for inner city children at risk
A1 - Price,Shannon
Y1 - 2009///
N1 - Dissertation Abstract: 2009-99020-341
English
Dissertation Abstract
KW - PsycInfo
KW - At Risk Populations
KW - Early Intervention
KW - Play Therapy

KW - Urban Environments
KW - Yoga
RP - NOT IN FILE
SP - 4439
JF - Dissertation Abstracts International: Section B: The Sciences and Engineering
VL - 69:
IS - 7-B
N2 - Our nation is filled with countless children who are at-risk and in need of support, yet are not getting the help that is required for them to succeed. One fifth of young children in the United States live in poverty (National Center for Children in Poverty, 2004). Children living in low-income neighborhoods are known to have a high incidence of behavioral problems, trauma exposure and stress (Brown, McQuaid, Farina & Winnick-Gelles, 2006; Stein et al., 2003; Ackerman, Brown, & Izard, 2004; Lengua, 2002). This paper proposes an early intervention strategy using a yoga-based play therapy model for inner city, economically disenfranchised children. The intention is that through secondary prevention, the YoPlay training program will help to mitigate the adverse impact of poverty, trauma and stress on the developing child by encouraging the use of movements that incorporate the mind and the body to improve self-regulation, self-esteem, coping strategies, social skills and socio-emotional growth. By educating early childcare providers, this program will provide the training, resources and support needed for teachers to help strengthen their children through play and yoga in the classroom. This paper reviews research literature that documents the devastating impact of poverty, trauma and stress on the healthy development of young children. In addition, research literature is presented that shows how, with proper support, children can be taught the skills necessary to become resilient in the face of overwhelming adversity. This literature outlines the importance of physical play, dance and yoga in enhancing social and emotional development as well as resilience. In particular, yoga designed specifically for young children is proposed as an effective way for children to build self-confidence and learn about self-regulation. A teacher-training manual is included in the methods section as a product of this project. During the training, teachers spend an entire day on their own personal development, and spend the next day learning body-based interventions targeting pre-school children. Research on the YoPlay training indicated an increased enthusiasm, playfulness and invigoration in the teachers. In addition, teachers reported that the YoPlay program helped children calm down, re-group, and get in touch with their bodies and emotions. By partnering with key childcare providers, the YoPlay program aims to foster the healthy development of young, at-risk children so they may reach their full potential and become productive, joyful adults with big dreams and the resilience to pursue them. (PsycINFO Database Record (c) 2012 APA, all rights reserved)
SN - 0419-4217
AD - Price, Shannon: Massachusetts School Of Professional Psychology, US
ER -

TY - JOUR
ID - 624
T1 - An exploration of the links between qualities of the parent-child relationship and romantic relational aggression
A1 - Melniker,Rachael F.
Y1 - 2009///
N1 - Dissertation Abstract: 2009-99020-337
English
Dissertation Abstract
KW - PsycInfo
KW - Aggressive Behavior
KW - Attachment Behavior
KW - Mother Child Relations
KW - Parental Attitudes
KW - Romance
KW - Relational Aggression
RP - NOT IN FILE
SP - 4433
JF - Dissertation Abstracts International: Section B: The Sciences and Engineering
VL - 69:

IS - 7-B
N2 - The purpose of this study is to explore the relationships between attachment style, parental behaviors and attitudes, and romantic relational aggression. A review of the literature underscored the continuity between the quality of one's early attachment relationships with parents/caregivers and the quality of subsequent relationships with both peers and romantic partners. Insecure attachment relationships have consistently been associated with a variety of negative outcomes, though the current study focuses exclusively on the development of aggressive behavior. Research conducted on the relationship between attachment and aggression has found that insecurely attached individuals are more likely to aggress against peers and romantic partners than their secure counterparts. Parental psychological control or overprotection has similarly been identified within the literature as a risk factor for the development of aggressive behavior. The current study explored the role that both attachment to parents and parental attitudes and behaviors play in the development of relationally aggressive behavior in romantic relationships. Participants in the current study were 154 heterosexual males and females, all 18 years of age and older, each of whom completed a survey based upon their relationships with both parents/caregivers and romantic partners. Statistical analyses revealed that relationships with fathers early in life play an important role in the development of relationally aggressive behavior in romantic relationships, while relationships with mothers did not emerge as significant predictors of romantic relational aggression. Individuals with more secure attachments to their fathers were less likely to aggress relationally against a romantic partner than were insecurely attached individuals. Similarly, fathers who demonstrated more warmth and caring had children who engaged in less romantic relational aggression than those with fathers who were colder and less supportive. This study provides additional insight into the antecedents of relationally aggressive behavior in romantic relationships and highlights the legacy even the earliest relationships leave. (PsycINFO Database Record (c) 2012 APA, all rights reserved)
SN - 0419-4217
AD - Melniker, Rachael F.: Massachusetts School Of Professional Psychology, US
ER -

TY - JOUR
ID - 625
T1 - Infant coping and maternal interactive behavior predict attachment in a Portuguese sample of healthy preterm infants. [References]
A1 - Fuertes,Marina; Lopes-dos-Santos,Pedro; Beeghly,Marjorie; Tronick,Ed
Y1 - 2009///
N1 - Peer Reviewed Journal: 2009-24481-007
English
Journal; Peer Reviewed Journal
KW - PsycInfo
KW - Attachment Behavior
KW - Coping Behavior
KW - Infant Development
KW - Mother Child Relations
KW - Premature Birth
RP - NOT IN FILE
SP - 320
EP - 331
JF - European Psychologist
VL - 14:
IS - 4
N2 - In this longitudinal study of a Portuguese sample of healthy preterm infants, the aim was to identify specific, independent predictors of infant-mother attachment status from a set of variables including maternal education, maternal representations' of infant temperament, infant regulatory behavior (coping), and mothers' interactive behavior in free play. The sample consisted of 48 medically low-risk preterm infants and their mothers who varied in education. When infants were 1 and 3 months (corrected age), mothers described their infants' temperament using a Portuguese temperament scale (Escala de Temperamento do Bebe). At 3 months (corrected age), infants' capacity to regulate stress (coping) was evaluated during Tronick's Face-to-Face Still-Face paradigm (FFSF). At 9 months (corrected age), mothers' interactive behaviors were evaluated during free play using the Crittenden's Child-Adult Relationship Experimental Index (CARE-Index). At 12 months

(corrected age), infants' attachment security was assessed during Ainsworth's strange situation. Sixteen (33.3%) infants were classified as securely attached, 17 (35.4%) as insecure-avoidant, and 15 (31.3%) as insecure-resistant. In bivariate analyses, multiple factors were significantly associated with attachment status. However, in hierarchical regression analyses, only infant coping and maternal responsiveness were significant predictors of attachment status. These findings suggest that both infant characteristics identifiable early in the first year, such as coping, and maternal characteristics such as sensitivity influence the process of attachment formation. (PsycINFO Database Record (c) 2012 APA, all rights reserved) (journal abstract)
SN - 1016-9040
AD - Fuertes, Marina: Child Development Unit, Boston, MA, US Lopes-dos-Santos, Pedro: University of Porto, Porto, Portugal Beeghly, Marjorie: Child Development Unit, Boston, MA, US Tronick, Ed: Child Development Unit, Boston, MA, US
ER -

TY - JOUR
ID - 626
T1 - Ethical issues relating to the banking of umbilical cord blood in Mexico. [References]
A1 - Serrano-Delgado,V.
Y1 - 2009///
N1 - Peer Reviewed Journal: 2009-21282-001
English
Journal; Peer Reviewed Journal
KW - PsycInfo
KW - Bioethics
KW - Biotechnology
KW - Laws
KW - Neural Regeneration
RP - NOT IN FILE
JF - BMC Medical Ethics
VL - 10 Aug 2009, ArtID 12.
N2 - Background: Umbilical cord banks are a central component, as umbilical cord tissue providers, in both medical treatment and scientific research with stem cells. But, whereas the creation of umbilical cord banks is seen as successful practice, it is perceived as a risky style of play by others. This article examines and discusses the ethical, medical and legal considerations that arise from the operation of umbilical cord banks in Mexico. Discussion: A number of experts have stated that the use of umbilical cord goes beyond the mere utilization of human tissues for the purpose of treatment. This tissue is also used in research studies: genetic studies, studies to evaluate the effectiveness of new antibiotics, studies to identify new proteins, etc. Meanwhile, others claim that the law and other norms for the functioning of cord banks are not consistent and are poorly defined. Some of these critics point out that the confidentiality of donor information is handled differently in different places. The fact that private cord banks offer their services as "biological insurance" in order to obtain informed consent by promising the parents that the tissue that will be stored insures the health of their child in the future raises the issue of whether the consent is freely given or given under coercion. Another consideration that must be made in relation to privately owned cord banks has to do with the ownership of the stored umbilical cord. Summary: Conflicts between moral principles and economic interests (non-moral principles) cause dilemmas in the clinical practice of umbilical cord blood storage and use especially in privately owned banks. This article presents a reflection and some of the guidelines that must be followed by umbilical cord banks in order to deal with these conflicts. This reflection is based on the fundamental notions of ethics and public health and seeks to be a contribution towards the improvement of umbilical cord banks' performance. (PsycINFO Database Record (c) 2012 APA, all rights reserved) (journal abstract)
SN - 1472-6939
AD - Serrano-Delgado, V. Moises: Obstetrics and Gynaecology Hospital with Family Medicine, Mexican Institute of Social Security, Mexico City, Mexico Novello-Garza, Barbara: Direccion de Prestaciones Medicas, Mexican Institute of Social Security, Mexico City, Mexico Valdez-Martinez, Edith: Health Research Council, Mexican Institute of Social Security, Mexico City, Mexico
ER -

TY - JOUR

ID - 628
T1 - Childhood body mass index in community context: Neighborhood safety, television viewing, and growth trajectories of BMI. [References]
A1 - Cecil-Karb,Rebecca; Grogan-Kaylor,Andrew
Y1 - 2009///
N1 - Peer Reviewed Journal: 2009-11363-001
English
Journal; Peer Reviewed Journal
KW - PsycInfo
KW - Childhood Play Behavior
KW - Neighborhoods
KW - Obesity
KW - Parental Attitudes
KW - Television Viewing
KW - Body Mass Index
KW - Safety
RP - NOT IN FILE
SP - 169
EP - 177
JF - Health & Social Work
VL - 34:
IS - 3
N2 - The United States is currently experiencing an epidemic of children who are overweight or obese. Recently, research on child obesity has begun to examine the relationship between neighborhood environments and the health behaviors of youths. The current study used growth curve analysis based on multilevel modeling to examine the relationship between parents' perceptions of neighborhood safety and children's body mass index (BMI). Parents' perceptions of neighborhood safety had a significant association with children's BMI, and this relationship was fully mediated by television viewing. The results of this study suggest that when parents perceive their neighborhood to be unsafe, they will restrict their children's outdoor activities and increase the likelihood of sedentary indoor activity. Policies aimed at reducing overweight and obesity in children should take into account the neighborhood contexts in which children live. (PsycINFO Database Record (c) 2012 APA, all rights reserved) (journal abstract)
SN - 0360-7283
AD - Cecil-Karb, Rebecca: School of Social Work, University of Michigan, Ann Arbor, MI, US Grogan-Kaylor, Andrew: School of Social Work, University of Michigan, Ann Arbor, MI, US
ER -

TY - JOUR
ID - 630
T1 - Reclaiming science: Evidence and truth in the age of persuasion. [References]
A1 - de,St
Y1 - 2009///
N1 - Electronic Collection: 2009-08971-001
English
Electronic Collection
KW - PsycInfo
KW - Childhood Development
KW - Fear
KW - Parenting Style
KW - Protective Factors
KW - Society
KW - Childhood Play Development
KW - Life Experiences
KW - Safety
KW - Social Deprivation
RP - NOT IN FILE

JF - PsycCRITIQUES
VL - 54:
IS - 43
N2 - Reviews the book, Reclaiming childhood: Freedom and play in an age of fear by Helene Guldberg (see record 2008-17614-000). In this book, the author addresses topics that are of great interest to parents, educators, and researchers. The take-home message of the book is that children in the United Kingdom and the United States today are worse off than were children from the last two generations because of societal shifts toward overprotection. Helicopter parenting, unreasonable safety requirements, and out-of-proportion fears regarding stranger danger, bullying, and Internet predators have led to a "cocooning" of children that deprives them of experiences that foster resiliency, curiosity, and self-efficacy. It is peculiar that we, both parents and developmental psychologists, were more likely to agree with this statement before we read the book than after reading the book. We chose to review this book, in part, so that we could become aware of the science that undergirds this claim. While it does, indeed, seem that children today are being partially paralyzed by fear-based parenting and overprotective policies, this book fails to convince us of that. The misuse of science and the clumsy attempt at persuasion leave us wondering whether and how children today are faring, given the changing sociohistorical realities they face. (PsycINFO Database Record (c) 2012 APA, all rights reserved)
SN - 1554-0138
ER -

TY - JOUR
ID - 631
T1 - The risk is that there is 'no risk': A simple, innovative intervention to increase children's activity levels. [References]
A1 - Bundy,Anita C.
Y1 - 2009///
N1 - Peer Reviewed Journal: 2009-06491-004
English
Journal; Peer Reviewed Journal
KW - PsycInfo
KW - Childhood Play Behavior
KW - Early Childhood Development
KW - Physical Activity
KW - Playgrounds
KW - Social Interaction
KW - Activity Level
KW - Creativity
KW - Safety
RP - NOT IN FILE
SP - 33
EP - 45
JA - International Journal of Early Years Education
VL - 17:
IS - 1
N2 - School playgrounds offer everyday opportunities for physically active and social play that combats obesity, develops skills, and promotes well-being. However, teachers' fear of the legal consequences of injury can elicit over-zealous risk reduction with the result that playgrounds lack challenge, and the potential benefits of play become limited. In this research, we trialled a simple, cost-effective strategy to encourage children to be more active and social on a school playground. Over 11 weeks, we made available materials with no fixed purpose (e.g. car tires, boxes) to a playground of children aged five to seven. Accelerometers showed children became significantly more active. Interviews with teachers suggested children also became more social, creative, and resilient. However, despite no incidence of injuries, teachers perceived an increased risk and encountered dilemmas regarding duty of care. We conclude that future interventions should address issues of 'surplus safety' at individual, school, system, and policy levels. (PsycINFO Database Record (c) 2012 APA, all rights reserved) (journal abstract)
SN - 0966-9760

AD - Bundy, Anita C.: Faculty of Health Sciences, University of Sydney, Lidcombe, NSW, Australia Luckett, Tim: Faculty of Health Sciences, University of Sydney, Lidcombe, NSW, Australia Tranter, Paul J.: University of New South Wales, Canberra, NSW, Australia Naughton, Geraldine A.: Australian Catholic University, Melbourne, VIC, Australia Wyver, Shirley R.: Macquarie University, Sydney, NSW, Australia Ragen, Jo: Faculty of Health Sciences, University of Sydney, Lidcombe, NSW, Australia Spies, Greta: Faculty of Health Sciences, University of Sydney, Lidcombe, NSW, Australia
ER -

TY - JOUR
ID - 632
T1 - Affordances for risky play in preschool: The importance of features in the play environment. [References]
A1 - Sandseter,Ellen Beate Hansen
Y1 - 2009///
N1 - Peer Reviewed Journal: 2009-05876-009
English
Journal; Peer Reviewed Journal
KW - PsycInfo
KW - Childhood Play Behavior
KW - Playgrounds
KW - Preschool Education
KW - Preschool Students
KW - Risk Taking
RP - NOT IN FILE
SP - 439
EP - 446
JF - Early Childhood Education Journal
VL - 36:
IS - 5
N2 - The purpose of this article is to qualitatively explore the affordances for risky play in two different preschool outdoor environments, an ordinary preschool playground and a nature playground, based on Gibson (The ecological approach to visual perception, 1979) theory of affordances and Heft's and Kyttea's (Heft in Children's Environ Qual 5(3) 29-37, 1988; Kytta in J Environ Psychol 22:109-123, 2002, Kytta in J Environ Psychol 24:179-198, 2004) extended work on this theory. Observations of risky play in two Norwegian preschools, one ordinary preschool (where play took place on an ordinary playground) and one nature and outdoor preschool (where play took place in a nature area) were conducted. In addition, the children were interviewed about their actualized affordances of risky play, their mobility license, and the constraints on risky play. The results show that both play environments afford an extensive amount of risky play among the children, and that the degree of mobility license tolerated by the staff is an important factor for the children to actualize these affordances. Differences in the qualities and features in the two play environments were found to have an impact on the degree of riskiness in the play situations. As such, the nature playground afforded a higher degree of risk in children's risky play. (PsycINFO Database Record (c) 2012 APA, all rights reserved) (journal abstract)
SN - 1082-3301
AD - Sandseter, Ellen Beate Hansen: Department of Physical Education, Queen Maud University, College of Early Childhood Education, Trondheim, Norway
ER -

TY - JOUR
ID - 633
T1 - Playful strategies to manage frustration: The turtle technique and beyond. [References]
A1 - Feindler,Eva L.
Y1 - 2009///
N1 - Book: 2009-04903-019
English
Book; Edited Book
KW - PsycInfo

KW - Aggressive Behavior
KW - Behavior Problems
KW - Frustration
KW - Play Therapy
KW - Anger Control
KW - Child Psychotherapy
KW - Clinicians
KW - Expressive Psychotherapy
RP - NOT IN FILE
SP - 401
EP - 422
JF - Drewes, Athena A [Ed]
VL - :
IS - 2009
N2 - (from the chapter) Aggressive behavior problems in young children are one of the most frequent referral issues for clinicians, and since early patterns may predict later antisocial behaviors, early interventions seem fundamental. Contextual risk factors have focused on parents who provide harsh discipline, poor monitoring, and inconsistent contingency management. Emotion regulation, in particular, the regulation of anger and frustration, influences the occurrence of cognitive and social skills necessary for prosocial functioning. Turtle Magic is a short children's story designed to highlight several main components of anger management for young children. Children's pretend play has long been proposed as a mode of social interaction that enhances the development of emotion regulation. Parent Child Interaction Therapy teaches parents behavior management strategies along with problem solving and play skills for enhancing the positive connections between parents and their children. Most likely, effective treatment would incorporate a number of play therapy strategies as they are quite effective in general for preschool children, would be disseminated in an individual or small-group format for children who are clinically referred, and would include a parent training component specific to anger management. Further, such strategies will help newly learned arousal management skills generalize across natural environments and will be better adapted to interactions with both peers and adults in those settings. Finally, parents and teachers play a crucial role in the implementation and maintenance of any therapeutic program for young children. (PsycINFO Database Record (c) 2012 APA, all rights reserved)
AD - Feindler, Eva L.: Long Island University, Brookville, NY, US
ER -

TY - JOUR
ID - 634
T1 - Parenting of 7-month-old infants at familial risk for ADHD during infant's free play, with restrictions on interaction. [References]
A1 - Landau,Rivka; Amiel-Laviad,Riki; Berger,Andrea; Atzaba-Poria,Naama; Auerbach,Judith
Y1 - 2009///
N1 - Peer Reviewed Journal: 2009-04272-006
English
Journal; Peer Reviewed Journal
KW - PsycInfo
KW - Attention Deficit Disorder with Hyperactivity
KW - Childhood Play Behavior
KW - Father Child Relations
KW - Mother Child Relations
KW - Parenting Style
KW - Risk Factors
RP - NOT IN FILE
SP - 173
EP - 182
JF - Infant Behavior & Development
JA - Infant behav
VL - 32:
IS - 2

N2 - Patterns of interaction of 34 mothers and fathers with their 7-month-old boys at familial risk for ADHD and 25 comparison families were studied during infant play with blocks. The parents were instructed to refrain from intervening as much as possible. Infants in the risk group did not differ from those in the comparison group in frequency of needing help or involving parents in play. Nonetheless, they received adequate responsivity from their mothers less often than infants in the comparison group. Mothers in the risk group were also more likely not to respond to these needs at all. Mothers in the comparison group were more physically intrusive. No group difference was found for maternal rebuilding of the infant's play. No group differences were found for any of father's behaviors. However, fathers in both groups rebuilt their infant's play more frequently than mothers, infants looked at them more often, and a larger number of infants involved the father in their play. (PsycINFO Database Record (c) 2012 APA, all rights reserved) (journal abstract)
SN - 0163-6383
AD - Landau, Rivka: Department of Psychology, Ben-Gurion University of the Negev, Beer Sheva, Israel Amiel-Laviad, Riki: Department of Psychology, Ben-Gurion University of the Negev, Beer Sheva, Israel Berger, Andrea: Department of Psychology, Ben-Gurion University of the Negev, Beer Sheva, Israel Atzaba-Poria, Naama: Department of Psychology, Ben-Gurion University of the Negev, Beer Sheva, Israel Auerbach, Judith G.: Department of Psychology, Ben-Gurion University of the Negev, Beer Sheva, Israel
ER -

TY - JOUR
ID - 636
T1 - Reclaiming childhood: Freedom and play in an age of fear. [References]
A1 - Guldberg,Helene
Y1 - 2009///
N1 - Book: 2008-17614-000
English
Book; Authored Book
(Created by PsycINFO) Acknowledgements Introduction Part I: The good, the bad, and the history: A balance sheet of modern childhood A childish panic about the next generation Cocooning children Childhood in historical perspective Part II: Freedom and child development Growing up: Why risk-taking is good for kids Play: What is it good for? The bullying bandwagon Virtual lives? Media, brands and the MySpace generation Part III: Taking real responsibility: The role of adult society Let parents be parents: The myth of infant determinism Let teachers be teachers-Not social workers and 'happiness counsellors' Let strangers be friends: How the 'stranger danger' panic is creating a hostile adult world Afterword Bibliography Index
KW - PsycInfo
KW - Childhood Development
KW - Childhood Play Behavior
KW - Early Experience
KW - Freedom
KW - Sociocultural Factors
KW - Expectations
KW - Fear
KW - Safety
KW - Uncertainty
RP - NOT IN FILE
SP - New
JF - (2009)
VL - Reclaiming childhood: Freedom and play in an age of fear. ix, 205 pp. New York, NY, US: Routledge/Taylor & Francis Group; US.
N2 - (from the book) Children are cooped up, passive, apathetic and corrupted by commerce ... or so we are told. Reclaiming Childhood confronts the dangerous myths spun today about childhood. Yes, modern children are losing out on many experiences past generations took for granted, but their lives have improved in so many other ways. Helene Guldberg exposes the stark consequences on child development of both our low expectations of fellow human beings and our safety-obsessed culture. She argues that, rather than projecting fears and uncertainties on to children, we need to allow them to grow and flourish, to balance sensible guidance with youthful independence. Reclaiming Childhood suggests ways we can work to improve a child's experience, as well as that of parents, teachers and 'strangers', simply by taking a step back from panic and doom-

mongering. (from the introduction) Unsupervised play is very important for children's social, emotional, cognitive, and physical development. Children need to be given space away from adults' watchful eyes-in order to play, experiment, take risks (within a sensible framework provided by adults), test boundaries, have arguments, fight, and learn how to resolve conflicts. (PsycINFO Database Record (c) 2012 APA, all rights reserved)
AD - Guldberg, Helene: Open U, Milton Keynes, England
ER -

TY - JOUR
ID - 641
T1 - Brief report: Behavioral risk factors for youth soccer (football) injury. [References]
A1 - Schwebel,David C.
Y1 - 2007///
N1 - Peer Reviewed Journal: 2007-06453-003
English
Journal; Peer Reviewed Journal
KW - PsycInfo
KW - Adolescent Attitudes
KW - Injuries
KW - Risk Factors
KW - Soccer
KW - Sports
RP - NOT IN FILE
SP - 411
EP - 416
JF - Journal of Pediatric Psychology
JA - J Pediatr Psychol.
VL - 32:
IS - 4
N2 - Objectives: By most reports, soccer (football) is among the most played and most popular sports in the world. This study prospectively examined behavioral risk factors for youth soccer injury. Method: Sixty 11- and 12-year-old boys who played on six teams in a suburban recreational soccer league were followed over the course of a season. Six predictors were assessed prior to the start of the season via self-report measures from coaches, parents, and the players themselves: inhibition, aggression, risk-taking, skill, experience playing soccer, and physical size. All games were videotaped, and tapes were reviewed to record players' collisions with other players, fouls, falls during the course of play, and injuries. Results: Greater skill and less experience playing soccer best predicted injury risk. Inhibition, aggression, and risk-taking did not emerge as predictors. Conclusion: Results are discussed with respect to previous research in youth sport and general pediatric injury risk. (PsycINFO Database Record (c) 2013 APA, all rights reserved) (journal abstract)
SN - 0146-8693
AD - Schwebel, David C.: Department of Psychology, University of Alabama at Birmingham, Birmingham, AL, US Banaszek, Mark M.: Department of Psychology, University of Alabama at Birmingham, Birmingham, AL, US McDaniel, McCall: Department of Psychology, University of Alabama at Birmingham, Birmingham, AL, US
ER -

TY - JOUR
ID - 645
T1 - The role of child temperament on head start preschoolers' social competence in the context of cumulative risk. [References]
A1 - Corapci,Feyza
Y1 - 2008///
N1 - Peer Reviewed Journal: 2007-19863-002
English
Journal; Peer Reviewed Journal
KW - PsycInfo

KW - Child Attitudes
KW - Personality
KW - Preschool Students
KW - Project Head Start
KW - Social Skills
KW - Childhood Play Behavior
KW - Peers
KW - Risk Factors
KW - Teachers
RP - NOT IN FILE
SP - 1
EP - 16
JF - Journal of Applied Developmental Psychology
VL - 29:
IS - 1
N2 - This study examined the main and interactive effects of cumulative risk and child temperament on teacher ratings of social competence and observer ratings of peer play in a sample of Head Start preschoolers. A cumulative risk index (CRI) was computed by summing the total number of risk factors for each family. There was a difference in the predictive power of the CRI when it consisted of 10 (including child temperament) versus 8 possible risk factors (excluding child temperament) for teacher ratings of social competence. The CRI based on 10 risk factors was related significantly and negatively to social competence. A significant interaction between inhibited child temperament and cumulative environmental risk revealed that highly inhibited children were perceived by their teachers as equally competent under both high- and low-risk, whereas less inhibited children received lower ratings as environmental risk increased. Less inhibited and less impulsive temperament emerged as promotive factors associated with high levels of interactive peer play and teacher ratings of social competence, respectively, regardless of the risk level. (PsycINFO Database Record (c) 2013 APA, all rights reserved) (journal abstract)
SN - 0193-3973
AD - Corapci, Feyza: Department of Psychological Sciences, Purdue University, West Lafayette, IN, US
ER -

TY - JOUR
ID - 646
T1 - Parent and teacher ratings of peer interactive play and social-emotional development of preschool children at risk. [References]
A1 - Gagnon,Sandra Glover; Nagle,Richard
Y1 - 2007///
N1 - Peer Reviewed Journal: 2007-07556-003
English
Journal; Peer Reviewed Journal
KW - PsycInfo
KW - Childhood Development
KW - Childhood Play Behavior
KW - Emotional Development
KW - Peer Relations
KW - Preschool Students
KW - Interpersonal Interaction
KW - Parental Attitudes
KW - Teacher Attitudes
RP - NOT IN FILE
SP - 228
EP - 242
JF - Journal of Early Intervention
VL - 29:
IS - 3

N2 - Estimates of agreement and consistency between parent and teacher ratings of peer-interactive play and social emotional development were examined in a sample of preschool children at risk for academic difficulties. Eighty-three pairs of parents and teachers of 4-year-old children completed the Penn Interactive Peer Play Scale (PIPPS) and 69 of these pairs also were interviewed with the Vineland Social-Emotional Early Childhood Scales (SEEC). Significant mean differences were found between parent and teacher scores on the PIPPS, but not on the SEEC. No noteworthy correlations were found between raters on the PIPPS, though low to moderate correlations were revealed between parent and teacher responses on the SEEC. Multivariate techniques revealed statistically significant and noteworthy correlations between parent and teacher responses on the SEEC but not on the PIPPS. Results are discussed within the context of conducting multifaceted, ecological assessments with preschool children. Implications for early childhood educators and interventionists are noted regarding the importance of gathering and interpreting multiple sources of information that contribute to an understanding of young children's behavior. (PsycINFO Database Record (c) 2012 APA, all rights reserved) (journal abstract)
SN - 1053-8151
AD - Gagnon, Sandra Glover: Department of Psychology, Appalachian State University, Boone, NC, US Nagle, Richard J.: Appalachian State University, Boone, NC, US Nickerson, Amanda B.: University at Albany, Albany, NY, US
ER -

TY - JOUR
ID - 647
T1 - Research to Reality: Applying Findings to Practice
A1 - Canham,Daryl L.
Y1 - 2006///
N1 - Peer Reviewed Journal: 2006-12946-009
English
Journal; Peer Reviewed Journal
KW - PsycInfo
KW - Attention Deficit Disorder with Hyperactivity
KW - Case Management
KW - Obesity
KW - Parental Attitudes
KW - School Nurses
KW - Early Intervention
KW - Family
KW - Health
KW - Preschool Students
RP - NOT IN FILE
SP - 296
EP - 298
JF - The Journal of School Nursing
VL - 22:
IS - 5
N2 - Presents two studies which apply findings to improve the practices of school nurses. The first study explored service use patterns and services needed for children with attention deficit/hyperactivity disorder (ADHD). This study presents several pertinent outcomes related to the school nurse role. School nurses need to target single-parent families and families with boys with ADHD, because these families may have a higher need for services. Development of case management models in the school setting is a major recommendation of this research study. The second study examined parental perceptions related to health status and play activities of a group of overweight toddlers and preschoolers. The purpose of this study was to examine parents' perceptions regarding their children's current health status; parental perceptions and understanding of the relationship between obesity and current and future health risks. School nurses play a major role in prevention and early intervention. School nurses who are associated with child care centers can screen preschoolers, identify those at risk for overweight and obesity, and refer families to appropriate resources. (PsycINFO Database Record (c) 2012 APA, all rights reserved)
SN - 1059-8405
AD - Canham, Daryl L.: School of Nursing, San Jose State University, San Jose, CA, US

ER -

TY - JOUR
ID - 648
T1 - Panic Symptoms and Disorder in Youth: What Role Does Anxiety Sensitivity Play? [References]
A1 - Hale,Lisa R.
Y1 - 2006///
N1 - Book: 2006-09562-005
English
Book; Edited Book
KW - PsycInfo
KW - Anxiety
KW - Panic Disorder
KW - Symptoms
KW - Anxiety Sensitivity
KW - Etiology
KW - Panic
RP - NOT IN FILE
SP - 131
EP - 162
JF - Velotis, Calvin M [Ed]
VL - :
IS - 2006
N2 - (from the chapter) There is growing recognition that panic symptoms and panic disorder are important clinical problems in youth, although there has been limited study and the developmental origins of these problems are largely unknown. Because little is know about the etiology of panic symptoms or panic disorder in children and adolescents, researchers have begun to investigate several processes. Anxiety sensitivity (AS), the fear of common anxiety symptoms and a possible cognitive risk factor for panic disorder, has been extensively studied in adults and has more recently been evaluated in adolescents and children. In investigations with clinical and nonclinical populations, AS has correlated with panic symptoms and panic severity. In an initial longitudinal investigation, increases in AS over time best predicted panic symptoms in youth. The developmental etiology of AS is largely unknown, but parent behavior has been implicated. AS may function as a cognitive risk factor for child and adolescent panic disorder and further study is warranted. (PsycINFO Database Record (c) 2012 APA, all rights reserved)
AD - Hale, Lisa R.: Department of Psychiatry and Behavioral Sciences, University of Kansas Medical Center, Kansas City, KS, US Calamari, John E.: Rosalind Franklin University of Medicine and Science, North Chicago, IL, US
ER -

TY - JOUR
ID - 649
T1 - PLAYSPACE: A preventive intervention for infants and young children at risk from postnatal depression. [References]
A1 - Kurzweil,Sonya
Y1 - 2008///
N1 - Peer Reviewed Journal: 2008-03761-002
English
Journal; Peer Reviewed Journal
KW - PsycInfo
KW - Childhood Play Behavior
KW - Childhood Play Development
KW - Group Psychotherapy
KW - Postpartum Depression
KW - Psychoanalysis
KW - At Risk Populations
KW - Intervention

KW - Prevention
KW - Socioeconomic Status
RP - NOT IN FILE
SP - 5
EP - 15
JF - International Journal of Mental Health Promotion
VL - 10:
IS - 1
N2 - Postnatal depression is known to have deleterious effects on child emotional, social and cognitive development, and yet there are few prevention programs and little research on prevention of psychopathology in populations of middle socio-economic status (SES). This paper presents a preventive intervention for this population of women, infants and young children. The intervention, referred to as PLAYSPACE, combines an ongoing, relational-developmental, psychoanalytically oriented therapy group for the women, with a teacher-led concurrent psychosocial play group in an engaging playspace for the children and, in addition for some women, individual relational-developmental psychotherapy with a concurrent individual child care option in the playspace. A previous article presented the adult group component and outcome research. In this article, the focus is child outcomes, the psychosocial play group and the individual child care component. (PsycINFO Database Record (c) 2012 APA, all rights reserved) (journal abstract)
SN - 1462-3730
AD - Kurzweil, Sonya: Department of Psychiatry, Harvard Medical School, Boston, MA, US
ER -

TY - JOUR
ID - 650
T1 - Examining resource and protective factors in the adjustment of Latino youth in low income families: What role does maternal acculturation play? [References]
A1 - Loukas,Alexandra; Suizzo,Marie-Anne; Prelow,Hazel
Y1 - 2007///
N1 - Peer Reviewed Journal: 2008-01922-010
English
Journal; Peer Reviewed Journal
KW - PsycInfo
KW - Acculturation
KW - Adjustment
KW - Parental Characteristics
KW - Protective Factors
KW - Risk Factors
KW - Internalization
KW - Juvenile Delinquency
KW - Lower Income Level
KW - Mother Child Relations
KW - Latinos/Latinas
RP - NOT IN FILE
SP - 489
EP - 501
JF - Journal of Youth and Adolescence
VL - 36:
IS - 4
N2 - This longitudinal study examined whether the risk and positive factors contributing to the delinquent behaviors and internalizing problems of 454 Latino adolescents varied across maternal linguistic acculturation and adolescent gender. Although the level of cumulative risk to which the 10-to-14-year old adolescents were exposed did not vary by maternal linguistic acculturation, the factors contributing to their subsequent adjustment 16 months later varied substantially. Multiple regression analyses showed that for boys, maternal monitoring offset cumulative risk effects in the high acculturation group, but was unrelated to adjustment in the low acculturation group. Social competence served a protective function for boys in the high acculturation group, but was detrimental for boys in the low acculturation group and mother-son relationship quality directly

predicted more subsequent delinquent behaviors among boys in the low acculturation group. Maternal monitoring was the only positive factor contributing to girls' adjustment, directly predicting fewer delinquent behaviors for all girls. (PsycINFO Database Record (c) 2012 APA, all rights reserved) (journal abstract)
SN - 0047-2891
AD - Loukas, Alexandra: Department of Kinesiology and Health Education, University of Texas at Austin, Austin, TX, US Suizzo, Marie-Anne: Department of Educational Psychology, University of Texas at Austin, Austin, TX, US Prelow, Hazel M.: Department of Psychology, University at Albany, State University of New York, Albany, NY, US
ER -

TY - JOUR
ID - 652
T1 - An ecological and developmental perspective on dropout risk factors in early adolescence: Role of school social workers in dropout prevention efforts. [References]
A1 - Jozefowicz-Simbeni,Debra M.
Y1 - 2008///
N1 - Peer Reviewed Journal: 2009-08088-006
English
Journal; Peer Reviewed Journal
KW - PsycInfo
KW - Childhood Development
KW - School Based Intervention
KW - School Dropouts
KW - Social Workers
KW - Ecology
KW - Prevention
KW - Risk Factors
RP - NOT IN FILE
SP - 49
EP - 62
JF - Children & Schools
VL - 30:
IS - 1
N2 - The role school social workers play within schools, the training they receive, and whether they are present in junior high schools are all factors that pose barriers and opportunities for school social workers' involvement in dropout prevention efforts during the middle school years. Such efforts are necessary because the unique challenges of early adolescence can bring about increased academic and behavioral problems and can eventually lead to school dropout. Unfortunately, few studies have examined dropout prevention in middle schools or junior high schools, and there has been little discussion of the role school social workers can play in dropout prevention efforts during this time of life. To highlight the ways that school social workers are needed and can be involved in dropout prevention in the middle schools, this article examines student and ecological risk factors associated with school dropout, the early adolescent life stage, dropout prevention approaches, and the role school social workers can play in multilevel dropout prevention efforts aimed at the middle school years. (PsycINFO Database Record (c) 2012 APA, all rights reserved) (journal abstract)
SN - 1532-8759
AD - Jozefowicz-Simbeni, Debra M. Hernandez: School of Social Work, Wayne State University, Detroit, MI, US
ER -

TY - JOUR
ID - 653
T1 - Preschoolers' heart rate and physical activity response to three different motivational climates: Mastery, performance, and unplanned free play
A1 - Parish,Loraine Elizabeth
Y1 - 2008///

N1 - Dissertation Abstract: 2008-99230-493
English
Dissertation Abstract
KW - PsycInfo
KW - Heart Rate
KW - Motivation
KW - Performance
KW - Physical Activity
KW - Physical Education
KW - Teachers
RP - NOT IN FILE
SP - 2198
JF - Dissertation Abstracts International Section A: Humanities and Social Sciences
VL - 69:
IS - 6-A
N2 - Although people of all ages benefit from regular, moderate intensity physical activity (U.S. Department of Health and Human Services, 1996) many children are showing less interest and participation in physical education and are adopting sedentary lifestyles (Sallis et al., 1992; U.S. Department of Health and Human Services, 1996). Incorporating regular, planned physical programs, as opposed to unplanned free play, into early childhood and preschool education programs may provide an effective means by which to engage young children in physical activity. In order to develop curricula that maximize engagement in physical activity it is important to identify teaching approaches that motivate young children to be physically activity. The purpose of the current study was to investigate the influence of three motivational environments (mastery-oriented climate [MC], performance-oriented climate [PC], and unplanned free play [FP] [i.e., high autonomy with limited instruction and equipment]), on the physical activity behaviors of 27 preschoolers (11 boys, 16 girls) between the ages of 3- and 5-years-old (M age = 4.5, SD = 1.1 years) and at risk for developmental delay and poor health. The physical activity environment was manipulated according to Ames' (1992a, 1992b) guidelines for creating a mastery-oriented and a performance-oriented climate. Participants engaged in six, 30-minute physical activity sessions for each of the three conditions during which physical activity was measured via heart rate monitoring and accelerometers and physical activity intensity level was categorized using PAHR > 50. As shown by the manipulation check results, the physical activity teachers created three different motivational physical activity climates. Analyses of the physical activity data indicated that heart rate and PAHR > 50 were not significantly different across the three conditions. Although accelerometer count did not differ between the MC and PC conditions, both conditions were significantly higher compared to the FP condition. Heart rate and accelerometer count did not change over the six physical activity sessions of each condition. Although PAHR > 50 differed between sessions during the MC and PC conditions, there was no clear trend of change over the six physical activity sessions for the FP condition. The results of this study provide preliminary insight into young children's physical activity engagement during different motivational climates. In conclusion, future research should to be conducted to better understand the impact of motivational climate on young children's physical activity. (PsycINFO Database Record (c) 2012 APA, all rights reserved)
SN - 0419-4209
AD - Parish, Loraine Elizabeth: Auburn U., US
ER -

TY - JOUR
ID - 654
T1 - "Will you play with me?": The relation of early aspects of maternal parenting to the later school social competence of children with disabilities
A1 - Young,Jessica Mercer
Y1 - 2008///
N1 - Dissertation Abstract: 2008-99080-160
English
Dissertation Abstract
KW - PsycInfo
KW - Developmental Disabilities
KW - Mother Child Relations

KW - Parenting Style
KW - Pediatrics
KW - Social Skills
RP - NOT IN FILE
SP - 7005
JF - Dissertation Abstracts International: Section B: The Sciences and Engineering
VL - 68:
IS - 10-B
N2 - This study seeks to extend knowledge of the relation between aspects of maternal parenting in early childhood to the later school social competence of children with disabilities. Children with developmental disabilities (DD) have more difficulty with social competence and exhibit more behavior problems than typically developing children. The question of which aspects of maternal parenting promote social competence is approached through a developmental-contextual systems model. Two key aspects of maternal parenting are posited to relate to the social competence and behavior problems of children with disabilities: (1) mother-child interaction and (2) maternal parenting-stress. The sample consisted of 146 families participating in the Early Intervention Collaborative Study, a longitudinal investigation of children with developmental disabilities (Down syndrome, motor impairment, or developmental delay) and their families. When the children were ages 2 and 3 mothers reported their levels of parenting-stress and mother-child dyads were observed during a teaching interaction. At age 5 children were observed in their classroom during peer-interaction. Teachers also reported on the children's classroom social competence. The findings reveal that mother-child dyads are at risk for less positive interactive styles as compared to typically developing dyads, as mothers of children with DD were found to interact with their children on average less positively and less contingently than mothers of children without disabilities. Despite this, mother-child interaction was important to the social competence of children with DD. The main findings indicate that at age 3 the quality of mother-child interaction was a key determinant of classroom social competence and behavior problems at age 5 predicting unique variance beyond the child's cognitive performance, type of disability, family socio-economic status, and parenting-stress. In contrast, no aspect of maternal parenting related to children's peer-related social competence. Collectively the findings suggest that interventions aimed at fostering positive mother-child interaction will target a relative weakness in parent-child interactions and simultaneously promote later classroom social competence and decrease behavior problems for children with developmental disabilities. (PsycINFO Database Record (c) 2012 APA, all rights reserved)
SN - 0419-4217
AD - Young, Jessica Mercer: Boston Coll., US
ER -

TY - JOUR
ID - 655
T1 - Play therapy techniques with very young at-risk children in child care settings. [References]
A1 - Robinson,Jane
Y1 - 2008///
N1 - Book: 2008-14952-009
English
Book; Edited Book
KW - PsycInfo
KW - At Risk Populations
KW - Attachment Behavior
KW - Early Intervention
KW - Mental Health
KW - Play Therapy
KW - Child Care
KW - Childhood Development
KW - Experimentation
KW - Program Evaluation
KW - Resilience (Psychological)
KW - Well Being
RP - NOT IN FILE

SP - 199
EP - 247
JF - Schaefer, Charles E [Ed]; Kelly-Zion, Sophronia [Ed]; McCormick, Judy [Ed]; Ohnogi, Akiko [Ed]
VL - :
IS - 2008
N2 - (from the chapter) At-risk children under the age of five are a large population, often forgotten, whose mental health needs are seldom met. Early intervention and prevention that focuses on attachment, resiliency, and play would help to strengthen their growth and development into functioning adults. This chapter will provide an overview of our All 'Bout Children, Inc. (ABC) multi-faceted intervention program, which focuses on at-risk young children, their families, and child care center staff. We will begin with a portrayal of a population of at-risk children, then move to a discussion of attachment, resiliency, and infant mental health and social emotional wellbeing. We will describe the program interventions, highlight child-centered play therapy and the rationale for its use, and conclude with discussion of program evaluation and research. (PsycINFO Database Record (c) 2012 APA, all rights reserved)
AD - Robinson, Jane: All 'Bout Children, West Palm Beach, FL, US Grobbel, Roxanne: All 'Bout Children, West Palm Beach, FL, US
ER -

TY - JOUR
ID - 656
T1 - Play therapy for very young children
A1 - Schaefer,Charles E [Ed]
Y1 - 2008///
N1 - Book: 2008-14952-000
English
Book; Edited Book
(Created by PsycINFO) Preface Part I: Major theoretical approaches * Dynamic play with very young children / Steve Harvey / 3-23 * Child-centered play therapy for very young children / Virginia Ryan and Sue Bratton / 25-66 * Filial play therapy for infants and toddlers / Hilda R. Glazer / 67-83 * Caregiver-toddler play therapy: An attachment-based approach / Helen E. Benedict / 85-102 * Ecosystemic play therapy with infants and toddlers and their families / Beth Limberg and Sue Ammen / 103-124 * Parent-child attunement therapy for toddlers: A behaviorally oriented, play-based parent training model / Stefan C. Dombrowski, Susan G. Timmer, and Nancy Zebell / 125-155 * Theraplay with zero- to three-year-olds / Evangeline Munns / 157-170 Part II: Applications to specific populations * Situational and story-stem scaffolding in psychodynamic play therapy with very young children / Theodore J. Gaensbauer and Kim Kelsay / 173-198 * Play therapy techniques with very young at-risk children in child care settings / Jane Robinson and Roxanne Grobbel / 199-247 * Issue-specific guided play to support infants, toddlers, and their families in health care settings: Rationale and interventions / Joy Goldberger and Anne Luebering Mohl / 249-277 * Watch, wait, and wonder: Infants as agents of change in a play-based approach to mother-infant psychotherapy / Mirek Lojkasek, Elisabeth Muir, and Nancy J. Cohen / 279-305 * Transdisciplinary play-based intervention with young children with disabilities / Toni W. Under, Keri Linos, and Kim Stokka / 307-337 * Dyadic play therapy for homeless parents and children / Ruth P. Newton / 339-365 * Developmental play therapy for very young children / Glenda F. Lester Short / 367-377 * Play-based intervention for very young children with autism: The PLAY Project / Richard Solomon / 379-401 Index About the editors and contributors
KW - PsycInfo
KW - Early Intervention
KW - Mental Health
KW - Play Therapy
KW - Parent Training
KW - Therapists
RP - NOT IN FILE
SP - Lanham, MD, US
JF - (2008)
VL - Play therapy for very young children. x, 424 pp. Lanham, MD, US: Jason Aronson; US.
N2 - (from the preface) Regardless of the theoretical orientation of the play therapist, Play Therapy for Very Young Children can help the clinician in conceptualizing an infant's or toddler's world and the specific play

therapy interventions that can be effectively utilized with these children that would benefit from professional support. The book weaves together play therapy and infant mental health, which seems to be a natural marriage of two disciplines. It is exciting to see two fields come together to share expertise with each other. A further goal is to provide a comprehensive guideline of the most beneficial effects of play therapy and play for the very young. These chapters are based on current research and practice, and on the solid foundation of knowledge that each author has, related to what is important for infants and toddlers and their families in the field of play therapy as an optimal form of early intervention. The book includes extensive discussions of developmental play therapy, attachment-based caregiver-toddler play therapy, filial play therapy, mother-infant play, behaviorally oriented play-based parent training, Theraplay, play-based interventions with young children with disabilities, child centered play therapy, Ecosystemic Play Therapy, Dynamic Play, play therapy with children in care settings, issue-specific guided play, Psychodynamic Play Therapy, play therapy with the young homeless, and play-based interventions with young children with autism. Professionals in the field of mental health, especially play therapists, will find this book useful for increase of knowledge, as well as for clinical intervention. Multiple leading professionals in the field of early intervention and play therapy have contributed the various chapters. Although many are based in the United States, some are from other countries, and the client populations vary. Thus, this book should have helpful play therapy interventions that can be adapted for use with very young children from different cultures around the world. (PsycINFO Database Record (c) 2012 APA, all rights reserved)
AD - Schaefer, Charles E.: Fairleigh Dickinson University, Teaneck, NJ, US Kelly-Zion, Sophronia: Judson Independent School District, San Antonio, TX, US McCormick, Judy: Health Services Executive, Limerick, Ireland Ohnogi, Akiko: Private Practice, Tokyo, Japan
ER -

TY - JOUR
ID - 657
T1 - So sexy so soon: The new sexualized childhood and what parents can do to protect their kids. [References]
A1 - Levin,Diane E; Kilbourne,Jean
Y1 - 2008///
N1 - Book: 2008-13706-000
English
Book; Authored Book
(Created by PsycINFO) Introduction: Changing times, changing needs, changing responses Never too young to be sexy: Living with children in today's sexualized world From Barbie to Bratz and beyond: Sexy sells Sexual development derailed: The toll on children The toll on parents, families, and schools Helping children through the minefields: What parents, families, and schools can do Working it out together: The power of connecting deeply with children The sexualized child enters adolescence: The floodgates open Helping teenagers through the minefields Creating a new cultural environment Acknowledgments Resources Notes Index
KW - PsycInfo
KW - Childhood Development
KW - Childrearing Practices
KW - Parent Child Relations
KW - Psychosexual Behavior
KW - Sexuality
KW - Communications Media
KW - Popular Culture
KW - Technology
RP - NOT IN FILE
SP - New
JF - (2008)
VL - So sexy so soon: The new sexualized childhood and what parents can do to protect their kids. x, 226 pp. New York, NY, US: Ballantine Books; US.
N2 - (from the jacket) Thong panties, padded bras, and risque Halloween costumes for young girls. T-shirts that boast "Chick Magnet" for toddler boys. Sexy content on almost every television channel, as well as in books, movies, video games, and even cartoons. Hot young female pop stars wearing provocative clothing and dancing suggestively while singing songs with sexual and sometimes violent lyrics. These products are marketed aggressively to our children; these stars are held up for our young daughters to emulate--and for our sons to see

as objects of desire. Popular culture and technology inundate our children with an onslaught of mixed messages at earlier ages than ever before. Corporations capitalize on this disturbing trend, and without the emotional sophistication to understand what they are doing and seeing, kids are getting into increasing trouble emotionally and socially; some may even engage in precocious sexual behavior. Parents are left shaking their heads, wondering: How did this happen? What can we do? So Sexy So Soonis an invaluable and practical guide for parents who are fed up, confused, and even scared by what their kids--or their kids' friends--do and say. Diane E. Levin, Ph.D., and lean Kilbourne, Ed.D., internationally recognized experts on early childhood development and the impact of the media on children and teens, understand that saying no to commercial culture--TV, movies, toys, Internet access, and video games--isn't a realistic or viable option for most families. Instead, they offer parents essential, age-appropriate strategies to counter the assault. For instance: 1) help your children expand their imaginations by suggesting new ways for them to play with toys--for example, instead of "playing house" with dolls, they might send their toys on a backyard archeological adventure; 2) Counteract the narrow gender stereotypes in today's media: ask your son to help you cook; get your daughter outside to play ball; 3) Share your values and concerns with other adults--relatives, parents of your children's friends--and agree on how you'll deal with TV and other media when your children are at one another's houses. Filled with savvy suggestions, helpful sample dialogues, and poignant true stories from families dealing with these issues. So Sexy So Soon provides parents with the information, skills, and confidence they need to discuss sensitive topics openly and effectively so their kids can just be kids. (PsycINFO Database Record (c) 2012 APA, all rights reserved)
AD - Levin, Diane E.: Wheelock College, Boston, MA, US Kilbourne, Jean: Wellesley Centers for Women, MA, US
ER -

TY - JOUR
ID - 661
T1 - A multicomponent intervention to increase peer-directed communication in Head Start children. [References]
A1 - Stanton-Chapman,Tina L.
Y1 - 2008///
N1 - Peer Reviewed Journal: 2008-07241-002
English
Journal; Peer Reviewed Journal
KW - PsycInfo
KW - Peers
KW - Project Head Start
KW - Reading
KW - Social Skills
KW - Language
KW - Pragmatics
KW - Vocabulary
RP - NOT IN FILE
SP - 188
EP - 212
JF - Journal of Early Intervention
VL - 30:
IS - 3
N2 - The effects of a multicomponent intervention strategy to increase peer-directed social communication in eight Head Start children at risk for poor language and social skill development were examined. The intervention consisted of three components: (a) a planning period, including reading a storybook that illustrated the play theme and use of the social pragmatic communication strategies, practice using vocabulary to be used in play, and choosing roles for the thematic activity; (b) a 10-min play session in which the interventionist coached children to interact while they played with the thematic related toys; and (c) a brief reporting period in which children reviewed their use of the social pragmatic strategies and specific vocabulary. A multiple-baseline design across dyads with pre- and postgeneralization probes was employed. Children increased their use of peer-directed communication over baseline levels, target vocabulary words, and other social pragmatic skills. (PsycINFO Database Record (c) 2012 APA, all rights reserved) (journal abstract)

SN - 1053-8151
AD - Stanton-Chapman, Tina L.: Vanderbilt University, Nashville, TN, US Kaiser, Ann P.: Vanderbilt University, Nashville, TN, US Vijay, Prathibha: Vanderbilt University, Nashville, TN, US Chapman, Carol: Vanderbilt University, Nashville, TN, US
ER -

TY - JOUR
ID - 662
T1 - Is exposure to media violence a public-health risk?
A1 - No,authorship indicated
Y1 - 2008///
N1 - Peer Reviewed Journal: 2008-04585-001
English
Journal; Peer Reviewed Journal
KW - PsycInfo
KW - Computer Games
KW - Mass Media
KW - Violence
KW - Public Health
RP - NOT IN FILE
SP - 1137
JF - The Lancet
VL - 371:
IS - 9619
N2 - For video game fans in the UK, this month sees the release of the much anticipated Manhunt 2 and Grand Theft Auto IV. The earlier versions of these so-called video nasties hit the headlines when their makers were taken to task over whether the violent nature of their games provoked real-life killings. The effects of violence in video games on negative behaviour in children and adolescents have been intensely studied and debated. There is a need for observational studies on what children actually play and how they play. Given that violence has different cultural contexts in the way it is portrayed, studies in children from ethnic minorities would be valuable in increasing our knowledge. Attention to identifying higher-risk groups, such as children with depression and behavioural and mental health problems, and those exposed to real-world violence, could yield possible markers for abnormal behaviour. (PsycINFO Database Record (c) 2012 APA, all rights reserved)
SN - 0140-6736
ER -

TY - JOUR
ID - 663
T1 - Play, aggression, the preschool child, and the family: A review of literature to guide empirically informed play therapy with aggressive preschool children. [References]
A1 - Davenport,Becky R.
Y1 - 2008///
N1 - Peer Reviewed Journal: 2008-04333-002
English
Journal; Peer Reviewed Journal
KW - PsycInfo
KW - Aggressive Behavior
KW - Behavior Problems
KW - Childhood Development
KW - Childhood Play Behavior
KW - Play Therapy
KW - Family
RP - NOT IN FILE
SP - 2
EP - 23
JF - International Journal of Play Therapy

VL - 17:
IS - 1
N2 - Preschool-aged children displaying high levels of aggression repeatedly have been shown to be at significant risk for continued behavior problems and other social and emotional challenges throughout their lifetimes. The present literature review seeks to summarize and integrate findings across the fields of developmental psychology, family studies, child development, and play therapy to inform play therapists' practices with this population. Specifically, findings from research in these fields hold substantial implications for empirically informed ways in which play therapists can include parents within play therapy processes. Affective quality of parent- child interactions emerges as the common theme across all existing literature and is emphasized as a critical focus for play-based therapeutic intervention. Topics for future research by play therapists focusing on this population are suggested. (PsycINFO Database Record (c) 2012 APA, all rights reserved) (journal abstract)
SN - 1555-6824
AD - Davenport, Becky R.: Department of Counseling and Human Services, St. Mary's University, San Antonio, TX, US
ER -

TY - JOUR
ID - 666
T1 - A "multitude" of solitude: A closer look at social withdrawal and nonsocial play in early childhood. [References]
A1 - Coplan,Robert J.
Y1 - 2007///
N1 - Peer Reviewed Journal: 2009-06146-006
English
Journal; Peer Reviewed Journal
KW - PsycInfo
KW - Childhood Play Behavior
KW - Early Childhood Development
KW - Social Isolation
KW - Timidity
RP - NOT IN FILE
SP - 26
EP - 32
JF - Child Development Perspectives
VL - 1:
IS - 1
N2 - It has long been argued that social withdrawal in early childhood is a risk factor for later socioemotional difficulties. However, in recent years, researchers have begun to make distinctions between types of social withdrawal in young children, including shyness, social disinterest, and social avoidance. In this article, we review the literature on multiple forms of social withdrawal in early childhood. In particular, we focus on (a) theoretical and empirical distinctions between shyness, social disinterest, and social avoidance; (b) links between these constructs and children's social and nonsocial play behaviors with peers; and (c) implications for children's psychosocial adjustment. As well, we provide suggestions for future research, particularly on the relatively understudied construct of social disinterest and the virtually unexplored phenomenon of social avoidance. (PsycINFO Database Record (c) 2012 APA, all rights reserved) (journal abstract)
SN - 1750-8592
AD - Coplan, Robert J.: Carleton University, Ottawa, ON, Canada Armer, Mandana: Carleton University, Ottawa, ON, Canada
ER -

TY - JOUR
ID - 667
T1 - Changes in parenting and mental health among mothers in early head start
A1 - Berkule,Samantha Brooke
Y1 - 2007///

N1 - Dissertation Abstract: 2007-99018-050
English
Dissertation Abstract
KW - PsycInfo
KW - Mental Health
KW - Mother Child Relations
KW - Parenting Skills
KW - Project Head Start
KW - Mothers
RP - NOT IN FILE
SP - 1958
JF - Dissertation Abstracts International: Section B: The Sciences and Engineering
VL - 68:
IS - 3-B
N2 - This study examined maternal mental health and dimensions of mother-child interaction in 46 low-income, Latino mothers who participated in a relationship-based Early Head Start (EHS) program with their infants and toddlers over the course of 1 years. Children were approximately 1-year-old at enrollment in EHS and the beginning of the study, and were approximately 2.5 years of age at the conclusion of the study. Observed dimensions of parenting behaviors were assessed during structured play interactions of mothers with their children. Maternal depressive symptoms were measured using the Center for Epidemiological Studies - Depression Scale (CES-D; Radloff, 1977). Parental stress and depression, and perceptions about social support were collected during self-report interview measures, using the Short Form of the Parenting Stress Index (PSI-SF; Abidin, 1986) and the Vaux Social Support Record (SSR; Vaux, 1985) respectively. Observational play measures and interview measures were collected at the time of enrollment in EHS (baseline) and again after 18 months of participation in EHS (outcome). Results showed significant increases in positive dimensions of parenting from enrollment to outcome, as reflected in maternal sensitivity, flexibility, autonomy support, positive affect, and significant decreases in maternal intrusiveness. In addition, changes in dimensions of mothers' positive parenting and intrusiveness during structured play with their children differed depending on the level of mental health risk of mothers at the time of enrollment in EHS, with depressed mothers showing more significant decreases in intrusiveness. In addition, after 18 months of participation in EHS, maternal depression and stress related to parenting were significantly reduced, and mothers reported a significant increase in the number of individuals who gave them advice. Despite a lack of detailed information concerning participants' attendance of EHS and utilization of its services, and a small participant sample size, these results are noteworthy. These findings highlight the importance of offering relationship-based interventions in order to improve parenting behaviors. These findings also illuminate the importance of screening mothers for depression, as well as offering mental health services and prevention models which address mental health issues, within the context of relationships. (PsycINFO Database Record (c) 2012 APA, all rights reserved)
SN - 0419-4217
AD - Berkule, Samantha Brooke: Yeshiva U., US
ER -

TY - JOUR
ID - 669
T1 - A review of the effects of sleep position, play position, and equipment use on motor development in infants. [References]
A1 - Pin,Tamis; Eldridge,Beverley; Galea,Mary
Y1 - 2007///
N1 - Peer Reviewed Journal: 2007-18585-014
English
Journal; Peer Reviewed Journal
KW - PsycInfo
KW - Childhood Play Behavior
KW - Infant Development
KW - Motor Development
KW - Sleep
RP - NOT IN FILE

SP - 858
EP - 867
JF - Developmental Medicine & Child Neurology
JA - Dev.Med Child Neurol.
VL - 49:
IS - 11
N2 - Since 1992, parents have been urged to place their infants on their back when asleep. The resulting lack of experience in a prone position appears to cause developmental delay in infants. Use of various infant equipment, except baby walkers, has not been examined thoroughly to establish their influence on the motor development of infants. The aim of this systematic review was to evaluate the effects of sleep and play positions, and use of infant equipment, on motor development. Nineteen studies with evidence at level II were selected against the selection criteria and scored against the Physiotherapy Evidence Database scale. Despite the generally poor methodological quality, the studies have consistently shown that there was transient delay in motor development for healthy term and low-risk preterm infants who were not exposed to the prone position or who did not use infant equipment. However, most of these infants walked unaided within a normal time frame. Limited evidence was found for the effect on more vulnerable infants. More rigorous longitudinal studies using outcome measures focusing on movement quality are recommended to understand any long-lasting influence on the motor skills in these infants. (PsycINFO Database Record (c) 2012 APA, all rights reserved) (journal abstract)
SN - 0012-1622
AD - Pin, Tamis: School of Physiotherapy, University of Melbourne, Melbourne, VIC, Australia Eldridge, Beverley: Physiotherapy Department, Royal Children's Hospital, Melbourne, VIC, Australia Galea, Mary P.: School of Physiotherapy, University of Melbourne, Melbourne, VIC, Australia
ER -

TY - JOUR
ID - 670
T1 - Affective disorders during childhood and adolescence. [German]. [References]
A1 - von Klitzing,Kai
Y1 - 2007///
N1 - Peer Reviewed Journal: 2007-17658-002
German
Journal; Peer Reviewed Journal
KW - PsycInfo
KW - Adolescent Development
KW - Affective Disorders
KW - Childhood Development
KW - Etiology
KW - Psychopathology
KW - Symptoms
RP - NOT IN FILE
SP - 287
EP - 304
JF - Kinderanalyse
VL - 15:
IS - 4
N2 - The role of emotional symptoms (depression, anxiety) in different phases of childhood and adolescent development are discussed from a developmental psychopathology perspective. Infants can become depressive as a result of a relational environment which is not good enough. Whether they can have primary depression is still an open question. On the other hand, feelings of depression and anxiety play an important role also in normal development. In preschool and early school children, play inhibition is a central symptom which can indicate the presence of depression and anxiety. In empirical studies, we found a tendency to exaggerated empathy in combination with rigid defence mechanisms and avoidance being prominent in many depressed children, especially girls. In adolescence, the decreasing cathexis of the primary objects and the insecure body concepts enhance the risk of becoming depressed. The role of interpersonal, intrapsychic, and biological factors as well as their interaction among each other in the aetiology of affective disorders in childhood and adolescence is discussed. (PsycINFO Database Record (c) 2012 APA, all rights reserved) (journal abstract)

SN - 0942-6051
AD - von Klitzing, Kai: Universitatsklinikum Leipzig, Klinik und Poliklinik fur Psychiatrie, Psychotherapie und Psychosomatik des Kindes- und Jugendalters, Leipzig, Germany
ER -

TY - JOUR
ID - 671
T1 - Teaching skills to second and third grade children to prevent gun play: A comparison of procedures. [References]
A1 - Kelso,Pamela D.
Y1 - 2007///
N1 - Peer Reviewed Journal: 2007-11879-002
English
Journal; Peer Reviewed Journal
KW - PsycInfo
KW - Childhood Play Behavior
KW - Firearms
KW - Injuries
KW - Teaching
KW - Behavior Modification
KW - Safety
KW - Self Report
RP - NOT IN FILE
SP - 29
EP - 48
JF - Education & Treatment of Children
VL - 30:
IS - 3
N2 - A posttest only control group design was used to investigate the effects of two programs to teach firearm injury prevention skills to second and third grade children. Children were taught the safety skills "Stop. Don't touch. Leave the area. Tell an adult." should they ever find a firearm. The effectiveness of the National Rifle Association's Eddie Eagle GunSafe Program (Level Two) was compared to a Behavioral Skills Training (BST) procedure in reducing gunplay behaviors. Participants were trained according to either the Eddie Eagle program or the BST procedure in one session lasting forty-five minutes to an hour. Self-report, role-play, and in-situ assessments were used to determine the effects of each treatment. Those children not demonstrating the target skills at assessment received an additional in-situ training session and a second assessment. Findings showed that (a) both programs were successful in teaching children to verbally state the target behaviors as compared to the control group; (b) the Behavioral Skills Training program and the Eddie Eagle program were equally effective in teaching children to role-play the skills, and the Behavioral Skills Training group was superior to the control group; (c) there were no differences among any of the conditions in regards to measures of in-situ assessment; and (d) children in each group varied on the amount of in-situ training sessions needed before displaying the target behavior. (PsycINFO Database Record (c) 2012 APA, all rights reserved) (journal abstract)
SN - 0748-8491
AD - Kelso, Pamela D.: North Dakota State University, Fargo, ND, US Miltenberger, Raymond G.: North Dakota State University, Fargo, ND, US Waters, Marit A.: North Dakota State University, Fargo, ND, US Egemo-Helm, Kristin: North Dakota State University, Fargo, ND, US Bagne, Angela G.: North Dakota State University, Fargo, ND, US
ER -

TY - JOUR
ID - 673
T1 - One-year internal consistency of the infant/toddler Home Inventory in a low risk infant group of Mexico City and its metropolitan zone. [Spanish]. [References]
A1 - Zanabria-Salcedo,Martha; Marquez-Caraveo,Maria Elena; Perez-Martinez,Antonio; Mendez-Ramirez,Ignacio
Y1 - 2007///

N1 - Peer Reviewed Journal: 2007-08665-009
Spanish
Journal; Peer Reviewed Journal
KW - PsycInfo
KW - Home Care
KW - Infant Development
KW - Psychometrics
KW - Risk Factors
KW - Test Reliability
KW - Inventories
RP - NOT IN FILE
SP - 67
EP - 73
JF - Salud Mental
VL - 30:
IS - 2
N2 - Summary: As the environment is a crucial source in an infant's development, it is important to assess the proximal environment where a variety of social relationships take place. The home concept and its influence on development led to the discovery and interrelations of several dimensions and today's emphasis relies on identifying the mechanisms of the variability of environment that exert an influence on the variability of development. The purpose of this investigation was to establish the internal consistency of the Infant/Toddler HOME Inventory in the version adapted by Cravioto through the assessment of the precision of its scoring in terms of internal consistency. Cohort: 62 infants and their mothers were contacted in the first 72 hours after delivery through a Research Program. Instrument: The Infant/Toddler HOME Inventory, version adapted by Cravioto, with 62 items in a binary format response was used. Subscale VII, Play Materials, asked about color, size, consistency or texture and type of toys. Results showed that alpha coefficient's values higher than 0.60 throughout the 12 months were observed in the VII Play Materials shoed a range of 0.64 and 0.84, and 0.60 and 0.83 for the Total Scale. Results showed the Infant/Toddler HOME Inventory had high internal consistency values in the Total Scale. Limitations in this study are not only this modification, but the size sample, and future research efforts should overcome this fact by trying to sample infants from different Mexican regions. This study points out the need to adapt, develop and evaluate psychometrically instruments that measure specific aspects of the environment of infant's homes. (PsycINFO Database Record (c) 2012 APA, all rights reserved)
SN - 0185-3325
AD - Zanabria-Salcedo, Martha: Departamento de Educacion y Comunicacion, Universidad Autonoma Metropolitan, Unidad Xochimilco, Tlalpan, Mexico Marquez-Caraveo, Maria Elena: Division de Investigacion, Hospital Psiquiatrico Infantil Dr. Juan N. Navarro, SSA, Mexico Perez-Martinez, Antonio: Egresado de la Maestria en Rehabilitacion Neurologica, Universidad Autonoma Metropolitana, Unidad Xochimilco, Tlalpan, Mexico Mendez-Ramirez, Ignacio: Instituto de Investigaciones en Matematicas Aplicadas y en Sistemas, Universidad Nacional Autonoma de Mexico, Mexico, Mexico
ER -

TY - JOUR
ID - 674
T1 - Parents-child role reversal in trilogue play: Case studies of trajectories from pregnancy to toddlerhood. [References]
A1 - Fivaz-Depeursinge,E; Frascarolo,
Y1 - 2007///
N1 - Peer Reviewed Journal: 2007-03646-002
English
Journal; Peer Reviewed Journal
KW - PsycInfo
KW - Childhood Development
KW - Parent Child Relations
KW - Role Taking
KW - Needs
KW - Risk Factors

RP - NOT IN FILE
SP - 17
EP - 31
JF - Attachment & Human Development
JA - Attach.Hum.Dev.
VL - 9:
IS - 1
N2 - Role reversal, whereby a child attempts to meet her parent's adult needs for parenting, intimacy, or companionship, has been identified as a risk factor for developmental disturbances. It has been defined from diverse perspectives as a child attachment strategy, a parent-toddler relational disturbance, and a boundary disturbance between parents and child. The recently discovered infant's triangular capacity, namely the sharing of her attention and affects with both parents, allows one to analyse the infant's contribution to early family dynamics. Role reversal was detected in 4 out of 45 father-mother-infant interactions observed in trilogue play from pregnancy to toddlerhood. The developmental trajectories towards role reversal are explored by means of case analyses. Results are compared with cases of problematic triangulation encountered in die same sample. In role reversal, family interactions are rigidly organized around a "two against one" coalition, whereby the normative hierarchy between parents and child is reversed. The child's triangular capacity is overactivated, controlling the tension between her parents by provocation-animation strategies. (PsycINFO Database Record (c) 2012 APA, all rights reserved) (journal abstract)
SN - 1461-6734
AD - Fivaz-Depeursinge, E.: University of Lausanne, Lausanne, Switzerland Frascarolo, F.: University of Lausanne, Lausanne, Switzerland Lopes, F.: University of Lausanne, Lausanne, Switzerland Dimitrova, N.: University of Lausanne, Lausanne, Switzerland Favez, N.: University of Lausanne, Lausanne, Switzerland
ER -

TY - JOUR
ID - 675
T1 - In Defense of Child's Play. [References]
A1 - Hawes,David J.
Y1 - 2007///
N1 - Electronic Collection: 2007-01907-001
English
Electronic Collection
KW - PsycInfo
KW - Child Psychology
KW - Childhood Development
KW - Childhood Play Behavior
KW - Educational Psychology
KW - Learning
RP - NOT IN FILE
JF - PsycCRITIQUES
VL - 52:
IS - 19
N2 - Reviews the book, Play = Learning: How Play Motivates and Enhances Children's Cognitive and Social-Emotional Growth, edited by Dorothy G. Singer, Roberta Michnick Golinkoff, and Kathy Hirsh-Pasek (see record 2006-10812-000). Play has been the subject of some of the most influential writings in child psychology, with iconic texts from figures such as Jean Piaget and Donald Winnicott establishing it as a major theme in both the developmental and clinical child literature. The volume essentially follows two broad premises. The first is that play for the sake of play is disappearing from childhood, much to the detriment of children's development and mental health. The second is that children's play has the potential to greatly inform early childhood practice and to be used more effectively in meeting academic and therapeutic needs both at the population level and with at-risk groups. A running theme throughout the book is the popular tendency (past and present) to dichotomize play and what has been termed the "real work" of learning. The book begins by examining the ways in which this conceptualization has shaped current practices in education and child rearing. An opening chapter by Edward Zigler, a founder of Head Start, provides an insightful backdrop for this discussion. The succeeding chapters of the book examine empirical evidence for the importance of play to specific cognitive and affective

domains of development, especially competencies key to school readiness and achievement. (PsycINFO Database Record (c) 2012 APA, all rights reserved)
SN - 1554-0138
ER -

TY - JOUR
ID - 676
T1 - Outdoor play spaces in Canada: The safety dance of standards as policy. [References]
A1 - Herrington,Susan
Y1 - 2007///
N1 - Peer Reviewed Journal: 2007-01307-006
English
Journal; Peer Reviewed Journal
KW - PsycInfo
KW - Childhood Play Development
KW - Government Policy Making
KW - Playgrounds
KW - Safety
KW - Child Day Care
RP - NOT IN FILE
SP - 128
EP - 138
JF - Critical Social Policy
VL - 27:
IS - 1
N2 - Over the past decade the outdoor play spaces designed for children in Canada have been largely shaped by fear and profit, rather than by what we know about children's play and development. Since the early 1980s the Canadian Standards Association (CSA) has played an increasingly important role in this transformation as their technical standards for children's outdoor play spaces have been gradually adapted as policy by local and regional agencies. While the CSA has historically regulated industrial and commercial projects that enable international trade and harmonization with countries such as the United States, their extension of influence to early childhood is problematic; particularly when applied to childcare centres. The following describes some of the issues that arise from the use of safety standards as policy, and the problems these standards cause when applied to childcare centres. (PsycINFO Database Record (c) 2012 APA, all rights reserved) (journal abstract)
SN - 0261-0183
AD - Herrington, Susan: School of Architecture and Landscape Architecture, University of British Columbia, Vancouver, BC, Canada Nicholls, Jamie: School of Architecture and Landscape Architecture, University of British Columbia, Vancouver, BC, Canada
ER -

TY - JOUR
ID - 677
T1 - Superheroes in play therapy with an attachment disordered child. [References]
A1 - Wenger,Carmela
Y1 - 2007///
N1 - Book: 2007-00887-010
English
Book; Edited Book
KW - PsycInfo
KW - Attachment Disorders
KW - Diagnosis
KW - Heroes
KW - Measurement
KW - Play Therapy
KW - Fantasy
RP - NOT IN FILE

SP - 193
EP - 211
JF - Rubin, Lawrence C [Ed]
VL - :
IS - 2007
N2 - (create) Carmela Wenger explores and demonstrates the usefulness of superheroes in the assessment, diagnosis, and treatment of a young male client with reactive attachment disorder. The focus of treatment was on improving the quality of interaction between Damien and his adoptive parents and siblings. The stages of play therapy with Damien paralleled the course of healthy development, moving from the establishment of trust to exploration, risk taking, and ultimately more mature levels of cognitive and affective processing. (PsycINFO Database Record (c) 2012 APA, all rights reserved)
ER -

TY - JOUR
ID - 678
T1 - The Play Paradox: Can Scientists Study Play Playfully? [References]
A1 - Ault,Ruth L.
Y1 - 2007///
N1 - Electronic Collection: 2006-22676-001
English
Electronic Collection
KW - PsycInfo
KW - Childhood Play Behavior
KW - Childhood Play Development
KW - Games
KW - Fantasy
KW - Toys
RP - NOT IN FILE
JF - PsycCRITIQUES
VL - 52:
IS - 2
N2 - Reviews the book, The Development of Play (3rd ed.) by David Cohen (see record 2006-06577-000). The chapters at the heart of the book cover traditional play topics. After the introductory chapter, Cohen briefly reviews historical writers' ideas about play, beginning with Aristotle, Plato, and Rousseau. Chapter 3, "Playing With Objects," gives an overview of Piaget's (1962) seminal work on imitation and the beginning of pretend play during the sensorimotor and preoperational periods and takes potshots at various empirical studies of how children play with toys (mostly as adjuncts to pretend play), because "the whole dynamics of the game are out of the children's control: adults run it" (p. 44). Chapter 4, "Playing With Other Children," likewise covers pretend play, with or without others; rough and tumble play among boys; and preschoolers' performance on appearance-reality and false belief tasks, which abilities children can learn through play (as well as through other types of interactions). Chapter 5, titled "Pretending," repeats many of the earlier themes. Throughout these early chapters, Cohen indicates how most play research follows three major traditional directions in ascribing cognitive, emotional (therapeutic), and social functions to play. He identifies additional aspects that ought to receive more research attention: cultural aspects of play, play among adults, and how television and the toy industry affect children's toy selection and play. In chapter 6 Cohen presents extended examples of the play of his sons at home when they were under age six. Apparently, this material formed much of his doctoral work and closely resembles the presentation in the previous two editions of The Development of Play. The first half of Chapter 7 reviews play therapy from the psychoanalytic perspective, which Cohen criticizes for not being empirically supported and for not enhancing the parent-child bond, because therapists routinely exclude parents from therapy sessions. The second half of Chapter 7 concerns the well-documented findings that children with autism play predominantly with objects, often in rigid, stereotyped ways, and lack pretend play. Cohen's examination of adult games in Chapter 8 starts with historical reasons why psychologists have neglected the topic. The four subsequent areas of Cohen's focus are sports (especially risky ones), computer and fantasy games, functionless knickknacks or consumer toys, and personal growth games. (PsycINFO Database Record (c) 2012 APA, all rights reserved)
SN - 1554-0138

ER -

TY - JOUR
ID - 679
T1 - Wanna play: Friendship skills for preschool and elementary grades
A1 - Ross,Ruth Herron; Roberts-Pacchione,Beth
Y1 - 2007///
N1 - Book: 2006-20765-000
English
Book; Authored Book
(Created by PsycINFO) (Abbreviated) Acknowledgments About the Authors Introduction: Welcome to the Wanna Play Program Guidelines for Curriculum Success How to Use This Book Social Interaction Checklist Unit 1: Discovering Friendship Unit 2: Making Eye Contact for Interaction Unit 3: Being a Friend Unit 4: Appropriate Body Behavior Unit 5: Interacting in a Group Unit 6: Playing Games Appendix A: Friendship Cards Appendix B: Worksheets
KW - PsycInfo
KW - Developmental Disabilities
KW - Friendship
KW - Individual Education Programs
KW - Social Skills
KW - Teaching
RP - NOT IN FILE
SP - Thousand
JF - (2007)
VL - Wanna play: Friendship skills for preschool and elementary grades. vii, 205 pp. Thousand Oaks, CA, US: Corwin Press; US.
N2 - (from the cover) Youngsters at risk or with developmental delays or disabilities may experience considerable difficulty in learning how to make friends. This practical teacher resource presents the Wanna Play Program, a curriculum devoted specifically to helping PreK-3 students develop the social skills they need to interact appropriately with individuals and groups. This parent- and teacher-friendly handbook provides over 60 lesson plans involving more than 300 games and activities. With dozens of reproducibles, it offers many instructional options for teachers, including: lesson plans outlining teaching concepts, objectives, and attitudinal approaches; links to Individual Education Program (IEP) goals and reporting categories; practical, flexible methods of engaging young students in developing friendship skills; and constructive advice for teaching children who exhibit difficult behavior but may not have been diagnosed with a disability. (PsycINFO Database Record (c) 2012 APA, all rights reserved)
AD - Ross, Ruth Herron: Wanna Play program, PA, US Roberts-Pacchione, Beth: Wanna Play program, PA, US
ER -

TY - JOUR
ID - 680
T1 - Examinations of social and non-social factors in the neurodevelopment of autism
A1 - Mccleery,Joseph Paul
Y1 - 2006///
N1 - Dissertation Abstract: 2006-99024-212
English
Dissertation Abstract
KW - PsycInfo
KW - Autism
KW - Brain
KW - Neural Development
KW - Social Processes
RP - NOT IN FILE
SP - 3484
JF - Dissertation Abstracts International: Section B: The Sciences and Engineering

VL - 67:
IS - 6-B
N2 - Autism is a pervasive developmental disorder characterized by deficits in social and communication skills, as well as restricted interests and repetitive behaviors, with symptom onset by 3 years of age. Recent studies have documented structural and functional abnormalities in a variety of brain regions associated with social processing and perception in this population. As a result, there is a growing belief that autism can be explained by impairment in social brain systems, and several neurodevelopmental models have been proposed to explain the social and communicative impairments of individuals with autism. The current research explores the role social and non-social factors play in the neurodevelopmental basis of autism in three studies of functional brain development. Study 1 examines social and non-social semantic integration in young children with autism by analyzing event-related potentials recorded during the processing of word and environmental sound meaning within a picture context. Results suggest that the neural integration of word, but not environmental sound, meaning is impaired in these children, providing support for a social/non-social distinction in this area of functioning. Experiments 2 and 3 explore the early functional development of brain systems involved in face and object processing in the first year of life in autism by studying neurodevelopmental risk in infant siblings of children diagnosed with autism. Specifically, experiment 2 employs event-related potentials to examine the neural correlates of early stages of face and object processing in 10-month old infants, and experiment 3 employs visual psychophysical measures to assess the integrity of the magnocellular and parvocellular visual pathways in 6-month old infants. The results of experiment 2 suggest that familial risk for autism is associated with abnormalities in face and object processing in the first year of life. The results of experiment 3 suggest that familial risk for autism is also associated with abnormalities in the subcortical magnocellular visual pathway, which is believed to provide critical input for face processing in the first several months of life. Together, these results provide new insight into the roles social and non-social factors may play in the neurodevelopment bases of autism. (PsycINFO Database Record (c) 2012 APA, all rights reserved)
SN - 0419-4217
AD - Mccleery, Joseph Paul: U California, San Diego, US
ER -

TY - JOUR
ID - 681
T1 - Violence exposure, play characteristics, and behavior in a sample of six-year-old children
A1 - Weigand Michener,Kristen Elizabeth
Y1 - 2006///
N1 - Dissertation Abstract: 2006-99014-233
English
Dissertation Abstract
KW - PsycInfo
KW - Behavior Problems
KW - Childhood Play Behavior
KW - Lower Income Level
KW - Violence
KW - Emotional States
KW - Imagination
KW - Parents
KW - Teachers
RP - NOT IN FILE
SP - 565
JF - Dissertation Abstracts International: Section B: The Sciences and Engineering
VL - 67:
IS - 1-B
N2 - Violence exposure is a prevalent problem, especially in low-income, urban communities. Children are at risk for exposure and the negative psychological and behavioral sequalae. Several studies have shown that victims of violent acts display more externalizing behavior problems whereas witnesses to violence display more internalizing behavior problems. In young children, play reflects a child's emotional state and behavioral strategies. In the present study, play characteristics, defined by aggressive affective themes, organization, and imagination were examined in the fantasy play of children exposed to violence. The purposes of the present

study were to: (1) examine aggressive affective themes, organization, and imagination of children exposed to violence utilizing a formalized measure of play; and (2) to examine the relationship between behavior observed during a standardized play assessment and child behavior as rated by parents and teachers; and (3) explore the relative strength of aggressive affective themes, organization, and imagination in play in predicting behavior problems. Contrary to initial hypotheses, there were few significant group differences on several of the constructs of play, including aggressive affect, organization and imagination. Significant findings included that the interaction of violence exposure type and severity of exposure predicted mean differences in organization of play. Higher frequencies of aggressive affect and total negative affect were significantly positively correlated with externalizing behavior problems, as reported by teachers. As in many previous studies, boys demonstrated significantly more aggressive affect than girls during the play task. Finally, neither gender, aggressive affect, quality of play organization, or their interactions accounted for significant, independent variance for any behavioral problems reported by caregivers or teachers. The study had several limitations, with the most salient being lack of sample variance. Future studies should address that as well as measure limitations. In a clinical context, the use of play to assess a child's ability to express and modulate different affective states in an imaginative, organized story in order to bolster coping and adjustment, emotional understanding, and interpersonal functioning can offer mechanisms to reduce symptoms and increase psychological wellness. (PsycINFO Database Record (c) 2012 APA, all rights reserved)
SN - 0419-4217
AD - Weigand Michener, Kristen Elizabeth: Kent State U., US
ER -

TY - JOUR
ID - 682
T1 - Parental stress, parenting behavior and observed parent-child interaction
A1 - Adams,Katrina L.
Y1 - 2006///
N1 - Dissertation Abstract: 2006-99012-084
English
Dissertation Abstract
KW - PsycInfo
KW - Dyads
KW - Parent Child Communication
KW - Parental Role
KW - Parents
KW - Stress
KW - Social Support
RP - NOT IN FILE
SP - 6911
JF - Dissertation Abstracts International: Section B: The Sciences and Engineering
VL - 66:
IS - 12-B
N2 - This study investigated the relationship between parental stress, social support, and directly observed parenting behavior and dyadic interaction in a non-clinical sample of 26 parent-child dyads, in which the child was age five or younger. This study also explored the differential impact of various types of stress on parenting behavior and dyadic interaction, including life stress as measured by the Social Readjustment Rating Scale - Revised (SRRS-R), parenting stress as measured by the Parenting Stress Index - Short Form (PSI-SF), and event stress as measured by the Impact of Event Scale - Revised (IES-R). Parenting behavior and dyadic interaction were assessed with selected variables from the Early Relational Assessment (ERA). Exploratory research questions addressed age and developmental trends, the relationship between parental stress and Parent Role Questionnaire (PRQ) ratings, and contextual differences in observed behavior. The findings of this research indicate that various types of stress differentially impact parent, child and dyadic behavior. Specifically, life stress was associated with decreased parental enjoyment, child positive affect, and mutual enjoyment/enthusiasm in both free and structured play. Contrary to expectations, higher event stress was related to more optimal parenting in this "low risk" sample, including increased mirroring, sensitivity/responsivity, flexibility and appropriate structuring of the interaction, as well as improved dyadic reciprocity and organization in free play, perhaps due to activation of an underlying parental reflective-self functioning variable. Parenting

stress was not found to be a strong predictor of parent, child or dyadic behavior. Further, higher social support was related to decreased life stress and better parenting behavior (i.e., enjoyment, positive affect, intrusiveness). The results of this study also suggest that even minimally structured play situations influence a dyad's interactive style. In this study, the PRQ was not significantly related to stress levels or parenting due to the extremely restricted range of ratings. Finally, this study found some evidence of gender and developmental trends in structured play. Clinical implications for work with parents-child dyads and families, limitations of this study, and directions for future research were also discussed. (PsycINFO Database Record (c) 2012 APA, all rights reserved)
SN - 0419-4217
AD - Adams, Katrina L.: Pace U., US
ER -

TY - JOUR
ID - 683
T1 - 'You can't wrap them up in cotton wool!' Constructing risk in young people's access to outdoor play. [References]
A1 - Jenkins,N.
Y1 - 2006///
N1 - Peer Reviewed Journal: 2006-23168-003
English
Journal; Peer Reviewed Journal
KW - PsycInfo
KW - Parental Attitudes
KW - Risk Factors
KW - Social Desirability
KW - Uncertainty
KW - Risk Assessment
KW - Accidents
KW - Family
KW - Injuries
KW - Judgment
KW - Morale
KW - Morality
RP - NOT IN FILE
SP - 379
EP - 393
JA - Health, Risk & Society
VL - 8:
IS - 4
N2 - Policy approaches towards risk in relation to young people's leisure activities are characterized by uncertainty. While some organizations have sought to emphasize the need to reduce or even eliminate the risk of 'unintentional injuries' in outdoor play, others highlight the dangers in excessively restricting young people's access to the outside world. Drawing on semi-structured interviews with families in the South Wales area, this paper argues that while parents are not in the grips of 'paranoia' (Furedi 2001) they are wrestling with competing sets of social expectations, as well as their own rational and emotive judgments. Young people tended to respond to their parents' concerns by demonstrating their street-wisdom, claiming sanctuary behind the notion of 'accident' and asserting their moral right to access the outside world. (PsycINFO Database Record (c) 2012 APA, all rights reserved) (journal abstract)
SN - 1369-8575
AD - Jenkins, N. E.: Glasgow University, Glasgow, United Kingdom
ER -

TY - JOUR
ID - 684
T1 - Review of Creative Play with Children at Risk
A1 - Hairsine,Jill

Y1 - 2006///
N1 - Peer Reviewed Journal: 2006-20720-010
English
Journal; Peer Reviewed Journal
KW - PsycInfo
KW - At Risk Populations
KW - Childhood Play Behavior
KW - Creativity
KW - Play Therapy
RP - NOT IN FILE
SP - 224
EP - 226
JF - Practice
VL - 18:
IS - 3
N2 - Reviews the book, Creative Play with Children at Risk by Sue Jennings (2005). Jennings uses the ideas of embodiment, projection and role to describe different kinds of play, and provides references to her earlier expositions of these ideas for those who wish to explore these more fully. The book presents itself in user-friendly format, spiral bound for ease of use, especially helpful for using the numerous photocopiable worksheets - a bonus for all busy practitioners - and with an end cover flap to more easily mark a page. This book could be used by natural and foster families, as well as by professional children's workers of all kinds, indeed for anyone who spends time with children in their daily lives, and is not solely relevant for children acknowledged to be especially at risk. The principles and ideas are important for all of us, young and old alike, in a world where childhood gets shorter every year and we are organized into so many formal activities we have no space to let our imaginations soar. It is easy to read and its tone is encouraging throughout. The author's passion for healthy and healing play seems quite contagious, prompting her readers to expand their repertoire of direct work skills and let their imaginations fly. (PsycINFO Database Record (c) 2012 APA, all rights reserved)
SN - 0950-3153
AD - Hairsine, Jill: Children and Lifelong Learning, STS, United Kingdom
ER -

TY - JOUR
ID - 685
T1 - My Magic Story Car: Video-Based Play Intervention to Strengthen Emergent Literacy of At-Risk Preschoolers. [References]
A1 - Bellin,Harvey F.
Y1 - 2006///
N1 - Book: 2006-10812-006
English
Book; Edited Book
KW - PsycInfo
KW - At Risk Populations
KW - Childhood Play Behavior
KW - Early Intervention
KW - Literacy Programs
KW - Preschool Students
KW - Early Childhood Development
KW - Imagination
KW - Lower Income Level
KW - Videotapes
RP - NOT IN FILE
SP - 101
EP - 123
JF - Singer, Dorothy G [Ed]; Golinkoff, Roberta Michnick [Ed]; Hirsh-Pasek, Kathy [Ed]
VL - :
IS - 2006

N2 - (from the chapter) This chapter focuses on My Magic Story Car, a video-based program that strengthens emergent literacy skills of at-risk preschool children from low-income families through one of the most effective available modalities--make-believe play. The program addresses its objective by interweaving three elements: (a) make-believe play as an intrinsically motivating modality for engaging preschoolers in learning activities, (b) empirical studies of the requisite skills of emergent literacy, and (c) video-based programs as an effective medium for empowering parents and caregivers of poor children with easily replicated, reliable interventions for implementation in any childcare setting. The approaches at the heart of the program outlined in this chapter have proven effective in democratizing and leveling the emergent literacy playing field for at-risk, disadvantaged preschoolers. (PsycINFO Database Record (c) 2012 APA, all rights reserved)
AD - Bellin, Harvey F.: Media Group of Connecticut, Inc., CT, US Singer, Dorothy G.: Department of Psychology, Yale University, New Haven, CT, US
ER -

TY - JOUR
ID - 686
T1 - Safety on the Playground: Mechanisms Through Which Adult Supervision Might Prevent Child Playground Injury. [References]
A1 - Schwebel,David C.
Y1 - 2006///
N1 - Peer Reviewed Journal: 2006-08533-005
English
Journal; Peer Reviewed Journal
KW - PsycInfo
KW - Accident Prevention
KW - Childhood Play Behavior
KW - Injuries
KW - Playgrounds
KW - Safety
RP - NOT IN FILE
SP - 141
EP - 149
JF - Journal of Clinical Psychology in Medical Settings
VL - 13:
IS - 2
N2 - Over 200,000 American children visit the emergency room annually after a playground injury. Among the most promising means to reduce playground injuries is through increased quantity and quality of playground supervision by adults. To implement empirically supported playground supervision interventions, it is essential to understand how supervision prevents injuries. Recent research suggests supervision likely prevents injuries through several different mechanisms; this paper discusses six: (a) Supervisors repetitively teach children playground rules; (b) supervisors recognize and stop children's dangerous behavior; (c) supervisors prevent children from behaving impulsively; (d) the presence of a supervisor causes children to behave differently; (e) supervisors change children's attribution of risk; and (f) supervisors have influence as modelers and persuaders of safe behavior. Theoretical implications for future work and applied implications for the development of playground safety interventions are discussed. (PsycINFO Database Record (c) 2012 APA, all rights reserved) (journal abstract)
SN - 1068-9583
AD - Schwebel, David C.: Department of Psychology, University of Alabama at Birmingham, Birmingham, AL, US
ER -

TY - JOUR
ID - 687
T1 - Sustained Withdrawal Behavior in Clinic-referred and Nonreferred Infants. [References]
A1 - Dollberg,Daphna; Feldman,Ruth; Keren,Miri; Guedeney,Antoine
Y1 - 2006///

N1 - Peer Reviewed Journal: 2006-07569-004
English
Journal; Peer Reviewed Journal
KW - PsycInfo
KW - Infant Development
KW - Mental Health
KW - Mother Child Relations
KW - Withdrawal (Defense Mechanism)
RP - NOT IN FILE
SP - 292
EP - 309
JF - Infant Mental Health Journal
VL - 27:
IS - 3
N2 - To examine the relations between infants' sustained withdrawal behavior and children's mental health status and maternal and child relational behavior, 36 clinic-referred and 43 control infants were evaluated. Families were visited at home, mother-child free play and feeding interactions were videotaped, and mothers completed self-report measures. Interactions were coded for sustained withdrawal using the Alarm Distress Baby Scale (ADBB; Guedeney and Fermanian, 2001) and for global relational patterns with the Coding of Interactive Behavior (CIB; Feldman, 1998). Higher ADBB scores were found for the referred group, with many infants (38.9%) scoring above the clinical cutoff (vs. 11.6% in the control group). More negative relational patterns were found for the withdrawn group in terms of higher maternal intrusiveness, lower reciprocity, and lower child involvement. Associations were found between maternal and child behavior during play and feeding and child sustained withdrawal behavior at play. Sustained withdrawal also was associated with unpredictable child temperament and lower sense of parental self-efficacy. Maternal depressive symptoms were higher in the referred group and correlated with maternal and child relational patterns. The findings contribute to the construct and discriminant validity of the CIB and the ADBB coding systems, and suggest that sustained withdrawal may serve as a risk indicator for early socioemotional disorders. (PsycINFO Database Record (c) 2012 APA, all rights reserved) (journal abstract)
SN - 0163-9641
AD - Dollberg, Daphna: Bar-Ilan University, Ramat-Gan, Israel Feldman, Ruth: Bar-Ilan University, Ramat-Gan, Israel Keren, Miri: Geha Psychiatric Hospital, Israel Guedeney, Antoine: Hospital Bichat-Claude Bernard, AP/HP Paris, Paris, France
ER -

TY - JOUR
ID - 688
T1 - Language Development Subcontexts in Head Start Classrooms: Distinctive Patterns of Teacher Talk During Free Play, Mealtime, and Book Reading. [References]
A1 - Gest,Scott D.
Y1 - 2006///
N1 - Peer Reviewed Journal: 2006-06160-005
English
Journal; Peer Reviewed Journal
KW - PsycInfo
KW - Childhood Play Behavior
KW - Classrooms
KW - Language Development
KW - Project Head Start
KW - Teachers
KW - Mealtimes
KW - Reading
RP - NOT IN FILE
SP - 293
EP - 315
JF - Early Education and Development

VL - 17:
IS - 2
N2 - Research findings. Language development subcontexts within 20 Head Start classrooms were studied by observing teachers' child-directed talk during free play, mealtime, and book reading. In each context, observers coded all child-directed statements, directives, and questions, noted instances of pretend talk and decontextualized talk, and rated the richness and sensitivity-responsiveness of teachers' talk. Rates of child-directed talk were similar across contexts, but the challenging features of teachers' talk varied substantially across contexts: pretend talk occurred almost exclusively during free play, decontextualized talk was most common during mealtime, and ratings of richness were greatest during book reading. Higher rates of pretend talk and decontextualized talk and higher ratings of richness were associated with higher ratings of sensitivity-responsiveness. Rates of child-directed talk did not differ reliably for Lead Teachers and Assistant Teachers. Implications for practice. Professional development efforts may focus productively on helping Head Start teachers to recognize natural opportunities for different types of challenging talk in different classroom contexts. In the majority of classrooms, encouraging teachers to engage in more child-directed talk carries little risk of reducing the quality of sensitive-responsive caregiving. Assistant Teachers play a major role in Head Start classroom language environments and should be included in professional development activities focused on children's language skills. (PsycINFO Database Record (c) 2012 APA, all rights reserved) (journal abstract)
SN - 1040-9289
AD - Gest, Scott D.: Department of Human Development and Family Studies, Pennsylvania State University, University Park, PA, US Holland-Coviello, Rebecca: Department of Human Development and Family Studies, Pennsylvania State University, University Park, PA, US Welsh, Janet A.: Prevention Research Center, Pennsylvania State University, PA, US Eicher-Catt, Deborah L.: Department of Communication Arts and Sciences, Pennsylvania State University, York, PA, US Gill, Sukhdeep: Department of Human Development and Family Studies, Pennsylvania State University, York, PA, US
ER -

TY - JOUR
ID - 689
T1 - Playful "Moments" in Psychotherapy. [References]
A1 - Terr,Lenore C.
Y1 - 2006///
N1 - Peer Reviewed Journal: 2006-05706-012
English
Journal; Peer Reviewed Journal
KW - PsycInfo
KW - Child Psychotherapy
KW - Psychotherapeutic Processes
KW - Childhood Play Behavior
KW - Creativity
KW - Imagination
KW - Psychiatrists
RP - NOT IN FILE
SP - 604
EP - 613
JF - Journal of the American Academy of Child & Adolescent Psychiatry
JA - J Am Acad.Child Adolesc Psychiatry
VL - 45:
IS - 5
N2 - A group of us previously published an article in this journal (Terr et al., 2005) exploring the reasons why some of our child patients dramatically turned around in their psychotherapies. We concluded that the realities of the doctor-patient relationship made a crucial contribution to youthful changes in treatment (how well the psychiatrist followed the child's lead, for example, how willing the psychiatrist was to be "real" when appropriate, and how thoroughly the doctor played out or discussed the child's facts and/or fantasies). In this article, a different group of us once again consider climactic "moments" in children's therapy; this time, we look specifically at how play, playfulness, and creative imagination affect the course of events in treatment. When the doctor's energies are purposefully put to playful pursuits, the young patient may almost instantly become

amenable to treatment. In the cases we present here, no matter what age the child was (three of these young people were teenagers and three were preschoolers), the psychiatrist's attention to play was immediately, implicitly appreciated. Each young patient willingly entered into the game, inside joke, or mutual adventure--no matter how lightly frolicking or darkly ironic it was. (PsycINFO Database Record (c) 2012 APA, all rights reserved)
SN - 0890-8567
AD - Terr, Lenore C.: U California, San Francisco, Fresno, CA, US Deeney, John M.: Oregon Health & Sciences U, Portland, OR, US Drell, Martin: Louisiana State U Medical School, New Orleans, LA, US Gaensbauer, Theodore J.: U Colorado, CO, US Massie, Henry: U California, San Francisco, Fresno, CA, US Minde, Klaus: McGill U, Montreal, PQ, Canada Stewart, George: U California, Berkeley, Berkeley, CA, US Teal, Stewart: U California, Davis, CA, US Winters, Nancy C.: Oregon Health & Sciences U, Portland, OR, US
ER -

TY - JOUR
ID - 690
T1 - The Educator's Role in Child and Adolescent Mental Health. [References]
A1 - Waller,Raymond J.
Y1 - 2006///
N1 - Book: 2006-03928-001
English
Book; Edited Book
KW - PsycInfo
KW - Adolescent Development
KW - Childhood Development
KW - Mental Health
KW - Roles
KW - School Based Intervention
KW - Classroom Management
KW - Mental Disorders
KW - Resilience (Psychological)
KW - Teachers
RP - NOT IN FILE
SP - 3
EP - 13
JF - Waller, Raymond J [Ed]
VL - :
IS - 2006
N2 - (from the chapter) Teachers face more challenges today than during any other time. Students present with more challenges than ever before, particularly in the area of mental health needs. Clear evidence suggests that a variety of mental problems are increasing in frequency, and access to and availability of services for students with mental health needs have not improved for decades. In the face of such need, it is easy for educators to accept the responsibility of this burden borne by so many of today's students. But teachers should resist the propensity to shoulder a burden that is outside the purview of their professional training and focus. Educators should acknowledge, however, that they can have a positive impact on children's mental health. Teachers can play an incalculable role in fostering psychological resilience in children, which can serve to protect them from exposure to a variety of risk factors. The strategies suggested in this chapter are classroom techniques that can assist in the development of these protective factors. They are techniques that have been found to be effective, while actually tending to decrease, rather than increase, one's workload. In addition, the impact of protective factors seems to be cumulative so that the more of these strategies a teacher implements, the more protection a student may cultivate. Teachers are among the most involved adults in the lives of children. In our society, physicians maintain a high level of professional prestige, whereas teaching has lost credibility. Ironically, a child may go through life and rarely, if ever, see a physician. The same cannot be said of educators. If a child begins to show symptoms of a mental health problem, teachers are likely to be the first professionals to see them. Thus, teachers can also play an indispensable role in recognition of problems, referral, participation in interdisciplinary planning, advocacy, and monitoring of their students and can do so without making a difficult job impossible. To assist in this, and to fill a void in most teacher training programs, this book is devoted to

helping teachers recognize the most common mental health issues seen in children and adolescents and to offer strategies on how they can help encourage and support all their students. (PsycINFO Database Record (c) 2012 APA, all rights reserved)
AD - Waller, Raymond J.: Piedmont College, Demorest, GA, US Bresson, Diane J.: J. H. House Elementary School, Conyers, GA, US Waller, Katherine S.: Piedmont College, Demorest, GA, US
ER -

TY - JOUR
ID - 691
T1 - Possible relations among congenital blindness, autistic features and maternal interaction style. [Portuguese]. [References]
A1 - de Sousa,Ana Delias; Bosa,Cleonice Alves; Hugo,Cristina Neves
Y1 - 2006///
N1 - Peer Reviewed Journal: 2006-02868-002
Portuguese
Journal; Peer Reviewed Journal
KW - PsycInfo
KW - Autism
KW - Blind
KW - Childhood Play Behavior
KW - Congenital Disorders
KW - Mother Child Relations
KW - Attention
KW - Stereotyped Behavior
RP - NOT IN FILE
SP - 355
EP - 364
JF - Estudos de Psicologia
VL - 22:
IS - 4
N2 - This study has examined the occurrence of autistic features in children with congenital blindness. Joint attention and symbolic play deficits, and stereotyped body movements were focused. Mother's interaction style was also investigated during playing episodes, while they were trying to engage their children. Eight child-mother dyads, 4 with congenital blindness and 4 sighted children were studied. A home-based interview about child's development and socio-demographic data has been performed, and a free-play session was conducted. Free-play sessions were used for coding both maternal and infant behaviors. The results, that are out of the literature expectations, have brought up only two blind children showed higher play deficits frequencies, and only one mother from the congenital blindness children group who had presented more maternal directive posture compared to the sighted children's mothers. It was also noted the presence of symbolic play in the congenital blindness group. These results suggest that children with congenital blindness may not be at risk, considering the autistic features development, as long as they are properly stimulated by their caregivers who are sensible to their cues and needs. (PsycINFO Database Record (c) 2012 APA, all rights reserved) (journal abstract)
SN - 0103-166X
AD - de Sousa, Ana Delias: Instituto de Psicologia, Universidade Federal do Rio Grande do Sul, Porto Alegre, Brazil Bosa, Cleonice Alves: Programa de Pos-Graduacao em Psicologia, Universidade Federal do Rio Grande do Sul, Porto Alegre, Brazil Hugo, Cristina Neves: Mestranda em Psicologia, Programa de Pos-Graduacao em Psicologia, Universidade Federal do Rio Grande do Sul, Porto Alegre, Brazil
ER -

TY - JOUR
ID - 692
T1 - Understanding Toddlers' In-Home Injuries: I. Context, Correlates, and Determinants. [References]
A1 - Morrongiello,Barbara A.
Y1 - 2004///

N1 - Peer Reviewed Journal: 2004-16924-002
English
Journal; Peer Reviewed Journal
KW - PsycInfo
KW - Child Attitudes
KW - Childhood Play Behavior
KW - Home Accidents
KW - Injuries
KW - Parent Child Relations
KW - Behavior
KW - Parental Attitudes
KW - Risk Taking
KW - SENSATION seeking
RP - NOT IN FILE
SP - 415
EP - 431
JF - Journal of Pediatric Psychology
JA - J Pediatr Psychol.
VL - 29:
IS - 6
N2 - Multimethod strategies (i.e., questionnaires, parents' observations, injury-event recording diaries, telephone and home interviews) were used to study in-home injuries experienced by toddlers over a 3-month period. Cuts, scrapes, and puncture wounds were the most common injuries. The majority of injuries affected children's limbs, and injuries most often occurred in the morning. Boys were injured most often in rooms designated for play, and a majority of their injuries followed from misbehavior. Girls were most often injured in nonplay areas of the home, with the majority of injuries occurring during play activities. Boys experienced more frequent and severe injuries than girls, although girls reacted more than boys to their injuries. Child factors relevant to injury included: risk taking, sensation seeking, and ease of behavior management. Temperament factors did not relate to child injury. Parent factors relevant to child injury included parents' beliefs about control over their child's health, protectiveness, and beliefs about child supervision. Regression analyses revealed that both child (i.e., risk taking) and parent (i.e., protectiveness) factors were significant determinants of child injury. (PsycINFO Database Record (c) 2013 APA, all rights reserved) (journal abstract)
SN - 0146-8693
AD - Morrongiello, Barbara A.: University of Guelph, Guelph, ON, Canada Ondejko, Lisa: University of Guelph, Guelph, ON, Canada Littlejohn, Amanda: University of Guelph, Guelph, ON, Canada
ER -

TY - JOUR
ID - 693
T1 - The influence of child attributes and social-situational context on school-age children's risk taking behaviors that can lead to injury. [References]
A1 - Morrongiello,Barbara A.
Y1 - 2005///
N1 - Peer Reviewed Journal: 2005-07129-006
English
Journal; Peer Reviewed Journal
KW - PsycInfo
KW - Emotions
KW - Interpersonal Influences
KW - Personality Traits
KW - Social Environments
KW - Elementary School Students
KW - Emotional Responses
KW - Fear
KW - Injuries
KW - Peer Relations

KW - SENSATION seeking
KW - Single Sex Environments
RP - NOT IN FILE
SP - 347
EP - 361
JF - Journal of Applied Developmental Psychology
VL - 26:
IS - 3
N2 - Many injuries occur to school-age children when they are allowed independence in decisions about risk taking during play. The present study examined the influence of child attributes (sensation seeking, typical emotional responses in risk situations) and social-situational context (peer presence) on risk taking. Children in the Experimental Group made decisions about risk taking with an unknown same-sex peer present and watching, while those in an age and sex-matched Control Group made decisions without a peer present. Results revealed that children who were high in sensation seeking and typically experienced excitement more than fear in risk situations engaged in greater risk taking. The presence of an observing peer, even though the peer was unknown to the child and said nothing, also was associated with greater risk taking. A regression analysis indicated that risk taking was predicted both from child attributes (sensation seeking) and emotion-based (fear) factors. Theoretical and practical implications of these findings are discussed. (PsycINFO Database Record (c) 2013 APA, all rights reserved) (journal abstract)
SN - 0193-3973
AD - Morrongiello, Barbara A.: Psychology Department, University of Guelph, Guelph, ON, Canada Sedore, Lisa: Psychology Department, University of Guelph, Guelph, ON, Canada
ER -

TY - JOUR
ID - 694
T1 - Impact of Fathers' Alcoholism and Associated Risk Factors on Parent-Infant Attachment Stability From 12 to 18 Months. [References]
A1 - Edwards,Ellen P.
Y1 - 2004///
N1 - Peer Reviewed Journal: 2004-22436-004
English
Journal; Peer Reviewed Journal
KW - PsycInfo
KW - Alcoholism
KW - Attachment Behavior
KW - Children of Alcoholics
KW - Parent Child Relations
KW - Risk Factors
RP - NOT IN FILE
SP - 556
EP - 579
JF - Infant Mental Health Journal
VL - 25:
IS - 6
N2 - This study examined short-term attachment stability and sought to identify predictors of stability and change within a sample characterized by fathers' alcoholism. Results suggest moderate stability of attachment classifications (60% for mothers, 53% for fathers) from 12 to 18 months. Higher paternal and maternal alcohol symptoms, maternal depression, and maternal antisocial behavior were found in families with stable insecure mother-infant attachment compared to those who were stable secure. Mother-infant stable insecurity was associated with higher levels of maternal negative affect expression during play. Father-infant stable insecurity was associated with lower levels of paternal positive affect expression and decreased sensitivity during play. Stable insecure children also had higher levels of negative affect during parent-infant interactions and higher negative emotionality during other episodes compared to stable secure children. Results indicate that infants who were insecure at both time points had the highest constellation of family risk characteristics. (PsycINFO Database Record (c) 2013 APA, all rights reserved) (journal abstract)

SN - 0163-9641
AD - Edwards, Ellen P.: Research Institute on Addictions, University at Buffalo, State University of New York, Buffalo, NY, US Eiden, Rina D.: Research Institute on Addictions, University at Buffalo, State University of New York, Buffalo, NY, US Leonard, Kenneth E.: Research Institute on Addictions, University at Buffalo, State University of New York, Buffalo, NY, US
ER -

TY - JOUR
ID - 696
T1 - Evaluation of behavioral skills training to prevent gun play in children. [References]
A1 - Miltenberger,Raymond G.
Y1 - 2004///
N1 - Peer Reviewed Journal: 2005-00038-008
English
Journal; Peer Reviewed Journal
KW - PsycInfo
KW - Childhood Development
KW - Childhood Play Behavior
KW - Firearms
KW - Prevention
KW - Safety
RP - NOT IN FILE
SP - 513
EP - 516
JF - Journal of Applied Behavior Analysis
JA - J Appl.Behav Anal.
VL - 37:
IS - 4
N2 - This study evaluated behavioral skills training (BST), in a multiple baseline across subjects design, for teaching firearm safety skills to 6 6- and 7-year-old children. Similar to previous research with 4- and 5-year-olds, half of the children acquired the safety skills following BST and half acquired the skills following BST plus in situ training. (PsycINFO Database Record (c) 2013 APA, all rights reserved) (journal abstract)
SN - 0021-8855
AD - Miltenberger, Raymond G.: North Dakota State University, Fargo, ND, US Flessner, Christopher: North Dakota State University, Fargo, ND, US Gatheridge, Brian: North Dakota State University, Fargo, ND, US Johnson, Brigitte: North Dakota State University, Fargo, ND, US Satterlund, Melisa: North Dakota State University, Fargo, ND, US Egemo, Kristin: North Dakota State University, Fargo, ND, US
ER -

TY - JOUR
ID - 698
T1 - Self-efficacy and parenting of high-risk infants: The moderating role of parent knowledge of infant development. [References]
A1 - Hess,Christine Reiner; Teti,Douglas
Y1 - 2004///
N1 - Peer Reviewed Journal: 2004-17945-002
English
Journal; Peer Reviewed Journal
KW - PsycInfo
KW - At Risk Populations
KW - Infant Development
KW - Knowledge Level
KW - Parenting Skills
KW - Self Efficacy
KW - Competence
KW - Mothers

RP - NOT IN FILE
SP - 423
EP - 437
JF - Journal of Applied Developmental Psychology
VL - 25:
IS - 4
N2 - The present study examined the independent and joint relations of parental self-efficacy beliefs and parent knowledge of child development to maternal behavioral competence among mothers of high-risk infants. Sixty-five mothers completed questionnaires regarding parental self-efficacy and knowledge of child development and were assessed for behavioral competence during a 10-min free-play session with their infants. There were no independent contributions of parental self-efficacy or parent knowledge of development in predicting parenting competence. However, the relation between parental self-efficacy and parenting competence was moderated by parent knowledge of development. Consistent with Bandura's [Psychol. Rev. 84 (1977) 191; Am. Psychol. 44 (1989) 1175] formulations, parental self-efficacy and parenting competence were positively associated when parental knowledge of development was high. By contrast, parental self-efficacy beliefs and parenting competence were inversely associated when knowledge of development was low. Mothers reporting high parental self-efficacy, but low knowledge of development, were the least sensitive with their infants in play interactions, suggesting that these mothers were naively confident about their parenting abilities. Implications for designing interventions for naively confident mothers are discussed. (PsycINFO Database Record (c) 2012 APA, all rights reserved) (journal abstract)
SN - 0193-3973
AD - Hess, Christine Reiner: Department of Psychology, University of Maryland, Baltimore County, Baltimore, MD, US Teti, Douglas M.: Human Development and Family Studies, Pennsylvania State University, University Park, PA, US Hussey-Gardner, Brenda: Division of Neonatology, Department of Pediatrics, University of Maryland, Baltimore, MD, US
ER -

TY - JOUR
ID - 700
T1 - Relationships between peer interactive play and social competence in at-risk preschool children. [References]
A1 - Gagnon,Sandra Glover; Nagle,Richard
Y1 - 2004///
N1 - Peer Reviewed Journal: 2004-10477-001
English
Journal; Peer Reviewed Journal
KW - PsycInfo
KW - Academic Failure
KW - At Risk Populations
KW - Childhood Play Behavior
KW - Psychosocial Development
KW - Social Interaction
KW - Childhood Development
KW - Emotional Development
KW - Peers
KW - Social Skills
RP - NOT IN FILE
SP - 173
EP - 189
JF - Psychology in the Schools
VL - 41:
IS - 2
N2 - Relationships between peer interactive play and social competence in a sample of preschool children (N = 85) considered at risk for academic difficulties were examined. Ratings of peer interactive play and social emotional development were collected from parents and teachers on the Penn Interactive Peer Play Scale and Vineland Social-Emotional Early Childhood Scale. Multivariate techniques revealed significant correlations

between parent and teacher ratings on the measures. Results are discussed within the context of conducting multifaceted, ecological assessments of preschool children. Implications for school psychologists are noted with regard to the important associations between peer interactive play and social-emotional development. The psychometric integrity of the PIPPS is further validated and extended beyond the initial standardization sample to include a sample of primarily Caucasian children living in a more rural area. (PsycINFO Database Record (c) 2012 APA, all rights reserved) (journal abstract)
SN - 0033-3085
AD - Gagnon, Sandra Glover: Appalachian State University, Boone, NC, US Nagle, Richard J.: U South Carolina, Columbia, SC, US
ER -

TY - JOUR
ID - 702
T1 - Identifying Factors that Relate to Children's Risk-Taking Decisions. [References]
A1 - Morrongiello,Barbara A.
Y1 - 2004///
N1 - Peer Reviewed Journal: 2004-19581-001
English
Journal; Peer Reviewed Journal
KW - PsycInfo
KW - Childhood Play Behavior
KW - Decision Making
KW - Friendship
KW - Risk Factors
KW - Risk Taking
KW - Dangerousness
KW - Dyads
KW - Human Sex Differences
KW - Parental Attitudes
KW - Peer Pressure
KW - Pleasure
RP - NOT IN FILE
SP - 255
EP - 266
JF - Canadian Journal of Behavioural Science/Revue canadienne des sciences du comportement
VL - 36:
IS - 4
N2 - Identified factors related to risk decisions and examined risk-taking in the context of best-friend dyads. Children were individually shown drawings of play situations, with different risk-level paths depicted. They gave ratings of their experience with the activities, provided fun and danger ratings for all paths, indicated the path they would take and why, and answered questions about the choice their best friend would make, and the path each parent would want them to take. Subsequently, a peer-influence session was contrived in which their best friend attempted to persuade them to select another path. Then, children made their final path choices. Greater experience with an activity was associated with greater risk-taking. Perceptions of danger negatively correlated with risk-taking decisions. For boys, fun ratings positively correlated with risk-taking decisions. Girls selected paths consistent with what they believed their parents would favour. Best friends were highly similar in risk-taking. When differences emerged, best friends were successful about half the time in their persuasion efforts. Rate of success positively correlated with friendship quality. (PsycINFO Database Record (c) 2012 APA, all rights reserved) (journal abstract)
SN - 0008-400X
AD - Morrongiello, Barbara A.: University of Guelph, Guelph, ON, Canada Dawber, Tess: University of Guelph, Guelph, ON, Canada
ER -

TY - JOUR
ID - 705

T1 - Chronicity of Maternal Depressive Symptoms, Maternal Sensitivity, and Child Functioning at 36 Months
Y1 - 2005///
N1 - Book: 2005-07172-010
English
Book; Edited Book
KW - PsycInfo
KW - Ability Level
KW - Major Depression
KW - Mother Child Relations
KW - Parental Characteristics
KW - Risk Factors
RP - NOT IN FILE
SP - 151
EP - 162
JF - The NICHD Early Child Care Research Network
VL - :
IS - 2005
N2 - (from the chapter) For this report we examine the link between maternal depression and mother-child interaction and their relations with children's functioning at 36 months. Specifically, we test a model that includes the chronicity of maternal depressive symptoms assessed from the birth of the study child to 36 months, and common co-occurring risk/protective factors such as household income, education, partner presence, and social support. We hypothesize that mothers who report more chronic symptoms of depression across this 3-year period will be less sensitive and responsive to their infants during play, and that the effects of the chronicity of depressive symptoms on mother-child interaction will be moderated by other risk/protective factors. Thus, women with chronic depressive symptoms who also have substantial resources and few psychosocial risks will be more sensitive with their babies than women who report chronic symptoms but also have few resources and little support from others. We also test the hypothesis that children whose mothers report chronic symptoms of depression will display more negative and less positive mood during play. Finally, our longitudinal data permit us to explore whether sensitive and responsive mother-child interaction changes over the first 36 months of life as a function of the chronicity of symptoms and other risk factors. (PsycINFO Database Record (c) 2012 APA, all rights reserved)
ER -

TY - JOUR
ID - 706
T1 - Home environments of 10-month-old infants selected by the WILSTAAR screen for pre-language difficulties. [References]
A1 - Alston,Enid; St James-Roberts,Ian
Y1 - 2005///
N1 - Peer Reviewed Journal: 2005-04988-002
English
Journal; Peer Reviewed Journal
KW - PsycInfo
KW - Home Environment
KW - Infant Development
KW - Language Disorders
KW - Screening Tests
RP - NOT IN FILE
SP - 123
EP - 136
JF - International Journal of Language & Communication Disorders
VL - 40:
IS - 2
N2 - Background and Aims: The Ward Infant Language Screening Test, Assessment, Acceleration and Remediation (WILSTAAR) comprises a programme for identifying and helping 8-10-month-old infants showing early signs of language and communication difficulty. The study addresses one of the queries raised by

critics of the programme by providing evidence about the backgrounds and home environments of infants who fail the WILSTAAR screening assessment. Methods and Procedures: Thirty 10-month-old infants who failed the screen ('at-risk' infants) and 30 gender- and age-matched infants who passed the screen ('not-at-risk' infants) were visited at home by a trained observer who collected information about the infants' physical and social environment and families' demographic features. Outcomes and Results: Parents of both groups were well educated, in stable relationships, and had no evidence of social or economic disadvantage. Measures of ambient sound level and television exposure did not support the hypothesis that the at-risk infants' environments involved high levels of noise. However, observations of spontaneous behaviour and a standardized Mother-Infant Play Task identified low amounts of social interaction in at-risk cases. During the play task, mothers of at-risk infants spent twice as much time without interacting with their infants. At-risk infants babbled one-third less than not-at-risk infants during 150-min observations of spontaneous behaviour. Conclusions: The findings point to deficiencies in social interaction and communication, involving both infant and parental contributions, as maintaining the infants' difficulties at this age. The core of the WILSTAAR intervention, of encouraging social communication between parents and infants, is not complex and does not need to be confined to at-risk cases. How this is best accomplished in the general community, and whether it should be included in national schemes such as Sure Start in the UK, are important topics for practitioners and policy-makers. (PsycINFO Database Record (c) 2012 APA, all rights reserved) (journal abstract)
SN - 1368-2822
AD - Alston, Enid: Thomas Coram Research Unit, Institute of Education, University of London, London, United Kingdom St James-Roberts, Ian: Thomas Coram Research Unit, Institute of Education, University of London, London, United Kingdom
ER -

TY - JOUR
ID - 709
T1 - The Use of Play and Narrative Story Stems in Assessing the Mental Health Needs of Foster Children. [References]
A1 - Hudd,Sheila
Y1 - 2005///
N1 - Book: 2005-07318-006
English
Book; Edited Book
KW - PsycInfo
KW - Foster Children
KW - Narratives
KW - Play Therapy
KW - Psychological Needs
KW - Psychotherapeutic Techniques
KW - Attachment Behavior
KW - Measurement
KW - Mental Health
KW - Storytelling
KW - Interpersonal Relationships
RP - NOT IN FILE
SP - 113
EP - 132
JF - Schaefer, Charles [Ed]; McCormick, Judy [Ed]; Ohnogi, Akiko [Ed]
VL - :
IS - 2005
N2 - (from the chapter) Attachment theory is a theory of personality development in close relationships and helps us understand why children who have suffered adverse relationships in their early years go on to find relationships difficult in the future. It is no surprise when parents of foster children consider some to be resistant to all efforts and attempts at closeness in any relationships and/or experience their behavior as extremely difficult to manage when they demonstrate a desperate need to manipulate and control their environments. While the use of narrative and story stems is not the only tool used in assessment, story stems are a useful tool as part of assessment and during any brief intervention which aims to gain clues to a child's functioning and

difficulties which will provide indications as to what type of care the child may need, and what type of support the foster parent may need. Once assessment has been completed, if the child's support systems are deemed to be secure, then play therapy can be offered. Play therapy goes on to continue to use story formats, actively help the child develop more adaptive coping strategies, challenge cognitive distortions, and rely less on negative behavior to communicate distress. This chapter in particular sets out to describe how play therapy techniques were used to assess and assist with understanding the difficulties of three foster children who were experiencing problems with their current placement, in school and relationships generally, to the extent that the placement was at severe risk of breakdown. (PsycINFO Database Record (c) 2012 APA, all rights reserved)
AD - Hudd, Sheila: William Harvey Child, Adolescent, and Family Clinic, Putney, England
ER -

TY - JOUR
ID - 710
T1 - The Developmental Psychopathology of Personality Disorders. [References]
A1 - Johnson,Jeffrey G.
Y1 - 2005///
N1 - Book: 2006-04904-015
English
Book; Edited Book
KW - PsycInfo
KW - Developmental Psychology
KW - Personality Disorders
KW - Psychopathology
KW - Susceptibility (Disorders)
RP - NOT IN FILE
SP - 417
EP - 464
JF - Hankin, Benjamin L [Ed]; Abela, John R
VL - Z [Ed]. :
IS - 2005
N2 - (from the chapter) A substantial body of research evidence has indicated that individuals with personality disorders (PDs) are more likely than other individuals to report a history of childhood adversities. Retrospective studies have also provided considerable evidence suggesting that specific combinations of childhood adversities may be differentially associated with risk for the development of specific types of PDs. Although strong inferences regarding causality are not possible based on retrospective data, prospective longitudinal studies have provided additional evidence supporting the hypothesis that specific combinations of childhood adversities are differentially associated with risk for specific PDs. These results dovetail with an accumulating body of evidence from genetic and neurobiological studies supporting the overall hypothesis that childhood abuse contributes to elevated risk for the development of PDs. However, many questions regarding the association between the childhood adversities and risk for PDs remain unanswered. Although there have been noteworthy advances in recent years, scientific understanding of the role that childhood adversities may play in the development of PDs remains somewhat limited. The evidence that is currently available suggests that childhood adversities may play an important role in the development of PDs and that certain combinations of childhood adversities may be differentially associated with risk for specific types of PDs. Increasingly, it will be important for future studies to investigate how childhood adversities may interact with genetic, prenatal, and other vulnerability factors to promote the development of PD symptoms. In addition to investigating the interaction of genetic and environmental factors, it will be important to investigate the three-way interaction of genetic, prenatal, and environmental factors. Such studies hold the promise of improving our understanding of the etiology of PDs and promoting advances in the prevention and treatment of these chronic and debilitating disorders. (PsycINFO Database Record (c) 2012 APA, all rights reserved)
AD - Johnson, Jeffrey G.: Columbia U, Coll of Physicians & Surgeons, Dept of Psychiatry, New York, NY, US McGeoch, Pamela G.: New School U, NY, US Caskey, Vanessa P.: Long Island U, Brooklyn, NY, US Abhary, Sotoodeh G.: Flinders Medical Ctr, Adelaide, SA, Australia Sneed, Joel R.: Columbia U, New York, NY, US Bornstein, Robert F.: Gettysburg Coll, Gettysburg, PA, US
ER -

TY - JOUR
ID - 711
T1 - Does lipreading help word reading: An investigation of the relationship between visible speech and early reading achievement
A1 - Hiramatsu,Sandra
Y1 - 2005///
N1 - Dissertation Abstract: 2005-99023-017
English
Dissertation Abstract
KW - PsycInfo
KW - Childhood Development
KW - Lipreading
KW - Reading Ability
KW - Reading Achievement
KW - Speech Perception
KW - Oral Communication
RP - NOT IN FILE
SP - 2153
JF - Dissertation Abstracts International Section A: Humanities and Social Sciences
VL - 66:
IS - 6-A
N2 - Numerous studies have acknowledged the importance of speech perception, phonological representation, phonological memory, phonological recall, and phonological awareness to reading. However, the role that visual articulatory cues (VAC) may play in these processes has rarely been investigated even though VAC are an integral aspect of speech perception. Watching a speaker's mouth and tongue enhances speech perception, particularly when hearing is challenged by noise, unfamiliar words, accents, or abstract listening situations, such as early classroom reading instruction. Word decoding requires accurate perception of phonemes and systematic mapping of phonemes to written representations. The more accurate these perceptions are, the greater the probability that what is "sounded out" will match recognizable spoken words. The purpose of this study was to explore the role that visible articulatory cues might play in the early reading achievement of normally functioning children. Specifically, this study investigated whether the availability of visible articulatory cues improved letter-sound perception and whether the ability to lipread had a relationship with children's identification of letter sounds, phonological awareness, pseudoword reading, and real word reading. Seventy-five kindergarten, first, and second grade students from at-risk socio-economic backgrounds responded to tests of the preceding attributes. Repeated measures ANOVA found that the presence of visible articulatory cues significantly contributed to subjects' accuracy in repeating letter sounds heard in the presence of noise. Multiple regression analyses revealed that lipreading helped to uniquely explain the variance in phonological awareness beyond the effects of print letter knowledge. Further regression analyses indicated that phonological awareness mediated the relationship between lipreading and pseudoword reading and real word reading. Study results supported a critical role for visible articulatory cues in the accuracy of letter sound perception and phonological awareness in an at-risk sample. Further investigation into the role of visible articulatory cues in early reading achievement and instruction is warranted. Findings also suggested that cognitive models of reading might be enhanced by the addition of visible articulatory cues. (PsycINFO Database Record (c) 2012 APA, all rights reserved)
SN - 0419-4209
AD - Hiramatsu, Sandra: U Washington, US
ER -

TY - JOUR
ID - 712
T1 - An examination of parent-child play as influenced in the development of aggression in preschool boys
A1 - Davenport,Becky Renae
Y1 - 2005///
N1 - Dissertation Abstract: 2005-99011-080
English
Dissertation Abstract

KW - PsycInfo
KW - Aggressive Behavior
KW - Behavior Problems
KW - Home Environment
KW - Preschool Students
KW - Parent Child Relations
KW - Recreation
RP - NOT IN FILE
SP - 4739
JF - Dissertation Abstracts International Section A: Humanities and Social Sciences
VL - 65:
IS - 12-A
N2 - Children displaying high levels of aggression have repeatedly been shown to be at significant risk for continued behavior problems and other social and emotional challenges throughout their lifetimes. The current project includes two papers that examine factors contributing to the development and maintenance of aggression in preschool-aged children, especially focusing on preschool boys. The first paper reviews theoretical and empirical literature addressing the development of aggressive behavior problems. Specific attention is given to influences within the child's family environment, including positive parental involvement, harsh discipline practices, and play activities of the child both individually and jointly with his or her parent or caregiver. The second paper presents results of an empirical investigation of the relationship between parent behaviors during parent-child interactions and teacher-reported problem behaviors in preschool boys. A stratified sample of 34 three- to five-year old boys and their primary caregivers were observed in their homes engaged in unstructured free-play and a problem-solving task. Behaviors of primary caregivers in each interaction were examined in relationship to teachers' ratings of the boys' externalizing behaviors in a child care setting. Parenting characteristics in the play, but not problem-solving, interaction were found to have a statistically significant relationship with boys' externalizing scores. Positive characteristics of the caregiver in play, such as warmth and sensitivity to the child, accounted for a statistically significant amount of variance in boys' teacher-reported externalizing scores beyond that accounted for by the negative characteristics, such as intrusiveness and hostility. The findings are discussed in relation to previous literature that has addressed both the development of behavior problems in young children and the role of parent-child play interactions in child development. Clinical implications for this population are also discussed. (PsycINFO Database Record (c) 2012 APA, all rights reserved)
SN - 0419-4209
AD - Davenport, Becky Renae: Iowa State U., US
ER -

TY - JOUR
ID - 714
T1 - Emotion regulation and adherence in children with diabetes
A1 - Dasari,Meena
Y1 - 2005///
N1 - Dissertation Abstract: 2005-99002-033
English
Dissertation Abstract
KW - PsycInfo
KW - Behavior
KW - Diabetes
KW - Emotional Control
KW - Emotional Responses
KW - Treatment Compliance
RP - NOT IN FILE
SP - 3702
JF - Dissertation Abstracts International: Section B: The Sciences and Engineering
VL - 65:
IS - 7-B

N2 - Emotion regulation refers to the ability to manage negative emotions in order to adaptively engage in one's environment. In school-age and clinical populations, better emotion regulation relates to important behavioral outcomes such as lower aggression levels and decreased risk of developing clinical disorders. However, little is known about the relationship of emotion regulation to behavioral functioning in pediatric populations. In particular, emotion regulation in children with diabetes may be a link to understanding adherence, an important behavioral outcome in pediatric populations. The primary purpose of the present study was to develop and to test a theoretical model examining the relationship of emotion regulation to adherence in school-age children with diabetes. A secondary purpose was to adapt existing measures to assess emotion regulation as a multi-dimensional construct. Participants included 83 children ages 6 to 12, their parents, and their nurse providers recruited from a diabetes clinic in a pediatric hospital. Multiple methods were used to assess emotion regulation (i.e., behavioral observations and ratings scales) and adherence (i.e., rating scales and electronic monitor readings). Each child was administered both a disappointment task and a play task. Parents completed rating scales on their child's emotion regulation and their treatment adherence, while nurses rated each child's adherence and collected electronic monitor readings. Using simultaneous and stepwise multiple regressions to analyze the proposed model, parent report of children's emotion regulation emerged as the best predictor of treatment adherence, independent of age and disease duration. Children who were perceived to have better emotion regulation ability were also more compliant with their diabetes regimen. Both disappointment task and play task variables did not consistently relate to adherence measures. Overall, the multiple measures of emotion regulation were reliable and factor analyses revealed several dimensions that underlie the construct of emotion regulation. Results of this study have clinical implications for children with diabetes as well as implications for future research examining emotion regulation in school-age pediatric populations. (PsycINFO Database Record (c) 2012 APA, all rights reserved)
SN - 0419-4217
AD - Dasari, Meena: Case Western Reserve U., US
ER -

TY - JOUR
ID - 717
T1 - Risk factors for alcohol and other drug disorders: A review. [References]
A1 - Marsh,A.
Y1 - 2005///
N1 - Peer Reviewed Journal: 2005-08137-003
English
Journal; Peer Reviewed Journal
KW - PsycInfo
KW - Alcohol Abuse
KW - Drug Abuse
KW - Mental Disorders
KW - Risk Factors
KW - Comorbidity
RP - NOT IN FILE
SP - 73
EP - 80
JF - Australian Psychologist
VL - 40:
IS - 2
N2 - The role of comorbid psychological disorders in alcohol and other drug disorders is considered in the context of other individual, family, peer and broader environmental risk factors. Although peer and environmental factors play an important role in all drug use, family factors, psychological difficulties and emotional distress play a crucial role in the transition from controlled to excessive drug use. Essentially, dysfunctional parent-child relationships and severe family disruption contribute to emotional distress and to a sense of alienation from conventional sources of reward (family, school, work, and intimate relationships). For people who are distressed or alienated, heavy drug involvement can be a way to manage negative affect, bond with others, and/or experience alternative source of reward. Even people who use drugs to manage negative affect, however, are also likely to use them in some circumstances such as to have fun and be social. Excessive

substance use also results in emotional distress, and it can be difficult to distinguish cause and consequence. (PsycINFO Database Record (c) 2012 APA, all rights reserved) (journal abstract)
SN - 0005-0067
AD - Marsh, A.: School of Psychology, Curtin University, Perth, WAU, Australia Dale, A.: School of Psychology, Curtin University, Perth, WAU, Australia
ER -

TY - JOUR
ID - 718
T1 - The Play's the Thing: A Clinical-Developmental Perspective on Video Games. [References]
A1 - Gelfond,Holly S; Salonius-Pasternak,Dorothy
Y1 - 2005///
N1 - Peer Reviewed Journal: 2005-07326-009
English
Journal; Peer Reviewed Journal
KW - PsycInfo
KW - Cognitive Development
KW - Computer Games
KW - Emotional Development
RP - NOT IN FILE
SP - 491
EP - 508
JF - Child and Adolescent Psychiatric Clinics of North America
VL - 14:
IS - 3
N2 - In this article, computer and video games are discussed as electronic play. Major perspectives on play and salient developmental issues are presented, along with similarities and differences between electronic play and other types of play. The authors consider possible benefits and risks associated with this type of play, with particular attention paid to cognitive and socioemotional development. Recommendations for clinicians in their work with children, adolescents, and parents are discussed, as are future directions for research. (PsycINFO Database Record (c) 2012 APA, all rights reserved) (journal abstract)
SN - 1056-4993
AD - Gelfond, Holly S.: Harvard Medical School, Center for Mental Health and Media, Waltham, MA, US Salonius-Pasternak, Dorothy E.: Department of Psychiatry, Massachusetts General Hospital, Boston, MA, US
ER -

TY - JOUR
ID - 719
T1 - Gaze-Shift Patterns of Young Children with Developmental Disabilities who are at Risk for being Nonspeaking. [References]
A1 - Arens,Kelli
Y1 - 2005///
N1 - Peer Reviewed Journal: 2005-06044-006
English
Journal; Peer Reviewed Journal
KW - PsycInfo
KW - Childhood Development
KW - Communication Skills
KW - Developmental Disabilities
KW - Eye Fixation
KW - Visual Attention
KW - At Risk Populations
RP - NOT IN FILE
SP - 158
EP - 170
JF - Education and Training in Developmental Disabilities

VL - 40:
IS - 2
N2 - Children with developmental disabilities often have difficulty with joint attention that can affect more advanced communication skills. This study evaluated the complexity of child engagement behaviors demonstrated by twenty-five pre-intentional children (age 9 to 25 months), who had developmental disabilities and were at risk for being nonspeaking. During free play with their parents, these children demonstrated infrequent and simple gaze shifts and focused more on individual objects or people than shared attention with parents during play. These children seldom engaged in coordinated attention behaviors such as shifting gaze back and forth between people and objects during their play with parents. Type and frequency of engagement behaviors are discussed relative to understanding the unique challenges for children with developmental disabilities that include motor and visual impairments. (PsycINFO Database Record (c) 2012 APA, all rights reserved) (journal abstract)
SN - 1547-0350
AD - Arens, Kelli: University of Nebraska, Lincoln, NE, US Cress, Cynthia J.: University of Nebraska, Lincoln, NE, US Marvin, Christine A.: University of Nebraska, Lincoln, NE, US
ER -

TY - JOUR
ID - 721
T1 - Role of Childhood Experiences in the Development of Maladaptive and Adaptive Personality Traits. [References]
A1 - Johnson,Jeffrey G.
Y1 - 2005///
N1 - Book: 2005-05013-013
English
Book; Edited Book
KW - PsycInfo
KW - Early Experience
KW - Personality Development
KW - Personality Disorders
KW - Personality Traits
RP - NOT IN FILE
SP - 209
EP - 221
JF - Oldham, John M [Ed]; Skodol, Andrew E [Ed]; Bender, Donna S [Ed]
VL - :
IS - 2005
N2 - (from the chapter) During the past century, clinical experience and research have provided considerable support for the hypothesis that interpersonal experiences during childhood and adolescence play an important role in personality development (e.g., Erikson 1963). Childhood adversities such as maladaptive parenting and childhood abuse and neglect may be likely to have an adverse impact on personality development because they interfere with or alter the trajectory of normative socialization processes during childhood and adolescence (Cohen 1999; Johnson et al. 2001a). Positive experiences during childhood and adolescence, such as parental warmth and support, may be likely to promote the development of adaptive traits such as trust, altruism, and optimism, due to social learning processes and development of a secure attachment style during childhood (e.g., Erikson 1963; Sroufe et al. 1999). This chapter presents a summary of research findings that are currently available regarding the role that childhood experiences may play in the development of maladaptive and adaptive personality traits. We begin by summarizing the evidence that is currently available from retrospective and prospective studies regarding the hypothesized association between childhood adversities and personality disorders. The association of childhood abuse and neglect with risk for the development of personality disorders is examined in particular depth because this association is of considerable interest to clinicians and because comparatively little information is available regarding other adversities that may contribute to the development of maladaptive traits. The chapter concludes with an examination of the available evidence regarding the association of positive childhood experiences with the development of adaptive traits. (PsycINFO Database Record (c) 2012 APA, all rights reserved)

AD - Johnson, Jeffrey G.: Columbia U, Coll of Physicians & Surgeons, Dept of Psychiatry, New York, NY, US Bromley, Elizabeth: West Los Angeles VA Mental Illness Research, Education, & Clinical Ctr (MIRECC), CA, US McGeoch, Pamela G.: The New School U, Dept of Psychology, New York, NY, US
ER -

TY - JOUR
ID - 722
T1 - The Primary Mental Health Project: A Play Intervention for School-Age Children. [References]
A1 - Johnson,Deborah B.
Y1 - 2005///
N1 - Book: 2005-02330-002
English
Book; Edited Book
KW - PsycInfo
KW - Adjustment Disorders
KW - Play Therapy
KW - Primary Mental Health Prevention
KW - School Adjustment
KW - School Based Intervention
RP - NOT IN FILE
SP - 13
EP - 30
JF - Reddy, Linda A [Ed]; Files-Hall, Tara M [Ed]; Schaefer, Charles E [Ed]
VL - :
IS - 2005
N2 - (from the book) In this chapter, the authors discuss a well-researched school-based preventative play intervention program, the Primary Mental Health Project (PMHP). PMHP, established in 1957, targets primary school-age children at risk for adjustment difficulties and has been implemented in over 2,000 schools worldwide. Under supervision, paraprofessionals are trained in child-centered play therapy principles and skills so they can conduct individual play sessions. Outcome evaluations of PMHP reveal that the children demonstrate significant improvements in adjustment in both the short and the long term. (PsycINFO Database Record (c) 2012 APA, all rights reserved)
AD - Johnson, Deborah B.: Children's Institute, Inc., US Pedro-Carroll, JoAnne L.: Children's Institute, Inc., US Demanchick, Stephen P.: University of Rochester, Rochester, NY, US
ER -

TY - JOUR
ID - 723
T1 - Responsiveness and attention during picture-book reading in 18-month-old to 24-month-old toddlers at risk. [References]
A1 - Fletcher,Kathryn L.
Y1 - 2005///
N1 - Peer Reviewed Journal: 2004-22506-005
English
Journal; Peer Reviewed Journal
KW - PsycInfo
KW - At Risk Populations
KW - Attention
KW - Childhood Development
KW - Language Development
KW - Reading Skills
KW - Literacy
RP - NOT IN FILE
SP - 63
EP - 81
JF - Early Child Development and Care

VL - 175:
IS - 1
N2 - The purpose of this research was to examine the spontaneous responsiveness and attention during picture-book reading in 18-month-old to 24-month-old children from at-risk backgrounds. Twenty-five, 18-month-old children in an early intervention program were randomly assigned to a read condition or play condition for six months. At each seventh session, all children, regardless of condition, were videotaped during reading. Videotapes were scored for children's spontaneous responses such as pointing and labeling as well as mean duration of joint attention. Primary caregivers completed language measures for children at 18 and 24 months of age. Overall, there was large variability in the level of children's spontaneous responses and attention during reading. Responsiveness increased from observation 3 to observation 4, whereas joint attention increased from observation 1 to observation 2. There were no differences in responsiveness and joint attention measures for the children in the read and play conditions. Measures of joint attention during reading from 18 to 24 months of age were related to language measures at 24 months. Implications for this research include a closer examination of individual differences in young children's participation in picture-book reading. (PsycINFO Database Record (c) 2012 APA, all rights reserved) (journal abstract)
SN - 0300-4430
AD - Fletcher, Kathryn L.: Ball State University, Muncie, IN, US Perez, Andreina: Research Triangle Institute, Research Triangle Park, NC, US Hooper, Corrie: University of Miami, Coral Gables, FL, US Claussen, Angelika H.: University of Miami, Coral Gables, FL, US
ER -

TY - JOUR
ID - 724
T1 - Rejection sensitivity and girls' aggression. [References]
A1 - Downey,Geraldine; Irwin,Lauren; Ramsay,Melissa; Ayduk,Ozlem
Y1 - 2004///
N1 - Book: 2005-15189-002
English
Book; Edited Book
KW - PsycInfo
KW - Aggressive Behavior
KW - Human Females
KW - Psychosocial Development
KW - Self Regulation
KW - Social Acceptance
KW - Adolescent Psychology
KW - Hostility
KW - Interpersonal Interaction
KW - Models
KW - Retaliation
KW - Self Concept
RP - NOT IN FILE
SP - 7
EP - 25
JF - Moretti, Marlene M [Ed]; Odgers, Candice L [Ed]; Jackson, Margaret A [Ed]
VL - :
IS - 2004
N2 - (from the chapter) Given the central importance of close relationships in women's lives (Gilligan, 1982; see Cross & Madson, 1997, for review), issues of acceptance and rejection appear to play a particularly salient role in women's interpersonal interactions (Purdie & Downey, 2000). It has been posited that maintaining harmonious intimate relationships is integral to women's self-concept (Baumeister & Sommer, 1997; Cross & Madson, 1997). Yet, some women react aggressively towards important others when the threat of rejection is perceived. Such reactions threaten the security and stability of the relationship, and, in turn, the woman's self-concept. What cognitive-affective processes might account for this distinctive pattern of female hostility? Our efforts to address this question have focused on the role that Rejection Sensitivity (RS) may play in helping explain why some women show high levels of relational, and even physical, aggression against the very people

they care about most. Our model also takes into account a resilience-generating process (i.e., self-regulation) that may play a potentially crucial role in breaking the link between RS and maladaptive behavior. Self-regulatory competency appears to protect RS women from the maladaptive consequences of the RS disposition. However, women who show a combination of both high RS and poor self-regulatory abilities appear to be particularly vulnerable to aggressive behavior toward significant others, as well as other personal and interpersonal difficulties. Although the mechanisms of the RS model hold true for both male and female populations, we have found that the context and resultant behavior can differ for women and men. In this chapter, our theoretical interpretation of RS and aggression, as well as our findings, refer to women. However, we want to clarify at the outset that most of these results also apply to men, but we have found that the results for men have generally been weaker and less consistent than for women. In this chapter, we first describe the RS model and the evidence that we have in support of the links in the model. We next describe the role of self-regulatory competency in moderating the maladaptive effects of RS. Whereas our focus is primarily on the links between RS and aggression, we also discuss how RS may place women at risk for other forms of harmful behavior. Specifically, we propose that, whereas the perception of the occurrence of rejection may unleash hostile retaliation, the threat of rejection may also prompt behavior that is intended to prevent rejection but instead puts the self or others in harm's way. For example, women may put themselves at risk for personal harm, as when they tolerate abusive behavior or engage in unprotected sex in order to maintain a relationship (Purdie & Downey, 2000). Furthermore, women may engage in antisocial behavior to maintain a relationship, such as concealing weapons or drugs for a romantic partner (Bedell, 1999). (PsycINFO Database Record (c) 2012 APA, all rights reserved)
AD - Downey, Geraldine: Dept of Psychology, Columbia U, New York, NY, US Irwin, Lauren: Dept of Psychology, Columbia U, New York, NY, US Ramsay, Melissa: Dept of Psychology, Columbia U, New York, NY, US Ayduk, Ozlem: Psychology Dept, U California, Berkeley, CA, US
ER -

TY - JOUR
ID - 725
T1 - Mothers' Child Abuse Potential as a Predictor of Maternal and Child Behaviors During Play-Time Interactions. [References]
A1 - Wilson,Steven R.
Y1 - 2004///
N1 - Peer Reviewed Journal: 2005-02140-002
English
Journal; Peer Reviewed Journal
KW - PsycInfo
KW - Child Abuse
KW - Childhood Play Behavior
KW - Mother Child Communication
KW - Mother Child Relations
RP - NOT IN FILE
SP - 395
EP - 421
JF - Communication Monographs
VL - 71:
IS - 4
N2 - This paper explores the effect of mothers' child abuse potential on maternal and child behaviors during play-time interactions. Forty-two mothers participated in a 10-min play period followed by a clean-up task along with one of their children (range = 3-8 years old), after which they completed Milner's (1994) Child Abuse Potential (CAP) Inventory. Mothers' behaviors were coded using a 21-category scheme, which was collapsed into five behavioral clusters: describing, directing, criticizing, soliciting/affirming, and silent play. Children were rated for levels of involvement (activity) and cooperation. Mothers' child abuse potential scores were inversely associated with their own rate of soliciting/affirming behaviors during the play period. Children of higher-risk mothers displayed higher levels of involvement combined with lower levels of cooperation compared children of lower-risk mothers. Implications for understanding and responding to child physical abuse are discussed. (PsycINFO Database Record (c) 2012 APA, all rights reserved) (journal abstract)
SN - 0363-7751

AD - Wilson, Steven R.: Purdue University, West Lafayette, IN, US Morgan, Wendy M.: Purdue University, West Lafayette, IN, US Hayes, Javette: California State University, Fullerton, CA, US Bylund, Carma: University of Iowa, Iowa City, IA, US Herman, Andrew: State University of New York, Geneseo, NY, US
ER -

TY - JOUR
ID - 727
T1 - Guest Editorial: On Behalf of Children
A1 - Chenfeld,Mimi Brodsky
Y1 - 2004///
N1 - Peer Reviewed Journal: 2004-21463-001
English
Journal; Peer Reviewed Journal
KW - PsycInfo
KW - Childhood Play Behavior
KW - Creativity
KW - Early Childhood Development
KW - Learning
KW - School Learning
RP - NOT IN FILE
SP - 141
EP - 142
JF - Early Childhood Education Journal
VL - 32:
IS - 3
N2 - On those rare occasions when I'm not running late to my movement sessions at the early childhood program where I've been hanging out for over thirty-three years, I love to poke around and watch kids at the start of their busy days. The lessons from today's early morning snoop-around are old, clear messages. They remind us that when children are given many opportunities to follow their own interests, they are excited! They are empowered! They share! Enthusiasm is contagious. All over this great land of ours, a land in the midst of tumultuous times-a high-pressured, test driven, statistics-centered culture, (education at a crossroads) there are teachers who breathe life into theories and terminology such as "language immersion," "integrated learning," "emergent curriculum"... While so many misguided folks advocate the downplaying of play, these educators value the vital place play holds in the natural and joyful ways children best learn. How lucky for those children whose teachers help keep them WHOLE, guiding, sharing, and celebrating the creative process, honoring their uniqueness, making room in their rooms for free spirited, cooperative, open-minded and open-ended adventures in learning. (PsycINFO Database Record (c) 2012 APA, all rights reserved)
SN - 1082-3301
ER -

TY - JOUR
ID - 728
T1 - Descriptive Analysis of Parent-Child Interactions in Young Children with or at Risk for Developmental Delay. [References]
A1 - Passey,Jennifer
Y1 - 2004///
N1 - Peer Reviewed Journal: 2004-20797-001
English
Journal; Peer Reviewed Journal
KW - PsycInfo
KW - Adaptive Behavior
KW - Behavior Problems
KW - Delayed Development
KW - Parent Child Relations
KW - Reinforcement
KW - At Risk Populations

RP - NOT IN FILE
SP - 233
EP - 246
JF - Behavioral Interventions
VL - 19:
IS - 4
N2 - Children with developmental delay (DD) are at risk for behavior problems, but little is known about natural contingencies of reinforcement that these children experience. The present study used descriptive analysis (antecedent-behavior-consequence observations) to study parent-child interactions of fourty-seven, 2-3 year-old children with or at risk for DD. Child adaptive and inappropriate behavior as well as their antecedents and consequences were observed across four conditions (free play, parent-directed play, mealtimes, parental distraction). When parents were engaged in another task (distraction), child appropriate behavior occurred less frequently than in the other conditions, and child inappropriate behavior occurred 63% of the time. A lack of parental attention and no activity were the most frequent antecedents for inappropriate behavior. Potential positive reinforcers appeared as consequences of inappropriate child behavior 77% of the time. These findings have implications for the development and prevention of serious behavior disorders. (PsycINFO Database Record (c) 2012 APA, all rights reserved) (journal abstract)
SN - 1072-0847
AD - Passey, Jennifer: Queen's University, Kingston, ON, Canada Feldman, Maurice: Brock University, St. Catharines, ON, Canada
ER -

TY - JOUR
ID - 729
T1 - Theorizing the Father-Child Relationship: Mechanisms and Developmental Outcomes. [References]
A1 - Paquette,Daniel
Y1 - 2004///
N1 - Peer Reviewed Journal: 2004-18780-001
English
Journal; Peer Reviewed Journal
KW - PsycInfo
KW - Childhood Development
KW - Father Child Communication
KW - Father Child Relations
KW - Mother Child Relations
KW - Parental Role
RP - NOT IN FILE
SP - 193
EP - 219
JF - Human Development
VL - 47:
IS - 4
N2 - The aim of this article is to propose a theorization of the father-child relationship based on our current understanding of attachment, interactions between fathers and their young children, and human-specific adaptations. The comparison of mother-child and father-child interactions suggests that fathers play a particularly important role in the development of children's openness to the world. Men seem to have a tendency to excite, surprise, and momentarily destabilize children; they also tend to encourage children to take risks, while at the same time ensuring the letter's safety and security, thus permitting children to learn to be braver in unfamiliar situations, as well as to stand up for themselves. But this dynamic can only be effective in the context of an emotional bond between father and child; this relationship is termed the father-child activation relationship, in contrast to the mother-child attachment relationship aimed at calming and comforting children in times of stress. The activation relationship is developed primarily through physical play. It is postulated, in particular, that father-child rough-and-tumble play encourages obedience and the development of competition skills in children. (PsycINFO Database Record (c) 2012 APA, all rights reserved) (journal abstract)
SN - 0018-716X
AD - Paquette, Daniel: Centre jeunesse de Montreal, University of Montreal, Montreal, PQ, Canada

ER -

TY - JOUR
ID - 731
T1 - Childhood obesity and consumption of fizzy drinks: Play outside to reduce childhood obesity. [References]
A1 - Sutton,Alan
Y1 - 2004///
N1 - Peer Reviewed Journal: 2004-16071-015
English
Journal; Peer Reviewed Journal
KW - PsycInfo
KW - Activity Level
KW - Exercise
KW - Obesity
KW - Public Health
KW - Risk Management
KW - Childhood Play Behavior
KW - Health Behavior
KW - Parent Child Relations
RP - NOT IN FILE
SP - 54
JF - BMJ: British Medical Journal
VL - 329:
IS - 7456
N2 - This letter to the editor comments on the 2004 article "Preventing childhood obesity by reducing consumption of carbonated drinks: cluster randomised controlled trial," by J. James et al. The rise in childhood obesity is paralleled by the rise in parental fears of the risks to children of being unaccompanied outdoors. I find it strange that the simple solution, to let children play outside more, is dismissed as impossible in our modern society. Stranger-danger is largely a myth promoted by the mass media, yet mere are few voices raised against it. Parents' refusal to allow free play for their children is now becoming embedded in a system of parental peer pressure whereby parents are afraid to allow children out for fear of being labeled uncaring (or worse) by their peers. (PsycINFO Database Record (c) 2012 APA, all rights reserved)
SN - 0959-8138
AD - Sutton, Alan: Westminster Play Association, London, United Kingdom
ER -

TY - JOUR
ID - 732
T1 - Social Play of Young Children At-Risk of Learning Difficulties: A situated performance? [References]
A1 - Van der Aalsvoort,Geerdina M.
Y1 - 2004///
N1 - Peer Reviewed Journal: 2004-15659-003
English
Journal; Peer Reviewed Journal
KW - PsycInfo
KW - At Risk Populations
KW - Childhood Play Behavior
KW - Learning Disabilities
RP - NOT IN FILE
SP - 151
EP - 169
JF - International Journal of Disability, Development and Education
VL - 51:
IS - 2

N2 - The question asked by this study was whether information on play behaviour of young children at-risk of learning difficulties could act as a diagnostic means of investigating emerging learning difficulties. A sociocultural perspective was taken to examine the role of interaction during the play of students in a regular primary school and in a special primary school using a multimethod approach. Groups of students from both schools were studied during eight 30-minute sessions over a period of 3 weeks. The groups were matched with respect to age and language development and then divided into 2 further groups of 3 students each per school. Each group was invited to build a zoo using toy animals and wooden blocks. The 32 sessions were videotaped, transcribed, and analysed microgenetically with measures involving the types of collaboration during play and metaplay. The results showed that the number of episodes and the most elaborate type of collaboration occurred more often in the regular primary school than in the special school, whereas the frequency of metaplay did not differ significantly between the schools. Fragments of discourse obtained during the sessions are presented and discussed. It is concluded from the findings that social play may emerge as a situated performance. (PsycINFO Database Record (c) 2012 APA, all rights reserved)
SN - 1034-912X
AD - Van der Aalsvoort, Geerdina M.: Leiden University, Leiden, Netherlands Van Tol, Annemarie M.: Leiden University, Leiden, Netherlands Karemaker, Arjette M.: Leiden University, Leiden, Netherlands
ER -

TY - JOUR
ID - 734
T1 - The development of symbolic play and language in toddlers with cleft palate. [References]
A1 - Snyder,Lynn E; Scherer,Nancy
Y1 - 2004///
N1 - Peer Reviewed Journal: 2004-13759-007
English
Journal; Peer Reviewed Journal
KW - PsycInfo
KW - Childhood Play Behavior
KW - Childhood Play Development
KW - Cleft Palate
KW - Gestures
KW - Language Development
RP - NOT IN FILE
SP - 66
EP - 80
JF - American Journal of Speech-Language Pathology
VL - 13:
IS - 1
N2 - This study compared the longitudinal performance of two groups of toddlers with palatal clefts and an age-matched group of children without palatal clefts on measures of elicited symbolic play at 18, 24, and 30 months. The results indicated that the group with isolated cleft palate differed significantly from both the cleft lip and palate group and the noncleft group on all but 1 play measure. Correlational analyses for each group indicated significant positive correlations between a number of the play variables at 18 months and productive vocabulary and MLU at 24 and 30 months of age. The findings suggest that assessment of early play gestures may assist clinicians in identifying children with clefts who are at risk for later language impairment. (PsycINFO Database Record (c) 2012 APA, all rights reserved) (journal abstract)
SN - 1058-0360
AD - Snyder, Lynn E.: University of Colorado, Boulder, CO, US Scherer, Nancy: East Tennessee State University, Johnson City, TN, US
ER -

TY - JOUR
ID - 735
T1 - Maternal support for play and language across early childhood. [References]
A1 - Hebert,Heather; Swank,Paul; Smith,Karen; Landry,Susan
Y1 - 2004///

N1 - Peer Reviewed Journal: 2004-10281-006
English
Journal; Peer Reviewed Journal
KW - PsycInfo
KW - Childhood Play Behavior
KW - Childrearing Practices
KW - Language Development
KW - Mother Child Relations
KW - Psychosocial Development
KW - At Risk Populations
KW - Parental Characteristics
KW - Premature Birth
RP - NOT IN FILE
SP - 93
EP - 113
JF - Early Education and Development
VL - 15:
IS - 1
N2 - Patterns of development in language and play for full term and preterm children from 6 to 54 months and the effects of maternal parenting strategies (i.e., maintaining attentional focus, use of directiveness) were examined. Significant risk differences in the growth of both language and play were found. The high risk children were more likely to show delays in both these skills. Maintaining had a positive influence on children's skills, while directiveness had a largely negative influence, especially at older ages. The relation between growth in play and language was stronger for the high risk, preterm, when compared to the low risk and full term, children, but only during the infancy and toddler period. Given that play and language development is more likely to be delayed for preterm, high-risk children and the relation between the development of these skills is stronger for high risk preterm children, particularly early, it would be important to provide the parents and educators with information about play and language development. For example, communicating the need to be consistent in using some interactive behaviors (e.g., maintaining) as children develop while modifying other behaviors (e.g., directiveness) would be essential. (PsycINFO Database Record (c) 2012 APA, all rights reserved) (journal abstract)
SN - 1040-9289
AD - Hebert, Heather: University of Texas Health Science Center, Houston, TX, US Swank, Paul: University of Texas Health Science Center, Houston, TX, US Smith, Karen: University of Texas Medical Branch, Galveston, TX, US Landry, Susan: University of Texas Health Science Center, Houston, TX, US
ER -

TY - JOUR
ID - 736
T1 - Sandplay, Art, and Play Therapy to Promote Anxiety Reduction. [References]
A1 - Carey,Lois
Y1 - 2004///
N1 - Book: 2004-00199-010
English
Book; Edited Book
KW - PsycInfo
KW - Anxiety Disorders
KW - Art Therapy
KW - Emotional Trauma
KW - Play Therapy
KW - Family Therapy
KW - Migraine Headache
KW - Separation Anxiety
KW - Terrorism
RP - NOT IN FILE
SP - 216

EP - 233
JF - Webb, Nancy Boyd [Ed]
VL - :
IS - 2004
N2 - (from the chapter) This chapter discusses creative techniques that are helpful in resolving disturbing responses to many situations of trauma. The case presented in this chapter is that of an anxious child whose symptom of migraine headaches was exacerbated by the events of 9/11. Alexander (age 10) was at risk because of several factors: (1) his history of migraine headaches; (2) his father's role in the World Trade Center search and rescue operations; (3) the exacerbation of a separation anxiety disorder. Art therapy, sandplay therapy, and family therapy were all employed in this child's treatment. Improvement was noted after the first few months, signified by the lessened frequency of his migraines and confirmed by what was revealed in the sand pictures. Alexander's resistance to attending therapy was ameliorated by decreasing the frequency of the sessions and by his recognition that his symptoms, though not fully resolved, were abating. Several pictorial examples of Alexander's journey toward healing are included in this chapter. These demonstrate how, through the use of these creative milieus, he did indeed accomplish some resolution of his fears. In so doing, he was able to reduce some of his symptoms. Alexander agreed to continue therapy with the objective of addressing his remaining anxieties. (PsycINFO Database Record (c) 2012 APA, all rights reserved)
AD - Carey, Lois: Private Practice, Upper Grandview, NY, US
ER -

TY - JOUR
ID - 737
T1 - Neighborhood playgrounds, fast food restaurants, and crime: Relationships to overweight in low-income preschool children. [References]
A1 - Burdette,Hillary L.
Y1 - 2004///
N1 - Peer Reviewed Journal: 2003-11128-008
English
Journal; Peer Reviewed Journal
KW - PsycInfo
KW - Lower Income Level
KW - Neighborhoods
KW - Obesity
KW - Overweight
KW - Crime
KW - Playgrounds
KW - Safety
RP - NOT IN FILE
SP - 57
EP - 63
JF - Preventive Medicine: An International Journal Devoted to Practice and Theory
VL - 38:
IS - 1
N2 - We examined the relationship between overweight in preschool children and three environmental factors--the proximity of the children's residences to playgrounds and to fast food restaurants and the safety of the children's neighborhoods. We hypothesized that children who lived farther from playgrounds, closer to fast food restaurants, and in unsafe neighborhoods were more likely to be overweight. This was a cross-sectional study of 7,020 low-income children, 36 through 59 months of age living in Cincinnati, OH. Overweight was defined as a measured body mass index >=95th percentile. Neighborhood safety was defined by the number of police-reported crimes per 1,000 residents per year in each of 46 city neighborhoods. Overall, 9.2% of the children were overweight, 76% black, and 23% white. The mean (+/-SD) distances from a child's home to the nearest playground and fast food restaurant were 0.31 miles, respectively. There was no association between child overweight and proximity to playgrounds, proximity to fast food restaurants, or level of neighborhood crime. Within a population of urban low-income preschoolers, overweight was not associated with proximity to playgrounds and fast food restaurants or with the level of neighborhood crime. (PsycINFO Database Record (c) 2012 APA, all rights reserved)

SN - 0091-7435
AD - Burdette, Hillary L.: Division of General and Community Pediatrics, Cincinnati Children's Hospital Medical Center, Cincinnati, OH, US Whitaker, Robert C.: Division of General and Community Pediatrics, Cincinnati Children's Hospital Medical Center, Cincinnati, OH, US
ER -

TY - JOUR
ID - 738
T1 - The Body Goes to the City project: Research on safe routes to school and playgrounds in Ferrara. [References]
A1 - Balzani,Marcello
Y1 - 2003///
N1 - Book: 2004-00314-023
English
Book; Edited Book
KW - PsycInfo
KW - Playgrounds
KW - Safety
KW - Schools
KW - Urban Environments
KW - Urban Planning
RP - NOT IN FILE
SP - 313
EP - 323
JF - Garcia Mira, Ricardo [Ed]; Sabucedo Cameselle, Jose M [Ed]; Romay Martinez, Jose [Ed]
VL - :
IS - 2003
N2 - (from the chapter) The research activity presented here is the fruit of a co-operation between an association (UISP Ferrara, The Body Goes to the City project), a faculty (the Faculty of Architecture of the University of Ferrara) and local authorities (La Citta bambina [Children's City] project). The common objective--to regain the possibility of children gaining autonomy with regards their personal mobility--has stimulated common strategies of intervention. These projects started from sociological research concerning liveability as well as safety, and have involved both school children and the whole neighbourhood, and were further developed in workshops in schools. Finally, the data collected in the studies and the workshops was adopted by town planners for the elaboration of the final project. The particularities of this research action are the common long-term intervention methodology, which--also thanks to the common training of operators with different professional backgrounds--allows the conservation of similar educational approaches and critical viewpoints; the adoption of the body as an analyser of the quality of life; the particular attention that the planners pay to the perceptive and qualitative survey of the neighbourhood. The results of the sociological research, which has clearly revealed the children's lack of autonomy in their everyday movements, are in some ways similar to those obtained in other studies. However, they appear even more worrying considering that they have been gathered in Ferrara, a city considered one of the most liveable cities in Italy and a "city for cyclists" on the European level. The word that characterises the results of the study is "limit". We reckon that as the situation persists, it shall not only modify the autonomy of the children, who increasingly adopt their parents' models of behaviour and do not perceive the city as a place where they can live and play, but, in the long run, even their very desires are limited. (PsycINFO Database Record (c) 2012 APA, all rights reserved)
AD - Balzani, Marcello: University of Ferrara, Italy Borgogni, Antonio: UISP Ferrara, Italy
ER -

TY - JOUR
ID - 739
T1 - The relationship between mother-child interaction, potential child abuse, and parenting stress of adolescent mothers in substitute care
A1 - Asencio,Diana Lea
Y1 - 2003///

N1 - Dissertation Abstract: 2003-95012-287
English
Dissertation Abstract
KW - PsycInfo
KW - Adolescent Mothers
KW - Child Abuse
KW - Mother Child Communication
KW - Childhood Play Behavior
KW - Mother Child Relations
KW - Observation Methods
KW - Parenting Skills
KW - Stress
RP - NOT IN FILE
SP - 6085
JF - Dissertation Abstracts International: Section B: The Sciences and Engineering
VL - 63:
IS - 12-B
N2 - The current study investigated differences in mother-child interactions within adolescent mothers, who are in substitute care, and their children. Videotaped interactions of three different contextual situations (i.e., routine caregiving, teaching, and child-directed free play) were coded via two observational systems to determine the usefulness of the interactional data for identifying dyads in need of intervention. In addition, two self-report measures of parenting stress and potential child abuse were compared to the observational systems in order to assess any correlations. Measures utilized included a demographic questionnaire, the Child Abuse Potential Inventory (CAPI), the Parenting Stress Inventory-Short Form (PSI-SF), the Budd Interaction Evaluation (BUDDIE), and the Mother-Child Interaction Scale (MCIS). Findings showed that the two observational methods were correlated for routine caregiving and teaching, but not for child-directed free play. However, no correlation was found between either self-report measure and the observational systems. The data did support some differences between the contextual situations for both observational systems. The differences, or lack thereof, found in the BUDDIE varied depending upon the type of formula used to calculate negative and positive attention proportions. In general, more negative attention was given during the teaching situation. However, the findings suggest more positive attention was given during child-directed free play. On the MCIS, significant differences were found between routine caregiving and teaching as well as between teaching and child-directed free play. The current research provides a contribution to the literature concerning adolescent mother-child interactions, potential for child maltreatment, and parenting stress among mothers who are in substitute care. In particular, it highlights the importance of using a multi-method approach when assessing this area. In addition, the findings indicated that adolescent mothers in substitute care may be at risk and require intervention. This research suggests that the contextual situation utilized during behavioral observations, as well as the manner in which data are calculated, impacts the findings. Due to the methodological limitations of this study and the concerns raised, future research is needed to support these findings more conclusively and to add to the relatively sparse literature for this specific population. (PsycINFO Database Record (c) 2012 APA, all rights reserved)
SN - 0419-4217
AD - Asencio, Diana Lea: Depaul U., US
ER -

TY - JOUR
ID - 740
T1 - Insufficient responsiveness in ambivalent mother-infant relationships: Contextual and affective aspects. [References]
A1 - Harel,Judith
Y1 - 2003///
N1 - Peer Reviewed Journal: 2003-07446-006
English
Journal; Peer Reviewed Journal
KW - PsycInfo
KW - Attachment Behavior

KW - Emotional Regulation
KW - Emotional States
KW - Mother Child Relations
KW - Separation Anxiety
KW - Dyads
KW - Emotional Control
KW - Mothers
RP - NOT IN FILE
SP - 371
EP - 383
JF - Infant Behavior & Development
JA - Infant behav
VL - 26:
IS - 3
N2 - The present study focused on mother-infant relationships across different situations in order to compare the ways secure and ambivalent-resistant dyads modulate positive and negative emotionality. Sixty-one Israeli mothers and their 12-month-old non-risk infants participated in a sequence of free play, Ainsworth Strange Situation, and a task involving filling out questionnaires, one of which referred to anxiety in the dyad. A comparison between the mothers of secure (n = 49) and ambivalent (n = 12) infants indicated that the latter group displayed less positive affect in play, reported more separation anxiety, but, at the same time, did not respond in a sufficient way to their child's bids for attention after experiencing a stressful separation. This pattern of results throws further light on the antecedents and correlates of ambivalent mother-child relationships. (PsycINFO Database Record (c) 2012 APA, all rights reserved) (journal abstract)
SN - 0163-6383
AD - Harel, Judith: Department of Psychology, University of Haifa, Haifa, Israel Scher, Anat: Department of Education, University of Haifa, Haifa, Israel
ER -

TY - JOUR
ID - 741
T1 - Increasing peer-directed social-communication skills of children enrolled in Head Start. [References]
A1 - Craig-Unkefer,Lesley A.
Y1 - 2003///
N1 - Peer Reviewed Journal: 2003-07280-001
English
Journal; Peer Reviewed Journal
KW - PsycInfo
KW - At Risk Populations
KW - Communication Skills
KW - Early Intervention
KW - Language Delay
KW - Social Skills
KW - Childhood Play Behavior
KW - Interpersonal Interaction
KW - Peer Relations
KW - Preschool Students
KW - Project Head Start
RP - NOT IN FILE
SP - 229
EP - 247
JF - Journal of Early Intervention
VL - 25:
IS - 4
N2 - The current study assessed the effects of an intervention to improve the social-communicative interactions of six children at-risk for delays in language and social skills enrolled in Head Start. A multiple-baseline design across three mixed-gender dyads was used to determine the effects of a three-component intervention on

children's verbal interactions and play. During the baseline, intervention, and generalization phases, children's social-communicative behaviors were videotaped, coded, and summarized. When the plan-play-report intervention was introduced in each dyad, children's social-communicative behaviors increased. Increases occurred in descriptive statements, requests, and language complexity and diversity. During the intervention, children also engaged in more complex play behaviors. Children generalized changes in social communication, interaction, and play to untrained peers from different classrooms. (PsycINFO Database Record (c) 2012 APA, all rights reserved) (journal abstract)
SN - 1053-8151
AD - Craig-Unkefer, Lesley A.: U Minnesota, Educational Psychology, Minneapolis, MN, US Kaiser, Ann P.: Vanderbilt U, Nashville, TN, US
ER -

TY - JOUR
ID - 742
T1 - Play outside! Contributions of the urban environment to the development and the well-being of the children. [French]. [References]
A1 - Jutras,Sylvie
Y1 - 2003///
N1 - Peer Reviewed Journal: 2003-07215-011
French
Journal; Peer Reviewed Journal
KW - PsycInfo
KW - Childhood Development
KW - Childhood Play Development
KW - Social Environments
KW - Urban Environments
KW - Well Being
KW - Childhood Play Behavior
KW - Intervention
KW - Neighborhoods
KW - Playgrounds
KW - Safety
KW - Social Issues
RP - NOT IN FILE
SP - 257
EP - 266
JF - Canadian Psychology/Psychologie canadienne
VL - 44:
IS - 3
N2 - This article brings together knowledge from environmental, community, developmental and health psychology for two purposes: The first is to examine the influence of the urban environment on children's wellness and on how children benefit from their environment to play and develop. The second objective presents recommendations regarding intervention, research and social policy for psychologists to follow favouring children's development within the urban environment. We focus on two urban environments: Spaces adjoining their residence provide children with stimulation, opportunities to run around, play and explore the physical and social world, develop personal identity, self-esteem and enhance their ability to deal with stress. The second urban environment we focus on are playgrounds, which can also serve children's physical, cognitive and social developmental needs. Physical, cognitive and psychological limitations make children vulnerable to traffic accidents. The article continues with the presentation of an action plan. There are a number of ways to increase place attachment and sense of belonging within the neighborhood so that children can play safely. (PsycINFO Database Record (c) 2012 APA, all rights reserved)
SN - 0708-5591
AD - Jutras, Sylvie: U Quebec a Montreal, Dept de psychologie, Montreal, PQ, Canada
ER -

TY - JOUR

ID - 743
T1 - The role of mother-child joint play in the early development of children's conduct problems: A longitudinal observational study. [References]
A1 - Gardner,Frances; Ward,Sarah; Burton,Jennifer; Wilson,Charlotte
Y1 - 2003///
N1 - Peer Reviewed Journal: 2003-06755-004
English
Journal; Peer Reviewed Journal
KW - PsycInfo
KW - Behavior Problems
KW - Mother Child Relations
KW - Psychosocial Development
KW - Recreation
KW - Early Childhood Development
RP - NOT IN FILE
SP - 361
EP - 378
JF - Social Development
VL - 12:
IS - 3
N2 - This observational study examined the relationship between mother-child spontaneous joint play and the development of conduct problems in preschoolers, using a short-term longitudinal design. The sample consisted of 60 children showing a range of levels of conduct problems, recruited at their third birthday through community health services in low SES areas. Spontaneous joint play and other mother-child activities were coded from naturalistic, unstructured observations in the home. Amount of time spent in joint play at age 3 predicted individual improvement in conduct problems at age 4, and importantly, this association was independent of initial level of child conduct problems and hyperactivity, social class, maternal depression, and frequency of negative mother-child interactions. The amount of time the child spent unoccupied and not interacting with mother independently predicted worsening conduct scores over time. However, time spent in other activities, including joint conversation and solitary play did not predict change in conduct scores over time. The results suggest that positive and proactive parenting processes such as joint play may make a unique contribution to the very early development of conduct problems, independent of other risk factors... (PsycINFO Database Record (c) 2012 APA, all rights reserved) (journal abstract)
SN - 0961-205X
AD - Gardner, Frances: U Oxford, Oxford, United Kingdom Ward, Sarah: U Oxford, Oxford, United Kingdom Burton, Jennifer: U Oxford, Oxford, United Kingdom Wilson, Charlotte: U Oxford, Oxford, United Kingdom
ER -

TY - JOUR
ID - 744
T1 - Differentiating among insecure mother-infant attachment classifications: A focus on child-friend interaction and exploration during solitary play at 36 months. [References]
A1 - McElwain,Nancy L.
Y1 - 2003///
N1 - Peer Reviewed Journal: 2003-05401-004
English
Journal; Peer Reviewed Journal
KW - PsycInfo
KW - Attachment Behavior
KW - Childhood Play Behavior
KW - Emotional Security
KW - Friendship
KW - Mother Child Relations
KW - Avoidance
KW - Early Childhood Development
KW - Exploratory Behavior

KW - Mothers
KW - Resistance
KW - Sensitivity (Personality)
RP - NOT IN FILE
SP - 136
EP - 164
JF - Attachment & Human Development
JA - Attach.Hum.Dev.
VL - 5:
IS - 2
N2 - Utilizing data from the NICHD Study of Early Child Care, the primary objective of the current report was to examine how avoidant and resistant mother-infant attachment classifications at 15 months were differentially associated with children's interaction with a same-sex friend and exploration during solitary play at 36 months. The added contributions of attachment security at 36 months and maternal sensitivity from 6 to 36 months to the prediction of child outcomes were also explored. As hypothesized, an avoidant attachment history was related to more instrumental aggression during child-friend interaction, whereas a resistant attachment history was associated with less self-assertion/control among friends and less attention and pretend play during exploration. Maternal sensitivity and concurrent attachment security also made unique contributions to the prediction of child outcomes at 36 months, although associations with 15-month attachment remained significant when these subsequent measures of the mother-child relationship were considered. Few differences emerged for the disorganized mother-infant attachment category in this relatively low-risk sample. Results underscore the need to differentiate between avoidant and resistant attachment groups and illustrate how... (PsycINFO Database Record (c) 2012 APA, all rights reserved) (journal abstract)
SN - 1461-6734
ER -

TY - JOUR
ID - 746
T1 - Facilitating peer-group entry in kindergartners with impairments in social communication. [References]
A1 - Beilinson,Jill Selber; Olswang,Lesley
Y1 - 2003///
N1 - Peer Reviewed Journal: 2003-00300-005
English
Journal; Peer Reviewed Journal
KW - PsycInfo
KW - Communication Disorders
KW - Communication Skills
KW - Communication Skills Training
KW - Social Interaction
KW - Early Intervention
KW - Peer Relations
RP - NOT IN FILE
SP - 154
EP - 166
JF - Language, Speech, and Hearing Services in Schools
VL - 34:
IS - 2
N2 - This series of case studies examined the efficacy of intervention designed to teach peer-group entry skills to kindergartners with social interaction and communication deficits. The participants were 3 kindergartners at the University of Washington Experimental Educational Unit (EEU) who were selected because of difficulty with peer-group entry and cooperative play as compared to other children in the classroom. The intervention program included direct treatment of the children by the primary researcher and teachers in the classroom. The intervention was modeled on research describing a sequential peer-entry hierarchy that incorporated the children moving from low risk strategies to high-risk strategies. Specifically, the treatment focused on teaching the children to use props to facilitate the production of high-risk verbal statements. Results demonstrated increases in (a) children's use of props and verbal statements to enter peer groups, (b) cooperative play, and (c) time spent

interacting with peers. Results also indicated that following treatment, the children's behaviors more closely resembled those of their comparison peers. (PsycINFO Database Record (c) 2012 APA, all rights reserved)
SN - 0161-1461
AD - Beilinson, Jill Selber: U Washington, Seattle, WA, US Olswang, Lesley B.: U Washington, Seattle, WA, US
ER -

TY - JOUR
ID - 747
T1 - Play and creation, the risk of the affect of collapse: The chess player by Stefan Zweig. [French]. [References]
A1 - Mellier,Denis
Y1 - 2002///
N1 - Peer Reviewed Journal: 2004-16657-010
French
Journal; Peer Reviewed Journal
KW - PsycInfo
KW - Chess
KW - Object Relations
KW - Recreation
KW - Separation Anxiety
RP - NOT IN FILE
SP - 265
EP - 283
JF - Psychologie Clinique et Projective
VL - 8 2002, pp. 265-283.
N2 - Play and creation have the same function: that of containing and symbolizing affects. Following the theory of M. Milner and D.W. Winnicott, we can say that the process depends on the creation of a zone of illusion shared by the subject and the outside world, as well as associative possibilities put into action through play or creation. The author then develops the hypothesis that such a process can paradoxically give birth to an experience of collapse. The observation of play in a very young child shows how his separation anxieties are gradually contained up to a point which leads him to experience paralysing anxiety. In the last short story of Stefan Zweig, a chess player risks collapse after have played a tournament. In this ultimate creation, the author may have staged a feeling which risked submerging him and for which he was said to have committed suicide. The persecutory rise of nazism, the dissention of his passionate connections and exile may have gradually broken him from his moorings, particularly his adhesive relationship to culture, and may have endangered the stability of his internal framework. (PsycINFO Database Record (c) 2012 APA, all rights reserved) (journal abstract)
SN - 1265-5449
AD - Mellier, Denis: Centre de Recherche en Psychologie et Psychopathologie Clinique, Institut de Psychologie, Universite Lumiere Lyon 2, Bron, France
ER -

TY - JOUR
ID - 748
T1 - Portraits of emergent literacy: A Vygotskian perspective on how children become more conscious of using a repertoire of strategies as part of a 'learning community'
A1 - Meaney,Wanda Magdalena
Y1 - 2002///
N1 - Dissertation Abstract: 2002-95011-075
English
Dissertation Abstract
KW - PsycInfo
KW - Early Childhood Development
KW - Literacy
RP - NOT IN FILE

SP - 4057
JF - Dissertation Abstracts International Section A: Humanities and Social Sciences
VL - 62:
IS - 12-A
N2 - When children's preconventional attempts at reading and writing are ignored or misunderstood as 'scribbles', these early attempts may be unnecessarily sacrificed for a well-intentioned adult's overemphasis on the importance of only conventions. The ethnography of this kindergarten classroom provided a 'thick description' of how emergent literacy is mediated through complex, interactive cognitive and social processes. In turn, social contexts such as classrooms influence children's opportunities to explore and grow in strategic control of their literacy development. Towards the end of kindergarten, I decided to extend the study by following eight of these children into grade one. The resultant intensive case studies are highly informative in covering a range of early literacy development profiles over time to highlight the variability in children's emergent literacy. Vygotsky's sociocultural theory of learning has been instrumental in helping us to understand how educators play a primary role in apprenticing students into the classroom culture. Children's emergent literacy is, therefore, an integral part of how they are supported in their interactions with both learners and teachers. The direct observation and documentation of actual classroom practices over time can serve as a powerful means for understanding teaching and learning as a social-communicative process. To plan for instruction accordingly, educators need to explore what hypotheses and ideas students bring to the classroom and how these understandings play out in the classroom. Key literacy events such as the journal writing center provide a revisitable trail for both children and teachers. The growth of young children into skilled participants in society is often accomplished through children's routine, and often tacit, guided participation in ongoing cultural activities, such as emergent literacy. Risk-taking is a critical strategy for children as they learn to read and write. By having routine ways of interacting with children such as the journal writing center we are in a better position to understand their tacit-knowledge and motives. Teachers have to encourage supported risk-taking, recognizing that for some children 'overcoming dependency on conversational partners', such as teachers and parents, may present the greatest hurdle in their literacy development. (PsycINFO Database Record (c) 2012 APA, all rights reserved)
SN - 0419-4209
AD - Meaney, Wanda Magdalena: U Calgary, Canada
ER -

TY - JOUR
ID - 749
T1 - The relationships among play, language development and parenting in children born prematurely with medical complications
A1 - Hebert,Heather Martin
Y1 - 2002///
N1 - Dissertation Abstract: 2002-95010-315
English
Dissertation Abstract
KW - PsycInfo
KW - Childhood Play Development
KW - Language Development
KW - Parent Child Relations
KW - Premature Birth
KW - Complications (Disorders)
RP - NOT IN FILE
SP - 5407
JF - Dissertation Abstracts International: Section B: The Sciences and Engineering
VL - 62:
IS - 11-B
N2 - Preterm low birth weight (LBW) infants have frequently been reported to have persistent impairments in their development of language, cognitive, and social skills (Hunt Cooper, & Tooley, 1988; Landry, Fletcher, Denson, & Chapieski, 1993). Play and language development, specifically, have been shown to be related in numerous studies with normal children (e.g., Bates, Bretherton, Shore, and McNew, 1983; Bornstein, Vibbert, Tal, and O'Donnell, 1992). The purpose of the current investigation was to explore preterm (of high or low

medical risk) and fullterm children's and toddlers' play and language development, and the specific parenting strategies that may facilitate or inhibit children's acquisition of these skills (i.e., maternal maintaining and structure). The participants were 365 urban and rural, low socioeconomic status (SES) families, recruited initially during 1990 and 1991 for a longitudinal study of parenting and developmental outcomes in preterm LBW children. The data was collected across five timepoints: when the children were 6, 12, 24, 40 and 54 months of age. Statistical analyses using growth curve modeling revealed that risk status predicted the children's level of both toyplay and language skills at all timepoints. However, risk status did not predict the children's rate of growth in either domain. Higher levels of maternal maintaining facilitated both play and language level across time. Higher levels of maternal structure predicted lower language levels across all timepoints. Higher levels of maternal structure predicted higher levels of toyplay at younger ages and lower levels of toyplay at older ages. Language and play levels at two and a half years were related, but the effect was moderated by risk status and SES. Preterm children from lower SES families had less of a relation between their toyplay and language scores than did preterm children from higher SES families or fullterm children. The major implications of these findings are: (1) evaluating the risk status of children born prematurely is critical in predicting cognitive outcomes and (2) maternal maintaining and structure have an important impact on the development of toyplay and language skills over the first four years of life. (PsycINFO Database Record (c) 2012 APA, all rights reserved)
SN - 0419-4217
AD - Hebert, Heather Martin: U Houston, US
ER -

TY - JOUR
ID - 750
T1 - A study of the effect of group family play on family relations for families with children considered at-risk for educational failure
A1 - Baldus,Carmen Colleen
Y1 - 2002///
N1 - Dissertation Abstract: 2002-95007-074
English
Dissertation Abstract
KW - PsycInfo
KW - Academic Failure
KW - Family Relations
KW - Play Therapy
RP - NOT IN FILE
SP - 3587
JF - Dissertation Abstracts International Section A: Humanities and Social Sciences
VL - 62:
IS - 10-A
N2 - This study investigated the difference in family health and parenting stress between families with children identified as at-risk for educational failure who participated in Group Family Play and families who did not participate. Family health was assessed through use of the health/competence, style, and conflict scales of the Beaver's Self Report Family Inventory (SFI), while parenting stress was measured by the Parenting Stress Index-3rd Edition (PSI). A total of 174 family members from 43 families participated in the study. Families were randomly assigned to one of the four groups comprising a Solomon Four Group Design. Groups 1 and 3 participated in an informational meeting and six sessions of Group Family Play, while Groups 2 and 4 attended the informational meeting, but did not participate in Group Family Play. Of the 43 families who participated, SFI data were completed on 121 individuals. Groups 1 and 2 took both pre- and posttests to determine differences in health/competence, style, conflict, and parenting stress. Groups 3 and 4 took only the posttest. A one-way analysis of variance (ANOVA) was computed to determine if there was a significant difference between the groups. The ANOVA did not detect a statistically significant difference at the .05 level on measures of health/competence, style, or conflict between families who participated in family play and families who did not participate. The ANOVA was not applied to the data received on the PSI due to a limited parent sample. Instead, a two-tailed t test was computed to look at the difference between the mean scores of parents who participated in Group Family Play (Groups 1 and 3 combined) and parents who did not participate (Groups 2 and 4 combined). The t test did not result in a statistically significant difference at the .05 level. Further research

is recommended to look at the use of Group Family Play for more defined populations and with different instrumentation. Also recommended is study of family play as a therapeutic technique used in conjunction with therapy. Recommendations concerning the study of family play and its relationship to gender, age, and population are also discussed. (PsycINFO Database Record (c) 2012 APA, all rights reserved)
SN - 0419-4209
AD - Baldus, Carmen Colleen: Western Michigan U., US
ER -

TY - JOUR
ID - 751
T1 - Short-term group play therapy with Chinese earthquake victims: Effects on anxiety, depression and adjustment. [References]
A1 - Shen,Yih Jiun
Y1 - 2002///
N1 - Peer Reviewed Journal: 2002-17897-003
English
Journal; Peer Reviewed Journal
KW - PsycInfo
KW - Adjustment
KW - Anxiety
KW - Group Psychotherapy
KW - Natural Disasters
KW - Play Therapy
KW - Assistance (Social Behavior)
KW - Chinese Cultural Groups
KW - Elementary Schools
KW - Major Depression
RP - NOT IN FILE
SP - 43
EP - 63
JF - International Journal of Play Therapy
VL - 11:
IS - 1
N2 - Investigated the effectiveness of short-term child-centered group play therapy in elementary school settings with Chinese children in Taiwan who experienced an earthquake in 1999. 65 children (aged 8-12 yrs) were screened. Children in the experimental group scored significantly lower on anxiety level and suicide risk after play therapy than did children in the control group. The effects of the treatment support previous studies of play therapy with American children. These findings reveal the possibility of disaster intervention services adopting Western helping techniques with school children of non-Western cultures. (PsycINFO Database Record (c) 2012 APA, all rights reserved)
SN - 1555-6824
AD - Shen, Yih-Jiun: Texas Tech U, Coll of Education, Div of Educational Psychology & Leadership, Lubbock, TX, US
ER -

TY - JOUR
ID - 752
T1 - An examination of the relationship between cycle training, cycle accidents, attitudes and cycling behaviour among children. [References]
A1 - Colwell,John
Y1 - 2002///
N1 - Peer Reviewed Journal: 2002-15828-002
English
Journal; Peer Reviewed Journal
KW - PsycInfo
KW - Accidents

KW - Child Attitudes
KW - Childhood Play Behavior
KW - Ground Transportation
KW - Recreation
KW - Education
KW - Human Sex Differences
KW - Injuries
KW - Safety
KW - Social Behavior
RP - NOT IN FILE
SP - 640
EP - 648
JF - Ergonomics
VL - 45:
IS - 9
N2 - Around 40% of 10 to 11-yr-old children receive cycle training every year in the UK, but concern has been expressed over the efficacy of training courses. One argument is that accidents occur too infrequently to be a viable evaluative criterion, and attitudes and behavior have been suggested as alternatives. A questionnaire that measured a number of variables including accidents, attitudes, and behavior was completed by 336 participants (aged 13-16 yrs) from 2 schools in the London Borough of Bromley. At least 1 cycling injury had been sustained by 58.3% of respondents, requiring hospital treatment in 19.1% of cases. Girls reported fewer accidents than boys. No relationship between training and accidents was found. A principal components analysis (PCA) of the attitudes items produced a "safe attitudes' factor. Girls displayed "safer' attitudes, but there was no evidence that training produced safer attitudes. A PCA of the cycling behavior scales produced 2 factors, "safe cycling' and "showing off.' Safe cyclists who obeyed basic safety rules were less likely to sustain cycle injuries, but showing off was not related to accidents. Girls were less likely to show off, but the safe behavior gender difference did not reach significance. Training did not relate to either factor. (PsycINFO Database Record (c) 2012 APA, all rights reserved)
SN - 0014-0139
AD - Colwell, John: De Montfort U, Dept of Psychology & Speech & Language Therapy, Leicester, United Kingdom Culverwell, Angus: London Borough of Bromley, Civic Ctr, Road Safety Unit, Bromley, United Kingdom
ER -

TY - JOUR
ID - 754
T1 - Improving the social communication skills of at-risk preschool children in a play context. [References]
A1 - Craig-Unkefer,Lesley A.
Y1 - 2002///
N1 - Peer Reviewed Journal: 2002-12813-001
English
Journal; Peer Reviewed Journal
KW - PsycInfo
KW - At Risk Populations
KW - Childhood Play Behavior
KW - Communication Skills
KW - Social Interaction
KW - Behavior Problems
KW - Language Delay
RP - NOT IN FILE
SP - 3
EP - 13
JF - Topics in Early Childhood Special Education
VL - 22:
IS - 1

N2 - Examined the effects of a 3-component intervention on the social-communicative interactions of 6 preschool children (aged 3yrs 5mo-3yrs 11mo) at risk for language delays and behavior problems. In a multiple baseline design across 3 dyads, children were taught to (1) plan their play, (2) use conversational social interaction strategies, and (3) self-evaluate their play interactions. The number of social communicative behaviors by each child increased following introduction of the intervention condition, as did their use of descriptive and request utterances during play sessions. Increases in linguistic complexity, diversity, and play complexity also were associated with the intervention. It is suggested that this intervention holds promise for improving social, linguistic, and play behaviors in preschoolers at risk for language delays and behavior problems. (PsycINFO Database Record (c) 2012 APA, all rights reserved)
SN - 0271-1214
AD - Craig-Unkefer, Lesley A: U Illinois, Chicago, IL, US Kaiser, Ann P.: Vanderbilt U, Nashville, TN, US
ER -

TY - JOUR
ID - 755
T1 - Parent-Infant Relationship Global Assessment Scale: A study of its predictive validity. [References]
A1 - Aoki,Yutaka
Y1 - 2002///
N1 - Peer Reviewed Journal: 2002-04012-002
English
Journal; Peer Reviewed Journal
KW - PsycInfo
KW - Infant Development
KW - Mother Child Relations
KW - Prediction
KW - Psychological Assessment
KW - Statistical Validity
KW - Behavior Problems
KW - Childhood Play Behavior
KW - Interpersonal Interaction
KW - Mental Health
KW - Rating Scales
RP - NOT IN FILE
SP - 493
EP - 497
JF - Psychiatry and Clinical Neurosciences
VL - 56:
IS - 5
N2 - The Parent-Infant Relationship Global Assessment Scale (PIRGAS; Zero to Three, 1994) provides a continuously distributed scale of infant-parent relationship adaptation, raging from "well-adapted' to "dangerously impaired.' The present study examined the predictive validity of the PIRGAS in a high-risk sample by coding relationship adaptation level from a single sample of 10 min of unstructured free play between mothers (aged 19-35 yrs) and their 20-mo-old infants and examining its relationship to subsequent interaction with mothers and behavior problems at 24 mo. Relationship adaptation assessed reliably from observations of only 10 min of free play between mothers and their infants at 20 mo of age using PIRGAS predicted subsequent mother-infant interaction in a laboratory based problem-solving paradigm (Crowell procedure) at 24 mo and internalizing symptomatology of Child Behavior Checklist at age 24 mo. These results contribute to the predictive validity of the PIRGAS as a measure of mother-infant relationship adaptation. (PsycINFO Database Record (c) 2012 APA, all rights reserved)
SN - 1323-1316
AD - Aoki, Yutaka: Tokai U School of Medicine, Dept of Psychiatry & Behavioral Science, Iseharashi Bouseidai, Japan Zeanah, Charles H.: Tulane U School of Medicine, Inst of Infant & Early Childhood Mental Health, New Orleans, LA, US Heller, Sherryl Scott: Tulane U School of Medicine, Inst of Infant & Early Childhood Mental Health, New Orleans, LA, US Bakshi, Sudha: Children's Hosp of Philadelphia, Philadelphia, PA, US
ER -

TY - JOUR
ID - 756
T1 - Childhood transformed? Risk perceptions and the decline of free play. [References]
A1 - O'Brien,Jonathon
Y1 - 2002///
N1 - Peer Reviewed Journal: 2002-02777-002
English
Journal; Peer Reviewed Journal
KW - PsycInfo
KW - Childhood Play Behavior
KW - Health
KW - Parental Attitudes
KW - Risk Perception
KW - Trends
KW - Occupational Therapists
KW - Parent Child Relations
KW - Recreation
RP - NOT IN FILE
SP - 123
EP - 128
JF - The British Journal of Occupational Therapy
VL - 65:
IS - 3
N2 - Occupational therapists have been concerned with the health-promoting aspects of children's play. The evidence suggests that children's freedom to play away from adult supervision has declined in recent years and that parental perception of risk may be a factor in this. This research used a qualitative approach to explore 6 parents' views on how their fear of risk might be shaping their children's play. The parents were aged 34-46 yrs and their children were 8 yrs old. The parents stated that their children were now more likely to take part in activities supervised by adults. The article shows that these findings may be linked to national trends and that occupational therapists should be concerned about the health implications of these changes. It is suggested that further research should take into account other factors that might affect children's play. (PsycINFO Database Record (c) 2012 APA, all rights reserved)
SN - 0308-0226
AD - O'Brien, Jonathon: Walton Ctr for Neurology & Neurosurgery, Liverpool, United Kingdom Smith, Jenny: U Brighton, School of Healthcare Professions, Eastbourne, ESX, United Kingdom
ER -

TY - JOUR
ID - 758
T1 - Cultural constraints on children's play. [References]
A1 - Lancy,David F.
Y1 - 2002///
N1 - Book: 2002-00366-003
English
Book; Edited Book
KW - PsycInfo
KW - Childhood Play Behavior
KW - Cooperation
KW - Human Sex Differences
KW - Safety
KW - Sociocultural Factors
RP - NOT IN FILE
SP - 51
EP - 60
JF - Roopnarine, Jaipaul L [Ed]

VL - :
IS - 2002
N2 - (from the chapter) Rather than considering strategies to increase, channel, or promote children's play, this chapter considers the many constraints that exist to curb it. There are numerous culture constrains on children's play. Imposing limits on where and how vigorously children may play in the interest of safety. Imposing chores earlier for girls than boys. Ensuring that play is appropriate to the child's sex. Providing a finite set of scripts which children may draw on in play. Promoting an ethos of cooperation, at least where children are concerned. The sheer physical size of the community influences the composition of the play group. Failing to provide a "benign environment", the society limits children's play time. All of which suggests that the benefits of play to children must be extensive and profound in order to overcome these pervasive attempts at restraint. (PsycINFO Database Record (c) 2012 APA, all rights reserved)
AD - Lancy, David F.: Utah State U, UT, US
ER -

TY - JOUR
ID - 760
T1 - The relation of medical risk and maternal stimulation with preterm infants' development of cognitive, language and daily living skills
A1 - Smith,Karen E; Landry,Susan
Y1 - 1996///
N1 - Peer Reviewed Journal: 1996-06597-008
English
Journal; Peer Reviewed Journal
KW - PsycInfo
KW - Cognitive Development
KW - Infant Development
KW - Language Development
KW - Mother Child Relations
KW - Premature Birth
KW - At Risk Populations
KW - Longitudinal Studies
RP - NOT IN FILE
SP - 855
EP - 864
JF - Child Psychology & Psychiatry & Allied Disciplines
VL - 37:
IS - 7
N2 - 212 very low birth-weight (VLBW) high-risk (HR) and low-risk (LR) preterm infants and 128 full-term infants (FT) from low socioeconomic homes were studied with their mothers at 6 and 12 mo of age. Infants' cognitive, language and daily living skills were evaluated in relation to mothers' warm sensitivity, use of strategies which maintained the infants' attention, and directiveness. Results show that VLBW HR Ss displayed lower cognitive, receptive language, and daily living scores at 6 mo, and slower rates of change over 6-12 mo of age for cognitive skills than VLBW LR and FT Ss. Higher levels of maternal attention-maintaining (AM) were positively related to infant development for all groups. During toy play, AM was most strongly related to expressive language skills for HR infants. In addition, AM during toy play and daily activities related positively to HR and LR infants' development of cognitive and language skills. (Japanese abstract). (PsycINFO Database Record (c) 2013 APA, all rights reserved)
SN - 0021-9630
AD - Smith, Karen E.: U Texas, Medical Branch, Dept of Pediatrics, Galveston, TX, US
ER -

TY - JOUR
ID - 761
T1 - Parenting and family support in Japan for 6- to 8- year-old children weighing under 1000 grams at birth
A1 - Itoigawa,Naosuke
Y1 - 1996///

N1 - Peer Reviewed Journal: 1996-06905-001
English
Journal; Peer Reviewed Journal
KW - PsycInfo
KW - Birth Weight
KW - Childhood Development
KW - Family Structure
KW - Parental Characteristics
KW - Parental Investment
KW - Fathers
KW - Premature Birth
RP - NOT IN FILE
SP - 477
EP - 490
JF - International Journal of Behavioral Development
VL - 19:
IS - 3
N2 - Explored paternal involvement in the caregiving of extremely low-birthweight (ELBW) children, specifically sociodemographic factors related to the family and individual biological factors related to ELBW. Possible developmental effects were also investigated. 28 children 6-8 yrs old with a mean birthweight of 845.5 g and a mean gestational age of 27.4 wks were administered developmental testing. Ss' parents completed questionnaires, an interview, and other measures. Paternal involvement in caregiving is related to family composition; cooperative fathers are more likely to come from families that have no older female sibling(s) and/or grandparent(s). Individual perinatal risk variables, the child's IQ, and paternal variables (age and socioeconomic status) were not related to paternal cooperativeness. Ss with noncooperative fathers prefer to play alone at 3-5 yrs of age, while Ss with cooperative fathers prefer to play with peers. (PsycINFO Database Record (c) 2013 APA, all rights reserved)
SN - 0165-0254
AD - Itoigawa, Naosuke: Osaka U, Faculty of Human Sciences, Dept of Comparative & Developmental Psychology, Osaka, Japan
ER -

TY - JOUR
ID - 763
T1 - Assessment of preschool play interaction behaviors in young low-income children: Penn Interactive Peer Play Scale
A1 - Fantuzzo,John W.
Y1 - 1995///
N1 - Peer Reviewed Journal: 1995-39411-001
English
Journal; Peer Reviewed Journal
KW - PsycInfo
KW - Childhood Play Behavior
KW - Peer Relations
KW - Rating Scales
KW - Test Construction
KW - Test Validity
KW - Blacks
KW - Preschool Students
KW - Urban Environments
RP - NOT IN FILE
SP - 105
EP - 120
JF - Early Childhood Research Quarterly
VL - 10:
IS - 1

N2 - Developed and validated the Penn Interactive Peer Play Scale (PIPPS), a teacher-rating instrument designed to assess the interactive play behaviors of preschool children. The PIPPS was designed (1) to differentiate children who demonstrate positive play relationships with peers from those who are less successful with peers, (2) to identify play strengths of resilient preschool children living in high risk urban environments, and (3) to inform early childhood intervention. 38 teachers from 5 representative urban Head Start centers completed the measure on 312 African-American children enrolled in Head Start. Exploratory factor analyses revealed 3 reliable underlying dimensions: Play Interaction, Play Disruption, and Play Disconnection. Concurrent validity was established by comparing the factor patterns of the PIPPS and the Social Skills Rating System. (PsycINFO Database Record (c) 2013 APA, all rights reserved)
SN - 0885-2006
AD - Fantuzzo, John W.: U Pennsylvania, Graduate School of Education, Philadelphia, US
ER -

TY - JOUR
ID - 764
T1 - Linking assessment to intervention for children with developmental disabilities or at-risk for developmental delay: The Developmental Play Assessment (DPA) instrument. [References]
A1 - Lifter,Karin
Y1 - 2000///
N1 - Book: 2001-01062-008
English
Book; Edited Book
KW - PsycInfo
KW - Autism
KW - Childhood Play Behavior
KW - Diagnosis
KW - Measurement
KW - Treatment
RP - NOT IN FILE
SP - 228
EP - 261
JF - Gitlin-Weiner, Karen [Ed]; Sandgrund, Alice [Ed]; Schaefer, Charles [Ed]
VL - :
IS - 2000
N2 - (from the book) Using a developmental foundation, K. Lifter's Developmental Play Assessment scale demonstrates how play behaviors can be employed as the basis for diagnostic statements leading to specific interventions. Lifter's technique and scale are particularly geared toward the assessment of children who are classified within the autistic-spectrum disorders. It is noted that most promising about this technique is its presentation of a play-based intervention for these typically difficult-to-treat youngsters. (PsycINFO Database Record (c) 2013 APA, all rights reserved)
AD - Lifter, Karin: Northeastern U, Dept of Counseling Psychology, Rehabilitation, & Special Education, Boston, MA, US
ER -

TY - JOUR
ID - 765
T1 - The impact of child abuse on children's play: A conceptual model. [References]
A1 - Cooper,Rodney J.
Y1 - 2000///
N1 - Peer Reviewed Journal: 2005-05340-005
English
Journal; Peer Reviewed Journal
KW - PsycInfo
KW - Child Abuse
KW - Childhood Development
KW - Childhood Play Behavior

KW - Occupational Therapy
KW - Sexual Abuse
RP - NOT IN FILE
SP - 259
EP - 276
JF - Occupational Therapy International
VL - 7:
IS - 4
N2 - The purpose of this article is to examine critically the impact of child abuse on children's play, using a descriptive model of children's play as a conceptual framework for this review of the literature. Play is recognized as a primary occupation of childhood by occupational therapists. When a child is exposed to an abusive or neglectful care environment his or her play development may be disrupted. Although the detrimental effects of physical, sexual or emotional abuse and neglect are well documented, relatively few studies have investigated the play of abused children. Reported effects suggest that abused or neglected children are more likely to show developmentally delayed play skills, decreased playfulness and behavioural disturbance in their play, which may place them at greater risk for learning and social problems. Occupational therapists often use play, including play therapy, to assess and treat abused children. This analysis highlights the need for occupational therapists to evaluate how play-based therapeutic approaches can best meet the developmental and emotional needs of abused and neglected children. Recommendations are made for occupational therapists to be more knowledgeable about child abuse and to address the need for research into the specific effects of child abuse on children's play. (PsycINFO Database Record (c) 2013 APA, all rights reserved) (journal abstract)
SN - 0966-7903
AD - Cooper, Rodney J.: University of Queensland, Brisbane, QLD, Australia
ER -

TY - JOUR
ID - 766
T1 - Psychological markers in the detection of autism in infancy in a large population
A1 - Baron-Cohen,Simon; Cox,Antony; Baird,Gillian; Sweettenham,John; Nighingale,Natasha
Y1 - 1996///
N1 - Peer Reviewed Journal: 1996-03077-005
English
Journal; Peer Reviewed Journal
KW - PsycInfo
KW - Attention
KW - Autism
KW - Childhood Play Behavior
KW - Delayed Development
KW - Gestures
KW - Followup Studies
KW - Psychodiagnosis
RP - NOT IN FILE
SP - 158
EP - 163
JF - The British Journal of Psychiatry
VL - 168:
IS - 2
N2 - 16,000 children in the southeast of England were screened for autism during their routine 18-mo-old developmental check-up, using the Checklist for Autism in Toddlers (CHAT). 12 consistently failed 3 key items (protodeclarative pointing, gaze-monitoring, and pretend play) that were predicted would identify Ss at risk for receiving a diagnosis of autism. Of these, 10 (83.3%) Ss received a diagnosis of autism. When the 10 autistic Ss were reassessed at 3.5 yrs of age, their diagnosis remained the same. Of 22 Ss who consistently failed either protodeclarative pointing and/or pretend play, none received a diagnosis of autism, but 15 (68.2%) received a diagnosis of language delay. (PsycINFO Database Record (c) 2012 APA, all rights reserved)
SN - 0007-1250
AD - Baron-Cohen, Simon: U Cambridge, Dept of Experimental Psychology, Cambridge, England

ER -

TY - JOUR
ID - 767
T1 - Can autism be detected at 18 months? The needle, the haystack, and the CHAT
A1 - Baron-Cohen,Simon; Allen,Jane; Gillberg,Christopher
Y1 - 1992///
N1 - Peer Reviewed Journal: 1993-29936-001
English
Journal; Peer Reviewed Journal
KW - PsycInfo
KW - At Risk Populations
KW - Autism
KW - Screening Tests
RP - NOT IN FILE
SP - 839
EP - 843
JF - The British Journal of Psychiatry
VL - 161 Dec 1992, pp. 839-843.
N2 - 41 toddlers (aged 17-21 mo), who were at high genetic risk for developing autism, and 50 randomly selected toddlers (aged 17-20 mo) were screened with the Checklist for Autism in Toddlers (CHAT). More than 80% of the Ss passed on all items, and none failed on more than 1 of pretend play, protodeclarative pointing, joint-attention, social interest, and social play. Four Ss in the high-risk group failed on 2 or more of these 5 key types of behavior. At follow-up at 30 mo of age, the 4 Ss who had failed on 2 or more of these key types of behavior at 18 mo received a diagnosis of autism by 30 mo. A shorter version of the CHAT is appended. (PsycINFO Database Record (c) 2012 APA, all rights reserved)
SN - 0007-1250
AD - Baron-Cohen, Simon: U London, Inst of Psychiatry, England
ER -

TY - JOUR
ID - 768
T1 - Preschool play behavior as a predictor of risk
A1 - Gallagher,Kathleen Patricia
Y1 - 2001///
N1 - Dissertation Abstract: 2001-95024-064
English
Dissertation Abstract
KW - PsycInfo
KW - At Risk Populations
KW - Childhood Play Behavior
KW - Risk Perception
RP - NOT IN FILE
SP - 2956
JF - Dissertation Abstracts International: Section B: The Sciences and Engineering
VL - 62:
IS - 6-B
N2 - This study piloted a preschool play behavior checklist (PPBC) designed to assess domains of children's play behaviors with the goal of identifying play behaviors that might differentiate between at-risk children and non at-risk children. The Caregiver-Teacher Report Form for Ages 1-5 (ACTRF) (Achenbach, 2000) was utilized to place 51 preschool children with a mean age of 4.3922 in either an "at-risk" group or a "non at-risk" group based on their respective T-Scores (a T-Score of 65 was the cut-off score used) on three indices: (1) Total Problem Score, (2) Internalizing Score, and (3) Externalizing Score. It was the hypothesis of this researcher that children who were classified as "at-risk" by the ACTRF would exhibit different play behaviors than children who were not classified as "at-risk" by the ACTRF. Three children received scores that placed them in the "at-risk" groups and all data were converted to nominal data, thereby confining statistical analysis to conducting chi

square analyses. A total of 28 play behaviors on the PPBC reached significance with at-risk scores on the Total Problem Score, Internalizing Score, and Externalizing Score of the ACTRF/Ages 1-5. The same 28 play behaviors on the PPBC were significant (p < .01) on all three scales of the ACTRF suggesting some reliability in their predictive utility despite inherent methodological flaws of the small at-risk group. In addition, two other items were significant (p < .05) with at-risk T-Scores on all three scales. Implications and suggestions for future research are discussed. (PsycINFO Database Record (c) 2012 APA, all rights reserved)
SN - 0419-4217
AD - Gallagher, Kathleen Patricia: Spalding U., US
ER -

TY - JOUR
ID - 769
T1 - Relationships between peer interactive play and school success in four year-old at-risk children
A1 - Gagnon,Sandra Glover
Y1 - 2001///
N1 - Dissertation Abstract: 2001-95022-018
English
Dissertation Abstract
KW - PsycInfo
KW - At Risk Populations
KW - Childhood Play Behavior
KW - Cognitive Ability
KW - School Readiness
RP - NOT IN FILE
SP - 2530
JF - Dissertation Abstracts International: Section B: The Sciences and Engineering
VL - 62:
IS - 5-B
N2 - The purpose of this study was to examine the relationships between peer interactive play and school success in a sample of four year-old children identified as at-risk for problems in school. Forty-three males and 42 females, ranging in age from 50 to 66 months (M = 57.74), participated in the study. Subjects were primarily Caucasian. Ratings of peer interactive play were collected from parents and teachers on the Penn Interactive Peer Play Scale (PIPPS; Fantuzzo, Coolahan, Mendez, McDermott, & Sutton-Smith, 1998; Fantuzzo, Mendez, & Tighe, 1998). Social-emotional functioning was assessed by the Vineland Social-Emotional Early Childhood Scale (SEEC; Sparrow, Balla, & Cicchetti, 1998), which was administered to parents and teachers. School success variables included measures of cognitive ability, including two subtests from the Differential Ability Scales (DAS; Elliott, 1990) and the School Readiness Composite from the Bracken Basic Concepts Scale, Revised (BBCS-R; Bracken, 1998). Simple correlations, canonical correlations, and multiple regression analyses were computed to analyze the data. Significant correlations were found between parent and teacher ratings on the PIPPS and their corresponding scores on the SEEC. Two scales on the teacher PIPPS correlated significantly with student scores on the DAS. Significant mean differences were found between parent and teacher ratings on the PIPPS. Findings are discussed with regard to situation specificity and interrater differences. Implications of these findings include the important contributions play makes to children's development of cognitive and social-emotional development. These findings provide evidence for the psychometric integrity of the PIPPS and suggest that psychologists should add measures of play to their assessment batteries with young children. Rating scales such as the PIPPS contribute to the ecological validity and multifaceted nature of preschool assessments. In addition, the utility of the PIPPS was extended beyond the initial standardization sample to include a sample of primarily Caucasian children living in a more rural area. (PsycINFO Database Record (c) 2012 APA, all rights reserved)
SN - 0419-4217
AD - Gagnon, Sandra Glover: U South Carolina, US
ER -

TY - JOUR
ID - 770

T1 - Young adolescent girls and older men: Issues of development, gender and abuse in sexual relations before the age of consent
A1 - Saul,Dale Hansen
Y1 - 2001///
N1 - Dissertation Abstract: 2001-95018-425
English
Dissertation Abstract
KW - PsycInfo
KW - Human Females
KW - Human Mate Selection
KW - Psychosexual Behavior
KW - Romance
KW - Sexual Abuse
RP - NOT IN FILE
SP - 1647
JF - Dissertation Abstracts International: Section B: The Sciences and Engineering
VL - 62:
IS - 3-B
N2 - This study explores how a history of abuse is a risk factor for early sexual experience with an older individual, of concern because of the potential for revictimization in a vulnerable population. The higher rates of STDs, pregnancy and childbearing among young abused adolescents with older sex partners indicate a need for better understanding of the psychosocial dynamics underlying this public health problem. To study these relationships, the life story narrative was selected as the method most likely to elicit context, meaning and thematic structure in participants' verbal and affective expression. Four girls, 15 and 16, identified as abused and having had a sexual relationship with an individual 4 or more years older, reflect upon their close relationships of childhood through the present and their impact on the girls' self-definition and expectations of others. As participants characterize the elements of their social world, the effects of abuse and the rationale for older partner involvement become evident through the coherence of their stories. The themes and patterns that emerge in these narratives help to delineate emotional and relational vulnerabilities in this age group and population and clarify the role romantic or sexual relationships with an older individual plays in adolescent risk-taking, self-repair and revictimization. (PsycINFO Database Record (c) 2012 APA, all rights reserved)
SN - 0419-4217
AD - Saul, Dale Hansen: The American U., US
ER -

TY - JOUR
ID - 771
T1 - Playmate selections of preschool children
A1 - Haynes,Gladys Verneal Oosting
Y1 - 2001///
N1 - Dissertation Abstract: 2001-95017-110
English
Dissertation Abstract
KW - PsycInfo
KW - Childhood Play Behavior
KW - Human Sex Differences
KW - Racial and Ethnic Differences
KW - Social Acceptance
RP - NOT IN FILE
SP - 907
JF - Dissertation Abstracts International Section A: Humanities and Social Sciences
VL - 62:
IS - 3-A
N2 - Social relationships are important in the development of young children's cognitive, language and more advanced social skills. Children who fail to develop appropriate social skills are at risk for future difficulties in interpersonal relationships, school failure and criminal behavior. Gender and race may be factors in the

development of social relationships. This study examined the selections of Caucasian and African American preschool children as they chose children "most like" them and selected playmates from photographs as well as during actual play. Overall, children were found to be more likely to identify with children of the same gender but were more likely to select familiar playmates of the same race. Children's selections of playmates from photographs predicted their selections of playmates during actual play only approximately half of the time. Findings also indicated that preschool children did not consistently prefer the same playmate but instead played with a small group of different playmates. Interventions were also implemented in an attempt to improve the status of preschool children least often chosen as playmates. Interventions used included modifications to the children's classroom environments or curriculum and peer-mediated interventions utilizing a modified peer training program. Neither intervention was found to significantly improve the number of times the least preferred children in a classroom were chosen as playmates during actual play. However, findings would suggest that interventions did result in the development of more advanced play skills. Significant correlations were found between the teacher's rankings of popularity and the number of times children were chosen as playmates during actual play as well as between teachers' rankings of popularity and the play checklist scores. Although significant, the correlation between children scores on the play checklist and the number of times they were selected as a playmate was very weak. Findings would suggest that factors other than social skills may influence children's status as a preferred playmate. (PsycINFO Database Record (c) 2012 APA, all rights reserved)
SN - 0419-4209
AD - Haynes, Gladys Verneal Oosting: U Nebraska - Lincoln, US
ER -

TY - JOUR
ID - 772
T1 - Latino mother-child dyads: Attachment, psychosocial factors, mother's perception of the child's temperament, and quality of dyadic competence
A1 - Bejarano,Anabel
Y1 - 2001///
N1 - Dissertation Abstract: 2001-95006-126
English
Dissertation Abstract
KW - PsycInfo
KW - Attachment Behavior
KW - Mother Child Relations
KW - Personality
KW - Psychosocial Factors
KW - Social Perception
KW - Dyads
KW - Latinos/Latinas
RP - NOT IN FILE
SP - 5050
JF - Dissertation Abstracts International: Section B: The Sciences and Engineering
VL - 61:
IS - 9-B
N2 - This study analyzed the mother-child relationship within a non-clinical sample of low socioeconomic status, Dominican and Puerto Rican mother-child dyads. The study examined the effects of stress, social support, maternal perception of the child's temperament, and attachment behaviors on dyadic competence for 23 dyads. Variables assessed shortly following the child's birth during the perinatal period, and at the child's age of 12 and 18 months, were used as predictors of dyadic competence in mother-child interaction during a free-play episode observed at the child's age of 18 months. The methodology included nonstandard, semi-structured stress and social support scales developed for a prior study on multi-risk mothers (Fiks, Johnson and Rosen, 1985). The stress scale measured the reported level of stress experienced in the areas of finances, living arrangements, children, family and friends. The size of the social support network was based on the number of individuals counted on by the mother, and available to help with financial, personal, and health/medical problems. The mothers' perception of her child's temperament was based on questions regarding the consistency of the child's eating and sleeping habits, activities enjoyed by the child, the degree of fussiness and ease of comforting the

child. Attachment behaviors (proximity- and contact-seeking, contact-maintaining, resistant, and avoidant behavior) and the amount of crying were assessed during two reunion episodes of a modified Strange Situation procedure at 12 months based on a 7-point dimensional rating scale (Ainsworth, Bell, Waters, and Wall, 1978). At the child's age of 18 months, the degree of mutual competence in dyadic interaction was measured by the Parent-Child Observation Guide (Bernstein, Percansky, and Hans, 1987). This scale is based on Goldberg's (1977) concept of mutual competence and rates behaviors of the mother and child that support or encourage mastery of developmental tasks. Maternal perception of the child as fussing alot at 18 months was highly predictive of less effective maternal competence in a free play situation. This dynamic relationship was stronger than that between maternal competence and stress, social support and attachment behaviors assessed at 12 and 18 months. However, stress from living arrangements and problems with friends and family during the perinatal period were predictive of maternal competence. The relationship between social support, rates of proximity seeking and contact maintenance behaviors and maternal competence failed to yield significant results. Although the mother and child's rates of competence were not concordant, it was found that mothers who had accurate expectations of the child for the child's age, who treated the child with respect, who helped the child stay interested in a toy or activity, and remained patient with the child had higher rates of maternal competence. For the child, rates of competence were most correlated with their ability to invite an interaction or play activity with the mother, and noticing their mothers smiling at them in several situations. In addition, high rates of proximity seeking behavior during the first reunion episode was a strong predictor of the child's competence. (PsycINFO Database Record (c) 2012 APA, all rights reserved)
SN - 0419-4217
AD - Bejarano, Anabel: City U New York, US
ER -

TY - JOUR
ID - 773
T1 - Depression as a risk factor for coronary artery disease in Type I diabetes mellitus: A study of possible mechanisms
A1 - Kinder,Leslie Smithline
Y1 - 2001///
N1 - Dissertation Abstract: 2001-95004-418
English
Dissertation Abstract
KW - PsycInfo
KW - Diabetes Mellitus
KW - Heart Disorders
KW - Major Depression
KW - Risk Factors
RP - NOT IN FILE
SP - 4465
JF - Dissertation Abstracts International: Section B: The Sciences and Engineering
VL - 61:
IS - 8-B
N2 - Recent evidence has suggested that depressive symptomatology is a risk factor for the development of coronary artery disease (CAD), although little is known about mechanisms that may explain this relationship. The Pittsburgh Epidemiology of Diabetes Complications (EDC) Study is an ongoing natural history study of 658 men and women with childhood onset Type I diabetes. Previous findings from the EDC study have shown an association between baseline depressive symptoms, assessed with the Beck Depression Inventory, and new cases of CAD, defined as angina, myocardial infarction, or CAD death, at 4- and 6-year follow-up. Univariate Cox proportional hazard modeling indicates that depressive symptomatology continues to predict the development of CAD at 10-year follow-up (RR = 1.040, CI = 1.009 - .071) and that the relationship between depressive symptomatology and CAD, as defined, is stronger in women than men (men RR = 1.029, CI = 0.984 - 1.076; women RR = 1.059, CI = 1.016 - 1.104). In the full sample, having ever smoked, white blood count, fibrinogen, neuropathy, and perceived health status statistically mediate the relationship between depressive symptomatology and incidence of CAD. In women, waist-hip ratio, neuropathy, and perceived health statistically mediate the relationship. These findings suggest that smoking and inflammatory processes may account for the depression-CAD relationship in Type I diabetic men and women. In women, Insulin Resistance

Syndrome, as marked by central adiposity, may play an important role as well. Finally, it is plausible that the severity of diabetic disease, characterized by perceptions of health and neuropathy, contributes to both depressive symptoms and the development of CAD. Psychosocial factors play an important role in CAD and Type I diabetes; our data suggest that a number of mechanisms, both behavioral and physiological, may explain the relationship between depressive symptomatology and incidence of CAD in Type I diabetes. (PsycINFO Database Record (c) 2012 APA, all rights reserved)
SN - 0419-4217
AD - Kinder, Leslie Smithline: U Pittsburgh, US
ER -

TY - JOUR
ID - 774
T1 - Perceived control over caregiving: Association with mothers' and fathers' observed interactions with their 12 month old infants
A1 - Guzell,Jacqueline Rose
Y1 - 2001///
N1 - Dissertation Abstract: 2001-95004-088
English
Dissertation Abstract
KW - PsycInfo
KW - Attribution
KW - Father Child Relations
KW - Mother Child Relations
KW - Dual Careers
RP - NOT IN FILE
SP - 4447
JF - Dissertation Abstracts International: Section B: The Sciences and Engineering
VL - 61:
IS - 8-B
N2 - Research suggests that parents who believe they have little control over negative caregiving outcomes tend to regard themselves as "power disadvantaged" (Bugental, Blue, & Lewis, 1990), which may lead to their efforts to restore balance by increasing their own controlling behaviors. The risk is that this behavior may escalate over time into coercive, denigrating and abusive behavior, especially when children are regarded as "difficult" or noncompliant by their parents. While previous research has demonstrated that low perceived control may be related to highly directive/controlling behavior in mothers of preschool or older children, the present study extends those findings by focusing on the attributions and behaviors of mothers and fathers of one-year-old infants. The 66 dual-earner families were drawn from the Penn State Health and Development Project, which examined home, daycare, and health factors that affected children's development. Videotaped mother-father-infant interactions during play in the family's home were coded for directive/controlling, sensitive, and detached behaviors that were assessed for their association with infant and parent characteristics, especially parents' perception of control over negative caregiving outcomes. The a priori hypothesis that perceived control would moderate the relationship between infant difficulty and parents' directive/controlling behavior during play was confirmed for mothers, but not for fathers. The group of mothers with low perceived control averaged over 50% more instances of directive/controlling behavior than other mothers and, in addition, mothers with low perceived control who rated their infants as difficult had the highest level of directive behavior. For fathers, low perceived control was associated with more directive/controlling behavior, regardless of whether or not their infants were perceived as difficult. In the present study, parents with low perceived control did not differ from other parents on their knowledge of child development, level of education, income, or religiosity. However, mothers with low perceived control scored significantly higher on a measure of categorical thinking than other mothers. Categorical thinking is indicative of the limited lens through which parents with low perceived control are believed to view difficult or unresponsive children, leading to script-like responses to stressful, negative parent-child caregiving situations and outcomes. (PsycINFO Database Record (c) 2012 APA, all rights reserved)
SN - 0419-4217
AD - Guzell, Jacqueline Rose: The Pennsylvania State U., US
ER -

TY - JOUR
ID - 775
T1 - Nonsocial play as a risk factor in social and emotional development. [References]
A1 - Cheah,Charissa S.
Y1 - 2001///
N1 - Book: 2001-18731-002
English
Book; Edited Book
KW - PsycInfo
KW - Childhood Play Behavior
KW - Childhood Play Development
KW - Emotional Development
KW - Psychosocial Development
KW - Risk Factors
KW - Childhood Development
KW - Cognitive Ability
KW - Emotional Control
KW - Parent Child Relations
KW - Personality Traits
KW - Problem Solving
KW - Social Skills
KW - Sociocultural Factors
RP - NOT IN FILE
SP - 39
EP - 71
JF - Goncu, Artin [Ed]; Klein, Elisa L [Ed]
VL - :
IS - 2001
N2 - (from the chapter) Examines the constructs of children's social and nonsocial play. In particular, this chapter discusses the developmental significance of social play and the potential risks associated with certain forms of nonsocial play. It is reported that social play is associated with positive markers of both social and cognitive competence. Similarly, solitary-passive behaviors were associated positively with competent problem solving, emotion regulation, and task persistence. Conversely, numerous risk factors associated with other types of nonsocial play were outlined. Next, the advantages and disadvantages of various methods used to study children's interactions and play were examined. Finally, the origins of individual and group variability found in social and nonsocial play, including dispositional factors, parental influences, and cultural contexts were discussed. The research presented suggests that cultural milieus and societal values create a framework within which these different behaviors may be evaluated. (PsycINFO Database Record (c) 2012 APA, all rights reserved)
AD - Cheah, Charissa S. L.: Yale U, Dept of Psychology, New Haven, CT, US
ER -

TY - JOUR
ID - 776
T1 - Children in play, story, and school
A1 - Goncu,Artin
Y1 - 2001///
N1 - Book: 2001-18731-000
English
Book; Edited Book
(Created by PsycINFO) Part I. Introduction * Children in play, story, and school: A tribute to Greta G. Fein / Artin Goncu and Elisa L. Klein Part II. Children in play * Play as improvisational rehearsal: Multiple levels of analysis in children's play / R. Keith Sawyer / 19-38 * Nonsocial play as a risk factor in social and emotional development / Charissa S. L. Cheah, Larry J. Nelson and Kenneth H. Rubin / 39-71 * Transforming the "play-oriented curriculum" and work in constructivist early education / Rheta DeVries / 72-106 * The adult as a tutor in fostering children's symbolic play / Anna Bondioli / 107-131 * Facilitating the narrative quality of

sociodramatic play / Rivka Glaubman, Gabi Kashi and Rina Koresh / 132-157 Part III. Children in story * Emotional breaches in play and narrative / Brian Sutton-Smith / 161-176 * "He was the best daddy": Postdivorce preschoolers' representations of loss and family life / Patricia Herman and Inge Bretherton / 177-203 * "I'm so glad I'm glad": The role of emotions and close relationships in children's play and narrative language / A. D. Pellegrini and Lee Galda / 204-219 * Playing "inside" stories / Lois A. Groth and Lynn Dietrich Darling / 220-237 Part IV. Children in school * Day care and the strange situation / Alison Clarke-Stewart, Virginia Allhusen and Frits Goossens / 241-266 * Processes in the formation of attachment relationships with alternative caregivers / Carollee Howes and Erin Oldman / 267-287 * Conceptual gaps in the lifespan theory of attachment / Nathan A. Fox and Yair Bar-Haim / 288-308 * Talking the talk: Constructivist teachers guiding children's problem solving / Michelle B. Patt and Artin Goncu / 309-332 * Visions and realities of achieving partnership: Parent-school relationships at the turn of the century / Douglas R. Powell / 333-357 * Contexts of quality: Implications for U.S. child-care policy / Elisa L. Klein and Louisa B. Tarullo Part V. Conclusions * Suppose there was a hill in Richmond Hill / Greta G. Fein Index
KW - PsycInfo
KW - Child Care
KW - Childhood Development
KW - Childhood Play Development
KW - Classroom Environment
KW - Storytelling
KW - Childhood Play Behavior
KW - Cognitive Development
KW - Early Experience
KW - Interpersonal Influences
KW - Language Development
KW - Learning
KW - Psychosocial Development
KW - Reading Development
KW - Social Interaction
RP - NOT IN FILE
SP - New
JF - (2001)
VL - Children in play, story, and school. x, 406 pp. New York, NY, US: Guilford Press; US.
N2 - (from the cover) This volume explores what play and story mean to young children, and how these vital aspects of development can best be supported in child care and educational settings. Vital connections are drawn between children's activities, their interpersonal relationships, and their emerging cognitive and affective capacities. Topics covered include promoting social play in the classroom, storytelling and literacy development, and the influences of early caregiving experiences on attachment and learning. Theoretical and methodological issues on those areas of research are addressed, as well as social policy implications. (PsycINFO Database Record (c) 2012 APA, all rights reserved)
AD - Goncu, Artin: U Illinois, Dept of Educational Psychology, Chicago, IL, US
ER -

TY - JOUR
ID - 777
T1 - Identifying sexually abused children in play therapy. [References]
A1 - Homeyer,Linda E.
Y1 - 2001///
N1 - Book: 2001-16195-010
English
Book; Edited Book
KW - PsycInfo
KW - Child Abuse
KW - Play Therapy
KW - Screening Tests
KW - Sexual Abuse
KW - At Risk Populations

KW - Victimization
RP - NOT IN FILE
SP - 131
EP - 154
JF - Landreth, Garry L [Ed]
VL - :
IS - 2001
N2 - (from the chapter) Children who have been (or are being) sexually abused may not be able to communicate that to others. This chapter describes the Play Therapy Screening Instrument for Child Sexual Abuse (PTSI-CSA), which can be used by the play therapist both in an attempt to understand children's play in the playroom as well as to comply with legal and ethical requirement to report abuse. The PTSI-CSA is an empirically researched screening instrument. The 15 items of play therapy behavior that make up the instrument can identify children who are at high risk of being sexually abused. Once a child is identified as high risk, the child can then be referred for a more formal sexual abuse evaluation. The user should be aware of the possible false negative and false positive findings. (PsycINFO Database Record (c) 2012 APA, all rights reserved)
AD - Homeyer, Linda E.: Southwest Texas State U, Dept of Educational Administration & Psychological Services, San Marcos, TX, US
ER -

TY - JOUR
ID - 778
T1 - Language development and symbolic play in children with and without familial risk for dyslexia. [References]
A1 - Lyytinen,Paula; Poikkeus,Anna-Maija; Laakso,Marja-Leena; Eklund,Kenneth; Lyytinen,Heikki
Y1 - 2001///
N1 - Peer Reviewed Journal: 2001-11200-014
English
Journal; Peer Reviewed Journal
KW - PsycInfo
KW - Childhood Play Behavior
KW - Dyslexia
KW - Genetics
KW - Language Delay
KW - Language Development
RP - NOT IN FILE
SP - 873
EP - 885
JF - Journal of Speech, Language, and Hearing Research
VL - 44:
IS - 4
N2 - Examined (a) whether children in families with a history of dyslexia were more likely to show delays in language development and (b) whether a delayed onset of expressive language predicted later language development. The language development of 200 children was analyzed longitudinally at 14, 24, 30, and 42 mo. Symbolic play was assessed at 14 mo. Half of the children were from families with a history of dyslexia (the Dyslexia Risk [DR] group) and others served as controls. Parental reports and structured tests were used to assess children. No differences emerged between the two groups in receptive language, symbolic play, or on the Bayley MDI. The maximum sentence length at 2 yrs and object naming and inflectional morphology skills at 3.5 yrs were higher for controls. Reynell receptive score at 2.5 yrs provided the greatest unique contribution to the prediction of the children's receptive and expressive language. Children's risk status provided a significant contribution to their expressive language at 3.5 yrs. Late talkers in the DR group differed from the others in that group in receptive and expressive language at 3.5 yrs. Children with a familial risk for dyslexia and with a history of late talking are at higher risk for delays in language acquisition. (PsycINFO Database Record (c) 2012 APA, all rights reserved)
SN - 1092-4388
AD - Lyytinen, Paula: U Jyvaskyla, Dept of Psychology, Jyvaskyla, Finland
ER -

TY - JOUR
ID - 779
T1 - Children's play. [References]
A1 - McArdle,Paul
Y1 - 2001///
N1 - Peer Reviewed Journal: 2001-10020-004
English
Journal; Peer Reviewed Journal
KW - PsycInfo
KW - Childhood Play Behavior
KW - Personality Development
KW - Well Being
RP - NOT IN FILE
SP - 509
EP - 514
JF - Child: Care, Health and Development
VL - 27:
IS - 6
N2 - This paper draws upon research from a number of disciplines to examine the significance of children's play. It concludes that play may well be central to normal personality development. However, its place in contemporary Western society is not secure, perhaps risking the development and well-being of urban and disadvantaged children in particular. (PsycINFO Database Record (c) 2012 APA, all rights reserved) (journal abstract)
SN - 0305-1862
AD - McArdle, Paul: Fleming Nuffield Unit, Newcastle upon Tyne, United Kingdom
ER -

TY - JOUR
ID - 780
T1 - Play between aggressive young children and their mothers. [References]
A1 - Landy,Sarah
Y1 - 2001///
N1 - Peer Reviewed Journal: 2001-06061-003
English
Journal; Peer Reviewed Journal
KW - PsycInfo
KW - Aggressive Behavior
KW - Attachment Behavior
KW - Childhood Play Behavior
KW - Conduct Disorder
KW - Mother Child Relations
KW - At Risk Populations
KW - Preschool Students
RP - NOT IN FILE
SP - 223
EP - 240
JF - Clinical Child Psychology and Psychiatry
VL - 6:
IS - 2
N2 - There is a growing number of pre-schoolers presenting to clinics with high levels of aggression. These children are at risk of developing conduct disorder, especially when the symptoms are severe. Treatment for older children with aggressive symptoms has often relied on teaching their parents behavior management techniques and has ignored affective and relationship issues. After presenting observations of the play interactions of aggressive pre-schoolers with their mothers and other relevant research findings, this article explores important aspects of the parent-child interaction and affective relationship. These aspects include the

mother's difficulty in tolerating and modulating the child's negative affect, reluctance to enter the play metaphor and failure to gradually move aggressive play to more pro-social themes. The importance of these interactions and affective relationship aspects and their contribution to the child's difficulty are discussed in light of their implications for treatment. Also, suggestions are made for caregivers working with these children to help them self-regulate and acquire appropriate cognitive strategies to contain the negative affects. (PsycINFO Database Record (c) 2012 APA, all rights reserved)
SN - 1359-1045
AD - Landy, Sarah: U Toronto, Dept of Psychiatry, Toronto, ON, Canada
ER -

TY - JOUR
ID - 781
T1 - Counselors coaching teachers to use play therapy in classrooms: The Play And Language to Succeed (PALS) early, school-based intervention for behaviorally at-risk children. [References]
A1 - Chaloner,W.
Y1 - 2001///
N1 - Book: 2001-01223-020
English
Book; Edited Book
KW - PsycInfo
KW - At Risk Populations
KW - Classroom Environment
KW - Play Therapy
KW - School Based Intervention
KW - Teacher Education
KW - Behavior Problems
KW - Early Intervention
KW - Coaching
RP - NOT IN FILE
SP - 368
EP - 390
JF - Drewes, Athena A [Ed]; Carey, Lois J [Ed]; Schaefer, Charles E [Ed]
VL - :
IS - 2001
N2 - (from the chapter) The incidence and severity of violence in schools has increased significantly over the past 30 yrs. Research indicates that impaired caregiver-child attachment is a primary risk factor for violence. The authors Play And Language to Succeed Model (PALS) trains school counselors, play therapists, and Pre-K-2 teachers to intervene early, using the three key elements of bonding, boundaries, and language to form positive attachments and teach social skills via play and other developmentally appropriate practices. The components that are necessary for successful implementation of PALS are (1) inclusive, classroom-based interventions by teachers; (2) individual and small group play therapy; (3) initial and ongoing districtwide staff development and support, (4) ongoing administrative and staff collaboration regarding program implementation; and (5) early, proactive behavioral screening, rating, and assessment. The case study in this chapter illustrated how potent a collaborative relationship among play therapists, counselors, and teachers can be in facilitating change with severely at-risk children while maximizing service delivery. (PsycINFO Database Record (c) 2012 APA, all rights reserved)
ER -

TY - JOUR
ID - 782
T1 - Play therapy for children of alcoholics. [References]
A1 - Emshoff,James G.
Y1 - 2001///
N1 - Book: 2001-01223-011
English
Book; Edited Book

KW - PsycInfo
KW - Alcoholism
KW - At Risk Populations
KW - Behavior Problems
KW - Parental Characteristics
KW - Play Therapy
KW - Children of Alcoholics
KW - Early Experience
RP - NOT IN FILE
SP - 194
EP - 215
JF - Drewes, Athena A [Ed]; Carey, Lois J [Ed]; Schaefer, Charles E [Ed]
VL - :
IS - 2001
N2 - (from the chapter) Discusses play therapy for children with alcoholic parents. It has been estimated that 26.8 million children of alcoholics (COAs) currently live in the US. Of these, over 11 million are younger than 18. COAs have also been shown to be at an increased risk for a wide range of behavioral and emotional problems, including addiction to alcohol and other drugs, depression, anxiety, school failure, delinquency, and difficulty getting along with their peers. This chapter emphasizes the potential benefits of using play therapy with COAs. Because of the numerous responsibilities that are often associated with living in a substance-abusing household, COAs typically receive fewer opportunities to engage in this fun than do other children. Play therapy allows these children to participate in activities that not only provide them with a relief from the pressures of being a COA, but can also be designed to address many of the risk factors these children experience. (PsycINFO Database Record (c) 2012 APA, all rights reserved)
AD - Emshoff, James G.: Georgia State U, Atlanta, GA, US
ER -

TY - JOUR
ID - 783
T1 - Child-centered play therapy for at-risk elementary school children. [References]
A1 - Post,Phyllis
Y1 - 2001///
N1 - Book: 2001-01223-006
English
Book; Edited Book
KW - PsycInfo
KW - At Risk Populations
KW - Behavior Problems
KW - Client Centered Therapy
KW - Play Therapy
KW - Elementary School Students
RP - NOT IN FILE
SP - 105
EP - 122
JF - Drewes, Athena A [Ed]; Carey, Lois J [Ed]; Schaefer, Charles E [Ed]
VL - :
IS - 2001
N2 - (from the chapter) At-risk elementary school children typically come to school with attention and behavior problems. Because these problems can detract from their academic and social development, cause persistent school misconduct, and affect other children negatively, helping these children become involved and successful in school is essential. Child-centered play therapy is one way to help at-risk children. In this chapter, the following topics are addressed: play therapy with at-risk children, the referral process, goals of child-centered play therapy with at-risk children, and consultations with caregivers and teachers. All of the case illustrations are based on the author's experience conducting child-centered play therapy in schools composed predominantly of at-risk children. (PsycINFO Database Record (c) 2012 APA, all rights reserved)

AD - Post, Phyllis: U North Carolina, Dept of Counseling, Special Education, and Child Development, Charlotte, NC, US
ER -

TY - JOUR
ID - 784
T1 - The role of friendship in psychological adjustment
A1 - Nangle,Douglas W.
Y1 - 2001///
N1 - Book: 2001-00673-000
English
Book; Edited Book
(Created by PsycINFO) (Abbreviated) Editor's notes [by] Douglas W. Nangle, Cynthia A. Erdley * Children's friendship experiences and psychological adjustment: Theory and research / Cynthia A. Erdley, Douglas W. Nangle, Julie E. Newman, Erika M. Carpenter / 5-24 * Friendship and peer rejection as predictors of adult adjustment / Catherine L. Bagwell, Michelle E. Schmidt, Andrew F. newcomb, William M. Bukowski / 25-49 * Children with attention-deficit/hyperactivity disorder: Peer relationships and peer-oriented interventions / Sylvie Mrug, Betsy Hoza, Alyson C. Gerdes / 51-77 * Peer group dynamics associated with iatrogenic effects in group interventions with high-risk young adolescents / Thomas J. Dishion, Francois Poulin, Bert Burraston / 79-92 * Friendship and the worlds of childhood / William M. Bukowski / 93-105 Index
KW - PsycInfo
KW - Childhood Development
KW - Emotional Adjustment
KW - Friendship
RP - NOT IN FILE
SP - San
EP - Bass
JF - (2001)
VL - The role of friendship in psychological adjustment. 112 pp. San Francisco, CA, US: Jossey-Bass; US.
N2 - (from the cover) The idea that children's peer relations play a critical role in their overall psychological adjustment is now assumed. As an index of popularity or status in the peer group, however, peer acceptance captures only one aspect of children's peer experiences. Friendships, close dyadic relationships, play a unique and crucial role in children's socioemotional development. The 5 chapters in this volume highlight different avenues of research aimed at deepening our understanding of the complex relationship between children's friendship experiences and psychological adjustment. This volume covers historical and conceptual issues involved in the study of friendship, as well as cutting-edge empirical research linking friendship and a range of outcome indices. (PsycINFO Database Record (c) 2012 APA, all rights reserved)
AD - Nangle, Douglas W.: U Maine, ME, US
ER -

TY - JOUR
ID - 785
T1 - Gender biases in children's appraisals of injury risk and other children's risk-taking behaviors. [References]
A1 - Morrongiello,Barbara A.
Y1 - 2000///
N1 - Peer Reviewed Journal: 2001-14627-004
English
Journal; Peer Reviewed Journal
KW - PsycInfo
KW - Child Attitudes
KW - Childhood Play Behavior
KW - Human Sex Differences
KW - Risk Perception
KW - Injuries
RP - NOT IN FILE
SP - 317

EP - 336
JF - Journal of Experimental Child Psychology
JA - J Exp.Child Psychol.
VL - 77:
IS - 4
N2 - Children ages 6, 8, and 10 yrs old were given tasks designed to assess their beliefs about risk of injury from activities. Children were asked to appraise the risk of injury for boys and girls engaging in various play behaviors and to judge the sex of the character in stories about children engaging in activities that result in injuries. Results revealed gender biases in children's appraisals of injury risk: Both boys and girls rated boys as having a lower likelihood of injury than girls even though the boys and girls were engaging in the exact same activities. Children also showed higher accuracy in identifying the sex of the character in stories of boys' injuries than girls' injuries, and accuracy improved with the participant's age. Overall, the results indicate that by the age of 6 yrs, children already have differential beliefs about injury vulnerability for boys and girls. Although boys routinely experience more injuries than girls, children rate girls as having a greater risk of injury than boys. With increasing age, school-age children develop a greater awareness of the ways in which boys and girls differ in risk-taking activities that lead to injury outcomes. (PsycINFO Database Record (c) 2012 APA, all rights reserved) (journal abstract)
SN - 0022-0965
AD - Morrongiello, Barbara A.: U Guelph, Psychology Dept, Guelph, ON, Canada
ER -

TY - JOUR
ID - 786
T1 - The effects of multiple risk factors including prenatal cocaine exposure on exploratory and symbolic play
A1 - Lundgren Kristine,Hong Tu
Y1 - 2000///
N1 - Dissertation Abstract: 2000-95018-337
English
Dissertation Abstract
KW - PsycInfo
KW - Childhood Play Behavior
KW - Childhood Play Development
KW - Cocaine
KW - Prenatal Exposure
KW - Risk Factors
KW - Exploratory Behavior
KW - Role Playing
RP - NOT IN FILE
SP - 1368
JF - Dissertation Abstracts International: Section B: The Sciences and Engineering
VL - 61:
IS - 3-B
N2 - According to the national estimates of women across socioeconomic groups, cocaine use during pregnancy affects between 10 and 20% of all children (Chasnoff, Landress & Barrett, 1990). With the growing number of children exposed to cocaine there has been a number of research studies focused on the potential developmental risks from prenatal cocaine-exposure. The results of these studies have been inconsistent and inconclusive due in part to the number of methodological difficulties encountered when investigating this population. There remains many unanswered questions regarding the overall development of children with prenatal cocaine-exposure. The purpose of this study was to provide descriptive information regarding early play development in a group of children with prenatal cocaine-exposure and associated risk factors. Their performance will be compared to an age matched control group from the same community with no history of drug exposure. The play assessment was based on a 20-minute sample of free play. The results suggest differences between the two groups as well as differences with the cocaine-exposed group. The major differences between the two groups were in the maturity of the play and the initiation of the play activities. The contribution of the multiple risk factors in the cocaine-exposed group will be discussed in relation to these

results. The findings from this study suggest that play development may be disrupted in children with cocaine-exposure and associated risk factors. (PsycINFO Database Record (c) 2012 APA, all rights reserved)
SN - 0419-4217
AD - Lundgren Kristine, Hong-Tu: Boston U., US
ER -

TY - JOUR
ID - 787
T1 - Perspective taking and adjustment in preschoolers
A1 - Pears,Katherine Chaffee
Y1 - 2000///
N1 - Dissertation Abstract: 2000-95004-152
English
Dissertation Abstract
KW - PsycInfo
KW - Adjustment
KW - Age Differences
KW - Aggressive Behavior
KW - Psychosocial Development
KW - Role Taking
KW - Childhood Development
KW - Social Skills
RP - NOT IN FILE
SP - 4244
JF - Dissertation Abstracts International: Section B: The Sciences and Engineering
VL - 60:
IS - 8-B
N2 - Anumber of theories attempt to explain social aggression in children. However, none of these theories satisfactorily accounts for both the development of social aggression and its maintenance across time and situations. In this dissertation, a theory positing perspective-taking as a within-child mechanism accounting for the continuity of social aggression is proposed. The theory predicts that perspective-taking (as indexed by social referencing, symbolic play, and other more traditional perspective-taking measures) should be related to social competence, particularly social aggression. The theory was tested in samples of children at risk for the development of problem behaviors and those not at-risk at three different ages: 21 months, 36 months, and 48 months. Social referencing was measured at 21 months, perceptual, motivational, cognitive and affective perspective-taking at 36 and 48 months, and symbolic play at 48 months. To assess children's social competence, and their social aggression in particular, parents and teachers rated children's externalizing, internalizing, and social behaviors at 48 months. Children's observed positive and aggressive behaviors with peers also were coded. Differences between risk groups were found on perspective-taking at 36 and 48 months but not on social referencing, symbolic play, or social competence. When longitudinal relations were examined, social referencing at 21 months predicted perspective-taking at 36 months and observed positive and aggressive behaviors at 48 months. Perspective-taking at 36 months predicted perspective-taking at 48 months but did not relate to symbolic play or social competence. However, perspective-taking at 48 months did predict symbolic play and some aspects of social competence at that same age. In particular, perspective-taking was related to social problems and internalizing behaviors, but not to externalizing behaviors. Finally, symbolic play was also correlated with social competence at 48 months, but again, the correlations were with social problems and internalizing rather than externalizing behaviors. Overall, the theory that perspective-taking is central to the continuity of social aggression received only mixed support. The implications of the findings for intervention in and prevention of social aggression are discussed. (PsycINFO Database Record (c) 2012 APA, all rights reserved)
SN - 0419-4217
AD - Pears, Katherine Chaffee: U Oregon, US
ER -

TY - JOUR
ID - 789

T1 - Just pretend: Participation in symbolic talk by children with histories of early corrective heart surgery
A1 - Ovadia,Rebecca; Hemphill,Lowry; Winner,Kendra; Bellinger,David
Y1 - 2000///
N1 - Peer Reviewed Journal: 2000-12247-002
English
Journal; Peer Reviewed Journal
KW - PsycInfo
KW - Childhood Play Behavior
KW - Heart Disorders
KW - Language Development
KW - Morphology (Language)
KW - Side Effects (Treatment)
KW - At Risk Populations
KW - Discourse Analysis
KW - Heart Surgery
KW - Language Delay
KW - Parent Child Relations
KW - Pretend Play
KW - Social Skills
KW - Syntax
KW - Vocabulary
RP - NOT IN FILE
SP - 321
EP - 340
JF - Applied Psycholinguistics
VL - 21:
IS - 3
N2 - Children with histories of early corrective heart surgery (ECHS) are at risk for language, cognitive, and motor delays. This study examined parent-child play in 30 children with ECHS (aged 4-5 yrs) and 30 typically developing children. Children were compared on basic language measures and on proportions of symbolic and nonsymbolic talk. Children with ECHS focused on concrete "here-and-now" talk and produced less symbolic talk than normative children. Only a third of the children with ECHS were able to produce story episodes. These findings reflect the ECHS children's relatively immature participation in joint pretense and their overreliance on earlier acquired strategies for pretend play. This style of participation may result from difficulty coordinating more complex social intentions with appropriate language forms. (PsycINFO Database Record (c) 2012 APA, all rights reserved)
SN - 0142-7164
AD - Ovadia, Rebecca: Harvard Graduate School of Education, Cambridge, MA, US
ER -

TY - JOUR
ID - 793
T1 - Parental influences on toddlers' injury-risk behaviors: Are sons and daughters socialized differently?
A1 - Morrongiello,Barbara A.
Y1 - 1999///
N1 - Peer Reviewed Journal: 2001-17644-002
English
Journal; Peer Reviewed Journal
KW - PsycInfo
KW - Childrearing Practices
KW - Human Sex Differences
KW - Parent Child Relations
KW - Risk Taking
KW - SOCIALIZATION
KW - Childhood Play Behavior
KW - Sex Roles

RP - NOT IN FILE
SP - 227
EP - 251
JF - Journal of Applied Developmental Psychology
VL - 20:
IS - 2
N2 - Examined mothers' and fathers' communications to sons and daughters (ages 2-4) in 48 families during both a free-play episode and their teaching the child a playground behavior (going down a firehouse-type pole) that posed some threat to safety because of the child's developmental status. The results revealed that mothers and fathers responded similarly to each other, but differently to sons and daughters. Coding of verbalizations revealed that sons received more directives, fewer explanations, and more questions communicating information about how to perform the pole task than daughters. Coding of physical contact revealed that parents applied more pressure to sons than daughters to attempt the pole task without physical assistance and provided more spontaneous physical assistance to daughters than sons, although there were no sex differences in children's playground skills or their abilities to complete the task independently. Coding of the free-play episode yielded results consistent with those from the teaching session. The results suggest that parents communicate to young children in ways that may promote greater risk taking by boys and greater perceived injury vulnerability among girls. (PsycINFO Database Record (c) 2012 APA, all rights reserved)
SN - 0193-3973
AD - Morrongiello, Barbara A.: U Guelph, Dept of Psychology, Guelph, ON, Canada
ER -

TY - JOUR
ID - 794
T1 - Impact of child-centered play therapy on the self-esteem, locus of control, and anxiety of at-risk 4th, 5th, and 6th grade students. [References]
A1 - Post,Phyllis
Y1 - 1999///
N1 - Peer Reviewed Journal: 2000-13732-001
English
Journal; Peer Reviewed Journal
KW - PsycInfo
KW - Anxiety
KW - At Risk Populations
KW - Internal External Locus of Control
KW - Play Therapy
KW - Self Esteem
KW - Academic Self Concept
RP - NOT IN FILE
SP - 1
EP - 18
JF - International Journal of Play Therapy
VL - 8:
IS - 2
N2 - Addresses the impact of child-centered play therapy on the self-esteem, locus of control, and anxiety level of at-risk 4th, 5th, and 6th grade children. The two groups of students, those who participated in play therapy and those who did not participate in play therapy, were administered the Coopersmith Self-Esteem Inventory, Intellectual Achievement Responsibility Scale-Revised, and State-Trait Anxiety Inventory for Children. While the results indicate that children participating in play therapy did not change, the students not participating in play therapy demonstrated a decrease in both self-esteem and locus of control over the course of the school year. These findings indicate that an intervention, such as play therapy, may be needed to prevent at-risk children from developing lower self esteem and from reducing their sense of responsibility for their academic successes and failures. (PsycINFO Database Record (c) 2012 APA, all rights reserved)
SN - 1555-6824
AD - Post, Phyllis: U North Carolina, Dept of Counseling, Special Education, & Child Development, Charlotte, NC, US

ER -

TY - JOUR
ID - 795
T1 - The delayed emergence of lexical morphology in preterm children: The case of verbs. [References]
A1 - Le Normand,Marie-Therese; Cohen,Henri
Y1 - 1999///
N1 - Peer Reviewed Journal: 2000-13141-005
English
Journal; Peer Reviewed Journal
KW - PsycInfo
KW - Language Delay
KW - Language Development
KW - Premature Birth
KW - Sociocultural Factors
KW - Verbs
KW - Age Differences
KW - At Risk Populations
KW - Lexical Access
KW - Morphology (Language)
RP - NOT IN FILE
SP - 235
EP - 246
JF - Journal of Neurolinguistics
VL - 12:
IS - 3-4
N2 - A linguistic analysis in low-risk preterm (PT) children was conducted to determine whether the acquisition of complex aspects of the lexicon, such as verbs, is influenced by their premature birth status and whether sociocultural factors play a modulating role in this aspect of cognitive development. 51 PT children, distributed in 3 birthweight groups-extremely low (780-1200 g), very low (1201-1500 g) and low (1501-2210 g)-were evaluated at 42 mo and 60 mo of age to assess their production of main, auxiliary, and non-finite verb types and tokens relative to that of 2 control groups of full-term children matched for age and socioeconomic level. The children's verb lexicon was collected during a 20-min controlled play context. Analyses of transcript data revealed first that the effect of sociocultural variables was independent of birth status or age. Further analyses revealed significant differences, indicating a marked increase in both verb type and token usage in the control children, whereas verb production in the 3 groups of PT children remained generally poorer. The data suggest that impaired language development is a cognitive consequence of prematurity independent of birthweight. (PsycINFO Database Record (c) 2012 APA, all rights reserved)
SN - 0911-6044
AD - Le Normand, Marie-Therese: Hopital de la Salpetriere, Neuropsychologie Clinique de l'Enfant, Batiment Pharmacie, Paris, France
ER -

TY - JOUR
ID - 796
T1 - Increasing the social-communicative skills of at-risk preschool age children in a play context.(language, intervention, head start)
A1 - Craig-Unkefer,Leslie Ann
Y1 - 1999///
N1 - Dissertation Abstract: 1999-95021-043
English
Dissertation Abstract
KW - PsycInfo
KW - At Risk Populations
KW - Childhood Play Behavior
KW - Language Delay

KW - Social Skills Training
KW - Verbal Communication
KW - Child Psychology
KW - Dyads
RP - NOT IN FILE
SP - 1445
JF - Dissertation Abstracts International Section A: Humanities and Social Sciences
VL - 60:
IS - 5-A
N2 - The purpose of the current study was to assess the effects of a social communication intervention on the peer interactions of preschool age children at-risk for delays in language and social skills. A multiple baseline design was used to determine the effects of an intervention on children's verbal interactions and play across three mixed gender dyads. The intervention was systematically and sequentially introduced to each dyad after baseline data were collected in order to demonstrate experimental control. During the baseline and intervention phases, children's social communicative behaviors were observed, recorded, and summarized. The observational data were used as the primary measure of behavioral change for the dyads; measures of language and play complexity were also observed. Generalization measures of language and play with untrained peers in another setting were assessed. Social validity was determined by comparison of baseline and intervention sessions of the participants. Results indicated an increase in use of social communicative behaviors for all of the participants and an increase in complex and interactive play. The results of the pre to post-intervention generalization measurement indicated that the participants increased in their use of social communicative behaviors to an untrained peer in another setting. There were also increases in complex and interactive play by the participants. (PsycINFO Database Record (c) 2012 APA, all rights reserved)
SN - 0419-4209
AD - Craig-Unkefer, Leslie Ann: Peabody Coll For Teachers Of Vanderbilt U., US
ER -

TY - JOUR
ID - 797
T1 - Empirical relationships between developmental competencies and peer play behaviors with African-American Head Start children
A1 - Mendez,Julia Laraine
Y1 - 1999///
N1 - Dissertation Abstract: 1999-95020-196
English
Dissertation Abstract
KW - PsycInfo
KW - Childhood Play Behavior
KW - Language Development
KW - Personality
KW - Self Management
KW - Social Skills
KW - Age Differences
KW - Blacks
KW - Childhood Development
KW - Human Sex Differences
KW - Project Head Start
RP - NOT IN FILE
SP - 1888
JF - Dissertation Abstracts International: Section B: The Sciences and Engineering
VL - 60:
IS - 4-B
N2 - School adaptation is an important national priority, particularly for children from low-income and minority backgrounds. Informing school readiness initiatives requires a detailed understanding of social competence among children at-risk for school difficulties. This study used a developmental theoretical framework and multivariate research methods to examine emerging capacities related to successful or

unsuccessful peer play interactions for African American children within their classroom setting. Social competence was investigated with 141 urban Head Start children by (a) determining the unique variance associated with each child capacity in the areas of temperament, language, and self regulation, (b) determining multivariate relationships between child capacities and peer play, and (c) exploring age and gender variability within peer play, temperament, language, and self regulation. Factor analysis showed seven out of eight child capacities could be distinctively measured, as indicated by adequate specific variance for each variable. Canonical analyses revealed two significant canonical variates, named Interactive Competence and Overactive-Disruption. The Competence variate was comprised of strong positive loadings for Play Interaction, Adaptability, Approach, and Receptive Language, and negative loadings for Activity Level and Play Disconnection. The Overactive-Disruption variate was comprised of strong positive loadings for Play Disruption and Activity Level. Repeated measures ANOVA showed an Age x Gender x Play effect, with older females receiving higher scores than younger males on the Play Interaction factor, and younger males scoring higher than older males and younger females on Play Disconnection. No significant differences were found for Play Disruption. An Age x Temperament effect revealed that younger children received higher scores for Activity than older children. Conversely, older children received higher scores for Approach than younger children. No significant age differences were found for Adaptability. A second interaction, Gender x Temperament, showed males scored higher than females on the Activity factor. No significant gender differences were found for the Adaptability or Approach factors. No significant interactions were found for language or self regulation. Implications for future research and promoting interactive peer play in the classroom were discussed. This study also informs developmentally appropriate curriculum, mental health policy, and early childhood intervention among poor preschool children. (PsycINFO Database Record (c) 2012 APA, all rights reserved)
SN - 0419-4217
AD - Mendez, Julia Laraine: U Pennsylvania, US
ER -

TY - JOUR
ID - 798
T1 - Prevention of violent behaviors through attachment via a play therapy and Montessori program
A1 - Nearing,Graciela Batista
Y1 - 1999///
N1 - Dissertation Abstract: 1999-95018-278
English
Dissertation Abstract
KW - PsycInfo
KW - Attachment Behavior
KW - Montessori Method
KW - Play Therapy
KW - Prevention
KW - Violence
RP - NOT IN FILE
SP - 1331
JF - Dissertation Abstracts International: Section B: The Sciences and Engineering
VL - 60:
IS - 3-B
N2 - A primary intervention program for preschoolers is designed with the objective of preventing the emergence of violent behaviors. By developing attachment to a play therapist and participating in a Montessori preschool program, the child at risk is helped to shape adaptive behaviors. The child born to low socio-economic and harsh family conditions is in peril of becoming aggressive, and in danger of developing pathological behaviors or behavior disorders. By designing a program that targets the ages when the child begins to make social contacts outside the home the possibility of influencing normal social skill is enhanced. This intensive program will take place for ages 3-6, during three consecutive scholastic calendar years in addition to fostering development of self-control and other positive social behaviors, the child is expected to be cognitively ready for formal school learning at the end of the intervention program. (PsycINFO Database Record (c) 2012 APA, all rights reserved)
SN - 0419-4217

AD - Nearing, Graciela Batista: Miami Inst. of Psychology of the Caribbean Ctr. For Advanced Studies, US
ER -

TY - JOUR
ID - 800
T1 - Play therapy for children exposed to violence: Individual, family, and community interventions. [References]
A1 - VanFleet,Rise; Lilly,John Paul; Kaduson,Heidi
Y1 - 1999///
N1 - Peer Reviewed Journal: 1999-11842-002
English
Journal; Peer Reviewed Journal
KW - PsycInfo
KW - At Risk Populations
KW - Play Therapy
KW - Violence
KW - Community Services
KW - Family Therapy
KW - Posttraumatic Stress Disorder
RP - NOT IN FILE
SP - 27
EP - 42
JF - International Journal of Play Therapy
VL - 8:
IS - 1
N2 - Outlines play therapy-related interventions that have been used to assist children who have been exposed to violence and are at risk for developing posttraumatic stress disorder (PTSD). Play therapy approaches for children exposed to violence, family play therapy for families affected by violent trauma, and a description of a community intervention that works with play therapists to ensure that children and families are empowered and supported following violence and abuse are included. (PsycINFO Database Record (c) 2012 APA, all rights reserved) (journal abstract)
SN - 1555-6824
AD - VanFleet, Rise: Family Enhancement & Play Therapy Ctr, Boiling Springs, PA, US
ER -

TY - JOUR
ID - 801
T1 - Let's talk and play: A study of poverty mothers and toddler daughters
A1 - Hess,Lucille J.
Y1 - 1999///
N1 - Peer Reviewed Journal: 1999-10710-001
English
Journal; Peer Reviewed Journal
KW - PsycInfo
KW - At Risk Populations
KW - Childhood Play Development
KW - Early Intervention
KW - Language Development
KW - Mother Child Communication
KW - Daughters
KW - Mothers
RP - NOT IN FILE
SP - 1
EP - 16
JF - Infant-Toddler Intervention
VL - 9:

IS - 1
N2 - Investigated whether early intervention initiated 0-3 mo after birth by family service workers result in facilitative play interaction for at-risk families and whether the toddlers of these families acquire early language within normal developmental expectations. The play behavior and language of 3 single mothers and their 12-l8 mo old daughters from an intervention program serving an at-risk infant population was assessed. The analysis of the play and oral language came from 4 30-min verbatim transcripts of in-home videotaped sessions as each parent talked and played with her child. Mean length of utterance and the proportion of the mother's total utterances for physical, social, and symbolic play and reading were scored. The utterances used to engage the child in play and provide positive and negative comments in each session assessed the quality of the interaction. Results show that the mothers engaged in more physical play, used a directive interaction style, and needed a high number of bids to keep their child's attention. The quality of talk and play improved when the mothers engaged in symbolic play and used pictures in books to talk to their children. Strategies to teach at-risk families are presented. (PsycINFO Database Record (c) 2012 APA, all rights reserved)
SN - 1053-5586
AD - Hess, Lucille J.: Indiana U Purdue U, Audiology & Speech Sciences, Ft Wayne, IN, US
ER -

TY - JOUR
ID - 802
T1 - Fear and exhilaration in response to risk: An extension of a model of injury risk in a real-world context
A1 - Cook,Scott; Peterson,Lizette; DiLillo,David
Y1 - 1999///
N1 - Peer Reviewed Journal: 1999-10598-001
English
Journal; Peer Reviewed Journal
KW - PsycInfo
KW - Childhood Play Behavior
KW - Emotional Responses
KW - Euphoria
KW - Fear
KW - Risk Taking
RP - NOT IN FILE
SP - 5
EP - 15
JF - Behavior Therapy
VL - 30:
IS - 1
N2 - Tested a model that predicts children's actual injury risk behavior from their current typical reported reactions of excitement vs fear in risky play situations. 130 4th graders were asked to report on their current typical levels of fear and excitement in response to common play situations, including those involving play in the water. A week or more later, the same children were observed during their turn at free play on the diving board of a local swimming pool. Reporting that current responses to risky play situations resulted in fear was related to lower rates of actual risky behavior and higher rates of protective behavior, whereas reporting current responses of exhilaration to risky situations was related to higher rates of some kinds of actual risky behavior. These relationships were even stronger when only water-related play situations were considered. The data are consistent with findings from observed behavior where the risk was academic, social, or sports-related failure rather than injury. This study documents that children's perceptions of their own current cognitive reactions to risky play situations predict their actual concurrent risky behavior in a potential physical injury situation. Thus, these findings have utility for injury prevention programs. (PsycINFO Database Record (c) 2012 APA, all rights reserved)
SN - 0005-7894
AD - Cook, Scott: U Missouri, Columbia, MO, US
ER -

TY - JOUR
ID - 803

T1 - Externalizing problems in two-year-olds: Implications for patterns of social behavior and peers' responses to aggression
A1 - Calkins,Susan D.
Y1 - 1999///
N1 - Peer Reviewed Journal: 1999-05699-002
English
Journal; Peer Reviewed Journal
KW - PsycInfo
KW - Aggressive Behavior
KW - At Risk Populations
KW - Behavior Problems
KW - Dyads
KW - Externalization
RP - NOT IN FILE
SP - 267
EP - 288
JF - Early Education and Development
VL - 10:
IS - 3
N2 - 48 2-yr-old children selected on the basis of parents' responses to 2 administrations of the Child Behavior Checklist for 2-3 yr olds was observed in peer interactions. 24 of these Ss displayed symptoms of aggressive/destructive (externalizing) problems that were in the borderline clinical range (labeled "high risk") and 24 Ss showed few such symptoms ("low risk"). The Ss were observed in matched dyads (1 high risk and 1 low risk child) across 4 tasks designed to vary in the degree of social participation they would elicit. Across all tasks, Ss in the high risk group displayed significantly and consistently more aggressive behavior than the Ss in the low risk group. High risk Ss did not differ from other Ss in terms of several indices of social and nonsocial play. When classified as high aggressive vs average vs low aggressive on the basis of laboratory behavior, Ss who displayed high amounts of aggression during the play sessions did not differ from less aggressive Ss on these indices of social play. The responses of non-aggressive dyad partners to aggressive acts indicated that Ss are responsive to aggression. Results are discussed in terms of the implications of early problematic behavior for later indices of maladjustment that include social competence and peer rejection. (PsycINFO Database Record (c) 2012 APA, all rights reserved)
SN - 1040-9289
AD - Calkins, Susan D.: U North Carolina, Dept of Psychology, Greensboro, NC, US
ER -

TY - JOUR
ID - 804
T1 - Early childhood assessment: A comparison of the Bayley Scales of Infant Development and play-based assessment in two-year old at-risk children
A1 - Kelly-Vance,Lisa; Needelman,Howard; Troia,Kim; Ryalls,Brigette Oliver
Y1 - 1999///
N1 - Peer Reviewed Journal: 1999-05622-001
English
Journal; Peer Reviewed Journal
KW - PsycInfo
KW - At Risk Populations
KW - Bayley Scales of Infant Development
KW - Childhood Play Behavior
KW - Cognitive Assessment
KW - Cognitive Development
RP - NOT IN FILE
SP - 1
EP - 15
JF - Developmental Disabilities Bulletin
VL - 27:

IS - 1
N2 - Practitioners are using a variety of assessment tools to measure cognitive functioning in young children. These measures are used for similar purposes yet the relationship among them is largely unknown. The purpose of this study was to analyze the relationship between two assessment approaches used to determine the cognitive functioning level in young children: the Bayley Scales of Infant Development-II and Play-Based Assessment. A sample of 38 2-yr-olds participating in a Neonatal Intensive Care Unit Follow-Up Clinic were evaluated on both measures. Results indicated a high correlation between the 2 techniques. A comparison of the 2 techniques is provided as well as recommendations as to when each might be used most effectively in early childhood assessment. (PsycINFO Database Record (c) 2012 APA, all rights reserved)
SN - 1184-0412
AD - Kelly-Vance, Lisa: U Nebraska, Psychology Dept, Omaha, NE, US
ER -

TY - JOUR
ID - 805
T1 - Persistent and chronic neglect in the context of poverty-When parents can't parent: Case of Ricky, age 3
A1 - Tonning,Louise M.
Y1 - 1999///
N1 - Book: 1999-04039-009
English
Book; Edited Book
KW - PsycInfo
KW - Child Abuse
KW - Child Neglect
KW - Play Therapy
KW - Poverty
KW - Violence
RP - NOT IN FILE
SP - 203
EP - 224
JF - Webb, Nancy Boyd [Ed]
VL - :
IS - 1999
N2 - (from the chapter) This chapter reports the case of a 3-yr-old African-American male born into chronic poverty who suffered abuse and neglect from birth, who witnessed the violent beatings of his half-brother, and whose mother was addicted to crack cocaine. Placed with his grandmother at 20 mo of age, he responded to the inconsistency of his attachment figures with resistance to contact. Treatment required the creation of a safe environment in which he could use play to work through his reactions to the violence he had witnessed and risk engaging in a new relationship, thereby developing a more versatile self. The modality used was child-centered play therapy. (PsycINFO Database Record (c) 2012 APA, all rights reserved)
AD - Tonning, Louise M.: Private Practice, Greenwich, CT, US
ER -

TY - JOUR
ID - 806
T1 - Why supportive relationships are essential
A1 - Pianta,Robert C.
Y1 - 1999///
N1 - Book: 1999-02145-001
English
Book; Authored Book
KW - PsycInfo
KW - Academic Achievement
KW - Childhood Development
KW - Classroom Environment
KW - Teacher Student Interaction

KW - At Risk Populations
KW - Psychosocial Development
KW - Risk Factors
KW - School Psychologists
RP - NOT IN FILE
SP - 7
EP - 23
JF - Pianta, Robert C
VL - :
IS - 1999
N2 - (from the book) Chapter 1 presents a conceptualization of the role that relationships can play for children, particularly for children who have the kinds of background experiences that tend to predict difficulty in school. For many children, relationships with adults are impoverished or conflictual and, in these cases, are a source of risk. Relationships with teachers are an essential part of classroom experience for all children and a potential resource for improving developmental outcomes. The concept of risk as a social process and the way in which a focus on child-teacher relationships can be used to reduce risk are introduced, as is the notion that the practice of psychology with children is closely tied to the management of contexts that provide resources for development. Schools are viewed as a central context in this respect. Psychologists working in school settings can play a major role in harnessing this potential resource for development. (PsycINFO Database Record (c) 2012 APA, all rights reserved)
AD - Pianta, Robert C.: U Virginia, Curry School of Education, Programs in Clinical & School Psychology, VA, US
ER -

TY - JOUR
ID - 807
T1 - The effects of modeling and experience on young children's persistence at a gambling game
A1 - Tremblay,George C.
Y1 - 1998///
N1 - Peer Reviewed Journal: 1999-05959-005
English
Journal; Peer Reviewed Journal
KW - PsycInfo
KW - Experience Level
KW - Gambling
KW - Observational Learning
KW - Persistence
RP - NOT IN FILE
SP - 193
EP - 210
JF - Journal of Gambling Studies
VL - 14:
IS - 2
N2 - Gambling is common among children and adolescents, but little is known about factors initiating or maintaining this behavior. 51 male and 51 female kindergarten and first grade children were invited to play a game involving repeated opportunities to select colored chips from a cup while blindfolded. Children playing for tangible incentives elected to play longer than those who were not. Seeing a videotaped model win or fail to win a large prize had no effect on persistence with the game. Playing again 1 wk later, children playing for incentives exhibited a more successful strategy, quitting sooner and with more winnings. The parameters of experiential vs observational learning are discussed, with implications for educating children about risk-taking. (PsycINFO Database Record (c) 2012 APA, all rights reserved) (journal abstract)
SN - 1050-5350
AD - Tremblay, George C.: U Missouri, Dept of Psychology, Columbia, MO, US
ER -

TY - JOUR

ID - 808
T1 - The effect of maternal depression in relation to dimensions of parenting in low income mothers
A1 - Albright,Martina Brandegee
Y1 - 1998///
N1 - Dissertation Abstract: 1998-95020-352
English
Dissertation Abstract
KW - PsycInfo
KW - Depression (Emotion)
KW - Lower Income Level
KW - Mother Child Relations
KW - Mothers
KW - Childhood Development
RP - NOT IN FILE
SP - 1909
JF - Dissertation Abstracts International: Section B: The Sciences and Engineering
VL - 59:
IS - 4-B
N2 - This study investigated the relationship between maternal depression and parenting dimensions in children's home environments in a sample of 53 poor White, African American and Latina mothers and their toddlers, 18-24 months of age. Mothers were divided into two groups, depressed and nondepressed using the CES-D depression inventory. Three aspects of the home environment that have been deemed important to childrens' cognitive and social development were the dependent measures: (1) Provision of Age Appropriate Play Materials, (2) Organization of the Home Environment, and (3) Quality of Mother-Child Interaction. The impact of certain risk factors, socioeconomic status, maternal IQ, and spouse support on the these three dimensions were also considered. Findings indicated that maternal depression is the most significant predictor of poor quality mother-child interaction while provision of play materials and organization of the home environment were found to be uninfluenced by depression. Depressed mothers were less sensitive, less engaged, more rigid, and less affectively warm than nondepressed mothers. Children of depressed mothers were less compliant, less affectively positive, less engaged and less gentle than children of nondepressed mothers. For depressed mothers and their children the quality of the mother-child interaction showed less mutual communication, less reciprocity, and less enjoyment than dyads with a nondepressed mother. The other three risk factors: socioeconomic status, maternal IQ and spouse support did not relate to any of the dimensions of the home environment except that more risk was detrimental to the quality of the mother-child interaction, given that one of the risks was depression. This study supports previous findings that maternal depression is a powerful predictor of problematic mother-child interaction, and that other dimensions of the home environment and specific risk factors were found to be unrelated to the mother-child interaction. Interventions targeted at maternal psychological well-being would create a more optimal mother-child interaction which would in turn enhance childrens' cognitive, social and emotional development. (PsycINFO Database Record (c) 2012 APA, all rights reserved)
SN - 0419-4217
AD - Albright, Martina Brandegee: New York U, US
ER -

TY - JOUR
ID - 809
T1 - Extracorporeal membrane oxygenation versus conventional treatment: Neurodevelopmental and social outcomes at 24 months
A1 - Landry,Susan H.
Y1 - 1998///
N1 - Peer Reviewed Journal: 1998-12900-004
English
Journal; Peer Reviewed Journal
KW - PsycInfo
KW - Artificial Respiration
KW - Childhood Development

KW - Neurology
KW - Respiratory Distress
KW - Social Skills
RP - NOT IN FILE
SP - 118
EP - 130
JF - Child Neuropsychology
VL - 4:
IS - 2
N2 - Compared the neurodevelopmental and social outcomes at 2 years of age for 25 children treated with extracorporeal membrane oxygenation (ECMO) to those of 16 children with severe respiratory illness treated with conventionally and to 29 matched, healthy full-term (FT) children. Mothers' parenting attitudes and children's mental, motor, language, exploratory play, and social competence skills were assessed. A retrospective chart review was also undertaken to further describe the groups with respect to the results of neuroimaging, audiologic, and neuromotor examinations. The FT group had significantly higher mental, motor, and language scores as compared to the ECMO and conventional groups. Groups did not differ on measures of behavior, social competence, exploratory play skills, or parental attitudes. Specifically for the ECMO group, there was a trend toward language being more problematic than other areas of development. In general, the data demonstrate that severe respiratory complications, regardless of type of treatment, place children at increased risk for neurodevelopmental difficulties. (PsycINFO Database Record (c) 2012 APA, all rights reserved)
SN - 0929-7049
AD - Landry, Susan H.: U Texas Medical School, Dept of Pediatrics, Houston, TX, US
ER -

TY - JOUR
ID - 810
T1 - The association of childhood play and adolescent-parent interactions with German adolescent leisure participation
A1 - Meschke,Laurie L.
Y1 - 1998///
N1 - Peer Reviewed Journal: 1998-12489-005
English
Journal; Peer Reviewed Journal
KW - PsycInfo
KW - Behavior
KW - Childhood Play Behavior
KW - Cross Cultural Differences
KW - Parent Child Relations
KW - Recreation
KW - Human Sex Differences
KW - Leisure Time
KW - Psychosexual Behavior
KW - Risk Taking
KW - Social Behavior
RP - NOT IN FILE
SP - 458
EP - 486
JF - Journal of Adolescent Research
VL - 13:
IS - 4
N2 - Macrosystem (nation and gender) and microsystem (childhood play and adolescent-parent interactions) correlates of adolescent leisure activities (risky, social-romantic, and creative-introspective) were investigated by means of questionnaires. The respondents included 221 adolescents from the former East Germany and 443 from West Germany aged 15-18 yrs. The leisure activities were each significantly related to both macro- and microsystem measures. Higher levels of risky leisure were related to West Germany residency, higher levels of childhood competitive play and creative activities, and lower levels of monitoring. Higher participation in

social-romantic leisure was related to being female and reporting higher levels of childhood competitive play. Higher levels of creative-introspective leisure were associated with being female, and reporting lower levels of childhood competitive play, higher childhood participation in cultural education and creative activities, and higher levels of shared interests with parents. Some topics for further research in the area of adolescent leisure are provided. (PsycINFO Database Record (c) 2012 APA, all rights reserved)
SN - 0743-5584
AD - Meschke, Laurie L.: Ohio State U, Human Development & Family Science, Columbus, OH, US
ER -

TY - JOUR
ID - 811
T1 - Cognitive-behavioral treatment of depression, Part IX: Confronting predisposing factors
A1 - Overholser,James C.
Y1 - 1998///
N1 - Peer Reviewed Journal: 1998-12383-004
English
Journal; Peer Reviewed Journal
KW - PsycInfo
KW - At Risk Populations
KW - Cognitive Therapy
KW - Major Depression
KW - Predisposition
KW - Biological Markers
KW - Psychosocial Factors
KW - Sociocultural Factors
RP - NOT IN FILE
SP - 289
EP - 305
JF - Journal of Contemporary Psychotherapy
VL - 28:
IS - 3
N2 - This article discusses the biological, psychological, and social/cultural factors that may predispose a person toward depressive episodes, and the implications for cognitive-behavioral treatment of depression. In order to address possible biological predispositions, it is important to assess for a positive family history of depression, evaluate family members' response to previous treatments, and refer for medications when needed. In order to address possible psychological predispositions, it is useful to evaluate long-standing personality styles, identify negative events from childhood, examine the client's relationship with his or her parents, evaluate the history of abuse, and identify early loss experiences. Cultural factors may play an influential role in the etiology of depression, and can be useful to incorporate into a broad treatment plan. However, cultural factors are difficult to modify through individual psychotherapy. Hence, the author states that they are unlikely to play a central role in cognitive-behavioral therapy for depression. It is concluded that therapists working with depressed clients should be prepared to confront a broad range of biological, psychological, and environmental factors that can create or perpetuate a client's risk for depression. (PsycINFO Database Record (c) 2012 APA, all rights reserved)
SN - 0022-0116
AD - Overholser, James C.: Case Western Reserve U, Dept of Psychology, Cleveland, OH, US
ER -

TY - JOUR
ID - 812
T1 - Risk factors for infant attrition and low engagement in experiments and free-play
A1 - Oates,John
Y1 - 1998///
N1 - Peer Reviewed Journal: 1998-11927-002
English
Journal; Peer Reviewed Journal

KW - PsycInfo
KW - Childhood Play Behavior
KW - Cognitive Processes
KW - Experimental Attrition
KW - Risk Assessment
RP - NOT IN FILE
SP - 555
EP - 569
JF - Infant Behavior & Development
JA - Infant behav
VL - 21:
IS - 4
N2 - Forty-three 2-month-old infants participated with their mothers in a laboratory study of factors associated with variations in infants' attrition and levels of attention/engagement in a cognitive-perceptual experiment, and in free-play with their mothers. Increased risk of attrition from the experiment was strongly associated with analgesic medication having been administered during labor and with more restricted maternal perceptions of infants' capacities. Attrition rates and levels of engagement varied independently, showing different patterns of association with explanatory variables. Infants' levels of engagement in the experiment and in free-play were correlated across settings and low levels were associated with a lack of maternal identification with the fetus during early pregnancy and more limited maternal perceptions of their infants' capacities, but were not associated with analgesic medication during labor. (PsycINFO Database Record (c) 2012 APA, all rights reserved) (journal abstract)
SN - 0163-6383
AD - Oates, John: Open U, School of Education, Ctr for Human Development & Learning, Milton Keynes, England
ER -

TY - JOUR
ID - 814
T1 - Arousal modulation in cocaine-exposed infants. [References]
A1 - Bendersky,Margaret
Y1 - 1998///
N1 - Peer Reviewed Journal: 1998-01885-015
English
Journal; Peer Reviewed Journal
KW - PsycInfo
KW - Cocaine
KW - Emotional Adjustment
KW - Mother Child Relations
KW - Prenatal Exposure
KW - Stress Reactions
KW - Social Interaction
RP - NOT IN FILE
SP - 555
EP - 564
JF - Developmental Psychology
JA - Dev.Psychol.
VL - 34:
IS - 3
N2 - The ability to modulate arousal is a critical skill with wide-ranging implications for development. In this study, the authors examined arousal regulation as a function of levels of prenatal cocaine exposure in 107 infants at 4 months of age using a "still-face" procedure. Facial expressions were coded. A greater percentage of heavily cocaine-exposed infants, compared with those who were unexposed to cocaine, showed less enjoyment during en face play with their mothers and continued to show negative expressions during the resumption of play following a period when the interaction was interrupted. This finding was independent of other substance exposure, neonatal medical condition, environmental risk, maternal contingent responsivity, and concurrent

maternal sensitivity and vocalizations. (PsycINFO Database Record (c) 2012 APA, all rights reserved) (journal abstract)
SN - 0012-1649
AD - Bendersky, Margaret: U Medicine & Dentistry of New Jersey-Robert Wood Johnson Medical School, Inst for the Study of Child Development, New Brunswick, NJ, US
ER -

TY - JOUR
ID - 815
T1 - The relation of change in maternal interactive styles to the developing social competence of full-term and preterm children
A1 - Landry,Susan H.
Y1 - 1998///
N1 - Peer Reviewed Journal: 1998-00500-010
English
Journal; Peer Reviewed Journal
KW - PsycInfo
KW - At Risk Populations
KW - Birth Weight
KW - Mother Child Relations
KW - Psychosocial Development
KW - Social Skills
KW - Childhood Development
KW - Longitudinal Studies
KW - Mothers
RP - NOT IN FILE
SP - 105
EP - 123
JF - Child Development
JA - Child Dev.
VL - 69:
IS - 1
N2 - Evaluated the changing nature of mothers' interactive behaviors to understand alterations in children's social development across 6, 12, 24, and 40 mo of age. Social skills were observed during daily activities and toy play in the home for 73 medically high risk and 114 low risk very low birthweight (VLBW) preterm and 112 full-term (FT) children. Variations in mothers' responses to children's changing capabilities predicted rates of change in children's social skills. For example, mothers who showed higher levels of maintaining measured across 6 to 40 mo had children who displayed greater increases in initiating, but this was more apparent in daily activities than toy play and for the VLBW children compared to the FT children. Those VLBW children at the highest degrees of biological risk displayed faster gains in initiating than the other groups when their mothers provided even greater levels of support. Results demonstrate the importance of using methodologies that test more complex models of growth when evaluating parent-child relations. (PsycINFO Database Record (c) 2012 APA, all rights reserved)
SN - 0009-3920
AD - Landry, Susan H.: U Texas Medical School, Dept of Pediatrics, Div of Developmental Pediatrics, Houston, TX, US
ER -

TY - JOUR
ID - 816
T1 - Applying the BeSafe method to product safety evaluation
A1 - Benedyk,Rachel
Y1 - 1998///
N1 - Peer Reviewed Journal: 1997-42652-001
English
Journal; Peer Reviewed Journal

KW - PsycInfo
KW - Human Factors Engineering
KW - Playgrounds
KW - Risk Management
KW - Safety
KW - Risk Assessment
KW - Consumer Behavior
KW - Evaluation
RP - NOT IN FILE
SP - 5
EP - 13
JF - Applied Ergonomics
VL - 29:
IS - 1
N2 - The BeSafe (Behavioural Safety) Method, has been developed by International Mining Consultants Ltd for assessing and reducing risk in industrial contexts. BeSafe is an ergonomic method of risk analysis and reduction, targeted at prevention of accidents due to human errors and violations, and emphasizing management strategies for action. Using the BeSafe Method, it may be possible to structure safety assessments of the use of products and target improvement measures. The authors tested the relevant parts of the method on children's play equipment in 24 playgrounds, and questionnaires were also administered to 348 7-11 yr olds and 15 adults. Useful results were obtained, suggesting that BeSafe could be adapted for other consumer products in a similar way. (PsycINFO Database Record (c) 2012 APA, all rights reserved)
SN - 0003-6870
AD - Benedyk, Rachel: U London, University Coll, Ergonomics & HCI Unit, London, England
ER -

TY - JOUR
ID - 817
T1 - The influence of peers on children's judgments about engaging in behaviors that threaten their safety
A1 - Christensen,Sally
Y1 - 1997///
N1 - Peer Reviewed Journal: 1999-01232-008
English
Journal; Peer Reviewed Journal
KW - PsycInfo
KW - Child Attitudes
KW - Choice Behavior
KW - Peer Pressure
KW - Risk Taking
KW - Safety
KW - Judgment
RP - NOT IN FILE
SP - 547
EP - 562
JF - Journal of Applied Developmental Psychology
VL - 18:
IS - 4
N2 - Examined peer influences on children's judgments about engaging in behaviors that threaten their physical safety. 77 Ss (aged 8.2-9.1 yrs) were shown pictures depicting play situations which involved different paths of travel, each of which posed different degrees of injury risk. Ss were asked to select the path they would take, and to assign a danger rating to reflect their beliefs about the likelihood of injury. A peer-influence session followed, in which a same-sex friend attempted to persuade the S to take another path. Following exposure to the friend's arguments, Ss made their final decision about a path of travel in each play situation. A number of other measures were used to determine factors that influence Ss' risk-taking decisions. Results revealed that friends were successful in their persuasion efforts: for 2 of the 3 play situations, a significant number of Ss who initially selected low-risk paths switched to more risky paths. Appraisal of danger related to initial path

decisions, but number of hazards identified and injury history did not significantly relate to initial decisions. The implications of these results for the understanding of injury-outcome processes are discussed. (PsycINFO Database Record (c) 2012 APA, all rights reserved)
SN - 0193-3973
AD - Christensen, Sally: U Guelph, Guelph, ON, Canada
ER -

TY - JOUR
ID - 818
T1 - Symbolic talk in preschoolers who have had infant heart surgery
A1 - Ovadia,Rebecca
Y1 - 1997///
N1 - Dissertation Abstract: 1997-95021-250
English
Dissertation Abstract
KW - PsycInfo
KW - Heart Surgery
KW - Infant Development
KW - Language Development
KW - Parent Child Communication
RP - NOT IN FILE
SP - 1586
JF - Dissertation Abstracts International Section A: Humanities and Social Sciences
VL - 58:
IS - 5-A
N2 - Most infants who are born with heart defects can be cured by surgical treatment, but may suffer learning difficulties, which are often identified only when they enter school. Research suggests that these potential adverse developmental outcomes are related to brain damage incurred during infant cardiac surgery. Currently, however, studies reveal conflicting results regarding the range of cognitive, motor, and language delays in children who have had early corrective heart surgery (ECHS). Often, these learning difficulties are subtle and not easily identified using standard cognitive tests. Nevertheless, given the prevalence of congenital heart disease and the wide range of developmental outcomes in children with ECHS, clinicians need tools for the early identification of children at risk for later academic difficulties. While language abilities in the preschool period may be especially vulnerable in ECHS children, there is presently little research on language outcomes in these children. Standard language measures appear to overlook potential areas of difficulty; however, tasks examining extended discourse competence seem to identify subtle but significant language difficulties. For example, a few studies suggest that ECHS children produce fewer narrative events, evaluations, story play episodes, and overall, less symbolic play talk than a normative group. In this project, the nature of these language difficulties is investigated during parent-child pretend play in four year old children with ECHS. This project is part of a larger research project at Children's Hospital in Boston, whose primary objective is to compare developmental sequelae of two surgical support techniques, total circulatory arrest (TCA) and low-flow bypass (LFB). Results show that ECHS children use predominantly early acquired verbal strategies to participate in pretend play with parents, including focus on concrete talk, production of few, mostly fragmented story episodes, and reliance on parents to initiate or maintain symbolic talk. These participation styles suggest difficulty with discourse skills. Children who had the TCA support technique showed worse status on symbolic talk outcomes than children who had LFB, and children who had early postoperative seizures showed worse performance than children who had no seizures. (PsycINFO Database Record (c) 2012 APA, all rights reserved)
SN - 0419-4209
AD - Ovadia, Rebecca: Harvard U, US
ER -

TY - JOUR
ID - 819
T1 - Infant space, infant time: Development of infant interactions with the physical environment, from 1 to 18 months
A1 - Pierce,Doris Emma

Y1 - 1997///
N1 - Dissertation Abstract: 1997-95006-181
English
Dissertation Abstract
KW - PsycInfo
KW - Childhood Play Development
KW - Cognitive Development
KW - Infant Development
KW - Motor Development
KW - Spatial Learning
RP - NOT IN FILE
SP - 5949
JF - Dissertation Abstracts International: Section B: The Sciences and Engineering
VL - 57:
IS - 9-B
N2 - This study describes the typical development of independent infant negotiations of the spatial and temporal dimensions of the physical environment of the home. The study's design was qualitative and longitudinal, using videotapes and maternal interviews from a cross-class sample of 18 typically-developing Caucasian infants followed from ages 1 to 18 months of age. The focus of data collection was self-directed play with the objects and spaces of the home. At the broadest level, this theory highlights three central characteristics of human occupation: instrumentality, intentionality, and the interactive dynamic between individual with context. Description of the emergence of the infant's capacity to instrumentally negotiate and manipulate the space and objects of the physical environment included ranging patterns, activity maps, the motor lens, the role of gaze and visual play, and a variety of types of stationary and mobile object interactions, including previously undescribed types of play such as dancing, climbing, propelling, driving, carries, and ferries. The infant's increasing intentionality was seen in developmental changes in the temporality of infant object play across three existing sequence lengths: the infant object interaction, phases within that interaction, and series of infant object interactions. Viewing this interaction across development revealed the lengthening of interactions, strengthening of the infant's ability to conceive and complete an action over time, and the extension of the infant's temporal horizons into past and future. Contextual influences on the infant's play in the home were described within sociocultural, spatial, temporal, and physiological domains. This occupational science study provides a developmental description of a set of human occupations in their simplest infant forms. As such, it contributes to the understanding of occupation, as well as promising potential to support the play-based interventions of occupational therapists working with children at risk for developmental delays. (PsycINFO Database Record (c) 2012 APA, all rights reserved)
SN - 0419-4217
AD - Pierce, Doris Emma: U Southern California, US
ER -

TY - JOUR
ID - 820
T1 - A prospective study of prenatal cocaine exposure: Language, play, and global cognitive abilities in 2-year-olds
A1 - Toth-Sadjadi,Susan Katalin
Y1 - 1997///
N1 - Dissertation Abstract: 1997-95006-113
English
Dissertation Abstract
KW - PsycInfo
KW - Childhood Play Development
KW - Cocaine
KW - Cognitive Ability
KW - Language Development
KW - Prenatal Exposure
KW - Cognitive Development
KW - Drug Dosages

RP - NOT IN FILE
SP - 5951
JF - Dissertation Abstracts International: Section B: The Sciences and Engineering
VL - 57:
IS - 9-B
N2 - The relationship between prenatal cocaine exposure and 2-year developmental outcome was examined in a sample of 200, 2-year-old children followed prospectively from birth. Adverse cocaine effects were hypothesized to emerge in highly-exposed children, rather than the whole group, and in the specific developmental abilities of language and play, rather than global cognitive skills. Cocaine-exposed toddlers (n = 100) were successfully group-matched with non-cocaine-exposed toddlers (n = 100) on selected maternal demographics, child characteristics, and other prenatal drug exposures. Woman in both groups completed 12 years of education, were in their mid-20's, primarily white, and similar on marijuana use and binge alcohol use during pregnancy (but greater parity and cigarette use for cocaine-using women). Prenatal cocaine use was gathered via self report on the day after delivery and later verified by maternal hair analysis. Productive child language was obtained from caregiver report and videotaped, caregiver-child interaction. Quality of play behaviors were coded from videotaped, child-alone play. Global cognitive skills were assessed with the Bayley. Total grams of cocaine used throughout pregnancy was the predictor in dose-response regression analyses, while group differences were compared with t-tests. Confounds not dealt with by study design (e.g., postnatal environment) were adjusted in statistical analyses. Neither dose-response nor group difference analyses revealed independent, adverse effects of prenatal cocaine exposure. For both groups, productive language was within normal age limits, global cognitive skills were in the low-average range, and the quality of most play behaviors was age- and context-appropriate. However, subtle signs of immature play behaviors (e.g., unelaborated play) were exhibited by both groups and a subset of children in both groups exhibited general cognitive delay. Findings suggest that early language, play, and general cognitive abilities are not adversely affected by cocaine, even for the most highly-cocaine-exposed children, but may be more related to moderating postnatal environmental factors. Results confirm the need to examine developmental outcome in terms of the complex interrelationships between cocaine exposure and the risk and protective factors in the postnatal environment. (PsycINFO Database Record (c) 2012 APA, all rights reserved)
SN - 0419-4217
AD - Toth-Sadjadi, Susan Katalin: U Washington, US
ER -

TY - JOUR
ID - 821
T1 - Pretense in pair play therapy: Examining the understanding of emotions in young at-risk children
A1 - Raya,Pamela Arboleda
Y1 - 1997///
N1 - Dissertation Abstract: 1997-95002-287
English
Dissertation Abstract
KW - PsycInfo
KW - At Risk Populations
KW - Comprehension
KW - Emotions
KW - Play Therapy
KW - Childhood Play Behavior
RP - NOT IN FILE
SP - 4751
JF - Dissertation Abstracts International: Section B: The Sciences and Engineering
VL - 57:
IS - 7-B
N2 - Young children who experience tremendous stresses in their lives tend to be at risk for poor interpersonal relationships. Research has shown that emotional understanding facilitates satisfactory social interactions. In this study, twelve young at-risk children's understanding of emotions is considered. All the children participated in a relational prevention-intervention program called Pair Play Therapy (PPT). To undertake this investigation, a system for examining how young children understand emotions through their pretend play, named the

Emotions in Pretense Coding Scheme (EPS), was developed first and its reliability established. Secondly, EPS was used to document the twelve children's pretend play over time and their elaborations on the Nice and Mean Interaction Scales stories. The pretense performed by these children was coded for (a) affective play themes, (b) emotions and their respective intensities, (c) the complexity of the emotion representations, and (d) the congruence between themes, emotions, and actual affective display. By compiling and comparing frequency differences between positive themes and negative themes, and frequency differences between positive emotions and negative emotions for each child, I identified three distinct groups of children. Analyses of the differences in representation preferences among the three groups were statistically significant (alpha =.05). Group 1 pretend play performances clearly showed a high predominance of negative themes and emotions, especially anger. Group 2 children showed an inclination towards fairly to moderately intense and frequent depictions of negative themes and emotions (except for more intense portrayals of fear compared to other emotions) in their play. Group 3 children tended to portray positive themes and positive emotions more often in their play. In the Nice and Mean Scales, the children, especially Group 1, tended to transform positive interaction stories into negative ones. In addition, the (PsycINFO Database Record (c) 2012 APA, all rights reserved)
SN - 0419-4217
AD - Raya, Pamela Arboleda: Harvard U, US
ER -

TY - JOUR
ID - 822
T1 - 101 favorite play therapy techniques
A1 - Kaduson,Heidi G.
Y1 - 1997///
N1 - Book: 1997-36793-000
English
Book; Edited Book
(Created by PsycINFO) Preface Section one: Fantasy techniques * The playing baby game / Charles E. Schaefer * Using guided imagery to augment the play therapy process / Kevin O'Connor * The rosebush / Violet Oaklander * Pretending to know how / S. Eileen Theiss Section two: Storytelling techniques * The feeling word game / Heidi Gerard Kaduson * The card story game / Norma Y. Leben * Storytelling with felts / Linda Mattingly * The box of buttons technique / Jo Ann L. Cook * Computer storytelling / N. E. Brewer * Using metaphors, fairy tales and storytelling in psychotherapy with children / Jamshid A. Marvasti * Art or verbal metaphors for children experiencing loss / Glenda F. Short * The guess my word story game / Mary Repp * The scarf story / Steve Harvey * Storytelling with objects / Jackie K. Frederiksen Section three: Expressive arts techniques * The before and after drawing technique / Donna Cangelosi * Feeling balloons / Glenda F. Short * Magic art / Ruby Walker * The yarn drawing game / Norma Y. Leben * Clayscapes / Lynn B. Hadley * Bad dreams / L. G. Agre * The personality pie / Tara M. Sinclair * Life maps / Glenda F. Short * Play art / Lynn B. Hadley * Gloop: Treating sensory deprivation / Neil Cabe * The clay squiggle technique / Richard Frankel * Expressive arts playdough / Lynn B. Hadley * Inner-reference / Aimee H. Short * Reworking / Sheri Saxe * A line down the middle of the page / Dolores M. Conyers * Create-a-community / Nancy H. Cochran * Outline drawings of boys and girls / Barbara A. Turner * The picture drawing game / Stanley Kissel * The color-emotive brain / Sheldon Berger and Jonna L. Tyler * Scribble art / Leslie Hartley Lowe * The feelings tree / Joyce Meagher * Design-a-Dad / Stazan K. Sina * Synthetic clay in play therapy / Martha D. Young Section four: Game play techniques * Checkers: Rules or no rules / L. G. Agre * Beat the clock / Heidi Gerard Kaduson * Pounding away bad feelings / Donna Cangelosi * The pick-up-sticks game / Barbara McDowell * The stealing game / Steve Harvey * Consequences: Reaching the oppositional defiant adolescent / Neil Cabe * Hide-and-seek in play therapy / John Allan and Mary Anne Pare * The spy and the sneak / Bria Bartlett-Simpson * Pool play: Helping children get out from behind the eight ball / Stanley Kissel * Tumbling feelings: Easing children into the counseling relationship / Christina Mattise * Make your own board game / Sandy Carter * Chess playing as a metaphor for life choices / Leslie Hartley Lowe Section five: Puppet play techniques * Battaro and the puppet house / Martha J. Harkin * The dowel finger puppet technique / Jo Ann L. Cook * Create-a-puppet / Anne Blackwell * Using a puppet to create a symbolic client / Carolyn J. Narcavage * Puppetry / Marie Boultinghouse * On the one handand then on the other / Christina Mattise Section Six: Play toys and objects techniques * Bodysox / Kimberly Dye * The me doll / Jessica Stone-Phennicie * Tearing paper / Kathy Daves * The mad game / Patricia Davidson * Sculpt-it / Michael Cascio * The magic carpet technique / Dolores M. Conyers * The snake / Alan Lobaugh * The baby bottle technique / Diane Murray * The T-shirt technique /

Nancy H. Cochran * The photo album technique / Sueanne Brown * The angry tower / Sheri Saxe * Balloons of anger / Tammy Horn * The worry can technique / Debbie S. Jones * The cardboard city / Berrell Mallery and Randall Martin * Fortune tellers / Judith Friedman Babcock * Jenga and a camera / Catherine G. Tierney * The anger shield / Teresa A. Glatthorn * Using self-made books to prepare children for predictable trauma or crisis / Kevin O'Connor * The angry feeling scale game / Joyce Meagher * Stomping feet and bubble popping / Cathy Wunderlich * Knocking down the walls of anger / Jennifer Leonetti * Figures / Sylvia Fisher Section Seven: Group play techniques * Elastablast / Kimberly Dye * Group building activity / Glenda F. Short * The captains of Avatar: A space adventure for children in transition / Tara M. Sinclair * Mr. Ugly / Mary May Schmidt * Therapeutic puppet group / Aileen Cunliffe * Group puppet show / Glenda F. Short * Mutual storytelling through puppet play in group play therapy / Mitch Jacobs Section eight: Other techniques * Using drawings of early recollections to facilitate life style analysis for children in play therapy / Harold M. Heidt * Self figures for sand tray / L. Jean Ley and Jean Howze * The worry list / Richard Sloves * My baby book / Karen Pitzen * The song flute or recorder / Robert W. Freeman * The starting over wedding gown ceremony / Patricia B. Grigoryev * Play therapy and pets / Mary-Lynn Harrison * The twelve-to-one technique / Mary May Schmidt * The therapist on the inside / Patricia B. Grigoryev * Play-by-play / David A. Snyder * The time line tape technique / Jo Ann L. Cook * Terminations utilizing metaphor / Teresa A. Glatthorn * The feelings center / Helen E. Benedict * The disposable camera technique / Jo Ann L. Cook * Use of animals in a play therapy setting / S. Eileen Theiss * The popcorn walk / Allan Gonsher * Co-play therapy / Ann C. Levinger
KW - PsycInfo
KW - Mental Disorders
KW - Play Therapy
KW - Psychotherapeutic Techniques
RP - NOT IN FILE
SP - Lanham, MD, US
JF - (1997)
VL - 101 favorite play therapy techniques. xvi, 401 pp. Lanham, MD, US: Jason Aronson; US.
N2 - (from the jacket) Building on children's natural inclinations to pretend and re-enact, play therapy is widely used in the treatment of psychological problems in childhood. This book incorporates methods developed to elicit the best responses from children by therapists representing cognitive-behavioral, psychodynamic, and other orientations, and selected for their practicality, specificity, and originality. Arranged for easy reference, each bearing a succinct description and targeted application, the interventions illustrated-including fantasy, storytelling, expressive arts, game play, puppet play, play toys and objects, and group play-have been used with success to address such common problems as low self-esteem and unresolved fear and anger as well as more serious difficulties arising from loss, abuse, and sexual trauma. (PsycINFO Database Record (c) 2012 APA, all rights reserved)
ER -

TY - JOUR
ID - 824
T1 - Family play therapy using a resiliency model. [References]
A1 - Seymour,John W.
Y1 - 1996///
N1 - Peer Reviewed Journal: 1997-05135-002
English
Journal; Peer Reviewed Journal
KW - PsycInfo
KW - Family Therapy
KW - Play Therapy
KW - Resilience (Psychological)
KW - Therapeutic Processes
KW - Treatment Planning
KW - Life Experiences
KW - Models
RP - NOT IN FILE
SP - 19
EP - 30

JF - International Journal of Play Therapy
VL - 5:
IS - 1
N2 - Children are at risk for current and future problems when they are faced with multiple life transitions. Their families are also significantly effected during these times of change and crisis. The authors describe a family play therapy approach for treating these children at risk, using M. Rutter's resiliency model as a framework for conceptualizing treatment planning and the therapeutic process. (PsycINFO Database Record (c) 2012 APA, all rights reserved)
SN - 1555-6824
AD - Seymour, John W.: East Texas State U, TX, US
ER -

TY - JOUR
ID - 825
T1 - Affect and behavior in the triadic play of parents and sons with attention deficit/hyperactivity disorder and normal comparison families: Effects of stimulant medication and predictions to peer status
A1 - Leddick,Katherine Howard
Y1 - 1996///
N1 - Dissertation Abstract: 1996-95009-267
English
Dissertation Abstract
KW - PsycInfo
KW - Attention Deficit Disorder
KW - Hyperkinesis
KW - Parent Child Relations
KW - Peer Relations
KW - Social Behavior
KW - CNS Affecting Drugs
KW - Emotional States
KW - Father Child Relations
KW - Interpersonal Interaction
KW - Mother Child Relations
RP - NOT IN FILE
SP - 6397
JF - Dissertation Abstracts International: Section B: The Sciences and Engineering
VL - 56:
IS - 11-B
N2 - Poor social adjustment in childhood is associated with negative outcomes in adolescence and adulthood ranging from continuing difficulties in social relationships to problems in occupational performance, arrests, and psychiatric status. For children with attention-deficit hyperactivity disorder (ADHD), difficulties in social relations are among the most salient aspects of their psychopathology. ADHD children engage in more negative interactions with their mothers, fathers, and peers and are more frequently rejected by peers. Little is known, however, about the playful interactions of ADHD boys with both parents or about family-peer linkages in this group of youngsters at risk. Past research indicates that family relationships, perhaps relationships with fathers in particular, may be important in understanding the course of disorder in ADHD boys. Some research also indicates that qualitative differences in parent-son relationships emerge when all three people are present. This study examines the affect and behavior of each family member in triadic, playful family interactions and children's peer status. Families (N = 70) are mothers, fathers, and boys who participated in one of two summer enrichment programs for ADHD and undiagnosed comparison boys between the ages of 6 and 12 years. Families of ADHD boys played twice on different days, and comparison families played once. Before the interactions, ADHD sons were administered stimulant medication (.3 mg/kg) in a counterbalanced, placebo-controlled, double-blind procedure. Few diagnostic group differences or medication effects emerged in family members' affect and interactive behavior in the playful context. However, strong associations were obtained between family measures and boys' peer status at the end of the 5-week summer programs. Associations were found between family measures and boys' peer acceptance, peer rejection, and social preference. Moreover,

diagnostic group differences and age effects emerged in t (PsycINFO Database Record (c) 2012 APA, all rights reserved)
SN - 0419-4217
AD - Leddick, Katherine Howard: U California, Los Angeles, US
ER -

TY - JOUR
ID - 826
T1 - The impact of low-risk prematurity on maternal behaviour and toddler outcomes
A1 - Barratt,Marguerite Stevenson; Roach,Mary
Y1 - 1996///
N1 - Peer Reviewed Journal: 1996-06905-007
English
Journal; Peer Reviewed Journal
KW - PsycInfo
KW - Infant Development
KW - Mother Child Communication
KW - Mother Child Relations
KW - Mothers
KW - Premature Birth
KW - Followup Studies
RP - NOT IN FILE
SP - 581
EP - 602
JF - International Journal of Behavioral Development
VL - 19:
IS - 3
N2 - Examined the impact of prematurity on patterns of maternal responsiveness, styles of maternal interaction, and toddlers' developmental outcomes. Mother-toddler free-play interaction and toddler developmental assessments were conducted at 12 and 20 mo of gestationally corrected age for 21 low-medical-risk preterm Ss and 21 full term Ss matched on the Social Status Index (A. B. Hollingshead, 1978). While maternal vocal and attentional responsiveness did not differ at 12 mo, mothers of full term Ss were more vocally responsive at 20 mo. Mothers of preterm Ss exhibited more control behavior when Ss were 12 and 20 mo old that did mothers of fullterm Ss. No differences in the cognitive and language performance of both groups at the ages of 12 and 20 mo were observed. In terms of play skills, the preterm Ss were more actively involved than the full term Ss. Despite the successful adjustment of these low-risk preterm Ss, maternal behavior was affected by the circumstances of preterm birth, even after 20 mo. (PsycINFO Database Record (c) 2012 APA, all rights reserved)
SN - 0165-0254
AD - Barratt, Marguerite Stevenson: U Wisconsin, Madison, WI, US
ER -

TY - JOUR
ID - 827
T1 - A play-focused intervention involving mothers of preschoolers
A1 - Esdaile,Susan A.
Y1 - 1996///
N1 - Peer Reviewed Journal: 1996-02401-005
English
Journal; Peer Reviewed Journal
KW - PsycInfo
KW - Childhood Development
KW - Childhood Play Behavior
KW - Mother Child Relations
KW - Occupational Therapy
KW - Stress

KW - Followup Studies
KW - Mothers
RP - NOT IN FILE
SP - 113
EP - 123
JA - American Journal of Occupational Therapy
VL - 50:
IS - 2
N2 - Studied the effectiveness of an occupational therapy (OT) intervention program for 35 mothers (mean age 28.4 yrs) of preschool children to enhance their interactions through play, reduce mothers' perceived stress, and increase their knowledge about child development. Ss were assigned either to a control group or to 2 intervention groups. In the interventions, Ss engaged in toy making and demonstrations, and were given information on play development and children's behavior. Maternal causal attributions for parent-child interaction outcomes, stress, self concept as educator, and children's behavior before, after, and at 18 mo followup were assessed. The program was rated positively by the mothers but did not result in major group differences. However, there was an increase in the self-concept of some mothers as educators of their children. OT can provide effective prevention programs for mothers and children at risk. (PsycINFO Database Record (c) 2012 APA, all rights reserved)
SN - 0272-9490
AD - Esdaile, Susan A.: Wayne State U, Coll of Pharmacy & Allied Health Professions, Dept of Occupational Therapy, Detroit, MI, US
ER -

TY - JOUR
ID - 829
T1 - The effects of prematurity and other perinatal factors on infants' mastery motivation
A1 - Harmon,Robert J.
Y1 - 1995///
N1 - Book: 1996-97609-009
English
Book; Edited Book
KW - PsycInfo
KW - Achievement Motivation
KW - Premature Birth
KW - Cognitive Development
KW - Literature Review
KW - Measurement
KW - Perinatal Period
RP - NOT IN FILE
SP - 237
EP - 256
JF - MacTurk, Robert H [Ed]; Morgan, George A [Ed]
VL - :
IS - 1995
N2 - (from the chapter) [reviews] recent studies of cognitive development and mastery motivation of perinatal risk infants, particularly premature infants review of cognitive development studies / review of studies of object play / review of mastery motivation studies / free play assessment / structured tasks assessment / adult report questionnaire (PsycINFO Database Record (c) 2012 APA, all rights reserved)
AD - Harmon, Robert J.: U Colorado, School of Medicine, Denver, CO, US
ER -

TY - JOUR
ID - 830
T1 - Learning to pretend: Preschoolers' use of meta-communication in sociodramatic play
A1 - Halliday-Scher,Kathy; Urberg,Kathryn
Y1 - 1995///

N1 - Peer Reviewed Journal: 1996-16930-001
English
Journal; Peer Reviewed Journal
KW - PsycInfo
KW - Age Differences
KW - Childhood Play Behavior
KW - Imagination
KW - Metacognition
KW - Verbal Communication
RP - NOT IN FILE
SP - 451
EP - 461
JF - International Journal of Behavioral Development
VL - 18:
IS - 3
N2 - Investigated the relation of children's differential use of verbal metacommunication to play theme, player's age, and episode duration, by observing the naturally occurring sociodramatic play of 30 preschoolers (aged 29-68 mo) in a university day care facility. Children's use of within-frame and out-of-frame metacommunication was unrelated to the duration of sociodramatic play. However, children's use of out-of-frame metacommunication was related to age. Older children used a greater percentage of out-of-frame metacommunication than did younger children. Furthermore, although there was no main effect of theme, there was an interaction between age and theme. Older children used less within-frame metacommunication than did younger children during adventure/fantastic episodes, but used an equivalent proportion during domestic/occupational episodes. (PsycINFO Database Record (c) 2012 APA, all rights reserved)
SN - 0165-0254
AD - Halliday-Scher, Kathy: Wayne State U, Dept of Psychology, Detroit, MI, US
ER -

TY - JOUR
ID - 831
T1 - Focused play therapy and non-directive play therapy: Can they be integrated?
A1 - Rasmussen,Lucinda A.
Y1 - 1995///
N1 - Peer Reviewed Journal: 1996-05499-001
English
Journal; Peer Reviewed Journal
KW - PsycInfo
KW - Child Abuse
KW - Play Therapy
KW - Sexual Abuse
KW - Victimization
RP - NOT IN FILE
SP - 1
EP - 20
JF - Journal of Child Sexual Abuse: Research, Treatment, & Program Innovations for Victims, Survivors, & Offenders
VL - 4:
IS - 1
N2 - Reviews the rationale for both nondirective and focused approaches to play therapy. Nondirective techniques can have some disadvantages when used with abused children. Being totally nondirective can place the child at risk for additional abuse or further inappropriate behaviors. Abuse-reactive children are unlikely to deal with the issues underlying their inappropriate sexual behaviors unless therapists confront these issues directly. A prescriptive approach is proposed that combines the rapport building component of nondirective play therapy with focused techniques (i.e., cognitive-behavioral therapy, metaphors, bibliotherapy, and art therapy). This integrated approach can meet the therapeutic needs of sexually abused and abuse-reactive children. (PsycINFO Database Record (c) 2012 APA, all rights reserved)

SN - 1053-8712
AD - Rasmussen, Lucinda A.: Primary Children's Medical Ctr, Child Protection Team, Salt Lake City, UT, US
ER -

TY - JOUR
ID - 832
T1 - The effect of maternal psychiatric disorder on responsivity in the mother-child interaction and on the development of children's coping behavior
A1 - Hughes,Susan Maureen
Y1 - 1995///
N1 - Dissertation Abstract: 1995-95015-002
English
Dissertation Abstract
KW - PsycInfo
KW - Coping Behavior
KW - Infant Development
KW - Mental Disorders
KW - Mother Child Relations
KW - Mothers
RP - NOT IN FILE
SP - 1127
JF - Dissertation Abstracts International: Section B: The Sciences and Engineering
VL - 56:
IS - 2-B
N2 - Since the turn of the century, there has been a general awareness that children raised by parents with psychiatric disorders remain at risk for psychopathology themselves. This research investigates the mother-child responsivity and coping behavior of high risk children. Two groups of mother-infant dyads (N = 60) (mothers with and without psychiatric disorders) were coded during a videotaped, semi-structured play interaction using the CARE-Index. Results indicated that mothers with psychiatric disorders were significantly less sensitive and more unresponsive with their children than normal mothers. Mothers with psychiatric disorders who had extreme levels of psychosocial stress were significantly less sensitive and more controlling than those mothers diagnosed as living under less stressful circumstances. Children of mothers with psychiatric disorders were significantly less cooperative with their mothers in the play interaction. The results indicated that children whose mothers had chronic psychiatric disorders demonstrated significantly less effective coping behavior on all subscales of the Early Coping Inventory. Implications for the identification and treatment of children at risk for psychiatric disorder are discussed. (PsycINFO Database Record (c) 2012 APA, all rights reserved)
SN - 0419-4217
AD - Hughes, Susan Maureen: Mcgill U, Canada
ER -

TY - JOUR
ID - 833
T1 - "Interactional style of at-risk and well-adapted children observed in two play settings: Competitive and cooperative": Erratum. [Italian]
A1 - Fonzi,Ada; Menesini,Ersilia; Gentili,Cristiana
Y1 - 1994///
N1 - Peer Reviewed Journal: 1996-85757-001
Italian
Journal; Peer Reviewed Journal
KW - PsycInfo
KW - At Risk Populations
KW - Competition
KW - Cooperation
KW - Problem Solving
KW - Games
KW - Interpersonal Interaction

KW - Oral Communication
RP - NOT IN FILE
SP - 119
JF - Ricerche di Psicologia
VL - 18:
IS - 4
N2 - Reports an error in a previously published article by A. Fonzi et al (Ricerche di Psicologia, 1994, Vol 18[2], 151-265). The article omitted a graphic depiction of competitive play, which is reproduced here. (The following abstract of this article originally appeared as 32:85623.) Studied the differences in behavior in competitive and cooperative situations between normal children and children classified as being "at risk" by school and government authorities because of behavioral or family problems. Differences in game-playing strategies and in verbal interactions between the 2 groups were assessed. Ss were 10 at-risk male children and 10 normal males (all aged 9-11 yrs). Ss were observed in 4 different game-playing situations: 2 competitive games and 2 cooperative games, 1 of each with members of the same group and members of the other group. (English abstract) (PsycINFO Database Record (c) 2012 APA, all rights reserved)
SN - 0391-6081
AD - Fonzi, Ada: U degli Studi, Florence, Italy
ER -

TY - JOUR
ID - 835
T1 - Encounters with aggressive peers in early childhood: Frequency, age differences, and correlates of risk for behaviour problems
A1 - Sinclair,Jamie J.
Y1 - 1994///
N1 - Peer Reviewed Journal: 1995-20982-001
English
Journal; Peer Reviewed Journal
KW - PsycInfo
KW - Age Differences
KW - Aggressive Behavior
KW - Human Sex Differences
KW - Peer Relations
KW - Family Socioeconomic Level
KW - Parental Characteristics
KW - Social Behavior
RP - NOT IN FILE
SP - 675
EP - 696
JF - International Journal of Behavioral Development
VL - 17:
IS - 4
N2 - Describes the range, types, and quality of social activity settings in which young children have contact with peers, and whether participation in these settings varies as a function of child sex and age (2-4 vs 4-5 yrs), and family demographic characteristics. Ss were 277 preschool-aged children. Each of 7 activity settings (e.g., neighborhood, organized play groups, day care) was rated for frequency of participation and frequency of exposure to aggressive peers. Ss had the greatest amount of peer contact and were exposed to aggressive peers most often in the neighborhood setting. In contrast, Ss participated least frequently in structured play group settings, and these settings were least likely to contain aggressive peers. Ss from lower SES and single-parent families were more likely to be involved in settings (especially neighborhoods) containing aggressive peers. (PsycINFO Database Record (c) 2012 APA, all rights reserved)
SN - 0165-0254
AD - Sinclair, Jamie J.: Auburn U, AL, US
ER -

TY - JOUR

ID - 836
T1 - Preschool children with behavioral problems: Observation in instructional and free play contexts
A1 - Del'Homme,Melissa Ann; Sinclair,Esther; Kasari,Connie
Y1 - 1994///
N1 - Peer Reviewed Journal: 1994-43172-001
English
Journal; Peer Reviewed Journal
KW - PsycInfo
KW - Academic Environment
KW - At Risk Populations
KW - Behavior Problems
KW - Recreation
KW - Social Behavior
KW - Lower Income Level
KW - Minority Groups
KW - Preschool Students
KW - Project Head Start
RP - NOT IN FILE
SP - 221
EP - 232
JF - Behavioral Disorders
VL - 19:
IS - 3
N2 - Examined task-related and social behaviors of teacher-identified preschool children at risk for behavior problems in 2 school settings, a structured instructional context and an unstructured free play context. Ss were 42 preschoolers from low-income, minority families attending local Head Start programs (21 from a teacher-identified problem behavior group and 21 from a nonidentified comparison group). The problem behavior group was composed of approximately equal numbers of Ss at risk for externalizing (EBPs) and internalizing behavioral problems. While all Ss exhibited more on-task behavior in the instructional context than in the free play context, Ss with EBPs exhibited less on-task behavior. EBPs also exhibited more aggressive behavior in the free play context, and along with comparison Ss, showed a strong tendency toward exhibiting more prosocial behavior than internalizers in the instructional context. (PsycINFO Database Record (c) 2012 APA, all rights reserved)
SN - 0198-7429
AD - Del'Homme, Melissa Ann: U California, Mental Retardation Research Ctr, Los Angeles, US
ER -

TY - JOUR
ID - 837
T1 - Investigation of familial and school-based risk factors for Hispanic Head Start children. [References]
A1 - Grossman,Judy
Y1 - 1994///
N1 - Peer Reviewed Journal: 1994-43081-001
English
Journal; Peer Reviewed Journal
KW - PsycInfo
KW - Family
KW - Project Head Start
KW - School Environment
KW - Latinos/Latinas
KW - Emotional Adjustment
KW - Learning
KW - Preschool Students
KW - Social Adjustment
RP - NOT IN FILE
SP - 456

EP - 467
JF - American Journal of Orthopsychiatry
JA - Am J Orthopsychiatry
VL - 64:
IS - 3
N2 - Studied the relation of familial and school-based risk factors to socioemotional and learning problems in 69 3-yr-old Hispanic children (37 girls and 32 boys) in Head Start programs. Measures included a semistructured maternal stress interview schedule and the Miller Assessment for Preschoolers. Socioemotional problems were found to relate most strongly to school-based solitary play, negative peer interaction, and maternal reports of child temperament, while associated learning problems related most strongly to school-based attention span. Solitary play was a significant predictor of socioemotional behavior for boys than for girls, while child temperament as it relates to goodness of fit was significant only for girls. Results underscore the importance of a multidimensional approach to the assessment of child behavior. (PsycINFO Database Record (c) 2012 APA, all rights reserved)
SN - 0002-9432
AD - Grossman, Judy: New York U School of Education, Dept of Occupational Therapy, US
ER -

TY - JOUR
ID - 838
T1 - The play of angry children: A study of ego defenses in the symbolic play of preschool children at-risk for conduct problems
A1 - Spencer-Sanchez,David Michael
Y1 - 1993///
N1 - Dissertation Abstract: 1995-74857-001
English
Dissertation Abstract
KW - PsycInfo
KW - At Risk Populations
KW - Behavior Problems
KW - Childhood Play Behavior
KW - Defense Mechanisms
KW - Ego
KW - Symbolism
RP - NOT IN FILE
SP - 2773
JF - Dissertation Abstracts International
VL - 54:
IS - 5-B
SN - 0419-4217
AD - Spencer-Sanchez, David Michael: Harvard U, MA, US
ER -

TY - JOUR
ID - 839
T1 - A comparative study of observed language behaviors of prekindergarten children at risk of academic failure in the context of a literacy-enriched play environment
A1 - Setthapun,Anong
Y1 - 1993///
N1 - Dissertation Abstract: 1994-74362-001
English
Dissertation Abstract
KW - PsycInfo
KW - At Risk Populations
KW - Childhood Play Behavior
KW - Curriculum

KW - Literacy
KW - Verbal Ability
KW - Language
RP - NOT IN FILE
SP - 3440
JF - Dissertation Abstracts International
VL - 53:
IS - 10-A
SN - 0419-4217
AD - Setthapun, Anong: Southern Illinois U, Carbondale, US
ER -

TY - JOUR
ID - 842
T1 - Play activities of at-risk and non-at-risk elementary students: Is there a difference?
A1 - Poidevant,John M.
Y1 - 1993///
N1 - Peer Reviewed Journal: 1994-23330-001
English
Journal; Peer Reviewed Journal
KW - PsycInfo
KW - Academic Failure
KW - At Risk Populations
KW - Childhood Play Behavior
KW - Elementary School Students
KW - Kindergarten Students
RP - NOT IN FILE
SP - 173
EP - 186
JF - Child Study Journal
VL - 23:
IS - 3
N2 - Examined characteristics of play activities of 26 at-risk and 23 non-at-risk students in kindergarten through Grade 3, using the Smilansky Scale for Evaluation of Dramatic and Sociodramatic Play. Group comparisons were made using the 6 subscales of the Smilansky Scale. The 2 groups showed significant differences on 3 scales: Make-Believe with Objects, Make-Believe with Action/Situations, and Verbal Communication. (PsycINFO Database Record (c) 2012 APA, all rights reserved)
SN - 0009-4005
AD - Poidevant, John M.: Cobb County Schools, Atlanta, GA, US
ER -

TY - JOUR
ID - 843
T1 - Parent-infant play as a window on infant competence: An organizational approach to assessment
A1 - Beeghly,Marjorie
Y1 - 1993///
N1 - Book: 1993-98052-003
English
Book; Edited Book
KW - PsycInfo
KW - Childhood Play Behavior
KW - Competence
KW - Parent Child Relations
KW - Infant Development
KW - Measurement
KW - Nutritional Deficiencies

RP - NOT IN FILE
SP - 71
EP - 112
JF - MacDonald, Kevin [Ed]
VL - :
IS - 1993
N2 - (from the chapter) parent-infant play has long been regarded by developmentalists as an important component of children's cognitive, socioemotional, and self-regulatory development / [argues] that parent-infant play also provides an excellent opportunity for assessing qualitative aspects of infant competence during the first two years of life / the theoretical framework guiding this approach to assessment is the organizational perspective to developmental psychopathology / following a brief review of this perspective, a working definition of infant competence will be provided in the context of the major developmental tasks facing infants during the first two years / selected parent-child play assessment paradigms which challenge infants and toddlers with respect to these stage-salient issues will then be described parent-toddler play in an illustrative risk population-toddlers with intrauterine growth retardation (IUGR) [resulting from maternal malnutrition]-will be examined in order to demonstrate the usefulness of parent-child play paradigms for assessing key dimensions of competence in these children (PsycINFO Database Record (c) 2012 APA, all rights reserved)
AD - Beeghly, Marjorie: Harvard Medical School, Boston, MA, US
ER -

TY - JOUR
ID - 845
T1 - Sibling play therapy with homeless children: An opportunity in the crisis
A1 - Hunter,Linda B.
Y1 - 1993///
N1 - Peer Reviewed Journal: 1993-30410-001
English
Journal; Peer Reviewed Journal
KW - PsycInfo
KW - Homeless
KW - Interpersonal Communication
KW - Play Therapy
KW - Self Esteem
KW - Siblings
KW - Development
RP - NOT IN FILE
SP - 65
EP - 75
JF - Child Welfare: Journal of Policy, Practice, and Program
VL - 72:
IS - 1
N2 - Homeless children face dangers to their development and self-esteem. Taking advantage of the opportunity offered by the gathering together in shelters of many high-risk families, an on-site social worker can make needed services accessible to parents and children. Play therapy, available on a flexible schedule to young children in small groups or individually, can address crises and long-term growth, helping to increase self-esteem, improve interpersonal skills, and restore relatedness. Empowering siblings to accept and nurture each other can help counteract isolation and interrupt destructive intergenerational cycles. Descriptions of interactions of families with different numbers of siblings illustrates how play therapy can be effective in addressing children's social and psychological needs. (PsycINFO Database Record (c) 2012 APA, all rights reserved)
SN - 0009-4021
AD - Hunter, Linda B.: Queen Lili'uokalani Children's Ctr, HI, US
ER -

TY - JOUR
ID - 846

T1 - The art of communicating with vulnerable children
A1 - Dale,Francis
Y1 - 1992///
N1 - Book: 1993-97901-010
English
Book; Edited Book
KW - PsycInfo
KW - At Risk Populations
KW - Interpersonal Communication
KW - Psychotherapeutic Processes
KW - Child Abuse
KW - Mental Disorders
KW - Play Therapy
RP - NOT IN FILE
SP - 185
EP - 209
JF - Varma, Ved Prakash [Ed]
VL - :
IS - 1992
N2 - (create) Examines the factors associated with communicating with children who are vulnerable to mental disorders and abuse, focusing on communication within the psychotherapeutic setting. Illustrates the communicative process through the case example of play therapy with a 7-yr-old boy. (from the chapter) barriers to communication / levels of communication [hearing the child, the therapeutic alliance, the initial contact, explaining and providing context, the therapeutic setting] / different forms of communication / communicating through play / primitive forms of communication (PsycINFO Database Record (c) 2012 APA, all rights reserved)
AD - Dale, Francis: Principal Child Psychotherapist, England
ER -

TY - JOUR
ID - 847
T1 - Risk-taking/gambling-like behavior in preschool children
A1 - Kearney,Christopher A.
Y1 - 1992///
N1 - Peer Reviewed Journal: 1993-05187-001
English
Journal; Peer Reviewed Journal
KW - PsycInfo
KW - Gambling
KW - Imitation (Learning)
KW - Risk Taking
KW - Social Learning
RP - NOT IN FILE
SP - 287
EP - 297
JF - Journal of Gambling Studies
VL - 8:
IS - 3
N2 - Examines social learning as a potential etiological factor of risk-taking behavior. Prior to playing a risk-taking game, 5 boys and 5 girls (aged 4-5 yrs) were exposed to a peer model who had ostensibly won a large prize, whereas 6 boys and 5 girls of similar age (controls) were exposed to a peer model who had won nothing. Ss in the experimental group initiated more risks to win the large prize in a high-risk situation than did children in the control group, indicating that modeling can enhance risk-taking behavior in young children. (PsycINFO Database Record (c) 2012 APA, all rights reserved)
SN - 1050-5350
AD - Kearney, Christopher A.: U Nevada, Las Vegas, US

ER -

TY - JOUR
ID - 848
T1 - The aggressive tendencies in small children: A checklist for determining aggressive behavior in free play situations. [Italian]
A1 - Venuti,Paola
Y1 - 1992///
N1 - Peer Reviewed Journal: 1992-88718-001
Italian
Journal; Peer Reviewed Journal
KW - PsycInfo
KW - Aggressive Behavior
KW - Behavioral Assessment
KW - Childhood Play Behavior
RP - NOT IN FILE
SP - 101
EP - 109
JF - Eta Evolutiva
VL - 41 Feb 1992, pp. 101-109.
N2 - Discusses the use of a children's behavior checklist to categorize and assess types of activity, risk situations, and aggressive behavior vs nonaggressive behavior. (PsycINFO Database Record (c) 2012 APA, all rights reserved)
SN - 0392-0658
AD - Venuti, Paola: U degli Studi Padua, Italy
ER -

TY - JOUR
ID - 849
T1 - A social partnership model for assessing early communication development: An intervention model for preconversational children
A1 - MacDonald,James D.
Y1 - 1992///
N1 - Peer Reviewed Journal: 1992-35930-001
English
Journal; Peer Reviewed Journal
KW - PsycInfo
KW - Delayed Development
KW - Early Intervention
KW - Interpersonal Communication
KW - At Risk Populations
KW - Language Delay
KW - Models
RP - NOT IN FILE
SP - 113
EP - 124
JF - Language, Speech, and Hearing Services in Schools
VL - 23:
IS - 2
N2 - Presents a model for assessing the interactive sources of communication development, which focuses on the reciprocal behaviors of adults and children as communication evolves from natural play, learning, and caregiving. The model integrates theoretical and empirical findings from a wide range of studies of the development of 1st relationships and is designed for parallel diagnostic and treatment purposes. The rationale and case illustrations are provided of the 5 developmental stages addressed by the model: social play, turn taking, nonverbal communication, language, and conversation. (PsycINFO Database Record (c) 2012 APA, all rights reserved)

SN - 0161-1461
AD - MacDonald, James D.: Ohio State U, Nisonger Ctr, Columbus, US
ER -

TY - JOUR
ID - 851
T1 - Maternal depression and locus of control orientation as predictors of dyadic play behavior
A1 - Houck,Gail M.
Y1 - 1991///
N1 - Peer Reviewed Journal: 1992-27441-001
English
Journal; Peer Reviewed Journal
KW - PsycInfo
KW - Childhood Play Behavior
KW - Internal External Locus of Control
KW - Major Depression
KW - Mother Child Relations
KW - Adolescent Mothers
KW - Mothers
RP - NOT IN FILE
SP - 347
EP - 360
JF - Infant Mental Health Journal
VL - 12:
IS - 4
N2 - Examined maternal locus of control orientation and depression in relation to joint negotiation and coordination of mother-child play at 20 mo. Ss were 51 high-social-risk mothers (aged 15-32 yrs) and children. Mothers completed Rotter's Internal-External Locus of Control Scale and the Beck Depression Inventory prenatally and at 20 mo; mother-child interaction was videotaped in a laboratory playroom at 20 mo. Tapes were coded to evaluate maternal control and sensitivity and child engagement and autonomy. Analyses of dyadic play behavior indicated that maternal controllingness was related to an external locus of control orientation. Child engagement during play was related to the resolution of mild maternal depression. (French, Spanish & Japanese abstracts) (PsycINFO Database Record (c) 2012 APA, all rights reserved)
SN - 0163-9641
AD - Houck, Gail M.: Oregon Health Sciences U, School of Nursing, Portland, US
ER -

TY - JOUR
ID - 852
T1 - Adolescent and adult mothers of handicapped children: Maternal involvement in play. [References]
A1 - Helm,James M.
Y1 - 1990///
N1 - Peer Reviewed Journal: 2001-09132-009
English
Journal; Peer Reviewed Journal
KW - PsycInfo
KW - Childhood Play Behavior
KW - Disorders
KW - Mother Child Relations
KW - Parental Attitudes
KW - Social Support
KW - Adolescent Mothers
KW - At Risk Populations
KW - Internal External Locus of Control
KW - Social Interaction
RP - NOT IN FILE

SP - 432
EP - 437
JF - Family Relations: An Interdisciplinary Journal of Applied Family Studies
VL - 39:
IS - 4
N2 - This study examines maternal involvement of adolescent (aged 14-19 yrs) and adult (aged 20-42 yrs) mothers with their handicapped and at-risk preschool children. Consistent with the literature on mothers of normally developing children, results indicate adolescent mothers of handicapped and at-risk children are rated as significantly less contingent and verbal, and provide a less positive affective climate than adult mothers. Regression techniques indicate perceived social support and locus of control were significant predictors of maternal involvement for adolescent and adult mothers respectively. (PsycINFO Database Record (c) 2012 APA, all rights reserved)
SN - 0197-6664
AD - Helm, James M.: North Carolina U, School of Medicine, NC, US
ER -

TY - JOUR
ID - 853
T1 - The Waterloo Longitudinal Project: Correlates and consequences of social withdrawal in childhood
A1 - Rubin,Kenneth H.
Y1 - 1990///
N1 - Peer Reviewed Journal: 1990-25024-001
English
Journal; Peer Reviewed Journal
KW - PsycInfo
KW - At Risk Populations
KW - Childhood Development
KW - Social Isolation
KW - Longitudinal Studies
RP - NOT IN FILE
SP - 73
EP - 90
JF - Infancia y Aprendizaje / Journal for the Study of Education and Development
VL - 49 1990, pp. 73-90.
N2 - Examined the risk status of childhood social withdrawal (CSW), using data from a longitudinal project that followed 77 children between the ages of 5 and 11 yrs. Younger Ss were observed in free play with their peers, and older Ss were evaluated using a class play method of peer assessment. CSW was relatively stable in the early school years and was associated with loneliness, nonclinical depression, and negative self-perceptions of competence in later childhood. Regarding development, children may arrive at a given "outcome" in different ways. Although some isolated and rejected children may be at risk for subsequent externalizing difficulties, others may be more at risk for later internalizing problems. (Spanish abstract) (PsycINFO Database Record (c) 2012 APA, all rights reserved)
SN - 0210-3702
AD - Rubin, Kenneth H.: U Waterloo, ON, Canada
ER -

TY - JOUR
ID - 855
T1 - Playgrounds at the crossroads: Policy and action research needed to ensure a viable future for public playgrounds in the United States
A1 - Moore,Robin C.
Y1 - 1989///
N1 - Book: 1989-97645-004
English
Book; Edited Book
KW - PsycInfo

KW - Environmental Planning
KW - Playgrounds
KW - Policy Making
KW - Childhood Development
KW - Safety
KW - Social Interaction
RP - NOT IN FILE
SP - 83
EP - 120
JF - Altman, Irwin [Ed]; Zube, Ervin H [Ed]
VL - :
IS - 1989
N2 - (from the chapter) purpose of this chapter is to provide a perspective on the current state of public playgrounds in the United States and to propose research and policy directions to support their improvement as viable places for child development main issues current in the field of practice and arising from a review of the empirical literature are discussed in depth / playground use / safety / play value of different types of setting / adventure play / site planning / characteristics of settings that support social integration (PsycINFO Database Record (c) 2012 APA, all rights reserved)
AD - Moore, Robin C.: North Carolina State U, School of Design, Raleigh, NC, US
ER -

TY - JOUR
ID - 856
T1 - Effects of group socialization procedures on the social interactions of preschool children
A1 - Brown,William H.
Y1 - 1988///
N1 - Peer Reviewed Journal: 1989-20576-001
English
Journal; Peer Reviewed Journal
KW - PsycInfo
KW - Autism
KW - Delayed Development
KW - Emotional Disturbances
KW - Group Instruction
KW - Peer Relations
KW - Preschool Students
KW - SOCIALIZATION
RP - NOT IN FILE
SP - 359
EP - 376
JF - Research in Developmental Disabilities
JA - Res.Dev.Disabil.
VL - 9:
IS - 4
N2 - Investigated the effects of group socialization procedures on the social behavior of preschoolers. Study 1 involved 20 Head Start students (aged 4 yrs 6 mo to 5 yrs 7 mo) with 2 target Ss (a developmentally delayed girl and a normally developing boy). Study 2 involved 17 Ss (aged 3 yrs 8 mo to 5 yrs 5 mo) at risk for handicapping conditions with 2 target Ss (an autistic and an emotionally disturbed boy). Group socialization procedures consisted of teachers using antecedent and consequent events to promote social interaction during children's games. Social behavior was assessed during group game periods (i.e., intervention sessions) and nonintervention play periods (i.e., generalization sessions). In both studies, during group game periods after intervention, target Ss increased their rates of both prompted and unprompted social interactions with peers. In nonintervention play periods, target Ss improved both the rate and the duration of their social responding with peers. (PsycINFO Database Record (c) 2012 APA, all rights reserved)
SN - 0891-4222

AD - Brown, William H.: Vanderbilt U George Peabody Coll for Teachers, Inst of Family & Child Development, Nashville, TN, US
ER -

TY - JOUR
ID - 857
T1 - Issue-specific play with infants and toddlers in hospitals: Rationale and intervention
A1 - Goldberger,Joy
Y1 - 1988///
N1 - Peer Reviewed Journal: 1989-02788-001
English
Journal; Peer Reviewed Journal
KW - PsycInfo
KW - Emotional Adjustment
KW - Hospitalized Patients
KW - Play Therapy
KW - Primary Mental Health Prevention
KW - At Risk Populations
KW - Family Members
KW - Social Support
RP - NOT IN FILE
SP - 134
EP - 141
JF - Children's Health Care
JA - Child Health Care
VL - 16:
IS - 3
N2 - Discusses planned, systematic play as a necessary facet of emotional and developmental support to hospitalized children (aged from 6 mo to 3 yrs) and their families and suggests categories and methods of issue-specific play interventions. (PsycINFO Database Record (c) 2012 APA, all rights reserved)
SN - 0273-9615
AD - Goldberger, Joy: Johns Hopkins Hosp Children's Ctr, Child Life Dept, Baltimore, MD, US
ER -

TY - JOUR
ID - 858
T1 - The diagnostic and therapeutic roles of children's rough-and-tumble play
A1 - Pellegrini,Anthony D.
Y1 - 1988///
N1 - Peer Reviewed Journal: 1989-02218-001
English
Journal; Peer Reviewed Journal
KW - PsycInfo
KW - Aggressive Behavior
KW - Childhood Play Behavior
KW - Play Therapy
KW - Psychodiagnosis
KW - At Risk Populations
KW - Health Care Services
KW - Physical Contact
RP - NOT IN FILE
SP - 162
EP - 168
JF - Children's Health Care
JA - Child Health Care
VL - 16:

IS - 3
N2 - Outlines the diagnostic and therapeutic roles of children's rough-and-tumble play (R&T). The diagnostic role of R&T is explicated by discussing a study of elementary school children's R&T and aggression. It is argued that observing R&T and aggression can help health care providers in predicting and preventing antisocial behaviors. Strategies for remediating children's aggressive play that are appropriate for home- or hospital-based intervention programs are described. (PsycINFO Database Record (c) 2012 APA, all rights reserved)
SN - 0273-9615
AD - Pellegrini, Anthony D.: U Georgia, Inst for Behavioral Research, Athens, US
ER -

TY - JOUR
ID - 860
T1 - Parental involvement in play interaction and caregiving roles in families with young high risk and handicapped children: A comparison of father and mother
A1 - Comfort,Marilee
Y1 - 1987///
N1 - Dissertation Abstract: 1988-54782-001
English
Dissertation Abstract
KW - PsycInfo
KW - At Risk Populations
KW - Childhood Play Behavior
KW - Parent Child Relations
KW - Parental Attitudes
KW - Parents
KW - Intellectual Development Disorder
RP - NOT IN FILE
SP - 627
JF - Dissertation Abstracts International
VL - 48:
IS - 3-A
SN - 0419-4217
AD - Comfort, Marilee: U North Carolina, Chapel Hill, US
ER -

TY - JOUR
ID - 861
T1 - Cognitive processes of at-risk and typically developing infants: Comparison of exploration, play, and problem solving
A1 - Cannella,Gaile S; Berkeley,Terry
Y1 - 1987///
N1 - Peer Reviewed Journal: 1988-19660-001
English
Journal; Peer Reviewed Journal
KW - PsycInfo
KW - At Risk Populations
KW - Childhood Play Development
KW - Cognitive Development
KW - Exploratory Behavior
KW - Problem Solving
RP - NOT IN FILE
SP - 269
EP - 286
JF - Child Study Journal
VL - 17:
IS - 4

N2 - Observed assimilation and accommodation with novel objects for 12 infants identified as mildly at-risk and 10 intellectually average (typical) infants (aged 8-18 mo). Typical Ss exhibited greater problem solving skills, whereas play behaviors were similar for both groups. Exploration and play were positively related to problem solving for the at-risk Ss and negatively related for the typical Ss. Behavior of younger and older typical Ss revealed a developmental progression from decreasing exploration to greater amounts of play. At-risk Ss did not follow this pattern; both types of activities increased with age. It is suggesed that, although at-risk infants are capable of developing higher levels of equilibrium, they need more exploration time to construct cognitive schema. (PsycINFO Database Record (c) 2012 APA, all rights reserved)
SN - 0009-4005
AD - Cannella, Gaile S.: St John's U, Jamaica, NY, US
ER -

TY - JOUR
ID - 862
T1 - Learning mathematics and science through play
A1 - Henniger,Michael L.
Y1 - 1987///
N1 - Peer Reviewed Journal: 1988-18386-001
English
Journal; Peer Reviewed Journal
KW - PsycInfo
KW - Childhood Play Behavior
KW - Mathematics Education
KW - Science Education
KW - Academic Achievement Motivation
KW - Divergent Thinking
KW - Elementary School Students
KW - Preschool Students
KW - Teaching Methods
RP - NOT IN FILE
SP - 167
EP - 171
JF - Childhood Education
VL - 63:
IS - 3
N2 - Presents examples of learning opportunities in science and mathematics available through children's play. Attitudes essential to learning in these disciplines that are developed through the opportunity to explore and create in a low-risk situation are described, including intellectual curiosity, willingness to engage in divergent thinking, and motivation to learn. (PsycINFO Database Record (c) 2012 APA, all rights reserved)
SN - 0009-4056
AD - Henniger, Michael L.: Central Washington U, Ellensburg, US
ER -

TY - JOUR
ID - 863
T1 - Impact of premature birth on the development of the infant in the family. [References]
A1 - Macey,Terri J.
Y1 - 1987///
N1 - Peer Reviewed Journal: 1988-14344-001
English
Journal; Peer Reviewed Journal
KW - PsycInfo
KW - Attachment Behavior
KW - Childrearing Practices
KW - Family Relations
KW - Mother Child Relations

KW - Premature Birth
KW - Cognitive Development
KW - Hospitalization
KW - Social Behavior
RP - NOT IN FILE
SP - 846
EP - 852
JF - Journal of Consulting and Clinical Psychology
VL - 55:
IS - 6
N2 - This article describes effects of the birth of a premature infant on the family system and focuses on how problems associated with premature birth such as infant illness, hospitalization, and immaturity can place the family at risk. New data are presented describing differences in the behavior of preterm and full-term infants and in the feelings of mothers toward their infants at 12 months. Mothers of preterm infants reported feeling overprotective toward them, were less willing to leave them with sitters, and believed that their birth had an initially negative effect on the family. Preterm infants showed less exploratory play and stayed closer to their mothers during free play. The fact that these differences are not reflected in patterns of attachment, a key measure of the mother-infant relationship, is discussed. (PsycINFO Database Record (c) 2012 APA, all rights reserved)
SN - 0022-006X
AD - Macey, Terri J.: U Colorado Health Sciences Ctr, Denver, US
ER -

TY - JOUR
ID - 864
T1 - Childhood gender identity change: Operant control over sex-typed play and mannerisms
A1 - Rekers,George A.
Y1 - 1976///
N1 - Peer Reviewed Journal: 1976-28418-001
English
Journal; Peer Reviewed Journal
KW - PsycInfo
KW - Case Report
KW - Human Males
KW - Operant Conditioning
KW - Psychosexual Behavior
KW - Transsexualism
KW - Behavior Therapy
KW - Sex Roles
RP - NOT IN FILE
SP - 51
EP - 57
JF - Journal of Behavior Therapy and Experimental Psychiatry
VL - 7:
IS - 1
N2 - Presents what is thought to be the 1st case of a successful change of childhood gender identity that is documented by pre- and postdiagnostic assessment procedures. Deviant sex-typed behaviors were modified in a 5-yr-old boy judged by an independent clinician to be a high risk for adult transsexualism. In Study 1, the child's mother was trained to reinforce "masculine" play behaviors and to extinguish "feminine" play behaviors in the clinic. This intra-S study introduced new procedures designed to maximize the generalization of the treatment effect to play in the alone condition. Study 2 was designed to modify the child's cross-gender mannerisms through the use of a response-cost and verbal prompt procedure. The cross-gender mannerism "flexed elbow" decreased as a function of the treatment. At the follow-up 25 mo after treatment, evaluation by an independent clinician indicated the therapeutic change to a male gender identity. (46 ref) (PsycINFO Database Record (c) 2013 APA, all rights reserved)
SN - 0005-7916

AD - Rekers, George A.: Fuller Theological Seminary, Graduate School of Psychology, Pasadena, CA
ER -

TY - JOUR
ID - 865
T1 - A study of the gross motor skills of cerebral palsied children in an adventure playground for handicapped children
A1 - Levitt,Sophie
Y1 - 1975///
N1 - Peer Reviewed Journal: 1976-24356-001
English
Journal; Peer Reviewed Journal
KW - PsycInfo
KW - Cerebral Palsy
KW - Childhood Play Development
KW - Motor Skills
KW - Play Therapy
KW - Playgrounds
RP - NOT IN FILE
SP - 29
EP - 43
JF - Child: Care, Health and Development
VL - 1:
IS - 1
N2 - Studied the play of 29 handicapped children (diagnoses included spastic quadriplegia, diplegia, triplegia, hemiplegia, athetoid quadriplegia, and ataxia) in an adventure playground in order to (a) investigate the relationship between therapy and its practical application in play and (b) examine how the playground can contribute to therapy. Results show that Ss did not take advantage of the opportunities in the playground to cover the full scope of their established motor skills. The behavior of adults, attitudes of the children, and the design of the playground and equipment are discussed as possible reasons for this finding. (PsycINFO Database Record (c) 2012 APA, all rights reserved)
SN - 0305-1862
AD - Levitt, Sophie: Wolfson Ctr, London, England
ER -

TY - JOUR
ID - 866
T1 - Children in four neighborhoods: The physical environment and its effect on play and play patterns
A1 - Berg,Mary
Y1 - 1980///
N1 - Peer Reviewed Journal: 1980-32305-001
English
Journal; Peer Reviewed Journal
KW - PsycInfo
KW - Childhood Play Behavior
KW - Environmental Effects
KW - Neighborhoods
KW - Recreation Areas
KW - Urban Planning
KW - Safety
KW - Social Interaction
KW - Socioeconomic Status
KW - Topography
RP - NOT IN FILE
SP - 320
EP - 348

JF - Environment and Behavior
VL - 12:
IS - 3
N2 - Compared children's play patterns across neighborhoods of different social status, terrains, levels of access to play space, and levels of municipally provided after-school services. In a California city, 764 11-12 yr olds were interviewed in their homes, while their parents completed a questionnaire that probed socialization priorities and childrearing practices affecting aspects of out-of-school life. It is argued that few neighborhoods are designed with the needs of young people in mind. By examining how children's play patterns in 4 neighborhoods are constrained or facilitated by the terrain, by the availability of "managed" and "unmanaged" play space, and by play problems associated with questions of safety and mobility, it is possible to see how planning and land-use decisions affect the everyday experience of the young. (29 ref) (PsycINFO Database Record (c) 2012 APA, all rights reserved)
SN - 0013-9165
AD - Berg, Mary: U California Schools of Law & Education, Berkeley
ER -

TY - JOUR
ID - 867
T1 - Children's play and urban playground environments: A comparison of traditional, contemporary, and adventure playground types
A1 - Hayward,D.
Y1 - 1974///
N1 - Peer Reviewed Journal: 1975-09396-001
English
Journal; Peer Reviewed Journal
KW - PsycInfo
KW - Playgrounds
KW - Recreation
KW - Social Interaction
RP - NOT IN FILE
SP - 131
EP - 168
JF - Environment and Behavior
VL - 6:
IS - 2
N2 - Studied children's activities and interactions in 3 playgrounds: a traditional equipment playground, a contemporary-design playground, and an adventure playground (which supplies play material, not play equipment). Observation, interviews, behavioral mapping, and behavior setting records were employed. The playgrounds had different predominant user groups, different patterns of use of areas and equipment, and different roles for and participation by play setting supervisors. Adults and preschoolers predominated at the traditional and contemporary playgrounds; 6-13 yr olds predominated at the adventure play area. At the 2 prebuilt playgrounds, use focused on the equipment, whereas at the adventure playground interactive play was common. The atmosphere of the playgrounds and the activities of users were shaped by a variety of factors, including opportunities and constraints of the physical environment, social influences, and freedom to make use of available opportunities. Information on users' choices and opinions relative to opportunities and freedom is presented, and implications for research and planning are discussed. (PsycINFO Database Record (c) 2012 APA, all rights reserved)
SN - 0013-9165
AD - Hayward, D. Geoffrey: Graduate School & University Ctr, City U New York
ER -

TY - JOUR
ID - 868
T1 - Social competence and children's sociometric status: The role of peer group entry strategies
A1 - Dodge,Kenneth A.
Y1 - 1983///

N1 - Peer Reviewed Journal: 1984-01060-001
English
Journal; Peer Reviewed Journal
KW - PsycInfo
KW - Peer Relations
KW - Social Approval
KW - Social Skills
KW - Status
RP - NOT IN FILE
SP - 309
EP - 336
JF - Merrill-Palmer Quarterly
VL - 29:
IS - 3
N2 - In Study 1, 50 popular, rejected, and neglected kindergartners were asked to initiate play with 2 same-age peers. In Study 2, naturally occurring peer group entry episodes were observed among 56 popular, rejected, neglected, average, and controversial 2nd-grade boys who were involved in free play. Observations of video records of these episodes revealed that the status groups differed in the frequency of their attempts at peer group entry, the probability of success of their attempts, and the behaviors that they used during entry. The entry behaviors were conceptualized and coded as tactics used by Ss to complete the task of entry. Successful entry attempts involved sequences of tactics that progressed from the use of low-risk tactics (such as waiting and hovering) to the use of high-risk tactics (such as statements and requests), while maintaining the frame of reference of the group. Findings are integrated with previous research to generate a model of social competence in children's peer-group entry behavior. (30 ref) (PsycINFO Database Record (c) 2012 APA, all rights reserved)
SN - 0272-930X
AD - Dodge, Kenneth A.: Indiana U, Bloomington
ER -

TY - JOUR
ID - 869
T1 - Risk-taking needs in children: An accommodating play environment
A1 - Jambor,Tom
Y1 - 1986///
N1 - Peer Reviewed Journal: 1989-21891-001
English
Journal; Peer Reviewed Journal
KW - PsycInfo
KW - Childhood Play Behavior
KW - Environmental Planning
KW - Playgrounds
KW - Risk Taking
RP - NOT IN FILE
SP - 22
EP - 25
JF - Children's Environments Quarterly
VL - 3:
IS - 4
N2 - Describes a play environment designed to acknowledge children's risk-taking needs. The playground, developed in a natural setting in Norway, was intended to allow children of various ages to participate at their own levels of ability in play involving decision making and social interaction. (PsycINFO Database Record (c) 2012 APA, all rights reserved)
SN - 0886-0505
AD - Jambor, Tom: U Alabama, School of Education, Birmingham, US
ER -

TY - JOUR

ID - 870
T1 - The influence of model infant-toddler group care on parent-child interaction at home
A1 - Edwards,Carolyn P.
Y1 - 1986///
N1 - Peer Reviewed Journal: 1988-13561-001
English
Journal; Peer Reviewed Journal
KW - PsycInfo
KW - Child Day Care
KW - Childhood Play Development
KW - Parent Child Relations
KW - Safety
RP - NOT IN FILE
SP - 317
EP - 332
JF - Early Childhood Research Quarterly
VL - 1:
IS - 4
N2 - Assessed the effects of daycare participation on parent-child interaction at home using a university-based, half-day model infant-toddler program with 38 2-22 mo olds and their parents (aged 21-42 yrs). Hypotheses concerned whether child-centered features of the physical and social environment were carried over by parents to the home. 19 matched pairs of center and noncenter children were followed for 8 mo. Parents showed few group differences during the 1st half of the study period. At study end, however, center homes were more child-centered with respect to play, safety, and dinner arrangements. Center parents scored higher in proximity and warmth and lower in teacher-avoided behaviors. Noncenter parents at study end scored higher in authority (limit setting) and communicating values and labels. (PsycINFO Database Record (c) 2012 APA, all rights reserved)
SN - 0885-2006
AD - Edwards, Carolyn P.: U Massachusetts School of Education, Amherst, US
ER -

TY - JOUR
ID - 871
T1 - Age-appropriateness and safety of a home merry-go-round for young children
A1 - Ridenour,Marcella V.
Y1 - 1986///
N1 - Peer Reviewed Journal: 1987-30974-001
English
Journal; Peer Reviewed Journal
KW - PsycInfo
KW - Age Differences
KW - Home Environment
KW - Playgrounds
KW - Safety
KW - Toys
RP - NOT IN FILE
SP - 271
EP - 278
JF - Perceptual and Motor Skills
VL - 63:
IS - 1
N2 - Summarized the guidelines for age-labeling of children's toy and playgrounds and compared home and public playground standards for the design of merry-go-rounds (MGRs). An investigation of home MGRs with 120 3-6 yr olds found that Ss had the motor and social interaction skills to operate a 2-person home MGR. Although the Ss were able to rotate the MGR successfully, a potential hazard was identified. (PsycINFO Database Record (c) 2012 APA, all rights reserved)
SN - 0031-5125

AD - Ridenour, Marcella V.: Temple U, Biokinetics Research Lab
ER -

TY - JOUR
ID - 873
T1 - Mother-child and father-child play
A1 - Roopnarine,Jaipaul L.
Y1 - 1985///
N1 - Peer Reviewed Journal: 1998-01826-004
English
Journal; Peer Reviewed Journal
KW - PsycInfo
KW - Childhood Play Behavior
KW - Fantasy
KW - Human Sex Differences
KW - Parent Child Relations
KW - Vocabulary
KW - Fathers
KW - Mothers
RP - NOT IN FILE
SP - 157
EP - 169
JF - Early Child Development and Care
VL - 20:
IS - 2-3
N2 - Examined parental initiations of fantasy with their preschool-aged sons and daughters and the relationship between the fantasy play initiations of parents and their children's receptive vocabulary skills. 36 mother-father-child triads (aged 25-45 yrs, aged 27-59 yrs, and aged 46-60 mo, respectively) participated. Parents interacted with their children for 10 minutes, and activities were coded as fantasy play (domestic or adventure themes), rough play, and joint positive. Results show that mothers were more likely to initiate fantasy and joint positive play compared to fathers, while fathers were more likely to initiate rough play compared to mothers. With one exception, mothers initiating more joint positive play with daughters than with sons, parental treatment of sons and daughters was very similar. (PsycINFO Database Record (c) 2012 APA, all rights reserved)
SN - 0300-4430
AD - Roopnarine, Jaipaul L.: Syracuse U, Dept of Child, Family & Community Studies, Syracuse, NY, US
ER -

TY - JOUR
ID - 874
T1 - A cross-setting assessment of family interaction and the prediction of school competence in children at risk
A1 - Cole,Robert E; Al-Khayyal,Manhal; Baldwin,Alfred
Y1 - 1984///
N1 - Book: 1997-36499-030
English
Book; Edited Book
KW - PsycInfo
KW - Academic Achievement
KW - At Risk Populations
KW - Family Relations
KW - Mental Disorders
KW - Offspring
KW - Childhood Play Behavior
KW - Parental Characteristics
KW - Recreation
KW - Rorschach Test
KW - Sons

RP - NOT IN FILE
SP - 388
EP - 392
JF - Watt, Norman F [Ed]; Anthony, E
VL - James [Ed]; Wynne, Lyman C [Ed]; Rolf, Jon E [Ed]. :
IS - 1984
N2 - (from the chapter) The development of a friendly, outgoing interpersonal style depends in part on behavior modeled upon and rewarded by a child's family. An examination of the frequency, reciprocity, and warmth of social interaction in families with children at risk for psychiatric disorders should help us understand why some of the children in our study of 4-, 7-, and 10-yr-old sons with 1 parent having schizophrenia, affective psychosis, or nonpsychotic mental disorders are more competent than others. The sample comes from the University of Rochester Child and Family Study (URCAFS). The URCAFS Project had a number of opportunities to observe our S families working and playing together. This includes both structured and unstructured tasks that involve various constellations of family members. This report presents some of the data from 2 of these tasks, the Free Play and the Family Rorschach. The Free Play is an unstructured task in which a subgroup of the family, the mother, father, and index child are asked to play together for 30 min as they might at home. The Consensus Rorschach is a structured task conducted in 2 phases, first with the marital pair, then with the whole family. In the first phase, the Spouse Rorschach, the parents, while alone, are given a Rorschach card and are asked to examine the card and reach a consensus about what the card looks like to them. Having completed this task, the parents are then instructed to explain the task to the children and to achieve consensus, as a family, about what a new card looks like. Thus, the Family Rorschach, the 2nd phase, includes clearly structured tasks: (1) parents teach the children; (2) family members solicit responses form 1 another; (3) family reaches consensus. The combined data from 2 such different settings provide a much more complete understanding of interaction in families than do data from only 1 setting and improve our predictions of how the children in these families will perform in school. (PsycINFO Database Record (c) 2012 APA, all rights reserved)
AD - Cole, Robert E.: U Rochester, Medical Ctr, Rochester, NY, US
ER -

TY - JOUR
ID - 875
T1 - Free Play family interaction and the behavior of the patient in Free Play
A1 - Baldwin,Clara P.
Y1 - 1984///
N1 - Book: 1997-36499-029
English
Book; Edited Book
KW - PsycInfo
KW - At Risk Populations
KW - Mental Disorders
KW - Offspring
KW - Parent Child Relations
KW - Recreation
KW - Affection
KW - Major Depression
KW - Parental Characteristics
KW - Psychosis
KW - Schizophrenia
KW - Sons
RP - NOT IN FILE
SP - 376
EP - 387
JF - Watt, Norman F [Ed]; Anthony, E
VL - James [Ed]; Wynne, Lyman C [Ed]; Rolf, Jon E [Ed]. :
IS - 1984
N2 - (from the chapter) Discussed the general features of the family interaction in the Free Play situation: the high rate of interaction with the child, and general predominance of parental initiations in the parent-child

interaction, the high correlation among all dyads on warmth. This led to the selection of 3 variables for study, rate of patient-child interaction, proportion of parental initiations to the child, and family warmth in at risk 4-, 7-, and 10-yr-old sons who have at least 1 parent with schizophrenia vs affective psychotics vs nonpsychotics. This population was taken from the University of Rochester Child and Family Study. Results suggest that: (1) there are major differences in family interaction depending on the age of the child; (2) the patient's rate of interaction with the child and proportion of interaction is lower than the spouse's rate (this holds for the 4- and 10-yr-olds separately but not for the 7-yr-olds); (3) the rate of patient-child interaction is highest for nonpsychotics, lower for affective psychotics, and least for schizophrenics; (4) patients with a low Global Assessment Score (GAS) have lower rates and lower proportions of interaction with their children and less warmth than patients with a higher GAS; and (5) patients who have been recently hospitalized have a lower rate than those hospitalized longer ago. (PsycINFO Database Record (c) 2012 APA, all rights reserved)
AD - Baldwin, Clara P.: U Rochester, Rochester, NY, US
ER -

TY - JOUR
ID - 876
T1 - Influence of inter-rung distance on self-prediction and success
A1 - Ridenour,Marcella V.
Y1 - 1983///
N1 - Peer Reviewed Journal: 1984-03735-001
English
Journal; Peer Reviewed Journal
KW - PsycInfo
KW - Jumping
KW - Motor Performance
KW - Playgrounds
KW - Prediction
KW - Safety
RP - NOT IN FILE
SP - 751
EP - 754
JF - Perceptual and Motor Skills
VL - 56:
IS - 3
N2 - 19 6-yr-old boys participated in a 2-part experiment on horizontal inter-rung distance. The 1st part was limited to self-prediction, and in the 2nd part Ss were asked to use a hanging-swing movement between pairs of horizontal rungs separated by 24-59 in. Analysis of videotapes indicated rung distance did not affect the accuracy of self-prediction, but it did influence success on the task and frequency of jumping. The percentage of successful trials decreased and the frequency of jumping movements increased with greater inter-rung distances. Results indicate that 6-yr-old boys have excellent self-prediction skills that are sometimes accompanied by poor and potentially hazardous movement choices. Implications for playground safety are discussed. (4 ref) (PsycINFO Database Record (c) 2012 APA, all rights reserved)
SN - 0031-5125
AD - Ridenour, Marcella V.: Temple U, Biokinetics Research Lab
ER -

TY - JOUR
ID - 877
T1 - Promoting social integration of young children at risk for learning disabilities
A1 - Odom,Samuel L.
Y1 - 1982///
N1 - Peer Reviewed Journal: 1983-08950-001
English
Journal; Peer Reviewed Journal
KW - PsycInfo
KW - Curriculum

KW - Mainstreaming (Educational)
KW - Predisposition
KW - Social Interaction
KW - Disorders
KW - Preschool Students
KW - Special Education
RP - NOT IN FILE
SP - 379
EP - 387
JF - Learning Disability Quarterly
VL - 5:
IS - 4
N2 - The Integrated Preschool Curriculum (IPC), designed to facilitate the social integration of young children at risk for learning disabilities into mainstreamed and integrated special education classes, was compared with a standard early childhood education model curriculum. Social interaction data during play were collected for 4 integrated and 2 nonintegrated special education classes (56 handicapped and 16 nonhandicapped Ss). Ss in the IPC classes exhibited significantly greater proportions of interactive and proximity play, while those in the contrast classes engaged in significantly greater proportions of isolate play. In the integrated classes implementing the IPC, a significantly greater proportion of the nonhandicapped Ss' interactive play involved handicapped Ss as compared to the integrated classes employing the contrast curriculum. In the IPC classes, handicapped Ss played with their nonhandicapped peers more often. Data indicate that the IPC successfully promoted social interaction between handicapped and nonhandicapped Ss in integrated special education classes. (42 ref) (PsycINFO Database Record (c) 2012 APA, all rights reserved)
SN - 0731-9487
AD - Odom, Samuel L.: U Pittsburgh School of Medicine, Western Psychiatric Inst & Clinic
ER -

TY - JOUR
ID - 878
T1 - Nonsocial play in preschoolers: Necessarily evil?
A1 - Rubin,Kenneth H.
Y1 - 1982///
N1 - Peer Reviewed Journal: 1982-27977-001
English
Journal; Peer Reviewed Journal
KW - PsycInfo
KW - Childhood Play Behavior
KW - Cognitive Ability
KW - Social Isolation
KW - Social Skills
RP - NOT IN FILE
SP - 651
EP - 657
JF - Child Development
JA - Child Dev.
VL - 53:
IS - 3
N2 - It has been suggested that children who play on their own, without interacting with peers, may be at risk for social, cognitive, and social-cognitive problems. Recently, however, the children's play literature has revealed that some forms of nonsocial activity are constructive and adaptive. The present study examined the social, cognitive, and social-cognitive correlates of nonsocial play. 122 4-yr-olds were observed for 20 min during free play. They were also administered a role-taking test, the Preschool Interpersonal Problem-Solving Test, and the Social Problem-Solving Task. Sociometric popularity and social competence (as rated by teachers using the Sociometric Rating Scale and the Preschool Behavior Questionnaire) were also assessed. Analyses indicated that nonsocial-functional (sensorimotor) and dramatic activities generally correlated negatively with

the measures of competence; parallel-constructive activities generally correlated positively with them. (26 ref) (PsycINFO Database Record (c) 2012 APA, all rights reserved)
SN - 0009-3920
AD - Rubin, Kenneth H.: U Waterloo, Canada
ER -

TY - JOUR
ID - 879
T1 - Development of symbolic play: Implications for the language specialist
A1 - McCune-Nicolich,Lorraine; Carroll,Shelley
Y1 - 1981///
N1 - Peer Reviewed Journal: 1982-25495-001
English
Journal; Peer Reviewed Journal
KW - PsycInfo
KW - Childhood Play Development
KW - Early Intervention
KW - Language Development
KW - Language Disorders
KW - Predisposition
RP - NOT IN FILE
SP - 1
EP - 15
JF - Topics in Language Disorders
VL - 2:
IS - 1
N2 - Discusses the development of symbolic play and language in preschool children in terms of the Piagetian transition from sensorimotor to representational thought. The development of early symbolization is reflected by the gradual separation of meaning from action and the integration of symbolic behaviors or meanings in sequences. The next step of this development is the ability to extend pretending beyond the child's own actions and experience. The symbolic play and language skills developed in the last 2 yrs form the basis of the more sophisticated sociodramatic play of the preschool and school-age child. Each of these stages is described in terms of the use of objects, prelanguage and language communication, and themes and roles. Child-centered play can provide both the context and the material for assessment and intervention with language-impaired and at-risk children as old as 5 yrs. Sociodramatic play with older children can lead to gains in both linguistic and social competence and can be adapted to various language intervention goals. (43 ref) (PsycINFO Database Record (c) 2012 APA, all rights reserved)
SN - 0271-8294
AD - McCune-Nicolich, Lorraine: Rutgers U Graduate School of Education, Graduate Faculty of Psychology, New Brunswick
ER -

TY - JOUR
ID - 880
T1 - The Children's Developmental Play Program: Physical activity designed to facilitate the growth and development of mildly handicapped children
A1 - Roswal,Glenn
Y1 - 1980///
N1 - Peer Reviewed Journal: 1981-31304-001
English
Journal; Peer Reviewed Journal
KW - PsycInfo
KW - Individualized Instruction
KW - Motor Skills
KW - Physical Activity
KW - Risk Taking

KW - Self Concept
KW - Disorders
KW - Physical Education
KW - Special Education
RP - NOT IN FILE
SP - 322
EP - 324
JF - Education & Training of the Mentally Retarded
VL - 15:
IS - 4
N2 - Describes a program that utilizes a fun approach to individualizing physical activities for developmentally disabled children. The fundamental concept is predicated on encouraging such children to master fine and gross motor skills while simultaneously improving their self-concept and optimizing risk-taking behaviors. The efficacy and significance of such a program is addressed. (12 ref) (PsycINFO Database Record (c) 2012 APA, all rights reserved)
SN - 0013-1237
AD - Roswal, Glenn: Jacksonville State U
ER -

TY - JOUR
ID - 881
T1 - The effects of a children's developmental play program on the self-concept, risk-taking, and motor proficiency of exceptional children
A1 - Roswal,Glenn M.
Y1 - 1979///
N1 - Dissertation Abstract: 1980-72861-001
English
Dissertation Abstract
KW - PsycInfo
KW - Childhood Play Behavior
KW - Gifted
KW - Psychomotor Development
KW - Risk Taking
KW - Self Concept
KW - Educational Programs
KW - Special Education
RP - NOT IN FILE
SP - 3189
JF - Dissertation Abstracts International
VL - 40:
IS - 6-A
SN - 0419-4217
AD - Roswal, Glenn M.: U Maryland
ER -

TY - JOUR
ID - 882
T1 - Games parents play with normal and high-risk infants
A1 - Field,Tiffany M.
Y1 - 1979///
N1 - Peer Reviewed Journal: 1979-33971-001
English
Journal; Peer Reviewed Journal
KW - PsycInfo
KW - Games
KW - Parent Child Relations

KW - Parents
KW - Predisposition
RP - NOT IN FILE
SP - 41
EP - 48
JF - Child Psychiatry and Human Development
VL - 10:
IS - 1
N2 - Game playing during early infant-parent interactions provided a context for learning conversational turntaking and contingent responsivity. The types and frequency of "universally" recognized infant games were observed during face-to-face interactions of 20 normal (i.e., term delivery) and 40 high risk (i.e., 20 preterm and 20 postterm) infant-parent dyads when the infants were 4 mo old. Approximately 6 different games were played for approximately 31% of the interaction time. Except during interactions with normal male infants mothers and fathers played games equally often. The high-risk infant-parent dyads engaged in game playing less frequently than the normal infant-parent dyads. These differences are discussed in the context of the high-risk infants being "difficult" babies and relatively less responsive. (16 ref) (PsycINFO Database Record (c) 2012 APA, all rights reserved)
SN - 0009-398X
AD - Field, Tiffany M.: U Miami School of Medicine, Mailman Ctr for Child Development, FL
ER -

TY - JOUR
ID - 883
T1 - Infant day care as a treatment intervention: A follow-up comparison study
A1 - Resch,Ruth C.
Y1 - 1977///
N1 - Peer Reviewed Journal: 1978-08397-001
English
Journal; Peer Reviewed Journal
KW - PsycInfo
KW - Child Day Care
KW - Childhood Play Development
KW - Infant Development
KW - Predisposition
KW - Psychopathology
KW - Followup Studies
KW - Primary Mental Health Prevention
KW - Separation Anxiety
KW - SOCIALIZATION
RP - NOT IN FILE
SP - 147
EP - 155
JF - Child Psychiatry and Human Development
VL - 7:
IS - 3
N2 - Evaluated the effects of early day care intervention for young children at risk for psychiatric and/or developmental pathology by comparing 2 groups of children: (a) 10 3-yr-olds entering regular day care who had been in an infant day care treatment from ages ranging from 3 to 18 mo, and (b) 14 normal matched 3-yr-old controls entering regular day care for the first time. All Ss were assessed for general pathology, play, socialization, and separation variables in arrival, play, and mealtime situations, and were naturalistically observed in the day centers. No significant differences between groups were found on any of the variables within situations or across situations, supporting the hypothesis that the treatment intervention supported major positive emotional developments and that the early separations were not detrimental in effect. Significant differences between the 2 groups on clusters of variables suggest patterns in coping and disturbance style specific to the control group. (PsycINFO Database Record (c) 2012 APA, all rights reserved)
SN - 0009-398X

AD - Resch, Ruth C.: State U New York, Downstate Medical Ctr
ER -

TY - JOUR
ID - 884
T1 - An empirical study of children's competitive play
A1 - Levy,Joseph
Y1 - 1976///
N1 - Peer-Reviewed Status-Unknown: 1978-03375-001
English
Journal; Peer-Reviewed Status-Unknown
KW - PsycInfo
KW - Achievement Motivation
KW - Childhood Play Behavior
KW - Competition
KW - Risk Taking
RP - NOT IN FILE
SP - 35
EP - 41
JF - Ontario Psychologist
VL - 8:
IS - 2
N2 - Examined the effect of high and low levels of achievement needs on risk-taking (RT) behavior in a competitive play situation. 144 boys in Grades 4-6 were divided into groups oriented toward high achievement (HA) and low achievement (LA) on the basis of the Children's Achievement Scale. RT was operationalized by the number of shots the S selected from 3 difficulty levels in a "ray gun" shooting contest and by the ability of the opponent selected by the S based on their viewing opponents of 3 different levels (trained confederates). As predicted, the LAs took more shots from the easy and hard categories, the HAs from the medium category. The expected similar differentiation in opponent selection was not found. Both groups tended to choose opponents of equal ability. (PsycINFO Database Record (c) 2012 APA, all rights reserved)
SN - 0030-3054
AD - Levy, Joseph: U Waterloo, Canada
ER -

TY - JOUR
ID - 885
T1 - Understanding child development through group techniques and play
A1 - Kernberg,Paulina F.
Y1 - 1975///
N1 - Peer Reviewed Journal: 1976-13183-001
English
Journal; Peer Reviewed Journal
KW - PsycInfo
KW - Childhood Development
KW - Empathy
KW - Group Counseling
KW - Recreation
KW - Teaching
RP - NOT IN FILE
SP - 409
EP - 419
JF - Bulletin of the Menninger Clinic
VL - 39:
IS - 5
N2 - Discusses the details of sessions and the results of a workshop on empathy with children, designed as an application of the Tavistock model. The workshop served as a laboratory where the following individual

differences were explored: coping mechanisms (including mechanisms of defense); aspects of socialization in different phases; and the crucial role of the teacher in a large group, as experienced by the participants in their workshop roles as "young students" under the consultant-teacher. A glimpse of what was necessary for empathic understanding of children was provided: i.e., to get in touch with the child within oneself. In the workshop climate, regressive phenomena could be expressed and the individual could risk a response. (15 ref) (PsycINFO Database Record (c) 2012 APA, all rights reserved)
SN - 0025-9284
AD - Kernberg, Paulina F.: Albert Einstein Coll of Medicine, Bronx Municipal Hosp Ctr, Yeshiva U
ER -

TY - JOUR
ID - 886
T1 - Predictions from play
A1 - Hutt,Corinne
Y1 - 1972///
N1 - Peer Reviewed Journal: 1972-31308-001
English
Journal; Peer Reviewed Journal
KW - PsycInfo
KW - Childhood Development
KW - Exploratory Behavior
KW - Personality
KW - Prediction
KW - Social Behavior
RP - NOT IN FILE
SP - 171
EP - 172
JF - Nature
VL - 237:
IS - 5351
N2 - Reexamined 48 7-10 yr. old Ss (from an original sample of 100 nursery schoolers) to determine the present manifestations of earlier-observed characteristics of curiosity and exploration. The earlier study showed that failure to explore was related to lack of adventure in boys and to difficulties in personality and social adjustment in girls. Results of the present tests indicate that failure to explore novelty was not necessarily associated with convergent thinking in girls, and that inventive play was positively associated with the propensity for divergent and creative thinking, particularly in boys. (17 ref.) (PsycINFO Database Record (c) 2012 APA, all rights reserved)
SN - 0028-0836
AD - Hutt, Corinne: U. Oxford, England
ER -

TY - JOUR
ID - 887
T1 - The importance of creative play
A1 - Michelman,Shirley
Y1 - 1971///
N1 - Peer Reviewed Journal: 1972-25390-001
English
Journal; Peer Reviewed Journal
KW - PsycInfo
KW - Adjustment
KW - Childhood Development
KW - Cognitive Processes
KW - Learning
KW - Social Behavior
RP - NOT IN FILE

SP - 285
EP - 290
JA - American Journal of Occupational Therapy
VL - 25
IS - 6
N2 - Discusses creative play as promoting problem-solving behavior which leads to learning, coping, and adaptation to change. The positive yields of the creative process are identified as curiosity, flexibility, improvisation, commitment, and the courage to risk. Developmental stages of creative play are described, as well as factors influencing creative behavior in both normal and handicapped children. (21 ref.) (PsycINFO Database Record (c) 2012 APA, all rights reserved)
SN - 0272-9490
AD - Michelman, Shirley: U. California, Center for Health Sciences, Los Angeles
ER -

TY - JOUR
ID - 888
T1 - Hysterical psychosis: Psychopathological aspects
A1 - Siomopoulos,V.
Y1 - 1971///
N1 - Peer Reviewed Journal: 1972-05275-001
English
Journal; Peer Reviewed Journal
KW - PsycInfo
KW - Case Report
KW - Fantasies (Thought Disturbances)
KW - Fantasy (Defense Mechanism)
KW - Hysteria
KW - Psychosis
RP - NOT IN FILE
SP - 95
EP - 100
JF - British Journal of Medical Psychology
VL - 44:
IS - 2
N2 - Hysterical psychosis represents regression to the specific form of thought activity involved in children's play. Reality testing in hysterical psychosis is not impaired, yet fantasy diffuses into reality. The 2 states, acted-out fantasy and reality, exist side by side, functioning in their own right and obeying their own rules of discourse. Play and the delusional ideas of hysterical psychosis constitute a microscopic picture in the area of thought processes of the massive splitting of consciousness that is identified, usually as a variant of conversion hysteria, as personality dissociation. Descriptions of auditory or visual hallucinations by hysterical psychotic patients represent not perceptualization of thought contents, but a linguistic shift from the frame of reference of thought content to that of sensory experience. Included are the case histories of a 33-yr-old female whose premorbid personality structure was considered to be of the hysterical type, and a 21-yr-old female with severe characterological difficulties who was considered to be a suicide risk. (PsycINFO Database Record (c) 2012 APA, all rights reserved)
SN - 0007-1129
AD - Siomopoulos, V.: U. Illinois, Medical School
ER -

TY - CHAP
ID - 889
T1 - Special guidance problems with migrant children. [References]
A1 - Wagoner,Lovisa C.
Y1 - 1947///

N1 - Book: 2009-00070-033
English
Book; Edited Book
KW - PsycInfo
KW - At Risk Populations
KW - Human Migration
KW - Psychosocial Development
KW - Risk Factors
KW - School Counseling
KW - Social Adjustment
KW - Sociocultural Factors
RP - NOT IN FILE
SP - 599
EP - 619
JF - Harms, Ernest [Ed]
N2 - (from the chapter) This chapter, incomplete as it is, bears out the findings of previous studies. The migrant child's position in the school is closely related to the position of his family in the community. Problems of social adjustment and emotional stability are temporary ones except in cases in which such difficulties are chronic and antedate the migration. Moving about may augment such inherent conditions, certainly it offers no cure. Delinquency always is greater in less favored areas. As yet it seems not to be increasing in proportion to population in spite of inadequate opportunities for play, bad housing conditions, too much money to spend. Of course, contact with different ways of life, acquaintance with different parts of the country have their value. Whether this equals that of a settled home, continuous schooling is certainly given to question. It must be remembered, however, that some families have bettered their condition; many children attend schools superior to any they have known heretofore. One significant fact that stands out is the general incooperation of child guidance procedures in the management of children. Another is the reiterated appreciation of family solidarity as the bulwark of the child's emotional and social stability. Generally speaking, the picture of the migrant child and his particular difficulties is an extremely complicated one, full of contradictory elements. Certain factors affecting the child are inevitably accompaniments of the way of life; others stem out of the particular family situation. The problems of the migrant child are not peculiar to his migratory status, but are those common to poverty, bad housing, lack of opportunity for play, and broken homes. (PsycINFO Database Record (c) 2012 APA, all rights reserved)
ER -

TY - JOUR
ID - 890
T1 - Playgrounds can be risky for children
Y1 - 2002/10//
N1 - Accession Number: 2003036288. Language: English. Entry Date: 20030221. Publication Type: journal article; brief item. Journal Subset: Biomedical; Online/Print; USA. NLM UID: 8303523
KW - Child Safety
KW - Play and Playthings
KW - Wounds and Injuries -- Prevention and Control -- In Infancy and Childhood
KW - Child
KW - CINAHL
RP - NOT IN FILE
SP - 23
EP - 23
JA - Orthopedics Today
VL - 22
IS - 10
SN - 0279-5647
UR - http://search.ebscohost.com/login.aspx?direct=true&db=cin20&AN=2003036288&site=ehost-live
ER -

TY - JOUR

ID - 891
T1 - Safeguarding kids from environmental hazards
Y1 - 2007/03//
N1 - Accession Number: 2009565037. Language: English. Entry Date: 20070720. Publication Type: journal article; CEU; pictorial; tables/charts. Journal Subset: Biomedical; Blind Peer Reviewed; Editorial Board Reviewed; Expert Peer Reviewed; Online/Print; Peer Reviewed; USA. Special Interest: Pediatric Care. No. of Refs: 29 ref. NLM UID: 8702030
KW - Child Safety
KW - Environmental Health
KW - Environmental Pollution -- Prevention and Control
KW - Hazardous Materials -- Adverse Effects
KW - Adolescence
KW - Asbestos -- Adverse Effects
KW - Carbon Monoxide Poisoning -- Prevention and Control
KW - Child
KW - Child Health -- Trends
KW - Child Safety -- Trends
KW - Child,Preschool
KW - Diagnosis,Differential
KW - Eating Behavior -- In Infancy and Childhood
KW - Education,Continuing (Credit)
KW - Environmental Exposure -- Adverse Effects
KW - Fish -- Adverse Effects
KW - Household Products -- Adverse Effects
KW - Lead Poisoning -- Prevention and Control
KW - Lead Poisoning -- Risk Factors
KW - Lung Diseases,Fungal -- Prevention and Control
KW - Mercury Poisoning -- Risk Factors
KW - Occupational Exposure -- Adverse Effects
KW - Passive Smoking -- Adverse Effects
KW - Passive Smoking -- Prevention and Control
KW - Play and Playthings -- Adverse Effects
KW - Radon -- Adverse Effects
KW - Seasons
KW - Skin Neoplasms -- Etiology
KW - Skin Neoplasms -- Prevention and Control
KW - Ultraviolet Rays -- Adverse Effects
KW - United States Environmental Protection Agency -- Standards
KW - Water Pollution -- Adverse Effects
KW - Water Pollution -- Prevention and Control
KW - CINAHL
RP - NOT IN FILE
SP - 64
EP - 78
JA - Contemporary Pediatrics
VL - 24
IS - 3
N2 - Pediatricians need to know how environmental toxicants affect kids' health, and where to turn for help
SN - 8750-0507
UR - http://search.ebscohost.com/login.aspx?direct=true&db=cin20&AN=2009565037&site=ehost-live
ER -

TY - JOUR
ID - 892
T1 - Chapter 5: Culturally Based Health and Illness Beliefs and Practices Across the Life Span
A1 - Anderson,N.L.R.

A1 - Andrews,M.
A1 - Bent,K.N.
A1 - Douglas,M.K.
A1 - Elhammoumi,C.V.
A1 - Keenan,C.
A1 - Kemppainen,J.K.
A1 - Lipson,J.G.
A1 - Thompson Martin,C.
A1 - Mattson,S.
Y1 - 2010/10/02/
N1 - Accession Number: 2010851439. Language: English. Entry Date: 20110107. Revision Date: 20130809. Publication Type: journal article; glossary; tables/charts. Supplement Title: Supplement. Journal Subset: Nursing; Peer Reviewed; USA. Special Interest: Nursing Education; Obstetric Care; Pediatric Care; Women's Health. NLM UID: 9001407
KW - Transcultural Nursing -- Education
KW - Education,Nursing
KW - Curriculum
KW - Health Beliefs
KW - Symptoms
KW - Medicine,Traditional
KW - Models,Theoretical
KW - Sick Role
KW - Transcultural Care
KW - Stigma
KW - Rituals and Ceremonies
KW - Religion and Religions
KW - Spiritual Care
KW - Health Services Accessibility
KW - Cultural Diversity
KW - Access to Information
KW - Health Information
KW - Health Status
KW - Childbirth
KW - Pregnancy
KW - Infant Care
KW - Child Rearing
KW - Parent-Child Relations
KW - Play and Playthings
KW - Child Care
KW - Child Health
KW - Ethnic Groups
KW - Hereditary Diseases
KW - Infant,Newborn
KW - Infant
KW - Child,Preschool
KW - Child
KW - Adolescence
KW - Sexuality
KW - Gender Identity
KW - Circumcision
KW - Female
KW - Male
KW - Child Abuse
KW - Anemia,Sickle Cell
KW - Risk Taking Behavior
KW - Acculturation

KW - Adult
KW - Middle Age
KW - GLBT Persons
KW - Reproductive Health
KW - Sexual Health
KW - Reproduction
KW - Acute Disease
KW - Trauma
KW - Family
KW - Sexually Transmitted Diseases
KW - Disabled
KW - Aged
KW - Chronic Disease
KW - Death
KW - Family Role
KW - Organ Procurement
KW - Organ Transplantation
KW - Culture
KW - Advance Care Planning
KW - Life Support Care
KW - Terminal Care
KW - Communication
KW - Discipline
KW - Human Rights
KW - Human Development
KW - CINAHL
RP - NOT IN FILE
SP - 152S
EP - 235s
JA - Journal of Transcultural Nursing
VL - 21
IS - 4
SN - 1043-6596
AD - University of California at Los Angeles, Los Angeles, CA USA
UR - http://search.ebscohost.com/login.aspx?direct=true&db=cin20&AN=2010851439&site=ehost-live
ER -

TY - JOUR
ID - 893
T1 - Affection workshop -- an intervention in primary health care and the contribution to the psycho-dramatic theory [Portuguese]
A1 - Araujo,J.S.
Y1 - 2005/01//
N1 - Accession Number: 2009151656. Language: Portuguese. Entry Date: 20060428. Revision Date: 20091218. Publication Type: journal article; research. Journal Subset: Biomedical; Double Blind Peer Reviewed; Editorial Board Reviewed; Expert Peer Reviewed; Mexico & Central/South America; Online/Print; Peer Reviewed. Special Interest: Pediatric Care; Public Health. No. of Refs: 8 ref
KW - Family Services
KW - Health Promotion
KW - Primary Health Care
KW - Psychodrama
KW - Adolescence
KW - Adult
KW - Brazil
KW - Child
KW - Child,Preschool

KW - Health Resource Utilization
KW - Play and Playthings
KW - Support,Psychosocial
KW - Human
KW - CINAHL
RP - NOT IN FILE
SP - 74
EP - 79
JA - Revista de Atencao Primaria a Saude
VL - 8
IS - 1
N2 - This study relates the experience of the Family Health Team (FHT) of the Jardim de Ala neighborhood in the city of Juiz de Fora, MG (1), beginning in February 2001, which covered micro-areas and population groups of higher social risk. The study was conducted by the Community Health Agents (CHA), under the supervision of the team's doctor and nurse, and was carried out using monthly meetings with children from the ages of 3 to 14, involving bingo games and snacks. The objective was to build a social support resource for these population groups that are submitted to greater social risk, in addition to improving the team/family link. The findings were an improvement in the link between the FHT and the families, and the perception, on the part of the FHT, that the users of the health services improved their utilization of preventive health activities. When we looked for theoretical support, we found reinforcement in J.L. Moreno's cluster theory for the proposal and the findings. We thus concluded: 1) games and play activities, applied by the CHAs are important tools to improve the link between the FHT and families under greater social risk; and 2) in addition to the theoretical propositions, the experience of the FHT at the Jardim de Ala demonstrated the need to study games as indirect facilitating tools of preventive health activities and also as psycho-social support
SN - 1516-7704
UR - http://search.ebscohost.com/login.aspx?direct=true&db=cin20&AN=2009151656&site=ehost-live
ER -

TY - JOUR
ID - 894
T1 - Children at risk: forensic nurses play a key role in caring for children living in methamphetamine labs
A1 - Bell,K.L.
Y1 - 2002/12//
N1 - Accession Number: 2009125793. Language: English. Entry Date: 20060324. Publication Type: journal article; pictorial. Journal Subset: Expert Peer Reviewed; Nursing; Online; Online/Print; Peer Reviewed; USA. No. of Refs: 19 ref
KW - Child Abuse
KW - Child Abuse -- Nursing
KW - Forensic Nursing
KW - Methamphetamine
KW - Substance Abuse
KW - Adult
KW - Child
KW - Child Development
KW - Child,Foster
KW - Child,Preschool
KW - Documentation
KW - Dopamine -- Metabolism
KW - Foster Home Care
KW - Home Environment
KW - Infant
KW - Methamphetamine -- Adverse Effects
KW - Methamphetamine -- Pharmacodynamics
KW - Methamphetamine -- Pharmacokinetics
KW - Nursing Assessment
KW - Parents

KW - Street Drugs
KW - Substance Abusers
KW - Substance Dependence
KW - United States
KW - CINAHL
RP - NOT IN FILE
SP - 1
JA - On the Edge
VL - 8
IS - 4
UR - http://search.ebscohost.com/login.aspx?direct=true&db=cin20&AN=2009125793&site=ehost-live
ER -

TY - THES
ID - 895
T1 - The emergence of purposeful reaching: developmental changes and constraints in hand and joint kinematics of early arm movements
A1 - Bhat,A.N.
Y1 - 2005///
N1 - Accession Number: 2009560967. Language: English. Entry Date: 20070427. Revision Date: 20091218. Publication Type: doctoral dissertation; research
KW - Arm -- In Infancy and Childhood
KW - Infant Development
KW - Movement -- In Infancy and Childhood
KW - Reaching -- In Infancy and Childhood
KW - Cross Sectional Studies
KW - Infant
KW - Infant Behavior
KW - Kinematics
KW - Play and Playthings
KW - Prospective Studies
KW - Shoulder
KW - Human
KW - CINAHL
RP - NOT IN FILE
EP - 138
PB - University of Delaware
N2 - Infants consistently reach for objects at three to five months of age. In the months before reaching, infants produce a variety of arm movements. The relationship between early arm movements and reaching is still unclear. The purpose of the present research was to determine changes in hand and joint kinematics of arm movements in the presence of a toy, in the weeks before reaching emerged. I conducted two different studies: a cross-sectional and a longitudinal study. During both the experiments, infants' arm movements were compared across two conditions: with and without a toy present. Kinematic analyses examined 3D hand, shoulder, and elbow motions. Infants showed multiple toy-oriented changes that were classified into three developmental phases. During the early phase infants scaled down their hand and joint motions in the presence of a toy and appeared to spend time visually exploring the toy. During the mid phase infants showed discontinuous and continuous toy-oriented changes. Discontinuous changes were limited to the mid phase and included an increase in movement number, speed, and smoothness. In general, infants scaled up their hand and joint motions and showed an "active period" wherein they explored their various joint motion combinations. Continuous changes existed across the mid and late phases and included an increase in hand's midline and forward positions and a decrease in distance between the hand and the toy. In addition, infants' ability to move closer to the toy was a result of toy-oriented changes in shoulder orientation and not the elbow or forearm. During the late phase infants moved closer to the toy and showed inconsistent or consistent toy contacts. The emergence of reaching showed increase in magnitude of continuous changes and additional increase in upward hand position and shoulder internal rotation orientation. These results suggest that there exists a systematic developmental trajectory for early arm movements. Early shoulder changes contribute to the emergence of reaching. Moreover, purposeful

reaching does not emerge de novo but is shaped from early arm movements. Together, these findings provide a normative database for future studies to identify patterns of infants at risk for reaching delays
SN - 0-542-29255-6
AV - UMI Order AAI3187605.
M1 - Ph.D.
UR - http://search.ebscohost.com/login.aspx?direct=true&db=cin20&AN=2009560967&site=ehost-live
ER -

TY - JOUR
ID - 896
T1 - Investigation of toy-noise exposure in children
A1 - Bittel,S.N.
A1 - Freeman,B.A.
A1 - Kemker,B.E.
Y1 - 2008/02//
N1 - Accession Number: 2009905511. Language: English. Entry Date: 20080627. Revision Date: 20091218. Publication Type: journal article; research; tables/charts. Journal Subset: Allied Health; Editorial Board Reviewed; Expert Peer Reviewed; Peer Reviewed; USA. Special Interest: Pediatric Care; Speech-Language Pathology/Audiology. No. of Refs: 17 ref. NLM UID: 8413380
KW - Environmental Exposure -- In Infancy and Childhood
KW - Hearing Loss,Noise-Induced -- Risk Factors
KW - Noise -- Standards
KW - Play and Playthings -- Standards
KW - Auditory Threshold -- Evaluation
KW - Child
KW - Child Safety -- Standards
KW - Hearing Loss,Noise-Induced -- Education
KW - Parents -- Education
KW - Product Labeling
KW - Product Surveillance
KW - Sound Spectrography -- Equipment and Supplies
KW - United States Occupational Safety and Health Administration
KW - Human
KW - CINAHL
RP - NOT IN FILE
SP - 10
EP - 18
JA - Seminars in Hearing
VL - 29
IS - 1
N2 - This study analyzed ear canal and open-field sound-pressure level measurements of 24 commercially available toys from five national retail stores. Only toys manufactured after creation of the American Society for Testing and Materials International (ASTM International) noise standard in 2003 were included. Sound-pressure level measurements were compared with the ASTM International standard to determine if toys put children at risk for acquiring noise-induced hearing loss (NIHL). The efficacy of this standard also was assessed. Results of this study indicate that many children's toys exceed the current recommended standards for safe noise levels. Children may be at risk for acquiring NIHL if exposed to these toys. A checks-and-balances system might encourage toy manufacturers to better adhere to the standard. Use of probe-microphone measurements in the creation of a new standard might be helpful. In addition to limiting maximum output levels, it might be prudent to require warning labels on all toys that produce noise at an intensity over a specified level. Current efforts to educate parents on the dangers of NIHL should be continued and expanded
SN - 0734-0451
AD - Nova Southeastern University, Fort Lauderdale, Florida
UR - http://search.ebscohost.com/login.aspx?direct=true&db=cin20&AN=2009905511&site=ehost-live
ER -

TY - JOUR
ID - 897
T1 - Readers panel. Risks and rewards
A1 - Brown,J.
A1 - Humm,C.
A1 - John,M.
A1 - Weeks,S.
Y1 - 2007/07/11/
N1 - Accession Number: 2009627945. Language: English. Entry Date: 20070831. Revision Date: 20080328. Publication Type: journal article; pictorial. Journal Subset: Double Blind Peer Reviewed; Europe; Expert Peer Reviewed; Nursing; Peer Reviewed; UK & Ireland. Special Interest: Pediatric Care. NLM UID: 8508427
KW - Child
KW - Child Safety
KW - Parent-Child Relations
KW - Play and Playthings
KW - CINAHL
RP - NOT IN FILE
SP - 26
EP - 27
JA - Nursing Standard
VL - 21
IS - 44
N2 - Should parents allow their children to play outdoors unsupervised, or is it too risky? We asked our readers panel
SN - 0029-6570
AD - Senior Clinical Governance Facilitator, Worcestershire Acute NHS Trust
UR - http://search.ebscohost.com/login.aspx?direct=true&db=cin20&AN=2009627945&site=ehost-live
ER -

TY - JOUR
ID - 898
T1 - Risky business: : dangerous games children play
A1 - Cartwright,C.
Y1 - 2009/03//
N1 - Accession Number: 2010270921. Language: English. Entry Date: 20090515. Revision Date: 20091204. Publication Type: journal article; abstract. Journal Subset: Canada; Editorial Board Reviewed; Nursing; Peer Reviewed. NLM UID: 101321312
KW - Games -- Adverse Effects -- In Adolescence
KW - Play and Playthings -- Adverse Effects -- In Adolescence
KW - Risk Taking Behavior -- In Adolescence
KW - Adolescence
KW - Neuroscience Nursing
KW - CINAHL
RP - NOT IN FILE
SP - 16
EP - 16
JA - Canadian Journal of Neuroscience Nursing
VL - 31
IS - 1
SN - 1913-7176
UR - http://search.ebscohost.com/login.aspx?direct=true&db=cin20&AN=2010270921&site=ehost-live
ER -

TY - JOUR
ID - 900
T1 - Language intervention and AAE-speaking children: issues and preliminary data

A1 - Craig-Unkefer,Lesley
A1 - Camarata,Stephen
Y1 - 2010/07//
N1 - Accession Number: 2010893077. Language: English. Entry Date: 20110114. Revision Date: 20110114. Publication Type: journal article; research; tables/charts. Journal Subset: Allied Health; Peer Reviewed; USA. Special Interest: Pediatric Care; Speech-Language Pathology/Audiology. Instrumentation: Peabody Picture Vocabulary Test (PPVT-3); Preschool Lanaguage Scale-3 (PLS-3) (Zimmerman et al); Expressive Vocabulary Test (EVT); Test for Auditory Comprehension of Language (TACL) (Carrow and Woolfolk). Grant Information: Grant 5P50DC003282-05 from the National Institute on Deafness and Other Communication Disorders of the National Institutes of Health. No. of Refs: 47 ref. NLM UID: 8201220
KW - Accents and Dialects
KW - Blacks
KW - Language Disorders -- In Infancy and Childhood
KW - Language Therapy -- Methods
KW - Child,Preschool
KW - Conversation
KW - Cultural Sensitivity
KW - Funding Source
KW - Grammar
KW - Human
KW - Illinois
KW - Interrater Reliability
KW - Language Tests
KW - Play and Playthings
KW - Pretest-Posttest Design
KW - Project Head Start -- Illinois
KW - Repeated Measures
KW - Role Models
KW - Speech and Language Assessment -- Methods
KW - Vocabulary
KW - CINAHL
RP - NOT IN FILE
SP - 205
EP - 222
JF - Topics in Language Disorders
VL - 30
IS - 3
N2 - Purpose: Facilitating language development in children with specific language impairment (SLI) who are learning African American English (AAE) as their first dialect requires clinicians to consider grammatical, lexical, and cultural differences. The purpose of this article is to examine 2 intervention methods that have an extensive history of validation for general American English -- imitation using discrete trials and conversational recast -- for use with children who speak AAE. Methods: Participants in the preliminary study were 4 pre-school-aged AAE-speaking children, who were at-risk for language delays and who used AAE forms. A within-participants design was employed. Results: Both interventions were associated with growth in contrastive and noncontrastive target structure use. All children increased production of specific language targets that were not observed in baseline samples in AAE or general American English forms, and language scores on posttreatment assessments increased in all participants. Discussion: Considerations include individualizing interventions on the basis of level of support and understanding the relationship between language learning and culture
SN - 0271-8294
AD - Vanderbilt University, Nashville, TN, USA
UR - http://search.ebscohost.com/login.aspx?direct=true&db=cin20&AN=2010893077&site=ehost-live
ER -

TY - JOUR
ID - 901
T1 - Parent directiveness in free play with young children with physical impairments

A1 - Cress,C.J.
A1 - Moskal,L.
A1 - Hoffmann,A.
Y1 - 2008/02//
N1 - Accession Number: 2009934097. Language: English. Entry Date: 20080711. Revision Date: 20120309. Publication Type: journal article; research; tables/charts. Journal Subset: Allied Health; Blind Peer Reviewed; Editorial Board Reviewed; Expert Peer Reviewed; Online/Print; Peer Reviewed; USA. Special Interest: Pediatric Care; Speech-Language Pathology/Audiology. Grant Information: Supported in part by Grant Number K08 DC00102-01A1 from the National Institute on Deafness and other Communicative Disorders, NIH. No. of Refs: 39 ref
KW - Alternative and Augmentative Communication
KW - Child Development Disorders
KW - Parental Behavior
KW - Parents of Disabled Children
KW - Age Factors
KW - Coding
KW - Communicative Disorders
KW - Correlational Studies
KW - Descriptive Statistics
KW - Female
KW - Funding Source
KW - Infant
KW - Male
KW - Mental Status
KW - Motor Skills Disorders
KW - Parent-Child Relations
KW - Play and Playthings
KW - Random Sample
KW - Human
KW - CINAHL
RP - NOT IN FILE
SP - 99
EP - 108
JF - Communication Disorders Quarterly
VL - 29
IS - 2
N2 - Children with disabilities are reported to experience a high degree of directive parent interaction compared to typically developing children because of poor communicative or task skills. This study examines relationships between parent behaviors (directiveness and contingency) and child skills (language and motor) for children with physical or neuromotor impairments at risk for being nonspeaking during undirected incidental play. The only correlation between parent behavior and child developmental status was between physical directiveness and child motor scores. Previous reports of high directiveness that increase with degree of disability might be attributable to task instructions during object play, in which high physical directiveness would be required for this population
SN - 1525-7401
AD - University of Nebraska-Lincoln
UR - http://search.ebscohost.com/login.aspx?direct=true&db=cin20&AN=2009934097&site=ehost-live
ER -

TY - JOUR
ID - 902
T1 - A positive behavioural intervention for toddlers: parent-child attunement therapy
A1 - Dombrowski,S.C.
A1 - Timmer,S.G.
A1 - Blacker,D.M.
A1 - Urquiza,A.J.

Y1 - 2005/03//
N1 - Accession Number: 2009130869. Language: English. Entry Date: 20070126. Revision Date: 20091218. Publication Type: journal article; research; tables/charts. Journal Subset: Allied Health; Europe; Online/Print; Peer Reviewed; UK & Ireland. Special Interest: Pediatric Care; Social Work. Instrumentation: Eyberg Child Behavior Inventory (ECBI)(Eyberg and Robinson); Emotional Availability Scales (EA)(Biringen et al); Child Behavior Checklist (CBCL) (Achenbach and Edelbrock); Parenting Stress Index (PSI-sf). No. of Refs: 45 ref. NLM UID: 9301476. Email: sdombrowski@rider.edu
KW - Child Abuse
KW - Child Behavior Disorders -- Therapy
KW - Mother-Infant Relations
KW - Parenting Education
KW - Adult
KW - California
KW - Checklists
KW - Child Behavior Checklist
KW - Child Behavior Disorders -- Complications
KW - Clinical Assessment Tools
KW - Communication
KW - Emotions
KW - Female
KW - Infant
KW - Infant Behavior
KW - Interrater Reliability
KW - Interviews
KW - Intraclass Correlation Coefficient
KW - Male
KW - Marriage -- Psychosocial Factors
KW - Nonparticipant Observation
KW - One-Shot Case Study
KW - Outcome Assessment
KW - Parenting
KW - Play and Playthings
KW - Reflection
KW - Reinforcement (Psychology)
KW - Repeated Measures
KW - Scales
KW - Human
KW - CINAHL
RP - NOT IN FILE
SP - 132
EP - 151
JA - Child Abuse Review
VL - 14
IS - 2
N2 - Parent-child attunement therapy (PCAT) is a promising intervention for toddlers (aged 12-30 months) who have experienced maltreatment. PCAT has two overall purposes: (1) to strengthen caregivers' relationship with their children; and (2) to facilitate caregivers' learning of appropriate child management techniques. PCAT represents an adaptation of parent-child interaction therapy (PCIT), which has been empirically documented in preschool and early elementary schoolchildren to improve behavioural adjustment and engender a stronger bond between caregiver and child. There is, however, a noted paucity of intervention research for toddlers, specifically maltreated toddlers. As toddlerhood represents a critical period for enhancing the relationship between caregivers and children and is a stage when youngsters are at increased risk for maltreatment, the objectives of PCAT become even more salient during the toddler years. The purpose of this study, therefore, is to introduce PCAT and then examine its effectiveness through a single case study of a 23-month-old maltreated toddler and his biological mother. Pre- and post-assessment measures included the Parenting Stress Index, the Dyadic Parent-Child Interaction Coding System (DPICS), the Achenbach Child Behavior Checklist (CBCL), the

Emotional Availability (EA) Scales and the Eyberg Child Behavior Inventory (ECBI). The results of this study demonstrate the effectiveness of PCAT in increasing the number of positive caregiver-toddler interactions and enhancing the overall quality of the caregiver-toddler relationship. Practitioners will be able to use the techniques described in this manuscript to improve the parent-toddler relationship and ameliorate many commonly experienced behavioural difficulties found among maltreatment-prone parent-toddler dyads. Therapeutic progress is easily charted so that effectiveness may be documented and termination of therapy may be easily discerned. Copyright - ª2005 John Wiley & Sons, Ltd
SN - 0952-9136
AD - 202 Memorial Hall, Rider University Lawrenceville, New Jersey
UR - http://search.ebscohost.com/login.aspx?direct=true&db=cin20&AN=2009130869&site=ehost-live
ER -

TY - JOUR
ID - 903
T1 - Physical activity, adiposity and urbanization level in children: results for the Italian cohort of the IDEFICS study
A1 - Donatiello,E.
A1 - Dello,Russo
A1 - Formisano,A.
A1 - Lauria,F.
A1 - Nappo,A.
A1 - Reineke,A.
A1 - Sparano,S.
A1 - Barba,G.
A1 - Russo,P.
A1 - Siani,A.
Y1 - 2013/08//
N1 - Accession Number: 2012366402. Language: English. Entry Date: 20131122. Publication Type: journal article; research; tables/charts. Journal Subset: Biomedical; Europe; Peer Reviewed; Public Health; UK & Ireland. Special Interest: Pediatric Care; Public Health. Grant Information: European Community, within the Sixth RTD Framework Programme Contract No. 016181 (FOOD).. NLM UID: 0376507
KW - Physical Activity -- In Infancy and Childhood
KW - Adipose Tissue -- In Infancy and Childhood
KW - Urban Population -- Italy
KW - Geographic Factors -- Italy
KW - Life Style -- Classification
KW - Human
KW - Funding Source
KW - Prospective Studies -- Italy
KW - Italy
KW - Obesity -- Risk Factors -- In Infancy and Childhood
KW - Child
KW - Child,Preschool
KW - Questionnaires
KW - Body Weights and Measures
KW - COMPARATIVE studies
KW - Rural Population
KW - Suburban Population
KW - Natural Environment
KW - Descriptive Statistics
KW - Play and Playthings -- Classification
KW - CINAHL
RP - NOT IN FILE
SP - 761
EP - 765
JA - Public Health (Elsevier)

VL - 127
IS - 8
SN - 0033-3506
AD - Epidemiology and Population Genetics, Institute of Food Sciences, CNR, Avellino, Italy
BIPS -- Institute for Epidemiology and Prevention Research GmbH, Bremen, Germany
UR - http://search.ebscohost.com/login.aspx?direct=true&db=cin20&AN=2012366402&site=ehost-live
ER -

TY - JOUR
ID - 904
T1 - As they grow. 4-5 years: little daredevil
A1 - Durocher,H.J.
Y1 - 2006/11//
N1 - Accession Number: 2009571647. Language: English. Entry Date: 20070525. Publication Type: journal article; pictorial. Journal Subset: Consumer Health; USA. Special Interest: Consumer Health; Pediatric Care. NLM UID: 9891378
KW - Child Behavior
KW - Parenting
KW - Play and Playthings -- In Infancy and Childhood
KW - Risk Taking Behavior -- In Infancy and Childhood
KW - Adult
KW - Child,Preschool
KW - CINAHL
RP - NOT IN FILE
SP - 219
EP - 220
JA - Parents (10836373)
VL - 81
IS - 11
SN - 1083-6373
UR - http://search.ebscohost.com/login.aspx?direct=true&db=cin20&AN=2009571647&site=ehost-live
ER -

TY - JOUR
ID - 905
T1 - Tales from the road: memorable experiences at work or play. Of fairs, fires, and family fun... this article originally appeared in Healthcare Traveler
A1 - Eason,J.A.
Y1 - 2007/03//
N1 - Accession Number: 2009546266. Language: English. Entry Date: 20070504. Revision Date: 20091030. Publication Type: journal article; pictorial. Journal Subset: Core Nursing; Nursing; Peer Reviewed; USA. NLM UID: 20010080R
KW - Careers in Nursing
KW - Traveling Health Professionals
KW - Adolescence
KW - California
KW - Family
KW - Female
KW - Male
KW - Registered Nurses
KW - CINAHL
RP - NOT IN FILE
SP - 55
EP - 55
JA - RN
VL - 70

IS - 3
N2 - When Robert Gilman, RN, received a wedding invitation from his cousin, he never realized it would turn into an opportunity to share an amazing adventure with his children
SN - 0033-7021
UR - http://search.ebscohost.com/login.aspx?direct=true&db=cin20&AN=2009546266&site=ehost-live
ER -

TY - JOUR
ID - 906
T1 - DataBase: research and evaluation results. A national study of neighborhood safety, outdoor play, television viewing, and obesity in preschool children
A1 - Evenson,K.
A1 - Aytur,S.
Y1 - 2006/09//
N1 - Accession Number: 2009338782. Language: English. Entry Date: 20061208. Revision Date: 20091218. Publication Type: journal article; abstract; commentary; research. Original Study: Burdette HL and Whitaker RC. A national study of neighborhood safety, outdoor play, television viewing, and obesity in preschool children. PEDIATRICS 2005 Sep; 116(3): 657-62 (research) . Journal Subset: Blind Peer Reviewed; Double Blind Peer Reviewed; Editorial Board Reviewed; Expert Peer Reviewed; Health Promotion/Education; Peer Reviewed; USA. Special Interest: Pediatric Care. NLM UID: 8701680
KW - Obesity -- Epidemiology -- In Infancy and Childhood
KW - Obesity -- Risk Factors -- In Infancy and Childhood
KW - Physical Activity
KW - Residence Characteristics
KW - Television
KW - Child,Preschool
KW - Human
KW - CINAHL
RP - NOT IN FILE
SP - 59
EP - 59
JF - American Journal of Health Promotion
JA - Am J Health Promot
VL - 21
IS - 1
SN - 0890-1171
UR - http://search.ebscohost.com/login.aspx?direct=true&db=cin20&AN=2009338782&site=ehost-live
ER -

TY - JOUR
ID - 907
T1 - Unsettling Representational Practices: Inhabiting Relational Becomings in Early Childhood Education
A1 - Fikile,Nxumalo
Y1 - 2012/07//
N1 - Accession Number: 2011887239. Language: English. Entry Date: 20130621. Revision Date: 20130621. Publication Type: journal article; pictorial. Journal Subset: Biomedical; Blind Peer Reviewed; Editorial Board Reviewed; Online/Print; Peer Reviewed; USA. Special Interest: Pediatric Care. No. of Refs: 59 ref. NLM UID: 7801928
KW - Cultural Diversity
KW - Education -- In Infancy and Childhood
KW - Race Relations
KW - Social Identity -- In Infancy and Childhood
KW - Social Justice
KW - Child
KW - Child Welfare
KW - Child,Preschool

KW - Play and Playthings
KW - Politics
KW - Schools,Nursery
KW - Stigma
KW - Students,Elementary
KW - Teaching Methods
KW - CINAHL
RP - NOT IN FILE
SP - 281
EP - 302
JA - Child & Youth Services
VL - 33
IS - 3-4
N2 - This article seeks to unsettle representational practices enacted through dominant multicultural pedagogical approaches in the early childhood classroom. Drawing from a research study in early childhood centers that investigated practitioners' and children's negotiations of racial difference, I explore how multicultural pedagogical approaches in early childhood spaces present a risk through the potential for static representations of difference and diversity. I argue that these approaches potentially reproduce inequalities and delimit ways of engaging with difference and diversity through prescribing identity and dampening capacities for certain bodies in certain spaces. I offer possibilities for movements away from pre-defined and prescriptive approaches toward complexified approaches that require close attunement to the emergence of material-discursive assemblages. Attention to relational becomings has the potential to open possibilities for socially just early childhood pedagogies that enact a micropolitical engagement with the material-discursive entanglements of everyday encounters
SN - 0145-935X
UR - http://search.ebscohost.com/login.aspx?direct=true&db=cin20&AN=2011887239&site=ehost-live
ER -

TY - JOUR
ID - 909
T1 - Medical malpractice and its consequences: does physician gender play a role?
A1 - Freeborn,D.K.
A1 - Levinson,W.
A1 - Mullooly,J.P.
Y1 - 1999/09//
N1 - Accession Number: 2000017878. Language: English. Entry Date: 20000301. Revision Date: 20091218. Publication Type: journal article; research; tables/charts. Journal Subset: Peer Reviewed; USA. Instrumentation: Tedium Index (Pines et al). No. of Refs: 40 ref. NLM UID: 9889882. Email: Don.Freeborn@kp.org
KW - Physicians
KW - Sex Factors
KW - Malpractice -- Legislation and Jurisprudence -- United States
KW - United States
KW - Risk Factors
KW - Health Maintenance Organizations
KW - Malpractice -- Psychosocial Factors
KW - SURVEYS
KW - Physicians,Women
KW - Burnout,Professional
KW - Hypothesis
KW - Ohio
KW - Northwestern United States
KW - Evaluation Research
KW - Professional Practice -- Methods
KW - Questionnaires
KW - Self Report
KW - Workload

KW - Summated Rating Scaling
KW - Coefficient Alpha
KW - Chi Square Test
KW - Analysis of Variance
KW - Independent Variable
KW - Logistic Regression
KW - Multiple Regression
KW - Descriptive Statistics
KW - P-Value
KW - Specialties,Medical
KW - Time Factors
KW - Odds Ratio
KW - Confidence Intervals
KW - Job Satisfaction
KW - Psychological Tests
KW - Child
KW - Aged
KW - Pregnancy
KW - Female
KW - Male
KW - Human
KW - CINAHL
RP - NOT IN FILE
SP - 201
EP - 214
JA - Journal of Gender, Culture, & Health
VL - 4
IS - 3
N2 - Research suggests that female physicians have a more patient-centered communication style, characterized by more collaboration with the patient and discussion of treatment options. These same communication characteristics have been associated with patient satisfaction and malpractice prevention. Few empirical studies, however, have examined whether gender affects malpractice risk. The primary objective of this study was to determine whether gender was associated with malpractice among health maintenance organization (HMO) physicians. A secondary objective was to examine how malpractice affected physicians' psychological well-being. Data came from mail surveys of physicians practicing in two group model HMOs. The average response rate was 80% (N = 608). Female physicians were significantly less likely than male physicians to have been sued. Only 26% of the female physicians had been sued compared with 49% of the male physicians. These differences remained after adjusting for physician age, time in practice, specialty, workload, and characteristics of physicians' patient panels. Sued physicians were significantly more at risk for burnout than never-sued physicians and were less satisfied with the HMO. These findings did not differ by gender
SN - 1087-3201
AD - Kaiser Center for Health Research, 3800 N. Interstate Avenue, Portland, Oregon 97227
UR - http://search.ebscohost.com/login.aspx?direct=true&db=cin20&AN=2000017878&site=ehost-live
ER -

TY - JOUR
ID - 911
T1 - Are children attending child play areas at risk of infectious diseases? What can be done?
A1 - Gauci,C.
A1 - Borg,D.
Y1 - 2007///
N1 - Accession Number: 2009521814. Language: English. Entry Date: 20070525. Revision Date: 20100409. Publication Type: journal article; research; tables/charts. Journal Subset: Europe; Health Promotion/Education; Online/Print; Peer Reviewed; Public Health; UK & Ireland. Special Interest: Public Health. No. of Refs: 14 ref. Email: charmaine.gauci@gov.mt
KW - Communicable Diseases -- Prevention and Control -- In Infancy and Childhood

KW - Infection Control
KW - Microbial Contamination
KW - Play and Playthings
KW - Accreditation
KW - Child
KW - Descriptive Statistics
KW - Environmental Monitoring
KW - Food Handling
KW - Health Knowledge
KW - Interviews
KW - Mediterranean Islands
KW - Parental Attitudes
KW - Parents
KW - Questionnaires
KW - Risk Assessment
KW - Sterilization and Disinfection
KW - SURVEYS
KW - Human
KW - CINAHL
RP - NOT IN FILE
SP - 22
EP - 28
JF - Journal of the Royal Society for the Promotion of Health
JA - J R SOC PROMOT HEALTH
VL - 127
IS - 1
N2 - Aims: Child play areas can pose a risk to children of infectious diseases. The prolonged presence of micro-organisms in the environment has already been established. In order to quantify this risk, specific studies are required when carrying out a risk assessment
Methods: In order to assess the microbiological hazard in play areas in Malta, a study was performed during 2005, which consisted of an examination of the hygienic practices carried out in play areas by means of face-to-face interviews; inspections of the premises; environmental sampling and testing, and a study on the awareness and attitudes of parents to risk factors (face-to-face interviews). Follow up studies were performed after recommendations on risk management were provided
Results: Overall, the hygienic practices in play areas were satisfactory. Of the premises 66% were licensed, 55% of the food handlers were registered, appropriate cooking and cooling facilities were available for most of the premises, temperature control records were available at 80% of premises, 60% were using disinfectant for cleaning play area surfaces and monitoring of refrigerated vehicles was carried out by 60%. There was an overall improvement after recommendations were given
At the first inspection, 67% of the premises were categorized as being fair and 33% as being good. On repeat inspection, after recommendations were made, the grading of the premises were: excellent 7%, satisfactory 33%, good 53% and fair 7%
The results of the environmental swabs taken had low counts of indicator organisms indicating a good overall hygienic condition
Parents stated that 58% of the areas were in good hygienic condition but lacked adequate hand-washing facilities for children. All parents agreed with the importance of hand hygiene and that infectious diseases can be transmitted via contaminated objects and from one person to another
Conclusion: An effective control strategy needs to be implemented involving all stakeholders to ensure that effective hygienic practices in play areas are available to protect children from infectious diseases
SN - 1466-4240
AD - Head of Disease Surveillance Unit, Disease Surveillance Unit, 37-39 Rue D'Argens, Msida, Malta
UR - http://search.ebscohost.com/login.aspx?direct=true&db=cin20&AN=2009521814&site=ehost-live
ER -

TY - JOUR
ID - 912

T1 - Interventions for childhood overweight and obesity: a place for parenting skills
A1 - Gibbons,K.
Y1 - 2007/09//
N1 - Accession Number: 2009657268. Language: English. Entry Date: 20080125. Publication Type: journal article; commentary. Original Study: GOLLEY RK, PERRY RA, MAGAREY A, and DANIELS L. Family-focused weight management program for five- to nine-year-olds incorporating parenting skills training with healthy lifestyle information to support behaviour modification. NUTR DIET 2007 Sep; 64(3): 144-50 . Journal Subset: Allied Health; Australia & New Zealand; Blind Peer Reviewed; Online/Print; Peer Reviewed. Special Interest: Nutrition; Pediatric Care. No. of Refs: 17 ref
KW - Obesity -- Therapy -- In Infancy and Childhood
KW - Parenting
KW - Weight Reduction Programs
KW - Australia
KW - Behavioral Changes
KW - Child
KW - Child Nutrition
KW - Obesity -- Risk Factors
KW - Parenting Education
KW - Play and Playthings
KW - Sleep
KW - Television
KW - CINAHL
RP - NOT IN FILE
SP - 142
EP - 143
JA - Nutrition & Dietetics
VL - 64
IS - 3
SN - 1446-6368
UR - http://search.ebscohost.com/login.aspx?direct=true&db=cin20&AN=2009657268&site=ehost-live
ER -

TY - JOUR
ID - 913
T1 - Myofascial Structural Integration: A Promising Complementary Therapy for Young Children With Spastic Cerebral Palsy
A1 - Hansen,Alexis
A1 - Price,Karen
A1 - Feldman,Heidi
Y1 - 2012/04//
N1 - Accession Number: 2011495669. Language: English. Entry Date: 20120420. Revision Date: 20120824. Publication Type: journal article; research; tables/charts. Journal Subset: Alternative/Complementary Therapies; Nursing; Online/Print; Peer Reviewed; USA. Special Interest: Pediatric Care; Physical Therapy. Instrumentation: Child Behavior Checklist (CBCL) (Achenbach and Edelbrock); Gross Motor Function Classification System (GMFCS); Gross Motor Function Measure (GMFM)-66; International Classification of Functioning, Disability, and Health. Grant Information: This study was conducted with support from Stanford University Medical School Medical Scholars funding program.. No. of Refs: 20 ref
KW - Muscle Spasticity -- Therapy
KW - Manual Therapy -- Psychosocial Factors
KW - Cerebral Palsy -- Complications
KW - Myofascial Release -- Methods
KW - Motor Skills Disorders -- Classification -- In Infancy and Childhood
KW - Rolfing -- Methods
KW - Funding Source
KW - Child
KW - Pilot Studies

KW - Human
KW - Male
KW - Female
KW - Child,Preschool
KW - Scales
KW - Play and Playthings
KW - Random Assignment
KW - Crossover Design
KW - Professional-Family Relations
KW - Muscle Relaxation -- Physiology
KW - Contracture -- Risk Factors
KW - Students,Medical
KW - Videorecording
KW - Child Behavior Checklist
KW - Descriptive Statistics
KW - Range of Motion
KW - International Classification of Functioning,Disability,and Health
KW - Treatment Outcomes
KW - CINAHL
RP - NOT IN FILE
SP - 131
EP - 135
JA - Journal of Evidence-Based Complementary & Alternative Medicine
VL - 17
IS - 2
SN - 2156-5872
AD - School of Medicine, Stanford University, Stanford, CA, USA
School of Medicine, Stanford University, Stanford, CA, USA hfeldman@stanford.edu
UR - http://search.ebscohost.com/login.aspx?direct=true&db=cin20&AN=2011495669&site=ehost-live
ER -

TY - JOUR
ID - 915
T1 - Can I Play? A Concept Analysis of Participation in Children with Disabilities
A1 - Hoogsteen,L.
A1 - Woodgate,R.L.
Y1 - 2010/11//
N1 - Accession Number: 2010828509. Language: English. Entry Date: 20101119. Revision Date: 20120727. Publication Type: journal article; case study; research; systematic review; tables/charts. Journal Subset: Allied Health; Double Blind Peer Reviewed; Editorial Board Reviewed; Expert Peer Reviewed; Online; Peer Reviewed; USA. Special Interest: Evidence-Based Practice; Occupational Therapy; Pediatric Care; Physical Therapy. No. of Refs: 84 ref. NLM UID: 8109120
KW - Play and Playthings -- In Infancy and Childhood
KW - Social Behavior -- Classification -- In Infancy and Childhood
KW - Child,Disabled
KW - Child
KW - Human
KW - Concept Analysis
KW - Social Alienation
KW - Skill Acquisition
KW - Psychological Well-Being
KW - Canada
KW - Models,Theoretical
KW - Conceptual Framework
KW - International Classification of Functioning,Disability,and Health
KW - Occupational Therapy Practice,Evidence-Based

KW - Physical Therapy Practice,Evidence-Based
KW - CINAHL Database
KW - Cochrane Library
KW - Medline
KW - Male
KW - Paraplegia
KW - Adolescence
KW - Female
KW - Interpersonal Relations
KW - Autonomy
KW - Quality of Life
KW - Occupation (Human)
KW - CINAHL
RP - NOT IN FILE
SP - 325
EP - 339
JA - Physical & Occupational Therapy in Pediatrics
VL - 30
IS - 4
N2 - Participation is essential to children's development. Children with disabilities are at risk for restricted participation. Despite wide use of the word participation, the definition remains vague. The eight steps of analysis by Walker and Avant (2005) were used to guide the concept analysis. The uses and defining attributes of participation were identified and cases formulated within the context of children with disabilities and health care professions. In order to participate, a child with disabilities must take part in something or with someone, they must have a sense of inclusion, control over what they are taking part in, and be working toward obtaining a goal or enhanced quality of life. Through participation children acquire new skills, have increased physical, emotional and social well-being and enhanced quality of life. It is hoped that health care professionals can use this definition to address the shortcomings of existing programs and develop measures to assess the attributes of participation
SN - 0194-2638
AD - Faculty of Nursing, University of Manitoba, Winnipeg, Manitoba R3T 2N2, Canada (E-mail: umhoogsl@cc.umanitoba.ca)
UR - http://search.ebscohost.com/login.aspx?direct=true&db=cin20&AN=2010828509&site=ehost-live
ER -

TY - JOUR
ID - 917
T1 - New Adventures with the Fire Ants
A1 - Huhtanen,Shelly
Y1 - 2011/11//
N1 - Accession Number: 2011401413. Language: English. Entry Date: 20120113. Revision Date: 20120224. Publication Type: journal article; anecdote; pictorial. Journal Subset: Consumer Health; USA. Special Interest: Consumer Health; Pediatric Care. NLM UID: 7702637
KW - Child,Disabled
KW - Autistic Disorder
KW - Play and Playthings
KW - Child
KW - Male
KW - Interpersonal Relations
KW - Teamwork
KW - CINAHL
RP - NOT IN FILE
SP - 28
EP - 28
JA - Exceptional Parent
VL - 41

IS - 11
SN - 0046-9157
UR - http://search.ebscohost.com/login.aspx?direct=true&db=cin20&AN=2011401413&site=ehost-live
ER -

TY - JOUR
ID - 918
T1 - Effect of sleep and play positions on infant development: reconciling developmental concerns with SIDS prevention
A1 - Hunter,J.G.
A1 - Malloy,M.H.
Y1 - 2002/03//
N1 - Accession Number: 2004030370. Language: English. Entry Date: 20040206. Revision Date: 20080725. Publication Type: journal article; case study; pictorial; tables/charts. Journal Subset: Nursing; Online/Print; USA. No. of Refs: 65 ref. NLM UID: 101126037
KW - Infant Development
KW - Infant Development Disorders -- Etiology
KW - Motor Skills Disorders -- Etiology -- In Infancy and Childhood
KW - Patient Positioning -- In Infancy and Childhood
KW - Play and Playthings -- In Infancy and Childhood
KW - Sleep -- In Infancy and Childhood
KW - Sudden Infant Death -- Prevention and Control
KW - Cranial Molding
KW - Infant
KW - Infant,Newborn
KW - Infant,Premature
KW - Male
KW - Posture -- In Infancy and Childhood
KW - Prone Position -- Adverse Effects -- In Infancy and Childhood
KW - Sudden Infant Death -- Epidemiology -- United States
KW - Sudden Infant Death -- Risk Factors
KW - Supine Position -- In Infancy and Childhood
KW - United States
KW - CINAHL
RP - NOT IN FILE
SP - 9
EP - 16
JA - Newborn & Infant Nursing Reviews
VL - 2
IS - 1
N2 - Preterm infants have traditionally been considered at risk for positional deformities and delayed or atypical development. During the last decade, however, similar problems have increasingly been documented in normal infants born at term gestation, with recommendations of supine sleeping for prevention of sudden infant death syndrome often implicated as a causal factor. This article seeks to inform and educate neonatal and pediatric nurses on positional and postural influences in normal and delayed infant development, including the relationship of sleep and play positions to developmental issues. An observant nurse who understands normal development, can recognize atypical postures, and is knowledgeable about potential developmental consequences is in a position to intervene at the earliest interaction with the family. The American Academy of Pediatrics (AAP) sleep position guidelines, however, must not be compromised in the process. Current AAP rationale and guidelines for prevention of sudden infant death syndrome are summarized to facilitate nursing efforts to correctly model and teach infant caregivers. Common-sense suggestions for hospital and community-based nurses to help parents avoid preventable developmental delay while reinforcing current sleep position guidelines are presented. Copyright 2002, Elsevier Science (USA). All rights reserved
SN - 1527-3369
AD - Department of Rehabilitation Services, Infant Special Care Unit, University of Texas Medical Branch, Galveston, TX

UR - http://search.ebscohost.com/login.aspx?direct=true&db=cin20&AN=2004030370&site=ehost-live
ER -

TY - JOUR
ID - 919
T1 - MINERVA
A1 - Jamal-Hanjani,Mariam
A1 - Beharry,Nigel
A1 - Samol,Jens
Y1 - 2011/07/02/
N1 - Accession Number: 2011244555. Language: English. Entry Date: 20110902. Revision Date: 20111111. Publication Type: journal article; diagnostic images. Journal Subset: Biomedical; Blind Peer Reviewed; Editorial Board Reviewed; Europe; Expert Peer Reviewed; Online/Print; Peer Reviewed; UK & Ireland. NLM UID: 101090866
KW - Stress Disorders,Post-Traumatic
KW - Dementia -- Risk Factors
KW - Cerebral Edema -- Complications
KW - Neoplasms -- Therapy
KW - Nanomedicine
KW - Spinal Fractures -- Surgery
KW - Domestic Violence -- Complications
KW - Child Behavior Disorders -- Etiology
KW - Play and Playthings -- Evaluation
KW - Arthroplasty,Replacement,Knee -- Evaluation
KW - Creutzfeldt-Jakob Syndrome -- Diagnosis
KW - Cardiovascular Diseases -- Mortality
KW - Physicians -- Evaluation
KW - Infant
KW - Child
KW - CINAHL
RP - NOT IN FILE
SP - 50
EP - 50
JA - BMJ: British Medical Journal (Overseas & Retired Doctors Edition)
VL - 343
IS - 7813
SN - 1759-2151
AD - Speicalist registrar in medical oncology, St George+,-+s Hospital Healthcare NHS Trust, London SW17 0QT
Consultant radiologist, St George+,-+s Hospital Healthcare NHS Trust, London SW17 0QT
Consultant medical oncologist, St George+,-+s Hospital Healthcare NHS Trust, London SW17 0QT
UR - http://search.ebscohost.com/login.aspx?direct=true&db=cin20&AN=2011244555&site=ehost-live
ER -

TY - JOUR
ID - 920
T1 - Group intervention as one facet of a multi-component intervention with high risk mothers and their babies
A1 - Jarrett,M.H.
A1 - Diamond,L.T.
A1 - El-Mohandes,A.
Y1 - 2000/07//
N1 - Accession Number: 2000055477. Language: English. Entry Date: 20000901. Revision Date: 20091218. Publication Type: journal article; research; tables/charts. Journal Subset: Biomedical; Peer Reviewed; USA. Grant Information: Grants (U18-HD30445, U18-HD30447, U18-HD30454, U18-HD30458, U18-HD30463, and U18-HD31206) from the NICHD and the NIH ORMH. No. of Refs: 36 ref. NLM UID: 8915767
KW - Support,Psychosocial

KW - Poverty
KW - Early Childhood Intervention
KW - Parenting Education
KW - Random Assignment
KW - Experimental Studies
KW - Support Groups
KW - Infant Development
KW - Research Subject Recruitment
KW - Mothers
KW - Play and Playthings
KW - Interviews
KW - Consumer Satisfaction -- Evaluation
KW - Adult
KW - Female
KW - Funding Source
KW - Human
KW - CINAHL
RP - NOT IN FILE
SP - 15
EP - 24
JA - Infants & Young Children: An Interdisciplinary Journal of Special Care Practices
VL - 13
IS - 1
N2 - The relationship between infants and parents is affected by the larger social system, including the correlates of poverty. Group intervention services have proven to be an effective means of informing and supporting parents of infants and young children. The Pride in Parenting Group Intervention Curriculum was developed as part of a multicomponent intervention with a population of indigent African American women and their infants. In exit interviews with 57 mothers, parent satisfaction was high for developmental and parenting information and for the support of the group and the intervention professionals. Copyright - ª2000 by Aspen Publishers, Inc
SN - 0896-3746
AD - Associate Professor, George Washington University, Washington, EC
UR - http://search.ebscohost.com/login.aspx?direct=true&db=cin20&AN=2000055477&site=ehost-live
ER -

TY - JOUR
ID - 922
T1 - Child and mother variables in the development of stuttering among high-risk children: a longitudinal study
A1 - Kloth,S.
A1 - Janssen,P.
A1 - Kraaimaat,F.
A1 - Brutten,G.J.
Y1 - 1998/11//
N1 - Accession Number: 1999027043. Language: English. Entry Date: 19990401. Revision Date: 20091218. Publication Type: journal article; research; tables/charts. Journal Subset: Allied Health; Peer Reviewed; USA. Instrumentation: Reynell Developmental Language Scales (RDLS) Dutch Version; Peabody Picture Vocabulary Test (PPVT) Dutch Version. Grant Information: Dutch Organization of Scientific Research (NWO-Psychon No. 560-268-038). No. of Refs: 31 ref. NLM UID: 7601744
KW - Fluency Disorders -- In Infancy and Childhood
KW - Fluency Disorders -- In Adulthood
KW - Mother-Child Relations
KW - Funding Source
KW - Parental Role
KW - Child,Preschool
KW - Risk Factors
KW - Case Control Studies

KW - Prospective Studies
KW - Adult
KW - Language Tests
KW - Videorecording
KW - Play and Playthings
KW - Speech Rate
KW - Analysis of Variance
KW - Descriptive Statistics
KW - Analysis of Covariance
KW - Repeated Measures
KW - Human
KW - CINAHL
RP - NOT IN FILE
SP - 217
EP - 230
JA - Journal of Fluency Disorders
VL - 23
IS - 4
SN - 0094-730X
AD - University of Utrecht, Utrecht, The Netherlands
UR - http://search.ebscohost.com/login.aspx?direct=true&db=cin20&AN=1999027043&site=ehost-live
ER -

TY - JOUR
ID - 923
T1 - Persistence and remission of incipient stuttering among high-risk children
A1 - Kloth,S.A.
A1 - Kraaimaat,F.W.
A1 - Janssen,P.
A1 - Brutten,G.J.
Y1 - 1999/12//
N1 - Accession Number: 2000025062. Language: English. Entry Date: 20000401. Revision Date: 20091218. Publication Type: journal article; research; tables/charts. Journal Subset: Allied Health; Peer Reviewed; USA. Instrumentation: Dutch Version of the Reynell Language Development Scale; Peabody Picture Vocabulary Test (PPVT). Grant Information: Dutch Organisation of Scientific Research (NWO-Psychon No.: 560-268-038). No. of Refs: 42 ref. NLM UID: 7601744
KW - Fluency Disorders -- In Infancy and Childhood
KW - Funding Source
KW - Risk Factors
KW - Prevalence
KW - Recovery
KW - Prospective Studies
KW - Child,Preschool
KW - Child
KW - Parental Attitudes
KW - Pretest-Posttest Design
KW - Language Tests
KW - Play and Playthings
KW - Mother-Child Relations
KW - Female
KW - Male
KW - Two-Way Analysis of Variance
KW - Repeated Measures
KW - Mean Length of Utterance
KW - Human
KW - CINAHL

RP - NOT IN FILE
SP - 253
EP - 265
JA - Journal of Fluency Disorders
VL - 24
IS - 4
SN - 0094-730X
AD - University of Utrecht, Dept of Phoniatrics, The Netherlands
UR - http://search.ebscohost.com/login.aspx?direct=true&db=cin20&AN=2000025062&site=ehost-live
ER -

TY - JOUR
ID - 924
T1 - Expressive Language Skills in Finnish Two-Year-Old Extremely- and Very-Low-Birth-Weight Preterm Children
A1 - Kunnari,Sari
A1 - Yliherva,Anneli
A1 - Paavola,Leila
A1 - Peltoniemi,Outi
Y1 - 2011/12//
N1 - Accession Number: 2011399577. Language: English. Entry Date: 20120127. Revision Date: 20120217. Publication Type: journal article; research; tables/charts. Journal Subset: Allied Health; Biomedical; Continental Europe; Editorial Board Reviewed; Europe; Expert Peer Reviewed; Online/Print; Peer Reviewed. Special Interest: Pediatric Care; Speech-Language Pathology/Audiology. Grant Information: Partly supported by a research grant from the Academy of Finland.. No. of Refs: 40 ref. NLM UID: 9422792
KW - Speech -- In Infancy and Childhood
KW - Communication Skills -- In Infancy and Childhood
KW - Language Development -- In Infancy and Childhood
KW - Child,Preschool
KW - Infant,Premature
KW - Infant,Very Low Birth Weight
KW - Human
KW - Finland
KW - Videorecording
KW - Play and Playthings
KW - Nonparametric Statistics
KW - Mann-Whitney U Test
KW - Funding Source
KW - Male
KW - Female
KW - Child
KW - CINAHL
RP - NOT IN FILE
SP - 5
EP - 11
JA - Folia Phoniatrica et Logopaedica
VL - 64
IS - 1
N2 - Objective: Preterm children with low birth weight are at greater risk of experiencing speech and language difficulties than full-term children. The aim of the current study was to investigate expressive language skills of Finnish-speaking preterm children with low birth weight [extremely-low-birth-weight (ELBW) children: n = 8; very-low-birth-weight (VLBW) children: n = 10] at 2 years of corrected age and to compare their language results with full-term controls (n = 18), using spontaneous speech samples. Methods: The children were video recorded in semistructured free-play sessions with their mothers. From these video samples, expressive vocabulary size and maximum sentence length (MSL) were analyzed. In addition, the possible effect of children's gender on language measures as well as associations between different language measures were

examined. Results: The results showed that there was no statistically significant difference between the preterm and full-term groups in the size of expressive vocabulary. In contrast, the MSL, which measures morphosyntactic skills, was significantly shorter in preterm children. A positive correlation was found between MSL and expressive vocabulary. Children's gender was not associated with language skills measured. Conclusion: The findings indicate that Finnish-speaking preterm children, especially ELBW children, experience difficulties in morphosyntactic skills. Copyright - ª2011 S. Karger AG, Basel
SN - 1021-7762
AD - Logopedics, Faculty of Humanities
Department of Pediatrics, University of Oulu, Oulu, Finland
UR - http://search.ebscohost.com/login.aspx?direct=true&db=cin20&AN=2011399577&site=ehost-live
ER -

TY - JOUR
ID - 925
T1 - Does a history of sexual abuse in childhood play a role in women's medical problems? A review
A1 - Laws,A.
Y1 - 1993/06//
N1 - Accession Number: 1993167699. Language: English. Entry Date: 19930901. Revision Date: 20090313. Publication Type: journal article; review; tables/charts. Journal Subset: Biomedical; Peer Reviewed; USA. No. of Refs: 72 ref. NLM UID: 9208978
KW - Child Abuse,Sexual
KW - WOMEN
KW - Health Status
KW - Adult Children
KW - Eating Disorders
KW - Pelvis
KW - Chronic Pain
KW - Substance Abuse
KW - Risk Taking Behavior
KW - Adult
KW - Middle Age
KW - Female
KW - CINAHL
RP - NOT IN FILE
SP - 165
EP - 172
JA - Journal of Women's Health
VL - 2
IS - 2
N2 - Purpose: To examine possible relations between childhood sexual abuse and women's medical problems. Data sources: Potential articles were retrieved by searching Medline and Psychlit databases using keywords which included incest, sexual abuse and assault, pain, alcohol and substance abuse, eating disorders, anorexia, bulimia, and obesity. Additional references were identified from bibliographies of these articles, from textbooks on child sexual abuse, and by experts in these fields. Study selection: Only those case series, case-control studies, and population-based surveys that were published in peer-reviewed journals were included. Results: Although available literature is sparse and many studies lack methodologic rigor, data suggest that a history of sexual abuse in childhood is common in women with a history of chronic pain (especially pelvic pain), functional bowel disorders, eating disorders, obesity, and alcohol abuse. Conclusions: Given the high prevalence of sexual abuse of girls and its possible relation to common medical problems often refractory to treatment, further research is needed both to substantiate associations and to guide therapies
SN - 1059-7115
AD - Dept Med, Stanford Univ School Med, Stanford, CA
UR - http://search.ebscohost.com/login.aspx?direct=true&db=cin20&AN=1993167699&site=ehost-live
ER -

TY - JOUR

ID - 926
T1 - Violence in bipolar disorder: what role does childhood trauma play?
A1 - Lee,A.M.R.
A1 - Galynker,I.I.
Y1 - 2010/11//
N1 - Accession Number: 2010918182. Language: English. Entry Date: 20110204. Revision Date: 20130614. Publication Type: journal article; tables/charts. Journal Subset: Biomedical; Online/Print; Peer Reviewed; USA. Special Interest: Psychiatry/Psychology. NLM UID: 9014543
KW - Child Abuse
KW - Trauma -- In Infancy and Childhood
KW - Violence -- Complications
KW - Bipolar Disorder -- Risk Factors
KW - Bipolar Disorder -- Therapy
KW - Risk Assessment
KW - Crisis Intervention
KW - Child
KW - CINAHL
RP - NOT IN FILE
SP - 32
EP - 34
JA - Psychiatric Times
VL - 27
IS - 11
SN - 0893-2905
AD - ECRIP research fellow
UR - http://search.ebscohost.com/login.aspx?direct=true&db=cin20&AN=2010918182&site=ehost-live
ER -

TY - THES
ID - 927
T1 - The effect of early intensive postural and movementtraining on head control, pre-reaching movements and early reaching behaviors
A1 - Lee,Hui Min
Y1 - 2010///
N1 - Accession Number: 2011284346. Language: English. Entry Date: 20110930. Revision Date: 20121005. Publication Type: doctoral dissertation; research. Special Interest: Pediatric Care; Physical Therapy
KW - Functional Training -- In Infancy and Childhood
KW - Movement
KW - Posture
KW - Reaching
KW - Treatment Outcomes
KW - Arm
KW - COMPARATIVE studies
KW - Head
KW - Human
KW - Infant,Newborn
KW - Kinematics
KW - Orientation
KW - Play and Playthings
KW - Prospective Studies
KW - Skill Acquisition
KW - Skill Retention
KW - Spatial Perception
KW - CINAHL
RP - NOT IN FILE
EP - 246

PB - University of Delaware
N2 - The current project investigated the effects of early postural and movement experiences on young infants' head control, pre-reaching movements and early reaching behaviors through multi-level measurements beginning when infants were 1 month old through when they were 4 months old. Results from the current project revealed that infants with training demonstrated more advanced head control and early reaching behaviors. Infants with training also displayed different control in pre-reaching movements which led to earlier successful reaching. Specifically, the training effects (1) were rapid; (2) widely spread across multiple variables and contexts; (3) continued months after training ended; and (4) displayed interesting sequential patterns. In head control, results showed that infants with training kept demonstrated better head control through out the period of investigation and used more advanced movement strategies for active head control after training stopped. In pre-reaching movements, results showed that infants with training had faster and smoother movements during training, different joint orientation from infants without training leading to successful reaching after training stopped, and continued moving hands more forward and closer to a midline toy from training period to reach onset. The training also displayed effects with temporal to spatial and proximal to distal patterns in arm kinematics. In addition, the training showed effects on both movements with and without the presence of a midline toy. These findings suggested that with training, infants further adapted pre-reaching movements that resulted in more advanced early reaching behaviors with earlier reaching onset, more toy contacts and longer contact duration. Results from the current project strongly support the importance of early experiences on the development of head control and reaching. Future work will quantify the effect of early postural and movement experiences on infants born at high risk for lifelong movement impairments such as those born preterm and with brain injuries
SN - 978-1-124-24096-1
AV - UMI Order AAI3423344.
M1 - Ph.D.
UR - http://search.ebscohost.com/login.aspx?direct=true&db=cin20&AN=2011284346&site=ehost-live
ER -

TY - JOUR
ID - 928
T1 - Differentiating Maternal Sensitivity to Infant Distress and Non-Distress
A1 - Leerkes,Esther
A1 - Weaver,Jennifer
A1 - O'Brien,Marion
Y1 - 2012/04//
N1 - Accession Number: 2011581737. Language: English. Entry Date: 20130913. Revision Date: 20131004. Publication Type: journal article; research; tables/charts. Commentary: Atkinson, Leslie. Strategic Decisions: Life History, Interpersonal Relations, Intergenerational Neurobiology, and Ethics in Parenting and Development. PARENTING SCI PRACT 2012 Apr-Sep; 12(2/3): 185-91 (commentary) . Journal Subset: Biomedical; Blind Peer Reviewed; Editorial Board Reviewed; Expert Peer Reviewed; Online/Print; Peer Reviewed; USA. Special Interest: Pediatric Care. Grant Information: Data collection for the NICHD SECCYD was supported by the Eunice Kennedy Shriver National Institute of Child Health and Human Development (NICHD) through a cooperative agreement (U10) calling for scientific collaboration between the grantees and NICHD staff. Data collection for the Infant Parent Project was supported by NICHD also (R03 HD048691) and by a New Faculty Grant and Summer Excellence Award from the Office of Sponsored Programs and seed money from the Human Environmental Sciences Center for Research at The University of North Carolina at Greensboro.. NLM UID: 101093743
KW - Maternal Behavior
KW - Stress
KW - Infant Behavior
KW - Parenting
KW - Human
KW - Secondary Analysis
KW - Cues
KW - Psychological Well-Being
KW - Prospective Studies
KW - Socioeconomic Factors

KW - Female
KW - Structural Equation Modeling
KW - Descriptive Statistics
KW - Emotions
KW - Infant
KW - Audiorecording
KW - Interviews
KW - Kappa Statistic
KW - Interrater Reliability
KW - Play and Playthings
KW - Funding Source
KW - CINAHL
RP - NOT IN FILE
SP - 175
EP - 184
JA - Parenting: Science & Practice
VL - 12
IS - 2/3
N2 - Drawing from a domain specificity perspective, we assert that maternal sensitivity to infant distress cues is distinct from maternal sensitivity to non-distress cues. We review evidence from prior research demonstrating that the two constructs have more unshared than shared variance and that sensitivity to infant distress is a unique predictor of infants' early emotional well-being when both types of sensitivity are examined as simultaneous predictors. In addition, we present new evidence to test the hypothesis that maternal sensitivity to infant distress and non-distress have different origins. We draw on data from a subset of mothers and infants who participated in Phase I of the National Institute of Child Health and Human Development Study of Early Child Care (Study 1) and from 101 motherâ€“infant dyads who participated in a longitudinal study of the origins of maternal sensitivity (Study 2). In both studies, maternal sensitivity to distress and non-distress were rated when infants were six months old. In both studies, socio-demographic risk (i.e., young, unmarried, low income mothers) was a stronger predictor of sensitivity to non-distress than of sensitivity to distress. In Study 2, mothers' emotional and cognitive responses to videotapes of crying infants during the prenatal period predicted maternal sensitivity during tasks designed to elicit infant fear and frustration but were unrelated to maternal sensitivity in a non-arousing free play context. Maternal sensitivity during infancy can be further divided into specific sub-types that have unique origins and unique effects on subsequent child well-being. Methodological, theoretical, and applied implications of such an approach are discussed
SN - 1529-5192
AD - Human Development and Family Studies, University of North Carolina, PO Box 26170, Greensboro, NC, 27402-6170, USA
Boise State University
The University of North Carolina, Greensboro
UR - http://search.ebscohost.com/login.aspx?direct=true&db=cin20&AN=2011581737&site=ehost-live
ER -

TY - JOUR
ID - 929
T1 - Early Intervention in a Child Care Setting Using Play and Family Therapy
A1 - Matte,Rebecca
A1 - Messmore,Carol
Y1 - 2013/07//
N1 - Accession Number: 2012196916. Language: English. Entry Date: 20130809. Revision Date: 20131011. Publication Type: journal article; case study; pictorial. Journal Subset: Biomedical; USA. Special Interest: Pediatric Care. NLM UID: 9879031
KW - Early Childhood Intervention
KW - Disruptive Behavior
KW - Behavior Therapy -- Methods
KW - Play Therapy
KW - Child,Preschool

KW - Male
KW - Florida
KW - Professional-Family Relations
KW - Family Therapy
KW - CINAHL
RP - NOT IN FILE
SP - 43
EP - 47
JA - Zero to Three
VL - 33
IS - 6
N2 - At an alarming rate, preschoolers are being expelled from child care centers because of disruptive behavior, and elementary schools are dealing with social-emotional behaviors that affect the entire classroom. The authors share the story of a child who would have been one of those expelled from child care and at risk in the elementary school classroom if he had not been referred to an early intervention program. Through the use of individual play therapy, group play therapy, and in-home family therapy, the child and his family were able to learn coping skills, improve the parent-child interaction, and increase self-awareness, all which benefited the family system as a whole
SN - 0736-8038
AD - Housing Partnership, Inc., Riviera Beach, Florida
Capella University
UR - http://search.ebscohost.com/login.aspx?direct=true&db=cin20&AN=2012196916&site=ehost-live
ER -

TY - JOUR
ID - 931
T1 - Playing it safe
A1 - Murphy,M.
Y1 - 2007/07//
N1 - Accession Number: 2009642191. Language: English. Entry Date: 20071019. Revision Date: 20100409. Publication Type: journal article; pictorial. Journal Subset: Europe; Health Promotion/Education; Online/Print; Peer Reviewed; Public Health; UK & Ireland. Special Interest: Pediatric Care; Public Health
KW - Child Safety
KW - Health Promotion
KW - Play and Playthings -- Adverse Effects -- In Infancy and Childhood
KW - Wounds and Injuries -- Etiology -- In Infancy and Childhood
KW - Wounds and Injuries -- Prevention and Control -- In Infancy and Childhood
KW - Adolescence
KW - Child
KW - Male
KW - Risk Taking Behavior
KW - Shoes
KW - Skating
KW - United Kingdom
KW - United States
KW - CINAHL
RP - NOT IN FILE
SP - 156
EP - 157
JF - Journal of the Royal Society for the Promotion of Health
JA - J R SOC PROMOT HEALTH
VL - 127
IS - 4
N2 - Every year over 35,000 children under 15 in the UK are admitted to hospital with injuries caused by toys. Accidents will happen, but are recent, potentially dangerous crazes such as Heelys, trampolines, scooters and even Nintendo Wiis contributing too much to the statistics? And how can we get the balance right by keeping

kids safe at play without wrapping them in cotton wool and discouraging physical activity? JRSH's Mandy Murphy finds out
SN - 1466-4240
UR - http://search.ebscohost.com/login.aspx?direct=true&db=cin20&AN=2009642191&site=ehost-live
ER -

TY - JOUR
ID - 932
T1 - Everyday uncertainty: parents' risk perceptions and childrent's free play... Occupational Therapy Australia, 24th National Conference and Exhibition, 29 June - 1 July 2011
A1 - Niehues,A.
A1 - Bundy,A.
Y1 - 2011/06/02/
N1 - Accession Number: 2011278281. Language: English. Entry Date: 20111209. Revision Date: 20111209. Publication Type: journal article; abstract; research. Supplement Title: Supplement. Journal Subset: Allied Health; Australia & New Zealand; Peer Reviewed. Special Interest: Occupational Therapy; Pediatric Care. NLM UID: 15420200R
KW - Child Safety
KW - Fear -- In Adulthood
KW - Health Promotion
KW - Obesity -- In Infancy and Childhood
KW - Parenting
KW - Play and Playthings
KW - Adult
KW - Child
KW - Constant Comparative Method
KW - Coping
KW - Human
KW - Perception
KW - Qualitative Studies
KW - Semi-Structured Interview
KW - Thematic Analysis
KW - CINAHL
RP - NOT IN FILE
SP - 42
EP - 42
JF - Australian Occupational Therapy Journal
JA - Aust Occup Ther J
VL - 58
SN - 0045-0766
UR - http://search.ebscohost.com/login.aspx?direct=true&db=cin20&AN=2011278281&site=ehost-live
ER -

TY - JOUR
ID - 934
T1 - Parent-child activity group treatment in preventive psychiatry
A1 - Olson,L.
A1 - Heaney,C.
A1 - Soppas-Hoffman,B.
Y1 - 1989/05//
N1 - Accession Number: 1989098474. Language: English. Entry Date: 19891101. Publication Type: journal article; case study. Journal Subset: Allied Health; Peer Reviewed; USA. No. of Refs: 31 ref. NLM UID: 8309883
KW - Pediatric Occupational Therapy
KW - Preventive Health Care
KW - Mental Disorders -- Prevention and Control

KW - Parent-Child Relations
KW - Child Development
KW - Play and Playthings
KW - Child,Preschool
KW - Adult
KW - Female
KW - CINAHL
RP - NOT IN FILE
SP - 29
EP - 43
JA - Occupational Therapy in Health Care
VL - 6
IS - 1
N2 - This article describes the role that occupational therapy played in a community outreach program instituted collaboratively by a major teaching hospital and a local day care center. The program focused on preschool children at risk for developing psychiatric disorders. A parent-child activity group was introduced and developed by an occupational therapist who was a member of the hospital staff. The frame of reference, the parent-child intervention including the use of play and group process, and the method of monitoring change in families are explained. A case study is also provided
SN - 0738-0577
UR - http://search.ebscohost.com/login.aspx?direct=true&db=cin20&AN=1989098474&site=ehost-live
ER -

TY - JOUR
ID - 935
T1 - Narrative production by children who are deaf or hard of hearing: the effect of role-play
A1 - Pakulski,L.A.
A1 - Kaderavek,J.N.
Y1 - 2003/06//
N1 - Accession Number: 2003134822. Language: English. Entry Date: 20031017. Revision Date: 20100402. Publication Type: journal article; research; tables/charts. Journal Subset: Allied Health; Blind Peer Reviewed; Editorial Board Reviewed; Expert Peer Reviewed; Peer Reviewed; USA. Special Interest: Pediatric Care; Speech-Language Pathology/Audiology. Instrumentation: Story Grammar Analysis Protocol. No. of Refs: 28 ref. NLM UID: 0417476
KW - Hearing Disorders -- In Infancy and Childhood
KW - Narratives -- In Infancy and Childhood
KW - Role Playing
KW - Adolescence
KW - Child
KW - Content Analysis
KW - Descriptive Statistics
KW - Female
KW - Grammar
KW - Male
KW - Nonparametric Statistics
KW - Quasi-Experimental Studies
KW - Questionnaires
KW - Storytelling
KW - Wilcoxon Signed Rank Test
KW - Human
KW - CINAHL
RP - NOT IN FILE
SP - 127
EP - 139
JA - Volta Review
VL - 103

IS - 3
N2 - Narrative ability is one of the best predictors of school success for children with a language impairment or learning disability. Much less is known about the development of narrative performance in children who are deaf or hard of hearing. Studies involving the development of text structure knowledge in children who are deaf or hard of hearing often focus on written or signed narrative productions. Children who are learning to listen and talk (auditory/oral or Auditory-Verbal approaches) are capable of benefitting from an auditory representation of the printed text. However, these children are at risk for language problems with respect to narrative productivity, fluency, lexical diversity, and grammatical complexity and accuracy. Repeated reading and role-playing are ways of targeting these skills explicitly in academically appropriate and contextually relevant situations
Story retellings were elicited from 14 children who are deaf or hard of hearing and are oral communicators following repeated readings of two stories and role-playing of one of the two stories. Results showed that children who are deaf or hard of hearing are capable of narrative production (story retelling) and that the sophistication and complexity of those retellings improve with role-play
SN - 0042-8639
AD - Assistant Professor of Audiology, University of Toledo, Toledo, OH
UR - http://search.ebscohost.com/login.aspx?direct=true&db=cin20&AN=2003134822&site=ehost-live
ER -

TY - THES
ID - 936
T1 - If I cry less I can fit in more play dates: mothers' experiences with time famine
A1 - Pontes,N.M.H.
Y1 - 2003///
N1 - Accession Number: 2005073993. Language: English. Entry Date: 20050506. Revision Date: 20091218. Publication Type: doctoral dissertation; research. Special Interest: Women's Health
KW - Family Health
KW - Health Status
KW - Mothers -- Psychosocial Factors
KW - Stress,Psychological
KW - Time
KW - Conceptual Framework
KW - Family Relations
KW - Female
KW - Interviews
KW - Maternal Role
KW - Participant Observation
KW - Phenomenological Research
KW - Role Stress
KW - Social Isolation
KW - Thematic Analysis
KW - Human
KW - CINAHL
RP - NOT IN FILE
EP - 235
PB - Columbia University
N2 - As time has become an increasingly valued resource of our postmodern world, many individuals are experiencing a phenomenon called "time famine," the chronic perceived shortage of time. Mothers are especially vulnerable to time famine as they assume multiple roles. Previous researchers hypothesized that these mothers are at increased risk for sickness and death. However, epidemiological studies do not support this premise primarily because these women tend to be younger and relatively free of chronic diseases
The purpose of this qualitative research was to explore six mothers' lived experiences of time famine and how it impacts their family health within a broad context of health and wellbeing. A multi-method approach using in-depth interviews, participant observations, and space activity profiles were used
Six themes emerged from these data: The women felt that their lives were characterized by (1) all work and no fun; they expressed feelings of being (2) rundown, (3) stressed, and (4) disconnected. They (5) consumed numerous goods and services in order to maintain a standard of living beyond their means. Their energies were

spent on (6) managing and concealing their psychological distress. Despite appearing healthy, in reality, they had significant problems with insomnia, sleep deprivation, depression, anxiety, disordered eating, chronic pain, and headaches
These participants purposed to provide a "better" childhood for their children than they themselves had experienced. Paradoxically, while giving the appearance of intense involvement with their children these mothers were frequently emotionally distant from both their children and spouse
The study concludes that (1) the participants lead lives that are consumed by the excessive consumption of goods and services to the extent that (2) they confuse dwelling with doing. Although they participate in many activities, they are often disconnected from the people and things around them. (3) As a society, our time has become increasingly privatized, whereby there is a disengagement from community and relationships that could otherwise function to buffer time famine. The resulting social isolation has far-reaching implications for society at large. A conceptual framework for time famine is described based on these phenomena and related literature about personal strivings, demoralization, and phenomenological geography
AV - UMI Order AAI3088407.
M1 - D.N.Sc.
UR - http://search.ebscohost.com/login.aspx?direct=true&db=cin20&AN=2005073993&site=ehost-live
ER -

TY - JOUR
ID - 937
T1 - The Active City of Liverpool, Active Schools and Sportslinx (A-CLASS) project
A1 - Ridgers,N.
A1 - Stratton,G.
A1 - Foweather,L.
A1 - Henaghan,J.
A1 - McWhannell,N.
A1 - Stone,M.R.
Y1 - 2006/04//
N1 - Accession Number: 2009244481. Language: English. Entry Date: 20070209. Revision Date: 20091218. Publication Type: journal article; algorithm; research. Journal Subset: Biomedical; Editorial Board Reviewed; Europe; Expert Peer Reviewed; Online/Print; Peer Reviewed; UK & Ireland. Special Interest: Pediatric Care. No. of Refs: 21 ref. NLM UID: 8805809
KW - Health Promotion
KW - Physical Activity -- In Infancy and Childhood
KW - Physical Education and Training
KW - Schools,Elementary
KW - Sports -- In Infancy and Childhood
KW - Body Composition
KW - Cardiovascular Risk Factors
KW - Child
KW - Child Development
KW - England
KW - Evaluation Research
KW - Interpersonal Relations
KW - Obesity
KW - Play and Playthings
KW - Program Development
KW - Students,Elementary
KW - Human
KW - CINAHL
RP - NOT IN FILE
SP - 26
EP - 29
JA - Education & Health
VL - 24
IS - 2

N2 - The A-CLASS Project is a unique multi-disciplinary project, which consists of multi-sports activity coaches who will be responsible for running after school and community sports clubs, and four research coaches who will analyse the effects of these programmes on the physical activity levels of Liverpool school children
SN - 0265-1602
AD - Research Institute for Sport and Exercise Sciences, Liverpool John Moores University, UK
UR - http://search.ebscohost.com/login.aspx?direct=true&db=cin20&AN=2009244481&site=ehost-live
ER -

TY - JOUR
ID - 938
T1 - Play hard, breathe easy: managing asthma in the camp setti;ng
A1 - Rodiquez,Jodie
Y1 - 2012/06//
N1 - Accession Number: 2011588350. Language: English. Entry Date: 20120928. Revision Date: 20130322. Publication Type: journal article; tables/charts. Journal Subset: Double Blind Peer Reviewed; Expert Peer Reviewed; Nursing; Peer Reviewed; USA. Special Interest: Pediatric Care. No. of Refs: 4 ref
KW - Asthma -- Nursing -- In Infancy and Childhood
KW - Camp Nursing
KW - Nursing Role
KW - Administration,Inhalation
KW - Adrenal Cortex Hormones -- Therapeutic Use
KW - Adrenergic Beta-Agonists -- Therapeutic Use
KW - Asthma -- Diagnosis
KW - Asthma -- Drug Therapy
KW - Asthma -- Education
KW - Asthma -- Prevention and Control
KW - Bronchodilator Agents -- Therapeutic Use
KW - Child
KW - Disease Exacerbation -- Prevention and Control
KW - Drug Administration -- Nursing
KW - Nebulizers and Vaporizers
KW - Staff Development
KW - CINAHL
RP - NOT IN FILE
SP - 10
EP - 13
JA - CompassPoint
VL - 22
IS - 2
N2 - Because asthma affects one out of 10 children in the US, it is imperative that camp nurses be knowledgeable and well equipped to manage asthma. Clinical assessment, trigger management, and medication administration are components in the daily care ofan asthmatic child. Further, it is critical that health center staff know the early and late signs of an asthma exacerbation and be able to manage those episodes, especially those which are emergent in nature. While kids with asthma can be fully active and partiCipate in all camp activities if managed correctly, children with asthma are at risk for serious consequences, even death, if not assessed and managed correctly
UR - http://search.ebscohost.com/login.aspx?direct=true&db=cin20&AN=2011588350&site=ehost-live
ER -

TY - JOUR
ID - 940
T1 - Emotional availability, parental self-efficacy beliefs, and child development in caregiver-child relationships with buprenorphine-exposed 3-year-olds
A1 - Salo,S.
A1 - Kivist+Ý,K.
A1 - Korja,R.

A1 - Biringen,Z.
A1 - Tupola,S.
A1 - Kahila,H.
A1 - Kivitie-Kallio,S.
Y1 - 2009/07//
N1 - Accession Number: 2010362200. Language: English. Entry Date: 20090911. Revision Date: 20120309. Publication Type: journal article; research; tables/charts. Journal Subset: Biomedical; Blind Peer Reviewed; Editorial Board Reviewed; Expert Peer Reviewed; Online/Print; Peer Reviewed; USA. Special Interest: Pediatric Care. Instrumentation: Emotional Availability Scales (EAS); Self-Efficacy for Parenting Tasks Index-Toddler Scale (SEPTI-TS); Bayley Scales of Infant and Toddler Development, Third Edition (Bayley-III). No. of Refs: 50 ref. NLM UID: 101093743
KW - Child Development
KW - Maternal Behavior
KW - Parent-Child Relations
KW - Self-Efficacy
KW - Buprenorphine -- Adverse Effects
KW - Caregivers
KW - Child,Preschool
KW - Coefficient Alpha
KW - COMPARATIVE studies
KW - Descriptive Statistics
KW - Female
KW - Finland
KW - Interrater Reliability
KW - Multiple Regression
KW - One-Way Analysis of Variance
KW - Play and Playthings
KW - Pregnancy
KW - Prenatal Exposure Delayed Effects
KW - Scales
KW - Substance Use Disorders
KW - Videorecording
KW - Human
KW - CINAHL
RP - NOT IN FILE
SP - 244
EP - 259
JA - Parenting: Science & Practice
VL - 9
IS - 3-4
N2 - Objective. The purpose was to compare emotional availability, maternal self-efficacy beliefs, and child developmental status in caregiver-child relationships with prenatally buprenorphineexposed and nonexposed 3-year-old children. Design. We compared prenatally buprenorphineexposed children living either with the biological mother (n = 7) or in foster care (n = 14) to nonexposed participants (n = 13). Emotional availability was coded from videotaped parentchild free-play interactions. Results. After controlling for covariates, buprenorphine-exposed children scored lower on maternal Sensitivity and Nonhostility and child Responsiveness and Involvement as well as lower on the Bayley Cognitive and Language scales than did nonexposed children. As compared to foster mothers, biological mothers scored lower on Sensitivity and Nonhostility and self-efficacy beliefs, and their children scored lower on Responsiveness and the Bayley Cognitive Scale. Regardless of group status, the parenting variables were meaningfully related to child socioemotional variables. Conclusions. Buprenorphine-exposed children experienced more environmental risks in emotional availability and parental self-efficacy and performed worse on the Bayley as compared to nonexposed children
SN - 1529-5192
UR - http://search.ebscohost.com/login.aspx?direct=true&db=cin20&AN=2010362200&site=ehost-live
ER -

TY - JOUR
ID - 941
T1 - Reliability and validity of a new instrument to measure tolerance of everyday risk for children... Occupational Therapy Australia, 24th National Conference and Exhibition, 29 June - 1 July 2011
A1 - Smith,A.
A1 - Bundy,A.
Y1 - 2011/06/02/
N1 - Accession Number: 2011277486. Language: English. Entry Date: 20111209. Revision Date: 20111209. Publication Type: journal article; abstract; research. Supplement Title: Supplement. Journal Subset: Allied Health; Australia & New Zealand; Peer Reviewed. Special Interest: Occupational Therapy; Pediatric Care. NLM UID: 15420200R
KW - Clinical Assessment Tools -- Evaluation
KW - Health Beliefs -- In Adulthood
KW - Instrument Validation
KW - Occupational Therapy
KW - Play and Playthings
KW - Risk Taking Behavior -- In Infancy and Childhood
KW - Adult
KW - Child
KW - Clinical Assessment Tools
KW - Coefficient Alpha
KW - Goodness of Fit Chi Square Test
KW - Human
KW - Questionnaires
KW - Reliability
KW - SURVEYS
KW - Validation Studies
KW - Validity
KW - CINAHL
RP - NOT IN FILE
SP - 8
EP - 8
JF - Australian Occupational Therapy Journal
JA - Aust Occup Ther J
VL - 58
SN - 0045-0766
UR - http://search.ebscohost.com/login.aspx?direct=true&db=cin20&AN=2011277486&site=ehost-live
ER -

TY - JOUR
ID - 943
T1 - Keeping the hospital environment safe for children
A1 - Stower,S.
Y1 - 2000/07//
N1 - Accession Number: 2001015657. Language: English. Entry Date: 20010223. Revision Date: 20110916. Publication Type: journal article; CEU; exam questions. Journal Subset: Double Blind Peer Reviewed; Editorial Board Reviewed; Europe; Expert Peer Reviewed; Nursing; Peer Reviewed; UK & Ireland. No. of Refs: 9 ref. NLM UID: 9013329
KW - Health Facility Environment
KW - Child Safety
KW - Pediatric Nursing
KW - Child
KW - Education,Continuing (Credit)
KW - Play and Playthings
KW - Security Measures

KW - Fires -- Prevention and Control
KW - Risk Assessment
KW - CINAHL
RP - NOT IN FILE
SP - 37
EP - 43
JA - Paediatric Nursing
VL - 12
IS - 6
N2 - This article is aimed at all healthcare staff working within a paediatric environment or environment where children are cared for. The purpose of the article is to heighten awareness of hazards that exist in a paediatric unit and how these can be managed effectively, and therefore, safely
SN - 0962-9513
AD - Senior Nurse Manager for Children's Services, Queen's Medical Centre, University Hospital, Nottingham
UR - http://search.ebscohost.com/login.aspx?direct=true&db=cin20&AN=2001015657&site=ehost-live
ER -

TY - JOUR
ID - 944
T1 - The role of occupational therapy within interdisciplinary pressure care in a Ukraine orphanage... Occupational Therapy Australia, 24th National Conference and Exhibition, 29 June - 1 July 2011
A1 - Swaine,J.
A1 - Nguyen,V.
A1 - Luong,A.
A1 - Safe,B.
A1 - Mawson,A.
Y1 - 2011/06/02/
N1 - Accession Number: 2011279782. Language: English. Entry Date: 20111209. Revision Date: 20111209. Publication Type: journal article; abstract. Supplement Title: Supplement. Journal Subset: Allied Health; Australia & New Zealand; Peer Reviewed. Special Interest: Occupational Therapy; Physical Therapy; Speech-Language Pathology/Audiology. NLM UID: 15420200R
KW - Child,Disabled
KW - Pressure Ulcer -- Prevention and Control -- In Infancy and Childhood
KW - Risk Assessment -- Methods
KW - Student Placement
KW - Students,Occupational Therapy
KW - Students,Physical Therapy
KW - Students,Speech-Language Pathology
KW - Child
KW - Conceptual Framework
KW - Multidisciplinary Care Team
KW - Needs Assessment
KW - Orphans and Orphanages -- Ukraine
KW - Play and Playthings
KW - Ukraine
KW - CINAHL
RP - NOT IN FILE
SP - 108
EP - 108
JF - Australian Occupational Therapy Journal
JA - Aust Occup Ther J
VL - 58
SN - 0045-0766
UR - http://search.ebscohost.com/login.aspx?direct=true&db=cin20&AN=2011279782&site=ehost-live
ER -

TY - JOUR
ID - 946
T1 - Advanced practice. Adolescent experimentation -- risky business or child's play?
A1 - Sweeney,C.T.
Y1 - 2002/02/25/
N1 - Accession Number: 2003082937. Language: English. Entry Date: 20030613. Revision Date: 20110916. Publication Type: journal article; case study. Journal Subset: Nursing; USA. No. of Refs: 9 ref. NLM UID: 9892045
KW - Risk Taking Behavior -- In Adolescence
KW - Risk Taking Behavior -- Prevention and Control -- In Adolescence
KW - Peer Pressure
KW - Pediatric Nursing
KW - Male
KW - Female
KW - Adolescence
KW - CINAHL
RP - NOT IN FILE
SP - 6
EP - 7
JA - Nursing Spectrum -- Philadelphia Tri -- State Edition
VL - 11
IS - 4
SN - 1074-858X
AD - Clinical Director, Urban Solutions, Philadelphia
UR - http://search.ebscohost.com/login.aspx?direct=true&db=cin20&AN=2003082937&site=ehost-live
ER -

TY - JOUR
ID - 947
T1 - RESEARCH NEWS
A1 - Tonks,Alison
Y1 - 2012/06/30/
N1 - Accession Number: 2011615485. Language: English. Entry Date: 20120727. Revision Date: 20121005. Publication Type: journal article; tables/charts. Journal Subset: Biomedical; Blind Peer Reviewed; Editorial Board Reviewed; Europe; Expert Peer Reviewed; Online/Print; Peer Reviewed; UK & Ireland. NLM UID: 101090866
KW - Female
KW - Vaginal Vault Prolapse -- Surgery
KW - Urinary Incontinence -- Epidemiology
KW - Loneliness
KW - Mortality -- Epidemiology
KW - Magnetics -- Adverse Effects
KW - Play and Playthings -- Adverse Effects
KW - Child Safety
KW - Atrial Fibrillation -- Drug Therapy
KW - Flecainide Acetate -- Therapeutic Use
KW - Tissue Plasminogen Activator -- Therapeutic Use
KW - Stroke -- Drug Therapy
KW - Warfarin -- Administration and Dosage
KW - Pregnancy
KW - Pregnancy,Ectopic -- Risk Factors
KW - Dietary Carbohydrates -- Administration and Dosage
KW - Energy Metabolism -- Evaluation
KW - CINAHL
RP - NOT IN FILE
SP - 14

EP - 15
JA - BMJ: British Medical Journal (Overseas & Retired Doctors Edition)
VL - 344
IS - 7863
SN - 1759-2151
UR - http://search.ebscohost.com/login.aspx?direct=true&db=cin20&AN=2011615485&site=ehost-live
ER -

TY - JOUR
ID - 948
T1 - The Early Start Denver Model: a case study of an innovative practice
A1 - Vismara,L.A.
A1 - Rogers,S.J.
Y1 - 2008/12//
N1 - Accession Number: 2010125265. Language: English. Entry Date: 20090116. Revision Date: 20120309. Publication Type: journal article; case study; research; tables/charts. Journal Subset: Allied Health; Blind Peer Reviewed; Editorial Board Reviewed; Peer Reviewed; USA. Special Interest: Evidence-Based Practice; Pediatric Care; Psychiatry/Psychology. Instrumentation: Mullen Scales of Early Learning (MSEL); Autism Diagnostic Observation Schedule (ADOS); Child Behavior Rating Scale (CBRS). Grant Information: Postdoctoral Training Award NIMH T32 MH073124; National Institute of Child Health and Human Development (No. U19HD35468), Collaborative Programs of Excellence in Autism; National Institute of Mental Health STAART Center No. U54 MH66399. No. of Refs: 41 ref. NLM UID: 8910748. Email: laurie.vismara@ucdmc.ucdavis.edu
KW - Autistic Disorder -- Therapy
KW - Communication -- Education
KW - Early Childhood Intervention
KW - Parenting Education
KW - Attention
KW - Autistic Disorder -- Symptoms
KW - Case Studies
KW - Child,Disabled
KW - Child,Preschool
KW - Fathers
KW - Female
KW - Funding Source
KW - Infant
KW - Male
KW - Mothers
KW - Outcomes of Education
KW - Parents
KW - Play and Playthings
KW - Psychological Tests
KW - Scales
KW - Social Behavior
KW - Summated Rating Scaling
KW - Human
KW - CINAHL
RP - NOT IN FILE
SP - 91
EP - 108
JF - Journal of Early Intervention
VL - 31
IS - 1
N2 - Intervention was implemented with an infant identified at 9 months of age with a behavioral profile consistent with autistic spectrum disorder. The intervention approach, the Early Start Denver model, consisted of a 12-week, 1.5-hr-per-week individualized parent-child education program. Results of this case study

demonstrated that the parent acquired multiple teaching tactics that were associated with growth in the infant's social communication behaviors. Improvements were also noted with respect to the severity of behavioral indicators related to autism between 18 and 24 months of age. Findings are discussed in relation to providing an intervention model that may equip parents with the necessary intervention skills to engage, communicate with, and teach their very young children with or at risk for autism
SN - 1053-8151
AD - Dept of Psychiatry and Behavioral Sciences, MIND Institute, 2825 50th Street, Sacramento, CA 95817
UR - http://search.ebscohost.com/login.aspx?direct=true&db=cin20&AN=2010125265&site=ehost-live
ER -

TY - JOUR
ID - 949
T1 - Toddlers with autism: developmental perspectives
A1 - Watson,L.R.
A1 - Baranek,G.T.
A1 - DiLavore,P.C.
Y1 - 2003/07//
N1 - Accession Number: 2009032387. Language: English. Entry Date: 20051007. Publication Type: journal article; review; tables/charts. Journal Subset: Biomedical; Peer Reviewed; USA. Special Interest: Pediatric Care. No. of Refs: 64 ref. NLM UID: 8915767. Email: Linda_Watson@med.unc.edu
KW - Autistic Disorder -- Diagnosis
KW - Child Behavior
KW - Child Development
KW - Affect
KW - Autistic Disorder -- Risk Factors
KW - Child,Preschool
KW - Communication
KW - Facial Expression
KW - Health Screening
KW - Parents -- Psychosocial Factors
KW - Play and Playthings
KW - Self Regulation
KW - SOCIALIZATION
KW - CINAHL
RP - NOT IN FILE
SP - 201
EP - 214
JA - Infants & Young Children: An Interdisciplinary Journal of Special Care Practices
VL - 16
IS - 3
N2 - Recent research has greatly expanded our knowledge about the early development of children with autism and related disorders. Familiarity with this literature will improve the ability of professionals to appropriately diagnose and intervene with young children with autism. This article reviews the literature pertaining to the development of children with autism under the age of 3 years. We examine findings on affective development, sensory processing and attention, praxis and imitation, communication, play, and motor features and stereotyped behaviors, and discuss the interrelationships among these different aspects of development. Screening and diagnostic tools with specific applicability to young children with autism are reviewed as well
SN - 0896-3746
AD - Division of Speech and Hearing Sciences, Department of Allied Health Sciences, CB # 7190, Medical School, Wing D, University of North Carolina at Chapel Hill, NC 27599-7120
UR - http://search.ebscohost.com/login.aspx?direct=true&db=cin20&AN=2009032387&site=ehost-live
ER -

TY - JOUR
ID - 950

T1 - Treating conduct problems and strengthening social and emotional competence in young children: the Dina Dinosaur treatment program
A1 - Webster-Stratton,C.
A1 - Reid,M.J.
Y1 - 2003/09//
N1 - Accession Number: 2004055023. Language: English. Entry Date: 20040409. Revision Date: 20060504. Publication Type: journal article; tables/charts. Journal Subset: Biomedical; Blind Peer Reviewed; Editorial Board Reviewed; Expert Peer Reviewed; Online/Print; Peer Reviewed; USA. Special Interest: Pediatric Care; Psychiatry/Psychology. No. of Refs: 57 ref. NLM UID: 9306047
KW - Behavior Therapy -- In Infancy and Childhood
KW - Child Behavior Disorders -- Therapy
KW - Social Skills Training -- In Infancy and Childhood
KW - Teaching Methods -- In Infancy and Childhood
KW - Aggression
KW - Anger
KW - Child
KW - Child,Preschool
KW - Emotions
KW - Fantasy
KW - Female
KW - Group Processes
KW - Male
KW - Play and Playthings -- Utilization
KW - Problem Solving
KW - Program Evaluation
KW - Reinforcement (Psychology)
KW - Role Playing
KW - Social Behavior
KW - Videorecording -- Utilization
KW - CINAHL
RP - NOT IN FILE
SP - 130
EP - 143
JA - Journal of Emotional & Behavioral Disorders
VL - 11
IS - 3
N2 - Young preschool and early-school-age children with early onset conduct problems are at high risk for school dropout, substance abuse, violence, and delinquency in later years. Consequently, developing treatment strategies for reducing conduct problems when aggression is in its more malleable form prior to age 8, and thus interrupting its progression, is of considerable benefit to families and society. This article describes a treatment program - the Dina Dinosaur Social, Emotional and Problem Solving Child Training Program - that was designed specifically with developmentally appropriate teaching methods for young children (ages 4 to 8 years) and based on theory related to the types of social, emotional, and cognitive deficits or excesses exhibited by children with conduct problems. The program emphasizes training children in skills such as emotional literacy, empathy or perspective taking, friendship and communication skills, anger management, interpersonal problem solving, school rules, and how to be successful at school. Emphasis is placed on ways to promote cross-setting generalization of the behaviors that are taught by involving parents and teachers in the treatment. A review of two randomized trials with this treatment approach and long-term results are provided
SN - 1063-4266
AD - Professor of Nursing, Clinical Child Psychologist, Director, Parenting Clinic, University of Washington
UR - http://search.ebscohost.com/login.aspx?direct=true&db=cin20&AN=2004055023&site=ehost-live
ER -

TY - JOUR
ID - 951

T1 - Older people as grandparents: how public policy needs to broaden its view of older people and the role they play within families
A1 - Wellard,Sarah
Y1 - 2012/12//
N1 - Accession Number: 2011784123. Language: English. Entry Date: 20130104. Revision Date: 20130510. Publication Type: journal article. Journal Subset: Allied Health; Europe; Nursing; Online/Print; Peer Reviewed; UK & Ireland. Special Interest: Gerontologic Care. No. of Refs: 28 ref
KW - Grandparents
KW - Intergenerational Relations
KW - Child Care
KW - Family
KW - Employment
KW - Psychological Well-Being
KW - Public Policy -- United Kingdom
KW - Quality of Life
KW - Caregivers
KW - United Kingdom
KW - Aged
KW - Public Assistance
KW - Child
KW - Grandchildren
KW - CINAHL
RP - NOT IN FILE
SP - 257
EP - 263
JA - Quality in Ageing & Older Adults
VL - 13
IS - 4
N2 - Purpose â€" The purpose of this paper is to provide a new perspective on the importance of grandparenting relationships and to highlight the continuing importance of intergenerational exchange across the life course. Design/methodology/approach â€" The paper draws on research from diverse fields including child welfare, gerontology and demography and journalistic sources, presenting an evidenced argument of the importance of grandparenting in contemporary families. Findings â€" The paper argues that the role of grandparents in family life is becoming more important as a result of demographic changes, and that grandparents offer a huge resource to families. Older people generally find their role as grandparents rewarding and that this contributes to wellbeing and reduced risk of loneliness. Originality/value â€" The paper provides a new perspective on the role of older people as grandparents
SN - 2044-1827
UR - http://search.ebscohost.com/login.aspx?direct=true&db=cin20&AN=2011784123&site=ehost-live
ER -

TY - JOUR
ID - 952
T1 - Helping families find a bright tomorrow
A1 - Wessling,S.
Y1 - 2006/07//
N1 - Accession Number: 2009282619. Language: English. Entry Date: 20061110. Revision Date: 20090313. Publication Type: journal article; pictorial. Journal Subset: Nursing; USA
KW - Early Childhood Intervention
KW - Family -- Psychosocial Factors
KW - Multidisciplinary Care Team
KW - Child,Preschool
KW - Developmental Disabilities -- Rehabilitation
KW - Home Visits
KW - Infant
KW - Massachusetts

KW - Play and Playthings
KW - CINAHL
RP - NOT IN FILE
SP - 18
EP - 21
JA - On Call
VL - 9
IS - 4
N2 - Early intervention focuses on young children who either have or are at risk for developmental delays while providing valuable support and assistance to the child's family
UR - http://search.ebscohost.com/login.aspx?direct=true&db=cin20&AN=2009282619&site=ehost-live
ER -

TY - JOUR
ID - 953
T1 - Play therapy with emotionally damaged adolescents
A1 - Wilson,K.
A1 - Ryan,V.
Y1 - 2002/08//
N1 - Accession Number: 2003105670. Language: English. Entry Date: 20030815. Revision Date: 20040123. Publication Type: journal article. Journal Subset: Biomedical; Blind Peer Reviewed; Europe; Peer Reviewed; UK & Ireland. No. of Refs: 31 ref. NLM UID: 9616901. Email: kate.wilson@nottingham.ac.uk
KW - Counseling -- Methods -- In Adolescence
KW - Parent-Child Relations
KW - Play Therapy -- In Adolescence
KW - Sexual Abuse -- Therapy -- In Adolescence
KW - Adult
KW - Female
KW - Male
KW - Professional-Client Relations
KW - Rehabilitation,Psychosocial -- Methods
KW - Treatment Outcomes
KW - Trust
KW - Adolescence
KW - CINAHL
RP - NOT IN FILE
SP - 178
EP - 192
JA - Emotional & Behavioural Difficulties
VL - 7
IS - 3
N2 - Adolescence is a critical period of development for all individuals, but particularly for those who have suffered earlier emotional difficulties or abuse. If these problems go unresolved, patterns of behaviour which become established during this period may be more difficult to change later on. However, more traditional talk therapies may be resisted by adolescents. This article argues that non-directive play therapy, given age-appropriate adaptations, offers an approach which, since it encompasses both play and verbal communication, is well suited to addressing adolescent concerns, particularly those of early adolescence. This argument is illustrated by two accounts of therapy with a boy and a girl, both in early adolescence, which show how a more traditional non-directive counselling approach was combined with play therapy by the adolescents themselves, allowing exploration of emotional difficulties on all levels of mental functioning. The cases show how traumatic or painful early memories may be reworked on a bodily and emotional level, without the feeling of over-intrusion risked by a directive or interpretive stance
SN - 1363-2752
AD - Professor of Social Work, Centre for Social Work, University of Nottingham, University Park, Nottingham NG7 2RD, UK
UR - http://search.ebscohost.com/login.aspx?direct=true&db=cin20&AN=2003105670&site=ehost-live

ER -

TY - GEN
ID - 954
T1 - Care of the Health of Boys in Girard, College, Philadelphia, Pennsylvania. Bulletin, 1914, No. 40. Whole Number 614
Y1 - 1914///
N1 - Last updated - 2013-06-04
KW - Pennsylvania
KW - ERIC,Resources in Education (RIE)
KW - At Risk Persons
KW - Play
KW - Dietetics
KW - MALES
KW - Lighting
KW - Nutrition
KW - Child Welfare
KW - Water
KW - Health Promotion
KW - Dental Health
KW - Sanitation
KW - Playgrounds
KW - Poverty
KW - Social Services
KW - Physicians
KW - Hygiene
KW - Physical Health
KW - ERIC
RP - NOT IN FILE
PB - United States Bureau of Education, Department of the Interior.
N2 - Girard College was founded and endowed by Stephen Girard, mariner and merchant, a native of France, who came to Philadelphia in 1776. After a long life of service to his adopted city, State, and country, he died in 1831, leaving the bulk of his estate in trust to the city of Philadelphia for the erection and maintenance of what is now known as Girard College. On a recent visit to Girard College, Philadelphia, the Secretary of the Interior, Commissioner P. P. Claxton, was so impressed with the evident good health of the boys in that institution and with the attention and care given to this important phase of the institution's work that he requested Dr. Cheesman A. Herrick, president of the college, to have prepared for the Bureau of Education some account of the health work of the college. President Herrick has very kindly complied with his request and has forwarded this manuscript to the bureau. Commissioner Claxton recommends that it be published as a bulletin of the Bureau of Education for distribution among the officers of orphanages, home schools, so-called industrial and reform schools, schools for the feeble-minded, State schools for the deaf and the blind, and other institutions in which children are detained and on which they depend for the care of physical health as well as for education. Sections of the bulletin include: (1) Girard College (Frank O. Zesinger); (2) Statement of the visiting physician; (3) Statement of the chief of nose, throat, and ear department; (4) Statement of the ophthalmologist; and (5) The dental department. (Contains 4 footnotes and 18 plates.) [Best copy available has been provided.]
UR - http://search.proquest.com/docview/1361846549?accountid=14701
ER -

TY - GEN
ID - 955
T1 - Zero to Three: Bulletin of the National Center for Clinical Infant Programs. Volume VII, Nos. 1-5, September, 1986-June 1987
Y1 - 1987/06//
N1 - Availability - Level 1 - Available online, if indexed January 1993 onward
SuppNotes - For volumes 8 and 9, see EC 221 095-096
Last updated - 2012-12-14

KW - ERIC,Resources in Education (RIE)
KW - Practitioners
KW - Preschool Education
KW - Parent Education
KW - Low Income Groups
KW - Play
KW - High Risk Persons
KW - Therapy
KW - Crisis Intervention
KW - Federal Legislation
KW - Psychotherapy
KW - Individual Differences
KW - Parent Child Relationship
KW - Disabilities
KW - Day Care
KW - Early Intervention
KW - Child Neglect
KW - Child Development
KW - Child Abuse
KW - Autism
KW - Infants
KW - ERIC
RP - NOT IN FILE
SP - 150
VL - 7
IS - 1
PB - National Center for Clinical Infant Programs, 733 15th St., N.W., Suite 912, Washington, DC 20005 ($18.00 per year).
N2 - Five bulletins of the National Center for Clinical Infant Programs include articles with the following titles and authors: "Infant Day Care: A Cause for Concern?" (Jay Belsky); "Model versus Modal Child Care for Children from Low-Income Families (Donna Wittmer); "Therapeutic Childcare at Merrywood School" (Maxine Siegel); "Individual Differences in Infants" (Lois Murphy et al.); "The 'Gourmet Baby' and the 'Little Wildflower'" (Edward Zigler and Mary Lang); "Infant-Parent Psychotherapy with an Autistic Toddler" (Barbara Kalmanson and Judith Pekarsky); "Parent Training with Young Autistic Children: A Report on the LEAP Model" (Philip Strain); "Point of View: Commenting on P.L. 99-457" (Linda Gilkerson et al.); "Selective Review of Infant Day Care Research: A Cause for Concern!" (Deborah Phillips et al.); "Risks Remain" (Jay Belsky); "Infant Mental Health and Child Abuse and Neglect: Reflections from an Infant Mental Health Practitioner" (Jeree Pawl); "Doll Play of Failure to Thrive Toddlers: Clues to Infant Experience" (Claire Haynes-Seman and Joan Suzuki Hart); "Abuse and Neglect in the Earliest Years: Groundwork for Vulnerability" (Brandt Steele); "Psychotherapy of the Violent Offender: A Recapitulation of Infant Development" (H. Joseph Horacek); "The Effectiveness of Crisis Intervention in Working with a Young Mother and Her Four Preschool Children" (Sarah Landy); "The Application of a Transactional Risk Model to Intervention with Multi-risk, Maltreating Families" (Dante Ciccheti and Sherre Toth); and "Toward Tenacity of Commitement: Understanding and Modifying Institutional Practices and Individual Responses that Impede Work with Multi-problem Families" (Barbara Fields). Issues typically also include publication reviews, program notes, and conference information. (DB)
UR - http://search.proquest.com/docview/63019921?accountid=14701
ER -

TY - GEN
ID - 956
T1 - Children and the Arts: A Source Book of Arts Experiences for School Age Child Care Programs Developed for Florida's At-Risk Children
Y1 - 1990/07/24/

N1 - Availability - Level 1 - Available online, if indexed January 1993 onward
SuppNotes - For a source book for pre-kindergarten programs, see PS 019 466
Last updated - 2012-12-14
KW - ERIC,Resources in Education (RIE)
KW - Teachers
KW - Administrators
KW - Practitioners
KW - Elementary Education
KW - Music Activities
KW - Dance
KW - At Risk Persons
KW - Dramatic Play
KW - Lesson Plans
KW - After School Programs
KW - School Age Day Care
KW - Learning Activities
KW - Elementary School Curriculum
KW - Art Activities
KW - ERIC
RP - NOT IN FILE
SP - 139
PB - Florida Department of Education, c/o Melvyne Lord, Educational Materials, Collins Building, Room B1, Tallahassee, FL 32399-0400 ($2.00; Florida residents must add 7% sales tax).
N2 - Designed to assist administrators and teachers conducting child care programs for school-age at-risk children, this curriculum guide outlines age-appropriate, process-oriented arts experiences. Content focuses on learning activities in the visual arts, dance/creative movement, drama, and music. Each section includes all or several of the following subsections: (1) comments on age-appropriate activities; (2) guidelines for implementing activities; (3) outlines of lessons; (4) suggestions concerning community resources; (5) guidelines on creating a space for activities; (6) recommendations on supplies; (7) lists of materials and bibliographic resources; and (8) sheet music. Lesson plans state objectives, list materials, advise on the preparation and demonstration of the activity, and discuss motivating children to participate. Individual sections also intermittently include suggestions on organizing an activity center and/or elaborating the activity in subsequent lessons. (RH)
UR - http://search.proquest.com/docview/62989970?accountid=14701
ER -

TY - GEN
ID - 957
T1 - Children and the Arts: A Source Book of Arts Experiences for Pre-Kindergarten Early Intervention Programs Developed for Florida's At-Risk Children
Y1 - 1990///
N1 - Availability - Level 1 - Available online, if indexed January 1993 onward
SuppNotes - For a source book for school age programs, see PS 019 467
Last updated - 2012-12-14
KW - Florida
KW - ERIC,Resources in Education (RIE)
KW - Teachers
KW - Administrators
KW - Practitioners
KW - Preschool Education
KW - Preschool Curriculum
KW - Music Activities
KW - Dance
KW - At Risk Persons
KW - Dramatic Play
KW - Lesson Plans

KW - Learning Activities
KW - Art Activities
KW - ERIC
RP - NOT IN FILE
SP - 101
PB - Florida Department of Education, c/o Melvyne Lord, Educational Materials, Collins Building, Room B1, Tallahassee, FL 32399-0400 ($2.00; Florida residents must add 7% sales tax).
N2 - Designed to assist administrators and teachers conducting prekindergarten early intervention programs for at-risk children, this curriculum guide outlines age-appropriate arts experiences that are process-oriented rather than product-oriented. After introductory statements, content focuses on learning activities in the visual arts, dance/creative movement, drama, and music. Each section includes all or several of the following subsections: (1) comments on age-appropriate activities; (2) guidelines for implementing activities; (3) outlines of lessons; (4) suggestions concerning community resources; (5) guidelines for creating a space for activities; (6) recommendations on supplies; (7) lists of materials and bibliographic resources; and (8) sheet music. Lesson plans provide objectives, list materials, advise on preparation and demonstration of the activity, and suggest methods to increase children's participation. Individual sections also intermittently include suggestions on modifying the lesson for older children, organizing an activity center, and/or elaborating the activity in subsequent lessons. (RH)
UR - http://search.proquest.com/docview/62975858?accountid=14701
ER -

TY - GEN
ID - 958
T1 - International High/Scope Registry Conference Proceedings (Ypsilanti, Michigan, May 8-11, 1996)
Y1 - 1996///
N1 - Availability - Level 2 - Produced in microfiche (1966-2003)
SuppNotes - For 1994 proceedings, see ED 383 406
Last updated - 2012-12-14
KW - China
KW - High Scope Model
KW - Project Head Start
KW - Romania
KW - ERIC,Resources in Education (RIE)
KW - Early Childhood Education
KW - Teacher Student Relationship
KW - High Risk Students
KW - Motor Development
KW - Motion
KW - Multicultural Education
KW - Professional Development
KW - Multiple Intelligences
KW - Conflict Resolution
KW - Student Centered Curriculum
KW - Classroom Techniques
KW - Curriculum Development
KW - Music
KW - Emergent Literacy
KW - Play
KW - Parent Teacher Cooperation
KW - Special Needs Students
KW - Painting (Visual Arts)
KW - Language Arts
KW - Foreign Countries
KW - Parent Student Relationship
KW - Freehand Drawing
KW - Behavior Modification

KW - Story Telling
KW - Active Learning
KW - ERIC
RP - NOT IN FILE
SP - 244
PB - High/Scope Educational Research Foundation, 600 North River Street, Ypsilanti, MI 48198-2898.
N2 - These conference proceedings provide summaries of 66 presentations on various aspects of the High/Scope active learning approach for high-risk students, as well as related topics. Topics addressed include: (1) real-life problem solving; (2) songs in daily routines; (3) open-ended art materials; (4) transition strategies; (5) recall ideas for special young learners; (6) adapting child interests into classroom activities; (7) outside play; (8) active learning and High/Scope approach for children with disabilities; (9) plan-do-review process; (10) proposal writing; (11) conflict resolution; (12) infant and toddler temperaments and exploration; (13) meeting parents' educational expectations; (14) integrating language arts; (15) developing emerging literacy goals; (16) using children's perceptions in center evaluation; (17) play; (18) computerized Child Observation Record (COR); (19) High/Scope program beyond grade three; (20) adult-child interaction; (21) painting and drawing; (22) storytelling; (23) new activity possibilities; (24) motor control; (25) behavior modification; (26) classroom transition; (27) parent child and parent teacher roles; (28) multicultural perspective in education; (29) Chinese and Romanian cultural activities; (30) Training of Trainers (TOT) project; (31) High/Scope Program Implementation Profile (PIP); (32) High/Scope at home; (33) Head Start staff development; (34) curriculum models; (35) observation/feedback; (36) multiple intelligences; (37) re-endorsement assessment strategies; (38) positive reinforcement; (39) movement and music series; and (40) small group activities. (SD)
UR - http://search.proquest.com/docview/62570640?accountid=14701
ER -

TY - RPRT
ID - 959
T1 - Full-Day Kindergarten: The Need for Quality
Y1 - 2008/08//
N1 - Last updated - 2013-03-04
KW - Colorado
KW - ERIC,Resources in Education (RIE)
KW - Early Childhood Education
KW - Kindergarten
KW - Preschool Education
KW - Vocabulary Development
KW - Program Effectiveness
KW - Play
KW - Cognitive Development
KW - State Legislation
KW - Young Children
KW - Cooperation
KW - School Schedules
KW - Vocabulary Skills
KW - Conflict Resolution
KW - Emotional Development
KW - Peer Relationship
KW - At Risk Students
KW - Early Intervention
KW - Social Development
KW - standards
KW - Learning Activities
KW - School Readiness
KW - Teaching Methods
KW - Educational Quality
KW - ERIC
RP - NOT IN FILE

SP - 4
PB - Colorado Children's Campaign. 1580 Lincoln Street Suite 420, Denver, CO 80203.
N2 - Because of the substantial impact on outcomes for children, states and school districts across the country are addressing issues surrounding early learning opportunities and school readiness for young children. Full-day kindergarten plays an important role in both. Colorado has made significant investments in full-day kindergarten as a means of providing high-quality education opportunities to young children, and better preparing them for success throughout their academic careers. Colorado has made a strong commitment to early learning opportunities through the Colorado Preschool Program (CPP) and, with House Bill 08-1388, a substantial investment in full-day kindergarten. CPP provides high-quality learning opportunities for preschool and kindergarten children who have the greatest social and economic need. As the Colorado Preschool Kindergarten Program (CPKP) shifts its full-day kindergarten slots to preschool, once again becoming the CPP, and CPKP standards are no longer required for full-day kindergarten, it is important for districts to consider the design and implementation of high-quality full-day kindergarten programs. Full-day kindergarten is also a key element of the commitment Colorado Governor Bill Ritter has made to the development of a preschool through postsecondary, or P-20, education system. A report from the National Institute for Early Education Research (NIEER) indicates children in full-day kindergarten programs spend more time engaged in valuable learning activities than children in half-day kindergarten programs. Results from empirical research suggest children in full-day kindergarten programs spend: (1) 57 minutes per day in self-selected activities, as opposed to 32 minutes per day for half-day kindergarten students; and (2) 16 percent less time in large-group, teacher-directed activities, and seven percent more time in child-initiated learning activities than half-day kindergarten children. Research has shown that both self-selected and child-initiated activities have long-term benefits on children's learning by promoting cognitive and social-emotional development. Center-based and child-directed play time helps children develop vocabulary skills, increase cooperation with peers, practice handling conflict and develop positive approaches to learning. (Contains 7 footnotes.)
UR - http://search.proquest.com/docview/1314311150?accountid=14701
ER -

TY - RPRT
ID - 960
T1 - WWC Quick Review of the Article "Promoting Broad and Stable Improvements in Low-Income Children's Numerical Knowledge Through Playing Number Board Games"
Y1 - 2008/08//
N1 - Last updated - 2012-12-14
KW - ERIC,Resources in Education (RIE)
KW - Early Childhood Education
KW - High Risk Students
KW - Play
KW - Family Influence
KW - Low Income
KW - Numeracy
KW - Improvement
KW - Correlation
KW - Number Concepts
KW - Computation
KW - Educational Games
KW - Disadvantaged Youth
KW - Preschool Children
KW - ERIC
RP - NOT IN FILE
SP - 1
PB - What Works Clearinghouse. P.O. Box 2393, Princeton, NJ 08543-2393.
N2 - This review examined a study designed to evaluate whether playing number board games improved numeracy skills of low-income preschoolers. The study included 136 pre-school children from 10 urban Head Start centers: 72 children were randomly selected to play a number board game with a trained experimenter; the remaining 64 children played a different version of the game using colors instead of numbers. Numeracy skills were assessed at the end of a two-week period and again nine weeks later. Study authors reported that children

who played the number game had better counting and number identification skills than children who played the color game. Children who played the number game were also better at picking the highest number from a pair of numbers and identifying positions on a number line. The effects persisted nine weeks after the game sessions ended. The review reports that the research described in the study is consistent with What Works Clearinghouse (WWC) evidence standards with reservations. The study was recognized as a well implemented randomized control trial. Cautionary statements regarding results are included. Twelve children from the initial sample of 136 were dropped from the analysis, including four who were assigned to play the number game and eight who were assigned to play the color game. In addition, children who played the number game had higher baseline scores on most outcome measures. Although these differences were not statistically significant, they may contribute to the higher scores reported in the follow-up period for children who played the number game. [The following report was the focus of this "Quick Review": Promoting Broad and Stable Improvements in Low-Income Children's Numerical Knowledge Through Playing Number Board Games. "Child Development," 79(2), 375-394. Geetha B. Ramani and Robert S. Siegler (EJ789398).]
UR - http://search.proquest.com/docview/61985338?accountid=14701
ER -

TY - GEN
ID - 961
T1 - Playgrounds & Toxic Threats
Y1 - 2010///
N1 - Last updated - 2012-12-14
KW - ERIC,Resources in Education (RIE)
KW - Parents
KW - Play
KW - Public Health
KW - Federal Legislation
KW - State Legislation
KW - Hazardous Materials
KW - Playgrounds
KW - Neighborhoods
KW - Risk Management
KW - School Safety
KW - Child Safety
KW - Laws
KW - ERIC
RP - NOT IN FILE
SP - 6
PB - Healthy Schools Network, Inc. 773 Madison Avenue 1st Floor, Albany, NY 12208. Tel: 518-462-0632: Fax: 518-462-0433; Web site: http://www.healthyschools.org
N2 - Neighborhood and school playgrounds are places that kids play, get fit and make friends. Individuals expect playgrounds to be safe and clean environments for children to play in. However, too many playgrounds across America have unsafe play equipment and are not well-maintained. Some may pose environmental health hazards such as the ones discussed in this guide. Children are more vulnerable to environmental health hazards because their bodies are still growing. Proportionally, they eat, drink, and breathe more per pound of body weight than adults. Children like to explore, which can expose them to more environmental threats than adults. But, they also do not know how to identify and protect themselves from these hazards. This guide discusses the following: what parents and schools can do to ensure safety in playgrounds; current federal and state laws governing toxic playground materials; and health concerns associated with each type of risk
UR - http://search.proquest.com/docview/815957889?accountid=14701
ER -

TY - GEN
ID - 962
T1 - Proceedings of the International Association for Development of the Information Society (IADIS) International Conference on Cognition and Exploratory Learning in Digital Age (CELDA) (Madrid, Spain, October 19-21, 2012)

Y1 - 2012/10//
N1 - Last updated - 2013-07-02
ER -

TY - JOUR
ID - 963
T1 - Students' Attitudes toward an After-School Physical Activity Programme
A1 - Agbuga,Bulent
A1 - Xiang,Ping
A1 - McBride,Ron
Y1 - 2013/02//
N1 - Last updated - 2013-07-02
SubjectsTermNotLitGenreText - 7840 126; 10181 730; 8033 730; 3736 9146 126; 309 8331; 728 6447; 3363 10278 8016 4542; 8535 6447; 5472 3629 6582; 7940 126; 4270 126; 715; 695 694 8016 4542
KW - Texas
KW - ERIC,Current Index to Journals in Education (CIJE)
KW - Grade 3
KW - Grade 4
KW - Grade 5
KW - Grade 6
KW - Elementary School Students
KW - Play
KW - Questionnaires
KW - After School Programs
KW - Attendance
KW - At Risk Students
KW - Physical Activities
KW - Positive Attitudes
KW - Student Attitudes
KW - Games
KW - Interviews
KW - Attitude Measures
KW - Extracurricular Activities
KW - ERIC
RP - NOT IN FILE
SP - 91
EP - 109
JF - European Physical Education Review
VL - 19
IS - 1
PB - SAGE Publications. 2455 Teller Road, Thousand Oaks, CA 91320.
N2 - Though considerable research on student attitudes has been conducted in physical education, little information exists concerning student attitudes toward after-school physical activity programmes. This study assessed students' attitudes toward their after-school physical activity programme located in southwest Texas, USA. Participants included 158 students in grades three to six, with ages ranging from eight to 12 years. They completed a five-item attitude questionnaire drawn from the attitude literature. Twenty students were also randomly selected and interviewed individually. Both the questionnaire and interview data indicated students in this study had positive attitudes toward their after-school physical activity programme. The interview data further revealed that students had positive attitudes primarily because they had fun, played a variety of games, and benefited from participation in the physical activities. Students' attitudes were also examined in relation to their attendance of the programme. No significant relationship was found between the two variables. (Contains 2 tables and 1 figure.)
SN - 1356-336X, 1356-336X
UR - http://search.proquest.com/docview/1373086109?accountid=14701
ER -

TY - BOOK
ID - 965
T1 - Improving Your Reflective Practice through Stories of Practitioner Research. Pen Green Books for Early Years Educators
A1 - Arnold,Cath
Y1 - 2012/05/21/
N1 - Last updated - 2012-12-14
KW - ERIC,Resources in Education (RIE)
KW - Practitioners
KW - Researchers
KW - Adult Education
KW - Early Childhood Education
KW - Preschool Education
KW - Teacher Researchers
KW - Preschool Teachers
KW - Play
KW - Young Children
KW - Teacher Improvement
KW - Research Projects
KW - Classroom Techniques
KW - Teacher Effectiveness
KW - Risk
KW - Adult Learning
KW - Imagination
KW - Leadership Effectiveness
KW - Physical Environment
KW - Video Technology
KW - Parent Participation
KW - Reflective Teaching
KW - Interpersonal Relationship
KW - Child Care Centers
KW - Educational Technology
KW - Photography
KW - Educational Research
KW - Teaching Methods
KW - ERIC
RP - NOT IN FILE
SP - 216
PB - Routledge, Taylor & Francis Group. 7625 Empire Drive, Florence, KY 41042.
N2 - "Improving Your Reflective Practice through Stories of Practitioner Research" shows how research has informed and created effective and valuable reflective practice in early years education, and offers depth to the arguments for a research-orientated stance to this vital field of study. This thought-provoking text explores and documents a variety of small-scale practitioner research projects from the home and early years settings. The stories are centred around real life for children, families and workers and offer practical ideas and support for early years students around the world. They engage in some of the most current debates in early childhood education today, such as: (1) how to support children as individuals; (2) how young children learn and how parents support their learning; (3) how to lead and facilitate change in a way that does not take power away from children, parents or workers; (4) how to support children in taking risks; and (5) how to support parents in returning to learning. Throughout this book, the "Pen Green" attitude to practitioner research is actively encouraged. This involves fostering curiosity, being open to the views of others, questioning the "taken for granted," making the implicit explicit and reflecting on one's daily work. Any practitioner research in early years education and care will draw inspiration from this accessible and supportive text. This book begins with an introduction by Cath Arnold. Chapters include: (1) Treasuring Millie: Millie's Use of Treasure Baskets (Elaine Grant); (2) One Child's Use of a Story as a Transitional Object (Gina Goody); (3) Young Children's Use of Digital Cameras to Share Home at Nursery: Bridges of Shared Understanding (Clare Knight); (4) "Is Breaking a Leg just Another Experience?"--Using Video to gain a Bi-cultural Perspective of Risk-Taking in the

Natural Environment (Suzanne Taylor); (5) How can we provide an optimal learning environment for young children's imaginative play? (David Westmore); (6) Knowing Individual Children Through Involving Their Parents (Janette Harcus); (7) Acorns to Oaks: Growing Leadership in Community Nurseries (Ana Sevilla); (8) Effective Leadership, Effective Learning--It's All About Relationships (Gill Allen); and (9) Learning to return: What supports adults to return to learning? (Anne Gladstone). Conclusion by Cath Arnold
SN - 9780415697293
UR - http://search.proquest.com/docview/1031154437?accountid=14701
ER -

TY - JOUR
ID - 966
T1 - Extending Classwide Social Skills Interventions to At-Risk Minority Students: A Preliminary Application of Randomization Tests Combined with Single-Subject Design Methodology
A1 - Bardon,Lisa A.
A1 - Dona,Daria Paul
A1 - Symons,Frank J.
Y1 - 2008/05//
N1 - Last updated - 2012-12-14
SubjectsTermNotLitGenreText - 278 1474 316 8016 4542 293 7160; 7940 126; 5470; 8721; 6716 8016 4542; 9925 3150; 5446 1970 1; 2941 2700 9804 9351 5964; 4420 5264; 2221 909; 695 694 8016 4542; 4954
KW - ERIC,Current Index to Journals in Education (CIJE)
KW - Elementary Education
KW - Grade 3
KW - Special Education
KW - Play
KW - Cooperation
KW - Intervention
KW - Referral
KW - Identification
KW - At Risk Students
KW - Minority Groups
KW - African American Children
KW - Interpersonal Competence
KW - Disproportionate Representation
KW - ERIC
RP - NOT IN FILE
SP - 141
EP - 152
JF - Behavioral Disorders
VL - 33
IS - 3
PB - Council for Children with Behavioral Disorders. Council for Exceptional Children, 1110 North Glebe Road, Arlington, VA 22201-5704.
N2 - Disproportionate representation of minority students in special education programs is a continuing issue of concern. A social emotional curriculum, the Promoting Alternative Thinking Strategies (PATHS), was implemented in a classwide and curricular-integrated format with initial effects evaluated for 3 third-grade African American children identified as at risk for special education referral. Effects were examined using direct observations of each target child's cooperative play in a multiple-baseline design across subjects with intervention onset randomized. Visual and randomization test analyses showed increased cooperative play associated with the PATHS intervention. The combined design and analysis may serve as a viable response-to-intervention approach for reducing referral assessment bias of at-risk minority students. Benefits and limitations of the analysis methods and the PATHS intervention are discussed. (Contains 1 figure.)
SN - 0198-7429, 0198-7429
UR - http://search.proquest.com/docview/742864174?accountid=14701
ER -

TY - JOUR
ID - 967
T1 - Is Attachment at Ages 1, 6 and 16 Related to Autonomy and Relatedness Behavior of Adolescents in Interaction towards Their Mothers?
A1 - Becker-Stoll,Fabienne
A1 - Fremmer-Bombik,Elisabeth
A1 - Wartner,Ulrike
A1 - Zimmermann,Peter
A1 - Grossmann,Klaus E.
Y1 - 2008///
N1 - Last updated - 2012-12-14
SubjectsTermNotLitGenreText - 6823 3932 8016 4542 7577 3816; 5118 11655 1474 316 8016 4542; 8996; 714 909; 197 316 8016 4542; 5348 8768; 11205; 312 5054 2842; 7736; 7537 3851 5449 8768; 1474 316 8016 4542; 694 8016 4542; 2267 10087 2574 3629 6582; 7940 126; 1443 5053 2787; 4109 4335; 7921; 7147 1849
KW - Adult Attachment Interview
KW - Germany
KW - ERIC,Current Index to Journals in Education (CIJE)
KW - At Risk Persons
KW - Interaction
KW - Play
KW - Mothers
KW - Correlation
KW - CHILDREN
KW - Vacations
KW - Risk
KW - Nonverbal Communication
KW - Foreign Countries
KW - Parent Child Relationship
KW - Planning
KW - Child Development
KW - Personal Autonomy
KW - Age Differences
KW - Adolescents
KW - Attachment Behavior
KW - Infants
KW - ERIC
RP - NOT IN FILE
SP - 372
EP - 380
JF - International Journal of Behavioral Development
VL - 32
IS - 5
PB - SAGE Publications. 2455 Teller Road, Thousand Oaks, CA 91320.
N2 - This study investigates whether attachment quality at ages 1, 6 and 16 is related to autonomy and relatedness behavior in adolescence. In a follow-up of the Regensburg Longitudinal Study, forty-three 16-year-old adolescents and their mothers were assessed in a revealed differences task and a planning a vacation task. Attachment was assessed during infancy using the Ainsworth Strange Situation and at age six with the reunion procedure. Adolescent attachment representation was assessed using the Adult Attachment Interview. Results provided no evidence for significant continuity between infant or childhood attachment behavior and adolescent attachment representation. Instability of attachment organization, however, was linked to a higher number of experienced risk factors. Substantial relations between adolescent attachment representation and adolescent autonomy and relatedness behavior were found in both interaction tasks with their mothers. Further, significant relations between attachment qualities at ages 1 and 6 and adolescent interaction behavior during the planning a vacation task at age 16 were found. Thus, independent of attachment stability or instability, both early attachment in infancy and childhood and concurrent attachment representation were significantly related to autonomy and relatedness behavior in adolescence. (Contains 4 tables.)

SN - 0165-0254, 0165-0254
UR - http://search.proquest.com/docview/61969486?accountid=14701
ER -

TY - JOUR
ID - 968
T1 - Practice Makes Poorer: Practice Gambling Modes and Their Effects on Real-Play in Simulated Roulette
A1 - Bednarz,Jana
A1 - Delfabbro,Paul
A1 - King,Daniel
Y1 - 2013/06//
N1 - Last updated - 2013-07-02
SubjectsTermNotLitGenreText - 141 9458 909 913 2877; 7729 909; 7940 126; 2035 4270 126; 2057 9651 6582; 8996
KW - ERIC,Current Index to Journals in Education (CIJE)
KW - Addictive Behavior
KW - Risk
KW - Computer Simulation
KW - Play
KW - Persistence
KW - Computer Games
KW - ERIC
RP - NOT IN FILE
SP - 381
EP - 395
JF - International Journal of Mental Health and Addiction
VL - 11
IS - 3
PB - Springer. 233 Spring Street, New York, NY 10013.
N2 - The aim of this study was to examine the role of free-play modes on gambling behaviour in computer-based roulette. Eighty participants were randomly allocated to one of four pre-exposure conditions: no exposure (control group), a loss condition, a break-even and a profit condition in which the return to player was greater than 100%. Behavioural persistence and betting behaviour was subsequently monitored in a period of regular roulette play. The results showed that players given opportunities for free-play sessions bet significantly more per spin and wagered more credits in total than the control group, although no significant group differences in behavioural persistence were observed. The results suggest that the role of free-play modes, as they commonly appear on some Internet gaming sites, may need to be investigated further because of their potential role in altering player perceptions of the activity and their influence on risk-taking behaviour
SN - 1557-1874, 1557-1874
UR - http://search.proquest.com/docview/1373083546?accountid=14701
ER -

TY - GEN
ID - 969
T1 - Among the Periodicals
A1 - Bell,Michael J.
Y1 - 1994///
N1 - Last updated - 2012-12-14
KW - Journal Articles
KW - Not at Risk Persons
KW - Outcome Based Education
KW - Story Problems
KW - Vygotsky (Lev S)
KW - ERIC,Current Index to Journals in Education (CIJE)
KW - At Risk Persons
KW - Elementary School Students

KW - Deafness
KW - Play
KW - Creative Thinking
KW - Periodicals
KW - Parent Child Relationship
KW - Childrens Literature
KW - Outcomes of Education
KW - ERIC
RP - NOT IN FILE
SP - 53
EP - 54
JF - Childhood Education
VL - 71
IS - 1
N2 - Reviews four journal articles on the following topics: (1) children's creative solutions to realistic and fanciful story problems; (2) Vygotskian perspectives on deafness and mother-child interaction; (3) outcomes-based education; and (4) the differences in play activities of at-risk and not-at-risk elementary school children. (TJQ)
UR - http://search.proquest.com/docview/62728970?accountid=14701
ER -

TY - THES
ID - 970
T1 - The Effects of the Project Approach on Children in Inclusive Early Childhood Classrooms
A1 - Beneke,Sallee Jean
Y1 - 2010///
N1 - Last updated - 2012-12-14
SubjectsTermNotLitGenreText - 11655 1474 316 8016 4542; 2877; 8299; 695 694 8016 4542; 5449 8768; 5748 11228 1703 5053 2787; 916 8234; 5015; 7940 126; 11325; 5072 9937 8331; 6734 8852 6582; 7234; 5472 3629 6582
IdentKeywords - Mean Length of Utterance
KW - Mean Length of Utterance
KW - ERIC,Resources in Education (RIE)
KW - Early Childhood Education
KW - Program Effectiveness
KW - Play
KW - Language Acquisition
KW - Young Children
KW - Mixed Methods Research
KW - Vocabulary
KW - Observation
KW - Behavior Problems
KW - At Risk Students
KW - Disabilities
KW - Inclusion
KW - Interviews
KW - Individualized Education Programs
KW - Interpersonal Relationship
KW - ERIC
RP - NOT IN FILE
SP - 240
N2 - Research on the impact of the Project Approach on young children with disabilities or children who are at-risk is limited. Mixed methods were used to study the impact of the Project Approach on the social interactions, challenging behaviors, and language development of eight focal children in two inclusive classrooms. Child participants were two children with IEPs and two children identified as at-risk from each class. Adult participants were six professionals who received high quality supports to implement the Project Approach.

Adults were interviewed prior to the beginning of the study and again mid-, and post-implementation. Choice time observations were videotaped twice per week over 14 weeks to assess the impact of the Project Approach on play levels and MLU[subscript m]. Results revealed that social interactions, challenging behaviors, vocabulary, MLU[subscript m], were positively impacted following implementation of the Project Approach. Limitations of the study and suggestions for research and practice are discussed. [The dissertation citations contained here are published with the permission of ProQuest LLC. Further reproduction is prohibited without permission. Copies of dissertations may be obtained by Telephone (800) 1-800-521-0600. Web page: http://www.proquest.com/en-US/products/dissertations/individuals.shtml.]
SN - 9781124636009
UR - https://www.ideals.illinois.edu/bitstream/handle/2142/18283/Beneke_Sallee.pdf?sequence=1
ER -

TY - GEN
ID - 971
T1 - Play as the Vehicle for Early Intervention with At-Risk Infants and Toddlers
A1 - Bergen,Doris
Y1 - 1991/04//
N1 - Availability - Level 1 - Available online, if indexed January 1993 onward
SuppNotes - Paper presented at the Annual Conference of the American Educational Research Association (Chicago, IL, April 3-7, 1991)
Last updated - 2012-12-14
KW - Developmental Delays
KW - Developmental Sequences
KW - Play Based Assessment
KW - ERIC,Resources in Education (RIE)
KW - Preschool Education
KW - Developmental Disabilities
KW - At Risk Persons
KW - Toddlers
KW - Special Education
KW - Play
KW - Young Children
KW - Evaluation Methods
KW - Prevention
KW - Parent Child Relationship
KW - Perceptual Motor Learning
KW - Early Intervention
KW - Child Development
KW - Pretend Play
KW - Infants
KW - ERIC
RP - NOT IN FILE
SP - 48
N2 - This paper reviews the current literature on the suggested uses of play for assessment, prevention, and intervention with special needs children. The paper also uses case studies to illustrate the ways play is being used to facilitate achievement of early intervention goals and discusses advantages and disadvantages of these uses of play. The play-based model of assessment involves observation of children's sensorimotor, symbolic, and social play by a transdisciplinary team. Parents are taught directive or playful techniques that minimize problems that arise from dysfunctional parent-child interaction patterns, and thus prevent developmental delays on the part of at-risk children. The use of play for educational intervention involves the development of play skills as a planned part of a curriculum and the incorporation of playful approaches for mastery of skills in other domains, such as language or social skills. This paper defines play and addresses questions about what constitutes play in play-based assessment, in parent-child social interaction, and in play intervention. Appendix 1 describes the three assessments used as examples in the text, and Appendix 2 presents additional examples of prevention and intervention through play. (BC)
UR - http://search.proquest.com/docview/63004775?accountid=14701

ER -

TY - JOUR
ID - 973
T1 - Play Therapy in Elementary Schools: A Best Practice for Improving Academic Achievement
A1 - Blanco,Pedro J.
A1 - Ray,Dee C.
Y1 - 2011///
N1 - Last updated - 2012-12-14
SubjectsTermNotLitGenreText - 3698 4542 8852 6582; 2207 4542 8852 6582; 7940 126; 28 96; 107 10789 6447; 4413 5264; 7941 10836 10839 8699; 3363 10278 8016 4542; 695 694 8016 4542; 8299; 7455; 98 4999; 1955 3629 6582; 5470
KW - ERIC,Current Index to Journals in Education (CIJE)
KW - Elementary Education
KW - Grade 1
KW - Program Effectiveness
KW - Elementary School Students
KW - Play
KW - Academic Achievement
KW - Intervention
KW - Experimental Groups
KW - Achievement Gains
KW - Outcomes of Treatment
KW - Comparative Analysis
KW - Play Therapy
KW - At Risk Students
KW - Control Groups
KW - Achievement Tests
KW - ERIC
RP - NOT IN FILE
SP - 235
EP - 243
JF - Journal of Counseling & Development
VL - 89
IS - 2
PB - American Counseling Association. 5999 Stevenson Avenue, Alexandria, VA 22304.
N2 - This pilot study of 1st graders who are academically at risk examined the effectiveness of child-centered play therapy (CCPT). The experimental group received biweekly, 30-minute play therapy sessions for 8 weeks. Findings indicated that these 1st graders participating in CCPT (n = 21) demonstrated a statistically significant increase on the Early Achievement Composite of the Young Children's Achievement Test (Hresko, Peak, Herron, & Bridges, 2000) when compared with children in the control group (n = 20). Results support using CCPT as an intervention for academic achievement
SN - 0748-9633, 0748-9633
UR - http://search.proquest.com/docview/881453660?accountid=14701
ER -

TY - GEN
ID - 974
T1 - Risk and Reality: Teaching Preschool Children Affected by Substance Abuse
A1 - Brady,Joanne P.
A1 - Grollman,Sharon
Y1 - 1994///
N1 - Availability - Level 1 - Available online, if indexed January 1993 onward
SuppNotes - For a related research review, see ED 397 986. Companion videotape not available from ERIC. Also funded by the Office of the Assistant Secretary for Elementary and Secondary Education (ED)
Last updated - 2012-12-14

KW - Risk Factors
KW - ERIC,Resources in Education (RIE)
KW - Practitioners
KW - Teachers
KW - Preschool Education
KW - Teacher Student Relationship
KW - High Risk Students
KW - Family Environment
KW - Parent Teacher Cooperation
KW - Classroom Environment
KW - Dramatic Play
KW - Cooperation
KW - Developmental Delays
KW - Educational Improvement
KW - Behavior Problems
KW - Classroom Techniques
KW - Learning Problems
KW - Fetal Alcohol Syndrome
KW - Student Needs
KW - Portfolio Assessment
KW - Student Evaluation
KW - Student Behavior
KW - ERIC
RP - NOT IN FILE
SP - 79
PB - Head Start Information and Publication Center, 1133 15th St., N.W., Suite 450, Washington, DC 20005 (guide: $2.50; videotape: $5).
N2 - In recent years, preschool and elementary school teachers have noted increases in behavior concerns and developmental delays, possibly caused by prenatal drug or alcohol exposure. This document notes that it is more important to ascertain the children's challenges than the causes. It describes the most promising practices identified for improving the learning of children at risk, whatever the reason. These are: (1) creating a nurturing classroom environment; (2) encouraging cooperative play; (3) facilitating classroom transitions and minimizing distractions; (4) helping children manage their behaviors; (5) conducting ongoing classroom assessment; and (6) building strong ties with families. The guide features a companion videotape. (Contains 43 resources.) (HTH)
UR - http://search.proquest.com/docview/62237343?accountid=14701
ER -

TY - GEN
ID - 975
T1 - How to Nurture Early Literacy
A1 - Britsch,Susan
Y1 - 1993///
N1 - Last updated - 2012-12-14
KW - ERIC,Current Index to Journals in Education (CIJE)
KW - Teachers
KW - Practitioners
KW - Early Childhood Education
KW - Teacher Role
KW - Teacher Student Relationship
KW - High Risk Students
KW - Play
KW - Language Acquisition
KW - Language Skills
KW - Language Arts
KW - Early Reading
KW - Prereading Experience

KW - Beginning Reading
KW - Freehand Drawing
KW - Emergent Literacy
KW - Early Experience
KW - ERIC
RP - NOT IN FILE
SP - 47
EP - 51
JF - Instructor
VL - 103
IS - 4
N2 - Presents some suggestions for nurturing and supporting emergent readers and writers (e.g., recognize drawing as an important step in early literacy, engage children in conversations about their daily experiences). Also presents an interview with two authors on the special literacy needs of students with major instabilities in their lives. (SM)
UR - http://search.proquest.com/docview/62795602?accountid=14701
ER -

TY - JOUR
ID - 977
T1 - Guided Dramatization: Fostering Social Development in Children with Disabilities
A1 - Brown,Mac H.
Y1 - 1993///
N1 - Last updated - 2012-12-14
SubjectsTermNotLitGenreText - 1375 7054 7848 2877 2118; Disabilities 2877; Dramatic Play 3005 9021 9651 6582; 3085 3150; 4723; 5449 8768; 5727 9306 5241; Mainstreaming 6250 7912; 7676 5449 8768; Preschool Children 8127 11655 1474 316 8016 4542; 8295; Social Development 9753 5053 2787; 9934 10278 8016 4542; Teacher Guidance 10522 4552
KW - Normal Children
KW - South Carolina
KW - ERIC,Current Index to Journals in Education (CIJE)
KW - Early Childhood Education
KW - Program Descriptions
KW - High Risk Students
KW - Teacher Guidance
KW - Special Needs Students
KW - Dramatic Play
KW - Mainstreaming
KW - Peer Relationship
KW - Laboratory Schools
KW - Cerebral Palsy
KW - Disabilities
KW - Social Development
KW - Preschool Children
KW - Interpersonal Relationship
KW - ERIC
RP - NOT IN FILE
SP - 68
EP - 71
JF - Young Children
VL - 48
IS - 2
N2 - Describes and illustrates a technique for using guided dramatization in intervention with special needs or at-risk children. Discusses social benefits of the technique. Maintains that the technique is useful for helping mainstreamed children socially integrate into the regular classroom. (BG)
SN - 0044-0728, 0044-0728

UR - http://search.proquest.com/docview/62872392?accountid=14701
ER -

TY - JOUR
ID - 978
T1 - Relations between Behavior Problems in Classroom Social and Learning Situations and Peer Social Competence in Head Start and Kindergarten
A1 - Bulotsky-Shearer,Rebecca J.
A1 - Dominguez,Ximena
A1 - Bell,Elizabeth R.
A1 - Rouse,Heather L.
A1 - Fantuzzo,John W.
Y1 - 2010/12//
N1 - Last updated - 2012-12-14
SubjectsTermNotLitGenreText - 916 8234; 7940 126; 2888 2883 8016 4542 11658 316; 7635 6886 10087 2574 3629 6582; 5649 5264; 5446 1970 1; 695 694 8016 4542; 7676 5449 8768; 3386 5053 2787; 1443 5053 2787; 11182 9306 5241; 8127 11655 1474 316 8016 4542; 312 5054 2842; 8099
KW - ERIC,Current Index to Journals in Education (CIJE)
KW - Early Childhood Education
KW - Kindergarten
KW - Preschool Education
KW - Play
KW - Urban Schools
KW - Behavior Problems
KW - Emotional Development
KW - Peer Relationship
KW - At Risk Students
KW - Interpersonal Competence
KW - Child Development
KW - Disadvantaged Youth
KW - Preschool Children
KW - Path Analysis
KW - Age Differences
KW - Predictor Variables
KW - ERIC
RP - NOT IN FILE
SP - 195
EP - 210
JF - Journal of Emotional and Behavioral Disorders
VL - 18
IS - 4
PB - SAGE Publications and Hammill Institute on Disabilities. 2455 Teller Road, Thousand Oaks, CA 91320.
N2 - The relations between early emotional and behavioral problems in classroom situations and peer social competence were examined for a representative sample of urban Head Start children. Behavior problems were assessed within the context of routine peer, teacher, and structured learning classroom situations early in the preschool year. Two path models were tested: (a) direct effects of preschool situational problems on peer social competence at the end of preschool and (b) direct and indirect effects of preschool situational problems on peer social competence at the end of kindergarten, accounting for preschool peer social competence and child demographic variables. Early problems in peer and socially mediated learning situations consistently predicted lower peer social competence in preschool and kindergarten. Problems in preschool peer situations directly and indirectly predicted greater disruptive play at the end of kindergarten, and problems in structured learning situations predicted lower interactive play at both time points. Implications for policy and practice are discussed. (Contains 1 note, 2 tables, and 2 figures.)
SN - 1063-4266, 1063-4266
UR - http://search.proquest.com/docview/822507674?accountid=14701
ER -

TY - JOUR
ID - 980
T1 - Designing a Children's Water Garden as an Outdoor Learning Lab for Environmental Education
A1 - Byrd,Renee K.
A1 - Haque,Mary Taylor
A1 - Tai,Lolly
A1 - McLellan,Gina K.
A1 - Knight,Erin Jordan
Y1 - 2007///
N1 - Last updated - 2012-12-14
SubjectsTermNotLitGenreText - 11421; 3521 3150; 1806 10278 8016 4542; 9552 3692 5882; 5895 3199 3760 5719; 4274 359 10669; 7458 3150; 5719 3760; 3692 5882; 7940 126; 4848 359 10669; 11416; 309 8331; 3363 10278 8016 4542; 695 694 8016 4542; 4070
KW - South Carolina
KW - ERIC,Current Index to Journals in Education (CIJE)
KW - Elementary Education
KW - Grade 3
KW - Grade 4
KW - Grade 5
KW - Higher Education
KW - Elementary School Students
KW - Floriculture
KW - Play
KW - Environmental Education
KW - After School Programs
KW - Laboratories
KW - Water Quality
KW - Horticulture
KW - Learning Laboratories
KW - Water
KW - Gardening
KW - At Risk Students
KW - Experiential Learning
KW - Service Learning
KW - College Students
KW - Outdoor Education
KW - ERIC
RP - NOT IN FILE
SP - 39
EP - 47
JF - Applied Environmental Education and Communication
VL - 6
IS - 1
PB - Routledge. , 325 Chestnut Street Suite 800, Philadelphia, PA 19106.
N2 - A Clemson University introductory landscape design class collaborated with South Carolina Botanical Gardens (SCBG) staff and coordinators of Sprouting Wings to design an exploratory Children's Garden within the SCBG. Service learning provides students with invaluable real-world experiences solving problems and interacting with clients while contributing to the local community. Project methodology included research, site selection and analysis, conceptual diagrams, preliminary designs, and full-color renderings of final designs. Based on children's desire for water play and on current issues in water quality and the need for environmental education, clients and students designed a Wonders of Water Garden that can be used as a model at other institutions. The design celebrates the beauty of water, and showcases the integral relationships among plants, wildlife, and water. The interactive garden will create awareness of current water quality issues, promote environmental stewardship, and serve as a model to teach visitors of all ages the vital role water plays in the environment. (Contains 1 table and 2 figures.)

SN - 1533-015X, 1533-015X
UR - http://search.proquest.com/docview/742883768?accountid=14701
ER -

TY - JOUR
ID - 982
T1 - Resilient Youths Use Humor to Enhance Socioemotional Functioning during a "Day in the Life"
A1 - Cameron,Leslie E.
A1 - Fox,Janice D.
A1 - Anderson,Michelle S.
A1 - Cameron,Catherine Ann
Y1 - 2010/09//
N1 - Last updated - 2012-12-14
SubjectsTermNotLitGenreText - 3967 5746 6111; 3083 316 8016 4542; 4923; 8901; 694 8016 4542; 8852 6582; 3990 7119 6362 11302; 9761 3518; 7940 126; 9015; 5445 1849; 4203 5449 8768; 3851 5449 8768
KW - ERIC,Current Index to Journals in Education (CIJE)
KW - At Risk Persons
KW - Play
KW - Research Methodology
KW - Early Adolescents
KW - Family Relationship
KW - Figurative Language
KW - Friendship
KW - Humor
KW - Resilience (Psychology)
KW - Interpersonal Communication
KW - Role
KW - Social Environment
KW - Films
KW - ERIC
RP - NOT IN FILE
SP - 716
EP - 742
JF - Journal of Adolescent Research
VL - 25
IS - 5
PB - SAGE Publications. 2455 Teller Road, Thousand Oaks, CA 91320.
N2 - In order to extend previous early years humor research into early adolescence, the authors adapted an innovative ecological research method such that at-risk adolescents could be filmed during an entire waking day in their life. Community youth advocates nominated one 15-year-old female and one 14-year-old male as doing well despite adverse circumstances. We examined the types and functions of these youths' humor within their social contexts. Their humor included joking, teasing, physical play, light tones, irony, sarcasm, and mocking/parody. Humor served many socioemotional roles, such as navigating complex socially sensitive topics and situations, and facilitating affiliation with friends and family. Humor assists in traversing challenging social terrain and can serve as a protective factor under risky circumstances. (Contains 3 tables.)
SN - 0743-5584, 0743-5584
UR - http://search.proquest.com/docview/754911578?accountid=14701
ER -

TY - GEN
ID - 984
T1 - Baby Talk. First Years Together. Project Enlightenment
A1 - Carothers,Linda
A1 - Wilson,Val
Y1 - 1986///

N1 - Availability - Level 2 - Produced in microfiche (1966-2003)
SuppNotes - For related documents, see EC 202 488-497. Document printed on colored paper
Last updated - 2012-12-14
KW - Early Intervention
KW - First Years Together (Project)
KW - ERIC,Resources in Education (RIE)
KW - Parents
KW - Parent Education
KW - Play
KW - High Risk Persons
KW - Discipline
KW - Pregnancy
KW - Hospitalized Children
KW - Safety Education
KW - Self Esteem
KW - Premature Infants
KW - Parent Child Relationship
KW - Infant Behavior
KW - Developmental Stages
KW - Child Development
KW - Activities
KW - ERIC
RP - NOT IN FILE
SP - 43
PB - Project Enlightenment, 501 S. Boylan Ave., Raleigh, NC 27603 ($3.00).
N2 - This series of 19 newsletters is designed to be distributed monthly to parents of premature or high risk infants on their child's adjusted age birthday from birth through 18 months. Each newsletter describes activities and behaviors of infants and toddlers appropriate to the month of age and discusses issues of concern to the parents. Regular topics include safety, discipline, and "premie notes," a section that addresses the particular characteristics or problems associated with prematurity and its effect on later development. For ages 3-12 months activities typical of infant behavior or which the baby will enjoy having the parent initiate are listed under the headings of moving, playing, thinking, and communicating. Other topics covered for specific ages include going home from the hospital (newborn), when to call the doctor (2 months), settling into a routine (3 months), the father's role (6 months), communicating (11 months), and imitation (12 months). Additional newsletters focus on such topics as thinking about another pregnancy, play as learning, setting limits for toddlers, and encouraging self-esteem. (VW)
UR - http://search.proquest.com/docview/63144819?accountid=14701
ER -

TY - RPRT
ID - 985
T1 - Prekindergarten Program Process Evaluation Report, 1990-91
A1 - Claus,Richard N.
A1 - Quimper,Barry E.
Y1 - 1991/02//
N1 - Availability - Level 1 - Available online, if indexed January 1993 onward
SuppNotes - For 1988-89 report, see ED 305 180; for 1989-90 report, see ED 316 317
Last updated - 2012-12-14
KW - Education Consolidation Improvement Act Chapter 1
KW - Saginaw City School System MI
KW - ERIC,Resources in Education (RIE)
KW - Preschool Education
KW - Program Effectiveness
KW - High Risk Students
KW - Play
KW - Cognitive Development

KW - Language Skills
KW - Program Improvement
KW - Classroom Observation Techniques
KW - Parent Participation
KW - Program Evaluation
KW - Compensatory Education
KW - Check Lists
KW - Psychomotor Skills
KW - Preschool Children
KW - School Readiness
KW - ERIC
RP - NOT IN FILE
SP - 30
N2 - During the first half of the 1990-91 school year, 446 children were served by the Chapter 1 Prekindergarten Program of the Saginaw, Michigan school district. A process evaluation was conducted to monitor the program and assess whether it was being implemented as planned. This year, the process evaluation consisted of half-day classroom observations of the classes of each of 5 new kindergarten teachers in 13 buildings. An activity checklist and observation instrument focused primarily on the implementation of the cognitive, psychomotor, and parent participation and education activities of the program, and secondarily on object labeling and language enhancement techniques in the classroom. Observations revealed that: (1) except for grouping and regrouping, and activities related to properties of objects, activities to meet daily objectives were taking place in all classrooms; (2) a record of parent participation was maintained in all classrooms; (3) labels were posted on objects throughout the classrooms in an effort to assist in word recognition; (4) teachers used language enhancement techniques, but with wide variation in frequency; and (5) a wide variation of methods for scheduling free play was observed. The program is operating as planned, but some areas could be improved. Recommendations for addressing these areas are suggested. Appendices provide a list of participants, a sample activity observation checklist, and a table displaying frequency of teachers' use of language enhancement techniques. (BC)
UR - http://search.proquest.com/docview/63003249?accountid=14701
ER -

TY - RPRT
ID - 986
T1 - Prekindergarten Program Process Evaluation Report, 1992-93
A1 - Claus,Richard N.
A1 - Quimper,Barry E.
Y1 - 1992/12//
N1 - Availability - Level 1 - Available online, if indexed January 1993 onward
SuppNotes - For the 1991-92 report, see ED 343 673
Last updated - 2012-12-14
KW - Education Consolidation Improvement Act Chapter 1
KW - Saginaw City School System MI
KW - ERIC,Resources in Education (RIE)
KW - Preschool Education
KW - Program Effectiveness
KW - High Risk Students
KW - Play
KW - Cognitive Development
KW - Language Skills
KW - Emotional Development
KW - Formative Evaluation
KW - Program Improvement
KW - Classroom Observation Techniques
KW - Parent Participation
KW - Program Evaluation
KW - Compensatory Education

KW - Check Lists
KW - Psychomotor Skills
KW - Preschool Children
KW - School Readiness
KW - ERIC
RP - NOT IN FILE
SP - 33
N2 - By December of the 1992-93 school year, 433 children were being served by the Chapter 1 Prekindergarten Program of the Saginaw, Michigan school district. A process evaluation was conducted to monitor the program and assess whether the program was being implemented as planned. The process evaluation consisted of a half-day classroom observation in 14 classrooms. An activity checklist and observation instrument were used to determine whether cognitive, psychomotor, parent participation, and education activities were being carried out and to explore classroom practices and teacher behaviors for encouraging pupils' language behavior. Observations revealed that: (1) activities to meet the program's daily objectives took place in all classrooms; (2) a record of parent participation was maintained in all classrooms; (3) labels were posted on objects throughout the classrooms to assist in word recognition; and (4) teachers used language production and enhancement techniques. Overall, the program was operating as planned. Suggestions for improving some areas of the program are offered. Appendixes include a list of program participants, copies of the activity checklist and observation instrument, and a table listing teachers' use of language production and enhancement techniques. (MM)
UR - http://search.proquest.com/docview/62869843?accountid=14701
ER -

TY - RPRT
ID - 987
T1 - Prekindergarten Program Process Evaluation Report, 1991-92
A1 - Claus,Richard N.
A1 - Quimper,Barry E.
Y1 - 1992/02//
N1 - Availability - Level 1 - Available online, if indexed January 1993 onward
SuppNotes - For 1988-89 report, see ED 305 180; for 1989-90 report, see ED 316 317, for 1990-91 report, see ED 330 449
Last updated - 2012-12-14
KW - Education Consolidation Improvement Act Chapter 1
KW - Saginaw City School System MI
KW - ERIC,Resources in Education (RIE)
KW - Preschool Education
KW - Program Effectiveness
KW - High Risk Students
KW - Play
KW - Cognitive Development
KW - Language Skills
KW - Emotional Development
KW - Formative Evaluation
KW - Program Improvement
KW - Classroom Observation Techniques
KW - Parent Participation
KW - Program Evaluation
KW - Compensatory Education
KW - Check Lists
KW - Psychomotor Skills
KW - Preschool Children
KW - School Readiness
KW - ERIC
RP - NOT IN FILE
SP - 30

N2 - By January of the 1991-92 school year, 428 children were being served by the Chapter 1 Prekindergarten Program of the Saginaw, Michigan school district. A process evaluation was conducted to monitor the program and assess whether the program was being implemented as planned. The process evaluation for this year consisted of a half-day classroom observation in 13 classrooms. An activity checklist and observation instrument were used to determine whether cognitive, psychomotor, parent participation, and education activities were being carried out and to explore classroom practices and teacher behaviors for encouraging pupils' language behavior. Observations revealed that: (1) activities to meet the program's daily objectives took place in all classrooms; (2) a record of parent participation was maintained in all classrooms; (3) labels were placed on objects throughout the classrooms to assist in word recognition; and (4) teachers used language enhancement techniques. Overall, the program was operating as planned. Suggestions for improving some areas of the program are offered. Appendixes include a list of program participants, copies of the activity checklist and observation instrument, and a table listing teachers' use of language enhancement techniques. (BC)
UR - http://search.proquest.com/docview/62929931?accountid=14701
ER -

TY - JOUR
ID - 988
T1 - Second-Impact Syndrome
A1 - Cobb,Sarah
A1 - Battin,Barbara
Y1 - 2004///
N1 - Last updated - 2012-12-14
SubjectsTermNotLitGenreText - 7940 126; 9241 9247 3417 8016 4542 5703 4908 8917 7207 4657 8267; 4630 5183 2877; 197 316 8016 4542; 4566; 1132 4890; 694 8016 4542; 704 7840 126; 698 8016 4542; 9015; 4659 4635 126; 8168; 4738 9419 10278 8016 4542; 1806 10278 8016 4542
KW - ERIC,Current Index to Journals in Education (CIJE)
KW - High Schools
KW - Higher Education
KW - Athletics
KW - At Risk Persons
KW - Play
KW - Guidelines
KW - Brain
KW - Head Injuries
KW - Health Promotion
KW - Prevention
KW - College Students
KW - Role
KW - High School Students
KW - School Nurses
KW - Adolescents
KW - Athletes
KW - ERIC
RP - NOT IN FILE
SP - 262
EP - 267
JF - Journal of School Nursing
JA - J Sch Nurs
VL - 20
IS - 5
PB - SAGE Publications. 2455 Teller Road, Thousand Oaks, CA 91320.
N2 - Sports-related injuries are among the more common causes of injury in adolescents that can result in concussion and its sequelae, postconcussion syndrome and second-impact syndrome (SIS). Students who experience multiple brain injuries within a short period of time (hours, days, or weeks) may suffer catastrophic or fatal reactions related to SIS. Adolescents are particularly susceptible to the dangers of SIS, and current return-to-play guidelines may be too lenient to protect a student from SIS. Any student with signs of a

concussion should receive medical evaluation and not be allowed to return to play in the current game or practice. The role of the school nurse includes being knowledgeable about management of head injuries and return-to-play guidelines, providing follow-up for athletes who have concussions, and providing education on prevention and management of head injuries. (Contains 5 tables.)
SN - 1059-8405, 1059-8405
UR - http://search.proquest.com/docview/742876358?accountid=14701
ER -

TY - JOUR
ID - 989
T1 - Outcomes and Stages of Child-Centered Play Therapy for a Child with Highly Disruptive Behavior Driven by Self-Concept Issues
A1 - Cochran,Jeff L.
A1 - Cochran,Nancy H.
A1 - Fuss,Angela
A1 - Nordling,William J.
Y1 - 2010///
N1 - Last updated - 2012-12-14
SubjectsTermNotLitGenreText - 1404 8697; 7940 126; 9466 9469 9451; 1432 909; 914 2094; 7941 10836 10839 8699; 916 8234; 9451; 7676 5449 8768; 5470; 694 8016 4542; 2302 6582; 7455; 6265 8016 4542; 2293
KW - Child Behavior Checklist
KW - ERIC,Current Index to Journals in Education (CIJE)
KW - At Risk Persons
KW - Play
KW - Child Behavior
KW - MALES
KW - Intervention
KW - Behavior Problems
KW - Outcomes of Treatment
KW - Peer Relationship
KW - Play Therapy
KW - Counseling Techniques
KW - Self Concept
KW - Behavior Modification
KW - Counseling Effectiveness
KW - Self Efficacy
KW - Check Lists
KW - ERIC
RP - NOT IN FILE
SP - 231
EP - 246
JF - Journal of Humanistic Counseling, Education and Development
VL - 49
IS - 2
PB - American Counseling Association. 5999 Stevenson Avenue, Alexandria, VA 22304.
N2 - Children with highly disruptive behavior present problems for their peers and are often a heavy burden to the schools, teachers, counselors, and other adults who care for them. Without successful intervention, such children certainly face lives of high risk, emotional pain, and ever-increasing difficulty; from a humanistic perspective, such an outcome would present the loss of personal, social, and academic potential. Aggressive disruptive behavior of children is the most common presenting problem area for psychotherapists, including play therapists. Play therapy, especially child-centered play therapy (CCPT), has demonstrated effectiveness across a wide range of children's mental health and behavioral problems, from developing self-efficacy to recovering from trauma and major clinical concerns. Descriptions in this case study of child-centered play therapy (CCPT) for a boy referred for highly disruptive behavior include observations of his process through stages of CCPT, teacher and parent ratings on the Child Behavior Checklist, and explanations of how CCPT worked for him. (Contains 2 tables.)

SN - 0735-6846, 0735-6846
UR - http://search.proquest.com/docview/762466740?accountid=14701
ER -

TY - GEN
ID - 992
T1 - Fool's Gold: A Critical Look at Computers in Childhood
A1 - Cordes,Colleen
A1 - Miller,Edward
Y1 - 2000///
N1 - Availability - Level 1 - Available online, if indexed January 1993 onward
Last updated - 2012-12-14
KW - Brain Development
KW - ERIC,Resources in Education (RIE)
KW - Early Childhood Education
KW - Elementary Education
KW - At Risk Persons
KW - Play
KW - Cognitive Development
KW - Computers
KW - Child Health
KW - Brain
KW - Childhood Needs
KW - Creativity
KW - CHILDREN
KW - Moral Development
KW - Computer Uses in Education
KW - Emotional Development
KW - Social Development
KW - Early Experience
KW - Educational Technology
KW - ERIC
RP - NOT IN FILE
SP - 105
PB - Alliance for Childhood, P.O. Box 444, College Park, MD 20741; Tel: 301-513-1777; Fax: 301-513-1777; e-mail: info@allianceforchildhood.net; Web Site: http://www.allianceforchildhood.net ($8.50).
N2 - Noting that computers are reshaping children's lives in profound and unexpected ways, this report examines potential harms and promised benefits of these changes, focusing on early childhood and elementary education. Chapter 1 argues that popular attempts to hurry children intellectually are at odds with the natural pace of human development. Chapter 2 presents information on the risks of using computers to children's physical health (including musculoskeletal injuries, vision problems, and obesity), emotional and social development (isolation, shifts toward computer-centered education, detachment from community, and the commercialization of childhood), creativity and intellectual development (impaired language and literacy, poor concentration, inability to tolerate frustration, plagiarism, and distraction from meaning), and moral development. Chapter 3 urges families and schools to recommit themselves to providing young children with the essentials of a healthy childhood, including strong bonds with caring adults, hands-on experiences with the physical world, time for unstructured play, exposure to the arts, and literacy activities. Chapter 4 discusses ways parents and teachers can help children achieve a technology literacy that also involves the capacity to think critically and use technology to serve personal, social, and ecological goals. Chapter 5 focuses on the costs of technology and argues that the national infatuation with computers in early childhood and elementary education is diverting scarce resources from children's real unmet needs. Chapter 6 concludes with recommendations, including a refocus on the essentials of a healthy childhood and an immediate moratorium on further introduction of computers in early childhood and elementary education. Each chapter contains reference notes. (KB)
UR - http://search.proquest.com/docview/62320480?accountid=14701
ER -

TY - RPRT
ID - 993
T1 - Early Intervention: Examples of Practice. Averroes Programmes for Children - An Experience to be Shared. Action Research in Family and Early Childhood Series. UNESCO Education Section Monograph No. 8
A1 - Cordus,Joyce
A1 - van Oudenhoven,Nico
Y1 - 1997/10//
N1 - Availability - Level 1 - Available online, if indexed January 1993 onward
Last updated - 2012-12-14
KW - Averroes Foundation (Netherlands)
KW - High Scope Model
KW - Netherlands
KW - UNESCO
KW - ERIC,Resources in Education (RIE)
KW - Early Childhood Education
KW - Preschool Education
KW - Program Descriptions
KW - Parent Education
KW - Caregiver Child Relationship
KW - Parenting Skills
KW - At Risk Persons
KW - Reading
KW - Play
KW - Action Research
KW - CHILDREN
KW - Foreign Countries
KW - Home Programs
KW - Transitional Programs
KW - Parent School Relationship
KW - Child Rearing
KW - Parent Participation
KW - Early Intervention
KW - ERIC
RP - NOT IN FILE
SP - 21
VL - ED-97/WS/55
PB - Early Childhood and Family Education Unit, ED/BAS/ECF, UNESCO, 7 Place de Fontenoy, 75352, Paris 07 SP, France.
N2 - Since 1995, UNESCO and the Averroes Foundation have collaborated to develop early childhood education programs. This report describes Averroes intervention programs currently used in the Netherlands with young at-risk children. Following a Foreword which describes various UNESCO early childhood activities, the report is divided into six parts. Part 1 provides background on early intervention program development in the Netherlands. Part 2 describes how intermediary agencies can connect effectively with researchers, policymakers, practitioners, and families. Part 3 describes the Averroes programs: (1) "Instapje," to improve the quality of caregiver-child interactions; (2) "Klimrek," a home-visit program to stimulate verbal interaction between parents and children; (3) "Spel ann Huis," a home-based program to stimulate play development in 1- to 4-year-olds; (4) "Opstapje," to facilitate transitions from home to elementary school for disadvantaged 2- to 4-year-olds; (5) "Opstap Opnieuw," a home-based extra-curriculum for disadvantaged 4- to 6-year-old children and their parents; (6) "Overstap," focusing on collaboration between school, parents, and children during the process of learning to read; (7) "Stap door," a continuation of the Overstap program for second and fifth graders; (8) "Stap rond," a parent education program for caregivers of children from birth to 6 years of age; and (9) "Kaleidoscoop," the Dutch version of the American High/Scope program. Part 4 discusses the limited value in using educational models as the basis for program development and presents key principles for developing intervention programs. Part 5 concerns lessons Averroes has learned in disseminating programs to large numbers. Part 6 discusses the relationship between home-based and center-based intervention strategies. (KB)

UR - http://search.proquest.com/docview/62542880?accountid=14701
ER -

TY - JOUR
ID - 995
T1 - What Happened to Kindergarten?
A1 - Curwood,Jen Scott
Y1 - 2007/08//
N1 - Last updated - 2012-12-14
SubjectsTermNotLitGenreText - 5649 5264; 11655 1474 316 8016 4542; 1443 5053 2787; 60 10031; 4723; 8233 1710; 7940 126; 10227 6996
KW - ERIC,Current Index to Journals in Education (CIJE)
KW - Teachers
KW - Kindergarten
KW - High Risk Students
KW - Play
KW - Young Children
KW - Student Needs
KW - Child Development
KW - Problem Solving
KW - Academic Standards
KW - ERIC
RP - NOT IN FILE
SP - 28
EP - 30
JF - Instructor
VL - 117
IS - 1
PB - Scholastic. 557 Broadway, New York, NY 10012.
N2 - This article deals with the increasing academic pressure facing five-year-old children who are in kindergarten schools in the U.S. Part of the reason kindergarten is becoming more and more academic is a growing understanding of the importance of early learning and the capabilities of young children. Proponents of ramping up standards in early elementary education tend to focus on the numbers: more children learning to read or do math sooner must be good. These achievements, however, may come at the expense of other skills children need to learn, such as self-reliance, problem-solving, and spatial thinking. According to Anne Stoudt, a kindergarten teacher, the risk is children who are already burned out on school by the time they reach third grade. Play is how children learn, so, there should be more of it in the upper grades, not less in the lower
SN - 1049-5851, 1049-5851
UR - http://search.proquest.com/docview/61957264?accountid=14701
ER -

TY - JOUR
ID - 996
T1 - Exposure to Media Violence and Other Correlates of Aggressive Behavior in Preschool Children
A1 - Daly,Laura A.
A1 - Perez,Linda M.
Y1 - 2009///
N1 - Last updated - 2012-12-14
SubjectsTermNotLitGenreText - 7940 126; 8372 9738 909; 11282 545 9738 909; 324 545 9738 909; 695 694 8016 4542; 8127 11655 1474 316 8016 4542; 8292; 6878 8751 10087 2574 3629 6582; 6363; 2267 10087 2574 3629 6582; 545 9738 909; 10717 126; 9457 909; 7546 5127; 312 5054 2842; 6823 3932 8016 4542 7577 3816; 3830 3518; 4290; 3896 7577 3816 8016 4542 6265; 11227 1849; 7234
KW - California
KW - Early Childhood Environment Rating Scale (Revised)
KW - ERIC,Current Index to Journals in Education (CIJE)
KW - Family Environment

KW - Play
KW - Multiple Regression Analysis
KW - Gender Differences
KW - Parent Influence
KW - Mothers
KW - Observation
KW - Correlation
KW - Fathers
KW - Violence
KW - Antisocial Behavior
KW - Mass Media Effects
KW - Verbal Communication
KW - At Risk Students
KW - Television Viewing
KW - Program Content
KW - Preschool Children
KW - Aggression
KW - Self Control
KW - Prosocial Behavior
KW - Age Differences
KW - ERIC
RP - NOT IN FILE
SP - 13
JF - Early Childhood Research & Practice
VL - 11
IS - 2
PB - Clearinghouse on Early Education and Parenting, University of Illinois at Urbana-Champaign. 51 Gerty Drive, Champaign, IL 61820.
N2 - This article examines the play behavior of 70 preschool children and its relationship to television violence and regulatory status. Linear regression analysis showed that violent program content and poor self-regulation were independently and significantly associated with overall and physical aggression. Advanced maternal age and child age and better self-regulation were independently and significantly associated with prosocial behavior. According to t-test analysis, two other statistically significant factors associated with overall aggression were gender and the lack of a father figure in the home. Analysis of t-tests showed a statistically significant relationship between children who watched violent content alone and verbal aggression. Multiple regression analysis established that poor self-regulation was the biggest predictor of overall aggression and that overall aggression was significantly related to gender. Results suggest limiting the amount of violent programming that preschool children see. Results also indicate that children benefit from interactive regulatory support. (Contains 5 tables.)
SN - 1524-5039, 1524-5039
UR - http://search.proquest.com/docview/61806862?accountid=14701
ER -

TY - JOUR
ID - 997
T1 - Relationships among Private Speech and Creativity in Head Start and Low-Socioeconomic Status Preschool Children
A1 - Daugherty,Martha
A1 - White,Stephen C.
Y1 - 2008///
N1 - Last updated - 2012-12-14
SubjectsTermNotLitGenreText - 2388 8409 5051; 2888 2883 8016 4542 11658 316; 11655 1474 316 8016 4542; 2391 10789 6447; 8127 11655 1474 316 8016 4542; 2385 1710; 10197 2472 2842 10187; 694 8016 4542; 7940 126; 2177 5127; 9960 1849; 9465 2898; 1710; 9835 10103; 6186 5019
KW - ERIC,Current Index to Journals in Education (CIJE)
KW - Early Childhood Education

KW - Context Effect
KW - At Risk Persons
KW - Play
KW - Speech Communication
KW - Young Children
KW - Low Income
KW - Socioeconomic Status
KW - Student Diversity
KW - Creativity
KW - Cognitive Processes
KW - Creative Thinking
KW - Creativity Tests
KW - Disadvantaged Youth
KW - Self Disclosure (Individuals)
KW - Preschool Children
KW - ERIC
RP - NOT IN FILE
SP - 30
EP - 39
JF - Gifted Child Quarterly
VL - 52
IS - 1
PB - SAGE Publications. 2455 Teller Road, Thousand Oaks, CA 91320.
N2 - The purpose of this study was to explore Vygotsky's notion of private speech as a cognitive self-regulatory process and how it related to creativity measures among at-risk children. Thirty-two Head Start and state-funded Pre-K children completed the Torrance creativity test Thinking Creatively in Action and Movement (TCAM). The children's private speech was collected in an open play context and while children completed structured logical--mathematical activities. Results revealed that both originality and fluency creativity were related to self-direction private speech and grand total private speech. Findings support that cognitive self-regulating private speech may be related to creative thinking and that private speech may offer a method for assessing early creative thinking in children from various cultural and economic backgrounds. (Contains 4 tables.)
SN - 0016-9862, 0016-9862
UR - http://search.proquest.com/docview/61971185?accountid=14701
ER -

TY - JOUR
ID - 1000
T1 - Rodent ultrasonic communication: Male prosocial 50-kHz ultrasonic vocalizations elicit social approach behavior in female rats (Rattus norvegicus)
A1 - Willadsen,M.
A1 - Seffer,D.
A1 - Schwarting,R.K.
A1 - Wohr,M.
Y1 - 2014/02//
N1 - Willadsen, Maria. Seffer, Dominik. Schwarting, Rainer K W. Wohr, Markus
Journal of comparative psychology (Washington, D.C. : 1983)
0414613, 8309850, dnq
IM
Journal Article
English
KW - Medline
KW - Rough and Tumble
RP - NOT IN FILE
SP - 56
EP - 64
JF - Journal of Comparative Psychology

JA - J Comp Psychol.
VL - 128
IS - 1
CY - United States
N2 - Rats emit distinct types of ultrasonic vocalizations (USV), which serve as situation-dependent affective signals with important communicative functions. Low-frequency 22-kHz USV typically occur in aversive situations, such as social defeat, whereas high-frequency 50-kHz USV can be observed in appetitive situations, like rough-and-tumble-play in juveniles or mating in adults. The 2 main USV types serve distinct communicative functions and induce call-specific behavioral responses in the receiver. While 22-kHz USV probably serve as alarm calls, 50-kHz USV appear to serve a prosocial communicative function in the sexual and the nonsexual context. In the sexual context, however, this view has recently been challenged by playback studies where only very limited behavioral changes were observed in response to prosocial 50-kHz USV. The aim of the present study was therefore to test whether female rats display social approach behavior in response to male prosocial 50-kHz USV by means of our established playback paradigm. To this aim, we exposed female rats to playback of the following 2 acoustic stimuli: (a) natural male 50-kHz USV and (b) time- and amplitude-matched white noise, with the latter serving as acoustic control for novelty-induced changes in behavior not linked to the communicative function of male prosocial 50-kHz USV. Our present findings show that female rats display high levels of social approach behavior in response to male prosocial 50-kHz USV, but not time- and amplitude-matched white noise, supporting the conclusion that male prosocial 50-kHz USV are likely to play an important role in establishing social proximity and possibly regulate mating behavior. (PsycINFO Database Record (c) 2014 APA, all rights reserved)
SN - 1939-2087
AD - Behavioral Neuroscience, Experimental and Biological Psychology, Philipps-University of Marburg., Behavioral Neuroscience, Experimental and Biological Psychology, Philipps-University of Marburg., Behavioral Neuroscience, Experimental and Biological Psychology, Philipps-University of Marburg., Behavioral Neuroscience, Experimental and Biological Psychology, Philipps-University of Marburg
UR - 24188619
ER -

TY - JOUR
ID - 1001
T1 - First Step to Success Early Intervention Program: A Study of Effectiveness with Native-American Children
A1 - Diken,Ibrahim H.
A1 - Rutherford,Robert B.
Y1 - 2005/11//
N1 - Last updated - 2012-12-14
SubjectsTermNotLitGenreText - 8299; 3090 5470; 10183 909; 7940 126; 4723; 465 10278 8016 4542 467 5046 3602; 916 8234; 914 2094; 10486 909; 7676 5449 8768
KW - ERIC,Current Index to Journals in Education (CIJE)
KW - Students
KW - Early Childhood Education
KW - Program Effectiveness
KW - High Risk Students
KW - Teacher Behavior
KW - Play
KW - Behavior Modification
KW - Early Intervention
KW - American Indian Students
KW - Student Behavior
KW - Behavior Problems
KW - Peer Relationship
KW - ERIC
RP - NOT IN FILE
SP - 444
EP - 465

JF - Education & Treatment of Children
VL - 28
IS - 4
PB - The Roscoe Ledger, P.O. Box 536, Chester & Latta Streets, Roscoe, PA 15477.
N2 - This study examined the effectiveness of the First Step to Success (FSS) early intervention program with four Native-American students, their teachers, and their parents on (a) targeted students' problem behaviors, (b) class-wide student behaviors, and (c) teacher behaviors. Participant teachers and parents were also interviewed to gather their perceptions of the FSS program. The results of direct observations of targeted students' play behaviors on the playground revealed that the FSS program had a significant positive affect on all participant students' social play behaviors. As soon as the intervention started, all participant students' social play behaviors significantly increased and their nonsocial behaviors decreased. All participant students showed higher levels of social play behaviors as soon as the intervention was initiated. Substantial decreases in problem behaviors were also reported by two teachers. Some positive changes in class-wide student behaviors and teacher behaviors were reported by the participant teachers. All but one parent reported significant changes in problem behaviors of targeted students. They all were highly satisfied with the program and rated it as easy to use. Limitations of the study and directions for future research are discussed
SN - 0748-8491, 0748-8491
UR - http://search.proquest.com/docview/62084121?accountid=14701
ER -

TY - JOUR
ID - 1002
T1 - Some Misunderstandings of School Readiness
A1 - Elkind,David
Y1 - 2008///
N1 - Last updated - 2012-12-14
SubjectsTermNotLitGenreText - 9261 8620; 5649 5264; 4413 5264; 7196; 6101; 2798; 6725; 7940 126; 4723; 8661 8623 11225 1 5792 9690; 6421 9690 1; 1443 5053 2787
KW - ERIC,Current Index to Journals in Education (CIJE)
KW - Elementary Education
KW - Grade 1
KW - Kindergarten
KW - High Risk Students
KW - Play
KW - Numeracy
KW - Reading Skills
KW - Mathematics Skills
KW - Misconceptions
KW - Developmental Stages
KW - Child Development
KW - Literacy
KW - School Readiness
KW - ERIC
RP - NOT IN FILE
SP - 49
EP - 52
JF - Exchange: The Early Childhood Leaders' Magazine Since 1978
IS - 180
PB - Exchange Press, P.O. Box 3249, Redmond, WA 98073-3249.
N2 - The phrase "school readiness" was, until recently, most often used in connection with a child's preparedness to meet the demands of a first grade classroom. With the contemporary push down of the curriculum, readiness is now taken to mean the child's preparedness to meet the demands of kindergarten. Whether in regard to first grade or kindergarten, this way of thinking assumes that school readiness resides entirely in the child's head. It also assumes that readiness is primarily academic, namely, knowing one's letters and numbers. In addition, readiness is often taken to mean that a child has acquired his or her knowledge of numbers and letters through one or another form of academic instruction. Finally, another interpretation of

readiness is that it is a matter of maturation and is related to age. While all of these ideas about readiness are understandable, they happen to be incorrect. In this article, the author discusses these misunderstandings of what readiness is all about
SN - 0164-8527, 0164-8527
UR - http://search.proquest.com/docview/61967371?accountid=14701
ER -

TY - GEN
ID - 1003
T1 - Every Child: The Magazine of the Australian Early Childhood Association, Inc., 2000
A1 - Elliott,Alison
Y1 - 2000///
N1 - Availability - Level 2 - Produced in microfiche (1966-2003)
SuppNotes - Published quarterly
Last updated - 2012-12-14
KW - Australia
KW - Brain Development
KW - Phonological Skills
KW - ERIC,Resources in Education (RIE)
KW - Early Childhood Education
KW - Teacher Student Relationship
KW - Motor Development
KW - Childhood Attitudes
KW - Physical Education
KW - Childhood Interests
KW - Parent School Relationship
KW - Emergent Literacy
KW - Work Environment
KW - Augmentative and Alternative Communication
KW - Enrichment
KW - Teaching Methods
KW - Teacher Researchers
KW - Preschool Teachers
KW - At Risk Persons
KW - Play
KW - Young Children
KW - Brain
KW - Adult Child Relationship
KW - Childhood Needs
KW - Physical Activities
KW - Foreign Countries
KW - Periodicals
KW - Games
KW - ERIC
RP - NOT IN FILE
SP - 146
VL - 6
IS - 1
PB - Publications Section, Australian Early Childhood Association, P.O. Box 105, Watson, ACT 2602, Australia ($47.20 Australian domestic per year; $50 Australian, overseas per year). Tel: +(02)-6241-6900; Fax: +(02)-6241-5547; e-mail: publishing@acea.org; Web site: http://www.aeca.org.au ($47.20, Australia; $50, Overseas).
N2 - This document is comprised of the four 2000 issues of a periodical published by the Australian Early Childhood Association focusing on the needs of children from birth to 8 years and on issues related to child care and early childhood education in Australia. Each issue features articles focusing on a particular theme along with editorials and regular columns related to research, child health, legal issues, ethics, and professional

development. The Autumn 2000 issue focuses on using brain research in the classroom and includes the following articles: (1) "Infant Brain Research: What Is It and What Does It Mean?" (Pam Linke); and (2) "Stress-Free Environments: Promoting Healthy Brain Development" (Heather Lawrence). The Winter 2000 issue has an Olympics and child health theme and includes the following articles: (1) "Young Children's Views of Australia and Australians" (Sue Dockett); and (2) "The Olympic Games: A Critical Look at What We Teach Children" (Sally Barnes). The Spring 2000 issue highlights visions for children, families, and communities and includes the following articles: (1) "Teaching Young Children: Specialist or Not?" (Glenda MacNaughton); and (2) "Visions of Children and Childhood" (Alison Elliott). The Summer 2000 issue focuses on current issues in early childhood research and practice and includes the following articles: (1) "Should We Teach Young Children To Read?" (Mimi Wellisch); and (2) "Augmentative Communication: Assisting Special Needs Children" (Lynette Duffy). Also part of each issue are a profile of a program providing services for children, book reviews, summaries of recent events in each of the Australian states, and information for use in parent newsletters. (KB)
UR - http://search.proquest.com/docview/62248533?accountid=14701
ER -

TY - JOUR
ID - 1004
T1 - Sexual Play: When Should You Be Concerned?
A1 - Essa,Eva L.
A1 - Murray,Colleen I.
Y1 - 1999///
N1 - Last updated - 2012-12-14
SubjectsTermNotLitGenreText - 694 8016 4542; 1432 909; 3085 3150; Play 7940 126; Preschool Children 8127 11655 1474 316 8016 4542; Preschool Teachers 8135 10591 8267 3417 8016 4542 5703 4908 8917; 9588; Sexuality 9591; 10482 730
KW - ERIC,Current Index to Journals in Education (CIJE)
KW - Early Childhood Education
KW - Sexuality
KW - Preschool Teachers
KW - At Risk Persons
KW - Play
KW - Child Behavior
KW - Teacher Attitudes
KW - Preschool Children
KW - Sexual Identity
KW - ERIC
RP - NOT IN FILE
SP - 231
EP - 234
JF - Childhood Education
VL - 75
IS - 4
N2 - Discusses the ambivalence felt by early-childhood teachers about young children's sexual behaviors. Focuses on the importance of context in understanding normal sexual development in young children and specific behaviors that might signal an underlying problem. Presents a continuum of young children's sexual behaviors, ranging from normal to pathological and provides tips for distinguishing inappropriate from normal sexual behavior. (KB)
SN - 0009-4056, 0009-4056
UR - http://search.proquest.com/docview/62484229?accountid=14701
ER -

TY - JOUR
ID - 1005
T1 - Adolescent opiate exposure in the female rat induces subtle alterations in maternal care and transgenerational effects on play behavior

A1 - Johnson,N.L.
A1 - Carini,L.
A1 - Schenk,M.E.
A1 - Stewart,M.
A1 - Byrnes,E.M.
Y1 - 2011///
N1 - Johnson, Nicole L. Carini, Lindsay. Schenk, Marian E. Stewart, Michelle. Byrnes, Elizabeth M
Frontiers in psychiatry
101545006
Journal Article
English
KW - Medline
KW - Rough and Tumble
RP - NOT IN FILE
SP - 29
JF - Frontiers in psychiatry Frontiers Research Foundation
JA - Front Psychiatr
VL - 2
CY - Switzerland
N2 - The non-medical use of prescription opiates, such as Vicodin() and MSContin(), has increased dramatically over the past decade. Of particular concern is the rising popularity of these drugs in adolescent female populations. Use during this critical developmental period could have significant long-term consequences for both the female user as well as potential effects on her future offspring. To address this issue, we have begun modeling adolescent opiate exposure in female rats and have observed significant transgenerational effects despite the fact that all drugs are withdrawn several weeks prior to pregnancy. The purpose of the current set of studies was to determine whether adolescent morphine exposure modifies postpartum care. In addition, we also examined juvenile play behavior in both male and female offspring. The choice of the social play paradigm was based on previous findings demonstrating effects of both postpartum care and opioid activity on play behavior. The findings revealed subtle modifications in the maternal behavior of adolescent morphine-exposed females, primarily related to the amount of time females' spend nursing and in non-nursing contact with their young. In addition, male offspring of adolescent morphine-exposed mothers (MOR-F1) demonstrate decreased rough and tumble play behaviors, with no significant differences in general social behaviors (i.e., social grooming and social exploration). Moreover, there was a tendency toward increased rough and tumble play in MOR-F1 females, demonstrating the sex-specific nature of these effects. Given the importance of the postpartum environment on neurodevelopment, it is possible that modifications in maternal-offspring interactions, related to a history of adolescent opiate exposure, plays a role in the observed transgenerational effects. Overall, these studies indicate that the long-term consequences of adolescent opiate exposure can impact both the female and her future offspring
SN - 1664-0640
AD - Department of Biomedical Science, Cummings School of Veterinary Medicine, Tufts University North Grafton, MA, USA
UR - 21713113
ER -

TY - JOUR
ID - 1006
T1 - The Relation Between Social Support, Infant Risk Status and Mother-Infant Interaction
A1 - Feiring,Candice
Y1 - 1987///
N1 - Last updated - 2012-12-14
SubjectsTermNotLitGenreText - 265 909; High Risk Persons 4722; Infants 5118 11655 1474 316 8016 4542; 5350 8852 6582; 5472 3629 6582; 6823 3932 8016 4542 7577 3816; Parent Child Relationship 7537 3851 5449 8768; 7940 126; Premature Infants 8112 5118 11655 1474 316 8016 4542; Social Networks 9783 7051; Social Support Groups 9813 4542
KW - ERIC,Current Index to Journals in Education (CIJE)
KW - Researchers

KW - Play
KW - High Risk Persons
KW - Premature Infants
KW - Parent Child Relationship
KW - Mothers
KW - Affective Behavior
KW - Social Support Groups
KW - Social Networks
KW - Interaction Process Analysis
KW - Interviews
KW - Infants
KW - ERIC
RP - NOT IN FILE
SP - 400
EP - 405
JF - Developmental Psychology
JA - Dev.Psychol.
VL - 23
IS - 3
N2 - Mothers from inner-city, poor families with high-risk infants were interviewed when their infants were three months of age concerning sources of support (e.g., fathers, relatives, friends) and types of support received (e.g., goods, services, advice, financial support). Observations of the mother-infant interaction in a free-play setting were also conducted.(Author/BN)
UR - http://search.proquest.com/docview/63278193?accountid=14701
ER -

TY - JOUR
ID - 1007
T1 - Posttraumatic Stress Disorder in Infants and Young Children Exposed to War-Related Trauma
A1 - Feldman,Ruth
A1 - Vengrober,Adva
Y1 - 2011/07//
N1 - Last updated - 2012-12-14
SubjectsTermNotLitGenreText - 9813 4542; 6823 3932 8016 4542 7577 3816; 8050 548 6531 2877; 5118 11655 1474 316 8016 4542; 714 909; 4109 4335; 2746 8415; 11405 2112; 10411; 8996; 8901; 2207 4542 8852 6582; 1651 4954; 1474 316 8016 4542; 7940 126; 9697; 11511 8415; 547 8415; 2452 909; 2267 10087 2574 3629 6582; 11465 8522; 5470
KW - Israel
KW - ERIC,Current Index to Journals in Education (CIJE)
KW - Play
KW - Anxiety
KW - Clinical Diagnosis
KW - Depression (Psychology)
KW - Well Being
KW - Mothers
KW - Intervention
KW - Crying
KW - Social Support Groups
KW - Correlation
KW - CHILDREN
KW - Risk
KW - Symptoms (Individual Disorders)
KW - Control Groups
KW - Foreign Countries
KW - Resilience (Psychology)
KW - War

KW - Sleep
KW - Withdrawal (Psychology)
KW - Posttraumatic Stress Disorder
KW - Attachment Behavior
KW - Infants
KW - ERIC
RP - NOT IN FILE
SP - 645
EP - 658
JF - Journal of the American Academy of Child & Adolescent Psychiatry
JA - J Am Acad.Child Adolesc Psychiatry
VL - 50
IS - 7
PB - Elsevier. 6277 Sea Harbor Drive, Orlando, FL 32887-4800.
N2 - Objective: Although millions of the world's children are growing up amidst armed conflict, little research has described the specific symptom manifestations and relational behavior in young children exposed to wartime trauma or assessed factors that chart pathways of risk and resilience. Method: Participants included 232 Israeli children 1.5 to 5 years of age, 148 living near the Gaza Strip and exposed to daily war-related trauma and 84 controls. Children's symptoms were diagnosed, maternal and child attachment-related behaviors observed during the evocation of traumatic memories, and maternal psychological symptoms and social support were self-reported. Results: PTSD was diagnosed in 37.8% of war-exposed children (n = 56). Children with PTSD exhibited multiple posttraumatic symptoms and substantial developmental regression. Symptoms observed in more than 60% of diagnosed children included nonverbal representation of trauma in play; frequent crying, night waking, and mood shifts; and social withdrawal and object focus. Mothers of children with PTSD reported the highest depression, anxiety, and posttraumatic symptoms and the lowest social support, and displayed the least sensitivity during trauma evocation. Attachment behavior of children in the Exposed-No-PTSD group was characterized by use of secure-base behavior, whereas children with PTSD showed increased behavioral avoidance. Mother's, but not child's, degree of trauma exposure and maternal PTSD correlated with child avoidance. Conclusions: Large proportions of young children exposed to repeated wartime trauma exhibit a severe posttraumatic profile that places their future adaptation at significant risk. Although more resilient children actively seek maternal support, avoidance signals high risk. Maternal well-being, sensitive behavior, and support networks serve as resilience factors and should be the focus of interventions for families of war-exposed infants and children. (Contains 3 figures and 2 tables.)
SN - 0890-8567, 0890-8567
UR - http://search.proquest.com/docview/964174678?accountid=14701
ER -

TY - JOUR
ID - 1008
T1 - Delivery Pain and the Development of Mother-Infant Interaction
A1 - Ferber,Sari Goldstein
A1 - Feldman,Ruth
Y1 - 2005///
N1 - Last updated - 2012-12-14
SubjectsTermNotLitGenreText - 7763 8409 5051; 7940 126; 6823 3932 8016 4542 7577 3816; 5118 11655 1474 316 8016 4542; 8996; 6447; 5348 8768; 2746 8415; 547 8415; 7537 3851 5449 8768; 1013; 7496; 8415; 312 5054 2842; 3170 28 96; 4109 4335
KW - Israel
KW - ERIC,Current Index to Journals in Education (CIJE)
KW - Interaction
KW - Measures (Individuals)
KW - Play
KW - Anxiety
KW - Depression (Psychology)
KW - Mothers
KW - Pain

KW - Educational Attainment
KW - Birth
KW - Risk
KW - Personality Traits
KW - Foreign Countries
KW - Psychological Patterns
KW - Parent Child Relationship
KW - Age Differences
KW - Infants
KW - ERIC
RP - NOT IN FILE
SP - 43
EP - 62
JF - Infancy
VL - 8
IS - 1
PB - Psychology Press. , 325 Chestnut Street Suite 800, Philadelphia, PA 19106.
N2 - This study examined delivery pain as a possible risk factor for the development of mother-infant interaction. Eighty-one mothers completed the Pain Catastrophizing Scale, the State-Trait Anxiety Inventory, and the Edinburgh Postnatal Depression Scale. A retrospective evaluation of labor pain was performed using the Visual Analog Scale at 2 days postpartum. Six weeks after birth the mothers were visited at home, completed measures of anxiety and depression, and were observed during a free play session with the infant. The mother's tendency to catastrophize pain predicted lower levels of mother-infant reciprocity at 6 weeks, controlling for maternal age, education, parity, epidural analgesia, pain perception, anxiety, and depression. Trait anxiety was related to lower maternal sensitivity. The mother's tendency to catastrophize pain was discussed in relation to the personality trait of exaggerated emotional perception of pain and its potential interference with the formation of the mother-infant relationship. (Contains 4 tables.)
SN - 1525-0008, 1525-0008
UR - http://search.proquest.com/docview/61871480?accountid=14701
ER -

TY - JOUR
ID - 1009
T1 - Parent Training: Can Intervention Improve Parent-Child Interactions?
A1 - Fleming,Darcy
A1 - McDonald,Linda
A1 - Drummond,Jane
A1 - Kysela,Gerald M.
A1 - Watson,Shelley L.
Y1 - 2005///
N1 - Last updated - 2012-12-14
SubjectsTermNotLitGenreText - 2888 2883 8016 4542 11658 316; 10621 3227 6582; 7940 126; 8127 11655 1474 316 8016 4542; 3090 5470; 7540 210 3150; 7537 3851 5449 8768; 8299; 1432 909; 5348 8768; 8058 3132 10103; 4109 4335; 7576 909; 4723
KW - Canada
KW - ERIC,Current Index to Journals in Education (CIJE)
KW - Early Childhood Education
KW - Parent Education
KW - Interaction
KW - Program Effectiveness
KW - High Risk Students
KW - Play
KW - Child Behavior
KW - Parenting Styles
KW - Foreign Countries
KW - Poverty

KW - Parent Child Relationship
KW - Early Intervention
KW - Disadvantaged Youth
KW - Preschool Children
KW - Teaching Methods
KW - ERIC
RP - NOT IN FILE
SP - 77
EP - 100
JF - Exceptionality Education Canada
VL - 15
IS - 2
PB - Exceptionality Education Canada. University of Alberta, Department of Educational Psychology, 6-102 Education North, Edmonton, AB T6G 2G5, Canada.
N2 - The aim of the present study was to determine the effect of the Natural Teaching Strategies (NTS) parent training intervention on parent and child behaviour during free play. A pretest/posttest experimental design was used with families recruited from Head Start programs. Measures of observed behaviour were used to determine the use of contingent responsive and scaffolding strategies by parents during free play with their preschooler. After six months of NTS training, it was found that parents were more positive and responsive to their children and episodes of joint engagement were longer, involving more reciprocity. Therefore, there is evidence that the NTS intervention has potential to support parents to engage their preschool aged children in longer more positive play interactions. (Contains 3 tables and 1 footnote.)
SN - 1183-322X, 1183-322X
UR - http://search.proquest.com/docview/62008064?accountid=14701
ER -

TY - JOUR
ID - 1010
T1 - Father Beliefs as a Mediator between Contextual Barriers and Father Involvement
A1 - Freeman,Harry
A1 - Newland,Lisa A.
A1 - Coyl,Diana D.
Y1 - 2008///
N1 - Last updated - 2012-12-14
SubjectsTermNotLitGenreText - 3090 5470; 2888 2883 8016 4542 11658 316; 7576 909; 1459; 8996; 8535 6447; 3896 7577 3816 8016 4542 6265; 7531 730; 874 8234; 7550 7615 909; 11655 1474 316 8016 4542; 9466 9469 9451; 7575 9690 1; 7552 8931; 10866 153 4398; 3864 8768; 7537 3851 5449 8768; 7940 126; 9822
KW - ERIC,Current Index to Journals in Education (CIJE)
KW - Early Childhood Education
KW - Parenting Skills
KW - Play
KW - Barriers
KW - Questionnaires
KW - Family Work Relationship
KW - Young Children
KW - Fathers
KW - SOCIALIZATION
KW - Risk
KW - Parent Responsibility
KW - Parenting Styles
KW - Parent Child Relationship
KW - Child Rearing
KW - Parent Participation
KW - Disadvantaged Youth
KW - Early Intervention
KW - Parent Attitudes

KW - Self Efficacy
KW - Time Management
KW - ERIC
RP - NOT IN FILE
SP - 17
JF - Early Child Development and Care
VL - 178
IS - 7
PB - Routledge. , 325 Chestnut Street Suite 800, Philadelphia, PA 19106.
N2 - Fathers' beliefs were examined as mediators between multiple risk factors and involvement practices with children age zero to five enrolled in Head Start or Early Head Start. A diverse sample of 101 fathers, living in rural Midwestern communities of the USA completed questionnaires assessing "mediators" (i.e. parenting efficacy, role beliefs, and responsibility to an intervention program), "barriers" (e.g. lack of time, energy, work constraints), and "father involvement" (i.e. physical play, didactic engagement, caregiving, socialisation, involvement in the program, and accessibility). In each of the regression models, father efficacy and beliefs reduced the influence of barriers and were significant unique predictors of father involvement. Findings suggest that fathers' beliefs are more proximal to parenting practices than is family context, which have implications for early intervention programs serving children in at-risk families. (Contains 2 tables and 1 note.)
SN - 0300-4430, 0300-4430
UR - http://search.proquest.com/docview/61957429?accountid=14701
ER -

TY - JOUR
ID - 1011
T1 - Double helix: reciprocity between juvenile play and brain development. [Review]
A1 - Cooke,B.M.
A1 - Shukla,D.
Y1 - 2011/10//
N1 - Cooke, Bradley M. Shukla, Deep
Developmental cognitive neuroscience
101541838
IM
Journal Article. Review
English
KW - Medline
KW - Rough and Tumble
KW - Adolescent
KW - Adolescent Behavior/ph [Physiology]
KW - Adolescent Behavior/px [Psychology]
KW - Animals
KW - Brain/gd [Growth & Development]
KW - Female
KW - Humans
KW - Male
KW - Nerve Net/gd [Growth & Development]
KW - Play and Playthings/px [Psychology]
KW - Sex Characteristics
KW - Social Behavior
RP - NOT IN FILE
SP - 459
EP - 470
JF - Developmental Cognitive Neuroscience
JA - Dev.Cogn Neurosci
VL - 1
IS - 4
CY - Netherlands

N2 - This review summarizes what is presently known about the function, sexual differentiation, and neural circuitry of juvenile rough-and-tumble play. Juvenile rough-and-tumble play is a unique motivated behavior that is widespread throughout the mammalian order and usually occurs more often in males. Immediate early gene studies indicate that cortical and subcortical circuits, many of which are sensitive to sex steroid hormones, mediate juvenile play. Sex differences in rough-and-tumble play are controlled in part by neonatal exposure to androgens or their estrogenic metabolites. Studies indicate that testicular androgens during play are also necessary to stimulate male-like levels of play initiation. The resemblance of rough-and-tumble play to aggression and sexual behavior has led some to question whether male-typical adult behavior is contingent upon the experience of play. Attempts to control the amount of play through social isolation show that social experience during adolescence is critical for male-typical adult behaviors to be expressed. This well-established finding, together with evidence that play induces neural plasticity, supports the hypothesis that juvenile play contributes to male-typical brain development that ultimately enables the expression of adult social and reproductive behavior. Copyright 2011 Elsevier Ltd. All rights reserved
SN - 1878-9307
AD - Neuroscience Institute, Georgia State University, Atlanta, GA 30303, United States. bcooke@gsu.edu
UR - 22436567
ER -

TY - JOUR
ID - 1012
T1 - Closing the circle between perceptions and behavior: a cybernetic view of behavior and its consequences for studying motivation and development. [Review]
A1 - Pellis,S.M.
A1 - Bell,H.C.
Y1 - 2011/10//
N1 - Pellis, Sergio M. Bell, Heather C
Developmental cognitive neuroscience
101541838
IM
Journal Article. Research Support, Non-U.S. Gov't. Review
English
KW - Medline
KW - Rough and Tumble
KW - Animals
KW - Behavior
KW - Animal/ph [Physiology]
KW - Humans
KW - Motivation/ph [Physiology]
KW - Perception/ph [Physiology]
KW - Play and Playthings/px [Psychology]
KW - Social Behavior
RP - NOT IN FILE
SP - 404
EP - 413
JF - Developmental Cognitive Neuroscience
JA - Dev.Cogn Neurosci
VL - 1
IS - 4
CY - Netherlands
N2 - The dynamic aspect of behavior is exaggerated during social interactions such as sex, combat and rough-and-tumble play where the movements of the two animals involved continually influence one another. The behavioral 'markers' abstracted from this stream can greatly influence the conclusions drawn about the effects of experimental procedures and how changes during development are interpreted. By using methods of analysis that treat behaving systems as being dynamic and governed by negative feedback processes, the behavioral markers that are abstracted can more accurately reflect the underlying mechanisms. Using examples from rats engaged in play fighting, serious fighting and food defense, it is shown that motivational from non-motivational

contributions to behavioral output and changes in that output with age can be discerned. For example, while sex differences in the frequency of initiating play by juvenile rats are shown to reflect differences in the motivation to engage in this behavior, sex differences in preferred motor patterns used during play do not. Rather, they reflect differences in perceptual and motor systems. Although an issue that is often neglected, we show that behavioral description, and the theoretical underpinnings of that description, is critical for the study of the mechanisms that produce and regulate behavior. Copyright 2011 Elsevier Ltd. All rights reserved
SN - 1878-9307
AD - Department of Neuroscience, University of Lethbridge, Lethbridge, Alberta, Canada. pellis@uleth.ca
UR - 22436563
ER -

TY - JOUR
ID - 1013
T1 - The Decline of Play and the Rise of Psychopathology in Children and Adolescents
A1 - Gray,Peter
Y1 - 2011///
N1 - Last updated - 2013-02-26
SubjectsTermNotLitGenreText - 8427 7637 6506 10669 8422 926 9351 5964; 197 316 8016 4542; 2746 8415; 7940 126; 4696; 1474 316 8016 4542; 10324 9458 909 2635; 694 8016 4542; 8415; 1443 5053 2787; 5449 8768; 8233 1710; 6532 4634
KW - ERIC,Current Index to Journals in Education (CIJE)
KW - At Risk Persons
KW - Play
KW - Depression (Psychology)
KW - Suicide
KW - Mental Health
KW - CHILDREN
KW - Helplessness
KW - Problem Solving
KW - Psychological Patterns
KW - Child Development
KW - Psychopathology
KW - Interpersonal Relationship
KW - Adolescents
KW - ERIC
RP - NOT IN FILE
SP - 443
EP - 463
JF - American Journal of Play
VL - 3
IS - 4
PB - The Strong. One Manhattan Square, Rochester, NY 14607.
N2 - Over the past half century, in the United States and other developed nations, children's free play with other children has declined sharply. Over the same period, anxiety, depression, suicide, feelings of helplessness, and narcissism have increased sharply in children, adolescents, and young adults. This article documents these historical changes and contends that the decline in play has contributed to the rise in the psychopathology of young people. Play functions as the major means by which children (1) develop intrinsic interests and competencies; (2) learn how to make decisions, solve problems, exert self-control, and follow rules; (3) learn to regulate their emotions; (4) make friends and learn to get along with others as equals; and (5) experience joy. Through all of these effects, play promotes mental health. (Contains 48 notes and 1 figure.)
SN - 1938-0399, 1938-0399
UR - http://search.proquest.com/docview/1312420929?accountid=14701
ER -

TY - JOUR
ID - 1014

T1 - Frequency-modulated 50 kHz ultrasonic vocalizations: a tool for uncovering the molecular substrates of positive affect. [Review]
A1 - Burgdorf,J.
A1 - Panksepp,J.
A1 - Moskal,J.R.
Y1 - 2011/10//
N1 - Burgdorf, Jeffrey. Panksepp, Jaak. Moskal, Joseph R
Neuroscience and biobehavioral reviews
oa7, 7806090
IM
Journal Article. Research Support, Non-U.S. Gov't. Review
English
KW - Medline
KW - Rough and Tumble
KW - Affect/ph [Physiology]
KW - Animals
KW - Brain/ph [Physiology]
KW - Breeding
KW - Humans
KW - Male
KW - Molecular Biology
KW - Pleasure/ph [Physiology]
KW - Rats
KW - Long-Evans
KW - Social Isolation
KW - Ultrasonics
KW - Vocalization
KW - Animal/ph [Physiology]
RP - NOT IN FILE
SP - 1831
EP - 1836
JF - Neuroscience & Biobehavioral Reviews
JA - Neurosci Biobehav.Rev
VL - 35
IS - 9
CY - United States
N2 - The evidence that frequency modulated (FM) 50 kHz ultrasonic vocalizations (USVs) reflect a positive emotional state in rats is reviewed. Positive emotional states in humans are measured by facial-vocal displays (e.g., Duchenne smiling and laughter), approach behavior, and subjective self-report of feeling states. In laboratory animals, only facial-vocal displays, along with approach behavior can be measured. FM 50 kHz USVs are uniquely elevated by hedonic stimuli and suppressed by aversive stimuli. Rates of FM 50 kHz USVs are positively correlated to the rewarding value of the eliciting stimulus. Additionally, playbacks of these vocalizations are rewarding. The neural and pharmacological substrates of 50 kHz USVs are consistent with those of human positive affective states. By experimentally eliciting FM 50 kHz USVs, the novel molecular underpinning of positive affect can be elucidated and may be similar to those in humans. In humans, positive emotional states confer resilience to depression and anxiety, as well as promote overall health. Using rough-and-tumble play induced hedonic USVs, we have identified insulin like growth factor I and the NR2B subunit of the NMDA receptor as playing a functional role in positive affective states. From this research, we have developed a promising new class of antidepressants that is entering phase II clinical trials for the treatment of depression. Copyright 2010 Elsevier Ltd. All rights reserved
SN - 1873-7528
AD - The Falk Center for Molecular Therapeutics, Department of Biomedical Engineering, The McCormick School of Engineering and Applied Sciences, Northwestern University, Evanston, IL 60201, USA. j-burgdorf@northwestern.edu
UR - 21144859
ER -

TY - JOUR
ID - 1015
T1 - Thinking Creatively Is Thinking Critically
A1 - Gruenfeld,Elizabeth
Y1 - 2010///
N1 - Last updated - 2012-12-14
SubjectsTermNotLitGenreText - 7940 126; 2432 1710; 2388 8409 5051; 2226 7921; 4109 4335; 1114 8193 8477; 309 8331; 3363 10278 8016 4542; 695 694 8016 4542; 4979 2388 8409 5051; 6120 4918 5964; 622 126
KW - Peru
KW - Argentina
KW - Massachusetts
KW - ERIC,Current Index to Journals in Education (CIJE)
KW - Elementary Education
KW - Grade 2
KW - Grade 3
KW - Elementary School Students
KW - Play
KW - Literature
KW - After School Programs
KW - Critical Thinking
KW - Creativity
KW - Imagination
KW - At Risk Students
KW - Foreign Countries
KW - Books
KW - Cooperative Planning
KW - Art Activities
KW - ERIC
RP - NOT IN FILE
SP - 71
EP - 83
JF - New Directions for Youth Development
JA - New Dir.Youth Dev.
IS - 125
PB - Jossey-Bass. , 111 River Street, Hoboken, NJ 07030-5774.
N2 - The Cartoneras projects aim to promote the celebration of language, culture, and creativity through a collaboration between top literary minds and cardboard collectors in Buenos Aires and Lima. They produce and publish beautiful books with hand-painted cardboard covers that speak of the wonderful literature inside. Inspired by those projects, the Paper Picker Press (PPP) program in Boston aims to engage higher-order thinking through an arts-based approach to rediscovering literature through play. PPP starts with the premise that a student who is thinking creatively is also thinking critically. Creative play is critical thinking. (Contains 20 notes.)
SN - 1533-8916, 1533-8916
UR - http://search.proquest.com/docview/742856405?accountid=14701
ER -

TY - JOUR
ID - 1016
T1 - Parental Perceptions of Neighborhood Processes, Stress, Personal Control, and Risk for Physical Child Abuse and Neglect
A1 - Guterman,Neil B.
A1 - Lee,Shawna J.
A1 - Taylor,Catherine A.
A1 - Rathouz,Paul J.
Y1 - 2009/12//

N1 - Last updated - 2012-12-14
SubjectsTermNotLitGenreText - 7040 1873 4542; 7940 126; 1454 545 9738 909 7006; 1429 545 9738 909; 6823 3932 8016 4542 7577 3816; 10162 1352 6752 9651 6582; 3602 8016 4542; 1459; 9761 3518; 7531 730; 8996; 8094; 5472 3629 6582; 1326 3629 6582 8836; 10152; 7537 3851 5449 8768
KW - ERIC,Current Index to Journals in Education (CIJE)
KW - Prediction
KW - Play
KW - Case Studies
KW - Structural Equation Models
KW - Mothers
KW - Neighborhoods
KW - Stress Variables
KW - Risk
KW - Ethnic Groups
KW - Parent Child Relationship
KW - Child Rearing
KW - Child Neglect
KW - Parent Attitudes
KW - Interviews
KW - Child Abuse
KW - Social Environment
KW - ERIC
RP - NOT IN FILE
SP - 897
EP - 906
JF - Child Abuse & Neglect: The International Journal
VL - 33
IS - 12
PB - Elsevier. 6277 Sea Harbor Drive, Orlando, FL 32887-4800.
N2 - Objective: This study set out to examine whether mothers' individual perceptions of their neighborhood social processes predict their risk for physical child abuse and neglect directly and/or indirectly via pathways involving parents' reported stress and sense of personal control in the parenting role. Methods: In-home and phone interview data were examined cross-sectionally from a national birth cohort sample of 3,356 mothers across 20 US cities when the index child was 3 years of age. Mothers' perceptions of neighborhood social processes, parenting stress, and personal control were examined as predictors, and three subscales of the Parent-To-Child Conflict Tactics Scale (CTS-PC) were employed as proxies of physical child abuse and neglect risk. Structural equation modeling (SEM) was employed to test direct and indirect pathways (via parenting stress and control) from perceived neighborhood processes to proxy measures of physical child abuse and neglect. Multiple group SEM was conducted to test for differences across major ethnic groups: African American, Hispanic, and White. Results: Although perceived negative neighborhood processes had only a mild direct role in predicting risk for physical child abuse, and no direct role on child neglect, these perceptions had a discernable indirect role in predicting risk via parenting stress and personal control pathways. Parenting stress exerted the clearest direct role on both physical abuse and neglect risk. This predictor model did not significantly differ across ethnic groups. Conclusions: Although neighborhood conditions may not play a clear directly observable role on physical child abuse and neglect risk, the indirect role they play underscores the importance of parents' perceptions of their neighborhoods, and especially the role they play via parents' reported stress and personal control. Practice implications: Such findings suggest that targeting parents' sense of control and stress in relation to their immediate social environment holds particular potential to reduce physical child abuse and neglect risk. Addressing parents' perceptions of their neighborhood challenges may serve to reduce parenting risk via improving parents' felt control and stress. (Contains 3 tables and 2 figures.)
SN - 0145-2134, 0145-2134
UR - http://search.proquest.com/docview/61821834?accountid=14701
ER -

TY - BOOK
ID - 1017

T1 - Partnering Dance and Education. Intelligent Moves for Changing Times
A1 - Hanna,Judith Lynne
Y1 - 1999/01//
N1 - Availability - Level 3 - Indexed only
Last updated - 2012-12-14
KW - ERIC,Resources in Education (RIE)
KW - Practitioners
KW - Students
KW - Teachers
KW - Elementary Secondary Education
KW - Higher Education
KW - High Risk Students
KW - Play
KW - Dance Education
KW - Stress Management
KW - Diversity (Student)
KW - CHILDREN
KW - Movement Education
KW - Sex Differences
KW - Cultural Differences
KW - Interdisciplinary Approach
KW - ERIC
RP - NOT IN FILE
SP - 256
PB - Human Kinetics, 1607 North Market Street, P.O. Box 5076, Champaign, IL 61825-5076; Tel: 217-351-5076; Tel: 800-747-4457 (Toll Free); Fax: 217-351-1549; E-mail: humank@hkusa.com; Web site: http://www.humankinetics.com ($30).
N2 - This book offers information and insights for university dance educators and their students, administrators, policy makers, parents, dance instructors, dancers, and dance enthusiasts. Part 1, "Understanding Dance Education," explains key features of the discipline of dance and how it is a performing, liberal, physical, and applied art. Its four chapters include (1) "Is Dance a Distinct Body of Knowledge?" (2) "The Power of Dance Well Taught," (3) "Survival of Dance Education," and (4) "Who Should Teach Dance?" Part 2, "Learning In, About, and Through Dance," describes how to teach social, academic, and career skills through dance. Its chapters include (5) "Teaching Academic, Citizenship, and Workplace Skills Through Dance," (6) "Dance Education for At-Risk Youth," (7) "Children's Dance at Play as a Teaching Tool," (8) "National Identity and Cultural Diversity in Dance Education," (9) "Dance Education and Gender," and (10) "Dance Education and Stress." A finale, "Overcoming Obstacles and Moving Forward," discusses hurdles to dance education's success and notes promising pathways to overcome these obstacles. The three appendixes include discussion questions, an outline of National Dance Education Standards, and dance education resources. (SM)
SN - 0880115114
UR - http://search.proquest.com/docview/62482446?accountid=14701
ER -

TY - JOUR
ID - 1018
T1 - Hazards faced by young designated drivers: in-car risks of driving drunken passengers
A1 - Rothe,P.J.
A1 - Carroll,L.J.
Y1 - 2009/06//
N1 - Rothe, Peter J. Carroll, Linda J
International journal of environmental research and public health
101238455
IM
Journal Article
English
KW - Medline

KW - Rough and Tumble
KW - Adolescent
KW - Adult
KW - Alberta
KW - Alcoholic Intoxication
KW - Automobile Driving
KW - Focus Groups
KW - Humans
KW - Risk Factors
KW - Young Adult
RP - NOT IN FILE
SP - 1760
EP - 1777
JF - International Journal of Environmental Research & Public Health [Electronic Resource]
JA - Int.J Environ.Res.Public Health
VL - 6
IS - 6
CY - Switzerland
N2 - This qualitative study explored the risk in the practice of young designated drivers transporting drunken peers. Young drivers 18-29 years old in Alberta, Canada participated in 12 focus groups (N = 146). Interviews were semi-structured. A key finding is that when highly intoxicated youth are driven by a designated driver who is a peer, they are likely to behave in ways that are unsafe. Unsafe actions of drunken passengers in the vehicle include physical "rough-housing" with the driver, creating stress for the driver that leads to high risk driving situations and disrupting safe driving through nausea and in-car vomiting
SN - 1660-4601
AD - Centre for Health Promotion Studies and Alberta Centre for Injury Control and Research, School of Public Health, University of Alberta, 4075 RTF, 8308-114 Street, Edmonton, Alberta T6G2E1, Canada. peter.rothe@ualberta.ca
UR - 19578459
ER -

TY - JOUR
ID - 1020
T1 - Autism Symptoms in Toddlers with Down Syndrome: A Descriptive Study
A1 - Hepburn,Susan
A1 - Philofsky,Amy
A1 - Fidler,Deborah J.
A1 - Rogers,Sally
Y1 - 2008/01//
N1 - Last updated - 2012-12-14
SubjectsTermNotLitGenreText - 811 7786 6531 2877; 2995 2118 2877 6540; 10883 11655 1474 316 8016 4542; 10411; 694 8016 4542; 7786 6531 2877; 2814 10789 6447; 1651 4954; 5445 1849; 7940 126; 5449 8768; 5446 1970 1
KW - ERIC,Current Index to Journals in Education (CIJE)
KW - At Risk Persons
KW - Toddlers
KW - Play
KW - Clinical Diagnosis
KW - Pervasive Developmental Disorders
KW - Symptoms (Individual Disorders)
KW - Interpersonal Competence
KW - Diagnostic Tests
KW - Interpersonal Communication
KW - Down Syndrome
KW - Interpersonal Relationship
KW - Autism

KW - ERIC
RP - NOT IN FILE
SP - 48
EP - 57
JF - Journal of Applied Research in Intellectual Disabilities
VL - 21
IS - 1
PB - Blackwell Publishing. 350 Main Street, Malden, MA 02148.
N2 - Background: Research suggests that children with Down syndrome may be at increased risk of having an autism spectrum disorder; however, previous studies have not utilized comprehensive, state-of-the-art diagnostic tools to address the question of comorbid autism and Down syndrome. Method: Comprehensive evaluations for autism were conducted in 20 2-year-old children with Down syndrome. The Autism Diagnostic Observation Schedule-Generic (ADOS-G) and the Autism Diagnostic Interview-Revised (ADI-R) were administered by experienced clinicians, who then determined if the child presented with significant symptoms of autism. Eighteen children participated in a follow-up evaluation at age 4 years. Results: Three children (15%) met criteria for an autism spectrum disorder at both time points, and two (10%) for autistic disorder. Case descriptions are provided. Several children presented with difficulties in communication and play, but did not show problems in core social relatedness. The ADOS-G, if used alone, over-identifies autism in toddlers with Down syndrome. Conclusions: Clinical judgement is critical in diagnosing autism in Down syndrome. Young children may show some signs of autism (particularly in the area of communication) and not have the disorder. Children who have comorbid Down syndrome and autism are particularly challenged, relative to their peers without autism
SN - 1360-2322, 1360-2322
UR - http://search.proquest.com/docview/61873655?accountid=14701
ER -

TY - JOUR
ID - 1021
T1 - Prenatal programming of sporting success: associations of digit ratio (2D:4D), a putative marker for prenatal androgen action, with world rankings in female fencers
A1 - Bescos,R.
A1 - Esteve,M.
A1 - Porta,J.
A1 - Mateu,M.
A1 - Irurtia,A.
A1 - Voracek,M.
Y1 - 2009/04//
N1 - Bescos, Raul. Esteve, Marc. Porta, Jordi. Mateu, Merce. Irurtia, Alfredo. Voracek, Martin
Journal of sports sciences
8405364, kbt
IM
Journal Article
English
KW - Medline
KW - Rough and Tumble
KW - Adolescent
KW - Adult
KW - Athletic Performance/ph [Physiology]
KW - Female
KW - Fetal Development
KW - Fingers/ah [Anatomy & Histology]
KW - Humans
KW - Organ Size
KW - Sex Characteristics
KW - Somatotypes
KW - Track and Field

KW - Young Adult
RP - NOT IN FILE
SP - 625
EP - 632
JF - Journal of Sports Sciences
JA - J Sports Sci
VL - 27
IS - 6
CY - England
N2 - Associations of the second-to-fourth digit ratio (2D:4D), a putative marker for prenatal androgen action, and of absolute finger length, a putative marker for pubertal-adolescent androgen action, with sport performance were examined in a multinational sample of 87 world-class women epee fencers. Lower (masculinized) digit ratios correlated, although not significantly so, with better current and highest past world rankings. These correlations were significant for right-hand 2D:4D with controls for the most salient factors for 2D:4D (ethnicity) and world rankings (years of international experience, height, and weight). Longer (masculinized) fingers correlated strongly with better current and highest past world rankings; these correlations became insignificant with the same controls. Replicating previous evidence for fencers, left-handedness was much more prevalent in this sample (21%) than in the female general population, and left-handers had somewhat, but not significantly so, lower 2D:4D as well as better world rankings than right-handers. These findings extend related evidence suggestive of prenatal programming of aptitude across a variety of sports, especially running and soccer. Some known extragenital effects of prenatal testosterone that contribute to the development of efficient cardiovascular systems, good visuospatial abilities, physical endurance and speed, and to the propensity for rough-and-tumble play, apparently promote sporting success in adult life
SN - 0264-0414
AD - Laboratory of Physiology, National Institute of Sports Sciences of Barcelona, Barcelona, Spain. raul.bescos@inefc.net
UR - 19308788
ER -

TY - JOUR
ID - 1022
T1 - Adult Attitudes and Beliefs regarding Play on Lana'i
A1 - Holmes,Robyn M.
Y1 - 2011///
N1 - Last updated - 2013-02-26
SubjectsTermNotLitGenreText - 7940 126; 1309 8016 4542; 1303 730; 1306 9015; 1440 1309 8016 4542; 1304 5449 8768; 1940 10380 3629 6582; 9748 1710; 8996; 4625 7491 7160 8016 4542 7492 5046 3602; 932 730; 2494; 9817 11212; 5472 3629 6582; 9020 1710; 2802 3242
KW - ERIC,Current Index to Journals in Education (CIJE)
KW - Elementary Secondary Education
KW - Caregiver Child Relationship
KW - Play
KW - Role Perception
KW - Hawaiians
KW - Child Caregivers
KW - Social Values
KW - Caregiver Attitudes
KW - Risk
KW - Caregivers
KW - Community Surveys
KW - Caregiver Role
KW - Developmentally Appropriate Practices
KW - Cultural Relevance
KW - Interviews
KW - Beliefs
KW - Social Cognition

KW - ERIC
RP - NOT IN FILE
SP - 356
EP - 384
JF - American Journal of Play
VL - 3
IS - 3
PB - The Strong. One Manhattan Square, Rochester, NY 14607.
N2 - This article describes how adult attitudes toward play on the Hawaiian island of Lana'i reflect the connection between play and culture. It is based on a study of ninety-two caregivers (parents, grandparents, and other adult custodians), primarily representing individuals of Filipino, part Hawaiian, and Japanese heritages. These respondents completed a survey about the value of play for their children, the types of play they encouraged or discouraged, and the extent and nature of their own involvement in their children's play. The caregivers acknowledged the importance of play for their children and its developmental benefits, particularly those connected to culture. In keeping with the more collectivist ideology of Pacific Rim cultures, the caregivers encouraged types of play that fostered social skills such as cooperation, sharing, and group play. They strongly discouraged types of play that might harm or injure children. And they acknowledged the need for caretakers to set aside time to participate in their children's play. (Contains 6 tables.)
SN - 1938-0399, 1938-0399
UR - http://search.proquest.com/docview/1312421327?accountid=14701
ER -

TY - JOUR
ID - 1023
T1 - The Outdoor Recess Activities of Children at an Urban School: Longitudinal and Intraperiod Patterns
A1 - Holmes,Robyn M.
Y1 - 2012///
N1 - Last updated - 2013-02-26
SubjectsTermNotLitGenreText - 7940 126; 11182 9306 5241; 7223 1096 7843 5051 2923 2877 1088; 7594 8216 9306 5241; 8684; 312 5054 2842; 4290; 4516 5348 8768; 290 293 7160 8016 4542 10278; 4758 10278 8016 4542 4759 7160; 6188 8016 4542; 7676 5449 8768; 5445 1849; 7840 126; 1974 909; 4270 126; 704 7840 126; 10576 5449 8768; 695 694 8016 4542; 2484 5127; 6175 1326 3629 6582 8836
KW - United States (Northeast)
KW - ERIC,Current Index to Journals in Education (CIJE)
KW - Elementary Education
KW - Elementary Secondary Education
KW - Grade 1
KW - Grade 2
KW - Grade 3
KW - Grade 4
KW - Grade 5
KW - Grade 6
KW - Grade 7
KW - Grade 8
KW - Athletics
KW - Low Income Groups
KW - Obesity
KW - Teacher Student Relationship
KW - Play
KW - Parochial Schools
KW - Gender Differences
KW - Recess Breaks
KW - Urban Schools
KW - Group Dynamics
KW - Longitudinal Studies
KW - Peer Relationship

KW - At Risk Students
KW - Physical Activities
KW - Cultural Influences
KW - Interpersonal Communication
KW - African American Students
KW - Hispanic American Students
KW - Games
KW - Competition
KW - Age Differences
KW - ERIC
RP - NOT IN FILE
SP - 327
EP - 351
JF - American Journal of Play
VL - 4
IS - 3
PB - The Strong. One Manhattan Square, Rochester, NY 14607.
N2 - Based on a study of 149 parochial-school students enrolled in kindergarten through eighth grade, this article explores children's outdoor recess activities in an urban setting--with a focus on how age, gender, and size of play group influence their outdoor play preferences--and examines changes in children's activity preferences over a single recess period. The majority of the children in the study have African American and Hispanic ethnic heritages and lower-socioeconomic backgrounds. Both boys and girls spent most of recess socializing with their peers, though their play varied by gender and age. Most girls spent the majority of their recess talking and socializing. Older boys engaged in physical, competitive activities such as sports and in larger boy groups. Younger children spent more time chasing each other in boy-girl groupings and in informal activities. Girls interacted with their teachers more than boys. The author argues that these and other findings from the study have applied value, expanding our understanding of how cultural and historical factors have shaped the play of American children and indicating that urban school children of African American and Hispanic heritages are at a greater risk for obesity. Such findings would be useful in shaping school policy regarding the duration of recess breaks, the types of activities that should be encouraged, and the impact the policy might have on children's overall health. (Contains 1 table and 11 figures.)
SN - 1938-0399, 1938-0399
UR - http://search.proquest.com/docview/1312420599?accountid=14701
ER -

TY - JOUR
ID - 1024
T1 - How Safe Are School and Park Playgrounds?
A1 - Hudson,Susan D.
A1 - Thompson,Donna
A1 - Olsen,Heather
Y1 - 2005/01/01/
N1 - Last updated - 2012-12-14
SubjectsTermNotLitGenreText - 1463 9074; 83 8168; 7943 8704 3760; 9273 9074; 7940 126; 5183 2877; 8704 3760; 8998 153 4398; 3557 6251; 7591 3760
KW - United States
KW - ERIC,Current Index to Journals in Education (CIJE)
KW - Elementary Education
KW - Recreational Facilities
KW - Play
KW - Injuries
KW - Playgrounds
KW - Equipment Maintenance
KW - Parks
KW - Risk Management
KW - School Safety

KW - Accident Prevention
KW - Child Safety
KW - ERIC
RP - NOT IN FILE
SP - 16
JF - Journal of Physical Education, Recreation & Dance (JOPERD)
VL - 76
IS - 1
PB - Journal of Physical Education, Recreation & Dance, American Alliance for Health, Physical Education, Recreation and Dance (AAHPERD), 1900 Association Drive, Reston, VA 20191. Tel: 800-213-7193, ext. 493 (Toll Free).
N2 - Playgrounds traditionally have been found in both schools and parks in America. Each year, parent-teacher associations and school administrations, as well as park and recreation departments, spend millions of dollars to provide playground structures. However, since 1981, HPER professionals and the public have become increasingly aware that these structures can also lead to injuries in children and pose a liability to the owner and operator of the play equipment. More than 200,000 children are injured annually in playground-related incidents that are serious enough for them to seek medical attention (Tinsworth & McDonald, 2001). Within the elementary school setting, 80 percent of injuries that occur at school happen on the playground (Bruya, 1998). One solution to this problem is to remove all play structures. After all, the reasoning goes, if no playground equipment is present, then the liability problem does not exist. However, this approach to the problem eliminates the physical, emotional, social, and intellectual development of children that can be gained from their interactions on play equipment. This zero approach also serves to devalue the play of children. This article reports on how SAFE America's school and park playgrounds are today, as found in the latest study. Specific topics that will be examined include a summary of the conditions of school and park playgrounds, a discussion of the four SAFE elements, and recommendations for change
SN - 0730-3084, 0730-3084
UR - http://search.proquest.com/docview/62138063?accountid=14701
ER -

TY - JOUR
ID - 1025
T1 - The Effects of a Buddy Skills Package on Preschool Children's Social Interactions and Play
A1 - Hughett,Kristy
A1 - Kohler,Frank W.
A1 - Raschke,Donna
Y1 - 2013/02//
N1 - Last updated - 2013-04-02
SubjectsTermNotLitGenreText - 2790; 3924 5348 8768; 7940 126; 8127 11655 1474 316 8016 4542; 3005 9021 9651 6582; 7676 5449 8768; 9685 5053 2787; 9753 5053 2787; 5449 8768; 7896 11317 10120; 8034 8762; 2221 909; 695 694 8016 4542
KW - ERIC,Current Index to Journals in Education (CIJE)
KW - Preschool Education
KW - Play
KW - Dramatic Play
KW - Cooperation
KW - Developmental Delays
KW - Skill Development
KW - Feedback (Response)
KW - Peer Relationship
KW - Positive Reinforcement
KW - At Risk Students
KW - Social Development
KW - Pictorial Stimuli
KW - Interpersonal Relationship
KW - Preschool Children
KW - ERIC

RP - NOT IN FILE
SP - 246
EP - 254
JF - Topics in Early Childhood Special Education
VL - 32
IS - 4
PB - SAGE Publications and Hammill Institute on Disabilities. 2455 Teller Road, Thousand Oaks, CA 91320.
N2 - The purpose of this study was to examine the impact of a buddy skills package on the social and play interactions between three preschoolers with developmental delays and their peers. Each child participated in sociodramatic play activities with two peers who exhibited age-appropriate social and play skills. Following baseline, the children participated in three sessions of training on the strategies of stay, play, and talk with your friends. The intervention consisting of teacher feedback, praise, and picture cards was then used to support children's social interactions and play. A multiple baseline design indicated that the package was effective for increasing children's cooperative play in the exchange of activity-related comments. In addition, each playgroup continued to demonstrate these positive outcomes during a maintenance condition. (Contains 2 tables and 1 figure.)
SN - 0271-1214, 0271-1214
UR - http://search.proquest.com/docview/1322246684?accountid=14701
ER -

TY - JOUR
ID - 1026
T1 - Playgroups as Protective Environments for Refugee Children at Risk of Trauma
A1 - Jackson,Dianne
Y1 - 2006/06//
N1 - Last updated - 2012-12-14
SubjectsTermNotLitGenreText - 7940 126; 9813 4542; 4109 4335; 9783 7051; 8733 6671 8016 4542; 4985 6671 8016 4542; 694 8016 4542; 2240 150 909; 11405 2112; 150 909; 10151; 7763 8409 5051; 9538 547 8415; 7537 3851 5449 8768; 11655 1474 316 8016 4542
KW - Australia
KW - ERIC,Current Index to Journals in Education (CIJE)
KW - At Risk Persons
KW - Play
KW - Young Children
KW - Immigrants
KW - Social Support Groups
KW - Separation Anxiety
KW - Stress Management
KW - Refugees
KW - Personality Traits
KW - Foreign Countries
KW - Adjustment (to Environment)
KW - War
KW - Parent Child Relationship
KW - Social Networks
KW - Coping
KW - ERIC
RP - NOT IN FILE
SP - 1
EP - 5
JF - Australian Journal of Early Childhood
VL - 31
IS - 2
PB - Early Childhood Australia. P.O. Box 7105, Watson, ACT 2602, Australia. Tel: +61-2-6242-1800; Fax: +61-2-6242-1818; e-mail: publishing@earlychildhood.org.au; Web site: http://www.earlychildhoodaustralia.org.au

N2 - Refugee families in Australia generally have a dual challenge--dealing with past traumatic experiences while facing the struggles of integrating with a new and unfamiliar society. For children and their families who have experienced war-related trauma and disruption, the resettlement process is being referred to as "secondary trauma" to highlight the problems refugees face under these circumstances (Fazel & Stein, 2002). This paper discusses the possible long-term effects of trauma and reports on a small study that investigated whether or not a supported playgroup used by refugee families in western Sydney could be considered a protective environment. Emerging evidence showed that, while families reported difficulties associated with resettlement, they experienced ongoing emotional support within the playgroup and developed new social networks which resulted in reduced levels of stress and anxiety. Children showed improvements in the home and playgroup environments which highlighted the significant role the group played in facilitating resilience. (Contains 1 table.)
SN - 0312-5033, 0312-5033
UR - http://search.proquest.com/docview/61924733?accountid=14701
ER -

TY - GEN
ID - 1027
T1 - Coordinating the Elusive Playground Triad: Managing Children's Risk-Taking Behavior, (While) Facilitating Optimal Challenge Opportunities, (within) a Safe Environment
A1 - Jambor,Tom
Y1 - 1995/10/10/
N1 - Availability - Level 1 - Available online, if indexed January 1993 onward
SuppNotes - Paper presented at the International Conference on Playground Safety (University Park, PA, October 10, 1995)
Last updated - 2012-12-14
KW - Playground Design
KW - Playground Equipment
KW - Risk Taking
KW - ERIC,Resources in Education (RIE)
KW - Risk
KW - Recreational Facilities
KW - Play
KW - Injuries
KW - Playgrounds
KW - Safety
KW - Childhood Needs
KW - School Safety
KW - CHILDREN
KW - Child Safety
KW - ERIC
RP - NOT IN FILE
SP - 9
N2 - While it is our responsibility as adults to protect our children from hazards that would inflict injury, it is also our responsibility to provide children with a safe play environment providing challenging opportunities to explore, practice, and reach personal levels of competence. Children learn their own play abilities and limitations through the trial and error of daily play encounters. Once personal competence and a sense of security are gained, children attempt higher levels of play involvement. Inventing new ways to play on boring equipment can lead to risk-taking that increases the chance of injury. Adults should be aware of children's understanding of play challenge. Toddlers have little understanding of potentially dangerous situations and must be closely monitored. Preschoolers should be given opportunities for play that lead to enhanced competence. By age 7, children are unlikely to exceed their ability and take unnecessary risk unless pressured by children or adults. However, some children are inherently more likely than others to be risk-takers at an early age. A constructive approach to risk-taking is to differentiate between developmental challenge and unnecessary risk or hazard. Most playground injuries are caused by hazards rather than poor judgment in risk-taking. The initial step in playground design or maintenance is to identify potential hazards. Completely safe playgrounds may be acceptable to adults but children are likely to reject them and seek challenge and risk in potentially dangerous

environments. The quality of children's play within adult contrived play spaces is questionable. We must balance safety regulations and the challenge and risk-taking needs of children. (KDFB)
UR - http://search.proquest.com/docview/62560954?accountid=14701
ER -

TY - GEN
ID - 1028
T1 - Developing Social Skills among Campers. Research Notes
A1 - Jordan,Debra J.
Y1 - 1994///
N1 - SuppNotes - Theme issue: "The Wealth of Diversity."
Last updated - 2012-12-14
KW - ERIC,Current Index to Journals in Education (CIJE)
KW - At Risk Persons
KW - Elementary School Students
KW - Child Behavior
KW - Camping
KW - Mainstreaming
KW - Intervention
KW - CHILDREN
KW - Peer Relationship
KW - Interpersonal Competence
KW - Disabilities
KW - Child Development
KW - Research
KW - Pretend Play
KW - Attitudes toward Disabilities
KW - ERIC
RP - NOT IN FILE
SP - 47
JF - Camping Magazine
VL - 67
IS - 1
N2 - Reviews research on the attitudes of nondisabled children toward disabled children in regular classroom settings, and on patterns of play among at-risk and non-at-risk children. Implications for camps include encouraging social skills development through an atmosphere of peer friendship development, and helping at-risk children develop verbal skills and appropriate social behaviors through play. (LP)
UR - http://search.proquest.com/docview/62732630?accountid=14701
ER -

TY - BOOK
ID - 1029
T1 - Vocabulary Instruction: Research to Practice. Second Edition
A1 - Kame'enui,Edward J.
A1 - Baumann,James F.
Y1 - 2012/04//
N1 - Last updated - 2012-12-14
KW - ERIC,Resources in Education (RIE)
KW - Teachers
KW - Early Childhood Education
KW - Elementary Education
KW - Elementary Secondary Education
KW - Preschool Education
KW - Intermediate Grades
KW - Vocabulary
KW - Student Diversity

KW - Reading Research
KW - At Risk Students
KW - Spelling
KW - Reading Instruction
KW - Educational Games
KW - Teaching Guides
KW - Literacy
KW - Preschool Children
KW - Multimedia Materials
KW - Learning Strategies
KW - Vocabulary Development
KW - Elementary School Students
KW - Class Activities
KW - Play
KW - Incidental Learning
KW - Second Language Learning
KW - Story Reading
KW - Spelling Instruction
KW - English Language Learners
KW - Grade 4
KW - Direct Instruction
KW - Student Evaluation
KW - Educational Assessment
KW - Reading Difficulties
KW - Morphemes
KW - ERIC
RP - NOT IN FILE
SP - 322
PB - Guilford Press. 72 Spring Street, New York, NY 10012.
N2 - This highly regarded work brings together prominent authorities on vocabulary teaching and learning to provide a comprehensive yet concise guide to effective instruction. The book showcases practical ways to teach specific vocabulary words and word-learning strategies and create engaging, word-rich classrooms. Instructional activities and games for diverse learners are brought to life with detailed examples. Drawing on the most rigorous research available, the editors and contributors distill what PreK-8 teachers need to know and do to support all students' ongoing vocabulary growth and enjoyment of reading. This second edition: (1) reflects the latest research and instructional practices; (2) contains a new section (five chapters) on pressing current issues in the field: assessment, authentic reading experiences, English language learners, uses of multimedia tools, and the vocabularies of narrative and informational texts; and (3) includes an expanded contributor panel with additional leading researchers. Contents of this book include: (1) Context for Vocabulary Instruction (Edward J. Kame'enui and James F. Baumann); (2) Direct and Rich Vocabulary Instruction Needs to Start Early (Margaret G. McKeown, Isabel L. Beck, and Cheryl Sandora); (3) Teaching Vocabulary in the Primary Grades: Vocabulary Instruction Needed (Andrew Biemiller); (4) Vocabulary Instruction for Young Children at Risk of Reading Difficulties: Teaching Word Meanings during Shared Storybook Readings (Michael D. Coyne, Ashley Capozzoli-Oldham, and Deborah C. Simmons); (5) Young Word Wizards!: Fostering Vocabulary Development in Preschool and Primary Education (Katherine A. Dougherty Stahl and Steven A. Stahl); (6) Teaching Prefixes: Making Strong Instruction Even Stronger (Michael F. Graves, Melanie Ruda, Gregory C. Sales, and James F. Baumann); (7) The Vocabulary-Spelling Connection and Generative Instruction: Morphological Knowledge at the Intermediate Grades and Beyond (Shane Templeton); (8) Teaching Word-Learning Strategies (James F. Baumann, Elizabeth Carr Edwards, Eileen Boland, and George Font); (9) Developing Word Consciousness: Lessons from Highly Diverse Fourth-Grade Classrooms (Judith A. Scott, Tatiana F. Miller, and Susan Leigh Flinspach); (10) Keep the "Fun" in Fundamental: Encouraging Word Consciousness and Incidental Word Learning in the Classroom through Word Play (Camille L. Z. Blachowicz and Peter Fisher); (11) Language Play: Essential for Literacy (Dale D. Johnson, Bonnie Johnson, and Kathleen Schlichting); (12) Vocabulary Assessment: Making Do with What We Have While We Create the Tools We Need (P. David Pearson, Elfrieda H. Hiebert, and Michael L. Kamil); (13) Reading and Vocabulary Growth (Anne E. Cunningham and Colleen Ryan O'Donnell); (14) Powerful Vocabulary Instruction for English Learners (Patrick C. Manyak); (15) Using

Multimedia to Support Generative Vocabulary Learning (Jill Castek, Bridget Dalton, and Dana L. Grisham); and (16) What Differences in Narrative and Informational Texts Mean for the Learning and Instruction of Vocabulary (Elfrieda H. Hiebert and Gina N. Cervetti)
SN - 9781462503988
UR - http://search.proquest.com/docview/1018479225?accountid=14701
ER -

TY - JOUR
ID - 1030
T1 - Changing What You Know and Do: The Parent-Child Psychotherapy Program
A1 - Kaplan,Betty Ann
A1 - Venza,James
Y1 - 2011/03//
N1 - Last updated - 2012-12-14
SubjectsTermNotLitGenreText - 7940 126; 714 909; 7577 3816 8016 4542; 8433 10839; 4537 10839; 3825 2291 4552; 694 8016 4542; 11655 1474 316 8016 4542; 1429 545 9738 909; 1454 545 9738 909 7006; 3830 3518; 2798; 2302 6582
KW - ERIC,Current Index to Journals in Education (CIJE)
KW - At Risk Persons
KW - Family Environment
KW - Play
KW - Young Children
KW - Group Therapy
KW - Family Counseling
KW - Counseling Techniques
KW - Psychotherapy
KW - Developmental Stages
KW - Child Neglect
KW - Child Abuse
KW - Parents
KW - Attachment Behavior
KW - ERIC
RP - NOT IN FILE
SP - 45
EP - 51
JA - Zero to Three
VL - 31
IS - 4
PB - Zero to Three. 2000 M Street NW Suite 200, Washington, DC 20036-3307.
N2 - The Parent-Child Psychotherapy Program (PPP) is a multifamily group therapy intervention for parents and young children at high risk for intergenerational patterns of neglect, abuse, and disorganized attachment. A "developmental and experiential model" that incorporates principles of attachment theory, the PPP addresses parent and child needs simultaneously in the context of structured activities, free play, and separate parent- and child-only groups. Therapeutic objectives aim to shift parents' internal working models of relationship toward increasing attachment security, to change parent-child behaviors to promote safety and exploration, to build a foundation for parent-therapist collaboration, and to propel children's healthy development in a safe, predictable, and emotionally attuned environment. The authors present the history of the PPP, its underlying assumptions, and specific interventions with a family
SN - 0736-8038, 0736-8038
UR - http://search.proquest.com/docview/881469509?accountid=14701
ER -

TY - GEN
ID - 1031
T1 - Family Living: Suggestions for Effective Parenting
A1 - Katz,Lilian G.

Y1 - 1989///
N1 - Availability - Level 1 - Available online, if indexed January 1993 onward
Last updated - 2012-12-14
KW - Infant Day Care
KW - Praise
KW - ERIC,Resources in Education (RIE)
KW - Parents
KW - Early Childhood Education
KW - Kindergarten
KW - Parent Education
KW - Parenting Skills
KW - Employer Supported Day Care
KW - High Risk Students
KW - Play
KW - Language Acquisition
KW - Young Children
KW - Individual Characteristics
KW - Academic Achievement
KW - Family Life
KW - Childhood Needs
KW - Creativity
KW - Elementary School Curriculum
KW - Emotional Experience
KW - Oral Language
KW - Behavior
KW - Parent Participation
KW - Annotated Bibliographies
KW - ERIC
RP - NOT IN FILE
SP - 121
PB - ERIC Clearinghouse on Elementary and Early Childhood Education, University of Illinois, 805 West Pennsylvania Avenue, Urbana, IL 61801 (Catalog No. 205, $11.75).
N2 - Suggestions for effective parenting of preschool children are provided in 33 brief articles on children's feelings concerning self-esteem; fear; adopted children; the birth of a sibling; death; depression; and coping with stress, trauma, and divorce. Children's behavior is discussed in articles on toddlers' eating habits, punishment and preschoolers, whining, making friends, bedtime, good manners, interpersonal conflict, and friendship. Articles on children's learning focus on preparation for preschool, what preschoolers should be learning, first days of preschool, exercising choice, and questions about sex. Parenting is discussed in articles on assessing preschoolers' development, nudity at home, traveling with a preschooler, monitoring television viewing, parental disagreements about child rearing, the only child, chores, sex differences, stress, and children's wish to sleep in their parents' bed. Also included are ERIC digests on involving parents in the education of their children, the escalating kindergarten curriculum, praise in the classroom, the nature of children's play, creativity in young children, infant day care, and language development. Resource lists cite ERIC documents and journal articles on employer-supported child care, at-risk children, and school success. A reprint of a computer search of the ERIC database on family living is included. (RH)
UR - http://search.proquest.com/docview/63030191?accountid=14701
ER -

TY - JOUR
ID - 1032
T1 - Links between play and dominance and attachment dimensions of dog-human relationships
A1 - Rooney,N.J.
A1 - Bradshaw,J.W.
Y1 - 2003///
N1 - Rooney, Nicola J. Bradshaw, John W S
Journal of applied animal welfare science : JAAWS

9804404
IM
Journal Article. Research Support, Non-U.S. Gov't
English
KW - Medline
KW - Rough and Tumble
KW - Animals
KW - Authoritarianism
KW - Behavior
KW - Animal
KW - Bonding
KW - Human-Pet
KW - Cooperative Behavior
KW - Dogs
KW - Female
KW - Humans
KW - Male
KW - Play and Playthings
KW - Questionnaires
RP - NOT IN FILE
SP - 67
EP - 94
JF - Journal of Applied Animal Welfare Science
JA - J Appl.Anim Welf.Sci
VL - 6
IS - 2
CY - United States
N2 - It is often claimed that certain behavioral problems in domestic dogs can be triggered by the games played by dog and caregiver (owner). In this study, we examine possible links between the types of games played and dimensions of the dog-owner relationship that are generally considered to affect such problems. Fifty dog-owner partnerships were filmed during 3-min play sessions in which the owner was allowed to choose the games played. All partnerships then undertook a 1-hr test designed to measure elements of behavior commonly ascribed to "dominance" and "attachment." Principal components analysis of the data produced 2 dominance-related factors (Amenability and Confident Interactivity) and 4 factors describing aspects of attachment (Nonspecific Attention Seeking, Preference for Owner, Preference for Unfamiliar Person, and Separation-Related Behavior). Amenability, in particular, varied significantly between breeds. In the study, we then compared types of games played to each of these factors. Dogs playing rough-and-tumble scored higher for Amenability and lower on Separation-Related Behavior than did dogs playing other types of games. Dogs playing tug-of-war and fetch scored high on Confident Interactivity. Winning or losing these games had no consistent effect on their test scores. If the dog started the majority of the games, the dog was significantly less amenable and more likely to exhibit aggression. The results suggest that how dogs play reflects general attributes of their temperament and relationship with their owner. This study provides no evidence that games play a major deterministic role on dominance dimensions of dog-human relationships, but the results suggest that playing games involving considerable body contact may affect attachment dimensions
SN - 1088-8705
AD - Anthrozoology Institute, University of Southampton
UR - 12909524
ER -

TY - JOUR
ID - 1033
T1 - Public health. Rough-and-tumble behind Bush's smallpox policy
A1 - Cohen,J.
A1 - Enserink,M.
Y1 - 2002/12/20/

N1 - Cohen, Jon. Enserink, Martin
Science (New York, N.Y.)
0404511, uj7
E, IM
News
English
KW - Medline
KW - Rough and Tumble
KW - Advisory Committees
KW - Bioterrorism/pc [Prevention & Control]
KW - Bioterrorism
KW - Clinical Trials as Topic
KW - Disease Outbreaks/pc [Prevention & Control]
KW - Health Personnel
KW - Health Policy
KW - Humans
KW - Immunization Programs
KW - Military Personnel
KW - Public Health
KW - Security Measures
KW - Smallpox/ep [Epidemiology]
KW - Smallpox/pc [Prevention & Control]
KW - Smallpox/tm [Transmission]
KW - Smallpox Vaccine/ae [Adverse Effects]
KW - Smallpox Vaccine/sd [Supply & Distribution]
KW - Smallpox Vaccine
KW - United States/ep [Epidemiology]
KW - United States Dept.of Health and Human Services
KW - Vaccination
RP - NOT IN FILE
SP - 2312
EP - 2316
JF - Science
VL - 298
IS - 5602
CY - United States
SN - 1095-9203
UR - 12493889
ER -

TY - JOUR
ID - 1034
T1 - Existential Motives in Play As Revealed in Gambling
A1 - Kusyszyn,Igor
Y1 - 1979/10//
N1 - Last updated - 2012-12-14
SubjectsTermNotLitGenreText - Adults 226 316 8016 4542; Existentialism 3676; Play 7940 126; Psychological Needs 8414 5058 6996; 8996; 9469 9451
KW - Gambling
KW - ERIC,Current Index to Journals in Education (CIJE)
KW - Risk
KW - Self Esteem
KW - Play
KW - Psychological Needs
KW - Adults
KW - Existentialism

KW - ERIC
RP - NOT IN FILE
SP - 59
EP - 60
JF - Journal of Physical Education and Recreation
VL - 50
IS - 8
N2 - Gambling as a sophisticated form of adult play reaffirms the individual's existence and confirms the individual's worth. (LH)
UR - http://search.proquest.com/docview/63709712?accountid=14701
ER -

TY - JOUR
ID - 1035
T1 - Low Birth Weight (LBW) Infants' Exploratory Behavior at 12 and 24 Months: Effects of Intraventricular Hemorrhage and Mothers' Attention Directing Behaviors
A1 - Landry,Susan H.
Y1 - 1993///
N1 - Last updated - 2012-12-14
SubjectsTermNotLitGenreText - 312 5054 2842; 694 8016 4542; Attention Control 723; 1019; Congenital Impairments 2118 2877; Exploratory Behavior 3711; 5118 11655 1474 316 8016 4542; Interaction 5348 8768; 6823 3932 8016 4542 7577 3816; Parent Child Relationship 7537 3851 5449 8768; 7719 5127; 7862 4634; 7940 126; 8112 5118 11655 1474 316 8016 4542; 10883 11655 1474 316 8016 4542
KW - Intraventricular Hemorrhage
KW - ERIC,Current Index to Journals in Education (CIJE)
KW - Birth Weight
KW - Interaction
KW - At Risk Persons
KW - Toddlers
KW - Play
KW - Mothers
KW - Exploratory Behavior
KW - Premature Infants
KW - Parent Child Relationship
KW - Attention Control
KW - Physical Health
KW - Perinatal Influences
KW - Age Differences
KW - Infants
KW - Congenital Impairments
KW - ERIC
RP - NOT IN FILE
SP - 237
EP - 249
JF - Research in Developmental Disabilities
JA - Res.Dev.Disabil.
VL - 14
IS - 3
N2 - This study of infants with low birthweight and significant or mild intraventricular hemorrhage showed that the association of mothers' interactive behaviors with infants' exploratory play responses was dependent on both the age and medical risk status of the child. The timing of mothers' attention-directing strategies and the level of structure they provided were important variables. (JDD)
SN - 0891-4222, 0891-4222
UR - http://search.proquest.com/docview/62889652?accountid=14701
ER -

TY - BOOK
ID - 1036
T1 - Too Safe for Their Own Good? Helping Children Learn about Risk and Lifeskills
A1 - Lindon,Jennie
Y1 - 1999///
N1 - Availability - Level 2 - Produced in microfiche (1966-2003)
Last updated - 2012-12-14
KW - Risk Assessment
KW - Risk Reduction
KW - Risk Taking Behavior
KW - ERIC,Resources in Education (RIE)
KW - Early Childhood Education
KW - Travel
KW - Caregiver Child Relationship
KW - Teacher Student Relationship
KW - Play
KW - Young Children
KW - Parent Teacher Cooperation
KW - Safety
KW - Child Safety
KW - Risk
KW - Accidents
KW - Self Care Skills
KW - Foreign Countries
KW - Risk Management
KW - Personal Autonomy
KW - Self Evaluation (Groups)
KW - ERIC
RP - NOT IN FILE
SP - 75
PB - National Early Years Network, 77 Holloway Road, London N7 8JZ, England, United Kingdom; Tel: 0207-607-9573; Fax: 0207-700-1105; email: NationalEarlyYearsNetwork@compuserve.com (8.50 British Pounds Sterling).
N2 - Because young children have a limited understanding of risks that surround them, many adults try to create a risk-free environment for them. Suggesting that this strategy may leave children without the skills to manage risk and may make it difficult for them to judge situations for themselves, this book provides a practical guide showing adults how they can share their skills with young children and teach them about safety in a caring, supervised environment. Chapter 1 discusses the use of risk assessment in early childhood settings. Chapter 2 examines what and how children learn about safety from birth to age 8 years. Chapter 3 discusses how adults can deal with accidents in a way that will support children's learning, focusing on a problem-solving approach and providing emotional support, while chapter 4 provides strategies adults can use to share their experience with children, including coaching and sharing skills. Chapter 5 details the opportunities for learning self-care and caring skills in early childhood settings, including hygiene, basic first aid, and fire and heat. Chapter 6 deals with ways of providing balance between safety and a decent challenge in physical play. Chapter 7 discusses the use of tools in early childhood activities such as crafts and gardening and ways to increase children's ability to take responsibility. Chapter 8 examines child safety during travel and their increasing ability for independent travel. Chapter 9 discusses sharing responsibility with parents regarding children's safety. Chapter 10 presents staff activities for ensuring safe, enjoyable play. (KB)
SN - 1870985532
UR - http://search.proquest.com/docview/62376279?accountid=14701
ER -

TY - JOUR
ID - 1037
T1 - Risk, Challenge and Safety: Implications for Play Quality and Playground Design
A1 - Little,Helen

A1 - Eager,David
Y1 - 2010/12//
N1 - Last updated - 2012-12-14
SubjectsTermNotLitGenreText - 7940 126; 9074; 7943 8704 3760; 5472 3629 6582; 4109 4335; 8127 11655 1474 316 8016 4542; 8996; 1443 5053 2787; 1432 909; 1434 3760; 8130 3085 3150; 3553
KW - Australia
KW - Structured Interviews
KW - ERIC,Current Index to Journals in Education (CIJE)
KW - Preschool Education
KW - Equipment
KW - Play
KW - Child Behavior
KW - Safety
KW - Risk
KW - Foreign Countries
KW - Playgrounds
KW - Child Development
KW - Interviews
KW - Preschool Children
KW - Child Care Centers
KW - ERIC
RP - NOT IN FILE
SP - 497
EP - 513
JA - European Early Childhood Education Research Journal
VL - 18
IS - 4
PB - Routledge. , 325 Chestnut Street Suite 800, Philadelphia, PA 19106.
N2 - In many countries playground design is driven more by safety than developmental benefits. As risk minimisation strategies become more stringent, many researchers and practitioners are questioning whether young children are able to take sufficient risks in play in order for their play experiences to be interesting and developmentally challenging. This study examined the outdoor play choices and risk-taking behaviour of 38 children (25 boys, 13 girls) aged between 48 and 64 months from Sydney, Australia. Semi-structured interviews examined children's play preferences and playground equipment usage. Observations of play explored engagement in risk-taking behaviour. Selection of equipment indicated a strong preference for challenge and excitement. However, the dominant observed play behaviours involved little risk suggesting equipment at these parks provided few opportunities to practice mastery of skills already in their behavioural repertoire, learning new skills or building on current capabilities. The findings have implications for play provision and playground design. (Contains 3 tables and 2 figures.)
SN - 1350-293X, 1350-293X
UR - http://search.proquest.com/docview/851231337?accountid=14701
ER -

TY - JOUR
ID - 1038
T1 - The rough and tumble of it. Hospitals flexing their muscles in contract disputes with insurers
A1 - Benko,L.B.
A1 - Bellandi,D.
Y1 - 2001/03//
N1 - Benko, L B. Bellandi, D
Modern healthcare
nfa, 7801798
H
Journal Article
English
KW - Medline

KW - Rough and Tumble
KW - Conflict (Psychology)
KW - Hospital Administration
KW - Insurance
KW - Hospitalization
KW - Negotiating
KW - Reimbursement Mechanisms
KW - United States
RP - NOT IN FILE
SP - 52
EP - 56
JF - Modern Healthcare
JA - Mod.Healthc.
VL - 31
IS - 12
CY - United States
SN - 0160-7480
UR - 11299955
ER -

TY - JOUR
ID - 1039
T1 - Relationship between Parents' Beliefs and Their Responses to Children's Risk-Taking Behaviour during Outdoor Play
A1 - Little,Helen
Y1 - 2010/10//
N1 - Last updated - 2012-12-14
SubjectsTermNotLitGenreText - 7940 126; 7554 9015; 7943 8704 3760; 7531 730; 932 730; 8996; 11655 1474 316 8016 4542; 1432 909; 728 6447; 8535 6447; 5472 3629 6582; 7234; 1463 9074; 9822; 4109 4335
KW - Australia
KW - ERIC,Current Index to Journals in Education (CIJE)
KW - Early Childhood Education
KW - Questionnaires
KW - Play
KW - Child Behavior
KW - Young Children
KW - Parent Role
KW - Observation
KW - SOCIALIZATION
KW - Child Safety
KW - Risk
KW - Foreign Countries
KW - Playgrounds
KW - Parent Attitudes
KW - Interviews
KW - Attitude Measures
KW - Beliefs
KW - ERIC
RP - NOT IN FILE
SP - 315
EP - 330
JA - Journal of Early Childhood Research
VL - 8
IS - 3
PB - SAGE Publications. 2455 Teller Road, Thousand Oaks, CA 91320.

N2 - Learning how to respond appropriately in risk situations comes not only from the child's direct experiences but also through the guidance of those around them. The role of parent practices in guiding children's decision-making in risky situations has mainly been investigated in experimental contexts. The present study examined children's risk-taking behaviour and parent responses in everyday outdoor play settings. Parents' own risk-taking beliefs and behaviours were assessed using the Attitudes Towards Risk Questionnaire (ATR). Interviews explored parents' attitudes towards children's risk-taking and parents (11 mothers, one father) were observed as they supervised their four- to five-year-old children on playground equipment at a park. Parents' ATR scores were predictive of children's risk-taking behaviour. Parents mainly supervised their child's play and provided encouragement and support to foster children's engagement in challenging use of the equipment. Parents rarely intervened to prevent children's risky play but provided advice on how to complete the activity safely. Parents emphasized that their responses to children's risk-taking are context dependent. (Contains 5 tables.)
SN - 1476-718X, 1476-718X
UR - http://search.proquest.com/docview/822506636?accountid=14701
ER -

TY - JOUR
ID - 1040
T1 - The Influence of Play Context and Adult Attitudes on Young Children's Physical Risk-Taking during Outdoor Play
A1 - Little,Helen
A1 - Wyver,Shirley
A1 - Gibson,Frances
Y1 - 2011/03//
N1 - Last updated - 2012-12-14
SubjectsTermNotLitGenreText - 7940 126; 7943 8704 3760; 5472 3629 6582; 8127 11655 1474 316 8016 4542; 8996; 2177 5127; 226 316 8016 4542; 5183 2877; 7234; 6816 7531 730; 8135 10591 8267 3417 8016 4542 5703 4908 8917; 10482 730; 9074; 8535 6447; 6447; 4109 4335
KW - Australia
KW - ERIC,Current Index to Journals in Education (CIJE)
KW - Preschool Education
KW - Preschool Teachers
KW - Context Effect
KW - Measures (Individuals)
KW - Questionnaires
KW - Play
KW - Injuries
KW - Safety
KW - Teacher Attitudes
KW - Mother Attitudes
KW - Observation
KW - Adults
KW - Risk
KW - Foreign Countries
KW - Playgrounds
KW - Interviews
KW - Preschool Children
KW - ERIC
RP - NOT IN FILE
SP - 113
EP - 131
JA - European Early Childhood Education Research Journal
VL - 19
IS - 1
PB - Routledge. , 325 Chestnut Street Suite 800, Philadelphia, PA 19106.
N2 - Many children naturally seek challenging physically active play which may involve injury-risk. Prior studies have attempted to describe the characteristics of risky play but to date none have considered factors that

impact on opportunities for risky play or the likely resultant outcomes. Using semi-structured interviews and naturalistic observations, this study aimed to investigate adult attitudes towards risk-taking and whether children's experiences of risky play differ according to the contexts in which the play takes place. Although the children's mothers and Early Childhood practitioners believed risky play was an important aspect of learning and development and encouraged this type of play, observations of children's play at a local playground and their Early Childhood centre revealed that these contexts provided limited opportunities for risky play. Regulatory factors and requirements for playground safety were identified as having a detrimental impact on the quality of play in these settings creating tension between adult beliefs about the benefits of risky play and its provision. (Contains 6 tables.)
SN - 1350-293X, 1350-293X
UR - http://search.proquest.com/docview/864940635?accountid=14701
ER -

TY - JOUR
ID - 1041
T1 - Early Childhood Teachers' Beliefs about Children's Risky Play in Australia and Norway
A1 - Little,Helen
A1 - Sandseter,Ellen Beate Hansen
A1 - Wyver,Shirley
Y1 - 2012///
N1 - Last updated - 2013-05-02
SubjectsTermNotLitGenreText - 11465 8522; 10621 3227 6582; 2784 4335; 4109 4335; 7940 126; 8135 10591 8267 3417 8016 4542 5703 4908 8917; 10482 730; 2362; 5933 8234; 8996; 3916 4398; 2653 1710; 9506 5472 3629 6582; 8127 11655 1474 316 8016 4542
KW - Australia
KW - Norway
KW - ERIC,Current Index to Journals in Education (CIJE)
KW - Early Childhood Education
KW - Preschool Education
KW - Preschool Teachers
KW - Legal Problems
KW - Play
KW - Well Being
KW - Teacher Attitudes
KW - Semi Structured Interviews
KW - Decision Making
KW - Risk
KW - Foreign Countries
KW - Developed Nations
KW - Federal Regulation
KW - Court Litigation
KW - Preschool Children
KW - Teaching Methods
KW - ERIC
RP - NOT IN FILE
SP - 300
EP - 316
JF - Contemporary Issues in Early Childhood
VL - 13
IS - 4
PB - Symposium Journals. P.O. Box 204, Didcot, Oxford, OX11 9ZQ, UK. Tel: +44-1235-818-062; Fax: +44-1235-817-275; e-mail: subscriptions@symposium-journals.co.uk; Web site: http://www.wwwords.co.uk/ciec
N2 - Positive risk-taking in the context of outdoor physical play is important for fostering children's optimal health and development. Despite this, there is mounting concern that many developmentally beneficial activities are now seen as dangerous and something to be avoided. However, perceptions of risk are very much subject to cultural interpretation, and the growing risk aversion evident in some developed Western societies, such as

Australia, the United Kingdom and the United States of America, is less apparent in other developed countries, notably some of the European and Scandinavian countries. To explore some of these cultural differences, early childhood practitioners from Australia and Norway were interviewed regarding their provision of outdoor play experiences for children and their attitudes towards risk-taking in play. Practitioners from both countries recognised the importance of risky play for children's development and well-being. However, differences in the extent to which children's risky play was supported were evident. Factors associated with the quality of the outdoor environment, regulatory requirements, and a litigious environment were identified as constraining teaching practice for the Australian practitioners. The findings have implications for the development of policy that supports teachers' pedagogical decision-making in providing developmentally challenging play environments for children. (Contains 1 table.)
SN - 1463-9491, 1463-9491
UR - http://search.proquest.com/docview/1347461119?accountid=14701
ER -

TY - JOUR
ID - 1042
T1 - Physical play and cognitive development: integrating activity, cognition, and education
A1 - Bjorklund,D.F.
A1 - Brown,R.D.
Y1 - 1998/06//
N1 - Bjorklund, D F. Brown, R D
Child development
0372725, d28
IM
Comment. Journal Article
English
KW - Medline
KW - Rough and Tumble
KW - Adolescent
KW - Adult
KW - Child
KW - Child Development
KW - Preschool
KW - Cognition
KW - Exercise
KW - Female
KW - Humans
KW - Infant
KW - Newborn
KW - Learning
KW - Male
KW - Motor Skills
KW - Play and Playthings
KW - Sex Factors
KW - SOCIALIZATION
KW - Space Perception
RP - NOT IN FILE
SP - 604
EP - 606
JF - Child Development
JA - Child Dev.
VL - 69
IS - 3
CY - UNITED STATES
N2 - We propose that humans may have evolved a special sensitivity to certain types of social information during rough-and-tumble play that facilitates social cognition. The cognitive benefits of physical play are

described as providing a break from demanding intellectual tasks, and are hypothesized to be related to gender differences in spatial cognition
SN - 0009-3920
AD - Department of Psychology, Florida Atlantic University, Boca Raton 33431, USA. DBjorklund@fau.edu
UR - 9680673
ER -

TY - JOUR
ID - 1043
T1 - Kindezi: A Distinctively Africentric Perspective on Early Childhood Education. Developmentally Appropriate Practice
A1 - Lowden,Frances Y.
Y1 - 2000///
N1 - Last updated - 2012-12-14
SubjectsTermNotLitGenreText - Afrocentrism 304; 1050; Culturally Relevant Education 2501 3150 8774 2494; 2802 3242; Early Childhood Education 3085 3150; 3211 4164 4770 4918 5964 9804 9351; Educational Philosophy 3236 4164 7807 4918 5964; 4723; 7940 126; Preschool Curriculum 8129 2515; Young Children 11655 1474 316 8016 4542
KW - Africa
KW - ERIC,Current Index to Journals in Education (CIJE)
KW - Early Childhood Education
KW - Preschool Curriculum
KW - High Risk Students
KW - Afrocentrism
KW - Play
KW - Culturally Relevant Education
KW - Young Children
KW - Developmentally Appropriate Practices
KW - Black Students
KW - Educational History
KW - Educational Philosophy
KW - ERIC
RP - NOT IN FILE
SP - 7
EP - 12
JF - Journal of Early Education and Family Review
VL - 8
IS - 1
N2 - Presents the Kindezian philosophy of early childhood education, a historical developmentally appropriate Africentric worldview. Describes the curriculum as predating Western curriculum development by centuries. Maintains that Kindezi affords children the possibility to develop their full potential and that it provides a framework on which to respond to educational needs of African children and the descendants of the African diaspora. (KB)
SN - 1084-6603, 1084-6603
UR - http://search.proquest.com/docview/62335699?accountid=14701
ER -

TY - JOUR
ID - 1044
T1 - Generalization of Parenting Skills: An Early Intervention Program
A1 - Lowry,Mark A.
A1 - Whitman,Thomas L.
Y1 - 1989///
N1 - Last updated - 2012-12-14
SubjectsTermNotLitGenreText - 150 909; 914 2094; Early Intervention 3090 5470; Generalization 4314 18 1710 5904; 4722; 5118 11655 1474 316 8016 4542; Interaction 5348 8768; Mothers 6823 3932 8016 4542 7577

3816; 7537 3851 5449 8768; Parent Education 7540 210 3150; Parenting Skills 7575 9690 1; 7576 909; 7940 126; 8301 3626; 9021 9651 6582; 10883 11655 1474 316 8016 4542
KW - Developmental Delays
KW - Early Intervention Programs
KW - Home Start Program
KW - Observational Studies
KW - ERIC,Current Index to Journals in Education (CIJE)
KW - Parent Education
KW - Parenting Skills
KW - Interaction
KW - Toddlers
KW - Play
KW - High Risk Persons
KW - Mothers
KW - Generalization
KW - Role Playing
KW - Parenting Styles
KW - Behavior Modification
KW - Adjustment (to Environment)
KW - Parent Child Relationship
KW - Early Intervention
KW - Program Evaluation
KW - Infants
KW - ERIC
RP - NOT IN FILE
SP - 45
EP - 65
JF - Child and Family Behavior Therapy
VL - 11
IS - 1
N2 - In a short-term, intensive parent-training program, mothers of developmentally delayed infants are shown how to teach their children toy play behaviors. This study evaluates intervention effects on parents and children, with a focus on maternal behaviors. Results indicate improved maternal teaching behavior and generalizability of positive parenting style. (AF)
UR - http://search.proquest.com/docview/63164062?accountid=14701
ER -

TY - GEN
ID - 1045
T1 - INDAP: Individualizing Developmentally Appropriate Practices for Young Children with Special Needs
A1 - Mahoney,Gerald
A1 - Wheeden,Abbie
A1 - Janas,Debra
Y1 - 1996///
N1 - Availability - Level 3 - Indexed only
Last updated - 2012-12-14
KW - ERIC,Resources in Education (RIE)
KW - Practitioners
KW - Teachers
KW - Early Childhood Education
KW - At Risk Persons
KW - High Risk Students
KW - Play
KW - Class Activities
KW - Individualized Instruction
KW - Classroom Environment

KW - Child Behavior
KW - Special Needs Students
KW - Individual Needs
KW - Curriculum Development
KW - Disabilities
KW - Developmentally Appropriate Practices
KW - Early Intervention
KW - Individualized Education Programs
KW - ERIC
RP - NOT IN FILE
SP - 106
PB - Family Child Learning Center, 143 Northwest Avenue, Building A, Tallmadge, OH 44278; phone: 330-633-2055 ($18, plus $3 shipping and handling. Make check payable to Children's Hospital Foundation; quantity discounts available).
N2 - The INDAP (Individualizing Developmentally Appropriate Practices) program provides teachers with ideas on how to design classroom environments and materials, activities and routines, and instructional groups and transitions while providing high quality programs to children with different strengths and needs. These guidelines provide information about the philosophy of the Developmentally Appropriate Practice (DAP) model of early childhood education and focuses on the processes of active engagement by employing four instructional strategies: (1) developmental match; (2) interest match; (3) behavioral style match; and (4) enjoyment. Following the introduction in chapter 1, chapter 2 focuses on understanding the DAP approach to early childhood education. Chapter 3 describes INDAP guidelines for an interactive match. Chapter 4 examines play and the concepts of active learning, exploration, manipulation, problem solving, and initiation. Chapter 5 focuses on curriculum and how observations of children can be used to plan developmentally appropriate learning experiences. Chapter 6 focuses on environment and materials, including how to arrange the classroom environment to more effectively address children's developmental needs, while chapter 7 describes characteristics of daily activities and opportunities that effectively promote children's learning. Chapter 8 focuses on behavior and how to help children manage themselves by giving them developmentally appropriate choices and helping them make good decisions. (SD)
UR - http://search.proquest.com/docview/62488285?accountid=14701
ER -

TY - JOUR
ID - 1046
T1 - Wendy or Tinkerbell? How the Underrepresentation of Girls Impacts Gender Roles in Preschool Special Education
A1 - Manwaring,Joanne S.
Y1 - 2008///
N1 - Last updated - 2012-12-14
SubjectsTermNotLitGenreText - 3932 8016 4542; 2941 2700 9804 9351 5964; 2877; 8127 11655 1474 316 8016 4542; 9776 9761 3518; 9925 3150; 10621 3227 6582; 4290; 9456 1620 3199 3760; 695 694 8016 4542; 10195 2787; 1716 8409 5051; 10496 1970 1; 2879 4954; 8135 10591 8267 3417 8016 4542 5703 4908 8917; 7940 126; 5748 11228 1703 5053 2787; 9753 5053 2787; 9685 5053 2787
KW - ERIC,Current Index to Journals in Education (CIJE)
KW - Preschool Education
KW - Preschool Teachers
KW - Language Acquisition
KW - Play
KW - Special Education
KW - Gender Differences
KW - Disability Identification
KW - Student Development
KW - Cognitive Style
KW - Skill Development
KW - Teacher Competencies
KW - Self Contained Classrooms

KW - At Risk Students
KW - Disabilities
KW - Social Development
KW - Social Isolation
KW - Females
KW - Preschool Children
KW - Disproportionate Representation
KW - Teaching Methods
KW - ERIC
RP - NOT IN FILE
SP - 60
EP - 65
JF - TEACHING Exceptional Children
VL - 40
IS - 5
PB - Council for Exceptional Children. 1110 North Glebe Road Suite 300, Arlington, VA 22201.
N2 - The scope of this article is to discuss the impact of underrepresentation of girls in separate, self-contained special education classes for preschool children in a large school district in the southeastern United States. On a regular basis, preschool girls identified with special needs are being placed in self-contained classes where there they may be the only girl. The underrepresentation of girls in preschool self-contained special education classes results in social isolation and places the girls at risk for appropriate language, social, academic, and play skills. Teachers need to be sure to teach to all of the learning styles in their classrooms and to promote social interactions between both genders to help alleviate the lack of same-gender opportunities for girls
SN - 0040-0599, 0040-0599
UR - http://search.proquest.com/docview/61861814?accountid=14701
ER -

TY - JOUR
ID - 1047
T1 - Internet Gambling Behavior in a Sample of Online Gamblers
A1 - McBride,Jessica
A1 - Derevensky,Jeffrey
Y1 - 2009/01//
N1 - Last updated - 2012-12-14
SubjectsTermNotLitGenreText - 5434 5147 7051 2045; 7940 126; 545 9738 909; 916 8234; 8535 6447; 4270 126; 5013; 7615 909; 694 8016 4542; 3018 909; 3049 909; 141 9458 909 913 2877; 6531 2877
KW - ERIC,Current Index to Journals in Education (CIJE)
KW - Drinking
KW - At Risk Persons
KW - Questionnaires
KW - Play
KW - Behavior Problems
KW - Antisocial Behavior
KW - Addictive Behavior
KW - Mental Disorders
KW - Participation
KW - Incidence
KW - Games
KW - Drug Use
KW - Internet
KW - ERIC
RP - NOT IN FILE
SP - 149
EP - 167
JF - International Journal of Mental Health and Addiction
VL - 7

IS - 1
PB - Springer. 233 Spring Street, New York, NY 10013.
N2 - The present study examined Internet gambling behavior in a sample of online gamblers. Participants (N = 563; 382 male; ages 18-over 65) were recruited from a banner placed in an online newsletter. Questionnaires were completed online and assessed demographic information, game-play patterns (e.g., frequency, duration, wagering), preferred type of play, and problem gambling (using the DSM-IV). In addition, participation in gambling-type games without money was also examined. Seventy-seven percent of respondents reported playing gambling-type games without money and 42% reported gambling on the Internet. Twenty-three percent of participants were identified as problem gamblers. Problem gamblers were significantly more likely than social gamblers to spend more time gambling per session, gamble alone, from school, or with a cell phone, gamble with more money, wager online while consuming alcohol or illicit drugs, and lose more money gambling online. These results hint at a disturbing and difficult relationship between the Internet and individuals with gambling problems
SN - 1557-1874, 1557-1874
UR - http://search.proquest.com/docview/61903785?accountid=14701
ER -

TY - JOUR
ID - 1048
T1 - Infant Special Education: Interactions with Objects
A1 - McCune,Lorraine
A1 - Ruff,Holly A.
Y1 - 1985///
N1 - SuppNotes - Theme Issue: Developmental Toys
Last updated - 2012-12-14
SubjectsTermNotLitGenreText - 1443 5053 2787; 2877; High Risk Persons 4722; Infants 5118 11655 1474 316 8016 4542; 6296 5258 3224; Object Manipulation 7225 8426 9690 1; Play 7940 126; 10436 7688 1710; 10910
KW - ERIC,Current Index to Journals in Education (CIJE)
KW - Practitioners
KW - Play
KW - Object Manipulation
KW - High Risk Persons
KW - Disabilities
KW - Child Development
KW - Toys
KW - Tactual Perception
KW - Manipulative Materials
KW - Infants
KW - ERIC
RP - NOT IN FILE
SP - 59
EP - 68
JF - Topics in Early Childhood Special Education
VL - 5
IS - 3
N2 - Research on object exploration by infants addresses milestones in the process by normally developing infants, including spontaneous manipulation strategies. Implications for developmentalists working with high-risk preterm infants are noted, including the need for direct assistance in manipulation and for selection of materials. (CL)
UR - http://search.proquest.com/docview/63309370?accountid=14701
ER -

TY - JOUR
ID - 1049
T1 - The Benefits of Using Sociodrama in the Elementary Classroom: Promoting Caring Relationships among Educators and Students

A1 - McLennan,Deanna Marie Pecaski
Y1 - 2008/04//
N1 - Last updated - 2012-12-14
SubjectsTermNotLitGenreText - 9021 9651 6582; 3190 3518; 2521 3184 2787; 3005 9021 9651 6582; 9469 9451; 9473; 8996; 3400 8415; 10576 5449 8768; 1571 9146 126
KW - ERIC,Current Index to Journals in Education (CIJE)
KW - Teachers
KW - Elementary Education
KW - Risk
KW - Teacher Student Relationship
KW - Class Activities
KW - Self Esteem
KW - Curriculum Development
KW - Educational Environment
KW - Dramatic Play
KW - Role Playing
KW - Self Expression
KW - Empathy
KW - ERIC
RP - NOT IN FILE
SP - 451
EP - 456
JF - Early Childhood Education Journal
VL - 35
IS - 5
PB - Springer. 233 Spring Street, New York, NY 10013.
N2 - In the current age of academic rigor, standards and accountability, the fostering of caring relationships in the classroom may not always be a priority. Expressing care for another person is a skill that can be taught and nurtured through a supportive educational environment. Sociodrama encourages each of its participants to develop self-confidence and self-expression through risk-taking and exploration in activities that explore real life personal feelings and situations. By using sociodrama as a means for exploring issues in the classroom it is possible to celebrate participants' individuality and to create caring and nurturing relationships among educators and students. This article examines the research describing the benefits of including sociodrama in the elementary curriculum
SN - 1082-3301, 1082-3301
UR - http://search.proquest.com/docview/61968793?accountid=14701
ER -

TY - JOUR
ID - 1050
T1 - Gender labels and play styles: their relative contribution to children's selection of playmates
A1 - Alexander,G.M.
A1 - Hines,M.
Y1 - 1994/06//
N1 - Alexander, G M. Hines, M
Child development
0372725, d28
IM
Comparative Study. Journal Article. Research Support, U.S. Gov't, P.H.S.
English
KW - Medline
KW - Rough and Tumble
KW - Age Factors
KW - Child Behavior
KW - Child
KW - Preschool

KW - Choice Behavior
KW - Female
KW - Humans
KW - Interview
KW - Psychological
KW - Male
KW - Photic Stimulation
KW - Play and Playthings
KW - Sex
KW - Sex Factors
KW - Task Performance and Analysis
RP - NOT IN FILE
SP - 869
EP - 879
JF - Child Development
JA - Child Dev.
VL - 65
IS - 3
CY - UNITED STATES
N2 - The relative contribution of gender labels and play styles (masculine or feminine) in playmate selection was evaluated in 60 children between the ages of 4 and 8 years using a novel interview measure. In the interview, when targets' gender labels and targets' play styles were presented as independent dimensions, children showed predicted sex differences in preferences for gender labels and for play styles (including toys, rough-and-tumble play, and activity level). However, when targets' gender labels and targets' play styles were presented as competing dimensions, boys of all ages chose female targets with masculine play styles over male targets with feminine play styles. In contrast, younger girls (4-5-year-olds) chose female targets with masculine play styles, whereas older girls (6-8-year-olds) chose male targets with feminine play styles. This suggests possible sex differences in the contribution of gender labels and of play styles in the development of children's preferences for same-sexed playmates
SN - 0009-3920
AD - University of California, Los Angeles
UR - 8045173
ER -

TY - JOUR
ID - 1051
T1 - Bob Bear: A Strategy for Improving Behaviors of Preschoolers Identified as At Risk or Developmentally Delayed
A1 - Michael,Meredith
A1 - Meese,Ruth L.
A1 - Keith,Stephen
A1 - Mathews,Rachel
Y1 - 2009///
N1 - Last updated - 2012-12-14
SubjectsTermNotLitGenreText - 5913 10830; 7940 126; 5470; 122 8836; 8127 11655 1474 316 8016 4542; 2790; 695 694 8016 4542; 914 2094; 5246 2768; 3264 3227 6582; 4232 3629 6582; 8130 3085 3150
KW - ERIC,Current Index to Journals in Education (CIJE)
KW - Teachers
KW - Preschool Education
KW - Educational Strategies
KW - Play
KW - Action Research
KW - Developmental Delays
KW - Intervention
KW - Instructional Design
KW - Functional Behavioral Assessment

KW - At Risk Students
KW - Behavior Modification
KW - Learning Theories
KW - Preschool Children
KW - ERIC
RP - NOT IN FILE
SP - 54
EP - 59
JF - TEACHING Exceptional Children
VL - 41
IS - 5
PB - Council for Exceptional Children. 1110 North Glebe Road Suite 300, Arlington, VA 22201.
N2 - Social learning theory, sociodramatic play, and the use of puppets and stuffed animals may be beneficial for improving social behaviors of preschoolers with and without disabilities. Therefore, this action research study is developed on the belief that a stuffed animal (Bob Bear) will enhance appropriate behaviors for preschool children when used by teachers as a part of their behavior management program. Teachers strategically harness sociodramatic play, helping preschoolers imagine that Bob Bear is a member of the class who can talk and display behaviors like all other children in the classroom. Thus, Bob Bear will be utilized daily as a teaching/learning tool to model, role play, and reinforce expected behaviors in the preschool classroom. The authors conclude that additional research is needed to determine whether or not this intervention strategy, used in this article for preschoolers identified as at risk or with developmental delays, might prove helpful with preschoolers having other exceptional needs. (Contains 3 figures and 1 table.)
SN - 0040-0599, 0040-0599
UR - http://search.proquest.com/docview/61878991?accountid=14701
ER -

TY - JOUR
ID - 1052
T1 - Prenatal exposure to diethylstilbestrol (DES): childhood play behavior and adult gender-role behavior in women
A1 - Lish,J.D.
A1 - Meyer-Bahlburg,H.F.
A1 - Ehrhardt,A.A.
A1 - Travis,B.G.
A1 - Veridiano,N.P.
Y1 - 1992/10//
N1 - Lish, J D. Meyer-Bahlburg, H F. Ehrhardt, A A. Travis, B G. Veridiano, N P
Archives of sexual behavior
8gr, 1273516
IM
Journal Article. Research Support, Non-U.S. Gov't. Research Support, U.S. Gov't, P.H.S.
English
KW - Medline
KW - Rough and Tumble
KW - Adolescent
KW - Adult
KW - Child
KW - Preschool
KW - Diethylstilbestrol/ae [Adverse Effects]
KW - Female
KW - Follow-Up Studies
KW - Gender Identity
KW - Humans
KW - Play and Playthings
KW - Pregnancy
KW - Prenatal Exposure Delayed Effects

RP - NOT IN FILE
SP - 423
EP - 441
JF - Archives of Sexual Behavior
JA - Arch.Sex Behav
VL - 21
IS - 5
CY - UNITED STATES
N2 - Data from lower mammals suggest a masculinizing or defeminizing influence of pre- or perinatal diethylstilbestrol (DES) exposure on various aspects of the sex-dimorphic behavior (including juvenile rough-and-tumble play) of genetic females. However, three previous studies on childhood play and adult gender-role behavior in human females have led to ambiguous results. In a follow-up study of 60 women with prenatal exposure to DES and 26 controls, we used psychometrically well-designed multi-item scales based on self-report inventories for the assessment of these aspects of behavior. No effects of DES could be demonstrated. We conclude that, at the doses studied, prenatal DES exposure in human females has not led to behavioral masculinization or defeminization of childhood play and adult gender-role behavior
SN - 0004-0002
AD - New York State Psychiatric Institute, New York
UR - 1417473
ER -

TY - JOUR
ID - 1053
T1 - Naltrexone open trial with a 5-year-old-boy. A social rebound reaction
A1 - Lensing,P.
A1 - Klingler,D.
A1 - Lampl,C.
A1 - Leboyer,M.
A1 - Bouvard,M.
A1 - Plumet,M.H.
A1 - Panksepp,J.
Y1 - 1992///
N1 - Lensing, P. Klingler, D. Lampl, C. Leboyer, M. Bouvard, M. Plumet, M H. Panksepp, J
Acta paedopsychiatrica
1ms, 0400661
IM
Journal Article
English
KW - Medline
KW - Rough and Tumble
KW - Attention Deficit Disorder with Hyperactivity/dt [Drug Therapy]
KW - Attention Deficit Disorder with Hyperactivity/px [Psychology]
KW - Autistic Disorder/dt [Drug Therapy]
KW - Autistic Disorder/px [Psychology]
KW - Child
KW - Preschool
KW - Humans
KW - Intellectual Disability/dt [Drug Therapy]
KW - Intellectual Disability/px [Psychology]
KW - Male
KW - Naltrexone/tu [Therapeutic Use]
KW - Personality Assessment
KW - Social Behavior
RP - NOT IN FILE
SP - 169
EP - 173

JF - Acta Paedopsychiatrica
JA - Acta Paedopsychiatr.
VL - 55
IS - 3
CY - GERMANY
N2 - The neurobiological rationale for an opiate antagonist pharmacotherapy of autism is presented. Naltrexone efficacy in decreasing autistic behaviour and in increasing social-affiliative behaviour was explored in a 5-year-old autistic boy. Naltrexone (0.5 mg/kg 3 times peer week) was effective in immediately decreasing gross motor activity and stereotyped behaviour and caused a delayed increase of crying, smiling and rough-and-tumble play. This single case presents preliminary evidence that a therapeutically valuable rebound reaction is possible and that the human opioid system modulates social-affective processes. The possibility of psychological factors being instrumental in achieving this effect is discussed as being suitable for future clinical trials
SN - 0001-6586
AD - School Psychology of Upper Austria, Linz
UR - 1414352
ER -

TY - JOUR
ID - 1054
T1 - Interpartner Conflict and Child Abuse Risk among African American and Latino Adolescent Parenting Couples
A1 - Moore,David R.
A1 - Florsheim,Paul
Y1 - 2008/04//
N1 - Last updated - 2012-12-14
SubjectsTermNotLitGenreText - 5470; 1429 545 9738 909; 324 545 9738 909; 8168; 2112; 7576 909; 1459; 8105; 197 316 8016 4542; 4759 7160 8016 4542; 293 7160 8016 4542; 3862 11282 545 9738 909; 5449 8768; 7940 126; 7537 3851 5449 8768; 694 8016 4542
KW - ERIC,Current Index to Journals in Education (CIJE)
KW - At Risk Persons
KW - Play
KW - African Americans
KW - Intervention
KW - Conflict
KW - Pregnancy
KW - Prevention
KW - Parenting Styles
KW - Hispanic Americans
KW - Parent Child Relationship
KW - Child Rearing
KW - Family Violence
KW - Interpersonal Relationship
KW - Aggression
KW - Child Abuse
KW - Adolescents
KW - ERIC
RP - NOT IN FILE
SP - 463
EP - 475
JF - Child Abuse & Neglect: The International Journal
VL - 32
IS - 4
PB - Elsevier. 6277 Sea Harbor Drive, Orlando, FL 32887-4800.
N2 - Objective: The goal of this study was to identify links between observed conflict interactions and risk for child abuse and harsh parenting among a multiethnic sample of adolescent mothers (14-19 years) and young fathers (14-24 years). Methods: Prior to childbirth (T1), observation-based relationship data were collected from

154 expectant adolescent couples as well as information about physical aggression between partners. Two years after childbirth (T2), data relevant to harsh disciplinary practices and child abuse-prone attitudes were collected from both young mothers and fathers. Multiple regression analyses were run to examine the correspondence between (a) couples' relationship quality prior to childbirth and (b) subsequent risk for harsh and potentially abusive parenting practices. Results: Findings indicated that interpartner violence prior to childbirth predicted physically punitive parenting behavior for fathers, but not for mothers. Young mothers and fathers observed to be more warmly engaged with each other during their pre-birth couple interactions (T1) reported lower rates of physically punitive parenting behavior with their children at T2. Couples' hostility at T1 predicted fathers' level of observed hostility toward his child during a structured play activity at T2. Conclusions: Results underscore the importance of addressing the quality of couples' relations as means of preventing dysfunctional parenting practices among adolescent mothers and their partners. Adolescent mothers and their partners are at heightened risk for engaging in dysfunctional parenting, including child abuse. Focusing on pregnant adolescents and their partners, this study sought to identify interpersonal predictors of child abuse risk. Although this study did not involve administering prevention or intervention services, the goal was to test hypotheses that would inform the development of programs for young at-risk couples. Practice implications: The decision to recruit young couples prior to childbirth was based on the presumption that this period of time could provide a window of opportunity to administer couple-based child abuse prevention programs. Consistent with previous research on marital relations and parenting, results of this study support the idea that efforts to develop and administer preventive-intervention programs targeting at-risk couples could help reduce the occurrence of harsh parenting behavior and abuse
SN - 0145-2134, 0145-2134
UR - http://search.proquest.com/docview/61969410?accountid=14701
ER -

TY - GEN
ID - 1055
T1 - Miniperformances, Many Stars! Playing with Stories
A1 - Morado,Carolyn
A1 - Koenig,Rosalie
A1 - Wilson,Alice
Y1 - 1999///
N1 - Last updated - 2012-12-14
KW - ERIC,Current Index to Journals in Education (CIJE)
KW - Primary Education
KW - High Risk Students
KW - Class Activities
KW - Dramatic Play
KW - Story Telling
KW - Creative Dramatics
KW - Childrens Literature
KW - Literacy
KW - ERIC
RP - NOT IN FILE
SP - 116
EP - 123
JF - Reading Teacher
VL - 53
IS - 2
N2 - Describes a collaborative compensatory program for at-risk kindergarten, first-, and second-grade students, in which literature, drama, music, and movement are woven together into miniperformances that develop and support literacy learning. Discusses the process of developing a miniperformance, and lists suggested books with stories for miniperformances. (SR)
UR - http://search.proquest.com/docview/62391837?accountid=14701
ER -

TY - JOUR

ID - 1056
T1 - Initial dyadic peer interaction of attention deficit-hyperactivity disorder and normal boys
A1 - Hubbard,J.A.
A1 - Newcomb,A.F.
Y1 - 1991/04//
N1 - Hubbard, J A. Newcomb, A F
Journal of abnormal child psychology
0364547, h33
IM
Journal Article. Research Support, Non-U.S. Gov't
English
KW - Medline
KW - Rough and Tumble
KW - Attention Deficit Disorder with Hyperactivity/di [Diagnosis]
KW - Attention Deficit Disorder with Hyperactivity/px [Psychology]
KW - Child
KW - Humans
KW - Interpersonal Relations
KW - Male
KW - Peer Group
KW - Play and Playthings
KW - Social Behavior
KW - Verbal Behavior
RP - NOT IN FILE
SP - 179
EP - 195
JF - Journal of Abnormal Child Psychology
JA - J Abnorm.Child Psychol.
VL - 19
IS - 2
CY - UNITED STATES
N2 - The present study assessed the frequency and patterns of play duration and verbal behavior of medicated attention deficit-hyperactivity disorder (ADHD) boys in an initial social encounter with a normal, same-age peer. Eight pairs each of previously unacquainted ADHD/normal boys and normal/normal boys were videotaped as they interacted in a free-play setting (N = 32). The ADHD/normal dyads engaged in more solitary play and less associative play than the normal/normal dyads. Sequential analyses of the ADHD/normal dyads' play patterns revealed that they had problems in their progression along the play hierarchy, in sustaining associative play, and in avoiding withdrawal after rough and tumble play. In comparison to the normal/normal dyads, the ADHD/normal dyads also differed in the quality of verbal interaction as seen in their lower levels of verbal reciprocity and affective expression. Process explanations for the problems ADHD boys display in an initial social encounter and the implications of these difficulties for diminished socialization opportunities were discussed
SN - 0091-0627
AD - Department of Psychology, University of Richmond, Virginia 23173
UR - 2056162
ER -

TY - JOUR
ID - 1057
T1 - Determinants of Children's Risk-Taking in Different Social-Situational Contexts: The Role of Cognitions and Emotions in Predicting Children's Decisions
A1 - Morrongiello,Barbara A.
A1 - Matheis,Shawn
Y1 - 2004///

N1 - Last updated - 2012-12-14
SubjectsTermNotLitGenreText - 5183 2877; 4390 7422; 8996; 2177 5127; 1710; 8415; 8094; 1707 6440; 8751 10087 2574 3629 6582; 2653 1710; 3899 8415; 7940 126; 10996 126
KW - ERIC,Current Index to Journals in Education (CIJE)
KW - Travel
KW - Prediction
KW - Context Effect
KW - Play
KW - Injuries
KW - Fear
KW - Decision Making
KW - Cognitive Measurement
KW - Regression (Statistics)
KW - Risk
KW - Cognitive Processes
KW - Psychological Patterns
KW - Goal Orientation
KW - ERIC
RP - NOT IN FILE
SP - 303
EP - 326
JF - Journal of Applied Developmental Psychology
VL - 25
IS - 3
PB - Elsevier. P.O. Box 045, New York, NY 10159-0945.
N2 - This study examined the contribution of cognitive and emotion-based factors in predicting school-age children's risk-taking decisions when the social-situational context did, and did not, pressure for risk-taking. Using drawings of play situations that depicted three possible paths of travel that varied in injury risk and pitted convenience (length of path) against level of risk (e.g., shortest path was most risky), we explored children's appraisals of danger and injury severity (i.e., cognitive measures) and anticipated fear and excitement (i.e., emotion measures) for each possible path of travel. Children also indicated which path they would take and gave a justification for this decision. Subsequently, they were informed of information that threatened loss of a desired goal unless one of the more convenient (faster), risky paths was selected. Children then repeated their path selections and the cognitive and emotion appraisals. When the situation pressured for greater risk-taking to attain a desired goal, children endorsed greater risk-taking the majority of the time, with convenience being the most frequently cited reason for this decision. Regression analyses indicated that both cognitive and emotion-based measures contributed to predict risk-taking decisions in both social-situational contexts. Theoretical and practical implications of these findings are discussed
SN - 0193-3973, 0193-3973
UR - http://search.proquest.com/docview/62079312?accountid=14701
ER -

TY - RPRT
ID - 1058
T1 - Interactional Patterns of Schizophrenic, Depressed and Well Mothers and Their Young Children
A1 - Musick,Judith S.
Y1 - 1979/09//
N1 - Availability - Level 1 - Available online, if indexed January 1993 onward
SuppNotes - Paper presented at the Annual Meeting of the American Psychological Association (87th, New York, NY, September 1-5, 1979)
Last updated - 2012-12-14
KW - ERIC,Resources in Education (RIE)
KW - Play
KW - Depression (Psychology)
KW - Mothers
KW - Schizophrenia

KW - Risk
KW - Rating Scales
KW - Parent Child Relationship
KW - Infant Behavior
KW - Interaction Process Analysis
KW - Psychopathology
KW - Preschool Children
KW - Infants
KW - ERIC
RP - NOT IN FILE
SP - 25
N2 - This study analyzes the interaction patterns and behavioral characteristics of mentally ill and well mothers and their young children. The children were between 1 and 4 years of age. The group of mentally ill mothers was comprised of 18 schizophrenic and psychotically depressed women each with a history of at least one psychiatric hospitalization. The well mothers were matched with two experimental groups (one receiving treatment in the home and the other receiving treatment in a special clinic program) on six variables: age and sex of youngest child, mothers age and marital status, race and socioeconomic status. The data were obtained--prior to intervention--from behavioral analyses of 72 five-minute videotaped sequences of feeding and unstructured play of each mother-child pair. Significant differences between the well and mentally ill mothers were found in only six of the 25 feeding categories and six of the 25 unstructured play categories. Only two of these 12 variables were significant in both feeding and unstructured play: consistency of maternal behavior over 5 minute segment, and amount of contact (touching, holding, gazing). The most significant difference between groups was found on the dyadic variable of reciprocity during feeding. The dyadic interaction of the mentally ill mothers and their children was characterized by fewer attempts by the mother to understand the child or to respond to the child's cues. (Author/SS)
UR - http://search.proquest.com/docview/63676943?accountid=14701
ER -

TY - JOUR
ID - 1059
T1 - Language Learning in Context: Teacher and Toddler Speech in Three Classroom Play Areas
A1 - O'Brien,Marion
A1 - Bi,Xiufen
Y1 - 1995///
N1 - Last updated - 2012-12-14
SubjectsTermNotLitGenreText - 694 8016 4542; Child Language 1451 5746; 1571 9146 126; Classroom Communication 1600 1849; 2790; High Risk Students 4723; Language Acquisition 5748 11228 1703 5053 2787; Language Usage 5800; 7940 126; 10527 5127; Teacher Student Relationship 10576 5449 8768; 10883 11655 1474 316 8016 4542
KW - ERIC,Current Index to Journals in Education (CIJE)
KW - Language Usage
KW - At Risk Persons
KW - Toddlers
KW - Teacher Student Relationship
KW - High Risk Students
KW - Teacher Influence
KW - Play
KW - Language Acquisition
KW - Class Activities
KW - Developmental Delays
KW - Classroom Communication
KW - Child Language
KW - ERIC
RP - NOT IN FILE
SP - 148
EP - 163

JF - Topics in Early Childhood Special Education
VL - 15
IS - 2
N2 - Patterns of teacher language used with at-risk toddlers in three different play contexts (doll/house, block/truck, and large motor) were studied within a naturally occurring classroom setting. Significantly different language-use patterns by teachers were found in each area, and these were associated with differences in the rate and nature of child language. (Author/SW)
SN - 0271-1214, 0271-1214
UR - http://search.proquest.com/docview/62758242?accountid=14701
ER -

TY - JOUR
ID - 1060
T1 - Specific cross-gender behaviour in boyhood and later homosexual orientation
A1 - Green,R.
A1 - Roberts,C.W.
A1 - Williams,K.
A1 - Goodman,M.
A1 - Mixon,A.
Y1 - 1987/07//
N1 - Green, R. Roberts, C W. Williams, K. Goodman, M. Mixon, A
The British journal of psychiatry : the journal of mental science
0342367, b1k
IM
Journal Article. Research Support, U.S. Gov't, P.H.S.
English
KW - Medline
KW - Rough and Tumble
KW - Child Behavior
KW - Child Development
KW - Gender Identity
KW - Homosexuality
KW - Humans
KW - Identification (Psychology)
KW - Male
KW - Role Playing
RP - NOT IN FILE
SP - 84
EP - 88
JF - British Journal of Psychiatry
JA - Br J Psychiatry
VL - 151
CY - ENGLAND
N2 - Data from a group of males aged 13 to 23, who as children exhibited extensive cross-gender behaviour, was analysed. In boyhood they frequently played with dress-up dolls, role-played as females, dressed in girls' clothes, stated the wish to be girls, primarily had girls as friends, and avoided rough-and-tumble play. The majority of the group evolved a bisexual or homosexual orientation; two types of behaviour, boyhood doll play and female role-playing, were found to be associated with later homosexual orientation. The findings suggest developmental associations between specific types of boyhood cross-gender behaviour and the objects of later sexual arousal
SN - 0007-1250
AD - Department of Psychiatry and Biobehavioral Science, UCLA School of Medicine
UR - 3676630
ER -

TY - GEN

ID - 1061
T1 - Facilitating and Learning at the Edge of Chaos: Expanding the Context of Experiential Education
A1 - Oekerman,Carl
Y1 - 1997/11//
N1 - Availability - Level 1 - Available online, if indexed January 1993 onward
SuppNotes - In: Deeply Rooted, Branching Out, 1972-1997. Annual AEE International Conference Proceedings; see RC 021 269
Last updated - 2012-12-14
KW - Complex Adaptive Systems
KW - System Dynamics
KW - ERIC,Resources in Education (RIE)
KW - Risk
KW - Play
KW - Systems Approach
KW - Experiential Learning
KW - Group Dynamics
KW - Creativity
KW - Organizational Development
KW - Role of Education
KW - Chaos Theory
KW - Models
KW - ERIC
RP - NOT IN FILE
SP - 7
N2 - Significant recent discoveries within a number of scientific disciplines, collectively referred to as the science of complexity, are creating a major shift in how human beings understand the complex, adaptive systems that make up the world. A complex adaptive system consists of networks of large numbers of agents that interact with each other and with their environment according to a set of rules. This set of rules contains two subsystems: a dominant, or legitimate, subsystem that encompasses the system's primary task, and a recessive, or shadow, subsystem that operates outside of the system's primary task, providing the arena for play, exploration of new behaviors, and creativity. The shadow subsystem also seeks to undermine or modify the dominant subsystem through change. These two subsystems coexist in dynamic tension, and when the system is operating in the narrow zone between order and chaos, called a phase transition, or "the edge of chaos," it is operating at its highest level of functioning. Here is where the system creates space for novelty, where the greatest information processing takes place, where risks are taken and new behavior is tried out. Five factors that determine whether a system can move into the edge of chaos are identified. Practitioners of experiential education, working with and within complex human systems, already intuitively understand many aspects of complex system dynamics, and are ideally suited to use their skills in broader applications. Organizations and institutions shifting to the new paradigm of complex systems will operate from a conceptual framework with which experiential educators are already skilled. Includes suggestions for further reading. (Author/TD)
UR - http://search.proquest.com/docview/62529490?accountid=14701
ER -

TY - JOUR
ID - 1062
T1 - Women's Refuges as Intervention Arenas for Children Who Experience Domestic Violence
A1 - Overlien,Carolina
Y1 - 2011///
N1 - Last updated - 2012-12-14
SubjectsTermNotLitGenreText - 5470; 3862 11282 545 9738 909; 3932 8016 4542; 4109 4335; 1474 316 8016 4542; 694 8016 4542; 916 8234; 3387 6531 2877; 2900 10157 3629 6582; 3378 4878 3760; 7940 126; 1443 5053 2787; 2291 4552; 3592 7807 4918 5964; 5472 3629 6582
KW - Norway
KW - ERIC,Current Index to Journals in Education (CIJE)
KW - At Risk Persons
KW - Play

KW - Intervention
KW - Emergency Shelters
KW - Counseling
KW - CHILDREN
KW - Behavior Problems
KW - Discourse Analysis
KW - Emotional Disturbances
KW - Foreign Countries
KW - Ethics
KW - Child Development
KW - Family Violence
KW - Interviews
KW - Females
KW - ERIC
RP - NOT IN FILE
SP - 375
EP - 391
JF - Child Care in Practice
VL - 17
IS - 4
PB - Routledge. , 325 Chestnut Street Suite 800, Philadelphia, PA 19106.
N2 - For over 30 years, children have been accompanying their mothers to women's refuges. We now know that these children are at risk of developing an array of behavioural and psychological difficulties. We also know the importance for children of keeping active and playing while at the refuge, and to be given the possibility of addressing and dealing with their own experiences of violence. However, we know very little about how children experience these interventions. This study analyses children's discourse about their everyday lives at refuges in Norway. The questions asked are: what interventions do children in refuges in Norway receive and how do the children experience those interventions? The analysis shows that the children focus on two forms of interventions as particularly helpful, activities (trips, "outings", and so on) and individual counselling. Furthermore, as the most important intervention for a child who experiences domestic violence is to remove him/her from a seriously violent environment, the refuge stay in itself can be perceived as an intervention. In the discussion, the author describes how and why refuges represent a unique opportunity for interventions with children. (Contains 4 notes.)
SN - 1357-5279, 1357-5279
UR - http://search.proquest.com/docview/964180715?accountid=14701
ER -

TY - JOUR
ID - 1063
T1 - Sex-typing behavior and sex-typing pressure in child/parent interaction
A1 - Jacklin,C.N.
A1 - DiPietro,J.A.
A1 - Maccoby,E.E.
Y1 - 1984/10//
N1 - Jacklin, C N. DiPietro, J A. Maccoby, E E
Archives of sexual behavior
8gr, 1273516
IM
Journal Article
English
KW - Medline
KW - Rough and Tumble
KW - Child
KW - Preschool
KW - Father-Child Relations
KW - Female

KW - Gender Identity
KW - Humans
KW - Identification (Psychology)
KW - Male
KW - Mother-Child Relations
KW - Parent-Child Relations
KW - Play and Playthings
KW - Sex Factors
KW - SOCIALIZATION
RP - NOT IN FILE
SP - 413
EP - 425
JF - Archives of Sexual Behavior
JA - Arch.Sex Behav
VL - 13
IS - 5
CY - UNITED STATES
N2 - The sex-typing of children and the sex-typing pressure of parents was investigated during free play in a home visit. There were 30 male and 24 female 45-month-olds observed with their mothers and fathers in separate free-play sessions during which an array of both sex-stereotyped and neutral toys were available. Behavioral observations were recorded for a variety of parent, child, and dyadic behaviors, including initiations of sex-typed play, total sex-typed play, and rough-and-tumble play. Children initiated sex-typed play and played with sex-appropriate toys. Father-child and mother-daughter dyads were more likely to engage in thematic play appropriate to the child's sex, while in mother-son dyads equal amounts of masculine and feminine play occurred. In addition father-son dyads displayed the highest levels of rough-and tumble play and arousal of child by parent. The results suggest that fathers are the discriminating influence on sex-appropriate play
SN - 0004-0002
UR - 6517683
ER -

TY - JOUR
ID - 1064
T1 - Politics and Play: Meditations on Rhetorical Bodily Performance
A1 - Pauliny,Tara
Y1 - 2013///
N1 - Last updated - 2013-07-02
SubjectsTermNotLitGenreText - 10820 4007 4918 5964; 4890; 7147 1849; 9588; 8009 2503; 7989; 7940 126; 4960; 6867; 3933; 6355; 2471 3518; 9769 5127; 694 8016 4542
KW - Ohio
KW - United States
KW - ERIC,Current Index to Journals in Education (CIJE)
KW - Adult Education
KW - Masculinity
KW - At Risk Persons
KW - Social Influences
KW - Cultural Context
KW - Play
KW - Popular Culture
KW - Sexual Identity
KW - Nonverbal Communication
KW - Human Body
KW - Theater Arts
KW - Ideology
KW - Multimedia Materials
KW - Political Power
KW - Femininity

KW - ERIC
RP - NOT IN FILE
SP - 179
EP - 191
JF - Research in Drama Education
VL - 18
IS - 2
PB - Routledge. , 325 Chestnut Street Suite 800, Philadelphia, PA 19106.
N2 - Focusing on the drag king performances of Christie Whisman, who lived and worked in Columbus, Ohio, USA, from 1999 to 2001, this essay argues that rhetorical bodily performances have the capacity not only to subvert normalised notions of identity categories, but that such performances can also create alternate pedagogical sites that persuasively and parodically connect politics to play. By exposing the interconnected nature of the material and theoretical body--the body constructed by physicality, and the body constructed through ideology--Whisman's work expands and deconstructs social categorisations that have material and psychic impact. It calls into question what constitutes masculinity and femininity, for example, and it provides an opportunity for the subject to answer back and have a hand in her own construction. By forwarding a political critique that highlights the symbiotic relationship between the body and identity, parody and persuasion, and subversion and reiteration, this essay argues that Whisman's work illustrates the political power of play. It notes that, while her performances remind us that categories of the body such as gender and even sex are certainly constructed and performed, the persuasive force of those performances is often attached to, in fact "performed by", an actual body. Her work asserts that it is this body--this flesh and blood, imagined and imagining, limited and possible--that intersects with actions and speech to create rhetorical and queer arguments; here, bodily matter brims with signification, resistance and, perhaps above all, subversive potential. In so doing, the arguments offered by rhetorical bodily performances often cross lines of race and class (given the spaces in which they are performed), subvert gender binaries, refuse to honour the artificial separation of politics and pop culture, and ultimately authorise the performing subject and her audience. (Contains 5 notes.)
SN - 1356-9783, 1356-9783
UR - http://search.proquest.com/docview/1373089187?accountid=14701
ER -

TY - JOUR
ID - 1065
T1 - An analysis and reduction of disruptive behavior on school buses
A1 - Greene,B.F.
A1 - Bailey,J.S.
A1 - Barber,F.
Y1 - 1981///
N1 - Greene, B F. Bailey, J S. Barber, F
Journal of applied behavior analysis
0174763, hdr
IM
Journal Article
English
KW - Medline
KW - Rough and Tumble
KW - Adolescent
KW - Behavior Therapy/mt [Methods]
KW - Child
KW - Child Behavior Disorders/rh [Rehabilitation]
KW - Feedback
KW - Florida
KW - Humans
KW - Motor Activity
KW - Noise/pc [Prevention & Control]
KW - Transportation
RP - NOT IN FILE

SP - 177
EP - 192
JF - Journal of Applied Behavior Analysis
JA - J Appl.Behav Anal.
VL - 14
IS - 2
CY - UNITED STATES
N2 - Thousands of children are injured or killed each year in school bus accidents. A significant number of these tragic incidents is precipitated by disruptive child behavior that distracts the drivers from their difficult task. Two experiments were conducted which addressed this problem. For both experiments an automated sound recording device (referred to as a Noise Guard) selectively responsive to frequencies above 500 Hz (i.e., unresponsive to bus drone) recorded both the duration and frequency of noise outbursts above a tolerable threshold. Additionally, an observer made in situ measurements of other disruptions including roughhousing and getting-out-of-seat. In the first experiment, following baseline measurements of these behaviors, middle-school students received feedback for noise outbursts. That is, when "Noise Guard" was activated, it in turn operated one of several lights on a panel visible to all passengers. Each day students were allowed to listen to high-appeal taped music while riding the bus and to participate in a raffle for prizes, provided the number of outbursts on the preceding day remained below a specified criterion indicated on the light panel. This intervention resulted in drastic reductions of noise outbursts with a concomitant reduction in other disruptive behaviors. Comparable results were obtained in the second experiment which eliminated the raffle from the intervention
SN - 0021-8855
UR - 7287600
ER -

TY - JOUR
ID - 1066
T1 - An Intervention to Promote Social Emotional School Readiness in Foster Children: Preliminary Outcomes from a Pilot Study
A1 - Pears,Katherine C.
A1 - Fisher,Philip A.
A1 - Bronz,Kimberly D.
Y1 - 2007///
N1 - Last updated - 2012-12-14
SubjectsTermNotLitGenreText - 9261 8620; 8658 8620; 5470; 5446 1970 1; 4151 9806 4911 9556; 3386 5053 2787; 4723; 8168; 8301 3626; 9457 909; 715; 1955 3629 6582; 7941 10836 10839 8699; 7455
KW - ERIC,Current Index to Journals in Education (CIJE)
KW - High Risk Students
KW - Intervention
KW - Attendance
KW - Foster Care
KW - Outcomes of Treatment
KW - Emotional Development
KW - Play Therapy
KW - Comparative Analysis
KW - Prevention
KW - Interpersonal Competence
KW - Reading Readiness
KW - Program Evaluation
KW - Self Control
KW - School Readiness
KW - ERIC
RP - NOT IN FILE
SP - 665
EP - 673
JF - School Psychology Review
VL - 36

IS - 4
PB - National Association of School Psychologists. 4340 East West Highway Suite 402, Bethesda, MD 20814.
N2 - Foster children are at great risk for poor school outcomes. Given that school readiness is a powerful predictor of later school success, the promotion of school readiness skills in foster children is an opportunity for preventive intervention. Results are presented from a preliminary evaluation of a program designed to improve school readiness in foster children. Twenty-four foster children were randomly assigned to the intervention or comparison conditions. The intervention consisted of therapeutic playgroups (twice weekly for 7 weeks during the summer) focusing on social competence and self-regulation skills. Attendance rates for the playgroups are reported. In addition, group differences on data collected before and after the intervention are reported. Intervention group children exhibited increased social competence and self-regulation. Comparison group children exhibited poorer performance in these domains over time. Results are discussed in terms of how the study has informed a current randomized efficacy trial of a school-readiness intervention. (Contains 1 table.)
SN - 0279-6015, 0279-6015
UR - http://search.proquest.com/docview/61947745?accountid=14701
ER -

TY - JOUR
ID - 1067
T1 - Risky Business: Making Inferences about Risk and Its Value. Book Review
A1 - Pellegrini,Anthony D.
Y1 - 1999///
N1 - SuppNotes - Reviews "Risk and Our Pedagogical Relation to Children: On the Playground and Beyond," by Stephen J. Smith
Last updated - 2012-12-14
SubjectsTermNotLitGenreText - 1108 8477; 1472 5058 6996; 1474 316 8016 4542; Play 7940 126; Playgrounds 7943 8704 3760; 8854 6996; Risk 8996
KW - ERIC,Current Index to Journals in Education (CIJE)
KW - Risk
KW - Book Reviews
KW - Play
KW - Research Needs
KW - Playgrounds
KW - Childhood Needs
KW - CHILDREN
KW - ERIC
RP - NOT IN FILE
SP - 435
EP - 438
JF - Early Childhood Research Quarterly
VL - 14
IS - 3
N2 - Evaluates the extent to which Smith's work adds to the theoretical and evidentiary basis for the role of playgrounds in children's education and development. Finds the basic construct of "risk" as presented in the book to be unfocused, and suggests that to make inferences from the work about the value of risk for children is premature. (HTH)
SN - 0885-2006, 0885-2006
UR - http://search.proquest.com/docview/62249384?accountid=14701
ER -

TY - RPRT
ID - 1068
T1 - Developmental and Clinical Aspects of Young Children's Play. WESTAR Series Paper #17
A1 - Pelz,Ruth
Y1 - 1982/10//
N1 - Availability - Level 1 - Available online, if indexed January 1993 onward
SuppNotes - Papers presented at a Symposium sponsored by The Playschool, a HCEEP model demonstration

program (Denver, CO, May 7-8, 1982)
Last updated - 2012-12-14
KW - ERIC,Resources in Education (RIE)
KW - Symbolic Learning
KW - Play
KW - Cognitive Development
KW - Young Children
KW - High Risk Persons
KW - Psychotherapy
KW - Disabilities
KW - Theories
KW - Child Development
KW - Infants
KW - ERIC
RP - NOT IN FILE
SP - 66
N2 - Five papers are presented from a 1982 conference on "Developmental and Clinical Aspects of Young Children's Play." In the first paper ("Cognitive Characteristics of Young Children's Play,") S. Rogers summarizes J. Piaget's theories on developmental stages, discusses the relationship of practice play to learning, and then focuses on the role of symbolic play, with special emphasis on research regarding play of handicapped children. D. Wolf ("Play as a Mirror for Development") considers play as a reflection of the child's development, symbolic growth, and social understanding; and as an indicator of individual differences. G. Farley examines "The Use of Play in Child Psychotherapy with Severely Emotionally Disturbed Preschoolers." I. Bretherton et al. focus on group and individual differences in child's play ("The Effect of Contextual Variation on Symbolic Play: Development from 20 to 28 Months"). In the final paper, "Development of Free Play Behavior in Infancy," R. Harmon and A. Glicken review studies on the free play behavior in normal, abused/neglected, and premature infants 12-18 months old. (CL)
UR - http://search.proquest.com/docview/63494453?accountid=14701
ER -

TY - JOUR
ID - 1069
T1 - Preschool Teachers' Fidelity in Implementing a Comprehensive Language-Rich Curriculum
A1 - Pence,Khara L.
A1 - Justice,Laura M.
A1 - Wiggins,Alice K.
Y1 - 2008/07//
N1 - Last updated - 2012-12-14
SubjectsTermNotLitGenreText - 5800; 3005 9021 9651 6582; 8127 11655 1474 316 8016 4542; 9974 7637 6506 10669; 8135 10591 8267 3417 8016 4542 5703 4908 8917; 10621 3227 6582; 694 8016 4542; 4723
KW - ERIC,Current Index to Journals in Education (CIJE)
KW - Practitioners
KW - Preschool Teachers
KW - Language Usage
KW - At Risk Persons
KW - High Risk Students
KW - Speech Language Pathology
KW - Dramatic Play
KW - Preschool Children
KW - Teaching Methods
KW - ERIC
RP - NOT IN FILE
SP - 329
EP - 341
JF - Language, Speech, and Hearing Services in Schools
VL - 39

IS - 3
PB - American Speech-Language-Hearing Association (ASHA). 10801 Rockville Pike, Rockville, MD 20852.
N2 - Purpose: This study examined preschool teachers' fidelity to the language-focused curriculum (LFC; B. Bunce, 1995), a comprehensive classroom curriculum designed to improve at-risk children's language outcomes through targeted improvements to a classroom's activity contexts (e.g., dramatic play, art, storybook reading) and instructional processes (e.g., teacher use of open-ended questions, recasts, and expansions). Specific aims included to (a) examine program differentiation by determining how measures of activity contexts and instructional processes differentiated treatment and comparison teachers, (b) determine treatment teachers' adherence to both activity contexts and instructional processes over an entire academic year, and (c) determine treatment teachers' reported quality of program delivery and comfort with curriculum implementation. Method: Fourteen preschool teachers were randomly assigned to implement the LFC or to maintain their prevailing curriculum. Fidelity was measured 3 times over an academic year using a curriculum fidelity checklist. Results: LFC teachers exhibited fidelity to activity contexts more readily than to instructional processes. Teacher use of language-focused instructional processes was relatively low even after a year of LFC implementation. Conclusion: This study supports the need for speech-language pathologists to work closely with preschool educators to implement the activity contexts and instructional processes associated with high-quality preschool language-learning environments
SN - 0161-1461, 0161-1461
UR - http://search.proquest.com/docview/61994339?accountid=14701
ER -

TY - JOUR
ID - 1070
T1 - The Development of Effortful Control in Children Born Preterm
A1 - Poehlmann,Julie
A1 - Schwichtenberg,J.A.
A1 - Shah,Prachi E.
A1 - Shlafer,Rebecca J.
A1 - Hahn,Emily
A1 - Maleck,Sarah
Y1 - 2010///
N1 - Last updated - 2012-12-14
SubjectsTermNotLitGenreText - 6175 1326 3629 6582 8836; 5118 11655 1474 316 8016 4542; 10883 11655 1474 316 8016 4542; 9457 909; 8105; 8996; 2746 8415; 1703 5053 2787; 8112 5118 11655 1474 316 8016 4542; 7940 126; 7537 3851 5449 8768; 8415; 6823 3932 8016 4542 7577 3816; 724 2877; 1701 1; 3821; 4634
KW - ERIC,Current Index to Journals in Education (CIJE)
KW - Family Characteristics
KW - Toddlers
KW - Play
KW - Cognitive Development
KW - Depression (Psychology)
KW - Mothers
KW - Health
KW - Longitudinal Studies
KW - Pregnancy
KW - Risk
KW - Cognitive Ability
KW - Psychological Patterns
KW - Parent Child Relationship
KW - Premature Infants
KW - Attention Deficit Disorders
KW - Self Control
KW - Infants
KW - ERIC
RP - NOT IN FILE
SP - 522

EP - 536
JF - Journal of Clinical Child and Adolescent Psychology
VL - 39
IS - 4
PB - Routledge. , 325 Chestnut Street Suite 800, Philadelphia, PA 19106.
N2 - This prospective longitudinal study examined emerging effortful control skills at 24- and 36-months postterm in 172 children born preterm (less than 36 weeks gestation). Infant (neonatal health risks), family (sociodemographic risks), and maternal risk factors (depressive symptoms, anger expressions during play interactions) were assessed at six time points across 3 years. In addition, children's emerging effortful control skills, cognitive development, and mother-reported behavior and attention problems were assessed at 24 and 36 months. Analyses documented links between effortful control skills, cognitive skills, and concurrent attention problems in children born preterm. The study also found that preterm children's effortful control skills improved over time. In addition, neonatal health risks, family sociodemographic risks, and angry parenting interactions were associated with less optimal effortful control skills. (Contains 2 tables and 1 figure.)
SN - 1537-4416, 1537-4416
UR - http://search.proquest.com/docview/754908508?accountid=14701
ER -

TY - JOUR
ID - 1071
T1 - Infant Physiological Regulation and Maternal Risks as Predictors of Dyadic Interaction Trajectories in Families with a Preterm Infant
A1 - Poehlmann,Julie
A1 - Schwichtenberg,J.A.
A1 - Bolt,Daniel M.
A1 - Hane,Amanda
A1 - Burnson,Cynthia
A1 - Winters,Jill
Y1 - 2011/01//
N1 - Last updated - 2012-12-14
SubjectsTermNotLitGenreText - 6562; 6823 3932 8016 4542 7577 3816; 8112 5118 11655 1474 316 8016 4542; 11655 1474 316 8016 4542; 8996; 9753 5053 2787; 5446 1970 1; 7888 994 6976 9351 5964; 4858 5241; 9835 10103; 7537 3851 5449 8768; 7940 126; 722; 8099; 6175 1326 3629 6582 8836
KW - ERIC,Current Index to Journals in Education (CIJE)
KW - Play
KW - Young Children
KW - Socioeconomic Status
KW - Mothers
KW - Physiology
KW - Longitudinal Studies
KW - Risk
KW - Interpersonal Competence
KW - Parent Child Relationship
KW - Premature Infants
KW - Social Development
KW - Attention
KW - Metabolism
KW - Predictor Variables
KW - Hospitals
KW - ERIC
RP - NOT IN FILE
SP - 91
EP - 105
JF - Developmental Psychology
JA - Dev.Psychol.
VL - 47

IS - 1
PB - American Psychological Association. Journals Department, 750 First Street NE, Washington, DC 20002-4242.
N2 - This longitudinal study examined predictors of rates of growth in dyadic interaction quality in children born preterm who did not experience significant neurological findings during neonatal intensive care unit (NICU) hospitalization. Multiple methods were used to collect data from 120 preterm infants (48% girls, 52% boys) and their mothers. Infant heart rate variability (HRV), gestational age, neonatal health, feeding route, and maternal socioeconomic (SES) risks were assessed at NICU discharge (mean of 36 weeks postconception). Mother-child interactions were observed at 4, 9, 16, and 24 months postterm and analyzed with hierarchical linear modeling. On average, children's quality of play, interest, and attention increased over time while their dysregulation and irritability decreased, whereas average maternal positive affect and involvement declined in quality (ps less than 0.05), although there was individual variation in rates of change. Mothers of infants with higher postfeeding HRV (i.e., vagal regulation) exhibited less decrease in positive affect and involvement between 4 months and 24 months, compared with mothers of infants with lower HRV (p less than 0.05). Although infants with higher postfeeding HRV showed less positive affect and communication at 4 months, they exhibited significantly greater increases in positive affect and social competence and decreases in dysregulation and irritability between 4 months and 24 months, compared with infants with lower HRV (ps less than 0.05). Dyads experiencing more SES risks showed less optimal interactions at 4 months; this difference remained as children grew older (ps less than 0.05). Results have implications for our understanding of social development in preterm infants. (Contains 2 footnotes, 6 tables, and 1 figure.)
SN - 0012-1649, 0012-1649
UR - http://search.proquest.com/docview/854552974?accountid=14701
ER -

TY - JOUR
ID - 1072
T1 - Effects of combat on a delinquent boy
A1 - Miller,Milton L.
Y1 - 1948///
N1 - Peer Reviewed Journal: 2013-38237-016
English
Journal; Peer Reviewed Journal
KW - PsycInfo
KW - Rough and Tumble
KW - Combat Experience
KW - Juvenile Delinquency
KW - Friendship
KW - Homosexuality
KW - Anxiety Sensitivity
RP - NOT IN FILE
SP - Apr
EP - 339
JF - American Journal of Orthopsychiatry
JA - Am J Orthopsychiatry
VL - .18
IS - 2
N2 - This article discusses the effects of combat on a delinquent boy, Nick. Nick, a gunner who was suffering from severe anxiety reactions, was seen at a convalescent center. From age seven to sixteen he had been the leader of a boys' gang, and at one time had spent a year in a reformatory for stealing. On release he went to work, his delinquent activities ceased, and he married happily. Nick served as a gunner on a Flying Fortress and went through many harrowing experiences. He returned wearing several rows of decorations, a national hero, with his picture in the newspapers but was in a state of severe depression. Now, in the convalescent hospital, his attitude toward those who tried to help him was defiant and belligerent. His relationship with the men overseas had been very warm and friendly just as with members of the gang he formerly led. At the same time his relationship with the boys was tinged with sublimated homosexuality-close friendships with much kidding, cajoling, and horseplay. He came to the psychiatrist's office for his second interview drinking a glass of milk

and began spontaneously to talk about his lost buddies. During a session under sodium pentothal sedation, Nick talked about some of the worst combat scenes. His attitude of great faithfulness toward his friends was outstanding, as was also his preoccupation with death. When this patient saw the reality of unleashed hostility in war, his own hostility was mobilized, he was terrified, torn between hostility and extreme guilt and self-destructive drives. (PsycINFO Database Record (c) 2013 APA, all rights reserved)
SN - 0002-9432
ER -

TY - RPRT
ID - 1073
T1 - Child-Centered Play Therapy: Working with At-Risk Youth in the Elementary School Setting
A1 - Post,Phyllis
A1 - Stopanio,Joe
A1 - Fielden,Ashley
Y1 - 1998///
N1 - Availability - Level 1 - Available online, if indexed January 1993 onward
Last updated - 2012-12-14
KW - ERIC,Resources in Education (RIE)
KW - Higher Education
KW - Intermediate Grades
KW - Play Therapy
KW - High Risk Students
KW - Graduate Students
KW - Elementary School Students
KW - Counselor Training
KW - ERIC
RP - NOT IN FILE
SP - 13
N2 - This paper details a project in which a counselor educator and 12 counseling graduate students, trained in child-centered play therapy, worked in an inner city school with 4th-6th grade students during the 1996-1997 academic year. The play therapy training undergone by the graduate students and the facility used for the therapy sessions are described. The paper presents issues in establishing the program. The following conclusions were drawn from the year-long experience: (1) working with at-risk children has the obvious advantage of access to the children, because they are in school for many days making it possible for counselors to reach children who would not attend counseling outside school; (2) play did seem to be an effective approach with these older at-risk children; (3) most of the children demonstrated interest in exploring a variety of the toys; and (4) child-centered play therapy was well accepted by these students. Communication with administrators, teachers and parents to help them understand more about play therapy and its value is essential; privacy is critical; and because the word "play" created difficulties, using the words "counseling" or "developmental counseling" may make the sessions seem more important to others. (MKA)
UR - http://search.proquest.com/docview/62542025?accountid=14701
ER -

TY - JOUR
ID - 1074
T1 - Strengthening Play through Father Involvement
A1 - Pruett,Kyle
Y1 - 2009/09//
N1 - Last updated - 2012-12-14
SubjectsTermNotLitGenreText - 7940 126; 11655 1474 316 8016 4542; 3896 7577 3816 8016 4542 6265; 7537 3851 5449 8768; 1443 5053 2787; 694 8016 4542; 3851 5449 8768
KW - ERIC,Current Index to Journals in Education (CIJE)
KW - Early Childhood Education
KW - At Risk Persons
KW - Play
KW - Young Children

KW - Parent Child Relationship
KW - Child Development
KW - Family Relationship
KW - Fathers
KW - ERIC
RP - NOT IN FILE
SP - 40
EP - 41
JA - Zero to Three
VL - 30
IS - 1
PB - Zero to Three. 2000 M Street NW Suite 200, Washington, DC 20036-3307.
N2 - In his essay exploring the latest research finding on the importance of men in the lives of young children, the author describes two ongoing empirical studies that are proving particularly instructive in understanding the significance of paternal contributions to improving young child outcomes. Both projects are encouraging direct paternal play engagement in at-risk populations and discovering positive outcomes for families
SN - 0736-8038, 0736-8038
UR - http://search.proquest.com/docview/61822285?accountid=14701
ER -

TY - JOUR
ID - 1075
T1 - Changing Antisocial Behavior Patterns in Young Boys: A Structured Cooperative Learning Approach
A1 - Quinn,Mary Magee
Y1 - 2002///
N1 - SuppNotes - Special Issue: Severe Behavior Disorders of Children and Youth
Last updated - 2012-12-14
SubjectsTermNotLitGenreText - 28 96; 545 9738 909; Behavior Disorders 913 2877; Behavior Modification 914 2094; Cooperative Learning 2225 5882; 3352 3368 3150; 4413 5264; 4723; 5248; 6265 8016 4542; Peer Teaching 7678 10621 3227 6582; 7940 126; 7943 8704 3760; Prevention 8168; 9753 5053 2787; 10867
KW - ERIC,Current Index to Journals in Education (CIJE)
KW - Elementary Education
KW - Grade 1
KW - Instructional Effectiveness
KW - High Risk Students
KW - Play
KW - Cooperative Learning
KW - MALES
KW - Peer Teaching
KW - Academic Achievement
KW - Antisocial Behavior
KW - Prevention
KW - Behavior Disorders
KW - Playgrounds
KW - Behavior Modification
KW - Social Development
KW - Time on Task
KW - ERIC
RP - NOT IN FILE
SP - 380
EP - 395
JF - Education and Treatment of Children
VL - 25
IS - 4
N2 - A six-week cooperative learning intervention using positive peer role models was used to teach interpersonal problem-solving skills to 42 first-grade boys at risk of antisocial behavior patterns. Participants

showed an increase in academic engaged time. However, the intervention failed to produce significantly lower rates of externalizing antisocial behaviors or negative playground interactions. (Contains references.) (Author/CR)
SN - 0748-8491, 0748-8491
UR - http://search.proquest.com/docview/62215046?accountid=14701
ER -

TY - JOUR
ID - 1076
T1 - Two Counseling Interventions to Reduce Teacher-Child Relationship Stress
A1 - Ray,Dee C.
Y1 - 2007/04//
N1 - Last updated - 2012-12-14
SubjectsTermNotLitGenreText - 3363 10278 8016 4542; 8127 11655 1474 316 8016 4542; 3366 9306 5241; 4723; 10197 2472 2842 10187; 7455; 2293; 10239 8234; 9195 9224 4552 2291; 7941 10836 10839 8699; 3281 10087 2574 3629 6582; 10482 730; 2302 6582; 10576 5449 8768; 2159 9945 8016 4542; 5051; 10151
KW - ERIC,Current Index to Journals in Education (CIJE)
KW - Counselors
KW - Elementary Education
KW - Teacher Student Relationship
KW - High Risk Students
KW - Elementary School Students
KW - Consultants
KW - Individual Characteristics
KW - Teacher Attitudes
KW - Student Diversity
KW - Stress Management
KW - Outcomes of Treatment
KW - Play Therapy
KW - Counseling Techniques
KW - Counseling Effectiveness
KW - Student Problems
KW - Preschool Children
KW - School Counseling
KW - Effect Size
KW - Elementary Schools
KW - ERIC
RP - NOT IN FILE
SP - 428
EP - 440
JF - Professional School Counseling
VL - 10
IS - 4
PB - American School Counselor Association. 1101 King Street Suite 625, Alexandria, VA 22314.
N2 - This article discusses a study investigating the impact of two school counseling interventions, child-centered play therapy (CCPT) and teacher consultation, on teacher-child relationship stress. CCPT and teacher consultation were conducted with 93 (pre-kindergarten to fifth grade) elementary school students across three elementary schools deemed to be at risk and ethnically diverse. Students were divided into three treatment groups: CCPT only, teacher consultation only, and a combined CCPT and teacher consultation group. Results demonstrated significant differences between pretesting and posttesting on the Index of Teaching Stress. All treatment groups were found to have statistically significant reduction in teacher and student problem characteristics with large effect sizes for total stress. (Contains 2 tables and 1 figure.)
SN - 1096-2409, 1096-2409
UR - http://search.proquest.com/docview/62038076?accountid=14701
ER -

TY - JOUR
ID - 1077
T1 - Gutter to Garden: Historical Discourses of Risk in Interventions in Working Class Children's Street Play
A1 - Read,Jane
Y1 - 2011/11//
N1 - Last updated - 2012-12-14
SubjectsTermNotLitGenreText - 11581 9744 8016 4542; 7940 126; 3085 3150; 1485; 2492 2472 2842; 5470; 4109 4335; 5649 5264; 694 8016 4542; 8462 7970; 125 9738 909; 11162 4335; 5015; 1541 10103
KW - United Kingdom
KW - ERIC,Current Index to Journals in Education (CIJE)
KW - Kindergarten
KW - At Risk Persons
KW - Cultural Pluralism
KW - Citizenship
KW - Play
KW - Intervention
KW - Early Childhood Education
KW - Public Policy
KW - Working Class
KW - Foreign Countries
KW - Urban Areas
KW - Inclusion
KW - Childrens Rights
KW - Activism
KW - ERIC
RP - NOT IN FILE
SP - 421
EP - 434
JF - Children & Society
VL - 25
IS - 6
PB - Wiley-Blackwell. 350 Main Street, Malden, MA 02148.
N2 - This article investigates interventions in the gutter play of British working class children in the first decade of the 20th century through their re-location within Free Kindergartens. In contemporary literature, the street child was viewed through a binary lens, as both "at risk" and "as risk", reflecting wider societal discourses in a period of rapidly developing social policy. The paper interrogates the motivations of free kindergarten activists from the standpoint of a range of theory and builds on recent papers discussing 21st century urban childhoods. The findings suggest both historical continuities and discontinuities in the theorisation of risk, which have implications for current social policy, urban design and early childhood education. The questions raised include issues of children's rights, citizenship, inclusion and cultural diversity
SN - 0951-0605, 0951-0605
UR - http://search.proquest.com/docview/964171426?accountid=14701
ER -

TY - JOUR
ID - 1078
T1 - Effects of Modern Educational Game Play on Attitudes towards Mathematics, Mathematics Self-Efficacy, and Mathematics Achievement
A1 - Ritzhaupt,Albert
A1 - Higgins,Heidi
A1 - Allred,Beth
Y1 - 2011///
N1 - Last updated - 2012-12-14
SubjectsTermNotLitGenreText - 6644 10278 8016 4542; 7940 126; 3132 10103; 10181 730; 3206 4270 126; 9466 9469 9451; 6411 96; 2074 2073 10675; 2328 4335; 10759 6388 2787; 4290; 8550 2842; 3268 10669; 695 694 8016 4542; 727 1387; 76; 11255 4270 126

KW - United States (Southeast)
KW - Elementary Secondary Education Act Title I
KW - ERIC,Current Index to Journals in Education (CIJE)
KW - Middle Schools
KW - Video Games
KW - Play
KW - Gender Differences
KW - Mathematics Achievement
KW - Middle School Students
KW - Counties
KW - Racial Differences
KW - Attitude Change
KW - Computer Uses in Education
KW - At Risk Students
KW - Student Attitudes
KW - Educational Games
KW - Access to Computers
KW - Self Efficacy
KW - Economic Status
KW - Educational Technology
KW - Test Construction
KW - ERIC
RP - NOT IN FILE
SP - 277
EP - 297
JF - Journal of Interactive Learning Research
VL - 22
IS - 2
PB - Association for the Advancement of Computing in Education. P.O. Box 1545, Chesapeake, VA 23327-1545.
N2 - This study investigated the effects of modern educational game playing on middle school students' attitudes towards mathematics, mathematics self-efficacy, and mathematics achievement. Two hundred twenty-five middle school students from four different Title 1 schools in two different counties in the southeastern United States were recruited. Students participated in a quasi-experimental pre-test and post-test design with 16-weeks of the game intervention and a minimum of one session of game play per week. The students' gender, socio-economic status (SES), ethnicity, frequency of game play, and frequency of computer use were examined as moderating variables and covariates. ANCOVA detected significant and positive changes in students' attitudes towards mathematics and mathematics self-efficacy. However, there was no significant change in students' mathematics achievement. A students' gender, SES, and frequency of computer use did not significantly interact with any of the criterion measures. Discussion and recommendations for future research are provided. (Contains 5 tables and 1 figure.)
SN - 1093-023X, 1093-023X
UR - http://search.proquest.com/docview/898323364?accountid=14701
ER -

TY - JOUR
ID - 1079
T1 - What Play Therapists Do within the Therapeutic Relationship of Humanistic/Non-Directive Play Therapy
A1 - Robinson,Sally
Y1 - 2011///
N1 - Last updated - 2012-12-14
SubjectsTermNotLitGenreText - 4109 4335; 7941 10836 10839 8699; 415 4657 3417 8016 4542 5703 4908 8917; 9015; 694 8016 4542; 10834 3518; 3387 6531 2877; 8415; 11465 8522; 2310 5449 8768; 11227 1849; 3400 8415; 636 10839; 9473; 3363 10278 8016 4542
KW - ERIC,Current Index to Journals in Education (CIJE)
KW - Elementary Education

KW - At Risk Persons
KW - Allied Health Personnel
KW - Elementary School Students
KW - Counselor Client Relationship
KW - Well Being
KW - Emotional Disturbances
KW - Play Therapy
KW - Verbal Communication
KW - Foreign Countries
KW - Psychological Patterns
KW - Art Therapy
KW - Role
KW - Self Expression
KW - Empathy
KW - Therapeutic Environment
KW - ERIC
RP - NOT IN FILE
SP - 207
EP - 220
JF - Pastoral Care in Education
VL - 29
IS - 3
PB - Routledge. , 325 Chestnut Street Suite 800, Philadelphia, PA 19106.
N2 - Play therapists are increasingly being employed in schools, yet there is confusion among many health, education and social care practitioners about the role of play therapists. This paper explains how play therapists position themselves and what they do through an examination of the therapeutic relationship between the therapist and child. It discusses the core conditions of congruence, acceptance and empathy with reference to recent research. Play therapists vary their practice in terms of verbal or non-verbal interaction, the tools in their playroom and how they physically place themselves. This paper argues for placing an emphasis on the non-verbal mirroring of the child, the incorporation of expressive media such as paint, clay and sand into the play room and the positioning of the therapist within the play space
SN - 0264-3944, 0264-3944
UR - http://search.proquest.com/docview/964173714?accountid=14701
ER -

TY - JOUR
ID - 1080
T1 - Bullying and coercion: School-based cognitive-behavioral interventions. [References]
A1 - Doll,Beth
Y1 - 2012///
N1 - Book: 2012-04950-012
English
Book; Edited Book
KW - PsycInfo
KW - Rough and Tumble
KW - Aggressive Behavior
KW - Cognitive Behavior Therapy
KW - Peer Relations
KW - School Based Intervention
KW - Bullying
KW - Coercion
KW - Peers
RP - NOT IN FILE
SP - 754
JF - Mennuti, Rosemary B [Ed]; Christner, Ray W [Ed]; Freeman, Arthur [Ed]
IS - 2012

N2 - (from the chapter) Peer aggression is an all-too-common occurrence in children's daily lives. The nature and frequency of children's peer conflicts shift and change across the developmental stages, such that participation in physical aggression is quite common among toddlers, and participation in verbal and relational aggression becomes quite common in adolescence. In this rough-and-tumble world of children's peer interactions, the ability to cope with and respond effectively to aggressive overtures is a critical adaptive skill. In some instances, however, the form and intensity of peer aggression make it unlikely that children can respond effectively, and it is unreasonable to expect them to do so. This chapter will describe one form of peer aggression-bullying-that is never adaptive, and is often quite damaging to the children who bully and to those who are bullied. Bullying will be described as a cognitive-behavioral phenomenon that is part of the broader framework of childhood aggression and that occurs within a social-ecological context. (PsycINFO Database Record (c) 2013 APA, all rights reserved)
AD - Doll, Beth: College of Education and Human Science, University of Nebraska-Lincoln, Lincoln, NE, US Swearer, Susan M.: University of Nebraska-Lincoln, Lincoln, NE, US Collins, Adam M.: School Psychology Program, University of Nebraska-Lincoln, Lincoln, NE, US Chadwell, Mindy R.: School Psychology Program, University of Nebraska-Lincoln, Lincoln, NE, US Dooley, Kadie: University of Nebraska-Lincoln, Lincoln, NE, US Chapla, Brooke A.: University of Nebraska-Lincoln, Lincoln, NE, US
ER -

TY - RPRT
ID - 1081
T1 - Playing and Learning Together: Patterns of Social Interaction in Handicapped and Nonhandicappped Children
A1 - Rogers-Warren,Ann
Y1 - 1980///
N1 - Availability - Level 1 - Available online, if indexed January 1993 onward
Last updated - 2012-12-14
KW - ERIC,Resources in Education (RIE)
KW - Preschool Education
KW - Teacher Role
KW - Moderate Mental Retardation
KW - Play
KW - High Risk Persons
KW - Social Behavior
KW - Mild Mental Retardation
KW - Mainstreaming
KW - Intervention
KW - Peer Relationship
KW - Interpersonal Competence
KW - Disabilities
KW - Down Syndrome
KW - ERIC
RP - NOT IN FILE
SP - 22
N2 - Two projects at the Kansas Early Childhood Institute investigated characteristics of social interaction by handicapped, at risk, and nonhandicapped children. The first project examined patterns of social interaction and play behavior among preschool children in an integrated classroom. A longitudinal observation of four mild to moderately retarded Down's syndrome children (5 to 6.5 years old) and four nonhandicapped Ss (4.5 to 5 years old) was conducted. Results indicated that handicapped and nonhandicapped children preferred the same types of activities. Handicapped children most frequently selected handicapped playmates, and nonhandicapped children usually selected nonhandicapped playmates. Handicapped Ss spent more time engaged in solitary play than nonhandicapped Ss. The second project described the specific social skills and interaction parameters exhibited by 15 normal, at risk, and handicapped preschoolers in a mainstreamed classroom. Handicapped children typically showed delays in social interaction skills commensurate with their general developmental delays; however, there was considerable variability across all types of Ss. Results suggest that altering either the settings or consequences for social interaction will result in increased interactions. Activities that bring the children into physical proximity and play equipment that require more than one child can enhance social

interactions. Directly prompting or instructing a child to interact with another child or to enter an activity are also frequently successful. In some cases, teacher praise or the contingent provision of some desirable activity or object when social interaction occurs are useful strategies. (Author/SEW)
UR - http://search.proquest.com/docview/63475360?accountid=14701
ER -

TY - JOUR
ID - 1083
T1 - Categorising Risky Play--How Can We Identify Risk-Taking in Children's Play?
A1 - Sandseter,Ellen Beate Hansen
Y1 - 2007/06//
N1 - Last updated - 2012-12-14
SubjectsTermNotLitGenreText - 7940 126; 11655 1474 316 8016 4542; 8135 10591 8267 3417 8016 4542 5703 4908 8917; 8996; 5472 3629 6582; 4109 4335; 8127 11655 1474 316 8016 4542; 7234; 8517 8836
KW - Structured Interviews
KW - Norway
KW - ERIC,Current Index to Journals in Education (CIJE)
KW - Early Childhood Education
KW - Preschool Education
KW - Qualitative Research
KW - Preschool Teachers
KW - Risk
KW - Foreign Countries
KW - Play
KW - Young Children
KW - Observation
KW - Interviews
KW - Preschool Children
KW - ERIC
RP - NOT IN FILE
SP - 237
EP - 252
JA - European Early Childhood Education Research Journal
VL - 15
IS - 2
PB - Routledge. , 325 Chestnut Street Suite 800, Philadelphia, PA 19106.
N2 - There is a growing debate on the balance between making sure our children are safe versus letting the children play in physically and emotionally stimulating and challenging environments. The focus is now on children's right to do risky play. There are no studies categorising risky play. The present study has aimed to do this. Qualitative observations of 38 children and semi-structured interviews with 8 children and 7 employees from two Norwegian preschools gave 6 categories of risky play: 1) Play with great heights; 2) Play with high speed; 3) Play with harmful tools; 4) Play near dangerous elements; 5) Rough-and-tumble play; and 6) Play where the children can "disappear"/get lost. The reliability of the analysed categories was tested through a second opinion made by an experienced preschool teacher, who has long and varied experience with children's play in preschools
SN - 1350-293X, 1350-293X
UR - http://search.proquest.com/docview/61885365?accountid=14701
ER -

TY - JOUR
ID - 1084
T1 - The archaeology of mind: Neuroevolutionary origins of human emotion. [References]
A1 - Panksepp,Jaak
Y1 - 2012///
N1 - Book: 2012-02256-000
English

Book; Authored Book
(Created by PsycINFO) Preface and acknowledgments Foreword by Daniel J. Siegel Ancestral passions The evolution of affective consciousness: Studying emotional feelings in other animals The SEEKING system: Brain sources of eager anticipation, desire, euphoria, and the quest for everything The ancestral sources of RAGE The ancestral roots of FEAR Beyond instincts: Learning and the affective foundations of memory LUSTful passions of the mind: From reproductive urges to romantic love Nurturing love: The CARE System Born to cry: The PANIC/GRIEF system and the genesis of life-sustaining social bonds PLAYful dreamlike circuits of the brain: The ancestral sources of social joy and laughter Toward a neurobiology of the soul: The core SELF and the genesis of primary-process feelings Brain emotional systems and affective qualities of mental life: From animal affects to human psychotherapeutics Philosophical reflections and complaints: Can we go from mice to men and back again? References Index
KW - PsycInfo
KW - Rough and Tumble
KW - Brain
KW - Emotions
KW - Mind
KW - Theory of Evolution
KW - Cognitive Neuroscience
KW - Affective Disorders
KW - Emotional Disturbances
KW - Emotional States
KW - Mental Disorders
RP - NOT IN FILE
SP - 562
JF - (2012)
VL - The archaeology of mind
N2 - (from the jacket) What makes us happy? What makes us sad? How do we come to feel a sense of enthusiasm? What fills us with lust, anger, fear, or tenderness? Traditional behavioral and cognitive neuroscience have yet to provide satisfactory answers. The Archaeology of Mind presents an affective neuroscience approach-which takes into consideration basic mental processes, brain functions, and emotional behaviors that all mammals share-to locate the neural mechanisms of emotional expression. It reveals-for the first time-the deep neural sources of our values and basic emotional feelings. This book elaborates on the seven emotional systems that explain how we live and behave. These systems originate in deep areas of the brain that are remarkably similar across all mammalian species. When they are disrupted, we find the origins of emotional disorders: (1) SEEKING: how the brain generates a euphoric and expectant response; (2) FEAR: how the brain responds to the threat of physical danger and death; (3) RAGE: sources of irritation and fury in the brain; (4) LUST: how sexual desire and attachments are elaborated in the brain; (5) CARE: sources of maternal nurturance; (6) GRIEF: sources of non-sexual attachments; (7) PLAY: how the brain generates joyous, rough-and-tumble interactions; and (8) SELF: a hypothesis explaining how affects might be elaborated in the brain The book offers an evidence-based evolutionary taxonomy of emotions and affects and, as such, a brand-new clinical paradigm for treating psychiatric disorders in clinical practice. (PsycINFO Database Record (c) 2012 APA, all rights reserved)
AD - Panksepp, Jaak: Center for the Study of Animal Well-Being, Department of Veterinary Comparative Anatomy, Pharmacology, and Physiology, College of Veterinary Medicine, Washington State University, Pullman, WA, US Biven, Lucy: Department of Psychotherapy, Child and Adolescent Mental Health Service, National Health Service, LEC, England
ER -

TY - JOUR
ID - 1085
T1 - Children's Expressions of Exhilaration and Fear in Risky Play
A1 - Sandseter,Ellen Beate Hansen
Y1 - 2009///
N1 - Last updated - 2012-12-14
SubjectsTermNotLitGenreText - 4109 4335; 11655 1474 316 8016 4542; 7940 126; 7841 909; 3899 8415; 8996; 5183 2877; 9015; 1463 9074; 9473; 2798; 8127 11655 1474 316 8016 4542; 8130 3085 3150

KW - Norway
KW - ERIC,Current Index to Journals in Education (CIJE)
KW - Early Childhood Education
KW - Preschool Education
KW - Play
KW - Injuries
KW - Young Children
KW - Fear
KW - Child Safety
KW - Risk
KW - Foreign Countries
KW - Developmental Stages
KW - Role
KW - Self Expression
KW - Preschool Children
KW - Physical Activity Level
KW - ERIC
RP - NOT IN FILE
SP - 92
EP - 106
JF - Contemporary Issues in Early Childhood
VL - 10
IS - 2
PB - Symposium Journals. P.O. Box 204, Didcot, Oxford, OX11 9ZQ, UK. Tel: +44-1235-818-062; Fax: +44-1235-817-275; e-mail: subscriptions@symposium-journals.co.uk; Web site: http://www.wwwords.co.uk/ciec
N2 - Children naturally seek and conduct exciting forms of play that involve a risk of physical injury (risky play). Even though several prior studies give descriptions of risky play, none of them deeply explore children's expressions of how they experience different kinds of risky play. This study aims to do that. The results from video observations of children's risky play in two Norwegian preschools reveal that children experience several emotions, expressed bodily, facially, and verbally, while engaging in risky play. Their experiences include both pure exhilaration and pure fear, and quite often both emotions are present at the same time. The findings also indicate that one of the main aspects of risky play is to keep the exhilaration bordering on the feeling of pure fear; but if pure fear occurs, the play ends with withdrawal. Suggested implications of the study are that risk taking should be acknowledged as an important part of children's play, and that children should be able to engage in challenging play adjusted to their individual sense of risk and urge for exhilaration. (Contains 1 table.)
SN - 1463-9491, 1463-9491
UR - http://search.proquest.com/docview/742865236?accountid=14701
ER -

TY - JOUR
ID - 1086
T1 - "It Tickles in My Tummy": Understanding Children's Risk-Taking in Play through Reversal Theory
A1 - Sandseter,Ellen Beate Hansen
Y1 - 2010///
N1 - Last updated - 2012-12-14
SubjectsTermNotLitGenreText - 7940 126; 3395 8930 909; 5472 3629 6582; 8996; 10181 730; 7802 7807 4918 5964; 3899 8415; 619 915; 728 6447; 4109 4335
KW - Structured Interviews
KW - Norway
KW - ERIC,Current Index to Journals in Education (CIJE)
KW - Preschool Education
KW - Arousal Patterns
KW - Risk
KW - Foreign Countries
KW - Play
KW - Student Attitudes

KW - Fear
KW - Emotional Response
KW - Attitude Measures
KW - Interviews
KW - Phenomenology
KW - ERIC
RP - NOT IN FILE
SP - 67
EP - 88
JA - Journal of Early Childhood Research
VL - 8
IS - 1
PB - SAGE Publications. 2455 Teller Road, Thousand Oaks, CA 91320.
N2 - "It tickles in my tummy" is one of the most frequent answers when children are asked to describe what they experience when engaging in risky play. Why do children take risks in spite of the fact that this can be a harmful and even fatal activity? This article aims to explore this issue. Semi-structured interviews of 23 pre-school children aged four and five years were analyzed on the basis of the concepts of paratelic and telic states in Reversal Theory, the paratelic state being an excitement-seeking mode, and the telic state being an anxiety-avoiding mode. The phenomenological structure of children's risky play was found to be characterized by children expressing the ambiguous nature of quick reversals between the paratelic and telic states, producing both pleasant and unpleasant emotions. This "walk on the dangerous edge" seemed to be one of the primary goals of engaging in risky play. As long as the paratelic state dominated the situation, children embraced the potential fear by arousal-increasing strategies and maintained play. If the telic state dominated the situation, children described arousal-avoidance strategies and ultimately withdrew from play. (Contains 1 figure.)
SN - 1476-718X, 1476-718X
UR - http://search.proquest.com/docview/61804170?accountid=14701
ER -

TY - BOOK
ID - 1089
T1 - Contemporary Perspectives in Literacy in Early Childhood Education. Contemporary Perspectives in Early Childhood Education
A1 - Saracho,Olivia N.
A1 - Spodek,Bernard
Y1 - 2002///
N1 - Availability - Level 2 - Produced in microfiche (1966-2003)
Last updated - 2012-12-14
KW - ERIC,Resources in Education (RIE)
KW - Early Childhood Education
KW - Teacher Role
KW - Preschool Teachers
KW - Context Effect
KW - At Risk Persons
KW - Play
KW - Family Environment
KW - Young Children
KW - Story Reading
KW - Educational Environment
KW - Research Needs
KW - Reading Instruction
KW - Parent Student Relationship
KW - Parent School Relationship
KW - Educational Practices
KW - Theories
KW - Emergent Literacy
KW - Parent Attitudes

KW - Parents as Teachers
KW - Beliefs
KW - Family Literacy
KW - ERIC
RP - NOT IN FILE
SP - 197
PB - Information Age Publishing, , 80 Mason Street, P.O. Box 4967, Greenwich, CT 06831 (paper: ISBN-1-930608-28-4, $29.95; cloth: ISBN-1-930608-29-2, $63.25).
N2 - This book identifies and analyzes research studies related to the critical skills, environments, and adult interactions that contribute to young children's literacy development. Following introductory remarks on the reformation that has emerged in the language and literacy education of young children, the chapters are: (1) "Teachers' Roles in Promoting Literacy-Related Play" (Olivia N. Saracho); (2) "Hypermediating Literacy Activity: How Learning Contexts Get Recognized" (Kris Gutierrez and Lynda Stone); (3) "Creating Opportunities for Discourse: Language and Literacy Development for At-Risk Children" (Barbara Wasik, Mary Alice Bond, and Annemarie Hindman); (4) "Storybook Reading: What We Know and What We Should Consider" (Jon Shapiro, Jim Anderson, and Ann Anderson); (5) "New Directions in Family Literacy: Building Effective Partnerships between Home and School" (Trevor H. Cairney); (6) "Engaging Children in the Appropriation of Literacy: The Importance of Parental Beliefs and Practices" (Susan Sonnenschein); (7) "Promising Perspectives and Practices in Family Literacy" (Olivia N. Saracho); and (8) "Contemporary Views of Research and Practice in Early Childhood Literacy Programs" (Olivia N. Saracho and Bernard Spodek). Each chapter contains references. (KB)
SN - 1930608284
UR - http://search.proquest.com/docview/62195721?accountid=14701
ER -

TY - JOUR
ID - 1090
T1 - Play and interpersonal processes. [References]
A1 - Jent,Jason F.
Y1 - 2011///
N1 - Book: 2011-14208-012
English
Book; Edited Book
KW - PsycInfo
KW - Rough and Tumble
KW - Childhood Play Behavior
KW - Pretend Play
KW - Social Skills
KW - Sociocultural Factors
RP - NOT IN FILE
SP - 352
JF - Russ, Sandra W [Ed]; Niec, Larissa N [Ed]
IS - 2011
N2 - (from the chapter) The aim of this chapter is to discuss the basic research on children's play and interpersonal processes within a context that facilitates the integration of the basic and applied fields. Specifically, we examine three questions: (1) How does play relate to important interpersonal processes? (2) What cultural factors influence the link between play and interpersonal processes? and (3) What relevance does the basic play research have for applied settings? In order to address these questions, we first briefly review the development of social play and present definitions of the play types that hold particular relevance to interpersonal functioning: pretend play and rough-and-tumble-play. The interpersonal processes manifested within play are vast and multifaceted. For the purposes of this chapter, we focus on perspective taking, empathy, emotion regulation, and interpersonal schemas. Next, we review cultural influences on play. Finally, we discuss the implications of current research for applied study, such as the development of prevention and intervention programs. (PsycINFO Database Record (c) 2012 APA, all rights reserved)

AD - Jent, Jason F.: U Miami, Miller School of Medicine, Miami, FL, US Niec, Larissa N.: Central Michigan U, Department of Psychology, Mt Pleasant, MI, US Baker, Sarah E.: Wayne State U, School of Medicine, Pediatric Prevention Research Center, Detroit, MI, US
ER -

TY - GEN
ID - 1091
T1 - Foster Care: Creating Interpersonal Change through Story-Telling and Family Play. Strategies to Support Client Assessment and Intervention
A1 - Schatz,Mona Struhsaker
A1 - Gaddis,Stephen R.
A1 - Zimmerman,Toni Schindler
Y1 - 1996/02//
N1 - Availability - Level 2 - Produced in microfiche (1966-2003)
SuppNotes - For related document, see CG 028 372
Last updated - 2012-12-14
KW - ERIC,Resources in Education (RIE)
KW - Adult Education
KW - Caregiver Child Relationship
KW - Parenting Skills
KW - At Risk Persons
KW - Play
KW - Foster Family
KW - Professional Training
KW - Social Services
KW - Personal Narratives
KW - Foster Children
KW - Interpersonal Relationship
KW - Youth
KW - ERIC
RP - NOT IN FILE
SP - 95
N2 - Story-telling and family play can exert powerful effects on children. Some beliefs, values, and personal life-style choices that relate to how play is created are examined. Using the primary concepts in "narrative therapy,""adventure-based programming," and "family play therapy," play is employed to create an empowerment framework to consider the unique lives of social services clients. This empowerment framework promotes a means for individuals to explore their own meaning and sense of self-expression, rather than holding on to negative and pathological interpretations of their ideas and behaviors. Topics covered include (1) the value of family play; (2) how safe and successful play creates a framework for play and introduces a number of beneficial games; (3) how human development and the value of stories introduces the "narrative therapy" approach to one's life stories; (4) ways in which foster children's stories focus on the ways foster children view their world; and (5) exercises for rewriting the foster child's story through play and creating stories based on love, trust and hope. Designed for collaborative training of foster parents and caseworkers, the manual includes materials for both the trainer and the trainee. "Play is Learning: A Supplement," and "Guidelines for Selecting Toys" Contains two supplements, along with resources, references, and an evaluation. (EMK)
UR - http://search.proquest.com/docview/62498209?accountid=14701
ER -

TY - GEN
ID - 1092
T1 - Interventions for Toddlers with Autism Spectrum Disorders: An Evaluation of Research Evidence
A1 - Schertz,Hannah H.
A1 - Reichow,Brian
A1 - Tan,Paulo
A1 - Vaiouli,Potheini
A1 - Yildirim,Emine

Y1 - 2012/09//
N1 - Last updated - 2013-02-26
KW - ERIC,Current Index to Journals in Education (CIJE)
KW - At Risk Persons
KW - Toddlers
KW - Play
KW - Well Being
KW - Case Studies
KW - Pervasive Developmental Disorders
KW - Intervention
KW - Literature Reviews
KW - Outcomes of Treatment
KW - Comparative Analysis
KW - Family Programs
KW - Parents
KW - Attention
KW - Autism
KW - Effect Size
KW - ERIC
RP - NOT IN FILE
SP - 166
EP - 189
JF - Journal of Early Intervention
VL - 34
IS - 3
PB - SAGE Publications. 2455 Teller Road, Thousand Oaks, CA 91320.
N2 - Recently emerging intervention studies for toddlers with autism spectrum disorders (ASD) were reviewed through a systematic assessment of intervention outcomes, research rigor, and intervention features. The review includes published peer-reviewed experimental studies of toddlers with high risk for or diagnosis of ASD in which the majority of interventions occurred before age 36 months. Of 20 identified research studies, 6 were group comparison studies, all of which showed small to large magnitudes of effect when a uniform metric was applied. Fourteen were single-case design (SCD) studies, all of which reported effects on a variety of outcomes. When grouped by area of intervention focus (communication, general development, family well-being, imitation, joint attention, and play), commonly identified needs within focus areas were for replication, common measures, and authentic practices. A majority of studies in most focus areas showed strong to acceptable levels of research rigor, though this is an area of ongoing need. (Contains 3 tables.)
UR - http://search.proquest.com/docview/1312422969?accountid=14701
ER -

TY - JOUR
ID - 1093
T1 - A Brief Program Improves Counseling of Mothers with Children Who Have Persistent Aggression
A1 - Scholer,Seth J.
A1 - Reich,Stephanie M.
A1 - Boshers,Robyn B.
A1 - Bickman,Len
Y1 - 2012/04//
N1 - Last updated - 2013-02-26
SubjectsTermNotLitGenreText - 8168; 8996; 5470; 6485 8260 3150; 3400 8415; 1664; 11655 1474 316 8016 4542; 6823 3932 8016 4542 7577 3816; 324 545 9738 909; 913 2877; 6866 10621 3227 6582; 8299; 2302 6582; 7940 126; 7662 6506 10669; 1955 3629 6582; 5922 10621 3227 6582; 4452 1806 10278 8016 4542; 6501 4452 1806 10278 8016 4542; 3281 10087 2574 3629 6582; 5472 3629 6582; 2894; 8490 8762
KW - ERIC,Current Index to Journals in Education (CIJE)
KW - Program Effectiveness
KW - Medical Students
KW - Graduate Students

KW - Play
KW - Young Children
KW - Pediatrics
KW - Punishment
KW - Mothers
KW - Multimedia Instruction
KW - Intervention
KW - Discipline
KW - Medical Education
KW - Comparative Analysis
KW - Risk
KW - Prevention
KW - Counseling Techniques
KW - Behavior Disorders
KW - Lecture Method
KW - Clinics
KW - Interviews
KW - Aggression
KW - Effect Size
KW - Empathy
KW - ERIC
RP - NOT IN FILE
SP - 991
EP - 1004
JF - Journal of Interpersonal Violence
VL - 27
IS - 6
PB - SAGE Publications. 2455 Teller Road, Thousand Oaks, CA 91320.
N2 - Objective: To assess whether a multimedia program can affect counseling behavior related to one of the strongest risk factors for violence later in life, persistent early childhood aggression. Method: The design was a controlled trial with unobtrusive measurement in a clinic setting. A researcher, pretending to be the mother of a 2 1/2 year old boy, called 19 pediatric residents during clinic hours and requested advice on how to manage her child's persistently hurtful behavior. The intervention was a 40-min lecture focusing on a multimedia program, "Play Nicely", which teaches accepted strategies for managing aggression in young children ages 1-7 years. Residents' responses were blindly assessed to determine the treatment effect of the intervention. Results: Compared with the control group (C), residents in the intervention (I) group were more likely to recommend setting the rule (I: 100% vs. C: 31%, p = .01), redirecting (I: 83% vs. C: 8%, p = 0.003), promoting empathy (I: 50% vs. C: 0%, p = 0.02), and more likely to discourage the use of physical punishment (I: 83% vs. C: 31%). These are the primary strategies encouraged by the intervention. The magnitude of the effect size was very large for each of these three strategies, ranging from d = 1.1 to 2.3. Conclusions: A brief intervention can improve the counseling behavior of primary care physicians regarding persistent childhood aggression. The findings have implications for child abuse prevention, violence prevention, medical education, and how to improve anticipatory guidance within primary care. (Contains 3 tables.)
SN - 0886-2605, 0886-2605
UR - http://search.proquest.com/docview/1312420854?accountid=14701
ER -

TY - THES
ID - 1094
T1 - An Examination of Play Behaviors in Young At-Risk Children
A1 - Schwabe,Adrienne
A1 - Craig-Unkefer,Lesley
Y1 - 2008///
N1 - Last updated - 2012-12-14
SubjectsTermNotLitGenreText - 7940 126; 8127 11655 1474 316 8016 4542; 1432 909; 2888 2883 8016 4542

11658 316; 694 8016 4542; 9956 9955 5752; 2790; 5748 11228 1703 5053 2787; 5446 1970 1; 5470
IdentKeywords - Preschool Language Scale; Child Behavior Checklist; Peabody Picture Vocabulary Test
KW - Preschool Language Scale
KW - Child Behavior Checklist
KW - Peabody Picture Vocabulary Test
KW - ERIC,Resources in Education (RIE)
KW - At Risk Persons
KW - Language Acquisition
KW - Play
KW - Child Behavior
KW - Interpersonal Competence
KW - Speech Acts
KW - Developmental Delays
KW - Disadvantaged Youth
KW - Intervention
KW - Preschool Children
KW - ERIC
RP - NOT IN FILE
SP - 42
N2 - This study examined the effects that a multi-component intervention had on play behaviors and mean length of utterance (MLU) of eight Head Start children at risk for language delays and social skill development. The intervention consisted of three components: (a) a planning period included reading a story book that illustrated the play theme and use of the social pragmatic communication strategies, practice using vocabulary to be used in play, and choosing roles for the thematic activity; (b) a 10-minute play session in which the interventionist coached children to interact while they played with the thematic related toys; and (c) a brief reporting period in which children reviewed their use of social pragmatic strategies and specific vocabulary. Increases in complex play and MLU were observed from baseline to intervention for all children. The increases however, were not consistent among dyads. (Contains 4 tables and 15 figures.)
UR - http://files.eric.ed.gov/fulltext/ED505793.pdf
ER -

TY - JOUR
ID - 1095
T1 - Behavioral Control in At-Risk Toddlers: The Influence of the Family Check-Up
A1 - Shelleby,Elizabeth C.
A1 - Shaw,Daniel S.
A1 - Cheong,JeeWon
A1 - Chang,Hyein
A1 - Gardner,Frances
A1 - Dishion,Thomas J.
A1 - Wilson,Melvin N.
Y1 - 2012///
N1 - Last updated - 2013-04-02
SubjectsTermNotLitGenreText - 5470; 916 8234; 7537 3851 5449 8768; 1432 909; 2207 4542 8852 6582; 7940 126; 9621 3816 8016 4542; 1309 8016 4542; 1459; 4815 6582; 9457 909; 7576 909; 914 2094; 3849 8331; 11655 1474 316 8016 4542; 694 8016 4542; 8535 6447; 7577 3816 8016 4542; 3395 8930 909; 1404 8697
KW - Child Behavior Checklist
KW - Eyberg Child Behavior Inventory
KW - ERIC,Current Index to Journals in Education (CIJE)
KW - At Risk Persons
KW - Questionnaires
KW - Play
KW - Young Children
KW - Child Behavior
KW - Home Visits
KW - Intervention

KW - Behavior Problems
KW - Caregivers
KW - Parenting Styles
KW - Control Groups
KW - Family Programs
KW - Behavior Modification
KW - Child Rearing
KW - Parent Child Relationship
KW - Check Lists
KW - Emotional Response
KW - Siblings
KW - Parents
KW - Self Control
KW - ERIC
RP - NOT IN FILE
SP - 288
EP - 301
JF - Journal of Clinical Child and Adolescent Psychology
VL - 41
IS - 3
PB - Routledge. , 325 Chestnut Street Suite 800, Philadelphia, PA 19106.
N2 - This study examines the role of one component of emotion regulation, behavioral control, in the growth of children's early behavior problems by examining whether increases in parental positive behavior support brought about by a family-centered intervention were associated with greater child behavioral control, and whether greater behavioral control at age 3 mediated the association between improvements in aspects of positive behavior support from ages 2 to 3 and decreases in growth of behavior problems from ages 2 to 4. The sample included 713 at-risk children (50% female) and their primary caregivers (50% European American, 28% African American, 13% biracial, 9% other) who were randomly assigned to the intervention or control group. Children had a mean age of 29.91 months at the initial assessment. Data were collected through home visits at child ages 2 to 4, which involved questionnaires for primary caregivers and structured and unstructured play activities for children with primary and alternative caregivers and siblings. Results indicated that the intervention improved parental positive behavior support and reduced growth of child behavior problems. One dimension of positive behavior support, proactive parenting, was modestly associated with behavioral control at age 3, which in turn was significantly associated with growth in behavior problems from ages 2 to 4, with greater behavioral control related to lower levels of growth in behavior problems. Results provide support for the notion that proactive parenting is an important factor in the development of children's behavioral control and that behavioral control plays an important role in the growth of behavior problems. (Contains 1 table, 3 figures and 1 footnote.)
SN - 1537-4416, 1537-4416
UR - http://search.proquest.com/docview/1322241891?accountid=14701
ER -

TY - JOUR
ID - 1096
T1 - The assessment of bullying. [References]
A1 - Cornell,Dewey G.
Y1 - 2010///
N1 - Book: 2010-06797-020
English
Book; Edited Book
KW - PsycInfo
KW - Rough and Tumble
KW - Victimization
KW - Bullying
KW - Verbal Abuse
RP - NOT IN FILE

SP - 614
JF - Jimerson, Shane R [Ed]; Swearer, Susan M [Ed]; Espelage, Dorothy L [Ed]
IS - 2010
N2 - (from the chapter) What is bullying? Bullying can generally be defined as the act of repeatedly humiliating a weaker person. Humiliation can be accomplished by physical intimidation or assault, or by verbal abuse that ridicules or demeans someone. A more subtle form of bullying involves excluding someone from social activities so that the person feels rejected and inferior to others. Thus bullying may be physical, verbal, or social. A bully is in a position of dominance or superiority over the victim. Especially among boys, dominance might be achieved because one boy is larger or stronger than the other, or because several boys outnumber their victim. Among girls, physical size may not matter as much as social status and popularity. In all cases, the bully intimidates the victim, who is made to feel inferior. Bullying does not occur between equals, which distinguishes it from ordinary conflict between peers. Because bullying is such a broad and abstract concept, it is difficult to distinguish from other forms of peer aggression and play. Consider the example of one boy who shoves another boy: If the aggressor is bigger than his victim, it might be bullying, but if the two boys are the same size, it is not bullying. If the action is done in a playful manner and the smaller boy is not hurt or distressed, the incident might be regarded as horseplay, but not bullying. And even when the bigger boy acts with malice, if the incident only happens once, the behavior might not be labeled bullying. (PsycINFO Database Record (c) 2012 APA, all rights reserved)
AD - Cornell, Dewey G.: Curry Programs in Clinical and School Psychology, University of Virginia, Charlottesville, VA, US Bandyopadhyay, Sharmila: Curry Programs in Clinical and School Psychology, University of Virginia, Charlottesville, VA, US
ER -

TY - RPRT
ID - 1097
T1 - Parenting through Play for School Readiness: Interim Report of Year One. Research Findings
A1 - Singer,Jerome L.
A1 - Singer,Dorothy G.
Y1 - 1998/09//
N1 - Availability - Level 1 - Available online, if indexed January 1993 onward
Last updated - 2012-12-14
KW - ERIC,Resources in Education (RIE)
KW - Preschool Education
KW - Parent Education
KW - Low Income Groups
KW - At Risk Persons
KW - Play
KW - Parent Influence
KW - Parent Role
KW - Academic Achievement
KW - Parent Child Relationship
KW - Parent Participation
KW - Disadvantaged Youth
KW - Program Evaluation
KW - Student Behavior
KW - Preschool Children
KW - School Readiness
KW - ERIC
RP - NOT IN FILE
SP - 48
N2 - A 2-year project was undertaken to develop, test, and distribute a video-based program to train low-income parents in engaging their 3- to 5-year-old children in highly motivating play techniques in order to enhance children's cognitive, social and motor skills for school readiness. An evaluation was conducted of the Year One phase of the training materials and procedures, and the effectiveness of the parent-training in leading to higher school readiness scores. The participants were the parents of 103 preschool children from inner city schools. The findings of the child assessments after 2 weeks of parent training indicate that children of parents

who participated in the program showed significantly higher scores than the control children in the Total School Readiness Test, particularly in the areas of vocabulary, nature knowledge, general information, and awareness of good manners. This suggests that types of play-training methods and materials developed for the project can impact the ability of low-income parents to enhance their children's cognitive and social skills. (Twelve appendices include a list of participating schools, data on the child care centers, and copies of testing instruments and data collection materials.) (JPB)
UR - http://search.proquest.com/docview/62494276?accountid=14701
ER -

TY - JOUR
ID - 1098
T1 - Parent-Child Interactions and Obesity Prevention: A Systematic Review of the Literature
A1 - Skouteris,Helen
A1 - McCabe,Marita
A1 - Ricciardelli,Lina A.
A1 - Milgrom,Jeannette
A1 - Baur,Louise A.
A1 - Aksan,Nazan
A1 - Dell'Aquila,Daniela
Y1 - 2012///
N1 - Last updated - 2012-12-14
SubjectsTermNotLitGenreText - 7223 1096 7843 5051 2923 2877 1088; 5470; 8168; 7537 3851 5449 8768; 6124 8477; 8099; 5350 8852 6582; 1096 7843 5051; 3103; 3102 2923 2877; 7940 126; 7575 9690 1; 7576 909; 4659 4635 126; 4660 7858 7862 4634; 4637 909; 1389 6582; 8852 6582; 6445 7230; 6446 6582; 8996; 7546 5127; 1443 5053 2787; 1446 4634
KW - ERIC,Current Index to Journals in Education (CIJE)
KW - Adult Education
KW - Early Childhood Education
KW - Elementary Secondary Education
KW - Parenting Skills
KW - Parent Influence
KW - Research Methodology
KW - Child Health
KW - Intervention
KW - Literature Reviews
KW - Measurement Techniques
KW - Health Promotion
KW - Body Weight
KW - Parenting Styles
KW - Parent Child Relationship
KW - Child Development
KW - Health Behavior
KW - Measurement Objectives
KW - Eating Disorders
KW - Predictor Variables
KW - Eating Habits
KW - Obesity
KW - Play
KW - Risk
KW - Prevention
KW - Interaction Process Analysis
KW - Change Strategies
KW - Health Related Fitness
KW - ERIC
RP - NOT IN FILE
SP - 153

EP - 174
JF - Early Child Development and Care
VL - 182
IS - 2
PB - Routledge. , 325 Chestnut Street Suite 800, Philadelphia, PA 19106.
N2 - Child obesity research has generally not examined multiple layers of parent-child relationships during weight-related activities such as feeding, eating and play. A literature review was conducted to locate empirical studies that measured parent-child interactions and child eating and child weight variables; five papers met the inclusion criteria and were included in the review. The findings of the review revealed that parent-child relationships are an important element in explaining the unhealthy trend of childhood obesity. We argue that prevention/intervention strategies must extend on the current models of parenting by targeting the family from a bi-directional perspective, and focusing, specifically, on the mutually responsive orientation that exists in the parent-child relationship. (Contains 1 table.)
SN - 0300-4430, 0300-4430
UR - http://search.proquest.com/docview/964179282?accountid=14701
ER -

TY - GEN
ID - 1099
T1 - Shaping the Future of Mathematics Education. Proceedings of the Annual Conference of the Mathematics Education Research Group of Australasia (33rd, Freemantle, Western Australia, Australia, July 3-7, 2010)
A1 - Sparrow,Len
A1 - Kissane,Barry
A1 - Hurst,Chris
Y1 - 2010/07//
N1 - Last updated - 2012-12-14
KW - ERIC,Resources in Education (RIE)
KW - Elementary Secondary Education
KW - Higher Education
KW - Elementary School Mathematics
KW - Course Content
KW - Indigenous Populations
KW - Play
KW - Numeracy
KW - Conferences (Gatherings)
KW - Teacher Education
KW - Secondary School Mathematics
KW - Mathematics Skills
KW - Computer Uses in Education
KW - Teacher Competencies
KW - Mathematics Education
KW - Problem Solving
KW - At Risk Students
KW - Mathematical Logic
KW - Foreign Countries
KW - Educational Change
KW - Mathematics Curriculum
KW - Preservice Teachers
KW - Mathematics Teachers
KW - Educational Technology
KW - Educational Research
KW - Teaching Methods
KW - ERIC
RP - NOT IN FILE
SP - 788

PB - Mathematics Education Research Group of Australasia. GPO Box 2747, Adelaide SA 5001, Australia. Tel: +61-8-8363-0288; Fax: +61-8-8362-9288; e-mail: sales@merga.net.au; Web site: http://www.merga.net.au/
N2 - These collected papers are a record of the proceedings of the 33rd Annual Conference of the Mathematics Education Research Group of Australasia entitled, "Shaping the Future of Mathematics Education." The conference is held in Fremantle, as was the previous conference a decade earlier. Keynote speakers are discussing issues that are emerging more sharply at the present time as the mathematics education community focuses on the education of Indigenous children, the scope of the mathematics curriculum and ways in which the content might be taught effectively, and the significant role of technologies in teaching and learning in the twenty-first century. Research papers are featured from mathematics educators from all states and territories of Australia, from colleagues in New Zealand, and from overseas--United Kingdom, Singapore, United States of America, India, Thailand, South Africa, and Indonesia. This set of proceedings includes abstracts and full papers for refereed research presentations, short communications of developing research, roundtable discussions, and symposia. Individual papers contain tables, footnotes, figures, references and appendices
SN - 9781920846251
UR - http://search.proquest.com/docview/881455631?accountid=14701
ER -

TY - BOOK
ID - 1100
T1 - Handbook of Research on the Education of Young Children. 2nd Edition
A1 - Spodek,Bernard
A1 - Saracho,Olivia N.
Y1 - 2006///
N1 - Last updated - 2012-12-14
KW - United States
KW - ERIC,Resources in Education (RIE)
KW - Early Childhood Education
KW - High Risk Students
KW - Inclusive Schools
KW - Play
KW - Motor Development
KW - Cognitive Development
KW - Young Children
KW - Multicultural Education
KW - Professional Development
KW - Child Care
KW - Creativity
KW - Bilingualism
KW - Moral Development
KW - Visual Arts
KW - Curriculum Development
KW - Interpersonal Competence
KW - Poverty
KW - Educational Policy
KW - Emergent Literacy
KW - Child Development
KW - Mathematical Concepts
KW - Feminism
KW - Educational Research
KW - ERIC
RP - NOT IN FILE
SP - 616
PB - Lawrence Erlbaum Associates. 10 Industrial Avenue, Mahwah, NJ, 07430.
N2 - This book is an essential reference on research in early childhood education not only in the United States but throughout the world. It provides a comprehensive overview of important contemporary issues and the information necessary to make judgments about these issues. The field has changed significantly since the

publication of the first edition of this "Handbook" in 1993, creating a need for an update. The "Handbook of Research on the Education of Young Children, Second Edition" is thus focused on research conducted over the past decade or so. The volume is organized into four parts: (1) Early Childhood Education and Child Development (New in this edition: Moral Development; The Development of Creativity); (2) Early Childhood Educational Curriculum (New in this edition: Movement or Dance Education; The Education of Linguistically and Culturally Diverse Children); (3) Foundations of Early Childhood Educational Policy (New in this edition: Childhood Poverty; The Education of Bilingual Children); and (4) Research and Evaluation Strategies for Early Childhood Education (New in this edition: Doing Historical Research in Early Childhood Education; Postmodern and Feminist Orientations). This book makes the expanding knowledge base related to early childhood education readily available and accessible. It is a valuable tool for all who work and study in the field. Following an introduction by B. Spodek and O. N. Saracho entitled, "A Researcher's Vade Mecum," this book presents the following chapters. Part I, Early Childhood Education and Child Development, includes: (1) Cognitive Development and the Education of Young Children (K. L. Seifert); (2) Young Children's Peer Relations and Social Competence (G. W. Ladd, S. L. Herald, and R. K. Andrews); (3) Children's Morality: Perspectives and Research (E. Johansson); (4) The Emotional Basis of Learning and Development in Early Childhood Education (S. A. Denham); (5) Motor Development in Young Children (D. L. Gallahue and J. C. Ozmun); and (6) The Development of Children's Creativity (M. A. Runco). Part II, Early Childhood Educational Curriculum, continues with: (7) Reconceptualizing Language Education in Early Childhood: Socio-Cultural Perspectives (J. Anderson, L. Moffatt, and J. Shapiro); (8) Emergent Literacy: Symbols at Work (S. E. Hill and S. Nichols); (9) The Literacy Education of Linguistically and Culturally Diverse Young Children: An Overview of Outcomes, Assessment, and Large-Scale Interventions (R. Rueda and D. B. Yaden, Jr.); (10) The Development of Young Children's Early Number and Operation Sense and Its Implications for Early Childhood Education (A. J. Baroody, M. L. Lai, and K. S. Mix); (11) Repositioning the Visual Arts in Early Childhood Education: A Decade of Reconsideration (C. M. Thompson); (12) The Dance of Learning (K. Bradley with M. Szegda); (13) The Musical Development and Education of Young Children (G. F. Welch); and (14) Pretend Play (R. D. Kavanaugh); and (15) Early Childhood Multicultural Education (P.G. Ramsey). Part III, Foundations of Early Childhood Educational Policy, contains: (16) Creating Play Environments for Early Childhood: Indoors and Out (J. A. Sutterby and J. Frost); (17) Childhood Poverty: Implications for School Readiness and Early Childhood Education (R. M. Ryan, R. C. Fauth, and J. Brooks-Gunn); (18) Effective Preschool Programs for Children at Risk of School Failure: A Best-Evidence Synthesis (B. Chambers, A. C. K. Cheung, and R. E. Slavin); (19) Educational Policy in the United States Regarding Bilinguals in Early Childhood Education (A. M. Wiese and E. E. Garcia); (20) Child Care for Young Children (C. Howes and K. Sanders); (21) Family Context in Early Childhood: A Look at Practices and Beliefs That Promote Early Learning (B. H. Fiese, T. Eckert, and M. Spagnola); (22) Early Childhood Teachers' Beliefs and Attitudes about Inclusion: What Does the Research Tell Us? (M. M. Ostrosky, B. M. Laumann, and W. Y. Hsieh); and (23) Preschool Teachers' Professional Development (O. N. Saracho and B. Spodek). Part IV, Research and Evaluation Strategies for Early Childhood Education, presents: (24) Alternative Means of Assessing Children's Learning in Early Childhood Classrooms (D. F. Gullo); (25) Evaluating the Quality of Early Childhood Educational Settings (R. Lambert, M. Abbott-Shim, and A. Sibley); (26) Integration, Innovation, and Evaluation in School-Based Early Childhood Services (J. Pelletier and C. Corter); (27) Qualitative Research: Paradigms and Possibilities (J. A. Hatch and G. Barclay-McLaughlin); (28) Feminist Issues in Early Childhood Scholarship (C. D. Thornton and L. S. Goldstein); (29) Beyond Certainties: Postmodern Perspectives, Research, and the Education of Young Children (S. Grieshaber and S. Ryan); (30) Early Childhood Education Research in Cross-National Perspective (J. L. Roopnarine and A. Metindogan); and (31) Historical Research in Early Childhood Education (B. S. F. Hinitz). [For the first edition of this book, see ED361107.]
SN - 0805847200
UR - http://search.proquest.com/docview/62095654?accountid=14701
ER -

TY - JOUR
ID - 1101
T1 - Competitive versus Cooperative Exergame Play for African American Adolescents' Executive Function Skills: Short-Term Effects in a Long-Term Training Intervention
A1 - Staiano,Amanda E.
A1 - Abraham,Anisha A.
A1 - Calvert,Sandra L.

Y1 - 2012/03//
N1 - Last updated - 2012-12-14
SubjectsTermNotLitGenreText - 7840 126; 5470; 197 316 8016 4542; 2207 4542 8852 6582; 7940 126; 11255 4270 126; 8426 9690 1; 9015; 1974 909; 3665 1710; 6186 5019; 7223 1096 7843 5051 2923 2877 1088; 293 7160 8016 4542; 2221 909; 1096 7843 5051; 3668 7840 126; 2267 10087 2574 3629 6582; 694 8016 4542; 7455
KW - ERIC,Current Index to Journals in Education (CIJE)
KW - At Risk Persons
KW - Obesity
KW - Video Games
KW - Play
KW - Cooperation
KW - Low Income
KW - African Americans
KW - Intervention
KW - Correlation
KW - Exercise
KW - Outcomes of Treatment
KW - Body Weight
KW - Physical Activities
KW - Control Groups
KW - Role
KW - Executive Function
KW - Psychomotor Skills
KW - Competition
KW - Adolescents
KW - ERIC
RP - NOT IN FILE
SP - 337
EP - 342
JF - Developmental Psychology
JA - Dev.Psychol.
VL - 48
IS - 2
PB - American Psychological Association. Journals Department, 750 First Street NE, Washington, DC 20002-4242.
N2 - Exergames are videogames that require gross motor activity, thereby combining gaming with physical activity. This study examined the role of competitive versus cooperative exergame play on short-term changes in executive function skills, following a 10-week exergame training intervention. Fifty-four low-income overweight and obese African American adolescents were randomly assigned to a competitive exergame condition, a cooperative exergame condition, or a no-play control group. Youths in the competitive exergame condition improved in executive function skills more than did those in the cooperative exergame condition and the no-play control group. Weight loss during the intervention was also significantly positively correlated with improved executive function skills. The findings link competitive exergame play to beneficial cognitive outcomes for at-risk ethnic minority adolescents
SN - 0012-1649, 0012-1649
UR - http://search.proquest.com/docview/1140138716?accountid=14701
ER -

TY - JOUR
ID - 1102
T1 - Children and play. [References]
A1 - Smith,Peter K.
Y1 - 2010///
N1 - Book: 2009-08420-000
English

Book; Authored Book
(Created by PsycINFO) (Abbreviated) Series editor's preface Acknowledgments An introduction to play A brief history of the study of play and of play theories Animal play: Definitions and descriptions Animal play: Theoretical perspectives on function Play in different cultures Physical activity play: Exercise play and rough-and-tumble Object play Pretend play: Description Pretend play: Theories and functions Some practical issues concerning play Concluding comments: Play and the middle way References Author index Subject index
KW - PsycInfo
KW - Rough and Tumble
KW - Childhood Development
KW - Childhood Play Behavior
KW - Early Childhood Development
KW - Age Differences
KW - Cross Cultural Differences
KW - Education
KW - Human Sex Differences
KW - Special Needs
RP - NOT IN FILE
SP - 256
EP - Blackwell
JF - (2010)
VL - Children and play. x, 256 pp.Wiley-Blackwell.
N2 - (from the cover) Using both contemporary and classic research, Smith examines how different age groups and sexes participate in a wide variety of play, including physical and rough-and-tumble play, play with objects, fantasy play and imaginary friends. The book gauges the function of play in early childhood education and the case for and against recess breaks in school. How play occurs in different societies and among various populations-including children with special needs-is also explored. With its comprehensive coverage of theoretical, cross-cultural, and evolutionary perspectives, Children and Play holds significant insights for parents, educators, and clinicians. (PsycINFO Database Record (c) 2012 APA, all rights reserved)
AD - Smith, Peter K.: Unit for School and Family Studies, Goldsmiths, University of London, London, United Kingdom
ER -

TY - RPRT
ID - 1103
T1 - The Effects of Prenatal Drug-Exposure on Toddlers' Temperament, Development and Play Behavior
A1 - Storkamp,Barbara J.
Y1 - 1993/03//
N1 - Availability - Level 1 - Available online, if indexed January 1993 onward
SuppNotes - Paper presented at the Biennial Meeting of the Society for Research in Child Development (60th, New Orleans, LA, March 25-28, 1993)
Last updated - 2012-12-14
KW - African Americans
KW - Fetal Drug Exposure
KW - ERIC,Resources in Education (RIE)
KW - Preschool Education
KW - At Risk Persons
KW - Toddlers
KW - Play
KW - Blacks
KW - Personality
KW - Foster Care
KW - Comparative Analysis
KW - Child Neglect
KW - Child Development
KW - Cocaine
KW - Child Abuse

KW - Drug Abuse
KW - ERIC
RP - NOT IN FILE
SP - 31
N2 - This study compared 17 toddlers identified as prenatally exposed to cocaine (along with marijuana, alcohol, or nicotine), with another group of 10 toddlers with no prenatal exposure. All subjects were African-American, 1-3 years of age, and in foster care. Toddlers were age- and gender-matched and compared on measures of temperament, development, growth and play behavior. There were no group differences in temperament, growth, and play behavior, but significant differences in development. The drug-exposed toddlers exhibited higher levels of development than the nonexposed toddlers. A majority of both groups, however, scored below age level on standard development measures. These results may be attributable to the fact that the comparison group was comprised of high-risk children placed in foster care primarily due to parental abuse and neglect. Findings also suggest that the effects of prenatal drug exposure are similar to developmental outcomes experienced by other high-risk groups of children. Contains 48 references. (Author/MDM)
UR - http://search.proquest.com/docview/62782699?accountid=14701
ER -

TY - JOUR
ID - 1104
T1 - Longitudinal Outcomes of Young High-Risk Adolescents with Imaginary Companions
A1 - Taylor,Marjorie
A1 - Hulette,Annmarie C.
A1 - Dishion,Thomas J.
Y1 - 2010/11//
N1 - Last updated - 2012-12-14
SubjectsTermNotLitGenreText - 7940 126; 3083 316 8016 4542; 2240 150 909; 694 8016 4542; 4979 2388 8409 5051; 6644 10278 8016 4542; 916 8234; 5472 3629 6582; 4203 5449 8768; 9808 10103; 7531 730; 1955 3629 6582; 4086 6175 1326 3629 6582 8836; 4738 9419 10278 8016 4542; 150 909; 194 5053 2787
KW - Child Behavior Checklist
KW - ERIC,Current Index to Journals in Education (CIJE)
KW - High Schools
KW - Middle Schools
KW - At Risk Persons
KW - Play
KW - Middle School Students
KW - Early Adolescents
KW - Adolescent Development
KW - Behavior Problems
KW - Comparative Analysis
KW - Friendship
KW - Imagination
KW - Followup Studies
KW - Adjustment (to Environment)
KW - Parent Attitudes
KW - Interviews
KW - Coping
KW - High School Students
KW - Social Status
KW - ERIC
RP - NOT IN FILE
SP - 1632
EP - 1636
JF - Developmental Psychology
JA - Dev.Psychol.
VL - 46
IS - 6

PB - American Psychological Association. Journals Department, 750 First Street NE, Washington, DC 20002-4242.
N2 - The creation and cultivation of an imaginary companion is considered to be a healthy form of pretend play in early childhood, but there tends to be a less positive view of older children who have them. To test the extent that having an imaginary companion in middle school is associated with positive or negative outcomes, an ethnically diverse sample of 152 middle school children at high risk for developing problem behaviors were interviewed about imaginary companions, coping styles, and problem behaviors. Although having a current imaginary companion (n = 13) was associated with using more positive coping strategies, peer nomination data indicated that these children had low social preference with peers. In addition, our data indicated that these children were perceived by their parents as having more problem behaviors compared with young adolescents who never had imaginary companions (n = 108) or children who had imaginary companions in the past (n = 31). However, a longitudinal follow-up at the end of high school indicated that the children who had imaginary companions in middle school showed greater positive adjustment on a multiple-indicator adjustment construct. (Contains 1 table.)
SN - 0012-1649, 0012-1649
UR - http://search.proquest.com/docview/889925708?accountid=14701
ER -

TY - JOUR
ID - 1105
T1 - Predictors of Social Skills for Preschool Children at Risk for ADHD: The Relationship between Direct and Indirect Measurements
A1 - Thomas,Lisa B.
A1 - Shapiro,Edward S.
A1 - DuPaul,George J.
A1 - Lutz,Gary J.
A1 - Kern,Lee
Y1 - 2011/04//
N1 - Last updated - 2012-12-14
SubjectsTermNotLitGenreText - 725 724 2877; 9791 8234; 7940 126; 3090 5470; 8127 11655 1474 316 8016 4542; 5446 1970 1; 8099; 694 8016 4542
KW - ERIC,Current Index to Journals in Education (CIJE)
KW - Preschool Education
KW - At Risk Persons
KW - Attention Deficit Hyperactivity Disorder
KW - Play
KW - Interpersonal Competence
KW - Early Intervention
KW - Social Problems
KW - Preschool Children
KW - Predictor Variables
KW - ERIC
RP - NOT IN FILE
SP - 114
EP - 124
JF - Journal of Psychoeducational Assessment
VL - 29
IS - 2
PB - SAGE Publications. 2455 Teller Road, Thousand Oaks, CA 91320.
N2 - The relationship between direct and indirect measurements of social skills and social problem behaviors for preschool children at risk for attention deficit hyperactivity disorder (ADHD) was examined. Participants included 137 preschool children, aged 3 to 5 years, at risk for ADHD, who were participating in a larger study examining the effects of early intervention for young children. Teachers rated the social skills and social problems of the participants. Direct observation data of participants were also collected at preschool during free play. Results support previous research on social skills assessment and suggest that indirect and direct measures

may not be measuring the same aspect of social skills. Thus, a variety of evaluation tools are necessary to comprehensively assess the social skills of preschool children with social challenges. (Contains 6 tables.)
SN - 0734-2829, 0734-2829
UR - http://search.proquest.com/docview/870283524?accountid=14701
ER -

TY - RPRT
ID - 1106
T1 - Where Our Children Play. Community Park Playground Equipment
A1 - Thompson,Donna
A1 - Bowers,Louis
Y1 - 1989///
N1 - Availability - Level 2 - Produced in microfiche (1966-2003)
Last updated - 2012-12-14
KW - ERIC,Resources in Education (RIE)
KW - Risk
KW - Recreational Facilities
KW - Recreation Legislation
KW - Questionnaires
KW - Play
KW - Community Surveys
KW - Playgrounds
KW - Safety
KW - Parks
KW - Equipment Evaluation
KW - Legal Responsibility
KW - ERIC
RP - NOT IN FILE
SP - 116
PB - American Alliance for Health, Physical Education, Recreation and Dance Publications, 1900 Association Drive, Reston, VA 22091.
N2 - This book describes the type and condition of playground equipment in community parks in the United States, and indicates the current status of safety of that environment. The results are then compared to play theories to explore the ways the environment stimulates play. Appropriate suggestions for change are made. Chapters cover the following topics: (1) the national survey of community park playground equipment; (2) results of the survey; (3) location, accessibility and equipment on park playgrounds; (4) swings, slides, and climbing equipment; (5) rotating, spring rocking, and seesaw equipment; (6) sand play containers, wading pools, signs, trees, and pathways; (7) litigation and playgrounds; and (9) plan of action: reflections and recommendations. Appendices include the instruments of the surveys conducted for this study. (JD)
UR - http://search.proquest.com/docview/63029438?accountid=14701
ER -

TY - JOUR
ID - 1107
T1 - The Evolution of a Children's Domestic Violence Counseling Group: Stages and Processes
A1 - Thompson,Heather E.
Y1 - 2011///
N1 - Last updated - 2012-12-14
SubjectsTermNotLitGenreText - 3862 11282 545 9738 909; 4516 5348 8768; 4513 2291 4552 4518; 11655 1474 316 8016 4542; 7941 10836 10839 8699; 2302 6582; 2293; 8068 7404; 8996; 547 8415; 9465 2898
KW - ERIC,Current Index to Journals in Education (CIJE)
KW - Risk
KW - Play Therapy
KW - Counseling Techniques
KW - Anxiety
KW - Young Children

KW - Power Structure
KW - Counseling Effectiveness
KW - Family Violence
KW - Self Disclosure (Individuals)
KW - Group Dynamics
KW - Group Counseling
KW - ERIC
RP - NOT IN FILE
SP - 178
EP - 201
JF - Journal for Specialists in Group Work
VL - 36
IS - 3
PB - Routledge. , 325 Chestnut Street Suite 800, Philadelphia, PA 19106.
N2 - The purpose of this qualitative case study is to illuminate the lived experiences of 4 young children between 6 and 7 years old who witnessed domestic violence while revealing the complex relationship between group process and stage development in their 18-week counseling group. Data revealed that processes occurring between and among group members shaped the evolution of the counseling group through a series of stages that are similar to those seen in adult counseling groups. The data also revealed developmental differences between group processes occurring between these young members and the ways in which group processes unfold in adult counseling groups. (Contains 1 figure.)
SN - 0193-3922, 0193-3922
UR - http://search.proquest.com/docview/889924468?accountid=14701
ER -

TY - JOUR
ID - 1108
T1 - Numeracy in the Early Years: Project Good Start
A1 - Thomson,Sue
Y1 - 2004///
N1 - Last updated - 2012-12-14
SubjectsTermNotLitGenreText - 10514 10486 909; 5046 8016 4542; 7196; 11162 4335; 10621 3227 6582; 8179 3352 3368 3150 3085; 4109 4335; 3085 3150; 9046 4335; 4954; 4723; 1326 3629 6582 8836; 4290; 7940 126; 8299
KW - Australia
KW - ERIC,Current Index to Journals in Education (CIJE)
KW - Teachers
KW - Early Childhood Education
KW - Primary Education
KW - Program Effectiveness
KW - High Risk Students
KW - Indigenous Populations
KW - Play
KW - Gender Differences
KW - Case Studies
KW - Numeracy
KW - Identification
KW - Rural Areas
KW - Teacher Effectiveness
KW - Foreign Countries
KW - Urban Areas
KW - Teaching Methods
KW - ERIC
RP - NOT IN FILE
SP - 14
EP - 17

JF - Australian Primary Mathematics Classroom
VL - 9
IS - 4
PB - Australian Association of Mathematics Teachers (AAMT). GPO Box 1729, Adelaide 5001, South Australia. Tel: +61-8-8363-0288; Fax: +61-8-8362-9288; e-mail: office@aamt.edu.au; Web site: http://www.aamt.edu.au
N2 - "Numeracy in the early years: Project good start" was a national project that commenced in 2001, and will conclude this year. The key objective of the project is to improve children's early numeracy outcomes by investigating the practices and learning experiences that support the numeracy development of a sample of children in the year before school and in their first year of formal schooling. Case studies of some of these children were also prepared and analysed. Children's before-school experiences (including at home, and in preschool and childcare settings) and first year of school experiences were examined, with a particular focus on factors affecting early numeracy development. The project investigated the effects of children's backgrounds (for example, geographically isolated, rural, low socioeconomic urban areas, and areas with high indigenous populations); the different numeracy programs that these children encounter; the beliefs of parents and practitioners regarding numeracy development and learning; effective teaching strategies in numeracy and the identification of "at risk" children in numeracy. (Contains 2 figures.)
SN - 1326-0286, 1326-0286
UR - http://search.proquest.com/docview/61906121?accountid=14701
ER -

TY - JOUR
ID - 1109
T1 - Mother-Child Interactions and Symbolic Play between Toddlers and Their Adolescent or Mentally Retarded Mothers
A1 - Unger,Olivia
A1 - Howes,Carollee
Y1 - 1988///
N1 - Last updated - 2012-12-14
SubjectsTermNotLitGenreText - 197 316 8016 4542; Early Parenthood 3092; 4722; Mental Retardation 6540 2877; Mothers 6823 3932 8016 4542 7577 3816; 7271 10839; Parent Child Relationship 7537 3851 5449 8768; Play 7940 126; Toddlers 10883 11655 1474 316 8016 4542
KW - ERIC,Current Index to Journals in Education (CIJE)
KW - Mental Retardation
KW - Toddlers
KW - Early Parenthood
KW - Play
KW - High Risk Persons
KW - Parent Child Relationship
KW - Occupational Therapy
KW - Mothers
KW - Adolescents
KW - ERIC
RP - NOT IN FILE
SP - 237
EP - 249
JF - Occupational Therapy Journal of Research
VL - 8
IS - 4
N2 - A study investigated the symbolic play development and mother-child interactions between toddlers and their adolescent or mentally retarded mothers. Differences in maternal behaviors were found between the not-at-risk control group and the mentally retarded and adolescent mothers. (JOW)
UR - http://search.proquest.com/docview/63183043?accountid=14701
ER -

TY - RPRT

ID - 1110
T1 - Developmental Lags in Preterm Infants from One to Three Years of Age
A1 - Ungerer,Judy A.
A1 - Sigman,Marian
Y1 - 1983///
N1 - Last updated - 2012-12-14
KW - Correction for Prematurity
KW - Developmental Differences
KW - Developmental Patterns
KW - Developmentally Delayed Persons
KW - Sensorimotor Development
KW - ERIC,Current Index to Journals in Education (CIJE)
KW - Comparative Analysis
KW - Play
KW - Language Acquisition
KW - High Risk Persons
KW - Premature Infants
KW - Child Development
KW - Longitudinal Studies
KW - ERIC
RP - NOT IN FILE
SP - 1217
EP - 1228
N2 - Assessments of play, sensorimotor, language, and general developmental skills were administered to two preterm and 20 full-term infants. Preterm infants showed some delays greater than would be predicted from biological maturity alone, but delays were not present for all skills and generally were not maintained beyond 2 years of age. (Author/RH)
UR - http://search.proquest.com/docview/63423370?accountid=14701
ER -

TY - JOUR
ID - 1111
T1 - Developmental Changes in the Relations between Inhibitory Control and Externalizing Problems during Early Childhood
A1 - Utendale,William T.
A1 - Hastings,Paul D.
Y1 - 2011///
N1 - Last updated - 2012-12-14
SubjectsTermNotLitGenreText - 324 545 9738 909; 6823 3932 8016 4542 7577 3816; 8127 11655 1474 316 8016 4542; 8996; 3777 6886 10087 2574 3629 6582; 9457 909; 916 8234; 1701 1; 5180; 7940 126; 7676 5449 8768; 10883 11655 1474 316 8016 4542; 6816 7531 730; 5649 5264; 10482 730; 6447; 1443 5053 2787; 2798
KW - ERIC,Current Index to Journals in Education (CIJE)
KW - Kindergarten
KW - Preschool Education
KW - Measures (Individuals)
KW - Toddlers
KW - Play
KW - Mothers
KW - Teacher Attitudes
KW - Mother Attitudes
KW - Inhibition
KW - Behavior Problems
KW - Factor Analysis
KW - Peer Relationship
KW - Risk
KW - Cognitive Ability

KW - Developmental Stages
KW - Child Development
KW - Preschool Children
KW - Aggression
KW - Self Control
KW - ERIC
RP - NOT IN FILE
SP - 181
EP - 193
JF - Infant and Child Development
VL - 20
IS - 2
PB - Wiley-Blackwell. 111 River Street, Hoboken, NJ 07030-5774.
N2 - Deficits in executive function, and in particular, reduced capacity to inhibit a dominant action, are a risk factor for externalizing problems (EP). Inhibitory control (IC) develops in the later preschool and early childhood periods, such that IC might not regulate EP in toddlers and younger preschoolers. Aggression was observed during peer play for 66 girls and 49 boys, from 2.75 to 6.00 years (M = 4.14, S.D. = 0.78). Mothers reported on children's IC and EP concurrently and 12 months later, and concurrent teacher reports of EP were also collected. Factor analysis supported aggregation of mother and teacher-reported EP and observed physical aggression into one measure of externalizing difficulties. Mothers reported lower IC for children with more externalizing difficulties, and the inverse relation between IC and externalizing difficulties strengthened over the toddler, preschool and kindergarten periods. Similar relations between IC and EP were observed 12 months later, and increases in IC also predicted reductions in EP over 1 year. These data demonstrate that the preschool years are a dynamic period of developmental change in the relations between IC and EP. (Contains 1 note, 3 tables, and 2 figures.)
SN - 1522-7219, 1522-7219
UR - http://search.proquest.com/docview/870287689?accountid=14701
ER -

TY - JOUR
ID - 1112
T1 - The role of child negative affect in the relations between parenting styles and play. [References]
A1 - Lagace-Seguin,Daniel G.
Y1 - 2006///
N1 - Peer Reviewed Journal: 2006-08797-002
English
Journal; Peer Reviewed Journal
KW - PsycInfo
KW - Rough and Tumble
KW - Child Attitudes
KW - Childhood Play Behavior
KW - Emotional States
KW - Parent Child Relations
KW - Parenting Style
KW - Preschool Teachers
KW - Psychosocial Development
RP - NOT IN FILE
SP - Jul
EP - 477
JF - Early Child Development and Care
VL - .176
IS - 5
N2 - Interactions between parenting styles and children's negative affect in the prediction of reticent, solitary-active, and rough-and-tumble play behaviours were examined. The present study involved 98 children, their mothers and their preschool teachers. Participants (53 boys and 45 girls) were a mean age of 3.83 years (standard deviation = 0.69). Mothers completed questionnaires pertaining to their disciplinary rearing styles and

their child's negative affect. Preschool/daycare teachers completed a measure concerning children's indoor-free play behaviours. Hierarchical multiple regression analyses revealed two significant interactions between parenting styles and children's negative affect in the prediction of play behaviours. For example, it was found that emotion coaching parenting was negatively related to rough-and-tumble play for children low in negative affect but not for children high in negative affect. Implications of these findings, and others, are discussed in terms of goodness of fit, overprotective parenting and their overall importance for children's social development. (PsycINFO Database Record (c) 2013 APA, all rights reserved) (journal abstract)
SN - 0300-4430
AD - Lagace-Seguin, Daniel G.: Mount Saint Vincent University, Halifax, NS, Canada d'Entremont, Marc-Robert L.: Mount Saint Vincent University, Halifax, NS, Canada
ER -

TY - JOUR
ID - 1113
T1 - Moving Forward? Addressing the Needs of Young At-Risk Students in the Dutch Education System
A1 - Van der Aalsvoort,Geerdina M.
Y1 - 2007///
N1 - Last updated - 2012-12-14
SubjectsTermNotLitGenreText - 9939 9306 5241; 3229 6996; 5018; 5891 2877; 4723; 4109 4335; 9925 3150; 3218 5939; 4954; 7940 126; 6175 1326 3629 6582 8836; 3363 10278 8016 4542; 8130 3085 3150; 3368 3150; 10482 730; 7531 730; 8027 2392 8697
KW - Netherlands
KW - Netherlands Antilles
KW - Suriname
KW - ERIC,Current Index to Journals in Education (CIJE)
KW - Elementary Education
KW - Elementary Secondary Education
KW - Preschool Education
KW - Secondary Education
KW - Elementary School Students
KW - High Risk Students
KW - Play
KW - Special Education
KW - Inclusive Schools
KW - Educational Legislation
KW - Learning Disabilities
KW - Teacher Attitudes
KW - Longitudinal Studies
KW - Identification
KW - Educational Needs
KW - Portfolios (Background Materials)
KW - Foreign Countries
KW - Parent Attitudes
KW - Special Schools
KW - ERIC
RP - NOT IN FILE
SP - 20
JF - Childhood Education
VL - 84
IS - 1
PB - Association for Childhood Education International. 17904 Georgia Avenue Suite 215, Olney, MD 20832.
N2 - Inclusive education in the Dutch education system has achieved new meaning in the last decade or so. Until 1998, the Netherlands recognized 19 types of special education. Then, two Educational Acts were passed, in 1998 and 2003, that decreased the types of special education by including measures to enhance inclusion of students with special educational needs in regular primary and secondary education. The authors first present information about the Dutch preschool and primary school system, followed by a description of the two laws

mentioned above. (The information applies to the Netherlands, the Netherlands Antilles, and Suriname.) They then present the main findings of three investigative studies. The first study was retrospective and it revealed teachers' and parents' views about students who had been referred to special schools in relationship to the students' portfolios. The second study was a literature search, conducted under orders of the Dutch government to clarify how identification of learning disabilities is organized in Europe and the United States. The third study was a longitudinal experimental study that included microgenetic data to analyze the social play of at-risk students taught in either regular or special primary schools
SN - 0009-4056, 0009-4056
UR - http://search.proquest.com/docview/61932060?accountid=14701
ER -

TY - JOUR
ID - 1114
T1 - The Microgenetic Emergence of Cooperative Play in 6-Year-Olds Developmentally At-Risk
A1 - Van der Aalsvoort,Geerdina M.
A1 - van der Leeden,Rien
Y1 - 2009///
N1 - Last updated - 2012-12-14
SubjectsTermNotLitGenreText - 7940 126; 5470; 4290; 2221 909; 5748 11228 1703 5053 2787; 10621 3227 6582; 695 694 8016 4542; 5797 11233 10789 6447; 9370 2572; 10087 2574 3629 6582; 5340 1718 10789 6447; 7146 1; 9835 10103; 3250; 8166 10789 6447; 3386 5053 2787; 7753; 3698 4542 8852 6582; 8299
KW - ERIC,Current Index to Journals in Education (CIJE)
KW - Language Tests
KW - Program Effectiveness
KW - Language Acquisition
KW - Play
KW - Gender Differences
KW - Socioeconomic Status
KW - Cooperation
KW - Personality
KW - Scores
KW - Experimental Groups
KW - Intervention
KW - Nonverbal Ability
KW - Emotional Development
KW - Pretests Posttests
KW - At Risk Students
KW - Statistical Analysis
KW - Educational Quality
KW - Intelligence Tests
KW - Teaching Methods
KW - ERIC
RP - NOT IN FILE
SP - 274
EP - 285
JF - International Journal of Educational Research
VL - 48
IS - 4
PB - Elsevier. 6277 Sea Harbor Drive, Orlando, FL 32887-4800.
N2 - Cooperative play was investigated by a controlled pre/post-test intervention design with 28 dyads of 6-year-old students developmentally at-risk. Selection was based upon cut-off scores on a language development test and a nonverbal IQ test, and same-sex pairs were matched within classrooms. Co-variables were: socio economic status, free play time in school and pedagogical quality of the classrooms. Each child was rated with respect to temperament. Play session 1 served as pretest for both research conditions. Then five play sessions of 20 min within 3 weeks took place in the experimental condition. Eight weeks after session 6 a play session took place in both research conditions serving as post-test. After microgenetic analyses of the data, ANCOVA's were

computed of time played in collaboration, and proportion of pretend play and of deep collaboration. The results revealed that the level of collaboration had improved dramatically in the experimental condition only. Moreover, the findings showed that the length of time dyads spent cooperating, and how deeply they collaborated on tasks, was related to how agreeable and emotionally stable they were. Gender effects also came forward. No long-term intervention effects were found. (Contains 8 tables and 1 figure.)
SN - 0883-0355, 0883-0355
UR - http://search.proquest.com/docview/742861322?accountid=14701
ER -

TY - JOUR
ID - 1115
T1 - Phonological Development in Very-Low-Birthweight Children: An Exploratory Study
A1 - Van Noort-Van Der Spek,Inge
A1 - Franken,Marie Christine
A1 - Wieringa,Marjan H.
A1 - Weisglas-Kuperus,Nynke
Y1 - 2010/06//
N1 - Last updated - 2012-12-14
SubjectsTermNotLitGenreText - 7825 6089 9804 9351 5964; 8112 5118 11655 1474 316 8016 4542; 1096 7843 5051; 694 8016 4542; 9970 2877; 1443 5053 2787; 1955 3629 6582; 7940 126; 6823 3932 8016 4542 7577 3816; 7537 3851 5449 8768; 5348 8768; 1703 5053 2787; 8426 9690 1; 5748 11228 1703 5053 2787; 9977 753 5792 9690 1; 7822 5792 9690 1
KW - Mean Length of Utterance
KW - ERIC,Current Index to Journals in Education (CIJE)
KW - Interaction
KW - At Risk Persons
KW - Language Acquisition
KW - Cognitive Development
KW - Play
KW - Speech Impairments
KW - Mothers
KW - Phonology
KW - Body Weight
KW - Comparative Analysis
KW - Phonological Awareness
KW - Speech Skills
KW - Parent Child Relationship
KW - Premature Infants
KW - Child Development
KW - Psychomotor Skills
KW - ERIC
RP - NOT IN FILE
SP - 541
EP - 546
JF - Developmental Medicine & Child Neurology
JA - Dev.Med Child Neurol.
VL - 52
IS - 6
PB - Wiley-Blackwell. 350 Main Street, Malden, MA 02148.
N2 - Aim: Very-low-birthweight (VLBW; birthweight less than 1500g and/or gestational age less than 32wks) children are at risk for speech problems. However, there are few studies on speech development in VLBW children at an early age. The aim of this study was to investigate phonological development in 2-year-old VLBW children. Method: Twenty VLBW children without major neurosensory impairment (7 males, 13 females; mean birthweight 971g, SD 315; mean gestational age 28wks, SD 1.81) and 20 term children (7 males, 13 females; mean birthweight 3503g, SD 416; mean gestational age 40wks, SD 1.26) were compared on measures of phonological development derived from 20-minute spontaneous speech samples of standardized

mother-child play interaction as well as on standardized tests of cognitive and psychomotor development, language, and behaviour. Results: VLBW children had significantly fewer acquired consonants (median 9, p = 0.02) and a significantly lower phonological mean length of utterance (pMLU; median 4.1, p less than 0.01) than term children (median acquired consonants 10, median pMLU 5.0). Interpretation: This study provides evidence for poor phonological development in even healthy VLBW children, compared with term-matched children, independent of their cognitive, psychomotor, and language development, and their behavioural functioning
SN - 0012-1622, 0012-1622
UR - http://search.proquest.com/docview/742872333?accountid=14701
ER -

TY - JOUR
ID - 1116
T1 - "Memories Are Made of This": Some Reflections on Outdoor Learning and Recall
A1 - Waite,Sue
Y1 - 2007/11//
N1 - Last updated - 2012-12-14
SubjectsTermNotLitGenreText - 7458 3150; 4109 4335; 3365 10591 8267 3417 8016 4542 5703 4908 8917; 3363 10278 8016 4542; 6519 1710; 1326 3629 6582 8836; 7747 8824 8477; 3250; 8135 10591 8267 3417 8016 4542 5703 4908 8917; 10380 3629 6582; 1433 9806 4911 9556; 5449 8768; 7940 126; 8996; 3692 5882; 9529 3685 853; 3395 8930 909; 226 316 8016 4542; 1474 316 8016 4542
KW - United Kingdom
KW - England
KW - ERIC,Current Index to Journals in Education (CIJE)
KW - Practitioners
KW - Elementary Education
KW - Preschool Teachers
KW - Elementary School Students
KW - Play
KW - Case Studies
KW - SURVEYS
KW - Child Care
KW - Adults
KW - Elementary School Teachers
KW - CHILDREN
KW - Sensory Experience
KW - Risk
KW - Memory
KW - Foreign Countries
KW - Experiential Learning
KW - Personal Narratives
KW - Emotional Response
KW - Interpersonal Relationship
KW - Outdoor Education
KW - Educational Quality
KW - ERIC
RP - NOT IN FILE
SP - 333
EP - 347
JF - Education 3-13
VL - 35
IS - 4
PB - Routledge. , 325 Chestnut Street Suite 800, Philadelphia, PA 19106.
N2 - Potential benefits for learning that the outdoors may hold have been brought into increased focus in the UK by the recent introduction of a manifesto for learning outside the classroom (DfES, "Learning outside the classroom: manifesto"; Nottingham, Department for Education and Skills, 2006). This article draws on two

recent studies of outdoor learning practices--a survey of 334 practitioners with children aged between 2 and 11, and a case study in a primary school in the West of England. The survey asked practitioners about their memories of outdoor experiences, and in the case study, the children talk of what they remember of their learning outdoors. With reference to relevant literature, the article reflects on how the quality of outdoor experience may sustain and support engagement and memory. (Contains 1 table and 1 note.)
SN - 0300-4279, 0300-4279
UR - http://search.proquest.com/docview/61899049?accountid=14701
ER -

TY - GEN
ID - 1117
T1 - Harvard Education Letter. Volume 28, Number 1, January-February 2012
A1 - Walser,Nancy
Y1 - 2012///
N1 - Last updated - 2012-12-14
KW - ERIC,Resources in Education (RIE)
KW - Elementary Secondary Education
KW - Program Effectiveness
KW - Play
KW - Pervasive Developmental Disorders
KW - School Culture
KW - Moral Development
KW - Skill Development
KW - At Risk Students
KW - Educational Environment
KW - Educational Games
KW - Interpersonal Competence
KW - Theater Arts
KW - Social Development
KW - Moral Values
KW - Autism
KW - Drama
KW - ERIC
RP - NOT IN FILE
SP - 8
PB - Harvard Education Press. 8 Story Street First Floor, Cambridge, MA 02138.
N2 - "Harvard Education Letter" is published bimonthly at the Harvard Graduate School of Education. This issue of "Harvard Education Letter" contains the following articles: (1) Using Theater to Teach Social Skills: Researchers Document Improvements for Children with Autism (Patti Hartigan); (2) The Family Model of Schooling Revisited: Few Teachers, Big Groups, and Lots of Flexibility (Ann Doss Helms); and (3) Promoting Moral Development in Schools (Richard Weissbourd)
UR - http://search.proquest.com/docview/964185289?accountid=14701
ER -

TY - JOUR
ID - 1118
T1 - Rough-and-tumble play: II: On the importance of providing boys with sufficient space to be boys. [Norwegian]. [References]
A1 - Eide-Midtsand,Nils
Y1 - 2007///
N1 - Peer Reviewed Journal: 2007-20035-002
Norwegian
Journal; Peer Reviewed Journal
KW - PsycInfo
KW - Rough and Tumble
KW - Childhood Play Behavior

KW - Human Males
KW - Child Psychotherapy
KW - Personality Correlates
RP - NOT IN FILE
SP - Dec
EP - 1474
JF - Tidsskrift for Norsk Psykologforening
VL - .44
IS - 12
N2 - The author explores the possibility that contemporary society moves in directions that make it increasingly difficult for boys to adjust. Gender differences in brain biology and behavioural predispositions make boys play and learn in different ways that girls. Like young males of other mammalian species boys are more prone to impulsivity, movement and rough-and-tumble play--a form of play that appears to boost maturational processes in their brain, but that is often misjudged as "bad behaviour" and discouraged. It seems that boys' natural ways are being increasingly pathologised, and that the epidemic of ADHD might well be regarded as a specific manifestation of this process. Also, boys may need other forms of therapy than do girls. (PsycINFO Database Record (c) 2012 APA, all rights reserved) (journal abstract)
SN - 0332-6470
AD - Eide-Midtsand, Nils: Avdeling for barn og ungdoms psykiske helse, Poliklinikken Sorlandet Sykehus HF, Kristiansand, Norway
ER -

TY - JOUR
ID - 1119
T1 - Rough-and-tumble play: Its functions in psychotherapy and in the mental development of children. [Norwegian]. [References]
A1 - Eide-Midtsand,Nils
Y1 - 2007///
N1 - Peer Reviewed Journal: 2007-20035-001
Norwegian
Journal; Peer Reviewed Journal
KW - PsycInfo
KW - Rough and Tumble
KW - Child Psychotherapy
KW - Childhood Development
KW - Childhood Play Behavior
KW - Cognitive Development
KW - Human Males
KW - Brain
KW - Trauma
RP - NOT IN FILE
SP - Dec
EP - 1466
JF - Tidsskrift for Norsk Psykologforening
VL - .44
IS - 12
N2 - Deprived and severely traumatised boys in psychotherapy often choose to express themselves through physical activities rather than words. They typically want to play roughhouse, and especially to play fight with the therapist. For various reasons the boys are often excluded from the normal rough-and-tumble play with other children. Excerpts from the therapy of a nine year old is presented, and illustrated with findings from the fields of psychology and brain research. It is suggested that rough-and-tumble play serves to further the development of regulatory brain mechanisms, essentially of the same kind as aimed at by play in psychotherapy. (PsycINFO Database Record (c) 2012 APA, all rights reserved) (journal abstract)
SN - 0332-6470
AD - Eide-Midtsand, Nils: Avdeling for barn og ungdoms psykiske helse, Poliklinikken Sorlandet sykehus HF, Kristiansand, Norway

ER -

TY - JOUR
ID - 1120
T1 - Ten Ways to Restrict Children's Freedom to Play: The Problem of Surplus Safety
A1 - Wyver,Shirley
A1 - Tranter,Paul
A1 - Naughton,Geraldine
A1 - Little,Helen
A1 - Sandseter,Ellen Beate Hansen
A1 - Bundy,Anita
Y1 - 2010///
N1 - Last updated - 2012-12-14
SubjectsTermNotLitGenreText - 7940 126; 9074; 11655 1474 316 8016 4542; 7943 8704 3760; 4109 4335; 3899 8415; 8996; 1955 3629 6582; 1443 5053 2787; 4185; 5183 2877; 3085 3150
KW - Australia
KW - Norway
KW - ERIC,Current Index to Journals in Education (CIJE)
KW - Early Childhood Education
KW - Play
KW - Injuries
KW - Young Children
KW - Fear
KW - Safety
KW - Comparative Analysis
KW - Risk
KW - Foreign Countries
KW - Playgrounds
KW - Child Development
KW - Freedom
KW - ERIC
RP - NOT IN FILE
SP - 263
EP - 277
JF - Contemporary Issues in Early Childhood
VL - 11
IS - 3
PB - Symposium Journals. P.O. Box 204, Didcot, Oxford, OX11 9ZQ, UK. Tel: +44-1235-818-062; Fax: +44-1235-817-275; e-mail: subscriptions@symposium-journals.co.uk; Web site: http://www.wwwords.co.uk/ciec
N2 - Play and playgrounds provide essential experiences for young children's growth, development and enjoyment of life. However, such play experiences are now limited for many children due to excessive fear of risk, or "surplus safety". In this article, the authors examine the pervasiveness of surplus safety in the lives of young children. They argue that restrictions now imposed on children's play to promote safety may, paradoxically, expose children to more serious short and longer term threats of illness and limit children's life opportunities. By comparing experiences from Australia and Norway, the authors demonstrate that surplus safety is not a necessary outcome of living in a modern Western society
SN - 1463-9491, 1463-9491
UR - http://search.proquest.com/docview/815959686?accountid=14701
ER -

TY - JOUR
ID - 1121
T1 - The Power of Play: A Literature-based after School Sports Program for Urban Youth
A1 - Zierk,Tom
Y1 - 2000///

N1 - Last updated - 2012-12-14
SubjectsTermNotLitGenreText - After School Programs 309 8331; Athletics 704 7840 126; 1443 5053 2787; 3368 3150; 4723; 5446 1970 1; Literacy 6101; Literature 6120 4918 5964; Play 7940 126; 8661 8623 11225 1 5792 9690; 11635 11615 11225 1 5792 9690; Youth Programs 11666 8331
KW - ERIC,Current Index to Journals in Education (CIJE)
KW - Elementary Secondary Education
KW - Athletics
KW - High Risk Students
KW - Play
KW - Literature
KW - After School Programs
KW - Writing Skills
KW - Reading Skills
KW - Youth Programs
KW - Interpersonal Competence
KW - Child Development
KW - Literacy
KW - ERIC
RP - NOT IN FILE
SP - 12
EP - 17
JF - Afterschool Matters
VL - 1
IS - 1
PB - Afterschool Matters, c/o Children & the Classics, 114 W. 17th St.,Suite 3R, New York, NY 10011;
N2 - A program that combines sports and literature can improve students' reading, writing, and comprehension skills, promoting links between children's personal development and self-esteem and forging links between sports, literature, and daily life. Describes one such program, Sports PLUS After School, noting: program goals, components, and structure; teachers as coaches; support components; and a typical week. (SM)
UR - http://search.proquest.com/docview/62342723?accountid=14701
ER -

TY - BOOK
ID - 1122
T1 - Annual report, 1970-71
Y1 - 1971///
N1 - Accession Number: SPH335981; Corporate Author: Children's Research Center. Motor Performance and Play Research Laboratory; Language: English; Description: 24 P.; Database Subset: SIRLS Database; Publication Type: Monograph or government document; Update Code: 19940101
KW - *CONTESTS
KW - *MOTIVATION (Psychology)
KW - *ACHIEVEMENT
KW - *PLAY
KW - *PSYCHOLOGY
KW - *RISK-taking (Psychology)
KW - Anxiety
KW - BIBLIOGRAPHY
KW - ABILITY
KW - CHILDREN
KW - SOCIAL psychology
KW - Social Support
KW - COACTION
KW - REVIEW
KW - SportDiscus
KW - Rough and Tumble
RP - NOT IN FILE

CY - Urbana, Illinois; United States
PB - University of Illinois. Motor Performance and Play Research Laboratory.
N2 - Contains abstracts of reports written by individuals at the Motor Performance and Research Laboratory
UR -
https://login.proxy.bib.uottawa.ca/login?url=http://search.ebscohost.com/login.aspx?direct=true&db=s3h&AN=SPH335981&site=ehost-live
ER -

TY - JOUR
ID - 1123
T1 - Clearing the Air
A1 - Attwood,Emily
Y1 - 2012/06//
N1 - Accession Number: 76921521; Author: Attwood, Emily email: emily@athleticbusiness.com. ; No. of Pages: 2; Language: English; Publication Type: Journal Article; Update Code: 20120620
KW - *PARKS
KW - *PLAYGROUNDS
KW - *PASSIVE smoking
KW - SMOKING policy
KW - NONSMOKING areas
KW - CIVIC leaders
KW - United States
KW - SportDiscus
RP - NOT IN FILE
SP - 185
EP - 186
JA - Athletic Business
VL - 36
IS - 6
N2 - The article reports on the move of parks and playgrounds in the U.S. to ban smoking. According to the Americans for Nonsmokers' Rights Association, more than 600 communities have implemented smoking bans in parks. They made the decision in response to the dangers of secondhand smoke in outdoor areas. Several programs have been launched to educate community leaders about the risks of tobacco use in parks and playground areas
SN - 0747315X
UR -
https://login.proxy.bib.uottawa.ca/login?url=http://search.ebscohost.com/login.aspx?direct=true&db=s3h&AN=76921521&site=ehost-live
ER -

TY - JOUR
ID - 1124
T1 - ARSENIC AND YOUR PLAYGROUND
A1 - Dolesh,Richard J.
Y1 - 2004/04//
N1 - Accession Number: 12860750; Author: Dolesh, Richard J.: 1 email: rdolesh@nrpa.org. ; Author Affiliation: 1 Senior Policy Associate, National Recreation and Park Association's Public Policy Office; No. of Pages: 6; Language: English; Publication Type: Journal Article; Update Code: 20040421
KW - *PARKS
KW - *PLAYGROUNDS
KW - WOOD preservatives
KW - ARSENIC
KW - ASSOCIATIONS,institutions,etc.
KW - United States
KW - SportDiscus
RP - NOT IN FILE

SP - 61
EP - 67
JA - Parks & Recreation
VL - 39
IS - 4
N2 - Reveals that the 2004 voluntary phase-out of arsenic-treated wood is giving park and recreation departments in the U.S. new reason to evaluate their parks. Definition of chromated copper arsenate; Information on the Environmental Working Group's 2001 report entitled "Poisoned Playgrounds"; Elements to consider in assessing risks and developing agency policies
SN - 00312215
UR -
https://login.proxy.bib.uottawa.ca/login?url=http://search.ebscohost.com/login.aspx?direct=true&db=s3h&AN=12860750&site=ehost-live
ER -

TY - JOUR
ID - 1125
T1 - Learning to teach science as inquiry in the rough and tumble of practice. [References]
A1 - Crawford,Barbara A.
Y1 - 2007///
N1 - Peer Reviewed Journal: 2007-05352-005
English
Journal; Peer Reviewed Journal
KW - PsycInfo
KW - Rough and Tumble
KW - Science Education
KW - Teacher Education
KW - Teaching
KW - Attitudes
KW - Classrooms
KW - Energy Expenditure
KW - Knowledge Level
RP - NOT IN FILE
SP - Apr
EP - 642
JF - Journal of Research in Science Teaching
VL - .44
IS - 4
N2 - This study examined the knowledge, beliefs and efforts of five prospective teachers to enact teaching science as inquiry, over the course of a one-year high school fieldwork experience. Data sources included interviews, field notes, and artifacts, as these prospective teachers engaged in learning how to teach science. Research questions included 1) What were these prospective teachers' beliefs of teaching science? 2) To what extent did these prospective teachers articulate understandings of teaching science as inquiry? 3) In what ways, if any, did these prospective teachers endeavor to teach science as inquiry in their classrooms? 4) In what ways did the mentor teachers' views of teaching science appear to support or constrain these prospective teachers' intentions and abilities to teach science as inquiry? Despite support from a professional development school setting, the Interns' teaching strategies represented an entire spectrum of practice--from traditional, lecture-driven lessons, to innovative, open, full-inquiry projects. Evidence suggests one of the critical factors influencing a prospective teacher's intentions and abilities to teach science as inquiry, is the teacher's complex set of personal beliefs about teaching and of science. This paper explores the methodological issues in examining teachers' beliefs and knowledge in actual classroom practice. (PsycINFO Database Record (c) 2012 APA, all rights reserved) (journal abstract)
SN - 0022-4308
AD - Crawford, Barbara A.: Department of Education, Cornell University, Ithaca, NY, US
ER -

TY - JOUR
ID - 1126
T1 - DOES HEIGHT MATTER?
A1 - Frost,Joe
A1 - Sutterby,John
A1 - Therrell,Jim
A1 - Brown,Pei San
A1 - Thornton,Candra
Y1 - 2002/05//
N1 - Accession Number: 6554089; Author: Frost, Joe Author: Sutterby, John Author: Therrell, Jim Author: Brown, Pei-San Author: Thornton, Candra ; No. of Pages: 9; Language: English; Publication Type: Journal Article; Update Code: 20020426
KW - *PLAYGROUNDS
KW - *EQUIPMENT & supplies
KW - *FALLS (Accidents)
KW - SAFETY measures
KW - United States
KW - SportDiscus
RP - NOT IN FILE
SP - 74
JA - Parks & Recreation
VL - 37
IS - 5
N2 - Discusses the risks of the designed fall height of several playground equipment in the U.S. Benefits of climbing for children; Details of the national guidelines and standards for heights and surfacing in the U.S.; Recommended fall height of climbing equipment and slides in the country; Factors which affect the potential injury resulting from falls; Discussion on playground safety
SN - 00312215
UR -
https://login.proxy.bib.uottawa.ca/login?url=http://search.ebscohost.com/login.aspx?direct=true&db=s3h&AN=6554089&site=ehost-live
ER -

TY - JOUR
ID - 1127
T1 - The Game Structure as a Critical Component in Life
A1 - Glassford,R.G.
Y1 - 1975///
N1 - Accession Number: SPH332353; Author: Glassford, R.G. ; Language: English; References: 18; Database Subset: SIRLS Database; Publication Type: Journal article; Update Code: 19940101; SIRC Article No.: 332353
KW - *GAMES
KW - *LIFESTYLES
KW - *PLAY
KW - Child Development
KW - SOCIALIZATION
KW - STRATEGY
KW - SportDiscus
RP - NOT IN FILE
SP - 3
JA - Katimavik
VL - 2
IS - 4
N2 - Discusses the concept of play and games as part of the great game' of life Games involving choice pathways, strategies, rules, goals, risks and trade-offs are considered to have a strong element in our culture. Considers the influence of unstructured play and rule-governed games on the development of young children

UR -
https://login.proxy.bib.uottawa.ca/login?url=http://search.ebscohost.com/login.aspx?direct=true&db=s3h&AN=SPH332353&site=ehost-live;http://articles.sirc.ca/search.cfm?id=332353
ER -

TY - JOUR
ID - 1128
T1 - Playing with Safety?
A1 - Johnston,Kevin
A1 - Bruya,Lawrence
A1 - Schappet,Jean
Y1 - 2004/09//
N1 - Accession Number: SPHS-1013666; Author: Johnston, Kevin Author: Bruya, Lawrence Author: Schappet, Jean ; No. of Pages: 1; Language: English; Parent Item: SPHP6376; General Notes: Perspectives.; Database Subset: Recreation and Leisure; Publication Type: Journal article; Material Type: PRINT; Update Code: 20060501; SIRC Article No.: S-1013666
KW - *WATER
KW - *PLAYGROUNDS
KW - *SAFETY
KW - standards
KW - AMERICAN Society for Testing & Materials
KW - SportDiscus
RP - NOT IN FILE
SP - 16
EP - 16
JA - Aquatics International
VL - 16
IS - 8
CY - United States
N2 - This article argues that the safety standards for water playgrounds being proposed by the ASTM (American Society for Testing & Materials) are not adequate, and do not take account of the special risks posed by this kind of play environment
SN - 10587039
UR -
https://login.proxy.bib.uottawa.ca/login?url=http://search.ebscohost.com/login.aspx?direct=true&db=s3h&AN=SPHS-1013666&site=ehost-live;http://articles.sirc.ca/search.cfm?id=S-1013666;http://www.hanley-wood.com/
ER -

TY - JOUR
ID - 1129
T1 - Evolutionary Foundations and Functions of Play: An Overview. [References]
A1 - Smith,Peter K.
Y1 - 2006///
N1 - Book: 2007-04529-002
English
Book; Edited Book
KW - PsycInfo
KW - Rough and Tumble
KW - Childhood Play Behavior
KW - Childhood Play Development
KW - Theory of Evolution
KW - History
KW - Pretend Play
RP - NOT IN FILE
SP - sociocultural, and
EP - 49

JF - Goncu, Artin [Ed]; Gaskins, Suzanne [Ed]
IS - 2006
N2 - (from the chapter) In this chapter I give an overview of how evolutionary theory has impacted the study of children's play. There have been distinct ups and downs in this influence since evolutionary theory originated, and I start with a historical account of this. I also mention the play ethos that influenced much research in the 20th century. I delineate what I regard as four important characteristics of an evolutionary approach, useful at the present time. I then discuss the relevance of such an approach to the main forms of play--physical activity and rough-and-tumble play, object play, and pretend and sociodramatic play. I take the opportunity to cross-refer to the following chapters by Mitchell (chap. 3, this volume, record 2007-04529-003), and Pellegrini (chap. 4, this volume, record 2007-04529-004), where appropriate. I end with a brief discussion of genetic and cultural evolution and of future directions in this field. (PsycINFO Database Record (c) 2012 APA, all rights reserved)
AD - Smith, Peter K.: Department of Psychology, Goldsmiths College, University of London, London, England
ER -

TY - JOUR
ID - 1130
T1 - Play and development: Evolutionary, sociocultural, and functional perspectives
A1 - Goncu,Artin
Y1 - 2006///
N1 - Book: 2007-04529-000
English
Book; Edited Book
(Created by PsycINFO) Preface List of contributors I. Introduction * An Integrative Perspective on Play and Development / Artin Goncu and Suzanne Gaskins / 3-17 II. Biological/ethological perspectives of play and development * Evolutionary Foundations and Functions of Play: An Overview / Peter K. Smith / 21-49 * Pretense in Animals: The Continuing Relevance of Children's Pretense / Robert W. Mitchell / 51-75 * The Development and Function of Rough-and-Tumble Play in Childhood and Adolescence: A Sexual Selection Theory Perspective / Anthony D. Pellegrini / 77-98 III. Social/cultural perspectives of play and development * On the Significance of Social Relationships in the Development of Children's Earliest Symbolic Play: An Ecological Perspective / Marc H. Bornstein / 101-129 * Guided Participation: How Mothers Structure and Children Understand Pretend Play / Angeline Lillard / 131-153 * Children's Play as Cultural Interpretation / Artin Goncu, Jyoti Jain, and Ute Tuermer / 155-178 * The Cultural Construction of Play / Suzanne Gaskins, Wendy Haight, and David F. Lancy / 179-202 IV. Applied perspectives of play and development * Hard Work for the Imagination / Paul L. Harris / 205-225 * Of Hobbes and Harvey: The Imaginary Companions Created by Children and Adults / Marjorie Taylor and Anne M. Mannering / 227-245 * The Interplay of Play and Narrative in Children's Development: Theoretical Reflections and Concrete Examples / Ageliki Nicolopoulou / 247-273 * Therapeutic Advantages of Play / Cindy Dell Clark / 275-293 Author index Subject index
KW - PsycInfo
KW - Rough and Tumble
KW - Childhood Play Behavior
KW - Childhood Play Development
KW - Evolutionary Psychology
KW - Sociocultural Factors
KW - Imagination
RP - NOT IN FILE
SP - sociocultural, and
JF - (2006)
VL - Play and development
N2 - (from the cover) Children's play is a universal human activity, and one that serves a significant purpose in personal development. Throughout this volume, the editors and contributors explore assumptions about play and its status as a unique and universal activity in humans. As a whole, Play and Development delves into three lines of research concerning this topic: examining these assumptions from an ethological perspective, exploring the view of research that shows play to be socially and culturally constructed, and looking at varied applications of play in the different contexts of childhood. Topics covered in this volume include: (a) evolutionary foundations and functions of play; (b) children's play as cultural interpretation; (c) the use of imagination in children's play; and (d) the imaginary companions created by children and adults. This volume will be of interest to scholars and

researchers in the fields of developmental psychology and cognitive development, and will be sought out by active researchers and practitioners in the field. (PsycINFO Database Record (c) 2012 APA, all rights reserved)
AD - Goncu, Artin: College of Education, University of Illinois at Chicago, Chicago, IL, US Gaskins, Suzanne: Department of Psychology, Northeastern Illinois University, Chicago, IL, US
ER -

TY - JOUR
ID - 1131
T1 - Young children's rough and tumble play: An exploratory study
A1 - Tannock,Michelle Therese
Y1 - 2006///
N1 - Dissertation Abstract: 2006-99021-102
English
Dissertation Abstract
KW - PsycInfo
KW - Rough and Tumble
KW - Childhood Development
KW - Childhood Play Behavior
KW - Parents
KW - Teachers
RP - NOT IN FILE
SP - 1645
JF - Dissertation Abstracts International Section A: Humanities and Social Sciences
VL - .67
IS - 5-A
N2 - This study explores, through observations and interviews, the rough and tumble play of young children in early childhood settings. The purpose of this study was to gain an understanding of how early childhood educators, parents, and young children interpret rough and tumble play. The study also identified the extent to which rough and tumble play is included or not included within early childhood settings. Participants in this study included 11 educators, 16 parents, and 16 children from four settings. Observations of the play of children and the responses of the educators to rough and tumble play were made at two settings. During 30:25 hours of observation, 110 incidents of rough and tumble play were recorded. Twenty-seven distinct rough and tumble play behaviours were exhibited during the observation period. Behaviours included components that had been identified as rough and tumble play in previous research and also additional behaviours that were not previously identified as elements of rough and tumble play. Results of the interviews of adults indicate that there is perceived value in rough and tumble play; the play needs to be supervised; the play is more acceptable at home rather than at daycare; adults are unaware of formal policies or guidelines for the play; and adults reject the notion that the play may be linked to aggressive behaviour. Results of the interviews with children indicate that adults place restrictions on the play; it is important that no one is hurt; there are gender differences; and while all the children were observed engaged in the play, 60% of the children stated that they do not engage in rough and tumble play at daycare. The results of this study will have implications for the understanding of child development. It may be that rough and tumble play evolves as children age; that children move into more, or less, complex play behaviours as they mature. This study might also have implications for early childhood education. The parents and educators conceded a lack of knowledge about rough and tumble play. This finding highlights the need for the development of teacher and parent education resources. (PsycINFO Database Record (c) 2012 APA, all rights reserved)
SN - 0419-4209
AD - Tannock, Michelle Therese: U Victoria, Canada
ER -

TY - JOUR
ID - 1132
T1 - Playground without risks would not interest children
A1 - Munch,A.
Y1 - 1979/11//

N1 - Accession Number: SPH70656; Author: Munch, A. ; Language: English; Database Subset: Recreation and Leisure; Publication Type: Journal article; Update Code: 19940901; SIRC Article No.: 70656
KW - *PLAYGROUNDS
KW - *SAFETY
KW - *EQUIPMENT & supplies
KW - CHILDREN
KW - LIABILITY
KW - SportDiscus
RP - NOT IN FILE
SP - 12
JA - Leisure Forum
VL - 6
IS - 5
CY - Canada
SN - 0704643X
UR -
https://login.proxy.bib.uottawa.ca/login?url=http://search.ebscohost.com/login.aspx?direct=true&db=s3h&AN=SPH70656&site=ehost-live;http://articles.sirc.ca/search.cfm?id=70656
ER -

TY - JOUR
ID - 1133
T1 - Harvard, soccer & over-scheduled families
A1 - Rosenfeld,A.
Y1 - 2004/03//
N1 - Accession Number: SPHS-944832; Author: Rosenfeld, A. ; No. of Pages: 4; Language: English; Parent Item: SPHP95851; References: 15; Database Subset: Indexing Partner Contribution; Publication Type: Journal article; Update Code: 20040701
KW - *COACHING (Athletics)
KW - *PLAY
KW - CHILDREN
KW - Child Rearing
KW - PARENT & child
KW - United States
KW - SportDiscus
RP - NOT IN FILE
SP - 15
EP - 18
JA - Youth Studies Australia
VL - 23
IS - 1
CY - Australia
N2 - The over-scheduled child-rearing style is currently being touted in the USA and in much of the rest of the developed world as the best way to raise children. However, this over-scheduled lifestyle may be damaging marriages; getting unhappy children diagnosed as learning disabled, ADD, bipolar and depressed; and causing adolescents to be involved with drugs, alcohol and premature sex (Luthar & Becker 2002). Renowned American child and adolescent psychiatrist Alvin Rosenfeld points out that the dangers and risks to children of this intensive lifestyle are known, but, he asks, "Is anyone listening"
SN - 10382569
UR -
https://login.proxy.bib.uottawa.ca/login?url=http://search.ebscohost.com/login.aspx?direct=true&db=s3h&AN=SPHS-944832&site=ehost-live
ER -

TY - JOUR
ID - 1134

T1 - Preschool teachers' exposure to classroom noise. [References]
A1 - Grebennikov,Leonid
Y1 - 2006///
N1 - Peer Reviewed Journal: 2006-02675-003
English
Journal; Peer Reviewed Journal
KW - PsycInfo
KW - Rough and Tumble
KW - Noise Effects
KW - Noise Levels (Work Areas)
KW - Preschool Teachers
KW - Classroom Behavior
RP - NOT IN FILE
SP - Mar
EP - 44
JA - International Journal of Early Years Education
VL - .14
IS - 1
N2 - This research examined exposure to classroom noise of 25 full-time teaching staff in 14 preschool settings located across Western Sydney. The results indicated that one teacher exceeded the maximum permissible level of daily noise exposure for employees under the health and safety legislation. Three staff approached this level and 92% of teachers were subjected to daily noise exposure which, if occurs repeatedly, is considered potentially harmful. Nine staff recorded peak noise rates in excess of the permitted limit. High levels of noise were evident when large numbers of students were located in confined areas, when they were involved in rough play or were distressed, when the students dropped heavy play equipment, and during music sessions. Further research on noise in early educational settings across different nations appears to be valuable for understanding both the scope of the problem and possible remedies. (PsycINFO Database Record (c) 2012 APA, all rights reserved) (journal abstract)
SN - 0966-9760
AD - Grebennikov, Leonid: University of Western Sydney, NSW, Australia
ER -

TY - JOUR
ID - 1135
T1 - APRENDIZAJE, JUEGO Y NINO: UN MODO DE ENTENDER LA EDUCACION FISICA. / Aprendizagem, jogo e crianca: um modo de entender a Educacao Fisica. / Learning, playing and children. A way to understand Physical Education
A1 - Rozengardt,Rodolfo
Y1 - 2005/05//
N1 - Accession Number: SPHS-999199; Author: Rozengardt, Rodolfo: 1 email: rozencar@ciudad.com.ar. ; Author Affiliation: 1 Maestrando en Metodologia de la Investigacion Cientifica. Rector del Instituto Superior de Educacion Fisica de La Pampa, Argentina Editor de la revista Educacion Fisica y Deportes, Papeles de investigacion; No. of Pages: 16; Language: Spanish; Parent Item: SPHP2646; References: 19; Publication Type: Journal article; Material Type: PRINT; Update Code: 20051201; SIRC Article No.: S-999199
KW - *PHYSICAL education & training
KW - *TEACHING
KW - *PLAY
KW - CHILDREN
KW - Learning
KW - PHILOSOPHY
KW - SportDiscus
RP - NOT IN FILE
SP - 111
EP - 126
JA - Revista Brasileira de Ciencias do Esporte
VL - 26

IS - 3
CY - Brazil
N2 - As Physical Education teachers, we conduct our daily work in reference to what we understand teaching may be, what we think the result of our work might be, and who the subjects with whom we interact may be and do. These frames of reference are historically constructed, and each of us continues to create and recreate them in our interactions with others, i.e., our social experience. Our teaching practice with young students also influences our capacity to maintain and change the prevailing values about who we think children are, and what they deserve in school and other contexts of institutional corporal practice. This work is an alert to the present risks involved in the fragmentation of childhood images, pointing out to a concept of corporal subject, and rescues the need to preserve the richness and potentiality of the world of the children by appreciating play and creative learning. Physical Education for children could be based on a pleasurable lively teaching without abandoning the focus on content. [ABSTRACT FROM AUTHOR]
En la tarea cotidiana con ninos, los profesores de Educacion Fisica actuamos animados por representaciones acerca de lo que ensenamos, los efectos de nuestras intervenciones de los sujetos con los que interactuamos. Esas imagenes se han conformado historicamente y cada uno de nosotros las elabora en una experiencia social. Nuestra propia practica con ninos incide en el mantenimiento o cambio de los valores dominantes acerca lo que los ninos son y merecen recibir en un contexto de practica corporal institucional. En el siguiente trabajo se advierte sobre los peligros actuales en torno a la fragmentacion la imagen de la infancia, destacando una concepcion de sujeto corporal y se rescata la necesidad de defender el mundo infantil en su riqueza y potencialidad mediante el valor del juego y el aprendizaje creativo. La Educacion Fisica con ninos podria sostenerse sobre un modo ludico de ensenanza, sin abandonar la centralidad otorgada al contenido. [ABSTRACT FROM AUTHOR]
SN - 01013289
UR -
https://login.proxy.bib.uottawa.ca/login?url=http://search.ebscohost.com/login.aspx?direct=true&db=s3h&AN=SPHS-999199&site=ehost-live;http://articles.sirc.ca/search.cfm?id=S-999199
ER -

TY - JOUR
ID - 1136
T1 - The riskiness of the playground
A1 - Smith,S.J.
Y1 - 1990/08//
N1 - Accession Number: SPH383596; Author: Smith, S.J. ; No. of Pages: 17; Language: English; Parent Item: SPHP968538; References: 38; Database Subset: Recreation and Leisure; Publication Type: Journal article; Update Code: 19951101; SIRC Article No.: 383596
KW - *PLAYGROUNDS
KW - *PLAY
KW - *SAFETY
KW - *EQUIPMENT & supplies
KW - CHILDREN
KW - Risk
KW - DESIGN
KW - SportDiscus
RP - NOT IN FILE
SP - 71
EP - 87
JA - Journal of Educational Thought
VL - 24
IS - 2
CY - Canada
N2 - Risk is described, not as a negative attribute of children's playground activity, but more positively, as challenge and adventure to which children can actively respond. Risk refers to something that is being accomplished by children, some intended activity which expands their sense of the world. Such a positive sense of risk is developed by showing that the riskiness of the playground is a reflection of our concerns for the safety of children and their proper supervision. I describe, in particular, how we might observe children closely and

how we might take responsibility for the risky situations in which children find themselves. In so doing, I present a way of understanding how we can bring an underlying sense of security to children's feelings for the playground and for the risks that can be taken there
Les activites des enfants, surtout sur les terrains de jeux, comportent un facteur de risque. C'est ce qui justifie, chez l'adulte responsable, la preoccupation pour la securite des enfants. Pourtant, du point de vue des enfants, la situation de risque peut etre une occasion d'apprendre a relever des defis, de gouter l'aventure et de s'ouvrir sur le monde. Pour que les enfants puissent profiter des activites des terrains de jeux, il faut trouver les moyens de leur fournir la surveillance qui leur assurera la securite dont ils ont besoin pour risquer de s'aventurer dans le monde
SN - 00220701
UR -
https://login.proxy.bib.uottawa.ca/login?url=http://search.ebscohost.com/login.aspx?direct=true&db=s3h&AN=SPH383596&site=ehost-live;http://articles.sirc.ca/search.cfm?id=383596
ER -

TY - JOUR
ID - 1137
T1 - Making Sense of Children's Play Spaces
A1 - Winther,N.R.
Y1 - 1986///
N1 - Accession Number: SPH338894; Author: Winther, N.R. ; Conference: North American Society for the Sociology of Sport. Seventh Annual Meeting. Las Vegas, Nevada. (October 29-November 1, 1986).; Language: English; References: 7; Database Subset: SIRLS Database; Publication Type: Journal article; Update Code: 19940101; SIRC Article No.: 338894
KW - *PLAY
KW - *SPORTS facilities
KW - CHILDREN
KW - CONSUMERS' preferences
KW - SportDiscus
RP - NOT IN FILE
SP - 10
JA - Unpublished Paper
N2 - Studies the favourite play spaces of children. Discusses some characteristics of children's play spaces: close to nature, located away from adult eyes, and rich with sensory stimulation. Notes the inclusion of pet animals, the desire to experience both aloneness and togetherness, and the attractiveness of the opportunity to take risks. Points out that children see the multiple possibilities of objects or situations as opportunities for play, and move freely between reality and fantasy. Emphasizes that adults who wish to create 'child-friendly' spaces must reflect on their own childhood experiences and try to understand how the world is seen through the eyes of children
UR -
https://login.proxy.bib.uottawa.ca/login?url=http://search.ebscohost.com/login.aspx?direct=true&db=s3h&AN=SPH338894&site=ehost-live;http://articles.sirc.ca/search.cfm?id=338894
ER -

TY - JOUR
ID - 1138
T1 - WORKMEN'S compensation; horseplay doctrine limited in California
Y1 - 1946/04//
N1 - Current legal thought; the lawyers' digest of law reviews
58720310R
OM
Journal Article
English
KW - Medline
KW - Rough and Tumble
KW - Industry

KW - Medicine
KW - Occupational Diseases
KW - Workers' Compensation
RP - NOT IN FILE
SP - 295
JF - Current legal thought; the lawyers' digest of law reviews
JA - Curr.Leg.Thought Lawyers.Dig.Law Rev
VL - 12
CY - Not Available
UR - 20987214
ER -

TY - JOUR
ID - 1139
T1 - Rough and tumble guide to laundering
Y1 - 1994/01//
N1 - Elderly care
bp9, 9310629
N
Journal Article
English
KW - Medline
KW - Rough and Tumble
KW - Laundering
RP - NOT IN FILE
SP - 14
EP - 15
JF - Elderly Care
JA - Elder.Care
VL - 6
IS - 1
CY - ENGLAND
SN - 1369-1856
UR - 8148826
ER -

TY - JOUR
ID - 1140
T1 - The effects of neonatal exposure to testosterone on the development of behaviour in female marmoset monkeys
A1 - Abbott,D.H.
A1 - Hearn,J.P.
Y1 - 1978/03/14/
N1 - Abbott, D H. Hearn, J P
Ciba Foundation symposium
d7x, 0356636
IM
Journal Article
English
KW - Medline
KW - Rough and Tumble
KW - Animals
KW - Newborn
KW - Behavior
KW - Animal/de [Drug Effects]
KW - Body Weight

KW - Callitrichinae
KW - Drug Implants
KW - Female
KW - Genitalia
KW - Female/ah [Anatomy & Histology]
KW - Female/de [Drug Effects]
KW - Haplorhini
KW - Humans
KW - Male
KW - Sexual Behavior
KW - Testosterone/pd [Pharmacology]
RP - NOT IN FILE
SP - 299
EP - 327
JF - Ciba Foundation Symposium
JA - Ciba Found.Symp.
IS - 62
CY - NETHERLANDS
N2 - Experimental investigations of sexual differentiation in primates have been mainly confined to the rhesus monkey, a highly polygamous species with marked anatomical and behavioural sexual dimorphism. The marmoset is a monogamous monkey which shows little anatomical or behavioural sexual dimorphism, and both sexes exhibit positive feedback in response to the administration of oestradiol. This monkey has a relatively short gestation period of 144 days and usually gives birth to dizygotic twins. These share a common placental circulation and hence develop as haematopoietic chimaeras. However, a female with a male co-twin is not adversely affected as a result of this and the freemartin condition does not occur. Since the newborn male experiences raised testosterone levels soon after birth it is possible that the 'critical period' for sexual marmosets were implanted with 25 mg of testosterone for 50 days after birth. After removal of the implants their genitalia remained partly masculinized and they initiated more masculine rough-and-tumble play than their controls. After puberty they exhibited a mixture of male and female sexual behaviour when presented with unfamiliar normal males and females during 15-minute behavioural trials. Four of the implanted females have also ovulated. Neonatal administration of testosterone therefore has had an organizing effect on female behaviour. Whether the marmoset is exceptional in this respect, or whether other primates which exhibit a male neonatal testosterone surge, such as man and the rhesus monkey, also undergo some postnatal behavioural differentation, remains to be seen
SN - 0300-5208
UR - 111908
ER -

TY - JOUR
ID - 1141
T1 - Giggle time--establishing the social connection--a program to develop the communication skills of children with autism, Aspergers syndrome and PDD
A1 - Attridge,Catherine
Y1 - 2005///
N1 - Peer Reviewed Journal: 2005-14164-009
English
Journal; Peer Reviewed Journal
KW - PsycInfo
KW - Rough and Tumble
KW - Aspergers Syndrome
KW - Autism
KW - Communication Skills
KW - Pervasive Developmental Disorders
KW - Social Skills
RP - NOT IN FILE
SP - Oct

EP - 314
JF - Child Language Teaching and Therapy
VL - .21
IS - 3
N2 - Reviews the book, Giggle time--establishing the social connection--a program to develop the communication skills of children with autism, Aspergers syndrome and PDD by S. A. Sonders (2002). This book is a child oriented, well structured, instruction book. It outlines the skills needed to teach the tools of: reciprocity, turn taking and joint attention, in young children. The reader is given the blue prints for many fast, physical and fun packed play sequences that can be tailored to and developed for individual children. The main body of the book, Chapters 1-4, is devoted to working with children who are at the more severe end of the autistic spectrum. Games are based around rough and tumble, deep pressure, sensory props and exaggerated, predictable words and phrases. The last section, Chapter 5, has games based on verbal play routines as a means to developing social reciprocity, receptive and expressive language. (PsycINFO Database Record (c) 2012 APA, all rights reserved)
SN - 0265-6590
ER -

TY - JOUR
ID - 1142
T1 - Rough-and-Tumble Social Play in Humans. [References]
A1 - Fry,Douglas P.
Y1 - 2005///
N1 - Book: 2005-09269-004
English
Book; Edited Book
KW - PsycInfo
KW - Rough and Tumble
KW - Aggressive Behavior
KW - Animal Play
KW - Childhood Play Behavior
KW - Social Behavior
RP - NOT IN FILE
SP - 308
JF - Pellegrini, Anthony D [Ed]; Smith, Peter K [Ed]
IS - 2005
N2 - (from the chapter) A great deal of play in humans involves interaction with others in the social world. Social play is diverse, involving physical games, running, jumping, and wrestling as well as a plethora of jointly created make-believe enactments of social scenes, activities, and rituals. Two or more children may assume particular roles during social play, such as "mother" and "children," "hunters" and "prey," "buyers" and "sellers," or "wedding guests," "bride," "groom," and "priest." As we will see later, the imitation of adult activities forms a prominent feature of social play across cultural settings. This chapter focuses on one form of social play called rough-and-tumble (R&T) in humans. The term "rough-and-tumble play" was originally used to describe play chasing, fleeing, and wrestling in rhesus monkeys (Harlow & Harlow, 1965), but R&T also occurs in many other animal species (Aldis, 1975; Einon & Potegal, 1991; Fagen, 1981; Pellis, Field, Smith, & Pellis, 1996; Smith, 1982). In primates, Jolly (1985, p. 406) notes, "chasing and wresting with peers is ubiquitous. Every species has rough-and-tumble." A number of features typify R&T in mammals: Threats are absent or infrequent, movements are free and easy, muscle tone is relaxed, biting is inhibited, play signals such as the play face and play vocalizations are evident, roles frequently reverse, dominance relations are relaxed, animals of different sizes are partners, and sequences of behavior vary (Aldis, 1975; Bekoff& Byers, 1981, 1985; Fagen, 1978, 1981; Pellis, 1984; Smith, 1982; Symons, 1978). (PsycINFO Database Record (c) 2012 APA, all rights reserved)
AD - Fry, Douglas P.: Department of Social Sciences, Abo Akademi University, Vaasa, Finland
ER -

TY - JOUR
ID - 1143

T1 - The nature of play: Great apes and humans
A1 - Pellegrini,Anthony D.
Y1 - 2005///
N1 - Book: 2005-09269-000
English
Book; Edited Book
(Created by PsycINFO) Part I: Background and Theory * Play in Great Apes and Humans / Anthony D. Pellegrini and Peter K. Smith / 3-12 * The Role of Play in the Evolution of Great Apes and Humans / Patrick Bateson / 13-24 Part II: Social Play * Social Play in the Great Apes / Kerrie P. Lewis / 27-53 * Rough-and-Tumble Social Play in Humans / Douglas P. Fry / 54-85 Part III: Object Play * Object Play in Great Apes: Studies in Nature and Captivity / Jacklyn K. Ramsey and William C. McGrew / 89-112 * Boys' and Girls' Uses of Objects for Exploration, Play, and Tools in Early Childhood / Anthony D. Pellegrini and Kathy Gustafson / 113-135 Part IV: Fantasy * Fantasy Play In Apes / Juan-Carlos Gomez and Beatriz Martin-Andrade / 139-172 * Social and Pretend Play in Children / Peter K. Smith / 173-209 Part V: Hunter-Gatherers and Pastoral Peoples * Play in Hunter-Gatherer Society / Yumi Gosso, Emma Otta, Maria de Lima Salum e Morais, Fernando Jose Leite Ribeiro, and Vera Silvia Raad Bussab / 213-253 * Farming, Foraging, and Children's Play in the Okavango Delta, Botswana / John Bock / 254-281 Part VI: Conclusion * Play in Great Apes and Humans: Reflections on Continuities and Discontinuities / Peter K. Smith and Anthony D. Pellegrini / 285-298 Index
KW - PsycInfo
KW - Rough and Tumble
KW - Animal Ethology
KW - Animal Play
KW - Childhood Play Behavior
KW - Developmental Psychology
KW - Primates (Nonhuman)
RP - NOT IN FILE
SP - 308
JF - (2005)
VL - The nature of play
N2 - (from the jacket) This uniquely integrative volume brings together leading experts in developmental psychology and animal behavior to provide a new perspective on the nature and functions of play. In an introductory chapter, distinguished ethologist Patrick Bateson describes how youthful exploration and games contribute to both individual development and group survival-not only in humans, but in other species as well. Parallel chapters then examine rough-and-tumble play, object play, and pretend or fantasy play in humans and great apes. Explored are the ways in which specific play behaviors generate skills and knowledge that are needed for successful functioning throughout life, as well as what they reveal about evolutionary processes. Topics covered include: How play fighting among both animals and humans enhances social cohesion; sex differences in play from infancy through adolescence; ways in which play fosters peer and parent-child relationships; how preschoolers learn to use objects as tools; and why imaginative play is so much more common in human children than in apes. While much of the knowledge on human play comes from industrialized Western societies, the book also features important chapters on hunter-gatherer and pastoral cultures. Comprehensive and up to date, this volume belongs on the desks of researchers and students in developmental psychology, comparative psychology, animal behavior, and evolutionary psychology, and will also be of interest to anthropologists. It is a richly informative text for advanced undergraduate- and graduate-level courses. (PsycINFO Database Record (c) 2012 APA, all rights reserved)
AD - Pellegrini, Anthony D.: Department of Educational Psychology, University of Minnesota, Twin Cities, MN, US Smith, Peter K.: School and Family Studies, Goldsmiths College, University of London, London, United Kingdom
ER -

TY - JOUR
ID - 1144
T1 - Play and the Regulation of Aggression. [References]
A1 - Peterson,Jordan B.
Y1 - 2005///

N1 - Book: 2005-09268-007
English
Book; Edited Book
KW - PsycInfo
KW - Rough and Tumble
KW - Aggressive Behavior
KW - Childhood Development
KW - Childhood Play Behavior
KW - Self Regulation
KW - SOCIALIZATION
KW - Aggressiveness
KW - Interpersonal Interaction
RP - NOT IN FILE
SP - 480
JF - Tremblay, Richard E [Ed]; Hartup, Willard W [Ed]; Archer, John [Ed]
IS - 2005
N2 - (from the chapter) This chapter concludes that the individual brings to the world a set of inborn motivations, including those that underlie aggression, and these motivations are brought under control--or not--as a consequence of socialization. This control appears at least twofold. The direct inhibition and regulation of aggression appears established as a consequence of rough-and-tumble (R&T) play and also appears associated, in principle, with the development of some forms of executive control. R&T play is different from exploratory activity and from aggression-two forms of behavior with which it can easily be confused. R&T play and exploratory activity share the fact that both are enjoyable. Formal behavioral analysis clearly discriminates R&T play from genuine aggression. Furthermore, it is clear that pathological socialization experiences, first in the context of the family and second in the context of early peer experiences (variants of the institutional sickness described by Rousseau) can produce and then reinforce in a child the conviction that the world is a cruel and sadistic place, fit only for interpretation through lenses colored by the desire for revenge. Finally, it is clear that complex processes of play, beginning with R&T play and culminating in the production of sophisticated, abstract socially shared frames of reference, play an important role in the modulation of aggression, both in regard to its inhibition and in regard to its integration into fully functional individual and social identities. (PsycINFO Database Record (c) 2012 APA, all rights reserved)
AD - Peterson, Jordan B.: Department of Psychology, University of Toronto, Toronto, ON, Canada Flanders, Joseph L.: Department of Psychology, McGill University, Montreal, PQ, Canada
ER -

TY - JOUR
ID - 1145
T1 - Recess: Its role in education and development. [References]
A1 - Pellegrini,Anthony D.
Y1 - 2005///
N1 - Book: 2005-07528-000
English
Book; Authored Book
(Created by PsycINFO) Preface Acknowledgments The Debate Over Recess: A Sad Tale of the Disjunctive Between Educational Policy and Scientific Research A Brief History of the Place of Play and Recess in American Schools The School Playground as a Venue for Children's Social Development The Two Worlds of the Playground: Gender Segregation at Recess Gender Differences in Preference for Outdoor Recess Children's Play and Rough-and-Tumble Play on the Playground Children's Games on the Playground at Recess The Role of Recess in Children's Cognitive Performance in Classrooms Summing Up: What Are the Implications of Recess for Children in School? References Author Index Subject Index
KW - PsycInfo
KW - Rough and Tumble
KW - Childhood Development
KW - Childhood Play Behavior
KW - Educational Programs
KW - Playgrounds

KW - Recreation
KW - Elementary Schools
RP - NOT IN FILE
SP - 202
JF - (2005)
VL - Recess
N2 - (from the preface) Writing a book about recess could be a very questionable endeavor for a serious academic psychologist. At first blush it seems to be a pretty trivial topic. It's the time during the school day when there's a break from what's typically considered the most serious work of the day--reading, writing, and arithmetic. Reflecting this trivial sort of tenor, it's also that time that kids will, perhaps only half-jokingly, say is their favorite part of the school day. There's even a cartoon TV show for kids called Recess! All of this reinforces the popular notion that recess is indeed trivial and certainly not worthy of serious academic and scientific attention. This perception has, I think, led to many schools questioning the role of recess in the school day. This orientation, I think, has done much more harm than good. If studying the role of recess during the school day seems questionable, so too, some people think, is the idea of taking recess away from kids in primary school. When people first hear of this state of affairs, a frequent initial reaction is disbelief--"You've got to be kidding!" This book attempts to broach these two views of recess; the perceived value of recess and the movement to eliminate or reduce the school recess period from the primary school day. Over the past 10 years, I have been contacted by a number of folk concerned with this trend. They know, in their hearts, that something is wrong here. When they speak with me, they ask for advice on how to counter the trend. I hope that this book helps them in this important job. (PsycINFO Database Record (c) 2012 APA, all rights reserved)
AD - Pellegrini, Anthony D.: University of Minnesota, MN, US
ER -

TY - JOUR
ID - 1146
T1 - A Qualitative Approach to Boys' Rough and Tumble Play: There Is More Than What Meets the Eye. [References]
A1 - Reed,Thomas L.
Y1 - 2005///
N1 - Book: 2005-06383-003
English
Book; Edited Book
KW - PsycInfo
KW - Rough and Tumble
KW - Childhood Play Behavior
KW - Human Males
KW - Personality Traits
KW - Aggressive Behavior
KW - Ethnography
KW - Prosocial Behavior
RP - NOT IN FILE
SP - MD, US
JF - McMahon, F
VL - F [Ed]; Lytle, Donald E [Ed]; Sutton-Smith, Brian [Ed].
IS - 2005
N2 - (from the chapter) The purpose of this ethnographic study was to examine and describe the way in which preadolescent boys in a natural setting express care for one another through rough and tumble (R&T) play. Caring, like playing, is a universal concept but not easily defined. Noddings (1990) calls caring the most significant form of human response. Caring, compassion, and connection are important for all people to understand and value (Noddings, 1990). Donaldson (1976) theorizes that care is expressed through R&T play. This research study is designed to test this theory and to answer the following questions: (1) How do boys set up the R&T experience? and (2) How do they express care for one another through a set of behaviors that appear to be aggressive in nature? (PsycINFO Database Record (c) 2012 APA, all rights reserved)
AD - Reed, Thomas L.: University of South Carolina, School of Education, Spartanburg, SC, US
ER -

TY - JOUR
ID - 1147
T1 - Play: An interdisciplinary synthesis. [References]
A1 - McMahon,F.
Y1 - 2005///
N1 - Book: 2005-06383-000
English
Book; Edited Book
(Created by PsycINFO) (Abbreviated) Editor's Note Acknowledgments Introduction I. Evolutionary Resonances for Play * Play, Five Gates of Evolution, and Paths to Art / Robert Fagen / 9-41 * Video Entertainment May Facilitate Recovery for Monkeys in a Clinical Setting / Peggy O'Neill-Wagner / 43-51 * A Qualitative Approach to Boys' Rough and Tumble Play: There Is More Than What Meets the Eye / Thomas L. Reed / 53-71 II. Cross Cultural Play Variations * Children's Play in Cross-Cultural Perspective: A New Look at the "Six Cultures" Study / Carolyn Pope Edwards / 81-96 * Children's Play and Toys in Changing Moroccan Communities / Jean-Pierre Rossie / 97-111 * At Work At Play: Training Hindu Nationalist Women / Kalyani Devaki Menon / 113-133 * Ludic Pathologies and Their Links to Play: Cultural and Neurocognitive Perspectives / Donald E. Lytte / 135-153 III. The Play Sytemics of Education * Play in Lower Primary School in Norway / Arne Trageton / 157-177 * The Mill Lake Hospital: Play in a Middle Classroom / Lynn Romeo and Kristie L. Andrews / 179-193 * Preschool Teachers' Conceptions of Children's "Chaotic Play" / Annica L. Lofdahl / 195-204 IV. The Variabilities Adult Leisure * Working to Play: College Student Athletes' Conceptions of Play and Work / Robin M. Holmes / 209-231 * Community Play: At the Mall / Felicia McMahon / 233-254 * Validity and Reliability of the Experience of Leisure Scales (TELS) / Cheryl R. H. Meakins, Anita C. Bundy and Jeffrey Gliner / 255-278 * Collecting as a Form of Play / Paola de Sanctis Ricciardone / 279-290 V. Conclusion: Playfulness and Tricksters Interstitial References Index About the Contributors Recent Titles in Play & Culture Studies Series
KW - PsycInfo
KW - Rough and Tumble
KW - Culture (Anthropological)
KW - Recreation
KW - Cross Cultural Differences
KW - Leisure Time
KW - Schools
KW - SOCIALIZATION
RP - NOT IN FILE
SP - MD, US
JF - (2005)
VL - Play
N2 - (from the introduction) This volume is the sixth in the present Play and Culture Studies series. The following volume is organized into four major sections: First (Part I), on the basic data about the evolution of play, second (Part II), then proceeds to evidence about universality from cross cultural comparisons, third (Part III), provides samples of the role of play in school socialization, and fourth (Part IV), concludes with some of the varying types of leisure as play to be found among adults. The references for this introduction and the interstitial material appear as a final bibliography. The other authors' references appear within each of their respective chapters. (PsycINFO Database Record (c) 2012 APA, all rights reserved)
AD - McMahon, F. F.: Syracuse University, Department of Anthropology, Syracuse, NY, US Lytle, Donald E.: California State University, Department of Kinesiology, Chico, CA, US Sutton-Smith, Brian: University of Pennsylvania, Department of Education, Philadelphia, PA, US
ER -

TY - JOUR
ID - 1148
T1 - Preschool Children's Pretend and Physical Play and Sex of Play Partner: Connections to Peer Competence. [References]
A1 - Colwell,Malinda J.
Y1 - 2005///

N1 - Peer Reviewed Journal: 2005-05502-007
English
Journal; Peer Reviewed Journal
KW - PsycInfo
KW - Rough and Tumble
KW - Childhood Play Behavior
KW - Human Sex Differences
KW - Peer Relations
KW - Social Skills
KW - Preschool Students
RP - NOT IN FILE
SP - Apr
EP - 509
JF - Sex Roles
VL - .52
IS - 7-8
N2 - This study was designed to examine associations between preschool children's pretend and physical play with same-sex, other-sex, and mixed sex peers and children's social competence with peers. Sixty predominately middle-class preschoolers (33 boys, 51 European-American) were observed on the playground at their school over a period of 4 months. Children's same-sex, other-sex, and mixed-sex peer play was observed, and teachers and peers provided assessments of children's social competence. Analyses revealed that children who engaged in more same-sex pretend play were better liked by peers and were viewed by teachers as being socially competent. In addition, girls who engaged in same-sex exercise play and boys who engaged in same-sex rough-and-tumble play were better liked by peers, whereas boys who engaged in rough-and-tumble play with other-sex peers were less liked by peers. The results suggest that child gender and gender of playmate are important factors in the association between pretend play and rough-and-tumble play and children's social competence with peers. (PsycINFO Database Record (c) 2012 APA, all rights reserved) (journal abstract)
SN - 0360-0025
AD - Colwell, Malinda J.: Department of Human Development and Family Studies, Texas Tech University, Lubbock, TX, US Lindsey, Eric W.: Department of Human Development and Family Studies, Texas Tech University, Lubbock, TX, US
ER -

TY - JOUR
ID - 1149
T1 - Play: Types and Functions in Human Development. [References]
A1 - Smith,Peter K.
Y1 - 2005///
N1 - Book: 2004-21781-011
English
Book; Edited Book
KW - PsycInfo
KW - Rough and Tumble
KW - Childhood Play Behavior
KW - Developmental Psychology
KW - Human Development
KW - Games
KW - Recreation
RP - NOT IN FILE
SP - 540
JF - Ellis, Bruce J [Ed]; Bjorklund, David F [Ed]
IS - 2005
N2 - (from the chapter) Play is often defined as activity that is both done for its own sake and is characterized by "means rather than ends"-the process of the play is more important than any end point or goal that is obvious to the participant or "naive" onlooker. These criteria contrast play with exploration (which may lead into play, as a child gets more familiar with a new toy or environment), and with work- or subsistence-related activities such

as feeding (which have a definite, obvious goal) and fighting (different from play fighting, as discussed later). Additional common characteristics of human play are flexibility (objects being put in new combinations, roles acted out in new ways), positive affect (children often smile and laugh in play, and say they enjoy it) and pretense (use of objects and actions in nonliteral ways). Although play may have no "obvious" goal, this does not mean that it has no positive benefits. Also, it does not mean that play has no evolutionary function(s). In fact, the ubiquity of forms of play throughout most mammal species, and humans in all cultures studied to date, and the time, energy, and sometimes danger costs of engaging in them, strongly suggest that selective pressures have acted to favor play behaviors, and that they have some functional value for the player, even if he or she is not fully aware of it. However, the range of functions for play, and strength of evidence for them, is still a matter of considerable debate. The following main types of play are well recognized: physical activity play (rhythmic stereotypies, exercise play; rough-and-tumble play); object play; and pretend play (including sociodramatic play as a particularly complex form). Of these, physical activity play and object play are seen widely in other species of mammals. Pretend and sociodramatic play are only seen in humans, apart from some possibly very elementary forms of pretense in great apes. A related concept in developmental psychology is that of games. Games with rules describe more organized forms of play in which there is some clear and publicly expressed goal (such as winning the game in a manner accepted by other players); games with rules are not reviewed furthering this chapter. (PsycINFO Database Record (c) 2012 APA, all rights reserved)
AD - Smith, Peter K.: Goldsmiths College, University of London, Department of Psychology, Unit for School and Family Studies, London, United Kingdom
ER -

TY - JOUR
ID - 1150
T1 - Relationships Among Motivational Climate, Satisfaction, Perceived Ability, and Fair Play Attitudes in Young Soccer Players. [References]
A1 - Boixados,Merce; Cruz,Jaume; Torregrosa,Miquel; Valiente,Lourdes
Y1 - 2004///
N1 - Peer Reviewed Journal: 2004-21395-001
English
Journal; Peer Reviewed Journal
KW - PsycInfo
KW - Rough and Tumble
KW - Motivation
KW - Satisfaction
KW - Soccer
KW - Sports (Attitudes Toward)
KW - Sport Psychology
RP - NOT IN FILE
SP - Dec
EP - 317
JF - Journal of Applied Sport Psychology
VL - .16
IS - 4
N2 - The present study examined relationships among perceptions of motivational climate, perceived ability, satisfaction and fair play attitudes in young soccer players. Participants were 472 male Catalan players (from 10 to 14 years). Results indicated that perceptions of a task-involving climate were positively associated with satisfaction in practices and self-referenced perceived ability and were inversely related to rough play attitudes and normative perceived ability. In addition, perceptions of an ego-involving motivational climate were related positively with normative perceived ability and with favorable attitudes towards winning in a soccer game. The differences across motivational climate profiles groups revealed that the highest level of acceptance of rough play was found in the subgroup with a low-task/high-ego orientation. In contrast, the lowest level of acceptance of rough play was found in the opposite subgroup of high-task/low-ego. The findings support the importance of creating a task-involving climate in sports. (PsycINFO Database Record (c) 2012 APA, all rights reserved) (journal abstract)
SN - 1041-3200

AD - Boixados, Merce: Universitat Oberta de Catalunya, Barcelona, Spain Cruz, Jaume: Universitat Autonoma de Barcelona, Spain Torregrosa, Miquel: Universitat Autonoma de Barcelona, Spain Valiente, Lourdes: Universitat Oberta de Catalunya, Barcelona, Spain
ER -

TY - JOUR
ID - 1151
T1 - Conceptualizing Fathers' Roles: Playmates and More. [References]
A1 - Tamis-LeMonda,Catherine S.
Y1 - 2004///
N1 - Peer Reviewed Journal: 2004-18780-002
English
Journal; Peer Reviewed Journal
KW - PsycInfo
KW - Rough and Tumble
KW - Childhood Development
KW - Father Child Communication
KW - Father Child Relations
KW - Mother Child Relations
KW - Parental Role
RP - NOT IN FILE
SP - Jul
EP - Aug
JF - Human Development
VL - .47
IS - 4
N2 - Comments on the article by Daniel Paquette (see record 2004-18780-001). Despite the exponential growth in fatherhood studies over the past several decades, research on fathers remains largely a-theoretical. Therefore the author's theorizing about co-dependencies between evolution and biology, styles of parent-child social engagements, attachment relationships, and developmental outcomes in children is a welcome contribution to the field. His description of the father-infant activation relationship is simultaneously controversial and provocative, and the opportunity to respond to, praise, criticize and analyze a well-articulated position is of enormous value. The central tenet of author's article is that fathers occupy a special place in children's development. Fathers arouse their children during bouts of rough-and tumble play, and this 'activation relationship' is fundamental to positive developmental outcomes in children. The author offers an intriguing evolutionary interpretation of the well documented finding that fathers engage in greater amounts of rough and tumble play relative to mothers. His theory incites reflection about the ways in which cultural values, tools, opportunities and practices intersect with biology. That in itself is an admirable feat. (PsycINFO Database Record (c) 2012 APA, all rights reserved)
SN - 0018-716X
AD - Tamis-LeMonda, Catherine S.: New York University, New York, NY, US
ER -

TY - JOUR
ID - 1153
T1 - Father-child relationship and openness to the world. [French]. [References]
A1 - Paquette,Daniel
Y1 - 2004///
N1 - Peer Reviewed Journal: 2004-15103-005
French
Journal; Peer Reviewed Journal
KW - PsycInfo
KW - Rough and Tumble
KW - Attachment Behavior
KW - Childhood Development
KW - Father Child Relations

RP - NOT IN FILE
SP - Apr
EP - 225
JF - Enfance
VL - .56
IS - 2
N2 - Here I propose to use the father-child "activation relationship" to explain the emotional bond of the child to his father. Contrary to the mother-child attachment relationship which permits the child to be calmed, the father-child activation relationship answers the child's need to be stimulated, to surpass himself in a context in which the child is confident of being protected from potential dangers. In a quality activation relationship, the child would learn to trust his own ability to deal with threats and strangeness of his physical and social environment, since his father would incitate him to take greater chances while exploring in a secure context. Fathers may play an indirect role via the emotional and physical support given to the mother during stressful periods, and a direct role in the development of exploration and autonomy as early as the second year of life during non-stressful periods. In particular, the father-child rough-and-tumble play during preschool years may prepare a child to assert himself in a competitive world, in a socialized manner without using aggression. (PsycINFO Database Record (c) 2012 APA, all rights reserved) (journal abstract)
SN - 0013-7545
AD - Paquette, Daniel: Institut de Recherche Pour le Developpement Social des Jeunes, Centre Jeunesse de Montreal et Departement de Psychologie, Universite de Montreal, Montreal, PQ, Canada
ER -

TY - JOUR
ID - 1154
T1 - Dolls
A1 - Zucker,Kenneth J.
Y1 - 2004///
N1 - Book: 2004-13881-014
English
Book; Edited Book
KW - PsycInfo
KW - Rough and Tumble
KW - Child Psychotherapy
KW - Childhood Development
KW - Gender Identity Disorder
KW - Treatment
RP - NOT IN FILE
SP - 334
JF - Spitzer, Robert L [Ed]; First, Michael B [Ed]; Gibbon, Miriam [Ed]; Williams, Janet B
VL - W [Ed].
IS - 2004
N2 - (from the chapter) Discusses the case of an 8-year-old boy for whom his parents seek treatment because "he wants to be a girl". The patient has a strong and persistent identification of himself as a female. He frequently states his desire to be a girl--not just play a female role. He is preoccupied with female stereotypical activity, preferring to play with girls and pretending that he is a girl, and frequently cross-dresses. He plays exclusively with stereotypically female toys such as dolls. When imitating characters from books and television, he always chooses female characters. There is also evidence of a persistent discomfort with being a boy. He rejects stereotypical male toys and activities and shows an aversion toward rough-and-tumble play. These are the characteristic features of Gender Identity Disorder (GID) as seen in a male. When the examiner provides feedback to the parents, they could be informed that the patient does appear to be unhappy about being a boy and that the goal of treatment would be to try and help him to feel more comfortable as a boy. Along similar lines, Rocky should be asked whether he would like to meet with a "talking doctor" to see if there are ways to help him feel happier about being a boy. The author's approach to treatment would be a combination of individual therapy with the patient, counseling with the parents, and specific recommendations for the parents to carry out in his naturalistic day-to-day environment. Individual therapy would be used with Rocky to explore his

fantasies that being a girl would make his life better. (PsycINFO Database Record (c) 2012 APA, all rights reserved)
AD - Zucker, Kenneth J.: Centre for Addiction and Mental Health-Clarke Division, Child, Youth, and Family Program, Gender Identity Service, Toronto, ON, Canada
ER -

TY - JOUR
ID - 1155
T1 - Attention deficit hyperactivity disorder in children: One consequence of the rise of technologies and demise of play? [References]
A1 - Armstrong,Thomas
Y1 - 2003///
N1 - Book: 2004-14727-007
English
Book; Edited Book
KW - PsycInfo
KW - Rough and Tumble
KW - Attention Deficit Disorder with Hyperactivity
KW - Childhood Play Behavior
KW - Frontal Lobe
KW - Neural Development
KW - Technology
KW - Computers
KW - Television
RP - NOT IN FILE
SP - 215
JF - Olfman, Sharna [Ed]
IS - 2003
N2 - (from the introduction) Presents evidence that current approaches to education that promote screen time and minimize opportunity for play are exacerbating the Attention Deficit Hyperactivity Disorder epidemic. The "rapid-fire" stimulation that children are exposed to increasingly on television and computers requires the brain to adapt information-processing strategies that become dysfunctional in contexts such as the classroom, in which--at increasingly younger ages--sustained attention is required. Armstrong also presents research that links physical play to frontal lobe development, which supports the capacities for organization, classification, synthesis, reflection, and cooperation. Diminished opportunities for rough-and-tumble play may thus be compromising frontal lobe development and exacerbating symptoms of hyperactivity, distractibility, and impulsivity. (PsycINFO Database Record (c) 2012 APA, all rights reserved)
ER -

TY - JOUR
ID - 1156
T1 - A Qualitative Investigation of Bullying: The Perspectives of Fifth, Sixth and Seventh Graders in USA Parochial School. [References]
A1 - Gamliel,Tiram
Y1 - 2003///
N1 - Peer Reviewed Journal: 2003-10267-004
English
Journal; Peer Reviewed Journal
KW - PsycInfo
KW - Rough and Tumble
KW - Intermediate School Students
KW - Middle School Students
KW - Schools
KW - Bullying
RP - NOT IN FILE
SP - Nov

EP - 420
JF - School Psychology International
VL - .24
IS - 4
N2 - Rarely have investigators qualitatively tapped the complex and sophisticated knowledge base of children about bullying. The present investigation was conducted in order to plumb the working knowledge of intermediate and middle school students about potential strategies for alleviating bullying. Fifth, sixth and seventh-graders (three males and three females) discussed the prevalence of bullying in their school, methods for dealing with bullying employed in the past and future coping strategies. Two of three female interviewees but none of the males reported experiencing bullying at the time of the interviews. While respondents struggled to articulate strategies for coping with bullying, most participants clearly expressed their desire to rationally work through problems with aggressors. When asked to rank order suggested strategies, participants selected violent or aggressive retaliation as the least effective and least desirable coping strategy. Additional themes emerging from the data include gender based experiential and interpretive differences, including the tendency for males to classify most potential conflicts as harmless horseplay. Interviewees portrayed chronic victims as passive and ineffectual when it came to dealing with aggression. (PsycINFO Database Record (c) 2012 APA, all rights reserved)
SN - 0143-0343
AD - Gamliel, Tiram: Richard P. Stadter Psychiatric Center, Grand Forks, ND, US Hoover, John H.: The Department of Special Education, St. Cloud State University, St. Cloud, MN, US Daughtry, Donald W.: Department of Counseling, University of North Dakota, ND, US Imbra, Christine M.: Department of Educational Leadership St. Cloud State University, St. Cloud, MN, US
ER -

TY - JOUR
ID - 1157
T1 - Rough-and-tumble play in human children. [References]
A1 - Scott,Eric
Y1 - 2003///
N1 - Peer Reviewed Journal: 2003-09794-006
English
Journal; Peer Reviewed Journal
KW - PsycInfo
KW - Rough and Tumble
KW - Aggressive Behavior
KW - Aggressiveness
KW - Childhood Play Behavior
KW - Human Sex Differences
RP - NOT IN FILE
SP - 2003, pp
EP - 2003,551
JF - Aggressive Behavior
JA - Aggress.Behav
VL - .29
IS - 6
N2 - Rough and tumble (R&T) play is a unique set of behaviors that can be reliably distinguished from aggression and other childhood activities. Although it may be the most fundamental form of play, it has received comparatively little experimental attention in the human species. Forty children, ages three to six, were allowed to play in pairs (10 male pairs, 10 female pairs) during a 30 minute videotaped session with no toys available while music played in the background during every other five-minute time period. The incidence frequency of children's play and related behavioral activities were scored using 20 behavioral categories. The major findings show only modest gender differences in the frequency of play behaviors in such controlled social encounters; the main difference is that boys engaged in slightly more physical play solicitations than girls. Music facilitated General Motor Activities (e.g., Running and Walking behaviors), but not specific R&T play actions (e.g., Wrestling, Ventral and Dorsal contacts). Finally, most play behaviors as well as general activities declined systematically over the course of each recording session. This experiment highlights how human social play can

be systematically studied in a controlled laboratory setting. (PsycINFO Database Record (c) 2012 APA, all rights reserved) (journal abstract)
SN - 0096-140X
AD - Scott, Eric: Indiana U School of Medicine, Indianapolis, IN, US Panksepp, Jaak: Bowling Green State U, Dept of Psychology, Bowling Green, OH, US
ER -

TY - JOUR
ID - 1158
T1 - Consequences of the intervention programme for developing Hellison's Personal and Social Responsibility on fair-play and self-control behaviours. [Spanish]. [References]
A1 - Cecchini,Jose A.
Y1 - 2003///
N1 - Peer Reviewed Journal: 2003-09589-017
Spanish
Journal; Peer Reviewed Journal
KW - PsycInfo
KW - Rough and Tumble
KW - Behavior
KW - Intervention
KW - Responsibility
KW - Self Control
KW - Social Responsibility
RP - NOT IN FILE
SP - Nov
EP - 637
JF - Psicothema
VL - .15
IS - 4
N2 - This study tested consequences obtained from the Intervention Programme for developing Hellison's Personal and Social Responsibility (1995) about fair-play behaviours and self-control among young students. The sample was formed by students from two state schools (N = 142), with an average age of 12.7 (experimental group = 72, control group = 70). The Intervention Programme was applied to the experimental group throughout ten one hour periods on indoor football initiation. The results showed, in experimental group, meaningful improvements in personal feedback, reward delay, criterial self-control, self-control process, opinions related to enjoyments, and sporting behaviours. Decreases in variables related to rough play, and un-sporting behaviours were observed. No meaningful changes were found in the control group. (PsycINFO Database Record (c) 2012 APA, all rights reserved)
SN - 0214-9915
AD - Cecchini, Jose A.: Universidad de Oviedo, Oviedo, Spain Montero, Javier: Universidad de Oviedo, Oviedo, Spain Pena, Jose V.: Facultad de Education, Universidad de Oviedo, Oviedo, Spain
ER -

TY - JOUR
ID - 1160
T1 - Prevalence of father-child rough-and-tumble play and physical aggression in preschool children. [References]
A1 - Paquette,Daniel; Carbonneau,Rene; Dubeau,Diane; Bigras,Marc; Tremblay,Richard
Y1 - 2003///
N1 - Peer Reviewed Journal: 2003-09190-006
English
Journal; Peer Reviewed Journal
KW - PsycInfo
KW - Rough and Tumble
KW - Aggressive Behavior
KW - Childhood Development

KW - Childhood Play Behavior
KW - Family
KW - Father Child Relations
KW - epidemiology
KW - Fathers
KW - Parental Characteristics
KW - Psychosocial Factors
RP - NOT IN FILE
SP - Jun
EP - 189
JF - European Journal of Psychology of Education
VL - .18
IS - 2
N2 - Three samples of Francophone subjects from Quebec are used to establish the prevalence of parent-child rough-and-tumble (RTP) according to different personal, social and family variables, and to verify if children who engage in more RTP with their father exhibit less physical aggression towards other children and are more competitive without resorting to aggression. Our results showed that 24% to 43% of fathers engaged in RTP with their children on a daily basis, and only 4% to 16% of fathers never do. Moreover, personal characteristics such as the age and sex of the participants seemed to have a greater influence on the frequency of parent-child RTP than variables related for example to work, socioeconomic conditions, or the living environment. The hypothesis that children who engage in more RTP with their fathers display less physical aggression towards their peers is invalidated here. We have concluded that it is important that not only RTP frequency, but also and especially indicators of RTP quality be used. Future observational studies of father-child RTP are required in order to do so. Finally, certain preliminary results support the hypothesis that father-child RTP fosters the development of the competition skills in children without using aggression. (PsycINFO Database Record (c) 2012 APA, all rights reserved) (journal abstract)
SN - 0256-2928
AD - Paquette, Daniel: IRDS, GRAVE-ARDEC, Canada Carbonneau, Rene: U Montreal, Montreal, PQ, Canada Dubeau, Diane: U Quebec en Outouais, GRAVE-ARDEC, PQ, Canada Bigras, Marc: U Sherbrooke, Sherbrooke, PQ, Canada Tremblay, Richard E.: U Montreal, Montreal, PQ, Canada
ER -

TY - JOUR
ID - 1161
T1 - Perceptions and Functions of Play and Real Fighting in Early Adolescence. [References]
A1 - Pellegrini,Anthony D.
Y1 - 2003///
N1 - Peer Reviewed Journal: 2003-08038-017
English
Journal; Peer Reviewed Journal
KW - PsycInfo
KW - Rough and Tumble
KW - Adolescent Attitudes
KW - Adolescent Development
KW - Aggressive Behavior
KW - Childhood Play Behavior
KW - Human Males
KW - Dominance
KW - Male Female Relations
RP - NOT IN FILE
SP - Sep
EP - Oct
JF - Child Development
JA - Child Dev.
VL - .74
IS - 5

N2 - The hypothesis is tested that adolescent boys' (mean age of 12.8 years) intrasexual rough-and-tumble play (R&T) is used for dominance and intersexual R&T is used to establish heterosexual relationships. In Study 1, boys' observed R&T was related to both dominance and aggression. In the first half of the school year, R&T occurred primarily between males, possibly to establish dominance. In the second half of the year, both boys and girls engaged in R&T, possibly to establish heterosexual relationships. Counter to the hypothesis, observed aggression increased across the year. In Study 2, youngsters viewed taped R&T bouts in which they were participants or nonparticipants. Participant, more than nonparticipant, males saw R&T as related to dominance whereas participant, more than nonparticipant, females saw it as playful. (PsycINFO Database Record (c) 2012 APA, all rights reserved) (journal abstract)
SN - 0009-3920
AD - Pellegrini, Anthony D.: U Minnesota, Department of Educational Psychology, Minneapolis, MN, US
ER -

TY - JOUR
ID - 1162
T1 - It's not what you know, it's who you know: Older siblings facilitate imitation during infancy. [References]
A1 - Barr,Rachel
Y1 - 2003///
N1 - Peer Reviewed Journal: 2003-00953-002
English
Journal; Peer Reviewed Journal
KW - PsycInfo
KW - Rough and Tumble
KW - Early Childhood Development
KW - Imitation (Learning)
KW - Siblings
RP - NOT IN FILE
SP - Jan
EP - 21
JA - International Journal of Early Years Education
VL - .11
IS - 1
N2 - Examined the effect of older siblings on imitation by infants. 300 parents provided diary records of imitation by their 12-, 15-, and 18-mo old infants. Results show that infants of all ages acquired 1 to 2 new behaviors daily through imitation. There were also age-related and sibling-related changes in the quality of behaviors acquired through imitation. Older infants imitated more multi-step sequences and substituted more objects during re-enactment than did younger infants. Infants with siblings imitated more behavior without explicit instruction. Imitation by infants with siblings was characterized by a higher level of pretence and rough-and-tumble play. (PsycINFO Database Record (c) 2012 APA, all rights reserved)
SN - 0966-9760
AD - Barr, Rachel: Georgetown U, Washington, DC, US Hayne, Harlene: U Otago, Dunedin, New Zealand
ER -

TY - JOUR
ID - 1163
T1 - Rough-and-tumble play from childhood through adolescence: Development and possible functions. [References]
A1 - Pellegrini,Anthony D.
Y1 - 2002///
N1 - Book: 2003-04408-022
English
Book; Edited Book
KW - PsycInfo
KW - Rough and Tumble
KW - Childhood Development
KW - Childhood Play Behavior

KW - Psychological Terminology
KW - Psychosocial Development
KW - Aggressive Behavior
RP - NOT IN FILE
SP - 665
JF - Smith, Peter K [Ed]; Hart, Craig H [Ed]
IS - 2002
N2 - (from the chapter) This chapter discusses the development and possible functions of rough-and-tumble play from childhood through adolescence. It defines rough-and-tumble play, discusses the developmental trajectory of rough-and-tumble play, and offers suggestions for future research. Conclusions emphasize that not all children seem to need the rough-and-tumble form of play to develop. (PsycINFO Database Record (c) 2012 APA, all rights reserved)
AD - Pellegrini, Anthony D.: U Minnesota, MN, US
ER -

TY - JOUR
ID - 1164
T1 - Blackwell handbook of childhood social development
A1 - Smith,Peter K.
Y1 - 2002///
N1 - Book: 2003-04408-000
English
Book; Edited Book
(Created by PsycINFO) List of contributors Introduction by the editors Part I: Historical overview * Historical perspectives on contemporary research in social development / W. Andrew Collins / 3-23 Part II: Influences on development: Disciplinary views * Behavioral genetics, shared and nonshared environment / Alison Pike / 27-43 * Evolutionary perspectives on social development / David F. Bjorklund and Anthony D. Pellegrini / 44-59 * Culture and social development: Explanations and evidence / Sara Harkness / 60-77 * A sociological approach to childhood development / Chris Jenks / 78-94 Part III: Influences on development: Causal factors * Temperament and social development / Ann Sanson, Sheryl A. Hemphill and Diana Smart / 97-115 * Sex differences in social behavior / Susan Golombok and Melissa Hines / 117-136 * Children's social development within the socialization context of childcare and early childhood education / Carollee Howes and Jolena James / 137-155 * Relative contributions of families and peers to children's social development / Ross D. Parke, Sandra D. Simpkins, David J. McDowell, Mina Kim, Colleen Killian, Jessica Dennis, Mary L. Flyr, Margaret Wild and Yumee Rah / 156-177 Part IV: The family context * Attachment in childhood / Joan Stevenson-Hinde and Karine Verschueren / 182-204 * Parent-child relationships / Alan Russell, Jacqueline Mize and Kerry Bissaker / 205-222 * Sibling relationships / Judy Dunn / 223-237 * Social development in different family arrangements / Dale F. Hay and Alison Nash / 237-261 Part V: The peer group * Peer acceptance and rejection in childhood / Shelley Hymel, Tracy Vaillancourt, Patricia McDougall and Peter D. Renshaw / 265-284 * Friends and enemies / Willard W. Hartup and Maurissa Abecassis / 286-306 * Emotional and social development in childhood / Susanne Denham, Maria von Salisch, Tjeert Olthof, Anita Kochanoff and Sarah Caverly / 308-328 * Social withdrawal and shyness / Kenneth H. Rubin, Kim B. Burgess and Robert J. Coplan / 330-352 Part VI: Social skills and social cognition * Social skills and interpersonal perception in early and middle childhood / Antonius H. N. Cillessen and Amy D. Bellmore / 356-374 * Social cognition / Carlie Lewis and Jeremy Carpendale / 376-393 * Children's interpersonal skills and relationships in school settings: Adaptive significance and implications for school-based prevention and intervention programs / Gary W. Ladd, Eric S. Buhs and Wendy Troop / 394-415 Part VII: Play * Understanding young children's pretend play in context / Artin Goncu, Michelle B. Patt and Emily Kouba / 418-437 * Rough-and-tumble play from childhood through adolescence: Development and possible functions / Anthony D. Pellegrini / 437-453 Part VIII: Helping and moral reasoning * Prosocial and helping behavior / Joan E. Grusec, Maayan Davidov and Leah Lundell / 457-474 * Children's social and moral reasoning / Charles C. Helwig and Elliot Turiel / 476-490 * Children's understanding of society / Martyn Barrett and Eithne Buchanan-Barrow / 492-512 Part IX:, Cooperation, competition, aggression, and bullying * Cooperation and competition / Jacques F. Richard, Ada Fonzi, Franca Tani, Fulvio Tassi, Giovanna Tomada and Barry H. Schneider / 515-532 * Sticks and stones and social exclusion: Aggression among girls and boys / Marion K. Underwood / 533-548 * Bullying in childhood / Ken Rigby / 549-568 Part X: Children with special needs * The development of social competence in children with disabilities / Karen E. Diamond / 572-587 *

Social development in children with specific language impairment and profound hearing loss / Bonnie Brinton and Martin Fujiki / 588-603 Index
KW - PsycInfo
KW - Rough and Tumble
KW - Childhood Development
KW - Psychosocial Development
RP - NOT IN FILE
SP - 665
JF - (2002)
VL - Blackwell handbook of childhood social development. xv, 665 pp. Malden
N2 - (create) This handbook describes the specifics of childhood social development. It considers all aspects of social development, including play, genetics, culture, family, peers, social skills and social cognition, moral reasoning, cooperation, competition, aggression, bullying, and children with special needs. The book also provides a historical overview of childhood social development and the various movements that have existed over time. (PsycINFO Database Record (c) 2012 APA, all rights reserved)
AD - Smith, Peter K.: Goldsmiths Coll, London, England Hart, Craig H.: Brigham Young U, Provo, UT, US
ER -

TY - JOUR
ID - 1165
T1 - Rough-and-tumble between parents and children and children's social competence
A1 - Mellen,Henry S.
Y1 - 2002///
N1 - Dissertation Abstract: 2002-95018-125
English
Dissertation Abstract
KW - PsycInfo
KW - Rough and Tumble
KW - Childhood Play Behavior
KW - Parent Child Relations
KW - Social Skills
RP - NOT IN FILE
SP - Sep
JF - Dissertation Abstracts International: Section B: The Sciences and Engineering
VL - .63
IS - 3-B
N2 - The purpose of this study was to determine whether rough-and-tumble was a dimension of parent-child physical play and whether parent-child rough-and-tumble was related to preschool children's social competence. Participants were 157 married parents and 56 preschool teachers from the suburbs of a large northeastern metropolis. The core hypothesis was that father-son rough-and-tumble would be related to sons' social competence. It was predicated on the example of playground rough-and-tumble, a predominantly male activity that has been linked with the social competence of schoolboys. Both parents completed the Physical Play Questionnaire. The PPQ was a measure developed by the researcher to quantify frequencies of parent-child physical play. Fathers, mothers, and teachers rated preschool children with the Social Competence and Behavior Evaluation. Additionally, fathers and mothers filled out the Ray Directiveness Scale. Parental directive behavior was believed to be a mediating variable. Rough-and-tumble emerged as one of four principal factors of physical play between parents and preschool children. Fathers and sons rough-and-tumbled approximately twice as much as mothers and daughters, respectively. Father-son rough-and-tumble was not associated with son's social competence, but father-son intimate physical play was. (Parental directiveness had no material effect on any relation). Additional analyses revealed that father-son rough-and-tumble behaviors that involved direct body contact were related moderately with sons' social competence and very strongly with 3-year-old sons' social competence. No kind of parental physical play was linked with daughters' social competence. The results of this study confirm that rough-and-tumble was both an intergenerational and domestic phenomenon. The quality of father-son rough-and-tumble was as critical to the contribution to sons' social competence as was the frequency. Because its benefits were both age- and gender-specific, rough-and-tumble emerged as an important factor for

future research into the developmental course of parent-child play and parental gender-role socialization of children. (PsycINFO Database Record (c) 2012 APA, all rights reserved)
SN - 0419-4217
AD - Mellen, Henry S.: Adelphi U, Inst Advanced Psychological Studies, US
ER -

TY - JOUR
ID - 1166
T1 - Beyond rough and tumble: Low-income fathers' interactions and children's cognitive development at 24 months. [References]
A1 - Shannon,Jacqueline D.
Y1 - 2002///
N1 - Peer Reviewed Journal: 2002-01103-001
English
Journal; Peer Reviewed Journal
KW - PsycInfo
KW - Rough and Tumble
KW - Cognitive Development
KW - Father Child Relations
KW - Lower Income Level
RP - NOT IN FILE
SP - Apr
EP - Jun
JF - Parenting: Science and Practice
VL - .2
IS - 2
N2 - Investigated associations between father-child interactions and children's cognitive status in low-income, ethnically diverse families. Ss were 65 inner-city fathers (aged 18-46 yrs) and their 24-mo-old children. Interactions were videotaped at home during semistructured free play, and mental scale scores on the Bayley Scales of Infant Development were obtained on children. The quality of interactions was assessed using Likert ratings of both the fathers and children. Factor analyses reveal 2 patterns of engagement in fathers (Responsive-Didactic and Negative-Intrusive) and 3 in children (Playful-Communicative, Social, and Regulated). 36 children scored within normal limits on the Mental Development Index and 29 scored in the delayed range. Together, fathers' and children's factor scores explained more than 25% of the variance in children's performance on the MDI. Logistic regressions indicate that fathers with high scores on the Responsive-Didactic factor were nearly 5 times more likely to have children within the normal range on the MDI than were low-scoring fathers. Definitions from the Caregiver-Child Affect,Responsiveness, and Engagement Scale are appended. (PsycINFO Database Record (c) 2012 APA, all rights reserved)
SN - 1529-5192
AD - Shannon, Jacqueline D.: New York U, Dept of Applied Psychology, New York, NY, US Tamis-LeMonda, Catherine S.: New York U, Dept of Applied Psychology, New York, NY, US London, Kevin: New York U, New York, NY, US Cabrera, Natasha: National Insts of Health, National Inst of Child Health & Human Development, Bethesda, MD, US
ER -

TY - JOUR
ID - 1167
T1 - Comparing pupil and teacher perceptions for playful fighting, serious fighting, and positive peer interaction. [References]
A1 - Smith,Peter K.
Y1 - 2002///
N1 - Book: 2002-00366-014
English
Book; Edited Book
KW - PsycInfo
KW - Rough and Tumble

KW - Aggressive Behavior
KW - Childhood Play Development
KW - Social Interaction
KW - Students
KW - Teachers
RP - NOT IN FILE
SP - social
EP - cognitive
JF - Roopnarine, Jaipaul L [Ed]
IS - 2002
N2 - (from the chapter) Compares children's and teacher's perspectives on playfighting and real fighting in primary schools in England. The study focused on boys, as they are more heavily involved in playfighting. The authors first report on the children's views on playfighting and then report some teacher views. Pupil-teacher agreement on playfighting, real fighting, and positive social interaction are compared, to see if agreement is less regarding playfighting than the other two domains. A majority of boys enjoy playfighting, thinking its quite fun and doing it quite often. They know the difference from real fighting quite clearly. Teachers have rather negative views of playfighting. They think that about one-third of playfights turn into real fights. Teachers agreed with boys on ratings of postive social interaction, and (moderately) on frequency of aggression, but did not agree on frequency of playfighting; they confounded frequency of playfighting with frequency of aggression. The authors conclude that it does seem important that teachers, and trainee teachers, get more information on rough-and-tumble play and playfighting on the basis of the existing research in order to combat their misconceptions. (PsycINFO Database Record (c) 2012 APA, all rights reserved)
AD - Smith, Peter K.: U London, Goldsmiths Coll, Unit for School & Family Studies, United Kingdom Smees, Rebecca: U London, Inst of Education, United Kingdom Pellegrini, Anthony D.: U Minnesota, Twin Cities Campus, MN, US Menesini, Ersilia: U Florence, Dept of Psychology, Florence, Italy
ER -

TY - JOUR
ID - 1168
T1 - Rough and tumble play in children: Research and theory
A1 - Boulton,Michael J.
Y1 - 1989///
N1 - Peer Reviewed Journal: 1990-14439-001
English
Journal; Peer Reviewed Journal
KW - PsycInfo
KW - Rough and Tumble
KW - Aggressive Behavior
KW - Childhood Development
KW - Childhood Play Behavior
KW - Age Differences
KW - Cross Cultural Differences
KW - Human Sex Differences
RP - NOT IN FILE
SP - 79
EP - 91
JF - Infancia y Aprendizaje / Journal for the Study of Education and Development
VL - .48 1989, pp. 79-91.
N2 - Reviews studies of rough-and-tumble (R&T) play among children and argues that there may be considerable variation between children of different cultural and social class backgrounds. R&T is primarily a friendly and playful activity that does not usually involve any intent to hurt, at least up to early adolescence. Its function remains unclear, but it appears to be part of normal development, and children who rough-and-tumble a lot are generally sociable but not especially aggressive. To this extent, there seems no reason to discourage R&T except in contexts where the noisy and vigorous activities might distract from other (e.g., educational) objectives. Discussed are age and sex differences in R&T, environmental influences on R&T, developmental

changes in the choice of partners in R&T, and R&T and aggression. (Spanish abstract) (PsycINFO Database Record (c) 2014 APA, all rights reserved)
SN - 0210-3702
AD - Boulton, Michael J.: U Sheffield, England
ER -

TY - JOUR
ID - 1169
T1 - Distinguishing serious playful fighting by children with learning disabilities and nondisabled children. [References]
A1 - Nabuzoka,Dabie
Y1 - 1999///
N1 - Peer Reviewed Journal: 1999-11886-005
English
Journal; Peer Reviewed Journal
KW - PsycInfo
KW - Rough and Tumble
KW - Aggressive Behavior
KW - Childhood Play Behavior
KW - Cognitive Ability
KW - Learning Disabilities
RP - NOT IN FILE
SP - Sep
EP - 890
JF - Journal of Child Psychology and Psychiatry
VL - .40
IS - 6
N2 - The ability to distinguish serious from playful fighting by 39 children with learning disabilities (LD; mean age = 9.3 and 11.8 yrs) and 21 non-LD children (mean age = 10.1 yrs) was examined. Ss watched a videorecording of 19 episodes of behavior originally developed and used by J. J. Boulton (1988), which assessed the ability to distinguish between rough-and-tumble play and serious fighting. Children with LD were generally able to make this distinction, with older children being more accurate. However, the performance of children with LD was not as high as that of non-LD children. On the other hand, the order in frequency of those criteria cited was similar for both non-LD children and children with LD. Physical actions of the participants, and inference about actions and/or intent were the most frequently cited criteria. These findings indicate that although children with LD use fewer social cues, and are less accurate in making judgements about the nature of behavioural episodes, the acquisition of the meaning of particular cues may follow the same sequence as for non-LD children. Possible delays in the social cognitive development of children with LD, and their implications for the social adjustment of these children, are discussed. (PsycINFO Database Record (c) 2013 APA, all rights reserved)
SN - 0021-9630
AD - Nabuzoka, Dabie: Sheffield Hallam U, School of Health & Community Studies, Sheffield, England
ER -

TY - JOUR
ID - 1171
T1 - How cigarettes are advertised in magazines: Special messages for special markets
A1 - Basil,Michael D.
Y1 - 1991///
N1 - Peer Reviewed Journal: 1991-29084-001
English
Journal; Peer Reviewed Journal
KW - PsycInfo
KW - Rough and Tumble
KW - Advertising
KW - Consumer Research

KW - Magazines
KW - Tobacco Smoking
RP - NOT IN FILE
SP - 1991, pp
EP - 91
JF - Health Communication
VL - .3
IS - 2
N2 - Extended content analyses of magazines by D. G. Altman et al (1987) to examine how the tobacco industry segments its audience based on demographics and smoking behavior and attempts to reach each through specialized magazines and copy. Data from a market research bureau on magazines representing 5 readership demographic categories (youth, women, Blacks, men, and general-adult) were used. The focus of the majority of ads appeared to depend on the segment's current rate of smoking and attributes of the product being offered. In accord with psychographic theories, significant content differences were found in magazines read by different market segments. Horseplay was targeted at female, poorer, and younger readers; overtly sexual appeals and coy model poses were aimed at female, Black, and poorer readers more often than at men or general audiences. (PsycINFO Database Record (c) 2013 APA, all rights reserved)
SN - 1041-0236
AD - Basil, Michael D.: Stanford U, CA, US
ER -

TY - JOUR
ID - 1175
T1 - Changing a schoolyard: Intentions, design decisions, and behavioral outcomes
A1 - Weinstein,Carol S; Pinciotti,Patricia
Y1 - 1988///
N1 - Peer Reviewed Journal: 1988-37338-001
English
Journal; Peer Reviewed Journal
KW - PsycInfo
KW - Rough and Tumble
KW - Childhood Play Behavior
KW - Environmental Planning
KW - Playgrounds
KW - School Environment
KW - Elementary School Students
KW - Kindergarten Students
RP - NOT IN FILE
SP - May
EP - 371
JF - Environment and Behavior
VL - .20
IS - 3
N2 - Describes the construction of a tire playground on an empty, fenced-in blacktop that had served as the schoolyard. Parents' and designer's goals for the playground and the way the playground design supported and reflected these goals are described. The eventual impact on children's (approximately 400 kindergartners through 3rd graders) behavior is documented: There were significant decreases in organized games, uninvolved behavior, and roughhousing, and significant increases in active and pretend play. Age and sex differences in play behavior are also described. (PsycINFO Database Record (c) 2012 APA, all rights reserved)
SN - 0013-9165
AD - Weinstein, Carol S.: Rutgers U Graduate School of Education, New Brunswick, NJ, US
ER -

TY - JOUR
ID - 1178
T1 - Differences of sex and sexual orientation in the human anterior commissure

A1 - Lasco,Mitchell Stuart
Y1 - 2001///
N1 - Dissertation Abstract: 2001-95016-246
English
Dissertation Abstract
KW - PsycInfo
KW - Rough and Tumble
KW - Brain
KW - Gender Identity
KW - Limbic System
KW - Sexual Development
KW - Sexual Orientation
KW - Human Sex Differences
RP - NOT IN FILE
SP - Aug
JF - Dissertation Abstracts International: Section B: The Sciences and Engineering
VL - .62
IS - 2-B
N2 - Connectivity within the limbic brain may be one possible biological influence on personality and early social-sexual development. Evidence suggests that the amygdala, a limbic structure implicated in mediating social behaviors, plays a role in determining a child's willingness to engage in social activities, including rough play. Building on Dayrl Bem's "Exotic Becomes Erotic" (EBE) theory of the origins of sexual orientation, a model of biological and social variables in eroto-sexual development is put forward. In EBE theory, biological factors influence adult sexual orientation only indirectly, by influencing personality. Interest in rough play is one example of a personality variable which is gender-typed by society and may thus influence the eroto-sexual development of the individual. The development of neuronal connections from the amygdala to other limbic brain structures (e.g. cingulate cortex and septal nuclei), leads to the normal development of greater social inhibition, and fear of strangers. Many of these connecting fibers pass through the anterior commissure (AC). By examining variability in the midsagittal cross-sectional area of the AC, the present study looked for correlations with gender and partner preference. Previous studies have reported differences in the AC associated with gender and partner preference, however, the studies conflict in their findings, and independent labs have failed to replicate any of the reports. Midsagittal AC measurements were taken from 57 presumed heterosexual males, 18 of whom were HIV positive, and 43 females, 9 of whom were HIV positive, and 20 HIV positive males with a known history of homosexual behavior. Unlike previous studies, there were no correlations between the AC measurements and either gender or partner-preference. Furthermore, there were no correlations found with the AC measurements and possibly confounding variables such as HIV status, age, or brain weight. The tremendous variability in midsagittal cross-sectional size of the AC is one consideration in these results. The lack of independent replication of the previous reports of AC differences associated with gender and sexual orientation, underscores the importance of replication before any findings are accepted as fact. (PsycINFO Database Record (c) 2012 APA, all rights reserved)
SN - 0419-4217
AD - Lasco, Mitchell Stuart: New York U., US
ER -

TY - JOUR
ID - 1179
T1 - The expression of care in the rough and tumble play of boys. [References]
A1 - Reed,Tom
Y1 - 2001///
N1 - Peer Reviewed Journal: 2001-14712-008
English
Journal; Peer Reviewed Journal
KW - PsycInfo
KW - Rough and Tumble
KW - Aggressive Behavior
KW - Childhood Play Behavior

KW - Friendship
KW - Intimacy
KW - Psychosocial Development
KW - Human Males
RP - NOT IN FILE
SP - Fal
EP - Win
JF - Journal of Research in Childhood Education
VL - .15
IS - 1
N2 - Examined pre-adolescent boys participating in rough and tumble play (RTP) and the ways in which they express care and intimacy as a result of their participation. Observations were conducted in a youth center for school-age children. Ss were 7 boys (aged 6-9 yrs) who were selected as a result of their sustained play in a game that encompassed RTP behaviors and exhibited evidence of caring. Methodology involved the Ss being videotaped while engaging in their favorite RTP in their natural surroundings. Ss were also asked to view the videotapes and offer their personal interpretation of the RTP experience. The criteria for identifying and recording an episode as RTP included reciprocal role taking, the play face, vigorous movement, and alternating between the roles of victim and victimizer. Ss were videotaped during a 10-day period (3 hrs each day). In all, 119 demonstrations of RTP were observed, taped, and analyzed. The results indicate that RTP is a means by which boys express care and intimacy for one another. Also, Ss were clear on where and when it was appropriate to express care and intimacy for one another. These findings suggest a need to reconsider the importance of RTP as 1 way boys express care, fondness, and friendship toward each other. (PsycINFO Database Record (c) 2012 APA, all rights reserved)
SN - 0256-8543
AD - Reed, Tom: U South Carolina, Spartanburg, SC, US
ER -

TY - JOUR
ID - 1180
T1 - The effect of network and public television programs on four and five year olds ability to attend to educational tasks
A1 - Geist,Eugene A.
Y1 - 2000///
N1 - Peer Reviewed Journal: 2001-14018-007
English
Journal; Peer Reviewed Journal
KW - PsycInfo
KW - Rough and Tumble
KW - Attention Span
KW - Classroom Behavior
KW - Education
KW - Television Viewing
RP - NOT IN FILE
SP - Dec
EP - 261
JF - Journal of Instructional Psychology
VL - .27
IS - 4
N2 - Examined the effect of network and public television programs on children's 1) ability to attend to a task, 2) children's time on task, and 3) engagement in rough and tumble play. 62 children (aged 4-5 yrs) were randomly assigned to 1 of 3 groups. One group watched Mister Rodgers' Neighborhood, a Public television program, one group watched The Mighty Morphin Power Rangers, a network television program, and one group watched no television for the treatment period and instead played with instructional materials. Results show that there was no significant difference on the attention variables between the Public television group and the instructional activities group. However, the network television program showed a significant difference with

both the other groups. No differences were found on the rough and tumble variable. (PsycINFO Database Record (c) 2012 APA, all rights reserved)
SN - 0094-1956
AD - Geist, Eugene A.: Ohio U, Athens, OH, US
ER -

TY - JOUR
ID - 1181
T1 - Preschool children's negotiation of intersubjectivity during rough-and-tumble play
A1 - Dewolf,Donna Michele
Y1 - 2000///
N1 - Dissertation Abstract: 2000-95010-393
English
Dissertation Abstract
KW - PsycInfo
KW - Rough and Tumble
KW - Childhood Play Behavior
KW - Negotiation
KW - Subjectivity
RP - NOT IN FILE
SP - May
JF - Dissertation Abstracts International: Section B: The Sciences and Engineering
VL - .60
IS - 11-B
N2 - This current study included an exploratory component, as well as, a confirmatory component. The exploratory component of the study involved developing an understanding of how children negotiate intersubjectivity during rough-and-tumble play (R&T play). Utilizing participant observation, the researcher described the context and the experiences of children as they jointly negotiated a shared understanding during R&T play. The researcher discovered, in R&T play, a structure and level of shared thinking among participants similar to other types of social play. The confirmatory component of the study involved conducting analyses on certain child characteristics as they related to participation in R&T play episodes. A portion of the findings from this aspect of the study corroborated findings from previous research. (PsycINFO Database Record (c) 2012 APA, all rights reserved)
SN - 0419-4217
AD - Dewolf, Donna Michele: The Louisiana State U And Agricultural And Mechanical Coll., US
ER -

TY - JOUR
ID - 1183
T1 - Hand signalling in dyadic proximal communication: Social strengths of children with autism who do not speak. [References]
A1 - Whittaker,Christopher A.
Y1 - 2000///
N1 - Peer Reviewed Journal: 2000-07682-003
English
Journal; Peer Reviewed Journal
KW - PsycInfo
KW - Rough and Tumble
KW - Autism
KW - Gestures
KW - Interpersonal Communication
KW - Learning Disabilities
RP - NOT IN FILE
SP - Feb
EP - 57
JF - Child Language Teaching and Therapy

VL - .16
IS - 1
N2 - A hand-signalling procedure was taught to 4 boys (aged 48-105 mo) with severe autism and learning disabilities, within dyadic proximal communication with an adult who was a stranger to them. Proximal communication involved rough and tumble play and imitation of the child, with frequent pause/burst phases to encourage him to initiate communication. It builds on the social strengths of the children and is discussed in relation to the prevailing deficit model of autism. High frequencies of hand signalling were shown by each child and sustained over time. A behavioral explanation was rejected in favor of a developmental one involving Piaget's concept of operational causality. (PsycINFO Database Record (c) 2012 APA, all rights reserved)
SN - 0265-6590
AD - Whittaker, Christopher A.: U Durham, School of Education, Durham, England
ER -

TY - JOUR
ID - 1184
T1 - The mystery of ticklish laughter
A1 - Harris,Christine R.
Y1 - 1999///
N1 - Peer-Reviewed Status-Unknown: 2001-17508-001
English
Journal; Peer-Reviewed Status-Unknown
KW - PsycInfo
KW - Rough and Tumble
KW - Laughter
KW - Pleasure
KW - Tactual Perception
KW - Tactual Stimulation
KW - Interpersonal Interaction
KW - Pain
KW - Physical Contact
KW - Reflexes
KW - Theory of Evolution
RP - NOT IN FILE
SP - 1999, pp
EP - 1999,351
JF - American Scientist
VL - .87
IS - 4
N2 - Investigated whether tickling is evolutionary, pleasure, pain, a social response, or a reflex. This is done by discussing the ontogeny, sociality and physiology of tickle and the laughter it induces. The author concludes by suggesting evolutionary psychological and physiological bases for tickling. These suggestions include: tickling may help facilitate the bond between parent and offspring; being ticklish in spots that are vulnerable in arm-to-arm combat confers an adaptive advantage by motivating individuals to protect these areas; and that the disconnection between positive outward expression (that encourages the tickling) and negative inward feeling (discomfort) motivates the growing child or ape to develop combat skills in much the same way that other rough-and-tumble play does. (PsycINFO Database Record (c) 2012 APA, all rights reserved)
SN - 0003-0996
AD - Harris, Christine R.: U California-San Diego, Dept of Psychology, La Jolla, CA, US
ER -

TY - JOUR
ID - 1185
T1 - Relationships between motivational climate and satisfaction, perceived ability and fairplay attitudes in young soccer players. [Spanish]
A1 - Angles,Merce
Y1 - 1999///

N1 - Peer Reviewed Journal: 2001-00061-002
Spanish
Journal; Peer Reviewed Journal
KW - PsycInfo
KW - Rough and Tumble
KW - Achievement
KW - Athletic Performance
KW - Goals
KW - Motivation
KW - Soccer
KW - Athletes
KW - Psychological Theories
RP - NOT IN FILE
SP - 1999, pp
EP - 64
JF - Revista de Psicologia Social Aplicada
VL - .9
IS - 1
N2 - Conducted a study to confirm the relationships proposed by motivation theory based on goal perspectives. 176 soccer players aged 9-14 yrs in Spain were administered the Perceived Motivational Climate in Sports Questionnaire (J. J. Seifriz et al, 1992) and questionnaires on satisfaction, perceived ability, and attitudes about fair play. Principal component factor analysis with varimax rotation and canonical correlation analysis were performed, and Pearson's correlation coefficient was calculated. The results support the motivational theory of achievement goals in sports. The perception of a task-associated motivational climate was related to a high level of satisfaction with training, with perceived ability more self-referenced than normative and with a favorable attitude towards enjoyment. However, the perception of an ego-associated motivational climate was related to the perception of ability based on normative criteria, with favorable attitudes towards rough play and victory in soccer games, a low level of satisfaction with training, and an unfavorable attitude towards enjoyment. (PsycINFO Database Record (c) 2012 APA, all rights reserved)
SN - 1131-6225
AD - Boixados i Angles, Merce: U Autonoma de Barcelona, Psicobiologia i Metodologia de les Ciencies de la Salut, Bellaterra, Spain
ER -

TY - JOUR
ID - 1186
T1 - Young children's conflicts and teachers' perspectives on them. [References]
A1 - Smith,Anne B.
Y1 - 1999///
N1 - Peer Reviewed Journal: 2000-07672-004
English
Journal; Peer Reviewed Journal
KW - PsycInfo
KW - Rough and Tumble
KW - Aggressive Behavior
KW - Childhood Play Behavior
KW - Conflict
KW - Peer Relations
KW - Teacher Student Interaction
KW - Child Care Workers
KW - Child Day Care
KW - Preschool Teachers
RP - NOT IN FILE
SP - 1999, pp
EP - 1999,348
JF - New Zealand Journal of Educational Studies

VL - .34
IS - 2
N2 - Examined infant peer conflicts and teachers' perspectives concerning conflict management. In Exp 1, 200 children (aged 1-26 mo) attending childcare centers were observed for the incidence, context, and nature of conflict. Results show that 2/3 of Ss were involved in a conflict episode within a 20-min period, with only a sixth of all conflicts involving hostile intentional aggression. No gender differences were observed in type, content, consequences, or strategy of dealing with conflict. In Exp 2, 30 early childhood teachers completed interviews regarding their views of child conflict. Results show that conflict was seen to be a very positive opportunity for children to learn, rough-and-tumble play was seen as moderately positive, and aggression was seen more negatively. Teachers were more likely to intervene when judging children to be physically at risk and believed that peer interactions needed careful monitoring. (PsycINFO Database Record (c) 2012 APA, all rights reserved)
SN - 0028-8276
AD - Smith, Anne B.: U Otago, Children's Issues Ctr, Dunedin, New Zealand
ER -

TY - JOUR
ID - 1187
T1 - Bargaining for advantage: Negotiation strategies for reasonable people
A1 - Shell,G.
Y1 - 1999///
N1 - Book: 2000-03854-000
English
Book; Authored Book
(Created by PsycINFO) Acknowledgments Introduction: It's your move Part I: The six foundations of effective negotiation The first foundation: Your bargaining style The second foundation: Your goals and expectations The third foundation: Authoritative standards and norms The fourth foundation: Relationships The fifth foundation: The other party's interests The sixth foundation: Leverage Part II: The negotiation process Step 1: Preparing your strategy Step 2: Exchanging information Step 3: Opening and making concessions Step 4: Closing and gaining commitment Bargaining with the devil without losing your soul: Ethics in negotiation Conclusion: On becoming an effective negotiator Appendix A: A note on your personal negotiation style Appendix B: Information-based bargaining plan Notes Selected bibliography Index
KW - PsycInfo
KW - Rough and Tumble
KW - Negotiation
KW - Strategies
RP - NOT IN FILE
SP - 286
JF - (1999)
VL - Bargaining for advantage
N2 - (from the cover) The author has taught thousands of business leaders, administrators, and other professionals how to survive and thrive in the sometimes rough-and-tumble world of negotiation. His systematic, step-by-step approach comes to life in this book, which combines lively storytelling, proven tactics, and reliable insights gleaned from the latest negotiation research. Shell's approach, which begins with a candid self-assessment of an individual's personal strengths and weaknesses, helps everyone from the inexperienced, anxious negotiator to the seasoned veteran. Readers will learn to (1) succeed even when they think they are short on bargaining power, (2) counter hardball tactics and tricks without compromising their ethics, (3) building trust in working relationships, (4) improve their leverage at each stage of the process, and (5) decide when to compromise. Laced with stories about some of the best negotiators of all time-including Benjamin Franklin, J. P. Morgan, Sony's Akio Morita, and Donald Trump-this how-to-guide gives readers the tools they need to become a skillful negotiator in every aspect of their lives. (PsycINFO Database Record (c) 2012 APA, all rights reserved)
AD - Shell, G. Richard: U Pennsylvania, Wharton School of Business, Philadelphia, PA, US
ER -

TY - JOUR

ID - 1188
T1 - Mothers' memories of childhood peer experiences: Associations to child-rearing behaviours and children's social competence
A1 - Mohr,Shelley Lynn
Y1 - 1999///
N1 - Dissertation Abstract: 1999-95024-013
English
Dissertation Abstract
KW - PsycInfo
KW - Rough and Tumble
KW - Childrearing Practices
KW - Early Memories
KW - Mother Child Relations
KW - Peer Relations
KW - Social Skills
KW - Mothers
RP - NOT IN FILE
SP - Jan
JF - Dissertation Abstracts International: Section B: The Sciences and Engineering
VL - .60
IS - 6-B
N2 - The present study evaluated the utility of a proposed model which specified potential associations from mothers' memories of childhood peer experiences (specific and general) to mothers' child-rearing behaviours in the domains of involvement in children's peer relationships and parenting style, and to children's social competence. Gender of child was also considered to influence these associations. Questionnaire data was gathered from 203 mother-(preschool)child dyads. Memories were measured by mothers' narrative recall of a specific peer experience, as well as by responses to a number of questions pertaining to mothers' general recall of peer acceptance, loneliness, as well as aggressive peer experiences. Measures of maternal depression, education, and mothers' value of children's sociability, and parenting style were also obtained. A two-factor measure of mothers' involvement in children's peer relations was yielded from Cohen's (1989) "Parental Involvement Checklist". Children's social competence was multiply determined by both mothers' and teachers' ratings. Results of multivariate analyses suggested that mothers who reported a specific peer memory were less likely to have children whom teachers rated as engaging in high levels of rough and tumble play. Additionally, children of mothers who reported specific anxious-lonely memories were the most popular amongst their peers, according to teachers. Results of hierarchical regression analyses suggested that mothers' aggressive memories were associated with authoritarian parenting behaviours, which were, in turn, associated with children's rough play, aggressive, and anxious behaviour. A trend was also observed for mothers with general memories of lonely peer experiences to be more directly involved in children's peer relations, which was, in turn, associated with children's reticent play, aggressive and anxious behaviour. Mothers' general memories of peer acceptance were positively associated with children's peer acceptance (according to mothers) while mothers' general aggressive memories tended to be positively associated with children's aggressive behaviour (according to mothers). Contrary to predictions, gender effects were not elucidated in these associations. Processes by which mothers' memories of childhood peer relationships may affect parental efficacy and childrearing behaviours were discussed. Additionally, the importance of discriminating specific peer memories from more general recall of peer memories, was discussed within this context, as well as an important consideration for future research. (PsycINFO Database Record (c) 2012 APA, all rights reserved)
SN - 0419-4217
AD - Mohr, Shelley Lynn: Carleton U., Canada
ER -

TY - JOUR
ID - 1189
T1 - Recreational activities of students with mental retardation in three types of schoolyards. [French]. [References]
A1 - Dore,Robert; Dion,Eric; Chapdelaine,Therese; Brunet,Jean-Pierre; Wagner,Serge
Y1 - 1999///

N1 - Peer Reviewed Journal: 1999-11887-003
French
Journal; Peer Reviewed Journal
KW - PsycInfo
KW - Rough and Tumble
KW - Childhood Play Behavior
KW - Mainstreaming (Educational)
KW - Playgrounds
KW - Special Education
KW - Elementary School Students
KW - Intellectual Development Disorder
RP - NOT IN FILE
SP - Jun
EP - 53
JF - Revue Francophone de la Deficience Intellectuelle
VL - .10
IS - 1
N2 - Studied the effect of the elementary school playground on the social and solitary activities of students with mental retardation who learn in regular classrooms, special classrooms in a regular school, or a special school. 26 moderately mentally retarded elementary school students (mean age 9 yrs) who attended regular classes (10 Ss), a special class in a regular school (8 Ss), or a special school (8 Ss) in Canada were observed during at least 1 recreational period lasting at least 15 min. One O was present in about 85% of cases, and 2 Os were present in about 15% of cases. Compared with Ss from special classrooms or a special school, Ss from regular classrooms had more opportunities to interact with normal peers during recess (although their school playground offered less play-related materials than playgrounds designed for mentally retarded students), were more involved in group play, and demonstrated less rough play and less solitary play. However, Ss in all 3 environments often were alone and unoccupied during recess. (PsycINFO Database Record (c) 2012 APA, all rights reserved)
SN - 0847-5733
AD - Dore, Robert: U Quebec a Montreal, Dept des Sciences de l'Education, Montreal, Canada
ER -

TY - JOUR
ID - 1190
T1 - Affective neuroscience: The foundations of human and animal emotions
A1 - Panksepp,Jaak
Y1 - 1998///
N1 - Book: 1999-02235-000
English
Book; Authored Book
(Created by PsycINFO) Part I: Conceptual background Affective neuroscience: History and major concepts Emotional operating systems and subjectivity: Methodological problems and a conceptual framework for the neurobiological analysis of affect The varieties of emotional systems in the brain: Theories, taxonomies, and semantics Neurostatics: The anatomy of the brain/mind Neurodynamics: The electrical languages of the brain Neurodynamics: Neurochemical maps of the brain Part II: Basic emotional and motivational processes Sleep, arousal, and mythmaking in the brain SEEKING systems and anticipatory states of the nervous system Energy is delight: The pleasures and pains of brain regulatory systems Nature red in tooth and claw: The neurobiological sources of rage and anger The sources of fear and anxiety in the brain Part III: The social emotions The varieties of love and lust: Neural control of sexuality Love and the social bond: The sources of nurturance and maternal behavior Loneliness and the social bond: The brain sources of sorrow and grief Rough-and-tumble play: The brain sources of joy Emotions, the higher cerebral processes, and the SELF: Some are born to sweet delight, some are born to endless night Appendix A: Bones, brains, and human origins Appendix B: The brain, language, and affective neuroscience Appendix C: Dualism in the neurosciences Notes Author index Subject index
KW - PsycInfo
KW - Rough and Tumble
KW - Brain

KW - Emotions
KW - Mind
KW - Motivation
KW - Neurobiology
KW - Mammals
KW - Neuroanatomy
KW - Neurochemistry
KW - Neurophysiology
RP - NOT IN FILE
SP - 466
JF - (1998)
VL - Affective neuroscience
N2 - (from the jacket) In this book, the author provides up-to-date information about the brain-operating systems that organize the fundamental emotional tendencies of all mammals. Presenting complex material in a readable manner, the book offers a comprehensive summary of the fundamental neural sources of human and animal feelings, as well as a conceptual framework for studying emotional systems of the brain. Panksepp approaches emotions from the perspective of basic emotion theory but does not fail to address the complex issues raised by constructionist approaches. These include relations to human consciousness and the psychiatric implications of this knowledge. Representing a synthetic integration of vast amounts of neurobehavioral knowledge, including relevant neuroanatomy, neurophysiology, and neurochemistry, this book makes an important contribution to understanding the biology of emotions. (PsycINFO Database Record (c) 2012 APA, all rights reserved)
AD - Panksepp, Jaak: Bowling Green State U, Bowling Green, OH, US
ER -

TY - JOUR
ID - 1191
T1 - Gender segregation in early childhood: A test of the play style compatibility hypothesis
A1 - Tietz,Julie Ann
Y1 - 1998///
N1 - Dissertation Abstract: 1998-95018-112
English
Dissertation Abstract
KW - PsycInfo
KW - Rough and Tumble
KW - Childhood Play Behavior
KW - Human Sex Differences
KW - Interpersonal Interaction
RP - NOT IN FILE
SP - Sep
JF - Dissertation Abstracts International: Section B: The Sciences and Engineering
VL - .59
IS - 3-B
N2 - Maccoby and Jacklin (1987) have suggested that gender segregation occurs for reasons other than a simple socialization explanation; rather, differences in interactional styles may cause children to prefer playmates of the same sex, leading to segregated play. Boys have often been found to display rough and tumble and verbally dominant behaviors during social interactions, whereas girls are more likely to engage in more socially skilled activities. According to the play style compatibility hypothesis, girls find boys' play style to be aversive and thus segregate according to gender during play. Cognitive factors, such as awareness of gender categories, are also thought to play a role. Research up to this point has been largely observational. The current study used an experimental manipulation to test the play style compatibility hypothesis. Preschool boys and girls were shown videotaped clips of other preschoolers playing in one of three play styles: (1) rough and tumble, (2) verbally dominating, or (3) friendly (neither rough and tumble nor verbally dominating). Participants were asked which of the videotaped models they preferred. Younger children were expected to base preferences on play styles; that is, females were expected to prefer models demonstrating friendly play, whereas males were expected to prefer models demonstrating rough and tumble or verbally dominating play. Older children were expected to

base preferences on gender; that is, females were expected to prefer female models, and males were expected to prefer male models because they have learned to associate preferred play styles with a particular gender. When comparing rough and tumble play versus friendly play, younger participants preferred rough and tumble play in females but friendly play in males; when comparing verbally dominant play versus friendly play, there was limited support for the hypothesis that younger females would prefer friendly play whereas younger males would prefer verbally dominant play. Older participants did display the predicted same-sex bias; furthermore, friendly play was liked more than the other two styles, suggesting socialization effects in this age group. (PsycINFO Database Record (c) 2012 APA, all rights reserved)
SN - 0419-4217
AD - Tietz, Julie Ann: Texas Tech U, US
ER -

TY - JOUR
ID - 1192
T1 - Stress and firefighters: An exploratory study
A1 - Ottlinger,James Michael
Y1 - 1998///
N1 - Dissertation Abstract: 1998-95004-125
English
Dissertation Abstract
KW - PsycInfo
KW - Rough and Tumble
KW - Coping Behavior
KW - Fire Fighters
KW - Occupational Stress
KW - Stress Management
RP - NOT IN FILE
SP - Feb
JF - Dissertation Abstracts International: Section B: The Sciences and Engineering
VL - .58
IS - 8-B
N2 - This study surveyed firefighters' perceptions of the causes of occupational stress. It also explored behavioral effects that could be experienced or exhibited by firefighters. The subjects of this research were 161 full-time firefighters and administrative fire department officers from six different fire departments. The results indicated that there was no significant relationship among the selected demographic variables (age, gender, rank, years of service, marital status, and level of education) and the causes of stress experienced and perceived by firefighters and the types of coping measures used by firefighters. Even though there was no significant relationship found, the study showed that stress does exist in the fire service today. The five most prominent perceived causes of stress were (a) a fellow firefighter dying on the job; (b) runs involving young people who are badly injured or killed; (c) after a fire, being second guessed by the authorities on the performance at the fire; (d) injuries to the other firefighters on the job; and (e) problems that continue to go on with nothing being done about them. The five most prominent mechanisms used to deal with stress were (a) horseplay, (b) sleep problems, (c) joking about and making fun of clients, (d) impatience with immature and ignorant people, and (e) complaining to each other about the other firefighters and the way they did the job. The findings point to a need to develop and utilize stress management and prevention programs designed to educate firefighters to stress-reduction techniques and help them to be aware of the signs and symptoms of stress. Improved communication skills are needed to avoid the pitfalls of withdrawal and excessive anxiety inherent in the firefighter occupation. (PsycINFO Database Record (c) 2012 APA, all rights reserved)
SN - 0419-4217
AD - Ottlinger, James Michael: Walden U, US
ER -

TY - JOUR
ID - 1193
T1 - Developmental changes in behavior and in steroid uptake by the male and female macaque brain
A1 - Michael,Richard P.

Y1 - 1998///
N1 - Peer Reviewed Journal: 1998-10117-004
English
Journal; Peer Reviewed Journal
KW - PsycInfo
KW - Rough and Tumble
KW - Animal Ethology
KW - Animal Sex Differences
KW - Neurochemistry
KW - Prenatal Development
KW - Sex Hormones
KW - Human Sex Differences
KW - Monkeys
RP - NOT IN FILE
SP - 1998, pp
EP - 1998,260
JF - Developmental Neuropsychology
JA - Dev Neuropsychol
VL - .14
IS - 2-3
N2 - Describes the sex differences in the development of play initiation, rough-and-tumble play, and mounting behavior in rhesus monkeys. The work of H. F. Harlow and colleagues (1959) demonstrated in rhesus monkeys the critical role of the early postnatal environment for the subsequent development of normal adult behavior; when infants are reared in social isolation, many aspects of their adult behavioral repertoire were disrupted.Even in adult primates reared in the wild, the behavioral effects of gonadal hormones are much influences by such variables as kinship, duration of familiarity with conspecific, partner preferences, individual differences, dominance rank, and the amount and quality of space available. These and other factors can modulate hormonal effects in humans, and knowledge of the role of gonadal hormones during nonhuman primate development may also be viewed in this broader perspective. Topics discussed in this paper include sex differences in the behavior of adult macaques, behavioral effects of prenatal androgen administration, and the developmental changes in hormone uptake by the primate brain. (PsycINFO Database Record (c) 2012 APA, all rights reserved)
SN - 8756-5641
AD - Michael, Richard P.: Emory U, School of Medicine/Georgia Mental health Inst, Dept of Psychiatry & Behavioral Sciences, Atlanta, GA, US
ER -

TY - JOUR
ID - 1194
T1 - Physical activity play: Consensus and debate
A1 - Pellegrini,A.
Y1 - 1998///
N1 - Peer Reviewed Journal: 1998-10043-006
English
Journal; Peer Reviewed Journal
KW - PsycInfo
KW - Rough and Tumble
KW - Childhood Play Behavior
KW - Childhood Play Development
KW - Developmental Age Groups
KW - Exercise
KW - Human Sex Differences
KW - Age Differences
KW - Cognitive Development
KW - Physical Activity
KW - Physical Development
KW - Psychosocial Development

KW - Stereotyped Behavior
RP - NOT IN FILE
SP - Jun
EP - 610
JF - Child Development
JA - Child Dev.
VL - .69
IS - 3
N2 - Replys to comments by J. A. Byers (see record 1998-10043-002), L. McCune (see record 1998-10043-003), D. F. Bjorklund and R. D. Brown (see record 1998-10043-004), and P. Rosenbaum (see record 1998-10043-005) on our article (see record 1998-10043-001) which considers the nature and possible developmental functions of physical activity play. In that article, we distinguish 3 kinds of physical activity play, with consecutive age peaks: rhythmic stereotypies peaking in infancy, exercise play peaking during the preschool years, and rough-and-tumble (R & T) play peaking in middle childhood. In this reply, we consider areas of consensus, including the value of an evolutionary perspective and the utility of exploring variations in physical activity play. We also consider areas of debate, including the nonplay/play distinction, functions of R & T, and the opportunities of juveniles for exercise training. We call for more directed research in this neglected area of children's behavior and development. (PsycINFO Database Record (c) 2012 APA, all rights reserved)
SN - 0009-3920
AD - Pellegrini, A. D.: U Minnesota, Dept of Educational Psychology, Minneapolis, MN, US
ER -

TY - JOUR
ID - 1195
T1 - Physical activity play in children with disabilities: A neglected opportunity for research?
A1 - Rosenbaum,Peter
Y1 - 1998///
N1 - Peer Reviewed Journal: 1998-10043-005
English
Journal; Peer Reviewed Journal
KW - PsycInfo
KW - Rough and Tumble
KW - Childhood Play Behavior
KW - Childhood Play Development
KW - Developmental Age Groups
KW - Exercise
KW - Human Sex Differences
KW - Age Differences
KW - Cognitive Development
KW - Physical Activity
KW - Physical Development
KW - Psychosocial Development
KW - Stereotyped Behavior
RP - NOT IN FILE
SP - Jun
EP - 608
JF - Child Development
JA - Child Dev.
VL - .69
IS - 3
N2 - Comments on A. D. Pellegrini and P. K. Smith's article (see record 1998-10043-001) which considers the nature and possible developmental functions of physical activity play. Pellegrini and Smith distinguish 3 kinds of physical activity play, with consecutive age peaks: rhythmic stereotypies peaking in infancy, exercise play peaking during the preschool years, and rough-and-tumble play peaking in middle childhood. Function is considered in terms of beneficial immediate and deferred consequences in physical, cognitive, and social domains. Pellegrini and Smith suggest that forms of physical activity play serve primarily immediate

developmental functions. Studies of the development of children with disorders of motor function afford opportunities, as yet unexploited, to understand the importance of motor function to overall child development. Pellegrini and Smith's review provides a challenge to developmentalists from many disciplines to use this natural model of developmental variation to move the field forward. (PsycINFO Database Record (c) 2012 APA, all rights reserved)
SN - 0009-3920
AD - Rosenbaum, Peter: McMaster U, Faculty of Health Sciences, Hamilton, ON, Canada
ER -

TY - JOUR
ID - 1197
T1 - Immediate and ultimate functions of physical activity play
A1 - McCune,Lorraine
Y1 - 1998///
N1 - Peer Reviewed Journal: 1998-10043-003
English
Journal; Peer Reviewed Journal
KW - PsycInfo
KW - Rough and Tumble
KW - Childhood Play Behavior
KW - Childhood Play Development
KW - Developmental Age Groups
KW - Exercise
KW - Human Sex Differences
KW - Age Differences
KW - Cognitive Development
KW - Physical Activity
KW - Physical Development
KW - Psychosocial Development
KW - Stereotyped Behavior
RP - NOT IN FILE
SP - Jun
EP - 603
JF - Child Development
JA - Child Dev.
VL - .69
IS - 3
N2 - Comments on A. D. Pellegrini and P. K. Smith's article (see record 1998-10043-001) which considers the nature and possible developmental functions of physical activity play. Pelligrini and Smith distinguish 3 kinds of physical activity play, with consecutive age peaks: rhythmic stereotypies peaking in infancy, exercise play peaking during the preschool years, and rough-and-tumble play peaking in middle childhood. I suggest that play has been difficult to define because it occurs as an aspect of many activities rather than being limited to a specific kind of activity; thus it rarely occurs in isolation. Classic theorists such as Piaget and Vygotsky emphasize representational play as play in its purist form, but both the immediate and ultimate functions of play can be discerned in the simplest physical activity play. (PsycINFO Database Record (c) 2012 APA, all rights reserved)
SN - 0009-3920
AD - McCune, Lorraine: Rutgers U, Dept of Educational Psychology, New Brunswick, NJ, US
ER -

TY - JOUR
ID - 1199
T1 - War toy play and aggression in Dutch kindergarten children
A1 - Hellendoorn,Joop
Y1 - 1997///

N1 - Peer Reviewed Journal: 1998-00197-005
English
Journal; Peer Reviewed Journal
KW - PsycInfo
KW - Rough and Tumble
KW - Aggressive Behavior
KW - Childhood Play Behavior
KW - Toys
KW - Human Sex Differences
KW - Parent Child Relations
RP - NOT IN FILE
SP - Nov
EP - 354
JF - Social Development
VL - .6
IS - 3
N2 - The play behavior of 54 Dutch children (aged 4-7 yrs) in the presence of a standard set of war toys and neutral toys was related to 5 groups of variables: parental attitude, possession of war toys at home, the habitual aggressiveness of the child in daily life, the characteristics of the toys themselves, and the play situation. Results showed that real object- or person-oriented aggression was rare and was not related to play with the war toys. Suppression of aggression by the experimental situation seemed unlikely. During play, the levels of involvement and fantasy were generally high. Regularly, playful fighting was observed, differentiated as Make-Believe-Aggression and Rough-and-Tumble. These 2 Play 'Aggression' factors, and Real Aggression, could be predicted from other variables, principally from gender, the influence of session partners, play involvement, type of war toy used, and parental attitude. (PsycINFO Database Record (c) 2012 APA, all rights reserved)
SN - 0961-205X
AD - Hellendoorn, Joop: Leiden U, Dept of Education, Leiden, Netherlands
ER -

TY - JOUR
ID - 1200
T1 - The expression of care in the rough and tumble play of pre-adolescent boys
A1 - Reed,Thomas L.
Y1 - 1997///
N1 - Dissertation Abstract: 1997-95009-052
English
Dissertation Abstract
KW - PsycInfo
KW - Rough and Tumble
KW - Childhood Play Behavior
KW - Human Males
KW - Interpersonal Interaction
RP - NOT IN FILE
SP - May
JF - Dissertation Abstracts International Section A: Humanities and Social Sciences
VL - .57
IS - 11-A
N2 - Rough and tumble (R&T) play is widely researched in relationship to social affiliation and the cognitive benefits associated with participation in this type of play. One less researched aspect of R&T play is the affective dimension, more specifically, the way in which boys care for one another through R&T play. The first part of this research examines the various ways in which boys participate in R&T play, how the R&T scenario is set up, who plays, what the rules are and how they are enforced. Boys were video taped while engaging in R&T play and during a game called Smear the Queer (Smear). Data gathered from observing the video taped segments of R&T play clearly suggests that R&T play is a way in which boys express care for one another, that Smear is a camouflage for intimacy, and that R&T play is more like a dance among the players than a helter-skelter mix of running, chasing, and tackling. The second part of this study involved interviewing the players

while they watched selected video segments of R&T play and expressions of care exhibited during R&T play. Analysis of the interview transcripts established that the boys were able to clearly delineate the difference between R&T play and aggression. Furthermore, the participants were able to validate the game of Smear as a way in which boys are able to express care for one another. Finally, the participants described R&T play as the vehicle in which they are able to experience physical intimacy with one another. It is concluded that R&T play is clearly misunderstood by the uninformed observer. The players are keenly aware that engagement in R&T play is a time that it is acceptable to hug, hold hands, and otherwise experience bodily contact not permitted outside the play arena. (PsycINFO Database Record (c) 2012 APA, all rights reserved)
SN - 0419-4209
AD - Reed, Thomas L.: U South Carolina, US
ER -

TY - JOUR
ID - 1201
T1 - Development of sex differences in preschool children: Social behavior during an academic year
A1 - Braza,Francisco; Braza,Paloma; Carreras,Maria Rosario; Munoz,Jose Manuel
Y1 - 1997///
N1 - Peer Reviewed Journal: 1997-07974-032
English
Journal; Peer Reviewed Journal
KW - PsycInfo
KW - Rough and Tumble
KW - Childhood Play Behavior
KW - Human Sex Differences
RP - NOT IN FILE
SP - Feb
EP - 188
JF - Psychological Reports
JA - Psychol.Rep.
VL - .80
IS - 1
N2 - Sex differences in activities of preschoolers were assessed during free play time from observation of the behavior of 31 children (23 girls, 8 boys). The differences were noted for the time girls and boys spent in the activities considered, boys spending more time in rough-and-tumble play and in agonistic activities and girls in organized games such as games with rules and role-play in addition to affiliative activities. Sex differences could be detected also in the distribution of time among various activities of the children during free-play in the 3 terms of the school year under consideration. (PsycINFO Database Record (c) 2012 APA, all rights reserved)
SN - 0033-2941
AD - Braza, Francisco: Estacion Biologica de Donana (CSIC), Sevilla, Spain
ER -

TY - JOUR
ID - 1202
T1 - Appreciating the Value of Recess and Playground Behavior: Educational Implications. [References]
A1 - Wandle,Caroline H.
Y1 - 1996///
N1 - Electronic Collection: 2004-17622-033
English
Electronic Collection
KW - PsycInfo
KW - Rough and Tumble
KW - Childhood Play Behavior
KW - Cognitive Development
KW - Psychosocial Development
KW - Playgrounds
KW - Psychologists

RP - NOT IN FILE
SP - Aug, 1996
JF - PsycCRITIQUES
VL - .41
IS - 8
N2 - Originally published in Contemporary Psychology: APA Review of Books, 1996, Vol 41(8), 806-808. The reviewer states that this book (see record 1994-98891-000) undertakes the important task of informing educators and psychologists about the crucial role of recess behavior in terms of both social and cognitive development. Unlike previous examinations, Pellegrini focuses on the child of the elementary and middle school years, thus extending our understanding beyond the more typical focus on the role of play for the preschool child. Of particular interest is his exploration of the role of play, especially rough play, for boys from elementary to early middle school. Furthermore, Pellegrini expands his study, touching briefly on the previously examined variables such as playground design and timing, to explore in more detail gender, age, and sociometric status as possible mediator variables. This work highlights the functions of recess and playground behaviors especially for boys in both cognitive and social development, providing valuable information for educators and psychologists. (PsycINFO Database Record (c) 2012 APA, all rights reserved)
SN - 1554-0138
AD - Wandle, Caroline H.: School Psychology Program, Tufts University, Medford, MA, US
ER -

TY - JOUR
ID - 1203
T1 - Self stories: Effects of children's emotional styles on their appropriation of self-schemata
A1 - Hencke,Rebecca Whitson
Y1 - 1996///
N1 - Dissertation Abstract: 1996-95021-252
English
Dissertation Abstract
KW - PsycInfo
KW - Rough and Tumble
KW - Emotional States
KW - Personality Traits
KW - Self Concept
RP - NOT IN FILE
SP - Nov
JF - Dissertation Abstracts International: Section B: The Sciences and Engineering
VL - .57
IS - 5-B
N2 - A model for children's acquisition of self-schemata is proposed. The proposed social-constructivist model of the self emphasizes both the importance of socio-cultural institutions as carriers of culture-specific ideal and possible selves and the importance of children as active agents re-creating within themselves the canonical models of their culture. The role of emotion in shaping the form of the individual's appropriated self-schemata by is recognized as critical to the process of self-appropriation. To test this model, 30 children participating in the Harvard Infant Study were interviewed at four years and again at five years of age, using the Peer Nice and Mean Scale (Fischer, Hencke and Hand, 1991/1993). Using the numerous behavioral observations of these children throughout infancy, three emotional styles were identified: the fearful, the shy, and the sociable. Comparisons of children's understanding of the social interactions shown to them were compared according to valence (Nice or Mean), presence of a character named after the child in the story, and amount of prompting required to elicit an answer from them. Results of the study supported the hypothesis that emotional style would bias children's performance on stories with negative valences and stories with a self character. Fearful and shy children showed a reluctance to retell or answer questions about the story involving meanness by the self-named character. Results are discussed in terms of the implications for development of this resistance to recognizing one's aggressive feelings and acts. In addition to delaying the development of a self-concept which recognizes both positive and negative aspects of oneself, it is suggested that the inability to tolerate an image of oneself as mean may lead to an unwillingness to participate in rough-and-tumble peer play, creating a risk of social

isolation and/or rejection, especially for boys. Larger studies, with children of varying ethnicities, are recomme (PsycINFO Database Record (c) 2012 APA, all rights reserved)
SN - 0419-4217
AD - Hencke, Rebecca Whitson: Harvard U, US
ER -

TY - JOUR
ID - 1204
T1 - How child sexual molesters groom adults to gain access to children
A1 - Van Dam,Carla Helene
Y1 - 1996///
N1 - Dissertation Abstract: 1996-95021-134
English
Dissertation Abstract
KW - PsycInfo
KW - Rough and Tumble
KW - Child Abuse
KW - Pedophilia
KW - Sex Offenses
KW - Sexual Abuse
RP - NOT IN FILE
SP - Nov
JF - Dissertation Abstracts International: Section B: The Sciences and Engineering
VL - .57
IS - 5-B
N2 - The literature on child sexual abuse and offender characteristics was surveyed. A specific case was presented to provide a framework for exploring the community dynamics that provide inadvertent support and enthusiasm for the molester, at the expense of the child victims. The dynamics of sexual abuse and the community response were examined. Definitions of terms were provided, and information about molesters was summarized. The social climate, both in society's attitudes towards women and children, as well as society's general denial around the topic was presented. The harm of sexual abuse was described, both in terms of the moral issues, the psychological effects, and the cultural concerns. Then, conditions necessary for sexual abuse to occur were covered, including the need for the molester to have a sexual interest in children, overcome resistance, groom the adults, and groom the child. Current prevention strategies and the limitations of those strategies were also summarized. Twenty-one men in treatment following child sexual molestation convictions were interviewed about the strategies they used to manage the adults in their environment, both to gain access to children and to maintain security. This "grooming" of the adults in the community appeared to occur and consistently included a number of behaviors. Participants described themselves as charming, nice, and likeable. Their helpfulness was exemplified by how they anticipated adults' needs, providing timely and appreciated assistance, while never seeming to accept assistance in return. All twenty-one molesters also engaged in behaviors described in the literature as part of the grooming of children. With younger children this involved tickling, roughhousing, and playing with them on their level. With adolescents it involved such sensual touching as caressing hair, and talking to them on their level. Both the tickling, roughhousing, and peer-like quality of the play, as well as the sensual touching and pee (PsycINFO Database Record (c) 2012 APA, all rights reserved)
SN - 0419-4217
AD - Van Dam, Carla Helene: The Union Inst, US
ER -

TY - JOUR
ID - 1205
T1 - A comparison of 8- and 11-year-old girls' and boys' participation in specific types of rough-and-tumble play and aggressive fighting: Implications for functional hypotheses
A1 - Boulton,Michael J.
Y1 - 1996///

N1 - Peer Reviewed Journal: 1996-05627-003
English
Journal; Peer Reviewed Journal
KW - PsycInfo
KW - Rough and Tumble
KW - Age Differences
KW - Aggressive Behavior
KW - Childhood Play Behavior
KW - Dominance
RP - NOT IN FILE
SP - 1996, pp
EP - 1996,287
JF - Aggressive Behavior
JA - Aggress.Behav
VL - .22
IS - 4
N2 - Investigated the role of rough play as practice for the development of real fighting skills and as a safe way to establish/display social dominance with 86 8- and 11-yr-olds. Boys engaged in more chase initiation activities, more bouts of brief rough-and-tumble play, more bouts of restraining and more bouts of hitting than girls. Data suggest rough play develops fighting skills and establishes dominance, although sex differences were not found for all types of rough play observed. No age differences were obtained. In all but one case, there were no differences in the extent to which 8/9- and 10/11-yr-old girls and boys employed wrestling, hitting and restraining, supporting the view that the practice fighting hypothesis is relevant throughout the 8-11-yr-old period. (PsycINFO Database Record (c) 2012 APA, all rights reserved)
SN - 0096-140X
AD - Boulton, Michael J.: Keele U, Psychology Dept, Keele, England
ER -

TY - JOUR
ID - 1206
T1 - Mother-infant, father-infant interaction and involvement in childcare and household labor among Taiwanese families
A1 - Sun,Li-Ching; Roopnarine,Jaipaul
Y1 - 1996///
N1 - Peer Reviewed Journal: 1996-01751-012
English
Journal; Peer Reviewed Journal
KW - PsycInfo
KW - Rough and Tumble
KW - Childrearing Practices
KW - Father Child Relations
KW - Household Management
KW - Mother Child Relations
KW - Human Sex Differences
KW - Parental Role
KW - Parents
KW - Sex Roles
RP - NOT IN FILE
SP - Jan
EP - Mar
JF - Infant Behavior & Development
JA - Infant behav
VL - .19
IS - 1
N2 - Mother-infant and father-infant interactions were observed in 25 Taiwanese homes, and parents provided estimates of their involvement in caregiving and household activities. Observations indicated that mothers held

infants more than did fathers and were more likely to feed, smile at, vocalize to, and engage in object play with them than were fathers. Fathers engaged in more rough play than did mothers. There were no sex-of-parent differences in soothing infants or displaying affection to them. Mothers and fathers treated males and females similarly. Infants smiled at, vocalized to, approached, and were more distressed in the presence of mothers than fathers. Data reveal sex-differentiated patterns of involvement in child care and household activities. Results are hypothesized to reflect Taiwanese societal rigidity in sex roles and filial piety. (PsycINFO Database Record (c) 2012 APA, all rights reserved)
SN - 0163-6383
AD - Sun, Li-Ching: Syracuse U, Syracuse, NY, US
ER -

TY - JOUR
ID - 1207
T1 - An indicator of pupil's movement satisfaction in the basic stage of education. [Arabic]
A1 - Ibraheem,Hashim
Y1 - 1995///
N1 - Peer-Reviewed Status-Unknown: 1997-86932-001
Arabic
Journal; Peer-Reviewed Status-Unknown
KW - PsycInfo
KW - Rough and Tumble
KW - Age Differences
KW - Motor Processes
KW - Satisfaction
KW - Elementary School Students
KW - Junior High School Students
RP - NOT IN FILE
SP - Feb
EP - 372
JF - Dirasat
VL - .22A
IS - 1
N2 - Movement is considered a fundamental way to express thoughts, concepts, feelings and self-concept in general. Therefore, the positive emotional condition that results from success is considered to be one of the major foundations of one's development. The purpose of this study was to determine the level of movement satisfaction for students in fourth, fifth and sixth grades, and to find differences among these grades in movement satisfaction. The descriptive approach was utilized with a random sample of 1,110 pupils, employing Tanner's Movement Satisfaction Scale, as adapted to the Arabic language. It was found that the level of movement satisfaction ranged between 80% and 86% for items such as dribbling, high jumping and kicking a ball; 70-79% for climbing, high-speed running and hanging; and 49-67% for lifting heavy objects, sudden stops while moving, rough play and balancing on one foot. More detailed results were presented and discussed, and their implicatons were presented. (PsycINFO Database Record (c) 2012 APA, all rights reserved)
SN - 0255-8033
AD - Ibraheem, Hashim: College of Physical Education, U of Jordan, Jordan
ER -

TY - JOUR
ID - 1208
T1 - War toy play and aggression in kindergartners. [Dutch]
A1 - Hellendoorn,J.
Y1 - 1995///
N1 - Peer Reviewed Journal: 1997-85166-001
Dutch
Journal; Peer Reviewed Journal
KW - PsycInfo
KW - Rough and Tumble

KW - Aggressive Behavior
KW - Childhood Play Behavior
KW - Parental Attitudes
KW - Teacher Attitudes
KW - Toy Selection
KW - Family Background
KW - Parents
KW - Teachers
RP - NOT IN FILE
SP - Nov
EP - 243
JF - Kind en Adolescent
VL - .16
IS - 4
N2 - Studied the relationship between war toy play and aggression in kindergartners, with consideration of the influence of family background, the attitudes of parents and teachers towards war toys, and the play situation. Human Ss: 168 normal male and female Dutch preschool and school-age children (aged 4-7 yrs) (kindergartners). Normal male Dutch adults (mean age 34 yrs) (children's fathers). Normal female Dutch adults (mean age 32 yrs) (children's mothers). Normal Dutch adults (children's teachers). The parents were administered a questionnaire on their family background and their attitudes towards war toys, and the teachers were interviewed about their attitudes towards war toys. The parents and teachers rated the children's aggressiveness with the Child Behavior Checklist. The children were observed in small-group play sessions in the presence of a standard set of toys, including various war toys. Four types of aggression were distinguished: real aggression, playful quasiaggression, fantasy aggression, and rough-and-tumble aggression. (English abstract) (PsycINFO Database Record (c) 2012 APA, all rights reserved)
SN - 0167-2436
AD - Hellendoorn, J.: Rijksuniversiteit Leiden, Vakgroep Orthopedagogiek, Netherlands
ER -

TY - JOUR
ID - 1209
T1 - Sissiness, tomboyism, sex-role, sex identity and orientation
A1 - McConaghy,Nathaniel
Y1 - 1995///
N1 - Peer Reviewed Journal: 1996-30406-001
English
Journal; Peer Reviewed Journal
KW - PsycInfo
KW - Rough and Tumble
KW - Gender Identity
KW - Heterosexuality
KW - Homosexuality
KW - Sex Roles
KW - Stereotyped Attitudes
KW - Femininity
KW - Masculinity
RP - NOT IN FILE
SP - Jun
EP - 283
JF - Australian and New Zealand Journal of Psychiatry
VL - .29
IS - 2
N2 - Investigated the relationship between homosexuality/heterosexuality, masculinity and femininity, and sex linked behaviors and degree of sexual identity in 66 males (mean age 19.5 yrs) and 51 females (mean age 19.8 yrs). Ss completed the Bem Sex-Role Inventory and the Sex-Linked Behaviors Questionnaire. Results show that 20% of the male and 28% of the female Ss reported current awareness of some degree of homosexual feeling.

The opposite sex linked "sissy" and "tomboyish" behaviors of male Ss correlated significantly with their reported ratio of homosexual to heterosexual feelings. High masculine scores in female Ss correlated strongly with several "tomboyish" behaviors. Masculine scores of male Ss showed a weaker association with liking of rough and tumble and contact sport than did those of female Ss. Feminine scores of female Ss showed the weakest relationships with the sex linked behaviors. (PsycINFO Database Record (c) 2012 APA, all rights reserved)
SN - 0004-8674
AD - McConaghy, Nathaniel: Prince of Wales Hosp, Dept of Psychiatry, Randwick, NSW, Australia
ER -

TY - JOUR
ID - 1210
T1 - The promotion and marketing of toys: Time to rethink the paradox?
A1 - Kline,Stephen
Y1 - 1995///
N1 - Book: 1995-98339-008
English
Book; Edited Book
KW - PsycInfo
KW - Rough and Tumble
KW - Childhood Play Behavior
KW - Imagination
KW - Toys
KW - Age Differences
KW - Human Sex Differences
KW - Television Advertising
RP - NOT IN FILE
SP - 306
JF - Pellegrini, Anthony D [Ed]
IS - 1995
N2 - (from the introduction) examines the role of toys and television advertising for toys on children's fantasy play / embeds [the] discussion of toys in the larger play literature, particularly that work studying symbolic play and rough-and-tumble play / reports interesting [sex and] age-differences for the influence of television on play (from the chapter) the psychological promotion of play / rethinking the play ethos / critiques of "free" toy play / the promotion of toys in America (PsycINFO Database Record (c) 2012 APA, all rights reserved)
ER -

TY - JOUR
ID - 1211
T1 - Boys' rough-and-tumble play and social competence: Contemporaneous and longitudinal relations
A1 - Pellegrini,Anthony D.
Y1 - 1995///
N1 - Book: 1995-98339-005
English
Book; Edited Book
KW - PsycInfo
KW - Rough and Tumble
KW - Aggressive Behavior
KW - Childhood Play Behavior
KW - Social Skills
KW - Activity Level
KW - Adaptability (Personality)
KW - Affiliation Motivation
KW - Cooperation
KW - Human Males
KW - Peer Relations

KW - Problem Solving
KW - Social Interaction
RP - NOT IN FILE
SP - 306
JF - Pellegrini, Anthony D [Ed]
IS - 1995
N2 - (from the chapter) in the 2 studies reported here, an attempt was made to identify aspects of R & T [rough-and-tumble play] which may serve a social-competence function in childhood / [study 1 examines] the relation between the flexibility dimension of R & T and flexibility in social problem-solving / determine the relation between 2 aspects of temperament-activity level and flexibility-and aspects of R & T / compare the social composition of boys' R & T groups with group composition during other forms of social interaction / [present] relations between specific dimensions of R & T and aspects of social competence / [Ss were 37 5th grade males] [study 2 examines] the social affiliative function served by R & T in a longitudinal context / the dimensions of affiliation examined included: engagement in cooperative games-with-rules, peer nominated popularity, and peer nominated dominance / 72 males [enrolled in grades K-5] participated in Study 2 (PsycINFO Database Record (c) 2012 APA, all rights reserved)
ER -

TY - JOUR
ID - 1212
T1 - The future of play theory: A multidisciplinary inquiry into the contributions of Brian Sutton-Smith
A1 - Pellegrini,Anthony D.
Y1 - 1995///
N1 - Book: 1995-98339-000
English
Book; Edited Book
(Created by PsycINFO) Introduction [by] Anthony D. Pellegrini Part I: Play as progress * Play, ethology, and education: A personal account / Peter K. Smith / 3-21 * Animal play, games of angels, biology, and Brian / Robert Fagen / 23-44 * Children's play and adult leisure / Garry Chick and Lynn A. Barnett / 45-69 Part II: Play as power * Competitive play on the plains: An analysis of games and warfare among native American warrior societies, 1800-1850 / John W. Loy and Graham L. Hesketh / 73-105 * Boys' rough-and-tumble play and social competence: Contemporaneous and longitudinal relations / Anthony D. Pellegrini / 107-126 * Aggressive toy play / Jeffrey Goldstein / 127-147 Part III: Play as fantasy * Toys and stories / Greta G. Fein / 151-164 * The promotion and marketing of toys: Time to rethink the paradox? / Stephen Kline / 165-185 * Imaginative play in childhood: Precursor of subjunctive thoughts, daydreaming, and adult pretending games / Jerome L. Singer / 187-219 * Social pretend with mother and sibling: Individual differences and social understanding / Lisa M. Youngblade and Judy Dunn / 221-239 Part IV: Play as self * Representing children's play: Anthropologists at work / Helen B. Schwartzman / 243-255 * Past play: Relics, memory, and history / Bernard Mergen / 257-274 Conclusion: The persuasive rhetorics of play [by] Brian Sutton-Smith Appendix: A list of Brian Sutton-Smith's play-related publications [by] F. F. McMahon
KW - PsycInfo
KW - Rough and Tumble
KW - Childhood Play Behavior
KW - Childhood Play Development
KW - Theories
RP - NOT IN FILE
SP - 306
JF - (1995)
VL - The future of play theory
N2 - (from the cover) This . . . book looks at the current status of play theory across the disciplines, literally from anthropology to zoology. The authors examine play phenomena from 4 perspectives [as studied by B. Sutton-Smith], play as progress; play as power; play as fantasy; and play as self. This book will serve as [a] reference tool, textbook, and source of inspiration for all those interested in the fascinating study of children's play. (PsycINFO Database Record (c) 2012 APA, all rights reserved)
AD - Pellegrini, Anthony D.: U Georgia, Athens, GA, US
ER -

TY - JOUR
ID - 1213
T1 - The new father: A study of attachment to primary caregiving fathers
A1 - Geiger,Brenda
Y1 - 1995///
N1 - Dissertation Abstract: 1995-95017-212
English
Dissertation Abstract
KW - PsycInfo
KW - Rough and Tumble
KW - Caregivers
KW - Father Child Relations
KW - Human Sex Differences
KW - Parent Child Relations
KW - Parental Role
RP - NOT IN FILE
SP - Sep
JF - Dissertation Abstracts International: Section B: The Sciences and Engineering
VL - .56
IS - 3-B
N2 - This research analyzes the unique and interactive effects of the gender of the caregiver and the primacy of the caregiver role on caregivers' and infants' behaviors. In 14 of the families included in this study, fathers had been the primary caregivers since their infants were at most 7 months old while mothers were employed full time. In 14 other families, fathers were employed full time while mothers were their infants' primary caregivers. All infants were less than 21 months of age, healthy, and mobile. Home videotaped observations were conducted on two separate occasions with each family. Each observation included three episodes. In episodes 1 and 2 parents took turns interacting alone with the infant in a nonstressful situation. In episode 3, infants were observed for 5 minutes while under stress in a modified version of the Strange Situation. A (gender of the caregiver) x (primacy of the caregiver role) analysis of variance was performed on all infants' and parents' attachment and affiliative behaviors coded under stressful and nonstressful conditions. No difference based on the caregiver gender or role was observed on any caregivers' affiliative behavior except for rough and tumble play for which a main gender effect was found. Primary caregiving fathers were found to engage in as much caregiving behavior as primary and secondary caregiving mothers, and to be the most affectionate caregivers. No caregiver gender or role effects were observed on most infants' affiliative behaviors in nonstressful conditions. The exception concerns the measure of synchrony. Infants' play interaction was found to be most synchronous with primary caregiving fathers. A main caregiver role effect was observed on the attachment behavior of infant's display of affection. A caregiver gender x role interaction effect was found on other measures of attachment--clinging, moving away and exploring. Under stress and when they had the choice between their parents, infants showed by (PsycINFO Database Record (c) 2012 APA, all rights reserved)
SN - 0419-4217
AD - Geiger, Brenda: State U New York at Albany, US
ER -

TY - JOUR
ID - 1215
T1 - A longitudinal study of boys' rough-and-tumble play and dominance during early adolescence
A1 - Pellegrini,Anthony D.
Y1 - 1995///
N1 - Peer Reviewed Journal: 1995-36435-001
English
Journal; Peer Reviewed Journal
KW - PsycInfo
KW - Rough and Tumble
KW - Adolescent Development
KW - Childhood Play Development

KW - Dominance
KW - Human Males
KW - Longitudinal Studies
RP - NOT IN FILE
SP - Jan
EP - Mar
JF - Journal of Applied Developmental Psychology
VL - .16
IS - 1
N2 - Longitudinally examined the extent to which 2 forms of rough-and-tumble play (R&T/Rough and R&T/Chase) varied across early adolescence and explored the role of each form of R&T in 82 12-13 yr old boys' dominance relations. Ss were observed at least 10 times per year for each of 2 yrs. The 2 forms of R&T were statistically independent of each other. R&T/Chase did not vary from Year 1 to Year 2 and was not related to dominance, but R&T/Rough declined across time and was related to dominance displays and aggression displays. (PsycINFO Database Record (c) 2012 APA, all rights reserved)
SN - 0193-3973
AD - Pellegrini, Anthony D.: U Georgia, Inst for Behavioral Research, Athens, US
ER -

TY - JOUR
ID - 1216
T1 - Gender identity disorder in boys: The interface of constitution and early experience
A1 - Coates,Susan W.
Y1 - 1995///
N1 - Peer Reviewed Journal: 1995-29278-001
English
Journal; Peer Reviewed Journal
KW - PsycInfo
KW - Rough and Tumble
KW - Early Experience
KW - Etiology
KW - Gender Identity
KW - Human Males
KW - Personality
KW - Disorders
RP - NOT IN FILE
SP - 1995, pp
EP - 38
JF - Psychoanalytic Inquiry
VL - .15
IS - 1
N2 - Clarifies the role of constitutional (CST) factors in the etiology of childhood gender identity disorder (GID). Clinical research into gender is reviewed, as well as the effect that prenatal hormones may have on gender role behavior. The work of S. J. Bradley (1985) suggests that children with GID have an inherited predisposition for affective disorder, manifested in early childhood in a proneness to anxiety states. GID boys are often averse to rough-and-tumble play, resembling other shy, inhibited boys who typically construct gender identities that are unconflicted. Thus the CST predisposition that lowers the threshold for a gender identity disturbance in boys may not be directly sex-linked, suggesting that the CST factor becomes associated with a disturbance in gender through an indirect route, specifically by causing the child to be especially vulnerable to disruptions in his primary attachment relationships. (PsycINFO Database Record (c) 2012 APA, all rights reserved)
SN - 0735-1690
AD - Coates, Susan W.: St Luke's/Roosevelt Hosp, Dept of Psychiatry, Childhood Gender Identity Ctr, New York, NY, US
ER -

TY - JOUR
ID - 1217
T1 - School recess and playground behavior: Educational and developmental roles
A1 - Pellegrini,Anthony D.
Y1 - 1995///
N1 - Book: 1994-98891-000
English
Book; Authored Book
(Created by PsycINFO) Acknowledgments Children on the playground at recess: What's so important? Playgrounds and children's behavior at recess Theory and method Dimensions of the playground and children's behavior: Implications for social cognition development The effects of recess timing on elementary school children's recess and classroom behavior Longitudinal relations between playground behavior and cognition: Explorations in social dimensions of cognition The rough-and-tumble play of primary school children: Contemporaneous and longitudinal relations Preference for outdoor play during early adolescence Longitudinal relations between social networks and adjustment to middle school Adolescent boys' rough-and-tumble play Conclusions and implications Index
KW - PsycInfo
KW - Rough and Tumble
KW - Childhood Development
KW - Childhood Play Behavior
KW - Playgrounds
KW - Cognitive Development
KW - Middle School Students
KW - Peer Relations
KW - Primary School Students
KW - Psychosocial Development
KW - School Environment
KW - Social Cognition
RP - NOT IN FILE
SP - 186
JF - (1995)
VL - School recess and playground behavior
N2 - (from the cover) The recess period represents a unique part of the school day. It is one of the few times when children can interact with their peers on their own terms with minimal adult intervention. Consequently, it represents one of the few places in primary and middle schools to observe spontaneous peer interaction. "School Recess and Playground Behavior" offers a programmatic examination of a neglected aspect of children's behavior and informs related literatures such as the educational, social-developmental, and cognitive-developmental literatures. (PsycINFO Database Record (c) 2012 APA, all rights reserved)
AD - Pellegrini, Anthony D.: U Georgia, Athens, GA, US
ER -

TY - JOUR
ID - 1218
T1 - The rough play of adolescent boys of differing sociometric status
A1 - Pellegrini,Anthony D.
Y1 - 1994///
N1 - Peer Reviewed Journal: 1995-09100-001
English
Journal; Peer Reviewed Journal
KW - PsycInfo
KW - Rough and Tumble
KW - Aggressive Behavior
KW - Childhood Play Behavior
KW - Dominance Hierarchy
KW - Sociometry
KW - Popularity

KW - Social Acceptance
RP - NOT IN FILE
SP - Sep
EP - 540
JF - International Journal of Behavioral Development
VL - .17
IS - 3
N2 - Chase and rough play (RP), 2 forms of rough-and-tumble play, were observed in 22 popular, 19 average, and 13 reject White adolescent boys (mean age for all groups 13 yrs) while they were on the playground during their school recess period. Sociometrically defined average and rejected Ss spent a significant portion of their time in RP when compared with popular Ss. RP was related to aggression and perspective-taking status for rejected Ss and was related to dominance status for all Ss. Rejected and average Ss chose to engage in RP with less dominant children. (PsycINFO Database Record (c) 2012 APA, all rights reserved)
SN - 0165-0254
AD - Pellegrini, Anthony D.: U Georgia, Inst for Behavioral Research, Athens, US
ER -

TY - JOUR
ID - 1220
T1 - Sex differences in toy play and use of video games
A1 - Goldstein,Jeffrey H.
Y1 - 1994///
N1 - Book: 1994-98307-006
English
Book; Edited Book
KW - PsycInfo
KW - Rough and Tumble
KW - Childhood Play Behavior
KW - Human Sex Differences
KW - Toys
KW - Computer Games
KW - Preferences
KW - Social Influences
RP - NOT IN FILE
SP - lay, and
EP - 129
JF - Goldstein, Jeffrey H [Ed]
IS - 1994
N2 - (from the introduction) differences in the play of boys and girls, and their different toy preferences, are discussed / biological and social origins of sex differences in play are considered, with a focus on aggressive play, war toys, and video games / [Ss in studies discussed were 4-16 yr olds] (from the chapter) children and adults view play differently / the role of prior experience / rough and tumble play / toy preference / social effects: isolation and sociability / school performance / cognitive effects / personality and psychopathology / aggression and video games (PsycINFO Database Record (c) 2012 APA, all rights reserved)
AD - Goldstein, Jeffrey H.: Utrecht U, Dept of Social & Organizational Psychology, Utrecht, Netherlands
ER -

TY - JOUR
ID - 1222
T1 - Boys' rough-and-tumble play, social competence and group composition
A1 - Pellegrini,A.
Y1 - 1993///
N1 - Peer Reviewed Journal: 1994-07550-001
English
Journal; Peer Reviewed Journal
KW - PsycInfo

KW - Rough and Tumble
KW - Childhood Play Behavior
KW - Peer Relations
KW - Personality
KW - Popularity
KW - Elementary School Students
KW - Problem Solving
KW - Social Interaction
RP - NOT IN FILE
SP - Sep
EP - 248
JF - British Journal of Developmental Psychology
VL - .11
IS - 3
N2 - Examined relations between aspects of 42 male 5th graders' rough-and-tumble play (RTP) and social competence. Ss were observed on the school playground for 20 wks and were asked to nominate peers whom they liked most and least, rank-order peers in terms of dominance, and solve hypothetical social problems. Teachers completed a temperament questionnaire for each S. Neither relative frequency of RTP nor the vigor dimension of RTP accounted for significant variance in Ss' popularity beyond other forms of reciprocal social interactions. Further, relative frequency of RTP was negatively, but not significantly, related to popularity. The flexibility dimension of RTP did account for significant variance in social problem solving. Both aspects (i.e., activity and flexibility) of temperament measured correlated significantly with the vigor level of RTP. (PsycINFO Database Record (c) 2012 APA, all rights reserved)
SN - 0261-510X
AD - Pellegrini, A. D.: U Georgia, Inst for Behavioral Research, Athens, US
ER -

TY - JOUR
ID - 1223
T1 - Gentle play partners: Mother-child and father-child play in New Delhi, India
A1 - Roopnarine,Jaipaul L.
Y1 - 1993///
N1 - Book: 1993-98052-011
English
Book; Edited Book
KW - PsycInfo
KW - Rough and Tumble
KW - Childhood Play Behavior
KW - Parent Child Relations
KW - Attachment Behavior
KW - Games
RP - NOT IN FILE
SP - 389
JF - MacDonald, Kevin [Ed]
IS - 1993
N2 - (from the chapter) hope to shed some light on adult-infant play in a society with a very lengthy history / while the major focus . . . is on early patterns of play between parents and infants in New Delhi, India, [the authors] provide some preliminary information on infant games, and play and infant stimulation / discuss the importance of rough play in the development of attachment to fathers (PsycINFO Database Record (c) 2012 APA, all rights reserved)
AD - Roopnarine, Jaipaul L.: Syracuse U, Coll of Human Development, Dept of Child & Family Studies, Syracuse, NY, US
ER -

TY - JOUR
ID - 1224

T1 - Parent-child play: Descriptions and implications
A1 - MacDonald,Kevin
Y1 - 1993///
N1 - Book: 1993-98052-000
English
Book; Edited Book
(Created by PsycINFO) Introduction: Parents and children playing [by] Kevin MacDonald * Dilemmas in adult play with children / Brian Sutton-Smith / 15-40 Part I. Theoretical perspectives * Parent-infant games as dynamic social systems / Alan Fogel, Evangeline Nwokah and Jeanne Karns / 43-70 * Parent-infant play as a window on infant competence: An organizational approach to assessment / Marjorie Beeghley / 71-112 * Parent-child play: An evolutionary perspective / Kevin MacDonald / 113-143 Part II. Mechanisms of parent-child play * Rough and tumble play: A fundamental brain process / Jaak Panksepp / 147-184 * Lessons from primate play / Maxine Biben and Steven J. Suomi / 185-196 * Parent-child physical play: Determinants and consequences / James Carson, Virginia Burks and Ross D. Parke / 197-220 * The necessary lightness of mother-child play / Phyllis Levenstein and John O'Hara / 221-237 * Mother-infant play and maternal depression / Jeffrey F. Cohn / 239-256 Part III. Cross-cultural perspectives * Peekaboo across cultures: How mothers and infants play with voices, faces, and expectations / Anne Fernald and Daniela K. O'Neill / 259-285 * Gentle play partners: Mother-child and father-child play in New Delhi, India / Jaipaul L. Roopnarine, Frank H. Hooper, Mohammad Ahmeduzzaman and Brad Pollack / 287-304 * "Mother, older sibling and me": The overlapping roles of caregivers and companions in the social world of two- to three-year-olds in Ngeca, Kenya / Carolyn Pope Edwards and Beatrice B. Whiting / 305-329 * Persistence of play and feeding interaction differences in three Miami cultures / Tiffany M. Field / 331-347 * Cultural differences in scaffolding pretend play: A comparison of American and Mexican mother-child and sibling-child pairs / Jo Ann M. Farver / 349-366 * The cultural context of mother-infant play in the newborn period / J. Kevin Nugent, Sheila Greene, Dorit Wieczoreck-Deering, Kathleen Mazor, John Hendler and Cynthia Bombardier / 367-386 Contributors Index
KW - PsycInfo
KW - Rough and Tumble
KW - Childhood Play Behavior
KW - Parent Child Relations
KW - Cross Cultural Differences
KW - Disorders
KW - Mental Disorders
RP - NOT IN FILE
SP - 389
JF - (1993)
VL - Parent-child play
N2 - (from the cover) This book provides the latest research and theory in the area of children's play with their parents. It includes discussions of the basic processes involved in parent-child play, parent-child play in atypical populations of children, and parent-child play in cross-cultural perspective. An opening section on basic processes provides a general background on the mechanisms involved in play and provides a foundation for the rest of the book. The section on atypical populations focuses on parent-child play among clinical populations, including Down syndrome children, premature children, hyperactive children, and economically-distressed families and families with depressed parents. It expands the context of the populations' data described in the first section and provides some additional insight into mechanisms. Finally, the book describes some of the enormous cross-cultural variations in play behavior. (PsycINFO Database Record (c) 2012 APA, all rights reserved)
AD - MacDonald, Kevin: California State U, Dept of Psychology, Associate Professor, Long Beach, CA, US
ER -

TY - JOUR
ID - 1225
T1 - Early play arousal, sex-typed play, and activity level as precursors to later rough-and-tumble play
A1 - McBride-Chang,Catherine; Jacklin,Carol
Y1 - 1993///

N1 - Peer Reviewed Journal: 1993-37143-001
English
Journal; Peer Reviewed Journal
KW - PsycInfo
KW - Rough and Tumble
KW - Activity Level
KW - Childhood Play Development
KW - Measurement
KW - Sex Roles
KW - Longitudinal Studies
RP - NOT IN FILE
SP - Apr
EP - 108
JF - Early Education and Development
VL - .4
IS - 2
N2 - Compared 2 ways of measuring rough-and-tumble play (RTP) and examined 3 precursors to this play. Early parental socializing influences (particularly from fathers), "boy" sex-typed play preferences, and activity level were hypothesized to be associated with later RTP. Objective measures of RTP at nursery school were moderately correlated with ratings of such play by parents and teachers at 1st grade. Arousal during play at 45 mo and extent of RTP at 18 mo by fathers, but not mothers, were correlated significantly with ratings of RTP at 1st grade. "Boy" sex-typed play was also significantly associated with 1st-grade RTP. These correlations were similar for both boys and girls. There were no correlates with nursery school RTP that were similar for both sexes. Activity levels were not consistently associated with later RTP behaviors. (PsycINFO Database Record (c) 2012 APA, all rights reserved)
SN - 1040-9289
AD - McBride-Chang, Catherine: U Southern California, US
ER -

TY - JOUR
ID - 1226
T1 - Commentary: At play in the public arena
A1 - Sutton-Smith,Brian
Y1 - 1992///
N1 - Peer Reviewed Journal: 2000-05448-008
English
Journal; Peer Reviewed Journal
KW - PsycInfo
KW - Rough and Tumble
KW - Childhood Play Behavior
RP - NOT IN FILE
SP - Oct
EP - 400
JF - Early Education and Development
VL - .3
IS - 4
N2 - Comments on the articles by C. H. Hart et al, A. D. Pelligrini, J. Roopnarine et al, M. J. Boulton, K. MacDonald, A. Costabile et al, and M. W. Watson and Y. Peng (see records 2000-05448-001, 2000-05448-002, 2000-05448-003, 2000-05448-004, 2000-05448-005, 2000-05448-006, 200005448-007, respectively) which explored issues of children's play. The present author contends that though valuable for their uniqueness, these papers are unfortunately largely devoid of historical consciousness. It is argued that the practical focus of these papers is upon the desirability of constraints upon play as a form of violence, whether manifest in play with toy guns, make-believe war themes, hyperactive recess behavior, or specific rough-and-tumble play. (PsycINFO Database Record (c) 2012 APA, all rights reserved)
SN - 1040-9289
AD - Sutton-Smith, Brian: U Pennsylvania, Graduate School of Education, Philadelphia, PA, US

ER -

TY - JOUR
ID - 1227
T1 - The relation between toy gun play and children's aggressive behavior
A1 - Watson,Malcolm W.
Y1 - 1992///
N1 - Peer Reviewed Journal: 2000-05448-007
English
Journal; Peer Reviewed Journal
KW - PsycInfo
KW - Rough and Tumble
KW - Aggressive Behavior
KW - Childhood Play Behavior
KW - Toys
KW - Weapons
RP - NOT IN FILE
SP - Oct
EP - 389
JF - Early Education and Development
VL - .3
IS - 4
N2 - To assess the relation between toy gun play and aggression, 36 3-5-yr olds were observed in free play in their daycare center and coded for amount of real aggression, pretend aggression, rough-and-tumble play, and nonaggressive pretend play. Based on a questionnaire completed by the parents, the children were also coded for the amount they played with toy guns in the home, the rated aggressive level of their preferred television programs, the rated aggressive level of their most preferred toys, and amount of parents' physical punishment of the children. Of all children, 56% played with toy guns in the home, most of whom were boys. Multiple regression analyses indicated that amount of parents' punishment strongly predicted real aggression, and amount of toy gun play strongly predicted real aggression in boys. However, when it came to pretend aggression, aggressive level of children's preferred toys was the strongest predictor, while toy gun play negatively predicted pretend aggression. Toy gun play did not predict nonaggressive pretend play, but parents' punishment negatively predicted nonaggressive pretend play. These results indicate that toy gun play and parental punishment are positively associated with a higher level of real aggression but not pretend aggression. (PsycINFO Database Record (c) 2012 APA, all rights reserved)
SN - 1040-9289
AD - Watson, Malcolm W.: Brandeis U, Dept of Psychology, Waltham, MA, US
ER -

TY - JOUR
ID - 1228
T1 - Attitudes of parents toward war play in young children
A1 - Costabile,Angela; Genta,Maria Luisa; Zucchini,Elisabetta; Smith,Peter
Y1 - 1992///
N1 - Peer Reviewed Journal: 2000-05448-006
English
Journal; Peer Reviewed Journal
KW - PsycInfo
KW - Rough and Tumble
KW - Childhood Play Behavior
KW - Cross Cultural Differences
KW - Parental Attitudes
KW - War
RP - NOT IN FILE
SP - Oct
EP - 369

JF - Early Education and Development
VL - .3
IS - 4
N2 - A questionnaire on attitudes toward war play was given to 316 Italian parents and 84 English parents of children aged 2-6 yrs. In both cultures, boys were significantly more likely to engage in war play, either social or solitary-most commonly weapon play, but also play with combat figures and acting out a combat role. Parental attitudes, television, and peers (including older siblings), appeared as common sources of influence. Parents expressed a range of views about how to respond to war play, varying from discouraging it (more common if their children did not engage in war play), to allowing it with limits, to allowing it unconditionally. There was more of a consensus that war toys should not be allowed in school and that playgroup supervisors and teachers should try to turn war play toward more constructive ends. Analysis of a range of attitudinal scales confirmed a general dimension of restrictiveness-permissiveness with regard to war play activities, linked to attitudes about rough-and-tumble play and pretend play in the Italian sample but not in the English sample. (PsycINFO Database Record (c) 2012 APA, all rights reserved)
SN - 1040-9289
AD - Costabile, Angela: U Calabria, Italy
ER -

TY - JOUR
ID - 1229
T1 - A time and a place for everything: A discrete systems perspective on the role of children's rough-and-tumble play in educational settings
A1 - MacDonald,Kevin
Y1 - 1992///
N1 - Peer Reviewed Journal: 2000-05448-005
English
Journal; Peer Reviewed Journal
KW - PsycInfo
KW - Rough and Tumble
KW - Childhood Development
KW - Childhood Play Behavior
KW - School Environment
RP - NOT IN FILE
SP - Oct
EP - 355
JF - Early Education and Development
VL - .3
IS - 4
N2 - Emphasizes the following points regarding the appropriate role of rough-and-tumble play (R & T) in educational settings. (1) There has been an important secular trend toward an increasing importance of adult supervision of children's play. As a result, children's R & T must be considered in the context of social values regarding the expected developmental significance of children's play. (2) R & T is an aspect of evolved systems that propel the children into enthusiastic interaction with their environment and can be reasonably supposed to have several beneficial influences on children's cognitive and social development. (3) R & T can be distinguished from aggression, and adult supervised R & T is potentially an important arena for learning the limits of appropriate R & T. (4) It is suggested that supervised educational settings should be concerned with socializing several discrete systems that underlie children's development, including the present emphasis on socializing children to be able to focus attention, inhibit behavior, and be neat and orderly. However, the purpose of the present paper is to present a case for socializing the systems underlying stimulus seeking, extroversion, sociability, and intellectual creativity as well. (PsycINFO Database Record (c) 2012 APA, all rights reserved)
SN - 1040-9289
AD - MacDonald, Kevin: California State U, Dept of Psychology, Long Beach, CA, US
ER -

TY - JOUR

ID - 1230
T1 - Rough physical play in adolescents: Does it serve a dominance function?
A1 - Boulton,Michael J.
Y1 - 1992///
N1 - Peer Reviewed Journal: 2000-05448-004
English
Journal; Peer Reviewed Journal
KW - PsycInfo
KW - Rough and Tumble
KW - Adolescent Attitudes
KW - Aggressive Behavior
KW - Childhood Play Behavior
KW - Dominance Hierarchy
RP - NOT IN FILE
SP - Oct
EP - 333
JF - Early Education and Development
VL - .3
IS - 4
N2 - In Exp 1, a questionnaire was designed to investigate secondary school pupils' participation in, and perceptions of, playful and aggressive fighting. It was administered to a sample of 13-16 yr old male and female pupils who attended 5 different schools. The majority of pupils (and significantly more males than females) reported that they did engage in playful fighting. Most pupils also indicated that it was possible to tell whether other students were engaged in playful as opposed to aggressive fighting, attesting to the view that playful and aggressive fighting should be seen as separate categories of behavior. Other results converged to indicate that, unlike during the childhood years, playful fighting may be used by some individuals (especially males) to establish and display dominance positions. The responses of some pupils also suggested that prowess in aggressive fighting was valued, in many cases because it deterred other pupils from directing unwanted behaviors at them. In Exp 2, more detailed individual interviews with 5 males and 5 females were carried out. These were used to illustrate some of the issues surrounding adolescents' participation in, and perceptions of, playful and aggressive fighting. (PsycINFO Database Record (c) 2012 APA, all rights reserved)
SN - 1040-9289
AD - Boulton, Michael J.: Sheffield City Polytechnic, Sheffield, England
ER -

TY - JOUR
ID - 1231
T1 - Parent-infant rough play: Its cultural specificity
A1 - Roopnarine,Jaipaul L.
Y1 - 1992///
N1 - Peer Reviewed Journal: 2000-05448-003
English
Journal; Peer Reviewed Journal
KW - PsycInfo
KW - Rough and Tumble
KW - Childhood Play Behavior
KW - Cross Cultural Differences
KW - Parent Child Relations
RP - NOT IN FILE
SP - Oct
EP - 311
JF - Early Education and Development
VL - .3
IS - 4
N2 - The salience of father-infant rough play in some industrialized and nonindustrialized countries around the world was reviewed, and data were presented on physical play interactions of 341 Indian mothers and fathers

with their year-old infants in the home. The authors' review of the parent-infant physical play literature failed to show that fathers had a uniformly greater propensity to engage in vigorous rough activity with infants than mothers did. The data from the observational study suggest that Indian parents are not vigorous play partners to infants. Mothers were more likely to engage in object-mediated play than were fathers. In total, the data reviewed do not support the contention that rough play is a major activity between fathers and infants across cultures. Rather, the data from parent-infant play in the nonindustrialized countries suggest that rough play may be culture specific, casting doubts about its biological underpinnings. (PsycINFO Database Record (c) 2012 APA, all rights reserved)
SN - 1040-9289
AD - Roopnarine, Jaipaul L.: Syracuse U, Child & Family Studies Dept, Syracuse, NY, US
ER -

TY - JOUR
ID - 1232
T1 - Children's perceptions of playfighting, playchasing and real fighting: A cross-national interview study
A1 - Smith,Peter K.
Y1 - 1992///
N1 - Peer Reviewed Journal: 1994-09111-001
English
Journal; Peer Reviewed Journal
KW - PsycInfo
KW - Rough and Tumble
KW - Aggressive Behavior
KW - Child Attitudes
KW - Childhood Play Behavior
RP - NOT IN FILE
SP - Sep
EP - 229
JF - Social Development
VL - .1
IS - 3
N2 - Examined rough-and-tumble play by looking at the views and beliefs held by children, in addition to perspectives from adult observers. A questionnaire was given to 192 8- and 11-yr-olds in England and southern Italy, as well as 72 English 4-6 yr olds, to obtain basic information on how Ss viewed playfighting and playchasing. Findings were generally similar for English and Italian children. Most boys and girls reported both liking, and taking part in, playchasing; liking and participation in playfighting was more divided, though more so for boys, with many children disliking the perceived risk of injury in playfighting. Most Ss reported that they could tell playfighting from a serious fight, though they thought it could lead to a serious fight, especially as a result of accidental injury. Many Ss reported that playfighting could improve one's strength or fighting skill and could be used to display strength or dominance to others. (PsycINFO Database Record (c) 2012 APA, all rights reserved)
SN - 0961-205X
AD - Smith, Peter K.: U Sheffield, England
ER -

TY - JOUR
ID - 1234
T1 - Rough-and-tumble play and social problem solving flexibility
A1 - Pellegrini,A.
Y1 - 1992///
N1 - Peer Reviewed Journal: 1993-05203-001
English
Journal; Peer Reviewed Journal
KW - PsycInfo
KW - Rough and Tumble
KW - Adaptability (Personality)

KW - Childhood Play Behavior
KW - Human Males
KW - Personality
KW - Problem Solving
KW - Social Interaction
RP - NOT IN FILE
SP - 1992, pp
EP - 26
JF - Creativity Research Journal
VL - .5
IS - 1
N2 - Investigated the relationship between boys" temperament and rough-and-tumble (R&T) play and determined the group composition of R&T, games, and other forms of reciprocal social interaction. The extent to which the vigor and flexibility components of R&T are related to social affiliation and social problem solving flexibility was explored. 13 sociometrically defined popular boys (aged 107-138 mo) were observed on their school playground during recess for 20 sessions. They nominated peers they liked most and least, ordered peers in terms of dominance, and solved hypothetical social problems. Teachers completed temperament questionnaires. R&T groups were similar to other social groupings in terms of number and dominance symmetry. The flexibility dimension of R&T was related to affiliation and social problem solving. Temperament was minimally associated with measures of behavior. (PsycINFO Database Record (c) 2012 APA, all rights reserved)
SN - 1040-0419
AD - Pellegrini, A. D.: U Georgia, Inst for Behavioral Research, Athens, US
ER -

TY - JOUR
ID - 1235
T1 - The social nature of play fighting and play chasing: Mechanisms and strategies underlying cooperation and compromise
A1 - Boulton,Michael J.
Y1 - 1992///
N1 - Book: 1992-98504-011
English
Book; Edited Book
KW - PsycInfo
KW - Rough and Tumble
KW - Aggressive Behavior
KW - Childhood Play Behavior
KW - Theory of Evolution
KW - Cooperation
RP - NOT IN FILE
SP - 666
JF - Barkow, Jerome H [Ed]; Cosmides, Leda [Ed]; Tooby, John [Ed]
IS - 1992
N2 - (from the chapter) suggest that there are important advantages in taking an evolutionary approach to the study of r/t [rough & tumble play] / evidence suggests that this form of play may be universal to all human cultures and that, as such, it may have been shaped by natural selection acting on physiological and psychological mechanisms, in order to provide some specific benefits / for humans, there is some evidence . . . that the benefits may be related to intraspecific fighting (with hunting and predator avoidance as less likely candidates) / other considerations suggest that these and other mechanisms that generate r/t have ensured that it is a cooperative activity in which participants with differing needs are willing to compromise (PsycINFO Database Record (c) 2012 APA, all rights reserved)
AD - Boulton, Michael J.: Sheffield City Polytechnic, School of Health & Community Studies, Psychology Section, Sheffield, England
ER -

TY - JOUR
ID - 1236
T1 - Sex typing in play and popularity in middle childhood
A1 - Moller,Lora C.
Y1 - 1992///
N1 - Peer Reviewed Journal: 1992-42650-001
English
Journal; Peer Reviewed Journal
KW - PsycInfo
KW - Rough and Tumble
KW - Childhood Play Behavior
KW - Human Sex Differences
KW - Peer Relations
KW - Popularity
KW - Sex Roles
KW - Social Acceptance
RP - NOT IN FILE
SP - Apr
EP - 353
JF - Sex Roles
VL - .26
IS - 7-8
N2 - Examined sex differences in play behavior across the early elementary school years as well as the relation between sex-typed play and peer acceptance. Ss were 86 Grade 2 children (37 males, 49 females) and 81 Grade 4 children (35 males, 46 females). Popularity was assessed using a rating-scale sociometric measure. Sex-typed behaviors were measured by observing the children at free play. Boys engaged in more aggressive and rough and tumble play as well as more functional, solitary-dramatic, and exploratory play and tended to be involved more in group play, whereas girls produced more parallel and constructive play as well as more peer conversations. In Grade 4, these differences were maximized so that boys produced more games-with-rules and girls exhibited more parallel-constructive activity. (PsycINFO Database Record (c) 2012 APA, all rights reserved)
SN - 0360-0025
AD - Moller, Lora C.: U Waterloo, ON, Canada
ER -

TY - JOUR
ID - 1237
T1 - Children, play, and development
A1 - Hughes,Fergus P.
Y1 - 1991///
N1 - Book: 1992-98389-000
English
Book; Authored Book
(Created by PsycINFO) (Abbreviated) Preface Historical and theoretical viewpoints * A definition of play Ethological and cultural perspectives The first two years of life * Exploration and play * Social aspects of infant and toddler play The preschool years: From two to five Play in later childhood and adolescence Gender differences in play * Toy selection * Fantasy play * Rough-and-tumble play * Games-with-rules Play in special populations * The physically handicapped child * The mentally handicapped child * The emotionally disturbed child * The child under stress: Play in the hospital Play and intellectual development * Language play and language development * Play, creativity, and problem solving The social benefits of play The uses of play in therapy Glossary Bibliography Subject index Author index
KW - PsycInfo
KW - Rough and Tumble
KW - Childhood Play Behavior
KW - Childhood Play Development
RP - NOT IN FILE

SP - lay, and
JF - (1991)
VL - Children, play, and development. xiv, 255 pp. Needham Heights, MA, US
N2 - (from the preface) That play is a central, all-encompassing characteristic of children's development is a major point throughout this book. The book is written primarily from the perspective of developmental psychology, and the goal was to blend the major theoretical perspectives on play with up-to-date reviews of the research literature. While the intended audience includes anyone interested in children's play, this is not a how-to book. Every effort was made to enhance the book's appeal by making it as readable as possible. However, it is hoped that the book will also challenge the reader. It is expected that this book will be used in college classrooms, either as the main text in courses on children's play or as a supplement in child development courses or in those dealing with early childhood education. (PsycINFO Database Record (c) 2012 APA, all rights reserved)
AD - Hughes, Fergus P.: U Wisconsin, Green Bay, WI, US
ER -

TY - JOUR
ID - 1238
T1 - Supporting the development of socially rejected children
A1 - Bullock,Janis R.
Y1 - 1991///
N1 - Peer Reviewed Journal: 1992-20734-001
English
Journal; Peer Reviewed Journal
KW - PsycInfo
KW - Rough and Tumble
KW - Social Acceptance
KW - Social Skills Training
RP - NOT IN FILE
SP - 15
EP - 23
JF - Early Child Development and Care
VL - .66 Jan 1991, pp. 15-23.
N2 - Describes peer status classifications and related behaviors for school children, focusing on rejected children, who tend to be unaccepted by peers in a variety of settings because they have trouble adapting to the children and play situation. Social skills interventions can be effective for young children who are having difficulty initiating, establishing, and maintaining interactions with peers. Social skills training techniques, including coaching, modeling, positive reinforcement, cognitive behavior modification, and rough and tumble play, are discussed. (PsycINFO Database Record (c) 2012 APA, all rights reserved)
SN - 0300-4430
AD - Bullock, Janis R.: Montana State U, Bozeman, US
ER -

TY - JOUR
ID - 1239
T1 - Partner preferences in middle school children's playful fighting and chasing: A test of some competing functional hypotheses
A1 - Boulton,Michael J.
Y1 - 1991///
N1 - Peer Reviewed Journal: 1992-01056-001
English
Journal; Peer Reviewed Journal
KW - PsycInfo
KW - Rough and Tumble
KW - Affection
KW - Age Differences
KW - Childhood Play Behavior

KW - Physical Strength
KW - Preferences
RP - NOT IN FILE
SP - May
EP - 193
JF - Ethology & Sociobiology
VL - .12
IS - 3
N2 - Observations of 86 middle school children (younger Ss with a mean age of 8.5 yrs and older Ss with a mean age of 11.5 yrs) participating in rough-and-tumble play (r/t) were related to sociometric data for liking and strength. Partners in r/t liked each other more than chance predicted, and tended to be closely matched for strength. Neither initiators of r/t episodes nor recipients were consistently perceived to be the strongest partner. Ss were also found to like their partners in a wide range of non-r/t activities significantly more than chance would predict. Thus children could simply choose to spend time with peers that they like, rather than r/t representing a special case. This view is supported by the finding that Ss had the same partners in a range of playground activities as they had for r/t. (PsycINFO Database Record (c) 2012 APA, all rights reserved)
SN - 0162-3095
AD - Boulton, Michael J.: U Sheffield, England
ER -

TY - JOUR
ID - 1241
T1 - A comparison of structural and contextual features of middle school children's playful and aggressive fighting
A1 - Boulton,Michael J.
Y1 - 1991///
N1 - Peer Reviewed Journal: 1991-30089-001
English
Journal; Peer Reviewed Journal
KW - PsycInfo
KW - Rough and Tumble
KW - Age Differences
KW - Aggressive Behavior
KW - Childhood Play Behavior
KW - Human Sex Differences
RP - NOT IN FILE
SP - Mar
EP - 145
JF - Ethology & Sociobiology
VL - .12
IS - 2
N2 - Compared features of rough-and-tumble of play (RTP) and aggressive fighting (AF) based on direct playground observations of 2 classes each of 8- and 11-yr-old children. Most bouts of RTP and AF were dyadic, but significantly more bouts of the former involved 3 or more participants. RTP contained more individual action patterns than AF, but the latter was more varied in form. Both wrestling and chasing were seen more in RTP than AF, but restraint in bouts of chasing was not more common in playful than aggressive chasing. Positive and neutral facial expressions were more characteristic of RTP, and negative facial expressions were more characteristic of AF. RTP initiations were more likely to receive an RTP response and less likely to receive an aggressive response than were aggressive initiations. (PsycINFO Database Record (c) 2012 APA, all rights reserved)
SN - 0162-3095
AD - Boulton, Michael J.: U Sheffield, England
ER -

TY - JOUR
ID - 1242

T1 - Antecedents of problem behaviors in children of depressed mothers
A1 - Zahn-Waxler,Carolyn; Iannotti,Ronald
Y1 - 1990///
N1 - Peer Reviewed Journal: 1991-21593-001
English
Journal; Peer Reviewed Journal
KW - PsycInfo
KW - Rough and Tumble
KW - Aggressive Behavior
KW - Behavior Problems
KW - Major Depression
KW - Offspring
KW - Mothers
RP - NOT IN FILE
SP - 1990, pp
EP - 1990,291
JF - Development and Psychopathology
VL - .2
IS - 3
N2 - Examined aggression in 2-yr-old children of 22 well and 22 depressed mothers in relation to problem behaviors at ages 5-6 yrs. Both normative (e.g., object struggles, rough play) and maladaptive (e.g., dysregulated, out-of-control behavior) forms of toddler aggression were identified in 2 or 3 play sessions. Dysregulated aggression predicted (1) externalizing problems reported by mothers at follow-up interviews when children were 5 yrs old and (2) children's reports of difficulties during a structured psychiatric interview at age 6 yrs. Problems were more frequent and continuity patterns more evident in children of depressed mothers. Early maladaptive aggression was a better predictor of later externalizing problems. Depressed mothers who used proactive childrearing approaches had children who showed fewer externalizing problems 3 yrs later. (PsycINFO Database Record (c) 2012 APA, all rights reserved)
SN - 0954-5794
AD - Zahn-Waxler, Carolyn: NIMH Lab of Developmental Psychology, Bethesda, MD, US
ER -

TY - JOUR
ID - 1244
T1 - Beyond the evil empire: Horseplay and aggression
A1 - Gergen,Mary
Y1 - 1990///
N1 - Peer Reviewed Journal: 1991-09624-001
English
Journal; Peer Reviewed Journal
KW - PsycInfo
KW - Rough and Tumble
KW - Aggressive Behavior
KW - Recreation
KW - Self Report
RP - NOT IN FILE
SP - 1990, pp
EP - 1990,398
JF - Aggressive Behavior
JA - Aggress.Behav
VL - .16
IS - 6
N2 - 75 male and 75 female undergraduates rated the extent to which they had engaged in horseplay and in aggressive acts with same-sex friends and boyfriends or girlfriends over the past 3 yrs. A broad range of playful and aggressive activities was reported. There was a strong relationship between the tendency to horseplay and to aggress. The relationship was strongest for males with male friends and for females with boyfriends. Having

steady relationships increased the likelihood that horseplay and aggressive acts would occur. Weekend alcohol consumption was related to playful activities; heavier use correlated with greater aggressive activities of males with males only. It may be useful to broaden the base for conceptualizing aggression to take into account the ways in which playful and friendly activities can facilitate those that are regarded as antisocial and aggressive. (PsycINFO Database Record (c) 2012 APA, all rights reserved)
SN - 0096-140X
AD - Gergen, Mary: Pennsylvania State U, Delaware County Campus, Media, US
ER -

TY - JOUR
ID - 1245
T1 - Rough-and-tumble play, aggression and dominance: Perception and behaviour in children's encounters
A1 - Smith,Peter K.
Y1 - 1990///
N1 - Peer Reviewed Journal: 1990-30723-001
English
Journal; Peer Reviewed Journal
KW - PsycInfo
KW - Rough and Tumble
KW - Affiliation Motivation
KW - Aggressive Behavior
KW - Childhood Play Behavior
KW - Social Skills
RP - NOT IN FILE
SP - Jul
EP - Oct
JF - Human Development
VL - .33
IS - 4-5
N2 - Examines rough-and-tumble play (RATP) and the criteria used for distinguishing RATP from fighting. Evidence is presented that children are able to use many of these criteria. RATP is considered in relation to dominance and affiliative relationships in children, and its functional significance in development is discussed. RATP may exercise social skills, but as a form of behavior it can also be used in socially manipulative ways, for example to inflict harm or achieve dominance. Implications for a social skills deficit model of aggressive behavior in childhood are considered. (PsycINFO Database Record (c) 2012 APA, all rights reserved)
SN - 0018-716X
AD - Smith, Peter K.: U Sheffield, England
ER -

TY - JOUR
ID - 1246
T1 - Characteristics of holding, patterns of play, and social behaviors between parents and infants in New Delhi, India. [References]
A1 - Roopnarine,Jaipaul L.
Y1 - 1990///
N1 - Peer Reviewed Journal: 1990-28112-001
English
Journal; Peer Reviewed Journal
KW - PsycInfo
KW - Rough and Tumble
KW - Human Sex Differences
KW - Parent Child Relations
RP - NOT IN FILE
SP - Jul
EP - 673
JF - Developmental Psychology

JA - Dev.Psychol.
VL - .26
IS - 4
N2 - Characteristics of holding, play, and social behaviors between 54 mothers and fathers and their 1-year-old infants were observed within the context of their extended families in New Delhi, India. Mothers picked up and held infants more than did fathers, and were more likely to feed and comfort them and to invest more time feeding and displaying affection to them than were fathers. When parents held infants they were more likely to display affection than to feed, comfort, or play with them. Fathers engaged in more rough play than did mothers, and mothers engaged in more peek-a-boo than did fathers. Mothers and fathers treated boys and girls quite similarly. Infants smiled at, vocalized to, and followed mothers more than they did fathers. Parents were generally preferred over relatives as social partners. The data point to the cultural specificity of certain parent-child activities. (PsycINFO Database Record (c) 2012 APA, all rights reserved)
SN - 0012-1649
AD - Roopnarine, Jaipaul L.: Syracuse U, NY, US
ER -

TY - JOUR
ID - 1247
T1 - Parental reports of early patterns of caregiving, play and discipline in India and Malaysia
A1 - Roopnarine,Jaipaul L.
Y1 - 1989///
N1 - Peer Reviewed Journal: 1998-01853-008
English
Journal; Peer Reviewed Journal
KW - PsycInfo
KW - Rough and Tumble
KW - Childrearing Practices
KW - Cross Cultural Differences
KW - Parent Child Relations
KW - Parental Role
KW - Child Discipline
KW - Dual Careers
KW - Fathers
KW - Human Sex Differences
KW - Mothers
RP - NOT IN FILE
SP - 109
EP - 120
JF - Early Child Development and Care
VL - .50 1989, pp. 109-120.
N2 - Explored early patterns of caregiving, discipline, and play between parents and their healthy 1-yr-old infants in New Delhi, India, and Kuching, Malaysia. The dual-provider families were from middle- to upper-middle-income backgrounds. The 19 Chinese Malaysian infants and the 19 Indian infants were within 4 wks of their 1st birthdays. Semi-structured interview responses indicate some redistribution of authority in terms of disciplining children, with mothers reporting a more central role. Men's participation with babies in terms of early caregiving did not depart greatly from traditional conceptions of men's roles in both cultures. Few fathers reported feeding, putting babies to bed, and changing the babies' nappies. Parents rarely engaged in rough stimulating play. Differences between the 2 cultures were noted in the use of physical punishment vs verbal feedback for discipline. (PsycINFO Database Record (c) 2012 APA, all rights reserved)
SN - 0300-4430
AD - Roopnarine, Jaipaul L.: Syracuse U, Syracuse, NY, US
ER -

TY - JOUR
ID - 1248

T1 - Individual and group similarities and dissimilarities in behaviors associated with peer status in experimental play groups. [French]
A1 - Boivin,Michel
Y1 - 1989///
N1 - Peer Reviewed Journal: 1991-71989-001
French
Journal; Peer Reviewed Journal
KW - PsycInfo
KW - Rough and Tumble
KW - Childhood Play Behavior
KW - Individual Differences
KW - Peer Relations
KW - Status
RP - NOT IN FILE
SP - 1989, pp
EP - 1989,349
JF - Science et Comportement
VL - .19
IS - 4
N2 - Studied the association of individual and group similarities and dissimilarities with peer status among school-age boys. Human subjects: 180 Canadian Black school-age boys (1st and 3rd grade). Ss were divided into 11 1st-grade and 19 3rd-grade groups, composed of 5-6 boys from different schools. Each group contained 1 popular, 1 ignored, 2 rejected, and 2 average boys. Playgroup interactions were videotaped on consecutive days; behaviors were recorded according to 5 preset categories: solitary play, prosocial interaction, rough and tumble play, reactive aggression, and dominant aggression. Stability of peer status and stability of group interaction scores were examined according to age, original status, and individual and group behavior profiles. Statistical tests were used. (English abstract) Developmental. (PsycINFO Database Record (c) 2012 APA, all rights reserved)
SN - 0841-7741
AD - Boivin, Michel: U Laval, Ecole de Psychologie, Quebec, Canada
ER -

TY - JOUR
ID - 1249
T1 - Roughhousing and fighting games among children from the perspective of comparative behavioral research: Implications for preschool and kindergarten education related to aggression and conflict. [German]
A1 - Gamber,Paul
Y1 - 1989///
N1 - Peer Reviewed Journal: 1991-70301-001
German
Journal; Peer Reviewed Journal
KW - PsycInfo
KW - Rough and Tumble
KW - Aggressive Behavior
KW - Childhood Development
KW - Childhood Play Behavior
KW - Early Intervention
KW - Preschool Students
RP - NOT IN FILE
SP - May
EP - 189
JF - Gruppendynamik
VL - .20
IS - 2
N2 - Reviews comparative behavioral research on the developmental functions of aggressive play, and discusses implications for educational interventions for nursery school and kindergarten. The research evidence

suggests that aggressive play has a socialization function in nonhuman as well as human primates and that roughhousing and play fighting do not promote aggression in children. It is recommended that preschool children be offered opportunities to engage in rough-and-tumble play and that social training be used to prevent aggressive outcomes. (English abstract) (PsycINFO Database Record (c) 2012 APA, all rights reserved)
SN - 0046-6514
AD - Gamber, Paul: Ruprecht-Karls-U Heidelberg, Germany
ER -

TY - JOUR
ID - 1250
T1 - Categorizing children's rough-and-tumble play
A1 - Pellegrini,Anthony D.
Y1 - 1989///
N1 - Peer-Reviewed Status-Unknown: 1991-09649-001
English
Journal; Peer-Reviewed Status-Unknown
KW - PsycInfo
KW - Rough and Tumble
KW - Childhood Play Behavior
KW - Aggressive Behavior
KW - Physical Contact
KW - Popularity
KW - Social Acceptance
RP - NOT IN FILE
SP - Feb
EP - 51
JF - Play & Culture
VL - .2
IS - 1
N2 - Addresses the categorization of rough-and-tumble (R&T) play and explicates a multimethod procedure for its categorization. Ethological and ethnographic methods are used with a sample of elementary school children, some of whom are defined as sociometrically popular and others as rejected. Results indicated that R&T is qualitatively different for each group to the extent that it is a playful, socially affiliative category for popular children but an aggressive category for rejected children. It may be that the ability of popular children to discriminate social information enables them to treat R&T and aggression as distinct categories. Social information processing capabilities may be an important determinant of children's social competence. (PsycINFO Database Record (c) 2012 APA, all rights reserved)
SN - 0894-4253
AD - Pellegrini, Anthony D.: U Georgia, Athens, US
ER -

TY - JOUR
ID - 1251
T1 - Sexuality in the workplace: The domination of men's sexuality
A1 - Collinson,David L.
Y1 - 1989///
N1 - Book: 1990-97260-006
English
Book; Edited Book
KW - PsycInfo
KW - Rough and Tumble
KW - Organizational Climate
KW - Sexuality
KW - Interpersonal Control
KW - Male Female Relations
KW - Organizational Structure

KW - Psychosexual Behavior
KW - Sexual Harassment
RP - NOT IN FILE
SP - CA, US
JF - Hearn, Jeff [Ed]; Sheppard, Deborah L [Ed]; Tancred-Sheriff, Peta [Ed]; Burrell, Gibson [Ed]
IS - 1989
N2 - (from the chapter) by examining in depth three empirical case studies, this chapter is intended to contribute to an understanding of the complex relationship between power and (hetero)sexuality in organizational practices / three different organizational settings that are characterized by contrasting degrees of sex segregation / all-male shop-floor context of a lorry[truck]-producing factory / 'white-collar' trade union which is . . . male dominated / major UK [United Kingdom] insurance company that employs a mixed workforce, in which women are largely confined to subordinate positions focusing on horseplay, sexual harassment and mutual sexuality, respectively, these studies illustrate the pervasiveness and dominance of men's sexuality in the workplace / illustrate the complex interrelationships which can emerge between men's definitions of male and female sexuality, and other organizational practices such as shop-floor resistance, the internal politics of trade unions, and workplace 'fiddles' [affairs] (PsycINFO Database Record (c) 2012 APA, all rights reserved)
AD - Collinson, David L.: U St Andrews, Dept of Management, Conoco Lecturer in Organizational Behaviour, St Andrews, Scotland
ER -

TY - JOUR
ID - 1252
T1 - Elementary school children's rough-and-tumble play
A1 - Pellegrini,A.
Y1 - 1989///
N1 - Peer Reviewed Journal: 1990-02917-001
English
Journal; Peer Reviewed Journal
KW - PsycInfo
KW - Rough and Tumble
KW - Age Differences
KW - Aggressive Behavior
KW - Childhood Play Behavior
KW - Elementary School Students
KW - Kindergarten Students
RP - NOT IN FILE
SP - Jun
EP - 260
JF - Early Childhood Research Quarterly
VL - .4
IS - 2
N2 - Four observers rated 48 male and 46 female kindergartners', 2nd graders', and 4th graders' rough-and-tumble play (RTP) with respect to aggression over an 8-mo period. Ss were observed during playground school recess periods. Ss' sociometric status, social problem solving, antisocial behavior, and ability to discriminate between RTP and aggression were measured. The incidence of RTP varied according to sex and playground location; RTP accounted for 11% of play behaviors. Boys engaged in significantly more RTP than did girls. Aggression rarely occurred and did not vary by age, sex, or playground location. RTP led to games with rules and was positively correlated, for boys, with social competence. Girls' RTP was positively related to antisocial behavior. (PsycINFO Database Record (c) 2012 APA, all rights reserved)
SN - 0885-2006
AD - Pellegrini, A. D.: U Georgia Inst for Behavioral Research, Athens, US
ER -

TY - JOUR
ID - 1253

T1 - What is a category? The case of rough-and-tumble play
A1 - Pellegrini,A.
Y1 - 1989///
N1 - Peer Reviewed Journal: 1990-01014-001
English
Journal; Peer Reviewed Journal
KW - PsycInfo
KW - Rough and Tumble
KW - Child Attitudes
KW - Childhood Play Behavior
KW - Popularity
KW - Social Acceptance
KW - Psychosocial Development
RP - NOT IN FILE
SP - Jul
EP - 341
JF - Ethology & Sociobiology
VL - .10
IS - 5
N2 - Interviewed 26 popular children and 16 rejected children in Grades K and 2 to determine the ways in which they differentiated and defined rough-and-tumble play (R&T) and real fighting. Prior to the interviews, which used 11 videotaped incidents, behavioral observations were made by 4 observers of children's playground behavior. Each child was observed a minimum of 112 times across the school year. Results indicate that R&T is a playful, social-interactive category for popular children, while rejected children probably do not differentiate R&T and aggression. R&T appears to serve a positive function for popular children (e.g., social affiliation). (PsycINFO Database Record (c) 2012 APA, all rights reserved)
SN - 0162-3095
AD - Pellegrini, A. D.: U Georgia, Athens, US
ER -

TY - JOUR
ID - 1254
T1 - Young children's social competence and their use of space in day-care centers
A1 - Legendre,Alain
Y1 - 1989///
N1 - Book: 1989-98373-013
English
Book; Edited Book
KW - PsycInfo
KW - Rough and Tumble
KW - Child Day Care
KW - Childhood Play Behavior
KW - Social Skills
KW - Competence
KW - Psychosocial Development
RP - NOT IN FILE
SP - 420
JF - Schneider, Barry H [Ed]; Attili, Grazia [Ed]; Nadel, Jacqueline [Ed]; Weissberg, Roger P
IS - 1989
N2 - (from the book) makes explicit the differences between real and feigned aggression (rough-and-tumble play) / outlines the possible positive contributions of rough-and-tumble play to prosocial development and psychological adjustment / makes use of ethological theories of social dominance . . . in exploring the functions of this type of play (PsycINFO Database Record (c) 2012 APA, all rights reserved)
AD - Legendre, Alain: Ctr National de la Recherche Scientifique, Lab de Psychobiologie de l'Enfant, Paris, France
ER -

TY - JOUR
ID - 1255
T1 - Social competence in developmental perspective
A1 - Schneider,Barry H.
Y1 - 1989///
N1 - Book: 1989-98373-000
English
Book; Edited Book
(Created by PsycINFO) Preface List of contributors Section I: Social competence in developmental perspective: Conceptual issues Introduction to Section I [by] Barry H. Schneider * Significance of peer relationship problems in childhood / Steven R. Asher and Jeffrey G. Parker / 5-23 * The role of competence in the study of children and adolescents under stress / Norman Garmezy / 25-39 * The nature of social action: Social competence versus social conformism / Louis Oppenheimer / 41-69 * Individual, differential, and aggregate stability of social competence / Jens Asendorpf / 71-90 * What to do while the kids are growing up: Changing instrumentation in longitudinal research (conversation summary) / Jane E. Ledingham * Socially competent communication and relationship development / Steve Duck / 91-106 * Measuring peer status in boys and girls: A problem of apples and oranges? / Tina Daniels-Beirness / 107-120 Section II: The emergence of social competence in early childhood Introduction to Section II [by] Jacqueline Nadel * Friendships in very young children: Definition and functions (conversation summary) / Carollee Howes * Communicating by imitation: A developmental and comparative approach to transitory social competence / Jacqueline Nadel and Anne-Marie Fontaine / 131-144 * Co-adaptation within the early peer group: A psychobiological study of social competence / F. F. Strayer / 145-174 * Development of communicative competencies in early childhood: A model and results / Pierre-Marie Baudonniere, Marie-Jose Garcia-Werebe, Juliette Michel and Jacqueline Liegeois / 175-193 Section III: Ongoing social development in middle childhood and adolescence Introduction to Section III [by] Barry H. Schneider Examining the impact of social behavior on peer status (conversation summary) [by] Sharon L. Foster * Self-perpetuating processes in children's peer relationships / Toon Cillessen and Tamara J. Ferguson / 203-221 * Types of aggressive relationships, peer rejection, and developmental consequences / John D. Coie, Christina Christopoulos, Robert Terry, Kenneth A. Dodge and John E. Lochman / 223-237 * The role of rough-and-tumble play in the development of social competence: Theoretical perspectives and empirical evidence / Peter K. Smith / 239-255 Section IV: Setting factors in children's social development: The influences of families and schools Introduction to Section IV [by] Grazia Attili * Young children's social competence and their use of space in day-care centers / Alain Legendre / 263-276 * Children's social competence and social supports: Precursors of early school adjustment? / Gary W. Ladd / 277-291 * Social competence versus emotional security: The link between home relationships and behavior problems in preschool / Grazia Attili / 293-311 * Maternal beliefs and children's competence / Kenneth H. Rubin, Rosemary S. L. Mills and Linda Rose-Krasnor / 313-331 Section V: Translating theory into practice: Social competence promotion programs Introduction to Section V: Challenges inherent in translating theory and basic research into effective social competence promotion programs [by] Roger P. Weissberg * Between developmental wisdom and children's social-skills training / Barry H. Schneider / 339-353 * Enhancing peer relations in school systems / Wyndol Furman, Ronita Giberson, Allison S. White, Leslie A. Gravin and Elizabeth A. Wehner / 355-369 * Promoting social competence in early adolescence: Developmental considerations / Marlene Zelek Caplan and Roger P. Weissberg / 371-385 Appendix: Research abstracts Subject index
KW - PsycInfo
KW - Rough and Tumble
KW - Competence
KW - Developmental Psychology
KW - Social Skills
RP - NOT IN FILE
SP - 420
JF - (1989)
VL - Social competence in developmental perspective. xix, 420 pp. New York, NY, US
N2 - (from the preface) The primary purpose of this book is to assist those interested in the scientific study of children's social competence in transcending the boundaries imposed both by geography and by selective exposure to the highly diverse schools of thought that have led to interest in this field. In Section I, entitled "Social Competence in Developmental Perspective: Conceptual Issues," the importance of social competence is

discussed and reaffirmed. Several important theoretical problems are addressed, including the definition of social competence and its relation to general adaptive ability. Important methodological dimensions, especially with respect to longitudinal investigations, are introduced. Section II is devoted to the "Emergence of Social Competence in Early Childhood." It contains deliberations as to the nature of the child's first social contacts and descriptions of the rapidly evolving forms of social exchange during the preschool years. Section III is a selective examination of "Ongoing Social Development in Middle Childhood and Adolescence." Special consideration is given to the impact of actual and pretend aggression on relations among peers. "Setting Factors in Children's Social Development: The Influences of Families and Schools" are considered in Section IV. Separate chapters within that section present evidence for the effects of families and schools on the social interaction of both preschoolers and school-age children. The final section of the book, "Translating Theory Into Practice: Social Competence Promotion Programs," focuses on applied interventions based on social development research. (PsycINFO Database Record (c) 2012 APA, all rights reserved)
AD - Schneider, Barry H.: U Ottawa, School of Psychology, Ottawa, ON, Canada
ER -

TY - JOUR
ID - 1256
T1 - War toys and childhood aggression
A1 - Sutton-Smith,Brian
Y1 - 1988///
N1 - Peer-Reviewed Status-Unknown: 1991-09665-001
English
Journal; Peer-Reviewed Status-Unknown
KW - PsycInfo
KW - Rough and Tumble
KW - Aggressive Behavior
KW - Childhood Play Behavior
KW - Toys
KW - War
RP - NOT IN FILE
SP - Feb
EP - 69
JF - Play & Culture
VL - .1
IS - 1
N2 - Evaluated 8 studies of the effects that play with war toys has on children's aggression. All of the studies were unreliable, but common findings across 7 studies were that (1) girls were not good Ss for such studies because they did not respond to war toys with aggressive behavior and (2) aggression aroused in the playroom did not carry over beyond it. This finding leads to the criticism that the studies were not sensitive to the different contexts in which they were carried out. The studies also failed to distinguish between real and playful aggression. The predominant function of war toys may be to serve as instruments in boys' playfighting, more often of the fantasy kind than of the rough-and-tumble variety. (PsycINFO Database Record (c) 2012 APA, all rights reserved)
SN - 0894-4253
AD - Sutton-Smith, Brian: U Pennsylvania, Graduate School of Education, Philadelphia, US
ER -

TY - JOUR
ID - 1257
T1 - Elementary-school children's rough-and-tumble play and social competence. [References]
A1 - Pellegrini,A.
Y1 - 1988///
N1 - Peer Reviewed Journal: 1989-08147-001
English
Journal; Peer Reviewed Journal
KW - PsycInfo

KW - Rough and Tumble
KW - Aggressive Behavior
KW - Childhood Play Behavior
KW - Social Skills
RP - NOT IN FILE
SP - Nov
EP - 806
JF - Developmental Psychology
JA - Dev.Psychol.
VL - .24
IS - 6
N2 - The intent of this study was to describe relations between elementary-school children's rough-and-tumble play and their social competence. Elementary-school children (Grades K, 2, and 4) were observed on the school playground during their recess periods. Results suggested that rough-and-tumble play for popular children led to games-with-rules, whereas it led to aggression for rejected children. Furthermore, popular children's rough-and-tumble was positively correlated with measures of social competence. These results are discussed in terms of the possible effects of the sociometric composition of rough-and-tumble play groups. (PsycINFO Database Record (c) 2012 APA, all rights reserved)
SN - 0012-1649
AD - Pellegrini, A. D.: U Georgia, Inst for Behavioral Research, Athens, US
ER -

TY - JOUR
ID - 1258
T1 - Rough-and-tumble play on the elementary school playground
A1 - Pellegrini,Anthony D.
Y1 - 1988///
N1 - Peer-Reviewed Status-Unknown: 1989-03238-001
English
Journal; Peer-Reviewed Status-Unknown
KW - PsycInfo
KW - Rough and Tumble
KW - Childhood Play Behavior
KW - Elementary Education
KW - Elementary School Students
RP - NOT IN FILE
SP - Jan
EP - 17
JF - Young Children
VL - .43
IS - 2
N2 - Defines rough-and-tumble (R&T) play among elementary school children, reviews a study of children's R&T play, and offers recommendations for incorporating such play into the curricula of young children. It is argued that R&T play has positive educational value. (PsycINFO Database Record (c) 2012 APA, all rights reserved)
SN - 0044-0728
AD - Pellegrini, Anthony D.: U Georgia, Athens, US
ER -

TY - JOUR
ID - 1260
T1 - Rough-and-tumble play: Developmental and educational significance
A1 - Pellegrini,Anthony D.
Y1 - 1987///

N1 - Peer Reviewed Journal: 1988-16584-001
English
Journal; Peer Reviewed Journal
KW - PsycInfo
KW - Rough and Tumble
KW - Childhood Development
KW - Childhood Play Behavior
KW - Education
RP - NOT IN FILE
SP - Win
EP - 43
JF - Educational Psychologist
VL - .22
IS - 1
N2 - Discusses the developmental and educational significance of rough-and-tumble play (RTP) in children. Ethological studies indicating a stable configuration of RTP behaviors in children are discussed. Problems in defining RTP, including the resemblance of RTP to aggression, are noted. Theories of RTP as a means of venting surplus activity are reviewed. The effects of gender differences and ecological variables (i.e., spatial density, curriculum organization, play props, indoor vs outdoor play spaces) on RTP are discussed. The functional significance of RTP is examined in terms of similarities between RTP and measures of social-cognitive development and educational achievement. (PsycINFO Database Record (c) 2012 APA, all rights reserved)
SN - 0046-1520
AD - Pellegrini, Anthony D.: U Georgia Graduate Studies Research Ctr, Inst for Behavioral Research, Athens, US
ER -

TY - JOUR
ID - 1261
T1 - Rough and tumble, friendship, and dominance in schoolchildren: Evidence for continuity and change with age
A1 - Humphreys,Anne P.
Y1 - 1987///
N1 - Peer Reviewed Journal: 1987-18420-001
English
Journal; Peer Reviewed Journal
KW - PsycInfo
KW - Rough and Tumble
KW - Age Differences
KW - Childhood Play Behavior
KW - Dominance
KW - Friendship
KW - Interpersonal Attraction
RP - NOT IN FILE
SP - Feb
EP - 212
JF - Child Development
JA - Child Dev.
VL - .58
IS - 1
N2 - Examined play-fighting and chasing activity among 7-, 9-, and 11-yr-olds (n = 29, 29, 36, respectively) in their school playground, where it took up some 10% of their time. Judging by characteristics of initiations and terminations, it tended to occur in a friendly, nonaggressive context. Sociometric information supported this interpretation. Observed rough-and-tumble partners liked each other better at all ages than chance predicted. With increasing age, the importance of strength relations in partner choice increased. 11-yr-olds tended to choose partners who were similar in strength to, but weaker than, themselves, while neither factor influenced 7-

and 9-yr-olds' choices significantly. Findings suggest that rough-and-tumble activity performs an affiliative function in young children, becoming important for dominance and fighting-skill development in older children, although immediate motivation remains nonaggressive. (PsycINFO Database Record (c) 2012 APA, all rights reserved)
SN - 0009-3920
AD - Humphreys, Anne P.: U Sheffield, England
ER -

TY - JOUR
ID - 1262
T1 - Aggressive and non-aggressive fighting in twelve-to-thirteen year old pre-adolescent boys
A1 - Neill,S.
Y1 - 1976///
N1 - Peer Reviewed Journal: 1976-29724-001
English
Journal; Peer Reviewed Journal
KW - PsycInfo
KW - Rough and Tumble
KW - Adolescent Development
KW - Aggressive Behavior
KW - Human Males
KW - Playgrounds
RP - NOT IN FILE
SP - Jul
EP - 220
JF - Child Psychology & Psychiatry & Allied Disciplines
VL - .17
IS - 3
N2 - 20 2-min ethological observations were made of each of 34 boys in a secondary school playground, covering all aspects of behavior, including fighting. Factor analyses were performed on individual behavior samples; the 2 main types of fighting were vigorous fighting, usually playful but often causing distress to the victim, and playful fighting of low intensity. Fighting of the 1st type was rare in young boys. It is suggested that the intermingling of rough play and aggression in older boys exposes aggression to social influences which in younger boys are confined to rough play. (19 ref) (PsycINFO Database Record (c) 2013 APA, all rights reserved)
SN - 0021-9630
AD - Neill, S. R.: U Warwick, Coventry, England
ER -

TY - JOUR
ID - 1264
T1 - Juvenile aggressivity and sissiness in homosexual and heterosexual males
A1 - Friedman,Richard C.
Y1 - 1980///
N1 - Peer Reviewed Journal: 1982-09723-001
English
Journal; Peer Reviewed Journal
KW - PsycInfo
KW - Rough and Tumble
KW - Aggressiveness
KW - Childhood Play Behavior
KW - Peer Relations
KW - Psychosexual Development
KW - Sex Roles
KW - Heterosexuality
KW - Human Males

KW - Male Homosexuality
RP - NOT IN FILE
SP - Jul
EP - 440
JF - Journal of the American Academy of Psychoanalysis
VL - .8
IS - 3
N2 - Attempted to replicate and extend a study by M. T. Saghir and E. Robins (1973) on childhood histories of aggressiveness in homosexual and heterosexual nonpatient males. In the present study, 17 23-48 yr old homosexual and 17 21-38 yr old heterosexual males with no history of gross psychopathology were interviewed in a semistructured format for an average of 20 hrs about types of play engaged in as children, sexual history, and emotional effects of their sexual orientation. Findings fail to replicate the similarity between one-third of the homosexuals and heterosexuals with regard to juvenile peer-group aggressivity reported by Saghir and Robins. No prehomosexual S had the type of juvenile aggressive encounters that the heterosexual Ss experienced. It is suggested that male-male peer aggressive competency learned after the juvenile period will not alter homosexual orientation, even though aggressive behavior styles may be adopted during adolescence and adulthood, and that regardless of the degree to which genetic, prenatal, and postnatal factors predispose a person to homosexuality, exposure to male-male peer aggression in a setting where a boy develops sex-typed aggressive skills during preadolescence will markedly diminish the likelihood that an exclusive, enduring homosexual orientation will occur. (PsycINFO Database Record (c) 2012 APA, all rights reserved)
SN - 0090-3604
AD - Friedman, Richard C.: New York Hosp-Cornell Medical Ctr, Westchester Div, White Plains
ER -

TY - JOUR
ID - 1265
T1 - Learning disabled children's understanding of social interactions of peers
A1 - Weiss,Elizabeth
Y1 - 1984///
N1 - Peer Reviewed Journal: 1985-12314-001
English
Journal; Peer Reviewed Journal
KW - PsycInfo
KW - Rough and Tumble
KW - Aggressive Behavior
KW - Blacks
KW - Learning Disabilities
KW - Peer Relations
KW - Social Perception
RP - NOT IN FILE
SP - Dec
EP - 615
JF - Journal of Learning Disabilities
JA - J Learn.Disabil.
VL - .17
IS - 10
N2 - Judgments of children who manifested social problems-physically aggressive and learning disabled (LD) males-were compared with judgments of children whose behavior was more appropriate. 111 Black, inner-city 11-15 yr olds were divided into 4 groups-nonaggressive learners in a mainstream setting, aggressive learners in classes for the emotionally disturbed (ED) or in day treatment, nonaggressive LD Ss in classes for the LD or resource rooms, and aggressive LD Ss in classes for LD/ED or in day treatment-and either viewed videotapes of similar children engaging in friendly, fighting, or horseplay interactions or heard verbal descriptions of the same events. No differences were found between aggressive and nonaggressive Ss, suggesting that aggressive perceivers do not necessarily "project" their own characteristics onto others. However, LD Ss, both aggressive and nonaggressive, found all scenes more unfriendly. Differences in responses to videotapes and to verbal descriptions alone suggest that conclusions about social perceptions obtained from one mode of stimulus

presentation may not generalize to other modes or to life situations. (12 ref) (PsycINFO Database Record (c) 2012 APA, all rights reserved)
SN - 0022-2194
AD - Weiss, Elizabeth: Mt Vernon Community Service Ctr, NY
ER -

TY - JOUR
ID - 1266
T1 - Concurrent and predictive correlates of sociometric status in kindergarten and Grade 1 children
A1 - Rubin,Kenneth H.
Y1 - 1983///
N1 - Peer Reviewed Journal: 1984-01110-001
English
Journal; Peer Reviewed Journal
KW - PsycInfo
KW - Rough and Tumble
KW - Peer Relations
KW - Social Approval
KW - Social Skills
KW - Status
KW - Prediction
RP - NOT IN FILE
SP - Jul
EP - 351
JF - Merrill-Palmer Quarterly
VL - .29
IS - 3
N2 - 72 kindergartners were assessed for sociometric status during free play and were administered measures of social problem solving, peer popularity and MA (as measured by the PPVT). One year later, all observations and measures were repeated with the addition of teacher ratings of social competence. Sociometric status was moderately stable from kindergarten to Grade 1. Status in kindergarten and Grade 1 was concurrently and positively correlated with the use of prosocial problem-solving strategies and positive social interactions and concurrently and negatively correlated with solitary-dramatic play. Kindergarten observations of rough-and-tumble play, solitary-functional and solitary-exploratory play, the proportion of negative peer exchanges, and agonistic social problem-solving strategies negatively predicted sociometric status in Grade 1. Kindergarten measures of parallel-constructive play, IQ, and the number of relevant social problem-solving strategies positively predicted sociometric status in Grade 1. Sociometric status in kindergarten negatively predicted solitary-functional and solitary-exploratory play; agonistic and bribe social problem-solving skills and teacher ratings of hostile-aggressive behavior. Kindergarten status positively predicted group play, game activities, and prosocial social problem-solving strategies in Grade 1. (34 ref) (PsycINFO Database Record (c) 2012 APA, all rights reserved)
SN - 0272-930X
AD - Rubin, Kenneth H.: U Waterloo, Canada
ER -

TY - JOUR
ID - 1268
T1 - Rough-and-tumble and aggression in schoolchildren: Serious play?
A1 - Neill,Sean R.
Y1 - 1985///
N1 - Peer Reviewed Journal: 1986-26927-001
English
Journal; Peer Reviewed Journal
KW - PsycInfo
KW - Rough and Tumble
KW - Aggressive Behavior

KW - Childhood Play Behavior
KW - Dominance
KW - Peer Relations
RP - NOT IN FILE
SP - Nov
EP - 1382
JF - Animal Behaviour
VL - .33
IS - 4
N2 - Argues that some types of play fighting may serve a serious purpose by allowing dominance conflicts to be resolved without disrupting social relationships. Such fights are likely to be most common at adolescence, when relative strength is changing, and between members of long-term peer groups. (15 ref) (PsycINFO Database Record (c) 2012 APA, all rights reserved)
SN - 0003-3472
AD - Neill, Sean R.: U Warwick, Coventry, England
ER -

TY - JOUR
ID - 1269
T1 - Rough-and-tumble play, fighting, and chasing in nursery school children
A1 - Smith,Peter K.
Y1 - 1985///
N1 - Peer Reviewed Journal: 1986-24231-001
English
Journal; Peer Reviewed Journal
KW - PsycInfo
KW - Rough and Tumble
KW - Aggressive Behavior
KW - Childhood Play Behavior
KW - Friendship
KW - Peer Relations
RP - NOT IN FILE
SP - 1985, pp
EP - 1985,181
JF - Ethology & Sociobiology
VL - .6
IS - 3
N2 - Examined the distinction between rough-and-tumble play (RTP) and aggressive fighting and chasing in a class of 26 preschoolers (aged 3 yrs 11 mo to 4 yrs 8 mo). Episodes differentiated by the structure of the behaviors also differed in the outcome for the Ss. RTP episodes had a larger number of participants, but a lower probability of adult intervention, than incidents of fighting. Regular partners for RTP were more likely to be liked and nominated as best friends. Replay of videotaped episodes showed that nursery staff and some of the Ss could reliably discriminate RTP from fighting; the types of criteria used included observation of the physical characteristics of actions, the verbal and physical expressions of the Ss involved, and information about the personalities of those involved. (21 ref) (PsycINFO Database Record (c) 2012 APA, all rights reserved)
SN - 0162-3095
AD - Smith, Peter K.: U Sheffield, England
ER -

TY - JOUR
ID - 1270
T1 - Gender differences: A biosocial perspective
A1 - Ehrhardt,Anke A.
Y1 - 1984///

N1 - Peer-Reviewed Status-Unknown: 1989-29120-001
English
Journal; Peer-Reviewed Status-Unknown
KW - PsycInfo
KW - Rough and Tumble
KW - Human Sex Differences
KW - Psychosexual Development
KW - Psychosocial Factors
KW - Sex Hormones
KW - Gender Identity
RP - NOT IN FILE
SP - 37
EP - 57
JF - Nebraska Symposium on Motivation
VL - .32 1984, pp. 37-57.
N2 - Proposes a dynamic interactional approach to the study of the influence of hormones on gender-related behaviors, taking constitutional and social-environmental factors into account. The main effect model, which is most often applied, postulates that one factor typically determines behavioral outcome. Principles of mammalian sexual differentiation are based on research on the interaction of hormones and behavior, with specific reference to the original so-called "central hypothesis" and "masculinization" and "defeminization." Examples from several areas of psychoendocrine research illustrate a biosocial perspective of gender identity and sex differences in behavior, such as rough-and-tumble play, nurturance, and parenting. Studies of females with prenatal exposure to sex hormones, such as estrogen diethylstilbestrol, point to influential factors on the expression of behavior within and between gender. (PsycINFO Database Record (c) 2012 APA, all rights reserved)
SN - 0070-2099
AD - Ehrhardt, Anke A.: Columbia U, New York State Psychiatric Inst, US
ER -

TY - JOUR
ID - 1272
T1 - Parent-infant play during the first year of life
A1 - Crawley,Susan B.
Y1 - 1984///
N1 - Peer Reviewed Journal: 1984-31078-001
English
Journal; Peer Reviewed Journal
KW - PsycInfo
KW - Rough and Tumble
KW - Childhood Play Behavior
KW - Childhood Play Development
KW - Father Child Relations
KW - Mother Child Relations
RP - NOT IN FILE
SP - Jan
EP - Mar
JF - Infant Behavior & Development
JA - Infant behav
VL - .7
IS - 1
N2 - Videotaped 72 7-, 10-, and 13-mo-old infants in their homes playing with either their father or their mother. As in previous research, it was found that more fathers used rough physical play with their infants than did mothers. For the most part, however, fathers and mothers exhibited the same developmental changes in their play. The proportion of parents using 2 stimulation forms of play (i.e., gentle and rough physical) was inversely related to increasing infant age. In contrast, more parents used 2 forms of play affording infant contribution of coordinated schemes (i.e., role games and pretend play) with 13-mo-olds than with 7-mo-olds. Similarly,

parents of older infants used coordinated object play more frequently than did parents of younger infants. Mothers decreased use of stimulation with objects and increased use of reading, while fathers used these behaviors infrequently, regardless of infant age. (11 ref) (PsycINFO Database Record (c) 2012 APA, all rights reserved)
SN - 0163-6383
AD - Crawley, Susan B.: U Illinois, Coll of Education, Chicago
ER -

TY - JOUR
ID - 1273
T1 - Social behavior patterns of nonhandicapped and developmentally disabled friend pairs in mainstream preschools
A1 - Strain,Phillip S.
Y1 - 1984///
N1 - Peer-Reviewed Status-Unknown: 1984-27295-001
English
Journal; Peer-Reviewed Status-Unknown
KW - PsycInfo
KW - Rough and Tumble
KW - Developmental Disabilities
KW - Friendship
KW - Interpersonal Interaction
KW - Preschool Students
KW - Mainstreaming (Educational)
RP - NOT IN FILE
SP - 1984, pp
EP - 28
JF - Analysis & Intervention in Developmental Disabilities
VL - .4
IS - 1
N2 - Observed during a 15-day period 15 paired nonhandicapped children and 15 dyads in which 1 child was developmentally disabled; all Ss were 38-56 mo of age and attended 10 mainstream preschools. Observational categories included reward-related activity, complimentary verbal statement, play organizer, sharing, physical assistance, rough and tumble play, affection, and conflict resolution. Results show that nonhandicapped Ss tended to select nonhandicapped friends of their own sex and age; some nonhandicapped Ss tended to select handicapped Ss older than themselves who were more advanced cognitively than other handicapped Ss and who were of the same sex. Nonhandicapped Ss directed many more initiations of reward-related activity, complimentary verbal statements, play organizers, and sharing behaviors toward nonhandicapped rather than handicapped Ss. Physical assistance, affection, and conflict resolution were directed more often toward handicapped Ss. Nonhandicapped Ss were far more likely to initiate positive social behaviors toward nonhandicapped others than were handicapped Ss. Nonhandicapped Ss responded positively at an equal level to the initiations of handicapped and nonhandicapped Ss. (35 ref) (PsycINFO Database Record (c) 2012 APA, all rights reserved)
SN - 0270-4684
AD - Strain, Phillip S.: U Pittsburgh School of Medicine
ER -

TY - JOUR
ID - 1274
T1 - Hanging tongues: A sociological encounter with the assembly line
A1 - Thompson,William E.
Y1 - 1983///
N1 - Peer-Reviewed Status-Unknown: 1985-05487-001
English
Journal; Peer-Reviewed Status-Unknown
KW - PsycInfo

KW - Rough and Tumble
KW - Blue Collar Workers
KW - Coping Behavior
KW - Interpersonal Interaction
KW - Job Characteristics
KW - Working Conditions
KW - Business Organizations
RP - NOT IN FILE
SP - Spr
EP - 237
JF - Qualitative Sociology
VL - .6
IS - 3
N2 - Conducted a 9-wk study of participant observation on an assembly line in the slaughter division of a large beef processing plant in the Midwest. Couched within a symbolic interactionist perspective, the study focused on the day-to-day activities of assembly line workers and examined the sociological aspects of the workers' interaction on the job and how they coped with the danger, strain, and monotony of the work. It also examined how the workers attempted to maintain their sense of self-worth despite the demeaning and dehumanizing aspects of their jobs. Findings show that (1) the workers had a subtle sense of unity among themselves; (2) they used techniques such as daydreaming, horseplay, and occasionally sabotage to protect their sense of self; and (3) consumer spending patterns among the workers seemed to "seal their fate" and make leaving the plant almost impossible. (33 ref) (PsycINFO Database Record (c) 2012 APA, all rights reserved)
SN - 0162-0436
AD - Thompson, William E.: Emporia State U
ER -

TY - JOUR
ID - 1275
T1 - The Northern California diet-behavior program: An empirical examination of 3,000 incarcerated juveniles in Stanislaus County Juvenile Hall
A1 - Schoenthaler,Stephen J.
Y1 - 1983///
N1 - Peer-Reviewed Status-Unknown: 1984-29660-001
English
Journal; Peer-Reviewed Status-Unknown
KW - PsycInfo
KW - Rough and Tumble
KW - Antisocial Behavior
KW - Diets
KW - Juvenile Delinquency
KW - Incarceration
KW - Sugars
RP - NOT IN FILE
SP - 1983, pp
EP - 1983,106
JF - International Journal of Biosocial Research
VL - .5
IS - 2
N2 - Retested the hypothesis that lower levels of violent crimes and antisocial acts can be effected by improved nutrition with juveniles incarcerated in a detention center for a 2-yr period. In a dietary modification, Ss were not allowed "junk" foods, which were frequently brought in by family and/or friends and used as rewards for good behavior. The facility's staff prepared nutritious snacks and treats, which did not contain as much sucrose or food additives and provided snacks such as popcorn and unsweetened orange juice rather than candy bars and soft drinks. During the following 12 mo, the incidence of serious antisocial behaviors in the male population (n = 2,005) declined 21% in contrast to the previous 12 mo, disruptions/horseplay declined 42%, and assaults/fights declined 25%. There were slight reductions in suicide attempts, verbal threats, and disobedience.

Possession of contraband was the only infraction to exhibit an increase (10%). A comparison group of 578 females on the diet found no significant differences in behavior. (15 ref) (PsycINFO Database Record (c) 2012 APA, all rights reserved)
SN - 0731-9169
AD - Schoenthaler, Stephen J.: California State Coll-Stanislaus, Turlock
ER -

TY - JOUR
ID - 1276
T1 - Types of offenses which can be reduced in an institutional setting using nutritional intervention: A preliminary empirical evaluation
A1 - Schoenthaler,Stephen J.
Y1 - 1983///
N1 - Peer-Reviewed Status-Unknown: 1984-13177-001
English
Journal; Peer-Reviewed Status-Unknown
KW - PsycInfo
KW - Rough and Tumble
KW - Antisocial Behavior
KW - Diets
KW - Juvenile Delinquency
KW - Sugars
RP - NOT IN FILE
SP - 1983, pp
EP - 84
JF - International Journal of Biosocial Research
VL - .4
IS - 2
N2 - Conducted a crossover double-blind experiment in attempting to reduce aggressive behavior in incarcerated juveniles by reducing their sugar intake. The incidence of antisocial behavior in the institution was lowered approximately 50% when contrasting the 12 mo before and after the nutritional revision. Over the 24-mo study, 276 incarcerated juveniles committed 934 infractions. During the 12 mo when Ss received a nutritionally superior diet, the incidence of assault was lowered 82%, theft was lowered 77%, horseplay was lowered 65%, refusal-to-obey-an-order was lowered 55%, general rule violations were lowered 23%, andd fighting was lowered 13%. The primary consideration in the dietary revision was to reduce sugar consumption by replacing soft drinks and junk food snacks with fruit juices and nutritional snacks, and eliminating high-sugar-content desserts and cereals. (6 ref) (PsycINFO Database Record (c) 2012 APA, all rights reserved)
SN - 0731-9169
AD - Schoenthaler, Stephen J.: California State Coll-Stanislaus, Turlock
ER -

TY - JOUR
ID - 1277
T1 - Sex differences in play at kindergarten
A1 - Halliday,Jan
Y1 - 1982///
N1 - Peer Reviewed Journal: 1983-29023-001
English
Journal; Peer Reviewed Journal
KW - PsycInfo
KW - Rough and Tumble
KW - Childhood Play Behavior
KW - Human Sex Differences
KW - Kindergarten Students
KW - Preferences
KW - Teacher Student Interaction

RP - NOT IN FILE
SP - Nov
EP - 170
JF - New Zealand Journal of Educational Studies
VL - .17
IS - 2
N2 - Examined sex differences in the preferred play activities of 11 boys and 11 girls (aged 4-5 yrs) who attended an urban kindergarten and the influence of teacher participation and location of activities on such differences. Ss were observed during 9 play sessions, and play activity was recorded using a time-sampling method. Sex differences in play generally conformed to previous research. Boys had a high preference for sandpit play and other energetic outdoor activities, while girls preferred quieter, indoor activities. However, there were more similarities between the activities chosen and avoided by each sex than has previously been reported. Also, boys showed a relatively low frequency of aggression and rough play. While girls tended to choose activities in which their teachers were involved, their most-preferred activity was climbing. Although the presence of female teachers seemed to maintain many of the girls' stereotyped choices of activity, this same presence appeared to extend the range of boys' choices beyond activities that were stereotypically male. (12 ref) (PsycINFO Database Record (c) 2012 APA, all rights reserved)
SN - 0028-8276
AD - Halliday, Jan: U Auckland, New Zealand
ER -

TY - JOUR
ID - 1278
T1 - Changes in interpersonal distances and categories of play behavior in the early weeks of preschool. [References]
A1 - Shea,John D.
Y1 - 1981///
N1 - Peer Reviewed Journal: 1981-28948-001
English
Journal; Peer Reviewed Journal
KW - PsycInfo
KW - Rough and Tumble
KW - Childhood Play Behavior
KW - Human Sex Differences
KW - Longitudinal Studies
KW - Nursery School Students
KW - Preschool Education
KW - Age Differences
RP - NOT IN FILE
SP - Jul
EP - 425
JF - Developmental Psychology
JA - Dev.Psychol.
VL - .17
IS - 4
N2 - Observations of 57 children were made using time lapse and moving film techniques in a preschool over a 10-wk period. Ss attended 5, 3, or 2 days/wk. Behavior was recorded in 5 categories: aggressive behavior, rough and tumble behavior, distance from the nearest child, distance from the teacher, and frequency of close proximity; a 6th category, play behavior, was related to the potential for social interaction in preschool play. Behavior was related to measures of attendance at preschool, preschool group, age, and sex via multiple regression analyses. Scores on all measures of social interaction except aggressive behavior increased with preschool experience. The 3 preschool groups showed different degrees of change, and this was directly related to the number of days of attendance at preschool. (20 ref) (PsycINFO Database Record (c) 2012 APA, all rights reserved)
SN - 0012-1649
AD - Shea, John D.: U Newcastle, Australia

ER -

TY - JOUR
ID - 1279
T1 - Rough and tumble play: A function of gender. [References]
A1 - DiPietro,Janet A.
Y1 - 1981///
N1 - Peer Reviewed Journal: 1981-07763-001
English
Journal; Peer Reviewed Journal
KW - PsycInfo
KW - Rough and Tumble
KW - Aggressive Behavior
KW - Childhood Play Behavior
KW - Human Sex Differences
RP - NOT IN FILE
SP - Jan
EP - 58
JF - Developmental Psychology
JA - Dev.Psychol.
VL - .17
IS - 1
N2 - Investigated rough and tumble play (RTP), a prosocial behavior whose expression and purpose varies as a function of gender, in 43 preschoolers, part of a longitudinal sample (targets), and 86 playmates (partners). A "playroom on wheels" was designed to maximize the amount of RTP displayed in same-sex triads. Observational coding techniques were devised to record various active and verbal behaviors, including a measure of activity level. Results indicate both quantitative and qualitative differences in the behaviors of the male and female triadic groups. Further, a robust sex difference in the amount and intensity of RTP was observed for both targets and partners. Analyses of the behavioral components indicated that RTP was distinct from other dominance-oriented or aggressive behaviors in this age group. A hypothesis concerning differential salience of interpersonal cues is presented to account for these findings, and speculations are made concerning the influence of RTP on subsequent development. (18 ref) (PsycINFO Database Record (c) 2012 APA, all rights reserved)
SN - 0012-1649
AD - DiPietro, Janet A.: Stanford U
ER -

TY - JOUR
ID - 1280
T1 - Convergence of parent and observer ratings of temperament with observations of peer interaction in nursery school
A1 - Billman,Jean
Y1 - 1980///
N1 - Peer Reviewed Journal: 1980-26485-001
English
Journal; Peer Reviewed Journal
KW - PsycInfo
KW - Rough and Tumble
KW - Nursery School Students
KW - Peer Relations
KW - Personality Measures
KW - Personality
KW - Social Interaction
KW - Observation Methods
KW - Psychosocial Development
RP - NOT IN FILE

SP - Jun
EP - 400
JF - Child Development
JA - Child Dev.
VL - .51
IS - 2
N2 - In a study with 78 34-64 mo olds, convergence between temperament ratings from 2 sources (mothers and observers) and observer codings of peer-peer interaction was assessed. Activity level, approach-withdrawal, intensity, distractibility, and sensory threshold correlated significantly with 1 or more variables in the N. G. Blurton-Jones (1972) peer-interaction categories. Ss classified as having more difficult temperaments exhibited more rough-and-tumble and aggressive behaviors than those with easier temperaments. Significant relationships between the Behavioral Style Questionnaire and the Teacher Temperament Questionnaire were demonstrated in 6 of the 7 categories common to both, in spite of the 4-6 mo period that separated the ratings. Results are discussed in terms of the relationship between temperament and social behavior in early childhood. (8 ref) (PsycINFO Database Record (c) 2012 APA, all rights reserved)
SN - 0009-3920
AD - Billman, Jean: Winona State U
ER -

TY - JOUR
ID - 1281
T1 - Re-thinking "banana time": Symbolic integration in a work setting
A1 - Handelman,Don
Y1 - 1976///
N1 - Peer-Reviewed Status-Unknown: 1980-08537-001
English
Journal; Peer-Reviewed Status-Unknown
KW - PsycInfo
KW - Rough and Tumble
KW - Business and Industrial Personnel
KW - Recreation
KW - Rites (Nonreligious)
KW - Work (Attitudes Toward)
KW - Working Conditions
KW - Alienation
KW - Occupational Stress
RP - NOT IN FILE
SP - Jan
EP - 448
JF - Urban Life
VL - .4
IS - 4
N2 - Presents a theoretical reinterpretation of D. F. Roy's observations of the repetitive sequences of ritual horseplay among 3 punchpress operators. Expressive action and work activity are posited as complementary modes of reaction to alienating work. Seemingly ridiculous rituals are seen to be symbolic constructions of the workers' interrelatedness. (21 ref) (PsycINFO Database Record (c) 2012 APA, all rights reserved)
AD - Handelman, Don: Hebrew U, Jerusalem, Israel
ER -

TY - JOUR
ID - 1282
T1 - One-hundred ten feminine and masculine boys: Behavioral contrasts and demographic similarities
A1 - Green,Richard
Y1 - 1976///

N1 - Peer Reviewed Journal: 1977-12673-001
English
Journal; Peer Reviewed Journal
KW - PsycInfo
KW - Rough and Tumble
KW - Demographic Characteristics
KW - Femininity
KW - Human Males
KW - Masculinity
KW - Sex Roles
KW - Family Background
RP - NOT IN FILE
SP - Sep
EP - 446
JF - Archives of Sexual Behavior
JA - Arch.Sex Behav
VL - .5
IS - 5
N2 - Reports interview data from an ongoing study in which 60 boys, referred for extensive cross-gender behavior, and their families were compared with a matched group of 50 boys showing typical gender/role behavior and their families. There were no significant differences between families on a variety of demographic variables, including ages or educational levels of mothers or fathers, number of children per family, ordering of younger and older siblings, ethnic background, religion, political party affiliation, or current marital status of the families. Compared to masculine boys, feminine boys participated less in sports and rough-and-tumble play; were more often voluntary loners or rejected by peers; related better to girls (vs boys) of the same age; were more frequent cross-dressers; reported that female-type dolls were their favorite toy (17%); had greater interest in play-acting and taking roles in fantasy games; commented more frequently on their mothers' clothing; more often verbalized the wish to grow up to be like their mothers or to have been born a girl (83%); more often preferred their mothers (masculine boys were divided in affection between parents or preferred their fathers); were more likely to have been hospitalized at least once; and were more often separated from their biological fathers before age 5. (PsycINFO Database Record (c) 2012 APA, all rights reserved)
SN - 0004-0002
AD - Green, Richard: State U New York, Stony Brook
ER -

TY - JOUR
ID - 1283
T1 - Reflection-impulsivity and behavioral problems in emotionally disturbed boys
A1 - Finch,A.
Y1 - 1976///
N1 - Peer Reviewed Journal: 1976-26146-001
English
Journal; Peer Reviewed Journal
KW - PsycInfo
KW - Rough and Tumble
KW - Behavior Problems
KW - Cognitive Style
KW - Emotional Disturbances
RP - NOT IN FILE
SP - Jun
EP - 274
JF - The Journal of Genetic Psychology: Research and Theory on Human Development
VL - .128
IS - 2
N2 - To investigate the difference in the behavioral problems presented by cognitively impulsive and reflective emotionally disturbed boys, the parents of 17 impulsive and 13 reflective males (mean age, 11.32 yrs; chosen by

scores on the Matching Familiar Figures Test) in residential treatment were asked to rate their sons' behavior. Results indicate that in contrast to reflective Ss, impulsives were more likely to talk of others blaming them unfairly, threaten to harm themselves, hit and bully other children, and be excessively rough in play. On the other hand, reflectives were more unwilling to talk with adults outside the family. Results are discussed in terms of supporting both previous research and J. Kagan and N. Kogan's (1970) "fear of failure" hypothesis. (PsycINFO Database Record (c) 2012 APA, all rights reserved)
SN - 0022-1325
AD - Finch, A. J.: Virginia Treatment Ctr for Children, Richmond
ER -

TY - JOUR
ID - 1284
T1 - Sex-related stereotyping of toddlers' behaviors
A1 - Fagot,Beverly I.
Y1 - 1973///
N1 - Peer Reviewed Journal: 1974-04887-001
English
Journal; Peer Reviewed Journal
KW - PsycInfo
KW - Rough and Tumble
KW - Childhood Play Development
KW - Human Sex Differences
KW - Sex Roles
KW - Social Perception
KW - Stereotyped Attitudes
RP - NOT IN FILE
SP - Nov
JF - Developmental Psychology
JA - Dev.Psychol.
VL - .9
IS - 3
N2 - Investigated whether 45 male and 57 female 20-25 yr olds who had little or no contact with young children would have stereotypes regarding sex-appropriate behavior for toddlers. Ss rated each of 38 behaviors of 2 yr olds as appropriate to either boys, girls, or both sexes. Only 6 behaviors were sex-typed by more than 5 Ss. Rough-house play, play with transportation toys, and aggressive behavior were considered masculine, whereas play with dolls, "dressing up," and looking in the mirror were considered feminine. Significantly more males sex-typed behaviors than did females. Reasons why girls show less sex-typing in their play behaviors than boys are briefly discussed. (PsycINFO Database Record (c) 2012 APA, all rights reserved)
SN - 0012-1649
AD - Fagot, Beverly I.: U. Oregon
ER -

TY - JOUR
ID - 1285
T1 - Aspects of social development in nursery school children with emphasis on introduction to the group
A1 - McGrew,W.
Y1 - 1972///
N1 - Book: 1973-26477-009
English
Book; Edited Book
KW - PsycInfo
KW - Rough and Tumble
KW - Childhood Development
KW - Group Participation
KW - Social Behavior
RP - NOT IN FILE

SP - 400
JF - Jones, N
VL - Blurton.
IS - 1972
N2 - Observed the social behavior of 12 3-5 yr. olds introduced singly into a nursery school containing 15 children during 5-day periods immediately after entry and 3 mo. later. Ss' behavior at entry varied greatly, but the group's reaction to the S was a more consistent neutral curiosity. During the 1st day, S was subdued and exhibited signs of ambivalence; nursery members were indifferent or friendly, but not hostile. Conspicuous changes in S's behavior occurred within 5 days of entry. Nervous exploration decreased and social approaches (including antagonism) increased, but participation in "rough-and-tumble play" was rare. Ss were behaviorally indistinguishable from the others after 3 mo. Unexpected daily consistencies were found in some behavioral pattern frequencies within morning observation periods. The usefulness of comparing social development in human and nonhuman primates is discussed. (26 ref.) (PsycINFO Database Record (c) 2012 APA, all rights reserved)
ER -

TY - JOUR
ID - 1286
T1 - Categories of child-child interaction
A1 - Jones,N.
Y1 - 1972///
N1 - Book: 1973-26477-006
English
Book; Edited Book
KW - PsycInfo
KW - Rough and Tumble
KW - Age Differences
KW - Aggressive Behavior
KW - Childhood Play Development
KW - Interpersonal Interaction
KW - Sex Linked Developmental Differences
RP - NOT IN FILE
SP - 400
JF - Jones, N
VL - Blurton.
IS - 1972
N2 - Observed the free play of 13 4-yr-olds and 12 2-yr-olds selected as being the oldest or the youngest of their sex in playgroups of mixed ages and sexes. The occurrence of 22 previously defined items of behavior (e.g., hit and smile) was continuously recorded during each of 15 5-min periods. Inter-S correlations of the behavior items occurring within each observation period revealed 3 main factors: "rough-and-tumble play vs. work," "aggression," and "social behavior." A less reliable "distress" dimension was also found. Comparisons of behavior between the age and sex groups and across Ss tended to dissect the aggression dimension. Ss who showed more rough-and-tumble play showed less aggression, but evidence suggests that this was because they were less often in aggression-provoking situations. Males did more wrestling and hitting during rough-and-tumble play than females. The amount of behavior directed toward the teacher decreased with age in males but not in females. The main dimension of between-S variation concerned all social behavior, age, and experience in the nursery. Working alone and watching children seemed to be younger forms of behavior. Subsidiary dimensions of between-S variation were play with toys vs. without toys and active or passive interaction. (PsycINFO Database Record (c) 2012 APA, all rights reserved)
ER -

TY - JOUR
ID - 1287
T1 - PERSONALITY CHARACTERISTICS OF THE CHILD ACCIDENT REPEATER
A1 - MANHEIMER,DEAN I.
Y1 - 1967///

N1 - Peer Reviewed Journal: 1967-10267-001
English
Journal; Peer Reviewed Journal
KW - PsycInfo
KW - Rough and Tumble
KW - Accident Prevention
KW - Accident Proneness
KW - Accidents
KW - Personality Development
KW - Personality
KW - Cerebrovascular Accidents
KW - Personality Traits
RP - NOT IN FILE
SP - 1967, pp
EP - 1967,513
JF - Child Development
JA - Child Dev.
VL - .38
IS - 2
N2 - FROM 8874 BOYS AND GIRLS AGES 4-18, 684 WERE SELECTED TO REPRESENT HIGH-, INTERMEDIATE-, OR LOW-ACCIDENT-LIABILITY CHILDREN, BASED ON RECORDS OF MEDICALLY ATTENDED INJURIES. USING DATA FROM INTENSIVE INTERVIEWS WITH MOTHERS SUPPLEMENTED WITH SCHOOL RECORDS, A STATISTICALLY SIGNIFICANT RELATION BETWEEN ACCIDENT LIABILITY AND INDEXES OF EXTRAVERSION, DARING, ROUGHHOUSING, AND OTHER TRAITS TENDING TO EXPOSE CHILDREN TO HAZARDS WAS FOUND. SIMILAR RELATIONS HELD FOR TRAITS SUCH AS POOR DISCIPLINE, AGGRESSIVENESS TOWARD PEERS, AND, FOR GIRLS, ATTENTION-SEEKING, WHICH COMPETE WITH THE CHILD'S ABILITY TO COPE WITH HAZARDS. OTHER TRAITS THAT MAY IMPAIR ABILITY TO COPE WITH HAZARDS WERE ALSO FOUND TO BE RELATED TO ACCIDENT LIABILITY (IMPULSIVITY, CARELESSNESS, AND UNRELIABILITY) AS WERE SEVERAL INDEXES DENOTING MALADJUSTMENT. (19 REF.) (PsycINFO Database Record (c) 2012 APA, all rights reserved)
SN - 0009-3920
AD - MANHEIMER, DEAN I.: LANGLEY PORTER NEUROPSYCHIATRIC INST., SAN FRANCISCO, CALIF
ER -

TY - JOUR
ID - 1288
T1 - The "buddy system" in the hospital treatment of alcoholics
A1 - Androes,L.
Y1 - 1966///
N1 - Peer Reviewed Journal: 1966-13322-001
English
Journal; Peer Reviewed Journal
KW - PsycInfo
KW - Rough and Tumble
KW - Alcohol Rehabilitation
KW - Alcoholism
KW - Hospitalized Patients
KW - Hospitals
RP - NOT IN FILE
SP - 1966, pp
EP - 1966,529
JF - Quarterly Journal of Studies on Alcohol
VL - .27
IS - 3

N2 - The buddy, or pairing, system was introduced to the group treatment program at a veterans administration hospital in 1963. During the 1st yr. 90 male patients participated in a trial of the buddy system. The mean age of the patients was 45 yr., most came from lower or middle class backgrounds, were unemployed, and reported approximately 15 yr. of heavy drinking. As the patients were voluntarily admitted to the 90-day inpatient program each was arbitrarily assigned to a fellow patient as a buddy. The successful pairs were friendly, close, mutually supportive, and shared personal problems which they were reluctant to present to a staff member. The unsuccessful pairs were mutually destructive or destructive toward the group as a whole, usually in the form of horseplay, undermining the confidence of the buddy, encouraging manipulation of or, most importantly, discouraging communication with the staff. It was concluded that the buddy system is a useful management device. The patients became more prompt in arriving at assignments and group meetings, and it became easier to maintain communication and organization within the program. (PsycINFO Database Record (c) 2012 APA, all rights reserved)
SN - 0033-5649
AD - Andoes, L.: Veterans Administration Hosp., Topeka, Kan
ER -

TY - JOUR
ID - 1289
T1 - Art therapy and aggression
A1 - Kramer,Edith
Y1 - 1958///
N1 - Book: 2009-02640-007
English
Book; Authored Book
KW - PsycInfo
KW - Rough and Tumble
KW - Aggressive Behavior
KW - Art Therapy
KW - Painting (Art)
KW - Conflict
KW - Personality
KW - Psychotherapeutic Processes
RP - NOT IN FILE
SP - 238
JF - Kramer, Edith
IS - 1958
N2 - (from the chapter) This chapter examines how art therapy is used to address aggression at the Wiltwyck School. It is noted that life at Wiltwyck School is colored by the prevalence of aggression. Even the boys' amiable small talk and play consists for the most part of insults, threats, profanity, and aggressive horseplay. The children's habitual response to all conflicts and adversity seems limited to aggressive acts, tantrums, and destructiveness. Because aggression is so ubiquitous and undifferentiated, it hides rather than reveals the children's personalities. It seemed, therefore, best to approach the specific problem of aggression in art therapy only after the reader had become more familiar with the children and their painting. (PsycINFO Database Record (c) 2012 APA, all rights reserved)
ER -

TY - JOUR
ID - 1290
T1 - Symbiosis and consensus as integrative factors in small groups
A1 - Gross,Edward
Y1 - 1956///
N1 - Peer Reviewed Journal: 1957-02698-001
English
Journal; Peer Reviewed Journal
KW - PsycInfo
KW - Rough and Tumble

RP - NOT IN FILE
SP - 174
EP - 179
JF - American Sociological Review
VL - .21 1956, pp. 174-179.
N2 - Data were gathered at an Air Defense Command base relative to group participation in 6 informal activities: eating lunch, drinking coffee, bull sessions, horseplay and joking on the job, leaving the air site, and spending time off the air site. Group composition was studied in each case. Results indicated that symbiotic groups (in which members were interdependent because each satisfied some important need of his fellow) tended to be composed of men of dissimilar or contrasting characteristics. Consensual groups (in which the members shared a goal or value or viewpoint) tended to be composed of men of like characteristics. (PsycINFO Database Record (c) 2012 APA, all rights reserved)
SN - 0003-1224
ER -

TY - JOUR
ID - 1291
T1 - Out-of-school living of eleven-year-old boys and girls from differing socioeconomic groups
A1 - Volberding,Eleanor
Y1 - 1949///
N1 - Peer Reviewed Journal: 1949-04713-001
English
Journal; Peer Reviewed Journal
KW - PsycInfo
KW - Rough and Tumble
RP - NOT IN FILE
SP - 348
EP - 353
JF - The Elementary School Journal
VL - .49 1949, pp. 348-353.
N2 - 80 eleven-year-old children from a midwestern community were grouped according to socioeconomic status and sex, all but one of the children falling into the lower-middle, upper-lower, and lower-lower classes. The lower-lower class children had smaller living space, lower activity in play, lower preferences for twilight, competitive group and rough play and listened to the radio less than those children from the higher groups. The upper-lower class child appeared to have more freedom than the middle-class child, and to have sufficient security and nourishment for strenuous living. The middle-class children had social confidence, security and social experience, and were more restricted in their behavior by parents. The lower-lower class children were insecure, non-aggressive, and lacking in prestige, their social experience and confidence being undermined by lack of status of the family group. (PsycINFO Database Record (c) 2012 APA, all rights reserved)
SN - 0013-5984
AD - Volberding, Eleanor: U. Utah, Salt Lake City
ER -

TY - JOUR
ID - 1292
T1 - Out-of-school behavior of eleven-year-olds
A1 - Volberding,E.
Y1 - 1948///
N1 - Peer Reviewed Journal: 1949-01706-001
English
Journal; Peer Reviewed Journal
KW - PsycInfo
KW - Rough and Tumble
RP - NOT IN FILE
EP - 441
JF - The Elementary School Journal

VL - .432-441 1948,
N2 - Data regarding out-of-school activities over a one-year period were obtained on 80 eleven-year-old children from a midwestern community. These data were collected by means of an individual map test, by interviews with adults and children, observation, questionnaires, compositions, and standardized tests. All the children belonged to small intimate play groups, and participated occasionally in the larger neighborhood play groups; the living space of the children was found to be quite restricted. The majority liked twilight play and competitive group play, but disliked rough play. Family social status was a differentiating factor in play preferences. Movies (western, action, comedy), roller skating, and radio (mysteries, comic programs, westerns) followed after play with other children in order of importance. Little recreational reading or club membership was reported. Similarities and differences compared with adolescent interests and activities are pointed out. (PsycINFO Database Record (c) 2012 APA, all rights reserved)
SN - 0013-5984
AD - Volberding, E.: U. Utah, Salt Lake City
ER -

TY - JOUR
ID - 1293
T1 - Growing up emotionally
A1 - Arlitt,Ada Hart
Y1 - 1938///
N1 - Book: 2008-17447-006
English
Book; Authored Book
KW - PsycInfo
KW - Rough and Tumble
KW - Adolescent Development
KW - Developmental Stages
KW - Emotional Development
KW - Love
RP - NOT IN FILE
SP - 242
EP - Hill
JF - Arlitt, Ada Hart
IS - 1938
N2 - (from the chapter) There are many phases of emotional development characteristic of the adolescent period. The child has passed from the stage of the gang to that of "the crowd." Where the gang went in search of adventure, the crowd usually just sits around and talks. The stage of crowd talking is followed after a brief period of "roughhousing." While the adolescent is engaged in rough play and teasing with people of the opposite sex, he tends to fall in love with people older than himself, usually at such a distance from his home that he cannot hope to meet them in person. The next level is the time of the puppy love affair. After the stage of puppy love children begin to make the selection of the person whom they intend to marry. Somewhere between the first puppy love affair and the choice of a permanent mate there occurs a period during which adolescents wish no interference with their activities and they will tell little of their day's activities. While not all children may go through all stages in this process, it is nevertheless not the parents' business to prevent the child from attaining mature development. (PsycINFO Database Record (c) 2012 APA, all rights reserved)
AD - Arlitt, Ada Hart: Graduate School of Arts and Sciences, University of Cincinnati, Cincinnati, OH, US
ER -

TY - JOUR
ID - 1295
T1 - Perspective and practices to address rough-and-tumble play in the school setting: A survey of school-wide positive behavior support teams in Utah
A1 - Basinger,Jason Christopher
Y1 - 2013///

N1 - Dissertation Abstract: 2013-99220-216
English
Dissertation Abstract
KW - PsycInfo
KW - Rough and Tumble
KW - Behavior Problems
KW - School Based Intervention
KW - Schools
KW - Bullying
KW - Teams
RP - NOT IN FILE
SP - No
JF - Dissertation Abstracts International: Section B: The Sciences and Engineering
VL - .74
IS - 5-B(E
N2 - Prior research of rough-and-tumble play (RTP) has shown mixed results-different definitions, varying functions, and positive and negative outcomes. Few researchers have studied interventions to address RTP in school settings. With unclear evidence of RTP outcomes and the extent school interventions are addressing RTP in school settings, this study explored the extent and effectiveness of intervention programs being implemented to prevent/reduce negative outcomes of RTP in elementary schools. A survey was created and conducted with 30 school problem-solving teams in a western state to obtain information concerning RTP in elementary school settings. Teams provided estimated percentages of RTP leading to beneficial and problematic behaviors, types of benefits or problems resulting from RTP, specific prevention/intervention programs that teams report implementing to address RTP concerns, percentage estimations of students responding to implemented interventions, and training needs to address interventions for RTP concerns. Survey responses showed higher estimated percentages for problematic outcomes than beneficial outcomes. Additionally, the greater number of problem items were endorsed by teams than the number of benefit items, suggesting RTP is more often problematic than beneficial. Interventions estimated to be effective in treating problematic RTP outcomes are reward systems, social skills trainings, active supervision, and bully prevention. These study findings are different from previous research, which concluded that RTP was harmless and/or beneficial to students, and might be due to setting differences (school vs. community). It might be estimated that schools should monitor or prevent RTP to avoid problems, such as aggression, bullying, and poor peer relationships. Programs frequently used by teams targeted skill acquisition through social skills training, anger management, and bully prevention. This study provides understanding to the extent RTP should be addressed in schools. School problem solving teams report that RTP can be problematic in school settings; however, it can be prevented with school-wide intervention, and intervened with individual and small-group interventions. (PsycINFO Database Record (c) 2013 APA, all rights reserved)
SN - 0419-4217
AD - Basinger, Jason Christopher: Utah State U., US
ER -

TY - JOUR
ID - 1296
T1 - Would a rose, by any other name, smell sweeter? [References]
A1 - Bentall,Richard P.
Y1 - 2013///
N1 - Peer Reviewed Journal: 2013-22213-021
English
Journal; Peer Reviewed Journal
KW - PsycInfo
KW - Rough and Tumble
KW - Diagnosis
KW - Psychosis
KW - Schizophrenia
KW - Stigma
RP - NOT IN FILE

SP - Jul
EP - 1562
JF - Psychological Medicine
VL - .43
IS - 7
N2 - Methods of psychiatric classification have numerous uses, ranging from the clinical (communication between clinicians, the facilitation of decisions about treatment), to the scientific (selecting participants for research into the aetiology and treatment of mental illness), through to the social and political (keeping statistics about mental health, developing mental health policy). Schizophrenia has been a contested label for many years not only because it is associated with stigma, but also because it fails to achieve any of the purposes for which it was originally designed. Even in the world of operationalized diagnostic criteria, different definitions of schizophrenia sometimes define different people as schizophrenic. In carefully conducted studies in which patients are followed up over time, patients sometimes move from one diagnosis to another within the psychosis spectrum and diagnostic shifts, for example between schizophrenia and bipolar disorder, are probably much more common in the rough and tumble of routine psychiatric care. To persuade the general public to be more accepting of people with mental illness, we must persuade them that psychosis arises, in part, understandably from adverse life experiences (while of course acknowledging that genetic factors must play some role), that it does not necessarily lead to violence, and that recovery is possible. Fortunately, there is a now a considerable volume of research that supports this position. (PsycINFO Database Record (c) 2013 APA, all rights reserved)
SN - 0033-2917
AD - Bentall, Richard P.: Institute of Psychology, Health & Society, Liverpool University, Liverpool, United Kingdom
ER -

TY - JOUR
ID - 1297
T1 - Presidential courage: Brave leaders and how they changed America 1789-1989. [References]
A1 - Beschloss,Michael
Y1 - 2007///
N1 - Book: 2007-09209-000
English
Book; Authored Book
(Created by PsycINFO) Preface A speedy death to general Washington! Kick this treaty to hell! The damnedest liar He may retire with undiminish'd glory Rivalries irritated to madness Oh, that I was a soldier! Rocks and quicksands on all sides The most splendid diamond in my crown I will kill it! Not a man to be forced I was born for the storm Who would have had the courage? I am going to be beaten Too angelic for this devilish rebellion A well-meaning baboon The country will be saved I see dynamite Black storm A rough-and-tumble man I upset them all We must protect the chief! Gloom personified Salute your Caesar? We have avoided a putsch No people except the Hebrews The right place at the right time How could this have happened? I am Cyrus! They never show their passion Go get him, Johnny boy! It's going to be a civil war A man has to take a stand We win and they lose! It left me greatly depressed Don't worry that I've lost my bearings A miracle has taken place Epilogue: Presidential courage Notes Sources Acknowledgments Index
KW - PsycInfo
KW - Rough and Tumble
KW - Courage
KW - History
KW - Leadership
KW - Politicians
RP - NOT IN FILE
SP - 430
JF - (2007)
VL - Presidential courage
N2 - (from the jacket) Michael Beschloss has brought us a brilliantly readable and inspiring saga about crucial times in America's history when a courageous President dramatically changed the future of the United States. With surprising new sources and a dazzling command of history and human character, Beschloss brings to life these flawed, complex men--and their wives, families, friends and foes. Never have we had a more intimate,

behind-the-scenes view of Presidents coping with the supreme dilemmas of their lives. As Beschloss shows in this gripping and important book, none of these Presidents was eager to incur ridicule, vilification or threats of political destruction and even assassination. But in the end, bolstered by friends and family, hidden private beliefs and, sometimes, religious faith, each ultimately proved himself to be, in Andrew Jackson's words, "born for the storm." (PsycINFO Database Record (c) 2012 APA, all rights reserved)
ER -

TY - JOUR
ID - 1298
T1 - The impractical man
A1 - Blackford,Katherine M.
Y1 - 1916///
N1 - Book: 2009-03134-008
English
Book; Authored Book
KW - PsycInfo
KW - Rough and Tumble
KW - Occupational Guidance
KW - Occupational Success
KW - Person Environment Fit
KW - Personality Traits
KW - Personality
KW - Arts
KW - Business
KW - Intelligence
KW - Occupational Aspirations
KW - Philosophies
KW - Sciences
KW - Working Conditions
RP - NOT IN FILE
SP - reprinted
EP - 221
JF - Blackford, Katherine M
VL - H; Newcomb, Arthur.
IS - 1916, reprinted 1920
N2 - (from the chapter) Using the life of Sydney Williams as an example, the authors describe the personality and intellect of impractical men. Although they have good intellects, and should be fitted to intellectual rather than manual labor, they often fail to find a good occupational match. When they fail, it is more often through their own incompetence; they usually dissipate their assets by impracticable schemes. The only hope for such men is to learn their limitations; to learn that, even though they may be ambitious for commercial success, they are utterly unqualified for it; that, although they may wish to do something in the way of production or selling, they have neither talent, courage, secretiveness, persistence, nor other qualities necessary for a success in these lines. They are too credulous. They are too impractical. They are too lacking in fighting qualities, and, therefore, too easily imposed upon. They are usually lazy physically and find disagreeable situations hard, so that they are out of place in the rough-and-tumble, strenuous, hurly-burly of business, manufacturing, or ordinary professional life. The authors provide a few stories to indicate what these men can do, do well, and what they can be happy and satisfied in doing: there is a real need for them in the world. These vocations include authors, scientists, philosophers, poets, actors, preachers, teachers, lecturers, and musicians. These people deal with abstractions, ideas, with theories, and with emotions. They may be very successful in the spinning of theories, in the working out of clever ideas, and in their appeal to the emotions of their fellowmen. They may write poetry which is the product of genius; they may devise profound philosophy. This is their realm. Here is where they are supreme, and it is in this kind of work they find an expression for all of their talent. (PsycINFO Database Record (c) 2012 APA, all rights reserved)
ER -

TY - JOUR

ID - 1299
T1 - Individual-group behavioral similarity and peer status in experimental play groups of boys: the social misfit revisited
A1 - Boivin,M.
A1 - Dodge,K.A.
A1 - Coie,J.D.
Y1 - 1995/08//
N1 - Boivin, M. Dodge, K A. Coie, J D
Journal of personality and social psychology
jn3, 0014171
IM
Journal Article. Research Support, Non-U.S. Gov't. Research Support, U.S. Gov't, P.H.S.
English
KW - Medline
KW - Rough and Tumble
KW - Child
KW - Child Behavior
KW - Humans
KW - Interpersonal Relations
KW - Male
KW - Peer Group
KW - Play and Playthings
KW - Social Behavior
RP - NOT IN FILE
SP - 269
EP - 279
JF - Journal of Personality & Social Psychology
JA - J Pers.Soc Psychol.
VL - 69
IS - 2
CY - UNITED STATES
N2 - This study evaluated individual-group similarity and dissimilarity hypotheses generally stipulating that the behavioral correlates of status are moderated by the peer group context in which they are displayed. Thirty play groups of 5 or 6 unacquainted same-age boys participated in five 45-min sessions. Five behaviors described group and individual characteristics: reactive aggression, proactive aggression, solitary play, rough-and-tumble play, and positive interactive behavior. Individual social preference scores were computed following a variant of the J. D. Coie and K. A. Dodge (1983) procedure. The behavioral correlates of emerging peer status were examined as a function of the group's behavioral norms. Evidence of a dissimilarity effect was found for solitary play and reactive aggression whereas positive interactive behavior followed a rule of similarity
SN - 0022-3514
AD - Ecole de Psychologie, Universite Laval Ste-Foy, Quebec, Canada
UR - 7643305
ER -

TY - JOUR
ID - 1300
T1 - Violent content enhances video game performance. [References]
A1 - Bosche,Wolfgang
Y1 - 2009///
N1 - Peer Reviewed Journal: 2009-23969-001
English
Journal; Peer Reviewed Journal
KW - PsycInfo
KW - Rough and Tumble
KW - Aggressive Behavior
KW - Computer Games

KW - Performance
KW - Violence
RP - NOT IN FILE
SP - 2009, pp
EP - 2009,150
JF - Journal of Media Psychology: Theories, Methods, and Applications
VL - .21
IS - 4
N2 - This study assesses the impact of violent video game content on players' game performance. According to the desensitization hypothesis (Carnagey, Anderson, & Bushman, 2007), violent content may elicit negative affective responses and inhibitions, which in turn should interfere with performance. On the other hand, the players might understand virtual violent acts as a digital form of rough-and-tumble play, associated with positive emotions and mobilization, which in turn should raise performance. To test these competing hypotheses on game performance, N = 50 males with no prior violent gaming experience were exposed to three different versions of a custom-made video game in which the actions to be performed were identical, though they were audio-visually presented to appear either nonviolent, moderately, or extremely violent. The results show no indication of an initial inhibition of aggressive behavior, that is, performance is elevated and remains so if the action is presented audio-visually as being violent. This supports the notion that being involved in violent video game activity is perceived as an essentially harmless acting-out of playful fighting behavior. (PsycINFO Database Record (c) 2012 APA, all rights reserved) (journal abstract)
SN - 1864-1105
AD - Bosche, Wolfgang: Department of Psychology, Technische Universitat Darmstadt, Darmstadt, Germany
ER -

TY - JOUR
ID - 1301
T1 - Violent video games prime both aggressive and positive cognitions. [References]
A1 - Bosche,Wolfgang
Y1 - 2010///
N1 - Peer Reviewed Journal: 2010-26441-001
English
Journal; Peer Reviewed Journal
KW - PsycInfo
KW - Rough and Tumble
KW - Aggressive Behavior
KW - Cognitions
KW - Computer Games
KW - Violence
RP - NOT IN FILE
SP - 2010, pp
EP - 2010,146
JF - Journal of Media Psychology: Theories, Methods, and Applications
VL - .22
IS - 4
N2 - Previous studies have shown that violent video games prime aggressive thoughts and concepts. Interestingly, positively valenced test stimuli are rarely used in this field, though they might provide useful information on the nature of the emotional response to virtual violence and its associative structure. According to the General Aggression Model (GAM) and its extensions (Carnagey, Anderson, & Bushman, 2007), normal negative reactions to violence are expected. Alternatively, playing violent video games might be construed as engaging in positively valenced playful fighting behavior. To test the potential of violent video games to prime positive concepts, N = 29 adult males played either a violent or a nonviolent video game for 20 minutes and were subsequently tested in a standard lexical decision task consisting of positive, aggressive, nonaggressive negative, and neutral target words. The data show that the violent video game primed aggressive concepts as expected, but also raised positive concepts, and did so independently of the participants' history of playing violent video games. Therefore, the results challenge the idea that violent video games inherently stimulate negative concepts only. (PsycINFO Database Record (c) 2012 APA, all rights reserved) (journal abstract)

SN - 1864-1105
AD - Bosche, Wolfgang: Technische Universitta Darmstadt, Darmstadt, Germany
ER -

TY - JOUR
ID - 1302
T1 - In praise of a rough-and-tumble life
A1 - Brenna,O.V.
Y1 - 2008/01//
N1 - Brenna, Oreste V
Trends in immunology
dzx, 100966032
IM
Comment. Letter
English
KW - Medline
KW - Rough and Tumble
KW - Allergens/im [Immunology]
KW - Environmental Exposure
KW - Humans
KW - Hypersensitivity/et [Etiology]
KW - Hypersensitivity/im [Immunology]
KW - Immune System/ph [Physiology]
KW - Skin/im [Immunology]
RP - NOT IN FILE
SP - 1
EP - 2
JF - Trends in Immunology
JA - Trends Immunol.
VL - 29
IS - 1
CY - England
SN - 1471-4906
UR - 18194757
ER -

TY - JOUR
ID - 1303
T1 - Juvenile horseplay purpura
A1 - Brockmann,K.
A1 - Diepold,K.
Y1 - 2002/10//
N1 - Brockmann, Knut. Diepold, Katharina
Archives of dermatology
6wu, 0372433
AIM, IM
Case Reports. Letter
English
KW - Medline
KW - Rough and Tumble
KW - Child
KW - Diagnosis
KW - Differential
KW - Female
KW - Humans
KW - Lumbar Vertebrae

KW - Purpura/di [Diagnosis]
KW - Purpura/et [Etiology]
KW - Vasculitis
KW - Leukocytoclastic
KW - Cutaneous/di [Diagnosis]
KW - Cutaneous/et [Etiology]
RP - NOT IN FILE
SP - 1384
JF - Archives of Dermatology
JA - Arch.Dermatol.
VL - 138
IS - 10
CY - United States
SN - 0003-987X
UR - 12374559
ER -

TY - JOUR
ID - 1304
T1 - Bored employees misbehaving: The relationship between boredom and counterproductive work behaviour. [References]
A1 - Bruursema,Kari
Y1 - 2011///
N1 - Peer Reviewed Journal: 2011-26444-001
English
Journal; Peer Reviewed Journal
KW - PsycInfo
KW - Rough and Tumble
KW - Boredom
KW - Employee Productivity
KW - Organizational Behavior
KW - Personnel
RP - NOT IN FILE
SP - Apr
EP - 107
JF - Work & Stress
VL - .25
IS - 2
N2 - In this study, the relationships among boredom proneness, job boredom, and counter-productive work behaviour (CWB) were examined. Boredom proneness consists of several factors, which include external stimulation and internal stimulation. Given the strong relationships between both the external stimulation factor of boredom proneness (BP-ext) and anger as well as the strong relationship between trait anger and CWB, we hypothesized that examining BP-ext would help us to better understand why employees commit CWB. Five types of CWB have previously been described: abuse against others, production deviance, sabotage, withdrawal and theft. To those we added a sixth, horseplay. Using responses received from 211 participants who were recruited by email from throughout North America (112 of them matched with co-workers), we found support for our central premise. Indeed, both BP-ext and job boredom showed significant relationships with various types of CWB. The boredom proneness factor also moderated the relationship between job boredom and some types of CWB, suggesting that a better understanding of boredom is imperative for designing interventions to prevent CWB. (PsycINFO Database Record (c) 2012 APA, all rights reserved) (journal abstract)
SN - 0267-8373
AD - Bruursema, Kari: Department of Psychology, Montclair State University, Upper Montclair, NJ, US Kessler, Stacey R.: Department of Management and Information Systems, School of Business, Montclair State University, Upper Montclair, NJ, US Spector, Paul E.: Department of Psychology, University of South, Tampa, FL, US
ER -

TY - JOUR
ID - 1305
T1 - Ischemic cerebellar infarct in a 5-year-old boy: sequela to minor back trauma
A1 - Byrd,L.R.
A1 - Vogel,H.L.
Y1 - 1996/04//
N1 - Byrd, L R. Vogel, H L
The Journal of the American Osteopathic Association
7503065, g90
IM
Case Reports. Journal Article
English
KW - Medline
KW - Rough and Tumble
KW - Anti-Inflammatory Agents
KW - Brain Ischemia/di [Diagnosis]
KW - Brain Ischemia/dt [Drug Therapy]
KW - Brain Ischemia/et [Etiology]
KW - Cerebellar Diseases/di [Diagnosis]
KW - Cerebellar Diseases/dt [Drug Therapy]
KW - Cerebellar Diseases/et [Etiology]
KW - Cerebrovascular Disorders/di [Diagnosis]
KW - Cerebrovascular Disorders/dt [Drug Therapy]
KW - Cerebrovascular Disorders/et [Etiology]
KW - Child
KW - Preschool
KW - Humans
KW - Magnetic Resonance Imaging
KW - Male
KW - Prednisolone/tu [Therapeutic Use]
KW - Wounds and Injuries/co [Complications]
RP - NOT IN FILE
SP - 245
EP - 249
JF - Journal of the American Osteopathic Association
JA - J Am Osteopath.Assoc.
VL - 96
IS - 4
CY - UNITED STATES
N2 - Minor trauma, a kick to the center of the back during roughhousing with an 8-year-old sibling, produced an ischemic cerebellar infarct in a 5-year-old boy. The boy was brought to medical attention because of vomiting and headache. He also had ataxia. Results of laboratory studies, structural examination, and cerebrospinal fluid analysis were noncontributory. An irregular, low-density region in the left cerebellar hemisphere of unknown cause was seen on computed tomography scans. Magnetic resonance imaging (MRI) revealed an area of signal alteration hyperintense on the T2-weighted image and mildly hyperintense on the proton-density image in the leftward cerebellar hemisphere. The distribution was most compatible with that of an acute superior cerebellar arterial infarction. No other abnormalities were detected. The patient was treated with oral prednisolone, recovered, and had no further episodes or sequelae. The authors recommend that post-traumatic stroke be considered early in the differential diagnosis of a child with the triad of symptoms seen in the patient described. They suggest that glucocorticoid therapy on a short-term basis may have a place in more rapid resolution of symptoms
SN - 0098-6151
AD - Grandview Hospital, Dayton, Ohio, USA
UR - 8935430
ER -

TY - JOUR
ID - 1306
T1 - Play preferences of typically developing children and children with developmental delays between ages 3 and 7 years. [References]
A1 - Case-Smith,Jane; Kuhaneck,Heather Miller
Y1 - 2008///
N1 - Peer Reviewed Journal: 2008-01654-004
English
Journal; Peer Reviewed Journal
KW - PsycInfo
KW - Rough and Tumble
KW - Childhood Play Behavior
KW - Delayed Development
KW - Measurement
KW - Preferences
KW - Age Differences
KW - Human Sex Differences
KW - Rating
RP - NOT IN FILE
SP - Win
EP - 29
JF - OTJR: Occupation, Participation and Health
VL - .28
IS - 1
N2 - This cross-sectional survey research investigated play preferences of children with and without developmental delays who were between 3 and 7 years old. Parents completed questionnaires regarding their child's play activity and context preferences. Valid results were obtained for 166 children, 83 of whom had developmental delays. Preference ratings were compared by gender, age, and delay status. Play preference did not differ by gender. Rough-and-tumble play and computer/video game play increased with age, whereas object exploration decreased. Children with developmental delays had higher preferences for rough-and-tumble play and object exploration and lower preferences for drawing and coloring, construction, and doll and action figurine play than typically developing children. This comparison of children's play preferences across ages, gender, and developmental status enhances our understanding of how these variables influence children's play. (PsycINFO Database Record (c) 2012 APA, all rights reserved) (journal abstract)
SN - 1539-4492
AD - Case-Smith, Jane: Division of Occupational Therapy, School of Allied Medical Professions, Ohio State University, Columbus, OH, US Kuhaneck, Heather Miller: Sacred Heart University, Fairfield, CT, US
ER -

TY - JOUR
ID - 1307
T1 - Effects of personal and social responsibility on fair play in sports and self-control in school-aged youths. [References]
A1 - Cecchini,Jose A.
Y1 - 2007///
N1 - Peer Reviewed Journal: 2008-00163-002
English
Journal; Peer Reviewed Journal
KW - PsycInfo
KW - Rough and Tumble
KW - Self Control
KW - Social Responsibility
KW - Sports
RP - NOT IN FILE
SP - Dec

EP - 211
JF - European Journal of Sport Science
VL - .7
IS - 4
N2 - In this study, we examined the impact of Hellison's (1995) model for the development of personal and social responsibility on fair play and self-control in youth. One hundred and eighty-six students from three public schools with a mean age of 13.6 years were assigned to one of three treatment groups: experimental group A (n = 63), experimental group B (n = 62), and a control group (n = 61). During 10 one-hour sessions of beginners' five-a-side soccer, group A went through five levels of the programme, whereas group B went through only four levels as the transferral phase was not introduced. Findings revealed that subsequent to the intervention programme, group A improved personal feedback, delayed gratification, self-control, and process self-regulation. Groups A and B showed an improvement in indicators of personal and social responsibility regarding enjoyment and sportsmanship and a decrease in variables related to the drive to win, rough play, contact fouls, and poor sportsmanship. No significant changes were observed in the control group. (PsycINFO Database Record (c) 2012 APA, all rights reserved) (journal abstract)
SN - 1746-1391
AD - Cecchini, Jose A.: Department of Education, University of Oviedo, Oviedo, Spain Montero, Javier: Department of Education, University of Oviedo, Oviedo, Spain Alonso, Alicia: Department of Education, University of Oviedo, Oviedo, Spain Izquierdo, Mikel: Studies, Research and Sport Medicine Centre, Government of Navarra, Spain Contreras, Onofre: Department of Physical Education, University of Castilla-LaMancha, Spain
ER -

TY - JOUR
ID - 1308
T1 - The perpetration of school violence in Taiwan: An analysis of gender, grade level and school type. [References]
A1 - Chen,Ji-Kang; Astor,Ron Avi
Y1 - 2009///
N1 - Peer Reviewed Journal: 2010-01084-002
English
Journal; Peer Reviewed Journal
KW - PsycInfo
KW - Rough and Tumble
KW - Grade Level
KW - Human Sex Differences
KW - School Environment
KW - School Violence
KW - Sociocultural Factors
RP - NOT IN FILE
SP - Dec
EP - 584
JF - School Psychology International
VL - .30
IS - 6
N2 - Using a nationally representative sample in Taiwan, this study aims to describe the prevalence of perpetration of school violence in Taiwan. The study explores how gender, age and school type relate to students' perpetration of violence in an Asian culture context. The sample included 14,022 students from elementary to high schools in grades 4 to 12. Students were given an anonymous questionnaire to report their violent behaviour in schools. The findings presented in this study provide insight on why perpetrators commit violent acts against their peers. Findings suggest the prevalence of perpetration may vary from setting to setting but the influences of age, gender and school type on violence tend to be similar in Taiwan to what theories expect based on studies done in Western cultures. A majority of perpetrators reported engaging in violence stemming from rough and tumble play or from situations where they were initially having fun. Findings suggest that promoting the quality of peer interactions could be a major violence prevention strategy. (PsycINFO Database Record (c) 2012 APA, all rights reserved) (journal abstract)

SN - 0143-0343
AD - Chen, Ji-Kang: Department of Social Work, Chinese University of Hong Kong, Hong Kong, China Astor, Ron Avi: Urban Social Development, University of Southern California, Schools of Social Work and Education, Los Angeles, CA, US
ER -

TY - JOUR
ID - 1309
T1 - In the beginning: American boyhood and the life stories of gay men. [References]
A1 - Cohler,Bertram J.
Y1 - 2009///
N1 - Book: 2009-01644-013
English
Book; Edited Book
KW - PsycInfo
KW - Rough and Tumble
KW - History
KW - Male Homosexuality
KW - Narratives
KW - Stereotyped Attitudes
KW - Childhood Play Behavior
KW - Interests
RP - NOT IN FILE
SP - 474
JF - Hammack, Phillip L [Ed]; Cohler, Bertram J [Ed]
IS - 2009
N2 - (from the chapter) Personal accounts of self-identified gay men writing or telling about their lives during the post-World War II period have emphasized rejection of such gender-stereotyped boyhood interests as "rough-and-tumble" play and "tough-guy" denial of feelings. These accounts often emphasize awareness of oneself as different from other boys and early childhood recognition of unusual interest in other boys and men. This chapter discusses the impact of the American "boy code" from the boy books of the nineteenth century to our own time upon present representations of boyhood in the accounts of men self-identifying as gay. These men represent four postwar generation-cohorts: (1) men who came of age in the 1960s, a time of nascent activism; (2) men who came of age in the 1970s with the emergence of the gay rights movement; (3) men who came of age in the 1980s with the appearance of the AIDS pandemic; and (4) men who came of age in the 1990s, when same-sex desire had become a recognized lifeway among urban men. (PsycINFO Database Record (c) 2012 APA, all rights reserved)
AD - Cohler, Bertram J.: Dept of Comparative Human Development, U Chicago, Chicago, IL, US
ER -

TY - JOUR
ID - 1310
T1 - Connections between the ivory tower and the multicolored world: linking abstract theories of social justice to the rough and tumble of affirmative action
A1 - Crosby,F.J.
A1 - Franco,J.L.
Y1 - 2003///
N1 - Crosby, Faye J. Franco, Jamie L
Personality and social psychology review : an official journal of the Society for Personality and Social Psychology, Inc
9703164
IM
Journal Article. Research Support, Non-U.S. Gov't
English
KW - Medline
KW - Rough and Tumble

KW - Humans
KW - Prejudice
KW - Psychological Theory
KW - Public Policy
KW - Social Justice
RP - NOT IN FILE
SP - 363
EP - 373
JF - Personality & Social Psychology Review
JA - Pers.Soc Psychol.Rev
VL - 7
IS - 4
CY - United States
N2 - This article seeks to combine the social psychologist's interest in articulating and testing concepts with the public policymaker's interest in the effective implementation of specific policies and programs. The first part of the article applies knowledge about distributive and procedural justice to understanding some of the opposition to affirmative action. The application also reveals some lacunae-specifically concerning rule change-in how social psychologists have looked at procedural justice issues. In the second part of the article, we discuss the problem of rule change and propose a set of conceptualizations about the conditions that govern people's reactions to rule change. We end by reflecting on some changes in our studies of procedural justice
SN - 1088-8683
AD - Department of Psychology, University of California, Santa Cruz 95064, USA. fjcrosby@ucsc.edu
UR - 14633472
ER -

TY - JOUR
ID - 1311
T1 - Molecular characterisation of rough strains of Vibrio cholerae isolated from diarrhoeal cases in India and their comparison to smooth strains
A1 - De,K.
A1 - Ramamurthy,T.
A1 - Faruque,S.M.
A1 - Yamasaki,S.
A1 - Takeda,Y.
A1 - Nair,G.B.
A1 - Nandy,R.K.
Y1 - 2004/03/12/
N1 - De, Keya. Ramamurthy, Thandavarayan. Faruque, Shah M. Yamasaki, Shinji. Takeda, Yoshifumi. Nair, G Balakrish. Nandy, Ranjan K
FEMS microbiology letters
fml, 7705721
IM
Journal Article. Research Support, Non-U.S. Gov't
English
KW - Medline
KW - Rough and Tumble
KW - Bacterial Proteins/bi [Biosynthesis]
KW - Bacterial Proteins/ge [Genetics]
KW - Bacteriophages/ge [Genetics]
KW - Bacteriophages/ph [Physiology]
KW - Cholera/mi [Microbiology]
KW - Cholera Toxin/bi [Biosynthesis]
KW - Cholera Toxin/ge [Genetics]
KW - DNA-Binding Proteins/bi [Biosynthesis]
KW - DNA-Binding Proteins/ge [Genetics]
KW - Diarrhea/mi [Microbiology]

KW - Feces/mi [Microbiology]
KW - Gene Expression Regulation
KW - Bacterial
KW - Genes
KW - Genomic Islands/ge [Genetics]
KW - Humans
KW - India
KW - Lysogeny
KW - Molecular Epidemiology
KW - O Antigens/an [Analysis]
KW - O Antigens/ge [Genetics]
KW - Ribotyping
KW - Transcription Factors/bi [Biosynthesis]
KW - Transcription Factors/ge [Genetics]
KW - Transcription
KW - Genetic
KW - Transduction
KW - Vibrio cholerae/cl [Classification]
KW - Vibrio cholerae/ge [Genetics]
KW - Vibrio cholerae/ip [Isolation & Purification]
KW - Virulence Factors/an [Analysis]
KW - Virulence Factors/ge [Genetics]
RP - NOT IN FILE
SP - 23
EP - 30
JF - FEMS Microbiology Letters
JA - FEMS Microbiol.Lett.
VL - 232
IS - 1
CY - Netherlands
N2 - Sixteen of the 18 Vibrio cholerae rough strains isolated from hospitalised diarrhoea patients were found to contain O1 serotype-specific (wbe) genes and all currently known virulence genes. Expression of the regulatory element ToxR was evident in these strains. Cholera toxin production ability of the rough strains was found to be higher (c. three- to five-fold) as compared to the smooth counterparts and this was transcriptionally regulated. Strains exhibiting the rough phenotype were more amenable to the uptake of CTXphi, which led us to consider that the rough phenotype could play a role in the generation of genetic diversity among V. cholerae strains
SN - 0378-1097
AD - Division of Microbiology, National Institute of Cholera and Enteric Diseases, P33 CIT Road, Scheme XM, Kolkata 700 010, India
UR - 15019730
ER -

TY - JOUR
ID - 1312
T1 - Dangerous horseplay
A1 - Dixon,B.
Y1 - 2010/11//
N1 - Dixon, Bernard
The Lancet infectious diseases
101130150
IM
Case Reports. Journal Article
English
KW - Medline
KW - Rough and Tumble
KW - Animals

KW - Communicable Disease Control/mt [Methods]
KW - Corynebacterium diphtheriae/ip [Isolation & Purification]
KW - Corynebacterium diphtheriae/py [Pathogenicity]
KW - Diphtheria/th [Therapy]
KW - Diphtheria/tm [Transmission]
KW - Diphtheria/ve [Veterinary]
KW - Horse Diseases/mi [Microbiology]
KW - Horse Diseases/th [Therapy]
KW - Horse Diseases/tm [Transmission]
KW - Horses
KW - Humans
KW - Male
KW - Zoonoses/mi [Microbiology]
KW - Zoonoses/tm [Transmission]
RP - NOT IN FILE
SP - 741
JF - The Lancet Infectious Diseases
JA - Lancet Infect.Dis.
VL - 10
IS - 11
CY - United States
SN - 1474-4457
AD - dixonadams@blueyonder.co.uk
UR - 21029983
ER -

TY - JOUR
ID - 1313
T1 - Peer status and aggression in boys' groups: developmental and contextual analyses
A1 - Dodge,K.A.
A1 - Coie,J.D.
A1 - Pettit,G.S.
A1 - Price,J.M.
Y1 - 1990/10//
N1 - Dodge, K A. Coie, J D. Pettit, G S. Price, J M
Child development
0372725, d28
IM
Journal Article. Research Support, U.S. Gov't, P.H.S.
English
KW - Medline
KW - Rough and Tumble
KW - Aggression/px [Psychology]
KW - Child
KW - Gender Identity
KW - Humans
KW - Leadership
KW - Male
KW - Peer Group
KW - Personality Development
KW - Play and Playthings
KW - Rejection (Psychology)
KW - Social Dominance
KW - Social Environment
KW - Sociometric Techniques
RP - NOT IN FILE

SP - 1289
EP - 1309
JF - Child Development
JA - Child Dev.
VL - 61
IS - 5
CY - UNITED STATES
N2 - The social transactions of popular, rejected, neglected, and average first- and third-grade boys were examined during their initial encounters with peers. 23 groups of 5 or 6 boys each were observed for 45-min free-play sessions conducted on 5 consecutive days, with sociometric interviews following each session. Social preference in the play groups correlated significantly with classroom social preference after the third and subsequent play sessions for the third graders, and after the fourth and subsequent sessions for the first graders. The observational coding system distinguished 4 types of aggressive behavior that were hypothesized to relate to peer status in different ways. The first, rough play, was not related to peer status. However, rejected boys at both ages displayed significantly higher rates of angry reactive aggression and instrumental aggression than average boys. The relation between bullying and peer status varied with the age of the child. Popular first graders engaged in more bullying than average first graders, but popular third graders did not differ from average in bullying. Other questions concerned the temporal relation between play group behaviors and social preference scores within the group. Socially interactive behaviors anteceded high preference by peers, and low preference in turn led to social isolation in subsequent sessions
SN - 0009-3920
AD - Vanderbilt University, Nashville, TN 37203
UR - 2245725
ER -

TY - JOUR
ID - 1314
T1 - Raising boys without men: How maverick moms are creating the next generation of exceptional men
A1 - Drexler,Peggy
Y1 - 2005///
N1 - Book: 2006-04190-000
English
Book; Authored Book
(Created by PsycINFO) Acknowledgments Introduction The Bad Rap against Mothers Good News from the New Home Front Tapping into Boy Power Finding Their Own Role Models Head-and-Heart Boys The Mental Work of Mothering Staying Connected Collected Families Conclusion: Only Connect Endnotes Index
KW - PsycInfo
KW - Rough and Tumble
KW - Family Structure
KW - Father Absence
KW - Parenting Style
KW - Single Mothers
KW - Sons
KW - Sex Role Attitudes
RP - NOT IN FILE
SP - 224
JF - (2005)
VL - Raising boys without men
N2 - (from the jacket) According to prevailing wisdom, a boy must be raised with a man in the house; otherwise, he's bound to be a failure. That same wisdom tells us that mothers left to their own devices will smother their sons and turn them into sissies, and that sons of unmarried mothers are destined to a life of crime. Peggy Drexler, Ph.D., wasn't quite convinced that this "common" knowledge was accurate or true, so she embarked on a groundbreaking study that compared boys from female-headed households with boys from traditional mom-and-dad families. The results were published in a reputable peer-reviewed journal, and they were so heartening that Dr. Drexler expanded her study into Raising Boys without Men, which is an examination of these boys and their amazing mothers. Rebutting confidently spouted opinions to the contrary,

Dr. Drexler's research shows that boys raised without fathers are socially savvy, generous, caring communicators, while still remaining extremely "boyish"--passionate about sports and adept at roughhousing with friends. These boys' maverick moms are pioneering a new form of parenting which rejects social judgments about family structure and gender stereotype and stresses the importance of communication, community, and love. These brave women have much to teach us about a better way to raise tomorrow's men. (PsycINFO Database Record (c) 2012 APA, all rights reserved)
AD - Drexler, Peggy: Weill Medical College of Cornell University, NY, US
ER -

TY - JOUR
ID - 1315
T1 - Laundry. The rough and tumble of tendering
A1 - Drummond,P.
Y1 - 1986/04/10/
N1 - Drummond, P
The Health service journal
8605800, g4f
H
Journal Article
English
KW - Medline
KW - Rough and Tumble
KW - Contract Services
KW - Financial Management
KW - Great Britain
KW - Laundry Service
KW - Hospital/og [Organization & Administration]
KW - State Medicine
RP - NOT IN FILE
SP - 488
EP - 489
JF - Health Service Journal
JA - Health Serv J
VL - 96
IS - 4994
CY - ENGLAND
SN - 0952-2271
UR - 10276766
ER -

TY - JOUR
ID - 1316
T1 - Unexpected consequences of rough play with friends
A1 - Edmiston,J.
Y1 - 2012/10//
N1 - Edmiston, Julie
JAAPA : official journal of the American Academy of Physician Assistants
9513102
T
Case Reports. Journal Article
English
KW - Medline
KW - Rough and Tumble
KW - Bronchi
KW - Bronchography
KW - Child

KW - Cough/et [Etiology]
KW - Foreign Bodies/et [Etiology]
KW - Foreign Bodies/ra [Radiography]
KW - Foreign Bodies/th [Therapy]
KW - Foreign Bodies
KW - Friends
KW - Humans
KW - Male
KW - Play and Playthings
RP - NOT IN FILE
SP - 59
EP - 60
JF - JAAPA
VL - 25
IS - 10
CY - United States
SN - 1547-1896
UR - 23115875
ER -

TY - JOUR
ID - 1317
T1 - Gender differences in temperament: a meta-analysis
A1 - Else-Quest,N.M.
A1 - Hyde,J.S.
A1 - Goldsmith,H.H.
A1 - Van Hulle,C.A.
Y1 - 2006/01//
N1 - Else-Quest, Nicole M. Hyde, Janet Shibley. Goldsmith, H Hill. Van Hulle, Carol A
Psychological bulletin
0376473, qe8
IM
Journal Article. Meta-Analysis. Research Support, Non-U.S. Gov't
English
KW - Medline
KW - Rough and Tumble
KW - Female
KW - Humans
KW - Male
KW - Personality
KW - Personality Development
KW - Psychological Theory
KW - Sex Factors
KW - Temperament
RP - NOT IN FILE
SP - 33
EP - 72
JF - Psychological Bulletin
JA - Psychol.Bull.
VL - 132
IS - 1
CY - United States
N2 - The authors used meta-analytical techniques to estimate the magnitude of gender differences in mean level and variability of 35 dimensions and 3 factors of temperament in children ages 3 months to 13 years. Effortful control showed a large difference favoring girls and the dimensions within that factor (e.g., inhibitory control: d = -.41, perceptual sensitivity: d = -0.38) showed moderate gender differences favoring girls, consistent with

boys' greater incidence of externalizing disorders. Surgency showed a difference favoring boys, as did some of the dimensions within that factor (e.g., activity: d = 0.33, high-intensity pleasure: d = 0.30), consistent with boys' greater involvement in active rough-and-tumble play. Negative affectivity showed negligible gender differences
SN - 0033-2909
AD - Department of Psychology, University of Wisconsin--Madison, Madison, WI 53706, USA. nmelse@wisc.edu
UR - 16435957
ER -

TY - JOUR
ID - 1319
T1 - Rough-and-tumble play and the development of physical aggression and emotion regulation: A five-year follow-up study. [References]
A1 - Flanders,Joseph L.
Y1 - 2010///
N1 - Peer Reviewed Journal: 2010-05206-001
English
Journal; Peer Reviewed Journal
KW - PsycInfo
KW - Rough and Tumble
KW - Aggressive Behavior
KW - Childhood Development
KW - Childhood Play Behavior
KW - Emotional Regulation
RP - NOT IN FILE
SP - May
EP - 367
JF - Journal of Family Violence
VL - .25
IS - 4
N2 - This is a follow-up to a study demonstrating that rough-and-tumble play was related to physical aggression in the preschool years. Fathers reported on the frequency of father-child rough-and-tumble play interactions, and the degree to which fathers were dominant in the play dyad was observed and coded from play interactions. In this follow-up study, school-aged children's physically aggressive behaviors and emotion regulation abilities were assessed with questionnaires 5 years later. Higher frequencies of father-child rough-and-tumble play in the preschool years were associated with more physical aggression and worse emotion regulation 5 years later for children whose fathers were less dominant, over and above the effects of physical aggression in the preschool years. Rough-and-tumble play was unrelated to these measures among children whose fathers were more dominant during play. This study shows that early rough-and-tumble play continues to be related to children's psychosocial adjustment over time, and that the effect remains moderated by the quality of the father-child relationship during play. (PsycINFO Database Record (c) 2012 APA, all rights reserved) (journal abstract)
SN - 0885-7482
AD - Flanders, Joseph L.: Department of Psychology, McGill University, Montreal, PQ, Canada Simard, Melissa: Department of Psychology, Concordia University, Montreal, PQ, Canada Paquette, Daniel: School of Psychoeducation, University of Montreal, Montreal, PQ, Canada Parent, Sophie: School of Psychoeducation, University of Montreal, Montreal, PQ, Canada Vitaro, Frank: School of Psychoeducation, University of Montreal, Montreal, PQ, Canada Pihl, Robert O.: Department of Psychology, McGill University, Montreal, PQ, Canada Seguin, Jean R.: Department of Psychiatry, University of Montreal, Montreal, PQ, Canada
ER -

TY - JOUR
ID - 1320
T1 - Rough-and-tumble play and the development of externalizing behaviour
A1 - Flanders,Joseph L.
Y1 - 2011///

N1 - Dissertation Abstract: 2011-99120-288
English
Dissertation Abstract
KW - PsycInfo
KW - Rough and Tumble
KW - Behavior Change
KW - Externalization
KW - Recreation
KW - Self Regulation
RP - NOT IN FILE
SP - 7760
JF - Dissertation Abstracts International: Section B: The Sciences and Engineering
VL - .71
IS - 12-B
N2 - Longitudinal research on externalizing disorders has demonstrated that the signs of this form of psychopathology emerge early in life. For example, physically aggressive behaviours can be first observed as early as 18 months of age. Most preschool-aged children develop the self-regulatory abilities to inhibit these problem behaviours and express their aggressive and competitive impulses in alternative, constructive ways. Some children do not develop these abilities and chronic psychosocial difficulties typically result. Several researchers have raised the possibility that Parent-child rough-and-tumble play (RTP) interactions give parents and children an opportunity to develop the child's self-regulatory abilities. Defined here as aggressive behaviours in a play context, RTP often challenges children to modulate their behaviour to ensure their play partner continues to enjoy the activity. In the work that follows, the hypothesis that parent-child RTP can influence the development of behaviour self-regulation is tested. The first study reported here shows that the frequency of mother-child RTP in the preschool is negatively associated with physical aggression later in development, though this effect depends on the presence of a father figure in the home. The second study validates the Rough-and-Tumble Play Scales (RTPS), designed to facilitate the study of RTP and the development of self-regulation. The third study uses observational techniques to highlight the importance of a dominant father in play interactions. RTP frequency was negatively associated levels of physical aggression in dyads in which the father was relatively more dominant. The reverse was true for dyads in which the father was relatively less dominant. Finally, the fourth study showed that this interaction between father-child RTP frequency and father dominance also predict several other aspects of self-regulatory functioning 5 years later. RTP is a highly common, natural, and enjoyable activity for many parents and their children. Collectively, these studies also suggest that under the right conditions, parent-child RTP can influence the development of a child's self-regulatory abilities. While further research is needed, these results hold promise for the development of new interventions for children at risk for externalizing problems and their parents. (PsycINFO Database Record (c) 2012 APA, all rights reserved)
SN - 0419-4217
AD - Flanders, Joseph L.: McGill U., Canada
ER -

TY - JOUR
ID - 1321
T1 - Rough and tumble play quality: Theoretical foundations for a new measure of father-child interaction. [References]
A1 - Fletcher,Richard; StGeorge,Jennifer; Freeman,Emily
Y1 - 2013///
N1 - Peer Reviewed Journal: 2013-18617-002
English
Journal; Peer Reviewed Journal
KW - PsycInfo
KW - Rough and Tumble
KW - Childhood Development
KW - Childhood Play Behavior
KW - Father Child Relations
KW - Cognitive Processes

KW - Father Child Communication
KW - Social Processes
RP - NOT IN FILE
SP - Jun
EP - 759
JF - Early Child Development and Care
VL - .183
IS - 6
N2 - Energetic, competitive, body-contact play (rough and tumble play (RTP)) is commonly observed among young children and is reported as an important feature of father-child relationships. Animal studies have demonstrated positive developmental effects of peer-peer play-wrestling, influencing cognitive and social outcomes. The purpose of this paper is to discuss the nature of RTP between father and child and its relationship to child development and to describe a theoretically informed measure of the quality of father-child RTP. (PsycINFO Database Record (c) 2013 APA, all rights reserved) (journal abstract)
SN - 0300-4430
AD - Fletcher, Richard: Family Action Centre, Faculty of Health, University of Newcastle, Callaghan, NSW, Australia StGeorge, Jennifer: Family Action Centre, Faculty of Health, University of Newcastle, Callaghan, NSW, Australia Freeman, Emily: Family Action Centre, Faculty of Health, University of Newcastle, Callaghan, NSW, Australia
ER -

TY - JOUR
ID - 1322
T1 - The rough and tumble life
A1 - Freeman,J.W.
Y1 - 2005/08//
N1 - Freeman, Jerome W
South Dakota journal of medicine
ujz, 0040162
IM
Editorial
English
KW - Medline
KW - Rough and Tumble
KW - Dissent and Disputes
KW - Humans
KW - Physician-Patient Relations
KW - Professional-Family Relations
RP - NOT IN FILE
SP - 307
EP - 308
JF - South Dakota Journal of Medicine
JA - S.D J Med
VL - 58
IS - 8
CY - United States
SN - 0038-3317
UR - 16425932
ER -

TY - JOUR
ID - 1323
T1 - Biology and the oedipus complex
A1 - Friedman,R.C.
A1 - Downey,J.I.
Y1 - 1995/04//

N1 - Friedman, R C. Downey, J I
The Psychoanalytic quarterly
qd5, 0226661
IM
Journal Article
English
KW - Medline
KW - Rough and Tumble
KW - Aggression/ph [Physiology]
KW - Animals
KW - Brain/ph [Physiology]
KW - Child
KW - Drive
KW - Female
KW - Freudian Theory
KW - Gender Identity
KW - Humans
KW - Instinct
KW - Male
KW - Oedipus Complex
KW - Play and Playthings
KW - Psychosexual Development/ph [Physiology]
KW - Testosterone/ph [Physiology]
RP - NOT IN FILE
SP - 234
EP - 264
JF - Psychoanalytic Quarterly
JA - Psychoanal.Q
VL - 64
IS - 2
CY - UNITED STATES
N2 - Recent observations in the behavioral and neurosciences have raised questions about the ubiquity of the oedipus complex as well as about its significance for psychological development. The authors argue that the construct Freud called the oedipus complex in males is best examined in its component parts. One component--the incestuous wish--does not occur in all individuals. Another component--the boy's urge to engage competitively with other male figures, including the father--does appear to be biologically based in testosterone's effect on the brain and to be manifested in childhood rough and tumble play behavior. It is proposed that reexamination of the oedipus complex in light of recent findings about the brain and behavior is indicated and that play, in particular, can usefully be considered as a separate developmental line
SN - 0033-2828
UR - 7652101
ER -

TY - JOUR
ID - 1324
T1 - Sexual differentiation of childhood play: a contemporary psychoanalytic perspective
A1 - Friedman,R.C.
A1 - Downey,J.I.
Y1 - 2014/01//
N1 - Friedman, Richard C. Downey, Jennifer I
Archives of sexual behavior
8gr, 1273516
IM
Journal Article
English
KW - Medline

KW - Rough and Tumble
RP - NOT IN FILE
SP - 197
EP - 211
JF - Archives of Sexual Behavior
JA - Arch.Sex Behav
VL - 43
IS - 1
CY - United States
N2 - Freud (1905/1953) anchored his theories of unconscious psychological functioning in observations and inferences about childhood sexuality. These ideas remain influential among psychoanalysts today. Much progress subsequently occurred in extra-psychoanalytic research in human sexuality. This included the discovery in 1959 of an entirely new area of psychology: the sexual differentiation of behavior (Phoenix, Goy, Gerall, & Young, 1959; Wallen, 2009). The observations that led to this new field originally concerned the effects of androgen administered prenatally to non-human animals. This early research was compatible with later studies of humans as well. Prenatal androgen influences both erotic and non-erotic behavior, including childhood rough-and-tumble play (RTP). We have previously emphasized the need to integrate this psychoneuroendocrine knowledge with psychoanalytic theory and practice (Friedman, 1988; Friedman & Downey, 2002, 2008a, 2008b). In this article, we discuss additional aspects of the relationship between sexually differentiated childhood play, particularly RTP, and gender differences in psychosexual development and functioning. These topics and other aspects of the sexual differentiation of behavior have been under-emphasized in psychoanalytic thought
SN - 1573-2800
AD - Department of Psychiatry, Cornell/Weill Medical College, New York, NY, USA, rcf2@columbia.edu
UR - 24366660
ER -

TY - JOUR
ID - 1325
T1 - Review of The role of play in human development
A1 - Galloway,David
Y1 - 2011///
N1 - Peer Reviewed Journal: 2011-01061-014
English
Journal; Peer Reviewed Journal
KW - PsycInfo
KW - Rough and Tumble
KW - Behavior Problems
KW - Childhood Play Behavior
KW - Human Development
KW - Learning
KW - Childhood Development
RP - NOT IN FILE
SP - Feb
JF - Child and Adolescent Mental Health
VL - .16
IS - 1
N2 - Reviews the book, The role of play in human development by A. D. Pellegrini (see record 2009-12009-000). Children's play, this book makes clear, can be a serious matter. Views are polarized between disciples who believe that all play is always good for all children, and target-driven practitioners who regard it as a distraction from achieving all-important learning outcomes. Pellegrini defines play as meeting two criteria: first, the means are more important than the ends; second, the orientation is non-functional, as in rough-and-tumble play, where there is no intention to hurt each other, although some aspects resemble genuine fighting. The discussion on the role of play in education should interest teachers who pick up the pieces after children have experienced failure and/or presented significant behavior problems at school. The author draws from a variety of disciplines in submitting theory, rhetoric and ideology to the test of empirical research. The book is well produced, referenced

and indexed and will be essential reading for researchers and practitioners with a particular interest in play, but the wealth of detail may make it less accessible to non-specialists. (PsycINFO Database Record (c) 2012 APA, all rights reserved)
SN - 1475-357X
AD - Galloway, David: School of Education, Durham University, Durham, United Kingdom
ER -

TY - JOUR
ID - 1326
T1 - "You got to have a heart of stone to work here": Coaching, teaching, and "building men" at Eastside High
A1 - Gilmer,Micah
Y1 - 2009///
N1 - Dissertation Abstract: 2009-99171-072
English
Dissertation Abstract
KW - PsycInfo
KW - Rough and Tumble
KW - Blacks
KW - Coaches
KW - Social Sciences
KW - Teaching
KW - Teaching Methods
KW - Coaching
RP - NOT IN FILE
SP - 924
JF - Dissertation Abstracts International Section A: Humanities and Social Sciences
VL - .70
IS - 3-A
N2 - This dissertation is the first study of this length to examine the ways adult African American men build community. It is also a new attempt to describe the pedagogical approaches these men use as educators, and to theorize how their life experiences and personal style impact their work in the classroom. The study centers on a group of African American football coaches, and expands from that critical site to the personal and professional lives of the educators on that staff. Though Black men are often assumed to be emotionally inexpressive, I find that the coaches I work with expressed their most intimate emotion to select groups of trusted partners. These individuals actively built communities of love and support through processes of racial vetting and personal character evaluation, and took extended periods of time to develop close friendships. After reviewing the ways in which the social sciences have generally regarded Black males with varying degrees of contempt, fear and pity, I examine the ways the game of football and the "consensual violence" the football community fostered help build, rather than deconstruct, personal bonds. I use examples of roughhousing and interpersonal confrontation as ways to talk about how, contrary to much of the scholarship on violence in sports, aggression can lead to intimacy. In similar fashion, the coach-educators of Eastside High approached teaching as an exercise underpinned by a need to be brutally honest, or "real," with their "kids." I found that these coaches were critical of their colleagues that insisted upon anything but honesty with students, and championed realistic expectations for students as a key to effective pedagogy. These educators also articulated the importance of engaging students from a communal perspective, particularly in an educational environment that at times can be openly hostile to "at risk" students. While these teachers and staff were often critical of youth culture in very specific ways, they also tried to create spaces for their students to express themselves, even in counter-cultural ways. Lastly, the coaches of Eastside High postulated teacher burnout as a pressing issue that should be at the center of considerations for educational reform. They argued that the testing regime had reduced their work as teachers to a numbers game, and insisted that the support of teachers should be central to systemic reform. (PsycINFO Database Record (c) 2012 APA, all rights reserved)
SN - 0419-4209
AD - Gilmer, Micah: Duke U., US
ER -

TY - JOUR

ID - 1327
T1 - Social influence on the display of sexually dimorphic behavior in rhesus monkeys: isosexual rearing
A1 - Goldfoot,D.A.
A1 - Wallen,K.
A1 - Neff,D.A.
A1 - McBrair,M.C.
A1 - Goy,R.W.
Y1 - 1984/10//
N1 - Goldfoot, D A. Wallen, K. Neff, D A. McBrair, M C. Goy, R W
Archives of sexual behavior
8gr, 1273516
IM
Journal Article. Research Support, U.S. Gov't, P.H.S.
English
KW - Medline
KW - Rough and Tumble
KW - Animals
KW - Female
KW - Gender Identity
KW - Humans
KW - Macaca mulatta
KW - Male
KW - Peer Group
KW - Play and Playthings
KW - Sex Factors
KW - Sexual Behavior
KW - Animal
KW - Social Dominance
KW - Social Environment
KW - Weaning
RP - NOT IN FILE
SP - 395
EP - 412
JF - Archives of Sexual Behavior
JA - Arch.Sex Behav
VL - 13
IS - 5
CY - UNITED STATES
N2 - Groups containing 5 or 6 infant rhesus monkeys and their mothers were formed when the infants were approximately 3 months old by random assignment from an available pool. There were 33 males and 38 females assigned to groups containing infants of both sexes (heterosexual groups); 15 males and 15 females were assigned to groups containing only infants of the same sex (isosexual groups). The social behavior of subjects in each group was observed and recorded during six 50-day periods from 3 months of age to 3 1/2 years of age. Two sexually dimorphic patterns, mounting and presenting, were recorded for each subject as indices of protosexual (i.e., patterns eventually used in adult mating behavior) development. In addition, rough play, a dimorphic response that is not protosexual, was also recorded. Both males and females in the isosexual condition were characterized by a partial inversion of the manifestation of protosexual behavior. Isosexual males showed statistically less foot-clasp mounting and more presenting than heterosexual males. Conversely, isosexually reared females showed statistically more mounting and less presenting than heterosexual females. The effect of rearing animals in same-sex groups was greater on heterotypical than on homotypical protosexual behavior. Among isosexual males, presenting responses deviated from the heterosexual male standard to a greater extent than did mounting. Among isosexual females, mounting behavior deviated from the heterosexual female standard more than presenting. Results suggested that presenting behavior by males was more easily augmented by isosexual rearing conditions than was mounting by females. At no time during the experiments did isosexual females mount at frequencies or at group proportions that were indistinguishable from those of heterosexually reared males. In contrast, males reared isosexually showed average frequencies of presenting that

equaled or exceeded means for females reared heterosexually. While mounting and presenting were both modified by same-sex rearing, rough-and-tumble-play frequencies were not influenced markedly in either sex. However, isosexual males did show statistically higher frequencies of rough play than heterosexual males during the final period of observation (3 to 3 1/2 years of age), and isosexual females showed less rough play than heterosexually reared females during the first year of life. Results support the conclusion that isosexual conditions can have a selective effect on the developmental expression of protosexual responses without altering the probability of display of other sexually dimorphic social behavior.(ABSTRACT TRUNCATED AT 400 WORDS)
SN - 0004-0002
UR - 6542772
ER -

TY - JOUR
ID - 1328
T1 - Methylphenidate and play skills in children with intellectual disability and ADHD. [References]
A1 - Handen,Benjamin L.
Y1 - 2009///
N1 - Peer Reviewed Journal: 2009-06362-001
English
Journal; Peer Reviewed Journal
KW - PsycInfo
KW - Rough and Tumble
KW - Attention Deficit Disorder with Hyperactivity
KW - Childhood Play Behavior
KW - Cognitive Impairment
KW - Drug Therapy
KW - Methylphenidate
KW - Comorbidity
KW - Side Effects (Drug)
KW - Social Behavior
RP - NOT IN FILE
SP - Jan
EP - Mar
JF - Journal of Mental Health Research in Intellectual Disabilities
VL - .2
IS - 1
N2 - Attention-deficit/hyperactivity disorder (ADHD) affects 9-12% of individuals with intellectual disability (ID). Although psychostimulant medication is often the primary treatment modality, little is known regarding the effects of such agents on social interactions and play in this population. Additionally, the role of ADHD symptoms in social and play deficits (beyond that accounted for by ID alone) is not well understood. The first aim of this study was to examine differences in play skills between children with ADHD and ID and non-ADHD/ID controls. The second aim was to determine what, if any, changes in social/play behavior would occur with the use of the stimulant, methylphenidate, among the participants with ADHD/ID. It was hypothesized that the ADHD/ID group would display significantly greater activity level, "rough" play, and more aggression than matched controls. Additionally, it was hypothesized that these deficits would evidence statistically significant improvement with pharmacologic treatment. This study replicates prior work conducted by the authors. (PsycINFO Database Record (c) 2012 APA, all rights reserved) (journal abstract)
SN - 1931-5864
AD - Handen, Benjamin L.: Department of Psychiatiy, University of Pittsburgh School of Medicine, Pittsburgh, PA, US Sagady, Amie E.: School of Public Health, University of Pittsburgh, Pittsburgh, PA, US McAuliffe-Bellin, Sarah: University of Pittsburgh Medical Center, Western Psychiatric Institute and Clinic, Pittsburgh, PA, US
ER -

TY - JOUR
ID - 1329

T1 - Physical play with boys of all ages. [References]
A1 - Harvey,Steve
Y1 - 2011///
N1 - Book: 2010-25612-004
English
Book; Edited Book
KW - PsycInfo
KW - Rough and Tumble
KW - Childhood Play Behavior
KW - Childhood Play Development
KW - Mental Health
KW - Physical Activity
KW - Psychotherapy
KW - Adolescent Development
KW - Childhood Development
KW - Father Child Relations
KW - Human Males
KW - Intervention
KW - Sons
RP - NOT IN FILE
SP - 324
JF - Haen, Craig [Ed]
IS - 2011
N2 - (from the chapter) This chapter will present how physically oriented play experiences can be incorporated into mental health intervention with boys to help increase their active participation. Developmental adaptations for boys from infancy through adolescence will be presented. Of special importance, the engagement of fathers in their son's play, when possible, will be discussed. Physical play is related to the natural rough and tumble play intrinsic to boys' experience growing up, especially with their fathers and brothers. Unfortunately, such full-body, physically interactive play is often overlooked in approaches to psychotherapy, including play therapy, with boys and their families. This chapter is meant to address this oversight. Some of the few approaches that use physical action will be presented as a way to describe elements that contribute to a positive outcome in interventions with boys. (PsycINFO Database Record (c) 2012 APA, all rights reserved)
AD - Harvey, Steve: Child and Adolescent Mental Health Service, New Plymouth, New Zealand
ER -

TY - JOUR
ID - 1330
T1 - Sex differences in rhesus monkey toy preferences parallel those of children
A1 - Hassett,J.M.
A1 - Siebert,E.R.
A1 - Wallen,K.
Y1 - 2008/08//
N1 - Hassett, Janice M. Siebert, Erin R. Wallen, Kim
Hormones and behavior
gb7, 0217764
IM
Journal Article. Research Support, N.I.H., Extramural. Research Support, Non-U.S. Gov't. Research Support, U.S. Gov't, Non-P.H.S.
English
KW - Medline
KW - Rough and Tumble
KW - Animals
KW - Brain/ph [Physiology]
KW - Child
KW - Choice Behavior/ph [Physiology]
KW - Depth Perception/ph [Physiology]

KW - Female
KW - Gonadal Steroid Hormones/ph [Physiology]
KW - Hierarchy
KW - Social
KW - Humans
KW - Macaca mulatta/ph [Physiology]
KW - Macaca mulatta/px [Psychology]
KW - Male
KW - Motor Activity/ph [Physiology]
KW - Orientation/ph [Physiology]
KW - Play and Playthings
KW - Pregnancy
KW - Prenatal Exposure Delayed Effects
KW - Psychomotor Performance/ph [Physiology]
KW - Sex Characteristics
KW - SOCIALIZATION
RP - NOT IN FILE
SP - 359
EP - 364
JF - Hormones & Behavior
JA - Horm.Behav
VL - 54
IS - 3
CY - United States
N2 - Sex differences in toy preferences in children are marked, with boys expressing stronger and more rigid toy preferences than girls, whose preferences are more flexible. Socialization processes, parents, or peers encouraging play with gender-specific toys are thought to be the primary force shaping sex differences in toy preference. A contrast in view is that toy preferences reflect biologically-determined preferences for specific activities facilitated by specific toys. Sex differences in juvenile activities, such as rough-and-tumble play, peer preferences, and infant interest, share similarities in humans and monkeys. Thus if activity preferences shape toy preferences, male and female monkeys may show toy preferences similar to those seen in boys and girls. We compared the interactions of 34 rhesus monkeys, living within a 135 monkey troop, with human wheeled toys and plush toys. Male monkeys, like boys, showed consistent and strong preferences for wheeled toys, while female monkeys, like girls, showed greater variability in preferences. Thus, the magnitude of preference for wheeled over plush toys differed significantly between males and females. The similarities to human findings demonstrate that such preferences can develop without explicit gendered socialization. We offer the hypothesis that toy preferences reflect hormonally influenced behavioral and cognitive biases which are sculpted by social processes into the sex differences seen in monkeys and humans
SN - 1095-6867
AD - Department of Psychology, Emory University, Atlanta, GA 30322, USA
UR - 18452921
ER -

TY - JOUR
ID - 1331
T1 - Who can do it? New science teachers with reform-based teaching strategies
A1 - Hick,Sarah Rachel
Y1 - 2009///
N1 - Dissertation Abstract: 2009-99050-192
English
Dissertation Abstract
KW - PsycInfo
KW - Rough and Tumble
KW - Educational Reform
KW - Science Education
KW - Sciences

KW - Teachers
KW - Strategies
KW - Teaching
RP - NOT IN FILE
SP - 3436
JF - Dissertation Abstracts International Section A: Humanities and Social Sciences
VL - .69
IS - 9-A
N2 - Despite consistent calls for pedagogical changes in the teaching of science since the 1989 publication of Science for All Americans (Rutherford & Ahlgren), most science teachers still teach in traditional ways. This is most surprisingly true even for new science teachers whose teacher education programs have emphasized reform-based instruction. In order to understand how reform-based teaching can be done by new teachers, I examined the experiences and beliefs of three reform-based new secondary science teachers. Research in teacher socialization has shown that three separate phases-"life history," teacher education, and in-service-shape-a teacher's beliefs and practices. Findings from this collective case study suggest that the ability to teach in reform-based ways in the "rough and tumble of practice" (Crawford, 2007) may be linked to a teacher having a "belief in" reform rather than a "knowledge of" reform. Findings from this study also provides evidence of teachers relying on their own learning style as a guide for teaching; drawing on authentic inquiry experiences in their instruction and their conceptions of the nature of science; and benefiting from having digital forms of lessons available, regardless of level of reform, to use as a springboard to crafting reform-based lessons. A possible link is explored between a disposition towards stewardship of the environment and disposition towards stewardship of children as learners. Recommendations are made for research, teacher education, and teacher in-service with regards to selection, preparation, and in-service support of new science teachers who can teach in reform-based ways. (PsycINFO Database Record (c) 2012 APA, all rights reserved)
SN - 0419-4209
AD - Hick, Sarah Rachel: U Minnesota, US
ER -

TY - JOUR
ID - 1332
T1 - Androgen and the development of human sex-typical behavior: rough-and-tumble play and sex of preferred playmates in children with congenital adrenal hyperplasia (CAH)
A1 - Hines,M.
A1 - Kaufman,F.R.
Y1 - 1994/08//
N1 - Hines, M. Kaufman, F R
Child development
0372725, d28
IM
Comparative Study. Journal Article. Research Support, U.S. Gov't, P.H.S.
English
KW - Medline
KW - Rough and Tumble
KW - Adrenal Hyperplasia
KW - Congenital/bl [Blood]
KW - Congenital/px [Psychology]
KW - Aggression
KW - Androgens/ph [Physiology]
KW - Case-Control Studies
KW - Child
KW - Child Behavior
KW - Child Development
KW - Preschool
KW - Choice Behavior
KW - Female
KW - Gender Identity

KW - Humans
KW - Interpersonal Relations
KW - Male
KW - Play and Playthings
KW - Psychosexual Development
KW - Sex Characteristics
KW - Videotape Recording
RP - NOT IN FILE
SP - 1042
EP - 1053
JF - Child Development
JA - Child Dev.
VL - 65
IS - 4
CY - UNITED STATES
N2 - We hypothesized that girls with congenital adrenal hyperplasia (CAH), who experience higher than normal levels of androgens prenatally, would show masculinization of behaviors that show sex differences. Therefore, we examined rough-and-tumble play and sex of preferred playmates in 3-8-year-old children with CAH and in unaffected 3-8-year-old male and female relatives. The hypothesized sex differences in rough-and-tumble play were seen, with unaffected boys showing more rough-and-tumble play than unaffected girls. However, CAH girls were similar to unaffected girls. Additionally, CAH boys showed reduced rough-and-tumble play. In contrast, sex of preferred playmates showed the hypothesized pattern of results. There were sex differences, with unaffected boys preferring boys and unaffected girls preferring girls. In addition, the preferences of girls with CAH were masculinized compared to those of unaffected girls. Results are discussed in terms of possible influences of social, hormonal, and illness factors
SN - 0009-3920
AD - Department of Psychiatry and Biobehavioral Sciences, University of California, Los Angeles, School of Medicine 90024-1759
UR - 7956464
ER -

TY - JOUR
ID - 1333
T1 - Peer interaction patterns among adolescents with autistic spectrum disorders (ASDs) in mainstream school settings
A1 - Humphrey,N.
A1 - Symes,W.
Y1 - 2011/07//
N1 - Humphrey, Neil. Symes, Wendy
Autism : the international journal of research and practice
9713494
IM
Journal Article
English
KW - Medline
KW - Rough and Tumble
KW - Adolescent
KW - Adolescent Behavior/px [Psychology]
KW - Analysis of Variance
KW - Case-Control Studies
KW - Child
KW - Child Development Disorders
KW - Pervasive/px [Psychology]
KW - Dyslexia/px [Psychology]
KW - Female
KW - Humans

KW - Interpersonal Relations
KW - Male
KW - Peer Group
KW - Schools
KW - Social Behavior
RP - NOT IN FILE
SP - 397
EP - 419
JF - Autism
VL - 15
IS - 4
CY - England
N2 - The aim of the current study was to document the peer interaction patterns of students with autistic spectrum disorders in mainstream settings. Structured observations of a group of 38 adolescents with ASD drawn from 12 mainstream secondary schools were conducted over a two-day period and data compared with those of school, age, and gender matched comparison groups of 35 adolescents with dyslexia and 38 with no identified special educational needs (the ASD and dyslexia groups were also matched on SEN provision). Frequency and duration of peer interaction behaviours were coded. In terms of duration, multivariate analyses of variance (MANOVAs) indicated that participants with ASD spent more time engaged in solitary behaviours, less time engaged in co-operative interaction with peers, and more time engaging in reactive aggression towards peers than either comparison group. In terms of frequency, similar patterns emerged, but additionally participants with ASD engaged in fewer instances of rough/vigorous play, and were subject to more instances of social initiation and instrumental verbal aggression by peers than either comparison group. The findings of the current study support the authors' theoretical model of peer group interaction processes for individuals with ASD, and have implications for both social skills training and the development of peer awareness and sensitivity. Limitations are noted
SN - 1461-7005
AD - School of Education, University of Manchester, Manchester, UK. neil.humphrey@manchester.ac.uk
UR - 21454385
ER -

TY - JOUR
ID - 1334
T1 - "Real Men Are Tough Guys": Hegemonic Masculinity and Safety in the Construction Industry. [References]
A1 - Iacuone,David
Y1 - 2005///
N1 - Peer Reviewed Journal: 2005-03171-006
English
Journal; Peer Reviewed Journal
KW - PsycInfo
KW - Rough and Tumble
KW - Business
KW - Health
KW - Masculinity
KW - Occupational Safety
KW - Alcohol Abuse
RP - NOT IN FILE
SP - Win
EP - 266
JF - The Journal of Men's Studies
VL - .13
IS - 2
N2 - Drawing on participant-observation research and 20 interviews with tradesmen, this article explores the link between masculinity and occupational health and safety (OH&S) in the Australian (state of Victoria) construction industry. The main contention of the article is that the hegemonic masculine construct most

commonly found on building sites serves to create a gender hierarchy, and this informal power matrix influences builders' perceptions of OH&S. Within this context, the article examines a range of topics including horseplay, alcohol consumption, the importance of risk and physical prowess, tradesmen's attitudes toward women, and one-upmanship. (PsycINFO Database Record (c) 2013 APA, all rights reserved) (journal abstract)
SN - 1060-8265
AD - Iacuone, David: RMIT University, Melbourne, VIC, Australia
ER -

TY - JOUR
ID - 1335
T1 - "Rough and tumble" play: Lessons in life. [References]
A1 - Jarvis,Pam
Y1 - 2006///
N1 - Peer Reviewed Journal: 2008-06798-038
English
Journal; Peer Reviewed Journal
KW - PsycInfo
KW - Rough and Tumble
KW - Childhood Development
KW - Childhood Play Behavior
KW - Human Sex Differences
KW - Narratives
RP - NOT IN FILE
SP - 330
EP - 346
JA - Evolutionary Psychology
VL - .4 2006, pp. 330-346.
N2 - This paper focuses upon the developmental role of Rough and Tumble (R&T) play with particular attention to the narratives that children use to underpin such activities, and to gender differences within these. The empirical research focused upon the R&T play of children in the early years department of a suburban primary school in Northern England. The children's playtime activities were ethnographically observed over a period of eighteen calendar months, encompassing five school terms. The effects of evolution, biology and culture are recognized in the approach taken to the analysis of data. Findings indicated that the narratives underpinning R&T play were socially complex and highly gendered, and that mixed gender R&T play in particular could be theorized to mirror and simplify aspects of complex, gendered adult interaction. (PsycINFO Database Record (c) 2012 APA, all rights reserved) (journal abstract)
SN - 1474-7049
AD - Jarvis, Pam: Carnegie Faculty of Sports and Education, Carnegie Hall, Leeds Metropolitan University, Leeds, United Kingdom
ER -

TY - JOUR
ID - 1336
T1 - Monsters, magic and Mr Psycho: A biocultural approach to rough and tumble play in the early years of primary school. [References]
A1 - Jarvis,Pam
Y1 - 2007///
N1 - Peer Reviewed Journal: 2007-13989-007
English
Journal; Peer Reviewed Journal
KW - PsycInfo
KW - Rough and Tumble
KW - Childhood Development
KW - Childhood Play Behavior
KW - Elementary Schools
KW - Narratives

KW - Attention
KW - Interpersonal Communication
RP - NOT IN FILE
SP - Jul
EP - 188
JF - Early Years: An International Journal of Research and Development
VL - .27
IS - 2
N2 - This paper focuses upon the developmental role of rough and tumble (R&T) play with particular attention to the narratives that children use to underpin such activities. A review of the literature suggests that current early years research and practice pays scant attention to children's outdoor free play activities. A piece of original research is described in which playtime activities were ethnographically observed over an 18-month period. A biocultural approach was used in the analysis of data, in an attempt to encompass the roles of biology, evolution and culture in human development. Findings indicate that the narratives underpinning R&T play were socially complex and highly gendered, and it is suggested that much developmental learning may occur within such free play activities. (PsycINFO Database Record (c) 2012 APA, all rights reserved) (journal abstract)
SN - 0957-5146
AD - Jarvis, Pam: Bradford College, West Yorkshire, United Kingdom
ER -

TY - JOUR
ID - 1337
T1 - Dangerous activities within an invisible playground: A study of emergent male football play and teachers' perspectives of outdoor free play in the early years of primary school. [References]
A1 - Jarvis,Pam
Y1 - 2007///
N1 - Peer Reviewed Journal: 2007-12897-002
English
Journal; Peer Reviewed Journal
KW - PsycInfo
KW - Rough and Tumble
KW - Childhood Play Behavior
KW - Human Males
KW - Primary School Students
KW - Soccer
KW - Teacher Attitudes
RP - NOT IN FILE
SP - Oct
EP - 259
JA - International Journal of Early Years Education
VL - .15
IS - 3
N2 - This research was carried out during a study which focused upon the rough and tumble play of children in the early years department of a suburban primary school in northern England. The child sample's playtime activities were ethnographically observed over a period of 18 calendar months, during which time interviews were also carried out with the children's class teachers and several other adults taking part in the children's daily school routines. It was found that the narratives created by a cohort of four-and-a-half- to six-and-a-half-year-old boys around their football (soccer) play were socially and symbolically complex, providing evidence that such play is a highly valuable developmental activity. However, the interview data indicated that owing principally to very low adult:child ratios in playground supervision, the focus of the adults was strongly directed towards negative aspects of outdoor free play. (PsycINFO Database Record (c) 2012 APA, all rights reserved) (journal abstract)
SN - 0966-9760
AD - Jarvis, Pam: Bradford College, Bradford, WYK, United Kingdom
ER -

TY - JOUR
ID - 1338
T1 - Tracing the Milky Way's Rough-and-Tumble Youth
A1 - Jayawardhana,R.
Y1 - 1992/11/27/
N1 - Jayawardhana, R
Science (New York, N.Y.)
0404511, uj7
Journal Article
English
KW - Medline
KW - Rough and Tumble
RP - NOT IN FILE
SP - 1439
EP - 1440
JF - Science
VL - 258
IS - 5087
CY - United States
SN - 0036-8075
UR - 17755103
ER -

TY - JOUR
ID - 1339
T1 - Children's play as a context for managing physiological arousal and learning emotion regulation. [References]
A1 - LaFreniere,Peter
Y1 - 2013///
N1 - Peer Reviewed Journal: 2013-35574-002
English
Journal; Peer Reviewed Journal
KW - PsycInfo
KW - Rough and Tumble
KW - Childhood Play Behavior
KW - Emotional Regulation
KW - Emotional States
KW - Physiological Arousal
KW - Age Differences
KW - Aggressive Behavior
KW - Human Sex Differences
RP - NOT IN FILE
SP - 2013, pp
EP - 2013,204
JF - Psihologijske Teme
VL - .22
IS - 2
N2 - In this paper I examine children's play as a context for managing physiological arousal and learning to regulate strong emotions. I define emotion regulation as the process by which children monitor and control their emotional states and their expression to adapt to different social situations or demands. Age trends and gender differences in emotion regulation problems and competencies are described. I then review the development of play, deprivation studies, and the biological functions of different forms of play in primates before discussing children's play. Vigorous social play benefits children by promoting the development of communication, perspective-taking and emotion regulation skills. For boys especially, rough-and-tumble play in early childhood provides a scaffold for learning emotion regulation skills related to managing anger and aggression. (PsycINFO Database Record (c) 2013 APA, all rights reserved) (journal abstract)

SN - 1332-0742
AD - LaFreniere, Peter: Department of Psychology, University of Maine, Orono, ME, US
ER -

TY - JOUR
ID - 1340
T1 - Review of Brain Gender. [References]
A1 - Leckman,James F.
Y1 - 2005///
N1 - Peer Reviewed Journal: 2005-06749-024
English
Journal; Peer Reviewed Journal
KW - PsycInfo
KW - Rough and Tumble
KW - Brain
KW - Gender Identity
KW - Hormones
KW - Human Body
KW - Human Sex Differences
KW - Biology
KW - Mental Rotation
KW - Violent Crime
RP - NOT IN FILE
SP - Spr
EP - 636
JF - Journal of the American Psychoanalytic Association
JA - J Am Psychoanal.Assoc.
VL - .53
IS - 2
N2 - Reviews the book "Brain Gender" by Melissa Hines (2004). Hines displays an impressive expertise in the biological and behavioral determinants of sex differences. From the outset she reminds us that men are more likely than women to be incarcerated for violent crimes, to become university professors, to hold positions of political power, and to have above-average incomes. She also reviews a large body of scientific literature that indicates that the magnitude of sex differences is most marked for, in descending order, core gender identity, sexual orientation, height, mental rotation of three-dimensional objects (males are generally better at this task), toy preferences, physical aggression, and rough-and-tumble play as children. Fascinated by these differences, she retraces her interest in exploring the role that biological factors, particularly the gonadal hormones androgen and estrogen, play in brain development. In sum, Hines finds little evidence to support claims that hormonal differences can account for the numerical predominance of men in jails, politics, or the academy, or their relative absence in caretaking roles. (PsycINFO Database Record (c) 2012 APA, all rights reserved)
SN - 0003-0651
AD - Leckman, James F.: Yale University Child Study Center, New Haven, CT, US
ER -

TY - JOUR
ID - 1341
T1 - Survey of accidents in a university day-care center
A1 - Lee,E.J.
A1 - Bass,C.
Y1 - 1990/01//
N1 - Lee, E J. Bass, C
Journal of pediatric health care : official publication of National Association of Pediatric Nurse Associates & Practitioners
jml, 8709735
N

Journal Article
English
KW - Medline
KW - Rough and Tumble
KW - Accidents/sn [Statistics & Numerical Data]
KW - Child Day Care Centers
KW - Child
KW - Preschool
KW - Female
KW - Humans
KW - Male
KW - Nurse Practitioners
KW - United States
KW - Universities
RP - NOT IN FILE
SP - 18
EP - 23
JF - Journal of Pediatric Health Care
JA - J Pediatr Health Care
VL - 4
IS - 1
CY - UNITED STATES
N2 - Accident reporting provides useful data for the analysis of injuries in day-care centers. This article reports the results of a survey study conducted to analyze accidental injuries in a large university day-care center in Southern California. Data were collected from individual student accident report forms filed by the teachers for the 1987 calendar year. A total of 103 accident reports were analyzed. Fifty-seven children ranging in age from 6 months to 6 years were involved in 103 accidents. The results of the study revealed that the highest number of accidents occurred among children 3 and 4 years of age. The majority of the accidents occurred during horseplay or while the children were running or fighting. Although no deaths or permanent disability occurred, this number of accidents do warrant the need for safety education
SN - 0891-5245
UR - 2299555
ER -

TY - JOUR
ID - 1342
T1 - Psychoanalytic preventions/interventions and playing "rough-and-tumble" games: Alternatives to medical treatments of children suffering from ADHD. [References]
A1 - Leuzinger-Bohleber,Marianne
Y1 - 2010///
N1 - Peer Reviewed Journal: 2011-00086-007
English
Journal; Peer Reviewed Journal
KW - PsycInfo
KW - Rough and Tumble
KW - Attention Deficit Disorder with Hyperactivity
KW - Intervention
KW - Prevention
KW - Psychoanalysis
KW - Therapeutic Processes
RP - NOT IN FILE
SP - 2010, pp
EP - 2010,338
JF - International Journal of Applied Psychoanalytic Studies
VL - .7
IS - 4

N2 - Much more clinical and empirical research is needed on all aspects of this serious spectrum of Attention deficit/hyperactivity disorders (ADHD). Nevertheless, given the current state of affairs, we reach the following conclusion: taking into account neurobiological, (unconscious) biographical as well as societal factors, we are convinced that contemporary psychoanalysis-due to its richness of conceptualization and clinical experience-offers a unique perspective on the complexities of ADHD and thus provides a means to achieve adequate prevention in families as well as in public institutions. More than other psychotherapeutic approaches, psychoanalysis offers suitable "corrective" experiences in the therapeutic relationships with these children and thus the possibility to "correct" or at least mitigate the effects of failed early development. Psychoanalytic treatment should be considered one of the promising therapeutic choices for treating children with ADHD. (PsycINFO Database Record (c) 2013 APA, all rights reserved)
SN - 1742-3341
ER -

TY - JOUR
ID - 1343
T1 - Pretend and physical play: Links to preschoolers' affective social competence. [References]
A1 - Lindsey,Eric W.
Y1 - 2013///
N1 - Peer Reviewed Journal: 2013-29322-004
English
Journal; Peer Reviewed Journal
KW - PsycInfo
KW - Rough and Tumble
KW - Childhood Play Behavior
KW - Preschool Students
KW - Social Skills
KW - Childhood Development
KW - Emotionality (Personality)
RP - NOT IN FILE
SP - Jul
EP - 360
JF - Merrill-Palmer Quarterly
VL - .59
IS - 3
N2 - This study investigated different forms of pretend and physical play as predictors of preschool children's affective social competence (ASC). Data were collected from 122 preschool children (57 boys, 65 girls; 86 European American, 9 African American, 17 Hispanic, and 10 other ethnicity) over a 2-year period. Children participated in emotion knowledge interviews, mothers rated children's emotion regulation skill, and observations were conducted of children's emotional expressiveness with peers in both Years 1 and 2. Naturalistic observations of children's peer play behavior were conducted to assess the proportion of time children spend in pretend and physical play in Year 1. Analyses revealed that sociodramatic play predicted children's emotional expressiveness, emotion knowledge, and emotion regulation 1 year later, after controlling for Year 1 ASC skills. Rough-and-tumble play predicted children's emotional expressiveness and emotion regulation 1 year later, whereas exercise play predicted only emotion regulation. Some associations between sociodramatic play and rough- and-tumble play and children's ASC were moderated by gender. (PsycINFO Database Record (c) 2013 APA, all rights reserved) (journal abstract)
SN - 0272-930X
AD - Lindsey, Eric W.: Penn State Berks, Reading, PA, US Colwell, Malinda J.: Texas Tech University, Lubbock, TX, US
ER -

TY - JOUR
ID - 1344
T1 - Preschool teachers' views of active play. [References]
A1 - Logue,Mary Ellin; Harvey,Hattie
Y1 - 2010///

N1 - Peer Reviewed Journal: 2010-11634-003
English
Journal; Peer Reviewed Journal
KW - PsycInfo
KW - Rough and Tumble
KW - Childhood Play Behavior
KW - Preschool Teachers
KW - Teacher Attitudes
KW - Human Sex Differences
RP - NOT IN FILE
SP - Jan
EP - 49
JF - Journal of Research in Childhood Education
VL - .24
IS - 1
N2 - This study surveyed 98 teachers of 4-year-olds about dramatic play in their classrooms and about their attitudes and practices about rough-and-tumble play. Gender differences emerged in the nature of dramatic play reported and in the ways in which teachers interacted with children engaged in different forms of dramatic play. Teachers also reported their coursework in early childhood education as the greatest influence on their beliefs and attitudes about rough-and-tumble play. The findings have implications for curriculum planning, school behavior policies, and teacher education programs. (PsycINFO Database Record (c) 2012 APA, all rights reserved) (journal abstract)
SN - 0256-8543
AD - Logue, Mary Ellin: University of Maine, Orono, ME, US Harvey, Hattie: University of Denver, Denver, CO, US
ER -

TY - JOUR
ID - 1345
T1 - "Rough and tumble" social interactions: An exploration of a new behavioral construct
A1 - Luckner,Amy Elizabeth
Y1 - 2008///
N1 - Dissertation Abstract: 2008-99020-379
English
Dissertation Abstract
KW - PsycInfo
KW - Rough and Tumble
KW - Constructivism
KW - Social Interaction
KW - Sociometric Tests
KW - Aggressive Behavior
KW - Antisocial Behavior
KW - Popularity
KW - Prosocial Behavior
RP - NOT IN FILE
SP - 4866
JF - Dissertation Abstracts International: Section B: The Sciences and Engineering
VL - .68
IS - 7-B
N2 - The purpose of this study was to investigate a new behavioral construct, "rough and tumble" social interactions, that encompasses behaviors that are superficially similar to relational aggression, but are playful in nature. This initial study examined the validity of the rough and tumble social interactions construct by investigating whether young adults differentiate between relational aggression and rough and tumble social interactions, and whether engagement in relational aggression and rough and tumble social interactions differentially predicts prosocial and antisocial behavior, and perceived and sociometric popularity. Participants, 144 sorority members and 109 fraternity members from a midwestern university, completed self-report and peer

nomination measures during one 30-45 minute session. Peer nominations of rough and tumble social interactions and relational aggression were moderately, positively correlated, demonstrating overlapping but distinct constructs. In addition, regression analyses revealed that after controlling for shared variance, peer nominations of relational aggression and rough and tumble social interactions differentially predicted prosocial behaviors, depression, and borderline features: relational aggression negatively predicted prosocial behavior and positively predicted depression and borderline features whereas rough and tumble social interactions positively predicted prosocial behavior, negatively predicted depression, and was not related to borderline features. Differential relations were also found for perceived and sociometric popularity. Relational aggression was negatively related to both variables whereas rough and tumble social interactions demonstrated positive relations. Overall, the results of this initial investigation suggest that rough and tumble social interactions is a valid construct, distinct from relational aggression, that provides unique information in the prediction of antisocial behaviors, prosocial behaviors, acceptance, and popularity. (PsycINFO Database Record (c) 2012 APA, all rights reserved)
SN - 0419-4217
AD - Luckner, Amy Elizabeth: U Minnesota, US
ER -

TY - JOUR
ID - 1346
T1 - Method for obtaining structure and interactions from oriented lipid bilayers
A1 - Lyatskaya,Y.
A1 - Liu,Y.
A1 - Tristram-Nagle,S.
A1 - Katsaras,J.
A1 - Nagle,J.F.
Y1 - 2001/01//
N1 - Lyatskaya, Y. Liu, Y. Tristram-Nagle, S. Katsaras, J. Nagle, J F
Physical review. E, Statistical, nonlinear, and soft matter physics
101136452
IM
Journal Article. Research Support, U.S. Gov't, Non-P.H.S.. Research Support, U.S. Gov't, P.H.S.
English
KW - Medline
KW - Rough and Tumble
KW - Lipid Bilayers/ch [Chemistry]
KW - Lipid Bilayers/me [Metabolism]
KW - Models
KW - Statistical
KW - Protein Structure
KW - Tertiary
KW - Scattering
KW - Radiation
KW - Thermodynamics
KW - X-Ray Diffraction/mt [Methods]
KW - X-Rays
RP - NOT IN FILE
SP - 011907
JF - Physical Review E
JA - Phys Rev E Stat.Nonlin.Soft.Matter Phys
VL - Statistical, Nonlinear, & Soft Matter Physics. 63
IS - 1 Pt 1
CY - United States
N2 - Precise calculations are made of the scattering intensity I(q) from an oriented stack of lipid bilayers using a realistic model of fluctuations. The quantities of interest include the bilayer bending modulus Kc, the interbilayer interaction modulus B, and bilayer structure through the form factor F(qz). It is shown how Kc and B may be obtained from data at large q(z) where fluctuations dominate. Good estimates of F(qz) can be made

over wide ranges of q(z) by using I(q) in q regions away from the peaks and for q(r) not equal0 where details of the scattering domains play little role. Rough estimates of domain sizes can also be made from smaller q(z) data. Results are presented for data taken on fully hydrated, oriented DOPC bilayers in the L(alpha) phase. These results illustrate the advantages of oriented samples compared to powder samples
SN - 1539-3755
UR - 11304287
ER -

TY - JOUR
ID - 1347
T1 - Experienced and expected similarity to same-gender peers: Moving toward a comprehensive model of gender segregation. [References]
A1 - Martin,Carol Lynn; Fabes,Richard
Y1 - 2011///
N1 - Peer Reviewed Journal: 2011-16716-012
English
Journal; Peer Reviewed Journal
KW - PsycInfo
KW - Rough and Tumble
KW - Attitude Similarity
KW - Childhood Development
KW - Experiences (Events)
KW - Heuristics
KW - Human Sex Differences
KW - Peers
RP - NOT IN FILE
SP - Sep
EP - 434
JF - Sex Roles
VL - .65
IS - 5-6
N2 - Gender segregation is often explained by children being interested in interacting with other children who behave similarly to themselves. Children's beliefs about girls and boys (i.e., their gender cognitions) may also play a role in gender segregation, but this idea has received little attention. In this study, we proposed a model of gender segregation that included similarity on gender-typed behavioral qualities (e.g., rough and tumble play) and gender cognitions concerning perceived similarity to same-gender others, and we assessed whether this more comprehensive heuristic model predicted observed peer interactions in young U.S. children (n = 74; M age = 51 m; middle-class families). A multi-method design was employed including observations of behavior and child reports of gender cognitions. Support was found for the linkages proposed in this comprehensive model for boys; partial support was found for girls. Specifically, the inclusion of gender cognitions was supported for both genders: gender cognitions about perceived similarity related to interactional partner choices for both girls and boys, and accounted for variance in observed partner choices even after behavioral similarity was included in the model. The traditional link concerning behavioral similarity on rough-and-tumble play predicted boys' but not girls' interactions. The findings extend knowledge about the role of social cognitions in social behavior, and are consistent with ideas proposed by gender schema theory and other constructivist theories. (PsycINFO Database Record (c) 2012 APA, all rights reserved) (journal abstract)
SN - 0360-0025
AD - Martin, Carol Lynn: School of Social and Family Dynamics, Arizona State University, Tempe, AZ, US Fabes, Richard A.: School of Social and Family Dynamics, Arizona State University, Tempe, AZ, US Hanish, Laura: School of Social and Family Dynamics, Arizona State University, Tempe, AZ, US Leonard, Stacie: School of Social and Family Dynamics, Arizona State University, Tempe, AZ, US Dinella, Lisa M.: School of Social and Family Dynamics, Arizona State University, Tempe, AZ, US
ER -

TY - JOUR
ID - 1348

T1 - Rough play: Pediatric concussions require early recognition & proper field treatment
A1 - Meredith,M.
Y1 - 1956/07//
N1 - Meredith, Mark
JEMS : a journal of emergency medical services
8102138, irc
H
Journal Article
English
KW - Medline
KW - Rough and Tumble
KW - Adolescent
KW - Athletic Injuries/di [Diagnosis]
KW - Athletic Injuries/th [Therapy]
KW - Brain Concussion/di [Diagnosis]
KW - Brain Concussion/th [Therapy]
KW - Child
KW - Emergency Medical Services
KW - Emergency Treatment/mt [Methods]
KW - Humans
RP - NOT IN FILE
SP - 52
EP - 54
JF - Journal of Emergency Medical Services
JA - J Emerg.Med Serv JEMS.
VL - 37
IS - 4
CY - United States
SN - 0197-2510
AD - Vanderbilt University Medical Center, USA. mark.meredith@vanderbilt.edu
UR - 22792623
ER -

TY - JOUR
ID - 1349
T1 - Is this the mob or healthcare? K.C. fraud has executives bemoaning prosecutors' rough-and-tumble tactics
A1 - Moore,J.D.,Jr.
Y1 - 1962/09/06/
N1 - Moore, J D Jr
Modern healthcare
nfa, 7801798
H
News
English
KW - Medline
KW - Rough and Tumble
KW - Aggression
KW - Criminal Law
KW - Fraud/lj [Legislation & Jurisprudence]
KW - Government Agencies/st [Standards]
KW - Humans
KW - Medicare/lj [Legislation & Jurisprudence]
KW - Missouri
KW - Social Justice
KW - United States
RP - NOT IN FILE

SP - 60
JF - Modern Healthcare
JA - Mod.Healthc.
VL - 29
IS - 25
CY - UNITED STATES
SN - 0160-7480
UR - 10538602
ER -

TY - JOUR
ID - 1350
T1 - [Inappropriately rough play behaviour and predatory attacks against people by a tomcat. A case report]. [German]
A1 - Morber,M.
A1 - Bartels,A.
A1 - Erhard,M.H.
Y1 - 2013///
N1 - Morber, M. Bartels, A. Erhard, M H
Tierarztliche Praxis. Ausgabe K, Kleintiere/Heimtiere
cye, 9717383
IM
English Abstract. Journal Article
German
KW - Medline
KW - Rough and Tumble
RP - NOT IN FILE
SP - 117
EP - 122
JF - Tierarztliche Praxis
JA - Tierarztl.Prax.Ausg K Klientiere.Heimtiere.
VL - Ausgabe K, Kleintiere/Heimtiere. 41
IS - 2
CY - Germany
N2 - The owner of a 6-months-old tomcat came to seek help because the cat had attacked her face on a near-daily basis. Through a detailed behavioural history, the cat's behaviour was diagnosed as human-directed predatory attack behaviour, play-related aggression and reduced motor as well as emotional self-control. Within a few weeks, behavioural therapy produced a significant improvement. After 5 months of therapy, the cat showed neither predatory attacks nor inappropriately rough or aggressive behaviour in play towards its owner or other humans
SN - 1434-1239
AD - Matina Morber, Tierarztliche Praxis, (Tatigkeitsschwerpunkt Verhaltenstherapie), Wendelsteinstrase 19, Erding. info@tier-verhalten-erding.de
UR - 23608967
ER -

TY - JOUR
ID - 1351
T1 - Detrusorectomy with Mitrofanoff stoma
A1 - Morecroft,J.A.
A1 - Searles,J.
A1 - MacKinnon,A.E.
Y1 - 1996/12//
N1 - Morecroft, J A. Searles, J. MacKinnon, A E
European journal of pediatric surgery : official journal of Austrian Association of Pediatric Surgery ... [et al] = Zeitschrift fur Kinderchirurgie

azo, 9105263
IM
Journal Article
English
KW - Medline
KW - Rough and Tumble
KW - Adolescent
KW - Appendix/su [Surgery]
KW - Child
KW - Preschool
KW - Female
KW - Follow-Up Studies
KW - Humans
KW - Ileum/su [Surgery]
KW - Male
KW - Postoperative Complications/pp [Physiopathology]
KW - Urinary Bladder/pp [Physiopathology]
KW - Urinary Bladder/su [Surgery]
KW - Urinary Bladder
KW - Neurogenic/pp [Physiopathology]
KW - Neurogenic/su [Surgery]
KW - Urinary Diversion/mt [Methods]
KW - Urodynamics/ph [Physiology]
RP - NOT IN FILE
SP - 30
EP - 31
JF - European Journal of Pediatric Surgery
JA - Eur.J Pediatr Surg.
VL - 6 Suppl 1
CY - GERMANY
N2 - The clinical and social benefits derived from bladder augmentation are well known. Most procedures involve the implantation of gastric or intestinal segments into the bladder. These have problems of the acid-haematuria syndrome, mucous production, urinary infection, stone formation and metabolic disturbances. Lang and colleagues presented their experiences in 1994 with detrusorectomy alone as an alternative procedure. We have performed detrusorectomy with Mitrofanoff stoma on 9 patients, protecting the denuded bladder mucosa with omentum. This represents 50% of the bladder reconstructions managed over an 18-month period. Follow-up urodynamics studies have demonstrated a compliant low-pressure system of good capacity in all but one case. Apart from this failure, all the rest are continent. One boy suffered a ruptured bladder due to rough play and another suffered excruciating bladder pain on drainage. He was managed by the instillation of bupivicaine (4 mg/kg) twice daily. Successful management of cases depends on intensive support of the outreach nursing service
SN - 0939-7248
AD - Department of Paediatric Surgery, Sheffield Children's Hospital, Western Bank, UK
UR - 9008817
ER -

TY - JOUR
ID - 1352
T1 - A novel NMDA receptor glycine-site partial agonist, GLYX-13, has therapeutic potential for the treatment of autism. [Review]
A1 - Moskal,J.R.
A1 - Burgdorf,J.
A1 - Kroes,R.A.
A1 - Brudzynski,S.M.
A1 - Panksepp,J.
Y1 - 2011/10//

N1 - Moskal, Joseph R. Burgdorf, Jeffrey. Kroes, Roger A. Brudzynski, Stefan M. Panksepp, Jaak
Neuroscience and biobehavioral reviews
oa7, 7806090
IM
Journal Article. Research Support, Non-U.S. Gov't. Review
English
KW - Medline
KW - Rough and Tumble
KW - Animals
KW - Autistic Disorder/dt [Drug Therapy]
KW - Autistic Disorder/ge [Genetics]
KW - Autistic Disorder/px [Psychology]
KW - Brain Chemistry/ge [Genetics]
KW - Brain Chemistry/ph [Physiology]
KW - Endorphins/ph [Physiology]
KW - Gene Expression/ph [Physiology]
KW - Humans
KW - Oligopeptides/tu [Therapeutic Use]
KW - Play and Playthings/px [Psychology]
KW - Rats
KW - Receptors
KW - Glycine/ag [Agonists]
KW - N-Methyl-D-Aspartate/ag [Agonists]
KW - Vocalization
KW - Animal/de [Drug Effects]
KW - Animal/ph [Physiology]
RP - NOT IN FILE
SP - 1982
EP - 1988
JF - Neuroscience & Biobehavioral Reviews
JA - Neurosci Biobehav.Rev
VL - 35
IS - 9
CY - United States
N2 - Deficits in social approach behavior, rough-and-tumble play, and speech abnormalities are core features of autism that can be modeled in laboratory rats. Human twin studies show that autism has a strong genetic component, and a recent review has identified 99 genes that are dysregulated in human autism. Bioinformatic analysis of these 99 genes identified the NMDA receptor complex as a significant interaction hub based on protein-protein interactions. The NMDA receptor glycine site partial agonist d-cycloserine has been shown to treat the core symptom of social withdrawal in autistic children. Here, we show that rats selectively bred for low rates of play-induced pro-social ultrasonic vocalizations (USVs) can be used to model certain core symptoms of autism. Low-line animals engage in less social contact time with conspecifics, show lower rates of play induced pro-social USVs, and show an increased proportion of non-frequency modulated (i.e. monotonous) ultrasonic vocalizations, compared to non-selectively bred random-line animals. Gene expression patterns in the low-line animals show significant enrichment in autism-associated genes and the NMDA receptor family was identified as a significant hub. Treatment of low-line animals with the NMDAR glycine site partial agonist GLYX-13 rescued the deficits in play-induced pro-social 50-kHz and reduced monotonous USVs. Thus, the NMDA receptor has been shown to play a functional role in autism, and GLYX-13 shows promise for the treatment of autism. We dedicate this paper to Ole Ivar Lovaas (May 8, 1927-August 2, 2010), a pioneer in the field of autism. Copyright 2011 Elsevier Ltd. All rights reserved
SN - 1873-7528
AD - Falk Center for Molecular Therapeutics, Department of Biomedical Engineering, Northwestern University, Evanston, IL 60201, USA. j-moskal@nothwestern.edu
UR - 21718719
ER -

TY - JOUR
ID - 1353
T1 - Solitary-functional play and solitary-pretend play: Another look at the construct of solitary-active behavior using playground observations. [References]
A1 - Nelson,Larry J.
Y1 - 2008///
N1 - Peer Reviewed Journal: 2008-15016-005
English
Journal; Peer Reviewed Journal
KW - PsycInfo
KW - Rough and Tumble
KW - Childhood Play Behavior
KW - Playgrounds
KW - Pretend Play
KW - Social Adjustment
RP - NOT IN FILE
SP - Nov
EP - 831
JF - Social Development
VL - .17
IS - 4
N2 - Although the construct of solitary-active behavior calls for the aggregation of solitary-functional play and solitary-pretend play, there is little empirical support for combining them into one construct. Furthermore, little work has been done in early childhood to examine these behaviors on the playground. The purpose of this study was to observe children's behavior on the playground to explore whether solitary-functional and solitary-pretend behaviors are related to one another and to other indices of social adjustment/maladjustment. Examining a sample of 361 preschoolers, results revealed that (1) solitary-functional and solitary-pretend play were not related, (2) solitary-functional play was associated with solitary-passive and reticent behaviors, as well as less social play, co-operative rough and tumble play, sociable/friendliness, assertiveness, and lower peer acceptance, and (3) solitary-pretend play was linked to lower peer acceptance and more social maladjustment, including venting, reactive aggression (but not proactive aggression), active exclusion, victimization, and being distractible. (PsycINFO Database Record (c) 2012 APA, all rights reserved) (journal abstract)
SN - 0961-205X
AD - Nelson, Larry J.: Brigham Young University, Provo, UT, US Hart, Craig H.: Brigham Young University, Provo, UT, US Evans, Cortney A.: Brigham Young University, Provo, UT, US
ER -

TY - JOUR
ID - 1354
T1 - Sufficient space for playing? I. The importance of playing in therapy and why is it put less into practice? [Danish]. [References]
A1 - Ostberg,Marit Andersen
Y1 - 2010///
N1 - Peer Reviewed Journal: 2011-13335-003
Danish
Journal; Peer Reviewed Journal
KW - PsycInfo
KW - Rough and Tumble
KW - Play Therapy
KW - Psychoanalysis
KW - Social Issues
RP - NOT IN FILE
SP - Oct
EP - 229
JF - Matrix: Nordisk Tidsskrift for Psykoterapi
VL - .27

IS - 3
N2 - The author took Andreas into play-therapy because he wasn't managing to make friends. Winnicott's theories of potential space and playing as healing in itself, was helpful. It was essential to resist being drawn into the unconscious game of rejection that was central to his social problems. During the second year the therapy evolved into role-playing as well as rough-and-tumble play, and Andreas succeeded in actually playing with the therapist. Shortly after he managed to make friends. It is regrettable that play-therapy is on the wane, but hardly surprising, given many different factors, e.g. the reduced attention the technique receives in the specialisation program for therapists and the general marginalization of psychoanalysis. (PsycINFO Database Record (c) 2012 APA, all rights reserved) (journal abstract)
SN - 0109-646X
AD - Ostberg, Marit Andersen: Boerum BUP, Vestre Viken HF, Rud, Norway
ER -

TY - JOUR
ID - 1355
T1 - [Phenomenologic and functional variability of violence among children]. [German]
A1 - Oswald,H.
A1 - Krappmann,L.
Y1 - 2000/01//
N1 - Oswald, H. Krappmann, L
Praxis der Kinderpsychologie und Kinderpsychiatrie
pj6, 0404246
IM
English Abstract. Journal Article
German
KW - Medline
KW - Rough and Tumble
KW - Child
KW - Dominance-Subordination
KW - Female
KW - Humans
KW - Male
KW - Motivation
KW - Peer Group
KW - Schools
KW - Social Environment
KW - Violence/px [Psychology]
RP - NOT IN FILE
SP - 3
EP - 15
JF - Praxis der Kinderpsychologie und Kinderpsychiatrie
JA - Prax.Kinderpsychol.Kinderpsychiatr.
VL - 49
IS - 1
CY - GERMANY
N2 - Examination of ten-year old children's everyday interactions with peers observed in natural settings of a school demonstrates that violence was present in many situations of help, sanctions, and rough-and-tumble play. Thorough analysis reveals diverse functions of violence in children's interactions: affirmation of reciprocity and justice, experience of agency and control, preservation of territories against intruders, clear messages, compensations of lacking capacities, and defense of the self
SN - 0032-7034
AD - Institut fur Padagogik, Universitat Potsdam
UR - 10783655
ER -

TY - JOUR

ID - 1357
T1 - Fathers and development: New areas for exploration. [References]
A1 - Paquette,Daniel; Coyl-Shepherd,Diana
Y1 - 2013///
N1 - Peer Reviewed Journal: 2013-18617-001
English
Journal; Peer Reviewed Journal
KW - PsycInfo
KW - Rough and Tumble
KW - Childhood Development
KW - Father Child Relations
KW - Parenting Style
RP - NOT IN FILE
SP - Jun
EP - 745
JF - Early Child Development and Care
VL - .183
IS - 6
N2 - This special issue presents seven articles exploring new avenues that promise a better understanding of the impact of fathering on child development. Collectively, these papers add to the research literature by presenting work that (1) examines key features of father child rough-and-tumble play and associations with children's development; (2) examines the link between rough-and-tumble play and the father-child activation relationship; (3) examines father involvement using newly developed observational measures which appropriately capture the unique characteristics of father- child interactions, including activative fathering, and associations with children's social-emotional development; (4) examines links between mothers' and fathers' perceptions of parental accessibility, division of household duties, parental roles, and child social adjustment; (5) examines father school-related involvement predictors and outcomes, controlling for levels of mother involvement; (6) examines associations between father perceptions of couple relationship quality, co-parenting, and father involvement, controlling for mother involvement and perceptions; and (7) examines mothers' and fathers' values in relation to children's social competence. This collection of studies explores the impact of father involvement using contemporary theories which examine the differential roles of mothers and fathers and connections between mother and father involvement (e.g. activation relationship theory, family systems theory). The collection also includes studies that have measured father involvement with tools designed specifically to assess fathering, including observational measures of the quality of father-child interactions. (PsycINFO Database Record (c) 2013 APA, all rights reserved)
SN - 0300-4430
AD - Paquette, Daniel: School of Psychoeducation, University of Montreal, Montreal, PQ, Canada Coyl-Shepherd, Diana D.: California State University at Chico, Chico, CA, US Newland, Lisa A.: University of South Dakota, Vermillion, SD, US
ER -

TY - JOUR
ID - 1358
T1 - Editorial
A1 - Parker-Rees,Rod
Y1 - 2007///
N1 - Peer Reviewed Journal: 2007-13989-001
English
Journal; Peer Reviewed Journal
KW - PsycInfo
KW - Rough and Tumble
KW - Childhood Development
KW - Culture (Anthropological)
KW - Evaluation
KW - Social Learning
KW - Teacher Attitudes

KW - Initiative
KW - Teachers
RP - NOT IN FILE
SP - Jul
EP - 101
JF - Early Years: An International Journal of Research and Development
VL - .27
IS - 2
N2 - The six papers published in this issue share a focus on ways in which young children's learning can be understood in terms of a dialogue between the phenomenological richness of 'real-life' experiences and the more formal, organized meaning structures which shape cultural priorities and which are represented in curricular targets and goals. The first paper, presents an evaluation of an Australian initiative to develop parents' and teachers' understanding of the learning potential of everyday activities and routines. The second paper emphasizes children's social agency in the process of developing different kinds of literacy. The third paper, presents rich ethnographic data from observations of 'everyday' interactions between young children and their grandparents in East London. The last two papers in this issue challenge this sort of narrow view of children's learning from different perspectives. The final paper, argues that children's rough and tumble play deserves greater recognition as an important medium for social learning. (PsycINFO Database Record (c) 2012 APA, all rights reserved)
SN - 0957-5146
AD - Parker-Rees, Rod: University of Plymouth, Plymouth, United Kingdom
ER -

TY - JOUR
ID - 1359
T1 - Physical activity play: the nature and function of a neglected aspect of playing. [Review] [150 refs]
A1 - Pellegrini,A.D.
A1 - Smith,P.K.
Y1 - 1998/06//
N1 - Pellegrini, A D. Smith, P K
Child development
0372725, d28
IM
Journal Article. Research Support, Non-U.S. Gov't. Review
English
KW - Medline
KW - Rough and Tumble
KW - Adolescent
KW - Adult
KW - Child
KW - Child Development
KW - Preschool
KW - Exercise/px [Psychology]
KW - Exercise
KW - Female
KW - Humans
KW - Infant
KW - Male
KW - Motor Skills
KW - Physical Endurance
KW - Physical Fitness
KW - Play and Playthings
KW - Sex Factors
RP - NOT IN FILE
SP - 577
EP - 598

JF - Child Development
JA - Child Dev.
VL - 69
IS - 3
CY - UNITED STATES
N2 - In this review, we consider the nature and possible developmental functions of physical activity play, defined as a playful context combined with a dimension of physical vigor. We distinguish 3 kinds of physical activity play, with consecutive age peaks: rhythmic stereotypies peaking in infancy, exercise play peaking during the preschool years, and rough-and-tumble play peaking in middle childhood. Gender differences (greater prevalence in males) characterize the latter 2 forms. Function is considered in terms of beneficial immediate and deferred consequences in physical, cognitive, and social domains. Whereas most theories assume that children's play has deferred benefits, we suggest that forms of physical activity play serve primarily immediate developmental functions. Rhythmic stereotypies in infancy are hypothesized to improve control of specific motor patterns. Exercise play is hypothesized to function primarily for strength and endurance training; less clear evidence exists for possible benefits for fat reduction and thermoregulation. In addition, there may be cognitive benefits of exercise play that we hypothesize to be largely incidental to its playful or physical nature. Rough-and-tumble play has a distinctive social component; we hypothesize that it serves primarily dominance functions; evidence for benefits to fighting skills or to emotional coding are more equivocal. Further research is indicated, given the potentially important implications for children's education, health, and development. [References: 150]
SN - 0009-3920
AD - Department of Educational Psychology, University of Minnesota, Minneapolis 55455, USA
UR - 9680672
ER -

TY - JOUR
ID - 1361
T1 - The Development and Function of Rough-and-Tumble Play in Childhood and Adolescence: A Sexual Selection Theory Perspective. [References]
A1 - Pellegrini,Anthony D.
Y1 - 2006///
N1 - Book: 2007-04529-004
English
Book; Edited Book
KW - PsycInfo
KW - Rough and Tumble
KW - Childhood Play Behavior
KW - Childhood Play Development
KW - Human Sex Differences
KW - Theories
KW - Animal Ethology
KW - Parental Investment
RP - NOT IN FILE
SP - sociocultural, and
EP - 98
JF - Goncu, Artin [Ed]; Gaskins, Suzanne [Ed]
IS - 2006
N2 - (from the chapter) In this chapter I integrate the ethological and developmental psychological play literatures. Indeed, such integration was called for explicitly by Piaget (1967): "There exists a parallelism, and a fairly close one, between biological doctrines of evolutionary variation and the particular theories of intelligence" (p. 11). To that end, I present a model for the development of rough-and-tumble play (R&T) utilizing both biological and developmental psychological principles. Specifically, I use sexual selection (Darwin, 1871) and parental investment theories (Trivers, 1972) as a way to explain the development and function of R&T during childhood and adolescence. I first define R&T. Next, I discuss basic tenets of sexual selection and parental investment theories and how these principles relate to gender differences in R&T. Third, I describe the context in which R&T develops by showing how sexually segregated peer groups in childhood is

the venue in which boys use highly vigorous and competitive forms of play. Fourth, I outline the development and functions of play during childhood and adolescence. As part of this discussion, I present two views of function (immediate and deferred benefits). I present cost-benefit analyses to argue that R&T probably serves an immediate function during childhood as well as an immediate function during adolescence. Specifically, during childhood R&T probably is used by boys as a way in which to affiliate with other boys and to condition cardiovascular and muscular systems. During adolescence, R&T continues to relate to boys' cardiovascular and muscular conditioning but also relates to dominance and aggression and is later used as a strategy to initiate heterosexual contact. (PsycINFO Database Record (c) 2012 APA, all rights reserved)
AD - Pellegrini, Anthony D.: Department of Educational Psychology, University of Minnesota-Twin Cities Campus, Minneapolis, MN, US
ER -

TY - JOUR
ID - 1362
T1 - The evolution of social play. [References]
A1 - Pellis,Sergio M.
Y1 - 2010///
N1 - Book: 2010-08663-030
English
Book; Edited Book
KW - PsycInfo
KW - Rough and Tumble
KW - Aggressive Behavior
KW - Animal Play
KW - Mammals
KW - Social Behavior
KW - Childhood Development
KW - Childhood Play Behavior
KW - Species Differences
KW - Theory of Evolution
RP - NOT IN FILE
SP - culture, and
EP - 431
JF - Worthman, Carol M [Ed]; Plotsky, Paul M [Ed]; Schechter, Daniel S [Ed]; Cummings, Constance A [Ed]
IS - 2010
N2 - (from the chapter) Researchers, both in human and non-human animal literature, continue to argue over the functions served by the rough-and-tumble play known as play-fighting (Power, 2000). During play-fighting, as one animal attacks another, the recipient may defend itself and then launch its own counterattack. The first attacker may then defend itself, and so on. The body targets contested can be derived from species-typical agonistic, predatory or socio-sexual behavior (Pellis, 1988,1993). Play-fighting is the most common form of play reported in non-human mammals (Pellis & Pellis, 1998a). In children freely interacting in the schoolyard or home, this type of play accounts for about 10% of all play (Smith, 2005), and, despite some minor cultural variations, has a basic theme that appears universal (Fry, 2005). Although play-fighting resembles serious fighting, a number of differences exist (Smith, 1997). Given that play-fighting can be distinguished from serious fighting, why do researchers emphasize different facets of the behavior and hence its potential to serve very different functions? In this chapter, we contend that to fully understand play-fighting, species differences must be taken seriously, and that this diversity can help unravel how play-fighting has evolved. Such an understanding can further our analysis of the functions play-fighting may serve. (PsycINFO Database Record (c) 2012 APA, all rights reserved)
AD - Pellis, Sergio M.: University of Lethbridge, Lethbridge, AB, Canada Pellis, Vivien C.: University of Lethbridge, Canadian Centre for Behavioural Neuroscience, Lethbridge, AB, Canada Reinhart, Christine J.: University of Lethbridge, Lethbridge, AB, Canada
ER -

TY - JOUR
ID - 1363

T1 - Gender variant and gender dysphoria in two young children. [References]
A1 - Perrin,Ellen; Smith,Nicola; Davis,Catherine; Spack,Norman; Stein,Martin
Y1 - 2010///
N1 - Peer Reviewed Journal: 2010-06258-011
English
Journal; Peer Reviewed Journal
KW - PsycInfo
KW - Rough and Tumble
KW - Gender Identity
KW - Human Sex Differences
KW - Major Depression
KW - Transgender
RP - NOT IN FILE
SP - Feb
EP - Mar
JF - Journal of Developmental and Behavioral Pediatrics
VL - .31
IS - 2
N2 - Alexandra is 5 1/2 years old, the youngest of four girls, and lives with her parents. She is described by her teachers as a thoughtful, imaginative girl with a good sense of humor, and strong literacy and math skills. Parents describe her typical mood as happy and easy going. She has many friends and engages in a variety of play activities. At 18 months, Alexandra insisted on wearing boys' underwear before she would complete toilet training. At 3 years, she told her parents that she "should have been a boy," and insisted on wearing boys' clothes. Alexandra now refuses to wear dresses and wears a coat and tie to formal occasions, which has led to conflicts with her grandparents. During make-believe play, Alexandra always chooses to play the male roles. She keeps her hair short and her dress and mannerisms are so masculine that people who do not know her assume that she is a boy. Alexandra makes frequent statements about being or wishing she were a boy. When asked directly she says "I'm a girl, but I'm a boy inside." Her parents have recently become alarmed because Alexandra has begun saying "I hate myself," and once said that when her breasts grow she will "slice them off." She has recently asked her parents to call her "Alex." There is no apparent bullying or teasing from other children. Alex's parents are understanding and supportive of her quandary, though they have questions and concerns about the long-term implications of these behaviors and choices. Chris is a 9-year-old boy, referred because of his preference for traditionally feminine clothing, toys, and activities. Chris's parents are divorced; he lives with his father and paternal grandparents, visiting his mother once a week. Chris met developmental milestones on time and always seemed "smart" and eager to learn. By 2 years of age, his family noticed that he was attracted to pretty and shiny things like dresses, sequins, and glitter, although he has always worn boys' clothing without complaint. During the next few years, he asked for Barbie dolls for his birthday and seemed fascinated by long blond hair. When coloring, he preferred pink and purple crayons and usually drew female characters, very decorated and with long hair. In comparison, his drawings of himself were small and plain. He occasionally said "I know I'm a boy, but I'm a girl in my head." His pediatrician advised the family to throw out the dolls and "girlish" toys, emphasize "male" activities, and insist that he draw boys as well as girls. Chris was so distraught by this that he was allowed to keep one Barbie doll, as long as he only played with it indoors and did not talk about the doll with friends. In kindergarten and first grade, Chris played mostly with girls and was not interested in sports or rough-and-tumble play. He was teased by other children and had few friends, mostly girls. He started to have sudden outbursts at home and at school that seemed beyond his control, sometimes kicking walls and punching himself in the head. At the beginning of third grade, Chris agreed to join the soccer team as his father wanted. His family no longer sees him playing with Barbies and he has agreed to a few play dates with new boys in his class. Chris is quieter than he used to be, with fewer outbursts. For his birthday, he privately asked his father's fiance for a blond long-haired wig, but when his father confronted him, he denied it. His father reports that "the phase" has finally passed. (PsycINFO Database Record (c) 2012 APA, all rights reserved) (journal abstract)
SN - 0196-206X
AD - Perrin, Ellen: Division of Developmental-Behavioral Pediatrics, Floating Hospital for Children, Tufts Medical Center, Boston, MA, US Smith, Nicola: Division of Developmental-Behavioral Pediatrics, Floating Hospital for Children, Tufts Medical Center, Boston, MA, US Davis, Catherine: Division of Developmental-Behavioral Pediatrics, Floating Hospital for Children, Tufts Medical Center, Boston, MA, US Spack, Norman:

Gender Management Service, Children's Hospital, Boston Harvard Medical School, Boston, MA, US Stein, Martin T.: Division of Child Development and Community Health, University of California San Diego, Rady Children's Hospital, San Diego, CA, US
ER -

TY - JOUR
ID - 1364
T1 - Sibling violence silenced: rivalry, competition, wrestling, playing, roughhousing, benign. [Review] [43 refs]
A1 - Phillips,D.A.
A1 - Phillips,K.H.
A1 - Grupp,K.
A1 - Trigg,L.J.
Y1 - 2009/04//
N1 - Phillips, Debby A. Phillips, Kate H. Grupp, Kitty. Trigg, Lisa J
ANS. Advances in nursing science
6e9, 7809992
IM, N
Journal Article. Review
English
KW - Medline
KW - Rough and Tumble
KW - Adolescent
KW - Aggression/px [Psychology]
KW - Attitude to Health/eh [Ethnology]
KW - Child
KW - Child Abuse/eh [Ethnology]
KW - Child Abuse/pc [Prevention & Control]
KW - Child Advocacy
KW - Child Behavior/eh [Ethnology]
KW - Preschool
KW - Competitive Behavior
KW - Conflict (Psychology)
KW - Denial (Psychology)
KW - Humans
KW - Nurse's Role/px [Psychology]
KW - Nurse's Role
KW - Nursing Methodology Research
KW - Play and Playthings/px [Psychology]
KW - Postmodernism
KW - Semantics
KW - Sibling Relations/eh [Ethnology]
KW - Sibling Relations
KW - Social Behavior
KW - Social Justice
KW - Violence/eh [Ethnology]
KW - Violence/pc [Prevention & Control]
RP - NOT IN FILE
SP - E1
EP - E16
JF - Advances in Nursing Science
JA - ANS Adv.Nurs Sci
VL - 32
IS - 2
CY - United States

N2 - In this article, sibling violence and the silence surrounding it is explicated through professional literature and research findings, exemplars from clinical practice, and statistics. Theoretical positions and discourse analysis have been used to help explain how regular broken bones, bruises, lacerations, and verbal humiliation can be minimized as normal sibling rivalry or roughhousing, which does not cause serious consequences. Nursing should be on the front lines of ending practices of violence. Recognizing sibling violence as such is part of this work and is a social justice issue. [References: 43]
SN - 1550-5014
AD - College of Nursing, Seattle University, 901 12th Ave, Box 22200, Seattle, WA 98122, USA. daphillip@seattleu.edu
UR - 19461218
ER -

TY - JOUR
ID - 1365
T1 - The overstimulation of everyday life: I. New aspects of male homosexuality
A1 - Phillips,S.H.
Y1 - 2001///
N1 - Phillips, S H
Journal of the American Psychoanalytic Association
h9z, 7505579
IM
Case Reports. Journal Article
English
KW - Medline
KW - Rough and Tumble
KW - Adaptation
KW - Psychological
KW - Adult
KW - Homosexuality
KW - Male/px [Psychology]
KW - Humans
KW - Male
KW - Psychoanalytic Theory
KW - Psychoanalytic Therapy
KW - Psychosexual Development
RP - NOT IN FILE
SP - 1235
EP - 1267
JF - Journal of the American Psychoanalytic Association
JA - J Am Psychoanal.Assoc.
VL - 49
IS - 4
CY - United States
N2 - Western heterosexual culture surrounds the homosexually inclined boy in a climate of erotic overstimulation that powerfully affects his development and adult sexual adaptation. This assertion is illustrated through a case presentation of a homosexual man who shared a bed with his brother from childhood into adolescence. Analysis of the patient's transference enactment--repeatedly falling asleep on the couch--gradually revealed the psychic impact of this everyday overstimulation: the creation of a tantalized inner world of longing. The regular occurrence and developmental understanding of adolescent homosexual boys' unrequited love affairs with adolescent heterosexual boys are described and explored. Finally, the overstimulation of everyday life is proposed as a new model for understanding certain behavioral aspects of male homosexuality, such as the avoidance of rough-and-tumble play in childhood and homosexual cruising
SN - 0003-0651
AD - Western New England Institute for Psychoanalysis, USA. Sidney.phillips@yale.edu
UR - 11809022
ER -

TY - JOUR
ID - 1366
T1 - Persistent vaginal bleeding in a patient with a broken Implanon
A1 - Pickard,S.
A1 - Bacon,L.
Y1 - 2002/10//
N1 - Pickard, Sally. Bacon, Lesley
The journal of family planning and reproductive health care / Faculty of Family Planning & Reproductive Health Care, Royal College of Obstetricians & Gynaecologists
101087687
IM
Case Reports. Journal Article
English
KW - Medline
KW - Rough and Tumble
KW - Adult
KW - Contraceptive Agents
KW - Female/ae [Adverse Effects]
KW - Desogestrel
KW - Equipment Failure
KW - Female
KW - Humans
KW - Menorrhagia/et [Etiology]
KW - Menstruation Disturbances/et [Etiology]
KW - Vinyl Compounds/ae [Adverse Effects]
RP - NOT IN FILE
SP - 207
EP - 208
JF - Journal of Family Planning & Reproductive Health Care
JA - J Fam.Plann.Reprod.Health Care
VL - 28
IS - 4
CY - England
N2 - A 29-year-old woman with an Implanon contraceptive device in situ presented with persistent and prolonged vaginal bleeding. The implant had been inserted 2 years previously; the patient had been happy with it and had been mainly amenorrhoeic with the occasional light period. She was concerned that the implant had broken during a game of 'rough and tumble' with her son in August 2000. Since the trauma to her arm her bleeding pattern had changed, and she began bleeding heavily for 3 weeks every month. The rod was removed and found to be fractured halfway across its width. A new Implanon device was inserted and the bleeding settled
SN - 1471-1893
AD - Department of Reproductive Health, Lewisham Primary Care Trust, London, UK.
s.pickard@macunlimited.net
UR - 12419064
ER -

TY - JOUR
ID - 1367
T1 - Toto's talking body. [References]
A1 - Poole,Gordon
Y1 - 2007///
N1 - Peer Reviewed Journal: 2007-12045-005
English
Journal; Peer Reviewed Journal
KW - PsycInfo
KW - Rough and Tumble

KW - Body Language
KW - Gestures
KW - Theatre
RP - NOT IN FILE
SP - 2007, pp
EP - 2007,253
JF - Gesture
VL - .7
IS - 2
N2 - The full-bodied, multi-faceted use of gesture by Italy's beloved vaudeville and cinema comedian Antonio De Curtis, in art Toto, is rooted in the rich gesture language of Naples. Toto's humble origins, the experience of poverty and deprivation, conditioned his art, as did his immersion as a mere child into the rough-and-tumble world of Neapolitan street theater. The teeming neighborhoods of Naples, a Baroque city, have made it into somewhat of a theatrum mundi, producing many fine actors and playwrights. The essay, illustrated by photograms from a few of Toto's films, suggests that Neapolitan gesture, whose purpose is communication, is stylized by Toto and transformed into artistry of a high order. (PsycINFO Database Record (c) 2012 APA, all rights reserved) (journal abstract)
SN - 1568-1475
AD - Poole, Gordon: Universita degli Studi "L'Orientale", Naples, Italy
ER -

TY - JOUR
ID - 1368
T1 - Reactive and proactive aggression in childhood: relations to peer status and social context dimensions
A1 - Price,J.M.
A1 - Dodge,K.A.
Y1 - 1989/08//
N1 - Price, J M. Dodge, K A
Journal of abnormal child psychology
0364547, h33
IM
Journal Article
English
KW - Medline
KW - Rough and Tumble
KW - Aggression/px [Psychology]
KW - Anger
KW - Child
KW - Preschool
KW - Cooperative Behavior
KW - Frustration
KW - Humans
KW - Interpersonal Relations
KW - Male
KW - Peer Group
KW - Play and Playthings
KW - Rejection (Psychology)
KW - Social Adjustment
RP - NOT IN FILE
SP - 455
EP - 471
JF - Journal of Abnormal Child Psychology
JA - J Abnorm.Child Psychol.
VL - 17
IS - 4
CY - UNITED STATES

N2 - Although there has been an accumulation of evidence to suggest a link between peer-directed aggression and social rejection, little attention has been given to the relations between specific subtypes of aggressive behavior and social rejection. The purpose of this investigation was to examine the relations between two subtypes of aggressive behavior (reactive and proactive aggression) and children's classroom peer status. The reciprocity of each of these subtypes of aggressive behavior and the social contexts in which these behaviors occur were also examined. Assessments of each of these forms of aggression among 70 boys (ages 5 and 6) were conducted using direct observations and teacher ratings. In general, directing reactive aggressive behavior toward peers was associated with social rejection, while utilization of instrumental aggression was positively related to peer status. The findings also indicated that directing proactive forms of aggression toward peers was related to being the target of proactive aggression. Finally, among older boys, both subtypes of aggression were more likely to occur during rough play than during any other type of play activity
SN - 0091-0627
AD - San Diego State University, California 92182
UR - 2794257
ER -

TY - JOUR
ID - 1369
T1 - A comparison of school injuries between children with and without disabilities
A1 - Ramirez,M.
A1 - Fillmore,E.
A1 - Chen,A.
A1 - Peek-Asa,C.
Y1 - 2010/09//
N1 - Ramirez, Marizen. Fillmore, Erin. Chen, Alex. Peek-Asa, Corinne
Academic pediatrics
101499145
IM
Comparative Study. Journal Article. Research Support, Non-U.S. Gov't. Research Support, U.S. Gov't, P.H.S.
English
KW - Medline
KW - Rough and Tumble
KW - Child
KW - Disabled Children/sn [Statistics & Numerical Data]
KW - Education
KW - Special
KW - Female
KW - Humans
KW - Learning Disorders/ep [Epidemiology]
KW - Los Angeles
KW - Male
KW - Retrospective Studies
KW - Schools
KW - Wounds and Injuries/ep [Epidemiology]
RP - NOT IN FILE
SP - 317
EP - 322
JF - Academic pediatrics
JA - Acad.Pediatr
VL - 10
IS - 5
CY - United States
N2 - OBJECTIVE: The aim of this study was to compare rates, nature, and mechanisms of school injuries in children with and without disabilities. METHODS: We conducted a retrospective cohort study with repeated measures of 269 919 children with and without disabilities who were enrolled in 35 adapted schools from a large urban school district. Reports of injuries sustained from 1994 to 1998 were collected by the district's

insurance division, and disability was assessed using special education guidelines determined by the California Department of Education. A generalized estimating equations model was used to estimate rate ratios, accounting for the repeated, nested nature of the data. RESULTS: Children with disabilities had more than double the rate of injury reported than children without disabilities (incidence density ratio [IDR] 2.3, 95% CI, 2.2-2.5). Almost one third of these injuries were due to fights, roughhousing, and assaults. Among all disabled children, those with orthopedic disabilities had the highest risk, with rates over 5 times that of children without disabilities (IDR 5.4, 95% CI, 4.4-6.6). Children with cognitive disabilities had comparatively lower rates of injury than children with physical disabilities. CONCLUSIONS: For children with disabilities, physical impairment may play a greater role than cognitive impairment in managing risk for injury at school. Individual education programs (IEP), developed for children in special education, could be tailored to include injury prevention strategies. Copyright 2010 Academic Pediatric Association. Published by Elsevier Inc. All rights reserved
SN - 1876-2867
AD - University of Iowa Injury Prevention Research Center, Department of Occupational and Environmental Health, College of Public Health, University of Iowa, Iowa City, Iowa 52242-5000, USA. marizen-ramirez@uiowa.edu
UR - 20674529
ER -

TY - JOUR
ID - 1370
T1 - Fair-play rules and injury reduction in ice hockey
A1 - Roberts,W.O.
A1 - Brust,J.D.
A1 - Leonard,B.
A1 - Hebert,B.J.
Y1 - 1996/02//
N1 - Roberts, W O. Brust, J D. Leonard, B. Hebert, B J
Archives of pediatrics & adolescent medicine
9422751, bwf
AIM, IM
Journal Article
English
KW - Medline
KW - Rough and Tumble
KW - Athletic Injuries/ep [Epidemiology]
KW - Athletic Injuries/et [Etiology]
KW - Hockey/in [Injuries]
KW - Hockey/st [Standards]
KW - Humans
KW - Incidence
KW - Male
KW - Minnesota/ep [Epidemiology]
KW - Prospective Studies
RP - NOT IN FILE
SP - 140
EP - 145
JF - Archives of Pediatrics & Adolescent Medicine
JA - Arch.Pediatr Adolesc Med
VL - 150
IS - 2
CY - UNITED STATES
N2 - OBJECTIVE: To determine the rate, type, and severity of injuries incurred and penalties assessed during the qualifying fair-play (points for playing without excessive penalties) and championship "regular" rules (winner advances) portions of a 1994 Junior Gold ice hockey tournament. DESIGN: A prospective evaluation of injuries by certified athletic trainers at the tournament site. SETTING: A community-organized, 3-day, 31-game tournament in Minnesota. PARTICIPANTS: Two hundred seventy-three male players, younger than 20 years

and in high school. MEASUREMENTS/MAIN RESULTS: Injuries were recorded by an on-site certified athletic trainer, and the penalties were tallied from the score sheets. The injury rates for the total number of injuries were 26.4 injuries per 1000 athlete exposures and 273.8 injuries per 1000 player hours. When only notable injuries (concussion, facial laceration, or moderate level of severity and above) were considered, the injury rates were 10 per 1000 athlete exposures and 103.9 per 1000 player hours. The ratio of notable fair-play to notable regular-rules injuries was 1:4.8. The number of penalties assessed per game averaged 7.1 penalties during fair-play rules and 13 penalties during the regular-rules competition. Penalties related to rough play and injury occurred four times more frequently during games with regular rules than those with fair-play rules. CONCLUSIONS: The fair-play concept can reduce injury rates, penalty rates, and severity of penalties and should be considered for ice hockey at all levels of play. The fair-play concept could be applied to other contact sports to reduce injury rates and rules infractions
SN - 1072-4710
AD - MinnHealth SportsCare Consultants, White Bear Lake, Minn, USA
UR - 8556117
ER -

TY - JOUR
ID - 1371
T1 - Gender differences in playful aggression during courtship in college students. [References]
A1 - Ryan,Kathryn M.
Y1 - 2005///
N1 - Peer Reviewed Journal: 2006-04126-011
English
Journal; Peer Reviewed Journal
KW - PsycInfo
KW - Rough and Tumble
KW - Aggressive Behavior
KW - Human Sex Differences
KW - Psychosexual Behavior
KW - Sexual Attitudes
KW - College Students
KW - Human Courtship
KW - Sexual Intercourse (Human)
RP - NOT IN FILE
SP - Oct
EP - 601
JF - Sex Roles
VL - .53
IS - 7-8
N2 - The current study explored gender differences in college students' definitions and enjoyment of playful force and aggression. Ninety-six female and 55 male college students who were predominantly White (96%) and heterosexual (97%) answered questions about playful force during sex and playful aggression at other times. A content analysis showed that the current definitions were very similar to those for children's rough-and-tumble play. However, a qualitative analysis found several potential gender differences. Physically aggressive men appeared to give aggressive definitions of playful force during sex. In addition, there was some disagreement among participants as to the acceptability of playful force during sex. Finally, there may be gender differences in the assumed agency of playful force and aggression. (PsycINFO Database Record (c) 2013 APA, all rights reserved) (journal abstract)
SN - 0360-0025
AD - Ryan, Kathryn M.: Lycoming College, Williamsport, PA, US Mohr, Sharon: Lycoming College, Williamsport, PA, US
ER -

TY - JOUR
ID - 1372
T1 - Wichern

A1 - Schaff,Philip
Y1 - 1857///
N1 - Book: 2009-21494-038
English
Book; Authored Book
KW - PsycInfo
KW - Rough and Tumble
KW - Christianity
KW - Religion
KW - Theology
RP - NOT IN FILE
SP - theology, and
EP - 418
JF - Schaff, Philip
IS - 1857
N2 - (create) We conclude these sketches with the most distinguished representative of practical Christianity in Germany, who converts the ideas of modern evangelical theology into deeds of charity, and goes forth from his study to the lanes of public life, the dens of misery, and the hells of vice, to do the work of the merciful Samaritan, and, as far as in him lies, to reclaim society to the gospel of peace. Topics covered in this chapter include character of Wichern; Christian philanthropy; the Rough House; personal interviews; the Church Diet and the Inner Mission; the Evangelical Conference and the Diaconate; Flieder and the deaconesses; Wichern in Prussia; and, concluding reflections on the prospects of Christianity in Germany. (PsycINFO Database Record (c) 2012 APA, all rights reserved)
AD - Schaff, Philip: Theological Seminary, Mercersburg, PA, US
ER -

TY - JOUR
ID - 1373
T1 - Henry Harrower and the turbulent beginnings of endocrinology
A1 - Schwartz,T.B.
Y1 - 1999/11/02/
N1 - Schwartz, T B
Annals of internal medicine
0372351, 5a6
AIM, IM, Q
Biography. Historical Article. Journal Article
English
KW - Medline
KW - Rough and Tumble
KW - Endocrinology/hi [History]
KW - History
KW - 19th Century
KW - 20th Century
KW - Humans
KW - Societies
KW - Medical/hi [History]
KW - United States
RP - NOT IN FILE
SP - 702
EP - 706
JF - Annals of Internal Medicine
JA - Ann.Intern.Med
VL - 131
IS - 9
CY - UNITED STATES

N2 - The emergence of new medical science in the mid-19th century was usually greeted with derision by "practical men" who saw their academic colleagues as elitist intellectuals whose work bore little or no relation to the rough-and-tumble aspects of patient care. This schism, which was nowhere greater than in the field of endocrinology, widened in 1891 when a myxedematous patient was dramatically restored to health after the administration of a thyroid extract. On the one hand, academicians-who saw this result as a triumphal example of the transference of laboratory studies to the bedside--were encouraged to pursue further studies in endocrine pathophysiology and pharmacology. On the other hand, medical practitioners began to believe that crude extracts from glands or other organs, when prescribed as orally administered mixtures, were effective for the treatment of most human ailments. The organotherapeutic forces were ably championed by Henry R. Harrower, MD, a manufacturer as well as a dispenser of organotherapeutic products. For some years, the claims of the organotherapists remained unchallenged. Finally, in 1921, Harvey Cushing, pioneer neurosurgeon and endocrinologist, launched a crushing assault on the purveyors of pluriglandular therapy. These attacks continued over ensuing years, and organotherapy fell into disrepute. Nevertheless, the assertions of "practical men" have not subsided; rather, we are now confronted by insistent claims for a bewildering array of herbal remedies, over-the-counter hormonal products, and alternative therapies
SN - 0003-4819
AD - Rush Medical College, Chicago, Illinois, USA
UR - 10577334
ER -

TY - JOUR
ID - 1374
T1 - Knife-related injuries treated in United States emergency departments, 1990-2008
A1 - Smith,G.A.
Y1 - 2013/09//
N1 - Smith, Gary A
The Journal of emergency medicine
ibo, 8412174
IM
Journal Article
English
KW - Medline
KW - Rough and Tumble
RP - NOT IN FILE
SP - 315
EP - 323
JF - Journal of Emergency Medicine
JA - J Emerg.Med
VL - 45
IS - 3
CY - United States
N2 - BACKGROUND: Knives cause more disabling injuries than any other type of hand tool. STUDY OBJECTIVES: This study investigates knife-related injuries requiring Emergency Department (ED) treatment among children and adults in the United States (US) from 1990 through 2008. METHODS: A retrospective analysis of data from the National Electronic Injury Surveillance System of the Consumer Product Safety Commission was conducted. RESULTS: An estimated 8,250,914 (95% confidence interval [CI] 7,149,074-9,352,755) knife-related injuries were treated in US EDs from 1990 to 2008, averaging 434,259 (95% CI 427,198-441,322) injuries annually, or 1190 per day. The injury rate was 1.56 injuries per 1000 US resident population per year. Fingers/thumbs (66%; 5,447,467 of 8,249,410) were injured most often, and lacerations (94%; 7,793,487 of 8,249,553) were the most common type of injury. Pocket/utility knives were associated with injury most often (47%; 1,169,960 of 2,481,994), followed by cooking/kitchen knives (36%; 900,812 of 2,481,994). Children were more likely than adults to be injured while playing with a knife or during horseplay ($p < 0.01$; odds ratio 9.57; 95% CI 8.10-11.30). One percent of patients were admitted to the hospital, and altercation-related stabbings to the trunk accounted for 52% of these admissions. CONCLUSIONS: Knives represent an important source of morbidity and mortality to people of all ages. Manufacturers should develop safer knife designs that incorporate features, such as improved opening and closing mechanisms on pocket

knives, to prevent these injuries. Other potential safety efforts include targeted educational interventions and changes in voluntary product safety standards and public policy. Copyright 2013 Elsevier Inc. All rights reserved
SN - 0736-4679
AD - Center for Injury Research and Policy, The Research Institute at Nationwide Children's Hospital, Columbus, Ohio, USA
UR - 23849364
ER -

TY - JOUR
ID - 1375
T1 - Evolutionary developmental psychology and socio-emotional development. [Spanish]. [References]
A1 - Smith,Peter K.
Y1 - 2003///
N1 - Peer Reviewed Journal: 2003-09642-004
Spanish
Journal; Peer Reviewed Journal
KW - PsycInfo
KW - Rough and Tumble
KW - Emotional Development
KW - Family Relations
KW - Human Development
KW - Peer Relations
KW - Psychosocial Development
KW - Adolescent Development
KW - Aggressive Behavior
KW - Childhood Development
KW - Developmental Psychology
KW - Evolutionary Psychology
KW - Human Sex Differences
KW - Infant Development
KW - Parent Child Relations
KW - Sibling Relations
RP - NOT IN FILE
SP - 2003, pp
EP - 2003,324
JF - Infancia y Aprendizaje / Journal for the Study of Education and Development
VL - .26
IS - 3
N2 - Evolutionary developmental psychology is relevant to understanding many aspects of socio-emotional development in infancy, childhood and adolescence. This article focuses on parental investment and paternity certainty, child abuse, grandparenting, and parent-offspring conflict; sibling relationships and theory of mind; children's peer groups, especially dominance, aggression, sex differences and pretend and rough-and-tumble play; and on a model linking early family experiences to peer behaviour, age of puberty and reproductive behaviour. The review illustrates some of the potential of evolutionary developmental psychology in providing a comprehensive framework for understanding many aspects of social behaviour, and of providing testable hypotheses. Evolutionary explanations should be seen as compatible with cultural and environmental explanations, that may provide the proximal mechanisms by which the more distal functions of these behaviours are typically realised in actual developmental process and experience. (PsycINFO Database Record (c) 2014 APA, all rights reserved) (journal abstract)
SN - 0210-3702
AD - Smith, Peter K.: Department of Psychology, Goldsmiths College, London, England
ER -

TY - JOUR
ID - 1376

T1 - Fathers' role in children's language development. [References]
A1 - Tamis-LeMonda,Catherine S; Baumwell,Lisa; Cabrera,Natasha
Y1 - 2013///
N1 - Book: 2012-30818-008
English
Book; Edited Book
KW - PsycInfo
KW - Rough and Tumble
KW - Father Child Communication
KW - Fathers
KW - Home Environment
KW - Language Development
KW - Parental Role
KW - Childhood Development
KW - Early Childhood Development
KW - Language
KW - Lower Income Level
RP - NOT IN FILE
SP - 486
JF - Cabrera, Natasha J [Ed]; Tamis-LeMonda, Catherine S [Ed]
IS - 2013
N2 - (from the chapter) The majority of research on parents' role in children's language development is based on observations of mother-child interactions. For the most part, fathers have not been included in such research, leaving a dearth of knowledge regarding their role in children's early language development. Although there has been a gradual movement towards including fathers in language research over the past two decades, this oversight is partly rooted in historically narrow characterizations of fathers as "breadwinners" and/or "rough-and-tumble playmates", as well as in a dominant research focus on associations between father "absence" or "presence" and children's behavior problems, delinquency, and school drop out. Moreover, mothers are generally more available to researchers than fathers; are expected to be more familiar with their children and to be children's principal communicative partners; and, are thought to influence their children's language development more so than fathers. Nonetheless, there exist several practical and theoretical reasons for broadening the scope of language development studies to include fathers. First, trends over the past several decades have resulted in rising numbers of mothers joining the labor market; changes to the distribution of household labor; and, major shifts in family structure. Second, it is particularly important to understand fathers' role in the language development of children living in low-income households. Finally, efforts to advance a deeper theoretical understanding of children's early language development in social context rests on documenting the full range of language "inputs" to the system (including mothers', fathers', and other adults' lexicons, syntax, pragmatics, and so forth) in relation to the full range of emerging language "products," including growth in children's receptive and productive vocabularies, syntactic and pragmatic skills. Including fathers in language research moves investigators a step closer to understanding the major "inputs" in children's language environments. (PsycINFO Database Record (c) 2014 APA, all rights reserved)
AD - Tamis-LeMonda, Catherine S.: New York University, New York, NY, US Baumwell, Lisa: New York University, New York, NY, US Cabrera, Natasha J.: University of Maryland, MD, US
ER -

TY - JOUR
ID - 1377
T1 - Rough and tumble play: An investigation of the perceptions of educators and young children. [References]
A1 - Tannock,Michelle T.
Y1 - 2008///
N1 - Peer Reviewed Journal: 2008-05090-009
English
Journal; Peer Reviewed Journal
KW - PsycInfo
KW - Rough and Tumble
KW - Child Attitudes

KW - Childhood Play Behavior
KW - Early Childhood Development
KW - Educational Programs
KW - Teacher Attitudes
RP - NOT IN FILE
SP - Feb
EP - 361
JF - Early Childhood Education Journal
VL - .35
IS - 4
N2 - This research investigated rough and tumble (R&T) play in two early childhood settings. Participants included 11 educators and 17 children (5 years old). The study focused on gaining an understanding of how early childhood educators and young children interpret R&T play. The results indicate that while there is perceived value in R&T related to the development of young children, educators are uncertain of how to manage the play. The results of this study demonstrate the need for early childhood programs to develop policies to guide how R&T is managed. (PsycINFO Database Record (c) 2012 APA, all rights reserved) (journal abstract)
SN - 1082-3301
AD - Tannock, Michelle T.: Special Education, University of Nevada, Las Vegas, Las Vegas, NV, US
ER -

TY - JOUR
ID - 1378
T1 - Bioethics: The citizen view and its perils. [References]
A1 - Timpane,John
Y1 - 2009///
N1 - Book: 2009-09394-005
English
Book; Edited Book
KW - PsycInfo
KW - Rough and Tumble
KW - Bioethics
KW - Political Issues
KW - Public Opinion
RP - NOT IN FILE
SP - 828
JF - Ravitsky, Vardit [Ed]; Fiester, Autumn [Ed]; Caplan, Arthur L [Ed]
IS - 2009
N2 - (from the chapter) Most people see bioethics as a set of political issues-matters of public policy they can vote up or down. They do not see them primarily as scientific issues because, to them, science is somewhat of a hermetically sealed-off realm, poorly explained, and none too responsive. Yes, these are outdated cliches about science; that doesn't matter. Many people see bioethical issues either as intensely personal matters-if the issues touch their own lives-or already politicized and partisanized matters (that is, issues for debate between the political parties), much the same as nonscientific policy issues. Bioethical issues get thrown in the ring and batted around as part of the rough-and-tumble of the democratic process in this country. So here are a few thoughts about what happens, or should happen, with bioethics in the public sphere. (PsycINFO Database Record (c) 2012 APA, all rights reserved)
AD - Timpane, John: Philadelphia Inquirer, Philadelphia, PA, US
ER -

TY - JOUR
ID - 1379
T1 - The experience of dog bites: a survey of veterinary science and veterinary nursing students
A1 - Wake,A.A.
A1 - Stafford,K.J.
A1 - Minot,E.O.
Y1 - 2006/06//

N1 - Wake, A A F. Stafford, K J. Minot, E O
New Zealand veterinary journal
0021406, obz
IM
Journal Article
English
KW - Medline
KW - Rough and Tumble
KW - Adolescent
KW - Adult
KW - Age Factors
KW - Animals
KW - Bites and Stings/co [Complications]
KW - Bites and Stings/ep [Epidemiology]
KW - Breeding
KW - Child
KW - Preschool
KW - Dogs
KW - Facial Injuries/ep [Epidemiology]
KW - Female
KW - Hand Injuries/ep [Epidemiology]
KW - Humans
KW - Male
KW - New Zealand
KW - Questionnaires
KW - Risk
KW - Rural Population/sn [Statistics & Numerical Data]
KW - Severity of Illness Index
KW - Sex Factors
KW - Wounds and Injuries/ep [Epidemiology]
KW - Wounds and Injuries/et [Etiology]
KW - Wounds and Injuries/pa [Pathology]
RP - NOT IN FILE
SP - 141
EP - 146
JF - New Zealand Veterinary Journal
JA - N.Z.Vet.J
VL - 54
IS - 3
CY - New Zealand
N2 - AIM: To investigate the circumstances of dogs biting people in New Zealand. METHODS: Veterinary science and veterinary nursing students enrolled at Massey University in 2003 completed a questionnaire detailing their lifetime experience of dog bites and their opinions of dog control legislation. RESULTS: The response rate was 100%. There were 228 completed responses to the survey, and 87 (38%) respondents had been bitten by a dog. Most bites caused minor injury, but 17 (20%) required medical attention. Male respondents were more likely to have been bitten. The highest proportion of respondents (27; 31%) had been bitten while between 6 and 10 years of age, and the hand was the part of the body most often injured. Eighteen (21%) bites involved a stranger's dog, but most bites were by a dog known to the victim. Respondents from rural areas were three times more likely to have been bitten than city dwellers. The victim's home was the place where most (31; 36%) bites occurred. Male dogs were responsible for a larger proportion of bites (38; 44%) than females (24; 28%); respondents were unsure about the sex of the remainder of the dogs (25; 29%) that bit them. Protecting the home, play, accidents, rough handling and pain were identified as the common reasons for dogs to bite. Most (46; 52%) respondents did not believe legislation could have prevented their bite. Only four (5%) bites were reported to authorities. CONCLUSION: Rural dwellers, people with more experience of dogs, children <11 years of age, and male respondents were bitten by dogs most often. Only legislation relating to the

fencing of dog owners' properties may have prevented a reasonable proportion (25; 29%) of the bites reported here
SN - 0048-0169
AD - Ecology Group, Institute of Natural Resources, Massey University, Private Bag 11222, Palmerston North, New Zealand
UR - 16751845
ER -

TY - JOUR
ID - 1380
T1 - Sexual differentiation of behaviour in monkeys: role of prenatal hormones. [Review] [45 refs]
A1 - Wallen,K.
A1 - Hassett,J.M.
Y1 - 2009/03//
N1 - Wallen, K. Hassett, J M
Journal of neuroendocrinology
brl, 8913461
IM
Journal Article. Review
English
KW - Medline
KW - Rough and Tumble
KW - Androgens/me [Metabolism]
KW - Animals
KW - Behavior
KW - Animal/ph [Physiology]
KW - Female
KW - Haplorhini/ph [Physiology]
KW - Humans
KW - Male
KW - Maternal Behavior/px [Psychology]
KW - Pregnancy
KW - Prenatal Exposure Delayed Effects
KW - Sex Characteristics
KW - Sex Differentiation/ph [Physiology]
KW - Sex Factors
KW - Testosterone/me [Metabolism]
KW - Vocalization
RP - NOT IN FILE
SP - 421
EP - 426
JF - Journal of Neuroendocrinology
JA - J Neuroendocrinol.
VL - 21
IS - 4
CY - England
N2 - The theoretical debate over the relative contributions of nature and nurture to the sexual differentiation of behaviour has increasingly moved towards an interactionist explanation that requires both influences. In practice, however, nature and nurture have often been seen as separable, influencing human clinical sex assignment decisions, sometimes with disastrous consequences. Decisions about the sex assignment of children born with intersex conditions have been based almost exclusively on the appearance of the genitals and how other's reactions to the gender role of the assigned sex affect individual gender socialisation. Effects of the social environment and gender expectations in human cultures are ubiquitous, overshadowing the potential underlying biological contributions in favour of the more observable social influences. Recent work in nonhuman primates showing behavioural sex differences paralleling human sex differences, including toy preferences, suggests that less easily observed biological factors also influence behavioural sexual differentiation in both monkeys and

humans. We review research, including Robert W. Goy's pioneering work with rhesus monkeys, which manipulated prenatal hormones at different gestation times and demonstrated that genital anatomy and specific behaviours are independently sexually differentiated. Such studies demonstrate that, for a variety of behaviours, including juvenile mounting and rough play, individuals can have the genitals of one sex but show the behaviour more typical of the other sex. We describe another case, infant distress vocalisations, where maternal responsiveness is best accounted for by the mother's response to the genital appearance of her offspring. Taken together, these studies demonstrate that sexual differentiation arises from complex interactions where anatomical and behavioural biases, produced by hormonal and other biological processes, are shaped by social experience into the behavioural sex differences that distinguish males and females. [References: 45]
SN - 1365-2826
AD - Department of Psychology and Yerkes National Primate Research Center, Emory University, Atlanta, GA 30322, USA. kim@emory.edu
UR - 19207815
ER -

TY - JOUR
ID - 1381
T1 - Coated kapok fiber for removal of spilled oil
A1 - Wang,J.
A1 - Zheng,Y.
A1 - Wang,A.
Y1 - 2013/04/15/
N1 - Wang, Jintao. Zheng, Yian. Wang, Aiqin
Marine pollution bulletin
0260231, dvz
IM
Journal Article. Research Support, Non-U.S. Gov't
English
KW - Medline
KW - Rough and Tumble
KW - Absorption
KW - Ceiba
KW - Environmental Remediation/mt [Methods]
KW - Oils/an [Analysis]
KW - Paraffin/an [Analysis]
KW - Petroleum/an [Analysis]
KW - Petroleum Pollution
KW - Soybean Oil/an [Analysis]
KW - Water Pollutants
KW - Chemical/an [Analysis]
RP - NOT IN FILE
SP - 91
EP - 96
JF - Marine Pollution Bulletin
JA - Mar.Pollut.Bull.
VL - 69
IS - 1-2
CY - England
N2 - Based on raw kapok fiber, two kinds of oil absorbers with high sorption capacity were prepared by a facile solution-immersion process. The coated polymer with low surface energy and rough fiber surface play important role in the retention of oil. The as-prepared fiber can quickly absorb gasoline, diesel, soybean oil, and paraffin oil up to above 74.5%, 66.8%, 64.4% and 47.8% of oil sorption capacity of raw fiber, respectively. The absorbed oils can be easily recovered by a simple vacuum filtration and the recovered coated-fiber still can be used for several cycles without obvious loss in oil sorption capacity. The thermodynamic study indicates that the adsorption process is spontaneous and exothermic, with complex physisorption and chemisorption. The results

suggest that the coated fiber can be used as a low-cost alternative for the removal of oil spilled on water surface.
Copyright 2013 Elsevier Ltd. All rights reserved
SN - 1879-3363
AD - Center of Eco-Material and Green Chemistry, Lanzhou Institute of Chemical Physics, Chinese Academy of Sciences, Lanzhou 730000, PR China
UR - 23419751
ER -

TY - JOUR
ID - 1382
T1 - Equine assisted therapy and theraplay. [References]
A1 - Weiss,Deborah
Y1 - 2009///
N1 - Book: 2009-08519-017
English
Book; Edited Book
KW - PsycInfo
KW - Rough and Tumble
KW - Animal Assisted Therapy
KW - Horses
KW - Play Therapy
KW - Therapists
RP - NOT IN FILE
SP - 300
JF - Munns, Evangeline [Ed]
IS - 2009
N2 - (from the chapter) In the 70s, horses began to take the role of "co-therapist" and the horses' special talents are now being written about. New methods are developing all over North America and Europe "Equine Assisted Therapy" has become a viable subgroup of Animal Assisted Therapy. Horses provide a powerful paradox. They are large, strong, fast animals that can quickly become dangerous and they are also vulnerable prey animals. Being carried on the back of a 1,200 pound vulnerable giant can certainly provide access to core personal issues. Partnering Theraplay with Irwin's horse training methods creates a powerful therapeutic tool. I developed the program "Horse Friends" to encompass these two modalities and it has since expanded to include activities from a number of equine trainers. With the encouragement of Dr. Evangeline Munns, who was director of Play Therapy Services at Blue Hills Child and Family Centre in Aurora at the time, I contacted the Ontario Trillium Foundation to see if they would be interested in funding such a venture. They were-and two years later the provincial funding agency had supported 35 children to participate in this pilot project. They continue to support our work and research to this day. The original program still exists under the name "Horse Play" and it is run by Horses At Heart Equestrian Adventures Inc. in conjunction with a number of regional child and family health service agencies. (PsycINFO Database Record (c) 2012 APA, all rights reserved)
AD - Weiss, Deborah: Theraplay Institute, IL, US
ER -

TY - JOUR
ID - 1383
T1 - Gunshot residue, ten years later
A1 - Wilber,C.G.
A1 - Lantz,R.K.
A1 - Sulik,P.L.
Y1 - 1991/09//
N1 - Wilber, C G. Lantz, R K. Sulik, P L
The American journal of forensic medicine and pathology
3hc, 8108948
IM
Case Reports. Journal Article
English

KW - Medline
KW - Rough and Tumble
KW - Antimony/an [Analysis]
KW - Barium/an [Analysis]
KW - Child
KW - Firearms
KW - Forensic Medicine/mt [Methods]
KW - Humans
KW - Jurisprudence
KW - Lead/an [Analysis]
KW - Male
KW - Microscopy
KW - Electron
KW - Scanning
KW - Paraplegia/et [Etiology]
KW - Skin/ch [Chemistry]
KW - Skin/pa [Pathology]
KW - Skin/ul [Ultrastructure]
KW - Wounds
KW - Gunshot/co [Complications]
KW - Gunshot/pa [Pathology]
RP - NOT IN FILE
SP - 204
EP - 206
JF - American Journal of Forensic Medicine & Pathology
JA - Am J Forensic Med Pathol.
VL - 12
IS - 3
CY - UNITED STATES
N2 - Gunshot residues may be central to a competent reconstruction of a shooting incident. When a young boy was shot in the neck by a playmate using a .22-caliber single-action revolver, permanent paralysis from mid-thorax downward ensued. Ten years later the victim sued the importer, the vendor, the German manufacturer, and the shooter's family. Investigative reports indicated "horseplay" and questionable emergency medical team care. Depositions were contradictory. The entry wound, removed at surgery, was fixed and processed for slides. The histopathologist referred to "black pigment granules" in the wound track. The 10-year-old slides and block were retrieved. The coverslip was removed from a representative slide that was examined under the scanning, x-ray dispersive microscope. The black granules contained amounts of lead, barium, and antimony far beyond any normal range. The firing range had to have been no greater than 6-12 in (15.24-30.48 cm). The case was promptly settled out of court
SN - 0195-7910
AD - Forensic Science Laboratory, Colorado State University, Fort Collins 80523
UR - 1750390
ER -

TY - JOUR
ID - 1384
T1 - Testing social acoustic memory in rats: effects of stimulus configuration and long-term memory on the induction of social approach behavior by appetitive 50-kHz ultrasonic vocalizations
A1 - Wohr,M.
A1 - Schwarting,R.K.
Y1 - 2012/09//
N1 - Wohr, Markus. Schwarting, Rainer K W
Neurobiology of learning and memory
9508166
IM

Journal Article. Research Support, Non-U.S. Gov't
English
KW - Medline
KW - Rough and Tumble
KW - Acoustic Stimulation
KW - Animals
KW - Appetitive Behavior/ph [Physiology]
KW - Behavior
KW - Animal/de [Drug Effects]
KW - Animal/ph [Physiology]
KW - Disease Models
KW - Animal
KW - Humans
KW - Male
KW - Memory
KW - Long-Term/de [Drug Effects]
KW - Long-Term/ph [Physiology]
KW - Muscarinic Antagonists/pd [Pharmacology]
KW - Rats
KW - Wistar
KW - Schizophrenia/pp [Physiopathology]
KW - Scopolamine Hydrobromide/pd [Pharmacology]
KW - Social Behavior
KW - Sound Spectrography
KW - Verbal Learning
KW - Vocalization
RP - NOT IN FILE
SP - 154
EP - 164
JF - Neurobiology of Learning & Memory
JA - Neurobiol.Learn.Mem.
VL - 98
IS - 2
CY - United States
N2 - Rats emit distinct types of ultrasonic vocalizations (USVs), which serve as situation-dependent affective signals. In appetitive situations, such as rough-and-tumble-play, high-frequency 50-kHz USVs occur, whereas low-frequency 22-kHz USVs can be observed in aversive situations, such as social defeat. USVs serve distinct communicative functions and induce call-specific behavioral responses in the receiver. While aversive 22-kHz USVs serve as alarm calls and induce behavioral inhibition, appetitive 50-kHz USVs have a pro-social communicative function and elicit social approach behavior, supporting the notion that they serve as social contact calls to (re)establish or maintain contact among conspecifics. The aim of the present study was to use the rat's ability to communicate in the ultrasonic range via high-frequency 50-kHz USVs in order to develop a test for social acoustic memory in rats with relevance for human verbal memory. Verbal learning and memory is among the seven cognitive domains identified as commonly deficient in human schizophrenia patients, but particularly difficult to model. We therefore tested whether the induction of social approach behavior by playback of appetitive 50-kHz USVs is dependent on (1) acoustic stimulus configuration and (2) social long-term memory, and whether (3) social long-term memory effects can be blocked by the administration of scopolamine, a muscarinic acetylcholine antagonist producing amnesia. Results show that social approach behavior in response to playback of natural 50-kHz USVs depends on acoustic stimulus configuration and occurs only when sound energy is concentrated to a critical frequency band in the ultrasonic range. Social approach behavior was detected during the first exposure to playback of 50-kHz USVs, whereas no such response was observed during the second exposure 1week later, indicating a stable memory trace. In contrast, when memory formation was blocked by i.p. administration of scopolamine (0.5mg/kg or 1.5mg/kg) immediately after the first exposure, rats displayed social approach behavior during the second exposure as well. Induction of social approach behavior in response to repeated playback of natural 50-kHz USVs may therefore

provide a new and rather unique approach for testing social acoustic memory in rats with relevance to human verbal memory. Copyright 2012 Elsevier Inc. All rights reserved
SN - 1095-9564
AD - Behavioral Neuroscience, Experimental and Physiological Psychology, Philipps-University of Marburg, Gutenbergstr. 18, 35032 Marburg, Germany. markus.woehr@staff.uni-marburg.de
UR - 22677211
ER -

TY - JOUR
ID - 1385
T1 - Affective communication in rodents: ultrasonic vocalizations as a tool for research on emotion and motivation
A1 - Wohr,M.
A1 - Schwarting,R.K.
Y1 - 2013/10//
N1 - Wohr, Markus. Schwarting, Rainer K W
Cell and tissue research
cqd, 0417625
IM
Journal Article. Research Support, Non-U.S. Gov't
English
KW - Medline
KW - Rough and Tumble
RP - NOT IN FILE
SP - 81
EP - 97
JF - Cell & Tissue Research
JA - Cell Tissue Res.
VL - 354
IS - 1
CY - Germany
N2 - Mice and rats emit and perceive calls in the ultrasonic range, i.e., above the human hearing threshold of about 20 kHz: so-called ultrasonic vocalizations (USV). Juvenile and adult rats emit 22-kHz USV in aversive situations, such as predator exposure and fighting or during drug withdrawal, whereas 50-kHz USV occur in appetitive situations, such as rough-and-tumble play and mating or in response to drugs of abuse, e.g., amphetamine. Aversive 22-kHz USV and appetitive 50-kHz USV serve distinct communicative functions. Whereas 22-kHz USV induce freezing behavior in the receiver, 50-kHz USV lead to social approach behavior. These opposite behavioral responses are paralleled by distinct patterns of brain activation. Freezing behavior in response to 22-kHz USV is paralleled by increased neuronal activity in brain areas regulating fear and anxiety, such as the amygdala and periaqueductal gray, whereas social approach behavior elicited by 50-kHz USV is accompanied by reduced activity levels in the amygdala but enhanced activity in the nucleus accumbens, a brain area implicated in reward processing. These opposing behavioral responses, together with distinct patterns of brain activation, particularly the bidirectional tonic activation or deactivation of the amygdala elicited by 22-kHz and 50-kHz USV, respectively, concur with a wealth of behavioral and neuroimaging studies in humans involving emotionally salient stimuli, such as fearful and happy facial expressions. Affective ultrasonic communication therefore offers a translational tool for studying the neurobiology underlying socio-affective communication. This is particularly relevant for rodent models of neurodevelopmental disorders characterized by social and communication deficits, such as autism and schizophrenia
SN - 1432-0878
AD - Behavioral Neuroscience, Experimental and Physiological Psychology, Philipps University of Marburg, Gutenbergstrasse 18, 35032, Marburg, Germany, markus.woehr@staff.uni-marburg.de
UR - 23576070
ER -

TY - JOUR
ID - 1386

T1 - A modified screening tool for autism (Checklist for Autism in Toddlers [CHAT-23]) for Chinese children
A1 - Wong,V.
A1 - Hui,L.H.
A1 - Lee,W.C.
A1 - Leung,L.S.
A1 - Ho,P.K.
A1 - Lau,W.L.
A1 - Fung,C.W.
A1 - Chung,B.
Y1 - 2004/08//
N1 - Wong, Virginia. Hui, Lai-Hing Stella. Lee, Wing-Cheong. Leung, Lok-Sum Joy. Ho, Po-Ki Polly. Lau, Wai-Ling Christine. Fung, Cheuk-Wing. Chung, Brian
Pediatrics
oxv, 0376422
AIM, IM
Journal Article
English
KW - Medline
KW - Rough and Tumble
KW - Attention
KW - Autistic Disorder/di [Diagnosis]
KW - China/eh [Ethnology]
KW - Cohort Studies
KW - Cost-Benefit Analysis
KW - Cross-Sectional Studies
KW - Humans
KW - Infant
KW - Mass Screening
KW - Play and Playthings
KW - Questionnaires
KW - Sensitivity and Specificity
KW - Social Behavior
RP - NOT IN FILE
SP - e166
EP - e176
JA - Pediatrics
VL - 114
IS - 2
CY - United States
N2 - BACKGROUND: There is a recent trend of a worldwide increase in the incidence of autistic spectrum disorder. Early identification and intervention have proved to be beneficial. The original version of the Checklist for Autism in Toddlers (CHAT) was a simple screening tool for identification of autistic children at 18 months of age in the United Kingdom. Children with an absence of joint attention (including protodeclarative pointing and gaze monitoring) and pretend play at 18 months were at high risk of autism. Section A of the CHAT was a self-administered questionnaire for parents, with 9 yes/no questions addressing the following areas of child development: rough and tumble play, social interest, motor development, social play, pretend play, protoimperative pointing (pointing to ask for something), protodeclarative pointing, functional play, and showing. Section B of the CHAT consisted of 5 items, which were recorded with observation of the children by general practitioners or health visitors. The 5 items addressed the child's eye contact, ability to follow a point (gaze monitoring), pretend (pretend play), produce a point (protodeclarative pointing), and make a tower of blocks. A 6-year follow-up study of >16,000 children screened with the CHAT at 18 months in the United Kingdom showed a sensitivity of only 0.40 and a specificity of 0.98, with a positive predictive value (PPV) of 0.26. Rescreening using the same instrument at 19 months for those who failed the 18-month screening yielded a higher PPV of 0.75. Therefore, children were likely to have autism if they failed the CHAT at 18 months and failed again at 19 months. It was estimated that consistent failure in 3 key questions (ie, protodeclarative pointing, gaze monitoring, and pretend play) at 18 months indicated an 83.3% risk of having autism. Because of

the poor sensitivity of the original CHAT for autism, a Modified Checklist for Autism in Toddlers (M-CHAT), consisting of 23 questions, with 9 questions from the original CHAT and an additional 14 questions addressing core symptoms present among young autistic children, was designed in the United States. The original observational part (ie, section B) was omitted. The M-CHAT was designed as a simple, self-administered, parental questionnaire for use during regular pediatric visits. The more questions children failed, the higher their risk of having autism. Two criteria were used to measure the sensitivity and specificity of M-CHAT. Criterion 1 used any 3 of the 23 questions, and criterion 2 used 2 of the 6 best questions that could be used to discriminate autism from other groups. The sensitivity and specificity for criterion 1 were 0.97 and 0.95 and those for criterion 2 were 0.95 and 0.99, respectively. M-CHAT had a better sensitivity than the original CHAT, because children up to 24 months of age were screened, with the aim of identifying those who might regress between 18 and 24 months. The 6 best questions of the M-CHAT addressed areas of social relatedness (interest in other children and imitation), joint attention (protodeclarative pointing and gaze monitoring), bringing objects to show parents, and responses to calling. Joint attention was addressed in the original CHAT, whereas the other areas were addressed only in the M-CHAT. To date, there has been no study of the application of either the original CHAT or the M-CHAT for Chinese populations. OBJECTIVES: CHAT-23 is a new checklist translated into Chinese, combining the M-CHAT (23 questions) with graded scores and section B (observational section) of the CHAT. We aimed to determine whether CHAT-23 could discriminate autism at mental ages of 18 to 24 months for Chinese children and to determine the best combination of questions to identify autism. METHODS: A cross-sectional cohort study was performed with 212 children with mental ages of 18 to 24 months. The children were categorized into 2 groups, ie, group 1 (N = 87) (autistic disorder: N = 53; pervasive developmental disorder: N = 33) and group 2 (N = 125) (nonautistic). The checklist included self-ad25) (nonautistic). The checklist included self-administered questionnaires with 23 questions (part A) and direct observations of 5 items by trained investigators (part B). We performed discriminant function analysis to We found that 7 key questions, addressing areas of joint attention, pretend play, social relatedness, and social referencing, were identified as discriminative for autism. For part A, failing any 2 of 7 key questions, ie, question 13 (does your child imitate you? [eg, you make a face; will your child imitate it?]), question 5 (does your child ever pretend, for example, to talk on the phone or take care of dolls, or pretend other things?), question 7 (does your child ever use his/her index finger to point, to indicate interest in something?), question 23 (does your child look at your face to check your reaction when faced with something unfamiliar?), question 9 (does your child ever bring objects over to you [parent] to show you something?), question 15 (if you point at a toy across the room, does your child look at it?), and question 2 (does your child take an interest in other children?), yielded sensitivity of 0.931 and specificity of 0.768. Failing any 6 of all 23 questions produced sensitivity of 0.839 and specificity of 0.848. For part B, failing any 2 of 4 items produced sensitivity of 0.736, specificity of 0.912, and PPV of 0.853. The 4 observational items were as follows: item B1: during the appointment, has the child made eye contact with you? item B2: does the child look across to see what you are pointing at? item B3: does the child pretend to pour out tea, drink it, etc?; item B4: does the child point with his/her index finger at the light? CONCLUSION: We found that integrating the screening questions of the M-CHAT (from the United States) and observational section B of the original CHAT (from the United Kingdom) yielded high sensitivity and specificity in discriminating autism at 18 to 24 months of age for our Chinese cohort. This new screening instrument (CHAT-23) is simple to administer. We found that a 2-stage screening program for autism can offer a cost-effective method for early detection of autism at 18 to 24 months. For CHAT-23, use of both the parental questionnaire and direct observation and use of the criterion of failing any 2 of 7 key questions yielded the highest sensitivity but a relatively lower specificity, whereas use of part B yielded the highest specificity but a lower sensitivity. We recommend identifying the possible positive cases with part A (parental questionnaire) and then proceeding to part B (observation) with trained assessors. The proposed algorithm for screening for autism is as follows. 1) The parents or chief caretakers complete a 23-item questionnaire when their children are 18 to 24 months of age. 2) The parents mail, fax, or hand this 23-item questionnaire to the local child health agency. 3) Clerical staff members check for and score failure, with the criteria of failing any 2 of 7 key questions or failing any 6 of 23 questions; if either criterion is met, then the staff members highlight the medical records of the suspicious cases. 4) Trained child health care professionals observe the children who failed any 2 of 7 key questions or any 6 of 23 questions. These identified patients are observed for 5 minutes for part B of the CHAT-23. 5) Any child who fails any 2 of 4 items requires direct referral to a comprehensive autism evaluation team, for early diagnostic evaluation and early intervention. The high sensitivity and specificity of the criteria observed in our study suggested that CHAT-23 might be used to differentiate children with autism. Additional international collaboration with the use of the CHAT, M-CHAT, and CHAT-23 could provide more prospective epidemiologic data, to establish whether there is a genuine increase in the worldwide incidence of autism

SN - 1098-4275
AD - Department of Paediatrics and Adolescent Medicine, University of Hong Kong, Hong Kong.
vcnwong@hkucc.hku.hk
UR - 15286253
ER -

TY - JOUR
ID - 1387
T1 - Epidemiology of trauma in a population of incarcerated youth
A1 - Woolf,A.
A1 - Funk,S.G.
Y1 - 1985/03//
N1 - Woolf, A. Funk, S G
Pediatrics
oxv, 0376422
AIM, IM
Journal Article. Research Support, Non-U.S. Gov't
English
KW - Medline
KW - Rough and Tumble
KW - Adolescent
KW - Child
KW - Female
KW - Humans
KW - Juvenile Delinquency
KW - Male
KW - North Carolina
KW - Prisoners
KW - Time Factors
KW - Wounds and Injuries/ep [Epidemiology]
RP - NOT IN FILE
SP - 463
EP - 468
JA - Pediatrics
VL - 75
IS - 3
CY - UNITED STATES
N2 - This study assessed the types, circumstances, frequency, and health consequences of trauma suffered by juvenile delinquents at a secure residential training school. A review of 369 medical records of 387 teenagers, aged 10 to 17 years, admitted to one such facility between 1978 and 1982 was made. Of the 369 students, 191 students (52%) suffered 391 separate trauma incidents serious enough to require a health care provider's attention. Circumstances surrounding the injuries included sports (36%), fights (20%), self-inflicted injuries (13%), suicide attempts (9%), incidents related to vocational studies (8%), and horseplay (3%). The most common types of injuries were musculoskeletal trauma, scratches, bruises and lacerations, and fractures. The usual sites of injury were the extremities, head, or neck. More than 50% of trauma incidents required a physician's attention initially or in follow-up; 28% of the injured were referred to an off-campus facility; 21% necessitated radiologic or laboratory studies; 4% required hospitalization; and one student died. The average trauma rate for teenagers in this setting was 1.2 injuries per person per year; the average rate of hospitalizations for trauma was 0.04 hospitalizations per person per year. These data call for further studies of trauma morbidity among adolescents in this and other settings and for innovative trauma prevention strategies
SN - 0031-4005
UR - 3975115
ER -

TY - JOUR
ID - 1388

T1 - The Development and Tendency of Western Research on Children' s Rough-and-Tumble Play. [Chinese]
A1 - Xinli,Zhang
Y1 - 2006///
N1 - Peer Reviewed Journal: 2006-07171-066
Chinese
Journal; Peer Reviewed Journal
KW - PsycInfo
KW - Rough and Tumble
KW - Childhood Development
KW - Childhood Play Behavior
KW - Experimentation
RP - NOT IN FILE
SP - Mar
EP - 498
JF - Psychological Science (China)
VL - .29
IS - 2
N2 - The origin, development, current situation and tendency of Western research on children's rough-and-tumble play were discussed. The concept, behavioural patterns, factors, and characteristics of children's age/gender etc. in rough-and-tumble play were expounded. The nature and function of rough-and-tumble play were also dealt with. The improving of methodology and expanding of research on children's rough-and-tumble play have been demonstrated. The integrative research of culture with ethological and psychological tradition is a new theoretical orientation, and presents a multidisciplinary research perspective. (PsycINFO Database Record (c) 2012 APA, all rights reserved) (journal abstract)
SN - 1671-6981
AD - Xinli, Zhang: Study Center of Education and Psychology of Ethnic Minorities in Southwest China, Southwest University, Chongqing, China
ER -

TY - JOUR
ID - 1389
T1 - Gender and environment as determinants of behavior in infant common baboons (Papio cynocephalus)
A1 - Young,G.H.
A1 - Bramblett,C.A.
Y1 - 1977/09//
N1 - Young, G H. Bramblett, C A
Archives of sexual behavior
8gr, 1273516
IM
Journal Article
English
KW - Medline
KW - Rough and Tumble
KW - Aggression
KW - Animals
KW - Newborn
KW - Behavior
KW - Animal
KW - Exploratory Behavior
KW - Female
KW - Haplorhini
KW - Humans
KW - Male
KW - Maternal Deprivation
KW - Motor Activity
KW - Papio

KW - Peer Group
KW - Play and Playthings
KW - Sex Factors
KW - Sexual Behavior
KW - Social Behavior
KW - Social Environment
KW - Species Specificity
KW - Stereotyped Behavior
KW - Vocalization
RP - NOT IN FILE
SP - 365
EP - 385
JF - Archives of Sexual Behavior
JA - Arch.Sex Behav
VL - 6
IS - 5
CY - UNITED STATES
N2 - Two groups of infant baboons were observed. The breast-fed group was housed in a gang cage with nursing mothers (n = 13) and the other motherless group was in a wire-cage nursery habitat (n = 20). Differences in behavior due to gender and environment were tested by analysis of variance. The results do not support the hypothesis that innate sex differences exist in baboons aged 0-3 months. Nursery-reared subjects had significantly higher scores for rough-and-tumble play, stereotypy, threat, avoid, explore, high tension, and nonaggressive social behaviors, but these behaviors are not significantly different between sexes in either groups
SN - 0004-0002
UR - 411455
ER -

TY - JOUR
ID - 1390
T1 - Exploration, Play and Social Development in Boys and Girls
A1 - Smith,P.K.
Y1 - 1986///
N1 - Accession Number: SPH335574; Author: Smith, P.K. ; Language: English; References: 61; Database Subset: SR; Publication Type: Book Analytic; Update Code: 19940101; SIRC Article No.: 335574
KW - *PLAY
KW - *SEX role
KW - Child Development
KW - SEX FACTOR
KW - STEREOTYPE
KW - SportDiscus
KW - Rough and Tumble
RP - NOT IN FILE
N2 - Considers three broad stages in the development of gender understanding in children: gender identity and labelling, gender stability, and gender constancy or consistency. Looks at children's understanding of sex-role stereotypes. Reviews the observations of social scientists regarding differences in the social and play behaviours of boys and girls. Discusses both immediate causal and functional/evolutionary explanations for the existence of differing sex roles. Illustrates an interactive developmental model for a typically sex-typed behaviour, using the example of rough-and-tumble play
UR -
https://login.proxy.bib.uottawa.ca/login?url=http://search.ebscohost.com/login.aspx?direct=true&db=s3h&AN=SPH335574&site=ehost-live;http://articles.sirc.ca/search.cfm?id=335574
ER -

TY - JOUR
ID - 1391

T1 - Making work play
A1 - Bowman,J.R.
Y1 - 1987///
N1 - Accession Number: SPH206329; Author: Bowman, J.R. ; Conference: Association for the Anthropological Study of Play. Meeting (11th : 1985 : Washington, D.C.).; Language: English; Parent Item: SPH203402; References: 29; Database Subset: SR; Publication Type: Book Analytic; Update Code: 19871201; SIRC Article No.: 206329
KW - *PLAY
KW - WORK
KW - Interviews
KW - SURVEYS
KW - Anthropology
KW - WORK ethic
KW - Observation
KW - SportDiscus
KW - Rough and Tumble
RP - NOT IN FILE
JA - Association for the Anthropological Study of Play.Meeting (11th : 1985 : Washington, D.C.)
CY - ;
N2 - Examines the ways in which work and play are interwoven during the work process. Describes the dualistic concept of work and play found in American culture. Presents a number of examples of adult play (pranks, horseplay, mock insults) which occur in social settings where individuals are working. Notes that while some of these episodes of playing on the job interrupt the ongoing work process, other examples describe workers successfully turning work into play while managing to accomplish work objectives. Concludes that the traditional way of viewing play and work as separate, polarized entities is far too simple, and that play and work must be seen as interactional events
UR -
https://login.proxy.bib.uottawa.ca/login?url=http://search.ebscohost.com/login.aspx?direct=true&db=s3h&AN=SPH206329&site=ehost-live;http://articles.sirc.ca/search.cfm?id=206329
ER -

TY - JOUR
ID - 1392
T1 - Should Horses 'Play' With Humans?
A1 - HAUG,LORE I.
Y1 - 2013/05//
N1 - Accession Number: 86857188; Author: HAUG, LORE I.: 1 ; Author Affiliation: 1 Texas Veterinary Behavior Services, Sugar Land, Texas; No. of Pages: 2; Language: English; Publication Type: Journal Article; Update Code: 20130410
KW - *HORSES -- Behavior
KW - *PLAY
KW - HUMAN-animal relationships
KW - SportDiscus
KW - Rough and Tumble
RP - NOT IN FILE
SP - 14
EP - 15
JA - Horse & Rider
VL - 52
IS - 5
N2 - The article provides an answer to a question of the playing behavior of horses with humans
SN - 00185159
UR -
https://login.proxy.bib.uottawa.ca/login?url=http://search.ebscohost.com/login.aspx?direct=true&db=s3h&AN=86857188&site=ehost-live
ER -

TY - JOUR
ID - 1393
T1 - Vertigo and social structure: notes on Hausa children's play
A1 - Olofson,H.
Y1 - 1977///
N1 - Accession Number: SPH67628; Author: Olofson, H. ; Conference: Association for the Anthropological Study of Play. Annual Meeting, 2d, Atlanta, 1976.; Language: English; Parent Item: SPH67617; References: 10; Database Subset: SR; Publication Type: Book Analytic; Update Code: 19940901; SIRC Article No.: 67628
KW - *GAMES
KW - *PLAY
KW - Societies
KW - Culture
KW - SOCIAL structure
KW - NIGERIA
KW - Child
KW - HAUSA
KW - SportDiscus
KW - Rough and Tumble
RP - NOT IN FILE
CY - ;
N2 - Data on the rough play of male children was elicted from an informant of the walled city of Zaria who had experienced such play himself. Provides notes on competitive games which (contrary to the assumptions of Caillois) have a strong component of vertigo. The function of vertigo and related play-symbols is analyzed as a factor in the socialization of male children into the Hausa world of social relations where there must be respect for age, strength, and superior social position. Vertigo in these examples is further explored in terms of a value for social and physical balance or stability which extends throughout Hausa culture
UR - https://login.proxy.bib.uottawa.ca/login?url=http://search.ebscohost.com/login.aspx?direct=true&db=s3h&AN=SPH67628&site=ehost-live;http://articles.sirc.ca/search.cfm?id=67628
ER -

TY - JOUR
ID - 1394
T1 - On Roughhousing and Throwing Your Children Around
A1 - Rader,Rick
Y1 - 2011/07//
N1 - Accession Number: 2011243768. Language: English. Entry Date: 20110909. Revision Date: 20140117. Publication Type: journal article; editorial. Journal Subset: Consumer Health; USA. Special Interest: Consumer Health; Pediatric Care. NLM UID: 7702637
KW - Serial Publications
KW - Parents of Disabled Children
KW - Play and Playthings -- Psychosocial Factors
KW - Child,Disabled -- Psychosocial Factors
KW - Child Development
KW - Parent-Child Relations
KW - Attitude to Disability
KW - CINAHL
KW - Rough and Tumble
RP - NOT IN FILE
SP - 4
EP - 4
JA - Exceptional Parent
VL - 41
IS - 7
SN - 0046-9157

UR - http://search.ebscohost.com/login.aspx?direct=true&db=cin20&AN=2011243768&site=ehost-live
ER -

TY - JOUR
ID - 1395
T1 - Rough Play: One of the Most Challenging Behaviors
A1 - Carlson,Frances M.
Y1 - 2011/07//
N1 - Last updated - 2012-12-14
SubjectsTermNotLitGenreText - 7940 126; 3085 3150; 916 8234; 11655 1474 316 8016 4542; 1432 909; 9254 7970; 10342 153 4398; 9017 6752 9651 6582 8016 4542; 10565 9015; 9753 5053 2787; 2221 909; 7676 5449 8768
KW - ERIC,Current Index to Journals in Education (CIJE)
KW - Early Childhood Education
KW - Preschool Education
KW - Teacher Role
KW - Play
KW - Child Behavior
KW - Young Children
KW - Cooperation
KW - Role Models
KW - Behavior Problems
KW - Peer Relationship
KW - School Policy
KW - Social Development
KW - Supervision
KW - ERIC
KW - Rough and Tumble
RP - NOT IN FILE
SP - 18
EP - 25
JF - Young Children
VL - 66
IS - 4
PB - National Association for the Education of Young Children. 1313 L Street NW Suite 500, Washington, DC 22205-4101.
N2 - Most children engage in rough play, and research demonstrates its physical, social, emotional, and cognitive value. Early childhood education settings have the responsibility to provide children with what best serves their developmental needs. One of the best ways teachers can support rough play is by modeling it for children. When adults model high levels of vigorous activity, the children in their care are more likely to play this way. Children also play more vigorously and more productively when their teachers have formal education or training in the importance of this type of play. Besides modeling, teachers can do three specific things to provide for and support rough play while minimizing the potential for injury: (1) prepare both the indoor and outdoor environment; (2) develop and implement policies and rules for rough play; and (3) supervise rough play so they can intervene when appropriate. When children successfully participate in big body play, it is "a measure of the children's social well-being and is marked by the ability of children to cooperate, to lead, and to follow." These abilities don't just support big body play; these skills are necessary for lifelong success in relationships
SN - 1538-6619, 1538-6619
UR - http://search.proquest.com/docview/1011399329?accountid=14701
ER -

TY - JOUR
ID - 1396
T1 - Observing Young Children's Rough-and-Tumble Play
A1 - Tannock,Michelle

Y1 - 2011/06//
N1 - Last updated - 2012-12-14
SubjectsTermNotLitGenreText - 7940 126; 11655 1474 316 8016 4542; 4290; 1443 5053 2787; 3085 3150; 1432 909; 7234; 2798; 1433 9806 4911 9556; 4109 4335; 1699 1595 7404
KW - Canada
KW - ERIC,Current Index to Journals in Education (CIJE)
KW - Early Childhood Education
KW - Coding
KW - Foreign Countries
KW - Play
KW - Child Behavior
KW - Young Children
KW - Gender Differences
KW - Developmental Stages
KW - Child Development
KW - Child Care
KW - Observation
KW - ERIC
KW - Rough and Tumble
RP - NOT IN FILE
SP - 13
EP - 20
JF - Australasian Journal of Early Childhood
VL - 36
IS - 2
PB - Early Childhood Australia. P.O. Box 7105, Watson, ACT 2602, Australia. Tel: +61-2-6242-1800; Fax: +61-2-6242-1818; e-mail: publishing@earlychildhood.org.au; Web site: http://www.earlychildhoodaustralia.org.au
N2 - This research investigated the rough-and-tumble play of 17 five-year-old children in two early childhood settings. The study resulted in an increased understanding of the forms of rough-and-tumble play displayed by young children. This study demonstrates that both boys and girls are engaged in a variety of rough-and-tumble play behaviours. Twenty-seven distinct rough-and-tumble play behaviours were exhibited during the observation period. They included eight components identified as rough-and-tumble play in previous research and also 19 behaviours not previously identified as elements of such. These elements indicate pre-operational forms of rough-and-tumble play. The results of this study have implications for the understanding of child development. It suggests that rough-and-tumble play evolves as children age; that children move into more complex play behaviours as they mature. (Contains 2 tables and 1 figure.)
SN - 1836-9391, 1836-9391
UR - http://search.proquest.com/docview/1031154668?accountid=14701
ER -

TY - JOUR
ID - 1397
T1 - Evolutionary Functions of Social Play: Life Histories, Sex Differences, and Emotion Regulation
A1 - LaFreniere,Peter
Y1 - 2011///
N1 - Last updated - 2013-02-26
SubjectsTermNotLitGenreText - 7940 126; 7782; 1446 4634; 4290; 3395 8930 909; 9457 909; 520; 1474 316 8016 4542; 990 6113 7104 8371 6120 4918 5964; 6124 8477; 1459; 9753 5053 2787; 1858 9690 1; 925 8836
KW - ERIC,Current Index to Journals in Education (CIJE)
KW - Animals
KW - Play
KW - Gender Differences
KW - Child Health
KW - Perspective Taking
KW - Literature Reviews

KW - CHILDREN
KW - Communication Skills
KW - Biographies
KW - Child Rearing
KW - Social Development
KW - Emotional Response
KW - Self Control
KW - Behavioral Science Research
KW - ERIC
KW - Rough and Tumble
RP - NOT IN FILE
SP - 464
EP - 488
JF - American Journal of Play
VL - 3
IS - 4
PB - The Strong. One Manhattan Square, Rochester, NY 14607.
N2 - Many research findings about animal play apply to children's play, revealing structural and functional similarities with mammals in general and primates in particular. After an introduction to life-history theory, and before turning to humans, the author reviews research about the two mammals in which play has been studied the most extensively: laboratory rats and monkeys. He looks at the development of play, deprivation studies, gender segregation, and the functions of gender-differentiated forms of play. The gender segregation and sex differences in play parenting and rough-and-tumble play observed in many primates are also evident in children. Vigorous social-play benefits all children physically by developing strong bones and muscles, by promoting cardiovascular fitness, and by encouraging exercise habits that help prevent obesity. Unsupervised play also helps hone the skills of communication, perspective taking, and emotion regulation. For boys especially, rough-and-tumble play in early childhood provides a scaffold for learning emotion-regulation skills related to managing anger and aggression
SN - 1938-0399, 1938-0399
UR - http://search.proquest.com/docview/1312421090?accountid=14701
ER -

TY - BOOK
ID - 1398
T1 - The Oxford Handbook of the Development of Play. First Edition. Oxford Library of Psychology
A1 - Pellegrini,Anthony D.
Y1 - 2010/11//
N1 - Last updated - 2012-12-14
KW - ERIC,Resources in Education (RIE)
KW - Parents
KW - Teachers
KW - Policymakers
KW - Cultural Context
KW - Learning
KW - Play
KW - Primatology
KW - Gender Differences
KW - Research Methodology
KW - Personality
KW - Observation
KW - Peer Relationship
KW - History
KW - Parent Child Relationship
KW - Educational Policy
KW - Theories
KW - Child Development

KW - Cultural Differences
KW - Games
KW - Interpersonal Relationship
KW - Autism
KW - Evolution
KW - Playground Activities
KW - ERIC
KW - Rough and Tumble
RP - NOT IN FILE
SP - 408
PB - Oxford University Press. 198 Madison Avenue, New York, NY 10016.
N2 - The role of play in human development has long been the subject of controversy. Despite being championed by many of the foremost scholars of the twentieth century, play has been dogged by underrepresentation and marginalization in literature across the scientific disciplines. "The Oxford Handbook of the Development of Play" marks the first attempt to examine the development of children's play through a rigorous and multidisciplinary approach. Comprising chapters from the foremost scholars in psychology, anthropology, and evolutionary biology, this handbook resets the landscape of developmental science and makes a compelling case for the benefits of play. Edited by respected play researcher Anthony D. Pellegrini, "The Oxford Handbook of the Development of Play" is both a scientific accomplishment and a shot across the bow for parents, educators, and policymakers regarding the importance of children's play in both development and learning. This book comprises 24 specially-commissioned chapters by the leading psychologists, anthropologists, and evolutionary biologists in the field of play. It discusses human play from an ethological perspective, examining its ontogeny, phylogeny (especially across primate species), proximal factors and functions. It also emphasizes the importance of play in the debate over the role of recess in the school curriculum. It is divided into seven parts. Part I, Introduction and Overview, contains the following: (1) Introduction (Anthony D. Pellegrini). Part II, Definitions, contains the following: (2) Defining and Recognizing Play (Gordon M. Burghardt); and (3) Cultural Variations in Beliefs about Play, Parent-Child Play, and Children's Play: Meaning for Childhood Development (Jaipaul L. Roopnarine). Part III, Theories, contains the following: (4) Theories of Play (Patrick Bateson); (5) Comparing and Extending Piaget's and Vygotsky's Understandings of Play: Symbolic play as Individual, Sociocultural, and Educational Interpretation (Artin Goncu and Suzanne Gaskins); (6) Gene X Environment Interactions and Social Play: Contributions from Rhesus Macaques (Khalisa N. Herman, Annika Paukner, and Stephen J. Suomi); (7) Playing at Every Age: Modalities and Potential Functions in Non-Human Primates (Elisabetta Palagi); (8) Play and Development (Robert M. Fagen); (9) The History of Children's Play in the United States (Howard P. Chudacoff); and (10) The Antipathies of Play (Brian Sutton-Smith). Part IV, Methods, contains the following: (11) The Cultural Ecology of Play: Methodological Considerations for Studying Play in Its Everyday Contexts (Jonathan R. H. Tudge, Jill R. Brown, and Lia B. L. Freitas); and (12) Observational Methods in Studying Play (Peter K. Smith). Part V, Dimensions of Play, contains the following: (13) Object Play and Tool Use: Developmental and Evolutionary Perspectives (David F. Bjorklund and Amy K. Gardiner); (14) The Development and Function of Locomotor Play (Anthony D. Pellegrini); (15) Not Just "Playing Alone": Exploring Multiple Forms of Nonsocial Play in Childhood (Robert J. Coplan); (16) Internalizing and Externalizing Disorders during Childhood: Implications for Social Play (David Schwartz and Daryaneh Badaly); (17) Gender and Temperament in Young Children's Social Interactions (Carol Lynn Martin, Richard A. Fabes, Laura D. Hanish); (18) Social Play of Children with Adults and Peers (Carollee Howes); (19) Rough-and-Tumble Play: Training and Using the Social Brain (Sergio M. Pellis and Vivien C. Pellis); (20) Children's Games and Playground Activities in School and Their Role in Development (Ed Baines and Peter Blatchford); (21) Mother-Child Fantasy Play (Angeline S. Lillard); (22) Origins and Consequences of Social Pretend Play (Robert D. Kavanaugh); (23) The Development of Pretend Play in Autism (Christopher Jarrold and Carmel Conn); and (24) Technology and Play (Jeffrey Goldstein). Part VI, Education, contains the following: (25) Playing around in School: Implications for Learning and Educational Policy (Kelly Fisher, Kathy Hirsh-Pasek, Roberta M. Golinkoff, Dorothy G. Singer, and Laura Berk). Finally, Part VII contains a conclusion by Anthony D. Pellegrini
SN - 0195393007
UR - http://search.proquest.com/docview/881462654?accountid=14701
ER -

TY - JOUR

ID - 1399
T1 - Preschool Teachers' Views of Active Play
A1 - Logue,Mary Ellin
A1 - Harvey,Hattie
Y1 - 2010///
N1 - Last updated - 2013-02-26
SubjectsTermNotLitGenreText - 2521 3184 2787; 7940 126; 10511 8331; 3005 9021 9651 6582; 3085 3150; 8135 10591 8267 3417 8016 4542 5703 4908 8917; 4290; 8127 11655 1474 316 8016 4542; 10576 5449 8768; 10579 10380 3629 6582; 10482 730; 8535 6447; 1432 909; 10183 909; 8466 4403 3417 8016 4542 5703 4908 8917 10591 8267; 8467 9306 5241; 10087 2574 3629 6582
KW - United States (Northeast)
KW - ERIC,Current Index to Journals in Education (CIJE)
KW - Early Childhood Education
KW - Preschool Education
KW - Preschool Teachers
KW - Teacher Student Relationship
KW - Questionnaires
KW - Play
KW - Child Behavior
KW - Gender Differences
KW - Dramatic Play
KW - Teacher Attitudes
KW - Public School Teachers
KW - Teacher Surveys
KW - Public Schools
KW - Curriculum Development
KW - Statistical Analysis
KW - Student Behavior
KW - Preschool Children
KW - Teacher Education Programs
KW - ERIC
KW - Rough and Tumble
RP - NOT IN FILE
SP - 32
EP - 49
JF - Journal of Research in Childhood Education
VL - 24
IS - 1
PB - Routledge. , 325 Chestnut Street Suite 800, Philadelphia, PA 19106.
N2 - This study surveyed 98 teachers of 4-year-olds about dramatic play in their classrooms and about their attitudes and practices about rough-and-tumble play. Gender differences emerged in the nature of dramatic play reported and in the ways in which teachers interacted with children engaged in different forms of dramatic play. Teachers also reported their coursework in early childhood education as the greatest influence on their beliefs and attitudes about rough-and-tumble play. The findings have implications for curriculum planning, school behavior policies, and teacher education programs. (Contains 3 tables and 5 figures.)
SN - 0256-8543, 0256-8543
UR - http://search.proquest.com/docview/881454279?accountid=14701
ER -

TY - JOUR
ID - 1400
T1 - Rough and Tumble Play 101
A1 - Carlson,Frances
Y1 - 2009///

N1 - Last updated - 2012-12-14
SubjectsTermNotLitGenreText - 7940 126; 3899 8415; 6725; 8684; 7676 5449 8768; 83 8168; 324 545 9738 909; 3190 3518; 10565 9015; 7840 126; 1443 5053 2787
KW - ERIC,Current Index to Journals in Education (CIJE)
KW - Teacher Role
KW - Physical Activities
KW - Educational Environment
KW - Misconceptions
KW - Play
KW - Fear
KW - Child Development
KW - Recess Breaks
KW - Aggression
KW - Accident Prevention
KW - Peer Relationship
KW - ERIC
KW - Rough and Tumble
RP - NOT IN FILE
SP - 70
EP - 72
JF - Exchange: The Early Childhood Leaders' Magazine Since 1978
IS - 188
PB - Exchange Press, P.O. Box 3249, Redmond, WA 98073-3249.
N2 - Many people fear that play-fighting or rough and tumble play is the same as real fighting. There is also a fear that this rough play will become real fighting if allowed to continue. Most of all, parents and teachers fear that during the course of rough and tumble play a child may be hurt. To provide for and allow children to play rough without injury, teachers need to understand how rough play is different from aggression, as well as about how to offer it in a safe and supportive environment. For young children, rough and tumble play is seemingly universal and dates back to the 12th century (Jarvis, 2007). This style of play incorporates a range of physical behaviors that range from running and chasing to tagging and wrestling. The author discusses the significance of rough and tumble play and outlines the physical benefits of rough and tumble play and its policies and rules
SN - 0164-8527, 0164-8527
UR - http://search.proquest.com/docview/61842162?accountid=14701
ER -

TY - JOUR
ID - 1401
T1 - Play Teaches What Testing Can't Touch: Humanity
A1 - Chmelynski,Carol
Y1 - 2006/11//
N1 - Last updated - 2012-12-14
SubjectsTermNotLitGenreText - 28 96; 9457 909; 2462 10120; 7847 5053 2787; 7223 1096 7843 5051 2923 2877 1088; 1485; 7940 126; 9769 5127; 8684; 9274 9126 7921; 3390 5333 8409 5051; 5446 1970 1
KW - Oregon
KW - ERIC,Current Index to Journals in Education (CIJE)
KW - Elementary Education
KW - Social Influences
KW - Obesity
KW - Play
KW - School Schedules
KW - Academic Achievement
KW - Recess Breaks
KW - Physical Development
KW - Emotional Intelligence
KW - Interpersonal Competence
KW - Cues

KW - Childrens Rights
KW - Self Control
KW - ERIC
KW - Rough and Tumble
RP - NOT IN FILE
SP - 10
EP - 13
JF - Education Digest: Essential Readings Condensed for Quick Review
VL - 72
IS - 3
PB - Prakken Publications. 832 Phoenix Drive, P.O. Box 8623, Ann Arbor, MI 48108.
N2 - Pressures to improve test scores and avoid lawsuits are causing many schools to cut back play time. According to the American Association for the Child's Right to play, nearly 40% of the nation's 16,000 school districts have either modified, deleted, or are considering deleting recess. Moreover, where playtime does still exist, it has become overly safe and highly predictable. But according to experts, play makes children more resistant to chronic illnesses since they become more physically active and fit. Also, rough-and-tumble play is how kids learn social skills, how they learn to inhibit aggression, and how to recognize aggressive facial cues. Lack of playtime increasing regulation of what playtime does exist contributes to the childhood obesity crisis, harms children by removing an outlet for creativity, and is a factor in impulse control problems like ADHD (attention deficit hyperactivity disorder)
SN - 0013-127X, 0013-127X
UR - http://search.proquest.com/docview/62019083?accountid=14701
ER -

TY - BOOK
ID - 1402
T1 - The Excellence of Play. Second Edition
A1 - Moyles,Janet
Y1 - 2005/08//
N1 - Last updated - 2012-12-14
KW - ERIC,Resources in Education (RIE)
KW - Students
KW - Practitioners
KW - Early Childhood Education
KW - Science Education
KW - Teacher Role
KW - Toddlers
KW - Class Activities
KW - Language Acquisition
KW - Play
KW - Young Children
KW - Special Needs Students
KW - Gender Differences
KW - Role Playing
KW - Educational Needs
KW - Music Education
KW - Mathematics Education
KW - Art Education
KW - Physical Activities
KW - Story Telling
KW - Student Evaluation
KW - Literacy
KW - Learning Strategies
KW - Infants
KW - ERIC
KW - Rough and Tumble

RP - NOT IN FILE
SP - 320
PB - Open University Press. The McGraw-Hill companies, Order Services, P.O. Box 182605, Columbus, OH 43218-2605.
N2 - This second edition of "The Excellence of Play" encapsulates all of the many changes that have taken place in early childhood in the last decade. It examines the vital importance of play as a tool for learning and teaching for children and practitioners, supporting all those who work in early childhood education and care in developing and implementing the highest quality play experiences for young children. All the contributors are experts in their fields and all are passionate about the excellence of play. While the importance of curriculum and assessment is retained and extended, this edition features many new contributions, including: Children as social and active agents in their own play; More background to current research on play theory and practice; Practitioners' roles in play and adults' enabling of play; Links with the Foundation Stage (including legislation and policy); Links with the first years of school and beyond Outdoor and physical play, including rough and tumble; Gender differences; Play and observation/assessment; Special Educational Needs and play. This book provides a powerful argument that a curriculum which sanctions and utilizes play is more likely to provide well-balanced citizens of the future, as well as happier and more learned children in the present. Following a foreword by Iram Siraj-Blatchford; and an introduction by Janet Moyles this book is divided into four parts. Part One, Setting the Play Context, contains the following chapters: (1) Play and Legislated Curriculum: Back to Basics--An Alternative View (Angela Anning); (2) Learning to Play, Playing to Learn--Babies and Young Children Birth to Three (Ann Langston and Lesley Abbott); and (3) Play and Special Needs (Theodora Papatheodorou). Part Two, Play, Language, and Literacy Development, continues with the following chapters: (4) Play, Storytelling and Creative Writing (David Whitebread and Helen Jameson); (5) Open-Ended Role Play: Supporting Creativity and Developing Identity (Pat Broadhead and Carey English); (6) Play, Literacy and Situated Learning (Nigel Hall); (7) Story-Making, Play and Gender (Jane Hislam); and (8) Fantasy Play and the Case for Adult Intervention (Neil Kitson). Part Three, Play and the Curriculum, presents the next six chapters: (9) Play Activity and Rough and Tumble Play (Peter K. Smith); (10) Outdoor Play and the Pedagogic Garden (Stephanie Harding); (11) Science and Play (Alan Howe and Dan Davies); (12) Mathematics and Play (Rose Craven Griffiths); (13) Art in the Early Years (Bernadette Duffy); and (14) Playing Music (Linda Pound). Part Four, Play Culture and Playful Practitioners, presents the final chapters: (15) Practitioners and Play: Reflecting in a Different Way (Sian Adams); (16) The Importance of Play in the Transition to School (Hilary Fabian and Aline-Wendy Dunlop); (17) Play in the Early Years: The Influence of Cultural Difference (Tricia David and Sacha Powell); and (18) Play, the Universe and Everything! (Tina Bruce). The book concludes with an afterword by Janet Moyles; and a list of references. [For the first edition of this book, see ED377984.]
SN - 0335217583
UR - http://search.proquest.com/docview/62087367?accountid=14701
ER -

TY - GEN
ID - 1403
T1 - Shaken Baby Syndrome: What Caregivers Need To Know
A1 - Wiggins,Paula
Y1 - 2000///
N1 - Last updated - 2012-12-14
KW - Shaken Baby Syndrome
KW - ERIC,Current Index to Journals in Education (CIJE)
KW - Play
KW - Parent Child Relationship
KW - Infant Behavior
KW - Child Caregivers
KW - Child Health
KW - Brain
KW - Crying
KW - Child Abuse
KW - Violence
KW - Infants
KW - ERIC

KW - Rough and Tumble
RP - NOT IN FILE
SP - 16
EP - 19
JF - Texas Child Care
VL - 23
IS - 4
N2 - Discusses the causes of shaken baby syndrome and how to recognize, respond to, and prevent it. Identifies horseplay to avoid and recommends never shaking baby even for apnea. Offers 12 tips for working with crying babies and includes ten discussion questions to test knowledge of the syndrome. (DLH)
UR - http://search.proquest.com/docview/62334008?accountid=14701
ER -

TY - GEN
ID - 1404
T1 - Child's Play: A Work-Family Issue
A1 - Kuchner,Joan F.
Y1 - 1998/04//
N1 - Availability - Level 1 - Available online, if indexed January 1993 onward
SuppNotes - Paper presented at the Annual National Coalition for Campus Children's Centers Conference (St. Louis, MO, April 1-4, 1998)
Last updated - 2012-12-14
KW - Daily Routines
KW - Family Activities
KW - Play Learning
KW - ERIC,Resources in Education (RIE)
KW - Preschool Education
KW - Preschool Teachers
KW - Play
KW - Parent Child Relationship
KW - Child Rearing
KW - Stress Management
KW - Preschool Children
KW - Parents
KW - ERIC
KW - Rough and Tumble
RP - NOT IN FILE
SP - 8
N2 - Many parents find that the daily stress of coping with the demanding realities of their work lives leaves them with little personal and family time; playing with their children may therefore fall to the bottom of the "to-do list." One of the tasks of early childhood professionals thus becomes helping parents understand the nature and value of play and guiding them toward an appreciation of how playful parent-child interactions may assist parents in reaching their own goals. This paper provides information for early childhood professionals to use toward that end. The paper discusses the role of play as: (1) part of a time management strategy to build relationships with their children, encouraging their success and their health--thereby meeting long-term goals; (2) reducing stress for parents and children; (3) facilitating school readiness; (4) nurturing communication skills that are part of emotional intelligence; (5) providing an avenue for emotional expression; (6) nourishing to the parent-child relationship; and (7) providing the basis for positive discipline, preventing power struggles. The paper concludes that by providing a forum for exploring ways to fit play into found time, early childhood professionals can help parents plan home environments to encourage play and realize how play can smooth rough spots in routines. (KB)
UR - http://search.proquest.com/docview/62377802?accountid=14701
ER -

TY - GEN
ID - 1405

T1 - Dealing with Hitting and Aggression in the Classroom
A1 - Saifer,Steffen
Y1 - 1996///
N1 - Last updated - 2012-12-14
KW - Hitting Behavior (Aggression)
KW - Rough and Tumble Play
KW - Toy Guns
KW - War Games (Children)
KW - War Toys
KW - ERIC,Current Index to Journals in Education (CIJE)
KW - Early Childhood Education
KW - Play
KW - Classroom Environment
KW - Change Strategies
KW - Discipline
KW - Aggression
KW - Behavior Problems
KW - Classroom Techniques
KW - ERIC
KW - Rough and Tumble
RP - NOT IN FILE
SP - 37
EP - 39
JF - NHSA Journal
VL - 15
IS - 1
N2 - Notes that while hitting-aggressive behavior is probably the greatest single behavior concern of teachers, children can be taught appropriate behavior for the classroom. Offers tips for dealing with: roughhousing; existing problems; grabbing toys; and war games, guns, or violent play. Suggests allowing children the choice of an alternative activity rather than employing "time out." (TJQ)
UR - http://search.proquest.com/docview/62691245?accountid=14701
ER -

TY - GEN
ID - 1406
T1 - Play: An Intergenerational Experience. Proceedings of IPA/USA, The American Affiliate of the International Association for the Child's Right To Play 1995 National Conference (Birmingham, Alabama, August 24-27, 1995)
A1 - Guddemi,Marcy
Y1 - 1996///
N1 - Availability - Level 3 - Indexed only
Last updated - 2012-12-14
KW - Historical Influences
KW - Play Theory
KW - Playground Design
KW - United Kingdom
KW - ERIC,Resources in Education (RIE)
KW - Teacher Role
KW - Kindergarten Children
KW - Reminiscence
KW - Play
KW - Intergenerational Programs
KW - CHILDREN
KW - Physical Education
KW - Foreign Countries
KW - Playgrounds

KW - Parent Child Relationship
KW - Childrens Rights
KW - Pretend Play
KW - ERIC
KW - Rough and Tumble
RP - NOT IN FILE
SP - 96
PB - Southern Early Childhood Association, P.O. Box 55930, Little Rock, AR 72215-5930 (SECA members, $8; Non-members, $9).
N2 - These proceedings contain reprints of 26 papers presented at the 1995 IPA/USA (The American Affiliate of the International Association for the Child's Right to Play) national conference. The aim of the conference was to bring professional and public awareness to intergenerational needs and models with regard to children's development and play environments. The proceedings are presented in five sections: (1) "Historical/Reflections," including papers on intergenerational support for play, memories of the elderly regarding play, and how intergenerational play differs in the 1920s, the 1950s, and the 1990s; (2) "Schools," including papers on teachers' role on the playground, kindergarten children's initial physical education experiences, and youngsters' construction of physical knowledge on swings; (3) "Home and Community," including papers on hospital play, parents and children as play partners, and planning and implementing a community play day; (4) "Design," including papers on community playgrounds, community-built gardens, and children's participation in designing outdoor play spaces; and (5) "Issues," including papers on rough and tumble play, playground safety, political action, and learning from programs in the United Kingdom. Each section is preceded by an introduction describing highlights of the articles in that section. Each article concludes with biographical information on the author(s). (KDFB)
SN - 0942388232
UR - http://search.proquest.com/docview/62580265?accountid=14701
ER -

TY - GEN
ID - 1407
T1 - Building in Opportunities for Gross Motor Development
A1 - Olds,Anita Rui
Y1 - 1994///
N1 - Last updated - 2012-12-14
KW - Play Learning
KW - Playground Equipment
KW - ERIC,Current Index to Journals in Education (CIJE)
KW - Early Childhood Education
KW - Physical Activities
KW - Learning
KW - Play
KW - Motor Development
KW - Special Needs Students
KW - Playgrounds
KW - Inservice Teacher Education
KW - Psychomotor Skills
KW - Playground Activities
KW - Physical Activity Level
KW - ERIC
KW - Rough and Tumble
RP - NOT IN FILE
SP - 31
EP - 50
JF - Child Care Information Exchange
IS - 97
N2 - This special training section includes four articles on the importance of movement and action to children's development. The articles are "From Cartwheels to Caterpillars: Children's Need to Move Indoors and Out";

"Kids Gotta Move: Adapting Movement Experiences for Children with Differing Abilities"; "Roughhousing as a Style of Play"; and "Moving Teachers to Move Children." (TJQ)
UR - http://search.proquest.com/docview/62818479?accountid=14701
ER -

TY - GEN
ID - 1408
T1 - Playful Teaching Workshop Sampler. Classroom Play Ideas for Early Childhood Educators
A1 - Battista,Donna
Y1 - 1994///
N1 - Availability - Level 1 - Available online, if indexed January 1993 onward
SuppNotes - Presented at the Annual Meeting of the Midwest Association for the Education of Young Children (Peoria, IL, April 20-23, 1994)
Last updated - 2012-12-14
KW - ERIC,Resources in Education (RIE)
KW - Teachers
KW - Practitioners
KW - Early Childhood Education
KW - Movement Education
KW - Physical Activities
KW - Play
KW - Class Activities
KW - Perceptual Development
KW - Piagetian Theory
KW - Cognitive Style
KW - ERIC
RP - NOT IN FILE
SP - 25
PB - Animated Learning Systems, 1110 Cross Creek, Valparaiso, IN 46383-2030 ($8).
N2 - Based on Piaget's theory "children learn best by doing," play offers a natural way of learning for young children. The "Playful Teaching" Workshop Sampler offers teachers several different movement activities for incorporation into academic classroom curricula. The play ideas were presented at national and regional teaching workshops. The publication concludes with the author's views on why high quality play curricula promote academic readiness for early learners. It is argued that purposeful play provides an effective avenue for advancing sensory and perceptual motor development and allows young children to begin to visualize learning as fun and to see themselves as capable learners. Play addresses the needs of all kinds of learners...the kinesthetic child, the tactile child, the auditory child, and the visual child. Different learning styles are encouraged through the freedom of playful movement activity. This sampler, organized into four sections, shares play ideas relating to: (1) the movement pattern of a snake; (2) the movement pattern of a horse; (3) basic balance concepts; and (4) ball manipulation. Each of the 20 activities provided includes descriptions of both necessary equipment and procedures. (LL)
UR - http://search.proquest.com/docview/62569413?accountid=14701
ER -

TY - RPRT
ID - 1409
T1 - A Descriptive Study of Multicultural Elementary Student Playground Behaviors and Their Relationship to Gender, Age, Race and Socioeconomic Status
A1 - Dahmes,Victoria M.
Y1 - 1993/11//
N1 - Availability - Level 1 - Available online, if indexed January 1993 onward
SuppNotes - Paper presented at the Annual Meeting of the Mid-South Educational Research Association (New Orleans, LA, November 10-12, 1993)
Last updated - 2012-12-14
KW - ERIC,Resources in Education (RIE)
KW - Elementary Education

KW - Elementary School Students
KW - Play
KW - Child Behavior
KW - Blacks
KW - Teacher Attitudes
KW - Ethnography
KW - Peer Relationship
KW - Whites
KW - Cross Cultural Studies
KW - Hispanic Americans
KW - Sex Differences
KW - Cultural Differences
KW - Asian Americans
KW - Interpersonal Relationship
KW - Age Differences
KW - Playground Activities
KW - ERIC
KW - Rough and Tumble
RP - NOT IN FILE
SP - 34
N2 - This paper examines the observable play behaviors of elementary school students to determine how children interact within and among cultural groups. Observations were conducted during the recess period at an urban elementary school attended by 623 boys and girls age 6 through 12. Recess periods and play areas were segregated by grade and sex. Data were collected through observations of playground behaviors and interviews of playground duty teachers. Results indicated that boys tended to engage in more rough and tumble play, whereas girls tended to engage in more passive activities such as jumping rope. Older boys tended to exclude younger boys from games they were playing, whereas older girls tended to include younger girls in their activities. Asian students kept to themselves more than any other group, often excluding non-Asians from their activities. Blacks, Hispanics, and Whites tended to mix freely and play in non-segregated groups. (MDM)
UR - http://search.proquest.com/docview/62803731?accountid=14701
ER -

TY - RPRT
ID - 1410
T1 - Early Play Arousal, Sex-Typed Play, and Activity Level as Precursors to Later Rough-and-Tumble Play
A1 - Bride-Chang,Catherine
A1 - Jacklin,Carol Nagy
Y1 - 1993///
N1 - Last updated - 2012-12-14
KW - ERIC,Current Index to Journals in Education (CIJE)
KW - Early Childhood Education
KW - Sex Role
KW - Play
KW - Young Children
KW - Parent Child Relationship
KW - Physical Activity Level
KW - ERIC
KW - Rough and Tumble
RP - NOT IN FILE
SP - 99
EP - 108
N2 - The extent of father's participation in rough-and-tumble (R&T) play with their children when the children were 18 months old, and children's early preferences for play sex-typed as boys' play, were related to levels of children's R&T play in first grade. (MDM)
UR - http://search.proquest.com/docview/62871270?accountid=14701
ER -

TY - JOUR
ID - 1411
T1 - Commentary: Play, Parenting, and Peer Partners: Keys to Understanding Children's Social Development?
A1 - Ladd,Gary W.
Y1 - 1992///
N1 - SuppNotes - Special Issue: Prosocial and Aggressive Play Behavior
Last updated - 2012-12-14
SubjectsTermNotLitGenreText - 915; Child Development 1443 5053 2787; 3085 3150; 7537 3851 5449 8768; 7672 5127; Peer Relationship 7676 5449 8768; 7940 126; Social Development 9753 5053 2787; 9822; Young Children 11655 1474 316 8016 4542
KW - Rough Play
KW - Toy Guns
KW - War Toys
KW - ERIC,Current Index to Journals in Education (CIJE)
KW - Early Childhood Education
KW - Play
KW - Young Children
KW - Parent Child Relationship
KW - Behavior Patterns
KW - Peer Influence
KW - Child Development
KW - Social Development
KW - SOCIALIZATION
KW - Peer Relationship
KW - ERIC
KW - Rough and Tumble
RP - NOT IN FILE
SP - 401
EP - 406
JF - Early Education and Development
VL - 3
IS - 4
N2 - A commentary on the articles in this special issue addresses the themes of the origins and consequences of children's adversarial social roles and behaviors; children's peer relationships as a context for social learning and development; and linkages between the family and peer systems. (LB)
SN - 1040-9289, 1040-9289
UR - http://search.proquest.com/docview/62849686?accountid=14701
ER -

TY - JOUR
ID - 1412
T1 - A Longitudinal Study of Popular and Rejected Children's Rough-and-Tumble Play
A1 - Pellegrini,D.A.
Y1 - 1991///
N1 - Last updated - 2012-12-14
SubjectsTermNotLitGenreText - 324 545 9738 909; 3352 3368 3150; Elementary School Students 3363 10278 8016 4542; 6175 1326 3629 6582 8836; Play 7940 126; Popularity 8012; 8233 1710; Rejection (Psychology) 8766 8415; Social Cognition 9748 1710; 10183 909
KW - Cognitive Flexibility
KW - Rough and Tumble Play
KW - Social Affiliation
KW - Social Problem Solving
KW - Sociometric Status
KW - ERIC,Current Index to Journals in Education (CIJE)
KW - Elementary Education

KW - Elementary School Students
KW - Rejection (Psychology)
KW - Play
KW - Popularity
KW - Social Cognition
KW - Student Behavior
KW - Longitudinal Studies
KW - Aggression
KW - Problem Solving
KW - ERIC
KW - Rough and Tumble
RP - NOT IN FILE
SP - 205
EP - 213
JF - Early Education and Development
VL - 2
IS - 3
N2 - Examined longitudinal associations between the rough-and-tumble play of popular and rejected children and measures of their social affiliation and social cognition. Results suggest that rough-and-tumble play for popular, but not rejected, children predicts aspects of social affiliation and social cognitive status. (Author/GLR)
SN - 1040-8289, 1040-8289
UR - http://search.proquest.com/docview/62928606?accountid=14701
ER -

TY - GEN
ID - 1413
T1 - School-Age Ideas and Activities for After School Programs
A1 - Haas-Foletta,Karen
A1 - Cogley,Michele
Y1 - 1990///
N1 - Availability - Level 2 - Produced in microfiche (1966-2003)
Last updated - 2012-12-14
KW - Social Problem Solving
KW - ERIC,Resources in Education (RIE)
KW - Elementary Education
KW - Printing
KW - Play
KW - Class Activities
KW - After School Programs
KW - Painting (Visual Arts)
KW - Horticulture
KW - Problem Solving
KW - Childrens Games
KW - Science Projects
KW - Handicrafts
KW - Day Care
KW - Cooking Instruction
KW - ERIC
KW - Rough and Tumble
RP - NOT IN FILE
SP - 174
PB - School-Age NOTES, P.O. Box 40205, Nashville, TN 37204 ($16.95).
N2 - This guide describes activities for school-age children in after-school day care programs. These activities may also be used in other settings. An introductory section discusses program philosophy, room arrangement, multicultural curriculum, program scheduling, summer programs and holiday care, field trips and special programs, age grouping, communication between parents and teachers, and conflict resolution. Sections of the

guide describe approximately 150 activities that relate to: (1) problem solving in general and social problem solving in particular; (2) rough-and-tumble play; (3) painting; (4) printing; (5) making cards and gifts; (6) making masks, puppets, and gifts; (7) candle making; (8) making three-dimensional artworks; (9) cooking art supplies from recipes; (10) craft projects involving nature; (11) planting and growing plants and vegetables; (12) science experiments; and (13) indoor and outdoor games. A resource list of more than 120 publications is appended. (BC)
SN - 0917505034
UR - http://search.proquest.com/docview/62890914?accountid=14701
ER -

TY - GEN
ID - 1414
T1 - The Positive Aspects of Aggressive Behavior in Young Children
A1 - Periolat,Janet
A1 - Nager,Nancy
Y1 - 1988/12//
N1 - Availability - Level 1 - Available online, if indexed January 1993 onward
Last updated - 2012-12-14
KW - Play Fighting
KW - ERIC,Resources in Education (RIE)
KW - Preschool Education
KW - Teacher Role
KW - Teacher Student Relationship
KW - Teacher Influence
KW - Teacher Behavior
KW - Play
KW - Child Behavior
KW - Childhood Needs
KW - Conflict Resolution
KW - Conflict
KW - Child Responsibility
KW - Teacher Responsibility
KW - Hostility
KW - Sex Differences
KW - Preschool Children
KW - Aggression
KW - ERIC
KW - Rough and Tumble
RP - NOT IN FILE
SP - 20
N2 - Not all fighting or aggression in young children is bad, and some kinds of teacher intervention may be beneficial. Play-fighting refers primarily to rough and tumble play and chasing, and several studies have shown that play and serious fighting can be clearly distinguished in young children. Numerous authors have pointed out the value and positive aspects of aggression. Aggressive behavior is often the most immediate way for a child to communicate his or her desires and needs, and teachers need to try to understand what children are communicating rather than judge their behavior. Play-fighting is a very common occurrence among boys, and seems to occur less with girls. Girls who display aggressive behavior have generally been socialized like boys. Boys often use aggressive behavior and play-fighting to make friends, exhibit frustration due to failure at making friends, and to become more assertive and overcome their fear of aggressive boys. It is crucial for adults and teachers to encourage children to respond to aggression by asserting their feelings with statements such as "Stop it" or "I don't like that," rather than by simply hitting back or running to the teacher. A child's self-esteem can build when encouraged to take responsibility for his or her own feelings. Teachers can play a positive role in assisting children to express and channel their aggressive behavior. (AS)
UR - http://search.proquest.com/docview/62698227?accountid=14701
ER -

TY - GEN
ID - 1415
T1 - Toy Making for Parents and Caregivers
A1 - Cortez,Carmen P.
Y1 - 1985/06//
N1 - Availability - Level 1 - Available online, if indexed January 1993 onward
SuppNotes - Paper presented at the Annual Conference of the Association for Childhood Education International (San Antonio, TX, June 19-22, 1985)
Last updated - 2012-12-14
KW - ERIC,Resources in Education (RIE)
KW - Parents
KW - Teachers
KW - Practitioners
KW - Instructional Materials
KW - Play
KW - Parent Child Relationship
KW - Child Caregivers
KW - Family Life
KW - Learning Processes
KW - Learning Activities
KW - Toys
KW - Psychomotor Skills
KW - Skill Development
KW - ERIC
KW - Rough and Tumble
RP - NOT IN FILE
SP - 79
N2 - Recognizing that toy-making activities have tremendous potential for developing good parent-child interactions, this paper examines (1) the why, where, when, how, and who of making toys at home; (2) the use of toys in play; and (3) the value of toys as tools for learning. The snags and obstacles of toy making as well as the challenges and benefits are mentioned. Most of the paper contains instructions for making the following toys: rolling can, sound canisters, toy drum with bean bags, wooden blocks, nesting cans, wall picture, feeling blocks, first picture book, chip can, happy/sad face pillow, shape puzzle, sequential cups, hobby horse, rag doll, and hand puppets. All instructions are accompanied by charts relating the toy to language learning, using the five senses, as well as modeling activities for parents. (HOD)
UR - http://search.proquest.com/docview/63298497?accountid=14701
ER -

TY - RPRT
ID - 1416
T1 - Children's Social-Cognitive Play Behavior: The Effects of Age, Gender, and Activity Centers
A1 - Pellegrini,Anthony D.
Y1 - 1983///
N1 - Availability - Level 1 - Available online, if indexed January 1993 onward
Last updated - 2012-12-14
KW - Dyads
KW - ERIC,Resources in Education (RIE)
KW - Preschool Education
KW - Play
KW - Cognitive Processes
KW - Social Behavior
KW - Sex Differences
KW - Learning Centers (Classroom)
KW - Preschool Children
KW - Age Differences
KW - Models

KW - ERIC
KW - Rough and Tumble
RP - NOT IN FILE
SP - 22
N2 - This study examined a model suggesting that personal variables (age and gender) and environmental variables (activity centers) affect social-cognitive aspects of children's play. A total of 80 children (32 boys and 48 girls) participated. Same-age and same-gender dyads from each of two age groups (4- and 5-year-olds) were observed playing in an experimental playroom. On two occasions, children played in a dramatic center and on two occasions in a blocks center. Social-cognitive aspects of play were scored according to a time-sampling procedure and were concurrently scored on a nested social-cognitive matrix with an additional listing for onlooking, unoccupied, and rough-and-tumble behaviors. Results were compatible with those suggested by the model. Specifically, play generally followed the themes suggested by the props in each of the two centers. Predicted age effects were supported to the extent that play became more complex with age. Gender interacted with age and activity center to cause variation in play. Main effects due to gender were, however, unreliable. Results were considered significant in that they document the main and interactive effects of personal and controlled environmental variables on preschoolers' play behaviors. (Author/RH)
UR - http://search.proquest.com/docview/63411951?accountid=14701
ER -

TY - GEN
ID - 1417
T1 - Metacommunication in Rough and Tumble Play
A1 - Donaldson,Fred
Y1 - 1976///
N1 - Last updated - 2012-12-14
KW - Metacommunication
KW - ERIC,Current Index to Journals in Education (CIJE)
KW - Elementary Education
KW - Play Therapy
KW - Play
KW - Nonverbal Communication
KW - Student Behavior
KW - ERIC
KW - Rough and Tumble
RP - NOT IN FILE
SP - 235
EP - 239
JF - Reading Improvement
VL - 13
IS - 4
N2 - Discusses the components of rough and tumble play, analyzing three play sequences with a brief summary of the metacommunication taking place in child behavior. (RB)
UR - http://search.proquest.com/docview/63931220?accountid=14701
ER -

TY - JOUR
ID - 1500
T1 - The freedom to explore: examining the influence of independent mobility on weekday, weekend and after-school physical activity behaviour in children living in urban and inner-suburban neighbourhoods of varying socioeconomic status
A1 - Stone,M.R.
A1 - Faulkner,G.E.
A1 - Mitra,R.
A1 - Buliung,R.N.
Y1 - 2014///

N1 - Stone, Michelle R. Faulkner, Guy Ej. Mitra, Raktim. Buliung, Ron N
The international journal of behavioral nutrition and physical activity
101217089
IM
Journal Article. Research Support, Non-U.S. Gov't
English
KW - Medline
KW - Unsupervised Play Addition
RP - NOT IN FILE
SP - 5
JF - International Journal of Behavioral Nutrition & Physical Activity
JA - Int.
VL - 11
CY - England
N2 - BACKGROUND: Children's independent mobility (CIM) is critical to healthy development in childhood. The physical layout and social characteristics of neighbourhoods can impact opportunities for CIM. While global evidence is mounting on CIM, to the authors' knowledge, Canadian data on CIM and related health outcomes (i.e., physical activity (PA) behaviour) are missing. The purpose of this study was to examine if CIM is related to multiple characteristics of accelerometry-measured PA behaviour (total PA, light PA, moderate-to-vigorous PA, time spent sedentary) and whether associations between CIM and PA behaviour systematically vary by place of residence, stratifying by gender and type of day/period (weekdays, after-school, weekend). METHODS: Participants were recruited through Project BEAT (Built Environment and Active Transport; http://www.beat.utoronto.ca). Children (n=856) were stratified into four neighbourhood classifications based on the period of neighbourhood development (urban built environment (BE) (old BE) versus inner-suburban BE (new BE)) and socioeconomic status (SES; low SES and high SES). Physical activity was measured via accelerometry (ActiGraph GT1M). CIM was assessed via parental report and two categories were created (low CIM, n=332; high CIM, n=524). A series of two-factor ANOVAs were used to determine gender-specific differences in PA for weekdays, weekend days and the after-school period, according to level of CIM, across four neighbourhood classifications. RESULTS: Children who were granted at least some independent mobility (high CIM) had more positive PA profiles across the school week, during the after-school period, and over the weekend; they were also less sedentary. The influence of CIM on PA behaviour was particularly salient during the after-school period. Associations of CIM with PA varied by gender, and also by neighbourhood classification. CIM seemed to matter more in urban neighbourhoods for boys and suburban neighbourhoods for girls. CONCLUSION: Our findings highlight the importance of independent mobility to multiple characteristics of children's PA behaviour across the week. Furthermore, they emphasize that independent mobility-activity relationships need to be considered by gender and the type of neighbourhood independent mobility is offered in. Future work will focus on developing a predictive model of CIM that could be used to inform decision-making around alleviating barriers to CIM
SN - 1479-5868
AD - School of Health and Human Performance, Faculty of Health Professions, Dalhousie University, 6230 South Street, PO Box 15000 Halifax, Nova Scotia B3H4R2, Canada. michelle.stone@dal.ca
UR - 24450739
ER -

TY - JOUR
ID - 1501
T1 - Associations between children's independent mobility and physical activity
A1 - Schoeppe,S.
A1 - Duncan,M.J.
A1 - Badland,H.M.
A1 - Oliver,M.
A1 - Browne,M.
Y1 - 2014///
N1 - Schoeppe, Stephanie. Duncan, Mitch J. Badland, Hannah M. Oliver, Melody. Browne, Matthew
BMC public health
100968562

IM
Journal Article
English
KW - Medline
KW - Unsupervised Play Addition
RP - NOT IN FILE
SP - 91
JA - BMC Public Health
VL - 14
IS - 1
CY - England
N2 - BACKGROUND: Independent mobility describes the freedom of children to travel and play in public spaces without adult supervision. The potential benefits for children are significant such as social interactions with peers, spatial and traffic safety skills and increased physical activity. Yet, the health benefits of independent mobility, particularly on physical activity accumulation, are largely unexplored. This study aimed to investigate associations of children's independent mobility with light, moderate-to-vigorous, and total physical activity accumulation. METHODS: In 2011 - 2012, 375 Australian children aged 8-13 years (62% girls) were recruited into a cross-sectional study. Children's independent mobility (i.e. independent travel to school and non-school destinations, independent outdoor play) and socio-demographics were assessed through child and parent surveys. Physical activity intensity was measured objectively through an Actiheart monitor worn on four consecutive days. Associations between independent mobility and physical activity variables were analysed using generalized linear models, accounting for clustered sampling, Actiheart wear time, socio-demographics, and assessing interactions by sex. RESULTS: Independent travel (walking, cycling, public transport) to school and non-school destinations were not associated with light, moderate-to-vigorous and total physical activity. However, sub-analyses revealed a positive association between independent walking and cycling (excluding public transport) to school and total physical but only in boys (b=36.03, p<0.05). Frequent independent outdoor play (three or more days per week) was positively associated with light and total physical activity (b=29.76, p<0.01 and b=32.43, p=0.03, respectively). No significant associations were found between independent outdoor play and moderate-to-vigorous physical activity. When assessing differences by sex, the observed significant associations of independent outdoor play with light and total physical activity remained in girls but not in boys. All other associations showed no significant differences by sex. CONCLUSIONS: Independent outdoor play may boost children's daily physical activity levels, predominantly at light intensity. Hence, facilitating independent outdoor play could be a viable intervention strategy to enhance physical activity in children, particularly in girls. Associations between independent travel and physical activity are inconsistent overall and require further investigation
SN - 1471-2458
AD - Central Queensland University, School of Human, Health and Social Sciences, Centre for Physical Activity Studies, Building 18, Bruce Highway, Rockhampton QLD 4702, Australia. s.schoeppe@cqu.edu.au
UR - 24476363
ER -

TY - JOUR
ID - 1502
T1 - The impact of parents' fear of strangers and perceptions of informal social control on children's independent mobility
A1 - Foster,S.
A1 - Villanueva,K.
A1 - Wood,L.
A1 - Christian,H.
A1 - Giles-Corti,B.
Y1 - 2014/03//
N1 - Foster, Sarah. Villanueva, Karen. Wood, Lisa. Christian, Hayley. Giles-Corti, Billie
Health & place
din, 9510067
T

Journal Article
English
KW - Medline
KW - Unsupervised Play Addition
RP - NOT IN FILE
SP - 60
EP - 68
JF - Health & Place
JA - Health Place
VL - 26
CY - England
N2 - Declines in children's independent mobility are commonly attributed to parents' fear of strangers, yet few empirical studies have investigated this relationship. We examined: (1) the impact of parents' fear of strangers on children's independent mobility; and (2) whether informal social control (i.e., parents' confidence that other residents would look out for local children) mitigated any association. Gender stratified logistic regression models tested these associations for 10-12 year-olds and their parents (n=1231) in Perth, Australia. For girls, parental fear of strangers was associated with lower odds of independent mobility (OR=0.71, p=0.002), and informal social control and other social and built environment variables had little attenuating influence. This pattern was consistent for boys; however odds ratios were lower and statistical significance weaker. Initiatives that target parental fears combined with interventions that normalise children's walking may help increase independent mobility. 2013 Elsevier Ltd. All rights reserved
SN - 1873-2054
AD - Centre for the Built Environment and Health, School of Population Health, The University of Western Australia, 35 Stirling Highway, Crawley, WA 6009, Australia. Electronic address: sarah.foster@uwa.edu.au., McCaughey Centre VicHealth Centre for Community Wellbeing, Melbourne School of Population and Global Health, University of Melbourne, Melbourne, VIC 3010, Australia. Electronic address: k.villanueva@unimelb.edu.au., Centre for the Built Environment and Health, School of Population Health, The University of Western Australia, 35 Stirling Highway, Crawley, WA 6009, Australia. Electronic address: lisa.wood@uwa.edu.au., Centre for the Built Environment and Health, School of Population Health, The University of Western Australia, 35 Stirling Highway, Crawley, WA 6009, Australia. Electronic address: hayley.christian@uwa.edu.au., McCaughey Centre VicHealth Centre for Community Wellbeing, Melbourne School of Population and Global Health, University of Melbourne, Melbourne, VIC 3010, Australia. Electronic address: b.giles-corti@unimelb.edu.au
UR - 24374289
ER -

TY - JOUR
ID - 1503
T1 - Independent mobility, perceptions of the built environment and children's participation in play, active travel and structured exercise and sport: the PEACH Project
A1 - Page,A.S.
A1 - Cooper,A.R.
A1 - Griew,P.
A1 - Jago,R.
Y1 - 2010///
N1 - Page, Angie S. Cooper, Ashley R. Griew, Pippa. Jago, Russell
The international journal of behavioral nutrition and physical activity
101217089
Journal Article
English
KW - Medline
KW - Unsupervised Play Addition
RP - NOT IN FILE
SP - 17
JF - International Journal of Behavioral Nutrition & Physical Activity
JA - Int.

VL - 7
CY - England
N2 - BACKGROUND: Independent mobility (IM) and perceptions of the built environment may relate differentially to children's participation in various physical activity contexts. This cross-sectional study investigated whether independent mobility and perceptions of the built environment in boys and girls were related to physical activity in three different contexts (outdoor play, structured exercise/sport, active commuting). METHODS: Thirteen hundred and seven 10-11 year old boys and girls from 23 schools in a large UK city completed a computerised questionnaire. Independent variables in logistic regression analyses were weekly self-reported frequency of participation in outdoor play, structured exercise/sport and mode of travel home from school. Dependent variables were perceptions of the environment (aesthetics, nuisance, safety, social norm, constraint, play space, accessibility), local and area independent mobility and linear distance from home to school. Analyses were adjusted for body mass index, minutes of daylight after school, level of neighbourhood deprivation and pubertal status. RESULTS: For boys, local independent mobility (Local-IM) was related to an increased likelihood of everyday participation in play (OR 1.58: 95% CI 1.19-2.10), structured exercise/sport (OR 1.42: 1.06-1.89) and active commuting (OR 1.40: 1.07-1.87) but was only related to active commuting for girls (OR1.49: 1.07-2.07). Boys and girls were more likely to report playing out every day if they had higher scores for Social Norm (Boys: OR 1.63 (1.12-2.37); Girls: OR 1.53 (1.01-2.31)) and, for girls only, more positive perceptions of traffic safety (OR 1.63: 1.14-2.34). Easy access to a range of destinations was the dominant predictor for taking part in structured exercise/sport everyday (Boys: OR 1.62 (1.01-2.66); Girls: OR 1.65 (1.07-2.53)). Shorter distance from home to school (OR 0.99: 0.98-0.99) and, for boys only, greater perceived accessibility (OR 1.87: 1.04-3.36) were significantly related to active commuting to school. CONCLUSIONS: Perceptions of the physical environment relate differently to different physical activity contexts and by gender. The only consistent correlate for outdoor play, structured ex/sport and active commuting was higher independent mobility to visit local destinations (Local-IM) for boys. Considering both the physical activity context and its independent correlates should improve the specificity of physical activity interventions in children
SN - 1479-5868
AD - Department of Exercise, Nutrition & Health Sciences, University of Bristol, Bristol, UK.
a.s.page@bris.ac.uk
UR - 20170504
ER -

TY - JOUR
ID - 1504
T1 - Independent mobility in relation to weekday and weekend physical activity in children aged 10-11 years: The PEACH Project
A1 - Page,A.S.
A1 - Cooper,A.R.
A1 - Griew,P.
A1 - Davis,L.
A1 - Hillsdon,M.
Y1 - 2009///
N1 - Page, Angie S. Cooper, Ashley R. Griew, Pippa. Davis, Laura. Hillsdon, Melvyn
The international journal of behavioral nutrition and physical activity
101217089
Journal Article
English
KW - Medline
KW - Unsupervised Play Addition
RP - NOT IN FILE
SP - 2
JF - International Journal of Behavioral Nutrition & Physical Activity
JA - Int.
VL - 6
CY - England

N2 - BACKGROUND: Children's independent mobility has fallen in recent years and may in part explain reported declines in physical activity in young people. This cross-sectional study investigated whether independent mobility in boys and girls was related to objectively measured physical activity. METHODS: Thirteen hundred and seven 10-11 year old boys and girls from 23 schools in a large UK city took part. Measures included objectively recorded physical activity (accelerometer (Actigraph GT1M)), height (m) and weight (kg), a newly developed scale for local (Local-IM) and area independent mobility (Area-IM), minutes of daylight after school, level of neighbourhood deprivation and pubertal status. RESULTS: Boys had greater Local-IM, Area-IM and physical activity (average weekday and weekend counts per minute) compared to girls. In linear regression analyses (adjusting for minutes of daylight after school, neighbourhood deprivation, pubertal status and body mass index) higher scores for Local-IM and Area-IM were significantly ($p < 0.01$) related to higher levels of physical activity on weekdays for boys and girls. For weekend physical activity, only Local-IM in girls remained significant ($p < 0.05$) in the model. CONCLUSION: Independent mobility appears to be an important independent correlate of weekday physical activity for both boys and girls
SN - 1479-5868
AD - Department of Exercise, Nutrition & Health Sciences, University of Bristol, Bristol, UK.
a.s.page@bris.ac.uk
UR - 19128458
ER -

TY - JOUR
ID - 1505
T1 - Practice considerations for the introduction and use of power mobility for children
A1 - Livingstone,R.
A1 - Paleg,G.
Y1 - 2014/03//
N1 - Livingstone, Roslyn. Paleg, Ginny
Developmental medicine and child neurology
0006761, e83
IM
Journal Article
English
KW - Medline
KW - Unsupervised Play Addition
RP - NOT IN FILE
SP - 210
EP - 221
JF - Developmental Medicine & Child Neurology
JA - Dev.Med Child Neurol.
VL - 56
IS - 3
CY - England
N2 - AIM: The aim of the study was to support clinicians in recommending and justifying power mobility for children of different ages and abilities, and with different needs. The study comprised three distinct parts: a literature review; a Delphi consensus; and clinical practice considerations. METHOD: A scoping review of eight electronic databases and manual searches carried out in February 2011 identified 15 themes or transferable messages among 27 articles meeting initial inclusion criteria and these formed the basis of a draft paper. Informal consensus at two international conference presentations refined and modified the paper to include 10 messages supported by 24 articles. The literature review was updated in May 2012 and a modified Delphi process sought to formalize the consensus process with an international panel of 16 expert clinicians and researchers using a priori criteria of 80% agreement. RESULTS: Evidence from studies was classified using the American Academy of Cerebral Palsy and Developmental Medicine guidelines, with evidence from most studies being classified as either level IV or level V, apart from one study each with evidence classified as level II and level III. Expert consensus on the content and wording of nine transferable messages may raise evidence overall to level III. INTERPRETATION: This paper suggests that power mobility may reasonably be considered as an effective and appropriate intervention for children lacking efficient, independent mobility from

around 12 months of age including children who may never become competent drivers and children lacking independent mobility only in early childhood. 2013 Mac Keith Press
SN - 1469-8749
AD - Sunny Hill Health Centre for Children, Vancouver, BC, Canada
UR - 23998510
ER -

TY - JOUR
ID - 1506
T1 - From playfulness and self-centredness via grand expectations to normalisation: a psychoanalytical rereading of the history of molecular genetics
A1 - Zwart,H.A.
Y1 - 2013/11//
N1 - Zwart, H A E
Medicine, health care, and philosophy
9815900, drk
E, IM
Journal Article. Research Support, Non-U.S. Gov't
English
KW - Medline
KW - Unsupervised Play Addition
RP - NOT IN FILE
SP - 775
EP - 788
JF - Medicine, Health Care & Philosophy
JA - Med Health Care Philos.
VL - 16
IS - 4
CY - Netherlands
N2 - In this paper, I will reread the history of molecular genetics from a psychoanalytical angle, analysing it as a case history. Building on the developmental theories of Freud and his followers, I will distinguish four stages, namely: (1) oedipal childhood, notably the epoch of model building (1943-1953); (2) the latency period, with a focus on the development of basic skills (1953-1989); (3) adolescence, exemplified by the Human Genome Project, with its fierce conflicts, great expectations and grandiose claims (1989-2003) and (4) adulthood (2003-present) during which revolutionary research areas such as molecular biology and genomics have achieved a certain level of normalcy--have evolved into a normal science. I will indicate how a psychoanalytical assessment conducted in this manner may help us to interpret and address some of the key normative issues that have been raised with regard to molecular genetics over the years, such as 'relevance', 'responsible innovation' and 'promise management'
SN - 1572-8633
AD - Institute for Science, Innovation and Society (ISIS), Department of Philosophy and Science Studies (Chair), Centre for Society and the Life Sciences (CSG), University of Nijmegen, Nijmegen, The Netherlands, h.zwart@science.ru.nl
UR - 23595614
ER -

TY - JOUR
ID - 1507
T1 - A qualitative study of 'fear' as a regulator of children's independent physical activity in the suburbs
A1 - O'Connor,J.
A1 - Brown,A.
Y1 - 2013/11//
N1 - O'Connor, Justen. Brown, Alice
Health & place
din, 9510067
T

Journal Article
English
KW - Medline
KW - Unsupervised Play Addition
RP - NOT IN FILE
SP - 157
EP - 164
JF - Health & Place
JA - Health Place
VL - 24
CY - England
N2 - A crisis discourse has developed within the health and physical activity literature that paints a disparaging picture about the decline of children's participation in active play and independent mobility. Fear as a moderator of children's mobility, in this case focused on independent active school travel (IAST), remains a poorly understood construct. Within the popular press and academic literature, middle class parents have been characterised as overly anxious towards culturally reinforced dangers whilst being ignorant of the benefits of IAST during childhood. Fear of strangers, dangers and risk-taking are portrayed as nebulous and irrational anxiety states that are contributing to another great fear, a fear that our children are getting fat. The study draws upon Tudor's, (2003), parameters of fear framework to explore parent negotiations of fear and risk associated with IAST. In depth individual and focus group interviews with parents (N=24) provide insight into meanings, rules and customs that govern the way in which fear is experienced and expressed within a middle class white suburb in Australia. Far from being irrational, and operating purely from drivers at a cultural level, parents were aware of the consequences of their decisions on a range of outcomes. Parents could articulate their own anxieties and in many cases the rational probabilities of anything bad happening. Fear was being constructed through complex relations, negotiated individually and collectively within a specific cultural and environmental context. In the process of their decision-making, there remained a constant negotiation between a desire to allow their child independence and the question 'what if?' Those seeking to mediate increased opportunities for children's IAST are encouraged to remove the rational/irrational focus that was redundant for parents in this study, and consider shifting the message toward the positive side of what is a highly contested decision making space for parents. 2013 Elsevier Ltd. All rights reserved
SN - 1873-2054
AD - Monash University Australia, Faculty of Education, Building A, McMahons Rd., Frankston, Melbourne, VIC 3199, Australia. Electronic address: justen.oconnor@monash.edu
UR - 24112962
ER -

TY - JOUR
ID - 1508
T1 - Effects of supervised exercise program on metabolic function in overweight adolescents
A1 - Meucci,M.
A1 - Cook,C.
A1 - Curry,C.D.
A1 - Guidetti,L.
A1 - Baldari,C.
A1 - Collier,S.R.
Y1 - 2013/11//
N1 - Meucci, Marco. Cook, Carol. Curry, Chelsea Diane. Guidetti, Laura. Baldari, Carlo. Collier, Scott Robert
World journal of pediatrics : WJP
101278599
IM
Journal Article
English
KW - Medline
KW - Unsupervised Play Addition
RP - NOT IN FILE
SP - 307

EP - 311
JF - World Journal of Pediatrics
JA - World J Pediatr
VL - 9
IS - 4
CY - China
N2 - BACKGROUND: Inactivity is a primary factor related to childhood obesity, yet aerobic exercise has been shown to prevent weight gain and improve fitness in adolescents. Moreover, children become less active during their summer break from school. This study compared the effects of 4 and 8 weeks of supervised summer activity versus an unsupervised summer break on metabolic function and fitness in adolescents. METHODS: Twenty-two adolescents were divided into 4-week (n=6, weight 48.1+14.9 kg, body fat 27.4+8.4%) and 8-week exercise groups (n=6, weight 43.4+10.9 kg, body fat 28.5+12.8%), that performed supervised, play-based physical activity, versus an age-matched 8 week control group that maintained their typical summer break (n=10, weight 41.7+10.0 kg, body fat 23.7+8.0%). Anthropometrics, resting energy expenditure (REE), resting heart rate (RHR) and peak aerobic capacity (VO(2peak)) were evaluated before and after the intervention (4 or 8 weeks). RESULTS: REE showed group differences in posttraining conditions (the 4-week group vs. the control group, 1220+169 vs. 1067+144 kcal/die, and the 8-week group vs. the control group, 1202+151 vs. 1067+144 kcal/die, P=0.047), but RHR decreased (pre-program vs. post program: 97+22 vs. 80+8 beat/min, P=0.001) and VO(2peak) significantly increased (pre-program vs. post program: 27.8+7.8 vs. 34.8+6.5 mL/kg/min, P=0.001) in the 8-week group compared to the control group. CONCLUSIONS: Eight weeks of supervised play-based activity increased REE and VO(2peak) in adolescents with concomitant decreases in RHR. These data suggest that this novel model of exercise prescription could be considered world-wide by clinicians to improve fitness base in adolescents and help to combat the growing epidemic of childhood obesity
SN - 1867-0687
AD - Health Science Department, University of Rome "Foro Italico", Boone, NC, USA
UR - 24235064
ER -

TY - JOUR
ID - 1509
T1 - Adolescents' empathy and prosocial behavior in the family context: a longitudinal study
A1 - Yoo,H.
A1 - Feng,X.
A1 - Day,R.D.
Y1 - 2013/12//
N1 - Yoo, Hana. Feng, Xin. Day, Randal D
Journal of youth and adolescence
0333507
IM
Journal Article. Research Support, Non-U.S. Gov't
English
KW - Medline
KW - Unsupervised Play Addition
RP - NOT IN FILE
SP - 1858
EP - 1872
JF - Journal of Youth & Adolescence
JA - J Youth Adolesc
VL - 42
IS - 12
CY - United States
N2 - Children's empathy and prosocial behavior play an important role in their social competence. Of the influential factors, research has demonstrated that parental behaviors and the quality of the parent-child relationship are important correlates of children's development of empathy and prosocial behavior. The current study examined the associations between different types of parental behaviors (i.e., parental knowledge, parental solicitation, and parental psychological control), "balanced connectedness" in the parent-child relationship,

which allows for both closeness and autonomy, and empathy and prosocial behavior in adolescents. The participants were 335 married couples (more than 80 % European American) and their adolescent child (49.0 % female; 10-13 years). Data were collected at three time points for parental behaviors, balanced parent-child connectedness, and adolescents' empathy and prosocial behavior, respectively. The results of structural equation modeling suggested that adolescents' perceptions of parental solicitation and parental psychological control may be associated with their empathy and prosocial behavior through their perceived balanced connectedness with parents. These findings suggest that enhancing balanced connectedness in the parent-child relationship may contribute to promoting empathy and prosocial behavior in adolescents over time. Further, this study suggests that parental solicitation may play a role in adolescents' empathic and prosocial development, possibly depending on the quality of the parent-child relationship
SN - 1573-6601
AD - Human Development and Family Science, The Ohio State University, 135 Campbell Hall, 1787 Neil Ave., Columbus, OH, 43210, USA, yoo.161@buckeyemail.osu.edu
UR - 23283695
ER -

TY - JOUR
ID - 1510
T1 - A new walking aid with axillary support for children with cerebral palsy: electromyographic evaluation
A1 - Botega,R.
A1 - Medola,F.O.
A1 - Santos,C.B.
A1 - Silva,A.T.
A1 - Iunes,D.H.
A1 - Purquerio,Bde M.
Y1 - 2013/11//
N1 - Botega, Renan. Medola, Fausto Orsi. Santos, Christiane B Abrao. Silva, Adriana Teresa. Iunes, Denise Hollanda. Purquerio, Benedito de Moraes
Disability and rehabilitation. Assistive technology
101255937
IM
Journal Article
English
KW - Medline
KW - Unsupervised Play Addition
RP - NOT IN FILE
SP - 507
EP - 510
JF - Disability & Rehabilitation Assistive Technology
JA - Disabil
VL - 8
IS - 6
CY - England
N2 - PURPOSE: To present a new walking aid with axillary support (WAAS) for children with cerebral palsy (CP) and to investigate its impact on the lower limb muscles activation in the gait of children with CP. METHOD: Six children with spastic diparesis due to CP performed a straight line walking course in two situations: with and without using the WAAS. Each course was repeated three times for both situations, and electromyography (EMG) activity of quadriceps, hamstrings, tibialis anterior and gastrocnemius were recorded during all trials. RESULTS: The use of WAAS significantly increased the EMG activity of the quadriceps and hamstrings during stance phase and tibialis anterior during swing phase, with no significant increase in gastrocnemius activation. CONCLUSION: The use of WAAS improved the synergism of lower limbs muscles of children with CP by reducing the coactivation of antagonistic muscles, especially during the swing phase of gait. Providing a walking aid with improved trunk stability may enable children with CP to improve muscular synergism and walking ability, thus favoring independent mobility, with possible gains in social participation and quality of life. IMPLICATIONS FOR REHABILITATION: Children with cerebral palsy have problems with the development of normal gait, as a result of the reduced motor control and the impaired muscle

synergism of the lower limbs. Standard walking aids do not provide proper trunk stability for this group of children. In a relatively small sample of children with cerebral palsy, a walking aid with axillary support was shown to be beneficial for the muscular synergism of the lower limbs during gait
SN - 1748-3115
AD - Department of Mechanical Engineering, Sao Carlos Engineering School, University of Sao Carlos , Sao Carlos (SP) , Brazil
UR - 23480125
ER -

TY - JOUR
ID - 1511
T1 - Safe play areas on farms in New South Wales
A1 - Depczynski,J.
A1 - Herde,E.
A1 - Fragar,L.
A1 - Lower,T.
Y1 - 2013/08//
N1 - Depczynski, Julie. Herde, Emily. Fragar, Lyn. Lower, Tony
The Australian journal of rural health
9305903, c0i
N
Journal Article. Research Support, Non-U.S. Gov't
English
KW - Medline
KW - Unsupervised Play Addition
RP - NOT IN FILE
SP - 220
EP - 224
JF - Australian Journal of Rural Health
JA - Aust J Rural Health
VL - 21
IS - 4
CY - Australia
N2 - OBJECTIVE: To assess the prevalence and security of fenced house yards on NSW farms and rural properties with a view to providing information to increase the development of safe play areas on farms. DESIGN: A cross-sectional stratified study using computer-assisted telephone interviewing. SETTING: Interviews were conducted in the four rural Area Health Services throughout February to December 2008. PARTICIPANTS: Randomly selected sample of 1117 adults living on a farm or rural property in the study areas. MAIN OUTCOME MEASURES: Self-reported issues involving fenced house yards or safe play areas for children. RESULTS: Overall, 79.8% of farm respondents reported that they had a fenced house yard or safe play area. For those respondents with a fenced house yard, 66.6% reported that it was secure enough to prevent /make it difficult for a young child to wander away unsupervised. Based on these figures, it is estimated that only 53.1% of all farms or rural properties have a secure fenced house yard or safe play area. There were statistically significant variations between geographic locations, with the North Coast (37.7%) being lower. CONCLUSIONS: This study illustrates a need across rural NSW to further promote, install or upgrade secure fenced house yards or safe play areas. While all geographic regions of the state could improve provision to protect children, there may also be some that are in need of more intensive intervention programs to enhance compliance. Such a proposition may also be relevant on a national level. 2013 The Authors. Australian Journal of Rural Health National Rural Health Alliance Inc
SN - 1440-1584
AD - Australian Centre for Agricultural Health and Safety, Moree, New South Wales, Australia
UR - 24033523
ER -

TY - JOUR
ID - 1512

T1 - Understanding the drive to escort: a cross-sectional analysis examining parental attitudes towards children's school travel and independent mobility
A1 - Mammen,G.
A1 - Faulkner,G.
A1 - Buliung,R.
A1 - Lay,J.
Y1 - 2012///
N1 - Mammen, George. Faulkner, Guy. Buliung, Ron. Lay, Jennifer
BMC public health
100968562
IM
Journal Article. Research Support, Non-U.S. Gov't
English
KW - Medline
KW - Unsupervised Play Addition
RP - NOT IN FILE
SP - 862
JA - BMC Public Health
VL - 12
CY - England
N2 - BACKGROUND: The declining prevalence of Active School Transportation (AST) has been accompanied by a decrease in independent mobility internationally. The objective of this study was to compare family demographics and AST related perceptions of parents who let their children walk unescorted to/from school to those parents who escort (walk and drive) their children to/from school. By comparing these groups, insight was gained into how we may encourage greater AST and independent mobility in youth living in the Greater Toronto and Hamilton Area, Canada. METHODS: This study involved a cross-sectional design, using data from a self-reported questionnaire (n =1,016) that examined parental perceptions and attitudes regarding AST. A multinomial logistic regression analysis was used to explore the differences between households where children travelled independently to school or were escorted. RESULTS: Findings revealed that unescorted children were: significantly older, the families spoke predominantly English at home, more likely to live within one kilometer from school, and their parents agreed to a greater extent that they chose to reside in the current neighborhood in order for their child to walk to/from school. The parents of the escorted children worried significantly more about strangers and bullies approaching their child as well as the traffic volume around school. CONCLUSIONS: From both a policy and research perspective, this study highlights the value of distinguishing between mode (i.e., walking or driving) and travel independence. For policy, our findings highlight the need for planning decisions about the siting of elementary schools to include considerations of the impact of catchment size on how children get to/from school. Given the importance of age, distance, and safety issues as significant correlates of independent mobility, research and practice should focus on the development and sustainability of non-infrastructure programs that alleviate parental safety concerns
SN - 1471-2458
AD - Faculty of Kinesiology and Physical Education, University of Toronto, 55 Harbord St, Toronto, ON, M5S 2W6, Canada
UR - 23051005
ER -

TY - JOUR
ID - 1513
T1 - Relationship between neurological injury and patterns of upright mobility in children with spinal cord injury
A1 - Chafetz,R.S.
A1 - Gaughan,J.P.
A1 - Calhoun,C.
A1 - Schottler,J.
A1 - Vogel,L.C.
A1 - Betz,R.
A1 - Mulcahey,M.J.

Y1 - 2013///
N1 - Chafetz, Ross S. Gaughan, John P. Calhoun, Christina. Schottler, Jennifer. Vogel, Lawrence C. Betz, Randal. Mulcahey, M J
Topics in spinal cord injury rehabilitation
9515174
Journal Article
English
KW - Medline
KW - Unsupervised Play Addition
RP - NOT IN FILE
SP - 31
EP - 41
JF - Topics in Spinal Cord Injury Rehabilitation
JA - Top Spinal Cord Inj Rehabil
VL - 19
IS - 1
CY - United States
N2 - BACKGROUND: The predictors and patterns of upright mobility in children with a spinal cord injury (SCI) are poorly understood. OBJECTIVE: The objective of this study was to develop a classification system that measures children's ability to integrate ambulation into activities of daily living (ADLs) and to examine upright mobility patterns as a function of their score and classification on the International Standards for Neurological Classification of Spinal Cord Injury (ISNCSCI) exam. METHODS: This is a cross-sectional, multicenter study that used a convenience sample of subjects who were participating in a larger study on the reliability of the ISNCSCI. A total of 183 patients between 5 and 21 years old were included in this study. Patients were asked if they had participated in upright mobility in the last month and, if so, in what environment and with what type of bracing. Patients were then categorized into 4 groups: primary ambulators (PrimA), unplanned ambulators (UnPA), planned ambulators (PlanA), and nonambulators. RESULTS: Multivariate analyses found that only lower extremity strength predicted being a PrimA, whereas being an UnPA was predicted by both lower extremity strength and lack of preservation of S45 pinprick sensation. PlanA was only associated with upper extremity strength. CONCLUSIONS: This study introduced a classification system based on the ability of children with SCI to integrate upright mobility into their ADLs. Similar to adults, lower extremity strength was a strong predictor of independent mobility (PrimA and UnPA). Lack of pinprick predicted unplanned ambulation, but not being a PrimA. Finally, upper extremity strength was a predictor for planned ambulation
SN - 1082-0744
AD - Shriners Hospitals for Children , Philadelphia, Pennsylvania
UR - 23678283
ER -

TY - JOUR
ID - 1515
T1 - Systematic reviews of bruising in relation to child abuse-what have we learnt: an overview of review updates. [Review]
A1 - Maguire,S.
A1 - Mann,M.
Y1 - 2013/03/07/
N1 - Maguire, Sabine. Mann, Mala
Evidence-based child health : a Cochrane review journal
101296879
IM
Journal Article. Research Support, Non-U.S. Gov't. Review
English
KW - Adolescent
KW - Child
KW - Child Abuse/di [Diagnosis]
KW - Preschool

KW - Contusions/di [Diagnosis]
KW - Contusions/pp [Physiopathology]
KW - Diagnosis
KW - Differential
KW - Evidence-Based Medicine
KW - Humans
KW - Infant
KW - Newborn
KW - Time Factors
KW - Wounds and Injuries/di [Diagnosis]
KW - Wounds and Injuries/pp [Physiopathology]
KW - Medline
KW - Unsupervised Play Addition
RP - NOT IN FILE
SP - 255
EP - 263
JF - Evidence-Based Child Health a Cochrane Review Journal
JA - Evid Based Child Health
VL - 8
IS - 2
CY - England
N2 - BACKGROUND: Dogma has long prevailed regarding the ageing of bruises, and whether certain patterns of bruising are suggestive or diagnostic of child abuse. OBJECTIVES: We conducted the first Systematic Reviews addressing these two issues, to determine the scientific basis for current clinical practice. There have been seven updates since 2004. METHODS: An all language literature search was performed across 13 databases, 1951-2004, using >60 key words, supplemented by 'snowballing' techniques. Quality standards included a novel confirmation of abuse scale. Updates used expanded key words, and a higher standard for confirmation of abuse. RESULTS: Of 1495 potential studies, only three met the inclusion criteria for ageing of bruises in 2004, confirming that it is inaccurate to do so with the naked eye. This was roundly rejected when first reported, generating a wave of new studies attempting to determine a scientifically valid method to age bruises, none of which are applicable in children yet. Regarding patterns of bruising that may be suggestive or diagnostic of abuse, we included 23 of 167 studies reviewed in 2004, although only 2 were comparative studies. Included studies noted that unintentional bruises occur predominantly on the front of the body, over bony prominences and their presence is directly correlated to the child's level of independent mobility. Bruising patterns in abused children, differed in location (most common site being face, neck, ear, head, trunk, buttocks, arms), and tended to be larger. Updates have included a further 14 studies, including bruising in disabled children, defining distinguishing patterns in severely injured abused and non-abused children, and importance of petechiae. CONCLUSIONS: Systematic Reviews of bruising challenged accepted wisdom regarding ageing of bruises, which had no scientific basis; stimulated higher quality research on patterns of bruises distinguishing abusive and non-abusive bruising patterns, and highlighted the benefits of regular updates of these reviews. Copyright 2013 The Cochrane Collaboration. Published by John Wiley & Sons, Ltd
SN - 1557-6272
AD - Early Years Research Programme, School of Medicine, Cardiff University, Wales, UK. sabinemaguire@gmail.com
UR - 23877882
ER -

TY - JOUR
ID - 1516
T1 - Associations of children's independent mobility and active travel with physical activity, sedentary behaviour and weight status: a systematic review. [Review]
A1 - Schoeppe,S.
A1 - Duncan,M.J.
A1 - Badland,H.
A1 - Oliver,M.
A1 - Curtis,C.

Y1 - 2013/07//
N1 - Schoeppe, Stephanie. Duncan, Mitch J. Badland, Hannah. Oliver, Melody. Curtis, Carey
Journal of science and medicine in sport / Sports Medicine Australia
c54, 9812598
IM
Case Reports. Journal Article. Research Support, Non-U.S. Gov't. Review
English
KW - Body Weight
KW - Child
KW - Exercise
KW - Humans
KW - Independent Living
KW - Randomized Controlled Trials as Topic
KW - Sedentary Lifestyle
KW - Travel
KW - Medline
KW - Unsupervised Play Addition
RP - NOT IN FILE
SP - 312
EP - 319
JF - Journal of Science & Medicine in Sport
JA - J Sci Med Sport
VL - 16
IS - 4
CY - Australia
N2 - UNLABELLED: Health benefits from children's independent mobility and active travel beyond school travel are largely unexplored. OBJECTIVES: This review synthesized the evidence for associations of independent mobility and active travel to various destinations with physical activity, sedentary behaviour and weight status. DESIGN: Systematic review. METHODS: A systematic search in six databases (PubMed, Scopus, CINAHL, SportDiscus, PsychInfo, TRIS) for papers published between January 1990 and March 2012 was undertaken, focussing on children aged 3-18 years. Study inclusion and methodological quality were independently assessed by two reviewers. RESULTS: 52 studies were included. Most studies focussed solely on active travel to and/or from school, and showed significant positive associations with physical activity. The same relationship was detected for active travel to leisure-related places and independent mobility with physical activity. An inverse relationship between active travel to school and weight status was evident but findings were inconsistent. Few studies examined correlations between active travel to school and self-reported screen-time or objectively measured sedentary behaviour, and findings were unclear. CONCLUSIONS: Studies on independent mobility suggested that children who have the freedom to play outdoors and travel actively without adult supervision accumulate more physical activity than those who do not. Further investigation of children's active travel to leisure-related destinations, measurement of diverse sedentary behaviour beyond simply screen-based activities, and consistent thresholds for objectively measured sedentary behaviour in children will clarify the inconsistent evidence base on associations of active travel with sedentary behaviour and weight status.
Copyright 2012 Sports Medicine Australia. Published by Elsevier Ltd. All rights reserved
SN - 1878-1861
AD - Central Queensland University, Institute for Health and Social Science Research, Centre for Physical Activity Studies, Rockhampton, QLD, Australia. s.schoeppe@cqu.edu.au
UR - 23219100
ER -

TY - JOUR
ID - 1517
T1 - Psychological needs, purpose in life, and problem video game playing among Chinese young adults
A1 - Wu,A.M.
A1 - Lei,L.L.
A1 - Ku,L.
Y1 - 2013///

N1 - Wu, Anise M S. Lei, Lamis L M. Ku, Lisbeth
International journal of psychology : Journal international de psychologie
0107305
IM
Journal Article
English
KW - Adult
KW - Asian Continental Ancestry Group/px [Psychology]
KW - Asian Continental Ancestry Group/sn [Statistics & Numerical Data]
KW - Female
KW - Goals
KW - Humans
KW - Interpersonal Relations
KW - Macau/ep [Epidemiology]
KW - Male
KW - Mental Competency
KW - Personal Autonomy
KW - Questionnaires
KW - Self Efficacy
KW - Video Games/px [Psychology]
KW - Video Games/sn [Statistics & Numerical Data]
KW - Medline
KW - Unsupervised Play Addition
RP - NOT IN FILE
SP - 583
EP - 590
JF - International Journal of Psychology
JA - Int J Psychol
VL - 48
IS - 4
CY - England
N2 - The negative impacts of excessive and problematic video game playing on both children and adults are attracting increasing concern. Based on self-determination theory (SDT; Ryan & Deci, 2000), this study hypothesized that the three basic psychological needs of autonomy, competence, and relatedness are positively associated with purpose in life, which in turn acts as a protective factor against problem video game playing among Chinese young adult players. Through a questionnaire survey with a sample of 165 Chinese adults aged between 18 and 30 years (mean age=22.7 years), we found that perceived autonomy, competence, relatedness, and purpose in life were all negatively correlated with problem game playing. The demographic and psychological factors explained 38% of the variances of problem game playing. Specifically, gender, perceived relatedness, and purpose in life emerged as the three most salient predictors of problem game playing among the Chinese young adults. The mediating role of purpose in life was evidenced and it was found that purpose in life mediated the influences of the psychological needs proposed by SDT on problem game playing. Moreover, young men were significantly more susceptible to problem game playing than their female counterparts. To conclude, psychological needs and purpose in life influenced Chinese young adults' vulnerability to problem game playing directly or indirectly. Intervention programs that encourage social involvement and voluntary work, as well as counseling service that helps clients to search for life purpose, are suggested for intervening in problem game playing among Chinese young adults
SN - 1464-066X
AD - Department of Psychology, University of Macau, Macao, China. anisewu@umac.mo
UR - 22506646
ER -

TY - JOUR
ID - 1518
T1 - Accidents due to falls from roof slabs
A1 - Rudelli,B.A.

A1 - Silva,M.V.
A1 - Akkari,M.
A1 - Santili,C.
Y1 - 2013///
N1 - Rudelli, Bruno Alves. Silva, Marcelo Valerio Alabarce da. Akkari, Miguel. Santili, Claudio
Sao Paulo medical journal = Revista paulista de medicina
100897261, dio
IM
Journal Article. Research Support, Non-U.S. Gov't
English
KW - Accident Prevention/mt [Methods]
KW - Accidental Falls/pc [Prevention & Control]
KW - Accidental Falls/sn [Statistics & Numerical Data]
KW - Accidents
KW - Home/pc [Prevention & Control]
KW - Home/sn [Statistics & Numerical Data]
KW - Adolescent
KW - Brain Injuries/pc [Prevention & Control]
KW - Child
KW - Preschool
KW - Female
KW - Housing
KW - Humans
KW - Infant
KW - Longitudinal Studies
KW - Male
KW - Play and Playthings/in [Injuries]
KW - Prospective Studies
KW - Risk Factors
KW - Medline
KW - Unsupervised Play Addition
RP - NOT IN FILE
SP - 153
EP - 157
JF - Sao Paulo Medical Journal = Revista Paulista de Medicina
JA - Sao Paulo Med J
VL - 131
IS - 3
CY - Brazil
N2 - CONTEXT AND OBJECTIVE Falls from the roof slabs of houses are accidents of high potential severity that occur in large Brazilian cities and often affect children and adolescents. The aims of this study were to characterize the factors that predispose towards this type of fall involving children and adolescents, quantify the severity of associated lesions and suggest preventive measures. DESIGN AND SETTING Descriptive observational prospective longitudinal study in two hospitals in the metropolitan region of Sao Paulo. METHODS Data were collected from 29 cases of falls from roof slabs involving children and adolescents between October 2008 and October 2009. RESULTS Cases involving males were more prevalent, accounting for 84%. The predominant age group was schoolchildren (7 to 12 years old; 44%). Leisure activities were most frequently being practiced on the roof slab at the time of the fall (86%), and flying a kite was the most prevalent game (37.9%). In 72% of the cases, the children were unaccompanied by an adult responsible for them. Severe conditions such as multiple trauma and traumatic brain injuries resulted from 79% of the accidents. CONCLUSION Falls from roof slabs are accidents of high potential severity, and preventive measures aimed towards informing parents and guardians about the dangers and risk factors associated with this type of accident are needed, along with physical protective measures, such as low walls around the slab and gates with locks to restrict free access to these places
SN - 1806-9460

AD - Department of Orthopedics and Traumatology, Hospital da Clinicas, Faculdade de Medicina da Universidade de Sao Paulo, Brazil. rudelli.bruno@yahoo.com.br
UR - 23903263
ER -

TY - JOUR
ID - 1519
T1 - Seasonal and locational variations in children's play: implications for wellbeing
A1 - Ergler,C.R.
A1 - Kearns,R.A.
A1 - Witten,K.
Y1 - 2013/08//
N1 - Ergler, Christina R. Kearns, Robin A. Witten, Karen
Social science & medicine (1982)
ut9, 8303205
IM
Journal Article. Research Support, Non-U.S. Gov't
English
KW - Child
KW - Child Welfare
KW - Female
KW - Geography
KW - Medical
KW - Humans
KW - Male
KW - New Zealand
KW - Play and Playthings/px [Psychology]
KW - Play and Playthings
KW - Residence Characteristics/sn [Statistics & Numerical Data]
KW - Seasons
KW - Medline
KW - Unsupervised Play Addition
RP - NOT IN FILE
SP - 178
EP - 185
JF - Social Science & Medicine
JA - Soc Sci Med
VL - 91
CY - England
N2 - Physical activity, through independent outdoor play, has come to the fore as a way to improve children's health through it fostering healthy mental and social as well as physiological development. However, in many high-income countries children's autonomous play opportunities have diminished due to urban intensification and declining parental license. Regardless of this trend, children's play varies across countries, cities, cultures and seasons. This paper offers new insights into the complexities of play as a vital aspect of children's wellbeing. Within the context of New Zealand - whose citizens generally regard themselves as outdoor people - this paper explores why 'play' might resonate differently across localities and seasons. We contrast the play affordances provided by Auckland's central city (dominated by apartment living) with Beach Haven, a suburban area. We employed a multi-method approach and included 20 children and their parents who were recruited through school and summer holiday programs embracing different gender and ethnicities to reflect the general cultural mix of the respective neighbourhoods. We advance two arguments. First, we suggest that the rarity of children playing outdoors unsupervised normalises supervised indoor play and reduces children's opportunities to see outdoor play as an alternative to interior or supervised pastimes. Second, we follow Bourdieu's theory of practice to argue that the regard parents and children have towards outdoor play reflects locally constituted beliefs about what is seasonally 'appropriate' children's activity. We found that extra-curricular activities and supervised excursions are undertaken in the central city all year around and only vary between social groups by the type of destination. In the suburb, independent outdoor play in summer represents children's main business

after school in ways that enhance their environmental literacy and potential future health gain. For others these symbolic values were replaced by safety concerns. In contrast, it seems that even in a relatively mild climate winter is the time to relax and stay indoors unless children have an outdoor habitus. We find that the determinants of seasonal outdoor play transcend modifiable barriers such as traffic and unsuitable play spaces as well as the inevitable issue of inclement weather. Copyright 2012 Elsevier Ltd. All rights reserved
SN - 1873-5347
AD - The University of Auckland, School of Environment, Private Bag 90219, Auckland 1142, New Zealand. c.ergler@auckland.ac.nz
UR - 23312793
ER -

TY - JOUR
ID - 1520
T1 - A place for play? The influence of the home physical environment on children's physical activity and sedentary behaviour. [Review]
A1 - Maitland,C.
A1 - Stratton,G.
A1 - Foster,S.
A1 - Braham,R.
A1 - Rosenberg,M.
Y1 - 2013///
N1 - Maitland, Clover. Stratton, Gareth. Foster, Sarah. Braham, Rebecca. Rosenberg, Michael
The international journal of behavioral nutrition and physical activity
101217089
IM
Journal Article. Research Support, Non-U.S. Gov't. Review
English
KW - Child
KW - Child Behavior
KW - Environment
KW - Exercise
KW - Health Behavior
KW - Humans
KW - Parent-Child Relations
KW - Parenting
KW - Parents
KW - Play and Playthings
KW - Sedentary Lifestyle
KW - Social Environment
KW - Television
KW - Video Games
KW - Medline
KW - Unsupervised Play Addition
RP - NOT IN FILE
SP - 99
JF - International Journal of Behavioral Nutrition & Physical Activity
JA - Int.
VL - 10
CY - England
N2 - The home environment is an important influence on the sedentary behaviour and physical activity of children, who have limited independent mobility and spend much of their time at home. This article reviews the current evidence regarding the influence of the home physical environment on the sedentary behaviour and physical activity of children aged 8-14 years. A literature search of peer reviewed articles published between 2005 and 2011 resulted in 38 observational studies (21 with activity outcomes, 23 with sedentary outcomes) and 11 experimental studies included in the review. The most commonly investigated behavioural outcomes were television watching and moderate to vigorous physical activity. Media equipment in the home and to a lesser

extent the bedroom were positively associated with children's sedentary behaviour. Physical activity equipment and the house and yard were not associated with physical activity, although environmental measures were exclusively self-reported. On the other hand, physical activity equipment was inversely associated with sedentary behaviours in half of studies. Observational studies that investigated the influence of the physical and social environment within the home space, found that the social environment, particularly the role of parents, was important. Experimental studies that changed the home physical environment by introducing a television limiting device successfully decreased television viewing, whereas the influence of introducing an active video game on activity outcomes was inconsistent. Results highlight that the home environment is an important influence on children's sedentary behaviour and physical activity, about which much is still unknown. While changing or controlling the home physical environment shows promise for reducing screen based sedentary behaviour, further interventions are needed to understand the broader impact of these changes. Future studies should prioritise investigating the influence of the home physical environment, and its interaction with the social environment, on objectively measured sedentary time and home context specific behaviours, ideally including technologies that allow objective measures of the home space
SN - 1479-5868
AD - School of Sport Science, Exercise and Health, University of Western Australia, 35 Stirling Highway, Crawley, WA 6009, Australia. clover.maitland@uwa.edu.au
UR - 23958282
ER -

TY - JOUR
ID - 1521
T1 - Parental physical activity, safety perceptions and children's independent mobility
A1 - Santos,M.P.
A1 - Pizarro,A.N.
A1 - Mota,J.
A1 - Marques,E.A.
Y1 - 2013///
N1 - Santos, Maria Paula. Pizarro, Andreia N. Mota, Jorge. Marques, Elisa A
BMC public health
100968562
IM
Journal Article. Research Support, Non-U.S. Gov't
English
KW - Adult
KW - Child
KW - Cross-Sectional Studies
KW - Female
KW - Health Knowledge
KW - Attitudes
KW - Practice
KW - Humans
KW - Independent Living
KW - Male
KW - Middle Aged
KW - Motor Activity
KW - Parent-Child Relations
KW - Perception
KW - Residence Characteristics
KW - Safety
KW - Sedentary Lifestyle
KW - Medline
KW - Unsupervised Play Addition
RP - NOT IN FILE
SP - 584
JA - BMC Public Health

VL - 13
CY - England
N2 - BACKGROUND: Parents are likely to be a basic influence on their children's behavior. There is an absence of information about the associations between parents' physical activity and perception of neighborhood environment with children's independent mobility.The purpose of this study is to examine the contribution of parental physical activity and perception of neighborhood safety to children's independent mobility. METHODS: In this cross-sectional study of 354 pupils and their parents, independent mobility, perceptions of neighborhood safety and physical activity were evaluated by questionnaire. Categorical principal components analyses were used to determine the underlying dimensions of both independent mobility and perceptions of neighborhood safety items. RESULTS: The strongest predictor of independent mobility was the parental perception of sidewalk and street safety (beta = 0.132). Parent's physical activity was also a significant predictor. The final model accounted for 13.0% of the variance. CONCLUSIONS: Parental perception of neighborhood safety and parents' self reported physical activity might be associated with children's independent mobility. Further research in this topic is needed to explore this possible association
SN - 1471-2458
AD - Research Centre in Physical Activity, Health and Leisure (CIAFEL), Faculty of Sport, University of Porto, Rua Dr. Placido Costa 91, Porto 4200-450, Portugal. msantos@fade.up.pt
UR - 23767778
ER -

TY - JOUR
ID - 1522
T1 - Independent mobility after early introduction of a power wheelchair in spinal muscular atrophy
A1 - Dunaway,S.
A1 - Montes,J.
A1 - O'Hagen,J.
A1 - Sproule,D.M.
A1 - Vivo,D.C.
A1 - Kaufmann,P.
Y1 - 2013/05//
N1 - Dunaway, Sally. Montes, Jacqueline. O'Hagen, Jessica. Sproule, Douglas M. Vivo, Darryl C De. Kaufmann, Petra
Journal of child neurology
ia2, 8606714
IM
Journal Article. Research Support, Non-U.S. Gov't
English
KW - Activities of Daily Living/cl [Classification]
KW - Activities of Daily Living/px [Psychology]
KW - Child
KW - Preschool
KW - Contracture/di [Diagnosis]
KW - Contracture/rh [Rehabilitation]
KW - Disability Evaluation
KW - Female
KW - Follow-Up Studies
KW - Humans
KW - Infant
KW - Male
KW - Motor Skills Disorders/di [Diagnosis]
KW - Motor Skills Disorders/rh [Rehabilitation]
KW - Muscular Dystrophies/cn [Congenital]
KW - Muscular Dystrophies/di [Diagnosis]
KW - Muscular Dystrophies/rh [Rehabilitation]
KW - Pilot Projects
KW - Spinal Muscular Atrophies of Childhood/di [Diagnosis]

KW - Spinal Muscular Atrophies of Childhood/rh [Rehabilitation]
KW - Wheelchairs
KW - Medline
KW - Unsupervised Play Addition
RP - NOT IN FILE
SP - 576
EP - 582
JF - Journal of Child Neurology
JA - J Child Neurol
VL - 28
IS - 5
CY - United States
N2 - Weakness resulting from spinal muscular atrophy causes severe limitations in functional mobility. The early introduction of power mobility has potential to enhance development and mitigate disability. These outcomes are achieved by simulating normal skill acquisition and by promoting motor learning, visuospatial system development, self-exploration, cognition, and social development. There are few reports on early power mobility in spinal muscular atrophy, and it is typically not prescribed until school age. The authors evaluated 6 children under age 2 years with neuromuscular disease (5 spinal muscular atrophy, 1 congenital muscular dystrophy) for power mobility. Parents recorded the practice hours necessary to achieve independence using the Power Mobility Skills Checklist. Four children achieved independence in all items on the checklist by 7.9 months (range: 73-458 days). Introduction of early power mobility is feasible in spinal muscular atrophy patients under age 2 years and should be introduced in late infancy when children typically acquire locomotor skills
SN - 1708-8283
AD - Neurology Department, Columbia University Medical Center, New York, NY 10032, USA. sld2120@columbia.edu
UR - 22772161
ER -

TY - JOUR
ID - 1523
T1 - The impact of recreational video game play on children's and adolescents' cognition
A1 - Blumberg,F.C.
A1 - Altschuler,E.A.
A1 - Almonte,D.E.
A1 - Mileaf,M.I.
Y1 - 2013///
N1 - Blumberg, Fran C. Altschuler, Elizabeth A. Almonte, Debby E. Mileaf, Maxwell I
New directions for child and adolescent development
100886823, dc2
IM
Journal Article
English
KW - Adolescent
KW - Adolescent Behavior
KW - Belgium
KW - Humans
KW - Netherlands
KW - Personal Autonomy
KW - Social Identification
KW - Medline
KW - Unsupervised Play Addition
RP - NOT IN FILE
SP - 41
EP - 50
JF - New Directions for Child & Adolescent Development

JA - New Dir.Child Adolesc Dev
VL - 2013
IS - 139
CY - United States
N2 - Current empirical findings show linkages between recreational video game play and enhanced cognitive skills, primarily among young adults. However, consideration of this linkage among children and adolescents is sparse. Thus, discussions about facilitating transfer of cognitive skills from video game play to academic tasks among children and adolescents remains largely uninformed by research. To inform this discussion, we review available research concerning the cognitive benefits of video game play among children and adolescents and their impressions of video games as learning tools as these impressions may impact their application of cognitive skills used during game play to academic tasks. Copyright 2013 Wiley Periodicals, Inc., A Wiley Company
SN - 1534-8687
AD - Division of Psychological & Educational Services, Fordham University Graduate School of Education, New York, USA. blumberg@fordham.edu
UR - 23483692
ER -

TY - JOUR
ID - 1524
T1 - The challenge of audience reception: a developmental model for educational game engagement
A1 - Sherry,J.L.
Y1 - 2013///
N1 - Sherry, John L
New directions for child and adolescent development
100886823, dc2
IM
Journal Article
English
KW - Adolescent
KW - Adolescent Behavior
KW - Belgium
KW - Humans
KW - Netherlands
KW - Personal Autonomy
KW - Social Identification
KW - Medline
KW - Unsupervised Play Addition
RP - NOT IN FILE
SP - 11
EP - 20
JF - New Directions for Child & Adolescent Development
JA - New Dir.Child Adolesc Dev
VL - 2013
IS - 139
CY - United States
N2 - According to educational gaming advocates, the engaging nature of games encourages sustained game play and enhanced attention to learning outcomes among players. Because children's and adolescents' play time varies by game genre, engagement with a game likely reflects the match between the genre and the player's preferences and needs. Youth learn which games are likely to promote satisfying psychological needs and yield positive experiences, which then informs their engagement with the games. A model is presented for research and development of educational games based on uses and gratifications theory from communication science, as well as developmental science and cognitive science findings. Copyright 2013 Wiley Periodicals, Inc., A Wiley Company
SN - 1534-8687
AD - Department of Communication at Michigan State University, Michigan, USA. jsherry@msu.edu

UR - 23483689
ER -

TY - JOUR
ID - 1525
T1 - Introduction: digital games as a context for cognitive development, learning, and developmental research
A1 - Blumberg,F.C.
A1 - Fisch,S.M.
Y1 - 2013///
N1 - Blumberg, Fran C. Fisch, Shalom M
New directions for child and adolescent development
100886823, dc2
IM
Introductory Journal Article
English
KW - Adolescent
KW - Adolescent Behavior
KW - Belgium
KW - Humans
KW - Netherlands
KW - Personal Autonomy
KW - Social Identification
KW - Medline
KW - Unsupervised Play Addition
RP - NOT IN FILE
SP - 1
EP - 9
JF - New Directions for Child & Adolescent Development
JA - New Dir.Child Adolesc Dev
VL - 2013
IS - 139
CY - United States
N2 - The authors present reasons why developmental psychologists should care about children's and adolescents' digital game play. These reasons may be identified as: a) digital game play is an integral aspect of children's and adolescents' lives; b) digital game play contributes to learning and cognitive development; and c) developmental research has the potential to contribute to effective educational game design. The authors expand on these reasons with the goal of introducing or reintroducing to developmental psychologists a rich and very relevant context in which to examine children's and adolescents' applied cognitive development. Copyright 2013 Wiley Periodicals, Inc., A Wiley Company
SN - 1534-8687
AD - Division of Psychological& Educational Services, Fordham University Graduate School of Education, New York, USA. blumberg@fordham.edu
UR - 23483688
ER -

TY - JOUR
ID - 1527
T1 - Individual, social and physical environmental correlates of 'never' and 'always' cycling to school among 10 to 12 year old children living within a 3.0 km distance from school
A1 - Ducheyne,F.
A1 - De,Bourdeaudhuij,I
A1 - Spittaels,H.
A1 - Cardon,G.
Y1 - 2012///
N1 - Ducheyne, Fabian. De Bourdeaudhuij, Ilse. Spittaels, Heleen. Cardon, Greet
The international journal of behavioral nutrition and physical activity

101217089
IM
Journal Article. Research Support, Non-U.S. Gov't
English
KW - Adolescent
KW - Automobile Driving
KW - Belgium
KW - Bicycling/sn [Statistics & Numerical Data]
KW - Child
KW - Cross-Sectional Studies
KW - Environment Design
KW - Female
KW - Health Knowledge
KW - Attitudes
KW - Practice
KW - Humans
KW - Logistic Models
KW - Male
KW - Multivariate Analysis
KW - Parents/px [Psychology]
KW - Questionnaires
KW - Residence Characteristics
KW - Risk Factors
KW - Schools
KW - Transportation
KW - Walking/sn [Statistics & Numerical Data]
KW - Medline
KW - Unsupervised Play Addition
RP - NOT IN FILE
SP - 142
JF - International Journal of Behavioral Nutrition & Physical Activity
JA - Int.
VL - 9
CY - England
N2 - BACKGROUND: Cycling to school has been identified as an important target for increasing physical activity levels in children. However, knowledge about correlates of cycling to school is scarce as many studies did not make a distinction between walking and cycling to school. Moreover, correlates of cycling to school for those who live within a distance, that in theory would allow cycling to school, stay undiscovered. Therefore, this study examined individual, social and physical environmental correlates of never and always cycling to/from school among 10 to 12 year old Belgian children living within a 3.0 km distance from school. METHODS: 850 parents completed a questionnaire to assess personal, family, behavioral, cognitive, social and physical environmental factors related to the cycling behavior of their children. Parents indicated on a question matrix how many days a week their child (1) walked, (2) cycled, was (3) driven by car or (4) public transport to and from school during fall, winter and spring. Multivariate logistic regression analyses were conducted to examine the correlates. RESULTS: Overall, 39.3% of children never cycled to school and 16.5% of children always cycled to school. Children with high levels of independent mobility and good cycling skills perceived by their parents were more likely to always cycle to school (resp. OR 1.06; 95% CI 1.04-1.15 and OR 1.08; 95% CI 1.01-1.16) and less likely to never cycle to school (resp. OR 0.84; 95% CI 0.78-0.91 and OR 0.77; 95% CI 0.7-0.84). Children with friends who encourage them to cycle to school were more likely to always cycle to school (OR 1.08; 95% CI 1.01-1.15) and less likely to never cycle to school (OR 0.9; 95% CI 0.83-1.0). In addition, children with parents who encourage them to cycle to school were less likely to never cycle to school (OR 0.78; 95% CI 0.7-0.87). Regarding the physical environmental factors, only neighborhood traffic safety was significantly associated with cycling: i.e., children were more likely to always cycle to school if neighborhood traffic was perceived as safe by their parents (OR 1.18; 95% CI 1.07-1.31). CONCLUSION: Individual, social and physical environmental factors were associated with children's cycling behavior to/from school. However,

the contribution of the physical environment is limited and highlights the fact that interventions for increasing cycling to school should not focus solely on the physical environment
SN - 1479-5868
AD - Department of Movement and Sports Sciences, Ghent University, Gent, Belgium. Fabian.Ducheyne@UGent.be
UR - 23228003
ER -

TY - JOUR
ID - 1528
T1 - Coaches' interpersonal style, basic psychological needs and the well- and ill-being of young soccer players: a longitudinal analysis
A1 - Balaguer,I.
A1 - Gonzalez,L.
A1 - Fabra,P.
A1 - Castillo,I.
A1 - Merce,J.
A1 - Duda,J.L.
Y1 - 2012///
N1 - Balaguer, Isabel. Gonzalez, Lorena. Fabra, Priscila. Castillo, Isabel. Merce, Juan. Duda, Joan L
Journal of sports sciences
8405364, kbt
IM
Journal Article. Research Support, Non-U.S. Gov't
English
KW - Adaptation
KW - Psychological
KW - Adolescent
KW - Child
KW - Humans
KW - Interpersonal Relations
KW - Longitudinal Studies
KW - Male
KW - Perception
KW - Personal Autonomy
KW - Personal Satisfaction
KW - Physical Education and Training
KW - Psychological Theory
KW - Questionnaires
KW - Soccer/px [Psychology]
KW - Social Control
KW - Informal
KW - Social Environment
KW - Stress
KW - Medline
KW - Unsupervised Play Addition
RP - NOT IN FILE
SP - 1619
EP - 1629
JF - Journal of Sports Sciences
JA - J Sports Sci
VL - 30
IS - 15
CY - England
N2 - This study entailed a longitudinal test of basic psychological needs theory, a sub-theory in the self-determination framework (Deci & Ryan, 2000), in young soccer players. We examined whether changes in

soccer players' perceptions of the coaches' interpersonal style (autonomy supportive and controlling) predicted changes in the players' need satisfaction/need thwarting, and in turn, variability in their reported subjective vitality and burnout over the course of a season. Young male soccer players (M = 12.58 + 0.54 years) completed a questionnaire at two time points in the season [n(T1) = 725; n(T2) = 597]. Changes in the players' perceptions of an autonomy supportive environment significantly predicted changes in psychological need satisfaction (positively) and in psychological need thwarting (negatively). Changes in psychological need satisfaction positively predicted changes in subjective vitality and negatively related to cross-time variation in global burnout scores. In contrast, changes in the players' perceptions of a controlling coach-created environment were positively associated with changes in psychological need thwarting that corresponded to increases in player burnout. Finally, results provided support for the assumed mediational roles of psychological need satisfaction and need thwarting in the social environment to well- and ill-being relationships
SN - 1466-447X
AD - Faculty of Psychology, University of Valencia, Valencia, Spain. Isabel.Balaguer@uv.es
UR - 23062028
ER -

TY - JOUR
ID - 1529
T1 - Young and free? A study of independent mobility among urban and rural dwelling Australian children
A1 - Carver,A.
A1 - Timperio,A.F.
A1 - Crawford,D.A.
Y1 - 2012/11//
N1 - Carver, Alison. Timperio, Anna F. Crawford, David A
Journal of science and medicine in sport / Sports Medicine Australia
c54, 9812598
IM
Journal Article. Research Support, Non-U.S. Gov't
English
KW - Adolescent
KW - Adult
KW - Australia/ep [Epidemiology]
KW - Bicycling/sn [Statistics & Numerical Data]
KW - Cellular Phone/sn [Statistics & Numerical Data]
KW - Child
KW - Cross-Sectional Studies
KW - Data Collection/mt [Methods]
KW - Female
KW - Humans
KW - Male
KW - Middle Aged
KW - Personal Autonomy
KW - Rural Population/sn [Statistics & Numerical Data]
KW - Schools/sn [Statistics & Numerical Data]
KW - Sex Factors
KW - Social Environment
KW - Urban Population/sn [Statistics & Numerical Data]
KW - Walking/sn [Statistics & Numerical Data]
KW - Medline
KW - Unsupervised Play Addition
RP - NOT IN FILE
SP - 505
EP - 510
JF - Journal of Science & Medicine in Sport
JA - J Sci Med Sport
VL - 15

IS - 6
CY - Australia
N2 - OBJECTIVES: To examine urban/rural differences in children's independent mobility; associations between mobility licences and children's independent mobility; and potential correlates of mobility licences. DESIGN: Cross-sectional study of 430 primary (48% boys; 72% urban) and 258 secondary schoolchildren (52% boys; 51.6% urban) and their parents. METHODS: Parents survey reported the mobility licences they granted to their children (e.g. allowing them to cross main roads on their own), access to outdoor playspaces and mobile phone ownership. Children survey reported their independent mobility on school journeys and on weekends. Differences were examined in mobility licences and independent mobility by sex, urban/rural setting and age-group. Regression analyses examined associations between mobility licences and independent mobility; and how access to playspaces, and mobile phone ownership, were associated with mobility licences. RESULTS: Overall, on average, boys were granted more mobility licences, than were girls, but there were no significant differences by urban/rural location. Variations in independent mobility by urban/rural locality were identified on the school journey but not on weekends. Boys attending urban primary schools had highest rates (44%) of walking/cycling independently to school; those attending rural secondary schools had the lowest (14%). Among urban boys and rural primary school-aged girls access to outdoor playspaces was associated with mobility licences. Mobile phone ownership was associated with mobility licences only among boys attending urban primary schools. CONCLUSIONS: Many Australian children in urban and rural areas lack independent mobility. Further research should examine social/physical environmental influences on parental restrictions, to inform interventions that aim to promote children's independent mobility. Copyright 2012 Sports Medicine Australia. Published by Elsevier Ltd. All rights reserved
SN - 1878-1861
AD - Centre for Physical Activity and Nutrition Research, School of Exercise and Nutrition Sciences, Deakin University, Australia. alison.carver@deakin.edu.au
UR - 22497720
ER -

TY - JOUR
ID - 1530
T1 - More than child's play: the potential benefits of play-based interventions for young children with ADHD
A1 - O'Neill,S.
A1 - Rajendran,K.
A1 - Halperin,J.M.
Y1 - 2012/10//
N1 - O'Neill, Sarah. Rajendran, Khushmand. Halperin, Jeffrey M
Expert review of neurotherapeutics
101129944
IM
Editorial. Research Support, N.I.H., Extramural
English
KW - Animals
KW - Attention Deficit Disorder with Hyperactivity/px [Psychology]
KW - Attention Deficit Disorder with Hyperactivity/th [Therapy]
KW - Child Behavior
KW - Child Development
KW - Child Psychology/ma [Manpower]
KW - Child Psychology/mt [Methods]
KW - Child Psychology/td [Trends]
KW - Child Psychology
KW - Child
KW - Preschool
KW - Family Relations
KW - Humans
KW - Parent-Child Relations
KW - Parents/ed [Education]
KW - Personal Autonomy

KW - Play Therapy
KW - Play and Playthings/px [Psychology]
KW - Medline
KW - Unsupervised Play Addition
RP - NOT IN FILE
SP - 1165
EP - 1167
JF - Expert Review of Neurotherapeutics
JA - Expert rev
VL - 12
IS - 10
CY - England
SN - 1744-8360
UR - 23082729
ER -

TY - JOUR
ID - 1531
T1 - Comparing participation in activities among children with disabilities
A1 - Masse,L.C.
A1 - Miller,A.R.
A1 - Shen,J.
A1 - Schiariti,V.
A1 - Roxborough,L.
Y1 - 2012/11//
N1 - Masse, Louise C. Miller, Anton R. Shen, Jane. Schiariti, Veronica. Roxborough, Lori
Research in developmental disabilities
8709782, rid
IM
Comparative Study. Journal Article. Research Support, Non-U.S. Gov't
English
KW - Adolescent
KW - Child
KW - Preschool
KW - Disability Evaluation
KW - Disabled Children/px [Psychology]
KW - Female
KW - Humans
KW - Leisure Activities
KW - Male
KW - Motor Activity
KW - Physical Education and Training
KW - Play and Playthings
KW - Recreation/px [Psychology]
KW - Social Participation
KW - Medline
KW - Unsupervised Play Addition
RP - NOT IN FILE
SP - 2245
EP - 2254
JF - Research in Developmental Disabilities
JA - Res.Dev.Disabil.
VL - 33
IS - 6
CY - United States

N2 - INTRODUCTION: Compared to typically developing peers, children with disabilities due to neurodevelopmental disorders and disabilities (NDD/D) and to chronic medical conditions (CMC) have reduced participation in activities. The extent to which these two groups of children have different levels of participation is unknown and was examined in this study. METHODS: The 2006 Participation and Activity Limitation Survey children dataset collected by Statistics Canada was analyzed. Children with disabilities due to NDD/D and CMC were identified following review and classification of all ICD-10 codes in the dataset by two pediatricians. Dependent variables were parent-reported child participation in supervised and unsupervised physical activities within and outside of school, educational activities, and social/recreational activities. Logistic regression analyses, with relevant covariates (child and familial characteristics), were used to analyze the data. RESULTS: Children with NDD/D were significantly more likely to take part in supervised and unsupervised physical activity at school than children with CMC ($p<0.001$). A similar trend was observed for participation in school outings, although the effect was not significant at $p<0.01$. Finally, a trend in the opposite direction was observed for educational activities, as children with NDD/D were less likely to take part in these activities than children with CMC. DISCUSSION: Finding decreased participation among children with CMC compared with NDD/D was not predicted a priori but has potential implications for their mental and physical health. CONCLUSIONS: Gaining a better understanding of the barriers to participation in physical activity may contribute to improving the overall health status of children with CMC. Copyright 2012 Elsevier Ltd. All rights reserved
SN - 1873-3379
AD - Department of Pediatrics, University of British Columbia, Vancouver, BC, Canada. lmasse@cfri.ubc.ca
UR - 22835684
ER -

TY - JOUR
ID - 1532
T1 - The Smart Wheelchair: is it an appropriate mobility training tool for children with physical disabilities?
A1 - McGarry,S.
A1 - Moir,L.
A1 - Girdler,S.
Y1 - 2012/09//
N1 - McGarry, Sarah. Moir, Lois. Girdler, Sonya
Disability and rehabilitation. Assistive technology
101255937
IM
Case Reports. Journal Article
English
KW - Activities of Daily Living
KW - Adolescent
KW - Cerebral Palsy/rh [Rehabilitation]
KW - Child
KW - Preschool
KW - Computer-Assisted Instruction/mt [Methods]
KW - Disabled Children/px [Psychology]
KW - Disabled Children/rh [Rehabilitation]
KW - Female
KW - Humans
KW - Male
KW - Quadriplegia/rh [Rehabilitation]
KW - Task Performance and Analysis
KW - Western Australia
KW - Wheelchairs
KW - Medline
KW - Unsupervised Play Addition
RP - NOT IN FILE
SP - 372
EP - 380

JF - Disability & Rehabilitation Assistive Technology
JA - Disabil
VL - 7
IS - 5
CY - England
N2 - PURPOSE: To describe the impact of a mobility training program using the Smart Wheelchair on the driving skills and psychosocial outcomes of children with physical disabilities. METHOD: A multiple case study design using mixed methods was used. Four children with physical disabilities were recruited through The Centre for Cerebral Palsy in Western Australia. The intervention was a 16 session Smart Wheelchair mobility training program. Data was collected using a quantitative driving skills assessment, field notes and qualitative parent interviews. RESULTS: Three out of four children gained independence in at least three driving skills or more, whilst one child was competent with verbal prompts. Three out of four mothers reported positive changes in their child's confidence, motivation and affect. CONCLUSIONS: The Smart Wheelchair has the ability to uncover learning potential and facilitate the recognition of abilities in children previously excluded from access to independent mobility. Given the significant limitation that restrictions in mobility pose to participation for children with physical disabilities, therapists must begin to understand the effectiveness of interventions such as the Smart Wheelchair. The descriptive findings of this study allow for future, more rigorous research, to be conducted on the effectiveness of the Smart Wheelchair as a mobility training tool
SN - 1748-3115
AD - Department of Occupational Therapy, School of Computing, Health and Science, Edith Cowan University, Perth, Australia. sarah.mcgarry@health.wa.gov.au
UR - 22124287
ER -

TY - JOUR
ID - 1534
T1 - Longitudinal associations between the quality of parent-child interactions and children's sleep at preschool age
A1 - Bordeleau,S.
A1 - Bernier,A.
A1 - Carrier,J.
Y1 - 2012/04//
N1 - Bordeleau, Stephanie. Bernier, Annie. Carrier, Julie
Journal of family psychology : JFP : journal of the Division of Family Psychology of the American Psychological Association (Division 43)
8802265, dlv
IM
Journal Article. Research Support, Non-U.S. Gov't
English
KW - Adult
KW - Child Development/ph [Physiology]
KW - Child
KW - Preschool
KW - Female
KW - Humans
KW - Infant
KW - Longitudinal Studies
KW - Male
KW - Parent-Child Relations
KW - Parenting/px [Psychology]
KW - Parents/px [Psychology]
KW - Prospective Studies
KW - Psychological Tests
KW - Sleep/ph [Physiology]
KW - Medline
KW - Unsupervised Play Addition

RP - NOT IN FILE
SP - 254
EP - 262
JF - Journal of Family Psychology
JA - J Fam Psychol
VL - 26
IS - 2
CY - United States
N2 - This study aimed to examine the prospective longitudinal links between the quality of mother-child and father-child interactions and preschoolers' sleep. Three dimensions of maternal interactive behavior were considered using 70 mother-child dyads, while the quality of father-child interactions was assessed using 41 of these families. Maternal mind-mindedness was assessed at 12 months during a mother-infant free-play sequence, maternal sensitivity was rated at 12 months based on observations performed throughout a home visit, maternal autonomy support was assessed at 15 months with a 10-min problem-solving situation, and the quality of father-child interactions was scored at 18 months, during father-child free play. Children's sleep was assessed at 3 and 4 years using a sleep diary completed by mothers during 3 consecutive days. Results indicated that, after controlling for family socioeconomic status and daycare attendance, the quality of both mother-infant and father-infant interactions was positively related to children's percentage of night-time sleep at preschool age. These findings add to previous literature in suggesting that early caregiving behavior by both mothers and fathers is related to subsequent child sleep. (c) 2012 APA, all rights reserved
SN - 1939-1293
AD - Department of Psychology, University of Montreal, P.O. Box 6128 Downtown Station, Montreal QC H3C 3J7, Canada
UR - 22369463
ER -

TY - JOUR
ID - 1536
T1 - Better walking performance in older children with cerebral palsy
A1 - Rodby-Bousquet,E.
A1 - Hagglund,G.
Y1 - 2012/05//
N1 - Rodby-Bousquet, Elisabet. Hagglund, Gunnar
Clinical orthopaedics and related research
0075674, dfy
AIM, IM
Journal Article. Research Support, Non-U.S. Gov't
English
KW - Activities of Daily Living
KW - Adolescent
KW - Age Factors
KW - Cerebral Palsy/di [Diagnosis]
KW - Cerebral Palsy/pp [Physiopathology]
KW - Child
KW - Preschool
KW - Cross-Sectional Studies
KW - Disability Evaluation
KW - Exercise Test
KW - Female
KW - Humans
KW - Male
KW - Motor Skills
KW - Muscle
KW - Skeletal/pa [Pathology]
KW - Skeletal/pp [Physiopathology]
KW - Walking

KW - Medline
KW - Unsupervised Play Addition
RP - NOT IN FILE
SP - 1286
EP - 1293
JF - Clinical Orthopaedics & Related Research
JA - Clin Orthop
VL - 470
IS - 5
CY - United States
N2 - BACKGROUND: Children with cerebral palsy (CP) often walk with a slower speed and a higher energy cost. Their walking performance and choice of mobility method may vary in different environments. Independent mobility is important for activity and participation. QUESTIONS/PURPOSES: We described walking performance at different distances and environments in relation to gross motor function, CP subtype, and age. PATIENTS AND METHODS: We performed a cross-sectional study including all 562 children 3 to 18 years with CP living in southern Sweden during 2008. Data were extracted from a Swedish CP register and healthcare program. The Functional Mobility Scale (FMS) was used for rating mobility at home (5 m), at school (50 m), and in the community (500 m). The FMS scores were analyzed in relation to Gross Motor Function Classification System (GMFCS) level, CP subtype, and age. RESULTS: In this population, 57% to 63% walked 5 to 500 m without walking aids and 4% to 8% used walking aids. We found a correlation between FMS and GMFCS. The walking performance varied between the subtypes from 96% to 98% in those with spastic unilateral CP to 16% to 24% in children with dyskinetic CP. An increased proportion of children walked independently on all surfaces in each successive age group. CONCLUSIONS: The overall walking performance increased up to 7 years of age, but the proportion of children walking independently on uneven surfaces was incrementally higher in each age group up to 18 years. The ability to walk on uneven surfaces is important for achieving independent walking in the community
SN - 1528-1132
AD - Department of Orthopaedics, Lund University, University Hospital, 221 85 Lund, Sweden.
elisabet.rodby_bousquet@med.lu.se
UR - 21445713
ER -

TY - JOUR
ID - 1537
T1 - Autonomy supportive environments and mastery as basic factors to motivate physical activity in children: a controlled laboratory study
A1 - Roemmich,J.N.
A1 - Lambiase Ms,M.J.
A1 - McCarthy,T.F.
A1 - Feda,D.M.
A1 - Kozlowski,K.F.
Y1 - 2012///
N1 - Roemmich, James N. Lambiase Ms, Maya J. McCarthy, Thomas F. Feda, Denise M. Kozlowski, Karl F
The international journal of behavioral nutrition and physical activity
101217089
IM
Controlled Clinical Trial. Journal Article. Research Support, N.I.H., Extramural
English
KW - Child
KW - Child Behavior
KW - Choice Behavior
KW - Energy Metabolism
KW - Environment
KW - Exercise/ph [Physiology]
KW - Exercise
KW - Female

KW - Humans
KW - Male
KW - Motivation
KW - Personal Autonomy
KW - Physical Exertion
KW - Play and Playthings
KW - Sex Factors
KW - Medline
KW - Unsupervised Play Addition
RP - NOT IN FILE
SP - 16
JF - International Journal of Behavioral Nutrition & Physical Activity
JA - Int.
VL - 9
CY - England
N2 - BACKGROUND: Choice promotes the experience of autonomy, which enhances intrinsic motivation. Providing a greater choice of traditional active toys may increase children's activity time. Mastery also increases intrinsic motivation and is designed into exergames, which may increase play time of a single exergame, reducing the need for choice to motivate activity compared to traditional active toys. Providing both choice and mastery could be most efficacious at increasing activity time. The energy expenditure (EE) of an active play session is dependent on the duration of play and the rate of EE during play. The rate of EE of exergames and the same game played in traditional fashion is not known. The purpose was to test the basic parameters of choice and mastery on children's physical activity time, activity intensity, and energy expenditure. METHODS: 44 children were assigned to low (1 toy) or high (3 toys) choice groups. Children completed 60 min sessions with access to traditional active toys on one visit and exergame versions of the same active toys on another visit. RESULTS: Choice had a greater effect on increasing girls' (146%) than boys' (23%) activity time and on girls' (230%) than boys' (minus 24%) activity intensity. When provided choice, girls' activity time and intensity were no longer lower than boys' activity time and intensity. The combination of choice and mastery by providing access to 3 exergames produced greater increases in physical activity time (1 toy 22.5 min, 3 toys 41.4 min) than choice alone via access to 3 traditional games (1 toy 13.6 min, 3 toys 19.5 min). Energy expenditure was 83% greater when engaging in traditional games than exergames. CONCLUSIONS: Boys and girls differ in their behavioral responses to autonomy supportive environments. By providing girls with greater autonomy they can be motivated to engage in physical activity equal to boys. An environment that provides both autonomy and mastery is most efficacious at increasing physical activity time. Though children play exergames 87% longer than traditional games, the rate of energy expenditure is 83% lower for exergames than traditional indoor versions of the same games
SN - 1479-5868
AD - Department of Pediatrics, School of Medicine and Biomedical Sciences, University at Buffalo, Buffalo, New York 14214-3000, USA. roemmich@buffalo.edu
UR - 22353207
ER -

TY - JOUR
ID - 1538
T1 - Adrenarche and middle childhood
A1 - Campbell,B.C.
Y1 - 2011/09//
N1 - Campbell, Benjamin C
Human nature (Hawthorne, N.Y.)
9010063
IM
Journal Article
English
KW - Adrenarche/ph [Physiology]
KW - Adrenarche/px [Psychology]
KW - Antioxidants/me [Metabolism]

KW - Brain/gd [Growth & Development]
KW - Child
KW - Child Behavior/ph [Physiology]
KW - Child Behavior/px [Psychology]
KW - Child Development/ph [Physiology]
KW - Dehydroepiandrosterone/me [Metabolism]
KW - Female
KW - Gender Identity
KW - Glucose/me [Metabolism]
KW - Humans
KW - Interpersonal Relations
KW - Male
KW - Medline
KW - Unsupervised Play Addition
RP - NOT IN FILE
SP - 327
EP - 349
JF - Human Nature
JA - Hum Nat
VL - 22
IS - 3
CY - United States
N2 - Middle childhood, the period from 6 to 12 years of age, is defined socially by increasing autonomy and emotional regulation, somatically by the development of anatomical structures for subsistence, and endocrinologically by adrenarche, the adrenal production of dehydroepiandrosterone (DHEA). Here I suggest that DHEA plays a key role in the coordinated development of the brain and body beginning with middle childhood, via energetic allocation. I argue that with adrenarche, increasing levels of circulating DHEA act to down-regulate the release of glucose into circulation and hence limit the supply of glucose which is needed by the brain for synaptogenesis. Furthermore, I suggest the antioxidant properties of DHEA may be important in maintaining synaptic plasticity throughout middle childhood within slow-developing areas of the cortex, including the insula, thamalus, and anterior cingulate cortex. In addition, DHEA may play a role in the development of body odor as a reliable social signal of behavioral changes associated with middle childhood
SN - 1936-4776
AD - Department of Anthropology, University of Wisconsin-Milwaukee, 290 Sabin Hall, 3413 N. Downer Ave., Milwaukee, WI 53211, USA. campbelb@uwm.edu
UR - 22388879
ER -

TY - JOUR
ID - 1540
T1 - Linking GPS and travel diary data using sequence alignment in a study of children's independent mobility
A1 - Mavoa,S.
A1 - Oliver,M.
A1 - Witten,K.
A1 - Badland,H.M.
Y1 - 2011///
N1 - Mavoa, Suzanne. Oliver, Melody. Witten, Karen. Badland, Hannah M
International journal of health geographics
101152198
IM
Journal Article. Research Support, Non-U.S. Gov't
English
KW - Algorithms
KW - Chi-Square Distribution
KW - Child
KW - Child Welfare

KW - Data Collection/is [Instrumentation]
KW - Data Collection/mt [Methods]
KW - Feasibility Studies
KW - Female
KW - Geographic Information Systems
KW - Health Services Research
KW - Humans
KW - Male
KW - Medical Records/sn [Statistics & Numerical Data]
KW - Public Health Practice
KW - Statistics as Topic
KW - Travel/sn [Statistics & Numerical Data]
KW - Medline
KW - Unsupervised Play Addition
RP - NOT IN FILE
SP - 64
JF - International Journal of Health Geographics [Electronic Resource]
JA - Int.J Health Geogr.
VL - 10
CY - England
N2 - BACKGROUND: Global positioning systems (GPS) are increasingly being used in health research to determine the location of study participants. Combining GPS data with data collected via travel/activity diaries allows researchers to assess where people travel in conjunction with data about trip purpose and accompaniment. However, linking GPS and diary data is problematic and to date the only method has been to match the two datasets manually, which is time consuming and unlikely to be practical for larger data sets. This paper assesses the feasibility of a new sequence alignment method of linking GPS and travel diary data in comparison with the manual matching method. METHODS: GPS and travel diary data obtained from a study of children's independent mobility were linked using sequence alignment algorithms to test the proof of concept. Travel diaries were assessed for quality by counting the number of errors and inconsistencies in each participant's set of diaries. The success of the sequence alignment method was compared for higher versus lower quality travel diaries, and for accompanied versus unaccompanied trips. Time taken and percentage of trips matched were compared for the sequence alignment method and the manual method. RESULTS: The sequence alignment method matched 61.9% of all trips. Higher quality travel diaries were associated with higher match rates in both the sequence alignment and manual matching methods. The sequence alignment method performed almost as well as the manual method and was an order of magnitude faster. However, the sequence alignment method was less successful at fully matching trips and at matching unaccompanied trips. CONCLUSIONS: Sequence alignment is a promising method of linking GPS and travel diary data in large population datasets, especially if limitations in the trip detection algorithm are addressed. 2011 Mavoa et al; licensee BioMed Central Ltd
SN - 1476-072X
AD - SHORE and Whariki Research Centre, School of Public Health, Massey University, Auckland, New Zealand. s.mavoa@massey.ac.nz
UR - 22142322
ER -

TY - JOUR
ID - 1541
T1 - Design of a novel mobility device controlled by the feet motion of a standing child: a feasibility study
A1 - Schoepflin,Z.R.
A1 - Chen,X.
A1 - Ragonesi,C.B.
A1 - Galloway,J.C.
A1 - Agrawal,S.K.
Y1 - 2011/10//
N1 - Schoepflin, Zachary R. Chen, Xi. Ragonesi, Christina B. Galloway, James C. Agrawal, Sunil K
Medical & biological engineering & computing

lpn, 7704869
IM
Evaluation Studies. Journal Article. Research Support, N.I.H., Extramural. Research Support, U.S. Gov't, Non-P.H.S.
English
KW - Cerebral Palsy/pp [Physiopathology]
KW - Cerebral Palsy/rh [Rehabilitation]
KW - Child
KW - Preschool
KW - Equipment Design
KW - Feasibility Studies
KW - Foot/ph [Physiology]
KW - Humans
KW - Male
KW - Man-Machine Systems
KW - Motor Skills
KW - Robotics/is [Instrumentation]
KW - Self-Help Devices
KW - Walking/ph [Physiology]
KW - Medline
KW - Unsupervised Play Addition
RP - NOT IN FILE
SP - 1225
EP - 1231
JF - Medical & Biological Engineering & Computing
JA - Med Biol Eng Comput
VL - 49
IS - 10
CY - United States
N2 - Self-generated mobility is a major contributor to the physical, emotional, cognitive, and social development of infants and toddlers. When young children have disorders that hinder self locomotion, their development is at risk for delay. Independent mobility via traditional power mobility devices may prevent this delay, but do little to encourage the child's development of gross motor skills. This research aims to develop a bio-driven mobile-assistive device that is controlled and driven by moving the feet, which may encourage the development of gross motor skills. In this study, system feasibility is shown by experiments on five typically developing toddlers and one special needs toddler with spastic cerebral palsy. Children were placed in the bio-driven device and instructed to navigate through a maze. All subjects were able to successfully complete the maze in multiple trials. In addition, two toddlers showed evidence of improved driving skill by completing the maze in shorter times in successive trials on a given testing day. The results suggest that such a device is feasible for purposeful driving. Recommendations are given for the device and protocol redesign for related future testing
SN - 1741-0444
AD - Mechanical Engineering, University of Delaware, Newark, USA
UR - 21850470
ER -

TY - JOUR
ID - 1542
T1 - "The moving body": a sustainable project to improve children's physical activity at kindergarten
A1 - Serpentino,C.
Y1 - 2011/10//
N1 - Serpentino, Carmela
International journal of pediatric obesity : IJPO : an official journal of the International Association for the Study of Obesity
101256330
IM

Journal Article
English
KW - Child
KW - Preschool
KW - Humans
KW - Motor Activity
KW - Play and Playthings
KW - Schools
KW - Self Concept
KW - SOCIALIZATION
KW - Medline
KW - Unsupervised Play Addition
RP - NOT IN FILE
SP - 60
EP - 62
JF - International Journal of Pediatric Obesity
JA - Int.J Pediatr Obes.
VL - 6 Suppl 2
CY - England
N2 - Physical activity promotes children's awareness of the body. Children, to experience and to learn, need to be physically active and their innate physical activity is playing. Playing together means building relationships with peers, means learning how to use tools and space, means addressing egocentric behaviour toward rules' respect; in summary, it means learning "social conviviality and respect". The aim of the project "The moving body" was to favour children's physical and cognitive development and their social relationships. Gross motor physical abilities were assessed in 270 children attending the kindergarten. The children were grouped according their level of motor abilities to promote learning through older children's imitation. Structured games and playing were alternated to free time, and words such as physical activity, movement, and so on, were never used. Every month the children were asked to draw their own body to assess their body shape perception and their ability in representing it. All the children improved their physical abilities as compared to the beginning of the project; they strengthened their creativity and fantasy, inventing new and imaginative games, became more confident in their own capacities, and they learned how to deal and overcome, with a greater autonomy, difficult tasks. The body shape perception, and its representation as drawing, showed, especially among children 3 years old, remarkable progress. The importance of physical activity and play in kindergarten activities was evidenced through the significant improvement of children's several specific motor and psychosocial competences
SN - 1747-7174
AD - Edmondo de Amicis School Institution Viale Abbadessa n92 CAP 72021 Francavilla Fontana, Brindisi, Italy. mariacarmelamaria@hotmail.it
UR - 21923300
ER -

TY - JOUR
ID - 1543
T1 - Parental and peer influences on physical activity among Scottish adolescents: a longitudinal study
A1 - Kirby,J.
A1 - Levin,K.A.
A1 - Inchley,J.
Y1 - 2011/08//
N1 - Kirby, Joanna. Levin, Kate A. Inchley, Jo
Journal of physical activity & health
101189457
IM
Journal Article
English
KW - Adolescent
KW - Child
KW - Exercise

KW - Female
KW - Humans
KW - Longitudinal Studies
KW - Male
KW - Parent-Child Relations
KW - Peer Group
KW - Regression Analysis
KW - Scotland
KW - Social Environment
KW - Medline
KW - Unsupervised Play Addition
RP - NOT IN FILE
SP - 785
EP - 793
JF - Journal of Physical Activity & Health
JA - J Phys Act.Health
VL - 8
IS - 6
CY - United States
N2 - BACKGROUND: This study investigated parental and peer influences on physical activity, examining gender and developmental differences during early-mid adolescence. METHODS: A 5-year longitudinal study tracking physical activity (measured by PAQ-C) among adolescents (n = 641) from final year of primary (P7) to fourth year of secondary school (S4). Peer support, peer socializing, parental support, and independent play were assessed. Logistic regression predicted physical activity, by year and gender, in relation to social influences. RESULTS: Boys reported higher physical activity, peer support, paternal support, and independent play than girls. Among both genders, peer, paternal, and maternal support decreased with age, whereas independent play increased. Time with friends was particularly important. Among high socializers (P7), odds of being active were over 3 times those of low socializers [boys: 3.53 (95% CI 1.77, 7.04), girls: 3.27 (95% CI 1.80, 5.92)]. Baseline physical activity was also a strong predictor among early secondary boys (OR 3.90 95% CI 2.10, 7.24) and girls (OR 4.15, 95% CI 2.00, 8.62). Parental support was less important than peer influences; only same-sex parental support remained significant in multivariables models. CONCLUSIONS: Parents and peers have important influences on adolescent physical activity. Significant gender and developmental effects are apparent through early-mid adolescence
SN - 1543-5474
AD - Child and Adolescent Health Research Unit, University of Edinburgh, United Kingdom
UR - 21832293
ER -

TY - JOUR
ID - 1544
T1 - Kids in the city study: research design and methodology
A1 - Oliver,M.
A1 - Witten,K.
A1 - Kearns,R.A.
A1 - Mavoa,S.
A1 - Badland,H.M.
A1 - Carroll,P.
A1 - Drumheller,C.
A1 - Tavae,N.
A1 - Asiasiga,L.
A1 - Jelley,S.
A1 - Kaiwai,H.
A1 - Opit,S.
A1 - Lin,E.Y.
A1 - Sweetsur,P.
A1 - Barnes,H.M.

A1 - Mason,N.
A1 - Ergler,C.
Y1 - 2011///
N1 - Oliver, Melody. Witten, Karen. Kearns, Robin A. Mavoa, Suzanne. Badland, Hannah M. Carroll, Penelope. Drumheller, Chelsea. Tavae, Nicola. Asiasiga, Lanuola. Jelley, Su. Kaiwai, Hector. Opit, Simon. Lin, En-Yi Judy. Sweetsur, Paul. Barnes, Helen Moewaka. Mason, Nic. Ergler, Christina
BMC public health
100968562
IM
Journal Article. Research Support, Non-U.S. Gov't
English
KW - Actigraphy/is [Instrumentation]
KW - Actigraphy/mt [Methods]
KW - Body Size
KW - Child
KW - Cities
KW - Cross-Sectional Studies
KW - Environment Design
KW - Exercise
KW - Female
KW - Focus Groups
KW - Humans
KW - Interviews as Topic
KW - Male
KW - New Zealand
KW - Research Design
KW - Sedentary Lifestyle
KW - Medline
KW - Unsupervised Play Addition
RP - NOT IN FILE
SP - 587
JA - BMC Public Health
VL - 11
CY - England
N2 - BACKGROUND: Physical activity is essential for optimal physical and psychological health but substantial declines in children's activity levels have occurred in New Zealand and internationally. Children's independent mobility (i.e., outdoor play and traveling to destinations unsupervised), an integral component of physical activity in childhood, has also declined radically in recent decades. Safety-conscious parenting practices, car reliance and auto-centric urban design have converged to produce children living increasingly sedentary lives. This research investigates how urban neighborhood environments can support or enable or restrict children's independent mobility, thereby influencing physical activity accumulation and participation in daily life. METHODS/DESIGN: The study is located in six Auckland, New Zealand neighborhoods, diverse in terms of urban design attributes, particularly residential density. Participants comprise 160 children aged 9-11 years and their parents/caregivers. Objective measures (global positioning systems, accelerometers, geographical information systems, observational audits) assessed children's independent mobility and physical activity, neighborhood infrastructure, and streetscape attributes. Parent and child neighborhood perceptions and experiences were assessed using qualitative research methods. DISCUSSION: This study is one of the first internationally to examine the association of specific urban design attributes with child independent mobility. Using robust, appropriate, and best practice objective measures, this study provides robust epidemiological information regarding the relationships between the built environment and health outcomes for this population
SN - 1471-2458
AD - School of Environment, The University of Auckland, Auckland, NZ. r.kearns@auckland.ac.nz
UR - 21781341
ER -

TY - JOUR

ID - 1545
T1 - Leisure, recreation, and play from a developmental context
A1 - Caldwell,L.L.
A1 - Witt,P.A.
Y1 - 2011///
N1 - Caldwell, Linda L. Witt, Peter A
New directions for youth development
101090644
IM
Journal Article
English
KW - Adolescent
KW - Adolescent Development/ph [Physiology]
KW - Age Factors
KW - Child
KW - Child Development/ph [Physiology]
KW - Female
KW - Goals
KW - Humans
KW - Leisure Activities/px [Psychology]
KW - Male
KW - Motivation
KW - Motor Activity
KW - Personal Autonomy
KW - Play and Playthings/px [Psychology]
KW - Recreation/ph [Physiology]
KW - Recreation/px [Psychology]
KW - Residence Characteristics
KW - Self Concept
KW - Social Identification
KW - Medline
KW - Unsupervised Play Addition
RP - NOT IN FILE
SP - 13
EP - 27
JF - New Directions for Youth Development
JA - New Dir.Youth Dev.
VL - 2011
IS - 130
CY - United States
N2 - Participation in activities and experiences defined as play, recreation,and leisure has important developmental implications for youth. Elements and characteristics of leisure experiences contribute directly to the development of identity, autonomy, competence,initiative, civic duty, and social connections. Whether in informal or formal, appropriately structured and organized programs,leisure experiences can help facilitate adolescent development in these areas. For example, one of the defining elements of leisure is that it is characterized by free choice and self-determination. Programs that promote leadership, choice, autonomy, and initiative can help adolescents deal with developmental challenges associated with this age group. Leisure experiences can also promote civic engagement and provide important peer-to-peer, peer to-adult, and peer-to-community connections. The social context of leisure is important to adolescent development in that it provides opportunities to learn empathy, loyalty, and intimacy in their group activities, as well as to negotiate with peers, resolve conflict,and work together for communal goals. In addition, adolescents often report positive emotional experiences in leisure, which can serve as a relief from the stress they feel in other areas of their lives and contribute to positive psychological adjustment and well-being. A case study is used to show how planned, purposive programs can be used as critical components of efforts to contribute to adolescent development.
Copyright 2011 Wiley Periodicals, Inc., A Wiley Company
SN - 1537-5781

AD - Pennsylvania State University, USA
UR - 21786407
ER -

TY - JOUR
ID - 1547
T1 - Effects of task organization on the independent play of students with autism spectrum disorders
A1 - Mavropoulou,S.
A1 - Papadopoulou,E.
A1 - Kakana,D.
Y1 - 2011/07//
N1 - Mavropoulou, Sophia. Papadopoulou, Eleni. Kakana, Domna
Journal of autism and developmental disorders
7904301, hgw
IM
Journal Article
English
KW - Behavior Therapy/mt [Methods]
KW - Child
KW - Child Development Disorders
KW - Pervasive/px [Psychology]
KW - Pervasive/th [Therapy]
KW - Communication
KW - Humans
KW - Male
KW - Play and Playthings
KW - Teaching
KW - Treatment Outcome
KW - Medline
KW - Unsupervised Play Addition
RP - NOT IN FILE
SP - 913
EP - 925
JF - Journal of Autism & Developmental Disorders
JA - J Autism Dev.Disord
VL - 41
IS - 7
CY - United States
N2 - The purpose of the study was to examine the impact of task organization, a component of Structured Teaching developed by Division TEACCH, on the independent play of children with autism spectrum disorders (ASD). On-task behavior, task accuracy, task performance and teacher prompting were measured across independent play sessions in the classroom. An ABAB design was implemented to evaluate the effects of task organization on the independent play skills of two young children with ASD. Results regarding on-task behavior, task accuracy and independence were variable and are discussed. The implications of findings on the use of task organization for increasing independence in children with ASD are discussed
SN - 1573-3432
AD - Department of Special Education, University of Thessaly, Argonafton & Filellinon, 38 221 Volos, Greece. smavrop@uth.gr
UR - 20960042
ER -

TY - JOUR
ID - 1548
T1 - Pediatric fire deaths in Ontario: retrospective study of behavioural, social, and environmental risk factors
A1 - Chen,Y.A.
A1 - Bridgman-Acker,K.

A1 - Edwards,J.
A1 - Lauwers,A.E.
Y1 - 2011/05//
N1 - Chen, Yingming Amy. Bridgman-Acker, Karen. Edwards, Jim. Lauwers, Albert Edward
Canadian family physician Medecin de famille canadien
blo, 0120300
IM
Journal Article
English
KW - Adolescent
KW - Child
KW - Child Mortality
KW - Child Welfare
KW - Preschool
KW - Cohort Studies
KW - Dangerous Behavior
KW - Female
KW - Fires/sn [Statistics & Numerical Data]
KW - Housing
KW - Humans
KW - Infant
KW - Newborn
KW - Male
KW - Retrospective Studies
KW - Risk Factors
KW - Safety Management
KW - Socioeconomic Factors
KW - Medline
KW - Unsupervised Play Addition
RP - NOT IN FILE
SP - e169
EP - e177
JF - Canadian Family Physician
JA - Can Fam Physician
VL - 57
IS - 5
CY - Canada
N2 - OBJECTIVE: To identify the predictors of residential fire deaths in the Ontario pediatric population using systematically collected data from the Office of the Chief Coroner. DESIGN: Retrospective cohort study. SETTING: Ontario. PARTICIPANTS: Children younger than 16 years of age who died in accidental residential fires in Ontario between January 1, 2001, and December 31, 2006. MAIN OUTCOME MEASURES: The study retrospectively reviewed the coroner's case files for 60 subjects who qualified according to the selection criteria. Reviewed documents included the coroner's investigation statements, autopsy reports, toxicology reports, fire marshal's reports, police reports, and Children's Aid Society (CAS) reports. Information on a range of demographic, behavioural, social, and environmental factors was collected. Statistical tests, including relative risk, relative risk confidence intervals, and chi(2) tests were performed to determine the correlation between factors of interest and to establish their significance. RESULTS: Thirty-nine fire events resulting in 60 deaths occurred between 2001 and 2006. Fire play and electrical failures were the top 2 causes of residential fires. More fires occurred during the night (midnight to 9 AM) than during the day (9 AM to midnight). Nighttime fires were most commonly due to electrical failures or unattended candles, whereas daytime fires were primarily caused by unsupervised fire play and stove fires. Smoke alarms were present at 32 of 39 fire events (82%), but overall alarm functionality was only 54%. Children from families with a history of CAS involvement were approximately 32 times more likely to die in fires. CONCLUSION: Risk factors for pediatric fire death in Ontario include smoke alarm functionality, fire play, fire escape behaviour, and CAS involvement. Efforts to prevent residential fire deaths should target these populations and risk factors, and primary care physicians should consider education around these issues as a primary preventive strategy for families with young children

SN - 1715-5258
AD - Faculty of Medicine, University of Toronto, Ontario
UR - 21571705
ER -

TY - JOUR
ID - 1549
T1 - A cross-sectional study of the individual, social, and built environmental correlates of pedometer-based physical activity among elementary school children
A1 - McCormack,G.R.
A1 - Giles-Corti,B.
A1 - Timperio,A.
A1 - Wood,G.
A1 - Villanueva,K.
Y1 - 2011///
N1 - McCormack, Gavin R. Giles-Corti, Billie. Timperio, Anna. Wood, Georgina. Villanueva, Karen
The international journal of behavioral nutrition and physical activity
101217089
IM
Journal Article. Research Support, Non-U.S. Gov't
English
KW - Canada
KW - Child
KW - Child Behavior
KW - Cross-Sectional Studies
KW - Facility Design and Construction
KW - Female
KW - Health Behavior
KW - Humans
KW - Leisure Activities
KW - Male
KW - Monitoring
KW - Ambulatory
KW - Motor Activity
KW - Parents
KW - Play and Playthings
KW - Questionnaires
KW - Residence Characteristics
KW - Sex Characteristics
KW - Social Facilitation
KW - Socioeconomic Factors
KW - Transportation
KW - Walking
KW - Medline
KW - Unsupervised Play Addition
RP - NOT IN FILE
SP - 30
JF - International Journal of Behavioral Nutrition & Physical Activity
JA - Int.
VL - 8
CY - England
N2 - BACKGROUND: Children who participate in regular physical activity obtain health benefits. Preliminary pedometer-based cut-points representing sufficient levels of physical activity among youth have been established; however limited evidence regarding correlates of achieving these cut-points exists. The purpose of this study was to identify correlates of pedometer-based cut-points among elementary school-aged children. METHOD: A cross-section of children in grades 5-7 (10-12 years of age) were randomly selected from the most

(n = 13) and least (n = 12) 'walkable' public elementary schools (Perth, Western Australia), stratified by socioeconomic status. Children (n = 1480; response rate = 56.6%) and parents (n = 1332; response rate = 88.8%) completed a survey, and steps were collected from children using pedometers. Pedometer data were categorized to reflect the sex-specific pedometer-based cut-points of >15000 steps/day for boys and >12000 steps/day for girls. Associations between socio-demographic characteristics, sedentary and active leisure-time behavior, independent mobility, active transportation and built environmental variables - collected from the child and parent surveys - and meeting pedometer-based cut-points were estimated (odds ratios: OR) using generalized estimating equations. RESULTS: Overall 927 children participated in all components of the study and provided complete data. On average, children took 11407 + 3136 steps/day (boys: 12270 + 3350 vs. girls: 10681 + 2745 steps/day; p < 0.001) and 25.9% (boys: 19.1 vs. girls: 31.6%; p < 0.001) achieved the pedometer-based cut-points.After adjusting for all other variables and school clustering, meeting the pedometer-based cut-points was negatively associated (p < 0.05) with being male (OR = 0.42), parent self-reported number of different destinations in the neighborhood (OR 0.93), and a friend's (OR 0.62) or relative's (OR 0.44, boys only) house being at least a 10-minute walk from home. Achieving the pedometer-based cut-points was positively associated with participating in screen-time < 2 hours/day (OR 1.88), not being driven to school (OR 1.48), attending a school located in a high SES neighborhood (OR 1.33), the average number of steps among children within the respondent's grade (for each 500 step/day increase: OR 1.29), and living further than a 10-minute walk from a relative's house (OR 1.69, girls only). CONCLUSIONS: Comprehensive multi-level interventions that reduce screen-time, encourage active travel to/from school and foster a physically active classroom culture might encourage more physical activity among children
SN - 1479-5868
AD - Department of Community Health Sciences, University of Calgary, Alberta, Canada. gmccorma@ucalgary.ca
UR - 21486475
ER -

TY - JOUR
ID - 1550
T1 - Mothers' reports of play dates and observation of school playground behavior of children having high-functioning autism spectrum disorders
A1 - Frankel,F.D.
A1 - Gorospe,C.M.
A1 - Chang,Y.C.
A1 - Sugar,C.A.
Y1 - 2011/05//
N1 - Frankel, Frederick D. Gorospe, Clarissa M. Chang, Ya-Chih. Sugar, Catherine A
Journal of child psychology and psychiatry, and allied disciplines
hp3, 0375361
IM
Journal Article. Research Support, N.I.H., Extramural. Research Support, Non-U.S. Gov't
English
KW - Adaptation
KW - Psychological
KW - Child
KW - Child Development Disorders
KW - Pervasive/px [Psychology]
KW - Family
KW - Female
KW - Humans
KW - Intelligence
KW - Interpersonal Relations
KW - Male
KW - Mothers
KW - Observation
KW - Peer Group
KW - Play and Playthings/px [Psychology]

KW - Schools
KW - Social Environment
KW - Medline
KW - Unsupervised Play Addition
RP - NOT IN FILE
SP - 571
EP - 579
JF - Journal of Child Psychology & Psychiatry & Allied Disciplines
JA - J Child Psychol.Psychiatry
VL - 52
IS - 5
CY - England
N2 - BACKGROUND: Children with high-functioning autism spectrum disorders (ASD) are generally included with typically developing peers at school. They have difficulties interacting with peers on the school playground. Previous literature suggests that having play dates in the home may be related to better peer acceptance at school. METHODS: This study examines the relationship between mother-reported play date frequency and amount of conflict, and peer interaction observed on the school playground for a sample of 27 boys and 4 girls meeting structured interview and observation criteria for ASD. Measures of intellectual functioning, adaptive behavior, and social skills were included in a stepwise regression analysis to account for their impact on relationships between maternal play date reports, general peer acceptance at school (as rated by the child's teacher) and observations of school playground behavior. RESULTS: Results revealed that children with autism spectrum disorders who had more play dates in their home tended to spend a greater amount of time engaged in behaviors such as mutual offering of objects, conversing and other turn-taking activities with peers on the school playground. They also received more positive responses to their overtures from peers. These relationships remained highly significant even after accounting for other demographic, general social, and cognitive variables. CONCLUSIONS: The present results suggest that play date frequency is strongly related to school playground behavior. Owing to the design of this study, future research must assess whether play dates in the home promote better peer relationships on the playground or the reverse. In either case, the assessment of play dates, as well as observation of spontaneous unsupervised social interactions, are important outcome measures to consider in social skills interventions for children with high-functioning ASD. 2010 The Authors. Journal of Child Psychology and Psychiatry 2010 Association for Child and Adolescent Mental Health
SN - 1469-7610
AD - UCLA Semel Institute for Neuroscience and Human Behavior, Los Angeles, CA, USA. ffrankel@ucla.edu
UR - 20860756
ER -

TY - JOUR
ID - 1551
T1 - What is the meaning and nature of active play for today's children in the UK?
A1 - Brockman,R.
A1 - Fox,K.R.
A1 - Jago,R.
Y1 - 2011///
N1 - Brockman, Rowan. Fox, Kenneth R. Jago, Russell
The international journal of behavioral nutrition and physical activity
101217089
IM
Journal Article. Research Support, Non-U.S. Gov't
English
KW - Bicycling
KW - Child
KW - Exercise
KW - Family
KW - Female
KW - Focus Groups

KW - Friends
KW - Great Britain
KW - Humans
KW - Leisure Activities
KW - Male
KW - Perception
KW - Play and Playthings
KW - Sedentary Lifestyle
KW - Sex Factors
KW - Social Values
KW - Medline
KW - Unsupervised Play Addition
RP - NOT IN FILE
SP - 15
JF - International Journal of Behavioral Nutrition & Physical Activity
JA - Int.
VL - 8
CY - England
N2 - BACKGROUND: Preventing the decline in physical activity which occurs around 10-11 years of age is a public health priority. Physically active play can make unique contributions to children's development which cannot be obtained from more structured forms of physical activity. Encouraging active play in children's leisure time has potential to increase physical activity levels while promoting optimal child development. Aspired wisdom states that contemporary British children no longer play outdoors, but systematic evidence for this is lacking. We need to build a more informed picture of contemporary children's play before we consider interventions to increase it. METHODS: Eleven focus groups were conducted with 77, 10-11 year old children from four primary schools in Bristol, UK. Focus groups examined: 1) children's perceptions of 'play'; 2) how much of their play is active play; and 3) contexts of children's active play. All focus groups were audio-taped and transcribed verbatim. Data were analysed using a thematic approach. RESULTS: Children's perceptions of play were broad and included both physically active and sedentary behaviours. Children reported that they frequently engaged in active play and valued both the physical and social benefits it provided. Whereas boys frequently reported having a 'kick about' or riding bikes as their preferred forms of active play, girls were less likely to report a specific activity. Additionally, boys reported greater independent mobility in their active play compared to girls. Finally, boys were more likely to report playing with neighbourhood friends but girls more frequently reported playing with family members. CONCLUSIONS: Promoting active play in children's leisure time may increase the physical activity of children, but interventions may need to be tailored according to gender
SN - 1479-5868
AD - Centre for Exercise, Nutrition & Health Sciences, School for Policy Studies, University of Bristol, Bristol, UK. Rowan.Brockman@bristol.ac.uk
UR - 21385336
ER -

TY - JOUR
ID - 1552
T1 - Young adolescents' independent mobility, related factors and association with transport to school. A cross-sectional study
A1 - Johansson,K.
A1 - Hasselberg,M.
A1 - Laflamme,L.
Y1 - 2010///
N1 - Johansson, Klara. Hasselberg, Marie. Laflamme, Lucie
BMC public health
100968562
IM
Journal Article. Research Support, Non-U.S. Gov't
English

KW - Adolescent
KW - Cluster Analysis
KW - Cross-Sectional Studies
KW - Female
KW - Humans
KW - Male
KW - Personal Autonomy
KW - Questionnaires
KW - Schools
KW - Sweden
KW - Transportation
KW - Medline
KW - Unsupervised Play Addition
RP - NOT IN FILE
SP - 635
JA - BMC Public Health
VL - 10
CY - England
N2 - BACKGROUND: Children's independent mobility differs between groups of adolescents, but knowledge is lacking on how mobility-limiting factors interact. This study explores the association between factors that can affect young adolescents' mobility, searching for typical patterns within a geographical area where mobility is both relatively high and promoted (in this case Stockholm County, Sweden). An additional question is how clusters of limiting factors and demographic attributes relate to active commuting to school. METHODS: A sample of 7th grade students (ca 13-14 years old) in Stockholm County, Sweden, answered a survey (n = 1008). A cluster analysis was performed on variables descriptive of the respondents and of potential limitations to their independent mobility, such as fears, coping, traffic situation in the neighbourhood and parent/child opinions on mobility (18 variables and 50 categories). Active commuting to/from school was compared using proportion (with 95% confidence intervals) by cluster. RESULTS: Five consistent and distinct clusters were identified. Among the most discriminating factors were fears experienced in the neighbourhood, strategies to cope with fear, type of housing and traffic environment. Girls were over-represented in the two clusters most typical of respondents experiencing fears (either several of these or darkness in particular) and boys in two others where housing (house vs. apartment) and neighbourhood conditions played a more determinant role. The proportion of active commuting among respondents was quite similar over clusters but was nonetheless higher in the cluster (over girls) reporting more fears and other factors limiting mobility. CONCLUSIONS: Whereas fears--and coping--are more typical of adolescent girls in the formation of the clusters, household and neighbourhood characteristics are more typical of boys. Broadly speaking, there seem to be two groups of girls with fears but these differ based on types of fear, ways of coping with fear and their living conditions. The association between the limitations to mobility and active commuting is unclear, the latter being higher among those disclosing a broader range of limiting factors, including fears
SN - 1471-2458
AD - Department of Public Health Sciences, Division of Global Health/IHCAR, Karolinska Institutet, Nobels vag 9, SE-171 77 Stockholm, Sweden. klara.johansson@ki.se
UR - 20969747
ER -

TY - JOUR
ID - 1554
T1 - Supervision of young children with fall injuries
A1 - Castro,Y.
A1 - Powell,E.C.
A1 - Sheehan,K.M.
Y1 - 2010/10//
N1 - Castro, Yessenia. Powell, Elizabeth C. Sheehan, Karen M
The Journal of trauma
kaf, 0376373
AIM, IM

Journal Article. Research Support, Non-U.S. Gov't
English
KW - Accidental Falls/pc [Prevention & Control]
KW - Accidental Falls/sn [Statistics & Numerical Data]
KW - Age Distribution
KW - Child
KW - Child Care
KW - Child Rearing
KW - Preschool
KW - Cohort Studies
KW - Emergency Service
KW - Hospital/sn [Statistics & Numerical Data]
KW - Female
KW - Humans
KW - Infant
KW - Male
KW - Play and Playthings
KW - Retrospective Studies
KW - Risk Factors
KW - Wounds and Injuries/di [Diagnosis]
KW - Wounds and Injuries/ep [Epidemiology]
KW - Wounds and Injuries/pc [Prevention & Control]
KW - Medline
KW - Unsupervised Play Addition
RP - NOT IN FILE
SP - S214
EP - S217
JF - Journal of Trauma-Injury Infection & Critical Care
JA - J Trauma
VL - 69
IS - 4 Suppl
CY - United States
N2 - BACKGROUND: Specific information about the supervision of young children with injuries related to falls is limited. In this study, we describe the supervision and physical environment of falls resulting in medical care in the emergency department. METHODS: We enrolled a convenience sample of 108 children younger than 7 years with fall injuries. RESULTS: The average age was 3 years, and 56% were male. Seventy-six (70%) were a fall from a height including 16 that involved stairs. Among caretakers in a nongroup setting (n = 95), most (61%) were supervising more than one child. The attention to the child was holding or playing with the child (13%), observing (45%), usually constantly, or listening for the child (19%); 9% reported no supervision at the time of the fall. Thirty-two percent stated they were touching or within reach of the child. Of falls indoors (n = 56), the supervisor was in the same room as the child for more than half of cases. There was no association between the number of children supervised and fall type (height vs. same level). When compared with those with same level falls, children with falls from a height were more often supervised with listening or no supervision (vs. observation, holding, or playing with the child) chi2, p = 0.004. CONCLUSIONS: Many children were supervised at the time of their fall. Most caretakers had visual contact, and up to a third were touching or within reach of the child. The strategies used in these apparently low-risk situations were insufficient to prevent the falls we report
SN - 1529-8809
AD - Children's Memorial Hospital, Department of Pediatrics, Northwestern University's Feinberg School of Medicine, Chicago, Illinois, USA
UR - 20938311
ER -

TY - JOUR
ID - 1555
T1 - Use of manual and powered wheelchair in children with cerebral palsy: a cross-sectional study

A1 - Rodby-Bousquet,E.
A1 - Hagglund,G.
Y1 - 2010///
N1 - Rodby-Bousquet, Elisabet. Hagglund, Gunnar
BMC pediatrics
100967804
IM
Journal Article. Research Support, Non-U.S. Gov't
English
KW - Activities of Daily Living
KW - Adolescent
KW - Cerebral Palsy
KW - Child
KW - Child Development
KW - Cross-Sectional Studies
KW - Female
KW - Humans
KW - Locomotion
KW - Male
KW - Wheelchairs
KW - Medline
KW - Unsupervised Play Addition
RP - NOT IN FILE
SP - 59
JF - BMC Pediatrics
JA - BMC Pediatr
VL - 10
CY - England
N2 - BACKGROUND: Mobility is important for the cognitive and psychosocial development of children. Almost one third of children with cerebral palsy (CP) are non-ambulant. Wheelchairs can provide independent mobility, allowing them to explore their environment. Independent mobility is vital for activity and participation and reduces the dependence on caregivers. The purpose of this study was to describe the use of manual and powered wheelchair indoors and outdoors in relation to the degree of independent wheelchair mobility or need for assistance in a total population of children with CP. METHODS: A cross-sectional study was performed including all children aged 3-18 years with CP living in southern Sweden during 2008. Data was extracted from a register and health care programme for children with CP (CPUP). There were a total of 562 children (326 boys, 236 girls) in the register. Information on the child's use of manual and powered wheelchair indoors and outdoors and the performance in self-propelling or need for assistance were analysed related to age, CP subtype and gross motor function. RESULTS: Wheelchairs for mobility indoors were used by 165 (29%) of the 562 children; 61 used wheelchair for independent mobility (32 using manual only, 12 powered only, 17 both) and 104 were pushed by an adult. For outdoor mobility wheelchairs were used by 228 children (41%); 66 used a wheelchair for independent mobility (18 using manual only, 36 powered only, 12 both) and 162 were pushed. The use of wheelchair increased with age and was most frequent in the spastic bilateral and dyskinetic subtypes. Most powered wheelchairs were operated by children at GMFCS level IV. CONCLUSION: In this total population of children with CP, aged 3-18 years, 29% used a wheelchair indoors and 41% outdoors. A majority using manual wheelchairs needed adult assistance (86%) while powered wheelchairs provided independent mobility in most cases (86%). To achieve a high level of independent mobility, both manual and powered wheelchairs should be considered at an early age for children with impaired walking ability
SN - 1471-2431
AD - Department of Orthopaedics, Lund University, University Hospital, 221 85 Lund, Sweden. elisabet.rodby_bousquet@med.lu.se
UR - 20712899
ER -

TY - JOUR
ID - 1556

T1 - A robotic wheelchair trainer: design overview and a feasibility study
A1 - Marchal-Crespo,L.
A1 - Furumasu,J.
A1 - Reinkensmeyer,D.J.
Y1 - 2010///
N1 - Marchal-Crespo, Laura. Furumasu, Jan. Reinkensmeyer, David J
Journal of neuroengineering and rehabilitation
101232233
IM
Case Reports. Journal Article. Randomized Controlled Trial. Research Support, U.S. Gov't, Non-P.H.S.. Validation Studies
English
KW - Aging
KW - Algorithms
KW - Automation
KW - Cerebral Palsy/rh [Rehabilitation]
KW - Child
KW - Preschool
KW - Dyskinesias/rh [Rehabilitation]
KW - Equipment Design
KW - Feasibility Studies
KW - Feedback
KW - Female
KW - Humans
KW - Insemination
KW - Artificial
KW - Pilot Projects
KW - Practice (Psychology)
KW - Robotics/is [Instrumentation]
KW - Robotics/mt [Methods]
KW - Time Factors
KW - Touch
KW - Treatment Outcome
KW - Wheelchairs
KW - Medline
KW - Unsupervised Play Addition
RP - NOT IN FILE
SP - 40
JF - Journal of Neuroengineering & Rehabilitation
JA - J Neuroengineering Rehabil
VL - 7
CY - England
N2 - BACKGROUND: Experiencing independent mobility is important for children with a severe movement disability, but learning to drive a powered wheelchair can be labor intensive, requiring hand-over-hand assistance from a skilled therapist. METHODS: To improve accessibility to training, we developed a robotic wheelchair trainer that steers itself along a course marked by a line on the floor using computer vision, haptically guiding the driver's hand in appropriate steering motions using a force feedback joystick, as the driver tries to catch a mobile robot in a game of "robot tag". This paper provides a detailed design description of the computer vision and control system. In addition, we present data from a pilot study in which we used the chair to teach children without motor impairment aged 4-9 (n = 22) to drive the wheelchair in a single training session, in order to verify that the wheelchair could enable learning by the non-impaired motor system, and to establish normative values of learning rates. RESULTS AND DISCUSSION: Training with haptic guidance from the robotic wheelchair trainer improved the steering ability of children without motor impairment significantly more than training without guidance. We also report the results of a case study with one 8-year-old child with a severe motor impairment due to cerebral palsy, who replicated the single-session training protocol that the non-disabled children participated in. This child also improved steering ability after training with

guidance from the joystick by an amount even greater than the children without motor impairment. CONCLUSIONS: The system not only provided a safe, fun context for automating driver's training, but also enhanced motor learning by the non-impaired motor system, presumably by demonstrating through intuitive movement and force of the joystick itself exemplary control to follow the course. The case study indicates that a child with a motor system impaired by CP can also gain a short-term benefit from driver's training with haptic guidance
SN - 1743-0003
AD - Mechanical and Aerospace Engineering Department, University of California, Irvine, CA, USA. laura.marchal@mavt.ethz.ch
UR - 20707886
ER -

TY - JOUR
ID - 1557
T1 - Child supervision practices for drowning prevention in rural Bangladesh: a pilot study of supervision tools
A1 - Callaghan,J.A.
A1 - Hyder,A.A.
A1 - Khan,R.
A1 - Blum,L.S.
A1 - Arifeen,S.
A1 - Baqui,A.H.
Y1 - 2010/07//
N1 - Callaghan, J A. Hyder, A A. Khan, R. Blum, L S. Arifeen, S. Baqui, A H
Journal of epidemiology and community health
i1p, 7909766
IM
Journal Article. Research Support, U.S. Gov't, Non-P.H.S.
English
KW - Adult
KW - Bangladesh
KW - Child
KW - Community Health Services/mt [Methods]
KW - Drowning/pc [Prevention & Control]
KW - Health Education
KW - Humans
KW - Infant Equipment
KW - Models
KW - Statistical
KW - Pilot Projects
KW - Process Assessment (Health Care)/st [Standards]
KW - Protective Devices
KW - Residence Characteristics
KW - Rural Population
KW - Medline
KW - Unsupervised Play Addition
RP - NOT IN FILE
SP - 645
EP - 647
JF - Journal of Epidemiology & Community Health
JA - J Epidemiol.Community Health
VL - 64
IS - 7
CY - England
N2 - BACKGROUND: Injuries are an increasing child health concern and have become a leading cause of child mortality in the 1-4 years age group in many developing countries, including Bangladesh. METHODS: Household observations during 9 months of a community-based pilot of two supervision tools-a door barrier and

a playpen-designed to assess their community acceptability in rural Bangladesh are reported in this article. RESULTS: Statistical analysis of 2694 observations revealed that children were directly supervised or protected by a preventive tool in 96% of visits. Households with a supervision tool had a significantly lower proportion of observations with the child unsupervised and unprotected than households without a tool. Families that received a playpen had 6.89 times the odds of using it at the time of the visit than families that received a door barrier. CONCLUSIONS: Interventions such as the playpen, when introduced to households through community-based programs, are accepted by parents. Field trials are urgently needed to establish the effectiveness of barrier-based interventions at reducing under-five drowning mortality rates in low-income countries like Bangladesh
SN - 1470-2738
AD - International Injury Research Unit, Department of International Health, Bloomberg School of Public Health, Johns Hopkins University, 615 North Wolfe Street, Baltimore, MD 21205, USA
UR - 20547700
ER -

TY - JOUR
ID - 1558
T1 - Social participation and independent mobility in children: the effects of two implementations of "we go to school alone"
A1 - Prezza,M.
A1 - Alparone,F.R.
A1 - Renzi,D.
A1 - Pietrobono,A.
Y1 - 2010/01//
N1 - Prezza, Miretta. Alparone, Francesca Romana. Renzi, Daniela. Pietrobono, Annalisa
Journal of prevention & intervention in the community
9702085
IM
Journal Article. Research Support, Non-U.S. Gov't
English
KW - Child
KW - Consumer Participation
KW - Humans
KW - Longitudinal Studies
KW - Personal Autonomy
KW - Program Evaluation
KW - Questionnaires
KW - Rome
KW - Schools
KW - Transportation
KW - Medline
KW - Unsupervised Play Addition
RP - NOT IN FILE
SP - 8
EP - 25
JF - Journal of Prevention & Intervention in the Community
VL - 38
IS - 1
CY - England
N2 - The aim of this research was to determine the outcomes of the "We go to school alone" program in two Districts of Rome through a longitudinal study involving 392 children (mean age = 8.37 years) and 270 parents. The outcomes of the program in the two Districts were very different. Only one resulted in an increase in children's autonomous mobility on the home-school journey, a reduction in the number of times a child was taken to school by car, and, even more important, in an increase in the general level of children's independent mobility in their neighborhood. The findings are discussed in terms of a process evaluation that enabled us to understand the differing results
SN - 1540-7330

AD - Department of Psychology, Sapienza University of Rome, 00185 Rome, Italy. miretta.prezza@uniroma1.i
UR - 20391051
ER -

TY - JOUR
ID - 1559
T1 - Community psychology in Italy: introduction and prospects
A1 - Santinello,M.
A1 - Martini,E.R.
A1 - Perkins,D.D.
Y1 - 2010/01//
N1 - Santinello, Massimo. Martini, Elvio Raffaello. Perkins, Douglas D
Journal of prevention & intervention in the community
9702085
IM
Introductory Journal Article
English
KW - Adolescent
KW - Child
KW - Consumer Participation
KW - Evaluation Studies as Topic
KW - Health Promotion
KW - Health Services Research
KW - Humans
KW - Italy
KW - psychology
KW - Social
KW - Public Policy
KW - Medline
KW - Unsupervised Play Addition
RP - NOT IN FILE
SP - 1
EP - 7
JF - Journal of Prevention & Intervention in the Community
VL - 38
IS - 1
CY - England
N2 - The history of community psychology in Italy is briefly reviewed. The field has developed extensively in universities and applied settings over the past 30 years. This issue presents 5 recent examples from different regions of Italy of preventive and other community psychological intervention studies. They include an evaluation of a program to increase the independent mobility of children walking to and from school, the ecological evaluation of child and adolescent residential care communities, participatory action-research with adolescents in schools and neighborhoods, evaluation of a participatory local health intervention planning process, and the description and evaluation of a collaborative, Internet-based community planning training program
SN - 1540-7330
AD - Department of Developmental and Social Psychology, University of Padova, 35131 Padova, Italy. massimo.santinello@unipd.it
UR - 20391050
ER -

TY - JOUR
ID - 1561
T1 - Knee-ankle-foot orthosis in children with duchenne muscular dystrophy: user views and adjustment
A1 - Garralda,M.E.
A1 - Muntoni,F.

A1 - Cunniff,A.
A1 - Caneja,A.D.
Y1 - 2006/07//
N1 - Garralda, M Elena. Muntoni, Francesco. Cunniff, Anna. Caneja, Angeles Diaz
European journal of paediatric neurology : EJPN : official journal of the European Paediatric Neurology Society
9715169, dgs
IM
Journal Article. Research Support, Non-U.S. Gov't
English
KW - Adaptation
KW - Psychological
KW - Adolescent
KW - Ankle
KW - Caregivers/px [Psychology]
KW - Child
KW - Data Interpretation
KW - Statistical
KW - Female
KW - Foot
KW - Humans
KW - Interview
KW - Knee
KW - Male
KW - Mental Disorders/ep [Epidemiology]
KW - Mental Disorders/px [Psychology]
KW - Muscular Dystrophy
KW - Duchenne/px [Psychology]
KW - Duchenne/rh [Rehabilitation]
KW - Orthotic Devices
KW - Parents/px [Psychology]
KW - Patient Satisfaction
KW - Questionnaires
KW - Risk
KW - Medline
KW - Unsupervised Play Addition
RP - NOT IN FILE
SP - 186
EP - 191
JF - European Journal of Paediatric Neurology
JA - Europ.J Paediatr Neurol
VL - 10
IS - 4
CY - England
N2 - BACKGROUND: The use of knee ankle foot orthoses (KAFOs) to prolong independent mobility is a widely used rehabilitation strategy for children with Duchenne muscular dystrophy (DMD). AIMS: To explore views and adjustment of families with a child with Duchenne muscular dystrophy to the use of KAFOs. METHODS: interviews with families of children aged 8-18 years with DMD; questionnaires on psychiatric adjustment (SDQ for children; GHQ for parents). RESULTS: In total, 17 parents and 9 children took part. Families experienced the introduction of KAFOs as a signal for illness deterioration and a re-awakening of the feelings experienced at diagnosis. Nevertheless, the majority expressed a positive attitude and over two-thirds satisfaction with KAFOs use. High psychiatric risk was found in 2/17 children (12%; expected 10%) and 7/17 main carers (41%; expected 20-30%). CONCLUSION: Most families were satisfied with KAFOs use, and its implementation was well tolerated especially by the children. However, mental distress was high in main carers who emphasized the importance of full preparation and support in this rehabilitation technique
SN - 1090-3798

AD - Child and Adolescent Psychiatry, Imperial College London, St. Mary's Campus, Norfolk Place, London W2 1PG, UK. e.garralda@imperial.ac.uk
UR - 16931076
ER -

TY - JOUR
ID - 1563
T1 - Imaging of American football injuries in children
A1 - Podberesky,D.J.
A1 - Unsell,B.J.
A1 - Anton,C.G.
Y1 - 2009/12//
N1 - Podberesky, Daniel J. Unsell, Bryan J. Anton, Christopher G
Pediatric radiology
owi, 0365332
IM
Journal Article
English
KW - Adolescent
KW - Child
KW - Preschool
KW - Female
KW - Football/in [Injuries]
KW - Humans
KW - Magnetic Resonance Imaging/mt [Methods]
KW - Male
KW - Tomography
KW - X-Ray Computed/mt [Methods]
KW - Wounds
KW - Nonpenetrating/di [Diagnosis]
KW - Medline
KW - Unsupervised Play Addition
RP - NOT IN FILE
SP - 1264
EP - 1274
JF - Pediatric Radiology
JA - Pediatr Radiol
VL - 39
IS - 12
CY - Germany
N2 - It is estimated that 3.2 million children ages 6 to 14 years participated in organized youth football in the United States in 2007. Approximately 240,000 children play football in the nation's largest youth football organization, with tackle divisions starting at age 5 years. The number of children playing unsupervised football is much higher, and the overall number of children participating in American football is increasing. Sports are the leading cause of injury-related emergency room visits for teenagers, and football is a leading precipitating athletic activity for these visits. Football is also the most hazardous organized sports in the United States. Though most pediatric football-related injuries are minor, such as abrasions, sprains, and strains of the extremities, football accounts for more major and catastrophic injuries than any other sport. Given football's popularity with children in the United States, combined with the high rate of injury associated with participation in this activity, radiologists should be familiar with the imaging features and injury patterns seen in this patient population
SN - 1432-1998
AD - Department of Radiology, Cincinnati Children's Hospital Medical Center, Cincinnati, OH 45229, USA. daniel.podberesky@cchmc.org
UR - 19774374
ER -

TY - JOUR
ID - 1564
T1 - Exploring the neighborhood: a web-based survey on the prevalence and determinants of fear among young adolescent girls and boys
A1 - Johansson,K.
A1 - Hasselberg,M.
A1 - Laflamme,L.
Y1 - 2009/07//
N1 - Johansson, Klara. Hasselberg, Marie. Laflamme, Lucie
International journal of adolescent medicine and health
8506960
IM
Journal Article. Research Support, Non-U.S. Gov't
English
KW - Adaptation
KW - Psychological
KW - Adolescent
KW - Adolescent Behavior
KW - Child
KW - Confidence Intervals
KW - Epidemiologic Factors
KW - Fear
KW - Female
KW - Humans
KW - Internet
KW - Logistic Models
KW - Male
KW - Multivariate Analysis
KW - Odds Ratio
KW - Parent-Child Relations
KW - Prevalence
KW - Psychometrics
KW - Questionnaires
KW - Residence Characteristics
KW - Safety/sn [Statistics & Numerical Data]
KW - Sex Factors
KW - Socioeconomic Factors
KW - Stress
KW - Psychological/ep [Epidemiology]
KW - Psychological/et [Etiology]
KW - Sweden/ep [Epidemiology]
KW - Medline
KW - Unsupervised Play Addition
RP - NOT IN FILE
SP - 347
EP - 359
JF - International Journal of Adolescent Medicine & Health
JA - Int J Adolesc Med Health
VL - 21
IS - 3
CY - England
N2 - Children's independent everyday mobility can be hindered by fears experienced in their neighborhood. The aim of this study is to assess the prevalence and determinants of such fears among boys and girls in early adolescence, a period when individual freedom is expected to be on the increase. A sample of 7th grade students (age-13 years) in Stockholm County, Sweden, during 2005/06 answered a survey in class (n = 1,008). The

relation that gender, housing, family characteristics, individual and peer negative experiences in the neighborhood, parental licensing, and length of stay in the neighborhood have with fear disclosure was assessed through multivariate logistic regression. A total of 60% of the girls and 40% of the boys reported experiencing fears in their neighborhood. Gender differences were significant for all of the most common fears, in particular darkness. When respondents or their friends had been chased, hit, or had something taken from them in their neighborhood, they were more likely to report fear (OR girls 2.3; 95% CI 1.6-4.5; boys 2.8; 95% CI 1.9-4.2). For girls, having one or more parents born outside Sweden was associated with fear. Boys nearly three times more often reported fear if (a) they thought their parents were negative toward adolescent independent mobility in the evening, or (b) they had lived longer than one year in their area. Many young adolescents admitted to experiencing fear in their neighborhood. Fears were more common among girls, and the types and determinants of fear seem to be gender specific
SN - 0334-0139
AD - Karolinska Institutet, Department of Public Health Sciences, Division of Global Health, Stockholm, Sweden. klara.johansson@ki.se
UR - 20014638
ER -

TY - JOUR
ID - 1565
T1 - Development of a cart for independent mobility assistance for non-ambulatory children
A1 - Kakimoto,A.
A1 - Suzuki,S.
A1 - Sekiguchi,Y.
Y1 - 2009///
N1 - Kakimoto, Akira. Suzuki, Shigenobu. Sekiguchi, Yukio
Conference proceedings : ... Annual International Conference of the IEEE Engineering in Medicine and Biology Society. IEEE Engineering in Medicine and Biology Society. Conference
101243413
IM
Journal Article
English
KW - Biomechanical Phenomena
KW - Biomedical Engineering
KW - Cerebral Palsy/pp [Physiopathology]
KW - Cerebral Palsy/rh [Rehabilitation]
KW - Child
KW - Preschool
KW - Equipment Design
KW - Female
KW - Humans
KW - Male
KW - Man-Machine Systems
KW - Motor Skills
KW - Self-Help Devices/sn [Statistics & Numerical Data]
KW - Self-Help Devices
KW - Software
KW - Wheelchairs
KW - Medline
KW - Unsupervised Play Addition
RP - NOT IN FILE
SP - 7273
EP - 7276
JF - Conference Proceedings:
JA - Conf.Proc IEEE Eng Med Biol Soc
VL - .. Annual International Conference of the IEEE Engineering in Medicine & Biology Society. 2009
CY - United States

N2 - Some parents of non-ambulatory children are not eager to allow their children to use powered wheelchairs because of apprehension of further deterioration of their functionality and the risk for accidents. The authors think that not all but some such children could develop their ability to operate powered wheelchairs and might expand their knowledge about the circumstances and interests in them. Thus we made a prototype cart for them eventually to experience motion by themselves. Based on a 6-wheeled chassis, the two middle wheels are driven with a traction roller drive system. We had been testing this prototype with a child with cerebral palsy for one year. Acquisition of skills in pushing switches and enjoyment of motion were confirmed. However, we encountered several problems such as difficulty in rolling on a carpet and in handling due to its weight. The ability to record moving speed and operation of input devices helps rehabilitation experts to conduct quantitative performance evaluation. To solve these problems, we manufactured another prototype. The second prototype was tested with another child with cerebral palsy for one year. The problems were solved. She acquired the operation skills necessary to operate the prototype with four switch operation
SN - 1557-170X
AD - Polytechnic University, 4-1-1 Hashimotodai Sagamihara, Kanagawa 229-1196, Japan.
kakimoto@uitec.ac.jp
UR - 19965102
ER -

TY - JOUR
ID - 1566
T1 - Encouraging walking for transport and physical activity in children and adolescents: how important is the built environment?. [Review] [1113 refs]
A1 - Giles-Corti,B.
A1 - Kelty,S.F.
A1 - Zubrick,S.R.
A1 - Villanueva,K.P.
Y1 - 2009///
N1 - Giles-Corti, Billie. Kelty, Sally F. Zubrick, Stephen R. Villanueva, Karen P
Sports medicine (Auckland, N.Z.)
8412297, spo
IM
Journal Article. Research Support, Non-U.S. Gov't. Review
English
KW - Adolescent
KW - Child
KW - Environment Design
KW - Exercise
KW - Female
KW - Health Promotion
KW - Humans
KW - Male
KW - Motivation
KW - Walking
KW - Medline
KW - Unsupervised Play Addition
RP - NOT IN FILE
SP - 995
EP - 1009
JF - Sports Medicine
JA - Sports Med
VL - 39
IS - 12
CY - New Zealand
N2 - In the post-World War II era, there have been dramatic changes to the environment that appear to be having a detrimental impact on the lifestyles and incidental physical activities of young people. These changes are not trivial and have the potential to influence not only physical health, but also mental health and child

development. However, the evidence of the impact of the built environment on physical activity to date is inconsistent. This review examines the evidence on the association between the built environment and walking for transport as well as physical activity generally, with a focus on methodological issues that may explain inconsistencies in the literature to date. It appears that many studies fail to measure behaviour-specific environmental correlates, and insufficient attention is being given to differences according to the age of study participants. Higher levels of out-of-school-hours physical activity and walking appear to be significantly associated with higher levels of urban density and neighbourhoods with mixed-use planning, especially for older children and adolescents. Proximate recreational facilities also appear to predict young people's level of physical activity. However, there are inconsistencies in the literature involving studies with younger children. Independent mobility increases with age. For younger children, the impact of the built environment is influenced by the decision-making of parents as the gatekeepers of their behaviour. Cross-cultural differences may also be present and are worthy of greater exploration. As children develop and are given more independent mobility, it appears that the way neighbourhoods are designed - particularly in terms of proximity and connectivity to local destinations, including schools and shopping centres, and the presence of footpaths - becomes a determinant of whether children are able, and are permitted by their parents, to walk and use destinations locally. If older children and adolescents are to enjoy health and developmental benefits of independent mobility, a key priority must be in reducing exposure to traffic and in increasing surveillance on streets (i.e. 'eyes-on-the-street') through neighbourhood and building design, by encouraging others to walk locally, and by discouraging motor vehicle use in favour of walking and cycling. Parents need to be assured that the rights and safety of pedestrians (and cyclists) - particularly child pedestrians and cyclists - are paramount if we are to turn around our 'child-free streets', now so prevalent in contemporary Australian and US cities. There remains a need for more age- and sex-specific research using behaviour- and context-specific measures, with a view to building a more consistent evidence base to inform future environmental interventions. [References: 1113]
SN - 1179-2035
AD - Centre for the Built Environment and Health, School of Population Health, University of Western Australia, Crawley, Western Australia, Australia. Billie.Giles-Corti@uwa.edu.au
UR - 19902982
ER -

TY - JOUR
ID - 1567
T1 - Sport and recreation-related injuries and fracture occurrence among emergency department attendees: implications for exercise prescription and injury prevention
A1 - Falvey,E.C.
A1 - Eustace,J.
A1 - Whelan,B.
A1 - Molloy,M.S.
A1 - Cusack,S.P.
A1 - Shanahan,F.
A1 - Molloy,M.G.
Y1 - 2009/08//
N1 - Falvey, E C. Eustace, J. Whelan, B. Molloy, M S. Cusack, S P. Shanahan, F. Molloy, M G
Emergency medicine journal : EMJ
b0u, 100963089
IM
Journal Article. Research Support, Non-U.S. Gov't
English
KW - Adolescent
KW - Adult
KW - Athletic Injuries/ep [Epidemiology]
KW - Athletic Injuries/pc [Prevention & Control]
KW - Child
KW - Preschool
KW - Emergency Service
KW - Hospital/sn [Statistics & Numerical Data]

KW - Exercise Therapy
KW - Female
KW - Fractures
KW - Bone/ep [Epidemiology]
KW - Bone/pc [Prevention & Control]
KW - Humans
KW - Ireland
KW - Male
KW - Prospective Studies
KW - Recreation
KW - Medline
KW - Unsupervised Play Addition
RP - NOT IN FILE
SP - 590
EP - 595
JF - Emergency Medicine Journal
JA - Emerg Med J
VL - 26
IS - 8
CY - England
N2 - OBJECTIVE: To investigate the epidemiology of sports and recreation-related injury (SRI) among emergency department (ED) attendees. DESIGN: Descriptive epidemiology study. SETTING: An Irish university hospital ED. PARTICIPANTS: All patients aged over 4 years attending a large regional ED, during a 6-month period, for the treatment of SRI were prospectively surveyed. ASSESSMENT OF RISK FACTORS: In all cases identified as SRI the attending physician completed a specifically designed questionnaire. It was postulated that recreation-related injury is a significant proportion of reported SRI. RESULTS: Fracture rate was highest in the 4-9-year age group (44%). On multivariate logistic regression the adjusted odds ratio (OR; 95% CI) of fracture was higher for children (vs adults) at 1.21 (1.0 to 1.45). The adjusted OR was higher for upper-limb 5.8 (4.5 to 7.6) and lower-limb injuries 1.87 (1.4 to 2.5) versus axial site of injury and for falls 2.2 (1.6 to 2.9) and external force 1.59 (1.2 to 2.1) versus an overextension mechanism of injury. In the same model, "play" was independently associated with fracture risk, adjusted OR 1.98 (1.2 to 3.0; p = 0.001) versus low-risk ball sports 1.0 (reference); an effect size similar to that seen for combat sports 1.96 (1.2 to 3.3; p = 0.01) and greater than that seen for presumed high-risk field sports 1.4 (0.9 to 2.0) CONCLUSION: Fall and subsequent upper-limb injury was the commonest mechanism underlying SRI fracture. Domestic "play" in all age groups at the time of injury accorded a higher fracture risk than field sports. Patient education regarding the dangers of unsupervised play and recreation represents a means of reducing the burden of SRI
SN - 1472-0213
AD - Department of Rheumatology, Sports and Exercise Medicine, Cork University Hospital, Wilton, Cork, Ireland. e.falvey@ireland.com
UR - 19625558
ER -

TY - JOUR
ID - 1568
T1 - Significant pediatric morbidity and mortality from intracranial ballistic injuries caused by nonpowder gunshot wounds. A case series
A1 - O'Neill,P.J.
A1 - Lumpkin,M.F.
A1 - Clapp,B.
A1 - Kopelman,T.R.
A1 - Matthews,M.R.
A1 - Cox,J.C.
A1 - Caruso,D.M.
A1 - Feiz-Erfan,I.
Y1 - 2009///

N1 - O'Neill, Patrick J. Lumpkin, Mary Foster. Clapp, Benjamin. Kopelman, Tammy R. Matthews, Marc R. Cox, Jordy C. Caruso, Daniel M. Feiz-Erfan, Iman
Pediatric neurosurgery
axe, 9114967
IM
Case Reports. Journal Article
English
KW - Adolescent
KW - Adult
KW - Age Distribution
KW - Child
KW - Preschool
KW - Fatal Outcome
KW - Female
KW - Firearms/sn [Statistics & Numerical Data]
KW - Humans
KW - Infant
KW - Male
KW - Morbidity
KW - Play and Playthings/in [Injuries]
KW - Registries
KW - Retrospective Studies
KW - Southwestern United States/ep [Epidemiology]
KW - Tomography
KW - X-Ray Computed
KW - Wounds
KW - Gunshot/mo [Mortality]
KW - Gunshot/ra [Radiography]
KW - Medline
KW - Unsupervised Play Addition
RP - NOT IN FILE
SP - 205
EP - 209
JF - Pediatric Neurosurgery
JA - Pediatr Neurosurg
VL - 45
IS - 3
CY - Switzerland
N2 - Nonpowder (ball-bearing and pellet) weapons derive their source of energy from compressed air or carbon dioxide. Such weapons are dangerous toys that cause serious injuries and even death to children and adolescents. A retrospective chart review study was undertaken to describe nonpowder gun injuries at a southwestern US urban level I adult and pediatric trauma center. Specific emphasis was placed on intracranial injuries. Over the past 6 years, a total of 29 pediatric and 7 adult patients were identified as having nonpowder firearm injuries. The patient population was overwhelmingly male (89.7%; mean age, 11 years). Overall, 17 out of 29 pediatric patients (56.8%) sustained serious injury. Nine patients (30.0%) required operation, 6 (20.7%) sustained significant morbidity, and there were 2 deaths (6.9%). Injuries to the brain, eye, head, and neck were the most common sites of injury (65.6%). Specific intracranial injuries in 3 pediatric patients are described that resulted in the death of 2 children. We suggest that age warning should be adjusted to 18 years or older for unsupervised use to be considered safe of these potentially lethal weapons. Copyright 2009 S. Karger AG, Basel
SN - 1423-0305
AD - Division of Burns, Trauma, and Surgical Critical Care, Department of Surgery, Maricopa Medical Center, Phoenix, Ariz., USA
UR - 19494565
ER -

TY - JOUR

ID - 1569
T1 - Parents' perceptions of postschool years for young adults with developmental disabilities
A1 - Bianco,M.
A1 - Garrison-Wade,D.F.
A1 - Tobin,R.
A1 - Lehmann,J.P.
Y1 - 2009/06//
N1 - Bianco, Margarita. Garrison-Wade, Dorothy F. Tobin, Romie. Lehmann, Jean P
Intellectual and developmental disabilities
101299965
IM
Journal Article
English
KW - Activities of Daily Living/px [Psychology]
KW - Adolescent
KW - Adult
KW - Community Health Services
KW - Education of Intellectually Disabled
KW - Female
KW - Health Services Needs and Demand
KW - Humans
KW - Male
KW - Mentally Disabled Persons/px [Psychology]
KW - Mentally Disabled Persons/rh [Rehabilitation]
KW - Parenting/px [Psychology]
KW - Patient Advocacy
KW - Personal Autonomy
KW - Rehabilitation
KW - Vocational
KW - Social Adjustment
KW - Social Environment
KW - Young Adult
KW - Medline
KW - Unsupervised Play Addition
RP - NOT IN FILE
SP - 186
EP - 196
JF - Intellectual & Developmental Disabilities
JA - Intellect Dev Disabil
VL - 47
IS - 3
CY - United States
N2 - This qualitative study investigated parents' perceptions of the various roles they played in their adult children's lives during the post-high school years. Individual face-to-face interviews were conducted with 9 families of young adults with developmental disabilities. Findings indicated that families perceived the complexity of their roles as balancing between advocating for their adult children's needs while promoting independence and self-determination. The roles parents assumed as their children entered into adult life were those of collaborators, decision makers, and program evaluators, role models, trainers, mentors and instructors, and systems change agents. Parents often felt they were the safety net for their children and the back-up plan for service agencies. Parents' quotes illustrated the complexity of the roles they played as their young adult children with developmental disabilities entered adulthood
SN - 1934-9491
AD - University of Colorado Denver, School of Education and Human Development, Denver, CO, USA. Margarita.Bianco@ucdenver.edu
UR - 19489664
ER -

TY - JOUR
ID - 1570
T1 - Coach autonomy support and quality of sport engagement in young soccer players
A1 - Alvarez,M.S.
A1 - Balaguer,I.
A1 - Castillo,I.
A1 - Duda,J.L.
Y1 - 2009/05//
N1 - Alvarez, Maria Sol. Balaguer, Isabel. Castillo, Isabel. Duda, Joan L
The Spanish journal of psychology
101095192
IM
Journal Article. Research Support, Non-U.S. Gov't
English
KW - Adolescent
KW - Athletic Performance
KW - Boredom
KW - Child
KW - Competitive Behavior
KW - Humans
KW - Internal-External Control
KW - Interpersonal Relations
KW - Male
KW - Models
KW - Psychological
KW - Motivation
KW - Personal Autonomy
KW - Personal Satisfaction
KW - Psychological Theory
KW - Questionnaires
KW - Self Concept
KW - Soccer/px [Psychology]
KW - Social Environment
KW - Social Perception
KW - Social Support
KW - Teaching
KW - Medline
KW - Unsupervised Play Addition
RP - NOT IN FILE
SP - 138
EP - 148
JF - Spanish Journal of Psychology
JA - Span J Psychol
VL - 12
IS - 1
CY - Spain
N2 - Based on the self-determination theory (Ryan & Deci, 2000), this study tested a model of the assumed sequential relationships between perceived autonomy support, psychological need satisfaction, self-determined motivation, and enjoyment/boredom. The hypothesized mediational roles of psychological need satisfaction and self-determined motivation were also studied. In a sample of 370 young male soccer players, path analysis results offered support for the proposed model. Total mediation was supported in the case of the psychological need satisfaction in the relationship between autonomy support and self-determined motivation, and partial mediation for self-determined motivation in the links between psychological need satisfaction and enjoyment (positive) and boredom (negative). Implications of autonomy-supportive behaviors provided by coaches for the quality of sport involvement among young athletes are discussed

SN - 1138-7416
AD - Universitat de Valencia, Spain
UR - 19476227
ER -

TY - JOUR
ID - 1572
T1 - Role of hip arthrodesis in current practice: long term results following conversion to total hip arthroplasty
A1 - Sirikonda,S.P.
A1 - Beardmore,S.P.
A1 - Hodgkinson,J.P.
Y1 - 2008/10//
N1 - Sirikonda, S P. Beardmore, S P. Hodgkinson, J P
Hip international : the journal of clinical and experimental research on hip pathology and therapy
9200413
IM
Journal Article
English
KW - Adolescent
KW - Adult
KW - Aged
KW - 80 and over
KW - Arthrodesis/ae [Adverse Effects]
KW - Arthroplasty
KW - Replacement
KW - Hip
KW - Back Pain/et [Etiology]
KW - Back Pain/pp [Physiopathology]
KW - Child
KW - Preschool
KW - Female
KW - Follow-Up Studies
KW - Health Status Indicators
KW - Hip Joint/pp [Physiopathology]
KW - Hip Joint/ra [Radiography]
KW - Hip Joint/su [Surgery]
KW - Humans
KW - Male
KW - Middle Aged
KW - Orthopedics/mt [Methods]
KW - Postoperative Complications/et [Etiology]
KW - Postoperative Complications/su [Surgery]
KW - Range of Motion
KW - Articular
KW - Reoperation
KW - Retrospective Studies
KW - Treatment Outcome
KW - Young Adult
KW - Medline
KW - Unsupervised Play Addition
RP - NOT IN FILE
SP - 263
EP - 271
JF - Hip International
JA - Hip int
VL - 18

IS - 4
CY - Italy
N2 - BACKGROUND: Hip arthrodesis was once considered as a preferred treatment option in young people with hip arthritis. The purpose of this retrospective study was to evaluate the long term outcome following conversion of hip arthrodesis to arthroplasty. The main indications for conversion were back pain and ipsilateral muscular or knee pain. METHODS: Sixty-eight procedures in 67 patients performed between 1963 and 2000 at Wrightington Hospital were evaluated. RESULTS: The mean age at the time of hip arthrodesis was 16.7 years (3 to 39 years). The mean age at the time of conversion was 48.7 years (24 to 74 years). The Merle d'Aubigne score improved from a mean of 8.81 pre-operatively to 13.6 at the latest follow-up. Complications included DVT (3 patients), transient foot drop (1 patient), and hepatitis following blood transfusion (1 patient). Seven patients required further revision surgery following conversion. CONCLUSIONS: Arthrodesis remains a sensible and safe treatment option maintaining independent mobility without multiple operations or significant bone loss for 20-30 years which subsequently can be successfully converted to an arthroplasty
SN - 1724-6067
AD - Department of Orthopaedics, Royal Liverpool and Broadgreen University Hospitals, Liverpool, UK. sirikonda.siva@rlbuht.nhs.uk
UR - 19097003
ER -

TY - JOUR
ID - 1574
T1 - Screen-viewing and the home TV environment: the European Youth Heart Study
A1 - Jago,R.
A1 - Page,A.
A1 - Froberg,K.
A1 - Sardinha,L.B.
A1 - Klasson-Heggebo,L.
A1 - Andersen,L.B.
Y1 - 2008/11//
N1 - Jago, Russell. Page, Angie. Froberg, Karsten. Sardinha, Luis B. Klasson-Heggebo, Lena. Andersen, Lars B
Preventive medicine
pm4, 0322116
IM
Journal Article. Research Support, Non-U.S. Gov't
English
KW - Adolescent
KW - Child
KW - Child Behavior
KW - Europe
KW - Female
KW - Health Status
KW - Housing
KW - Humans
KW - Life Style
KW - Male
KW - Obesity
KW - Parent-Child Relations
KW - Personal Autonomy
KW - Television/ut [Utilization]
KW - Medline
KW - Unsupervised Play Addition
RP - NOT IN FILE
SP - 525
EP - 529
JA - Preventive Medicine

VL - 47
IS - 5
CY - United States
N2 - OBJECTIVE: Examine if home environmental factors are associated with screen-viewing. METHODS: Data are for 2670, 3rd and 9th grade participants in Denmark, Portugal, Estonia and Norway collected between 1997 and 2000. Outcomes were spending >2 h after-school watching television (TV) and >1 h per day playing computer games. Child Autonomy and the home TV Environment were exposures. RESULTS: Each unit increase in Child Autonomy was associated with 9% increase in risk of watching more than 2 h of TV per day after school and a 19% increase in risk of spending more than an hour per day playing computer games. TV Environment was associated with a 31% per unit increase in risk of watching >2 h of TV after school and 11% increase in risk of spending >1 h playing computer games. CONCLUSIONS: A family environment in which after-school TV viewing is part of the home culture and homes where children have more autonomy over their own behavior are associated with an increased risk of watching >2 h of TV per day after school and spending more >1 h per day playing computer games. The home screen-viewing environment and Child Autonomy may be malleable targets for changing screen-viewing
SN - 1096-0260
AD - Department of Exercise, Nutrition and Health Sciences, University of Bristol, Tyndall Avenue, Bristol BS81TP, UK. russ.jago@bris.ac.uk
UR - 18722400
ER -

TY - JOUR
ID - 1575
T1 - Shopping cart injuries, entrapment, and childhood fatality
A1 - Jensen,L.
A1 - Charlwood,C.
A1 - Byard,R.W.
Y1 - 2008/09//
N1 - Jensen, Lisbeth. Charlwood, Cheryl. Byard, Roger W
Journal of forensic sciences
i5z, 0375370
IM
Case Reports. Journal Article
English
KW - Accidents
KW - Asphyxia/et [Etiology]
KW - Asphyxia/pa [Pathology]
KW - Consumer Product Safety
KW - Female
KW - Forensic Pathology
KW - Humans
KW - Infant
KW - Neck Injuries/et [Etiology]
KW - Neck Injuries/pa [Pathology]
KW - Medline
KW - Unsupervised Play Addition
RP - NOT IN FILE
SP - 1178
EP - 1180
JF - Journal of Forensic Sciences
JA - J Forensic Sci
VL - 53
IS - 5
CY - United States
N2 - Shopping carts may be associated with a variety of injuries, particularly in toddlers and young children. These usually relate to falls from carts or to tip-overs. Injuries that are sustained include hematomas/contusions,

abrasions, lacerations, fractures, and fingertip amputations. Fatal episodes are uncommon and are usually due to blunt craniocerebral trauma from falls. A case involving a 19-month-old girl is reported who became entrapped when she inserted her head through the side frame of a cart that had been removed from a supermarket and left at her home address. Death was caused by neck compression. Although rare, the potential for lethal entrapment during unsupervised play means that the presence of stray shopping carts at private residences and in public places, including playgrounds and parks, is of concern. Strategies, such as coin deposits, should be encouraged to assist in the return of such carts to supermarkets
SN - 1556-4029
AD - Discipline of Pathology, University of Adelaide, Frome Road, Adelaide 5005, Australia
UR - 18624893
ER -

TY - JOUR
ID - 1577
T1 - Inflatable bouncer-related fractures in children
A1 - Avoian,T.
A1 - Choi,P.D.
A1 - Manjra,N.
A1 - Weiss,J.
Y1 - 2008/09//
N1 - Avoian, Tigran. Choi, Paul D. Manjra, Nahid. Weiss, Jennifer
Journal of pediatric orthopedics
hsw, 8109053
IM
Journal Article
English
KW - Accidents
KW - Adolescent
KW - Adult
KW - Age Factors
KW - Child
KW - Preschool
KW - Databases
KW - Factual
KW - Female
KW - Fractures
KW - Bone/ep [Epidemiology]
KW - Bone/et [Etiology]
KW - Bone/pc [Prevention & Control]
KW - Humans
KW - Infant
KW - Male
KW - Parenting
KW - Play and Playthings
KW - Retrospective Studies
KW - Risk Factors
KW - Sex Factors
KW - Trauma Centers/sn [Statistics & Numerical Data]
KW - Upper Extremity/in [Injuries]
KW - Medline
KW - Unsupervised Play Addition
RP - NOT IN FILE
SP - 656
EP - 659
JF - Journal of Pediatric Orthopedics
JA - J Pediatr Orthop

VL - 28
IS - 6
CY - United States
N2 - BACKGROUND: Inflatable bouncers or moonbouncers are very popular in private and public settings and are usually perceived as very safe attractions, but are associated with frequent fractures in children. To date, there are no publications in the medical literature about these types of injuries. The purpose of this study was to show skeletal injuries related to inflatable bouncer use, describe their characteristics, and determine possible risk factors and preventive measures. METHODS: Demographic data and injury characteristics were analyzed for all patients who were treated for inflatable bouncer-associated injuries in the pediatric fracture clinic of a level I trauma center from October 2002 to March 2007. RESULTS: Forty-nine patients were treated for inflatable bouncer-related fractures. Children ranged in age from 1.5 to 15 years old (mean age, 7.8 years) with a male-female ratio of approximately 3:1. The most commonly injured region was the upper extremity (65.5%, n = 32). The most commonly injured area was the elbow (31%, n = 15), and the most common single diagnosis was supracondylar humerus fracture (22%, n = 11). Diaphyseal long bone fractures were found in 18% (n = 9) of the patients and nondiaphyseal in 71% (n = 35). One patient (2%) had an open fracture. Mechanisms of injury included collision of 1 person with another (67%), falling out of a bouncer onto a hard object outside the device (19%), and twisting motion to the leg (14%). There was no adult supervision in many of the incidents (43%), and the presence of different-aged children inside the jumper took place in 52% cases. CONCLUSIONS: Inflatable bouncers can cause serious orthopaedic injuries. Children playing in the bouncer should be placed in small groups according to their size and should be closely supervised at all times. STUDY DESIGN: Case series. Level IV evidence
SN - 1539-2570
AD - University of Southern California, Los Angeles, CA, USA. avoian@usc.edu
UR - 18724203
ER -

TY - JOUR
ID - 1578
T1 - The cost of Child Health Days: a case study of Ethiopia's Enhanced Outreach Strategy (EOS)
A1 - Fiedler,J.L.
A1 - Chuko,T.
Y1 - 2008/07//
N1 - Fiedler, John L. Chuko, Tesfaye
Health policy and planning
8610614, f9q
H
Evaluation Studies. Journal Article. Research Support, Non-U.S. Gov't
English
KW - Anthelmintics/sd [Supply & Distribution]
KW - Bedding and Linens/sd [Supply & Distribution]
KW - Child
KW - Child Health Services/ec [Economics]
KW - Cost Allocation
KW - Ethiopia
KW - Health Care Costs/sn [Statistics & Numerical Data]
KW - Health Priorities
KW - Health Status Indicators
KW - Humans
KW - Immunization Programs/ec [Economics]
KW - Measles/ec [Economics]
KW - Measles/pc [Prevention & Control]
KW - Organizational Case Studies
KW - Preventive Health Services/ec [Economics]
KW - Program Evaluation
KW - Quality-Adjusted Life Years
KW - Regional Health Planning/ec [Economics]

KW - Value of Life/ec [Economics]
KW - Vitamin A/sd [Supply & Distribution]
KW - Medline
KW - Unsupervised Play Addition
RP - NOT IN FILE
SP - 222
EP - 233
JF - Health Policy & Planning
JA - Health Policy Plan.
VL - 23
IS - 4
CY - England
N2 - Child Health Days (CHDs) are twice-annual campaign-style events designed to increase the coverage of vitamin A and one or more other child health services. Although more than two dozen countries have had a CHD, little has been published about them. This paper presents an activity-based costing study of Ethiopia's version of CHDs, the Enhanced Outreach Strategy (EOS). The December 2006 round reached more than 10 million beneficiaries at an average cost per beneficiary of US$0.56. When measles is added, the cost of the package doubles. Given the way the distribution day delivery system and the service package are structured, there are economies of scope. Because most of the costs are determined by the number of delivery sites and are independent of the number of beneficiaries, other things equal, increasing the beneficiaries would reduce the average cost per beneficiary. Taking into account only the mortality impact of vitamin A, EOS saved 20,200 lives and averted 230,000 DALYs of children 6-59 months. The average cost per life saved was US$228 and the cost per DALY averted was equivalent to 6% of per capita GDP (US$9), making the EOS cost-effective, according to WHO criteria. While CHDs are generally construed as a temporary strategy for improving coverage of supply-constrained systems, inadequate attention has been paid to demand-side considerations that suggest CHDs have an important role to play in changing care-seeking behaviour, in increasing community organization and participation, and in promoting district autonomy and capacity. Recognition of these effects suggests the need for decisions about where and when to introduce, and when to end, a CHD to take into account more than 'just' health sector considerations: they are more broadly about community development. UNICEF played a key role in initiating the EOS and finances 68% of costs, raising concern about the programme's long-term sustainability
SN - 0268-1080
AD - Social Sectors Development Strategies, Boston, MA, USA. jfiedler@ssds.net
UR - 18562457
ER -

TY - JOUR
ID - 1579
T1 - Differential effects of an adult observer's presence on sex-typed play behavior: A comparison between gender-schematic and gender-aschematic preschool children
A1 - Wilansky-Traynor,P.
A1 - Lobel,T.E.
Y1 - 2008/08//
N1 - Wilansky-Traynor, Pamela. Lobel, Thalma E
Archives of sexual behavior
8gr, 1273516
IM
Journal Article
English
KW - Adult
KW - Child
KW - Preschool
KW - Choice Behavior
KW - Female
KW - Gender Identity
KW - Humans

KW - Individuality
KW - Male
KW - Motivation
KW - Observation
KW - Personality Assessment
KW - Play and Playthings
KW - Social Conformity
KW - Social Environment
KW - SOCIALIZATION
KW - Stereotyping
KW - Medline
KW - Unsupervised Play Addition
RP - NOT IN FILE
SP - 548
EP - 557
JF - Archives of Sexual Behavior
JA - Arch.Sex Behav
VL - 37
IS - 4
CY - United States
N2 - The present study examined the differential effect of an adult observer's presence on the sex-typed play behavior of gender schematic and aschematic preschoolers. A total of 116 Israeli preschoolers (M age = 64.9 months) participated in the study. Children were classified as either gender schematic or aschematic based upon responses to a computerized measure of different sex stereotype components. Children's play behavior with gender typical and atypical, attractive and unattractive, toys was videotaped. An observer was present for half the children's play and absent for the other half's play. Observation status affected the aschematic, but not the schematic, children's play with gender typical toys. For example, observed aschematic boys spent a greater percent of time playing with the unattractive masculine toys compared to unobserved aschematic boys. This difference was not apparent for schematic boys. Additionally, a difference found for schematic boys was not apparent in schematic girls, i.e., when unobserved, schematic boys tended to spend a greater percent of time playing with the unattractive masculine toy than aschematic boys. Further, some differences were found for unattractive, and not attractive, toys. For instance, observed aschematic boys spent a greater percent of time playing with the unattractive masculine toy than did the unobserved aschematic boys. This gap was not found for the attractive masculine toy. Results are discussed with reference to the accessibility and complexity of gender schemas
SN - 0004-0002
AD - Child, Youth, and Family Program, Centre for Addiction and Mental Health, 250 College St., Toronto, ON, Canada, M5T 1R8. Pamela_Wilansky@camh.net
UR - 18299975
ER -

TY - JOUR
ID - 1581
T1 - Infants in group care: their interactions with professional caregivers and parents across the second year of life
A1 - Gevers Deynoot-Schaub,M.J.
A1 - Riksen-Walraven,J.M.
Y1 - 2008/04//
N1 - Gevers Deynoot-Schaub, Mirjam J J M. Riksen-Walraven, J Marianne
Infant behavior & development
7806016
IM
Comparative Study. Journal Article
English
KW - Age Factors
KW - Caregivers/px [Psychology]

KW - Child Day Care Centers/mt [Methods]
KW - Female
KW - Humans
KW - Infant
KW - Infant Behavior/px [Psychology]
KW - Infant Care/mt [Methods]
KW - Infant Care
KW - Male
KW - Parent-Child Relations
KW - Parents/px [Psychology]
KW - Play and Playthings/px [Psychology]
KW - Medline
KW - Unsupervised Play Addition
RP - NOT IN FILE
SP - 181
EP - 189
JF - Infant Behavior & Development
JA - Infant behav
VL - 31
IS - 2
CY - United States
N2 - Seventy children were observed during structured play with their primary professional caregivers and three peers in the child care center and with their primary caregiving parents at home at 15 and 23 months of age. The same structured play tasks were used in the two settings and the quality of the children's interactions with caregivers and parents was rated using the same 7-point scales. As expected, the quality of caregiver-child interactions significantly increased between 15 and 23 months. At 15 months, the quality of caregiver-child interactions was significantly lower than the quality of parent-child interactions, particularly with regard to caregiver supportive presence and respect for the child's autonomy. At 23 months, however, the quality of caregiver-child interactions was no longer lower and in some respects even higher than the quality of parent-child interactions. At both ages, the children expressed more negativity towards their parents than towards their professional caregivers
SN - 1934-8800
AD - Department of Educational Sciences, University of Amsterdam, The Netherlands
UR - 18048100
ER -

TY - JOUR
ID - 1584
T1 - Effects of caregiver-child interactions on play occupations among young children institutionalized in Eastern Europe
A1 - Daunhauer,L.A.
A1 - Coster,W.J.
A1 - Tickle-Degnen,L.
A1 - Cermak,S.A.
Y1 - 2007/07//
N1 - Daunhauer, Lisa A. Coster, Wendy J. Tickle-Degnen, Linda. Cermak, Sharon A
The American journal of occupational therapy : official publication of the American Occupational Therapy Association
7705978, 3o4
IM
Journal Article. Research Support, Non-U.S. Gov't. Research Support, U.S. Gov't, P.H.S.
English
KW - Caregivers
KW - Child Behavior/px [Psychology]
KW - Child
KW - Preschool

KW - Emotions
KW - Female
KW - Humans
KW - Infant
KW - Interpersonal Relations
KW - Male
KW - Orphanages
KW - Pediatrics
KW - Play and Playthings
KW - Romania
KW - Medline
KW - Unsupervised Play Addition
RP - NOT IN FILE
SP - 429
EP - 440
JA - American Journal of Occupational Therapy
VL - 61
IS - 4
CY - United States
N2 - OBJECTIVE: We investigated whether children institutionalized in an orphanage would engage in more developmentally competent play with their caregivers as opposed to playing alone and whether specific qualities of caregiver-child interactions were associated with more developmentally competent play. METHOD: Twenty-six children, ages 10 to 38 months, participated in independent play sessions and in a play session with a caregiver. Interrater reliability for coding play performance was established using the weighted kappa statistic (M = .82). Twelve pediatric experts rated both child and caregiver behaviors for the interactive sessions (mean effective reliability with intraclass correlations = .89). RESULTS: The children demonstrated more developmentally competent play when interacting with a caregiver than when playing alone, t (25) = -1.88, p < .04, one-tailed. The effect size was moderate (d = .53). Longer periods of institutionalization were associated with less improvement in play performance from independent to interactive play sessions (r = -.51, p < .01). Successful engagement for the child was associated with having a caregiver who provided more structure and assistance and who was directive and encouraging (r = .82, .75, .75, and .64, respectively). CONCLUSION: Caregivers facilitated more developmentally competent participation in play with children residing in an orphanage, despite the fact that these interactions occurred in an environment vulnerable to many challenges not typical of an exclusive caregiver-child relationship. Findings are discussed in the context of environmental challenges and occupational therapy practice
SN - 0272-9490
AD - Department of Occupational Therapy, College of Applied Human Sciences, Colorado State University, Fort Collins, CO 80523-1573, USA. ldaun@cahs.colostate.edu
UR - 17685176
ER -

TY - JOUR
ID - 1585
T1 - Associations between youth assets and sexual activity: does adult supervision play a role?
A1 - Harris,L.
A1 - Oman,R.F.
A1 - Vesely,S.K.
A1 - Tolma,E.L.
A1 - Aspy,C.B.
A1 - Rodine,S.
A1 - Marshall,L.
A1 - Fluhr,J.
Y1 - 2007/07//
N1 - Harris, L. Oman, R F. Vesely, S K. Tolma, E L. Aspy, C B. Rodine, S. Marshall, L. Fluhr, J
Child: care, health and development
7602632, c9x

IM
Comparative Study. Journal Article. Research Support, U.S. Gov't, P.H.S.
English
KW - Adolescent
KW - Adolescent Behavior
KW - Adolescent Psychology
KW - Adult
KW - Female
KW - Humans
KW - Male
KW - Parent-Child Relations
KW - Parents
KW - Risk-Taking
KW - Sex Education/mt [Methods]
KW - Sexual Behavior
KW - Sexually Transmitted Diseases/pc [Prevention & Control]
KW - Social Environment
KW - United States
KW - Urban Health
KW - Medline
KW - Unsupervised Play Addition
RP - NOT IN FILE
SP - 448
EP - 454
JF - Child: Care, Health & Development
JA - Child Care Health Dev.
VL - 33
IS - 4
CY - England
N2 - BACKGROUND: Youth participation in sexual risk behaviours continues to be a critically important public health topic. Additionally, as youth are frequently being left alone during the day without adult supervision, there are increased opportunities for sexual risk-taking behaviour. This study examined how the relationships of nine youth assets and sexual activity may vary according to the stratification of youth into two groups: self-care and supervised. METHODS: Data were collected through at-home, in-person interviews from a random sample of inner-city youth (mean age = 15.4 years; 51.5% female; 48.8% White; 22.4% Black; 18.5% Hispanic; 10% Native American) and their parents (n = 1079 parent/youth pairs). Nine youth assets were analysed using multiple logistic regression. Examples of assets youth may possess are: positive role models, family communication, school connectedness, constructive use of time and aspirations for the future. The item used to assess sexual intercourse was 'Have you ever had sexual intercourse ("done it", "had sex", "made love", "gone all the way")?'. Asset/risk behaviour associations that were unique to one of the two strata were the focus of the study. RESULTS: Thirty-seven per cent of youth spent two or more hours per day home alone. Youth who were supervised had a greater number of unique significant associations between assets and sexual activity than youth who were in the self-care group. CONCLUSIONS: Youth in supervised settings may be less likely to participate in sexual activity because of the presence of assets. Certain assets may also be important in deterring sexual activity for youth who are in self-care
SN - 0305-1862
AD - Department of Health Administration and Policy, College of Public Health, University of Oklahoma Health Sciences Center, Oklahoma City, OK 73190, USA. lindsey-harris@ouhsc.edu
UR - 17584401
ER -

TY - JOUR
ID - 1587
T1 - Estimated maturity status and perceptions of adult autonomy support in youth soccer players
A1 - Cumming,S.P.
A1 - Battista,R.A.

A1 - Martyn,S.
A1 - Ewing,M.E.
A1 - Malina,R.M.
Y1 - 2006/10//
N1 - Cumming, Sean P. Battista, Rebecca A. Martyn, Standage. Ewing, Martha E. Malina, Robert M
Journal of sports sciences
8405364, kbt
IM
Journal Article
English
KW - Adolescent
KW - Age Factors
KW - Body Mass Index
KW - Child
KW - Female
KW - Humans
KW - Interpersonal Relations
KW - Linear Models
KW - Male
KW - Parent-Child Relations
KW - Personal Autonomy
KW - Physical Education and Training
KW - Predictive Value of Tests
KW - Puberty
KW - Research Design
KW - Sex Factors
KW - Soccer/sn [Statistics & Numerical Data]
KW - Soccer
KW - Social Perception
KW - Social Support
KW - Medline
KW - Unsupervised Play Addition
RP - NOT IN FILE
SP - 1039
EP - 1046
JF - Journal of Sports Sciences
JA - J Sports Sci
VL - 24
IS - 10
CY - England
N2 - In this study, we examined the relations between biological maturity status, body mass index, age, and perceptions of adult autonomy support in the context of youth soccer. A total of 70 female and 43 male soccer players, aged 9 - 15 years, completed three adult-specific versions (i.e. mother, father, coach) of the perceived autonomy support subscale from the Interpersonal Style Scale. The participants' percent predicted adult stature was used as an estimate of biological maturity status. Multiple linear regression analyses revealed that advanced maturity status in male players predicted lower perceptions of autonomy support from the coach. Maturity status was unrelated to perceptions of autonomy support from the coach in female soccer players, and paternal and maternal autonomy support in male and female players. Age and body mass index were unrelated to perceptions of adult (i.e. coach, mother, father) autonomy support in male and female players
SN - 0264-0414
AD - Department of Psychology, University of Washington, Seattle, WA 98195-1525, USA.
seanscot@u.washington.edu
UR - 17115515
ER -

TY - JOUR

ID - 1588
T1 - [Links between life events, traumatism and dementia; an open study including 565 patients with dementia]. [French]
A1 - Charles,E.
A1 - Bouby-Serieys,V.
A1 - Thomas,P.
A1 - Clement,J.P.
Y1 - 2006/10//
N1 - Charles, E. Bouby-Serieys, V. Thomas, P. Clement, J-P
L'Encephale
efb, 7505643
IM
English Abstract. Journal Article
French
KW - Aged
KW - 80 and over
KW - Alzheimer Disease/di [Diagnosis]
KW - Alzheimer Disease/ep [Epidemiology]
KW - Alzheimer Disease/px [Psychology]
KW - Female
KW - France
KW - Health Surveys
KW - Humans
KW - Life Change Events
KW - Male
KW - Mental Status Schedule
KW - Retrospective Studies
KW - Risk Factors
KW - Statistics as Topic
KW - Stress Disorders
KW - Post-Traumatic/di [Diagnosis]
KW - Post-Traumatic/ep [Epidemiology]
KW - Post-Traumatic/px [Psychology]
KW - Medline
KW - Unsupervised Play Addition
RP - NOT IN FILE
SP - 746
EP - 752
JF - Encephale
VL - 32
IS - 5 Pt 1
CY - France
N2 - SUMMARY: Ageing is due to a progressive loss of the person's adaptation capability, whereas during this period environmental aggression increases. In the elderly, life events re-present a psychological traumatism that overwhelms the old person and related family, disrupting and fragilising homeostatic balance. A number of authors have suggested a possible link between life traumatisms and the dementia processes. The aim of this study is to reveal the presence of life traumatisms preceding the apparition of the dementia syndrome. METHOD: This is a retrospective and comparative work based on the PIXEL study on complaints and demands from the principle informal caregivers of Alzheimer patients. It includes 565 patients presenting the criterion of dementia as defined by the DSM IV, and questionnaires filled out by the principle caregivers. One item of the questionnaire referred to life events which could have played a part in the development of the disorder. In a second stage, the reported events were classified into 4 distinct categories: loss, repeated or prolonged stress, psychotraumatism and depression-inducing events. The statistics were produced using SAS and Stat 10 software. Student's test, ANOVA and chi2-test were used. RESULTS: 372 caregivers answered the first item (65%); 76 of them believed there was no event while 296 related the disorder to one or several life events (79% of responders, 52% of the sample). These results confirm Persson and Clement's study which evidenced a higher

frequency of stressing life events for subjects afflicted with dementia as compared with older people without any psychic disorder. Reported events and their respective frequency: spouse death (15.39%), parents' death (15%), familial difficulty (10.08%), anaesthesia (8.49%), child's death (4.42%), somatic disturbance (4%), depression (3.89%), retirement (3.89%), financial problems (2.65%), loneliness (2.65%), removal (1.76%), fall (1%), alcohol (0.8%), traumatism (0.53%), spouse care (0.35%), leaving for home care, storm and caregiver change in life (0.17%). Regrouping the data: 82.71% of the answers can be connected to prolonged stress, 62.85% to real or symbolic loss, 39% to a psychotraumatic event and finally, most of these events can potentially induce depression. DISCUSSION: Some of the events refer to difficulties concerning people close to the subject (death of a close relation, hospitalisation of husband or wife) in a period when the loss of autonomy or handicap means greater dependence on surrounding people. General anaesthesia is mentioned by 48 caregivers. In fact, this usually implies surgery, the presence of organic pathology or the need for hospitalisation, which we know has a destabilizing effect in the elderly. It is therefore not surprising that 23 answers mentioned somatic disorders (4% of the sample). According to Leger, it's mostly a loss type event which is implied in the elderly. Such losses would induce a weakening of cognitive stimulation which could decompensate an infraclinical dementia or accelerate an emerging dementia process. Antecedents of depressive illness are considered as an element of risk for the development of Alzheimer's disease. Depression is spontaneously mentioned by 22 caregivers. We must add the many reported life events which are well known to induce depression in older people. Most of the events considered in this study are liable to provoke manic mood swings. Depression resulting from life events can be considered either as an affection occurring along with dementia or as the aggravating factor of an infraclinical process or, finally, as an additional factor of vulnerability. With older people, many events may constitute a trauma because of the proximity of death and because of their sudden onset (fall, hospitalisation, somatic illness). Several studies have pointed out that a particularly traumatic event could enhance the risk of dementia. Life events associated with chronic or repeated stress are characterized by their permanence or their repetition. According to a general psychosomatic biological pattern, psychic distress will engender a series of degradations or an acute or chronic response to an early trauma. According to this hypothesis, prolonged exposure to an excess of glucocorticosteroids at the time of a disadaptative stress would have deleterious effects on the hippocampus. Indeed, the hippocampus plays a part in a number of functions affected by dementia such as memory, learning process and emotional adjustment. This study takes into account stress factors ("stressors") but not factors influencing their impact on the subject such as an individual predisposition (genetic, psychopathologic, coping abilities) and social support. The force of the impact of these events on older people and what is really experienced by them remain unknown. This study strengthens a number of others evincing an unusual frequency of life events in dementia processes. According to an integrative pattern, repeated or prolonged stress could be a deciding factor in the degenerative process or a factor of decompensation with older people presenting a genetic, biological or psychological vulnerability to dementia. The impact of such life events would vary according to the subject's pre-morbid personality, coping abilities and the support he/she can rely on. CONCLUSION: Some life events may be involved in the dementia process as shown by the results of this study, but this relationship does not imply direct causality. It's difficult to appreciate whether these results are not a consequence of the greater attention paid to the patient after the appearance of the first symptoms, leading to a closer observation. Stress could trigger the degenerative process. This argues for the necessity of an early diagnosis taking into account a traumatic event of life either precocious or late
SN - 0013-7006
AD - Service Hospitalo-Universitaire de Psychiatrie Adulte (Professeur Clement), Centre Hospitalier Esquirol, 15, rue du docteur Marcland, 87025 Limoges cedex
UR - 17099599
ER -

TY - JOUR
ID - 1589
T1 - Law, pregnancy and sport: what are the repercussions when a pregnant lady plays?
A1 - Werren,J.
Y1 - 2006/08//
N1 - Werren, Julia
Journal of law and medicine
9431853
IM

Journal Article. Legal Cases
English
KW - Australia
KW - Female
KW - Guidelines as Topic
KW - Humans
KW - LIABILITY
KW - Legal
KW - Organizational Policy
KW - Organizations/lj [Legislation & Jurisprudence]
KW - Organizations/st [Standards]
KW - Personal Autonomy
KW - Pregnancy
KW - Pregnant Women
KW - Prenatal Injuries/pc [Prevention & Control]
KW - Prenatal Injuries
KW - Risk Assessment
KW - Risk-Taking
KW - Sports/lj [Legislation & Jurisprudence]
KW - Medline
KW - Unsupervised Play Addition
RP - NOT IN FILE
SP - 45
EP - 63
JF - Journal of Law & Medicine
JA - J Law Med
VL - 14
IS - 1
CY - Australia
N2 - This article reflects on the issue of pregnancy and sport that was brought to the fore in Gardner v National Netball League (2001) 182 ALR 408; [2001] FMCA 50 and Gardner v All Australia Netball Association Ltd (2003) 174 FLR 452. It suggests that these cases did not provide a definitive discussion of the tortious liability implications that initially led Netball Australia to introduce a ban on pregnant players. In an attempt to fill some of these gaps, other case law that deals with liability of sporting organisations and prenatal injury is discussed. The article primarily focuses on whether the unborn child when born alive will have an action against her or his mother as a result of injury occasioned while the mother was playing sport when pregnant. This examination is undertaken in light of recent Australian tort reform as well as changes in policy direction. The article summarises the legal position of the parties involved in sport--sporting organisations, medical practitioners, other participants and the pregnant mother--and argues that, with reference to the guidelines and case law, in only a very small number of cases would liability be found against the sporting organisation or pregnant mother as a result of injuries incurred prenatally
SN - 1320-159X
AD - Faculty of Law, University of New England. jwerren@une.edu.au
UR - 16937781
ER -

TY - JOUR
ID - 1590
T1 - Reading and spelling abilities in children with severe speech impairments and cerebral palsy at 6, 9, and 12 years of age in relation to cognitive development: a longitudinal study
A1 - Dahlgren,Sandberg A.
Y1 - 2006/08//
N1 - Dahlgren Sandberg, Annika
Developmental medicine and child neurology
0006761, e83
IM

Comparative Study. Journal Article. Research Support, Non-U.S. Gov't
English
KW - Adolescent
KW - Case-Control Studies
KW - Cerebral Palsy/co [Complications]
KW - Cerebral Palsy/px [Psychology]
KW - Child
KW - Child Development
KW - Child Language
KW - Preschool
KW - Cognition
KW - Developmental Disabilities/et [Etiology]
KW - Developmental Disabilities/px [Psychology]
KW - Disabled Children/px [Psychology]
KW - Dystonia/co [Complications]
KW - Dystonia/px [Psychology]
KW - Female
KW - Follow-Up Studies
KW - Humans
KW - Intelligence
KW - Male
KW - Matched-Pair Analysis
KW - Reading
KW - Severity of Illness Index
KW - Speech Articulation Tests
KW - Speech Disorders/co [Complications]
KW - Speech Disorders/px [Psychology]
KW - Statistics
KW - Nonparametric
KW - Verbal Behavior
KW - Medline
KW - Unsupervised Play Addition
RP - NOT IN FILE
SP - 629
EP - 634
JF - Developmental Medicine & Child Neurology
JA - Dev.Med Child Neurol.
VL - 48
IS - 8
CY - England
N2 - Development of literacy skills was studied in six children (one male, five females) with severe speech impairments and cerebral palsy (CP). These skills were related to intellectual development, phonological abilities, and short-term memory. Three of the children were diagnosed with dystonia, and three with diplegia. They had no, or severely restricted, independent mobility (Gross Motor Function Classification System Level IV for four children and Level V for two), and severe fine motor problems, including difficulty with pointing. As they had no intelligible speech, the Bliss system was the primary communication mode. Assessments were made at approximately 6, 9, and 12 years of age. The results revealed that the children had difficulties acquiring literacy skills, although intellectual level and phonological ability predicted otherwise. Positive development during the first 3 years was followed by an arrest. A conspicuous decrease in IQ points was also found. Thus, phonological ability does not seem to have the same predictive power for literacy development in children with severe speech impairments and CP as in typically developing children. Further studies are needed to clarify the role of phonological abilities, working memory, and strategies used in literacy acquisition in these children. Such studies might also clarify the importance of articulatory abilities in early literacy acquisition
SN - 0012-1622
AD - Department of Psychology, Goteborg University, Box 500, SE-405 30 Goteborg, Sweden. Annika.Dahlgren.Sanberg@psy.gu.se

UR - 16836773
ER -

TY - JOUR
ID - 1591
T1 - Mother-child play and emerging social behaviors among infants from maltreating families
A1 - Valentino,K.
A1 - Cicchetti,D.
A1 - Toth,S.L.
A1 - Rogosch,F.A.
Y1 - 2006/05//
N1 - Valentino, Kristin. Cicchetti, Dante. Toth, Sheree L. Rogosch, Fred A
Developmental psychology
0260564, cn8
IM
Journal Article. Research Support, N.I.H., Extramural
English
KW - Adult
KW - Child Abuse
KW - Cognition
KW - Family
KW - Female
KW - Humans
KW - Infant
KW - Maternal Age
KW - Mother-Child Relations
KW - Play and Playthings
KW - Social Behavior
KW - Medline
KW - Unsupervised Play Addition
RP - NOT IN FILE
SP - 474
EP - 485
JF - Developmental Psychology
JA - Dev.Psychol.
VL - 42
IS - 3
CY - United States
N2 - Mother-child play of 12-month-old infants (N=130) from maltreating (N=78) and non-maltreating (N=52) families was analyzed as a context that integrates infants' developing social and cognitive skills. Play was coded from semistructured and unstructured play paradigms. No group differences were found in infants' play maturity. Infants from abusing families demonstrated more imitative play than infants from non-maltreating families, and engaged in less independent play than infants from both neglecting and non-maltreating families, suggesting a delay in emerging social behaviors. Mothers from abusing and non-maltreating families differed in attention directing behaviors. Maternal behaviors predicted child play style variables, but did not mediate the effects of maltreatment. Findings discuss the influence of an early maltreating environment upon the development of the emergent self. Implications for early intervention are underscored. ((c) 2006 APA, all rights reserved)
SN - 0012-1649
AD - Mt. Hope Family Center, University of Rochester, Rochester, NY 14608, USA. valentik@psych.rochester.edu
UR - 16756439
ER -

TY - JOUR
ID - 1592

T1 - Smart wheelchairs: A literature review. [Review] [45 refs]
A1 - Simpson,R.C.
Y1 - 2005/07//
N1 - Simpson, Richard C
Journal of rehabilitation research and development
8410047, jrd
IM
Historical Article. Journal Article. Review
English
KW - Activities of Daily Living
KW - Adult
KW - Child
KW - Communication Aids for Disabled
KW - Electronics/is [Instrumentation]
KW - Electrooculography
KW - Equipment Design
KW - History
KW - 20th Century
KW - 21st Century
KW - Humans
KW - Infrared Rays
KW - Robotics/is [Instrumentation]
KW - Software
KW - Therapy
KW - Computer-Assisted/is [Instrumentation]
KW - Ultrasonics/cl [Classification]
KW - User-Computer Interface
KW - Wheelchairs/cl [Classification]
KW - Wheelchairs/hi [History]
KW - Wheelchairs
KW - Medline
KW - Unsupervised Play Addition
RP - NOT IN FILE
SP - 423
EP - 436
JF - Journal of Rehabilitation Research & Development
JA - J Rehabil Res Dev
VL - 42
IS - 4
CY - United States
N2 - Several studies have shown that both children and adults benefit substantially from access to a means of independent mobility. While the needs of many individuals with disabilities can be satisfied with traditional manual or powered wheelchairs, a segment of the disabled community finds it difficult or impossible to use wheelchairs independently. To accommodate this population, researchers have used technologies originally developed for mobile robots to create "smart wheelchairs." Smart wheelchairs have been the subject of research since the early 1980s and have been developed on four continents. This article presents a summary of the current state of the art and directions for future research. [References: 45]
SN - 1938-1352
AD - Department of Rehabilitation Science and Technology, University of Pittsburgh, Pittsburgh, PA 15238-2887, USA. ris20@pitt.edu
UR - 16320139
ER -

TY - JOUR
ID - 1593
T1 - Relations between parents' interactive style in dyadic and triadic play and toddlers' symbolic capacity

A1 - Keren,M.
A1 - Feldman,R.
A1 - Namdari-Weinbaum,I.
A1 - Spitzer,S.
A1 - Tyano,S.
Y1 - 2005/10//
N1 - Keren, M. Feldman, R. Namdari-Weinbaum, I. Spitzer, S. Tyano, S
The American journal of orthopsychiatry
3r6, 0400640
IM
Comparative Study. Journal Article
English
KW - Adult
KW - Aggression/px [Psychology]
KW - Child
KW - Preschool
KW - Creativity
KW - Father-Child Relations
KW - Female
KW - Gender Identity
KW - Humans
KW - Intelligence
KW - Male
KW - Marriage/px [Psychology]
KW - Mother-Child Relations
KW - Parenting/px [Psychology]
KW - Play and Playthings
KW - Symbolism
KW - Medline
KW - Unsupervised Play Addition
RP - NOT IN FILE
SP - 599
EP - 607
JF - American Journal of Orthopsychiatry
JA - Am J Orthopsychiatry
VL - 75
IS - 4
CY - United States
N2 - Play has a major role in the evaluation and treatment of young children referred to mental health clinicians. The present study examined parental correlates of preschoolers' symbolic play during dyadic and triadic play interactions. Boys' play contained more aggressive themes, and girls' contained more nurturing themes. Mothers displayed more caring themes during play with both sons and daughters, and fathers displayed more repair and construction themes. Mothers' and fathers' facilitative- creative interaction style in dyadic play predicted the level of the child's symbolic play. Co-parenting style marked by cooperation and autonomy predicted symbolic play during a triadic family session. Child intelligence predicted symbolic play beyond the parent's style during triadic but not dyadic interactions. The findings have implications for early intervention directed at increasing symbolic play in young children
SN - 0002-9432
AD - Community Infant Mental Health Unit, Sackler Medical School, Petah Tiqwa, Israel. ofkeren@internet-zahav.net
UR - 16262517
ER -

TY - JOUR
ID - 1594
T1 - Poor neighborhoods: safe playgrounds

A1 - Powell,E.C.
A1 - Ambardekar,E.J.
A1 - Sheehan,K.M.
Y1 - 2005/09//
N1 - Powell, Elizabeth C. Ambardekar, Erin J. Sheehan, Karen M
Journal of urban health : bulletin of the New York Academy of Medicine
c5l, 9809909
IM
Journal Article. Research Support, Non-U.S. Gov't
English
KW - Chicago
KW - Child
KW - Consumer Product Safety
KW - Humans
KW - Play and Playthings
KW - Poverty Areas
KW - Urban Population
KW - Waste Products
KW - Wounds and Injuries/pc [Prevention & Control]
KW - Medline
KW - Unsupervised Play Addition
RP - NOT IN FILE
SP - 403
EP - 410
JF - Journal of Urban Health
JA - J Urban Health
VL - 82
IS - 3
CY - United States
N2 - Although unstructured physical play is helpful to child development and physical activity is important to obesity prevention, up-to-date information about playgrounds and playground hazards in urban areas is limited. Local data are needed to identify problems and target interventions. The aim of this study was to describe the hazards in playgrounds located in low-income (median dollars 28,728-38,915) and very low-income (median dollars 18,266-18,955) Chicago neighborhoods. Using a standardized on-site survey (National Program for Playground Safety), two investigators reviewed seventy-eight public playgrounds for hazards related to playground design, safe surfaces, supervision, and equipment design and maintenance. The design of 56 playgrounds (72%) posed no hazards. One playground lacked protection from motor vehicles, and 21 had minor flaws. One playground had an asphalt surface; all others had protective surfaces, usually wood chips. The chips were too thin in many places, and in 15 playgrounds (19%), at least one concrete footing was exposed. Trash was a common surface hazard (68%). Although most equipment was safe (swings of soft materials and appropriate platform barriers), many pieces needed repairs. Equipment maintenance hazards included gaps (44%) and missing (38%) or broken parts (35%). In 13 of 39 playgrounds (33%) where children were observed playing, one or more were unsupervised. Playgrounds in very low-income neighborhoods more often had trash in the fall zone and exposed footings (P<.01 for each); there were no differences between low and very low-income neighborhoods in playground design or equipment maintenance. We conclude that playgrounds in low-income Chicago neighborhoods are of good design and have appropriate surfaces. Needed improvements include attention to wood chip depth, the removal of trash from the fall zone, and equipment repairs. Greater adult supervision is warranted
SN - 1099-3460
AD - Division of Pediatric Emergency Medicine, Box 62, Children's Memorial Hospital, 2300 Children's Plaza, Chicago, IL 60614, USA. epowell@northwestern.edu
UR - 16033929
ER -

TY - JOUR
ID - 1595

T1 - The compound thoracodorsal perforator flap in the treatment of combined soft-tissue defects of sole and dorsum of the foot
A1 - Van,Landuyt K.
A1 - Hamdi,M.
A1 - Blondeel,P.
A1 - Monstrey,S.
Y1 - 2005/04//
N1 - Van Landuyt, K. Hamdi, M. Blondeel, Ph. Monstrey, S
British journal of plastic surgery
b0w, 2984714r
IM
Case Reports. Journal Article
English
KW - Adolescent
KW - Adult
KW - Aged
KW - Child
KW - Preschool
KW - Female
KW - Foot Injuries/pa [Pathology]
KW - Foot Injuries/su [Surgery]
KW - Humans
KW - Infant
KW - Male
KW - Microcirculation
KW - Microsurgery/mt [Methods]
KW - Middle Aged
KW - Reconstructive Surgical Procedures/mt [Methods]
KW - Soft Tissue Injuries/pa [Pathology]
KW - Soft Tissue Injuries/su [Surgery]
KW - Surgical Flaps/bs [Blood Supply]
KW - Surgical Flaps
KW - Medline
KW - Unsupervised Play Addition
RP - NOT IN FILE
SP - 371
EP - 378
JF - British Journal of Plastic Surgery
JA - Br J Plast Surg
VL - 58
IS - 3
CY - England
N2 - Nine cases of massive soft-tissue loss of the foot were reconstructed by means of a compound (chimera) thoracodorsal artery perforator (TAP) flap, which reconstituted the different functional units (dorsum, heel, instep, weight-bearing surface). In each case, the flap consisted of a skin component isolated on its perforator in combination with a portion of latissimus dorsi muscle and/or serratus fascia, all pedicled on the thoracodorsal vessels. The pedicle length allows up to 4-6 cm of independent mobility of the skin island. The mobility of the various flap components allows the various functional units of the foot to be reconstructed without relying on multiple flaps or anastomoses. The pedicle length was sufficient to be able to perform the anastomosis out of the zone of injury. In some cases the skin island was harvested along with intercostal nerve branches, this gave us the potential to develop a sensate flap. The indications and advantages of this reconstructive method are discussed
SN - 0007-1226
AD - Department of Plastic Surgery, University Hospital Gent, De Pintelaan 185, B-9000 Gent, Belgium. koenraad.vanlanduyt@ugent.be
UR - 15780233

ER -

TY - JOUR
ID - 1596
T1 - The effects of maternal depression and parental conflict on children's peer play
A1 - Hipwell,A.E.
A1 - Murray,L.
A1 - Ducournau,P.
A1 - Stein,A.
Y1 - 2005/01//
N1 - Hipwell, A E. Murray, L. Ducournau, P. Stein, A
Child: care, health and development
7602632, c9x
IM
Journal Article. Research Support, Non-U.S. Gov't
English
KW - Adult
KW - Aggression/px [Psychology]
KW - Child Behavior/px [Psychology]
KW - Child of Impaired Parents
KW - Child
KW - Preschool
KW - Conflict (Psychology)
KW - Cooperative Behavior
KW - Depression
KW - Postpartum/px [Psychology]
KW - Female
KW - Humans
KW - Infant
KW - Male
KW - Mother-Child Relations
KW - Mothers/px [Psychology]
KW - Parents/px [Psychology]
KW - Peer Group
KW - Personal Autonomy
KW - Play and Playthings/px [Psychology]
KW - Prospective Studies
KW - Regression Analysis
KW - Sex Factors
KW - Statistics as Topic
KW - Medline
KW - Unsupervised Play Addition
RP - NOT IN FILE
SP - 11
EP - 23
JF - Child: Care, Health & Development
JA - Child Care Health Dev.
VL - 31
IS - 1
CY - England
N2 - BACKGROUND: Little is known about the relative effects of exposure to postnatal depression and parental conflict on the social functioning of school-aged children. This is, in part, because of a lack of specificity in the measurement of child and parental behaviour and a reliance on children's reports of their hypothetical responses to conflict in play. METHODS: In the course of a prospective longitudinal study of children of postnatally depressed and well women, 5-year-old children were videotaped at home with a friend in a naturalistic dressing-up play setting. As well as examining possible associations between the occurrence of

postnatal depression and the quality of the children's interactions, we investigated the influence of parental conflict and co-operation, and the continuity of maternal depression. The quality of the current mother-child relationship was considered as a possible mediating factor. RESULTS: Exposure to postnatal depression was associated with increased likelihood, among boys, of displaying physical aggression in play with their friend. However, parental conflict mediated the effects of postnatal depression on active aggression during play, and was also associated with displays of autonomy and intense conflict. While there were no gender effects in terms of the degree or intensity of aggressive behaviours, girls were more likely to express aggression verbally using denigration and gloating whereas boys were more likely to display physical aggression via interpersonal and object struggles. CONCLUSIONS: The study provided evidence for the specificity of effects, with strong links between parental and child peer conflict. These effects appear to arise from direct exposure to parental conflict, rather than indirectly, through mother-child interactions
SN - 0305-1862
AD - Department of Psychiatry, School of Medicine, University of Pittsburgh, Pittsburgh, PA 15213, USA. hipwellae@upmc.edu
UR - 15658962
ER -

TY - JOUR
ID - 1598
T1 - [Transitional objects in the Maghreb]. [French]
A1 - Farad-Bensenouci,S.
Y1 - 2004/12//
N1 - Farad-Bensenouci, Samia
Soins. Pediatrie, puericulture
9604503, cjc
N
Journal Article
French
KW - Africa
KW - Northern/eh [Ethnology]
KW - Anxiety
KW - Separation/eh [Ethnology]
KW - Separation/pc [Prevention & Control]
KW - Child
KW - Child Behavior/eh [Ethnology]
KW - Child Psychology
KW - Preschool
KW - Cultural Diversity
KW - Humans
KW - Object Attachment
KW - Personal Autonomy
KW - Play and Playthings/px [Psychology]
KW - Medline
KW - Unsupervised Play Addition
RP - NOT IN FILE
SP - 25
JF - Soins
JA - Soins Pediatr Pueric.
VL - Pediatrie, Puericulture.
IS - 221
CY - France
SN - 1259-4792
AD - Service de medecine interne A, CHRU de Tours (37)
UR - 15636207
ER -

TY - JOUR
ID - 1599
T1 - Pamidronate treatment of less severe forms of osteogenesis imperfecta in children
A1 - Zacharin,M.
A1 - Kanumakala,S.
Y1 - 2004/11//
N1 - Zacharin, Margaret. Kanumakala, Shankar
Journal of pediatric endocrinology & metabolism : JPEM
bu0, 8510464, cef, 9508900
IM
Clinical Trial. Journal Article
English
KW - Absorptiometry
KW - Photon
KW - Adolescent
KW - Alkaline Phosphatase/bl [Blood]
KW - Body Height
KW - Body Weight/ph [Physiology]
KW - Bone Density/de [Drug Effects]
KW - Bone Density/ph [Physiology]
KW - Bone and Bones/me [Metabolism]
KW - Child
KW - Preschool
KW - Cohort Studies
KW - Diphosphonates/tu [Therapeutic Use]
KW - Female
KW - Humans
KW - Male
KW - Osteoblasts/de [Drug Effects]
KW - Osteoclasts/de [Drug Effects]
KW - Osteogenesis Imperfecta/co [Complications]
KW - Osteogenesis Imperfecta/dt [Drug Therapy]
KW - Osteogenesis Imperfecta/ra [Radiography]
KW - Pain/ep [Epidemiology]
KW - Pain/et [Etiology]
KW - Puberty/ph [Physiology]
KW - Medline
KW - Unsupervised Play Addition
RP - NOT IN FILE
SP - 1511
EP - 1517
JF - Journal of Pediatric Endocrinology
JA - J Pediatr Endocrinol
VL - 17
IS - 11
CY - England
N2 - BACKGROUND: Bisphosphonate therapy improves bone quality in children with severe osteogenesis imperfecta (OI). Children with milder phenotypes also have prepubertal fractures, bone pain and reduced bone mass, predisposing them to adult osteoporosis. OBJECTIVE: To evaluate treatment effects of pamidronate in children with mild phenotypes of OI. METHODS: Open label, 2-year observational study of 18 patients, using pamidronate, with clinical, biochemical and radiological monitoring. RESULTS: Over 2 years, bone pain decreased from 16 to 1 patient and disturbed sleep from 12 children to 0. Independent mobility improved from 10 to 17 children. Fracture incidence decreased from 1.6 to 0.5 fractures/child/year. Surgical interventions decreased from a mean 1.3 procedures/patient to 0 in the second year of treatment. Growth velocity remained stable at a mean 4.8 cm/year. Mean lumbar vertebral bone mineral density improved by 40.8%, from 0.375 to 0.528 g/cm2 ($p < 0.0001$), z-score from -3.77 to -2.44 ($p < 0.0001$). Mean vertebral height improved by 17.3%,

from 15.6 to 18.38 mm (p = 0.07); plasma alkaline phosphatase decreased from 222 to 169 U/l (p = 0.0009) and urinary deoxypyridinoline crosslinks decreased from 26.7 to 21.8 nmol/mmol creatinine (p = 0.21). Two children with vitamin D insufficiency were concurrently treated. A significant association (r = -0.6, p = 0.008) was shown between age at start of treatment and percentage change in BMD after 2 years. CONCLUSIONS: Pamidronate treatment improves bone quality in children with mild types of OI. It ameliorates clinical symptoms, improves mobility, reduces fracture frequency and thus improves quality of life and in future is likely to reduce the severity and consequences of adult osteoporosis by improved peak bone mass in these children
SN - 0334-018X
AD - Department of Endocrinology and Diabetes, Royal Children 's Hospital, Melbourne, Australia.
margaret.zacharin@rch.org.au
UR - 15570988
ER -

TY - JOUR
ID - 1600
T1 - Social competence in young children with inflicted traumatic brain injury
A1 - Landry,S.H.
A1 - Swank,P.
A1 - Stuebing,K.
A1 - Prasad,M.
A1 - Ewing-Cobbs,L.
Y1 - 2004///
N1 - Landry, Susan H. Swank, Paul. Stuebing, Karla. Prasad, Mary. Ewing-Cobbs, Linda
Developmental neuropsychology
dlx, 8702038
IM
Comparative Study. Journal Article. Research Support, U.S. Gov't, P.H.S.
English
KW - Analysis of Variance
KW - Brain Injuries/pp [Physiopathology]
KW - Brain Injuries/px [Psychology]
KW - Child
KW - Preschool
KW - Cognition/ph [Physiology]
KW - Competitive Behavior/ph [Physiology]
KW - Demography
KW - Developmental Disabilities
KW - Female
KW - Follow-Up Studies
KW - Humans
KW - Infant
KW - Male
KW - Motor Activity/ph [Physiology]
KW - Neuropsychological Tests/sn [Statistics & Numerical Data]
KW - Play and Playthings
KW - Prospective Studies
KW - Reproducibility of Results
KW - Social Behavior
KW - Trauma Severity Indices
KW - Verbal Behavior/ph [Physiology]
KW - Medline
KW - Unsupervised Play Addition
RP - NOT IN FILE
SP - 707
EP - 733

JF - Developmental Neuropsychology
JA - Dev Neuropsychol
VL - 26
IS - 3
CY - United States
N2 - As infants develop skills that allow for increasing independence in social and cognitive domains, they acquire the ability to identify goals, sequence behaviors to carry out goals, and to flexibly use strategies for attaining goals in both social and independent play contexts. Little is known about how brain injury in young children may disrupt the precursors to such executive processes. In this study, we examined social and cognitive competence in 25 infants ages 3 to 23 months who sustained moderate to severe traumatic brain injury (TBI) secondary to physical abuse and in 22 healthy community comparison children. Children with TBI were evaluated an average of 1.6 months after the injury. A toy-centered activity with the examiner was used to capture joint attention and social behavior and an exploratory toy play situation was used to measure independent goal-directed play. The inflicted TBI group showed significant reduction in both social and cognitive domains relative to the comparison group. Canonical correlation analyses disclosed that inflicted TBI was associated with reduction in (a) initiation of social interactions, (b) responsiveness to interactions initiated by the examiner, (c) positive affect, and (d) compliance. The groups performed comparably on indexes of gestural and verbal communication and for the occurrence of negative affect. Joint attention was an area of vulnerability for the TBI group in both social initiation and response contexts. Although general cognitive and motor scores were lower in the inflicted TBI group, the complexity of independent toy play did not differ across groups. Early brain injury causes significant disruption in behaviors regulating initiation and responsiveness in social contexts. Longitudinal follow-up will characterize the long-term consequences of early disruption in joint attention and other behaviors on the development of social and cognitive precursors to executive processes
SN - 8756-5641
AD - Division of Developmental Pediatrics, University of Texas Health Science Center at Houston, 77030, USA. susan.h.landry@uth.tmc.edu
UR - 15525566
ER -

TY - JOUR
ID - 1601
T1 - Improving functional skills and physical fitness in children with Rett syndrome
A1 - Lotan,M.
A1 - Isakov,E.
A1 - Merrick,J.
Y1 - 2004/11//
N1 - Lotan, M. Isakov, E. Merrick, J
Journal of intellectual disability research : JIDR
9206090, bga
IM
Journal Article
English
KW - Activities of Daily Living/px [Psychology]
KW - Child
KW - Education of Intellectually Disabled
KW - Exercise/px [Psychology]
KW - Feasibility Studies
KW - Female
KW - Humans
KW - Motor Skills
KW - Physical Fitness
KW - Rett Syndrome/px [Psychology]
KW - Rett Syndrome/rh [Rehabilitation]
KW - Social Behavior
KW - Statistics as Topic
KW - Treatment Outcome

KW - Medline
KW - Unsupervised Play Addition
RP - NOT IN FILE
SP - 730
EP - 735
JF - Journal of Intellectual Disability Research
JA - J Intellect Disabil Res
VL - 48
IS - Pt 8
CY - England
N2 - BACKGROUND: To investigate the feasibility of a physical exercise programme with treadmill for persons with Rett syndrome (RS) in order to promote fitness and health. METHODS: A daily training programme on a treadmill was designed for four females with RS over a period of 2 months with tests performed in three intervals, at time 1, 2 and 3, 2 months apart with intervention taking place between tests 2 and 3. Participants were four girls with RS aged 8.5-11 years (mean: 10 years) attending the educational facility Beit Issie Shapiro, Raanana, Israel, all with independent mobility and with typical characteristics of RS stage III. The training took place at the educational facility, on a 1400 model treadmill (Trimline, capable of very low speeds < 0.5 k/h), with very long side rails. Special low side rails were adapted to the treadmill in order to fit the height of the children and velcro straps were added to assist in safely placing the hands. Pulse was monitored constantly during exercise by an A3 polar pulse belt. Pulse measurements at rest during training were considered as evaluators of aerobic physical condition. Functional measurement was based on a scale specially established for the present study. The scale was a 31-item motor-functioning tool that measures the ability of participants to knee walk and knee stand, to get up to a standing position, duration of walking different paths, and to go up and down stairs and slopes. RESULTS: The study showed that physical fitness of the children at the end of the training programme had improved considerably (P < 0.05). Tests showed that general functional abilities had improved considerably (P < 0.0001). Although all items of the functional ability measure showed impressive positive change, some of the 31 items on it showed statistically significant improvement (knee walking, going up and down stairs and speed of walking for 25 m. Pearson correlation showed high linkage (r = -0.76) between functional improvement and change in physical fitness. CONCLUSIONS: Physical fitness programme executed on a daily basis is capable of improving functional ability of children with RS. Nonprofessional personnel can execute such a programme under supervision of a qualified physical therapist
SN - 0964-2633
AD - Zvi Quittman Residential Center, The Millie Shime Campus, Elwyn Jerusalem, Israel
UR - 15494062
ER -

TY - JOUR
ID - 1602
T1 - A paediatric trauma study of scooter injuries
A1 - Fong,C.P.
A1 - Hood,N.
Y1 - 2004/04//
N1 - Fong, Christina P H. Hood, Natalie
Emergency medicine Australasia : EMA
101199824
IM
Journal Article
English
KW - Adolescent
KW - Athletic Injuries/ep [Epidemiology]
KW - Athletic Injuries/pc [Prevention & Control]
KW - Child
KW - Preschool
KW - Female
KW - Fractures
KW - Bone/ep [Epidemiology]

KW - Humans
KW - Incidence
KW - Male
KW - Play and Playthings/in [Injuries]
KW - Prospective Studies
KW - Risk Factors
KW - Soft Tissue Injuries/ep [Epidemiology]
KW - Statistics
KW - Nonparametric
KW - Victoria/ep [Epidemiology]
KW - Medline
KW - Unsupervised Play Addition
RP - NOT IN FILE
SP - 139
EP - 144
JF - Emergency Medicine Australasia
JA - Emerg Med Australas
VL - 16
IS - 2
CY - Australia
N2 - OBJECTIVE: To investigate the incidence and describe the nature of non-motorized scooter related injuries in children presenting to the ED. SETTING: Paediatric ED of a metropolitan tertiary referral hospital. METHODS: A prospective observational study of patients aged under 19 years presenting with injuries sustained while using a non-motorized scooter. Clinicians recorded the data in the patient record. Main outcome measures: type of injury sustained; period of experience on the scooter; the use of protective gear; the presence of adult supervision; the place of accident; and the patient outcome. RESULTS: Sixty-two eligible patients were recruited over an 18 month period. The incidence of scooter- related injuries was 1.3% of all paediatric trauma presentations. There was a fall in scooter injury presentations over the study period; however, this was not statistically significant. The most common injury sustained using a scooter was an upper limb fracture (41.9%). Closed head injury comprised 8.1% of all scooter related injuries. The majority of patients were not wearing protective gear and were unsupervised at the time of their accident. Most patients (79%) were managed in the ED and discharged. CONCLUSIONS: There has been no significant change in scooter injury presentations over the two summer periods of 2000 and 2001. Children presenting to the ED with a scooter related injury tend to be primary school aged, which may have implications on scooter design, age recommendations and safety guidelines
SN - 1742-6731
AD - Department of Emergency Medicine, Monash Medical Centre, Melbourne, Victoria, Australia
UR - 15239729
ER -

TY - JOUR
ID - 1604
T1 - Autonomy, sanity and moral theory
A1 - Law,I.
Y1 - 2003///
N1 - Law, Iain
Res publica (Liverpool, England)
100973395
E
Journal Article
English
KW - Adult
KW - Child
KW - Coercion
KW - Ethical Theory
KW - Humans

KW - Mental Competency
KW - Mentally Ill Persons
KW - Models
KW - Theoretical
KW - Personal Autonomy
KW - Treatment Refusal
KW - Medline
KW - Unsupervised Play Addition
RP - NOT IN FILE
SP - 39
EP - 56
JF - Res Publica
VL - 9
IS - 1
CY - England
N2 - The concept of autonomy plays at least two roles on moral theory. First, it provides a source of constraints upon action: because I am autonomous you may not interfere with me, even for my own good. Second, it provides a foundation for moral theory: human autonomy has been thought by some to produce moral principles of a more general kind. This paper seeks to understand what autonomy is, and whether the autonomy of which we are capable is able to serve these roles. We would naturally hope for a concept of autonomy that is value-neutral rather than value-laden. That is to say, we would want the judgement that I am autonomous to depend wholly on, say, structural features of my psychology, and in no way to require us to make ethical judgements, or other value judgements. Being value-neutral is perhaps desirable in a concept of autonomy serving the first role, and plausibly indispensible in one playing the second. I shall argue, however, that value-neutral conceptions of autonomy are impoverished and out of line with our intentions; set out and defend an explicitly value-laden conception of autonomy; and explore the implications of such a view for the ability of autonomy to play the roles mentioned above
SN - 1356-4765
AD - Department of Philosophy, University of Birmingham, Edgbaston, Birmingham, B15 2TT, UK.
lawir@hhs.bham.ac.uk
UR - 15199941
ER -

TY - JOUR
ID - 1606
T1 - Rates of pediatric injuries by 3-month intervals for children 0 to 3 years of age
A1 - Agran,P.F.
A1 - Anderson,C.
A1 - Winn,D.
A1 - Trent,R.
A1 - Walton-Haynes,L.
A1 - Thayer,S.
Y1 - 2003/06//
N1 - Agran, Phyllis F. Anderson, Craig. Winn, Diane. Trent, Roger. Walton-Haynes, Lynn. Thayer, Sharon
Pediatrics
oxv, 0376422
AIM, IM
Journal Article. Multicenter Study. Research Support, U.S. Gov't, P.H.S.
English
KW - Accidental Falls/mo [Mortality]
KW - Accidental Falls/sn [Statistics & Numerical Data]
KW - Accidents
KW - Traffic/mo [Mortality]
KW - Traffic/sn [Statistics & Numerical Data]
KW - Age Distribution
KW - Battered Child Syndrome/ep [Epidemiology]

KW - Battered Child Syndrome/mo [Mortality]
KW - Burns/ep [Epidemiology]
KW - Burns/mo [Mortality]
KW - Child Abuse/mo [Mortality]
KW - Child Abuse/sn [Statistics & Numerical Data]
KW - Child
KW - Hospitalized/sn [Statistics & Numerical Data]
KW - Preschool
KW - Death Certificates
KW - Drowning/ep [Epidemiology]
KW - Drowning/mo [Mortality]
KW - Foreign Bodies/ep [Epidemiology]
KW - Foreign Bodies/mo [Mortality]
KW - Health Care Surveys/sn [Statistics & Numerical Data]
KW - Humans
KW - Infant
KW - Newborn
KW - Near Drowning/ep [Epidemiology]
KW - Poisoning/ep [Epidemiology]
KW - Poisoning/mo [Mortality]
KW - Wounds and Injuries/ep [Epidemiology]
KW - Wounds and Injuries/mo [Mortality]
KW - Medline
KW - Unsupervised Play Addition
RP - NOT IN FILE
SP - e683
EP - e692
JA - Pediatrics
VL - 111
IS - 6 Pt 1
CY - United States
N2 - OBJECTIVE: Mortality and morbidity data on childhood injury are used to construct developmentally appropriate intervention strategies and to guide pediatric anticipatory counseling on injury prevention topics. Effective anticipatory guidance depends on detailed injury data showing how risks change as children develop. Conventional age groupings may be too broad to show the relationship between children's development and their risk of various causes of injury. Previous studies revealed differences in overall rates and specific causes of injury by year of age. However, single year of age rates for children younger than 4 years may not reflect the variations in risk as a result of rapid developmental changes. This study was designed to analyze injury rates for children younger than 4 years by quarter-year intervals to determine more specifically the age period of highest risk for injury and for specific causes. METHODS: We used data from 1996-1998 California hospital discharges and death certificates to identify day of age and external cause of injury (E-code) for children younger than 4 years. The number of California residents for each day of age was estimated from US Census of estimates of California's population by year of age for the midpoints (1996-1998). Rates were calculated by 3-month intervals. We grouped the E-codes into major categories that would be particularly relevant for developmentally related risks of injury specific to young children. The categorization took into account physical, motor, behavioral, and cognitive developmental milestones of children 0 to 3 years. RESULTS: There were a total of 23,173 injuries; 636 resulted in death. The overall annual rate for children aged 0 to 3 years was 371/100,000. Beginning at age 3 to 5 months, the overall rate of injury rapidly increased with increased age, peaking at 15 to 17 months. The mean injury rate calculated for each single year of age did not reflect the variation and the highest rate of injury by quarter year of age for children younger than 1 year, 1 year, and 2 years. The leading major causes of injury in descending order were falls, poisoning, transportation, foreign body, and fires/burns. The overall rate of the major category of falls exceeded poisoning, the second leading cause of injury, by a factor of 2. Age-related differences were detected within each major cause of injury. For children 0 to 12 months of age, there was a different leading cause of specific injury for each 3-month period: other falls from height (0-2 months), battering (3-5 months), falls from furniture (6-8 months), and nonairway foreign body (9-11 months). Hot liquid and vapor injuries were the leading specific causes for children 12 to 17 months.

Poisoning by medication was the leading specific cause of injury for all age groups from 18 to 35 months and exceeded poisoning by other substances. Pedestrian injury was the leading specific cause of injury for all age groups from 36 to 47 months. Fall from furniture has the highest rates of specific causes of falls from age 3 to 47 months. Fall from stairs peaked at age 6 to 8 months and 9 to 11 months. Fall from buildings was highest at 24 to 26 months. Poisoning by medication peaked at age 21 to 23 months, but poisoning by other substances peaked at 15 to 17 months. The motor vehicle occupant injury rates were fairly stable over the age span of this study. The pedestrian injury rate increased beginning at age 12 to 14 months and by 15 to 17 months was double that of the motor vehicle occupant. Foreign body had a marked peak at age 9 to 11 months. Both battering and neglect rates were highest among infants 0 to 2 and 3 to 5 months. Bathtub submersions had a narrow peak at age 6 to 11 months. Other submersions peaked at 12 to 14 months and remained high until 33 to 35 months. CONCLUSIONS: We departed from usual groupings of E-codes and devised groupings that would be reflective of age-related developmental characteristics. Differences in rates by narrow age groups for young children can be related to developmental achievements, w can be related to developmental achievements, which place the child at risk for specific causes of injury. We found marked variability in both rates and leading causes of injury by 3-month interval age groupings that were masked by year of age analyses. Children aged 15 to 17 months had the highest overall injury rate before age 15 years. This coincides with developmental achievements such as independent mobility, exploratory behavior, and hand-to-mouth activity. The child is able to access hazards but has not yet developed cognitive hazard awareness and avoidance skills. A remarkable finding was the high rate of battering injury among infants 0 to 5 months, suggesting the need to address potential child maltreatment in the perinatal period. Poisoning was the second major leading cause of injury; more than two thirds were medication. Cultural factors may influence views of medications, storage practices, use of poison control system telephone advice, and risk of toddler poisoning. The pedestrian injury rate doubled between 12 and 14 months and 15 and 17 months and exceeded motor vehicle occupant injury rates for each 3-month interval from 15 to 47 months. Pedestrian injury has not received sufficient attention in general and certainly not in injury prevention counseling for children younger than 4 years. Anticipatory guidance for pedestrian injury should be incorporated before 1 year of age. Effective strategies must be based on the epidemiology of childhood injury. Pediatricians and other pediatric health care providers are in a unique position to render injury prevention services to their patients. Integrating injury prevention messages in the context of developmental assessments of the child is 1 strategy. These data can also be used for complementary childhood injury prevention strategies such as early intervention programs for high-risk families for child abuse and neglect, media and advocacy campaigns, public policies, and environmental and product design
SN - 1098-4275
AD - University of California, Center for Health Policy and Research, Pediatric Injury Prevention Research Group, Irvine, California 92697-5800, USA. pagran@uci.edu
UR - 12777586
ER -

TY - JOUR
ID - 1607
T1 - Factors of significance for mobility in children with myelomeningocele
A1 - Norrlin,S.
A1 - Strinnholm,M.
A1 - Carlsson,M.
A1 - Dahl,M.
Y1 - 2003///
N1 - Norrlin, S. Strinnholm, M. Carlsson, M. Dahl, M
Acta paediatrica (Oslo, Norway : 1992)
bgc, 9205968
IM
Journal Article. Research Support, Non-U.S. Gov't
English
KW - Activities of Daily Living
KW - Caregivers
KW - Child
KW - Cognition Disorders/et [Etiology]
KW - Cognition Disorders/pp [Physiopathology]

KW - Cognition Disorders/rh [Rehabilitation]
KW - Female
KW - Hand/pp [Physiopathology]
KW - Hand Strength/ph [Physiology]
KW - Humans
KW - Locomotion/ph [Physiology]
KW - Male
KW - Meningomyelocele/co [Complications]
KW - Meningomyelocele/pp [Physiopathology]
KW - Meningomyelocele/rh [Rehabilitation]
KW - Nervous System Diseases/et [Etiology]
KW - Nervous System Diseases/pp [Physiopathology]
KW - Nervous System Diseases/rh [Rehabilitation]
KW - Self-Help Devices
KW - Severity of Illness Index
KW - Medline
KW - Unsupervised Play Addition
RP - NOT IN FILE
SP - 204
EP - 210
JF - Acta Paediatrica
JA - Acta Paediatr
VL - 92
IS - 2
CY - Norway
N2 - AIM: To investigate neurological impairment, hand function and cognitive function in a group of children with myelomeningocele, in order to identify factors of significance for independent mobility and the physical assistance required for mobility in daily activities. METHODS: The study material comprised 32 children, aged 6-11 y and without mental retardation. Mobility was assessed with the Pediatric Evaluation of Disability Inventory, scored as caregiver assistance. Statistical differences and correlations between the caregiver assistance scores and the selected variables were analysed. RESULTS: Nine children scored independent mobility. The independent children had better hand coordination (p = 0.004) and walking ability (p = 0.01), lower cele levels (p = 0.011), higher performance IQ (p = 0.027), better visuospatial function (p = 0.029) and executive function (p = 0.037) than the others. The caregiver assistance scores were lower for both the children with early and severe symptoms of brainstem dysfunction and the children with scoliosis. Statistically significant correlations were found between the need for caregiver assistance and reduced walking ability, high cele level, poor hand strength and coordination, visuospatial function, executive function and performance IQ. In the subgroup of children who needed a wheelchair or walking aids, hand strength was the only variable significantly correlated with caregiver assistance (r(s) = 0.703, p = 0.000). CONCLUSION: Most of the children were dependent on others in daily activities. Impaired hand function and cognitive function were significant for mobility, and this has implications for the therapy programme in children with myelomeningocele
SN - 0803-5253
AD - Folke Bernadottehemmet, Children's Hospital, Department of Women's and Children's Health, Section of Paediatrics, Uppsala University, Uppsala, Sweden. simone.norrlin@ped.uas.lul.se
UR - 12710647
ER -

TY - JOUR
ID - 1608
T1 - Lack of human herpesvirus 8 and Epstein-Barr virus in Kikuchi's histiocytic necrotizing lymphadenitis
A1 - George,T.I.
A1 - Jones,C.D.
A1 - Zehnder,J.L.
A1 - Warnke,R.A.
A1 - Dorfman,R.F.
Y1 - 2003/02//

N1 - George, Tracy I. Jones, Carol D. Zehnder, James L. Warnke, Roger A. Dorfman, Ronald F
Human pathology
gec, 9421547
IM
Case Reports. Journal Article
English
KW - Adolescent
KW - Adult
KW - Child
KW - DNA
KW - Viral/an [Analysis]
KW - Female
KW - Herpesvirus 4
KW - Human/ge [Genetics]
KW - Human
KW - Herpesvirus 8
KW - Histiocytic Necrotizing Lymphadenitis/vi [Virology]
KW - Humans
KW - In Situ Hybridization
KW - Male
KW - Polymerase Chain Reaction
KW - RNA
KW - Medline
KW - Unsupervised Play Addition
RP - NOT IN FILE
SP - 130
EP - 135
JF - Human Pathology
JA - Hum Pathol
VL - 34
IS - 2
CY - United States
N2 - Kikuchi's histiocytic necrotizing lymphadenitis is a self-limited disorder that typically involves the cervical lymph nodes of young women. Although a viral etiology has been postulated, a definitive viral agent has not been identified. Recent reports have suggested that human herpesvirus 8 (HHV 8) or Epstein-Barr virus (EBV) may play an etiologic role. We investigated the presence of HHV 8 and EBV in archival tissue from 34 cases of Kikuchi's histiocytic necrotizing lymphadenitis. We examined 29 cases for HHV 8 using a nested polymerase chain reaction (PCR) on paraffin-embedded or frozen tissue, and 24 cases for EBV RNA using in situ hybridization (ISH) for EBER1. Controls included reactive lymph nodes from 8 adult women presenting with cervical or axillary lymphadenopathy. The study patients included 7 men and 27 women with a mean age of 28 years. All patients were previously healthy without evidence of immunocompromise and presented with cervical, axillary, or inguinal lymphadenopathy. Two cases exhibited EBV RNA by ISH; this was confirmed by PCR for EBV DNA. HHV 8 DNA was not amplified by nested PCR in any of the cases of Kikuchi's histiocytic necrotizing lymphadenitis or reactive lymph nodes; control PCR demonstrated the presence of amplifiable DNA in all cases. These findings suggest that HHV 8 and EBV do not play causative roles in Kikuchi's histiocytic necrotizing lymphadenitis. Copyright 2003, Elsevier Science (USA). All rights reserved
SN - 0046-8177
AD - Department of Pathology, Stanford University Medical Center, CA 94305, USA
UR - 12612880
ER -

TY - JOUR
ID - 1609
T1 - Fatal unintentional traumatic asphyxia in childhood
A1 - Byard,R.W.
A1 - Hanson,K.A.

A1 - James,R.A.
Y1 - 2003/01//
N1 - Byard, R W. Hanson, K A. James, R A
Journal of paediatrics and child health
arp, 9005421
IM
Journal Article
English
KW - Accidents
KW - Adolescent
KW - Asphyxia/et [Etiology]
KW - Asphyxia/mo [Mortality]
KW - Child
KW - Preschool
KW - Female
KW - Humans
KW - Male
KW - Medical Audit
KW - South Australia/ep [Epidemiology]
KW - Wounds and Injuries/co [Complications]
KW - Medline
KW - Unsupervised Play Addition
RP - NOT IN FILE
SP - 31
EP - 32
JF - Journal of Paediatrics & Child Health
JA - J Paediatr Child Health
VL - 39
IS - 1
CY - Australia
N2 - OBJECTIVE: The present study was undertaken to examine specific features of unintentional traumatic asphyxial deaths in childhood. METHODS: Coronial files and records at the Forensic Science Centre in Adelaide, South Australia, were examined over a 35-year period from 1966 to 2000 for all cases of traumatic asphyxial death occurring in children under the age of 17 years. RESULTS: Six cases of unintentional fatal traumatic asphyxia were identified. All of the victims were boys with an age range of 2-15 years (mean 6.8 years) and all were found dead at the scene. Fatal traumatic asphyxia resulted from entrapment beneath a chest of drawers, beneath a table tennis table, between a pile of wooden pallets and a metal fence, between a conveyor belt and its frame, and under a motor vehicle (in two cases). CONCLUSIONS: Fatal traumatic asphyxia in childhood is a rare event, with younger children commonly being trapped by furniture or by industrial equipment while playing, and older children being trapped under motor vehicles in similar circumstances to adult traumatic asphyxial deaths. Unsupervised play of young children around heavy and potentially unstable pieces of furniture may be dangerous, particularly if more than one child is present. Unsupervised play of young children in industrial yards should be avoided
SN - 1034-4810
AD - Forensic Science Centre, Adelaide, South Australia, Australia. byard.roger@saugov.sa.gov.au
UR - 12542809
ER -

TY - JOUR
ID - 1610
T1 - Penetrating needle injury of the eye causing cataract in children
A1 - Rabiah,P.K.
Y1 - 2003/01//
N1 - Rabiah, Peter K
Ophthalmology
oi5, 7802443

IM
Journal Article
English
KW - Adolescent
KW - Cataract/et [Etiology]
KW - Child
KW - Preschool
KW - Endophthalmitis/et [Etiology]
KW - Eye Injuries
KW - Penetrating/co [Complications]
KW - Female
KW - Humans
KW - Infant
KW - Lens
KW - Crystalline/in [Injuries]
KW - Male
KW - Needlestick Injuries/co [Complications]
KW - Retrospective Studies
KW - Visual Acuity
KW - Medline
KW - Unsupervised Play Addition
RP - NOT IN FILE
SP - 173
EP - 176
JF - Ophthalmology
VL - 110
IS - 1
CY - United States
N2 - PURPOSE: To review the presentation, management, and outcome of children with cataract caused by ocular needle penetration. DESIGN: Retrospective, noncomparative interventional case series. PARTICIPANTS: Forty-two children with cataract caused by ocular needle penetration. INTERVENTION: Cataract surgery. MAIN OUTCOME MEASURES: Best-corrected postoperative visual acuity. RESULTS: Injuries were unintentional and occurred during unsupervised play. The type of needle involved was hypodermic in 24 cases, sewing in 7, and undetermined in 11. Endophthalmitis developed in 14 cases and retinal detachment in 6. Endophthalmitis occurred in 12 cases (50%) of hypodermic needle injury but in no case of sewing needle injury. With a mean postoperative follow-up of 2.3 years, the best-corrected visual acuity was 20/40 or better in 19 cases, 20/50 to 20/80 in 7, 20/100 to counting fingers in 6, light perception in 1, no light perception in 6, and undetermined in 3. Eyes with endophthalmitis and/or retinal detachment had a worse visual prognosis. CONCLUSIONS: Ocular penetration causing cataract occurred in children during unsupervised play with inadequately stored or disposed of hypodermic or sewing needles. Endophthalmitis occurred frequently in injuries caused by hypodermic needles but not in those caused by sewing needles. Visual outcome after management was good in approximately half of the cases especially if endophthalmitis or retinal detachment did not develop
SN - 0161-6420
AD - Department of Ophthalmology, King Khaled Eye Specialist Hospital, Riyadh, Saudi Arabia
UR - 12511363
ER -

TY - JOUR
ID - 1611
T1 - Self in context: autonomy and relatedness in Japanese and U.S. mother-preschooler dyads
A1 - Dennis,T.A.
A1 - Cole,P.M.
A1 - Zahn-Waxler,C.
A1 - Mizuta,I.
Y1 - 2002/11//

N1 - Dennis, Tracy A. Cole, Pamela M. Zahn-Waxler, Carolyn. Mizuta, Ichiro
Child development
0372725, d28
IM
Comparative Study. Journal Article
English
KW - Adult
KW - Attention
KW - Child
KW - Preschool
KW - Cross-Cultural Comparison
KW - Culture
KW - Ego
KW - Female
KW - Humans
KW - Male
KW - Mother-Child Relations
KW - Personal Autonomy
KW - Play and Playthings
KW - Socioeconomic Factors
KW - Videotape Recording
KW - Medline
KW - Unsupervised Play Addition
RP - NOT IN FILE
SP - 1803
EP - 1817
JF - Child Development
JA - Child Dev.
VL - 73
IS - 6
CY - United States
N2 - Cultural differences and similarities in socialization during two contrasting laboratory tasks were examined in 30 Japanese mothers and their preschoolers, both temporarily residing in the United States, and 30 U.S. mothers and their preschoolers (age: M = 55.8 months, SD = 4.9). Mother and child actions, speech, emotion, and attention were coded from videotaped observations during a free play task and waiting task. Cross-cultural comparisons showed that U.S. mothers had more conversations that emphasized individual experiences, more often acted as playmates and used joint attention, maintained more physical distance, showed more positive emotions, and made more positive responses to child accomplishment. In contrast, Japanese mothers had more conversations that emphasized shared experiences, showed more divided attention, and maintained social role distinctions. Similar, but fewer cultural differences emerged for children. However, maternal and child characteristics also varied by task context. The results suggested an emphasis on autonomy in U.S. dyads and an emphasis on relatedness in Japanese dyads, but the interactions with task context revealed the coexistence of autonomy and relatedness
SN - 0009-3920
AD - New York University School of Medicine, Child Study Center, NY 10016, USA. dennit01@med.nyu.edu
UR - 12487495
ER -

TY - JOUR
ID - 1613
T1 - Car window entrapment and accidental childhood asphyxia
A1 - Byard,R.W.
A1 - James,R.A.
Y1 - 2001/04//
N1 - Byard, R W. James, R A
Journal of paediatrics and child health

arp, 9005421
IM
Case Reports. Journal Article
English
KW - Accidents
KW - Asphyxia
KW - Automobiles
KW - Child
KW - Preschool
KW - Equipment Safety
KW - Fatal Outcome
KW - Humans
KW - Male
KW - Neck Injuries
KW - South Australia
KW - Medline
KW - Unsupervised Play Addition
RP - NOT IN FILE
SP - 201
EP - 202
JF - Journal of Paediatrics & Child Health
JA - J Paediatr Child Health
VL - 37
IS - 2
CY - Australia
N2 - A 3-year-old boy was found hanging from a partly opened car window. Attempted resuscitation was unsuccessful. Postmortem reconstruction of events leading up to death indicated that the boy had slipped while standing on his tricycle and entrapped his head as he was attempting either to look into the car, or to climb in through the window. While it is not always possible to completely secure childhood environments, open cars should not be regarded as safe places for young children to play in or around when an adult is not in attendance. To minimize the chance of injury or death to children from parked cars, young children should be removed from vehicles and all windows and doors securely closed and locked, if they are to be left in the vicinity of the car to play unsupervised
SN - 1034-4810
AD - Forensic Science Centre, Adelaide, South Australia, Australia. byardr01@forensic.sa.gov.au
UR - 11328481
ER -

TY - JOUR
ID - 1614
T1 - A multivariate analysis of the revised Conners' Teacher Rating Scale with low-income, urban preschool children
A1 - Fantuzzo,J.
A1 - Grim,S.
A1 - Mordell,M.
A1 - McDermott,P.
A1 - Miller,L.
A1 - Coolahan,K.
Y1 - 2001/04//
N1 - Fantuzzo, J. Grim, S. Mordell, M. McDermott, P. Miller, L. Coolahan, K
Journal of abnormal child psychology
0364547, h33
IM
Journal Article. Research Support, U.S. Gov't, P.H.S.
English
KW - African Americans/px [Psychology]

KW - Attention Deficit Disorder with Hyperactivity/di [Diagnosis]
KW - Attention Deficit Disorder with Hyperactivity/px [Psychology]
KW - Child Behavior Disorders/di [Diagnosis]
KW - Child Behavior Disorders/px [Psychology]
KW - Child
KW - Preschool
KW - Early Intervention (Education)
KW - Female
KW - Humans
KW - Male
KW - Personality Assessment/sn [Statistics & Numerical Data]
KW - Poverty/px [Psychology]
KW - Psychometrics
KW - Reproducibility of Results
KW - Social Behavior
KW - Urban Population
KW - Medline
KW - Unsupervised Play Addition
RP - NOT IN FILE
SP - 141
EP - 152
JF - Journal of Abnormal Child Psychology
JA - J Abnorm.Child Psychol.
VL - 29
IS - 2
CY - United States
N2 - The present investigation addressed the utility of the revised Conners' Teachers Rating Scale (CTRS-28) with low-income urban preschool children. CTRS-28 ratings for a large sample of preschool children from an urban Head Start program were analyzed using exploratory factor analysis. Analyses yielded a 3-factor structure: Conduct problems, Hyperactivity, and Passivity. Further analyses cross-validated this structure for males and females and supported its integrity. Multimethod, multisource validity analyses substantiated the CTRS-28 dimensions. The Play Disruption factor of the parent and teacher Penn Interactive Peer Play Scales (PIPPS) provided convergent validity for the Conduct and Hyperactivity factors of the CTRS-28, whereas the Play Interaction factor revealed divergent validity. The Play Disconnection factor of the PIPPS validated the CTRS-28 Passivity factor. The Q-Sort Emotional Regulation scale provided divergent validity for the Conduct and Hyperactivity factors and likewise the Q-Sort Autonomy scale provided divergent validity for the Passivity factor. Age and sex differences were assessed across the 3 factors of the derived preschool structure. A main effect was found for sex and age indicating that boys displayed higher levels of Hyperactivity and Passivity problems than girls did. Similarly, 4-year-old children demonstrated higher levels of Passivity problems than did 5-year-old children
SN - 0091-0627
AD - Graduate School of Education, University of Pennsylvania, Philadelphia 19104-6216, USA. johnf@gse.upenn.edu
UR - 11321629
ER -

TY - JOUR
ID - 1616
T1 - Parental attitudes toward BB and pellet guns
A1 - Damore,D.T.
A1 - Ramundo,M.L.
A1 - Hanna,J.P.
A1 - Dayan,P.S.
Y1 - 2000/05//
N1 - Damore, D T. Ramundo, M L. Hanna, J P. Dayan, P S
Clinical pediatrics

dhe, 0372606, 8407647
AIM, IM
Comparative Study. Journal Article
English
KW - Adolescent
KW - Adult
KW - Age Distribution
KW - Analysis of Variance
KW - Attitude to Health
KW - Chi-Square Distribution
KW - Child
KW - Preschool
KW - Female
KW - Humans
KW - Incidence
KW - Male
KW - New York City/ep [Epidemiology]
KW - Parent-Child Relations
KW - Play and Playthings
KW - Prospective Studies
KW - Questionnaires
KW - Risk Factors
KW - Sampling Studies
KW - Sex Distribution
KW - Statistics
KW - Nonparametric
KW - Urban Population
KW - Wounds
KW - Gunshot/ep [Epidemiology]
KW - Gunshot/et [Etiology]
KW - Medline
KW - Unsupervised Play Addition
RP - NOT IN FILE
SP - 281
EP - 284
JF - Clinical Pediatrics
JA - Clin.Pediatr (Phila)
VL - 39
IS - 5
CY - UNITED STATES
N2 - As well as describing our pediatric BB and pellet gun injuries and the circumstances surrounding these injuries, we also evaluated parental perceptions of the dangers of BB and pellet guns. A convenience sample of three groups of parents and their children presenting to a Midwest, urban, children's hospital emergency department was prospectively enrolled. The three groups of parents included the injured group, which consisted of the parents whose children had been injured by BB or pellet guns; the gun group, which consisted of the parents who allowed their children to possess BB or pellet guns but had not sustained injury from these guns; and the no gun group, which consisted of the parents who did not allow their children to have these guns. All parents completed a survey concerning their attitudes toward BB and pellet guns. Twenty-eight parents completed questionnaires in each of the three groups. Most BB and pellet gun injuries occurred in adolescent males at home without adult supervision and were inflicted by a friend or by themselves. The injured group and the no gun group viewed BB and pellet guns as significantly more dangerous than the gun group. Parents who allow their children to have BB or pellet guns appear to misperceive their potential for injury by allowing their children to use these guns in an unsafe manner. Clinicians must educate parents about the significant potential for injury of nonpowdered guns
SN - 0009-9228
AD - Children's Hospital Medical Center of Akron, Ohio, USA

UR - 10826075
ER -

TY - JOUR
ID - 1617
T1 - Monkeybar injuries: complications of play
A1 - Waltzman,M.L.
A1 - Shannon,M.
A1 - Bowen,A.P.
A1 - Bailey,M.C.
Y1 - 1999/05//
N1 - Waltzman, M L. Shannon, M. Bowen, A P. Bailey, M C
Pediatrics
oxv, 0376422
IM
Journal Article
English
KW - Age Distribution
KW - Age Factors
KW - Athletic Injuries/ep [Epidemiology]
KW - Child
KW - Preschool
KW - Craniocerebral Trauma/ep [Epidemiology]
KW - Data Collection
KW - Equipment Design
KW - Female
KW - Fractures
KW - Bone/ep [Epidemiology]
KW - Hospitalization/sn [Statistics & Numerical Data]
KW - Humans
KW - Infant
KW - Male
KW - Massachusetts/ep [Epidemiology]
KW - Play and Playthings/in [Injuries]
KW - Retrospective Studies
KW - Medline
KW - Unsupervised Play Addition
RP - NOT IN FILE
SP - e58
JA - Pediatrics
VL - 103
IS - 5
CY - UNITED STATES
N2 - BACKGROUND: Playground equipment resulted in >200 000 injuries from 1990 to 1994, according to the Consumer Product Safety Commission; 88% were attributable to climbers (monkeybars/jungle gyms [MB/JGs]), swings, and slides. Equipment-specific injury requiring emergency department (ED) evaluation has not been reported previously. OBJECTIVE: To describe the spectrum of significant MB/JG-related injuries. METHODS: A 2-year retrospective chart review was performed using the computerized charting system at a large urban Children's Hospital/Regional Pediatric Trauma Center with 50 000 ED visits per year. A telephone survey also was conducted after the chart review to obtain additional information concerning the injury location, the surface type below the equipment, and the presence of adult supervision. RESULTS: A total of 204 patients were identified. Mean age was 6.2 years (range, 20 months to 12 years); 114 (56%) were male. A seasonal variation was noted with June to August accounting for 43% of visits. Injuries included fractures in 124 (61%), contusions in 20 (10%), neck and back strains in 17 (8%), lacerations in 16 (8%), closed head injuries in 10 (5%), abdominal trauma in 5 (3%), genitourinary injuries in 5 (3%), and miscellaneous injuries in the remainder. Among fractures, 90% were fractures of the upper extremity; 48 (40%) were supracondylar fractures. One child

sustained a C7 compression fracture. Abdominal injuries included 1 child who sustained a splenic laceration. All genitourinary injuries (2 vaginal hematomas, 1 vaginal contusion, 1 penile laceration, and 1 urethral injury) were from straddle-type injuries. Fifty-one (25%) patients were admitted to the hospital. Of these, 47 (92%) required an operative procedure (orthopedic reduction or vaginal examination under anesthesia). Analysis of the telephone data revealed that the surface did not influence the injury type. Of the 79 fractures, 30 occurred on "soft surfaces." Injury type was associated significantly with chronologic age. Younger children (1 to 4 years of age) sustained more long-bone fractures than did older children. The presence of adult (at least 18 years of age) supervision, did not influence the occurrence of fractures. CONCLUSIONS: These data suggest that 1) a significant proportion (25%) of MB/JG-related injuries that are evaluated in the ED require hospitalization; 2) most of the injuries resulting in admission will require operative intervention (92%); 3) the surface below the equipment has no influence on the type or severity of the injury; 4) younger children are more likely to sustain long-bone fractures than are older children; and 5) adult supervision does not influence the injury pattern. These data identify the need for additional investigation of means of making MB/JGs safer for child use
SN - 1098-4275
AD - Children's Hospital, Boston, Division of Emergency Medicine, Harvard Medical School, Boston MA 02115, USA. waltzmanvm@a1.tch.harvard.edu
UR - 10224202
ER -

TY - JOUR
ID - 1618
T1 - Perceived visual ability for independent mobility in persons with retinitis pigmentosa
A1 - Turano,K.A.
A1 - Geruschat,D.R.
A1 - Stahl,J.W.
A1 - Massof,R.W.
Y1 - 1999/04//
N1 - Turano, K A. Geruschat, D R. Stahl, J W. Massof, R W
Investigative ophthalmology & visual science
gwi, 7703701
IM
Journal Article. Research Support, U.S. Gov't, P.H.S.
English
KW - Activities of Daily Living
KW - Adolescent
KW - Adult
KW - Child
KW - Preschool
KW - Contrast Sensitivity/ph [Physiology]
KW - Humans
KW - Infant
KW - Middle Aged
KW - Questionnaires
KW - Regression Analysis
KW - Reproducibility of Results
KW - Retina/pp [Physiopathology]
KW - Retinitis Pigmentosa/pp [Physiopathology]
KW - Visual Acuity/ph [Physiology]
KW - Medline
KW - Unsupervised Play Addition
RP - NOT IN FILE
SP - 865
EP - 877
JF - Investigative Ophthalmology & Visual Science
JA - Invest Ophthalmol Vis Sci
VL - 40

IS - 5
CY - UNITED STATES
N2 - PURPOSE: To determine the distribution of perceived ability for independent mobility in people who are at various stages of retinitis pigmentosa (RP). METHODS: A questionnaire was developed to ask subjects to rate how difficult they found each of 35 mobility situations if they had no assistance. The scale was 1 (no difficulty) to 5 (extreme difficulty). In each of 127 subjects, the Rasch analysis, a latent trait analysis, was used to convert the ordinal difficulty ratings into interval measures of perceived visual ability for independent mobility. RESULTS: Content validity of the questionnaire was shown by good separation indexes (4.55 and 8.0) and high reliability scores (0.96 and 0.98) for the person and the item parameters. Construct validity was shown with model fit statistics. Criterion validity of the questionnaire was shown by good discrimination among mobility-related behavior such as "limit independent travel," "always ask for accompaniment," "use a mobility aid," and "have a fear of falling." The mobility situation shown to require the least visual ability was "moving about in the home"; the situation requiring the most was "walking at night." Bivariate regression analysis determined that for every decade of disease progression, perceived visual ability for mobility decreased by approximately 0.5 logit, which was slightly less than 10% of the total range in the study sample. A linear combination of the visual function measures, log minimum angle of resolution, log contrast sensitivity, and log retinal area accounted for 57% of the variability in the person measure. CONCLUSIONS: The patient-based assessment, developed to determine difficulty across a range of mobility situations, is a valid way to measure perceived ability for independent mobility. This latent trait varies systematically with the progression of RP and with visual function measures
SN - 0146-0404
AD - Wilmer Eye Institute, The Johns Hopkins University School of Medicine, Baltimore, MD, USA
UR - 10102283
ER -

TY - JOUR
ID - 1619
T1 - Living with dementia: communicating with an older person and her family
A1 - Long,A.
A1 - Slevin,E.
Y1 - 1999/01//
N1 - Long, A. Slevin, E
Nursing ethics
b6m, 9433357
E, N
Case Reports. Journal Article
English
KW - Adaptation
KW - Psychological
KW - Adult
KW - Adult Children
KW - Aged/px [Psychology]
KW - Beneficence
KW - Communication
KW - Dehumanization
KW - Dementia/nu [Nursing]
KW - Dementia/px [Psychology]
KW - Ethical Analysis
KW - Family/px [Psychology]
KW - Female
KW - Humans
KW - Male
KW - Middle Aged
KW - Nurse-Patient Relations
KW - Nursing Methodology Research
KW - Nursing Staff

KW - Hospital/px [Psychology]
KW - Nursing Theory
KW - Paternalism
KW - Personal Autonomy
KW - Professional-Family Relations
KW - Medline
KW - Unsupervised Play Addition
RP - NOT IN FILE
SP - 23
EP - 36
JF - Nursing Ethics
JA - Nurs Ethics
VL - 6
IS - 1
CY - ENGLAND
N2 - This article is designed to explore and examine the key components of communication that emerged during the interactional analysis of a role play that took place in the classroom. The 'actors' were nurses who perceived the interaction to reflect an everyday encounter in a hospital ward. Permission to tape the interaction was sought and given by all persons involved. The principal 'players' in the scenario were: the patient, a 70-year-old-woman who had been admitted with dementia, her son and daughter, and the nurse in charge of the ward. The fundamental dynamics of the use of power and restriction, truth telling, family stress, interpersonal conflict, ageism, sexism, empathy and humanism surfaced during the analysis. The findings show that therapeutic communication should be the foundation on which nursing should stand. The article continues with an exploration of the theoretical frameworks that guided the analysis of interaction and concludes by suggesting tentatively some meaningful implications for nursing practice. It plans to furnish provocative new insights into the sometimes covert communication dynamics occurring within the nurse-patient relationship. Finally, it aims to generate discussion on this little-charted realm of human social interaction
SN - 0969-7330
AD - University of Ulster, Newtonabbey, NI
UR - 10067554
ER -

TY - JOUR
ID - 1620
T1 - Cognitive predictors of young children's readiness for powered mobility
A1 - Tefft,D.
A1 - Guerette,P.
A1 - Furumasu,J.
Y1 - 1999/10//
N1 - Tefft, D. Guerette, P. Furumasu, J
Developmental medicine and child neurology
0006761, e83
IM
Journal Article. Research Support, U.S. Gov't, Non-P.H.S.. Research Support, U.S. Gov't, P.H.S.
English
KW - Child Development
KW - Child
KW - Preschool
KW - Cognition/cl [Classification]
KW - Disabled Children
KW - Female
KW - Humans
KW - Infant
KW - Male
KW - Motor Skills/cl [Classification]
KW - Patient Selection

KW - Prognosis
KW - Regression Analysis
KW - Wheelchairs
KW - Medline
KW - Unsupervised Play Addition
RP - NOT IN FILE
SP - 665
EP - 670
JF - Developmental Medicine & Child Neurology
JA - Dev.Med Child Neurol.
VL - 41
IS - 10
CY - ENGLAND
N2 - Independent mobility in early childhood has been associated with the development of various cognitive and psychosocial skills. However, children with physical disabilities are not always able to move independently and may be at risk for delays in these areas. Early provision of powered mobility can offer young children an opportunity for independent mobility. Despite this, there is little information to help determine when a young child has the cognitive skills necessary to operate a powered wheelchair safely. This current research aims to identify these skills. A cognitive assessment battery and a wheelchair mobility training and assessment program were developed. Twenty-six children with physical disabilities between the ages of 20 and 36 months were evaluated on the cognitive assessment and participated in the wheelchair training and assessment program. A stepwise regression analysis was used to determine which of the cognitive skills predicted wheelchair mobility performance. The cognitive domains of spatial relations and problem solving were found to be significant and accounted for 57% of the variance in wheelchair skills. Developmental cut-off points on these scales as they relate to wheelchair skills are presented and clinical applications are discussed
SN - 0012-1622
AD - Rehabilitation Engineering Research Center on Technology for Children with Orthopedic Disabilities, Rancho Los Amigos Medical Center, Downey, California, USA. donitatefft@ranchorep.org
UR - 10587042
ER -

TY - JOUR
ID - 1621
T1 - A playroom observation procedure to assess children with mental retardation and ADHD
A1 - Handen,B.L.
A1 - McAuliffe,S.
A1 - Janosky,J.
A1 - Feldman,H.
A1 - Breaux,A.M.
Y1 - 1998/08//
N1 - Handen, B L. McAuliffe, S. Janosky, J. Feldman, H. Breaux, A M
Journal of abnormal child psychology
0364547, h33
IM
Journal Article. Research Support, U.S. Gov't, P.H.S.
English
KW - Attention Deficit Disorder with Hyperactivity/cl [Classification]
KW - Attention Deficit Disorder with Hyperactivity/di [Diagnosis]
KW - Attention Deficit Disorder with Hyperactivity/px [Psychology]
KW - Attention Deficit and Disruptive Behavior Disorders/cl [Classification]
KW - Attention Deficit and Disruptive Behavior Disorders/di [Diagnosis]
KW - Attention Deficit and Disruptive Behavior Disorders/px [Psychology]
KW - Child
KW - Female
KW - Humans
KW - Intellectual Disability/cl [Classification]

KW - Intellectual Disability/di [Diagnosis]
KW - Intellectual Disability/px [Psychology]
KW - Intelligence
KW - Male
KW - Observer Variation
KW - Personality Assessment/sn [Statistics & Numerical Data]
KW - Personality Assessment
KW - Play and Playthings
KW - Psychometrics
KW - Sensitivity and Specificity
KW - Social Behavior
KW - Social Environment
KW - Medline
KW - Unsupervised Play Addition
RP - NOT IN FILE
SP - 269
EP - 277
JF - Journal of Abnormal Child Psychology
JA - J Abnorm.Child Psychol.
VL - 26
IS - 4
CY - UNITED STATES
N2 - Forty-two children (ages 6 to 12 years old) with moderate mental retardation to borderline intellectual functioning were studied in a laboratory playroom setting to determine whether children identified as ADHD (attention deficit hyperactivity disorder) or controls differed on activity and attentional measures. Children with ADHD were further divided into ADHD + conduct problems (ADHD + CD) and ADHD-only subgroups (with an ADHD-combined group comprising children of both subgroups). An interval recording system was used to code observations of independent play and a restricted academic task. Results indicated that the ADHD-combined group was significantly more vocal and engaged in a significantly greater number of toy changes than controls during independent play. Significant group differences were also noted during the restricted academic task, with the ADHD-combined and ADHD + CD groups more off-task and engaging in a greater number of toy touches than controls. Discriminant analyses found independent play measures to predict group membership in 70 percent of cases (ADHD-combined vs. controls), but in only 64 percent of cases using measures from the restricted academic task. No significant findings resulted when the ADHD subjects were further divided into two subgroups. Despite some inconsistent findings, such laboratory-based observations may be of value in the diagnosis of ADHD in children with moderate mental retardation to borderline intellectual functioning
SN - 0091-0627
AD - University of Pittsburgh School of Medicine, Pennsylvania 15213, USA
UR - 9700519
ER -

TY - JOUR
ID - 1622
T1 - Golf clubs: hidden home hazard for children
A1 - Ridenour,M.V.
Y1 - 1998/06//
N1 - Ridenour, M V
Perceptual and motor skills
ozb, 0401131
IM
Journal Article
English
KW - Accidents
KW - Home/sn [Statistics & Numerical Data]
KW - Adolescent
KW - Adult

KW - Age Factors
KW - Child
KW - Preschool
KW - Craniocerebral Trauma/ep [Epidemiology]
KW - Craniocerebral Trauma/mo [Mortality]
KW - Death Certificates
KW - Golf/in [Injuries]
KW - Golf
KW - Humans
KW - Play and Playthings/in [Injuries]
KW - United States/ep [Epidemiology]
KW - Medline
KW - Unsupervised Play Addition
RP - NOT IN FILE
SP - 747
EP - 753
JA - Perceptual & Motor Skills
VL - 86
IS - 3 Pt 1
CY - UNITED STATES
N2 - Different accident patterns for golf equipment were noted for adults and children. Children are more likely than adults to be hit by a golf club as bystanders who entered the swing zone of a golf club. Previous publications investigating accidents involving golf clubs and over 2000 incidents involving emergency room treatment of children injured by golf clubs were reviewed. Most injuries occurred when unsupervised children played with golf clubs at home. When studies limit their focus to accident victims, who are currently being treated in the emergency room, hospital, or outpatient facility, no deaths are reported. A separate analysis of death certificates of children between the ages of 4 and 14 years also identified death as a possible consequence as 19 deaths have been reported to the National Injury Information Clearinghouse. Based on these three sources of data, golf clubs should not be used by children unsupervised given the potential for serious and permanent head injury and death. Recommendations for parents and manufactures regarding the safety of golf clubs are provided
SN - 0031-5125
AD - Biokinetics Research Laboratory, Temple University, Philadelphia, PA 19122, USA
UR - 9656265
ER -

TY - JOUR
ID - 1624
T1 - [The Dutch poliomyelitis epidemic 1992/'93; invalidity and rehabilitation]. [Dutch]
A1 - van Asbeck,F.W.
Y1 - 1996/06/01/
N1 - van Asbeck, F W
Nederlands tijdschrift voor geneeskunde
nuk, 0400770
IM
English Abstract. Journal Article. Research Support, Non-U.S. Gov't
Dutch
KW - Activities of Daily Living
KW - Adolescent
KW - Adult
KW - Child
KW - Preschool
KW - Disability Evaluation
KW - Disease Outbreaks
KW - Female
KW - Humans

KW - Infant
KW - Locomotion
KW - Male
KW - Muscle Contraction
KW - Netherlands/ep [Epidemiology]
KW - Poliomyelitis/ep [Epidemiology]
KW - Poliomyelitis/rh [Rehabilitation]
KW - Medline
KW - Unsupervised Play Addition
RP - NOT IN FILE
SP - 1178
EP - 1182
JF - Nederlands Tijdschrift voor Geneeskunde
JA - Ned Tijdschr Geneeskd
VL - 140
IS - 22
CY - NETHERLANDS
N2 - OBJECTIVE: To describe epidemiologic characteristics and neurological and functional recovery of the victims of the 1992/'93 Dutch poliomyelitis epidemic compared to the 1971 and 1978 epidemics. DESIGN: Descriptive research. SETTING: Rehabilitation Centre 'De Hoogstraat', Utrecht, The Netherlands. METHODS: Two written questionnaires were sent out: one to the doctors attending the 71 registered patients five months after the end of the epidemic, the second 30 months after the end of the epidemic to the doctors of the patients with initial neurological impairments. Once every three months, muscular strength (MRC criteria), activities of daily life (ADL; using the Functional Independence Measure) and mobility (according to Hoffer) were assessed for 13 adult patients with moderately severe and severe disabilities. RESULTS: The questionnaire response was 100%. The median age increased from 5-9 years in 1971 to 10-14 years in 1978 to 20 years old in 1992/'93. Due to the 1992/'93 epidemic adults (> or = 20 years) had more moderately severe and severe sequelae than people below 20 years of age (p < 0.00I). In the 1978 epidemic these differences were not significant. Muscular strength improved until 30 months after the onset of the disease although no improvement was seen in 10/13 patients after 18 months. The ability to cope independently with the ADL improved until 18 months; ten patients became almost independent. Mobility improved until 12 months. Three patients remained wheelchair bound, 1 became an exercise walker, 5 household walkers and 4 community walkers. CONCLUSION: Poliomyelitis is no longer a paediatric disease in the Netherlands. In future epidemics it should be borne in mind that most disablement probably will occur among adults. After an attack of acute poliomyelitis, muscular strength can continue to improve for up to two and a half years, while ADL usually remain stable after one and a half years and mobility after one year
SN - 0028-2162
AD - Revalidatiecentrum 'De Hoogstraat', Utrecht
UR - 8692353
ER -

TY - JOUR
ID - 1625
T1 - Whose life is it anyway? A study in respect for autonomy
A1 - Norden,M.
Y1 - 1995/06//
N1 - Norden, M
Journal of medical ethics
j1d, 7513619
E, IM
Journal Article
English
KW - Beneficence
KW - Drama
KW - Ethical Analysis
KW - Ethicists

KW - Ethics
KW - Medical
KW - Humans
KW - Literature
KW - Modern
KW - Moral Obligations
KW - Paternalism
KW - Personal Autonomy
KW - Quadriplegia/px [Psychology]
KW - Resource Allocation
KW - Right to Die
KW - Value of Life
KW - Medline
KW - Unsupervised Play Addition
RP - NOT IN FILE
SP - 179
EP - 183
JF - Journal of Medical Ethics
JA - J Med Ethics
VL - 21
IS - 3
CY - ENGLAND
N2 - Brian Clark's drama, Whose Life Is It Anyway?, explores the difficulties of applying the principle of respect for autonomy to real-life circumstances. In the play a permanently disabled patient, who wishes to be allowed to die, raises moral questions about the adequacy of the autonomous agent, respect for the autonomy of others, the authority of the law, the allocation of society's resources, and the intrinsic value of human life. After a brief review of the story and definition of respect for autonomy, this paper cites passages from the play that dramatize the tension between respect for autonomy and these other moral concerns. There follows a review of relevant commentary from the classicists Kant and Mill and the modernists Childress and Gillon. The study concludes that although classical and contemporary philosophers have clarified and elaborated upon the relationship between ethical principles, they have not provided definitive guidelines
SN - 0306-6800
AD - Marymount University, Arlington, Virginia, USA
UR - 7674285
ER -

TY - JOUR
ID - 1626
T1 - Maternal sensitivity in the second year: gender-based relations in the dyadic balance of control
A1 - Biringen,Z.
A1 - Robinson,J.L.
A1 - Emde,R.N.
Y1 - 1994/01//
N1 - Biringen, Z. Robinson, J L. Emde, R N
The American journal of orthopsychiatry
3r6, 0400640
IM
Journal Article
English
KW - Female
KW - Gender Identity
KW - Humans
KW - Infant
KW - Internal-External Control
KW - Language Development
KW - Male

KW - Maternal Behavior
KW - Mother-Child Relations
KW - Personality Development
KW - Play and Playthings
KW - Medline
KW - Unsupervised Play Addition
RP - NOT IN FILE
SP - 78
EP - 90
JF - American Journal of Orthopsychiatry
JA - Am J Orthopsychiatry
VL - 64
IS - 1
CY - UNITED STATES
N2 - In an investigation of gender-based differences in autonomy and relatedness in the mother-toddler dyad, gender-specific stylistic differences were found in mothers' sensitivity toward their toddlers. In the context of greater sensitivity, boys appeared to control the flow of interactions (initiation and maintenance) to a greater extent than did girls
SN - 0002-9432
AD - Department of Psychology, University of Colorado, Boulder
UR - 8147430
ER -

TY - JOUR
ID - 1627
T1 - Integrated study of 100 patients with Xp21 linked muscular dystrophy using clinical, genetic, immunochemical, and histopathological data. Part 3. Differential diagnosis and prognosis
A1 - Nicholson,L.V.
A1 - Johnson,M.A.
A1 - Bushby,K.M.
A1 - Gardner-Medwin,D.
A1 - Curtis,A.
A1 - Ginjaar,I.B.
A1 - den Dunnen,J.T.
A1 - Welch,J.L.
A1 - Butler,T.J.
A1 - Bakker,E.
Y1 - 1993/09//
N1 - Nicholson, L V. Johnson, M A. Bushby, K M. Gardner-Medwin, D. Curtis, A. Ginjaar, I B. den Dunnen, J T. Welch, J L. Butler, T J. Bakker, E. et al
Journal of medical genetics
j1f, 2985087r
IM
Journal Article. Research Support, Non-U.S. Gov't
English
KW - Adolescent
KW - Adult
KW - Aged
KW - Blotting
KW - Western
KW - Child
KW - Preschool
KW - Cohort Studies
KW - Creatine Kinase/bl [Blood]
KW - Diagnosis
KW - Differential

KW - Dystrophin/bi [Biosynthesis]
KW - Dystrophin/ge [Genetics]
KW - Female
KW - Frameshift Mutation
KW - Gene Deletion
KW - Genetic Linkage
KW - Genotype
KW - Humans
KW - Immunohistochemistry
KW - Male
KW - Middle Aged
KW - Muscular Dystrophies/di [Diagnosis]
KW - Muscular Dystrophies/ge [Genetics]
KW - Muscular Dystrophies/me [Metabolism]
KW - Muscular Dystrophies/pa [Pathology]
KW - Phenotype
KW - Prognosis
KW - Severity of Illness Index
KW - X Chromosome
KW - Medline
KW - Unsupervised Play Addition
RP - NOT IN FILE
SP - 745
EP - 751
JF - Journal of Medical Genetics
JA - J Med Genet
VL - 30
IS - 9
CY - ENGLAND
N2 - This report is the third part of a trilogy from a multidisciplinary study which was undertaken to investigate gene and protein expression in a large cohort of patients with well defined and diverse clinical phenotypes. The aim of part 3 was to review which of the analytical techniques that we had used would be the most useful for differential diagnosis, and which would provide the most accurate indication of disease severity. Careful clinical appraisal is very important and every DMD patient was correctly diagnosed on this basis. In contrast, half of the sporadic BMD patients and all of the sporadic female patients had received different tentative diagnoses based on clinical assessments alone. Sequential observations of quantitative parameters (such as the time taken to run a fixed distance) were found to be useful clinical indicators for prognosis. Intellectual problems might modify the impression of physical ability in patients presenting at a young age. Histopathological assessment was accurate for DMD but differentiation between BMD and other disorders was more difficult, as was the identification of manifesting carriers. Our data on a small number of women with symptoms of muscle disease indicate that abnormal patterns of dystrophin labelling on sections may be an effective way of differentiating between female patients with a form of limb girdle dystrophy and those carrying a defective Xp21 gene. Dystrophin gene analysis detects deletions/duplications in 50 to 90% of male patients and is the most effective non-invasive technique for diagnosis. Quantitative Western blotting, however, would differentiate between all Xp21 and non-Xp21 male patients. In this study we found a clear relationship between increased dystrophin abundance (determined by densitometric analysis of blots) and clinical condition, with a correlation between dystrophin abundance and the age at loss of independent mobility among boys with DMD and intermediate D/BMD. This indicates that blotting is the most sensitive and accurate technique for diagnosis and prognosis
SN - 0022-2593
AD - Muscular Dystrophy Group Research Laboratories, Newcastle General Hospital, Newcastle upon Tyne, UK
UR - 8411069
ER -

TY - JOUR
ID - 1628

T1 - Functional significance of dystrophin positive fibres in Duchenne muscular dystrophy
A1 - Nicholson,L.V.
A1 - Johnson,M.A.
A1 - Bushby,K.M.
A1 - Gardner-Medwin,D.
Y1 - 1993/05//
N1 - Nicholson, L V. Johnson, M A. Bushby, K M. Gardner-Medwin, D
Archives of disease in childhood
6xg, 0372434
AIM, IM
Journal Article. Research Support, Non-U.S. Gov't
English
KW - Adolescent
KW - Age Factors
KW - Blotting
KW - Western
KW - Child
KW - Dystrophin/me [Metabolism]
KW - Humans
KW - Male
KW - Muscles/me [Metabolism]
KW - Muscles/pp [Physiopathology]
KW - Muscular Dystrophies/me [Metabolism]
KW - Muscular Dystrophies/pp [Physiopathology]
KW - Medline
KW - Unsupervised Play Addition
RP - NOT IN FILE
SP - 632
EP - 636
JF - Archives of Disease in Childhood
JA - Arch.Dis.Child
VL - 68
IS - 5
CY - ENGLAND
N2 - The age when boys lose the ability to walk independently is one of the milestones in the progression of Duchenne muscular dystrophy (DMD). We have used this as a measure of disease severity in a group of 30 patients with DMD and six patients with intermediate Duchenne/Becker dystrophy (D/BMD). Dystrophin analysis was performed on tissue sections and western blots of muscle biopsy specimens from these patients and the relationships that were found between clinical severity and abundance of dystrophin labelling are reported. All patients with intermediate D/BMD had dystrophin labelling that was detected on sections and blots. Weak dystrophin labelling was found in sections from 21/30 DMD cases and on blots in 18/30 cases. Two non-exclusive patterns of dystrophin labelling were observed on sections: very clear labelling on a small percentage of fibres (usually < 1%) or very weak labelling on a much higher proportion (about 25%). The mean age at loss of mobility among the DMD patients with no dystrophin labelling on tissue sections was 7.9 years (range 6.3-9.5) while the mean age among those with some labelling was 9.9 years (range 8.0-11.9); this is a significant difference. Quantitative estimates of dystrophin abundance were obtained from densitometric analysis of dystrophin bands on blots. In the whole group of 36 patients, a significant positive relationship was found between the abundance of dystrophin and the age at loss of independent mobility. It is concluded that even the very low concentrations of dystrophin found in DMD patients may have some functional significance
SN - 1468-2044
AD - Muscular Dystrophy Group Research Laboratories, Newcastle General Hospital
UR - 8323331
ER -

TY - JOUR
ID - 1629

T1 - The relation of mothers' controlling vocalizations to children's intrinsic motivation
A1 - Deci,E.L.
A1 - Driver,R.E.
A1 - Hotchkiss,L.
A1 - Robbins,R.J.
A1 - Wilson,I.M.
Y1 - 1993/04//
N1 - Deci, E L. Driver, R E. Hotchkiss, L. Robbins, R J. Wilson, I M
Journal of experimental child psychology
2985128r, i2n
IM
Journal Article. Research Support, U.S. Gov't, P.H.S.
English
KW - Adult
KW - Child
KW - Child Behavior
KW - Communication
KW - Female
KW - Humans
KW - Male
KW - Mother-Child Relations
KW - Mothers
KW - Motivation
KW - Parenting
KW - Play and Playthings
KW - Verbal Behavior
KW - Medline
KW - Unsupervised Play Addition
RP - NOT IN FILE
SP - 151
EP - 162
JF - Journal of Experimental Child Psychology
JA - J Exp.Child Psychol.
VL - 55
IS - 2
CY - UNITED STATES
N2 - Twenty-six mother-child dyads played together in a laboratory setting. Play sessions were surreptitiously videotaped (with mothers' permission), and each maternal vocalization was transcribed and coded, first into 1 of 24 categories and then ipso facto into one of three supercategories--namely, controlling, autonomy supportive, and neutral. The degree of mothers' controllingness was calculated as the percentage of vocalizations coded as controlling. This index was correlated with the intrinsic motivation of their 6- or 7-year-old children, as assessed primarily by the free-choice behavioral measure and secondarily by a child self-report measure of interest and liking for the task. Both correlations were significantly negative, thereby suggesting that the robust laboratory findings of a negative relation between controlling contexts and individuals' intrinsic motivation are directly generalizable to the domain of parenting. Results are discussed in terms of the processes that undermine intrinsic motivation and the means through which parental controllingness is communicated
SN - 0022-0965
AD - Department of Psychology, University of Rochester, NY 14627
UR - 8501424
ER -

TY - JOUR
ID - 1630
T1 - Teaching children with autism appropriate play in unsupervised environments using a self-management treatment package
A1 - Stahmer,A.C.

A1 - Schreibman,L.
Y1 - 1992///
N1 - Stahmer, A C. Schreibman, L
Journal of applied behavior analysis
0174763, hdr
IM
Journal Article. Research Support, U.S. Gov't, P.H.S.
English
KW - Autistic Disorder/px [Psychology]
KW - Autistic Disorder/th [Therapy]
KW - Behavior Therapy/mt [Methods]
KW - Child
KW - Female
KW - Follow-Up Studies
KW - Generalization (Psychology)
KW - Humans
KW - Internal-External Control
KW - Language Development Disorders/px [Psychology]
KW - Language Development Disorders/th [Therapy]
KW - Male
KW - Play and Playthings
KW - Self Stimulation
KW - Social Environment
KW - Stereotyped Behavior
KW - Medline
KW - Unsupervised Play Addition
RP - NOT IN FILE
SP - 447
EP - 459
JF - Journal of Applied Behavior Analysis
JA - J Appl.Behav Anal.
VL - 25
IS - 2
CY - UNITED STATES
N2 - The present study used a self-management treatment package to teach 3 children with autism, who exhibited inappropriate play behaviors, to play appropriately in the absence of a treatment provider. After self-management training, generalization and maintenance of the behavior change were assessed. Because of the detrimental effects of self-stimulation (arm flapping, spinning toys, twirling, etc.) on learning, the relationship between self-stimulatory behaviors and appropriate play was measured. Results indicated that the children learned to exhibit appropriate play skills in unsupervised settings, appropriate play skills generalized to new settings, and 2 of the children maintained their gains at 1-month follow-up. In addition, self-stimulatory behaviors decreased as appropriate play increased. Treatment implications of these findings are discussed
SN - 0021-8855
AD - University of California, San Diego 92093
UR - 1634432
ER -

TY - JOUR
ID - 1631
T1 - A regional survey of technical aids used by handicapped children in day care and at school
A1 - Korpela,R.A.
A1 - Koivikko,M.J.
Y1 - 1992/01//
N1 - Korpela, R A. Koivikko, M J
Disability and rehabilitation
9207179, a8i

IM
Journal Article
English
KW - Adolescent
KW - Child
KW - Child Day Care Centers
KW - Preschool
KW - Disabled Persons
KW - Female
KW - Finland
KW - Humans
KW - Infant
KW - Intellectual Disability/rh [Rehabilitation]
KW - Mainstreaming (Education)
KW - Male
KW - Orthopedic Equipment
KW - Rehabilitation/is [Instrumentation]
KW - Schools
KW - Self-Help Devices
KW - Medline
KW - Unsupervised Play Addition
RP - NOT IN FILE
SP - 16
EP - 22
JF - Disability & Rehabilitation
JA - Disabil Rehabil
VL - 14
IS - 1
CY - ENGLAND
N2 - Normalization, which underlines mainstreaming, is widely accepted in paediatric rehabilitation, but its success depends on the availability of special services, including proper technical aids for day care and school. This study aimed to evaluate the need for and use of technical aids in various forms of day care and education. Handicapped children used basic technical aids. In day care children needed more technical aids to promote independent mobility and playing, and in school they needed more computers and adaptations and software for these computers. The arrangement of technical aids for handicapped children requires good cooperation between the children's parents and various professionals
SN - 0963-8288
AD - Department of Paediatric Neurology, Tampere University Hospital, Finland
UR - 1534028
ER -

TY - JOUR
ID - 1632
T1 - Discipline. [Review] [17 refs]
A1 - Christophersen,E.R.
Y1 - 1992/06//
N1 - Christophersen, E R
Pediatric clinics of North America
oum, 0401126
AIM, IM
Journal Article. Research Support, U.S. Gov't, P.H.S.. Review
English
KW - Adaptation
KW - Psychological
KW - Adolescent
KW - Ambulatory Care/mt [Methods]

KW - Child
KW - Child Behavior
KW - Child Rearing
KW - Preschool
KW - Communication
KW - Humans
KW - Learning
KW - Parenting
KW - Parents/px [Psychology]
KW - Pediatrics/mt [Methods]
KW - Play and Playthings
KW - Social Values
KW - Teaching
KW - Medline
KW - Unsupervised Play Addition
RP - NOT IN FILE
SP - 395
EP - 411
JF - Pediatric Clinics of North America
JA - Pediatr Clin.North Am
VL - 39
IS - 3
CY - UNITED STATES
N2 - Discipline is one topic that virtually every ambulatory care pediatrician is frequently asked about. Although there is a tendency to provide parents with short, glib answers to questions about discipline, the literature on time-in, self-quieting skills, and independent play skills has rarely been addressed in the pediatric literature. It is, rather, covered in the research literature--a literature that is not exactly easily accessed by the pediatrician. [References: 17]
SN - 0031-3955
AD - Behavioral Pediatrics Division, Children's Mercy Hospital, Kansas City, Missouri
UR - 1574351
ER -

TY - JOUR
ID - 1633
T1 - Rural Youth Disability Prevention Project Survey: results from 169 Iowa farm families
A1 - Hawk,C.
A1 - Gay,J.
A1 - Donham,K.J.
Y1 - 1991///
N1 - Hawk, C. Gay, J. Donham, K J
The Journal of rural health : official journal of the American Rural Health Association and the National Rural Health Care Association
jx4, 8508122
H
Journal Article. Research Support, Non-U.S. Gov't
English
KW - Accident Prevention
KW - Adolescent
KW - Adolescent Health Services/sn [Statistics & Numerical Data]
KW - Agriculture/sn [Statistics & Numerical Data]
KW - Child
KW - Evaluation Studies as Topic
KW - Family Health
KW - Health Knowledge
KW - Attitudes

KW - Practice
KW - Health Services Needs and Demand/sn [Statistics & Numerical Data]
KW - Humans
KW - Iowa/ep [Epidemiology]
KW - Pilot Projects
KW - Preventive Health Services/og [Organization & Administration]
KW - Questionnaires
KW - Safety
KW - Medline
KW - Unsupervised Play Addition
RP - NOT IN FILE
SP - 170
EP - 179
JF - Journal of Rural Health
JA - J Rural Health
VL - 7
IS - 2
CY - UNITED STATES
N2 - Agriculture is now the most hazardous occupation in the United States and it is the only one in which children not only comprise a significant part of the work force, but also live and play at the work site. Annually, 23,500 pediatric agricultural injuries are reported, with nearly 300 fatalities (Rivara, 1985). The Rural Youth Disability Prevention Project was designed to use innovative, community-oriented methods to address the unique problems of child safety in agriculture. Toward this end, a survey instrument was designed to gather data both to assist in program development and to serve as a pretest for the subsequent evaluation. Analysis of these data indicated several issues to target for intervention efforts. One is lack of supervision--more than 40 percent of children who operate equipment do so unsupervised. Approximately 30 percent of children more than 3 years old play alone in work areas, and 80 percent of these children play near machinery in operation. Another issue is operation of farm machinery by very young children--respondents' children began operating equipment at an average age of 12 years. Coupling this with the finding that the parents believe their children are not capable of operating equipment until age 15 exemplifies the most important issue, the disparity between parents' levels of safety knowledge and safety behavior. Using the survey data to increase local involvement, efforts are being directed toward facilitating an ongoing, community-sponsored intervention program to empower farm families to effect their own solutions
SN - 0890-765X
AD - College of Medicine, University of Iowa, Iowa City 52240
UR - 10116776
ER -

TY - JOUR
ID - 1634
T1 - [Surgery of juvenile chronic arthritis]. [French]
A1 - Touzet,P.
A1 - Prieur,A.M.
A1 - Merckx,J.
Y1 - 1991///
N1 - Touzet, P. Prieur, A M. Merckx, J
Pediatrie
a4b, 0401127
IM
English Abstract. Journal Article
French
KW - Adolescent
KW - Anesthesia
KW - General
KW - Arthritis/co [Complications]
KW - Arthritis/rh [Rehabilitation]

KW - Arthritis/su [Surgery]
KW - Biopsy/ae [Adverse Effects]
KW - Humans
KW - Joint Diseases/et [Etiology]
KW - Joint Diseases/th [Therapy]
KW - Postoperative Period
KW - Preoperative Care
KW - Spinal Diseases/et [Etiology]
KW - Spinal Diseases/th [Therapy]
KW - Synovial Membrane/su [Surgery]
KW - Medline
KW - Unsupervised Play Addition
RP - NOT IN FILE
SP - 173
EP - 187
JF - Pediatrie
JA - Pediatrie (Bucur)
VL - 46
IS - 2
CY - FRANCE
N2 - A number of surgical procedures can be proposed in juvenile chronic arthritis (JCA). These are particularly useful in two circumstances: 1), during the early stages of the disease, as local treatment for hyperplasic synovium (intra-articular injection or synovectomy) in order to avoid further articular destruction; 2) in the late stages of the disease with the aim of restoring function to as near normal as possible (osteotomy, arthrodesis, arthroplasty, tendon repair). However, in all circumstances, such surgery must be decided upon by a team consisting of paediatrician and orthopaedic surgeon. Procedures are often multiple and require careful preparation including psychological support, nutritional, medical and orthopaedic care. And should be followed by intensive and specific physiotherapy. When these conditions are fulfilled, the operative procedures may restore nearly normal joint function in severe forms of JCA, thus allowing children and adolescents to maintain good physical, intellectual and psychological development through independent mobility
SN - 0031-4021
AD - Unite fonctionnelle de rhumatologie pediatrique, CHU Necker-Enfants-Malades, Paris, France
UR - 1646434
ER -

TY - JOUR
ID - 1635
T1 - Fire hydrant play: injuries and their prevention
A1 - Joffe,M.
A1 - Torrey,S.B.
A1 - Baker,M.D.
Y1 - 1991/06//
N1 - Joffe, M. Torrey, S B. Baker, M D
Pediatrics
oxv, 0376422
AIM, IM
Journal Article
English
KW - Adolescent
KW - African Americans
KW - Child
KW - Preschool
KW - Emergencies
KW - Epidemiologic Methods
KW - Female
KW - Hispanic Americans

KW - Hot Temperature
KW - Humans
KW - Injury Severity Score
KW - Male
KW - Philadelphia
KW - Play and Playthings
KW - Poverty
KW - Seasons
KW - Urban Population
KW - Wounds and Injuries/ep [Epidemiology]
KW - Wounds and Injuries/eh [Ethnology]
KW - Wounds and Injuries/et [Etiology]
KW - Wounds and Injuries/pc [Prevention & Control]
KW - Medline
KW - Unsupervised Play Addition
RP - NOT IN FILE
SP - 900
EP - 903
JA - Pediatrics
VL - 87
IS - 6
CY - UNITED STATES
N2 - A total of 86 children treated for injuries that occurred while playing in water from fire hydrants are described. Patients were urban (100%), minority (97%) children with few alternative means for keeping cool. Injuries occurred on extremely hot summer days (mean maximum temperature 36.3 degrees C [97.5 degrees F]). Laceration of the foot on broken glass was the most common injury and was prevented by wearing footwear (P less than .001). Motor vehicles caused all serious injuries. Sprinkler attachments on the hydrants were associated with significantly fewer motor vehicle-related injuries (P less than .001) and water pressure-related injuries (P = .02). Adults were present at more than 90% of injury scenes, but had no effect on the safety of fire hydrant play. Public policy should be directed toward increasing the availability of alternative means for keeping cool, increasing the number of hydrants equipped with sprinklers, and reducing the amount of broken glass in the streets. Public education targeting adults to remove glass from the street, insist that children wear footwear, and open only those hydrants that have sprinklers could further reduce injuries to urban children who play in water from fire hydrants
SN - 0031-4005
AD - Sections of Emergency Medicine, St Christopher's Hospital for Children, Temple University School of Medicine, Philadelphia, PA 19134-1095
UR - 2034497
ER -

TY - JOUR
ID - 1636
T1 - Causes of fatal childhood accidents involving head injury in northern region, 1979-86
A1 - Sharples,P.M.
A1 - Storey,A.
A1 - ynsley-Green,A.
A1 - Eyre,J.A.
Y1 - 1990/11/24/
N1 - Sharples, P M. Storey, A. Aynsley-Green, A. Eyre, J A
BMJ (Clinical research ed.)
8900488, bmj, 101090866
AIM, IM
Journal Article. Research Support, Non-U.S. Gov't
English
KW - Accidental Falls
KW - Accidents

KW - Traffic
KW - Adolescent
KW - Child
KW - Child Behavior
KW - Preschool
KW - Craniocerebral Trauma/et [Etiology]
KW - Craniocerebral Trauma/mo [Mortality]
KW - England/ep [Epidemiology]
KW - Humans
KW - Infant
KW - Play and Playthings
KW - Poverty Areas
KW - Retrospective Studies
KW - Trauma Severity Indices
KW - Medline
KW - Unsupervised Play Addition
RP - NOT IN FILE
SP - 1193
EP - 1197
JF - BMJ
VL - 301
IS - 6762
CY - ENGLAND
N2 - OBJECTIVE: To examine the causes and circumstances surrounding fatal accidents involving head injuries in children in the Northern region. DESIGN: Retrospective review of the hospital case notes, necropsy reports, and records of the coroners' inquests. SETTING: Northern Regional Health Authority. PATIENTS: All 255 children aged less than 16 years who died with a head injury during 1979-86. MAIN OUTCOME MEASURES: Cause of injury and circumstances of accident according to reports of inquests; injury severity score; number of fatal accidents and mortality per 100,000 children in 10 groups of local authority wards ranked according to their score on the overall deprivation index; and distance of site of accident from child's home. RESULTS: Of the 255 children who died after a head injury, 136 (53%) children were playing at the time of the accident. 195 (76%) children sustained the head injury in road traffic accidents, 135 as pedestrians, 35 as cyclists, and 25 as passengers in a vehicle. In 120 accidents in child pedestrians the primary cause of accident was the unsafe behaviour of the child. 172 (67%) accidents occurred within one to two km of the child's home and 153 (63%) between 3 pm and 9 pm. The mortality was significantly related to social deprivation; excluding eight children injured while on holiday in the region, 15-fold decrease in mortality was recorded between the local authority wards that ranked highest on the overall deprivation index and those that ranked lowest (14.0/100,000 children, group 10 v 0.9/100,000, group 1 respectively, p less than 0.00001). CONCLUSIONS: The finding that most accidents occurred in children living in deprived areas who were playing unsupervised near their home suggests that childhood mortality might be appreciably reduced if children at play were protected from traffic, particularly in socially deprived areas
SN - 0959-8138
AD - Department of Child Health, University of Newcastle upon Tyne
UR - 2261557
ER -

TY - JOUR
ID - 1637
T1 - From acting out to interactive play
A1 - Willock,B.
Y1 - 1990///
N1 - Willock, B
The International journal of psycho-analysis
gsl, 2985179r
IM

Journal Article
English
KW - Acting Out
KW - Aggression/px [Psychology]
KW - Antisocial Personality Disorder/px [Psychology]
KW - Child
KW - Child Behavior Disorders/px [Psychology]
KW - Ego
KW - Humans
KW - Object Attachment
KW - Personality Development
KW - Play Therapy/mt [Methods]
KW - Psychoanalytic Therapy/mt [Methods]
KW - Medline
KW - Unsupervised Play Addition
RP - NOT IN FILE
SP - 321
EP - 334
JF - International Journal of Psycho-Analysis
JA - Int.J Psychoanal.
VL - 71
IS - Pt 2
CY - ENGLAND
N2 - Many of the most problematic behaviours characteristic of severely disturbed, aggressive, conduct-disordered children can be understood in terms of developmental arrests and distortions which have occurred on a crucial, early developmental line related to the mastery of separation anxiety and the integration of autonomy. A clinical investigation of psychoanalytic treatment of hyperaggressive children demonstrates the utility of this conceptualization for understanding the nature of their profound ego defects as psychologically treatable, developmental distortions in the realm of object relations, self-structure and ego development. This mode of understanding enables one to specify the point in development where ego distortion occurred, comprehend the nature of the psychopathology, plan effective treatment and evaluate therapeutic progress
SN - 0020-7578
AD - C.M. Hincks Treatment Centre, Toronto, Ontario, Canada
UR - 2365551
ER -

TY - JOUR
ID - 1638
T1 - Disabled access to information technology--a portable adaptable, multipurpose device
A1 - Thornett,C.E.
A1 - Langner,M.C.
A1 - Brown,A.W.
Y1 - 1990/05//
N1 - Thornett, C E. Langner, M C. Brown, A W
Journal of biomedical engineering
k6k, 7906074
IM
Journal Article
English
KW - Activities of Daily Living
KW - Adolescent
KW - Child
KW - Communication Aids for Disabled
KW - Equipment Design
KW - Female
KW - Humans

KW - Male
KW - Microcomputers
KW - Quality of Life
KW - Self-Help Devices
KW - Software
KW - User-Computer Interface
KW - Medline
KW - Unsupervised Play Addition
RP - NOT IN FILE
SP - 205
EP - 208
JF - Journal of Biomedical Engineering
JA - J Biomed Eng
VL - 12
IS - 3
CY - ENGLAND
N2 - The technology exists to allow severely physically disabled people to have a far more independent lifestyle than many handicapped people enjoy today. To use an ever increasing range of sophisticated domestic equipment, without special modifications, an individually tailored control system and a universal accessing device are required. A portable multipurpose device allows multiply handicapped, non-speaking people a means of operating a variety of general-purpose or specialized pieces of electronic equipment at home and elsewhere, safely and independently. The device also provides a means of communication through the medium of computer-computer communication over telephone lines and will interface to a powered chair for independent mobility. Various features are provided which are not available from conventional environmental control systems. Through a specialized adjustable multipurpose switch system and a universal control device the handicapped person should be able to enjoy the independence afforded by the intelligent home of the future
SN - 0141-5425
AD - Chailey Heritage Hospital, Lewes, Sussex, UK
UR - 2140870
ER -

TY - JOUR
ID - 1639
T1 - Cognitive play of mentally retarded preschoolers: observations in the home and school
A1 - Malone,D.M.
A1 - Stoneman,Z.
Y1 - 1990/03//
N1 - Malone, D M. Stoneman, Z
American journal of mental retardation : AJMR
ame, 8800463
IM
Journal Article. Research Support, U.S. Gov't, Non-P.H.S.
English
KW - Cerebral Palsy/px [Psychology]
KW - Child
KW - Preschool
KW - Cognition Disorders/px [Psychology]
KW - Developmental Disabilities/px [Psychology]
KW - Down Syndrome/px [Psychology]
KW - Education of Intellectually Disabled
KW - Fragile X Syndrome/px [Psychology]
KW - Humans
KW - Intellectual Disability/px [Psychology]
KW - Play and Playthings
KW - Social Adjustment
KW - Social Environment

KW - Medline
KW - Unsupervised Play Addition
RP - NOT IN FILE
SP - 475
EP - 487
JF - American Journal of Mental Retardation
JA - Am J Ment.Retard.
VL - 94
IS - 5
CY - UNITED STATES
N2 - A within-subjects design was used to examine the cognitive play of 12 mainstreamed mentally retarded preschool boys during group free-play at school and in an independent play situation at home. Children played with available toys at school and with three designated toy sets at home. Cognitive and communicative abilities were established through administration of the Battelle Developmental Inventory. Children not only played more in the home condition, but this play was more sophisticated than that in the school condition. Increased differences between home and school performance were positively associated with expressive communicative developmental age. Peak play did not differ between the two settings. Children not only sequenced play activities more at home, but these sequences were longer and more complex than those at school
SN - 0895-8017
AD - Georgia University Affiliated Program, University of Georgia, Athens 30602
UR - 1690555
ER -

TY - JOUR
ID - 1640
T1 - Interaction of normal children with anatomical dolls
A1 - Sivan,A.B.
A1 - Schor,D.P.
A1 - Koeppl,G.K.
A1 - Noble,L.D.
Y1 - 1988///
N1 - Sivan, A B. Schor, D P. Koeppl, G K. Noble, L D
Child abuse & neglect
can, 7801702
IM
Journal Article. Research Support, Non-U.S. Gov't
English
KW - Aggression/px [Psychology]
KW - Attention
KW - Child
KW - Child Abuse
KW - Sexual/px [Psychology]
KW - Preschool
KW - Female
KW - Gender Identity
KW - Humans
KW - Male
KW - Play and Playthings
KW - Sexual Behavior
KW - Social Environment
KW - Medline
KW - Unsupervised Play Addition
RP - NOT IN FILE
SP - 295
EP - 304
JF - Child Abuse & Neglect

JA - Child Abuse Negl.
VL - 12
IS - 3
CY - ENGLAND
N2 - Anatomical (sexually explicit) rag dolls are frequently used in interviews of children suspected of being sexually abused. Abused children have been noted clinically to be aggressive and sometimes sexually precocious in their doll play, and testimony to that effect is often accepted in the courtroom as pertinent evidence. However, to date, normative data on the play behaviors with the sexually explicit dolls have been unavailable. This pilot study provides empirical information on the play interaction of a relatively large number of normal (nonreferred) children with anatomical dolls. One hundred forty-four children (ages 3 to 8 years) were observed in a playroom containing these special dolls during three conditions: (1) with an adult present, (2) without an adult, and (3) with the dolls undressed. The observations showed that nonreferred children found these dolls no more interesting than other toys. Little aggression and no explicit sexual activity were observed. In contrast to clinical observation of abused children, the doll play of nonreferred children is unlikely to be characterized by aggression or sexual concerns; thus these behaviors when observed in interaction with these dolls should be taken seriously
SN - 0145-2134
AD - Department of Pediatrics, University of Iowa, Iowa City
UR - 3167619
ER -

TY - JOUR
ID - 1641
T1 - Powered mobility vehicles as aids in independent locomotion for young children. Suggestion from the field
A1 - Chiulli,C.
A1 - Corradi-Scalise,D.
A1 - Donatelli-Schultheiss,L.
Y1 - 1988/06//
N1 - Chiulli, C. Corradi-Scalise, D. Donatelli-Schultheiss, L
Physical therapy
0022623, p6w
AIM, IM
Case Reports. Journal Article
English
KW - Child
KW - Preschool
KW - Disabled Persons
KW - Female
KW - Humans
KW - Locomotion
KW - Male
KW - Motor Skills
KW - Quadriplegia/rh [Rehabilitation]
KW - Scoliosis/rh [Rehabilitation]
KW - Self-Help Devices
KW - Medline
KW - Unsupervised Play Addition
RP - NOT IN FILE
SP - 997
EP - 999
JF - Physical Therapy
JA - Phys Ther
VL - 68
IS - 6
CY - UNITED STATES

N2 - Both vehicles are good training devices for future mobility. The independent mobility provided by this equipment gave each child a boost of self-esteem and independence
SN - 0031-9023
AD - Department of Physical Therapy, United Cerebral Palsy Association of Nassau County, Inc., Rossevelt, NY 11575
UR - 2967503
ER -

TY - JOUR
ID - 1642
T1 - Play therapy in health care settings: promises never kept?. [Review] [27 refs]
A1 - Phillips,R.D.
Y1 - 1988///
N1 - Phillips, R D
Children's health care : journal of the Association for the Care of Children's Health
czx, 8101257
H
Journal Article. Review
English
KW - Anxiety
KW - Child
KW - Child Care
KW - Hospitalized/px [Psychology]
KW - Preschool
KW - Communication
KW - Evaluation Studies as Topic
KW - Humans
KW - Internal-External Control
KW - Play Therapy
KW - Medline
KW - Unsupervised Play Addition
RP - NOT IN FILE
SP - 182
EP - 187
JF - Children's Health Care
JA - Child Health Care
VL - 16
IS - 3
CY - UNITED STATES
N2 - This article examines the variety of claims made about the effectiveness of play therapy in health care settings. The claims are organized into four categories of "promises": (a) mastery, autonomy, and control; (b) cooperation and communication; (c) coping with anxiety and fear; and (d) learning and information giving. The empirical questions within each promise are identified and relevant experimental research is discussed. The conclusion is that play therapy has not yet delivered on its promises in health care settings. [References: 27]
SN - 0273-9615
UR - 10302208
ER -

TY - JOUR
ID - 1643
T1 - Effects of powered mobility on self-initiated behaviors of very young children with locomotor disability
A1 - Butler,C.
Y1 - 1986/06//
N1 - Butler, C
Developmental medicine and child neurology
0006761, e83

IM
Case Reports. Journal Article. Research Support, Non-U.S. Gov't. Research Support, U.S. Gov't, Non-P.H.S.. Research Support, U.S. Gov't, P.H.S.
English
KW - Child
KW - Preschool
KW - Communication
KW - Disabled Persons
KW - Exploratory Behavior
KW - Female
KW - Humans
KW - Infant
KW - Locomotion
KW - Male
KW - Wheelchairs
KW - Medline
KW - Unsupervised Play Addition
RP - NOT IN FILE
SP - 325
EP - 332
JF - Developmental Medicine & Child Neurology
JA - Dev.Med Child Neurol.
VL - 28
IS - 3
CY - ENGLAND
N2 - This study reports the effects of powered mobility on the self-initiated behavior of six children with various disabilities who, between 23 and 38 months of age, learnt to use motorized wheelchairs in less than three weeks. Using a multiple baseline design, two-hour observation periods were video-recorded at 10-day intervals before and after they achieved independent mobility. Frequency of self-initiated interaction with objects, spatial exploration and communication with care-giver were analyzed. Three children increased all three types of behavior; one increased in two types but decreased in interaction with objects; and two increased in spatial exploration only
SN - 0012-1622
UR - 2941328
ER -

TY - JOUR
ID - 1644
T1 - Correlates of mastery-related behavior: a short-term longitudinal study of infants in their second year
A1 - Frodi,A.
A1 - Bridges,L.
A1 - Grolnick,W.
Y1 - 1985/10//
N1 - Frodi, A. Bridges, L. Grolnick, W
Child development
0372725, d28
IM
Journal Article
English
KW - Affect
KW - Age Factors
KW - Child Behavior
KW - Child Rearing
KW - Female
KW - Humans
KW - Infant

KW - Longitudinal Studies
KW - Male
KW - Mother-Child Relations
KW - Motivation
KW - Object Attachment
KW - Play and Playthings
KW - Medline
KW - Unsupervised Play Addition
RP - NOT IN FILE
SP - 1291
EP - 1298
JF - Child Development
JA - Child Dev.
VL - 56
IS - 5
CY - UNITED STATES
N2 - The present study examined correlates of mastery-related behavior across the infant's second year of life. Maternal control style was quantified on a control to support-of-autonomy continuum, infant-mother attachment was assessed in the Strange Situation, and mastery-related behavior was observed in a toy play session at 12 and 20 months. Infants whose mothers were supportive of their autonomy displayed greater task-oriented persistence and competence during play than did infants of more controlling mothers; securely attached and avoidant infants tended to exhibit greater persistence at tasks than anxious-ambivalent babies, and ambivalent babies were the most negative in affect
SN - 0009-3920
UR - 4053744
ER -

TY - JOUR
ID - 1645
T1 - Concerns of acutely ill, chronically ill, and healthy preschool children
A1 - Ritchie,J.A.
A1 - Caty,S.
A1 - Ellerton,M.L.
Y1 - 1984/12//
N1 - Ritchie, J A. Caty, S. Ellerton, M L
Research in nursing & health
7806136, raq
IM, N
Comparative Study. Journal Article. Research Support, Non-U.S. Gov't
English
KW - Acute Disease/px [Psychology]
KW - Child Behavior
KW - Child Psychology
KW - Child
KW - Hospitalized/px [Psychology]
KW - Preschool
KW - Chronic Disease/px [Psychology]
KW - Female
KW - Humans
KW - Interviews as Topic
KW - Male
KW - Play and Playthings
KW - Sampling Studies
KW - Medline
KW - Unsupervised Play Addition
RP - NOT IN FILE

SP - 265
EP - 274
JF - Research in Nursing & Health
JA - Res.Nurs Health
VL - 7
IS - 4
CY - UNITED STATES
N2 - The study was designed to determine if the concerns of 2- to 5-year-old children hospitalized for chronic illness differ from the concerns of other children. Interviews were conducted with 32 short-stay chronically ill, 10 long-stay chronically ill, 20 acutely ill, and 20 healthy children. Narrative recordings were analyzed using a categorical system of 10 developmental and hospital-related concerns. Differences between groups in mean proportion of concerns expressed were examined for each interview using analysis of variance techniques. The major concerns expressed by each group on all interviews were Autonomy and Exploration and, in the chronically ill children, Intrusion. Increased age was associated with increased expression of Intrusion in all groups except the long-stay chronic illness group
SN - 0160-6891
UR - 6570056
ER -

TY - JOUR
ID - 1646
T1 - [Sample of high school students with a demonstration of intervening factors in school achievement and failure]. [French]
A1 - Piquilloud,C.
Y1 - 1984///
N1 - Piquilloud, C
Schweizer Archiv fur Neurologie, Neurochirurgie und Psychiatrie = Archives suisses de neurologie, neurochirurgie et de psychiatrie
uds, 8709012
IM
English Abstract. Journal Article
French
KW - Achievement
KW - Adaptation
KW - Psychological
KW - Adolescent
KW - Defense Mechanisms
KW - Humans
KW - Intelligence
KW - Learning Disorders/px [Psychology]
KW - Personality Development
KW - Underachievement
KW - Medline
KW - Unsupervised Play Addition
RP - NOT IN FILE
SP - 251
EP - 262
JF - Schweizer Archiv fur Neurologie, Neurochirurgie und Psychiatrie
JA - Schweiz.Arch Neurol Neurochir Psychiatr
VL - 134
IS - 2
CY - SWITZERLAND
N2 - This research investigates the relationship and link between intellectual cathexis, school achievement and personality functioning in a representative sample of secondary school students aged 12 to 15 years. The level of structure of the personality plays a most significant role. Good school achievement does not necessarily depend on an optimal psychological equilibrium. In fact, the most determining factor seems to be the part played by the

defense mechanisms--particularly those which aim at controlling libidinal drives--developed and elaborated during the period of latency. A certain intellectual potential, however, is a necessary but not sufficient condition for school achievement - indeed, an end of school certificate i.e. the Swiss "certificat de maturite" could be obtained by students of average intelligence. The capacity of maintaining an active intellectual investment seems to be an equally determining factor. This quality of functioning contains an oedipal component - particularly the ability of taking one's stand and of competing - as well as a pre-oedipal one - the narcissistic integrity, allowing one to preserve one's self-esteem facing difficulties. Contrary to our hypothesis, the quality of subject-environment interaction with family, peers, teachers, plays a relatively minor role. A more important aspect concerns the effects and results of relations established during childhood such as the interiorization of parental expectations, the quality of the superego, the degree of internal autonomy allowing compromise with external demands, etc. Underlying conflicts can intervene at several levels. One of these levels could result in the subject's incapacity to utilize his own resources and these imply consequences ranging from difficulties in basic school achievement to non-acquisition of cognitive mecanisms and under-development of symbolization. Psychological testing is a means of identifying the underlying difficulties and determining their nature. Remediating methods include psycho-pedagogical or psychotherapeutic intervention or, in some cases, both
SN - 0036-7273
UR - 6463609
ER -

TY - JOUR
ID - 1647
T1 - A specialized walking frame for children with cerebral palsy
A1 - Myles,J.W.
Y1 - 1983/11//
N1 - Myles, J W
Journal of pediatric orthopedics
hsw, 8109053
IM
Journal Article
English
KW - Cerebral Palsy/rh [Rehabilitation]
KW - Child
KW - Humans
KW - Orthopedic Equipment
KW - Walkers
KW - Medline
KW - Unsupervised Play Addition
RP - NOT IN FILE
SP - 620
EP - 621
JF - Journal of Pediatric Orthopedics
JA - J Pediatr Orthop
VL - 3
IS - 5
CY - UNITED STATES
N2 - To help severely physically handicapped children with cerebral palsy gain some independent mobility, a special walking frame of great stability has been evolved. It incorporates a variable-resistance roller and automatic reversing brake, and can be folded
SN - 0271-6798
UR - 6655062
ER -

TY - JOUR
ID - 1648
T1 - The effect of two body positions on head righting in severely disabled individuals with cerebral palsy
A1 - O'Brien,M.

A1 - Tsurumi,K.
Y1 - 1983/10//
N1 - O'Brien, M. Tsurumi, K
The American journal of occupational therapy : official publication of the American Occupational Therapy Association
7705978, 3o4
IM
Clinical Trial. Controlled Clinical Trial. Journal Article
English
KW - Adolescent
KW - Adult
KW - Cerebral Palsy/pp [Physiopathology]
KW - Child
KW - Female
KW - Head
KW - Humans
KW - Male
KW - Movement
KW - Posture
KW - Probability
KW - Regression Analysis
KW - Wheelchairs
KW - Medline
KW - Unsupervised Play Addition
RP - NOT IN FILE
SP - 673
EP - 680
JA - American Journal of Occupational Therapy
VL - 37
IS - 10
CY - UNITED STATES
N2 - This study examines the association between head righting and two commonly used therapeutic positions: semiprone on an inclined prone positioner and upright short-sitting in an adapted wheelchair. The subjects were 26 individuals with cerebral palsy and multiple handicaps who had difficulty maintaining active head control in a vertical orientation. Such head control is necessary for attending to learning tasks in the classroom and activities of daily living in the clinic. The frequency and duration of head righting during a feeding task was measured by means of a mercury switch system mounted on a headband and attached to an electromechanical digit counter and a clock. Measurements of duration and frequency beyond the predetermined range of functional head righting plus measurements on five control variables were analyzed by four statistical methods: t-test on the means, simple correlation, cannonical correlation, and regression analysis. The control variables were age, mobility level, degree of hypertonicity, level of seizure activity, and level of reflex maturity. Results indicate that, in this severely handicapped population, head righting while in the adapted wheelchair is no different from head righting while in the semiprone position. One control variable, level of independent mobility, was positively correlated with the length of time that subjects were able to maintain head righting in the semiprone position
SN - 0272-9490
UR - 6638143
ER -

TY - JOUR
ID - 1649
T1 - Powered mobility for very young disabled children
A1 - Butler,C.
A1 - Okamoto,G.A.
A1 - McKay,T.M.
Y1 - 1983/08//

N1 - Butler, C. Okamoto, G A. McKay, T M
Developmental medicine and child neurology
0006761, e83
IM
Journal Article. Research Support, Non-U.S. Gov't. Research Support, U.S. Gov't, Non-P.H.S.. Research Support, U.S. Gov't, P.H.S.
English
KW - Child
KW - Preschool
KW - Disabled Persons
KW - Female
KW - Humans
KW - Infant
KW - Male
KW - Wheelchairs
KW - Medline
KW - Unsupervised Play Addition
RP - NOT IN FILE
SP - 472
EP - 474
JF - Developmental Medicine & Child Neurology
JA - Dev.Med Child Neurol.
VL - 25
IS - 4
CY - ENGLAND
N2 - Nine motor-disabled children with normal intelligence and varying degrees of disability were studied to see if competent control of a motorized wheelchair is attainable between 20 and 39 months of age. Each child used a conventional motorized wheelchair with adaptive seating. Parents supervised the learning at home. Eight children drove safely and independently within 1.7 to 12 hours accumulated over a one to seven week period. Parents reported that this independent mobility stimulated their child's social, emotional and intellectual behavior
SN - 0012-1622
UR - 6225687
ER -

TY - JOUR
ID - 1650
T1 - BB and pellet guns--toys or deadly weapons?
A1 - Harris,W.
A1 - Luterman,A.
A1 - Curreri,P.W.
Y1 - 1983/07//
N1 - Harris, W. Luterman, A. Curreri, P W
The Journal of trauma
kaf, 0376373
AIM, IM
Case Reports. Journal Article
English
KW - Abdominal Injuries/et [Etiology]
KW - Adolescent
KW - Child
KW - Preschool
KW - Humans
KW - Intestinal Perforation/et [Etiology]
KW - Liver/in [Injuries]
KW - Male

KW - Pancreas/in [Injuries]
KW - Play and Playthings
KW - Stomach/in [Injuries]
KW - Wounds
KW - Gunshot/co [Complications]
KW - Medline
KW - Unsupervised Play Addition
RP - NOT IN FILE
SP - 566
EP - 569
JF - Journal of Trauma-Injury Infection & Critical Care
JA - J Trauma
VL - 23
IS - 7
CY - UNITED STATES
N2 - BB and pellet weapons are not included in gun control laws and are often sold as children's toys. Injuries caused by these weapons have been considered trivial unless they involve vulnerable surface organs such as the eye. The purpose of this study was to review the management of six cases of pellet or BB gun injuries that required abdominal exploration at the University of South Alabama Medical Center from January 1980 through June 1982. Five of the six patients had significant internal injuries including perforations of the stomach, jejunum, liver, and pancreas. The ballistics of pneumatic weapons are reviewed. The muzzle velocities of many of these weapons necessitate that wounds caused by these weapons be handled with the same principles as for any small-caliber, low-velocity (less than 1,200 feet/second) weapons. Public education programs are urgently needed to educate parents as to the potential danger involved in purchasing these weapons for unsupervised use as toys by children
SN - 0022-5282
UR - 6876209
ER -

TY - JOUR
ID - 1651
T1 - Gaze and mutual gaze of preschool children in conversation
A1 - Krantz,M.
A1 - George,S.W.
A1 - Hursh,K.
Y1 - 1983/01//
N1 - Krantz, M. George, S W. Hursh, K
The Journal of psychology
jtz, 0376332
IM
Journal Article
English
KW - Attention
KW - Child Behavior
KW - Child
KW - Preschool
KW - Communication
KW - Female
KW - Fixation
KW - Ocular
KW - Humans
KW - Interpersonal Relations
KW - Male
KW - Play and Playthings
KW - Social Behavior
KW - Speech

KW - Medline
KW - Unsupervised Play Addition
RP - NOT IN FILE
SP - 9
EP - 15
JF - Journal of Psychology
JA - J Psychol
VL - 113
IS - 1st Half
CY - UNITED STATES
N2 - The study was designed to describe the functional significance of gaze and mutual gaze in the context of the free-play conversation of preschool children. It was hypothesized that mutual gaze is not a fortuitous event in young children but a result of a gaze-action-gaze-reaction sequential process. Eleven high- and 11 low-friendship dyads were formed on the basis of the frequency of interaction in freeplay. Each dyad was videotaped for 15-minute play sessions. The videotapes were analyzed for the occurrence of gaze-related utterances and the occurrence of mutual gaze. When the speaker gazed at the listener the contingent probability of gaze response in the listener was 45% (as compared to a base rate of 3% gaze-related utterances). When the listener gazed at the speaker, the probability of gaze response was 38%. Gaze-related utterances were longer, more likely to result in a relevant response from the listener and less likely to result in nonresponse than utterances unaccompanied by gaze. Friendship between dyad members had no effect on any of the measures. It was concluded that gaze and mutual gaze are systematic components of the conversational behavior of preschool children
SN - 0022-3980
UR - 6834291
ER -

TY - JOUR
ID - 1652
T1 - Beyond informed consent: ethical and philosophical considerations in using behavior modification or play therapy in the treatment of enuresis
A1 - Noll,R.B.
A1 - Seagull,A.A.
Y1 - 1982///
N1 - Noll, Robert B. Seagull, Arthur A
Journal of clinical child psychology
co7, 7603095
E
Journal Article
English
KW - Behavior
KW - Behavior Control
KW - Child
KW - Conditioning
KW - Operant
KW - Freedom
KW - Humans
KW - Informed Consent
KW - Mental Disorders
KW - Patient Participation
KW - Personal Autonomy
KW - Psychotherapy
KW - Medline
KW - Unsupervised Play Addition
RP - NOT IN FILE
SP - 44
EP - 49
JF - Journal of Clinical Child Psychology

JA - J Clin.Child Psychol.
VL - 11
IS - 1
CY - United States
SN - 0047-228X
UR - 11649460
ER -

TY - JOUR
ID - 1653
T1 - Behavioral assessment of peer interaction and social functioning in institutional and structured settings
A1 - Michelson,L.
A1 - Dilorenzo,T.M.
Y1 - 1981/07//
N1 - Michelson, L. Dilorenzo, T M
Journal of clinical psychology
htj, 0217132
IM
Journal Article
English
KW - Adolescent
KW - Affective Symptoms/px [Psychology]
KW - Child
KW - Child Behavior Disorders/px [Psychology]
KW - Institutionalized/px [Psychology]
KW - Female
KW - Humans
KW - Interpersonal Relations
KW - Learning Disorders/px [Psychology]
KW - Male
KW - Peer Group
KW - Play and Playthings
KW - Professional-Patient Relations
KW - Social Adjustment
KW - Medline
KW - Unsupervised Play Addition
RP - NOT IN FILE
SP - 499
EP - 504
JF - Journal of Clinical Psychology
JA - J Clin Psychol
VL - 37
IS - 3
CY - UNITED STATES
N2 - Observed 18 children in a short-term psychiatric facility over a 5-month period using a peer interaction coding system. The direct behavioral observation method was adapted and revised from existing scales to increase reliability and efficiency for use by institutional staff. Response codes included Adaptive Peer Interaction, Maladaptive Peer Interaction, Solitary Independent Play, and Response to Staff. Reliability was assessed on 35% of the total coded intervals, which yielded an overall reliability coefficient of 94.3%. In addition, reliability coefficients were calculated for each individual component behavior, an extremely important but often ignored statistic, and all yielded reliabilities greater than 90%. Normative rates for children on the psychiatric unit also are presented, and discussion is provided concerning the application of this coding system in clinics, schools, and institutional settings
SN - 0021-9762
UR - 7263875
ER -

TY - JOUR
ID - 1654
T1 - Injury and its correlates among 1-year-old children. Study of children with both normal and low birth weights
A1 - McCormick,M.C.
A1 - Shapiro,S.
A1 - Starfield,B.H.
Y1 - 1981/02//
N1 - McCormick, M C. Shapiro, S. Starfield, B H
American journal of diseases of children (1960)
3gs, 0370471
AIM, IM
Journal Article. Research Support, Non-U.S. Gov't
English
KW - Birth Weight
KW - Demography
KW - Humans
KW - Infant
KW - Low Birth Weight
KW - Newborn
KW - Locomotion
KW - Random Allocation
KW - Risk
KW - Sampling Studies
KW - Socioeconomic Factors
KW - United States
KW - Wounds and Injuries/ep [Epidemiology]
KW - Medline
KW - Unsupervised Play Addition
RP - NOT IN FILE
SP - 159
EP - 163
JF - American Journal of Diseases of Children
JA - Am J Dis Child
VL - 135
IS - 2
CY - UNITED STATES
N2 - Factors associated with injury in the first year of life were examined using data on a random sample of infants in eight regions in the United States. By 1 year of age, 8.6% of the 4,989 infants surveyed had had an injury for which medical care was sought, although serious sequelae (eg, death and developmental delay) were infrequent. The data suggest that the achievement of independent mobility, eg, walking, was a major factor in the risk of injury. Other risk factors included very young mothers and isolated mothers, but not socioeconomic status, illness in the infant, or low birth weight. However, identification of small groups at very high risk is not possible, so that health professionals should be prepared to counsel all new parents early about injuries
SN - 0002-922X
UR - 7193407
ER -

TY - JOUR
ID - 1655
T1 - Development of spontaneous manding in language deficient children
A1 - Simic,J.
A1 - Bucher,B.
Y1 - 1980///

N1 - Simic, J. Bucher, B
Journal of applied behavior analysis
0174763, hdr
IM
Journal Article
English
KW - Child
KW - Cues
KW - Education of Intellectually Disabled
KW - Female
KW - Generalization (Psychology)
KW - Humans
KW - Language Development
KW - Language Development Disorders/rh [Rehabilitation]
KW - Language Disorders/rh [Rehabilitation]
KW - Male
KW - Medline
KW - Unsupervised Play Addition
RP - NOT IN FILE
SP - 523
EP - 528
JF - Journal of Applied Behavior Analysis
JA - J Appl.Behav Anal.
VL - 13
IS - 3
CY - UNITED STATES
N2 - Six institutionalized children, aged 7-11, with little or no spontaneous vocal manding, were trained to request food items under appropriate natural conditions when snacks were presented. "I want a" was appropriate when an adult presented food in the playroom. "Out" was appropriate when the items were displayed in the hallway, across a half-door barrier from the child. A sequence of steps was trained, through increasingly naturalistic setting and cuing conditions. The two mands were trained in sequence, not concurrently. To encourage "spontaneous" productions, no vocal cuing was provided by the adult. After criterion performance in each step, several probe sessions were conducted for various cuing conditions, adults, and settings. Probes after imitation training showed no spontaneous manding. Thus, failure of manding was not due to production difficulties. In probes after training for "approximately" natural cues, most children showed little transfer to the natural cues. This implies that training for the specific appropriate cues may often be required. However, good transfer generally occurred across persons, and from training room to playroom. Probes also showed that most children did not use one of the trained mands in the stimulus conditions that were appropriate for the other mand. Thus, adding a second mand did not generally disrupt use of the first. However, significant disruption occurred for two children. Finally, at the end of training, extinction training was given for one mand in one setting. Performance of the other mand was little affected. In sum, the appropriate form of a mand depends on specific stimulus and setting characteristics, and these characteristics must be considered in training
SN - 0021-8855
UR - 7430009
ER -

TY - JOUR
ID - 1656
T1 - Documenting the value of supervised play in a pediatric ambulatory care clinic
A1 - Williams,Y.B.
A1 - Powell,M.
Y1 - 1980///
N1 - Williams, Y B. Powell, M
Journal of the Association for the Care of Children's Health
kek, 8101321
H

Journal Article
English
KW - Child
KW - Child Behavior
KW - Documentation/mt [Methods]
KW - Evaluation Studies as Topic
KW - Hospital Bed Capacity
KW - 500 and over
KW - Humans
KW - Indiana
KW - Outpatient Clinics
KW - Hospital/og [Organization & Administration]
KW - Pediatrics
KW - Play Therapy
KW - Medline
KW - Unsupervised Play Addition
RP - NOT IN FILE
SP - 15
EP - 20
JF - Journal of the Association for the Care of Childrens Health
JA - J Assoc Care Child Health
VL - 9
IS - 1
CY - UNITED STATES
N2 - This study tested the hypothesis that supervision of play in a pediatric ambulatory clinic would increase positive resonses and decrease negative responses in outpatients. During the control week, children played unsupervised in the unaltered clinic waiting area; selected behaviors were recorded then and during the experimental week, in which changes introduced in the clinical environment facilitated supervised play. Significant increases in positive responses and significant decreases in negative responses were noted during the experimental week (X2=474.4; p. <. 001.). A second study demonstrated that significant changes were not caused by environment alteration alone. The resultant outpatient program and its benefits are described
SN - 0274-8916
UR - 10248699
ER -

TY - JOUR
ID - 1657
T1 - Help-seeking mothers and their children
A1 - Kogan,K.L.
Y1 - 1978///
N1 - Kogan, K L
Child psychiatry and human development
d2b, 1275332
IM
Journal Article. Research Support, U.S. Gov't, P.H.S.
English
KW - Attitude
KW - Behavior Therapy
KW - Child
KW - Child Behavior Disorders/th [Therapy]
KW - Preschool
KW - Counseling
KW - Female
KW - Humans
KW - Male
KW - Maternal Behavior

KW - Mother-Child Relations
KW - Mothers
KW - Play and Playthings
KW - Verbal Behavior
KW - Medline
KW - Unsupervised Play Addition
RP - NOT IN FILE
SP - 204
EP - 218
JF - Child Psychiatry & Human Development
JA - Child Psychiatry Hum Dev
VL - 8
IS - 4
CY - UNITED STATES
N2 - Forty-three mothers sought help with management of childhood behaviors. Presenting complaints in half or more of the families included stubbornness, talking back to parents, disobedience and other traits indicative of parent-child interaction difficulties. Pretreatment behavioral observations revealed that mothers shared common characteristics of being low in positive warmth and high in negative and oppositional responses. The children were observed to share common traits of little independent play, frequent frustration, and either ignored their mothers or were verbally and physically oppositional. Behavioral counselling and monitored rehearsal served to reverse many of the behaviors. Almost all mothers reported reduction in problem behaviors. Mothers varied widely in attributing usefulness to different program components
SN - 0009-398X
UR - 688804
ER -

TY - JOUR
ID - 1658
T1 - Observations of young normal and handicapped children
A1 - Phemister,M.R.
A1 - Richardson,A.M.
A1 - Thomas,G.V.
Y1 - 1978/07//
N1 - Phemister, M R. Richardson, A M. Thomas, G V
Child: care, health and development
7602632, c9x
IM
Journal Article
English
KW - Adolescent
KW - Attention
KW - Child
KW - Child Day Care Centers
KW - Child Development
KW - Preschool
KW - Disabled Persons
KW - Hospital Units
KW - Humans
KW - Infant
KW - Intellectual Disability/px [Psychology]
KW - Interpersonal Relations
KW - Play and Playthings
KW - Self Stimulation
KW - Medline
KW - Unsupervised Play Addition
RP - NOT IN FILE

SP - 247
EP - 259
JF - Child: Care, Health & Development
JA - Child Care Health Dev.
VL - 4
IS - 4
CY - UNITED STATES
N2 - An observational study was made of 1-2-year-old children, and of mentally handicapped children functioning at a similar level, to determine the extent to which they involved themselves in play with toys and other objects and the extent to which their day was "empty". Observations of normal children were made in their homes and in day nurseries; observations of mentally handicapped children were made at home, in day care centres (special schools) and in the schools and wards of subnormality hospitals. In no setting were children interacting with adults for more than 20% of their non-routine time. These findings seem to justify concern over independent play with toys and other objects. The handicapped children in all settings had "emptier" days than the normal children, this being accounted for by greater amounts of inattentive handling of objects and self-stimulation and lesser amounts of social interaction and attentive play. However, attentive play scores for the handicapped children varied markedly with setting. In appropriate circumstances, there can be as much spontaneous contact with objects in handicapped children as in normal children at a similar development level. Despite current concern over the effects of day care on the development of very young children, normal children in day nurseries did not differ from normal children at home, either in attentive play scores or in the proportion of nonroutine time spent interacting with adults
SN - 0305-1862
UR - 149618
ER -

TY - JOUR
ID - 1659
T1 - Surgery of the knee in children with spina bifida
A1 - Birch,R.
Y1 - 1976///
N1 - Birch, R
Developmental medicine and child neurology. Supplement
0006762
IM
Journal Article
English
KW - Child
KW - Preschool
KW - Humans
KW - Knee/ab [Abnormalities]
KW - Knee/su [Surgery]
KW - Orthopedics
KW - Spinal Dysraphism/co [Complications]
KW - Medline
KW - Unsupervised Play Addition
RP - NOT IN FILE
SP - 111
EP - 113
JF - Developmental Medicine & Child Neurology - Supplementum
JA - Dev Med Child Neurol (Suppl)
IS - 37
CY - UNITED STATES
N2 - The achievement of independent mobility by children with spina bifida is often hampered by deformities of the knee joints. This report reviews the results of surgical treatment in 34 spina-bifida children with knee deformities. The surgical procedures are described and it is concluded that surgery is justified in a proportion of cases with fixed-knee deformities

SN - 0419-0238
UR - 797602
ER -

TY - JOUR
ID - 1660
T1 - MATERNAL RESPONSES TO THE INFANT'S EMERGING AUTONOMY AS ILLUMINATED BY HOME OBSERVATIONS: A CASE REPORT
A1 - MCCOLLUM,A.T.
Y1 - 1964/07//
N1 - MCCOLLUM, A T
Journal of the American Academy of Child Psychiatry
7505568, h54
OM
Journal Article
English
KW - Child Psychology
KW - Infant
KW - Infant Care
KW - Infant Nutritional Physiological Phenomena
KW - Parent-Child Relations
KW - Play and Playthings
KW - Pregnancy
KW - psychology
KW - Medline
KW - Unsupervised Play Addition
RP - NOT IN FILE
SP - 516
EP - 535
JF - Journal of the American Academy of Child Psychiatry
JA - J Am Acad Child Psychiatry
VL - 3
CY - UNITED STATES
SN - 0002-7138
UR - 14179096
ER -

TY - JOUR
ID - 1661
T1 - Permissiveness, permission, and aggression: the effect of adult presence or absence on aggression in children's play
A1 - SIEGEL,A.E.
A1 - KOHN,L.G.
Y1 - 1959/03//
N1 - SIEGEL, A E. KOHN, L G
Child development
0372725, d28
OM
Journal Article
English
KW - Behavior
KW - Projective Techniques
KW - Medline
KW - Unsupervised Play Addition
RP - NOT IN FILE
SP - 131

EP - 141
JF - Child Development
JA - Child Dev.
VL - 30
IS - 1
CY - Not Available
SN - 0009-3920
UR - 13629745
ER -

TY - JOUR
ID - 1662
T1 - I like my dog, does my dog like me?
A1 - Rehn,Therese
A1 - Lindholm,Ulrika
A1 - Keeling,Linda
A1 - Forkman,Bj”rn
Y1 - 2014/01//
KW - PsycInfo
KW - Unsupervised Play Addition
KW - Dogs
KW - affectional bond
KW - Behavior
KW - attachment style
KW - Animal Ethology
KW - Attachment Behavior
KW - Affection
RP - NOT IN FILE
SP - 65
EP - 73
JF - Applied Animal Behaviour Science
VL - 150
CY - Netherlands
PB - Elsevier Science
U1 - Sponsor: Swedish Research Council for Environment, Agricultural Sciences and Spatial Planning; Sweden. Recipients: No recipient indicated
N2 - In this study, the possibility of there being an association between how an owner perceives his/her relationship to their dog and the way the dog experiences the relationship to its owner was investigated using two well-established methods within the anthrozoology literature. Twenty dog-owner dyads participated in the Strange Situation Procedure (SSP), to evaluate the bond on the dog's part, and the Monash Dog Owner Relationship Scale (MDORS) was used to investigate the strength of the relationship from the owner's view. Six attachment variables were created based on changes in the dogs' behaviour when it was with the owner vs. when it was with the stranger in the SSP. These were: changes in exploration, passive behaviour, independent play, social play, physical contact and tail wagging. The magnitude of these changes in the dogs' behaviour was then tested for correlations with the owners' scores in the MDORS. Only two correlations were found and both were with the MDORS subscale that measures "Dog-owner interaction". They suggested that owners who interact more frequently with their dog, have dogs showing more proximity-seeking behaviour upon reunion (? = 0.56, P = 0.01, N = 20) and less independent play behaviour (? = ?0.52, P = 0.02, N = 20). This might be a consequence of dogs being positively reinforced for close interaction by the owner, or these dogs may have developed an attachment style similar to insecurely attached children. No correlation was found between any of the six attachment variables in the SSP and the overall MDORS score, the MDORS subscale "Perceived costs" and, more importantly, no correlation to "Perceived emotional closeness". In summary, there is no support from this study for the view that the strength of the relationship an owner feels to his/her dog is mirrored in the strength of the bond of the dog to its owner. (PsycINFO Database Record (c) 2014 APA, all rights reserved) (journal abstract)
SN - 0168-1591

AD - Rehn, Therese, Department of Animal Environment and Health, Swedish University of Agricultural Sciences Box 7068, SE-750 07, Uppsala, Sweden, Therese.Rehn@slu.se
UR - http://search.ebscohost.com/login.aspx?direct=true&db=psyh&AN=2013-40463-001&site=ehost-live;Therese.Rehn@slu.se
L3 - 10.1016/j.applanim.2013.10.008
ER -

TY - CHAP
ID - 1663
T1 - Pretend play and creativity: An overview
A1 - Russ,Sandra W.
Y1 - 2014///
N1 - Accession Number: 2013-18606-002. First Author & Affiliation: Russ, Sandra W.; Case Western Reserve University, Cleveland, OH, US. Release Date: 20131111. Publication Type: Book, (0200); Authored Book, (0240); . Media Covered: Print. Document Type: Chapter. Book Type: Textbook/Study Guide. ISBN: 1-4338-1561-3, Hardcover, 1433815613; 978-1-4338-1561-4, Hardcover, 9781433815614. Language: English. Major Descriptor: Cognitive Processes; Creativity; Pretend Play. Classification: Psychosocial & Personality Development (2840) . Population: Human (10); . Age Group: Childhood (birth-12 yrs) (100) Adolescence (13-17 yrs) (200) Adulthood (18 yrs & older) (300) . Intended Audience: Psychology: Professional & Research (PS) . Tests & Measures: Affect in Play Scale [Appended]; Creative Achievement Questionnaire [Appended] DOI: 10.1037/t05569-000; . Page Count: 22
KW - PsycInfo
KW - Unsupervised Play Addition
KW - Pretend Play
KW - Creativity
KW - cognitive & affective processes
KW - creative processes
KW - Cognitive Processes
RP - NOT IN FILE
SP - 7
EP - 28
T2 - Pretend play in childhood: Foundation of adult creativity
CY - Washington, DC US
PB - American Psychological Association
N2 - (from the chapter) Creativity and pretend play are both multidimensional. Many cognitive and affective processes are involved, and there are differences across individuals for each process. There is overlap among processes that occur in pretend play and processes that occur in creativity. This introductory chapter first reviews definitions of creativity, with a focus on creative products and creative people. It emphasizes the cognitive and affective processes involved in creativity and considers how different creative processes are involved in different stages of creative production. Next, it reviews definitions of pretend play, with a focus on cognitive and affective processes in pretend play. Finally, it presents a model showing the overlap between pretend play and adult creativity. This chapter provides only an overview of processes that occur in both the pretend play of the child and the creative acts of the adult; later chapters present the theories and research in greater depth. When we think about creativity, we encounter different aspects of the construct. First there is the creative product-the actual artwork or scientific discovery that the field labels as being creative. There is also the person who is the maker of the creative product. What are the internal processes that enable an individual to think in a creative fashion? The distinction between product and process is an important one (Golann, 1963; Mackinnon, 1962). (PsycINFO Database Record (c) 2013 APA, all rights reserved) (chapter)
SN - 1-4338-1561-3
UR - http://search.ebscohost.com/login.aspx?direct=true&db=psyh&AN=2013-18606-002&site=ehost-live
L3 - 10.1037/14282-002
ER -

TY - JOUR
ID - 1664

T1 - Go out and play, but mean it: Using frame analysis to explore recent news media coverage of the rediscovery of unstructured play
A1 - Bishop,Ronald
Y1 - 2013/12//
KW - PsycInfo
KW - Unsupervised Play Addition
KW - news media coverage
KW - unstructured play
KW - newspapers
KW - Magazines
KW - journalists
KW - Mass Media
RP - NOT IN FILE
SP - 510
EP - 520
JF - The Social Science Journal
VL - 50
IS - 4
CY - Netherlands
PB - Elsevier Science
N2 - A frame analysis was conducted on newspaper articles, news magazine articles, and the transcripts of broadcast segments on the need for and rediscovery of unstructured play that appeared between January 1, 2005 and December 31, 2010. This time frame was selected to coincide with the recent revival of interest in play, both among scholars and the public. The eight frames that emerged from the coverage reveal that the spontaneity in play valued by children and endorsed by scholars is absent from recent descriptions by journalists of play. Journalists did not frame play as a respite for children from daily life, or an "interlude" (Huizinga, 1971, p. 21) undertaken to escape the real world. Play as portrayed through these frames must be productive-a "useful pastime" (Chudacoff, 2007, p. 222). Descriptions of play by journalists support the "rhetoric of progress" discussed by Sutton-Smith. Play is seen as the "optimal state of being for children" (Gentry, 2009, p. 14A), rather than something a child simply wants to do for fun. Few of the sources consulted by journalists favor letting kids go off in an unsupervised fashion. Kids can play, but only if they do so in clearly defined spaces and according to clearly laid out schedules. (PsycINFO Database Record (c) 2014 APA, all rights reserved) (journal abstract)
SN - 0362-3319
AD - Bishop, Ronald, Department of Culture and Communication, Drexel University 569 Hemingway Drive, Hockessin, DE, US, 19707, rcbsam@comcast.net
UR - http://search.ebscohost.com/login.aspx?direct=true&db=psyh&AN=2013-42164-012&site=ehost-live;rcbsam@comcast.net
L3 - 10.1016/j.soscij.2013.07.014
ER -

TY - JOUR
ID - 1665
T1 - Psychological essentialism among Korean children and adults: A modified replication study
A1 - Kim,Geunyoung
Y1 - 2013/10//
KW - PsycInfo
KW - Unsupervised Play Addition
KW - psychological essentialism development
KW - Age Differences
KW - cultural factors
KW - Personality
KW - Psychological Development
KW - Sociocultural Factors
RP - NOT IN FILE
SP - 809

EP - 817
JF - International Journal of Psychology
JA - Int J Psychol
VL - 48
IS - 5
CY - United Kingdom
PB - Taylor & Francis
U1 - Sponsor: Sogang University; Korea. Grant: 201110077.01. Other Details: Research Grant. Recipients: No recipient indicated
N2 - Developmental psychologists have shown interest in the development of psychological essentialism among children; that is, a belief that certain psychological characteristics (such as personality) are relatively stable and unchanging. Although previous studies have shown that children are essentialistic about human traits, and the coherence among various essentialism dimensions increases with age, moderating cultural factors in the development of essentialism, especially among Asian children, have received little attention. Using the methodologies of Gelman, Heyman, and Legare (2007), levels of psychological essentialism among Korean children and adults were measured, and compared with the original US data. Results demonstrated cross-cultural similarity in the development of coherence in essentialistic thought, and difference in the level of essentialism among adult participants. The present findings imply that different cultural values between Asians and Westerners can play a role in the developmental trajectory of psychological essentialism. (PsycINFO Database Record (c) 2014 APA, all rights reserved) (journal abstract)
SN - 1464-066X
AD - Kim, Geunyoung, Department of Psychology, Sogang University 35 Baekbeom-ro (Sinsudong), Mapo-gu, Seoul, Korea, 121-742, kimg@sogang.ac.kr
UR - http://search.ebscohost.com/login.aspx?direct=true&db=psyh&AN=2013-32364-007&site=ehost-live;kimg@sogang.ac.kr
L3 - 10.1080/00207594.2012.693182
ER -

TY - JOUR
ID - 1666
T1 - Freedom, flow and fairness: Exploring how children develop socially at school through outdoor play
A1 - Waite,Sue
A1 - Rogers,Sue
A1 - Evans,Julie
Y1 - 2013/09//
KW - PsycInfo
KW - Unsupervised Play Addition
KW - Freedom
KW - fairness
KW - outdoor play
KW - social interactions
KW - Classroom Behavior
KW - Childhood Development
KW - Childhood Play Behavior
KW - Social Interaction
KW - Recreation
RP - NOT IN FILE
SP - 255
EP - 276
JF - Journal of Adventure Education and Outdoor Learning
VL - 13
IS - 3
CY - United Kingdom
PB - Taylor & Francis

U1 - Sponsor: Economic and Social Research Council; United Kingdom. Grant: RES-000-22-3065. Date: From January, 2009 to May, 2011. Other Details: 'Opportunities Afforded by the Outdoors for Alternative Pedagogies in the Transition between Foundation Stage and Year 1'. Recipients: Waite, Sue; Rogers, Sue; Evans, Julie
N2 - In this article, we report on a study that sought to discover micro-level social interactions in fluid outdoor learning spaces. Our methodology was centred around the children; our methods moved with them and captured their social interactions through mobile audio-recording. We argue that our methodological approach supported access to negotiations beyond adult gaze whilst acknowledging some associated ethical and practical dilemmas. Outdoor contexts were characterised by lower levels of adult presence and control and were associated with freedom to engage in sustained inter-child play and interaction. We theorise how opportunities to practice pro-social behaviour contribute to children's social cohesion in the classroom and what adults can learn from such instances in order to support children's social development and learning. Children's conversations are analysed through conceptual lenses of cultures of play, schooling and society. (PsycINFO Database Record (c) 2013 APA, all rights reserved) (journal abstract)
SN - 1754-0402
AD - Waite, Sue, School of Education, Faculty of Health, Education and Society, Plymouth University , Plymouth, United Kingdom, PL4 8AA, sjwaite@plymouth.ac.uk
UR - http://search.ebscohost.com/login.aspx?direct=true&db=psyh&AN=2013-28756-007&site=ehost-live;sjwaite@plymouth.ac.uk
L3 - 10.1080/14729679.2013.798590
ER -

TY - JOUR
ID - 1667
T1 - Child-friendly urban structures: Bullerby revisited
A1 - Broberg,Anna
A1 - Kytt„Marketta
A1 - Fagerholm,Nora
Y1 - 2013/09//
KW - PsycInfo
KW - Unsupervised Play Addition
KW - environmental child friendliness
KW - Built Environment
KW - Urban Areas
KW - emotional affordances
KW - Physical Environment
KW - Childhood Development
KW - Emotional Development
KW - Urban Environments
RP - NOT IN FILE
SP - 110
EP - 120
JF - Journal of Environmental Psychology
VL - 35
CY - Netherlands
PB - Elsevier Science
U1 - Sponsor: Tekes. Recipients: No recipient indicated;Sponsor: Finnish Funding Agency for Technology and Innovation; Finland. Recipients: No recipient indicated;Sponsor: Finnish Ministry of Education and Culture; Finland. Recipients: No recipient indicated
N2 - Definitions of environmental child friendliness offer broad criteria that are not easy to study or assess. We suggest that due to this broadness, these definitions have produced surprisingly few attempts to evaluate how child-friendly various types of physical environments are. The purpose of this study is to analyse how the structure of the built environment contributes to environmental child friendliness. We define child friendliness by two central criteria: children's possibilities for independent mobility and their opportunities to actualize environmental affordances. We study how built environment qualities condition environmental child friendliness in place-based ways by asking children and youth in Turku, Finland, to tell about their meaningful places and their mobility to these. The data consists of over 12,000 affordances, localized by the respondents.

This experiential and behavioural place-based knowledge is combined with objectively measured data on residential and building density, and quantity of green structures. Moderate urban density seems to have child-friendly characteristics such as an ability to promote independent access to meaningful places and the diversity of affordances. We find that affordances situated on residential areas are likely to be reached alone, whereas access to affordances situated in densely built urban cores is less independent. The proportion of green structures is not associated with independent access. The diversity of affordances is highest in areas that are densely populated and not very green. Green areas are important settings for doing things, and green structures around emotional affordances increase the likelihood of liking the place significantly. Combining children's place-based experiences with information derived from objective measurable qualities of the physical environment provides a valuable methodological contribution to studies on environmental child friendliness, and the two proposed criteria of child friendliness are supported by this study. There is no one environment that is child-friendly, but different environments have different uses and meanings. (PsycINFO Database Record (c) 2013 APA, all rights reserved) (journal abstract)
SN - 0272-4944
AD - Broberg, Anna, Department of Surveying and Planning, Aalto University P.O. Box 12200, 00076, Aalto, Finland, anna.broberg@aalto.fi
UR - http://search.ebscohost.com/login.aspx?direct=true&db=psyh&AN=2013-27385-013&site=ehost-live;ncfage@utu.fi;marketta.kytta@aalto.fi;anna.broberg@aalto.fi
L3 - 10.1016/j.jenvp.2013.06.001
ER -

TY - JOUR
ID - 1669
T1 - Where do children travel to and what local opportunities are available? The relationship between neighborhood destinations and children's independent mobility
A1 - Villanueva,Karen
A1 - Giles-Corti,Billie
A1 - Bulsara,Max
A1 - Timperio,Anna
A1 - McCormack,Gavin
A1 - Beesley,Bridget
A1 - Trapp,Georgina
A1 - Middleton,Nicholas
Y1 - 2013/08//
KW - PsycInfo
KW - Unsupervised Play Addition
KW - CHILDREN
KW - destinations
KW - independent mobility
KW - Neighborhoods
KW - Travel
KW - Commuting (Travel)
KW - Physical Activity
KW - Physical Mobility
RP - NOT IN FILE
SP - 679
EP - 705
JF - Environment and Behavior
VL - 45
IS - 6
CY - US
PB - Sage Publications
U1 - Sponsor: National and Medical Research Council. Grant: 403933. Recipients: No recipient indicated;Sponsor: Sponsor name not included. Other Details: Ad Hoc Scholarships. Recipients: Villanueva, Karen; Trapp, Georgina;Sponsor: National Health and Medical Research Council. Grant: 458668. Other Details: Capacity Building Grant. Recipients: Trapp, Georgina;Sponsor: National Health and Medical Research Council.

Grant: 1004900. Other Details: Principal Fellow Award. Recipients: Giles-Corti, Billie;Sponsor: VicHealth. Grant: 2004 0536. Other Details: Public Health Research Fellowship. Recipients: Timperio, Anna;Sponsor: Alberta Heritage Foundation. Other Details: Award for Medical Research Postdoctoral Fellowship. Recipients: McCormack, Gavin
N2 - Associations between access to local destinations and children's independent mobility (IM) were examined. In 2007, 10- to 12-year-olds (n = 1,480) and their parents (n = 1,314) completed a survey. Children marked on a map the destinations they walked or cycled to (n = 1,132), and the availability of local destinations was assessed using Geographic Information Systems. More independently mobile children traveled to local destinations than other children. The odds of IM more than halved in both boys and girls whose parents reported living on a busy road (boys, OR = 0.48; girls, OR = 0.36) and in boys who lived near shopping centers (OR = 0.18) or community services (OR = 0.25). Conversely, the odds of IM more than doubled in girls living in neighborhoods with well-connected low-traffic streets (OR = 2.32) and increased in boys with access to local recreational (OR = 1.67) and retail (OR = 1.42) destinations. Creating safe and accessible places and routes may facilitate children's IM, partly by shaping parent's and children's feelings of safety while enhancing their confidence in the child's ability to use active modes without an adult. (PsycINFO Database Record (c) 2013 APA, all rights reserved) (journal abstract)
SN - 1552-390X
AD - Villanueva, Karen, Centre for the Built Environment and Health (M707), School of Population Health, The University of Western Australia 35 Stirling Highway, Crawley, WA, US, 6009, karen.villanueva@uwa.edu.au
UR - http://search.ebscohost.com/login.aspx?direct=true&db=psyh&AN=2013-23864-001&site=ehost-live;karen.villanueva@uwa.edu.au
L3 - 10.1177/0013916512440705
ER -

TY - JOUR
ID - 1671
T1 - The development of organized visual search
A1 - Woods,Adam J.
A1 - G”ksun,Tilbe
A1 - Chatterjee,Anjan
A1 - Zelonis,Sarah
A1 - Mehta,Anika
A1 - Smith,Sabrina E.
Y1 - 2013/06//
KW - PsycInfo
KW - Unsupervised Play Addition
KW - visual search development
KW - guiding behavior
KW - Childhood Development
KW - Visual Search
RP - NOT IN FILE
SP - 191
EP - 199
JF - Acta Psychologica
VL - 143
IS - 2
CY - Netherlands
PB - Elsevier Science
U1 - Sponsor: National Institutes of Health; US. Grant: T32NS007413; R01 DC008779. Recipients: No recipient indicated
N2 - Visual search plays an important role in guiding behavior. Children have more difficulty performing conjunction search tasks than adults. The present research evaluates whether developmental differences in children's ability to organize serial visual search (i.e., search organization skills) contribute to performance limitations in a typical conjunction search task. We evaluated 134 children between the ages of 2 and 17 on separate tasks measuring search for targets defined by a conjunction of features or by distinct features. Our

results demonstrated that children organize their visual search better as they get older. As children's skills at organizing visual search improve they become more accurate at locating targets with conjunction of features amongst distractors, but not for targets with distinct features. Developmental limitations in children's abilities to organize their visual search of the environment are an important component of poor conjunction search in young children. In addition, our findings provide preliminary evidence that, like other visuospatial tasks, exposure to reading may influence children's spatial orientation to the visual environment when performing a visual search. (PsycINFO Database Record (c) 2013 APA, all rights reserved) (journal abstract)
SN - 0001-6918
AD - Woods, Adam J., Department of Neurology, University of Pennsylvania 3710 Hamilton Walk, room 319, Philadelphia, PA, US, 19104, woodsaj@gmail.com
UR - http://search.ebscohost.com/login.aspx?direct=true&db=psyh&AN=2013-16319-006&site=ehost-live;adwoods@mail.med.upenn.edu
L3 - 10.1016/j.actpsy.2013.03.008
ER -

TY - JOUR
ID - 1672
T1 - Evaluation of learning outcomes using an educational iPhone game vs. traditional game
A1 - Furi¢,David
A1 - Gonz lez-Gancedo,Santiago
A1 - Juan,M.Carmen
A1 - Segu ¡,Ignacio
A1 - Rando,Noem ¡
Y1 - 2013/05//
KW - PsycInfo
KW - Unsupervised Play Addition
KW - educational evaluation
KW - learning outcomes
KW - Educational Games
KW - iPhone games
KW - traditional games
KW - subject preferences
KW - Computer Games
KW - Curriculum
KW - Educational Measurement
KW - School Learning
KW - Preferences
RP - NOT IN FILE
SP - 1
EP - 23
JF - Computers & Education
VL - 64
CY - Netherlands
PB - Elsevier Science
U1 - Sponsor: Sponsor name not included. Grant: TIN2009-14319-C02. Other Details: Spanish APRENDRA Project. Recipients: No recipient indicated
N2 - In this paper, we present an initial study to determine the subject preferences for educational computer games for children, in which 150 education professionals participated. From the results of this first study, we have developed an iPhone game for transmitting knowledge as part of multiculturalism, solidarity and tolerance following established learning theories, several design principles, and the objectives and competences of the Spanish law for primary education. We also report on a second study to determine whether the iPhone game has better learning outcomes than a traditional game by analyzing the participation of 84 children ranging in age from 8 to 10 years old. The frequency of playing with consoles or computer games was also taken into account in this second study, and the worldwide trend of previous studies has been corroborated. For learning outcomes, the results did not show significant differences between the two groups. However, 96% of the children indicated that they would like to play with the iPhone game again, and 90% indicated that they preferred the experience

with the iPhone game over the traditional one. From these results, we can conclude that the children achieved similar knowledge improvements using both the autonomous game (iPhone game) and the custom, guided game (traditional game). This could facilitate versatility in the learning process since the learning activity could be performed at any place and time without requiring supervision. Therefore, it could be a useful tool in the learning process and help teachers to fulfill students' training needs. (PsycINFO Database Record (c) 2013 APA, all rights reserved) (journal abstract)
SN - 0360-1315
AD - Juan, M.-Carmen, Instituto Universitario de Automatica e Informatica Industrial, Universitat Politecnica de Valencia Camino de Vera, s/n., 46022, Valencia, Spain, mcarmen@dsic.upv.es
UR - http://search.ebscohost.com/login.aspx?direct=true&db=psyh&AN=2013-09432-002&site=ehost-live;mcarmen@ai2.upv.es
L3 - 10.1016/j.compedu.2012.12.001
ER -

TY - CHAP
ID - 1674
T1 - Cognitive-behavioral-ecological intervention to facilitate social-emotional understanding and social interaction in youth with high-functioning ASD
A1 - Bauminger-Zviely,Nirit
Y1 - 2013///
N1 - Accession Number: 2013-32785-010. First Author & Affiliation: Bauminger-Zviely, Nirit; Graduate Program in Special Education, School of Education, Bar-Ilan University, Ramat-Gan, Israel. Release Date: 20131125. Publication Type: Book, (0200); Edited Book, (0280); . Media Covered: Print. Document Type: Chapter. ISBN: 978-1-4625-1048-1, Hardcover, 9781462510481. Language: English. Major Descriptor: Cognitive Ability; Cognitive Behavior Therapy; Pervasive Developmental Disorders; Social Interaction; Social Skills. Minor Descriptor: Ability Level; Autism; Intervention. Classification: Developmental Disorders & Autism (3250) . Population: Human (10); . Age Group: Childhood (birth-12 yrs) (100) School Age (6-12 yrs) (180) Adolescence (13-17 yrs) (200) . Intended Audience: Psychology: Professional & Research (PS) . Tests & Measures: Social Responsiveness Scale; Nonverbal Accuracy 2-Child Facial Expressions; Problem Solving Measure; Emotion Inventory Measure; Affective Matching Measure; Loneliness Rating Scale; Self Perception Profile of Children; Autism Diagnostic Interview-Revised; Social Skills Rating System; . Methodology: Empirical Study; Quantitative Study. References Available: Y.. Page Count: 30
KW - PsycInfo
KW - Unsupervised Play Addition
KW - cognitive-behavioral-ecological intervention
KW - socio-intervention
KW - Social Interaction
KW - high-functioning youth
KW - autism spectrum disorder
KW - Cognitive Ability
KW - Cognitive Behavior Therapy
KW - Pervasive Developmental Disorders
KW - Social Skills
KW - Ability Level
KW - Autism
KW - Intervention
RP - NOT IN FILE
SP - 226
EP - 255
T2 - CBT for children and adolescents with high-functioning autism spectrum disorders
A2 - Scarpa,Angela
A2 - Williams White,Susan
A2 - Attwood,Tony
CY - New York, NY US
PB - Guilford Press

N2 - (from the chapter) Social functioning-more specifically, interacting with peers and understanding social situations-is a lifelong struggle for individuals with autism spectrum disorders (ASD) at different levels of functioning. Billstedt, Gillberg, and Gillberg (2007) recently demonstrated the persistence of the social-communicative reciprocity deficit in ASD from early childhood into adulthood. In their study, 15 of 22 symptoms of deficient social interaction skills were still present in adulthood for half or more of their sample. Major difficulties appeared in adults' reciprocal peer interactions, which were infrequent as well as inappropriate, including inappropriate emotional responses to peers; a one-sided, self-centered social approach; infrequent physical expressions of affection; inappropriate conventions; and poor or unfocused eye contact. Predictors of better adult outcomes were speech before 5 years and higher IQ; higher-functioning individuals scored better. Indeed, ASD research on children has also linked IQ with peer interaction outcomes. For example, children with an IQ over 85 had more friendsthan those with an IQ under 85 (Mazurek & Kanne, 2010), and higher Verbal IQ correlated with better dyadic friendship qualities such as responsiveness and coordinated play (e.g., Bauminger et al., 2008).To set the stage for the proposed intervention guidelines, I first review the multidimensional social deficit in HFASD, as well as recent multimodal social intervention studies designed specifically for HFASD that adopt cognitive-behavioral therapy (CBT) principles. Next, I present a multidimensional social intervention for HFASD that combines CBT with ecological treatment (Bauminger, 2002, 2007a, 2007b), emphasizing its practical aspects and research supporting its efficacy. Finally, I end with suggestions for future research in the area of social intervention for school-age children with HFASD. (PsycINFO Database Record (c) 2013 APA, all rights reserved) (chapter)
SN - 978-1-4625-1048-1
UR - http://search.ebscohost.com/login.aspx?direct=true&db=psyh&AN=2013-32785-010&site=ehost-live
ER -

TY - THES
ID - 1675
T1 - A cross-cultural investigation of the threat-superiority effect: Evidence for perceptual biases shaped by experience and natural selection
A1 - Penkunas,Michael James
Y1 - 2013///
N1 - Accession Number: 2013-99200-389. First Author & Affiliation: Penkunas, Michael James; U California, Davis, US. Other Journal Titles: Dissertation Abstracts International. Release Date: 20131104. Publication Type: Dissertation Abstract, (0400); . Media Covered: Electronic. Document Type: Dissertation. Dissertation Number: AAI3544793. ISBN: 978-1-267-75975-7, 9781267759757. Language: English. Major Descriptor: Age Differences; Cross Cultural Differences; Threat. Minor Descriptor: Reaction Time; South Asian Cultural Groups; Visual Search; Visual Attention; Executive Function. Classification: General Psychology (2100) . Population: Human (10); Male (30); Female (40); . Location: US. Age Group: Childhood (birth-12 yrs) (100) Preschool Age (2-5 yrs) (160) Adulthood (18 yrs & older) (300) . Methodology: Empirical Study; Quantitative Study
KW - PsycInfo
KW - Unsupervised Play Addition
KW - experiences
KW - threat
KW - Visual Attention
KW - reaction time
KW - Aging
KW - Age Differences
KW - Cross Cultural Differences
KW - South Asian Cultural Groups
KW - Visual Search
KW - Executive Function
RP - NOT IN FILE
JF - Dissertation Abstracts International: Section B: The Sciences and Engineering
VL - 74
IS - 4-B(E)
CY - US
PB - ProQuest Information & Learning

N2 - The threat-superiority effect is characterized by the preferential recruitment of visual attention by potentially threatening visual features over nonthreatening visual features. This perceptual phenomenon may be the historical product of natural selection shaping the human visual system to detect rapidly the various ecological dangers in complex settings. While previous investigations into visual biases in detecting dangerous animals have focused primarily on snakes, a host of predators besides snakes have also acted as persistent sources of selection on human ancestors. Chapter 1 replicated and expanded upon previous research on the threat-superiority effect using visual-search tasks in three experiments. The first experiment presented American preschool-age children and adults with 3 x 3 matrices of either a single snake target embedded in lizard images or a single lizard target embedded in snake images. Twenty-four trials were presented using a touch-screen enabled LCD monitor. Children's reaction times to detect the target animals were analyzed using multiple regression with age, sex, target animal type, and age by target type interaction as predictor variables. Adult RT data were analyzed using two-factor randomized group analyses of variance and tests of simple effects with sex and target type as independent variables. In this experiment, both children and adults were faster at detecting target snake images juxtaposed randomly in matrices of visually similar lizards than target lizard images juxtaposed randomly in matrices of snakes. This study provided a more stringent test for the advanced detection of snakes by comparing the salience of snakes to visually similar lizards. In the second experiment, I provided the first evidence that American preschool-age children and adults preferentially detected target images of lions over images of antelope. This ability in children and adults possibly reflects a predisposition for detecting large-bodied felids, which fossil record suggests have hunted hominins for the past 7 million years. In the third experiment, American preschool-age children and adults were presented with target cows embedded in matrices of horses and the converse. The results of this experiment showed that adult women preferentially detected target images of horses over target images of cows, an effect that was not present for adult men and preschool children of either sex. To complement this experiment, a child-adapted Stroop-like test examined the executive function of children as a predictor of reaction time on the visual search task. The inclusion of the executive function test did not improve the predictive power of the regression model, indicating that the age variable may be acting as a proxy measure for the cognitive skills normally acquired during early childhood. The combined results of the three experiments in Chapter 1 indicate that the human visual system is indeed sensitive to snake images as well as historical predators other than snakes. Nevertheless, evidence of a bias for horse detection suggests that experience plays a role in the development of visual biases across the life span. To examine the role of experience in the acquisition of biases in animal detection, three to eight year-old children from two distinct populations in southern India were tested on the three visual-search tasks described in Chapter 1. Chapter 2 provides evidence that the visual biases towards snakes and lions are not unique to American participants, but instead might constitute a reliably developing feature of the human visual system. This multi-site study included two populations of children in southern India who have very different experiences with dangerous wildlife. (PsycINFO Database Record (c) 2013 APA, all rights reserved)
SN - 0419-4217
UR - http://search.ebscohost.com/login.aspx?direct=true&db=psyh&AN=2013-99200-389&site=ehost-live
ER -

TY - JOUR
ID - 1676
T1 - Adult mediation of children's television viewing experiences as a catalyst for learning and development: A case study, using Play School
A1 - van Vliet,Helen
A1 - Harrison,Cathie
A1 - Anderson,Tracy
Y1 - 2013/01//
KW - PsycInfo
KW - Unsupervised Play Addition
KW - adult mediation
KW - children's television viewing experiences
KW - Learning
KW - Development
KW - behavioral responses
KW - Childhood Development
KW - Mediation

KW - Responses
KW - Television Viewing
KW - Behavior
RP - NOT IN FILE
SP - 17
EP - 36
JF - Early Child Development and Care
VL - 183
IS - 1
CY - United Kingdom
PB - Taylor & Francis
U1 - Sponsor: ACU/Australian Broadcasting Corporation (ABC); Australia. Other Details: ACU Industry Research Incentive Scheme (IRIS). Recipients: No recipient indicated
N2 - The television programme Play School is screened free to air in Australia on weekdays. Adult presenters guide child viewers through experiences of play and learning. An online survey assessed behavioural responses of adult co-viewers both during and after the programme to ascertain how adult co-viewers were participating in, or support the viewing experience of child viewers. Results revealed a large number and variety of behavioural responses from adults both during and after viewing. These responses can be viewed as positive as they appear to facilitate learning and development through shared interactions between adult and child. This research begins to elucidate how adult behaviours during and after viewing television may optimise the child's learning experience. (PsycINFO Database Record (c) 2013 APA, all rights reserved) (journal abstract)
SN - 1476-8275
AD - van Vliet, Helen, School of Education, Australian Catholic University Strathfield Campus, 25A Barker Road, Strathfield, Sydney, NSW, Australia, 2135, helen.vanvliet@acu.edu.au
UR - http://search.ebscohost.com/login.aspx?direct=true&db=psyh&AN=2012-34710-002&site=ehost-live;helen.vanvliet@acu.edu.au
L3 - 10.1080/03004430.2011.653810
ER -

TY - CHAP
ID - 1677
T1 - Adoptive parenting of teenagers and young adults
A1 - White,Joseph
Y1 - 2013///
N1 - Accession Number: 2013-21144-009. First Author & Affiliation: White, Joseph; Private Practice, Austin, TX, US. Release Date: 20131007. Publication Type: Book, (0200); Edited Book, (0280); . Media Covered: Print. Document Type: Chapter. ISBN: 978-1-118-10912-0, Paperback, 9781118109120; 978-1-118-41615-0, Electronic, 9781118416150; 978-1-118-41891-8, Electronic, 9781118418918; 978-1-118-61169-2, Electronic, 9781118611692. Language: English. Major Descriptor: Adolescent Development; Adopted Children; Adoptive Parents; Adult Development; Parent Child Relations. Minor Descriptor: Adoption (Child); Attachment Behavior; Identity Formation; Independence (Personality); Separation Individuation. Classification: Childrearing & Child Care (2956) Psychosocial & Personality Development (2840) . Population: Human (10); Male (30); Female (40); . Age Group: Adolescence (13-17 yrs) (200) Adulthood (18 yrs & older) (300) Young Adulthood (18-29 yrs) (320) . Intended Audience: Psychology: Professional & Research (PS) . Page Count: 12
KW - PsycInfo
KW - Unsupervised Play Addition
KW - adoptive parenting
KW - Adolescents
KW - young adults
KW - parent-child relationship
KW - identity development
KW - Autonomy
KW - individuation
KW - attachment
KW - Adolescent Development
KW - Adopted Children

KW - Adoptive Parents
KW - Adult Development
KW - Parent Child Relations
KW - Adoption (Child)
KW - Attachment Behavior
KW - Identity Formation
KW - Independence (Personality)
KW - Separation Individuation
RP - NOT IN FILE
SP - 169
EP - 180
T2 - Working with adoptive parents: Research, theory, and therapeutic interventions
A2 - Brabender,Virginia M.
A2 - Fallon,April E.
CY - Hoboken, NJ US
PB - John Wiley & Sons Inc
N2 - (from the chapter) This chapter addresses adoptive parenting issues associated with parenting adolescents. Parenting of adolescents and young adults can present challenges for any parent-either adoptive or birth-due to the developmental characteristics of youth at this age. These typical characteristics can interact with the difficult history of some adopted children, resulting in further challenges to the parent-child relationship. Parenting adolescents and young adults, whether adopted or nonadopted, presents inherent challenges associated with the major tasks of this developmental period, including the development of identity and the drive for independence and autonomy. The difficult (or missing) history of some adopted children can result in further challenges to the parent-child relationship. Both the facts of the adoption history and their perceived meaning play a critical role in the development of identity in the adopted child. Healthy individuation can be affected by both the quality of attachment and the degree to which birth families normalize or pathologize behaviors associated with the drive for autonomy and independence. (PsycINFO Database Record (c) 2013 APA, all rights reserved) (chapter)
SN - 978-1-118-10912-0
UR - http://search.ebscohost.com/login.aspx?direct=true&db=psyh&AN=2013-21144-009&site=ehost-live
ER -

TY - BOOK
ID - 1678
T1 - Free to learn: Why unleashing the instinct to play will make our children happier, more self-reliant, and better students for life
A1 - Gray,Peter
Y1 - 2013///
N1 - Accession Number: 2012-32884-000. First Author & Affiliation: Gray, Peter; Department of Psychology, Boston College, Boston, MA, US. Release Date: 20130318. Correction Date: 20130415. Publication Type: Book, (0200); Authored Book, (0240); . Media Covered: Print. ISBN: 0-465-02599-4, Hardcover, 0465025994; 978-0-465-02599-2, Hardcover, 9780465025992; 0-465-03791-7, PDF, 0465037917; 978-0-465-03791-9, PDF, 9780465037919. Language: English. Major Descriptor: Childhood Development; Childhood Play Behavior; Internal External Locus of Control; Learning Environment; Nontraditional Education. Minor Descriptor: Peer Relations; Problem Solving; Resilience (Psychological); School Learning; Schools; Student Engagement. Classification: Curriculum & Programs & Teaching Methods (3530) . Population: Human (10); . Age Group: Childhood (birth-12 yrs) (100) . Intended Audience: Psychology: Professional & Research (PS) . Tests & Measures: Rotter Internal External Locus of Control Scale; Taylor Manifest Anxiety Scale DOI: 10.1037/t00936-000; Minnesota Multiphasic Personality Inventory; Narcissistic Personality Inventory DOI: 10.1037/t00001-000; Interpersonal Reactivity Index DOI: 10.1037/t01093-000; . References Available: Y
KW - PsycInfo
KW - Unsupervised Play Addition
KW - school
KW - CHILDREN
KW - Play
KW - Learning
KW - Development

KW - life control
KW - Problem Solving
KW - Peers
KW - emotional resilience
KW - Childhood Development
KW - Childhood Play Behavior
KW - Internal External Locus of Control
KW - Learning Environment
KW - Nontraditional Education
KW - Peer Relations
KW - Resilience (Psychological)
KW - School Learning
KW - Schools
KW - Student Engagement
RP - NOT IN FILE
CY - New York, NY US
PB - Basic Books
N2 - (from the jacket) Our children spend their days being passively instructed, and made to sit still and take tests-often against their will. We call this imprisonment schooling, yet wonder why kids become bored and misbehave. Even outside of school children today seldom play and explore without adult supervision, and are afforded few opportunities to control their own lives. The result: anxious, unfocused children who see schooling-and life-as a series of hoops to struggle through. In Free to Learn, developmental psychologist Peter Gray argues that our children, if free to pursue their own interests through play, will not only learn all they need to know but will do so with energy and passion. Children come into this world burning to learn, equipped with the curiosity, playfulness, and sociability to direct their own education. Yet we have squelched such instincts in a school model originally developed to indoctrinate, not to promote intellectual growth. To foster children who will thrive in today's constantly changing world, we must entrust them to steer their own learning and development. Drawing on evidence from anthropology, psychology, and history, Gray demonstrates that free play is the primary means by which children learn to control their lives, solve problems, get along with peers, and become emotionally resilient. This capacity to learn through play evolved long ago, in hunter-gatherer bands where children acquired the skills of the culture through their own initiatives. And these instincts still operate remarkably well today, as studies at alterative, democratically administered schools show. When children are in charge of their own education, they learn better-and at lower cost than the traditional model of coercive schooling. A brave, counterintuitive proposal for freeing our children from the shackles of the curiosity-killing institution we call school, Free to Learn suggests that it's time to stop asking what's wrong with our children, and start asking what's wrong with the system. It shows how we can act-both as parents and as members of society-to improve children's lives and promote their happiness and learning. (PsycINFO Database Record (c) 2013 APA, all rights reserved) (jacket)
SN - 0-465-02599-4
UR - http://search.ebscohost.com/login.aspx?direct=true&db=psyh&AN=2012-32884-000&site=ehost-live
ER -

TY - JOUR
ID - 1683
T1 - Refugee and asylum seeker children
A1 - Bhugra,Dinesh
Y1 - 2012/05//
KW - PsycInfo
KW - Unsupervised Play Addition
KW - Refugees
KW - asylum seeking children
KW - health needs
KW - Child Welfare
KW - Health Service Needs
KW - Mental Health
KW - Physical Health

KW - Psychiatric Hospitals
RP - NOT IN FILE
SP - 1
EP - 2
JF - Arab Journal of Psychiatry
VL - 23
IS - 1
CY - Jordan
PB - Arab Federation of Psychiatrists
N2 - There are several million refugees across the globe. Children and adolescent refugees and asylum seekers, especially if they are unaccompanied, have specific needs to ensure that their mental health is preserved. Political reasons, war, religious factors, gender and sexual orientation may all play a role in creating 'push' factors for these individuals to migrate to other countries. They have specific physical and mental health needs. Policy makers need to get the right advice from psychiatrists who are also important in clinical assessment and management of these individuals. Clinicians can play a significant role in caring for such a vulnerable group. (PsycINFO Database Record (c) 2012 APA, all rights reserved) (journal abstract)
SN - 1016-8923
AD - Bhugra, Dinesh, Health Service and Population Research Department, Institute of Psychiatry, King's College London De Crespigny Park, London, United Kingdom, SE5 8AF, dinesh.bhugra@kcl.ac.uk
UR - http://search.ebscohost.com/login.aspx?direct=true&db=psyh&AN=2012-22033-001&site=ehost-live;dinesh.bhugra@kcl.ac.uk
ER -

TY - JOUR
ID - 1684
T1 - Commentary on Patrizio Campanile's 'I had twenty-five piercings and pink hair when...'
A1 - Imber,Ruth
Y1 - 2012/04//
KW - PsycInfo
KW - Unsupervised Play Addition
KW - Adolescent Development
KW - transformative experience
KW - Subjectivity
KW - transitional hysteria
KW - Psychoanalysis
KW - Personality Development
KW - Hysteria
KW - Life Changes
KW - Experiences (Events)
RP - NOT IN FILE
SP - 429
EP - 436
JF - The Psychoanalytic Quarterly
VL - 81
IS - 2
CY - US
PB - Psychoanalytic Quarterly
N2 - Comments on an article by Patrizio Campanile (see record 2012-16795-006). Campanile's engaging article is a useful contribution to building cross cultural bridges. Campanile's paper is concerned with the psychic strains and conflicts often encountered in successfully navigating the challenges of attaining adolescence. His main clinical presentation is a dramatic description of a pubescent 12 year old girl entangled in the arduous task of leaving childhood and entering young adulthood. The methodology presented illustrates the author's eagerness to make swift and timely use of what he suspects are the unconscious dynamics at play for the patient, without a long, drawn-out exploration of the process by which she arrived at her current condition. From my analytic "subcultural" viewpoint which, embedded in it as I am, I believe to be progressive and valid-there is too much emphasis here on the idea of discrete stages and less on the fluid back and forth that is more likely to

characterize our investment in self and other throughout our lives. While I doubt that Campanile would disagree with this belief in fluidity, his adherence to the language of libido and narcissism lends itself to what to me seems an outmoded way of viewing psychic life. There is some irony in this as I also believe that there is much in his work as described here that is quite up to date and clinically useful. (PsycINFO Database Record (c) 2013 APA, all rights reserved)
SN - 2167-4086
AD - Imber, Ruth, 451 West End Avenue, Suite 1-J, New York, NY, US, 10024, rimberphd@aol.com
UR - http://search.ebscohost.com/login.aspx?direct=true&db=psyh&AN=2012-16795-008&site=ehost-live;rimberphd@aol.com
L3 - 10.1002/j.2167-4086.2012.tb00500.x
ER -

TY - THES
ID - 1685
T1 - The effect of music on transitions and spoken redirections in a preschool classroom
A1 - Gadberry,David L.
Y1 - 2012///
N1 - Accession Number: 2012-99050-194. First Author & Affiliation: Gadberry, David L.; U Kansas, US. Other Journal Titles: Dissertation Abstracts International. Release Date: 20120528. Publication Type: Dissertation Abstract, (0400); . Media Covered: Electronic. Document Type: Dissertation. Dissertation Number: AAI3458216. ISBN: 978-1-124-69859-5, 9781124698595. Language: English. Major Descriptor: Classrooms; Music; Preschool Education; Teachers; Training. Classification: Educational Psychology (3500) . Population: Human (10); . Age Group: Adulthood (18 yrs & older) (300) . Methodology: Empirical Study; Quantitative Study. Page Count: 1
KW - PsycInfo
KW - Unsupervised Play Addition
KW - preschool classroom
KW - Teachers
KW - music education training
KW - Classrooms
KW - Music
KW - Preschool Education
KW - Training
RP - NOT IN FILE
JF - Dissertation Abstracts International Section A: Humanities and Social Sciences
VL - 72
IS - 9-A
CY - US
PB - ProQuest Information & Learning
N2 - The purpose of this study was to investigate the effect of music on transition duration for preschool-age children. Particularly, the focus was on transitioning between a preferred activity to a non-preferred activity, which was free play to cleaning up the toys from free play. Classroom teachers, without formal music education training, were given instruction in using musical transitions with both live and recorded music. Teachers were recorded singing without accompaniment for use during the recorded music treatment, and the teachers provided unaccompanied singing during the live music treatment. An ABAC reversal design was used to examine the baseline measurements (A) and the music treatments (B, C). Three classes were observed and treated using a single case design. Data collection consisted of the overall length of the transition in sections and behavioral differences for the teachers, which were the number of spoken redirections. The data concerning the first research question, whether there were differences in the transition lengths between the baseline and music conditions were shortest during the live music treatment in two of the three classrooms. The data addressing the second research question, which was whether there were differences in the number of spoken redirections, also showed the fewest redirections during the music treatments in two of the three classes. Of these two, one exhibited the fewest redirections during the recorded music treatment, and the other exhibited the fewest redirections during the live music treatment. (PsycINFO Database Record (c) 2012 APA, all rights reserved)
SN - 0419-4209
UR - http://search.ebscohost.com/login.aspx?direct=true&db=psyh&AN=2012-99050-194&site=ehost-live

ER -

TY - THES
ID - 1686
T1 - Intergenerational transmission of health-promoting behaviors: Examining participation in physical activity between middle-aged mothers and their younger adult children
A1 - Stahl,Sarah T.
Y1 - 2012///
N1 - Accession Number: 2012-99120-332. First Author & Affiliation: Stahl, Sarah T.; West Virginia U., US. Other Journal Titles: Dissertation Abstracts International. Release Date: 20120827. Publication Type: Dissertation Abstract, (0400); . Media Covered: Electronic. Document Type: Dissertation. Dissertation Number: AAI3476509. ISBN: 978-1-124-89735-6, 9781124897356. Language: English. Major Descriptor: Age Differences; Exercise; Health Promotion; Physical Activity; Transgenerational Patterns. Minor Descriptor: Mothers. Classification: General Psychology (2100) . Population: Human (10); Female (40); . Location: US. Age Group: Adulthood (18 yrs & older) (300) Young Adulthood (18-29 yrs) (320) Thirties (30-39 yrs) (340) Middle Age (40-64 yrs) (360) . Methodology: Empirical Study; Quantitative Study. Page Count: 1
KW - PsycInfo
KW - Unsupervised Play Addition
KW - intergenerational transmission
KW - health promoting behavior
KW - Physical Activity
KW - middle aged mothers
KW - younger adult children
KW - Age Differences
KW - Exercise
KW - Health Promotion
KW - Transgenerational Patterns
KW - Mothers
RP - NOT IN FILE
JF - Dissertation Abstracts International: Section B: The Sciences and Engineering
VL - 72
IS - 12-B
CY - US
PB - ProQuest Information & Learning
N2 - Research has provided considerable evidence that participation in regular physical activity is associated with numerous physical and mental health benefits (Penedo & Dahn, 2005). Despite public health efforts to increase the activity levels adults, only 25% of the U.S. adult population is regularly active and nearly 60% remains sedentary (US Department of Health and Human Services [USDHHS], 2008). A small, but growing, area of research has examined physical activity from an intergenerational or dyadic perspective that considers how involvements in close, personal relationships influence levels of physical activity. In a sample of middle-aged mothers and their younger adult children, the present study had three primary objectives: (a) to examine the relations among well-known predictors of physical activity in younger adulthood and midlife, (b) to examine the relations between individual characteristics and interpersonal variables on physical activity within mother-child dyads, and (c) to examine whether mothers influenced their daughters more strongly than their sons. Data from 48 mother-child dyads between the ages of 18 and 57 were collected via an online survey. Findings from the first research objective indicate an adequate fit of the model to the data for middle-aged mothers (?[sup]2[/sup] (df = 2; N = 48) = 2.938, p = .230) and younger adults (?[sup] 2[/sup] (df = 3; N = 48) = .288, p = .962). With regard to the second research objective, results indicated an adequate fit of the model to the data ?[sup]2[/sup] (df = 6; N = 48) = 5.057, p = .537. The hypothesized model explained 2.4% of variance in younger adults' physical activity and 17.5% of variance in middle-aged mothers' physical activity. In addition, standardized beta weights provided support for one actor effect, as mothers' internal health locus of control was positively associated with physical activity. (? = .42). Research objective three was not supported. Findings from this study may help inform the design of future health interventions. Specifically, the results suggest that personal relationships, such as the relationship one has with a family member, may play a role in understanding participation in physical activity. (PsycINFO Database Record (c) 2012 APA, all rights reserved)
SN - 0419-4217

UR - http://search.ebscohost.com/login.aspx?direct=true&db=psyh&AN=2012-99120-332&site=ehost-live
ER -

TY - JOUR
ID - 1687
T1 - Integrating intention and context: Assessing social cognition in adults with Asperger syndrome
A1 - Baez,Sandra
A1 - Rattazzi,Alexia
A1 - Gonzalez-Gadea,Mar ą L.
A1 - Torralva,Teresa
A1 - Vigliecca,Nora Silvana
A1 - Decety,Jean
A1 - Manes,Facundo
A1 - Ibanez,Agustin
Y1 - 2012/11//
KW - PsycInfo
KW - Unsupervised Play Addition
KW - integrating intention
KW - Social Cognition
KW - Asperger syndrome
KW - theory of mind
KW - Executive Function
KW - Aspergers Syndrome
KW - Intention
RP - NOT IN FILE
JF - Frontiers in Human Neuroscience
VL - 6
CY - Switzerland
PB - Frontiers Research Foundation
U1 - Sponsor: CONICET, FONDECYT. Grant: 1130920. Recipients: No recipient indicated;Sponsor: INECO Foundation. Recipients: No recipient indicated
N2 - Deficits in social cognition are an evident clinical feature of the Asperger syndrome (AS). Although many daily life problems of adults with AS are related to social cognition impairments, few studies have conducted comprehensive research in this area. The current study examined multiple domains of social cognition in adults with AS assessing the executive functions (EF) and exploring the intra and inter-individual variability. Fifteen adult's diagnosed with AS and 15 matched healthy controls completed a battery of social cognition tasks. This battery included measures of emotion recognition, theory of mind (ToM), empathy, moral judgment, social norms knowledge, and self-monitoring behavior in social settings. We controlled for the effect of EF and explored the individual variability. The results indicated that adults with AS had a fundamental deficit in several domains of social cognition. We also found high variability in the social cognition tasks. In these tasks, AS participants obtained mostly subnormal performance. EF did not seem to play a major role in the social cognition impairments. Our results suggest that adults with AS present a pattern of social cognition deficits characterized by the decreased ability to implicitly encode and integrate contextual information in order to access to the social meaning. Nevertheless, when social information is explicitly presented or the situation can be navigated with abstract rules, performance is improved. Our findings have implications for the diagnosis and treatment of individuals with AS as well as for the neurocognitive models of this syndrome. (PsycINFO Database Record (c) 2013 APA, all rights reserved) (journal abstract)
SN - 1662-5161
AD - Ibanez, Agustin, Laboratory of Experimental Psychology and Neuroscience, Institute of Cognitive Neurology Pacheco de Melo 1860, 1860, Buenos Aires, Argentina, aibanez@ineco.org.ar
UR - http://search.ebscohost.com/login.aspx?direct=true&db=psyh&AN=2012-33123-001&site=ehost-live;aibanez@ineco.org.ar
L3 - 10.3389/fnhum.2012.00302
ER -

TY - CHAP

ID - 1688
T1 - Developing motivation through peer interaction: A cross-cultural analysis
A1 - Medina,Jose Sanchez
A1 - Martinez,Virginia
Y1 - 2012///
N1 - Accession Number: 2011-27520-006. First Author & Affiliation: Medina, Jose Sanchez. Release Date: 20120312. Publication Type: Book, (0200); Edited Book, (0280); . Media Covered: Print. Document Type: Chapter. ISBN: 978-0-521-76742-2, Hardcover, 9780521767422. Language: English. Major Descriptor: Childhood Development; Childhood Play Behavior; Motivation; Peer Relations; Psychological Development. Minor Descriptor: Conflict Resolution; Cross Cultural Differences; Culture (Anthropological); History; Preschool Students; Psychological Theories; Social Values; Socialization; Sociocultural Factors; Vygotsky (Lev). Classification: Developmental Psychology (2800) . Population: Human (10); Male (30); . Age Group: Childhood (birth-12 yrs) (100) Preschool Age (2-5 yrs) (160) . Intended Audience: Psychology: Professional & Research (PS) . References Available: Y.. Page Count: 18
KW - PsycInfo
KW - Unsupervised Play Addition
KW - Psychological Development
KW - Motivation
KW - Peers
KW - interactions
KW - Culture
KW - Play
KW - CHILDREN
KW - motives
KW - History
KW - Theories
KW - Vygotsky
KW - values
KW - Cultural Differences
KW - Research
KW - pre-schoolers
KW - Conflict Resolution
KW - socialisation
KW - Childhood Development
KW - Childhood Play Behavior
KW - Peer Relations
KW - Cross Cultural Differences
KW - Culture (Anthropological)
KW - Preschool Students
KW - Psychological Theories
KW - Social Values
KW - SOCIALIZATION
KW - Sociocultural Factors
KW - Vygotsky (Lev)
RP - NOT IN FILE
SP - 97
EP - 114
T2 - Motives in children's development: Cultural-historical approaches
A2 - Hedegaard,Mariane
A2 - Edwards,Anne
A2 - Fleer,Marilyn
CY - New York, NY US
PB - Cambridge University Press
N2 - (from the chapter) This chapter focuses on how peer interactions in play constitute a setting which contributes to the children's acquisition of motives. We also argue that the study of peer interactions can shed light on two gaps which persist in the explanation of psychological development within the cultural-historical

theory (Vygotsky, 1978; Wertsch, 1985). The first is that, although this theoretical approach has placed great emphasis on the social and cultural construction of psychological development, in this construction the role of adults such as caregivers and teachers is highlighted and that of peers has been neglected. The adults are seen as holding the keys to the cultural adult world, organising the interactions and regulating children's behaviours in ways which give access to the cultural system of values and cultural norms. The second gap is, as Stetsenko and Arievitch (2004) point out, that although the cultural-historical theory recognises the active role of individuals in the appropriation of tools and systems of motives and cultural values, it has paid much less attention to the active role of individuals in creating and changing culture itself. Hedegaard (2005, 2008) has shown that the child is not a passive participant in these exchanges. The position, the way of behaving, and the degree of engagement of children in everyday practices can transform the practices substantially, playing a leading role in how these practices are developed. By focusing on peer interaction without adult direct supervision, we are able to analyse the active role of children acting in a setting in which they take over the values and norms of the adult world. To illustrate this argumentation, we present a research on peer interaction in two different cultures. We will use Corsaro's (1997) concept of peer-cultures to analyse how children create and recreate the adult cultures in their interactions. The distinction between individualistic and collectivist cultures will be the backbone on which we will build our argument. Based on observational studies of pre-schoolers' behaviour during free playtime at school, we show that the way children play and resolve their conflicts reflects and recreates the society's motive system. We argue that peer interactions are a distinctive setting for socialisation and development in which children acquire the motives to act in relation to their adult world of reference. (PsycINFO Database Record (c) 2012 APA, all rights reserved) (chapter)
SN - 978-0-521-76742-2
UR - http://search.ebscohost.com/login.aspx?direct=true&db=psyh&AN=2011-27520-006&site=ehost-live
ER -

TY - CHAP
ID - 1689
T1 - 'Please sir! Can I come out of the closet and into the classroom?': British low culture and representations of queer masculinities in education
A1 - Jachimiak,Peter Hughes
Y1 - 2012///
N1 - Accession Number: 2012-16023-013. First Author & Affiliation: Jachimiak, Peter Hughes; Cardiff School of Creative and Cultural Industries, University of Glamorgan, Wales. Translated Book Series Title: Explorations of educational purpose. Release Date: 20120820. Publication Type: Book, (0200); Edited Book, (0280); . Media Covered: Print. Document Type: Chapter. ISBN: 978-94-007-2551-5, Hardcover, 9789400725515; 978-94-007-2552-2, PDF, 9789400725522. Language: English. Major Descriptor: Homosexuality; Masculinity; Popular Culture; Schools; Television. Minor Descriptor: Classrooms. Classification: Mass Media Communications (2750) Sexual Behavior & Sexual Orientation (2980) . Population: Human (10); . Location: Great Britain. Intended Audience: Psychology: Professional & Research (PS) . References Available: Y.. Page Count: 21
KW - PsycInfo
KW - Unsupervised Play Addition
KW - British low culture
KW - queer masculinities
KW - Education
KW - Popular Culture
KW - cultural pedagogy
KW - Homosexuality
KW - Masculinity
KW - Schools
KW - Television
KW - Classrooms
RP - NOT IN FILE
SP - 225
EP - 245
VL - 21
T2 - Queer masculinities: A critical reader in education
A2 - Landreau,John C.

A2 - Rodriguez,Nelson M.
CY - New York, NY US
PB - Springer Science + Business Media
T3 - Explorations of educational purpose; Vol 21; 1875-4449 (Print);
N2 - (from the chapter) This proposed chapter considers representations of queer masculinities in popular culture as cultural pedagogy. As such, it aims to make explicit the pedagogical role of both the presence and, perhaps more importantly, the absence of queer masculinities in the cultural representations of schools, schooling, and schoolchildren. Offering quintessentially British case studies that possess global resonances, I intend to draw from what Leon Hunt (1998) refers to as "'[b]ehind the school desk' fiction" (p. 75)-such as weekly comics and mass-market "pulp" novels-all illicitly read by schoolchildren during school hours. Additionally, this chapter will examine television sitcoms, dramas, films, and so on that are aimed at not only children and young adults but also adults and present (misrepresent even) the schoolchildren, schools, its environments, and ideologies. For example. Please Sir!, a highly popular ITV television series of the 1970s, now available via nostalgia-oriented Network DVD, or the more "honest" and "gritty" Grange Hill, recently decommissioned by the BBC after its 30-year run. Produced during an era when the low end of popular culture tended to be openly racist, sexist, and homophobic, this chapter insists that such texts, as per hegemonic cultural pedagogy, implicitly reproduce the heterosexual status quo, while simultaneously suppressing homosexuality. By examining the intersections between the categories/concepts of queer masculinities and education, the aim of this chapter (in line with the broader aims of this reader) is to propose an understanding of the range and layers of meanings and practices of those intersections as well as offering the possibility for imaginatively reconstructing the categories/concepts themselves. Indeed, this chapter encourages-instead of the unquestioned absence of queer masculinities from the classroom-the introduction of non-differential attitudes (while, at the same time, being appreciative of "difference"), not only within schools and schooling but, perhaps more importantly, cultural representations of education more generally. In turn, it is hoped that this will help to advance the meanings and/or practices of queer masculinity in education, especially as this plays out within the context of popular culture as a contested site of cultural pedagogy. (PsycINFO Database Record (c) 2012 APA, all rights reserved) (chapter)
SN - 978-94-007-2551-5
AD - Jachimiak, Peter Hughes, Cardiff School of Creative and Cultural Industries, University of Glamorgan , Wales, phjachim@glam.ac.uk
UR - http://search.ebscohost.com/login.aspx?direct=true&db=psyh&AN=2012-16023-013&site=ehost-live;phjachim@glam.ac.uk
L3 - 10.1007/978-94-007-2552-2_14
ER -

TY - JOUR
ID - 1690
T1 - The effects of a conversation prompt procedure on independent play
A1 - Conallen,Kevin
A1 - Reed,Phil
Y1 - 2012/01//
KW - PsycInfo
KW - Unsupervised Play Addition
KW - independent play
KW - conversation prompt procedure
KW - Teaching
KW - autism spectrum disorders
KW - Autism
KW - Pervasive Developmental Disorders
KW - Conversation
KW - Recreation
RP - NOT IN FILE
SP - 365
EP - 377
JF - Research in Autism Spectrum Disorders
VL - 6

IS - 1
CY - Netherlands
PB - Elsevier Science
N2 - This study used a multiple baseline design (ABCAD) to determine whether teaching children with autistic spectrum disorders (ASD) to tact private events would function as a conditioned reinforcer for teaching non-preferred play activities. In this study, 10 children, aged between 5.3 and 8.9 years of age, were taught to tact a set of private events (e.g., fun, bored, easy, hard) after engaging in selected preferred, and non-preferred, play activities. These `typical' language exchanges were built into existing individual activity play schedules, and were designed to prompt a conversational unit after a play period of up to 10 min. This conversational unit was designed to serve as a conditioned reinforcer for the activity under observation. The results show that having access to a set of tacts for putatively private events could function as a conditioned reinforcer when teaching non-preferred play activities, reduced off-task play behavior, while revealing a measurable increase in spontaneous language, emitted both during the targeted play sessions. (PsycINFO Database Record (c) 2012 APA, all rights reserved) (journal abstract)
SN - 1750-9467
AD - Reed, Phil, Department of Psychology, Swansea University Singleton Park, Swansea, United Kingdom, SA2 8PP, P.Reed@swansea.ac.uk
UR - http://search.ebscohost.com/login.aspx?direct=true&db=psyh&AN=2011-25855-043&site=ehost-live;P.Reed@swansea.ac.uk
L3 - 10.1016/j.rasd.2011.06.010
ER -

TY - JOUR
ID - 1691
T1 - Park-based physical activity among children and adolescents
A1 - Floyd,Myron F.
A1 - Bocarro,Jason N.
A1 - Smith,William R.
A1 - Baran,Perver K.
A1 - Moore,Robin C.
A1 - Cosco,Nilda G.
A1 - Edwards,Michael B.
A1 - Suau,Luis J.
A1 - Fang,Kunsheng
Y1 - 2011/09//
KW - PsycInfo
KW - Unsupervised Play Addition
KW - Physical Activity
KW - Childhood Development
KW - Adolescent Development
KW - Environment
KW - Parks
KW - Recreation Areas
RP - NOT IN FILE
SP - 258
EP - 265
JA - American Journal of Preventive Medicine
VL - 41
IS - 3
CY - Netherlands
PB - Elsevier Science
N2 - Background: Availability of parks is associated with higher levels of physical activity among children and adolescents. Few studies examine actual park use and park-based physical activity in these populations. Purpose: This study examined associations among individual, park, and neighborhood environmental characteristics and children's and adolescent's park-based physical activity. Methods: Data were collected in 2007 on 2712 children in 20 randomly selected parks in Durham NC. The System for Observing Play and

Recreation in Communities (SOPARC) provided measures of physical activity. Hierarchic regression analysis assessed associations among individual, park, and neighborhood environmental characteristics and children's park-based physical activity. Data were analyzed in 2010. Results: Of the 2712 children observed, 34.2% and 13.2% were engaged in walking or vigorous physical activity. Environmental features of parks were associated with activity levels whereas neighborhood characteristics were not. Physical activity was negatively associated with gender (girls) (p = 0.003); presence of a parent (p < 0.0001); presence of nonparental adult (p = 0.006); and an interaction involving the 0-5 years age group and style of play (p = 0.017). Higher level of physical activity was associated with presence of other active children (p < 0.0001); courts (e.g., basketball); and an interaction between number of recreation facilities and formal activities (p = 0.004). Conclusions: These social factors and design features should be considered in order to stimulate higher levels of park-based physical activity among children and adolescents. (PsycINFO Database Record (c) 2012 APA, all rights reserved) (journal abstract)
SN - 0749-3797
AD - Floyd, Myron F., Department of Parks, Recreation and Tourism Management, North Carolina State University Biltmore Hall 4008J, Box 8004, Raleigh, NC, US, 27695-8004, mffloyd@ncsu.edu
UR - http://search.ebscohost.com/login.aspx?direct=true&db=psyh&AN=2011-18803-006&site=ehost-live;mffloyd@ncsu.edu
L3 - 10.1016/j.amepre.2011.04.013
ER -

TY - JOUR
ID - 1695
T1 - Social engagement leads 2?year?olds to overestimate others' knowledge
A1 - Moll,Henrike
A1 - Carpenter,Malinda
A1 - Tomasello,Michael
Y1 - 2011/05//
KW - PsycInfo
KW - Unsupervised Play Addition
KW - social engagement
KW - Knowledge Level
KW - playing behavior
KW - Interpersonal Interaction
KW - 2-year-old children
KW - Childhood Play Behavior
KW - Early Childhood Development
KW - Social Behavior
RP - NOT IN FILE
SP - 248
EP - 265
JF - Infancy
VL - 16
IS - 3
CY - United Kingdom
PB - Wiley-Blackwell Publishing Ltd.
N2 - Previous research has found that young children recognize an adult as being acquainted with an object most readily when the child and adult have previously engaged socially with that object together. In the current study, we tested the hypothesis that such social engagement is so powerful that it can sometimes lead children to overestimate what has been shared. After having shared two objects with an adult in turn, 2-year-old children played with a third object the adult could not see. In three out of four conditions, the adult remained co-present and/or communicated to the child while she played with the third object. Children falsely perceived the adult as being acquainted with the third object when she remained co-present (whether or not she also communicated) but not when she clearly terminated the interaction by disengaging and leaving. These results suggest that when young children are engaged with a co-present person they tend to overestimate the other's knowledge. (PsycINFO Database Record (c) 2012 APA, all rights reserved) (journal abstract)
SN - 1532-7078

AD - Moll, Henrike, Department of Psychology, Max Planck Institute for Evolutionary Anthropology Deutscher Platz 6, 04103, Leipzig, Germany, moll@eva.mpg.de
UR - http://search.ebscohost.com/login.aspx?direct=true&db=psyh&AN=2011-07099-002&site=ehost-live;moll@eva.mpg.de
L3 - 10.1111/j.1532-7078.2010.00044.x
ER -

TY - JOUR
ID - 1697
T1 - Evidence-based practices addressed in community-based children's mental health clinical supervision
A1 - Accurso,Erin C.
A1 - Taylor,Robin M.
A1 - Garland,Ann F.
Y1 - 2011/05//
KW - PsycInfo
KW - Unsupervised Play Addition
KW - child mental health services
KW - clinical supervision
KW - evidence-based care
KW - behavioral problems
KW - Clinical Practice
KW - Community Mental Health Services
KW - Professional Supervision
KW - Behavior Problems
KW - Evidence Based Practice
KW - Mental Health
RP - NOT IN FILE
SP - 88
EP - 96
JF - Training and Education in Professional Psychology
VL - 5
IS - 2
CY - US
PB - American Psychological Association
U1 - Sponsor: National Institutes of Health. Grant: F31MH083399. Recipients: Accurso, Erin C.;Sponsor: National Institutes of Health. Grant: R01MH66070. Recipients: Garland, Ann F.
N2 - Clinical supervision is the principal method of training for psychotherapeutic practice, however there is virtually no research on supervision practice in community settings. Of particular interest is the role supervision might play in facilitating implementation of evidence-based (EB) care in routine care settings. This study examines the format and functions of clinical supervision sessions in routine care, as well as the extent to which supervision addresses psychotherapeutic practice elements common to EB care for children with disruptive behavior problems, who represent the majority of patients served in publicly funded routine care settings. Supervisors (n = 7) and supervisees (n = 12) from four publicly funded community-based child mental health clinics reported on 130 supervision sessions. Supervision sessions were primarily individual in-person meetings lasting one hour. The most common functions included case conceptualization and therapy interventions. Coverage of practice elements common to EB treatments was brief. Despite the fact that most children presenting to public mental health services are referred for disruptive behavior problems, supervision sessions are infrequently focused on practice elements consistent with EB treatments for this population. Supervision is a promising avenue through which training in EB practices could be supported to improve the quality of care for children in community-based "usual care" clinics. (PsycINFO Database Record (c) 2012 APA, all rights reserved) (journal abstract)
SN - 1931-3926
AD - Accurso, Erin C., 3020 Children's Way, MC 5033, San Diego, CA, US, 92123, eaccurso@casrc.org
UR - http://search.ebscohost.com/login.aspx?direct=true&db=psyh&AN=2011-09655-006&site=ehost-live;eaccurso@casrc.org
L3 - 10.1037/a0023537

ER -

TY - CHAP
ID - 1701
T1 - Behavioral treatment of autism
A1 - Kodak,Tiffany
A1 - Grow,Laura L.
Y1 - 2011///
N1 - Accession Number: 2011-22103-026. First Author & Affiliation: Kodak, Tiffany; Munroe-Meyer Institute, University of Nebraska Medical Center, Omaha, NE, US. Release Date: 20120402. Publication Type: Book, (0200); Edited Book, (0280); . Media Covered: Print. Document Type: Chapter. Book Type: Handbook/Manual. ISBN: 978-1-60918-468-1, Hardcover, 9781609184681; 978-1-60918-503-9, PDF, 9781609185039. Language: English. Major Descriptor: Autism; Developmental Disabilities; Early Intervention; Models; Special Education. Minor Descriptor: Adaptive Behavior; Behavior Problems; Childhood Development; Generalization (Learning); Intervention; Operant Conditioning; Pervasive Developmental Disorders; Reinforcement; Stimulus Control. Classification: Behavior Therapy & Behavior Modification (3312) Special & Remedial Education (3570) . Population: Human (10); . Location: US. Age Group: Childhood (birth-12 yrs) (100) Preschool Age (2-5 yrs) (160) School Age (6-12 yrs) (180) Adolescence (13-17 yrs) (200) . Intended Audience: Psychology: Professional & Research (PS) . Tests & Measures: Assessment of Basic Learning and Language Skills-Revised; Verbal Behavior Milestones Assessment and Placement Program; . References Available: Y.. Page Count: 15
KW - PsycInfo
KW - Unsupervised Play Addition
KW - autism spectrum disorders
KW - educational programming
KW - Early Intervention
KW - Adaptive Behavior
KW - reduce problem behavior
KW - Operant Conditioning
KW - Reinforcement
KW - stimulus control
KW - Generalization
KW - Models
KW - Autism
KW - Developmental Disabilities
KW - Special Education
KW - Behavior Problems
KW - Childhood Development
KW - Generalization (Learning)
KW - Intervention
KW - Pervasive Developmental Disorders
RP - NOT IN FILE
SP - 402
EP - 416
T2 - Handbook of applied behavior analysis
A2 - Fisher,Wayne W.
A2 - Piazza,Cathleen C.
A2 - Roane,Henry S.
CY - New York, NY US
PB - Guilford Press
N2 - (from the chapter) Autism spectrum disorders (ASDs) are pervasive developmental disorders that are evident in early childhood. The diagnosis of an ASD is based on specific diagnostic criteria established by the Diagnostic and Statistical Manual of Mental Disorders, and encompasses a number of disorders, including autism, Asperger's syndrome, Rett's disorder, childhood disintegrative disorder, and pervasive developmental disorder not otherwise specified. Early and intensive behavioral intervention (EIBI) is an approach to educational programming for children with ASDs. Intensive behavior intervention is characterized by a

comprehensive, hierarchically arranged curriculum implemented for several years, with the goal of improving the overall functioning level of the child. The procedures utilized to increase adaptive behavior and reduce problem behavior are based on principles of operant conditioning (e.g., reinforcement, stimulus control, generalization). Numerous learning opportunities are presented with carefully programmed instructional and reinforcement procedures. Multiple areas of functioning are targeted in a developmental sequence to improve several broad skill areas. Educational targets for EIBI programming often include preacademic and academic skills, language, social skills, and independent play skills, among others. A number of early intervention models based on the principles of ABA were developed to provide an educational curriculum for teaching individuals with ASDs. The models described below have demonstrated effectiveness in studies published in peer-reviewed journals, and have since been published as commercially available treatment manuals. Each model is reviewed in detail, followed by a description of key differences. Finally, recommendations are provided for integrating the models to maximize learning. (PsycINFO Database Record (c) 2012 APA, all rights reserved) (chapter)
SN - 978-1-60918-468-1
UR - http://search.ebscohost.com/login.aspx?direct=true&db=psyh&AN=2011-22103-026&site=ehost-live
ER -

TY - CHAP
ID - 1702
T1 - Intellectual disabilities
A1 - Alexander,Katie
Y1 - 2011///
N1 - Accession Number: 2012-32980-009. First Author & Affiliation: Alexander, Katie; Model Asperger Program, Ivymount School, Inc., Rockville, MD, US. Release Date: 20130128. Correction Date: 20140120. Publication Type: Book, (0200); Edited Book, (0280); . Media Covered: Print. Document Type: Chapter. ISBN: 0-8036-1704-6, Hardcover, 0803617046; 978-0-8036-1704-9, Hardcover, 9780803617049. Language: English. Major Descriptor: Occupational Therapists; Occupational Therapy; Intellectual Development Disorder. Minor Descriptor: Developmental Disabilities; Drug Therapy; Environmental Effects; Human Sex Differences; Sociocultural Factors. Classification: Mental Retardation (3256) Occupational & Vocational Rehabilitation (3384) . Population: Human (10); . Intended Audience: Psychology: Professional & Research (PS) . References Available: Y.. Page Count: 12
KW - PsycInfo
KW - Unsupervised Play Addition
KW - intellectual disabilities
KW - Developmental Disabilities
KW - Occupational Therapists
KW - Occupational Therapy
KW - gender influences
KW - cultural influence
KW - medication
KW - environmental factors
KW - Intellectual Development Disorder
KW - Drug Therapy
KW - Environmental Effects
KW - Human Sex Differences
KW - Sociocultural Factors
RP - NOT IN FILE
SP - 111
EP - 122
T2 - Occupational therapy in mental health: A vision for participation
A2 - Brown,Catana
A2 - Stoffel,Virginia C.
CY - Philadelphia, PA US
PB - F A Davis
N2 - (from the chapter) Occupational therapists have the opportunity to work with individuals with developmental disabilities in many contexts. Because many developmental disabilities are identified early in life, occupational therapists working in early intervention are among the first to develop and share a

comprehensive understanding of a child. They work alongside the family and other caregivers to identify barriers to participation and, through client-centered intervention, foster optimal participation, minimize disability, and promote a solid foundation on which future progress can be built. Occupational therapists may also work in school or community settings, serving students later in childhood. The therapeutic process remains the same, but the social and task expectations of the many contexts in which children live, play, and learn shift as a child gets older. The transition from adolescence into adulthood presents a new context that is rich with opportunity to foster community participation. Occupational therapists who work in early childhood, school systems, and mental health are likely to serve an individual because of his or her developmental disability. However, an occupational therapist in settings such as physical rehabilitation may serve an individual with a developmental disability primarily due to physical impairment, for example, from trauma; in this case, the presence of an intellectual disability will have a significant affect on intervention decisions. In all cases, each individual with a developmental disability will have his or her own preferences, strengths, difficulties, expectations, and goals. This chapter highlights the heterogeneity of intellectual disabilities. Although the diagnosis is simple and straightforward, there are a multitude of causes and the resulting severity and impact on occupational functioning varies widely among people with intellectual disabilities. This chapter also provides information on the prevalence, gender- and culture-specific influences, medications, and environmental factors that affect occupational performance. (PsycINFO Database Record (c) 2014 APA, all rights reserved) (chapter)
SN - 0-8036-1704-6
UR - http://search.ebscohost.com/login.aspx?direct=true&db=psyh&AN=2012-32980-009&site=ehost-live
ER -

TY - JOUR
ID - 1704
T1 - Quantifying the effects of normal ageing on white matter structure using unsupervised tract shape modelling
A1 - Bastin,Mark E.
A1 - Mu¤oz Maniega,Susana
A1 - Ferguson,Karen J.
A1 - Brown,Laura J.
A1 - Wardlaw,Joanna M.
A1 - MacLullich,Alasdair M.J.
A1 - Clayden,Jonathan D.
Y1 - 2010/05//
KW - PsycInfo
KW - Unsupervised Play Addition
KW - normal aging
KW - white matter structure
KW - unsupervised tract shape modeling
KW - brain atrophy
KW - Magnetic Resonance Imaging
KW - tractography
KW - Aging
KW - Brain Size
KW - Corpus Callosum
KW - White Matter
KW - Cerebral Atrophy
KW - Cognitive Ability
RP - NOT IN FILE
SP - 1
EP - 10
JF - NeuroImage
VL - 51
IS - 1
CY - Netherlands
PB - Elsevier Science

U1 - Sponsor: Medical Research Council. Other Details: Clinician Scientist Fellowship. Recipients: Ferguson, Karen J.; MacLullich, Alasdair M. J.;Sponsor: Scottish Funding Council (SFC); Scotland. Other Details: Through the SINAPSE Collaboration (Scottish Imaging Network. A Platform for Scientific Excellence). Recipients: Wardlaw, Joanna M.;Sponsor: Medical Research Council, Centre for Cognitive Ageing and Cognitive Epidemiology. Recipients: No recipient indicated;Sponsor: National Institutes of Health; US. Grant: R01 EB004155-03. Recipients: No recipient indicated
N2 - Quantitative tractography may provide insights into regional heterogeneity of changes in white matter structure in normal ageing. Here we examine how brain atrophy and white matter lesions affect correlations between tract shape, tract integrity and age in a range of frontal and non-frontal tracts in 90 non-demented subjects aged over 65 years using an enhanced version of probabilistic neighbourhood tractography. This novel method for automatic single seed point placement employs unsupervised learning and streamline selection to provide reliable and accurate tract segmentation, whilst also indicating how the shape of an individual tract compares to that of a predefined reference tract. There were significant negative correlations between tract shape similarity to reference tracts derived from a young brain white matter atlas and age in genu and splenium of corpus callosum. Controlling for intracranial and lateral ventricle volume, the latter of which increased significantly with age, attenuated these correlations by 40% and 84%, respectively, indicating that this age-related change in callosal tract topology is significantly mediated by global atrophy and ventricular enlargement. In accordance with the "frontal ageing" hypothesis, there was a significant positive correlation between mean diffusivity (<D>) and age, and a significant negative correlation between fractional anisotropy (FA) and age in corpus callosum genu; correlations not seen in splenium. Significant positive correlations were also observed between <D> and age in bilateral cingulum cingulate gyri, uncinate fasciculi and right corticospinal tract. This pattern of correlations was not, however, reproduced when those subjects with significant white matter lesion load were analyzed separately from those without. These data therefore suggest that brain atrophy and white matter lesions play a significant role in driving regional patterns of agerelated changes in white matter tract shape and integrity. (PsycINFO Database Record (c) 2012 APA, all rights reserved) (journal abstract)
SN - 1053-8119
AD - Bastin, Mark E., Medical and Radiological Sciences (Medical Physics), University of Edinburgh, Western General Hospital Crewe Road, Edinburgh, United Kingdom, EH4 2XU, Mark.Bastin@ed.ac.uk
UR - http://search.ebscohost.com/login.aspx?direct=true&db=psyh&AN=2010-05166-001&site=ehost-live;Mark.Bastin@ed.ac.uk
L3 - 10.1016/j.neuroimage.2010.02.036
ER -

TY - THES
ID - 1706
T1 - The variable child: The vulnerabilities of children and youth in the Canadian refugee determination system
A1 - Ballucci,Dale
Y1 - 2010///
N1 - Accession Number: 2010-99110-152. First Author & Affiliation: Ballucci, Dale; U Alberta, Canada. Other Journal Titles: Dissertation Abstracts International. Release Date: 20100802. Publication Type: Dissertation Abstract, (0400); . Media Covered: Electronic. Document Type: Dissertation. Dissertation Number: AAINR54034. ISBN: 978-0-494-54034-3, 9780494540343. Language: English. Major Descriptor: Decision Making; Legal Decisions; Refugees; Social Processes; Susceptibility (Disorders). Classification: Social Processes & Social Issues (2900) . Population: Human (10); . Location: Canada. Age Group: Childhood (birth-12 yrs) (100) . Methodology: Empirical Study; Quantitative Study. Page Count: 1
KW - PsycInfo
KW - Unsupervised Play Addition
KW - vulnerabilities
KW - Canadian refugee determination system
KW - legal decision-making
KW - Decision Making
KW - Legal Decisions
KW - Refugees
KW - Social Processes
KW - Susceptibility (Disorders)
RP - NOT IN FILE

JF - Dissertation Abstracts International Section A: Humanities and Social Sciences
VL - 70
IS - 12-A
CY - US
PB - ProQuest Information & Learning
N2 - The Variable Child concerns the legal decision-making process in unaccompanied child refugee applications, and the role that conceptions of childhood play in the process. I examine when particular types of knowledge are drawn upon by legal actors, as well as the effects of the claim-making practices that create meaning, or `truth effects', in legal decision-making. I identify how legal actors exercise discretion by investigating how facts are constructed with different ideas about children's competence, abilities and knowledge. The "Unaccompanied Child Refugee Evidentiary and Procedure Guidelines", which governs legal decisions, has embedded within it various, sometimes competing, conceptions of the child and childhood. These multiple notions create considerable discretionary space for refugee officers to make decisions about individual cases. My examination of legal decisions reveals a strategic use of vulnerable and/or responsible conceptions of childhood. Another strategy used to establish facts in these cases is to exclude the cultural differences of childhood-both these practices are accomplished through employing several different knowledge moves. Refugee officers invoke vulnerable and/or responsible constructions of childhood to displace the impact of other/alternative constructions of childhood, namely Chinese ideas of parental relations. This avoids the potential for legal decisions to set standards for similar cases in the future. Childhood studies have documented how different axes of scholarly inquiry produce different understandings, typologies, and knowledges of the child and childhood. What remains understudied is how competing knowledges of the child and childhood are applied, negotiated, and formalized in legal decision-making. My study investigates how power relations constitute particular constructions of childhood, and the consequences these relations have for children's lives. Unlike examining childhood as contextual, I document how variable understandings of the child and childhood are constituted, institutionalized, and normalized through the law. My study examines the complexities of legal decision-making, a process that is often black-boxed. I also trace which conceptions of childhood are drawn upon to substantiate legal claims, and how a social context for the child and childhood emerges. By examining the relations of law in the context of children, my work contributes to the growing area of childhood studies and socio-legal practices. (PsycINFO Database Record (c) 2012 APA, all rights reserved)
SN - 0419-4209
UR - http://search.ebscohost.com/login.aspx?direct=true&db=psyh&AN=2010-99110-152&site=ehost-live
ER -

TY - THES
ID - 1707
T1 - Teaching mothers to use promised consequences with their children
A1 - Owen,Daniela Jade
Y1 - 2010///
N1 - Accession Number: 2010-99180-196. First Author & Affiliation: Owen, Daniela Jade; State U New York at Stony Brook, US. Other Journal Titles: Dissertation Abstracts International. Release Date: 20101129. Publication Type: Dissertation Abstract, (0400); . Media Covered: Electronic. Document Type: Dissertation. Dissertation Number: AAI3399762. ISBN: 978-1-109-66304-4, 9781109663044. Language: English. Major Descriptor: Dyads; Mother Child Relations; Mothers; Teaching. Classification: Social Psychology (3000) . Population: Human (10); Female (40); . Age Group: Childhood (birth-12 yrs) (100) . Methodology: Empirical Study; Quantitative Study. Page Count: 1
KW - PsycInfo
KW - Unsupervised Play Addition
KW - teaching mothers
KW - CHILDREN
KW - promised consequences
KW - Dyads
KW - child actions
KW - Mother Child Relations
KW - Mothers
KW - Teaching
RP - NOT IN FILE

JF - Dissertation Abstracts International: Section B: The Sciences and Engineering
VL - 71
IS - 3-B
CY - US
PB - ProQuest Information & Learning
N2 - Noncompliance in early childhood is the top reason why parents seek out mental health services for young children (Chamberlain & Smith, 2003; Schuhmann, Durning, Eyberg, & Boggs, 1996) and, if untreated, early childhood noncompliance has been found to develop into more profound problems (e.g., Campbell, 1991; McMahon, 1994). Promised consequences-consequences for noncompliance stated with a directive prior to child action-are associated with greater likelihood of child compliance (Owen, Slep, & Heyman, in press). Seventy-five mother-child dyads were recruited to participate in this promised consequences training study. Dyads were randomly assigned to a modeling only condition (MO), a modeling plus directed interaction condition (MDI), or a no training control condition (C). Mothers practiced using promised consequences during a 35-minute mother-child interaction, including a clean-up and an independent play period. Mothers in the two experimental conditions delivered promised consequences more than mothers in the control condition, and mothers in the MDI condition were more likely to follow through with promised consequences than mothers in the control condition. Children did not comply differently based on condition. Mothers in the training experimental conditions reported greater efficacy expectancies for their abilities to use promised consequences and greater outcome expectancies for their children's compliance with promise consequences than mothers in the control condition. Neither efficacy expectancies nor outcome expectancies mediated the relationship between condition and maternal use of promised consequences during the mother-child interaction. Promised consequences may be a beneficial disciplinary technique to add to parent training programs because it is both portable and adaptable to several situations. Teaching promised consequences by using a modeling plus directed interaction method is recommended for more complete learning in a brief training format. Further exploration of how promised consequences work and whether they offer a long term benefit are recommended. (PsycINFO Database Record (c) 2012 APA, all rights reserved)
SN - 0419-4217
UR - http://search.ebscohost.com/login.aspx?direct=true&db=psyh&AN=2010-99180-196&site=ehost-live
ER -

TY - JOUR
ID - 1708
T1 - Subglottal resonances and distinctive features
A1 - Lulich,Steven M.
Y1 - 2010/01//
KW - PsycInfo
KW - Unsupervised Play Addition
KW - subglottal resonances
KW - distinctive features
KW - Phonetics
KW - vowels
KW - acoustics
KW - speech production
KW - Oral Communication
KW - Speech Characteristics
KW - Vibration
RP - NOT IN FILE
SP - 20
EP - 32
JF - Journal of Phonetics
VL - 38
IS - 1
CY - Netherlands
PB - Elsevier Science
U1 - Sponsor: National Institutes of Health; US. Grant: DC00075; T32DC000038. Recipients: No recipient indicated

N2 - This paper addresses the phonetic basis of the distinctive feature [ñback]. The second subglottal resonance (Sg2) is known to fall near the boundary between [?back] and [+back] vowels, and it has been claimed that Sg2 actually defines this distinction. In this paper, new evidence in support of this hypothesis is presented from 14 adult and 9 child speakers of American English, in which accelerometer recordings of subglottal acoustics were made simultaneously with speech recordings. The first three formants and the second subglottal resonance were measured, and both Sg2 and F3-3.5 bark were tested as boundaries between front and back vowels in the F2-dimension. It was found that Sg2 provides a reliable boundary between front and back vowels for children of all ages, as well as for adults, whereas F3-3.5 bark provides a similarly reliable boundary only for older children and adults. Furthermore, a study of connected speech in one adult male indicated that Sg2 forms a boundary between front and back vowels in such speech as well as in laboratory speech. Some implications for quantal theory and landmark theory are discussed, as well as the possibility that subglottal resonances might play a broader role in speech production. (PsycINFO Database Record (c) 2012 APA, all rights reserved) (journal abstract)
SN - 0095-4470
AD - Lulich, Steven M., Speech Communication Group, MIT 77 Massachusetts Ave., Cambridge, MA, US, 02139, lulich@speech.mit.edu
UR - http://search.ebscohost.com/login.aspx?direct=true&db=psyh&AN=2010-04601-006&site=ehost-live;lulich@speech.mit.edu
L3 - 10.1016/j.wocn.2008.10.006
ER -

TY - CHAP
ID - 1711
T1 - Social-cognitive models and skills
A1 - Erdley,Cynthia A.
A1 - Rivera,Michelle S.
A1 - Shepherd,Elizabeth J.
A1 - Holleb,Lauren J.
Y1 - 2010///
N1 - Accession Number: 2010-00310-002. First Author & Affiliation: Erdley, Cynthia A.; Department of Psychology, University of Maine, Orono, ME, US. Release Date: 20100329. Publication Type: Book, (0200); Edited Book, (0280); . Media Covered: Print. Document Type: Chapter. Book Type: Handbook/Manual. ISBN: 978-1-4419-0608-3, Paperback, 9781441906083; 978-1-4419-0609-0, PDF, 9781441906090. Language: English. Major Descriptor: Models; Social Cognition; Social Skills. Minor Descriptor: Intervention; Measurement; Psychosocial Development; Social Skills Training. Classification: Social Psychology (3000) Developmental Psychology (2800) . Population: Human (10); . Age Group: Childhood (birth-12 yrs) (100) Adolescence (13-17 yrs) (200) Adulthood (18 yrs & older) (300) . Intended Audience: Psychology: Professional & Research (PS) . References Available: Y.. Page Count: 15
KW - PsycInfo
KW - Unsupervised Play Addition
KW - social cognitive models
KW - social cognitive skills
KW - Social Behavior
KW - social skills intervention
KW - assessment
KW - Development
KW - Models
KW - Social Cognition
KW - Social Skills
KW - Intervention
KW - Measurement
KW - Psychosocial Development
KW - Social Skills Training
RP - NOT IN FILE
SP - 21
EP - 35

T2 - Practitioner's guide to empirically based measures of social skills
A2 - Nangle,Douglas W.
A2 - Hansen,David J.
A2 - Erdley,Cynthia A.
A2 - Norton,Peter J.
CY - New York, NY US
PB - Springer Publishing Co
N2 - (from the chapter) Researchers studying social competence have been interested not only in specific social skills but also in the types of social-cognitive processes that might underlie individuals' behavioral choices. A variety of theories propose that individual differences in social information processing skills may help explain why people confronted with the same social situation may choose to act in very different ways. For example, two children may be teased by a peer. One child may perceive this as harmless play and may laugh, whereas another child may interpret this as mean and threatening and may choose to act aggressively toward the peer. Many theorists (e.g., Crick & Dodge, 1994; Dodge, 1986; Ladd & Crick, 1989; Lemerise & Arsenio, 2000) suggest that distortions or deficiencies in social information processing may lead to maladaptive behavior. Thus, in addition to focusing on improving specific social behaviors in social skills intervention programs, it seems that social-cognitive variables can be an important target for treatment as well (e.g., Guerra & Slaby, 1990; Hudley & Graham, 1993). In fact, social-cognitive processes can be viewed as social skills themselves (see Chapter 1, for further discussion). In this chapter, several social-cognitive models will be reviewed and the ways in which social-cognitive variables have been assessed in children, adolescents, and adults will be presented. (PsycINFO Database Record (c) 2012 APA, all rights reserved) (chapter)
SN - 978-1-4419-0608-3
AD - Erdley, Cynthia A., Department of Psychology, University of Maine 5742 Little Hall, Orono, ME, US, 04469, cynthia.erdley@umit.maine.edu
UR - http://search.ebscohost.com/login.aspx?direct=true&db=psyh&AN=2010-00310-002&site=ehost-live;cynthia.erdley@umit.maine.edu
L3 - 10.1007/978-1-4419-0609-0_2
ER -

TY - CHAP
ID - 1712
T1 - Links between children's independent mobility, active transport, physical activity and obesity
A1 - Whitzman,Carolyn
A1 - Romero,Vivian
A1 - Duncan,Mitch
A1 - Curtis,Carey
A1 - Tranter,Paul
A1 - Burke,Matthew
Y1 - 2010///
N1 - Accession Number: 2010-10942-013. First Author & Affiliation: Whitzman, Carolyn; Planning Institute of Australia, Australia. Translated Book Series Title: BMJ books. Release Date: 20110905. Publication Type: Book, (0200); Edited Book, (0280); . Media Covered: Print. Document Type: Chapter. ISBN: 978-1-4051-5889-3, Paperback, 9781405158893; 978-1-4443-1852-4, PDF, 9781444318524. Language: English. Major Descriptor: Childhood Development; Environmental Psychology; Obesity; Physical Mobility; Public Health. Minor Descriptor: Public Transportation. Classification: Promotion & Maintenance of Health & Wellness (3365) . Population: Human (10); . Age Group: Childhood (birth-12 yrs) (100) . Intended Audience: Psychology: Professional & Research (PS) . References Available: Y.. Page Count: 8
KW - PsycInfo
KW - Unsupervised Play Addition
KW - children's independent mobility
KW - child obesity rates
KW - environmental psychology
KW - transportation planning
KW - social geography
KW - Public Health
KW - Childhood Development

KW - Obesity
KW - Physical Mobility
KW - Public Transportation
RP - NOT IN FILE
SP - 105
EP - 112
T2 - Preventing childhood obesity: Evidence, policy, and practice
A2 - Waters,Elizabeth
A2 - Swinburn,Boyd
A2 - Seidell,Jacob
A2 - Uauy,Ricardo
PB - Wiley-Blackwell
T3 - BMJ books
N2 - (from the chapter) Children's independent mobility means the freedom of those under 18 years to move around in public spaces without adult accompaniment. The phrases "battery reared" and "free range" refer to two diametrically opposed approaches to children's upbringing. "Free range" children have traditionally been allowed, and indeed expected, to walk, cycle, or take public transport on their own for everyday trips between home, school, parks, shops and friends' houses. They have also been allowed, in previous generations, to independently explore their immediate neighborhood and the broader city with increasing confidence as they mature. Internationally, the past 40 years have seen a number of inter-related trends: radically declining levels of children's independent mobility (CIM), increased parental use of cars to transport children to school and play, decreasing everyday physical activity of both children and adults, and increasing child obesity rates. There is a relatively small and recent interdisciplinary research on children's independent mobility, primarily reflecting the disciplines of transportation planning, social geography, environmental psychology and public health. The purpose of this chapter is to explore the links between these various phenomena, to detail the complex factors behind these trends, and to examine policy changes that show promise in addressing these interrelated problems. It draws on research published in English, most of which has been conducted in Western Europe, North America and Australasia. Research websites (e.g., Active Living Research, health-evidence.ca, UNESCO Child Friendly Cities) were searched for additional literature regarding promising policies and practices. (PsycINFO Database Record (c) 2012 APA, all rights reserved) (chapter)
SN - 978-1-4051-5889-3
UR - http://search.ebscohost.com/login.aspx?direct=true&db=psyh&AN=2010-10942-013&site=ehost-live
L3 - 10.1002/9781444318517.ch13
ER -

TY - JOUR
ID - 1713
T1 - €ocukluktan eri?kinli?e obsesif kompulsif bozuklukta hatali De?erlendirme ve inan‡ alanlari
A1 - Pi?gin,?rem
A1 - ™zen,Dilek
Y1 - 2010///
KW - PsycInfo
KW - Unsupervised Play Addition
KW - faulty appraisals
KW - belief domains
KW - Obsessive Compulsive Disorder
KW - developmental periods
KW - cognitive control model
KW - Attitudes
KW - Cognitive Appraisal
KW - Developmental Stages
KW - Cognitive Control
RP - NOT IN FILE
SP - 117
EP - 131
JF - Psikiyatride G • ncel Yakla??mlar

VL - 2
IS - 1
CY - Turkey
PB - Cukurova University Faculty of Medicine
N2 - Among cognitive models attempting to explain the etiology of Obsessive Compulsive Disorder (OCD), models such as Inflated Responsibility Model, Misinterpretation of Significance Theory and Cognitive Control Model are currently considered to be valid in many aspects in understanding adulthood OCD. Embracing these models that underline various cognitions in adult OCD, the presence of six faulty appraisals and belief domains can be noticed: inflated responsibility; overimportance of thoughts; excessive concern about the importance of controlling one's thoughts, overestimation of threat, intolerance of uncertainty and perfectionism. Previous studies indicated a difference between early onset OCD and late onset OCD with regards to the presence of pure compulsions, insidious onset of symptoms. Examining faulty assessment and belief domains related with OCD reveals that overimportance of thoughts, intolerance of uncertainty and perfectionism is not only limited to adulthood, but also observed during childhood and/or adolescence periods. Nevertheless, inflated responsibility, excessive concern about the importance of controlling one's thoughts and overestimation of threat found in childhood and adolescence period is not as pronounced and striking as observed with adults. Considering the facts that OCD symptoms and related areas of faulty appraisals and belief domains differ amongst various age groups, early diagnosis and intervention will be critical in terms of the course of treatment for obsessive compulsive disorder. The purpose of this review is to briefly examine three current cognitive models proposed for OCD and evaluate six faulty appraisals and belief domains considered to play a role in the understanding of OCD with respect to developmental periods. (PsycINFO Database Record (c) 2012 APA, all rights reserved) (journal abstract)
SN - 1309-0674
AD - Pi?gin, ?rem, Buyuk Camlica Cad. 23/3 Kisikli-Uskudar, Istanbul, Turkey, irempisgin@hotmail.com
UR - http://search.ebscohost.com/login.aspx?direct=true&db=psyh&AN=2011-16920-007&site=ehost-live;irempisgin@hotmail.com
ER -

TY - JOUR
ID - 1714
T1 - Patterns of home- and classroom-based toy play of preschoolers with and without intellectual disabilities
A1 - Malone,Michael
Y1 - 2009/12//
KW - PsycInfo
KW - Unsupervised Play Addition
KW - home based toy play
KW - classroom-based toy play
KW - preschoolers
KW - intellectual disabilities
KW - Mental Retardation
KW - Childhood Play Behavior
KW - Classroom Behavior
KW - Preschool Students
KW - Toys
KW - Intellectual Development Disorder
RP - NOT IN FILE
SP - 333
EP - 347
JF - International Journal of Disability, Development and Education
VL - 56
IS - 4
CY - United Kingdom
PB - Taylor & Francis
N2 - Children with and without intellectual disabilities were observed playing with toys during both home-based independent play and classroom-based freeplay situations. Categorical and sequential play was analysed for within- and between-group patterns. Within-group patterns during classroom freeplay were similar for both

groups. There were no significant differences among home-based categorical play variables for children with intellectual disabilities; however, children without intellectual disabilities engaged in significantly more constructive play than other home-based categorical play types. Between-group analyses of home-classroom difference scores revealed greater variability in play for children with intellectual disabilities than children without intellectual disabilities. The analyses presented complement and extend extant work on contextually-based variation of children's toy play supporting a more positive ability profile for children with intellectual disabilities than that engendered by classroom-based observations. Results have implications for (a) perceptions of and attitudes toward children with intellectual disabilities held by stakeholders, and (b) how intervention targets are determined. (PsycINFO Database Record (c) 2012 APA, all rights reserved) (journal abstract)
SN - 1465-346X
AD - Malone, Michael, College of Education, University of Cincinnati, Cincinnati PO Box 210105, Cincinnati, OH, US, 45221-0105, d.michael.malone@uc.edu
UR - http://search.ebscohost.com/login.aspx?direct=true&db=psyh&AN=2009-22197-003&site=ehost-live;d.michael.malone@uc.edu
L3 - 10.1080/10349120903306558
ER -

TY - JOUR
ID - 1715
T1 - Review of 'Children at play: An American history'
A1 - Benzaqu,n,Adriana S.
Y1 - 2009///
KW - PsycInfo
KW - Unsupervised Play Addition
KW - Childhood Play Behavior
KW - American history
KW - Childhood Development
KW - History of Psychology
RP - NOT IN FILE
SP - 75
EP - 77
JF - Journal of the History of the Behavioral Sciences
VL - 45
IS - 1
CY - US
PB - John Wiley & Sons
N2 - Reviews the book, "Children at play: An American history" by Howard P. Chudacoff (see record 2007-12982-000). Howard P. Chudacoff's history of children's play in the United States since colonial times is largely motivated by his concern about the state of play and childhood in the present. In seven chapters-one covering the period 1600-1800, one on each half of the nineteenth century, two chapters on the first half of the twentieth century, and two on the period from 1950 to the present-the book develops two arguments. The first argument has to do with the very definition of play, which, the author claims, means different things for adults and for children. The second argument is historical: After a "golden age" in the first half of the twentieth century, unstructured, independent play has become increasingly rare, primarily as a result of tightening adult control, extensive commercialization, and anxiety about children's safety and future. Chudacoff's conclusion is that despite many present scholars' and cultural critics' worries regarding the state of children's play, "the long view of history suggests that things are never as bad, or as good, as they may seem". (PsycINFO Database Record (c) 2012 APA, all rights reserved)
SN - 1520-6696
UR - http://search.ebscohost.com/login.aspx?direct=true&db=psyh&AN=2009-01075-013&site=ehost-live
L3 - 10.1002/jhbs.20356
ER -

TY - JOUR
ID - 1716
T1 - Sleep polygraphic study of children and adolescents with narcolepsy/cataplexy

A1 - Ferri,Raffaele
A1 - Franceschini,Christian
A1 - Zucconi,Marco
A1 - Drago,Valeria
A1 - Manconi,Mauro
A1 - Vandi,Stefano
A1 - Poli,Francesca
A1 - Bruni,Oliviero
A1 - Plazzi,Giuseppe
Y1 - 2009/09//
KW - PsycInfo
KW - Unsupervised Play Addition
KW - sleep polygraphs
KW - CHILDREN
KW - Adolescents
KW - narcolepsy
KW - cataplexy
KW - rapid eye movement sleep
KW - non rapid eye movement sleep
KW - cyclic alternating pattern
KW - NREM Sleep
KW - Polygraphs
KW - Rapid Eye Movement
KW - Persistence
KW - Sleep
KW - Sleep Wake Cycle
RP - NOT IN FILE
SP - 523
EP - 538
JF - Developmental Neuropsychology
JA - Dev Neuropsychol
VL - 34
IS - 5
CY - United Kingdom
PB - Taylor & Francis
N2 - The alterations of the Cyclic Alternating Pattern (CAP) recently found in narcoleptic adult patients suggest the presence of an impaired modulation of the fluctuations of the arousal level during their non-rapid eye movement (NREM) sleep, possibly because of the persistence of neurophysiological mechanisms typical of rapid eye movement (REM) sleep. The same mechanism might play a role in the occurrence of leg movement (LM) activity during sleep characterized by low levels of periodicity. The aim of this study was to evaluate CAP and sleep LM activity in a group of children and adolescents with narcolepsy, to interpret the results under a developmental point of view and integrate this new information with data already available for adults. Thirteen young patients with narcolepsy/ cataplexy were consecutively recruited for this study, together with 13 age- and sex-matched normal controls. Nocturnal polysomnography was carried out after a night of adaptation in a sleep laboratory room; sleep stages, CAP, and LMs were scored and evaluated following standard criteria. Narcoleptic patients showed shorter sleep onset and REM sleep latency, higher number of stage shifts and awakenings per hour of sleep, and higher percentage of wakefulness after sleep onset; CAP rate was found to be decreased in all NREM sleep stages (in particular CAP A1 subtypes) in narcoleptic patients who also showed significant higher values of all types of LMs (periodic or isolated), during both REM and NREM sleep; however, the most evident differences were found during REM sleep. The results of this study confirm that the sleep microstructure and LM activity changes observed in adulthood are already present and detectable in childhood and might have a role in the already known impaired prefrontal functioning of these subjects. The well-established orexin deficiency might be the unifying factor playing a major role in the modulation of CAP and LMs during sleep in children and adolescents with narcolepsy/cataplexy. (PsycINFO Database Record (c) 2012 APA, all rights reserved) (journal abstract)
SN - 1532-6942

AD - Ferri, Raffaele, Sleep Research Centre, Department of Neurology I.C., Oasi Institute for Research on Mental Retardation and Brain Aging (IRCCS) Via C. Ruggero 73, 94018, Troina, Italy, rferri@oasi.en.it
UR - http://search.ebscohost.com/login.aspx?direct=true&db=psyh&AN=2010-11445-002&site=ehost-live;rferri@oasi.en.it
L3 - 10.1080/87565640903133699
ER -

TY - JOUR
ID - 1717
T1 - Children discover the spectral skeletons in their native language before the amplitude envelopes
A1 - Nittrouer,Susan
A1 - Lowenstein,Joanna H.
A1 - Packer,Robert R.
Y1 - 2009/08//
KW - PsycInfo
KW - Unsupervised Play Addition
KW - Speech Perception
KW - sine wave speech
KW - children's speech perception
KW - perceptual object formation
KW - Foreign Language Learning
KW - Language
KW - Oral Communication
RP - NOT IN FILE
SP - 1245
EP - 1253
JF - Journal of Experimental Psychology: Human Perception and Performance
VL - 35
IS - 4
CY - US
PB - American Psychological Association
U1 - Sponsor: National Institute on Deafness and Other Communication Disorders. Grant: R01 DC000633. Recipients: No recipient indicated
N2 - Much of speech perception research has focused on brief spectro-temporal properties in the signal, but some studies have shown that adults can recover linguistic form when those properties are absent. In this experiment, 7-year-old English-speaking children demonstrated adultlike abilities to understand speech when only sine waves (SWs) replicating the 3 lowest resonances of the vocal tract were presented, but they failed to demonstrate comparable abilities when noise bands amplitude-modulated with envelopes derived from the same signals were presented. In contrast, adults who were not native English speakers but who were competent 2nd-language learners were worse at understanding both kinds of stimuli than native English-speaking adults. Results showed that children learn to extract linguistic form from signals that preserve some spectral structure, even if degraded, before they learn to do so for signals that preserve only amplitude structure. The authors hypothesize that children's early sensitivity to global spectral structure reflects the role that it may play in language learning. (PsycINFO Database Record (c) 2012 APA, all rights reserved) (journal abstract)
SN - 1939-1277
AD - Nittrouer, Susan, Department of Otolaryngology-Head & Neck Surgery, College of Medicine, Ohio State University Cramblett Hall, Room 4126, 456 West 10th Avenue, Columbus, OH, US, 43210, nittrouer.1@osu.edu
UR - http://search.ebscohost.com/login.aspx?direct=true&db=psyh&AN=2009-11357-018&site=ehost-live;nittrouer.1@osu.edu
L3 - 10.1037/a0015020
ER -

TY - JOUR
ID - 1720
T1 - Saccades in adult Niemann-Pick disease type C reflect frontal, brainstem, and biochemical deficits

A1 - Abel,L.A.
A1 - Walterfang,M.
A1 - Fietz,M.
A1 - Bowman,E.A.
A1 - Velakoulis,D.
Y1 - 2009/03//
KW - PsycInfo
KW - Unsupervised Play Addition
KW - Niemann Pick disease
KW - Frontal Lobe
KW - brainstem
KW - biochemical deficits
KW - supranuclear palsy
KW - Psychosis
KW - cognitive deficits
KW - saccades
KW - Cognitive Impairment
KW - Eye Movements
KW - Picks Disease
KW - Brain Stem
RP - NOT IN FILE
SP - 1083
EP - 1086
JF - Neurology
VL - 72
IS - 12
CY - US
PB - Lippincott Williams & Wilkins
N2 - Background: The autosomal recessive disorder Niemannn-Pick type C (NPC) presents in adulthood with psychosis or cognitive deficits associated with supranuclear gaze palsies. While saccadic innervation to the extraocular muscles is generated in the brainstem, the frontal lobes play an integral role in the initiation of volitional saccades and the suppression of unwanted reflexive saccades. No study has examined the frontally driven volitional control of saccadic eye movements in NPC. Objective: To examine self-paced and antisaccades as well as reflexive saccades in adult patients with NPC, a disorder known to affect brainstem and frontal cortical function. Methods: Three biochemically confirmed adult patients with NPC were compared with 10 matched controls on horizontal saccadic and antisaccadic measures using an infrared limbus eye tracker. Patients' cholesterol esterification and filipin staining, Mini-Mental State performance, and NPC symptom level were rated. Results: Reflexive saccade latency ranged from shorter to longer than normal, reflexive saccade gain was reduced, asymptotic peak velocity was reduced, fewer self-paced saccades were generated, and increased errors on antisaccades were made by patients compared to controls. Patients with more severe biochemical, cognitive, and symptom deficits performed most poorly on brainstem and frontal ocular motor measures. Paradoxically, less severe illness was associated with an abnormally reduced saccadic latency. Conclusions: Ocular motor measures provide an index of disease severity in Niemannn-Pick type C (NPC) and may be a useful adjunct for monitoring the illness progress and medication response. Reduced saccadic latency may result from inadequate fixation input from abnormally functioning frontal eye fields in NPC. (PsycINFO Database Record (c) 2012 APA, all rights reserved) (journal abstract)
SN - 1526-632X
AD - Walterfang, M., Royal Melbourne Hospital Level 2, John Cade Building, VIC, Australia, 3050, mark.walterfang@mh.org.au
UR - http://search.ebscohost.com/login.aspx?direct=true&db=psyh&AN=2009-04324-011&site=ehost-live;mark.walterfang@mh.org.au
L3 - 10.1212/01.wnl.0000345040.01917.9d
ER -

TY - THES
ID - 1722

T1 - An investigation of the effects of make-believe play training on the development of self-regulation in head start children
A1 - Ogan,Amy T.
Y1 - 2009///
N1 - Accession Number: 2009-99200-042. First Author & Affiliation: Ogan, Amy T.; Illinois State U., US. Other Journal Titles: Dissertation Abstracts International. Release Date: 20100125. Publication Type: Dissertation Abstract, (0400); . Media Covered: Electronic. Document Type: Dissertation. Dissertation Number: AAI3353104. ISBN: 978-1-109-09374-2, 9781109093742. Language: English. Major Descriptor: Childhood Play Behavior; Classrooms; Self Regulation. Minor Descriptor: Ability; Preschool Education. Classification: Educational Psychology (3500) . Population: Human (10); . Age Group: Childhood (birth-12 yrs) (100) Preschool Age (2-5 yrs) (160) . Methodology: Empirical Study. Page Count: 1
KW - PsycInfo
KW - Unsupervised Play Addition
KW - Self Regulation
KW - head start children
KW - Classrooms
KW - self regulatory skills
KW - play based preschool programs
KW - Childhood Play Behavior
KW - ABILITY
KW - Preschool Education
RP - NOT IN FILE
JF - Dissertation Abstracts International: Section B: The Sciences and Engineering
VL - 70
IS - 4-B
CY - US
PB - ProQuest Information & Learning
N2 - In recent years, an emphasis on structured pre-academic time has shifted the focus away from play-based preschool programs. According to Vygotsky, play-based classrooms may actually be more beneficial to helping young children develop self-regulatory skills necessary to succeed in the elementary school years. Children from impoverished backgrounds often lag behind their peers in self-regulation and may benefit the most from play training. This was a short-term longitudinal study investigating the effects of play training on preschooler's self-regulation. Forty-five preschoolers attending a Head Start program were randomly divided into two groups. One group received eight weekly 15-min play training sessions followed by 5 minutes of freeplay. Trained research assistants guided the child's play and encouraged self-regulation skills. The other group participated in eight weekly 15-min freeplay only sessions. Freeplay for both groups was coded for activity type, self-regulation displayed, adult involvement, and child vocalizations. In addition, all children were administered a pretest and two posttest batteries of self-regulation tasks. It was hypothesized that children attending the play training sessions would show greater pre- to posttest gains in self-regulation, engage in more make-believe play, and display more self-regulation during freeplay than their peers who participated only in freeplay sessions. Contrary to expectation, results indicated that children who participated in freeplay only sessions showed more gains in self-regulation and spent more time in make-believe play than their peers who received play training. Few instances of self-regulation were observed during the freeplay sessions, however, as predicted more prosocial/rule following behavior was observed in the children who attended play training sessions. The findings suggest that play training sessions where an adult guides the child's play may actually constrict children's spontaneous freeplay. Instead, children may benefit more from play sessions with an adult present to gently support and encourage the play. (PsycINFO Database Record (c) 2012 APA, all rights reserved)
SN - 0419-4217
UR - http://search.ebscohost.com/login.aspx?direct=true&db=psyh&AN=2009-99200-042&site=ehost-live
ER -

TY - JOUR
ID - 1723
T1 - Play, cognition and self-regulation: What exactly are children learning when they learn through play?
A1 - Whitebread,David
A1 - Coltman,Penny

A1 - Jameson,Helen
A1 - Lander,Rachel
Y1 - 2009///
KW - PsycInfo
KW - Unsupervised Play Addition
KW - Play
KW - Metacognition
KW - self-regulation
KW - problem-solving
KW - CHILDREN
KW - Childhood Play Behavior
KW - Childhood Play Development
KW - Problem Solving
KW - Self Regulation
RP - NOT IN FILE
SP - 40
EP - 52
JF - Educational and Child Psychology
VL - 26
IS - 2
CY - United Kingdom
PB - British Psychological Society
N2 - This paper explores the particular aspects of learning which might be supported through playful activity and reviews research and theory which link children's play, and particularly pretence or symbolic play, to the development of metacognitive and self-regulatory skills. Three studies are reported, one observational and two experimental, which have explored this relationship. The observational study involved the video-recording of 582 metacognitive or self-regulatory 'events' within Foundation Stage settings. The two experimental studies replicated in different learning domains the classic study of Sylva, Bruner and Genova (1976), which contrasted the problem-solving performance of 3- to 5-year-old children who had experienced a 'taught' and 'play' condition. Evidence from the present studies reported and other studies supports the view that play, and particularly pretence or symbolic play, which might be with objects or other children, is particularly significant in its contribution to the development of children as metacognitively skilful, self-regulated learners. Evidence from the observational study indicated that child-initiated playful activities, in small groups without adult supervision, supported the greatest proportion of self-regulatory behaviours. The experimental studies suggested that the experience of the 'play' condition was particularly effective in preparing the children for effortful, problem-solving or creative tasks which require a high level of metacognitive and self-regulatory skill. Metacognitive and self-regulatory development is crucially important in the development of academic skills which involve intentional learning, problem-solving and creativity. An understanding of the relationship between pretend or symbolic play and self-regulation is also helpful in providing clear guidelines for adults working with young children as regards their role in supporting and encouraging play in educational contexts. (PsycINFO Database Record (c) 2012 APA, all rights reserved) (journal abstract)
SN - 0267-1611
AD - Whitebread, David, University of Cambridge 184 Hills Road, Cambridge, United Kingdom, CB2 8PQ, dgwl004@cam.ac.uk
UR - http://search.ebscohost.com/login.aspx?direct=true&db=psyh&AN=2009-16552-005&site=ehost-live;dgwl004@cam.ac.uk
ER -

TY - CHAP
ID - 1724
T1 - Historical memories
A1 - Blatz,Craig W.
A1 - Ross,Michael
Y1 - 2009///
N1 - Accession Number: 2010-05180-009. First Author & Affiliation: Blatz, Craig W.; Department of Psychology, University of Waterloo, Waterloo, ON, Canada. Release Date: 20100517. Publication Type: Book,

(0200); Edited Book, (0280); . Media Covered: Print. Document Type: Chapter. ISBN: 978-0-521-75892-5, Paperback, 9780521758925; 978-0-521-76078-2, Hardcover, 9780521760782. Language: English. Major Descriptor: False Memory; History; Memory. Minor Descriptor: Ingroup Outgroup. Classification: Learning & Memory (2343) . Population: Human (10); . Intended Audience: Psychology: Professional & Research (PS) . References Available: Y.. Page Count: 15
KW - PsycInfo
KW - Unsupervised Play Addition
KW - historical memories
KW - ingroup
KW - skewed memories
KW - False Memory
KW - History
KW - Memory
KW - Ingroup Outgroup
RP - NOT IN FILE
SP - 223
EP - 237
T2 - Memory in mind and culture
A2 - Boyer,Pascal
A2 - Wertsch,James V.
CY - New York, NY US
PB - Cambridge University Press
U1 - Sponsor: Natural Sciences and Engineering Research Council. Other Details: Doctoral fellowship. Recipients: Blatz, Craig W.
N2 - (from the chapter) After the attacks on the World Trade Center and Pentagon on September 11, 2001, the Princeton historian Bernard Lewis explained the thinking and behavior of Muslims to the American public in a New Yorker article (Lewis, 2001). Lewis wrote that, unlike Americans, Muslims know and care about history: In current American usage, the phrase "that's history" is commonly used to dismiss something as unimportant, of no relevance to current concerns. ... Middle Easterner's perception of history is nourished from the pulpit, by the schools, and by the media, and although it may be-indeed, often is-and inaccurate, it is nevertheless vivid and powerfully resonant, (p. 51) Lewis implies that Americans' (and presumably most Westerners') historical memories are not "nourished from the pulpit, by the schools, and by the media." He further implies that Americans' historical memories are accurate, even if they do not care much about history. In this chapter, we dispute each of these claims. Muslims are not unique in their use or abuse of history. People's memories of the histories of the national, ethnic, or religious groups to which they belong (ingroups) are often tilted in favor of their ingroups and against other groups. We present evidence that historical memories are skewed, in part, because children and adults are presented with selective and biased depictions of the past. Educators, religious leaders, politicians, and media all play an important role by influencing the knowledge available. We also show that people's response to the information available further affects their historical memories. People's memories are influenced by how they attend to, process, and retrieve the information that they receive. Individuals create historical memories that depict their valued ingroups favorably, even after reading unfavorable information. We reason that favorable memories of ingroups satisfy important group and individual needs. We conclude by discussing a recent surge in government apologies for past injustices. Government redress is important because it can possibly satisfy group and individual concerns, and contribute to more balanced historical memories. (PsycINFO Database Record (c) 2012 APA, all rights reserved) (chapter)
SN - 978-0-521-75892-5
AD - Blatz, Craig W., Department of Psychology, University of Waterloo 200 University Avenue West, Waterloo, ON, Canada, N2L 3G1, cwblatz@uwaterloo.ca
UR - http://search.ebscohost.com/login.aspx?direct=true&db=psyh&AN=2010-05180-009&site=ehost-live;cwblatz@uwaterloo.ca
L3 - 10.1017/CBO9780511626999.013
ER -

TY - JOUR
ID - 1725
T1 - Crisis and review of residential child care. Its role in child protection

A1 - Bravo,Amaia
A1 - Del Valle,Jorge F.
Y1 - 2009///
KW - PsycInfo
KW - Unsupervised Play Addition
KW - residential child care
KW - child protection
KW - Child Abuse
KW - Mental Health
KW - Child Care
KW - Protective Services
KW - Residential Care Institutions
RP - NOT IN FILE
SP - 42
EP - 52
JF - Papeles del Psic¢logo
VL - 30
IS - 1
CY - Spain
PB - Revista Del Colegio Oficial De Psicologos
N2 - Residential care continues to play an important role in Spain in the provision of care for children and adolescents in situations of abuse or neglect. Some newly-emerging issues, such as the arrival of unaccompanied asylum-seeking children, the increase in child-to-parent violence, or the need for therapeutic attention in children with emotional and mental health problems, have given rise to substantial changes in the structure and objectives of children's homes. Currently, child care work in children's homes is focused on intervention with adolescents and the new problems described. Although resources for younger children continue to exist, these lend to disappear as foster care becomes adequately established. Since the 1990s, the specialized child care model involving differentiated resources has been implemented in a highly inequitable way across Spain's different Autonomous Regions. As will be discussed in the present article, there remain many difficulties to overcome and challenges to be met in the area of residential child care. (PsycINFO Database Record (c) 2012 APA, all rights reserved) (journal abstract)
SN - 0214-7823
AD - Bravo, Amaia, Departamento de Psicologia, Grupo de Investigacion en Familia e Infancia Plaza Feijoo s/n, 33003, Oviedo, Spain, amaiabravo@uniovi.es
UR - http://search.ebscohost.com/login.aspx?direct=true&db=psyh&AN=2009-22396-005&site=ehost-live;amaiabravo@uniovi.es
ER -

TY - JOUR
ID - 1729
T1 - Effects of powered mobility on self-initiated behaviours of very young children with locomotor disability (1986)
A1 - Rosenbaum,Peter
Y1 - 2008/09//
KW - PsycInfo
KW - Unsupervised Play Addition
KW - powered mobility
KW - self-initiated behaviours
KW - locomotor disability
KW - motorized wheelchairs
KW - spatial exploration
KW - Communication
KW - caregiver
KW - Exploratory Behavior
KW - Interpersonal Communication
KW - Locomotion

KW - Mobility Aids
KW - Movement Disorders
KW - Caregivers
RP - NOT IN FILE
JF - Developmental Medicine & Child Neurology
JA - Dev.Med Child Neurol.
VL - 50
IS - 9
CY - United Kingdom
PB - Wiley-Blackwell Publishing Ltd.
N2 - This article presents a commentary of Butler (see record 1987-25503-001) which was previously published as "Effects of powered mobility on self-initiated behaviours of very young children with locomotor disability" in Dev Med Child Neurol 1986; 28; 325-32. This study reports the effects of powered mobility on the self-initiated behaviour of six children with various disabilities who, between 23 and 38 months of age, learnt to use motorized wheelchairs in less than three weeks. Using a multiple baseline design, two-hour observation periods were video-recorded at 10-day intervals before and after they achieved independent mobility. Frequency of self-initiated interaction with objects, spatial exploration and communication with caregiver were analyzed. Three children increased all three types of behaviour; one increased in two types but decreased in interaction with objects; and two increased in spatial exploration only. (PsycINFO Database Record (c) 2012 APA, all rights reserved)
SN - 1469-8749
UR - http://search.ebscohost.com/login.aspx?direct=true&db=psyh&AN=2008-12970-002&site=ehost-live
ER -

TY - JOUR
ID - 1730
T1 - Effects of prenatal ethanol exposure on regulation of basal hypothalamic-pituitary-adrenal activity and hippocampal 5-HT[sub]1A[/sub] receptor
A1 - Sliwowska,J.H.
A1 - Lan,N.
A1 - Yamashita,F.
A1 - Halpert,A.G.
A1 - Viau,V.
A1 - Weinberg,J.
Y1 - 2008/09//
KW - PsycInfo
KW - Unsupervised Play Addition
KW - prenatal ethanol exposure
KW - regulation
KW - basal hypothalamic-pituitary-adrenal activity
KW - hippocampal receptor
KW - gonadal hormones
KW - serotonin Type 1A receptor
KW - Ethanol
KW - Hypothalamic Pituitary Adrenal Axis
KW - Neural Receptors
KW - Prenatal Exposure
KW - Serotonin
KW - Gonadotropic Hormones
KW - Hippocampus
KW - Rats
RP - NOT IN FILE
SP - 1111
EP - 1123
JF - Psychoneuroendocrinology
VL - 33

IS - 8
CY - Netherlands
PB - Elsevier Science
N2 - Prenatal ethanol exposure, like other early adverse experiences, is known to alter hypothalamic-pituitary-adrenal (HPA) activity in adulthood. The present study examined the modulatory effects of the gonadal hormones on basal HPA regulation and serotonin Type 1A receptor (5-HT[sub]1A[/sub]) mRNA levels in adult female rats prenatally exposed to ethanol (E) compared to that in females from pair-fed (PF) and ad libitum-fed control (C) conditions. We demonstrate, for the first time, long-lasting consequences of prenatal ethanol exposure for basal corticosterone (CORT) regulation and basal levels of hippocampal mineralocorticoid (MR), glucocorticoid (GR) and serotonin Type 1A (5-HT[sub]1A[/sub]) receptor mRNA, as a function of estrous cycle stage: (1) basalCORT levelswere higher in E compared to C females in proestrus but lower in E and PF compared to C females in estrus; (2) there were no differences among groups in basal levels of adrenocorticotropin (ACTH), estradiol or progesterone; (3) hippocampal MR mRNA levels were decreased in E compared to PF and C females across the estrus cycle, with the greatest effects in proestrus, whereas E (but not PF or C) females had higher hippocampal GR mRNA levels in proestrus thanin estrousanddiestrus; (4)5-HT[sub]1A[/sub] mRNA levels were increased in E compared to PF and C females in diestrus. That alterations were revealed as a function of estrous cycle stage suggests a role for the ovarian steroids in mediating the adverse effects of ethanol. Furthermore, it appears that ethanol-induced nutritional effects may play a role in mediating at least some of the effects observed. The resetting of HPA activity by early environmental events could be one mechanism linking early life experiences with long-term health consequences. Thus, changes in basal CORT levels, a shift in the MR/GR balance and alterations in 5-HT[sub]1A[/sub] receptor mRNA could have important clinical implications for understanding the secondary disabilities, such as an increased incidence of depression, in children with FASD. (PsycINFO Database Record (c) 2012 APA, all rights reserved) (journal abstract)
SN - 0306-4530
AD - Sliwowska, J. H., Department of Cellular and Physiological Sciences, The University of the British Columbia 2350 Health Sciences Mall, Vancouver, BC, Canada, V6T 1Z3, sliwowsk@interchange.ubc.ca
UR - http://search.ebscohost.com/login.aspx?direct=true&db=psyh&AN=2008-11644-010&site=ehost-live;sliwowsk@interchange.ubc.ca
L3 - 10.1016/j.psyneuen.2008.05.001
ER -

TY - THES
ID - 1734
T1 - First step or last chance: At-risk youth, alternative schooling and juvenile delinquency
A1 - Free,Janese Lynette
Y1 - 2008///
N1 - Accession Number: 2008-99170-526. First Author & Affiliation: Free, Janese Lynette; Northeastern U., US. Other Journal Titles: Dissertation Abstracts International. Release Date: 20081020. Publication Type: Dissertation Abstract, (0400); . Media Covered: Electronic. Document Type: Dissertation. Dissertation Number: AAI3304117. Language: English. Major Descriptor: Criminal Behavior; Criminal Justice; Juvenile Delinquency; Schools. Minor Descriptor: Social Behavior; Violence. Classification: Health & Mental Health Treatment & Prevention (3300) . Population: Human (10); . Age Group: Adolescence (13-17 yrs) (200) Adulthood (18 yrs & older) (300) . Methodology: Empirical Study; Quantitative Study. Page Count: 1
KW - PsycInfo
KW - Unsupervised Play Addition
KW - schooling
KW - Juvenile Delinquency
KW - criminal justice system
KW - Criminal Behavior
KW - Criminal Justice
KW - Schools
KW - Social Behavior
KW - Violence
RP - NOT IN FILE
JF - Dissertation Abstracts International Section A: Humanities and Social Sciences

VL - 69
IS - 3-A
CY - US
PB - ProQuest Information & Learning
N2 - Schools are meant to be places where children can go to learn, play and socialize. While this remains true the vast majority of the time, some school children now face other issues such as assaults, robbery, weapons and even violence while in the classroom. As a result, policies such as the Gun Free Schools Act of 1994, were implemented in an attempt to deter students from bringing weapons to school by promising a swift, severe punishment with no exceptions for breaking the law. The implementation of this bill, along with other key influential factors, led to an increase in the number of at-risk students suspended or expelled from school, frequently leaving them with few options. The combination of out of school at-risk youth, at home alone or on the streets, with little or no supervision has proven to be a dangerous situation. To date we have found few answers to solving this social problem - academically or practically. This dissertation adds to the current research and literature by examining one option to addressing the dilemma that often leads youth down a hazardous path - namely, alternative schooling. These non-traditional schools offer at-risk youth, who are not otherwise successful in conventional schools, a "second chance" at literacy, pro-social behaviors and high school diplomas to help get their lives on a more positive track. As a result of months of participant observation, teacher interviews and student school records, this dissertation analyzed how an alternative education influences at-risk students who attended an alternative school in New England. The key questions guiding this research addressed why students attend alternative schools, how these specialized schools differ from traditional schools and how alternative students are influenced by learning in a non-traditional educational setting. Lastly, this dissertation not only identified the key factors influencing alternative students, but also discussed which of those factors enhanced students' development and which factors inhibited it. The purpose of this study is to further the academic literature and practical understanding of alternative schools, the at-risk youth who attend them and what factors influence these students' development and academic progress. If alternative schools are to be considered a possible catalyst for positive change, then we must first understand their positive and negative effects on students, behaviorally and academically. This dissertation concludes by offering several significant policy recommendations based on the key findings regarding alternative schooling from a macro-sociological perspective, as well as a micro-sociological perspective. This research will hopefully assist future researchers and professionals to better understand the potential alternative schools have for being students' "first step" toward positive life outcomes or their "last chance" before becoming uneducated, unemployed and possibly involved in the criminal justice system. (PsycINFO Database Record (c) 2012 APA, all rights reserved)
SN - 0419-4209
UR - http://search.ebscohost.com/login.aspx?direct=true&db=psyh&AN=2008-99170-526&site=ehost-live
ER -

TY - THES
ID - 1735
T1 - Finding Sally: Personal encounters of the divine feminine through the archetype of the Angel
A1 - Udouj,Anne F.
Y1 - 2008///
N1 - Accession Number: 2008-99160-285. First Author & Affiliation: Udouj, Anne F.; Pacifica Graduate Inst., US. Other Journal Titles: Dissertation Abstracts International. Release Date: 20080929. Publication Type: Dissertation Abstract, (0400); . Media Covered: Electronic. Document Type: Dissertation. Dissertation Number: AAI3302075. Language: English. Major Descriptor: Archetypes; Childhood Development; Femininity. Classification: Developmental Psychology (2800) . Population: Human (10); . Methodology: Empirical Study; Quantitative Study. Page Count: 1
KW - PsycInfo
KW - Unsupervised Play Addition
KW - personal encounters
KW - divine feminine
KW - archetype
KW - Angel
KW - childhood imaginary
KW - Archetypes
KW - Childhood Development

KW - Femininity
RP - NOT IN FILE
JF - Dissertation Abstracts International: Section B: The Sciences and Engineering
VL - 69
IS - 2-B
CY - US
PB - ProQuest Information & Learning
N2 - My finding Sally is a soul journey that sought my banished childhood imaginary companion. Early childhood necessity prompted a cognizant relationship with my Angel, and I discovered an enigmatic imaginary playmate named Sally. I then had to close my imaginary door because Sally was not real or visible to adults. Sally's luminous presence vanished when I said she could no longer come to play under the maple tree. The complexities of this loss led to further woundings that have taken a lifetime to understand, grieve, and ultimately heal. Finding Sally through the archetype of the Angel involves a study of the thinly veiled imaginal space of grace, transcendence, and archetypal Angelic energy that separates the worlds of spirit and matter (Corbin, 1984/1995). I address the Angelic function of the guardian spirits of animals, ancestors, and Mary (Mother Mary and Mary Magdalene) as the Wisdom of Sophia in scholarly research, dreams, and imagination. Research modalities include the heuristic approach of Moustakas (1990), Corbin's (1984/1995) spiritual hermeneutic of ta'wil (bringing back), Bachelard's (1960/1969) notion of doubling, poetic reverie, and word dreaming, along with the Jungian shadow. I employ an erotic method of alchemical hermeneutics of mutual seduction and loving engagement with the subject (Romanyshyn & Goodchild, 2003) and spiral through the myths of Inanna, Cinderella, Vasilisa, and the Shulamite Bride of the Song of Songs as they are poetically addressed in a circling (Addison, 1989) and continually deepening process by which I connect the dots left as glimmers of Angel. I engage the Angel through the mundus imaginalis (Corbin, 1984/1995) and imagine a watery carriage with wheels of fire to transport me as I find Sally through numinous dream images, synchronicities, the imagination as reality (Avens, 1980), and deep unconscious ancestral memories of Other. Kabbalah as perception of beauty in seeking the Angel, an icon known as the Mandylion of Edessa, and the iconic restoration of my mother's antique doll are tended. Imaginal glimpses into Jerusalem as mystical Bride, Chartres Cathedral as living stone, elemental nature spirits, the Black Virgin, Mary, and Elijah are all viewed through an Angelic lens. Maya Angelou, Kali, Black Sarah, St. Nicholas, and other dazzling shadow figures emanate from the stars, with sock monkeys and Holy Fools as Angel in a labyrinthine process. I surprisingly discover my banished imaginary Sally to be a very real and numinous emanation of the Judaic Shekhinah (Scholem, 1991), and a final surprise from the Angel is discovering my Jewish ancestral roots, and how these ancestral memories have colored my dreams with starry footprints in the night. Sally continues to lead me in an ongoing engagement with the Divine Feminine. Thank you, Sally. You are love. (PsycINFO Database Record (c) 2012 APA, all rights reserved)
SN - 0419-4217
UR - http://search.ebscohost.com/login.aspx?direct=true&db=psyh&AN=2008-99160-285&site=ehost-live
ER -

TY - CHAP
ID - 1740
T1 - Working with adult survivors of sexual and physical abuse
A1 - White,Beverly
Y1 - 2007///
N1 - Accession Number: 2007-07356-021. First Author & Affiliation: White, Beverly; Phillips-White Associates, Philadelphia, PA, US. Release Date: 20070924. Publication Type: Book, (0200); Edited Book, (0280); . Media Covered: Print. Document Type: Chapter. Book Type: Handbook/Manual. ISBN: 0-8261-0215-8, Hardcover, 0826102158; 978-0-8261-0215-7, Hardcover, 9780826102157. Language: English. Major Descriptor: Child Abuse; Cognitive Behavior Therapy; Physical Abuse; Sexual Abuse; Social Casework. Minor Descriptor: Early Experience; Schema; Survivors. Classification: Cognitive Therapy (3311) . Population: Human (10); Male (30); Female (40); Outpatient (60); . Age Group: Adulthood (18 yrs & older) (300) Young Adulthood (18-29 yrs) (320) Thirties (30-39 yrs) (340) . Intended Audience: Psychology: Professional & Research (PS) . References Available: Y.. Page Count: 23
KW - PsycInfo
KW - Unsupervised Play Addition
KW - adult survivors
KW - childhood sexual & physical abuse

KW - social work
KW - Cognitive Behavior Therapy
KW - schema-focused approach
KW - Child Abuse
KW - Physical Abuse
KW - Sexual Abuse
KW - Social Casework
KW - Early Experience
KW - Schema
KW - Survivors
RP - NOT IN FILE
SP - 467
EP - 489
T2 - Cognitive behavior therapy in clinical social work practice
A2 - Ronen,Tammie
A2 - Freeman,Arthur
CY - New York, NY US
PB - Springer Publishing Co
N2 - (from the chapter) Working with adult survivors of childhood sexual and physical abuse is broad and complex. Social workers especially play important roles as service providers to many adult survivors who present as clients or patients. The challenge for workers is to effectively implement evidence-based interventions, such as cognitive behavior therapy (CBT), that will enhance the success rate of treatment for these often complex and difficult clients. Case illustrations are included of a 29-yr-old male and a 32-yr-old female. (chapter)
Topics include: literature review, working with difficult clients using CBT (basic treatment strategies, stages of change) and methods of treatment (schema-focused approach). (PsycINFO Database Record (c) 2012 APA, all rights reserved) (chapter)
SN - 0-8261-0215-8
UR - http://search.ebscohost.com/login.aspx?direct=true&db=psyh&AN=2007-07356-021&site=ehost-live
ER -

TY - CHAP
ID - 1741
T1 - The crisis of parental deployment in military service
A1 - Herzog,Joseph R.
A1 - Everson,R.Blaine
Y1 - 2007///
N1 - Accession Number: 2007-14176-011. First Author & Affiliation: Herzog, Joseph R.; College of Social Work, University of South Carolina, Columbia, SC, US. Release Date: 20080331. Publication Type: Book, (0200); Edited Book, (0280); . Media Covered: Print. Document Type: Chapter. Book Type: Textbook/Study Guide. ISBN: 1-59385-495-1, Hardcover, 1593854951; 978-1-59385-495-9, Hardcover, 9781593854959. Language: English. Major Descriptor: Family Relations; Family; Military Deployment; Stress; Trauma. Minor Descriptor: Childhood Development; Developmental Stages; Military Personnel; Models; Play Therapy. Classification: Military Psychology (3800) . Population: Human (10); Male (30); Female (40); . Location: US. Age Group: Childhood (birth-12 yrs) (100) Neonatal (birth-1 mo) (120) Infancy (2-23 mo) (140) Preschool Age (2-5 yrs) (160) School Age (6-12 yrs) (180) . Intended Audience: Psychology: Professional & Research (PS) . References Available: Y.. Page Count: 21
KW - PsycInfo
KW - Unsupervised Play Addition
KW - crisis
KW - military families
KW - Family Life
KW - family-systems-based approach
KW - family stressors
KW - child stressors
KW - parental deployment

KW - combat-related trauma
KW - play therapy model
KW - Developmental Stages
KW - Family Relations
KW - Family
KW - Military Deployment
KW - Stress
KW - Trauma
KW - Childhood Development
KW - Military Personnel
KW - Models
KW - Play Therapy
RP - NOT IN FILE
SP - 228
EP - 248
T2 - Play therapy with children in crisis: Individual, group, and family treatment (3rd Ed.)
A2 - Webb,Nancy Boyd
CY - New York, NY US
PB - Guilford Press
N2 - (from the chapter) In the aftermath of September 11, 2001, the U.S. military has been increasingly involved in conflicts around the world as part of the globalized war on terror. The family lives of soldiers, sailors, or air personnel involve numerous associated stressors and strains, including frequent relocations and lengthy separations that create incongruency between work and family life. Military families often consist of members from various regions of the world and/or different ethnic backgrounds, and one or both parents may have had previous marriages or relationships. In today's military, it is not uncommon for a child's father, mother, or both parents to deploy. This deployment may be of short duration (such as for a training mission) or for a longer period (such as for sea duty, peacekeeping missions, unaccompanied tours [those without family members], or extended tours in combat zones). Parental separation can have a profound impact on the family in general and on children in particular. The family member(s) in the military may view the deployment as business as usual, but children experience this separation as loss of daily contact with their parent(s). This loss is compounded by the potentially dangerous duty in which the parent(s) may be engaged. This chapter provides an overview of a family-systems-based approach with children in military families. We begin by discussing pertinent issues for families and children within the military context. In particular, we discuss the family and child stressors specifically associated with the deployment cycle, as well as the occurrence of combat-related trauma in service members and secondary traumatic stress in their family members. Next, an evolving dynamic model for play therapy is discussed by developmental stages and phases of treatment. Finally, we review two cases that illustrate this approach to play therapy with children in military families. (PsycINFO Database Record (c) 2012 APA, all rights reserved) (chapter)
SN - 1-59385-495-1
UR - http://search.ebscohost.com/login.aspx?direct=true&db=psyh&AN=2007-14176-011&site=ehost-live
ER -

TY - CHAP
ID - 1742
T1 - Naturally occurring mentoring relationships involving youth
A1 - Spencer,Ren,e
Y1 - 2007///
N1 - Accession Number: 2007-00535-007. First Author & Affiliation: Spencer, Ren,e; School of Social Work, Boston University, Boston, MA, US. Release Date: 20071105. Publication Type: Book, (0200); Edited Book, (0280); . Media Covered: Print. Document Type: Chapter. Book Type: Handbook/Manual. ISBN: 1-4051-3373-2, Hardcover, 1405133732; 978-1-4051-3373-9, Hardcover, 9781405133739. Language: English. Major Descriptor: Adolescent Development; Childhood Development; Mentor; Role Models; Interpersonal Relationships. Minor Descriptor: Cognitive Processes; Individual Differences. Classification: Group & Interpersonal Processes (3020) . Population: Human (10); . Age Group: Childhood (birth-12 yrs) (100) School Age (6-12 yrs) (180) Adolescence (13-17 yrs) (200) Adulthood (18 yrs & older) (300) . Intended Audience: Psychology: Professional & Research (PS) . References Available: Y.. Page Count: 19

KW - PsycInfo
KW - Unsupervised Play Addition
KW - naturally occurring mentoring relationships
KW - Youth
KW - Adults
KW - relationship formation & development
KW - support
KW - psychological processes
KW - Individual Differences
KW - very important people
KW - Role Models
KW - Adolescent Development
KW - Childhood Development
KW - Mentor
KW - Interpersonal Relationships
KW - Cognitive Processes
RP - NOT IN FILE
SP - 99
EP - 117
T2 - The Blackwell handbook of mentoring: A multiple perspectives approach
A2 - Allen,Tammy D.
A2 - Eby,Lillian T.
CY - Malden
PB - Blackwell Publishing
N2 - (from the chapter) When successful adults who have overcome difficult life circumstances are asked to reflect on the major influences in their lives, many recall a caring adult whose presence during their youth made all the difference. These familiar stories of what have come to be called natural mentor relationships are receiving increasing attention by researchers of child development and practitioners working to intervene in the lives of vulnerable and troubled youth. Natural mentoring relationships may evolve out of a range of roles that adults play in the lives of youth, including extended family members, family friends, neighbors, teachers, coaches, after-school program staff, and religious group leaders. These relationships may be longstanding, spanning many years, or more short-term with brief encounters at critical junctures leaving a lasting impression on a young person. Natural mentor relationships appear to be far more prevalent than formal ones. Yet, natural mentor relationships have received relatively scant attention in the developmental literature to date. The research literature on natural mentors is diffuse, as the topic has been approached from a variety of angles. The literature on youth's relationships with unrelated adults, work that has grown out of social network research, has served as the foundation for the consideration of natural mentoring relationships. More recently, focused attention has been paid to what are being called adult very important people (VIPs) in the lives of youth. The growing interest in mentoring more generally has contributed to an increase in these studies of adults who serve as a guide or role model to young people in some fashion. As these adults are often family members the use of the term nonparental rather than unrelated adult has been increasing. Other studies use the term natural mentors to refer to influential adults, related and unrelated, in the lives of youth. In this chapter, research from each of these traditions is synthesized to present a picture of our current understanding of natural mentoring relationships between youth and adults. First, what is known about the prevalence of natural mentoring relationships is reviewed. This is followed by discussions of the development of natural mentoring relationships, the types of support these relationships offer, and what are thought to be some of the key psychological processes occurring in these important connections. Individual differences that appear to contribute to the formation of natural mentoring relationships are then considered and, finally, the conclusion of this chapter is devoted to detailing areas for further research. (PsycINFO Database Record (c) 2012 APA, all rights reserved) (chapter)
SN - 1-4051-3373-2
UR - http://search.ebscohost.com/login.aspx?direct=true&db=psyh&AN=2007-00535-007&site=ehost-live
ER -

TY - BOOK
ID - 1743

T1 - Children at play: An American history
A1 - Chudacoff,Howard P.
Y1 - 2007///
N1 - Accession Number: 2007-12982-000. First Author & Affiliation: Chudacoff, Howard P.; Brown University, Providence, RI, US. Release Date: 20070910. Publication Type: Book, (0200); Authored Book, (0240); . Media Covered: Print. ISBN: 0-8147-1664-4, Hardcover, 0814716644; 978-0-8147-1664-9, Hardcover, 9780814716649. Language: English. Major Descriptor: Childhood Play Behavior; History. Minor Descriptor: Childhood Development. Classification: Psychosocial & Personality Development (2840) . Population: Human (10); . Location: US. Age Group: Childhood (birth-12 yrs) (100) . Intended Audience: General Public (GP)
KW - PsycInfo
KW - Unsupervised Play Addition
KW - History
KW - children's play
KW - Childhood Play Behavior
KW - Childhood Development
RP - NOT IN FILE
CY - New York, NY US
PB - New York University Press
N2 - (from the jacket) Today many parents can seem overly involved with their children's activities--from never letting them out of their sight, to managing their every activity morning to night--while just a couple decades ago children more often were left to play without adult supervision. But for all the attention from adults, we know little about how children actually play, and even less about play from a child's point of view. In this fascinating and enlightening book, Howard Chudacoff presents the first history of children's play in the U.S. and ponders what it tells us about ourselves. Through expert investigation in primary sources--including dozens of children's diaries, hundreds of autobiographical recollections of adults, and a wealth of child-rearing manuals--along with wide-ranging readings of the work of educators, journalists, market researchers, and scholars--Chudacoff digs into the "underground" of play. Ranging over the last 300 years, he contrasts the activities that genuinely occupied children's time with what adults thought children ought to be doing. Filled with intriguing stories and rich insights, Children at Play provides a chronological history of play in the U.S. from the viewpoint of children themselves. Focusing on youngsters between the ages of about six and twelve, it highlights the transformation of "child's play," paying attention not only to the activities of the cultural elite but to those of working-class children, of slaves, and of Native Americans. In addition, the author considers the findings, observations, and theories of numerous social scientists along with those of fellow historians. (PsycINFO Database Record (c) 2012 APA, all rights reserved) (jacket)
SN - 0-8147-1664-4
UR - http://search.ebscohost.com/login.aspx?direct=true&db=psyh&AN=2007-12982-000&site=ehost-live
ER -

TY - BOOK
ID - 1744
T1 - Adolescents, media, and the law: What developmental science reveals and free speech requires
A1 - Levesque,Roger J.R.
Y1 - 2007///
N1 - Accession Number: 2007-09555-000. First Author & Affiliation: Levesque, Roger J. R.. Release Date: 20070917. Publication Type: Book, (0200); Authored Book, (0240); . Media Covered: Print. ISBN: 978-0-19-532044-2, Hardcover, 9780195320442. Language: English. Major Descriptor: Adolescent Development; Communications Media; Law (Government). Minor Descriptor: Behavior; Body Image; Health; Legal Processes; Mass Media; Self Concept; Sexuality; Tobacco Smoking; Violence. Classification: Developmental Psychology (2800) Mass Media Communications (2750) . Population: Human (10); . Age Group: Adolescence (13-17 yrs) (200) . Intended Audience: Psychology: Professional & Research (PS) . References Available: Y
KW - PsycInfo
KW - Unsupervised Play Addition
KW - media
KW - law
KW - adolescent identity & behavior & health

KW - Adolescent Development
KW - Prosocial Behavior
KW - Violence
KW - body images
KW - Smoking
KW - Sexuality
KW - free speech rights
KW - legal approaches
KW - Communications Media
KW - Law (Government)
KW - Behavior
KW - Body Image
KW - Health
KW - Legal Processes
KW - Mass Media
KW - Self Concept
KW - Tobacco Smoking
RP - NOT IN FILE
CY - New York, NY US
PB - Oxford University Press
T3 - American Psychology-Law Society series
N2 - (from the foreword) In this book Roger Levesque focuses on the impact of media on adolescents. As he notes in his introductory chapter, today's adolescents are inundated with a wide variety of media, more so than any prior generation. Television, video games, movies, Internet, and MP3 players play an increasing dominant role in the lives of many youth. Unlike prior generations, adolescents are considerably more autonomous, typically interacting with the media with little or no adult supervision. Media clearly play an important role in shaping adolescent identity, behavior, and health. Levesque's perspective is that the media can have both positive and negative effects on adolescent behavior and he is interested in understanding these effects in the context of adolescent development. He cautions us to not view the relationship of media and adolescent development in a narrow and simplistic manner. Indeed, while he acknowledges that the research on media effects has provided useful information about its impact, he is clear that the research falls short in aiding our understanding of the multiple factors that shape adolescent development. Professor Levesque notes that the media's impact on prosocial behavior may be more powerful than its negative impact. Unfortunately, a substantial amount of the research focuses on the negative effects. He presents concise, clear summaries of what the research tells us about the impact of the media in four areas: violence, body images, smoking, and sexuality. He identifies gaps in our knowledge and provides directions for future research. He points to many contradictory findings and notes that we have insufficient information about long-term effects. His conclusion that there is evidence of negative effects in each of these areas will not be surprising to most. This review of research is important in that it supports the conclusion that society should be concerned about the impact of media on adolescent development. It provides the basis for his analysis of how society should respond to this concern. He notes that the dominant response has been censorship to restrict access of adolescents to certain media. Parents are, of course, expected to be the primary mechanism for restricting access. As Levesque points out, this may be viable with young children but generally fails with adolescents. Following a stimulating and thoughtful discussion of free speech rights, he concludes that the current legal approaches to limit adolescent rights also fail to address the concerns, and he concludes that the legal system's adult-centered approach has negatively impacted the constitutional rights of adolescents. Levesque proposes a creative alternative, one that would not restrict adolescent access but rather would foster self-governance and increased civic participation. Rather than focusing on media as a source of negative influence, his approach seeks to promote the development of competency through access to information. His analysis and recommendations will be of interest to adolescents, parents, teachers, school administrators, community leaders, and policy makers, and I expect they will provide the foundation for stimulating and valuable discussion about the role of media in our society. (PsycINFO Database Record (c) 2012 APA, all rights reserved) (foreword)
SN - 978-0-19-532044-2
UR - http://search.ebscohost.com/login.aspx?direct=true&db=psyh&AN=2007-09555-000&site=ehost-live
ER -

TY - JOUR
ID - 1745
T1 - Contextually influenced patterns of play-developmental age associations for preschoolers with and without mental retardation
A1 - Malone,D.Michael
Y1 - 2006/12//
KW - PsycInfo
KW - Unsupervised Play Addition
KW - play-developmental age associations
KW - preschoolers
KW - Mental Retardation
KW - Childhood Development
KW - Childhood Play Behavior
KW - Intellectual Development Disorder
RP - NOT IN FILE
SP - 215
EP - 225
JF - Early Childhood Education Journal
VL - 34
IS - 3
CY - Germany
PB - Springer
N2 - The purpose of this paper is to present data demonstrating the differential pattern of association among play behaviors and developmental age that emerge for children with and without mental retardation across play situations. Seventeen preschool children with mental retardation and 17 preschool children without mental retardation were matched on developmental age and sex and observed during both home-based independent play and classroom-based freeplay. Results are presented for (a) between-group comparisons of home-based and classroom-based categorical-developmental age and sequential play-developmental age correlational patterns, (b) between setting comparisons of home-based and classroom-based categorical-developmental age and sequential play-developmental age correlational patterns within each group, and (c) comparisons of the pattern of categorical play-developmental age and sequential play-developmental age correlations within both setting and group. Results suggest both group and setting variation in the play-developmental age correlational patterns. Implications for multi-contextual understanding of children's play and the use of play in early childhood assessment are discussed. (PsycINFO Database Record (c) 2012 APA, all rights reserved) (journal abstract)
SN - 1573-1707
AD - Malone, D. Michael, Early Childhood Education, University of Cincinnati , Cincinnati, OH, US, 45221-0105, d.michael.malone@uc.edu
UR - http://search.ebscohost.com/login.aspx?direct=true&db=psyh&AN=2007-07685-004&site=ehost-live;d.michael.malone@uc.edu
L3 - 10.1007/s10643-006-0134-7
ER -

TY - JOUR
ID - 1746
T1 - Differential expression of toy play by preschoolers with and without mental retardation
A1 - Malone,D.Michael
Y1 - 2006///
KW - PsycInfo
KW - Unsupervised Play Addition
KW - differential expression
KW - toy play
KW - preschoolers
KW - Mental Retardation
KW - developmental age
KW - play ability
KW - Childhood Play Behavior

KW - Developmental Age Groups
KW - Preschool Students
KW - Toys
KW - Classrooms
KW - Parents
KW - Intellectual Development Disorder
RP - NOT IN FILE
SP - 117
EP - 131
JF - Journal of Research in Childhood Education
VL - 21
IS - 2
CY - US
PB - Assn for Childhood Education International
N2 - Analyses of the peak and predominant toy play of 17 preschool children with mental retardation and 17 preschool children without mental retardation are presented. Groups were matched on developmental age and sex and observed during both a home-based independent play situation and classroom-based free play. Results are presented for 1) a comparison of predominant play between setting for each group, 2) a comparison of peak play between setting for each group, 3) between-group analyses of predominant and peak play, and 4) a comparison of predominant and peak play. The results provide additional support for, and extend efforts to understand, contextual variation of children's toy play by demonstrating an advantage of home-based play over classroom-based play for both groups as well as an advantage of children with mental retardation over children without mental retardation during the home-based play situation. These data can be used to promote positive perceptions of, and attitudes toward, children with mental retardation among both parents and teachers, and to promote the use of a comprehensive, multi-contextual understanding of children's play ability and potential that is necessary for developing effective interventions. (PsycINFO Database Record (c) 2012 APA, all rights reserved) (journal abstract)
SN - 0256-8543
AD - Malone, D. Michael, College of Education, University of Cincinnati One Edwards Center, Room 2150-U, P.O. Box 210105, Cincinnati, OH, US, 45221-0105
UR - http://search.ebscohost.com/login.aspx?direct=true&db=psyh&AN=2007-12057-001&site=ehost-live
L3 - 10.1080/02568540609594583
ER -

TY - JOUR
ID - 1747
T1 - Randomised controlled trial of a parenting intervention in the voluntary sector for reducing child conduct problems: outcomes and mechanisms of change
A1 - Gardner,Frances
A1 - Burton,Jennifer
A1 - Klimes,Ivana
Y1 - 2006/11//
KW - PsycInfo
KW - Unsupervised Play Addition
KW - video based intervention
KW - parenting skill
KW - community
KW - cognitive behavioral program
KW - voluntary sector
KW - conduct problems
KW - Behavior
KW - clinically referred children
KW - home observation
KW - Problem Solving
KW - Behavior Problems
KW - Cognitive Behavior Therapy

KW - Group Psychotherapy
KW - Parenting Skills
KW - Behavior Change
KW - Community Mental Health Services
KW - Consumer Satisfaction
KW - Intervention
KW - Videotape Instruction
RP - NOT IN FILE
SP - 1123
EP - 1132
JF - Journal of Child Psychology and Psychiatry
VL - 47
IS - 11
CY - United Kingdom
PB - Blackwell Publishing
N2 - Background: To test effectiveness of a parenting intervention, delivered in a community-based voluntary-sector organisation, for reducing conduct problems in clinically-referred children. Methods: Randomised controlled trial, follow-up at 6, 18 months, assessors blind to treatment status. Participants--76 children referred for conduct problems, aged 2-9, primarily low-income families, randomised to treatment vs. 6-month wait-list group. Retention was 93% at 6 months, 90% at 18 months. Interventions--Webster-Stratton Incredible Years video-based 14-week group programme, teaches cognitive-behavioural principles for managing behaviour, using a collaborative, practical, problem-solving approach. Primary outcomes--child problem behaviour by parent-report (Eyberg) and home-based direct observation; secondary outcomes--observed positive and negative parenting; parent-reported parenting skill, confidence and depression. Results: Post-treatment improvements were found in child problem behaviour, by parent-report (effect size (ES) .48, p=.05) and direct observation (ES .78, p=.02); child independent play (ES .77, p=.003); observed negative (ES .74, p=.003) and positive (ES .38, p=.04) parenting; parent-reported confidence (ES .40, p=.03) and skill (ES .65, p=.01), using ANCOVA to control for baseline scores. Maternal depression did not change. Consumer satisfaction was high. At 18-month follow-up, although no randomised comparison was possible, changes appeared to maintain, with no significant change toward baseline level on any measure. Change in observed positive parenting appeared to mediate change in child problem behaviour (p <.025). Conclusions: Findings suggest that a group-based cognitive-behavioural parenting programme, delivered by well-trained and supervised staff, can be effective in a community voluntary-sector setting, for reducing conduct problems and enhancing parenting skills. Change in parenting skill appears to be a key mechanism for change in child behaviour. Findings have implications for feasibility of translating evidence-based programmes, even for clinically-referred conduct problems, into less specialised community settings, likely to have lower costs and be more accessible for families. (PsycINFO Database Record (c) 2012 APA, all rights reserved) (journal abstract)
SN - 1469-7610
AD - Gardner, Frances, Centre for Evidence-Based Intervention, Department of Social Policy & Social Work, Oxford University 32, Wellington Square, Oxford, United Kingdom, OX1 ER, frances.gardner@socres.ox.ac.uk
UR - http://search.ebscohost.com/login.aspx?direct=true&db=psyh&AN=2006-20991-006&site=ehost-live;frances.gardner@socres.ox.ac.uk
L3 - 10.1111/j.1469-7610.2006.01668.x
ER -

TY - THES
ID - 1750
T1 - Encouraging independent play: Verbal versus nonverbal reinforcement
A1 - Smith,Raegan B.
Y1 - 2006///
N1 - Accession Number: 2006-99024-032. First Author & Affiliation: Smith, Raegan B.; Oklahoma State U., US. Other Journal Titles: Dissertation Abstracts International. Release Date: 20070312. Publication Type: Dissertation Abstract, (0400); . Media Covered: Electronic. Document Type: Dissertation. Dissertation Number: AAI3220241. Language: English. Major Descriptor: Childhood Development; Nonverbal Reinforcement; Positive Reinforcement; Verbal Reinforcement. Minor Descriptor: Feedback; Parents. Classification: Health & Mental Health Treatment & Prevention (3300) General Psychology (2100) . Population: Human (10); . Age

Group: Childhood (birth-12 yrs) (100) Preschool Age (2-5 yrs) (160) . Methodology: Empirical Study; Quantitative Study. Page Count: 1
KW - PsycInfo
KW - Unsupervised Play Addition
KW - nonverbal reinforcement
KW - Positive Reinforcement
KW - Young Children
KW - Childhood Development
KW - Feedback
KW - Parents
KW - verbal reinforcement
RP - NOT IN FILE
JF - Dissertation Abstracts International: Section B: The Sciences and Engineering
VL - 67
IS - 6-B
CY - US
PB - ProQuest Information & Learning
N2 - Two types of positive reinforcement were compared to examine their effectiveness in encouraging independent play in young children. A within-subjects design was used with mothers and their 30- to 42-month-old children. Experimental conditions varied according to type of positive feedback parents were instructed to give, such that praise and nonverbal affection strategies were used in alternate conditions and counterbalanced for order of presentation. Paired samples mean comparisons indicated no difference in children's behaviors across study conditions. However, differences were found between the first and second halves within each condition. Lower rates of independent play were found in the latter halves of both conditions. In the nonverbal condition, higher amounts of solicitation for attention and negative affect were found in the second half of the condition. In addition, correlation analyses suggested relations between child behavior and adjustment problems and observed behavior under various conditions of the study. (PsycINFO Database Record (c) 2012 APA, all rights reserved)
SN - 0419-4217
UR - http://search.ebscohost.com/login.aspx?direct=true&db=psyh&AN=2006-99024-032&site=ehost-live
ER -

TY - CHAP
ID - 1751
T1 - Media Use by Infants and Toddlers: A Potential for Play
A1 - Weber,Deborah S.
Y1 - 2006///
N1 - Accession Number: 2006-10812-009. First Author & Affiliation: Weber, Deborah S.; Fisher-Price Child Research Department, US. Release Date: 20061113. Publication Type: Book, (0200); Edited Book, (0280); . Media Covered: Print. Document Type: Chapter. ISBN: 0-19-530438-1, Hardcover, 0195304381; 978-0-19-530438-1, Hardcover, 9780195304381. Language: English. Major Descriptor: Childhood Play Behavior; Early Childhood Development; Infant Development; Mass Media; Parent Child Relations. Minor Descriptor: Television Viewing; Videotapes. Classification: Developmental Psychology (2800) Mass Media Communications (2750) . Population: Human (10); . Age Group: Childhood (birth-12 yrs) (100) . Intended Audience: Psychology: Professional & Research (PS) . References Available: Y.. Page Count: 23
KW - PsycInfo
KW - Unsupervised Play Addition
KW - media viewing
KW - Infants
KW - Toddlers
KW - Play
KW - parent-child interactions
KW - Television
KW - videos
KW - Childhood Play Behavior
KW - Early Childhood Development

KW - Infant Development
KW - Mass Media
KW - Parent Child Relations
KW - Television Viewing
KW - Videotapes
RP - NOT IN FILE
SP - 169
EP - 191
T2 - Play = learning: How play motivates and enhances children's cognitive and social-emotional growth
A2 - Singer,Dorothy G.
A2 - Golinkoff,Roberta Michnick
A2 - Hirsh-Pasek,Kathy
CY - New York, NY US
PB - Oxford University Press
N2 - (from the chapter) Can the media serve as a tool for parents and caregivers to facilitate play among infants and toddlers? A common belief is that television and videos take the place of spending time with others and playing. Another concern is that parents use television as a "babysitter." This may conjure up an image of children spending numerous hours passively watching television or videos with no adult presence or interaction. Is this in fact what is really occurring? Can and do television and videos actually encourage parent-child interactions? Can children use media as a model to incorporate in their imitative play? Do the media enhance play or detract from play? The amount of time that very young children spend watching television and video programs is increasing, and unfortunately this most likely detracts from the amount of time they spend playing. We now have data indicating the media-viewing habits of infants and toddlers, what factors influence these media-viewing habits, and the effects of such early viewing on the development of cognitive and social-emotional skills. This chapter discusses research regarding infant and toddler media and places it in the context of children's play and parent-child interactions. (PsycINFO Database Record (c) 2012 APA, all rights reserved) (chapter)
SN - 0-19-530438-1
UR - http://search.ebscohost.com/login.aspx?direct=true&db=psyh&AN=2006-10812-009&site=ehost-live
ER -

TY - JOUR
ID - 1752
T1 - Playschool in pictures: Children's photographs as a research method
A1 - Einarsdottir,Johanna
Y1 - 2005/08//
KW - PsycInfo
KW - Unsupervised Play Addition
KW - children's photographs
KW - playschool
KW - child perception
KW - educational setting
KW - child research
KW - research methods
KW - Child Attitudes
KW - Childhood Play Behavior
KW - Early Childhood Development
KW - Photographs
KW - Preschool Education
KW - Cameras
KW - Methodology
KW - Preschool Students
RP - NOT IN FILE
SP - 523
EP - 541
JF - Early Child Development and Care

VL - 175
IS - 6
CY - United Kingdom
PB - Taylor & Francis
N2 - The paper focuses on children's photographs as a method to use in research with children. Studies using photographs with children are reviewed and compared and a study conducted in one Icelandic playschool is described. The playschool was involved in a project where the purpose was to look at the ways children think about their early childhood educational setting and to develop methods for listening to children's perspectives. The paper describes and compares two approaches where cameras were used. One group used digital cameras to take pictures in their playschool while they showed the researcher important places and things in the playschool. The other group was given disposable cameras that they could use unsupervised for a period of time. The results show that using cameras and children's photos is a notable method to use when seeking children's perspectives on their life in an early childhood setting. (PsycINFO Database Record (c) 2012 APA, all rights reserved) (journal abstract)
SN - 1476-8275
UR - http://search.ebscohost.com/login.aspx?direct=true&db=psyh&AN=2005-07650-005&site=ehost-live
L3 - 10.1080/03004430500131320
ER -

TY - JOUR
ID - 1753
T1 - Symbolic play in congenitally blind children
A1 - Bishop,Martin
A1 - Hobson,R.Peter
A1 - Lee,Anthony
Y1 - 2005///
KW - PsycInfo
KW - Unsupervised Play Addition
KW - symbolic play
KW - congenitally blind children
KW - Scaffolding
KW - social relations
KW - Verbal Ability
KW - Blind
KW - Recreation
KW - Interpersonal Relationships
RP - NOT IN FILE
SP - 447
EP - 465
JF - Development and Psychopathology
VL - 17
IS - 2
CY - United Kingdom
PB - Cambridge University Press
N2 - There is controversy over the existence and nature of blind children's limitations in symbolic play. In this study we tested 13 5- to 9-year-old congenitally blind children for the ability to symbolize when an adult provided scaffolding for their play. The blind children were selected on the basis that they did not have the syndrome of autism, and they comprised two groups matched for age (MA) and verbal ability on the Wechsler Intelligence Scale for Children who contrasted in their ability to engage in social relations. We also tested a group of sighted children who were MA matched with the more socially able blind children. On the basis of an hypothesis about the social-developmental sources of symbolic play deficits in congenitally blind children, we predicted differences between the socially able and socially impaired groups of blind children in the following respects: the attribution of symbolic meanings to play materials, the ascription of individual roles to play figures, and the anchorage of play in the scenario as presented by the adult. The results accorded with these predictions. Whereas the more socially able blind children showed symbolic play that was very similar to that of sighted children, the MA- and IQ-matched socially impaired blind children were limited in the three aspects of

their play. On the other hand, even the socially impaired children showed instances of symbolizing. The findings suggest a way to reconcile conflicting reports of symbolic play deficits in young blind children, and may be relevant for explaining the association between autism and congenital blindness. (PsycINFO Database Record (c) 2012 APA, all rights reserved) (journal abstract)
SN - 1469-2198
AD - Hobson, R. Peter, Adult Department, Tavistock Clinic 120, Belsize Lane, London, United Kingdom, NW3 5BA, r.hobson@ucl.ac.uk
UR - http://search.ebscohost.com/login.aspx?direct=true&db=psyh&AN=2005-05658-008&site=ehost-live;r.hobson@ucl.ac.uk
L3 - 10.1017/S0954579405050212
ER -

TY - THES
ID - 1754
T1 - The developmental status and play occupations of young children institutionalized in Eastern Europe
A1 - Daunhauer,Lisa Ann
Y1 - 2005///
N1 - Accession Number: 2005-99012-162. First Author & Affiliation: Daunhauer, Lisa Ann; Boston U., US. Other Journal Titles: Dissertation Abstracts International. Release Date: 20051011. Publication Type: Dissertation Abstract, (0400); . Media Covered: Electronic. Document Type: Dissertation. Dissertation Number: AAI3157364. Language: English. Major Descriptor: Childhood Play Development; Infant Development; Institutionalization. Minor Descriptor: Age Differences; Childhood Development; Childhood Play Behavior; Cognitive Ability; Mental Health; Orphanages. Classification: Developmental Psychology (2800) . Population: Human (10); Male (30); Female (40); . Location: Eastern Europe. Age Group: Childhood (birth-12 yrs) (100) Infancy (2-23 mo) (140) Preschool Age (2-5 yrs) (160) . Methodology: Empirical Study; Quantitative Study. Page Count: 1
KW - PsycInfo
KW - Unsupervised Play Addition
KW - developmental status
KW - play occupations
KW - mental performance
KW - prematurity
KW - Orphanages
KW - cognitive functioning
KW - Infant Development
KW - Institutionalization
KW - Childhood Play Development
KW - Age Differences
KW - Childhood Development
KW - Childhood Play Behavior
KW - Cognitive Ability
KW - Mental Health
RP - NOT IN FILE
JF - Dissertation Abstracts International: Section B: The Sciences and Engineering
VL - 65
IS - 12-B
CY - US
PB - ProQuest Information & Learning
N2 - This three-part investigation examined the effects of institutionalization on development using both standardized measures and naturalistic observations. In Study I, 93 infants and young children (50 males, 43 females) between 1 and 46 months of age were assessed using the Bayley Scales of Infant Development (BSID-II). Results indicated that the study group performed significantly below the standardization sample on both the BSID-II mental and psychomotor domains (t(92) = -26.91, p le; .001, and t(92) = -25.97, p le; .001 respectively). Linear multiple regressions indicated that prematurity and current weight-for-age best predicted mental performance, while current weight best predicted psychomotor performance, with prematurity approaching significance (R = .45, F(6, 84) = 3.36, p le; .01, and R = .36, F(6, 84) = 1.87, p le; .10 respectively).

A subsample (n = 20) was re-assessed (M = 10 months later, SD = .98). Paired t-tests indicated no significant differences between the subsample's first and second performances, however, there was a medium effect size for an increase in the mental score over time (d = .46). Correlations between first and second assessments were r = .08 (mental domain) and r = .52 (psychomotor domain). Standardized assessments used with children living in orphanages may provide a narrow view of a child's strengths and abilities. Therefore, Study II investigated the relationship between the institutionalized children's independent play skills and cognitive functioning. Twenty-six infants and young children between 10 and 38 months of age were assessed with the BSID-II, followed by video-taped play sessions. Results indicated that when compared to a typical American sample matched for age, significantly more study participants than the comparison group demonstrated play skills below chronological age on levels of symbolic play, but generally not below mental developmental age. In Study III, the same study group was video-taped interacting with a caregiver in order to assess the effects of caregiver interactions on play skills. The children demonstrated higher-level play skills in the interactive condition than when playing alone (t(25) = -1.88, p le; .07, d = .53). Two caregiver interaction patterns emerged during the childrens' highest levels of play: social-emotional support and task support. Success for the child was strongly associated with having a caregiver who provided more structure/assistance, and who was directive, mutually engaged, and encouraging. This project highlights the importance of considering factors other than length of time institutionalized when evaluating the developmental status of this population. (PsycINFO Database Record (c) 2012 APA, all rights reserved)
SN - 0419-4217
UR - http://search.ebscohost.com/login.aspx?direct=true&db=psyh&AN=2005-99012-162&site=ehost-live
ER -

TY - THES
ID - 1755
T1 - The role that highlights play in providing information about depth and shape
A1 - Elieff,Chryle Ann
Y1 - 2005///
N1 - Accession Number: 2005-99010-242. First Author & Affiliation: Elieff, Chryle Ann; U Minnesota, US. Other Journal Titles: Dissertation Abstracts International. Release Date: 20050829. Publication Type: Dissertation Abstract, (0400); . Media Covered: Electronic. Document Type: Dissertation. Dissertation Number: AAI3154039. Language: English. Major Descriptor: Depth Perception; Form and Shape Perception; Photographs; Visual Perception. Minor Descriptor: Observers. Classification: Developmental Psychology (2800) . Population: Human (10); . Age Group: Childhood (birth-12 yrs) (100) Neonatal (birth-1 mo) (120) Infancy (2-23 mo) (140) Preschool Age (2-5 yrs) (160) School Age (6-12 yrs) (180) Adolescence (13-17 yrs) (200) Adulthood (18 yrs & older) (300) . Methodology: Empirical Study. Page Count: 1
KW - PsycInfo
KW - Unsupervised Play Addition
KW - highlights
KW - depth perception
KW - shape perception
KW - high quality photographs
KW - specular reflections
KW - Form and Shape Perception
KW - Photographs
KW - Visual Perception
KW - Observers
RP - NOT IN FILE
JF - Dissertation Abstracts International: Section B: The Sciences and Engineering
VL - 65
IS - 11-B
CY - US
PB - ProQuest Information & Learning
N2 - Little research has been done to explore the role that highlights, specular reflections present on the surface of objects, play in providing information about depth and shape to the observer. One way to explore whether their presence on objects and high-quality photographs of objects provides information about object shape is by exploring how they are perceived and subsequently acted upon by adults and children. This paper reports data

from four experiments demonstrating the role that highlights play in the perception of depth. Specifically, I ask whether highlights play a role in the perception of depth and, if so, whether the information is learned through experience with the environment. In the first experiment, adults were presented monocularly and binocularly with objects and high-quality photographs of those objects both with and without highlights and were allowed to manually act on the displays. In a second study, adults were presented with the displays from Experiment 1 as well as a photograph of a chrome plated cylinder with the reflections of the surrounds in the normal position or rotated 90 degrees. They were asked to draw and answer questions concerning their perception of the displays. In the third experiment, pre-schoolers were shown the same photographs of the cylinder and were asked to reach into a box and select one of two objects inside the box that matched the photograph. In the fourth study, a grasping task similar to Experiment 1 was used with nine-month-old infants. Results suggest that highlights may serve as a monocular depth cue for adults and some pre-schoolers, but there was little evidence to suggest that infants were sensitive to highlights. This may suggest that the ability to use specular reflections as information for shape may require experience associating specular reflections with three-dimensionality and that pre-schoolers already have that ability. (PsycINFO Database Record (c) 2012 APA, all rights reserved)
SN - 0419-4217
UR - http://search.ebscohost.com/login.aspx?direct=true&db=psyh&AN=2005-99010-242&site=ehost-live
ER -

TY - CHAP
ID - 1756
T1 - Assessment of Sibling Relationships Using Play, Art, and Stories
A1 - Kirk,Kate
Y1 - 2005///
N1 - Accession Number: 2005-07318-008. First Author & Affiliation: Kirk, Kate. Release Date: 20060103. Publication Type: Book, (0200); Edited Book, (0280); . Media Covered: Print. Document Type: Chapter. Book Type: Handbook/Manual. ISBN: 0-7657-0122-7, Hardcover, 0765701227. Language: English. Major Descriptor: Art; Measurement; Play Therapy; Sibling Relations; Storytelling. Minor Descriptor: Psychological Assessment. Classification: Art & Music & Movement Therapy (3357) . Population: Human (10); Male (30); Female (40); . Age Group: Childhood (birth-12 yrs) (100) Preschool Age (2-5 yrs) (160) School Age (6-12 yrs) (180) . Intended Audience: Psychology: Professional & Research (PS) . Tests & Measures: Play Therapy Observational Instrument; Sibling Relationship Checklists; Kinetic Family Drawing; Attachment Story Completions Task; Draw A Person: A Quantitative Scoring System; Family Relations Test: Children's Version; . Methodology: Nonclinical Case Study. References Available: Y.. Page Count: 17
KW - PsycInfo
KW - Unsupervised Play Addition
KW - assessment
KW - sibling relationships
KW - Play
KW - Art
KW - stories
KW - Measurement
KW - Play Therapy
KW - Sibling Relations
KW - Storytelling
KW - Psychological Assessment
RP - NOT IN FILE
SP - 159
EP - 175
T2 - International handbook of play therapy: Advances in assessment, theory, research, and practice
A2 - Schaefer,Charles
A2 - McCormick,Judy
A2 - Ohnogi,Akiko
CY - Lanham, MD US
PB - Jason Aronson
N2 - The author works as an independent play therapist assessing sibling relationships for court and welfare agencies. (The names, ages, and sex of the children in this group have been changed to preserve confidentiality.)

She chose to write up a case study of an assessment she carried out some time ago. The children had been separated from each other and their parents for several months when she was appointed by the court to carry out an assessment of their relationship. The children were removed from their family of origin because of severe and chronic neglect that years of intervention from the statutory agencies could not change. In addition, all four children had witnessed long-standing domestic violence and two of them had been sexually abused. The author describes the referral, assessment methodology, sibling assessment sessions, and provides a summary of the sibling relationships. (PsycINFO Database Record (c) 2012 APA, all rights reserved)
SN - 0-7657-0122-7
UR - http://search.ebscohost.com/login.aspx?direct=true&db=psyh&AN=2005-07318-008&site=ehost-live
ER -

TY - JOUR
ID - 1757
T1 - Response to trauma in Haitian youth at risk
A1 - Douyon,Richard
A1 - Marcelin,Louis Herns
A1 - Jean-Gilles,MichŠle
A1 - Page,J.Bryan
Y1 - 2005///
KW - PsycInfo
KW - Unsupervised Play Addition
KW - Trauma
KW - at risk population
KW - social relations
KW - sociodemographic information
KW - Self Report
KW - Haitian youth
KW - undesirable behavior
KW - At Risk Populations
KW - Behavior
KW - Demographic Characteristics
KW - Emotional Trauma
KW - Experiences (Events)
KW - Criminal Behavior
RP - NOT IN FILE
SP - 115
EP - 138
JF - Journal of Ethnicity in Substance Abuse
VL - 4
IS - 2
CY - US
PB - Haworth Press
N2 - In order to characterize undesirable behavior (drug use, fighting, criminal activity) among Haitian youth at risk and determine the relationship between traumatic experience and that kind of behavior, investigators recruited 292 Haitian youths via networks of informal social relations in two zones of Miami/Dade County strongly identified with Haitian ethnicity. Each recruit responded to an interview schedule eliciting sociodemographic information and self-reported activities, including involvement in youth-dominated groups. They also reported traumatic experience. Clinicians administered CAPS to a subset of those respondents who self-reported traumatic experience. Staff ethnographers selected respondents for in-depth interviews and family studies to provide contextual depth for findings of the interview schedule and the CAPS assessments. Although traumatic experience may still play a role in mental health outcomes among children, childhood victimization among Haitian children does not appear to be related to the drug use and undesirable behaviors associated with unsupervised youth, including formation of gangs. (PsycINFO Database Record (c) 2012 APA, all rights reserved) (journal abstract)
SN - 1533-2659

AD - Page, J. Bryan, University of Miami, Department of Anthropology 102 Merrick (LC2005), Coral Gables, FL, US, 33124, Bryan.page@miami.edu
UR - http://search.ebscohost.com/login.aspx?direct=true&db=psyh&AN=2005-15549-006&site=ehost-live;Bryan.page@miami.edu
L3 - 10.1300/J233v04n02_06
ER -

TY - CHAP
ID - 1758
T1 - After-school sport for children: Implications of a task-involving motivational climate
A1 - Duda,Joan L.
A1 - Ntounumis,Nikos
Y1 - 2005///
N1 - Accession Number: 2005-01368-014. First Author & Affiliation: Duda, Joan L.; School of Sport and Exercise Sciences, University of Birmingham, United Kingdom. Release Date: 20050214. Publication Type: Book, (0200); Edited Book, (0280); . Media Covered: Print. Document Type: Chapter. ISBN: 0-8058-4430-9, Hardcover, 0805844309; 0-8058-4431-7, Paperback, 0805844317. Language: English. Major Descriptor: Athletic Participation; Athletic Performance; Motivation; Sports; Teams. Minor Descriptor: After School Programs. Classification: Psychosocial & Personality Development (2840) Sports (3720) . Population: Human (10); Male (30); Female (40); . Location: US. Age Group: Childhood (birth-12 yrs) (100) School Age (6-12 yrs) (180) Adolescence (13-17 yrs) (200) Adulthood (18 yrs & older) (300) Young Adulthood (18-29 yrs) (320) . Intended Audience: Psychology: Professional & Research (PS) . References Available: Y.. Page Count: 20
KW - PsycInfo
KW - Unsupervised Play Addition
KW - sport activities
KW - after-school sport program
KW - athletic teams
KW - motivational climate
KW - Athletic Participation
KW - Athletic Performance
KW - Motivation
KW - Sports
KW - Teams
KW - After School Programs
RP - NOT IN FILE
SP - 311
EP - 330
T2 - Organized activities as contexts of development: Extracurricular activities, after-school and community programs
A2 - Mahoney,Joseph L.
A2 - Larson,Reed W.
A2 - Eccles,Jacquelynne S.
CY - Mahwah, NJ US
PB - Lawrence Erlbaum Associates Publishers
N2 - (from the chapter) Only about 14% of all children and adolescents who participate in sport in the United States are members of interscholastic athletic teams (which take place before or after classes but within the school setting). The largest number of youth participants are engaged in an agency-sponsored sport program. Indicating a potential growing significance of after-school sport in young people's lives, this increase in engagement in organized, community sport programs in recent years is coupled with a decreased involvement in spontaneous, unsupervised, free-play types of activities (Ewing & Seefeldt, 2002). One cannot help but wonder about the possible implications of such differential trends for how youngsters are now being socialized via their experiences in the physical domain. Moreover, as those who are more physically able are more likely to feel at home in organized sport programs (perhaps due to their more competitive features; Roberts, 1984), the ramifications of the enhanced attractiveness of after-school sport activities (in contrast to the seemingly diminished appeal of informal physical activities and games) for the development and long-term involvement of all children has yet to be determined. (PsycINFO Database Record (c) 2012 APA, all rights reserved) (chapter)

SN - 0-8058-4430-9
UR - http://search.ebscohost.com/login.aspx?direct=true&db=psyh&AN=2005-01368-014&site=ehost-live
ER -

TY - JOUR
ID - 1759
T1 - Gibt es eine Kinder- und Jugendlichenpsychoanalyse?
A1 - Nicola‹dis,Graziella
Y1 - 2005///
KW - PsycInfo
KW - Unsupervised Play Addition
KW - child psychoanalysis
KW - adolescent psychoanalysis
KW - Adolescent Psychotherapy
KW - Child Psychotherapy
KW - Psychoanalysis
RP - NOT IN FILE
SP - 368
EP - 377
JF - Zeitschrift f • r Psychoanalytische Theorie und Praxis/ Journal for Psychoanalytical Theory and Practice
VL - 20
IS - 4
CY - Germany
PB - Stroemfeld Verlag
N2 - Many psychoanalysts still ask if child and adolescent analysis really exist. Arguing their doubts they refer to problems with the frame and to the fact that games, drawing and acting play a more important role than talking and free associations of the adult analysis. They point out that in contrast to the adult analysis the presence of parents and their interference with the cure are real. Is there a transference neurosis, an analytic process? How does one interpret with a child? How important is the age difference that can be experienced as a seduction after all?. (PsycINFO Database Record (c) 2012 APA, all rights reserved) (journal abstract)
SN - 0169-3395
AD - Nicola‹dis, Graziella, Route de Malagnon 32, CH-1208, Geneve, Switzerland, g.nicolaidis@bluewin.ch
UR - http://search.ebscohost.com/login.aspx?direct=true&db=psyh&AN=2006-08992-003&site=ehost-live;g.nicolaidis@bluewin.ch
ER -

TY - JOUR
ID - 1760
T1 - Early Focused Attention Predicts Outcome for Children Born Prematurely
A1 - Lawson,Katharine R.
A1 - Ruff,Holly A.
Y1 - 2004/12//
KW - PsycInfo
KW - Unsupervised Play Addition
KW - early focused attention
KW - Attention
KW - cognitive function
KW - causal attention
KW - very low birth weight
KW - Birth Weight
KW - Cognitive Ability
KW - Premature Birth
KW - Selective Attention
RP - NOT IN FILE
SP - 399
EP - 406

JF - Journal of Developmental and Behavioral Pediatrics
VL - 25
IS - 6
CY - US
PB - Lippincott Williams & Wilkins
N2 - There is evidence that early focused, but not casual, attention to objects reflects concurrent regulation of attention and active learning. Because attentional abilities are of particular relevance in preterm infants, we evaluated whether early focused attention would be a better predictor of later attention and cognitive function than casual attention in 55 children born at very low birth weight. Participants were tested initially at 7 months and then at 2, 3, and/or 4/5 years of age. Focused attention was defined as the duration of concentrated examination of objects during independent play. Outcome measures were maternal ratings on standard attention-deficit hyperactivity disorder rating scales and standardized cognitive assessments. Results indicate that 7-month focused attention was predictive of reported problems in hyperactivity/ impulsivity at age 4/5 years and cognitive abilities at 2, 3, and 4/5 years; casual attention measures were not related to these outcomes. Early focused attention appears continuous with later attentional skills in at-risk infants and is related to cognitive abilities through the preschool years. (PsycINFO Database Record (c) 2012 APA, all rights reserved) (journal abstract)
SN - 1536-7312
AD - Lawson, Katharine R., Fisher Landau Center, Albert Einstein College of Medicine 1165 Morris Park Avenue, Bronx, NY, US, 10461, krlawson@aecom.yu.edu
UR - http://search.ebscohost.com/login.aspx?direct=true&db=psyh&AN=2005-01036-003&site=ehost-live;krlawson@aecom.yu.edu
L3 - 10.1097/00004703-200412000-00003
ER -

TY - JOUR
ID - 1761
T1 - The Role of Shame in Predicting Adult Anger and Depressive Symptoms Among Victims of Child Psychological Maltreatment
A1 - Harper,Felicity W.K.
A1 - Arias,Ileana
Y1 - 2004/12//
KW - PsycInfo
KW - Unsupervised Play Addition
KW - shame
KW - adult anger
KW - child maltreatment
KW - victims
KW - depressive symptoms
KW - Gender Differences
KW - Anger
KW - Child Abuse
KW - Major Depression
KW - Victimization
KW - Human Sex Differences
RP - NOT IN FILE
SP - 367
EP - 375
JF - Journal of Family Violence
VL - 19
IS - 6
CY - Germany
PB - Springer
N2 - Previous research on child maltreatment and adult outcomes has failed to consider affective reactions to the maltreatment, which may play a critical role in victim outcomes. One such affective reaction--shame--may help to explain this relationship. In the context of maltreatment, feelings of shame are seen as a natural

extension of the helplessness experienced by many victims of child maltreatment [Finkelhor, D., and Browne, A. (1986). Initial and long-term effects: A conceptual framework. In Finkelhor, D. (ed.), A Sourcebook on Child Sexual Maltreatment, Sage, Newbury Park, CA, pp. 180- 198]. The current study examined the moderating role of shame in the relationship between victim reactions to child psychological maltreatment and adult anger and depressive symptoms. Results showed that shame moderated between child psychological maltreatment and adult anger for men but not for women, whereas shame moderated between child psychological maltreatment and depressive symptoms for adult women. Presence of gender-related differences suggests that gender should be considered in the design and development of therapeutic techniques for the treatment and prevention of anger and depression in adult survivors of child psychological maltreatment. (PsycINFO Database Record (c) 2012 APA, all rights reserved) (journal abstract)
SN - 1573-2851
AD - Arias, Ileana, Division of Violence Prevention, National Center for Injury Prevention and Control, Centers for Disease Control and Prevention 4770 Buford Highway, N.E., Mailstop K60, Atlanta, GA, US, 30341, iarias@cdc.gov
UR - http://search.ebscohost.com/login.aspx?direct=true&db=psyh&AN=2005-02609-004&site=ehost-live;iarias@cdc.gov
L3 - 10.1007/s10896-004-0681-x
ER -

TY - JOUR
ID - 1764
T1 - Working with other adults: What teachers need to know
A1 - Calder,Isobel
A1 - Grieve,Ann
Y1 - 2004/06//
KW - PsycInfo
KW - Unsupervised Play Addition
KW - trained teachers
KW - classroom
KW - teacher education courses
KW - teacher's concepts
KW - Teacher Role
KW - professional identity
KW - Classrooms
KW - Concepts
KW - Preservice Teachers
KW - Student Teachers
KW - Teacher Education
KW - Role Perception
RP - NOT IN FILE
SP - 113
EP - 126
JF - Educational Studies
VL - 30
IS - 2
CY - United Kingdom
PB - Taylor & Francis
N2 - This paper discusses the concepts which trained teachers carry into the classroom from their initial teacher education courses and argues that these outmoded concepts must be altered so that the roles which other adults play can be understood by teachers. There is a common perception that teaching is a solitary activity carried out by a trained adult with a group of learners. The reality is that there is often another adult present. The paper is set in the context of classroom assistants working in Scottish primary mainstream classrooms. It concerns the way in which teachers and other adults work together to support children with behavioural difficulties. Although the data is peculiarly Scottish, the lessons learned have a wider national and international currency, for it seeks to explain the changes necessary in teachers' thinking about collaborative working. This paper argues that continuing professional development must be provided to allow teachers already in the field to consider the

effect on their professional identity produced by the presence of other adults. Most crucially, it is argued that initial teacher education must acknowledge the changes happening in mainstream classes and must include personnel and performance management training in courses for student teachers. (PsycINFO Database Record (c) 2012 APA, all rights reserved)
SN - 1465-3400
AD - Calder, Isobel, Professional Development Unit, University of Strathclyde, Faculty of Education 76 Southbrae Drive, Glasgow, United Kingdom, G13 1PP, isobel.calder@strath.ac.uk
UR - http://search.ebscohost.com/login.aspx?direct=true&db=psyh&AN=2004-11171-002&site=ehost-live;isobel.calder@strath.ac.uk
L3 - 10.1080/0305569032000159778
ER -

TY - JOUR
ID - 1765
T1 - The extent of children's independent mobility and the number of actualized affordances as criteria for child-friendly environments
A1 - Kytt„Marketta
Y1 - 2004/06//
KW - PsycInfo
KW - Unsupervised Play Addition
KW - independent mobility
KW - actualized affordances
KW - child-friendly environments
KW - Childhood Development
KW - Environmental Planning
KW - Independence (Personality)
KW - Person Environment Fit
KW - Physical Mobility
RP - NOT IN FILE
SP - 179
EP - 198
JF - Journal of Environmental Psychology
VL - 24
IS - 2
CY - Netherlands
PB - Elsevier Science
N2 - Diversity of environmental resources and access to play and exploration have been regarded as the two central criteria of a child-friendly environment. The former has been operationalized in this article by the number of actualized, positive affordances and the latter by the degree of independent mobility. A hypothetical model in which the degree of independent mobility and the number of actualized affordances covary in four varying types of children's environments was constructed. The latter are called Bullerby. Wasteland, Cell, and Glasshouse. The subjects were 8-9-year-old children, who were studied by using individual interviews and questionnaires. The results indicate that all of the hypothesized environment types appeared in the data. Each neighborhood had a unique combination of affordances and independent mobility in terms of the model. The Bullerby type of setting abounded in the Finnish communities. The Cell, Wasteland and Glasshouse were the most common types of environment in the Belarushian data. In general, the proportion of Bullerby-type settings decreased and the glasshouse-type increased as the degree of urbanization augmented. The models and measures applied need further elaboration and testing in different environments and with varying groups of children. (PsycINFO Database Record (c) 2012 APA, all rights reserved)
SN - 0272-4944
AD - Kytt„ Marketta, Centre for Urban and Regional Studies, Helsinki University of Technology P.O. Box 9300, 02015, Finland, marketta.kytta@hut.fi
UR - http://search.ebscohost.com/login.aspx?direct=true&db=psyh&AN=2004-95273-003&site=ehost-live;marketta.kytta@hut.fi
L3 - 10.1016/S0272-4944(03)00073-2
ER -

TY - JOUR
ID - 1766
T1 - Observers' proficiency at identifying pretense acts based on behavioral cues
A1 - Richert,Rebekah A.
A1 - Lillard,Angeline S.
Y1 - 2004/04//
KW - PsycInfo
KW - Unsupervised Play Addition
KW - observer proficiency
KW - discrimination
KW - pretense behavior
KW - pretense vs real acts
KW - behavioral cues
KW - Development
KW - pretender behavior
KW - pretend content
KW - pretense interpretation
KW - CHILDREN
KW - Adults
KW - Behavior
KW - Cues
KW - Faking
KW - Reality
KW - Childhood Development
KW - Developmental Age Groups
RP - NOT IN FILE
SP - 223
EP - 240
JF - Cognitive Development
VL - 19
IS - 2
CY - Netherlands
PB - Elsevier Science
N2 - Discriminating what is pretense from what is real is a fundamental problem in development. Research has addressed the proficiency with which adults and children discriminate between play fighting and real fighting, and yet none (to our knowledge) has investigated discrimination of other kinds of pretense and real acts. In addition, little is known about what aspects of pretender behavior (as opposed to pretend content) might cue pretense interpretations. In two experiments, 8-20 s clips showing pretense and real snack behaviors were presented to adult and child participants. All participants distinguished between pretense and real behaviors at better than chance level. Furthermore, certain features (specific looking patterns and mistimed behaviors) were most prominent in the videotapes that were most often correctly identified. This provides empirical support for the suggestion that these cues, as opposed to more commonly cited cues, like smiles, might serve as important indicators of pretense for children and adults. (PsycINFO Database Record (c) 2012 APA, all rights reserved) (journal abstract)
SN - 0885-2014
AD - Lillard, Angeline S., Department of Psychology, University of Virginia 102 Gilmer Hall, P.O. Box 400400, Charlottesville, VA, US, 22904-4400, lillard@virginia.edu
UR - http://search.ebscohost.com/login.aspx?direct=true&db=psyh&AN=2004-14578-005&site=ehost-live;lillard@virginia.edu
L3 - 10.1016/j.cogdev.2004.01.001
ER -

TY - JOUR
ID - 1767
T1 - The role of historical intuitions in children's and adults' naming of artifacts

A1 - Gutheil,Grant
A1 - Bloom,Paul
A1 - Valderrama,Nohemy
A1 - Freedman,Rebecca
Y1 - 2004/02//
KW - PsycInfo
KW - Unsupervised Play Addition
KW - CHILDREN
KW - Adults
KW - artifacts
KW - object naming
KW - historical intuition
KW - History
KW - Intuition
KW - Naming
KW - Object Recognition
KW - Childhood Development
RP - NOT IN FILE
SP - 23
EP - 42
JF - Cognition
VL - 91
IS - 1
CY - Netherlands
PB - Elsevier Science
N2 - It is commonly assumed that artifacts are named solely on the basis of properties they currently possess; in particular, their appearance and function. The experiments presented here explore the alternative proposal that the history of an artifact plays some role in how it is named. In three experiments, children between the ages of 4 and 9 years and adults were presented with familiar artifacts whose appearance and function were then radically altered. Participants were tested as to whether they believed that the modified objects were still members of the artifact kind. Results indicate that object history becomes increasingly important over the course of development. (PsycINFO Database Record (c) 2012 APA, all rights reserved) (journal abstract)
SN - 0010-0277
AD - Gutheil, Grant, ggutheil@skidmore.edu
UR - http://search.ebscohost.com/login.aspx?direct=true&db=psyh&AN=2004-10619-002&site=ehost-live;ggutheil@skidmore.edu
L3 - 10.1016/S0010-0277(03)00165-3
ER -

TY - JOUR
ID - 1768
T1 - The role of entrenchment in children's and adults' performance on grammaticality judgment tasks
A1 - Theakston,Anna L.
Y1 - 2004/01//
KW - PsycInfo
KW - Unsupervised Play Addition
KW - entrenchment
KW - grammaticality judgment tasks
KW - overgeneralization errors
KW - verb argument structure
KW - Age Differences
KW - children vs adults
KW - Cognitive Generalization
KW - Errors
KW - Grammar
KW - Judgment

KW - Verbs
RP - NOT IN FILE
SP - 15
EP - 34
JF - Cognitive Development
VL - 19
IS - 1
CY - Netherlands
PB - Elsevier Science
N2 - Between the ages of 3 and 7 years, children have been observed to produce verb argument structure overgeneralization errors. A number of recent studies have begun to find evidence that the precise distributional properties of the input may provide an important part of the explanation for why children retreat from overgeneralization errors. This study evaluates the role of entrenchment in constraining argument structure overgeneralization errors using a grammaticality judgment task. Five-yr-old, 8-yr-old, and adult Ss were presented with examples of argument structure errors containing high and low frequency verbs matched for semantic class and asked to indicate whether, or the extent to which they found the sentences to be grammatical. The data show that across all groups, sentences with argument structure errors containing low frequency verbs were judged to be significantly more grammatical than those containing high frequency verbs. These findings provide further support for the entrenchment hypothesis and suggest that verb frequency plays an important and continuing role in determining a speaker's choice of verb argument structure. (PsycINFO Database Record (c) 2012 APA, all rights reserved)
SN - 0885-2014
AD - Theakston, Anna L., Department of Psychology, University of Manchester Oxford Road, Manchester, United Kingdom, M13 9PL, theaksto@psy.man.ac.uk
UR - http://search.ebscohost.com/login.aspx?direct=true&db=psyh&AN=2004-11189-002&site=ehost-live;theaksto@psy.man.ac.uk
L3 - 10.1016/j.cogdev.2003.08.001
ER -

TY - THES
ID - 1770
T1 - Childhood sexual abuse and adult defensive functioning
A1 - Callahan,Kelley Lynn
Y1 - 2004///
N1 - Accession Number: 2004-99004-399. First Author & Affiliation: Callahan, Kelley Lynn; Adelphi U, Inst Advanced Psychological Studies, US. Other Journal Titles: Dissertation Abstracts International. Release Date: 20040802. Publication Type: Dissertation Abstract, (0400); . Media Covered: Electronic. Document Type: Dissertation. Dissertation Number: AAI3103522. Language: English. Major Descriptor: Adult Attitudes; Child Abuse; Defense Mechanisms; Sexual Abuse. Classification: Health & Mental Health Treatment & Prevention (3300) . Population: Human (10); . Age Group: Childhood (birth-12 yrs) (100) Adulthood (18 yrs & older) (300) . Methodology: Empirical Study. Page Count: 1
KW - PsycInfo
KW - Unsupervised Play Addition
KW - childhood sexual abuse
KW - adult defensive functioning
KW - Adult Attitudes
KW - Child Abuse
KW - Defense Mechanisms
KW - Sexual Abuse
RP - NOT IN FILE
JF - Dissertation Abstracts International: Section B: The Sciences and Engineering
VL - 64
IS - 8-B
CY - US
PB - ProQuest Information & Learning

N2 - Although numerous studies have documented symptoms experienced by survivors of childhood sexual abuse (CSA), few studies have sought to investigate the dynamics that contribute to particular symptom presentations in adulthood. A growing body of empirical evidence suggests that defense mechanisms play an integral role in development. Based on a developmental model of psychopathology addressing the effects of CSA on self and social functioning (Cole & Putnam, 1992), this study sought to investigate differences in adult defensive functioning between outpatients with and without a history of CSA. Participants were 71 treatment-seeking individuals enrolled in an ongoing treatment process and outcome study (Hilsenroth, 2000). Clinicians utilized the Abuse Dimensions Inventory, Sexual Abuse Behaviors scale (ADI; Chaffin, Wherry, Newlin, Crutchfield, & Dykman, 1997), to rate the presence, absence, and severity of CSA experiences (CSA group n = 23; non-CSA group n = 48). Clinicians rated participants' defensive functioning using the Defensive Functioning Scale (DFS; American Psychiatric Association, 1994). A personality disorder index was also utilized to characterize personality psychopathology in a dimensional manner. An external rater also rated participants' CSA experiences, defensive functioning, and personality psychopathology. Interrater reliability for the ADI and personality disorder index was excellent. Interrater reliability on the DFS was largely in the good to excellent range. The CSA group used the Major Image-Distorting level of defense, an immature level of defense characterized by the distortion/misattribution of self-image or the image of others (APA, 1994), significantly more than the non-CSA group. Post-Hoc analysis of individual defenses within the Major Image-Distorting level suggested a significantly greater use of projective identification by the CSA group than the non-CSA group. Similarly, Major Image-Distorting was the only level of defense related to abuse severity, such that more severe abuse was associated with greater use of Major Image-Distorting defenses. The CSA and non-CSA group were not found to significantly differ in personality disorder psychopathology. Findings suggest that the use of specific immature defense mechanisms may contribute to difficulties in maintaining a complex, coherent, and regulated sense of self and problems in interpersonal relations that are frequently seen in clinical samples of adult survivors of CSA. (PsycINFO Database Record (c) 2012 APA, all rights reserved)
SN - 0419-4217
UR - http://search.ebscohost.com/login.aspx?direct=true&db=psyh&AN=2004-99004-399&site=ehost-live
ER -

TY - JOUR
ID - 1771
T1 - Robot-mediated joint attention in children with autism: A case study in robot-human interaction
A1 - Robins,Ben
A1 - Dickerson,Paul
A1 - Stribling,Penny
A1 - Dautenhahn,Kerstin
Y1 - 2004///
KW - PsycInfo
KW - Unsupervised Play Addition
KW - robot-human interaction
KW - CHILDREN
KW - Autism
KW - Rehabilitation
KW - Therapy
KW - Education
KW - robot-mediated joint attention
KW - Attention
KW - Human Computer Interaction
KW - Robotics
RP - NOT IN FILE
SP - 161
EP - 198
JF - Interaction Studies: Social Behaviour and Communication in Biological and Artificial Systems
VL - 5
IS - 2
CY - Netherlands
PB - John Benjamins

N2 - Interactive robots are used increasingly not only in entertainment and service robotics, but also in rehabilitation, therapy and education. The work presented in this paper is part of the Aurora project, rooted in assistive technology and robot-human interaction research. Our primary aim is to study if robots can potentially be used as therapeutically or educationally useful 'toys'. In this paper we outline the aims of the project that this study belongs to, as well as the specific qualitative contextual perspective that is being used. We then provide an in-depth evaluation, in part using Conversation Analysis (CA), of segments of trials where three children with autism interacted with a robot as well as an adult. We focus our analysis primarily on joint attention which plays a fundamental role in human development and social understanding. Joint attention skills of children with autism have been studied extensively in autism research and therefore this behaviour provides a relevant focus for our study. In the setting used, joint attention emerges from natural and spontaneous interactions between a child and an adult. We present the data in the form of transcripts and photo stills. The examples were selected from extensive video footage for illustrative purposes, i.e. demonstrating how children with autism can respond to the changing behaviour of their co-participant, i.e. the experimenter. Furthermore, our data shows that the robot provides a salient object, or mediator for joint attention. The paper concludes with a discussion of implications of this work in the context of further studies with robots and children with autism within the Aurora project, as well as the potential contribution of robots to research into the nature of autism. (PsycINFO Database Record (c) 2012 APA, all rights reserved) (journal abstract)
SN - 1572-0381
AD - Robins, Ben, Adaptive Systems Research Group, University of Hertfordshire College Lane, Hatfield, Hertfordshire, United Kingdom, AL10 9AB, B.1.Robins@herts.ac.uk
UR - http://search.ebscohost.com/login.aspx?direct=true&db=psyh&AN=2005-01243-001&site=ehost-live;B.1.Robins@herts.ac.uk
L3 - 10.1075/is.5.2.02rob
ER -

TY - JOUR
ID - 1772
T1 - Childhood Adversity and Later Mortality in an Urban African American Cohort
A1 - Juon,Hee Soon
A1 - Ensminger,Margaret E.
A1 - Feehan,Michael
Y1 - 2003/12//
KW - PsycInfo
KW - Unsupervised Play Addition
KW - Mortality
KW - childhood factors
KW - Foster Care
KW - African American Children
KW - family factors
KW - adversity
KW - Childhood Development
KW - Early Experience
KW - Family Background
KW - Mortality Rate
KW - Risk Factors
KW - Blacks
KW - Urban Environments
RP - NOT IN FILE
SP - 2044
EP - 2046
JF - American Journal of Public Health
JA - Am J Public Health
VL - 93
IS - 12
CY - US
PB - American Public Health Assn

N2 - In this article, we examine family and childhood factors in relation to the risk of later mortality. Data were obtained from the children's teachers and mothers when the children were 6 years old and from study participants themselves at ages 16-17 years and 32-34 years. Information on death was obtained from family members and neighbors. Four family types were defined by the adults who were present in the household. First-grade teachers rated the children's behavior on scales indicating aggressive and shy behavior. A 4-point measure ranging from shy to aggressive was used. Psychological symptoms were measured by the 38-item Mother's Symptom Inventory (MSI). Of the 1242 first graders, 1040 were located for adult follow-up. Of the 202 not located, 51 were known to be alive; the status of the remaining 151 was unknown. Of the 1091 cohort members whose status was known at follow-up, 44 had died by the time of the adult assessment. Among the 44 deaths, homicide was the most frequent identifiable cause of death. These findings raise the question whether foster care is responsible for or plays a causal role in those deaths. Policies for foster care could be designed that might decrease the risk. (PsycINFO Database Record (c) 2012 APA, all rights reserved)
SN - 1541-0048
AD - Juon, Hee-Soon, Department of Health Policy and Management, Johns Hopkins Bloomberg School of Public Health 624 N. Broadway, Baltimore, MD, US, 21205, hjuon@jhsph.edu
UR - http://search.ebscohost.com/login.aspx?direct=true&db=psyh&AN=2003-10418-002&site=ehost-live;hjuon@jhsph.edu
L3 - 10.2105/AJPH.93.12.2044
ER -

TY - JOUR
ID - 1773
T1 - Video technology and persons with autism and other developmental disabilities: An emerging technology for PBS
A1 - Sturmey,Peter
Y1 - 2003///
KW - PsycInfo
KW - Unsupervised Play Addition
KW - Video Technology
KW - autistic children
KW - Developmental Disabilities
KW - Teaching
KW - Autism
KW - Teaching Methods
KW - Technology
KW - Videotape Instruction
RP - NOT IN FILE
SP - 3
EP - 4
JF - Journal of Positive Behavior Interventions
VL - 5
IS - 1
CY - US
PB - PRO-ED
N2 - Introduces four articles (see records 2002-08658-004, 200208658-005, 200208658-006, 200208658-007, and 200208658-008) which demonstrate that video technology can be a powerful tool for teaching appropriate social behavior, independent play, and academic tasks to children with autistic disorder. (PsycINFO Database Record (c) 2012 APA, all rights reserved)
SN - 1538-4772
UR - http://search.ebscohost.com/login.aspx?direct=true&db=psyh&AN=2002-08658-003&site=ehost-live
L3 - 10.1177/10983007030050010401
ER -

TY - CHAP
ID - 1775
T1 - Projects and policies for childhood in Italy

A1 - Rissotto,Antonella
Y1 - 2003///
N1 - Accession Number: 2004-00314-022. First Author & Affiliation: Rissotto, Antonella; Institute of Cognitive Sciences and Technologies, National Research Council, Rome, Italy. Release Date: 20060221. Correction Date: 20100329. Publication Type: Book, (0200); Edited Book, (0280); . Media Covered: Print. Document Type: Chapter. ISBN: 0-88937-282-9, Hardcover, 0889372829. Language: English. Conference Information: International Association for People-Environment Studies Conference. Major Descriptor: Child Care; Community Development; Community Involvement; Government Policy Making; Urban Environments. Minor Descriptor: Childhood Play Development; Human Rights. Classification: Community & Environmental Planning (4050) . Population: Human (10); Male (30); Female (40); . Location: Italy. Age Group: Childhood (birth-12 yrs) (100) Preschool Age (2-5 yrs) (160) School Age (6-12 yrs) (180) Adolescence (13-17 yrs) (200) . Intended Audience: Psychology: Professional & Research (PS) . Methodology: Empirical Study. References Available: Y.. Page Count: 13
KW - PsycInfo
KW - Unsupervised Play Addition
KW - urban environment
KW - children's empowerment
KW - independent mobility
KW - children's participation
KW - network of cities
KW - legislative actions
KW - evaluation of Children's City project
KW - Child Care
KW - Community Development
KW - Community Involvement
KW - Government Policy Making
KW - Urban Environments
KW - Childhood Play Development
KW - Human Rights
RP - NOT IN FILE
SP - 299
EP - 311
T2 - Culture, environmental action and sustainability
A2 - Garc ┬a Mira,Ricardo
A2 - Sabucedo Cameselle,Jos‚ M.
A2 - Romay Mart¡nez,Jos‚
CY - Ashland, OH US
PB - Hogrefe & Huber Publishers
N2 - (from the chapter) This contribution has two aims. First to briefly describe the cultural and legislative context produced in Italy by the ratification of the United Nations Convention on the Rights of the Child. Second to present some of the outcomes of the Children's City project. This project was started in 1991 and today it involves 46 cities in Italy, several in Argentina and a few in Spain. It is aimed at improving the urban environment from the perspective of children. The study of actions carried out by the Italian cities in order to promote children's participation and children's autonomous mobility highlights the positive and negative aspects of the project. The main limits of the project are connected with the following factors: project's dependence on temporary tasks of politicians; the emphasis given to the educational dimension of interventions which shifts attention away from the city's transformation and from children's empowerment; the experimental character of the interventions which results in reduced impact on the urban environment and on the child population. The main positive aspects of the project are related to changes in the perception of childhood, which motivate administrators to make innovative choices and encourages the engagement of larger components of the community. (PsycINFO Database Record (c) 2012 APA, all rights reserved) (chapter)
SN - 0-88937-282-9
AD - Rissotto, Antonella, Institute of Cognitive Sciences and Technologies, National Research Council Viale K. Marx 15, 00137, Rome, Italy, rissotto@ip.rm.cnr.it
UR - http://search.ebscohost.com/login.aspx?direct=true&db=psyh&AN=2004-00314-022&site=ehost-live;rissotto@ip.rm.cnr.it

ER -

TY - CHAP
ID - 1776
T1 - Encouraging independent play skills
A1 - Christophersen,Edward R.
A1 - Mortweet,Susan L.
Y1 - 2003///
N1 - Accession Number: 2003-07192-010. First Author & Affiliation: Christophersen, Edward R.; Children's Mercy Hosp, Kansas City, MO, US. Release Date: 20031027. Publication Type: Book, (0200); Authored Book, (0240); . Media Covered: Print. Document Type: Chapter. ISBN: 1-55798-924-9, Paperback, 1557989249. Language: English. Major Descriptor: Childhood Development; Childhood Play Behavior; Parent Training. Classification: Childrearing & Child Care (2956) . Population: Human (10); . Age Group: Childhood (birth-12 yrs) (100) Adulthood (18 yrs & older) (300) . Intended Audience: General Public (GP) . References Available: Y.. Page Count: 11
KW - PsycInfo
KW - Unsupervised Play Addition
KW - independent play skills
KW - Development
KW - self-entertainment
KW - CHILDREN
KW - Parent Training
KW - Childhood Development
KW - Childhood Play Behavior
RP - NOT IN FILE
SP - 195
EP - 205
T2 - Parenting that works: Building skills that last a lifetime
CY - Washington, DC US
PB - American Psychological Association
N2 - (from the chapter) "Independent play skills" refers to a child's ability to entertain himself for extended periods of time without any help from an adult. Children with well-developed independent play skills derive enjoyment simply from their activities, with no need or expectation of the involvement of others or an external reward. Independent play skills enable children to do long homework assignments, to work in their seats at school, to complete independent projects, to read a long book, and to master a hobby or talent. In addition to developing a child's ability to concentrate, stick with tasks, and enjoy time alone, independent play skills also have other benefits for the child and her parents. Children with independent play skills usually play better with other children than those who do not. This chapter describes how parents can encourage the development of independent plays skills in their children. The authors provide general guidelines as well as age-specific suggestions from infancy through school age. (PsycINFO Database Record (c) 2012 APA, all rights reserved) (chapter)
SN - 1-55798-924-9
UR - http://search.ebscohost.com/login.aspx?direct=true&db=psyh&AN=2003-07192-010&site=ehost-live
ER -

TY - CHAP
ID - 1777
T1 - Accessibility or obstacles? Children's independent mobility and valuation of the outdoor environment
A1 - Heurlin-Norinder,Mia
Y1 - 2003///
N1 - Accession Number: 2003-00929-012. First Author & Affiliation: Heurlin-Norinder, Mia; Stockholm Inst of Education, The Dept of Society, Culture & Learning, Research Group in Environmental Psychology & Education, Stockholm, Sweden. Release Date: 20031014. Publication Type: Book, (0200); Edited Book, (0280); . Media Covered: Print. Document Type: Chapter. ISBN: 0-88937-263-2, Paperback, 0889372632. Language: English. Major Descriptor: Aesthetic Preferences; Community Development; Commuting (Travel); Psychosocial Factors; Spatial Organization. Minor Descriptor: Child Attitudes; Safety. Classification:

Community & Environmental Planning (4050) . Population: Human (10); . Age Group: Childhood (birth-12 yrs) (100) Preschool Age (2-5 yrs) (160) School Age (6-12 yrs) (180) . Intended Audience: Psychology: Professional & Research (PS) . Methodology: Empirical Study; Quantitative Study. References Available: Y.. Page Count: 12
KW - PsycInfo
KW - Unsupervised Play Addition
KW - Child Attitudes
KW - neighborhood design preferences
KW - Psychosocial Factors
KW - independent mobility
KW - Safety
KW - commuting
KW - Aesthetic Preferences
KW - Community Development
KW - Commuting (Travel)
KW - Spatial Organization
RP - NOT IN FILE
SP - 161
EP - 172
T2 - People, places, and sustainability
A2 - Moser,Gabriel
A2 - Pol,Enric
A2 - Bernard,Yvonne
A2 - Bonnes,Mirilia
A2 - Corraliza,Jos, Antonio
A2 - Giuliani,Maria Vittoria
CY - Ashland, OH US
PB - Hogrefe & Huber Publishers
N2 - (from the chapter) The author shows that children's design preferences for neighbourhoods are related to their degree of independent mobility and how they evaluate their environment. Analyses point to differences concerning the interaction with the physical environment and social aspects of the neighbourhood environment, in terms of affordances and individual evaluations. In the first study 732 children (8 and 11 years old) in 9 schools filled in questionnaires concerning, for example, how they had come to school on that particular day, how they used to travel to activities after school, if they were allowed to visit friends and go out and play without being followed by any adults. The children also made drawings showing their way to school. The aim of the second, qualitative study was to get a deeper understanding of differences that were found in the first study, but also the meaning of the neighbourhood's planning and characteristics related to children's independent mobility. Traffic-separated areas seem to be very positive for children's independent mobility, but they are not enough if you want the neighbourhood to be stimulating, developing and safe. There is more to it. (PsycINFO Database Record (c) 2012 APA, all rights reserved) (chapter)
SN - 0-88937-263-2
AD - Heurlin-Norinder, Mia, Stockholm Inst of Education, The Dept of Society, Culture & Learning, Research Group in Environmental Psychology & Education Box 34103, S-100 26, Stockholm, Sweden
UR - http://search.ebscohost.com/login.aspx?direct=true&db=psyh&AN=2003-00929-012&site=ehost-live
ER -

TY - CHAP
ID - 1778
T1 - Rural children's play in the natural environment
A1 - Beach,Betty A.
Y1 - 2003///
N1 - Accession Number: 2003-06677-010. First Author & Affiliation: Beach, Betty A.; University of Maine, Farmington, ME, US. Translated Book Series Title: Play & culture studies, Vol. 5. Release Date: 20031020. Publication Type: Book, (0200); Edited Book, (0280); . Media Covered: Print. Document Type: Chapter. ISBN: 1-56750-684-4, Hardcover, 1567506844. Language: English. Major Descriptor: Childhood Play Behavior; Rural Environments. Minor Descriptor: Childhood Play Development; Recreation. Classification: Psychosocial &

Personality Development (2840) . Population: Human (10); Male (30); Female (40); . Location: US. Age Group: Childhood (birth-12 yrs) (100) . Intended Audience: Psychology: Professional & Research (PS) . Methodology: Empirical Study; Qualitative Study. References Available: Y.. Page Count: 12
KW - PsycInfo
KW - Unsupervised Play Addition
KW - rural children
KW - rural communities
KW - natural habitat
KW - Play
KW - Childhood Play Behavior
KW - Rural Environments
KW - Childhood Play Development
KW - Recreation
RP - NOT IN FILE
SP - 183
EP - 194
T2 - Play and educational theory and practice
A2 - Lytle,Donald E.
CY - Westport, CT US
PB - Praeger Publishers/Greenwood Publishing Group
T3 - Play & culture studies, Vol. 5
N2 - As children's play becomes increasingly domesticated and regimented by adults, limited to boundaries established and overseen by adults, pressured by commercial interests, and subject to intensifying demands for academic achievement, it is reasonable to inquire whether unfettered play still exists. The current study was undertaken to expand the knowledge of rural children's encounters with their natural habitat. The author reports on 100 hrs of naturalistic observation of children under the age of 12 at play in 2 rural communities, semi-structured interviews with 15 adults, and analysis of local historical and municipal documents. Historical and municipal documents were analyzed to create a more complete understanding of the conditions and changing context of play in the 2 communities. Results show that rural children are still visibly roaming their communities in a free-ranging manner, generally unaccompanied by adults. It is concluded that in perhaps the ultimate irony, adults may be busily circumscribing children's play while simultaneously remembering their own unrestrained childhoods with nostalgia. (PsycINFO Database Record (c) 2012 APA, all rights reserved)
SN - 1-56750-684-4
UR - http://search.ebscohost.com/login.aspx?direct=true&db=psyh&AN=2003-06677-010&site=ehost-live
ER -

TY - BOOK
ID - 1779
T1 - Parenting that works: Building skills that last a lifetime
A1 - Christophersen,Edward R.
A1 - Mortweet,Susan L.
Y1 - 2003///
N1 - Accession Number: 2003-07192-000. First Author & Affiliation: Christophersen, Edward R.; Children's Mercy Hosp, Kansas City, MO, US. Release Date: 20031027. Publication Type: Book, (0200); Authored Book, (0240); . Media Covered: Print. ISBN: 1-55798-924-9, Paperback, 1557989249. Language: English. Major Descriptor: Parent Child Relations; Parent Training; Parenting Skills. Minor Descriptor: Behavior Modification; Child Discipline; Coping Behavior; Parent Child Communication. Classification: Childrearing & Child Care (2956) . Population: Human (10); . Age Group: Childhood (birth-12 yrs) (100) Adulthood (18 yrs & older) (300) . Intended Audience: General Public (GP)
KW - PsycInfo
KW - Unsupervised Play Addition
KW - Parenting
KW - Parenting Skills
KW - Feedback
KW - Communication
KW - Discipline

KW - behavior management
KW - parent coping
KW - child coping
KW - long-term plan
KW - Parent Child Relations
KW - Parent Training
KW - Behavior Modification
KW - Child Discipline
KW - Coping Behavior
KW - Parent Child Communication
RP - NOT IN FILE
CY - Washington, DC US
PB - American Psychological Association
N2 - (from the introduction) Takes the position that, from the beginning, we are training our children to be adults--adults with whom we would like to interact regularly, and whose company we anticipate and enjoy. Effective parenting requires that you have a long-term plan for what you want to teach your child about appropriate behavior and that you have daily interactions with your child that foster the characteristics you find important. The basic approach in this book involves helping you use your time, energy, and introspection to decide now what skills you want your child to have in 10 years. The book is divided into sections on parenting skills and on child skills. Part I discusses building your skills as a parent, including encouraging appropriate behaviors in your child, providing your child with positive feedback, communicating with your child, using effective discipline strategies, and developing vital parent coping skills. Part II provides suggestions for managing your child's behavior in public, choosing toys and games for your child, and helping your child cope with divorce. Part III discusses building your child's skills, with particular reference to self-quieting skills and independent play skills. Part IV addresses problems with bedtime, toileting, aggression, and whining and tantrums. (PsycINFO Database Record (c) 2012 APA, all rights reserved) (introduction)
SN - 1-55798-924-9
UR - http://search.ebscohost.com/login.aspx?direct=true&db=psyh&AN=2003-07192-000&site=ehost-live
ER -

TY - JOUR
ID - 1780
T1 - The curriculum experiences of pre-school children in Northern Ireland: Classroom practices in terms of child-initiated play and adult-directed activities
A1 - Guimaraes,Sofia
A1 - McSherry,Kathleen
Y1 - 2002/06//
KW - PsycInfo
KW - Unsupervised Play Addition
KW - Curriculum
KW - child play
KW - adult direction
KW - class activity
KW - preschools
KW - private day nursery
KW - nursery school
KW - playgroups
KW - reception class
KW - preschoolers
KW - Childhood Play Behavior
KW - Preschool Education
KW - Preschool Students
KW - Teaching
KW - Nursery Schools
RP - NOT IN FILE
SP - 85

EP - 94
JA - International Journal of Early Years Education
VL - 10
IS - 2
CY - United Kingdom
PB - Taylor & Francis
N2 - Examined the curriculum approaches, in relation to child-initiated play vs adult-directed activities, experienced by children across different types of pre-school provision in Northern Ireland. Observations were carried out in 71 pre-school settings including private day nurseries, nursery schools/classes, playgroups, and reception classes. Each center was observed for a complete session during a 'typical' day. A record was taken of the activities in which the children took part and their contexts/settings were noted, as were the number of adults and children present, the nature of the adult involvement together with the time spent on the activities. The children observed were between 3 and 4 yrs of age. The results show that the type of center was associated with the types of activities promoted in terms of adult- vs child-initiated activities. The pre-school curriculum offered in most reception settings was strongly based on an adult-directed approach while most nursery classes/schools, playgroups, and private day nurseries adopted a child-initiated approach. The results are discussed in relation to what constitutes a developmentally appropriate pre-school curriculum. (PsycINFO Database Record (c) 2012 APA, all rights reserved)
SN - 1469-8463
UR - http://search.ebscohost.com/login.aspx?direct=true&db=psyh&AN=2002-15121-001&site=ehost-live
L3 - 10.1080/09669760220141971
ER -

TY - JOUR
ID - 1781
T1 - A script-based symbolic play intervention for children with developmental delay
A1 - Nevile,Maryann
A1 - Bachor,Dan G.
Y1 - 2002///
KW - PsycInfo
KW - Unsupervised Play Addition
KW - symbolic play intervention
KW - script based
KW - developmental delay
KW - CHILDREN
KW - independent play
KW - peer play
KW - Childhood Play Behavior
KW - Delayed Development
KW - Intervention
KW - Schema
KW - Symbolism
KW - Peers
RP - NOT IN FILE
SP - 140
EP - 172
JF - Developmental Disabilities Bulletin
VL - 30
IS - 2
CY - Canada
PB - JP Das Development Disabilities Centre
N2 - We implemented a single-case research design, using an A B A post check format, to examine the effects of a script-based symbolic play intervention in two contexts (independent play and playing with peers) with five boys who exhibited developmental delay and were situated in an inclusive classroom setting. Four of the five boys produced gains in mean performance over baseline levels in both play contexts and in each phase of the study. Participants also maintained treatment effects two months after the intervention. Symbolic play skills

occurred more frequently in independent play than in shared play. The positive effects of modeling and transition training to promote generalization between contexts were supported. A relationship was observed between the frequency of language production and symbolic play. Diversity of language decreased with increased word production for two participants while four of the participants exhibited a trend toward more mature social interaction with peers. (PsycINFO Database Record (c) 2012 APA, all rights reserved) (journal abstract)
SN - 1184-0412
AD - Bachor, Dan G., Department of Educational Psychology and Leadership Studies, Faculty of Education, University of Victoria , Victoria, BC, Canada, V8W 3N4, dbachor@uvic.ca
UR - http://search.ebscohost.com/login.aspx?direct=true&db=psyh&AN=2003-07551-002&site=ehost-live;dbachor@uvic.ca
ER -

TY - CHAP
ID - 1782
T1 - The various roles of municipal leaders
A1 - Ouellette,Mark
A1 - Kyle,John E.
Y1 - 2002///
N1 - Accession Number: 2003-02186-008. First Author & Affiliation: Ouellette, Mark; National League of Cities' Inst for Youth, Education, and Families, Washington, DC, US. Translated Book Series Title: New directions for youth development: Theory practice research. Release Date: 20030825. Publication Type: Book, (0200); Edited Book, (0280); . Media Covered: Print. Document Type: Chapter. Book Type: Reference Book. ISBN: 0-7879-6337-2, Paperback, 0787963372. Language: English. Major Descriptor: After School Programs; Child Self Care; Leadership; Politicians; Urban Environments. Minor Descriptor: Adolescent Development; Childhood Development; Community Services. Classification: Community & Social Services (3373) . Population: Human (10); . Age Group: Childhood (birth-12 yrs) (100) Adolescence (13-17 yrs) (200) . Intended Audience: Psychology: Professional & Research (PS) . Page Count: 8
KW - PsycInfo
KW - Unsupervised Play Addition
KW - after-school hours
KW - lack of supervision
KW - CHILDREN
KW - Adolescents
KW - empty house
KW - community-wide strategies
KW - municipal officials
KW - Leadership
KW - After School Programs
KW - Child Self Care
KW - Politicians
KW - Urban Environments
KW - Adolescent Development
KW - Childhood Development
KW - Community Services
RP - NOT IN FILE
SP - 125
EP - 132
T2 - Youth development and after-school time: A tale of many cities
A2 - Noam,Gil G.
A2 - Miller,Beth M.
CY - San Francisco, CA US
PB - Jossey-Bass
T3 - New directions for youth development: Theory practice research
N2 - (from the chapter) An estimated 8 million children between the ages of 5 and 14 yrs go home to an empty house on a regular basis. Many of these children are left alone for as many as 4 hours a day. For cities and towns

across America, these unsupervised hours after school mean both heightened risk and missed opportunities. Leadership from municipal officials is usually required to forge a community-wide strategy that works for all children. Municipal officials can play an important role in developing a community-wide after-school system and serve as catalysts for far-reaching efforts that address key challenges: promoting partnerships, building public will, assessing local resources and needs, improving quality, broadening access, and developing financing strategies. (PsycINFO Database Record (c) 2012 APA, all rights reserved) (chapter)
SN - 0-7879-6337-2
UR - http://search.ebscohost.com/login.aspx?direct=true&db=psyh&AN=2003-02186-008&site=ehost-live
ER -

TY - JOUR
ID - 1783
T1 - The influence of psychosocial and environmental factors on children's independent mobility and relationship to peer frequentation
A1 - Prezza,Miretta
A1 - Pilloni,Stefania
A1 - Morabito,Carmela
A1 - Sersante,Cinzia
A1 - Alparone,Francesca Romana
A1 - Giuliani,Maria Vittoria
Y1 - 2001/11//
KW - PsycInfo
KW - Unsupervised Play Addition
KW - Demographic Characteristics
KW - psychosocial characteristics
KW - environmental factors
KW - independent urban mobility
KW - Play
KW - CHILDREN
KW - Mothers
KW - Childhood Play Behavior
KW - Independence (Personality)
KW - Psychosocial Factors
KW - Urban Environments
RP - NOT IN FILE
SP - 435
EP - 450
JF - Journal of Community & Applied Social Psychology
VL - 11
IS - 6
CY - US
PB - John Wiley & Sons
N2 - The first aim of this study was to investigate whether demographic characteristics of children (i.e. age, sex, birth order), mothers' psychosocial characteristics (i.e. fear of crime, neighbourhood relations, sense of community, perceived risk of traffic) and environmental factors (i.e. living in a new/old neighbourhood, in a building with a condominium courtyard, near a park or in a private street) influence the independent urban mobility of 7-12-yr-old children. The second aim was to verify whether children who are more independent meet their peers more frequently for indoor and outdoor play. A semi-structured interview, the Italian Sense of Community Scale and a Neighbourhood Relations scale were administered to 251 mothers (mean age 39 yrs) living in Rome. The results indicate that more independent children are older, male, live in apartment buildings with courtyards, near parks and in new neighbourhoods. Also, their mothers have more neighbourhood relations. Regarding the second aim, it emerged that children who are more independent play more often with their peers, both indoors and outdoors. (PsycINFO Database Record (c) 2012 APA, all rights reserved)
SN - 1099-1298
AD - Prezza, Miretta, Dept of Psychology, U 'La Sapienza' Via dei Marsi, 78, 00185, Rome, Italy, miretta.prezza@uniroma1.it

UR - http://search.ebscohost.com/login.aspx?direct=true&db=psyh&AN=2001-09820-003&site=ehost-live;miretta.prezza@uniroma1.it
L3 - 10.1002/casp.643
ER -

TY - THES
ID - 1784
T1 - Training family therapists to work with children in family therapy: A modified Delphi study
A1 - Sori,Catherine Ellen Ford
Y1 - 2001/01//
N1 - Accession Number: 2001-95023-090. First Author & Affiliation: Sori, Catherine Ellen Ford; Purdue U., US. Other Journal Titles: Dissertation Abstracts International. Release Date: 20020417. Publication Type: Dissertation Abstract, (0400); . Media Covered: Electronic. Document Type: Dissertation. Dissertation Number: AAI3018272. Language: English. Major Descriptor: Family Therapy; Marriage Counseling; Psychotherapy Training. Classification: Health & Mental Health Treatment & Prevention (3300) . Population: Human (10); . Age Group: Adulthood (18 yrs & older) (300) . Methodology: Empirical Study. Page Count: 1
KW - PsycInfo
KW - Unsupervised Play Addition
KW - Family Therapy
KW - marriage therapy
KW - child focused therapy training
KW - Delphi methodology
KW - Marriage Counseling
KW - Psychotherapy Training
RP - NOT IN FILE
JF - Dissertation Abstracts International Section A: Humanities and Social Sciences
VL - 62
IS - 6-A
CY - US
PB - ProQuest Information & Learning
N2 - The field of marriage and family therapy must address the needs of all family members, including young children. Yet research indicates that many MFTs do not feel adequately trained or comfortable to work with children, and many exclude children from family therapy sessions. The purpose of this study was to gather a consensus of recommendations from experts in the field on how to improve child-focused training. A modified Delphi methodology was used to collect both quantitative and qualitative data. Based on previous research, six open-ended questions were posed on Delphi I. Four panelists participated in qualitative interviews to clarify responses. Delphi II contained panelists responses to Delphi I and comments from panelist interviews for some items. Following Delphi II, six more qualitative interviews were conducted to address discrepancies in the data. Finally, a qualitative interview was conducted with Salvador Minuchin, who reflected on these results and offered his thoughts on this topic. Panelists believed that children should be active participants in family therapy sessions for both child and adult presenting problems. They should only be excluded during adult sexual discussions, or initially when parents are discussing how to share sensitive information. Child-focused course content should emphasize developmental issues, practical techniques to engage children and families, theoretical issues, play therapy theory, MFT treatment for child disorders, and specific issues that impact children and families. Panelists only recommended one reference for child coursework. Therapist attributes and relational skills are essential skills for MFTs to develop. Panelists recommend both deductive and inductive training methods and highlight the role of supervision to increase therapist comfort and skills with children. Therapist playfulness and creativity are important considerations in training, but no specific play therapy techniques were recommended. Minuchin discussed these findings in light of historical aspects of training and recent developments in the field. Implications for training programs and for the field are discussed. (PsycINFO Database Record (c) 2012 APA, all rights reserved)
SN - 0419-4209
UR - http://search.ebscohost.com/login.aspx?direct=true&db=psyh&AN=2001-95023-090&site=ehost-live
ER -

TY - JOUR

ID - 1785
T1 - Played out? Passive behavior by children with Down syndrome during unstructured play
A1 - Linn,Margaret Inman
A1 - Goodman,Joan F.
A1 - Lender,Winifred Lloyds
Y1 - 2000///
KW - PsycInfo
KW - Unsupervised Play Addition
KW - passivity
KW - child play behavior
KW - Down Syndrome
KW - Childhood Play Behavior
KW - Down's Syndrome
KW - Passiveness
RP - NOT IN FILE
SP - 264
EP - 278
JF - Journal of Early Intervention
VL - 23
IS - 4
CY - US
PB - Council for Exceptional Children
N2 - Investigated the duration, frequency, and trajectory of passive behavior during play in children with Down syndrome. 14 children with Down syndrome (mean age 54 mo) were observed during 30-60-min independent play sessions, with passive episodes noted. Results show that Down syndrome Ss spent more time in passive behavior and shifted from play to passivity and back to play more often than did control Ss during the initial 30 min of play. No difference between Down syndrome Ss and controls was found for the time spent in social activities, but Down syndrome Ss exhibited greater frequency of social behavior. Down syndrome Ss spent more time in passivity as time progressed over the play period, whereas controls showed a decline in amount of time spent passively as the play period progressed. (PsycINFO Database Record (c) 2012 APA, all rights reserved)
SN - 1053-8151
UR - http://search.ebscohost.com/login.aspx?direct=true&db=psyh&AN=2001-00593-006&site=ehost-live
L3 - 10.1177/10538151000230040601
ER -

TY - JOUR
ID - 1786
T1 - Types of preschool friendships
A1 - Kerns,Kathryn A.
Y1 - 2000/09//
KW - PsycInfo
KW - Unsupervised Play Addition
KW - preschool friendship types
KW - 25-72 mo olds
KW - 1 yr followup
KW - Friendship
KW - Peer Relations
RP - NOT IN FILE
SP - 311
EP - 324
JF - Personal Relationships
VL - 7
IS - 3
CY - United Kingdom
PB - Blackwell Publishing

N2 - Utilized observational data collected from preschool friends and cluster analysis to derive friendship types. 50 friend pairs (aged 25-72 mo) were videotaped in a 1-hour free-play session, and observers rated several qualities of the interaction using the Dyadic Relationships-Q set (K. A. Park and E. Waters, 1986). Additionally, relationship stability was assessed in a follow up phone call 1 year after the experiment, and child-mother attachment was assessed using the Stevenson-Hinde's 75-item version of the Attachment Q-set (B.E. Vaughn et al, 1992). Cluster analysis suggested that both 3 and 5 cluster solutions produced meaningful groupings. For both solutions, the largest cluster included friend pairs that were harmonious, responsive, and interactive during play. A second type of friendship was characterized by harmony but relatively independent play. A disjointed friendship type, in which friends were low on measures of coordinated interaction and positive interaction, was the most stable over a 1 year period and tended to contain at least one insecurely attached child. Discussion focuses on how analysis of friendship type can be used to extend our understanding of the nature and socialization functions of friendship. (PsycINFO Database Record (c) 2012 APA, all rights reserved)
SN - 1475-6811
UR - http://search.ebscohost.com/login.aspx?direct=true&db=psyh&AN=2000-02582-006&site=ehost-live
L3 - 10.1111/j.1475-6811.2000.tb00019.x
ER -

TY - JOUR
ID - 1787
T1 - Preventing dog bites in children: Randomised controlled trial of an educational intervention
A1 - Chapman,Simon
A1 - Cornwall,John
A1 - Righetti,Joanne
A1 - Sung,Lynne
Y1 - 2000/06//
KW - PsycInfo
KW - Unsupervised Play Addition
KW - educational program designed to instill precautionary behavior around dogs & prevent bites
KW - 7-8 yr olds
KW - Educational Programs
KW - Interspecies Interaction
KW - Prevention
KW - Dogs
RP - NOT IN FILE
SP - 1512
EP - 1513
JF - BMJ: British Medical Journal
VL - 320
IS - 7248
CY - United Kingdom
PB - BMJ Publishing Group
N2 - "Prevent-a-Bite" is an educational program for primary school children designed to instill precautionary behavior around dogs, assuming that this might reduce the incidence of bites. A randomized controlled trial of the efficacy of the intervention was conducted in 346 Australian children (aged 7-8 yrs). The intervention consisted of a 30 min lesson conducted by a dog handler. The handler and dog demonstrated various "dos and don'ts" of behavior around dogs and the Ss practiced patting the dog in the correct manner and precautionary and protective body posture to adopt when approached or knocked over by a dog. A resource kit for teachers, included activities to be undertaken before and after the demonstration, was also distributed. Seven to 10 days after participating in the program, Ss were let out to play unsupervised in the school grounds where a docile Labrador dog was tethered 5 meters away from its owner. The Ss were not told that the dog was there and were videotaped by a hidden camera for 10 min. Ss who had received the intervention displayed appreciably greater precautionary behavior than Ss who had not received the intervention. The educational program increased appreciably the precautionary behavior of young children around strange dogs in the short term. (PsycINFO Database Record (c) 2012 APA, all rights reserved)
SN - 0959-8138
UR - http://search.ebscohost.com/login.aspx?direct=true&db=psyh&AN=2001-03038-005&site=ehost-live

L3 - 10.1136/bmj.320.7248.1512
ER -

TY - THES
ID - 1788
T1 - Interactive media: An analysis of children's computer and video game use
A1 - Scantlin,Ronda Mae
Y1 - 2000/06//
N1 - Accession Number: 2000-95012-270. First Author & Affiliation: Scantlin, Ronda Mae; U Texas At Austin, US. Other Journal Titles: Dissertation Abstracts International. Release Date: 20010228. Publication Type: Dissertation Abstract, (0400); . Media Covered: Electronic. Document Type: Dissertation. Dissertation Number: AAI9956920. Language: English. Major Descriptor: Age Differences; Childhood Play Behavior; Computer Games; Human Sex Differences; Racial and Ethnic Differences. Classification: Developmental Psychology (2800) . Population: Human (10); Male (30); Female (40); . Location: US. Age Group: Childhood (birth-12 yrs) (100) Infancy (2-23 mo) (140) Preschool Age (2-5 yrs) (160) School Age (6-12 yrs) (180) . Methodology: Empirical Study. Page Count: 1
KW - PsycInfo
KW - Unsupervised Play Addition
KW - age & sex & ethnicity
KW - categories of computer & video game use & context of play
KW - 1-12 yr olds
KW - Age Differences
KW - Childhood Play Behavior
KW - Computer Games
KW - Human Sex Differences
KW - Racial and Ethnic Differences
RP - NOT IN FILE
JF - Dissertation Abstracts International: Section B: The Sciences and Engineering
VL - 60
IS - 12-B
CY - US
PB - ProQuest Information & Learning
N2 - Children vary in the amount of time they spend playing computer and video games and in their preference for particular types of game content. The purpose of this study was to describe time-use patterns of 572 children 1-12 years of age. Game categories (educational, sports, sensorimotor, strategy, and other) and context of play (secondary activities, location of play, and who was with the child when playing) were analyzed by child age, sex, and ethnicity. Total minutes of interactive game play increased as a function of age, with boys' playing time higher and rising faster than girls' playing time. Younger children played more minutes of educational games than did older children, and girls more than boys. African American children reported little educational play at any age. Generally, sensorimotor and sports play increased as a function of age, with boys reporting significantly more minutes than girls at all ages. African American children spent a greater proportion of their time playing games whose titles were "unknown" than did White children. All groups of children spent about 60 percent of their game time focused only on interactive games, and about 30 percent of their game time communicating with others while playing. About 80 percent of the time spent playing games occurred in children's own homes. The youngest children, 1-5 years, spent a higher proportion of their time with adults present (20 percent), while the 9-12 year olds spent less time with adults (8 percent) and relatively more time alone (44 percent). Children of all ages, sexes, and ethnic groups spent 32-38 percent of their game time playing with other youths. (PsycINFO Database Record (c) 2012 APA, all rights reserved)
SN - 0419-4217
UR - http://search.ebscohost.com/login.aspx?direct=true&db=psyh&AN=2000-95012-270&site=ehost-live
ER -

TY - JOUR
ID - 1789
T1 - Gedanken • ber Beginn und Abbruch einer Kinderanalyse
A1 - Stork,Jochen

Y1 - 1999/12//
KW - PsycInfo
KW - Unsupervised Play Addition
KW - spiritual aspects of journey of discovery
KW - psychoanalytic work
KW - Psychoanalysis
KW - Psychotherapeutic Processes
KW - Spirituality
RP - NOT IN FILE
SP - 378
EP - 410
JF - Kinderanalyse
VL - 7
IS - 4
CY - Germany
PB - Verlag-Klett-Cotta Stuttgart
N2 - Comments on the spiritual aspects of the journey of discovery represented by psychoanalytic work, whether with a child or with an adult. The present author thinks of a journey in which the psychoanalyst plays the role of the mystagogue, simultaneously entering the unknown, enigmatic world of a human heart, even though it may lead the guide to cast a dangerous glance at the world of folly. This essay describes the analysis of a 6 1/2-yr old boy (he is in his 9th yr when the analysis is interrupted), who brings a wealth of fantasy material to the initial stages of the analysis. This material can be subsumed under the problematics of the castration complex. The forced termination, approximately 100 sessions into this analysis, was not adequate to elaborating the analysand's pathologies; to cope with these unconscious problems, an omnipotent introject is annually incorporated. (PsycINFO Database Record (c) 2012 APA, all rights reserved)
SN - 0942-6051
UR - http://search.ebscohost.com/login.aspx?direct=true&db=psyh&AN=2001-06320-004&site=ehost-live
ER -

TY - JOUR
ID - 1791
T1 - The social development of children with severe learning difficulties: A case study of an inclusive educational initiative between two primary schools in Oxfordshire, UK
A1 - Dew-Hughes,Denise
A1 - Blandford,Sonia
Y1 - 1999/08//
KW - PsycInfo
KW - Unsupervised Play Addition
KW - Student Attitudes
KW - student socialization
KW - severe learning difficulties
KW - Mainstreaming
KW - Special Education
KW - Interpersonal Interaction
KW - Teacher Student Interaction
KW - Play
KW - Caregivers
KW - Learning Disabilities
KW - Mainstreaming (Educational)
KW - SOCIALIZATION
KW - Childhood Play Behavior
RP - NOT IN FILE
SP - 1
EP - 11
JF - Down Syndrome: Research & Practice
VL - 6

IS - 1
CY - United Kingdom
PB - Down Syndrome Educational Trust
N2 - Compared the attitudes and socialization of children with severe learning difficulties in an inclusive mainstream and special school. Six severely learning disabled 3rd grade students attending either mainstream or special schools were observed during academic activity, personal interactions, teacher/student interactions and times of unsupervised work or play. Observations concerned Ss' dependence on adults, differences in language and behavior at the 2 schools, and whether learning-disabled Ss preferred the company of adult Ss during recreation time. Caregivers completed interviews. Results show that Ss in mainstream schools managed longer periods of time on task without adult intervention, worked more independently, and used between task time more effectively than did Ss in special schools. Ss in mainstream schools took personal responsibility for movement between classes and were exposed to more sophisticated vocabulary than were special school Ss. It is concluded that the social development of severe learning disabled children is better served in mainstream, integrated schools than in special schools. (PsycINFO Database Record (c) 2012 APA, all rights reserved)
SN - 1753-7606
AD - Dew-Hughes, Denise, Oxford Brooks U, School of Education , Wheatley, England, OX33 1HX
UR - http://search.ebscohost.com/login.aspx?direct=true&db=psyh&AN=2001-11477-001&site=ehost-live
L3 - 10.3104/case-studies.92
ER -

TY - JOUR
ID - 1792
T1 - Sex differences in behavior: The debate continues
A1 - Power,Thomas G.
Y1 - 1999/08//
KW - PsycInfo
KW - Unsupervised Play Addition
KW - Sex Differences
KW - human behavioral development
KW - peer experiences
KW - gender segregation
KW - sex-typed behavior patterns
KW - CHILDREN
KW - adult roles
KW - Childhood Development
KW - Human Development
KW - Human Sex Differences
KW - Sex Roles
KW - Peer Relations
RP - NOT IN FILE
SP - 333
EP - 335
JF - PsycCRITIQUES
VL - 44
IS - 4
CY - US
PB - American Psychological Association
N2 - Originally published in Contemporary Psychology: APA Review of Books, 1999, Vol 44(4), 333-335. Reviews the book "The Two Sexes: Growing Up Apart, Coming Together" by E. E. Maccoby (see record 1998-07927-000). Maccoby provides a well-articulated presentation of her most recent view of the origins and implications of sex differences in human behavioral development. She argues that sex differences in behavior are a consequence of experiences with peers--especially experiences in gender-segregated groups. Maccoby additionally argues that the tendency to choose same-sex playmates provides experiences that shape the development of sex-typed behavior patterns. Maccoby's analysis is likely to stimulate further research and debate on the origins and implications of sex differences in human development. It is also consistent with numerous contextual theories of sex differences in behavior that argue that one should not view sex differences

in behavior behavior in individual terms, but instead in the context of specific relationships and social contexts (e.g., Deaux & Major, 1987). Finally, in a more systematic way Maccoby begins to bridge the gap between the literatures on sex differences in children's behavior and gender differences in adult roles. Criticisms presented by this reviewer include the fact that Maccoby devotes so little space to the indirect effects of nonparent adults who have a significant impact on children's play activities--those in the media and those who create and market children's toys. An additional criticism is that although the animal and cross-cultural data are provocative, they do not support the role of gender-segregated peer groups as strongly as a reading of the present volume would indicate. (PsycINFO Database Record (c) 2012 APA, all rights reserved)
SN - 1554-0138
AD - Power, Thomas G., Department of Psychology, University of Houston 101 Heyne, Houston, TX, US, 77204-5341, tpower@uh.edu
UR - http://search.ebscohost.com/login.aspx?direct=true&db=psyh&AN=2004-17593-034&site=ehost-live;tpower@uh.edu
L3 - 10.1037/002051
ER -

TY - JOUR
ID - 1793
T1 - Credenze delle madri slovene su metodi educativi, apprendimento e gioco infantili
A1 - Gril,Alenka
A1 - Puklek,Melita
Y1 - 1999/06//
KW - PsycInfo
KW - Unsupervised Play Addition
KW - beliefs about childrearing & children's learning & independent play & personality traits
KW - mothers of 12-30 mo olds
KW - Childhood Play Behavior
KW - Childrearing Attitudes
KW - Learning
KW - Mothers
KW - Personality Traits
KW - Cognitive Development
KW - Parental Attitudes
RP - NOT IN FILE
SP - 53
EP - 60
JF - Et… Evolutiva
VL - 63
CY - Italy
PB - Giunti Gruppo Editoriale SPA
N2 - Studied the beliefs of mothers in Slovenia about child development, child rearing, and independent play with toys. 40 mothers of boys or girls, aged 12-30 mo, were administered an open questionnaire on child rearing and children's learning, independent play, and personality traits. 23 Ss had a university degree, and 17 were secondary school graduates. 56% of Ss believed that children start learning immediately after birth. 40% of Ss maintained that play was not distinct from other childhood activities. Two-thirds of Ss referred to stereotypical sex differences in personality types. 40% of Ss believed that both parents gave emotional support to their child. Ss used mainly verbal educational and child-rearing strategies with their child. (PsycINFO Database Record (c) 2012 APA, all rights reserved)
SN - 0392-0658
UR - http://search.ebscohost.com/login.aspx?direct=true&db=psyh&AN=1999-11421-004&site=ehost-live
ER -

TY - THES
ID - 1794
T1 - Painful medical procedures in children with cancer: The impact of reframing previous experiences in distress

A1 - Chen,Edith
Y1 - 1999/03//
N1 - Accession Number: 1999-95006-172. First Author & Affiliation: Chen, Edith; U California, Los Angeles, US. Other Journal Titles: Dissertation Abstracts International. Release Date: 19991201. Publication Type: Dissertation Abstract, (0400); . Media Covered: Electronic. Document Type: Dissertation. Dissertation Number: AAM9906093. Language: English. Major Descriptor: Child Psychology; Distress; Leukemias; Medical Diagnosis; Pain Perception. Classification: Health & Mental Health Treatment & Prevention (3300) Cognitive Processes (2340) . Population: Human (10); . Age Group: Childhood (birth-12 yrs) (100) Preschool Age (2-5 yrs) (160) School Age (6-12 yrs) (180) Adolescence (13-17 yrs) (200) Adulthood (18 yrs & older) (300) Young Adulthood (18-29 yrs) (320) . Methodology: Empirical Study. Page Count: 1
KW - PsycInfo
KW - Unsupervised Play Addition
KW - reframing previous experiences in distress
KW - future procedural distress for lumbar punctures
KW - 3-18 yr olds diagnosed with leukemia
KW - Child Psychology
KW - Distress
KW - Leukemias
KW - Medical Diagnosis
KW - Pain Perception
RP - NOT IN FILE
JF - Dissertation Abstracts International: Section B: The Sciences and Engineering
VL - 59
IS - 9-B
CY - US
PB - ProQuest Information & Learning
N2 - Previous intervention research studying children's procedural distress has focused on teaching coping skills to help the child deal with upcoming procedures. However, research into conditioned responses to repeated aversive events suggests that cognitive factors such as memory and evaluation of one's reactions to an event play a central role in determining the magnitude of a conditioned response such as fear. Additionally, research into children's eyewitness testimony suggests that children's memories of events are shaped by discussions with adults afterward. The present study tested a novel intervention aimed at positively reframing children's memories of previous medical procedures in order to reduce future procedural distress. Fifty children diagnosed with leukemia (25 treatment and 25 attention control, ages 3-18) were observed for three consecutive lumbar punctures (LPs; baseline, post-intervention, and follow-up) and were interviewed to assess their memories for these LPs. At post-treatment children in the treatment group showed reductions in self-report and physiological indices of anticipatory distress, while children in the control group showed no such reductions. At follow-up children in the treatment group reported and were rated by the technician to have less anxiety and pain during the LP and displayed reductions in other physiological indices of distress. Children in the attention control group showed no such reductions. The memory interview results revealed that after intervention, children in the treatment group had less exaggeration in negative memory compared to the attention control group, and that exaggerations in negative memory were associated with greater distress during LPs. These results suggest that children's negative memories influence LP-related distress and that targeting them can be an effective way of reducing both anticipatory and procedural distress. (PsycINFO Database Record (c) 2012 APA, all rights reserved)
SN - 0419-4217
UR - http://search.ebscohost.com/login.aspx?direct=true&db=psyh&AN=1999-95006-172&site=ehost-live
ER -

TY - CHAP
ID - 1795
T1 - Cognitive functioning in depressed children and adolescents: A developmental perspective
A1 - Gotlib,Ian H.
A1 - Sommerfeld,Beth Kaplan
Y1 - 1999///

N1 - Accession Number: 2000-07252-006. First Author & Affiliation: Gotlib, Ian H.; Stanford U, Dept of Psychology, Stanford, CA, US. Release Date: 20000401. Publication Type: Book, (0200); Edited Book, (0280); . Media Covered: Print. Document Type: Chapter. ISBN: 0-7657-0188-X, Paperback, 076570188X. Language: English. Major Descriptor: Cognitive Processes; Major Depression; Theories. Minor Descriptor: Adolescent Development; Childhood Development; Epidemiology. Classification: Affective Disorders (3211) . Population: Human (10); . Age Group: Childhood (birth-12 yrs) (100) Adolescence (13-17 yrs) (200) Adulthood (18 yrs & older) (300) . Intended Audience: Psychology: Professional & Research (PS) . Methodology: Literature Review. Page Count: 42
KW - PsycInfo
KW - Unsupervised Play Addition
KW - developmental perspective on epidemiology & nature & cognitive theories of depression in children & adolescents
KW - Cognitive Processes
KW - Major Depression
KW - Theories
KW - Adolescent Development
KW - Childhood Development
KW - epidemiology
RP - NOT IN FILE
SP - 195
EP - 236
T2 - Depressive disorders in children and adolescents: Epidemiology, risk factors, and treatment
A2 - Essau,Cecilia Ahmoi
A2 - Petermann,Franz
CY - Lanham, MD US
PB - Jason Aronson
N2 - (from the chapter) In this chapter, the authors address the following 2 questions: Are the cognitive factors and processes that have been found to be associated with depression in adulthood also present in children who are depressed? If they are, what role do they play in childhood depression? The authors begin their examination of these questions by discussing the nature of depression in children. In this context, they describe epidemiologic data demonstrating that depression does indeed exist in childhood. They continue with a description of the major cognitive theories of depression. This description provides a framework for presenting the results of investigations examining the tenets of cognitive theories, both in adults and in children and adolescents. In presenting these data, the authors also address the appropriateness of applying cognitive theories to children. Throughout the literature review, they relate the findings obtained with children to normal developmental processes. (PsycINFO Database Record (c) 2012 APA, all rights reserved) (chapter)
SN - 0-7657-0188-X
UR - http://search.ebscohost.com/login.aspx?direct=true&db=psyh&AN=2000-07252-006&site=ehost-live
ER -

TY - THES
ID - 1796
T1 - The effects of sibling-implemented training on social behaviors of autistic children
A1 - Sullivan,Cristin Lea
Y1 - 1999/01//
N1 - Accession Number: 1999-95024-362. First Author & Affiliation: Sullivan, Cristin Lea; Western Michigan U., US. Other Journal Titles: Dissertation Abstracts International. Release Date: 20000607. Publication Type: Dissertation Abstract, (0400); . Media Covered: Electronic. Document Type: Dissertation. Dissertation Number: AAI9935414. Language: English. Major Descriptor: Autism; Siblings; Social Behavior. Classification: Health & Mental Health Treatment & Prevention (3300) . Population: Human (10); Male (30); . Age Group: Adulthood (18 yrs & older) (300) . Methodology: Empirical Study. Page Count: 1
KW - PsycInfo
KW - Unsupervised Play Addition
KW - sibling-implemented Pivotal Response Training
KW - social behaviors
KW - male autistic children

KW - Autism
KW - Siblings
KW - Social Behavior
RP - NOT IN FILE
JF - Dissertation Abstracts International: Section B: The Sciences and Engineering
VL - 60
IS - 6-B
CY - US
PB - ProQuest Information & Learning
N2 - The present study investigated the effectiveness of sibling-implemented Pivotal Response Training (PRT) on the social behaviors of five autistic children. Using a multiple-baseline design across sibling pairs, participants were trained to use PRT while playing with their brother with autism. Measures of play skills and social behavior were assessed during baseline, training, sibling-independent play, and two month follow-up sessions. Also, parents were asked to rate randomly selected video-tape segments from baseline and sibling-independent play sessions. The results indicated that siblings were able to learn effectively and implement PRT strategies following training. All of the autistic children demonstrated an increase in maintaining interactions and a decrease in non-engagement following training. Parent ratings demonstrated the social validity of positive behavior changes. (PsycINFO Database Record (c) 2012 APA, all rights reserved)
SN - 0419-4217
UR - http://search.ebscohost.com/login.aspx?direct=true&db=psyh&AN=1999-95024-362&site=ehost-live
ER -

TY - JOUR
ID - 1797
T1 - Knocking the Girls Off the Basketball Court: Unnatural Selection through Taste and Unexamined Assumptions of Fairness on an Unsupervised Playground
A1 - McGrath,Daniel J.
A1 - Kuriloff,Peter J.
Y1 - 1999///
KW - PsycInfo
KW - Unsupervised Play Addition
KW - Athletics
KW - sixth grade girls
KW - school community
KW - fairness
KW - unstructured play
KW - sex discrimination
KW - basketball
KW - unsupervised play
KW - gender relations
KW - Playgrounds
KW - sixth grade boys
KW - Elementary School Students
KW - Human Females
KW - Justice
RP - NOT IN FILE
SP - 41
EP - 65
JF - The School Community Journal
VL - 9
IS - 2
CY - US
PB - Academic Development Institute
N2 - This paper offers a look inside one school community. It relates the story of a group of athletic sixth grade girls who controlled a central niche on the playground of a progressive elementary school for most of a school year, only to be displaced by a few highly competitive sixth grade boys. Data are from observations of

unstructured play included in the school's curriculum. The boys were able to dominate play sites because their taste for large, highly structured, and hierarchical games was implicitly favored by the school's rules for access to sites and activities. The study has implications for understanding gender relations among children, as well as gender and access to resources. (PsycINFO Database Record (c) 2012 APA, all rights reserved) (journal abstract)
SN - 1059-308X
UR - http://search.ebscohost.com/login.aspx?direct=true&db=psyh&AN=2003-07882-003&site=ehost-live
ER -

TY - JOUR
ID - 1798
T1 - Goal-directed behavior in children with Down syndrome: The role of joint play situations
A1 - Landry,Susan H.
A1 - Miller-Loncar,Cynthia L.
A1 - Swank,Paul R.
Y1 - 1998/10//
KW - PsycInfo
KW - Unsupervised Play Addition
KW - goal-directed behaviors during joint play interaction with their mothers
KW - 4-7 yr olds with Down syndrome
KW - Childhood Play Behavior
KW - Down's Syndrome
KW - Goals
KW - Interpersonal Interaction
KW - Mother Child Relations
RP - NOT IN FILE
SP - 375
EP - 392
JF - Early Education and Development
VL - 9
IS - 4
CY - US
PB - Lawrence Erlbaum
N2 - Children's goal-directed behaviors were examined in independent play sessions before and after a joint-play interaction with their mothers for a group of children with Down syndrome (n?=?22) and a control group of mental and language age matched typically developing children (n?=?24). While both groups showed comparable amounts of time spent in independent goal-directed play during the pre-session and similar play responses with their mothers during the joint-play session, only the control children significantly increased their goal-directed behavior from the pre- to the post-session. Maternal behaviors that provided information about how to use the toy predicted increases from the pre- to post-sessions in independent goal-directed play but only for the control children. These findings suggest that children with Down syndrome may have more difficulty transferring the goal-directed play behaviors they can use with support from their mothers to an independent play situation. Findings are discussed in relation to early education program design for children with Down syndrome. (PsycINFO Database Record (c) 2012 APA, all rights reserved)
SN - 1040-9289
UR - http://search.ebscohost.com/login.aspx?direct=true&db=psyh&AN=1998-12630-003&site=ehost-live
L3 - 10.1207/s15566935eed0904_4
ER -

TY - JOUR
ID - 1800
T1 - Variability in the play of preschoolers with cognitive delays across different toy sets
A1 - Malone,D.Michael
A1 - Langone,John
Y1 - 1998/06//
KW - PsycInfo

KW - Unsupervised Play Addition
KW - doll vs vehicle vs mixed toy set
KW - play behavior
KW - 2-5 yr old preschool males vs females with cognitive delays
KW - Childhood Play Behavior
KW - Cognitive Ability
KW - Delayed Development
KW - Human Sex Differences
KW - Toys
RP - NOT IN FILE
SP - 127
EP - 142
JF - International Journal of Disability, Development and Education
VL - 45
IS - 2
CY - United Kingdom
PB - Taylor & Francis
N2 - The variability in play associated with different toy sets was examined in 30 preschool children (aged 2-5 yrs) with cognitive delays. 12 dependent variables representing categorical and sequential play were coded from videotaped home-based independent play sessions using a continuous 15-sec interval coding procedure. The authors chose a mixed toy set, a doll toy set, and a vehicle toy set to address the issue of general social stereotypes of children's play. Sex by toy set interaction effects emerged for exploration, pretend, ordered multischeme sequences, and global play sophistication. Main effects across toy sets were noted for nonplay, functional play, construction, overall proportion of play, time spent sequencing activities, and length of play sequences. The pattern of play observed within each toy set was strikingly similar across each of the 3 toy sets. Implications for practice are discussed. (PsycINFO Database Record (c) 2012 APA, all rights reserved)
SN - 1465-346X
UR - http://search.ebscohost.com/login.aspx?direct=true&db=psyh&AN=1998-04373-001&site=ehost-live
L3 - 10.1080/1034912980450202
ER -

TY - THES
ID - 1801
T1 - The relation between disruptive behavior problems, self-worth and perceived competence in middle childhood
A1 - Usher,Barbara Ann
Y1 - 1998/05//
N1 - Accession Number: 1998-95010-272. First Author & Affiliation: Usher, Barbara Ann; U Maryland Coll Park, US. Other Journal Titles: Dissertation Abstracts International. Release Date: 19990801. Publication Type: Dissertation Abstract, (0400); . Media Covered: Electronic. Document Type: Dissertation. Dissertation Number: AAM9816535. Language: English. Major Descriptor: Behavior Problems; Competence; Conduct Disorder; Self Concept; Self Perception. Minor Descriptor: Mother Child Relations. Classification: Developmental Psychology (2800) Personality Psychology (3100) . Population: Human (10); . Age Group: Childhood (birth-12 yrs) (100) School Age (6-12 yrs) (180) . Methodology: Empirical Study; Longitudinal Study. Page Count: 1
KW - PsycInfo
KW - Unsupervised Play Addition
KW - global self-worth & perceived competence & behavior problems & mother's social support
KW - 9-11 yr olds at risk for conduct disorder
KW - 5 yr study
KW - Behavior Problems
KW - Competence
KW - Conduct Disorder
KW - Self Concept
KW - Self Perception
KW - Mother Child Relations
RP - NOT IN FILE

JF - Dissertation Abstracts International: Section B: The Sciences and Engineering
VL - 58
IS - 11-B
CY - US
PB - ProQuest Information & Learning
N2 - Self-concept plays a critical role in motivating behavior and mediating affect in the lives of children, adolescents, and adults. The present correlational study examined global self-worth and perceived competence among children who exhibit disruptive behavior problems. Disruptive behavior problems comprise the most common referrals for child and adolescent mental health services. They are known to be stable over time, difficult to treat, and predictive of poor outcome in adolescence and adulthood. This study examined (a) the relation of behavior problems with self-worth and perceived competence, (b) discrepancy between children's and teacher's perceptions of competence, (c) the role of maternal and social support in relation to self-worth and behavior problems, (d) how self-conscious emotions are related to self-worth and behavior problems, and (e) children's attributions for their self-judgments. Subjects were 73 children, ages 9 to 11, who had been participating in a longitudinal study of children at risk for conduct disorder since they were 4- to 5-years-old. Mothers' and teachers' ratings of children's behavior problems were available for each of three data collection points. The main findings suggested that mothers' and teachers' behavior problem ratings were not associated with children's sense of global self-worth, but they were associated with children's negative evaluations in specific domains. The significance of associations was not consistent across domains. Comparison of children's and teachers' perceptions of children's competence revealed that children who underestimated their competence in one or more of the specific domains reported lower global self-worth. However, children who overestimated their own behavioral conduct were rated higher on externalizing problems by their mothers and teachers. As expected, children who perceived higher maternal and social support reported higher self-worth and competence. The importance of specific sources of support varied among the domains. Perceptions of maternal acceptance and support were more strongly associated with self-worth for children with externalizing and attention problems. (PsycINFO Database Record (c) 2012 APA, all rights reserved)
SN - 0419-4217
UR - http://search.ebscohost.com/login.aspx?direct=true&db=psyh&AN=1998-95010-272&site=ehost-live
ER -

TY - JOUR
ID - 1802
T1 - Il segreto e il sequestro. Dall'oggetto nascosto all'oggetto prigioniero
A1 - D'Andrea,Anna
Y1 - 1998/05//
KW - PsycInfo
KW - Unsupervised Play Addition
KW - psychoanalytic perspective of role of secrecy to object in narcissistic personalities
KW - children & adults
KW - Narcissistic Personality Disorder
KW - Object Relations
KW - Psychoanalytic Theory
KW - Secrecy
RP - NOT IN FILE
SP - 293
EP - 305
JF - Psichiatria dell'infanzia e dell'adolescenza
VL - 65
IS - 3
CY - Italy
PB - Edizioni Borla
N2 - Presents a psychoanalytic discussion of the role of secrecy in relation to the object in narcissistic personality structures. Theoretical references and clinical reports of children and adults are presented to show how secrecy can have the significance of conservation of the object (which is concealed so as to be protected and kept apart) in some situations and the significance of omnipotent control of the object (which is placed under constraint so as to be made harmless) in other situations. The possibility is discussed that secrecy may be

transformed into sequestration and may play a pathological role in some behaviors typical of narcissistic personalities. (English abstract) (PsycINFO Database Record (c) 2012 APA, all rights reserved)
SN - 0393-361X
UR - http://search.ebscohost.com/login.aspx?direct=true&db=psyh&AN=1999-00270-003&site=ehost-live
ER -

TY - JOUR
ID - 1803
T1 - The effect of reinforcer preference on functional analysis outcomes
A1 - Lalli,Joseph S.
A1 - Kates,Kelly
Y1 - 1998///
KW - PsycInfo
KW - Unsupervised Play Addition
KW - functional analysis & concurrent-schedule assessments
KW - identification of reinforcer preference during problem behavior situations
KW - 2.5-3.5 yr old males with developmental delays
KW - Behavior Problems
KW - Behavioral Assessment
KW - Delayed Development
KW - Reinforcement
RP - NOT IN FILE
SP - 79
EP - 90
JF - Journal of Applied Behavior Analysis
JA - J Appl.Behav Anal.
VL - 31
IS - 1
CY - US
PB - Journal of Applied Behavior Analysis
N2 - Combined functional analyses and concurrent-schedule assessments to identify reinforcer preference during situations in which problem behavior may have been multiply controlled. Participants were 3 children (aged 2.5-3.5 yrs) with developmental delays who engaged in problem behavior during toy play with another child and 1 adult present, suggesting that problem behavior may have been maintained by adult attention or access to tangible reinforcement. Thus, conditions were designed to test attention and access-to-toys hypotheses. Initial functional analyses suggested multiple control. Subsequent concurrent-schedule assessments identified preference between the reinforcers, and treatments were based on these findings. Findings are discussed regarding the assessment of potentially multiply controlled problem behavior. (PsycINFO Database Record (c) 2012 APA, all rights reserved)
SN - 1938-3703
UR - http://search.ebscohost.com/login.aspx?direct=true&db=psyh&AN=1998-01324-005&site=ehost-live
L3 - 10.1901/jaba.1998.31-79
ER -

TY - THES
ID - 1804
T1 - Preschool children's use of race and ethnicity in everyday interaction
A1 - Van Ausdale,Debra
Y1 - 1998/01//
N1 - Accession Number: 1998-95001-053. First Author & Affiliation: Van Ausdale, Debra; U Florida, US. Other Journal Titles: Dissertation Abstracts International. Release Date: 19980101. Publication Type: Dissertation Abstract, (0400); . Media Covered: Electronic. Document Type: Dissertation. Dissertation Number: AAM9800197. Language: English. Major Descriptor: Childhood Play Behavior; Racial and Ethnic Attitudes; Social Cognition. Minor Descriptor: Early Childhood Development. Classification: Developmental Psychology (2800) . Population: Human (10); . Age Group: Childhood (birth-12 yrs) (100) . Page Count: 1
KW - PsycInfo

KW - Unsupervised Play Addition
KW - Preschool children's use of race and ethnicity in everyday interaction
KW - Childhood Play Behavior
KW - Racial and Ethnic Attitudes
KW - Social Cognition
KW - Early Childhood Development
RP - NOT IN FILE
JF - Dissertation Abstracts International Section A: Humanities and Social Sciences
VL - 58
IS - 7-A
CY - US
PB - ProQuest Information & Learning
N2 - This study is a participant observation exploration of the how very young children construct, understand and use race and ethnicity during unsupervised play. It challenges conventional theoretical orientations that proceed from the assumption that children cannot understand racial and ethnic concepts because of the children's stage of cognitive development. The purpose of this study is to analyze and describe how, from a very early age, race and ethnicity become central to very young children's social knowledge of the world. (PsycINFO Database Record (c) 2012 APA, all rights reserved)
SN - 0419-4209
UR - http://search.ebscohost.com/login.aspx?direct=true&db=psyh&AN=1998-95001-053&site=ehost-live
ER -

TY - CHAP
ID - 1805
T1 - Guilt in the classroom
A1 - Williams,Christopher
Y1 - 1998///
N1 - Accession Number: 1997-36487-011. First Author & Affiliation: Williams, Christopher; Cornell U, Medical Coll, Dept of Public Health, Inst for Prevention Research, New York, NY, US. Release Date: 19980401. Publication Type: Book, (0200); Edited Book, (0280); . Media Covered: Print. Document Type: Chapter. ISBN: 0-12-148610-9, Hardcover, 0121486109. Language: English. Major Descriptor: Academic Achievement; Classroom Behavior; Classroom Discipline; Guilt. Classification: Classroom Dynamics & Student Adjustment & Attitudes (3560) . Population: Human (10); . Intended Audience: Psychology: Professional & Research (PS) . Page Count: 11
KW - PsycInfo
KW - Unsupervised Play Addition
KW - role of guilt in behavior within & outside classroom & educational performance & differences in guilt across academic track & guilt induction as discipline technique
KW - Academic Achievement
KW - Classroom Behavior
KW - Classroom Discipline
KW - Guilt
RP - NOT IN FILE
SP - 233
EP - 243
T2 - Guilt and children
A2 - Bybee,Jane
CY - San Diego, CA US
PB - Academic Press
N2 - (from the chapter) Begin by summarizing ways in which guilt may be adaptive in the classroom environment. The author then explores the role guilt may play in the regulation and control of classroom conduct, in fostering academic achievement, and in determining participations in risky, unsupervised out-of-school activities. The author then turns to differences observed in guilt as a function of academic track. The author closes by suggesting conditions under which teachers and parents may effectively use and induce guilt feelings among young people to raise scholastic and socioemotional competence. (PsycINFO Database Record (c) 2012 APA, all rights reserved) (chapter)

SN - 0-12-148610-9
UR - http://search.ebscohost.com/login.aspx?direct=true&db=psyh&AN=1997-36487-011&site=ehost-live
L3 - 10.1016/B978-012148610-5/50012-6
ER -

TY - BOOK
ID - 1806
T1 - Inuit morality play: The emotional education of a three-year-old
A1 - Briggs,Jean L.
Y1 - 1998///
N1 - Accession Number: 1998-07639-000. First Author & Affiliation: Briggs, Jean L.. Release Date: 19980701. Publication Type: Book, (0200); Authored Book, (0240); . Media Covered: Print. ISBN: 0-300-07237-6, Hardcover, 0300072376. Language: English. Major Descriptor: Early Childhood Development; Education; Interpersonal Interaction; Recreation; Socialization. Minor Descriptor: Childrearing Practices; Drama; Emotional Development; Inuit. Classification: Psychosocial & Personality Development (2840) . Population: Human (10); Female (40); . Location: Canada. Age Group: Childhood (birth-12 yrs) (100) Preschool Age (2-5 yrs) (160) Adulthood (18 yrs & older) (300) . Intended Audience: Psychology: Professional & Research (PS)
KW - PsycInfo
KW - Unsupervised Play Addition
KW - growing up & emotional education & socialization through dramatic play interactions
KW - 3 yr old Inuit girl
KW - Early Childhood Development
KW - Education
KW - Interpersonal Interaction
KW - Recreation
KW - SOCIALIZATION
KW - Childrearing Practices
KW - Drama
KW - Emotional Development
KW - Inuit
RP - NOT IN FILE
CY - New Haven, CT US
PB - Yale University Press
N2 - (from the jacket) Inuit adults often playfully present small children with difficult, even dangerous, choices and then dramatize the consequences of the child's answers. They are enacting in larger-than-life form the plots that drive Inuit social life-testing, acting out problems, entertaining themselves, and, most of all, bringing up their children. (jacket)
J. L. Briggs takes readers through 6 mo of dramatic interactions in the life of Chubby Maata, a 3-yr-old girl growing up in a Baffin Island hunting camp. The book examines the issues that engaged the child-belonging, possession, love-and shows the process of growing. Briggs questions the nature of "sharedness" in culture and assumptions about how culture is transmitted. She suggests that both cultural meanings and strong personal commitment to one's world can be (and perhaps must be) acquired not by straightforwardly learning attitudes, rules, and habits in a dependent mode but by experiencing oneself as an agent engaged in productive conflict in emotionally problematic situations. Briggs finds that dramatic play is an essential force in Inuit social life. It creates and supports values; engenders and manages attachments and conflicts; and teaches and maintains an alert, experimental, constantly testing approach to social relationships. (PsycINFO Database Record (c) 2012 APA, all rights reserved) (jacket)
SN - 0-300-07237-6
UR - http://search.ebscohost.com/login.aspx?direct=true&db=psyh&AN=1998-07639-000&site=ehost-live
ER -

TY - JOUR
ID - 1807
T1 - Adult and infant perception of two English phones
A1 - Pegg,Judith E.

A1 - Werker,Janet F.
Y1 - 1997/12//
KW - PsycInfo
KW - Unsupervised Play Addition
KW - English phonetic discrimination
KW - 6-8 vs 10-12 mo olds vs adults
KW - Age Differences
KW - Auditory Discrimination
KW - Developmental Age Groups
KW - Phonemes
KW - Speech Perception
RP - NOT IN FILE
SP - 3742
EP - 3753
JF - Journal of the Acoustical Society of America
VL - 102
IS - 6
CY - US
PB - Acoustical Society of American
N2 - Previous research has shown that young infants easily discriminate both native and non-native consonant contrasts, but by 10-12 mo of age infants perform like adults and easily discriminate only native consonant contrasts. The present study was designed to determine what kind of experience is required to maintain discrimination of native consonants. To address this question, English listeners of 3 ages (6-8 mo, 10-12 mo, and adults) were presented with a phonetic difference. This distinction occurs in English but is not phonemic. If passive exposure is sufficient to maintain discrimination, all age groups should discriminate the 2 sounds. However, if phonological status plays a role, then older infants and adults should fail. In Exp 1, English adults judged the 2 sounds to be equally good instances of the same phonemic category. In an AX procedure in Exp 2, English adults discriminated the 2 sounds better than chance but worse than native phonemic levels. In the Conditioned Head Turn procedure in Exp 3, adults and 6- to 8-mo-old infants discriminated the sounds but 10- to 12-mo-old infants did not. Taken together, these results are most consistent with the hypothesis that phonological status plays a role in maintaining discrimination of phonetic information. (PsycINFO Database Record (c) 2012 APA, all rights reserved)
SN - 0001-4966
UR - http://search.ebscohost.com/login.aspx?direct=true&db=psyh&AN=1997-42754-015&site=ehost-live
L3 - 10.1121/1.420137
ER -

TY - JOUR
ID - 1808
T1 - 'Skal vi lage pulver?' Om spr†klige gjentakelser i lek-et bidrag til hypoteser om spr†kutvikling hos sm† barn
A1 - Aukrust,Vibeke G.
Y1 - 1997/09//
KW - PsycInfo
KW - Unsupervised Play Addition
KW - repetition during play as verbally mediating activity
KW - male 2 yr olds
KW - 4 mo study
KW - implications for hypotheses on language acquisition
KW - Childhood Play Behavior
KW - Imitation (Learning)
KW - Language Development
RP - NOT IN FILE
SP - 192
EP - 211
JF - Nordisk Psykologi

VL - 49
IS - 3
CY - Denmark
PB - Hans Reitzels Forlag
N2 - Investigated if children's extensive use of different forms of repetition have the function of verbally mediating activity. Human Ss: Two normal male preschool children (aged 2 yrs). Ss were videofilmed during dyadic play of a game named by the Ss as "making powder" once or twice a wk for 4 mo. The significance of repetition during play was examined in relationship to self-repetition, immediate repetition of the peer and delayed repetition of non-present adults to determine if repetition played a factor in directing the activity. (English abstract) (PsycINFO Database Record (c) 2012 APA, all rights reserved)
SN - 0029-1463
UR - http://search.ebscohost.com/login.aspx?direct=true&db=psyh&AN=1997-43376-001&site=ehost-live
ER -

TY - THES
ID - 1809
T1 - Family correlates of sibling relationships in young adulthood
A1 - Chebra,Janice Marie
Y1 - 1997/04//
N1 - Accession Number: 1997-95007-194. First Author & Affiliation: Chebra, Janice Marie; The Ohio State U, US. Other Journal Titles: Dissertation Abstracts International. Release Date: 19970101. Publication Type: Dissertation Abstract, (0400); . Media Covered: Electronic. Document Type: Dissertation. Dissertation Number: AAM9710543. Language: English. Major Descriptor: Attachment Behavior; Family Relations; Rites (Nonreligious); Sibling Relations. Minor Descriptor: Age Differences; Human Sex Differences; Systems Theory. Classification: Developmental Psychology (2800) . Population: Human (10); Male (30); Female (40); . Age Group: Childhood (birth-12 yrs) (100) Adolescence (13-17 yrs) (200) Adulthood (18 yrs & older) (300) Young Adulthood (18-29 yrs) (320) . Methodology: Empirical Study. Page Count: 1
KW - PsycInfo
KW - Unsupervised Play Addition
KW - Family correlates of sibling relationships in young adulthood
KW - Attachment Behavior
KW - Family Relations
KW - Rites (Nonreligious)
KW - Sibling Relations
KW - Age Differences
KW - Human Sex Differences
KW - Systems Theory
RP - NOT IN FILE
JF - Dissertation Abstracts International Section A: Humanities and Social Sciences
VL - 57
IS - 10-A
CY - US
PB - ProQuest Information & Learning
N2 - Social scientists increasingly recognize the importance that siblings play throughout the life course and the intrinsic and extrinsic benefits that this special kinship bond may provide. Although evidence suggests that the context of the sibling relationship changes over time, most of the research has ignored the significance of the sibling bond throughout early and middle adulthood. The present study explores the relationship of family satisfaction, family strength, and family ritual to the quality of sibling relationships for college-age young adults. This investigation offers an important test of family systems theory by evaluating the interdependence of subsystems within the larger family system. The sample was drawn from college students enrolled in introductory family courses at two Midwestern universities. Participants were undergraduates between the ages of 18-23; unmarried or never married; had no children; had at least one living sibling; and were not a twin. The final sample consisted of 283 females and 25 males (N=308). Participants responded to a series of self-report measures included in the Sibling Survey to assess demographic and personal information about the subject, their family and siblings. Significant findings suggest the following conclusions regarding the significance of sibling and family relationships during young adulthood. First, young adults positively evaluated closeness to the

sibling nearest in age. Based on retrospective reports, sibling relationships were closer in childhood and young adulthood than in adolescence. Secondly, there was a positive relationship among family strength, family satisfaction, family ritual and sibling warmth. Third, low levels of family strength and family satisfaction were related to higher sibling conflict and sibling rivalry. Fourth, high ritual routine was positively related to total sibling conflict; whereas high ritual meaning was negatively related to total sibling conflict. Fifth, high ritual routine was related to greater paternal sibling rivalry. Finally, sister-sister dyads reported higher levels of total sibling warmth, intimacy, emotional support, affection, knowledge, and admiration than sister-brother dyads. However, no statistically significant differences were found between gender dyad (sister-sister or sister-brother) and sibling conflict or sibling rivalry. (PsycINFO Database Record (c) 2012 APA, all rights reserved)
SN - 0419-4209
UR - http://search.ebscohost.com/login.aspx?direct=true&db=psyh&AN=1997-95007-194&site=ehost-live
ER -

TY - CHAP
ID - 1810
T1 - Metacommunication as a source of indeterminism in relationship development
A1 - Fogel,Alan
A1 - Branco,Angela Uchoa
Y1 - 1997///
N1 - Accession Number: 1997-97399-003. First Author & Affiliation: Fogel, Alan; U Utah, Salt Lake City, UT, US. Release Date: 19970901. Publication Type: Book, (0200); Edited Book, (0280); . Media Covered: Print. Document Type: Chapter. ISBN: 0-8058-1805-7, Hardcover, 0805818057; 0-8058-1806-5, Paperback, 0805818065. Language: English. Major Descriptor: Communication; Determinism; Interpersonal Interaction; Psychosocial Development. Minor Descriptor: Dialectics; Parent Child Relations; Peer Relations; Systems Theory. Classification: Psychosocial & Personality Development (2840) Philosophy (2630) . Population: Human (10); . Age Group: Childhood (birth-12 yrs) (100) Infancy (2-23 mo) (140) Preschool Age (2-5 yrs) (160) . Intended Audience: Psychology: Professional & Research (PS) . Page Count: 28
KW - PsycInfo
KW - Unsupervised Play Addition
KW - dialectical & determinism & indeterminism dynamic systems perspectives on relationship development & metacommunication in parent-infant & early childhood peer interaction
KW - Communication
KW - Determinism
KW - Interpersonal Interaction
KW - Psychosocial Development
KW - Dialectics
KW - Parent Child Relations
KW - Peer Relations
KW - Systems Theory
RP - NOT IN FILE
SP - 65
EP - 92
T2 - Dynamics and indeterminism in developmental and social processes
A2 - Fogel,Alan
A2 - Lyra,Maria C.D.P.
A2 - Valsiner,Jaan
CY - Hillsdale, NJ England
PB - Lawrence Erlbaum Associates, Inc
N2 - (from the chapter) the premise of this chapter is that interpersonal relationships are developing systems of communication, systems that generate meaning in the form of emotion, commitment, and memories / relationships construct their own life histories and move through developmental phases, such as getting acquainted, establishing trust and intimacy, maintenance, decline, and rejuvenation or termination / take a dialectical perspective on relationship continuity and change, focusing on the everyday communication processes that constitute the direct contacts between participants / examine relationship development from the perspective of determinism and indeterminism / review the literature on dynamic systems theory to suggest how indeterminacy can arise in a developing system and, in particular, how it may arise in relationship development /

explore the relative roles of communication and metacommunication in relationship development (chapter) communication includes all forms of everyday coactions between participants, including sharing, cooperation, conflict, debate, social play, and conversation / in metacommunication, participants evaluate their own communication process / review the literature on adult relationships and present data from our own work on parent-infant and preschool peer play / hypothesize that metacommunication may be one source of indeterministic change in interpersonal relationships, primarily because it constrains the future course of a relationship by making explicit the connection of the future with the past and the present / indeterminism can arise with respect to the timing of metacommunication, its topic and scope, and its impact on action (PsycINFO Database Record (c) 2012 APA, all rights reserved) (chapter)
SN - 0-8058-1805-7
UR - http://search.ebscohost.com/login.aspx?direct=true&db=psyh&AN=1997-97399-003&site=ehost-live
ER -

TY - CHAP
ID - 1811
T1 - Kinesiology and sensorimotor function
A1 - Rosen,Sandra
Y1 - 1997///
N1 - Accession Number: 1997-36844-005. First Author & Affiliation: Rosen, Sandra; San Francisco State U, Programs in Visual Impairment, San Francisco, CA, US. Release Date: 19980401. Publication Type: Book, (0200); Edited Book, (0280); . Media Covered: Print. Document Type: Chapter. Book Type: Textbook/Study Guide. ISBN: 0-89128-946-1, Hardcover, 0891289461. Language: English. Major Descriptor: Perceptual Motor Development; Perceptual Motor Processes; Vision Disorders. Classification: Vision & Hearing & Sensory Disorders (3299) . Population: Human (10); . Age Group: Childhood (birth-12 yrs) (100) Adulthood (18 yrs & older) (300) . Intended Audience: Psychology: Professional & Research (PS) . Page Count: 30
KW - PsycInfo
KW - Unsupervised Play Addition
KW - kinesiology & sensorimotor development & functioning
KW - children & adults who are visually impaired
KW - Perceptual Motor Development
KW - Perceptual Motor Processes
KW - Vision Disorders
RP - NOT IN FILE
SP - 170
EP - 199
T2 - Foundations of orientation and mobility (2nd ed.)
A2 - Blasch,Bruce B.
A2 - Wiener,William R.
A2 - Welsh,Richard L.
CY - New York, NY US
PB - American Foundation for the Blind
N2 - (from the chapter) This chapter explores sensorimotor development and functioning in children and adults who are visually impaired. This consideration includes the role of sensorimotor skills in independent mobility and the impact of visual impairment on their development. Kinesiology, the study of movement, provides the structure for categorizing, analyzing, understanding, and communicating about sensorimotor skills. (PsycINFO Database Record (c) 2012 APA, all rights reserved) (chapter)
SN - 0-89128-946-1
UR - http://search.ebscohost.com/login.aspx?direct=true&db=psyh&AN=1997-36844-005&site=ehost-live
ER -

TY - CHAP
ID - 1812
T1 - The child in the adult: The contribution of child analysis to the psychoanalysis of adults
A1 - Anderson,Robin
Y1 - 1997///

N1 - Accession Number: 1997-97199-007. First Author & Affiliation: Anderson, Robin. Release Date: 19970701. Publication Type: Book, (0200); Edited Book, (0280); . Media Covered: Print. Document Type: Chapter. ISBN: 0-8236-1055-1, Hardcover, 0823610551. Language: English. Major Descriptor: Age Differences; Child Psychotherapy; Psychoanalysis. Classification: Psychoanalytic Therapy (3315) . Population: Human (10); . Age Group: Childhood (birth-12 yrs) (100) Adulthood (18 yrs & older) (300) . Intended Audience: Psychology: Professional & Research (PS) . Page Count: 12
KW - PsycInfo
KW - Unsupervised Play Addition
KW - child vs adult psychoanalysis
KW - Age Differences
KW - Child Psychotherapy
KW - Psychoanalysis
RP - NOT IN FILE
SP - 414
EP - 425
T2 - The contemporary Kleinians of London
A2 - Schafer,Roy
CY - Madison, CT US
PB - International Universities Press, Inc
N2 - (from the book) provides an . . . overview of the contemporary approach to child analysis, and it [includes] sessions drawn from the analysis of a 4-yr-old boy / develop [a] comparison of child and adult analysis / shows how the child's play sessions are paralleled by the adult's largely verbal, though also gestural and attitudinal productions / argues that, because each adult brings an internal world to the consulting room, he or she will enact the internal child through the verbal and other means of communication used in the sessions / analysts familiar with child analysis are bound to see a more subtle and perhaps less raw version of the child's inner world in the adult's production / presents illustrative material from the analysis of a man (PsycINFO Database Record (c) 2012 APA, all rights reserved) (book)
SN - 0-8236-1055-1
UR - http://search.ebscohost.com/login.aspx?direct=true&db=psyh&AN=1997-97199-007&site=ehost-live
ER -

TY - JOUR
ID - 1813
T1 - An assessment protocol for selective mutism: Analogue assessment using parents as facilitators
A1 - Schill,Melissa T.
A1 - Kratochwill,Thomas R.
A1 - Gardner,William I.
Y1 - 1996///
KW - PsycInfo
KW - Unsupervised Play Addition
KW - parent facilitated assessment protocol for identification of maintaining variables & formulation of treatment recommendations
KW - 8 yr old female with selective mutism
KW - Client Characteristics
KW - Elective Mutism
KW - Oral Communication
KW - Behavior Therapy
KW - Parental Attitudes
RP - NOT IN FILE
SP - 1
EP - 21
JF - Journal of School Psychology
VL - 34
IS - 1
CY - Netherlands
PB - Elsevier Science

N2 - Presents an assessment protocol for conducting a functional analysis of maintaining environmental variables for children with selective mutism. The S was an 8-yr-old girl in 1st grade who met Diagnostic and Statistical Manual of Mental Disorders-IV (DSM-IV) criteria for selective mutism. A modified version of the Functional Diagnostic Protocol was used during an initial interview with the S's parents to develop a hypothesis about environmental and personal variables that may be contributors to the disorder. The S's speech rates were measured across a continuum of high- and low-demand situations in which various behavior strategies were implemented to increase speech. Speech was best maintained in the presence of unfamiliar adults when it was required for play in a family game or when fading techniques were used. Based on this data, treatment recommendations deemed suitable by both the S and her parents were generated for use at school and home. (PsycINFO Database Record (c) 2012 APA, all rights reserved)
SN - 0022-4405
UR - http://search.ebscohost.com/login.aspx?direct=true&db=psyh&AN=1996-00617-001&site=ehost-live
L3 - 10.1016/0022-4405(95)00023-2
ER -

TY - JOUR
ID - 1814
T1 - You can be the baby brother but you aren't born yet: Preschool girls' negotiation for power and access in pretend play
A1 - Sheldon,Amy
Y1 - 1996///
KW - PsycInfo
KW - Unsupervised Play Addition
KW - facets of empowerment in conflict negotiation & social facilitation during role playing conversation
KW - 3-5 yr old females
KW - Childhood Play Behavior
KW - Conflict
KW - Conversation
KW - Negotiation
KW - Social Facilitation
KW - Empowerment
KW - Role Playing
RP - NOT IN FILE
SP - 57
EP - 80
JF - Research on Language and Social Interaction
VL - 29
IS - 1
CY - US
PB - Lawrence Erlbaum
N2 - Examined the discourse skills used by some White, middle-class advantaged preschool girls to manage their disagreements. The authors show how their management of opposition is a jointly constructed achievement. As such, it requires the coordination of high levels of sociolinguistic skill to arrive at cooperative as well as self-serving, competitive, or discordant ends. The episode discussed here is a sustained and discontinuous oppositional example of verbally engineered social ostracism, which is fairly common in preschoolers' interactions, especially during pretend play. Ss were 36 3-5 yr old girls videotaped in unsupervised play at their daycare center. The events examined are likened to a theatrical production, with roles played both on and off stage. The double-voiced nature of 3 girls' interactions can be described as simultaneously self-serving and socially savvy as one S tries to exclude another, who in turn tries to negotiate a role for herself in the play scenario. An analysis of their interactions highlights the linguistic effectiveness of the double-voiced method, through the use of a falsetto vocal tone, in exerting control and managing conflict while remaining civil and socially facilitative. (PsycINFO Database Record (c) 2012 APA, all rights reserved)
SN - 1532-7973
UR - http://search.ebscohost.com/login.aspx?direct=true&db=psyh&AN=1999-05145-003&site=ehost-live
L3 - 10.1207/s15327973rlsi2901_4
ER -

TY - JOUR
ID - 1815
T1 - Gender differences in the object-oriented play of preschoolers with cognitive delays
A1 - Malone,D.Michael
A1 - Langone,John
Y1 - 1995///
KW - PsycInfo
KW - Unsupervised Play Addition
KW - Sex
KW - object oriented play
KW - preschoolers with cognitive delays
KW - Childhood Play Behavior
KW - Cognitive Development
KW - Delayed Development
KW - Human Sex Differences
RP - NOT IN FILE
SP - 302
EP - 314
JF - Journal of Early Intervention
VL - 19
IS - 4
CY - US
PB - Council for Exceptional Children
N2 - Gender differences in the play of preschool children with cognitive delays were investigated. 12 dependent variables representing categorical and sequential play were coded from observations of 18 boys and 12 girls playing during an independent-play session at home. Boys and girls were observed to play differentially with 3 toy sets (mixed, doll, and vehicle) with which they were presented. Overall, boys engaged in more functional play than girls and were more sophisticated when playing with the vehicle toy set. Girls, however, engaged in more constructive play than boys and demonstrated a tendency for greater sophistication with respect to the doll toys. (PsycINFO Database Record (c) 2012 APA, all rights reserved)
SN - 1053-8151
UR - http://search.ebscohost.com/login.aspx?direct=true&db=psyh&AN=1996-03965-005&site=ehost-live
L3 - 10.1177/105381519501900405
ER -

TY - JOUR
ID - 1816
T1 - Methylphenidate in children with mental retardation and ADHD: Effects on independent play and academic functioning
A1 - Handen,Benjamin L.
A1 - McAuliffe,Sarah
A1 - Janosky,Janine
A1 - Feldman,Heidi
Y1 - 1995/06//
KW - PsycInfo
KW - Unsupervised Play Addition
KW - Methylphenidate
KW - independent play & academic on task behavior
KW - mentally retarded 6.1-12.5 yr olds with attention deficit hyperactivity disorder
KW - Attention Deficit Disorder
KW - Childhood Play Behavior
KW - Mental Retardation
KW - Time on Task
KW - Drug Therapy
KW - Hyperkinesis

RP - NOT IN FILE
SP - 91
EP - 103
JF - Journal of Developmental and Physical Disabilities
VL - 7
IS - 2
CY - Germany
PB - Springer
N2 - 22 children (aged 6.1-12.5 yrs) with mental retardation and attention deficit hyperactivity disorder (ADHD) participated in a double-blind, placebo-controlled study of methylphenidate involving .3 and .6 mg/kg medication doses and a placebo. Clinic-based observations of independent play and a restricted academic task were conducted to assess drug response. An interval recording system was used to code behaviors such as length of play, physical movement, intensity of play, and toy pickups during a 10-min independent play observation. On-task behavior, fidgetiness, out-of-seat behavior, and touching toys were coded during a 10-min restricted academic task. Results indicate significant changes across a number of dependent measures with the use of methylphenidate. Both play and restricted academic tasks may serve as appropriate clinic-based means of assessing stimulant medication efficacy in children with mental retardation and ADHD. (PsycINFO Database Record (c) 2012 APA, all rights reserved)
SN - 1573-3580
UR - http://search.ebscohost.com/login.aspx?direct=true&db=psyh&AN=1995-44827-001&site=ehost-live
L3 - 10.1007/BF02684955
ER -

TY - JOUR
ID - 1817
T1 - Crisis intervention with survivors of natural disaster: Lessons from Hurricane Andrew
A1 - Shelby,Janine S.
A1 - Tredinnick,Michael G.
Y1 - 1995/05//
KW - PsycInfo
KW - Unsupervised Play Addition
KW - crisis intervention issues & therapeutic responses
KW - mental health workers assisting survivors of Hurricane Andrew
KW - Crisis Intervention Services
KW - Natural Disasters
KW - Counseling
RP - NOT IN FILE
SP - 491
EP - 497
JF - Journal of Counseling & Development
VL - 73
IS - 5
CY - US
PB - American Counseling Assn
N2 - Presents anecdotal evidence from mental health work with Hurricane Andrew survivors in south Florida and discusses implications for general disaster counseling. The mental health workers served a predominantly lower SES group with diverse ethnic identities, all of whom had been left homeless by the 1992 hurricane. Interventions were based on the belief that enhancing clients' perceived power would restore or improve predisaster functioning. Issues presented by adults included helplessness and a sense of loss, both of which were dealt with through individual counseling, stress reduction techniques, and helping the adults increase their sense of mastery. Children experienced regression and anxiety and were treated through play therapy, drawing, and positive reframing. The effects of multicultural issues are noted. (PsycINFO Database Record (c) 2012 APA, all rights reserved)
SN - 1556-6676
UR - http://search.ebscohost.com/login.aspx?direct=true&db=psyh&AN=1996-92364-001&site=ehost-live
L3 - 10.1002/j.1556-6676.1995.tb01784.x

ER -

TY - JOUR
ID - 1818
T1 - Contextual variation of correspondences among measures of play and developmental level of preschool children
A1 - Malone,D.Michael
A1 - Stoneman,Zolinda
A1 - Langone,John
Y1 - 1994///
KW - PsycInfo
KW - Unsupervised Play Addition
KW - independent home vs classroom free play & cognitive & communicative developmental age
KW - categorical & sequential play patterns
KW - 24-70 mo olds with developmental delays
KW - Childhood Play Behavior
KW - Childhood Play Development
KW - Delayed Development
KW - Home Environment
KW - School Environment
KW - Cognitive Development
KW - Communication Skills
KW - Developmental Age Groups
RP - NOT IN FILE
SP - 199
EP - 215
JF - Journal of Early Intervention
VL - 18
IS - 2
CY - US
PB - Council for Exceptional Children
N2 - Convergent assessment in a multicontext design was used to examine the development of 22 preschool children (aged 24-70 mo) with cognitive delays (range 10-53 mo). The children were observed during both an independent-play condition at home and a group free-play condition at school. Both categorical and sequential play activity were coded. Cognitive and communicative performance levels were established through the administration of the Battelle Developmental Inventory. Data were examined for patterns of association within and across contexts. Differential patterns of association among play variables emerged across contexts. Assessed developmental age was more predictive of play behaviors demonstrated in the independent-play condition than in the classroom free-play condition. Findings highlight the need to consider contextual variation associated with play-based assessment and program development. (PsycINFO Database Record (c) 2012 APA, all rights reserved)
SN - 1053-8151
UR - http://search.ebscohost.com/login.aspx?direct=true&db=psyh&AN=1995-21823-001&site=ehost-live
L3 - 10.1177/105381519401800207
ER -

TY - JOUR
ID - 1819
T1 - L'uso regolativo del linguaggio nell'interazione precoce fra pari
A1 - Lavelli,Manuela
Y1 - 1994/02//
KW - PsycInfo
KW - Unsupervised Play Addition
KW - utterances during play activity with peers with vs without adults
KW - 18-30 mo olds
KW - Italy

KW - Oral Communication
KW - Peer Relations
KW - Childhood Play Behavior
RP - NOT IN FILE
SP - 41
EP - 52
JF - Et… Evolutiva
VL - 47
CY - Italy
PB - Giunti Gruppo Editoriale SPA
N2 - Studied the nature and functions of the utterances produced by 18-30-mo-old toddlers in interactions both with peers and with adults; and assessed the development of functions with age, socially regulative functions of utterances, differences in interaction with peers and with adults, and differences in verbal-communication behavior between males and females. Human Ss: Six normal male and female Italian infants and preschool children (aged 1-2 yrs). At the ages of 18, 21, 24, 27, and 30 mo, Ss were videotaped in 10-min sessions of play activity with 3 peers both with and without the presence of an adult. Their utterances were analyzed for the relevant information. (English abstract) (PsycINFO Database Record (c) 2012 APA, all rights reserved)
SN - 0392-0658
UR - http://search.ebscohost.com/login.aspx?direct=true&db=psyh&AN=1994-87336-001&site=ehost-live
ER -

TY - JOUR
ID - 1820
T1 - Analysis of state organizational patterns among students with profound disabilities
A1 - Guess,Doug
A1 - Siegel-Causey,Ellin
A1 - Roberts,Sally
A1 - Guy,Barbara
Y1 - 1993///
KW - PsycInfo
KW - Unsupervised Play Addition
KW - interaction between behavior state & endogenous & exogenous variables
KW - multiply handicapped 4-16 yr olds
KW - implications for intervention & education
KW - Consciousness States
KW - Environment
KW - Multiple Disabilities
KW - Physiology
KW - Profound Mental Retardation
KW - Special Education Students
RP - NOT IN FILE
SP - 93
EP - 108
JF - Journal of the Association for Persons with Severe Handicaps
VL - 18
IS - 2
CY - US
PB - Assn for Persons with Severe Handicaps
N2 - Reviews recent research on state behavior among children and youth with profound disabilities and presents a model depicting interactions of state with endogenous and exogenous variables. Applications of the model are illustrated through case studies of 4 students (aged 4-16 yrs) with profound disabilities, including mental retardation, lack of independent mobility, and congenital anomalies. Results indicate that persons with profound disabilities demonstrate significant individual differences in their state organization. Nevertheless, they show extremely low occurrences of those responses subsumed under the awake active-alert state that allow for the behavioral diversity, fluency, and richness important to adaptive performance in complex environments. (PsycINFO Database Record (c) 2012 APA, all rights reserved)

SN - 0274-9483
UR - http://search.ebscohost.com/login.aspx?direct=true&db=psyh&AN=1994-05612-001&site=ehost-live
ER -

TY - JOUR
ID - 1821
T1 - Comunicazione verbale e non verbale nell'interazione del bambino con i coetanei e con gli adulti
A1 - Lavelli,Manuela
Y1 - 1993/04//
KW - PsycInfo
KW - Unsupervised Play Addition
KW - age
KW - verbal vs nonverbal communication with peers vs adults
KW - 18-30 mo olds
KW - Italy
KW - Age Differences
KW - Nonverbal Communication
KW - Social Interaction
KW - Verbal Communication
RP - NOT IN FILE
SP - 281
EP - 297
JF - Giornale Italiano di Psicologia
VL - 20
IS - 2
CY - Italy
PB - Societ… editrice il Mulino
N2 - Studied and compared the use of verbal and nonverbal means of communication by 18 young children in communicating with their peers and with adults, and assessed changes in patterns of communication from the ages of 18 mo to 30 mo. Ss, who were equally divided into age groups of 18 mo, 21 mo, 24 mo, 27 mo, and 30 mo, were observed and videotaped in play groups of 3 each with and without the presence of an adult. Ss' communication behaviors were scored for instances of verbal and nonverbal communication. (English abstract) (PsycINFO Database Record (c) 2012 APA, all rights reserved)
SN - 0390-5349
UR - http://search.ebscohost.com/login.aspx?direct=true&db=psyh&AN=1994-85096-001&site=ehost-live
ER -

TY - CHAP
ID - 1822
T1 - Simple phobia in adults
A1 - Stanley,Melinda A.
A1 - Beidel,Deborah C.
Y1 - 1993///
N1 - Accession Number: 1993-98371-009. First Author & Affiliation: Stanley, Melinda A.; U Texas, Medical School, Dept of Psychiatry & Behavioral Sciences, Assistant Professor, Houston, TX, US. Translated Book Series Title: General psychology series, Vol. 171. Release Date: 19940201. Publication Type: Book, (0200); Edited Book, (0280); . Media Covered: Print. Document Type: Chapter. Book Type: Handbook/Manual. ISBN: 0-205-14583-3, Hardcover, 0205145833. Language: English. Major Descriptor: Behavior Therapy; Behavioral Assessment; Phobias. Classification: Neuroses & Anxiety Disorders (3215) Behavior Therapy & Behavior Modification (3312) . Population: Human (10); . Intended Audience: Psychology: Professional & Research (PS) . Page Count: 15
KW - PsycInfo
KW - Unsupervised Play Addition
KW - presents information on assessment & treatment of simple phobias in adulthood
KW - Behavior Therapy
KW - Behavioral Assessment

KW - Phobias
RP - NOT IN FILE
SP - 137
EP - 151
T2 - Handbook of behavior therapy with children and adults: A developmental and longitudinal perspective
A2 - Ammerman,Robert T.
A2 - Hersen,Michel
CY - Needham Heights, MA US
PB - Allyn & Bacon
T3 - General psychology series, Vol. 171
N2 - (from the chapter) although simple phobias may be common in the general population, they are rarely seen in clinical settings / it is likely that many of those in the community who meet diagnostic criteria have successfully arranged their lives so that their phobic symptoms do not interfere with daily functioning / if these fears are present in adulthood, there appears to be little likelihood of their spontaneous remission / those patients who do present to clinics often have additional clinical symptomatology which may play an important role in treatment response / [presents information on] the literature on the assessment and treatment of simple phobias (chapter)
behavioral assessment strategies / behavior therapy approaches / pharmacological treatments (PsycINFO Database Record (c) 2012 APA, all rights reserved) (chapter)
SN - 0-205-14583-3
UR - http://search.ebscohost.com/login.aspx?direct=true&db=psyh&AN=1993-98371-009&site=ehost-live
ER -

TY - JOUR
ID - 1823
T1 - Neuropsychological deficits in sexual offenders: Implications for treatment
A1 - Lang,Reuben A.
Y1 - 1993///
KW - PsycInfo
KW - Unsupervised Play Addition
KW - brain pathology
KW - sex offenders
KW - treatment implications
KW - Neuropathology
KW - Sex Offenses
RP - NOT IN FILE
SP - 181
EP - 200
JF - Sexual & Marital Therapy
VL - 8
IS - 2
CY - United Kingdom
PB - Taylor & Francis
N2 - Provides a brief overview of the growing body of evidence that a proportion of adult sexual offenders present with some localized form of brain pathology, often left temporal lobe, that may, in part, explain the presence of gender dysphoria and the attraction to deviant behavior patterns in sexually anomalous men who prefer child surrogate partners or unsuspecting women. The techniques employed rely on social skill training, psychodramatic role plays (using a doll or mannequin), stress reduction and anger management, empathy training, guided visualization, neurolinguistic programming, cognitive restructuring, sketches of covert fantasies or deviant actions for group discussion, and victim-perpetrator role reversal, selectively applied within the context of a relapse prevention model for adult sexual offenders. (PsycINFO Database Record (c) 2012 APA, all rights reserved)
SN - 0267-4653
UR - http://search.ebscohost.com/login.aspx?direct=true&db=psyh&AN=1994-02046-001&site=ehost-live
L3 - 10.1080/02674659308408193
ER -

TY - CHAP
ID - 1824
T1 - Play: Its role in child analysis, its fate in adult analysis
A1 - Mahon,Eugene J.
Y1 - 1993///
N1 - Accession Number: 1993-98417-010. First Author & Affiliation: Mahon, Eugene J.; Columbia U, Coll of Physicians & Surgeons, Assistant Clinical Professor of Psychiatry, New York, NY, US. Release Date: 19940301. Publication Type: Book, (0200); Edited Book, (0280); . Media Covered: Print. Document Type: Chapter. ISBN: 0-300-05438-6, Hardcover, 0300054386. Language: English. Major Descriptor: Childhood Play Behavior; Psychoanalysis; Psychotherapeutic Processes. Classification: Psychoanalytic Therapy (3315) . Population: Human (10); . Age Group: Childhood (birth-12 yrs) (100) Preschool Age (2-5 yrs) (160) Adulthood (18 yrs & older) (300) . Intended Audience: Psychology: Professional & Research (PS) . Methodology: Empirical Study. Page Count: 11
KW - PsycInfo
KW - Unsupervised Play Addition
KW - role of play in child & adult psychoanalysis
KW - 5 & 23 yr old males
KW - Childhood Play Behavior
KW - Psychoanalysis
KW - Psychotherapeutic Processes
RP - NOT IN FILE
SP - 172
EP - 182
T2 - The many meanings of play: A psychoanalytic perspective
A2 - Solnit,Albert J.
A2 - Cohen,Donald J.
A2 - Neubauer,Peter B.
CY - New Haven, CT US
PB - Yale University Press
N2 - Discusses the role of play in child and adult psychoanalysis and presents examples of its use in the analyses of a five-yr-old boy and a twenty-three-yr-old man. (PsycINFO Database Record (c) 2012 APA, all rights reserved)
SN - 0-300-05438-6
UR - http://search.ebscohost.com/login.aspx?direct=true&db=psyh&AN=1993-98417-010&site=ehost-live
ER -

TY - JOUR
ID - 1825
T1 - Consultation with adult children of aging parents
A1 - Shulman,Bernard H.
A1 - Sperry,Len
Y1 - 1992/12//
KW - PsycInfo
KW - Unsupervised Play Addition
KW - Adlerian perspective on consultation issues
KW - adult children of aging parents
KW - Adlerian Psychotherapy
KW - Adult Offspring
KW - Aging
KW - Individual Psychotherapy
KW - Professional Consultation
RP - NOT IN FILE
SP - 427
EP - 431
JF - Individual Psychology: Journal of Adlerian Theory, Research & Practice

VL - 48
IS - 4
CY - US
PB - North American Society of Adlerian Psychology
N2 - Reviews the concerns adult children (AC) present concerning their aging parents. Adequate nutrition, decent housing, economic stability, and access to appropriate medical care are essential concerns for both the elderly and their caregivers. Of considerable concern to caregivers are many psychosocial issues. Aging parent-adult child relationships tend to follow 1 of 4 patterns depending on the status the aging parents retain and the role the AC play. These 4 patterns are status-equality, status-quo, status-conflict, and status-reversal. A consultant can be most useful in building better relationships between parents and AC by helping them see beyond the roles they have previously played so that they can view each other as fellow human beings. (PsycINFO Database Record (c) 2012 APA, all rights reserved)
SN - 0277-7010
UR - http://search.ebscohost.com/login.aspx?direct=true&db=psyh&AN=1993-38418-001&site=ehost-live
ER -

TY - JOUR
ID - 1826
T1 - Preterm infants' affective responses in independent versus toy-centered play with their mothers
A1 - Garner,Pamela W.
A1 - Landry,Susan H.
Y1 - 1992///
KW - PsycInfo
KW - Unsupervised Play Addition
KW - independent vs toy centered play with mother
KW - affective responses
KW - preterm medically high vs low risk 6 mo olds
KW - Childhood Play Behavior
KW - Emotional Responses
KW - Premature Birth
KW - Toys
KW - At Risk Populations
KW - Mother Child Relations
RP - NOT IN FILE
SP - 219
EP - 230
JF - Infant Mental Health Journal
VL - 13
IS - 3
CY - US
PB - John Wiley & Sons
N2 - The affective behavior of 33 medically high-risk (HR) infants, 40 low-risk (LR) infants, and a control group of 44 full-term (FT) infants was compared at age 6 mo during a 5-min independent and a 5-min toy-centered play session with their mothers. It was predicted that HR infants would show less positive affect and more negative affect in toy-centered (vs independent) play and joint attention (vs face-to-face) interactions than would the LR or FT infants. HR infants displayed fewer smiles across the independent and mother toy-centered play condition and across face-to-face and joint attention interactions than LR or FT infants. The mother's presence facilitated the expression of positive affect for all 3 infant groups. (French, Spanish & Japanese abstracts) (PsycINFO Database Record (c) 2012 APA, all rights reserved)
SN - 1097-0355
UR - http://search.ebscohost.com/login.aspx?direct=true&db=psyh&AN=1993-14169-001&site=ehost-live
L3 - 10.1002/1097-0355(199223)13:3<219::AID-IMHJ2280130308>3.0.CO;2-9
ER -

TY - JOUR
ID - 1828

T1 - Situational variation in problem behavior at home and school in attention deficit disorder with hyperactivity: A factor analytic study
A1 - Altepeter,Thomas S.
A1 - Breen,Michael J.
Y1 - 1992/05//
KW - PsycInfo
KW - Unsupervised Play Addition
KW - factor analysis of Home vs School Situations Questionnaires
KW - assessment of variability in problem behaviors
KW - 5-12 yr olds with attention deficit disorder with hyperactivity
KW - Attention Deficit Disorder
KW - Factor Structure
KW - Hyperkinesis
KW - Questionnaires
KW - Social Environments
KW - Behavior Problems
KW - Home Environment
KW - School Environment
RP - NOT IN FILE
SP - 741
EP - 748
JF - Journal of Child Psychology and Psychiatry
VL - 33
IS - 4
CY - United Kingdom
PB - Blackwell Publishing
N2 - Assesses situational variation in problem behaviors of children with attention deficit disorder with hyperactivity (ADD/H). Data were collected on 163 clinic-referred children (aged 5-12 yrs). Principal-component factors were computed for the Home/School Situations Questionnaires (HSQ/SSQ). For the HSQ, 4 factors emerged (nonfamily transactions, custodial transactions, task performance transactions, and isolate play). For the SSQ, 3 factors emerged (unsupervised settings, task performance, and special events). Factors were similar to those previously derived with nonreferred children. To assess discriminative power, factors were compared (t-tests) to previous data. All 7 factors significantly discriminated ADD/H from nonreferred children. These data suggest that the factors add to the utility of the HSQ/SSQ in assessing ADD/H. (PsycINFO Database Record (c) 2012 APA, all rights reserved)
SN - 1469-7610
UR - http://search.ebscohost.com/login.aspx?direct=true&db=psyh&AN=1992-33605-001&site=ehost-live
L3 - 10.1111/j.1469-7610.1992.tb00909.x
ER -

TY - JOUR
ID - 1829
T1 - Maternal behavior and infant attention
A1 - Lawson,Katharine R.
A1 - Parrinello,Roseanne
A1 - Ruff,Holly A.
Y1 - 1992/04//
KW - PsycInfo
KW - Unsupervised Play Addition
KW - Maternal Behavior
KW - infant's attention to objects during joint play
KW - full term vs preterm 12 mo olds & their mothers
KW - Attention
KW - Childhood Play Behavior
KW - Mother Child Relations
KW - Mothers

KW - Premature Birth
RP - NOT IN FILE
SP - 209
EP - 229
JF - Infant Behavior & Development
JA - Infant behav
VL - 15
IS - 2
CY - Netherlands
PB - Elsevier Science
N2 - Investigated associations between maternal behavior and 12-mo-old infants' attention to objects during a period of joint play. 90 full-term and 59 preterm Ss were presented with a set of toys for independent play; their mothers then joined them for play with the same toys. Focused exploration of the objects was higher and active inattention was lower during the interaction than during independent play. Children were divided into high-, average-, and low-attending groups on the basis of their independent focused attention and active inattention; interaction with the mother resulted in the greatest increases in focused attention and greatest decreases in active inattention for the low attenders. Analyses showed that focused attention and active inattention during the interaction were related to both the child's spontaneous tendency to be attentive and specific maternal behaviors and characteristics. (PsycINFO Database Record (c) 2012 APA, all rights reserved)
SN - 0163-6383
UR - http://search.ebscohost.com/login.aspx?direct=true&db=psyh&AN=1993-01092-001&site=ehost-live
L3 - 10.1016/0163-6383(92)80024-O
ER -

TY - JOUR
ID - 1830
T1 - Social pretend play among friends and familiar preschoolers
A1 - Werebe,Maria G.
A1 - BaudonniŠre,Pierre Marie
Y1 - 1991/12//
KW - PsycInfo
KW - Unsupervised Play Addition
KW - partner familiarity
KW - organization & duration & content of spontaneous social pretend play
KW - 3-5 yr olds in triads of 2 friends & 1 familiar partner
KW - France
KW - Childhood Play Behavior
KW - Familiarity
KW - Peer Relations
KW - Pretend Play
KW - Friendship
RP - NOT IN FILE
SP - 411
EP - 428
JF - International Journal of Behavioral Development
VL - 14
IS - 4
CY - US
PB - Lawrence Erlbaum
N2 - Assessed, among 60 boys and 60 girls (aged 3-5 yrs), the role of partner familiarity in the organization, duration, and content of spontaneous social pretend play in a triadic situation. Each triad of classmates was made up of a dyad of friends, plus familiar partner. Findings show that friends preferred each other as a partner in fantasy play, and play between friends was longer and richer than play with the 3rd partner. Girls spent nearly twice as much time in fantasy as boys. The use of 2 sets of identical objects for 3 children without adult presence, constituted a powerful paradigm to evidence the effect of the degree of familiarity in children's

interaction in general, and in pretend play in particular. (PsycINFO Database Record (c) 2012 APA, all rights reserved)
SN - 1464-0651
UR - http://search.ebscohost.com/login.aspx?direct=true&db=psyh&AN=1992-19531-001&site=ehost-live
ER -

TY - JOUR
ID - 1831
T1 - Effects of maternal presence on sibling behavior
A1 - Musun-Miller,Linda
Y1 - 1991/04//
KW - PsycInfo
KW - Unsupervised Play Addition
KW - maternal presence vs absence
KW - interactions & other social behavior
KW - 4-9 yr old sibling pairs
KW - Interpersonal Interaction
KW - Mother Child Relations
KW - Sibling Relations
KW - Social Behavior
RP - NOT IN FILE
SP - 145
EP - 157
JF - Journal of Applied Developmental Psychology
VL - 12
IS - 2
CY - Netherlands
PB - Elsevier Science
N2 - Analyzed videotapes of 20 sibling pairs (aged 4-9 yrs) interacting in a laboratory playroom for 15 min with their mother present and 15 min with her absent. There were significant differences in the behavior emitted by siblings depending on maternal presence/absence. When mothers were present, siblings engaged in more attending, helping, and interacting. When mothers were absent, siblings engaged in more disapproval, independent play, ignoring, negative physical contact, talking, and teasing. Maternal presence also interacted significantly with sex of each individual child and the sex of the sibling pair. Maternal presence may play a facilitative role in promoting prosocial behavior, whereas her absence is associated with a wider variety of behavior and a greater incidence of antagonistic behavior by siblings. (PsycINFO Database Record (c) 2012 APA, all rights reserved)
SN - 0193-3973
UR - http://search.ebscohost.com/login.aspx?direct=true&db=psyh&AN=1992-01098-001&site=ehost-live
L3 - 10.1016/0193-3973(91)90008-R
ER -

TY - JOUR
ID - 1832
T1 - Urban children's access to their neighborhood: Changes over three generations
A1 - Gaster,Sanford
Y1 - 1991/01//
KW - PsycInfo
KW - Unsupervised Play Addition
KW - Demographic Characteristics
KW - children's access & use of urban public neighborhood space
KW - Adults
KW - 1915-76
KW - Childhood Play Behavior
KW - Neighborhoods
KW - Urban Environments

RP - NOT IN FILE
SP - 70
EP - 85
JF - Environment and Behavior
VL - 23
IS - 1
CY - US
PB - Sage Publications
N2 - Investigated a New York City neighborhood to determine changes in its children's use of local public space between 1915 and 1976. 29 adults were interviewed, and such archival sources as US census reports were consulted for demographic changes. Substantial changes were detected in (1) the age at which children were first allowed outdoors without supervision, (2) the number and quality of settings visited, (3) the number and nature of environmental obstacles, (4) the number and nature of parent-imposed restrictions, and (5) the number of professionally supervised activities undertaken. Both the degree to which the neighborhood environment supported children's play and children's access to their neighborhood have declined substantially since the 1940s. (PsycINFO Database Record (c) 2012 APA, all rights reserved)
SN - 0013-9165
UR - http://search.ebscohost.com/login.aspx?direct=true&db=psyh&AN=1991-14071-001&site=ehost-live
L3 - 10.1177/0013916591231004
ER -

TY - JOUR
ID - 1834
T1 - Lies and truth: A study of the development of the concept
A1 - Strichartz,Abigail F.
A1 - Burton,Roger V.
Y1 - 1990/02//
KW - PsycInfo
KW - Unsupervised Play Addition
KW - development of "lie" & "truth" concepts
KW - nursery school vs preschool students vs 1st vs 5th graders vs adults
KW - test of prototype usage
KW - Age Differences
KW - Childhood Development
KW - Concept Formation
KW - Truth
KW - Deception
KW - Honesty
RP - NOT IN FILE
SP - 211
EP - 220
JF - Child Development
JA - Child Dev.
VL - 61
IS - 1
CY - United Kingdom
PB - Blackwell Publishing
N2 - Examined the developmental questions of when children begin to use the terms lie and truth, how they understand them, and when their understanding approaches that of adults. 150 Ss in 5 groups (nursery schoolers, preschoolers, 1st graders, 5th graders, and adults) were presented a series of 8 short puppet plays that varied the presence or absence of the 3 prototype elements: factuality of a statement, the speaker's belief in the factuality or falsity of the statement, and the speaker's intent to deceive the listener. Responses of 5th graders were transitional between those of the younger children and adults. These results replicate L. Coleman and P. Kay's (1981) finding that adults decide whether a statement is a lie by comparing it against a prototype defined by the elements of factuality, intent, and belief, and that they consider the speaker's belief system the most important element of the prototype. (PsycINFO Database Record (c) 2012 APA, all rights reserved)

SN - 1467-8624
UR - http://search.ebscohost.com/login.aspx?direct=true&db=psyh&AN=1990-14417-001&site=ehost-live
L3 - 10.2307/1131060
ER -

TY - CHAP
ID - 1835
T1 - Developmental and psycholinguistic considerations
A1 - Wishart,J.G.
A1 - Tomporowski,P.D.
A1 - Lewis,A.
A1 - Carpenter,B.
A1 - Binzley,A.
A1 - Polomsky,P.
A1 - Shah,P.
A1 - Walker,D.M.
A1 - Lloyd,L.L.
Y1 - 1990///
N1 - Accession Number: 1990-97940-005. Partial author list. First Author & Affiliation: Wishart, J. G.; U Edinburgh, Dept of Psychology, Edinburgh Ctr for Research in Child Development, Edinburgh, Scotland. Release Date: 19900101. Publication Type: Book, (0200); Edited Book, (0280); . Media Covered: Print. Document Type: Chapter. ISBN: 0-415-01363-1, Hardcover, 0415013631. Language: English. Major Descriptor: Human Development; Psycholinguistics; Intellectual Development Disorder. Minor Descriptor: Attention; Communication; Imagery; Learning. Classification: Mental Retardation (3256) . Population: Human (10); . Intended Audience: Psychology: Professional & Research (PS) . Page Count: 58
KW - PsycInfo
KW - Unsupervised Play Addition
KW - Mental Retardation
KW - Human Development
KW - Psycholinguistics
KW - Intellectual Development Disorder
KW - Attention
KW - Communication
KW - Imagery
KW - Learning
RP - NOT IN FILE
SP - 249
EP - 306
T2 - Key issues in mental retardation research: Proceedings of the Eighth Congress of the International Association for the Scientific Study of Mental Deficiency (IASSMD)
A2 - Fraser,William Irvine
CY - Florence, KY US
PB - Taylor & Frances/Routledge
N2 - (from the book) [book section covering several chapters] (book)
"Learning to Learn: The Difficulties Faced by Infants and Young Children with Down's Syndrome" / J. G. Wishart / reports results from a series of interlinked, longitudinal studies of early learning in Down's Syndrome (DS) / the first set of studies monitored the development of a concept of objects / the second used operant learning techniques to examine learning styles used at different stages in development (book)
"Sustained Attention in Mentally Retarded Persons" / P. D. Tomporowski / evaluate research studies which have been conducted to assess the sustained attention of retarded individuals / describe findings obtained in our laboratory and discuss the results of these studies in context of current theories of sustained attention (book)
"Discourse, in an Integrated School Setting, Between Six- and Seven-Year-Old Non-Handicapped Children and Peers with Severe Learning Difficulties / A. Lewis and B. Carpenter / examine some aspects of discourse between non-handicapped (NH) six and seven year olds, and peers with severe learning difficulties (SLD) / hypothesized that talk by the NH children to SLD partners would contain characteristics found in talk by NH children to younger NH children (book)

"Self-Talk in the Institutionalized Retarded: Naturalistic Observations" / V. A. Binzley, P. Polomsky and P. Shah / given the developmental delay and poor self-regulation noted in some retarded adults, the present study was undertaken to determine if the developmental phenomenon of self-talk occurs in this population (book)
"Theory into Practice: Inculcating Initial Notions about Language Intervention with Children with Mental Handicap" / D. M. Walker / describes the establishment and initial results of a programme to enhance the communication skills of mentally handicapped non-verbal children using a combination of group and individual treatments (book)
"The Role of Iconicity in Augmentative and Alternative Communication-Symbol Learning" / L. L. Lloyd and D. R. Fuller / discussed the role iconicity plays in the initial acquisition of augmentative and alternative communication (AAC) symbols (PsycINFO Database Record (c) 2012 APA, all rights reserved) (book)
SN - 0-415-01363-1
UR - http://search.ebscohost.com/login.aspx?direct=true&db=psyh&AN=1990-97940-005&site=ehost-live
ER -

TY - JOUR
ID - 1836
T1 - Vyvoj hry u d?t ¡p?edsskoln ¡m v?ku
A1 - Antelmanov ,D sa
A1 - Severov ,Marie
Y1 - 1990///
KW - PsycInfo
KW - Unsupervised Play Addition
KW - age
KW - developmental trends in cognitive vs practical vs symbolic free play activities
KW - 2-6 yr olds
KW - 9 mo study
KW - Childhood Play Behavior
KW - Early Childhood Development
KW - Longitudinal Studies
RP - NOT IN FILE
SP - 99
EP - 106
JF - Psychol ¢gia a Patopsychol ¢gia Die?a?a
VL - 25
IS - 2
CY - Slovak Republic
PB - Vyskumny Ustav Detskij Psychologie a Patopsychologie
N2 - Studied the free play of 32 children divided at the beginning of the study in 4 age groups of 2, 3, 4, and 5 yrs. Each group of 4 boys and 4 girls was observed for 30 min periods for 9 mo, at regular 4-mo intervals. The developmental trend of free play activities was measured for the duration of the activities and classified qualitatively into 3 categories: (1) cognitive, (2) practical (manipulations, movements, social communications), and (3) symbolic. The children were involved in all 3 play categories independently or within mutual social activities. Results show that with age children's ability to stay involved in one play became stronger, the number of practical manipulative plays was lowered, symbolic plays became more frequent, the number of independent plays diminished, and the number of social plays rose. (Russian & English abstracts) (PsycINFO Database Record (c) 2012 APA, all rights reserved)
SN - 0555-5574
UR - http://search.ebscohost.com/login.aspx?direct=true&db=psyh&AN=1993-87128-001&site=ehost-live
ER -

TY - BOOK
ID - 1837
T1 - Incest-related syndromes of adult psychopathology
A1 - Kluft,Richard P.
Y1 - 1990///

N1 - Accession Number: 1990-98769-000. First Author & Affiliation: Kluft, Richard P.; Temple U, School of Medicine, Clinical Professor of Psychiatry, Philadelphia, PA, US. Release Date: 19900101. Publication Type: Book, (0200); Edited Book, (0280); . Media Covered: Print. Book Type: Conference Proceedings. ISBN: 0-88048-160-9, Hardcover, 0880481609. Language: English. Conference Note: This book is based on a symposium held at the 140th Annual Meeting of the American Psychiatric Association in Chicago, Illinois, May 1987. Major Descriptor: Early Experience; Incest; Psychopathology. Minor Descriptor: Child Abuse; Sexual Abuse; Victimization. Classification: Behavior Disorders & Antisocial Behavior (3230) . Population: Human (10); . Age Group: Adulthood (18 yrs & older) (300) . Intended Audience: Psychology: Professional & Research (PS)
KW - PsycInfo
KW - Unsupervised Play Addition
KW - discusses the clinical psychopathology seen in adult patients who suffered childhood incestuous exploitation
KW - Early Experience
KW - Incest
KW - Psychopathology
KW - Child Abuse
KW - Sexual Abuse
KW - Victimization
RP - NOT IN FILE
A2 - Kluft,Richard P.
CY - Washington, DC US
PB - American Psychiatric Association
N2 - (from the introduction) "Incest-Related Syndromes of Adult Psychopathology" attempts to consolidate findings that bear on the clinical presentations of adult patients who have suffered childhood incestuous exploitation. Although there are many accounts of incest now available from the lay literature (Armstrong 1983; Bass and Davis 1988; Bass and Thornton 1983), there is much less about how incest presents in such a manner that it and its impact can be minimized, discounted, or denied or appear to play such a peripheral role in a clinical situation that its importance remains unrecognized. Because the study of the incestuous experiences of boys and their long-term consequences is a rather new and undeveloped field of inquiry in comparison to the exploration of the incestuous experiences of girls and their sequelae, "Incest-Related Syndromes of Adult Psychopathology" focuses almost exclusively on the plight of the female victim of incest. (introduction)
(from the jacket) This book explores the connections between incest and somatoform disorders, disturbances of the self, problems in cognitive functioning, borderline psychopathology, the dissociative disorders, posttraumatic symptoms, vulnerability to revictimization, and other crucial issues. (PsycINFO Database Record (c) 2012 APA, all rights reserved) (jacket)
SN - 0-88048-160-9
UR - http://search.ebscohost.com/login.aspx?direct=true&db=psyh&AN=1990-98769-000&site=ehost-live
ER -

TY - JOUR
ID - 1838
T1 - Finding the Music in Psychoanalytic Study
A1 - Davis,Harold B.
Y1 - 1989/10//
KW - PsycInfo
KW - Unsupervised Play Addition
KW - ego psychology
KW - Psychoanalysis
KW - CHILDREN
KW - Play
KW - Childhood Development
KW - Childhood Play Development
KW - Ego
RP - NOT IN FILE
SP - 937

EP - 938
JF - PsycCRITIQUES
VL - 34
IS - 10
CY - US
PB - American Psychological Association
N2 - Originally published in Contemporary Psychology: APA Review of Books, 1989, Vol 34(10), 937-938. Reviews the book, "The Psychoanalytic Study of the Child, Vol. 42" by Albert J. Solnit and Peter B. Neubauer (Eds.) (1987). The current volume devotes its first section to Psychoanalytic Views of Play. The other sections are Clinical Papers, Freud and Theory Building, Applied Psychoanalysis, and a single entry under the title Psychoanalytic Education. Although the articles are primarily concerned with children, as befitting the title of the book, their relevance to adults is ever present. This volume is a collection of essays (i.e., personal attempts to explain observable phenomena). As such, it will serve well those committed to the concepts of classical psychoanalysis and ego psychology. It will also be helpful to all psychoanalytically oriented people, regardless of specific orientation, who can gain from the insights and inferences of experienced clinicians. (PsycINFO Database Record (c) 2012 APA, all rights reserved)
SN - 1554-0138
UR - http://search.ebscohost.com/login.aspx?direct=true&db=psyh&AN=2006-06491-039&site=ehost-live
L3 - 10.1037/030672
ER -

TY - JOUR
ID - 1839
T1 - Variables associated with frequency of rumination in a boy with profound mental retardation
A1 - Humphrey,Frederick J.
A1 - Mayes,Susan D.
A1 - Bixler,Edward O.
A1 - Good,Carl
Y1 - 1989/09//
KW - PsycInfo
KW - Unsupervised Play Addition
KW - environmental & interpersonal & temporal variables & frequency of rumination
KW - profoundly mentally retarded 12.8 yr old male
KW - Case Report
KW - Environment
KW - Ingestion
KW - Interpersonal Interaction
KW - Intellectual Development Disorder
KW - Activity Level
RP - NOT IN FILE
SP - 435
EP - 447
JF - Journal of Autism and Developmental Disorders
VL - 19
IS - 3
CY - Germany
PB - Springer
N2 - Investigated the relationships between frequency of rumination in a boy (chronological age 12 yrs 9 mo; mental age 11.5 mo) with profound mental retardation and a variety of environmental, interpersonal, and temporal variables during all waking hours over a 4-wk period. Low levels of rumination were associated with periods of special education programming (vs nonschool hours), individual attention (vs group activities and independent play), and time spent with caretakers who like the child (vs those who like him less). There was a mealtime effect (decreasing rumination as time elapsed following meals) and a time of day effect (increasing rumination as the day progressed). (PsycINFO Database Record (c) 2012 APA, all rights reserved)
SN - 1573-3432
UR - http://search.ebscohost.com/login.aspx?direct=true&db=psyh&AN=1990-01749-001&site=ehost-live

L3 - 10.1007/BF02212941
ER -

TY - JOUR
ID - 1840
T1 - Pretend play with mothers and siblings: Relations between early performance and partners
A1 - Dale,Naomi
Y1 - 1989/09//
KW - PsycInfo
KW - Unsupervised Play Addition
KW - cooperative pretend play
KW - 2 yr olds playing with mother vs 4-5 yr old sibling
KW - Childhood Play Behavior
KW - Mother Child Relations
KW - Pretend Play
KW - Sibling Relations
RP - NOT IN FILE
SP - 751
EP - 759
JF - Journal of Child Psychology and Psychiatry
VL - 30
IS - 5
CY - United Kingdom
PB - Blackwell Publishing
N2 - Investigated whether 9 2-yr-old infants' cooperative pretend play varies when playing with their mother and 4-5 yr old sibling. Results suggest marked differences in frequency of production of various transformational types in each social setting, and one type of play only appears in games with the sibling. Possible facilitation by the play partner is discussed. In the difference between the children's social and independent play, mothers seemed either to have no effect on their child's play or enhance what has generally been considered a fairly low level of pretend play. (PsycINFO Database Record (c) 2012 APA, all rights reserved)
SN - 1469-7610
UR - http://search.ebscohost.com/login.aspx?direct=true&db=psyh&AN=1990-04222-001&site=ehost-live
L3 - 10.1111/j.1469-7610.1989.tb00787.x
ER -

TY - JOUR
ID - 1841
T1 - Music of the self and others: Longitudinal observations on musical giftedness
A1 - Pruett,Kyle D.
Y1 - 1989///
KW - PsycInfo
KW - Unsupervised Play Addition
KW - development of musical giftedness
KW - 22 yr old females observed since 8 & 9 yrs
KW - Gifted
KW - Musical Ability
KW - Longitudinal Studies
RP - NOT IN FILE
SP - 87
EP - 100
JF - The Psychoanalytic Study of the Child
VL - 44
CY - US
PB - Yale University Press

N2 - Presents findings from an ongoing observational study of musical giftedness as it was being experienced by young music-makers at various developmental stages, focusing on the transition from adolescence to adulthood. Examples are presented of 2 girls' (observed from the ages of 8 and 9 to 22 yrs) experience of the simultaneous exhibition, enjoyment, and expansion of the musical gift while dealing with the unique problem of their body serving as the medium to express that gift. Topics include notions of the self, the role of significant others, "play" in the playing of music, creativity as generativity, and the role of normal narcissism in the life of the young musician. (PsycINFO Database Record (c) 2012 APA, all rights reserved)
SN - 0079-7308
UR - http://search.ebscohost.com/login.aspx?direct=true&db=psyh&AN=1990-09418-001&site=ehost-live
ER -

TY - JOUR
ID - 1843
T1 - The theraplay approach to the self-contained patient
A1 - Jernberg,Ann M.
Y1 - 1988///
KW - PsycInfo
KW - Unsupervised Play Addition
KW - theraplay treatment approach
KW - intimacy avoidance due to early disruption in attachment & bonding experience
KW - self contained 14 yr old male & adult female
KW - Client Characteristics
KW - Intimacy
KW - Psychotherapeutic Techniques
KW - Attachment Behavior
KW - Early Experience
KW - Play Therapy
KW - Social Isolation
RP - NOT IN FILE
SP - 85
EP - 93
JF - Psychotherapy Patient
VL - 4
IS - 3-4
CY - US
PB - Haworth Press
N2 - Discusses intimacy-avoidance as a result of a disruption in the process of parent-child attachment due to adoption, temperamental mismatch, parental exploitation, or other forms of parental unavailability. Therapists may assist patients in overcoming their self-containing life resolve by determining their patients' early attachment history and by relating to them as parent to small child on the level they presumably stopped their emotional growth. Cases of 2 self-contained patients (a 14-yr-old boy and an adult female) are presented. Theraplay, a treatment modality based on normal parent-infant interaction, is described as it relates to the 2 cases. (PsycINFO Database Record (c) 2012 APA, all rights reserved)
SN - 0738-6176
UR - http://search.ebscohost.com/login.aspx?direct=true&db=psyh&AN=1989-33805-001&site=ehost-live
L3 - 10.1300/J358v04n03_09
ER -

TY - JOUR
ID - 1844
T1 - The holding/letting go dialectic as a factor in the recovery of learning
A1 - Greenhalgh,Paul
Y1 - 1987///
KW - PsycInfo
KW - Unsupervised Play Addition
KW - holding & letting go of disturbing feelings & behavior

KW - recovery of learning
KW - junior high school student
KW - Behavior Problems
KW - Emotional Disturbances
KW - School Counseling
KW - School Learning
KW - Junior High School Students
RP - NOT IN FILE
SP - 37
EP - 43
JF - Maladjustment & Therapeutic Education
VL - 5
IS - 3
CY - United Kingdom
PB - Taylor & Francis
N2 - Explores the holding and letting go of disturbing feelings and behavior that interfere with learning. It is suggested that children need to experience holding of these feelings and that they need to let go of them in order to explore their relations with the unconscious. These processes are illustrated through the case of an English boy in the 2nd year of junior school. Disturbing feelings and behavior are held through the processes of providing a mirror, a container, and a safety net. The process of letting the child go into necessary imagination and play is made possible by providing a relaxed, reliable setting where the child can forget the presence of adults and still internalize their availability. The qualities of adult presence that make this possible are described. (PsycINFO Database Record (c) 2012 APA, all rights reserved)
SN - 0264-4614
UR - http://search.ebscohost.com/login.aspx?direct=true&db=psyh&AN=1989-06818-001&site=ehost-live
ER -

TY - JOUR
ID - 1845
T1 - Generalized reduction of disruptive behavior in unsupervised settings through specific toy training
A1 - Santarcangelo,Suzanne
A1 - Dyer,Kathleen
A1 - Luce,Stephen C.
Y1 - 1987///
KW - PsycInfo
KW - Unsupervised Play Addition
KW - specific toy training
KW - disruptive behavior & generalization & maintenance of appropriate play
KW - autistic 5-13 yr olds
KW - Autism
KW - Behavior Modification
KW - Behavior Problems
KW - Childhood Play Behavior
KW - Generalization (Learning)
KW - Differential Reinforcement
KW - Toys
RP - NOT IN FILE
SP - 38
EP - 44
JF - Journal of the Association for Persons with Severe Handicaps
VL - 12
IS - 1
CY - US
PB - Assn for Persons with Severe Handicaps
N2 - In Exp I, 2 autistic students (aged 5 and 9 yrs) were exposed to training consisting of differential reinforcement of appropriate play, verbal feedback and prompts for inappropriate play or disruptive behaviors,

and a specific toy training condition that involved extrinsic reinforcement of specific components of toy play. This training resulted in decreases in disruptive behaviors accompanied by generalization and maintenance of appropriate play in an unsupervised setting. In Exp II, 2 additional autistic Ss (aged 6 and 13 yrs) exposed to the specific toy training procedure showed similar decreases in disruptive behaviors accompanied by generalization and maintenance of play skills. (PsycINFO Database Record (c) 2012 APA, all rights reserved)
SN - 0274-9483
UR - http://search.ebscohost.com/login.aspx?direct=true&db=psyh&AN=1988-20955-001&site=ehost-live
ER -

TY - JOUR
ID - 1846
T1 - Outpatient treatment of pica in a developmentally delayed child
A1 - Kalfus,Grace R.
A1 - Fisher-Gross,Susan
A1 - Marvullo,Mary A.
A1 - Nau,Paul A.
Y1 - 1987///
KW - PsycInfo
KW - Unsupervised Play Addition
KW - overcorrection & differential reinforcement
KW - pica
KW - nonverbal developmentally delayed 4 yr old
KW - Delayed Development
KW - Differential Reinforcement
KW - Overcorrection
RP - NOT IN FILE
SP - 49
EP - 63
JF - Child & Family Behavior Therapy
VL - 9
IS - 3-4
CY - US
PB - Haworth Press
N2 - Evaluated the effectiveness of overcorrection and differential reinforcement to reduce pica in a 4-yr-old nonverbal developmentally delayed boy. Parents and classroom staff were trained as mediators of treatment by a behavioral consultant. Despite the effectiveness of the procedure, treatment mediators terminated intervention due to its aversive nature. Therefore, an alternative program consisting of positive reinforcement and time-out was implemented according to a reversal design. Pica was measured via direct observation and parental monitoring during a variety of activities, including fine motor tasks, gross motor play, and independent play. Results indicate clear reductions in pica as a result of both interventions. (PsycINFO Database Record (c) 2012 APA, all rights reserved)
SN - 1545-228X
UR - http://search.ebscohost.com/login.aspx?direct=true&db=psyh&AN=1988-36589-001&site=ehost-live
L3 - 10.1300/J019v09n03_04
ER -

TY - JOUR
ID - 1847
T1 - Communicating with persons with severe handicaps: Roles of parents and professionals
A1 - MacDonald,James D.
A1 - Gillette,Yvonne
Y1 - 1986///
KW - PsycInfo
KW - Unsupervised Play Addition
KW - solo & social play & nonverbal communication & language & conversation as stages in communication development

KW - strategies for parents & teachers for facilitative interaction
KW - children with severe language delays
KW - Developmental Stages
KW - Interpersonal Interaction
KW - Language Delay
KW - Communication Skills
KW - Conversation
KW - Nonverbal Communication
KW - Parent Child Relations
KW - Teacher Student Interaction
KW - Verbal Communication
RP - NOT IN FILE
SP - 255
EP - 265
JF - Journal of the Association for Persons with Severe Handicaps
VL - 11
IS - 4
CY - US
PB - Assn for Persons with Severe Handicaps
N2 - Presents guidelines for adult-child interactions, based on research with a new model for teaching social interaction and communication to children with severe language delays. Five stages of communication development between adult and child are identified, and 4 major developmental areas are described in terms of the establishment of social-communication habits. For each of these areas (social play, communication, language, and conversation), specific problems and strategies for enhancing communication development are discussed. Implications of the model for service delivery, for reevaluation of the roles of parents and professionals, and for understanding the importance of social interaction in the development of language and communication are noted. A screening tool for identifying problems and strengths and preparing training goals is presented. (PsycINFO Database Record (c) 2012 APA, all rights reserved)
SN - 0274-9483
UR - http://search.ebscohost.com/login.aspx?direct=true&db=psyh&AN=1988-11624-001&site=ehost-live
ER -

TY - JOUR
ID - 1848
T1 - Unstructured play in hospital settings: An internal locus of control rationale
A1 - Bolig,Rosemary
A1 - Fernie,David E.
A1 - Klein,Elisa L.
Y1 - 1986///
KW - PsycInfo
KW - Unsupervised Play Addition
KW - Locus of Control
KW - unstructured play
KW - Hospitalized Children
KW - implications for role of adults
KW - Childhood Play Behavior
KW - Coping Behavior
KW - Hospitalized Patients
KW - Internal External Locus of Control
KW - Roles
RP - NOT IN FILE
SP - 101
EP - 107
JF - Children's Health Care
JA - Child Health Care
VL - 15

IS - 2
CY - US
PB - Lawrence Erlbaum
N2 - Presents a locus-of-control rationale for unstructured play in hospital settings and presents implications for adults' roles in young children's play that enhance their internal perception of control. (PsycINFO Database Record (c) 2012 APA, all rights reserved)
SN - 1532-6888
UR - http://search.ebscohost.com/login.aspx?direct=true&db=psyh&AN=1988-05557-001&site=ehost-live
L3 - 10.1207/s15326888chc1502_8
ER -

TY - JOUR
ID - 1850
T1 - Mom or Dad says I shouldn't: Supervised and unsupervised children's knowledge of their parents' rules for home safety
A1 - Peterson,Lizette
A1 - Mori,Lisa
A1 - Scissors,Cathy
Y1 - 1986/06//
KW - PsycInfo
KW - Unsupervised Play Addition
KW - knowledge of & adherence to parents' perceptions of worrisome problems & rules for home safety
KW - supervised vs unsupervised 8-10.6 yr olds
KW - implications for child accident prevention & health & home security
KW - Home Environment
KW - Parental Absence
KW - Parental Attitudes
KW - Safety
KW - Permissive Parenting
KW - Knowledge Level
RP - NOT IN FILE
SP - 177
EP - 188
JF - Journal of Pediatric Psychology
JA - J Pediatr Psychol.
VL - 11
IS - 2
CY - United Kingdom
PB - Oxford Univ Press
N2 - 32 parents of schoolchildren (aged 8 yrs to 10 yrs 7 mo) completed a questionnaire concerning what at-home problems were important and worrisome and what rules parents had generated concerning potential problem situations; the questionnaire also suggested several rules that parents and children might or might not adhere to, asking for acceptance or rejection of these rules. The children were asked the same questions in an interview. The parents' responses to the questionnaire showed that they rated emergency situations, encounters with strangers, and play location as presenting important and worrisome problems; that they had formulated multiple rules concerning their children's behavior in these situations; and that they believed their children were aware of these rules. However, the children's interview responses indicated that children who were left unsupervised once, 2-3 times, or more than 4 times a week, as well as children who were never left alone, were largely unaware of their parents' rules. Implications for child accident prevention, child health, and child home security are discussed. (14 ref) (PsycINFO Database Record (c) 2012 APA, all rights reserved)
SN - 1465-735X
UR - http://search.ebscohost.com/login.aspx?direct=true&db=psyh&AN=1986-26932-001&site=ehost-live
L3 - 10.1093/jpepsy/11.2.177
ER -

TY - JOUR

ID - 1851
T1 - The role of attentiveness, mobility history, and separation of hiding sites on Stage IV search behavior
A1 - Horobin,Karen
A1 - Acredolo,Linda
Y1 - 1986/02//
KW - PsycInfo
KW - Unsupervised Play Addition
KW - visual attentiveness & search behavior & duration of independent mobility
KW - performance on object permanence task
KW - 8-10 mo olds
KW - Attention
KW - Object Permanence
KW - Perceptual Development
KW - Visual Search
RP - NOT IN FILE
SP - 114
EP - 127
JF - Journal of Experimental Child Psychology
JA - J Exp.Child Psychol.
VL - 41
IS - 1
CY - Netherlands
PB - Elsevier Science
N2 - Investigated the relationship between visual attentiveness, search behavior, and duration of independent mobility for 56 8-10 mo olds presented with 3 versions of the Stage IV object permanence task. The number and spatial separation of hiding sites was manipulated to explore the role of these factors on visual attention and search performance. In a repeated-measures design, perseverative search errors were less likely on both a 2- and a 6-location task with equally wide separation of the A and B hiding sites than on a 2-location task with A and B close together, thus indicating that spatial separation of sites is a more important contributor to successful search than number of hiding sites alone. Visual attentiveness was significantly associated with correct search at B in all 3 versions of the task. A significant developmental relationship was found between the length of time Ss had been independently mobile and visual attentiveness during the hiding procedure. Findings are discussed in terms of the transition to self-produced mobility and expanding spatial experience in the 2nd half of the 1st yr and how visual attentiveness and search performance might improve as a function of such changes. (14 ref) (PsycINFO Database Record (c) 2012 APA, all rights reserved)
SN - 0022-0965
UR - http://search.ebscohost.com/login.aspx?direct=true&db=psyh&AN=1986-16921-001&site=ehost-live
L3 - 10.1016/0022-0965(86)90054-8
ER -

TY - JOUR
ID - 1852
T1 - Action field and language use in child-child interactions
A1 - Bokus,Barbara
Y1 - 1986///
KW - PsycInfo
KW - Unsupervised Play Addition
KW - initiation of peer interaction
KW - perceived self & peer action field & indicative vs descriptive utterances
KW - dyads of 3-5 yr olds
KW - Interpersonal Communication
KW - Peer Relations
KW - Social Interaction
KW - Social Perception
RP - NOT IN FILE
SP - 197

EP - 206
JF - Polish Psychological Bulletin
VL - 17
IS - 3-4
CY - Poland
PB - Blackhorse Publishing
N2 - Investigated how 24 dyads of children (aged 3-5 yrs) perceived play situations in which they initiated social interaction. Ss presented to an adult their own action field and that ascribed to the other child in ongoing play. Observations were made of nonverbal and verbal modes used in presenting the 2 action fields, either as 1 shared by both or as 2 separate fields. The manner in which each S initiated ensuing social interactions in the play conditions was analyzed (i.e., how the initiator drew the partner into his/her activity). Results show Ss used 2 types of utterances: indicative or descriptive. It is concluded that the child takes into account the partner as agent of activity and this alters the child's use of language. (PsycINFO Database Record (c) 2012 APA, all rights reserved)
SN - 1641-7844
UR - http://search.ebscohost.com/login.aspx?direct=true&db=psyh&AN=1988-23061-001&site=ehost-live
ER -

TY - JOUR
ID - 1853
T1 - The influence of home, dispositional, and setting variables on the proximity of preschool children to activities, peers, and adults
A1 - Giblin,Paul T.
A1 - Tzelepis,Angela
Y1 - 1985/09//
KW - PsycInfo
KW - Unsupervised Play Addition
KW - home environment & personality
KW - proximity to activities & peers & adults
KW - preschoolers
KW - Activity Level
KW - Home Environment
KW - Interpersonal Interaction
KW - Peers
KW - Personality Traits
RP - NOT IN FILE
SP - 319
EP - 331
JF - The Journal of Genetic Psychology: Research and Theory on Human Development
VL - 146
IS - 3
CY - US
PB - Heldref Publications
N2 - Observed 25 preschool children and 7 adult female caregivers during scan sampling of morning free-play periods. Children's movements, locations, and proximities to adults, peers, and activities were recorded for 15 min each day for 5 days. Adult caretakers also rated Ss' dependency and autonomous achievement striving, and parents completed questionnaires assessing the Ss' home environments. Data indicate that proximities to activities were related to the presence of adults and peers. Adults were proximal to small-motor activities, and peers were proximal to fantasy activities. Dependency and autonomy also were related to proximities to activities. Dependent children were unlikely to be proximal to reading activities but were often near large-motor activities. Autonomous children spent little time near eating and bathroom areas. Home environments varied most notably on the frequency of adult involvement in child activities. High adult involvement at home was correlated negatively with autonomy. (22 ref) (PsycINFO Database Record (c) 2012 APA, all rights reserved)
SN - 1940-0896
UR - http://search.ebscohost.com/login.aspx?direct=true&db=psyh&AN=1986-19668-001&site=ehost-live
L3 - 10.1080/00221325.1985.9914461

ER -

TY - JOUR
ID - 1854
T1 - The social cognitive ecology of preschool classrooms: Contextual relations revisited
A1 - Pellegrini,Anthony D.
Y1 - 1984/09//
KW - PsycInfo
KW - Unsupervised Play Addition
KW - adult vs peer presence
KW - social cognitive aspects of play behavior
KW - 3 & 4 yr old preschool students
KW - Childhood Play Behavior
KW - Classroom Environment
KW - Cognitive Processes
KW - Preschool Students
RP - NOT IN FILE
SP - 321
EP - 332
JF - International Journal of Behavioral Development
VL - 7
IS - 3
CY - US
PB - Elsevier Science, Inc.
N2 - 10 3- and 10 4-yr-olds were observed in their preschool classrooms during free play to examine the extent to which social cognitive aspects of their play behaviors varied according to 2 classroom contextual variables: presence in different learning centers and participants (number of children and adults present) in those centers. Results indicate that Ss behaved differently according to these variables. Ss engaged in lower-order social-cognitive behaviors in art centers than they did in blocks and housekeeping centers. Adult presence was related to less mature forms of play, whereas peer presence was related to more mature forms of play. Data indicate that peer presence and adult absence relate to more mature forms of children's social cognitive play and support Piagetian notions of peer interaction and cognitive development. (21 ref) (PsycINFO Database Record (c) 2012 APA, all rights reserved)
SN - 1464-0651
UR - http://search.ebscohost.com/login.aspx?direct=true&db=psyh&AN=1985-10788-001&site=ehost-live
L3 - 10.1016/S0163-6383(84)80383-5
ER -

TY - JOUR
ID - 1855
T1 - Overjustification: A self-perception perspective
A1 - Newman,Joan
A1 - Layton,Bruce D.
Y1 - 1984/09//
KW - PsycInfo
KW - Unsupervised Play Addition
KW - internal interest & reward magnitude
KW - preference for interesting vs uninteresting toy
KW - 1st & 2nd graders
KW - Motivation
KW - Preferences
KW - Rewards
KW - Toys
KW - Self Perception
KW - Toy Selection
RP - NOT IN FILE

SP - 419
EP - 425
JF - Personality and Social Psychology Bulletin
VL - 10
IS - 3
CY - US
PB - Sage Publications
N2 - Self-perception theory predicts a decrease in preference for a rewarded activity (the overjustification effect) only when 2 conditions are met: Internal interest in the activity is high, and the reward is perceived as more than adequate justification for performance. To test this prediction, internal interest was established that was uncontaminated by external factors. Such baseline measures were provided by unrequired, unsupervised, and unrewarded play with target toys. In a 2?x?3 factorial design, 60 1st- and 2nd-grade children played with an interesting or uninteresting toy and were given 1 of 3 levels of reward for their play. An additional 22 1st and 2nd graders provided a baseline measure of internal interest in the toys by playing with them alone and unrewarded. The predicted interaction of internal interest and reward size was found on behavioral indices of play following the reward period. Compared to the baseline, increasing reward size was associated with a decrease in preference for the interesting toy but not for the uninteresting one. (12 ref) (PsycINFO Database Record (c) 2012 APA, all rights reserved)
SN - 1552-7433
UR - http://search.ebscohost.com/login.aspx?direct=true&db=psyh&AN=1985-22387-001&site=ehost-live
L3 - 10.1177/0146167284103010
ER -

TY - JOUR
ID - 1856
T1 - Short-term treatment outcome using parents as co-therapists for their own autistic children
A1 - Short,Andrew B.
Y1 - 1984/07//
KW - PsycInfo
KW - Unsupervised Play Addition
KW - training & use of parents as co-therapists in developmental treatment
KW - short term treatment outcome
KW - autistic 2.3-7.5 yr olds
KW - Autism
KW - Behavior Therapy
KW - Parent Training
KW - Treatment Effectiveness Evaluation
RP - NOT IN FILE
SP - 443
EP - 458
JF - Journal of Child Psychology and Psychiatry
VL - 25
IS - 3
CY - United Kingdom
PB - Blackwell Publishing
N2 - Evaluated the effects of E. Schopler and R. J. Reichler's (see record 1972-05273-001) developmental treatment for autistic children, which uses parents as co-therapists. 15 children (aged 2.3-7.5 yrs) were followed during waiting and treatment periods. Direct behavioral observations, a blind rating of family stress based on interviews with mothers, and 2 questionnaires were used to assess outcome. All data were collected during home visits. The therapeutic training skills of the parents focused on developmental assessment of skills, communication at a level appropriate for the child, presenting appropriate tasks, presenting materials in a teaching setting, and the effective use of reinforcement and time-out. Significant treatment effects were found for appropriate child behavior and parental involvement. Treatment effects were not shown for inappropriate child behavior and the measure of family stress and adaptation. Data are presented that suggest a positive change with treatment in parent-child interaction patterns. A lack of change in independent play is noted.

Questionnaire responses indicated that parents saw the treatment as helpful. (46 ref) (PsycINFO Database Record (c) 2012 APA, all rights reserved)
SN - 1469-7610
UR - http://search.ebscohost.com/login.aspx?direct=true&db=psyh&AN=1984-32110-001&site=ehost-live
L3 - 10.1111/j.1469-7610.1984.tb00163.x
ER -

TY - JOUR
ID - 1857
T1 - A multimethod assessment and intervention with a socially rejected child
A1 - Petersen,Nancy J.
A1 - Moe,Glen L.
Y1 - 1984///
KW - PsycInfo
KW - Unsupervised Play Addition
KW - multimethod intervention
KW - Social Skills
KW - socially rejected 9 yr old Mexican American female
KW - Social Acceptance
KW - Social Skills Training
KW - Case Report
KW - Mexican Americans
RP - NOT IN FILE
SP - 391
EP - 396
JF - School Psychology Review
VL - 13
IS - 3
CY - US
PB - National Assn of School Psychologists
N2 - Describes a 9-yr-old Mexican-American female whose attempts to control other children led to negative peer interactions, social rejection and isolation, and excessive interactions with adults. Significant improvements were achieved through a multimethod intervention that combined structured teaching sessions, role playing and feedback, observation of a social model, supervised and unsupervised play, and generalization exercises. (8 ref) (PsycINFO Database Record (c) 2012 APA, all rights reserved)
SN - 0279-6015
UR - http://search.ebscohost.com/login.aspx?direct=true&db=psyh&AN=1985-04742-001&site=ehost-live
ER -

TY - JOUR
ID - 1858
T1 - 'Call the police, okay?': Social comparison by young children during play in preschool
A1 - Chafel,Judith A.
Y1 - 1984/03//
KW - PsycInfo
KW - Unsupervised Play Addition
KW - cognitive uncertainty & social comparison
KW - elicitation of understanding from peer
KW - 3-5 yr olds
KW - Childhood Play Behavior
KW - Peer Relations
KW - Social Comparison
RP - NOT IN FILE
SP - 201
EP - 216
JF - Early Child Development and Care

VL - 14
IS - 3-4
CY - United Kingdom
PB - Taylor & Francis
N2 - Describes and analyzes several examples of data drawn from naturalistic observations of 3-, 4-, and 5-yr-old children enrolled in nursery schools serving predominantly White, middle-class families to examine situations in which young children use social comparison to elicit understanding from a peer due to cognitive uncertainty. These situations are described as requests for information, agreement, permission, compliance, verbal responsiveness, and attention. Peer exchanges indicated that the Ss were socially competent. On the whole, they accurately sized up a social situation and successfully elicited verbal feedback from a peer that could be used to check their understanding. Since Ss were able to sustain cohesive verbal exchanges unsupervised by adults, it is contended that Piaget's global construct of egocentrism in young children needs revision. Implications for the use of tag expression across a wide variety of activity settings and developmental ages and for teacher behaviors are discussed. (34 ref) (PsycINFO Database Record (c) 2012 APA, all rights reserved)
SN - 1476-8275
UR - http://search.ebscohost.com/login.aspx?direct=true&db=psyh&AN=1985-06347-001&site=ehost-live
L3 - 10.1080/0300443840140303
ER -

TY - JOUR
ID - 1859
T1 - 'Conversazioni' tra bambini di due anni. Intenzioni comunicative e scambi dialogici
A1 - Morra Pellegrino,Maria L.
A1 - Scopesi,Alda
A1 - D'Aniello,Paola
Y1 - 1984/02//
KW - PsycInfo
KW - Unsupervised Play Addition
KW - adult presence in group play
KW - communicative intentions & dialogue
KW - 2 yr olds
KW - Childhood Play Behavior
KW - Conversation
KW - Interpersonal Influences
KW - Peer Relations
RP - NOT IN FILE
SP - 52
EP - 65
JF - Et… Evolutiva
VL - 17
CY - Italy
PB - Giunti Gruppo Editoriale SPA
N2 - Studied 26 small groups of 2-yr-olds (3 Ss in each group) by observing their play for 20 min; Ss were acquainted with each other and were accompanied by a caretaker who participated in their play activity and conversation during the 1st half of the observation and then left the group. Verbal interactions were taped, and context and nonverbal behavior were noted. Analysis showed that Ss used different verbal styles when they communicated with an adult than when they communicated with peers, and they produced more sequential dialog when playing by themselves without an adult; when an adult was present, Ss talked more to her than to peers. (26 ref) (PsycINFO Database Record (c) 2012 APA, all rights reserved)
SN - 0392-0658
UR - http://search.ebscohost.com/login.aspx?direct=true&db=psyh&AN=1984-31112-001&site=ehost-live
ER -

TY - CHAP
ID - 1860

T1 - Moral reasoning and behavior
A1 - Kaus,Cheryl R.
A1 - Lonky,Edward
A1 - Roodin,Paul
Y1 - 1984///
N1 - Accession Number: 1987-98579-007. First Author & Affiliation: Kaus, Cheryl R.. Translated Book Series Title: Guidance and counseling series. Release Date: 19970801. Publication Type: Book, (0200); Edited Book, (0280); . Media Covered: Print. Document Type: Chapter. Book Type: Handbook/Manual. ISBN: 0-8077-2884-5, Paperback, 0807728845. Language: English. Major Descriptor: Adolescent Development; Moral Development; Morality; Reasoning. Minor Descriptor: Activism; Cheating; Cognitive Development; Juvenile Delinquency; Psychosocial Factors; Theories. Classification: Psychosocial & Personality Development (2840) . Population: Human (10); . Age Group: Adolescence (13-17 yrs) (200) . Intended Audience: Psychology: Professional & Research (PS) . Page Count: 51
KW - PsycInfo
KW - Unsupervised Play Addition
KW - theories of & factors affecting moral development & reasoning & behavior
KW - Adolescents
KW - Adolescent Development
KW - Moral Development
KW - Morality
KW - Reasoning
KW - Activism
KW - Cheating
KW - Cognitive Development
KW - Juvenile Delinquency
KW - Psychosocial Factors
KW - Theories
RP - NOT IN FILE
SP - 231
EP - 281
T2 - Experiencing adolescents: A sourcebook for parents, teachers, and teens
A2 - Lerner,Richard M.
A2 - Galambos,Nancy L.
CY - New York, NY US
PB - Teachers College Press
T3 - Guidance and counseling series
N2 - (from the chapter) [discuss] how the transitions from childhood to adolescent morality came about and . . . exactly what makes the morality of adolescents somewhat different in character from that of adults / [present] an overview of the major theoretical orientations which have served to guide and direct the psychological investigation of moral development / describes how adolescents think about morality, and the role their reasoning may play in influencing moral behavior / examine 3 areas of adolescent moral behavior: academic cheating, juvenile delinquency, and student activism / explore the influence of individual, family, and situational factors on these behaviors / [explore the question] what role can our schools play in attempting to influence moral development / present overviews of 2 current approaches to moral education: values clarification and the cognitive-developmental approa (chapter)
frameworks to understand morality [psychoanalytic view, social learning view, cognitive developmental view / the impact of parents / influence of parenting style on morality / impact of peers on morality / characteristics of moral reasoning in adolescence [idealistic thinking, social thought, reasoning about law and justice, relativistic thinking] / religious orientation (PsycINFO Database Record (c) 2012 APA, all rights reserved) (chapter)
SN - 0-8077-2884-5
UR - http://search.ebscohost.com/login.aspx?direct=true&db=psyh&AN=1987-98579-007&site=ehost-live
ER -

TY - JOUR
ID - 1864
T1 - Children's reponses to moral and social conventional transgressions in free-play settings

A1 - Nucci,Larry P.
A1 - Nucci,Maria S.
Y1 - 1982/10//
KW - PsycInfo
KW - Unsupervised Play Addition
KW - responses to moral & social conventional transgressions during unsupervised free play
KW - male vs female 7-10 vs 11-14 yr olds
KW - Age Differences
KW - Antisocial Behavior
KW - Childhood Play Behavior
KW - Human Sex Differences
KW - Morality
KW - Responses
RP - NOT IN FILE
SP - 1337
EP - 1342
JF - Child Development
JA - Child Dev.
VL - 53
IS - 5
CY - United Kingdom
PB - Blackwell Publishing
N2 - Observed 20 7-24 yr olds' responses to naturally occurring moral and social conventional transgressions during unsupervised free play. Findings paralleled results of previous observational studies conducted in adult-governed (school) contexts by the authors (e.g., see record 1982-20767-001). It was found that Ss responded to both moral and conventional forms of transgression. Responses of both the younger (7-10 yr old) and older (11-24 yr old) Ss to moral transgressions revolved around the intrinsic (hurtful and unjust) consequences of acts on victims. Ss' responses to conventional breaches, in contrast, focused on aspects of the social order (i.e., rules, normative expectations). Sex differences in the use of specific forms of response to moral and conventional breaches were found. (14 ref) (PsycINFO Database Record (c) 2012 APA, all rights reserved)
SN - 1467-8624
UR - http://search.ebscohost.com/login.aspx?direct=true&db=psyh&AN=1983-03231-001&site=ehost-live
L3 - 10.2307/1129024
ER -

TY - JOUR
ID - 1865
T1 - Second order effects of peers on mother-toddler interaction
A1 - Rubenstein,Judith L.
A1 - Howes,Carollee
A1 - Pedersen,Frank A.
Y1 - 1982/04//
KW - PsycInfo
KW - Unsupervised Play Addition
KW - presence of adult vs 13-29 mo old peer
KW - mother-infant interaction
KW - mothers & their 19 mo olds
KW - Mother Child Relations
KW - Peers
KW - Social Influences
RP - NOT IN FILE
SP - 185
EP - 194
JF - Infant Behavior & Development
JA - Infant behav
VL - 5

IS - 2
CY - Netherlands
PB - Elsevier Science
N2 - Investigated the influence of adult and toddler peers on mother-toddler interaction in the natural home environment. Eight 19-mo-olds and their mothers were observed in 4 circumstances on 4 different days: (1) at home by themselves, (2) with a toddler peer (aged 13-29 mo), (3) with an adult peer, and (4) with a toddler peer and an adult peer. In the presence of a toddler peer, toddlers made fewer instrumental and distal social bids to their mothers. Presence of an adult peer or mother without a toddler peer increased Ss' tactile and proximal bids to mother. Maternal affect expression, both positive and negative, was intensified in the absence of the peers. Higher-level reciprocal toddler peer play was more frequent in the absence of the adult peer. Findings are interpreted in terms of different types of second order effects. (20 ref) (PsycINFO Database Record (c) 2012 APA, all rights reserved)
SN - 0163-6383
UR - http://search.ebscohost.com/login.aspx?direct=true&db=psyh&AN=1983-03317-001&site=ehost-live
L3 - 10.1016/S0163-6383(82)80027-1
ER -

TY - JOUR
ID - 1866
T1 - Can television teach prosocial behavior?
A1 - Lovelace,Valeria O.
A1 - Huston,Aletha C.
Y1 - 1982///
KW - PsycInfo
KW - Unsupervised Play Addition
KW - TV programs
KW - conflict resolution strategy in transmission of prosocial ideas & behavior
KW - CHILDREN
KW - Conflict Resolution
KW - Prosocial Behavior
KW - Television
RP - NOT IN FILE
SP - 93
EP - 106
JF - Prevention in Human Services
VL - 2
IS - 1-2
CY - US
PB - Haworth Press
N2 - Reviews the effect of TV on children's learning and prosocial (PS) behavior and discusses the advantages and disadvantages of 3 methods of presenting PS content on TV. TV programs can transmit PS ideas and behavior to children from about age 3 yrs onward. The most effective method of conveying a PS message may be to present the PS behavior without any contrasting conflict or antisocial behavior. The conflict resolution strategy can also effectively convey PS behavior if there are a variety of models showing PS actions, if the PS resolution is given sufficient time and attention, and if viewing conditions are adequate. Because learning is often situation-specific, more generalized effects will result from showing ordinary people in a variety of everyday situations working together, helping each other, and being sensitive to each other. Dramatic story formats appear better suited than brief didactic bits for influencing children's behavior. In many cases, postviewing rehearsal, discussion, and related play can enhance the effects of the TV program. A 3rd technique, the presentation of unresolved conflict, can be useful in classroom or therapeutic situations where an adult can guide postviewing and discussion and activity, but it has unknown effects in unsupervised circumstances. (48 ref) (PsycINFO Database Record (c) 2012 APA, all rights reserved)
SN - 0270-3114
UR - http://search.ebscohost.com/login.aspx?direct=true&db=psyh&AN=1983-27684-001&site=ehost-live
L3 - 10.1300/J293v02n01_08
ER -

TY - JOUR
ID - 1868
T1 - Reciprocal influence in the social interactions of mothers and three-year-old children from different socioeconomic backgrounds
A1 - Farran,Dale C.
A1 - Haskins,Ron
Y1 - 1980/09//
KW - PsycInfo
KW - Unsupervised Play Addition
KW - socioeconomic background
KW - social interaction during free play
KW - mothers & 3 yr olds
KW - Childhood Play Behavior
KW - Mother Child Relations
KW - Mothers
KW - Socioeconomic Status
RP - NOT IN FILE
SP - 780
EP - 791
JF - Child Development
JA - Child Dev.
VL - 51
IS - 3
CY - United Kingdom
PB - Blackwell Publishing
N2 - Observed the social interactions between 51 3-yr-olds and their mothers during a 20-min free-play laboratory session. These dyads were from 2 socioeconomic groups differing in income, education, and occupation. Middle-income dyads spent twice as much time in mutual play as low-income dyads; low-income mothers spent more time reading to themselves, and their children were more often in independent play or no clear activity. Results do not support previous work showing low-income mothers to be more controlling than middle-income mothers. A series of profile analyses using conditional probabilities indicated nearly identical dyadic processes in both groups of mothers and children. Controlling and interactive behaviors of mothers were inextricably linked to the presenting behaviors of children for both groups. Socioeconomic background had the effect of altering the quantity of certain behaviors but not fundamentally changing the pattern of mother-child interactions. (25 ref) (PsycINFO Database Record (c) 2012 APA, all rights reserved)
SN - 1467-8624
UR - http://search.ebscohost.com/login.aspx?direct=true&db=psyh&AN=1981-03168-001&site=ehost-live
L3 - 10.2307/1129465
ER -

TY - JOUR
ID - 1869
T1 - Effects of adult-directed activity, number of toys, and sex of child on social and exploratory behavior in young children
A1 - Vlietstra,Alice G.
Y1 - 1980/07//
KW - PsycInfo
KW - Unsupervised Play Addition
KW - sex & number of toys & amount of adult direction in preschool environment
KW - manipulative vs exploratory vs social activities
KW - 2.3-3.8 yr olds
KW - Childhood Play Behavior
KW - Classroom Environment
KW - Human Sex Differences
KW - Nursery School Students

KW - Preschool Education
KW - Exploratory Behavior
KW - Social Interaction
RP - NOT IN FILE
SP - 231
EP - 238
JF - Merrill-Palmer Quarterly: Journal of Developmental Psychology
VL - 26
IS - 3
CY - US
PB - Wayne State University Press
N2 - 73 2.3-3.10 yr old children from preschools with high or low levels of adult-directed activity were presented with bowls with lids to be opened that contained various common objects. An adult presented the bowls, under either adult-directed or child-directed conditions. One or 5 toys were present, and the S could choose to play with them at any time. Observers recorded the amount of time spent (a) opening bowls, (b) in social interaction, (c) manipulating objects from the bowls, and (d) playing with the other toys. High levels of adult direction increased the amount of time spent with the objects and bowls, and it had a stronger effect on social interaction than on manipulation of the objects. Levels of adult direction in the schools had similar effects. Ss spent more time with toys when 5 toys were available and in a 2nd session, when the task was no longer novel. Males were more exploratory than females, who engaged in more social interaction. Results should be considered in designing preschool environments. (10 ref) (PsycINFO Database Record (c) 2012 APA, all rights reserved)
SN - 1535-0266
UR - http://search.ebscohost.com/login.aspx?direct=true&db=psyh&AN=1980-33366-001&site=ehost-live
ER -

TY - JOUR
ID - 1870
T1 - The effects of adult presence on the prosocial behavior of preschool children
A1 - Barton,Edward J.
A1 - Olszewski,Mark J.
A1 - Madsen,Jennifer J.
Y1 - 1979///
KW - PsycInfo
KW - Unsupervised Play Addition
KW - behavioral training in sharing & subsequent influence of peer presence & peer smiling & peer praise
KW - unprogrammed generalization of physical sharing over time
KW - preschoolers
KW - Behavior Therapy
KW - Interpersonal Influences
KW - Peers
KW - Response Generalization
KW - Sharing (Social Behavior)
KW - Observers
RP - NOT IN FILE
SP - 271
EP - 286
JF - Child Behavior Therapy
VL - 1
IS - 3
PB - Haworth Press, Inc.
N2 - Investigated the influence of adult presence, peer smiling, and peer praise on the unprogrammed generalization of physical sharing over time. During free play 3 groups of 5 preschoolers were taught to share toys via a behavioral training package composed of instructions, modeling, behavior rehearsal, prompts, and praise. Immediately after free play, Ss were given art materials and observed in the presence of different adults. For all Ss, regardless of the adult presence, treatment facilitated sharing in both the free-play and the art-activity

settings. In addition, 2 groups continued to share in both settings after the training. The presence of an adult was necessary for unprogrammed maintenance of treatment gains in both settings whereas peer smiling and peer praise were not. Observer presence suppressed physical sharing and smiling before, during, and after treatment. (25 ref) (PsycINFO Database Record (c) 2012 APA, all rights reserved)
SN - 0162-1416
UR - http://search.ebscohost.com/login.aspx?direct=true&db=psyh&AN=1981-05987-001&site=ehost-live
L3 - 10.1300/J473v01n03_05
ER -

TY - JOUR
ID - 1871
T1 - When adult therapists work with children: Differential treatment considerations
A1 - Goggin,James E.
A1 - Goggin,Eileen B.
Y1 - 1979/06//
KW - PsycInfo
KW - Unsupervised Play Addition
KW - ethical principles & professional standards & diagnostic & treatment approaches
KW - treatment of children by adult therapists
KW - Child Psychotherapy
KW - Professional Ethics
KW - Professional Standards
KW - Psychotherapeutic Techniques
KW - Psychotherapists
KW - Psychodiagnosis
RP - NOT IN FILE
SP - 330
EP - 337
JF - Professional Psychology
VL - 10
IS - 3
CY - US
PB - American Psychological Association
N2 - Too few mental health professionals have special training in child therapy. Most children referred for psychotherapy must be treated by practitioners whose training has been essentially with adults. In the present article, ethical principles and professional standards that relate to the issue of adult therapists treating children are examined. Selected diagnostic and treatment approaches used with children are described. Differential treatment considerations revolving around the issues of play therapy vs talk therapy, open-ended therapy vs directive therapy, and the role of parental involvement are briefly examined. (9 ref) (PsycINFO Database Record (c) 2012 APA, all rights reserved)
SN - 0033-0175
UR - http://search.ebscohost.com/login.aspx?direct=true&db=psyh&AN=1980-32900-001&site=ehost-live
L3 - 10.1037/0735-7028.10.3.330
ER -

TY - JOUR
ID - 1872
T1 - The structural characteristics of monologues in the speech of normal children: Syntactic nonconversational aspects
A1 - Craig,Holly K.
A1 - Gallagher,Tanya M.
Y1 - 1979/03//
KW - PsycInfo
KW - Unsupervised Play Addition
KW - language developmental stage

KW - syntactic characteristics & mean length & sequencing patterns of nonconversational monolog vs dialog utterances
KW - 2-3 yr old children
KW - Age Differences
KW - Language Development
KW - Speech Characteristics
KW - Syntax
KW - Verbal Communication
KW - Response Duration
RP - NOT IN FILE
SP - 46
EP - 62
JF - Journal of Speech & Hearing Research
VL - 22
IS - 1
CY - US
PB - American Speech-Language-Hearing Assn
N2 - The monolog-dialog language samples collected by T. Gallagher and H. Craig (see record 1979-08115-001) from 9 normal children, 3 at each of R. Brown's (1973) language Stages 1, 2, and 3, were analyzed syntactically. The original procedure consisted of the collection of a 2-hr language sample from each child containing alternations of dialog with the examiner and monolog produced during independent play. Results indicate that the syntactic categories that occurred in monolog speech were similar to those appearing in dialog speech, but the percentage frequencies differed. Monolog subsamples can be characterized syntactically as highly structured sequences of revision behavior reflecting a basic metalinguistic performative. (19 ref) (PsycINFO Database Record (c) 2012 APA, all rights reserved)
SN - 0022-4685
UR - http://search.ebscohost.com/login.aspx?direct=true&db=psyh&AN=1980-25276-001&site=ehost-live
ER -

TY - JOUR
ID - 1873
T1 - Sequential analysis: Another approach to describing the stream of behaviour in children's interactions
A1 - Murray,John P.
A1 - Hayes,Alan J.
A1 - Smith,Jacqui E.
Y1 - 1978/12//
KW - PsycInfo
KW - Unsupervised Play Addition
KW - sequential analysis of peer model influence
KW - group interactions & aggression & individual differences & social learning effects
KW - male vs female 4-4.5 yr olds
KW - Childhood Play Behavior
KW - Group Dynamics
KW - Interpersonal Interaction
KW - Peer Relations
KW - Social Learning
KW - Aggressive Behavior
KW - Analysis
KW - Human Sex Differences
KW - Individual Differences
RP - NOT IN FILE
SP - 207
EP - 215
JF - Australian Journal of Psychology
VL - 30
IS - 3

CY - United Kingdom
PB - Taylor & Francis
N2 - A modification of B. M. Caldwell's (1969) APPROACH coding scheme was used to analyze sequentially the influence of peer models in group interactions. 20 4-4«yr old preschool children from upper-middle to lower-upper income levels were equally assigned to 4 mixed-sex groups. Following exposure to active and arousing televised material, videotaped records of 10 min free play were obtained unobtrusively. For 2 of the groups a female adult was present for the 1st half of the play period, while for the others the adult entered after 5 min had elapsed. Analysis revealed intergroup and individual differences in the frequency of aggressive, prosocial, and neutral behaviors, with aggression the most frequently occurring class of behavior. The level of aggressive interaction increased in the 2nd half of the play period, and while the boys' aggressive behavior appeared independent of the presence or absence of the adult, the girls were significantly more aggressive when the adult was absent. Simultaneous and time-lagged correlation analyses explicated the sequential influence of each individual upon the group, and 4 styles of interaction were identified: actors, leaders, followers, and individualists. It is proposed that actors and leaders are most likely to exert a prime modeling influence on the observing followers, with the individualist being the least susceptible to social learning effects in the peer group. (17 ref) (PsycINFO Database Record (c) 2012 APA, all rights reserved)
SN - 1742-9536
UR - http://search.ebscohost.com/login.aspx?direct=true&db=psyh&AN=1980-11798-001&site=ehost-live
L3 - 10.1080/00049537808256373
ER -

TY - JOUR
ID - 1876
T1 - Structural characteristics of monologues in the speech of normal children: Semantic and conversational aspects
A1 - Gallagher,Tanya M.
A1 - Craig,Holly A.
Y1 - 1978/03//
KW - PsycInfo
KW - Unsupervised Play Addition
KW - stage of language development
KW - semantic & conversational aspects of monologues
KW - preschool age children
KW - Conversation
KW - Developmental Stages
KW - Language Development
KW - Semantics
KW - Speech Characteristics
RP - NOT IN FILE
SP - 103
EP - 117
JF - Journal of Speech & Hearing Research
VL - 21
IS - 1
CY - US
PB - American Speech-Language-Hearing Assn
N2 - Investigated semantic and conversational aspects of monologues in the speech of normal children. Ss were 9 children, 3 each at R. Brown's Language Stages 1, 2, and 3 (mean ages 26, 26, and 35 mo, respectively). A 2-hr language sample, containing alternations of dialogue with the examiner and monologue produced during independent play, was collected from each S. Results indicate that the semantic and conversational categories that occurred in monologue speech were similar to those that appeared in contextually matched dialogue speech but the proportional frequencies differed. Monologue subsamples were generally short in length and had a sequencing pattern that was highly consistent and semantically organized. Results are discussed in terms of Piagetian cognitive theory and pragmatic language models. (22 ref) (PsycINFO Database Record (c) 2012 APA, all rights reserved)
SN - 0022-4685

UR - http://search.ebscohost.com/login.aspx?direct=true&db=psyh&AN=1979-08115-001&site=ehost-live
ER -

TY - JOUR
ID - 1878
T1 - Some characteristics of intonation in infants' single-word utterances
A1 - Kishi,Manabu
Y1 - 1978/01//
KW - PsycInfo
KW - Unsupervised Play Addition
KW - intonation characteristics in single-word utterances
KW - Infants
KW - Infant Vocalization
KW - Speech Characteristics
KW - Words (Phonetic Units)
RP - NOT IN FILE
SP - 25
EP - 35
JF - Journal of Child Development
VL - 14
CY - Japan
PB - Japan Society for Research in Child Development
N2 - Two contrasting viewpoints have been presented in the literature as to the relation between single-word utterances and intonation. First, intonation plays some syntactic roles, and single-word utterances can be regarded as "holophrases." Second, intonation is only an imitation of adults' utterances. The present study recorded the single-word utterances and contexts observed with 5 infants, aged 10 mo 6 days to 14 mo 8 days at 1st observation. Intonation pattern was classified into 13 types, and duration, mean variance rate, and frequency range were calculated. Variations of intonation among the repetitional utterances and among the differences of context were compared. Results show agreement in type of intonation among repetitional utterances, but no accord in terms of quantitative aspects. In the utterances that functioned as demand, question, and hail, 71% were "rise" at the end of utterances and 85% were "non-rise." Findings indicate that infants use intonation actively and communicate their intentions with it. (PsycINFO Database Record (c) 2012 APA, all rights reserved)
SN - 0449-2293
UR - http://search.ebscohost.com/login.aspx?direct=true&db=psyh&AN=1979-28270-001&site=ehost-live
ER -

TY - JOUR
ID - 1879
T1 - The relation of classroom structure to social behavior, imaginative play, and self-regulation of economically disadvantaged children
A1 - Huston-Stein,Aletha
A1 - Friedrich-Cofer,Lynette
A1 - Susman,Elizabeth J.
Y1 - 1977/09//
KW - PsycInfo
KW - Unsupervised Play Addition
KW - classroom structure
KW - social behavior & imaginative play & self-regulation
KW - economically disadvantaged 2-5 yr olds in urban Head Start classes
KW - Childhood Play Behavior
KW - Classroom Environment
KW - Disadvantaged
KW - Imagination
KW - Project Head Start
KW - Classroom Behavior

KW - Self Regulation
RP - NOT IN FILE
SP - 908
EP - 916
JF - Child Development
JA - Child Dev.
VL - 48
IS - 3
CY - United Kingdom
PB - Blackwell Publishing
N2 - Defined structure as the amount of adult-directed activity in preschool classes and examined the relation of structure to naturally occurring behavior in 13 urban Head Start classes containing 141 2-5 yr olds. Ss in high structure classes engaged in less prosocial behavior to peers, less imaginative play, and less aggression than Ss in low structure classes but had slightly more friendly peer interactions. Ss in high structure classes were more attentive in circle time and helped to clean up more after free play, but they did not show more independent task persistence. The latter finding suggests that high levels of adult direction produce conformity when adults are present but do not facilitate independent task-oriented behavior. (PsycINFO Database Record (c) 2012 APA, all rights reserved)
SN - 1467-8624
UR - http://search.ebscohost.com/login.aspx?direct=true&db=psyh&AN=1978-21888-001&site=ehost-live
L3 - 10.2307/1128340
ER -

TY - JOUR
ID - 1880
T1 - The exploratory nature of children's social relations
A1 - Haskett,G.J.
Y1 - 1977/04//
KW - PsycInfo
KW - Unsupervised Play Addition
KW - novel toy presentation
KW - social behavior toward adult toy giver & relation to exploratory behavior
KW - 34-61 mo olds
KW - Childhood Play Behavior
KW - Exploratory Behavior
KW - Social Interaction
KW - Stimulus Novelty
KW - Toys
RP - NOT IN FILE
SP - 101
EP - 113
JF - Merrill-Palmer Quarterly: Journal of Developmental Psychology
VL - 23
IS - 2
CY - US
PB - Wayne State University Press
N2 - Baseline measures of 4 types of social behavior were obtained by observing a child playing with familiar toys in the presence of 2 adults. The 8 Ss ranged in age from 34 to 61 mo. After 10 min of play with familiar toys, the S was presented with a novel toy by one of the adults. Social behaviors toward that adult were significantly increased. Discussion of the findings centers on the similarity of social and exploratory behavior. (21 ref) (PsycINFO Database Record (c) 2012 APA, all rights reserved)
SN - 1535-0266
UR - http://search.ebscohost.com/login.aspx?direct=true&db=psyh&AN=1978-09717-001&site=ehost-live
ER -

TY - THES

ID - 1881
T1 - The role of different environmental conditions on leisure time activity of the severely developmentally disabled
A1 - Wehman,Paul H.
Y1 - 1977/03//
N1 - Accession Number: 1978-21328-001. First Author & Affiliation: Wehman, Paul H.; U Wisconsin, Madison. Other Journal Titles: Dissertation Abstracts International: Section B: The Sciences and Engineering. Release Date: 19780701. Publication Type: Dissertation Abstract, (0400); . Media Covered: Electronic. Document Type: Dissertation. Language: English. Major Descriptor: Childhood Play Behavior; Experimental Instructions; Imitation (Learning); Toys. Minor Descriptor: Intellectual Development Disorder. Classification: Mental Retardation (3256) . Population: Human (10); . Page Count: 2
KW - PsycInfo
KW - Unsupervised Play Addition
KW - toy proximity vs modeling vs instructions plus modeling
KW - independent play
KW - severely developmentally disabled
KW - Severe Mental Retardation
KW - Childhood Play Behavior
KW - Experimental Instructions
KW - Imitation (Learning)
KW - Toys
KW - Intellectual Development Disorder
RP - NOT IN FILE
SP - 5751
EP - 5752
JF - Dissertation Abstracts International
VL - 37
IS - 9-A
CY - US
PB - ProQuest Information & Learning
SN - 0419-4217
UR - http://search.ebscohost.com/login.aspx?direct=true&db=psyh&AN=1978-21328-001&site=ehost-live
ER -

TY - JOUR
ID - 1882
T1 - Can TV stimulate imaginative play?
A1 - Singer,Jerome L.
A1 - Singer,Dorothy G.
Y1 - 1976///
KW - PsycInfo
KW - Unsupervised Play Addition
KW - viewing of "Mister Rogers' Neighborhood" program with vs without adult presence & involvement vs make-believe training with adult using exercises & games
KW - imaginative play
KW - 3-4.5 yr olds in day care center
KW - Child Day Care
KW - Childhood Play Behavior
KW - Curriculum
KW - Imagination
KW - Television Viewing
RP - NOT IN FILE
SP - 74
EP - 80
JF - Journal of Communication
VL - 26

IS - 3
CY - United Kingdom
PB - Blackwell Publishing
N2 - Examined the extent to which the imaginative play of young children is enhanced by adult intervention. 60 3-4.5 yr old Ss from a daycare center were divided into 4 equal-size groups relatively equated by age, sex, IQ, and predisposition to imaginative play. Group A watched selected segments of the Mister Rogers program for 30 min/day for 2 wks. The viewing situation of Group B was similar to that of Group A, except that an adult involved herself with the ongoing program. Group C spent each 30 min session with an adult in a previously prepared sequence of exercises and games and watched no TV. The exercises and games had certain basic concepts in common with the Mister Rogers segments. Group D, the controls, followed the normal nursery school routine, watched no TV, and received no make-believe training. Each S was observed in spontaneous play on 2 occasions 2 wks pre- and 2 wks post-experiment. Significant differences were found between the groups in imaginativeness of play. Group D showed a decrease in imaginative play, while all the other groups showed a modest increase. The linearity of the increase from Group D through Groups A, B, and C was significant. A similar linear increase was found for positive affect. Results suggest that young children are more susceptible to the influence of an adult who can direct and respond to them than to that of a TV program. (PsycINFO Database Record (c) 2012 APA, all rights reserved)
SN - 1460-2466
UR - http://search.ebscohost.com/login.aspx?direct=true&db=psyh&AN=1979-04660-001&site=ehost-live
L3 - 10.1111/j.1460-2466.1976.tb01907.x
ER -

TY - JOUR
ID - 1883
T1 - Assessing learning effects of an educational toy
A1 - Vagt,Gerhard
A1 - M • ller,Eberhard
Y1 - 1976///
KW - PsycInfo
KW - Unsupervised Play Addition
KW - playing vs not playing with commercial learning toy advertised as promoting perceptual skills
KW - Perceptual Development
KW - 4-6 yr olds at recreational center
KW - Childhood Play Development
KW - Educational Toys
RP - NOT IN FILE
SP - 44
EP - 50
JF - Zeitschrift f • r Entwicklungspsychologie und P„dagogische Psychologie
VL - 8
IS - 1
CY - Germany
PB - Hogrefe Verlag GmbH & Co. KG
N2 - Assessed the effects on children 4-6 yrs old at a recreational center of playing for 3 wks with a commercial learning toy advertised as promoting perceptual skills. Control Ss participated only in the usual activities of the center. Pre- and posttest scores revealed no significant differences in performance between the groups. It is concluded that learning toys which provide opportunities for play unaccompanied by structured training procedures fail to accelerate perceptual development. (PsycINFO Database Record (c) 2012 APA, all rights reserved)
SN - 0049-8637
UR - http://search.ebscohost.com/login.aspx?direct=true&db=psyh&AN=1978-07707-001&site=ehost-live
ER -

TY - JOUR
ID - 1884
T1 - Sex differences in antisocial behavior: Does research methodology produce or abolish them?

A1 - Caplan,Paula J.
Y1 - 1975///
KW - PsycInfo
KW - Unsupervised Play Addition
KW - Research Methodology
KW - sex differences in antisocial behavior
KW - Antisocial Behavior
KW - Experimental Methods
KW - Human Sex Differences
RP - NOT IN FILE
SP - 444
EP - 460
JF - Human Development
VL - 18
IS - 6
CY - Switzerland
PB - Karger
N2 - Classifies the literature on sex differences in antisocial behavior into 2 categories: those studies which demonstrate boys to be more antisocial and those which show no difference. It is pointed out that both a failure experience and the presence of an adult during the dependent measure are conducive to the 1st type of outcome, whereas success or no-failure experiences and the absence of an adult are conducive to the 2nd type. These elements of the experiments may play a greater role in producing or abolishing sex-differential behavior than do sex differences in personality that are alleged to be pervasive or even innate; a mechanism for that is explicated. (5 p ref) (PsycINFO Database Record (c) 2012 APA, all rights reserved)
SN - 1423-0054
UR - http://search.ebscohost.com/login.aspx?direct=true&db=psyh&AN=1976-28930-001&site=ehost-live
L3 - 10.1159/000271505
ER -

TY - JOUR
ID - 1885
T1 - Parent-child interactions as the therapy target
A1 - Seitz,Sue
A1 - Riedell,Gail
Y1 - 1974/12//
KW - PsycInfo
KW - Unsupervised Play Addition
KW - experimental language therapy program
KW - parent-child interactions
KW - severely retarded Hindu child
KW - Parent Child Relations
KW - Speech Therapy
KW - Intellectual Development Disorder
RP - NOT IN FILE
SP - 295
EP - 304
JF - Journal of Communication Disorders
VL - 7
IS - 4
CY - Netherlands
PB - Elsevier Science
N2 - Presents results of an experimental language therapy program for a severely retarded Hindu child and her parents. The treatment target was the parent-child interactions. Immediate results show changes in the desired direction in these interactions, with some of these changes being documented over 3 mo. Immediate improvement was seen in nonverbal child behaviors such as positively responding to parents and engaging in independent play such that parents viewed the child as more competent and were able to support independent

play and reduce their physical contact and interruptive behavior. Improvement in the child's verbal behavior was reported in follow-ups at 3 mo and again at 1 yr. It is argued that parent-child interactions constitute the child's language environment, and that it is the quality of this environment that will most affect the verbal behavior of the slow developing child. (24 ref) (PsycINFO Database Record (c) 2012 APA, all rights reserved)
SN - 0021-9924
UR - http://search.ebscohost.com/login.aspx?direct=true&db=psyh&AN=1977-13362-001&site=ehost-live
L3 - 10.1016/0021-9924(74)90012-4
ER -

TY - JOUR
ID - 1886
T1 - Cross-modal matching by spastic children
A1 - Jones,Bill
A1 - Alexander,Richard
Y1 - 1974/02//
KW - PsycInfo
KW - Unsupervised Play Addition
KW - within- & cross-modal matching of visual & auditory spatiotemporal patterns
KW - perceptual & perceptual-motor deficits
KW - children with spastic quadriplegia
KW - Auditory Perception
KW - Paralysis
KW - Perceptual Disturbances
KW - Perceptual Motor Processes
KW - Visual Perception
RP - NOT IN FILE
SP - 40
EP - 46
JF - Developmental Medicine & Child Neurology
JA - Dev.Med Child Neurol.
VL - 16
IS - 1
CY - US
PB - Mac Keith Press
N2 - Tested the hypothesis that failure of intersensory integration is responsible for various perceptual and perceptual-motor deficits in cerebral palsy patients. In a controlled study, 20 children with spastic quadriplegia were tested for within- and cross-modal matching of visual and auditory spatiotemporal patterns. The spastic group and the matched normal group were equally deficient in all 4 tasks. It is felt that cross-modal matching has only limited applicability in analyzing perceptual deficit in the brain-damaged; inexperience in independent mobility would account for their poor performance. (French & German summaries) (PsycINFO Database Record (c) 2012 APA, all rights reserved)
SN - 1469-8749
UR - http://search.ebscohost.com/login.aspx?direct=true&db=psyh&AN=1974-32780-001&site=ehost-live
L3 - 10.1111/j.1469-8749.1974.tb02709.x
ER -

TY - JOUR
ID - 1887
T1 - Familial variables related to the expression of violent aggression in preschool age children
A1 - Abramson,Paul R.
Y1 - 1973/06//
KW - PsycInfo
KW - Unsupervised Play Addition
KW - familial variables
KW - expression of violent aggression
KW - black preschoolers & their mothers

KW - Aggressive Behavior
KW - Blacks
KW - Family Structure
KW - Mother Child Relations
RP - NOT IN FILE
SP - 345
EP - 346
JF - The Journal of Genetic Psychology: Research and Theory on Human Development
VL - 122
IS - 2
CY - US
PB - Heldref Publications
N2 - Explored familial variables related to the expression of violent aggression in 122 black preschool children and their mothers. The family composition variables were assessed by interviews with the mothers, and violent aggression was assessed by children's doll play. Children who expressed violent aggression were significantly more likely to have older male siblings or another adult present in the household than children who did not express violent aggression. There were no significant main effects for Sex, and the Violent Aggression * Sex interaction effects were nonsignificant. (PsycINFO Database Record (c) 2012 APA, all rights reserved)
SN - 1940-0896
UR - http://search.ebscohost.com/login.aspx?direct=true&db=psyh&AN=1974-00963-001&site=ehost-live
ER -

TY - JOUR
ID - 1889
T1 - Looking and approach behavior of psychotic and normal children as a function of adult attention or preoccupation
A1 - Churchill,Don W.
A1 - Bryson,Carolyn Q.
Y1 - 1972/03//
KW - PsycInfo
KW - Unsupervised Play Addition
KW - adult attention & preoccupation
KW - looking & approach behavior
KW - 3-7 yr.old autistics & early childhood schizophrenics
KW - Attention
KW - Autism
KW - Childhood Schizophrenia
KW - Interpersonal Interaction
KW - Approach Behavior
RP - NOT IN FILE
SP - 171
EP - 177
JF - Comprehensive Psychiatry
VL - 13
IS - 2
CY - Netherlands
PB - Elsevier Science
N2 - Studied 14 hospitalized 3-7 yr. olds who had been diagnosed as autistic (5 boys, 3 girls) or early childhood schizophrenic (3 boys, 3 girls). A control group consisted of 14 nonhospitalized Ss matched for age, sex, and socioeconomic status. Each S was observed 3 times/day, 1 day/wk, for 3 consecutive wk. in a room containing a 1-way mirror, a small cubicle seating 1 adult, and 4 zones containing sets of identical toys. An S was left in the room 1st with no adult present, and then with an unfamiliar female who was seated in the cubicle in an attentive and then a preoccupied manner. Measures of approach and attention behavior were recorded. All groups spent the largest amount of time in Zone 2 (where they could see and be seen by the adult), and most Ss looked more at the attentive than the preoccupied adult. Gross differences between psychotic and normal Ss were found in

style of play and interpersonal contact. No support was found for the hypothesis that adult attention drives away autistic or schizophrenic children. (20 ref.) (PsycINFO Database Record (c) 2012 APA, all rights reserved)
SN - 0010-440X
UR - http://search.ebscohost.com/login.aspx?direct=true&db=psyh&AN=1973-29122-001&site=ehost-live
L3 - 10.1016/0010-440X(72)90023-5
ER -

TY - JOUR
ID - 1890
T1 - Interpersonal relationships in a gypsy community in the Paris district
A1 - Flavigny,H.
Y1 - 1972/01//
KW - PsycInfo
KW - Unsupervised Play Addition
KW - individual vs.group influences
KW - child rearing & interpersonal relationships
KW - permanent gypsy community
KW - Childrearing Practices
KW - Interpersonal Interaction
KW - Racial and Ethnic Groups
KW - Social Influences
KW - Interpersonal Relationships
RP - NOT IN FILE
SP - 63
EP - 80
JF - Revue de Neuropsychiatrie Infantile et d'Hygiene Mentale de l'Enfance
VL - 20
IS - 1
N2 - Presents an ethnological description of a permanent gypsy community of 691 persons with emphasis on child rearing, by a team of social scientists well known to the community. The young child is treated permissively in all areas including eating and sleeping schedules, sex play, and toilet training. He is also ignored by adults who provide no supervision or specially adapted diet or play area. It is suggested that members of the group do not act as individuals but as group members, probably because of the confusion of ego and superego, resulting in very strong group ties and minimal contact with the outside community. (English, German, & Spanish summaries) (PsycINFO Database Record (c) 2012 APA, all rights reserved)
SN - 0035-1628
UR - http://search.ebscohost.com/login.aspx?direct=true&db=psyh&AN=1973-10965-001&site=ehost-live
ER -

TY - JOUR
ID - 1891
T1 - Effects of adult and peer observers on boys' and girls' responses to an aggressive model
A1 - Martin,Marian F.
A1 - Gelfand,Donna M.
A1 - Hartmann,Donald P.
Y1 - 1971/10//
KW - PsycInfo
KW - Unsupervised Play Addition
KW - imitative & total aggressive responding to model depicting aggressive behavior sequence solitary free play vs.play with observer present
KW - 5-7 yr.olds
KW - Aggressive Behavior
KW - Imitation (Learning)
KW - Peer Relations
KW - Social Behavior
RP - NOT IN FILE

SP - 1271
EP - 1275
JF - Child Development
JA - Child Dev.
VL - 42
IS - 4
CY - United Kingdom
PB - Blackwell Publishing
N2 - Studied the effects in 100 5-7 yr. olds of individually observing a model depicting a sequence of aggressive behavior. Subsequent ratings of Ss' imitative and total aggressive responding during solitary free play or with an O present indicated that (a) boys displayed more aggressive behavior than girls in all O conditions, (b) peer Os facilitated aggressive responding, and (c) Ss' aggressive responding increased from beginning to end of the free play session when a permissive adult was present. (PsycINFO Database Record (c) 2012 APA, all rights reserved)
SN - 1467-8624
UR - http://search.ebscohost.com/login.aspx?direct=true&db=psyh&AN=1972-22712-001&site=ehost-live
L3 - 10.2307/1127810
ER -

TY - JOUR
ID - 1892
T1 - Behavior modification in a profoundly retarded child: A case report
A1 - Miller,Harold R.
A1 - Patton,Mary E.
A1 - Henton,Karel R.
Y1 - 1971/07//
KW - PsycInfo
KW - Unsupervised Play Addition
KW - Behavior Modification
KW - self-feeding & verbal command response & increased standing ability & environmental responsiveness & independent mobility
KW - profoundly retarded 7 yr.old boy
KW - Behavior Therapy
KW - Case Report
KW - Food Intake
KW - Learning
KW - Treatment
KW - Intellectual Development Disorder
RP - NOT IN FILE
SP - 375
EP - 384
JF - Behavior Therapy
VL - 2
IS - 3
CY - US
PB - Association for Advancement of Behavior Therapy
N2 - Describes 3 behavior modification programs used with a profoundly retarded 7-yr-old boy. The programs developed a self-feeding operant, taught S to respond appropriately to a verbal command, and greatly increased his ability to stand without support. Side effects included an apparent increase in S's responsiveness to his environment, and independent mobility by crawling or using a walker. Observational follow-up information indicates that S's behavior gains have been maintained in his home. Questions raised by the training techniques used are considered. (PsycINFO Database Record (c) 2012 APA, all rights reserved)
SN - 0005-7894
UR - http://search.ebscohost.com/login.aspx?direct=true&db=psyh&AN=1972-23167-001&site=ehost-live
L3 - 10.1016/S0005-7894(71)80072-2
ER -

TY - JOUR
ID - 1893
T1 - Generalization of the control of screaming behavior in an emotionally disturbed, retarded female
A1 - Reiss,Steven
A1 - Redd,William H.
Y1 - 1970///
KW - PsycInfo
KW - Unsupervised Play Addition
KW - control of screaming behavior
KW - social isolation & positive social reinforcement
KW - emotionally disturbed retarded female 10 yr.old
KW - Case Report
KW - Mental Disorders
KW - Social Isolation
KW - Social Reinforcement
KW - Treatment
KW - Intellectual Development Disorder
RP - NOT IN FILE
SP - 741
EP - 742
JF - Proceedings of the Annual Convention of the American Psychological Association
VL - 5
IS - Pt. 2
CY - US
PB - American Psychological Association
N2 - Suppressed the screaming behavior of a 10-yr-old emotionally disturbed, retarded female by means of brief social isolation contingent upon screaming and positive social reinforcement for appropriate play behavior. 5 adult females employed these techniques during successive 20-min training sessions. Each of these adults acquired stimulus control of the child's behavior. That is, the child did not scream in the presence of these adults. The number of training sessions required to obtain stimulus control decreased for each successive adult. After 40 training sessions involving 3 adults, stimulus control generalized to unfamiliar adults. (PsycINFO Database Record (c) 2012 APA, all rights reserved)
UR - http://search.ebscohost.com/login.aspx?direct=true&db=psyh&AN=1970-19050-001&site=ehost-live
ER -

TY - JOUR
ID - 1894
T1 - Thematic role playing of small children
A1 - Zemskova,A.
Y1 - 1969///
KW - PsycInfo
KW - Unsupervised Play Addition
KW - thematic role-playing as stage in child's development
KW - Childhood Development
KW - Roles
RP - NOT IN FILE
SP - 17
EP - 20
JF - Doshkol'Noe Vospitanie
VL - 42
IS - 10
N2 - Presents examples of role-playing as a "new stage in the child's development, as transition to independent play." (PsycINFO Database Record (c) 2012 APA, all rights reserved)
UR - http://search.ebscohost.com/login.aspx?direct=true&db=psyh&AN=1970-14308-001&site=ehost-live
ER -

TY - JOUR
ID - 1895
T1 - Vocalization in infants during the first year of life and its connection with various forms of behavior
A1 - Kononova,I.M.
Y1 - 1968///
KW - PsycInfo
KW - Unsupervised Play Addition
KW - vocalizations during various forms of behavior
KW - 1st yr.of life
KW - Auditory Stimulation
KW - Social Interaction
KW - Stimulation
KW - Verbal Communication
RP - NOT IN FILE
SP - 119
EP - 127
JF - Voprosy Psychologii
VL - 14
IS - 5
CY - Russia
PB - Voprosy Psikhologii
N2 - Vocalization and behavior were recorded in 70 2-12 mo. old infants during all periods of wakefulness. The situations producing vocalizations were interaction with an adult, presence of an adult, interaction with another child, play activity, other motor activities, and no observable causes. Vocalizations were most frequent in social interaction. As the frequency of social interaction with an adult decreased, vocalizations increased on those occasions when the adult was merely physically present. Adding motor activity to social interaction increased vocalization; it decreased, however, when new motor acts were being learned. (23 ref.) (PsycINFO Database Record (c) 2012 APA, all rights reserved)
SN - 0042-8841
UR - http://search.ebscohost.com/login.aspx?direct=true&db=psyh&AN=1969-03744-001&site=ehost-live
ER -

TY - JOUR
ID - 1896
T1 - LONG-TERM EFFECTS OF MULTIPAROUS AND PRIMIPAROUS MONKEY MOTHER REARING
A1 - Mitchell,G.D.
A1 - Ruppenthal,G.C.
A1 - Raymond,E.J.
Y1 - 1966///
KW - PsycInfo
KW - Unsupervised Play Addition
KW - Behavior
KW - PRIMIPAROUS & MULTIPAROUS MOTHERED OFFSPRING
KW - Animal Social Behavior
KW - Mother Child Relations
KW - Monkeys
RP - NOT IN FILE
SP - 781
EP - 791
JF - Child Development
JA - Child Dev.
VL - 37
IS - 4
CY - United Kingdom
PB - Blackwell Publishing

N2 - 4 ADOLESCENT RHESUS MONKEYS BORN OF PRIMIPAROUS MOTHERS WERE COMPARED WITH 4 ADOLESCENTS OF MULTIPAROUS MOTHERS. THEIR SOCIAL AND NONSOCIAL BEHAVIORS WHEN PAIRED WITH ADULT, AGE-MATE, AND JUVENILE STRANGERS WERE RECORDED. THE PRIMIPAROUS-MOTHERED ADOLESCENTS PLAYED LESS WITH THEIR PARTNERS, DISPLAYED FEWER SPONTANEOUS EMOTIONALITY INDEXES, EXHIBITED LESS SELF-DIRECTED BEHAVIOR, AND WERE MORE DISTURBED IN THE PLAYROOM. THE MULTIPAROUS-MOTHERED MONKEYS WERE OBSERVED TO EXPLORE THEMSELVES ORALLY MORE FREQUENTLY IN THE PRESENCE OF ADULT STRANGERS AND WERE MORE HOSTILE TOWARD THEM. YET, THE PROGENY OF THE EXPERIENCED MOTHERS WERE MUCH MORE RELAXED AND PLAYFUL, PARTICULARLY THE MALES. IT IS CONCLUDED THAT (1) "INDIVIDUAL PECULIARITIES" WITH REGARD TO PLAY PERSIST IN THE PRIMIPAROUS-MOTHERED MALES AT PUBERTY; (2) SUBTLE BUT SIGNIFICANT DIFFERENCES BETWEEN THE GROUPS APPEAR EITHER AS A CONSEQUENCE OF AGE OR OF THE MORE DEMANDING STIMULUS STRANGER SITUATION; AND (3) THE PROGENY OF THE MORE REJECTING MOTHERS (MORE EXPERIENCED MOTHERS) ARE MORE HOSTILE, BUT THIS HOSTILITY IS NEITHER BRUTAL NOR ABNORMAL. (PsycINFO Database Record (c) 2012 APA, all rights reserved)
SN - 1467-8624
UR - http://search.ebscohost.com/login.aspx?direct=true&db=psyh&AN=1967-02639-001&site=ehost-live
L3 - 10.2307/1126601
ER -

TY - JOUR
ID - 1897
T1 - Avoidance of feminine toys by kindergarten boys: The effects of adult presence or absence, and an adult's attitudes towards sex-typing
A1 - Kobashigawa,A.
A1 - Arakaki,K.
A1 - Awaguni,A.
Y1 - 1966///
KW - PsycInfo
KW - Unsupervised Play Addition
KW - feminine toys
KW - kindergarten boys
KW - Adult Attitudes
KW - sex typing
KW - Childhood Play Behavior
KW - Sex Roles
KW - Toy Selection
RP - NOT IN FILE
SP - 96
EP - 103
JF - Japanese Journal of Psychology
VL - 37
IS - 2
CY - Japan
PB - Japanese Psychological Assn
N2 - An experiment was conducted to investigate the avoidance of feminine toys by 60 kindergarten boys. 1 group of Ss observed an E who expressed "strict" attitudes toward inappropriately sex-typed behaviors by another child; the 2nd group observed an E who expressed "permissive" attitudes; and the 3rd group observed an E who expressed "neutral" attitudes. Following this, «the Ss were observed while playing with feminine and neutral toys in the presence of E, and the remainder in the absence of E. Ss in the adult-present condition showed a stronger avoidance of feminine toys than Ss in the adult-absent condition. The adult's expressed strict attitudes toward sex-typing strengthened the avoidance of the feminine toys in Ss while the permissive attitudes had a disinhibitory effect on inappropriately sex-typed responses. (PsycINFO Database Record (c) 2012 APA, all rights reserved)
SN - 0021-5236

UR - http://search.ebscohost.com/login.aspx?direct=true&db=psyh&AN=1966-13084-001&site=ehost-live
L3 - 10.4992/jjpsy.37.96
ER -

TY - JOUR
ID - 1898
T1 - Social-class differences in mothers' expectations for children in Indonesia
A1 - Thomas,R.Murray
A1 - Surachmad,Winarno
Y1 - 1962///
KW - PsycInfo
KW - Unsupervised Play Addition
KW - Social Class
KW - & CHILD-REARING IN INDONESIA
KW - CULTURES & COUNTRIES
KW - INDONESIA
KW - CHILD-REARING PRACTICES
KW - SOCIAL CLASS DIFFERENCES IN
KW - Child Rearing
KW - SOCIAL CLASS DIFFERENCES
KW - IN INDONESIA
KW - No terms assigned
RP - NOT IN FILE
SP - 303
EP - 307
JF - The Journal of Social Psychology
VL - 57
IS - 2
CY - US
PB - Heldref Publications
N2 - A comparison of child-raising practices of 100 upper-middle-class with 100 lower-class mothers in Bandung, Indonesia, showed upper-middle mothers expected earlier weaning, reading, and writing. Lower-class mothers expected earlier counting. Differences in expectation for unsupervised play and for a child's feeding himself were not significant. (PsycINFO Database Record (c) 2012 APA, all rights reserved)
SN - 1940-1183
UR - http://search.ebscohost.com/login.aspx?direct=true&db=psyh&AN=1963-04843-001&site=ehost-live
L3 - 10.1080/00224545.1962.9710928
ER -

TY - JOUR
ID - 1900
T1 - Aggressive behavior of young children in the absence of an adult
A1 - Siegel,Alberta Engvall
Y1 - 1957///
KW - PsycInfo
KW - Unsupervised Play Addition
KW - Play
KW - SUPERVISION VS.NONSUPERVISION OF
KW - ADULT ABSENCE EFFECT ON
KW - Guilt
KW - IN PLAY WITH ADULT ABSENCE
KW - childhood
KW - ANXIETY IN
KW - & ADULT
KW - ABSENCE
KW - Aggression

KW - ADULT ABSENCE &
KW - BY CHILD
KW - No terms assigned
RP - NOT IN FILE
SP - 371
EP - 378
JF - Child Development
JA - Child Dev.
VL - 28
CY - United Kingdom
PB - Blackwell Publishing
N2 - "The social play of like-sexed pairs of children, in a situation free of adult supervision. was observed in 2 sessions a week apart. The incidence of aggression and of anxiety and guilt in play decreased from session I to session II. This finding is opposed to earlier findings concerning session differences in aggression in doll play. this disparity may be accounted for by the presence or absence of an adult in the play sessions: In the presence of an adult experimenter, young children may abdicate superego functions to him, whereas in the absence of any adult their own internalized standards increasingly restrict expression of unacceptable drives, with a consequent reduction in anxiety and guilt." (PsycINFO Database Record (c) 2012 APA, all rights reserved)
SN - 1467-8624
UR - http://search.ebscohost.com/login.aspx?direct=true&db=psyh&AN=1959-03446-001&site=ehost-live
ER -

TY - BOOK
ID - 1901
T1 - Children of today and tomorrow
A1 - Dukes,Ethel
A1 - Hay,Margaret
Y1 - 1949///
N1 - Accession Number: 1950-04517-000. First Author & Affiliation: Dukes, Ethel. Release Date: 19500901. Publication Type: Book, (0200); . Media Covered: Print. Language: English. Major Descriptor: No terms assigned. Classification: Developmental Psychology (2800)
KW - PsycInfo
KW - Unsupervised Play Addition
KW - TEXTBOOKS
KW - Child
KW - CARE
KW - PARENT
KW - -CHILD
KW - RELATIONS
KW - TEXTBOOK
KW - Child Care
KW - CHILDHOOD & ADOLESCENCE
KW - No terms assigned
RP - NOT IN FILE
CY - Oxford England
PB - Macmillan
N2 - An approach to problems of "emotional health" which attempts to correct current excessive emphasis on environmental events (and consequent excessive parent guilt feelings.) "In parenthood and in the teaching profession adults are presented with a child who possesses a certain psychic and physical constitution. It is their task (a) to recognize it; (b) to make the best of it." Summaries of play therapy sessions with various kinds of children are included. These include the "hypersensitive," the "dull," the "gifted," the "power-seeking," the "power-rejecting" child, and others. The concluding section deals with the parent-child relationship. Originally published in Great Britain. 32 references. (PsycINFO Database Record (c) 2012 APA, all rights reserved)
UR - http://search.ebscohost.com/login.aspx?direct=true&db=psyh&AN=1950-04517-000&site=ehost-live
ER -

TY - JOUR
ID - 1902
T1 - Freiwillige Schweiger und sprachscheue Kinder
A1 - Spieler,J.
Y1 - 1941///
KW - PsycInfo
KW - Unsupervised Play Addition
KW - Speech
KW - Fear
KW - Child
KW - CHILD (ART
KW - Intelligence
KW - Language
KW - Learning
KW - Play
KW - THOUGHT)
KW - MENTAL TESTS
KW - No terms assigned
RP - NOT IN FILE
SP - 39
EP - 43
JF - Zeitschrift f • r Kinderforschung
VL - 49
N2 - A study of 43 cases of children who, after the development of normal speech, remained in a state of complete or almost complete speechlessness for a prolonged period of time revealed the following: (1) Practically all these children had suffered some sudden and violent nervous or emotional shock which precipitated the attack of speechlessness. Some had been attacked by vicious dogs or other animals; others had suffered severe falls; other, extreme frights or emotional trauma as through the death of a parent. (2) The majority were below par physically. (3) There were more than the usual number of unstable persons in the direct ancestry. Two cases are briefly described by way of illustration. Of these, one talked freely when alone with other children but could not be induced to speak in the presence of adults. The other, as far as could be determined, never spoke at all. The refusal to speak is a means whereby the child symbolically escapes from a social world that has proved too terrifying for him to cope with. (PsycINFO Database Record (c) 2012 APA, all rights reserved)
UR - http://search.ebscohost.com/login.aspx?direct=true&db=psyh&AN=1942-04610-001&site=ehost-live
ER -

TY - JOUR
ID - 1903
T1 - A b□n”ss,g gyermeki megit,l,se
A1 - Nagy,M.V.
Y1 - 1939///
KW - PsycInfo
KW - Unsupervised Play Addition
KW - CHILD (ART
KW - Intelligence
KW - Language
KW - Learning
KW - Play
KW - THOUGHT)
KW - MORAL
KW - Judgment
KW - Child
KW - CHILDHOOD AND ADOLESCENCE
KW - No terms assigned
RP - NOT IN FILE

SP - 116
EP - 127
JF - Psychol.Stud.Univ.Bp.
VL - 3
N2 - This is a report of experiments conducted by the author at the Jean Jacques Rousseau Institute in Geneva on 30 school children aged 7-14 years, most of them aged 11-12 years, by the method of Piaget. It was found that the judgment of guilt goes through 3 developmental stages: (1) Moral law is obligatory only in the presence of adults. (2) Irrespective of the situation, the moral act is judged as the violation of a rule. According to the rigidity of this conception the judgment of the child knows no pardon. Identifying himself with the rule, he condemns the sinner. (3) The action is judged according to the motives of the subject; an offense is considered an act against the conscience of the subject and the interests of society. Rigid judgment is replaced by evaluation which leads to the conception of forgiveness. (PsycINFO Database Record (c) 2012 APA, all rights reserved)
UR - http://search.ebscohost.com/login.aspx?direct=true&db=psyh&AN=1941-03202-001&site=ehost-live
ER -

TY - JOUR
ID - 1904
T1 - Leisure time activities of New York's lower west side
A1 - Robinson,R.
Y1 - 1936///
KW - PsycInfo
KW - Unsupervised Play Addition
KW - LEISURE
KW - CITY CHILD
KW - Child
KW - CITY
KW - SOCIAL FUNCTIONS OF THE INDIVIDUAL
KW - No terms assigned
RP - NOT IN FILE
SP - 484
EP - 493
JF - Journal of Educational Sociology
VL - 9
CY - US
PB - American Sociological Assn
N2 - Diary schedules covering a period of four days in early spring were obtained from 1100 junior high school children (75% foreign parentage). On analysis it was found that the most frequently reported leisure time activities were, in order, listening to radio (chiefly entertainment features), attending motion pictures (84% of which were considered unsuited to children), reading (tabloid newspapers, five-cent magazines, adventure story books) and spending time outdoors (84% unsupervised play on the streets). (PsycINFO Database Record (c) 2012 APA, all rights reserved)
UR - http://search.ebscohost.com/login.aspx?direct=true&db=psyh&AN=1936-04582-001&site=ehost-live
L3 - 10.2307/2262324
ER -

TY - JOUR
ID - 1905
T1 - P„dagogen erliegen dem Fluche der L„cherlichkeit
A1 - Zulliger,H.
Y1 - 1934///
KW - PsycInfo
KW - Unsupervised Play Addition
KW - TEACHER ADJUSTMENT
KW - RIDICULE OF
KW - RIDICULE OF TEACHER
KW - Educational Psychology

KW - No terms assigned
RP - NOT IN FILE
SP - 286
EP - 295
JF - Zeitschrift f□r Psychoanalytische P„dagogik
VL - 8
N2 - In the humorous productions of all races Jews, politicians, mothers-in-law and teachers play an important part. These individuals are somehow considered dangerous and uncomfortable, and ridicule is a socially acceptable method of expressing condemnation. In part the traditional poverty of teachers is responsible; further, their disinclination to manual work, the manifoldness of their interests, their desire to instruct others and their habit of simplified thinking, the endlessness of their task, the uncertainty of results, and the uselessness of much they teach are contributory factors. Though teachers try to counteract this by participating in political movements, parent-teacher and other associations, and by giving lectures, they remain in a class by themselves. In their presence adults watch their manners and grammar. Disappointment results from the conflict between the desire to consider the teacher superhuman and the knowledge of his shortcomings. Compared to the ideal, reality is "ridiculous." This is aggravated by the teacher's taking the parents' place early in the child's life and the subsequent discovery of his lack of authority. The child finds that he has been afraid of something non-existent, a mere shadow. (PsycINFO Database Record (c) 2012 APA, all rights reserved)
UR - http://search.ebscohost.com/login.aspx?direct=true&db=psyh&AN=1935-04827-001&site=ehost-live
ER -

TY - JOUR
ID - 1906
T1 - Die Gestaltungseinheit im Spiel des Grundsch • lers. Eine morphologische Untersuchung auf Grund von Beobachtungen im Schullandheim
A1 - Schultze,W.
Y1 - 1933///
KW - PsycInfo
KW - Unsupervised Play Addition
KW - Play
KW - Child
KW - CHILDHOOD AND ADOLESCENCE
KW - No terms assigned
RP - NOT IN FILE
SP - 1
EP - 68
JF - Archiv f • r die Gesamte Psychologie
VL - 87
CY - Germany
PB - Bouvier Verlag Herbert Grundmann
N2 - This study deals with play in boys 9-10 years old. It describes their activity with especial reference to the attraction for the boys and closure in themselves of play units. Work and play can be distinguished only as regards the realms in which they occur. Play is found in the realm of sheer joy of life. Playing is either spontaneous activity (sand pile, imitation, play-acting, tag, etc.) or it is to a greater or less extent determined by rules not originating with the players (games both with and without specific toys, competitions, etc.). In both cases, but especially the former, the world or ordinary objects frequently undergoes radical changes to suit the play. A chair is a horse, an automobile, the counter of a fish market or a fortress as occasion demands. In all their play none of the children seemed disposed to aid his fellows-even those on his "side" in a game of war. Most pronounced, however, were the influences exerted upon the players by the occurrence of changes internal to the games themselves. Thus pirates quickly became chauffeurs if the piratical cave pierced through an embankment to become a tunnel. Sometimes an additional player would change a small group into an army. Occasionally two independent play groups would find themselves brought together (e.g. each wishing to use the same spade) and a new game combining them all would emerge. The creative productivity of play is stressed. (PsycINFO Database Record (c) 2012 APA, all rights reserved)
UR - http://search.ebscohost.com/login.aspx?direct=true&db=psyh&AN=1933-06039-001&site=ehost-live
ER -

TY - JOUR
ID - 1907
T1 - Leadership among preschool children
A1 - Parten,M.B.
Y1 - 1933/01//
KW - PsycInfo
KW - Unsupervised Play Addition
KW - Sex Differences
KW - Leadership
KW - Preschool Children
KW - Play
KW - Childhood Play Behavior
KW - Early Childhood Development
KW - Human Sex Differences
RP - NOT IN FILE
SP - 430
EP - 440
JF - The Journal of Abnormal and Social Psychology
VL - 27
IS - 4
CY - US
PB - American Psychological Association
N2 - Observations of the spontaneous play of nurseryschool children by the one-minute sampling method showed: that leadership may be consistently recorded by this method; that 60 one-minute samples of behavior furnish reliable measures of leadership when appropriate weights are assigned to the various categories of leadership; and that even at the preschool age there are two definite types of leaders, the "diplomat" and the "bully." The former, by indirect suggestions, controls a large number of children; the latter employs brute force in "bossing" the small group he has chosen for his "gang." Independent play is most characteristic of all ages, but decreases in frequency as the children grow older. Sex differences in leadership are negligible. Leaders somewhat exceed non-leaders in intelligence. There was a trend toward development of leadership as the school year advanced. (PsycINFO Database Record (c) 2012 APA, all rights reserved)
SN - 0096-851X
UR - http://search.ebscohost.com/login.aspx?direct=true&db=psyh&AN=1933-05490-001&site=ehost-live
L3 - 10.1037/h0073032
ER -

TY - CHAP
ID - 1908
T1 - Social behavior in infancy and childhood
A1 - Anderson,John E.
Y1 - 1932///
N1 - Accession Number: 2008-17075-008. First Author & Affiliation: Anderson, John E.. Release Date: 20081215. Correction Date: 20111010. Publication Type: Book, (0200); Edited Book, (0280); . Media Covered: Print. Document Type: Chapter. Book Type: Classic Book; Textbook/Study Guide. Language: English. Major Descriptor: Childhood Development; Infant Development; Psychosocial Development; Social Behavior; Social Processes. Classification: Psychosocial & Personality Development (2840) . Population: Human (10); . Age Group: Childhood (birth-12 yrs) (100) . Intended Audience: Psychology: Professional & Research (PS) . Page Count: 9
KW - PsycInfo
KW - Unsupervised Play Addition
KW - Social Behavior
KW - infancy
KW - childhood
KW - Social Development
KW - social life

KW - Social Attitudes
KW - Childhood Development
KW - Infant Development
KW - Psychosocial Development
KW - Social Processes
RP - NOT IN FILE
SP - 73
EP - 81
T2 - Psychology today: Lectures and study manual
A2 - Bingham,Walter V.
CY - Chicago, IL US
PB - University of Chicago Press
N2 - (from the chapter) Present-day research on the origins of social behavior not only goes back into the early life of the adult for knowledge of the antecedents of his present behavior, but also studies groups of children as they grow in order to observe the process of social development. The beginnings of social life are found in infancy. Almost from birth onward, the infant is more interested in persons than in things. Between the ages of two and five there is a decrease in the tendency to maintain parallel activities and an increase in co-operative or group play. Independent play decreases with age and that definite social attitudes, such as leadership, become more marked. Older children prefer to be in larger and larger groups of their fellows. From these examples of modern researches which are establishing the principles underlying the orderly development of social behavior, we may move on to the factors affecting its development. (PsycINFO Database Record (c) 2012 APA, all rights reserved) (chapter)
UR - http://search.ebscohost.com/login.aspx?direct=true&db=psyh&AN=2008-17075-008&site=ehost-live
L3 - 10.1037/13342-008
ER -

TY - BOOK
ID - 1909
T1 - Anger in young children
A1 - Goodenough,F.L.
Y1 - 1931///
N1 - Accession Number: 1932-01577-000. First Author & Affiliation: Goodenough, F. L.. Release Date: 19320401. Publication Type: Book, (0200); . Media Covered: Print. Language: English. Major Descriptor: No terms assigned. Classification: Developmental Psychology (2800)
KW - PsycInfo
KW - Unsupervised Play Addition
KW - Child
KW - Anger
KW - CHILDHOOD AND ADOLESCENCE
KW - No terms assigned
RP - NOT IN FILE
CY - Oxford England
PB - Univ. of Minnesota
N2 - The report of an investigation, the aim of which was to study the frequency, duration, causes and methods of handling anger outbursts in children in the home. There is a brief review of the literature and an orientation of the present study in the literature. The author's tables show that the frequency of outbursts reaches its peak during the second year; after the second year the outbursts are more frequent and violent for the boys than for the girls. The duration of the outbursts varies but slightly during the first eight years, though the outbursts seem to take on subtle aspects as the age increases. Intrinsic factors showing a distinct relationship to frequency of outbursts are: "child's state of health,. length of time since last meal; and possibly fatigue"; extrinsic factors are size and composition of family group, and the presence of adult or child visitors in the home. There is a marked diurnal variation in the frequency with which anger is shown. The longest outbursts occur when the child is in bed, having his bath, or going to bed; the most frequent, during solitary play or play with playmates, or when the child is in bed. Among the two infants in the study the major sources of anger were conflicts over routine physical habits, problems of social relationship and minor physical discomforts. The relative effectiveness of the different methods of control was determined both statistically and subjectively. The author points out the

importance of consistency in discipline, of preventive as against corrective methods of control, of using the daily schedule only as a tool, of the spirit of tolerance and the feeling of security in the home. A 23-page bibliography. (PsycINFO Database Record (c) 2012 APA, all rights reserved)
UR - http://search.ebscohost.com/login.aspx?direct=true&db=psyh&AN=1932-01577-000&site=ehost-live
ER -

TY - BOOK
ID - 1910
T1 - A study of the unsupervised behavior of a group of institutional children
A1 - Smith,M.
Y1 - 1931///
N1 - Accession Number: 1932-00473-000. First Author & Affiliation: Smith, M.. Release Date: 19320101. Publication Type: Book, (0200); . Media Covered: Print. Language: English. Major Descriptor: No terms assigned. Classification: Developmental Psychology (2800)
KW - PsycInfo
KW - Unsupervised Play Addition
KW - Child
KW - Behavior
KW - CHILDHOOD AND ADOLESCENCE
KW - No terms assigned
RP - NOT IN FILE
CY - Oxford England
PB - Marshall & Bruce
N2 - 46 children (25 boys and 21 girls) ranging in age from four to nine were the subjects and were studied in the play room of the receiving home in Nashville, Tenn. The written records of observation by several trained observers showed a reliability of .94. 20 five-minute periods of observation were sufficient to show consistency in the behavior of the child. The behavior was classified under 62 categories. Results show a reliable sex difference in attacking and being attacked and in "giving." The older age groups showed a reliable difference in surpassing the younger in pattern play and active social behavior. Reliable relationships were sought between the different activities and between the activities and age, weight and height. (PsycINFO Database Record (c) 2012 APA, all rights reserved)
UR - http://search.ebscohost.com/login.aspx?direct=true&db=psyh&AN=1932-00473-000&site=ehost-live
ER -

TY - JOUR
ID - 1911
T1 - The 'negative phase' in relation to the behavior of pubescent girls
A1 - Hurlock,E.B.
A1 - Sender,S.
Y1 - 1930///
KW - PsycInfo
KW - Unsupervised Play Addition
KW - Adolescence
KW - NEGATIVE PHASE
KW - CHILDHOOD AND ADOLESCENCE
KW - No terms assigned
RP - NOT IN FILE
SP - 325
EP - 340
JF - Child Development
JA - Child Dev.
VL - 1
CY - United Kingdom
PB - Blackwell Publishing
N2 - The authors report a study of "negative phase" (a behavior period of two to nine months in length which precedes puberty, according to Charlotte B • hler of Vienna) as it is described in the literature, as it is reported in

a questionnaire sent to teachers, matrons of homes and others, and as it appears in court records of juvenile sex delinquencies. The new data of this study were collected in various sections of the city of New York. The authors advance the following conclusions: (1) "that generally restlessness and instability are concomitants of puberty in girls, but whether this disquietude becomes marked enough to be outstanding depends upon a number of factors, the most important of which are environment and home training. The girls reported showing the behavior characteristic of this phase were from inferior social and economic levels; they lived in poor, congested neighborhoods; were unsupervised in their play and conduct; and suffered from deplorable and undesirable home conditions. . (2) The occurrence of sexual delinquency, it would seem, may be conditioned by a great many factors: insufficient supervision by parents, subnormal intelligence, bad companionship, physical inferiority, debasing surroundings in the home and neighborhood rather than physiological development." The authors lay emphasis upon considering the peculiar personality of the individual which is played upon by emotional appeal as being the fundamental factor in the situation rather than immediate environment or low mental caliber. (PsycINFO Database Record (c) 2012 APA, all rights reserved)
SN - 1467-8624
UR - http://search.ebscohost.com/login.aspx?direct=true&db=psyh&AN=1931-02500-001&site=ehost-live
L3 - 10.2307/1125785
ER -

TY - CHAP
ID - 1912
T1 - Twelve Months versus Eighteen Months
A1 - Gesell,Arnold
Y1 - 1925///
N1 - Accession Number: 2006-07528-022. First Author & Affiliation: Gesell, Arnold; Yale U, Yale Psycho-Clinic, New Haven, CT, US. Release Date: 20060626. Publication Type: Book, (0200); Authored Book, (0240); . Media Covered: Print. Document Type: Chapter. Book Type: Classic Book. Language: English. Major Descriptor: Age Differences; Childhood Development. Classification: Developmental Psychology (2800) . Population: Human (10); . Age Group: Childhood (birth-12 yrs) (100) Infancy (2-23 mo) (140) . Intended Audience: Psychology: Professional & Research (PS) . Page Count: 5
KW - PsycInfo
KW - Unsupervised Play Addition
KW - Age Differences
KW - twelve month olds
KW - eighteen month olds
KW - Development
KW - Childhood Development
RP - NOT IN FILE
SP - 251
EP - 255
T2 - The mental growth of the pre-school child: A psychological outline of normal development from birth to the sixth year, including a system of developmental diagnosis
CY - New York, NY US
PB - MacMillan Co
N2 - Compared behaviors of twelve- vs eighteen-month-olds on a variety of measures. The examiners observed the children's initial reactions to the laboratory setting, when objects were placed before them, when an object was hidden, and when presented with paper and crayon. Independent play was also observed. (PsycINFO Database Record (c) 2012 APA, all rights reserved)
UR - http://search.ebscohost.com/login.aspx?direct=true&db=psyh&AN=2006-07528-022&site=ehost-live
L3 - 10.1037/11012-022
ER -

TY - CHAP
ID - 1913
T1 - Eighteen Months versus Two Years
A1 - Gesell,Arnold
Y1 - 1925///

N1 - Accession Number: 2006-07528-023. First Author & Affiliation: Gesell, Arnold; Yale U, Yale Psycho-Clinic, New Haven, CT, US. Release Date: 20060626. Publication Type: Book, (0200); Authored Book, (0240); . Media Covered: Print. Document Type: Chapter. Book Type: Classic Book. Language: English. Major Descriptor: Age Differences; Childhood Development. Classification: Developmental Psychology (2800) . Population: Human (10); . Age Group: Childhood (birth-12 yrs) (100) Infancy (2-23 mo) (140) Preschool Age (2-5 yrs) (160) . Intended Audience: Psychology: Professional & Research (PS) . Page Count: 6
KW - PsycInfo
KW - Unsupervised Play Addition
KW - eighteen month olds
KW - two year olds
KW - Age Differences
KW - Development
KW - Childhood Development
RP - NOT IN FILE
SP - 256
EP - 261
T2 - The mental growth of the pre-school child: A psychological outline of normal development from birth to the sixth year, including a system of developmental diagnosis
CY - New York, NY US
PB - MacMillan Co
N2 - (from the chapter) Compared behaviors of eighteen-month-olds to two-year-olds on a variety of measures. The examiners observed the children's initial reactions to the laboratory setting, reactions to having objects placed before them, interactions with examiner, imitative behavior, reactions to being presented with paper and crayon, and reactions to lights. Independent play and children's reactions to each other were also examined. (PsycINFO Database Record (c) 2012 APA, all rights reserved) (chapter)
UR - http://search.ebscohost.com/login.aspx?direct=true&db=psyh&AN=2006-07528-023&site=ehost-live
L3 - 10.1037/11012-023
ER -

TY - CHAP
ID - 1914
T1 - Two Years versus Three Years
A1 - Gesell,Arnold
Y1 - 1925///
N1 - Accession Number: 2006-07528-024. First Author & Affiliation: Gesell, Arnold; Yale U, Yale Psycho-Clinic, New Haven, CT, US. Release Date: 20060626. Publication Type: Book, (0200); Authored Book, (0240); . Media Covered: Print. Document Type: Chapter. Book Type: Classic Book. Language: English. Major Descriptor: Age Differences; Childhood Development. Classification: Developmental Psychology (2800) . Population: Human (10); . Age Group: Childhood (birth-12 yrs) (100) Preschool Age (2-5 yrs) (160) . Intended Audience: Psychology: Professional & Research (PS) . Page Count: 5
KW - PsycInfo
KW - Unsupervised Play Addition
KW - two year olds
KW - three year olds
KW - Age Differences
KW - Development
KW - Childhood Development
RP - NOT IN FILE
SP - 262
EP - 266
T2 - The mental growth of the pre-school child: A psychological outline of normal development from birth to the sixth year, including a system of developmental diagnosis
CY - New York, NY US
PB - MacMillan Co
N2 - (from the chapter) Compared behaviors of two- versus three-year olds on a variety of measures. The examiners observed the children's initial reactions to the laboratory setting, behaviors when objects were placed

before them, performance on a multiple choice and command test, performance on a paper-folding task, drawing, and picture completion. Independent play and children's reactions to each other were also observed. (PsycINFO Database Record (c) 2012 APA, all rights reserved) (chapter)
UR - http://search.ebscohost.com/login.aspx?direct=true&db=psyh&AN=2006-07528-024&site=ehost-live
L3 - 10.1037/11012-024
ER -

TY - JOUR
ID - 1916
T1 - Standing for a More Flexible and Functional Future
A1 - Labandz,Stephanie
Y1 - 2013/11//
KW - SportDiscus
KW - Unsupervised Play Addition
KW - *BOTULINUM toxin
KW - *PREVENTION
KW - *SPASTICITY
KW - *BACLOFEN
KW - *NERVOUS system
KW - *SURGERY
KW - *STANDING position
KW - *CHILDREN with disabilities
KW - *REHABILITATION
KW - *CHILDREN
KW - THERAPEUTIC use
KW - CONTRACTURE (Pathology)
KW - Product Design
KW - AIDS for people with disabilities
KW - CEREBRAL palsied children
RP - NOT IN FILE
SP - 16
EP - 19
JA - Rehab Management: The Interdisciplinary Journal of Rehabilitation
VL - 26
IS - 9
N2 - The article reflects on how combining stander use with adjunct treatments help in reducing the impact of joint contractures on independent mobility in children. Further is discussed how joint contractures occur and its negative impact on motion and function. Topics discussed include selective dorsal rhizotomy, intrathecal baclofen and the role of Botulinum Toxin in preventing contraction of the targeted muscle
SN - 08996237
UR -
https://login.proxy.bib.uottawa.ca/login?url=http://search.ebscohost.com/login.aspx?direct=true&db=s3h&AN=92994469&site=ehost-live
ER -

TY - JOUR
ID - 1918
T1 - Building GREAT SKILLS in 2 to 3 Year Olds
A1 - Lawrence,Emma
Y1 - 2013/09//
KW - SportDiscus
KW - Unsupervised Play Addition
KW - *SWIMMING for children -- Training
KW - *SWIMMING instruction
KW - *FLOATING (Swimming)
KW - *SWIMMING

KW - *SWIMMING techniques
KW - *KICKING (Swimming)
KW - SAFETY measures
RP - NOT IN FILE
SP - 45
EP - 47
JA - Swimming in Australia
VL - 30
IS - 1
N2 - The article discusses swimming and water safety teaching for two- to three-year-old children. It notes that by two years of age, children can exhibit independent mobility in deep water with supervision by adults. It relates how children can be taught several types of floating including assisted floating, unassisted streamline floating and back floating. It stresses the importance of physical manipulation when teaching propulsive skills such as kicking and paddling
SN - 14452545
UR -
https://login.proxy.bib.uottawa.ca/login?url=http://search.ebscohost.com/login.aspx?direct=true&db=s3h&AN=90498531&site=ehost-live
ER -

TY - JOUR
ID - 1921
T1 - INDEPENDENT MOTION: Exploring advances in pediatric mobility
A1 - Smith,Rick
Y1 - 2009/06//
KW - SportDiscus
KW - Unsupervised Play Addition
KW - *WHEELCHAIRS
KW - *CHILDREN -- Health
KW - *CHILD development
KW - *PEDIATRICS
KW - DESIGN & construction
KW - POWER tools
RP - NOT IN FILE
SP - 16
EP - 19
JA - Rehab Management: The Interdisciplinary Journal of Rehabilitation
VL - 22
IS - 5
N2 - The article focuses on the modern and power wheelchairs offering greater independence and advantages of mobility in very young clients in the U.S. It examines the benefits of such wheelchairs in the self-confidence, self-help skills, and social and visual perceptual skills development of a particular client. It also explores the safety methods and the possible negative effects of such wheelchairs in the exercise habit of children
SN - 08996237
UR -
https://login.proxy.bib.uottawa.ca/login?url=http://search.ebscohost.com/login.aspx?direct=true&db=s3h&AN=39878689&site=ehost-live
ER -

TY - JOUR
ID - 1922
T1 - Predicting mobility gains among children with cerebral palsy after application of botulinum toxin A. / Predizendo ganhos de mobilidade em crianA as com paralisia cerebral apA3s aplicaA□Aœo de toxina botulA-nica A
A1 - Cury,V.C.R.
A1 - Mancini,M.C.

A1 - Fonseca,S.T.
A1 - Melo,A.P.P.
A1 - Sampaio,R.F.
A1 - Tirado,M.G.A.
Y1 - 2009/01//
KW - SportDiscus
KW - Unsupervised Play Addition
KW - *CHILDREN with disabilities
KW - *BOTULINUM toxin
KW - *MOTOR ability in children
KW - *ACTIVITIES of daily living
KW - *GAIT in humans
KW - CEREBRAL palsied children
KW - ORIENTATION & mobility
KW - botulinum toxin type A
KW - Cerebral Palsy
KW - mobility limitations
KW - Motor Skills
KW - habilidades motoras
KW - limitaA Aæes da mobilidade
KW - paralisia cerebral
KW - toxina botulA-nica tipo A
RP - NOT IN FILE
SP - 44
EP - 51
JA - Brazilian Journal of Physical Therapy / Revista Brasileira de Fisioterapia
VL - 13
IS - 1
N2 - Background: Botulinum toxin A (BTA) is commonly used to treat children with cerebral palsy (CP). However, the variables measured before BTA application and associated with motor function and independent mobility, known as predictors of functional outcomes, have not been well defined. Objective: To identify clinical predictors of gains in functional motor skills and independence of mobility among children with CP, three and six months after BTA application. Methods: This was a convenience sample of children with spastic CP (n=35). Measurements of quantitative gains in motor skills and independence of mobility were taken three and six months after BTA application. These outcomes were observed through repeated applications of two functional tests: GMFM (Gross Motor Function Measure) and PEDI (Pediatric Evaluation of Disability Inventory). These tests evaluated gross motor function while sitting or standing and during transitions between these postures, and also during dynamic activities such as walking, running and jumping, along with the children's independence in mobility tasks. The independent variables included the children's characteristics such as age, severity, topographic diagnosis, neuromuscular-skeletal parameters (range of motion and spasticity), gait quality and performance in the functional tests before BTA. Results: Four predictive models were developed (RAý between 0.58 and 0.83; p<0.05) through the use of CART analysis: two at three months and two at six months after BTA application. The results indicated that children with better gait quality, smaller repertoire of functional motor skills, less independence of locomotion and age below four years and six months before BTA presented greater gains in motor skills and independence in mobility. Conclusion: The results identified significant clinical parameters that can predict functional outcomes of BTA applications among children with CP. ABSTRACT FROM AUTHOR
SN - 14133555
UR - https://login.proxy.bib.uottawa.ca/login?url=http://search.ebscohost.com/login.aspx?direct=true&db=s3h&AN=36889615&site=ehost-live
ER -

TY - JOUR
ID - 1923
T1 - STRIKING A BALANCE

A1 - Kirkner,Amy
A1 - Dworak,Patti
Y1 - 2009/01//
KW - SportDiscus
KW - Unsupervised Play Addition
KW - *CHILDREN with disabilities
KW - *MEDICAL rehabilitation
KW - *EQUIPMENT & supplies
KW - *PEOPLE with disabilities
KW - *PREVENTION
KW - *SKIN
KW - *WHEELCHAIRS
KW - *REHABILITATION
KW - ORIENTATION & mobility
KW - CARE
KW - BEDSORES
KW - CARE & hygiene
RP - NOT IN FILE
SP - 18
EP - 21
JA - Rehab Management: The Interdisciplinary Journal of Rehabilitation
VL - 22
IS - 1
N2 - The article focuses on the mobility systems to be applied in the rehabilitation of pediatric patients with disabilities. It notes that therapist should ensure that pediatric patients have independent mobility at the earliest age possible. It also notes that skin care for patients should be considered to prevent bedsores and irritation. Wheelchairs are also suggested to be effective in ensuring the security of patients. Partnership between therapist and parents of the patients is also discussed
SN - 08996237
UR -
https://login.proxy.bib.uottawa.ca/login?url=http://search.ebscohost.com/login.aspx?direct=true&db=s3h&AN=36133985&site=ehost-live
ER -

TY - JOUR
ID - 1924
T1 - Why a Pediatric Mobile Prone Stander?
A1 - Davis,Dan
Y1 - 2006///Winter
KW - SportDiscus
KW - Unsupervised Play Addition
KW - *POSTURE
KW - *CHILDREN with disabilities
KW - *PEOPLE with disabilities
KW - *SUPINE position
KW - EXCEPTIONAL children
KW - HELPLESSNESS (Psychology)
KW - ORIENTATION & mobility
RP - NOT IN FILE
SP - 33
EP - 45
JA - Palaestra
VL - 22
IS - 1

N2 - Focuses on the implementation of several positioning options for children with physical disabilities. Susceptibility of children with special health care needs to learned helplessness; Advantages of independent mobility to children with disabilities; Information on supine support
SN - 87565811
UR -
https://login.proxy.bib.uottawa.ca/login?url=http://search.ebscohost.com/login.aspx?direct=true&db=s3h&AN=20305063&site=ehost-live
ER -

TY - JOUR
ID - 1925
T1 - Determinants of functional independence and quality of life in children with spina bifida
A1 - Schoenmakers,M.A.G.C.
A1 - Uiterwaal,C.S.P.M.
A1 - Gulmans,V.A.M.
A1 - Gooskens,R.H.J.M.
A1 - Helders,P.J.M.
Y1 - 2005/09//
KW - SportDiscus
KW - Unsupervised Play Addition
KW - *SPINA bifida
KW - *PATIENTS
KW - *QUALITY of life
KW - *LIFE skills
KW - *MUSCLE strength
KW - *ACTIVITIES of daily living
KW - NEUROMUSCULAR diseases in children
KW - NEURAL tube -- Abnormalities
KW - MYELOMENINGOCELE
RP - NOT IN FILE
SP - 677
EP - 685
JA - Clinical Rehabilitation
VL - 19
IS - 6
N2 - Objective: To investigate determinants of functional independence and study which functional abilities were determinants for 'health-related quality of life' in children with myelomeningocele. Design: Cross-sectional study by means of clinical assessment, 'disability' measurement and questionnaires. Uni- and multivariate logistic regression models were used to investigate factors that were determinants for these outcomes. Results were expressed as odds ratios (OR) and 95% confidence intervals (95% CI). Setting: Outpatient spina bifida clinic at a university hospital. Subjects: One hundred and twenty-two children with myelomeningocele. Mean age 7.9; range 1ƒ_"18 years. Main measures: Functional independence as measured by the Pediatric Evaluation of Disability Inventory (PEDI), and quality of life as measured by the Spina Bifida Health Related Quality of Life Questionnaire. Results: Lesion level below L3 (OR 0.4, 95% CI 0.1ƒ_"1.0), mental status of IQ ƒ% • 80 (OR 4.2, 95% CI 1.2ƒ_"14.9), having no contractures in lower extremities (OR 3.4, 95% CI 1.3ƒ_"8.8), and having normal strength of knee extensor muscles (OR 4.1, 95% CI 1.4ƒ_"11.5) were most strongly associated with independence in self-care. Mental status (OR 16.1, 95% CI 2.8ƒ_"93.9), having no contractures in lower extremities (OR 1.5, 95% CI 1.4ƒ_"5.3), and normal strength in knee extensors (OR 11.0, 95% CI 1.3ƒ_"97.0) were the most important determinants for independence in mobility. Concerning functional abilities, being independent with regard to mobility was the most important determinant for 'health-related quality of life' (OR 5.3, 95% CI 1.6ƒ_"17.4). Conclusions: In children with myelomeningocele, good muscle strength, mental ability and being independent in mobility appeared to be much more important for daily life function and quality of life than other medical indicators of the disorder. ABSTRACT FROM AUTHOR
SN - 02692155

UR -
https://login.proxy.bib.uottawa.ca/login?url=http://search.ebscohost.com/login.aspx?direct=true&db=s3h&AN=17705802&site=ehost-live
ER -

TY - JOUR
ID - 1927
T1 - Inter-rater reliability in assigning ICF codes to children with disabilities
A1 - Ogonowski,Julie A.
A1 - Kronk,Rebecca A.
A1 - Rice,Carryn N.
A1 - Feldman,Heidi M.
Y1 - 2004/03/18/
KW - SportDiscus
KW - Unsupervised Play Addition
KW - *DISABILITIES
KW - *HEALTH self-care
KW - *ACTIVITIES of daily living
KW - *HEALTH behavior
KW - *HEALTH
RP - NOT IN FILE
SP - 353
EP - 361
JF - Disability & Rehabilitation
JA - Disabil Rehabil
VL - 26
IS - 6
N2 - Purpose : The purpose of this study was to determine if independent raters would arrive at similar decisions about which codes from the Activities and Participation component of the International Classification of Functioning, Disability, and Health (ICF) to assign to children with disabilities based on the results of standard paediatric functional assessment measures. Method : Children (Nƒ_S=ƒ_S60), 9 months to 17.75 years old, with a range of disabilities were assessed using either the Vineland Adaptive Behavior Scales (n ƒ_S=ƒ_S20), the Pediatric Evaluation of Disability Inventory (PEDI) (n ƒ_S=ƒ_S20), or the School Function Assessment (SFA) (n ƒ_S=ƒ_S20). Two raters independently determined which of 40 codes from the Activities and Participation component of the ICF applied to each child based on items and standard scores from the assessment measure. The Cohen's kappa statistic was used as the measure of inter-rater reliability. Results : The kappa statistic was ⩾ƒ_S0.70, the criterion for good agreement in this study, for 23/40 codes using the Vineland, 17/40 codes using the PEDI, and 17/40 codes using the SFA. The mean kappa statistic reached ⩾ƒ_S0.70 for the Self care domain, using all three tools. The mean kappa statistic also reached ⩾ƒ_S0.70 for Learning and Applying Knowledge and Mobility using the Vineland. Conclusions : Independent raters reached high rates of agreement when assessment test items were structured developmentally and corresponded to a single ICF code. For domains other than Self care, alternative assessment strategies may be necessary to improve assigning ICF codes to children with disabilities. ABSTRACT FROM AUTHOR
SN - 09638288
UR -
https://login.proxy.bib.uottawa.ca/login?url=http://search.ebscohost.com/login.aspx?direct=true&db=s3h&AN=12511442&site=ehost-live
ER -

TY - JOUR
ID - 1928
T1 - Lower Limb Orthotic Management of Duchenne Muscular Dystrophy: A Literature Review
A1 - Stevens,Phillip M.
Y1 - 2006/10//
KW - SportDiscus
KW - Unsupervised Play Addition

KW - *ORTHOPEDICS
KW - *PEOPLE with disabilities
KW - *LEG
KW - *ORTHOPEDIC apparatus
KW - *MUSCULAR dystrophy
KW - *HORSES -- Paces,gaits,etc.
KW - *BIOMECHANICS
KW - *WALKING
KW - *WHEELCHAIR sports
KW - *FRACTURES
KW - *ADRENOCORTICAL hormones
KW - Risk
KW - Research
KW - Child
KW - STANCE
KW - REVIEW
KW - Biomechanics
RP - NOT IN FILE
SP - 111
EP - 119
JA - Journal of Prosthetics & Orthotics (JPO)
VL - 18
IS - 4
CY - ;
N2 - Although currently incurable, Duchenne muscular dystrophy remains treatable. The characteristic gradual loss of functional muscle and the concurrent developments of progressive contracture are often indications for orthotic interventions. As the disease progresses through the abbreviated life of the young man, he will encounter three functional stages: independent ambulation, assisted ambulation, and wheelchair mobility. Although controversy continues as to the appropriate role of orthoses during each of these stages, some generalities may be gleaned from a review of published literature. Specific patterns of weakness, accommodation, and contracture development characterize the initial stage of independent ambulation. Orthotic intervention is often confined to nighttime splints to slow the development of equinus contracture. As weakness and contracture progress, balance becomes increasingly precarious, and independent ambulation is ultimately precluded. Many authors have suggested that a degree of ambulation may be maintained during this phase by combinations of surgery, knee-ankle-foot orthoses and aggressive rehabilitation. The popularity of such procedures has declined since its peak in the 1970s and 1980s. Appropriate timing, patient selection, and rehabilitation appear to be essential in obtaining optimal outcomes. Weakness and contracture continue to progress until even assisted ambulation is precluded and wheelchair confinement ensues. Some authors have suggested a limited role of orthotic intervention in the form of postoperative positional ankle-foot orthoses to prevent recurrence of deformities. The relevance of corticosteroids, fracture incidence, and cognitive ability are also discussed. [ABSTRACT FROM AUTHOR]
SN - 10408800
UR - https://login.proxy.bib.uottawa.ca/login?url=http://search.ebscohost.com/login.aspx?direct=true&db=s3h&AN=SPHS-1037931&site=ehost-live;http://articles.sirc.ca/search.cfm?id=S-1037931;http://www.lww.com/
L2 - http://articles.sirc.ca/search.cfm?id=S-1037931
ER -

TY - JOUR
ID - 1929
T1 - From the Editor: Look In the Mirror
A1 - Roberts,Rachel
Y1 - 2007/04//
KW - SportDiscus
KW - Unsupervised Play Addition
KW - *OUTDOOR recreation for children

KW - *OVERWEIGHT children
KW - *PHYSICAL fitness for children
KW - *OBESITY
KW - *PLAY
KW - *RECREATION
KW - *PARKS
KW - EDITORIALS
RP - NOT IN FILE
SP - 10
EP - 10
JA - Parks & Recreation
VL - 42
IS - 4
N2 - The author introduces the issue's focus on childhood health issues, including obesity, and opportunities for park and recreation agencies to respond. Unsupervised play is becoming less common, so adults need to take steps to encourage play. The author urges adults to model active outdoor behavior, to play with children, and to teach kids that outdoor play is fun
SN - 00312215
UR -
https://login.proxy.bib.uottawa.ca/login?url=http://search.ebscohost.com/login.aspx?direct=true&db=s3h&AN=24715815&site=ehost-live
ER -

TY - JOUR
ID - 1930
T1 - Making SURFACING a Play Asset
A1 - Schappet,Jean
A1 - Malkusak,Antonio
A1 - Bruya,Lawrence
Y1 - 2003/02//
KW - SportDiscus
KW - Unsupervised Play Addition
KW - *PLAYGROUNDS
KW - *PLAY
KW - *PEOPLE with disabilities
KW - LEGAL status,laws,etc.
KW - United States
RP - NOT IN FILE
SP - 60
JA - Parks & Recreation
VL - 38
IS - 2
N2 - Stresses that an integrated playground will support children's need for independent, self-directed play. Elements of play; Criteria for pathways that lead to the playground; Categories of surfacing materials; Significance of the Americans with Disabilities Act in the U.S
SN - 00312215
UR -
https://login.proxy.bib.uottawa.ca/login?url=http://search.ebscohost.com/login.aspx?direct=true&db=s3h&AN=9207827&site=ehost-live
ER -

TY - JOUR
ID - 1931
T1 - A gender perspective on adolescents' perceived safety and security when moving outdoors
A1 - Johansson,K.
A1 - Laflamme,L.

A1 - Eliasson,M.
Y1 - 2010/09/02/
KW - SportDiscus
KW - Unsupervised Play Addition
RP - NOT IN FILE
SP - A230
EP - A230
JA - Injury Prevention
VL - 16
N2 - Adolescents in Sweden can move relatively independently in public space. Girls are, however, in general more limited than boys, for instance due to perceived insecurity in the environment. This study explores Swedish girls and boys understandings of threats to their outdoor safety in connection with their everyday independent mobility, and also how they see the potential threat of traffic. Focus group discussions were conducted with 25 boys and 25 girls, 15ƒ_"16 years old. Transcripts were analyzed with discourse analysis. To girls, rape was seen as a main threat (and paedophiles to younger children of either sex). Both boys and girls described gangs of aggressive adolescent boys as a major threat, for boys in terms of provocations and violence but for girls gangs were a more ambiguous threat. Traffic was described as unpredictable and fast, often because of irresponsible car drivers. However, many adolescents felt they can compensate for drivers irresponsible behaviour by being careful themselves. In conclusion, sexual and physical violence were seen as major threats and were in general clearly gendered. Traffic, though sometimes threatening, was less prominent, and not described as gendered. To improve safety and security in public space is a matter of gender equity, and needs to take into account both girls higher levels of fear and boys often higher risks of injury. ABSTRACT FROM PUBLISHER
SN - 13538047
UR - https://login.proxy.bib.uottawa.ca/login?url=http://search.ebscohost.com/login.aspx?direct=true&db=s3h&AN=66043155&site=ehost-live
ER -

TY - JOUR
ID - 1932
T1 - Developing fundamental movement skills in outdoor settings : three case studies of children playing
A1 - Taggart,A.
A1 - Keegan,L.
Y1 - 1997///
KW - SportDiscus
KW - Unsupervised Play Addition
KW - *PLAY
KW - Case Studies
KW - Child
KW - MOTOR SKILL
RP - NOT IN FILE
SP - 11
EP - 17
JA - ACHPER Healthy Lifestyles Journal
VL - 44
IS - 4
CY - ;
N2 - Recently research has shown that many children are not developing competence in fundamental movement skills. For example, it has been suggested that Victorian children in Grade 2 have major developmental deficits in throwing, catching, striking and kicking. It is clear that early schooling experiences must provide equitable and outcome producing play experiences for children if they are to develop fundamental movement skills that form that basis of future participation in physical activity. Three comparative case studies examined the opportunities that pre-primary children (5 year-olds) have to engage in fundamental movement skills in an outdoor play settings. Direct observation of individuals and small groups allowed for the analysis of the fundamental movement skills performed by children. Results indicated that many children are not engaging in

the manipulative skills of throwing, catching, hitting and kicking while running, jumping and climbing were the predominant skills performed. Further findings indicate the variability in the practice of fundamental movement skills at the different centres relate to time allocation, teacher/adult presence in the play setting, playground design and equipment usage/availability
SN - 13210394
UR -
https://login.proxy.bib.uottawa.ca/login?url=http://search.ebscohost.com/login.aspx?direct=true&db=s3h&AN=SPH455193&site=ehost-live;http://articles.sirc.ca/search.cfm?id=455193
L2 - http://articles.sirc.ca/search.cfm?id=455193
ER -

TY - JOUR
ID - 1933
T1 - The impact of play on development: a meta-analysis
A1 - Fisher,E.P.
Y1 - 1992/05//
KW - SportDiscus
KW - Unsupervised Play Addition
KW - *PLAY
KW - Child Development
KW - REVIEW
KW - Child
RP - NOT IN FILE
SP - 159
EP - 181
JF - Play & Culture
VL - 5
IS - 2
CY - ;
N2 - This paper reviews the results of 46 studies conducted since 1974 on the importance of play to childhood development. A circumscribed area of the literature was inventoried that treats play as the independent variable. A quantitative synthesis of data was achieved using techniques of meta-analysis. Methodological and substantive features were systematically coded, and each study weighted for quality of design. Effect size findings provide convincing evidence of the impact of play, which appears to promote improved performance outcomes in both cognitive-linguistic and affective-social domains. Notable differences were discovered among the effects of different types of play, and especially robust results were obtained for child-oriented sociodrama
SN - 08944253
UR -
https://login.proxy.bib.uottawa.ca/login?url=http://search.ebscohost.com/login.aspx?direct=true&db=s3h&AN=SPH340580&site=ehost-live;http://articles.sirc.ca/search.cfm?id=340580
L2 - http://articles.sirc.ca/search.cfm?id=340580
ER -

TY - JOUR
ID - 1934
T1 - Children's play as an indicator of cross-cultural and intra-cultural difference
Y1 - 1970///
KW - SportDiscus
KW - Unsupervised Play Addition
KW - *PLAY
KW - *GAMES
KW - *SPORTS
KW - CROSS-cultural studies
KW - Methodology
KW - Research
KW - Japan

KW - United States
KW - Child
KW - SEX FACTOR
RP - NOT IN FILE
CY - ;
N2 - A method of analyzing cross-cultural data on children's play as an index to differential cultural impact was proposed and tested. The hypotheses were: 1) that children's play will show a developmental sequence independent of the culture, and 2) that differences between sexes and between rural-urban groups reflect cross-cultural rather than developmental differences. The hypotheses were supported
UR -
https://login.proxy.bib.uottawa.ca/login?url=http://search.ebscohost.com/login.aspx?direct=true&db=s3h&AN=SPH20813&site=ehost-live;http://articles.sirc.ca/search.cfm?id=20813
L2 - http://articles.sirc.ca/search.cfm?id=20813
ER -

TY - JOUR
ID - 1935
T1 - Active Kids
A1 - Thompson,Dixie L.
Y1 - 2006/07//
KW - SportDiscus
KW - Unsupervised Play Addition
KW - *PHYSICAL fitness
KW - *CARDIOVASCULAR fitness
KW - *STRESS (Physiology)
KW - *PLAY
KW - *OBESITY
KW - *CHRONIC diseases
KW - TEENAGERS
KW - Child
RP - NOT IN FILE
SP - 4
EP - 4
JA - ACSM's Health & Fitness Journal
VL - 10
IS - 4
CY - ;
N2 - A discussion on how adults can take an active role in fighting the rise in obesity and chronic disease in children and adolescents through physical activity is provided. The following tips for adults are presented: encourage regular physical activity, discourage sedentary behaviours and poor food choices, and become a community advocate
SN - 10915397
UR -
https://login.proxy.bib.uottawa.ca/login?url=http://search.ebscohost.com/login.aspx?direct=true&db=s3h&AN=SPHS-1027904&site=ehost-live;http://articles.sirc.ca/search.cfm?id=S-1027904;http://www.lww.com
L2 - http://articles.sirc.ca/search.cfm?id=S-1027904
ER -

TY - JOUR
ID - 1936
T1 - Adapting Equipment for Children With Disabilities
Y1 - 1989/01//
KW - SportDiscus
KW - Unsupervised Play Addition
KW - *DEVELOPMENTAL disabilities
KW - *PLAY

KW - CEREBRAL palsied children
KW - EXCEPTIONAL children
KW - Toys
RP - NOT IN FILE
SP - 13
EP - 13
JA - Adapted Physical Activity Quarterly
VL - 6
IS - 1
N2 - This article summarizes the research paper "Making toys accessible for children with cerebral palsy," by C. Schaeffler, in "Teaching Exceptional Children." The author lists and describes adaptations of toys that have been found to be most effective in enhancing active independent use of play materials for children with cerebral palsy
SN - 07365829
UR -
https://login.proxy.bib.uottawa.ca/login?url=http://search.ebscohost.com/login.aspx?direct=true&db=s3h&AN=20724807&site=ehost-live
ER -

TY - JOUR
ID - 1937
T1 - Improving the independent play of severely mentally retarded children
A1 - Hopper,C.
A1 - Wambold,C.
Y1 - 1981///
KW - SportDiscus
KW - Unsupervised Play Addition
KW - *PEOPLE with disabilities
KW - *MENTAL retardation
KW - *PLAY
KW - *TEACHING
KW - ABILITY
KW - Child
KW - DISABILITIES-Mental retardation - activities
RP - NOT IN FILE
CY - ;
UR -
https://login.proxy.bib.uottawa.ca/login?url=http://search.ebscohost.com/login.aspx?direct=true&db=s3h&AN=SPH116432&site=ehost-live;http://articles.sirc.ca/search.cfm?id=116432
L2 - http://articles.sirc.ca/search.cfm?id=116432
ER -

TY - JOUR
ID - 1938
T1 - Continuity and discontinuity in play conditioning: the adult-child connection
A1 - Kelly-Byrne,D.
Y1 - 1984///
KW - SportDiscus
KW - Unsupervised Play Addition
KW - *PLAY
KW - ADULTHOOD
KW - Interpersonal Relations
KW - COMPARATIVE studies
KW - MARRIAGE
KW - Child
RP - NOT IN FILE

CY - ;
N2 - Examines play as a continuous and a discontinuous behaviour. Presents descriptions of adult play which differ from those commonly held by culture and suggests that they are similar to descriptions of a child's play behaviour amongst friends. Examines similarities in the play of the two age groups. Argues that what might be discontinuity in the culture is not the behaviour we call play but rather the attitudes toward the play of children and adults
UR -
https://login.proxy.bib.uottawa.ca/login?url=http://search.ebscohost.com/login.aspx?direct=true&db=s3h&AN=SPH143260&site=ehost-live;http://articles.sirc.ca/search.cfm?id=143260
L2 - http://articles.sirc.ca/search.cfm?id=143260
ER -

TY - JOUR
ID - 1939
T1 - The Neglected Factor-Play
A1 - Feitelson,D.
A1 - Ross,G.S.
Y1 - 1973///
KW - SportDiscus
KW - Unsupervised Play Addition
KW - *ACHIEVEMENT
KW - *PLAY
KW - CREATIVE ability
KW - Culture
KW - ABILITY
KW - Child
RP - NOT IN FILE
SP - 202
EP - 223
JA - International Journal of Child Psychotherapy
VL - 1
IS - 3July
N2 - Investigates whether modelling is an essential prerequisite for the emergence of thematic play and whether there is a link between level of thematic play and performance on conventional creativity tests. Twenty-four Ss were first assessed on level of thematic play and innovative and exploratory behavior. They were then assigned to one of four treatment groups- individual play tutoring, access to play props unaccompanied by tutoring, individual tutoring in music, no intervention. Finally, level of thematic play and performance on creativity tests were assessed again. Results were in the predicted directions
UR -
https://login.proxy.bib.uottawa.ca/login?url=http://search.ebscohost.com/login.aspx?direct=true&db=s3h&AN=SPH331425&site=ehost-live;http://articles.sirc.ca/search.cfm?id=331425
L2 - http://articles.sirc.ca/search.cfm?id=331425
ER -

TY - JOUR
ID - 1942
T1 - Four-part choral synthesis system for investigating intonation in a cappella choral singing
A1 - Howard,David
A1 - Daffern,Helena
A1 - Brereton,Jude
Y1 - 2013/10//
KW - CINAHL
KW - Unsupervised Play Addition
KW - Voice Quality -- Evaluation
KW - Singing -- Evaluation
KW - Computers and Computerization -- Utilization

KW - Singing -- Psychosocial Factors
KW - Human
KW - Funding Source
KW - Larynx -- Physiology
KW - Performing Arts
KW - Speech -- Organizations
KW - Singing -- Classification
KW - Video Games
KW - Radio Frequency Identification
KW - Graphical User Interface
KW - Speech Acoustics
KW - Data Analysis Software
KW - Audiorecording
KW - Female
KW - Young Adult
KW - Students,Undergraduate
KW - Electronic Spreadsheets
RP - NOT IN FILE
SP - 135
EP - 142
JA - Logopedics Phoniatrics Vocology
VL - 38
IS - 3
U2 - PMID: 23855264
N2 - Accurate tuning is an important aspect of singing in harmony in the context of a choir or vocal ensemble. Tuning and 'pitch drift' are concerning factors in performance for even the most accomplished professional choirs when singing a cappella (unaccompanied). In less experienced choirs tuning often lacks precision, typically because individual singers have not developed appropriate listening skills. In order to investigate accuracy of tuning in ensemble singing situations, a chorally appropriate reference is required against which frequency measurements can be made. Since most basic choral singing involves chords in four parts, a four-part reference template is used in which the fundamental frequencies of the notes in each chord can be accurately set. This template can now be used in experiments where three of the reference parts are tuned in any musical temperament (tuning system), in this case equal and just temperaments, and played over headphones to a singer to allow her/his tuning strategy to be investigated. This paper describes a practical implementation of a four-part choral synthesis system in Pure Data (Pd) and its use in an investigation of tuning of notes by individual singers using an exercise originally written to explore pitch drift in a cappella choral singing
SN - 1401-5439
AD - Audio Laboratory, Department of Electronics, University of York, Heslington, York, YO10 5DD, UK
UR - http://search.ebscohost.com/login.aspx?direct=true&db=cin20&AN=2012309973&site=ehost-live
ER -

TY - JOUR
ID - 1944
T1 - Independent Mobility is Key to Overall Child Development
A1 - Kuntzler,Patrice
Y1 - 2013/07//
KW - CINAHL
KW - Unsupervised Play Addition
KW - Child,Disabled
KW - Child Development
KW - Assistive Technology Devices -- In Infancy and Childhood
KW - Attitude to Disability
KW - Child
RP - NOT IN FILE
SP - 53
EP - 55

JA - Exceptional Parent
VL - 43
IS - 7
SN - 0046-9157
AD - Vice President, Program Development, Henry Viscardi School, The Viscardi Center in Albertson, NY
UR - http://search.ebscohost.com/login.aspx?direct=true&db=cin20&AN=2012476392&site=ehost-live
ER -

TY - JOUR
ID - 1947
T1 - Substance use in young adults with pediatric-onset spinal cord injury
A1 - Hwang,,M
A1 - Chlan,,K,M
A1 - Vogel,,L,C
A1 - Zebracki,,K
Y1 - 2012/07//
KW - CINAHL
KW - Unsupervised Play Addition
KW - Spinal Cord Injuries -- In Infancy and Childhood
KW - Substance Abuse -- Epidemiology -- In Adulthood
KW - Cross Sectional Studies
KW - United States
KW - Interviews
KW - Human
KW - Adult
KW - Child
KW - Structured Questionnaires
KW - Clinical Assessment Tools
KW - Scales
KW - Questionnaires
KW - Chi Square Test
KW - Mann-Whitney U Test
KW - Step-Wise Multiple Regression
KW - Logistic Regression
KW - Descriptive Research
KW - Male
KW - Female
KW - Funding Source
RP - NOT IN FILE
SP - 497
EP - 501
JF - Spinal Cord
VL - 50
IS - 7
U2 - PMID: 22370762
N2 - Study design:Cross-sectional surveyObjectives:To assess the prevalence of substance use in young adults with pediatric-onset spinal cord injury (SCI) and its relationship with demographic factors, and medical and psychosocial outcomes.Setting:Young adults living in the United States who received pediatric SCI care at one of three SCI programs at the Shriners Hospitals for Children.Methods:Individuals aged 21-25 years who had sustained SCI before the age of 19 were interviewed with a structured questionnaire including standardized outcome measures: FIM, Satisfaction with Life Scale (SWLS), Short-Form 12 Health Survey (SF-12), Patient Health Questionnaire-9 (PHQ-9) Depression Scale, and Craig Handicap Assessment and Recording Technique (CHART).Results:Sample included 215 individuals with a mean age at interview of 23.3 (s.d.=0.9) years and mean age at injury of 13.2 (s.d.=4.9) years. In all, 24% had a college degree, 36% were employed and 12% were married. Regular substance use was reported by 28% for tobacco, 55% for alcohol and 11% for marijuana. Tobacco use was associated with depressive symptoms and unemployment; alcohol use was associated with

having a college degree, single status and independent mobility; and marijuana use was associated with not having a college degree. There were no significant associations between substance use and injury-related factors or life satisfaction.Conclusion:Substance use in young adults with pediatric-onset SCI was associated with factors such as education, employment, marital status and depressive symptoms. Clinicians caring for youth with SCI should counsel patients and caregivers regarding the use of substances and potential associations with outcomes in adulthood
SN - 1362-4393
AD - 1] Marquette University, Milwaukee, WI, USA [2] Shriners Hospitals for Children, Chicago, IL, USA
Shriners Hospitals for Children, Chicago, IL, USA
1] Shriners Hospitals for Children, Chicago, IL, USA [2] Rush Medical College, Chicago, IL, USA
UR - http://search.ebscohost.com/login.aspx?direct=true&db=cin20&AN=2011600643&site=ehost-live
ER -

TY - JOUR
ID - 1948
T1 - Rethinking passive transport: Bus fare exemptions and young people's wellbeing
A1 - Jones,A.
A1 - Steinbach,R.
A1 - Roberts,H.
A1 - Goodman,A.
A1 - Green,J.
Y1 - 2012/05//
KW - CINAHL
KW - Unsupervised Play Addition
KW - Motor Vehicles -- Economics
KW - Personal Satisfaction
KW - Transportation -- Economics
KW - Adolescence
KW - Child
KW - Female
KW - Focus Groups
KW - Human
KW - Interviews
KW - England
KW - Male
KW - Public Health
KW - Transportation -- Methods
RP - NOT IN FILE
SP - 605
EP - 612
JF - Health & Place
JA - Health Place
VL - 18
IS - 3
U2 - PMID: 22321902
N2 - Much recent public health research has emphasised the health impacts for young people of 'active travel' modes, typically defined as walking and cycling. Less research has focused on public transport modes. Drawing on qualitative data, we examine the links between bus travel and wellbeing in London, where young people currently have free bus travel. Our findings indicate that bus travel can be both a physically and socially active experience for young people. We suggest a more nuanced understanding of 'active travel' is now needed, alongside greater attention to urban public transport networks as key sites that impact on important determinants of wellbeing such as independent mobility and social inclusion
SN - 1353-8292
AD - Department of Health Services Research & Policy, London School of Hygiene & Tropical Medicine, 15-17 Tavistock Place, London WC1H 9SH, UK
UR - http://search.ebscohost.com/login.aspx?direct=true&db=cin20&AN=2011510212&site=ehost-live

ER -

TY - JOUR
ID - 1950
T1 - Use of the Wii Fit system for the treatment of balance problems in the elderly: a feasibility study
A1 - Brown,Riki
A1 - Burstin,Arie
A1 - Sugarman,Heidi
Y1 - 2011/04//
KW - CINAHL
KW - Unsupervised Play Addition
KW - Balance Training,Physical -- In Old Age
KW - Video Games
KW - Accidental Falls -- Epidemiology
KW - Aged,80 and Over
KW - Case Studies
KW - Clinical Assessment Tools
KW - Female
KW - Human
KW - Pilot Studies
KW - Questionnaires
KW - Scales
KW - Stroke -- Complications
KW - Treatment Outcomes
RP - NOT IN FILE
SP - 32
EP - 32
JA - Journal of the Israeli Physical Therapy Society (JIPTS)
VL - 13
IS - 1
N2 - Background: The rate of falls among patients following a stroke is twice the rate seen in an age and gender matched population. In recent years, the use of virtual reality (VR) for the treatment of balance problems in the elderly has become increasingly widespread. This paper is a report of the feasibility and outcome of using a low-cost, commercially available gaming system - Wii Fit - for balance training after stroke. Methods: The patient was an 86 year-old woman five weeks after stroke, who was unable to walk without close supervision, even with a walker, due to poor balance and a tendency to fall. In addition to standard physical therapy, the patient participated in four training sessions with the Wii Fit system; each training session included four different games. The outcome measures were the Berg Balance Scale (BBS), the Functional Reach and Lateral Reach tests, Timed Up & Go test (TUG), computerized posturography, and the Short Feedback Questionnaire (SFQ). The tests were done before the intervention and on the last day of training. The SFQ was done during the third training session. Results: The patient greatly enjoyed the training sessions and felt that she was receiving very 'up-to-date' treatment. She did not experience any discomfort such as nausea or dizziness while using the system. Following combined Wii and standard treatment, the patient demonstrated improved antero-posterior symmetry of stance, and there was a 10 second improvement in her TUG score. When released from hospital, she was able to walk with a walker with minimal supervision. Conclusions: This initial pilot study indicates that the Wii Fit gaming system has the potential to be used in clinical settings in order to improve balance. A full clinical trial is necessary in order to examine this premise
AD - Beit Rivka Geriatric Rehabilitation Center Petach Tikva, Israel
Research Center for the Health Professions, Ono Academic College, Kiryat Ono, Israel
UR - http://search.ebscohost.com/login.aspx?direct=true&db=cin20&AN=2011037884&site=ehost-live
ER -

TY - THES
ID - 1951
T1 - Motion-Based Video Games for Stroke Rehabilitation with Reduced Compensatory Motions
A1 - Alankus,Gazihan

Y1 - 2011///
N1 - Accession Number: 2012158634. Language: English. Entry Date: 20130628. Publication Type: doctoral dissertation; research. Special Interest: Physical Therapy
KW - CINAHL
KW - Unsupervised Play Addition
KW - Stroke -- Rehabilitation
KW - Video Games -- Utilization
KW - Evaluation Research
KW - Exercise Positions
KW - Feedback
KW - Home Rehabilitation
KW - Human
KW - Recovery
RP - NOT IN FILE
EP - 238
PB - Washington University in St. Louis
N2 - Stroke is the leading cause of long-term disability among adults in industrialized nations, with 80% of people who survive strokes experiencing motor disabilities. Recovery requires daily exercise with a high number of repetitions, often without therapist supervision. Motion-based video games can help motivate people with stroke to perform the necessary exercises to recover. We explore the design space of video games for stroke rehabilitation using Wii remotes and webcams as input devices, and share the lessons we learned about what makes games therapeutically useful. We demonstrate the feasibility of using games for home-based stroke therapy with a six-week case study. We show that exercise with games can help recovery even 17 years after the stroke, and share the lessons that we learned for game systems to be used at home as a part of outpatient therapy. As a major issue with home-based therapy, we identify that unsupervised exercises lead to compensatory motions that can impede recovery and create new health issues. We reliably detect torso compensation in shoulder exercises using a custom harness, and develop a game that meaningfully uses both exercise and compensation as inputs. We provide in-game feedback that reduces compensation in a number of ways. We evaluate alternative ways for reducing compensation in controlled experiments and show that using techniques from operant conditioning are effective in significantly reducing compensatory behavior compared to existing approaches
SN - 978-1-267-09807-8
AV - UMI Order AAI3489930.
M1 - Ph.D.
UR - http://search.ebscohost.com/login.aspx?direct=true&db=cin20&AN=2012158634&site=ehost-live
ER -

TY - JOUR
ID - 1954
T1 - Online risks obstructing safe Internet access for students
A1 - Lazarinis,F.
Y1 - 2010///
KW - CINAHL
KW - Unsupervised Play Addition
KW - Internet
KW - Safety
KW - Students,College -- Greece
KW - Access to Information
KW - Advertising
KW - Bullying
KW - Child Abuse,Sexual
KW - discrimination
KW - Gambling
KW - Greece
KW - Human
KW - Language

KW - Photography
KW - Pornography
KW - Racism
KW - Search Engines
KW - Stereotyping
KW - Terrorism
KW - Theft
KW - Video Games
KW - Violence
RP - NOT IN FILE
SP - 157
EP - 170
JA - Electronic Library
VL - 28
IS - 1
N2 - Purpose -- The purpose of this paper is to provide an overall insight into the factors which undermine safe internet access for students. Safer internet access for students is an issue raised during recent years as the web becomes increasingly more accessible to children. However, recent research studies and initiatives demonstrated a number of potential online risks for students. To be able to protect children from potentially hazardous content it is first necessary to have a good understanding of the existing online threats. Design/methodology/approach -- Initially, prior works on online safety problems for children are reviewed and a list of online risks is assembled based on the previous studies and on information gathered from web sites dedicated to safer internet access. Then, based on this listing, an access log of unsupervised internet access of high school students is analyzed to understand which of these risks can be identified. Findings -- The review of previous works on the topic of safer internet access produces a list of diverse problems for unsupervised internet access for children. The results of the log files evaluation show that several of the identified online risks existed in the students' visited sites and that the students are able to bypass the applied restrictions to visit their preferred sites. Originality/value -- The list of online risks can be used by researchers, teachers, parents, educational technologists, web designers, school administrators and libraries to obtain a clearer view of the internet safety issues. Further, this list could be used for directing future research projects on the social consequences of internet usage for children
SN - 0264-0473
AD - Lecturer, Technological Educational Institute of Mesolonghi, Mesolonghi, Greece
UR - http://search.ebscohost.com/login.aspx?direct=true&db=cin20&AN=2010610758&site=ehost-live
ER -

TY - THES
ID - 1955
T1 - Towards a Framework for Post-Stroke Interactive and Entertaining Rehabilitation with Reactive Objects
A1 - Alamri,Atif
Y1 - 2010///
N1 - Accession Number: 2012250397. Language: English. Entry Date: 20130913. Publication Type: doctoral dissertation; research. Special Interest: Physical Therapy
KW - CINAHL
KW - Unsupervised Play Addition
KW - Rehabilitation -- Methods
KW - Stroke -- Rehabilitation
KW - Disabled
KW - Exercise
KW - Human
KW - Stroke -- Complications
KW - Stroke -- Mortality
KW - Video Games
KW - Virtual Reality
RP - NOT IN FILE
EP - 125
PB - University of Ottawa (Canada)

N2 - A stroke has been considered one of the leading causes of death in the world. Survivors may suffer minor or major disabilities in their cognitive and motor capabilities, and as a result, they are unable to carry out their usual daily life activities. Thus, they go through a rehabilitation process to recover their motor abilities up to a certain extent. Virtual Reality (VR) technologies have been used to provide entertaining environments for stroke patients to relearn basic motor function movements. Haptic technologies incorporated with virtual environments allow patients to feel and touch virtual objects

In this thesis, we present a new framework based on Augmented Reality (AR) technology that can increase the patients involvement in the rehabilitation exercise, and at the same time, measure the patients performance without the direct supervision of a therapist. We called this system SIERRA, for post-Stroke Interactive and Entertaining Rehabilitation with ReActive objects

The framework uses AR technology to provide a natural exercise environment and motivating virtual objects. It adopts the Serious Games concept to provide patients with a more entertaining environment for treatments. We seamlessly superimpose virtual objects into a real environment and patients can interact with them in motivating game scenarios using a tangible object. In the framework, the tangible object is three-dimensionally tracked by using vision analysis algorithms and the movement of the tangible object is mapped on a virtual avatar that can interact with the virtual environment. Since vibrotactile actuators are attached to this tangible object, the patient can experience haptic/tactile feedback as well as audiovisual feedback. The usability study we conducted with stroke patients shows that the serious games with vibrotactile feedback offers advantages both in terms of improving the interest of patients for the game and in dealing with the realism of the games

SN - 978-0-494-87855-2
AV - UMI Order AAINR87855.
M1 - Ph.D.
UR - http://search.ebscohost.com/login.aspx?direct=true&db=cin20&AN=2012250397&site=ehost-live
ER -

TY - JOUR
ID - 1956
T1 - Investigation of mobility levels in visually impaired children: a pilot study [Turkish]
A1 - Uysal,S.A.
A1 - Aki,E.
Y1 - 2009/12//
KW - CINAHL
KW - Unsupervised Play Addition
KW - Physical Mobility -- Evaluation
KW - Rehabilitation of Vision Impaired
KW - Child
KW - Female
KW - Human
KW - Male
KW - Pilot Studies
KW - Questionnaires
KW - Task Performance and Analysis
RP - NOT IN FILE
SP - 201
EP - 206
JA - Fizyoterapi Rehabilitasyon
VL - 20
IS - 3

N2 - Purpose: The aim of this pilot study was to investigate the mobility level functions in children with visual impairment. Material and methods: The study included 30 normal vision subjects whose ages were 9.36A ñ2.39 years, 30 low vision subjects whose ages were 9.42A ñ2.37 years and 30 total blind children whose ages were 9.41A ñ2.18 years. Independent Mobility Questionnaire was performed on all the children. Results: When the levels of mobility was compared among the groups, children with normal vision got significantly better test scores than the low vision and total blind groups (p<0.05). Nevertheless, significant differences were not found between low vision and blind children's mobility levels. Conclusion: According to the results of this study, we found that mobility skills were affected by every level of loss of visual function. Visual input and experiences

which will be used during life are especially gained in the first 8-10 years and children in our study group were still in their learning stage. Therefore, inadequate visual sensory inputs could cause mobility problems which could affect their lives. Also the Independent Mobility Questionnaire is a useful evaluation tool for visually impaired children as it can be applied in a short time and includes different mobility activities. As the visually impaired children need help in their activities, the questionnaire was not found to be sensitive enough to evaluate the activities that need help. However, no alternative method exists in the literature, so we believe that the questionnaire is important as it can provide information about visually impaired children's mobility levels
SN - 1300-8757
AD - Hacettepe University, Faculty of Health Sciences, Department of Physical Therapy and Rehabilitation, Ankara, Turkey
UR - http://search.ebscohost.com/login.aspx?direct=true&db=cin20&AN=2010610730&site=ehost-live
ER -

TY - JOUR
ID - 1958
T1 - The effects of the Norsk Funktion-walking orthosis on the walking ability of children with cerebal palsy and severe gait impairment
A1 - Kuenzle,C.
A1 - Brunner,R.
Y1 - 2009/07//
KW - CINAHL
KW - Unsupervised Play Addition
KW - Cerebral Palsy -- Complications
KW - Cerebral Palsy -- Physiopathology
KW - Orthoses
KW - Walking
KW - Adolescence
KW - Balance,Postural
KW - Chi Square Test
KW - Child
KW - Child,Preschool
KW - Clinical Assessment Tools
KW - Data Analysis Software
KW - Female
KW - Funding Source
KW - Male
KW - Nonexperimental Studies
KW - Outcome Assessment
KW - Prospective Studies
KW - Questionnaires
KW - Torso
KW - Two-Way Analysis of Variance
KW - Wilcoxon Rank Sum Test
KW - Human
RP - NOT IN FILE
SP - 138
EP - 144
JA - Journal of Prosthetics & Orthotics (JPO)
VL - 21
IS - 3
N2 - The purpose of this study was 1) to test whether an orthosis, which provides postural stability of the trunk and guides the leg movements during walking (Norsk Funktion-walker orthosis [NFWO]), would enable nonambulant children with cerebral palsy with poor or no leg coordination and with little or no trunk control to walk on their own, 2) to investigate if there is an increase in motor function and activity while using a NFWO, and 3) defining requirements for a successful provision. Ninety-three children (39 girls, 54 boys; mean age 7.6 years; 67 with bilateral spastic, 10 with dyskinetic, 10 with mixed, 6 with ataxic cerebral palsy; Gross Motor

Function classification System level 4: 45, level 5: 48) were provided with a NFWO. The following assessments were carried out immediately before and 3 months after using the NFWO: WeeFIM walking score; independence rating by parents or caregivers; aims or expectations of parents or caregivers; at a mean interval of 265 days after provision: mean daily walking distance (meters). With the NFWO, 78 children (84%) became ambulatory, 10 children (11%) used it exclusively as a dynamic standing frame only, and 5 children (5%) returned the NFWO. The mean daily walking distance was 99 m (2-463 m). The mean WeeFIM walking score of 1.99 (SD 0.83) without the NFWO increased to 4.42 (SD 1.00) with the NFWO indoors and to 3.71 (SD 1.24) (p < 0.001) outdoors. Independence rating by parents or caregivers reflected a highly significant increase in independent mobility with the NFWO compared with locomotion without ambulatory aides (p < 0.001) and of bilateral hand function (p < 0.001). No significant increase in the mobility was found when comparing former mobility aides (wheelchair, tricycle) with the NFWO. The ability to cross obstacles did neither significantly increase with the NFWO. Successful indication for a NFWO depends on 1) the child's motivation to walk and the support of the environment to achieve independent walking mobility through the use of this assistive tool, 2) the ability for selective reciprocal leg movements, and 3) no flexion contractures of hips and knees above 20[degrees] and a foot dorsiflexion of at least neutral-0[degrees]. The NFWO proved to be a useful ambulatory aid in the children with cerebral palsy with severe gait impairment to increase independent mobility
SN - 1040-8800
AD - Department of Rehabilitation and Development, East Swiss Children's Hospital, Claudiusstr. 6, CH-9006 Saint Gallen, Switzerland
UR - http://search.ebscohost.com/login.aspx?direct=true&db=cin20&AN=2010374210&site=ehost-live
ER -

TY - JOUR
ID - 1959
T1 - Children's active free play in local neighborhoods: a behavioral mapping study
A1 - Veitch,J.
A1 - Salmon,J.
A1 - Ball,K.
Y1 - 2008/10//
KW - CINAHL
KW - Unsupervised Play Addition
KW - Child Behavior
KW - Motor Activity
KW - Play and Playthings
KW - Residence Characteristics
KW - Child
KW - Environment
KW - Female
KW - Male
KW - Social Class
KW - Human
RP - NOT IN FILE
SP - 870
EP - 879
JF - Health Education Research
JA - Health Educ Res.
VL - 23
IS - 5
U2 - PMID: 18039726
N2 - Many Australian children are more sedentary than they should be, and almost one in five are currently overweight or obese. Some children may face difficulties finding opportunities to be active, having poor access to safe public open spaces or having low independent mobility limiting their access to places to play. This study aimed to examine children's access to places in their neighborhood for active free play and how these vary by age, sex and socioeconomic status (SES). Behavioral maps of the local neighborhood were completed by children (8-12 years) from five primary schools across different areas of Melbourne. Children living in low SES outer-urban neighborhoods had to travel greater distances to access local parks compared with those in inner-

urban mid and high SES areas. One-third (32%) of children reported an independent mobility range of <100 m from home. In conclusion, for some children opportunities to engage in active free play in the local neighborhood may be limited due to lack of parks in close proximity to home and restricted independent mobility. It is important to collaborate with local governments, urban planners and community groups to improve access to neighborhood parks and to promote a sense of neighborhood safety
SN - 0268-1153
AD - Centre for Physical Activity and Nutrition Research, Deakin University, Burwood, Victoria 3125, Australia. jveitch@deakin.edu.au
UR - http://search.ebscohost.com/login.aspx?direct=true&db=cin20&AN=2010076322&site=ehost-live
ER -

TY - JOUR
ID - 1960
T1 - Focused attention in toddlers: measurement, stability, and relations to negative emotion and parenting
A1 - Gaertner,B.M.
A1 - Spinrad,T.L.
A1 - Eisenberg,N.
Y1 - 2008/07//
KW - CINAHL
KW - Unsupervised Play Addition
KW - Attention -- In Infancy and Childhood
KW - Emotions
KW - Infant Behavior
KW - Parenting
KW - Adult
KW - Child,Preschool
KW - Descriptive Statistics
KW - Factor Analysis
KW - Female
KW - Funding Source
KW - Infant
KW - Male
KW - Mother-Child Relations
KW - Play and Playthings
KW - Prospective Studies
KW - Questionnaires
KW - Socioeconomic Factors
KW - Human
RP - NOT IN FILE
SP - 339
EP - 363
JA - Infant & Child Development
VL - 17
IS - 4
N2 - This longitudinal study examined individual differences and correlates of focused attention when toddlers were approximately 18 months old (T1; n=256) and a year later (T2; n=230). Toddlers' attention and negative emotionality were reported by mothers and non-parental caregivers and rated globally by observers. Toddlers' focused attention also was observed during two mother-child interactions and an independent play task. Measures of maternal emotional support and control were obtained via self-report and observation. Some contemporaneous relations among indices of toddlers' attention were obtained, particularly for observed measures. Moreover, all measures of attention demonstrated stability across time. Negative emotionality was negatively related to toddlers' observed attention at both ages, whereas maternal praise had positive concurrent associations. Maternal control was negatively related to observed attention at T2 and also predicted longitudinally, but only for children who initially had low or moderate attention. The findings suggest that individual differences in focused attention evidence stability early in life but can be influenced by adult socialization. Copyright Ac 2008 John Wiley & Sons, Ltd

SN - 1522-7227
AD - School of Social and Family Dynamics, Arizona State University, Tempe, AZ, USA
UR - http://search.ebscohost.com/login.aspx?direct=true&db=cin20&AN=2010002477&site=ehost-live
ER -

TY - JOUR
ID - 1962
T1 - Rehabilitation methods for the burn injured individual
A1 - Spires,M.C.
A1 - Kelly,B.M.
A1 - Pangilinan,P.H.,Jr.
Y1 - 2007/11//
KW - CINAHL
KW - Unsupervised Play Addition
KW - Burns -- Rehabilitation
KW - Skin -- Injuries
KW - Amputation -- Utilization
KW - Burns -- Classification
KW - Burns -- Physiopathology
KW - Child,Preschool
KW - Cicatrix -- Prevention and Control
KW - Exercise
KW - Pediatrics
KW - Skin -- Pathology
KW - Trauma Severity Indices
KW - Human
RP - NOT IN FILE
SP - 925
JF - Physical Medicine & Rehabilitation Clinics of North America
JA - Phys Med Rehabil Clin N Am
VL - 18
IS - 4
U2 - PMID: 17967369
N2 - Physiatrists play a critical role in managing the medical and functional consequences of serious burn injuries. Goals of rehabilitation include wound healing, scar prevention, hypertrophic scarring suppression, full range of motion, strengthening, and independent mobility and activities of daily living. This article is an overview of burn rehabilitation principles and patient management. The ultimate rehabilitation goal is independence in all spheres of an individual's life. Achievement of independence depends on the commitment of the injured individual and the entire health care team
Copyright Ac 2007 by Elsevier Inc
SN - 1047-9651
AD - Department of Physical Medicine and Rehabilitation, University of Michigan Health System, 325 E. Eisenhower, Suite 100, Ann Arbor, MI 48108, USA. mcspires@umich.edu
UR - http://search.ebscohost.com/login.aspx?direct=true&db=cin20&AN=2009920549&site=ehost-live
ER -

TY - JOUR
ID - 1963
T1 - The clinical decision-making process of prescribing power mobility for a child with cerebral palsy
A1 - Huhn,K.
A1 - Guarrera-Bowlby,P.
A1 - Deutsch,J.E.
Y1 - 2007/09//
KW - CINAHL
KW - Unsupervised Play Addition
KW - Cerebral Palsy

KW - Decision Making,Clinical
KW - Wheelchairs,Powered
KW - Child
KW - Female
KW - Physical Therapy
RP - NOT IN FILE
SP - 254
EP - 260
JF - Pediatric Physical Therapy
JA - PEDIATR
VL - 19
IS - 3
U2 - PMID: 17700355
N2 - PURPOSE:: Powered mobility has been shown to be an effective method for children with disabilities to achieve independent mobility. The purpose of this case report is to describe the physical therapist's clinical decision making related to power mobility for a child with multiple disabilities. CASE DESCRIPTION:: Power wheelchair evaluation for a nine-year-old child was conducted using Furumasu's tasks for wheelchair readiness moving through a doorway, maneuvering through three cones, and driving in a hallway. Ongoing team assessment with family consultation informed clinical decision-making. OUTCOMES:: A mid-wheel-drive chair afforded improved performance on Furumasu's tasks compared with a rear-wheel-drive chair. SUMMARY:: This case describes the clinician's role in prescribing power wheelchairs to affect the user's functional skills, as well as how, in the absence of evidence, clinical experience and patients' needs can guide clinical decision-making
SN - 0898-5669
AD - Rivers Lab, School of Health Related Professions, University of Medicine and Dentistry of New Jersey, Newark, New Jersey
UR - http://search.ebscohost.com/login.aspx?direct=true&db=cin20&AN=2009656931&site=ehost-live
ER -

TY - JOUR
ID - 1965
T1 - Mobility challenges in individuals with a spinal cord injury with increased body weight
A1 - Fitzgerald,S.G.
A1 - Kelleher,A.R.
Y1 - 2007/03//
KW - CINAHL
KW - Unsupervised Play Addition
KW - Obesity
KW - Physical Mobility
KW - Spinal Cord Injuries
KW - Activities of Daily Living
KW - Adolescence
KW - Adult
KW - Body Weight
KW - Child
KW - Disabled
KW - Equipment Design
KW - Functional Status
KW - Health Promotion
KW - Physical Activity
KW - Social Environment
KW - Therapeutic Exercise
KW - Wheelchairs
RP - NOT IN FILE
SP - 54
EP - 63

JF - Topics in Spinal Cord Injury Rehabilitation
JA - Top Spinal Cord Inj Rehabil
VL - 12
IS - 4
N2 - For the individual with a spinal cord injury (SCI), independent mobility is crucial. For the individual with an SCI who is also obese, independent mobility is especially challenging. This article focuses on the issues surrounding mobility and obesity. Using the International Classification of Functioning, Disability and Health as a framework, mobility issues are discussed in terms of the body and functioning, activities and participation, and environmental factors. Although challenging to prescribe, mobility options do exist for the individual who is obese and uses a wheelchair
SN - 1082-0744
AD - Associate Professor, Department of Rehabilitation Science and Technology, University of Pittsburgh
UR - http://search.ebscohost.com/login.aspx?direct=true&db=cin20&AN=2009539436&site=ehost-live
ER -

TY - JOUR
ID - 1967
T1 - Independent mobility for children with disabilities
A1 - Fernandes,T.
Y1 - 2006/07//
KW - CINAHL
KW - Unsupervised Play Addition
KW - Assistive Technology Devices -- In Infancy and Childhood
KW - Child Development
KW - Child,Disabled
KW - Wheelchairs -- In Infancy and Childhood
KW - Cerebral Palsy
KW - Child
KW - Equipment Design
KW - Information Resources
KW - Muscular Dystrophy
KW - Philanthropy
KW - Rehabilitation,Pediatric
KW - United Kingdom
KW - Wheelchairs,Powered
RP - NOT IN FILE
SP - 329
JA - International Journal of Therapy & Rehabilitation
VL - 13
IS - 7
N2 - It is essential that children with disabling conditions such as cerebral palsy or muscular dystrophy are helped to be independently mobile from an early age. If not, they can develop 'learned helplessness', where the child will become passive and dependent upon others, and will not grow and develop to his or her potential. Independent mobility can be achieved through the provision of customized vehicles such as wheelchairs or tricycles. The UK charity Whizz-Kidz provides this equipment, which is often not available through the NHS, to help children to 'realize their true potential'. This article aims to describe the different types of mobility equipment available to help children with disabilities to move around and develop their skills, and offers some examples of different products on the market
SN - 1741-1645
UR - http://search.ebscohost.com/login.aspx?direct=true&db=cin20&AN=2009242596&site=ehost-live
ER -

TY - JOUR
ID - 1968
T1 - Toward a better understanding of playfulness in adults
A1 - Guitard,P.

A1 - Ferland,F.
A1 - Dutil,A%
Y1 - 2005///
KW - CINAHL
KW - Unsupervised Play Addition
KW - Attitude to Life -- In Adulthood
KW - Play and Playthings -- In Adulthood
KW - Adult
KW - Audiorecording
KW - Constant Comparative Method
KW - Creativeness
KW - Female
KW - Funding Source
KW - Grounded Theory
KW - Interview Guides
KW - Male
KW - Middle Age
KW - Purposive Sample
KW - Semi-Structured Interview
KW - Triangulation
KW - Wit and Humor
KW - Human
RP - NOT IN FILE
SP - 9
EP - 22
JA - OTJR: Occupation, Participation & Health
VL - 25
IS - 1
N2 - Playfulness is normally associated with children and remains poorly documented in adult-hood. Studies have confirmed that playfulness is present in adulthood but do not provide any information on its specific nature. An enhanced comprehension of playfulness in adulthood is needed to determine its possible use in occupational therapy intervention with an adult clientele. A qualitative study led to a conceptual definition of playfulness in adulthood. Playfulness can be defined as an internal predisposition characterized by creativity, curiosity, pleasure, sense of humor, and spontaneity. This attitude extends to all life situations and modifies how adults perceive, evaluate, and approach situations. Playfulness enables adults to distance themselves from others, from situations, and from conventions to approach situations with an open mind to find original solutions to problems, to confront difficulties, and to accept failure. Therefore, it may be an important tool in occupational therapy to develop and enhance adults' ability to perform occupations
SN - 1539-4492
AD - Occupational Therapy Program, University of Ottawa, Ottawa, Ontario, Canada
UR - http://search.ebscohost.com/login.aspx?direct=true&db=cin20&AN=2005057515&site=ehost-live
ER -

TY - JOUR
ID - 1970
T1 - Accidental burials in sand: a potentially fatal summertime hazard
A1 - Zarroug,A.E.
A1 - Stavlo,P.L.
A1 - Kays,G.A.
A1 - Rodeberg,D.A.
A1 - Moir,C.R.
Y1 - 2004/06//
KW - CINAHL
KW - Unsupervised Play Addition
KW - Accidents
KW - Asphyxia -- Etiology

KW - Play and Playthings
KW - Adolescence
KW - Child
KW - Male
RP - NOT IN FILE
SP - 774
EP - 776
JF - Mayo Clinic Proceedings
JA - Mayo Clin Proc
VL - 79
IS - 6
U2 - PMID: 15182092
N2 - Accidental burial in sand is a tragically unrecognized risk associated with a popular childhood recreational activity. We describe 4 boys, aged 10 to 13 years, who were accidentally buried by sand. One boy died after his self-made tunnel in a sandbox collapsed. In a separate incident at a construction site, 1 boy died, and 2 were injured after a 30-foot sandpile collapsed as they ran down the embankment; all 3 were buried by the sand. In both incidents, play was unsupervised, and burial was sudden and complete. The calculated weight of the sand exceeded the expected maximal muscle effort of the chest, leading to traumatic asphyxiation secondary to restrictive compression of the chest. Only 15 accidental burials have been reported in the literature. To our knowledge, this is the first report describing children who died of respiratory asphyxia due to overwhelming thoracic compression after sand burial. Greater awareness by public health and safety officials at beaches, sandboxes, sandpiles, and natural play areas may prevent potentially lethal accidents
SN - 0025-6196
AD - Department of Surgery, Mayo Clinic College of Medicine, Rochester, MN
UR - http://search.ebscohost.com/login.aspx?direct=true&db=cin20&AN=2004168521&site=ehost-live
ER -

TY - JOUR
ID - 1971
T1 - Development pilot: a toy robot for physically disabled children
A1 - Prazak,B.
A1 - Kronreif,G.
A1 - Hochgatterer,A.
A1 - FA¬rst,M.
Y1 - 2004/06//
KW - CINAHL
KW - Unsupervised Play Addition
KW - Child,Disabled
KW - Robotics
KW - Play and Playthings
KW - Equipment Design
RP - NOT IN FILE
SP - 131
EP - 136
JA - Technology & Disability
VL - 16
IS - 3
N2 - The aim of this project is to explore how children with physical disabilities could use a toy robot system for autonomous playing. Children with physical disabilities have difficulties interacting with the material environment and they have fewer possibilities for autonomous play. This paper reports the developmental process of a robot system and results of the first user trials with this system and the target group. Working with this toy robot system allows children with physical disabilities to independently manipulate the real objects in a play situation. Future development is outlined
SN - 1055-4181
AD - Research Group on Rehabilitation and Inclusion, ARC Seibersdorf research GmbH, Viktor Kaplan Str. 2, A-2700 Wr. Neustadt, Austria

UR - http://search.ebscohost.com/login.aspx?direct=true&db=cin20&AN=2005077350&site=ehost-live
ER -

TY - JOUR
ID - 1972
T1 - Paintball injuries in children: more than meets the eye
A1 - Listman,D.A.
Y1 - 2004//02/
KW - CINAHL
KW - Unsupervised Play Addition
KW - Eye Injuries -- Etiology -- In Infancy and Childhood
KW - Eye Protective Devices -- Standards
KW - Play and Playthings
KW - Adolescence
KW - Child
KW - Emergency Service
KW - Epidemiological Research
KW - Eye Hemorrhage -- Epidemiology
KW - Eye Hemorrhage -- Etiology
KW - Eye Injuries -- Epidemiology
KW - Eye Injuries -- Prevention and Control
KW - Female
KW - Firearms
KW - Incidence
KW - Male
KW - Retina -- Injuries
KW - Retinal Detachment -- Epidemiology
KW - Retinal Detachment -- Etiology
KW - Human
RP - NOT IN FILE
SP - e15
EP - e18
JA - Pediatrics
VL - 113
IS - 1
U2 - PMID: 14702489
N2 - OBJECTIVE: To describe the increasing incidence of ocular injuries in the pediatric population caused by paintballs. The awareness of this trend will help the physician who treats the child to give appropriate care as well as educate physicians, who counsel children, about this growing mechanism of pediatric injuries. METHODS: Previously unpublished data from the US Consumer Product Safety Commission were analyzed to find the frequency of ocular injuries in children. A review of the English-language literature was also conducted to describe trends with regard to age, sex, location at the time of injury, use of eye protection, types of injuries sustained, and long-term visual outcome. RESULTS: The incidence of paintball eye injuries treated in emergency departments has risen from an estimated 545 in 1998 to >1200 in 2000. The proportion of these injuries that occurred in the pediatric population may be well over 40%. These injuries are seen predominantly in boys, a growing proportion of whom are playing informally in unsupervised settings and not wearing eye protection. The cases previously reported include large numbers of hyphemas, retinal detachments, cataracts, corneal abrasions, vitreous hemorrhages, and commotio retina. Many of the patients in these cases sustained permanent visual impairment, with 43% having best vision at follow-up of 20/200 or worse. CONCLUSIONS: Pediatric eye injuries caused by paintballs are an unrecognized cause of severe injury and permanent visual loss. The advances in eye protection for participants in "war games" have had little if any effect on the youngest population. Children and teens are unlikely to wear eye protection voluntarily when playing at undesignated or unsupervised locations. Changes should be made to restrict availability of these very dangerous but easily acquired guns and ammunition. Practitioners must counsel young people and their caregivers to avoid injuries by wearing appropriate eye protection. Parents should supervise the use of paintball equipment as they would other activities that involve high-speed projectiles

SN - 0031-4005
AD - Department of Pediatrics, St Barnabas Hospital, 4422 Third Ave, Mills 4, Bronx, NY 10457
UR - http://search.ebscohost.com/login.aspx?direct=true&db=cin20&AN=2005073898&site=ehost-live
ER -

TY - JOUR
ID - 1974
T1 - Complementary and alternative medicine use in families of children with cerebral palsy
A1 - Hurvitz,E.A.
A1 - Leonard,C.
A1 - Ayyangar,R.
A1 - Nelson,V.S.
Y1 - 2003/06//
KW - CINAHL
KW - Unsupervised Play Addition
KW - Alternative Therapies -- Utilization -- In Infancy and Childhood
KW - Cerebral Palsy -- Therapy
KW - Adolescence
KW - Chi Square Test
KW - Child
KW - Child,Preschool
KW - Data Analysis Software
KW - Data Analysis,Statistical
KW - Descriptive Research
KW - Descriptive Statistics
KW - Female
KW - Hydrotherapy
KW - Infant
KW - Logistic Regression
KW - Male
KW - Massage
KW - Michigan
KW - Odds Ratio
KW - P-Value
KW - Parents -- Psychosocial Factors
KW - Survey Research
KW - T-Tests
KW - Two-Tailed Test
KW - Human
RP - NOT IN FILE
SP - 364
EP - 370
JF - Developmental Medicine & Child Neurology
JA - Dev.Med Child Neurol.
VL - 45
IS - 6
U2 - PMID: 12785436
N2 - In order to assess patterns of usage of complementary and alternative medicine (CAM) in families of children with cerebral palsy (CP), 213 families with a child (0 to 18 years) with CP were recruited at the university medical center in Ann Arbor, MI, USA as part of a descriptive survey. Two hundred and thirty-five surveys were distributed. Mean age of the child was 8 years 6 months (SD 4y : 9mo) and 56% of the sample was male with 35% full-time independent ambulators, while the rest used an assistive device or a wheelchair. Fifty-four percent were in special education classrooms. Families were given a survey on functional status of the child with CP, CAM usage of the child and the parent, factors influencing the decision to use CAM, demographics, and clinical information. Of the families, 56%, used one or more CAM techniques. Massage therapy (25%) and aquatherapy (25%) were the most common. Children of families that used CAM were significantly younger (7y

: 9mo, SD 4y : 7mo) than non-users (9y : 6mo, SD 4y : 6mo: t-test p < 0.01 two-tailed). Children with quadriplegic CP, with spasticity, and those who could not walk independently were more commonly exposed to CAM (Pearson's chi2 [P(chi)2] p = 0.01 two-tailed; for mobility, odds ratio [OR] of 2.5 with regression). Mothers with a college degree had a greater tendency to use CAM for their child than those without (P(chi)2 p = 0.01 two-tailed). Fathers of children who used CAM were older than fathers of those who did not (37y : 9mo versus 33y : 2mo, p = 0.04 two-tailed). There was no significant difference between groups for mother's age, father's education, income, or for population of home town. Parents who used CAM for themselves were more likely to try CAM for their child (70% versus 47%, OR 2.1), and were much more likely to be pleased with the outcome (71% versus 42%, OR 3.5). Child's age (younger), lack of independent mobility, and parental use of CAM were the most significant predictive factors identified via logistic regression
SN - 0012-1622
AD - Dept of PM&R, CS Mott Hospital F7822, Ann Arbor, MI 48109-0230
UR - http://search.ebscohost.com/login.aspx?direct=true&db=cin20&AN=2004060024&site=ehost-live
ER -

TY - JOUR
ID - 1975
T1 - Creating a game for sexuality and aging: the Sexual Dysfunction Trivia Game
A1 - Skinner,K.D.
Y1 - 2000/07//
KW - CINAHL
KW - Unsupervised Play Addition
KW - Games
KW - Sexual Dysfunction,Male -- Education
KW - Sexual Dysfunction,Female -- Education
KW - Nursing Staff,Hospital -- Education
KW - Staff Development -- Methods
KW - Aged
KW - Sexual Dysfunction,Male -- In Old Age
KW - Sexual Dysfunction,Female -- In Old Age
KW - Pilot Studies
RP - NOT IN FILE
SP - 185
EP - 189
JA - Journal of Continuing Education in Nursing
VL - 31
IS - 4
U2 - PMID: 11261164
N2 - Background: Older adults often present with signs and symptoms of sexual dysfunction, and nurses must possess the necessary knowledge to address this issue. Therefore, The Sexual Dysfunction Trivia Game was designed to educate staff nurses about sexual dysfunction and the aging process. Method: A pilot study was conducted. Five staff nurses played The Sexual Dysfunction Trivia Game. Each nurse completed a pretest before playing the game and a posttest after playing the game. Results: After playing the board game, the nurses were more knowledgeable about what the physical examination includes, what the laboratory tests are, and what the treatment options are for sexual dysfunction. Conclusion: Based on the pretest and posttest findings, The Sexual Dysfunction Trivia Game appears to be an effective teaching tool to educate staff nurses about sexual dysfunction in the older adult. However, more studies are needed to measure its effectiveness
SN - 0022-0124
AD - Nurse Practitioner, West Roxbury Veterans Administration Hospital, West Roxbury, MA
UR - http://search.ebscohost.com/login.aspx?direct=true&db=cin20&AN=2000076802&site=ehost-live
ER -

TY - JOUR
ID - 1976
T1 - Selective dorsal rhizotomy: efficacy and safety in an investigator-masked randomized clinical trial
A1 - McLaughlin,J.F.

A1 - Bjornson,K.F.
A1 - Astley,S.J.
A1 - Graubert,C.
A1 - Hays,R.M.
A1 - Roberts,T.S.
A1 - Price,R.
A1 - Temkin,N.
Y1 - 1998/04//
KW - CINAHL
KW - Unsupervised Play Addition
KW - Rhizotomy -- Methods
KW - Cerebral Palsy -- Surgery
KW - Physical Therapy
KW - Combined Modality Therapy
KW - Muscle Spasticity
KW - Clinical Assessment Tools
KW - Cerebral Palsy -- Therapy
KW - Treatment Outcomes
KW - Spinal Nerve Roots -- Pathology
KW - Funding Source
KW - Clinical Trials
KW - Single-Blind Studies
KW - Questionnaires
KW - T-Tests
KW - Chi Square Test
KW - Fisher's Exact Test
KW - Mann-Whitney U Test
KW - Two-Tailed Test
KW - Child,Preschool
KW - Child
KW - Adolescence
KW - Male
KW - Female
KW - Human
RP - NOT IN FILE
SP - 220
EP - 232
JF - Developmental Medicine & Child Neurology
JA - Dev.Med Child Neurol.
VL - 40
IS - 4
U2 - PMID: 9593493
N2 - The objective of this single-center investigator-masked randomized clinical trial was to investigate the efficacy and safety of selective dorsal rhizotomy (SDR) in children with spastic diplegia. Forty-three children with spastic diplegia were randomly assigned on an intention-to-treat basis to receive SDR plus physical therapy (PT), or PT alone. Thirty-eight children completed follow-up through 24 months. Twenty-one children received SDR (SDR+PT group) and 17 received PT (PT Only group). SDR was guided with electrophysiological monitoring and performed by one experienced neurosurgeon. All subjects received equivalent PT. Spasticity was quantified with an electromechanical torque measurement device (spasticity measurement system [SMS]). The Gross Motor Function Measure (GMFM) was used to document changes in functional mobility. Primary outcome measures were collected at baseline, 6, 12, and 24 months by evaluators masked to treatment. At 24 months, the SDR+PT group exceeded the PT Only group in mean reduction of spasticity by SMS measurement (-8.2 versus +5.1 newton meters/radian, P=0.02). The SDR+PT group and the PT Only group demonstrated similar improvements in independent mobility on the GMFM (7.0 versus 7.2 total percent score, P=0.94). Outcomes on secondary variables were consistent with primary outcomes. There were no serious adverse events. We conclude that SDR is safe and reduces spasticity in children with spastic diplegia. SDR plus PT and

equivalent PT without SDR result in equal improvements in independent mobility at 24 months. SDR may not be an efficacious treatment for children with mild spastic diplegia
SN - 0012-1622
AD - Children's Hospital and Regional Medical Center, CH-47, PO Box 5371, Seattle, WA 98105-0371. E-mail: jfmcl@u.washington.edu
UR - http://search.ebscohost.com/login.aspx?direct=true&db=cin20&AN=1999066189&site=ehost-live
ER -

TY - JOUR
ID - 1977
T1 - Improvements in child pedestrian safety: have they been gained at the expense of other health goals?
A1 - Dixey,R.
Y1 - 1998/03//
KW - CINAHL
KW - Unsupervised Play Addition
KW - Child Safety -- Trends
KW - Health Promotion -- Trends
KW - Parents -- Psychosocial Factors
KW - Funding Source
KW - Survey Research
KW - Questionnaires
KW - Data Analysis,Statistical
KW - Data Analysis Software
KW - Semi-Structured Interview
KW - Transportation -- Trends
KW - Child
KW - Male
KW - Female
KW - Human
RP - NOT IN FILE
SP - 60
EP - 69
JA - Health Education Journal
VL - 57
IS - 1
N2 - Data collected in 1995 and 1996 in seven Leeds primary schools in relatively disadvantaged areas show high rates of accompanied journeys to and from school. Parents responded to the perceived danger to children from traffic and other causes by increasing surveillance of children on journeys and at play. This paper presents the findings of the Leeds study and asks whether gains in terms of reductions in child pedestrian accidents have been at the expense of children's independent mobility and parents' time. Restrictions on children have consequences for their independent mobility and therefore on levels of physical activity, freedom and opportunities for learning
SN - 0017-8969
AD - Faculty of Health and Environment, Leeds Metropolitan University, Calverley Street, Leeds, LS1 3HE
UR - http://search.ebscohost.com/login.aspx?direct=true&db=cin20&AN=1998053424&site=ehost-live
ER -

TY - JOUR
ID - 1978
T1 - Using a creative teaching process with adult patients
A1 - Duffy,B.
Y1 - 1997/02//
KW - CINAHL
KW - Unsupervised Play Addition
KW - Patient Education
KW - Teaching Methods

KW - Home Nursing,Professional
KW - Learning -- In Adulthood
KW - Games
KW - Adult
RP - NOT IN FILE
SP - 102
EP - 108
JA - Home Healthcare Nurse
VL - 15
IS - 2
U2 - PMID: 9146150
N2 - Teaching is an integral component of the home care nurse's role in every visit. Even though many teaching materials are at the nurse's disposal, not everyone feels comfortable teaching in the home setting. A simple teaching process specifically geared toward adult learners is presented as well as strategies and games that the home care nurse can use to make the teaching process effective and efficient
SN - 0884-741X
AD - Interim HealthCare, South Daytona, Florida
UR - http://search.ebscohost.com/login.aspx?direct=true&db=cin20&AN=1997016824&site=ehost-live
ER -

TY - JOUR
ID - 1979
T1 - Children's Thinking Styles, Play, and Academic Performance
A1 - Holmes,Robyn M.
A1 - Liden,Sharon
A1 - Shin,Lisa
Y1 - 2013///
KW - ERIC
KW - Unsupervised Play Addition
KW - Group Embedded Figures Test
KW - ERIC,Current Index to Journals in Education (CIJE)
KW - Middle Schools
KW - Program Effectiveness
KW - Play
KW - Hawaiians
KW - Aptitude Treatment Interaction
KW - Middle School Students
KW - Filipino Americans
KW - Academic Achievement
KW - Standardized Tests
KW - Correlation
KW - Cognitive Style
KW - Cultural Influences
KW - Preferences
KW - Extracurricular Activities
RP - NOT IN FILE
SP - 219
EP - 238
JF - American Journal of Play
VL - 5
IS - 2
PB - The Strong. One Manhattan Square, Rochester, NY 14607.
N2 - Based on the study of seventy-four middle school children of mostly Filipino and part Hawaiian heritages, this article explores the relationships of children's thinking styles, play preferences, and school performance. Using the Group Embedded Figures Test, the Articulation of the Body Scale, and written responses to three questions, the authors found significant relationships between children's field-independent or field-dependent

thinking styles and play preferences; play preferences and academic performance; thinking styles and academic performance; and thinking styles and cultural setting. They also discovered that children's preferences for sports related, both positively and negatively, to their scores on state-mandated tests for language and math; that children who preferred unstructured play activities tended to achieve academic success; and that cultural values were correlated to thinking style. The authors argue that their study has applied value for educators because it relates children's play preferences to other aspects of their life experiences, which can help school policy makers decide the extracurricular activities and the types of play they should encourage. (Contains 9 figures.)
SN - 1938-0399, 1938-0399
UR - http://search.proquest.com/docview/1373090682?accountid=14701
ER -

TY - JOUR
ID - 1980
T1 - Does Pretend Play Matter? Searching for Evidence: Comment on Lillard et al. (2013)
A1 - Bergen,Doris
Y1 - 2013/01//
KW - ERIC
KW - Unsupervised Play Addition
KW - ERIC,Current Index to Journals in Education (CIJE)
KW - Early Childhood Education
KW - Preschool Education
KW - Play
KW - Young Children
KW - Research Methodology
KW - Child Development
KW - Value Judgment
KW - Research Design
RP - NOT IN FILE
SP - 45
EP - 48
JF - Psychological Bulletin
JA - Psychol.Bull.
VL - 139
IS - 1
PB - American Psychological Association. Journals Department, 750 First Street NE, Washington, DC 20002-4242.
N2 - Lillard et al. (2013) have done a thorough review of published pretend play research from the past 50 plus years. However, they did not thoroughly address the reasons why this body of research has such flaws as well as the contradictory or minimal findings that call into question strongly held views and published assertions regarding the importance of the role of pretend play in fostering children's developmental progress. This comment addresses 3 aspects of the problematic nature of play research: First, I suggest that the research methods in most of these studies were unable to capture genuine pretend play, instead measuring "playful work." Second, I encourage rigorous research designs to better capture genuine examples of the pretend play phenomenon in order to gain deeper insights into these relationships. Third, I speculate on why pretend play development, as a valued behavior independent of its influence on other areas of development, is rarely undertaken and provide suggestions regarding this research direction
SN - 0033-2909, 0033-2909
UR - http://search.proquest.com/docview/1413414963?accountid=14701
L2 - http://sfx.scholarsportal.info/ottawa?url_ver=Z39.88-2004&rft_val_fmt=info:ofi/fmt:kev:mtx:journal&genre=article&sid=ProQ:ProQ%3Aericshell&atitle=Does+Pretend+Play+Matter%3F+Searching+for+Evidence%3A+Comment+on+Lillard+et+al.+%282013%29&title=Psychological+Bulletin&issn=00332909&date=2013-01-01&volume=139&issue=1&spage=45&au=Bergen%2C+Doris&isbn=&jtitle=Psychological+Bulletin&btitle=&rft_id=info:eric/EJ1006430
ER -

TY - JOUR
ID - 1981
T1 - Teaching Pretend Play to Young Children with Autism
A1 - Barton,Erin E.
A1 - Pavilanis,Rachel
Y1 - 2012/03//
KW - ERIC
KW - Unsupervised Play Addition
KW - ERIC,Current Index to Journals in Education (CIJE)
KW - Teachers
KW - Early Childhood Education
KW - Prompting
KW - Play
KW - Young Children
KW - Intervention
KW - Feedback (Response)
KW - Imagination
KW - Cues
KW - Social Development
KW - Inclusion
KW - Autism
KW - Teaching Methods
RP - NOT IN FILE
SP - 5
EP - 17
JF - Young Exceptional Children
VL - 15
IS - 1
PB - SAGE Publications. 2455 Teller Road, Thousand Oaks, CA 91320.
N2 - Play is a "critical" intervention goal for children with autism in inclusive settings because it sets the occasion for social interactions and communication with peers, caregivers, and teachers, and provides an authentic context for embedding instruction. Furthermore, several research studies have found "pretend" play to be a predictor of social and language skills, particularly for young children with autism. By teaching children with autism to engage in pretend play, teachers are providing opportunities for learning and independence within natural, social settings. Also, because it is flexible and child directed, play provides opportunities for embedding child-focused instruction within and across meaningful activities in natural settings with typical peers. In this article the authors aim to define pretend play (based on recent reviews of the literature; Barton, 2010; Barton & Wolery, 2008) and provide strategies for promoting the independent pretend play skills of children with autism in inclusive classrooms. (Contains 1 figure and 3 tables.)
SN - 1096-2506, 1096-2506
UR - http://search.proquest.com/docview/968112982?accountid=14701
L2 - http://sfx.scholarsportal.info/ottawa?url_ver=Z39.88-2004&rft_val_fmt=info:ofi/fmt:kev:mtx:journal&genre=article&sid=ProQ:ProQ%3Aericshell&atitle=Teaching+Pretend+Play+to+Young+Children+with+Autism&title=Young+Exceptional+Children&issn=10962506&date=2012-03-01&volume=15&issue=1&spage=5&au=Barton%2C+Erin+E.%3BPavilanis%2C+Rachel&isbn=&jtitle=Young+Exceptional+Children&btitle=&rft_id=info:eric/EJ958518
ER -

TY - JOUR
ID - 1982
T1 - "Some Kids Climb up; Some Kids Climb down": Culturally Constructed Play-Worlds of Children with Impairments
A1 - Burke,Jenene
Y1 - 2012///
KW - ERIC

KW - Unsupervised Play Addition
KW - Australia
KW - ERIC,Current Index to Journals in Education (CIJE)
KW - Elementary Education
KW - Social Influences
KW - Elementary School Students
KW - Self Determination
KW - Play
KW - Observation
KW - CHILDREN
KW - Foreign Countries
KW - Cultural Influences
KW - Social Attitudes
KW - Playgrounds
KW - Disabilities
KW - Evidence
KW - Photography
RP - NOT IN FILE
SP - 965
EP - 981
JF - Disability & Society
VL - 27
IS - 7
PB - Routledge. , 325 Chestnut Street Suite 800, Philadelphia, PA 19106.
N2 - In this paper, the author explores how children with impairments can act as self-monitoring, autonomous individuals in their play in a community playground. In addition, the notion of children with impairments as creative agents in their play is examined. The evidence presented in this paper is derived from the views and perspectives of children with impairments as playground users, and was collected from the children's photographic scrapbooks and the researcher's own observations of children's play in a naturalistic playground setting. The theoretical perspective for this paper draws on the emerging "social model of childhood disability" and provides scope to attempt to understand the culturally constructed play-worlds of children with impairments. (Contains 13 figures.)
SN - 0968-7599, 0968-7599
UR - http://search.proquest.com/docview/1312420188?accountid=14701
L2 - http://sfx.scholarsportal.info/ottawa?url_ver=Z39.88-2004&rft_val_fmt=info:ofi/fmt:kev:mtx:journal&genre=article&sid=ProQ:ProQ%3Aericshell&atitle=%22Some+Kids+Climb+up%3B+Some+Kids+Climb+down%22%3A+Culturally+Constructed+Play-Worlds+of+Children+with+Impairments&title=Disability+%26+Society&issn=09687599&date=2012-01-01&volume=27&issue=7&spage=965&au=Burke%2C+Jenene&isbn=&jtitle=Disability+%26+Society&btitle=&rft_id=info:eric/EJ985305
ER -

TY - JOUR
ID - 1984
T1 - Mother-Child Play and Maltreatment: A Longitudinal Analysis of Emerging Social Behavior from Infancy to Toddlerhood
A1 - Valentino,Kristin
A1 - Cicchetti,Dante
A1 - Toth,Sheree L.
A1 - Rogosch,Fred A.
Y1 - 2011/09//
KW - ERIC
KW - Unsupervised Play Addition
KW - ERIC,Current Index to Journals in Education (CIJE)
KW - Low Income Groups
KW - Toddlers

KW - Play
KW - Imitation
KW - Parent Influence
KW - Mothers
KW - Social Behavior
KW - Correlation
KW - Longitudinal Studies
KW - Behavior Problems
KW - Imagination
KW - Cognitive Processes
KW - Interpersonal Competence
KW - Parent Child Relationship
KW - Early Intervention
KW - Social Development
KW - Child Abuse
KW - Age Differences
KW - Infants
RP - NOT IN FILE
SP - 1280
EP - 1294
JF - Developmental Psychology
JA - Dev.Psychol.
VL - 47
IS - 5
PB - American Psychological Association. Journals Department, 750 First Street NE, Washington, DC 20002-4242.
N2 - Mother-child play of maltreating and nonmaltreating families was analyzed when infants were 12 months old (Time 1), and 2 years old (Time 2), as a context to examine children's developing cognitive and social skills. At Time 1, infants from abusing families demonstrated less independent and more imitative behavior during play than did infants from neglecting and nonmaltreating families, suggesting a delay in emerging social behaviors. In this longitudinal follow-up, mother-child play was reassessed 1 year later (N = 78), with a focus on children's engagement in nonplay and pretend play and on children's abilities to initiate social exchanges and respond to parental requests. Play and social behavior were coded from semistructured and unstructured play paradigms at both time points. Maternal attention-directing behavior and limit setting also was assessed. At Time 2, children from abusing, neglecting, and nonmaltreating families did not differ in cognitive play complexity. However, children from abusing families engaged in less child-initiated play than did children from neglecting and nonmaltreating families, demonstrating less socially competent behavior. Longitudinal analyses revealed child initiated play at Time 2 was negatively associated with abuse and with maternal physical attention directing behavior at Time 1. Child negative reactivity at Time 2 was positively associated with Time 1 maternal physical behavior and child imitation and with Time 2 maternal controlling behavior. Implications for early intervention efforts are emphasized. (Contains 8 tables.)
SN - 0012-1649, 0012-1649
UR - http://search.proquest.com/docview/964178359?accountid=14701
L2 - http://sfx.scholarsportal.info/ottawa?url_ver=Z39.88-2004&rft_val_fmt=info:ofi/fmt:kev:mtx:journal&genre=article&sid=ProQ:ProQ%3Aericshell&atitle=Mother-Child+Play+and+Maltreatment%3A+A+Longitudinal+Analysis+of+Emerging+Social+Behavior+from+Infancy+to+Toddlerhood&title=Developmental+Psychology&issn=00121649&date=2011-09-01&volume=47&issue=5&spage=1280&au=Valentino%2C+Kristin%3BCicchetti%2C+Dante%3BToth%2C+Sheree+L.%3BRogosch%2C+Fred+A.&isbn=&jtitle=Developmental+Psychology&btitle=&rft_id=info:eric/EJ945351
ER -

TY - JOUR
ID - 1988
T1 - "Good-Fit" Teacher-Child Play Interactions and the Subsequent Autonomous Play of Preschool Children
A1 - Trawick-Smith,Jeffrey

A1 - Dziurgot,Traci
Y1 - 2011///
KW - ERIC
KW - Unsupervised Play Addition
KW - ERIC,Current Index to Journals in Education (CIJE)
KW - Early Childhood Education
KW - Preschool Education
KW - Preschool Teachers
KW - Teacher Student Relationship
KW - Play
KW - Interrater Reliability
KW - Teacher Response
KW - Goodness of Fit
KW - Interviews
KW - Adults
KW - Preschool Children
KW - Models
RP - NOT IN FILE
SP - 110
EP - 123
JF - Early Childhood Research Quarterly
VL - 26
IS - 1
PB - Elsevier. 6277 Sea Harbor Drive, Orlando, FL 32887-4800.
N2 - The purpose of this study was to test a model of adult-child play interactions in preschool classrooms, based on the work of Vygotsky and neo-Vygotskian scholars. The model predicts that adults will tailor the play support they provide to the immediate needs of individual children, and that this will lead to subsequent independent play. Classroom interactions between eight preschool teachers and 32 students were videotaped, transcribed, and analyzed over a six-month period. Interviews were conducted with the eight adult participants to confirm and elucidate findings from observed classroom behaviors. Distinct types of play support needed by children and given by teachers were identified. These were operationally defined; definitions were used to create a coding system that was found to have high inter-rater reliability. Sequential analyses were conducted to determine goodness-of-fit between teacher responses and children's needs. Behavioral outcomes of a good fit were also examined. Findings support the model tested here. Teachers often responded to children's play with behaviors matching the level of support needed. Good-fit interactions more frequently led to autonomous subsequent play than poor-fit interactions. (Contains 5 tables and 2 figures.)
SN - 0885-2006, 0885-2006
UR - http://search.proquest.com/docview/822506346?accountid=14701
L2 - http://sfx.scholarsportal.info/ottawa?url_ver=Z39.88-2004&rft_val_fmt=info:ofi/fmt:kev:mtx:journal&genre=article&sid=ProQ:ProQ%3Aericshell&atitle=%22Good-Fit%22+Teacher-Child+Play+Interactions+and+the+Subsequent+Autonomous+Play+of+Preschool+Children&title=Early+Childhood+Research+Quarterly&issn=08852006&date=2011-01-01&volume=26&issue=1&spage=110&au=Trawick-Smith%2C+Jeffrey%3BDziurgot%2C+Traci&isbn=&jtitle=Early+Childhood+Research+Quarterly&btitle=&rft_id=info:eric/EJ906817
ER -

TY - JOUR
ID - 1989
T1 - Thinking about Evolution: Combinatorial Play as a Strategy for Exercising Scientific Creativity
A1 - Wingate,Richard J.
Y1 - 2011///
KW - ERIC
KW - Unsupervised Play Addition
KW - ERIC,Current Index to Journals in Education (CIJE)

KW - Higher Education
KW - Science Education
KW - Thinking Skills
KW - Undergraduate Students
KW - Zoology
KW - Play
KW - Case Studies
KW - Brain
KW - Models
KW - Creative Teaching
KW - Scientists
KW - Cognitive Processes
KW - Classification
KW - Tests
KW - Biomedicine
KW - Universities
RP - NOT IN FILE
SP - 50
EP - 53
JF - Journal of Biological Education
VL - 45
IS - 1
PB - Routledge. , 325 Chestnut Street Suite 800, Philadelphia, PA 19106.
N2 - An enduring focus in education on how scientists formulate experiments and "do science" in the laboratory has excluded a vital element of scientific practice: the creative and imaginative thinking that generates models and testable hypotheses. In this case study, final-year biomedical sciences university students were invited to create and justify a taxonomy of selected vertebrates on the basis of their brain organisation, as part of an exercise exploring the evolution of embryonic development. While raising a number of issues surrounding the context and methods of comparative zoology, this exercise also invoked a set of cognitive processes that can neither be adequately characterised as role-play nor critical thinking. By contrast, the act of formulating and justifying taxonomy identifies a style of creative thought that is a prerequisite for hypothesis formation. A defining characteristic of this exercise is that it engages activities that are independent of disciplinary perspective. This flexibility in approach may provide a route through to defining what qualifies as a creative teaching exercise in science. (Contains 1 figure.)
SN - 0021-9266, 0021-9266
UR - http://search.proquest.com/docview/898323655?accountid=14701
L2 - http://sfx.scholarsportal.info/ottawa?url_ver=Z39.88-2004&rft_val_fmt=info:ofi/fmt:kev:mtx:journal&genre=article&sid=ProQ:ProQ%3Aericshell&atitle=Thinking+about+Evolution%3A+Combinatorial+Play+as+a+Strategy+for+Exercising+Scientific+Creativity&title=Journal+of+Biological+Education&issn=00219266&date=2011-01-01&volume=45&issue=1&spage=50&au=Wingate%2C+Richard+J.&isbn=&jtitle=Journal+of+Biological+Education&btitle=&rft_id=info:eric/EJ937398
ER -

TY - JOUR
ID - 1990
T1 - Parents as Play Date Facilitators for Preschoolers with Autism
A1 - Jull,Stephanie
A1 - Mirenda,Pat
Y1 - 2011/01//
KW - ERIC
KW - Unsupervised Play Addition
KW - Canada
KW - British Columbia
KW - ERIC,Current Index to Journals in Education (CIJE)
KW - Social Influences

KW - Program Effectiveness
KW - Interaction
KW - Play
KW - MALES
KW - Cooperation
KW - Parent Role
KW - Intervention
KW - Skill Development
KW - Friendship
KW - Foreign Countries
KW - Interpersonal Competence
KW - Affective Behavior
KW - Reinforcement
KW - Parent Attitudes
KW - Preschool Children
KW - Parents
KW - Interpersonal Relationship
KW - Autism
RP - NOT IN FILE
SP - 17
EP - 30
JF - Journal of Positive Behavior Interventions
VL - 13
IS - 1
PB - SAGE Publications and Hammill Institute on Disabilities. 2455 Teller Road, Thousand Oaks, CA 91320.
N2 - Teaching children with autism to interact with their typically developing peers can be a challenge. Previous research has documented that there are many effective ways to teach social interaction; however, interventions in this regard are usually implemented by professionals. The purpose of this study was to assess the effectiveness of parent-implemented contextually supported play dates. Two parents were taught to design cooperative play arrangements to facilitate social interactions between their children with autism and typically developing peers in their homes. Two independent reversal designs were used to demonstrate functional relationships between parent-implemented, contextually supported play dates and an increase in synchronous reciprocal interactions for both participants. Social validity, both immediately after the intervention and 1 year later, was also high for both parents; however, there was no consistent impact on participant, confederate, or parent affect during the study. The results are discussed with reference to previous research, future directions, limitations, and implications for practice. (Contains 4 tables and 2 figures.)
SN - 1098-3007, 1098-3007
UR - http://search.proquest.com/docview/851229391?accountid=14701
L2 - http://sfx.scholarsportal.info/ottawa?url_ver=Z39.88-2004&rft_val_fmt=info:ofi/fmt:kev:mtx:journal&genre=article&sid=ProQ:ProQ%3Aericshell&atitle=Parents+as+Play+Date+Facilitators+for+Preschoolers+with+Autism&title=Journal+of+Positive+Behavior+Interventions&issn=10983007&date=2011-01-01&volume=13&issue=1&spage=17&au=Jull%2C+Stephanie%3BMirenda%2C+Pat&isbn=&jtitle=Journal+of+Positive+Behavior+Interventions&btitle=&rft_id=info:eric/EJ909164
ER -

TY - JOUR
ID - 1991
T1 - Affordances in Outdoor Environments and Children's Physically Active Play in Pre-School
A1 - Storli,Rune
A1 - Hagen,Trond Loge
Y1 - 2010/12//
KW - ERIC
KW - Unsupervised Play Addition
KW - ERIC,Current Index to Journals in Education (CIJE)
KW - Early Childhood Education

KW - Preschool Education
KW - Physical Activities
KW - Play
KW - Physical Environment
KW - Environmental Influences
KW - Playgrounds
KW - School Culture
KW - Preschool Children
RP - NOT IN FILE
SP - 445
EP - 456
JA - European Early Childhood Education Research Journal
VL - 18
IS - 4
PB - Routledge. , 325 Chestnut Street Suite 800, Philadelphia, PA 19106.
N2 - The purpose of this article is to quantitatively and qualitatively explore children's physically active play outdoors in a traditional playground and natural (nature) environment and discuss how these environments influence children's physical activity. Fjortoft has previously explored the relationship between environmental affordances and children's play and development. She suggests that environmental complexity and diversity in nature are highly associated with increased play opportunities and activities. To explore the interactive relationship between children and their material environments, Kytta has theoretically specified and clarified this relationship in the view of ecological perceptual psychology based on Gibson's theory of affordances. To understand how affordances in the play environment influence 3- to 5-year-old children's (n = 16) level of physical activity play, accelerometers were used to measure physically active play, and Heft's functional taxonomy of children's outdoor play environment was utilised in observing how the children actualised the potential affordances in free play. The results show there is no difference in the level of physically active play between the traditional playground environment and the natural environment in this study. Strong correlations of level of physically active play on an individual basis were found among the different days. This indicates that the children's physically active behaviour on an individual basis is similar from day-to-day, relatively independent of the environment. In analysing these results for the potential benefits of physically active play in pre-school, constraints in the individual (personal characteristics), the physical environment (complexity, diversity) and the sociocultural environment (pre-school culture) are discussed. (Contains 1 note, 4 tables, and 3 figures.)
SN - 1350-293X, 1350-293X
UR - http://search.proquest.com/docview/851229943?accountid=14701
L2 - http://sfx.scholarsportal.info/ottawa?url_ver=Z39.88-2004&rft_val_fmt=info:ofi/fmt:kev:mtx:journal&genre=article&sid=ProQ:ProQ%3Aericshell&atitle=Affordances+in+Outdoor+Environments+and+Children%27s+Physically+Active+Play+in+Pre-School&title=European+Early+Childhood+Education+Research+Journal&issn=1350293X&date=2010-12-01&volume=18&issue=4&spage=445&au=Storli%2C+Rune%3BHagen%2C+Trond+Loge&isbn=&jtitle=European+Early+Childhood+Education+Research+Journal&btitle=&rft_id=info:eric/EJ908420
ER -

TY - JOUR
ID - 1992
T1 - Teaching Children with Autism to Play a Video Game Using Activity Schedules and Game-Embedded Simultaneous Video Modeling
A1 - Blum-Dimaya,Alyssa
A1 - Reeve,Sharon A.
A1 - Reeve,Kenneth F.
A1 - Hoch,Hannah
Y1 - 2010/08//
KW - ERIC
KW - Unsupervised Play Addition
KW - ERIC,Current Index to Journals in Education (CIJE)
KW - Program Effectiveness

KW - Video Games
KW - Video Technology
KW - Play
KW - Scheduling
KW - Interpersonal Competence
KW - Leisure Time
KW - Reinforcement
KW - Role Models
KW - Autism
KW - Teaching Methods
RP - NOT IN FILE
SP - 351
EP - 370
JF - Education and Treatment of Children
VL - 33
IS - 3
PB - West Virginia University Press. P.O. Box 6295, West Virginia University, Morgantown, WV 26506.
N2 - Children with autism have severe and pervasive impairments in social interactions and communication that impact most areas of daily living and often limit independent engagement in leisure activities. We taught four children with autism to engage in an age-appropriate leisure skill, playing the video game Guitar Hero II[TM], through the use of (a) an activity schedule to set up, turn on, and turn of the game and system, (b) simultaneous video modeling embedded in the game to teach manipulation of the Guitar Hero II[TM] controller to play the game, and (c) the training of multiple exemplars of songs to develop a generalized repertoire of playing Guitar Hero II[TM]. A multiple-probe design across participants was used to evaluate the effectiveness of the training package. All of the participants successfully learned to play Guitar Hero II[TM] and playing skills generalized to a song and a setting not used during training. (Contains 2 tables and 3 figures.)
SN - 0748-8491, 0748-8491
UR - http://search.proquest.com/docview/757168659?accountid=14701
L2 - http://sfx.scholarsportal.info/ottawa?url_ver=Z39.88-2004&rft_val_fmt=info:ofi/fmt:kev:mtx:journal&genre=article&sid=ProQ:ProQ%3Aericshell&atitle=Teaching+Children+with+Autism+to+Play+a+Video+Game+Using+Activity+Schedules+and+Game-Embedded+Simultaneous+Video+Modeling&title=Education+and+Treatment+of+Children&issn=07488491&date=2010-08-01&volume=33&issue=3&spage=351&au=Blum-Dimaya%2C+Alyssa%3BReeve%2C+Sharon+A.%3BReeve%2C+Kenneth+F.%3BHoch%2C+Hannah&isbn=&jtitle=Education+and+Treatment+of+Children&btitle=&rft_id=info:eric/EJ893791
ER -

TY - JOUR
ID - 1993
T1 - A Turkish View on Fathers' Involvement in Children's Play
A1 - Ivrendi,Asiye
A1 - Isikoglu,Nesrin
Y1 - 2010/04//
KW - ERIC
KW - Unsupervised Play Addition
KW - Turkey
KW - ERIC,Current Index to Journals in Education (CIJE)
KW - Employment Level
KW - Socioeconomic Influences
KW - Foreign Countries
KW - Play
KW - Gender Differences
KW - Family Structure
KW - Parent Child Relationship
KW - Parent Participation
KW - Statistical Analysis

KW - Parent Attitudes
KW - Fathers
RP - NOT IN FILE
SP - 519
EP - 526
JF - Early Childhood Education Journal
VL - 37
IS - 6
PB - Springer. 233 Spring Street, New York, NY 10013.
N2 - The purpose of this study is to examine fathers' participation in and views about play. This study was conducted in the southwestern part of Turkey and its sample consisted of 97 fathers who had a child attending randomly chosen public early childhood institutions. The Parents' Participation and Views on Play instrument was developed and used to collect data. Independent sample t tests and a series of repeated measures analysis of variance procedures were conducted to assess the influence of independent variables on the fathers' participation in and views on play. The results of the study indicated that fathers frequently participated in their children's play, and they hold positive views about play. Fathers' socio-demographic characteristics such as income, working status, family type and children's gender influenced their participation and views about play. Results are discussed in relation to the literature on parent-child play and suggestions for further research are presented
SN - 1082-3301, 1082-3301
UR - http://search.proquest.com/docview/742867211?accountid=14701
L2 - http://sfx.scholarsportal.info/ottawa?url_ver=Z39.88-2004&rft_val_fmt=info:ofi/fmt:kev:mtx:journal&genre=article&sid=ProQ:ProQ%3Aericshell&atitle=A+Turkish+View+on+Fathers%27+Involvement+in+Children%27s+Play&title=Early+Childhood+Education+Journal&issn=10823301&date=2010-04-01&volume=37&issue=6&spage=519&au=Ivrendi%2C+Asiye%3BIsikoglu%2C+Nesrin&isbn=&jtitle=Early+Childhood+Education+Journal&btitle=&rft_id=info:eric/EJ878269
ER -

TY - JOUR
ID - 1994
T1 - Approaching Complexity through Planful Play: Kindergarten Children's Strategies in Constructing an Autonomous Robot's Behavior
A1 - Levy,T.S.
A1 - Mioduser,D.
Y1 - 2010/04//
KW - ERIC
KW - Unsupervised Play Addition
KW - ERIC,Current Index to Journals in Education (CIJE)
KW - Early Childhood Education
KW - Kindergarten
KW - Robotics
KW - Programming
KW - Play
KW - Young Children
KW - Cybernetics
KW - Learning Processes
KW - Investigations
KW - Student Behavior
KW - Spatial Ability
KW - Educational Technology
KW - Learning Strategies
RP - NOT IN FILE
SP - 21
EP - 43
JF - International Journal of Computers for Mathematical Learning
VL - 15

IS - 1
PB - Springer. 233 Spring Street, New York, NY 10013.
N2 - This study investigates how young children master, construct and understand intelligent rule-based robot behaviors, focusing on their strategies in gradually meeting the tasks' complexity. The wider aim is to provide a comprehensive map of the kinds of transitions and learning that take place in constructing simple emergent behaviors, particularly for young children. Six kindergarten children participated individually in the study along five sessions. Regarding modes of engagement, it was found that the children conducted intensive and extended playful investigations of the robot's behaviors, interacting with it in a variety of ways; it was also found that their constructions were planful and anticipatory, as they could simulate how the behaviors play out even prior to running their programs. Three kinds of transitions were found in the children's comprehension of the system: one involved adaptation to the formal language; the second, coordination of multiple spatial perspectives; and the third involved a shift from viewing rules as one-time events to their view as recurring and continual descriptions of a process. Finally, it was found that the children employed two strategies to reduce the amount of information in the system: "pruning" involved ignoring part of the logical structure and focusing on another; "fusing" involved coalescing several rules or functions into one. These results are discussed with respect to previous literature on children's programming and with regards to understanding and supporting young children's learning through their construction of adaptive autonomous behaviors
SN - 1382-3892, 1382-3892
UR - http://search.proquest.com/docview/742872036?accountid=14701
L2 - http://sfx.scholarsportal.info/ottawa?url_ver=Z39.88-2004&rft_val_fmt=info:ofi/fmt:kev:mtx:journal&genre=article&sid=ProQ:ProQ%3Aericshell&atitle=Approaching+Complexity+through+Planful+Play%3A+Kindergarten+Children%27s+Strategies+in+Constructing+an+Autonomous+Robot%27s+Behavior&title=International+Journal+of+Computers+for+Mathematical+Learning&issn=13823892&date=2010-04-01&volume=15&issue=1&spage=21&au=Levy%2C+T.+S.%3BMioduser%2C+D.&isbn=&jtitle=International+Journal+of+Computers+for+Mathematical+Learning&btitle=&rft_id=info:eric/EJ883931
ER -

TY - JOUR
ID - 1996
T1 - Preschool Children's Outdoor Play Area Preferences
A1 - Holmes,Robyn M.
A1 - Procaccino,Jill K.
Y1 - 2009/12//
KW - ERIC
KW - Unsupervised Play Addition
KW - ERIC,Current Index to Journals in Education (CIJE)
KW - Early Childhood Education
KW - Play
KW - Gender Differences
KW - Playgrounds
KW - MALES
KW - Naturalistic Observation
KW - Decision Making
KW - Preschool Children
KW - Playground Activities
RP - NOT IN FILE
SP - 1103
EP - 1112
JF - Early Child Development and Care
VL - 179
IS - 8
PB - Routledge. , 325 Chestnut Street Suite 800, Philadelphia, PA 19106.
N2 - This study explores preschool children's outdoor play preferences. The sample was 40 (20 male, 20 female) primarily European-American three and four year olds. Data were collected via naturalistic observation and analyzed using repeated measures ANOVAs and MANOVAs. The independent variable was sex of child;

dependent variable was play space (riding, sandbox, slide, jungle gym, open space, castle/play house, swings). Findings revealed a significant effect of sex of child on choice of play space--boys preferred to play on the jungle gym and swings, whereas girls preferred the sandbox more than boys. These findings both support and contradict existing literature. Future research should pursue children's use of microspaces in their play environments. (Contains 1 figure and 3 tables.)
SN - 0300-4430, 0300-4430
UR - http://search.proquest.com/docview/61836494?accountid=14701
L2 - http://sfx.scholarsportal.info/ottawa?url_ver=Z39.88-2004&rft_val_fmt=info:ofi/fmt:kev:mtx:journal&genre=article&sid=ProQ:ProQ%3Aericshell&atitle=Preschool+Children%27s+Outdoor+Play+Area+Preferences&title=Early+Child+Development+and+Care&issn=03004430&date=2009-12-01&volume=179&issue=8&spage=1103&au=Holmes%2C+Robyn+M.%3BProcaccino%2C+Jill+K.&isbn=&jtitle=Early+Child+Development+and+Care&btitle=&rft_id=info:eric/EJ863093
ER -

TY - JOUR
ID - 1997
T1 - Safe Play Areas for Prevention of Young Children Drowning in Farm Dams
A1 - Depczynski,Julie
A1 - Fragar,Lyn
A1 - Hawkins,Antonia
A1 - Stiller,Laurence
Y1 - 2009/09//
KW - ERIC
KW - Unsupervised Play Addition
KW - Australia
KW - ERIC,Current Index to Journals in Education (CIJE)
KW - Program Effectiveness
KW - Death
KW - Play
KW - Young Children
KW - SURVEYS
KW - Intervention
KW - Rural Areas
KW - Water
KW - Child Safety
KW - Mass Media Effects
KW - Foreign Countries
KW - Recreational Activities
KW - Agricultural Occupations
KW - Accident Prevention
RP - NOT IN FILE
SP - 50
EP - 57
JF - Australasian Journal of Early Childhood
VL - 34
IS - 3
PB - Early Childhood Australia. P.O. Box 7105, Watson, ACT 2602, Australia. Tel: +61-2-6242-1800; Fax: +61-2-6242-1818; e-mail: publishing@earlychildhood.org.au; Web site: http://www.earlychildhoodaustralia.org.au
N2 - Injuries are the leading cause of death to young children in rural Australia, with drowning in farm dams being a major risk. This paper assesses the impact of an intervention to increase safe play areas on farms to prevent unsupervised access by young children to water bodies and other hazards. Surveys of 1,117 adult farmers attending Ag Quip Agricultural Field Days in NSW between 2003 and 2007 were used to identify the child security level of fences and changes in the percentage of farms with fenced house yards. Over this four-year period, the percentage of people who had a fenced yard on their farm ranged from 75 per cent to 79 per

cent with no significant fluctuations. However, those with a safe play area who stated the area was difficult/almost impossible for a child 0-5yrs to breach, ranged from 40 per cent to 55 per cent over the period, with a statistically significant positive trend (X2=13.46 df=4 p[less than or equal] 0.01). Actions to improve child safety on farms and recall of child farm safety messages in the media in association with programs promoting securely fenced house yards are also discussed. In order to affect further reduction in child drowning incidents, there is an ongoing need to promote improved security of fenced house yards on farms in Australia. (Contains 2 tables and 5 figures.)
SN - 1836-9391, 1836-9391
UR - http://search.proquest.com/docview/61810683?accountid=14701
L2 - http://sfx.scholarsportal.info/ottawa?url_ver=Z39.88-2004&rft_val_fmt=info:ofi/fmt:kev:mtx:journal&genre=article&sid=ProQ:ProQ%3Aericshell&atitle=Safe+Play+Areas+for+Prevention+of+Young+Children+Drowning+in+Farm+Dams&title=Australasian+Journal+of+Early+Childhood&issn=18369391&date=2009-09-01&volume=34&issue=3&spage=50&au=Depczynski%2C+Julie%3BFragar%2C+Lyn%3BHawkins%2C+Antonia%3BStiller%2C+Laurence&isbn=&jtitle=Australasian+Journal+of+Early+Childhood&btitle=&rft_id=info:eric/EJ869666
ER -

TY - JOUR
ID - 1998
T1 - Discipline and Play with/in a Mandated Literacy Curriculum
A1 - Kontovourki,Stavroula
A1 - Siegel,Marjorie
Y1 - 2009/09//
KW - ERIC
KW - Unsupervised Play Addition
KW - New York
KW - No Child Left Behind Act 2001
KW - ERIC,Current Index to Journals in Education (CIJE)
KW - Elementary Education
KW - Writing Instruction
KW - Beginning Reading
KW - Play
KW - Independent Reading
KW - Curriculum
KW - Kindergarten
KW - Literacy
KW - Discipline
KW - Inquiry
KW - Teaching Methods
RP - NOT IN FILE
SP - 30
EP - 38
JF - Language Arts
VL - 87
IS - 1
PB - National Council of Teachers of English. 1111 West Kenyon Road, Urbana, IL 61801-1096.
N2 - In this article, the authors explore a young child's lived experience of discipline and play with/in a mandated balanced literacy curriculum. Mandating balanced literacy presents an interesting case of disciplining literacy because it is rooted in the progressive tradition of meaning-and-process pedagogies. As such, it avoids the most obvious forms of regulation and control found in scripted lessons, yet imposes particular meanings of literacy, teaching, and learning on teachers and children. Drawing on data from a year-long inquiry of one kindergarten classroom, the authors demonstrate how the mandated curriculum disciplined Hector and his teacher but also how they played with/in this curriculum. The slash between "with" and "in" serves to highlight the fact that Hector never escaped the limits of the mandated curriculum. Sometimes he was "in" the curriculum, so fully disciplined by it that he sought out recognition as a successful reader and writer. Other times, he played

within the spaces made available by independent reading and writing time. In these cases, he untethered school literacy from its individualistic and monomodal roots, but his play remained "within" the bounds of the curricular routines. The fluency with which Hector shifted from a writer at play to a student at work points to the complexities of policy mandates and their enactment in classrooms. (Contains 1 figure.)
SN - 0360-9170, 0360-9170
UR - http://search.proquest.com/docview/61842604?accountid=14701
L2 - http://sfx.scholarsportal.info/ottawa?url_ver=Z39.88-2004&rft_val_fmt=info:ofi/fmt:kev:mtx:journal&genre=article&sid=ProQ:ProQ%3Aericshell&atitle=Discipline+and+Play+with%2Fin+a+Mandated+Literacy+Curriculum&title=Language+Arts&issn=03609170&date=2009-09-01&volume=87&issue=1&spage=30&au=Kontovourki%2C+Stavroula%3BSiegel%2C+Marjorie&isbn=&jtitle=Language+Arts&btitle=&rft_id=info:eric/EJ856168
ER -

TY - GEN
ID - 1999
T1 - Interactive Computer Play in Rehabilitation of Children with Sensorimotor Disorders: A Systematic Review
A1 - Sandlund,Marlene
A1 - McDonough,Suzanne
A1 - Hager-Ross,Charlotte
Y1 - 2009/03//
N1 - Last updated - 2012-12-14
KW - ERIC
KW - Unsupervised Play Addition
KW - ERIC,Current Index to Journals in Education (CIJE)
KW - Program Effectiveness
KW - Play
KW - Motivation
KW - Motion
KW - Rehabilitation
KW - Case Studies
KW - Computers
KW - Intervention
KW - Literature Reviews
KW - Cerebral Palsy
KW - Sensory Integration
KW - Child Development
KW - Spatial Ability
KW - Psychomotor Skills
KW - Physical Disabilities
RP - NOT IN FILE
SP - 173
EP - 179
JF - Developmental Medicine & Child Neurology
JA - Dev.Med Child Neurol.
VL - 51
IS - 3
PB - Wiley-Blackwell. 350 Main Street, Malden, MA 02148.
N2 - The aim of this review was to examine systematically the evidence for the application of interactive computer play in the rehabilitation of children with sensorimotor disorders. A literature search of 11 electronic databases was conducted to identify articles published between January 1995 and May 2008. The review was restricted to reports of intervention studies evaluating the impact of interactive computer play on motor rehabilitation in children. For each study the quality of the methods and the strength of the evidence were assessed by two independent reviewers using the guidelines of the American Academy for Cerebral Palsy and Developmental Medicine. A total of 74 articles were identified, of which 16 met the inclusion criteria. Three

studies were randomized controlled trials (RCTs) and half were case series or case reports. Areas investigated were movement quality, spatial orientation and mobility, and motivational aspects. Thirteen studies presented positive findings. Two of the three RCTs investigating movement quality and one level III study examining spatial orientation showed no significant improvements. Interactive computer play is a potentially promising tool for the motor rehabilitation of children but the level of evidence is too limited to assess its value fully. Further and more convincing research is needed
UR - http://search.proquest.com/docview/61863398?accountid=14701
L2 - http://sfx.scholarsportal.info/ottawa?url_ver=Z39.88-2004&rft_val_fmt=info:ofi/fmt:kev:mtx:journal&genre=article&sid=ProQ:ProQ%3Aericshell&atitle=Interactive+Computer+Play+in+Rehabilitation+of+Children+with+Sensorimotor+Disorders%3A+A+Systematic+Review&title=Developmental+Medicine+%26+Child+Neurology&issn=00121622&date=2009-03-01&volume=51&issue=3&spage=173&au=Sandlund%2C+Marlene%3BMcDonough%2C+Suzanne%3BHager-Ross%2C+Charlotte&isbn=&jtitle=Developmental+Medicine+%26+Child+Neurology&btitle=&rft_id=info:eric/EJ849941
ER -

TY - JOUR
ID - 2000
T1 - What Kids Don't Get to Do Anymore and Why
A1 - Sutterby,John A.
Y1 - 2009///
KW - ERIC
KW - Unsupervised Play Addition
KW - ERIC,Current Index to Journals in Education (CIJE)
KW - Mortality Rate
KW - Play
KW - Anxiety
KW - Leisure Time
KW - Parent Role
KW - Child Safety
KW - Caregivers
KW - Parenting Styles
KW - Access to Information
KW - Child Rearing
KW - Child Development
KW - Parent Attitudes
RP - NOT IN FILE
SP - 289
JF - Childhood Education
VL - 85
IS - 5
PB - Association for Childhood Education International. 17904 Georgia Avenue Suite 215, Olney, MD 20832.
N2 - Since 1981, the amount of time children spend with each other or alone without adult supervision has declined, especially in the area of outdoor free play. Free time for children has declined 16% between 1981 and 1997. A number of researchers have examined societal changes over the last century, especially from the mid-1970s to today. According to Stearns (2003), parental anxiety has risen throughout the 20th century, as children are perceived as both more valuable and more vulnerable. With falling mortality rates for children and increases in parental advice came the idea that if anything bad happened to the child, then the parents were somehow responsible. In this article, the author takes a recent historical look at the role of parents in keeping children safe. The author explores the increase in parental anxiety, the role of information in creating this anxiety, and the resultant consequences for children. The author concludes with a call for caregivers to use both common sense and research, rather than media hype, to guide their child-rearing practices
SN - 0009-4056, 0009-4056
UR - http://search.proquest.com/docview/61861551?accountid=14701
L2 - http://sfx.scholarsportal.info/ottawa?url_ver=Z39.88-2004&rft_val_fmt=info:ofi/fmt:kev:mtx:journal&genre=article&sid=ProQ:ProQ%3Aericshell&atitle=What+Ki

ds+Don%27t+Get+to+Do+Anymore+and+Why&title=Childhood+Education&issn=00094056&date=2009-01-01&volume=85&issue=5&spage=289&au=Sutterby%2C+John+A.&isbn=&jtitle=Childhood+Education&btitle=&rft_id=info:eric/EJ848631
ER -

TY - JOUR
ID - 2001
T1 - Embedding Sensory Preference into Toys to Enhance Toy Play in Toddlers with Disabilities
A1 - DiCarlo,Cynthia F.
A1 - Schepis,Maureen M.
A1 - Flynn,Linda
Y1 - 2009///
KW - ERIC
KW - Unsupervised Play Addition
KW - ERIC,Current Index to Journals in Education (CIJE)
KW - Program Effectiveness
KW - Sensory Experience
KW - Toddlers
KW - Play
KW - Perceptual Development
KW - Disabilities
KW - Child Development
KW - Stimuli
KW - Sensory Training
KW - Toys
RP - NOT IN FILE
SP - 188
EP - 200
JF - Infants and Young Children
VL - 22
IS - 3
PB - Lippincott Williams & Wilkins. 351 West Camden Street, Baltimore, MD 21201.
N2 - Playing with toys as a means of environmental engagement has long been considered important in early child development (D. J. Messer, D. Rachford, M. E. McCarthy, & L. J. Yarrow, 1987; M. Wolery, M. G. Werts, & M. Holcombe, 1994). However, children with highly significant disabilities often engage in toy play less frequently than their peers without disabilities (P. M. Blasco, D. B. Bailey, & M. A. Burchinal, 1993; C. F. DiCarlo, D. H. Reid, & S. B. Stricklin, 2003; M. B. Langley, 1985) and frequently need specialized support to promote toy play (J. J. Ivory & J. A. McCollum, 1999; M. B. Langley, 1985; S. Sandall, 2003). In this study, preference assessment technology was used to identify preferred sensory attributes of very young children. Once identified, those attributes were embedded into a toy previously identified as nonpreferred. The initial paired-item presentation consisted of a rotation of 4 toys from the natural environment using a child-directed approach, which consisted of choice, prompting, and praise for completion of functional toy play. An additional condition added the child's preferred sensory attribute to a nonpreferred toy using the above-mentioned procedures. Results indicated that the addition of a preferred sensory attribute increased selection of that toy with 2 of 3 children and resulted in increased independent functional toy play for all 3 children
SN - 0896-3746, 0896-3746
UR - http://search.proquest.com/docview/742874687?accountid=14701
L2 - http://sfx.scholarsportal.info/ottawa?url_ver=Z39.88-2004&rft_val_fmt=info:ofi/fmt:kev:mtx:journal&genre=article&sid=ProQ:ProQ%3Aericshell&atitle=Embedding+Sensory+Preference+into+Toys+to+Enhance+Toy+Play+in+Toddlers+with+Disabilities&title=Infants+and+Young+Children&issn=08963746&date=2009-01-01&volume=22&issue=3&spage=188&au=DiCarlo%2C+Cynthia+F.%3BSchepis%2C+Maureen+M.%3BFlynn%2C+Linda&isbn=&jtitle=Infants+and+Young+Children&btitle=&rft_id=info:eric/EJ884516
ER -

TY - JOUR

ID - 2003
T1 - Do Children Prefer Contingencies? An Evaluation of the Efficacy of and Preference for Contingent versus Noncontingent Social Reinforcement during Play
A1 - Luczynski,Kevin C.
A1 - Hanley,Gregory P.
Y1 - 2009///
KW - ERIC
KW - Unsupervised Play Addition
KW - ERIC,Current Index to Journals in Education (CIJE)
KW - Preschool Education
KW - Contingency Management
KW - Social Reinforcement
KW - Interaction
KW - Play
KW - Child Behavior
KW - Behavior Modification
KW - Attitude Measures
KW - Preschool Children
KW - CHILDREN
KW - Interpersonal Relationship
RP - NOT IN FILE
SP - 511
EP - 525
JF - Journal of Applied Behavior Analysis
JA - J Appl.Behav Anal.
VL - 42
IS - 3
PB - Society for the Experimental Analysis of Behavior. , Kansas University, 1000 Sunnyside Avenue, Lawrence, KS 66045-2133.
N2 - Discovering whether children prefer reinforcement via a contingency or independent of their behavior is important considering the ubiquity of these programmed schedules of reinforcement. The current study evaluated the efficacy of and preference for social interaction within differential reinforcement of alternative behavior (DRA) and noncontingent reinforcement (NCR) schedules with typically developing children. Results showed that 7 of the 8 children preferred the DRA schedule; 1 child was indifferent. We also demonstrated a high degree of procedural fidelity, which suggested that preference is influenced by the presence of a contingency under which reinforcement can be obtained. These findings are discussed in terms of (a) the selection of reinforcement schedules in practice, (b) variables that influence children's preferences for contexts, and (c) the selection of experimental control procedures when evaluating the effects of reinforcement. (Contains 6 figures.)
SN - 0021-8855, 0021-8855
UR - http://search.proquest.com/docview/61833643?accountid=14701
L2 - http://sfx.scholarsportal.info/ottawa?url_ver=Z39.88-2004&rft_val_fmt=info:ofi/fmt:kev:mtx:journal&genre=article&sid=ProQ:ProQ%3Aericshell&atitle=Do+Children+Prefer+Contingencies%3F+An+Evaluation+of+the+Efficacy+of+and+Preference+for+Contingent+versus+Noncontingent+Social+Reinforcement+during+Play&title=Journal+of+Applied+Behavior+Analysis&issn=00218855&date=2009-01-01&volume=42&issue=3&spage=511&au=Luczynski%2C+Kevin+C.%3BHanley%2C+Gregory+P.&isbn=&jtitle=Journal+of+Applied+Behavior+Analysis&btitle=&rft_id=info:eric/EJ859306
ER -

TY - RPRT
ID - 2005
T1 - Using Stimulus Pairing to Enhance Observational Learning of Peer Initiations for a Child with Autism in a Community Play Group
A1 - Silla,Vanessa A.
A1 - Vesloski,Mary J.

Y1 - 2008/04/11/
N1 - Last updated - 2012-12-14
KW - ERIC
KW - Unsupervised Play Addition
KW - ERIC,Resources in Education (RIE)
KW - Observational Learning
KW - Case Studies
KW - Intervention
KW - Outcomes of Treatment
KW - Play Therapy
KW - Associative Learning
KW - Child Development
KW - Stimuli
KW - Association (Psychology)
KW - Peer Groups
KW - SOCIAL psychology
KW - Autism
RP - NOT IN FILE
SP - 23
N2 - The importance of play in child development and the barriers that individuals with autism face regarding play skills requires us to identify specific interventions which can assist in the development of such skills. Stimulus pairing, which has been documented as a procedure by which an event comes to elicit a response by being paired with an event that elicits that response (Chance, 1998), may be of benefit during play activities. This study attempted to evaluate the influence of the use of Stimulus pairing procedures to pair typical children with preferred tangibles and activities. This study also sought to identify whether observational learning of play skills can be enhanced through the use of such pairing procedures. Stimulus pairing was applied in this study by pairing typically developing peers with preferred tangibles (edibles, toys, etc.) and activities. Following preference assessments, each child with autism was paired with a typically developing peer who possessed primary reinforcers. Data was collected on the number and quality of social interactions between the children with autism and their peers across a series of ten 90-minute play sessions. The duration of time spent on activities initiated by children with autism was also examined. Findings for one particular play group participant, Caitlyn, were analyzed to see if there were any significant changes in her peer play behaviors. Although Caitlyn did not engage in any independent peer approaches of requests during the first nine sessions of the play group, on the tenth session she spent nearly half the play group time engaged with peers in activities she initiated. Implications of the findings and recommendations for future research are discussed. (Contains 1 figure and 2 tables.)
UR - http://search.proquest.com/docview/61970216?accountid=14701
ER -

TY - JOUR
ID - 2006
T1 - Language Outcome in Autism: Randomized Comparison of Joint Attention and Play Interventions
A1 - Kasari,Connie
A1 - Paparella,Tanya
A1 - Freeman,Stephanny
A1 - Jahromi,Laudan B.
Y1 - 2008/02//
KW - ERIC
KW - Unsupervised Play Addition
KW - ERIC,Current Index to Journals in Education (CIJE)
KW - Early Childhood Education
KW - Preschool Education
KW - Comparative Analysis
KW - Control Groups
KW - Language Acquisition
KW - Play

KW - Expressive Language
KW - Intervention
KW - Preschool Children
KW - Attention
KW - Autism
RP - NOT IN FILE
SP - 125
EP - 137
JF - Journal of Consulting and Clinical Psychology
VL - 76
IS - 1
PB - American Psychological Association. Journals Department, 750 First Street NE, Washington, DC 20002-4242.
N2 - This study reports results of a randomized controlled trial aimed at joint attention (JA) and symbolic play (SP) in preschool children with autism, with prediction to language outcome 12 months later. Participants were 58 children (46 boys) with autism between 3 and 4 years of age. Children were randomized to a JA intervention, an SP intervention, or control group. Interventions were conducted 30 min daily for 5-6 weeks. Assessments of JA skills, SP skills, mother-child interactions, and language development were collected at 4 time points: pre- and postintervention and 6 and 12 months postintervention by independent testers. Results indicate that expressive language gains were greater for both treatment groups compared with the control group, and results could not be explained by differences in other interventions in which children participated. For children beginning treatment with the lowest language levels, the JA intervention improved language outcome significantly more than did the SP or control interventions. These findings suggest clinically significant benefits of actively treating JA and SP skills in young children with autism
SN - 0022-006X, 0022-006X
UR - http://search.proquest.com/docview/61964798?accountid=14701
L2 - http://sfx.scholarsportal.info/ottawa?url_ver=Z39.88-2004&rft_val_fmt=info:ofi/fmt:kev:mtx:journal&genre=article&sid=ProQ:ProQ%3Aericshell&atitle=Language+Outcome+in+Autism%3A+Randomized+Comparison+of+Joint+Attention+and+Play+Interventions&title=Journal+of+Consulting+and+Clinical+Psychology&issn=0022006X&date=1996-01-01&volume=&issue=&spage=125&au=Saracho%2C+Olivia+N.&isbn=&jtitle=&btitle=Preschool+Children%27s+Cognitive+Styles+and+Play+Behaviors.&rft_id=info:eric/EJ533033
ER -

TY - JOUR
ID - 2007
T1 - Development of Symbolic Play through the Use of Virtual Reality Tools in Children with Autistic Spectrum Disorders: Two Case Studies
A1 - Herrera,Gerardo
A1 - Alcantud,Francisco
A1 - Jordan,Rita
A1 - Blanquer,Amparo
A1 - Labajo,Gabriel
A1 - De Pablo,Cristina
Y1 - 2008///
KW - ERIC
KW - Unsupervised Play Addition
KW - ERIC,Current Index to Journals in Education (CIJE)
KW - Imagination
KW - Computer Simulation
KW - Play
KW - Case Studies
KW - Pervasive Developmental Disorders
KW - Intervention
KW - CHILDREN
KW - Autism

KW - Teaching Methods
RP - NOT IN FILE
SP - 143
EP - 157
JF - Autism: The International Journal of Research and Practice
VL - 12
IS - 2
PB - SAGE Publications. 2455 Teller Road, Thousand Oaks, CA 91320.
N2 - Difficulties in understanding symbolism have been documented as characteristic of autistic spectrum disorders (ASDs). In general, virtual reality (VR) environments offer a set of potential advantages for educational intervention in ASD. In particular, VR offers the advantage, for teaching pretend play and for understanding imagination, of it being possible to show these imaginary transformations explicitly. This article reports two case studies of children with autism (aged 8:6 and 15:7, both male), examining the effectiveness of using a VR tool specifically designed to work on teaching understanding of pretend play. The results, confirmed by independent observers, showed a significant advance in pretend play abilities after the intervention period in both participants, and a high degree of generalization of the acquired teaching in one of them. (Contains 4 figures and 3 tables.)
SN - 1362-3613, 1362-3613
UR - http://search.proquest.com/docview/61956596?accountid=14701
L2 - http://sfx.scholarsportal.info/ottawa?url_ver=Z39.88-2004&rft_val_fmt=info:ofi/fmt:kev:mtx:journal&genre=article&sid=ProQ:ProQ%3Aericshell&atitle=Development+of+Symbolic+Play+through+the+Use+of+Virtual+Reality+Tools+in+Children+with+Autistic+Spectrum+Disorders%3A+Two+Case+Studies&title=Autism%3A+The+International+Journal+of+Research+and+Practice&issn=13623613&date=2008-01-01&volume=12&issue=2&spage=143&au=Herrera%2C+Gerardo%3BAlcantud%2C+Francisco%3BJordan%2C+Rita%3BBlanquer%2C+Amparo%3BLabajo%2C+Gabriel%3BDe+Pablo%2C+Cristina&isbn=&jtitle=Autism%3A+The+International+Journal+of+Research+and+Practice&btitle=&rft_id=info:eric/EJ786682
ER -

TY - JOUR
ID - 2008
T1 - Infant and Mother-Infant Play and the Presence of the Television
A1 - Masur,Elise Frank
A1 - Flynn,Valerie
Y1 - 2008///
KW - ERIC
KW - Unsupervised Play Addition
KW - ERIC,Current Index to Journals in Education (CIJE)
KW - Practitioners
KW - Researchers
KW - Questionnaires
KW - Play
KW - Research Methodology
KW - Parent Child Relationship
KW - Mothers
KW - Television
KW - Toys
KW - Infants
RP - NOT IN FILE
SP - 76
EP - 83
JF - Journal of Applied Developmental Psychology
VL - 29
IS - 1
PB - Elsevier. 6277 Sea Harbor Drive, Orlando, FL 32887-4800.

N2 - Forty-eight middle-class mothers answered questionnaires about their 11-through 18-month-old infants' typical television watching and interest, the frequency and duration of their independent play with toys and dyadic play with and without toys, and whether the television was typically on or not on in the room at the time. Mothers reported that most infants had little interest in and spent little time actively watching television. However, mothers reported the television was typically turned on in the room at least half the time during independent play with toys for 44% of infants and during dyadic play with toys for 53% of infants. These findings provide evidence relevant to researchers' and practitioners' concerns regarding the potential negative impact of television's presence on infant development, mother-child interaction, and the ecological validity of observational research methods involving play or interactions in the absence of a television
SN - 0193-3973, 0193-3973
UR - http://search.proquest.com/docview/61961676?accountid=14701
L2 - http://sfx.scholarsportal.info/ottawa?url_ver=Z39.88-2004&rft_val_fmt=info:ofi/fmt:kev:mtx:journal&genre=unknown&sid=ProQ:ProQ%3Aericshell&atitle=The+Effect+of+Dramatic+Play+on+the+Story+Retellings+of+Second+Grade+Children.&title=&issn=&date=1983-04-01&volume=&issue=&spage=10&au=Galda%2C+Lee%3BMiller%2C+Michael&isbn=&jtitle=&btitle=&rft_id=info:eric/ED233812
ER -

TY - JOUR
ID - 2009
T1 - The Bubble-Wrap Generation: Children Growing Up in Walled Gardens
A1 - Malone,Karen
Y1 - 2007/09//
KW - ERIC
KW - Unsupervised Play Addition
KW - Australia
KW - Germany
KW - ERIC,Current Index to Journals in Education (CIJE)
KW - Early Childhood Education
KW - Elementary Education
KW - Middle Class
KW - Environmental Education
KW - Play
KW - Competence
KW - Anxiety
KW - Safety
KW - CHILDREN
KW - Personality Traits
KW - Parenting Styles
KW - Physical Environment
KW - Foreign Countries
KW - Child Development
KW - Freedom
RP - NOT IN FILE
SP - 513
EP - 527
JF - Environmental Education Research
VL - 13
IS - 4
PB - Routledge. , 325 Chestnut Street Suite 800, Philadelphia, PA 19106.
N2 - Children in middle class Australia, and many western countries around the world experience restricted opportunities to engage in free play in their neighbourhood streets and parks. The impact of this has been a drastic decrease in children's independent mobility and environmental play. Recent research has focused on the physical environment of neighbourhoods' in an attempt to understand how they might be designed to encourage children's play and increase their environmental learning. This paper explores these same issues but from a

different perspective. That is, to explain some of the changes in childhood environmental behaviours I explore children and parent relationships, in particular, the phenomena of 'bubble-wrapping' children to appease the anxieties of some middle class parents. I acknowledge when making these claims that parenting is a complex and highly individual enterprise with many variables, therefore this paper is only seeking to draw attention to a broad pattern of behaviour of a select group within society. I will conclude with an overview of some of the key attributes and competencies that are being lost when children lack independent mobility in their neighbourhoods and will subsequently argue why opening the front gates of our middle class suburbs is important to enhance children's environmental learning. (Contains 5 figures and 2 notes.)
SN - 1350-4622, 1350-4622
UR - http://search.proquest.com/docview/62057431?accountid=14701
L2 - http://sfx.scholarsportal.info/ottawa?url_ver=Z39.88-2004&rft_val_fmt=info:ofi/fmt:kev:mtx:journal&genre=article&sid=ProQ:ProQ%3Aericshell&atitle=The+Bubble-Wrap+Generation%3A+Children+Growing+Up+in+Walled+Gardens&title=Environmental+Education+Research&issn=13504622&date=2007-09-01&volume=13&issue=4&spage=513&au=Malone%2C+Karen&isbn=&jtitle=Environmental+Education+Research&btitle=&rft_id=info:eric/EJ775883
ER -

TY - JOUR
ID - 2010
T1 - Effects of an Individual Work System on the Independent Functioning of Students with Autism
A1 - Hume,Kara
A1 - Odom,Sam
Y1 - 2007/07//
KW - ERIC
KW - Unsupervised Play Addition
KW - ERIC,Current Index to Journals in Education (CIJE)
KW - Instructional Effectiveness
KW - Play
KW - Intervention
KW - Outcomes of Treatment
KW - Educational Environment
KW - Independent Study
KW - Visual Aids
KW - Cues
KW - Work Environment
KW - Autism
KW - Time on Task
KW - Teaching Methods
RP - NOT IN FILE
SP - 1166
EP - 1180
JF - Journal of Autism and Developmental Disorders
VL - 37
IS - 6
PB - Springer. 233 Spring Street, New York, NY 10013.
N2 - This study examined the effects of a work system on the independent work and play skills of students with autism. Work systems, an element of structured teaching developed by Division TEACCH, are organized sets of visual information that inform a student about participation in work or play areas. A single subject withdrawal of treatment design, with replications across three participants, was used to assess the on-task behavior and work completion skills of the students in classroom and employment settings as a result of the intervention. Observational data indicated that all students showed increases in on-task behavior, increases in the number of tasks completed or play materials utilized, and reduction of teacher prompts. The results were maintained through the 1-month follow-up
SN - 0162-3257, 0162-3257

UR - http://search.proquest.com/docview/62048787?accountid=14701
L2 - http://sfx.scholarsportal.info/ottawa?url_ver=Z39.88-2004&rft_val_fmt=info:ofi/fmt:kev:mtx:journal&genre=article&sid=ProQ:ProQ%3Aericshell&atitle=Effects+of+an+Individual+Work+System+on+the+Independent+Functioning+of+Students+with+Autism&title=Journal+of+Autism+and+Developmental+Disorders&issn=01623257&date=2007-07-01&volume=37&issue=6&spage=1166&au=Hume%2C+Kara%3BOdom%2C+Sam&isbn=&jtitle=Journal+of+Autism+and+Developmental+Disorders&btitle=&rft_id=info:eric/EJ767554
ER -

TY - JOUR
ID - 2011
T1 - Current Fear of Crime, Sense of Community, and Loneliness in Italian Adolescents: The Role of Autonomous Mobility and Play during Childhood
A1 - Prezza,Miretta
A1 - Pacilli,Maria Giuseppina
Y1 - 2007/03//
KW - ERIC
KW - Unsupervised Play Addition
KW - Italy
KW - UCLA Loneliness Scale
KW - ERIC,Current Index to Journals in Education (CIJE)
KW - community
KW - Play
KW - Crime
KW - Mobility
KW - Gender Differences
KW - Fear
KW - Structural Equation Models
KW - CHILDREN
KW - Foreign Countries
KW - Psychological Patterns
KW - Personal Autonomy
KW - Adolescents
KW - Predictor Variables
RP - NOT IN FILE
SP - 151
EP - 170
JF - Journal of Community Psychology
VL - 35
IS - 2
PB - John Wiley & Sons, Subscription Department, 111 River Street, Hoboken, NJ 07030-5774.
N2 - A structural equation model was used to examine the role of autonomous mobility and play in public and semipublic places in childhood to predict adolescents' sense of community, fear of crime, and, through the mediation of these two last psychosocial factors, feelings of loneliness. Participants included 789 Italian students (469 females and 320 males; mean age 16.62, SD = 0.69). The instruments used were the Italian Sense of Community Scale, the Neighborhood Relations Scale, and the UCLA Loneliness Scale. A questionnaire was also administered to investigate the adolescents' current fear of crime and their autonomous mobility when they were children by asking them to recall their play habits and independent mobility at 8-10 years of age. The structural equation model proved satisfying: Higher autonomous mobility and higher use of public places for play in childhood predicted less intense fear of crime and a stronger sense of community in adolescence. Furthermore, a better relationship with community predicted less pervasive feelings of loneliness. The simultaneous multigroup analysis showed that the final model was comparable for both girls and boys with only one difference. (Contains 3 tables, 9 footnotes and 2 figures.)
SN - 0090-4392, 0090-4392
UR - http://search.proquest.com/docview/968117717?accountid=14701

L2 - http://sfx.scholarsportal.info/ottawa?url_ver=Z39.88-2004&rft_val_fmt=info:ofi/fmt:kev:mtx:journal&genre=article&sid=ProQ:ProQ%3Aericshell&atitle=Current+Fear+of+Crime%2C+Sense+of+Community%2C+and+Loneliness+in+Italian+Adolescents%3A+The+Role+of+Autonomous+Mobility+and+Play+during+Childhood&title=Journal+of+Community+Psychology&issn=00904392&date=2007-03-01&volume=35&issue=2&spage=151&au=Prezza%2C+Miretta%3BPacilli%2C+Maria+Giuseppina&isbn=&jtitle=Journal+of+Community+Psychology&btitle=&rft_id=info:eric/EJ958157
ER -

TY - JOUR
ID - 2012
T1 - A Pilot Study of the Effects of a Social-Pragmatic Intervention on the Communication and Symbolic Play of Children with Autism
A1 - Keen,Deb
A1 - Rodger,Sylvia
A1 - Doussin,Kim
A1 - Braithwaite,Michelle
Y1 - 2007///
KW - ERIC
KW - Unsupervised Play Addition
KW - ERIC,Current Index to Journals in Education (CIJE)
KW - Program Effectiveness
KW - Measures (Individuals)
KW - Play
KW - Young Children
KW - Interpersonal Competence
KW - Early Intervention
KW - Stress Variables
KW - Autism
KW - Communication Skills
RP - NOT IN FILE
SP - 63
EP - 71
JF - Autism: The International Journal of Research & Practice
VL - 11
IS - 1
PB - SAGE Publications. 2455 Teller Road, Thousand Oaks, CA 91320.
N2 - The acquisition of social communication skills is a major challenge faced by children with autism. This pilot study investigated the effects of the Stronger Families Project, a social-pragmatic intervention, on the communication and symbolic abilities of 16 children aged 2-4 years with autism. Standardized measures of the child's communication and symbolic behaviour were conducted by independent observers at a university clinic pre and post-intervention, and parents were interviewed to determine the impact of variables such as maternal stress and competence on the child's social communication. Changes in some communication and symbolic behaviours occurred following the Stronger Families Project intervention according to parent report. However, improvements based on ratings by independent observers were not significant. Results are discussed in relation to the assessment of changes in the social communication and symbolic play of children with autism following intervention using clinical observation and parent report. (Contains 2 tables.)
SN - 1362-3613, 1362-3613
UR - http://search.proquest.com/docview/62040416?accountid=14701
L2 - http://sfx.scholarsportal.info/ottawa?url_ver=Z39.88-2004&rft_val_fmt=info:ofi/fmt:kev:mtx:journal&genre=article&sid=ProQ:ProQ%3Aericshell&atitle=A+Pilot+Study+of+the+Effects+of+a+Social-Pragmatic+Intervention+on+the+Communication+and+Symbolic+Play+of+Children+with+Autism&title=Autism%3A+The+International+Journal+of+Research+%26+Practice&issn=13623613&date=2007-01-01&volume=11&issue=1&spage=63&au=Keen%2C+Deb%3BRodger%2C+Sylvia%3BDoussin%2C+Kim%3B

Braithwaite%2C+Michelle&isbn=&jtitle=Autism%3A+The+International+Journal+of+Research+%26+Practice&btitle=&rft_id=info:eric/EJ747743
ER -

TY - JOUR
ID - 2013
T1 - Using Video Modeling for Generalizing Toy Play in Children with Autism
A1 - Paterson,Claire R.
A1 - Arco,Lucius
Y1 - 2007///
KW - ERIC
KW - Unsupervised Play Addition
KW - Australia
KW - ERIC,Current Index to Journals in Education (CIJE)
KW - Elementary Education
KW - Elementary School Students
KW - Instructional Effectiveness
KW - Play
KW - MALES
KW - Videotape Recordings
KW - Correlation
KW - CHILDREN
KW - Verbal Communication
KW - Foreign Countries
KW - Toys
KW - Autism
KW - Teaching Methods
KW - Modeling (Psychology)
RP - NOT IN FILE
SP - 660
EP - 681
JF - Behavior Modification
VL - 31
IS - 5
PB - SAGE Publications. 2455 Teller Road, Thousand Oaks, CA 91320.
N2 - The present study examined effects of video modeling on generalized independent toy play of two boys with autism. Appropriate and repetitive verbal and motor play were measured, and intermeasure relationships were examined. Two single-participant experiments with multiple baselines and withdrawals across toy play were used. One boy was presented with three physically unrelated toys, whereas the other was presented with three related toys. Video modeling produced increases in appropriate play and decreases in repetitive play, but generalized play was observed only with the related toys. Generalization may have resulted from variables including the toys' common physical characteristics and natural reinforcing properties and the increased correspondence between verbal and motor play. (Contains 3 tables and 4 figures.)
SN - 0145-4455, 0145-4455
UR - http://search.proquest.com/docview/62048715?accountid=14701
L2 - http://sfx.scholarsportal.info/ottawa?url_ver=Z39.88-2004&rft_val_fmt=info:ofi/fmt:kev:mtx:journal&genre=article&sid=ProQ:ProQ%3Aericshell&atitle=Using+Video+Modeling+for+Generalizing+Toy+Play+in+Children+with+Autism&title=Behavior+Modification&issn=01454455&date=2007-01-01&volume=31&issue=5&spage=660&au=Paterson%2C+Claire+R.%3BArco%2C+Lucius&isbn=&jtitle=Behavior+Modification&btitle=&rft_id=info:eric/EJ771584
ER -

TY - JOUR
ID - 2014
T1 - Making Play Happen: Ideas for Mounting a Campaign to Increase Healthy Play in Your Community

A1 - Oliver,Susan J.
A1 - Klugman,Edgar
Y1 - 2007///
KW - ERIC
KW - Unsupervised Play Addition
KW - ERIC,Current Index to Journals in Education (CIJE)
KW - Early Childhood Education
KW - Play
KW - Young Children
KW - Interpersonal Competence
KW - Goal Orientation
KW - Child Development
KW - Social Development
KW - Cooperative Planning
KW - Creativity
KW - Outcome Based Education
KW - Emotional Development
KW - Classroom Techniques
RP - NOT IN FILE
SP - 34
EP - 37
JF - Exchange: The Early Childhood Leaders' Magazine Since 1978
VL - 176
PB - Exchange Press, Inc. P.O. Box 3249, Redmond, WA 98073-3249. Tel: 800-221-2864; Fax: 425-867-5217; e-mail: info@ChildCareExchane.com; Web site: www.childcareexchange.com
N2 - Much has been written in recent years about early childhood play, documenting its role in healthy development, detailing the risks children face if they do not have access to play, and offering implementation tips to educators to use in communicating about play to administrators and parents. For those ready to move forward in their own communities to impact change that will ensure that today's children have sufficient access to healthy play, Oliver and Klugman discuss a number of basic steps: (1) Conceptualizing a local campaign on play to raise parental and preschool awareness, increase community safety for unsupervised or lightly supervised play, set priorities and identify best chances for success; (2) Joining forces with those who can bring understanding of the target audience, attention, leadership, necessary skill sets and funding to the project; (3) Establishing clear campaign objectives with defined goals, realistic timeline and measurement tools; (4) Doing one's homework by identifying potential competition, advocates/detractors, developing counterpoint to anticipated "anti" positions and compiling easy-to-understand statistics from well-defined inquiries; (5) Creating message points that attract attention, establish the problem, provide the solution and issue a call to action and (6) Developing an execution and implementation plan, based on available media and events best suited to the target community. Oliver and Klugman acknowledge that the challenge to understand how much learning happens through play and to convince parents, administrators, policy makers and others to make play as important as mathematics, literacy and science is large. The authors balance this argument by pointing out the payoffs for children, such as acquiring the social and emotional skills that help make them school-ready. Children also maintain their natural motivation to be engaged, curious learners in an outcome-oriented educational context
SN - 0164-8527, 0164-8527
UR - http://search.proquest.com/docview/62042419?accountid=14701
L2 - http://sfx.scholarsportal.info/ottawa?url_ver=Z39.88-2004&rft_val_fmt=info:ofi/fmt:kev:mtx:journal&genre=article&sid=ProQ:ProQ%3Aericshell&atitle=Making+Play+Happen%3A+Ideas+for+Mounting+a+Campaign+to+Increase+Healthy+Play+in+Your+Community&title=Exchange%3A+The+Early+Childhood+Leaders%27+Magazine+Since+1978&issn=01648527&date=2007-01-01&volume=176&issue=&spage=34&au=Oliver%2C+Susan+J.%3BKlugman%2C+Edgar&isbn=&jtitle=Exchange%3A+The+Early+Childhood+Leaders%27+Magazine+Since+1978&btitle=&rft_id=info:eric/EJ769528
ER -

TY - JOUR
ID - 2016

T1 - A Longitudinal Study of Belief and Desire State Discourse during Mother-Child Play and Later False Belief Understanding
A1 - Symons,Douglas K.
A1 - Fossum,Kristin Lee
A1 - Collins,B.T.
Y1 - 2006/11//
KW - ERIC
KW - Unsupervised Play Addition
KW - ERIC,Current Index to Journals in Education (CIJE)
KW - Cognitive Development
KW - Play
KW - Mothers
KW - Socioeconomic Status
KW - Correlation
KW - Longitudinal Studies
KW - Discourse Analysis
KW - Cognitive Processes
KW - theory of mind
KW - Parent Child Relationship
KW - Role
KW - Child Language
RP - NOT IN FILE
SP - 676
EP - 692
JF - Social Development
VL - 15
IS - 4
PB - Wiley-Blackwell. 350 Main Street, Malden, MA 02148.
N2 - There is considerable interest in the role of mental state language in theory of mind development. This study examines cognitive and desire state discourse of 43 mothers during play interactions with their two-year-old children and theory of mind as indicated by a battery of false belief tasks around the age of five. Desire state comments of mothers during free play which were appropriate to the conversational context were significantly related to children's later theory of mind, and this relation was independent of other confounding variables, including a home-based measure of maternal sensitivity, and socio-economic status, and an estimate of child language. The child's desire state language was concurrently related to the mother's desire state language used appropriately, and the child's cognitive state language was related to the mother's appropriate cognitive state language. Although cognitive state language was not predictive of later theory of mind, this may be because this kind of language plays a later role in theory of mind development
SN - 0961-205X, 0961-205X
UR - http://search.proquest.com/docview/964170536?accountid=14701
L2 - http://sfx.scholarsportal.info/ottawa?url_ver=Z39.88-2004&rft_val_fmt=info:ofi/fmt:kev:mtx:journal&genre=article&sid=ProQ:ProQ%3Aericshell&atitle=A+Longitudinal+Study+of+Belief+and+Desire+State+Discourse+during+Mother-Child+Play+and+Later+False+Belief+Understanding&title=Social+Development&issn=0961205X&date=2006-11-01&volume=15&issue=4&spage=676&au=Symons%2C+Douglas+K.%3BFossum%2C+Kristin-Lee+M.%3BCollins%2C+B.+T.&isbn=&jtitle=Social+Development&btitle=&rft_id=info:eric/EJ942069
ER -

TY - JOUR
ID - 2017
T1 - Joint Attention and Symbolic Play in Young Children with Autism: A Randomized Controlled Intervention Study
A1 - Kasari,Connie
A1 - Freeman,Stephanny
A1 - Paparella,Tanya
Y1 - 2006/06//

KW - ERIC
KW - Unsupervised Play Addition
KW - ERIC,Current Index to Journals in Education (CIJE)
KW - Program Effectiveness
KW - Play
KW - Control Groups
KW - Young Children
KW - Parent Child Relationship
KW - Mothers
KW - Early Intervention
KW - Interaction Process Analysis
KW - Autism
KW - Outcomes of Treatment
RP - NOT IN FILE
SP - 611
EP - 620
JF - Journal of Child Psychology and Psychiatry
VL - 47
IS - 6
PB - Wiley-Blackwell. 350 Main Street, Malden, MA 02148.
N2 - Background: Delays and deficits in joint attention and symbolic play constitute two important developmental problems in young children with autism. These areas of deficit have been well studied in autism but have rarely been the focus of treatment efforts (see Kasari, Freeman, & Paparella, 2001). In this study, we examine the efficacy of targeted interventions of joint attention and symbolic play. Methods: Participants were 58 children with autism aged 3 and 4 years (46 boys). Children were randomized to a joint attention intervention, a symbolic play intervention, or control group. Interventions were conducted 30 minutes daily for 5-6 weeks. Both structured assessments of joint attention and play skills and mother-child interactions were collected pre and post intervention by independent assessors. Results: Results indicate that both intervention groups improved significantly over the control group on certain behaviors. Children in the joint attention intervention initiated significantly more showing and responsiveness to joint attention on the structured joint attention assessment and more child-initiated joint attention in the mother-child interaction. The children in the play group showed more diverse types of symbolic play in interaction with their mothers and higher play levels on both the play assessment and in interaction with their mothers. Conclusions: This randomized controlled trial provides promising data on the specificity and generalizability of joint attention and play interventions for young children with autism. Future studies need to examine the long-term effects of these early interventions on children's development
SN - 0021-9630, 0021-9630
UR - http://search.proquest.com/docview/964185204?accountid=14701
L2 - http://sfx.scholarsportal.info/ottawa?url_ver=Z39.88-2004&rft_val_fmt=info:ofi/fmt:kev:mtx:journal&genre=article&sid=ProQ:ProQ%3Aericshell&atitle=Joint+Attention+and+Symbolic+Play+in+Young+Children+with+Autism%3A+A+Randomized+Controlled+Intervention+Study&title=Journal+of+Child+Psychology+and+Psychiatry&issn=00219630&date=2006-06-01&volume=47&issue=6&spage=611&au=Kasari%2C+Connie%3BFreeman%2C+Stephanny%3BPaparella%2C+Tanya&isbn=&jtitle=Journal+of+Child+Psychology+and+Psychiatry&btitle=&rft_id=info:eric/EJ950297
ER -

TY - JOUR
ID - 2019
T1 - Children's Categorisation of Play and Learning Based on Social Context
A1 - Howard,Justine
A1 - Jenvey,Vickii
A1 - Hill,Carly
Y1 - 2006/05//
KW - ERIC
KW - Unsupervised Play Addition
KW - Australia

KW - ERIC,Current Index to Journals in Education (CIJE)
KW - Preschool Education
KW - Context Effect
KW - Play
KW - Young Children
KW - Early Childhood Education
KW - Teacher Attendance
KW - Association Measures
KW - Peer Relationship
KW - Group Activities
KW - Foreign Countries
KW - Cues
KW - Pictorial Stimuli
KW - Attitude Measures
KW - Social Environment
RP - NOT IN FILE
SP - 15
JF - Early Child Development and Care
VL - 176
IS - 3
PB - Customer Services for Taylor & Francis Group Journals, 325 Chestnut Street, Suite 800, Philadelphia, PA 19106.
N2 - While much time has been spent deliberating about the definition of play, little emphasis has been placed on what children themselves perceive as play. The aim of the present study was to examine social context as a cue for children's perceptions of play and learning. Ninety-two children aged between four and six years (mean four years nine months) participated in the study and completed the Activity Apperception Story Procedure. Children were asked to sort photographic stimuli into those they believed depicted play/not play and learning/not learning. Each of the stimuli were identified by independent raters as containing one of the following social cues; teacher absence (solitary activity, parallel activity or cooperative activity) or teacher presence (teacher involved activity or teacher directed activity). Findings revealed that children associated teacher absence with play. More specifically, children made links between play and the presence of peers (parallel and cooperative activity). Findings are discussed in relation to play in the early years curriculum, differences in children's early educational experiences and the importance of understanding children's perceptions of play
SN - 0300-4430, 0300-4430
UR - http://search.proquest.com/docview/62096949?accountid=14701
L2 - http://sfx.scholarsportal.info/ottawa?url_ver=Z39.88-2004&rft_val_fmt=info:ofi/fmt:kev:mtx:journal&genre=article&sid=ProQ:ProQ%3Aericshell&atitle=Children%27s+Categorisation+of+Play+and+Learning+Based+on+Social+Context&title=Early+Child+Development+and+Care&issn=03004430&date=2006-05-01&volume=176&issue=3&spage=15&au=Howard%2C+Justine%3BJenvey%2C+Vickii%3BHill%2C+Carly&isbn=&jtitle=Early+Child+Development+and+Care&btitle=&rft_id=info:eric/EJ729198
ER -

TY - JOUR
ID - 2021
T1 - Developing Citizenship Through Supervised Play: The Civics Institute of Ireland Playgrounds, 1933-75
A1 - Kernan,Margaret
Y1 - 2005/11//
KW - ERIC
KW - Unsupervised Play Addition
KW - Ireland
KW - ERIC,Current Index to Journals in Education (CIJE)
KW - Play
KW - Foreign Countries
KW - Playgrounds

KW - Citizenship Education
KW - Service Learning
KW - Learning Experience
KW - Intervention
KW - Civics
KW - Interviews
KW - CHILDREN
KW - Teaching Methods
RP - NOT IN FILE
SP - 675
EP - 687
JF - History of Education
VL - 34
IS - 6
PB - Customer Services for Taylor & Francis Group Journals, 325 Chestnut Street, Suite 800, Philadelphia, PA 19106.
N2 - Prompted by a concern regarding the large numbers of unsupervised children playing on the streets of Dublin in the 1920s and 1930s, the Civics Institute of Ireland (referred to subsequently as the Civics Institute) established 10 playgrounds where children aged between four and 14 years could play after school hours and during school holidays. The playgrounds were open throughout the whole year and the children played under the guidance of play leaders. In the present article it is proposed that the motives of the organizers of the Civics Institute playgrounds can be assessed in terms of their educative social purpose, in addition to the dual need of protecting children from the perceived dangers of the city street, and safeguarding the street from crowds of unsupervised playing children. Once the children were removed from the street, the playgrounds came to be identified by the organizers as a focus of intervention and investment for the future needs of a newly independent Gaelic Ireland. In this regard, it is suggested that it was the intention of the Civics Institute that the pedagogical practices of the playgrounds would work for the development of a spirit of citizenship and patriotism. Thus, supervised play was perceived as the medium by which the particular attitudes and behaviours of good citizenship could be developed. The present article considers the play in the Civics Institute playgrounds as represented by multiple perspectives. These include "the voice of officialdom" represented through the various papers and documents of the Civics Institute such as annual reports, minute books, correspondence, newspaper clippings and accounts of ex-Civics Institute Committee members. Ex-play leaders and assistant play leaders who were able to provide first-hand accounts of the day-to-day pedagogical practice in the playgrounds were also interviewed in the course of conducting the research. The author also had the opportunity of capturing the voice of "the child player", albeit through the memoried childhood of a small number of ex-playground attendees. These first-hand accounts provide an additional dimension in helping to understand the player's experiences in the playgrounds. (Contains 56 footnotes.)
SN - 0046-760X, 0046-760X
UR - http://search.proquest.com/docview/62154034?accountid=14701
L2 - http://sfx.scholarsportal.info/ottawa?url_ver=Z39.88-2004&rft_val_fmt=info:ofi/fmt:kev:mtx:journal&genre=article&sid=ProQ:ProQ%3Aericshell&atitle=Developing+Citizenship+Through+Supervised+Play%3A+The+Civics+Institute+of+Ireland+Playgrounds%2C+1933-75&title=History+of+Education&issn=0046760X&date=2005-11-01&volume=34&issue=6&spage=675&au=Kernan%2C+Margaret&isbn=&jtitle=History+of+Education&btitle=&rft_id=info:eric/EJ721963
ER -

TY - JOUR
ID - 2022
T1 - The Validity of Head Start Teacher Assistant Report of Children's Peer Play Competencies
A1 - McWayne,Christine
A1 - Sekino,Yumiko
A1 - Fantuzzo,John
Y1 - 2005/03//
KW - ERIC
KW - Unsupervised Play Addition

KW - Parent Reports
KW - ERIC,Current Index to Journals in Education (CIJE)
KW - Preschool Teachers
KW - Play
KW - Academic Achievement
KW - Test Validity
KW - Urban Schools
KW - Correlation
KW - Factor Analysis
KW - Peer Relationship
KW - Teacher Aides
KW - Early Intervention
KW - Disadvantaged Youth
KW - Construct Validity
KW - Preschool Children
RP - NOT IN FILE
SP - 103
EP - 120
JF - NHSA Dialog
VL - 8
IS - 1
PB - Routledge. , 325 Chestnut Street Suite 800, Philadelphia, PA 19106.
N2 - This study examined the validity of teacher assistant report of children's classroom peer play behaviors using the Penn Interactive Peer Play Scale (PIPPS). Participants for the examination of construct validity were 344 urban Head Start children. Exploratory factor analyses resulted in a reliable three-factor solution, corroborating the dimensions of interactive peer play previously found with teacher and parent reports. Factor-matching analyses yielded high congruence between the teacher-assistant structure and the credentialed-teacher and parent structures. Additionally, concurrent validity was demonstrated with a subsample of 270 children by significant relationships found between the PIPPS dimensions and teacher observations of children's classroom learning competencies and an independent assessment of children's early academic success. The results affirm that teacher assistants in Head Start are a reliable and valid source of information on children's interactive, peer play competencies. (Contains 2 tables.)
SN - 1524-0754, 1524-0754
UR - http://search.proquest.com/docview/61881351?accountid=14701
L2 - http://sfx.scholarsportal.info/ottawa?url_ver=Z39.88-2004&rft_val_fmt=info:ofi/fmt:kev:mtx:journal&genre=article&sid=ProQ:ProQ%3Aericshell&atitle=The+Validity+of+Head+Start+Teacher+Assistant+Report+of+Children%27s+Peer+Play+Competencies&title=NHSA+Dialog&issn=15240754&date=2005-03-01&volume=8&issue=1&spage=103&au=McWayne%2C+Christine%3BSekino%2C+Yumiko%3BFantuzzo%2C+John&isbn=&jtitle=NHSA+Dialog&btitle=&rft_id=info:eric/EJ834008
ER -

TY - JOUR
ID - 2023
T1 - Assessing Peer Entry and Play in Preschoolers at Risk for Maladjustment
A1 - Brotman,Laurie Miller
A1 - Gouley,Kathleen Kiely
A1 - Chesir-Teran,Daniel
Y1 - 2005///
KW - ERIC
KW - Unsupervised Play Addition
KW - ERIC,Current Index to Journals in Education (CIJE)
KW - Preschool Education
KW - Evaluation Methods
KW - High Risk Students
KW - Rating Scales

KW - Play
KW - Student Adjustment
KW - Parent Child Relationship
KW - Psychopathology
KW - Preschool Children
KW - Psychometrics
KW - Antisocial Behavior
KW - Peer Relationship
RP - NOT IN FILE
SP - 671
EP - 680
JF - Journal of Clinical Child and Adolescent Psychology
VL - 34
IS - 4
PB - Lawrence Erlbaum Associates, , Journal Subscription Department, 10 Industrial Avenue, Mahwah, NJ 07430-2262.
N2 - This study evaluated the psychometric properties of an observational rating system for assessing preschoolers' peer entry and play skills: Observed Peer Play in Unfamiliar Settings (OPPUS). Participants were 84 preschoolers at risk for psychopathology. Reliability and concurrent validity are reported. The 30-min paradigm yielded reliable indexes of engaging and disruptive behavior. Adequate interrater reliability and stability were obtained with minimally trained observers. OPPUS scores were related in expected ways to independent observations of disruptive and engaging behavior during parent-child interactions. OPPUS scores were associated with parent-rated self-control and internalizing behaviors. OPPUS scores were associated with parent ratings of play skills for children with school experience and assertiveness for children without school experience. The OPPUS is a useful, brief method for assessing peer entry and play behaviors in preschoolers
SN - 1537-4416, 1537-4416
UR - http://search.proquest.com/docview/62147562?accountid=14701
L2 - http://sfx.scholarsportal.info/ottawa?url_ver=Z39.88-2004&rft_val_fmt=info:ofi/fmt:kev:mtx:journal&genre=article&sid=ProQ:ProQ%3Aericshell&atitle=Assessing+Peer+Entry+and+Play+in+Preschoolers+at+Risk+for+Maladjustment&title=Journal+of+Clinical+Child+and+Adolescent+Psychology&issn=15374416&date=2005-01-01&volume=34&issue=4&spage=671&au=Brotman%2C+Laurie+Miller%3BGouley%2C+Kathleen+Kiely%3BChesir-Teran%2C+Daniel&isbn=&jtitle=Journal+of+Clinical+Child+and+Adolescent+Psychology&btitle=&rft_id=info:eric/EJ722582
ER -

TY - JOUR
ID - 2024
T1 - Effects of Punishment and Response-Independent Attention on Severe Problem Behavior and Appropriate Toy Play
A1 - Doughty,Shannon S.
A1 - Poe,Susannah G.
A1 - Anderson,Cynthia M.
Y1 - 2005///
KW - ERIC
KW - Unsupervised Play Addition
KW - ERIC,Current Index to Journals in Education (CIJE)
KW - Developmental Disabilities
KW - Toddlers
KW - Play
KW - Injuries
KW - Punishment
KW - MALES
KW - Intervention
KW - SOCIALIZATION

KW - Behavior Problems
KW - Self Destructive Behavior
KW - Reinforcement
KW - Severe Disabilities
KW - Toys
KW - Interpersonal Relationship
KW - Autism
RP - NOT IN FILE
SP - 91
EP - 98
JF - Journal of Early and Intensive Behavior Intervention
VL - 2
IS - 2
PB - Joseph Cautilli, Ph.D. & The Behavior Analyst Online Organization. 535 Queen Street, Philadelphia, PA 19147-3220.
N2 - Problem behavior can interfere with learning, the development of appropriate skills, and socialization in persons with developmental disabilities. In severe cases, problem behavior could result in life-threatening injury. For one 21-month-old participant diagnosed with autism engaging in severe problem behavior for whom reinforcement-based interventions had failed, punishment was implemented. After the efficacy of punishment alone was evaluated, it was implemented concurrent with response-independent attention. Problem behavior was suppressed and appropriate toy play emerged as a function of these combined interventions. Thus, punishment in combination with response-independent attention was an effective intervention for severe, attention-maintained problem behavior. (Contains 1 figure.)
SN - 1554-4893, 1554-4893
UR - http://search.proquest.com/docview/61863434?accountid=14701
ER -

TY - JOUR
ID - 2025
T1 - Increasing Pretend Toy Play of Toddlers with Disabilities in an Inclusive Setting
A1 - DiCarlo,Cynthia F.
A1 - Reid,Dennis H.
Y1 - 2004///
KW - ERIC
KW - Unsupervised Play Addition
KW - ERIC,Current Index to Journals in Education (CIJE)
KW - Early Childhood Education
KW - Toddlers
KW - Play
KW - Disabilities
KW - Toys
KW - Teaching Methods
RP - NOT IN FILE
SP - 197
JF - Journal of Applied Behavior Analysis
JA - J Appl.Behav Anal.
VL - 37
IS - 2
PB - Department of Applied Behavioral Science, 1000 Sunnyside Ave., KU, Lawrence, KS 66045-2133.
N2 - We evaluated a program for increasing pretend toy play of 2-year-old children with disabilities in an inclusive classroom. Classroom personnel implemented the program, which involved choices of classroom centers containing toys that tend to occasion pretend play in toddlers without disabilities, along with prompting and praise. Increases occurred in independent pretend-play rates among all 5 participating toddlers. Results are discussed regarding the importance of promoting toy play of very young children with disabilities that is similar to the type of play of their nondisabled peers, and the need to identify critical program components that are applicable in inclusive settings

SN - 0021-8855, 0021-8855
UR - http://search.proquest.com/docview/62124650?accountid=14701
L2 - http://sfx.scholarsportal.info/ottawa?url_ver=Z39.88-2004&rft_val_fmt=info:ofi/fmt:kev:mtx:journal&genre=article&sid=ProQ:ProQ%3Aericshell&atitle=Increasing+Pretend+Toy+Play+of+Toddlers+with+Disabilities+in+an+Inclusive+Setting&title=Journal+of+Applied+Behavior+Analysis&issn=00218855&date=2004-01-01&volume=37&issue=2&spage=197&au=DiCarlo%2C+Cynthia+F.%3BReid%2C+Dennis+H.&isbn=&jtitle=Journal+of+Applied+Behavior+Analysis&btitle=&rft_id=info:eric/EJ696378
ER -

TY - JOUR
ID - 2026
T1 - Literacy Is Child's Play: Making Sense in Khwezi Park
A1 - Prinsloo,Mastin
Y1 - 2004///
KW - ERIC
KW - Unsupervised Play Addition
KW - South Africa (Cape Town)
KW - ERIC,Current Index to Journals in Education (CIJE)
KW - Semiotics
KW - Play
KW - Foreign Countries
KW - Emergent Literacy
KW - Ethnography
KW - Games
KW - Creativity
KW - CHILDREN
KW - Literacy Education
KW - Peer Relationship
RP - NOT IN FILE
SP - 291
EP - 304
JF - Language and Education
VL - 18
IS - 4
PB - Multilingual Matters, Frankfurt Lodge, Clevedon Hall, Victoria Road, Clevedon, UK BS21 7HH. Tel: +44 1275-876519; Fax: +44 1275-871673; e-mail: info@multilingual-matters.com; Web site: http://www.multilingual-matters.com/multi/journals.asp.
N2 - The paper draws on ethnographic data on children's game-playing in Khwezi Park outside Cape Town. This unsupervised, child-choreographed play is seen as a site of meaning-making and identity work where children draw on a range of resources and influences to take and make meaning. These resources for semiosis and interaction are multilingual and multi-modal, and are sourced from social domains that are local, regional and global. This site of play is shown to be itself a distinctive domain where these children can mediate and model for each other the semiotics, practices and resources of school, local and popular culture, religion, mass media and home. These resources allow them to experiment as meaning-makers and sign-makers under conditions of peer feedback in a situated context where there is both contained specificity and freedom to innovate. Such sustained peer-play is seen to be a resource for this particular group of children, encouraging them to be inventive and reflexive in developing their sign-making potentials. The children's social semiotic activity is seen as productive of meanings that are seen as both hybrid in their sources, domain-specific and linked to the children's performances in other contexts, particularly that of school-based reading and writing
SN - 0950-0782, 0950-0782
UR - http://search.proquest.com/docview/62057649?accountid=14701
L2 - http://sfx.scholarsportal.info/ottawa?url_ver=Z39.88-2004&rft_val_fmt=info:ofi/fmt:kev:mtx:journal&genre=article&sid=ProQ:ProQ%3Aericshell&atitle=Literacy+Is+Child%27s+Play%3A+Making+Sense+in+Khwezi+Park&title=Language+and+Education&issn=09500782&date=2004-01-

01&volume=18&issue=4&spage=291&au=Prinsloo%2C+Mastin&isbn=&jtitle=Language+and+Education&btitle=&rft_id=info:eric/EJ728274
ER -

TY - JOUR
ID - 2027
T1 - Child Culture, Play and Child Development
A1 - Thyssen,Sven
Y1 - 2003/12//
KW - ERIC
KW - Unsupervised Play Addition
KW - Denmark
KW - ERIC,Current Index to Journals in Education (CIJE)
KW - Early Childhood Education
KW - Context Effect
KW - Foreign Countries
KW - Play
KW - Young Children
KW - Cultural Influences
KW - Child Development
KW - Child Care
KW - Interpersonal Relationship
KW - School Culture
RP - NOT IN FILE
SP - 589
EP - 612
JF - Early Child Development and Care
VL - 173
IS - 6
PB - Routledge. , 325 Chestnut Street Suite 800, Philadelphia, PA 19106.
N2 - The article discusses child development in the context of child life in day care. It traces children's development of an independent, autonomous life and their creation of culture where play is a central feature. The culture produced by the children is analysed on the basis of classical notions of culture. Furthermore the emergence and development of play in the life of the child is traced and the particular way of thinking represented in play is analysed: thinking related to creating drama with pretend figures on a "stage." The potentials for learning inherent in this particular way of thinking are discussed with reference to the child's learning in the context of early instruction. (Contains 5 figures and 1 note.)
SN - 0300-4430, 0300-4430
UR - http://search.proquest.com/docview/62000545?accountid=14701
L2 - http://sfx.scholarsportal.info/ottawa?url_ver=Z39.88-2004&rft_val_fmt=info:ofi/fmt:kev:mtx:journal&genre=article&sid=ProQ:ProQ%3Aericshell&atitle=Child+Culture%2C+Play+and+Child+Development&title=Early+Child+Development+and+Care&issn=03004430&date=2003-12-01&volume=173&issue=6&spage=589&au=Thyssen%2C+Sven&isbn=&jtitle=Early+Child+Development+and+Care&btitle=&rft_id=info:eric/EJ770725
ER -

TY - JOUR
ID - 2028
T1 - Weak Central Coherence, Poor Joint Attention, and Low Verbal Ability: Independent Deficits in Early Autism
A1 - Morgan,Bronwyn
A1 - Maybery,Murray
A1 - Durkin,Kevin
Y1 - 2003///
KW - ERIC

KW - Unsupervised Play Addition
KW - theory of mind
KW - Visual Attention
KW - ERIC,Current Index to Journals in Education (CIJE)
KW - Developmental Disabilities
KW - Comparative Analysis
KW - Symptoms (Individual Disorders)
KW - Young Children
KW - Verbal Ability
KW - Theories
KW - Pervasive Developmental Disorders
KW - Pretend Play
KW - Autism
KW - Attention
KW - Predictor Variables
RP - NOT IN FILE
SP - 646
EP - 656
JF - Developmental Psychology
JA - Dev.Psychol.
VL - 39
IS - 4
N2 - Compared preschoolers with autism spectrum disorders with a matched control group to investigate whether weak central coherence could account for deficits in two behaviors purported to tap capabilities fundamental to a theory of mind: joint attention and pretend play. Found that pretend play did not differentiate the two groups. Weak central coherence, poor joint attention, and low verbal ability contributed significantly and independently to predicting autism group membership. (Author/KB)
SN - 0012-1649, 0012-1649
UR - http://search.proquest.com/docview/62167930?accountid=14701
L2 - http://sfx.scholarsportal.info/ottawa?url_ver=Z39.88-2004&rft_val_fmt=info:ofi/fmt:kev:mtx:journal&genre=article&sid=ProQ:ProQ%3Aericshell&atitle=Weak+Central+Coherence%2C+Poor+Joint+Attention%2C+and+Low+Verbal+Ability%3A+Independent+Deficits+in+Early+Autism.&title=Developmental+Psychology&issn=00121649&date=2003-01-01&volume=39&issue=4&spage=646&au=Morgan%2C+Bronwyn%3BMaybery%2C+Murray%3BDurkin%2C+Kevin&isbn=&jtitle=Developmental+Psychology&btitle=&rft_id=info:eric/EJ678194
ER -

TY - GEN
ID - 2030
T1 - Choosing Play Materials for Primary School Children (Ages 6-8). NAEYC Resources in Focus: Selected Excerpts
A1 - Bronson,Martha B.
Y1 - 2003///
N1 - Last updated - 2012-12-14
KW - ERIC
KW - Unsupervised Play Addition
KW - National Association Educ of Young Children
KW - Play Materials
KW - ERIC,Current Index to Journals in Education (CIJE)
KW - Primary Education
KW - Instructional Materials
KW - Play
KW - Young Children
KW - Organizations (Groups)
KW - Selection
RP - NOT IN FILE

SP - 24
EP - 25
JF - Young Children
VL - 58
IS - 3
N2 - Presents guidelines for selecting materials for 6- to 8-year-olds in primary school settings involved in four categories of play: social and fantasy play; exploration and mastery play; music, art, and movement play; and gross motor play. Asserts that providing a variety of materials for children's independent activities pursued alone or with peers is one dimension of effectively responding to individual differences within the classroom. (KB)
UR - http://search.proquest.com/docview/62243452?accountid=14701
L2 - http://sfx.scholarsportal.info/ottawa?url_ver=Z39.88-2004&rft_val_fmt=info:ofi/fmt:kev:mtx:journal&genre=article&sid=ProQ:ProQ%3Aericshell&atitle=Choosing+Play+Materials+for+Primary+School+Children+%28Ages+6-8%29.+NAEYC+Resources+in+Focus%3A+Selected+Excerpts.&title=Young+Children&issn=15386619&date=2003-01-01&volume=58&issue=3&spage=24&au=Bronson%2C+Martha+B.&isbn=&jtitle=Young+Children&btitle=&rft_id=info:eric/EJ672020
ER -

TY - JOUR
ID - 2031
T1 - Making Sense of Outdoor Pretend Play
A1 - Perry,Jane P.
Y1 - 2003///
KW - ERIC
KW - Unsupervised Play Addition
KW - ERIC,Current Index to Journals in Education (CIJE)
KW - Early Childhood Education
KW - Outdoor Activities
KW - Teacher Student Relationship
KW - Play
KW - Learning Centers (Classroom)
KW - Pretend Play
KW - Preschool Children
RP - NOT IN FILE
SP - 26
EP - 30
JF - Young Children
VL - 58
IS - 3
N2 - Focuses on the independent outdoor pretend play of preschool children. Describes the complex learning occurring in a university child study center's play yard and in outdoor learning centers, how teachers complement children's play, and the importance of space. Illustrates the pattern of pretend play: initiation, negotiation, and enactment. Includes a sample diagram of an outdoor setting for preschool and primary grades and suggestions for recording observations. (KB)
SN - 1538-6619, 1538-6619
UR - http://search.proquest.com/docview/62245477?accountid=14701
L2 - http://sfx.scholarsportal.info/ottawa?url_ver=Z39.88-2004&rft_val_fmt=info:ofi/fmt:kev:mtx:journal&genre=article&sid=ProQ:ProQ%3Aericshell&atitle=Making+Sense+of+Outdoor+Pretend+Play.&title=Young+Children&issn=15386619&date=2003-01-01&volume=58&issue=3&spage=26&au=Perry%2C+Jane+P.&isbn=&jtitle=Young+Children&btitle=&rft_id=info:eric/EJ672021
ER -

TY - GEN
ID - 2032

T1 - A Validation Study of the Penn Interactive Peer Play Scale with Urban Hispanic and African American Preschool Children
A1 - Castro,Marcelo
A1 - Mendez,Julia L.
A1 - Fantuzzo,John
Y1 - 2002///
N1 - Last updated - 2012-12-14
KW - ERIC
KW - Unsupervised Play Addition
KW - ERIC,Current Index to Journals in Education (CIJE)
KW - Preschool Education
KW - Play
KW - School Psychology
KW - Test Validity
KW - Black Students
KW - Hispanic American Students
KW - Factor Structure
KW - Preschool Children
KW - Psychometrics
KW - Peer Relationship
RP - NOT IN FILE
SP - 109
EP - 127
JF - School Psychology Quarterly
VL - 17
IS - 2
N2 - Investigates the psychometric properties of a Spanish and English version of the Penn Interactive Peer Play Scale (PIPPS) when employed with Spanish- and English-speaking teachers and students. The independent emergence of comparable Spanish and English PIPPS factor structures provides initial support for use of this measure in research with Hispanic preschool children from low-income backgrounds. (Contains 65 references and 3 tables.) (GCP)
UR - http://search.proquest.com/docview/62302670?accountid=14701
L2 - http://sfx.scholarsportal.info/ottawa?url_ver=Z39.88-2004&rft_val_fmt=info:ofi/fmt:kev:mtx:journal&genre=article&sid=ProQ:ProQ%3Aericshell&atitle=A+Validation+Study+of+the+Penn+Interactive+Peer+Play+Scale+with+Urban+Hispanic+and+African+American+Preschool+Children.&title=School+Psychology+Quarterly&issn=10453830&date=2002-01-01&volume=17&issue=2&spage=109&au=Castro%2C+Marcelo%3BMendez%2C+Julia+L.%3BFantuzzo%2C+John&isbn=&jtitle=School+Psychology+Quarterly&btitle=&rft_id=info:eric/EJ650127
ER -

TY - JOUR
ID - 2033
T1 - A Critical Approach to Will Hobbs'"Bearstone" from a Play Perspective
A1 - Thompson,Edgar H.
A1 - Sluss,Dorothy
Y1 - 2000///
KW - ERIC
KW - Unsupervised Play Addition
KW - Piaget (Jean)
KW - Smilanskys Cognitive Play Categories
KW - ERIC,Current Index to Journals in Education (CIJE)
KW - Secondary Education
KW - Literary Criticism
KW - Childrens Games
KW - Literary Devices
KW - Literature Appreciation

KW - Child Behavior
KW - Dramatic Play
KW - Adolescent Literature
KW - Novels
RP - NOT IN FILE
SP - 8
EP - 10
JF - ALAN Review
VL - 27
IS - 2
N2 - Claims there is a missing link between the two seemingly independent stories in Will Hobbs' novel "Bearstone." This missing link can be found through looking at four kinds of play that occur in the novel. (NH)
SN - 0882-2840, 0882-2840
UR - http://search.proquest.com/docview/62425615?accountid=14701
L2 - http://sfx.scholarsportal.info/ottawa?url_ver=Z39.88-2004&rft_val_fmt=info:ofi/fmt:kev:mtx:journal&genre=article&sid=ProQ:ProQ%3Aericshell&atitle=A+Critical+Approach+to+Will+Hobbs%27%22Bearstone%22+from+a+Play+Perspective.&title=ALAN+Review&issn=08822840&date=2000-01-01&volume=27&issue=2&spage=8&au=Thompson%2C+Edgar+H.%3BSluss%2C+Dorothy&isbn=&jtitle=ALAN+Review&btitle=&rft_id=info:eric/EJ604677
ER -

TY - JOUR
ID - 2035
T1 - A Factor Analysis of Pre-school Children's Play Strategies and Cognitive Style
A1 - Saracho,Olivia N.
Y1 - 1999///
KW - ERIC
KW - Unsupervised Play Addition
KW - ERIC,Current Index to Journals in Education (CIJE)
KW - Early Childhood Education
KW - Higher Education
KW - Play
KW - Cognitive Development
KW - Social Behavior
KW - Child Development
KW - Observation
KW - Preschool Children
KW - Cognitive Style
KW - Factor Analysis
RP - NOT IN FILE
SP - 165
EP - 180
JF - Educational Psychology: An International Journal of Experimental Educational Psychology
VL - 19
IS - 2
N2 - Explores children's play as social behavior in relation to cognitive style and identifies the social factors underlying both young children's play and cognitive style. Indicates that field-dependent children participated more in social play activities, while field-independent children engaged in more nonsocial play activities. Discusses the implications for practice and research. Includes references. (CMK)
SN - 0144-3410, 0144-3410
UR - http://search.proquest.com/docview/62315448?accountid=14701
L2 - http://sfx.scholarsportal.info/ottawa?url_ver=Z39.88-2004&rft_val_fmt=info:ofi/fmt:kev:mtx:journal&genre=article&sid=ProQ:ProQ%3Aericshell&atitle=A+Factor+Analysis+of+Pre-school+Children%27s+Play+Strategies+and+Cognitive+Style.&title=Educational+Psychology%3A+An+Intern

ational+Journal+of+Experimental+Educational+Psychology&issn=01443410&date=1999-01-01&volume=19&issue=2&spage=165&au=Saracho%2C+Olivia+N.&isbn=&jtitle=Educational+Psychology%3A+An+International+Journal+of+Experimental+Educational+Psychology&btitle=&rft_id=info:eric/EJ625621
ER -

TY - RPRT
ID - 2037
T1 - Early Supplemental Feeding and Spontaneous Play in West Java, Indonesia
A1 - Walka,Helen
A1 - Pollitt,Ernesto
A1 - Triana,Nina
A1 - Jahari,Abas B.
Y1 - 1997/04//
N1 - Availability - Level 1 - Available online, if indexed January 1993 onward
SuppNotes - Paper presented at the Biennial Meeting of the Society for Research in Child Development (62nd, Washington, DC, April 3-6, 1997)
Last updated - 2012-12-14
KW - ERIC
KW - Unsupervised Play Addition
KW - Functional Play
KW - Indonesia (West Java)
KW - Manipulative Play
KW - Nutritional Supplements
KW - ERIC,Resources in Education (RIE)
KW - Experiments
KW - Comparative Analysis
KW - Toddlers
KW - Play
KW - Foreign Countries
KW - Sex Differences
KW - Pretend Play
KW - Nutrition
KW - Infants
RP - NOT IN FILE
SP - 13
N2 - This study examined the effects of nutritional supplements on the duration and level of spontaneous play of 55 mildly to moderately malnourished toddlers living within the tea plantations of West Java, Indonesia. Infants were randomly assigned by their day care centers to one of three supplement groups: (1) energy and micronutrient supplements; (2) micronutrient plus skim milk; and (3) skim milk alone. After 6 months of supplementation, the children (age 24 months) were videotaped for 30 minutes playing with toys in their home with their caregiver present. The videotapes were coded for duration and frequency of four developmental levels of play behavior: manipulative, relational, functional, and symbolic play. Duration of time off-task, waiting before playing, breastfeeding, and time in social interaction were also coded. Findings indicated that girls demonstrated longer play duration and higher play level, independent of supplementation. There were consistent effects of supplementation on duration and interest in play. Children in the energy plus micronutrient supplementation group played with more toys, had longer activity spans, were less likely to breastfeed, and were less likely to engage in social interaction outside of play than children in other groups. Girls benefited from the supplementation more than boys. (Author/AS)
UR - http://search.proquest.com/docview/62498998?accountid=14701
ER -

TY - JOUR
ID - 2038
T1 - Young Children's Understanding of Pretense Expressions of Independent Agency
A1 - Kavanaugh,Robert D.
A1 - Eizenman,Dara R.

A1 - Harris,Paul L.
Y1 - 1997///
KW - ERIC
KW - Unsupervised Play Addition
KW - ERIC,Current Index to Journals in Education (CIJE)
KW - Toddlers
KW - Cognitive Development
KW - Infant Behavior
KW - Sex Differences
KW - Intention
KW - Pretend Play
KW - Age Differences
RP - NOT IN FILE
SP - 764
EP - 770
JF - Developmental Psychology
JA - Dev.Psychol.
VL - 33
IS - 5
N2 - Studied 2-year olds' understanding of pretense expressions of independent agency in scenarios in which a doll acted as the agent of a series of pretend events. Found no gender differences in the doll's imaginary intentions, but older toddlers performed reliably better than younger. Episodes requiring enacting conclusions to events that began with doll-directed actions were difficult for children to comprehend. (Author/KB)
SN - 0012-1649, 0012-1649
UR - http://search.proquest.com/docview/62555265?accountid=14701
L2 - http://sfx.scholarsportal.info/ottawa?url_ver=Z39.88-2004&rft_val_fmt=info:ofi/fmt:kev:mtx:journal&genre=article&sid=ProQ:ProQ%3Aericshell&atitle=Young+Children%27s+Understanding+of+Pretense+Expressions+of+Independent+Agency.&title=Developmental+Psychology&issn=00121649&date=1997-01-01&volume=33&issue=5&spage=764&au=Kavanaugh%2C+Robert+D.%3BEizenman%2C+Dara+R.%3BHarris%2C+Paul+L.&isbn=&jtitle=Developmental+Psychology&btitle=&rft_id=info:eric/EJ561705
ER -

TY - RPRT
ID - 2039
T1 - Preschool Children's Cognitive Styles and Play Behaviors
A1 - Saracho,Olivia N.
Y1 - 1996///
N1 - Last updated - 2012-12-14
KW - ERIC
KW - Unsupervised Play Addition
KW - ERIC,Current Index to Journals in Education (CIJE)
KW - Play
KW - Child Behavior
KW - Field Dependence Independence
KW - Behavior Patterns
KW - Preschool Children
KW - Cognitive Style
KW - Age Differences
RP - NOT IN FILE
SP - 125
EP - 148
N2 - Tested 2,400 three- to five-year olds for cognitive style and observed their play behaviors. Repeated measures multivariate analysis indicated that field-independent children engaged in more play than did field-dependent children. Significant interaction was found for age, cognitive style, and play behaviors. Older field-

independent children exhibited more field independence and play behaviors than did younger field- dependent children. (HTH)
UR - http://search.proquest.com/docview/62599053?accountid=14701
L2 - http://sfx.scholarsportal.info/ottawa?url_ver=Z39.88-2004&rft_val_fmt=info:ofi/fmt:kev:mtx:book&genre=unknown&sid=ProQ:ERIC&atitle=&title=Preschool+Children%27s+Cognitive+Styles+and+Play+Behaviors.&issn=00094005&date=1996-01-01&volume=&issue=&spage=125&au=Saracho%2C+Olivia+N.&isbn=&jtitle=&btitle=Preschool+Children%27s+Cognitive+Styles+and+Play+Behaviors.&rft_id=info:eric/EJ533033
ER -

TY - RPRT
ID - 2040
T1 - Constructing Knowledge about Play: Case Method and Teacher Education
A1 - Klein,Amelia
Y1 - 1996///
N1 - Availability - Level 2 - Produced in microfiche (1966-2003)
SuppNotes - In: Playing for Keeps: Supporting Children's Play. Topics in Early Childhood Education, Volume 2; see PS 025 050
Last updated - 2012-12-14
KW - ERIC
KW - Unsupervised Play Addition
KW - Case Analysis
KW - Case Histories
KW - ERIC,Resources in Education (RIE)
KW - Early Childhood Education
KW - Higher Education
KW - Play
KW - Case Studies
KW - Teacher Education
KW - Constructivism (Learning)
KW - Teaching Methods
KW - Models
KW - Case Method (Teaching Technique)
RP - NOT IN FILE
SP - 11
N2 - This paper explores the case method as a model for teaching education students about play. The case method may contribute to an examination of the barriers to children's play expression within school and create more effective classroom practice. Within the model, students can consider cases which have been carefully constructed from actual practice and designed to illuminate the complexity of human situations. Through discussion, analysis, and collaborative problem solving, the case method provides an interactive forum in which students can construct knowledge about the nature, value, and purpose of play in early education. Play education should be guided by a framework building upon and integrating: (1) theoretical knowledge regarding play; (2) practical skills applied in the classroom setting; and (3) dispositions toward play and children's learning. The case method facilitates self-governed learning; produces cognitive dissonance; requires students to apply theories, use analytical skills, and create innovative approaches to resolving problems; and fosters independent thinking and reflection. It is important to develop with care the written narrative and case questions, to conduct the case discussion, and teach case writing to students. A play case bank may be built with teacher- and student-written cases, videos, films, and books. (KDFB)
UR - http://search.proquest.com/docview/62593127?accountid=14701
L2 - http://sfx.scholarsportal.info/ottawa?url_ver=Z39.88-2004&rft_val_fmt=info:ofi/fmt:kev:mtx:book&genre=unknown&sid=ProQ:ERIC&atitle=&title=Constructing+Knowledge+about+Play%3A+Case+Method+and+Teacher+Education.&issn=&date=1996-01-01&volume=&issue=&spage=11&au=Klein%2C+Amelia&isbn=&jtitle=&btitle=Constructing+Knowledge+about+Play%3A+Case+Method+and+Teacher+Education.&rft_id=info:eric/ED405103
ER -

TY - RPRT
ID - 2041
T1 - The Relationship of Preschool Children's Cognitive Style to Their Play Preferences
A1 - Saracho,Olivia N.
Y1 - 1994///
N1 - Last updated - 2012-12-14
KW - ERIC
KW - Unsupervised Play Addition
KW - Analytic Ability
KW - Manipulative Play
KW - Play Style
KW - ERIC,Current Index to Journals in Education (CIJE)
KW - Preschool Education
KW - Play
KW - Interpersonal Competence
KW - Field Dependence Independence
KW - Dramatic Play
KW - Sex Differences
KW - Preschool Children
KW - Cognitive Style
RP - NOT IN FILE
SP - 21
EP - 33
N2 - Three hundred preschoolers aged 3 through 5 were tested for their cognitive style and their preferences for different play areas. Results indicated that field-independent (FI) children (socially detached with high analytical abilities) preferred physical and block play more than field-dependent (FD) children (socially adept and sensitive) and that FI children engaged more in manipulative play than did FD children. (MDM)
UR - http://search.proquest.com/docview/62813773?accountid=14701
L2 - http://sfx.scholarsportal.info/ottawa?url_ver=Z39.88-2004&rft_val_fmt=info:ofi/fmt:kev:mtx:book&genre=unknown&sid=ProQ:ERIC&atitle=&title=The+Relationship+of+Preschool+Children%27s+Cognitive+Style+to+Their+Play+Preferences.&issn=03004430&date=1994-01-01&volume=&issue=&spage=21&au=Saracho%2C+Olivia+N.&isbn=&jtitle=&btitle=The+Relationship+of+Preschool+Children%27s+Cognitive+Style+to+Their+Play+Preferences.&rft_id=info:eric/EJ479930
ER -

TY - RPRT
ID - 2043
T1 - Preschool Children's Use and Perceptions of Outdoor Play Areas
A1 - Cullen,Joy
Y1 - 1993///
N1 - Last updated - 2012-12-14
KW - ERIC
KW - Unsupervised Play Addition
KW - Australia (Perth)
KW - ERIC,Current Index to Journals in Education (CIJE)
KW - Early Childhood Education
KW - Childhood Attitudes
KW - Physical Activities
KW - Play
KW - Foreign Countries
KW - Teacher Attitudes
KW - Sex Differences
KW - Creativity
KW - Preschool Children
KW - Playground Activities

RP - NOT IN FILE
SP - 45
EP - 56
N2 - Forty preschool children were observed in outdoor play areas and interviewed about their perceptions of the observed play. The children's teachers were also interviewed. It was found that a significant negative correlation existed between physical play and creative play and that the majority of children perceived that outdoor play was independent and did not require assistance from the teacher. (MDM)
UR - http://search.proquest.com/docview/62795110?accountid=14701
L2 - http://sfx.scholarsportal.info/ottawa?url_ver=Z39.88-2004&rft_val_fmt=info:ofi/fmt:kev:mtx:book&genre=unknown&sid=ProQ:ERIC&atitle=&title=Preschool+Children%27s+Use+and+Perceptions+of+Outdoor+Play+Areas.&issn=03004430&date=1993-01-01&volume=&issue=&spage=45&au=Cullen%2C+Joy&isbn=&jtitle=&btitle=Preschool+Children%27s+Use+and+Perceptions+of+Outdoor+Play+Areas.&rft_id=info:eric/EJ473193
ER -

TY - JOUR
ID - 2044
T1 - "You Can't Be Grandma; You're a Boy": Events within the Thematic Fantasy Play Context That Contribute to Story Comprehension
A1 - Williamson,Peter A.
A1 - Silvern,Steven B.
Y1 - 1992///
KW - ERIC
KW - Unsupervised Play Addition
KW - Metaplay
KW - Productive Language Competence
KW - Retelling
KW - ERIC,Current Index to Journals in Education (CIJE)
KW - Primary Education
KW - Kindergarten Children
KW - Play
KW - Language Acquisition
KW - Story Telling
KW - Story Grammar
KW - Piagetian Theory
KW - Comprehension
KW - Story Reading
KW - Pretend Play
KW - Fairy Tales
RP - NOT IN FILE
SP - 75
EP - 93
JF - Early Childhood Research Quarterly
VL - 7
IS - 1
N2 - Examined the contribution of play, metaplay, and productive language competence in the thematic-fantasy play context to kindergartners' comprehension of familiar and unfamiliar stories. Results indicated that metaplay and productive language competence are independent constructs and both contribute to comprehension, whereas play does not. (GLR)
SN - 0885-2006, 0885-2006
UR - http://search.proquest.com/docview/62935649?accountid=14701
L2 - http://sfx.scholarsportal.info/ottawa?url_ver=Z39.88-2004&rft_val_fmt=info:ofi/fmt:kev:mtx:journal&genre=article&sid=ProQ:ProQ%3Aericshell&atitle=%22You+Can%27t+Be+Grandma%3B+You%27re+a+Boy%22%3A+Events+within+the+Thematic+Fantasy+Play+Context+That+Contribute+to+Story+Comprehension.&title=Early+Childhood+Research+Quarterly&issn=08852006&date=1992-01-

01&volume=7&issue=1&spage=75&au=Williamson%2C+Peter+A.%3BSilvern%2C+Steven+B.&isbn=&jtitle=Early+Childhood+Research+Quarterly&btitle=&rft_id=info:eric/EJ446317
ER -

TY - RPRT
ID - 2047
T1 - Playing with Fear: Children's Play and Its Role in Expressing Fears
A1 - Heibeck,Tracy H.
Y1 - 1989/04//
N1 - Availability - Level 1 - Available online, if indexed January 1993 onward
SuppNotes - Paper presented at the Biennial Meeting of the Society for Research in Child Development (Kansas City, MO, April 27-30, 1989)
Last updated - 2012-12-14
KW - ERIC
KW - Unsupervised Play Addition
KW - ERIC,Resources in Education (RIE)
KW - Preschool Education
KW - Play
KW - Fear
KW - Affective Behavior
KW - Videotape Recordings
KW - Preschool Children
KW - Aggression
KW - Emotional Experience
KW - Maturity (Individuals)
RP - NOT IN FILE
SP - 44
N2 - Preschool children's fears of animals and the dark were investigated in an effort to examine how preschoolers use play as a medium for expressing fearful emotions. A total of 48 children participated in the study. Interviews with parents were used to identify 12 preschoolers who were anxious about the dark and 12 who were anxious about dogs; 24 children were rated by parents as not being anxious about these experiences. Each child was invited to create stories about topics suggested by the experimenter. Some topics included benign themes popular in children's play; other themes reflected fears of dogs and darkness. Each child's videotaped play session was coded by three independent judges on measures assessing the maturity, duration, imaginativeness, aggression, concentration of play, and other factors. Children who were anxious about dogs played less constructively and at a less mature level in regards to that topic than did children not anxious about dogs. But children anxious about the dark were able to engage in well-developed pretend play when playing about benign themes and about the dark. These children tended to include more aggression in their play, especially when the story involved the dark. It is suggested that findings have implications for the use of play in therapy with children. (Author/RH)
UR - http://search.proquest.com/docview/63128908?accountid=14701
L2 - http://sfx.scholarsportal.info/ottawa?url_ver=Z39.88-2004&rft_val_fmt=info:ofi/fmt:kev:mtx:book&genre=unknown&sid=ProQ:ERIC&atitle=&title=Playing+with+Fear%3A+Children%27s+Play+and+Its+Role+in+Expressing+Fears.&issn=&date=1989-04-01&volume=&issue=&spage=44&au=Heibeck%2C+Tracy+H.&isbn=&jtitle=&btitle=Playing+with+Fear%3A+Children%27s+Play+and+Its+Role+in+Expressing+Fears.&rft_id=info:eric/ED307995
ER -

TY - JOUR
ID - 2048
T1 - Cognitive Style in the Play of Young Children
A1 - Saracho,Olivia N.
Y1 - 1989///
KW - ERIC
KW - Unsupervised Play Addition
KW - Child Behavior

KW - ERIC,Current Index to Journals in Education (CIJE)
KW - Play
KW - Young Children
KW - Individual Differences
KW - Field Dependence Independence
KW - Social Behavior
KW - Sex Differences
KW - Cognitive Style
RP - NOT IN FILE
SP - 65
EP - 76
JF - Early Child Development and Care
VL - 51
N2 - Assessed cognitive styles of 300 children aged 3-5 years and explored social factors in children's play based on their cognitive style. Field dependent children engaged more often in activities with others and with concrete objects, while field independent children were more involved in restructuring the environment. (RJC)
UR - http://search.proquest.com/docview/63068426?accountid=14701
L2 - http://sfx.scholarsportal.info/ottawa?url_ver=Z39.88-2004&rft_val_fmt=info:ofi/fmt:kev:mtx:journal&genre=article&sid=ProQ:ProQ%3Aericshell&atitle=Cognitive+Style+in+the+Play+of+Young+Children.&title=Early+Child+Development+and+Care&issn=&date=1989-01-01&volume=51&issue=&spage=65&au=Saracho%2C+Olivia+N.&isbn=&jtitle=Early+Child+Development+and+Care&btitle=&rft_id=info:eric/EJ407422
ER -

TY - JOUR
ID - 2049
T1 - Negotiation and Enactment in Social Pretend Play: Relations to Social Acceptance and Social Cognition
A1 - Doyle,Anna Beth
A1 - Connolly,Jennifer
Y1 - 1989///
KW - ERIC
KW - Unsupervised Play Addition
KW - Enactment
KW - Negotiation Process
KW - ERIC,Current Index to Journals in Education (CIJE)
KW - Play
KW - Young Children
KW - Interpersonal Competence
KW - Social Cognition
KW - SOCIALIZATION
KW - Peer Acceptance
RP - NOT IN FILE
SP - 289
EP - 302
JF - Early Childhood Research Quarterly
VL - 4
IS - 3
N2 - Observed 62 children, 4- and 5-years-old, to determine the relation between negotiation and enactment in social pretend play and to indices of social acceptance and cognition. Negotiation and enactment were positively correlated and predicted peer social acceptance. Enactment predicted peer acceptance independent of negotiation. (RJC)
UR - http://search.proquest.com/docview/63058067?accountid=14701
L2 - http://sfx.scholarsportal.info/ottawa?url_ver=Z39.88-2004&rft_val_fmt=info:ofi/fmt:kev:mtx:journal&genre=article&sid=ProQ:ProQ%3Aericshell&atitle=Negotiation+and+Enactment+in+Social+Pretend+Play%3A+Relations+to+Social+Acceptance+and+Social+Cognition.&title=Early+Childhood+Research+Quarterly&issn=&date=1989-01-

01&volume=4&issue=3&spage=289&au=Doyle%2C+Anna-Beth%3BConnolly%2C+Jennifer&isbn=&jtitle=Early+Childhood+Research+Quarterly&btitle=&rft_id=info:eric/EJ402838
ER -

TY - JOUR
ID - 2050
T1 - The Factorial Structure of Three- to Five-Year-Old Children's Social Behavior: Cognitive Style and Play
A1 - Saracho,Olivia N.
Y1 - 1989///
KW - ERIC
KW - Unsupervised Play Addition
KW - Preschool Embedded Figures Test
KW - ERIC,Current Index to Journals in Education (CIJE)
KW - Early Childhood Education
KW - Play
KW - Field Dependence Independence
KW - Social Development
KW - Factor Analysis
KW - Peer Relationship
RP - NOT IN FILE
SP - 21
EP - 28
JF - Journal of Research and Development in Education
VL - 22
IS - 4
N2 - Results are reported from a study which explored which play behaviors are characteristic of the field dependent and field independent cognitive styles. Social factors underlying cognitive style and play are identified. Subjects were 300 3- to 5-year-old children. Implications for teachers and researchers are discussed. (IAH)
UR - http://search.proquest.com/docview/63052034?accountid=14701
L2 - http://sfx.scholarsportal.info/ottawa?url_ver=Z39.88-2004&rft_val_fmt=info:ofi/fmt:kev:mtx:journal&genre=article&sid=ProQ:ProQ%3Aericshell&atitle=The+Factorial+Structure+of+Three-+to+Five-Year-Old+Children%27s+Social+Behavior%3A+Cognitive+Style+and+Play.&title=Journal+of+Research+and+Development+in+Education&issn=&date=1989-01-01&volume=22&issue=4&spage=21&au=Saracho%2C+Olivia+N.&isbn=&jtitle=Journal+of+Research+and+Development+in+Education&btitle=&rft_id=info:eric/EJ398431
ER -

TY - GEN
ID - 2052
T1 - Cognitive/Developmental Characteristics of Play and Their Relation to Ego Resiliency: A Longitudinal Study
A1 - Gordon,Debra Ellen
Y1 - 1987/04//
N1 - Availability - Level 1 - Available online, if indexed January 1993 onward
SuppNotes - Paper presented at the Biennial Meeting of the Society for Research in Child Development (Baltimore, MD, April 23-26, 1987)
Last updated - 2012-12-14
KW - ERIC
KW - Unsupervised Play Addition
KW - Ego Resiliency
KW - ERIC,Resources in Education (RIE)
KW - Researchers
KW - Play

KW - Cognitive Development
KW - Adjustment (to Environment)
KW - Sex Differences
KW - Pretend Play
KW - Preschool Children
KW - Longitudinal Studies
KW - Coping
KW - CHILDREN
KW - Adolescents
RP - NOT IN FILE
SP - 22
N2 - This study links cognitive development and psychological adaptation by relating play attributes, assessed from a predominantly cognitive-developmental perspective, to "ego resiliency," which is a measure of flexibility in coping with stress. Subjects were 104 participants in a longitudinal study (Block & Block, 1980); 78 were given solitary free play assessments at age 3, and all 104 were administered similar play assessments at age 11. Numerous scaled play ratings were grouped into two play dimensions for each age level: "Symbolic Elaboration" and "Intercoordination/Organization." Ego resiliency scores were derived from independent personality Q-sorts at ages 3, 11, 14, and 18 years. For both sexes, concurrent relationships between play dimensions and ego resiliency were examined at age 3 and age 11. Furthermore, play activities of 3- and 11-year-olds were related longitudinally to ego resiliency at ages 11, 14, and 18. Consistency of play behavior from age 3 to age 11 was explored. A five-page reference list is included. (Author/PCB)
UR - http://search.proquest.com/docview/63263683?accountid=14701
L2 - http://sfx.scholarsportal.info/ottawa?url_ver=Z39.88-2004&rft_val_fmt=info:ofi/fmt:kev:mtx:journal&genre=unknown&sid=ProQ:ProQ%3Aericshell&atitle=Cognitive%2FDevelopmental+Characteristics+of+Play+and+Their+Relation+to+Ego+Resiliency%3A+A+Longitudinal+Study.&title=&issn=&date=1987-04-01&volume=&issue=&spage=22&au=Gordon%2C+Debra+Ellen&isbn=&jtitle=&btitle=&rft_id=info:eric/ED284677
ER -

TY - GEN
ID - 2053
T1 - A Practical Guide to Play, Piaget, and Language in Preprimary Hearing Impaired Programs
A1 - Smith,Dawn
Y1 - 1987/04//
N1 - Availability - Level 1 - Available online, if indexed January 1993 onward
Last updated - 2012-12-14
KW - ERIC
KW - Unsupervised Play Addition
KW - Detroit Public Schools MI
KW - ERIC,Resources in Education (RIE)
KW - Teachers
KW - Practitioners
KW - Preschool Education
KW - Teacher Role
KW - Play
KW - Language Acquisition
KW - Hearing Impairments
KW - Cognitive Development
KW - Class Activities
KW - Classroom Environment
KW - Audiolingual Skills
KW - Classroom Techniques
KW - Classroom Design
KW - Demonstration Programs
KW - Family School Relationship

KW - Classroom Observation Techniques
KW - Interpersonal Competence
KW - Diagnostic Tests
KW - Developmental Stages
KW - Curriculum Guides
KW - Social Development
KW - Piagetian Theory
RP - NOT IN FILE
SP - 572
N2 - This detailed curriculum guide describes a model program (the Preschool Hearing Impaired Support Center) implemented in the Detroit Public Schools' preprimary classes for the hearing impaired. The auditory-oral approach is based on the developmental philosophy of Jean Piaget and on principles of regular early childhood education programs. Section I discusses the program's overall philosophy and theoretical orientation. Section II, on the classroom environment, describes physical space arrangements, materials, and accessories, while section III covers the daily schedule, lesson plans and planning, teacher planning, and time management. Section IV explains the role of the adult; it includes strategies for facilitating learning and describes keys for teacher success. Detailed curricular objectives for cognitive, language, speech, auditory, and social development are outlined in section V. Content and materials for independent, small group, and large group activities are described in section VI. In section VII, sample objectives and examples of teacher-child interactions are used to demonstrate the integration of cognitive, language, and social learning throughout the curriculum. Section VIII presents sample observation checklists, summarizes evaluation instruments considered most useful for teachers, and includes a teacher's guide to analyzing play observations. Section IX presents objectives and specific activities for school-home-school carryover. The final section of the guide is comprised of sample forms. (JW)
UR - http://search.proquest.com/docview/63163549?accountid=14701
L2 - http://sfx.scholarsportal.info/ottawa?url_ver=Z39.88-2004&rft_val_fmt=info:ofi/fmt:kev:mtx:journal&genre=unknown&sid=ProQ:ProQ%3Aericshell&atitle=A+Practical+Guide+to+Play%2C+Piaget%2C+and+Language+in+Preprimary+Hearing+Impaired+Programs.&title=&issn=&date=1987-04-01&volume=&issue=&spage=572&au=Smith%2C+Dawn&isbn=&jtitle=&btitle=&rft_id=info:eric/ED286293
ER -

TY - JOUR
ID - 2054
T1 - Independent and Social Play among Profoundly Mentally Retarded Adults: Training, Maintenance, Generalization, and Long-Term Follow-Up
A1 - Singh,Nirbhay N.
A1 - Millichamp,Jane C.
Y1 - 1987///
KW - ERIC
KW - Unsupervised Play Addition
KW - ERIC,Current Index to Journals in Education (CIJE)
KW - Severe Mental Retardation
KW - Prompting
KW - Play
KW - Followup Studies
KW - Interpersonal Competence
KW - Leisure Time
KW - Generalization
KW - Training Methods
KW - Adults
KW - Interpersonal Relationship
KW - Normalization (Handicapped)
RP - NOT IN FILE
SP - 23
EP - 34
JF - Journal of Applied Behavior Analysis

JA - J Appl.Behav Anal.
VL - 20
IS - 1
N2 - Verbal prompting and graduated physical guidance procedures were found to substantially increase independent and social play in two interrelated experiments with eight profoundly mentally retarded adults. Independent and social play remained in the subjects' repertoire for 12 months following termination of programmed maintenance. (Author/JW)
UR - http://search.proquest.com/docview/63266040?accountid=14701
L2 - http://sfx.scholarsportal.info/ottawa?url_ver=Z39.88-2004&rft_val_fmt=info:ofi/fmt:kev:mtx:journal&genre=article&sid=ProQ:ProQ%3Aericshell&atitle=Independent+and+Social+Play+among+Profoundly+Mentally+Retarded+Adults%3A+Training%2C+Maintenance%2C+Generalization%2C+and+Long-Term+Follow-Up.&title=Journal+of+Applied+Behavior+Analysis&issn=&date=1987-01-01&volume=20&issue=1&spage=23&au=Singh%2C+Nirbhay+N.%3BMillichamp%2C+Jane+C.&isbn=&jtitle=Journal+of+Applied+Behavior+Analysis&btitle=&rft_id=info:eric/EJ352498
ER -

TY - RPRT
ID - 2056
T1 - Negotiation and Enactment in Collaborative Pretend Play: Relations to Social Acceptance and Social Cognition
A1 - Doyle,Anna Beth
Y1 - 1986/12/16/
N1 - Availability - Level 1 - Available online, if indexed January 1993 onward
Last updated - 2012-12-14
KW - ERIC
KW - Unsupervised Play Addition
KW - Canada
KW - Negotiation Processes
KW - Quebec (Montreal)
KW - ERIC,Resources in Education (RIE)
KW - Preschool Education
KW - Foreign Countries
KW - Generalization
KW - Communication (Thought Transfer)
KW - Social Cognition
KW - Pretend Play
KW - Preschool Children
KW - Peer Acceptance
RP - NOT IN FILE
SP - 24
N2 - Examined were the relationships of children's (1) negotiation of play context and (2) enactment of pretend episodes to (3) children's social adjustment, social acceptance and social cognition. It was predicted that negotiation and enactment of pretend play would be relatively independent, and that enactment would be strongly related to indices of social competence. Participants were 30 boys and 32 girls 4- to 5-years-old who attended three day care centers located in urban residential areas of Montreal. Frequencies of pretend play negotiation and enactment were recorded during morning indoor free play periods. Each child was observed for two 20-second focal intervals per play period, for a total of 52 intervals over 1 month. Pretend negotiation and enactment were assessed by recording their occurrence every 5 seconds during each 20-second observation interval. Six measures of social competence were collected: a teacher rating of social adjustment, one teacher and two peer measures of peer popularity, and two measures of social cognitive skill. Findings indicated that negotiation and enactment were positively correlated. In addition, negotiation and enactment correlated with indices of peer popularity. Enactment was generally more highly correlated with popularity than negotiation was. Despite significant between-center variability, findings were generally replicated across centers. (Forty-four references are provided). (Author/RH)
UR - http://search.proquest.com/docview/63252205?accountid=14701

L2 - http://sfx.scholarsportal.info/ottawa?url_ver=Z39.88-2004&rft_val_fmt=info:ofi/fmt:kev:mtx:book&genre=unknown&sid=ProQ:ERIC&atitle=&title=Negotiation+and+Enactment+in+Collaborative+Pretend+Play%3A+Relations+to+Social+Acceptance+and+Social+Cognition.&issn=&date=1986-12-16&volume=&issue=&spage=24&au=Doyle%2C+Anna-Beth&isbn=&jtitle=&btitle=Negotiation+and+Enactment+in+Collaborative+Pretend+Play%3A+Relations+to+Social+Acceptance+and+Social+Cognition.&rft_id=info:eric/ED279412
ER -

TY - JOUR
ID - 2057
T1 - Children's Play and Self-Education
A1 - Gehlbach,Roger D.
Y1 - 1986///
KW - ERIC
KW - Unsupervised Play Addition
KW - Play Learning
KW - ERIC,Current Index to Journals in Education (CIJE)
KW - Practitioners
KW - Early Childhood Education
KW - Elementary Secondary Education
KW - Play
KW - Cognitive Development
KW - Independent Study
KW - Educational Administration
KW - Simulation
KW - Autoinstructional Aids
RP - NOT IN FILE
SP - 203
EP - 213
JF - Curriculum Inquiry
VL - 16
IS - 2
N2 - Children's instructional play and adults' self-education are activities characterized by learning without conventional teaching and by interactional variables. Because both child and adult perceive that they have learned "on their own," play as a form of "curriculum" may serve as an important simulation for later, self-directed learning. (29 references) (CJH)
UR - http://search.proquest.com/docview/63333915?accountid=14701
L2 - http://sfx.scholarsportal.info/ottawa?url_ver=Z39.88-2004&rft_val_fmt=info:ofi/fmt:kev:mtx:journal&genre=article&sid=ProQ:ProQ%3Aericshell&atitle=Children%27s+Play+and+Self-Education.&title=Curriculum+Inquiry&issn=&date=1986-01-01&volume=16&issue=2&spage=203&au=Gehlbach%2C+Roger+D.&isbn=&jtitle=Curriculum+Inquiry&btitle=&rft_id=info:eric/EJ337461
ER -

TY - JOUR
ID - 2058
T1 - Managing Small Group Instruction in an Integrated Preschool Setting
A1 - O'Connell,Joanne Curry
Y1 - 1986///
KW - ERIC
KW - Unsupervised Play Addition
KW - ERIC,Current Index to Journals in Education (CIJE)
KW - Practitioners
KW - Preschool Education
KW - Play
KW - Individualized Instruction

KW - Disabilities
KW - Mainstreaming
RP - NOT IN FILE
SP - 166
EP - 171
JF - TEACHING Exceptional Children
VL - 18
IS - 3
N2 - A structured small group instructional setting helps to teach mainstreamed handicapped preschoolers the skills necessary to interact with the classroom materials without direct supervision. Examples are cited of individualized play activities with puzzles, paint, and play dough. (CL)
UR - http://search.proquest.com/docview/63324082?accountid=14701
L2 - http://sfx.scholarsportal.info/ottawa?url_ver=Z39.88-2004&rft_val_fmt=info:ofi/fmt:kev:mtx:journal&genre=article&sid=ProQ:ProQ%3Aericshell&atitle=Managing+Small+Group+Instruction+in+an+Integrated+Preschool+Setting.&title=Teaching+Exceptional+Children&issn=&date=1986-01-01&volume=18&issue=3&spage=166&au=O%27Connell%2C+Joanne+Curry&isbn=&jtitle=Teaching+Exceptional+Children&btitle=&rft_id=info:eric/EJ331510
ER -

TY - RPRT
ID - 2059
T1 - Father Involvement, Sex-Typing and the Toy Play of Children
A1 - Adubato,Susan A.
Y1 - 1985/08//
N1 - Availability - Level 2 - Produced in microfiche (1966-2003)
SuppNotes - Paper presented at the Annual Convention of the American Psychological Association (93rd, Los Angeles, CA, August 23-27, 1985)
Last updated - 2012-12-14
KW - ERIC
KW - Unsupervised Play Addition
KW - ERIC,Resources in Education (RIE)
KW - Researchers
KW - Sex Role
KW - Play
KW - Parent Influence
KW - Parent Child Relationship
KW - Sex Differences
KW - Father Attitudes
KW - Toys
KW - Fathers
KW - CHILDREN
RP - NOT IN FILE
SP - 18
N2 - Children are encouraged early in life to adopt socially appropriate sex-role activities and behaviors. Parents influence their children's behavior and attitudes through the toys they choose for the children and the toy play they sanction. The term androgynous is used to indicate the blending of masculine and feminine characteristics. This study examines the effects of the degree of father involvement on sex-role orientation of the fathers and the children and on the child's sex-typed toy play. It was hypothesized that increased father involvement would reduce sex-role classification. Participants included 90 4-year-old children enrolled in day care and their parents who were of middle to upper middle socioeconomic status. Both parents filled out independent descriptions of typical weeks of their children. From this, the families were classified as high or low in father involvement. Using the Children's Sex-Role Inventory (CSRI), children were classified as androgynous, masculine, feminine, or undifferentiated based on their toy preferences. Results suggested no direct relationship between father involvement and cross-sexed toy play. The study is limited for two reasons:

the total degree of father involvement was low and most of the children spent a large portion of their week in day care. Further study might examine fathers who are prime caregivers. (ABL)
UR - http://search.proquest.com/docview/63322589?accountid=14701
ER -

TY - JOUR
ID - 2060
T1 - Increasing Simple Toy Play in Profoundly Mentally Handicapped Children. I. Training to Play
A1 - Murphy,G.
Y1 - 1985///
KW - ERIC
KW - Unsupervised Play Addition
KW - ERIC,Current Index to Journals in Education (CIJE)
KW - Severe Mental Retardation
KW - Play
KW - Behavior Modification
KW - Training Methods
KW - Toys
RP - NOT IN FILE
SP - 375
EP - 388
JF - Journal of Autism and Developmental Disorders
VL - 15
IS - 4
N2 - Twenty profoundly retarded children (mean chronological age 14 years) were trained to play with ordinary toys, half by behavior modification and half by a control procedure. Operant training led to some increases in total toy contact but was not significantly more effective than the control technique in promoting independent constructive play. (Author/CL)
UR - http://search.proquest.com/docview/63313023?accountid=14701
L2 - http://sfx.scholarsportal.info/ottawa?url_ver=Z39.88-2004&rft_val_fmt=info:ofi/fmt:kev:mtx:journal&genre=article&sid=ProQ:ProQ%3Aericshell&atitle=Increasing+Simple+Toy+Play+in+Profoundly+Mentally+Handicapped+Children.+I.+Training+to+Play.&title=Journal+of+Autism+and+Developmental+Disorders&issn=&date=1985-01-01&volume=15&issue=4&spage=375&au=Murphy%2C+G.&isbn=&jtitle=Journal+of+Autism+and+Developmental+Disorders&btitle=&rft_id=info:eric/EJ329695
ER -

TY - BOOK
ID - 2061
T1 - Play, Language, and Stories: The Development of Children's Literate Behavior
A1 - Galda,Lee
A1 - Pellegrini,Anthony D.
Y1 - 1985///
N1 - Availability - Level 3 - Indexed only
Last updated - 2012-12-14
KW - ERIC
KW - Unsupervised Play Addition
KW - ERIC,Resources in Education (RIE)
KW - Preschool Education
KW - Language Usage
KW - Language Research
KW - Language Acquisition
KW - Role Playing
KW - Early Reading
KW - Discourse Analysis
KW - Knowledge Level

KW - Parent Child Relationship
KW - Family Role
KW - Social Development
KW - Preschool Children
KW - Linguistics
KW - Play
KW - Language Skills
KW - Reading Skills
KW - Second Language Learning
KW - Reading Comprehension
KW - Language Processing
KW - Beginning Reading
KW - Cognitive Processes
KW - Speech Acts
KW - Sociolinguistics
KW - Child Language
KW - Structural Analysis (Linguistics)
RP - NOT IN FILE
SP - 188
PB - Ablex Publishing Corporation, 355 Chestnut St., Norwood, NJ 07648 ($29.95).
N2 - The question of the relationship between children's play and more formal, literate uses of language is explored in the 9 studies described in this volume. Chapter titles and authors are as follows: (1) "The Influence of Discourse Content and Context on Preschoolers' Use of Language" (Lucia A. French, Joan Lucariello, Susan Seidman, and Katherine Nelson); (2) "Being and Becoming: The Interface of Language and Family Role Knowledge in the Pretend Play of Young African American Girls" (Vonnie C. McLoyd, Shirley Aisha Ray, and Gwendolyn Etter-Lewis); (3) "Narratives in Preschoolers' Sociodramatic Play: The Role of Knowledge and Communicative Competence" (Jacqueline Sachs, Jane Goldman, and Christine Chaille); (4) "The Origins of Autonomous Texts in Play Boundaries" (Dennis P. Wolf and James Pusch); (5) "Relations Between Preschool Children's Symbolic Play and Literate Behavior" (Anthony D. Pellegrini); (6) "Preschool Children's Narratives: Linking Story Comprehension, Production, and Play Discourse" (Marilyn Guttman and Carl H. Frederiksen); (7) "Play Beyond Play: Its Role in Formal Informative Speech" (Mary Ann Evans); (8) "Narrative Play in Second Language Learning" (Shirley Brice Heath with Hey-Kyeong Chin); and (9) "Assessing Children's Knowledge About Book Reading" (Catherine Snow, Debra Nathan, and Rivka Perlman). Author and subject indexes are included. (EL)
SN - 0893912921
UR - http://search.proquest.com/docview/63293095?accountid=14701
L2 - http://sfx.scholarsportal.info/ottawa?url_ver=Z39.88-2004&rft_val_fmt=info:ofi/fmt:kev:mtx:book&genre=book&sid=ProQ:ERIC&atitle=&title=Play%2C+Language%2C+and+Stories%3A+The+Development+of+Children%27s+Literate+Behavior.&issn=&date=1985-01-01&volume=&issue=&spage=188&au=Galda%2C+Lee%3BPellegrini%2C+Anthony+D.&isbn=0893912921&jtitle=&btitle=Play%2C+Language%2C+and+Stories%3A+The+Development+of+Children%27s+Literate+Behavior.&rft_id=info:eric/ED265555
ER -

TY - JOUR
ID - 2062
T1 - Improving Independent Engagement of Nonambulatory Multihandicapped Persons through the Systematic Analysis of Leisure Materials
A1 - Jones,Michael L.
Y1 - 1984///
KW - ERIC
KW - Unsupervised Play Addition
KW - ERIC,Current Index to Journals in Education (CIJE)
KW - Play
KW - Institutionalized Persons
KW - Environmental Influences

KW - Multiple Disabilities
KW - Leisure Time
KW - Severe Disabilities
KW - Intervention
KW - Toys
KW - Behavior Change
RP - NOT IN FILE
SP - 313
EP - 332
JF - Analysis and Intervention in Developmental Disabilities
VL - 4
IS - 4
N2 - Research conducted in an institutional living environment during times when program activities were minimal and clients were typically inactive showed that empirically based toy selection, construction, and modification, and environmental engineering to ensure convenient and constant material availability increased Ss' active engagement with toys. (Author/CL)
UR - http://search.proquest.com/docview/63380467?accountid=14701
L2 - http://sfx.scholarsportal.info/ottawa?url_ver=Z39.88-2004&rft_val_fmt=info:ofi/fmt:kev:mtx:journal&genre=article&sid=ProQ:ProQ%3Aericshell&atitle=Improving+Independent+Engagement+of+Nonambulatory+Multihandicapped+Persons+through+the+Systematic+Analysis+of+Leisure+Materials.&title=Analysis+and+Intervention+in+Developmental+Disabilities&issn=&date=1984-01-01&volume=4&issue=4&spage=313&au=Jones%2C+Michael+L.&isbn=&jtitle=Analysis+and+Intervention+in+Developmental+Disabilities&btitle=&rft_id=info:eric/EJ320989
ER -

TY - RPRT
ID - 2063
T1 - When Play Is Learning: A School Designed for Self-Directed Education
A1 - Gray,Peter
A1 - Chanoff,David
Y1 - 1984///
N1 - Last updated - 2012-12-14
KW - ERIC
KW - Unsupervised Play Addition
KW - Sudbury Valley School MA
KW - ERIC,Current Index to Journals in Education (CIJE)
KW - Elementary Secondary Education
KW - Free Schools
KW - Play
KW - Educational Principles
KW - Independent Study
KW - Open Education
KW - Cultural Education
KW - Nontraditional Education
KW - Private Schools
KW - Student Centered Curriculum
RP - NOT IN FILE
SP - 608
EP - 611
N2 - The school described here operates on the principle that play for the sake of play, without unsolicited adult intervention, entails the acquisition of skills and knowledge. Sudbury Valley School promotes cultural acquisition in a manner harmonious with the child's biological self-education system, without age segregation, grading, or academic requirements. (MJL)
UR - http://search.proquest.com/docview/63457179?accountid=14701

L2 - http://sfx.scholarsportal.info/ottawa?url_ver=Z39.88-2004&rft_val_fmt=info:ofi/fmt:kev:mtx:book&genre=unknown&sid=ProQ:ERIC&atitle=&title=When+Play+Is+Learning%3A+A+School+Designed+for+Self-Directed+Education.&issn=&date=1984-01-01&volume=&issue=&spage=608&au=Gray%2C+Peter%3BChanoff%2C+David&isbn=&jtitle=&btitle=When+Play+Is+Learning%3A+A+School+Designed+for+Self-Directed+Education.&rft_id=info:eric/EJ298056
ER -

TY - RPRT
ID - 2064
T1 - Competence, Gender and Preschooler's Free Play Choices When a Microcomputer Is Present in the Classroom
A1 - Lipinski,Judith M.
Y1 - 1984///
N1 - Availability - Level 1 - Available online, if indexed January 1993 onward
SuppNotes - Product of the Children and Technology Project (CAT)
Last updated - 2012-12-14
KW - ERIC
KW - Unsupervised Play Addition
KW - Choice Behavior
KW - ERIC,Resources in Education (RIE)
KW - Preschool Education
KW - Time Factors (Learning)
KW - Play
KW - Microcomputers
KW - Competence
KW - Sex Differences
KW - Preschool Children
RP - NOT IN FILE
SP - 22
N2 - The proliferation of microcomputers in early childhood education has raised concerns about the computer's effects on the preschool curriculum. Of particular concern is the possibility that children will interact with the computer to the exclusion of other important activities. To examine this issue, a two-study project investigated children's activity choices in a 1-hour free-play period. Gender, competence, and free-play areas were the independent variables. The first study involved a class of 4-year-old children at a university-based nursery school; a class of 4-year-olds attending a university day care center participated in the second study. A microcomputer and age-appropriate software were placed in each classroom according to the following schedule of treatment weeks: baseline, computer-noninteractive (omitted in the second study), computer-interactive, and computer-interactive/teacher-interactive. The presence of the microcomputer intitially disrupted children's free-play choices. After several weeks, children's free-play patterns returned to baseline levels. In the first study, it was found that girls spent more time at the computer than did boys; however, no significant sex differences were found in the second study. In both, children judged to have high and medium levels of competence spent equal amounts of time at the computer. (Author/RH)
UR - http://search.proquest.com/docview/63444909?accountid=14701
L2 - http://sfx.scholarsportal.info/ottawa?url_ver=Z39.88-2004&rft_val_fmt=info:ofi/fmt:kev:mtx:book&genre=unknown&sid=ProQ:ERIC&atitle=&title=Competence%2C+Gender+and+Preschooler%27s+Free+Play+Choices+When+a+Microcomputer+Is+Present+in+the+Classroom.&issn=&date=1984-01-01&volume=&issue=&spage=22&au=Lipinski%2C+Judith+M&isbn=&jtitle=&btitle=Competence%2C+Gender+and+Preschooler%27s+Free+Play+Choices+When+a+Microcomputer+Is+Present+in+the+Classroom.&rft_id=info:eric/ED243609
ER -

TY - RPRT
ID - 2065
T1 - The Effect of Dramatic Play on the Story Retellings of Second Grade Children
A1 - Galda,Lee

A1 - Miller,Michael
Y1 - 1983/04//
N1 - Availability - Level 1 - Available online, if indexed January 1993 onward
SuppNotes - Paper presented at the Annual Meeting of the American Educational Research Association (Montreal, Canada, April 11-14, 1983)
Last updated - 2012-12-14
KW - ERIC
KW - Unsupervised Play Addition
KW - Constructs
KW - Piagetian Theory
KW - ERIC,Resources in Education (RIE)
KW - Researchers
KW - Grade 2
KW - Primary Education
KW - Elementary School Students
KW - Competence
KW - Language Skills
KW - Dramatic Play
KW - Narration
RP - NOT IN FILE
SP - 10
N2 - Effects of dramatic play on children's narrative competence were investigated among 36 second-graders. Children were read a familiar folktale and then "played" the story, discussed it, or drew a picture about it. They were then asked to retell the story either to an informed or to a naive listener. It was hypothesized that (1) more "evaluative" elaborative devices would be found in stories retold to a naive listener than in stories told to an informed listener and (2) more such devices would be found in the retellings of children in the play condition than in the other two conditions. Independent variables were sex, listener, and condition. Dependent variables were number of words, number of evaluative clauses, number of evaluative syntactic devices, and ratios of evaluative devices to number of words. Narrative competence was assessed by measuring the number of evaluative devices present in the retellings, and data were analyzed using a three-way multiple analysis of variance. The finding of no significant effects may be seen as support for Piaget's model of play, in which effects of play decrease in school-age children. The lack of significant results also indicates that narrative competence may not be a unitary construct. Subjects exhibited differences in the cohesive features of their language but not in their use of evaluative markers. It was concluded that various instruments may measure markedly different aspects of narrative competence. (RH)
UR - http://search.proquest.com/docview/63409096?accountid=14701
L2 - http://sfx.scholarsportal.info/ottawa?url_ver=Z39.88-2004&rft_val_fmt=info:ofi/fmt:kev:mtx:book&genre=unknown&sid=ProQ:ERIC&atitle=&title=The+Effect+of+Dramatic+Play+on+the+Story+Retellings+of+Second+Grade+Children.&issn=&date=1983-04-01&volume=&issue=&spage=10&au=Galda%2C+Lee%3BMiller%2C+Michael&isbn=&jtitle=&btitle=The+Effect+of+Dramatic+Play+on+the+Story+Retellings+of+Second+Grade+Children.&rft_id=info:eric/ED233812
ER -

TY - RPRT
ID - 2066
T1 - The Effect of Dramatic Play on Children's Generation of Cohesive Text
A1 - Pellegrini,D.A.
Y1 - 1983/04//
N1 - Availability - Level 1 - Available online, if indexed January 1993 onward
SuppNotes - Paper presented at the Annual Meeting of the American Educational Research Association (Montreal, Canada, April 11-14, 1983)
Last updated - 2012-12-14
KW - ERIC
KW - Unsupervised Play Addition
KW - ERIC,Resources in Education (RIE)
KW - Primary Education

KW - Kindergarten Children
KW - Elementary School Students
KW - Oral Language
KW - Freehand Drawing
KW - Dramatic Play
KW - Discussion
KW - Story Telling
KW - Recall (Psychology)
KW - Verbal Ability
KW - Child Language
RP - NOT IN FILE
SP - 25
N2 - The extent to which children's cohesive oral text varied as a function of listener status and the extent to which children could be trained to generate decontextualized oral text was examined in a study of children in kindergarten through second grade. Independent variables were children's age; exposure to a play, discussion, or drawing condition; and the naive or informed status of the audience to which a story was retold. The dependent measure consisted of individual children's oral texts, generated on a recall task. Recall was scored for cohesion according to endophoric or exophoric reference and ellipsis. Endophorics are defined as linguistically encoded ties between the presupposed element and the presupposing element (e.g., "John's here. He'll leave later."). Exophorics rely on context or unencoded assumptions to convey meaning (e.g., the statement "It's nice," when "it" is defined by the speaker's pointing to an object). Reference is composed of three subcategories: pronominals, demonstratives, and comparatives. Ellipsis is also composed of three subcategories: nominal, verbal, and causal ellipsis. Children assigned to same-age groups of four were read children's books on three occasions. Following the readings, subjects were exposed to their respective treatments, then were asked to retell the story to a naive or informed listener. Results supported the proposition that children's register is affected by participants in discourse. Additionally, dramatic play appeared to be a context within which children learn to put meaning into words. (RH)
UR - http://search.proquest.com/docview/63513794?accountid=14701
L2 - http://sfx.scholarsportal.info/ottawa?url_ver=Z39.88-2004&rft_val_fmt=info:ofi/fmt:kev:mtx:book&genre=unknown&sid=ProQ:ERIC&atitle=&title=The+Effect+of+Dramatic+Play+on+Children%27s+Generation+of+Cohesive+Text.&issn=&date=1983-04-01&volume=&issue=&spage=25&au=Pellegrini%2C+D.+A.&isbn=&jtitle=&btitle=The+Effect+of+Dramatic+Play+on+Children%27s+Generation+of+Cohesive+Text.&rft_id=info:eric/ED227964
ER -

TY - JOUR
ID - 2067
T1 - Solitary Play: Social Immaturity or Autonomous Achievement Striving?
A1 - Barnes,M.G.
Y1 - 1981/09//
KW - ERIC
KW - Unsupervised Play Addition
KW - Autonomy (Personal)
KW - Solitary Play
KW - ERIC,Current Index to Journals in Education (CIJE)
KW - Early Childhood Education
KW - Play
KW - Social Development
KW - Preschool Children
KW - Maturity (Individuals)
RP - NOT IN FILE
SP - 12
EP - 15
JF - Australian Journal of Early Childhood
VL - 6
IS - 3

N2 - Traces the concept of solitary play through a review of the available research, queries whether solitary play is an indicator of social immaturity or autonomous achievement striving, and calls for further research in this area. (Author/MP)
UR - http://search.proquest.com/docview/63561380?accountid=14701
L2 - http://sfx.scholarsportal.info/ottawa?url_ver=Z39.88-2004&rft_val_fmt=info:ofi/fmt:kev:mtx:journal&genre=article&sid=ProQ:ProQ%3Aericshell&atitle=Solitary+Play%3A+Social+Immaturity+or+Autonomous+Achievement+Striving%3F&title=Australian+Journal+of+Early+Childhood&issn=&date=1981-09-01&volume=6&issue=3&spage=12&au=Barnes%2C+M.+G.&isbn=&jtitle=Australian+Journal+of+Early+Childhood&btitle=&rft_id=info:eric/EJ255247
ER -

TY - RPRT
ID - 2068
T1 - Play Styles in Early Childhood: Continuity and Change as a Function of Sex
A1 - Fagot,Beverly I.
Y1 - 1979/06//
N1 - Availability - Level 1 - Available online, if indexed January 1993 onward
SuppNotes - Paper presented at the meeting of the International Society for the Study of Behavioural Development (Lund, Sweden, June 25-29, 1979)
Last updated - 2012-12-14
KW - ERIC
KW - Unsupervised Play Addition
KW - ERIC,Resources in Education (RIE)
KW - Social Reinforcement
KW - Teacher Influence
KW - Play
KW - MALES
KW - Behavior Patterns
KW - Peer Influence
KW - Sex Differences
KW - Females
KW - Preschool Children
KW - SOCIALIZATION
KW - Sex Stereotypes
RP - NOT IN FILE
SP - 7
N2 - The continuity of play styles of preschool children over a 1-year period was observed and related to teacher and peer reinforcement patterns influenced by sex stereotypes. Sixty-seven children, aged 18 months to 58 months, were observed in a natural setting using a behavior checklist consisting of 33 child behavior categories and 15 teacher- and peer-reaction categories. Each child in the study was observed for at least 40 hours, with 12 observations per hour. Data from the upper and lower 25% of the children on each of seven factors (derived from a factor analysis of the 33 behavior scores) were examined for continuity between the first and last 10 weeks of the children's participation in the study. The results indicate that certain play styles show stability for all children, while others change over time. The behaviors which change are those behaviors for which peers or teachers are giving an unusual amount of feedback to one group of children. For example, teachers had an extremely high rate of initiation activity towards passive children. Also, peers successfully exerted pressure to change a child's behavior, as in the case of boys who were initially low on male-preferred or high on female-preferred activities. Peers did not, however, exert negative pressure on girls who were high on the male-preferred activities or low on the female-preferred activities. Such girls show high stability in play style even though their behavior does not fit the traditional stereotypes. On the other hand, teachers gave girls with high activity levels more negative feedback, and these girls show low stability on this factor. Participation in solitary activities appears to be independent of feedback. (Author/SS)
UR - http://search.proquest.com/docview/63659453?accountid=14701
L2 - http://sfx.scholarsportal.info/ottawa?url_ver=Z39.88-2004&rft_val_fmt=info:ofi/fmt:kev:mtx:book&genre=unknown&sid=ProQ:ERIC&atitle=&title=Play+Styles+i

n+Early+Childhood%3A+Continuity+and+Change+as+a+Function+of+Sex.&issn=&date=1979-06-01&volume=&issue=&spage=7&au=Fagot%2C+Beverly+I.&isbn=&jtitle=&btitle=Play+Styles+in+Early+Childhood%3A+Continuity+and+Change+as+a+Function+of+Sex.&rft_id=info:eric/ED175562
ER -

TY - RPRT
ID - 2069
T1 - An Investigation of the Effects of Forms of Imaginative Play on Language Development and Language Comprehension in Young Children
A1 - Yawkey,Margaret L.
A1 - Yawkey,Thomas D.
Y1 - 1979///
N1 - Availability - Level 1 - Available online, if indexed January 1993 onward
Last updated - 2012-12-14
KW - ERIC
KW - Unsupervised Play Addition
KW - ERIC,Resources in Education (RIE)
KW - Primary Education
KW - Language Research
KW - Play
KW - Language Acquisition
KW - Young Children
KW - Dramatic Play
KW - Comprehension
KW - Language Processing
KW - Oral Language
KW - Developmental Stages
KW - Sex Differences
KW - Child Language
RP - NOT IN FILE
SP - 25
N2 - A study investigated the effects of symbolic play treated as a mediator for increasing language comprehension and facilitating oral language growth. The study included two aspects of language: language comprehension and language development. Independent variables were forms of play--puppet action, body action, abstract (imagined) action, and no action (control); age; and sex. Dependent measures were cloze comprehension, 10-item measures for language comprehension, and the students' connected discourse scored t-units for language development. Subjects were 80 five-year-olds and 80 seven-year-olds. After no significant main or interaction effects on preassessment language measure between the factors were found and after the treatments were completed, major findings were as follows: (1) on the cloze measure of language comprehension, children in the body action play group received significantly higher mean scores than did those in the abstract action play and control groups; (2) on the question measure of language comprehension, five-year-olds in the puppet action and body action play groups received significantly higher mean scores than did the five-year-olds in the abstract action and play groups; and (3) on the t-unit measure of language development, the seven-year-olds received significantly higher mean scores than did the five-year-olds. There were no significant effects for sex. (Author/HTH)
UR - http://search.proquest.com/docview/63217995?accountid=14701
L2 - http://sfx.scholarsportal.info/ottawa?url_ver=Z39.88-2004&rft_val_fmt=info:ofi/fmt:kev:mtx:book&genre=unknown&sid=ProQ:ERIC&atitle=&title=An+Investigation+of+the+Effects+of+Forms+of+Imaginative+Play+on+Language+Development+and+Language+Comprehension+in+Young+Children.&issn=&date=1979-01-01&volume=&issue=&spage=25&au=Yawkey%2C+Margaret+L.%3BYawkey%2C+Thomas+D.&isbn=&jtitle=&btitle=An+Investigation+of+the+Effects+of+Forms+of+Imaginative+Play+on+Language+Development+and+Language+Comprehension+in+Young+Children.&rft_id=info:eric/ED284226
ER -

TY - GEN

ID - 2070
T1 - Structural Elements of Symbolic Play of Preschool Children
A1 - Gowen,Jean W.
Y1 - 1978/08//
N1 - Availability - Level 1 - Available online, if indexed January 1993 onward
SuppNotes - Paper presented at the Annual Meeting of the American Psychological Association (86th, Toronto, Canada, August 28-September 1, 1978)
Last updated - 2012-12-14
KW - ERIC
KW - Unsupervised Play Addition
KW - ERIC,Resources in Education (RIE)
KW - Preschool Education
KW - Semiotics
KW - Imagination
KW - Play
KW - Symbolic Language
KW - Research
KW - Pretend Play
KW - Preschool Children
KW - Infants
RP - NOT IN FILE
SP - 17
N2 - The structural elements of the spontaneous symbolic play of 2- and 3-year-olds were compared with those of 4- and 5-year-olds. All 32 children studied were enrolled in a private day care center. Each child was observed during free play in the center for 15 minutes. Three categories of signifiers (animate, inanimate and imaginary) and five modes of representation (enactment, object substitution, symbolic construction, preparatory behavior and comments) were identified. The data were coded in four phases. In the first phase of coding, incidents of symbolic play were identified. During the other three phases of coding, the structural elements, i.e. the signifieds, signifiers, and modes of representation, were identified, coded and counted. Seven transcripts were selected at random to be coded by two independent coders to yield reliability data. Percentage of agreement among coders ranged from 80 to 84 percent. Results suggest that younger children used more symbolic construction, preparation and comments as modes of representation and more animate and imaginary signifiers. Implications of results for Piaget's and Vvgotsky's theories of symbolic play are discussed. (Author/RH)
UR - http://search.proquest.com/docview/63752500?accountid=14701
L2 - http://sfx.scholarsportal.info/ottawa?url_ver=Z39.88-2004&rft_val_fmt=info:ofi/fmt:kev:mtx:journal&genre=unknown&sid=ProQ:ProQ%3Aericshell&atitle=Structural+Elements+of+Symbolic+Play+of+Preschool+Children.&title=&issn=&date=1978-08-01&volume=&issue=&spage=17&au=Gowen%2C+Jean+W.&isbn=&jtitle=&btitle=&rft_id=info:eric/ED164127
ER -

TY - GEN
ID - 2071
T1 - The Effects of Action and Violence in Television Programs on the Social Behavior and Imaginative Play of Preschool Children
A1 - Huston-Stein,Aletha
Y1 - 1978/03//
N1 - Availability - Level 1 - Available online, if indexed January 1993 onward
SuppNotes - Paper presented at the meeting of the Southwestern Society for Research in Human Development (Dallas, TX, March 1978). For a related document, see IR 008 192
Last updated - 2012-12-14
KW - ERIC
KW - Unsupervised Play Addition
KW - ERIC,Resources in Education (RIE)
KW - Television Viewing

KW - Psychological Studies
KW - Television Research
KW - Pretend Play
KW - Preschool Children
KW - Aggression
KW - Violence
RP - NOT IN FILE
SP - 18
N2 - The independent contributions of action and violence in television programs to children's attention and social behavior were investigated. Pairs of preschool children were assigned to one of four television conditions (1) high action-high violence, (2) high action-low violence, (3) low action-low violence, or (4) no television. Action was defined as rapid movement by characters or objects; violence was physical aggression by characters. Visual attention was greater in high action than in low action programs; there were no differences in attention as a function of violence when action was controlled. Children were observed in free play sessions before and after viewing. Those who saw low action-low violence television or no television increased in imaginative, fantasy play; those who saw high action-high violence decreased in imaginative play; the high action-low violence group fell in between. There was some tendency for aggressive behavior to follow the opposite pattern--higher aggression following high action-high violence or high action-low violence than after low action-low violence or no television. There were no differences in activity level as a function of treatment. These results were interpreted as supporting arousal theory more strongly than observational learning theory. (Author/JEG)
UR - http://search.proquest.com/docview/63689795?accountid=14701
L2 - http://sfx.scholarsportal.info/ottawa?url_ver=Z39.88-2004&rft_val_fmt=info:ofi/fmt:kev:mtx:journal&genre=unknown&sid=ProQ:ProQ%3Aericshell&atitle=The+Effects+of+Action+and+Violence+in+Television+Programs+on+the+Social+Behavior+and+Imaginative+Play+of+Preschool+Children.&title=&issn=&date=1978-03-01&volume=&issue=&spage=18&au=Huston-Stein%2C+Aletha&isbn=&jtitle=&btitle=&rft_id=info:eric/ED184524
ER -

TY - RPRT
ID - 2073
T1 - Fantasy Play in Child Psychotherapy
A1 - Stollak,Gary E.
Y1 - 1978///
N1 - Availability - Level 1 - Available online, if indexed January 1993 onward
SuppNotes - Paper presented at the Annual Convention of the American Psychological Association (Toronto, Ontario, Canada, August, 1978)
Last updated - 2012-12-14
KW - ERIC
KW - Unsupervised Play Addition
KW - Self Presentation
KW - ERIC,Resources in Education (RIE)
KW - Training
KW - Research Projects
KW - CHILDREN
KW - Developmental Psychology
KW - Creative Expression
KW - Therapists
KW - Maturation
KW - Helping Relationship
KW - Behavior Patterns
KW - Supervision
KW - Pretend Play
KW - Child Psychology
RP - NOT IN FILE
SP - 87

N2 - Relationships among adult and young children's behavior in extended play encounters were examined. One group of normal six-year-old children completed 20 play encounters, while a second group of clinic-referred children completed 15 play encounters. Each child encountered either a trained/supervised or an untrained/unsupervised college undergraduate. Analyses of videotapes made of the "non-directive" play encounters indicated that the children who encountered trained/supervised undergraduates emitted greater numbers of fantasy behaviors, especially those indicative of mature, coping and adaptive functioning than did children encountering untrained/unsupervised undergraduates. Adult involvement in play and allowing the child self-direction seemed key behaviors in eliciting such child behavior. (Author/JLL)
UR - http://search.proquest.com/docview/63746686?accountid=14701
ER -

TY - RPRT
ID - 2074
T1 - Enhancing Imaginative Play in Preschoolers: Television and Live Adult Effects
A1 - Singer,Jerome L.
A1 - Singer,Dorothy G.
Y1 - 1974///
N1 - Availability - Level 1 - Available online, if indexed January 1993 onward
Last updated - 2012-12-14
KW - ERIC
KW - Unsupervised Play Addition
KW - Mister Rogers Neighborhood
KW - ERIC,Resources in Education (RIE)
KW - Teacher Influence
KW - Play
KW - Cognitive Development
KW - Responses
KW - Individual Characteristics
KW - Learning Processes
KW - Emotional Development
KW - Imagination
KW - Self Concept
KW - Sex Differences
KW - Television Research
KW - Preschool Children
KW - Educational Television
KW - Aggression
KW - Teaching Methods
RP - NOT IN FILE
SP - 38
N2 - The present study was designed to explore the possibility that exposure to the "Misterogers' Neighborhood" program might increase the likelihood of spontaneous imaginative play in preschool children who watched the program over a period of two weeks. The specific focus of this investigation was to determine whether a well-produced professional program would be more effective in enhancing imaginative play than instruction from a live adult. The study involved four varied conditions: (1) a non TV-viewing control group observed in spontaneous play on two occasions separated in time by a period comparable to that taken up by the experimental conditions; (2) a group who watched the Misterogers' show daily over a two-week period; (3) a group who watched the same show daily in the company of an adult who interacted with the children about content of the performance; and (4) a group who saw no television at school but received a comparable daily time period of fantasy game-playing and practice in imagery with an adult teacher. An analysis of the matrix of intercorrelations between the independent and dependent variables of the study seems to suggest that children in the 3- to 4-year-old age group remain most susceptible to influence by a concerned adult in their presence who can engage them directly and provide them with immediate feedback for their own responses. It is therefore likely that at the very least, television's prosocial or optimal cognitive benefits may have to depend on some mediation by an adult. (Author/CS)
UR - http://search.proquest.com/docview/64045586?accountid=14701

ER -

TY - GEN
ID - 2075
T1 - The Effects of Viewed Aggression on the Group Play of Children
A1 - Ross,Lee B.
Y1 - 1972/05//
N1 - Availability - Level 1 - Available online, if indexed January 1993 onward
SuppNotes - Paper presented at Midwestern Psychological Association Meeting, May 4-6, 1972, Cleveland, Ohio
Last updated - 2012-12-14
KW - ERIC
KW - Unsupervised Play Addition
KW - ERIC,Resources in Education (RIE)
KW - Groups
KW - Television Viewing
KW - Play
KW - Behavior
KW - Television
KW - Group Behavior
KW - CHILDREN
KW - Aggression
KW - Violence
RP - NOT IN FILE
SP - 25
N2 - The present investigation extended the methodology to investigate the effects of viewed aggression on groups of two and four children. The present study employed a 2 x 2 x 3 factorial design, using as independent variables sex of the child, group size (two or four children), and cartoon condition (aggressive cartoon, nonaggressive cartoon, and no cartoon). Forty-eight boys and 48 girls of kindergarten age were randomly assigned to the experimental groups. A factor analysis of the dependent measures identified four factors, accounting for 85 percent of the total variance, each of which responded differently to the independent variables. The finding that (a) transgressive-aggressive was not affected by viewing aggressive cartoons, and (b) that normative-aggression loads on the same factor as normative-play, rejects the assumption that aggression is a single class of behavior and rejects the hypothesis that the effect of viewing aggressive models reduce inhibitions against aggression. The results are interpreted in terms of factors affecting levels of activity. (Author)
UR - http://search.proquest.com/docview/64241319?accountid=14701
ER -

TY - GEN
ID - 2076
T1 - The Influence of Ability Level and Materials on Classificatory and Imaginative Behavior in Free Play
A1 - Phinney,Jean
Y1 - 1972///
N1 - Availability - Level 1 - Available online, if indexed January 1993 onward
Last updated - 2012-12-14
KW - ERIC
KW - Unsupervised Play Addition
KW - ERIC,Resources in Education (RIE)
KW - Preschool Education
KW - Research Reports
KW - Play
KW - Cognitive Development
KW - Observation
KW - Creativity
KW - Manipulative Materials

KW - Imagination
KW - Cognitive Ability
KW - Object Manipulation
KW - Classification
KW - Preschool Children
KW - Age Differences
RP - NOT IN FILE
SP - 37
N2 - A dissertation proposal involved a study to observe spontaneous behavior of children in interaction with materials in order to gain understanding of the factors that influence classificatory and imaginative behavior in free play. Children at two levels of ability in terms of classification skills were observed in interaction with materials at two levels of complexity during four play sessions. Materials were two related sets of specially designed blocks defined as simple or complex on the basis of the variations and the possible combinations among individual members of the set. The 3- to 6-year-olds were to play in pairs to simulate the social play typical of children in natural situations. Observation focused on free classification, creative or design behavior, imaginative behavior or variety of themes. A final measure was used to determine the effect of interaction with the materials on classification ability. Results consisted of observational data from the experimental sessions and posttest scores. Non-definitive results indicated replicating the experiment with modifications. It was not feasible to use pairs as the experimental unit, so one child at a time was observed. Interaction between ability and complexity of blocks was found in testing, but not in observed behavior. No interaction effects with age or ability were observed. A revised study is proposed involving age as the independent variable and redesigned materials. (KM)
UR - http://search.proquest.com/docview/64172231?accountid=14701
ER -

TY - GEN
ID - 2077
T1 - Play Behavior and Efficacy in Ghetto Four-Year-Olds: Organization and Psychosexual Content of Play
A1 - Borowitz,Gene H.
Y1 - 1970///
N1 - Availability - Level 1 - Available online, if indexed January 1993 onward
Last updated - 2012-12-14
KW - ERIC
KW - Unsupervised Play Addition
KW - Efficacy
KW - Play Session Behavior Scale (Hirsch et al)
KW - Psychosexual Content of Play Scale (Borowitz)
KW - ERIC,Resources in Education (RIE)
KW - Interaction
KW - Behavior Rating Scales
KW - Play
KW - Ghettos
KW - Interpersonal Competence
KW - Sex Differences
KW - Child Development
KW - Black Youth
KW - Observation
KW - Preschool Children
KW - Behavioral Science Research
RP - NOT IN FILE
SP - 31
N2 - Effectance, defined as the child's independent, effective interaction with people and things, is studied through observation of the play of 36 preschool low income black children. The children were observed and filmed in semi-structured play sessions, primarily with family dolls, doll furniture, and small baby bottle. Instruments used were: (1) the Play Session Behavior Scale, which includes 22 variables defining organization, involvement, and interpersonal responsiveness in play, and (2) the Psychosexual Content of Play Scale, which

includes 13 variables defining the amount of content reflecting each of the five early psychosexual stages of development. It is concluded that: (a) Effective functioning as a learner at age 4 requires skills which became crystallized during the Anal Retentive stage, such as the ability to persist in tasks, take pleasure in accomplishments, and look for rules and order; and (b) Optimal functioning as a learner at age 4 is facilitated by skills arising out of the Phallic stage, such as curiosity, self-assertion, and an emerging sense of where one fits in the social order. Statistical tables and an appendix consisting of the instruments and rater's manual used comprise almost half of the document. (Author/NH)
UR - http://search.proquest.com/docview/64302405?accountid=14701
ER -

TY - GEN
ID - 2078
T1 - Characterization of the Effect of Space, Materials, and Teacher Behavior on Preschool Children's Free Play Activity Patterns. Research Report No. 1
A1 - Holt,Carol
A1 - Boger,Robert P.
Y1 - 1969/11//
N1 - Availability - Level 1 - Available online, if indexed January 1993 onward
Last updated - 2012-12-14
KW - ERIC
KW - Unsupervised Play Addition
KW - ERIC,Resources in Education (RIE)
KW - Instructional Materials
KW - Play
KW - Research Needs
KW - Classroom Observation Techniques
KW - Behavior Patterns
KW - Recreational Activities
KW - Space Utilization
KW - Individual Activities
KW - Preschool Children
RP - NOT IN FILE
SP - 22
N2 - The object of this study was to determine the extent to which children displayed predictable, recurring patterns of behavior when engaged in self-selected activities. Subjects were four girls and three boys, 4 years of age, all from middle class urban families. The classroom's free play space was divided into four equal areas. Toys were divided into four groups: art materials, blocks, dramatic toys, and manipulative toys and books. Each day the toy groups were randomly assigned to the four areas, with each free play session being videotaped simultaneously from two directions. Observer recordings of each child's movements and the toys he used were summarized, coded, and analyzed. Significant differences were found in the time spent by children in specific physical locations. These differences were related to the nature of the area and the proximity of the play materials to the teacher. Children tended to choose play materials that could be moved to another area of the room. Differences in the nature of independent and group play were related to materials and location. (DR)
UR - http://search.proquest.com/docview/64341700?accountid=14701
ER -

TY - JOUR
ID - 2079
T1 - Teaching Object Permanence: An Action Research Study
A1 - Bruce,Susan M.
A1 - Vargas,Claudia
Y1 - 2013///
KW - ERIC
KW - Unsupervised Play Addition
KW - ERIC,Current Index to Journals in Education (CIJE)
KW - visual impairments

KW - Action Research
KW - Disabilities
KW - Direct Instruction
KW - Human Body
KW - Multiple Disabilities
KW - Cues
KW - CHILDREN
KW - Object Permanence
RP - NOT IN FILE
SP - 60
EP - 64
JF - Journal of Visual Impairment & Blindness
VL - 107
IS - 1
PB - American Foundation for the Blind. 11 Penn Plaza Suite 300, New York, NY 10001.
N2 - "Object permanence," also known as "object concept" in the field of visual impairment, is one of the most important early developmental milestones. The achievement of object permanence is associated with the onset of representational thought and language. Object permanence is important to orientation, including the recognition of landmarks. Independent mobility (such as crawling) improves visual attentiveness, which is critical to the achievement of later object permanence tasks. Direct instruction can accelerate the mastery of object permanence in children with disabilities and is more effective than general stimulation programs. This article presents an action research study on teaching object permanence to a child with multiple disabilities and visual impairment. It illustrates some of the principles of assessment and instruction described in Bruce and Vargas (2012). The Institutional Review Board at Boston College approved this study, which was part of a set of action research studies on pivotal milestones that were conducted with children with multiple disabilities. (Contains 2 figures.)
SN - 0145-482X, 0145-482X
UR - http://search.proquest.com/docview/1413413998?accountid=14701
L2 - http://sfx.scholarsportal.info/ottawa?url_ver=Z39.88-2004&rft_val_fmt=info:ofi/fmt:kev:mtx:journal&genre=article&sid=ProQ:ProQ%3Aericshell&atitle=Teaching+Object+Permanence%3A+An+Action+Research+Study&title=Journal+of+Visual+Impairment+%26+Blindness&issn=0145482X&date=2013-01-01&volume=107&issue=1&spage=60&au=Bruce%2C+Susan+M.%3BVargas%2C+Claudia&isbn=&jtitle=Journal+of+Visual+Impairment+%26+Blindness&btitle=&rft_id=info:eric/EJ1008202
ER -

TY - JOUR
ID - 2080
T1 - Disorganized Attachment and Inhibitory Capacity: Predicting Externalizing Problem Behaviors
A1 - Bohlin,Gunilla
A1 - Eninger,Lilianne
A1 - Brocki,Karin Cecilia
A1 - Thorell,Lisa B.
Y1 - 2012/04//
KW - ERIC
KW - Unsupervised Play Addition
KW - ERIC,Current Index to Journals in Education (CIJE)
KW - Attention Deficit Hyperactivity Disorder
KW - Play
KW - Young Children
KW - Pervasive Developmental Disorders
KW - Longitudinal Studies
KW - Inhibition
KW - Behavior Problems
KW - Emotional Development
KW - Symptoms (Individual Disorders)

KW - Toys
KW - Autism
KW - Attachment Behavior
RP - NOT IN FILE
SP - 449
EP - 458
JF - Journal of Abnormal Child Psychology
JA - J Abnorm.Child Psychol.
VL - 40
IS - 3
PB - Springer. 233 Spring Street, New York, NY 10013.
N2 - The aim of the present study was to investigate whether attachment insecurity, focusing on disorganized attachment, and the executive function (EF) component of inhibition, assessed at age 5, were longitudinally related to general externalizing problem behaviors as well as to specific symptoms of ADHD and Autism spectrum disorder (ASD), and callous-unemotional (CU) traits. General externalizing problem behaviors were also measured at age 5 to allow for a developmental analysis. Outcome variables were rated by parents and teachers. The sample consisted of 65 children with an oversampling of children with high levels of externalizing behaviors. Attachment was evaluated using a story stem attachment doll play procedure. Inhibition was measured using four different tasks. The results showed that both disorganized attachment and poor inhibition were longitudinally related to all outcome variables. Controlling for initial level of externalizing problem behavior, poor inhibition predicted ADHD symptoms and externalizing problem behaviors, independent of disorganized attachment, whereas for ASD symptoms no predictive relations remained. Disorganized attachment independently predicted CU traits
SN - 0091-0627, 0091-0627
UR - http://search.proquest.com/docview/968107987?accountid=14701
L2 - http://sfx.scholarsportal.info/ottawa?url_ver=Z39.88-2004&rft_val_fmt=info:ofi/fmt:kev:mtx:journal&genre=article&sid=ProQ:ProQ%3Aericshell&atitle=Disorganized+Attachment+and+Inhibitory+Capacity%3A+Predicting+Externalizing+Problem+Behaviors&title=Journal+of+Abnormal+Child+Psychology&issn=00910627&date=2012-04-01&volume=40&issue=3&spage=449&au=Bohlin%2C+Gunilla%3BEninger%2C+Lilianne%3BBrocki%2C+Karin+Cecilia%3BThorell%2C+Lisa+B.&isbn=&jtitle=Journal+of+Abnormal+Child+Psychology&btitle=&rft_id=info:eric/EJ957952
ER -

TY - JOUR
ID - 2081
T1 - The Effects of Prompts and a Group-Oriented Contingency on Out-of-School Physical Activity in Elementary School-Aged Students
A1 - Hastie,Peter A.
A1 - van der Mars,Hans
A1 - Layne,Todd
A1 - Wadsworth,Danielle
Y1 - 2012/04//
KW - ERIC
KW - Unsupervised Play Addition
KW - Alabama
KW - ERIC,Current Index to Journals in Education (CIJE)
KW - Elementary Education
KW - Grade 4
KW - Educational Strategies
KW - Instructional Effectiveness
KW - Equipment
KW - Play
KW - Rural Areas
KW - Physical Activities
KW - Physical Education

KW - Behavior Modification
KW - Reinforcement
KW - Teaching Methods
KW - Physical Activity Level
RP - NOT IN FILE
SP - 131
EP - 145
JF - Journal of Teaching in Physical Education
VL - 31
IS - 2
PB - Human Kinetics, 1607 North Market Street, Champaign, IL 61820.
N2 - This study examined the effectiveness of three conditions in which 48 fourth-grade students were prompted to be physically active out of school. Using an alternating treatments design (Cooper, Heron, & Heward, 2007) the three intervention conditions included: (a) Baseline: No prompting of students, (b) Teacher Prompts: Verbal prompt to "remember to do something active after school today", and (c) Teacher Prompts and group-oriented contingencies: Verbal prompts with an index card where students could record their activity to earn bonus points as part of a team challenge. Graphically plotted pedometer data depicting data paths, variability, and trends within and across three conditions showed that students were more active outside of school only when the contingent reinforcement (c) was in place. This suggests that using prompts and group-oriented contingencies within Sport Education appears to be an effective and authentic context for promoting independent (i.e., free play) out-of-school time physical activity. (Contains 2 tables and 1 figure.)
SN - 0273-5024, 0273-5024
UR - http://search.proquest.com/docview/1140128052?accountid=14701
L2 - http://sfx.scholarsportal.info/ottawa?url_ver=Z39.88-2004&rft_val_fmt=info:ofi/fmt:kev:mtx:journal&genre=article&sid=ProQ:ProQ%3Aericshell&atitle=The+Effects+of+Prompts+and+a+Group-Oriented+Contingency+on+Out-of-School+Physical+Activity+in+Elementary+School-Aged+Students&title=Journal+of+Teaching+in+Physical+Education&issn=02735024&date=2012-04-01&volume=31&issue=2&spage=131&au=Hastie%2C+Peter+A.%3Bvan+der+Mars%2C+Hans%3BLayne%2C+Todd%3BWadsworth%2C+Danielle&isbn=&jtitle=Journal+of+Teaching+in+Physical+Education&btitle=&rft_id=info:eric/EJ978086
ER -

TY - JOUR
ID - 2082
T1 - Young Children's Explorations: Young Children's Research?
A1 - Murray,Jane
Y1 - 2012///
KW - ERIC
KW - Unsupervised Play Addition
KW - England
KW - ERIC,Current Index to Journals in Education (CIJE)
KW - Early Childhood Education
KW - Teacher Role
KW - Play
KW - Young Children
KW - Ethnography
KW - Child Care
KW - Epistemology
KW - Educational Environment
KW - Time
KW - Foreign Countries
KW - Discovery Processes
KW - Naturalistic Observation
RP - NOT IN FILE
SP - 1209

EP - 1225
JF - Early Child Development and Care
VL - 182
IS - 9
PB - Routledge. , 325 Chestnut Street Suite 800, Philadelphia, PA 19106.
N2 - "Exploration" is recognised as research behaviour; anecdotally, as an early years' teacher, I witnessed many young children exploring. However, young children's self-initiated explorations are rarely regarded as research by adult researchers and policy-makers. The exclusion of young children's autonomous explorations from recognition as research conflicts with "new sociology" perspectives positioning children as social actors. These tensions have driven a small-scale interpretive study, developed with children aged four to eight years in three "good" schools in England to investigate (1) Do children aged four to eight years in three Early Childhood Education and Care settings explore?; (2) If so, what are their explorations and what effects and affects them?; and (3) Do young children's explorations count as epistemology? Findings indicate that in settings where "free-flow" play characterised practice, four- to five-year-old children engaged in exploration, but its quality was affected by several factors, including variable levels of children's autonomy. Seven- to eight-year-old children in a teacher-directed setting explored less than the four- to five-year-old children, but were frequently observed "off-task", pursuing self-initiated explorations. (Contains 2 tables and 2 figures.)
SN - 0300-4430, 0300-4430
UR - http://search.proquest.com/docview/1322247108?accountid=14701
L2 - http://sfx.scholarsportal.info/ottawa?url_ver=Z39.88-2004&rft_val_fmt=info:ofi/fmt:kev:mtx:journal&genre=article&sid=ProQ:ProQ%3Aericshell&atitle=Young+Children%27s+Explorations%3A+Young+Children%27s+Research%3F&title=Early+Child+Development+and+Care&issn=03004430&date=2012-01-01&volume=182&issue=9&spage=1209&au=Murray%2C+Jane&isbn=&jtitle=Early+Child+Development+and+Care&btitle=&rft_id=info:eric/EJ993789
ER -

TY - JOUR
ID - 2083
T1 - Influence of Previous Knowledge, Language Skills and Domain-Specific Interest on Observation Competency
A1 - Kohlhauf,Lucia
A1 - Rutke,Ulrike
A1 - Neuhaus,Birgit
Y1 - 2011/10//
KW - ERIC
KW - Unsupervised Play Addition
KW - ERIC,Current Index to Journals in Education (CIJE)
KW - Elementary Secondary Education
KW - Play
KW - Competence
KW - Language Skills
KW - Research Methodology
KW - Higher Education
KW - Observation
KW - CHILDREN
KW - English (Second Language)
KW - Biological Sciences
KW - Scientific Methodology
KW - Sciences
KW - Age Differences
RP - NOT IN FILE
SP - 667
EP - 678
JF - Journal of Science Education and Technology
VL - 20

IS - 5
PB - Springer. 233 Spring Street, New York, NY 10013.
N2 - Many epoch-making biological discoveries (e.g. Darwinian Theory) were based upon observations. Nevertheless, observation is often regarded as "just looking" rather than a basic scientific skill. As observation is one of the main research methods in biological sciences, it must be considered as an independent research method and systematic practice of this method is necessary. Because observation skills form the basis of further scientific methods (e.g. experiments or comparisons) and children from the age of 4 years are able to independently generate questions and hypotheses, it seems possible to foster observation competency at a preschool level. To be able to provide development-adequate individual fostering of this competency, it is first necessary to assess each child's competency. Therefore, drawing on the recent literature, we developed in this study a competency model that was empirically evaluated within learners (N = 110) from different age groups, from kindergarten to university. In addition, we collected data on language skills, domain-specific interest and previous knowledge to analyse coherence between these skills and observation competency. The study showed as expected that previous knowledge had a high impact on observation competency, whereas the influence of domain-specific interest was nonexistent. Language skills were shown to have a weak influence. By utilising the empirically validated model consisting of three dimensions ("Describing", "Scientific reasoning" and "Interpreting") and three skill levels, it was possible to assess each child's competency level and to develop and evaluate guided play activities to individually foster a child's observation competency
SN - 1059-0145, 1059-0145
UR - http://search.proquest.com/docview/964172571?accountid=14701
L2 - http://sfx.scholarsportal.info/ottawa?url_ver=Z39.88-2004&rft_val_fmt=info:ofi/fmt:kev:mtx:journal&genre=article&sid=ProQ:ProQ%3Aericshell&atitle=Influence+of+Previous+Knowledge%2C+Language+Skills+and+Domain-Specific+Interest+on+Observation+Competency&title=Journal+of+Science+Education+and+Technology&issn=10590145&date=2011-10-01&volume=20&issue=5&spage=667&au=Kohlhauf%2C+Lucia%3BRutke%2C+Ulrike%3BNeuhaus%2C+Birgit&isbn=&jtitle=Journal+of+Science+Education+and+Technology&btitle=&rft_id=info:eric/EJ941467
ER -

TY - RPRT
ID - 2084
T1 - Evaluation of Breakthrough's "America 2049" Game
A1 - Diamond,James
A1 - Brunner,Cornelia
Y1 - 2011/10//
N1 - Last updated - 2013-07-02
KW - ERIC
KW - Unsupervised Play Addition
KW - Brazil
KW - Australia
KW - Canada
KW - Estonia
KW - France
KW - Germany
KW - India
KW - INDONESIA
KW - Pakistan
KW - Romania
KW - Singapore
KW - United Arab Emirates
KW - United Kingdom
KW - United States
KW - ERIC,Resources in Education (RIE)
KW - Cultural Pluralism
KW - Instructional Effectiveness
KW - Play

KW - World Views
KW - Moral Issues
KW - Critical Thinking
KW - Simulation
KW - Civil Rights
KW - Decision Making
KW - Foreign Countries
KW - Educational Games
KW - Ethnic Diversity
KW - Social Justice
KW - Civics
KW - United States History
KW - Selection
KW - Futures (of Society)
RP - NOT IN FILE
SP - 50
PB - Education Development Center, 55 Chapel Street, Newton, MA 02458-1060.
N2 - Breakthrough, a global human rights organization, produced "America 2049," an alternate-reality game set in a dystopian future in which the United States is on the verge of breaking apart because of an inability to tolerate diversity and promote human rights. During the 12-week game launch, players uncovered artifacts related to the persistent struggle for human rights in American history, while also watching an unfolding narrative about oppression and the loss of human rights in the future. Players could decide whether to support the activities of "Divided We Fall," a group dedicated to preserving national unity, or the "Council on American Heritage," an organization pushing for dissolution. By using a narrative-driven, episodic game (the narrative was released in weekly installments during the 12-week launch), Breakthrough engaged game players on human rights issues and instances of social injustice in a different way. Rather than simply telegraphing positions on issues, game play in "America 2049" permits some level of individual agency by enabling players to decide whether and how to align their in-game personas with opposing factions in the context of an unfolding drama. Based on personal or game-related goals, players can choose to support either side (pro- or anti-human rights) in the conflict without affecting their score. The goal was not to promote "good" or pro-human rights behavior during game play, but rather to encourage players to play with possibilities within a human rights-focused narrative and consider how societal choices about human rights could influence the future. To investigate the game's effectiveness as a tool to mobilize real-world action around the issues confronted in the game, Breakthrough engaged the Education Development Center, Inc. (EDC) as an independent evaluator to evaluate the game. Two research questions framed the evaluation: (1) Do players indicate a willingness to reconsider issues or become active around them following game play? (And when they do indicate willingness, which aspects of the game do they say are influential?); and (2) Is there a relationship between the categories of "moral reasoning" players use in the game and their willingness to reconsider issues? Findings include: (1) 93% (97/104) of survey respondents described their political leanings toward the issues in the game as either "Very liberal," "Liberal," or "Moderate"; (2) 89% (48/54 comments) of the post-game survey comments about the game were positive; (3) 86% (89/104) of players who completed the follow-up survey indicated at least some willingness to become active at some point in the future on an issue they encountered in the game; (4) 58% (60/104) of respondents reported that they played serious games "Never" or "A few times a year"; (5) 47% (49/104) of the survey respondents cited one of two game features as most influential in prompting them to reconsider the issues: the game's overarching narrative and the specific experiences of the non-player characters; and (6) There is no discernible relationship between the specific choices a player makes during game play and her or his willingness to consider becoming active on issues (as indicated on the survey), nor is there a relationship between choices and a player's reported "play style". To continue to capitalize on players' positive experiences during the game launch, Breakthrough may wish to consider the following recommendations for future game development: (1) Consider introducing other game elements that might bring more players "into the fold" earlier; (2) Consider following up with players who did not persist; (3) Structure dialogues about human rights; and (4) Allow players to experience the consequences of game play choices. Appended are: (1) Choice-point rationales; (2) Follow-up survey text (originally online at surveymonkey.com); (3) List of levels and themes/issues addressed in the game narrative; and (4) Main characters, groups, and plot points in "America 2049". (Contains 7 figures and 4 tables.)
UR - http://search.proquest.com/docview/1373088506?accountid=14701

L2 - http://sfx.scholarsportal.info/ottawa?url_ver=Z39.88-2004&rft_val_fmt=info:ofi/fmt:kev:mtx:book&genre=unknown&sid=ProQ:ERIC&atitle=&title=Evaluation+of+Breakthrough%27s+%22America+2049%22+Game&issn=&date=2011-10-01&volume=&issue=&spage=50&au=Diamond%2C+James%3BBrunner%2C+Cornelia&isbn=&jtitle=&btitle=Evaluation+of+Breakthrough%27s+%22America+2049%22+Game&rft_id=info:eric/ED543350
ER -

TY - JOUR
ID - 2085
T1 - "Games Are Made for Fun": Lessons on the Effects of Concept Maps in the Classroom Use of Computer Games
A1 - Charsky,Dennis
A1 - Ressler,William
Y1 - 2011/04//
KW - ERIC
KW - Unsupervised Play Addition
KW - ERIC,Current Index to Journals in Education (CIJE)
KW - Grade 9
KW - Educational Strategies
KW - Instructional Effectiveness
KW - Video Games
KW - Computer Simulation
KW - Class Activities
KW - Play
KW - Instructional Materials
KW - Scaffolding (Teaching Technique)
KW - Control Groups
KW - Educational Games
KW - Concept Mapping
KW - Learning Motivation
KW - Concept Teaching
KW - Student Motivation
RP - NOT IN FILE
SP - 604
EP - 615
JF - Computers & Education
VL - 56
IS - 3
PB - Elsevier. 6277 Sea Harbor Drive, Orlando, FL 32887-4800.
N2 - Does using a computer game improve students' motivation to learn classroom material? The current study examined students' motivation to learn history concepts while playing a commercial, off-the-shelf computer game, Civilization III. The study examined the effect of using conceptual scaffolds to accompany game play. Students from three ninth-grade classrooms were assigned to one of three groups: one group used an expert generated concept map, one group constructed their own concept maps, and a control group used no map. It was predicted that the use of concept maps would enhance the educational value of the game playing activity, in particular students' motivational levels; however, the opposite happened. Students who used a concept map showed lower motivation on the task relative to their baseline motivation for regular classroom instruction. In contrast, the levels of motivation in playing the game, for students in the control group, met or exceeded their levels of motivation during regular classroom instruction. These results suggest that using a conceptual scaffold can decrease students' motivation to learn classroom material through game play, perhaps because conceptual maps can (a) focus students' attention on the difficulty of learning the concepts and on the extrinsic rewards for playing the game and (b) make game play less autonomous, less creative, and less active. All of these can negate the primary property that provides playing its principal potential pedagogical power: fun. (Contains 4 tables and 8 figures.)
SN - 0360-1315, 0360-1315
UR - http://search.proquest.com/docview/851228990?accountid=14701

L2 - http://sfx.scholarsportal.info/ottawa?url_ver=Z39.88-2004&rft_val_fmt=info:ofi/fmt:kev:mtx:journal&genre=article&sid=ProQ:ProQ%3Aericshell&atitle=%22Games+Are+Made+for+Fun%22%3A+Lessons+on+the+Effects+of+Concept+Maps+in+the+Classroom+Use+of+Computer+Games&title=Computers+%26+Education&issn=03601315&date=2011-04-01&volume=56&issue=3&spage=604&au=Charsky%2C+Dennis%3BRessler%2C+William&isbn=&jtitle=Computers+%26+Education&btitle=&rft_id=info:eric/EJ908621
ER -

TY - JOUR
ID - 2086
T1 - The Fun Culture in Seniors' Online Communities
A1 - Nimrod,Galit
Y1 - 2011/04//
KW - ERIC
KW - Unsupervised Play Addition
KW - ERIC,Current Index to Journals in Education (CIJE)
KW - Play
KW - Well Being
KW - Gender Differences
KW - Leisure Time
KW - Social Behavior
KW - Ethnography
KW - Aging (Individuals)
KW - Computer Mediated Communication
KW - Older Adults
KW - Participation
KW - Games
KW - Coping
KW - Interpersonal Relationship
KW - Internet
RP - NOT IN FILE
SP - 226
EP - 237
JF - Gerontologist
VL - 51
IS - 2
PB - Oxford University Press. Great Clarendon Street, Oxford, OX2 6DP, UK. Tel: +44-1865-353907; Fax: +44-1865-353485; e-mail: jnls.cust.serv@oxfordjournals.org; Web site: http://gerontologist.oxfordjournals.org
N2 - Purpose of the study: Previous research found that "fun on line" is the most dominant content in seniors' online communities. The present study aimed to further explore the "fun culture" in these communities and to discover its unique qualities. Design and Methods: The study applied an online ethnography (netnography) approach, utilizing a full year's data from 6 leading seniors' online communities. The final database included about 50,000 posts. Results: The majority of posts were part of online social games, including cognitive, associative, and creative games. The main subjects in all contents were sex, gender differences, aging, grandparenting, politics, faith, and alcohol. Main participatory behaviors were selective timing, using expressive style, and personalization of the online character. Although most participants were "lurkers," the active participants nurtured community norms and relationships, as reflected in the written dialogues. Implications: In a reality of limited alternatives for digital games that meet older adults' needs and interests, seniors found an independent system to satisfy their need for play. Seniors' online communities provided a unique form of casual leisure, whose nature varied among different groups of participants. The fun culture seemed to offer participants many desired benefits, including meaningful play, liminality and communitas, opportunity to practice and demonstrate their abilities, and means for coping with aging. Therefore, it may have positive impact on seniors' well-being and successful aging
SN - 0016-9013, 0016-9013
UR - http://search.proquest.com/docview/864938974?accountid=14701

L2 - http://sfx.scholarsportal.info/ottawa?url_ver=Z39.88-2004&rft_val_fmt=info:ofi/fmt:kev:mtx:journal&genre=article&sid=ProQ:ProQ%3Aericshell&atitle=The+Fun+Culture+in+Seniors%27+Online+Communities&title=Gerontologist&issn=00169013&date=2011-04-01&volume=51&issue=2&spage=226&au=Nimrod%2C+Galit&isbn=&jtitle=Gerontologist&btitle=&rft_id=info:eric/EJ918785
ER -

TY - JOUR
ID - 2087
T1 - Impaired Attention to the Eyes of Attachment Figures and the Developmental Origins of Psychopathy
A1 - Dadds,Mark R.
A1 - Jambrak,Jasmin
A1 - Pasalich,Dave
A1 - Hawes,David J.
A1 - Brennan,John
Y1 - 2011/03//
KW - ERIC
KW - Unsupervised Play Addition
KW - ERIC,Current Index to Journals in Education (CIJE)
KW - Play
KW - Fear
KW - MALES
KW - Mothers
KW - Correlation
KW - Fathers
KW - Behavior Problems
KW - Antisocial Behavior
KW - Sons
KW - Personality Traits
KW - Nonverbal Communication
KW - Recognition (Psychology)
KW - Parent Child Relationship
KW - Human Body
KW - Emotional Response
KW - Psychopathology
KW - Intervals
KW - Evidence
KW - Attachment Behavior
KW - Empathy
RP - NOT IN FILE
SP - 238
EP - 245
JF - Journal of Child Psychology and Psychiatry
VL - 52
IS - 3
PB - Wiley-Blackwell. 350 Main Street, Malden, MA 02148.
N2 - Background: A pervasive failure to attend and respond to emotionally salient stimuli is a core feature of psychopathy. We hypothesise that this begins early in life and is expressed most importantly as a failure to attend to core emotional features (viz., the eyes) of attachment figures. The current study tested whether impaired eye contact is a characteristic of children with antisocial behaviour and callous-unemotional (CU) traits in real life settings. Methods: Conduct problem males were assessed on levels of CU traits and observed in free play and "emotion talk" scenarios with their parents. Eye contact was measured for each dyad (child to mother, child to father, mother to child, father to child) as a proportion of intervals in which the child and parent interacted. Results: Levels of eye contact were reciprocated in mother-son and father-son dyads, but males with high CU traits showed consistent impairments in eye contact towards their parents. Mothers of high CU boys did not show impairments; however, fathers of high CU boys showed similar impairment. Levels of eye contact

were also associated with independent measures of fear recognition, and general empathy in the boys. Conclusions: The findings provide the first evidence that impairments in eye contact, previously shown during computer tasks, characterise psychopathic traits in young males
SN - 0021-9630, 0021-9630
UR - http://search.proquest.com/docview/881455373?accountid=14701
L2 - http://sfx.scholarsportal.info/ottawa?url_ver=Z39.88-2004&rft_val_fmt=info:ofi/fmt:kev:mtx:journal&genre=article&sid=ProQ:ProQ%3Aericshell&atitle=Impaired+Attention+to+the+Eyes+of+Attachment+Figures+and+the+Developmental+Origins+of+Psychopathy&title=Journal+of+Child+Psychology+and+Psychiatry&issn=00219630&date=2011-03-01&volume=52&issue=3&spage=238&au=Dadds%2C+Mark+R.%3BJambrak%2C+Jasmin%3BPasalich%2C+Dave%3BHawes%2C+David+J.%3BBrennan%2C+John&isbn=&jtitle=Journal+of+Child+Psychology+and+Psychiatry&btitle=&rft_id=info:eric/EJ928626
ER -

TY - JOUR
ID - 2088
T1 - Structured Work Systems: Supporting Meaningful Engagement in Preschool Settings for Children with Autism Spectrum Disorders
A1 - Carnahan,Christina
A1 - Harte,Helene
A1 - Schumacher Dyke,Karin
A1 - Hume,Kara
A1 - Borders,Christy
Y1 - 2011/03//
KW - ERIC
KW - Unsupervised Play Addition
KW - ERIC,Current Index to Journals in Education (CIJE)
KW - Preschool Education
KW - Play
KW - Leisure Time
KW - Student Participation
KW - Pervasive Developmental Disorders
KW - Group Activities
KW - Job Skills
KW - Student Needs
KW - Visual Stimuli
KW - Hygiene
KW - Preschool Children
KW - Autism
KW - Time on Task
KW - Teaching Methods
RP - NOT IN FILE
SP - 2
EP - 16
JF - Young Exceptional Children
VL - 14
IS - 1
PB - SAGE Publications. 2455 Teller Road, Thousand Oaks, CA 91320.
N2 - Key characteristics of autism include differences in communication, social interaction, and restricted or repetitive activities and interests (American Psychiatric Association, 2000). Because of these differences, active engagement is challenging for many young children with autism spectrum disorders (ASD). However, such engagement in a variety of activities is crucial for promoting positive future outcomes (Iovannone, Dunlap, Huber, & Kincaid, 2003). This article suggests structured work systems (SWS) as one tool that may help promote engagement for children with ASD in inclusive preschool settings. SWS is a visually structured intervention used to organize a series of activities for individuals with autism (Hume & Odom, 2007; Mesibov, Shea, & Schopler, 2005). SWS addresses individual child needs and promotes participation in the classroom,

home, or community setting. SWS can be used to organize small-group activities, play and leisure activities, academic tasks, personal hygiene activities, household responsibilities, and vocational work jobs. SWS supports independent participation and activity completion and increases on-task behavior for young children with autism (Hume & Odom, 2007). (Contains 2 tables and 3 figures.)
SN - 1096-2506, 1096-2506
UR - http://search.proquest.com/docview/864939825?accountid=14701
L2 - http://sfx.scholarsportal.info/ottawa?url_ver=Z39.88-2004&rft_val_fmt=info:ofi/fmt:kev:mtx:journal&genre=article&sid=ProQ:ProQ%3Aericshell&atitle=Structured+Work+Systems%3A+Supporting+Meaningful+Engagement+in+Preschool+Settings+for+Children+with+Autism+Spectrum+Disorders&title=Young+Exceptional+Children&issn=10962506&date=2011-03-01&volume=14&issue=1&spage=2&au=Carnahan%2C+Christina%3BHarte%2C+Helene%3BSchumacher+Dyke%2C+Karin%3BHume%2C+Kara%3BBorders%2C+Christy&isbn=&jtitle=Young+Exceptional+Children&btitle=&rft_id=info:eric/EJ917323
ER -

TY - JOUR
ID - 2089
T1 - When Is Creativity? Intrinsic Motivation and Autonomy in Children's Artmaking
A1 - Jaquith,Diane B.
Y1 - 2011/01//
KW - ERIC
KW - Unsupervised Play Addition
KW - ERIC,Current Index to Journals in Education (CIJE)
KW - Teachers
KW - Studio Art
KW - Play
KW - Motivation
KW - Incentives
KW - Creativity
KW - Personal Autonomy
KW - Childrens Art
RP - NOT IN FILE
SP - 14
EP - 19
JF - Art Education
VL - 64
IS - 1
PB - National Art Education Association. 1916 Association Drive, Reston, VA 20191.
N2 - The title of this article borrows loosely from the philosopher Nelson Goodman, whose classic essay "When is Art" addresses context and symbolic function. The discussion in this article concerns an entirely different matter: identifying moments when a learner's creativity is sparked in school art programs. The word "creativity" usually enters conversations with students, teachers, parents, and administrators as a generic term for children's overall artistic output. Now school systems are rapidly incorporating 21st-century skills into their curricula, including creativity skills. In order to implement these skills in classrooms, teachers need to know what is and what is not creative work. There is great joy in the surprises that emerge when learners direct their own artmaking. This is the gift found in teaching--to watch learners take control and become independent thinkers and doers. When is creativity "not" in school art programs? Creativity is not found in rote exercises and prescribed assignments. Creativity is compromised by external motivations that supply students with excessive information, reward closed systems, and place undue emphasis on grades and deadlines. Creativity can arise when students respond to visual culture around them, particularly when they can integrate meaningful connections with popular culture into their own work. Creativity abounds when a student thinks divergently, ponders, intuits, perceives, infers, plays, makes mistakes, and embraces ambiguity. Creativity in school art programs thrives when learners are intrinsically motivated and have full autonomy to problem find and solve, defer, revise, redirect, and work at their own pace. In this article, the author lists some strategies that can help encourage creativity during art class. (Contains 7 figures.)
SN - 0004-3125, 0004-3125

UR - http://search.proquest.com/docview/898323559?accountid=14701
L2 - http://sfx.scholarsportal.info/ottawa?url_ver=Z39.88-2004&rft_val_fmt=info:ofi/fmt:kev:mtx:journal&genre=article&sid=ProQ:ProQ%3Aericshell&atitle=When+Is+Creativity%3F+Intrinsic+Motivation+and+Autonomy+in+Children%27s+Artmaking&title=Art+Education&issn=00043125&date=2011-01-01&volume=64&issue=1&spage=14&au=Jaquith%2C+Diane+B.&isbn=&jtitle=Art+Education&btitle=&rft_id=info:eric/EJ935011
ER -

TY - JOUR
ID - 2090
T1 - Can Unpredicted Outcomes Be Intended? The Role of Outcome-Beliefs in Children's Judgments of Intention
A1 - Liao,Yu
A1 - Li,Hong
A1 - Deak,Gedeon O.
Y1 - 2011///
KW - ERIC
KW - Unsupervised Play Addition
KW - ERIC,Current Index to Journals in Education (CIJE)
KW - Experiments
KW - Play
KW - Young Children
KW - Intention
KW - Games
KW - Beliefs
KW - Bias
RP - NOT IN FILE
SP - 106
EP - 117
JF - Cognitive Development
VL - 26
IS - 2
PB - Elsevier. 6277 Sea Harbor Drive, Orlando, FL 32887-4800.
N2 - An adult-like concept of intention includes a deliberate action to achieve a goal and a belief that one's action (if successful) will cause the desired outcome. For example, good outcomes caused by accident or by chance are not believed to be caused intentionally. In two experiments, we asked whether children understand this connection between intentions and outcomes. Children played two games in which actions could produce unintended outcomes (i.e., causes were unplanned). Children sometimes received a desirable reward independent of intention. In Experiment 1, 4- and 5-year-olds mistakenly claimed they had intended the desirable outcome even when it was unexpected. Four-year-olds judged that they had not intended a deliberate action if it did not yield a rewarding outcome. Experiment 2 demonstrates that 6-year-olds seldom make these errors. The results suggest that 4- and 5-year-old children have not yet attained an adult-like concept of intention. Their inaccurate judgments regarding their intentions, given a rewarding yet unexpected outcome, can be explained by a positivity bias
SN - 0885-2014, 0885-2014
UR - http://search.proquest.com/docview/870288748?accountid=14701
L2 - http://sfx.scholarsportal.info/ottawa?url_ver=Z39.88-2004&rft_val_fmt=info:ofi/fmt:kev:mtx:journal&genre=article&sid=ProQ:ProQ%3Aericshell&atitle=Can+Unpredicted+Outcomes+Be+Intended%3F+The+Role+of+Outcome-Beliefs+in+Children%27s+Judgments+of+Intention&title=Cognitive+Development&issn=08852014&date=2011-01-01&volume=26&issue=2&spage=106&au=Liao%2C+Yu%3BLi%2C+Hong%3BDeak%2C+Gedeon+O.&isbn=&jtitle=Cognitive+Development&btitle=&rft_id=info:eric/EJ923271
ER -

TY - JOUR
ID - 2091
T1 - Child-Child Interactions and Positive Social Focus among Preschool Children
A1 - Naerland,Terje
A1 - Martinsen,Harald
Y1 - 2011///
KW - ERIC
KW - Unsupervised Play Addition
KW - Norway
KW - ERIC,Current Index to Journals in Education (CIJE)
KW - Preschool Education
KW - Prediction
KW - Interaction
KW - Play
KW - Observation
KW - Correlation
KW - Peer Relationship
KW - Foreign Countries
KW - Video Technology
KW - Interpersonal Competence
KW - Preschool Children
KW - Social Status
KW - Age Differences
KW - Predictor Variables
RP - NOT IN FILE
SP - 361
EP - 370
JF - Early Child Development and Care
VL - 181
IS - 3
PB - Routledge. , 325 Chestnut Street Suite 800, Philadelphia, PA 19106.
N2 - This study is based on video-recorded observations of 64 children during free play at their nursery. A measure of "social focus" in the preschool, regarded as an indicator of social status, was constructed from the amount of positive and neutral contacts children received from their peers. Only six children often received positive or neutral contacts from their peers. The majority of the children were relatively seldom focused on by the others. A total of 16 contact categories were observed which reflected the content, manner and social function of the contacts. Twelve of the contact categories were significantly correlated with social focus. A "contact score" was constructed based on the child-child contacts that were significantly correlated with social focus. The contact score predicted to a large extent social status assigned to the child by other children, sharing about 60% variance with social focus. Age did not have a significant independent contribution to the prediction of social focus, when the contact score and age were tested together in a variance analysis. This is indicative of a strong relationship between social behaviour and social status among preschool children. The relation between social focus, age and social behaviour is discussed. (Contains 4 tables.)
SN - 0300-4430, 0300-4430
UR - http://search.proquest.com/docview/870284984?accountid=14701
L2 - http://sfx.scholarsportal.info/ottawa?url_ver=Z39.88-2004&rft_val_fmt=info:ofi/fmt:kev:mtx:journal&genre=article&sid=ProQ:ProQ%3Aericshell&atitle=Child-Child+Interactions+and+Positive+Social+Focus+among+Preschool+Children&title=Early+Child+Development+and+Care&issn=03004430&date=2011-01-01&volume=181&issue=3&spage=361&au=Naerland%2C+Terje%3BMartinsen%2C+Harald&isbn=&jtitle=Early+Child+Development+and+Care&btitle=&rft_id=info:eric/EJ922716
ER -

TY - JOUR
ID - 2092
T1 - Language Competence and Social Focus among Preschool Children

A1 - Naerland,Terje
Y1 - 2011///
KW - ERIC
KW - Unsupervised Play Addition
KW - ERIC,Current Index to Journals in Education (CIJE)
KW - Kindergarten
KW - Preschool Education
KW - Measures (Individuals)
KW - Language Acquisition
KW - Play
KW - Language Skills
KW - Scores
KW - Pragmatics
KW - Peer Relationship
KW - Video Technology
KW - Preschool Children
KW - Social Status
KW - Age Differences
RP - NOT IN FILE
SP - 599
EP - 612
JF - Early Child Development and Care
VL - 181
IS - 5
PB - Routledge. , 325 Chestnut Street Suite 800, Philadelphia, PA 19106.
N2 - This study explores how various aspects of language competence are related to social focus among preschoolers. The study presented is based on video-recorded observation of 64 children, aged 11-61 months, during free play at their kindergarten. A measure of social focus in the preschool, regarded as an indicator of social status, was constructed based on the amount of positive and neutral contacts children received from their peers. The majority of the children were relatively seldom focused on by the others. From the transcription of the utterances, three measures of language competence were derived: a score to reflect the child's pragmatic skills, a score to reflect the range of categories the children have available in their characterisations of the world, and a score of mean length of utterance. All measures of language competence predicted social status when in combination with age. However, only the measure of pragmatic skills had an independent contribution to social focus when the variance of age was removed. When language competence is controlled for, age does not predict social status among preschool children. The relation between social focus in the kindergarten, age, and the three measures of language competence is discussed. (Contains 6 tables and 3 figures.)
SN - 0300-4430, 0300-4430
UR - http://search.proquest.com/docview/870283964?accountid=14701
L2 - http://sfx.scholarsportal.info/ottawa?url_ver=Z39.88-2004&rft_val_fmt=info:ofi/fmt:kev:mtx:journal&genre=article&sid=ProQ:ProQ%3Aericshell&atitle=Language+Competence+and+Social+Focus+among+Preschool+Children&title=Early+Child+Development+and+Care&issn=03004430&date=2011-01-01&volume=181&issue=5&spage=599&au=Naerland%2C+Terje&isbn=&jtitle=Early+Child+Development+and+Care&btitle=&rft_id=info:eric/EJ924044
ER -

TY - JOUR
ID - 2093
T1 - Character Toys as Psychological Tools
A1 - Smirnova,Elena O.
Y1 - 2011///
KW - ERIC
KW - Unsupervised Play Addition
KW - Russia (Moscow)
KW - ERIC,Current Index to Journals in Education (CIJE)

KW - Early Childhood Education
KW - Preschool Education
KW - Play
KW - Young Children
KW - psychology
KW - Personality
KW - Mental Health
KW - Criteria
KW - Child Safety
KW - Evaluation Methods
KW - Imagination
KW - Foreign Countries
KW - Self Concept
KW - Ethics
KW - Developmental Stages
KW - Child Development
KW - Role
KW - Toys
RP - NOT IN FILE
SP - 35
EP - 43
JA - International Journal of Early Years Education
VL - 19
IS - 1
PB - Routledge. , 325 Chestnut Street Suite 800, Philadelphia, PA 19106.
N2 - The main characteristic of children's play is its mental aspect--the fact that it is based on thoughts and feelings and not on objective reality. During imaginary play, children go beyond the limits of reality, and toys are tools that help them to do this. Children need character toys--toys that play the role of companion or partner--in the early stages of development of play. Young children (under seven years) are not yet able to establish distance from their playthings and hence identify with dolls and absorb their characteristics. For young children, dolls and soft toys become an "alternative self", and in order to be a "good" psychological tool, they should be open to a child's inner world. "Interactive" toys are quite different from this in that they have an independent life of their own. An observational study of 50 children aged 5 to 5 1/2 showed that although they were intensely interested in an interactive toy, their play was at the level of simple functional play, and that the toy evoked no imaginary involvement and no play storylines, even though the children were clearly capable of these higher levels of play. The Moscow Toy and Game Centre (at the Moscow City University of Psychology and Education) have developed methods for evaluating toys, and the key criteria for evaluation are given here. The first step of evaluation is an "ethical and aesthetic filter" that screens out toys that could be a psychological or pedagogical danger. After this, the central criterion is whether the toy will allow children to project their inner world into it. Other criteria include the balance of detailed and schematised imagery and the tendency of toys (such as interactive toys) to encourage simple manipulation over the development of rich storylines
SN - 0966-9760, 0966-9760
UR - http://search.proquest.com/docview/889924867?accountid=14701
L2 - http://sfx.scholarsportal.info/ottawa?url_ver=Z39.88-2004&rft_val_fmt=info:ofi/fmt:kev:mtx:journal&genre=article&sid=ProQ:ProQ%3Aericshell&atitle=Character+Toys+as+Psychological+Tools&title=International+Journal+of+Early+Years+Education&issn=09669760&date=2011-01-01&volume=19&issue=1&spage=35&au=Smirnova%2C+Elena+O.&isbn=&jtitle=International+Journal+of+Early+Years+Education&btitle=&rft_id=info:eric/EJ933308
ER -

TY - JOUR
ID - 2094
T1 - Children's Leadership Strategies in Early Childhood
A1 - Mawson,Brent
Y1 - 2011///

KW - ERIC
KW - Unsupervised Play Addition
KW - New Zealand
KW - ERIC,Current Index to Journals in Education (CIJE)
KW - Early Childhood Education
KW - Play
KW - Young Children
KW - Individual Characteristics
KW - Gender Differences
KW - Case Studies
KW - Student Participation
KW - Observation
KW - Group Dynamics
KW - Transcripts (Written Records)
KW - Leadership Styles
KW - Performance Factors
KW - Leadership
RP - NOT IN FILE
SP - 327
EP - 338
JF - Journal of Research in Childhood Education
VL - 25
IS - 4
PB - Routledge. , 325 Chestnut Street Suite 800, Philadelphia, PA 19106.
N2 - Young children's leadership is an undertheorized and underresearched topic. Much of the research literature involves paired children working on adult-set tasks that are aimed to provide data on specific aspects of children's collaborative work. This article describes and discusses an investigation into children's leadership styles within independent collaborative play episodes. The research project involved observing children's independent collaborative play in a daylong early childhood setting one morning a week for 5 months. It was an interpretivist study, and the researcher did not participate in any way in the children's play, which was documented using field notes and digital photographs. Although clear gender differences in leadership styles were evident, the predominant boy leader only used assertive strategies, whereas the predominant girl leaders used physical assertive strategies rather than relational strategies to exert their control. It is suggested that individual cultural experiences and contextual factors play a greater part in children's leadership in early childhood education than current gender-focused explanations would suggest. (Contains 2 tables.)
SN - 0256-8543, 0256-8543
UR - http://search.proquest.com/docview/964187226?accountid=14701
L2 - http://sfx.scholarsportal.info/ottawa?url_ver=Z39.88-2004&rft_val_fmt=info:ofi/fmt:kev:mtx:journal&genre=article&sid=ProQ:ProQ%3Aericshell&atitle=Children%27s+Leadership+Strategies+in+Early+Childhood&title=Journal+of+Research+in+Childhood+Education&issn=02568543&date=2011-01-01&volume=25&issue=4&spage=327&au=Mawson%2C+Brent&isbn=&jtitle=Journal+of+Research+in+Childhood+Education&btitle=&rft_id=info:eric/EJ948803
ER -

TY - JOUR
ID - 2095
T1 - Learning to Drive a Wheelchair in Virtual Reality
A1 - Inman,Dean P.
A1 - Loge,Ken
A1 - Cram,Aaron
A1 - Peterson,Missy
Y1 - 2011///
KW - ERIC
KW - Unsupervised Play Addition
KW - ERIC,Current Index to Journals in Education (CIJE)

KW - Program Effectiveness
KW - Computer Simulation
KW - Drills (Practice)
KW - CHILDREN
KW - Computer Uses in Education
KW - Skill Development
KW - Assistive Technology
KW - Memory
KW - Vignettes
KW - Severe Disabilities
KW - Visual Perception
KW - Physical Disabilities
RP - NOT IN FILE
SP - 21
EP - 34
JF - Journal of Special Education Technology
VL - 26
IS - 3
PB - Technology and Media Division of the Council for Exceptional Children. 2900 Crystal Drive Suite 1000, Arlington, VA 22202.
N2 - This research project studied the effect that a technology-based training program, WheelchairNet, could contribute to the education of children with physical disabilities by providing a chance to practice driving virtual motorized wheelchairs safely within a computer-generated world. Programmers created three virtual worlds for training. Scenarios 1 and 2 promoted independent exploration, discovery, cause and effect relationships, and visual memory (skills prerequisite to independent mobility that orthopedically impaired children often lack due to lack of experience. Scenario 3 provided a more structured environment to establish appropriate street-crossing skills on a crosswalk with a traffic light. The project evaluated the children's progress in actual reality. Results from a multivariate repeated measures design suggest all four skills improved as a function of training time. Examination of individual data suggests that children spent most of their time in the first or second worlds in the early stages but, as their skills improved, they began to spend proportionally more time in the third (more difficult) street-crossing program. All those who completed the study showed gains in driving skills as indexed in actual reality. This is significant and supports the contention that children with severe orthopedic disabilities can acquire important functional skills in virtual reality without taking the risks of learning to drive in the real world and the cost of obtaining an actual wheelchair while they learn. (Contains 1 table and 6 figures.)
SN - 0162-6434, 0162-6434
UR - http://search.proquest.com/docview/1361846129?accountid=14701
L2 - http://sfx.scholarsportal.info/ottawa?url_ver=Z39.88-2004&rft_val_fmt=info:ofi/fmt:kev:mtx:journal&genre=article&sid=ProQ:ProQ%3Aericshell&atitle=Learning+to+Drive+a+Wheelchair+in+Virtual+Reality&title=Journal+of+Special+Education+Technology&issn=01626434&date=2011-01-01&volume=26&issue=3&spage=21&au=Inman%2C+Dean+P.%3BLoge%2C+Ken%3BCram%2C+Aaron%3BPeterson%2C+Missy&isbn=&jtitle=Journal+of+Special+Education+Technology&btitle=&rft_id=info:eric/EJ1001790
ER -

TY - THES
ID - 2096
T1 - Games as Artistic Medium: Interfacing Complexity Theory in Game-Based Art Pedagogy
A1 - Patton,Ryan Matthew
N1 - Last updated - 2012-12-14
SubjectsTermNotLitGenreText - 129 11129 2351 2515; 628 5330 4770 4918 5964 9804 9351; 9814; 7940 126; 11255 4270 126; 122 8836; 2035 4270 126; 3206 4270 126; 2041 10666; 10279 625 3150; 625 3150; 10423 4786 6582; 7234; 2340; 3363 10278 8016 4542; 6644 10278 8016 4542; 3268 10669; 2525; 5472 3629 6582
KW - ERIC
KW - Unsupervised Play Addition
KW - ERIC,Resources in Education (RIE)

KW - Elementary Education
KW - Elementary Secondary Education
KW - Middle Schools
KW - Elementary School Students
KW - Studio Art
KW - Video Games
KW - Play
KW - Course Descriptions
KW - Systems Approach
KW - Action Research
KW - Middle School Students
KW - Observation
KW - Computer Literacy
KW - Art Education
KW - Social Systems
KW - Educational Games
KW - Computer Games
KW - Art History
KW - Activity Units
KW - Interviews
KW - Curriculum Implementation
KW - Educational Technology
RP - NOT IN FILE
SP - 258
N2 - Having computer skills, let alone access to a personal computer, has become a necessary component of contemporary Western society and many parts of the world. Digital media literacy involves youth being able to view, participate in, and make creative works with technologies in personal and meaningful ways. Games, defined in this study as structured play, provided the foundation for many of the works from 20th century art movements, such as Dadaism, Surrealism, Situationism, and Fluxus. I argue that these artists used games as methods to explore and expose rules and systems in ways of understanding the world through art. I describe how these artworks embodied complexity thinking in their use of game making methods to expose social, political, economic, and environmental systems. The game-based art pedagogy derived from this art history, also draws from the features of game-like unit operations (Bogost, 2006), strategies and tactics (de Certeau, 1997), and infinite play (Carse, 1987) to foster a critical aesthetic. Complexity thinking (or complexity theory), represents a way for constructing meaning that involves the integration of multiple types of systems, including dynamic models, closed-looped systems, and the ability to transfer one model of a system to another situation or phenomenon. Emergent behavior is supported in the complex systems modeled in video games such as "SimCity" and "Civilization." Much of game-based art pedagogy research centers on students learning by playing games. Learning history or other factual data in the form of games has value, however using games in this way does not encapsulate games as an artistic medium for creative purposes, only as a means for teaching. That is, while students created video games in a variety of classroom environments over the last fifteen years, typically it was done to learn subjects like math, computer science, or to develop language skills. In my action research study, I began with the premise based on my prior teaching experience, that video game creation was an attainable goal by youth, and a valuable studio project in the art classroom to understand complexity in social systems, and learn an art history of games as artworks. I recruited youth (ages of 8-13) and taught them how to make games in four iterations of a game creation course. The make-up of the courses comprised one class of middle school girls, two classes of elementary school children, and one class of middle school boys and girls. Each class met during a five-day course, learning concepts and methods of game development by playing and making physical, board, and video games. New curricular elements for the research included a physical game activity, a mobile game using 2-D barcodes, a tabletop game connecting the video game instruction, and game cards written as independent programmable unit operations. Students made video games that used the concepts of move, avoid, release, and contact (MARC) as a method I designed for exploring complexity thinking. I observed and recorded the participants' game making process; collected their games, journals, and pre and post surveys; and from these observations and feedback, I reviewed and revised the curriculum for each class. I interviewed the other course instructors who used the curriculum that I developed providing additional insight to the pedagogy, delivery of the curriculum, and student learning. Three months after the courses ended, a sample

set of students and parents took part in follow-up interviews regarding the impact of the course. Because games, specifically digital games (also called video games), are seen as potentially corrupting to children, I gathered parental input on their child's involvement. At the center of this study's curriculum, playful, game-like methods were used to create game-based artworks. Students critiqued games using detailed, expressive language to describe how games work, critically aware of how commercial games differ in complexity. From their game making experiences, students gained confidence and knowledge finding game structures in everyday life and how to make programmable media like video games. This study argues that learning through game-based art pedagogy, students begin to understand complexity thinking by producing digital media as a form of artistic expression, and as a form of preparation for future learning in and beyond a 4-12th grade art curriculum. [The dissertation citations contained here are published with the permission of ProQuest LLC. Further reproduction is prohibited without permission. Copies of dissertations may be obtained by Telephone (800) 1-800-521-0600. Web page: http://www.proquest.com/en-US/products/dissertations/individuals.shtml.]
SN - 9781124980409
UR - http://search.proquest.com/docview/1140130376?accountid=14701
L2 - http://sfx.scholarsportal.info/ottawa?url_ver=Z39.88-2004&rft_val_fmt=info:ofi/fmt:kev:mtx:dissertation&genre=unknown&sid=ProQ:ERIC&atitle=&title=Games+as+Artistic+Medium%3A+Interfacing+Complexity+Theory+in+Game-Based+Art+Pedagogy&issn=&date=2011-01-01&volume=&issue=&spage=&au=Patton%2C+Ryan+Matthew&isbn=9781124980409&jtitle=&btitle=&rft_id=info:eric/ED534484
ER -

TY - JOUR
ID - 2098
T1 - Early Social Experience and Individual Differences in Infants' Joint Attention
A1 - Gaffan,Elizabeth A.
A1 - Martins,Carla
A1 - Healy,Sarah
A1 - Murray,Lynne
Y1 - 2010/05//
KW - ERIC
KW - Unsupervised Play Addition
KW - ERIC,Current Index to Journals in Education (CIJE)
KW - Cognitive Development
KW - Play
KW - Depression (Psychology)
KW - Socioeconomic Status
KW - Mothers
KW - Social Experience
KW - Mental Health
KW - Individual Differences
KW - Parent Child Relationship
KW - Researchers
KW - Toys
KW - Neurology
KW - Infants
RP - NOT IN FILE
SP - 369
EP - 393
JF - Social Development
VL - 19
IS - 2
PB - Wiley-Blackwell. 350 Main Street, Malden, MA 02148.
N2 - Fifty-nine healthy infants were filmed with their mothers and with a researcher at two, four, six and nine months in face-to-face play, and in toy-play at six and nine months. During toy-play at both ages, two indices of joint attention (JA)--infant bids for attention, and percent of time in shared attention--were assessed, along with

other behavioural measures. Global ratings were made at all four ages of infants' and mothers' interactive style. The mothers varied in psychiatric history (e.g., half had experienced postpartum depression) and socioeconomic status, so their interactive styles were diverse. Variation in nine-month infant JA--with mother and with researcher--was predicted by variation in maternal behaviour and global ratings at six months, but not at two or four months. Concurrent adult behaviour also influenced nine-month JA, independent of infant ratings. Six-month maternal behaviours that positively predicted later JA (some of which remained important at nine months) included teaching, conjoint action on a toy, and global sensitivity. Other behaviours (e.g., entertaining) negatively predicted later JA. Findings are discussed in terms of social-learning and neurobiological accounts of JA emergence
SN - 0961-205X, 0961-205X
UR - http://search.proquest.com/docview/61797842?accountid=14701
L2 - http://sfx.scholarsportal.info/ottawa?url_ver=Z39.88-2004&rft_val_fmt=info:ofi/fmt:kev:mtx:journal&genre=article&sid=ProQ:ProQ%3Aericshell&atitle=Early+Social+Experience+and+Individual+Differences+in+Infants%27+Joint+Attention&title=Social+Development&issn=0961205X&date=2010-05-01&volume=19&issue=2&spage=369&au=Gaffan%2C+Elizabeth+A.%3BMartins%2C+Carla%3BHealy%2C+Sarah%3BMurray%2C+Lynne&isbn=&jtitle=Social+Development&btitle=&rft_id=info:eric/EJ876357
ER -

TY - JOUR
ID - 2099
T1 - Training, Sharing or Cheating? Gamer Strategies to Get a Digital Upper Hand
A1 - Mortensen,Torill Elvira
Y1 - 2010///
KW - ERIC
KW - Unsupervised Play Addition
KW - Norway
KW - ERIC,Current Index to Journals in Education (CIJE)
KW - Programming
KW - Play
KW - Puzzles
KW - Task Analysis
KW - Fathers
KW - Computer Assisted Design
KW - Information Technology
KW - Foreign Countries
KW - Parent Child Relationship
KW - Internet
KW - Game Theory
KW - Learning Strategies
RP - NOT IN FILE
SP - 79
EP - 89
JF - E-Learning and Digital Media
VL - 7
IS - 1
PB - Symposium Journals. P.O. Box 204, Didcot, Oxford, OX11 9ZQ, UK. Tel: +44-1235-818-062; Fax: +44-1235-817-275; e-mail: subscriptions@symposium-journals.co.uk; Web site: http://www.wwwords.co.uk/elea
N2 - Digital game-players devote a large amount of their time to discovering rules hidden in the code and discoverable through empirical study, experiments, and developing or rediscovering the mathematical formulae governing the code. They do this through their own independent play as they test areas, gear and abilities, through data mining using "add-ons", and through joint efforts outside of the game where they analyse and discuss the results from different gaming situations and create theories on how their game world works. This article shows how gamers are attracted to solving of the code puzzle and share their efforts in sites dedicated to efficient gaming, and also discusses the activity gamers call "theory crafting"--"rule mining"--, in the light of gaming as a learning strategy. (Contains 2 notes.)

SN - 2042-7530, 2042-7530
UR - http://search.proquest.com/docview/742876829?accountid=14701
L2 - http://sfx.scholarsportal.info/ottawa?url_ver=Z39.88-2004&rft_val_fmt=info:ofi/fmt:kev:mtx:journal&genre=article&sid=ProQ:ProQ%3Aericshell&atitle=Training%2C+Sharing+or+Cheating%3F+Gamer+Strategies+to+Get+a+Digital+Upper+Hand&title=E-Learning+and+Digital+Media&issn=20427530&date=2010-01-01&volume=7&issue=1&spage=79&au=Mortensen%2C+Torill+Elvira&isbn=&jtitle=E-Learning+and+Digital+Media&btitle=&rft_id=info:eric/EJ882674
ER -

TY - THES
ID - 2100
T1 - The Effects of Peer-Mediated Intervention in Promoting Social Skills for Children with Disabilities
A1 - Harris,Kathleen I.
N1 - Last updated - 2012-12-14
SubjectsTermNotLitGenreText - 8127 11655 1474 316 8016 4542; 3924 5348 8768; 9738 909; 8299; 7940 126; 5470; 2877; 8099; 5446 1970 1; 9753 5053 2787; 7672 5127; 7676 5449 8768; 10136 5752; 5015; 6250 7912
KW - ERIC
KW - Unsupervised Play Addition
KW - ERIC,Resources in Education (RIE)
KW - Preschool Education
KW - Program Effectiveness
KW - Play
KW - Social Behavior
KW - Mainstreaming
KW - Intervention
KW - Feedback (Response)
KW - Peer Relationship
KW - Interpersonal Competence
KW - Story Telling
KW - Disabilities
KW - Peer Influence
KW - Inclusion
KW - Social Development
KW - Preschool Children
KW - Predictor Variables
RP - NOT IN FILE
SP - 186
N2 - Peer-mediated intervention (PMI), a strategy those working in preschool inclusive environments can use, creates opportunities for peers to assume instructional roles to promote positive social behaviors for children with disabilities. The purpose of the study was threefold: first, to examine peer mediators' use of PMI during baseline and intervention; second, to examine the effects of PMI in increasing positive social behaviors and decreasing negative social behaviors of the target children; and third, to examine whether the social skills for target children improved from baseline to intervention following implementation of PMI. A multiple baseline design across participants was used to examine the effectiveness of PMI with three target children. Six children served as peer mediators and were responsible for implementing PMI strategies. Dependent variables were identified measuring child attributes essential to the social area of development (i.e., positive social behaviors) and those that were not desired or considered socially acceptable (i.e., negative social behaviors). Turn taking was selected as the essential positive social behavior for all child participants. Teacher participants trained peer mediators to administer the independent variable, peer-mediated intervention, specifically peer prompting, peer initiating, and/or giving feedback with the support of a social story during free play. Overall, results of the study indicate peer mediators implemented PMI successfully and with fidelity and little training. Target children increased positive social behaviors and decreased negative social behaviors from baseline to intervention. In addition, target children stayed the same or increased their social skills from baseline (pre) to intervention (post). Findings contribute to the literature regarding the effectiveness of PMI as an evidence-based practice for increasing positive social behaviors and decreasing negative social behaviors in the social area of development

for young children in preschool inclusive settings. [The dissertation citations contained here are published with the permission of ProQuest LLC. Further reproduction is prohibited without permission. Copies of dissertations may be obtained by Telephone (800) 1-800-521-0600. Web page: http://www.proquest.com/en-US/products/dissertations/individuals.shtml.]
SN - 9781124164724
UR - http://search.proquest.com/docview/889930853?accountid=14701
L2 - http://sfx.scholarsportal.info/ottawa?url_ver=Z39.88-2004&rft_val_fmt=info:ofi/fmt:kev:mtx:dissertation&genre=unknown&sid=ProQ:ERIC&atitle=&title=The+Effects+of+Peer-Mediated+Intervention+in+Promoting+Social+Skills+for+Children+with+Disabilities&issn=&date=2010-01-01&volume=&issue=&spage=&au=Harris%2C+Kathleen+I.&isbn=9781124164724&jtitle=&btitle=&rft_id=info:eric/ED522616
ER -

TY - JOUR
ID - 2101
T1 - What Are Our Boundaries and where Can We Play? Perspectives from Eight- to Ten-Year-Old Australian Metropolitan and Rural Children
A1 - MacDougall,Colin
A1 - Schiller,Wendy
A1 - Darbyshire,Philip
Y1 - 2009/02//
KW - ERIC
KW - Unsupervised Play Addition
KW - Australia
KW - ERIC,Current Index to Journals in Education (CIJE)
KW - Childhood Attitudes
KW - Play
KW - Rural Schools
KW - Social Planning
KW - Safety
KW - Freedom
KW - Public Policy
KW - Supervision
KW - Metropolitan Areas
RP - NOT IN FILE
SP - 189
EP - 204
JF - Early Child Development and Care
VL - 179
IS - 2
PB - Routledge. , 325 Chestnut Street Suite 800, Philadelphia, PA 19106.
N2 - This study took place in an inner metropolitan Adelaide school and a rural school on Kangaroo Island off the South Australian coast. We compare 33 eight- to 10-year-old children's accounts of what the area is like for them. What are the rules and boundaries and who sets them? Metropolitan children were found to have tighter boundaries and required adult supervision to use facilities that rural children could use unsupervised. Rural children negotiated freedom of movement by considering broad principles about safety. Findings increase our understanding of how children perceive movement within their communities, and suggest policies and environmental changes to increase freedom of movement. Study findings raise concerns about the way the environment is designed for social planning, and the importance of children's engagement and interaction with the natural environment. (Contains 4 tables and 5 figures.)
SN - 0300-4430, 0300-4430
UR - http://search.proquest.com/docview/61900982?accountid=14701
L2 - http://sfx.scholarsportal.info/ottawa?url_ver=Z39.88-2004&rft_val_fmt=info:ofi/fmt:kev:mtx:journal&genre=article&sid=ProQ:ProQ%3Aericshell&atitle=What+Are+Our+Boundaries+and+where+Can+We+Play%3F+Perspectives+from+Eight-+to+Ten-Year-

Old+Australian+Metropolitan+and+Rural+Children&title=Early+Child+Development+and+Care&issn=03004430&date=2009-02-01&volume=179&issue=2&spage=189&au=MacDougall%2C+Colin%3BSchiller%2C+Wendy%3BDarbyshire%2C+Philip&isbn=&jtitle=Early+Child+Development+and+Care&btitle=&rft_id=info:eric/EJ827304
ER -

TY - JOUR
ID - 2102
T1 - Maternal Scaffolding of Children's Problem Solving and Children's Adjustment in Kindergarten: Hmong Families in the United States
A1 - Stright,Anne Dopkins
A1 - Herr,Maysee Yang
A1 - Neitzel,Carin
Y1 - 2009/02//
KW - ERIC
KW - Unsupervised Play Addition
KW - ERIC,Current Index to Journals in Education (CIJE)
KW - Kindergarten
KW - Prediction
KW - Thinking Skills
KW - Play
KW - Child Behavior
KW - Persistence
KW - Parent Background
KW - Mothers
KW - Educational Attainment
KW - Problem Solving
KW - Scaffolding (Teaching Technique)
KW - Student Adjustment
KW - Parent Child Relationship
KW - Child Rearing
KW - Hmong People
KW - Beliefs
RP - NOT IN FILE
SP - 207
EP - 218
JF - Journal of Educational Psychology
VL - 101
IS - 1
PB - American Psychological Association. Journals Department, 750 First Street NE, Washington, DC 20002-4242.
N2 - The study examined relations between maternal scaffolding of children's problem solving and children's adjustment in kindergarten in Hmong families living in the United States. Mothers and their children (63 dyads) were visited the summer before kindergarten. Mothers' years in the United States, age, education, reasoning skills, and parenting beliefs were assessed. Maternal scaffolding (cognitive support, directiveness of instruction, praise, and criticism) was coded while mothers helped their children with school-like tasks. Children's reasoning skills, conscientiousness, autonomous behavior, and task persistence in kindergarten were reported by teachers at the end of kindergarten (54 children). Maternal cognitive support of children's problem solving predicted children's reasoning skills in kindergarten even after controlling for maternal education and reasoning skills. Maternal directive instruction positively predicted children's conscientious behavior and negatively predicted children's autonomous behaviors after controlling for maternal education and parenting beliefs. (Contains 3 tables.)
SN - 0022-0663, 0022-0663
UR - http://search.proquest.com/docview/61902119?accountid=14701
L2 - http://sfx.scholarsportal.info/ottawa?url_ver=Z39.88-2004&rft_val_fmt=info:ofi/fmt:kev:mtx:journal&genre=article&sid=ProQ:ProQ%3Aericshell&atitle=Maternal

+Scaffolding+of+Children%27s+Problem+Solving+and+Children%27s+Adjustment+in+Kindergarten%3A+Hmong+Families+in+the+United+States&title=Journal+of+Educational+Psychology&issn=00220663&date=2009-02-01&volume=101&issue=1&spage=207&au=Stright%2C+Anne+Dopkins%3BHerr%2C+Maysee+Yang%3BNeitzel%2C+Carin&isbn=&jtitle=Journal+of+Educational+Psychology&btitle=&rft_id=info:eric/EJ829240
ER -

TY - JOUR
ID - 2103
T1 - RESNA Position on the Application of Power Wheelchairs for Pediatric Users
A1 - Rosen,Lauren
A1 - Arva,Julianna
A1 - Furumasu,Jan
A1 - Harris,Michele
A1 - Lange,Michelle L.
A1 - McCarthy,Elisabeth
A1 - Kermoian,Rosanne
A1 - Pinkerton,Heather
A1 - Plummer,Teresa
A1 - Roos,Jodi
A1 - Sabet,Andrina
A1 - Vander Schaaf,Paula
A1 - Wonsettler,Terri
Y1 - 2009///
KW - ERIC
KW - Unsupervised Play Addition
KW - ERIC,Current Index to Journals in Education (CIJE)
KW - Assistive Technology
KW - age
KW - Cognitive Ability
KW - Attitudes
KW - Readiness
KW - Vision
KW - Child Development
KW - Physical Mobility
KW - CHILDREN
KW - Intellectual Development
KW - Child Safety
RP - NOT IN FILE
SP - 218
EP - 226
JF - Assistive Technology
VL - 21
IS - 4
PB - Taylor & Francis, 325 Chestnut Street Suite 800, Philadelphia, PA 19106.
N2 - This document, approved by the Rehabilitation Engineering & Assistive Technology Society of North America (RESNA) Board of Directors in March 2007, shares typical clinical applications and provides evidence from the literature supporting the use of power wheelchairs for children. It is RESNA's position that age, limited vision or cognition, behavioral issues, and the ability to walk or propel a manual wheelchair short distances should not, in and of themselves, be used as discriminatory factors against power mobility (PM) for children. RESNA recommends early utilization of PM for the appropriate candidates as medically necessary to promote psychosocial development, reduce learned helplessness, and facilitate social and educational integration and independence. It is RESNA's position that early utilization of PM for the appropriate child enhances independence, improves psychosocial development, and enables children to grow to become productive and integrated members of society. Without efficient, independent mobility, children may develop learned helplessness and experience delays in both physical and cognitive domains. Mobility should be effortless and

provide children with the opportunity to attend to and fulfill all daily tasks as typically expected from their nondisabled peers. Age, limited vision or cognition, difficulty of accessing controls, parental concerns, and the ability to utilize other means of mobility for very short distances should not, in and of themselves, eliminate the child as a candidate for PM
SN - 1040-0435, 1040-0435
UR - http://search.proquest.com/docview/881462592?accountid=14701
L2 - http://sfx.scholarsportal.info/ottawa?url_ver=Z39.88-2004&rft_val_fmt=info:ofi/fmt:kev:mtx:journal&genre=article&sid=ProQ:ProQ%3Aericshell&atitle=RESNA+Position+on+the+Application+of+Power+Wheelchairs+for+Pediatric+Users&title=Assistive+Technology&issn=10400435&date=2009-01-01&volume=21&issue=4&spage=218&au=Rosen%2C+Lauren%3BArva%2C+Julianna%3BFurumasu%2C+Jan%3BHarris%2C+Michele%3BLange%2C+Michelle+L.%3BMcCarthy%2C+Elisabeth%3BKermoian%2C+Rosanne%3BPinkerton%2C+Heather%3BPlummer%2C+Teresa%3BRoos%2C+Jodi%3BSabet%2C+Andrina%3BVander+Schaaf%2C+Paula%3BWonsettler%2C+Terri&isbn=&jtitle=Assistive+Technology&btitle=&rft_id=info:eric/EJ930338
ER -

TY - JOUR
ID - 2104
T1 - Biochemistry De-Natured--How Unstructured Outdoor Play Can Support Later Learning
A1 - White,Harold B.
Y1 - 2009///
KW - ERIC
KW - Unsupervised Play Addition
KW - ERIC,Current Index to Journals in Education (CIJE)
KW - Elementary Secondary Education
KW - Higher Education
KW - Play
KW - Folk Culture
KW - Biochemistry
KW - Problem Based Learning
KW - Leisure Time
KW - Academic Achievement
KW - Epistemology
KW - Experiential Learning
KW - Relevance (Education)
KW - College Graduates
KW - Informal Education
KW - Low Achievement
KW - Grade Inflation
KW - Outdoor Education
KW - Discovery Learning
RP - NOT IN FILE
SP - 369
EP - 370
JF - Biochemistry and Molecular Biology Education
VL - 37
IS - 6
PB - John Wiley & Sons, Subscription Department, 111 River Street, Hoboken, NJ 07030-5774.
N2 - The concurrent improvement in average grades earned by college graduates would seem to indicate better quality students, although some observers attribute that to grade inflation. From the author's perspective derived from almost four decades of teaching at one institution, students are intellectually as good as ever; however, the author sees a big decrease in their common knowledge and awareness of natural history and the out-of-doors that often provide the context for future learning. A substantial proportion of today's college students spent their free time as children indoors watching television or interacting with a computer screen in air-conditioned comfort during the summer and with central heating in the winter. They grew up separated from what were

everyday experiences a generation or two ago. In this article, the author contends that students' experiences in unsupervised outdoor play in their childhood lay the groundwork for future learning in any science. The author stresses that students would have an easier time with biochemistry if it were not "denatured," and urges teachers to help students make connections between the classroom and the world outside
SN - 1470-8175, 1470-8175
UR - http://search.proquest.com/docview/61821215?accountid=14701
L2 - http://sfx.scholarsportal.info/ottawa?url_ver=Z39.88-2004&rft_val_fmt=info:ofi/fmt:kev:mtx:journal&genre=article&sid=ProQ:ProQ%3Aericshell&atitle=Biochemistry+De-Natured--How+Unstructured+Outdoor+Play+Can+Support+Later+Learning&title=Biochemistry+and+Molecular+Biology+Education&issn=14708175&date=2009-01-01&volume=37&issue=6&spage=369&au=White%2C+Harold+B.&isbn=&jtitle=Biochemistry+and+Molecular+Biology+Education&btitle=&rft_id=info:eric/EJ867632
ER -

TY - JOUR
ID - 2105
T1 - What's Age Gotta Do with It? Understanding the Age-Identities and School-Going Practices of Mexican Immigrant Youth in New York City
A1 - Martinez,Isabel
Y1 - 2009///
KW - ERIC
KW - Unsupervised Play Addition
KW - United States
KW - Mexico
KW - ERIC,Current Index to Journals in Education (CIJE)
KW - Secondary Education
KW - Mexicans
KW - Undocumented Immigrants
KW - Play
KW - Immigration
KW - Immigrants
KW - Role Conflict
KW - Educational Needs
KW - Labor
KW - Adolescent Attitudes
KW - Foreign Countries
KW - Developmental Stages
KW - Education Work Relationship
RP - NOT IN FILE
SP - 34
EP - 48
JF - High School Journal
VL - 92
IS - 4
PB - University of North Carolina Press. 116 South Boundary Street, P.O. Box 2288, Chapel Hill, NC 27515-2288.
N2 - Recent reports of out-of-school immigrant youth have brought attention to predominantly Mexican and Central American immigrant youth who immigrate to the United States and do not enroll in formal schooling (Fry, 2002; Hill and Hayes, 2007). Many arrive to the United States unaccompanied, joining their older, undocumented counterparts in becoming part of the undocumented labor queue (Esquivel, 2007). New York City is one of the more recent destinations for these immigrant youth, with Mexicans leading all immigrant groups in terms of the highest percentage of school-age youth not in school. This article examines how these youth understand their life stages, both pre-immigration in Mexico and post-immigration in New York City, as well as the behaviors and actions they undertake in both contexts that lead to earlier and more rapid transitions

to adulthood across the transnational social space. Most often considering themselves adults, these youth remain outside of formal high schools to meet their own labor and life demands. (Contains 3 footnotes.)
SN - 0018-1498, 0018-1498
UR - http://search.proquest.com/docview/61877296?accountid=14701
L2 - http://sfx.scholarsportal.info/ottawa?url_ver=Z39.88-2004&rft_val_fmt=info:ofi/fmt:kev:mtx:journal&genre=article&sid=ProQ:ProQ%3Aericshell&atitle=What%27s+Age+Gotta+Do+with+It%3F+Understanding+the+Age-Identities+and+School-Going+Practices+of+Mexican+Immigrant+Youth+in+New+York+City&title=High+School+Journal&issn=00181498&date=2009-01-01&volume=92&issue=4&spage=34&au=Martinez%2C+Isabel&isbn=&jtitle=High+School+Journal&btitle=&rft_id=info:eric/EJ837055
ER -

TY - JOUR
ID - 2106
T1 - Holding in Mind Conflicting Information: Pretending, Working Memory, and Executive Control
A1 - Albertson,Kathleen
A1 - Shore,Cecilia
Y1 - 2008/10//
KW - ERIC
KW - Unsupervised Play Addition
KW - ERIC,Current Index to Journals in Education (CIJE)
KW - Early Childhood Education
KW - Play
KW - Young Children
KW - Recall (Psychology)
KW - Identification
KW - Preschool Children
KW - Short Term Memory
RP - NOT IN FILE
SP - 390
EP - 410
JF - Journal of Cognition and Development
VL - 9
IS - 4
PB - Psychology Press. , 325 Chestnut Street Suite 800, Philadelphia, PA 19106.
N2 - Preschoolers' recall of the true and pretend identities of an object in pretense was examined along with a battery of executive functioning and working memory tasks. We expected that children would retain separate identities, as well as a link between them, after observing episodes of pretense, and that memory for pretense would be related to executive functioning and working memory. Children (aged 37-59 months) recalled the true identity of an object better than its pretend identity. Children's recall of at least one identity was correlated with executive functioning and "dual" working memory tasks, independent of age and verbal ability. Memory for both identities was only correlated with executive functioning. The findings are generally supportive of the claim that children form separate representations of the true and pretend identities of objects. The results extend findings of well-established relations between false belief tasks and executive functioning and working memory. (Contains 3 tables.)
SN - 1524-8372, 1524-8372
UR - http://search.proquest.com/docview/61897310?accountid=14701
L2 - http://sfx.scholarsportal.info/ottawa?url_ver=Z39.88-2004&rft_val_fmt=info:ofi/fmt:kev:mtx:journal&genre=article&sid=ProQ:ProQ%3Aericshell&atitle=Holding+in+Mind+Conflicting+Information%3A+Pretending%2C+Working+Memory%2C+and+Executive+Control&title=Journal+of+Cognition+and+Development&issn=15248372&date=2008-10-01&volume=9&issue=4&spage=390&au=Albertson%2C+Kathleen%3BShore%2C+Cecilia&isbn=&jtitle=Journal+of+Cognition+and+Development&btitle=&rft_id=info:eric/EJ827765
ER -

TY - JOUR
ID - 2108
T1 - Learning by Looking: Infants' Social Looking Behavior across the Transition from Crawling to Walking
A1 - Clearfield,Melissa W.
A1 - Osborne,Christine N.
A1 - Mullen,Molly
Y1 - 2008/08//
KW - ERIC
KW - Unsupervised Play Addition
KW - ERIC,Current Index to Journals in Education (CIJE)
KW - Interaction
KW - Physical Activities
KW - Motor Development
KW - Play
KW - Developmental Stages
KW - Visual Stimuli
KW - Psychomotor Skills
KW - Interpersonal Relationship
KW - Age Differences
KW - Social Environment
KW - Infants
RP - NOT IN FILE
SP - 297
EP - 307
JF - Journal of Experimental Child Psychology
JA - J Exp.Child Psychol.
VL - 100
IS - 4
PB - Elsevier. 6277 Sea Harbor Drive, Orlando, FL 32887-4800.
N2 - This study investigated how infants gather information about their environment through looking and how that changes with increases in motor skills. In Experiment 1, 9.5- and 14-month-olds participated in a 10-min free play session with both a stranger and ambiguous toys present. There was a significant developmental progression from passive to active social engagement, as evidenced by younger infants watching others communicate more and older infants making more bids for social interaction. Experiment 2 examined longitudinally the impact of age and walking onset on this progression. The transition to independent walking marked significant changes in how often infants watched others communicate and made active bids for social interaction. Results suggest that infants transition from passive observers as crawlers to active participants in their social environment with the onset of walking. (Contains 4 figures and 1 table.)
SN - 0022-0965, 0022-0965
UR - http://search.proquest.com/docview/61999582?accountid=14701
L2 - http://sfx.scholarsportal.info/ottawa?url_ver=Z39.88-2004&rft_val_fmt=info:ofi/fmt:kev:mtx:journal&genre=article&sid=ProQ:ProQ%3Aericshell&atitle=Learning+by+Looking%3A+Infants%27+Social+Looking+Behavior+across+the+Transition+from+Crawling+to+Walking&title=Journal+of+Experimental+Child+Psychology&issn=00220965&date=2008-08-01&volume=100&issue=4&spage=297&au=Clearfield%2C+Melissa+W.%3BOsborne%2C+Christine+N.%3BMullen%2C+Molly&isbn=&jtitle=Journal+of+Experimental+Child+Psychology&btitle=&rft_id=info:eric/EJ804148
ER -

TY - JOUR
ID - 2109
T1 - The Effects of Praising Qualifying Autoclitics on the Frequency of Reading
A1 - Hubner,M.M.
A1 - Austin,J.
A1 - Miguel,F.C.
Y1 - 2008///

KW - ERIC
KW - Unsupervised Play Addition
KW - Brazil
KW - ERIC,Current Index to Journals in Education (CIJE)
KW - Positive Reinforcement
KW - Verbal Communication
KW - Foreign Countries
KW - Play
KW - Independent Reading
KW - Reading Fluency
KW - Intervention
KW - Reading Motivation
KW - Outcomes of Treatment
RP - NOT IN FILE
SP - 55
EP - 62
JF - Analysis of Verbal Behavior
VL - 24
PB - Association for Behavior Analysis International. 1219 South Park Street, Kalamazoo, MI 49001.
N2 - In the current study, tacts with positive qualifying autoclitics for reading were reinforced in order to determine if this procedure would increase the time spent reading by participants. Participants included 5 children, between 9-10 years old. Participants were individually exposed to 4 free operant sessions during which they were instructed to independently choose play activities. During pre- and post-treatment conditions, no consequences were provided for choosing reading. In addition, 4 treatment sessions were conducted on days separate from the free operant session days, during which the experimenter praised each positive reading-related statement emitted by the participant. Following treatment sessions, 4 out of 5 participants increased the time allocated to reading, suggesting that reading could be increased when praise is delivered contingent upon positive reading-related verbalizations. (Contains 5 figures.)
SN - 0889-9401, 0889-9401
UR - http://search.proquest.com/docview/61806021?accountid=14701
L2 - http://sfx.scholarsportal.info/ottawa?url_ver=Z39.88-2004&rft_val_fmt=info:ofi/fmt:kev:mtx:journal&genre=article&sid=ProQ:ProQ%3Aericshell&atitle=The+Effects+of+Praising+Qualifying+Autoclitics+on+the+Frequency+of+Reading&title=Analysis+of+Verbal+Behavior&issn=08899401&date=2008-01-01&volume=24&issue=&spage=55&au=Hubner%2C+M.+M.%3BAustin%2C+J.%3BMiguel%2C+F.+C.&isbn=&jtitle=Analysis+of+Verbal+Behavior&btitle=&rft_id=info:eric/EJ869174
ER -

TY - JOUR
ID - 2110
T1 - Reciprocity of Prosocial Behavior in Japanese Preschool Children
A1 - Fujisawa,Keiko K.
A1 - Kutsukake,Nobuyuki
A1 - Hasegawa,Toshikazu
Y1 - 2008///
KW - ERIC
KW - Unsupervised Play Addition
KW - Japan
KW - ERIC,Current Index to Journals in Education (CIJE)
KW - Preschool Education
KW - Friendship
KW - Play
KW - Helping Relationship
KW - Child Behavior
KW - Correlation
KW - Preschool Children

KW - Prosocial Behavior
KW - Peer Relationship
RP - NOT IN FILE
SP - 89
EP - 97
JF - International Journal of Behavioral Development
VL - 32
IS - 2
PB - SAGE Publications. 2455 Teller Road, Thousand Oaks, CA 91320.
N2 - This study investigated the reciprocity of prosocial behavior among 3- and 4-year-old Japanese preschool children during free-play time. Matrix correlation tests revealed positive correlations between the frequencies of object offering given and received within dyads and between the frequencies of helping given and received within dyads. These results suggest that young children reciprocate prosocial behavior spontaneously. Positive correlations were also found between the frequencies of object offering and helping behavior exchanged within dyads, suggesting that children exchanged the two types of prosocial behaviors (i.e., "interchanged"). The interchange was independent of both reciprocity within object offering and reciprocity within helping behavior in 4-year-olds. Friends reciprocated object offerings more frequently than non-friends, suggesting that friendship affects the quantitative aspect of reciprocity. These data provide refined evidence of reciprocity among children and also suggest that reciprocity becomes more complicated as children grow older. (Contains 5 tables.)
SN - 0165-0254, 0165-0254
UR - http://search.proquest.com/docview/61956911?accountid=14701
L2 - http://sfx.scholarsportal.info/ottawa?url_ver=Z39.88-2004&rft_val_fmt=info:ofi/fmt:kev:mtx:journal&genre=article&sid=ProQ:ProQ%3Aericshell&atitle=Reciprocity+of+Prosocial+Behavior+in+Japanese+Preschool+Children&title=International+Journal+of+Behavioral+Development&issn=01650254&date=2008-01-01&volume=32&issue=2&spage=89&au=Fujisawa%2C+Keiko+K.%3BKutsukake%2C+Nobuyuki%3BHasegawa%2C+Toshikazu&isbn=&jtitle=International+Journal+of+Behavioral+Development&btitle=&rft_id=info:eric/EJ793689
ER -

TY - JOUR
ID - 2112
T1 - Use of Songs to Promote Independence in Morning Greeting Routines for Young Children with Autism
A1 - Kern,Petra
A1 - Wolery,Mark
A1 - Aldridge,David
Y1 - 2007/08//
KW - ERIC
KW - Unsupervised Play Addition
KW - ERIC,Current Index to Journals in Education (CIJE)
KW - Early Childhood Education
KW - Music Therapy
KW - Play
KW - Inclusive Schools
KW - Young Children
KW - Autism
KW - Singing
KW - Teaching Methods
KW - Peer Relationship
RP - NOT IN FILE
SP - 1264
EP - 1271
JF - Journal of Autism and Developmental Disorders
VL - 37
IS - 7

PB - Springer. 233 Spring Street, New York, NY 10013.
N2 - This study evaluated the effects of individually composed songs on the independent behaviors of two young children with autism during the morning greeting/entry routine into their inclusive classrooms. A music therapist composed a song for each child related to the steps of the morning greeting routine and taught the children's teachers to sing the songs during the routine. The effects were evaluated using a single subject withdrawal design. The results indicate that the songs, with modifications for one child, assisted the children in entering the classroom, greeting the teacher and/or peers and engaging in play. For one child, the number of peers who greeted him was also measured, and increased when the song was used
SN - 0162-3257, 0162-3257
UR - http://search.proquest.com/docview/62052752?accountid=14701
L2 - http://sfx.scholarsportal.info/ottawa?url_ver=Z39.88-2004&rft_val_fmt=info:ofi/fmt:kev:mtx:journal&genre=article&sid=ProQ:ProQ%3Aericshell&atitle=Use+of+Songs+to+Promote+Independence+in+Morning+Greeting+Routines+for+Young+Children+with+Autism&title=Journal+of+Autism+and+Developmental+Disorders&issn=01623257&date=2007-08-01&volume=37&issue=7&spage=1264&au=Kern%2C+Petra%3BWolery%2C+Mark%3BAldridge%2C+David&isbn=&jtitle=Journal+of+Autism+and+Developmental+Disorders&btitle=&rft_id=info:eric/EJ771532
ER -

TY - JOUR
ID - 2113
T1 - "THOMAS" Training: An Early Years Intervention for Children with an Autistic Spectrum Disorder (ASD)
A1 - Medhurst,Belinda
A1 - Beresford,Jayne
Y1 - 2007/03//
KW - ERIC
KW - Unsupervised Play Addition
KW - United Kingdom
KW - ERIC,Current Index to Journals in Education (CIJE)
KW - Early Childhood Education
KW - Program Effectiveness
KW - Play
KW - Young Children
KW - Training
KW - Pervasive Developmental Disorders
KW - Intervention
KW - Skill Development
KW - Antisocial Behavior
KW - Foreign Countries
KW - Interpersonal Competence
KW - Interpersonal Communication
KW - Early Intervention
KW - Program Evaluation
KW - Autism
RP - NOT IN FILE
SP - 1
EP - 17
JF - Educational Psychology in Practice
VL - 23
IS - 1
PB - Routledge. , 325 Chestnut Street Suite 800, Philadelphia, PA 19106.
N2 - The "THOMAS" course (The Hampshire Outline for Meeting the needs of under fives on the Autistic Spectrum) is a four-day training programme to enhance the learning of young children with impairments in social understanding, communication and play by increasing the use of appropriate interventions. It has been supporting families and professionals in the early years settings for over five years and feedback has been exceptionally positive. This article reviews the extent to which training skills have been embedded in the long-

term. Results indicate that the training is still as effective a year on, with many techniques, including visual structure and behaviour management, seen as highly effective interventions. Furthermore, there is an indication that course participants may experience an increase in confidence that enables them to become more independent and generate their own solutions as skills and knowledge become embedded over time. (Contains 6 tables.)
SN - 0266-7363, 0266-7363
UR - http://search.proquest.com/docview/62040365?accountid=14701
L2 - http://sfx.scholarsportal.info/ottawa?url_ver=Z39.88-2004&rft_val_fmt=info:ofi/fmt:kev:mtx:journal&genre=article&sid=ProQ:ProQ%3Aericshell&atitle=%22THOMAS%22+Training%3A+An+Early+Years+Intervention+for+Children+with+an+Autistic+Spectrum+Disorder+%28ASD%29&title=Educational+Psychology+in+Practice&issn=02667363&date=2007-03-01&volume=23&issue=1&spage=1&au=Medhurst%2C+Belinda%3BBeresford%2C+Jayne&isbn=&jtitle=Educational+Psychology+in+Practice&btitle=&rft_id=info:eric/EJ764161
ER -

TY - JOUR
ID - 2114
T1 - A Meta-Analytic Investigation of Therapy Modality Outcomes for Sexually Abused Children and Adolescents: An Exploratory Study
A1 - Hetzel-Riggin,Melanie D.
A1 - Brausch,Amy M.
A1 - Montgomery,Brad S.
Y1 - 2007/02//
KW - ERIC
KW - Unsupervised Play Addition
KW - ERIC,Current Index to Journals in Education (CIJE)
KW - Program Effectiveness
KW - psychology
KW - Group Therapy
KW - Behavior Problems
KW - Outcomes of Treatment
KW - Play Therapy
KW - Symptoms (Individual Disorders)
KW - Counseling Techniques
KW - Psychological Patterns
KW - Meta Analysis
KW - Behavior Modification
KW - Counseling Effectiveness
KW - Cognitive Restructuring
KW - Sexual Abuse
KW - Child Abuse
KW - Adolescents
RP - NOT IN FILE
SP - 125
EP - 141
JF - Child Abuse & Neglect: The International Journal
VL - 31
IS - 2
PB - Elsevier. 6277 Sea Harbor Drive, Orlando, FL 32887-4800.
N2 - Objective: The purpose of the current study was to investigate the independent effects of different treatment elements on a number of secondary problems related to childhood and adolescent sexual abuse, as well as investigate a number of different moderators of treatment effectiveness. Method: Twenty-eight studies that provided treatment outcome results for children and adolescents who had been sexually abused were included in the meta-analysis. Different aspects of psychological treatment, such as specific treatment modalities (individual, cognitive-behavioral, etc.) or secondary problems (behavior problems, psychological distress, etc.) were investigated. Results: The overall mean weighted effect size for the meta-analysis was

d=0.72 (SE=0.02). The results indicate that psychological treatment after childhood or adolescent sexual abuse tended to result in better outcomes than no treatment. There was significant heterogeneity in the effectiveness of the various psychological treatment elements. Play therapy seemed to be the most effective treatment for social functioning, whereas cognitive-behavioral, abuse-specific, and supportive therapy in either group or individual formats was most effective for behavior problems. Cognitive-behavioral, family, and individual therapy seemed to be the most effective for psychological distress, and abuse-specific, cognitive-behavioral, and group therapy appeared to be the most effective for low self-concept. Conclusions: The choice of therapy modality should depend on the child's main presenting secondary problem. Further research should be conducted investigating other possible moderators and secondary problem outcomes
SN - 0145-2134, 0145-2134
UR - http://search.proquest.com/docview/62033316?accountid=14701
L2 - http://sfx.scholarsportal.info/ottawa?url_ver=Z39.88-2004&rft_val_fmt=info:ofi/fmt:kev:mtx:journal&genre=article&sid=ProQ:ProQ%3Aericshell&atitle=A+Meta-Analytic+Investigation+of+Therapy+Modality+Outcomes+for+Sexually+Abused+Children+and+Adolescents%3A+An+Exploratory+Study&title=Child+Abuse+%26+Neglect%3A+The+International+Journal&issn=01452134&date=2007-02-01&volume=31&issue=2&spage=125&au=Hetzel-Riggin%2C+Melanie+D.%3BBrausch%2C+Amy+M.%3BMontgomery%2C+Brad+S.&isbn=&jtitle=Child+Abuse+%26+Neglect%3A+The+International+Journal&btitle=&rft_id=info:eric/EJ755893
ER -

TY - JOUR
ID - 2115
T1 - Learning about Life--Through Books!
A1 - Neuman,Susan B.
Y1 - 2007///
KW - ERIC
KW - Unsupervised Play Addition
KW - ERIC,Current Index to Journals in Education (CIJE)
KW - Teachers
KW - Early Childhood Education
KW - Kindergarten
KW - Preschool Education
KW - Preschool Teachers
KW - Reading Material Selection
KW - Young Children
KW - Dramatic Play
KW - Academic Achievement
KW - Childrens Literature
KW - Written Language
KW - Story Reading
KW - Reader Text Relationship
KW - Reading Aloud to Others
KW - Creative Thinking
KW - Parent Participation
KW - Emergent Literacy
RP - NOT IN FILE
SP - 34
EP - 43
JF - Early Childhood Today
VL - 21
IS - 4
PB - Scholastic. 557 Broadway, New York, NY 10012.
N2 - The early years are a time of joy and a period of great learning for young children. They are beginning to interact with print and experience the delights of being read to. This article presents the experiences of a kindergarten teacher during her class' independent reading time. Aside from just plain fun, children can acquire a wide range of knowledge and skills about the world and about written language. "Why", "how", and "what" is

read to young children has enormous significance in terms of the role books will play in school achievement and in enriching children's lives. The significance of reading books includes the following: (1) building skills; (2) making connections; and (3) supporting creative thinking. A good selection of books from traditional, fantasy, information, poetry and rhyme, predictable, and alphabet is important for young children. This article presents recommendations for reading to children. In addition, choosing books for children at different ages and stages, sharing books age by age, and home-school connection are described. (Contains 5 resources and 4 online resources.)
SN - 1070-1214, 1070-1214
UR - http://search.proquest.com/docview/62047493?accountid=14701
L2 - http://sfx.scholarsportal.info/ottawa?url_ver=Z39.88-2004&rft_val_fmt=info:ofi/fmt:kev:mtx:journal&genre=article&sid=ProQ:ProQ%3Aericshell&atitle=Learning+about+Life--Through+Books%21&title=Early+Childhood+Today&issn=10701214&date=2007-01-01&volume=21&issue=4&spage=34&au=Neuman%2C+Susan+B.&isbn=&jtitle=Early+Childhood+Today&btitle=&rft_id=info:eric/EJ754265
ER -

TY - JOUR
ID - 2116
T1 - Mental Playmates: Siblings, Executive Functioning and Theory of Mind
A1 - McAlister,Anna
A1 - Peterson,Candida C.
Y1 - 2006/11//
KW - ERIC
KW - Unsupervised Play Addition
KW - ERIC,Current Index to Journals in Education (CIJE)
KW - Play
KW - Multiple Regression Analysis
KW - Language Aptitude
KW - Developmental Psychology
KW - Imagination
KW - Cognitive Processes
KW - theory of mind
KW - Interpersonal Communication
KW - Siblings
KW - Preschool Children
KW - Age Differences
KW - Predictor Variables
RP - NOT IN FILE
SP - 733
EP - 751
JF - British Journal of Developmental Psychology
VL - 24
IS - 4
PB - British Psychological Society. St Andrews House, 48 Princess Road East, Leicester, LE1 7DR, UK. Tel: +44-116-254-9568; Fax: +44-116-227-1314; e-mail: enquiry@bps.org.uk; Web site: http://www.bpsjournals.co.uk
N2 - This study assessed the theory of mind (ToM) and executive functioning (EF) abilities of 124 typically developing preschool children aged 3 to 5 years in relation to whether or not they had a child-aged sibling (i.e. a child aged 1 to 12 years) at home with whom to play and converse. On a ToM battery that included tests of false belief, appearance-reality (AR) and pretend representation, children who had at least 1 child-aged sibling scored significantly higher than both only children and those whose only siblings were infants or adults. The numbers of child-aged siblings in preschoolers' families positively predicted their scores on both a ToM battery (4 tasks) and an EF battery (2 tasks), and these associations remained significant with language ability partialled out. Results of a hierarchical multiple regression analysis revealed that independent contributions to individual differences in ToM were made by language ability, EF skill and having a child-aged sibling. However, even though some conditions for mediation were met, there was no statistically reliable evidence that EF skills

mediated the advantage of presence of child-aged siblings for ToM performance. While consistent with the theory that distinctively childish interaction among siblings accelerates the growth of both ToM and EF capacities, alternative evidence and alternative theoretical interpretations for the findings were also considered
SN - 0261-510X, 0261-510X
UR - http://search.proquest.com/docview/964170587?accountid=14701
L2 - http://sfx.scholarsportal.info/ottawa?url_ver=Z39.88-2004&rft_val_fmt=info:ofi/fmt:kev:mtx:journal&genre=article&sid=ProQ:ProQ%3Aericshell&atitle=Mental+Playmates%3A+Siblings%2C+Executive+Functioning+and+Theory+of+Mind&title=British+Journal+of+Developmental+Psychology&issn=0261510X&date=2006-11-01&volume=24&issue=4&spage=733&au=McAlister%2C+Anna%3BPeterson%2C+Candida+C.&isbn=&jtitle=British+Journal+of+Developmental+Psychology&btitle=&rft_id=info:eric/EJ941085
ER -

TY - JOUR
ID - 2118
T1 - Cooperation and Conversations about the Mind: A Study of Individual Differences in 2-Year-Olds and Their Siblings
A1 - Hughes,Claire
A1 - Fujisawa,Keiko K.
A1 - Ensor,Rosie
A1 - Lecce,Serena
A1 - Marfleet,Rachel
Y1 - 2006/03//
KW - ERIC
KW - Unsupervised Play Addition
KW - ERIC,Current Index to Journals in Education (CIJE)
KW - Early Childhood Education
KW - Family Characteristics
KW - Play
KW - Video Technology
KW - theory of mind
KW - Sibling Relationship
KW - Young Children
KW - Individual Differences
KW - Gender Differences
KW - Verbal Ability
KW - Siblings
KW - Developmental Psychology
RP - NOT IN FILE
SP - 53
EP - 72
JF - British Journal of Developmental Psychology
VL - 24
IS - 1
PB - British Psychological Society. St Andrews House, 48 Princess Road East, Leicester, LE1 7DR, UK. Tel: +44-116-254-9568; Fax: +44-116-227-1314; e-mail: enquiry@bps.org.uk; Web site: http://www.bpsjournals.co.uk
N2 - Sibling relationships appear important in fostering young children's growing theory-of-mind skills, but the quality of sibling interactions has rarely been investigated directly in relation to children's mental-state awareness (e.g. as indexed by talk about perceptions, desires, feelings, cognitions, i.e. inner state talk; IST). This study explored individual differences in young siblings' IST in relation to (i) child characteristics (verbal ability and theory-of-mind task performance); (ii) family characteristics (sib-ship size; social disadvantage, maternal well-being); and (iii) the quality of the children's play with their siblings (frequencies of pretence and reciprocal play). A socially diverse sample of 111 2-year-olds was filmed at home playing with a sib (mean age =4.92 years; SD=1.67 years); these sessions were coded from video for reciprocal play and then transcribed and coded for IST and pretence. Verbal ability was assessed for both sibs; in addition, 2-year-olds completed theory-of-

mind tasks. Age and verbal ability were correlated with frequencies of overall talk, IST, pretence and reciprocal play. (Theory-of-mind scores were also correlated with 2-year-olds' IST, but this relation fell below significance once verbal ability was controlled). Significant independent relations were found between IST and children's quality of play, even when age, verbal ability and overall rates of talk were controlled. Although there were no gender contrasts in mean rates or in the variety of IST, gender differences were found in their correlates: in particular, context effects were significantly more important for girls than for boys. Individual differences in children's IST reflect multiple factors, including the dynamic features of children's reciprocal play
SN - 0261-510X, 0261-510X
UR - http://search.proquest.com/docview/964173440?accountid=14701
L2 - http://sfx.scholarsportal.info/ottawa?url_ver=Z39.88-2004&rft_val_fmt=info:ofi/fmt:kev:mtx:journal&genre=article&sid=ProQ:ProQ%3Aericshell&atitle=Cooperation+and+Conversations+about+the+Mind%3A+A+Study+of+Individual+Differences+in+2-Year-Olds+and+Their+Siblings&title=British+Journal+of+Developmental+Psychology&issn=0261510X&date=2006-03-01&volume=24&issue=1&spage=53&au=Hughes%2C+Claire%3BFujisawa%2C+Keiko+K.%3BEnsor%2C+Rosie%3BLecce%2C+Serena%3BMarfleet%2C+Rachel&isbn=&jtitle=British+Journal+of+Developmental+Psychology&btitle=&rft_id=info:eric/EJ941019
ER -

TY - GEN
ID - 2119
T1 - Activity Schedules, Computer Technology, and Teaching Children with Autism Spectrum Disorders
A1 - Stromer,Robert
A1 - Kimball,Jonathan W.
A1 - Kinney,Elisabeth M.
A1 - Taylor,Bridget A.
Y1 - 2006///
N1 - Last updated - 2012-12-14
KW - ERIC
KW - Unsupervised Play Addition
KW - ERIC,Current Index to Journals in Education (CIJE)
KW - Practitioners
KW - Researchers
KW - Elementary Secondary Education
KW - Play
KW - Scheduling
KW - Pictorial Stimuli
KW - Learning Activities
KW - CHILDREN
KW - SOCIALIZATION
KW - Computer Uses in Education
KW - Autism
KW - Skill Development
KW - Communication Skills
RP - NOT IN FILE
SP - 14
EP - 24
JF - Focus on Autism and Other Developmental Disabilities
VL - 21
IS - 1
PB - PRO-ED, 8700 Shoal Creek Boulevard, Austin, TX 78757-6897.
N2 - A review of selected literature suggests that integrating multimedia computer supports with activity schedules can be an effective way to teach students to manage their work, play, and skill-building activities independently. Activity schedules originally were a means of promoting independent execution of previously learned responses by using pictures and words in notebooks or lists to cue a student's performance of a sequence of activities. As activity schedules subsequently became more technologically elaborate, they also evolved as a

means of expanding existing repertoires. Preliminary studies illustrate how activity schedules delivered on the computer may engender new learning via the videos, sounds, dialogue, images, and words employed as instructional stimuli. For the researcher, the blend of computer and notebook activity schedules provides a framework for studies on teaching play, socialization, and communication. For the practitioner, use of activity schedules addresses pressing needs to teach generative and functional skills
UR - http://search.proquest.com/docview/62014949?accountid=14701
L2 - http://sfx.scholarsportal.info/ottawa?url_ver=Z39.88-2004&rft_val_fmt=info:ofi/fmt:kev:mtx:journal&genre=article&sid=ProQ:ProQ%3Aericshell&atitle=Activity+Schedules%2C+Computer+Technology%2C+and+Teaching+Children+with+Autism+Spectrum+Disorders&title=Focus+on+Autism+and+Other+Developmental+Disabilities&issn=10883576&date=2006-01-01&volume=21&issue=1&spage=14&au=Stromer%2C+Robert%3BKimball%2C+Jonathan+W.%3BKinney%2C+Elisabeth+M.%3BTaylor%2C+Bridget+A.&isbn=&jtitle=Focus+on+Autism+and+Other+Developmental+Disabilities&btitle=&rft_id=info:eric/EJ757992
ER -

TY - JOUR
ID - 2120
T1 - Conditioned Observation of Books and Accelerated Acquisition of Textual Responding by Preschool Children
A1 - Tsai,Hshin hui
A1 - Greer,Douglas R.
Y1 - 2006///
KW - ERIC
KW - Unsupervised Play Addition
KW - ERIC,Current Index to Journals in Education (CIJE)
KW - Early Childhood Education
KW - Preschool Education
KW - Play
KW - Conditioning
KW - Responses
KW - Books
KW - Reinforcement
KW - Observation
KW - Toys
KW - Preschool Children
RP - NOT IN FILE
SP - 35
EP - 61
JF - Journal of Early and Intensive Behavior Intervention
VL - 3
IS - 1
PB - Joseph Cautilli, Ph.D. & The Behavior Analyst Online Organization. 535 Queen Street, Philadelphia, PA 19147-3220.
N2 - We report an experiment investigating the effects of conditioning books as reinforcers for observing responses on the learning of textual responses by pre-school children. The independent variable was the acquisition of conditioned reinforcement of observing responses and choice of book stimuli in free play settings where children could choose to play with toys or look at books. Prior to the conditioning procedures, the children played with toys and did not look at books in free time in 4 preconditioning 5-minute free-play sessions. During the treatment we conducted simultaneous stimulus conditioning procedures until looking at books became the preferred free-play activity. The dependent variable consisted of the numbers of learn-units-to mastery of textual responses before and after conditioning books as reinforcers for observing responses. Three boys and one girl (ages 2 years and 9 months to 4 years) participated in the experiment in a pre and post learn-units-to-criterion and simultaneous matched-pairs design with a time-lagged component. Prior to reinforcement conditioning, we matched children in pairs based on learn units they required to master a sets of 5 counterbalanced word sets. One child in each of 2 pairs received book conditioning initially, and another child received an equal number of conditioning trials with toys, as a control conditions, and then book conditioning.

The results showed that (1) all four children required fewer learn-units-to-criterion on textual responses after books were conditioned as reinforcers for choice and observing and those who received the book conditioning first performed best. (2) Three of the four children maintained preference for books at 33%, 83%, and 100% of time in free play probes at 1 month. (3) There were no maintenance effects on accuracy of textual responding. (Contains 2 tables and 6 figures.)
SN - 1554-4893, 1554-4893
UR - http://search.proquest.com/docview/61859823?accountid=14701
ER -

TY - JOUR
ID - 2121
T1 - The Effects of a Stimulus-Stimulus Pairing Procedure on the Acquisition of Conditioned Reinforcement on Observing and Manipulating Stimuli by Young Children with Autism
A1 - Longano,Jennifer M.
A1 - Greer,Douglas R.
Y1 - 2006///
KW - ERIC
KW - Unsupervised Play Addition
KW - ERIC,Current Index to Journals in Education (CIJE)
KW - Play
KW - Conditioning
KW - Child Behavior
KW - Young Children
KW - MALES
KW - Reinforcement
KW - Stimuli
KW - Observation
KW - Intervals
KW - Autism
RP - NOT IN FILE
SP - 62
EP - 80
JF - Journal of Early and Intensive Behavior Intervention
VL - 3
IS - 1
PB - Joseph Cautilli, Ph.D. & The Behavior Analyst Online Organization. 535 Queen Street, Philadelphia, PA 19147-3220.
N2 - In 2 multiple baseline experiments, we tested stimulus-stimulus pairing effects on acquisition of conditioned reinforcement for observing and manipulating stimuli and stereotypy/passivity. In Experiment I we studied a 5 year-old male with autism and we collected data using continuous 5-sec whole interval recording in 5 min sessions in which the student emitted appropriate play, and partial intervals of stereotypy, or passivity. Experiment 2 tested the effects of same procedure on independent work by 2 male participants with autism. The dependent variables were: intervals in which students worked independently, percentage of correct responses, and worksheet completion. Results from both experiments showed significant increases in numbers of intervals students emitted the target behaviors and decreases in stereotypy and passivity. (Contains 5 tables and 6 figures.)
SN - 1554-4893, 1554-4893
UR - http://search.proquest.com/docview/61857702?accountid=14701
ER -

TY - JOUR
ID - 2122
T1 - Kevin: "I Gotta Get to the Market": The Development of Peer Relationships in Inclusive Early Childhood Settings
A1 - Kim,Sonja de Groot
Y1 - 2005/12//

KW - ERIC
KW - Unsupervised Play Addition
KW - ERIC,Current Index to Journals in Education (CIJE)
KW - Early Childhood Education
KW - Inclusive Schools
KW - Young Children
KW - Dramatic Play
KW - Social Adjustment
KW - Mainstreaming
KW - Physical Development
KW - Peer Relationship
KW - Friendship
KW - Verbal Communication
KW - Nonverbal Communication
KW - Paraprofessional School Personnel
KW - Physical Disabilities
RP - NOT IN FILE
SP - 163
EP - 169
JF - Early Childhood Education Journal
VL - 33
IS - 3
PB - Springer. 233 Spring Street, New York, NY 10013.
N2 - This study describes the lived experience of Kevin, a young child with physical disabilities who is placed in an inclusive classroom, a classroom where children with and without disabilities are educated together, with extra support provided for the child with disabilities. Children with disabilities who are placed in inclusive early childhood settings tend to be rejected or neglected more than their typically developing peers and are involved more with the adults in the classroom than with their peers. Kevin's experiences are framed in the context of the current emphasis on inclusive practices. The study documents that adult assistance and curriculum activities both play an important role in either facilitating or constraining social interaction with peers. When the assigned assistant functions as the child's playmate and mostly chooses table activities, both of these seem to limit opportunities for social interaction with peers. This study also documents that, without the assistant present, the child makes independent choices such as where and with whom to play. The dramatic play area serves as a conduit for meaningful verbal and nonverbal communication with peers. At the same time, Kevin's strong desire to play in this area seems to be the impetus for physical development--he walks alone for the very first time. Suggestions are given for teacher facilitation of dramatic play
SN - 1082-3301, 1082-3301
UR - http://search.proquest.com/docview/62090674?accountid=14701
L2 - http://sfx.scholarsportal.info/ottawa?url_ver=Z39.88-2004&rft_val_fmt=info:ofi/fmt:kev:mtx:journal&genre=article&sid=ProQ:ProQ%3Aericshell&atitle=Kevin%3A+%22I+Gotta+Get+to+the+Market%22%3A+The+Development+of+Peer+Relationships+in+Inclusive+Early+Childhood+Settings&title=Early+Childhood+Education+Journal&issn=10823301&date=2005-12-01&volume=33&issue=3&spage=163&au=Kim%2C+Sonja+de+Groot&isbn=&jtitle=Early+Childhood+Education+Journal&btitle=&rft_id=info:eric/EJ747223
ER -

TY - RPRT
ID - 2123
T1 - Behavioral and Social Effects of Inclusion at the Preschool Level: Exploring an Integrated Early Childhood Classroom
A1 - Brennan,Heather R.
Y1 - 2005/07//
N1 - Last updated - 2012-12-14
KW - ERIC
KW - Unsupervised Play Addition
KW - ERIC,Resources in Education (RIE)

KW - Preschool Education
KW - Inclusive Schools
KW - Play
KW - Young Children
KW - Mainstreaming
KW - Early Childhood Education
KW - Educational History
KW - Skill Development
KW - Regular and Special Education Relationship
KW - Peer Relationship
KW - Disabilities
KW - Social Development
KW - Student Behavior
KW - Modeling (Psychology)
RP - NOT IN FILE
SP - 49
N2 - The move to a more integrated setting for preschool classrooms has become a primary choice in the education of young children. The current research suggests that inclusion at the preschool level has a social impact on the behaviors of young children. Studies examining child play preference and attitudes towards children with disabilities have encouraged more research on inclusive preschool classrooms from a social development perspective. This study focuses on the types of behaviors and and social play initiated by general education students in an integrated preschool setting. The setting for this study is a inclusive classroom where the day is divided between inclusion and independent class time. An analysis of data collected through observations in a preschool classroom indicated that those behaviors initiated by general education students were typically cooperative in nature with a higher frequency of modeling behavior occurring during those interactions initiated by a general education student towards a classmate with special needs. This research project is the first stage in an avenue of research focusing on the field of early childhood inclusion and the effects of environment on student social development. Appended are: (1) Parental?Assent?Form; (2) Social and Behavioral Interactions Preschool Inclusive Classroom Observation Log; (3) Survey?on?Preschool?Inclusion; and (4) Data Analysis-Tally Sheet/Frequency of Behaviors. (Contains 2 tables.) [M.S. Capstone Project, Keuka College.]
UR - http://search.proquest.com/docview/62082546?accountid=14701
ER -

TY - JOUR
ID - 2124
T1 - Peer-Mediated Treatment of Socially Withdrawn Maltreated Preschool Children: Cultivating Natural Community Resources
A1 - Fantuzzo,John
A1 - Manz,Patricia
A1 - Atkins,Marc
A1 - Meyers,Raymond
Y1 - 2005/05/01/
KW - ERIC
KW - Unsupervised Play Addition
KW - ERIC,Current Index to Journals in Education (CIJE)
KW - Early Childhood Education
KW - Peer Mediation
KW - Program Effectiveness
KW - Play
KW - Classroom Environment
KW - Community Resources
KW - Withdrawal (Psychology)
KW - Disadvantaged Youth
KW - Intervention
KW - Preschool Children

RP - NOT IN FILE
SP - 320
EP - 325
JF - Journal of Clinical Child and Adolescent Psychology
VL - 34
IS - 2
PB - Lawrence Erlbaum Associates, , Journal Subscription Department, 10 Industrial Avenue, Mahwah, NJ 07430-2262.
N2 - This study evaluated the effectiveness of Resilient Peer Treatment (RPT). This is a peer-mediated, classroom-based intervention for socially withdrawn, maltreated preschool children. It examined whether the RPT impact generalized from the treatment setting to larger classroom context. Eighty-two maltreated and nonmaltreated, socially withdrawn Head Start children were randomly assigned to either RPT or attention-control (AC) conditions. Data were collected by teachers and independent observers blind to both maltreatment status and treatment condition. Treatment resulted in higher levels of collaborative peer play interactions in the treatment setting posttreatment for both the maltreated and nonmaltreated children. Results documented generalization of the treatment impact to classroom free-play sessions. These findings were supported by teacher ratings of interactive peer play and social skills
SN - 1537-4416, 1537-4416
UR - http://search.proquest.com/docview/62140615?accountid=14701
L2 - http://sfx.scholarsportal.info/ottawa?url_ver=Z39.88-2004&rft_val_fmt=info:ofi/fmt:kev:mtx:journal&genre=article&sid=ProQ:ProQ%3Aericshell&atitle=Peer-Mediated+Treatment+of+Socially+Withdrawn+Maltreated+Preschool+Children%3A+Cultivating+Natural+Community+Resources&title=Journal+of+Clinical+Child+and+Adolescent+Psychology&issn=15374416&date=2005-05-01&volume=34&issue=2&spage=320&au=Fantuzzo%2C+John%3BManz%2C+Patricia%3BAtkins%2C+Marc%3BMeyers%2C+Raymond&isbn=&jtitle=Journal+of+Clinical+Child+and+Adolescent+Psychology&btitle=&rft_id=info:eric/EJ684201
ER -

TY - JOUR
ID - 2125
T1 - Dramatic Playing beyond the Theory of Multiple Intelligences
A1 - Guss,Faith Gabrielle
Y1 - 2005/02/01/
KW - ERIC
KW - Unsupervised Play Addition
KW - ERIC,Current Index to Journals in Education (CIJE)
KW - social life
KW - Play
KW - Dramatic Play
KW - Child Development
KW - Preschool Children
KW - Multiple Intelligences
KW - Developmental Psychology
RP - NOT IN FILE
SP - 43
EP - 54
JF - Research in Drama Education
VL - 10
IS - 1
PB - Customer Services for Taylor & Francis Group Journals, 325 Chestnut Street, Suite 800, Philadelphia, PA 19106.
N2 - Related to aspects of drama and theatre education, I search beyond the findings about symbolic play set forth by Dr Howard Gardner in "Frames of mind. The theory of multiple intelligences". Despite the inspiration for and solidarity with arts educators that emanate from his theory, I sensed that it did not provide a full picture of the complex aesthetic and reflective skills manifested by young children in their playing. I refer to symbolic

play as "play-drama", viewing it as autonomous drama performed in the socio-cultural context of children's collective playing. The individual child-orientation in Gardner's developmental psychology study does not address the child-cultural dimension. Yet there is much to be learned for arts educators about the significance for the children of their cultural-aesthetic ways of being together. At the conclusion of the research, I view the capacity for expert dramatic playing as an executive capacity, a dramatic intelligence, which coordinates the deployment of the multiple artistic symbols that constitute play-drama. Children's skills go beyond what Gardner proposes as interpersonal and intrapersonal. The skills can be compared with those of a first-draft auteur, who masters the artistic positions and form-languages of a dramatist, dramaturg, director, actor, dancer/choreographer, storyteller, musician/composer, scenographer, props person, light designer and sound designer. In their aesthetic social life, play-ensembles enter and exit the drama-cultural mode on and off throughout the long hours in day-care
SN - 1356-9783, 1356-9783
UR - http://search.proquest.com/docview/61831420?accountid=14701
L2 - http://sfx.scholarsportal.info/ottawa?url_ver=Z39.88-2004&rft_val_fmt=info:ofi/fmt:kev:mtx:journal&genre=article&sid=ProQ:ProQ%3Aericshell&atitle=Dramatic+Playing+beyond+the+Theory+of+Multiple+Intelligences&title=Research+in+Drama+Education&issn=13569783&date=2005-02-01&volume=10&issue=1&spage=43&au=Guss%2C+Faith+Gabrielle&isbn=&jtitle=Research+in+Drama+Education&btitle=&rft_id=info:eric/EJ691398
ER -

TY - JOUR
ID - 2126
T1 - Inquiry-Based Environments for the Inclusion of Students With Exceptional Learning Needs
A1 - Rapp,Whitney H.
Y1 - 2005///
KW - ERIC
KW - Unsupervised Play Addition
KW - ERIC,Current Index to Journals in Education (CIJE)
KW - Play
KW - Educational Environment
KW - Inclusive Schools
KW - Scaffolding (Teaching Technique)
KW - Independent Study
KW - Special Needs Students
KW - Parent School Relationship
KW - Museums
KW - Parent Participation
KW - Teaching Methods
KW - Inquiry
RP - NOT IN FILE
SP - 296
EP - 310
JF - Remedial & Special Education
VL - 26
IS - 5
PB - PRO-ED, , 8700 Shoal Creek Boulevard, Austin, TX 78757-6897.
N2 - A qualitative study of children's museums' successful inquiry-based learning environments is described, focusing on four students with various exceptional learning needs. Benefits for the students in terms of scaffolded instruction, meaningful and contextualized activities, self-regulated learning, the establishment of learning communities, play, and parental involvement are noted. A discussion of the generalization of learning to the classroom and of positive attitudes and children's museums as inclusive, supplemental environments is included. Implications for future practice involve school-museum partnerships and individualized programming for all learners
SN - 0741-9325, 0741-9325
UR - http://search.proquest.com/docview/62147792?accountid=14701

L2 - http://sfx.scholarsportal.info/ottawa?url_ver=Z39.88-2004&rft_val_fmt=info:ofi/fmt:kev:mtx:journal&genre=article&sid=ProQ:ProQ%3Aericshell&atitle=Inquiry-Based+Environments+for+the+Inclusion+of+Students+With+Exceptional+Learning+Needs&title=Remedial+%26+Special+Education&issn=07419325&date=2005-01-01&volume=26&issue=5&spage=296&au=Rapp%2C+Whitney+H.&isbn=&jtitle=Remedial+%26+Special+Education&btitle=&rft_id=info:eric/EJ722324
ER -

TY - JOUR
ID - 2127
T1 - Walking and Eating Behavior of Toddlers at 12 Months Old
A1 - Koda,Naoko
A1 - Akimoto,Yuko
A1 - Hirose,Toshiya
A1 - Hinobayashi,Toshihiko
A1 - Minami,Tetsuhiro
Y1 - 2004/12//
KW - ERIC
KW - Unsupervised Play Addition
KW - ERIC,Current Index to Journals in Education (CIJE)
KW - Early Childhood Education
KW - Eating Habits
KW - Physical Activities
KW - Toddlers
KW - Play
KW - Foreign Countries
KW - Gender Differences
KW - Nursery Schools
RP - NOT IN FILE
SP - 9
JF - Early Child Development and Care
VL - 174
IS - 7
PB - Customer Services for Taylor & Francis Group Journals, 325 Chestnut Street, Suite 800, Philadelphia, PA 19106.
N2 - Locomotive and eating behavior of 52 toddlers was observed at 12 months old in a nursery school and investigated in relation to the acquisition of independent walking. The toddlers who acquired walking ate more by themselves using the hands than the toddlers who did not start walking. This suggested that acquisition of walking was associated with eating behavior. However, onset of walking did not immediately influence the toddlers' interest in other persons or skilful ability to use tools. Sex differences in the toddlers' behavior were minor, but the teachers showed sex bias to the toddlers' behavior
SN - 0300-4430, 0300-4430
UR - http://search.proquest.com/docview/62126120?accountid=14701
L2 - http://sfx.scholarsportal.info/ottawa?url_ver=Z39.88-2004&rft_val_fmt=info:ofi/fmt:kev:mtx:journal&genre=article&sid=ProQ:ProQ%3Aericshell&atitle=Walking+and+Eating+Behavior+of+Toddlers+at+12+Months+Old&title=Early+Child+Development+and+Care&issn=03004430&date=2004-12-01&volume=174&issue=7&spage=9&au=Koda%2C+Naoko%3BAkimoto%2C+Yuko%3BHirose%2C+Toshiya%3BHinobayashi%2C+Toshihiko%3BMinami%2C+Tetsuhiro&isbn=&jtitle=Early+Child+Development+and+Care&btitle=&rft_id=info:eric/EJ681509
ER -

TY - JOUR
ID - 2128
T1 - Psychometric Properties of the STAT for Early Autism Screening
A1 - Stone,Wendy L.

A1 - Coonrod,Elaine E.
A1 - Turner,Lauren M.
A1 - Pozdol,Stacie L.
Y1 - 2004/12//
KW - ERIC
KW - Unsupervised Play Addition
KW - ERIC,Current Index to Journals in Education (CIJE)
KW - Verbal Communication
KW - Measures (Individuals)
KW - Play
KW - Young Children
KW - Imitation
KW - Diagnostic Tests
KW - Interrater Reliability
KW - Autism
KW - Psychometrics
KW - Screening Tests
RP - NOT IN FILE
SP - 691
EP - 701
JF - Journal of Autism and Developmental Disorders
VL - 34
IS - 6
PB - Springer. 233 Spring Street, New York, NY 10013.
N2 - The STAT is an interactive screening measure for autism that assesses behaviors in the areas of play, communication, and imitation skills. In Study 1, signal detection procedures were employed to identify a cutoff score for the STAT using developmentally matched groups of 2-year-old children with autism and with nonspectrum disorders. The resulting cutoff yielded high sensitivity, specificity, and predictive values for the development sample as well as for an independent validation sample. Study 2 examined psychometric properties of the STAT and revealed acceptable levels of interrater agreement, test-retest reliability, and agreement between STAT risk category and ADOS-G classification. The STAT demonstrates strong psychometric properties and shows promising utility as a Level 2 screening measure for autism
SN - 0162-3257, 0162-3257
UR - http://search.proquest.com/docview/62075385?accountid=14701
L2 - http://sfx.scholarsportal.info/ottawa?url_ver=Z39.88-2004&rft_val_fmt=info:ofi/fmt:kev:mtx:journal&genre=article&sid=ProQ:ProQ%3Aericshell&atitle=Psychometric+Properties+of+the+STAT+for+Early+Autism+Screening&title=Journal+of+Autism+and+Developmental+Disorders&issn=01623257&date=2004-12-01&volume=34&issue=6&spage=691&au=Stone%2C+Wendy+L.%3BCoonrod%2C+Elaine+E.%3BTurner%2C+Lauren+M.%3BPozdol%2C+Stacie+L.&isbn=&jtitle=Journal+of+Autism+and+Developmental+Disorders&btitle=&rft_id=info:eric/EJ735531
ER -

TY - JOUR
ID - 2130
T1 - The Effects of the Webster-Stratton Parenting Program on Maltreating Families: Fostering Strengths
A1 - Hughes,J.R.
A1 - Gottlieb,L.N.
Y1 - 2004/10//
KW - ERIC
KW - Unsupervised Play Addition
KW - ERIC,Current Index to Journals in Education (CIJE)
KW - Parent Education
KW - Parenting Skills
KW - Control Groups
KW - Play

KW - Home Visits
KW - Mothers
KW - Child Rearing
RP - NOT IN FILE
SP - 1081
EP - 1097
JF - Child Abuse and Neglect: The International Journal
VL - 28
IS - 10
PB - Elsevier Customer Service Department, 6277 Sea Harbor Drive, Orlando, FL 32887-4800.
N2 - Objective:: This study examined the effects of the Webster-Stratton parenting program on the parenting skills of maltreating mothers and on the autonomy of their children (3-8 years). Method:: A randomized controlled trial was used. Twenty-six maltreating families were randomly assigned to one of two conditions: the 16-hour weekly intervention group, or the 4-month wait list control group. Pre- and post-intervention independent assessments included a 2-hour home visit involving videotaped mother-child interactions during two prescribed, 10-minute play activities. Results:: Compared to the control group, treatment mothers demonstrated significant improvement in involvement and marginally significant improvement in autonomy-support, but no improvement in structure. Treatment group children showed no significant improvement in autonomy when compared to control group children. Conclusions:: This parenting program proved effective with maltreating parents. The lack of demonstrated effect on children may reflect the need for a larger and more sustained treatment dose and/or the need to include parent-child interaction opportunities in the program. At the same time, while treatment gains were limited to the parent, the high treatment adherence rate (92% attended six or more of the eight program sessions) and low attrition rate (n=1) indicate that the treatment gains may hold potential for more thorough examination
SN - 0145-2134, 0145-2134
UR - http://search.proquest.com/docview/62123793?accountid=14701
L2 - http://sfx.scholarsportal.info/ottawa?url_ver=Z39.88-2004&rft_val_fmt=info:ofi/fmt:kev:mtx:journal&genre=article&sid=ProQ:ProQ%3Aericshell&atitle=The+Effects+of+the+Webster-Stratton+Parenting+Program+on+Maltreating+Families%3A+Fostering+Strengths&title=Child+Abuse+and+Neglect%3A+The+International+Journal&issn=01452134&date=2004-10-01&volume=28&issue=10&spage=1081&au=Hughes%2C+J.R.%3BGottlieb%2C+L.N.&isbn=&jtitle=Child+Abuse+and+Neglect%3A+The+International+Journal&btitle=&rft_id=info:eric/EJ698085
ER -

TY - JOUR
ID - 2131
T1 - "Flexible Control": Towards a Conception of Personal Autonomy for Postmodern Education
A1 - Aviram,Roni
A1 - Yonah,Yossi
Y1 - 2004/02//
KW - ERIC
KW - Unsupervised Play Addition
KW - ERIC,Current Index to Journals in Education (CIJE)
KW - Play
KW - Postmodernism
KW - Educational Objectives
KW - Games
KW - Democracy
KW - Personal Autonomy
KW - Self Control
KW - Instructional Design
KW - Educational Philosophy
RP - NOT IN FILE
SP - 3
EP - 17

JF - Educational Philosophy and Theory
VL - 36
IS - 1
PB - Wiley-Blackwell. 350 Main Street, Malden, MA 02148.
N2 - This article offers a way to salvage the ideal of the autonomous person from the predicament besetting it and to reclaim it as a worthy and respectable ideal. Carefully maneuvering around this ideal, jettisoning its obsolete qualities while reaffirming its sound ones, the authors offer outlines for a conception of personal autonomy suitable for postmodern democratic education. A key concept which is introduced and developed in this article is that of "flexible control". The authors show that this concept can assist in avoiding the pitfalls encumbering the traditional concept of personal autonomy, while allowing to salvage its propitious aspects for use in postmodern democratic education. This concept has three main features: (1) it recognizes the capacity of the agent to exercise different levels of control; (2) these levels do not indicate distinct and closed phases, but are placed on a continuous temporal axis; and (3) the agent may shift from a minimal level of control, or from absence of control, to a maximal level of control, as the circumstances may decree. The authors argue that when the necessary qualifications are made, the ideal of the autonomous person and the idea of self-control associated with it can, firstly, accommodate the previously mentioned and well-grounded concerns, and secondly, be unproblematically adopted as a legitimate goal of education. (Contains 1 table and 8 notes.)
SN - 0013-1857, 0013-1857
UR - http://search.proquest.com/docview/1140145711?accountid=14701
L2 - http://sfx.scholarsportal.info/ottawa?url_ver=Z39.88-2004&rft_val_fmt=info:ofi/fmt:kev:mtx:journal&genre=article&sid=ProQ:ProQ%3Aericshell&atitle=%22Flexible+Control%22%3A+Towards+a+Conception+of+Personal+Autonomy+for+Postmodern+Education&title=Educational+Philosophy+and+Theory&issn=00131857&date=2004-02-01&volume=36&issue=1&spage=3&au=Aviram%2C+Roni%3BYonah%2C+Yossi&isbn=&jtitle=Educational+Philosophy+and+Theory&btitle=&rft_id=info:eric/EJ980314
ER -

TY - BOOK
ID - 2132
T1 - Environmental Print in the Classroom: Meaningful Connections for Learning to Read
A1 - Prior,Jennifer
A1 - Gerard,Maureen R.
Y1 - 2004///
N1 - Last updated - 2012-12-14
KW - ERIC
KW - Unsupervised Play Addition
KW - ERIC,Resources in Education (RIE)
KW - Kindergarten
KW - Curriculum Development
KW - Play
KW - Reading Instruction
KW - Educational Games
KW - Child Development
KW - Emergent Literacy
KW - Relevance (Education)
KW - Early Childhood Education
KW - Student Evaluation
KW - Media Specialists
KW - Literacy Education
RP - NOT IN FILE
SP - 144
PB - International Reading Association, Headquarters Office, 800 Barksdale Rd., P.O. Box 8139, Newark, DE 19714-8139.
N2 - Find out how environmental print can be used as an instructional literacy tool in the kindergarten classroom. This book: (1) Describes games, activities, and centers that use environmental print; (2) Shows how to use environmental print to create a curriculum and assess students print awareness and sight word vocabulary;

and (3) Gives recommendations for helping parents assist their children in learning to read using environmental print. Although kindergarten students are the focus of the book, ideas for preschoolers and for more advanced learners can also be found. Following a foreword, the following chapters are included: (1) "Early Literacy Development and Environmental Print: Making the Connection"; (2) "Play and Environmental Print in the Early Years"; (3) "Implementing an Environmental Print Curriculum"; (4) "Games and Activities for Independent Use"; (5) "Assessment and Evaluation: Documenting Development"; and (6) "Getting Started: Recommendations for Teachers and Parents"; Appended are: (1) "Description of Authors' Studies"; and (2) "Survey and Survey Results", followed by references and an index
SN - 0872075532
UR - http://search.proquest.com/docview/62115544?accountid=14701
L2 - http://sfx.scholarsportal.info/ottawa?url_ver=Z39.88-2004&rft_val_fmt=info:ofi/fmt:kev:mtx:book&genre=book&sid=ProQ:ERIC&atitle=&title=Environmental+Print+in+the+Classroom%3A+Meaningful+Connections+for+Learning+to+Read&issn=&date=2004-01-01&volume=&issue=&spage=144&au=Prior%2C+Jennifer%3BGerard%2C+Maureen+R.&isbn=0872075532&jtitle=&btitle=Environmental+Print+in+the+Classroom%3A+Meaningful+Connections+for+Learning+to+Read&rft_id=info:eric/ED488977
ER -

TY - BOOK
ID - 2133
T1 - Teaching and Learning in Preschool: Using Individually Appropriate Practices in Early Childhood Literacy Instruction
A1 - Venn,Elizabeth Claire
A1 - Jahn,Monica Dacy
Y1 - 2004///
N1 - Availability - Level 2 - Produced in microfiche (1966-2003)
Last updated - 2012-12-14
KW - ERIC
KW - Unsupervised Play Addition
KW - Risk Taking
KW - Transitional Activities
KW - Vygotsky (Lev S)
KW - Zone of Proximal Development
KW - ERIC,Resources in Education (RIE)
KW - Practitioners
KW - Teachers
KW - Preschool Education
KW - Performance Based Assessment
KW - Teacher Student Relationship
KW - Play
KW - Individualized Instruction
KW - Integrated Curriculum
KW - Individual Needs
KW - Student Centered Curriculum
KW - Classroom Techniques
KW - Models
KW - Preschool Curriculum
KW - Reading Instruction
KW - Individual Differences
KW - Bibliographies
KW - Parent School Relationship
KW - Developmentally Appropriate Practices
KW - Emergent Literacy
KW - Learning Activities
KW - Student Educational Objectives
KW - Educational Quality

RP - NOT IN FILE
SP - 336
PB - International Reading Association, Order Department, P.O. Box 6021, Newark, DE 19714-6021 ($30.95).
N2 - This book presents a preschool framework that integrates literacy activities into content area lessons while embedding instruction within adult-child social interactions and realistic, playful activities tailored to each child's individual needs. Chapter 1 of the book delineates the theory and rationale behind the framework, and outlines essential preschool components. Chapter 2 discusses three critical teacher competencies necessary for quality preschool instruction. Chapter 3 outlines a variety of formal and informal assessments and curriculum outcomes as they relate to lesson design and instruction. Chapter 4 addresses children's social and emotional development and includes information on classroom management, self-regulation, risk taking, and dramatic play. Chapter 5 discusses the critical role of oral language development and phonological awareness in early literacy. Chapter 6 explores the different reading components of shared reading, read-alouds, grouping students based on zone of proximal development (ZPD), independent reading, print concepts, and listening comprehension. Chapter 7 focuses on the vital areas of early writing, including language experience and shared, scaffolded, ZPD, and independent writing. The central theme of Chapter 8 is learning about print, including letter knowledge and sound-symbol correspondence. Chapter 9 describes the content areas of mathematics, social studies, and science, and highlights the importance of free exploration, discovery time, literacy links, and thematic integration. Chapter 10 describes music, movement, and the creative arts as they relate to literacy. Chapter 11 covers gross and fine motor development and health and safety topics. Chapter 12 presents information about transitioning families comfortably from the home to the school culture. The book concludes with recommendations for improving early childhood preservice and inservice teacher education. Six appendices supplement the text with lesson plans, outco
SN - 0872075354
UR - http://search.proquest.com/docview/62184682?accountid=14701
L2 - http://sfx.scholarsportal.info/ottawa?url_ver=Z39.88-2004&rft_val_fmt=info:ofi/fmt:kev:mtx:book&genre=book&sid=ProQ:ERIC&atitle=&title=Teaching+and+Learning+in+Preschool%3A+Using+Individually+Appropriate+Practices+in+Early+Childhood+Literacy+Instruction.&issn=&date=2004-01-01&volume=&issue=&spage=336&au=Venn%2C+Elizabeth+Claire%3BJahn%2C+Monica+Dacy&isbn=0872075354&jtitle=&btitle=Teaching+and+Learning+in+Preschool%3A+Using+Individually+Appropriate+Practices+in+Early+Childhood+Literacy+Instruction.&rft_id=info:eric/ED481894
ER -

TY - JOUR
ID - 2134
T1 - Predicting Language Outcome in Infants with Autism and Pervasive Developmental Disorder
A1 - Charman,Tony
A1 - Baron-Cohen,Simon
A1 - Swettenham,John
A1 - Baird,Gillian
A1 - Drew,Auriol
A1 - Cox,Antony
Y1 - 2003/07//
KW - ERIC
KW - Unsupervised Play Addition
KW - ERIC,Current Index to Journals in Education (CIJE)
KW - Prediction
KW - Toddlers
KW - Language Acquisition
KW - Play
KW - Imitation
KW - Clinical Diagnosis
KW - Language Skills
KW - Pervasive Developmental Disorders
KW - Correlation
KW - Longitudinal Studies

KW - Intelligence Quotient
KW - Individual Differences
KW - Interpersonal Communication
KW - Preschool Children
KW - Autism
KW - Attention
KW - Infants
RP - NOT IN FILE
SP - 265
EP - 285
JF - International Journal of Language & Communication Disorders
VL - 38
IS - 3
PB - Taylor & Francis, 325 Chestnut Street Suite 800, Philadelphia, PA 19106.
N2 - Background: To examine longitudinal associations between diagnosis, joint attention, play and imitation abilities and language outcome in infants with autism and pervasive developmental disorder. Methods and Procedures: Experimental measures of joint attention, play and imitation were conducted with a sample of infants with autism spectrum disorder at age 20 months. Language outcome was assessed at age 42 months. A within-group longitudinal correlational design was adopted. Outcomes and Results: Language at 42 months was higher for children with a diagnosis of pervasive developmental disorder than for children with a diagnosis of autism. Language at follow-up was also positively associated with performance on experimental measures of joint attention and imitation, but not with performance on experimental measures of play and "goal detection" at 20 months, nor with a non-verbal intelligence quotient, although these associations were not examined independent of diagnosis. However, floor effects on the measure of play at 20 months and the small sample size limit the conclusions that can be drawn. Conclusions: Individual differences in infant social-communication abilities as well as diagnosis may predict language outcome in preschoolers with autism spectrum disorders. Attention should be directed at assessing these skills in 2- and 3-year-old children referred for a diagnosis of autism spectrum disorder. Imitation and joint attention abilities may be important targets for early intervention. (Contains 2 tables and 2 figures.)
SN - 1368-2822, 1368-2822
UR - http://search.proquest.com/docview/61990055?accountid=14701
L2 - http://sfx.scholarsportal.info/ottawa?url_ver=Z39.88-2004&rft_val_fmt=info:ofi/fmt:kev:mtx:journal&genre=article&sid=ProQ:ProQ%3Aericshell&atitle=Predicting+Language+Outcome+in+Infants+with+Autism+and+Pervasive+Developmental+Disorder&title=International+Journal+of+Language+%26+Communication+Disorders&issn=13682822&date=2003-07-01&volume=38&issue=3&spage=265&au=Charman%2C+Tony%3BBaron-Cohen%2C+Simon%3BSwettenham%2C+John%3BBaird%2C+Gillian%3BDrew%2C+Auriol%3BCox%2C+Antony&isbn=&jtitle=International+Journal+of+Language+%26+Communication+Disorders&btitle=&rft_id=info:eric/EJ770321
ER -

TY - GEN
ID - 2135
T1 - Strategies To Improve Perceived Competence in Children with Visual Impairments
A1 - Shapiro,Deborah R.
A1 - Lieberman,Lauren J.
A1 - Moffett,Aaron
Y1 - 2003///
N1 - Last updated - 2012-12-14
KW - ERIC
KW - Unsupervised Play Addition
KW - ERIC,Current Index to Journals in Education (CIJE)
KW - Practitioners
KW - Elementary Secondary Education
KW - Self Evaluation (Individuals)
KW - Athletics

KW - visual impairments
KW - Self Esteem
KW - Self Concept
KW - Interpersonal Competence
KW - Teaching Methods
RP - NOT IN FILE
SP - 69
EP - 80
JF - RE:view
VL - 35
IS - 2
N2 - This article suggests strategies to improve perceived competencies in children with visual impairments. Strategies to increase perceptions of athletic competence include introducing role models and using behavior assessments. Guided discovery and peer tutoring are suggested teaching techniques. Strategies to improve social competence include increasing independent mobility, facilitating social interaction during lunch, and teaching that initiative is part of empowerment. (Contains references.) (DB)
UR - http://search.proquest.com/docview/62179325?accountid=14701
L2 - http://sfx.scholarsportal.info/ottawa?url_ver=Z39.88-2004&rft_val_fmt=info:ofi/fmt:kev:mtx:journal&genre=article&sid=ProQ:ProQ%3Aericshell&atitle=Strategies+To+Improve+Perceived+Competence+in+Children+with+Visual+Impairments.&title=RE%3Aview&issn=08991510&date=2003-01-01&volume=35&issue=2&spage=69&au=Shapiro%2C+Deborah+R.%3BLieberman%2C+Lauren+J.%3BMoffett%2C+Aaron&isbn=&jtitle=RE%3Aview&btitle=&rft_id=info:eric/EJ677087
ER -

TY - JOUR
ID - 2136
T1 - Caregiver and Peer Responses to Children with Language and Motor Disabilities in Inclusive Preschool Programs
A1 - Harper,Lawrence V.
A1 - McCluskey,Karen S.
Y1 - 2002///
KW - ERIC
KW - Unsupervised Play Addition
KW - ERIC,Current Index to Journals in Education (CIJE)
KW - Early Childhood Education
KW - Caregiver Child Relationship
KW - Comparative Analysis
KW - Teacher Student Relationship
KW - Play
KW - Inclusive Schools
KW - Special Needs Students
KW - Disabilities
KW - Social Behavior
KW - Preschool Children
KW - Peer Relationship
RP - NOT IN FILE
SP - 148
EP - 166
JF - Early Childhood Research Quarterly
VL - 17
IS - 2
N2 - Compared free-play social behaviors of preschoolers with special needs in two inclusive preschool programs with those of typically-developing classmates, matched by age and sex. Found that children with no/little productive language spent more time in solitary pursuits than others and seldom initiated interactions.

Children who were incapable of independent locomotion depended on adults for initiating activity changes and social exchanges. (Author/KB)
SN - 0885-2006, 0885-2006
UR - http://search.proquest.com/docview/62209254?accountid=14701
L2 - http://sfx.scholarsportal.info/ottawa?url_ver=Z39.88-2004&rft_val_fmt=info:ofi/fmt:kev:mtx:journal&genre=article&sid=ProQ:ProQ%3Aericshell&atitle=Caregiver+and+Peer+Responses+to+Children+with+Language+and+Motor+Disabilities+in+Inclusive+Preschool+Programs.&title=Early+Childhood+Research+Quarterly&issn=08852006&date=2002-01-01&volume=17&issue=2&spage=148&au=Harper%2C+Lawrence+V.%3BMcCluskey%2C+Karen+S.&isbn=&jtitle=Early+Childhood+Research+Quarterly&btitle=&rft_id=info:eric/EJ654404
ER -

TY - RPRT
ID - 2137
T1 - Literacy Skills, Occupational Assignment and the Returns to Over- and Under-Education. International Adult Literacy Survey
A1 - Boothby,Daniel
Y1 - 2002/01//
N1 - Last updated - 2012-12-14
KW - ERIC
KW - Unsupervised Play Addition
KW - Canada
KW - General Educational Development Tests
KW - ERIC,Resources in Education (RIE)
KW - Adult Education
KW - Adult Literacy
KW - Statistics
KW - Play
KW - Literature
KW - MALES
KW - Academic Achievement
KW - SURVEYS
KW - Adults
KW - On the Job Training
KW - Outcomes of Education
KW - Evaluation Methods
KW - Foreign Countries
KW - Educational Development
KW - Vocational Education
KW - Females
KW - Labor Force Development
KW - Education Work Relationship
RP - NOT IN FILE
SP - 48
PB - Human Resources and Skills Development Canada. Service Canada, Ottawa, ON K1A 0J9, Canada.
N2 - This study uses data from the Canadian panel of the International Adult Literacy Survey to examine the relations between schooling, literacy and occupational assignment and to determine the extent to which returns to over- and under-education are in fact returns to literacy skills. Two measures of required training time for the job are used, both of which are based on detailed occupation. One is the General Educational Development (GED) level of the occupation; the other is the sum of the GED and Specific Vocational Preparation (SVP) times. Regression analysis of the required training time of individuals' jobs shows that literacy skills are an important determinant of occupational assignment by skill level, once schooling is taken into account. Skills acquired through on-the-job training may also play an important role in occupational assignment. The research literature on returns to over-education and under-education examines the relation between workers' skills, as measured by their level of schooling, and the skill requirements of their job. The "typical" findings in this body of research are 1) that over-educated workers (schooling greater than required by their job) earn more than

workers in jobs with comparable educational requirements but with the (lower) schooling levels that match these requirements; 2) that over-educated workers earn less than workers with comparable schooling in jobs which require this level of schooling; and 3) that under-educated workers (schooling less than required by their job) earn more than comparably educated workers in jobs which match their schooling, but less than workers in jobs with comparable educational requirements whose schooling matches these requirements. We find this pattern of returns to over-education and under-education for women and men in our sample using regression analysis of the (log of) earnings of full-time workers. When measures of literacy skill are added to these regressions, the estimated coefficients of both over-education and under-education decrease in absolute value for men and the estimated coefficients of under-education increase for women. When a measure of literacy use at work is added, this variable has a positive coefficient; and there are further decreases in the absolute values of the coefficients of both over- and under-education for both women and men. We conclude that literacy skills play a significant role in occupational assignment, independent of the role of schooling, that the return to under-education for both women and men is in large part a return to above average literacy skills for their level of schooling, and that for men, the return to over-education is in large part a return to literacy skills which are above average for their jobs. This would seem to indicate that employers are capable of determining their employees' literacy skills by more accurate means than simply depending on the level of schooling as an indicator of literacy skills. Appendices include: (1) Sample Restrictions and Effects on Sample Size; (2) Issues Concerning the Functional Form of the Earnings Equations; and (3) The Relation between Education, Literacy and Literacy Scores. (Contains 8 tables and 27 endnotes
UR - http://search.proquest.com/docview/881457159?accountid=14701
ER -

TY - GEN
ID - 2138
T1 - Outdoor Experiences for Young Children. ERIC Digest
A1 - Rivkin,Mary S.
Y1 - 2000/12//
N1 - Availability - Level 1 - Available online, if indexed January 1993 onward
Last updated - 2012-12-14
KW - ERIC
KW - Unsupervised Play Addition
KW - ERIC Digests
KW - School Yards
KW - ERIC,Resources in Education (RIE)
KW - Early Childhood Education
KW - Primary Education
KW - Outdoor Activities
KW - Facility Requirements
KW - Play
KW - Young Children
KW - Playgrounds
KW - Experiential Learning
KW - Developmentally Appropriate Practices
KW - Child Development
RP - NOT IN FILE
SP - 4
PB - ERIC/CRESS, P.O. Box 1348, Charleston, WV 25325-1348; Tel: 800-624-9120 (Toll Free). Full text at Web site: http://www.ael.org/eric/digests/edorc007.htm.
N2 - This digest examines the value of outdoor experience for young children, reasons for its decline, ways to enhance school play spaces, and aspects of developmentally appropriate outdoor environments. Young children appear to benefit from being outdoors and especially need the broad experiential base provided by being outdoors. The richness and novelty of the outdoors stimulates brain development and function. The knowledge gained outdoors provides a foundation for literacy and science learning and can help children to learn to care for the environment. Nevertheless, children now spend most of their time indoors. Unsupervised outdoor play is rare for preschoolers and declining for primary-age children due to various adult fears about safety; the lack of suitable places to play, especially in urban areas; and such indoor attractions as television and computers.

Improvements to school yard design and maintenance can allow the use of the outdoor environment to enhance curriculum, demonstrate the value of stewardship to young children, offer opportunities for student projects, create habitats for birds and other creatures, and increase the use of school grounds by teachers and students. Suggestions are offered for designing developmentally appropriate outdoor play spaces for infants, toddlers, preschoolers, and primary children. (Contains 24 references.) (SV)
UR - http://search.proquest.com/docview/62348703?accountid=14701
ER -

TY - RPRT
ID - 2139
T1 - Assistive Technology Training for Early Childhood Personnel. Final Report
Y1 - 2000///
N1 - Availability - Level 1 - Available online, if indexed January 1993 onward
SuppNotes - Project period: 8/1/94 through 7/31/00
Last updated - 2012-12-14
KW - ERIC
KW - Unsupervised Play Addition
KW - ERIC,Resources in Education (RIE)
KW - Early Childhood Education
KW - Play
KW - Theory Practice Relationship
KW - On the Job Training
KW - Assistive Devices (for Disabled)
KW - Staff Development
KW - Computer Uses in Education
KW - Participant Satisfaction
KW - Inservice Teacher Education
KW - Disabilities
KW - Change Strategies
KW - Interdisciplinary Approach
KW - Augmentative and Alternative Communication
KW - Technical Assistance
KW - Technology
RP - NOT IN FILE
SP - 128
N2 - This document reports on the activities and achievements of a federally supported project in Arizona to provide inservice early childhood personnel with the necessary competencies and strategies to facilitate the inclusion of assistive technology for young children with disabilities. The 5-year project focused on three major assistive technology components: adaptive play, computers, and augmentative communication devices. The project relied upon an inter-agency, inter-network design to develop and field test a personnel training model that focused on the formulation and implementation of interdisciplinary partnerships. Major project activities included: (1) inservice training of interdisciplinary personnel teams; (2) independent replication of assistive technology methods and procedures by trained personnel teams; and (3) development, field-testing, and dissemination of training materials. Over 150 early childhood personnel participated in some form of project activities. Project evaluation indicated that on-site technical assistance was more beneficial in helping participants implement the ideas presented in the training sessions than were follow-up workshops. Individual sections of the report describe project activities, project evaluation methods, and project findings. Eight appendices present project evaluation data and comprise the bulk of the document. (Contains 24 references.) (DB)
UR - http://search.proquest.com/docview/62331841?accountid=14701
ER -

TY - GEN
ID - 2143
T1 - Real Literacy in a School Setting: Five-Year-Olds Take on the World
A1 - Hall,Nigel

Y1 - 1998///
N1 - Last updated - 2012-12-14
KW - ERIC
KW - Unsupervised Play Addition
KW - ERIC,Current Index to Journals in Education (CIJE)
KW - Primary Education
KW - Play
KW - Childrens Writing
KW - Literacy
KW - Models
RP - NOT IN FILE
SP - 8
EP - 17
JF - Reading Teacher
VL - 52
IS - 1
N2 - Summarizes the autonomous literacy paradigm often used in schools. Describes an alternative model, ideological literacy, which situates literacy practices within cultural values and practices. Shows how this functioned in a class of 4.5 to 5.5 year-olds, in which children's world of play was linked to the wider world and in which literacy had an embedded role. (SR)
UR - http://search.proquest.com/docview/62458355?accountid=14701
L2 - http://sfx.scholarsportal.info/ottawa?url_ver=Z39.88-2004&rft_val_fmt=info:ofi/fmt:kev:mtx:journal&genre=article&sid=ProQ:ProQ%3Aericshell&atitle=Real+Literacy+in+a+School+Setting%3A+Five-Year-Olds+Take+on+the+World.&title=Reading+Teacher&issn=00340561&date=1998-01-01&volume=52&issue=1&spage=8&au=Hall%2C+Nigel&isbn=&jtitle=Reading+Teacher&btitle=&rft_id=info:eric/EJ571642
ER -

TY - RPRT
ID - 2144
T1 - Promoting Generalized Social Interactions Using Sociodramatic Scripts in an Integrated Preschool Setting
A1 - Gronna,Sarah S.
Y1 - 1996/04//
N1 - Availability - Level 1 - Available online, if indexed January 1993 onward
SuppNotes - Paper presented at the Annual Conference of the American Educational Research Association (New York, NY, April 8-12, 1996)
Last updated - 2012-12-14
KW - ERIC
KW - Unsupervised Play Addition
KW - Guided Practice
KW - ERIC,Resources in Education (RIE)
KW - Preschool Education
KW - Puppetry
KW - Play
KW - Inclusive Schools
KW - Scripts
KW - Peer Relationship
KW - visual impairments
KW - Interpersonal Competence
KW - Disabilities
KW - Interpersonal Communication
KW - Learning Activities
KW - Behavior Change
KW - Prosocial Behavior
RP - NOT IN FILE

SP - 17
N2 - Many children with and without disabilities fail to develop the requisite social skills necessary to obtain a level of social competence that facilitates positive peer interactions and relationships. This study was designed to test a combination of training and generalization techniques to promote independent social interactions for children with severe visual impairment. Methods used were sociodramatic script training using puppets, social skills training, systematic instruction, and the generalization techniques of naturally occurring contingencies and multiple exemplars. The subjects included a 30-month-old girl with severe esotropia and four of her classmates without disabilities. All of the subjects were enrolled in an integrated preschool. Results demonstrated that the visually impaired child learned the target behaviors and generalized their use to free-play activities with her peers. The study supports the use of puppet script training using sociodramatic scripts to teach social skills to young children with visual impairments and suggests that script training sessions immediately followed by free play activities with group participants promotes the generalization of taught social skills without teacher prompting in the generalized setting. In addition, teaching sociodramatic scripts related to actual social skills such as greetings, respondings and conversations probably facilitated generalization since the skills taught in the scripts were more applicable to the natural environment. Further studies should incorporate additional students and group participants for puppet script training and expand data collection to all subjects. Contains 17 references. (MOK)
UR - http://search.proquest.com/docview/62676546?accountid=14701
ER -

TY - RPRT
ID - 2145
T1 - Maternal Scaffolding in the Child's Zone of Proximal Development: Cultural Perspectives
A1 - Kermani,Hengameh
A1 - Brenner,Mary E.
Y1 - 1996/04//
N1 - Availability - Level 1 - Available online, if indexed January 1993 onward
SuppNotes - Paper presented at the Annual Conference of the American Educational Research Association (New York, NY, April 8-12, 1996)
Last updated - 2012-12-14
KW - ERIC
KW - Unsupervised Play Addition
KW - Cultural Studies
KW - Dyads
KW - Iranian Americans
KW - Strategy Training
KW - Zone of Proximal Development
KW - ERIC,Resources in Education (RIE)
KW - Thinking Skills
KW - Play
KW - Young Children
KW - Mothers
KW - Learning Processes
KW - Cross Cultural Studies
KW - Scaffolding (Teaching Technique)
KW - Teaching Skills
KW - Parent Child Relationship
KW - Anglo Americans
KW - Child Development
KW - Cultural Differences
KW - Parents as Teachers
RP - NOT IN FILE
SP - 12
N2 - This study examined cultural differences in the amount and type of maternal scaffolding of children's learning and their effects of scaffolding on children's independent performance across two distinct activities: goal-directed versus free play. Twenty Iranian-American and 20 Anglo-American mothers with their preschool

children participated in this study. Analyses of maternal scaffolding revealed that Iranian mothers were more directive in their teaching strategies than Anglo-American mothers were in the goal-directed activity. These differences disappeared in free play. Maternal sensitivity was examined in relation to task demand and children's level of competence. No cultural differences were observed between the two groups of mothers. However, both groups altered their teaching strategies to adjust to the task demands as well as to the children's level of competence. An examination of children's independent performance revealed no cultural differences. Both groups of children performed equally well. The results of this study highlight the importance of culture, nature of task, and child's effect in mothers' construction and application of teaching strategies. (Author/MOK)
UR - http://search.proquest.com/docview/62675859?accountid=14701
ER -

TY - RPRT
ID - 2146
T1 - Children Thinking Mathematically Beyond Authoritative Identities
A1 - Macmillan,Agnes
Y1 - 1995///
N1 - Last updated - 2012-12-14
KW - ERIC
KW - Unsupervised Play Addition
KW - ERIC,Current Index to Journals in Education (CIJE)
KW - Primary Education
KW - Play
KW - Young Children
KW - Mathematical Concepts
KW - Observation
KW - Personal Autonomy
KW - Social Environment
RP - NOT IN FILE
SP - 111
EP - 131
N2 - Reports a study of mathematical interactions and developing attitudes of children in transition from preschool to school. Two episodes of construction play were analyzed to suggest a model of autonomous learning. Access to self-regulatory social relations was closely linked to accessibility of mathematical meanings. Contains 59 references. (FDR)
UR - http://search.proquest.com/docview/62673800?accountid=14701
L2 - http://sfx.scholarsportal.info/ottawa?url_ver=Z39.88-2004&rft_val_fmt=info:ofi/fmt:kev:mtx:book&genre=unknown&sid=ProQ:ERIC&atitle=&title=Children+Thinking+Mathematically+Beyond+Authoritative+Identities.&issn=10332170&date=1995-01-01&volume=&issue=&spage=111&au=Macmillan%2C+Agnes&isbn=&jtitle=&btitle=Children+Thinking+Mathematically+Beyond+Authoritative+Identities.&rft_id=info:eric/EJ525146
ER -

TY - GEN
ID - 2147
T1 - Early Childhood Corner: The Role of Self-Directed Discovery Time in the Development of Mathematics Concepts
A1 - Andrews,Angela Giglio
Y1 - 1995///
N1 - Last updated - 2012-12-14
KW - ERIC
KW - Unsupervised Play Addition
KW - Mathematics Activities
KW - ERIC,Current Index to Journals in Education (CIJE)
KW - Teachers
KW - Practitioners
KW - Primary Education

KW - Elementary School Mathematics
KW - Play
KW - Mathematics Instruction
KW - Independent Study
KW - Learning Activities
KW - Mathematics Education
RP - NOT IN FILE
SP - 116
EP - 120
JF - Teaching Children Mathematics
VL - 2
IS - 2
N2 - Provides vignettes of self-directed discovery time in a preprimary classroom to see how to generate mathematically valuable experiences. (MKR)
UR - http://search.proquest.com/docview/62655649?accountid=14701
L2 - http://sfx.scholarsportal.info/ottawa?url_ver=Z39.88-2004&rft_val_fmt=info:ofi/fmt:kev:mtx:journal&genre=article&sid=ProQ:ProQ%3Aericshell&atitle=Early+Childhood+Corner%3A+The+Role+of+Self-Directed+Discovery+Time+in+the+Development+of+Mathematics+Concepts.&title=Teaching+Children+Mathematics&issn=10735836&date=1995-01-01&volume=2&issue=2&spage=116&au=Andrews%2C+Angela+Giglio&isbn=&jtitle=Teaching+Children+Mathematics&btitle=&rft_id=info:eric/EJ514082
ER -

TY - BOOK
ID - 2148
T1 - Let's Begin Reading Right: Developmentally Appropriate Beginning Literacy. Third Edition
A1 - Fields,Marjorie V.
A1 - Spangler,Katherine L.
Y1 - 1995///
N1 - Availability - Level 3 - Indexed only
Last updated - 2012-12-14
KW - ERIC
KW - Unsupervised Play Addition
KW - ERIC,Resources in Education (RIE)
KW - Teachers
KW - Practitioners
KW - Early Childhood Education
KW - Writing Instruction
KW - Beginning Reading
KW - Play
KW - Reading Instruction
KW - Classroom Environment
KW - Beginning Writing
KW - Developmentally Appropriate Practices
KW - Emergent Literacy
KW - Theory Practice Relationship
KW - Whole Language Approach
RP - NOT IN FILE
SP - 398
PB - Prentice-Hall, Order Processing, P.O. Box 11071, Des Moines, IA 50336-1071 ($27 plus shipping/handling).
N2 - Combining developmentally appropriate early childhood practice with current views of emergent literacy and whole language instruction, this book is designed to help pre-service and in-service teachers incorporate new views of literacy into their classrooms. The book includes extensive examples from actual classrooms to assist readers in understanding the teaching ideas presented. It is also designed to help the teacher of young

children understand and validate the importance of play in the child's literacy development and to take into account how children construct knowledge. Chapters in the book are: (1) Understanding Emergent Readers and Writers; (2) Encouraging Emergent Literacy through Play and Experiences; (3) Encouraging Emergent Literacy through Oral Language and Story Time; (4) Assisting Emergent Readers; (5) Assisting Emergent Writers; (6) Supporting Independent Writers; (7) Creating a Classroom for Readers; (8) Assessing Growth in Literacy; (9) Selecting Early Literacy Materials; and (10) Constructing Your Understanding. Contains approximately 500 references. (RS)
SN - 0023372834
UR - http://search.proquest.com/docview/62745832?accountid=14701
L2 - http://sfx.scholarsportal.info/ottawa?url_ver=Z39.88-2004&rft_val_fmt=info:ofi/fmt:kev:mtx:book&genre=book&sid=ProQ:ERIC&atitle=&title=Let%27s+Begin+Reading+Right%3A+Developmentally+Appropriate+Beginning+Literacy.+Third+Edition.&issn=&date=1995-01-01&volume=&issue=&spage=398&au=Fields%2C+Marjorie+V.%3BSpangler%2C+Katherine+L.&isbn=0023372834&jtitle=&btitle=Let%27s+Begin+Reading+Right%3A+Developmentally+Appropriate+Beginning+Literacy.+Third+Edition.&rft_id=info:eric/ED375381
ER -

TY - RPRT
ID - 2149
T1 - Once Upon a Classroom
A1 - Obersinner,Kathy
Y1 - 1994/12//
N1 - Availability - Level 1 - Available online, if indexed January 1993 onward
Last updated - 2012-12-14
KW - ERIC
KW - Unsupervised Play Addition
KW - Juneau Borough School District AK
KW - ERIC,Resources in Education (RIE)
KW - Grade 1
KW - Primary Education
KW - Qualitative Research
KW - Elementary School Students
KW - Dramatic Play
KW - Role Playing
KW - Conflict Resolution
KW - Conflict
KW - SOCIALIZATION
KW - Story Telling
KW - Behavior Patterns
KW - Social Development
KW - Interpersonal Relationship
RP - NOT IN FILE
SP - 17
N2 - This research project explored how children's ability to exchange ideas and resolve conflict was influenced by regularly setting aside time to dictate and act out stories. Research methods for this 3-month project included audiotapes, a questionnaire, and the stories from 23 first-grade children. Observations looked specifically at conflict resolution, roles and interactions among storytellers, and the sense of community manifested in play. Observations indicated that the children became more autonomous in exchanging ideas and listening to one another's potential solutions, as the children encountered issues that arose in the natural context of acting out their stories. As the children devised a workable structure for storytelling, they began to assume greater ownership as the process became more familiar, directing their comments and questions to one another. As they acted in one another's stories, each child's view of the world was assimilated by his or her peers. Children's story plays formed a distinctive and colorful quilt of reoccurring patterns that merged into new designs as the children shared their attempts to make sense of their world. (WP)
UR - http://search.proquest.com/docview/62745655?accountid=14701

ER -

TY - THES
ID - 2150
T1 - Social Cognition in Preschoolers: Social Perception Social Knowledge, and Relationship Goals and Expectations
A1 - Meece,Darrell Wesley
N1 - Last updated - 2012-12-14
SubjectsTermNotLitGenreText - 2462 10120; 7940 126; 9738 909; 8127 11655 1474 316 8016 4542; 9748 1710; 11225 1; 5446 1970 1; 5449 8768; 6752 9651 6582; 1710; 3681
IdentKeywords - Alabama
KW - ERIC
KW - Unsupervised Play Addition
KW - Alabama
KW - ERIC,Resources in Education (RIE)
KW - Cognitive Processes
KW - Play
KW - Interpersonal Competence
KW - Verbal Ability
KW - Social Behavior
KW - Cues
KW - Social Cognition
KW - Interpersonal Relationship
KW - Preschool Children
KW - Expectation
KW - Models
RP - NOT IN FILE
SP - 144
N2 - The purpose of the present study was to test the utility of a model of young children's social cognition as a predictor of preschoolers' social competence with same-age peers. The model investigated in this study proposes that three, relatively independent, domains of social-cognitive processes are pertinent to young children's peer relations. The first of these processing skills, social knowledge, was conceptualized as a repertoire of social strategies. The second skill, social perception, was conceptualized as both the accurate encoding of relevant social cues and the positive interpretation of social cues that have been encoded. The final element of this model of social cognition is young children's relationship goals and expectations, or the extent to which preschoolers would like to play with other children and feel that other children would like to play with them. Subjects in this study were 34 4- and 5-year-old children who completed multiple assessments that were developed or adapted to measure each of these domains with preschoolers. Results indicated that measures of each of the three hypothesized domains were associated with measures of children's peer competence. Furthermore, measures of aspects of two of the domains - the quality of social knowledge and positive interpretation of social cues - independently predicted teacher-rated peer competence when the subjects' general verbal ability was statistically controlled. These results are interpreted as evidence that multiple, at least partially independent, social cognitive processes may guide children's social behavior. Associations among measures of the three hypothesized domains of social cognition suggest that the hypothesis that these domains are independent may not be entirely adequate. Furthermore, the pattern of associations among measures of these domains and measures of children's verbal ability and peer competence provide some evidence that a two-factor model may provide a more accurate and parsimonious description of young children's social cognitive processing. (Contains 11 tables.) [Master Theses, Auburn University.]
UR - http://search.proquest.com/docview/61920176?accountid=14701
ER -

TY - GEN
ID - 2151
T1 - Rethinking Table Activities. Australian Early Childhood Resource Booklets No. 5
A1 - Cullen,Joy
Y1 - 1991/12//

N1 - Availability - Level 1 - Available online, if indexed January 1993 onward
Last updated - 2012-12-14
KW - ERIC
KW - Unsupervised Play Addition
KW - Australia
KW - ERIC,Resources in Education (RIE)
KW - Teachers
KW - Practitioners
KW - Early Childhood Education
KW - Play
KW - Class Activities
KW - Dramatic Play
KW - Student Centered Curriculum
KW - Preschool Curriculum
KW - Foreign Countries
KW - Learning Centers (Classroom)
KW - Learning Activities
KW - Creative Activities
KW - Preschool Children
KW - Learning Strategies
RP - NOT IN FILE
SP - 19
PB - Australian Early Childhood Association, , P.O. Box 105, Watson, Australian Capital Territory 2602, Australia ($6.25 Australian).
N2 - This booklet discusses the use of structured table activities in preschool programs in Australia and alternatives to these activities, such as free play. The booklet notes that structured table activities tend to be teacher-directed, passive, non-creative, and focus on goals set by the teacher. They often do not address the learning requirements of young children, who need to feel competent and independent, pursue their own interests, represent their own experiences, focus on processes rather than products, and interact with peers and adults. The booklet recommends programming designed to meet young children's needs, that is, child-centered, active, creative, and focused on goals set by the child. Indicators of children's use of independent learning strategies, interest in activities, and use of creative processes are also examined. The booklet concludes with guidelines for the appropriate use of table activities. (MDM)
SN - 1863230289
UR - http://search.proquest.com/docview/62769389?accountid=14701
ER -

TY - GEN
ID - 2152
T1 - Touch the Future: Discovering Abilities through Technology for Living, Learning, Working and Playing. Southeast Regional Conference (3rd, Atlanta, Georgia, April 10-12, 1991)
Y1 - 1991/04//
N1 - Availability - Level 1 - Available online, if indexed January 1993 onward
Last updated - 2012-12-14
KW - ERIC
KW - Unsupervised Play Addition
KW - ERIC,Resources in Education (RIE)
KW - Practitioners
KW - Administrators
KW - Elementary Secondary Education
KW - Technological Advancement
KW - Financial Support
KW - Play
KW - Independent Living
KW - Employment
KW - Linking Agents

KW - Assistive Devices (for Disabled)
KW - Computer Uses in Education
KW - Accessibility (for Disabled)
KW - Disabilities
KW - Computer Oriented Programs
KW - Technology Transfer
KW - Educational Technology
RP - NOT IN FILE
SP - 245
N2 - This packet of materials was originally intended for participants in a 1991 conference on assistive technology for the disabled. After a detailed listing of the conference schedule, individual sections provide abstracts, biographical sketches, and summaries concerning the following conference topics: blending, computer labs, family, grants and funding, interactive labs, learning, living, playing, and working. Also briefly described are posters presented at the conference, the video theater, and exhibitor displays. Among topics covered are: computer programs for the traumatically brain injured, resources for employment, impact of developmental disability on a family, developing conversational interaction with the "Unicorn Board", the Alliance in Technology Access resource network, seating and mobility, supported employment and assistive technology for the severely disabled, curriculum based computerized measurement, low vision aids, the Technology Related Assistance for Individuals with Disabilities Act, setting up an Assistive Technology Demonstration Center, telecommunication in special education, the Kurzweil Personal Reader, technology and the aging, adventure programming for the physically disabled, computer access for the visually impaired, portable electronic writing aids, augmentative communication, funding, and adapted bikes. (DB)
UR - http://search.proquest.com/docview/62906430?accountid=14701
L2 - http://sfx.scholarsportal.info/ottawa?url_ver=Z39.88-2004&rft_val_fmt=info:ofi/fmt:kev:mtx:journal&genre=unknown&sid=ProQ:ProQ%3Aericshell&atitle=Touch+the+Future%3A+Discovering+Abilities+through+Technology+for+Living%2C+Learning%2C+Working+and+Playing.+Southeast+Regional+Conference+%283rd%2C+Atlanta%2C+Georgia%2C+April+10-12%2C+1991%29.&title=&issn=&date=1991-04-01&volume=&issue=&spage=245&au=&isbn=&jtitle=&btitle=&rft_id=info:eric/ED341182
ER -

TY - JOUR
ID - 2153
T1 - Creating Quality Curriculum Yet Not Buying Out the Store
A1 - Lanser,Shirley
A1 - McDonnell,Laura
Y1 - 1991///
KW - ERIC
KW - Unsupervised Play Addition
KW - City and Country School NY
KW - Pratt (Caroline)
KW - ERIC,Current Index to Journals in Education (CIJE)
KW - Preschool Education
KW - Teacher Role
KW - Program Descriptions
KW - Play
KW - Preschool Curriculum
KW - Outdoor Activities
KW - Instructional Materials
KW - Educational Practices
KW - Parent Attitudes
KW - Preschool Children
KW - Playground Activities
KW - Educational Philosophy
RP - NOT IN FILE
SP - 4

EP - 9
JF - Young Children
VL - 47
IS - 1
N2 - Describes the educational philosophy and procedures of New York City's City and Country School, where two through seven year olds use primary materials to enrich cognitive, emotional, social, and physical growth. The school promotes deep involvement, independent learning, and increased attention span. (BB)
SN - 0044-0728, 0044-0728
UR - http://search.proquest.com/docview/62909936?accountid=14701
L2 - http://sfx.scholarsportal.info/ottawa?url_ver=Z39.88-2004&rft_val_fmt=info:ofi/fmt:kev:mtx:journal&genre=article&sid=ProQ:ProQ%3Aericshell&atitle=Creating+Quality+Curriculum+Yet+Not+Buying+Out+the+Store.&title=Young+Children&issn=00440728&date=1991-01-01&volume=47&issue=1&spage=4&au=Lanser%2C+Shirley%3BMcDonnell%2C+Laura&isbn=&jtitle=Young+Children&btitle=&rft_id=info:eric/EJ436425
ER -

TY - RPRT
ID - 2154
T1 - Parenting in Cross-Cultural Perspective: The United States, France, and Japan
A1 - Bornstein,Marc H.
Y1 - 1991///
N1 - Availability - Level 1 - Available online, if indexed January 1993 onward
Last updated - 2012-12-14
KW - ERIC
KW - Unsupervised Play Addition
KW - France
KW - Japan
KW - Nurturance
KW - United States
KW - ERIC,Resources in Education (RIE)
KW - Behavior Development
KW - Play
KW - Stimulation
KW - Speech Communication
KW - Mothers
KW - Social Behavior
KW - Cross Cultural Studies
KW - Foreign Countries
KW - Parent Child Relationship
KW - Infant Behavior
KW - Child Rearing
KW - Cultural Differences
KW - Infants
RP - NOT IN FILE
SP - 23
N2 - In this study of prominent characteristics of parenting in the United States, France, and Japan, 72 mother-infant dyads were examined. The study focused on three prominent interactive domains of visual and vocal exchange between mother and baby (nurturing, social exchange, and didactic stimulation), examining their frequency of occurrence and patterns of covariation. Findings revealed significant main effects of culture, with American mothers showing significantly higher rates of object stimulation than French or Japanese mothers. American and Japanese mothers showed equal rates of social stimulation, while American mothers engaged in more social stimulation than French mothers. Maternal behaviors appeared to be largely independent of coherence among parenting behaviors. The data indicated that mothers from the different cultures differed most in the degree to which they stimulated, spoke to, and responded to their infants vis-a-vis the object and social

worlds. Mothers were most similar in terms of the degree to which they engaged their infants in tactile kinesthetic play and responded in a nurturing or an imitative manner. (Contains 5 pages of references.) (SH)
UR - http://search.proquest.com/docview/62553215?accountid=14701
ER -

TY - GEN
ID - 2155
T1 - The Important Early Years: Intelligence through Movement Experiences
A1 - Diem,Liselott
Y1 - 1991///
N1 - Availability - Level 2 - Produced in microfiche (1966-2003)
SuppNotes - Originally published in German, 1986
Last updated - 2012-12-14
KW - ERIC
KW - Unsupervised Play Addition
KW - ERIC,Resources in Education (RIE)
KW - Parents
KW - Play
KW - Motor Development
KW - Young Children
KW - Perceptual Development
KW - Parent Role
KW - Creative Development
KW - Health Education
KW - Intellectual Development
KW - Childrens Games
KW - Parent Child Relationship
KW - Perceptual Motor Learning
KW - Neonates
KW - Playground Activities
RP - NOT IN FILE
SP - 99
PB - Publications Sales, AAHPERD, 1900 Association Drive, Reston, VA 22091.
N2 - It is generally recognized that early childhood experiences are extremely important for human development. The development of rational thinking, attention span, and ability to memorize in the first years of life are very much dependent upon motor experiences gained through exploring and comprehending. Early learning experiences should not be forced upon the child, but should develop through self-discovery, play with others, and personal challenges. Uninhibited movement experiences, rather than regimented or forced activities, produce the essential and lasting curiosity and thirst for knowledge necessary for human creativity. Within this context, the 28 chapters of this book discuss the following topics: spontaneity and sureness of movement; keen perceptual awareness; orientation in space; rational visualization and creativity in movement; comprehension of situations and independent reaction. The content covers prenatal through age 8, with a heavy focus on preprimary years. The book provides a variety of suggestions on how to promote a child's own competence, starting with the first year of life. (AMH)
SN - 0883144913
UR - http://search.proquest.com/docview/62992121?accountid=14701
ER -

TY - RPRT
ID - 2156
T1 - Childhood Antecedents of Achievement in Professional Women
A1 - Coats,Patricia Boyne
A1 - Overman,Steve
Y1 - 1989/11//
N1 - Availability - Level 1 - Available online, if indexed January 1993 onward
SuppNotes - Paper presented at the Annual Meeting of the Mid-South Educational Research Association (Little

Rock, AR, November 8-10, 1989)
Last updated - 2012-12-14
KW - ERIC
KW - Unsupervised Play Addition
KW - ERIC,Resources in Education (RIE)
KW - Professional Personnel
KW - Play
KW - Business
KW - Leisure Time
KW - Background
KW - Career Choice
KW - SOCIALIZATION
KW - Individual Development
KW - Childhood Interests
KW - Nontraditional Occupations
KW - Employed Women
KW - Females
RP - NOT IN FILE
SP - 21
N2 - While the trend for increasing numbers of women to enter the labor market is clear and career opportunities for women have expanded, women continue to be employed in occupations that are traditionally female and low-paying. This study examined childhood preferred play materials, structured and free play activities, and leisure time activities of women presently in traditional and nontraditional professions to investigate the antecedents of women's choices of careers in traditional versus nontraditional professions, with special focus on business as a profession. Independent variables antecedent to professional career choices focused on childhood socialization experiences through age 12 and included demographic variables relating to the subjects' childhood environment, family structure, parent characteristics, parental encouragement, and play. Subjects were 110 adult women from Mississippi, of whom 47 were in traditional professions, 25 were in moderately nontraditional professions, 15 were in highly nontraditional professions, and 23 were in business professions. Chi square and t-tests revealed significant differences among subgroups on several independent variables. Professional business women had participated more in competitive sports as children and (along with women in other nontraditional professions) reported more male playmates and fewer female playmates than did women in traditional professions. Women in nontraditional professions received different forms of parental encouragement than did women in traditional professions. (NB)
UR - http://search.proquest.com/docview/63069529?accountid=14701
L2 - http://sfx.scholarsportal.info/ottawa?url_ver=Z39.88-2004&rft_val_fmt=info:ofi/fmt:kev:mtx:book&genre=unknown&sid=ProQ:ERIC&atitle=&title=Childhood+Antecedents+of+Achievement+in+Professional+Women.&issn=&date=1989-11-01&volume=&issue=&spage=21&au=Coats%2C+Patricia+Boyne%3BOverman%2C+Steve&isbn=&jtitle=&btitle=Childhood+Antecedents+of+Achievement+in+Professional+Women.&rft_id=info:eric/ED312588
ER -

TY - JOUR
ID - 2157
T1 - Making Toys Accessible for Children with Cerebral Palsy
A1 - Schaeffler,Carol
Y1 - 1988///
KW - ERIC
KW - Unsupervised Play Addition
KW - ERIC,Current Index to Journals in Education (CIJE)
KW - Practitioners
KW - Parents
KW - Accessibility (for Disabled)
KW - Play
KW - Cerebral Palsy
KW - Material Development

KW - Toys
KW - Psychomotor Skills
KW - Assistive Devices (for Disabled)
KW - Media Adaptation
RP - NOT IN FILE
SP - 26
EP - 28
JF - TEACHING Exceptional Children
VL - 20
IS - 3
N2 - Techniques for modifying toys for children with cerebral palsy to enhance active, independent use of play materials are described. The most successful techniques include stabilization; creating boundaries; and use of grasping aids, manipulation aids, and switches. Good positioning is emphasized to maximize controlled and relaxed movement and visual contact. (VW)
UR - http://search.proquest.com/docview/63214748?accountid=14701
L2 - http://sfx.scholarsportal.info/ottawa?url_ver=Z39.88-2004&rft_val_fmt=info:ofi/fmt:kev:mtx:journal&genre=article&sid=ProQ:ProQ%3Aericshell&atitle=Making+Toys+Accessible+for+Children+with+Cerebral+Palsy.&title=Teaching+Exceptional+Children&issn=&date=1988-01-01&volume=20&issue=3&spage=26&au=Schaeffler%2C+Carol&isbn=&jtitle=Teaching+Exceptional+Children&btitle=&rft_id=info:eric/EJ367414
ER -

TY - JOUR
ID - 2158
T1 - Early Readers: Important Emotional Considerations
A1 - Werner,Patricia Holden
A1 - Strother,JoAnna
Y1 - 1987///
KW - ERIC
KW - Unsupervised Play Addition
KW - ERIC,Current Index to Journals in Education (CIJE)
KW - Early Childhood Education
KW - Play
KW - Family Environment
KW - Young Children
KW - Emotional Development
KW - Early Reading
KW - Play Therapy
KW - Recreational Reading
KW - Independent Reading
KW - Parent Child Relationship
KW - Emotional Problems
KW - Social Development
RP - NOT IN FILE
SP - 538
EP - 543
JF - Reading Teacher
VL - 40
IS - 6
N2 - Enumerates ways to help early readers without negatively affecting their development in other areas, such as: (l) providing an atmosphere rich in environment print and positive role models, (2) using encouragement rather than praise as a means of reinforcing their progress, (3) exhibiting respect for the child, (4) eliminating criticism and minimizing mistakes, and (5) avoiding "premature structuring." (NKA)
UR - http://search.proquest.com/docview/63265011?accountid=14701

L2 - http://sfx.scholarsportal.info/ottawa?url_ver=Z39.88-2004&rft_val_fmt=info:ofi/fmt:kev:mtx:journal&genre=article&sid=ProQ:ProQ%3Aericshell&atitle=Early+Readers%3A+Important+Emotional+Considerations.&title=Reading+Teacher&issn=&date=1987-01-01&volume=40&issue=6&spage=538&au=Werner%2C+Patricia+Holden%3BStrother%2C+JoAnna&isbn=&jtitle=Reading+Teacher&btitle=&rft_id=info:eric/EJ346942
ER -

TY - JOUR
ID - 2159
T1 - The Effects of Television Form and Violent Content on Boys' Attention and Social Behavior
A1 - Potts,Richard
Y1 - 1986///
KW - ERIC
KW - Unsupervised Play Addition
KW - ERIC,Current Index to Journals in Education (CIJE)
KW - Researchers
KW - Television Viewing
KW - Play
KW - Attention Span
KW - MALES
KW - Behavior Patterns
KW - Social Behavior
KW - Toys
KW - Television Research
KW - Preschool Children
KW - Aggression
KW - Violence
RP - NOT IN FILE
SP - 1
EP - 17
JF - Journal of Experimental Child Psychology
JA - J Exp.Child Psychol.
VL - 41
IS - 1
N2 - Reports on an experiment that examined the independent effects of television content (violence) and television formal features (action) on preschool boys' attention to programs and their postviewing social behavior. Shows rapid character action facilitated visual attention to the programs and that television action level had no systematic effects on subjects' behavior. (HOD)
UR - http://search.proquest.com/docview/63334144?accountid=14701
L2 - http://sfx.scholarsportal.info/ottawa?url_ver=Z39.88-2004&rft_val_fmt=info:ofi/fmt:kev:mtx:journal&genre=article&sid=ProQ:ProQ%3Aericshell&atitle=The+Effects+of+Television+Form+and+Violent+Content+on+Boys%27+Attention+and+Social+Behavior.&title=Journal+of+Experimental+Child+Psychology&issn=&date=1986-01-01&volume=41&issue=1&spage=1&au=Potts%2C+Richard&isbn=&jtitle=Journal+of+Experimental+Child+Psychology&btitle=&rft_id=info:eric/EJ336378
ER -

TY - RPRT
ID - 2160
T1 - A Study of the Behavior of Children in a Preschool Equipped with Computers
A1 - Klinzing,Dene G.
Y1 - 1985/04//
N1 - Availability - Level 1 - Available online, if indexed January 1993 onward
SuppNotes - Paper presented at the Annual Meeting of the American Educational Research Association (69th, Chicago, IL, March 31-April 4, 1985)
Last updated - 2012-12-14

KW - ERIC
KW - Unsupervised Play Addition
KW - Solitary Play
KW - ERIC,Resources in Education (RIE)
KW - Preschool Education
KW - Teacher Role
KW - Play
KW - Microcomputers
KW - Laboratory Schools
KW - Class Activities
KW - Cooperation
KW - Sex Differences
KW - Interaction Process Analysis
KW - Preschool Children
KW - Age Differences
RP - NOT IN FILE
SP - 44
N2 - A study was conducted: (1) to compare the popularity of computer stations with nine other activity stations; (2) to determine the differences in the type of play displayed by the children in preschool and note the type of play displayed at the computer stations versus the other activity stations; (3) to determine whether the preschool activities, particularly the computer activities, were teacher directed; (4) to describe the communication interactions; and (5) to determine whether children's choices of computer activities varied by sex. For 2 weeks, four observers gathered data in a classroom for 4-year-olds and a classroom for 5-year-olds. Both classrooms had housekeeping, art, language arts, blocks, cooking, science, and manipulative activities. The classroom for 4-year-olds had an Apple IIe computer, while the classroom for 5-year-olds had a PLATO terminal, a Turtle robot, and an Apple IIe computer. The children were free to choose among a number of software programs. Several measures were taken: (1) frequency counts of the number of children at each station; (2) identification of play behavior (using Parton's Play Classifications); (3) instances of teacher-directed play; (4) verbal interaction (using Flanders' Interaction Analysis Scale); and (5) frequency counts of the number of male and female children at computer stations. Results indicated that, while 4-year-old males used the computer extensively, other children seemed to engage in a wide variety of activities, including computer use. While activities generally were not teacher-directed, teachers did tend to direct the computer activities of the 4-year-olds and to use more praise in those activities. (These effects might have been due to the computer's relative novelty in that classroom.) The computers seemed to encourage both cooperative and independent play. (CB)
UR - http://search.proquest.com/docview/63361030?accountid=14701
L2 - http://sfx.scholarsportal.info/ottawa?url_ver=Z39.88-2004&rft_val_fmt=info:ofi/fmt:kev:mtx:book&genre=unknown&sid=ProQ:ERIC&atitle=&title=A+Study+of+the+Behavior+of+Children+in+a+Preschool+Equipped+with+Computers.&issn=&date=1985-04-01&volume=&issue=&spage=44&au=Klinzing%2C+Dene+G.&isbn=&jtitle=&btitle=A+Study+of+the+Behavior+of+Children+in+a+Preschool+Equipped+with+Computers.&rft_id=info:eric/ED255320
ER -

TY - GEN
ID - 2161
T1 - Where Service Begins: Working with Parents to Provide Early Intervention. Considerations for the Culturally Different
A1 - Rogow,Sally M.
Y1 - 1985/04//
N1 - Availability - Level 1 - Available online, if indexed January 1993 onward
SuppNotes - Paper presented at the Annual Convention of the Council for Exceptional Children (63rd, Anaheim, CA, April 15-19, 1985)
Last updated - 2012-12-14
KW - ERIC
KW - Unsupervised Play Addition
KW - ERIC,Resources in Education (RIE)
KW - Play

KW - Young Children
KW - Home Programs
KW - Family School Relationship
KW - Disabilities
KW - Intervention
KW - Cultural Differences
KW - Chinese Americans
KW - Family (Sociological Unit)
RP - NOT IN FILE
SP - 14
N2 - The difficulties faced by culturally different parents of multiply handicapped young children are considered, and examples of families of Chinese origin are offered to illustrate the impact of cultural styles and beliefs. The structure of the Chinese family is analyzed, its reaction to professionals and handicapped people explained. Guidelines for providing services to culturally different families are offered and three major goals of home intervention noted: (1) helping the parents to recognize the importance of working with the child, (2) encouraging independent behavior and helping the child to achieve specific developmental goals, and (3) demonstrating the value and importance of adult/child interactions. The use of play as an intervention medium is stressed. (CL)
UR - http://search.proquest.com/docview/63386274?accountid=14701
ER -

TY - RPRT
ID - 2162
T1 - Accelerating Language Learning in Young Children
A1 - Roth,Froma P.
Y1 - 1984///
N1 - Last updated - 2012-12-14
KW - ERIC
KW - Unsupervised Play Addition
KW - ERIC,Current Index to Journals in Education (CIJE)
KW - Language Research
KW - Psycholinguistics
KW - Play
KW - Language Acquisition
KW - Cognitive Development
KW - Young Children
KW - Child Language
KW - Grammar
RP - NOT IN FILE
SP - 89
EP - 107
N2 - Examined effects of direct intervention on language learning. Using a toy manipulation task, 18 children aged 3;6 to 4;6 were systematically taught linguistic structures beyond their developmental grasp. Solid improvement was found in the experimental conditions; no significant improvement was noted in control conditions, showing that the language learning process is somewhat independent of cognitive development. (SL)
UR - http://search.proquest.com/docview/63454865?accountid=14701
L2 - http://sfx.scholarsportal.info/ottawa?url_ver=Z39.88-2004&rft_val_fmt=info:ofi/fmt:kev:mtx:book&genre=unknown&sid=ProQ:ERIC&atitle=&title=Accelerating+Language+Learning+in+Young+Children.&issn=&date=1984-01-01&volume=&issue=&spage=89&au=Roth%2C+Froma+P.&isbn=&jtitle=&btitle=Accelerating+Language+Learning+in+Young+Children.&rft_id=info:eric/EJ295254
ER -

TY - JOUR
ID - 2163
T1 - A Simple Thing Like a Swing

A1 - Brenner,Suzanne
Y1 - 1983/04//
KW - ERIC
KW - Unsupervised Play Addition
KW - ERIC,Current Index to Journals in Education (CIJE)
KW - Preschool Education
KW - Downs Syndrome
KW - Play
KW - Parent Materials
KW - Parent Child Relationship
KW - Parent Attitudes
KW - Playground Activities
RP - NOT IN FILE
SP - 11
EP - 13
JA - Exceptional Parent
VL - 13
IS - 2
N2 - The mother of a Down's syndrome four-year-old describes how a slight adaptation in a playground swing allowed her son to experience the joy of independent play. (CL)
UR - http://search.proquest.com/docview/63531825?accountid=14701
L2 - http://sfx.scholarsportal.info/ottawa?url_ver=Z39.88-2004&rft_val_fmt=info:ofi/fmt:kev:mtx:journal&genre=article&sid=ProQ:ProQ%3Aericshell&atitle=A+Simple+Thing+Like+a+Swing.&title=Exceptional+Parent&issn=&date=1983-04-01&volume=13&issue=2&spage=11&au=Brenner%2C+Suzanne&isbn=&jtitle=Exceptional+Parent&btitle=&rft_id=info:eric/EJ285360
ER -

TY - GEN
ID - 2164
T1 - "I Got Religion!"--Evangelism in Language Teaching
A1 - Maley,Alan
Y1 - 1983///
N1 - Availability - Level 1 - Available online, if indexed January 1993 onward
SuppNotes - In: Savignon, Sandra J., Ed. and Berns, Margie S., Ed. Communicative Language Teaching: Where Are We Going? Urbana, Language Learning Laboratory, University of Illinois at Urbana-Champaign, 1983; see FL 016 358
Last updated - 2012-12-14
KW - ERIC
KW - Unsupervised Play Addition
KW - ERIC,Resources in Education (RIE)
KW - Teacher Role
KW - Affective Objectives
KW - Student Role
KW - Play
KW - Communicative Competence (Languages)
KW - Teacher Attitudes
KW - Language Teachers
KW - Educational Trends
KW - Group Dynamics
KW - Humanistic Education
KW - Second Language Instruction
KW - Teaching Methods
KW - Learning Strategies
RP - NOT IN FILE
SP - 9

N2 - There is a risk of second language teachers becoming emotionally overcharged about innovative methods and techniques. Innovators promoting particular methods often claim comprehensive answers to instructional problems, and in spite of an apparent concern for scientific respectability, new approaches take on some characteristics of religious movements. But panaceas do not exist, and pedagogical movements should be kept in perspective. Among the positive results of new approaches are changes in the roles of teacher and learner, emphasis on group supportiveness, relaxation and reduction of threat to the learner, realization of the hidden capacity of the human brain, enhancement of the role of play in learning, changes in attitudes about error, emphasis on building inner learner criteria, and recognition of the value of creative silence. However, some of the approaches are also contradictory or inconsistent. Teachers must learn to exercise independent judgment about teaching methods and not accept them wholesale. (MSE)
UR - http://search.proquest.com/docview/63209603?accountid=14701
L2 - http://sfx.scholarsportal.info/ottawa?url_ver=Z39.88-2004&rft_val_fmt=info:ofi/fmt:kev:mtx:journal&genre=article&sid=ProQ:ProQ%3Aericshell&atitle=%22I+Got+Religion%21%22--Evangelism+in+Language+Teaching.&title=&issn=&date=1983-01-01&volume=&issue=&spage=9&au=Maley%2C+Alan&isbn=&jtitle=&btitle=&rft_id=info:eric/ED278231
ER -

TY - GEN
ID - 2165
T1 - Conceptual Development and Early Multiword Speech
A1 - Shore,Cecilia
Y1 - 1982/03//
N1 - Availability - Level 1 - Available online, if indexed January 1993 onward
SuppNotes - Paper presented at the International Conference on Infant Studies (Austin, TX, March 1982)
Last updated - 2012-12-14
KW - ERIC
KW - Unsupervised Play Addition
KW - Blocks
KW - Multiword Speech
KW - symbolic play
KW - ERIC,Resources in Education (RIE)
KW - Language Usage
KW - Language Research
KW - Concept Formation
KW - Play
KW - Language Acquisition
KW - Child Language
KW - Infants
RP - NOT IN FILE
SP - 17
N2 - The purposes of this study were to investigate (1) the level of development of four target vocal and gestural symbols (Doggie, Cup, Car, and Fiffin, a novel concept), and (2) the relationship of symbolic maturity to the use of symbols in combinations. Thirty infants (15 boys and 15 girls), between 82 and 91 weeks of age, were observed for approximately 45 minutes in a laboratory playroom setting. During the sessions a number of tasks were administered which were designed to assess the children's use of words and conventional gestures, as well as their ability to combine words and gestures. For each target concept, four potential exemplars were presented: a realistic exemplar, an "unusual" exemplar, a perceptually similar member of the same superordinate category, and an object often found with the target object, or related by contiguity. A total of 16 trials were ordered semi-randomly so that no two objects from the same category or level of membership were presented together. The child's utterances during these and other interactions with the experimenter were transcribed. The data were used to obtain several measures of multiword use, including the longest utterance in different content words, the mean length of utterance (MLU) in content words on the five longest utterances, and MLU in morphemes on the five longest utterances. The children's play and language were transcribed by two independent coders. Only actions and utterances agreed upon by both coders were entered into analyses. Results are discussed. (Author/RH)
UR - http://search.proquest.com/docview/63566469?accountid=14701

L2 - http://sfx.scholarsportal.info/ottawa?url_ver=Z39.88-2004&rft_val_fmt=info:ofi/fmt:kev:mtx:journal&genre=unknown&sid=ProQ:ProQ%3Aericshell&atitle=Conceptual+Development+and+Early+Multiword+Speech.&title=&issn=&date=1982-03-01&volume=&issue=&spage=17&au=Shore%2C+Cecilia&isbn=&jtitle=&btitle=&rft_id=info:eric/ED214640
ER -

TY - GEN
ID - 2166
T1 - Cognitive Correlates of Early Multiword Speech
A1 - Shore,Cecilia
Y1 - 1982/03//
N1 - Availability - Level 1 - Available online, if indexed January 1993 onward
SuppNotes - Paper presented at the International Conference on Infant Studies (Austin, TX, March 1982)
Last updated - 2012-12-14
KW - ERIC
KW - Unsupervised Play Addition
KW - Blocks
KW - Multiword Speech
KW - symbolic play
KW - ERIC,Resources in Education (RIE)
KW - Language Research
KW - Cognitive Ability
KW - Play
KW - Cognitive Development
KW - Language Skills
KW - Child Language
KW - Semantics
KW - Infants
RP - NOT IN FILE
SP - 11
N2 - Relationships between "combinatorial" abilities in language, symbolic play, blockbuilding, and non-semantic action sequences were explored in a study of 30 infants between 82 and 91 weeks of age. Subjects were observed in a laboratory playroom setting for approximately 45 minutes. During this time, a number of tasks were administered which were aimed at assessing the child's ability to combine his or her actions into sequences. For each of four target objects, two scenarios were modeled: one involved an appropriate exemplar of a target object (Doggie, Cup, Car, or Fiffin, a novel concept), and the other involved an inappropriate object which was substituted for the target object (e.g., a flashlight as a doggie). Additionally, the experimenter modeled meaningless actions and constructed four block structures. The children's utterances during the entire session were transcribed. Data on infants'"combinatorial" abilities were obtained in five domains: symbolic play with appropriate objects, symbolic play with inappropriate objects, non-semantic action sequences, blockbuilding, and language. Two independent transcripts were made of each child's utterances and play. Only those behaviors agreed upon by two raters were included in the analyses. Results are discussed. (Author/RH)
UR - http://search.proquest.com/docview/63566866?accountid=14701
L2 - http://sfx.scholarsportal.info/ottawa?url_ver=Z39.88-2004&rft_val_fmt=info:ofi/fmt:kev:mtx:journal&genre=unknown&sid=ProQ:ProQ%3Aericshell&atitle=Cognitive+Correlates+of+Early+Multiword+Speech.&title=&issn=&date=1982-03-01&volume=&issue=&spage=11&au=Shore%2C+Cecilia&isbn=&jtitle=&btitle=&rft_id=info:eric/ED214641
ER -

TY - RPRT
ID - 2167
T1 - Summary of a Study of Father Fantasies of Preschool Children with Nonresident Fathers
A1 - Michaels,Carol S.
Y1 - 1981/08//
N1 - Availability - Level 1 - Available online, if indexed January 1993 onward
SuppNotes - Paper presented at the Annual Meeting of the American Psychological Association (89th, Los

Angeles, CA, August 24-28, 1981)
Last updated - 2012-12-14
KW - ERIC
KW - Unsupervised Play Addition
KW - ERIC,Resources in Education (RIE)
KW - Mothers
KW - Parent Role
KW - Fatherless Family
KW - Fathers
KW - Childhood Attitudes
KW - Fantasy
KW - Sex Differences
KW - Parent Attitudes
KW - Pretend Play
KW - Preschool Children
KW - Coping
KW - One Parent Family
RP - NOT IN FILE
SP - 11
N2 - Preschool children's fantasy views of their fathers who had left home before the children were 3 years of age were investigated by trained observers during three separate 20-minute doll play sessions conducted within 12 consecutive school days. Ninety-six pairs of black, white, and hispanic mothers and children ranging in age from 5 to approximately 6.5 years participated in the study. Observers recorded each child's doll play responses on precoded record sheets. Independent variables considered in the study were child's age at father's departure, gender, quantity of contact, ethnicity, and maternal attitudes toward the nonresident father. A total of 18,846 fantasies were produced by the children and nearly 46 per cent included the absent father, while the present mother was included in 43 per cent of the fantasies. All of the children's father fantasies were categorized into "good,""bad" and "silhouette" fantasies. The "good" father fantasies, signifying affectionate, heroic, sexual, happy interactions with the father doll, accounted for 21 per cent of the father fantasies and were significantly associated with the maternal attitude variable. "Bad" fantasies, signifying aggressive, authoritarian, withdrawing, and sad interactions with the father doll were relatively few in number (8.6 per cent) and could be accounted for by several variables of which gender was the most important. The high proportion of "silhouette" fantasies, signifying passive, stereotyped, and unimaginitive doll interactions, (15.1 per cent) suggests that such fantasies, being relatively free from strong emotion and personal conflict, comprise an important element in the children's play. Conclusions drawn from the results are discussed. (Author/RH)
UR - http://search.proquest.com/docview/63559480?accountid=14701
L2 - http://sfx.scholarsportal.info/ottawa?url_ver=Z39.88-2004&rft_val_fmt=info:ofi/fmt:kev:mtx:book&genre=unknown&sid=ProQ:ERIC&atitle=&title=Summary+of+a+Study+of+Father+Fantasies+of+Preschool+Children+with+Nonresident+Fathers.&issn=&date=1981-08-01&volume=&issue=&spage=11&au=Michaels%2C+Carol+S.&isbn=&jtitle=&btitle=Summary+of+a+Study+of+Father+Fantasies+of+Preschool+Children+with+Nonresident+Fathers.&rft_id=info:eric/ED208987
ER -

TY - JOUR
ID - 2168
T1 - Effects of Maternal Interference on the Attachment and Exploratory Behavior of One-Year-Olds
A1 - Gray,Madelyn D.
Y1 - 1979/12//
KW - ERIC
KW - Unsupervised Play Addition
KW - Exploratory Behavior
KW - Maternal Interference
KW - ERIC,Current Index to Journals in Education (CIJE)
KW - Play
KW - Parent Child Relationship
KW - Mothers

KW - Attachment Behavior
KW - Infants
RP - NOT IN FILE
SP - 1211
EP - 1214
JF - Child Development
JA - Child Dev.
VL - 50
IS - 4
N2 - Results from a study of 40 one-year-old children and their mothers showed that maternal interference with children's independent object play had no short-term effect on infant social initiatives to mother, responsiveness to mother's social bids, or exploratory play. (JMB)
UR - http://search.proquest.com/docview/63736465?accountid=14701
L2 - http://sfx.scholarsportal.info/ottawa?url_ver=Z39.88-2004&rft_val_fmt=info:ofi/fmt:kev:mtx:journal&genre=article&sid=ProQ:ProQ%3Aericshell&atitle=Effects+of+Maternal+Interference+on+the+Attachment+and+Exploratory+Behavior+of+One-Year-Olds.&title=Child+Development&issn=&date=1979-12-01&volume=50&issue=4&spage=1211&au=Gray%2C+Madelyn+D.&isbn=&jtitle=Child+Development&btitle=&rft_id=info:eric/EJ226086
ER -

TY - GEN
ID - 2169
T1 - Considerations for Integration of Physically Handicapped and Non-Handicapped Preschool Children
A1 - Cormack,Elizabeth O.
Y1 - 1979/04//
N1 - Availability - Level 1 - Available online, if indexed January 1993 onward
SuppNotes - Paper presented at the Annual International Convention, The Council for Exceptional Children (57th, Dallas, Texas, April 22-27, 1979, Session T-59)
Last updated - 2012-12-14
KW - ERIC
KW - Unsupervised Play Addition
KW - Spina Bifida
KW - ERIC,Resources in Education (RIE)
KW - Preschool Education
KW - Program Descriptions
KW - Social Experience
KW - Mainstreaming
KW - Blindness
KW - Cerebral Palsy
KW - Environmental Influences
KW - Educational Experience
KW - Adjustment (to Environment)
KW - Perceptual Motor Learning
KW - Peer Influence
KW - Developmental Stages
KW - Physical Disabilities
KW - Congenital Impairments
RP - NOT IN FILE
SP - 15
N2 - Eight physically handicapped (blind, spina bifida, cerebral palsy, or genetic defect) preschool children and eight children with normal sensory motor abilities (mean age of both groups 3 1/2 years) participated in a preschool demonstration project designed to promote equal educational and social experiences in an integrated preschool through the use of environmental adaptation and multisensory media. Independent mobility, developmental skill levels, and number and quality of peer interactions were measured. The surroundings were designed to promote independent mobility and peer interaction and included a number of sitting and working

options, such as bolsters, and carpet squares, as well as floor level easels and tables of various shapes and heights. Results of the assessment indicated increased social interactions, accelerated developmental rates, and improved independence. (PHR)
UR - http://search.proquest.com/docview/63766651?accountid=14701
L2 - http://sfx.scholarsportal.info/ottawa?url_ver=Z39.88-2004&rft_val_fmt=info:ofi/fmt:kev:mtx:journal&genre=unknown&sid=ProQ:ProQ%3Aericshell&atitle=Considerations+for+Integration+of+Physically+Handicapped+and+Non-Handicapped+Preschool+Children.&title=&issn=&date=1979-04-01&volume=&issue=&spage=15&au=Cormack%2C+Elizabeth+O.&isbn=&jtitle=&btitle=&rft_id=info:eric/ED171024
ER -

TY - RPRT
ID - 2170
T1 - The Relation of Children's Activity Preference to Sex-Typed Behavior
A1 - Carpenter,Jan C.
Y1 - 1978/08//
N1 - Availability - Level 1 - Available online, if indexed January 1993 onward
SuppNotes - Paper presented at the Annual Meeting of the American Psychological Association (86th, Toronto, Canada, August 28-September 1, 1978)
Last updated - 2012-12-14
KW - ERIC
KW - Unsupervised Play Addition
KW - Structured Activities
KW - Unstructured Activities
KW - ERIC,Resources in Education (RIE)
KW - Preschool Education
KW - Sex Role
KW - Play
KW - Classroom Research
KW - Sex Differences
KW - Conformity
KW - Feedback
KW - Preschool Children
KW - SOCIALIZATION
KW - Models
RP - NOT IN FILE
SP - 15
N2 - The relationship between aspects of preschool socialization experiences and sex-typed behavior was investigated in this study. Three hypotheses were tested: (1) that preschool play activities differ consistently in structure level, (2) that there are sex differences in participation in activities classified as high or low structure, and (3) that children of both sexes are more compliant in highly structured activities and show more novel use of materials and self-initiated activity changes in low structure activities. The behaviors and activities of 83 children, ages 2 1/2 to 5, and their teachers in five preschool classrooms were coded by trained observers during daily free-choice activity periods. The activities were coded for degree of structure by the amounts of individual and group feedback provided by the teacher. An additional index of structure consisted of a parent questionnaire concerning availability of an adult model in the home for 20 analogous activities. Preschool activities were found to differ in level of structure on all indices. Boys participated more than girls in the low-structure activities, and girls participated more than boys in the high-structure activities for all indices. Compliance occurred more often in the high- than low-structure activities as indexed by feedback, but the reverse occurred on the index of modeling. Novel behavior occurred more often in low-structure activities on the index of individual feedback. Compliance and novelty were a function of activity, not of sex. Sex differences independent of activity were found only for self-initiated activity. (Author/SS)
UR - http://search.proquest.com/docview/63765708?accountid=14701
L2 - http://sfx.scholarsportal.info/ottawa?url_ver=Z39.88-2004&rft_val_fmt=info:ofi/fmt:kev:mtx:book&genre=unknown&sid=ProQ:ERIC&atitle=&title=The+Relation

+of+Children%27s+Activity+Preference+to+Sex-Typed+Behavior.&issn=&date=1978-08-01&volume=&issue=&spage=15&au=Carpenter%2C+Jan+C.&isbn=&jtitle=&btitle=The+Relation+of+Children%27s+Activity+Preference+to+Sex-Typed+Behavior.&rft_id=info:eric/ED170052
ER -

TY - GEN
ID - 2171
T1 - Social Skills Assessment and Curricula
A1 - Bair,Anne G.
Y1 - 1976/11//
N1 - Availability - Level 1 - Available online, if indexed January 1993 onward
SuppNotes - Paper presented at the Conference, Breaking Affective Barriers for the Handicapped, Virginia Council on Health and Medical Care (Roanoke, Virginia, November 21-23, 1976) ; Appendix may not reproduce clearly due to print quality
Last updated - 2012-12-14
KW - ERIC
KW - Unsupervised Play Addition
KW - ERIC,Resources in Education (RIE)
KW - Early Childhood Education
KW - Handicapped Children
KW - Play
KW - Curriculum
KW - Social Development
KW - Toys
RP - NOT IN FILE
SP - 46
N2 - Described is an independent toy play curriculum for young handicapped children. It is explained that toys were analyzed according to functions in three classifications: functional toy skills (toys designed to elicit simple motor actions of short duration); physical toy skills (toys designed to elicit gross motor action); and constructive toy skills (toys designed to elicit the child's imagination and organization). The curriculum includes information on pretesting and instruction, as well as progress profiles and instructional profiles which list behavioral objectives and criteria for each toy. Also included is information on nine other curricula for social, communication, self help and occupational development of handicapped children. (CL)
UR - http://search.proquest.com/docview/63899950?accountid=14701
ER -

TY - GEN
ID - 2172
T1 - In Defense of Playfulness
A1 - Dillon,Stephen V.
Y1 - 1975///
N1 - Last updated - 2012-12-14
KW - ERIC
KW - Unsupervised Play Addition
KW - ERIC,Current Index to Journals in Education (CIJE)
KW - Junior High Schools
KW - Humanistic Education
KW - Play
KW - Classroom Environment
KW - Goal Orientation
KW - Psychological Needs
KW - Psychological Characteristics
KW - Elementary Schools
KW - Discovery Learning
KW - Achievement Need
KW - Models

RP - NOT IN FILE
SP - 206
EP - 213
JF - Elementary School Journal
VL - 75
IS - 4
N2 - Discusses how "Messing About" may lead to exploratory learning and, ultimately, to steady growth in productive thought and problem-solving ability. Emphasizes the role of classroom organization and teacher-models of competence in facilitating autonomous interest. (CS)
UR - http://search.proquest.com/docview/64077596?accountid=14701
L2 - http://sfx.scholarsportal.info/ottawa?url_ver=Z39.88-2004&rft_val_fmt=info:ofi/fmt:kev:mtx:journal&genre=unknown&sid=ProQ:ProQ%3Aericshell&atitle=In+Defense+of+Playfulness&title=Elementary+School+Journal&issn=&date=1975-01-01&volume=75&issue=4&spage=206&au=Dillon%2C+Stephen+V.&isbn=&jtitle=Elementary+School+Journal&btitle=&rft_id=info:eric/EJ109714
ER -

TY - RPRT
ID - 2173
T1 - Transformations and Codes in Early Pretending
A1 - Fein,Greta G.
Y1 - 1974/09//
N1 - Availability - Level 1 - Available online, if indexed January 1993 onward
SuppNotes - A briefer version of this paper was presented at the Annual Meeting of the American Psychological Association (82nd, New Orleans, Louisiana, Aug. 30-Sept. 3, 1974)
Last updated - 2012-12-14
KW - ERIC
KW - Unsupervised Play Addition
KW - Piaget (Jean)
KW - ERIC,Resources in Education (RIE)
KW - Early Childhood Education
KW - Behavior Development
KW - Cognitive Development
KW - Imitation
KW - Responses
KW - Literature Reviews
KW - Imagination
KW - Infant Behavior
KW - Behavior Patterns
KW - Toys
KW - Pretend Play
KW - Infants
RP - NOT IN FILE
SP - 37
N2 - Evidence which suggests that pretend activities become increasingly independent of the presence of realistic objects is examined in this paper. Results of research on pretend behavior in children 1 1/2 - 2 years of age are described and analyzed. Striking changes in pretend behavior are shown to occur during the second year of life. Pretend play is treated as a paradigm for understanding how children represent objects and relations. It is argued that the child can pretend that one thing is another when he can represent objects (or object activities) as prototypes and when he can use the core properties of prototypes to transform representations of immediate physical stimulation. The analysis suggests a three-phase sequence in the development of pretending based on a progression from the analogue mapping of representations to the selection and, eventually, the production of the core properties of object representations. (Author/ED)
UR - http://search.proquest.com/docview/63978710?accountid=14701
ER -

TY - RPRT
ID - 2174
T1 - Home-Based Educational Curricula for Mothers and Infants
A1 - Apfel,Nancy
A1 - Brion,LaRue
Y1 - 1974///
N1 - Availability - Level 2 - Produced in microfiche (1966-2003)
SuppNotes - Not available in hard copy due to sections of marginal legibility of original document; Journal reprints and flyers of the original document are copyrighted and therefore not available. They have not been included in the pagination
Last updated - 2012-12-14
KW - ERIC
KW - Unsupervised Play Addition
KW - Curriculum Research in Infant Education
KW - ERIC,Resources in Education (RIE)
KW - Preschool Education
KW - Parent Education
KW - Interaction
KW - Play
KW - Language Acquisition
KW - Home Visits
KW - Intervention
KW - Observation
KW - Instructional Materials
KW - Home Programs
KW - Parent Child Relationship
KW - Child Rearing
KW - Social Development
KW - Pretend Play
KW - Infants
RP - NOT IN FILE
SP - 526
VL - OCD-CB-98
N2 - This package contains five home-based intervention curricula for families with children between 12 and 30 months of age. Three independent curricula (each emphasizing children's language, play or social development) enlist the mother's aid as observer, teacher and researcher, and promote a three-way interaction among mother, child, and home-visitor. The Mother Only curriculum combines the three independent curricula into a single program in which the primary interaction is between the mother and home-visitor, to the exclusion of the child (who should benefit from the education the mother receives). Likewise, the Baby Only curriculum is in combination form, but with the mother excluded from the intervention program. Each curriculum contains materials for a program of 32 visits by a trained home visitor over an 18-month period. The goals, home-visitor role, patterns of visits, and problems encountered in testing the language, play, and social development curricula are briefly discussed. (ED)
UR - http://search.proquest.com/docview/63970153?accountid=14701
ER -

TY - RPRT
ID - 2175
T1 - Toward the Development of a Technique to Measure the Racial Awareness-Attitudes of Three- to Five-Year-Old Children
A1 - Scanlan,Peter A.
A1 - Dokecki,Paul R.
Y1 - 1973/05//
N1 - Availability - Level 1 - Available online, if indexed January 1993 onward
Last updated - 2012-12-14
KW - ERIC

KW - Unsupervised Play Addition
KW - Demonstration and Research Ctr Early Child Educ
KW - ERIC,Resources in Education (RIE)
KW - Early Childhood Education
KW - Racial Identification
KW - Play
KW - Research Methodology
KW - Socioeconomic Status
KW - Racial Attitudes
KW - Geographic Regions
KW - Racial Differences
KW - Literature Reviews
KW - Measurement Techniques
KW - Social Science Research
KW - Perception
KW - Sex Differences
KW - Preschool Children
KW - Age Differences
RP - NOT IN FILE
SP - 28
N2 - This paper presents a comprehensive review of research literature in the area of interracial awareness development in preschool children and highlights key issues for future research. An historical look at the study of racial attitudes reveals three major research techniques. The first type utilizes pictures, line drawings, or actual photos. The chief drawback of this technique is that it relies heavily on the child's linguistic ability and does not allow the use of unstructured play. A second technique involves the use of puzzles, doll assembly, or picture insets. This method is suitable for measuring racial awareness but not attitudes. A third technique involves doll-play and seems to offer the most promise for future investigators of racial awareness attitudes in young children. The independent variables shown to be significant which must be considered for future studies are age, race, sex, social class, shade of skin color, contact, region of the country, and race of the interviewer. (MS)
UR - http://search.proquest.com/docview/63919095?accountid=14701
ER -

TY - BOOK
ID - 2176
T1 - I Can Do It. Project Enlightenment
A1 - Coley,Elise D.
Y1 - 1973///
N1 - Availability - Level 2 - Produced in microfiche (1966-2003)
SuppNotes - For related documents, see EC 202 488-497
Last updated - 2012-12-14
KW - ERIC
KW - Unsupervised Play Addition
KW - ERIC,Resources in Education (RIE)
KW - Parents
KW - Preschool Education
KW - Parent Education
KW - Play
KW - Family Environment
KW - Home Programs
KW - Parent Role
KW - Child Development
KW - Skill Development
RP - NOT IN FILE
SP - 49
PB - Project Enlightenment, 501 S. Boylan Ave., Raleigh, NC 27603 ($4.00).

N2 - This book is designed for use by parents at home to help them encourage the development of necessary skills in their preschool-age children through play and other daily living experiences. The activities are presented as a series of short paragraphs written in the first person from the child's perspective with a box that the child can check when he or she accomplishes the task described. The experiences are intended to strengthen the use of the child's five senses, enhance the acquisition of language and prereading skills, provide opportunities for conceptual growth through reasoning and independent observation, and encourage social growth and development. Specific activities take advantage of routine household chores such as grocery shopping, cooking, and cleaning as well as outside play and visits to community facilities (e.g., parks, schools, fire stations) as potential learning opportunities. The three sections group the activities by those occurring inside the house, those immediately outside the house, and those in the neighborhood. A section at the end of the book reviews each of the skill areas that are important to a young child's growth in terms of how the skills are acquired and how they contribute to later development. (VW)
UR - http://search.proquest.com/docview/63165892?accountid=14701
L2 - http://sfx.scholarsportal.info/ottawa?url_ver=Z39.88-2004&rft_val_fmt=info:ofi/fmt:kev:mtx:book&genre=book&sid=ProQ:ERIC&atitle=&title=I+Can+Do+It.+Project+Enlightenment.&issn=&date=1973-01-01&volume=&issue=&spage=49&au=Coley%2C+Elise+D.&isbn=&jtitle=&btitle=I+Can+Do+It.+Project+Enlightenment.&rft_id=info:eric/ED293293
ER -

TY - GEN
ID - 2177
T1 - A History of the Children's Theatre Association of Baltimore, Maryland from 1943-1966
A1 - Kraus,Joanna Halpert
Y1 - 1972///
N1 - Availability - Level 3 - Indexed only
SuppNotes - Ed.D. Dissertation, Columbia University
Last updated - 2012-12-14
KW - ERIC
KW - Unsupervised Play Addition
KW - Childrens Theatre Association MD
KW - Maryland (Baltimore)
KW - ERIC,Resources in Education (RIE)
KW - Play
KW - Speech Communication
KW - Creative Dramatics
KW - Research Projects
KW - Theater Arts
KW - Child Development
KW - Humanities
KW - Drama
RP - NOT IN FILE
SP - 408
PB - University Microfilms, A Xerox Company, Dissertation Copies, Post Office Box 1764, Ann Arbor, Michigan 48106 (Order No. 72-19,518, MFilm $4.00, Xerography $10.00)
N2 - A descriptive history is presented of the Children's Theatre Association of Baltimore, an independent community children's theater, focusing on the years 1943-1966, the period in which founder Isabel Burger was in office. The Association's educational goals, influence, and contribution to the community are stressed. A major portion of the research was obtained from unpublished sources. Separate chapters of the study discuss the expansion of artistic activity, community influence, administrative policy, national and international activities of the Association's founder, and Burger's professional activities and the status of the organization since her retirement. Appendices include a chronological listing of all productions and the forms sent to past participants. The study demonstrates that the Theatre's goal of human development through the use of drama and the dynamic personality of the founder had great influence. (LH)
UR - http://search.proquest.com/docview/64186166?accountid=14701

L2 - http://sfx.scholarsportal.info/ottawa?url_ver=Z39.88-2004&rft_val_fmt=info:ofi/fmt:kev:mtx:journal&genre=unknown&sid=ProQ:ProQ%3Aericshell&atitle=A+History+of+the+Children%27s+Theatre+Association+of+Baltimore%2C+Maryland+from+1943-1966.&title=&issn=&date=1972-01-01&volume=&issue=&spage=408&au=Kraus%2C+Joanna+Halpert&isbn=&jtitle=&btitle=&rft_id=info:eric/ED067682
ER -

TY - RPRT
ID - 2178
T1 - Project CHILD
A1 - Robison,Helen F.
Y1 - 1970/10//
N1 - Availability - Level 1 - Available online, if indexed January 1993 onward
Last updated - 2012-12-14
KW - ERIC
KW - Unsupervised Play Addition
KW - ERIC,Resources in Education (RIE)
KW - Program Descriptions
KW - Auditory Discrimination
KW - Teacher Behavior
KW - Play
KW - Language Acquisition
KW - Teacher Education
KW - Intervention
KW - Teacher Effectiveness
KW - Preschool Curriculum
KW - Curriculum Development
KW - Educational Change
KW - Disadvantaged Youth
KW - Compensatory Education
KW - Teaching Methods
RP - NOT IN FILE
SP - 113
N2 - This document described Project CHILD, a program of educational change and curriculum development for disadvantaged prekindergarten and kindergarten children. The historical part of this report indicates that the project began in 1966 with a small-scale study of teacher behavior and children's responses in a few classrooms in a Harlem school district. This study led to the development of a reserach instrument and suggested the overwhelming need for program improvement. In 1966-'67 the curriculum was compiled in rough form and implemented on a very limited basis. Data from this period focuses on language development, a major emphasis of the curriculum. 1967-'68 was a year of broadening the implementation effort, as four teachers used the program with in-depth training. The curriculum was revised during this year, as it became a 2-year intervention approach. The field study was backed up by much data collection, including a detailed project to determine intervention effects on auditory discrimination. By 1968-'69 the program had developed a comprehensive curriculum package, and the report focuses on problems encountered in changing teachers' methods and attitudes. A content analysis of the curriculum discusses internal inconsistencies on theoretical issues, language arts training, views of play, teaching strategies, motor development, and behavioral objectives. Appendix A is an independent assessment of the curriculum, Appendix B describes the goals, procedures, and history of the parent project, and Appendix C is a description of the curriculum prepared for an educational conference. (DP)
UR - http://search.proquest.com/docview/64100542?accountid=14701
ER -

TY - JOUR
ID - 2179
T1 - Drama Education in the Age of AIDS
A1 - Singh,Lorraine

Y1 - 2012/10//
KW - ERIC
KW - Unsupervised Play Addition
KW - Kwazulu
KW - South Africa (Natal)
KW - ERIC,Current Index to Journals in Education (CIJE)
KW - Higher Education
KW - Student Teacher Attitudes
KW - Cultural Context
KW - Didacticism
KW - Drama Workshops
KW - Dramatic Play
KW - World Views
KW - Preservice Teacher Education
KW - Controversial Issues (Course Content)
KW - Skill Development
KW - Health Promotion
KW - Sexuality
KW - Student Experience
KW - Foreign Countries
KW - Social Attitudes
KW - Teaching Skills
KW - Educational Practices
KW - Acquired Immunodeficiency Syndrome (AIDS)
KW - Females
KW - Knowledge Base for Teaching
KW - Critical Theory
KW - Teaching Methods
RP - NOT IN FILE
SP - 21
EP - 29
JF - Perspectives in Education
VL - 30
IS - 3
PB - Perspectives in Education. Faculty of Education, University of the Free State, Bloemfontein 9301, South Africa. Fax: +27-51-401-7044; e-mail: pie@ufs.ac.za; Web site: http://search.sabinet.co.za/pie
N2 - This article arose out of my involvement in an undergraduate drama module at the School of Education, University of KwaZulu-Natal, where I made use of workshop theatre methodologies to explore how second-year drama students construct knowledge and develop sociocultural understandings of critical issues in society. The workshop theatre project described in this article aimed to allow drama students to explore and expose the myths, practices and world view associated with the sexual behaviour of young people in two townships and on two campuses. The purpose was to ensure that, as prospective teachers, these students are prepared with knowledge and skills to engage their future learners on this topic in a relevant and effective manner. The article draws on the theories of applied drama and argues for workshopping as a cogent means of researching and representing social issues. The data-gathering process undertaken by students, and the plays constructed from the analysis are described. I conclude with a discussion on how the methodology reflected the beliefs and attitudes of the participants in a contextualised manner, exposing a flaw in their image of the sexually liberated and independent young woman. I also show how the approach allowed me to develop an emancipatory pedagogy which endows students with authority
SN - 0258-2236, 0258-2236
UR - http://search.proquest.com/docview/1347459520?accountid=14701
L2 - http://sfx.scholarsportal.info/ottawa?url_ver=Z39.88-2004&rft_val_fmt=info:ofi/fmt:kev:mtx:journal&genre=article&sid=ProQ:ProQ%3Aericshell&atitle=Drama+Education+in+the+Age+of+AIDS&title=Perspectives+in+Education&issn=02582236&date=2012-10-01&volume=30&issue=3&spage=21&au=Singh%2C+Lorraine&isbn=&jtitle=Perspectives+in+Education&btitle=&rft_id=info:eric/EJ998096

ER -

TY - JOUR
ID - 2180
T1 - Computer Use within a Play-Based Early Years Curriculum
A1 - Howard,Justine
A1 - Miles,Gareth E.
A1 - Rees-Davies,Laura
Y1 - 2012///
KW - ERIC
KW - Unsupervised Play Addition
KW - Wales (South)
KW - Kruskal Wallis Test
KW - Mann Whitney U Test
KW - ERIC,Current Index to Journals in Education (CIJE)
KW - Early Childhood Education
KW - Computer Use
KW - Information Technology
KW - Foreign Countries
KW - Play
KW - Computers
KW - Learner Engagement
KW - Semi Structured Interviews
KW - Preschool Children
KW - Computer Uses in Education
KW - Computer Literacy
RP - NOT IN FILE
SP - 175
EP - 189
JA - International Journal of Early Years Education
VL - 20
IS - 2
PB - Routledge. , 325 Chestnut Street Suite 800, Philadelphia, PA 19106.
N2 - Early years curricula promote learning through play and in addition emphasise the development of computer literacy. Previous research, however, has described that teachers feel unprepared to integrate Information and Communication Technology (ICT) and play. Also, whereas research has suggested that effective computer use in the early years is associated with adult direction, further research suggests adult presence can inhibit play and reduce children's engagement. Focusing on 12 settings following the Welsh play-based Foundation Phase, this paper explores teachers' experiences of integrating computer use into classroom practice, children's levels of engagement with computer activities and how playful children perceive computer use to be. Teachers confidently delivered a variety of computing experiences. Children consistently rated these activities as play regardless of adult presence and demonstrated moderate to high levels of engagement. Findings and the features of observed practice are discussed in relation to the teachers' role as a play partner and the successful co-construction of the play-based curriculum. (Contains 3 tables and 1 figure.)
SN - 0966-9760, 0966-9760
UR - http://search.proquest.com/docview/1238188138?accountid=14701
L2 - http://sfx.scholarsportal.info/ottawa?url_ver=Z39.88-2004&rft_val_fmt=info:ofi/fmt:kev:mtx:journal&genre=article&sid=ProQ:ProQ%3Aericshell&atitle=Computer+Use+within+a+Play-Based+Early+Years+Curriculum&title=International+Journal+of+Early+Years+Education&issn=09669760&date=2012-01-01&volume=20&issue=2&spage=175&au=Howard%2C+Justine%3BMiles%2C+Gareth+E.%3BRees-Davies%2C+Laura&isbn=&jtitle=International+Journal+of+Early+Years+Education&btitle=&rft_id=info:eric/EJ983545
ER -

TY - JOUR
ID - 2182
T1 - Program-Level Influences on the Measurement of Early Communication for Infants and Toddlers in Early Head Start
A1 - Greenwood,Charles R.
A1 - Buzhardt,Jay
A1 - Walker,Dale
A1 - Howard,Waylon J.
A1 - Anderson,Rawni
Y1 - 2011/06//
KW - ERIC
KW - Unsupervised Play Addition
KW - ERIC,Current Index to Journals in Education (CIJE)
KW - Early Childhood Education
KW - Geographic Location
KW - Program Effectiveness
KW - Toddlers
KW - Play
KW - Individualized Family Service Plans
KW - Socioeconomic Status
KW - Decision Making
KW - Outcomes of Education
KW - Communication Skills
KW - Evaluation Methods
KW - Program Improvement
KW - Program Implementation
KW - Disadvantaged Youth
KW - Early Intervention
KW - Program Evaluation
KW - Total Communication
KW - Limited English Speaking
KW - Infants
RP - NOT IN FILE
SP - 110
EP - 134
JF - Journal of Early Intervention
VL - 33
IS - 2
PB - SAGE Publications. 2455 Teller Road, Thousand Oaks, CA 91320.
N2 - Measurement in early childhood is an increasingly large-scale endeavor addressing purposes of accountability, program improvement, child outcomes, and intervention decision making for individual children. The Early Communication Indicator (ECI) is a measure relevant to intervention decision making for infants and toddlers, including response to intervention approaches. The widespread use of the ECI is growing in multiple programs and states. Local program staff members collect ECI data and, with their program directors, manage their own system of ECI measurement. Program-level implementations represent independent ECI measurement replications, and the success of each potentially influences the quality of data produced and, ultimately, the validity of the inferences made thereof. The purpose of this research was to examine program-level influences on child-level ECI total communication growth and 36-month outcomes in a large sample of children, including those with individual family service plans served by multiple Early Head Start programs in two states. Results indicated variation in programs' sociodemographic composition, ECI implementation quality, ECI total communication growth, and 36-month outcomes. Program-level sociodemographic composition was found not to be an influence on ECI growth or 36-month outcomes, whereas state location and implementation quality were. Implications are discussed. (Contains 6 notes, 7 tables, and 3 figures.)
SN - 1053-8151, 1053-8151
UR - http://search.proquest.com/docview/964170388?accountid=14701

L2 - http://sfx.scholarsportal.info/ottawa?url_ver=Z39.88-2004&rft_val_fmt=info:ofi/fmt:kev:mtx:journal&genre=article&sid=ProQ:ProQ%3Aericshell&atitle=Program-Level+Influences+on+the+Measurement+of+Early+Communication+for+Infants+and+Toddlers+in+Early+Head+Start&title=Journal+of+Early+Intervention&issn=10538151&date=2011-06-01&volume=33&issue=2&spage=110&au=Greenwood%2C+Charles+R.%3BBuzhardt%2C+Jay%3BWalker%2C+Dale%3BHoward%2C+Waylon+J.%3BAnderson%2C+Rawni&isbn=&jtitle=Journal+of+Early+Intervention&btitle=&rft_id=info:eric/EJ938926
ER -

TY - JOUR
ID - 2183
T1 - Effects of Conditioning Voices as Reinforcers for Listener Responses on Rate of Learning, Awareness, and Preferences for Listening to Stories in Preschoolers with Autism
A1 - Greer,Douglas R.
A1 - Pistoljevic,Nirvana
A1 - Cahill,Claire
A1 - Du,Lin
Y1 - 2011///
KW - ERIC
KW - Unsupervised Play Addition
KW - ERIC,Current Index to Journals in Education (CIJE)
KW - Preschool Education
KW - Learning
KW - Play
KW - Responses
KW - Intervention
KW - Verbal Stimuli
KW - Adults
KW - Behavior Problems
KW - Auditory Stimuli
KW - Conditioning
KW - Story Telling
KW - Reinforcement
KW - Intervals
KW - Preschool Children
KW - Autism
KW - Listening Skills
RP - NOT IN FILE
SP - 103
EP - 124
JF - Analysis of Verbal Behavior
VL - 27
PB - Association for Behavior Analysis International. 1219 South Park Street, Kalamazoo, MI 49001.
N2 - We used a delayed non-concurrent pre- and post-intervention probe design to test the effects of a voice conditioning protocol (VCP) with 3 preschoolers with autism on (a) rate of acquisition of listener curricular objectives, (b) observing voices and the presence of adults across 3 settings, (c) selecting to listen to adults tell stories in free play setting, and (d) the occurrence of stereotypy in the story setting. The VCP conditioned voices as reinforcers for listening to recordings of voices via stimulus-stimulus pairing, which resulted in the children listening to audio recordings of voices in 90% of intervals in 5-min concurrent-operant preference tests. After voices became conditioned reinforcers, all 3 children's learning accelerated; 2 children's observing responses increased in the 3 settings; and 2 children selected to listen to stories and also showed decreased stereotypy in the story setting. The data suggest that conditioned reinforcement for observing responses may be a verbal behavior developmental cusp that acts to accelerate learning that involves listening, and that the cusp may be induced using the VCP. (Contains 4 tables and 6 figures.)
SN - 0889-9401, 0889-9401
UR - http://search.proquest.com/docview/1011399513?accountid=14701

L2 - http://sfx.scholarsportal.info/ottawa?url_ver=Z39.88-2004&rft_val_fmt=info:ofi/fmt:kev:mtx:journal&genre=article&sid=ProQ:ProQ%3Aericshell&atitle=Effects+of+Conditioning+Voices+as+Reinforcers+for+Listener+Responses+on+Rate+of+Learning%2C+Awareness%2C+and+Preferences+for+Listening+to+Stories+in+Preschoolers+with+Autism&title=Analysis+of+Verbal+Behavior&issn=08899401&date=2011-01-01&volume=27&issue=&spage=103&au=Greer%2C+Douglas+R.%3BPistoljevic%2C+Nirvana%3BCahill%2C+Claire%3BDu%2C+Lin&isbn=&jtitle=Analysis+of+Verbal+Behavior&btitle=&rft_id=info:eric/EJ959195
ER -

TY - JOUR
ID - 2184
T1 - Differences in Practitioners' Understanding of Play and How This Influences Pedagogy and Children's Perceptions of Play
A1 - McInnes,Karen
A1 - Howard,Justine
A1 - Miles,Gareth
A1 - Crowley,Kevin
Y1 - 2011///
KW - ERIC
KW - Unsupervised Play Addition
KW - United Kingdom
KW - ERIC,Current Index to Journals in Education (CIJE)
KW - Early Childhood Education
KW - Teacher Role
KW - Play
KW - Role Perception
KW - Aptitude Treatment Interaction
KW - Child Caregivers
KW - Teacher Attitudes
KW - Protocol Materials
KW - Observation
KW - Foreign Countries
KW - Individual Differences
KW - Educational Practices
KW - Interaction Process Analysis
KW - Learning Activities
KW - Interviews
RP - NOT IN FILE
SP - 121
EP - 133
JF - Early Years: An International Journal of Research and Development
VL - 31
IS - 2
PB - Routledge. , 325 Chestnut Street Suite 800, Philadelphia, PA 19106.
N2 - This paper presents research that examines the links between practitioners' understanding of play and its relationship to learning, their pedagogic interactions with children and children's own perceptions of their play. Previous research has shown a mismatch between practitioners' understanding of play and their practice. This research identifies how differences in understanding of play, especially the role of the adult, by practitioners in two unrelated settings are associated with variation in pedagogic interactions emphasising choice and control. Furthermore, it is argued that these differences are reflected in differences in children's use of the cue of adult presence as a defining feature of play and not-play activities. (Contains 2 tables.)
SN - 0957-5146, 0957-5146
UR - http://search.proquest.com/docview/889927638?accountid=14701
L2 - http://sfx.scholarsportal.info/ottawa?url_ver=Z39.88-2004&rft_val_fmt=info:ofi/fmt:kev:mtx:journal&genre=article&sid=ProQ:ProQ%3Aericshell&atitle=Differences+in+Practitioners%27+Understanding+of+Play+and+How+This+Influences+Pedagogy+and+Children%27s+

Perceptions+of+Play&title=Early+Years%3A+An+International+Journal+of+Research+and+Development&issn=09575146&date=2011-01-01&volume=31&issue=2&spage=121&au=McInnes%2C+Karen%3BHoward%2C+Justine%3BMiles%2C+Gareth%3BCrowley%2C+Kevin&isbn=&jtitle=Early+Years%3A+An+International+Journal+of+Research+and+Development&btitle=&rft_id=info:eric/EJ932620
ER -

TY - JOUR
ID - 2185
T1 - Sharing Attention and Activities among Toddlers: The Spatial Dimension of the Setting and the Educator's Role
A1 - Musatti,Tullia
A1 - Mayer,Susanna
Y1 - 2011///
KW - ERIC
KW - Unsupervised Play Addition
KW - Italy
KW - ERIC,Current Index to Journals in Education (CIJE)
KW - Preschool Education
KW - Preschool Teachers
KW - Teacher Role
KW - Interaction
KW - Toddlers
KW - Play
KW - Cognitive Development
KW - Child Behavior
KW - Classroom Environment
KW - Early Childhood Education
KW - Child Care
KW - Classroom Design
KW - Video Technology
KW - Foreign Countries
KW - Environmental Influences
KW - Educational Practices
KW - Child Development
KW - Learning Activities
KW - Psychomotor Skills
RP - NOT IN FILE
SP - 207
EP - 221
JA - European Early Childhood Education Research Journal
VL - 19
IS - 2
PB - Routledge. , 325 Chestnut Street Suite 800, Philadelphia, PA 19106.
N2 - The study proposes an analysis of the processes through which the material and symbolic features of the setting and the activities of the educators interact to determine the children's experience in an early educational centre. This analysis is of particular interest with regard to both educational practice and understanding developmental processes during children's second year of life. During this period, important changes occur in children's interaction with the environment in its physical and social components, on the basis of their cognitive development as well as of their acquired capacity to independent locomotion. Two examples will be presented that illustrate how the educators' activities and the spatial arrangement of the setting combine and interact with toddlers' recently acquired independent locomotion in sustaining their cognitive engagement and social encounters during their daily routine in an educational centre. The discussion of the examples opens new perspectives for understanding early sociality in a group situation and for innovating educational practices. (Contains 3 figures.)
SN - 1350-293X, 1350-293X

UR - http://search.proquest.com/docview/889928342?accountid=14701
L2 - http://sfx.scholarsportal.info/ottawa?url_ver=Z39.88-2004&rft_val_fmt=info:ofi/fmt:kev:mtx:journal&genre=article&sid=ProQ:ProQ%3Aericshell&atitle=Sharing+Attention+and+Activities+among+Toddlers%3A+The+Spatial+Dimension+of+the+Setting+and+the+Educator%27s+Role&title=European+Early+Childhood+Education+Research+Journal&issn=1350293X&date=2011-01-01&volume=19&issue=2&spage=207&au=Musatti%2C+Tullia%3BMayer%2C+Susanna&isbn=&jtitle=European+Early+Childhood+Education+Research+Journal&btitle=&rft_id=info:eric/EJ933040
ER -

TY - JOUR
ID - 2186
T1 - The Influence of Father Education Programs on the Levels of Father Involvement with Children: An Experimental Study
A1 - Taskin,Necdet
A1 - Erkan,Semra
Y1 - 2009///
KW - ERIC
KW - Unsupervised Play Addition
KW - Turkey
KW - ERIC,Current Index to Journals in Education (CIJE)
KW - Parent Education
KW - Program Effectiveness
KW - Foreign Countries
KW - Play
KW - Nonparametric Statistics
KW - Parent Child Relationship
KW - Child Care
KW - Interviews
KW - CHILDREN
KW - Fathers
KW - Pretests Posttests
RP - NOT IN FILE
SP - 136
EP - 148
JF - Hacettepe University Journal of Education
VL - 37
PB - Hacettepe University. Faculty of Education, Beytepe, Ankara 06800, Turkey. Tel: +90-312-2978583; Fax: +90-312-2992083; e-mail: efdergi@hacettepe.edu.tr; Web site: http://www.efdergi.hacettepe.edu.tr
N2 - This study aims to reveal the effects of father education programs on the levels of their involvement with their children aged 2 to 9. The study group comprised 14 fathers who participate in a father education program. The study employed the pre-test-posttest design. Data were collected by using Father Interview Forms. The independent variable of the study is the Father Education Program activity implemented on the study group between the pre- and posttest. Starting from one week after the fathers were given the Father Interview Form as a pretest, the fathers took participated a 10-week education. One week after the education program was complete, the same test was administered once again to the fathers as a posttest. Data obtained from the study were analyzed with the Non-Parametric Wilcoxon Signed Rank Test. The findings have shown that the father education program increased "the frequency of fathers becoming involved with their children's games, going out with their children, having verbal interactions with them, teaching them new things, becoming involved with their daily care, and looking after them at special times". (Contains 7 tables.)
SN - 1300-5340, 1300-5340
UR - http://search.proquest.com/docview/61808843?accountid=14701
L2 - http://sfx.scholarsportal.info/ottawa?url_ver=Z39.88-2004&rft_val_fmt=info:ofi/fmt:kev:mtx:journal&genre=article&sid=ProQ:ProQ%3Aericshell&atitle=The+Influence+of+Father+Education+Programs+on+the+Levels+of+Father+Involvement+with+Children%3A+An+Experimental+Study&title=Hacettepe+University+Journal+of+Education&issn=13005340&date=2009-01-

01&volume=37&issue=&spage=136&au=Taskin%2C+Necdet%3BErkan%2C+Semra&isbn=&jtitle=Hacettepe+University+Journal+of+Education&btitle=&rft_id=info:eric/EJ871025
ER -

TY - JOUR
ID - 2188
T1 - Social Grooming in the Kindergarten: The Emergence of Flattery Behavior
A1 - Fu,Genyue
A1 - Lee,Kang
Y1 - 2007/03//
KW - ERIC
KW - Unsupervised Play Addition
KW - ERIC,Current Index to Journals in Education (CIJE)
KW - Kindergarten
KW - Social Influences
KW - Play
KW - Child Behavior
KW - Developmental Psychology
KW - Psychological Patterns
KW - Freehand Drawing
KW - Interpersonal Communication
KW - Social Development
KW - Preschool Children
KW - Age Differences
KW - Social Environment
RP - NOT IN FILE
SP - 255
EP - 265
JF - Developmental Science
VL - 10
IS - 2
PB - Wiley-Blackwell. 350 Main Street, Malden, MA 02148.
N2 - The present study examined the emergence of flattery behavior in young children and factors that might affect whether and how it is displayed. Preschool children between the ages of 3 and 6 years were asked to rate drawings produced by either a present or absent adult stranger (Experiments 1 and 2), child stranger (Experiments 2 and 3), classmate, or the children's own teacher (Experiment 3). Young preschoolers gave consistent ratings to the same drawing by the person regardless of whether the person was absent or present. In contrast, many older preschoolers gave more flattering ratings to the drawing when the person was present than in the person's absence. Also, older preschoolers displayed flattery regardless of whether the recipient was an adult or a child. However, they displayed flattery to a greater extent towards familiar individuals than unfamiliar ones, demonstrating an emerging sensitivity to social contexts in which flattery is used. These findings suggest that preschoolers have already learned not to articulate bluntly their true feelings and thoughts about others. Rather, they are able to manipulate their communications according to social context
SN - 1363-755X, 1363-755X
UR - http://search.proquest.com/docview/61855508?accountid=14701
L2 - http://sfx.scholarsportal.info/ottawa?url_ver=Z39.88-2004&rft_val_fmt=info:ofi/fmt:kev:mtx:journal&genre=article&sid=ProQ:ProQ%3Aericshell&atitle=Social+Grooming+in+the+Kindergarten%3A+The+Emergence+of+Flattery+Behavior&title=Developmental+Science&issn=1363755X&date=2007-03-01&volume=10&issue=2&spage=255&au=Fu%2C+Genyue%3BLee%2C+Kang&isbn=&jtitle=Developmental+Science&btitle=&rft_id=info:eric/EJ849122
ER -

TY - JOUR
ID - 2189

T1 - Reflecting on "Project Katrina" and Developmentally Appropriate Practices: A Graduate Student's Perspective
A1 - Schellhaas,Andree
A1 - Burts,Diane C.
A1 - Aghayan,Carol
Y1 - 2007/01//
KW - ERIC
KW - Unsupervised Play Addition
KW - ERIC,Current Index to Journals in Education (CIJE)
KW - Early Childhood Education
KW - Preschool Education
KW - Writing for Publication
KW - School Community Relationship
KW - Teacher Student Relationship
KW - Graduate Students
KW - Play
KW - Integrated Curriculum
KW - Peer Relationship
KW - Laboratory Schools
KW - Developmentally Appropriate Practices
KW - Child Development
KW - Natural Disasters
KW - Preschool Children
KW - Teaching Methods
RP - NOT IN FILE
SP - 77
EP - 81
JF - Journal of Early Childhood Teacher Education
VL - 28
IS - 1
PB - Routledge. , 325 Chestnut Street Suite 800, Philadelphia, PA 19106.
N2 - This article describes the independent study project of a student who was a graduate assistant in a child development laboratory preschool when Hurricanes Katrina and Rita struck the Gulf Coast area. Through her experiences with "Project Katrina" she deepens her understanding of developmentally appropriate practices as she learns firsthand about the project approach and writing an article for publication in a professional journal
SN - 1090-1027, 1090-1027
UR - http://search.proquest.com/docview/61882899?accountid=14701
L2 - http://sfx.scholarsportal.info/ottawa?url_ver=Z39.88-2004&rft_val_fmt=info:ofi/fmt:kev:mtx:journal&genre=article&sid=ProQ:ProQ%3Aericshell&atitle=Reflecting+on+%22Project+Katrina%22+and+Developmentally+Appropriate+Practices%3A+A+Graduate+Student%27s+Perspective&title=Journal+of+Early+Childhood+Teacher+Education&issn=10901027&date=2007-01-01&volume=28&issue=1&spage=77&au=Schellhaas%2C+Andree%3BBurts%2C+Diane+C.%3BAghayan%2C+Carol&isbn=&jtitle=Journal+of+Early+Childhood+Teacher+Education&btitle=&rft_id=info:eric/EJ828428
ER -

TY - JOUR
ID - 2190
T1 - The Funeral of Froggy the Frog: The Child as Dramatist, Designer, and Realist
A1 - Cummins,Lauren
Y1 - 2004/07//
KW - ERIC
KW - Unsupervised Play Addition
KW - ERIC,Current Index to Journals in Education (CIJE)
KW - Death
KW - Play
KW - Childrens Literature

KW - Emotional Response
KW - Art Expression
KW - Creativity
KW - Coping
KW - Drama
RP - NOT IN FILE
SP - 87
EP - 91
JF - Young Children
VL - 59
IS - 4
PB - National Association for the Education of Young Children. 1313 L Street NW Suite 500, Washington, DC 22205-4101.
N2 - One sunny afternoon, six-year-old Zachary and his friend John Michael, four and a half, discovered a dead frog in a bag of clay in the garage. Zachary proposed, "Let's have a funeral for the frog." This is how the funeral drama of Froggy the Frog began. This article describes the play experiences of Zachary and John Michael as designers, dramatists, and realists and addresses their specific play experience with death through the funeral of Froggy. In this article, the author explains the creative and artistic aspect of producing the funeral, and how they expressed their emotions and questions. She presents tips for adults in helping children understand and cope with death, as well as children's books on death and dying. (Contains a list of 23 resources.)
SN - 1538-6619, 1538-6619
UR - http://search.proquest.com/docview/62022109?accountid=14701
L2 - http://sfx.scholarsportal.info/ottawa?url_ver=Z39.88-2004&rft_val_fmt=info:ofi/fmt:kev:mtx:journal&genre=article&sid=ProQ:ProQ%3Aericshell&atitle=The+Funeral+of+Froggy+the+Frog%3A+The+Child+as+Dramatist%2C+Designer%2C+and+Realist&title=Young+Children&issn=15386619&date=2004-07-01&volume=59&issue=4&spage=87&au=Cummins%2C+Lauren&isbn=&jtitle=Young+Children&btitle=&rft_id=info:eric/EJ751303
ER -

TY - JOUR
ID - 2191
T1 - Young Children's Play Qualities in Same-, Other-, and Mixed-Sex Peer Groups
A1 - Fabes,Richard A.
A1 - Martin,Carol Lynn
A1 - Hanish,Laura D.
Y1 - 2003///
KW - ERIC
KW - Unsupervised Play Addition
KW - Cross Sex Interaction
KW - Cross Sex Peers
KW - Dyads
KW - Same Sex Interaction
KW - Same Sex Peers
KW - ERIC,Current Index to Journals in Education (CIJE)
KW - Context Effect
KW - Comparative Analysis
KW - Play
KW - Gender Issues
KW - Peer Influence
KW - Sex Differences
KW - Preschool Children
KW - Peer Groups
RP - NOT IN FILE
SP - 921
EP - 932

JF - Child Development
JA - Child Dev.
VL - 74
IS - 3
N2 - This study examined how children's play varied as function of child's sex, play partner's sex, and whether they played in dyads or groups. Observation data revealed that boys' same-sex play was more active-forceful, farther from presence of adults, and more stereotyped than girls', a pattern exaggerated in groups. Mixed-sex play was more likely than other configurations to occur near adults and was intermediate in active-forceful and stereotyped activities. (Author/KB)
SN - 0009-3920, 0009-3920
UR - http://search.proquest.com/docview/62165882?accountid=14701
L2 - http://sfx.scholarsportal.info/ottawa?url_ver=Z39.88-2004&rft_val_fmt=info:ofi/fmt:kev:mtx:journal&genre=article&sid=ProQ:ProQ%3Aericshell&atitle=Young+Children%27s+Play+Qualities+in+Same-%2C+Other-%2C+and+Mixed-Sex+Peer+Groups.&title=Child+Development&issn=00093920&date=2003-01-01&volume=74&issue=3&spage=921&au=Fabes%2C+Richard+A.%3BMartin%2C+Carol+Lynn%3BHanish%2C+Laura+D.&isbn=&jtitle=Child+Development&btitle=&rft_id=info:eric/EJ676633
ER -

TY - RPRT
ID - 2192
T1 - Learning through Drama
A1 - Jensen,Ina
A1 - Rechis,Ruth
A1 - Luna,Don J.
Y1 - 2002/04//
N1 - Availability - Level 1 - Available online, if indexed January 1993 onward
SuppNotes - In: "Early Childhood Literacy: Programs & Strategies To Develop Cultural, Linguistic, Scientific and Healthcare Literacy for Very Young Children & their Families, 2001 Yearbook"; see PS 030 591
Last updated - 2012-12-14
KW - ERIC
KW - Unsupervised Play Addition
KW - Corpus Christi Independent School District TX
KW - Texas (Corpus Christi)
KW - Texas A and M University Corpus Christi
KW - ERIC,Resources in Education (RIE)
KW - Early Childhood Education
KW - Program Descriptions
KW - Class Activities
KW - Young Children
KW - Dramatic Play
KW - Bilingual Education
KW - Childrens Literature
KW - College School Cooperation
KW - Classroom Techniques
KW - Movement Education
KW - Physical Activities
KW - Laboratory Schools
KW - Developmentally Appropriate Practices
KW - Child Development
KW - Child Development Centers
KW - Drama
KW - Teaching Methods
RP - NOT IN FILE
SP - 11

N2 - This chapter is part of a book that recounts the year's work at the Early Childhood Development Center (ECDC) at Texas A & M University-Corpus Christi. Rather than an "elitist" laboratory school for the children of university faculty, the dual-language ECDC is a collaboration between the Corpus Christi Independent School District and the university, with an enrollment representative of Corpus Christi's population. The chapter describes the ECDC's Learning through Drama program, in which children explore weekly concepts through movement and drama. Examples include movement and drama activities related to children's books, mime, field trips, geography, movies, and math, language, and science skills. The chapter includes a list of children's books. (EV)
UR - http://search.proquest.com/docview/62194954?accountid=14701
ER -

TY - RPRT
ID - 2193
T1 - Dialogical Drama with Puppets and Children's Creation of Sense
A1 - Bredikyte,Milda
Y1 - 2000/08//
N1 - Availability - Level 1 - Available online, if indexed January 1993 onward
SuppNotes - Paper presented at the European Conference on Quality in Early Childhood Education (EECERA) (10th, London, England, August 29-September 1, 2000)
Last updated - 2012-12-14
KW - ERIC
KW - Unsupervised Play Addition
KW - ERIC,Resources in Education (RIE)
KW - Preschool Education
KW - Thinking Skills
KW - Puppetry
KW - Class Activities
KW - Young Children
KW - Dramatic Play
KW - Scripts
KW - Creative Development
KW - Creative Expression
KW - Comparative Analysis
KW - Creative Thinking
KW - Classroom Research
KW - Acting
KW - Story Telling
RP - NOT IN FILE
SP - 26
N2 - Dialogical drama with puppets is a specific method of revealing basic cultural values for preschool children. For this study, an interactive performance of a puppet show for young children was videotaped, along with a later performance of the same show by the children. The study compared the techniques used by the adult performers and the children and the similarities and differences in the fairy tale plot between the adults' and children's performances. The study also attempted to clarify the possibilities for children's creative expression in this type of activity. Episodes of the adults' and children's presentations were compared regarding composition, texts, addressees, and means of expression. Analysis indicated that the puppet show performed by the children was not a repetition but an independent creative activity that helped the children to shape the process of their own understanding and to discover sense hidden in the fairy tale; sensitive and skillful adult participation seemed key to the quality of the children's creative learning experience. (Contains 10 references.) (EV)
UR - http://search.proquest.com/docview/62346362?accountid=14701
ER -

TY - BOOK
ID - 2194
T1 - Making Make-Believe: Fun Props, Costumes and Creative Play Ideas
A1 - Kohl,MaryAnn F.

Y1 - 1999///
N1 - Availability - Level 2 - Produced in microfiche (1966-2003)
SuppNotes - Illustrations by K. Whelan Dery
Last updated - 2012-12-14
KW - ERIC
KW - Unsupervised Play Addition
KW - Costumes
KW - Hats
KW - Masks
KW - Play Learning
KW - Play Materials
KW - ERIC,Resources in Education (RIE)
KW - Early Childhood Education
KW - Puppetry
KW - Play
KW - Class Activities
KW - Young Children
KW - Parent Materials
KW - Dramatic Play
KW - Recipes (Food)
KW - Role Playing
KW - Childrens Literature
KW - Creativity
KW - Imagination
KW - Books
KW - Learning Activities
KW - Pretend Play
KW - Cooking Instruction
KW - Art Activities
RP - NOT IN FILE
SP - 383
PB - Gryphon House, Inc., 10726 Tucker Street, Beltsville, MD 20705; Tel: 800-638-0928 (Toll Free); Fax: 301-595-0051; Web site: gryphonhouse.com ($14.95).
N2 - Intended to encourage children to develop their imaginations naturally, with only a small amount of help from adults, this book presents over 125 activities for creative and pretend play for young children. The book's introduction describes the importance and benefits of pretend play, the adult's role, and needed materials and space. The activities are presented in seven chapters: (1) "Play Settings and Imagination Spaces"; (2) "Little Scenes and Mini-Play"; (3) "Puppets and Sets"; (4) "Hats, Costumes, and Masks"; (5) "Storybook Play & Pretend," with activities related to popular children's books; (6) "Let's Play Make-Believe"; and (7) "Cooking, Games, and Other Activities Too Fun To Miss." Each activity includes drawings to illustrate the project and icons to assist readers in evaluating the project with regard to type, time needed for preparation, and ease of use. In addition, each activity lists the materials needed, lists the steps for completion, notes which steps require adult supervision, and presents additional ideas related to the activity. Three indices organize activities by type, materials used and skill level, and project name. (KB)
SN - 0876591985
UR - http://search.proquest.com/docview/62480235?accountid=14701
L2 - http://sfx.scholarsportal.info/ottawa?url_ver=Z39.88-2004&rft_val_fmt=info:ofi/fmt:kev:mtx:book&genre=book&sid=ProQ:ERIC&atitle=&title=Making+Make-Believe%3A+Fun+Props%2C+Costumes+and+Creative+Play+Ideas.&issn=&date=1999-01-01&volume=&issue=&spage=383&au=Kohl%2C+MaryAnn+F.&isbn=0876591985&jtitle=&btitle=Making+Make-Believe%3A+Fun+Props%2C+Costumes+and+Creative+Play+Ideas.&rft_id=info:eric/ED430689
ER -

TY - RPRT
ID - 2195
T1 - Zone of Proximal Development and the World of the Child

A1 - Seng,Seok Hoon
Y1 - 1997/11//
N1 - Availability - Level 1 - Available online, if indexed January 1993 onward
SuppNotes - Paper presented at the Educational Research Association Conference (Singapore, November 24-26, 1997)
Last updated - 2012-12-14
KW - ERIC
KW - Unsupervised Play Addition
KW - Mediated Instruction
KW - Vygotsky (Lev S)
KW - Zone of Proximal Development
KW - ERIC,Resources in Education (RIE)
KW - Early Childhood Education
KW - Academic Aptitude
KW - Scaffolding (Teaching Technique)
KW - Foreign Countries
KW - Cognitive Development
KW - Learning Theories
KW - Child Development
KW - Pretend Play
KW - Educational Research
KW - Teaching Methods
RP - NOT IN FILE
SP - 15
N2 - This paper examines Lev Vygotsky's theory concerning the zone of proximal development (ZPD) in children and its relevance to early childhood education. As per Vygotsky's "Mind in Society" (1978), ZPD is the difference between a child's "actual development level as determined by independent problem solving" and the "potential development as determined through problem solving under adult guidance or in collaboration with more capable peers." The paper focuses on the different nature of ZPD in the context of real world and pretend world activities of young children and how these activities differ in terms of parameters and motivations. Various studies on the application of ZPD to early childhood education are reviewed, especially in regard to guided participation, scaffolding, mediated learning, and other instructional techniques. The paper concludes by noting that while a Vygotskian perspective has theoretical and concrete value for early childhood education, further investigation is needed to extend Vygotsky's conceptions and to clarify their educational implications. Contains 14 references. (MDM)
UR - http://search.proquest.com/docview/62545083?accountid=14701
ER -

TY - RPRT
ID - 2196
T1 - The Development of Private Speech among Two- to Five-Year-Olds in the Naturalistic Preschool Setting
A1 - Kirby,Kathleen Campano
Y1 - 1997/04//
N1 - Availability - Level 1 - Available online, if indexed January 1993 onward
SuppNotes - Paper presented at the Biennial Conference of the Society for Research in Child Development (62nd, Washington, DC, April 3-6, 1997)
Last updated - 2012-12-14
KW - ERIC
KW - Unsupervised Play Addition
KW - Private Speech
KW - Vygotsky (Lev S)
KW - ERIC,Resources in Education (RIE)
KW - Preschool Education
KW - Context Effect
KW - Play
KW - Developmental Stages

KW - Preschool Children
KW - Age Differences
RP - NOT IN FILE
SP - 11
N2 - In this study, preschoolers were observed in the naturalistic preschool context to determine the development of private speech over the preschool years and contextual influences on its occurrence among 3- to 5-year-olds. Thirty-nine preschoolers were observed in their preschool classroom 4 times in 10-minute segments during free choice play activities. Each observation was coded for the child's type of play, the goal of the activity, adult presence, peer involvement, private speech, and social speech. The results showed that each preschooler emitted some type of private speech over the course of the investigation and that the total amount of private speech declined linearly as age increased. This age-related decline in private speech, with the highest incidence at age 2, suggests that Vygotsky's hypothesis of a curvilinear development trend needs to be modified. Children's changing activities during free choice periods in the preschool setting may have influenced this trend. (Author/HTH)
UR - http://search.proquest.com/docview/62509872?accountid=14701
ER -

TY - JOUR
ID - 2197
T1 - The Relation between Individual Differences in Fantasy and Theory of Mind
A1 - Taylor,Marjorie
A1 - Carlson,Stephanie M.
Y1 - 1997///
KW - ERIC
KW - Unsupervised Play Addition
KW - False Beliefs
KW - Perceived Reality
KW - Representational Thinking
KW - theory of mind
KW - ERIC,Current Index to Journals in Education (CIJE)
KW - Childhood Attitudes
KW - Cognitive Processes
KW - Cognitive Development
KW - Fantasy
KW - Perspective Taking
KW - Pretend Play
KW - Preschool Children
RP - NOT IN FILE
SP - 436
EP - 455
JF - Child Development
JA - Child Dev.
VL - 68
IS - 3
N2 - Examined relation between early fantasy/pretense and knowledge about mental life in 3- and 4-year olds. Found that performance on theory of mind tasks was significantly intercorrelated when effects of verbal intelligence and age were statistically controlled. Individual differences in fantasy/pretense were related to theory of mind performance in 4-year olds, independent of verbal intelligence. (Author/KB)
SN - 0009-3920, 0009-3920
UR - http://search.proquest.com/docview/62507383?accountid=14701
L2 - http://sfx.scholarsportal.info/ottawa?url_ver=Z39.88-2004&rft_val_fmt=info:ofi/fmt:kev:mtx:journal&genre=article&sid=ProQ:ProQ%3Aericshell&atitle=The+Relation+between+Individual+Differences+in+Fantasy+and+Theory+of+Mind.&title=Child+Development&issn=00093920&date=1997-01-01&volume=68&issue=3&spage=436&au=Taylor%2C+Marjorie%3BCarlson%2C+Stephanie+M.&isbn=&jtitle=Child+Development&btitle=&rft_id=info:eric/EJ549520

ER -

TY - GEN
ID - 2198
T1 - Peer Conflicts in the Classroom. ERIC Digest
A1 - Wheeler,Edyth J.
Y1 - 1994/09//
N1 - Availability - Level 1 - Available online, if indexed January 1993 onward
Last updated - 2012-12-14
KW - ERIC
KW - Unsupervised Play Addition
KW - ERIC Digests
KW - ERIC,Resources in Education (RIE)
KW - Teachers
KW - Parents
KW - Practitioners
KW - Teacher Student Relationship
KW - Play
KW - Young Children
KW - Classroom Environment
KW - Adult Child Relationship
KW - Conflict Resolution
KW - Conflict
KW - Peer Relationship
KW - Verbal Ability
KW - Sex Differences
KW - Social Development
KW - Age Differences
RP - NOT IN FILE
SP - 3
N2 - Recent theory and research suggest that peer conflict contributes to children's development and represents an important form of social interaction. Research has identified structural features of children's conflict as issues, such as control of objects or physical space; strategies, including aggressive and nonaggressive physical and verbal tactics; and outcomes, such as an unresolved situation, an adult-imposed solution, one child's submission to another, or a mutually agreed-upon solution. Studies of children's conflicts indicate that age makes a difference in conflict resolution; the role of gender, however, is not as clear. Children's conflicts during play are influenced by the play setting, the children's prior relationships, and the presence of adults. Disputes are more likely to occur in closed than open play areas, children who were playing together before a conflict are more likely to resolve the conflict than children who were not playing together, and children more often generate their own solutions to conflicts when an adult is absent than when an adult is present. Implications for teachers and parents can be drawn from the research on children's conflicts. Teachers should: (1) be aware of children's intentions in an apparent conflict; (2) let children who are verbally competent try to resolve their conflict themselves; (3) note whether children were playing together before the conflict; (4) make play spaces accessible and provide ample play materials; and (5) help children, in conflicts characterized by physical strategies, to find words to use to express their feelings. (BC)
UR - http://search.proquest.com/docview/62820186?accountid=14701
ER -

TY - GEN
ID - 2199
T1 - Self Talk in Normal and Autistic Children
A1 - Kerr,Kaye M.
Y1 - 1993/03//
N1 - Availability - Level 1 - Available online, if indexed January 1993 onward
SuppNotes - Paper presented at the Society for Research in Child Development (New Orleans, LA, March 25-

28, 1993)
Last updated - 2012-12-14
KW - ERIC
KW - Unsupervised Play Addition
KW - Self Talk
KW - ERIC,Resources in Education (RIE)
KW - Preschool Education
KW - Play
KW - Foreign Countries
KW - Speech Habits
KW - Videotape Recordings
KW - Adults
KW - Preschool Children
KW - Interpersonal Relationship
KW - Speech
KW - Autism
RP - NOT IN FILE
SP - 17
N2 - This investigation compared self-talk use in five preschool autistic children (ages 57 to 86 months) and in matched chronological age and mental age peer groups. Videotape recordings of the children during free play with and without an adult were coded for the following self-talk categories: (1) private, mastery speech; (2) stereotypic repetitions; (3) word play; (4) self-regulative speech; (5) utterances; and (6) mouthing of words. All three groups exhibited fewer instances of self-talk in the presence of adults than without an adult. The autistic children demonstrated a similar pattern of self-talk with adults as did their control peers. Autistic children showed less consistency in behaviors between the adult present and adult absent situation than did controls; this was not felt to reflect their lack of response to adults in their environment, but rather to the high production of utterances and private mastery talk when they were not with an adult. (Contains six figures/tables.) (JDD)
UR - http://search.proquest.com/docview/62773526?accountid=14701
ER -

TY - RPRT
ID - 2200
T1 - Developmental Changes in Toddlers' Social Orientation and Affect during Mastery Play
A1 - Jennings,Kay Donahue
Y1 - 1993/03//
N1 - Availability - Level 1 - Available online, if indexed January 1993 onward
SuppNotes - Paper presented at the Biennial Meeting of the Society for Research in Child Development (60th, New Orleans, LA, March 25-28, 1993)
Last updated - 2012-12-14
KW - ERIC
KW - Unsupervised Play Addition
KW - Internalization
KW - Mastery Motivation
KW - Mastery Task
KW - ERIC,Resources in Education (RIE)
KW - Preschool Education
KW - Toddlers
KW - Cognitive Ability
KW - Play
KW - Cognitive Development
KW - Attention Span
KW - Mastery Learning
RP - NOT IN FILE
SP - 14
N2 - This study explored how toddlers' increasing social sophistication and increasing ability to coordinate attention between the social and object realm are reflected in toddlers' mastery play. A total of 57 toddlers (24

boys and 33 girls from a wide range of socio-economic backgrounds) between the ages of 15 and 35 months participated in 7 mastery tasks initially presented by an adult examiner. The study examined developmental changes in three areas: (1) regulation of attention to tasks and people; (2) regulation of affect during mastery play; and (3) internalization of mastery standards. It was found that younger toddlers, as expected, required more adult prompts to maintain their task focus, but were able to coordinate their attention to objects and persons about as well as older toddlers. Younger toddlers also had more difficulty regulating negative affect to frustrations inherent in mastery tasks, although responses to failure were generally similar across age groups. Evidence of internalization of culturally defined standards for mastery, such as pride in task completion, was found for even the youngest toddlers. The results suggest greater sophistication in young toddlers' mastery play than was previously thought. (MDM)
UR - http://search.proquest.com/docview/62781763?accountid=14701
ER -

TY - RPRT
ID - 2201
T1 - Anatomical Doll Play among Young Children: A Follow-Up of Sexual Demonstrators and Doll Avoiders
A1 - Boat,Barbara W.
A1 - Everson,Mark D.
Y1 - 1989/08//
N1 - Availability - Level 1 - Available online, if indexed January 1993 onward
SuppNotes - Paper presented at the Annual Meeting of the American Psychological Association (97th, New Orleans, LA, August, 1989)
Last updated - 2012-12-14
KW - ERIC
KW - Unsupervised Play Addition
KW - Anatomically Correct Dolls
KW - Comfort Index
KW - Sex Knowledge
KW - ERIC,Resources in Education (RIE)
KW - Preschool Education
KW - Play
KW - Mothers
KW - Videotape Recordings
KW - Individual Development
KW - Test Reliability
KW - Knowledge Level
KW - Followup Studies
KW - Diagnostic Tests
KW - Sexual Abuse
KW - Preschool Children
KW - Early Experience
KW - Child Abuse
RP - NOT IN FILE
SP - 15
N2 - As one phase of an extensive research project, normative data on the anatomical doll play of 209 nonreferred 2- through 5-year-old children were collected which revealed extreme, non-normative responses among some 4- and 5-year-olds. Later, a follow-up study was conducted to investigate the consistency of non-normative behavior over time and to explore children's knowledge of genital intercourse, as well as sources of that knowledge. In the normative study, 5 percent of subjects (Demonstrators) clearly placed the dolls in intercourse positions and 8 percent (Avoiders) refused to touch the unclothed dolls. Involving 40 of the original participants, including 10 Demonstrators and 10 Avoiders, follow-up occurred 16 months after the initial study and matched Demonstrator and Avoider subjects to 20 controls on gender, race, and socioeconomic status. Findings revealed changes over time in Demonstrators' and Avoiders' behaviors that may be explained by cultural, maturational, and socialization factors. Most Demonstrators indicated that they had learned about intercourse from watching sexually explicit videos. Implications for children's testimony concerning sexual abuse and for understanding children's behaviors are explored. It is concluded that anatomical dolls can never be

a definitive test of child sexual abuse, that the presence of an adult interviewer appears to have an inhibiting effect on children's exploratory behaviors with the dolls, that even 6- and 7-year-olds may need professional support in disclosing sexual abuse, and that further research is needed. (RH)
UR - http://search.proquest.com/docview/63062623?accountid=14701
ER -

TY - GEN
ID - 2203
T1 - Strawberry Shortcake and Other Jumping Rope Ideas
A1 - Adams,Polly K.
A1 - Taylor,Michaell K.
Y1 - 1982///
N1 - Availability - Level 1 - Available online, if indexed January 1993 onward
Last updated - 2012-12-14
KW - ERIC
KW - Unsupervised Play Addition
KW - Jump Roping
KW - PF Project
KW - Rhyme
KW - ERIC,Resources in Education (RIE)
KW - Teachers
KW - Practitioners
KW - Elementary Education
KW - Sequential Approach
KW - Play
KW - Aerobics
KW - Physical Fitness
KW - Guidelines
KW - Physical Development
KW - Cardiovascular System
KW - Exercise
KW - Childrens Games
KW - Physical Activities
KW - Cultural Influences
KW - Recreational Activities
KW - Games
KW - Playground Activities
RP - NOT IN FILE
SP - 14
N2 - Information, guidelines, and activities for jumping rope are given. A short history of jumping rope explains how it evolved from a spring ritual for men to a play activity involving mostly young girls. Physical and cultural reasons are given as to why jumping rope has been more a sport for girls than for boys. Research studies are noted which show that jumping rope is a good cardiopulmonary activity that is being included in present-day adult fitness routines. It is pointed out that jumping rope provides an excellent source of release from tension and stress, and is an effective vehicle for achieving and maintaining appropriate fitness levels. Advice is given on selecting commercial ropes, making jump ropes, fitting the rope to the jumper, storing and cleaning ropes, and integrating rope jumping into the rest of the curriculum. The remainder of this document contains 39 jump rope activities arranged in a skill-building sequence which includes: (1) beginner rope exercises; (2) single jumping exercises; (3) double exercises; and (4) exercises using rhythms and chants for children to recite while jumping. Concluding this document are 14 references to journal articles and books. (EM)
UR - http://search.proquest.com/docview/63423599?accountid=14701
L2 - http://sfx.scholarsportal.info/ottawa?url_ver=Z39.88-2004&rft_val_fmt=info:ofi/fmt:kev:mtx:journal&genre=unknown&sid=ProQ:ProQ%3Aericshell&atitle=Strawberry+Shortcake+and+Other+Jumping+Rope+Ideas.&title=&issn=&date=1982-01-

01&volume=&issue=&spage=14&au=Adams%2C+Polly+K.%3BTaylor%2C+Michaell+K.&isbn=&jtitle=&btitle=&rft_id=info:eric/ED242386
ER -

TY - RPRT
ID - 2204
T1 - The Effects of a Playgroup Experience on Mother-Child and Father-Child Interaction
A1 - Vandell,Deborah Lowe
Y1 - 1978/03//
N1 - Availability - Level 1 - Available online, if indexed January 1993 onward
SuppNotes - An earlier version of this paper was presented at the Southwestern Society for Research in Human Development Conference (Dallas, Texas, March 17-18, 1978)
Last updated - 2012-12-14
KW - ERIC
KW - Unsupervised Play Addition
KW - ERIC,Resources in Education (RIE)
KW - Play
KW - Responses
KW - Mothers
KW - social relations
KW - Fathers
KW - Parent Child Relationship
KW - Infant Behavior
KW - Child Rearing
KW - Intergroup Relations
KW - Parent Attitudes
KW - Interaction Process Analysis
KW - Research
KW - Preschool Children
KW - Early Experience
KW - Peer Groups
RP - NOT IN FILE
SP - 31
N2 - The purpose of this study was to compare the mother-child and father-child interaction of six toddlers who were completely home reared with the interactions of six toddlers who were participants in a daily 3-hour playgroup. For the comparisons, both mother-child and father-child interactions were videotaped in a semi-structured laboratory setting. The first observation was made immediately before the beginning of the playgroup experience, with subsequent observations being made after half of the toddlers had been in a playgroup three and six months. While there were no differences in the playgroup and homecare toddlers before the playgroup experience, significant differences were found after the playgroup experience. The playgroup toddlers became proportionally more active in their social interactions. They also became more responsive to the interaction initiations of their parents. Significant differences were also found in parental behavior as a function of the playgroup experience. The parents of the playgroup children became significantly less controlling. In addition to playgroup/homecare differences, significant differences were also found in mother-child and father-child interaction independent of the playgroup experience. (Author/CM)
UR - http://search.proquest.com/docview/63842740?accountid=14701
L2 - http://sfx.scholarsportal.info/ottawa?url_ver=Z39.88-2004&rft_val_fmt=info:ofi/fmt:kev:mtx:book&genre=unknown&sid=ProQ:ERIC&atitle=&title=The+Effects+of+a+Playgroup+Experience+on+Mother-Child+and+Father-Child+Interaction.&issn=&date=1978-03-01&volume=&issue=&spage=31&au=Vandell%2C+Deborah+Lowe&isbn=&jtitle=&btitle=The+Effects+of+a+Playgroup+Experience+on+Mother-Child+and+Father-Child+Interaction.&rft_id=info:eric/ED153726
ER -

TY - GEN
ID - 2205
T1 - Social Studies: The Child's Point of View

A1 - Wheeler,Ronald
A1 - Losito,William F.
Y1 - 1978///
N1 - Last updated - 2012-12-14
KW - ERIC
KW - Unsupervised Play Addition
KW - ERIC,Current Index to Journals in Education (CIJE)
KW - Elementary Education
KW - Concept Formation
KW - Play
KW - Anthropology
KW - Human Relations
KW - Social Studies
KW - Observation
KW - Interpersonal Relationship
KW - CHILDREN
KW - Peer Relationship
RP - NOT IN FILE
SP - 378
EP - 380
JF - Social Education
VL - 42
IS - 5
N2 - Excerpts from observational diaries kept by education students acting as anthropologists studying the conceptual tools of young children at home and in school. Notes describe children's rules for activities and games; individual projects; and reactions to social studies concepts presented from an "adultcentric" point of view. (AV)
UR - http://search.proquest.com/docview/63882134?accountid=14701
L2 - http://sfx.scholarsportal.info/ottawa?url_ver=Z39.88-2004&rft_val_fmt=info:ofi/fmt:kev:mtx:journal&genre=unknown&sid=ProQ:ProQ%3Aericshell&atitle=Social+Studies%3A+The+Child%27s+Point+of+View&title=Social+Education&issn=&date=1978-01-01&volume=42&issue=5&spage=378&au=Wheeler%2C+Ronald%3BLosito%2C+William+F.&isbn=&jtitle=Social+Education&btitle=&rft_id=info:eric/EJ178580
ER -

TY - RPRT
ID - 2206
T1 - The Effects of Age, Sex, and Adult Presence on Preschool Children's Sex-Typed Toy Play Behavior
A1 - Garrett,Candace Schau
Y1 - 1977/08//
N1 - Availability - Level 1 - Available online, if indexed January 1993 onward
Last updated - 2012-12-14
KW - ERIC
KW - Unsupervised Play Addition
KW - ERIC,Resources in Education (RIE)
KW - Sex Role
KW - Play
KW - Research Projects
KW - Sex Differences
KW - Child Development
KW - Toys
KW - Preschool Children
RP - NOT IN FILE
SP - 23
PB - C. S. Garrett, Department of Educational Foundations, College of Education, University of New Mexico, Albuquerque, NM 87131

N2 - Subjects were 26 female three-, four- and five-year-old children who free-played four different sets, each containing a culturally-defined feminine, masculine and neutral toy. A same- or opposite-sex adult was either present or absent during play. Results included the following: (1) girls played longer than boys with feminine and neutral toys, while boys played longer than girls with masculine toys; (2) there was a linear increase with age in the length of time both sexes played with feminine toys, but no age differences were found for masculine or neutral toys; and (3) there were no effects due to adult presence. These results are discussed in terms of sex-role development and methodological needs in psychometric and observational sex-typing research. (Author)
UR - http://search.proquest.com/docview/63745971?accountid=14701
L2 - http://sfx.scholarsportal.info/ottawa?url_ver=Z39.88-2004&rft_val_fmt=info:ofi/fmt:kev:mtx:book&genre=unknown&sid=ProQ:ERIC&atitle=&title=The+Effects+of+Age%2C+Sex%2C+and+Adult+Presence+on+Preschool+Children%27s+Sex-Typed+Toy+Play+Behavior.&issn=&date=1977-08-01&volume=&issue=&spage=23&au=Garrett%2C+Candace+Schau&isbn=&jtitle=&btitle=The+Effects+of+Age%2C+Sex%2C+and+Adult+Presence+on+Preschool+Children%27s+Sex-Typed+Toy+Play+Behavior.&rft_id=info:eric/ED160910
ER -

TY - GEN
ID - 2207
T1 - Play, Recreation and Leisure for People Who Are Deaf-Blind
A1 - Nesbitt,John A.
Y1 - 1975/04//
N1 - Availability - Level 1 - Available online, if indexed January 1993 onward
SuppNotes - Based on papers and preceedings of: National Institute on Program Development and Training in Recreation for Deaf-Blind Children, Youth and Adults (University of Iowa, April 29 - May 1, 1974)
Last updated - 2012-12-14
KW - ERIC
KW - Unsupervised Play Addition
KW - Parent Resources
KW - ERIC,Resources in Education (RIE)
KW - Athletics
KW - Play
KW - Camping
KW - Leisure Time
KW - Play Therapy
KW - Exceptional Child Services
KW - Rehabilitation Counseling
KW - Deaf Blind
KW - Recreation
KW - Advocacy
KW - Recreational Activities
KW - Severe Disabilities
KW - Resource Materials
KW - Games
KW - Playground Activities
RP - NOT IN FILE
SP - 49
N2 - Based on position papers and proceedings of the National Institute on Program Development and Training in Recreation for Deaf-Blind Children, Youth, and Adults, the document presents approximately 50 brief articles for use by parents, teachers, rehabilitation personnel, and therapeutic recreation personnel. Sections cover the following topics (sample article topics in parentheses): perspectives on recreation (suggestions for activities and games); play, recreation, and leisure (arts and crafts, camping, and sports); leisure education (role of the school); special considerations in recreation (problems and issues); consumerism and advocacy for recreation (strategies for advocacy); evaluation in recreation (evaluation of motor skills); guidelines on recreation (recreation's contribution to rehabilitation and education); administration of recreation (coordinating

development of services); and sources of information and assistance (regional centers, and information and research centers). Included is a listing of contributors with brief background sketches. (IM)
UR - http://search.proquest.com/docview/63893240?accountid=14701
ER -

© 2015 by the authors; licensee MDPI, Basel, Switzerland. This article is an open access article distributed under the terms and conditions of the Creative Commons Attribution license (http://creativecommons.org/licenses/by/4.0/).
